# Validation of a Diagnostic Microarray for Human Papillomavirus: Coverage of 102 Genotypes

**DOI:** 10.4061/2011/756905

**Published:** 2011-05-18

**Authors:** Sarah Tuttleton Arron, Peter Skewes-Cox, Phong H. Do, Eric Dybbro, Maria Da Costa, Joel M. Palefsky, Joseph L. DeRisi

**Affiliations:** ^1^Department of Dermatology, University of California San Francisco, San Francisco, CA 94143, USA; ^2^Biological and Medical Informatics Program, University of California San Francisco, San Francisco, CA 94143, USA; ^3^Department of Medicine, University of California San Francisco, San Francisco, CA 94143, USA; ^4^Department of Biochemistry and Biophysics, University of California San Francisco, San Francisco, CA 94143, USA; ^5^Howard Hughes Medical Institute, Chevy Chase MD 20115-6789, USA

## Abstract

Papillomaviruses have been implicated in a variety of human diseases ranging from common warts to invasive carcinoma of the anogenital mucosa. Existing assays for genotyping human papillomavirus are restricted to a small number of types. Here, we present a comprehensive, accurate microarray strategy for detection and genotyping of 102 human papillomavirus types and validate its use in a panel of 91 anal swabs. This array has equal performance to traditional dot blot analysis with the benefits of added genotype coverage and the ability to calibrate readout over a range of sensitivity or specificity values.

## 1. Introduction

Papillomaviruses are a group of nonenveloped, epitheliotropic DNA viruses that infect the skin and mucous membranes of humans and animals. There are over 100 different human papillomaviruses (HPV), which are associated with disease of the skin, mucous membranes, and aerodigestive tract. HPV infection leads to lesions ranging from common and genital warts to laryngeal papillomatosis, to epithelial cancers, particularly cervical carcinoma and a subset of head and neck squamous cell carcinoma (HNSCC). Detection of papillomavirus is usually based on molecular assays for viral nucleic acid [1].

There is considerable sequence diversity within the family *papillomaviridae*. The HPVs fall into five genera: alphapapillomavirus (*α*-PV), betapapillomavirus (*β*-PV), gammapapillomavirus (*γ*-PV), mupapillomavirus (*μ*-PV), and nupapillomavirus (*ν*-PV). The mucosal *α*-PV are the best described, due to their association with genital malignancy. There are thirteen types of high-risk mucosal *α*-PV associated with cervical and anal intraepithelial neoplasia: 16, 18, 31, 33, 35, 39, 45, 51, 52, 56, 58, 59, and 68. It has recently been recommended that HPV 66 be added to this high-risk group [2]. HPV testing is becoming increasingly important as a screening tool, in combination with cytology, for HPV-associated neoplasia [3, 4].

Most commercial assays for alphapapillomaviruses are limited to the high-risk types, which are relevant for use in the clinical setting but provide limited information in the research setting. The current FDA-approved tests for HPV are the Digene Hybrid Capture 2 (HC2) test and the Cervista HPV16/18 and HR test. HC2 combines antibody capture and chemiluminescent signal detection to detect 13 high-risk HPV types (Digene Corporation, Gaithersburg, Md, USA), while the Cervista HR uses invader chemistry to detect 14 high risk types (Hologic, Inc., Bedford, Mass, USA) [5]. While both of these assays have good sensitivity and specificity for cervical neoplasia, neither specifically genotypes the HPV infection.

There are currently no genotyping assays that cover multiple HPV genera. The majority of laboratory tests use PCR amplification with degenerate or multiplex primer sets followed by genotype readout with sequencing or blot, or bead-based Multiplexing [6–13]. A number of groups have developed microarray strategies for detection of HPV, but these have been limited to targeted PCR and array readout of a specific subset of HPV types [14–23].

The obstacle to a comprehensive detection strategy is the sequence diversity of HPVs. Standard PCR assays are sensitive, but it is difficult to design primers to detect a broad range of papillomavirus types. Here, we present a microarray strategy for the detection and genotyping of 102 HPV types representing all genera and validate its use in a large panel of anal swabs from an HPV cohort study.

## 2. Materials and Methods

### 2.1. Sample Material

Ninety-one samples were assayed, drawn from a previously reported cohort study of HPV infection in the anal canal of homosexual and bisexual men [24]. The study was approved by the University of California, San Francisco Committee on Human Research, and informed consent was obtained from all subjects. As reported in that study, all samples were typed for HPV with MY09/MY11 consensus HPV-L1 primers [9] followed by dot blot with type-specific biotinylated probes for HPV types 6, 11, 16, 18, 26, 31, 32, 33, 35, 39, 40, 45, 51, 52, 53, 54, 55, 56, 58, 59, 61, 66, 68, 69, 70, 73, 82 variant, 83, and 84, as well as the following 10 HPV types together in a probe mixture: 2, 13, 34, 42, 57, 62, 64, 67, 72, and 82 [24]. These samples were drawn from a population with more multiplicity of types than a typical cervical sample set from healthy women.

### 2.2. Array Design

60 nt oligonucleotide probes were designed based on the full sequences of the 102 full-length HPV genomes listed in GenBank on January 6, 2009 (see Supplementary File 1 in Supplementary Material available online at doi: 10.406/2011/756905). Genotype-specific probes were designed using ArrayOligoSelector [25], a design package that addresses several design considerations including uniqueness, sequence complexity, self-annealing as a measure of secondary structure, and GC content (http://derisilab.ucsf.edu/index.php?page=software). Ten probes were selected for each genome with a target GC content of 30% and a maximum binding energy cutoff of −45 kcal/mol. Sequences containing AT regions longer than 20 bp and less than 10% of non-AT nucleotides were excluded from probe selection. A second set of genotype-specific probes were generated using a tiling strategy, with an offset of 20 nt across the whole genome and an offset of 5 nt over the L1 region, the region traditionally used for genotyping. These probes were filtered for a predicted binding energy of <−45 kcal/mol with their source HPV species and a predicted binding energy of >−10 kcal/mol against all other HPV species. All probes were filtered against nonspecific binding to the human genome. 

Additional probes were designed to cover conserved regions in each genus using a variation on a previously described strategy [26]. Each fully sequenced genome was divided into overlapping 60 nt segments offset by 30 nt, and a pairwise nucleotide BLAST [27] was performed between each potential probe and each viral genome. The top-ranked probe was selected, and the process was repeated iteratively on each sequence lacking significant homology to the selected probe until each genome was covered by at least five conserved probes with a predicted binding energy of <−45 kcal/mol. All probes were filtered against nonspecific binding to the human genome. 

Overall, 14,161 60-mer oligonucleotide probes and their corresponding reverse complements were designed. The array was synthesized on the Agilent 8x60K SurePrint G3 custom array platform (Agilent Technologies, Santa Clara, Calif, USA). Each of the 28,322 probes was printed in duplicate (Supplementary File 2).

### 2.3. Array Hybridization

DNA was randomly amplified and labeled with Cy3 using an adaptation of a previously described protocol without the reverse transcription step [28]. In parallel, HeLa cell DNA was used as a positive amplification and hybridization control, and water was used as a negative control. Array hybridization was performed with the Agilent Gene Expression Hybridization Kit, adapted from manufacturer's specifications with the following modification: nuclease-free water substituted for fragmentation buffer. Arrays were washed using Agilent Wash Buffer Kit, according to manufacturer's specifications, and scanned using Agilent Scan Control A.8.1.3 software. Data were extracted from the scanned array image using Agilent Feature Extraction version 10.5.1.1 Software.

### 2.4. Array Analysis

Values from replicate spots on the array were averaged for analysis, and data were analyzed using the E-Predict algorithm [29]. An E-matrix was generated from theoretical energy profiles for each of the 102 full-length HPV genomes in the design set, matched against each of the array probes. E-Predict compares this theoretical E-matrix profile to the observed hybridization pattern on an array to generate a similarity score and rank order of likelihood for each genome. E-Predict was run with unit vector plus quadratic matrix normalization, unit vector array normalization, and a dot product distance metric.

A set of uninformative oligonucleotides were defined as probes with significant autofluorescence when hybridized to water and probes with cross-hybridization to human DNA in a testing set of HPV-negative specimens. These were excluded from E-Predict analysis.

### 2.5. Sequence Genotyping of HPV

*α*-HPV PCR was performed using the nested primer sets MY09-MY011 and GP5-GP6, as previously described in [9, 13]. The PCR products were visualized by agarose gel electrophoresis, and amplicons of correct size were gel purified using the PureLink Quick Gel Extraction Kit (Invitrogen, Carlsbad, Calif, USA). Gel-purified DNA was inserted into pCR2.1 TOPO vector (Invitrogen) and transformed into chemically competent TOP10 *E. coli *(Invitrogen), according to manufacturer's specifications. For each DNA sample, 12 transformant colonies were selected for PCR insert amplification using M13 forward-reverse primers followed by sequencing on the ABI 3130xl Genetic Analyzer (Applied Biosystems, Carlsbad, Calif, USA).

### 2.6. Statistical Analysis

A receiver-operating characteristic (ROC) curve was generated to assess the similarity score for each of the 102 possible HPV genotype outcomes on array with the actual genotype(s) detected by sequencing of the 91 samples, for a total of 9282 tests. Sensitivity and specificity for the array were calculated with similarity score cutoff values of 0.035 and 0.1. Sensitivity and specificity were calculated for the blot based on the 29 types covered, for a total of 2639 tests. Agreement and Cohen's kappa coefficients were calculated for the blot versus the array for the 29 types covered on the blot. All statistical analysis was performed with Stata 11 (StataCorp LP, College Station, Tex, USA).

## 3. Results

28,322 60-mer oligonucleotide probes were designed and printed on the array. An E-matrix was generated to reflect the theoretical hybridization energies of each genome in the design set to each oligonucleotide on the array. This matrix was used for E-Predict analysis of biological samples hybridized to the array. 

**Figure 1:**
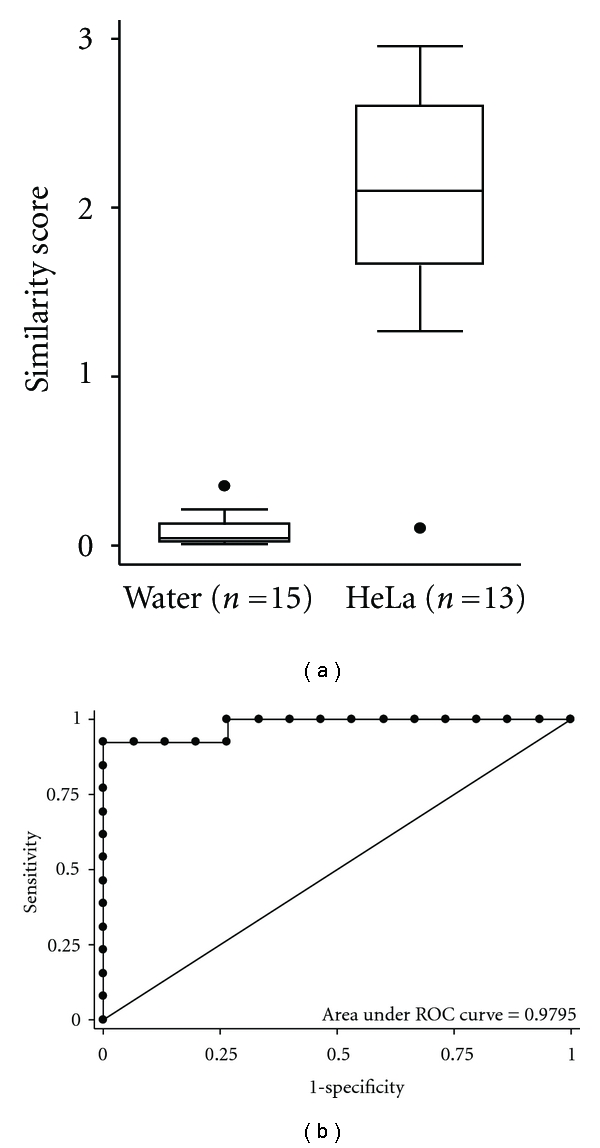
Array performance for HPV18. (a) Clear demarcation among similarity scores between positive and negative samples. (b) Receiver-operating characteristic curve for HPV18.

Performance of the array for one representative HPV type, HPV18, is demonstrated in Figure 1. The HeLa cervical cancer cell line, which contains integrated HPV18, was used as a positive control, and water was used as a negative control. The range of similarity scores for HPV18 showed a clear demarcation between positive and negative controls (Figure 1(a)), and a ROC curve was generated with an area under the curve of 0.9795 (Figure 1(b)), a near-perfect classification.

**Figure 2:**
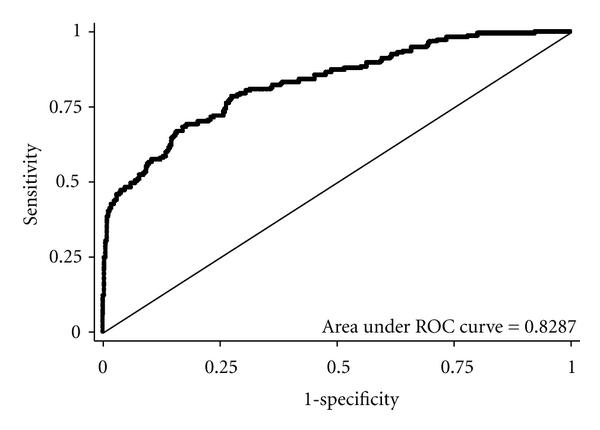
Receiver operating characteristic curve of the microarray for 91 anal swab samples tested for 102 HPV genotypes.

To characterize the performance of the array across all genomes, DNA from 91 anal swab samples was randomly amplified and hybridized to the array. For each specimen run on the array, a similarity score was generated for each of the 102 HPV genotypes in the E-matrix, generating 9,282 observations. These results were compared to a gold standard of PCR and sequencing. A ROC curve was generated with an area under the curve of 0.8287 (Figure 2).

Based on the ROC curve, two similarity score cut-points were generated for sensitivity and specificity calculations (Table 1). A score of 0.035 yielded a sensitivity of 0.72 and a specificity of 0.76 for the 102 genotypes in the E-matrix. A score of 0.1 yielded a sensitivity of 0.41 and a specificity of 0.99 for the same 102 types.

Each of the samples had previously been genotyped for HPV with a PCR and blot assay that covers 29 individual types and a probe mixture that detects ten additional types but does not discriminate between them. Compared to PCR and sequencing, the blot had a sensitivity of 0.41 and a specificity of 0.98 for the 29 specific types covered, similar to the array cut-point of 0.1. For the 29 types covered on the blot, the two assays had an agreement of 96.6%, kappa 0.6, representing good agreement [30]. 

Among the 91 samples, 157 instances of 34 HPV types were detected by PCR and sequencing. The types detected were HPV 6, 11, 12, 16, 18, 26, 31, 33, 34, 35, 40, 44, 45, 52, 53, 54, 55, 56, 58, 59, 61, cand62, 66, 67, 70, 71, 72, 81, 82, 83, 84, cand85, 97, and 102. Among those, there were 22 instances of 11 types which were not specifically covered by the blot strategy. Eighteen of these 22 infections (82%) were detected on the microarray (Table 2).

## 4. Discussion

We demonstrate the performance of a comprehensive microarray for detection and genotyping of HPV and validate this system on a large panel of anal swab samples. Previous microarrays for HPV genotyping were designed to read out multiplex or degenerate PCR amplification and targeted a small number of HPV types. The main benefit of this system is its accuracy for broad range of HPV genotypes, as demonstrated by an area under the ROC curve of 0.8287. Because the nucleic acid amplification is not reliant on degenerate or multiplex PCR, any HPV type present in the sample may be detected.

In this sample set, an *s* value cutoff of 0.1 yielded a sensitivity of 0.41 and specificity of 0.99. This cutoff favors specificity over sensitivity, a feature common to many HPV assays in clinical use. This is based on the prevalent assumption that extremely low levels of HPV in a sample are likely not clinically relevant. One example of this in practice is the blot assay used to characterize this sample set in a previously reported study [24]. Our array performance was similar to that of the blot assay (sensitivity 0.41, specificity 0.98) with the benefit of 61 additional HPV types covered on the array. In this cohort of 91 samples, there were 22 instances of 11 HPV types not present on the blot, of which 82% were detected by the microarray. This demonstrates the benefit of additional type coverage in a genotyping strategy.

As well as covering more HPV types, our assay is more flexible than the standard array readout system. Because the similarity score is a continuous variable, the cutoff can be tailored to the needs of the assay system. The similarity score cutoff that optimized both sensitivity and specificity was at a score of 0.035 (Figure 2). This yielded a sensitivity of 0.71 and specificity of 0.76 compared with PCR and sequencing.

We validated the performance of this microarray by a comparison to a gold standard of sequence genotyping. No previous study has reported microarray sensitivity and specificity with this type of validation. Some studies reported limits of detection and the absence of cross-hybridization but did not address true sensitivity and specificity [16, 20, 21]. Other studies compared array performance to other targeted HPV assays rather than to a gold standard of sequencing. Thus, the authors were only able to report interassay agreement or Cohen's kappa. Ermel et al. compared performance of *α*-PV detection with the Digene Hybrid Capture II Assay, the Roche Linear Array Assay, and the Kurabo GeneSquare Assay and reported a sample proportion of agreement ranging from 0.86 to 0.89 between the three assays. In two studies assessing multiplex PCR and array primer extension (APEX), Gheit et al. compared their arrays to reverse line blot or to one-step PCR and reverse hybridization with overall kappas of 0.55 and 0.5. In those studies, only a few samples were selected for sequencing to confirm array detection of types not seen on the other assays [14, 15]. In our study, we measured a sample proportion of agreement of 0.97 between the array and blot, with a kappa of 0.6. This level of agreement demonstrates the excellent performance of our system compared to previously reported arrays.The drawback to comparing assays with different types of coverage is that the agreement can only be assessed for the overlapping types, which underestimates the utility of a comprehensive array such as this one.

A number of arrays targeting the high-risk *α*-PV have been evaluated by calculating the sensitivity and specificity of the test for a cytologic diagnosis of neoplasia, which may be clinically relevant for a screening diagnostic but does not address the performance of the assay in genotyping HPV [18, 19, 22, 23, 31]. Future studies may address the sensitivity and specificity of our assay for cervical or anal intraepithelial neoplasia.

The theoretical E-matrix of energy profiles used to generate similarity scores was created from the 102 HPV genomes used for oligonucleotide probe design. However, the E-matrix can be expanded to include additional papillomavirus genomes if desired, allowing for an updated genotyping strategy without the synthesis of additional probes. This flexibility will lend itself to future studies as the number of complete papillomavirus sequences continues to expand. As vaccination for HPV types 6, 11, 16, and 18 increases, we may see a shift in HPV prevalence and the rise of less common types. This assay is well suited to detect these trends.

Overall, this microarray provides a comprehensive and accurate solution for the detection and genotyping of a broad spectrum of HPV types. It will lend itself to high-throughput applications such as screening of tumor cohorts and large epidemiologic studies.

## Supplementary Material

Supplementary File 1: HPV genomes used for probe design. This list includes the 102 full-length HPV genomes listed in GenBank on January 6, 2009.
Supplementary File 2: Oligonucleotide probes included on the array.

## Figures and Tables

**Table 1:** Comparison of array performance to blot. *P* values: Fisher's exact test.

Test	Blot		Array (*s* ≥ 0.1)		Array (*s* ≥ 0.035)	
Sequence	Positive	Negative	Positive	Negative	Positive	Negative
Test result +	55	52	63	115	112	2163
Test result −	80	2452	94	9010	45	6962
Sensitivity	.407		.401		.713	
Specificity	.979		.987		.763	
*P* value	<.0001		<.0001		<.0001	

**Table 2:** Array detection of 11 HPV types not covered by the blot.

HPV type	Array positive	Array negative
12	0	1
34	1	0
44	1	0
cand62	2	1
67	1	0
71	2	0
72	3	0
81	5	1
cand85	1	1
97	1	0
102	1	0

Total	18	4
