# Supplementary material for: Validation of a Diagnostic Microarray for Human Papillomavirus: Coverage of 102 Genotypes
Source: J Nucleic Acids. 2011 May 18;2011:756905. doi: 10.4061/2011/756905 (PMC3139178; doi:10.4061/2011/756905)
Supplement: Supplementary file 1 — HPV genomes used for probe design. This list includes the 102 full-length HPV genomes listed in GenBank on January 6, 2009. Supplementary File 2: Oligonucleotide probes included on the array. [file 756905.f1.pdf]

```
>gi|9627100|ref|NC_001526.1|_Human_papillomavirus_-_16,_complete_genome
ACTACAATAATTCATGTATAAAACTAAGGGCGTAACCGAAATCGGTTGAACCGAAACCGG
TTAGTATAAAAGCAGACATTTTATGCACCAAAAGAGAACTGCAATGTTTCAGGACCCACA
GGAGCGACCCAGAAAGTTACCACAGTTATGCACAGAGCTGCAAACAACTATACATGATAT
AATATTAGAATGTGTGTACTGCAAGCAACAGTTACTGCGACGTGAGGTATATGACTTTGC
TTTTCGGGATTTATGCATAGTATATAGAGATGGGAATCCATATGCTGTATGTGATAAATG
TTTAAAGTTTTATTCTAAAATTAGTGAGTATAGACATTATTGTTATAGTTTGTATGGAAC
AACATTAGAACAGCAATACAACAAACCGTTGTGTGATTTGTTAATTAGGTGTATTAACTG
TCAAAAGCCACTGTGTCCTGAAGAAAAGCAAAGACATCTGGACAAAAAGCAAAGATTCCA
TAATATAAGGGGTCGGTGGACCGGTCGATGTATGTCTTGTTGCAGATCATCAAGAACACG
TAGAGAAACCCAGCTGTAATCATGCATGGAGATACACCTACATTGCATGAATATATGTTA
GATTTGCAACCAGAGACAACTGATCTCTACTGTTATGAGCAATTAAATGACAGCTCAGAG
GAGGAGGATGAAATAGATGGTCCAGCTGGACAAGCAGAACCGGACAGAGCCCATTACAAT
ATTGTAACCTTTTGTTGCAAGTGTGACTCTACGCTTCGGTTGTGCGTACAAAGCACACAC
GTAGACATTCGTACTTTGGAAGACCTGTTAATGGGCACACTAGGAATTGTGTGCCCCATC
TGTTCTCAGAAACCATAATCTACCATGGCTGATCCTGCAGGTACCAATGGGGAAGAGGGT
ACGGGATGTAATGGATGGTTTTATGTAGAGGCTGTAGTGGAAAAAAAAACAGGGGATGCT
ATATCAGATGACGAGAACGAAAATGACAGTGATACAGGTGAAGATTTGGTAGATTTTATA
GTAAATGATAATGATTATTTAACACAGGCAGAAACAGAGACAGCACATGCGTTGTTTACT
GCACAGGAAGCAAAACAACATAGAGATGCAGTACAGGTTCTAAAACGAAAGTATTTGGTA
GTCCACTTAGTGATATTAGTGGATGTGTAGACAATAATATTAGTCCTAGATTAAAAGCTA
TATGTATAGAAAAACAAAGTAGAGCTGCAAAAAGGAGATTATTTGAAAGCGAAGACAGCG
GGTATGGCAATACTGAAGTGGAAACTCAGCAGATGTTACAGGTAGAAGGGCGCCATGAGA
CTGAAACACCATGTAGTCAGTATAGTGGTGGAAGTGGGGGTGGTTGCAGTCAGTACAGTA
GTGGAAGTGGGGGAGAGGGTGTTAGTGAAAGACACACTATATGCCAAACACCACTTACAA
ATATTTTAAATGTACTAAAAACTAGTAATGCAAAGGCAGCAATGTTAGCAAAATTTAAAG
AGTTATACGGGGTGAGTTTTTCAGAATTAGTAAGACCATTTAAAAGTAATAAATCAACGT
GTTGCGATTGGTGTATTGCTGCATTTGGACTTACACCCAGTATAGCTGACAGTATAAAAA
CACTATTACAACAATATTGTTTATATTTACACATTCAAAGTTTAGCATGTTCATGGGGAA
TGGTTGTGTTACTATTAGTAAGATATAAATGTGGAAAAAATAGAGAAACAATTGAAAAAT
TGCTGTCTAAACTATTATGTGTGTCTCCAATGTGTATGATGATAGAGCCTCCAAAATTGC
GTAGTACAGCAGCAGCATTATATTGGTATAAAACAGGTATATCAAATATTAGTGAAGTGT
ATGGAGACACGCCAGAATGGATACAAAGACAAACAGTATTACAACATAGTTTTAATGATT
GTACATTTGAATTATCACAGATGGTACAATGGGCCTACGATAATGACATAGTAGACGATA
GTGAAATTGCATATAAATATGCACAATTGGCAGACACTAATAGTAATGCAAGTGCCTTTC
TAAAAAGTAATTCACAGGCAAAAATTGTAAAGGATTGTGCAACAATGTGTAGACATTATA
AACGAGCAGAAAAAAAACAAATGAGTATGAGTCAATGGATAAAATATAGATGTGATAGGG
TAGATGATGGAGGTGATTGGAAGCAAATTGTTATGTTTTTAAGGTATCAAGGTGTAGAGT
TTATGTCATTTTTAACTGCATTAAAAAGATTTTTGCAAGGCATACCTAAAAAAAATTGCA
TATTACTATATGGTGCAGCTAACACAGGTAAATCATTATTTGGTATGAGTTTAATGAAAT
TTCTGCAAGGGTCTGTAATATGTTTTGTAAATTCTAAAAGCCATTTTTGGTTACAACCAT
TAGCAGATGCCAAAATAGGTATGTTAGATGATGCTACAGTGCCCTGTTGGAACTACATAG
ATGACAATTTAAGAAATGCATTGGATGGAAATTTAGTTTCTATGGATGTAAAGCATAGAC
CATTGGTACAACTAAAATGCCCTCCATTATTAATTACATCTAACATTAATGCTGGTACAG
ATTCTAGGTGGCCTTATTTACATAATAGATTGGTGGTGTTTACATTTCCTAATGAGTTTC
CATTTGACGAAAACGGAAATCCAGTGTATGAGCTTAATGATAAGAACTGGAAATCCTTTT
TCTCAAGGACGTGGTCCAGATTAAGTTTGCACGAGGACGAGGACAAGGAAAACGATGGAG
ACTCTTTGCCAACGTTTAAATGTGTGTCAGGACAAAATACTAACACATTATGAAAATGAT
AGTACAGACCTACGTGACCATATAGACTATTGGAAACACATGCGCCTAGAATGTGCTATT
TATTACAAGGCCAGAGAAATGGGATTTAAACATATTAACCACCAAGTGGTGCCAACACTG
GCTGTATCAAAGAATAAAGCATTACAAGCAATTGAACTGCAACTAACGTTAGAAACAATA
TATAACTCACAATATAGTAATGAAAAGTGGACATTACAAGACGTTAGCCTTGAAGTGTAT
TTAACTGCACCAACAGGATGTATAAAAAAACATGGATATACAGTGGAAGTGCAGTTTGAT
GGAGACATATGCAATACAATGCATTATACAAACTGGACACATATATATATTTGTGAAGAA
GCATCAGTAACTGTGGTAGAGGGTCAAGTTGACTATTATGGTTTATATTATGTTCATGAA
```

GGAATACGAACATATTTTGTGCAGTTTAAAGATGATGCAGAAAAATATAGTAAAAATAAA
GTATGGGAAGTTCATGCGGGTGGTCAGGTAATATTATGTCCTACATCTGTGTTTAGCAGC
AACGAAGTATCCTCTCCTGAAATTATTAGGCAGCACTTGGCCAACCACCCCGCCGCGACC
CATACCAAAGCCGTCGCCTTGGGCACCGAAGAAACACAGACGACTATCCAGCGACCAAGA
TCAGAGCCAGACACCGGAAACCCCTGCCACACCACTAAGTTGTTGCACAGAGACTCAGTG
GACAGTGCTCCAATCCTCACTGCATTTAACAGCTCACACAAAGGACGGATTAACTGTAAT
AGTAACACTACACCCATAGTACATTTAAAAGGTGATGCTAATACTTTAAAATGTTTAAGA
TATAGATTTAAAAAGCATTGTACATTGTATACTGCAGTGTCGTCTACATGGCATTGGACA
GGACATAATGTAAAACATAAAAGTGCAATTGTTACACTTACATATGATAGTGAATGGCAA
CGTGACCAATTTTTGTCTCAAGTTAAAATACCAAAAACTATTACAGTGTCTACTGGATTT
ATGTCTATATGACAAATCTTGATACTGCATCCACAACATTACTGGCGTGCTTTTTGCTTT
GCTTTGTGTGCTTTTGTGTGTCTGCCTATTAATACGTCCGCTGCTTTTGTCTGTGTCTAC
ATACACATCATTAATAATATTGGTATTACTATTGTGGATAACAGCAGCCTCTGCGTTTAG
GTGTTTTATTGTATATATTATATTTGTTTATATACCATTATTTTTAATACATACACATGC
ACGCTTTTTAATTACATAATGTATATGTACATAATGTAATTGTTACATATAATTGTTGTA
TACCATAACTTACTATTTTTTCTTTTTTATTTTCATATATAATTTTTTTTTTGTTTGTT
TGTTTGTTTTTTAATAAACTGTTATTACTTAACAATGCGACACAAACGTTCTGCAAAACG
CACAAAACGTGCATCGGCTACCCAACTTTATAAAACATGCAAACAGGCAGGTACATGTCC
ACCTGACATTATACCTAAGGTTGAAGGCAAAACTATTGCTGAACAAATATTACAATATGG
AAGTATGGGTGTATTTTTTGGTGGGTTAGGAATTGGAACAGGGTCGGGTACAGGCGGACG
CACTGGGTATATTCCATTGGGAACAAGGCCTCCCACAGCTACAGATACACTTGCTCCTGT
AAGACCCCCTTTAACAGTAGATCCTGTGGGCCCTTCTGATCCTTCTATAGTTTCTTTAGT
GGAAGAAACTAGTTTTATTGATGCTGGTGCACCAACATCTGTACCTTCCATTCCCCCAGA
TGTATCAGGATTTAGTATTACTACTTCAACTGATACCACACCTGCTATATTAGATATTAA
TAATACTGTTACTACTGTTACTACACATAATAATCCCACTTTCACTGACCCATCTGTATT
GCAGCCTCCAACACCTGCAGAAACTGGAGGGCATTTTACACTTTCATCATCCACTATTAG
TACACATAATTATGAAGAAATTCCTATGGATACATTTATTGTTAGCACAAACCCTAACAC
AGTAACTAGTAGCACACCCATACCAGGGTCTCGCCCAGTGGCACGCCTAGGATTATATAG
TCGCACAACACAACAGGTTAAAGTTGTAGACCCTGCTTTTGTAACCACTCCCACTAAACT
TATTACATATGATAATCCTGCATATGAAGGTATAGATGTGGATAATACATTATATTTTTC
TAGTAATGATAATAGTATTAATATAGCTCCAGATCCTGACTTTTTGGATATAGTTGCTTT
ACATAGGCCAGCATTAACCTCTAGGCGTACTGGCATTAGGTACAGTAGAATTGGTAATAA
ACAAACACTACGTACTCGTAGTGGAAAATCTATAGGTGCTAAGGTACATTATTATTATGA
TTTAAGTACTATTGATCCTGCAGAAGAAATAGAATTACAAACTATAACACCTTCTACATA
TACTACCACTTCACATGCAGCCTCACCTACTTCTATTAATAATGGATTATATGATATTTA
TGCAGATGACTTTATTACAGATACTTCTACAACCCCGGTACCATCTGTACCCTCTACATC
TTTATCAGGTTATATTCCTGCAAATACAACAATTCCTTTTGGTGGTGCATACAATATTCC
TTTAGTATCAGGTCCTGATATACCCATTAATATAACTGACCAAGCTCCTTCATTAATTCC
TATAGTTCCAGGGTCTCCACAATATACAATTATTGCTGATGCAGGTGACTTTTATTTACA
TCCTAGTTATTACATGTTACGAAAACGACGTAAACGTTTACCATATTTTTTTTCAGATGT
CTCTTTGGCTGCCTAGTGAGGCCACTGTCTACTTGCCTCCTGTCCCAGTATCTAAGGTTG
TAAGCACGGATGAATATGTTGCACGCACAAACATATATTATCATGCAGGAACATCCAGAC
TACTTGCAGTTGGACATCCCTATTTTCCTATTAAAAAACCTAACAATAACAAAATATTAG
TTCCTAAAGTATCAGGATTACAATACAGGGTATTTAGAATACATTTACCTGACCCCAATA
AGTTTGGTTTTCCTGACACCTCATTTTATAATCCAGATACACAGCGGCTGGTTTGGGCCT
GTGTAGGTGTTGAGGTAGGTCGTGGTCAGCCATTAGGTGTGGGCATTAGTGGCCATCCTT
TATTAAATAAATTGGATGACACAGAAAATGCTAGTGCTTATGCAGCAAATGCAGGTGTGG
ATAATAGAGAATGTATATCTATGGATTACAAACAAACACAATTGTGTTTAATTGGTTGCA
AACCACCTATAGGGGAACACTGGGGCAAAGGATCCCCATGTACCAATGTTGCAGTAAATC
CAGGTGATTGTCCACCATTAGAGTTAATAAACACAGTTATTCAGGATGGTGATATGGTTC
ATACTGGCTTTGGTGCTATGGACTTTACTACATTACAGGCTAACAAAAGTGAAGTTCCAC
TGGATATTTGTACATCTATTTGCAAATATCCAGATTATATTAAAATGGTGTCAGAACCAT
ATGGCGACAGCTTATTTTTTTATTTACGAAGGGAACAAATGTTTGTTAGACATTTATTTA
ATAGGGCTGGTACTGTTGGTGAAAATGTACCAGACGATTTATACATTAAAGGCTCTGGGT
CTACTGCAAATTTAGCCAGTTCAAATTATTTTCCTACACCTAGTGGTTCTATGGTTACCT

CTGATGCCCAAATATTCAATAAACCTTATTGGTTACAACGAGCACAGGGCCACAATAATG
GCATTTGTTGGGGTAACCAACTATTTGTTACTGTTGTTGATACTACACGCAGTACAAATA
TGTCATTATGTGCTGCCATATCTACTTCAGAAACTACATATAAAAATACTAACTTTAAGG
AGTACCTACGACATGGGGAGGAATATGATTTACAGTTTATTTTTCAACTGTGCAAAATAA
CCTTAACTGCAGACGTTATGACATACATACATTCTATGAATTCCACTATTTTGGAGGACT
GGAATTTTGGTCTACAACCTCCCCCAGGAGGCACACTAGAAGATACTTATAGGTTTGTAA
CCCAGGCAATTGCTTGTCAAAAACATACACCTCCAGCACCTAAAGAAGATGATCCCCTTA
AAAAATACACTTTTTGGGAAGTAAATTTAAAGGAAAAGTTTTCTGCAGACCTAGATCAGT
TTCCTTTAGGACGCAAATTTTTACTACAAGCAGGATTGAAGGCCAAACCAAAATTTACAT
TAGGAAAACGAAAAGCTACACCCACCACCTCATCTACCTCTACAACTGCTAAACGCAAAA
AACGTAAGCTGTAAGTATTGTATGTATGTTGAATTAGTGTTGTTTGTTGTGTATATGTTT
GTATGTGCTTGTATGTGCTTGTAAATATTAAGTTGTATGTGTGTTTGTATGTATGGTATA
ATAAACACGTGTGTATGTGTTTTTAAATGCTTGTGTAACTATTGTGTCATGCAACATAAA
TAAACTTATTGTTTCAACACCTACTAATTGTGTTGTGGTTATTCATTGTATATAAACTAT
ATTTGCTACATCCTGTTTTTGTTTTATATATACTATATTTTGTAGCGCCAGGCCCATTTT
GTAGCTTCAACCGAATTCGGTTGCATGCTTTTTGGCACAAAATGTGTTTTTTTAAATAGT
TCTATGTCAGCAACTATGGTTTAAACTTGTACGTTTCCTGCTTGCCATGCGTGCCAAATC
CCTGTTTTCCTGACCTGCACTGCTTGCCAACCATTCCATTGTTTTTTACACTGCACTATG
TGCAACTACTGAATCACTATGTACATTGTGTCATATAAAATAAATCACTATGCGCCAACG
CCTTACATACCGCTGTTAGGCACATATTTTTGGCTTGTTTTAACTAACCTAATTGCATAT
TTGGCATAAGGTTTAAACTTCTAAGGCCAACTAAATGTCACCCTAGTTCATACATGAACT
GTGTAAAGGTTAGTCATACATTGTTCATTTGTAAAACTGCACATGGGTGTGTGCAAACCG
ATTTTGGGTTACACATTTACAAGCAACTTATATAATAATACTAA
>gi|12084981|ref|NC_002644.1|_Human_papillomavirus_type_71,_complete_genome
TTGTTCTACTTCTTACTCATTATTATAAATTATAATGTTTGTATAAGAAATATAGGTGTA
ACCGAAAACGGTGTAACCGAAATGGGTGCATATATAAAGCAATGCTTGGTTCAGCAGATT
TAGCTATGTCCAGCGGGGACGCATACCCCACCAACCTCTTCAGACTGTGTAACCAGTACG
ACGTGGACCTGCAGGACCTGAACCTAACCTGCATATTCTGCAGAACAATTAACAGACGTG
GAAGTCGTGGCCTTGCATATAAGGAGCTGAAAGTTGTGTGGAGAAGTGGTTTTCCGTTTG
CTGCATGTGCCTGCTGTTTGGAAATAGCTGGAAAACTAAGGCAACTTAGATATTGGCAAT
TTTCAGGCTTTGCAAACACAGTGGAATTAGACACCGGAACGCCAGTTACAGAGCAACTAA
TACGGTGCTACGTGTGTCACAAGCCATTGTGTAGTGTGGAAAAAGAAAGAATAATTACAG
AAGGCAGGCGATTTCATAAAATAGCAGGCCATTGGCGCGGTGCTTGCCTACAGTGTTGGA
AACCATGCGAGGCCAACAATGTACCTTAAAGACATTGTCCTGCAGCTACAGCCAGAGGTT
GTTGACCTGTATTGTCACGAGCAATTTGCCAGCTCAGACGAGGAAGATAATAGGGTGGAC
GGTGAGCAACCCACAGAACCAGCACAGCAGGCATATAGGGTGGTTTCATACTGTGGTAGG
TGCTGTCGTGCAGTTAGGCTTGTGGTGGAAAGCGACGAAGCAGACATAAGAGCGCTTCAA
CAGCTGTTACTGGGCACACTGACAATAGTGTGTCCCATCTGCGTGTAGCTGCCATGGCCG
ACTGTGAAGGTACAGGTACTGATGATGATGGGGGCGAAGCGGGAACAGGCGCGGGAGGGT
GGTTTTTTGTAGAAGCTATTGTGGACAGATGTACCGGCCCACAGCCATCCAGTGATGAGG
ATGAGGATGACAATGATACTGGGGAAGATATGGTGGATTTTATAAATGACAGGCATGCAG
GGGACGGACAGGAAGTGGCAGCAGAGGTGTACAGACAGCAAGAAGCATTAGATGACGAGG
CAATTGTGCAGCCCCTAAAACGAAAGTTTCTTGCAAGTCCTTTGTCTGCAGGGGTGTGTG
TAGACAAAGAGTTAAGCCCGCGGCTAGATGCTATATCCATAGGCAGGGAATCCCAAAAAG
CAAAACGAAGGTTATTTGAACTACAGGACAGTGGGTATGGCAATACGCAAGTGGATACTG
AAGCGGCAGGAAACCAGGTACCAAGGGACGGGACGCCAGGGGGGCTGCACACAGAACAGG
AGGAGGAGCGTCAGGGGGGGGATGGGGCGGCAGGCATAGACAGTACACCAGCACACACAG
GAACAAATACAGTGTTGGCTTTGTTAAACTCCAGTAATCGAAGGGCAACACTGCTAGGTA
AGTTTAAAGACTTATATGGGTTATCATATATGGAATTGGTACGGCAATTTAAAAGTAATA
AAACAACATGTTTGGACTGGGTAGTATGTGCGTTTGGTGTGTATTGCACGGTTGCGGAGG
GTGTAAAAAACCTTGATACAGCAACACTGTGAATACGCACACATACAACAACAAACATGTT
CCTGGGGAGTGGTTATATTAATGCTGTTGCGATATAAGTGTGCTAAAAACAGAGATACCG
TGGCAAAGGGATTAAGCATGTTATTAAATATACCAGAAACAAACATGCTAATAGAACCAC
CAAAAATAAGAAGTACGCCTGCTGCATTATATTGGTTTAGGGCGAGCATGGGAAACGCAA
GTGACATATTCGGGGAAACGCCAGAATGGATAGTTAGACAAACTGTGGTAGGACACAGCA

TGGAGGAATGCCAGTTTCAGTTATCAGTAATGGTGCAATGGGCATATGACCATGATATAA
CAGATGAAAGTATATTGGCATATGAATATGCCCGTCTGGCTGATGTAGATAGTAATGCAG
CAGCCTTTTTAGCAAGCAATTGTCAAGCCAAATATGTGAAAGATGCATGCACAATGTGTA
GACATTATAAACGAGCAGAGCAGGCACAAATGTCTATGTCACAATGGATATCATTTAGAA
GTGCTAAAATAACAGAAGAAGGGGATTGGCGAACAATAGTAAAATATTTAAGACACCAGG
ATATAGAATTTATTACATTTATTATAGCATTAAAAAATTTTTTAAAGGGTATACCAAAGA
AAAGCTGTTTAGTATTTTATGGGCCTGCAGACACTGGCAAATCCTATTTTTGCATGAGCC
TATTACGGTTTTTGGGTGGGGTTGTCATTTCCTATGCCAATTCCAGCAGCCATTTTTGGT
TGCAGCCGTTAGCTGATGCAAAATTAGGACTAATAGATGATGTAACCCCTAATTGCTGGA
GCTATATAGATGTATATCTAAGAAATGCATTAGACGGTAATCAAATATGTATAGATAGAA
AACACAGGCCATTACTGCAGTTAAAGTGTCCCCCATTGTTAATAACAACAAATACCAATC
CGTTAGAGGAGGAACGATGGAAGTTTTTACGTAGTAGACTGCAGCTGTTTACATTTAAAA
ATGCATTTCCTTTAAATTCAAAAGGGGACCCTATGTATCCACTAAATGATGCAAACTGGA
AATGCTTTTTTCAAAGGTTGTGGGCTCGGTTAGACTTACACGAGCAGGACGAGCAGGAGG
ACAATGGAGACACTGGCCAGCCGTTTAGATGCGTGCCAGGAGACGTTACTAGAACTGTAT
GAAAAAGATAGTGACCAGCTACAGGACCAAATTAAACACTGGCAACACGTGCGATGGGAA
AATGTGTTGTTATTTAAGGCAAGGGAAGCAGGAATTACTCATCTTGCCCACCAGGTGGTG
CCTGTGCTTGGTATTGCTAAAGCCAAAGCTTGTAAAGCAATTGAAATTCAGTTGGCATTA
AAGACATTACTTAACAGTCCCTATAGCAACGAACGATGGACATTGCGTGACACGAGCCAG
GAAATGTGGGACGCAGTGCCTAAGCAATGCTGGAAAAAAAAAGGCTACACTGTAGAAGTG
CGATACGATTGCAAAGAGGAAAAGACAATGTGTTACACATGTTGGAGGGAAATATATGTG
CAAAACAGTACAAATGAGACATGGGAAAAAGTGTGTGGCCTGGTGGACCATGCGGGCATA
TACTATTTACACGATGGGATACGTGTAGACTGTGTATTATTCTCCAAGGAAGCAGTAATA
TATGGGGACACAGGCATCTGGGAAGTACATGTGGGTTCAAGGGTGATTTATGATGCATTC
GACTCCTCTGTGTCTAGCACCCAGGACACCGAGCAAGACCAAGTACCCACTATTAAACCT
ACTGACCACGGACCCGACTCGCACCCCCAACAGGCCTCCACCACCACCCAAGTGCTGGGC
ACCAACGAAACCCAAGTGTCGACCCCGCCATTTAAGCGACAGCGACTCGGAGACAGACAG
CGGACCCTTGAGCAGCCCGATTCTACAAAAGCACCACAGCAGCTGGCACGTGTCAACCTT
AGGACCCAGTGTGACACTGACGGCGCACACGAGCACGGGAGGACACGTGACTGTAACAGT
GCACCTGTAATACACCTTAGAGGTGAAGCCAATAAACTAAAGTGTTTAAGGTATAGGTTG
CAAAAACATAAATCTGTACTGTTTGCCAAAGCATCCTCCACGTGGCATTGGGCCACTGGC
ACAGAGGACAATACATGTAAAACAACATTTGTAACATTGTGGCATGATAGTGTGGAACAG
CGGGCACAATTTCTAGCCACTGTACATATTCCTAAGGGCATAGAGGCCTTACCAGGATAT
ATGTCATTGTTTGCATAATCTTTGTAAATATTGTATATATTGTATCTATTGTATAGACGC
GGAACGTTAAGGGTACACACCTACCTGTAGTGCTGGAGCCCTTTCTATGCTGGGTGTCTG
CATGGACCTATGCACTACTACTACTAATTAGCTTTTGGCTGTCTATTTTATCTTCTCTTA
CTGCCTTTTTAATTTTTTTTGTTACTGTGTTTCTTGGGTTTCTAGCACTATATATACAGG
CAGCAGCGTCCCTTACCTAACTGTGACTTGTGACTACCACACAACCAGCCAATACTGCTA
CTACGTGTACATAACCTATCCATTTGTGTTATAGATTGCATATATGTATCCTGTTGTGGT
AAAGGATTCCCAAGGCGGACATTATGATATTGTGGTGTGGGGCCCTGATGATGTAGATGT
ATTGTTTGTGTTTTTAGTGTTGGTGTGTCTTATGTTGCTTCTGTTTTTGTTACGGTTGAT
GCAGTAGGTACCCCCCCTTTTGTATTGCCCTGTTTATACATATACATATACATATTGTTT
TTATTTGGTTTTTGTTTTTGTTTTTTTGTGTGTGCCTGTGTGTGTAAATAAACACATTTA
CAATGCCACGTGCACGGCGTCGCAAACGTGCTTCAGTTACACAGCTATATCAGTCCTGTA
AGCTAACAGGCACATGTCCCCCTGATGTTATTAATAAGGTGGAGCACAATACCTTGGCTG
ATAAAATATTACGCTGGGGTAGTTTAGGAATATTTTTGGGAGGTTTGGGCATTGGCACAG
GGTCTGGCACCGGTGGGCGCACAGGCTATATTCCCATTGGTACACGTCCTCCTACTGTTG
TGGATGTAGGACCTCCTGCACGCCCCCCTGTGGTTATAGAAACAGTAGGGGCCTCTGATC
CATCTATTGTGTCTTTGGTAGAGGATTCTAGTATTATAGAAGCAGGAGCACCATATCCTA
ACTTTACTGGCACAGGTGGGTTTGAGGTCACTACAGCATCCACTACTACTCCTGCTGTGT
TAGACATTACTCCAGGTAACACTGTGCAGGTTAGTAGCAGTAGTTTTACTAACCCATCCT
TTACTGAACCTGCCTTAGTGGAGCCCCCTCAAACAGGTGAGGTTTCGGGACATATTTTGG
TTAGTACCTCTACATCTGGCACCCATGGCTATGAGGAAATACCCATGCAAACATTTGCGT
CGGAGGGAACAGGCAATGAACCTATAAGTAGTACACCTATTCCTGGGGTACGCAGGTTAG
CAGGCCCTCGCCTGTATAGTAGGGCCTATCAGCAGGTGCGGGTGGATGAATCCACATTTC

TTCGCCACCCTGCATCTATGGTTACATATGACAACCCTGTGTATGACCCAGAGGAAACTA
TAATATTTGAACATCCTAGCATACACCAGGCTCCTGATCCAGCATTTATGGATATTGTGG
CCTTGCACAGGCCGGCCCTTACTGCCCGTAAAGGTACGGTACGTTTCAGTCGTTTAGGAC
AAAAATCTACCCTTCGCACCCGTAGTGGTAAACAAATAGGGGCGCGGGTACATTTTTATC
ATGACATTAGCCCTATACAACCCACCGAACACTTAGAACTGCAGCCACTAGGGCGGGCCT
TACAACAAGAACCTATTGACACATTATATGACATATATCTGACACAGATTATTCCAATGA
TACTGTCATTCAACCTACTTCTGTGTCCAGCAGGCCTACACCTACTACTATACCCCTCTG
TAACTGCCACATCAGCCGTGTCTGCCTCTCGCACACAAAATGTTACAGCACCTTTGTCTG
CAGGAGCAGATGTTCCAGTGTTTGATGGCCCTGACATTGATTTTTCCACCTCCCATGCCA
CTACTCCTACTCCTGTAGTGCCGTCCATTGCACCTCCCAGTTCTTTTATTGTGTATGGAA
CTGAGGTATTATTTAATGCCTAGTTATATATTTTTTCCTAAAAAACTAAACGTGTCCACT
ATTTTTTTGCAGATGGCTTTGTGGCGGCCTAGTGACAGCAAGGTATACCTGCCTCCTGCC
CCCCGTATCCAAGTTCTCAGCACCGACGACTATGTTACCAGAACAAAACTATTTTATTAT
GCTGGTAGTTCTAGATTACTTACTGTTGGCCATCCATATTTTCCTATTCGCCAGGCAAGT
GGTAAAAATCGTATAGTTGTCCCCAAAGTGTCTGGATACCAATATAGAGTGTTTCGTGTG
CGGCTACCCGACCCAAATAAATTTGGACTACCTGATGCTTCATTATACAATCCCGATACC
CAGCGCTTAGTGTGGGCTTGTAAGGGTCTTGAGGTAGGCCGCGGACAGCCTTTAGGAATA
GGTGTTAGTGGCCATCCATTGTTTAACAAGCTTAATGACACTGAAAATGCCACGCTGTTT
GATGTTAATCCTGGTGAGGATACTCGGGATAATGTTTCTATGGATTATAAACAAACACAA
CTATGTATTATTGGTTGCAAGCCTCCCTTAGGTGAACATTGGGCAAAAGGTACTCCATGC
AGTGGCGCTTCAGCTGCCGCTGGTAGTTGCCCCCCACTTGAACTTGCCAGTACTGTTATA
CAGGATGGTGATATGGTAGACACAGGTTTTGGGGCAATGGATTTTGCAGCCCTGCAAACA
AATAGGTCTGACGTTCCCTTGGATATTGTTACTACAACATGTAAATATCAGATTATTTCG
AATGGCTGCAGAGCCATTGGTGATCGCATGTTTTTCTTGCGCCGAGAGCAATGTTTTAAG
ACATTTTATAATAGGCAGGCACACCTAGGCAGGGTTCCTGATGACTATTATTTAAAGGTT
CACCTTCTACCTTTCGTGCTTCCCCTACAAGCTCTCTTTATGCATCCACACCTAGTGGGT
CCATGGTTACCTCAGAGTCACAGTTGTTTAACAAGCCTTACTGGCTACAACGTGCACAGG
GCACAAACAATGGCATTTGTTGGGCAATCTGCTTTTTGTAACAGTTGTGACACATCACGT
AGTACAAATATGTCCATCTGTGCTACCAAAACTGTTGAGTCTACATATAAAGCCTCTAGT
TTCATGGAATATTTGAGACATGGAGAAGAATTTGATTTGCAATTTATATTTCAACTATGT
GTTATTAATTTAACAGCTGAAATTATGGCCTACTTACATGGCATGGATGCTACATTACTG
GAGGACTGGAATTTTGGTTCCTTACCACCTCCTACTGCTAGTCTTGGTGATACCTACCGC
TTTTTACAGTCTCAGGCCATAACCTGTCAGAAAAACAGTCCTCCTCCTGCAGAAAAAAAG
GACCCCTATGCAGATCTTACATTTTGGGAGGTGGATTTAAAGGAGCGGTTTTCACTAGAA
TTGGATCAGTTTCCATTGGGGCGCAAGTTTTTGCTGCAAAGTGGCACCCGCTCGCGGCCT
ACTGCATTGTCCCGTAAAAGGGTTGCAGCATCTACCACATCCACCGCCCCCAAACGTAAA
CGTGTTAAACGCTCCCGGTAGTAGTGGTGTGTGTGTGTGTGAGTATGTATGTGTATGTGT
GTGTGTGTGTGTGTGTGTGTGTGTGTGTGCATGTTTATATATGTATATGTGTTTGTTGTG
TATATTGTATGTTGTTGTGTACGTGTGTATATACTGTGCGACCCCCTGGCTTCCTGTATG
CATGTTTGTGTTGTTAATAAATGCGTGTCATGTATGTGTGTTATTTGGTTGCACCCCTGT
GAGTAAGTGTGCAAGCAGATATAACCCCCCCTGTCCGGTGACGCCTTACTGACCAGTGAC
CATGCCCTGCCTTTGGCCCCTGGTTATATTTAAGGAACGAACCGTTTTCGGTTGCCTAAA
ATGGCCGCCGTTTTGGCTGGCGGCCGCCGTTTTGGCGGGCTACTATACAATGGGTAATCC
TTGTATTGCTTCATATCCTTTCCAACACAGGTGTGCATACCATCATAAGGTGTGCCTGGC
AGTTATTTGGCACACTATAAATGCATGTTATAATTAACTTTAATACATGTGTTACTCACC
GTGCAATACCCATTGTTTGGGGCATATAGTTGTGCTGACTATGTTCGCCTAAGTACGTTT
TTGGCAACAGCAGTGTAGTTTCTGTCCAAGAATGTGTCTGCTAACTTTTATAATGTACTT
AGCAACATGGTTTATACACACCTAATCCGGTTGTTCC
>gi|9628574|ref|NC_001694.1|_Human_papillomavirus_-_61,_complete_genome
TAACAATCATATATGTAAAAAGTAGGTTGGCACCGAAAACGGTCGGACCGACCTGGGTAC
ATATATAAAGAAACCGTAGGGTCAGCAAAGCACACTCATCTATGGGACCGTGCAATCCAA
CTAATATTTTCCTGCTCTGCAAGGACTACGAGGTGGACTTTGAGGATTTAAGGCTGACAT
GTGTATTTTGCAAAAATGAATTAACAACAGAAGAATTGCTGGCGTTTGCACTAAAGGAGC
TAAGCATTGTGTGGAGACATAACTGGCCATTTGGAGTATGCGCACCATGCTTGGCACGTG
AAGTAAAAGTGAGGGAGCTGCGACATTGGGACCATTCCTGTTACGGACCCACTGTGGAAC

AGACAACAGGACGGTCATTAGCTGAACTATATATACGGTGCCATGCATGCAGCAAACCGT
TAAGTATACAGGAAAAGGAGCATCAGGTACAGGCATACATCCACTTCCACTATATAGCTG
GACAGTGGACGGAAGGTGTTGCCAGTGTAGAGGGCCATGCACGGCCAGGTGGCAACCAT
AAAGGACATAGTCCTTGAAGAGCGTCCTGAGGTGGTTGACCTACATTGCAATGAGCAGTT
ATTAGACAGCTCAGAGTCAGAGGAGGAGGATAGTGTGCGTGAGCAACTTGTTGAACAAGC
ACAGCAGGCCTACAGGGTGGTTACTACCTGTGGCATTTGTAAGTGTCCAGTTAGGCTGGT
GGTGCAGTGCGGAGACGCAGACCTGAAGGTGCTACATGAACTACTGCTGGGCGACTTGTC
CATAGTGTGTCCTGGCTGCGCATAAGGGACATGGCTGACAGTGAAGGTACAGAAAGCGGG
GATGGGACCGAGGCCGCGGAACGCGCAGGGGGGTGGTTTCTGGTAGAAGCCGTGGTAGAC
CGCACCACAGGATACCAGGTGTCCAGTGATGAGGAGGACAATAGCATTGACACAGGGGAA
GACCTAGTAGACTTCATAGATACAAGGCGCCCCGGGGATGGGCAGGAAGTGCCGCTTGCG
TTGTTCGTTCAACAAAATGCACAGGATGACGCTGCAACGGTGCAGGCACTAAAACGAAAG
TATACATGTAGCCCTGCAAGCAGCACCTGTGTGTCCTTGGTGGACAGTGAATTAAGTCCC
CGGCTGGACGCCATACGGATACACCGGGGACAGGACAGGGCTAGGAGAAGGCTGTTTGAG
CAAGATAGTGGCTATGGCCATACGCAAGTGGAAATTGGAGCATCAGAAAGTCAGGTACCG
GGGGATGCGCAACATGAGGGGGGGGGGGAATCCGTGCAGGAAGCAGAGGAGGAGCGTGGG
GGGGGGGACGGAGAGGCCGAGGCCACAGGTAACCAGGAAACGCAAGCGCAGGAGCAGGCG
GCAGACATATTAGAGGTGTTTAAGGTTAGTAATTTAAAAGCAAAATTACTGTACAAATTC
AAGGACCTATTTGGACTAGCATTTGGGGAGCTGGTAAGAAATTTTAAAAGTGATAAGTCA
ATATGTGGGGACTGGGTAATATGTGCGTTTGGTGTATACCATGCTGTGGCAGAGGCTGTA
AAAACCTTAATACAACCTATATGTGTGTATGCACACATACAAATACAGACATGCCAGTGG
GGAATGGTAATATTAATGCTTGTGCGATACAAATGTGGGAAGAGCAGGGAAACAGTAGCA
CACAGCATGGGAAAACTGTTAAACATACCGGAAAGACAGATGCTAATTGAACCACCAAAG
ATTAGAAGCGCACCGTGCGCACTATATTGGTATAGAACAGCCATGGGAAACGCCAGCGAG
GTGTATGGCGAAACACCTGAATGGATAGTAAGACAAACAGTGGTAGGACATGCAATGCAA
GAAGCACAGTTTAGTTTGTCCATGTTAGTGCAATGGGCATATGACAATGACATAACAGAT
GAAAGTGTGCTGGCCTATGAATATGCATTGCTGGGAAATGAGGACCCAAATGCGGCGGCC
TTCTTGGCCAGTAACTGCCAAGCAAAGTATATTAAAGATGCAATTACAATGTGTAAGCAT
TATAGACGTGCAGAGCAGGCAAAAATGACAATGGCACAGTGGATTACACATAGAGGCCGC
AAGGTGGCAGACACAGGGGATTGGAAGGCTATTGTAAAGTATTTAAGATATCAACAGGTA
GAATTTGTACCATTTATTAGTGCCCTTAAATTGTTTCTAAAGGGGGTACCAAAAAAAAGC
TGCATGGTATTTTACGGACCAAGCGACACAGGGAAGTCGCTATTTTGCATGAGCCTACTA
AATTTTCTGGGAGGAGCTGTGATTTCATATGTAAATTCAAGCAGCCATTTTTGGTTATCA
CCTTTAGCAGATACTAAAGTGGGACTACTGGACGATGCCACCTATCAGTGCTGGCAATAC
ATAGATACATATTTACGGACAGTATTAGATGGCAATGCCATAAGCATAGATAGGAAACAC
AGAAATTTAACACAGCTGAAGTGTCCTCCATTAATGATAACAACAAATATAAATCCACTA
GAGGATCCTACGTTTAAATACCTGCACAGTAGAATAGTGGTGTTTCAGTTTTTACATAAG
TGTCCACTAAACAGTAATGGGGACCCAGTATATACCCTGAATAATGAAAATTGGAAATCA
TTTTTCCGAAGGTCCTGGGCACGCATAGAGGGATCTGATCAACAGGAGGAGGAGGAGGAG
GAGGATGAGGATGGAGTCACTAGCCGACCGTTTAGATGCGTGCCAGGAGAAATTACTAGA
CCTCTATGAAAAAGATAGCAACAAGCTTGAGGACCAAATATTGCATTGGCACTATGTGCG
TTTGGAAAATGCAATGTTATTTAAGGCACGACAAGCAGGACTAACGCGTGTGGGCCACCA
GATGGTGCCAACACTTAGTGTTACAAAAGGCAAAGCACATAAAGCAATTGAAGTACATCT
GTCATTGCAAGGGTTACAAACAAGTGCATATGCGCATGAACCCTGGACATTGCAAACCAC
CTCTTTGGAAATGTGGAATACGCAGCCACAACGATGCTGGAAGAAAAAAGGACGCAGACT
AACAGTTAAATTTGATGGCGAGGACCACAAAGCAGTGGAATACGTTAGTTGGGGGTATAT
TTATGTACAAAGCACAGAAACTGATCTGTGGTATAAGGTTCCTGGAAAAGTGTCATATAA
GGGCCTATATTATGAAATGGAGGGACAGGAACACTATTATGTGACATTTGCACAGGAGGC
CCAAAAATATGGGGAAACAGGCAAATGGGAGGTACATATGGGAAACACAGTGATTTATGA
ACCCTGCGCCTCTGTATCTAGTACCCAGGACGCCGTGCGAGAAGTATCCACTGCTGAAAC
TGCTGGACACCTGCGGGACAACACCACACAGACCACCACCACCCCCACGTGCGTGGGCCC
CACCCAGACATCCACCTCGGTGCAGACGCCGCCTCATAAGCGACAGCGACTCCACAGAGA
CAGAGAGCAGCAGCCCGACACAACACAAAAAGACAACCACAAGCGGGTGGACAGTACTGA
CCAGTGGATCAACGGTCACCGTAACAGCACAGAAACAGGGGACAACTGTGACAGTTACAG
TTCACCTGTAATACACCTAAAAGGTGATCCAAATAAATTAAAGTGTTTTCGGTATAGGTT

ACAACATTCAGTACCTGAGCTGTTTGACAAAGCATCATCCACATGGCATTGGGCCGGTGG
ACAAAGTACAACCAGAGCAGCATTTGTAACATTGTGGTATGTTAATGTGGAACAACGAAA
ACAATTTTTAAATCGTGTAACCATTCCAAAGGGTATACAAGCCACTGCAGGCTATATGTC
AATGTGTATATAAGTGTGTGTATACAAAAACATAAATACTGTGTTGCAAGTATTGAGCCA
GCTACAGCTATTTACATACCACACCTGCATTATTGTGTAAACCATAAACAATCCTGGACG
AGCTTGTGTGTTTGATAGATGGATGGTGTACGATACTCCTGCTGCTTATACTTTTCTGGC
TTTCCTATCTTTCCTCACTAAGCGCATTTTTGGTGTTTTTCTGTGCCATATATGTAGGAT
TGTTGTGCATATACATGCAAGTGTTGTGGTATATGGAATAACTGTGGCGCCACCAGCCAT
ACACTGCTGCTCATATGTAAATAGTGTTCCTTGTGTCTGTTGTTCGTTTGCAATATGTAT
CCCCTTGTAGTTAGTTCCCCTCAGGGGTTTGCATTACTGGTTGTGGATCAGCAAAACTGC
GGATTGTTATTTTTGTTTTTGCTATTACTGCTTATTGCAATACTGTTGTACAGGCTTTTA
CATTAGGTGGTGTACCTTGTGTGTTTTCCCTCCCCACAGTTTTTACTTTGTTATATTTT
TATATGTTTATGGCCTAATAAACATTATGGCTCTTAAACGTCGCAAGCGTGCATCTGCAA
CTGACCTCTATCGTACATGTAAACAATCAGGCACATGTCCCCCTGATGTTGTTCCAAAGG
TGGAAGGGGATACCCTTGCTGACCGCATTTTAAAGTGGGCTAGTTTGGGGGTTTTCTTTG
GGGGATTGGGTATAGGCACCGGCTCTGGTACTGGGGGTCGTACTGGGTATGTGCCTATTG
GCACCCGGCCTCCTACTGTAGTGGATATTGGCCCTGTGTCCCGCCCCCCTGTAGTTATTG
ATCCAGTGGGTGCTGCCGATCCCTCTATAGTTACCCTTGTAGAGGAATCCAGCGTCATTG
AGGCTGGGGCTACAGTCCCTACATTTTCCGGGTCCGGGGGCTTCAATGTCACGTCGTCTT
CTACTACCACCCCGGCTGTGTTGGACATTACTCCATCGGGTGGGTCTGTGCAGGTTAGCA
GCACCAGTTTTATTAATCCTTTATTCACTGAGCCTTCTATTATTGAACCTCCTCAGGCAG
GGGACCTTGCCGGCCATGTCATTTCCAGTACCCCTACCGCTGGGTCCCACAGTTTTGAGG
AAATCCCAATGCACACCTTTGCTACCTCTGAGGGTCCTGGAAGTAGTACCCCGCTGCCTG
GCATCCGTCGCCTTGCACGGCCCCGCCTGAATTTATACAGTAAGGCTAACCAACAAATTA
AGGTTGCTAACCCTACATTTATGTCTGATCCAGCCTCCTTAATTACATATGACAATCCTA
TATTTGATCCTGAGGAAACTATTATATTTGAACATCCTAGTATATATACACCTCCTGACC
CGGACTTTTTAGATATTGTGTCCTTACACAGGCCCGCGCTCACATCCCGCCAGGGAACTG
TACGGTTTAGTCGCCTTGGTCAACGTGCCACCTTGCGTACCCGTAGTGGTAGGCGCATTG
GCGCTCGGGTACACTTTTACCATGACATTAGTCCCATTCCATCGGATGCTGTGGAGTTAC
AGCCTTTGGTGCCGTCCTCGTCCCCTAGTATAACATATGATATATATGCTGACCCTGAGG
TGTTGGATCTTCCTGCACAACATACACAACCCACACTTACAGTACAGGGCCCTTCCCTCT
CTGCTGCATCTGCATCTACCAAGGTACACAATGTCACTGTTCCACTGGCCACAGGGCTTG
ATACGCCTGTGACATCTGGCCCTGATGTTGATTTTGCACATGCTCCTGCGCCTGTACCAG
CTGTACCATATGTACCTGCCACCCATCCACATTCTATATATATTCAGGGCTCTGATTTTT
ATTTGTTGCCTGCTTATGTGTTTTTTCCTAAACGTCGTAAACGTGTACCCTATTCTTTTT
CAGATGGCTTTGTGGCGGCCTGGTGACGGCAAGGTATACCTGCCTCCCACTCCTGTGTCT
AAAGTTATCAGTACTGACCGTTATGTGCAACGCACCAACTTATTTTATTATGGTGGCAGT
TCCCGTCTGCTTACTGTAGGACATCCCTATTGTAGTTTGCAGCTTGATGGGCTGCAGGGC
AAGAAAAACACTATCCCCAAGGTGTCTGGCTATCAATATAGGGTGTTTAGGGTACAGCTT
CCTGACCCTAATAAATTTGCTTTGCCTGATGGCACCTTGTATAATCCTGATACTGAGCGC
ATGGTGTGGGCCTGCAGGGGCATTGAGGTTGGTAGGGGGCAGCCATTGGGGGTTGGTACT
AGTGGTCACCCGCTGTACAATCGCCTGGATGACACTGAAAACACCACATTACTTGTTGCA
GAGTCCTCTGACAGTCGTGACAATGTGTCTGTTGATTATAAGCAGACCCAGCTACTTATT
GTAGGTTGCAAACCCCCTATTGGGGAACACTGGACAAAGGGTACTGCTTGTGCTAACCCC
GCGCCCCGGCCCACTGACTGTCCTCCATTGGAATTTACAAATACCACCATACAGGATGGC
GATATGGTGGAAACAGGCTATGGGGCCATTGATTTTGCTGCATTGCAGGAAAATAAATCT
GAAGTGCCATTGGATATTTGCACCACTATATGCAAGTATCCTGATTATTTACAAATGGCT
GCTGAACCATATGGAGACTGTATGTTTTTTTGTTTACGCAGGGAACAAATGTTTGCTCGC
CACTTTTTCAATCGCCAGGGTGTCATGGGTGAGGCACTGCCTGACTCTTATTATCTTAAG
GGTGCGAATGACAAGGCGGCCCCTGGTAGTTATATTTATTCCCCTACGCCTAGTGGCTCT
ATGGTGTCCTCTGATTCCCAATTATTTAATAAGCCATATTGGCTGCAACGTGCCCAGGGC
CACAACAATGGTATTTGTTGGTTTAATGAATTGTTTGTAACCGTTGTGGATACCACCCGC
AGTACTAATTTAACCATTTGTACTGCTACATCCCCCCCTGTATCTGAATATAAAGCCACA
AGCTTTAGGGAATATTTGCGCCATACAGAGGAGTTTGATTTGCAATTTATTTTTCAGTTA
TGTAAAATACATTTAACCCCTGAAATTATGGCCTACCTACATAATATGAATAAGGCCTTG

TTGGATGACTGGAACTTTGGTGTGGTACCACCACCCTCTACCAGTTTAGAAGACACATAT
AGGTTTTTGCAGTCCAGAGCTATTACATGTCAGAAGGGTGCTGCTGCCCCGCCGCCCAAG
GAGGATCGCTATGCCAAGTTATCCTTTTGGACTGTTGATTTACGAGACAAGTTTTCCACT
GATTTGGATCAGTTTCCTTTGGGGCGCAAGTTTTTATTGCAGGCCGGTCCCCGCTCAGTA
TCTGTGTCACGCAAACGTGCTGCCCCCAGCTCTACCCCTACATCCTCCCCTGCTACAAAA
CGCAAAAAGCGTAAACAGTAGTGATGTTTGTCTGTATGCTGTTGTTGTGCATGTGTTGTT
TTGTATTGTGCTGTACTGGCCTTGTGCATGTGCTGTTTGTATGGTGCCTGGTTTGTATGA
CTGTATTTGCTATGGTTGTGTATTGTGTGACCCTGTACTGACCCTTGTCCCCTGTGTTGT
GTATCTGTGGAATGTGTGTTACACTGTGTGTGCATTAATAAACTGTGTGTCATGTGTGTG
TGGTGTCCGGTGCACCCTGTGAGTAAGTGTGTTTATTGTTGCACGCCTTATTTTGTGCAG
CTTCCCCATTTCTGTGTTCCTTGTGTTCGGGTAGCCATTTTGTGTTGCCGACCGTTTTCG
GTCTCCCGCCTTTTTTGTTGCCGACCGGTTTCGGTCGCCCGCCTTTTTGGTTGTGGCACT
ATGCCACTGTACACATACCACATCCTGTTTAAACAAACTTTATGTGTTGCACCTGGGTGT
GTTTGACAGGTTGCTAACACACACAGTTTGGAACAATGCTCCATCTATCTTTTTTGACAT
GTTGTGCAATTACTGTGCTTTTAGGCACATATTTTGTGCTGACTACTTTCCCTTAATTGC
TGGTTTGGCATTGCTTGTATACTTTTCTAGCCAGGAAACTGTCTTGCAACTTTGGGCATG
TTTTACACACATTATGTTACAAAGCCGATCCGGTCGCTCCCTATGTCTCATTCTTTTTAT
GATAGTTTA
>gi|9627389|ref|NC_001595.1|_Human_papillomavirus_type_7,_complete_genome
TGTTTAATAATTGTACAGTTACTAAAGGGCGTAACCGAAAACGGTCCGACCGAAAACGGT
ACATATAAAAACCAACCCAAAAAACCTGATCTGGGGCCACTATGTCTGCACGTTGCGGCT
CCACAGCTAGAACTTTATTTGAATTATGTGACCAGTGCAATATAACATTGCCTACGTTGC
AAATTAATTGCATATTTTGTAACAGCATTTTACAAACAGCTGAGGTGCTGGCCTTTGCAT
TTAGAGAGTTATATGTAGTGTGGCGCAACGACTTTCCTTTTGCAGCGTGTGTAAAGTGTT
TAGAATTTTATGGAAAAGTGAATCAGTATAGGAACTTTAGATACGCTGCATATGCACCAA
CAGTGGAAGAAGAAACAGGATTAACAATTTTAGAAGTGAGAATAAGATGCTGCAAATGCC
ACAAACCATTGTCTCCTGTGGAAAAAACCAACCACATTGTTAAGAAGACACAGTTTTTA
AACTGCAAGATTCGTGGACAGGGTACTGTCTGCACTGTTGGAAGAAATGCATGGAGAAAG
GCCAACGCTCGGAGACATCGTGTTAGACTTGCAACCCGAACCAGTAAGTTTAAGTTGCAA
CGAGCAATTAGACAGCTCAGACTCAGAAGATGACCATGAACAAGACCAACTAGACAGCTC
ACACAATAGACAGCGTGAGCAACCCACGCAACAGGACTTGCAAGTAAATTTGCAATCATT
TAAAATAGTAACACATTGTGTATTTTGTCACTGTTTAGTTCGCCTAGTAGTCCATTGTAC
TGCTACTGATATAAGGCAGGTTCATCAGTTGCTGATGGGCACACTAAATATAGTGTGCCC
CAACTGTGCAGCTACAGCGTGACAACAATGGCAGACGATTCAGGTACAGAGGATGTGGGG
TCTGGGTGTTCAGGATGGTTTTTAGTTGAGGCTGTGGTAGATAAACAAACAGGTGATGTA
GTTTCAGAAGATGAAGATGAGGATGCTATAGAGGACAGTGGATATGATATGGTAGATTTT
ATTAATGATACTGTAGTAAGTGAACATGAAGAACTAAGTAATGCACAGGCTTTGTTACAT
GCACAACAGACATGTGCAGATGCTGTAGAGTTGTGTGAGCTAAAACGAAAGTACATTAGT
CCATATGTAAGTCCTATTCAGTGCTCAGAACCGTCCGTGGACGGGGATTTAAGTCCAAGG
CTGCATGCCATAAAGCTTGGCGGCGGTAAAAAGGCTAAAAGGCGGTTGTTTGAGCGATTG
GAGCAGCGAGACAGTGGCTATGGCTATTCACAAGTGGAAACAACAGAGACACAGGTAGAG
GAAGAACATGGCGAACCGGAAGGTATAGAGGGGGGCAGTGGGAGGGCTGCGACAGTTGAA
ACGGAAGCGGTTGAAGTGCTAGAAGAAAGCAGTGATGTTATACAGCAACTTAGTCCGCGT
ACACAGGTGGTAGAGCTGTTTAAATGCAAGGATTTAAATGCTAAACTGTGTGGTAAGTTT
AAGGAACTTTTTGGAGTGGGCTTTCACGATTTGGTTAGACAGTTTAAAAGTGATAAATCA
ACGTGTACAGATTGGGTGTACGCAGTGTTTGGGGTTAATCCCACTATAGCAGAAGGCTTT
CATACATTATTAAAAGGACAGGCATTATACTTACATACACAGTGGACAACGTGTAGATGG
GGTATGGTATTGCTTGCATTGTGTAGATATAAGGTAGCAAAAAATAGAGAAACAGTAGTG
CGGCAGCTTGCCAAAATGTTAAATGTACCAGATAATCAACTAATGGTACAACCACCTAAA
TTACAAAGTTCTGCAGCGGCTTTATTTTGGTTTAGATCAGGAATGGGTAATGGAAGTGAG
GTGTCTGGCACAACACCGGAATGGATAGCTAAACAAACAATGTTGGAACATAGTTTTGCT
GAAGCACAGTTTAGTTTAACTCAGATGGTGCAGTGGGCATATGATAATGGGCATACAGAT
GAATGTGAAATAGCATATTATTATGCACAAATAGCAGATATAGATGCAAATGCAGCAGCG
TTTTTAAAAAGTAACAATCAAGCTAAATATGTTAGAGATTGTGCAGCTATGTGTAAGCAT
TATAGGTTGGCAGAAATGAGGCGGATGTCAATGGCAGACTGGATAAAGCATAGAGGTGAA

AAATGTGATGAAGGAGATTGGAAGCCTATAGTTAAACTACTAAGATATCAACATATAGAC
ATAATTGTGTTTTTAGCTGCGTTGAAAAAATGGCTACATGGAATACCAAAAAAAAATTGT
ATTTGTATTGTTGGACCTCCAGATACTGGAAAGTCATGTTTTGGTATGAGTTTGATGCAT
TTTTTGCAAGGTACTATAATTTCATTTGTTAATTCGTGTAGCCATTTTTGGTTGCAATCA
TTAGTGGACGCAAAAGTAGCTATGTTAGATGATGTGACTTCTGCATGCTGGGCCTATATG
GATACACACATGAGAAATTTATTAGATGGAAACCCAACAAGTATAGATAGAAAACATAAA
TCATTGGCTGTGATTAAATGTCCTCCATTATTGTTAACATCAAATATAAACATTAAACAT
GATTGCAAATATCAATATTTACAGAGTAGAGTGACAGTGTTTGAATTTCCAAATCCATTT
CCATTTGACAGCAACGGAAATGCTGTGTATGAATTAAGTGATGCAAATTGGAACTCCTTT
TTTAAAAGGTTGGCGTCCAGTTTAGAGCTGCAGACAACAGAGGACGAGGATGGAGAAACT
AGCCAGGCGCCTAGATTTGTGCCAGGAACAGTTGTTAGAACTTTATGAACAAGACAGCAA
ACAGCTACAGCACCATATATTGCACTGGAAATATATACGTTATGAAAGTGTAATATATTA
TACAGCAAGACAAATGGGCATTAAACGTCTGGGCCACCAGGTGGTGCCAAGTTTAGATGT
GTCAAAAGCCAAAGCCCATGCAGCAATTGAAATGCAAATGTGTCTAGAATCTTTGCAAAC
TACTGAATATAACTTAGAGCCATGGACGTTACAGGACACAAGTCAAGAACTATGGCTTGC
AGAACCAAAGAAATGTTTTAAAAAAGGAGGAAAGACAGTAGAAGTTAGATTTGACTGTAA
TGAACATAATGCAATGCATTATACTCTATGGACTGCAGTATATGTACAGGTGGAGGATAC
ATGGACAAAGGTTGAAGGCCAGGTGGACCACAGAGGCCTATTTTATACAGTGCATGGGTG
CACAACATATTATGTAGACTTTGGAAAGGAAGCACATACATATGGGAAAACAAATGACTG
GACTGTTATTGTGGGTTCACGCGTTATATGTTCTCCTAGTACTGTCGAAGGGCTACCCAT
TGTTGCGCCTGTTGACATCAGACATCCCGCGGCCACCGACGCCACCGACGCCACCAAGGT
GCACGACGCCCCCTACGCCCTGCCCGCGTCGACCACCAAAGTATACAACGACAGCCACGC
ACCGCCCCGAAAGCGAAGGAGAGACGGAGACTTGTCCATCAGTGCAGTGGACGGATGTAG
TGGAAGAAAATACGTGGACACTGGAAACAGAGCACGCTCGCCTGATATTGAAAGCAACAA
CAAAATCAGGAACAGTGGTGGAGGTCATTCTACACCTATAATACAACTGGAAGGTGATGC
CAATTGTTTAAAGTGTTTTAGATATAGGCTAACAAAGGTTAGCCATTTATATACAAATTC
TTCAACTACATGGAGGTGGACTACAGAATCTAGAACAAATAAAAATGCCATTATAACATT
AACATATAGTAGTGTACACCAACGGTCACAATTTCTAGCACTTGTAAAAATACCTAAAAC
TATTAAACATAGTTTAGGCATGTTAACTATAATGTAAATATGTTTGTATATTTGTAAAAA
ACATATGTATGGAACGCAACTGTGAAGGGTAATGCATATGCATTTACCACACAACCAGCC
AAACTGCTGTTATTGGTGTTAATAGAGTCAACGCTTGGCATACTAATAGTATATATACTC
TGTCTTATAACTGCAATTTTAATATGCATGCATATACCTGATTTCTGTGTCTGGAGCTCT
TTGATGGCCACTATTTCTATTCTTTGCTTTATAACGTGGGGTGCACTAACATCTATTATT
AACGTATTTTTTTAGTGTTGTTAGTGTGGTATTTGCCTGCACTGTTCCTTCATATGTCA
ATTGTGTATGCTATACAACAAGAACAACAATAACAATAATTGCAAATATGTTAACCTGTA
AATTAGATGATGGTGATACATGGATGGCATTATGGATGTTACTTACATTTATAACTGTAT
TGTTACTGTTATTGGCGTTTCATTGTAGGACATTATATGTATACAAATATAGTAAGTAAA
ATACTGTGTATTAAATAAATATTTTTATATTTGCAGCGCCTTGTTATGGTGTCCAGCAGG
CCCCGTAGGCGTAAGCGGGCGTCCGCAACACAGTTATATCAAACATGCAAGGCAGCAGGC
ACCTGTCCACCAGATGTTGTTAATAAGGTGGAGCAAACAACCGTTGCAGATCAAATTTTA
AAATGGGGCAGCATGGGAGTGTTTTTTGGCGGTCTTGGCATAGGTTCAGGCTCTGGCTCA
GGCGGCCGTGCTGGCTATGTGCCTTTGTCTACAGGTTCCCGTGCAATACCTCCTAAATCA
TTAGCTCCAGATGTTATTGCTAGGCCGCCTGTTGTGGTGGATACTGTCGCCCCCACTGAT
CCATCCATTGTATCTTTAATTGAGGAAAGTAGTATTATTCAGTCTGGGGCTCCTTCGCCA
GTAATTCCCACAGAGGGTGGGTTTTCAATAACATCATCAGGTACAGATGTCCCTGCAATT
TTAGATATATCTTCTACTAATACAGTACATGTTACATCTACCACACACCATAACCCCATA
TTTACTGATCCTTCGGTTGTGCAGCCTATTCCACCTGTAGAGGCTAGTGGTCGTATCATT
GTGTCGCATTCCTCTATTACTACTGGTGCAGCTGAAGAAATACCTATGGACACATTTGTT
GTTCATAGTGATCCACTGTCCAGTACACCTGTGCCTGGTGTGTCAGCGCGGCCTAAAGTT
GGGCTATATAGCAAAGCTTTGCAGCAAGTAGAAATAGTAGATCCAACATTTATGTCCACC
CCTCAACGTTTAATTACTTATGACAATCCTGTATTTGACAACATTGAAGATACACTACAT
TTTGAACAGCCTTCTATTCATAACGCACCAGATCCTGCCTTTATGGATATCATTACTTTA
CATAGGCCTGCCTTGACCTCTAGGCGTGGTGTGGTACGTTTTAGTAGGGTGGGTCAACGT
GGAACCATGTATACACGTCGTGGTACCCGTATTGGTGGTCGTGTACACTTTTTTAAAGAT
ATTAGTCCTATAGCTTCATCTGAAGAAATTGAATTGCACCCTCTAGTGGCCTCACCTAAT

AACAGTGACCTTTTTGATGTTTATGCAGATATAGATGATATTGATGAAAATATATTATAT
TCTACTATAGACAATAATACACCAACTTCTACCTATTCCTTGTATCCAGGTAATTCTACA
CGCATAGCAAATACATCTATACCTCTTGCCACAATTCCTGATACATTCTTAACATCTGGT
CCTGACATAGTGTTTCCTTCTGTTCCTGCAGGTACACCATATTTGCCTGTGTCACCTTCT
ATACCTGCCATATCTGTACTGATTCGCGGTACTGATTATTATTTGAATCCTGCATACTAT
TTCAGAAAACGCCGAAAGCGCATATTAGCATATTAGGATGTGGCAACTTAATGAAAACCA
AGTGTATTTACCACCGCCCACGCCTGTTGCTACAATTGTTAGCACAGATGAGTACGTGCA
ACGCACCAGTTTATATTATCATGCAGGTAGTACCAGGTTATTAACCATAGGACATCCATA
TTTTGAATTGAAAAAGCCTAATGGCGATGTATCGGTGCCTAAAGTGTCTGGACATCAATA
CAGAGTGTTTAGAGTACGCTTGCCCGACCCTAATAAATTTGGATTATCAGACACGTCTTT
ATTTAATTCTGAAACCCAACGCCTTGTATGGGCCTGTGTTGGTGTTGAGGTCGGTCGAGG
TCAGCCATTAGGTGTAGGCATTAGTGGTCATCCATACTTTAATAAAGATGAAGATGTGGA
AAACTCGTCTGTATATGGAACAGTACCTGGTCAGGACAGCAGAGAAAATGTTGCTATGGA
TTATAAACAAACTCAGTTATGTATTGTAGGCTGTACTCCTCCTATTGGAGAATATTGGGG
TATGGGTACACCGTGCAATGCTTCTAAAGTGTCTCCTGGTGACTGTCCTGTACTAGAATT
AAAAAGTGAAGTTATTGAGGATGGCGACATGGTTGATGCAGGCTTTGGTGCCATGGATTT
TGCATCATTGCAGGCCAATAAAAGCGATGTGCCTTTAGATTTATGTACATCTATTAGTAA
ATACCCAGATTATTTAGGAATGGCTGCAGAACCGTATGGTAATAGTTTATTTTTTTTTCT
TAGAAGAGAACAAATGTTTGTTAGGCACTTTTTTAATAGGGCAGGAACTACTGGAGACAG
TGTTCCAAATGATTTATATATAACAGGTTCATCTAATCGCGCTTCTATTGCAGGCAGTAT
TTATTATTCCACACCAAGTGGCTCTCTAGTTACCTCTGATTCTCAGATTTTTAATAAACC
TTTGTGGATACAAAAGGCCCAGGGTCATAACAATGGCATTTGTTTTGGCAATCAGTTATT
TGTTACAGTTGTAGATACTACTCGTAGCACAAATTTAACATTATGTGCTGCTACACAATC
GCCCACACCAACACCATATGACAATAGTAAGTTTAAAGAATATTTACGTCATGGGGAAGA
GTTTGATTTACAGTTTATTTTTCAGTTATGTGTTATTACATTAAATGCAGAGGTTATGAC
ATATATACATGCTATGGATTCTTCCTTATTAGATGATTGGAATTTTAAAATTGGTCCTCC
AGCGTCTGCAACCTTGGAAGATACTTATAGGTTTCTTACCAATAAAGCCATAGCATGTCA
GCGTGATGCACCCCCAAAAGAAAAGGAGGATCCATATAAAAAATATAAATTTTGGGAAGT
AAATTTAACAGAAAAATTTTCATCTCAGTTAGATCAATTTCCATTAGGACGTAAGTTTCT
TATGCAGGCAGGCCTACGCACAGGGCCTAAGTTTAAATCCAGGAAGCGACCTGCCCCTAC
CTCTTCTTCCTCTTCTGGGTCAGTCACCCCCAAACGTAAGAAAACAAAACGATGATTGTG
TATGTGTGTGTGTGTGATACTCCCTTGCACTCCCTTATGTGTTGTTACTCTGGAATGTAT
GTATGTGTTGTTTATGTATGTGTGTTTGTATATGTTGTTTATGTGTGAGAATGTATTTGT
GTGTATGAATGTAATGTATTGTTGTTGTGTTTGTTAATAATAAATATATTGTGTGTTGTG
TGGTAAAGTACTTTCTTTGTTTTAAAAGTTACTTATTAAACTCACTTATTTTAAAATGTC
TGCTCTGCACACTGCAACCGTTTTCGGTCGCGGTTGGCAACTCATTACATTTGTCAGCAT
GTTTTTATAACATGTTTAAAATTGTTAGCTTTATATAACTATATAAATCCTTCAATTTCC
ACCCATAACCGTTTCCAGTCTCGGTTGGCAAGTCACCATGTTTGTCAGCATATTTGCATT
GCATGTTTCAAATTGCTAGGTCAAAGTTCCCTGCCAAAAATGCCGCCCAATATGTACTAT
TAGGGTGAGGTTGCCACACCTTTAATTACACTTTTATTGCACTGTTACTCATCTTATTTT
ATACGCTTCCAAACTTGCTTTTAGGCACATAGTTTTACTGCATAAACATTTAGCTAATAG
CAGTTTGGCACCACATAACACTATGGTTAACAAAATACACATTACTCATGGTACACACCT
GCAAACCGCTTTCGGTTGCTACACGTTTTATTACTTTCTAGTTATTA
>gi|9627257|ref|NC_001576.1|_Human_papillomavirus_type_10,_complete_genome
TTATAAACTATAATCTAGACAATAATAAATAGGGAGGGACCGAATACGGTGCGACCGAAT
GGGGTACATATAAAACAAGGCCCGTAGCATCTGCAGAAGCTATGTCCATGGGTGCACAGG
AACCCAGAAACATATTGCTTTTGTGTAGAAATTGTGGAATACCTTTGGAGGACCTTCGCC
TGTGCTGTATATTTTGCACAAAACAGCTGACCGCAGCGGAATTGGCAGCATTTGCACTTA
GAGAATTATATTTGGTGTGGAGAGCGGGAGTGCCATACGGTGCCTGTGCACGGTGTTTAC
TCCTACAGGGCATTGTACGACGCCTAAAATATTGGGACTATTCATATTATGTAGAAGGTG
TGGAAGAGGAGACCAAACAATCTATATATACACAGCTGATCAGATGCTACATGTGTCACA
AACCGCTGGTAAGGGAAGAAAAAGACAGACATCGTAACGAACGGCGACGACTGCACAAAA
TATCAGGGTACTGGAGAGGTAGTTGTGAGTATTGCTGGTCACGATGCACGGTCCGCATCC
CACAGTAAAAGATATCGAATTGAGTCTTGCACCAGAGGATATCCCTGTATGCAATGTGCA
ATTAGATGAAGAAGATTATACAGATGCGGTGGAACCAGCACAACAAGCGTATAGGGTGGT

AACAGAATGTACAAAGTGTAGTTTACCACTGCGACTGGTGGTAGAGTGCAGCCACGCAGA
TATAAGGGCACTGGAACAGCTGCTACTAGGCACATTGAAGCTCGTGTGTCCTCGCTGCGT
GTAACAGGACATGGACGATAATACAGGTACAGAGGGGGGCGCATGTTCCGAATCGGAACG
GGCGGGTGGATGGTTTATAGTGGAAGCCATTGTAGATAGGCGGACAGGCGATCCAATATC
TAGTGATGATGATGAGGAGGAGGACGAAGCAGGGGAAGACTTTGTAGACTTTATAGATGA
TACTAGGTCGCTAGGGGATGGACAGGAAGTGGCACAGGAACTGTTCCAGCAGCAGACAGC
TGCAGATGACGATGTAGCTGTGCAGACTGTAAAACGAAAGTTTGCACCCAGTCCTTATTT
CAGCCCCGTGTGTGAGCAAGCCAGCATAGAACATGAACTAAGCCCAAGGCTAGACGCCAT
AAAGCTGGGGAGACAGTCAGCAAAGGCCAAACGTCGGCTGTTTGAGCTACCGGACAGTGG
CTATGGCCAAACACAGGTGGATACGGAATCGGGACCAAAACAGGTACAGGGCAGTAGTGA
AACGCAAGATGGCCGACAGGATGATGATGAGGGGAGTGTGGTACAGAGCACACTTGACAC
AGGCAACCAAAATGGCCGCCAGAACAATGATGAGGGGAGTGGGAGGAATGTGGGGGAACA
TGGCAGCCAAGAGGAGGAGCGTGCAGGAGGGGATGGGGAGGAATCTGACTTACAAAGTAC
AAGCACTGGCAAGGGAGCAGGTGGCGTGGTAGAAATATTAAGAGCCAGCAATAAGAAAGC
AACTCTACTGGGTAAGTTTAAAGAACAGTTTGGGTTGGGGTATAACGAACTAATTAGGCA
CTTTAAAAGTGATAGAACATCGTGTGCCGACTGGGTGGTGTGTGTGTTCGGGGTGTTTTG
CACAGTGGCAGAGGGCATAAAGACCCTAATACAACCATTGTGTGATTATGCACACATACA
AGTGCTACCATGCCAATGGGGAATGACAGTGCTTATGCTGGTACGGTACAAACGTGCCAA
AAACAGGGAAACAGTGGCTAAAGGCTTAAGCACATTATTAAATGTACCGGAAAGCCAGAT
GTTAATTGAACCACCAAAATTACGAAGTGGTCCAGCAGCGTTGTATTGGTACAAGACTAG
TATGTCCAGCTGTAGCGACGTGTATGGGGAAACACCAGAGTGGATAGTCAGGCAGACAAT
GGTGGGACATGCAATGGAGGATGCGCAGTTTAGCCTTTCAGAGATGGTGCAGTGGGCATA
TGACCATGATATTACAGACGAGAGCACGCTGGCATACGAGTATGCACTGATTGCTGACAC
AGATTCAAATGCAGCTGCGTTTCTAAGTAGCAATTGTCAGGCTAAATATCTAAAGGATGC
ATGCACAATGTGCAGACATTATAAAAGAGGAGAACAGGCGCGCATGAGCATGTCAGAATG
GATATGGTTTAGAGGCGACAAAGTACAGGGAGATGGAGATTGGAAACCAATAGTTCAATT
TTTAAGATATCAGGATGTTGAATTTATCCCATTCCTATGTGCCTTTAAAACATTCCTACA
AGGAGTACCAAAAAAAAGTTGTTTAGTGTTTTATGGACCAGCAGACACTGGCAAATCATA
CTTTTGTATGAGCTTACTTAGATTTTTGGGGGGGGCTGTTATATCCTATGCTAACTCAAG
CAGCCATTTTTGGTTGCAGCCATTATCTGAAGCCAAAATAGGACTGTTAGATGATGCAAC
TAGTCAGTGTTGGAACTATATTGACACTTATTTAAGAAATGCCTTAGATGGCAACCAAAT
ATGTGTAGACAGAAAGCATAGAGCATTGTTACAGCTAAAATGCCCTCCACTATTAATAAC
AACAAATATAAATCCATTGACGGATGAAAGATGGAAGTTTTTGCGCAGCAGATTGCAGCT
CTTCACATTTAAAAACCCTTTTCCAGTGACAACACAAGGAGAACCAATGTATACATTAAA
TGATCAAAATTGGAAATGCTTTTTTCGAAGGTTATGGGCACGTTTAAGCCTTACCGATCC
TGAAGACGAGGAGGAGCATGGAAACCCTAGCGAACCGTTTAGATGCGTGCCAGGACAAAA
TGCTAGAACTATATGAAAAGGATAGCGACAAACTTGAGGACCAGATCACGCATTGGCACT
TATTGCGTGTAGAAAATGCTTTGCTGTACAAAGCAAGAGAATGTGGACTGACACATATTG
GCCATCAGGTGGTGCCACCTCTTAGTGTAACTAAAGCCAAGGCACGCAATGCCATTGAAG
TGCATGTAGCTTTACAGCAATTGCAAGAAAGTGCCTATGCACACGAACCCTGGACATTGC
GGGACACATCACGTGAAATGTGGGACACTGCTCCTAAAGGGTGCTGGAAAAAAAGGGGGA
TAACTGTTGAAGTCAGATATGATGGAGACGAATCTAAAGCCATGTGCTATGTACAATGGA
GGGAACTTTATGTGCAGAACTATAGTGACGATAGATGGGTGAAGGTGCCAGGAAAAGTCT
CATACGAGGGTCTATATTATACACATGAAAATATGAACATATATTATGTGAATTTCAAGG
ATGACGCTTGTGTATATGGGGAAACAGGCAAATGGGAGGTACATGTGGGAGGCAAAGTAA
TTCACCATGATGCATTTGACCCTGTATCTAGCACACGAGAAATATCCACTCCTGGACCTG
TGTGCACCAGTAACACCACCCCAGCGTCCACCCAAGCCCAGGTGGGCGCGTCCGAGGGAC
CGGAACAAAAGCGACAGCGACTCGAGGCGGTCGACGGACAGCACCAGCAGCAGCGACAAG
GGTCCAAAGATTCCACCCAGAAGGCCGCGGAACGAGCGGGTGGACAAGTGGACAGTGACA
GGACCCGGTTGTGTGACACTAGAAGTGCACACCCCGTCCGGCACCCAAGTGACCCTGACT
GTGCACCTGTAATACACCTACGAGGTGATCCTAACAGTTTAAAATGCTTTAGATATAGAT
TACACCACGGAAAAAGGAAACTATACTCACGGTCATCCTCCACATGGAGGTGGTCTTGTG
AGTCAGAAAACCAGGCAGCGTTTGTAACGCTTTGGTATACCAGCGATACACAGCGTACTG
AATTTCTTAATGTTGTAAAGGTTCCCCCTGGCATACAAGTGATTTTGGGGTATATGTCAA
TATTCTAATATGTCAGATATTTCTATACATATATAGATCTATAGGGTGATTGTACATTCT

GGATTTTTACTTGTGTCGACTACATTGCTGGGCTTTTTTTGTGCTGCTGCTGTGTTTGTT
TTGGCTGTGTGTGCTTCCCGCGCTGACGTGCTATCTGGCATTTGTGCTTTGTGTGTACTT
GGGCCTTATAGCATTATATTTACAAATTGTGACACGTATTGTACAGGTCAACACATAGGT
CTCACAATGTATCCTCTTATATTAAGAGATCACACTGGTGACCATCCTGTATTGTTGTTT
GAGCCGGGGGACGTGTATTTACTACTGTGTGTAATCATTTTTGTTATAATAGCACTGTTT
GTGTTATATAGACATCTTGGTGTATTGTAACATAATATGTGGTAGCCTGTACGGATCCCA
CATAGGGGGTTGTATATCATATGTTGTTGTTGTGTGTACCCTAGTGTGCTATTGTGGCAC
CATTCTTTTTCTATTTTTGTTTTTTTTTTTACAGTTAAATAAAGCAACCATGGTGGCACA
ACGTGCAAGGCGTCGCAAGCGTGCATCCGCCACACAGCTTTATAGGACCTGCAAGGCCTC
AGGCACATGCCCCCCAGATGTTATTCCCAAAGTAGAGGGCACCACCTTGGCAGATCGCAT
TTTGCAGTGGGGTAGCCTTGGTGTATATTTGGGGGGCCTAGGCATTGGAACTGGGTCTGG
TACTGGGGGTCGCACGGGGTATGTTCCTATCAGTACACGACCTGGCACTGTTGTGGATGT
TAGTGTTCCTGCCAGGCCTCCTGTTGTTATTGAGCCTGTAGGCCCGTCAGATCCTTCCAT
TGTAAATTTGTTGGAGGACTCCAGCATTATTAATTCAGGGTCCACCATACCTACATTTTC
TGGTACTAGTGGCTTTGAAGTTACATCCTCTGCCACAACCACCCCAGCTGTGTTGGACAT
CACACCTGCCAGTGAGAATGTGGTTATTAGTAGTACAAACTTTACAAATCCTGCATTCAC
AGAGCCGTCCCTTGTAGAAGTTCCACAGAGTGGTGAGGTTTCGGGACACATACTTATAAG
CACACCTACAGCTGGCACCCATGGATATGAGGAAATACCCATGGACACGTTTGCTTCTTC
GGGTACTGGCACGGAACCCATAAGCAGTACCCCTGTGCCTGGTGTCAGTAGAATAGCAGG
CCCACGCTTATATAGCAGGGCTAACACACAGGTCAAGGTGTCCGATCCTGCATTTCTGTC
ACGTCCTTCCTCCTTGTTAACATTTGACAATCCTGTGTTTGAACCGGAGGATGAAACAAT
CATATTCGAACGTCCGTACTCTCCCTCACGGGTGCCGGACCCTGATTTCCTTGACATTGT
GCGTCTGCATCGCCCTGCATTAACATCTCGCAGGGGTACTGTACGTTTCAGCCGCCTGGG
TCAAAAGTTCAGCATGCGCACACGCAGTGGCAAGGGTATTGGAGCCCGGGTACATTACTA
TCAGGACCTTAGCCCTATAGCCCCAATCGAGGACATTGAAATGGAACCTTTACTTGCTCC
AGCTGCCTCTGACACTATTTATGACATTTTTGCTGATGTTGATGATGGTGATGTTGCTTT
TACAGAGGGATATCGTAGTACCACACAGTCCAGGGGATATAATACCACTTCCCCTTTGTC
TTCTACACTTTCCACTAAGTATGGAAATGTAACAATTCCCTTTGTGTCTCCTGTTGATGT
AACTTTACATACTGGGCCTGATATTGTACTACCTACTTCAGCACAGTGGCCATATGTGCC
CCTGTCACCTGCTGACACCACCCATTATGTGTACATAGATGGCGGGGATTTCTATCTTTG
GCCTGTTACCTTTCACTTCTCCCGACATCGTCGCCGTAAACGTGTCTCATATTTTTTTGC
AGATGGCACTCTGGCGCTCTAGTGACAACTTGGTGTACCTGCCTCCCACTCCCGTGTCTA
AAGTTCTCAGCACGGACGACTATGTGACACGCACCAACATTTACTATTATGCAGGCACTT
CACGGTTGCTTACTGTAGGCCATCCATATTTTCCTATACCTAAGTCAAGTAACAATAAGG
TAGATGTCCCCAAGGTATCTGCATTTCAATATAGGGTGTTTCGGGTGCGGTTGCCTGACC
CTAATAAGTTTGGACTGCCTGACGCCCGCATATATAACCCTGACGCCGAGCGACTGGTCT
GGGCTTGTACTGGGGTTGAGGTAGGTCGTGGACAGCCACTGGGTGTGGGGCTCAGTGGAC
ACCCTCTATATAATAAGCTAGAGGACACAGAAAACTCTAATATAGCACATGGGCCAATTG
GTCAGGATTCACGGGACAATATTTCTGTTGATAATAAGCAAACACAGCTATGTATTATTG
GTTGTACACCTCCAATGGGAGAGCATTGGGGCAAGGGAACCCCGTGCAGGAACCCACCTG
CACAGGGCGATTGCCCTCCCCTGGAGCTTATAACTTCCCCTATTCAGGATGGTGATATGG
TGGACACTGGCTATGGGGCCATGGACTTTACTGCTTTACAATTAAATAAGTCTGACGTGC
CTATAGATATTTGCCAATCTACTTGTAAATATCCAGATTATTTGGGAATGGCCGCGGAGC
CTTATGGCGACAGCATGTTCTTTTACTTGCGCAGGGAACAACTGTTTGCAAGACATTTCT
TTAATCGGGCTAGTGCTGTTGGAGACGCCATCCCAGACACTTTCATATTAAAGAGCAACG
GTGGGGGGCGAGACGTTGGTAGTGCTGTGTATAGCCCCACACCCAGTGGGTCCATGGTAA
CGTCTGAGGCTCAATTGTTTAATAAGCCATATTGGCTGCGGCGGGCCCAGGGGCACAACA
ATGGTATATGCTGGGCTAACCAATTGTTTGTTACTGTGGTAGACACGACTCGCAGTACCA
ATATGTGCTTGTGTGTTCCTTCTGAGGCCTCCCCTGCCACTACGTATGACGCCACCAAAT
TTAAAGAATATTTGAGGCACGGAGAGGAATATGATTTGCAGTTCATTTTTCAGTTGTGTA
AGGTAACATTGACCCCGGATATTATGGCCTATTTGCACACCATGAATAGTAGTTTATTGG
AGGATTGGAACTTTGGGTTAACTTTGCCACCGTCCACTAGCTTGGAGGACACATATAGAT
TTTTGTCCTCTTCAGCCATTACTTGTCAGAAAGATACACCCCCCACCGAGAAGCAGGATC
CCTATGCAAAACTTAACTTTTGGGACGTAGATCTTAAGGATAGGTTTTCCCTGGACCTGT
CTCAGTTTCCCCTGGGACGAAAATTTTTGCTGCAGCTGGGTGTACGTTCTCGCTCCGCCG

TCTCCGTCCGCAAACGCCCGGCGACTTCCGCGACAGGATCCACGGCTGCAAAAAGAAAAA
GAACTAAGAAATAACTGCATGTTTGTGTTAAATGTATGTGCATATTTGTGTATGTGGTTG
GCTGTTAATAGGTGTTTGTAATGTCTATGTATGTGTGTATGTATGTAATGTGTTTGTATG
TTATGAGCATGTATGTGTGTATGTGTAAATAAAGTTTGTCACATAGTTTTATATTTTTTA
ATTTGTGTAATTGCTGTTCCTGTGAGTAAGTAAGGTTGTTCTAGGTCAGGAGACCGATTT
CGGTACAAGATGGCCGCCTTTCCAGGTGTGCACACCACCAATTAGTCATGCTGATCTATA
TCCTGCGACCTGCCCTGTCACGCCATTTTTTTGGCTAAGATTGTATAGTTTCTATAGTTT
AGTTTATTGCTGTATCATGCTTTCTGGCACGGCAAACTGTCTCCATTGCAAATTTAACAG
CTTCTGGGCACCAACTTATTATGACTACTTTCACATAATTACTGTTCTGGCTGCGTTTTC
TAGGTTGCCTTGCCAATAAAATGTGCTTCCAAATCTCCACCAAGACACACCTAATCCGGT
CGCTGCTTGCTTTCTAGCTTTAATTAATGCAGTTGCTACACGTCTCTTTCTAACTATAA
>gi|9627145|ref|NC_001531.1|_Human_papillomavirus_-_5,_complete_genome
AACGGTAAGTTGCAATTTCCTTGTACCAGGTGCGGTATTGGGATTTCACAATTATAATGG
TTGTTGCCAACTACCATAGGCATATTCAAGTTTTTGCCTGTATCGTTTTCGTATCCTGTA
ATAATATCCAATATATGTATACATAAATAAATATATATATATATAAGTGTCTAAGATTGG
GTTCTTCTGTAATCAGGCAATGGCTGAGGGAGCCGAACACCAACAGAAACTGACAGAAAA
AGATAAGGCAGAATTACCTTTAAGTATTAGAGACTTAGCTGAAGCCTTAGGCATCCCTGT
GATTGATTGTTTAATACCTTGCAATTTCTGTGGCAACTTTCTAAATTATTTGGAAGCTTG
TGAATTCGACTACAAAAGGCTTAGTCTAATTTGGAAAGATTATTGTGTGTTTGCGTGCTG
TCGCGTATGCTGTGGCGCCACTGCAACTTATGAATTTAACCAATTTTATGAGCAGACAGT
GTTAGGAAGAGATATTGAATTAGCTTCAGGACTTTCAATATTTGATATTGATATCAGGTG
TCAAACTTGCTTAGCATTTCTTGACATTATAGAAAAGTTAGATTGCTGTGGCAGAGGCCT
TCCCTTTCATAAGGTGAGGAACGCCTGGAAGGGAATCTGTAGGCAGTGTAAGCATTTTTA
TCATGATTGGTAAAGAGGTCACCGTGCAAGATATTATTCTGGAGCTCAGTGAGGTGCAGC
CCGAAGTGCTACCAGTTGACCTGTTTTGTGAAGAGGAATTACCAAACGAGCAGGAAACGG
AGGAGGAGCCTGACAACGAAAGGATCTCTTACAAAGTTATAGCTCCGTGCGGTTGCAGGA
ACTGTGAGGTCAAGCTTCGCATTTTTGTCCACGCCACAGAATTTGGTATTAGAGCTTTCC
AACAGCTACTGACCGGAGATCTGCAGCTCCTGTGCCCTGACTGTCGCGGAAACTGCAAAC
ATGACGGATCCTAATTCTAAAGGTAGTACATCTAAAGAAGGGTTTGGTGATTGGTGTTTA
TTGGAAGCTGACTGTAGTGATGTAGAAAATGATTTGGGACAATTATTTGAGAGAGATACA
GACTCTGATATATCGGATTTGTTAGATGATACTGAACTGGAGCAGGGCAATTCCCTGGAA
CTATTTCATCAACAGGAGTGTGAGCAGAGCGAGGAGCAATTGCAAAAACTAAAACGAAAG
TATCTTAGTCCAAAAGCTGTCGCACAGCTTAGTCCGCGACTTGAGTCAATTTCATTGTCA
CCCCAGCAGAAGTCTAAGCGAAGGCTCTTTGCAGAGCAGGACAGCGGACTCGAGCTGACT
TTAAACAATGAAGCTGAAGATGTTACTCCTGAGGTGGAGGTACCGGCTATTGACTCTCGG
CCGGATGACGAGGGAGGTTCAGGGGACGTAGATATACATTACACTGCATTGTTGCGTTCT
AGCAACAAAAAAGCTACATTAATGGCTAAGTTTAAAGAGTCGTTTGGAGTAGGTTTTAAT
GAATTGACACGGCAATTCAAAAGCCACAAAACCTGCTGTAAGGACTGGGTTGTCTCTGTA
TATGCAGTGCATGATGATCTATTTGAAAGCTCAAAGCAGCTATTGCAACAGCATTGTGAC
TATATCTGGGTCCGTGGGATAGGTGCAATGTCATTATACCTATTGTGTTTTAAGGCGGGA
AAAAATCGCGGGACAGTTCATAAGTTAATTACCTCAATGTTAAATGTGCATGAACAGCAA
ATATTGTCTGAGCCGCCAAAATTGAGAAATACAGCCGCTGCATTGTTCTGGTATAAGGGT
TGTATGGGATCGGGGGCGTTTAGCCATGGACCATATCCTGATTGGATTGCCCAACAAACT
ATATTAGGTCACAAAAGTGCTGAGGCAAGTACTTTTGATTTTTCAGCAATGGTCCAATGG
GCATTTCATAATCACTTATTAGACGAAGCAGATATAGCATACCAGTATGCAAGGCTTGCT
CCCGAAGACGCGAATGCAGTAGCTTGGCTTGCACATAACAACCAGGCCAAATTTGTGAGA
GAATGTGCATATATGGTACGATTTTATAAGAAGGGACAAATGAGAGACATGAGTATATCT
GAATGGATATACACTAAAATCAATGAAGTAGAAGGGGAAGGGCACTGGTCAGATATAGTA
AAGTTTATTAGATACCAAAATATAAACTTTATTGTATTCCTAACTGCATTAAAAGAATTC
CTACACTCAGTGCCAAAAAAAAATTGCATTTTAATTTATGGTCCTCCAAATTCTGGAAAG
TCATCATTTGCAATGTCATTAATAAGAGTGTTGAAGGGTAGAGTGTTGTCATTTGTAAAT
TCTAAAAGTCAGTTTTGGCTGCAACCCCTTTCAGAGTGCAAGATAGCTCTATTGGATGAT
GTAACAGACCCTTGTTGGATATACATGGATACATATTTAAGAAATGGCTTGGATGGACAT
TATGTTTCATTAGATTGTAAATATAGAGCCCCAACGCAAATGAAATTTCCCCCATTATTA
TTAACATCTAACATTAATGTGCATGGGGAAACTAATTATAGATATTTACACACTACAATA

AAAGGATTTGAATTTCCAAATCCTTTTCCTATGAAAGCAGATAATACACCTCAGTTCGAA
CTAACTGACCAAAGCTGGAAATCTTTTTTTACAAGGCTTTGGACACAATTAGACCTGAGT
GATCAAGAAGAGGAGGGCGAGGATGGAGAATCTCAGCGAGCGTTTCAATGCTCTGCAAGA
TCAGCTAATGAACATTTATGAAGCTGCAGAACAAACATTGCAGGCACAAATTAAACATTG
GCAAACCTTACGAAAAGAACCTGTATTACTCTACTATGCTAGGGAGAAAGGTGTTACAAG
GCTTGGATATCAACCTGTGCCTGTAAAGGCAGTATCAGAAACAAAGGCTAAAGAAGCCAT
AGCAATGGTGCTGCAGCTTGAGTCACTACAGACATCTGATTTTGCTCATGAGCCATGGAC
TCTAGTTGATACCAGCATAGAAACATTTAGAAGCGCTCCAGAAGGTCACTTCAAAAAAGG
CCCCCTCCCTGTAGAAGTTATTTATGACAATGATCCAGATAATGCCAATTTGTATACAAT
GTGGACCTATGTGTATTATATGGATGCGGATGATAAGTGGCATAAGGCAAGAAGTGGGGT
GAATCACATTGGCATTTATTATTTACAAGGAACTTTTAAAAACTATTATGTACTGTTTGC
TGACGATGCGAAAAGATATGGTACAACTGGAGAATGGGAAGTAAAAGTTAATAAGGAAAC
TGTGTTTGCTCCTGTCACCAGCTCCACGCCTCCAGGGTCGCCAGGAGGACAAGCAGACAC
AAACACCACCCCCGCGACCCCCACCACCTCCACAACCGCCGTTGACTCCACGTCCAGACA
GCTCACCCACATCAAAACAGCCACAACAAACCGAAACCAGAGGAAGAAGGTACGGACGGAG
GCCCTCCAGCAAGTCAAGGAGATCGCAAACGCAGCAAAGGCGATCAAGGTCCCGACACCG
GTCCCGGTCTCGGTCCCGGTCGCGGTCCAAGTCCCAAACCCACACCACTCGGTCCACCAC
CAGGTCCCGGTCCACGTCGCTCACCAAGACTCGGGCCCTTACAAGCAGATCGCGATCCAG
AGGAAGGTCCCCAACCACCTGCAGAAGGGGAGGTGGAAGGTCACCCAGGCGGCGATCAAG
GTCACCCTCCACCTCCTCCTCCTGCACCACACAACGGTCACAGCGGGCACGAGCCGAAAG
TTCAACAACCAGAGGGGCCCGAGGGTCGAGAGGGTCACGAGGAGGGAGCCGTGGGGGGAG
AGGGCGGCGACGAGGAAGGTCATCCTCCTCCTCCTCCCCCGCCCACAAACGGTCACGAGG
GGGGTCTGCTAAGCTCCGTGGCGTCTCTCCTGGTGAAGTGGGAGGGTCACTTCGATCAGT
TAGTTCAAAGCATACAGGACGACTTGGAAGATTACTGGAAGAAGCTCGCGACCCCCCAGT
AATCATTGTCAAAGGGGCGGCTAACACACTGAAAAATGTCCGCAACAGAGCTAAAATTAA
ATACATGGGACTGTTTAGGTCATTTAGTACTACCTGGTCATGGGTGGCAGGAGATGGCAC
TGAGCGTCTAGGCAGGCCCAGAATGCTCATTAGCTTTTCTTCCTATACTCAAAGGAGAGA
TTTTGATGAAGCGGTGCGATACCCCAAAGGAGTTGATAAGGCCTATGGCAACCTGGACAG
TCTTTAACATTTACTAATGCTGCTTTTGCTACTAACATACTAACATACCCTAGCATTTTA
TATTTTTTTTTACATTTTGTATTTGCTATGGCGCGTGCAAAAACGGTCAAGCGAGACTCT
GTAACTCATATTTACCAAACCTGCAAACAGGCAGGCACTTGCCCCCCTGATGTTATTAAT
AAAGTGGAACAAACAACAGTTGCTGACAATATTTTAAAATATGGCAGTGCTGGTGTATTT
TTTGGTGGCCTTGGTATTAGTACAGGCCGAGGAACTGGGGGTGCTACAGGGTACGTGCCA
CTTGGGGAAGGTCCTGGTGTCCGTGTCGGAGGAACCCCCACGGTTGTAAGGCCTTCCTTG
GTTCCTGAAACAATCGGGCCCGTTGATATTTTGCCCATTGATACAGTTAACCCCGTGGAA
CCTACAGCATCATCCGTGGTCCCTCTAACTGAGTCCACAGGCGCTGATTTACTTCCAGGT
GAAGTAGAAACAATTGCTGAAATCCATCCTGTACCTGAGGGGCCATCAGTGGATACCCCT
GTAGTTACCACTAGCACAGGTTCCAGTGCTGTTTTAGAGGTTGCCCCAGAGCCTATTCCT
CCAACACGGGTCAGGGTTTCACGCACACAGTATCACAATCCATCTTTTCAAATAATAACT
GAGTCTACTCCAGCACAAGGGGAATCGTCTCTTGCAGATCACGTTTTGGTGACATCGGGT
TCTGGGGGGCAACGAATAGGGGGTGATATAACTGACATAATTGAGTTAGAGGAAATTCCT
AGTAGGTATACATTTGAAATTGAAGAACCAACTCCTCCACGCCGCAGCAGTACTCCATTG
CCACGCAATCAATCTGTAGGCCGTAGGAGGGGTTTCTCTTTGACTAATAGACGTTTAGTA
CAGCAGGTACAAGTGGACAATCCATTGTTTCTAACTCAACCATCTAAGTTAGTTCGTTTT
GCATTTGATAATCCTGTTTTTGAGGAAGAAGTGACTAATATATTTGAAAATGATCTGGAT
GTCTTTGAAGAACCTCCAGACAGAGATTTTCTTGATGTTAGGGAATTGGGACGTCCACAA
TATTCTACAACACCAGCGGGATATGTTAGAGTAAGCAGGTTGGGGACTCGAGCCACTATT
CGCACTCGCTCTGGTGCACAGATAGGGTCGCAAGTCCATTTTTACAGAGATCTTAGCTCT
ATTAATACTGAAGATCCTATTGAATTACAATTATTAGGCCAACATTCAGGTGATGCTACT
ATAGTCCACGGACCTGTTGAAAGCACATTTATAGATATGGATATTTCTGAAAATCCATTA
TCTGAAAGCATTGAAGCATATTCACATGATTTATTATTAGATGAAACGGTGGAAGATTTC
AGTGGGTCTCAGCTGGTTATAGGTAATCGAAGGAGCACAAACTCTTACACTGTTCCTAGG
TTTGAAACTACAAGAAATGGTTCATACTATACACAAGACACAAAGGGATATTATGTTGCA
TATCCAGAGTCACGTAATAATGCAGAAATCATTTATCCTACACCTGATATTCCTGTAGTC
ATTATACACCCTCATGACAGTACAGGGGACTTTTATTTACATCCCAGTCTTCACAGGCGC

AAACGTAAAAGAAAATATTTGTGATTTGCATTCGAGATGGCAGTGTGGCACTCGGCTAAT
GGTAAAGTATATCTTCCACCATCGACACCGGTGGCCAGAGTCCAAAGCACCGATGAATAC
ATTCAAAGAACAAATATCTACTATCATGCATTTAGTGACAGATTGTTAACTGTAGGTCAT
CCTTATTTCAATGTATACAATATTAATGGTGATAAGCTTGAGGTTCCTAAGGTTTCAGGA
AATCAACACAGAGTATTTCGCCTAAAATTACCAGATCCTAACAGATTTGCATTACCTGAT
ATGTCTGTTTACAACCCTGACAAAGAACGTTTGGTTTGGGCCTGTAGAGGCTTAGAAATA
GGTAGGGGCCAGCCATTAGGTGTACGGAGTACTGGTCACCCTTATTTCAATAAAGTAAAA
GATACAGAAAACAGTAATGCATACATAACATTTTCTAAAGATGACAGACAGGATACATCT
TTTGATCCTAAACAGATCCAAATGTTTATTGTAGGATGCACACCTTGCATAGGAGAGCAT
TGGGATAAAGCTGTTCCATGTGCAGAAAATGATCAGCAAACTGGCCTTTGTCCTCCTATT
GAACTAAAAAACACATATATACAAGATGGTGATATGGCAGACATAGGTTTTGGGAACATG
AATTTTAAGGCACTTCAAGATAGTAGATCAGATGTCAGTTTAGACATCGTCAATGAAACT
TGCAAGTATCCAGATTTTTTAAAGATGCAAAACGATATTTATGGCGATGCGTGCTTTTTT
TATGCTCGTAGGGAGCAATGTTATGCCAGACACTTTTTTGTTAGAGGGGGAAAAACTGGT
GATGACATTCCACGTGCACAAATTGACAATGGTACATACAAAAATCAGTTTTACATTCCA
GGGGCTGATGGCCAAGCTCAAAAGACTATAGGAAATTCCATGTATTTCCCAACTGTTAGT
GGCTCATTAGTATCCAGTGATGCTCAATTGTTTAACAGGCCCTTCTGGCTCCAAAGAGCC
CAAGGTCATAATAATGGCATCCTGTGGGCTAATCAAATGTTTATCACAGTGGTTGACAAC
ACAAGAAATACTAATTTCAGTATTTCTGTATATAATCAGGCTGGAGCACTAAAAGATGTT
GCAGACTATAATGCAGATCAATTTAGAGAATATCAAAGACATGTAGAAGAATATGAAATA
TCTTTAATTCTACAACTCTGTAAGGTTCCTTTAAAGGCACAGGTATTGGCACAGATCAAT
GCAATGAACTCTTCGTTATTGGAGGATTGGCAGTTAGGATTTGTTCCCACTCCTGATAAT
CCAATTCAGGACACCTACAGATATATTGACTCTTTGGCTACACGGTGTCCAGATAAGAAT
CCTCCGAAAGAAAAGGAAGACCCTTATAAGGGCTTACATTTTTGGGATGTAGATTTAACT
GAAAGATTGTCATTAGATTTAGATCAATATTCCTTAGGCAGAAAATTTTTATTCCAAGCT
GGGTTACAACAAACGACCGTTAACGGTACAAAAGCAGTGTCTTATAAAGGGTCTAATAGA
GGAACAAAACGCAAACGTAAAAATTGAGGTCTGACCGAAAGTGGTACATTTTTATAAACT
TTTACACAGTATTCAAGGAATGTTTGTTTACTCTGACTAAGTATAAGTCTTCCAAGGATA
CCGACCGCACCCGGTACACTCAGTCAAGTTGTTGCCAATATAGAATCAGATCAGTGCCAA
ACACACCGTCTTGGACTCAGAACAGACCGTGTTCGTTATAACATGCTCGGATTAGGGACC
TCCCCAAAGAAGATTTAATCTACAATCGCTTTTGGCAATCGCATTTGGCACTGCTAAAAG
ACCGTT
>gi|9626597|ref|NC_001457.1|_Human_papillomavirus_type_4,_complete_genome
GTCTGTAATGATAGTTGGCAACAATCATTACTTATAGCTATATATAACCGGAAGAGATAC
ATATAAAAAGGGACAGTGCATTTCTACTAAATCCTGTCCAGATGGCAGATGGCAGACCTG
CAACCTTGGACGACTTCTGCAGACGATTCGACATTTCCTTTTTTGATTTGCGCCTTACTT
GTATTTTTTGTTCTCATACTGTCGATCTTGCGGATCTTGCTTTATTCTATCTTAAGAAAC
TTAGTTTAGTATTTAGAGGAAATTGTTATTATGCATGTTGTTCTGAATGCTTAAGATTAA
GTGCACTGTTTGAACAAGAGAATTATTTTCAATGTTCTATTAAAGCTGTACATTTGGAGG
AAATTGCTCAGAAAAAGATTAAGGAAATTTGCATTAGATGCATTTGCTGCCTTAGATTAC
TTGATATTGTTGAGAAATTAGATTTATTATACTCTGACGAGACTTGCTATTTAATAAGGG
GTTTGTGGAGGGGCTATTGCAGAAATTGTATTAGGAAACAATGAGAGGAGCAGCGCCCAC
GGTTGCAGATCTTAATTTAGAACTAAATGACTTAGTGTTACCAGCAAACCTGCTGAGTGA
GGAGGTCTTGCAATCTTCAGATGATGAGTATGAGATTACAGAGGAGGAGTCGGTGGTTCC
ATTTAGAATAGACACCTGTTGCTATAGATGTGAAGTTGCTGTAAGAATTACATTGTATGC
TGCTGAGCTCGGACTACGGACCTTGGAACAACTTCTTGTAGAAGGAAAGCTGACGTTTTG
CTGCACCGCTTGTGCAAGAAGTCTTAACAGAAATGGCAGATAAAGGTACAGACAATTTTG
ACTTAGAAGGGAATAATTGGTATATTGTCCATGAAGCAGAATGCACTGACAGTATAGATA
CGTTGGATGATTTATGCGACGAAAGTAATGACGATTCAAACATTTCTAACTTAATTGATG
ACGATGTCGTTGATCAGGGGAATTCCCTTGCGCTGTACAATGCACAAATAAATGAGGATT
GTGACAATGCACTAGCACACCTAAAACGAAAGTATAACAAAAGTCCAGAGCAGGCAGTCG
CTGAATTGAGTCCGCAGTTGCAGGCTGTGAAAATAACTCCTGAAAGACACAGCAAAAGGA
GATTATTTCAGGACAGTGGGATTTTCGAAGATGAAGCTGAAAATTCTCTTACACAGGTAG
AATCCGAGAGCCAGGCTGGACCTTCTAGCCAAGATGGCGGCGGAGATATTAATTTGTTGT
TGTTACAAAGTAGTAACAGGAGGGCAACAATGCTAGCAAAGTTTAAAGAATGGTATGGGG

TCTCATACAATGAAATAACAAGAATTTATAAAAGTGATAAATCTTGTAGTGATAATTGGG
TAATAGTTATTTTTAGAGCTGCTGTTGAAGTATTAGAAAGTTCAAAGATTGTTTTAAAGC
AGCATTGTACATATATTCAAGTTAAGATCTTTGGATTTTCAGCTTTATATTTAGTACAGT
TTAAAAGTGCGAAAAGTAGAGAAACTGTACAAAAGTTGATGTGTTCTATATTAAATATCC
AAGAATATCAAATGTTATGTGATCCTCCAAAATTACGAAGTGTACCCACAGCATTATACT
TTTATAAGCATGCTATGTTAACAGAGAGTTCTGTTTTTGGACAAACACCGGATTGGATCG
CAAAACAAACTCTCGTAAGTCATCAAGCAGCAACTACTGCAGAGACTTTTGAGTTATCTA
GAATGGTTCAGTGGGCATACGATAATAATTATGTGGATGAATGTGACATTGCTTATCACT
ATGCAATGTACGCAGAGGAGGATGCAAATGCTGCTGCTTATTTAAAAAGTAATAATCAAG
TAAAGCATGTACGAGATTGTAGTACAATGGTCAGGATGTATAAAAGATATGAAATGAGAG
ATATGTCAATGTCAGAATGGATTTATAAATGTTGTGATGAATGTTCTGAAGAAGGAGATT
GGAAGCCAATCTCACAGTTTTTAAAATATCAAGGTGTTAATATATTATCCTTTCTTATAG
TGCTTAAATCATTTTTAAAAGGTATTCCAAAAAAAAACTGTATAGTTATTCATGGTCCAC
CAGATACAGGAAAATCATTATTTTGTTATTCTTTTATAAAATTTTTAAAAGGAAAAGTAG
TTTCATATGTAAATAGAAGTAGCCATTTTTGGTTGCAGCCTCTGATGGATTGCAAGGTAG
GATTTATGGATGATGCTACCTATGTGTGCTGGACATATATAGATCAAAATTTAAGGAATG
CATTAGATGGTAATCCAATGTGTATTGACGCTAAACACAGAGCACCACAACAATTAAAAT
TACCACCAATGCTAATAACGTCAAATATTGATATTAAACAGGAACAATCTTTAATGTATT
TACACAGTAGAATACAGTGTTTTAATTTTCCTAACAAAATGCCTATTTTAGATGATGGTA
GTCCTATGTATACATTTACTGACGGTACTTGGAAATCTTTTTTCCAAAAGCTTGGCAGAC
AATTAGAATTAACAGATCCTGAAGAGGAAAACAATGGAGTCCCTAGTCGCACGTTTCGAT
GCACTTCAAGAAGCAATTCTGACTCATATTGAGTCACAGGAGAGCACTTTGGAATCCCAA
ATCCAATATTGGGAAAAATATCAGAAAAGAAAATGCTATAATGCATTATGCTCGAAAACAA
GGCCTAACCAAATTAGGTCTACAACCACTTCCTACACTAGCAGTAACTGAATACAATGCA
AAGCAAGCTATTCAGATACATTTAACTTTGCAATCATTGTTAAAATCTCCCTTTGCATCT
GAACGGTGGACATTGACAGATGTTAGTGCAGAACTGATAAATACCTCTCCACAAAACTGT
TTAAAAAAGGGAGGTTATGATGTTGCTGTGTGGTTTGATAATGATAGACAGAATGCAATG
CTGTACACAAATTGGGACTTTTTATATTATCAAGATATGAATGAACAGTGGCACAAAGTT
AAAGGTGAAGTGGATTATGATGGCTTATACTTTACAGACCATACGGGAGAAAGAGCTTAT
TTTACATTATTTAGCTCTGATGCTCAAAGATTTAGCAGAACTGGACTGTGGACTGTGCAT
TTTAAAACCCAAGTTATTTCCTCCCCTATTGTTAGCTCTACATACTCCTCCTCCTTCGAC
ACTGAGGAACAACAGTTACCCGGGCCCTCCACCAGCTACTCCGAAGTTACCGAGCAGGCG
AGCCCTACTCGAAGGAGGAAACCGAGGAAATCCGACGCGACCTCCACCACGTCCCCTGAA
ACCGAGGGAGTACGACTACGACGAAGACGACGAGAAGGAAAATCAGGGCCCGGGTCAGGA
GAAACCCCCCGCAAAAGAAGAAGAGGAGGAGGAAGAGGAGGAGGAGAGACCGAATTGGGA
TCTGCACCATCTCCTGCAGAAGTGGGGAGCAGACATCGACAAGTTGAAAGACAAGGTCTG
TCGCGACTTGGACTCTTACAAGCAGAAGCTAGGGATCCGCCTATGATATTGTTAAAGGGC
ACAGCAAATTCTTTGAAATGTTGGAGATATAGAAAAGTTAACTCAAATTGCTGCAACTTC
TTATTCATGAGTACTGTTTGGAACTGGGTTGGAGATTGCTCACATAATCATAGTCGCATG
CTTATTGCATTTGATAGCACTGACCAAAGAGACGCTTTTGTAAAACACAACCTTTTTCCT
AAACTGTGTACATATACCTACGGCTCATTGAATAGTTTATAAAATGCAAAGCTTGAGTAG
AAGGAAAAGAGATTCAGTTCCAAATCTTTATGCAAAATGTCAACTGTCTGGCAATTGCCT
ACCTGATGTAAAAAATAAAGTAGAAGCTGATACTCTTGCTGATCGTTTGCTGAGATGGTT
GGGAAGTGTAATATACCTAGGAGGCTTGGGTATTGGTACTGGGAGAGGTAGTGGGGGGTC
AACTGGGTATAATCCAATTGGAGCTCCAAGTAGAGTCACACCTAGTGGTACTTTAGTAAG
GCCTACAGTGCCTGTGGAAAGTTTGGGACCCTCAGAAATAATCCCAATAGATGCAATAGA
CCCAACAACATCTTCTGTTGTGCCATTAGAGGATCTGACCATCCCAGATGTCACAGTAGA
TAGTGGAGATACAAGAGGAATAGGGGAGACTACTCTTCAGCCTGCACAAGTAGATATTTC
AACATCACATGACCCTATATCAGATGTCACTGGTGCTAGCAGCCACCCTACAATCATATC
TGGCGAGGATAACGCCATTGCAGTGTTAGATGTGTCCCCTATAGAACCTCCCACAAAACG
GATAGCATTGGCAACTAGGGGAGCCTCAGCAACTCCACATGTAAGTGTCATATCTGGCAC
AACCGAATTCGGTCAGTCATCTGATCTGAATGTATTTGTGAATGCCACATTTTCAGGCGA
TTCCATTGGTTATACAGAAGAAATTCCATTAGAACCGTTGAACCCCTTTCAAGAATTCGA
AATAGAAAGCCCTCCAAAAACTAGTACACCACGTGACGTTTTAAATCGTGCAATAGGAAG
AGCACGGGATTTATATAATAGAAGGGTTCAGCAAATACCTACTAGGAACCCAGCTTTACT

GACACAGCCTTCCCGCGCAATAGTATTTGGATTTGAAAATCCCGCCTTTGATGCTGACAT
CACTCAAACATTTGAGCGGGATTTAGAACAGGTTGCAGCAGCTCCAGATGCTGACTTTGC
AGACATAGTCACTATAGGGCGTCCAAGGTTTTCAGAGACAGATGCTGGTCAAATTAGAGT
TAGCAGGCTTGGACGCCGAGGCACAATAAAAACTAGAAGTGGTGTGCAAATTGGGCAGGC
GGTTCATTTTTATTACGACCTAAGTACAATAGATACTGCTGATGCTATTGAATTATCTAC
TTTAGGTCAACATTCAGGAGAACAAAGCATTGTTGATGCTATGATAGAAAGCAGCTTAAT
AGATCCTTTTGAAATGCCCGATCCTACTTTTACAGAAGAACAACAGCTTTTAGATCCACT
TACAGAAGATTTTAGTCAGTCACACTTGGTGCTTACTAGTAGCAGACGTGGGACATCATT
TACTATACCTACAATACCACCTGGATTAGGTCTTAGAATTTATGTAGATGATGTAGGTTC
TGATTTATTTGTTTCCTATCCAGAATCTAGAGTAATACCTGCTGGAGGTTTACCAACTGA
GCCATTTGTTCCTCTAGAACCAGCTTTGTTATCTGATATATTTAGTACGGATTTTGTATA
TCGTCCTAGTTTATATCGCAAGAAACGGAAACGATTAGAAATGTTTTAATTGTTTTGCAG
GAACATGTCGAGTTGGTTATCTACAACGGGTAAAGTCTACTTACCTCCAGCTCAACCTGT
GGCAAGAGTTTTGGAAACTGACGAATATATCACTGGAACATCTCTGTATTTCCACGCTGG
TACAGAAAGGCTTTTAACTGTAGGCCATCCTTATTTTCCAGTGAAAGATGTACAGGAACC
TCACAAAGTATTAGTTCCTAAGGTTTCAGGAAGTCAATTTAGAGTGTTTAGATTCAATTT
GCCAGACCCAAACAGATTTGCTTTAATTGATAATGGCTTTTATGATTCTGATCATGAACG
CCTAGTATGGAAACTGAGGGGAATAGAAATAGGAAGAGGAGGACCGCTTGGTATAGGTAC
TACAGGTCATCCTTTATATAATAAGTTTGGAGACACAGAAAATCCTAATGGCTACAAAAA
GCAATCAGATGATAATAGACAGGATGTCTCTTTAGACCCAAAACAAACACAGATGTTTAT
TATAGGTTGCACTCCTGCAATAGGTGAACATTGGGATAAAGCTGAACCTTGTCCCAGCCC
TGCTCCGCAACAGGGAGATTGCCCACCAATAGAGCTTGTAAATTCATACATTCAAGATGG
AGATATGTGTGACATTGGATTTGGGGCTTTCAATTTTAAAGCTTTGCAGGCTGATAAATC
TAGTGCTCCTTTGGATGTCATTGCCACAGTTTGTAAATGGCCAGATTTTTTAAAAATGGG
GAAAGATATCTATGGAGATAGCTTGTTTTTCTTTGGAAGAAGAGAACAACTATATGCCAG
ACATTTCTTTGTCAGAGCAGGCACCATGGGAGATGCTCTACCAGAACCTTTTGAAGCTAC
CTCAGATTATTTTATTGGTGCTCAAAACCAACAAGATCAGTACACTTTAGGACCTCATAT
TTATGTAGGGACCCCTAGTGGCTCTTTAGTATCCAGTGAATCCCAGTTGTTTAATCGACC
GTATTGGTTAAACAGAGCTCAGGGTACAAATAATGGAATTTGTTGGGATAATCAGTTGTT
TGTTACTCTTGTAGATAACACTCATAATACAAACTTTACAATTTCTGTGAAGTCAGATGG
TGCTAATGACAATTATCAGTATAAAGCTAGTGATTTTAAACAGTACCTCAGACATATAGA
GGAGTTTGAAATGGAATTTATATTTCAACTTTGTAAAGTTCCTCTAACTGCAGATGTTAT
GGCTCATTTAAATGTAATGAATCCTAATATTTTGGATAATTGGCAGTTAAATTTTGTTCC
ACCACCTCCCTCTGGAATTGAGGATCAATATAGATTTTTGCAATCTAGAGCTACAAGATG
CCCTACACAGACCCCTGCAACTGAAAAAGAAGATCCATATAAAGATTTGTCTTTTTGGGT
TGTTGATTTAAGTGAAAGATTTTCCAGTGAATTGAGCCAATTTTCCTTAGGCAGGCGGTT
TTTATATCAAAGTGGTTTAATTAATGGTTCTCTAAAACGTAAAAGAATAATAAGTTCTTC
TCATGCACAAACTAATACCAAACGTTCTGCCAAACGAAAACGGTCTCTGAAATAACAATG
TGAACTCTTCTGGAATGTTTTATTCTGCCAGGAAAACCTTCAACTGAGCCAAATTATTAT
ATAATCGTTCTTAATCTCAAAATTGAGCTAATTATATAAGATTTGCAAACGTGTATGTAT
CTGTTTTTGTGAACTATAGTGAAATAAACTGCCACATACTTGCCAGTGTCCAGTCTCTCT
GAGTCATTTGGTCAACATGCGTCCGCACCCCAATAATTATTTGCATACACAGATCAGTAG
GAGAGGCGCCAAGACGGACATATCCTCTTCAAATTTCCTTAAAATTATTGAATTTAACAA
CTGTAAGCTACAAAAGACCGTTATCGTTTCCTCTAACCTTGGGAAAAAGGTGAGTGAAAG
TTTTATTGCACCTTTTGTGAGTCAATTTGTCTGGCGGCGCTGAACGAATTTGGCTGTCAG
CCTTTGCACCGGGAGTGGTGGAAAATAGTTTCT
>gi|9626069|ref|NC_001357.1|_Human_papillomavirus_-_18,_complete_genome
ATTAATACTTTTAACAATTGTAGTATATAAAAAAGGGAGTAACCGAAAACGGTCGGGACC
GAAAACGGTGTATATAAAAGATGTGAGAAACACACCACAATACTATGGCGCGCTTTGAGG
ATCCAACACGGCGACCCTACAAGCTACCTGATCTGTGCACGGAACTGAACACTTCACTGC
AAGACATAGAAATAACCTGTGTATATTGCAAGACAGTATTGGAACTTACAGAGGTATTTG
AATTTGCATTTAAAGATTTATTTGTGGTGTATAGAGACAGTATACCCCATGCTGCATGCC
ATAAATGTATAGATTTTTATTCTAGAATTAGAGAATTAAGACATTATTCAGACTCTGTGT
ATGGAGACACATTGGAAAAACTAACTAACACTGGGTTATACAATTTATTAATAAGGTGCC
TGCGGTGCCAGAAACCGTTGAATCCAGCAGAAAAACTTAGACACCTTAATGAAAAACGAC

GATTTCACAACATAGCTGGGCACTATAGAGGCCAGTGCCATTCGTGCTGCAACCGAGCAC
GACAGGAACGACTCCAACGACGCAGAGAAACACAAGTATAATATTAAGTATGCATGGACC
TAAGGCAACATTGCAAGACATTGTATTGCATTTAGAGCCCCAAAATGAAATTCCGGTTGA
CCTTCTATGTCACGAGCAATTAAGCGACTCAGAGGAAGAAAACGATGAAATAGATGGAGT
TAATCATCAACATTTACCAGCCCGACGAGCCGAACCACAACGTCACACAATGTTGTGTAT
GTGTTGTAAGTGTGAAGCCAGAATTGAGCTAGTAGTAGAAAGCTCAGCAGACGACCTTCG
AGCATTCCAGCAGCTGTTTCTGAACACCCTGTCCTTTGTGTGTCCGTGGTGTGCATCCCA
GCAGTAAGCAACAATGGCTGATCCAGAAGGTACAGACGGGGAGGGCACGGGTTGTAACGG
CTGGTTTTATGTACAAGCTATTGTAGACAAAAAAACAGGAGATGTAATATCAGATGACGA
GGACGAAAATGCAACAGACACAGGGTCGGATATGGTAGATTTTATTGATACACAAGGAAC
ATTTTGTGAACAGGCAGAGCTAGAGACAGCACAGGCATTGTTCCATGCGCAGGAGGTCCA
CAATGATGCACAAGTGTTGCATGTTTTAAAACGAAAGTTTGCAGGAGGCAGCACAGAAAA
CAGTCCATTAGGGGAGCGGCTGGAGGTGGATACAGAGTTAAGTCCACGGTTACAAGAAAT
ATCTTTAAATAGTGGGCAGAAAAAGGCAAAAAGGCGGCTGTTTACAATATCAGATAGTGG
CTATGGCTGTTCTGAAGTGGAAGCAACACAGATTCAGGTAACTACAAATGGCGAACATGG
CGGCAATGTATGTAGTGGCGGCAGTACGGAGGCTATAGACAACGGGGGCACAGAGGGCAA
CAACAGCAGTGTAGACGGTACAAGTGACAATAGCAATATAGAAAATGTAAATCCACAATG
TACCATAGCACAATTAAAAGACTTGTTAAAAGTAAACAATAAACAAGGAGCTATGTTAGC
AGTATTTAAAGACACATATGGGCTATCATTTACAGATTTAGTTAGAAATTTTAAAAGTGA
TAAAACCACGTGTACAGATTGGGTTACAGCTATATTTGGAGTAAACCCAACAATAGCAGA
AGGATTTAAAACACTAATACAGCCATTTATATTATATGCCCATATTCAATGTCTAGACTG
TAAATGGGGAGTATTAATATTAGCCCTGTTGCGTTACAAATGTGGTAAGAGTAGACTAAC
AGTTGCTAAAGGTTTAAGTACGTTGTTACACGTACCTGAAACTTGTATGTTAATTCAACC
ACCAAAATTGCGAAGTAGTGTTGCAGCACTATATTGGTATAGAACAGGAATATCAAATAT
TAGTGAAGTAATGGGAGACACACCTGAGTGGATACAAAGACTTACTATTATACAACATGG
AATAGATGATAGCAATTTTGATTTGTCAGAAATGGTACAATGGGCATTTGATAATGAGCT
GACAGATGAAAGCGATATGGCATTTGAATATGCCTTATTAGCAGACAGCAACAGCAATGC
AGCTGCCTTTTTAAAAAGCAATTGCCAAGCTAAATATTTAAAAGATTGTGCCACAATGTG
CAAACATTATAGGCGAGCCCAAAAACGACAAATGAATATGTCACAGTGGATACGATTTAG
ATGTTCAAAAATAGATGAAGGGGGAGATTGGAGACCAATAGTGCAATTCCTGCGATACCA
ACAAATAGAGTTTATAACATTTTTAGGAGCCTTAAAATCATTTTTAAAAGGAACCCCCAA
AAAAAATTGTTTAGTATTTTGTGGACCAGCAAATACAGGAAAATCATATTTTGGAATGAG
TTTTATACACTTTATACAAGGAGCAGTAATATCATTTGTGAATTCCACTAGTCATTTTTG
GTTGGAACCGTTAACAGATACTAAGGTGGCCATGTTAGATGATGCAACGACCACGTGTTG
GACATACTTTGATACCTATATGAGAAATGCGTTAGATGGCAATCCAATAAGTATTGATAG
AAAGCACAAACCATTAATACAACTAAAATGTCCTCCAATACTACTAACCACAAATATACA
TCCAGCAAAGGATAATAGATGGCCATATTTAGAAAGTAGAATAACAGTATTTGAATTTCC
AAATGCATTTCCATTTGATAAAAATGGCAATCCAGTATATGAAATAAATGACAAAAATTG
GAAATGTTTTTTTGAAAGGACATGGTCCAGATTAGATTTGCACGAGGAAGAGGAAGATGC
AGACACCGAAGGAAACCCTTTCGGAACGTTTAAGTTGCGTGCAGGACAAAATCATAGACC
ACTATGAAAATGACAGTAAAGACATAGACAGCCAAATACAGTATTGGCAACTAATACGTT
GGGAAAATGCAATATTCTTTGCAGCAAGGGAACATGGCATACAGACATTAAACCACCAGG
TGGTGCCAGCCTATAACATTTCAAAAAGTAAAGCACATAAAGCTATTGAACTGCAAATGG
CCCTACAAGGCCTTGCACAAAGTCGATACAAAACCGAGGATTGGACACTGCAAGACACAT
GCGAGGAACTATGGAATACAGAACCTACTCACTGCTTTAAAAAAGGTGGCCAAACAGTAC
AAGTATATTTTGATGGCAACAAAGACAATTGTATGACCTATGTAGCATGGGACAGTGTGT
ATTATATGACTGATGCAGGAACATGGGACAAAACCGCTACCTGTGTAAGTCACAGGGGAT
TGTATTATGTAAAGGAAGGGTACAACACGTTTTATATAGAATTTAAAAGTGAATGTGAAA
AATATGGGAACACAGGTACGTGGGAAGTACATTTTGGGAATAATGTAATTGATTGTAATG
ACTCTATGTGCAGTACCAGTGACGACACGGTATCCGCTACTCAGCTTGTTAAACAGCTAC
AGCACACCCCCTCACCGTATTCCAGCACCGTGTCCGTGGGCACCGCAAAGACCTACGGCC
AGACGTCGGCTGCTACACGACCTGGACACTGTGGACTCGCGGAGAAGCAGCATTGTGGAC
CTGTCAACCCACTTCTCGGTGCAGCTACACCTACAGGCAACAACAAAAGACGGAAACTCT
GTAGTGGTAACACTACGCCTATAATACATTTAAAAGGTGACAGAAACAGTTTAAAATGTT
TACGGTACAGATTGCGAAAACATAGCGACCACTATAGAGATATATCATCCACCTGGCATT

GGACAGGTGCAGGCAATGAAAAAACAGGAATACTGACTGTAACATACCATAGTGAAACAC
AAAGAACAAAATTTTTAAATACTGTTGCAATTCCAGATAGTGTACAAATATTGGTGGGAT
ACATGACAATGTAATACATATGCTGTAGTACCAATATGTTATCACTTATTTTTTTATTTT
GCTTTTGTGTATGCATGTATGTGTGCTGCCATGTCCCGCTTTTGCCATCTGTCTGTATGT
GTGCGTATGCATGGGTATTGGTATTTGTGTATATTGTGGTAATAACGTCCCCTGCCACAG
CATTCACAGTATATGTATTTTGTTTTTTATTGCCCATGTTACTATTGCATATACATGCTA
TATTGTCTTTACAGTAATTGTATAGGTTGTTTTATACAGTGTATTGTACATTGTATATTT
TGTTTTATACCTTTTATGCTTTTTGTATTTTTGTAATAAAAGTATGGTATCCCACCGTGC
CGCACGACGCAAACGGGCTTCGGTAACTGACTTATATAAAACATGTAAACAATCTGGTAC
ATGTCCACCTGATGTTGTTCCTAAGGTGGAGGGCACCACGTTAGCAGATAAAATATTGCA
ATGGTCAAGCCTTGGTATATTTTTGGGTGGACTTGGCATAGGTACTGGCAGTGGTACAGG
GGGTCGTACAGGGTACATTCCATTGGGTGGGCGTTCCAATACAGTGGTGGATGTTGGTCC
TACACGTCCCCCAGTGGTTATTGAACCTGTGGGCCCCACAGACCCATCTATTGTTACATT
AATAGAGGACTCCAGTGTGGTTACATCAGGTGCACCTAGGCCTACGTTTACTGGCACGTC
TGGGTTTGATATAACATCTGCGGGTACAACTACACCTGCGGTTTTGGATATCACACCTTC
GTCTACCTCTGTGTCTATTTCCACAACCAATTTTACCAATCCTGCATTTTCTGATCCGTC
CATTATTGAAGTTCCACAAACTGGGGAGGTGGCAGGTAATGTATTTGTTGGTACCCCTAC
ATCTGGAACACATGGGTATGAGGAAATACCTTTACAAACATTTGCTTCTTCTGGTACGGG
GGAGGAACCCATTAGTAGTACCCCATTGCCTACTGTGCGGCGTGTAGCAGGTCCCCGCCT
TTACAGTAGGGCCTACCAACAAGTGTCAGTGGCTAACCCTGAGTTTCTTACACGTCCATC
CTCTTTAATTACATATGACAACCCGGCCTTTGAGCCTGTGGACACTACATTAACATTTGA
TCCTCGTAGTGATGTTCCTGATTCAGATTTTATGGATATTATCCGTCTACATAGGCCTGC
TTTAACATCCAGGCGTGGGACTGTTCGCTTTAGTAGATTAGGTCAACGGGCAACTATGTT
TACCCGCAGCGGTACACAAATAGGTGCTAGGGTTCACTTTTATCATGATATAAGTCCTAT
TGCACCTTCCCCAGAATATATTGAACTGCAGCCTTTAGTATCTGCCACGGAGGACAATGA
CTTGTTTGATATATATGCAGATGACATGGACCCTGCAGTGCCTGTACCATCGCGTTCTAC
TACCTCCTTTGCATTTTTTAAATATTCGCCCACTATATCTTCTGCCTCTTCCTATAGTAA
TGTAACGGTCCCTTTAACCTCCTCTTGGGATGTGCCTGTATACACGGGTCCTGATATTAC
ATTACCATCTACTACCTCTGTATGGCCCATTGTATCACCCACGGCCCCTGCCTCTACACA
GTATATTGGTATACATGGTACACATTATTATTTGTGGCCATTATATTATTTTATTCCTAA
GAAACGTAAACGTGTTCCCTATTTTTTTGCAGATGGCTTTGTGGCGGCCTAGTGACAATA
CCGTATATCTTCCACCTCCTTCTGTGGCAAGAGTTGTAAATACCGATGATTATGTGACTC
CCACAAGCATATTTTATCATGCTGGCAGCTCTAGATTATTAACTGTTGGTAATCCATATT
TTAGGGTTCCTGCAGGTGGTGGCAATAAGCAGGATATTCCTAAGGTTTCTGCATACCAAT
ATAGAGTATTTAGGGTGCAGTTACCTGACCCAAATAAATTTGGTTTACCTGATACTAGTA
TTTATAATCCTGAAACACAACGTTTAGTGTGGGCCTGTGCTGGAGTGGAAATTGGCCGTG
GTCAGCCTTTAGGTGTTGGCCTTAGTGGGCATCCATTTTATAATAAATTAGATGACACTG
AAAGTTCCCATGCCGCCACGTCTAATGTTTCTGAGGACGTTAGGGACAATGTGTCTGTAG
ATTATAAGCAGACACAGTTATGTATTTTGGGCTGTGCCCCTGCTATTGGGGAACACTGGG
CTAAAGGCACTGCTTGTAAATCGCGTCCTTTATCACAGGGCGATTGCCCCCCTTTAGAAC
TTAAAAACACAGTTTTGGAAGATGGTGATATGGTAGATACTGGATATGGTGCCATGGACT
TTAGTACATTGCAAGATACTAAATGTGAGGTACCATTGGATATTTGTCAGTCTATTTGTA
AATATCCTGATTATTTACAAATGTCTGCAGATCCTTATGGGGATTCCATGTTTTTTTGCT
TACGGCGTGAGCAGCTTTTTGCTAGGCATTTTTGGAATAGAGCAGGTACTATGGGTGACA
CTGTGCCTCAATCCTTATATATTAAAGGCACAGGTATGCCTGCTTCACCTGGCAGCTGTG
TGTATTCTCCCTCTCCAAGTGGCTCTATTGTTACCTCTGACTCCCAGTTGTTTAATAAAC
CATATTGGTTACATAAGGCACAGGGTCATAACAATGGTGTTTGCTGGCATAATCAATTAT
TTGTTACTGTGGTAGATACCACTCCCAGTACCAATTTAACAATATGTGCTTCTACACAGT
CTCCTGTACCTGGGCAATATGATGCTACCAAATTTAAGCAGTATAGCAGACATGTTGAGG
AATATGATTTGCAGTTTATTTTTCAGTTGTGTACTATTACTTTAACTGCAGATGTTATGT
CCTATATTCATAGTATGAATAGCAGTATTTTAGAGGATTGGAACTTTGGTGTTCCCCCCC
CCCCAACTACTAGTTTGGTGGATACATATCGTTTTGTACAATCTGTTGCTATTACCTGTC
AAAAGGATGCTGCACCGGCTGAAAATAAGGATCCCTATGATAAGTTAAAGTTTTGGAATG
TGGATTTAAAGGAAAAGTTTTCTTTAGACTTAGATCAATATCCCCTTGGACGTAAATTTT
TGGTTCAGGCTGGATTGCGTCGCAAGCCCACCATAGGCCCTCGCAAACGTTCTGCTCCAT

CTGCCACTACGTCTTCTAAACCTGCCAAGCGTGTGCGTGTACGTGCCAGGAAGTAATATG
TGTGTGTGTATATATATATACATCTATTGTTGTGTTTGTATGTCCTGTGTTTGTGTTTGT
TGTATGATTGCATTGTATGGTATGTATGGTTGTTGTTGTATGTTGTATGTTACTATATTT
GTTGGTATGTGGCATTAAATAAAATATGTTTTGTGGTTCTGTGTGTTATGTGGTTGCGCC
CTAGTGAGTAACAACTGTATTTGTGTTTGTGGTATGGGTGTTGCTTGTTGGGCTATATAT
TGTCCTGTATTTCAAGTTATAAAACTGCACACCTTACAGCATCCATTTTATCCTACAATC
CTCCATTTTGCTGTGCAACCGATTTCGGTTGCCTTTGGCTTATGTCTGTGGTTTTCTGCA
CAATACAGTACGCTGGCACTATTGCAAACTTTAATCTTTTGGGCACTGCTCCTACATATT
TTGAACAATTGGCGCGCCTCTTTGGCGCATATAAGGCGCACCTGGTATTAGTCATTTTCC
TGTCCAGGTGCGCTACAACAATTGCTTGCATAACTATATCCACTCCCTAAGTAATAAAAC
TGCTTTTAGGCACATATTTTAGTTTGTTTTTACTTAAGCTAATTGCATACTTGGCTTGTA
CAACTACTTTCATGTCCAACATTCTGTCTACCCTTAACATGAACTATAATATGACTAAGC
TGTGCATACATAGTTTATGCAACCGAAATAGGTTGGGCAGCACATACTATACTTTTC
>gi|206902429|ref|NC_011279.1|_Human_papillomavirus_FA75/KI88-
03,_complete_genome
AACGGTAAGTTTTTATTTGTTTGAACCGCTGCCGGTTGCTATATTACTCATTTATCTGAT
TGTGGTTTACAACAACCATTCCTTAATTAAATACATGTGACCGCTTGCGTTAGATGTAAA
CCTATAAAATTACTTGAAGAATATTAGAGACACATATACTGATGGAGAGGCCTAGAACAG
TACAAGTTCTGAGTCGGCATTTAGAAATACCAATAGAAGATTTATTGGTACCATGCAAGT
TTTGTGAGCGCTTTTTAACATATATAGAACTTTTGCAATTTGATTACAAACAATTGCAAC
TCATTTGGATTGAAGATTTAGTGTATGCTCTTTGCTCTAGTTGTGCATATGCATCTGCTG
CATTTGAATACAAAAATTATTTAGAAGGCACAGTCAAAGGCAGAGAAATAGAGCAGGTGA
CTCAACAGTCTGTAGGCTTTATTACTATCAGATGCATTTATTGTTTAAAGCATCTAGACC
TTTTAGAAAAGTTAGACAATTGTGCTAGGCATCAGCAGTTTTATAAGGTGAGAGGTAATT
GGAAGGGATTGTGCAGACATTGTTGGTCGATAGAATGATTGGGAAAGAAGTTACTATTCC
AGATATTGTTCTGGAACTGCAAGAGCTTGTCCAGCCCATTGACCTGCATTGCTACGAAGA
ATTACCAGAGCTTCCAGATGTTCCTAGTGAAGATTCAGAGGAGGAGCAAGTAGAGCGCAT
CCCATACAAGATAGTTGCTCCTTGTGGTGGTTGTGGCGCTCGCCTTCGACTATTTGTGAT
TGCTACCTTCTTTGGAATCCGCACACAGCAAGAACTTTTAATTCAAGAAGTGCAACTTGT
GTGTCCTCAGTGCCGAGAGGTCATTCGCAATGGCGGACAAGGACCATAAAGGTATTGATG
CTAAGGAAGGCTGTAGTGATTGGTTTTTAGTAGAAGCTGAATGTTCAGATACTAGTGATC
TAGAAAATGATTTAGAAAACTTATTTAATGAAGGGGCAGACTCAGATATTTCTAATTTAA
TTGATGATGGGGACATTGAACAGGGGAATTCCCGCGAATTGCTATGCCAGCAAGAAGCAG
AGGAAAGCGAGCAACAGACACAATTGCTAAAACGAAAGTACCTCAGTCCTAAAGAGAAAG
AGATCCTGCAGCTCAGTCCGCAGCTGGAGTCAATCTCCCTGTCGCCCCATCATAAATCTA
AAAAACGGCTGTTTGTGGAGCAAGATAGTGGACTAGAGCTTTCACTAAATGAAGTTGAAG
ATCTTTCTGAAGAGGTGGAGGTACCGGCGATTACTCCAGCGCCGGCAGCGCAGGGTGGTG
AGGGAGAGGGAAACTTGCATTATAAAGCGCTGTTAAAGTGCAGCAATGCAAAAGCAACAT
TGTTAAGTAAATTTAAGGATGCGTTTGGAGTGAGTTTTAATGAGCTCACAAGGCACTATC
AAAGTAATAAGACATGTTGTAGGGATCGGGTTGTTAGTATTTATGCAGTGAAAGATGATC
TGTTAGAAGGGTCTAAGCATTTATTACAAAAGCATTGTTCATATATTTGGATGCATGTTT
TACAACCAATGTCATTATTTTTACTGTGTTTTAATGCTGGGAAAAGTAGAGAAACTGTAT
TTAGATTACTATCTTCTATTTTGCAAGTAAATGAAATTCAAACATTGTCTGAACCACCTA
AGTGGCGTAGTGTAATAGCGGCATTGTATTGGTATAAAGGAAGTATGAATCCAAATATTT
ATACTTTTGGAACATATCCTGATTGGATAGTGACACAAACTATGATTAGCCATCAATCTT
TGGAGGCCACGCAATTTTCTTTATCTGACATGATACAGTGGGCATTTGATAATGATTATA
TAGACGATGCAGATATAGCTTACAACTATGCTAAACTGTCTGATACAAATAGTAATGCAA
AAGCATTTTTAGATAGTAATAGTCAAGCAAAGTATGTTAGAGAATGTGCTTTAATGGTTA
AACATTATAAACGTGGGCAAATGAGGGAAATGTCAATATCAAGTTGGATTCATACACGCC
TTTTATCAGTAGAAGGAGAAGGTCATTGGTCTCATATTGTAAAATTTATCAGATATCAAA
ACTTAAATTTTATTATGTTTTTAGATAAGTTTAGGACATTTTTGCAGAATACACCTAAAA
GGAATTGCATTTTATTTCATGGGCCTCCTGATTCAGGGAAATCTATGTTTACAATGTCTT
TAATTCGTGTGTTAAAAGGAAAAGTGTTATCATTTGCTAATTCTAGAAGTCATTTTTGGT
TGCAACCACTGTCAGATGCTAAAATAGCACTGCTAGACGATGCTACATATGAGTGTTGGA
ATTATTTTGATACATTCTTAAGAAATGGCATTGATGGAAACCCAGTCAGTCTAGATATAA

AACACAAAGCTCCTTTGCAAATACAATTTCCACCTTTAATGATAACATCTAATATAGATA
TTAATAAAGAAGATAGATATAGGTATTTGCAAAGTAGAATCATGTCATTTGAATTTCCTA
ATAAATTTCCTTTTGATAGTAATAATAAACCTCAATTTCTTTTAACTGACCAAAGCTGGA
AATCTTTTTTTGAAAGGCTTTGGACACAATTAGAGCTCAGTGATCAAGAAGACGAGGGAG
AGGATGGAAACTCTCAGCGCACGTTTCAATGCACTGCAAGAGAAGTTAATGGACATTTAT
GAATCAGGTGTAGAAACGTTGGAAGCACAAATTGAGCACTGGCAATTATTAAGACAAGAG
CAGGTGCTTTTAAATTATGCAAGGAGGAATGGTGTTATGCGCCTAGGATATCAACCTGTA
CCAAGTCTAGCAACGTCAGAGACTAAAGCCAAGGATGCAATTCGCATGGTTATACTTTTA
CAAAGTTTACAAACTTCTGAGTATGCTGATGAACCGTGGAACTTGGTGCAGACTAGCCTT
GAAACTGTTAGAAGTCCTCCTGAAAATTGTTTTAAAAAGGGACCAAAAAATATTGAGGTT
ATTTTTGATGGAAATCCTGAAAATCTTATGATTTATACTGTATGGACATGGATATATTAT
CAAAACTCAGATGATAAGTGGCAAAAGGTAGAAGGTGCTGTGGACTATGAGGGTGCTTAC
TATACAGAGGGAAAACATAAGCACTACTACCTTAGATTTGAAGCAGATGCTAACAGATTT
AGCACTACTGGACTGTGGGAAGTGCATGTTAACCAAGATACTGTGTTTACTCCTGTCACT
AGCTCTTCGCCGCCATCTGGAAGCAGGGTATCCCTTCTGCCCTCCGACACCACATCCCTC
CAGCCCTCTTCCAGCGTCTCCACCACACAAAAACGATACGCACGAACCGGATCTAGCCCT
ACAGCCACCGCCCGGCGGCAGAGGAAAAGAGAAAGACAAGAAGGAGACGGAGAAGGAACG
CAGGCGACATCGAGACAACGTGTCAGATCAAGATCAAGACGCTCCAGCAGAGGGAGGAGG
CAAGAAACCTCAGTCAAACGAGGGAAAGGGCGAGGAGGAGGGTCACGAGGGAGAAGAGAA
CCCACCCAACGAGACACCTCCTCCAGGGGAGGGAGAAGTAGAAGGCGGCCCCAAACACGG
TCCAGGTCCAGATCAAGAGGGTCTTCTAATGAGCGTGGCCTCTTGCCTTCAGAAGTGGGA
AGATCAGTACAATCAGTTGGTTCAAAACATCTTGGTAGACTTGAGCGATTATTGGAAGAG
GCTGCAGATCCCCCAGTAATTCTGTTTCGTGGGGAGGCTAATCAGCTTAAATGCTATCGC
TACAGGGTGCATAAAAGGCATAAGGGTGGCTACAAATATGTCAGTAGTACTTGGTCATGG
ATCACAGAACATAACAATGACAGAGTGGGACGCGCTCGGATGCTTGTTCTGTTTTCATCA
ACAGAACAAAGGGAAGCATTTCTAGACACTATGAAATTACCTCAGGGTGTTGATTGGTCC
TATGGGAATCTTGATAATTTTTAATTAACTAACCCTTTTTACTAATTGCATGCTTTTACA
CTAATATACTAATCTGCTAATCTGCTATGGCTCGTGCACGCAGAACAAAACGTGCTTCTG
TTACTGATATTTACAGGGGTTGCAAGGCTGCAGGTACTTGCCCTCCTGATGTTGTAAATA
AAGTTGAACAAAGCACAATAGCAGATAAAATTTTAAAATATGGAAGTGCTGCTGTGTTTT
TTGGTGGGTTGGGAATAAGTACAGGCCGCGGTACTGGTGGCACAACAGGATATGTACCTC
TTGGAGAAGGCCCTGGGGTGCGCGTTGGTGGTGCGCCCACAGTGGTACGCCCCGGGGTCA
TACCTGAAGTTATTGGTCCAACTGAGTTAATACCTATTGATACAGTCACTCCAATTGACC
CTACAGCACCCTCTGTAGTCACCTTAACTGACAGCTCTGCTGTTGACCTTTTACCTGGTG
AAATAGAGACAATTGCAGAAGTGCATCCAGTCCCAGTTCCAAGTGCACCAGAAATTGACA
CTCCTGTGGTCACTGGCGGGCGAAATTCATCTGCTGTGTTAGAGGTGGCTGATCCTAGTC
CTCCCACACGTACTAGAGTATCTAGAACACAATACCATAACCCATCTTTTCAAATAATAT
CTGAGTCTACACCAATAGCTGGGGAGTCTTCATTATCAGATCATATATTGGTGTTTGAAA
ACACAGGGGGTCAAGCTATAGGTGGTACAAGAGAAGAAATTGAACTACAATCATTACCTT
CCAGGTATAGCTTTGAAATAGAAGAGGCCACTCCTACTCGTCAAACCAGCACACCTATTC
AAAGAGGTAGGCAAGCTGTTTCTGCTTTACGTAGGGCCTTGTACAATCGAAGATTAACTC
AACAGATAGCTGTAGAAGACCCTTTATTTTTATCCAAACCATCTAAGCTAGTAAAATTTC
AGTTTGATAATCCTGTATTTGAAGAAGATATTACCCAGATATTTGAAAGGGATATAGAAC
AAGTTGAAGAACCTCCTGACAGACAGTTTCTGGATGTAGCAAAATTAGGTAGGCCTACAT
ATTCTGAAACTCCTCAAGGCTATGTTAGGGTAAGTAGGCTAGGGCGACGCGCTTCCCTAA
GGACTAGGAGTGGTACTCAGGTTGGTGCACAGGTTCATTTCTACAGGGACCTTAGTACAA
TTAACACTGAGGAGCCCATAGAATTACAACTTTTAGGAGAACATTCTGGGGATTCTACAA
TTGTACAAGGGCCTGTTGAAAGTACACTTATAGATGTTAATATTAATGAAACACCTAGCC
TTTTGGAAACTTCAGAATTTAATTCTGAAGATTTATTACTTGATGATGCTGTGCAGGACT
TTAGTGGGTCTCAATTAGTGTTTGGTACATCTAGGAGGTCAACTTCATCTGTTACTGTAC
CTAGATTCAGTTCTCCTAGGGAAACAAGTTTATATATTCAAGATATTGAAGGTTATAATG
TAGCTTATCCAGAGTCACGAGAAGTACCTGAAATAATTTATCCACAGCCTGATATTCCAG
CTGTTGTTATACATATAGCAGATGCTTCTGGGGATTTTTATTTACATCCCAGTTTACAGA
AACGAAAACGCAAGCGAGCTTATTTATAATTTTTTTCAGATGTCTCTCTGGCTTCCTGCA
AGTGGTAAGGTATATTTGCCACCATCAACTCCAGTAGCTAAGGTGTTAAGCACTGATGAA

TATGTAGAAAGAACTAACATATTTTATCACGCAACTAGTGATCGCCTTTTAACAGTAGGA
CATCCATATTTTGATGTTACAAATTCTGATGGCTCCAAAATAGAAGTTCCCAAAGTGTCA
GGAAATCAATTTAGGGCCTTTAGAATGTTATTTCCTGATCCAAATAGATTTGCATTAGCA
GATATGTCCATATATAACCCAGAAAAGGAAAGATTGGTTTGGGCTTGTAAAGGAATAGAA
ATTAGCAGAGGTCAGCCTTTAGGTGTAGGTACATGTGGTCATCCATTATTTAATAAAGTG
AAAGATACAGAAAATAGCAATAGCTATCAGACAGGGTCCACAGATGACAGACAAAATACA
TCCTTTGATCCTAAACAAGTTCAAATGTTTATTATTGGATGTACACCATGTTTAGGAGAA
TACTGGGATAAAGCTGAGCCATGTGAAAATGCAGGAGATCAACAGGGAAAATGTCCACCT
ATAGAACTGAAAAATTCTGTGATTCAAGATGGCGATATGATGGACATTGGATTTGGAAAT
ATAAATTTTAAAACTCTATCCCTGAATAGATCAGATGTTAGCTTAGATTTAGTAGACGAA
ATTGCTAAATATCCAGATTTTCTAACTATGGCAAATGATGTGTATGGAGACTCATGTTTC
TTTTTTGCAAGAAGGGAACAGTGCTATGCAAGACATTATTTTACCAGAGGAGGTAATGTT
GGAGACGCTACTCCAGATGGAGCAAATAATCAAGATCATAAATTTTATTTACCTCCTAAG
TCATCACAAAGTCAAACACTAGGAAATTCAATTTACTTTCCTACTGTTAGTGGTTCTCTA
GTCTCTTCAGATGCTCAGTTATTTAATAGGCCTTTTTGGTTACAAAAAGCTCAAGGCCAT
AACAATGGTATACTATGGGGAAATCAAATGTTTGTCACTGTTGCTGATAACACTAGAAAC
ACTAACTTTACCATTTGTGTACCTTCAGATAATGGTGCTATAACTGAGTATGATTCTAGC
AAATTTAGAGAATTTTTAAGGCACGTGGAAGAGTATCAAATATCTGTAATATTACAACTG
TGTAAAGTATCACTGCAACCTGATGTGCTAGCCCAGATCAATGCAATGAATTCAGGTATA
TTAGAAGATTGGCAGTTAGGATTTGTACCAACTCCTGACAATGCAGTACATGACACCTAT
AGATTTATAAATTCCTCAGCCACTAAATGTCCAGATAAGGTTCCTGCAAAAGATAGAGAG
GATCCATTTGCTCAATATTTCTTTTGGAGAGTAGATATGACTGAAAAATTATCATTAGAT
TTAGATCAATATCCTTTAGGAAGAAAGTTTTTGTTCCAAGCAGGATTACAGAACACCAGG
GTCAAATCATTAAAGCGTAGCAGATCTGTTTCTCGGGGTACTGCAAAACGACGACGGACA
TAACGACCGAAATCGGTTTGTATTTAATAAATAAATATTCTAACCAAAATGGTATGTGAA
GCATTTTTTAAACCGTCTTCGTGACTAAACCGAACAAGTCAACACCAGCAACCGCGCCCG
GTATAATCTGCTTATAAATCGCAGCCGGTACTTCACCTTACAAAGACTTGGCAGTCTGTC
TAGGAACACCGCAGGCGCCCGCCAAGATTCTATTGAAGATATCTTGGCAGAACATCAATT
TGGCCTCACTTCTACACCGAT
>gi|9627396|ref|NC_001596.1|_Human_papillomavirus_type_9,_complete_genome
CCGCAGGCAACCGCCAATTTCACTGCCAAGGTTCGTTGGCAGACCGTCCTGGCTTCAAAA
CGACCGATAACGGTAAGTCTTGGCACGTAGGTGGTTATTTGATCGTTGGGATGATTGTGG
TTAACAACAATCTACATACACATTTTCATATGACCGCCTTCGTTAATAAGCTTATATAGA
CATAAATATATAAGGTGCCATGTATTTAACAGAGCAGATTATGGACAGGCCAAAACCTAG
AACAGTAAAGGAACTAGCAGACACTCTTGTGATTCCTTTAATAGATTTGTTGATACCTTG
TAAATTTTGCAATAGATTTTTATCTTATTTTGAGCTACTTAATTTTGATCACAAGTGTTT
ACAGCTTATTTGGACAGAGGAGGATTTGGTGTATGGACTCTGTAGTAGCTGTGCTTATGC
GTCTGCACAGTTAGAATTTACACATTTTTTTCAATTTGCTGTAGTTGGAAAAGATATAGA
AACTGTAGAAGGAACAGCTATTGGAAATATTTGTATTAGGTGTCGCTACTGTTTTAAGTT
ATTAGACTTAGTGGAGAAGTTAGCTACATGCTATAAGTTTGAGCAGTTTTATAAGGTCAG
AAACAGCTGGAAAGGATTGTGCAGACACTGTGGGTCGGTAGAATGATTGGGAAAGAAGCT
ACTATACCAGAGGTGGTTCTAGAACTGCAAGAGCTTGTCCAACCCACTGCTGACCTGCAT
TGTTACGAAGAATTGACAGAAGAACCTGCAGAGGAGGAGCAGTGTCTCACTCCCTACAAG
ATCGTAGCTGGCTGTGGTTGCGGTGCAAGACTTCGTTTATACGTGCTTGCTACAAATTTA
GGAATTCGAGCGCAACAGGAACTTTTGCTGGGTGATATACAACTGGTGTGTCCGGAGTGC
CGAGGCAGACTTCGCCATGAGTGACAATAAAGGTACTAAATTAGATCCTAAAGAATGCTG
TAGTGCTTGGTTATCGTTAGAAGCAGAATGCTCTGATTCTAGTTTAGATGGTGATTTGGA
AAAATTATTTGACGAAGGGACAGACTCTGATATTTCAGACCTAATAGATGATGGAGATGC
TGTACAGGGAAACTCCCGCGAACTGTTTTGCCAGCAAGAGAGTGAGGAAAGCGAGCAACA
AACACAATTGCTAAAACGAAAGTATATCAGTCCCCAAGCTGTTTTGCAGCTTAGCCCTCA
ACTGGAGTCTATCTCTTTGTCGCCTCAGCATAAACCTAAAAGGAGATTATTTGAACAAGA
CAGCGGACTAGAATGTTCTGTAAATGAAGCTGAAGATCTTTCTGAAACACAGGTGGAAGA
GGTACCGGCCAATCCACCAACAACAGCTCAGGGAACTAAGGGCTTGGGAATTGTTAAAGA
TTTACTTAAACATAGCAATGTGAAAGCTGTATTAATGGCTAAGTTTAAAGAGGCGTTTGG
TGTGGGGTTTGCTGAGCTAACAAGACAATATAAAAGTAACAAAACATGCTGTAGAGATTG

GGTAATTGCTGTGTATGCTGTGAATGATGACTTAATTGAAAGCTCTAAACAATTGTTATT
GCAGCATTGTGCTTATATTTGGCTACATTATATGCCACCAATGTGTTTATATTTATTATG
TTTTAACGTAGGCAAAAGTAGAGAAACTGTATGTAGACTATTAAGCACTTTGCTGCAAGT
ATCTGAAGTGCAATTATTAAGTGAGCCTCCAAAGTTGCGAAGTGTGTGTGCTGCATTATT
TTGGTATAAAGGAAGTATGAACCCTAATGTATACGCACATGGTGCGTATCCTGAATGGAT
ACTTACACAAACACTAATTAATCACCAATCTGCAAATGCTACACAATTTGACTTATCGAC
AATGATACAATTTGCCTATGATCATGAATATTTTGATGAAGCTACCATTGCATATCAATA
TGCAAAGCTGGCTGAAACAGATGCTAATGCCAGGGCTTTTTTACAAAGTAACAGTCAAGC
CAGACTAGTAAAAGAATGTGCAACCATGGTGAGACATTACATGAGGGGAGAGATGAAAGA
AATGAGTATGTCCACATGGATACATAGAAAACTGCTTACAGTGGAAAGCAATGGGCAATG
GTCAGATATAGTACGGTTTATTAGATACCAGGATATTAATTTTATTGAATTTCTAACAGT
ATTTAAAGCATTTCTGCAAAACAAACCAAAGCAAAACTGTTTATTATTTCATGGACCACC
TGACACGGGAAAATCAATGTTTACAATGTCACTAATATCTGTGTTAAAAGGAAAGGTACT
GTCATTTGCCAATTGCAAAAGTACTTTTTGGCTACAACCTATAGCTGATACTAAACTTGC
TTTAATTGATGATGTAACACATGTGTGTTGGGAATATATAGATCAGTACTTAAGGAATGG
ATTGGATGGCAATTATGTATGTTTAGATATGAAACATAGAGCACCTTGTCAAATGAAATT
TCCACCCCTTATGTTAACGTCTAACATAGATATTACTAAAGACCAAAAGTACAAATATTT
GCACAGCAGAGTTAAATCCTTTGCTTTCAATAACAAATTTCCACTTGATGCTAATCACAA
ACCACAATTTGAACTTACTGACCAAAGCTGGAAATCTTTTTTTAAAAGGCTTTGGACACA
GTTAGATCTGAGTGATCAAGAAGACGAGGGAGAGGATGGAAACTCTCAGCGCACGTTTCA
ATGCACTGCAAGAGACTTTAATGGACCTGTATGAATCAGGTCGAGAGGATCTACAAAGTC
AGATTGACCACTGGCAGACTTTAAGACAAGAGCAAATACTTTTGCATTATGCCAGGAAAA
ATGGAGTTATGCGATTGGGGTACCAACCTGTACCTCCGTTGGCTACCAGTGAACAGAAAG
CTAAAGATGCTATTGGCATGGTTTTACTATTGCAAAGCCTTCAAAGATCAGCTTATGGAC
AGGAACCTTGGACACTGGCACAAACTAGTCTTGAGGCGGTACGCAGTCCACCTGCATATG
CCTTTAAAAAGGGTCCACAAAATATTGAAGTAGTTTATGATGGAGATCCTGATAATGTTA
TGAGCTATACTATATGGAACTTTATATATTATCAGACTGTTAATGATACTTGGGAAAAAG
TTCAAGGTCACGTGGATTATTTTGGAGCCTATTACTTTGAAGGGACTGTAAAAACATATT
ATATTAACTTTGACAAAGATGCAGCCAGGTATGGCAGAACTGGCGTTTGGGAAGTGCATG
TTAACAAGGACATTGTGTTTGCCCCTGTTACTAGCTCTTCGCCACCAACTGGAGACGGGG
GAGAGACCTCCAAGCACACCCTTTCCAGGTCGGGGTCGCCAACAACATCGCGACTCCCTG
CCACCACCGTGCCCACCGGAGGATCCAGGACATCATCCCGACGATACCAACGAAAAGCCT
CTAGCCCCACCACCAGGAAGAAAAGACAGAGACAAGGAGAAGGAGAAGGAGAAGGAGAAG
GAGAAGAAACCAACTACAGGAGACAAAGGTCCAGATCCAAGGGTCGAACAGAAACCGAAA
GGGGAGGGGAGCGACGGAGACGAGGAAGGTCCTCCTCCGCAGACTCCACTACCCCCACCG
ACAGGCGAAGGGGAAGGGGAGGTGGAAGGGGGCCCACGACCAGGTCCCAGTCCCGTTCCC
GTTCCCGCTCCCACTCCCGGTCGCGGTCCCGAGGAGGGACTGCTTCCAGGGTTGGCGTCT
CGCCTGATGAAGTGGGAACACGAGTTCGATCAGTTGGTGCAGGACATCACGGGAGACTTG
CACGATTACTGGCTGAGGCTAAAGACCCCCCATTAATGCTGTTGCGTGGCGACGCCAATG
TGCTTAAGTGCTATCGCTTTCGGGAACGCAAAAAAAAAAGAGGCTTAGTAAAATATTATA
GTACTACGTGGTCATGGGTAGGGGAAGACAGTTGTGATAGAGTTGGAAGAGCGCGAATGA
TTTTAGCCTTTGACACATATGAGCACAGACAACAATTCATTAGGACTATGAAATTACCAC
CTACAGTAGATTGGTCTTTAGGAAATGTTGATGATCTGTAAGCTTTACTAACGCTAACGC
TGGCATTGCTACTAACCCATACTAACTAACAAACCCATACTAACTAACATGGTTCGTGCA
AAACGTACTAAACGTGCCTCTGTTACAGATATATACAGAGGCTGCAAAGCTGCTGGTACA
TGTCCACCAGATGTAATTAATAAAGTGGAGCACACAACTATTGCTGATAAAATTTTGCAA
TATGGAAGTGCTGGTGTGTTTTTCGGGGGCTTGGGAATAAGTACAGGCCGTGGCACTGGT
GGTGCCACTGGCTATGTTCCATTAGGGGAAGGGCCAGGAGTCCGTGTAGGTGGCACCCCC
ACTATAGTTCGCCCTGGGGTGATACCTGAAATAATTGGCCCAACTGATCTAATTCCTTTA
GACACAGTCAGACCAATTGACCCCACAGCACCCAGTATTGTCACAGGCACTGACAGCACT
GTTGACCTTTTACCTGGTGAAATAGAATCAATTGCTGAGATACACCCAGTACCAGTGGAC
AATGCTGTAGTAGATACTCCAGTTGTAACAGAAGGTAGAAGAGGCTCGTCTGCCATTTTA
GAGGTGGCTGACCCAAGCCCTCCTATGCGAACCCGTGTTGCACGAACTCAATACCATAAT
CCAGCTTTTCAAATTATTTCTGAGTCTACACCTATGTCAGGTGAATCTTCCTTAGCAGAT
CATATTATAGTTTTTGAAGGATCTGGGGGCCAGCTAGTAGGTGGTCCTAGGGAATCATAC

ACAGCATCTTCTGAAAACATAGAATTACAAGAATTTCCTAGTAGATATAGTTTTGAAATA
GATGAAGGAACACCTCCTCGGACTAGTACACCTGTCCAAAGAGCAGTACAATCATTATCT
AGTCTGCGTAGAGCTCTATATAACAGACGTCTTACAGAACAAGTGGCTGTGACAGATCCA
TTATTTTTAAGTAGGCCTTCTCGTTTAGTTCAATTTCAGTTTGATAATCCAGCATTTGAA
GATGAGGTCACACAAATATTTGAAAGAGATCTAAGTACTGTTGAGGAGCCTCCAGATAGG
CAATTTTTAGATGTACAACGCCTTAGTAGGCCTTTATATACAGAAACACCTCAGGGATAT
GTTCGGGTTAGTAGACTAGGCCGAAGAGCAACAATCCGCACACGTAGTGGTGCACAGGTG
GGCGCACAGGTTCATTTCTACAGGGACTTAAGCACCATTAACACAGAAGAACCTATAGAA
ATGCAATTATTGGGGGAACACTCAGGTGACAGTACCATAGTACAAGGCCCAGTTGAAAGT
TCAATTGTTGATGTTAATATTGATGAACCTGATGGTTTGGAGGTGGGAAGACAGGAAACC
CCTTCTGTTGAAGATGTGGATTTTAATTCTGAAGACTTACTGTTAGATGAGGGTGTAGAA
GATTTTAGTGGGTCTCAGCTAGTCGTTGGCACACGCCGCAGTACAAATACATTAACAGTG
CCACGCTTTGAAACTCCAAGGGACACTAGTTTTTATATTCAGGATATACAAGGCTACACA
GTGTCCTATCCCGAGTCTAGACAAACCACAGATATAATTTTTCCACATCCTGACACCCCC
ACAGTAGTAATCCACATCAATGATACATCAGGAGATTATTATTTACACCCAAGTCTCCAA
AGGAAAAAACGCAAACGCAAATATTTATAATTTTGTTTTTGCAGATGTCATTGTGGCTTC
CAGCAAGTGGTAAGGTATATTTGCCACCAGCAACACCAGTGGCGAGAGTTCAAAGCACCG
ATGAATATGTGGAAAGAACAAATATTTTTTATCATGCAATTAGTGACCGTTTGCTAACAG
TGGGTCATCCATATTATGATGTCCGCTCAGGCGACGGACAAAGGATTGAAGTCCCTAAAG
TGTCTGGTAATCAGTATCGGGCCTTTAGAATTAGCTTACCTGATCCAAATAGGTTTGCTT
TAGCAGATATGTCAGTTTATAATCCTGATAAGGAACGTCTAGTTTGGGCCTGTAGAGGTA
TTGAAATAGGCAGAGGACAACCTTTAGGGGTTGGAACATCAGGTCACCCATTATTTAATA
AGGTTAGAGACACAGAAAACTCTAGCAATTATCAAGGCACAACAATGGATGACAGGCAAA
ACACATCTTTTGACCCCAAACAGGTACAAATGTTCATTATAGGATGTATTCCATGCTTAG
GAGAACACTGGGATAAAGCCAAAGTGTGTGAAAAGGATGCTAATAATCAACTAGGCTTAT
GTCCTCCTATAGAATTAAGAAACACAGTAATTGAGGATGGGGACATGTTTGATATTGGAT
TTGGAAATATCAACAATAAGGAACTGTCCTTTAATAAGTCTGATGTAAGCTTAGATATTG
TTGATGAAACCTGCAAATATCCAGACTTTCTAACAATGGCAAATGATGTTTATGGAGATG
CATGTTTCTTTTTTGCAAGAAGAGAACAATGTTATGCCAGGCATTATTATGTTAGAGGAG
GTTCAGTTGGTGACGCTGTTCCTGATGGTGCAGTAAACCAGGATCATAATTTCTTTTTGC
CAGCAAAAAGTGATCAACAACAACGAACAATAGCTAATTCCACCTACTATCCTACAGTAA
GTGGGTCATTAGTAACTTCAGATGCTCAATTGTTTAATAGGCCATTTTGGCTCCAAAGAG
CACAAGGTCACAACAATGGCATTTTATGGGGTAATCAGATATTTGTTACAGTGGCAGACA
ATACACGTAACACCAATTTTACCATTAGTGTGTCTACAGAGGCAGCTCAAACAGAAGAAT
ATAATGCCAATAATATTAGAGAATATTTAAGACATGTTGAAGAATATCAGATTTCATTAA
TCTTACAGTTGTGTAAAGTGCCTTTAGTAGCTGAAGTATTATCCCAGATAAATGCAATGA
ACTCAGGTATTTTAGAGGATTGGCAATTAGGGTTTGTTCCAACTCCTGAAAATGCTGTTC
ATGATATCTACAGATATATTGATTCAAAAGCCACAAAATGCCCAGATGCTGTTGAGCCTA
CAGAAAAAGAAGATCCCTTTGCCAAATACTCATTTTGGAAAGTGGATCTAACTGAAAGAT
TATCGTTGGATCTTGATCAATATCCTTTAGGTAGAAAATTTCTTTTTCAAGCTGGTTTGC
AAACACGAAAACGTCCTATTAAAACATCTGTTAAAACATCTAAAAATGCTAAGAGAAGGC
GAACCTAACCGATATCGGTTTCCAATAAAATTTAAGTTATCCAATTTGGTATGTGAAGCA
TTTTTTAACCATCTTCGTGACTAAACCGTACAAGTCAACACAGAGCGACCGCACCCGGTT
TATCTGATTATAAAGTGCACCTGGTGCAATTTGAACAATACTATCGTGGAATCA
>gi|186461202|gb|EU410349.1|_Human_papillomavirus_type_111,_complete_genome
AACGGTAAGCTTTGGCACGTAGGTGGTTGGTTGATCGTAAGAGTGATTGTGGTTAACAAC
AATCAGAGTTTACACTTACATGTGACCGCTTGCGTTTTCTTTTACATATATACATAAATA
TACACCCTATGGTTGTATTTAATAGGGAGAGCTCAATGGATAGGCCAAAGCCTAGGACAG
TAAAGGAACTAGCAGACACACTAGCAATTCCTGTAATAGATTTATTATTACCATGCAACT
TTTGCAATAGATTCTTATCTTACTTTGAGTTGCTGAGTTTTGATTATAAGTATTTGCAAC
TGATATGGACTGTTGAAGACTTAGTATATGCTATATGTAGTAGTTGTGCTTTTGCTTCAG
CACAGTTTGAATTTATGAAATATTTTGAATACTCTGTTGTAGGAAAACAGATAGAAACTG
TAGCAAACCAGCCTATTGGAAATATTACTGTTAGGTGTCAATATTGTTTGAAAAAATTGG
ATCTGGTAGAGAAGCTAGGAGTGTGCTACAAACAACAATATTTTCATAGAGTTAGGGATA
ATTGGAAAGGATTGTGCAGACATTGTGGGTCGATAGAATGATTGGGAAACAACCCACTAT

ACCAGACGTAGTTCTTGAGCTGCAAGAGCTTGTCCAACCCACTGACCTGCATTGCTACGA
AGAATTGACAGAAGAGCCTGCAGAGGAGGAGCAGTGCCTCACTCCTTACAAAATTGTAGT
TGCTTGCAATTGTGGTACAAGACTTCGCTTATACGTCTTTGCTACGGAACTTGGAATAAG
AGCCCAACAAGAACTATTACTGGGTGAAGTACAGCTTGTGTGTCCAGAGTGCCGCGAGAG
TCTTCGCCATGAGTGATGATAAAGGTACTAAATATGATGCTAAAGAAGGATGTAGTGAAT
GGTTGTCTTTAGAAGCAGAATGCTCTGATTCTAGTTTAGATGGTGATTTGGAAAAACTGT
TTGACGAAGGGACAGACTCTGATAATTCAGACTTTATAGATGATGGAGATGCTATTCAGG
GAAACTCCCGCGAACTGTTTTGCCAGCAAGAGTGTGAGGAAAGCGAACAGCAAACACAAT
TGCTAAAACGAAAGTATATCAGTCCTACAGCTGTTTTGCAGCTTAGTCCTCAATTGGAGT
CTATCTCTTTGTCACCACAGCATAAAACTAAGAGGAGATTATTTGAACAAGACAGCGGAC
TAGAGAGTTCAATAAATGAAGCTGAAGATCATTCTCAAACACAGGTGGAAGAGGTACCAG
CCATTGTTCCACCAACAACAGCTCAGGGAACTAAGGGTTTGGAGATTGTAAAAGAATTAC
TTAAATCTAATAATGTGAAAGCTGTGTTACTGGCTAAATTTAAAGAGGCATTTGGAGTTG
GGTTTATGGAACTTACAAGACAATATAAAAGTAATAAAACATGTTGTAGAGATTGGGTAA
TTAGTATTTATGCAGCTAATGGCGATCTTGTAGAAGGTTCTAAGCAATTGTTGATACAGC
ACTGTGGTTATATTTGGTTACAACAAATGCCTCCTATGTGTTTATATTTACTGTGTTTTA
ATGTTGGTAAAAGTAGAGAAACTGTACTACGATTATTAAGCACAATATTACAAGTATCAG
AAGTGCAGGTATTGGCAGATCCTCCTAAATTGCGCAGCGTGTTGGCTGCACTGTTTTGGT
ACAAAGGAAGTATGAATCCTAATGTGTATGCTCACGGTACATACCCAGATTGGATATTAG
CACAAACAATGATCAATCATCAAAATGCTGATGCTACTCAATTTGATTTATCCACCATGA
TACAATTTGCTTATGATAATCAATATGTAGAAGAAGCAATAATTGCCTATCAGTATGCCA
AACTAGCAGATACAGATGCAAATGCTAGAGCATTTCTACAAAGTAATAGCCAAGCTAGAA
TTGTCAGGGAATGTGCTACTATGGTAAGACATTATATTCGAGGGGAGATGCGAGAGATGA
GTATGTCAACTTGGATTCACAAACAATTATTGTCTGTGGAAGGGGAAGGGCATTGGTCAA
ATATTGTGAAATTCGTTAGATATCAAGGAATAAATTTTATAGATTTTCTTTCTGTATTTA
CTAAGTTTTTACATAATAGGCCTAAGCAAAACTGCATATTGCTTCATGGACCACCAGATA
CTGGTAAATCTATGTTTACCATGTCTCTCATTAATGTTCTTAAAGGTAAAGTGCTCTCTT
TTGCAAATTGTAGAAGTACTTTCTGGCTGCAACCACTTATTGAAGCTAAAATTGCATTAA
TTGATGATGTGACAGATGTATGTTGGGATTATATTGATAATTATTTGCGTAATGGATTAG
ATGGCAATGTTGTCTGTTTAGACATAAAACATAGAGCACCTTGTCAAGTAAAATTTCCCC
CACTATTATTAACATCAAACATAGATATATCCAAGGAGCAGAAGCATAAATATTTACATA
GCAGAATTAAATGCTTTGCTTTTACTAACAAGTTCCCCTTTGATGCTGACCATAAACCAC
AGTTTGAACTTACTGACCAAAGCTGGAAATCTTTTTTTGAAAGGCTTTGGACACAGTTAG
AGCTCTGATCAAGAAGACGAGGGAGAGGATGGAAACACTCAGCGAACGTTTCAATGCACT
GCAAGAGACTTTAATGGGCCTGTATGAATCAGGTCGTGAGGATCTACAAAGTCAGATTGA
ACATTGGCAAGCATTGAGACAAGAGCAAGTTTTATTGTACTTTGCTCGAAAAAATGGAGT
CATGCGCATAGGGTATCGACCCGTGCCTCCTTTAGCCACCAGTGAAAGTAAAGCTAAAGA
CGCAATTGGCATGGTTATTTTGTTGCAAAGCCTCCAAAAATCTAAATATGGCCAGGAGCC
TTGGACACTTGTACAAACTAGTCTTGAGACTGTCCGCAGCCCCCCTGCAAATTGTTTTAA
AAAAGGGCCTTACAATATTGAAGTAATCTTTGATGGCGATCCAGAGAATCTAATGAGCTA
TACAGTATGGAAAGATATATATTATCAAACTGTGACTGACTCTTGGGAAAAAGTAGAAGG
CCAAGTGGATTATTTCGGAGCCTTTTATTTTGAAGGGGCTTTGAAAACTTATTATATTGA
CTTTGAAAGTGATGCAGCCAGATATAGCACAACTGGCAAATGGGAAGTACATGTTAACAA
GAATGTTATGTTTGCCCCTGTCACTAGTTTTTCGCCACCTCCTGGAGACGGGGCCAGTGG
AGAGGCCTCCGGCAACACCTCATCCAGGCCGCAATCACCAGCGCGGGTTCCCTCCACCTT
GTCCTCCCAACGGCCACTCACCAGAACATCCCGACGATACAGAAGAAAAGCCTCTAGCCC
CACCACCACGCGGCAAGAAAGACAAAGAAAAAGACAAAAAGCCATTACAAGGAAATCAAG
GTCCAGATCAAGGGGTCGAACAGAAACCCACCGGGGAGGGCGAGGTGGAAGGGCATCCTC
CGCAGACTCCGACAGCTCCACCAACGGGCAGCGGGGAAGGGGAGGAGGAAAGGGGCCCAC
CACAAGATCCCAATCCCGTTCCCGATCCCGGTCCCGGTCGCAGTCCCACACCCGAAGGGC
TACTTCCAGCACTGGCGTCTCGCCTGCTGATGTGGGATCAGGAGTTCGATCGGTTGGTCC
AGAACATCACGGACGACTTGCACGGTTATTGGCAGAGGCTAAAGACCCTCCAGTAGTAAT
ATTGCGTGGAGATGCTAATGTGCTAAAATGCTATCGCTTCAGAGCTCGCAAAAAACATCA
AGGTTTAGTTAAATATTATAGTACAACATGGTCATGGGTAGGTGGGGACTGCTGTGATAG
AGTAGGCAGATCTAGAATGCTTTTAGCTTTTGATACATACAAACATCGAGAACAGTTTAT

CACTACTATGAAATTACCACCTAAGGTAGACTGGTCATTTGGACATTTAGATGATTTATA
ATTTACTTACTAACTTATGCATTGCTACTAACACACTAACAAATAATGGCTAGGGCAAAA
CGTGTAAAACGTGCCTCTGTCACTGATATATACAGAGGTTGCAAAGCAGCTGGTACCTGT
CCTCCAGATGTACTTAATAAAGTGGAACAAACTACTATAGCAGATCAAATTTTAAAATAT
GGAAGTGCTGGTGTTTTTTTTGGTGGGTTGGGAATAAGCACAGGGCGTGGTACCGGTGGT
ACCACAGGATATGTCCCTTTGGGTGAAGGCCCAGGAGTACGCATAGGTGGAACTCCCACT
GTGGTTCGCCCTGGGGTCATTCCAGAGGTAATAGGCCCTGCAGAGTTAATACCTATAGAC
ACAGTCAGACCCATTGACCCTACAGCTCCAAGTATTGTCACTGGTGTCGACACCACAGTT
GACCTTTTACCTGGTGAAATAGAATCTATTGCTGAGATACACCCAGTACCTATAGATAAT
GTAGAAATAGATACACCAGTTGTTACAGGAGGACGGGGCTCTTCAGCCATTTTAGAAGTG
GCTGATCCCAGTCCTCCTGTGCGAACCCGTGTTACACGAACACAGTATCATAACCCTTCC
TTTCAAATTATCTCTGAATCAACACCAACAAGTGGAGAATCGTCTTTAGCAGACCATGTT
ATTGTGTTTGAAGGCTCTGGAGGACAGTTGGTTGGAGGTGCTCGAGAACCCTCCATAGTT
ACTGAAACTATAGAACTGCAAGAATTACCTTCTAGATACAGTTTTGAAATCGATGAAGGT
ACCCCACCTCGTACCAGTACTCCTGTGCAACGAGCAGTGCAATCACTTAGTACTTTAAGA
AGAGCACTGTACAATAGACGTTTGACAGAGCAGGTAGCTGTAACTGACCCTTTATTCTTA
ACAAAACCTTCACGTCTTGTACACTTTCAGTTTGATAATCCTGCATTTGAAGAAGAAGTT
ACCCAAATATTTGAAAGGGATGTGGATCAAATTGAAGAACCACCTGATAGACAGTTTTTG
GATGTAGCAAAATTAAGTAGGCCTTTATATACTGAAACACCTCAAGGATATGTTAGAGTT
AGTAGACTGGGTCGCAGAGCCTCCATACGCACGCGTAGTGGAGCACAGGTGGGAGCTCAA
GTACATTTTTACAGGGATTTAAGTAGCATTAACACTGAAGCACCAATAGAAATGGAATTA
TTAGGTGAACATTCTGGAGATAGCACTATTTTTCAAGGACCAGTAGAGAGTTCTTTAGTA
GATGTTAATATTGATGAACCTGACGCCTTGGTTGTAGGCAGACCGGAAACCCCTCCATTA
GAAGATGAAATAGATTTTAATTCAGAAGATTTGTTATTGGAGGAAGGGGTAGAAGATTTT
AGTGGCTCACAACTTGTAGTAGGTACTAGGCGAAGCACAAATACATTAACAGTTCCCCGC
TTTGAAACACCTCGTGACACTAGTTTTTATATACAGGACTTTCAGGGCTATACAGTCTCA
TACCCAGAATCTAGAAACACTGAAAATATTATTTTTCCTCATCCAGAAGCTCCCACAGTT
GTGATTCACATATCAGATACTTCTGGTGATTATTATTTACATCCTAGTTTACAAAAACGA
AAACGTAAGCGCAAACGAAAATATCTATAATTTCTTTGCAGATGTCATTGTGGCTGCCTG
CAAGCGGTAAGGTATATTTGCCACCATCTACACCAGTGGCTCGCGTACAAAGCACCGATG
AATATGTGGAAAGAACCAATATTTTTTACCATGCAGTTAGTGATCGTCTGCTGACAGTGG
GGCATCCATATTTTGATGTTCGCTCCACTGATGGGCAACGCATTGAGGTTCCTAAAGTAT
CAGGCAATCAATATAGGGCCTTTAGAATAACACTACCAGATCCCAATCGTTTTGCCTTAG
CTGATATGTCTGTATATAACCCTGAAAAAGAAAGGTTAGTATGGGCCTGCAGAGGTCTTG
AAATAGGACGTGGTCAACCACTAGGTGTAGGTACATCAGGACATCCATTATTTAATAAGG
TTCGTGACACTGAAAATTCCAGTAATTATCAAGCTGTGACACAAGATGATAGACAAAATA
CCTCATTTGATCCTAAGCAAGTACAAATGTTTATAATAGGATGTGTTCCCTGTATGGGAG
AATACTGGGACAAAGCAAAACCCTGTGATGATGCAGGAAACCAGCAAGGCTTATGCCCGC
CAATTGAATTAAAGAATTCTGTAATAGAGGATGGAGACATGTTTGATATAGGATTTGGTA
ATATCAATAATAAGGAATTATCTTTTAATAAGTCAGATGTTAGTTTAGATATTGTCAATG
AAGTATGTAAATACCCTGACTTCCTAACTATGGCTAATGACGTCTATGGGGACGCTTGTT
TCTTTTTTGCCAGAAGGGAGCAATGTTATGCCAGACATTACTTTGTTAGAGGAGGCAATG
TAGGAGATGCAATTCCAGATGCAGCAGTGCAACAAAATCACAATTATTATCTGCCAGCAC
AGGGTAATCAACAACAAAACATTTTGGGTAACTCCATTTATTATCCTACTGTTAGTGGTT
CTCTTGTTACTTCAGATGCGCAGCTATTTAATAGACCATTTTGGTTGCAACGTGCACAAG
GCCACAACAATGGTATTTTATGGGGAAATCAAATGTTTATAACAGTAGCAGATAATACCA
GAAACACTAATTTTACTATAAGCGTATCCACAGAAGCTAATGCACAGCAATATAATGCTA
GTAATATTAGAGAATATTTAAGACATGTAGAAGAATATCAATTGTCTTTGATTCTTCAAC
TATGTAAAGTTTCTTTAGTTCCAGAAGTTTTATCTCAAATCAATGCAATGAATTCGAATA
TTTTGGAAGATTGGCAATTGGGATTTGTACCAACACCAGATAATTCTGTGCATGACACAT
ATAGATATATTAATTCAAAGGCTACTAAATGTCCAGATGCTGTAGAACCTAAAGAGAGAG
AAGATCCATTTGCTAAATATACATTTTGGAATGTAGATTTATCAGACAAACTGTCCTTAG
ATTTAGATCAATACCCTCTAGGGCGCAAATTTTTATTCCAGGCTGGTTTACAAACACGAA
AACGGACTTTAAAACCTTCGTCTGTGAAATCTACTAAATCTGCAAAACGCAGACGCACCT
AACCGATTTCGGTTTTTCAATAAAATGTAAGTAATCCATATTGGTATGTGAAGCATTTTT

TAACCATCTTCGTGACTAAACCGTACAAGTCAACACAGGGCAACCGCGCCCGGTTTTATC
TGATTATAAACCGCACCAGGTGCAGTTTTAAACAATGCTATCGTGGAATCACCGGAGGCG
CCCGCCACTTTGACTGCCAAGTTTTACTTGGCTGTACACCGATTTGGCTTCAACTGCGAC
CGAT
>gi|186461194|gb|EU410348.1|_Human_papillomavirus_type_110,_complete_genome
AACGGTAAGATTTTTATCTATTAACCGTAGGCGTTACTTTCTGATTGGTAAGACTGATTG
TAGTTAACAACAATCTTGATTTATAGCTACATGTAACCGCATGCGTTACAATTTAGGTAC
ATCTACATAAATATATTTCTCACACCTATCACATACTATTTAAGAGGTTAGATGGCCAGG
CCAAAGCCTTTGACAGTACAACAGCTTGCAGATACATTATGCATTCCTGTTTTAGACATA
TTAATACCGTGTAGATTTTGTACTAGGTTTTTATCATATATAGAATTATTAGGTTTTGAT
TATAAAGGGTTACAACTTATTTGGACTGAGGAAGATTTTGTTTTTGCATGCTGTAGTAGT
TGTGCTCATGCTACAGCGCAATATGAATTTACAAGGTTTTATGAACATTCTGTAAATGGA
CGTGAAATAGAAATAGTAGAGCAAAAAGGAATTGGGGAAGTGGTTGTGCGTTGCCAATAT
TGTTTAAAACGTCTAGATCTAATAGAGAAGCTAGAAGTTTGTTGTAAGCATCAGCAATTT
CATAGGGTAAGAGGCAATTGGAAAGGAGTGTGCAGACATTGTGGGTCGATAGGATGATTG
GGAAACAAGCTACTATACCAGACATTGTTCTGGAATTGCAAGAGCTTGTTCAGCCCACTG
CTGACCTGCATTGCTACGAGGAGTTAACTGAAGAAGAAACAGAGGAGGAGTCACGTCATA
CACCTTACAAGATTGTTGCTGCTTGTGGCCTTTGTGGTGCGCGACTTCGGCTTTTTGTGC
TTGCAACAGTTTTTGGAATTCGTACTCAGCAAGAAATTTTGCTTGGTGAAGTACAGCTGC
TGTGTCCAGAGTGTCGGGAGAAGCTTCGCCATGAGTAACGATAAAGGTACAGAAAATGAT
GCCAAACAAGGATGTAGTGATTGGTTTTTAGAGGAAGCAGAGTGCTCTGATGCTAGTTTA
GATGGTGATTTGGAAAAGTTATTTGAAGAAGGTGCAGATACAGACATTTCTGATTTTATA
GATGATGAAAACACTATTCAGGGAAACTCCCGCGAATTGTTATGCCTGCAGGAAAGCGAG
GAGAGCGAGCAACAGATACAATTGCTAAAACGAAAGTATTTCAGTTCACAAGAGGTTTTG
CAGCTTAGCCCACGACTGCAGTCTATCAGTATATCGCCACAGCATAAATCTAAAAGGAGA
TTATTTGAACAAGACAGCGGACTAGATCTTTCTCTAAATGAAGCTGAAGATAATTCTCAG
ACATTGGAGGTGGCGGAGGTACCGGCGTCCATAGTTGCGCCGGCAGCTCAAAGTGCTGAG
GGTTTGGGTATTGTTAAAGACTTACTTAAATGCAGCAATGTAAAGGCTGTATTATTGGCC
AAATTTAAGGAATCATTTGGTGTAGGATTTATGGAATTGACAAGACAATATAAAAGTAAT
AAGACTTGTTGTAGAGACTGGGTAGTTACAGCTTATGCTGTGAAGGATGAACTAATAGAA
AGCTCTAAACAATTGCTTCTGCAGCATTGTGCTTATATATGGCTACAAAATATGCCACCT
ATGACTTTATATTTATTGTGTTTTAATGTAGGTAAAAGCAGAGACACTGTTTGTAGATTG
CTAATGAGTCTCTTGCAAATTGGAGAGATTCAATTAATAGCTGAGCCACCAAAGTTAAGA
AGTGTATTGGCAGCACTATTTTGGTATAAAGGAAGCATGAATCCTAATGTATATGCTCAT
GGTGAATATCCAGAGTGGATAATGGCACAAACTATGATAAATCATCAAACTGCAGACGCT
ACTCAATTTGATTTGTCTAGTATGATACAATTTGCTTATGATAATGAATATTTAAATGAA
GATGAAATAGCATACCATTATGCTAAATTGGCTGATACAGACGCAAATGCTAGAGCTTTT
CTGCAACATAATAGCCAAGCTCGTTTTGTTAGAGAATGTGCTATTATGGTGAGACATTAT
AAACGTGGTGAAATGAAAGAAATGTCAATGTCTACCTGGATACATAGAAAATTACTAACA
GTAGAAGGCGAAGGTCATTGGTCGCATATAGTTAAATTTTTACGGTTTCAGGAAATTAAT
TTTATTTACTTTCTTGATGTATTTAAAGCTTTTTTGCATAATACTCCTAAACAAAATTGT
ATACTAATACATGGACCTCCAGACACAGGAAAGTCTATGTTTACTATGTCTTTATTAAGA
GTACTGAAAGGTAAAGTGTTATCATTTGTAAATTGTAAAAGTACATTTTGGTTGCAACCT
GTAGCAGACACAAAACTTGCATTGATAGATGATGTTACAGATGTATGTTGGGACTATATA
GATAAATATCTTAGAAATGTATTAGATGGGAATGTTGTTTGTCTGGATATGAAACATAAA
GCTCCATGCCAATTAAAATTTCCTCCTTTATTATTAACATCCAATATGGATATAATGAAA
GAAGAAAAATATAGATATTTGCATAGTAGACTTAGGAGCTTTGCATTTCCAAATAAGTTT
CCTTTTGATAATGACCATAAACCACAATTTCAACTTACTGACCAAAGCTGGAAATCTTTT
TTTGAAAGGCTTTGGAAACAGTTAGATCTCAGTGATCAAGAAGAAGAGGGAGACGATGGA
AACTCTCAGCGATCGTTTCAATGCACTGCAAGACAAATTAATGGACATTTATGAGTCTGG
TCAAGATGACCTAGAAACTCAAATATTGCATTGGAAATATTTAAGAGAGGAGCAAGTGCT
TTTGTATTATGCCAGGAGAAATGGCATAATGAGATTGGGGTATCAGTTGGTACCACCATT
AGCTACCAGTGAAGCTAAAGCAAAAGATGCAATTGGCATGATGCTCCTTTTGCAAAGTCT
GCAAAGGTCTCCTTATGCTTCTGAGTCATGGACATTAATGCAGACTAGTTTGGAAGCAGT
GAGAAGTCCACCTGCGAACTGCTTTAAGAAAGGTCCATACAATATTGAAGTAATGTATGA

CAGTGATCCAGAGAATCTAATGTCATACACATTATGGTCATTCATTTATTATCAAAGAGT
AGATGATACTTGGAACAAAGTGGAAGGCCATGTGGACTATAATGGTGCTTATTATTATGA
AGGCAATTTAAAAACCTATTATATTGAGTTTGAAAGTGATGCTGCCAGATATGGCAAAAC
TGGACAATGGGAAGTGCATGTTAACAAGGACACTATCTTTGCTCCTGTCACTAGCACTTC
GCCGGGAACTGGAGGAGGGGACTCCGACCAAACCTCCGTCCACTCCGTATCCCGGGCGTC
ATCAACCGTCGCGCGACGGTCTGCCTCCCCCCTGCCCACCCGAACCACCACACAACGGTC
ACGAACCGAAAGCAGACGGTACCAACGAAAAGAATCTAGCCCTACATCCACCGCCACCCG
GCAACAAAAAAGACAGCGAGAAGAAGAAACCAACAGGCGATCAAGGTCCAGCTCCCGGGG
GCAAGAAGCAGACTGGGGAGGGGATCGACGCAGAAGGAGGGGAGGAGACTCCTCCACCAG
GAGCCACTCCTCCAACAGGGCAAGGGGAGGGAGAAGTGGAGGGAGGCCCAGAACAAGGTC
CCAATCTCGCTCCAAATCAAGGTCGAGATCCAGAGGCCCTTCTACCAGGCGTGGCGTCTC
GCCTGCTGAGGTGGGAGAGTCACTTCGATCAGTTGGTCGACGACATCATGACCGACTTGA
AAGGCTACTGGACGAGGCTAAAGACCCCCCAGTAGTTTTGTTGCGCGGCGCTGCTAACAC
CTTAAAGTGCTATCGCTACAGAGCTAAAAAGAAGTATAGTGCTTTAGTGAAATACTTTAG
CACTACATGGTCTTGGGTTGGGGGCGACAATAATGATAGAATAGGGCGTGCTAGAATGCT
ATTAGCTTTTAGCTCAAATACACAAAGAGAATTGTTTTTAAAAGTAATGAAATTGCCACC
TAATGTAGATTGGTCTTTAGGATCTTTAGATGATTTATAAACTACTAATAACCTACTAAC
TTGCTTTGCTACTAACACAACTAATGTTCCATGGCTCGTGCACGTAGAACAAAGCGTGCA
TCTGTAACAGACATCTACAGGGGCTGCAAGCAAGCTGGTACTTGCCCTCCTGATGTTATT
AATAAAGTGGAACAAACTACTATAGCAGATAAAATTTTAAAATATGGCAGTGCTGGTGTC
TTTTTTGGCGGGTTGGGAATTGGCACAGGCCGTGGAACAGGCGGTGCCACAGGATATGTC
CCTTTGGGGGAAGGACCTGGTGTACGTGTGGGCGGCACTCCCACCATAGTCCGCCCTGGG
GTCATTCCTGAATTGATTGGACCAACAGATGTAATACCAATTGACACAGTCACACCAATT
GACCCTACAGCACCCAGTATTGTCACAATAACTGACACTACAGCTGTTGACCTTTTACCC
AGTGAAATAGAAACTATTGCTGAGGTGCATCCTGTCCCTACAGATAATGTGCCAGTAGAC
ACACCAGTTGTTTCAGGTGGCCGCGGTTCTGCAGCTGTACTAGAAGTTGCAGATCCTAGC
CCTCCTGTGCGTTCAAGAATTTCTCGGACACAATACCAGAATCCATCCTTTCAAATTATT
ACAGAATCTACACCATTGCTAGGCGAATCTTCTATGGCAGATCATGTTTTAGTATTTGAA
GGTGCAGGTGGACAAAGTATTGGTGGCACTAGACAGGCTGCCATTGATACAGCACAGGAA
AGTATTGAGCTACAATCATGGCCTAGCAGATATAGCTTTGAAATTGAGGAAGGTACTCCA
CCACGAACAAGTACACCAGTACAACGTGCAGTAGAATCTCTGTCTAACCTTAGAAGGGCT
TTATACAACAGAAGATTAACTGAACAGGTAGCAGTAACAGATCCTTTATTTTTAAGCAGG
CCTTCTCGTTTGGTGCAATTTCAATTTGATAATCCTGCTTTTGATGAAGAAGTGACACAA
TTGTTTGAAAGAGATGTAGAAAATTTTGAGGAGCCACCTGATAGACAATTTTTAGATGTG
GTAAGATTAGGCCGTCCCACAGTCGCTGAAACACCTCAAGGTTATTTAAGAGTCAGTCGG
CTTGGACGGCGTGCAACCATTAGAACTCGCAGTGGTGCTCAAGTAGGAACACAAGTACAC
TTTTACAGAGATTTAAGCACAATTGATTCTGAAGCTCCTATTGAAATGCAAATACTTGGT
GAACATTCAGGAGACAGCACAATTATTCAGGGTCCCGTGGAAAGTTCCTTTGTAGATGTT
AATATAGACGAGCCAGATCCTTTAAATGTTGGGCAGTTTGAATCAATGGAAATAGAAGAA
CCAGATTATAATTCTGCTGATTTTTTACTAGATGACCATGTAGATGATTTTAGTGGTTCA
CAATTGGTATTTGGAAATCCTAGACGCAGCACAACTTCAGTGACTGTACCGCGATTTGAA
ACTCCAAGAGATACAGGTTTTTATATACAAGATATTCAAGGCCATACTGTTGCTTACCCA
GAATCTAGAGACAACACTGAAATTATACTCCCACTACCTGATACTCCAACTGTGGTCATA
CACATCAGTGACACTAGTGGTGATTATTATTTACATCCCAGTTTACAAACACGAAAACGA
AGACGCAAACGCAAATATTTGTAATTGTTTTACAGATGACCTTGTGGCTTCCTGCTTCGG
GTAAAGTATACTTGCCACCATCGCCACCGGTAGCACGTGTCCAAAGCACCGATGAATATG
TGGAGCGAACAAATGTGTTTTATCATGCAAGCAGTGATCGCTTGTTAATAGTGGGACACC
CTTATTATGATGTTAGATCAGGAGATGGCGCAAAAATAGAAGTCCCTAAGGTATCAGGAA
ATCAATACAGAGCATTCCGGGTGCAATTGCCTGATCCTAATAGGTTTGCATTAGCAGACA
TGTCTGTATATAATCCAGATAAAGAACGTTTAGTGTGGGCCTGTAGGGGTGTTGAAATAG
GCCGTGGGCAACCTTTAGGGGTAGGTACCAGTGGTCATCCCTTATTTAATAAAGTTAGAG
ATACTGAAAATTCTAGCAATTATCAAGCAGGCTCTACTGATGACAGGCAGAATACTTCAT
TTGATCCTAAACAGGTTCAAATGTTTGTTATTGGTTGTGTGCCCTGTATGGGAGAGCATT
GGGACAAAGCTCCTGTTTGTGAATCAGAAGCAGGTAATCAGTTAGGTTTATGTCCTCCAT
TAGAATTAAAAAACTCAGTAATAGAAGATGGAGATATGTTTGACATAGGCTTTGGAAACA

TCAATAATAAAGTGCTATCATTTAATAGGTCAGATGTTAGTTTAGATATAGTTAATGAAA
TTTGCAAATATCCTGATTTTTTAACTATGGCAAATGATGTTTATGGAGATGCCTGCTTTT
TCTTTGCTAGACGAGAACAGTGTTATGCTAGGCATTACTTTGTTAGAGGGGGTGCTGTAG
GGGATGCAATACCTGATGGTGCAGTGAATCAAAATCATAACTATTTCTTGCCAGCAAAGC
AAGATCAACAACAACGCACACTAGCAAACTCTACATATTTTCCAACAGTAAGTGGTTCTC
TAGTTACTTCAGATGCTCAGTTATTTAATAGACCATTTTGGTTACAAAGAGCACAAGGCC
ATAATAATGGCATATTGTGGGGAAATCAAATGTTTATAACTGTTGCTGATAATACTCGTA
ATACTAATTTTTCAATTAGTGTTACTACAGAAGCTGGAGCAACTGAATACAATTCACAAA
ATATAAGAGAATTTCTTAGACATGTAGAGGAATATCAACTTTCTTTAATACTGCAATTAT
GTAAGGTACCCTTAAAAGCAGAGGTTTTAACACAAATTAATGCAATGAACTCAGGTATCT
TAGAAGAGTGGCAGTTAGGATTTGTTCCTACACCAGATAATTCAGTACATGATACTTATA
GGTATATAAACTCAAAAGCTACAAAATGTCCAGATGCTGTAGTTCCTAAGGAAAAAGAAG
ATCCATTTGCTAAATATTCTTTTTGGCACGTAGATTTAACTGAAAAACTATCATTAGATT
TAGATCAGTATCCTCTTGGTAGAAAATTTTTATTTCAGGCTGGTTTACAAAGTCGACCTC
GCTCTGTAAGGACATCTGTTAAGGTCTCTAAGGGTACCAAACGCAAACGGTCGTGACCGA
TATCGGTGTCAATAAATTTTATAACCAATCTGGTATGTGAAGTATGTTTTTTACCGTGTT
CGTGACTAAACCGTGATTGTTACTGCCAAGACCGCGCCCGGTTAATCAGATTATAAAGTG
CACCTGGTGCGATTGGATCTAGCTTTTGTGGAAGCACCGCAGGCAACCGCCAAAACCAGT
GCCAAGTCTCTTGGCAGAACATTTTCTTGGAAGACGCACCGCT
>gi|126131393|gb|EF422221.1|_Human_papillomavirus_type_107,_complete_genome
GTTAATCAGATTATATATCGCTGTCGGTGCCAAACAAGACAAAGACTTGGCTGGATCTCG
AGGAAACACCGCAGGCGCCCGCCAAAAGTCTGCCAAGTTTTCTTGGCAGAACATCCATTT
GGCTGCAACTGTCACCGATAACGGTAAGTATTTGTTTTGAGCACCGGTGCCGGTTGCTAA
ATTACTCATATGGTGATTGTGGTTTACAACCATCATCTTGACATATTACATGTAACCGCA
TGTGTAGTATCTAATATAAAAATATTTTGTGCTTCTACAACTGTTCAGATGCTCATGGAC
AGGCCAAGGACAATCGTACAGCTTACACAGCATCTTAATATACCAGTGGAAGACTTACTG
GTACCTTGCAAATTTTGCAAACGATTCTTAACTTTTATAGAACTTTTACAATTTGATTAT
AAAAATTTACAATTAATTTGGCATGAAAATTTAGTATATGGTTGTTGTTGCAGCTGTGCT
TATGCATCAGCAGCGTTTGAATTTAAAAATCATTTTGAATTTCAGGTTGTAGGTAAGCAG
ATTGAGGAGATCACTCAGCAAAGTATTGGATTTATTAATATTAGATGTGTGTTTTGTTTA
AAGAATTTGGATTTGTTGGAGAAGTTAGACAATTGTGCTAGGCATCAGCAGTTTTTTAAG
GTGAGAGGTAATTGGAAGGGATTGTGTAGACATTGTGAGGCGATAGAATGATTGGGAAAG
AAGTAACTATTCCAGATATAGTTCTGGAAGAGCTGCAAGAGCTTGCTCAGCCCATTGACC
TGCATTGTTACGAAGAGTTACCAGAGCTTCCAGATGCTACTAGTGAAGAATCAGAGGAGG
AGCCTGAACGCATCCCTTACAAAATTGTAGTACCTTGTTCTGGGTGTCCAGTGAAACTTC
GTCTCTACGTTTTTGCAACCCATTTTGGCATTCGGGCATTGCAGGATCTTTTGCTTCAAG
AAGTCCAACTGATCTGTCCACAGTGTCGAGAGGCTATTCGCCATGGAGGACAATAAAGGT
ACTGATGCAGGTGCAGGATGTAGTGATTGGTTTTTGGTAGAAGCTGACTGTTCCGATACT
AGTGATTTAGAAAACGATTTAGAAAAACTGTTTAATGAAGAGACAGATTCAGATATTTCT
GATTTAATTGATGATGGGGATATTGAGCAGGGAAACTCCCGCGAATTGCTATGCCAGCAA
GAAACAGAGGAGAGCGAGCAACAAGTGCAATTGCTAAAACGAAAGTATTTCAGTCCTAAA
GCTATTTCACAGCTTAGTCCGCGGCTGCAGTCCATCTCCCTGTCGCCCCAACATAAATCT
AAAAAACGTCTGTTTGTGGAGCAGGACAGCGGCTTAGAGCTCTCACTAAATGAAGTTGAA
GATATTACTGAAGAGGTGGAGGTACCGGCGAGTGCTTCAGTGCCGGCAGCACAGGGTGGA
AAGGGAGTGGGAGAGATGCATTTTAAACAGCTGTTAAAATCTAGTAATGTTAAAGCTACT
TTAATGGGAAAATTTAAGGACGCGTTTGGGGTAGGATTCAATGAACTGACTAGACAATAT
AAGAGCAATAAGACGTGTTGCAAAGATTGGGTTGTAACCATATACTGTGTTCAAGATGAT
TTATTAGAAGCTTCTAAGATATTGTTACAAAAACATTGTAATTATATATGGATGCATGTA
CTACAGCCTATGACATTATATTTATTATGTTTTAATGCTGGTAAAAGCAGAGAAACTGTA
TGTAGATTATTAAGTTCCATTCTGCAAATTGATGATATGCAGGCATTATTAGAGCCTCCC
AGATTGCGAAGTGTGTTGTCTGCATTATTTTGGTACAAAGGCAGTATGAATCCCAATGTA
TATTCATTTGGTACGTATCCAGACTGGATAGTAGCTCAAACTATGATAAGTCATCAAGCA
GCAGAGGCTACACAGTTTTCATTATCAAGAATGGTACAGTGGGCTTTTGACAATGAGCAT
TTAGAAGAGGCTGATATAGCATACAATTATGCTAAATTAGCTGAAACTGATAGTAATGCA
AAAGCCTTTTTGGATAGTAATAGTCAAGCCAACTTTGTAAGACAGTGTGCTTTGATGGTG

AGGCATTATAAACGAGGGCAAATGAGAGATATGTCAATGTCATGTTGGATTCATACCCGT
CTTATGTCTGTAGAAGGAGAAGGACATTGGTCAGAAATAGTAAAATTTATAAGATATCAA
AACTTAAATTTTATAATGTTTTTGGATAAATTTCGTACGTTTTTAAAGAATACTCCAAAA
AGAAATTGTATGTGCTTTTATGGTCCACCAGATTCTGGTAAATCTATGTTTACTATGTCC
TTAATAAATGTATTGAAAGGAAGAGTACTTTCCTTTGCAAACTCCAGAAGCCAGTTTTGG
TTGCAGCCTTTGTCAGAAACTAAGCTTGCTTTGCTAGATGATGCTACACAAGAATGCTGG
AATTATATTGATACATTTTTAAGAAATGGTGTAGATGGTAATTATGTATCTTTAGACATT
AAACACAGGGCTCCGTTACAGATCAAATTTCCTCCATTAATGATTACCACAAATATGAAT
ATACTAAAGGAGGAAAAGTACAGGTATCTACATACTAGAATAGAATTTTTTGAATTCCCA
AACAAATTTCCATTTGATAATAATAACAAGCCACAATTTCACCTAACTGACCAAAGCTGG
AAATCTTTTTTTGAAAGGCTTTGGACACAATTAGAGCTGAGTGACCAAGAAGACGAGGGA
GAGGAGGATGGAGACTCTGAGCGAACGTTTCACTGCACTGCAAGACAAGTTAATGGACAT
ATATGAATCAGGCCTAGAAACATTGGAAGTCCAAATTGAGCACTGGCAACTCTTAAGACA
AGAGCAGGTGCTTCTGAACTTTGCTAGGCGAAATGGTATTTTGCGACTGGGATATCAACC
AGTCCCAAGTCTTGCAACTTCAGAGACCAAAGCCAAAGATGCAATTGGCATGGTACTTAT
GTTACAAAGTTTGCAGAACTCCAAATATGCCGAGGAAACATGGAGTTTGGTGCAAACTAG
CCTTGAAACTGTTCGAAGTCCTCCTGAAAATTGCTTTAAAAAGGAACCACAGACTATTGA
AGTGGTGTTTGATGGAGATCCTGAAAATCGTATGATATACACAGTATGGAACTGGATTTA
TTTTCAGAATACAGAGGACATATGGCAAAAAGTTAAAGGACATGTGGACTATGAGGGTGC
CTTTTACATGGAAGGCACACACAAACATTATTATATAAAGTTTGAAAATGATGCTAACAG
ATTTAGCGCTACAGGACTTTGGGAAGTTCATGTTAACCAAGATACTGTGTTTACTCCTGT
TACTAGCTCTACGTCGCAAGCTGGAAGCAGACTATCCCCGCTGCCCTCTGACCCCACATC
CCGACGGCCCAGCCCATCCATCTCCACAGTCACCACCAAGCAGCGACGGTACGGGCGAAA
AAGATCTAGCCCTACAGCCACCACCACCGGGAGGCAGAGGCAGAGAACGCGAGAGACACA
GCAAAAAACCAAAAGAACCAGAAGCAGGAGCAGAACAAAATCAAGCACCCGTTCAAGAAA
CCGCAGCCGCAGCCGCAGCCGCAGCAGCGGAAGGCAAGACACCGACGCCACCAAAGGGCG
AGGGCAAGGTGGAAGGGGACGGGAACGGGAACGGGAACGGGAACGAGAACGGGAACGAGC
AGACACCTCTAACAGAACCTCCCGAGGGGGAGGGCGAAGTGGAAGGAGGCCGCAAACCCG
GTCAAGATCCAGATCCGGAGGATCTCCCGACAGGGTTGGCATTGCGCCTTCTGAAGTGGG
AAGAACATTACAATCTGTTGGTACAACAGGTCATGGGCGACTTGGAAGGTTACTGGATGA
GGCTAAAGACCCCCCAGTAATTTTGTTAAGAGGGGAAGCAAACCAGCTTAAATGCTATCG
CTTTAGAGCACGCAAAAAGCACAAAGGAAACTATAAGTATTTTAGTAGTACCTGGTCCTG
GATAGGGGAACATAGTAATGATAGAATAGGTAGAGCCCGAATGTTAGTTAGTTTTACATC
CACACACCAACGTGAAATGTTTTTAGATACTATGAAATTACCACAAGGTGTAGACTGGTC
ATATGGGAATTTTGACAGCTTATAATACTAACCTTTTTGCTATTTTTAACTGCTGCCTAG
CTACTAACATACTAACATGGCGCGTGCGCGCAGAACTAAACGTGCCTCTGTCACAGATAT
ATACAAAGGCTGCAAAGCTGCTGGCACATGCCCTCCAGATGTTATTAATAAAGTTGAAAA
CAAAACTATAGCAGATAAAATATTGCAATATGGTAGTGCAGCTGTTTTTTTTGGGGGCCT
GGGTATAAGCACAGGTAAAGGCACTGGTGGAACAACAGGTTATGTGCCTTTGGGAGAAGG
CCCTGGTGTGCGTGTAGGTGGCACTCCAACAGTGGTTCGCCCTGGGGTCATACCTGAAGT
TATTGGTCCCACTGAAGTAATACCAATTGATACAGTCACTCCAATTGACCCTGCAGCACC
TTCCATAGTTAATCTGACTGACAGCTCTGCAGTTGACCTTTTACCTGGTGAAATAGAAAC
TATTGCAGAAGTGCATCCTGTTCCTGTGCCAGATACAGAAATAGACACACCTGTTGTAAC
AGGCGGTCGAGGTTCCTCTGCTGTCCTAGAGGTGGCAGACCCCAGCCCCCCAATACGCAC
ACGTGTGTCACGTACCCAGTATCACAATCCATCCTTTCAGATAATATCCGAATCTACACC
TATTGCTGGTGAGGCATCCCTGTCAGACCAAGTGTTAGTGTTTGAAAATACTGGAGGACA
AACAATCGGTGGGATTCGGGAAGAAATTGAATTGCAACCCTTACCTTCTAGGTATAGTTT
TGAAATAGAAGAAGCAACACCACCAAGACAAACCAGCACACCTATTGAAAGAGGTAGGCA
AGCGCTTTCCTCCATTCGAAGAGCCCTGTATAATAGGCGCTTAACACAGCAGGTGCCGGT
GGAAGATCCATTATTTTTTAGTAGGCCATCTAAACTTGTAAGATTTCAATTTGATAATCC
AACATTTGAGGAGGAAGTAACACAAATATTTGAAAGGGATTTAGAATCTGTTGAAGAACC
TCCTGACAGGCAATTTTTGGATGTTGCCAAGTTAGGTAGGCCTCTATATTCCGAAACCCC
ACAAGGCTATATACGGGTCAGTAGATTAGGCAACAGAGCCTCTTTACGAACCAGAAGCGG
TGCACAAGTAGGGGCACAAGTACACTTTTATAAAGATGTTAGTACCATTGATTCTGAAGC
TCCTATTGAACTACAATTACTGGGGGAACATTCAGGAGACACCAGTATTATCCAAGGCC

TGTTGAAAGTACACTGGTGGATATTAATGTAAGGGATACTCCAGAATTGCTTGAAAGTTC
TGAATTTAACTCTCAGGATCTACTTATTGATGATGCTATAGAGGATTTTAGTGGGTCACA
GCTAGTGTTTGGCAACCCTAGACGGTCTACCACTTCTGTCACTGTACCCAGATTTAGTTC
TCCAAGGGAAACTACTTTATATGTGCAGGATATACAAGGTTACACTGTAGCATACCCAGA
ATCACGTGATAGGCCAGTAATAATTTATCCTCAACCTGAGATTCCTGCAGTTGTTATCCA
TTTTGGGGAATCAGGTGCTGATTACTATTTACATCCCCATTTACAGCGACGCAAGCGCAA
GCGAGCTTATTTATAATTGTTTTTCAGATGTCTTTGTGGCTTCCTGCAACTGGTAAAGTA
TATTTGCCACCATCAACTCCGGTGGCACGGGTGCTAAGTACCGATGAGTACGTGGAGCGG
ACCAATATATTTTATCACGCCACTAGTGACAGATTATTAACAGTAGGACATCCATATTTT
GATGTTCGGTCGTCTGATGGCTCTAAAGTAGAAGTACCTAAAGTGTCAGGAAATCAATTT
AGGGCATTTCGAATGCTGTTTCCAGATCCCAATCGATTTGCATTAGCAGACATGTCAGTA
TACAATCCTGACAAGGAAAGGCTGGTATGGGCCTGTGCAGGTATAGAAATTAATCGAGGA
CAACCATTAGGTGTTGGTACAACTGGTCATCCTTTGTTCAATAAGGTAAAGGACACTGAA
AATACAAATAGCTATCAAAACAGTTCAACAGATGATAGACAAAACATTTCATTTGACCCT
AAACAAGTACAGATGTTTGTTATAGGATGCACACCATGTTTAGGGGAATATTGGGATAGG
GCACTGCCATGCGATGATGCTGGTGACCAACAAGGTAAATGTCCGCCCATTGAATTAAAA
AATTCTTTTATAGAAGATGGGGATATGATAGATATTGGGTTTGGCAATATAAATAACAAG
ACATTATCAGTAAACAAATCAGATGTTAGTTTAGATTTAGTAAATGAAATAGCTAAATAT
CCAGATTTTTTAACAATGGCTAATGATGTGTATGGCGATTCTTGCTTTTTTTTTGCCAGG
AGAGAACAATGTTATGCTAGACATTATTTCACTAGAGGAGGAGCTGTGGGTGATGCTATA
CCTGATACAACAACTAATCAAGATCACAAATACTATATAGCACCTAAGAGTGGACAATCC
CAAAGTCCTTTGGGTAATTCTATTTACTATCCCACCGTTAGTGGCTCCTTAGTTTCTTCT
GATGCACAGCTCTTTAACAGACCCTTCTGGTTGAAACGTGCACAAGGCCACAATAATGGT
ATTCTATGGGGAAACCAAATGTTTGTTACTGTTGCAGACAATACAAGGAACACCAATTTT
ACTATAAGTGTGCCTAGTCAAAATGGACCCCTTACAGAATATGATGCCAATAATATCAGG
GAATTTTTAAGGCATGTTGAGGAATATCAAATATCTGTAATACTGCAACTTTGTAAAGTG
TCTTTACAACCAGATGTGTTAGCCCAAATTAATGCTATGAATTCAGGCATTTTGGAAGAC
TGGCAATTAGGTTTTGTACCAACACCAGATAATTCAGTTCATGACACTTATAGATTTATT
AATTCTGCTGCTACTAAATGTCCTGACAAGGTTGCTCCTAAAGAAAAGGAAGATCCTTTT
GCTCAATACTTTTTCTGGAGAGTTGATATGACAGAAAAATTATCTTTGGATTTAGATCAG
TATCCTTTAGGACGTAAATTCCTATTCCAAACAACACTACCTGCCTCTCGAAAACGTGCA
GCGCCGACCACCAAATCGGTTGCAAAGCATAGCAAACGAAAACGTACTTGACCGAAATCG
GTCCCTCTCAATAAACAAGTATTCTAACTAAAGTGGTATGTGAAGCATTTTTTAACCGTG
TTCGTGACTAAACCGAACAAGTCAACACCAGCAACCGCACCCGGTTAATCAGATTATATA
TCGCTGTCGGTGCCAAACAAGACAAAGACTTGGCTGGATCTCGAGGAAACACCGCAGCGC
CCGCCAAAAGTCTGCCAAGTTTTCTTGGCAGAACATCCATTTGGCTGCAACTGTCACCGA
TA
>gi|9628566|ref|NC_001693.1|_Human_papillomavirus_type_60,_complete_genome
TATAGGATATACACTGATTGTTGGCAACTATCATTAAATCATAAAAAAAATATGACCAGA
AGAGGTATATATAAATAGCTGAGACGTTCGTAAACTTTAGGATGCAGATGGAAGAAGACA
GGTTTCCAACAACAGTGGCTGATTATTGCTCTGAATTTGATATTCCTTTAAAAGATCTTA
AGTTAAAATGTGTATTTTGTAGATTTTACTTAACTGAACAGCAGTTGGCTGCATTTTATA
TTAAGAATTTAAAGTTAGTTTGGAAAAACCGTTATTGCTTTGCTTGTTGTACTCCGTGTC
TTAGACTTACTGCTAAATTTGAAGCTGAAAACTATTTTCAGTGTATGTGTAAAGGAGAAG
TATTAGAAGTTCTAACTCGTATTCCTTTGAGTTCTCTTTCTGTACGCTGTTTTGATTGCC
TTACATTGTTATCTTTTGCAGAAAAAATAGATTGTATAATTAGTGGTCAAAACTTTTATC
TTGTAAGGGGTCGCTGGAGATCATATTGTAGAAATTGCATTGAGAAATGATTGGTAATCA
GCCTAATGTTAATAACCTTGATGTAAATTTGGAGGAGTTGGTTTTACCTGTCAGTCTTTT
AGCTGATGAGGAGTTGTCACCCGATGGTGATCCTGAGGAGGAGGAGCACTATCCCTATAC
AATAGACACCTGTTGCAAACCTTGTGGAGCAGGTGTTAGATTTACTATTATTGCTACTCC
TTCTGCTGTAATAACTCTTCGTCAACTATTGCTTCAAGAAGTGTTCCTGACCTGTTTGAG
GTGCTCCAGATCCCTTTTTCGACATGGCAGATCCTAATAAAGGTATTAATTCTCTTGAAT
TAAATGAGGGGCATAGCGAATGGTATGTTGTGACAGAAGCTGAGTGTATTAATAGTTTGG
ATACAATGGAAGAGCTATTTGAAGAAAGTACAGATGGATCTATTGTGTCCAATCTGATAG
ACGATTCCGAGGAACTGGAGGAGGGAAATTCCCTGGCACTCTACAATGAACAGTTAACAG

AGGATTGCAATAGAGCTATTTTAGCGCTAAAACGAAAGTTAACTAAAACACCTTTGAAAA
GCCAGGACAGAACGGTTGCTGACCTAAGTCCAAGGTTGGAAGCTGTCACAATTTCACCTC
AGAGACAAAGCAAAAGGAGATTATTTGAGGACAGTGGATTAGGAGAAGATGAAGCTACAA
ATTCTATTGAAAAAAAGGTAGTTTCGAACTCTTTAGAGAGTAATGAAAGTGGGACCTTGG
TTGTGGAAACGGACAGTATATTTCGCAGTACTAATAGAAAAGCCACTTTATTGGCTAAAT
TTAAAGAATACTTTGGTGTAGCATATGGAGATTTAACGCGACCGTTTAAAAGTGATAGAT
CATGTTGTGAAAACTGGGTTATAAGTGTGTGTGCCGCTGCAGAAGAAGTAATAGAAGCTT
CAAAAACAGTTATGCAGCAACATTGTGATTTTTTACAGGTTATTTCATATGGGTTTTATG
CTTTATATTTGGTAAAATTTAAAACAGCAAAAAGTAGAGATACTATAATGAAATTATTTT
CTTTAACATTGAATGTACAAGAACAACAATTAATGTGTGATCCACCAAAATCTAGAAGTA
CCCCAACAGCTCTTTATTTTTATAGAAGGTCATTTGGAAATGCATCGTTTATTTATGGAC
CGTTTCCAGATTGGTTAGCAAAATTAACTATGTTAGACCATGAATCCGCCGCGAGCTCAG
AACAGTTTGAACTTGCTCAAATGATTCAATTTGCTTATGACAATAACTTGACAACTGAAT
CTGAAATAGCTTATAAATATGCATTGTTAGCTGATTCTGATGCTAATGCAGCTGCATTTT
TAAAAAGTAATCAACAGGTAAAATATGTACGAGACTGCTATGCAATGTTAAGATATTATA
AAAGACAAGAAATGAAAGATATGTCAATTTCTGAGTGGATATGGAAATGTTGCGATGATT
GTAATCAAGAGGGCAACTGGAAATTAATAGCACAATTTTTACGTTATCAAGAAGTTAATT
TCATTTCATTTTTATGTGCATTAAAGACATTGTTTAAAGGTATTCCAAAAAGAAATTGTT
TAGTGTTTTGGGGACCTCCAGATACAGGAAAGTCTTATATTTGTTCTTCACTTACACGGT
TTATGCAGGGAAAAGTGGTATCTTTCATGAATAGACACAGTCAATTTTGGCTACAACCTT
TACAAGATTGTAAGCTTGGATTCTTAGATGATGCTACATTTCAATGTTGGCAATACATGG
ATGTAAATATGCGAAATGCATTAGATGGTAATCATATTTCATTAGATTTAAAACATAAAG
CTCCTTTACAAATAAAATTACCTCCTTTATTAATAACTACCAATGTTGATGTTGAAAATG
AAGCTAGCTTAATGTATTTAAAGAGTAGACTAGTATTTTTTAAGTTTCCTAACAAATTAC
CTTTAAAAGAAAATGATGAAGTACTTTATGAAATTACTGATGCATCATGGAAATGTTTTT
TTATCAAATTTGCAAGCCATTTAGAGCTGACAGCCAGAGGAGACGAGCAGCATGAATCAG
GCCGATCTGACCGAGCGTTCCGATGCACTGCAGGAACAAATACTGAATCTATATGAGCAA
GATTCAAAGGACATTCAGGCCCAAATACAATATTGGGATTTAAATAGAAAACTATATGTC
ACATACTATTATGCAAGAAAAGAAGGCTATTCGCATTTGGGTCTTCAACCCTTACCAGCT
TTGCAAGTATCAGAGTACAAAGCCAAGCAGGCAATCGAAATGGGATTGTTATTAACCAGT
TTAAGCAAATCCCAATATGCTTCAGAACTATGGGGTTTAACAGATACAAGTGCAGAATTG
TTATTAACCCCTCCAAGAAATACATTTAAAAAGAAGGGATATACTGTAAATGTGTGGTTT
GATAATAATGAAAATAATACATTTCCCTATACAAATTGGGAATATATTTACTATCAGGAT
GATATTGAACAATGGCACAGGACCAGAGGAGAGGTGGACTATAATGGACTTTATTTCACA
GAAAATAATGGAAATAGAGCATATTTTCTCCTATTTGATAGTGATGCACAAACATATTCA
CAAACTGGGACATGGACAGTGCATTATAAAAACCAAATTATTTCTGCTCCTGTTACCAGC
TCCTCAAAACAATCCTCCGACGACTACACTTCCAAAGCCGGGCAGCAACCCCCACTTCTTC
GCCTCATCCTCATCGCCGACTACTACCGACGGAGGACAGACCTCACAAGAGGGAGTCTCT
AGCTCTACCACGTCGCCGAGTGCTGTTCGATTACGACGCCGAAGATCCAACGAGCAACAA
AGAGAACTATCCTCCAGAGAGTCGCCCCGTACCAAAAGACGCAGAGTACCCGACGAAGTC
GACCGACAGAGTGCCGTGGGGTCTGCCCCAACTGCTGAAGAGGTGGGAAGCAGACATAGA
TCTCTTCCTAGAAGCGGTATATCAAGACTTGCAAGACTTCAAGGAGAAGCTCGGGATCCT
CCAATCCTGCTAATTAAAGGTCTAGCAAACTCCCTTAAATGTTGGAGATATCGATTGAAA
AAGTATACACGTTATTTTAAATGTATGAGTACAGTATTTAGATGGGTAGACATAGACGTA
CCAGAATCTTCTAGACATAAATTGTTAGTTGTTTTTAATGATACCACACAACGAGATGTT
TTTATGAAATTAGTAACTTTGCCAAGAGGTTGTACATATACATTTGGAACATTAAACTCT
TTGTAATATTTGTAAAACAAAACAAAATGTATGCTAGAGTAAAGCGTGTTAAAAGAGACT
CTGTTGAAAACTTATATAAACAATGTCAACTTGGTGCTGACTGCCCTCCTGATGTACGAA
ATAAAGTAGAAGGAACCACACTTGCAGATCGATTATTACAAATATTTGGAAGTATCTTAT
ACCTGGGGAATTTGGGTATAGGGACAGGTAAAGGATCGGGGGGTGCTACAGGCTATACAC
CTCTTGGTACTGCAAGAGTGCCAGCGTCTACACCTGGAACAGTTATAAAACCCACACGAC
CGTTTTCGGTTCCTTTAGATCCAATAGGTTCTGGAATTCCATCGCAACCTGTAGGAGGTC
GGTTACCTGTGGACATTATAGATGCCAGTGCTTCCTCTATTATACCCTTGCAGGAGGTCC
TGCCAGAGACCACCATTATAGTAGGCGGTGACAGCGGTCCTGGATTGGGTGCAAGTGAAA
TTGATATAGTTTCAGAACCAAGACCAGATGTGGTCGGTGTTGATACTCAACCAACAGTAT

ATACCTCCATTGATAATACAGTTGCTACATTAGACATTACACCAGCTACACCACCAGTGA
AGAAAATAATATTAGACCCTATAAGTTCAGGTTCTGAAGGTGCTGCAGCAATAACATTTT
CAGATATAAGTGCTGCAGATTTAAATGTATTTGTTGATCCTCAGGGAGCAGGAGATAGAA
TAAGTTTTGGAGAAGAAATAGAGTTAGGCCCTATTAATCAGCCTGCTCAATTTGAAATAG
AAGAACCTCCACGTACCAGTACACCTGGGGAGGGATTTCAACGTGTTACAACACGGGCAA
GGGAATTATATAATAGATTTGTGCAGCAACAGCCAACACAAAATATTGACTTCTTAGGAC
GTCCTTCCCGCGCTGTACAATTTGAATTTGAAAATCCCGCCTTTTTTAATGATGAGGTGA
CTATGCAGTTTGAGCAAGATTTGCAAGAGGTGGCTGCAGCGCCAGACCAGGATTTTGCAG
ATGTCAGGGAATTAGGACGAGCTCGGTTTTCAGAAACATCTGCAGGAACAATACGAGTCA
GCAGATTAGGAACAAAAGGAACAATGAAAACACGAAGTGGACTAACTATTGGTCAGAAAG
TTCACTTTTATTTTGATATTAGTGATATTCCAGCTGCAGAAACCATACAACTAAGAACAT
TAGGCGAGTCTTCCCACGATTTTTCTGCAGTAGATAATATTACTGAAAGTACATATATTA
ATTTAACTGAAACTACAAATGAAGGCCTAATACCTGACAATATACTAGAGGATGAATTTA
CAGAAAACTTTAATAATGCACAGCTGATATTTGCAACAATAGATGAAGGAGAATCTATGA
TCATGCCCACAATTCCTCCAGGTGTAGCTTTAAAGTTATTTATTCCAGAAATAGCTGCAA
GCGTGTTAAATGTGGTTCATCCTTCTTCTGAGTGGACTATTTTGATTCCTAATGTTCCAG
ATGAAATTATTCAGCCTGCTATGGCAGTAGATGTTTATGATGACTTTTATTTACATCCTC
ATCTTCTTAGGCGACGCAAACGAAAACGGTTGGATTTTTTTAATGTTTTACAGATGGCT
CTTTGGTTGCAGACAGCTGGACAATTGTATCTTCCACCAAGCAAGCCTGTTGCTCGTGTA
CTCAGTACGGATGAATATGTACAGCCAACAAATTTAGTTTTTCATACAGGAACTGACAGA
ATGCTAATTGTAGGACACCCTTATTTTGATATTATAGACAGTGGTTCAAATAATATTACT
GTACCTAAATGTAGTGGAAATCAGTTTAGAGTTATGCGACTACTATTTCCAGATCCAAAC
AAATTTGCAATGATAGACAGAGCTGTCTTTAACCCAGAAAGAGAGAGATTAGTCTGGAGA
TTGGAAGGCCTTGAAATTGGTAGAGGTGGGCCTTTAGGAATTGGAACTTCTGGCCATCCA
TTATTTAATAAATATGGAGATACAGAAAATCCTGCCGCATATCCTTTAAAGCAGAATAAT
GGTGATGACAATAGAATGGATGTTTCAATGGATCCAAAACAAATGCAATTGTTTATTGTA
GGATGTAAACCAGCCACTGGTGAACACTGGGATATTGCTAAACCTTGTGATCCTGCTCCA
GCAAAAGGATCCTGTCCCCCAATTAAATTAACACAGTCTATAATTCAGGACGGTGAAATG
TGCGATACAGGCTTTGGAAATGCAAATTTTATTACCTTGCAAGAAGATAAATCAGGTGTT
CCTCTAGATATTACCAATGAAATTTGTAAATATCCTGATTTACTAAAAATGACAAAAGAT
ATCTATGGAGATGCAGTATTTTTCTTTGGTAAACGTGAACAAATTTATTCCAGGCATTAT
TTTGTAAGAGGAGGGATAGATGGAGATAGTTTGCCAGATTCTGGATATTACTTAGCACCA
CAAACTGATAAACCTCAAAATAACCTTGGTGGTTATAGTTACTTCCCTACACCAAGTGGT
TCTGTAGCTTCTAGTGATAATCAACTTTTTAATAGACCTTATTGGCTTCACAGAGCCCAA
GGTGCAAATAATGGTATTTGTTGGGGTAACCAATTATTCATTACTATAGTAGATAATACT
CGAAATACTAATTTATCTATTTCAGTTTACAAACAAGATGCTGCTATTGATAACAGATAT
AAATATAAACAGGAAGATTTTAGACAGTATTTACGCCATACTGAAGAATATGAAGTAGAG
TTAATTCTGAGACTCTGTAAAGTGCCTTTGAATCCAGATGTTCTAGCTCATTTAAATGTA
ATGGACAAAAATATTTTAGAGGATTGGCAACTTTCATTTGTACCTCCACCACCACAAGGT
ATTGAAGATGCTTACAGATATATAATGTCTCAGGCAACAATGTGTCCAACAGATGTTCCT
AATACTGAAAGGGAAGACCCTTATAAACAGTATACATTTTGGACAATAGATTTGCAAGAA
CGTTTTTCTAACGAATTATCCCAGTTTTCTCTTGGAAAAAGATATTTATATCAGTATGGC
TTACTTAACGGACGAAAACGGTCTGCTAGTAGTTTTGTAACGAAGAAATCAAAAACTGTG
AAACGTAAAAGAACTAAATAAATACATATATATATTATATGATTAATGAAGGATATATTA
ATTAATAATGCTGCTGTTGTAAAATGAAGGATTAATAAATTGCACATCAGCAAGTGTCTG
ACTCACGGGGTCTACATTTTTGTCTACCGCGCCTTCATAATTATCTTGGCATTGCATATC
GAGACTATAAGAAACAATCACTTGGCACACTCTCGGATTTAACAAGCATTGCTGGACACT
TACCAACCGTAACTGGTTAAAAAGAGCGCCAAAGGGGAAACAGGCAACCGTTTTCGTTC
TCCTGAAGGAACGTTTTCCAGGTAAGTACATGTTCATACAAAGTTTTGACTCACTCTCTT
TGTTAGAAACCATTTCCGTTCTGTGGATATGCGCCAGACCAAAAACGGTTGCC
>gi|9628550|ref|NC_001691.1|_Human_papillomavirus_type_50,_complete_genome
TCTATCTAATAATAGTTGCCAACAATTATTAGACATAGAGACGTGACAACCGACTACGGT
GCATATAAAAGAGCTGATCAGCACAGATTTGAAGGAGACTGATGGAGCCTCAGAGAGCTA
AAAATTTGGTGGACTATTGCAAGCAACAACAGATTAGTTTTTTGAATTAGAGCTACAGT
GCTTGTTTTGTAAATTTGTTATTACTTTGCCTGACTTAGCTAGCTTTCATTGTAAAAAGT

TAGCTCTTGTTTATAGAGATGGTATTGCTTTTGCTGCGTGCGCTAAATGCTTAAGATTGT
CTGCTATCTTTGAAAATGAGAGATATTATACTTGCTCTATAAAAGCTTACTTGCTATCTG
ATTTGATTGGTAGACCTTTGAGTGAAATCGCAATTCGCTGTGAAAATTGTATGTGTTTGT
TGGATTATATTGAAAAGTACGATTGTATTTGTCACGGTGGTTATTTTCATTTAGTTAGAG
GTAATTGGAGGGGCTGTTGTAGAAATTGTTACGAACATGAGGGGTGATAAACCAACTATT
CCTGATATTGTTTTAGAAGAGCTTGTTTTGCCTGTGAATTTGCTTAGTGACGAATCTATT
GAAACTGATGACATAGCAGAATCTGAAAGGTCACCATTCAAGATTGATTCTACGTGTAAG
CATTGTCATTGTCGCGTTAGACTGTGTTGTGTCGCCACCGACGCAGCTATCCGTGTGTTT
GAGTGCCTTCTTCAATCAGAATTTTCTTTTCTGTGTCTTAAGTGCTCCAAAGAATTGCTA
CGAAATGGCAGAACTTAAAGGTACAGATAATAATTCTAATGTAGAAATTATTAATGAGTT
GTTTGATAATGAAGCTGTCTGTGATGATGATTCTTTTCAGGAATTGTTTGATGAAAGTAC
GGATGAATCTACTATCTCTAATTTAATAGATGACTCTGAAAACGTTGTGCAGGGGAATTC
CCATGCATTACTAAATGCACAGCTTTCAGAGGAATATGACAAAGACCTGGTTACAGTAAA
ACGAAAGTTTTATGCCACCCCGGAAAAGCTCGCCTCTGATTTGAGTCCGAGACTTTCAGC
GGTGCATATAACACCAGAAAGACAATCCAAAAGGCGTTTATTTAGAGACAGTGGGATTGA
AGATGAAGCTGAAAATTCTATTGTTCAGGTACAGGATGATAGTTCGAATGTGGCGGGAAA
CAAAAATGGCGCTGATGCGGAGTTGTATTCGTTGCTTCATAGCAATAATCGTAGAGCTGC
TTTGCTTTGTAAGTTTAAAGAAAAATATGGCATTCCATTTAATGAAATTACTAGAACTTT
TAAAAGTAATAAGAGTTGTACACAAAATTGGATTATTGTAGTTTTTGCCTGTGCAGAAGA
TTTAATTGAAGCTTCTAAAACCACGATGCAAAATCATGTTTCCTATTTACAAATGATTAC
TTCTGATTTTTCAGCTTTGTATATTATTTGCTTTAAGGCTGCTAAGAGCAGAGAGACTGT
TGTTAAATTGATTAATAGCTTGCTAAATACAAAAGAGGAACAAGTACTATGTGATCCTCC
TAAAATAAAAAGTATGGCTGCAGCATTATATTGCTATAAAAAGGTAATAGCAGATACCTG
TTATAAATATGGAGACTTTCCTGACTGGATAGCCACACATACTGTTATAAATCATCAGCT
AGCAACTGCAGACAGCTTTAAATTTAGTGATATGGTACAGTGGGCATACGATAATGATAT
GCTGGATGAAGCTGCAATTGCTTACAATTATGCGTGCTATGCAAGTGAAAATGAAAATGC
AGCTGCATTTTTACAAACTAATAGTCAATTAAAGTATGTTAAGGAATGTTGTGCAATGGT
CAGGTTATACAAAAAGCAAGAAATGAGGAATATGACTATGCCAGAATGGATTAAATCATG
TTTCACAAATAATTATAATTCTGATGATTGGAAAGTTATTGTAAGATATTTAAAATATCA
AAATATTAACTTTTTAGAATTTTTGTTAGCTTTAAAGTTACTTTTAAAAGGCATACCTAA
AAAAATGTGCTTAGTTATTTATGGACCACCAGATACAGGCAAGTCATACTTTTGTTATCA
ATTTATTCAGTTTATGAGAGGAAAAGTTGTATCCTTCATGAACAAAAATAGTCATTTTTG
GTTAATGCCTTTATTAGATTCAAAAATAGGATTTTTAGATGATGCAACTCAATGTTGTTG
GATGTATTTGGACACACACACATGAGAAATGCATTTGATGGTAATGCAGTTTCTGTTGATGT
GAAACATAAAAATTTACAACAAATAGTTTTACCACCTATGTTAATAACAACAAATTGTGA
TGTATGCAGAGATCCAACGTTTATGTACTTAAGGAGTAGATTAACATGTTTTAACTTTCC
AAATAAGTTACCATTGTATGAAAATGGAGAACCAAAATTTAAATTTACTGATAATTGCTG
GACGTCTTTTTTTAGCAAGTTTTGGAAGCACTTAGATTTACCTGAAGATGACCCAGATGG
AGACACAGGAAACTCTGAGCGCACGTTTTCTTGCACAACAAGACATTCAATTGAATCTGA
TTGAAAAAGATTCAAAAAATTTAAAAGACCATATAGACTATTGGGAATCTATGAGAAAAG
AACAAGTGCTTGCATTTTATGCAAAAAAAGAAAATATGAGCAGGCTTGGATTACAACCTC
TTCCTCCTGCTAAGGTTTCTGAGCAAAAAGCAAAAGATGCAATTAGAATTCAATTACTTT
TGCAATCCTTGTATAAGTCTGATTTTGGGTCTGAACCATGGACTTTATCAGAATGTAGTT
TAGAAATGTTAAATGCACCTCCTAGAAATTGTTTTAAAAAACAACCGTTTACTGTAACTG
TGCAATTTGACAATGATCCTAAAAATGTGTATCCTTATATATGTTATGAATATATATATT
ATCAAGATGATAGAGACAAATGGCATAAAGTTAAAGGTCTAGTGGATCACAATGGCCTTT
ATTTTAAAGAAGTGACTGGTGATTCAGTATACTTTAAACTCTTTCAACCTGATGCCACTG
TATATGGCAAATCTGGACAATGGACTGTTATATTTAAAAACAAAACTATTCATTCCTCTG
TCACTAGCTCCTCAAGGAGTGCGTTTGGGCCTGCTGACGAACAGCCCGGGCCTTCCACAT
CGTACGACAAGAGCCAACAGGAAAGATCTGGAAGCGGTCAACCAAAAGCCCTACAGGACA
CCGAACCACCCACCTCGACATCAACAGTACGACTTCGACGAGGACGACGAGAAAGAGAAC
ACCATTCCTACAGACACAGAAAGTCACAATCAGAATTGGGAGCCGACTCTGCGCCAACTC
CTGAAGAAGTGGGAAGAAGATCTCACACAGTTGCAGCGCATGGTCTGTCAAGACTTAGAC
GATTACAAGAGGAAGCTCGGGATCCACCAGTCCTAATTATTACAGGTCAACAGAATAATC
TTAAATGTTGGAGGTATAGGTTCTCACAAAAATATGCAGATCTCTATGAGTGTTGTAGCT

CTGCATGGAAGTGGTTGGGCCCAAAATCAGAGGGATACAGGGGTGATGCTAAATTGCTTA
TTGCTTTTAAAAATCCTGAACAAAGGCTATCTTTTTTAAACACTGTTGGATTACCCAAAA
ATACTACGTACTCTATGGGACATTTGGACTCTCTATAATGCTTCGCAGACGCAAACGGGC
AAGCCCTACGGACTTATATAGATCATGCTTACAAGGGGGGGATTGCATACCTGATGTTCA
AAATAAATTTGAAGGCAACACAATCGCAGATTGGTTATTGAAAATATTTGGAGGTTTAGT
ATACTTTGGCAATTTGGGAATTGGAACAGGAAGAGGAACTGGGGGTACTTTTGGCTATAG
ACCTTTTGGGGCCCCTGGGTCTGGAAGGCCAACTCAGGAACTACCTATCGCAAGACCAAA
TGTTGTTATAGATCCATTAGGGCCAGCGCCTATTGTACCAGTAGATCCCTCTGCAGCTTC
AATAGTTCCATTAGTAGAAGGCGCCCCTGATGTTGGATTTGCAGCTCCAGATGCTGGACC
TGCAGCAGGTGGAACTGATATTGAATTATATACTATAACAAATTCCACAACAGATGTTGG
TGCTGTTGGTGGTGGGCCCACTGTTACCTCTAACGAAGAATTTGAAGTAGCTGTTATAGA
TGCACAACCAATTGCACCCTATCCAAAGCAATTATTATATGACTCTACTATAGCAGCAAC
TTTTGAAACACAGATTAATCCCTTCATAAATCCAGACATAAATAATGTTAATGTATTAGT
TGACCCCAGCTTTGCAGGAGACACTGTAGGTGACTACTTTTATGAAGAGATACCTTTGGA
ACGTTTAGATATTCAAACCTTTGACATTTTAGAGCCACCTACTGAAAGCACGCCCACCCA
ATTAGGTAATAGGTTTGTTTCTAGGGCAAGAGATCTGTACAGTCGATTTGTTGCACAGCA
GCCAATATCTGAGCCAGATTTTTTGAGTCAACCTTCCCGCCTTGTTCAGTTTGAATATAG
AAATCCCGCCTTTGACCCAGATGTGAGCCTATACTTTGAGCGGGATTTGGAAGGCCTCAG
GGCAGCGCCATTGCAGGAATTTGCTGATGTAGTTTATTTAGGCAGGCCAAGAGTATCCAG
CACCTCAGAAGGTACAATAAGAGTAAGTAGATTAGGTACCAGAGCAGCTTTAACAACACG
TAGTGGTCTTTCTGTGGGACCTCAGGTACACTTTTATATGGATTTAAGTGATATACCTCC
AGAGGATTCTATAGAGTTGCATACATTAAACGTTACACCACAAACAAGTACAATTGTAGA
TGATATATTAGCAACAACTACATTTGATGATCCTGCAAACTCCTTATTTACACAGTTTAA
TGAAGATGTATTAACAGACGATGTTGAACATAATTTTACAGAGTCTCATTTAGTTATACC
AGCTACTGATGAAGAAAATGACACAGCAATAAATATTATTAATTTAAGAAATATTCCTCT
TACTGTGGGCATGAATTCAGGTGACATATCTACTACATTATCAGATTATAATATTTTAGA
TGCATCCTTGATAGTAAAATCAAATGTTTCTGAACAGCCCCTTTTTGTTTTAGATTATTC
AGATTATGATTTACATCCAGGTCTTCTTCCAAAAAGAAGACGCATAGATTATTTTTAATT
TATTTTACAGATGGCTCATTGGTCCTCAACCTCTGGAAAGTTATACCTTCCACCAAGTAC
TCCTGTTGCCAGAGTTTTAAGCACCGATGAATATGTGAAAGAAACTGATGTTTACTTTCA
TGCACGCAGTGAGCGTTTACTAATTGTGGGACACCCATATTATGATATAGAAGATGGTGG
AGATATCAAAGTTCCTAAAGTATCAGCAAATCAATACAGAGTTTTTCGTTGTGAATTACC
AGATCCTAACAAGTTTGCTCTAATTGATACTACATTGTATAACTCAGATACAGAACGTTT
GGTTTGGAAACTAGTAGGTATAGAAGTCGGGAGAGGTGGGCCTTTAGGTGTTGGATCTAC
TGGTCATCCCTTGTTTAATAAGGTTGGAGACACAGAAAATCCTAGCTTTTACTTAGGTCC
ACAAGAAAAAGATGAAAGACAAAACTTGTCTATAGATCCTAAACAAACACAGTTATTGAT
TGTAGGCTGCAAACCAGCTGTTGGTGAATATTGGGATTTAGCAGAACCTTGTGACAAAAA
CAGCTTAAATAATGGCAAGTGTCCTCCAATACAACTAGTAAACAGTTATATTCAGGATGG
TGATATGGGAGATATAGGATTTGGTAATGCAAACTTTCCTAAACTACAACAGGACAGAGC
AGGGGTCCCTTTAGATATAGTGGATTCAATCAGCTTATGGCCTGATCTATTAAAAATGAC
TAAAGATGTCTATGGGGACCATGTGTTTTTCTATGCAAAGCAAGAGCAATTATATGCCAG
ACATTTGTTTACCCATGCAGGACCAATTGGGGAACCTATACCTAATGTCTCAGGAGTTTA
TAATTATGCAGTAAACCCTAATCAACCAGAGCAAAATCGTAGAACCAATATTGGTTCCTA
CTTATATTTTACTACACCAAGTGGATCTTTAAATACAAGCAGTTCACAGTTATTTAATAG
ACCATATTGGATTCGCAGAGCTCAGGGTACGAACAATTGTATTTGTTGGGGTAATGAGGT
ATTTGTTACAGTATTTGATAACACCAGAAACATTAACTTTAATATTAGTGTTAAGAAAGA
TGTCAATCCTTTGGATCCTTTAAATGTAGCAAGTTCTTATATGTACTCAAAGGATGATTT
TAATCAGTACAGCCGTCATACTGAAGAATATGAATTAGAATTTATATTTCAGTTATGTAA
AGTAGGATTGGATGCTGATATATTAGCTCATCTTAATGTAATGGACCCCAGAATTTTGGA
AAATTGGCAATTAGCTTATGTTCCACCAGCTCCCAGTGGTATAGGCGATACTTACAGATA
CTTAAAATCTGATGCTACAAAATGTCCTGCAAAAGACAGTAGCGCCGAAGTAGTAGACCC
TTATAAAGAATATACTTTTTGGAATGTCAACCTTACAGAAAAGTTTTCGTCTGAACTTGA
TCAGTATGCCCTAGGACGAAAATTTTTGTTTCAGACTGGATTATTAAAGAGAAGAGTTAG
AACTGATTACACTGTTGCTACAGTTTCCAAACCAAACAAGAGAAAACGTACCAGATAAGC
TGCTGACATGCTTTCTTGGCTTTACTGTGAATATTGTACTGTGAATTCTATACTGTGAAT

GCTTTAGATCTGAGTCTTGAACATTTCACAAAATGAATAAACAAATAAAGTTAATGTACA
GGTGTGTGTTGTCTTTGAGTCAGAGTTATTTACTCCCGCCCTTTGTAGTATAAATATGTT
GCACCTCTGTGATCAATCATGTTGACCAGCTGGACGTGCTGTACACCGGTCACTTTAAGA
GAGACACGACCGTTTTCGGTAAGTTTGTATGCCTTTAGGGAATATAAAGCGACCGAAGTC
GGTACTTGCTAACAAAGATAAAATCTCACGAGTTAGAACAAACCTCTGAGTCACCTCAGT
GCAGCTGGCAGAAGAAAGTATACCGATATCGGTTGCTATGTTTT
>gi|9628542|ref|NC_001690.1|_Human_papillomavirus_type_48,_complete_genome
TCTGACGCATAATAATTGTTGGCAACAATCGTGCATAATAACTTAGGAACCGAGACAGTA
TCATATAAATACTGCTGAACAGTAGATTTCTTCAGAAGCTGATGGAGCCACAATTTCCTA
CTGATTTGGATTCATACTGCAAATATTTTAACATAAGCTTTTTTGACTTAGTTCTTAAGT
GTATCTTTTGTAAATTTTCTGTATCTATTGTTGATCTTGCTTCTTTTCATAACAAACGAC
TGTCTGTAATTTGGAGAGATAATACACCATTTGCATGTTGTACTAAATGTTTAAGATTAA
CTGCATTATATGAAAAAGATAATTTTTTTGTCTGTACTGCAAAAAGTCATTTGTTAACTG
GTTTAGTTAAAAAGGAATTGTCAGATATTAATATTAGGTGCCAACATTGTTATTCATTTT
TAGATTATTTAGAAAAGTTGTACCATTTATATAATGATGTTGATTTTCTGTTAATTCGCG
GGACTTGGAGAGGTGTTTGTAGAAATTGTATCAGCCATGAGGGGAGATAAGGCTACTATT
CCTGACATAGAGTTAGAAGAGCTTGTTTTGCCTGCTAACTTAATTTCTGACGAGTCATTG
TCACCAGATGCTACTGCAGAGGAGGAGTTTTGTCCGTACCGGATAGACTCCAAGTGTCAC
AATTGTGGCTGTCGCATAAGGGTGACAGTCGCTGCAACTGAATTTGGAATACGTTGTTTT
GAGCAACTGTTACTGAAAGAGCTTTGTTTATTTTGTCCTGCGTGCTCCAGACAACTTCCC
CGCAATGGCAGATCATAAAGGTACTGATAATATTGATCATAATGATGTTTTAGATGGGTC
TTGGTGTTTAATAACTGAAGCTGAATGTGAGGACGATACGTTAGTGGATTTGTTTGAGGA
GAGCACAAATGACTCTGTAGTTTCGAATCTGTTAGACGATTCCGAAAGTATAATTCAGGG
GAATTCTGAGGAAAGTGACCGCTGTATTCAGGAGCTAAAAAGAAAGTTAAATGTTACTCC
TGAAAAACAGATCTCAGAGCTAAGTCCGCGGTTATCGGCAGTGCATATAACGCCTGAGAG
AGCATCTAAGCGGAGACTGTTTAATGATAGTGGAGTTGTTGAAGATGAAGCTGAAAGTAA
TACTATTCAGGTAGATTCACTGTTGGTGCAAAAAGACGCGGGAAATCAAAATGGCGCTGA
GTGTGAACTTAATTCTATTTTGCGTAGTAATAATATCAGAGCTACTGTACTTTGCAAATT
TAAAGATAAATTTGGGGTTTCATTTAATGAGCTTACAAGAAGTTTTAAAAGTGATAAGAC
ATGTACACCAAATTGGGTAATAACAGCAATAGGTATTAGAGAAGATTTACGAGATGCTTG
TAAAGTTTTATTACAGCAACATGTTGAGTTTTTAGAAATGATTTGCAATGATTTTTCTGT
GCTACTATTGGTAGAATTTAAGGTAACTAAAAATAGAGAAACGGTTTTAAAGTTAATGTG
TAGTATGTTAAATGCTAAAGAAGAACAAATTTTGTGTGAACCGCCAAAATTGAAAAGTAC
AGCTGCAGCTTTATATTTCTATAAGAAGATTATAACTGATACATGTTTTAAATATGGCAC
TTTGCCTTCCTGGGTTAGTAGATTAACTATAGTAGAACATCAGTTAGCTTCAGCAGACAC
ATTTTCATTATCTGAAATGGTACAATGGGCTTATGACAATGATTTTACTGAAGAAGCATC
TGTTGCTTATAATTATGCATGTTATGCCACAGAAAATACAAATGCAGCAGCCTTTTTAGC
CAGTAATATGCAAGTTAAGTATGTTAAAGATTGTGTAGCAATGGTTAGAATGTATAAAAG
ACAAGAAATGAAATCAATGACAATGTCAGAGTGGATTTCCAAATGTTGTAAAGAAGAAAC
TATTGGTGAAGAGTGGAAAGAAATTGTACAGTTTTTAAAATATCAGGGAGTAAACTTTTT
AGAATTTTTAATAGCTTTAAAACAATTTTTTAAATGCACTCCTAAAAAGATGTGTATAGT
AATATATGGTCCACCAGATACTGGAAAGTCAATGTTTTGTTTTAAATTAGTACAATTTTT
GAAAGGTCAAGTTGTATCTTATATTAACAAATCGAGTCAGTTTTGGTTAATGCCATTACA
AGATGCTAAAATTGGTTTGTTAGATGATGCCACACATAATTGTTGGATTTATTTAGACAC
ATATTTGAGAAATGCATTTGATGGTAACACGTTTTGTTTAGATATTAAACATAAAAACCT
GCAACAGACAAAACTTCCACCTATGATAATAACTACTAATGTTAATGTAACAACTGATGA
ATCTCTATTTTATTTGAGAAGTAGACTTACGTGTTTCAATTTTCCGAATAAGTTACCAAT
GTCTGACAAAGATGAACCTTTGTTTACCATTTCTGACAAAAGCTGGACCTGTTTTTTTAG
AAAGTTTTGGAATCAATTAGAACTTCAAGAAGATGCAGCCAGAGACCCAGGAGAGCCTGA
GCACCCGTTTTGCTGCACAGCAAGAAATTCAGTTGACTTTGATTGAAAAAGAATCTTATG
ATCTTAAAGATCATTTAGCATACTGGAAAGCTGTAAGATTAGAAAATGTTATTGCCTATT
ATGCTAGAAAAGAACATATTACTAAATTAGGTCTGCAGCCATTACCAACATTAGCTGTAA
CTGAATATAAAGCAAAAGAAGCAATTAATATACAGCTGCTTATTCAAAGTTTATTGAAAT
CAGAGTTTGCTTTGGAAAGATGGACTCTTGCAGAAACTAGTGCAGAAACTATAAACAGTT
CCCCCAGAAATTGTTTCAAAAAAGTACCTTTCATTGTAAATGTATGGTTTGATAATGATG

AAAGAAATTCTTTTCCTTATACCTGTTGGGATTTTATATATTATCAAGATGACCAAAACA
AATGGCACAAGACTGAAGGGCTGGTTGATCATAATGGATGTTATTATGTAGATCTAAATG
GTGATTTTGTATACTTTACTTTATTTCAACCTGATGCTGTAAAATATGGAAAAACTGGAC
TATGGACAGTTAGATTTAAAAACAAAACTATTTCTGCCTCTGTTACTAGCTCCTCGAGAA
ATACAAATCCCTCTTCTGAAAGCAGGGTCGGGCTCTCGACCTCCAGCAGCTCGGAGAGCC
CTCGAAGGAGACCGAGCATCTCAGAAAACTCCAACACCGAGTCGCCCACCTCCTCGACAT
CCAGACTACGAGAGCGACGACGACGAGAACCGAGAGAATCTGGAACCACCGACACCACAC
CCAGAAGACGAGGAACAAAGAGGAAATTGGGGTCCGACTCTGCACCAACTCCTTCGGAAG
TGGGATCAAGATCTACAACGCTTGCAAGACACGGTTACTCACGACTTGGACGATTACAGG
AAGAAGCTCGGGATCCGCCATTAGTGTTATTTACAGGTCAACAAAATAATTTGAAATGTT
GGAGAAATCGCTGTACTACAAAATATGCTAGTTTATTTTTATGCTTCAGCTCTGTTTGGA
AGTGGCTTGGTCCTAATTCTGATGGGGGTGCTGCAAAAGTGTTAGTTGCATTTAAAAGTG
ATGCCCAAAGACAAGTGTTCTTAAACACAGTTCATATTCCTAAAGGAACTACTATAACTC
TGGGAAGACTTGACAGTTTATAATGTCCTTACGTAGAAGAAAAAGAGCAAGTCCTACTGA
TCTTTATAAAACATGCTTGCAAGGGGGGGACTGCATTCCTGATGTTAAAAATAAATTTGA
AAATTCTACTATTGCAGATTGGTTATTAAAAATATTTGGAAGTTTGGTATATTTTGGAAA
TTTGGGAATAGGATCTGGAAAAGGGTCTGGGGGATCATTTGGATATAGACCATTAGGATC
CGCAGGAAGTGGAAGACCAGCCACAGACTTACCAGTGACTAGACCTAATGTTGTGATAGA
ACCTATAGGTCCTCAAAGTATAGTACCCATTGATCCTGGAGCGTCATCTATAGTCCCTCT
TGTTGAGGGAGGGCCTGATATATCTTTTATTGCACCAGATGCAGGTCCAGGTATAGGAGG
TGAGGATATTGAACTATTCACCTTTAGAGATCCAGCAACTGATGTAGGTGGTGTTAGTGG
AGGTCCTACTACTATTTCTACAGAAGAAAGTGAGACAGCTATAATAGATGCTTTACCAAG
TGCCACAACTCCCAAACAGTTGTTTTATGATTCTTATACACAAACTATCTTGCAAACACA
GGTAAACCCATTTTTAAATAATGCTATTAGTGATACTAATGTGTTTGTAGATCCATTATT
TGCAGGAGAGACAATTGGGGACAACATATTTGAAGAGATACCCTTGCAGAATTTAAATTT
CAGTTTTCCGCGGGAAAGTACACCTGTTAAACCTGGGAGGGGTTTACGAACACCAGCTCA
AAGATCTTACAGTAGATTTATGGAACAGTACCCAATCCAAGCTCCGGAATTTCTTAGTCA
GCCTTCTCGATTGGTGCAGTTTGAGTTTGAAAATCCCGCCTTTGATCCAGATATTAGTAT
ACAGTTCCAGCGTGATGTAAATAGCCTAGAGGCTGCGCCAAATCCAGCTTTTGCTGACAT
TGCTTATTTAAGTAGGCCGCACATGTCTGCTACATCAGAAGGATTAGTCAGAGTCAGCAG
AATTGGATCTCGAGCAGTATTACAAACCAGGAGTGGATTAACTATAGGCCCTAAAGTACA
TTATTATATGGATTTGTCTGCAATATCAACAGAAGCTATAGAATTGCAAACCTTTGCAGA
TTCTGGACATGTTCACACAATAGTTGATGATTTCTTATCAGTTACTGCTTTAGATGATCC
AGCAAATATAGCTGATATAAATTATACAGAAGATGATTTATTAGATCCTTTACTTGAAAA
CTTTAATAATTCACATATTACAGTACAAGGGGTTGATGAGGAAGGAGAAACAGTTGCTCT
TCCAATTCCTTCAATTACTAATTCTTCCAAAACTTTTGTTACAGACATTGCAGAAAATGG
TCTGTTTGCAAATGATACAGATAGTCTTTTAACCCCAGCAAGCACTATTGTACCTGCTAT
TAATTGGTTCCCTTTATTTGATAGTTACTCAGACTTTGCTTTAGATCCCTTTTTTATTCC
ACGTAAGAAACGACGCTTAGATATCCTTTAATGTTTTTCAGATGGCTCTGTGGTCTGCAG
TCCCTGGCAAGGTTTACCTACCTCCTAGTGCACCAGTAGCTCGTGTGTTGCGCACTGATG
AATACGTGCAAGAAACGGATGTATATTTTTATACAAGCACTGAAAGACTTTTAATAGTTG
GTAATCCTTATTTTGATGTTGAAAACAGAGACACTATAACAGTACCTAAAGTTTCTGCTA
ATCAATACCGAGTATTTAGATGTAAACTTCCAGATCCTAACAAGTTTGCTCTAGTTGATA
AAAATTTATATAATTCAGATAAGGAACGTTTAGTTTGGAAACTTGTTGGTTTGGAAGTAG
GCAGAGGGGGGTCCTCTTGGCGTAGGATCTACAGGTCATCCTTTACTAAATAAGATAGGGG
ACACAGAAAATCCTAGCTTTTATTTAGGAGAACAAACAAAGGATGAGAGACAAAATGTTT
CCATGGATCCTAAGCAGAGTCAGATACTAATAGTTGGTTGTGCTCCAGCCACTGGAGAAT
ACTGGGATTTAGCTAAACCATGCAATGATTTGGAAAACGGGGCTGCACCACCAATTCAAC
TAGTTAATACTGTTATTCAGGATGGTGATATGGGAGATATAGGCTTTGGGGCTGCTAATT
TTCCTAAGTTGATGCAGGATCGTGCTGGCGTCCCTCTAGAGTTAATAGATTCTATTAGTA
TATGGCCAGATTTTTTAAAGATGACCAAAGATATTTATGGAGACTCTGTCTTCTTTTTTG
GTAAACGGGAACAATGTTATGCTCGTCATCTTTTTGCAAGAGCTGGCCAAATGGGGGAGC
CTATACCAACAGAAAATGGAGTATATTATATAACTCCTGATTCTGCCGATCAAAACAACA
GATCTTCTCATTTAGGATCTTCTGTGTATTTTACAACACCAAGTGGATCCTTGAATACTA
GCGATTCTCAGTTATTTAATAGGCCTTATTGGCTTAGACGAGCCCAGGGAACAAATAATG

GCATTTGTTGGGGAAATGAGCTGTTTATTACAGTTTTTGATAATACTCATAATGTGAATT
TTACTATTAGTGTTAAGAATGATAAAACTGCATTAACTGAAAACTACATAGATAATGGTT
ACAAATATAATAATGCAGATTTTAAACAATATCTTCGACATACAGAAGAATATGAAATAG
AGTTGGTTTTTCAGTTATGCAAAGTGAACTTGACTGCAGATGTTCTGGCACATTTACACG
TTATGAATCCCAGAATATTAGAGGAGTGGCAATTAGCTTTTGTTCCACCAGCTCCAACGG
GCATTGAAGATACTTACAGGTATATCAAATCCATGGCTACTAAATGCCCTACTGCTGAAC
CAGAAGAAGACACAGATCCATACAAAGCTTACAGTTTCTGGACTTTAGATATGACAGAGC
GCTTCTCGTCTGATTTAAGTCAGTTTTCCTTGGGTCGAAAATTTTTATATCAAACTGGTT
TGTTAAATGGTAAACGAGCTAGAACAGACTATACAGCTGCAGGATCTAGTACCAGATCTA
CAAAGCGTAGGAGAGTAAGATAGTAAAGATGTAGAATGTTTACATTATTAATACTGTGAA
TCAAACAAACTGTGATCAATAAACACTGTGAATATTTTACCGTGGTGTCATTTATACGCC
TCCATTGGGTGGGTTCTGACCTATAAACTCCTGGGTCAGTCATTTGGTCAGCAGACGCTG
AGGAGCAGCAGACGATTCAGACGTCCATTTGTGAGACGAAGACCGTTTTCGGTAAGTTTC
ATAGCCGCCAGGTATTGTTTGTACCGATACAGTTACACCTTAAAATGTACTGCCAACTTT
TACAAGTTCAGACAGAGATCAACAAGATATCTTAAAACAGTACCTAAAGCGGTACTGTAC
CGTTTGTGGTATGTTGATGC
>gi|9628486|ref|NC_001683.1|_Human_papillomavirus_type_24,_complete_genome
GCGCCAAGTTTCTTGGCACTCGCACAGCACCGTTAACGGTAAGTGTTTTGTATACTGTAC
CGGGCGCGATACCAAATATGCCACCTGCGCAATTGTTGTTAGCAACTACTTTCATTACCT
AACAAGTATTTTCATGCACCGTTTCCGTATCACTGTGAATGTATTTATTGATTAGACATA
TAAAAAGGAAGACATCTTGATGGCTCAACCAGGTAAACCTCAGTCAGTGTTAGAACTTAG
TAGATTATTAAATATACCATTAGACGATTGTGTTGTACCATGTAACTTTTGCAAAAGATT
TCTAAGTTATACAGAGTTAACTGACTTTGACACCAAATGCTTAAGTTTGATTTGGAAAGA
CGATTTTGTGTTTGCATGTTGTAGATATTGTTGTGTTGCTACAGCAGCATTTGAATTTGA
AAATTATTTTGTAGAGAGTGTGATAGGTTGGGAAATAGAACAAAAAGAAAATACACCTCT
TTCGGACATTATTGTAAGGTGTCACCATTGCTTAAAATTACTTAATCAAATTGAAAAGCT
TGATATCTGTGGAAGATCTGAATTATTTCATAAAGTGAGGAGGGGCTGGAAAGGACTCTG
TAGGCAGTGTAAGCAGATATAATGATTGGAAAAGAGGTCACTCTACAAGACATTGTCTTA
GAGTTGACTGAGCCACAGACTGTTGATTTGCACTGTGAAGAGGAGTTGCCAGAGCAGGAT
ACGGAGGTGGAGCCTGAAAGAAGAGCTTACAAAATAATACTTTGTTGCGGCGGCGGTTGT
GGAACCCGGCTTCGATTATTTGTAGCAGCAACACAGTTTGGAATACGTGGCCTGCAAGAC
TTACTGCTAGAAGAGGTGGTCATTTTGTGTCCCGACTGCCGTAACAGCGATCTGCAGCAT
GGCGGACAATAAAGGTAGTGAAACAGACGGGTTGCATGAATGGTGCTTATTAGAAGCTGA
CTGTAGTGATATTGAAAATGATTTGGACACATTGCTTGAGCAAAATTCAGATTCTGATGT
ATCAGATCTTATAAGTAATGATGGGGACTTGGAACAGGGAAACTCCCGAGAACTGTTTCA
ACAGCAGGAGTTAGAGGAGAGCAATGCTTTGTTGCAAAGTCTAAAACGAAAGTATATTAG
TCCTAAAGCTGTATTACAGCTGAGTCCACAACTTGAATCCATTTCGCTGTCATCAGACCA
TAAAACTAAGAGGAAATTGTTTGCTGAGCAGGACAGTGGAGTAGAATTGACCCTTACAAA
TGAAACTGAAGATGTTACTACATTGGTGCAGCAGGAGGAAGAGGTACCAGCTTTAGAGAC
ATCTTCCACCAGTAACTTAAGGAAGGAAGATAATGCACATTATAAAGAACTTATGCGATG
CAGTAACTTAAAAGCTACCTTACTATCAAAATTTAAAAATGCTTTTGGTGTAAGCTTTGT
TGAACTGACTCGCCAGTTCAGAAGTAATAAGACTTGTTGTAATGATTGGGTGGTAGCAAT
ATATGGTGTAAATTATGATTTATTTGAAAGTTCTAAGCAATTACTACAACAGCATTGTGA
TTATATTTGGGTTACAGAAATGTCTGCAATGTTTTTATATCTGTTGTGTTTCAAGGCTGG
CAAAAATAGACAAACTGTTATAAGGTTATTAGTGTCTATGTTATATGTGGCAGAGGAGCA
AATTTTATCAGAACCTCCAAAATTGCGAAGTACAGTGTCAGCTTTATTTTGGTATAAAGG
TAGCTCAAATGCTGCTACCTTTACACATGGGTCTTATCCTAAATGGATTATAGAACAAAC
ACTGATAGGACATCAAACAGGAGAAGCTGCAACGTTTGACATGTCCACAATGGTACAATG
GGCTTTTGATAATGATCTCACAGAGGAAGCTGACATAGCTTTTCAATATGCAAAGCTGGC
ACCTGATGATGTAAATGCAACTGCTTGGTTGGCTCACAATAATCAGGCGCGTTTTGTAAG
AGAATGTGCTAATATGGTTAGATATTATAAAAAAGGTCAGATGCGAGAAATGTCTATGTC
GGCATGGATCCACTTTAAATTAGAGCAAGTAGAAGGGGAAGGGCAGTGGTCAACTATTGT
AAAATTTATTAGGTATCAGGGTATAAATTTTATTAGTTTTTTAAGTGCTTTAAAAGATTT
TTTACATGGTAAACCTAAAAAGAATTGTTTATTAATATATGGCCCTCCAAATACTGGAAA
ATCAGCATTTACTATGTCATTAATTAAAGTGTTGCATGGCAGAGTAATATCATTTGTAAA

TTCTAAAAGTCACTTTTGGTTGCAACCAATGTCTGAAGCCAAAATAGCTTTACTTGATGA
TGCTACTGATCCATGTTGGATATATATGGATACATATTTAAGAAATGGTCTTGATGGACA
CTTAGTTTCATTAGATTGTAAGCATAAAGCACCTATACAAATACGCTTTCCTCCATTACT
TATAACATCTAATATTAATGCAATGGCAGAACCTAACTATAGATATTTACACAGTAGGTT
AGTAGCGTTTGAATTTCCTAATCCCTTTCCTATGAAAGATGACGATACCCCTGAATTTGA
ACTTACTGACCAAAGCTGGAAATCTTTTTTTAAAAGGCTTTGGAGACAATTAGACCTCAG
TGACCAAGAAGACGAGGGAGAGGATGGAGAACCTGAAAAAGCGTTTCGATGTGTTGCAAG
ATCTACTAATGAACATTTATGAACAAGGCAGTGATACACTAGAATCACAAATTGAACATT
GGCAGGCCTTGCGAAGAGAGGCAGTGTTACTTTATTATGCTAGACAAAATGGGGTGCTGC
GGCTGGGTTACTTACCAGTTCCACCACTAGCTACCTCAGAAGCCAAAGCTAAACAGGCTA
TTAGTATGGTGTTGCAGCTGCAATCTTTACAACAATCTCCTTATGGCACAGAAAAGTGGA
CACTGGTGGACACAAGCATAGAAACCTTTAAAAATACTCCAGAGAACCATTTTAAAAAAG
GACCTATAAATGTGGAGGTTATATATGATGGTGATCCGGACAATGCCAATTTGTATACTA
TGTGGAAATATGTATATTATATGGATGATAATGACCAGTGGCAAAAAACTGAAAGTGGCG
CCAATCATACAGGCATATATTATTTAATAGGGGAGTTTAAACATTATTATGTGCTGTTTG
CTGATGATGCCAATAGATACAGTAAATCTGGCCAATGGGAGGTCAGGATTAATAAGGAAA
CTGTGTTTGCCCCTGTCACCAGCTCCACACCACCCGACTCCCCAGGAGGGTCCCGAGAAC
TACCCGGATCCACCGCTAACTCCAAGGCCTCAAGCCCAACCCAACAGCCACAACAAGCCT
GTAGTGACGAAACAACCAAGCGGAAGAGGTACGGGCGAAGGGAGTCAAGCCCCACTGACT
CCAGATGCAGACGACGATCCTCGTCCCGGCAAAAGAAGCAAGGGCGACGAGCACGGTCCC
GCACCCGGTCGCGCTGCAGCTCCACTCAAACTCGATCTAGATCCACCTCAAGGAGGTCCA
GATCAACCTCCAGGGGCAACAGGAGGTGTAGGGGAGACACCCCCAGAGGGCAACGAGGAG
TCTCAACCTCCTCCAGGGGAAGGGGAAGGGGCAGTAGAAGGTCATCCTCCTCCTCCTCCC
CCACCCCCAGAACCAAAGCCTCACAACGGGGATGCGACACACGGTCTGTTAGGGACAGTG
GCATCTCTCCTGGGGACGTGGGAAGAAAGCTTCAGACAGTTAGTGGAAGAAATTCAGGAA
GACTTGGACGATTATTGGAGGAGGCTCTCGATCCCCCAGTAATTTTACTTCGTGGGGGTG
CTAACACATTAAAATGTTTTCGCAACAGGGCAAAGCTTAGATATAGAGGACATTATAAAG
CATTTAGCACATCTTGGTCATGGGTAGCTGCAGATGGCACAGAGCGTCTAGGCAGGTCCA
GATTGCTCGTCAGTTTTACCAGTTTTAAGCAGCGAAGTGGGTTTCTCGACCTAGTAAGAT
TTCCTAAGGGTGTAGATTGGTCACTGGGAAGTTTTGATAAACTGTAAGTACTAACATAGC
TTTTGCTACTAACACACATATAACCTATTTTTACTTTTTGTATGCTTTGCAATGGTGCGT
GCTAAAAGAACAAAACGAGATTCTGCTACTAATATTTACAGAACATGCAAGCAGGCGGGC
ACATGTCCACCTGATGTTATTAATAAAGTGGAACAGTCAACCATTGCTGATAATATACTT
AAATATGGAAGTGCTGGTGTCTTTTTTGGTGGCCTGGGTATTAGTACAGGCCGAGGGACT
GGAGGCACCACAGGATATGTGCCATTGGGTGAAGGTACTGGAGTTCGTGTGGGCAGTACA
CCCACAGTCGTTCGGCCTGCCCTTGTGCCTGAAGTAATAGGTCCTGCTGACTTATTACCT
GTTGATACAATAGCCCCTGTTGACCCAGCATCGTCATCTATAGTTCCTCTTACTGAATCA
TCAGGTGTAGACCTTTTACCTGGTGAAATAGAAACAATAGCAGAAGTACATCCTATACCT
GATGTGCCCACATTTGATACTCCAGTAGTGACAACAAGCAAAGGCTCTAGTGCCATTTTA
GAAGTAGCTCCTGAGCCTACTCCACCGACACGTGTTCGTGTCAGCAGAACACAATACCAT
AATCCAGCATTTCATATTATTACAGAATCTACACCAAGTCAAGGTGAGAGTTCATTGTCA
GACGAAATAATTGTCGCCTCTGGTGCAGGTGGACAATCAGTAGGCGTTTCTGAAAATATA
GAACTGCAGGATTTATCAAATAGATATTCTTTTGAAATAGAAACACCAACACCACCAAGG
CGTAGCAGTACCCCATTACAAAGAGCTACACAAGCATTTAGACAAAGATCACTTACAAAT
AGGAGACTGTTACAACAAGTGCCTGTTGAAGACCCTTTGTTTTAACTCAACCATCTAAA
TTGGTTAGATTTGCATTTGAGAATCCAGCTTTTGAAGAGGAAGTAACACAGGTATTTGAA
CAAGACCTTGCAGGTTTTGTAGAGCCTCCTAACAGAGATTTTTAGATATTGCAGAACTG
GGAAGGCCTAGATTTTCTGAAACACGCGAGGGTTATGTTAGGTTAAGCAGATTGGGTCGT
AGAGCAACTATTAGAACAAGGGCAGGAACACAAATAGGAGCACAAGTACATTTTTATAAA
GATCTAAGTTCTATTAATACTGAAGCTCCTATTGAGTTGGATCTTTTAGGGCAGCATTCT
GGGGATGCAACTATAGTTCATGGGACTGTAGAAAGCACATTTATAGATACTAATATAGAG
GAAAATCCTTTAGCTGAACAAATGGAGTTGGAAATTGATACTTATCCTGAAGCTCATTCA
TTTGATGCTTTGTTAGATGAAGCAACAGACGATTTTAGTGGTTCACAGTTAGTTATAGGC
AATAGAAGATCCACTACATCATATACTGTTCCTAGATTTGAATCCCCAAGAAATTCTTCT
TATTATGTACAGGATTTGCAGGGATATTATGTAGCCTATCCTGAATCTCGCGATAAAATA

GAACTTATTTATCCCTCACCCACATTACCTGCAGTTGTCATACATACAGAAGATAGTAGT
GGGGACTTTTATTTACATCCTAGTTTATTGCAAAGGCGCAGACGCAAACGAAAATATTTG
TGATTTTTACAGATGTCGGTGTGGTTGCCAGCCAGTGGTAAGGTATATTTGCCACCATCA
ACACCTGTTGCGAAAGTTCAAAGCACGGATGAATACATACAGAGAACAAACATCTTCTAT
CATGCTTATAGTGACCGCCTATTAACTGTAGGACACCCATATTTCAATGTTTACAACAAT
GATGGCACAGTATTAGAGGTCCCTAAGGTTTCAGGAAATCAACACAGAGTTTTTAGGCTT
AAATTACCAGACCCTAATAGATTTGCTTTAGCAGATATGTCCGTATATAATCCAGAGAAG
GAAAGATTGGTATGGGGTTGCAGAGGAGTAGAAATAGGTAGAGGACAACCATTAGGTGTT
GGGACAAGTGGACATCCATTATTTAACAAAGTGAATGACACAGAAAACCCTGTATCATAT
AGGACACAAGCATCGTCCACAGATGATAGACAAAATACCTCATTTGATCCTAAACAAATT
CAAATGTTTATTATAGGTTGTGCACCCTGCATAGGAGAACATTGGGAAGTAGCTGAGAGG
TGTGCTGGTGATAATAATGATGCTGGTAGATGTCCACCTATTAAGTTAGTAAATTCAGTA
ATTCAAGATGGTGATATGGCAGATATTGGTTATGGGAATTTAAATTTTAGAACACTACAA
CAAAGTAGATCAGACGTAAGTTTGGATATTGTAAATGAAACCTGTAAATATCCAGACTTT
TTAAAAATGCAAAATGATGTTTATGGAGATTCTTGTTTTTTCTTTGCTCGCCGTGAGCAA
TGTTATGCAAGACATTTTTTTGTACGAGGAGGTAAACCAGGGGATGACATACCTGGTGAG
CAAATTGATGCAGGGACCTACAAAAATGACTTTTACATTCCAGCAGCCACAGGTCAGACA
CAAAAAAATATTGGCAACTCAATGTATTTCCCAACAGTCAGTGGCTCATTGGTATCTAGT
GATGCTCAATTATTTAATAGACCATTTTGGTTACAGCGTGCACAAGGTCATAATAATGGA
ATTTGTTGGGCTAATCAATTGTTCATTACGGTAGTAGATAACACCAGGAACACCAATTTT
AGTATAAGTGTATACACTGAAAATGGCAAAGTAACAGATATTAACGAGTATGATGCTAAT
AAATTTAGGGAATATCAGAGACATGTAGAAGAGTATGAAATTTCACTTATACTGCAGCTA
TGTAAAATCCCTTTAAAAGCAGATGTGTTAGCACAGATCAATGCTATGAATCCATCTCTA
TTGGAAGAGTGGCAGTTAGGATTTGTGCCTGCACCAGACAATCCTCTTCAAAGTACCTAC
AGATATATAGAAAGTTTGGCAACACCTTGTCCAGATAAAGTAGCTCCAAAAGAAAGAGAA
GATCCTTATGCCCCTTATACGTTTTGGAATGTAGATTTATCTGAACGCTTATCTTTGGAA
CTGGATCAATATTCCTTAGGACGAAAGTTCCTGTTTCAAGCGGGTCTTGTACAAAAAACA
TCTAAAAAAACATCTAATGTATCCAAGGGGACCAAACGAAAACGCACGTAAAATAAAGGC
TGTTTACCGTTTTCGGTACAATATATGTGTATTCCAAGAATGCTTGTTATTCATGTGCGT
GACTAATTTGAACTTTGGCTTGAATGTCAACCGCACCCGGTACAAGTAGATAAAATCTTG
CTACCACAGCAGACACAAAGGAATCACTGTCGGTCTCAACACGCTCGGATTTGGCGCATT
CAACCGTTTTTG
>gi|9626063|ref|NC_001356.1|_Human_papillomavirus_-_1,_complete_genome
GTTAACTACCATCATTCATTATTCTAGTTACAACAAGAACCTAGGAGTTATATGCCAGAA
GTAAGCCTATAAAATACACAGGTAAGACTCTGCACAGGACCAGATGGCGACACCAATCCG
GACCGTCAGACAGCTTTCCGAAAGCCTCTGTATCCCATATATTGATGTTTTATTGCCTTG
TAATTTTTGTAATTATTTTTTGTCTAATGCTGAGAAGCTGCTTTTTGATCATTTTGATTT
GCATCTTGTCTGGAGAGACAATTTGGTGTTTGGATGCTGTCAAGGGTGTGCTAGAACTGT
TAGCCTATTGGAGTTTGTTTTATATTATCAGGAGTCTTATGAGGTACCGGAAATAGAAGA
AATTTTGGACAGACCTTTATTGCAAATTGAACTCCGTTGTGTTACATGCATAAAAAAACT
GAGTGTTGCTGAAAAATTGGAGGTTGTGTCAAACGGAGAAAGAGTGCATAGAGTTAGAAA
CAGACTTAAAGCAAAGTGTAGTTTGTGTCGCTTGTATGCTATATAACAATGGTGGGCGAA
ATGCCAGCACTAAAGGACCTGGTTCTTCAACTTGAACCAAGCGTCCTAGATTTAGATCTT
TATTGTTACGAGGAGGTGCCTCCTGATGACATAGAGGAGGAGTTAGTGTCGCCTCAGCAA
CCTTATGCTGTCGTTGCTTCCTGTGCCTATTGCGAGAAACTGGTTCGATTGACCGTCCTC
GCGGATCACAGCGCCATTAGACAGCTGGAGGAACTCCTTCTGCGATCTTTGAACATCGTG
TGCCCACTGTGCACCCTACAGCGACAGTAAAATGGCAGATAATAAAGGTACTGAAAACGA
TTGGTTTTTGGTGGAGGCGACAGATTGTGAGGAAACGTTAGAGGAAACCTCACTTGGTGA
CCTAGATAATGTTTCTTGTGTTAGCGACTTATCTGATTTATTAGACGAGGCGCCGCAAAG
CCAGGGGAATTCCCTGGAATTGTTCCACAAGCAAGAATCGCTGGAAAGCGAACAGGAACT
TAATGCTTTAAAACGAAAGTTACTTTACAGTCCTCAGGCGAGAAGCGCGGACGAAACAGA
CATTGCTAGCATTAGTCCTAGATTAGAAACTATTTCTATTACAAAGCAAGACAAAAAAAG
GTATCGAAGGCAACTGTTTTCTCAGGATGATAGTGGTTTAGAGCTATCGCTGCTTCAGGA
TGAAACTGAAAATATTGATGAATCGACACAGGTAGATCAACAGCAGAAAGAACATACTGG
GGAAGTTGGGGCCGCTGGGGTGAACATTTTGAAAGCTAGTAATATCCGCGCCGCATTATT

AAGCAGATTTAAAGATACGGCTGGCGTCAGTTTTACAGACCTGACGCGGTCGTACAAGAG
CAACAAAACCTGTTGTGGAGATTGGGTTTTGGCAGTTTGGGGTGTCCGTGAAAATTTAAT
TGACAGTGTAAAAGAATTATTGCAAACCCATTGTGTGTATATTCAATTGGAACATGCAGT
AACTGAAAAAAAATAGATTTTTATTTTTATTGGTACGATTTAAAGCCCAGAAAAGTAGAGA
GACTGTGATAAAACTTATAACCACAATTCTTCCAGTTGATGCTAGCTATATTTTGTCTGA
GCCTCCAAAATCAAGAAGTGTGGCTGCTGCATTATTTTGGTATAAAAGATCTATGTCTTC
AACTGTTTTTACATGGGGTACAACTTTGGAGTGGATTGCACAGCAAACCCTTATTAATCA
TCAGTTAGATTCCGAAAGTCCCTTTGAGCTTTGTAAAATGGTTCAGTGGGCCTATGATAA
TGGACATACAGAAGAGTGTAAAATTGCATATTATTATGCTGTTTTAGCAGATGAGGATGA
AAATGCAAGGGCATTTCTAAGCTCTAATTCACAGGCAAAATATGTGAAAGACTGTGCACA
AATGGTAAGACACTATTTACGTGCTGAGATGGCACAAATGTCTATGTCAGAGTGGATTTT
TAGAAAACTAGATAATGTAGAAGGTTCTGGTAATTGGAAAGAAATTGTAAGATTTTTAAG
ATTTCAAGAAGTTGAATTTATAAGCTTTATGATTGCATTTAAAGATTTGTTATGTGGTAA
GCCAAAGAAAAACTGTTTGTTAATATTTGGACCTCCAAATACAGGAAAATCAATGTTTTG
TACAAGTTTATTAAAGTTGTTAGGAGGGAAAGTGATTTCATACTGTAACAGTAAAAGTCA
GTTTTGGTTGCAGCCTCTGGCTGATGCTAAGATAGGGCTATTAGATGATGCAACAAAGCC
ATGTTGGGATTATATGGACATTTATATGAGAAATGCATTGGATGGTAACACTATTTGTAT
TGATTTAAAACATAGAGCTCCTCAACAAATTAAATGCCCACCTTTACTTATTACTAGTAA
TATTGATGTTAAATCAGATACCTGTTGGATGTATTTGCATAGTAGAATATCAGCTTTTAA
ATTTGCTCATGAGTTTCCATTTAAAGACAATGGTGATCCAGGATTTTCCTTAACAGACGA
AAATTGGAAATCTTTCTTTGAAAGGTTTTGGCAACAGTTAGAATTAAGTGACCAAGAAGA
CGAGGGAAACGATGGAAAACCTCAGCAGTCGCTTAGACTTACTGCAAGAGCAGCTAATGA
ACCTATATGAACAGGACAGTAAATTGATAGAAGATCAAATTAAGCAGTGGAATCTAATTA
GACAAGAACAAGTTCTTTTCCATTTCGCCAGAAAAAATGGGGTAATGAGAATTGGATTGC
AGGCAGTTCCATCTTTAGCGTCCTCACAGGAGAAGGCAAAGACAGCTATTGAAATGGTGT
TACATTTAGAGTCTTTAAAGGACTCACCTTATGGCACAGAGGATTGGTCACTTCAAGACA
CTAGCAGAGAGCTGTTTTTGGCACCCCCAGCTGGCACCTTCAAGAAGAGTGGCAGCACAC
TTGAGGTTACCTATGACAATAACCCTGATAATCAGACAAGGCACACAATTTGGAATCATG
TGTATTATCAAAATGGGGACGATGTATGGAGAAAAGTATCCAGTGGTGTTGATGCTGTAG
GAGTGTACTATTTAGAACACGATGGCTATAAAAATTATTATGTGTTATTTGCTGAGGAGG
CCTCTAAGTACAGCACAACAGGACAATATGCTGTAAATTACAGGGGTAAAAGGTTTACAA
ATGTTATGTCTTCCACTAGCTCCCCAAGGGCTGCTGGGGCTCCTGCAGTACACTCCGACT
ACCCAACCCTATCCGAGAGTGACACCGCCCAGCAATCGACGTCCATCGACTACACCGAAC
TCCCAGGACAGGGGGAGACCTCGCAGGTCCGACAAAGACAGCAGAAAACACCTGTACGCA
GACGGCCTTACGGACGGCGAAGATCCAGAAGTCCCAGAGGTGGAGGACGAAGAGAAGGAG
AATCAACGCCCTCTAGGACACCCGGATCTGTCCCTTCTGCGCGAGACGTTGGAAGTATAC
ACACAACGCCTCAAAAGGGACATTCTTCAAGACTTAGACGACTTCTGCAGGAAGCTTGGG
ATCCACCCGTGGTCTGTGTAAAAGGGGGTGCCAATCAGCTTAAGTGTCTCAGGTACAGAC
TTAAAGCATCTACTCAAGTTGACTTTGACAGCATAAGCACCACATGGCATTGGACAGATA
GAAAAAACACCGAGAGGATAGGTAGTGCTAGAATGTTAGTAAAGTTTATTGATGAGGCTC
AACGAGAGAAGTTTCTTGAGAGAGTTGCTTTGCCCAGATCAGTGTCTGTGTTTTTGGGAC
AGTTTAATGGGTCTTAAAATTAATGGAAGTTGATTTTGCTTGGACGTGTGTACATAGTCC
CTGTATATATTCCCCTCCTACCCCCACATACCTTGAAGCTTGCAACATTGTAACAAATGT
ATCGCCTACGTAGAAAACGCGCTGCCCCCAAAGATATATACCCCTCATGCAAAATATCAA
ACACCTGCCCACCTGACATTCAAAATAAAATTGAGCATACAACAATTGCTGATAAAATAT
TGCAATATGGCAGTCTGGGAGTTTTTTTGGGAGGTTTGGGCATTGGAACAGCCAGAGGCT
CTGGAGGAAGAATTGGTTATACTCCCCTCGGTGAGGGTGGTGGGGTTAGAGTTGCTACTC
GTCCAACTCCAGTAAGGCCTACAATACCTGTGGAAACAGTAGGCCCCAGTGAAATTTTCC
CCATAGATGTTGTAGATCCTACAGGCCCTGCTGTTATTCCCCTACAAGATTTAGGTAGAG
ACTTCCCAATACCAACTGTGCAGGTTATTGCAGAAATTCACCCTATTTCTGACATACCAA
ACATTGTTGCATCTTCAACAAATGAAGGAGAATCTGCCATATTAGATGTGTTACGAGGGA
ATGCAACCATACGCACTGTTTCAAGAACACAATACAATAACCCCTCTTTCACTGTTGCAT
CTACATCTAATATAAGTGCTGGAGAAGCATCAACATCAGATATTGTATTTGTTAGCAATG
GTTCAGGTGACAGGGTGGTGGGCGAGGATATCCCCTTGGTAGAATTAAACTTAGGCCTTG
AAACAGACACATCTTCTGTTGTACAAGAAACAGCATTTTCCAGCAGCACACCAATTGCTG

AAAGACCCTCTTTTAGGCCCTCAAGATTCTATAATAGGCGTCTATATGAACAGGTGCAAG
TACAAGACCCTAGGTTCGTTGAGCAGCCACAGTCAATGGTCACTTTTGATAATCCAGCAT
TTGAGCCAGAGCTTGATGAGGTGTCTATTATCTTCCAAAGAGACTTAGATGCTCTTGCTC
AGACACCAGTGCCTGAATTTAGAGATGTAGTTTATCTGAGCAAGCCCACATTTTCGCGGG
AACCAGGGGGACGGTTAAGGGTTAGCCGCCTTGGCAAAAGTTCAACTATTCGTACACGCC
TGGGCACAGCAATTGGCGCCAGAACCCACTTTTTCTATGATTTAAGTTCTATTGCTCCAG
AAGACTCAATTGAATTATTGCCTTTAGGTGAGCATAGTCAAACAACAGTCATTAGTTCCA
ACTTAGGTGACACAGCATTTATACAAGGTGAGACAGCAGAGGATGACTTAGAAGTTATCT
CTTTAGAAACACCACAATTATATTCAGAAGAAGAGCTTTTAGACACAAACGAAAGTGTGG
GCGAAAATTTGCAACTTACTATTACTAACTCAGAGGGTGAGGTTTCTATACTAGATTTAA
CACAAAGCAGAGTCAGGCCACCTTTTGGCACTGAAGATACTAGCTTGCATGTATATTACC
CAAATTCTTCTAAAGGGACTCCAATAATTAATCCTGAAGAATCATTTACACCTTTGGTTA
TTATAGCTCTTAACAACTCAACAGGGGATTTTGAGTTACATCCTAGTCTTAGAAAGCGTC
GTAAAAGAGCTTATGTATAATGTTTTTCAGATGGCTGTCTGGTTACCAGCGCAGAATAAG
TTCTATCTTCCTCCCCAGCCCATCACTAGAATCCTGTCCACTGATGAATATGTAACCAGA
ACCAATCTCTTCTACCATGCAACATCTGAACGTCTACTGCTGGTCGGACATCCTTTGTTT
GAGATCTCCAGTAATCAAACTGTAACTATACCAAAAGTGTCACCAAATGCATTTAGAGTT
TTTAGGGTGCGTTTTGCTGATCCAAATAGATTTGCATTTGGGGATAAGGCAATTTTTAAT
CCAGAAACAGAAAGATTAGTTTGGGGCCTAAGAGGGATAGAGATAGGTAGAGGCCAGCCT
TTAGGTATAGGAATAACGGGCCACCCTCTTTTAAATAAGTTAGATGATGCAGAAAATCCA
ACAAATTATATTAATACTCATGCAAATGGAGATTCTAGACAAAATACTGCTTTTGATGCA
AAACAGACACAAATGTTCCTCGTCGGCTGTACTCCTGCTTCAGGTGAACACTGGACAAGT
AGTCGTTGCCCAGGGGAACAAGTGAAACTTGGGGACTGCCCCAGGGTGCAAATGATAGAG
TCTGTCATAGAAGATGGTGACATGATGGATATTGGTTTTGGGGCTATGGATTTTGCTGCT
TTACAGCAAGACAAGTCTGATGTCCCTTTAGATGTTGTTCAAGCAACATGCAAATATCCT
GATTATATCAGAATGAACCATGAAGCCTATGGCAACTCTATGTTTTTTTTTGCACGTCGC
GAGCAAATGTATACCAGGCACTTTTTTACTCGCGGGGGTTCGGTGGGTGATAAGGAGGCA
GTCCCACAAAGCCTGTATTTAACAGCAGATGCTGAACCAAGAACAACTTTAGCAACAACA
AATTATGTAGGCACACCAAGTGGCTCTATGGTTTCATCTGATGTCCAATTGTTTAATAGA
TCTTACTGGCTTCAGCGATGTCAAGGCCAGAATAATGGCATTTGCTGGAGAAACCAGTTA
TTTATTACAGTTGGAGATAATACCAGAGGAACAAGTTTATCTATCAGTATGAAAAACAAT
GCAAGTACTACATATTCCAATGCTAATTTTAATGATTTTCTAAGACATACTGAAGAATTT
GATCTTTCTTTTATAGTTCAGCTTTGTAAAGTAAAGTTAACTCCCGAAAATCTAGCCTAC
ATTCATACAATGGACCCTAATATTTTAGAGGATTGGCAACTATCTGTATCTCAACCACCT
ACCAATCCTCTAGAAGATCAATATAGGTTTTTAGGGTCTTCCTTGGCAGCAAAATGTCCA
GAACAGGCGCCTCCTGAGCCCCAGACTGATCCTTATAGTCAATATAAATTCTGGGAAGTC
GATCTCACAGAAAGGATGTCCGAACAATTAGACCAATTTCCACTAGGAAGGAAATTTCTA
TATCAAAGTGGCATGACACAACGTACTGCTACTAGTTCCACCACAAAGCGCAAAACAGTG
CGTGTATCTACGTCAGCCAAGCGCAGGCGTAAGGCTTAGTATATATTATATATAACTATA
TTTATTAGTAGATTATTTATTATATATTTTTATATTTTTATACTTTTTATACTTGTTTAG
TTCTAAATAGACATGTAAGATTTACATTAGTATAAGTAGGCATGTATTTACATAAAATAG
TCTTGGAAACCTTTTATTAGTGAACCATCATTTACAATAGTGACATCATAGTTCATCTGC
AATTGCTATTCCATCGTTCTTCACATATTCTACAGTAGTGTTCTCTAGATTGTATTGCTA
TTTTCCTGTTAGGCAAACAACAACATCTGTACATGGACCAAACAACCCACTTTCATTTTA
TTGTGCTGCATATATTCCAGATTGTTGAGGATTTATTTGTTTAGACTCCGGTGCATTATA
CACAAGTGTGCATTTTTTGTGTTCTCTGATTGATTGTGTGTTATTTTCCTGCAATATGCA
ATAAAAGTGAGCTGTCCTTTCTTTTTGTTAATCCCTCCCTACTCCAATAAAAAATCCCTA
CCCCTAAAATCTGTTTGTGCTGGTTTTATTAATAATTGCGCTCTTTTATATAATAAGTAC
TATTAACACCGCACCCGTTGTGGCTAATCCCTTATGGTATTTAAAAGACTACACCTACAG
GATGTATTGTCTTCATTGTTTATGGTTTACCGCGCTCCAAAGACGGTTTGCCCAAAGACG
GTTTGCCAACCGCGGTTAGGACTTGTTTCAATTTGCTGCCAAACTTATCTGGTCGTGCTC
CAACGGGTTTCCTGCCAAGCACCTAAAACGGTAGGTGTGTACTCTTTTCAAGAATTAACA
AAGGAGATTTCTCCCGCCAAATTAGTTTCGAGCGACCGAATTCGGTCGTAAAAATCTAAA
GTGATGATTGTTGTT
>gi|9626032|ref|NC_001352.1|_Human_papillomavirus_-_2,_complete_genome

ATAATGTATAACTATAATCCTTTATTTAAAAATAGGGTGTGACCGAAAACGGTCAGACCG
AATTCGGTTGTATATAAACAGAAGCAGGATGCACACAAGGGCAGGGATGTCTGAGGAGAA
TCCATGCCCTAGGAACATCTTTTTGCTTTGCAAAGAGTATGGTTTGGAGCTAGAGGATTT
GCGATTGCTCTGTGTATGGTGCAAACGGCCGTTATCAGAGGCTGACATATGGGCATTTGC
AATAAAAGAACTGTTTGTAGTGTGGAGAAAGGGCTTCCCATTTGGAGCCTGCGGAAAATG
CCTGATTGCAGCAGGAAAACTTAGACAATACAGACATTGGCATTACTCATGCTACGGAGA
CACAGTGGAGACTGAGACAGGAATACCCATACCTCAGCTGTTTATGAGATGCTATATTTG
CCATAAGCCCCTGAGCTGGGAGGAGAAGGAGGCATTACTAGTTGGAAACAAGCGTTTCCA
CAACATATCAGGCCGGTGGACGGGACATTGCATGAACTGCGGGTCATCATGCACGGCAAC
CGACCCAGCCTCAAGGACATTACACTAATATTGGATGAAATACCCGAAATTGTTGACCTA
CATTGCGACGAGCAATTTGACAGCTCAGAAGAAGAGAATAACCATCAACTGACAGAACCA
GATGTGCAGGCCTACGGGGTGGTAACTACCTGCTGTAAGTGTGGCAGAACCGTCCGGCTG
GTGGTTGAGTGCGGACAAGCAGACCTAAGAGAGCTGGAACAGCTGTTCTTGAAGACGCTG
ACTCTAGTGTGCCCTCACTGCGCCTAGCGTTATGGAGGATTCCGAAGGTACCGACGGGAC
CGAGGAGGACGGGTGCCGGGCAGGGGGGTGGTTTCATGTGGAGGCCATTATAACACACGG
CCAGAGGCAGGTATCCAGTGACGAGGACGAGGACGAAACAGAGACAGGGGAGGATTTAGA
CTTTATAGACAATAGGGTTCCCGGAGATGGGCAGGAAATTCCCTTGCAGCTATATGCACA
ACAAACCGCTCAGGATGACGAAGCAACAGTGCAGGCCCTAAAACGAAAGTTTGTGGCCAG
TCCTTTGTCTGCATGCTCATGCATAGAGAATGATTTAAGTCCCAGATTAGATGCAATCTC
CCTAAACAGAAAGTCAGAAAAGGCGAAGAGGCGCTTATTCGAGACAGAACCACCAGACAG
TGGGTATGGCAATACGCAGATGGTTGTTGGAACGCCAGAGGAGGTAACGGGGGATGAGGA
AAGCCAAGGGGGGCGGCCGGTGGAGGATCAGGAGGAGGAGCGTCAAGGGGGAGACGGAGA
GGCAGATCTAACTGTACACACTCCACAGTCAGGAACAGATGCGGCGGGTAGCGTGCTGAC
CTTACTAAGAAGTAGCAATCTGAAGGCGACGTTGCTGAGTAAGTTTAAGGACCTGTTTGG
GGTGGGATTCTATGAACTGGTCAGACAGTTCAAAAGCAGCAAGACAGCATGTGCAGACTG
GGTCGTCTGCGCCTATGGTGTGTATTATGCTGTAGCGGAGGGTCTAAAGAAATTAATACA
GCCACATACACAATATGCACATATACAGGTACAGACCAGCTCGTGGGGCATGGTGGTCTT
TATGCTGCTGCGATACAACTGTGCAAAAAACAGGGACTCAGTGTCCAAGAACATGAGCAT
GCTGCTAAACATTCCCGAAAAGCATATGCTCATAGAACCACCAAAACTGAGAAGTACCCC
TGCCGCCTTATACTGGTACAAGACGGCCATGGGCAACGGAAGTGAGGTATATGGGGAAAC
ACCAGAATGGATTGTTAGACAGACGTTGGTAGGACATAGCATGGAAGACGAACAGTTCAG
ACTGTCAGTTATGGTACAGTATGCATATGACCATGACATTGTAGAGGAAAGTGTGCTTGC
ATTTGAGTATGCACAACTAGCAGATGTGGATGCCAATGCAGCAGCATTTCTAAACAGTAA
CTGTCAGGCCAAGTACGTGAAGGACGCAGTGACAATGTGCAGGCACTATAAGCGTGCAGA
GAGAGAACAGATGAGTATGTCACAGTGGATAACATTCAGAGGAAATAAGGTATCAGAGGA
AGGGGACTGGAAGCCCATAGTCAGGTTTCTAAGACATCAAGGGGTAGAGTTTGTGTCGTT
CCTAGCTGCCTTTAAATTGTTCCTAAAAGGCGTGCCAAAGAAAAATTGTATAGTGTTCTA
TGGACCTGCAGACACAGGCAAATCATATTTTTGCATGAGCTTGTTGCAGTTCCTAGGCGG
CGCTGTTATCTCATATGCTAATTCTAGCAGCCATTTTTGGCTTCAACCTTTATCAGATAG
TAAGATAGGGTTACTGGACGACGCAACACCCCAGTGTTGGAGTTACATAGATATATATTT
AAGAAATCTTTTGGATGGACACCCAGTGAGCATAGACAGAAAGCACAAAACTTTGCTGCA
GCTTAAGTGTCCACCCCTAATGATAACAACCAACACCAATCCTCTAGAGGAGGACAGATG
GAAATATTTGCGCAGCAGGCTGACAGTGTTTACATTTAAGAATCCATTTCCTTTTGCAAG
TCCGGGAGAGCCCCTGTACCCGATAAATAATGCAAACTGGAAATGCTTTTTCCAAAGGTC
GTGGTCCCGCTTAGACCTAAACAGTCCAGAGGAGCAGGACGACAATGGAAACACTGGCGA
ACCGTTTAGATGCGTGCCAGGAGACGTTGCTAGAACTGTATGAAAAGGATAGCAACAAAC
TTGAGGATCAGATTAAGCATTGGGCGCAGGTCCGGCTAGAAAATGTCATGCTGTTTAAGG
CCCGAGAATGTGGAATGACACGAGTCGGCTGTACAGCTGTGCCTGCCCTCACCGTGTCAA
AAGCTAAGGCATGTCAGGCCATAGAGGTACAGCTGGCATTACAGACATTGATGCAGAGTG
CCTATAGCACGGAGGCATGGACCCTACGAGACACGTGTCTGGAGATGTGGGACGCACCTC
CAAAGAAATGCTGGAAAAAAAAAGGACAATCAGTATTAGTGAAATTTGATGGCAGCAGTG
ACAGAGACATGATATATACAAGCTGGGGATTCATTTATGTGCAGGACACTATCACTGATT
CCTGGCATAAGGTGCCAGGGCAGGTGGACGAACTGGGATTATATTATGTGCACGATGGTG
TACGTGTTAACTATGTGGACTTTGGAACAGAGTCCTTGACCTATGGGGTCACCGGGACGT
GGGAGGTGCACGTGGCTGGGACTGTTATTCACCATACATCCGCATCTGTGTCTAGCACCC

AGGCCAGCGCCTCGGACGACGAACCACTATCCCCTATTAGAACTGCTGTATCCCCAGTCC
CAGCCCCAGTCGCAGCCTCAGCAGAATCAACAGGAGCAGGAAGAGCAGCTCCGCCCACCC
AAGCGTTGTGCTCCGCCCAGGCGCCAACGAGTCCGCCGGCCAAGCGCCAGCGTGTCATCG
TCGGACAGCAGCATCCCCGGCCCGACTCTACGCGAACGGTCGGAGAGGGGGAAGTGGAGT
GTTACAACAAGCGGAGCATCAGTGACTCTAACCGCACAGACCCCAGGTGGGGCCACGGTG
ACACTGACTCTGTGCCTGTAATCCACCTGAGAGGTGATGCAAATTGTTTAAAGTGCTTCA
GATACAGGGTGCAAAAACATAAAGACGTACTGTATGCCAGGGTGTCCTCCACGTGGCACT
GGGCGGGTGGGAACGGTGATAAGACAGCCTTTGTAACACTGTGGTACACCAGCGTTGAAC
AGCGTACAGAGTTCCTGACAAGAGTCAGTATACCTAAGGGATTGATAGCATTGCCAGGGT
ATATGTCTGCATTTGTATAATCCTACATGCTTGTATAAACATATGGTCCAATACATTTCA
AGGCCTGCCTCCGCAACACAGCCCTGGACTACTTTCTCTGCGTGGTTGCAGGGTGGACAC
ATCTGCTTGTGCTACTGCTCTTCCTGTGGCTCTCTCAACTAACCCCCCTTGTGGCCTATC
TGGTGTTCTTTTTCTGTGTCTATCTGGGGCTGTGGTTGATATATGTGCAGGCCTTTTGGT
TTTTACCATAGTCGTTATTATTTCGCCATACGTTGCTGCTAGCTTGTATACATAGTCTAT
ATACCCATTGTGTGAGATTTGCAATGTACCCTGTTGTGTATAAGGGATCTGAGGGAACAT
ATCCTGTGGTACTGTGGGGTCATGATGATGTTCAATGTCTGTTGGTGATTCTTATCCTAA
TCGCCTTTTTATTGTTGATGTTTTATGTCCGTTTGTTAAACCACACCTAACACCCCCACT
TTTTTATATTGTTTTGATACATTTTCATTTTGATACATTTGTGTTTTTTTTGTATTTGCT
GCGTTTTAATAAACGTGCAACCATGTCTATACGTGCCAAGCGTCGAAAGCGCGCCTCCCC
CACAGACCTCTATCGTACCTGCAAGCAGGCAGGTACCTGCCCCCCAGACATTATCCCAAG
AGTGGAACAGAACACTTTAGCAGATAAAATCCTTAAGTGGGGCAGTTTAGGTGTGTTTTT
TGGGGGTCTAGGTATAGGCACCGGCAGCGGCACAGGGGGGCGTACTGGGTACATTCCTGT
AGGTTCGCGACCCACCACTGTAGTTGACATTGGTCCAACGCCCAGGCCGCCTGTTATCAT
TGAACCTGTGGGGGCCTCTGAACCCTCTATTGTCACTTTGGTGGAGGACTCTAGCATCAT
TAACGCAGGAGCGTCACATCCCACCTTTACTGGTACTGGTGGCTTCGAAGTGACAACCTC
CACCGTTACAGACCCCGCCGTCTTGGATATCACCCCCTCAGGTACCAGTGTGCAGGTCAG
CAGCAGTAGCTTTCTTAACCCACTATACACTGAGCCAGCTATTGTGGAGGCTCCCCAAAC
AGGGGAAGTATCTGGCCATGTACTTGTTAGTACAGCCACCTCAGGGTCTCATGGCTATGA
GGAAATACCAATGCAGACGTTTGCCACGTCGGGGGGCAGCGGTACAGAGCCTATCAGTAG
CACACCCCTCCCTGGCGTGCGGAGAGTTGCCGGACCCCGCCTGTACAGTAGAGCCAATCA
GCAAGTGCAAGTCAGGGATCCTGCGTTTCTTGCAAGGCCTGCTGATCTAGTAACATTTGA
CAATCCTGTGTATGACCCAGAGGAAACTATAATATTTCAGCATCCAGACTTGCATGAGCC
ACCGGATCCTGATTTTTTGGACATAGTGGCGTTGCATCGTCCCGCCCTCACGTCCAGAAG
GGGTACTGTCCGTTTTAGTAGGTTGGGACGCAGGGCTACACTCCGCACCCGTAGTGGTAA
ACAAATTGGGGCACGGGTGCACTTCTATCATGATATTAGCCCTATAGGTACTGAGGAGTT
GGAGATGGAGCCACTGTTGCCCCCAGCTTCTACTGATAACACAGATATGTTATATGATGT
TTATGCTGATTCGGATGTCCTTCAGCCATTGCTTGATGAGTTACCCGCCGCCCCTCGCGG
TTCACTCTCTCTGGCTGACACTGCTGTGTCTGCCACCTCCGCATCTACACTACGGGGGTC
CACTACTGTCCCTTTATCAAGTGGTATTGATGTGCCTGTGTACACCGGTCCTGACATTGA
ACCACCCAATGTTCCTGGCATGGGACCTCTGATTCCTGTGGCTCCATCCTTACCATCGTC
TGTGTACATATTTGGGGGAGATTATTATTTGATGCCAAGTTATGTCTTGTGGCCTAAACG
ACGTAAACGTGTCCACTATTTCTTTGCAGATGGCTTTGTGGCGGCCTAATGAAAGCAAGG
TATACCTACCTCCAACACCTGTTTCAAAGGTGATCAGTACGGATGTCTATGTCACGCGGA
CTAATGTGTATTACCATGGTGGCAGTTCTAGGCTTCTCACTGTGGGTCATCCATATTACT
CTATAAAGAAGAGTAATAATAAGGTGGCTGTGCCCAAGGTATCTGGGTACCAATATCGTG
TATTTCACGTGAAGTTGCCAGATCCAAATAAGTTTGGCCTGCCCGATGCTGATTTGTATG
ATCCAGATACCCAGAGACTTCTGTGGGCGTGCGTGGGAGTAGAGGTGGGCCGTGGGCAGC
CTTTGGGTGTGGGTGTGTCTGGTCACCCATATTACAATAGACTGGATGACACTGAAAATG
CACACACACCTGATACAGCTGATGATGGCAGGGAAAACATTTCTATGGATTATAAACAGA
CACAGCTGTTCATTCTGGGCTGCAAACCCCCTATTGGTGAGCACTGGTCTAAGGGTACCA
CCTGTAATGGGTCTTCTGCTGCTGGTGACTGCCCGCCCCTCCAATTTACTAACACAACTA
TTGAGGACGGGGATATGGTTGAAACAGGGTTCGGTGCCTTGGATTTTGCCACTCTGCAGT
CAAATAAGTCAGATGTTCCTTTGGATATTTGTACCAATACCTGTAAATATCCTGATTATC
TGAAGATGGCTGCAGAGCCTTATGGTGATTCTATGTTCTTCTCGCTGCGTAGGGAACAAA
TGTTCACTCGTCATTTTTTCAATCTGGGTGGTAAGATGGGTGACACCATCCCGGATGAGT

TATACATTAAAAGTACCTCAGTTCCAACTCCAGGCAGTCATGTTTATACTTCCACTCCTA
GTGGCTCTATGGTGTCCTCTGAACAACAGTTGTTTAATAAGCCTTACTGGCTACGGAGGG
CCCAAGGGCACAACAATGGTATGTGCTGGGGCAATAGGGTCTTTCTGACTGTGGTGGACA
CCACACGTAGCACTAATGTATCTCTGTGTGCCACTGAGGCGTCTGATACTAATTATAAGG
CTACCAATTTTAAGGAATATCTCAGGCATATGGAGGAATATGATTTGCAGTTCATCTTCC
AACTGTGCAAGATAACCCTTACTCCTGAAATTATGGCCTATATACATAATATGGATCCCC
AGTTGTTAGAGGATTGGAACTTCGGTGTACCCCCTCCGCCGTCTGCCAGTTTACAGGATA
CCTATAGATATTTGCAGTCCCAGGCTATTACATGTCAAAAACCTACACCTCCTAAGACCC
CTACCGATCCCTATGCCTCCCTGACCTTTTGGGATGTGGATCTCAGTGAAAGTTTTTCCA
TGGATCTGGACCAATTTCCCTTGGGTCGCAAGTTTTTGCTGCAGCGGGGGGCTATGCCTA
CCGTGTCTCGCAAGCGCGCCGCTGTTTCGGGGACCACGCCGCCCACTAGTAAACGAAAAC
GGGTAAGGCGTTAGCTCTCAGTGTCGCATCATTTCCTCTGTTCTACTTTTTACATATTAT
TTTGTTGTCTGTAATATGTTTATGTTGTTGTTGTGCTTATATTACATGTATACATGTATG
GTATGTATCCCCTCCCGTATGAATAAACGTGTGTCATGTGTTGTGTGTTCTGTAACTGTA
CGTTCTGGTGCACAGATTTCTGCACCCCATCGCCTTGTGTGTAGCCCCCAGTTTCATGCA
ACCGTTTTCGGTTGCGTGCAGTTTCGGTCGGCGCCGTTGCCAACCCAGCTTAATCCTTTA
ATTGCTCTCATCCTAAAGTGTTATCTGTGCCAGCGACGATGAGTTTGGATTTTGGTTGTT
TAATGCTTTTTCTTTTCAGTTTTTCCTTTGTTTGTGCCAGGCCGCGAGAGGGCGTGCACA
TTCCTAGGCTGATTATCTTAATGTGTTTGGCACATCTTTGTACTGCGTCTGCAGAAAAAC
CTGCAGCAACAGCACTTTGGGCGCGTCGTTTTTGCAGCCAACTTTCACTTGCCAACTTGC
CTTGCCGCGCATTCCAAGAAACACACCTATTCCGGTCGCAATGTCTACTATGTGTGGTTT
>gi|30315616|ref|NC_004761.1|_Human_papillomavirus_RTRX7,_complete_genome
AACGGTAAGTCTCGCCCTTGTACCAGGTGCGGTACGATTTCTCAATAGCTCCTTCAATAA
CAGGTTGTTATTGCCAACTACCATCATCACTTTCAAGTTTTTGCTTGTATCGTTTTCGTA
TCATCTCCTATTTGTATATAATTAAATAAATAAATATATATAATGTTTGAGGCTTGGTTA
TTTTGCAACTGTGATTGGGACAAATGGCTCAGCAGACTACACCAGACCAACAGAAACCTA
AAGAGGAAACATTTGAACTGCCCACAACTATTAAAGAGTTAGCTGACCTCTTACAAATTC
CTTTAGTTGATTGCTTAGTGCCTTGCAACTTCTGTGGAAGGTTCTTAGATTTTTTGGAGG
TTTGCGAGTTTGACAAAAAGCAGCTAACATTGATTTGGAAAGACTACTTTGTTTCTGCTT
GCTGTCGAAGTTGTTGTGTAGCTACTGCAACTTTCGAATTCAATGAATATTACCAGCAAA
CAGTTTTAGGTAGAGATATAGAGCTAGCTACAGGAAAGTCAATATTTGAATTGAAAATCA
GGTGTCAGACTTGCTTATCATTTTTGAGCACCATTGAAAAATTAGATAGCTGTGGTCGAG
GCCTTCCGTTCCACAAAGTGAGAGACGGCTGGAAAGGAGTGTGCAGGCACTGTAAGCATT
TGTATAATGATCGGTAAAGAGGTCACTGTGCAAGACTTTATTTTGGAGCTTAGTGAGGTG
CAGCCTGAAGTGATACCAGTTGACCTGCTTTGTGAAGAGGAATTACCAAACGAACAGGAA
ACGGAGGAGGAGTCAAATATCGACAGAACCGTGTTCAAAGTCATTGCACCGTGTGGCTGC
AGCTTCTGTGAGGTCAATCTTCGTGTTTTTGTCAACGCAACTGAACTTGGTATTAGAACC
CTGCAGGAACTACTGACCAGCGATCTGCAGCTGCTGTGTCCAGAGTGCCGTGGTAACTGC
AAGCATGGCGGATCCTAAAGGTAGTACATCTAAAGAAGGGTTAGGTGATTGGTGTATTTT
GGAAGCTGACTGTAGTGATTTAGAAAATGATTTTGAACAGTTATTTGAGCAAGATGCAGA
CTCAGATGTATCGGACTTGCTAGATAATGGTGAACTGGAACAGGGCAATTCCCTGGAACT
ATTTCATCAACAAGAGTGTAAGCAGAGCGAGGAGCAATTACAAATACTAAAACGAAAGTA
TCTTAGTCCAAAAGCTGTCGCGCAGCTTAGTCCGCGTCTTGAGTTGATGTCATTGTCACC
TCAGCAGAAATCCAAGCGAAGGCTCTTTGCAGAGCAGGACAGCGGACTCGAGCTATCTCT
AAACAATGAAGCTGAAGATAATGCTCCTGAGGTGGAGGTACCGGCTCTAGACTCTGGGCC
GGTAGACGAGGGAGGAACAGGGGATGTAGATATTGATTATTTAGCCTTGTTGCGTTCCAG
CAACAGAAAAGCCACATTAATGGCGAAATTTAAAGACGCGTTTGGGGTAGGCTTTAATGA
ACTAACTCGCCAGTTTAAAAGCTACAAAACCTGTTGTAATCATCGGGTTTTAGCTGTGTA
TGCAGTTCATGATGATCTATTTGAAAGCTCAAAGCAGCTGTTGCAACAGCACTGTGACTA
TATCTGGGTCCGTGGGATAGCAGCTATGACACTTTACCTATTGTGTTTTAAGGCGGGAAA
GAATCGCGGTACTGTGCATAAGTTACTTACGTCAATGTTAAATGTGCAAGAGCAGCAAAT
ATTGTCTGAGCCTCCTAAGTTAAGAAATACAGCTGCGGCATTGTTTTGGTATAAAGGTGG
TATGGGGTCAGGAGCATTTACATATGGTAAATATCCTGATTGGATTGCGCAGCAAACAGT
ACTTGGCCACCAAAATGCGGAGGCAAGCACATTTGATTTTTCAGTGATGGTACAATGGGC
ATTTGATAATAATCACGTAGACGAAGCAGATATTGCTTACCAATATGCAAGGCTTGCACC

AGAAGATAGTAATGCAGTAGCCTGGCTTGCTCATAATAGCCAAGCAAAGTTTGTGCGAGA
CTGTGCAGCAATGGTACGCTTTTATAAAAATTTACAAATGAGAGAAATGTCCATGTCTGA
ATGGATATACACAAGGATAAACGAGGTGGAAGGAGAAGGACATTGGTCATCTATTGTTAA
ATTTTTAGGATATCAAGGAGTTAATTTTATAATGTTTTTAGCAGCATTGAAAAATTTTTT
GCATGCTGTACCAAAACAGAATTGCATATTAATACATGGGCCTCCTAACTCTGGAAAGTC
ATCTTTTGCTATGTCATTGATAAAAGTATTGAAAGGGAGAGTATTGTCTTTTGTAAATTC
CAGAAGTCAATTTTGGTTACAACCTCTTGGAGAGTGTAAAATTGCCTTGATAGATGATGT
AACAGATCCTTGTTGGCTATATATGGACACATATTTACGAAATGGTTTGGATGGCCATTT
TGTGTCACTAGACTGTAAATACAAAGCGCCTGTGCAAACGAAGTTTCTTCCATTATTGCT
CACATCAAATATTAATGTGCATGAGGAAACAAATTACAGATATTTACATAGTAGAATAAA
AGGATTTGAATTTCCAAATCCTTTTCCAATGAAATCAGACAACACACCTCAATTTGAGTT
AACTGACCAAAGCTGGAAATCTTTTTTTGAAAGGCTTTGGACACAATTAGAACTGAGTGA
CCAAGAAGAGGAGGAGGAGGGCGAACATGGAGAAACTCAGCGAGCGTTTCAATGTTCTGC
AAGATCAGCTAATGAACATATATGAAGCTGCAGAACACACACTTGAGACACAAATTGCAC
ATTGGACACTTTTGCGAAGAGAAGCTGTATTGCTTTACGTTGCTAGGCAAAAGGGTATCA
CAAGGCTTGGATACCAACCTGTGCCTCCATTGGCAGTCTCTGAAGGAAAAGCAAAGGAGG
CCATAGGGATAATGCTGCAACTGCAATCTCTGCAAAAGTCTGAGTATGCTTCAGAGCCCT
GGACATTAGTGGACACAAGTGCAGAAACTTATAGGAATGCTCCAGAACAACATTTTAAAA
AAGGCCCAATACCTGTGGAGGTCATTTATGATAAGGAGTCCGGAAATGCAAATGTTTATA
CTATGTGGAAGTTTGTATATTACATGGATCCAGAGGATGTATGGCATAAAACTACAAGTG
GTGTAAACCACAATGGTATCCACTATTTACATGGTGACTTTAAACACTATTATGTGTTGT
TTGCTGATGGTGCACGTACATATAGTAAAACAGGATATTGGGAAGTGAAAGTTAATAAGG
AAACTGTGTTTGCTCCTGTCACCAGCTCCACACCTCCCGGATCTCCAGGACAAACAGACA
CAAACACCACCACCAAGGCCCCCGCCACCTCCTCTGACTCCACGACCAGATCCAGTGACA
AACAGTCACAACAAGCCGCAACCAAGAGGAAAGGATTCGGACGACGACCATCCAGTAGAA
CAAGGCGACAAGAAACGCAGCAAAGGCGATCAAGGTCGCGATACCGCTCCCGGTCTCACT
CCCGGTCGCACTCCCAGTCCGATACCAGGGCCATTGCCACCTCCCTATCCAGGTCCTCCA
GGTCCCCGTCGGTCTCCAAGACTAGGAGTCGGAGGTCAGAATCCAGATCACAATCCCAGA
AGTACCTCCAGCCGAAGGCAGGTCGAGGGTCACCCACCGACACCACCACCAGGCGTCGGA
GATCCCGATCAAAGTCATCCTCCACCTCCACCTCTTCCTCCTCCCAACGGTCACAACGGG
GAGGGAGAGACAGAGGGGAAAGAGGAAGTAGGAGCTGTAGGGGGTGCGGTAGGGGAACAG
GAAAGCGACGGGGTAGATCATCCTCCACCTCCCCACCCTCAAAACGGTCTCGACCAAAGT
CTAGCAGGCTCCGTGGCGTGTCTCCTGACGAAGTGGGAAGATCTCTTCAATCTGTTAGTT
CAAAACATAGAGGAAGACTTGGGAGATTATTGGAAGAGGCTCTCGACCCCCCAGTAATAC
TTTGCAAGGGGGGGGCAAACACATTAAAATGCTTTCGCAACAGAACTAGAGGGAAATACA
CGGGGCTGTTCAGAGCCATTAGCACAACATGGTCTTGGGTGGCTGGAGATAGCACTGAGC
GTCTAGGCAGGCCCAGAATGCTCATTAGCTTTTCTTCAGATGGTCAGAGAAAACATTTTG
ATGAGACTGTGAAATATCCGAAAGGGGTTGAGACATCATATGGCAACTTGGACAGCCTAT
AACACTACTAACATTGCTTTGCTACTAACACCACTAACATTACAGAAATAGTATAGTTTA
ACATTTTTTTACTTTTTTATTGCTATGGTGCGTGCTAAGCGGGTCAAGCGGGTCAAGCGT
GACTCTGTAACTCATATATACCAAACCTGCAAACAAGCAGGCACTTGCCCCCCTGATGTT
CTTAATAAAGTGGAGCAAACAACAGTTGCTGACAATATTCTTAAATATGGCAGTGCTGGT
GTATTTTTTGGAGGCCTGGGCATAGGAACCGGCCGTGGTACAGGAGGCGTGACAGGGTAC
ACGCCACTGTCTGAAGGGCCTGGTATCCGTGTTGGTGGTACCCCCACAGTTGTACGGCCC
TCACTTGTTCCTGAAAGTATTGGTCCAGCAGATTTGCTGCCAATAGACACTATCAATCCT
GTGGAGTCCACTGCATCATCGGTGGTCCCATTGACTGAATCTTCAGCAGCTGATTTGCTT
CCAGGGGAAGTTGAAACAATAGCTGAGATACATCCTGTTCCTGAAGGTCCCATAGTTGAC
TCGCCTGTTGTGACCACCAGCAGAGGCTCAAGTGCCATACTAGAGGTTGCTCCAGAGCCT
ACACCACCTACACGTGTTCGTGTGGCACGCACACAATATCATAACCCATCTTTTCAAATA
TTAACAGAATCAACACCTACTCAGGGTGAAAGTTCTTTAGCAGATCATATATTAGTTACA
TCTGGTTCAGGGGGTCAAACCATAGGTGGTAATGTTACAGACATTATTGAGTTACAAGAA
TTACCCAGTAGATATTCTTTTGAAATAGAAGAGCCAACTCCACCACGGCGCAGTAGCACA
CCACTTCAAAGAGCACTATCCATTGGCAGGCGTAGGGGGCCTTCTCTAACAAATAGAAGG
TTAGTTCAGCAAGTACAAGTAGACAATCCTTTATTTGTTTCTCGACCATCCAAATTAGTA
AGGTTTGCTTTTGATAATCCTGTATTTGAGGAGGATGTTACAAATATTTTTGAGCAGGAT

GTAGATACGTTTGAAGAGCCCCCTGATAGGGATTTTTAGACATTCGCAAACTGGGACGA
CCCCAATACTCAACAACACCTGCAGGTTATGTTAGGGTAAGTCGACTCGGCACCCGGGCC
ACTATTCGCACACGTTCAGGTGCTCAAATAGGATCTCAAGTACATTTCTATAGAGATTTA
AGTTCAATAGATTCTGAGGACCCTATTGAGCTACAGTTATTAGGTCAACATTCTGGGGAT
GCGACCATCATACAAGGTACTGTGGAAAGTACGTTCATAGACATGGATACAGCAGAAAAT
CCTTTGTCTGAGAGTATTGAGGCGCATTCTAATGATCTGCTACTTGATGAAGCTGTTGAG
GATTTTAGTGGCTCTCAATTAGTTATAGGAAATAGACGTAGTACTACATCATATACTGTC
CCATGGTTTGAAACAACAAGAAACAGCTCCTATTATGTGCAGGACACACAAGGTTATTAT
GTGGCTTATCCTGAGAGTCGAGATACTGCAGAAATAATTTATCCTACACCAGACATTCCC
GTGGTGGTCATTCATACCCATGATAGCAGTGGTGACTTTTATTTGCATCCTAGTCTTCGA
CGGGCAAGGGTAAGAGAAAATATTTGTGATTTGCTTACAGATGGCTGTGTGGCATTCGGC
TAACGGTAAGGTCTATCTACCACCATCAACACCAGTGGCCAGGGTGCAAAGCACGGATGA
ATACATTCAAAGAACAAACATCTACTATCATGCCAATACTGACAGACTGCTCACTGTAGG
CCATCCATATTTTAATGTGTATGATAATACTGGGAAAAAATTGGAAGTTCCTAAAGTATC
AGGAAACCAACATAGGGTATTTCGTTTGAAATTACCAGATCCAAACAGATTTGCTTTGGC
TGACAAGTCTGTATACAACCCCGATAAAGAAAGGCTGGTATGGGGTTGCAGAGGGTTGGA
AATCAGTAGAGGACAGCCTTTGGGAGTTGGCAGTACTGGACATCCATATTTTAATAAGGT
AAAAGACACGGAAAACACTAACAATTATACCACAGGCTCTACAGATGATAGGCAGAACAC
ATCTTTTGACCCCAAACAAATTCAGATGTTTATAGTAGGTTGTACACCTTGTGTGGGAGA
GCATTGGGAAAAGGCCTTACCTTGTGGAGATGCGCCTGCAGATAATGGTATTTGCCCTCC
AATTGAATTAAAAAACACTTTTATTGAAGATGGAGACATGGCAGATATTGGTTTTGGCAA
TATGAATTTCAAAACTTTACAGCAGAATAGGGCTGATGTCAGTCTGGATATAGTTAATGA
AACTTGCAAATATCCAGACTTTTTGAAAATGCAAAATGATGTCTATGGCGATGCGTGTTT
TTTCTATGCTCGCAGGGAGCAGTGTTATGCCAGACATTTTTTTGTTAGGGGGCGCAAGAC
AGGTGACAATATTCCTGACGCATATATTGATGATGGCAATATGAAAAATTCTTTTTACAT
ACCAGCAGCTTCAAATCAATCTCAAAAGGATATTGGTAATGCTATGTATTTCCCAACAGT
CAGTGGCTCACTAGTCTCTAGTGATGCTCAATTGTTTAACAGGCCCTTCTGGCTGCAAAG
AGCACAGGGTCATAATAATGGCATTCTGTGGGCTAATCAGATGTTTGTCACAGTTGTAGA
CAACACGCGAAACACCAATTTCAGTATATCTATATCTAGTGAAAATCAAGATATACAGCA
AATACAATCATATGACTCACAAAAGTTTAGGGAATATTTAAGGCACGTAGAAGAATATGA
AATTTCTATTATTTTACAGTTGTGTAAGATTCCACTACAAGCAGAAGTTTTAGCACAAAT
AAATGCAATGAACCCCTCCTTACTAGAGGATTGGCAGTTAGGATTTGTGCCAACTCCCGA
TAATCCTATCCAGGACACATACAGATTTATTGATTCCTTAGCTACCAGGTGTCCCGATAA
AAATCCACCAAAGGAAAAACCTGATCCTTATGAAAAATTAACATTCTGGAATGTAGACCT
AACTGAAAGATTATCTTTAGACTTGGATCAATATTCTTTAGGTCGCAAGTTTTTGTTCCA
AGCTGGATTGCAGCAAACGACCGTTAACGGTACAAAGCCTTCAACGTATAGAAGTTCTGT
ACGGGGGATCAAAAGAAAACGCAAAAACTAACAATTACCGAATTCGGTACATTTGTATCA
ACTTTTACACAGTATTCAAGGAATGTCTGTTTACTCTGACTAAGTATAACTCTACCTAGG
AAACCAACCGCGCCCGGTACATAAAGGTGAGTTGGTGCCAAATTAGATTCAGTGTGGTGC
CAAGACATACCGTGTTGGTTCTAACATGCTCGGATTAGGTCGACCGCCAAGGAACCTTTA
GTGTGCCAATAGCTTACAGCAGTCCAGATTTGGCACATTTTGTGGACCGAT
>gi|22138122|ref|NC_004104.1|_Human_papillomavirus_type_90,_complete_genome
ATGACCAACGGAGACTACTGCCCAAGAAATATCTTCCTATTGTGCAAAGTCTATGAACTG
GACCTGCAGGACCTGAACATAACCTGCGTGTTTTGCGGAACAGCATTAACAGAGGAGGAG
GTTGTATCGTTTGCATACAAGGAGCTACAAGTTGTGTGGAGAAAGCATTTTCCATTTGCA
GCATGTCCTGCGTGTCTGGAAGCAGCCGGGAAACTAAGGCAATTTAGATACTGGCAGTAT
TCTAGCTATGCACCCACAGTGGAATATGAAACAGGAGTGCCTATTTTAGAATTGCTTATT
AGATGCTACCTGTGCCATAAACCACTGTGCAGTGAGGAAAAGGAACGGCTTATCAGTGAA
GGCAAACGGTTCCATAAAATAGCTGGGCTTTGGCGAGGCGCTTGCCTGCAGTGTTGGAAA
CCATGCATGGCCAACGACCAACCTTAAAGGACATATTTCTAGAGCTGGATCAGCCTGATG
CGGTTGACCTACATTGTAACGAGCAATTAGCCAGCTCAGAAGAGGAGGATAATAGGGAGG
ACGGTGAGCAACCTACACAACCTACAGAACCAGCACAGCAAGCATATAGGGTGGTAACAA
GTTGTTGTAGGTGTTGCTGTACAGTAAGGCTGGTGGTGGAGAGTGGACACGCAGAAATAA
GACAACTCCAGGAGCTTCTGTTGGGCGCACTGCATATAGTGTGTCCGCTCTGCGCATAAC
TGCTATGGCCGACGGTGAAGGTACAAACAATGGGGACGATAGTGAGGAGGGACGGAGCGC

ATGCGGGTGGTTTTGGGTGGAAGCCACAGTGGACAGAACCACGGGACAGCAAATATCTAG
TGATGAGGACGAGGAGGACGGTGACAGCGGACAGGACATGGTTGATTTTATTGATAATAG
TAGGCATCCCGGGGATGGACAGGAAGTTGCACTTGAGTTATACAGACAGCAGGAGGCACA
AGATGACGAGGCCTTTGTGCAGGCCCTAAAACGAAAGTATCTAGCTAGTCCGTTGGCCGC
CGGGGTATGTGTAGACAAAGACCTAAGTCCACGGCTGGATGCCATAACACTAGGCAGAAG
CTCACAAAAAGCAAAACGTAGACTGTTTGAGCTGCAAGACAGTGGGTATGGCAATACGCA
AGTGGATATTGACGCGACAGAGGACCAGGTAGTAGGGGAAAGCAGGGGGGGTGCAGGCAC
AGGGGGGGTACAGGAAACGGAGGAGGAGGAGCGACAAGGGGGGGATGGTGCGCCGGGTGC
AGTTACAGCACCGGCACAATCAGGTACAGATGCTATCCTGTCTCTGCTTAAAACAAGTAA
CCTAAGGGCCACATTACTAGGTAAGTTTAAAGATATATATGGGTTGTCTTTTATGGAGCT
AGCAAGACAGTTTAAAAGTAATAGGACCACGTGTTTGGACTGGGTAATATGTGCATTTGG
CGTGTACTGCACAGTTGCAGAGGGTGTCAAAACCCTTATACAGCAACACTGTCAATATGC
ACACATACAGGTGCAAACCAGTAACTGGGGAATGGTGGTCCTAATGCTAGTGAGATATAA
TTGTGCCAAGAACAGGGACACGGTGGCAAAATGTATGGGCAGGTTACTAAATATACCAGA
GCAACATATGCTCATAGAACCTCCCAAAATAAGACATCCAGCGGCTGCACTATATTGGTA
TAAAGCAGGCATGGGCAATGCTAGTGAAATAATTGGAGAAACACCAGAATGGATTGTAAG
ACAAACAGTTGTGGGGCACAGTCTAGAAGAATGCCAGTTTCAGTTATCAGTAATGGTGCA
ATGGGCATATGACCATGATATAACAGACGAAAGTATATTGGCATATGAATATGCAAAATT
GGCAGATGTAGATGGTAATGCAGCGGCATTCCTAGCAAGCAACTGCCAAGCAAAATATGT
AAAGGATGCATGCACAATGTGCAGGCATTACAAACGGGCAGAGCAGGCACAAATGACAAT
GTCAGAATGGATAAGATTTAGAAGTGCAAAGATATCAGAGGAGGGCGACTGGAGACCCAT
AGTACGGTTCCTAAGACACCACGACATAGAGTTTATTACCTTTGTAATATCGCTGAAAAA
CTTTTTAAAAGGCATACCAAAAAAATGTTGTATAGTAATATATGGACCTGCAGACACAGG
TAAATCATACTTTTGTATGAGCCTGTTGCGCTTCCTAGGAGGGGTTGTGATTTCCTATGC
CAACTCCACCAGCCATTTTTGGCTGCAGCCATTATGTGATGCCAAAATAGGGCTTATAGA
TGATGTTACCCCCCAGTGCTGGAGCTATATAGATACCTATTTGCGAAATGCACTGGATGG
CAATCAGGTATGCGTAGACAGAAAACATAGGCCTTTGCTGCAGCTAAAATGTCCACCACT
GTTAATGACAACAAATACCAATCCCTTAGAGGAAGACAGGTGGAAATTTTTACGTAGTAG
ACTGCAGCTGTTTACCTTTCCTAATGCATTTCCTGTAAATCAGAAAGGGGATCCCTTGTA
TACACTAAATGATGCAAACTGGAAATGCTTCTTCCAAAGGTTGTGGGCTCGGTTAGACTT
ACACGAGCAGGACGACCAGGAGGACAATGGAGACATTGGCCAGCCGTTTAAATGCGTGCC
GGGAGAAGTTGCTAGAACTGTATGAAACAGATAGCGACCAGCTGCAGGACCAAATTATGC
ACTGGGGACTTGTGCGATTGGAAAATGTTATTATGTATAAGGCACGAGAAGCGGGAATGT
TGCACCTAAACCACCAGGTGGTGCCAGTGCAATTGATTGCAAAAGCAAAGGCATGTAAAG
CTATTGAAATGCAGTTGGCATTAAAGACATTGCTAAAAAGCCCCTATGGCACGGAACCTT
GGACATTACGTGACACAAGCCAGGAAATGTGGGACGCAGCACCCAAGCAATGTTGGAAAA
AAAAGGGCTACACCGTAGACGTTAAGTTTGATGGCGAGGACACAAAAACTATGTGCTACA
CATGCTGGAGGTATATATATGTACAAAGCGGTGATGATGATACATGGACCAAGGTGTCTG
GCCATGTGGACCATGCAGGACTATACTTTACACACGAAGGGACACGTGTAACCTATGTTA
GGTTTGCACAGGAAGCACAGACATATGGGAACACTGGCACCTGGGAGGTGCATGTGGGAG
GACAGCTAATTTATGATGCATTCGACTCCTCTGTGTCTAGCACCCAGGACACCGACCAAG
AACCGCTATCCTCTACTAGACCTACTGACCCCCCATACACCGACGAACCCGACGCCACCA
ACCCCGCCCAGGTGTTGGGCCCCGCGTCGCCACACGTGCCGCCGCCGCATAAGCGTCAGC
GACTCGGACACGGATATCAGCATCCCGACTCCACAGAAGACAGGATCCAGCGGCTGGCAC
GTGACAACACAGGGGACCAGTGTAACCCTGACAGCGCAAAGCAGCACGGGAACATCCGTG
TCGGTGACAGTGCACCTGTAACACACCTCCGAGGTGATCCAAACAAATTAAAGTGTTTAA
GGTATAGATTGCACAAACATAAATCTGTACTGTTTAGCAAAGTAACCTCCACCTGGCGAT
GGTCAACGTGCAGTAACACCACCAATGAAAAATCAGCCTTTGTAACCCTGTGGCATGATA
GTGCGGAACAAAGGGCAACCTTCCTATCAACCATAACCTTACCTAAAGACATTGAAGCAG
TACTGGGGTACATGACTATGTTAGCATAATATACTGGTATATTGTAAATATATGTAAACT
GTGTACAGTGCGGAACGTTAAGGGACATCTGTTGTATACAGTGTGGAGACCTTTATTTGC
TACTTGTCTGCCTGGACACAACTGCTGCTAATACTAATCTTCTTTTGGCTGTCTATCCTT
CCTGGCGTTACTGCTTTTCTTATTGTATTCCTTACTGTGTTTCTAGGCCTCCTGATCATA
TACATACAGGCGTTGCAGTCCCTTGACCAACTGTGATATCACCACACAACCAGCCAAGCC
TGCTGCTCAATTGTATATATTCCTCTTGTGTTGTAGTGTGCAATAGTAGTATGTATCCTG

TTGTAGTACGTGATAGAGATGGGGGGGAATATCCTGTAGTACTGTGGACACCTGCAGATG
TGCAGGTGTTATCCCTGTTGTTGCTTTTCGTGTGTCTTATTTTGTTTATTTTTTACCTGC
GTCTGTTGCAGTAGTTACCTTACCCATTGTACCTATATGTATTGTATACACAGTGTATGT
AGACACATGTAGTGGGTTTGTGGGTGTCATACATTTATTGGGTGTGGGGTATTCATTATA
GTGGTGTGGTTTGGTGTTTTGTTTTTGGCTTTTTGTATGGTTTGTATTTTTCATTTTTTT
ACATTATATTGCTAAATAAATTTTAGTATGCATCGCTCTAAACGTCGCAAGCGTGCCTCC
GCTACCCAATTATATCAAACCTGCAAATTGGCAGGTACCTGTCCCTCCGATGTGATCAAT
AAGGTGGAACACACCACACTTGCAGACAAAATCCTGCAATGGGGTAGCTTGGGTGTCTTC
TTTGGGGGTTTGGGCATTGGAACAGGGTCCGGCACTGGTGGGCGCACTGGATACATCCCT
ATTGGGACCCGACCCCCCACTGTTGTGGATGTTGGCCCTCCTGCCCGCCCCCCTGTCGTT
ATTGAACCCGTGGGGGCATCCGATCCTTCTATTGTCACATTGGTGGAGGATTCCAGCATA
ATTGCATCGGGCTCTCCCCATCCCACATTCACAGGTACCGGGGGGTTTGAGGTTACAACA
GCCTCTACCACAACCCCTGCAGTGTTGGACATAACTCCATCCGGTGGCAATGTGCAGGTT
AGCAGTAGTAGCTTCATTAATCCTTTGTTTACTGAGCCTGCTATAGTGGAACCTCCGCAG
GCAGGAGAGGTAACAGGCCATGTTTTAGTTAGTACCCCTACAGCTGGATCCCATGGCTAT
GAGGAAATCCCTATGCAAACTTTTGCTGTACGGGGTACTGGTGATGAACCTATTATTAGT
ACCCCGTTGCCAGGGGTCCGTCGTGTGGCTGGGCCCCGGTTGTACAGCAGGGCATACCAA
CAAGTACATGTACAGGACCCCAGGTTTTTATCACAGCCTGAAACACTTGTTACCTATGAC
AATCCTGTATTTGACACAGAGGAAACCATACTATTTGAGCATCCTAGTATTCATCAGGTC
CCGGATCCTGACTTTTTGGATATTGTGGCTCTACACAGACCCGCCCTTACTGCACGCAGG
GGTACCGTCCGGTACAGCCGTCTGGGTCAGCGTGCCACATTAAAAACCCGCAGTGGCAAG
CGCATAGGTGCCACAGTACATTTTTATCAGGATTTAAGCCCTATTGCACCTGTTGCTGAC
GAGCTGGAAATGCAACCTTTGGTTTCTGACACTCCTGATTATCTTGATTCTCTATATGAT
ATATATGCTGACACTGCTTCTGTGTCCCGTCACCGCACCTTGACCCCTACACGCCCCTCC
ACACCTTTACAGGCTCCATCAGTGACTGCATCGTCTGCCCTATCATCTGCAGCATCCAAT
ACCACTGTGCCTCTATCTACAGGCCTGGATATTCCTGTCTTTAGTGGGCCTGACAGTGCC
CTCCCGGATTCCCATGCTGTTTGGCCCGTGCCCCCTGCCCCTCCCGGTGTTGTTCCTGGC
TCTGTTCTTGTAAATGGGTCCACATATTATTTGTTGCCCCCATTAGGTTTGCTACCTAAA
AAACGTAAACGTTTTCCATATTTTTTTGCAGATGGCAATGTGGAAGCCTAGTGACAACAA
GGTATACCTGCCTCCTGCCCCTGTGTCCAAGGTCCTCAGCACCGACGCCTATGTATCCAG
AACAAACATATATTATTATGCAGGCAGTTCCCGACTGTTAACTGTTGGCCATCCTTATTT
TGCTATCAAAAAGCAATCAGGAAAAAACCCTATAGTGGTTCCCAAGGTGTCTGGATATCA
ATATAGGGTGTTTAGGGTACGTTTGCCTGATCCCAATAAGTTTGGCCTTCCTGATGCATC
GCTATACAATCCTGACTCGCAGCGCCTTGTATGGGCCTGTACAGGTGTTGAGGTTGGCAG
GGGACAGCCTTTAGGCGTTGGGGTAAGTGGCCACCCGTTGTACAACCGCCTGTATGACAC
TGAAAACACCAATTTATATGATGTTGTGCCTGGCGATGACACCCGGGACAATCTTACTAT
GGACTATAAGCAAACCCAGCTATTTATTATAGGGTGCAAACCTCCTTTAGGCGAGCACTG
GGCAAAGGGTACCCCATGCAATATGTCTAATGTACAGGCTGGGGATTGCCCTCCTATAGA
ACTTAAATCTTCCACAATTCAGGATGGGGACATGATAGATACTGGTTTTGGTGCTCTTGA
TTTTGCAGCGTTGCAAACCAATAAGTCTGATGTGCCTTTAGATATTGTAACCACTACATG
CAAGTACCCTGATTTTTTGCAGATGGCAGCTGAGCCATATGGTGACCTTATGTTTTTCTG
TTTACGTAGGGAACAGATGTTTATAAGGCACTTTTATAACAGAGCAGGTACTATGGGCGA
CGCAATTCCCGATGGCTTTGTGCTAAAAGGAACATCTTCTACCTCTCGTGGCACTCCTAC
AAGCTCTGTATATGGGTCCACGCCTAGTGGGTCCATGGTTACCTCTGAATCCCAGTTGTT
TAACAAGCCCTACTGGCTGCAACGAGCACAGGGCCCTAACAATGGTATCTGCTGGGGTAA
TCAGCTTTTTGTAACTGTGGTTGATACTACACGTAGCACCAATATGACTATTTGTGCCAC
ACAAACACCCTCTGACACATACAAGGCTTCCAATTTTAAAGAGTACATGCGCCATGGCGA
GGAATTTGATTTGCAGTTTATTTTCCAGTTATGTGTTATTAATTTAACTACAGAGGTTAT
GGCCTATCTGCATGGTATGAATGCTACGTTACTGGAAGACTGGAATTTCGGTTCCTTGCC
CCCTCCCACTGCTAGTTTGGGCGATACCTACCGGTTCCTACAGTCCCAGGCTATTTCCTG
TCAAAAACCTTCTGCTGTCCCTACAGAGAACAAGGACCCTTATGCAGGGCTTACATTTTG
GGAGGTGGATTTGTCTGAACGTTTTTCCCTTGAGCTAGACCAATTCCCTCTTGGCAGGAA
ATTTTTGCTGCAGCGTGGCACCCGGGTACGGTCGTCCCCTGTGTCCCGCAAGCGTCCCGC
ACCCTCTACCGCCCCCTCTACAAAACGTAAACGGTCCAAACGTTCCTAAGCATGTTATGT
GTGTGTCTGTTGTATGCTGATGTTTACTGTGTCTGTACACGGGTTCCTTGTACTGTTGTG

TATGTATGGGTGGCCCCCTTTTGTTATGTATGTTTCCTTATGCATGTTTGTGTGCATGTT
TTGTATGTTAATAAATGTGTGTGTCATGTGTTGTGTGTGTCCGGTTGCGCCTTGTGAGTC
ACATAACATATATGTGTGGTGTCCTGTATGTGACCCGGCCGTCCCCTAGCCTTTAATACC
CCCGGACCGATTTCGGTCGCTCCCGGTTTCGGTCGTGCACAACATGGCGTTTTGTTGGCA
CTGTTTGCAAAACTGTTAGCTGTTTCGCGCCACATTTTGCAGGTGTGCATGCTTGTCTAG
GGTGTGCCTGACAGTTAGTTGGCCAGCTATACATTTCTTTCACAATATTTATTGTCTGTG
TTACTCACACTCCAATAAATATACTTTTAGGCACATAGTTTTATGCTGACTGCTTTCACT
TAAGTGCTTATCTGGCAGGCAAATTTGCTTTTCTATTCAACAATGTGTCTTGTAAATCCT
ACTGTACAACTTCAAACATGTTTGTAGCACACCTAATCCGGTTGCTTCCCCTGTACATCT
TACTCATTATTATAAACTATAATCGTTCTATTACAAAATAGGAGGGACCGAAAACGGTCC
AACCGAAATGGGTACATATAAAAAGGATATGTTAACTTCAGCACAAAATAGCC
>gi|9628437|ref|NC_001676.1|_Human_papillomavirus_-_54,_complete_genome
TAACTACAACCATGATTTATAAAATAAGGGAGGGACCGAAAACGGTTCAACCGAAACCGG
TACATATAAAAGCGGTTGTAGAAAACAGTTATTTGGGGGCAATGTCTGCTACTGAACCCC
ACACGGACCAGCCGCGTACTCTAGCTGATTTGTGCAAGGTATGCAATATTCCTATGCATA
GTTTGCAACTTCCTTGTGCCTTTTGCAAGAAGACGGTGTGTACAGCAGAGATTTATGCAT
TTCAATATAAGGACCTATTTGTGGTGTGGAGACACGGCTTTCCACATGCTGCATGTGCAC
TGTGCCTAGAACTGCACGGGCAAATAAATTATAGAAGGCATCGCGACCGTGCGTGCCTGT
GGGAAACAGTGGAACAAGAGTGTGGAAAGCCATTGGAAGAAATATTTATAAGGTGCTGGC
TGTGCCATAAACCTTTGTGCAACGTGGAAAAGCAACGGCACGTAGATTACAACAGGCGAT
TCCACTGTGTTAGAGGCTATTGGAAGGGTAGGTGTCTACATTGCTGGAAGCCATAATGCA
TGGAAATGTGGCTACAATTGAGGATATAGTCCTTGATTTAAAACCAGAACCGTTTGACCT
GTACTGCAGGGAGCAATTAGAAGACTCTGATGCAGAAGATGAGACAGCAGTAACACAACC
TGACAAACAAGCATTTAAGGTGTTAAGCCAGTGTGGAGGTGTGTGTTGTAAGACAGTACG
GCTATGTGTGTATAGCACGCACACAGGCATAAGGGTACTGCAGGAACTGCTTCATCAGGA
CGCACTGCAAATAGTGTGTCCCACCTGTGCTTCACGACTATAACGGCATGGCGGATAACC
AAGGTACAGAGGAGGAGGGGACTGGGTGTAATGGATGGTTTTTTGTGGAAGCAATTGTAG
AACGTAAAACAGGGGATATTATTTCAGATGATGAGCCTGAGGATGTGGAGGACAGTGGGC
TTGATATGGTGGACTTTATTGATAATAGTGTGTCACAGGTAGAGGGGCAGGAAAATCCAC
AGGCATTGTTACATGCCCAACAGCTGCAGGCAGATGTAGAGGCAGTGCAACAATTAAAAC
GAAAGTATATAGGCAGTCCGTATGTAAGTCCTGTTGCAAACAGCGAACCCTGTGTAGAAA
AGGACCTAAGCCCCCGGCTAGGGGCTATATCCCTAGGACGGCGGTCAGCCAAAGCCAAAC
GACGGCTGTTTGATAAGGCCCAACCGCCGCCAAATGGCCATACTGACGTGGAAGCTGCGG
TGGAGGTAAATACCGAGGGGACAGATGAAACAGAGACAGACCAGGTGCAGACAGTATCTG
GGGAAACAACTACAGATAGCCTAGGAAGGCAGCAAATTACAGAATTAATACATAACACAA
ATATTCGTGTAGCATTGTTTGGTATGTTTAAAGACCTATATGGATTAAGTTTTATGGACC
TAGCACGGCCATTTAAAAGTGACAAAACAGTGTGTACCGATTGGGTTATTGCAGCATTTG
GAATATATCATGGTATTACAGATGGATTTAAAACATTGCTAGAGCCACATTGTTTGTATG
GCCATATCCAATGGCTAACATGTAGGTGGGGCATGGTATTATTATTATTAACAAGATTTA
AATGTGGCAAAAACAGATTAACAGTAAGTAAATGTTTAGGAATGTTATTAAATATACCAG
AAACCCAAATGTTAATAGATCCTCCAAAATTACGGACGCCTGCAGCAGCTTTGTATTGGT
ATAGACAGGGGCTGTCCAATGCAAGTGAAATATTTGGTACACCCCCGGAATGGCTGGCCA
GACAAACTGTAATTGAATATAGCTTAGCAGACAGCCAGTTTGATTTATCTAAAATGGTAC
AATGGGCATATGACCATAATTATATTGATGACAGTATTATTGCCCTGGAATATGCTAAAT
TAGCTGATATAGATGAAAATGCGGCTGCCTTTCTAGGAAGTAATTGTCAAGCTAAGTATG
TAAAAGACTGTGGAACAATGTGTAGACATTATATAAGGGCACAAAAAATGCAAATGACAA
TGTCACAATGGATTAAACATCGTTGTGATTTAGTAGAGGAGGAAGGTGAGTGGAAGGAAA
TAGTACGATTCCTTAGATATCAACATGTGGATTTTATATCTTTTATGATAGCATTAAAAC
AATTTTTACAAGGCATACCAAAACACAATTGTATATTACTGTATGGACCTCCAGACACAG
GAAAATCTAATTTTGCCATGAGTTTAATTAGCTTTTTAGGAGGTGTAGTGCTATCATATG
TTAATTCTAGTAGCCATTTTTGGTTAGAACCTTTGGCAGATGCCAAAATAGCTATGCTAG
ACGATGCTACAACACAATGCTGGAACTATATGGACATTTATATGCGCAATGCATTAGATG
GCAATCCTATGTGCTTTGATAGAAAACATCGAGCTATGGTGCAAACAAAATGTCCTCCAC
TAATAGTGACCTCCAACATAAATGCTAGTACAGACGACAGATGGCGCTACCTACACAGTA
GAGTAAAATGTTTTTGTTTTCCCAATAGATTTCCATTTGATAGTAATGGAAACCCTGTGT

ATGATTTAAGTAATAAAAATTGGAAATCATTCTTTAAAAGGTCATGGTCACGTTTAGCGC
TGAACGACAACGATAACGAGGAGGAGGAGAATGGAGACCCTAGCAACACGTTTAGATGTG
TGCCAGGAAAGGCTTCTAGACCTATATGAAAAAGATAGTAATAAACTAGAGGACCAAATA
GAACATTGGAAATGCATACGCCTGGAGTGTGCATTGCAATACAAAGCCCGGGAAATGGGA
TACAAAGTGTTACAGCACCAGGCACTGCCTGCACTTGCTGTGTCAAAGGGCAAAGGGCAC
AAGGCCATTGAACTGCAACTTGCATTAGAGACATTGCAAAAGACAGTATACAGCACCGAA
CCGTGGACATTACAGGACACATGCCTGGAACGGTGGAATGCACCACCAACTGGTTGCTTA
AAAAGACGTGGACAAACAGTAGATGTTATATTTGATGGGCATCAGGATAACACAATGCAA
TATGTAATGTGGGGAGATATTTATTATCAAAACTGTGATGGGGAGGGATGGACTAAGGTG
TGCAGTAATATAGATGCAATGGGTATTTATTATATGGATGCAGAACACAAAGTGTATTAT
GTGGATTTTAAAAAGGAAGCATCTAAGTATGGGGAATATGGACAGTGGGAGGTGCGTATG
GGCAGCAGCATCATTTTTTCTCCTGCATCTGTATCTAGTACCGAAGAGGCATTGTCAATA
TCCTCTACTGGCACTGCTGAACACACCAGACCAGCCAATTCCACACCACGTACCGACAAC
TCCACAAAAGCAATCCCGTGCACGCCGCCGCCTAGAAAACGAGCTAGAGTCTACAGCACA
GACCAGCAACCACACAGCACCTCAGACCCCGTGGGCTGTGACAACGACAGGCACATCAGT
GACGATAACAACAAGAACCAAGGACGGCACACAAGTAGTGGTGACACTACACCTATAGTG
CACTTTAAAGGTGAACCTAATACATTAAAATGTTTTAGGCAAAGAATACAAAAGTATAAA
CATTTGTTTGAACAAGCATCCTCAACATGGCATTGGGCATGTGTACCAGGTACCACTAAG
AATAGGGGCATTGTAACATTAACCTATAGTAGTGTAGAACAAAGGCAACAATTTTTAGTA
ACTGTTCGCATTCCACCTAGTATATCTATGTCATTAGGGGTAATGTCATTATAACAGATA
ATCATTGTAAAGTTTGTGATTGTACATACTATATTGTAAAACAACCAGCCAATACTGCTG
CTATGTGTATATAGTGAGGTATGTATGGTGAGTATGGTAGTGAGTGCATGCAGGCCTGCA
GTGACTGAGGTGAGGTGTGTGTGTAGGTGTGTGTAGTGTATGCAGTAATAATGTTGTATA
CAGTAAGTTGGTTAACATGTTGGTTTTTATTATCCATGTTATTATGGCAATGTATTACAA
CAGCATTTACTTTTTTTCTTGTAGCCTTTATTCTATTGTGGGTACCTGCCCTGTGTGTTT
ATGTGCGCCTTGCCAGGCTTCTTGAACTTGACCTTCACTTTACATAAGGTGTTACTGTGC
ACACCTACTGTGGATGCCCTGTGTAACCTGCTTACCATAGTATAATAAATATTGTGTAGA
TATATTTGTGTATTATGGCTAAAGCAAGAGCCCCACGCCGGAAACGTGCATCTGCAACAC
AATTATATCAAACATGCAAGGCCTCTGGCACATGCCCTTCAGATGTAATTCCTAAGGTAG
AGGGTACAACTATAGCAGATCAAATCCTACGATGGGGCAGTATGGGCGTGTTTTTTGGGG
GCTTGGGAATAGGTACTGGTAGTGGCACAGGGGGCCGCACAGGGTATATACCACTTGGAC
GACCCTCTACTACCCTTGAACCAGGTCCTCCTGTACGTCCCGCAGGGGCTGTAGAAACAG
TAGCACCCTCTGACCCTTCCATTGTGTCTTTAGTAGAGGAATCTAGTGTTGTGGATGTTG
GGGCACCAACCCCTACCATTCCATCACAGGGTGGGTTTGAAATAGCCACATCTAGTGATG
CTACCCCTGCTATATTAGATGTCACCTCCACTACCACCCCTATTAGGGTGTCTATAACAT
CCCATGATAATCCTATATATACAGAGCCATCATTATTAGACCCCCCCCCTCCAGTACAAA
TGGATGGTCGTGTTTTAGTATCTACCTCTACTTTGCAATCGTCCACTGCTGAAAATATAC
CTATGGACACTTTTATAATTATGCAGGACCATATAGGCACAACAACTAGCACACCTATAC
CAAGGCCGCCTGCACGCCCACGCTTAGGTTTATATTCCCGTGCGTTACAGCAAGTGCCTG
TACAGGACCCTGCCTTTTTACAACAGCCTTCCAGTCTTATAACATATGACAATCCTGTAT
ATGAGGGTAACCCAGATGTTACCCTACACTTTGAGCAGCCCACAATACATAATGCACCTG
ATCCCGCCTTTATGGACATATTTGCTTTACATAGACCAGCACTTACAACTCGTAGGGGGG
TTGTACGTTATAGTAGGGTGGGCGACAGGGCCACATTACACACACGTAGTGGCCTGCAAC
TTAAACCTCGTGTGCATTTTTTTCAGGACTTAAGCCCTATTGCTCATGTCCCTGAGGAAA
TAGAATTACACCCTTTAATATCTGCAAACAATACAAGCATTAACAATGGTCTTTACTCAG
ATATATATGATGTATATGCTGACACAGATTTTGCTGACACTGGTGGTTTTTCCTCTTCTA
CTGTTTCACATAGTTCTGTACAAACAGCCCTACAAACTACATCTATACCATCTCAGTATG
GTAACACCACAGTTCCCCTAACTGCCTCATCGCCATATACACCTATACCCACATCCTTTA
GGCCCTCATCAGGTCACACCCCATTTGTTCCCGCACGTCCTATATTTCCACAAACACCCA
TTGCTGTTAATGGTGGGGATTTTTACCTGCATCCTAGTTATACTTATGTACGCAAACGCC
GTAAACGTTTCCCATATTTTCTTGCAGATGGCTATGTGGCGGCCTAGCGAAAACAAAGTA
TACCTGCCTCCTACCCCAGTTTCTAAGGTTGTCAGCACGGATGAATATGTGACTCGCACA
AGCATATACTATCATGCAAGCAGCTCTAGATTATTGGCTGTTGGACATCCATATTTTAAA
GTACAAAAAACCAATAATAAGCAAAGTATTCCTAAAGTATCAGGATATCAATATAGGGTG
TTTAGGGTGCAACTACCTGATCCTAATAAGTTTGGACTGCCTGATCCTAGTTTATATAAT

CCTGAGACACAACGCTTAGTATGGGCATGCACAGGTGTGGAGGTTGGTAGGGGCCAGCCA
TTGGGTCTTGGCCTAAGTGGTCATCCATTATTAAATAAATTGGATGATACTGAAAATGCA
CCTAAATATGTTGGTGCAGGAGCTGACAATAGGGAAAATGTTAGCATGGACTATAAACAA
ACACAGTTATGTATTTTGGGCTGCACACCACCTATAGGTGAACACTGGGCTAAAGGCAAC
CTATGTACACCTAATACATTGGCTGCTGGTGACTGTCCTCCTTTGGAATTAGTAAATTCA
TATATACAGGATGGTGATATGGTAGATATAGGATTTGGGGCTATGGATTTTAAAACCCTA
CAAACCTCAAAAAGTGAGGTACCCCTTGATGTAGCTACCTCAATTTGTAAATATCCTGAT
TACCTTAAAATGGCTGCAGAGGCATATGGGGACAGTTTATTTTTTTACTTAAGGCGGGAA
CAAATGTTTGTTAGGCATATGTTAAATAGGGCAGGTACCATGGGTGAGCCTGTACCTAAT
GACTTATACATTAAGAAATCCTCAGGTAACCTTGACAGTTCTATTTATGCTGCAACTCCT
AGTGGCTCTATGGTAACATCTGAATACCAAATATTTAATAAGCCATACTGGTTACAACGG
GCCCAGGGTCAAAACAATGGTATTTGTTGGGGCAATCAGGTGTTTTTAACAGTTGTAGAT
ACCACCCGTAGTACTAACCTAACATTGTGTGCTACAGCATCCACGCAGGATAGCTTTAAT
AATTCTGACTTTAGGGAGTATATTAGACATGTGGAGGAATATGATTTACAGTTTATATTT
CAGTTATGTACCATAACCCTTACAGCAGATGTTATGGCCTATATTCATGGAATGAATCCC
ACTATTCTAGAGGACTGGAACTTTGGTATAACCCCCCCAGCTACAAGTAGTTTGGAGGAC
ACATATAGGTTTGTACAGTCACAGGCCATTGCATGTCAAAAGAATAATGCCCCTGCAAAG
GAAAAGGAGGATCCTTACAGTAAATTTAATTTTTGGACTGTTGACCTTAAGGAACGATTT
TCATCTGACCTTGACCAGTTTCCCTTGGGTCGCAAGTTTTTACTACAGGCTGGCCTACGT
GCACGTCCGCGCCTTCGGCCTGTAAAGCGTGCAGCCCCTTCCTCCTCTAAGGGTACAGCG
CGCAAGCGTGCTAAAACTAAAAGGTAACAGTGTTGTATGTTGTGTTGTTATGTGTTGTTG
TGTTTGTACCTTGTCTGTGTGTATGTTGTGTATGTATTATGTTATGTGTGGAATGTTTGT
GTGTCCTATTTATGTTGTGACTCGTATGTTATATATGTTGTATGTTTTCCTGTATTATAT
ATAATAAAGACTTTGGTTGCACCCTATGAGTAACTTTTGTGTGATGTTGTTTGCTACGTG
CTTAGTAGCCTTTACCTTTTCACCTTTGGTGTCCATTTTGTATAATCTCCATTTTATATA
ACCGAAACCGTTTTCGGTTGCTGTTGGCACTAAAACTGTGTTTTAAGCACTTTGTATCGT
ACATCTAATCTTTTGGGCGCCGCCACATCCTGCCCTGGTAGTTTTGCCAGCACATCTGCA
CACCCACACATTTATAACTGTCAGAACAACTTCACTTTCCATTGTGTAAAAACACGCCCT
TTGCATTCCAACATTGATTTATATATTTTTTACATAATAAAACATGCTAGTAGGCACATA
TTTTAGCTGTGTAAACTTAGTAAGTGCATAGTTGGCAAACTAAAACATAGGTGTATGCCA
GGTACGTGTCTGTACAACAGTGACTAATTCAGGAAACCGATTTCGGTTGCTCACCCATTA
TCTTTTTTATCATTATTAT
>gi|9627377|ref|NC_001593.1|_Human_papillomavirus_type_53,_complete_genome
GAAAGTAACAATCCTACTTTTATAGACAGGGAGTAACCGAAATAGGTTTAGGACCGAAAA
CGGTACATATAAAAGCACTGTGTACAACACCCAGGACATCCATGGATCGTCAGTTATTTG
AAAATACAGAAGAGCGACCACGTACATTGCACCAGCTATGTGAAGTTGTGAATAAACCAT
TGCTGGAGCTGCAACTTGGCTGTGTGTTCTGCAAGAAGGCATTGACAGCGTCAGAGGTAT
ATAATTTTGCATATACAGATCTAAGAGTAGTGTATAGAGACGGGTATCCGTATGGAGTGT
GCAAATTCTGTTTGCTATTTTATAGTAAGGTCCGAAAATTAAGATATTACAATTGTTCAG
TGTACGGGGCTAGCCTGGAAGCACTAACTAAAAAAAAGTTATCTGATTTATCAATAAGGT
GCTACAGATGTCAACATCCGTTGACACCAGAGGAAAAACAGTTACACTGTGACTATAAGA
AACGGTTTCACAAAATTTCACATATGTGGACCGGGTCGTGCCTGACATGCTGGAGACACA
CAACAGCAACTGAATCAGCAGTATAATCATGCACGGTAACGTACCAACACTTCCACAATA
TATTATAGAACTTATACCACAAACTGAGATTGACCTGCAATGCCATGAGCAATTGAACAG
CTCAGAGGATGAGGATGAGGATGAAGTAGACCATCTGCAGGAGCAGCCACAGCAAGCTAG
ACGGGACGAACAACATCCTTGTTACCTAATTGAAACACAGTGTTGTAGGTGTGAGTCGTT
GGTGCAGTTGGCTGTTCAGAGTTCAACAAAAGAGCTGCGTATTTTACAACAAATGCTTAT
GGGCACAGTGGAGCTTGTGTGCCCCCTCTGCGCAACAAGGCGATAACTGCAATGGCGTCA
CCTGAAGGTACAGATGATGAGGGGGGATGCCGGGGATGGTTTCACGTGGAGGCAATAGTA
AAAAAACGTACAGGGGATGTAATATCTGAAGATGAAACAGACGAGGAAAGCACCGAATCT
GATTTGGATGGGTTTATAGACAATAGTAATATAATATCTACACAGGCAGAAAGGGAGACA
GCTCAGCAGTTGTTACATGCCAAAACACACATGCAGATACACAGACGCTGCAGAAATTAA
AACGAAAGTATTTAGGCAGTCCATTAGGTGATATTAGTAATCAGCAAACAGTGTGCCGGG
AAGCTGTAAAACGAAGGCTAATTGATACAGAAGTGCCGGACAGCGGGTATGGCAATACTT
TGGAAACGTGGAAGCAACGCAACAGGTACAGGAACAGTATGTAAGGGAGGCAAGTGGCGG

GAAAGAGCAAAATGGAGGCTCACAACATAGTGTGTGTAGCAGAGATGGGAGCATAGGGTC
AGGAAGTGATATGGATGTGGATAGACAGGATATAATGCCACTGCAACAAATACAAGATAT
ATTAAAATGTAGTAATGTGCAGGCCAAGCTATATTGTAAATTTAAAGATATATTTGGCAT
ACCATTTTCAGAGTTGGTACGAACATTTAAAAGTGATAGTACATGTTGTCATGATTGGAT
ATGTGCTATATTTGGAGTAAATGAACATTAGCAGAGGCATTAAAAACAATTATTAAAACA
CAGTGCATATATTACCATATGCAATGTTTAACATGTACATGGGGCGTGGTTATATTATTG
TTAATTAGATATACGTGTGGCAAAAACAGAAAAACAATTGTAAAGTCTTTAGCATCCATA
TTAAATGTACCAACTGAACAAATGCTTGTGCAGCCTCCAAAAATACGTAGCCCTGCGGTT
GCATTATACTTTTATAAAACATCTATATCCAACATTAGTGACGTGTATGGGAGTACACCA
GAATGGATAGAAAGACAAACACAGTTACAACATAGCTTTGAGGACTGTCAATTTGAACTA
TCTAAAATGGTGCAGTGGGCATTTGACAATGAAGTAACCGATGATAGTCAAATAGCATTT
CATTATGCACAGTTAGCAGATGTAGACAGTAATGCACAAGCATTTTTAAAAAGTAATATG
CAAGCAAAATATGTAAAAGACTGTGGAATAATGTGTAGACACTATAAACGGGCACAACAA
CAACAGATGAATATGAAACAATGGATAAAGCATGTATGTAGCAAGGTGGATGATGGTGGG
GACTGGAAGCCAATAGTACAGTTCCTAAGGTATCAAGGGGTCGAGTTTATTTCATTTTTA
AGTTATTTTAAATTATTTTTACAAGGTACACCAAAACATAATTGTTTAGTTATTTATGGT
CCTCCAAACACGGGTAAATCATGTTTTGCTATGAGTCTTATAAACTTTTTTCATGGGTCA
GTCATTTCATATGTAAATTCACACAGTCACTTTTGGTTACAACCGTTAGACAATACTAAA
TTAGGTATGCTAGATGATGCCACAGAGGCATGTTGGAAATATATTGATGAATACCTAAGA
AATTTATTAGATGGCAATCCTGTAAGCTTAGATAGAAAACATAAGCAACTAGTACAAATA
AAGTGTCCACCTGTACTAATAACAACTAATATAAATCCAATGCAAGATGCAAAGCTACGG
TATTTGCACAGTAGAATACATGTGTTACAGTTTTTAAATCCATTTCCAATTGATGTGAAT
GGAAATCCTGTATACCAATTAAATAATGCAAACTGGAAATGTTTTTTTGAAAGGACATGG
TCCAGATTAGATTTGGATAACGACGAAGACAAGGAAAACGATGGAGACGCTATGCCAACG
TTTAGATGCGTGCCAGGAGAAAATTCTAGACTGTTTTGAAAGAGATAGCAAAAATATTAC
AGACCATATAGACTACTGGAAAGCTGTGCGACAAGAAAATGTAATATATTATAAGGCAAG
AGAAAATAATATGACTAAACTAGGCCACCAGGTGGTGCCCTGTTTACAAGTGTGTAAAGC
AAAGGCATGCGTTGCTATAGAACTACAAATAGCATTGGAATCACTTTGTAAAACAGAATA
TAATATGGAAGAGTGGACATTAAGGGATGTATGTGAAAGTATGTGGTATACAGAACCCAA
ACAGTGTTTTAAAAAACAAGGACAACATATAGAAGTGTGGTTTGATGGCAGCAAGGACAA
TCGGGCTGAATATGTTGTGTGGAAATGGGTATATTATTGTGGGGAGGATGGGTGGTGTAA
AGTGTCTTCTGCAGTAAGCTATGAGGGCATATATTATATACATGACGGCCATAAAACGTA
TTATACAAACTTTAAAGACGAGGCCACCAAATATGGGTGTAAAGGCACATGGGAAGTGCA
TATGGGAAAACAAAGTATTTATTGTCCCGACTCTGTGTCCAGTACCTTTAGATCCAACGT
ATCCTCTGTTGAAACTGTTAACGAATACTACTCCCATAAGACCCCCACCACCTCCACGCC
CGTGGGCACCTACGAAGCCTCATCATCCCTGCGGCCGGGAAAACGTCCCAGAACCACAGA
GCCCGACAGTACTGACTCCACCACACAGTCCACTACCACAGCCAGAGAGTCCTACGCAGA
GTGTGTCGCAAGGAACACAGACAACACAAACAACAACACCAGAAAACACCTCCCTGGTGG
AGCTTCGTGTAACAACACCGAAATCGACAGTGGTTATAAGACTGCACCTGTAGTACATAT
AAAAGGTGAAGCAAACAGACTAAAGTGTTTAAGGTATCGGTTTCAAAAACATAAACAACT
GTTTGTTACTGTATCATCCACCTATCATTGGACAAATGTAAACTGTGCTGTAAATAATAG
TTATATTACTGTTGTATATAAAGATGAAACCCAACGCCAAAAATTTTTGGATATTGTAAA
AATACCCCCTAGTGTATCACTGGTACTGGGACATATGACATGTGTTGATATGTAATGTTA
ATGTAATTGTTACATTATGTAATATTGTATTACAATAATACCTGGTATATACAATACTAC
ACACGCCATATATGCTGCTAGGCCTGTAAACTTGCAACCATTGTGTTGTGTGTTAGTTTT
GTGTTTTTGCTTTGCTGCTTTGTGTGCCTGTTTGTCTGCTTTAATGTGCCGCTATTGCAG
TCTGCCTCTGTGTTTGCCTCGTGTATATTACTTATACTTTTATTTTGGTTTGTTGTGGCA
TCATCCTACATAACCACATTTACACTATATATACTTTTTTTTTACTTCCCTTTATTATGT
TTCTATAGCCATGCTGTGTGGTTAATTAACACACAATAAATCACAGTTTTTTTGTATTGT
ATGTATTTGTGTGTTATATATAATGGTTGCCCACCGGGCACGCAGACGTAAGCGGGCATC
AGCAACGCAGCTATATCAAACATGTAAACAATCTGGCACATGTCCTGAGGATGTTATTAA
CAAAATTGAACACAAAACCTGGGCTGATAAAATATTGCAATGGGGTAGTTTATTTACATT
TTTTGGAGGCCTTGGTATTGGCACTGGCTCTGGCACTGGGGGGCGTACAGGATATATTCC
TTTGGGTACTAGGCCTTCCACTGTTGTTGATGTAACCCCTGCTCGGCCCCCTATTGTTGT
AGAATCGGTAGGCCCCACTGACCCTTCAATTGTTACATTGGTTGAAGAATCCAGTGTTAT

TGAGTCTGGAGCTTCGTTCCCTAATTTTACAGGTACTGCAGGATTTGAAGTAACATCCTC
CTCAACTACCACACCTGCAGTATTAGATATAACACCCACCTCTACCTCTGTACATGTTAG
TTCTACTACATATACTAATCCCACATTTGTAGACCCTCCTGTTATTGAGGTCCCCCAAAC
TGGTGAGGTATCTGGGAATATATTGATAAGTACCCCAACATCTGGGGTTCATAGTTATGA
GGAAATACCTATGCAAACATTTGCTGTTCAGGGTACAGGCAATGAACCTATCAGTAGTAC
TCCTATTCCTGGTCTTAGGCGTATAGCTGCCCCACGTTTATATAAAAAAGCCTTTCAACA
GGTTAAGGTCACAGATCCTGCATTTCTTCACAAACCTGAAACTTTAATTAATGTTGATAA
TCCCATATTTCAAAATGCTGACACAACGTTAACCTTTTCACCATCAGGTGTGGCACCTGA
TCCTGATTTTTTAGACATTGTGGCTTTACACAGACCTGCCTTTACAACACGTAGAGGTGG
TGTGCGTTTTAGTAGGCTTGGCACAAAGGCCACTATGCGCACCAGAAGTGGCAAACAAAT
AGGTGCTCGTGTACATTATTATTATGATGTGAGTCCTATTACACAGACTGAAGAAATTGA
AATGCAGCCATTATTGTCTACAGATAATACATTTGATGGCCTATATGATATTTATGCAAA
CATAGATGATGAGGCACCTGTGTCATCTCGTTTTTCTATTGCTACACCTTCTAGGTTGCC
TACCAACACTGTTCCTTTGTCTTTTAGTGGTAGTACCTCTAATGTTACAATACCCTTTGG
TACATCTTGGGATGTTCCCATCTATTCAGGTCCCGATGTAGTGTTGCCCACAGGACCCCC
TACATGGCCTTATGCACCTCAATCTCCTTTTGATACCACCCATGATGTAGTTATACAGGG
CTCCACATTTGCGTTATGGCCTGTTTATTTTTTGAAACGTAGGCGTCGTAAACGTATTCC
CTATTTTCTTGCAGATGGCGGTGTGGCGGCCTAGTGACAGCAAGGTTTATCTGCCTCCTA
CCCCTGTATCAAAGGTCATTACAACGGATGCCTATGTAAAACGCACCACTATATTTTATC
ATGCTGGAAGCTCTCGCTTGCTTACCGTGGGACATCCTTATTACCCCATTTCTAAATCTG
GTAAAGCAGACATCCCTAAGGTGTCTGCATTTCAGTATAGGGTGTTTAGAGTACGCCTCC
CAGATCCTAATAAGTTTGGCCTTCCTGATACAAATATATTTAATCCTGACCAGGAGCGGC
TTGTATGGGCCTGTGTAGGGCTAGAGATTGGCCGTGGACAGCCTTTAGGTGTTGGTGTAA
GTGGCCATCCACTGTTCAATAGGTTGGATGACACTGAAAGCTCTAGTATAGCTATTCAGG
ATACTGCCCCGGACAGTAGGGATAATGTTTCAGTGGATCCTAAACAAACACAGTTATGCA
TTATTGGCTGTGCACCCGCTATAGGTGAACACTGGACAAAGGGTACGGCCTGTCGTTCCA
CACCTACTACAGCGGGCGACTGCCCCCCATTGGAACTTATCAATTCACCTATTGAGGATG
GAGACATGGTGGACACAGGTTTTGGTGCATTAAACTTTAAAGCTTTGCAGGAATCTAAAT
CTGATGTGCCATTGGATATTGTACAATCCACATGTAAATATCCCGATTATTTGAAAATGA
GTGCAGATGCCTATGGGGATTCTATGTGGTTTTATTTACGTAGGGAACAGTTGTTTACCA
GACATTTTTTTAATAGGGCAGGCGTTATTGGTGAGGAAATACCTAATGACTTATATATTA
AGGGTAGTAATGGCAGGGACCCGCCCCCTAGCTCTGTATATGTTGCTACACCTAGTGGGT
CTATGATAACTTCAGAGGCTCAATTGTTTAATAAGCCATATTGGCTGCAACGTGCCCAGG
GACATAATAATGGCATCTGTTGGAACAATCAGTTATTTGTAACTGTTGTGGATACCACCA
GGAATACAAACATGACTCTTTCCGCAACCACACAGTCTATGTCTACATATAATTCAAAGC
AAATTAAACAGTATGTTAGACATGCAGAGGAATATGAATTACAATTTGTGTTTCAACTAT
GTAAAATATCCCTGTCTGCTGAGGTTATGGCCTATTTACATACTATGAATTCTACCTTAC
TGGAAGACTGGAATATAGGTTTGTCGCCTCCTGTTGCCACTAGCTTAGAGGACAAATACA
GATATGTGAAAAGTGCAGCTATAACCTGTCAAAAGGATCAGCCCCCTCCTGAAAAGCAGG
ACCCACTATCTAAATATAAATTTTGGGAGGTCAATTTGCAAAACAGTTTTTCTGCTGATT
TGGATCAGTTTCCTCTTGGCAGGAAGTTTTTAATGCAGGTTGGGGTCCGTACTAAACCGC
CTGTATCCTCTAAAAAACGCTCTGCTTCTACTACATCTACCTCAGCCCCCTCCTCCAAGC
GCAAACGCAAATAGTATGTTGTGTGTCTGTATTGTGTATGTATGGTTGTATTATGTACTA
TTTATTTATTGTGTATACTGTATGTGTGTATTTGTTGTCTGTGTTGTATGACTGTATGTA
TGTGTAATGTTTGCATGTTGTTAATAAATATGAATGAGTGTTACTTTTACGCGTGGTTGC
ATAAACTAAGGCGCGGTGGTGTCCCTAGGCAGTTGGGTGGCATGTTAGGTGGCGTCCCTG
TTTATATACCAATCTCCACCCTGTATTATTAATATATGTACTGTTATCATGCAGTTACCT
ATACCACATATAGTATAGCTTATAGGTATCCATTTTAGTTTGGCGCCTTTTTGGTACCCT
CCATTTTGCCTTGCAACCGGTTTCGGTTGTGCATATCTTGTAAATACCACAAGCATATTC
AGCAGAACTGTTAATCCTTTGGCATAGTGCCGTTTCCTGTGTTTAATGTTTTTTTGTTTT
ATACACTATGCCTTACCTATTAGTCACTTACTGTAGGCTGCCAACTATGCTTTTACCTGC
ATACCTTAGTGCTTTTGGGCACACATTTTTATTGCAAACATCTGCACTTTAGTGTTTTGG
CTTGCAGCATACGTTCTGCTAGCCAAGTATCTGTCTAGTAAACCAGGTGTGCACCTATTA
CTCATGCACCGCAACCGTTTACGGTTTTGCAGCAACAGGCCTTTTTTATAATTATT
>gi|9627305|ref|NC_001583.1|_Human_papillomavirus_type_26,_complete_genome

TAACAATTATATGTTACTAAAAGGGTGTAACCGAAAACGGTTGCAACCGAAACCGGTACA
TATAAAAGTAAAAGGCTAGCTACGTGCAAAACAGCTATGTTCGAGGATCCTAGAGAACGA
CCCAGAACGCTACATGAGCTATGTGAAAGCTTGAATACTACTTTGCAAAATTTGCAGGTA
CAGTGTGTATATTGCAAGGAAACCTTACAATGGGCTGATGTATATAATTTTGCAATTTGT
GACCTAAGAGTAGTATATAGAGATAGGAGTCCGTATGCTGCATGCAAAAGATGTGTAATA
TTTTATTCAAAAATAACAGAGTATAGACGCTATACATGTTCTGTGTATGGTGCAACATTA
GAAGCCTTAACTAAAAAAAGTTTATGTAATTTGTTAATAAGGTGTCATAGATGTCAAATG
CCATTGGGGCCAGAAGAAAAACAAAGAATTGTGGATGAAAAGCGACGATTTCACGAAATA
GCAGGGCAGTGGAAAGGGTTGTGTACAAATTGTTGGAGACCAAGGCGCCAAACAGAAACA
CAAGTGTAAAGAACAATGCATGGAAACATAATTAATATTGAAGATGTAATACTAGATCTG
GTGCCGCAACCCGAAATTGACCTACGCTGCTACGAACAATTGGACTATGAACAATTTGAC
AGCTCAGATGAGGATGAAACAGATAATATGCGTGACCAGCAGGCCAGACAAGCTGGACAA
GAAGTGTGTTACAGAATTGAAGCACAATGTTGTATGTGTAATAGTATAGTGCAGCTAGCT
GTGCAGAGCAGTCGACAGAACGTTCGAGTGCTGGAGCAGATGTTAATGGAAGACGTGTCC
TTGGTGTGCCATCAGTGTGCTGCACAGTAAACCTGCAATGGACTGTGAAGGTACAAATGA
GGAGGGGCGGGGGTGTACAGGGTGGTTTTCAGTAGAAGCTATAGTGGAAAAACATACAGG
GGACACAATATCAGATGATGAAACAGACAATAGTAGTGATACAGGGTCGGACCTAATAGG
ATTTATAGATGATAGTAGTATAAGTGATTATGCAGAACAGGAGGTAACCCAGGCATTGTT
TCAGGCACAACAAAAACAGGCAAATACAAAGGCAGTGCGCAATTTAAAACGAAAGTTACT
AGGTAGTCAGAACAGCCCGTTGCAAGACATAACAAATCAACACAGACAGCAAAGCGACAG
TCAGCAGAATACACACCAAGTAAATAATTCACAGGCCAAAAGGAGAGCCGTGGACAGTGT
ACCGGACAGCGGGTATGGCTATACTGAAGTGGAAACTCTTACGCCCGTACAGGTAGATAA
ACAATATGAAGAAAATGGCGGGTTGCCTAGTGTGTGTAGTCAGGGGGGGTCAAATGCCTC
AGTGGAAGATATCGATGTAGACACACATGTAAACAGTGTTACACAAATATGTGAATTATT
AAAATGTAGTAATGTAAAAGCAGCATTGTTAAGTAAATTTAAAACAGTATATGGTGTAAG
TTTTGCAGAACTAGTACGGGTGTTTAAAAGTGACAAAACATGCTGTTCAGATTGGGTGTG
TGCAGCATTCGGTGTGGCAGGCTCTGTAGCAGAAAGTATTAAATCATTAATACAACAATA
TTGTTTATATTATCATATACAATGTTTAACATGTAATTGGGGAGTAATAGTACTAATGCT
AGTGCGCTTTACATGTGCAAAAAACAGAACAACAATTAAAAACTGCCTATGTATGTTATT
AAATGTGCCAGAAACGCAATTACTAATTGAACCACCAAAATTGCGAAGTACAGCAGTAGC
ATTATATTTTTATAAAACAGGGTTGTCCAATATAAGTGAGACATATGGAGATACACCAGA
ATGGATAGTACGACAAACACAATTAGAACATAGTTTTGATGATGCTACATTTGATTTATC
AAAAATGGTGCAATGGGCGTTCGATCATGACATAACAGATGATAGTGAAATTGCATTTAA
ATATGCACAGTTAGCTGACATAGATAGTAATGCAGCTGCCTTTTTAAAAAGCAATTGTCA
GGCAAAATATGTAAAAGACTGTGCAACCATGACTAGACATTATAAAAGAGCACAGAAACG
ATCTATGTGTATGTCACAATGGCTACAATATAGATGTTCTAAAATAGAAGAGGGCGGGTC
GTGGAAGGAAATTGCCAAATTTTTAAGGTTTCAACATGTAAACTTTATTTATTTTTTACA
AGTGTTAAAACAGTTTTTAAAGGGTACCCCAAAGCACAATTGTATAGTAATATATGGACC
GCCAAATACTGGTAAGTCACAGTTTGCAATGAGTTTTATAAAATTTATGCAAGGGTCAGT
CATTTCATATGTAAATTCAAATAGCCATTTTTGGCTGCAGCCTTTAGAAGATGCAAAAGT
TGCAGTATTAGATGATGCTACATATAGCTGCTGGTTATATATTGATAAATATTTACGTAA
CTTTTTAGATGGAAATCCCTGTTGTATAGACAGAAAACATAGAAGCCTACTGCAAGTTAC
ATGCCCCCCATTAATAATTACCTCAAATATAAATCCTCAAGAAGATAACTCACTTTTGTA
TTTACATAGTAGAGTAACAGTGATACCATTTCCAAATACATTTCCATTTGACAGCAATGG
GAATCCTGTATATGCATTGACTGATGTAAATTGGAAAAGCTTTTTTTCCACCACCTGGTC
CAGATTAGATTTGGAGGAGGACGCGGACAAAGAAAATGGAGAACCTTTGCCAGCGTTTAA
ATGCGTGCCAGGAGAAAATACTAGACTATTATGAACTGGACAGTAATAAATTAACTGATC
AAATTGATTATTGGAAACTGGTACGATATGAATGTGCAATATTTTATAAAGCTCGTGAAG
GAAACATGCAATGTATAAACCACCAGGTGGTGCCCTCTACTGTTGTGTGTAAACAAAAGG
CATGGCAGGCAATTGAAATACATATAGCATTGCAGTCGTTAATAAACACGGACTATAATA
CAGAAGCTTGGACAATGCGAGACACAAGCTATGAAATGTATATGACAGAACCTAAACATT
GTTTTAAAAAAGAAGGAACAACGGTAACAGTGGTATTTGATTGTAATAAGGAAAATACAA
TGGATTATATTAGGTGGAAATATGTGTATTATAAAACTGATATAGGGTGGTGTAAAGGTA
CTGGAGATGTTGATGCAAAAGGGATATATTATACACAAGGGGCATATAAGCAGTATTACG
TGGACTTTAAACAAGAGGCGGAAAAATATGGACAGGTGTGCAATGGGCTGTACATGTGT

GTGGTCAGGTAATCTGTTGTCCTGAATTTGTATCTAGTACCTGCAGCAGCAACCAAATAT
CCACTGCTAAAACTGCTGAGCCAGTATCAAACGCCACCACCCAGACCACCGAAGCCTACG
TGCCCGTGGGCACCAAGGAAACCGAGGCGCCATACCCAGGAAAGCGACGACGACTCAGTG
GACCTGACACCACCGTCACCACAGTCACCACTGTCACCACAGCTGCCACACAGCCCGGAC
AGTCAGTGGACTATACAAACAACAACCTACACAGTACAAGTGGAGGCCATCACCCGGGAA
GGGACACGAGTAGTGACCAAACTGTGTTTATAGTACACCTAAAAGGTGATACAAATAGTT
TAAAATGTTTAAGATATAGATTTAAAAAGCATAAAGGATTGTATTGCAATGTATCATCTA
CCTGGCATTGGACCAGTAATGATACCAATCAACAAGGCATTGTAACAATTACCTTTAACA
GTATAACACAACGTAATAATTTTTTAACAACTGTTAAAATACCACAAAGTATAACTTCAA
CATTGGGAATAATGTCATTGTAATATAGTGTATATTTTACCAACACACAAGCCAATATGT
GCTGCTAACACACCTATATACCTGTAATCATTATTCCTTTTATAGTTTATGTGTTTGTGC
TTTGCGTGTGTGTGTGTGTGTTGCTGTGTTGTTTGTTGCCACTTTTGCTTTCCATTTATG
TGTTTGCAGCCTCGCTATTATTAGTGTTTTGTTTTTGGTTTGTGGTATCTACATCATATA
TAACTACCTATATTGTGTATATTTGCTTATTTTTTATACCTGCTTGTTTTTTACATTTTT
ATACTGTAATGGTAATTGCTACTTAGTCCCTTTAATAAACGTGTTACAATGGTAGCTGTC
CGTGCCCCTCGACGCAAACGAGCATCAGCTACAGACTTATACAAAACATGTAAGGCCGCT
GGTACGTGCCCTCCTGATGTTATTCCTAAAATTGAAGGTTCTACCCTTGCTGATAAGATA
TTACAATGGAGTGGTTTGGGAATATTTTTAGGTGGTCTTGGTATAGGTACAGGAACTGGG
TCTGGTGGGCGTACTGGATACATTCCCCTAGGAGGGGGTGGTAGACCCTCTGTTGTGGAT
ATCGGCCCTACCCGTCCGCCCATTATTATTGAACCTGTGGGTCCTACAGAACCTTCTATA
GTTACTTTGGTGGAGGAATCTAGTATTATACAATCTGGAGCCCCTATACCTACATTTAGT
GGTGGCAATGGCTTTGAACTTACCACATCCTCTGCAACAACACCTGCTGTGTTGGACATC
ACCCCCTCTGCTGGTACTGTACATGTAACAAGTACCAATATACAAAATCCATTATATATT
GAACCCCCTATAGATATACCACAGGCCGGGGAAGCATCAGGTCATATATTTACTACAACG
TCCACAGCTGGCACACATAGTTATGAAGAAATTCCAATGGAAGTATTTGCTTCTACTAAT
GGAACAGGATTAGAACCTATTAGTAGTACACCTATTCCTGGTATACAACGAGTGTCAGCT
CCTCGTTTGTATAGTAAGGCCTATCAACAGGTAAAGGTTACAGATCCCAATTTTATTGGT
AATCCCTCCACATTTGTTACCTTTGATAATCCTGCATATGAACCTATAGATGAAACACTT
ACATATGCTTCCAGTAGTACTGTAGCACCTGACCCCGATTTTTTGGACATTATTGCATTG
CATCGTCCGGCCCTTACATCTCGCAAAGGTACTGTACGCTATAGTAGGTTGGGTCAAAAG
GCCACTATGAAAACACGTAGTGGAAAACAAATTGGAGCTACAGTACATTATTATCATGAT
ATTAGTCCTATACAGTCTTTTGCTGAACACGAAGAAATTGAATTGCAGCCTTTACATACA
TCTACCCATTCATCTGCACCTTTGTTTGATATATATGCAGACCCTGATACAGTTCCTAGC
ATACATACGCCGCGCATGTCATATTCCCCTACAACATTACCAGTTCCAAGATATGCCTCC
AATGTGTTTTCCTCTATTAATACTTCCACTACCAATGTTACTGTGCCTTTATCCACCTCA
TTTGAACTACCTGTATATAGTGGGTCAGACATTTACACGCCCACATCTTCCCCGACATGG
CCATCATTGCCCCCCCCACCCACCACTAACTTACCTGCAATAGTTGTGCATGGGGATAAT
TATTATTTATGGCCCTATATTTATTTAATCCATAAACGCCGTAAACGTATGCCTTATTTT
TTTTCAGATGGCTTTGTGGCGTACTAGTGACAGCAAGGTATATCTTCCTCCCACCCCTGT
GTCTCGGGTTGTCAACACGGATGAATATGTAACTCGCACCGGCATATATTATTATGCGGG
CAGCTCTCGTTTATTAACATTAGGACATCCATATTTTTCCATACCTAAAACTGGCCAAAA
GGCCGAAATTCCTAAGGTATCTGCCTATCAGTACAGGGTATTTAGAGTGCACCTACCTGA
TCCTAATAAATTTGGATTGCCTGATCCACAGTTATATAATCCTGACACAGAACGCCTGGT
GTGGGCCTGTGTTGGTGTTGAAGTTGGTAGAGGACAGCCATTAGGCATTGGCCTTAGTGG
ACATCCTTTGTTTAATAAGTTGGATGATACCGAAAACTCTCATTTGGCTACTGTAAATGC
AGACACTGACAACAGGGACAATGTTTCAGTTGATAATAAACAAACACAGTTATGTATTAT
AGGTTGTACACCGCCCTTGGGAGAGCACTGGGGTATTGGCACTATATGTAAAAATACACA
GACACAACGTGGGGATTGCCCCCCCTTAGAATTAATTTCCAGCATTATTGAGGATGGCGA
TATGATTGATACAGGCTTTGGAGCTATGGATTTTACTGCCTTACAGGCTACCAAATCAGA
TGTGCCCATTGATATTAGTCAATCCACATGTAAATATCCTGATTATCTTAAAATGTCTGC
AGATACATATGGAAACAGCATGTTTTTTTTTCTTCGCCGGGAACAATTATTTGCCAGACA
TTTTTATAATAAGGCGGGGGCTGTTGGGGATGCTATACCCACCACTTTGTATATTAAAGG
TGCTGAATCAGGCAGGGAGCCCCCTACATCTTCTATTTATTCTGCTACACCTAGTGGCTC
TATGGTTACTTCGGATGCACAACTATTTAATAAGCCATACTGGTTACAACGTGCACAGGG
TCATAATAATGGTATCTGTTGGGGCAATCAATTGTTTGTTACCTGTGTTGATACCACCCG

CAGTACTAACCTTACCATTAGTACATTATCTGCAGCATCTGCATCCACTCCATTTAAACC
ATCTGATTATAAACAATTTATAAGACATGGCGAAGAATATGAATTACAATTTATATTTCA
GTTGTGTAAAATAACACTTACAACAGATGTTATGGCTTACATACATTTAATGAATGCCTC
CATATTGGAGGATTGGAATTTTGGACTAACCTTACCTCCCACTGCTAGTTTGGAAGATGC
CTATAGGTTTATTAAAAACTCTGCTACTACCTGTCAGCGTAACGCCCCTCCTGTGCCAAA
GGAAGATCCTTTTCAAAAATTTAAATTTTGGGATGTAGATTTAAAAGAAAAATTTTCTAT
TGATTTGGATCAATTTCCACTAGGGCGTAAGTTTATGTTACAGGCCGGCATACAACGGCG
GCCGAAACTAGGCACCAAACGTCCCTTATCTTCTACCTCTTCCTCTACCAAACGCAAAAA
ACGTAAACTTACTAAATAATTCCGCATGTTTGTGTGTGTGTATATGTTTGTGGTATGTTA
TGTAAGGTGTGTTTGTATGTGTGTACAACTGTATGTAATGTAAAGGCGTGTTTTGTATGT
ATGTATTATATGTATGCATGGTTATGTGTTTTCCTGTTTGTATGCATACTTGTTATTTAA
TAAAGTATGAATGTGTCTTCATGCATGGTTACATGTCTTTACTACACTATTTGTCATTTG
TTTTACCCCTGAGGTAATGGGAGGAACCTTAGGTGGTGTCCCTTATAATTATTATATTAC
ACAAGTTTACCTTTTATATGTATTTCACTAAACTTTTGTAGTGTTATATTTTTACTTTTT
ATATTTTCTATTACTATCTATTGTCCCAACCGTTTTCGGTCGTTCCTTATTTTAGTTTTT
ATCCAACTTTCATGCTGTATCCTGCAGGAACAGTTAATCCTTTGGCAGACAACACATCCT
GCCTCCTACGCTTGGCTTGCCATTTTGGCACTATAAGTGGCGCGCCTGTATTAGTCACAT
ATATTTAAACAATACTTACATAAGCACTTTTTCTTACATTATAATAAAACTGCTGTTAGG
CACATATTTTTATTTTATTTTATAGGTCTTTTAAGTGCATAGTTGGCTAACATATACACT
TTTGTTTGCCAACTATGTGTCTGACACATACTGTTGTAACCCATAGTTAAACACAGGTGT
GTATGTAACCGAAATGTGTTTTGTTAATGCATGCAAGTTTCTTTATAATAACTTT
>gi|167600365|ref|NC_010329.1|_Human_papillomavirus_type_88,_complete_genome
ATAGTTGGCAACAATCACCTCTGCAGAAAATATATAACCGGCAGTGGTACTGCAGATTAT
TATTTTTTGCCGAAGTATGCAGATGGAGGATTCTTATCTACCAAAACGGCTAGATGACTT
CTGTTCGATTTTTAATATTTCTCTTTTTGATGTGCATCTTCCTTGCATATTTTGTGGCTA
TATTTTGGACCTACAGCAGTTGGGTTCGTTCTATCAGAAACAGCTTAGTTTGGTCTGGAG
GAGCGGTGCTTGTTTTGCGTGTTGTGTCCCTTGTTCTAGGCTTTCTGCTAGGTATGAGGC
TGAACGCTTTTACCGCTGCTCTGTAAAGGGAATTCATTTTGAGGATTTCCTAAGAAAGAA
TCTAGCTGATGTGGTATGCCGATGCTATGAATGTATGTCATTGCTTGATTTACCTGAAAA
ATTAGATTGTATCTTTAGGGGTGAGTGTTTCCATCTTGTGAGAAACACTTGGAGGGGAAC
GTGCAGAAATTGTTGTAGAAAATGATTGGGAAGGAGCCCTCAATTAATGATCTAGAAATA
AAGCTGGAAGAACATGTATTGCCTGCTAACCTTTTAAGCAACGAGGTTTTGTCATCTGAT
GAGGAGACCCAGGAGGAGGAGGAGCGAGAACCTTTCCAAATAGATACCAGTTGCGCCTTC
TGTGAAGCAGGTGTAAGGGTATTTGTTCTAGCTTCACCAGCCGGAATTCGCACGCTTCAG
CAGCTTTTACTTGCAGAGATTTCCATAAGTTGCCCTGGGTGTTCCAGGAACAACTTTCGA
CATGGCAGACCCCAATAAAGGTACTGATACTACTGATAATTTTGACTGTAATTCTGAATG
GTTACTTGTAGATGAAGCAGAATGTGTAGACGATTTAGATGTACTTGATGAATTGTTAGA
AGAAAGTACGCAATGTTCAACGGTTTCAAACTTGATTGATGATGACCCAGTTGATATTGA
GGATCAGGGAAATTCCCTGGCCTTATTCAACAGTCAGGTAACAGAGGATTGTAACACAGC
TATAAGCGCCCTAAAACGAAAGTTAATCAAAAGTCCGCAGCTGCAGACTGTTGCTGAGTT
GAGTCCAAGGCTTCAAGCAGTTTCAATTTCACCCAAAAAACAAAGCAAAAGACGCTTATT
CGATGACAGTGGCATAGGAGAAGATGAAGCTCAAAATACTTCTCAGGTAGCGTTGGACTC
CTTAGAAAGCTCCTTAGAGACATCGGGAGCTGGGCTAACTATTAACCTTTTACATTGCAG
CAACCGACAGGCAACCGTCCATCTAAAATTTAAAGAACTCTATGGTGTATCATTTAAAGA
ACTAACTAGAAATTTTAAAAGTGATAAGTCTTGTTCCAATAGCTGGGTAATTGCTGTGTA
TAATGCTGTAGAAGAAGTTCTGGAGGCATCAAAGGTGCAGCTTCAGAAGCATGTAGAGTT
CTTACAGTTAATAGTGTATGGATTTCATGGGTTGTATCTAATTGTATTTAAAAGCACTAA
AAATAGAGATACTGTATCTAAATTGTTTACGGAAATGTTAAATGTTAATGCTTTACAATT
AATGTGTGATCCACCAAGGACAAGAAGTGTTCCGGTAGCGTTATTTTTTTATAGAAAAAG
TTTGGGAAATGCTTCTTTTATGTATGGAGAAGTTCCAGATTGGATTAAAAAACAAACCTT
GGTAGAGCATCAGTCTGCGTCAGCAGCAGAGACATTTGATTTAGCTCTATGGTTCAATG
GGCATATGATAATGAATTCACAGAAGAAGCAGAAATTGCCTATCAATATGCTTTGGCAGC
AGATGAAGATCCAAATGCCGAGGCATTTTTAAAACATAATAATCAGTATAAATTTGTAAA
AGATTGTGCCGCAATGGTAAAAATGTATAGAAGGTATGAATTGAGACAAATGTCAATGGC
TGAATGGATAGATAAATGTTGTTCAAAATGTACCGAAAATGGTAACTGGAAAATAATTGC

TACATTTCTGAAACATCAGAATGTACAATTTTTATCATTTCTCATTGCATTGAAACCTTT
CTTACAGGGTGTTCCAAAGAAGAATTGTCTGGTGTTTGTTGGGCCCCCTGACACTGGGAA
ATCATATTTCTGCTTCTCATTAATACGTTTTTTTGGGGGAAAAGTTGTGTCCTTTATGAA
TAGAAATAGTCATTTCTGGTTACAACCATTGCAAGATACTAAATTAGGCTTCCTAGATGA
TGCAACACATCCATGTTGGGTGTATATGGATATTAACCTTCGGAATGGACTAGATGGTAA
TCTTGTATCTCTTGATGCAAAGCATAGAGCTCCAATGCAAATGAAATTACCTCCTTTACT
TGTTACCACTAACGTGGATGTAATGGAGGATCAATCCTTAAGATATCTGCATAGTAGACT
AGTATGTTTTAAATTCCCTAACAAATTACCACTGAATGACGATGGCTCCCTTGTATATGA
AATAACGAATGATACATGGGCCTGTTTTTTTAGAAAATTTGCTACCCAGTTAAACCTGGT
CTTAGAAGATGGCGAGAATGGAAACACAGGAAACTCTGACAGAACGTTTCGTTGCACTGC
AAGATGCCATATTGAATCTAATTGAAAGGGGAGAAACTGATTTAAGATCCCAAATACAAT
ATTGGGAGCTGGTTAGAAAAGAGCAGGTGATCCTTTATTATGCTAGAAAGAGTGGCTATA
ATAGACTTGGCCTGCAGCCTACTCCAGCACCAGCGGTTTCAGAATACAATGCTAAACAAG
CTATCCATCTCCAGCTTATGTTAAAATCCCTTGAAAAATCAAAATTTGCAAAAGAGCCAT
GGTCATTAACTGATGCAAGTGCAGAGCTTGTAAATACTCCTCCTAGGGACTGCTTCAAAA
AAGGAGGATTTACAGTAACTGTGTACTTTGATAATGATCGAGAAAACAGCTTTCCCTACA
CACAATGGGAACATATTTATTATCAGGACCAGAACGAGCAGTGGCATAAAGTGCCAGGTG
GTGTTGATCACAATGGGCTGTACTATGATGAGGAAAATACCAATGAAAGGGTATATTTCC
TTTTATTTGAGCCTGAGTCTCAAAAATATGGCAGTAGTGGACAATGGACTGTGCATTATA
AAAATACTACTGTTTCTGCCTCTGCCACCAGCTCTTCTAGGCGGTCCTCACCCATCTCCA
CCAAAACCGATTTCGACGCCACAACCGCCGGCAACACCACGACCTCTGCCCCGCAGAGAA
GTCCAAGGAAGCGACTACAAGAGGCGGTCAGCTCAACCACCTCCCCGCCGGCCCACAATC
TTCGATCTCCAGGAAGAGGACGAGGAGAAGGAGAACGAACCTCCGGAGCAAAGAGAAGAA
GAACAGCCACCGACGGAAACACTCTCGGAGAATCTGTCCCGTCTCCTTCGCAGGTGGGAT
CAAGACATAGAGCACCTGAAAGATCTGGTCTCTCGCGACTTGGACGACTTCAAGCGGACG
CTTGGGATCCCCCATTGATCATTATTAAAGGTCCTGCAAATACCCTTAAATGCTGGAGAA
ATAGAATGAAAAAAAATAGTTCCTCCAATTTAGTTTGCAGCTCAGTCTGGAGATGGATAG
ATAGTACCACCCATGAAAACAGCCGCATGCTTGTAGCATTTCAAAATACTGCTGAAAGAA
CTCGATTTCTAAATTCAGTGACTTTGCCAAAGGGTACAACCTACGCATTTGGTTACCTTG
ATTCATTGTAACTATGTATAAAACCAGAAGTAAACGTGACACTGCTGAGAATTTGTATAG
ACACTGTAAAGCTACTGGCAACTGTCCTCCAGATGTAGAAAATAAAATAGAAGGCAATAC
ATTAGCAGATCGATTGCTGCGCATTTTTGGTAGTGTTATATACCTGGGGGGTCTGGGTCT
TGGTACAGGGGAGGGCACAACAGGTATTAGACCTATAGAAGCTCCTGTTGAAACAGTTCG
CCCAGATATAACCGTAGAGAGACCCACAGTAAGACCAAGACCACAAAGACCTACCACTTT
CGGTACACCTATAGATAGAATTGGATCTGCTGATATCACTCCTAATGTAGTTAAGCCCAC
AGAATCCTCTATCGTGCCCCTAAATGAAAGTGGGATTCCAGACCCTACAATTATAGATTC
TGCAACAGGGGGTGGTGAGGGTCTAGGAGAATATGACATATTGACCACTGTAGATCCAAA
TGAGACCCTCGGAGCTACAGGGGGGCATCCTACCACTTCTGGTACACTTAATAATGAAAC
AGCAATCTTAGACATCAGTCCCTATGAACCTCCTCCAAAACGTTTTGCACTTGCCCCAAG
TGTGCATGCAGAAGCAGACATTACTATTATAGAGAGCTCACTACCTACAGAATCAAACAT
AAATGTGTTTGTAGATGCTAATATTACAGGTGAAATTGTTGGTGAAGAAATCCCATTAGA
GCCTATAAATTCTATAGAAGAATTCGAAATAGAGGCTGGGCGCCAGACTAGTACTCCTAG
GGAAGCTGTTGAAAGATTTCTTGGGCGGGCCAGAAGCTTATATAATCGTTACATTCAACA
AATCAGAACAGACAATGTTGACTTTTTAACCCGGCCTTCACGCGCGGTTCAATTTGAATT
TGAAAATCCCGCCTTTACTGGTGATGTAAGTCTTGAATTTGCGCGGGATGTTGCTGAAAT
AACAGCGGCTCCTGATCCAGATTTTGCAGATATAATTAGGTTGGGCCGTCCCATATTTTC
TGAAACACCAGGTGGAACAGTGCGAGTCAGCAGATTGGGAACAAAAGGAGCGATTAGTAC
CAGAAGCGGTACCATAATAGGTCCTCGTGTGCACTACTACTTTGATCTTAGTGCAATTGA
ACCTATAGAGCCTGATGTTATTGAGCTTTCTAATTTAGGGGAGTTTAGTGGGGAAAGTAC
AATTGTAGATTCAATTTTATCTGGGCATACAGTAGATCCTATAGCACCTTTTGAGTCAAC
ATTTTCTATTGCAGATCTAGAAGACCCTTTACTAGAGGACTTTAGTAATAGTCACTTATT
TGTGCATTTTGAGGAAGAGGATGAACTTATATCTGTTCCTACTCTTCCTCCTGGCGCAGC
AATAAAAGCTTTTGTAGATGATTATGCAGATATTATAGTTTCATATCCTGAAATGGTAAA
TGTAAACAAAATTGAAATCCCTGCAACTACATTAGTGCCCTCTGAACCAGACATTAGACT
AGATTGGTTTTCTCCAGATTATGATTTGCACCCTTCTTTACTTCGTAGACGACGCAAACG

CAAACGCAATATGTTTTGATGTTTTGCAGATGGCATTATGGCAACAACAAACTGGGAAGC
TGTTCCTTCCGCCGGCTAAACCTGTGGCAAAAATATTATCTACAGATGATTATGTTATTG
GAACTCAGTTACATTTTCATGCTGGCACTGATCGTCTGTTAACAGTAGACCATCCCTACT
ATGAAATTAGGAATGATGGAGATCCCAACAAAATTGAGATTCCTAAGGTGTCTGCATGCC
AATATCGAGTGTTTAGGCTGAAATTGCCTGATCCAAATAAATTTGCCTTAATAGATCAGT
CAATTTTCAATCCTGAACGTGAACGTTTAGTTTGGAAATTAAAAGGTATTCAAATTGGTA
GAGGTGGACCACTAAATGTAGGTACTGCAGGTCATCCTTTATTTAACAAAGCTCCAGATA
CTGAAAATCCTAATGATTATCCACCAGCTCAAACAGATGAAGACAGACGTAACGTGTCTA
TGGACCCCAAACATACCCAATTATTTATAATTGGTTGCATTCCTGCAGATGGTGAATATT
GGGATGTTGCTAAACCATGTGTTGGAGATCAGGCGAATGCAGGAGACTGTCCACCTATAC
AGTTAGTTAATGCTGTTATTCAGGACGGAACAATGGGTGACACAGGTTTTGGGGCAGCAA
ATTTTAAGAACTTTTTGCAAGACAAAGCAGGTGTTCCTCTGGATATTATTAATGATATAT
GTATGTATCCAGACTTCCTTAAAATGGACAAAGATGTTTATGGCGATAAATGTTTCTTTT
TTGGAAAACGTGAGCAGTCGTATGCTCGCCATTTTATGGCTCGTGAAGGTAAAATGGGAG
ATGCCATACCAGAGGGAAATAAATACTTATTGCCACCTACAAATGCTGTGAATTTTAAAA
TGGGATCTCATGTTTATTTCCCTACTGCTAGTGGATCACTTACTACTAGTGATTCTAACT
TATTTAACAGACCTTATTGGCTACAGAGATCTTTAGGTGCAAATAATGGAATATGTTGGG
GGAATCAGTTATTTGTTACTGTTCTTGATAACAGCAGGAATACTAATTTTACTCTTTCCA
TTTATAATCAGGGAGGAGCTATTGGAAATCAGTATACTTATAAATCTCAAGATTTTACTC
AGTATACTCGACATGCAGAGGAATACGAATTGGAAGCTATATTCCAGCTTTGTAGAGTTC
CTTTGGAGCCTGATATTTTAGCTCACTTAAATGTTATGAATCCTAATATATTAGATGAAT
GGCAATTAGCTTTTGTGCCTCCTCCTCCTCAGAGTATAGAGGACAATTATAGATATATAA
CATCTTTGGCAACAAGATGTCCTACTGAAAATCCTGCTCCAGAAATACCTGACCCTTATA
AAGATTATAATTTTTGGTTAGTTGATTTGCAAGAGAAGTTCTCCAGTGAACTTGACCAAT
TTAGCTTAGGTCGCAAATTTTTGTTTCAAAGTGGTTTGCTGCCCACAAAACGTGTCAGGA
CAGTTGCCAATAGTTCTGTTAGAAATACTTCCAGAACAGTAAAAAGAAAACGAACACGAT
AATTGCATATTTAAGATAATTACATCCTGAAGCCCATAAAACTGTGAAATAATTGCTGTG
ACTCTTGGATCAACTTGGTACCAACTTGCCTGATAATACTTGAAGGAATGTATTGATTAA
ATAAATTGCTGCTATACCATCAGTTGAGTCATGGGGTCGTCATTTATGGTCTCCACCCGT
CCACTCGACCTTATAAGTGTGGTCAGCAGGCTGGACAGGTAGTATTGTCCAGCGAAGTAA
TCCAGCTGTCAAACCATTAAAGGTAAGTTGTTTGGAACATAGCCGTCTCTCGAACGGAAG
CGGTACAAAATGTGCCCTAAAGGCCTTTTAATAACCGTCTCTGGTATTTTGGCGCCTTTT
TTTTCGCGTGACTGCAAGTTCAGACACTCATGTATTGAGTCAGACACATGGCAGAATATG
TTTTTGGCTGCCTGCCTATGTACCGGGAGTGGTGACCGGGAGTGGTGTTCACAGACATTA
ACAATG
>gi|222386|dbj|D90400.1|PPH58_Human_papillomavirus_type_58_complete_genome
CTAAACTATAATGCCAAATCTTGTAAAAACTAGGGTGTAACCGAAAACGGTCTGACCGAA
ACCGGTGCATATATAAAGCAGACATTTTTTGGTAGGCTACTGCAGGACTATGTTCCAGGA
CGCAGAGGAGAAACCACGGACATTGCATGATTTGTGTCAGGCGTTGGAGACATCTGTGCA
TGAAATCGAATTGAAATGCGTTGAATGCAAAAAGACTTTGCAGCGATCTGAGGTATATGA
CTTTGTATTTGCAGATTTAAGAATAGTGTATAGAGATGGAAATCCATTTGCAGTATGTAA
AGTGTGCTTACGATTGCTATCTAAAATAAGTGAGTATAGACATTATAATTATTCGCTATA
TGGAGACACATTAGAACAAACACTAAAAAAGTGTTTAAATGAAATATTAATTAGATGTAT
TATTTGTCAAAGACCATTGTGTCCACAAGAAAAAAAAAGGCATGTGGATTTAAACAAAAG
GTTTCATAATATTTCGGGTCGTTGGACAGGGCGCTGTGCAGTGTGTTGGAGACCCCGACG
TAGACAAACACAAGTGTAACCTGTAACAACGCCATGAGAGGAAACAACCCAACGCTAAGA
GAATATATTTTAGATTTACATCCTGAACCAACTGACCTATTCTGCTATGAGCAATTATGT
GACAGCTCAGACGAGGATGAAATAGGCTTGGACGGGCCAGATGGACAAGCACAACCGGCC
ACAGCTAATTACTACATTGTAACTTGTTGTTACACTTGTGGCACCACGGTTCGTTTGTGT
ATCAACAGTACAACAACCGACGTACGAACCCTACAGCAGCTGCTTATGGGCACATGTACC
ATTGTGTGCCCTAGCTGTGCACAGCAATAAACACCATCTGCAATGGATGACCCTGAAGGT
ACAAACGGGGTAGGGGCGGGCTGTACTGGCTGGTTTGAGGTAGAAGCGGTAATAGAACGA
AGAACAGGAGATAATATTTCAGATGATGAGGACGAAACAGCAGACGATAGTGGTACAGAT
TTAATAGAGTTTATAGATGATTCAGTACAAAGTACTACACAGGCAGAAGCAGAGGCAGCC
CGAGCGTTGTTTAATGTACAGGAAGGGGTGGACGATATAAATGCTGTGTGTGCACTAAAA

CGAAAGTTTGCAGCATGCTCAGAAAGTGCTGTAGAGGACTGTGTGGACCGGGCTGCAAAT
GTGTGTGTATCGTGGAAATATAAAAATAAAGAATGCACACACAGAAAACGAAAAATTATT
GAGCTAGAAGACAGCGGATATGGCAATACTGAAGTGGAAACTGAGCAGATGGCACACCAG
GTAGAAAGCCAAAATGGCGACGCAGACTTAAATGACTCGGAGTCTAGTGGGGTGGGGGCT
AGTTCAGATGTAAGCAGTGAAACGGATGTAGACAGTTGTAATACTGTTCCATTACAAAAT
ATTAGTAATATTCTACATAACAGTAATACTAAAGCAACGCTATTATATAAATTCAAAGAA
GCTTATGGAGTAAGTTTTATGGAATTAGTTAGACCATTTAAAAGTGATAAAACAAGCTGT
ACAGATTGGTGTATAACAGGGTATGGAATAAGTCCCTCCGTAGCAGAAAGTTTAAAAGTA
CTAATTAAACAGCACAGTATATATACACACCTACAATGTTTAACGTGTGACAGAGGAATT
ATATTATTATTGTTAATTAGATTTAAATGTAGCAAAAATAGATTAACTGTGGCAAAATTA
ATGAGTAATTTACTATCAATTCCTGAAACATGTATGATTATCGAGCCACCAAAATTACGA
AGTCAAGCATGTGCCTTATATTGGTTTAGAACAGCAATGTCAAATATAAGTGATGTGCAA
GGGACAACACCAGAATGGATAGATAGATTAACAGTGTTACAGCATAGCTTTAATGATGAT
ATATTTGATTTAAGTGAAATGATACAATGGGCATATGATAATGACATTACAGATGATAGT
GACATTGCATATAAATATGCACAGTTAGCAGATGTTAATAGTAATGCAGCAGCATTTTTA
AGAAGCAATGCACAAGCAAAAATAGTAAAAGACTGTGGCGTTATGTGCAGACATTATAAA
AGAGCAGAAAAGCGTGGTATGACAATGGGACAATGGATACAAAGTAGGTGTGAAAAAACA
AATGATGGAGGTAATTGGAGACCAATAGTACAATTTTTAAGATATCAAAATATTGAATTT
ACAGCATTTTTAGTTGCATTTAAACAGTTTTTACAAGGTGTACCAAAAAAAAGTTGTATG
TTACTGTGTGGCCCAGCAAATACAGGGAAATCATATTTTGGAATGAGTTTAATACATTTT
TTAAAAGGATGCATTATTTCATATGTAAATTCCAAAAGTCATTTTTGGTTGCAGCCATTA
TCAGATGCTAAACTAGGTATGATAGATGATGTAACAGCCATAAGCTGGACATATATAGAT
GATTATATGAGAAATGCATTAGATGGTAACGACATTTCAATAGATGTAAAACATAGGGCA
TTAGTACAATTAAAATGTCCACCATTAATAATTACCTCAAATACAAATGCAGGCAAAGAT
TCACGATGGCCATATTTGCACAGTAGACTAACAGTATTTGAATTTAACAATCCATTTCCA
TTTGATGCAAATGGTAATCCAGTGTATAAAATAAATGATGAAAATTGGAAATCCTTTTTC
TCAAGGACGTGGTGCAAATTAGGCTTAATAGAGGAAGAGGACAAGGAAAACGATGGAGGA
AATATCAGCACGTTTAAGTGCAGTGCAGGACAAAATCCTAGACATATACGAAGCTGATAA
AAATGATTTAACATCACAAATTGAACATTGGAAACTAATACGCATGGAGTGTGCTATAAT
GTATACAGCCAGACAAATGGGAATATCACATTTGTGCCACCAGGTGGTGCCGTCATTGGT
AGCATCAAAGACTAAAGCGTTTCAAGTAATTGAACTGCAAATGGCATTAGAGACATTAAA
TGCATCACCATATAAAACAGATGAATGGACATTGCAACAAACAAGCTTAGAAGTGTGGTT
ATCAGAGCCACAAAAATGCTTTAAAAAAAAAGGCATAACAGTAACTGTACAATATGACAA
TGATAAAGCAAACACAATGGATTATACAAATTGGAGTGAAATATATATTATTGAGGAAAC
AACATGTACTTTGGTAGCAGGAGAAGTTGACTATGTGGGGTTGTATTATATACATGGCAA
TGAAAAGACGTATTTTAAATATTTTAAAGAGGATGCAAAAAAGTACTCTAAAACACAATT
ATGGGAGGTACATGTGGGTAGTCGGGTAATTGTATGTCCTACATCTATACCTAGTGATCA
AATATCCACTACTGAAACTGCTGACCCAAAGACCACCGAGGCCACCAACAACGAAAGTAC
ACAGGGGACAAAGCGACGACGACTCGATTTACCAGACTCCAGAGACAACACCCAGTACTC
CACAAAGTATACAGACTGCGCCGTGGACAGTAGACCACGAGGAGGAGGACTACACAGTAC
AACTAACTGTACATACAAAGGGCGGAACGTGTGTAGTTCTAAAGTTTCACCTATCGTGCA
TTTAAAAGGTGACCCAAATAGTTTAAAATGTTTAAGATATAGATTAAAACCATTTAAAGA
CTTATACTGTAATATGTCATCCACATGGCATTGGACCAGTGATGACAAAGGTGACAAAGT
AGGAATTGTTACTGTAACATACACAACGGAAACACAACGACAACTGTTTTTAAACACTGT
TAAAATACCACCCACTGTGCAAATAAGTACTGGTGTTATGTCATTGTAATTGTATTGTAC
AATTACTGTATGTAAACCACAAGCCAATATGTGCTGCTAAGTGTATATACAATGATATTA
CCTATTTTTGTTGTTTGTTTTATACTGTTTTTATGCTTGTGCATTTTTTTGCGGCCATTG
GTGCTATCTATTTCTATATATGCTTGGTTGCTGGTGTTGGTGTTGCTGCTTTGGGTGTCT
GTGGGGTCGGCTCTACGAATTTTTTTCTGTTACTTAATATTTTTATATATACCAATGATG
TGTATTAATTTTCATGCACAATACTTAACCCAACAAGACTAACTGTATACTGGTTCTGCA
CATGGTGGTATGGTATTGTAAATATTTACTGTTGTGTGTGTTGTTTTTATTATTTTTATA
CATTTACTAATAAATACTTTTATATTTTTAGCACTGTCTTATTATGAGACACAAACGGTC
TACAAGGCGCAAGCGTGCATCTGCTACACAACTTTACCAAACATGCAAGGCCTCAGGCAC
CTGCCCACCTGATGTTATACCCAAAGTTGAAGGCACTACTATAGCAGATCAAATATTACG
ATATGGTAGCTTAGGGGTGTTTTTTGGAGGTTTAGGCATTGGTACAGGGTCGGGTACAGG

TGGCAGGACTGGATATGTGCCCCTTGGTAGTACCCCACCGTCTGAGGCTATACCTTTACA
GCCCATACGTCCCCCAGTTACCGTTGATACTGTGGGGCCTTTGGATTCTTCTATTGTATC
TTTAATAGAGGAATCTAGTTTTATAGACGCCGGTGCACCAGCCCCATCAATTCCCACTCC
ATCTGGTTTTGATATTACCACCTCTGCAGATACTACACCTGCAATACTTAATGTTTCCTC
TATTGGAGAATCATCTATACAAACTGTTTCTACACATTTAAATCCCTCCTTTACTGAGCC
ATCCGTACTCCGCCCTCCTGCACCTGCAGAGGCCTCTGGACATTTAATATTTTCCTCTCC
TACTGTTAGCACACATAGTTATGAAAACATACCAATGGATACCTTTGTTATTTCTACTGA
CAGTGGCAATGTCACGTCTAGCACACCCATTCCAGGGTCTCGCCCTGTGGCACGCCTTGG
TTTATACAGTCGCAACACCCAACAAGTTAAGGTTGTTGACCCTGCTTTTTTAACATCTCC
TCATAGACTTGTAACATATGATAATCCAGCATTTGAAGGCTTTAACCCTGAGGACACATT
GCAGTTTCAACATAGTGACATATCGCCTGCTCCTGATCCTGATTTTCTAGATATTGTTGC
ATTACACAGACCTGCATTAACCTCTCGCAGGGGTACTGTACGTTATAGTAGGGTTGGGCA
AAAGGCTACACTTCGTACTCGCAGTGGAAAGCAAATAGGGGCTAAAGTACATTACTACCA
AGACTTAAGTCCCATACAGCCTGTCCAGGAACAGGTACAACAGCAGCAACAATTTGAATT
ACAATCTTTAAATACTTCTGTTTCTCCCTATAGTATTAATGATGGACTTTATGATATTTA
TGCTGACGATGCTGATACTATACATGATTTTCAGAGTCCTCTGCACTCACATACGTCCTT
TGCCACCACACGTACCAGTAATGTGTCCATACCATTAAATACTGGATTTGACACTCCTCT
TGTGTCATTGGAACCTGGTCCAGACATTGCATCTTCTGTAACATCTATGTCTAGTCCATT
TATTCCTATATCTCCACTAACTCCTTTTAATACCATAATTGTGGATGGTGCTGATTTTAT
GTTGCACCCTAGCTATTTTATTTTGCGTCGCAGACGTAAACGTTTTCCATATTTTTTTGC
AGATGTCCGTGTGGCGGCCTAGTGAGGCCACTGTGTACCTGCCTCCTGTGCCTGTGTCTA
AGGTTGTAAGCACTGATGAATATGTGTCACGCACAAGCATTTATTATTATGCTGGCAGTT
CCAGACTTTTGGCTGTTGGCAATCCATATTTTTCCATCAAAAGTCCCAATAACAATAAAA
AAGTATTAGTTCCCAAGGTATCAGGCTTACAGTATAGGGTCTTTAGGGTGCGTTTACCTG
ATCCCAATAAATTTGGTTTTCCTGATACATCTTTTTATAACCCTGATACACAACGTTTGG
TCTGGGCATGTGTAGGCCTTGAAATAGGTAGGGGACAGCCATTGGGTGTTGGCGTAAGTG
GTCATCCTTATTTAAATAAATTTGATGACACTGAAACCAGTAACAGATATCCCGCACAGC
CAGGGTCTGATAACAGGGAATGCTTATCTATGGATTATAAACAAACACAATTATGTTTAA
TTGGCTGTAAACCTCCCACTGGTGAGCATTGGGGTAAAGGTGTTGCCTGTAACAATAATG
CAGCTGCTACTGATTGTCCTCCATTGGAACTTTTTAATTCTATTATTGAGGATGGTGACA
TGGTAGATACAGGGTTTGGATGCATGGACTTTGGTACATTGCAGGCTAATAAAAGTGATG
TGCCTATTGATATTTGTAACAGTACATGCAAATATCCAGATTATTTAAAAATGGCCAGTG
AACCTTATGGGGATAGTTTGTTCTTTTTTCTTAGACGTGAGCAGATGTTTGTTAGACACT
TTTTTAATAGGGCTGGAAAACTTGGCGAGGCTGTCCCGGATGACCTTTATATTAAAGGGT
CCGGTAATACTGCAGTTATCCAAAGTAGTGCATTTTTTCCAACTCCTAGTGGCTCTATAG
TTACCTCAGAATCACAATTATTTAATAAGCCTTATTGGCTACAGCGTGCACAAGGTCATA
ACAATGGCATTTGCTGGGGCAATCAGTTATTTGTTACCGTGGTTGATACCACTCGTAGCA
CTAATATGACATTATGCACTGAAGTAACTAAGGAAGGTACATATAAAAATGATAATTTTA
AGGAATATGTACGTCATGTTGAAGAATATGACTTACAGTTTGTTTTTCAGCTTTGCAAAA
TTACACTAACTGCAGAGATAATGACATATATACATACTATGGATTCCAATATTTTGGAGG
ACTGGCAATTTGGTTTAACACCTCCTCCGTCTGCCAGTTTACAGGACACATATAGATTTG
TTACCTCCCAGGCTATTACTTGCCAAAAAACAGCACCCCCTAAAGAAAAGGAAGATCCAT
TAAATAAATATACTTTTTGGGAGGTTAACTTAAAGGAAAAGTTTTCTGCAGATCTAGATC
AGTTTCCTTTGGGACGAAAGTTTTTATTACAATCAGGCCTTAAAGCAAAGCCCAGACTAA
AACGTTCGGCCCCTACTACCCGTGCACCATCCACCAAACGCAAAAAGGTTAAAAAATAAT
TGTTGTGGTACTTACACTATTTTATTATACATGTTTGTTTGTTTTATGTATGTGTTGTCT
GTTTGTTTATGTTTGTGTATATGTTGTATGTGTTATGTGTCATGTTTGTGTACATGTTCT
ATGTCCTTGTCAGTTTCCTGTTTCTGTATATATGTAATAAACTATTGTGTGTATTGTAAA
CTATTTGTATTGTTTGGGTGTATCTATGAGTAAGGTGCTGTCCCTAAATTGCCCTACCCT
GCCCTGCCTATTATGCATACCTATGTAATAGTATTTGTATGATATGTATTTTATAGTTTT
TAACAGTACTGCCTCCATTTTACTTTACCTCCATTTTGTGCATGTAACCGATTTCGGTTG
CTGGCACAAACGTGTTTTTTTTAAACTACAATTTAAACAATACAGTTAATCCTTTCCCTT
CCTGCACTGCTTTTGCCTATACTTGCATATGTGACTCATATATACATGCAGTGCAGTTGC
AAAATGTTTAATTATACTCATAGTTTAAACATGCTTATAGGCACATATTTTAACTTACTT
TCAATGCTTAAGTGCAGTTTTGGCTTGCACAATAGTTTGTTATGCCAAACTATGTCTTGT

AAAAGTGACTCACTAACATTTATTGCCAGGTGTGGACTAACCGTTTTGGGTCACATTGTT
CATGTTTCAACATTTTATATAATA
>gi|109390389|ref|NC_008189.1|_Human_papillomavirus_type_101,_complete_genome
ATGAGAGGACAAAAGGCAACTATTGGAGATGTAAAGTTAGAACTTAGCCCTTTAGTGCTA
CCTGCAAACTTATTATGTGATGAATTAGTAACCGAGGAGGAGGAGCAGCAGGACGAGCAA
GCTTTAAGCCCTTATAGAATTGTGACAGATTGTGGCAGTGTCACAAAACCTTAGCATTA
CATGTACTAGCTACGGACGGGGCAATAAGGTCTGTGGAACAACTGCTGTGTGGAGACCTG
GAATTGGTGTGTGCAAGCTGCATAAGAGCCCAGCGCCATGGAGGACAAAGACAATAAAGG
TACATCCCCTCACATTTGTGACTATAATGCAATAGACTTTATTGACGCAGAAGCGGCATG
TGAGGAGTTAGACCCTTTGGAAGCCGTATTGGAAGAAAGTGATGGGACAGATATTTCAGA
CCTAATAGACAATACGGAGGAGCCAGATGAGGGAAATTCCCTAGCATTGTTAAATAAGCA
GCAGCTAGAGGACGACACGCAGCAGCTTAAGATTCTAAAACGAAAGTACTTTAGTCCTAG
CCCAAAGCAACAAGCAGTAATAGATTTGAGCCCAAGACTGCAACAGTTGCGTGTATCGTC
CCAAGGATCCAGTAAAAGACGGCTGGTATTTGAGGACAGTGGACTAGGCAATGAAACTGA
AGATTCTGTTACGGAGGTGGCAAAGGTAGCGTCGGAGCAGCCATTAAGCTGCAACCCCGG
GGAGCCCGAAACCAATGAAGACGTCAGCACAGATGTAGAGACTAGATCTGGGGGGCCATG
TGGCGGCACAACAACCAGTAACAATAATAGTAACAACAATTGTATAGACCTATTGCATAG
TAGTAATAGAGAAGCCACTGCATATGCAAAATTTAAAGCCACATTTGATGTGGGATATAA
GGAATTAACAAGACCATTCATAAGTAATAAAAGCTGTTGCTGCAGTTGGATAGCGGGGAT
ATTTGGGGTGGTAGCAGAAATACTGGAGGCAGCAAAAACATTACTGCAACCACATTGTGA
ATACCTGCAAATTATAAATCCAAGCATGGGGACGGGCGTTACAGTAATGATGTTGTTTCA
GTTTTATGCTGCAAAATGTCGGGATACTGTAATAAATTTATTGTGTAATGTATTGCATGT
ACGGGAATGGCAAATTATAACTAATCCACCAAAGCATAGGAGTGTTGCTGTAGCATTATA
CTTTTATAAAACAAGTATGTCTAATGTTAGCTATTGTTATGGAGCAATGCCAGAGTGGAT
TAAAAAACAAACATTGGTTAATCATCAACAGGAGTCAGACAACTTTGAATTTGCAACTAT
GGTGCAATGGGCATATGATAATAATATTAGAGATGAGGCAGAAGTTGCTTATGGATATGC
AAGCCTTGCAGACGACGATACAAATGCAGCTGCATGGCTAAAATGTAATAATCAATTCAA
ATATGTAAAAGACTGTGTACAAATGGTTGCAATGTATAAAAGGTATGAAATGAGAAATAT
GACAATAGGACAGTGGCTAGTGAAATGTGGAGAACGTGTAACAGAAGAAGGCAATTGGAA
AAATATTATTAATTTTTTAAAATATCAGGAAATATCTATAGTGGCATTTTTAACAACATT
AAGATACTTCCTACAAGGAAGGCCAAAAAAAAACTGTTTAGCAATATGGGGACCGCCTGA
TACGGGCAAATCAATGTTTTGCTATAGCTTAATAAAATATACCCAAGGTAAGGTGGTATC
GTTTGTAAATAGCAGAAGCCAATTTTGGTTGCAACCACTAGTAGATGGGAAAATAGGGCT
AATAGATGATGCAACATTTGCTTGCTATCAATATATGGATGTATATATGAGGAATGGACT
GGATGGAAATGCAGTGTCAGTAGATGTAAAGCATAAGGCACCAATACAATTAAAACTACC
GCCATTACTTCTAACCTCTAACATTGAAGTACATGCAGAAGCCCCATTAAAATATTTACA
TAGCAGAATTCAAGAATACAAATTTCCAAATAAATTGCGTTTAGATGCAAATGGAAATCC
AATAATAACAATTACTGATGCAGATTGGAAATCTTTTTTTAGTAAACTGTGGAAGCAATT
AGACCTGGGCGATCCAGACGACAGCTCAGATGGAGACCCTGGTCGACCGTTTCGATGCTG
TGCAAGACAGGCTACTGGGGATTTATGAAGCAGGCCGCACAGACATAGATGGACAAATAG
TACATTGGGACCTTGTAAGGCAGGAATATGTATTACTGCACTATGCCAGAAAAAACGGTA
TTAGCAATTTGGGCATACAAAAAGTACCGTCATTGGCCACATCTGAAAGCAAGGCCAAAC
AGGCTATATTAATGGGCATAGTGCTAAAAAGTCTAAAGAACTCTGCATTTGGAAATGAAC
AATGGACATTGACAGATACAAGCTATGAACTGTTTATGACACCTCCATCGCATACATTTA
AAAAACAAGGAAAAACCATAGAGGTGTGGTATGGAAATAATGAGGACAATGCAGTGCCAT
ACACGTTATGGACCGCTATTTATTACCAAGACGCACAGGACAATTGGTACAAGGCCCGTG
GACAGGTATCTGCAGACGGCCTGTACTATATAGACTGGGAAGGAAATAAACATTTCTATG
TGCGGTTTGCAGATGAGGCAACACAATTTGATGCAGCAAATGAATGGCAAGTACGAACTG
AAAGTGAAATAATATCTAATTCTGTTACCAGCACCCCCCCACGGAGCGACCCGAGGCCTA
CTGGATCTTCTGCCATCACCTATGAAACTTCCAGACACGACACGAGGAGGACTAGGAGAT
CTACTAAAAGACCCCGACCGTCCCCCAGCCCATCCGCGACATCGGCGACCGAAAGCGATA
CCCCTGGGCACCATACCAGAAGCCGACGAATCCAACAAAGAAAACATAAATCCCAACGAC
CCAGGTGGGGATCCGAGTCTCCTATCCAGTCTCCTTCATCAGTGGGGCAAGGACATCGAC
TGCCTAATAGACACTATCGTTCGCGACTTGCAAGATTACAAGACGAGGCTAGGGATCCGC
CAGTAATACTTATTAAAGGGCCTGCAAATACTACCAAATGTTGGAGGTATAGAATGAAAC

AAAATTATAGGGCGCTATTTGAAAGTATAAGCACTGCATTTTCATGGGTAGGTGATAGGG
GCTCAGATAGGGTAGGGTGTAGTAGGGTATTAGTAGCATTTAGAAATAATAGCCAACGGG
AAAGGTTTTTACATACTGTGAAGTTACCAAAGGGTACACAGTTTTCATTTGGAAACCTGG
AATCCCTGTAACCACACATGCCAGTAATGCTGCCTATTTGTAAATAGCATCACATCTGCC
TCTTGTGATTTTGTGTACCTTGTATATTATACTTCTTTTGTACTGCTTTGCTACACTTGT
ATAATCAATGGAGCCTCCACATGCACGTAGGCGAAAGCGTGACACAGTTGAAAATATATA
CAGGCATTGTGCCCTGTCTGGTACATGCCCTGATGATGTTAAAAATAAAGTGGAAAATAA
TACCATTGCAGATAGGATATTAAAATGGATTGCAAGTATTGTGTACTTTGGGGGCCTGGG
TATAGGTACTGGCAGGGGCTCCGGGGGATCTATGGGATATACCCCTGTAGGGGCTGGGGG
GGGTAGGGTTACGTCCCAGGGTACTGTACTACGTCCTGGGGTTGTTTTGGATCCTGTAGG
ACCATCTGATATTGTACCCATTGATCCTATATCACCTACTGCATCCTCTATTGTTCCTTT
GGAGACTGTACCTGAAACACAAATACCTGATGTTCCTGTGCGACCCCCTGTTGGAGGTGA
ATTGGAGGTTACCGTTGGTACTGACCCTGTATCAGATATTACTATAGAGGCCACCTCGCC
TGCTATTACACCCATAGACCCCACAAGTGCCGTTATTGAGGCACAACCTCCTACATCTAC
TCCTAAGCGTGTTTCAGTAACAAGCACACGGCATGTTAATCCTTCTTACATATCGGTATA
TGGCCATCCCACAGACCCTGTAACACAGGCTGGTGCTGAGGTGTTTGTAGGGGCAGCTGT
TGGTGAGGGACGGGTTATTAGTATAGGTGAATCTATACCTTTAGACACATTTGTTGAAAC
AGGAGGGTCTTCTACATTTTCTATTGAGGAGGCTGTTACGCCCCCCACGGACCAGTACCCC
TCGTGTATTTCAAAGGGCCTTTCAACGTGCACGGGACCTTTATAACAGACGTGTACAACA
AGTACAAACACACAATATGGCTTTTCTTGGGCGCCCCAGGCAGGCGGTAACATTTGAATT
TGAAAATCCCGCCTTTCAGGATGAGGTCACGCAGCAGTTTGAACAGGATTTACAGTCTGT
TGCTGCAGCGCCTGACCCTGATTTTGCTGACATTGTAACATTGGGACGGCCTCGCTTTTC
TGAAACAGCAGAGGGGCGCGTGCGTCTTAGTCGCCTAGGTCGCCGGGGCACTATTGTAAC
ACGCAGTGGATTACAAATAGGATCCAATGTGCATTTTTATTATGATTTCAGCTCTATAGA
TGCTGGCTCAGTTGAATTGCAACCACTTGGTCAGCAATCTGGGGTGGCCTCTATTGTTGA
TGGCTTAGCATCTGCTAACATTAATGGCAGTGCTGAAACATCTTTTATAGAAGGTCATAC
ACTTGTTGGCCCTACGGGTGAGGCTGACTTGTATGACACGTACTCAGAAGAGTTTGGAAG
TTCTCGATTGTTACTTACACCTCAGGCAGTTCCTGCTGTTCAGGTCCCTCCTTTGGTTCG
TGTCCTGACACTTCCTCCAGGCACAGCCCTACAAATCTTACTCTCAGATGTAGGGGGTCT
TGTACCTTTTTCTGTGCCTTCTGTACATCCTGTTATTCCTGGCACTGTGTTTCCAGATGT
GTCTGAACCTCCTATTGTTGTGGATGGTTTTGATGCATTTGATACATTTTATTTGCATCC
AAGTCACAGACGCAAACGCAAGCGCAAACGGTCTGGTTTTTAATGTTTTGCAGATGGCTG
GTTTGTGGCTACCTCAGACTGGTAAAATATACCTACCTCCAGCTCCTCCTGTGTCTAGGA
GTCTTACCACCGATGAATATGTTGTGGAAACATCTGTATTCTTCCATGCATCCAGTGATC
GTCTAGTAACTGTGGGTCACCCCTATTTTCCCGTTATAAAACCAGGCAATCACACCGTTG
AGGTTCCTAAGGTTTCTGGCAACCAGTTTAGGGCTTTTCGCCTACGTCTCCCAGATCCCA
ATAAGTTTGCTTTAATTGACAAACAATTGTATAATGTTGAATCGGAGCGCCTCGTGTGGC
GTCTTAGAGGAATTGATATTGGCAGAGGAGGGCCTTTAGGTATTGGTTCTACTGGGCATC
CTTTATTTAATAAGTTAAAGGATACTGAAAATCCTAATGGATATCAAAAGGGTGGTGCTA
AGGACGCTAGACAAAATGTTTCTATGGATCCTAAACAAACACAGTTGTTTGTTGTTGGTT
GCACCCCTTGTAAAGGTGAACACTGGGATGTTGCTCCTGCATGTGACAAAAATCCTCCTA
AAGGCACCTGTCCTCCTTTACAGTTGGTGTCCTCTGTTATTCAGGATGGGGATATGTGTG
ATATTGGCTTTGGTGCCATGAATTTTGCTGCCTTGCAGGCTGATCACTCAGGGGTACCCC
TAGATATTGTTGCAGATACTTGTAAATGGCCAGACTTTCTTGGTATGGCCAATGATAGGT
TTGGGGATGGTATGTTTTTCTTTGGCCGCCGCGAGCAGGTGTATGCACGTCATTATTTCA
CCCATAATGGCACTGTTGGTGACAGTGTTCCAGAGGAGGGGTTTGAGGGTAATGGTAAAA
AGTATTTTATTGGTGCTGAGCAAGGCCAGGACCAGCATCAGCTGGCTCCCTCTTTATATT
TTGCTACACCTAGTGGGTCCTTAGCATCTAGTGACTCCCAGTTATTTAGTAGGCCCTACT
GGATTGAACGGGCTCAGGGTGCTAATAATGCTGTATTATGGAATAATAATGTATTTGTTA
CTTTGGTTGATAATACCCACAATGTGAATTTTACTATTAATGTTGCTACAGAAGATAATA
TTCAGTCCTATGATTCTGCAAAGTACAAGCACTACCTCAGACATGTGGAGGAATTTGAAA
TTTCTCTCATTGTGCAGCTCTGTAAAGTGTCTCTTACTCCCGATGTATTGGCACACATAC
ACTTAATGAATCCCTCTATTCTGGATGAATGGCAATTAGGTATTGCGGCTCCACCGTCTA
CCTCTATTGAAACCACTTACAGATATATTGATTCTCTTGCTACAATGTGTCCCACAGCAG
AGCCTCCTAAGGAAAAGGAAGATCCATATGACAAAATGTCTTTTTGGGTGGTTGATTTAT

CAGACAGAATGTCCTCTGAATTAGGGGCCTATCCTCTTGGTAGGCGCTTTTTACATCAAA
CAGGGTCTGCCCGGCCTGCGCGCCCCCTAAAGCGACCCCGTCCTTCTACGCCTACCAGGC
CTACCTCCAGGTCTTCCAAACGCCGACGCAGGCAAGTTTAAACATTATATGTATTTTACA
ACATTCCTACCTCTGTACATTTTACATTTCTATACTCTTATAATAAATAAAGTATGTAGC
ATGTCAACATTGTCGTTGTATGAGTTATTTCTTCTGACTCAGGGCAGTATTTTGGCCACA
CCTGGGCTTTTAGTATTTGGCTTCCGGCCGGCGGACGTGTAGGGGCTCTGCTACATATTT
TTCGTTTTTTATTGCTTTTGGAGGCACCGGTATCGGTTGCTGGTACATTTGCTGCCAATT
TTGCCAGCAGGTATGTTCATGTTTTAACAAATGTATTACTCATGTGTTTTGCAACGTTTT
AGGTACTGGCTACATTCCACAACCGGGAGCGGTGCTTTTACAGTGCCTTTTTCTCTTATT
GTTGTTGGCAACAATCATCTATTCCGTTTGTAACATGAACCGGGAGTGGTGTGGGCAGTG
TCTATATATACAGCAGGGGGCCTATACAACGGTCCTTTTGCGACCGCCTGTGTGCATGTA
CTGGGAGGTAGACTGGGGACTGTGTAATGTTATGAATATTGTACCTATATTACTGCTTAT
GTGGCAATTGTATATTTGTGTTGTGTTTTTAATTACTGTGCTTAGCCTTATTAAGATATT
TTTATTGTTGGGAATACGGGTATAGGGACACGGCCAGTTGTAATATAATAGTACAAACT
>gi|109390382|ref|NC_008188.1|_Human_papillomavirus_type_103,_complete_genome
ATGAGGGGAAAGAAACCAACAATTAAAGATATTAACTTAGAACTACATAGTTTAGTATTA
CCACAAAATTTACTCTGTGGTGAAGAACTTGTGGAGGAGGAGGAGGAGGAGCAGGAAGAG
CAGGATAAGTGTCCTTACCGGGTGGTGACAGATTGCAAGTATTGTGAGAAGTCATTGTGT
ATGTATGTGGTAGCCACGGAGGAGGGCATTAGAAACTTTGAACAACAGCTGTGTGGAAAC
CTGGATATTGTGTGTCCAGCCTGTGCAAAGCAACGCCAGCGAAATGGAGGACAAAGACAC
TAAAGGTACATCTTGCTTTACTGACATTACAGCAAATGCTTGTGAGTTTATAGAAACAGA
AGCGACCTGTGAGGATGGTTTAGATTCATTGGAAGCATTATTTGAAGAGAGCGATGGGAC
AGATATTTCAGAACTAATAGAAAATATTGATGAACCTGATAGGGGGAATCCCCAAGCACT
GTTAAATAGGCAACAGCTAGAAGAAGACAGTCAGCTACTAACAGTGCTAAAACGAAAGTA
TGTTAGTCCTAGCCCGAAGCAGCGGGTAATAGATTTAAGCCCACGACTTGAAGCGCTGTG
TGTGTCGCCACGAGGATCCAGCAGTAAAAGACGACTATTTGAGGACAGTGGCTTAGGACA
TGAGCCTGAAGATTCTAATGCTGAGGTGGAAAAGGTAGCGTCGGAGGCATTGACTAATTC
AGGGACATTGACTAATGGGGATGAAAATGCTGTATGTGCAACGCAGGATGGCGACACACG
GTGCCTGGATATTTTACACAGCAGTAATAGACAAGCTACTGCTTTAACTAAATTTAAAAA
TGTTTTTGGAGTGGGATATAAGGAAATAACCAGGCCATTTCAAAGTAATAAGTCATGTTG
TCATAGCTGGGTAGCAGCAATATTTGGTGTTGTAGCAGAAATGTTGGAAGCGGCTAAGAC
GCTATTTAAAGTGCATTGTGATTATTTACAAATTATTAATCCTAGTATGGGTACAGGAGT
TACTGTATTATGCTTGTTTGAGTTTTCAAGTTCTAAATGTAGAGATACTGTACAAAAACT
GATTAGCAATGTGCTAAACGTGCAGGAACATCAAATAATAACAGATCCTCCGCGACATAG
AAGTGTACCAGTAGCATTGTTTTTTTATAAACAAAGCATGTCTAATACAAGTTTTGTACA
TGGAACAATGCCTGACTGGCTTAAAAGACAAACTATGCTAAACCACCAGCAGGAGGCAGA
CTCATTTGAACTGTCTCATATGGTGCAATGGGCATATGACAATAATATTTGGGATGAAGC
AGAATTAGCCTATCAATATGCATGTTTAGCAGATGTGGAACCAAATGCAGCAGCATGGCT
AAAAAGCAACCAGCAATATAAGTATGTGTCAGATTGTGCAAAAAATGGTTAGAATGTACAA
AAAGTATGAAATGCAACAAATGAGTATGGCACAGTGGATAAAAAAATGTGGAGAGCATAT
AGAAACAGACGGAGACTGGAAAAAAATTATAAACCTATTAAAATATCAGGAAATATCAGT
AATAGCATTTTTAACAAGTTTTAGAATGTTTTTAAAGGGACAACCAAAGAAAAACTGTAT
AGCACTATGGGGACCTCCAGATACTGGCAAATCTATGTTTTGCTACAGCCTAATTAGATA
TGTAAAAGGTAAAGTAGTATCATATGCTAATAGTAAAAGTCAGTTTTGGTTACAACCACT
AACAGATGCTAAATTGGGGCTTATAGACGATGCTACATTTCCATGTTTCCAATTTATGGA
TGTGTATATGAGAAGTGCTTTAGATGGAAACGAGGTGTCTGTGGATTGTAAGCATAGGGT
ACCAGTGCAAATTAAATTACCTCCATTACTTGTTACCTCCAACATAGATATGCATTCGGA
GGCCTCTTTAAAATACCTGCAGAGTAGAATTACAAGTTTTAAGTTTCCACATAAACTTCC
ATTAGATACAAATGGAAATCCAATATTTATTATAACTGACACAGATTGGAAATGTTTTTT
TAGCAAGCTGGAGCAGCAGTTAGACCTTTGTGACCCAGAAGACAGTACAAATGGAGACCC
TGGTGGAGCGTTTCGATGCTGTCCAAGAGAAACTACTGGGGCTTTATGAGGCCGGACGCT
CAGACCTGGAATCCCAAATACAACACTGGGATCTTGTAAGGCAGGAGCATGTACTGCTAA
ACTATGCCAGAAGGCAGGGCATACAAACATTGGGCCTACAAAAAGTGCCCCCATTGGCAG
CATCTGAAAGCAAAGCCAAAACTGCTATTATGATGCAGTTGGTGCTGAAAAGCCTAAAGA
AATCCCAATATGGGCAGGAACCATGGACATTAACAGATACAAGCCATGAACTATTTACCA

CAGCACCAGAGAATACATTTAAAAAAGGAGGTGTAACTGTGGAAGTGTTATTTGACAACG
AGGAAGAAAAAGCTATGCCATATACACTATGGAGACTAATATATTATCAAGATGCAGATG
AGCTGTGGCATAAAACACATGGACAGGTAGACGCGGATGGCCTGTACTTTATAGACATGG
TAAAGCAAAAACACTATTATGTGCATTTTACCAAGGAAGCAGTAAAATATGGACAAAAAG
GGATGTGGCAAGTGAGGACTGGAACTGATGTATTATTTGGTCCTTCTTCTATCGCTAGCA
CTTCGACGGGGGCCGAGCAAGGTGACACCACCGCACAAGTCACCCGAAGCGCCAGAAAAG
GCAAACGACGAAGACATTCCAGAAGGCAAACCCCCTCGACCACCTCGACGACCACAGAGA
CCGACTCAGAGGATTCGGCTTCCGAATATAGAAGAAGAAAGCGAAGACACAGAGAACGAA
AACGATCCAAGCACAGGTGGGACACAGGTTCAGAATCTTCTACTGACACTGCTGCAGCAG
TGGGATCGCGACATCGAAGCCCTGATAGAAACTATCCGTCAAGACTTGCAAGGCTACAAG
CGGAGGCTTGGGATCCCCCAATATTATTAGTTAAAGGGCCTTCAAATACATTGAAATGTT
GGAGGTACAGGTGTAGAATAAAATATAGAGGCTTATTTCAGTGTATTAGCACAGCTTTTA
CATGGGTGGCGGATGCGGGTCCACAATTAGGAAATGCTAGAATAATAGTAGGATTTACTA
ATACATCACAAAGGGAAAAGTTTATACAAACTGTGAAATTACCTAAAGGCACAAGTAGTG
CATTAGGAAACCTTGATTCATTTTAAGCCAATAATGCTGCCAATGGTCCCCTGTATATTA
TCCTAACCCTTACATAGGTATAAGTGTATGTACTATATATGTTGTAAATAGCTTACATGG
ATGTACAACCGAAAAAGCGACAAAAACGGGATACTGTGGAAAATATATATAAACATTGTG
CAGGGTCAGGCACATGTCCTGAGGATGTTAAAAATAAAGTAGAGCAAACAACATTAGCAG
ACAAACTGTTAAAATGGATTGCCAGTGTGGTTTACTTTGGGGGTCTGGGTATAGGCACAG
GTAAGGGTACTGGTGGGGCCTCTGGCTATGTTCCCATTGGGTCGGGGTCTGCAGGTGGGG
GTCGGGTAACATCACAGGGTACTGTAGTACGTCCGGGTGTTGTGGTAGATCCTATTGGGC
CTCCTGATATAGTTCCTATAGATGCAGTTACACCACAGTCCTCCTCTGTGGTACCATTAG
AGGTAATACCTGAGATTGTTGGCGACCCTACGCCCCCTGATATATCTACTCCTAATATTA
TTACTGTAACAGATCCTGTATCTGACATAACACTAGACACCACCTCTCCTGCTGTTGCTA
CTATTGACCCCACCAGTGCTATACTGGAGGTGCAGCCCACTGCATCAACACCTAAAAGGG
TCTCTGTAGGCAGTAGCAGACATGTTACGCCATCATACATCTCTGTATATGGGCATCCTA
CAGACCCTGTTACACAATCTGCAGCTGAGGTGTTTATAGGGGGTGCTGTAACAGAAGGAT
CTGTTGTCAATATTGGGGAATCTATACCATTAGAAACATTTGTGGAAACACAGGGGTCTG
CCACATTTGATATTCAACCTACTGCAGAGCCACCACGTACTAGTACCCCTCGGGCATTTC
AAAGAGCATTCCAAAGGGCTCGTGAATTATATAATAGGCGGGTTCAACAAGTTAGAACTA
GGAACATAGATTTCCTAAACCGCCCACGACAGGCGGTTACTTTTCAATTTGAAAATCCCG
CCTTTGAGGATGAAGTTAGTCTTGTATTTGAACAGGATTTAAATGCCTTAGCTACTGCTG
CACCAGATCCTGATTTTGCTGATGTTGTGCGTTTAGGTAGGCCTACTTTATCTGAAACTC
CTGAAGGCCACATTAGACTAAGCAGGTTGGGTCAACGTGGCACTATTCGTACAAGAAGTG
GCCTGCAAATAGGTCCACATGTACATTATTATTATGATTTAAGTAGTATCCACCCTGACT
CATTTGAATTGCAGCCTTTAGGTCAGCATTCCGGTGACACAGGTATTATTGATGCAGCAA
CAGCACAATCTATTGTTGCAGACACAGCTGAATCTAGTTTTATAGAAGGTCAAACATTAA
TAGGCCCACAGCTAGACAATGAGCTGCTTGATTCTACAACCGAGGATTTTAGCTCCTCTC
GTCTCCGATTTGCAGGGGTTCACCATGGCATCCAGTTTCCCTCTTTAGATATTGTTACTT
TACCACCTGGTGCTGCATTACAATTTGTTGTCACTGCATCCGCTGGTAAACATAATACTA
CAACTATTATTCCACAATATATCCCCACTACACCTTTTATTCCTTCTGTGGTAGTAGATT
CCTTTGTTACTTCTACTACATTTTATTTACATCCTGGACTGTCTCGCAAACGCAAACGTT
CGCATATGTTTTAATATTTTGCAGATGTCTTTGTGGCTGCCACAGTCCGGTAAACTTTAT
CTACCACCTTCTCAGCCAGTGGCTAAAATACTGCCTACTGATGAATACGTTGTGGAAACG
TCTATTTTCTTCCATGCCTCCAGCGACCGCCTGCTTACTGTAGGACACCCATATTTTCCA
ATTACAAAAGTGGGTGAAGGTACAATTGTTGTACCTAAGGTATCTGGTAATCAATTCAGG
GCCTTTCACATCCTTTTACCTGATCCTAACAAGTTGGCACTTGTGGATTCCAATATATAT
AATGCCGAGTCAGAACGGCTTGTTTGGAAGTTACGGGGTCTGGATATTGGTCGTGGTGGA
CCCCTTAGCGTTGGCTCTTCTGGACACCCTTTATTTAATAAACTTAAGGATACTGAAAAT
CCTAATAGTTATGTTAAAACAGGAAAGGAAAACAGACAAAGTGTCTCTATGGACCCAAAG
CAAACTCAGCTATTTGTAGTAGGCTGTACTCCTTGTTTGGGGGAGCATTGGGATGCTGCA
AAGCCATGTGCAGATGCTAGGCAGGATATGGGTGCATGTCCTCCTTTAGAGCTTGTTAGT
TCCATTATTGAGGATGGGGATATGTGTGATATTGGCTTTGGTGCAATGAATTTTGCTACA
TTGCAGGAGGACCATTCAGGTGTACCCTTGGACATTGTAGCTTCTAAATGTAAATGGCCT
GATTTTCTTAGAATGGCAAATGACACATATGGCAATCAAATGTTTTTCTTTGGAAGACGT

GAGCAGTTGTATGCTCGCCACTTTTTTGCTAGGGATGGTGTCATGGGCGACAGTATACCT
GAGGCTGAATTTGAGGGTGTTAAGCACACCTATATTTTACAGGGGGCGTCTGACAAAGAC
CAAAATACAGCCTCCTCGTCTGTTTACTATGCAACCCCCAGTGGATCACTGGTTTCTAGT
GATTCTCAGTTGTTTGGCAGACCATACTGGATACAAAAGGCTCAGGGCCCTAATAATAGT
GTGTGTTGGAGAAATAATTTATTTATTACATTGGTTGATAACACACACAATGTAAATTTT
ACAATAAATGTTAAGTCAGACGCTGGCGTTGCACAGCAATATGCTGCCACCAGCTATAAA
CATTATCTGCGACATGTAGAGGAATTTGAGATTGCTCTTATATTGCAATTATGCAAGGTT
CCTCTCACAGCTGATGTATTAGCCCATATACATGTAATGGATCCAGCTATAATTGAGGAC
TGGCAGCTAGGGTTTGTACCACCGCCTCCTTCTTCTATTGGAGATACATACAGATTTTTA
ACATCTTTAGCCACACGCTGCCCTGCTGCAGAACCTCCCAAGGAAAAACAGGACCCATAT
GATAAATTATCTTTTTGGAAGGTGGATCTTACAGAACGTTTGTCCTCTGATCTTGACAGA
TATCCTTTGGGTCGCAGATTTTTATCTCAAACTGGGGTTAGCTCGTCTTCTACCTCCTCT
AGAAAGCGGTCTCGTGTAGAGACCCCTACCCGCCCAGTACGGTCTGCTAAAAAACGTAGA
CGGGGGGTTTAATCTATGGCATTACCTGTGTTGCACATGCACTTTATATTTTTGTTCTTG
TATCAACTGCAGTACTGTGTTAACTTGTCTTGTACATTCCTTAACTTATATTACATTTAA
GGGTATTAAATATATTTGTCATGGTACCGGTCTCTGCGTGATTTGTTATTGAGTCATGTA
AGGTCAATAGCCACACCTGGCTTTTAGTCTTTGGCTTCCGTCCGGCTTGGACGTGCAGGG
GCTATACACACTGTTTTTTTGGATACTTCTTTAATAAAGCTTTCTTTCAGTGAAACCGAT
AACGGTCGCCAACTCCGTTGCCAACTTTCAGGTATGTACTTTGCAAGTTTCAACAACAAG
ATTACTCAGCCTTCCAGCAACCGAATTAGTTATCTGGCCGTTGGCCACGACCGGCAGCGA
TGTTTTGTAATATTCTTGATCCTATGATTGTTGTTGGCAACTACCACCTATTCCGTTTGC
TTTACTAACCGGGAGAGGTGTATACTATATAAAGTAGAAGCTGTATTGGGCAGTTCTCTT
TTGCGTGGTTGTACCCCTGTGTGGTGGATGGTGGTGGGCTCTGGGAAATGAATATTGTTC
CATTCTTGTTATTTGTATGGCAATTGTATTTATGCTGTGCGTTTGTTTTAACTGTAATAT
GCATGTTAAAAATATTTTTATGTTTAGCTATTAGAGTGTAACTATATATATGCGGGATTT
GGCCAGTACTTGTTGTATGTATAGTGTATAAAAGGTGTGTAAATACAGTAGCGGTGTGAT
AAA
>gi|50253426|ref|NC_005134.2|_Human_papillomavirus_type_96,_complete_genome
TAAGGTATGTGAGTAATTTTTGAACCGCCCCTGACTAATCTAAACATGCTAACCGCACCC
GGTATAAGCTATAAAATATCCTGAGGTGATGCAGTATCTGATCCTGGCAGTCACTCCTGG
AACAACACCGATCCAGTGGCTTTGCCAAAAGCAGTTTAAACCTGCCAAGAAAAGCCCGCC
AAAACGTCTGCCAACTTTGGAACCGTTAACGGTAAGTTTCGCAACTCTCTCGGTTGCCAA
AAGTTTTATCTTGAGAGGTTGTTGGCAATTACAACCTGCCACTCATAAACAAGTAACCGC
CTGCGTTTCTAGGACATTTTATATACAGACATACAGATCTCTGTTCATTGTTGAGATGGA
TTTAAAGACAGTGCAGCAACTTAGAAGTCATTTGGGCATTCCTACTGAAGACCTGTTGTT
AGCCTGTAACTTCTGTAAGAAATTTTTAACCTTTGAGGAGCTATTAGGATTTGATAGCAA
GACTTTAAATCTTATTTGGAAGGAAGGTTATGCATATGCGTGTTGTTTCAATTGTGCTAG
AACAGTAGCCAAAGTAGAATTTGAACATTTTTATGAAAAAACAGTCAAGGGACGAGAAAT
AGAATCTCGCAGTGGCAGTTTACTTTGTTGTATTGTTGTTAGATGCCAGTTTTGTTTGCG
ACTGTTAGATTATTTAGAAAAGTTAAATATTTGTGGGTATCAAGAAAACTTTTATTTAGT
AAGAGGAGGTTGGAAAGGCATTTGCAGATATTGTAGAGAGATATGATTGGGAAAGAAGCA
ACTATACCAGACATAAAGCTGGAGCTGCAAGACCTTGTCCTGCCCATTGACCTGCATTGT
GACGAAGAGTTGACAGAAGAGCAGTCAGAAAACCTGTCAGAATCTACAGTAGCAGAGGTG
GAGCCTCAGAGAACTCTGTACAAGATTGTAGTACCGTGTGGAGGCTGTGATTCCCGGCTG
AAAATCTACGTTTCTGCTACACAGTTTGGAATTCGTTCTTTAGAAGACTTGCTGCTAGAA
GAACCTTCTTTGCTGTGTCCTGCTTGCAGAAATGGCAGATAATAGAGGTATTGATCCTAA
AGAAGGGTGTAGTAATTGGTTTTCTCTTGAAGCAGACTGTAGTGATTTAGATGATGATTT
GGAAAAGCTGTTTGAAGAAGGGACAAATTCAGATTTATCTGATTTAATTGATGATGGGGA
TGCTGTTGATAATGCGAATGAGCAGGGAATTTCCCGAGATCTCTTTCGCCAGCAGGGGAG
TGAGGAGTTTGAGCAGCAAATACAGGACTTAAAACGAAAGTATTTCAGTCCGAAAGAGTT
GCAGCAGCTTAGCCCTCGTTTGGAAGCAATACCTTTGTCGCCGCAACACAAATGTAAAAA
GCGGCTATTTATGGAACAGGACAGCGGGCTTGAGTTATCATTAACTCATGAGTGTGAAGA
TACTGCTCCGGAGGTGGAGGTACCCCCAAATACTGACGGCACAGCACGGACAGAAGTTCA
GGCGAGCAATGAGCATTATAGACTATTGTTAAAATGCAGTAATCTCAGAGCTACAATGCT
CAGCAAATTTAAAAACAGCTTTGGGGTGGGGTTTATGGAACTGTGTAGGAAATTTAATAG

CAATAAGACTTGTTGTCGGGACTGGGTCGTAACTGCATATGGAGTTAAAGAGGAATTATT
AGAAGGATGTAAGCAACTGTTACAAGAACATTGTGGTTATATATGGCTTCATACACTATG
TCCAATGTCACTCTTTCTATTGTGTTTTAAAACTGGTAAAAGCAGAGATACAGTTGTAAG
ATTGTTACAAAGGATGTTAAGTATTCATAAAGAGCAACTCTTAACTGAACCACCAAAGTT
ACGAAGTGTTATGGCTGTACTTTACTGGTATAAGGGCAGTATGAATCCAAATATCTATGC
ATTTGGGGAGTATCCAGACTGGATTGTTCAGCAAACGATGATTAGTCACCATGAGGGCGA
TAATTTGCAATTTGAATTGTCTCCTATGGTACAATGGGCCTATGACAATGACTATATCGA
AGATTCTGACATTGCATATAACTATGCTAAATTAGCTGACGAAGACATAAATGCTCGCGC
GTTTTTAGCTCATAATAACCAAGCAAAAATTGTTAGAGATTGTGCTTGGATGGTAAGACA
TTACAAACGAGGTGAAATGAGATATATGTCTATTTCTAAATGGATATGGTATAAATTAAA
AAAAGTAGAAAATGGCGGCCACTGGTCAAACATAGTAAAATTTGTTAGATTCCAAGGTAT
AAATTTTATAATGTTTTTAGATGCCTTCAAACACTTCCTTTTGTCTAGAACAAAAAAAAA
CTGTATTTTATTTTATGGTCCTTCTGATTGTGGCAAAACGATGTTTTGCATGTCACTAAT
TAAAGCTCTCGGTGGTAGGGTGATTTCATTTGCCAATGCAAAAAGTCAATTTTGGTTACA
ACCTTTAACGGAAAGCAAGATTGCTATGTTAGATGATGCTACAGAAGCTTGTTGGAATTA
TTTAGATACCTATTTAAGAAATGGTTTAGATGGGAATTGGGTTAGTGTTGATTGCAAACA
TAAAGCTCCCATACAGATTAGATTTCCTCCATTATTGATTACCTCCAATTATGATATTTT
AAAAAATGAGAGGTATAGATTTTTGGTTAGCAGAATTAAAATATTTGAATTTAAGAATAA
ATTTCCTTTTAATGAAGATGGTACCCCTATGTTTGAACTTACTGACCAAAGCTGGAAATC
TTTTTTTCAAAGGCTTTGGAAGCAGTTAGATCTAAGTGACCAAGAAGACGAGGGGGAGGA
TGGAGGCTCTGAACCAGCGTTTCAATGCTCTAGAAGACCAGCTCATGGACATATATGAAC
AAGGGAGTGATAAATTAGAAGATCAAATAAAACATTGGAACTTTTTAAGAAAAGAACAGA
TTTTATTGCATTATGCCAGGAGGCGAGGCATTATGCGTTTGGGGTACACTCCTGTTCCTA
GTTTAGCAGCTTCAGAAACAAAAGCTAAAGATGTTATTTACATGGTTCTTCAACTAGAGA
AATTAAGAGACTCACCTTACAAAGATGAACCTTGGACATTGATTAACACCAGTTTAGAAA
CATATAGAGCTCCTCCTGTTAACTGCTTTAAAAAAGGTCCTCAAAATGTAGAGGTTATTT
TTGATGCAGATCCTGAAAATATTATGTTATACACATCCTGGCAGTTCATTTACTTTGAAG
ACACTGATGGACATTGGCAAAAAACTGAAGGGCGTATTGATTATGCTGGTTTATATTATT
TGGAAGGGCAATTAAGACATTATTACGTGGAATTTAAAGTAGATGCTCGCAGATTTGGAA
CTAGTGGAGAATGGGAAGTGCGTTTTAACGGGGAAACTATCTTTGCTCCTGTTACCAGCT
CCTCGCCGTCCTCATACGAAGAGGTCAGAGAACGGGCTGAGCCCCCCGACGTCCCCGAGG
TCGGAACCGCCCCTGACTCCGGCACCCTACAGACTGGGAGACCTGGAACCGAAACCGCCA
AAGGGCGAAGGTACGGACGAAAAGAATCTAGTCCTACAGTTGCCTCCCTCAGGGTCAGGC
AAAAAGTATCACGATCTACTACACGGAAAGCCGAAGAACGACGGTCCCGGAGTCCATCGT
CAGGGACAGAGCAAGCCGGTGCACGGGGACGGGGACAACGGGGAACCAGGGCCCGTTCCA
GAACCAGAAGCAGGAGCAGGCGCCGGAGCAGGAGCAGCAGCAGAGCCAGAAGCAGGAGGG
CAAGGGAACACTCAGAATCGAGGGGAGGGAGAGGTAGAGGGGCGTACGCCACAAGATCCA
GAAGTAGATCTGCAGAGTGCAGATCTATTGACCAGTGTGGCATTCCAGCTTCACAAGTGG
GAAGGTCAGTTCAATCAGTTGGTAGAAACAATTCTTCGAGACTTAAGAGATTACTGGATG
AAGCTCTCGACCCCCCAGTGATATTGTTAAGAGGGGAACCAAATACTTTAAAATGTTATC
GCTACAGGGTGAAAGACAAACTTAGAGGCTCTTTTGATAAAATTAGTACAACATGGTCAT
GGGTGGCTTCACAAGGTAATACACGCATAGGTAGAGCTCGCATGCTGCTTAGCTTTGTTT
CAGAAGATCAGAGGGAAAAATTTATCAATGTTATGAAATTACCTAAGGGTGTTGATTGGT
CCTTTGGTAGCTTTGATAGTCTTTAACTACTAACATTTCATTTTTTTATACTAATAACTA
ACCCACTTTATATATTGTATGGCGCGCGCACGTAGAGTAAAGCGTGATTCTGTTACAAAT
ATTTACAGGGGCTGTAAGGCAGCTGGCACATGCCCCCCTGATGTTATTAATAAAGTTGAA
CAAAAAACTATTGCTGACCAAATTTTAAAGTATGGCAGCACCGCTGCGTTTTTTGGTGGG
TTGGGTATTAGTACAGGCAAAGGAACTGGAGGCAGTACTGGTTATGTCCCTTTGCCTGAA
GGACCTGCACCTGGTGTTCGCGTGGGTGGTACACCAACTGTGGTGCGCCCCGGGGTCATT
CCAGAAGCGATTGGTCCTACTGATATAATACCTTTGGATACAGTCAACCCTATTGACCCT
GTTGCACCTTCAGTTGTCCCTCTTACAGACACAGGACCTGATTTGTTGCCAGGAGAAATT
GAGACCATTGCTGAGGTACATCCTGTGTCAGATGTAACACCTGTTGACACACCAGTGGTG
ACAGGTGGTAGAGGCTCGAGTGCAGTATTAGAGGTTGCTGACCCAAGTCCTCCCACTCGT
GCACGTGTCAGTAGAACACAATATCATAACCCAGCTTTTCAAATAATATCTGAAACAACA
CCAACAACTGGGGAAGCGTCGTTATCTGACCAAATCATTGTACAATCAGGTTCTGGAGGA

CAAAATATTGGTGGTAGTGGGCCTTCTGTGGAAATAGAATTAGAAGAGTTCCCCACAAGA
TATTCATTTGAAATAGAAGAGCCAACCCCTCCTAGAAAAACTAGTACACCTGTAAGAATG
GCTCAGCAGGCCTCACGAGCTTTACGTAGAGCTTTATACAATCGTAGATTAACACAACAG
GTTTCTGTAGAAAATCCTCTATTTTTACAACAGCCTTCTAAATTAGTTACTTTTCAATTT
GATAACCCTGCATATGAGGAGGAAATAACACAAATATTTGAGAGGGATTTAAGCTCCATT
GAAGAACCTCCAGATAGACAATTTATGGATGTTGTTAAATTAGGTAGGCCTACATATGCT
GAAACACCAGAAGGTTACATTAGAGTCAGTAGACTTGGGAAACGAGCAACCATCAGAACA
CGCTCTGGAGCACAGGTTGGCACTCAAGTTCACTTTTACAGAGATATAAGCACTATTGAC
ACAGAACCCTCCATTGAATTGCAACTGTTAGGGGAACATTCTGGGGATGCTAGTATTGTT
CAAGGCCCAGTAGAAAGTACATTTGTTAATATGGATGTACAAGAAATTCCTACTTTGGAG
GAAGTGCCAGAATTACATTCTGAAGATGTGCTATTAGAGGAGGCATTAGAAGACTTTAGT
GGAGCACAATTAGTTTTTGGAAATTCTAGAAGATCAAATGTAATAACTATTCCTAGATTT
GAGACTCCAAGAGAGATTAATATTTATACACCAGATTTAGATGGATATTACATATCATAT
CCAGAAACAAGGAATATTCCAGAAGTTATATACACTGAGCCAGACACGACTCCAACAATA
ATAATTCATACAGAGGATTTCAGTGGTGATTATTATTTACATCCAAGTTTGAGACGAAGA
AAAAGAAAACGAGCCTATTTGTAATTTTTTTGCAGATGTCATCATTGTGGTTGTCAACAA
CGGGTAAGGTCTATTTACCACCATCAACACCAGTTGCCAGGGTGCAAAGCACGGACTCCT
ACATACAAAGAACAAACATCTATTATCATGCTAATACTGACCGCCTGTTAACAGTAGGAC
ATCCTTATTTTGATGTGAGGAAAAATAATGGAGATCATGAAGTGTTAGTTCCCAAGGTGT
CAGGTAATCAGTACAGGGCCTTTAGGGTACACTTACCGGATCCTAACAGATTTGCTCTAG
CTGACATGTCAGTGGTAAATCCTGATAGGGAGCGTTTGGTATGGGCTGTTAGAGGAATGG
AAATTGGTCGTGGACAGCCATTAGGTGTAGGTACATCAGGACATCCATTATTTAACAAGG
TGAAAGACACGGAAAATCCAAATGGCTATAATACAGGTGGAAAGGATGATAGGGTGAATA
CATCCTTTGATCCCAAACAAATTCAAATGTTTGTTTTGGGTTGTATACCCTGCTTGGGGG
AACATTGGGACAAGGCCTTACCTTGTGTAGAAAATCCTCCTGATCAGGGAGCGTGTCCAC
CTCTAGAATTAAAAAATACTATTATTGAAGATGGGGACATGGGAGACATAGGGTTTGGAA
ATCTTAATTTTAAAACATTATCAGTCACTAAGTCTGATGTTAGTCTGGATATTGTTAATG
AAATTTGCAAGTATCCAGATTTCTTAAAAATGGCTAATGATGTGTATGGCAATGCTTGCT
TCTTTTATGCCAGAAGAGAACAATGTTATGCCAGACATATGTTTTGTAGAGGTGGGTCAG
TAGGAGACAGTATTCCAGATGATGCAGTTGGAGAAGACAACCATTATTATTTAAAGGCTG
CCAGTGATCAAAACAGAGATACAATGGCAAGTTCCATTTACACTCCCACAGTCAGTGGAT
CTTTAGTTTCTACAGATGCACAGATTTTCAATAGGCCTTTTTGGCTGCAAAGGGCTCAAG
GCCATAATAATGGTATTTGCTGGGGTAATCAAATCTTTCTCACAGTAATAGATAATACCA
GGAATACTAATTTCTGTATCAGTGTCTCCTCAAATGATCAGGCATTACAGGAATACAATA
CTGCAAACTTTAGAGAATATTTGAGACATGTAGAAGAGTATGAATTATCCTTTATATTAC
AATTATGTAAAGTTCCATTAGAGCCAGAAGTATTAGCACAAATTAATGCTATGAATGCAG
ACATTTTAGAAGATTGGCAATTAGGTTTTGTTCCTTCTCCTGACAATCCCATCAATGATA
CATATAGATACATACATTCAGCAGCCACACGGTGTCCAGATAAAACTACACCTAAAGAAA
AAGCAGATCCCTTTGCAGGTTATCACTTTTGGGATGTTGATTTGTCTGAAAAGTTATCAT
TAGATTTAGATCAGTATTCTCTGGGACGTAAATTCTTATTTCAAGCCAACCTGCAAAACA
AAAGAGTTAACAGAGGGGTTACTGTAACCGGGAGGGCTACAACCTCAAGAGGTACAAAAC
GAAAACGACGCTGACCGCTATCGGTTCGGGGACCTTCAATAAACATATTTGGAACGTT
>gi|37089393|gb|AY382778.1|_Human_papillomavirus_type_93,_complete_genome
CACCAAGTATTGCACCTGGATGATTGTTGTTGGCAACAACCTTTATTACTCAACAAGTAT
TTTCTTGCACCGATTTCGTAGCACCTGTTTATAAGTATTGATATATGGTATATAAGGCAG
ACATCTTAATGGCGGCAGTAAGTAAACCTCAGACCATTACAGAGCTTGCTAGGTGTCTTG
GTATACCGTTAGAAGATGCTTTGGTGCCTTGTAATTTTTGTAAAAAGTTTTTAACATATT
TGGAAGTCGTTGAATTTGATGTAAAACAACTTTGTCTAATTTGGAAAGAAGATTTAGTGT
TTGGCTGTTGCAGATGTTGTTGTATAGCATCAGCCACGTATGAATTTGATAATTACTTGA
ATGATAGTGTTGTTGGCTGGGCAATAGAAGTTAAGGAAAAAAAGCCACTCTCTGAAATTA
TAGTGAGGTGTCGCCATTGTATGAAAAAATTAGATCAAATTGAAAAGCTTGATATCTGTG
GTAGATCTCTATTGTTTTACAAAGTGAGGAGGGGCTGGAAAGGACTCTGTAGGCAGTGTA
AGTAGATATAATGATTGGAAAAGAGGTCACTCTGCAAGACATTGTGTTAGGGTTGACACC
ACCAGAGCCTGTTGCATTGAATTGTGAAGAGGAGTTACCAACCGAGCAGGATACGGAGGA
GGAGCCTGCAAGAACAGCTTTCAAGATTATTGTGTTCTGCGGCGGAGGCTGTGGATCCCG

ACTTCGAATATTTGTTGCTGCCACAGAATTTGGAGTTCGTTGCTTTCAGCAATTGCTAGT
TAAGGAGTTGGATCTTCTGTGTCCTAACTGCCGAAACAGCGACCTGCAGCATGGCGGACA
ATAAAGGTAGTGGTCCTGAAGGGTTAAGTGATTGGTGTTTATTAGAAGCGGAATGTAGTG
ATATTGAAAATGATTTGGAAACATTATATGAACAAAATGCAGATTCAGATGTTTCTGATT
TATTAAATGATGATGAGGACTTGGAGCAGGGAAATTCCAGAGAACTATTTCAACAACAAG
AGTTAGAAGACAGCGAGACTTTATTGCAATCATTAAAGCGAAAGTATATTAGTCCCAGGG
CTGTTTTACAATTAAGTCCTCAACTTGAATCTATTTCGCTGTCATCACAGCATAAAAGTA
AAAGGAAACTGTTTGCTGAGCAGGACAGTGGAGTAGAGCTGAACTTAACAAATGAAGTTG
AAGATATTGTTACACAGGTGGAACAAAAGGAGGAGGTACCGACAGCATCCACAAACTTGA
GGGAGGGGGAAAATGTACATTACAAGGAACTTATGAGATGTAGTAATTTGAAAGCTACAT
TACTTGCAAAATTCAAAAGTGCTTTTGGTGTTAGTTTTGCAGAGTTGACGCGGCAGTATA
AAAGTAATAAGACATGTTGTAACGATTGGGTGATTGCAATTTATGGTGTGAATAATGATT
TGTTTGAAGGTTCCAAAAAATTGCTGCAACAGCACTGTGATTATATTTGGCTCACTGAAA
TGTCTGCAATGTATTTATATTTATTATGTTTTAAAGCTGGGAAGAATAGGCATACTGTGA
TAAGATTATTAATCTCTATGCTACATGTTGCAGAGGAACAAATACTGTCTGAACCACCAA
AGCTTAGAAGCACTGTAGCAGCTTTGTTTTGGTATAAAGGCAGTTCCAACTCGGGAACAT
TTACCCATGGTTCATATCCTAAATGGATAGTAGAACAAACATTAATAGGACATCAGTCTG
GAGAAGCGGCAACATTTGATATGTCAACTATGGTACAATGGGCGTTTGATAATAATCTTA
CAGAAGAGGCTGACATAGCTTTTCAATATGCTAAACTAGCACCTGATGATGTTAATGCAA
CTGCCTGGTTGGCACATAATAATCAAGCTAGATTTGTTAGAGAAGTTGCTGCAATGGTCA
GGTTTTATAAAAAAGGACAAATGAGAGAAATGAGTATGTCTGCATGGATACATTTTAGAT
TAGAGCAAGTAGAGGGAGAAGGACATTGGTCCTCTATAGTAAAATTTATTAGATACCAAG
GTATTAATTTTATAAGTTTTCTCTCTGCTTTGAAGGACTTTTTGCATGCTAAACCAAAAA
AAAATTGTTTGCTTATATATGGACCGCCGAATACTGGCAAATCAGCATTCACAATGTCAT
TAATTAAAGTATTAAATGGAAGAGTAATATCCTTTGTTAATTCTAAAAGTCATTTTTGGT
TACAACCTATGTCTGAGTGTAAAATTGCTTTATTAGATGATGCAACAGATCCATGTTGGG
TTTATATGGATACATATTTAAGGAATGGTCTTGATGGACATTTAGTATCATTAGATTGTA
AACATAGAGCACCAATGCAAATTAAATTTCCTCCACTTTTAATAACTTCTAATATTAATG
CAATGGCTGAATCAGCATATAGATATTTACATAGTAGACTTGTAGCTTTTGAATTTCCTA
ATCCTTTTCCTATGAAAGATGATGATACCCCTGAATTTGAGCTAACTGACCAAAGCTGGA
AATCTTTTTTTACAAGGCTTTGGACACAATTAGAACTGAGTGACCAAGAAGACGAGGGAG
AGGATGGAGACTCTGAGAGAACGTTTCGATGTGTTACAAGATCAACTAATGAACATTTAT
GAAGTTGCTAGTGATACCCTAGAAGCACAAATAGAACATTGGCGGCTTCTTCGTAAAGAA
GCTGTGCTGTTATACTTTGCTAGGCAAAATGGTGTTTTAAGACTTGGGTATCAGCCAGTA
CCACCACTTGCAACATCTGAAGCAAAAGCCAAAGAGGCAATTGGCATGATGTTACAGCTG
CAGTCATTACAAAAGTCAGCCTATGCATCTGAAAAGTGGACTTTGGTGGATACCAGCATA
GAGACCTTTAAAAATACTCCAGAAAATCATTTTAAAAAAGGACCTGTAAATGTGGAAGTG
ATTTATGATGGTGACCCTGACAATGCAAATTTGTATACAATGTGGAAATATGTGTATTAT
TTGGACTCTGAGGACAGATGGCAAAAAACTGAAAGTGGGGCAAATCATACAGGCATTTAT
TATACTATAAAGGATTTCAAGCATTACTATACTTTGTTTGCTGACGATGCAAAAAAATAT
GGTAAATCTGGCCAGTGGGAGGTCAGAATTAATAAGGAAACTGTGTTTGCTCCTGTTACT
AGCTCCACGCCGCCAGAATCCCCAGGACGAGAAAGAGCTTCCCCAGAGTCCACCGCTCAC
ACCAAGACCACCGCCCGTTCCAGCGCCTCCCCCGACCACGAACTCCCACAACAAACCAGT
GACGAAACCAACCGAAAGCGGAGGTACGGAAGGCGAGAATCAAGTCCCACAGAGTCCAAA
CGAAAGCGACGACGGTCCTCGTCCAGGCAAAAGAAGCAAGGGCGAAGGTCGCGGTCCAGA
ACAACATCCTCTCAATCTCGATCTCGGTCGCGATCCTCTTCCTCTAGAGGATCAAGGGGG
TCACGGGGACGAACCCCCAGAGGGAGGGGAAAACACACAACCCGGGGCAGGGGGAGAGGG
AGTGGAAGGAAAGGTGACAGAAGGGGAAGGAGAAGTCGAAGGTCATCCTCCTCTTCCTCC
CCCACCATCTGCACCAGATCAGCCTCACAAACCCGATCAAAACAATCCAAGTGTGCTAGG
GACGGTGGCATATCTCCTGGGGACGTGGGAAGATCAGTTCAGACAGTTAGTGGACGAAAT
ACAGGGAGACTTGGAAGATTATTGGAAGAAGCTCGCGATCCCCCAGTAATTTTAATTAGA
GGGGAAGCAAATACAGTAAAATGCTTTAGGAATCGGGCAAAACAAAAGTATAAAGGCTTA
TGCAAGGCATTTAGCACAACATGGTCATGGGTTGCCGCAGATGGCACAGAGCGTCTAGGC
AGGTCCAGAGTGCTCGTAAGTTTTACCAGCCATACACAGCGCAGCTCTTTTTTAAAGGTA
GTTAAGTTTCCAAAAGGAGTAGATTGGTCTTTAGGCAACTTAGATAAACTATAATACTAA

CTTGCTTTTGCTACTAACATATAACCAAGCTTTCTTTGTTTTTGTATATTCTTGCAATGG
TGCGTGCCAAAAGAACAAAGAGAGATTCTGCAACTAATATTTACAGAACATGCAAGCAGG
CGGGAACTTGTCCCACTGATGTCATTAATAAAGTGGAACAAACAACCATTGCTGACAATA
TTTTAAGATATGGCAGTGCTGGTGTGTTCTTTGGTGGCCTTGGAATCAGTACCGGCCGTG
GTACAGGAGGTGCAACTGGATACGTCCCCTTAAGTGAAGGGCCTGTAAGAGTGGGTAGTA
CACCCACTGTAGTTAGGCCTGCTTTGGTTCCTGAAGTTATTGGGCCTGCAGAATTGCTAC
CCGTAGATACCATAGCTCCTGTAGAACCATCCTCATCTTCAATTGTTCCATTAACAGAAA
CTTCTGGTGTAGATTTATTACCAGGAGAAATTGAATCTATTGCAGAAGTACATCCTGTAC
CTGATGTTGCCAGTTTTGATACACCAGTTGTTACCACCAGTAAAGGTTCCAGTGCAATTT
TAGAAGTTGCTCCCGATCCAACTCCTCCTACACGTGTACGTGTAAGCAGAACACAATATC
ATAATCCTTCTTTTCAAATTATGACAGAGTCTACACCTACACAAGGTGAAAGTTCTCTAT
CTGATCAAATTTTTGTGGCTTCTGGTTCTGGTGGACAGTCAGTAGGTAGAGCTGAAGAAA
TCGAGTTGCAAGAATTCCCTAGTAGATATTCATTTGAAATAGAAACACCTACTCCACCAA
GGAGAAGTAGCACTCCTTTACAAAGACTTAGACAAGCTGTAAGGGGCAGATCTTTAACAA
ATAGACGGTTAATACAACAAGTAAATGTAGAAGATCCTTTGTTTTTATCACAGCCATCTA
AATTGGTTAGATTTTCTTTTGATAATCCAGCATTTGAAGAAGAAGTGACACAGCAATTTG
AGCAAGATTTAGATGATTTTATAGAACCACCAAACAGAGATTTTCTTGATATTCAAACTC
TCAGTAGGCCTCAATATTCTGAAACTCCTGCAGGATATATTAGAGTAAGCAGATTGGGAA
AACGAGCCACTATTCGTACACGCTCGGGGGCCCAAATTGGGTCACAGGTACACTTTTATA
AAGATCTCAGTTCCATTGATACTGAAGCACCTATTGAATTAGATTTACTGGGACAACGTT
CTGGTGATGCCACTATAGTTCAAGGGACTGTAGAAAGCACATTTATAGATGTCAATGTTG
ATGACAATCCTTTATCAGAGTTGGAATCATACCCTGAGGCACATACAGAGGATGCATTAT
TAGATGAAGCAGTAGAAGATTTCAGTGGTTCTCAATTGGTAATTGGCAATAGAAGATCTA
CCTCTACTTACACAGTACCTCGCTTTGAAACCCCAAGAAATGCTACATATTACACACAAG
ACTTAAAAGGTTATTATGTATCATATCCAGAATCTAGAGATGATTTGCAAATTATTTATC
CCTCTCCAGATCTCCCAGCAGTTGTAATTCATACATATGATAATAGTGGGGATTTTTATT
TACACCCAAGTTTACTACGGAGACGCAGACGCAAACGTAAATATTTGTGATTTTTACAGA
TGTCGGTGTGGTTACCTGCCAGTGGTAAGGTATATTTGCCACCTTCAACACCAGTTGCCA
GAGTACAAAGCACGGATGAATATGTACAAAGGACCAATATTTTCTATCATGCATATAGTG
ACAGATTATTAACAGTAGGTCACCCATATTACAATGTTTACAATAATAATGGAACAGTAT
TGGAAGTACCAAAGGTTTCAGGTAATCAGCACAGGGTGTTTAGATTGAAATTACCAGACC
CAAATAGGTTTGCATTAGCTGATATGTCTGTTTATAACCCGGAAAAGGAAAGGTTAGTTT
GGGCCTGTAGAGGCTTGGAAATAGGGCGGGGCCAACCTTTAGGTGTTGGTACTACTGGGC
ATCCATTATTTAATAAGGTAAATGATACAGAAAATCCTTTAAATTACAGAACTCAGGCAT
TTTCTACTGATGATAGACAAAACCTATCATTTGATCCAAAACAAATACAAATGTTCATAA
TAGGTTGCACACCATGTGTAGGTGAACACTGGGATGTTGCACCTAAATGTGATGGAGCCA
ATGACCAAAATGGGATTTGCCCACCTATACAATTAGTAAACACTGTTATTCAAGATGGGG
ATATGGCAGATATCGGGTATGGTAATCTTAATTTTAAGGCTTTGCAGGAAAATAGATCTG
ATGTTAGTCTTGACATTGTTAATGAAACCTGCGAATATCCTGATTTTTTAAAGATGCAAA
ATGATGTTTATGGGGATTCTTGCTTTTTTTTTGCTCGCAGAGAGCAATGTTATGCCAGAC
ACTTTTTTGTAAGAGGTGGCAACGTAGGAGATGACATTCCTGGTGAACAAATAGACGCAG
GCACATATAAAAATGATTTTTACATTCCAGGAGCAACAGGTCAGACACAAAATAAAATAG
GTAACTCCATGTATTTCCCAACAGTTAGTGGCTCATTAGTGTCTAGTGATGCTCAGTTGT
TTAATAGGCCCTTCTGGCTCCAACGCGCACAAGGTCATAATAATGGTATTTGTTGGGCAA
ACCAATTGTTTATTACAGTTGTAGACAACACAAGAAACACAAACTTCAGTATTAGTGTTT
ATACAGACCCTGGGAAAATAAGGGATATAAATGATTATGATGCTAACAAATTTAGAGAAT
ATCAAAGACATGTGGAAGAATATGAAATTTCCTTGATATTACAATTATGTAAAATACCTT
TAAAATCAGAAGTACTGGCTCAAATAAATGCAATGAACCCCTCACTGCTAGAGGAATGGC
AATTAGGGTTTGTCCCAACTCCAGACAATCCGTTACAGAGTACTTATCGATATATTAATA
GTTTAGCTACACCATGTCCCGATAAGGTTCCACCAAAGGAAAAGGAAGATCCTTATGCAC
CGTATACATTTTGGAATGTAGATTTAACTGAAAGATTATCATTAGATCTAGATCAATACT
CCTTAGGGCGCAAATTTCTGTATCAGGCAAATCTTGTACAATCACCAGCTAAACGGTCAT
CATCTATTTCTCGAGGAACTAAAAGAAAAAGAAGCAAATAATTTTACCACTTTTGGTACA
ATAAAGAGTATATCAAACAGTATTCCAAGAATGCTTGTATTCATATGTCTGACTAATTTG
AACTTTGGCTAGAATGTGAACCGCGCCCGATACAGATAGATAACATCTTGCAACCACAGC

AGAGACAATGAGAACCTTGTAGGTTCCAACACGCTCGGATTTGGCGCATCCTACCACATT
TGGCGCCTTTTCTGCTGGCAAGAAAACTGCACCGTTAACGGTAAGTTTTTTGTTTATGTA
CCAGGCAAGA
>gi|27531786|ref|NC_004500.1|_Human_papillomavirus_type_92,_complete_genome
TATTGTTGCCAACAATCATCACGCCATAGAAAAACACGTAACCGCCTGCGTTATAATACA
AACAGCTAGTATATAAATACAGGCAGTGAAAGTGTTCCCATCACAATGGCAAAACCTCCT
TCGGTGCAGGAACTTAGAAGACAGTTAGATATTCCATTGGAGGACATTTTATTGCATTGT
AATTTTTGTGAGGCTTTTTTAACATTTGAGGAGCTACTGCAATTTGATGCAAAAAATTTA
AATTTAATTTGGAAGGAGAATTATGCTTATGCTTGCTGTGGTGCTTGTGCTAAACAAGTA
GCAGCAATAGAAACAAAACATTTTTATGAGTATAGTGTACAAGGAAAGGATGCTATAGAA
AGGGACTCAGGTAGTCTTTTGTGTTGTTTAACTGTTAGATGTAAGTTTTGTTTAAGACAT
TTGGATTACTTAGAGAAATTGGCAGTTTGTGCATCAGGCATTCCTTTTGATAGAGTTAGA
GGAGCTTGGAAGGCAGTGTGTAGGTTTTGTACAGAGATATGATTGGGAAACAGGCTACTA
TACCAGATATTGTGCTGGATCTGCAAGACCTTGTCCAGCCCATTGACCTGCATTGTGACG
AAGACTTGTCAGAAAATCAGGAGGAGGAGCCTGCACCTCAAAGAATAGACTACAAGATAG
TTTCCTCGTGTGGTGGCTGCGGAATTAAGCTTCGAATTTTTGCATCGTGTACCCAATTTG
GAATTAGAACTCTGCAAGACCTACTTCTTGAAGAAATAGCGTTGCTGTGTCCTGACTGCA
AAAATGGCAGATAAAGGTATAGATCCTAAAGAAGGCTGTAGTACTTGGTGTTTAATAGAA
GCTGATTGTAGTGATGTAGATGGGGATTTTGAAAAGTTATTTGACAAAGACACAGACTCA
GATATTTCAGATTTATTAGATGATGGGGACCTTGGGGACGCAGAATTGGGAAATCCCCAA
GAGCTGCTGTGCCTGCAGGAGAGAGAGGAGAGCGATCTACAGCTGCAGCAGTTAAAACGA
AAGTATTTTAGTCCTAAAGCTGTTTTACAGCTTAGTCCACAATTGGAATCTATTACTATT
TCGCCTCAACGCAAAAGTAAGAGGCGACTGTTCGAGGAACAGGACAGCGGACTTGAGCTT
TCTTTAACTCATGAAGCTGAAGATTCTGTTGCGGAAGTGGAGGTACCGGGGTCAAAAGAT
GACGTCCCAGAAACTGTTTCTGCTACAGCAGAAACTAAGGGAAGCCAAAACAAAGAACAT
TACAAACAGTTACTACAGTGCAGCAATGCGCGGGCTACATTGCTTAGTAAATTTAAAGCT
GCTTTTGGTGTTAGCTTTACAGAGTTAACCAGAAGATACAAAAGTGATAATACATGTTGC
AGAGACTGGGCAATTATTGTCTATGGGTTGCAGGATGAAATTATTGAAGGCTCAAAGCAT
TTATTTCAGCAGCATTGTGAATATATTTGGTTGCATGTTTTATCTCCAATATCTTTGTAT
TTACTATGTTTTAAAACTGGAAAAAGCAGAAATACTGTAAAGAACTTGTTGATGTCCATT
TTAAATGTTGGGGATGCACAGCTTATAGCTGATCCACCCCAGATTCGCAGCGTAGTAGCA
GCTTTGTTTTGGTACAAAGAATCTATGAATAAAAATGTATATACCCATGGAGAATACCCA
GAGTGGATAGCAAATCAAACATTGCTTTCTCATCAGGAATATGAAACACAGCAATTTGAT
TTAAGTAGAATGATTCAGTGGGCATATGATAATGAATATACTGAGGACTCAGATATTGCT
TATCATTATGCAAAATTAGCAGATGAAGATTCAAATGCTCGCGCCTTTTTAGCTCATAAC
AGTCAGGCAAAATTTGTTAGAGAATGTGGACAGATGGTAAGGCATTATAAAAGAGGAGAA
ATGAAAAATATGAGTATGTCAGCCTGGATTTATACTAGATTGAAATCAATTGAAGGACCA
GGCCATTGGTCAGACATTGTTAAATTTATACGATTTCAGCAGATTAATTTTATAATGTTT
CTAGATGTATTCAAGCAATTTCTTGCCTCAGTACCTAAAAGAAATTGTTTATTAATTTAT
GGTGCACCTGATTGTGGCAAATCAATGTTTTGTATGTCTTTAATAAAGGCCTTAAAGGGA
AAAGTTATATCGTTTGTAAATGCTAGAAGTCAATTTTGGTTATCTCCATTAGTAGAATCT
AAAATTGCACTACTAGATGATGCCACCGAGTGCTGTTGGAATTATATTGATAATTATTTA
AGAAATGGAATAGATGGTAACATGGTTAGTGTGGATTGTAAGCATAAAAATCCGGTCCAA
ATTAGATTTCCACCATTATTGATTACATCAAATAATAATATAATGTCTGATCCAAAGTAT
AAATATCTGCATAGTAGAATTAAAGCATTTGAGTTTGTAAATAAGTTTCCATTTAAGGAC
GATGGCAGTCCCTTGTTTGAACTTACTGACCAAAGCTGGAAATCTTTTTTTCAAAGGCTT
TGGAGGCAATTAGATCTAAGTGACCAAGAAGACGAGGGTGAGGATGGAGGCTCTCAGCGA
CCGTTTCAATGCACTGCAAGACAAGTTAATGACAATTTATGAAAGAGCTAGTGAATCCTT
AAAAGATCAAATTGAACATTGGAACTTGTTAAGACAGGAGCAGGTGTTATTTCATTATGC
CAGACAAAGAGGAGTATTGCGCCTTGGTTATCAGCCAGTACCTGCATTAACTATTTCAGA
GGCTAAAGCTAAGGAAGCCATTGCTATGGTTTTACATTTAGAAGCATTGCAAAGATCACC
TTACAAAAATGAAAAATGGACATTAGTAAATACAAGTGTAGAAACGTTTCGCACACCCCC
AGAAAATTGTTTTAAAAAGGGCCCTAAGACTATTGAAATAGTGTATGATGGCAATCCTGA
AAATACAATGTTATACACTATTTGGACACATATATATTTTGAAGATGACGAAGGCAACTG
GCAAAAGACTGAGGGACATTTGGACTATCATGGTGCCTATTTTATGGATGGATTAAATAA

ACAATACTATATCAGATTTGCTCAAGACGCACGCAGATTTAGTGAAACTGGAGAATGGGA
AGTTAAGTTTAACAACGAAATTTTGTTTGCTCCTGTTACCAGCTCCACCAACTCCGAAGA
AGAAAGGGACCGACCCGCCCCTGCCACAGACCCCGGCTCCCTTTCGCAGACATCCGGAGG
ACAGTCCCCTGTACCCACTCAACGGAAGCAACCATCTAAAGGAAGGTACGGGCGAAAAGA
CTCTGGTGCTACAACCGCCTCCAGGGGGATCCAAAGACGACCGAAAGCGTCACCGAGGAG
ATCACGATCGCGGTCAGGATCGAGATCGGGATCACAAGGAGACGCGCGGACCCTCCTCAC
AGTCAGACGCGGAGAACGGGAACGGGGACAAGGAAGGGGACAAGGAAGCCGGGGTCGGGG
AAGGAGCGGGGACAGAAGCAGGAGCGGGAGCAGAAGCAGGAGCGGGAGCAGGAGAAGGAG
CAGGAGCAGGGGCAGGAGCAGGAACGGAAGGGAACGGGGGAGAGCAGCCTCCAGAGGCCG
TAGAGGGTACAGCAACAGGAGGTCAAGATCCAAATCTGTTGGCACAAGTGGCATACCACC
TGAGCAAGTGGGAAGCAGCCTACAAGGTGTTGGTAGACAACATAGTGGACGACTTGCGAG
ATTATTGGACGACGCTAGGGATCCCCCAGTAATTTTGTTGAAAGGACAAGCCAATACTCT
TAAGTGTTATCGCTACAGGGCTAAAGAAAAGTATAAAGGCTATTATGATTGCTTCAGTAC
TACATGGTCATGGGTCAGTGCAGGTAGCAACGATAGAATAGGACGCTCTAGAATGATTAT
TAGCTTTACCAGTAAATCTCAAAGACAAATGTTTTTAAGTATTATGAAATTACCAAAGGG
CGTTGATTGGTCTCTTGGGTGCTTTGACTCTATTTAACACACTAACCTTTCTAGTTTTTT
TACTAACACATACGTTTCAATAGAATTGTTAATGCATGGCTCGCGCACGCAGAACAAAGC
GTGATTCTGTTACTAATATTTACAGGACCTGCAAAGCAGCAGGCACCTGCCCCCCTGATG
TTGTTAATAAAGTGGAGCAGACTACTGTAGCTGATCAAATTTTAAAATATGGCAGCACTG
GTGTATTTTTTGGAGGTTTGGGAATTGGTACAGGAAGAGGCACTGGTGGTAGCACTGGAT
ATGGGCCATTAGGTGAAGGGACGAGTGTAAGAGTTGGAAATACACCCACAGTTATTAGGC
CTGCTTTGGTGCCTGAGGCTATAGGACCAAGTGAACTAATACCTATTGACAGTGTCAATC
CTATTGACCCCAGTGCTTCTTCTATCATACCTTTAACAGAGTCAACAGGTCCTGACCTCT
TACCAGGTGAAATAGAAACAATTGCAGAGGTGCATCCTGCCCCTGACATACCTACAGTAG
ATACACCAGTGGTGACTGGGGGCAGAAACTCGAATGCTGTTCTGGAGGTGGCTGATCCAA
GTCCACCCACACGAAACAGAGTTAGTAGAACACAATATAACAATCCTGCATTTCAAATCA
TATCTGAAACTACACCAAGTGCGGGGGAAACGTCCCTATCAGACCAAATTGTTGTTCAGT
CATTTGATGGTGGACAATATATAGGTGGTAACCCACCTCCGCGATCAGTAGTTGAAATAG
AATTACAAGAAATTCCCTCACAATATTCTTTTGAAATCGAAGAGCCAACCCCACCTAGGC
AAACAAGCACTCCTGTCAGACAGGCACAACAAATGGCCTCAGCATTACGGAGGGCTTTAT
ACAATAGAAGGTTCACACAGCAGGTTCAAGTGGAAGATCCAATGTTTTATAGTAGACCTT
CCAGGTTAGTTAGGTTTCAATTTGATAATCCTGTATTTGAAGAAGAAGTTACTCAGGTGT
TTGAAAGAGACCTAGAAACTATAGAAGAGCCTCCTGATAGACAATTTTTAGATGTACAAA
AACTTGGTAGGCCTACCTATGCTGAAACACCTGCAGGCTATATAAGGGTTAGCAGACTTG
GCAAACGAGCTACTATAAGGACCAGGTCTGGAACTACAATAGGCGGTCAGGTACATTTTT
TTAGGGATATTAGCAGTATTGATACTCAACCTTCTATTGAACTGCAAGTTCTTGGGGAAC
ATTCTGGCGATGCTACAATAGTCCAGGGTCCTGTGGAAAGTACGTTCGTAAATATTGATT
TGGAAGAGTTACCTAATTTAGAGGAAAATGTACACCTAGAATCTGATGATATACTTATTG
ATGAAGCTATAGAGGATTTTAGTGGTGCCCAATTAGTGTTTGGAAATTCTAGAAGATCAA
ATACTGTTACATTACCTCGCTTTGAAACTGTAAGGGAAACTTCTTTATATACTGTAGATT
TAGATGGATTCCATGTGTCTTATCCTGAGAGTAGAGCGTATCCAGAAGTTATTCCTACAG
AACCAGATAATACCCCAACAGTAATAATTCACACAGAAGATTTTAGTGGTGATTATTATC
TACATCCTAGCTTAAAATGGAAGAAACGAAAACGGGCCTATTTATAATTTTTTGCAGATG
TCCTATTGGCTTCCAGCAAATGGTAAGGTATACTTACCCCCTTCAACACCGGTTGCAAGG
GTACAAAGTACGGATGAATTTGTTCAAAGGACCAACATCTTTTATCATGCAAATAGTGAT
CGCCTGCTGACAGTTGGACACCCCTTATTTTGAAGTGAGAAGCTCAGTTGATCCACATGAT
GTATTAGTGCCTAAGGTGTCAGGGAATCAGTTTAGAGCTTTTCGACTGAAATTACCAGAT
CCTAATAGATTTGCTTTAGCTGACATGTCTGTTTATAATCCAGACAAGGAAAGGCTTGTT
TGGGGCTGCAGGGGATTGGAAATAGGGCGAGGGCAGCCTTTAGGTGTTGGCACCACAGGT
CATCCATTATTTAATAAGGTATTGGACACTGAAAATCCAAATAAGTACAATACTGGAACA
AAGGATGACAGAGTAAACACATCTTTTGATCCAAAGCAAATTCAGTTATTTGTTTTAGGA
TGTACACCATGCTTAGGTGAACATTGGGACACAGCCTTACCATGTGCTGAAAAGCAACCA
GATACTGGGGGATGCCCACCATTAGAGTTAAAAAACACTGTTATCTCTGATGGAGATATG
GTTGACATAGGCTTCGGTAATATGAATTTTAAGGCCTTATCAGTAACCAAATCTGACGTA
AGTTTGGATATAGTAGACTCCACATGTAAATATCCAGACTTTTTAAAGATGTCAAATGAT

GTATATGGCAACTCATGTTTTTTCTATGGACGACGAGAACAATGTTATGTTAGGCATATG
TTTGTGCGCGGTGGTGTTGTGGGTGATACCATCCCAGATGCAGTTGTAAATGAAGACCAT
AACTTTATGTTACCTGCAGCATCCAGTGACCAGTCTAGAAGTCAAATTGCCAGTTCTATC
TATTTCCCTACTGTTAGTGGGTCTTTGGTATCCACTGATGCACAATTATTTAATCGGCCA
TATTGGTTACAAAGAGCACAAGGCCACAACAATGGTATTTGCTGGAGTAATGAACTGTTT
CTGACAGTTTGTGATAATACCAGGAATACTAACTTTAATATTAGTGTCCCTAAGGAAGGT
GGTCAAATAACCGACTATGATTCACAAAAGATTAGAGAATACACTAGACATGTTGAAGAA
TATGAAATATCACTAATATTACAATTATGTAAAATTCCTTTGGAAGCTGAGATATTAGCT
CAAATTAATGCAATGAATCCAAATATTTTGGAGGACTGGCAGTTAGGATTTGTTCCTACT
CCAGATAACCCTATTCAGGATGCATACAGATTTATTCATTCTAAAGCAACACCTTGTCCA
GATAAAGCACAACCTAAAGAAAGAGAAGATCCATGGGCCCCATATACATTTTGGGTTGTA
GACTTAACTGAAAAATTATCTTTAGATTTAGATCAATATTCATTGGGTAGAAAATTTTTA
TTTCAAGCTGGATTAACTAATACATCTGTTAATGGTCTTAAAAGAACTAGAAGCAGTTCT
CAAAGAGGTACTAAACGAAAAAGAAAAAGTAACTAAAACGGTCAGTATTCTTTATTGAAA
ATAAAATTTTTGGAACTCATGTGTTATGAGTAATGATTATTATCTATTCTGACTAACTCA
AACATGTTAACCGCGCCCGGTGTATTCAATATAAACGCTGATGGTACAAGTTGTCAAGGA
ACTTGGCAGTCTGAACTACAGTGGTGCCAACACCTGGAAGGCACACAAGATTTGCGCGCC
AAAACTACTTGGCAGAACATTTCACCGATAACGGTAAGATTTTATCTTTAACCGGGTGCG
GTCGTTGGGTTACTGTTTAGG
>gi|9627363|ref|NC_001591.1|_Human_papillomavirus_type_49,_complete_genome
CCACATTCGTTCCAGCTACATTTTGGCGCCAACTCTTTGGCAGCAACACCAGAACGATAA
CGGTAAGTTTCAATCGGGCGCGGTCACATTATACTTAGTCATCTCTTGTGGTTGTTAACA
ACAATCTTGAAACAGATATACATGTAACCGCTTGCGTGCTGTACTTTCTTTATTCTTGGA
AAGAATACAGACAGGACACATGGCTAGACCTGTTAAGGTATGTGAGCTAGCCCACCACTT
AAATATACCTATTTGGGAAGTTTTGCTTCCTTGTAATTTTTGCACGGGGTTTCTAACATA
TCAGGAGTTGTTAGAATTTGACTATAAAGACTTTAATTTGCTGTGGAAAGACGGATTTGT
CTTTGGTTGTTGTGCAGCTTGTGCCTATAGATCAGCATATCACGAGTTTACTAATTATCA
CCAAGAAATTGTCGTAGGCATCGAAATAGAAGGACGAGCAGCGGCTAATATTGCTGAGAT
AGTAGTCAGATGTCTCATTTGCCTTAAGAGGCTAGATTTGTTGGAAAAGCTTGATATTTG
TGCACAGCACAGAGAGTTTCACAGAGTTAGAAATAGGTGGAAAGGGGTGTGTAGACATTG
CAGAGTTATAGAATGATTGGGAAAGAAGTTACAATACCAGATATAATACTACAAGAAGAG
TTTGGCCAGCCCATTGACCTGCAATGCTACGAGAATCTAACAGCTGAAGCGCCAGCTGAA
CAAGAGTTGGAGGCAGAGGAGGAGCTTATCCAAGGCATCCCTTACAAAGTTATTGCTACT
TGTGGCGGCGGATGCGGTGCCAGACTGCGAGTCTTCGTGTTAGCCACTGACGCTGCTATT
AGAAGTTTCCAAGAACTGCTTCTGGAGGAACTGCAATTCTTGTGTCCTCAGTGTCGTGAA
GAAATTCGGAATGGCGGACGATAAAGGTACTGATCCCAAAGAAGGGTGTAGCGAGTGGTT
TATAGATAATGAAGCAGACTGTAGTGATTTAGAAAATGATTTGGAACAATTATTTGATGA
AAGCCCAAAGTCCAATATTTCAAATTTGTTAAATGATGAGGAGGATGTGGAGCAGGGAAA
TTCGCGAGATCTGCTTCGCCAGCAGGAATTTGAGGAGAGCGCGGAGCAAGTACAAAAGTT
AAAACGAAAGTATTTCAGTCCTAAAGCAGTTCAACAACTTAGCCCACGGTTGCAGTCTAT
GTCAATATCTCCGCGACAAAAGTCTAAACGAAGGCTATTTGAGGAGGACAGCGGGCTGGA
ATTATCGGGGCTCGAACAGTCTTTGACTAATGAAATTGAAGATACTCCTGCGGAGCTGGA
GGTACCGGCGGCAACGCCGGCAGAGCAGGGTGGTCAGGGAGAGGGCAATTTGCATTATAA
AGAGTTAATGCGATGCAATAATAGTCGTGCAAAATTATTAAGTAAAGTCAAGGAATATTT
TGGTGTGGGTTTTTATGAGTTAGCTAGACAGTATAAAAGTGATAAAACATGCTGTAAAGA
TTGGGTAATTGCAGCCTATGGCGTGCGAGAAGAGCTGGTAGAAAGTGCAAAACAATTACT
TTTAAATCATTGTTCCTATGTGTGGATAAATATAAATGGGATTATGACTTTATATTTACT
GTGTTTTAATCATGCAAAGAGTAGAGAAACTGTTGGTAGATTGCTTATGTCAATACTGGA
TGTACAATTATTGCAATTAATTTGTGAACCACCAAAACTAAGAAGTGTGGTGTCAGCACT
ATACTGGTACAAAGGCAGTATGGACTCATCTGTGTATGCTCATGGAGCCTATCCTGATTG
GATTGTAAATCAGACCATGATAAGTCATCAGGCAGCAGCAGATGCTATGCAATTTGACCT
TTCTGAAATGATACAATGGGCCTATGATAGCGATCTCACAGATGAAGCTGACATTGCATA
TCTTTATGCTAAAATGGCAAATAGTGACTCTAATGCAAGAGCTTGGTTAGCACATAATAA
TCAGGCAAGGTACTTAAGAGAATGTGCTCAAATGGTTAGACATTACAGACGGGGAGAAAT
GAGGGATATGAGTATGTCTGAGTGGATACATCACAGAATACAACAAGTAGAAGGGGAAGG

CCATTGGTCTGAAATAGTTAAGTTTATAAGATTTCAAGAAATAAACTTTATAATATTTCT
GGATGCATTTAAACAGTTTATACATGGCAAACCTAAAAAAAGCTGTTTATTAATACATGG
GCCGCCGGACTGTGGCAAGTCAATGTTTGCTATGTCATTATTAAAAGTTTTAAAAGGCAA
GGTAATTTCATTTGTAAATGCAAAAAGTCAATTTTGGCTGTCTCCACTTTCAGAATGTAA
AATAGGGCTGTTGGATGATGCTACCGATCCTTGTTGGCAATATATAGATACATATTTAAG
AAATGGTCTCGATGGAAATGTTGTAAGTGTGGATTGCAAACATAAAACCCCTATGCAAAT
TAGGTTCCCACCATTGTTAATAACTTCAAATTATAATATTAAAGCTAATGATAAATATAA
GTTTTTGTACAGTAGAATTGCAATATTTGAATTTAAACATAAGTTCCCATTCAAAGAGGA
TGGTACCCCTGTATTTCAACTTACTGACCAAAGCTGGAAATCTTTTTTTGAAAGGCTTTG
GACACAATTAGAGCTCAGTGACCCAGAAGACGAGGCAGACAATGGAGGCACTCAACGCTC
GTTTCAATGTACTACAAGAGATGTTAATGGACATTTATGAATCAGGGAAAGAGGATCTTG
AAACACAAATAGAACATTGGAAACTGTTAAGACAGGAACAAGCTTTATTATTTTTTGCAC
GTAAACACAGCATAATGAGACTGGGGTATCAACCCGTACCTCCGATGGCAGTATCTGAAA
CCAAAGCCAAACAAGCTATTGGCATGATGCTAACTTTGCAAAGCTTGCAAAAGTCTCCTT
TTGGAAAAGAAAAGTGGACTTTAGTAAACACAAGTCTTGAAACATACAATGCACCACCAG
CACAGTGCTTTAAAAAAGGTCCTTATAATATAGAAGTTATATTTGATGGAGATCCTGAAA
ATCTAATGGTATATACTGCTTGGAAAGAGATTTATTTTGTAGACTCAGATGATATGTGGC
AAAAGGTGCAAGGTGAGGTGGATTATGCAGGTGCATATTATAAGGATGGAACTATCAAAC
AGTATTATGTTACCTTCGCTGATGATGCTGTTAGATATGGGACATCTGGACAATATGAAG
TCCGCATTAACAACGAAACTGTGTTTGCTCCTGTTACTAGCTCCACCCCACCATCCACGG
GGCTACGAGAATCCTCCAACGCCAGCCCCGTTCACGACACCGTCGACGAGACACCCACCA
GCACCCACAGCAACCACCACCACCTTCAGCACCACCACAGCCACAGCCACAGCCACAGGAG
CACCTGAACTCTCATCCAAAACCGGTACCAGGAAAGGAAGGTACGGGCGAAAAGACTCTA
GTCCTACAGCAGCCTCCAACTCCAGGAAAGAGGTCTCGCGACGACGATCCAGGTCTAGAA
CCAGGACCCGCAGACGGGAAGCGAGCACCTCAAGGTCCCAAAAAGCCAGCCGTTCCAGAT
CCAGATCCAGATCCACTTCCAGAGGATCCAGAGGGTCCGGAGGATCTGTCACAACCTCCA
GAGATTCCAGCCCCAAGAGAACCCGCAGGGGCAGAGGGAGGGGAGGGAGAAGTAGAAGGT
CACCCACCCCCACCTCCACCAGTAAACGGGAAAGAAGGCGCAGCCGGTCAAGGGGGGGAG
AGCCTGTTTCTGGAGGGGTTGGCATCTCGCCTGACAAGGTGGGATCAAGAGTACAAACAG
TTAGTGGACGACATCTTGGACGACTTGGAAGGTTACTGGAGGAGGCTAGCGATCCTCCAG
TAATACTTTTGCGAGGAGACCCAAATATTTTAAAATGTTACAGATACAGAGATAAGAAGC
GTAAATTAGGTTTAGTAAAACATTATAGTACCACCTGGTCATGGGTTGGTGTAGATGGCA
ATGAAAGAATAGGTAGATCACGTATGCTTTTAAGTTTTACTTCAAACAGCACTAGATCAC
AGTATGTTAAAATTATGAAGCTCCCTAAAGGTGTGGAATGGTCTTTTGGTAATTTTGATA
AGCTTTAACATTTTGCTAACATACTAACGGTGCTTGCACTACTAACACATTAATCTTTTA
ACATTTTTATATTGCTTTTTTATTTTTATATAATGGTGCGTGCTCGCAGAACAAAGCGAG
ATTCTGTAACAAACATTTACAGAACCTGCAAACAGGCAGGAAACTGTCCTCCGGATGTTG
TTAATAAAGTGGAACAAACTACAATTGCTGACCAAATATTAAAATTTGGCAGCACTGGTG
TGTTTTTTGGTGGTTTGGGAATAGGTACAGGCCGTGGTACCGGTGGCAGTACTGGCTATG
TACCTATAGGTGAAGGCCCAGCAATACGTGTTGGGGGCACTCCAAGTGTTGTTCGTCCAG
GTATACTCCCTGAGGCTATTGGTCCGGCGGATATCATTCCTATTGATACTGTCAATCCAA
TTGATCCAAATGCATCATCTGTGGTCCCACTCACTGACACAGGACCTGATTTGCTACCTG
GGACAATTGAGACTATTGCAGAAGTGAACCCTGCCCCAGATATTCCTAGAGTTGACACAT
CTGTTGTCACAACAAGCAGAGGCTCCAGTGCTGTATTGGAGGTTGCCTCTGAACCCACAC
CACCCACTCGCACCAGAATTTCCAGAACACAGTACCATAATCCCTCTTTTCAAATATTAA
CTGAATCTACACCCTCTTTGGGAGAATCTGCATTAACTGATCATGTTGTTGTTACTAGTG
GTTCTGGTGGTCAACCAATAGGTGGAGTTACACCAGTTGAAATAGAATTACAAGAACTTC
CTAGCAGATATACTTTTGAAATAGAGGAACCTACACCACCAAGACGCTCTAGTACCCCAC
TACGCAACATCACACAAGCTGTAGGAAATTTAAGAAGATCACTATATAATAGGCGACTTA
CTCAACAAGTAAATGTCCAGGATCCATTATTCTTACAACAGCCCTCACGTTTAGTTCGCT
TTGCCTTTGATAATCCTGTGTTTGAAGAAGAAGTTACACAAATATTTGAAAGGGACGTAG
CAGCTGTAGAAGAACCTCCAGACAGAGACTTTTTAGATATAGCAAAATTAAGCCGCCCTC
TTTACTCTGAAACACCACAGGGATATGTCAGGGTAAGCCGCTTAGGTAATAGGGCTTCTA
TTAGAACACGTAGTGGAGCTACAGTAGGGGCTCAAGTGCATTTTTATACAGATCTTAGCA
CAATCGATGCAGAGGAGTCTATAGAGTTATCACTATTAGGGGAACATTCTGGTGATGCTA

CTATTGTCCAAGGCCCAGTAGAAAGCTCATTTGTAGATTTAAATGTTCAGGAACTGCCTC
AAGTAATAGAAGTAGACCCAGAACCTACTTTCCACTCTGATGATTTGCTACTGGATGAGC
AAAATGAAGATTTTTCTGGCTCCCAGTTAGTTTATGGTAGTGGCAGGCGTTCTACCACAT
TTACTGTACCCCGCTTCTCTACTCCCAGATCTGATACCTTTTATGTACAAGATTTGGAAG
GTTATGCTGTGTCATATCCTGAACGAAGGAATTATCCAGAAATTATTTATCCTCAACCCG
ATTTGCCAACTGTAATAATTCATACTGCAGATACCTCTGGGGACTTCTATTTACATCCAA
GCCTTCGCAGGCGAAAACGTAAACGCACTTATTTATGATATTTCTTTCAGATGACCTCGC
TATGGTTACCTGCAACTGGTAAGGTATATCTACCACCTTCAACACCTGTGGCAAGGGTAC
AAAGCACGGATGAATACATTCAGAGGACAGACATCTACTATCATGCTAATAGTGATCGAT
TGTTAACTGTAGGACATCCATATTTTGATGTGAGAGATACAGCAGACAATTCTAAAATTT
TAGTACCAAAGGTTTCAGGTAATCAATATCGAGCCTTTAGATTACTATTACCAGATCCCA
ACAGATTTGCACTAGTAGATATGAATATATATAACCCAGAAAAGGAAAGATTAGTATGGG
CCTGTAGAGGCTTAGAAATTGGTCGTGGCCAGCCTTTAGGTGTTGGTACAACAGGACATC
CATTGTTTAACAAAGTCAAAGATACTGAAAATGCTAATAACTATATAGTAACTTCTAAAG
ATGATAGACAGGATACTTCATTTGACCCTAAACAGGTACAAATGTTTATCATAGGTTGTA
CTCCTTGTATGGGTGAGTACTGGGACGCTGCTAAACCTTGTGATGCAGATGCTGGTCAGG
GTAAATGCCCTCCATTAGAATTAATCAATTCAGTTATACAAGATGGTGATATGATTGATA
TAGGTTTTGGTAATATCAATAATAAGACATTATCTGTTAACAGATCTGATGTCAGTTTGG
ATATAGTAAATGACATTTGCAAGTATCCTGATTTTTTAAAGATGGCAAATGACATATATG
GGGATGCTTGTTTCTTCTATGCTAGACGTGAACAATGTTATGCCAGGCACTTCTTTGTTA
GAGGTGGTAATGTAGGGGATGCGATACCCAATACTGCTGTAGGTCAGGATAACAATTACA
TATTACCTGCAGCAAGTCAACAGGCCCAAAATACTCTTGGCAGCTCCATCTATTTCCCTA
CCGTCAGTGGCTCTTTGGTATCTACTGATGCGCAGCTATTCAATAGACCTTTTTGGTTAC
AAAGAGCACAGGGTCACAACAATGGAATTTGCTGGGAGAATCAGCTTTTTATAACAGTGG
CTGATAATACCAGAAATACCAATTTTACTATTAGTGTAAGTACGGATGGCCAGACACCTA
CAGAATATGACAGTACCAAGGTTAGAGAATTTTTAAGACATGTAGAGGAATATGAAATTT
CAATTATATTACAATTGTGTAAGGTACCTTTAGAACCGGAAGTCCTGGCACAAATCAATG
CTATGAATTCTTCTATATTGGAAAATTGGCAATTGGGATTTGTTCCTACCCCTGATAATC
CTATACATGACACATATAGGTATCTTACATCACAGGCAACACGATGCCCTGACAAACAAC
CTGCTCCAGAAAGGAAAGATCCATATGAGCAGTATAACTTTTGGACTGTAGATTTAACAG
AAAAACTGTCTTTGGATTTGGATCAATATTCTTTAGGAAGAAAGTTTTTATTTCAAGCTG
GGCTACAACGGGCTTCTAGAGTGTCTAAATCCTCTGCTGCTAGAGCTTCCACACGGGGTA
TTAAACGAAAACGGAGATGACCGTTTTCGGTTGCTGGGTCTTATAATAAAATATTTTATA
AACTGTTTTGGTATGTGAGGCATGTTTTAACCGAGTTCGTGACTAAGATTGATTAACCCA
CCTGCAACCGCACCCGGTTAATCAGATTATAAAGGTGCGCCGGTGTTCACCTCTGGCTAC
TTGGCAGTTACAAGTTCACCTCTGCCAGAAGTGTGTTTTTGCCAAGACATTTGCCAAGTA
>gi|9627334|ref|NC_001587.1|_Human_papillomavirus_type_34,_complete_genome
ACTATAATCCTACTATAAAATATAGGGTGTAACCGAAAACGGTTGCAACCGATATCGGTG
CATATATAAGTGCTGCAGTACACTGCTGGACAGATTGGGAAATGTTTTTTCCCAATCCTG
AGGAACGGCCATACAAGCTACCAGCCTTATGTGAAGAGGTCAACATTTCAATACATGAAA
TAGAATTGGACTGTGTGTATTGCGAACGACAACTGTACAGATGTGAGGTATATGATTTTA
TATTTAGAGATTTATGTGTTGTATATAGAAAGGGGAAACCACTTGGGGTATGTCAACCGT
GTTTACTGTTTTACTCAAAGGTTAGACAATATAGAAGATATAACCAATCAGTGTATGGAC
GGACGTTAGAGAATTTAACTAACAAACAGTTGTGTAATATTTTAATAAGGTGCGGAAAAT
GCCAAAAACCACTGTGTCCATTGGAAAAGCAAAGGCATGTAGATGAAAACAAACGGTTTC
ACCAAATAGCGGATCAGTGGACCGGACGCTGTACACAGTGCTGGAGACCATCTGCAACAG
TGGTGTAAGAATGCATGGAAAAAAACCAAGTGTGCAGGACATTGTGTTAGATCTGAAACC
AACGACCGAGACTGACCTTACATGTTACGAGTCATTAGACAACTCAGAGGATGAGGATGA
AACAGACAGCCATCTAGAAAGACAAGCTGAGCAAGCCTGGTACAGAATAGTTACTGATTG
CAGCAGATGTCAGTCCACAGTGTGTCTTACCATTGAGAGCACACACGCTGACCTATTAGT
GTTAGAAGACCTGCTTATGGGTGCACTAAAAATTGTGTGCCCCAACTGTTCCAGACGCCT
ATAACAGAAGATGGCTGATTCAGGTAATTGGGAAGGGAGGTGTTCGGGATGGTTTAATGT
AGAAGCCATTGTAGAAAGGAAAACAGGGGATGCAATACCAGCAGATGAAAATTATGATGG
GGACGATACAGAGGATTCTGAAATGGGGGATTTTATTGATAATGCACACATATCTAATAT
ATATTCACAGCAGGAAATTGCACAGGCATTATATCACTCACAGCAAGTTAATGCAGACAA

TGAGGCTATACGTGTTCTAAAACGAAAGTTTGCAGGTAGTGCTGGCAGTAGCCCAGACAG
TAAAAGACATGAATTGAAACACAAACAGCGTAGTCCACATATATTGACGATAAGGGACAC
TAATACTACATCTACACACCTATTGTGTGAGGAACAAGACAGCGGATATGGCAATACTGA
AGTGGAAACGTACGAGAGACAGGTACCGGGGCCGGGGGGATGTTTACAAAGTACTAGCAG
TAGTAACAACGGCAGCCAAATGGCGTCGCCAGGGGAAACAAATAGTGGGTCCAGTAGCAT
TTCAAATATGGATATAGACATGGAAAGCACACCTATAACGGACATTACAAACATATTAAA
AAGTAGTAATGTAAAGGCAACATTATTAGCAAAATTTAAAGAGGTATATGGATTAAGTTA
TATGGAATTAGTAAGGCCTTATAAAAGTGATAAAACACAGTGTCAGGACTGGGTATGTGC
AGTGTTTGGGGTAGCGCCATCATTGGCAGAAAGTTTAAAATCATTACTAACCCAATATTG
CCTATACATACATCTACAATGTTTAACATGTTCGTGGGGTATAATAGTATTATTGTTAGC
AAGATTTAAGTGCAATAAAAATAGATTGACAGTACAAAAATTATTACATGGGTTATTAAA
TGTAACACAGGAATATATGTTAATAGAACCACCTAGACTAAGAAGCACGCCATGTGCATT
ATACTGGTACAGAACTAGCCTATCAAACATTAGTGAAACGGTGGGAGAAGTACCCGAATG
GATTAAAAGACAAACAGTAGTACAGCACAGCTTAGAGGACTGTCAATTTGACCTATCTCA
AATGGTACAGTGGGCATTTGATAATGACATAACAAATGACTGTGAAATAGCATATAAATA
TGCATTATTAGCATCTGAGGATAGCAATGCTGCTGCATTTTTAAAAAGCAATGCACAAGC
AAAATATGTTAAGGATTGTGGAACAATGTGTAGACATTATAAAGCTGCAGAACGTAAACA
AATGACTATGTCACAATGGATTACACATAGATGTGATTTAATAGATGATGGAGGAAACTG
GAAACATATTGTGCAATTTTTAAGATATCAGCAGGTTGAATTTGTACCGTTTTTAATTGC
TTTAAAACAATTTTTAAAGGGTATACCAAAACAAAATTGTATAGTTATATATGGACCACC
AGATACAGGAAAGTCACATTTTGGAATGAGTTTAATGCAGTTTATGCAAGGTGTGGTTAT
TTCATATGTAAATTCCAATAGTCATTTTTGGTTATCGCCATTGGCTGATGCAAAAATGGC
ATTATTAGATGATGCAACACCTGCATGCTGGACATATATTGATAGATATTTAAGAAATGC
ATTAGATGGCAATCCTATGTGTTTAGACAGAAAACATAAACATTTATTACAAATTAAATG
TCCTCCATTACTAATAACATCAAATACAAATCCTAAAGCAGATGATACCTGGAAATATTT
GCACAGTAGAATGAAAGTGTTTACGTTTTCAAATCCATTTCCATTTGACAGTAATGGAAA
TCCACTATACCAACTTACTAATGAAAACTGGAAGGCATTTTTTACAAAGACGTGGTCAAA
ACTAGATTTAACAGAGGACGACGACAAGGAAAATGATGGAGACACTGTGCAAACGTTTAA
GTGCGTGTCAGGACGCAATCCTAGAACTGTATGAACGTGATAGTATACATTTAAGTGATC
ATATTGATCACTGGAAACACGTGCGACTGGAAAATGTATTATTACATAAGGCACGTGAAA
TGGGACTGCAATCAGTTAACCAACAAGCGGTGCCAAGCCTTGCAGTATCACGATCCAAAG
GGCATAATGCAATTGAACTACAATTAGCCCTAGAAAGTTTAAATGAATCAAGCTATAACA
CAGAAGAATGGACATTACAACAGACAAGTTGGGAACAGTGGGTAACGGACCCAAAACAAT
GTTTTAAAAAAGGTGGAAAAACAGTAGAAGTTAGATATGACTGTGACAAGGACAACACCA
TGCAATATGTGGTATGGACATTTGTGTATTATTGGTTGGAAGGCAAGTGGTATAAAGTGA
GTAGCCATGTAGATTATAATGGTATATATTATGAAACACAGGACAATGAAAAGGTATATT
ATACACAATTTGACAGAGATGCAAAACGATATGGGGTTAAAGGAATATGGGATGTATGTA
TGGGCGGTAAGGTAATATGTTTTGCTCCTGTATTTAGCCCGTGTGAAGTATCCACTCCTG
AAATTGTTAGACCCCTGCACACAAGCAACAGCAGCAACGCACAGGACGCGGGTGTGCCAA
CACGGAAACGGCATAGACAGTGTGACCCAGACGAGGGGCCCTTGGACTTTGTACATAACC
TACAGCCCACAACAGACTCATCGACCCAGTGTACTCTACATAATGTTGCGCCAATAGTAC
ATTTAAAAGGTGACAAAAACAGTTTAAAATGCTTAAGATATAGGATGCATAAAGGGTATT
CACATTTGTTTAATAATGTAACAACTACATGGCATTGGACCAATAATACAAATAGTAAAT
GTGGTGTAATTACATTTATGTTTTCCAGTACATCCCAACAAAAACAATTTTTACAATGTG
CTAAAATACCACCAACTATATCAGTGTCATCAGGGTATATGTCCATATAACACAAATATG
TAACATTGGTTACAATTGTATACATTGGTATACTGTATAATGTCATGATCATTTTTATAT
TTGTAATTTTGTTTGTCTTTGGCTTTTATATGTGCCTGTCTGTGTCGCTTGCAGTGTCTG
TGTGTGTTTATGCATGGTTATTGCTTTTGATTATAATTACTTTTTTACATGTGTCCCAAT
CCCTGTTAAAAGTATATATATTATATGTGTGTGTGTTTTATATTCCTATGGCACTGGTAC
ATTACCATGCTACCTTGCAAGTAACATAAATATTTTCATATATATACAATAAATTTATTG
TTTGTGTACCAGTGTGTAGTTTACCATGCGTCGCAAGCGTGACACACATATAAGACGTAA
ACGTGCATCAGCCACACAATTATACAAAACATGTAAGCAAAGTGGCACATGTCCCCCTGA
CATAATTCCAAAGGTTGAGGGCAATACTTTAGCTGACCAAATATTAAAATATGGTAGCAT
TGGGGTATTTTTTGGGGGTTTGGGTATTGGTAGCGGGTCAGGAACCGGGGGTCGTACAGG
ATATGTGCCATTACCCACAACTACTCCATCTAGACCAGTTGAAATTCCTTTGCAACCTAC

CAGACCTCCTGTTATTACATCTGTAGGGGCCTCTGATTCTTCTATAGTTTCATTAGTGGA
GGAGTCAAGTTTTATTGAAGCTGGTGTGCCAGGTCCTACCTCCATAGTGCCTTCTAGTTC
TGGATTTAATGTAACAACATCTGTGGACAGTACACCTGCTATTATAGATGTGGCAACTAT
AAGTGACACTACACAAGTATCTGTTAGTACATTTAACAATCCAACTTTTACTGACCCATC
CGTGTTGCAACCTCCTCCACCCTTAGAGGCCTCTGGCAGACTTTTATTTTCAAATGATAC
TGTAACTACTCATTCTTATGAAAATATACCTCTTGACACATTTGTAGTTACAACAGATAA
CAATAGTATTGTTAGTAGTACGCCCATTCCAGGGAGGCACCCTCCTGCACGCTTAGGGCT
TTATGGACGTGCTATACAACAGGTTAAGGTTGTAGACCCTGCTTTTGTAACAACGCCTAC
ACGTTTGGTAACATATGATAATCCTGCCTTTGAGGGCCTGCAAGATACCACATTAGAATT
TCAGCACAGTGACTTGCATAATGCTCCTGACTCTGATTTTTTAGATATTGTAAAGTTACA
TAGGCCTGCTTTAACTGCTAGAAAAACTGGCATACGTGTTAGTAGACTAGGACAACGTGC
CACTATGTTTACTAGAAGTGGCAAACGTATAGGTGGTAGGGTACATTTTTATCATGATTT
AAGTCCAATACCTACGGAAAATATTGAACTGCAGCCTTTACTGCCTTCTGCATCTGCTAC
TGTAACAGATGCTAATGGCATTAATGATGGGCTATATGATGTATTGTTAGATAATAATGT
AGATATTACTGAAGTGGAAACACCTACTGGTACAAACACACAAAGTGTTTTTGCAAGTGA
GATTTCCACTACAACTGCAAATACCACTATTCCTTTAAATGCTGGTTTGGATACACATCC
TGGCCCTGATATTGCTTTACCTGTACCTACTGCAGAAACCATTTTTACCCCAACTGTACC
TGTACAGCCTTCTGGTCCTATATATATATATATGGGTCCGATTTTATATTGCATCCTAGCTT
GTATGTCATTCCACGCAAACGTAAACGTTTGTCATATTTTTTTGCAGATGTGGCGACCTA
CTGAGGCAAAGGTATACCTGCCCCCTGTGTCTGTATCTAAAGTTGTAAGCACTGAAGAGT
ATGTAACACGCACAAATATATATTATTATGCAGGTAGTACACGCTTGCTGGCAGTAGGAC
ATCCCTATTATCCTATAAAGGATACTAATGGGAAACGTAAGATTGCTGTACCTAAAGTTT
CAGGTTTGCAATACAGGGTATTTAGAATACGTTTGCCAGATCCCAATAAATTTGGGTTCC
CAGATGCATCTTTTTATAATCCTGACAAGGAGCGGTTGGTTTGGGCCTGTGCAGGCGTTG
AGGTTGGACGCGGACAGCCATTAGGTATAGGTACTAGTGGTAATCCTTTTATGAATAAAC
TTGAGGATACTGAAAATGCTGCAAAATATATTGGTGGAAACATAGCAGATAGTAGGGAGT
GTATGTCAGTAGATTATAAACAAACACAGTTATGTATTGTAGGCTGTAAGCCTCCCTTAG
GGGAACATTGGGGTACAGGCACACCATGTGGTACCCAAAATGCTGGGGAATGTCCTCCGC
TTGAATTAAAGAACACCACTATACAGGATGGTGATATGATTGATGTGGGCTTTGGTGCCA
TGGATTTTAAGGCCTTACAGGCAAATAAAAGTGATGTGCCCATTGATATTTCCAACACTA
TATGTAAATATCCAGATTATCTAGGCATGGCCGCAGATCCCTATGGCGATTCTATGTGGT
TTTATATTCGTAGGGAACAAATGTTTGTTAGACACCTATTTAACAGGGCAGGTACTGTAG
GCGATGCTATTCCAGATGACTTAATGATTAAGGGTACAGGCAATACTGCATCGCCATCCA
GTTGTGTGTTTTATCCTACTCCTAGTGGGTCTATGGTATCCTCAGATGCACAAATTTTTA
ATAAGCCTTATTGGTTGCAAAAGGCCCAGGGACAAAACAATGGCATTTGCTGGCATAATC
AACTGTTTTTAACTGTTGTAGATACTACTAGAAGCACAAACTTTTCAGTTTGTGTAGGTA
CACAATCCACAAGTACAACTGCACCATATGCAAACAGTAATTTTAAGGAATACCTCAGAC
ATGCAGAAGAGTATGACCTGCAGTTTGTGTTTCAGTTATGCAAAATCAATTTAACTACAG
ATGTAATGACATATATACATTCTATGAGTTCTAGTATATTGGAACAGTGGAATTTTGGTC
TTACACCACCGCCTTCAGGTACTTTAGAAGAAACATATAGATATGTTACTTCACAGGCCA
TTACATGTCAGCGTCCGCAACCTCCTAAGGAAACAGAGGACCCATATGGTAAAATGACAT
TTTGGGAGGTAGACCTTAAAGAAAAATTTTCTGCAGAATTAGATCAGTTTGCCTTAGGTA
GAAAGTTTTTATTACAACTAGGTATGCGTGCCCGTCCTAGGTTACAGGCCTCTAAACGGT
CTGCACCTTCATCATCTAGTACAGCACCTAAGAAAAAACGTGCAAAACGTATTTAATTAA
ATGTATGTGTGTTGTGTTTGTGTATGTGTATTGTGTGTTTGTATATGTTTGTGTATGTGT
ATTGTGTGTTTGTATATGTTATGTAATGTATGTATTATTGTATTGTTGTATGTGGTGTGG
TATTGTATGGCATATGGTGTTGTACATGTGTGTGTATGTATGTATGTATGTGTTTTATGT
TCAATAAACTATATATGTTGTGTGAGTGGTTTTTAATAGCCACTGTGTCTCCATTTTGTT
CATTCGCCATTTTATATATTTTAAAGTTATTCTGCGACCGCTTTCGGTTGCCTATACTAT
AACATGTTTTGGTCTGTTTGTGCTGTTTTAGCACAAAGGTTTAATGTGTTTTGGCTTCCT
GCAGGCAACTTGGATTGTACTCAAGCACTTAGGATTAACATGCTTCCTGCTTATGTTTCA
TCCTACGCAAGGTCATACACGTTTAAGGCGCCACCTGGCAGTTACTCATGTGTCTGCAAT
TGTTACTACAATGTTTTGCACACACACATTTTTTACCCACCCTCTCATAAAATTGCTTTTAA
TCACATACTTGTACTATGTAAACAGTGTACCTTGGCAGAACATTGTATTTTACATGCCAG
GTAATTGTTGAAAACTGACTAAGCAGTGCTTACTCATTTTGCACCTGGACTGTTAAACCG

TTTTGGATCACACAGTGTACCAACCCTTTTATAATTATAAAAA
>gi|9627327|ref|NC_001586.1|_Human_papillomavirus_type_32,_complete_genome
TAATCTTTGAATTATAAAAAAGTAGGGAGGAACCGATATCGGTTTAACCGAAAACGGTGC
ATATATAAACCACCCTGGGCAGTGGTCCTTGTTAAGGCAGAATGGCAAGTACTTCTGCCT
CATCACAGCCAAGTACATTATACCAATTGTGCAAAGATTTTGGGCTGACCCTGCGGAATT
TACAAATCTGCTGTATTTGGTGTAAAAACCACTTAACCAGTGCTGAAGCGTATGCATATC
ATTTTAAAGATTTGCACGTAGTGTGGAAGAAAGGCTTTCCATATGCCGCCTGTGCCTTCT
GCTTAGAATTTTATTCTAAAGTGTGTGCACTGCGACACTACGACAGATCAGCATTTTGGC
ATACAGTAGAACAAGAAACAGGACTACTGTTGGAAGAACAAATAATTCGCTGTGCTATAT
GTCAAAAGCCTTTATCGCCAAGTGAGAAAGATCATCATATTTATAACGGACGGCATTTCA
GATTCATTTTAAATAGGTGGACGGGTCGCTGTACCCAGTGCAGAGAATAATGCGTGGAAA
CGCACCAACGCTAAAGGACATTATTTTGTATGACCTGCCAACGTGTGACCCGACAACGTG
CGACACACCGCCGGTTGACCTGTATTGTTATGAACAATTTGACACCTCAGATGAAGATGA
TGAAGACGATGACCAACCTATAAAACAGGACATACAACGTTACAGAATAGTGTGTGGTTG
TACACAGTGTGGACGGTCAGTTAAACTTGTTGTCAGTAGTACAGGCGCGGACATACAACA
GCTGCATCAGATGCTTCTGGACACACTGGGCATTGTGTGTCCATTGTGTGCCTGCGTGGA
GTGACTGCAATGGCGGATGATACAGGTACAGAGGAGGGGCTAGGGTGTTCTGGTTGGTTT
TCTGTAGAAGCAATAGTAGAAAGGACTACAGAAAATACTATATCAGACGATGAGGATGAA
AATGTAGAGGACAGCGGGTTGGACCTCGTAGATTTTGTAGACGACAGCAGAATAATACCT
ACAAATCAATTAAAGGCGCAGGCATTATTAAATAGGCAACAAGCACATGCAGATAAGGAG
GCAGTACAGGCACTAAAACGAAAGTTATTAGGCAGTCCATATGAAAGTCCCGCCAGTGAT
TTACAGGAGAGCATAAACAAAGAGCTAAGCCCTAGGCTTGGTGGATTACAGTTATGTCGG
GGGTCCCAAGGGGCCAAACGACGACTATTTCAATCATTGGAAAATCGCGACAGTGGATAT
GGCTATTCTGAAGTGGAAATACGGCAGGAACAGGTAGAAAATGGACATGGCGCCCCAGAC
GGGAGTATGGGTAACGGGGGGGGCATGGGCAGTGTACATGGGGTGCAGGAAAATCAGGAA
ATAGGCACAAATACGCCTACAACAAGGGTGGTGGAATTGCTTAAGTGTAAGAACTTGCAA
GCAACATTGTTAGGTAAGTTTAAAGAGCTGTTTGGATTGTCATTTGGTGATTTAGTAAGA
CAATTTAAAAGTGACAAAAGTAGTTGTACAGATTGGGTAGTTGCAGCATTTGGTGTACAT
CATAGTATTGCAGAAGGCTTTAATACACTAATTAAAGCAGAGGCGTTATATACACACATA
CAATGGTTAACCTGTACCTGGGGAATGGTATTGTTAATGCTAATTAGATTTAAATGTGGC
AAGAATCGCACCACAGTGTCTAAAGGAATGTGCAAACTATTAAATATACCTGCTAATCAA
CTGTTAATAGAGCCGCCACGATTACAAAGTGTGGCAGCAGCAATATATTGGTTTCGAGCA
GGAATATCTAATGCCAGTGTAGTAACCGGGGAAACACCCGAATGGATACAAAGACAAACA
ATTGTAGAACATTGCTTTGCAGATACACAGTTTAATTTAACAGAAATGGTGCAATGGGCA
TATGATAATGATTTAACCGAAGATAGTGACATTGCATATGAATATGCCCAACGTGCTGAC
ACAGATAGTAATGCAGCTGCATTTTTAAAAAGCAACTGTCAGGCAAAATATGTAAAAGAT
TGTGGAATTATGTGTAGACACTATAAAAAAGCACAAATGAAACGTATGTCAATGCCACAG
TGGATTAAACATAGAAGTGAAAGAACTGGCGATAATGGTGATTGGAGACCTATAGTAAAA
TTTATTAGATATCAAGGAATAGATTTTTTAACATTTATGTCTGCATTCAAAAAATTTTTG
CATAATATACCAAAGAAAAGCTGTTTAGTATTAATTGGTCCGCCAAATACAGGAAAATCA
CAGTTTGGAATGAGTTTAGTAAAGTTTTTAGCAGGAACAGTAATTTCCTTTGTAAATTCA
CACAGCCATTTTTGGTTGCAGCCATTAGACAGTGCAAAAATAGCAATGCTAGATGATGCA
ACACCTCCATGTTGGACATACTTAGATACATATTTAAGAAATTTACTAGATGGCAATCCA
TGCAGTATTGATAGAAAGCATAAAGCATTAACAGTTGTTAAATGTCCACCATTAATAATA
ACATCAAATACAGATATTAGAACAGAAGACAGATGGAAATACTTATATAGTAGAATTAGT
TTGTTTGAATTTCCAAACCCATTTCCATTAGATAAAAATGGAAATCCTGTATATGTGTTA
AATGATGAAAATTGGAAATCATTTTTTCAAAGGTTGTGGTCCAGCTTAGAATTTCAAGAA
TCAGAGGACGAGGAAGAAAATGGAGACACTGGCCAAACGTTTAGATGCGTGCCAGGAACA
GTTGTTAGAACTGTATGAGGAAGATAGTAAACATTTAGAAAAACATGTGCAGCACTGGAA
GTGTTTACGCATAGAAGCAGCCTTATTATTTAAGGCTCGTGAAATGGGCTATGCACAAGT
AGGACATCAAATAGTGCCAGCACTGGAAATATCCAGGGCCAAGGCCCACGTTGCAATTGA
AATTCAATTGGCGTTAGAGACATTATTGCAGTCCACATTTGGTACAGAACCATGGACATT
GCAAGAGACAAGTTATGAAATGTGGCATGCGGAGCCCAAAAAGTGTTTAAAAAAACAGGG
ACGCACTGTGGAGGTTGTATTCGATGGAAATCCTGAGAATGCAATGCATTATACAGCATG
GACATTTATATACGTGCAAACACTAGATGGCACATGGTGTAAAGTATACGGACACGTATG

CTATGCAGGACTATACTACATTGTGGACAACATGAAACAGTTTTATTGTAACTTTAAAAA
TGAGGCAAAAAAATATGGGGTAACCGGACAATGGGAGGTACATGATGGCACTCAGGTGAT
TGTTTCTCCTGCATCCATATCTAGCACCACAACCACCGAAGCAGAGGTATCCTCTTCTGG
ACTTACTGAATTGGTACAAACCACCGACCTATACAACACCACACCTACACCCACAACCAT
CACAAGGAGTAACTGCGACCCAGACGGCACAGACGGAATATTATACAAAGACCCCACCCC
GACCACCCCGCCGCGAAAACGATACCGACAGTCTTTGCAGCCACCAACAAAGCACCTGCA
GCACTACGGCGTCACAAACGTACCCGTGGACCCCGGATCGCAAAGGGTCACATCTGACAA
TAACAATAACCAGCGACGGAACCCGTGTGGAAATCAGACTACGCCAGTAATACACTTACA
AGGTGATCCTAATTGCCTAAAGTGTTTAAGGTGGAGGTTAAAGAAAAATTGTTCTCACTT
ATTTACTCAAGTGTCATCCACATGGCACCTGACAGAAAAAGACTATACACGTGACTCAAA
GGATGGTATAATAACAATTCATTATTATAATGAGGAACAACGAGATAAGTTTTTAAGTAC
TGTAAAACTTCCTCCTGGTATTAAATCTTGCATTGGGTACATGTCAATGTTACAATTTAT
GTAGTGTGTACATATGTACAAACATATATAGGGGAACATTACTGTGAGTCCACTATTGTG
TGGGACAACCGGCCAGACACTGCTGCTATTATTGTTTATAGTTGTTGCTGCGTGTGTGTT
GTGTGTGTGGATTAGTTTAATAGACCCCCCATATCCCTTGTGGGCCTCCTGTCTTGCTAG
CTATCTAATATTAGTACTATTGTCCTGGTTGCAGCTACTAACATCTGTGCAATTTTTTTT
TTTAGCTTTGCTTGTTGTTGTGTTTCCTGCCTTTTTACTAACCCTATTAATACACTTTGC
ACTACATTAATGTAGAAGGTGTACATACAATAGTATAGCTGTGTGTGTGTGGTGTGCATG
GTATACACAATAATTGTACATACGTGATAATATGGTACATTGTACTTTAGAAAATGGCGA
TGTGTGGCTGCTGCTGTGGTTTTTAGCCACAGTGTTTATTTGTATATTAGTACTGTTGTT
AAACTACTGCAGACATACGTGTGTACATTGCTGTAAATAAAGTTGGGTTTTTGTATTGTT
TGTACCAAAGTGTATTATCAATGCCACCACACCGAAGTCGCAGACGCAAACGGGCATCTG
CTACACAGTTATATCAAACATGTAAGGCCTCTGGGACATGCCCACCCGATGTAATTCCTA
AGATTGAAGGTCGCACGTGGGCAGATCAAATATTAAAATGGGGCAGCACTGGTGTGTTTT
TTGGGGGCCTTGGCATAGGTACCGGTGCTGGGTCAGGTGGGCGCACAGGGTATGTGCCTA
TAGGAACACGACCGCCTGTTGTTGCAGAGCCGGGCCCTGCAATACGTCCTCCAGTGGTTG
TTGACACTATTGGGCCTACTGACCCATCTGTAATTTCCTTATTGGAAGAGTCTGCAGTTA
TTGATTCTAGCATACCAGTGCCTACCGACACGTCTCATGGTGGGTTTAATATTACGTCCT
CTGCAAGTGGCCCGTCCTCTACACCTGCTGTATTGGACATTTCTCCTCCCACCAATACTA
TAAGGGTTGCATCTACTACGTCCCACAATCCAGTATACAGTGATCCTTTTACTTTACGGC
CTTCTTTGCCAGTAGAGGGTAATGGTAGACTCTTAACATCCCACCCAACAATAGCCCCTC
ATTCGTATGAGGAAATTCCTATGGACACTTTTGTGGTATCCACAGATACAAGTAATACTG
TTACCAGTACCCCTATTCCTGGACCTCGCCCTACTATGCGCCTTGGTTTATATACCAGGG
TCACTCAACAACGTCCAGTTGCTACTACAACATTTTTAACATCTCCCGAGCGTTTGGTAA
CCTATGACAACCCTGCATATGAGGGTCCTGCTGAGGGTACGTTGGAATTTGAACATCCCA
CCATTCATGAGGCTCCTGATTCTGATTTTATGGATATTATTGCATTACACCGTCCTGTGC
TATCTGCTAGGCAGGGCACTGTCCGTGTCAGTCGTATTGGGCAACGGGCTTCCTTGCAAA
CACGTAGTGGGGCTCGTATTGGGTCTAGGGTACATTTTTTCCATGATATTAGCCCAATCA
CTAGGCCATCAGAGGCTATAGAATTGCAACCTTTAGGCTCCTCCTCCACAGCTGTATCTA
CTACTGCTTCATCCGCAATTAATGATGGCCTGTTTGATGTTTATGTTGACCCTGACATAC
CTCCCTCACACGCGTTACCGCCCCTACGGTCCCCCACACACGTGTCAACTGTTTCTTTAA
CTAGTCTTGGTAGTGTTCCTGCACAAACTGCAAATACAACTGTCCCTCTTTCCTTACCTA
CAAATATTAATGTAGGCCCTGACCTTTCACCTCCTGAGTCTCCGCCCTTTATTAGTACAC
GTCCTGTATCACCTTCTTTTGACTCTGTTATGGTATTAGGATGGGATTTTATATTGCATC
CCAGTTATATGTGGCGTAAGCGCCGTAAACCTGTACCATATTTTTTTGCAGATGTCCGTG
TGGCGGCCTAGTGACAACAAGGTTTATCTGCCTCCTCCTCCTGTTTCCAAGGTGGTCAGC
ACAGATGAATATGTGCAACGTACCAACTACTTTTATCATGCCAGCAGTTCTAGGCTTTTG
GCTGTTGGGCATCCATATTATACTATTAAGAAGACACCCAATAGAACATCTATTCCAAAG
GTGTCTGGATTGCAGTATAGAGTATTTAGGGTTAGGCTTCCAGACCCTAATAAATTTACA
TTACCTGAAACAAACTTATATAATCCTGAAACACAACGTATGGTGTGGGCCTGTGTGGGT
TTGGAGGTTGGCCGTGGACAGCCTTTAGGTGTTGGTCTTAGTGGGCATCCTTTATTAAAT
AGATTGGATGATACTGAAAATGGGCCTAGATATGCTGCAGGGCCTGGAACTGATAATAGA
GAAAATGTATCTATGGATTGTAAACAAACCCAATTGTGTTTGGTGGGTTGTAAACCTGCC
ATTGGCGAGCATTGGGGTAAGGGTGCTGCTTGCTCTGCACAATCAAATGGCGACTGCCCA
CCTTTGGAATTACAAAACAGTGTTATTCAGGATGGTGATATGGCAGATGTAGGGTTTGGA

GCAATGGACTTTAGTGCTTTGCAAACTTCAAAAGCTGAGGTGCCATTAGATATTATGAAC
TCCATTAGTAAATATCCTGACTATTTAAAAATGTCTGCAGAGGCCTATGGCGACAATATG
TTTTTCTTTTTGAGACGGGAACAAATGTTTGTTCGTCACTTGTTTAATAGGGCAGGAACC
CTTGGTGAACCTGTTCCTGAGGACATGTATATAAAAGCTTCTAATGGTGCTTCTGGCAGA
AATAATTTAGCTAGTAGTATTTATTATCCAACTCCCAGTGGTTCTATGGTCACCTCTGAT
GCACAAATATTTAATAAACCATATTGGTTACAGCAGGCACAAGGCCACAATAATGGTATA
TGTTGGGGTAATCAAGTGTTTCTAACTGTTGTGGATACTACCCGTAGTACTAACATGACT
GTGTGTGCTACTGTAACAACTGAAGACACATACAAGTCTACTAACTTTAAGGAATATCTA
CGCCATGCAGAGGAATATGATATACAGTTTATATTTCAATTGTGCAAAATTACATTATCT
GTAGAGGTTATGTCATATATCCACACCATGAATCCTGACATACTAGACGATTGGAATGTT
GGTGTAGCTCCACCGCCCTCTGGTACTTTAGAAGATAGTTATAGATTTGTGCAGTCTCAG
GCCATACGATGTCAAGCTAAGGTAACAGCACCTGAAAAAAAGGATCCTTTTTCTGACTAT
TCATTTTGGGAAGTAAATTTATCTGAAAAGTTTTCTAGTGATTTAGATCAGTTTCCATTG
GGTAGGAAGTTTTTACTGCAAGCTGGGTTACGTGCAAGACCTAAACTTACAGCAGTAAAA
CGAACAGCATCTTCCAGTCAAAAGTCTTCTTCTCCTGCAAAACGCAGAAAAACACGTAAA
TAATATTTTTTCCTGTGTGTATGTGTTGTGCAACTTGTGTGTGGTGTGCATGTATTGTGT
ATATGTGTGTTTCGGTGTGCATATTAATAAAGTATGATTGTGTTCATTGTATGTTGTTGT
ACCACCATTTTATTTTTCAATCCTCCATTTTAGTACATGCAACCGAAATCGGTTGCCGTT
GGATAATGTCCATTAAATTTAGCAAGCACATGCAGTTTTTACTGCCAAATATATGTACTG
CCAAATGGTATTGCTAAGTAGCAAAATGTTTTTACATACATAACATACACGCCCTTTTGC
ACAAGCATGTTTTAGAAAGGTTGGCATAGCTTTGCATTTACTTATTTCCTTCTTCCTTTG
TTCATGTTATGACTACTGTATTTGTTATGTATAATTAAAATGCTTTTAGGCACATATTTG
TGGGTTTTGGCACAGTACTTTACAAGTTACTCTGGCCTAGACAAGACTAGTGCTTTGTCA
TGCCTTATATAACTAAGTATTAGTCACTGACTGCATCTAATTAAACTGTCAACCGAAATC
GGTGCATAAATCCTAACTTGTTCTGTTGTTATTATAAAGTA
>gi|9626605|ref|NC_001458.1|_Human_papillomavirus_type_63,_complete_genome
GTTAACAACTATCAGGCGATTCTCTAGTTCTAACACGAACGTTTACGGTCGTTGCCAGCT
TTTTCCTTATAAAACTCTGGTGGGAATTTCTCTTGGGACAGATGGACCTGACATCTGTAC
ATTCGGTTCGGGATCTGAGTTCTGCTCTCCGTATCCCATTTATTGATTTGGTTGTTCCTT
GCAATTTTTGCTTGAAATTTCTTACAAATGCTGAAAAATTGCTGTTTGATTATTTTGACT
TGCATCTTATCTGGCGAGATAATTTCGTGTTTGCTTGTTGTCAGTGCTGTGCTAGGCATG
TTAGTCTGCTTGAGTTTATGCTTTATTATCAGGAGTCTTTCGAGGTATCTGAAGTAGAAG
AATTACTTAATCAACCTCTTGTAAATATTGGTTTAAGGTGTGTTACATGCACAAAAAAAC
TGACTGTTTCAGAAAAGTTAGCTGTTGTTTCTGCTGGAGAAAGAGTTCATAAAGTAAGGA
ACAAATTCAAAGCAAAGTGCAGTTTGTGCAGACTCTACATTATATAGTTTGTGCAGACTC
TATATAATTAACAATGGTGGGAGAGCAGCCAAATATAGGTGATTTGGTGAGTCAAGAAGA
ACCAAGCGTCCTAGATCTAAATTGTTATGAGGATATACCTGCTGAGGAGGAGGAGTCTGA
ATATCCATATGCAATTGTGCTTCCTTGTGGTTTGTGCGATCAGCTGTTAAGGCTGACCTG
CGTTTCTGACCTGTCTACTCTTACGCGTCTGGAGGAGCTGCTGTTAGGCTCACTGAGGAT
CGTGTGTCCCCTGTGTGCCATTCGACACCAACGACACTAAGATGACCGACAGAGGTACAA
ATAATGATGATTGGTATATTGTGGATGAGGCAGAATGTCGGGATGATGATGAGAGCGAAT
TGGAGGATTTGGAGGACACCTATAATTCATTGTTTAATAGATCTGAAAGTGACATATCAG
ATCTATTAGACGATACGCAGCAAAGTCAGGGAAATTCCCTGGAACTGTTCCACTTACAGG
AGCACTTGCAGAACGAGCAGGACCTAAATACCCTAAAACGAAAGTACTTAAACAGTCCTC
CGCAGGCAAGTGCCACAGAGACTGCCTGCAATAGCCTCAGTCCCAGATTGGAATCTATAA
CAATTTCGCAGAGGGAAAAAAAGGCAAGAAAGCAACTATTTACACAAAATGACAGTGGCA
TAGAGTTATCGCTATGCCAGGATGAAGTTGACAATATTAACGAAGCGCTTCAGGAGCAGG
TAGACATCGTACAGTCTCTGGGAGGTGGGGTGCGTGACTGTATAGGAGTGGACATTTTGA
AATGCAGTAATACAAGATCTGCTCTACTTGCCAAATTTAAAGACACAGTAGGTGTCAGTT
TTACTGACCTCACCAGAGCATACAAAAACAACAAGACATGCTGTAGTTACTGGGTCATAG
CAGTGTGGGGAGTAACATCTACGTCTGTGGACGTTGTGAAAACTGTATTCCAAGTTCAGT
GTAATTATATGCATGTAGAACATTGTTTAACTGAAAAAAATAAGTTTCTAATTGTATTAG
CTGGCTTTAAAGCTCAAAAAAGTAGAGAAACAGTGTTAAATCTCGTAACTAGCAGTTTGA
ATGTGCAAAGTAATTACATAATGGCTGAACCACCAAAAAATAGAAGTATGGCGGCAGCGT
TATATTGGTATAGGAGATCTATGTCTCCAGCTGTATATACCTGGGGAGAAATGCCAGATT

GGATGGCGCAGCAGACATTGTTGAATCATCAATTAGCATCAGAAAAGCATTTTGAATTGT
CACAAATGGTACAATGGGCTTATGATAATGGCTATACAGATGAAAGTGATATTGCATACT
ATTATGCTATTTTAGCAGAAGAAGATGAAAATGCAAAAGCATTCTTGGCTTCTAATGCAC
AAGCAAAATATGTTAAGGACTGTGCTAGAATGGTTAGTCATTACAAAAGGGCAGAAATGA
GTAGTATGTCTATGTCAGCATGGATTTATAAAAGACTGGAGGAAGTTGAAAATGGTGGTG
ACTGGAAACATATTGTAAAGTTCTTGAGGTTTCAAGAAGTAGAATTTATAAGTTTCATGA
TAGCATTTAAGGAATTGTTAAGTGGTAAACCAAAGAAAAATTGTCTTGTAATATATGGTC
CACCAAATACTGGTAAATCTATGTTTTGTATGAGTTTGTTGAGAGTATTAAAAGGAAAAG
TAATATCTTATGTAAATAGCAAAAGTCAATTTTGGTTGCAACCACTAGCTAGCACTAAAA
TAGCATTATTAGATGATGCAACAAAACCAGCATGGGATTATATTGATTTATTTTTGAGAA
ATGCTTTAGATGGGAATCCTATTTGTGTAGATCTGAAACATAAGGCACCACAACAAATAA
AATGTCCTCCACTTATGATAACTTCTAATATAAATGTTAAGGCTGATGTATGTTGGATGT
ATTTACATAGTAGGATAACATGTTTTGAATTTAAACAACCTTTTCCATTTGATGAAAATG
GTCAACCGGCATTTTCCTTAACAGACATCAATTGGAAATCTTTTTTTGAAAGGTTTTGGA
GCCAGTTAGACTTAAGTGACCAAGAAGACGAGGAGAGTGATGGAAAGCCTCAACAACCGC
TTAGACTGGCTACAAGAGCAGCTTCTAACTCTATATGAGAAAGACAGTAAAGATATTGAA
GATCAGATAATGCAGTGGAATCTACTTAGACAGGAACAAGTGTTATTCCACTATGCCCGA
AAAAAGGGAATAATGCGACTTGGCCTGCAAGTTGTGCCTTCCCTTGCAGCTTCCCAGGAT
AAAGCAAAAACAGCTATAGAAATGACTCTTTATCTTAGTGGCCTCAGAGACTCACAATAT
GGTTCTGAACAGTGGTCTTTACAAGATACTAGCAGAGAAATCTTTTTAGCACCACCAGAT
CATACATTCAAAAAGGGAGGGCAAACAATTGAGGTAATCTATGATGAGGATCCCAATAAT
AGCACCAGACATACTGTATGGCGCCATATATATTATCAAAACGGTGATAACAGATGGAGA
AAAGCAGCTAGTGATGTAGATGTTCATGGTGTGTTTTATTTAGAATATGATGGTGTCAAA
AACTACTATGTTGACTTTCAAGAAGAGGCCAATCGATATAGCAAAACAGGTCGATATACT
GTTCAATATGAGGGTAAAAGGTTCACAAATGTTATGTCTCCTGTCAATAGCTCCCCACTA
CGGACTTCTGGGTCTCCTACAGACACCAACCCAGCCACCCAAGGACAATCCACCCAAACT
GCCAGAAAAGCAGAGACGAAGGGGTCGAGACACCACCCGAAATCGCCGGCTGTTCGCAAG
CGACGGCCCTACGGACGAAGAAGGTCCAGAAGTCCCAGAGATACCACCCTCAGACGAGGA
GAAGGAGAATCGGCCAGAGCCTCTGCCGGTAGTGGAGAACGGGTGGCATTCATTTCTCCG
GGAGACGTTGGAACATCAACTAGGTCGCCTCCAAAGGGAGGTCAATCAAGACTTCGAAGA
CTTATACAGGAGGCTCGGGATCCACCCATAATTTGTCTGAAGGGGGGCCCTAATCAACTT
AAGTGCTTAAGGTATAGGATTAAAGCTTCAAATTCATCTGACTTTGAAAGTATCAGTACT
ACATGGCATTGGGTACATAATAAATGCACAGATAGAGTAGGTCATGCACGTATGCTGGTG
CGTTTTATATCAACAGAACAACGTGACCGATTTTTAGATAAGGTGGTGGTGCCTAAATCT
GTTTCTGTTATTTTAGGGGCATTTGACGGTTCCTAAGGGTGGGTGTTGGGGTATATTTTG
TAATCATGTTAAGAGTACGTAAACGACGAGCTGCTCCACAAGATATTTATCCTGCTTGTA
AGGTTGCAAACAATTGCCCCCCTGATATACAAAATAAAATTGAACAAACAACAGTTGCTG
ACAAGATTTTACAATATGGGAGTTTGGGAATATTCCTGGGAGGTTTGGGTATTGGTACTG
GCAAGGGTGGGGGTGGCCGGTATGGTTATACACCTCTAGGGGACAGTGGTGCGGTGCGAG
TTGGTGGCAGAAGTACACCTGTAAGACCAACAGTACCTGTGGAGACTGTAGGACCAAGGG
ATATATTACCTATAGATTCATTGGATCCTTTAGGGCCCTCAGTCATTGAACTAGAAGATA
TTCCAGCCACAACAGTGGAAGTAGTGGCTGAAGTGCATCCCATATCTGATACTCCACAAA
TACCGGCACCTACTACTGATGAATCTAGTTCAGCTGTTCTTCATATTCCACAAGAAAGTC
CTGCTGCACGTACAATCACACGTTCCCAATACAATAATCCTTTATTCAGGATCACAGCTA
GTGCAGACATAGCATCAGGTGAAGCTTCAGCATCTGATAATATTTTTATAGATGTAGATA
CGCCGGGTCAAATAGTAGGACAAGAAATACCACTAGTTAATTTTGATATGGGACCTATAT
CTACTGAAGGTGAGCTTGAAACTGAGTTCACAACTAGTACACCAAGAACCACACAAGTAC
AGGAAAGGCCTACACGTTTCTATAATAGACGCTATTATGAACAAGTGCCAGTTACTGCAC
CTGAATTTATCACAAGGCCTGCTTCCTTAGTTACTTTTGAGAATCCTGCATTTGAAAGGA
GTGTTTCTTTGATTTTTGAACAAGATTTAGAAGATATTTTAAATGCTCCTGATCAGGATT
TTAGAGACATTGTTTATTTAAGCAGACCAACATACAGTCGTGCCCCTGATGGCCGCATGC
GCCTAAGCCGCCTGGGACGCAGAGCCACTATAAGTACCAGAAGTGGTGTTACTATAGGTG
CTCAATCACACTTTTATATGGATATTAGCTCTATCTCCTCAAATGATGGCATTGAGTTAC
AAACACTGGGTGAAGCTTCTGGCGAGACTGTGGTGCAAAGTTCTCTTGCTGCATCGGATC
CTATTGAAGCAGAACATTCATTCATTGAACCAGCACCATCTATAGATAGTTATGATATTG

TTTCACTTCAGTCTGAGACTTATTCAGATGAACATTTGTTAGATATGTATGAACCTGTAG
GTTCTTCCTTGCAATTACAAATATCAGACGTCAGAGGTCGGCCAACTGTTATTGATATTC
CCTTTAGACCCCGCAGGCCTCCATTAGGTCCTATAAATGCTGGTGTTGATATCTATAGTC
CAACTGCTAGTGTTGGATCACCTACTATAAATCCTACTGATCTTGACATTCCATTAATTA
TTATACATTTAGATAATTCAACAGGGGATTATGATTTACATCCAAGTTTGCGTAAACGTC
GCAAATTAGTTCATATTTGATATTTTACAGATGGCTGTTTGGCTTCCTGCCCAGAATAAG
TTTTACCTTCCTACCCAACCGATCACCAAGATTCTAAGCAGCGATGATTATGTGTCTCGC
ACCAACATCTTCTATCACGCTACCAGTGATCGACTGCTCATTGTGGGACACCCGCTCTAT
GAGGTTACCCGTGCAAATGATAACACTATGACTGTGCCTAAAGTTTCTCCAAATCAGTAT
AGAGTCTTTCGTGTTAGATTTCCAGATCCTAACCGATTTGCCTTTGGAGATAAGGATATT
TTTGACCCAGAAACTGAGAGACTAGTTTGGGGTCTTAGAGGCATAGAAATCGGTAGGGGT
CAACCATTAGGTGTGGGTATTTCAGGCAATCCATTATTAAATAGGTTTGATGATGCTGAA
AATCCTAGCAGATATAATAATACACATGCAACTGGTGATAATAGGCAAAATGTTGCTTTT
GATGCAAAACAAACCCAAATGTTTCTAATTGGCTGTACACCAGCCACTGGGGAACACTGG
TCAATAGCTCGACGCTGTGCAGGAACACAGTTTCAGCTTGGAGATTGTCCTCCTATAGAA
TTAGTTAACACAGTTATTGAGGATGGTGATATGTTTGACATAGGTCTAGGTGCTATGGAC
TTTGGTTCTTTGCAAGCAAACAAAGCAGATGCTCCTTTGGATATTGCAGGCACTGTCTGC
AAATATCCAGATTATATTAAAATGGGACAGGAAGTACATGGTAATTCTCTGTTTTTCTTT
GCTCGCAGAGAACAAATGTATTTAAGGCATGTATTTACACATGCTGGAATTGTTAGTGAA
AAAGAGAAAGTCCCTACCAGTGCATATATTGCTGCTAAAGCCGAGCAACCCCAAAATACT
ATTGCTACAGATAATTATTTTGTAGCTCCCAGTGGATCTTTAGTGTCCTCTGATGTGCAA
ATTTTTAATAGGCCCTATTGGTTACAACGTTCTCAAGGACAGAACAATGGTATCTGTTGG
AGAAATGAGTTATTTGTAACTGTAGCTGATAATACCAGAGGAACCACGATGAATATAAAT
GTTCTTAACAAAGCAACCCCTGAGACTTATGATAGCGCAGATTATAATGAGTATACTCGC
CATGTGGAGGAATATGAGTTATCCTTTATAGTTCAGCTTTGTAAGGTAAAACTAACACCT
GAAAATTTAGCATTTTTGCATAATATGGATCCAACAATTATCGATTCCTGGCAGTTAACA
GTTTCTCAACCTCCTGCAAATGCTATAGAGGACAAGTATAGATTTATTGAATCATTAGCA
ACAAAATGTCCTGATAACGTGCCCCCACCCACTCCTACTGATCCTTACAAAGATTTACGT
TTTTGGGATGTAGACCTCAGTGAGCGAATGTCGGAGCAGCTTGATCAATTTCCTTTAGGC
CGCAAATTTTTGTATCAAAGTGGTCTTGCACAGCGTTCTGTTCCAAAAACTGTGAATTTC
AGAAAACGTAGATCCTCCAATACTACTGTGGCCAAACGGAGGCGACGGGCCTGAATATAC
ATGTGAATGTTGAATTATATAATGTGAATTGTGAATTCTTGACTTTGGCACTTGCACTTT
ATTCTTGGCATACTGATACTTGAAACTTGTTCAATGCTTGAAGGTTACACACCTGTACAG
TATTGTTAATAAACGTTTATGCTGCTGTCATTTACCTGTCTTCGAGTCATTATTGCCTAG
TCATATAGCCTCATGACTTGGCATGCAATTGGTATGTGGCAGATACTTCAAACAGGATAC
TGGTATCCTTTTTGGCGCGCGCGCGAATTTTGAAGTTACCACTGTTCCAACTTGTTCTGA
GACGTCTGGATCTGATCCCGACCGCTGTCGTTACTGCCAAAGACGAAAGTGGTAGGCGCG
AACCGTTTGTGGTTTCCCTGGGGCTAGCAGAAACTCTTTAGGTTGCGACCGTTTTCGGTC
GGGCCAATAATCTCTTTCGATCGTTGTT
>gi|9626041|ref|NC_001354.1|_Human_papillomavirus_type_41,_complete_genome
ACAATCATAATCATCGCCCTTTCGTGTTATTTCTTGTAACGAATTCGTTACAAAACACAC
ACACAGTATATAAGATAGAGGAACGGATTGGTACACCACAGATGGCATCAACAAGCGGTG
TGGGATCCGTCGGGCCTGCAAGCTGTTGCGAGACGCAGAAGCCACATACCATACGGGAGT
TGTGTTTGGCGCAGCAGATAACTTATCCATGCATACAGCTCTGCTGCCATTATTGCTATA
AGATCCTTAGCGTATTGGATATTTACGCTTTCGACCAGAGCTGTCTGTACTTATCCTGGG
GAGAAGGGGGGCCAACGGGTATTTGTTCTCAGTGTACTAGAGTGCTTGCAAGGCTGGAGT
TCACTGCACGGCACGAAGTGTCTTGTGCAGCCAGCCGTCTGCCGCACTTTATAGGACAGA
GCCTCAGCGACCTTGAGGTGAGGTGTGTGAGGTGCCTAGCTCTTCTACAATCTGTGGAAA
AGGATTACATATTGCGGGAAGACTTGTCTGTGCATAGAATTGGCGGGATCTGGAGGGGAA
CTTGTGTTCGATGTATGGTAGGACTGTATTAGCTGTGAGACTAATATACTGTTTGCTGTA
TTGTATTGCTGTAATCGTGCGTAAATTGCTATACCCTGTAATAATGAGAGGGAATAGTGT
TGACCTGCAAGAAATTGTGCTTGTTCAGCAGGGGGAGGTACCTGAGAATGCTGCAGTGCA
TTCAGGGGAGCATTCTGATGATGAGGGTGAGAGCGAGGAGGAGGAGCGGGAACAGGTGCA
GCAAGTCCCCACACCCAGGAGAACATTATACCTGGTAGAGAGTCAGTGTCCATTTTGCCA
GGCTATCATACGATTTGTATGCGTAGCAAGCAACACTGGGATACGGAATCTACAGGCACT

CCTGGTCAACAGTCACCTTGACCTCGCTTGTCACGCCTGTGTCGAGCAGAATGGCGTCCA
GGGTCTCAGACACCGGCAATGGCAATGAAAACAAAGAGAATGAAGGTACAGTGGCATCTG
ATCATTCTGAGGCGCGTTGTAGCTATATATTATTTGAGGCTGAATGTAGCGATGGCGGGG
ACGATGAGGAAAGTATGGAGGATAGCTTGGTGGAAGACCTTGTGGATGATGCTTCTGTGC
ATCAGGGAAATTCCTTGTCGCTGTTTCATGCCCAAACTGTCGAGGAATACGAGGGAGAGA
TCCAGAGCCTAAAACGAAAGTTTATCCTGAGTCCCTTGCATAGGGATGTGGCAGAACTAA
GCCCGCGTCTGGCGGGTGTTTCCCTGGAAGAAAACCGTGGGAAAAAGGCTCGCAAATCTC
TGTTCCACGATGACAGTGGCATAGACAGCAGCGCAGTGGAAGTCTCCCAGCTATCTAGTA
CGCCATCAGCTCCAGGGCCAGACATCCGGCTGCCTAAACCCTCAGATATAGATCTAGAGC
CACTGTTCCAAAGCCGCCAGCGCTGTACGCATATGTATAGCAAATTTAAAGCTGTGTACG
GGGTTAGCTTTACAGATATAACCAGGCCATTCAAAAGCGACAAAACAACATCACAGCATT
GGGTTGTGGCCGCCTACTATTTAGCTTTTGATAGTGAGATAAGTGCTATGGAGGTTTTGC
TGCGACAACAATGCCAATTTTTATACATTGACAACAATGATGGCATTATACTGTTCTTCC
TGGAATACAACGTGCAGAAATCTAGGACTACAGTGTACAATTGGTTCACAGCCAATTTCC
ATTATAATGAAAATAGAATGCTAGCTAATCCGCCAAGGACACGAAACATGCCTGCTGCTT
TATTCTTCTATCATAGATTTATGGGTACAGGGGGTATAAAACATGGCGCAATGCCAGAAA
TAATTGTAAACCAGTGCGTGGTGTCTAATCAGCAGACAGACACCTTTGAATTATCACGTA
TGGTACAGTGGGCACTGGACAACGATCTGCAAGATGAACATATGTTAGCTTTAGAGTATG
CTTTGCTTGCTGAAAGTGATGGCAATGCGCGGGCTTTTTTAAAGCAGAATAATCAGCCAA
TGATAGTGAAGAATTGTAGCATAATGGTTAGACACTACAAGACAGCGCTGGTCGCAAAAA
TGTCTATTTCACAGTATGTGAATAAGCGGTGTCTGGACCATGGGGAAGCTGATGAAAACA
GCTGGCGGGGAATTGTGCATTTTCTGAGGTATCAAGGTCAGGAATTCCTGCCCTTCATGT
GTAAAATGCACAATTTCCTACACCATAGACCAAAGAAATCAACACTTGTATTATGTGGAC
CGTCGGACACAGGCAAATCATATTTTGCCAATGGTCTTAACAAATTTTTGGATGGACACG
TGCTGAGCTTTGTCAGCAATGGGTCACATTTTTGGTTATCACCATTACGTGGGGCACGGT
GCTGTCTAATAGACGATGCGACCCTCACGTTTTGGAGGTACGCGGACCAAAACATGAGGG
CACTGCTAGATGGATATGAGATTTCCATTGATGCAAAACACAGAAACCCAATGCAAACTA
GAGCACCACCATTAATAATAACCACAAATGAGGACATTATGCGATTAGATGAATTCAAAT
ATCTGCAAACCAGAACAATGTATGTGTACTTTAACAAGCCATTTCCTCTTAAAGGAAATG
GGCAACCGTTATATTACATTGATGGTTATACATGGAACTCTTTTTTTAGGAAATTTTGGC
GTCACCTAAATCTAAAAGACCCTGAGGATGAGTCAGATGGAGAGACTCCTGGAACGATTA
GACTATATACAAGAGCAGATACTGACACTATATGAGAAAGATAGTGTTGACCTAGAGGAT
CATATAAGGCTATGGAATCTGCTAAGGAGGGAAAATGCAATCTGGTATGTACTCAGACAG
GAAGGACACGCAAGGGTCGGCGGCAGAGCGGTGCCGGCAATGACGGTATCGGAAGCCAAT
GCCAAATTCGCAATAGAAATGCAGATAAAGCTAGAATCACTAAAGGCCAGTCCCTATGCG
GCCGAGGGCTGGTCATTGCAAGAAACCACCAAGGAACGGTACTTGGCTGAACCGTCTCGG
ACATTTAAGAAATTAGGGCAGCCAGTTACCCTAATGTTTGACAATGATCCCGAAAACCTT
ACAGAAGTTGTATTGTGGAAATGGGTTTATTATATTACACCAACAGATGAATGGTATAAA
GCTAGAGGTGGCATTGATGACACTGGTATATACTACATTGACCACGAGTCTGTTAAAATG
TACTATGTGAGATTTGACATGGAAGCGGAGAACTTTAGCGAGACAGGCACTGTCACCTAC
CGGCTAGGCAGCGCCCTGGTAAATGTACCTGAACCTGTAACTGTTACCGACAGCTCCTCC
ACGAGGGAGAGAACCCCAAAGGTACTACGACCGCAGGGGTCGAGACGACGCAGAAACGAG
GAAACGGGGGAGCCGGTCGCCCCAGCCCCTAAGCGAAGACGAGGAGCTTACGGACGCAGA
TCCTCCCCGAAGGCCCAACGCAGGACCGCGGCGTCGCCTGTTTCTAGAGGAAACGGAGGA
TCGTCTGACTTCACTTCTGGAGAGTCTGACGAAGGACATCGAGTCAGACATAGAGCACTT
CGAAAGAAAACTGCGGGTGTTGCTCCAGCAGAAGGACACTATCTAGTTGGCGCCAAAGGT
CCAGTGAATAGCCTGCGGTGCTTAAGGTACAAATGGAAAAACAAGTATAGCGGTGACATA
ATGTATCTGGGGACTACTTTCACATGGACGGAGTCTGACGGGACAGAACGGTGTGGGTCG
GGGCGCTTTTTTTGTGCTTTCTCTAATGAAACAAAAAGAGAAAAGTTCCTCAAATCTGTC
AAGATTCCTAAAAACATTGGGCTGTTTCGCGCACACGCAGAAAAGCTGTGACCTGTGTAT
CATTAAACAATGCTTGCTAGGCAAAGGGTTAAACGCGCTAATCCTGAACAACTGTATAAG
ACATGCAAAGCAACGGGGGGCGATTGTCCACCCGATGTTATTAAACGCTATGAGCAAACT
ACACCTGCTGATAGTATATTAAAGTATGGGAGTGTAGGGGTTTTCTTTGGCGGTCTGGGC
ATTGGCACAGGACGTGGTGGCGGTGGCACAGTGCTTGGGGCTGGGGCAGTTGGGGGACGC
CCGTCCATATCCAGTGGTGCAATTGGTCCCCGGGATATTTTGCCAATTGAATCAGGGGGG

CCTTCACTGGCAGAGGAAATACCTCTGCTTCCCATGGCACCCCGTGTGCCAAGGCCTACA
GATCCCTTTCGGCCGTCAGTGCTGGAAGAGCCTTTTATTATAAGGCCTCCTGAACGCCCA
AACATTTTGCATGAGCAGCGTTTCCCTACAGACGCTGCACCATTTGACAATGGCAACACA
GAAATCACAACCATTCCTAGCCAATATGATGTTAGTGGGGGAGGGGTTGACATTCAGATA
ATTGAACTCCCTAGTGTGAATGACCCCGGTCCCTCGGTTGTTACCCGCACACAATACAAC
AATCCAACGTTTGAGGTGGAGGTGTCCACTGACATTAGTGGAGAAACCTCATCAACGGAC
AACATTATTGTAGGAGCTGAAAGCGGTGGCACATCCGTAGGTGACAATGCTGAACTGATA
CCTTTGCTAGATATATCCCGGGGGGACACAATTGACACAACAATACTTGCCCCTGGCGAG
GAGGAGACTGCCTTTGTGACCAGCACTCCTGAACGTGTGCCTATACAGGAGCGATTACCT
ATTAGGCCCTATGGCAGACAGTATCAGCAAGTGCGAGTTACCGACCCTGAATTTTTAGAC
AGCGCTGCAGTACTTGTCTCTTTAGAGAATCCAGTGTTTGATGCAGACATTACTCTCACG
TTTGAGGATGATCTGCAGCAGGCACTACGTAGTGACACAGACCTGCGGGACGTGCGTCGC
CTCAGTAGACCTTATTACCAGAGGCGCACTACTGGCCTTCGTGTTAGTCGCCTGGGGCAA
CGTCGGGGTACTATATCCACGCGCTCTGGTGTTCAGGTAGGCTCCGCTGCTCATTTTTTC
CAGGACATTAGTCCAATCGGCCAGGCTATTGAGCCAATTGATGCAATTGAACTAGATGTA
CTGGGTGAGCAATCCGGTGAGGGGACTATTGTGAGAGGAGACCCTACGCCTTCTATTGAG
CAAGACATAGGACTAACCGCTTTGGGGGACAACATTGAAAATGAATTGCAGGAAATAGAT
TTATTAACTGCGGATGGTGAAGAAGACCAGGAGGGCAGAGACCTGCAGTTGGTATTTTCC
ACTGGCAATGATGAGGTGGTTGATATTATGACTATACCTATACGTGCAGGCGGGGATGAC
AGGCCTTCAGTATTTATTTTTAGCGATGATGGCACTCACATTGTCTATCCTACTAGCACA
ACAGCCACCACCCCACTCGTGCCTGCACAGCCCAGCGATGTGCCCTACATTGTTGTTGAC
TTGTATAGTGGAAGTATGGATTATGATATACATCCTAGCCTGTTGCGCAGGAAACGTAAA
AAACGCAAACGTGTTTATTTTTCAGATGGCCGTGTGGCTTCCAGGCCCAAATAGATTTTA
CTTACCCCCTCAACCTATACAACGGACATTGAACACAGAGGAATACGTGAGACGCACCAG
TACTTTCCTCCATGCTGCCACTGACCGTTTGCTTACTGTTGGACATCCATTTTACAATAT
TACTAATGCGGATGGCAAAGAGGTGGTCCCTAAAGTTTCCTCTAATCAGTTCAGGGCCTT
CCGTGTCCGTTTCCCAAATCCCAATACCTTTGCATTTTGTGATAAGTCCCTTTTTAACCC
TGACAAGGAGCGTCTGGTCTGGGGTATTCGTGGGATTGAGGTTTCTAGGGGACAGCCCTT
AGGTATTGGTGTAACAGGGAACCCTTTTTTTAATAAGTTTGATGATGCTGAAAATCCCTA
CAATGGTATAAACAAAAATAACATTACTGACCAAGGTTCAGACTCAAGGTTGAGCATTGC
ATTTGACCCTAAGCAAACACAGCTGCTGATAGTAGGTGCTAAACCTGCAAAGGGTGAGTA
CTGGGACGTTGCTGCAACATGTGAAAACCCTCCACTGACCAAAGCAGATGACAAATGTCC
TGCTCTAGAGCTTAAGTCCTCATACATTGAGGATGCAGACATGAGTGACATAGGCCTGGG
AAACTTGAATTTTTCTACACTGCAGAGAAACAAATCCGATGCCCCATTAGATATTGTGGA
TTCTATCTGCAAATATCCTGACTACCTGCAAATGATAGAAGAACTATATGGAGACCACAT
GTTTTTCTATGTGCGGCGTGAAGCTCTGTATGCTAGGCATATAATGCAACACGCGGGCAA
GATGGATGCTGAGCAATTTCCCACTTCTCTGTACATAGACTCCTCTGTAGAAGGTGAGAA
ATTAAATTCCTTGCAGCGCACTGATAGGTATTTCATGACACCCAGCGGCTCCCTGGTAGC
TACTGAGCAGCAGCTGTTTAACAGGCCCTTTTGGCTGCAGAGATCCCAGGGCCATAACAA
TGGCATACTGTGGCACAACGAGGCCTTTGTAACATTGGTTGACACTACCAGGGGAACTAA
CTTTACCATCAGTGTTCCTGAGGGGGATGCTTCTTCATATAACAATTCTAAGTTTTTTGA
GTTTTTAAGGCACACCGAGGAGTTTCAGCTTGCCTTTATTCTACAGCTGTGTAAGGTAGA
CCTTACCCCTGAGAATTTGGCTTACATACACACAATGGATCCATCCATTATTGAAGACTG
GCATTTAGCTGTCACTTCACCTCCCAATTCTGTACTGGAGGATCATTATAGGTACATACT
GTCCATTGCAACTAAATGTCCCTCTAAGGATGCAGATGATACCTCCACTGACCCATACAA
AGATCTTAAGTTTTGGGAGGTTGATCTACGGGATCGTATGACAGAGCAATTGGACCAGAC
TCCCCTTGGCAGGAAGTTTTTGTTTCAAACTGGTATCACTCAGTCATCATCAAATAAGCG
GGTGTCCACGCAGTCTACTGCCCTTACTACCTACAGGCGGCCTACTAAGCGCCGCCGGAA
GGCTTAAACGAATTGCTGGTATTGTGGTGCGGTGTCCTCGACGGTCCATGTGTCATCTTA
TAATCACTTGGTCAGTCCAGGGTACACCACTCCATTATCTATTTACTTCGCATGTATTTC
TCTGTTATGTTCCTGTATGGGTTATGAATGTGTTAATAAAATATGTTGGTAACGCTGTGC
ACGGGTTTGTTCACGTTCATGTCTCATGATTTGGCACCCCTGTATTCCCGCCGCCGCCCG
GGGGATCGCAGATATAATCCCCAAACCCAAAGCGTTCCAACATTGGCAAACGTCTCTGGC
CCCGATACAACTGAAACGGTCTGTCTTGCCAATAGCCCCATCTGGCGGGGATTCAACTGA
AACGGTGTGTACTGCCAAGTAACATTTTTGTTATTGGAACGCCTCCGGTGCTGGCGGAAG

CGCAAGGATTTAGGCGCGAAGACAGTTTTATTGCCAAAACCTTTTGGTTGCTGCCAATAG
CAGGCGTGGTCTCAACGAATTCGTTGCGGCAATAGGTATGTACCATGGTTATGA
>gi|1491683|emb|X94164.1|_Human_papillomavirus_type_72_E6,_E7,_E1A,_E1B,_E2,_E4,
_L2,_and_L1_genes
ATTACTAACAATAATACATGTAAAAAAGTAAGACAAGACCGAAAACGGTCCGACCGACAT
AGGTACATATATAAGGGAACTGTGAACTCAGCAAATCAGCAATGCCTATGGGACTGCACA
ATCCAACTAATATTTGGTTGCTGTGCAAGGAAATTGAGGTGGACCTAGAAGATTTACGGA
TTACCTGCATATTTTGCAAAAATGAATTAACAACAGAAGAATTGCTGGCGATTGCAATAA
AGGAGCTGCAGATTGTGTGGCGGGACAACTGGCCATTTGGAGTCTGCGCACCATGCCTTG
CAAGAGCAACTAAAGTGAGGGAGCTACGATACTGGACGTATTCGGGCTACGGACCCACTG
TGGAACAGGAAACAGGCAAATCATTAGCAGAACTATATATAAGGTGCCATGCATGCTGCA
AACCCCTAAGCTGTCAGGAAAAGGAATATCAGGTGCAGACAGGAATCCACTTCCACAAGA
TAAGCGGACTGTGGACGGGAAGGTGCTGCCAGTGTAGAGGGGCATGCACGGCCAGGTGGC
AACCATAAAGGACATTGTCCTTCAGGAACTTCCTGATGTGGTTGACCTACACTGCAATGA
GCAGTTACTAGACAGCTCAGAGTCAGAGTCAGAGGATGAGAGGGACGGTGTTGGTGTGCA
GGAGCAACTTGTAGAACAAGCACAGCAGGCCTACGGGGTGGTTACTACCTGTGGCAGGTG
CTACCGTCCAGTTAGGCTGGTGGTGGAGTGCAGAGACGCAGACGTGAAGGCGCTACAACA
ACTACTGCTGGACAATTTGTCCATAGTGTGTCCTCGCTGCGCATAAGGGACATGGCCAAC
TGCGAAGGTACTGAACGGGGGGATGGGGACGAGGATGCGAATCGCGCGGGCGGATGGTTT
TTGGTTGAGGCCATAGTGGAGCAAACCACAGGGTACCAAGAGTCCAGTGATGAGGACGAA
AACAGTGAGGACAGGGGAGAAGATCTGGTAGACTTTATAGACACAAGATCCTTAGGGGAT
GGGCAGGAAGTGCCGTTAGATTTGTTCGTGCAACAAAATGCACGGGATGACGCTGCAACC
GTGCAGGCCCTAAAACGAAAGTATACATGTAGCCCAGCAAGCAGCTCGTGTGTGTCTTTG
GTGGACAGTGAGTTAAGTCCCCGACTGGACGCCATAAGCATAAACCGGGGACACGACAGG
GCTAGAAGAAGGCTGTTTGACCAAGACAGTGGCTATGGCCATACGCAGGTGGATATTGGA
GCACCAGAAAGCCAGGTATCGGGGGGTACACAGCATACAAAGGGGGGAGGCGGCGCCGTT
CAGGAAGCGGAAGAGGAGCGTGTGGGGGGGGATGGTGAGGCGCAGTGTAGTGCACAGACA
CAGCAAACGCCAGAGAGAGCAGCAGACGTACTAGAAATATTTAAGGTTAGTAATTTGCGT
GTCACATTACTGCATAAATTTAAAGAGCTATTTGGACTAGCATATGGGGATCTGGTAAGA
CAATTTAAAAGCGATAAATCAATATGTGGGGATTGGGTAGTATGTGCATTTGGGGTATAT
CATGCAGTGGCAGAGGCAGTAAAGACGTTAATACAACCCATATGTCTGTATGCACATATA
CAAATACAGACGTGTCAATGGGGGATGGTAATTTTAATGCTGGTGCGGTATAAATGTGGC
AAGAGTAGGGAGACAGTGGCACACAGCATGAGCACGCTGCTAAATATACCTGAAAAGCAA
ATGCTTATTGAACCACCAAAAATTAGAAGTGGACCATGTGCCCTATACTGGTATAGAACA
GCAATGGGAAATGGCAGCGAGGTGTACGGGGAAACCCCAGAATGGATAGTAAGACAAACA
GTAGTGGGGCATGCAATGCAAGAGACACAGTTTAGCCTTTCTACCTTAGTACAGTGGGCA
TATGACAATGACATAACAGATGAGAGCGAGCTAGCATATGACTACGCAATGCTAGGTAAT
GAGGACCCAAATGCAGCAGCATTTTTAGCAAGCAACTGCCAGGCAAAGTATATTAAGGAT
GCAATTACAATGTGCAAACATTATAAACGTGCAGAACAGGCACGAATGTCTATGACACAG
TGGATAGCACATAGGGGGCGCAAGGTGGCAGATTCAGGTGACTGAGAGAAATAGTAAAAT
ATTTAAGATATCAAAGGGTTGAATTTGTAACATTTATGGGAGCATTAAAGCTATTTTTAA
AAGGGGTACCAAAAAAAAGCTGTATGGTATTCTATGGGCCAAGTGACACCGGAAAGTCAT
TGTTTTGTATGAGTTTACTTAAGTATTTAGGGGGAGCAGTAATTTCATATGTAAATTCAG
GAAGCCATTTTTGGTTATCACCACTGGTAGACGCCAAAGTAGGGTTGTTAGATGATGCAA
CATACCAGTGCTGGCAATATATAGATACATACCTACGAACAGTGTTAGATGGAAATGCTA
TAAGCATAGATAGAAAACATAGAAATTTAACACAGTTGAAGTGTCCACCACTTATGATAA
CAACAAATATAAATCCATTGGAAGACCAGGCATTTAAATATTTGCACAGTAGAATAGTGT
TGTTTAAATTTATGCATAAGTGCCCATTAAAAAGCAACGGTGATCCCGTATATACCCTAA
ATAATGAAAATTGGAAATCGTTTTTCCAAAGGTCCTGGGCACGTATAGAGGGACCTGACG
AACAGGAGGAGGAGGAGGATGAGGATGGAAGCACTAGCCGACCGTTTAGATGCGTGCCAG
GAGAAATTGCTAGACCTTTATGAAAAAGATAGCGACAAGCTTGAGGACCAAATATTGCAT
TGGCACTATGTGCGTCTGGAACATGCAATGTTATTTAAGGCACGACAAGCAGGACTTACC
CATGTAGGCCACCAGGTGGTACCAACACTTAGTGTTACAAAAGGCAAAGCACATCAGGCA
ATTGAAGTGCACCTGTCACTGCAAGGGTTGCAAAACAGTGCGTATGCGCAAGAACCATGG
ACATTACAGAACACCTCACTGGAAATGTGGAATGCACACCCACAACGGTGTTGGAAGAAA

AAAGGACGCACAATAACAGTTAAATTTGATTGCGAGGACCTAAAAGCAGTGGAGTATGTG
AGCTGGGGGTGTATTTATGTGCAAAGTACAGAGGACGAACAGTGGTATAAAGTACAAGGA
CATGTGTCATATCATGGGCTATATTATGAATTTCAGGGTCAGAAACAGTACTATGTAACA
TTTGGACACGAAGCCAGAAAATATGGGGACACAAACACATGGGAGGTACATGTGGGAAGT
ACAGTGATTTATGAACCCTGCGCCTCTGTATCTAGCACCCAGGACACCGTGCGAGAAGTA
CCCACTGTTGAAACTGTTGGGCGACTGCCAGACGCCACCAAATCCACCGCCACCGCCACG
TGCGTGGGCCCCGCCCAGACATCCTCCTCAGTGCAGACGCCGCCTTGTAAGCGACAGCGA
CTCCACAGAGACGGATTGCAGCAGCAGCCCGACTCTACGGAAAGAGACATCTGCAGGCAA
CGGCGTGACAGTGCTGACCAGTGGGTCAACCGTGACAGTGACTGCACACAACAAGCAAGG
GACATCTGTAACAGTCACGGTGCACCTATAATACATTTAAAAGGTGAACCAAATAAGTTA
AAGTGTTTTCGGTATAGGCTTCAGCAGTCAGTGCCTAACTTGTTTTTAAAAGCATCCTCT
ACATGGCATTGGGCCTGTGGGGGTGACACAACAAAATGTGCATTTGTAACACTGTGGTAT
GTGGATACTGACCAACGGACACAATTTTTAAGTCGTGTGAACATTCCAAAGGGGATACAA
GCCACTGCTGGCTATATGTCAATGTGTATATAATGTTTGTTGCGATGGCAACCAGTGTAT
AGAACCACACCTGCAACATTATGTAAGGCAGAAGCAATCCTGGATATACTTGTGTGTTTG
ATATCTGGGTGGTGTACTGTGCTGTTGCTGCTTATTATTTTCTGGCTTTCCTATCTTTCT
GCACTAAGTGCTTTTTTGGTGTTTGTGTGTGTTATATATCTAGGATTGTTTTGTATATAT
ATGCAGGTGATGTGGTACATAGGTGACTTATAATCCACCCAGCCATTACATGCTGCTATT
GTGTAAATAGTGTTCCTTGTGTATCTTGTATGTAATATGTATCCTGTTGTAGTGGGCAAT
ACGGATGGGGGTGCATTAATTGTACTACGAGACGATAATTGTGGATTGTGGTTCTTCTTG
TGTATGTTAATAATCATTGTAGTGTTGCTATATAGGTTGCTACACTGATCCCTTCCTTTT
GTGTATTCCCACCTCCTTTTTATTTTTGTTTTGTTTTGTTTTTGTTTTTTATTTTTTTGC
ATTTTTATAATAAACATTATCTGCCAAAATGACCCAAGCTGTAAGGCGTCGCAAACGTGC
CTCTGCAACGGACCTGTATCGCACATGCAAACAGGCGGGTACCTGCCCTCCTGATGTTAT
ACCAAAGGTGGAGGGTGACACCCCTTGCTGATAGGTTCCTGAAGTGGGCCAGTTTAGGGGT
GTTCTTTGGTGGGTTAGGCATAGGCACGGGTTCAGGCACCGGTGGGCGCACTGGCTATGT
GCCTATAGGTACTCGCCCTCCCACTGTTGTGGATATAGGCCCTACAACACGCCCGCCTGT
TGTTATTGAGCCCGTGGGGGCCGCAGACCCTTCCATAGTCACCCTTGTGGAAGAATCCAG
CGTTGTGGAAGCCGGTGCCACCGTTCCCACTTTTACTGGGTCTGGTGGCTTTGAGGTTAC
CACGTCCTCAACTACTACCCCTGCTGTTTTAGACATTACACCCTCTGGAGCGTCTGTCCA
AGTTAGCAGTAGTAGCTTTACAAATCCCTTATTTACTGAACCGTCCATTATTGAACCTCC
ACAGGCCGGGGACCTTTCAGGCCATGTATTCACTAGCACACCCACATCTGGGTCGCATAG
CTTTGAGGAAATACCCATGCACACATTTGCAACTCATAGCAGTACTAGCACAGACCCCTT
TAGTAGTACCCCTTTGCCTGGTGTTCGTCGCCTTGCACAGCCCCGCTTAGGATTGTATAG
CAAGGCTAATCAACAGGTTAGGGTTACTAACCCTGCCTTTTTGTCTCGACCCCAGTCTCT
TGTTACTTATGACAACCCTGTGTATGATCCAGAGGAAACTATTATTTTTGAGCATCCTAG
TATATATACCCCTCCTGATCCTGACTTTTTGGATATTATTTCCTTACATAGGCCTGCCCT
TACAGCCCGCCAGGGTACAGTACGGGTCAGCCGGTTGGGTCAACGTGCTACCTTGCGTAC
ACGTAGTGGCAAACGCATTGGCGCTCGGGTACACTTTTATCAGGACATTAGCCCCATTTC
ATCTGATACTATTGAAATGCAATCCTTGGCCTCCTCTACGCAGCCAGACATAACATATGA
CATTTATGCTGACCCTGATTTAGGGGAACCCCCGCCGCGTGCTTCTGTGTCTTCTACATC
ATTGCACAGCCCGTCCCTGTCTGCAGCGTCTGCTGTTTCTGCCAAGTATGACAATGTAAC
AGTTCCCTTGTCCTTAGGGCCACACATCCCTGCCTCCTCTGGCCCTGACATTGATTTGTC
CTTTGCTCCTGCCCCTGTACCTACAATGCCTCTTGTACCCTCTACGCATCCACATTCTAT
TTATGTTGAGGGCTTTGATTTTTATTTGTTGCCTGCATATATCTTTTTTCCTAAACGTCG
TAAACGTGTGCCCTATTCTTTTGCAGATGGCTTTGTGGCGGCCTGGTGACGGCAAGGTAT
ACCTGCCTCCCAATCCTGTTTCTAAGGTTCTCAGTACTGATCGCTATGTCCAACGCACCA
ACCTCTATTATTATGGTGGCAGTTCTCGTCTACTAACTGTAGGACATCCTTACTGTGCCA
TACCTCTCAACGGACAGGGCAAAAAAAACACCATTCCTAAGGTTTCGGGGTATCAATACA
GGGTGTTTAGAGTAAAACTTCCTGATCCCAATAAATTTGCTTTGCCTGATGGCACACTTT
ACAATCCAGATACTGAACGGCTGGTATGGGCCTGTCGTGGCATTGAGGTTGGTAGGGGCC
AGCCCCTTGGTGTTGGCACTAGCGGTCACCCCTTGTATAATCGCTTGGATGACACTGAAA
ACACTTCCTTACTTGTGGCTGACAATTCTGACAGTCGGGACAATGTATCTGTTGACTACA
AACAGACCCAATTGCTTATTATAGGGTGCAAGCCTCCCATTGGTGAGCATTGGACCAAGG
GCACTCCTTGTGCAGGCTCTAATTCTCAGCCAACTGACTGCCCCCCTTTAGAATTTACAA

ATTCCACTATACAGGATGGTGACATGGTGGAAACAGGCTATGGTGCCATAGATTTTGCTA
CCCTTCAGGAAAATAAATCAGAAGTGCCTTTGGATATTTGCACCACCACCTGCAAATATC
CTGACTATTTGCAAATGGCTGCTGAACCATATGGTGATTGTATGTTTTTTTGTCTTCGCA
GGGAACAAATGTTTGCACGCCATTTTTTTAATAGGCAGGGCACAATGGGTGAGGCACTAC
CAGCCAGTTTATATCTTAAAGGTGCCTCGGGTAGCGACAGGGTGACACCTGGTAGTTATA
TTTATTCTCCCACCCCCAGCGGCTCTATGGTGTCCTCTGATGCACAATTATTTAATAAGC
CCTATTGGCTACAGCGCGCCCAGGGTCACAACAATGGCATCTGTTGGTTTAATGAGCTTT
TTGTGACAGTTGTAGATACTACTCGCAGTACTAATGTAACTATTTGTACTGCCACAGCGT
CCTCTGTATCAGAATATACAGCTTCTAATTTTCGTGAGTATCTTCGCCACACTGAGGAAT
TTGATTTGCAGTTTATATTTCAACTGTGTAAAATTCACTTAACTCCTGAAATTATGGCCT
ACTTGCACAATATGAATAAGGCCTTATTGGATGACTGGAATTTTGGTGTGGTGCCTCCTC
CTTCTACCAGTTTGGATGATACCTATAGGTTTTTGCAGTCTCGTGCCATTACCTGTCAAA
AGGGGGCTGCCACCCCTCCTCCTAAAGAAGATCCATATGCTAACTTATCCTTTTGGACTG
TGGATTTAAAGGACAAATTTTCCACTGACTTGGATCAGTTTCCTTTAGGGCGCAAGTTTC
TGTTACAGGTTGGTTCTAGGGCTGTTTCAGTGTCACGCAAACGTGCTGCCCCACCAAGCT
CTACCTCGACCCCCGCCCCTACTAAACGTAAAAAGCGCAAAAAGTAACATGTCATACTGT
TTGTGTGGTGTATGTGTGTATGTGTGCAATGCATGCATGTGTGTTTCTGTTGTTGTTTGT
GTGTACATGTTTGTACTATGTTATGTTGTTGTATGTTTTTTGTATGGCCCCTGCCCCCGT
GTTGTGTATGTATGTGGAATGTGTGTTATGTGTTGTGCATTAATAAAGCGTGTGTCATGT
GTGTGTGTGTGTCCGGTGAACCCTGTGAGTAAGTGTGTGTTTGCACGTTGTCCTACTTCC
TACACTTTGTTTTGTGTCACCTTTGTATGCCCTTTACTGTACTCCATTTTATATTTTCTC
CATTTTGTATTCGCGACCGTTTTCGGTCTCCCGCCTTTTCGGTCGTGGCGCCGTGCCACT
GTACATAGAAACTATGCATTGTGCTTTCCTCCCACATCCTGTTTCAACAAACCTTATCCA
CATCTGGGTGTGCCTGACAGGTTTCTGGCACATACATTTTCCATAGTTATGTGTTTCCTG
ACTCATTTTACAATAGATATGCTTTTAGGCACATATTTTATGCTGACTACTTTCTCCTAA
TTGCTGTTTTGGCTACCTTTCTAGGTGTTGTAGCCAAGTATGTGTCTTGCAACTATGGGC
AAGCCCTTTACAAACGTGTTAAAACATTCTACTCCGGTCGCTCCCCTATGTCTCATGGTT
TTATAGTT
>gi|71726694|gb|DQ080080.1|_Human_papillomavirus_type_97,_complete_genome
ATGGCGCGATTTGAAGATCCAAGCAAACGGCCCTACAAGTTGCCAGACCTGTGCACAGAA
TTGAATACTTCATTACCTGAAATAGAGATAAGCTGTGTATATTGCAAAACAACCTTGGAA
CGCACAGAGGTATATGAATTTGCATTTAAGGATTTATTTGTAGTGTATAGAGACTGTATA
GCATATGCTGCATGTACTAAATGTTTAACCTTTTTTTCTAAAATTAGAGAATTACGATAT
TATTCACATTCAGTGTATGGAGATACATTAGAAAAAATTACTAATACTGGATTATACAAT
TTGTTAATAAGGTGTTTGCAGTGCCAGAAACCGTTGAATCCAGCAGACAAATATAAACAT
CTTAAAGACAAACGACGATTCCACCACATATCTGGTTATTATAGAGGCCAGTGCAATTCG
TGTTACAACCAATCACGGCAAGAAAGGCTTTCCAGACGTAGAGAAACACAAGTATAATAA
TAAGTATGCATGGACCGAAACCAACATTACAAGAAATTATATTGCATTTGCAACCCCAAA
ATGAAATAAATTCGGCTGACCTTCAATGTTATGAGCACCTAAGTGACTTAGAGGAAGAAT
ACGATGAAACAGATGATGTTAATCATCGACAACTACCAGCCCGACGAGCTGAACCACAGC
GTCACACAATTGTGTGTATGTGTTGTAAATGTGAGAGCAGACTTCAGTTTGTGGTAGAAA
GCTCCGCCGACGACCTGCGGTCACTGCAGCAGCTGTTTTTGAGCACCCTGTCCTTTGTGT
GTCCTTGGTGTGCAACCCACCAGTAATCTACAATGGAAGATTCACAAGGTACAGACGGGG
AGGGTACGGGATGTAATGGCTGGTTTTATGTACAAGCAATAGTAAACAAAAAAACAGGAG
ATATAATATCAGAGGATGAGGATGAAAATGCAACAGACACAGGGTCGGATATGGTAGATT
TTATTGATACACAATTAACTATTTGTGAGCAGGCAGAGCTAGAGACAGCACAAGCATTGT
TCCATGCGCAGGAAGTAGACAATGATGCACAGGTGTTGCATGTTTTAAAACGAAAGTATG
GAACAGAAAGTAAAGAAAACAGCCCATTAGGGGAGCAGCCAGGTGTGGATACAGATTTAA
GTCCACGATTACAAGAAATATCATTAAATAGTGGGCAGAAAAAAGCAAAACGACGTCTGT
TTCAGATAGCAGACAGTGGCTATGGCTGTTCTGAAGTGGAAGCGGCAGAGACTCAGGTAA
CCGTAAACACAAATATAAATAGAGAACATGGCGGCAGTTTATGTAGTAGTGGGAGTAGTA
GTGGGTGTATGGATGGTAATCATGATAATAGTAATATAGAAAATGAAAATCCACAATTAG
TAATTGCAGAATTAAAAGACCTATTAAAGGCAAATAATAAAAAAGCTGCAATGTTAGCTG
TATTTAAAGAAACATATGGGCTATCATTTGCAGATTTAGTAAGAACGTTTAAAAGTGATA
AAACAACGTGTACAGATTGGGTTACAGCTATATTTGGAGTAAATCCAACAATTGCAGAAG

GATTTAAAACGTTAATACAGCCATGTACATTATATGCTCACATTCAATGTTTAGACTGTA
AATGGGGAGTATTAATATTAGCATTATTGCGCTATAAATGTGGAAAAAATAGACTAACAG
TTGCAAAAGGATTAAGTACATTATTGCACGTACCTGAAACCTGTATGTTAATTGAACCAC
CAAAGCTGCGAAGTAGCGTTGCAGCATTATATTGGTATAGAACAGGAATATCTAATATTA
GTGAAGTAATAGGAGACACACCTGAGTGGATACAAAGACTTACAATTATACAGCATGGTG
TAGATGATAGTATTTTTGATTTATCAGAAATGATACAATGGGCATTTGATAACGACTTTA
CAGACGAAAGTGATATAGCATATGAATATGCTCAATTAGCAGATTGCAATAGCAATGCAG
CTGCATTTTTAAAAAGTAATTGCCAAGCCAAATATTTAAGAGATTGTGCAGTTATGTGCA
GACATTATAAACGAGCCCAAAAGCGCCAAATGAATATGTCACAGTGGATTAAATATAGAT
GTTCAAAAATAGACGAAGGAGGGGACTGGAGGCCAATAGTACAATTTTTAAGATTTCAAG
GAATAGAATTTATTACCTTTTTGGGAGCACTGAAAGCTTTTTTAAAGGGAACACCTAAAA
AAAACTGTATAGTAATACATGGACCAGCAAATACAGGTAAATCATATTTTGGAATGAGCT
TTATACATTTTATACAAGGTGCAATAATATCATTTGTAAATTCAAATAGCCATTTTTGGT
TAGAACCGTTAGCGGATGCCAAGGTAGCCATGTTGGATGATGCAACAAACACATGTTGGA
CATATTTTGATAACTATATGAGAAATGCTTTAGATGGAAATCCTATAAGTATAGATAGAA
AGCACAAGCCATTACTACAGTTAAAATGTCCGCCTATATTATTAACCTCAAATATTAATC
CAGCTATAGATAACAGATGGCCATATTTAGAAAGTAGAGTGACAGTGTTTACATTTCCAA
ATGCATTTCCATTTGATAAGAATGGTAATCCAGTGTATGAAATAAATGACAAAAATTGGA
AATGTTTTTTTGAAAGGACATGGTCCAGATTAGATTTGCAAGAGGAAGATGACGATGCAG
ACACCGAAGGAAACACTTTCGGAACGTTTAAGTGTGTTACAGGACAAAATCCTAGACCAC
TATGAAAATGACAGTAAAGACATACATAGCCAAATTAATTATTGGCAACTTATACGTTTG
GAAAATGCAATATTCTTTGCAGCAAGGGAACATGGTATACAGACATTAAACCACCAGGTG
GTGCCAGCCTTTAACATTTCAAAAAACAAAGCATATAAAGCAATTGAACTGCAGATGGCC
CTAAAGGGCCTTGCACAAAGTCAGTATAACACAGAGGAATGGACACTGCAAGATACATGC
GAGGAACTGTGGAATACAGAGCCTACACAGTGTTTTAAAAAAAGTGGGCAAACAGTGGAA
GTATATTATGACTGCAACAAAGACAACTGTATGTCTTATGTAGCATGGAAATGTGTGTAC
TATATGACTGAGGCAGGAACATGGGATAAAACAGAAGCATGTGTAAGTCACTGGGGACTC
TATTATATGAAAGAAGGAATGAAAACATTTTATGTGCAATTTGAACATGAAAGTAAAAAA
TATGGTAACAGTAATAAGTGGGAAGTGCATTTTGGGGGCGTTGTAATTGATTGTAATGAC
TCTATGTGCAGTACCAGTGACGAAGTATCCGCTACTCAGATTGTTAGACCACTACAACAC
GCCTCCACGTCCTACACCAAAGCCCCGTCCGTGGGCCCCGCAAAAACCCAAACGCCGGCT
ACTAAACGACCTGGACAGTGTGGACTTCCGGAGCAACACAGTGGACGTGTCACCAACCAC
GTGCACAACCCACTACTCTGTGCAACTACATCTGGAAGCAACAAACGACGGAAGTTGTGT
AGTGGTAACACTACGCCTATAATACACTTAAAAGGTGATAAAAACAGTTTAAAATGTTTG
CGGTATCGATTTAAAAAATATAATCAATACTATAGTTCTATATCATCAACCTGGCATTGG
ACAGATTCCTGTAATAAAAAAGCTGGTATATTAACTGTAATGTATGATAGTGAGCATCAA
CGAACCAAATTTTTGGATAGTGTTCCTATTCCTAACAGTGTACAAATATTGGTGGGATAC
ATGACAATGTAATATGTATATTGTATACAATGTGGTAAACAAATGTTAACATTTGTGTTT
ATATTTTGCTTTTGTGTGTGCCTTTATGTGTGTTGCAGTGTCCCGCTTTTGCAATCTGTT
TATGTTTGTGCGTTTGCATGGCTGTTGGTGTTTTTGTTTATTGTGGTTATAACAACCCCT
GTAACTGCATTTACAGCATATGTATGTTTTTTTTTATTGCCTATGTTTTTATTACACATA
CATGCTAGATATACATAACATTACTTGTATATACTGTATTGTACAGTATCTTTTGTATTG
CATAGTTTTTTTATACTTGTTTTGTATTATTGTTTACTGTTGTAATAAATATGGTATCCC
ACCGTGCAGCTCGACGCAAACGGGCCTCTGCAACTGATTTATATAAAACATGTAAACAAT
CTGGCACATGTCCGCCTGATGTTATTAACAAAGTAGAAGGTACTACATTGGCAGATAAAA
TTTTACAGTGGTCCAGCCTTGGTATATTTTTGGGTGGTCTTGGGATAGGTACTGGTAGTG
GTACTGGGGGTCGTACAGGGTATATTCCTTTAGGTGGGCGTCCTAATACTGTTGTGGATG
TAGGTCCCACTCGTCCTCCTGTAATTATTGAACCTGTAGGCCCTACTGACCCTTCCATTG
TTACCTTGGTGGAGGAGTCAAGTGTGGTTACATCTGGTGCGCCTGTTCCAACATTTACTG
GCACATCTGGGTTTGAAATAACATCTTCTGGTACTACTACACCTGCTGTGTTAGACATAA
CACCTACTTCTGGGTCTGTTTCTATTTCTACAACCAATTTACCAATCCAGCATTTTCAG
ACCCTTCACTTATAGAGGTACCTCAAACCGGGGAGGTATCAGGTAATATATTTGTTGGTA
CACCAACGTCAGGAACACATGGATATGAAGAAATACCTTTGCAAACATTTGCATCTCATG
GGTCAGGTAATGAACCAATTAGTAGTACCCCATTGCCTAATATTAGGCGTGTGGCAGGTC
CCCGCCTTTATAGTAAGGCTAATCAACAGGTTCGTGTGTCTACTTCACAGTTTTTAACAC

ATCCCTCCTCTTTAATTACATATGACAACCCTGCCTTTAATCCTATTGACTCTACATTAA
CATTTGAACCTACTAGTAATGTTCCCGATTCTGATTTTATGGACATTATTCGTTTACATA
GACCTGCTTTAACATCCAGGCGTGGTACTGTTAGGTATAGTAGATTAGGTCAAAGGGCAA
CTATGTTTACTCGTAGTGGCAAACAAATAGGTGCTAGGGTACATTTTTATCATGACTTAA
GCCCTATCTCTGCTACAGAAAATATTGAAATGCAGCCTTTAATTTCTGCAACAGACGACA
GCAACTTATTTGATGTTTATGCTGACATTCCTACTACATCTGACACTAGTGTTACTGCTA
ACACAGCATTTACATACCCTAAATACTATTCTACTGTATCGTCAGTCTCTTCTTATGGTA
ACCTTACTGTTCCTTTAACTTCCTCATGGGATGTGCCTGTATATACAGGCCCAGATATTG
TATTACCTTCTAGTACTTCTGTATGGCCTTTTATAACACCTACATCTACGTCCACGACAC
ACTATATAGGTATAAATGGTACGCATTACTATTTATGGCCCTTATATTATTTTCTTCCTA
AAAAACGTAAACGTGTTCCCTATTTTTTTGCAGATGGCTTACTGGCGGCCTAGTGACAGT
ACGGTTTATCTGCCACCTCCTTCTGTGTCCAGAGTTGTCAACACTGATGATTATGTGTCT
CGCACAAGCATATTTTATCATGCTGGCAGTTCTAGATTATTAGCTGTAGGCAATCCTTAT
TTTAAAGTTTCTAAAGGTGCAGGTAGTAGGCAAGATGTTCCCAAAGTATCTGCCTACCAA
TATAGAGTGTTTAGGGTAACTTTGCCAGACCCTAATAAATTTGGGTTACCTGACTCTACT
ATTTACAATCCCGAAACACAACGTCTTGTGTGGGCCTGTGTTGGTGTTGAAATAGGCCGT
GGACAACCTTTAGGTATTGGCCTAAGTGGCCATCCTTTATATAACAAATTAGATGACACT
GAAAGTTCACATGCTGCTACAGCTGTTGTTACACAGGATGTTAGAGATAATGTATCAGTA
GATTATAAACAAACACAGTTATGTATACTAGGTTGTGTACCTGCTATTGGTGAACATTGG
GCTAAAGGTACACCATGTAAATCTACTCACATACAGCAAGGTGACTGTCCTCCTTTAGAG
CTTAAAAATACAGTGTTGGAGGATGGCGATATGGTAGATACAGGTTATGGTGCCATGGAC
TTTAGTACATTACAGGATACTAAATGTGAAGTGCCATTAGACATTTGTCAATCTATTTGT
AAATATCCTGATTATTTACAAATGTCTAATGATCCATATGGCGATTCTATGTTTTTTTGT
TTACGGCGTGAACAACTGTTTGCTAGGCATTTTTGGAACAGAGCTGGAACAATGGGTGAC
ACTATTCCACAGGAGTTATATATTAAAGGAACTGGTATGCGTGAAACTCCTGGCAGTTTT
GTATATTCTCCGTCTCCCAGTGGCTCTATTATTAACACAGAATCACAGTTGTTTAATAAG
CCATATTGGTTGCATAGGGCCCAGGGCCATAACAATGGTATTTGTTGGCATAATCAATTA
TTTGTTACTGTGGTGGACACAACACGCAGTACTAATTTAACACTGTGTGCTTCTACACAA
AATGGCGTAGCTACCACATATGACCCTACAAAATATAAACAGTATAGTAGACATGTGGAA
GAGTATGATTTACAGTTTATTTTTCAATTGTGTACTATTACTTTATCTGCAGATGTAATG
TCTTATATACATAGTATGAATAGCAGTATTTTGGAAAACTGGAATTTTGGTGTCCCACCG
CCACCCACTACCAGTTTAGTAGATACATATCGCTTTGTACAGTCTGTTGCTATAACCTGT
CAAAAGGATACTACTCCCGCTGAGAAAAAGGATCCTTATGATAACTTAAAATTTTGGAAT
GTAGATTTAAAAGAAAGGTTTTCATTAGATTTAGATCAATATCCTCTTGGACGCAAATTT
TTAGTTCAATCTGGATTACGTCGTAGGCCTACCATAGGACCCCGCAAACGTTCTGCCCCG
TCTGCCCCTACTACTTCCAAACCTGCTAAGCGTTTACGTGTCCGTCGTAAAAAATAGTAT
GTATGTATCTATGTTTATGTTTGTGTATGTATGTATGTGTGCATTTTAGCATGTATGTAT
GTTATTTGTATGTTGTTGTACATCCTTGTATGTGTTATGTGGTATTAAATAAAATATGTT
TGGTAGTTTCCTGTATGGTTGCACCCTGGTGAGTAACAAATAGTGTTTGTGTTCGTGGTA
TGAGTGTATTAATTTATTTACTTTTATATTTTACATTTTAAAGTACCATCCATTTTATAT
TACAATTCTCCATTTTAATGTGCAACCGTTTTCGGTTGCCCTTGGCTTACCTTTGTACAT
TCTGGCACTGTTGCAAACATTACCTTTGTAGATTCTGGCACTGTTGCAAACATTAATCTT
AAATACATTACTGTACTGGCACTATTGCAAATGTTAATCTCACATACAATAGTCATACAT
ATTTTCACCACTGGCACGTATTTTGGCGCACACTTTGGCGCACCTGGTATTAGTCATTTT
CCTGTCCAGGTGCACTACAACAATTACTTCCACATTTGCATTTACGCCCTCTTTAATAAA
AATGCTTTTAGGCACATAGTTTTGTTAGTTTGTACTTGTACTACTTGTATTATTGGCCTG
TGCAACTACTTTTACATCCAACAATCTGTCTACTCTTCTTATAAAGTATAAAATGACTCA
GTTATGTATACATAGGTGTAGCAACCGAAAAAGGTTGGGCAGTACAACCCATACTTTTCA
TTAATACTTTTAACAATAATGTTATATAAAAATGGGTGTAACCGAAAACGGTTGCAACCG
AAACTGGTGTATATAAAAGACCTGCAAAAATGTGCACTACAGG
>gi|22095322|gb|AF436128.1|_Human_papillomavirus_-_cand89,_complete_genome
ATGCCCGGACCGCCATATCCAAAAAACATATTTGTGTTGTGCAAAGACTGCGACGTGGAT
TTAGAGGATCTACGGCTTACATGCATATATTGCACCAAGGAGCTTACAACATCAGAAGTG
CTGTCCTTTGCACTAAAGGAACTGGATATAGTGTGGAAGTGGCAGCTGCCCCATGGTGTA
TGTGCACCGTGCTTAAGTAGAGCCGCCAAGACACGGGAGCTACGACACTGGAACTATTCA

AGCTACGGAACAACAGTGGAGCAGGAGACCAACACACCGTTGGCCCAGCTGTATATGAGA
TGCTATATATGCTGTAAGCCGTTAGGCAGCCAGGAAAAGGACTATATGGTGTATTTTAAC
GAGCGATTTCATAAAATATCTGGAGAGTGGACAGGAAAGTGTTGCAACTGCAGGGTAATA
TGCACGGCCGCACGCCGACCATCCAGGATATAGTTCTGGAAGAACGTCCAGAACCAATTA
GCCTACAATGTGAGGAAGAAATGCTAGACAGCTCAGATGAGGAAGATGCTAGGGATTGTA
TGCATGAGCAACAGCAACTGGCAGAACGAGCACAGCAGGCCTTTAGGGTGGTAACTACGT
GTGGTGTGTGCTATCGTGCAGTTAGGTTAGTAGTATTGTCTGACGACGCACATCTAAGAC
ATCTACAACAGCTGCTGCTGAATGACCTGTCCATAGTGTGTCCGGGCTGCGCGTAAGGAC
ATGGCAGACCACGAAGGTACTAACAGTGGGGACGAAAGCGATGCAGCAACACGGGCGGGC
GGGTGGTTTCTGGTAGAGGCCATAGTGGAGAACACAACAGGGGACCAGGTGTCCAGTGAT
GAGGATGAGGATGACACCTGTGTAGATACTGGGGAAGATCTTGTAGACTTTATAGTAAAT
GAAAGGCACCCGGGGGATGGGCAGGAAGTGCCGTTAGCACTGTATGTGCACCAAAATGCA
CAGGATGACGCTGCAGCAGTGCAGGCACTAAAACGAAAGTTTACCTATAGCCCGGCATCC
AGTACCGGCGTGTCATGGGTAGATAAGGAATTAAGTCCCCGGTTGGATGCCATAAACCTA
AACAGGGGCCACGAAAAAGCCAGGCGACGGTTGTTTGAACGGGACAGTGGGTATGGCAAT
ACGCAGGTGGATACGGGAGCATCCGAAAGTGAGGTATTGGGGGACACAGACAACACCGGG
GGGGAGGGCGCTGCGCAGGACGCAGGGGAGGAGGAACGCGGGGGGGGGGATGGGGAATCA
CAGCCGTCTGCAAATTTACAACAGACGCAGGAGGGAACGCAAGGTGTGCTGGAGCTGCTT
AAGGCGAGCAACATAAGGGCCACCATACTGAGTAAGTTTAAGGAACTATTTGGACTATCC
TACTATGACCTTGTAAGGCAATTTAAAAGTGATAAGTCTACATGTGGGGACTGGGTTGTG
TGTGGCTTTGGGGTGTACCATGCAGTGGCGGAAGCTGTAAAAACATTGCTACAGCCACAC
TGTGTGTATGCACACATACAGCTGCAAACATGTAATTGGGGCATGGTTGTGCTTGCATTA
GTAAGGTTTAAATGTGGCAAAAACAGGGAGTCAGTGGCCCATTGCATGGCAACACTGTTA
AATATACCTGACAGGCACATGTTAATAGAACCACCAAAAATACGTAGCGGGCCATGTGCA
CTATATTGGTATAGAACAGCAATGGGAAATGCAAGCGAAGTCTTTGGGGAAACACCAGAA
TGGATAGTAAGGCAGACAGTAATAGGGGAGGCAATGAAGGAAACCCAGTTTAGTCTATCA
ACATTAGTACAATGGGCCTATGACAATGATATTACAGATGAAAGTCAGCTAGCATATGAG
TATGCACTATTAGGAAACGAGGACCCAAATGCAGCAGCCTTTTTAGCTAGTAATTGCCAA
GCTAAATATATTAAAGATGCTATAACAATGTGTAAGCATTACAAACGGGCAGAACAGGCA
CGCATGACAATGGCACAATGGATTAAATATAGAGGCAGCAAAGTACCAGACACAGGCGAC
TGGAGACATATAGTAAAATTTCTAAGATACCAAAATGTGGAATTTATTACATTTATGAGT
GCATTTAAGCACTTCCTAAAGGGCACACCAAAGAAAAGTTGTATGGTGTTCTATGGGCCA
AGTGACACAGGGAAATCCCTGTTTTGCATGAGTTTATTGCATTACCTTGGGGGAGCAGTA
ATTTCATTTGTAAATTCCAGCAGCCATTTTTGGCTGTCCCCGTTGGTGGATGCCAAAGTA
GGATTGCTAGACGATGCCACAATGCAATGCTGGACATACATAGATGTATATTTGCGGAGC
ATATTGGATGGTAACGCAATAAGTATAGATAGAAAACATAGAAACCTAACACAACTAAAA
TGTCCACCCTTAATGATAACCACCAATGTGGACCCCTTAGCAGATGATACATTAAAATAT
TTAAAAAGTAGAATAGTAGTGTTTAGATTTCTAAACAAATGTCCAATGAACGCAAACGGG
GAGCCAGTATATACATTAAATAATGAAACATGGAAATCATTTTTTCAAAGGTCTTGGGCA
CGTTTAGACTTACAACCTGAGGAGGAGGAGGAGGAGGAGGAGGAAAATGGCATCGCTAGC
CGACCGTTTAGATGCGTGCCAGGAGAGGTTAATAGACCTTTATGAAAAGGATAGCAACAA
GCTACAGGACCAATTATCACATTGGCAGTACGTGCGTCTGGAATATGCTATTTTATATAA
AGCACGCCAAGCAGGACTAACAAGTGTAGGCCACCAGGTGGTGCCACCACTTAATGTAAC
CAAAGAAAAGGCCAGTCAGGCCATTACCATACATTTGTCATTGCAAAGTTTAAATGCCAG
TGCATATAAAGATGAACCCTGGACACTGCAGCATACATCACTGGACATGTGGAACGCCCC
CCCCAAGGGGTGCTGGAAGAAAAAAGGACATACAGTAACTGTTAAATACGATGGAGAGGA
CCAAAAGGCCATGGAATATGTTTCTTGGGGTGAAATATACATACAATGTCCAGAAACAGA
CATGTGGTACAAGGTGGCCGGACATGTGAATGCCACAGGGCTATATTACGAGGTGGAGGG
CTGTAGACACTACTATGTGAACTTTACAAAGGAGGCAAAGCACTATGGGGATACAGGCGC
GTGGGAGGTGCATTGGGGAAGCACTGTTATTTACCATGCATGCGCATCTGTATCTAGTAC
CCAGGACCTTGTGCGAGAAGTACCCTCTGCTGCAGTTGGTGACCGGCTACACCACCCCAC
CACACCCACTTCCTGCACCGACGGTGTGGGCACCACCGAGACAGCCCCCACGGTGCAGAC
GCCGCCTTATAAGCGACAGCGACTCCACAGAGACGGAGTGCAGCAGCCCACCACAACAGC
ACAAAAGCACGTGGACAGTAGCCACAGGGGGCTCCACAGTAACCCTGACGGCACAGAACG
ACTCGGGATCGCGCATTGTTGTAACACTGCACCTGTAATACACCTACAAGGTGAAGCCAA

CAAACTAAAATGTTTTAGATATAGATTAAAGCAAACAGTACCTGACCTATACCTACGTGC
GTCCTCCACATGGCATTGGGCCTGTGGGGGGCCCACCACTAAGGCTACCTTTGTGACACT
GTGGTATTCTAGTGCTGAACAACGGGCACAATTTCTTGCACGTGTGCATATACCAAAGGG
TATACAGGCTATGCAAGGTCATATGTCCATGTTTATGTAACCACCTTACATGTAATATTT
GGTACAGTGTATTGTGTATATACAATGTAAATAATGTTTGTACTGCATAGCTGAGCCTAC
CATAGTCCTGTGCCACATATATTGTACATTGCAATAGTAACCCTGGATATTATACTACGT
CTTATATGTTTGTGGAGTGATATACTTGTGCTGCTTATTTGCTTTTGGCTTTCGCATTTG
CCTGCATTAACAGCTTTCCTTGTGTTGTTGGGTATTGTGTATGTAGGGTTACTACTTCTT
TATGTGCAAGTATTGTGGTATATAGGACTATAACCACACACCAGCCAATACTGCTGCTAC
CTGTATATACATCCCATTGCTTTATACACATGTTCCCCGTAGAGGTACGCGGTCGGGGGG
GGGGGTACGACATGGTGGTATTTGACAGACATGATGTTGGGTTACTAGGCATATTGTTGT
TACTTATTGCTCTTGTATTAATTTTGTTATACATTCGTTTGCTACTACATTATTGAACAT
GCTTTTATAGTGCATTCCCATACCTGTATTTGGTACACTGCTATAGGATTGTGTTTTATA
TTTTTATATATACAATAAATACATTGTTGCAATGACCCACCCTGTGCGGCGTCGTAAACG
GGCTTCGGTCACAGACCTGTATCGCACATGTAAGGCCACAGGCACATGTCCCTCTGATGT
TATACCTAAGGTAGAGGGCAATACATTGGCTGACAAAATATTGAAATGGGCCAGTCTTGG
TGTGTTTTTTGGTGGCCTAGGCATTGGCACGTCCTCTGGCACTGGAGGCCGTACTGGCTA
TGTGCCCTTGGGTACCCGCCCTCCTACTGTTGTGGATGTAGGCCCTACTGCACGCCCGCC
TGTGGTAATTGAACCTGTAGGTGCAGGCGAGCCCTCCATTGTTAACCTTGTGGAGGACTC
TAGCATTGTTGAGTCAGGGGCTGTCTTGCCCAATTTTACAGGCACCGATGGCTTTGAGAT
TACCACCTCCTCCACTACCACCCCTGCCGTGTTGGACATCACCCCCTCAGGTGCTACTGT
ACAAGTTAGTAGCACCAGTTATAGCAATCCTGCATTTACCGAGCCCTCCATTATTGAGCC
CCCGCAGCATGGCAGTGTTTCTGGCCATGTGTTTACTAGTACTCCCACCTCGGGGTCCCA
CACATTTGAGGAAATACCTATGAGCACATTTGCCACCTCTGGGGACGTGGACGCTGGTCC
TATAAGCAGTACCCCAGTACCTGGCGTACGTAGGATTGCAGGGCCTCGCCTTAACCTATA
TAGTAAGGCCACTCAACAGGTTCCTGTATCTGATTCTGCCTTTGTGTCCCGTCCTGCATC
ATTTGTCACATATGATAATCCTGTGTATGATCCTGAGGAAACTATTATATTTGAACATCC
TGATTTACATGCACCTCCAGATCCTGATTTTCTGGATGTTGTGGCCTTACATCGCCCTGC
CCTTACGTCTCGCAAAGGCACTGTACGTTTTAGTCGTGTAGGACAGCGTGCTACCTTAAA
AACACGTAGTGGCAAACAAATAGGGGCCCGGGTACACTTTTACCATGATGTAAGTCCCAT
TTCGGCTGCAGAATCCATTGAGATGCAGCCATTATTACCTTCCCCTGACCTTACACAGCC
TGTGTATGATATATATGCTGACACTACACATTTGGACTCTGTGTTGGCAGAACGCCCCTT
TCCCGCCTATTCCTCTGCCAATACCACCTTTTCTGGCCCGACCGTTACTGCTACATCTGC
TGTATCGTCTCAATACACAAATGTTACTGTTCCTTTGTCTCAGGGGTTTGACGTTCCTGC
TGTTACAGGGCCTGACATAGCATTTCCCACTGCCCCTAGTGTCCCCCCCATATTCCCCTC
ATATACCCTTGGCCCCTCCACAGGCTATATATATTCACGGTACGGATTTTTATTTAGTAC
CCAATTATGTGTTTTTTCCTAAAAGGCGTAAACGTGTCCCCTATTCTTTTGCAGATGGCT
TTGTGGCGGCCTGGTGACGGCAAGGTATACCTGCCTCCCACTCCTGTGTCCAAAGTTTTA
AGTACGGACCGCTATGTCCAGCGTACCAACCTGTACTATTATGGAGGCAGCTCCCGCCTT
ATTACAGTTGGCCACCCTTATTATACTGTACAGGTCAATGGTGCTAACAAAAAGGCCAAC
ATACCTAAGGTATCAGGGTATCAATACAGGGTATTTAGGGTACGGTTGCCTGATCCAAAT
AAGTTTGGCCTTCCGGACGCTTCCTTATATAATCCTGACACTGAGCGGCTGGTGTGGGCC
TGCAGGGGCATTGAGGTGGGCCGGGGTCAGCCCTTGGGGGTTGGTACAAGTGGCCACCCG
TTATACAACCGTTTGGATGACACTGAAAATGCTTCCTTATTGGTTGCTGCAGATGTTGAC
AGTAGGGATAATATATCTGTTGACTATAAACAAACACAGCTTCTTATCATTGGCTGTAAG
CCTCCTATTGGGGAGCACTGGGCCAAGGGTACTGTGTGCTCTAGTGTTGTGCCTCAGCAG
GGGGATTGCCCACCTTTGCAGTTGCTAAATACTACTATTGAGGATGGCGATATGGTGGAA
ACAGGCTATGGAGCCATGGACTTTTCCAAACTGCAGGAATCTAAATCGGAGGTGCCTATT
GATATTTGTGACTCCACCTGCAAGTATCCCGATTATTTGCAAATGGCTGCAGAGCCATAT
GGGGATTGTATGTTTTTTTGTTTGCGGCGGGAGCAAATGTTTGCTCGCCACTTTTTTAAT
AGGCAGGGTGTTATGGGTGAGGACCTGCCAAAAACCTTGTATATGCCTGGGTCTGCTGCT
AATAACAGAAATACACTTACTAGCTATGTATATGCCCCAACGCCCAGTGGGTCTATGGTT
ACATCTGATTCCCAGTTATTTAATAAGCCTTATTGGCTGCACCGTGCACAGGGCCATAAT
AATGGTATTTGTTGGTTTAATGAGTTGTTTGTTACTGTGGTGGATACCACCCGTAGTACC
AACCTTACCATTTGTGCTGCTTCCCAGTCTGGCACAGAATACAGTTCTACACGCTTTAAG

GAATATTTAAGACACACTGAGGAATATGACCTACAGTTTATATTCCAACTATGTAAGATA
CACCTAACGCCTGAGATAATGTCCTATTTACACAATATGAATGACACATTGTTAGATGAA
TGGAACTTTGGTGTCATTCCCCCTCCCTCCACTAGTTTGGATGATACCTATCGCTTTCTT
ACCTCTCGGGCCATTACATGTCAAAAGGGCACTGCTGCCCCAGAACCTAAAAAGGATCCA
TATGATAAGTTATCCTTTTGGGATGTGGATCTTAAGGAACGTTTGTCCACTGATCTAGAC
CAGTTCCCTTTGGGTCGCAAGTTTTTATTACAATTGGGCGGCCGCCCCTCGTCTGTACCT
CGCAAGCGTGCTGCCCCCGTGTCTACCTCCAAAGCCCCCAAACGCAAAAAGGCCAAACGG
TAATTTATACTCTGTGTGTTGTGTGTGCTGTATTTGGTTTTGTATATTATGTTATGTGGT
GCCCTCTGTGTGTGTATGTATTGTATAACTTATGTATGTATCCCTACTGTTGTGTAGTGT
GTGTCCTCTATGTACCCCTGTGTAGTGTGTGTCCTGTATGTAACCCCTATGTACTGTGTG
TCCTGTATGTAACCCCTATTGCACACATGTGTGTGTATGTATTTATGGAATGTGTGTGTT
GGTTTGTGCATTAATAAACTATGTGTCACTGTGGCCCTGTGTCCAGCTGCGCCCTATGAG
ATATGTATTCGGCCATTTCCTTTCCCACCCAGCGTCCATTTTGAGTTCAGCGACCGGTTT
CGGTCTCCCGCGTTTTTGTTTTTGTTATGGCGCCATGCCACTTTACACAAAGACTTGTAA
TCTTTTATACTTCCTGCGTAGGCGGCTACATGTTCCACACCTGGGAGTGCCTGACATTTA
TTTGGCTACAAATACATTGTGCTGTGCACAATTTATGTACTTTCATTACTCATTTTGCAA
TAATACTGCTGTTAGGCACATAGTTTTGCTGACTACTTTCCCCTAATTACACTTTTGGCT
TGAAAACCTGCCCACGCATTCAACTATGTGTCTTGTAACCCTTGTTTTATTGCTTTTAAA
CATGTTTCATGCCATACCCATACCGGTCGCTCCCTATGTGTCATTCTTTACTTATTATTA
TATATTACAATCATAAGACTTAAAAATAAGGAGGGACCGAAATCGGTGCACCGAGATAGG
TACATATAAAAAGGATTTGGGCAGGAAGCACAGTAGCA
>gi|85827580|gb|DQ344807.1|_Human_papillomavirus_type_13,_complete_genome
GTTTCTAACAATCTTAAGTTTAAAAAATAGGTGGGACCGAAAACGGTTTTAACCGAAAAC
GGTGATATATAAACCAGCCCCAAAATTGAGCAAGCGGGGCATAATGGAAAGTGCAAATGC
CTCCACGCCTGCAAAAACGATAGACCAGTTGTGCAAGGAGTGCAACCTTTCTATGCACAG
CTTGCAAATTCTATGCGTGTTCTGCAGGAAAACCCTGTCCACGGCAGAGGTTTATTCATT
TCAGTATAAGAGTTTATATATAGTGTGGCGAGGATCGTTTCCATTTGCGGCTTGTGCATG
CTGCTTAGAAATACAAGGAAAGATTAACCAGTTTAGGCATTTTGACTTCGCGGGATTTGC
TGTAACAGTTGAAGAAGACACAAAGCAGTCAATTTTGGATGTGCTAATTCGCTGCTATTT
ATGCCACAAACCATTGTGTGAAGTGGAGAAACTAAGACATATTTTGCAGAAGGCACGATT
TATTAAATTAAACAGCAGTTGGAAAGGCCGCTGTTTTCATTGCTGGTCATCATGCATGGA
CAATATACTACCTTAAAAGACATTGTTTTAGACCTGACTCCTGACCCTGTAGGTCTACAT
TGCAATGAGCAATTAGACAGCTCAGAAGACGAGGTGGACGAACAAGCCACGCAAGCCACG
CAACATAGCACACTATTACAATGCTACCAAATACTAACGTCCTGTAGTAAATGTTGTAGC
AACGTCCGGCTGGTGGTGGAGTGTACAGGACCTGACATTCACGACCTACACGACCTACTG
CTGGGCACGCTGAATATAGTGTGCCCTTTGTGTGCACCAAAAAGCTAACTACGATGGCAG
AGGATACAGGTACTGATAATGAGGGGACGGATGCTCAGGATGGTTTTTAGTAGAGGCTG
TAGTAGAACAAACAACTGGGCAACAAATATCAGATGATGAGGATGAAACAGTGGAAGATA
GTGGGTTGGATATGGTGGATTTTATAGATGACAGACCTATTACACACAATTCCTTGGAAG
CACAGGCATTGTTAAACGAGCAGGAGGCGGATGCTCATTATGCGGCTGTGCAGGACCTAA
AACGAAAGTATTTAGGCAGTCCATATGTTAGTCCCCTAGGACATATTGAACAGTCAGTGG
ACTGTGATATAAGTCCTCGATTGGACGCTATAAAATTAAGTAGAAATTCTAAAAAAGTAA
AGCGACGGCTGTTTCAATCAAGGGAAATAACGGACAGTGGATATGGCTATTCTGAAGTGG
AAGCTGGAACGCAGGTAGAGAGAAATGGCGAACCGGAAAATGATTGTGGGGGTGATGGAC
ACGGAAGGGACAAAGAGGGGGAGGGACAGGTGCACACGGAAGTGCACACAGGCAGCCAGA
TAGAAGAGCACACAGGGACCACGCGGGTGTTAGAACTACTTAAATGTAAGGATGTAAGGG
CTACATTGTATGGTAAGTTTAAAGACTGTTATGGGTTATCATTTACAGATTTAAGTAGAC
AATTTAAAAGTGATAAATCAACATGTGGGGACTGGGTGGTTGCGGCATTTGGTATACATC
ATAGTGTATCAGAGGCATTTGAAAAGTTAATGCAGCCATTAACAACATATATGCATATAC
AATGGCTTACAAATGCATGGGGGATGGTATTGTTAGTATTAATAAGATTTAAAGTAAATA
AAAGTAGATGCACAGTGGCGCGAACACTGGCAACCTTTCTTAATATTCCTGAGGACCACA
TGTTAATTGAACCTCCCAAAATACAAAGCAGTGTGGCAGCATTATACTGGTTTAGAACAG
GTATTTCTAATGCTAGTATAGTAACTGGTGAAACACCAGAATGGATAAAAAGGCAAACAA
TTGTAGAGCATGGACTTGCAGATAATCAATTTAAATTAACTGAAATGGTGCAGTGGGCAT
ATGATAATGATTTTTGTGATGAAAGCGAAATAGCATTTGAATATGCACAACGAGGAGATT

TTGATTCAAATGCCAGGGCATTTTTAAATAGTAATTGTCAGGCAAAATATGTAAAAGATT
GTGCAACAATGTGCAAGCATTATAAAAATGCAGAAATGAAAAAAATGTCTATGAAACAAT
GGATAACATATAGAAGTAAAAAAATAGAGGAAGCAGGAAATTGGAAACCAATAGTACAAT
TTTTAAGGCATCAAAATATAGAATTTATTCCATTTTTAAGTAAATTAAAATTGTGGCTTC
ATGGCACGCCAAAGAAAAACTGTATTGCAATAGTGGGGCCACCAGATACAGGCAAATCAT
GTTTTTGCATGAGCTTAATTAAGTTTTTAGGGGGCACAGTAATTAGTTATGTAAATTCAA
GTAGCCATTTTTGGCTGCAGCCATTATGTAATGCAAAGGTAGCTTTGCTAGATGATGCAA
CGCAGTCATGCTGGGTATATATGGACACATACATGAGAAATTTATTAGATGGCAATCCAA
TGAGCATTGATAGAAAACATAAGTCTTTAGCATTAATAAAATGTCCGCCATTATTAGTAA
CATCTAATGTAGACATTACCAAAGATGACAAATATAAATATTTGTATAGTAGAGTAACAA
CACTTACATTTCCAAATCCATTCCCTTTTGACAGAAATGGGAATGCAGTATATGAGTTGT
CTGATGCAAACTGGAAATGTTTTTTTACAAGATTGTCAGCAAGCCTAGATATACAGGACT
CTGAGGACGAGGACGATGGAGACAATAGCCAAGCATTTAGATGCGTGCCAGGAACAAGGG
CGAATTCGTTTAAACATGTATGAAGAAAATAGTAATGAACTTAAAAAACATATACAACAT
TGGAAATGCTTAAGGTACGAAAGTGTACTTTTACACAAAGCACGCCAAATGGGCCTAAGC
CACATTGGATTACAAGTGGTGCCACCATTGACAGTATCACAAGCTAAGGGACACGAGGCA
ATTGAAATGCAAATGACTTTAGAGACATTACTAGAGTCTGAGTTTGGTATGGAACCATGG
ACCTTACAAGATACAAGTCGTGAAATGTGGCTAACACCCCCAAAACGCTGTTTTAAGAAA
CAGGGACAAACTGTGGAAGTAAAATATGACTGTAATACAGACAATAGAATGGATTATGTG
TCGTGGACATACATATATGTGTTTGACACAGATAAATGGACAAAGGTGAAAGGAATGGTA
GATTATAAAGGGTTGTACTACATACATGGAAATTTGAAAACATATTATATAGAGTTTGAA
AAGGAGGCTAAAAAATATGGGGAAACGTTACAATGGGAAGTATGTATTGGCAGCACAGTC
ATATGTTCTCCTGCATCTGTATCTAGTACTGTACAAGAAGTATCCATTGCTGGGCCTGCT
TCATACTCCACCACCACCTCCACACAGGCCTCCACCGCAGTGTCCTGCAGCGCCCCGGAA
GAATGTGTGCAAGCGCCGCCTTGTAAACGACAACGAGGACCTTCACGTCCCATTGGAAAC
CCCCAGAACACACAAAGCATTGTGTGTGTCACAGACGACGACACCGTGGACAGTGCAAAC
AACAACATCAACGTTAACCATTACAACAATAACAAAGGACGGGACAACAGTTACTGTGCA
GCTACGCCTATAGTTCAATTACAAGGTGACTCTAATTGTCTAAAGTGTTTTCGATATAGA
TTACATGAGAAATATAAAAATTTATTTTTGTTAGCATCATCTACATGGCATTGGACCGCC
CCTAATAATTCACAAAAACATGCACTGGTAACCTTAACCTATGTAAATGAACAACAAAGA
CAAGACTTTTTAAACACTGTAAAAATACCTGCAACCATAACACATAAACTAGGTTTTATG
TCATTGCAATTGTTGTAACAGCATATATTGTATGTAAATATTTGTTGTGTGTGTGTATAT
ATTGTACATGGAATTTATACCTGTGGATGTTACTACACAGGAAACCAGCAAGTCATTACT
GCCACTTGTAATTGCACTTACAGTGTGTGTAGTTAGCATTATAACAATATTGTGGATATC
AGAGTTTTTGGTGTACACAAACGTTTTAGTACTAACATTAATTTTATATATACTTTTGTG
GCTTTTACTAACAACTCCCTTGCAATTCTATTTACTAACCCTGTCTCTTTGCTTTCTTCC
TGCGTTGTGTGTACACCAATATATTTTACAAACACAAGAATAATTATACACAATGTTAAC
CTGTACTTTTGATGATGGTGACACATGGTTGCTATTATGGTTAATTTTATCATTTATTGT
AGCCATTCTAGGGTTACTGTTGCTGTATATAAGAACTGGACATATGCATTGCCAGTGCTG
GAGTAAATAAGTGGTTTTATATTTTGTGTGTATTCATTTAATTATGGCACATAGTAGGGC
TCGCAGACGCAAACGCGCTTCAGCTACACAACTATATCAAACTTGTAAGGCTTCTGGAAC
ATGTCCTCCAGATGTTATACCAAAGGTTGAACAAAACACTCTTGCAGATAAAATATTAAA
GTGGGGCAGTTTAGGAGTATTTTTTGGGGGGCTTGGCATTGGCACAGGCTCTGGTACTGG
CGGTAGGACTGGCTATGTACCAGTAGGATCCACCCCACGCCCTGCCATATCATCTGGGCC
TACTGCACGTCCTCCTATTGTTGTTGATACTGTTGGGCCTACAGACCCTTCTATTGTATC
TTTGGTAGAGGAATCAGCTATTATTAATTCTGGAGTGCCTGACCCTTTGCCTCCGGTTCA
TGGGGGTTTTGAAATTACCACATCTCAATCAGCCACTCCAGCAATATTGGATGTGTCTGT
TACAACACAAAACACTACGTCCACAAGTATATTTAGAAATCCTGTTTTTTCAGAACCTTC
TATTACACAATCTCAACCTTCTATTGAAAGTGGTGCACACGTGTTTATATCGCCATCTAC
TATTTCCCCTCATTCTACAGAAGACATTCCTTTAGATACATTTATTGTATCTTCCTCAGA
TAGTAATCCTGCATCAAGCACCCCTGTTCCAGCAACTGTTGCACGTCCACGTCTAGGCCT
TTACAGTAGGGCCTTACATCAAGTACAGGTTACTGATCCTGCCTTTTTTTCGTCGCCCCA
ACGCCTTATAACCTTTGATAACCCAACATATGAAGGTGAAGATATAAGTTTGCAGTTTGC
CCACAATACCATTCATGAACCCCCTGATGAGGCATTTATGGATATTATAAGACTACATAG
GCCAGCCATAACATCACGGCGTGGTCTTGTTAGGTTTAGTCGAATTGGTCAGAGGGGGTC

TATGTATACTCGAAGCGGCAAGCATATAGGTGGAAGGGTCCATTTCTTTAAGGATATTTC
TCCTATATCTGCAGCTGCAGAACAAATAGAATTACACCCCCTTGTGGCTGCTGCACAGGA
TCACAGTGGTTTGTTTGATATTTATGCAGAACCTGACCCTGACCCTGTGGCTGTAAACAC
CTCTGGGTCATTGTCTTCTGCCTCCACACCATTTGCGCAATCTTCTTTGTCTTCCGCCCC
ATGGGGTAATACTACTGTTCCTCTTTCACTACCAGGTGATATATTTATACAGCCTGGTCC
TGACATAACATTCCCAACTGCACCTACAGTAACACCTTATAATCCTGTTACGCCTATTTT
ACCTACAGGTCCTGTTTTTATTACTGCTTCTGGATTTTATTTATATCCTACATGGTATTT
TACACGCAAACGCCGTAAACGTGTTTCCTTGTTTTTTACAGATGTGGCGGCCTAGTGACA
ACAAACTATATGTGCCTCCTCCCGCCCCTGTATCAAAAGTAATTACTACGGATGCCTATG
TTACACGTACCAACATATTTTATCATGCTAGCAGTTCTAGACTACTTGCAGTGGGAAATC
CTTATTTTCCTATTAAGAAACAAAACAAAACTGTTGTCCCTAAGGTATCTGGTTATCAGT
TTAGGGTATTTAAAGTTGTATTACCTGACCCTAATAAATTTGCCCTGCCTGACACATCTA
TATTTGACTCAACTAGTCAACGCTTAGTGTGGGCCTGCACAGGTTTAGAGGTTGGTAGGG
GTCAACCCTTAGGTGTTGGTATTAGTGGTCATCCATTATTAAATAAATATGATGATGTGG
AAAATTCTGCAAGTTATGCTGCCAATCCTGGCCAGGATAATAGGGTTAATGTGGCTATGG
ACTATAAACAAACACAGTTATGTTTAGTGGGCTGTGCACCTCCTTTAGGTGAACATTGGG
GACAGGGCAAGCAATGTACTGGTGTAAATGTACAACCTGGAGATTGCCCTCCTTTAGAAT
TAATTAGTAGTGTAATTCAGGATGGTGACATGGTAGATACAGGATTTGGAGCCATGAATT
TTGAGGAATTGCAATCTAATAAATCTGATGTGCCACTAGACATATGCACGTCCACATGCA
AATATCCTGACTATTTACAAATGGCTGCGGATCCTTATGGAGACAGATTATTTTTTTATT
TGCGAAAGGAACAAATGTTTGCAAGGCATTTCTTTAACAGGGCAGGCTCTGTTGGTGAAC
TAATCCCAGCAGAATTATATGTTAAGGGTAGTAATACACTTTCTAATAGTATTTACTATA
ATACTCCCAGTGGCTCTCTTGTGTCTTCCGAGGCCCAGTTGTTTAATAAACCTTATTGGT
TACAAAAGGCCCAGGGACACAATAATGGTATATGTTGGGGCAATCACTTGTTTGTTACTG
TAGTTGATACTACACGCAGTACTAACATGACTGTGTGTGCAGCCACTACATCATCTCTTT
CAGACACATATAAGGCCACAGAATATAAACAGTACATGCGACATGTAGAAGAATTTGATT
TACAATTTATTTTTCAATTGTGCACTATTAAATTAACTGCAGAGGTTATGTCATATATTC
ATACTATGAATCCTACAATTCTAGAAGACTGGAACTTTGGGCTATCTCCCCCTCCTAATG
GAACATTGGAAGACACATATAGATATGTACAATCTCAGGCCATAACGTGTCAAAAGCCTA
CACCTGATAAAGAAAAACAGGATCCGTATGCGGGTCTTAGTTTTTGGGAGGTTAATCTTA
AGGAAAAGTTTTCTAGTGAACTAGATCAGTATCCCCTTGGCAGAAAGTTTTTATTACAAA
CAGGCGTTCAGTCTAGGTCACCTATTCGTGTAGGTAGAAAACGTGCTGCATCTACATCTA
CTGCCACACCTACTACACGTAAAAAAGCTAAAAGGAAATAATAGTTTGTTTATGATTGTG
TATGTATGTCACGTTTGTTTGTACTGTATATATGTTGTGTACTGTATGTGTAATGTTGTA
TGTATGTGCATGTTACTTATTAAAGAATGTGTGTGTGTGTTTGTATGCAATAAATTTAAT
CTGTGGTGTCCTGTTCCACCCTATGAGTAAGTGGTATGTTGTGTCTCGTGTGTTGTTTTG
TATACTATACTATAACAGTAGTGCAACCATTTTGTAACTTTTCTTACATTTTACGTCTCC
ATATTAAGTGCAACCGATTTCGGTTGCTATTGTTTCTGCAACCGATTTGTTGCAGCACGC
TGTTTATATAATCTTACCTACCGCCTGCCAAAATTATCCACCGCTTGCCAAAATCACCCA
CACACCTGGCGTTGCTAGGGCGCGGTTATATATGTTTACTAAATCTTACTAATCTTTCTA
TCACTCATTTTACCTTTATAACAATACTTTTGCTTTTCAAGTACATTTTTCTACTTACTA
GCCAATGCCTGAAAGGTTTTTTGGCTACCAGCACTACATTTTTGTACAGTTAATGTTACA
TGTATAAAATGAGTAACCTAAGGTCACACACCTGCAAACCGGTATCGGTTGAAACACACC
CTCTATAGTTCCTTATTATA
>gi|71726718|gb|DQ080083.1|_Human_papillomavirus_type_102,_complete_genome
ATGTCAAGTGCGCGCTACCCCACCAACATTTTTGTGCTGTGCAAAGAATGTGAAGTGGAT
TTGGAGGACTTACGACTGCTTTGCATTTATTGCACAACTGAACTGACCGCAGCAGAAGTA
TTGTCCTTTGCAATTAAGGAGCTGTGTATTAAGTGGGACCACGAACTGCCCTACGGAGCC
TGTGCACAGTGTTTGTGCAGAGCAGCTAAAGTGCGGGAACTACGGCACTGGAACTACTCC
AGCTACGGCGCAACGGTTGAAGAAGAAACCCGCACAGCATTACCACAGCTGTATATGAGA
TGCTATATGTGCCTGAAGCCGTTGTGTAGTCACGAAAAGGAATATTTGGTGCAAACAGGC
GATAGGTTTCATAAAATAGCAGGGGAGTGGACAGGAAAGTGCTGCAACTGTAGGGTATCA
TGCACGGCCACGCAGCAACGATAGCAGATATAGTTTTAGAAGAATTACCAGATGTAATTG
ACCTGTATTGTAATGAGCAAGCAATAGACAGCTCAGAGGAGGAGGACGAGAGGGATTGTG
TGCGTGACCAACTGGCAGAACAAGCAAAGCAGGCCTATAGGGTGGTAACAAAGTGTGGTA

TGTGTGGCCAGTCACTGAGACTGGCAGTGCTGTGCGGAGACACAGACATAAGACGTCTAC
AGGAGCTTCTGGTGAATGCGGTGGACATAGTGTGTCCCAGCTGCGCATAAGGACATGGCA
CATGCAGAAGGTACAGATAATGGGGAATTTAATACTACAACAAACGGGGCAGGGGGGTGG
TTTGTGGTGGAAGCCATAGTTGACAGAACAACAGGGGACCAGGTGTCCAGTGATGAGGAT
GAGGAGGGAACAGATAATGGGGAAGACCTTGTAGATTTCATAGAACGATATCCAGGGGAT
GGGCAGGAAGTTCCGTTAGAGCTGTTTGTACAACAAACTGCAAATGATGACGCTGCTGCA
GTGCAAGCCCTAAAACGAAAGTTTGTAGGTAGTCCCGCATCAAGCTCCTGTGCGACATGG
GTGGACAGTGAACTAAGTCCTAGGTTAGACGCCATTAAAATAGATAGGAGACAGGAAAAA
GCTAGACGAAGGTTGTTTGACCAAGACAGTGGGTATGGCAATACGCAGGTGGAAGCTGGA
ACATTGGAAAGCCAGGTACCAGGGGATAATGGCGTCGCGGGGGGGCAGGTGGCGGCACAG
GAAATGGGGGAGGAGAACAGGGAGGGGGGGGACGGCGAATGTGAACCGTCTGTGCAGGTA
CAGCAACAGCAAACGCAGGACAAAGCGCCAGAGCTGCTGGAGCTGTTTAAAGGCAGTAAT
GTTAGAGCCGCCATATTAAGTAAGTTTAAAGATTTATTTGGGCTATCATTTTATGATTTG
GTAAGGCAGTTTAAAAGTGACAAATCCATATGTGGGGATTGGGTGGTGTGCGCCTTTGGG
GTGTACTATGCAGTAGCAGAAGCAGTAAAAACATTAATACAGCCGCAGTGTATATATGCA
CACATACAAATACAAACCAGCCAATGGGGAATGGTTGTGTTGCTACTGGTAAGATTTAAA
TGTGGCAAAAGTAGAGAAACAGTGGCAAAATATATGGGCACGGTACTAAATGTACCGGAA
AAGCATATGCTAATTGAACCACCAAAAATACGTAGTGGTCCATGTGCCCTATATTGGTAT
AGGACAGCCATGGGCAATGCATGTGAAGTGATTGGGGAAACACCGGAATGGATAGTACGA
CAAACAGTGGTAGGGCATGCCATGGGAGAAACTCAATTTAGTTTGTCAGTGCTTGTGCAG
TGGGCATATGATAATGATATACAGGATGAAAGTGATTTAGCATATGAATATGCAAAATTA
GGTAATGAAGATGCTAATGCAGCTGCATTTTTAGCCAGCAATTGCCAAGCAAAGTACATA
AAAGATGCAATGACAATGTGCAGGCTATATAGACGTGCAGAACAGGCCAGAATGAGCATG
GCACAGTGGATAGTGCACAGAGGACGCAAGGTGGCAGACACGGGGGATTGGAAACATATT
GTAAAATTCTTAAGATACCAAAGAGTAGAATTTATTACATTTATTAGTGCATTTAAGTTG
TTTCTAAAAGGGATACCAAAAAAAAGCTGCTTAGTATTTTATGGACCTAGTGATACAGGA
AAATCATTGTTTTGCATGAGTTTATTGCAATACCTAGGGGGCGCAGTAATATCTTTTGTA
AATTCAAGTAGCCATTTCTGGTTGTCCCCGTTAGCAGATGCAAAAATAGGCCTGTTAGAT
GATGCAACAGGACAATGCTGGACCTATATTGATGTATACCTGCGTAGCATATTAGATGGA
AATCCCATAAGTATAGACAGAAAACATAGAACACTGACACAACTAAAATGTCCACCGTTA
ATGATAACAACTAATGTAGATCCGTTGGCAGACGAGTCACTAAAATATTTACGTAGCAGA
ATAACAGTGTTTAAGTTTATGAATAAATGTCCAGTAACAGCCAGCGGGGAACCAGTGTAT
ACACTTAATAATGAAACGTGGAAATCATTTTTCCAAAGGTCCTGGGCACGTTTAGAATTG
CATCCAGAGGAGGAGGAGGAGGAGGAGGAGGATGGCATCGCTAGCCGACCGTTTAGATGC
GTGCCAGGAGATGCTAATAGACCTCTATGAAAAAGATAGCAACAAATTGGAGGACCAGTT
GTTGCATTGGCAATGTGTGCGTCGGGAACATGCAATATTATTTAGGGCAAGAGAAGCAGG
ACACACCAGTGTAGGCCACCAGGTGGTGCCACCACTTAGTGTAACCAAAACAAAGGCATG
TCAGGCCATTGCAGTGCACCTATCATTGCAGAGCCTAAATAACAGTGACTTTAAGTATGA
ACCGTGGACACTACAGGATACATCCATGGACATGTGGAACACACCCCCACAAGGGTGCTG
GAAGAAAAAAGGATGTACAGTGACAGTTAAATTTGATAACAGTGACTGTAATGAAATGGA
CTATGTCAGCTGGGGACACATATATGTAAATAAGGAGGACACAGACACATGGTATAAAGT
GCCAGGCCGCATAGATTATGCGGGGCTGTATTATGAGGTGCAGGGATGCAAACATTACTA
TGTGGCGTTTGCAAAGGAGGCCCTAAAATATGGGACAAAAAACATATGGGAAGTGCATGT
GGGCAACAATGTTATTCACCAACCATGCGACTCTGTATCTAGCACCCAGGACACCGTGTC
AGAAGTACCCCTTGCTGCAACTTGTGACAGGCTACCACACGCCGACGAACCCACCACCAG
CCCCGAGGTGTTGGGCACCACCCAGAGCACCCCCCAAGTGCAGGCGCCGCCTTGTAAGCG
ACAGCGACTCTACGGAGACGGAGCAGAGCAGCCCGACGCCACCGAAAAACAACATCAAAA
GCGGCTGGACACTAGTACCCACGGATTCTACGGTGACACTGAGTGCCCAAAACGCCCAAG
GGACAACAGTGACAGTAACAATACACCTGTAATCCACTTACAAGGTGAAGCCAATAAGTT
AAAGTGTTTTCGATACAGATTACACAAGTTTGTACCAACATTGCTTGTGGGGGCCACGTC
CACGTGGCACTGGACGTGCGGGGAGACAGCCACTAAAGCAACCTTTGTTACCTTGCGGTA
TAGCAGTGTAGAACAACGTAACCTGTTTTTAAGTAGGGTAACAATTCCAAAAGGTATTAA
AGCAATGCAAGGCCATATGTCCATGTGCTTCTAAACATATTGGGTGTATGTACATATATG
TATATATGCTGTAATACTTACATTGTATAGGTGTATACAAATTGTGTTTGCAGTACACTG
AGCCACATATTACCAATCCACCCCCACACCTGGTAAAGTGTGCACACAGTTACAACCCTG

GATCTTGTTTTATGCTTTATATGTTGGTGGTGTGACATACTTGTGCTTCTTGTTTGCTTT
TGGCTTTCGTACCTTTCTCCCTGTGCTGCCTTTGTTGTGTTTATGTGCATTATATACCTA
GGGTTGTTTTTAGTGTACCTTCAGGTACTGTGGTCCAGTGGATTGTTATAACCACACAAC
CAGCCATACACTGCTGCTATTTGTATATAGTTTGTTCCTTGTGTATTGTAGGTGCATTAT
GTATCCATTGGAAACACGACGGGGGGGGCCGTCCTATGATGTGATTGTTTTTGGGCAAGC
TGATACTGGATTACTGTTAATAATTTTAATTTTGATCATCATAATTATTATCCTGCTGTA
TATGAGGCTTATGCACATGTAGACTCCTTACTGTGTTCCTTTTGTATTCCCATACATTTT
TGATATTACTTTTTGTATTGTTTTATACACAGCACTTAATAAATACATTTAATTACACAA
TGTCCACTGTGGTTAGGCGACGCAAACGAGCGTCCGCAACAGATTTGTATCGGACCTGCA
AAGCTGCAGGTACCTGTCCCCCAGATGTTATTCCTAAGGTTGAAGGTGATACATGGGCCG
ACAGACTGTTAAAATGGGCCAGCTTGGGTGTGTTTTTTGGACGTCTTGGAATTGGTACAT
CCTCTGGTACAGGGGGTCGTACAGGCTATATACCGTTGGGTACCCGTCCTCCCACGGTGG
TAGATGTTGGTCCTACAGCACGCCCCCCTGTGGTTATTGAACCTGTGGGCGCCGGGGAGC
CTTCTATTGTACAGCTTGTAGAGGATTCCAGTGTTATTGAGGCCGGGGCTACTGTGCCTA
CATTTACAGGCACACATGGGTTTGAGGTCACTAGCTCTTCCACCACTACCCCTGCTGTGC
TAGATATTACCCCTTCGTCCACCACCGTACAGGTTAGCAGCACTAGCTTTACTAATCCCT
TGTTTACAGAACCTTCTATAATTGAGGCTCCACAACATGGGGACATTACTGGACATGTGT
TTACAAGCACGCCCACATCTGGCACTCATGGCTTTGAGGAAATACCCATGCAAACCTTTG
GCACCTCCTATGGTACAGGCCAGGAACCTATAAGTAGCACTCCCATTCCAGGTGTGCGAC
GTGTTGCAGGCCCTCGCATTGGCCTTTACAGTCGTGCGACAGACCAAGTTAAAGTATCTG
ATTCTGCCTTTATATCCCGCCCAGGCTCTTTTGTAACATATGACAATCCTGTATTTGACC
CCGAGGAAACAATCATATTTGAACACCCTAGTTTACATGCTCCACCCGATCCTGACTTTC
TGGATATTGTGGCATTGCACAGGCCTGCCTTAACAACTAAGCAAGGTGCTGTTCGGGTTA
GTCGTATAGGTCAAAGGGCCACATTACGCACCCGTAGTGGCAAACGCATTGGTGCCCGGG
TGCATTTTTACCATGATATTAGTCCTATTGCAGGTGCAGAGCATATTGAACTTCGCCCCT
TGCAGCCTTCCATTGCTACTGCCCCAGGGGACTCCTTATATGATATTTATGCTGATAATG
GCCACTTGGACTCCTTGGCGTCTCCACATGTTTCATATACTTCTTCCACGTCTGTTCCTT
TGCAATCTACAAATATTTCAGCTACATCTGTGGTCTCTGCGCGGTATGACAATGTCACAG
TACCACTCTCTTCTGGGTTTGATTTGCCTGCATATACAGGCCCTGACATTCCAATTGCCA
CTCCTACTACCAGTGTGCTGCATGTTTCCCAGTCCTCTAATGTTCCTTCCCAATCTGTGT
TTGTGGATGGTACAGACTATTTTTTATTGCCTAATTACTTATTCTTTCCTAAAAAACGTA
AACGTGTGCACTATTCTTTTGCAGATGGCTTTGTGGCGACATGGTGACGGCAAAACATAC
TTGCCTCCCACTCCTGTGTCTAAGGTTATGAGCACGGACCGTTATGTTACACGCACCAGC
CTATTTTATTATGGTGGCAGCTCCAGACTTCTTACTGTGGGACACCCATACTATCCTGTG
CAGGTTAATGGGCAGGGCAAAAAGGCAAACATACCTAAGGTGTCTGGGTATCAGTACAGG
GTATTTCGCGTTAAATTACCTGATCCTAATAAATTTGCTCTTCCTGATACTGCTTTATAT
AATCCAGACACTGAACGCATGGTGTGGGCCTGCCGTGGTGTAGAGGTGGGTCGTGGCCAG
CCTCTTGGCGTTGGCACCAGTGGCCACCCTTTATATAATAGACTGGATGACACTGAAAAC
ACCCCATTACTTGCTGCAGCAGACGCCGATAGACGGGACAATGTTTCTGTAGATTACAAA
CAAACCCAACTACTTATTATTGGGTGTAAACCCCCTATTGGGGAACACTGGGCTAAAGGC
ACTGTTTGCACTGGAGTGTCCTCTAAGGCTGGCGATTGCCCCCCCTTACAGTTTGTAAAT
TCTACAATTCAGGATGGCGACATGGTTGAAACAGGGTACGGTGCCATGGATTTTGCCAAT
TTACAGGAATCTAAGTCAGAGGTGCCTATTGATATTTGCACAGGTATTTGTAAATATCCT
GATTATTTACAAATGGCGGCTGAGCCCTACGGAGACTGTATGTTTTTTTGCCTCCGTAGG
GAACAAATGTTTGCCAGGCATTTCTTTAACAGACATGGAACCATGGGCGAGGAACTCCCT
GCCTCCTATTACATACCTGGCACCTCTGGAAATAATAGACAAACTCCTACCAGTTATGTA
TATGCCCCAACTCCTAGTGGGTCCATGGTGACATCGGATTCACAACTGTTTAATAAGCCT
TACTGGCTTCAACGGGCCCAGGGACACAATAATGGCATTTGTTGGTTTAATGAGGTATTT
GTAACAGTTGTAGATACTACCCGTAGCACCAATATTACTATTTCTGCAGCCACATCACAG
TCCGGTGAATACCAGGCCTCTAACTTTAAGGAATACCTACGCCACACAGAAGAATATGAT
TTACAATTTATCTTTCAGCTGTGCAAAATACGTATTACACCCGAAATTATGGCCTACCTC
CATGGTATGAATGAATCTTTGCTAGATGACTGGAATTTTGGTGTATTGCCCCCTCCTTCC
ACCAGCCTTGATGATACATATAGGTATTTGCAGTCCCGTGCTATTACCTGCCAAAAGGGG
GTTTCTGCTCCTGACCCCAAGAAGGATCCATATGATGGCCTTGCTTTTTGGGAGGTGGAT
CTCAAAGACAAACTGTCTATGGATCTGGATCAATTTCCTTTGGGGCGTAAGTTTTTGTTG

CAGCTGGGGTCCCGTTCTGTTTCTGTGTCTCGTAAGCGCCCTGCCTCTACATCCACGTCT
GCCCCTTCTAAAAAAAAAGTAAAAAAACGTAAGTAGCGTTATACTTATGTGTTGTACGTG
TGTGTTGTGTATGTGTGTGTATACATATGTTGCCTATGTGTTCGTGTATGTATGCATGTT
TGTTTGTTACATGTTTGTGTGTTCTATGTGTATGTATTTGTGGAATGTATGTGTTGTTTG
TGCATTAATAAACAGTGTGTCACTGTGGTCCAGTGTCCGGTTGCACCCCGTGAGTAAGCC
CTCCACACGTCCTGCACCGCCTTACAGTTCCTGACCCCTTATGTACCAGTTCCTTTTGTG
TTGCTGCGACCGGTTTCGGTCTCCCCAATTCCTTTTGTGTTGCTGCGACCGGTTTCGGTC
TCCCGCCTTTTCGGTCTCTACAGTGGCATGCTGCCACAACACACAATAGTAAGTAATCCT
GTCTTACGCATATCCTGCATACATTGTGCCAATGTTCCACACCTGGGAGTGCCTGACAGT
TTGTTGGCTGCACAACGTGGTTGCCAGACAATATTTATGTTTTACTCATACTCATCCTGC
AAAAAATATGCTTTTAGGCATGTACTTTGGCTGACTACTTTCCCCTAATTACTTTTTTGG
CCATGCAAACTGCTTGTGTGGCCAACTATGTGTCTTGTAAAGCAGGACTAACAGTTACAA
ACTTGTTGCACACACCTAATCCGGTTACTACCTACACAGCAATTTCTTTTATAATAATTT
ATAACAATCATACCTATAAAAAAGTAGAGAGCAACCGGAATCGGTCGGACCGAGGGGTAT
ACATATAAAAGGGAAGTGTGTGCTCAGCAAAGCTAGCT
>gi|71726710|gb|DQ080082.1|_Human_papillomavirus_type_106,_complete_genome
ATGGGTACATATAAAAGAGATATGTTGCTTCAGCACAAAATAGCCATGACCAACGGAGAC
TGCTGTCCAAAGAATATCTTCCTATTGTGCAAAGCCTATCAACTGGACCTGCAGGACCTG
AACATAACTTGCGTGTTCTGCAAAACAGCACTAACGGAGGCGGAGGTTGTATCGTTTGCA
TGTAAGGAGCTTAAAGTTGTGTGGAGAAAACAGTTTCCATTTGCAGCGTGCTCGGCGTGC
CTGAAAGCAGCTGGAAAACTTAGACAATTTAGATACTGGGACTACTCCAGCTATGCACCC
ACAGTGGAGTATGAAACAGGGATACCAATACTAGAGCTGTTTATTAGATGCTACCTGTGC
CATGCACCGTTGTGCACTGTGGAAAAGGAACGGCTAATAACAGAAGGCAAACGGTTCCAT
AAAATAGCCGGGCACTGGCGAGGCGCTTGCCTGCAGTGTTGGACATCATGCATGGCCAAC
AACCAACCTTAAAGGACATCCTGTTAGAATTGGATGAGCCAGATGTTGTTGACCTGCATT
GTAACGAGCAATTTGCCAGCTCAGAAGAGGAGGATAATAGGGAGGACGGTGAGCAACCTA
CAGAACCAGCACAGCAGGCATACAGGGTATTAACATGCTGTTGTAGGTGTGGGCGTGCAG
TAAGGCTGGTGGTGGAGAGTGGACACGCAGAACTCAGAGTGCTGCAGGAGCTTCTGTTGG
GTGCACTGGGCATAGTGTGTCCGCACTGCGCATAACTGCTATGGCCGACTGCGAAGGTAC
CAATGATGGGGACGATGTGGAGGAGGGACGGGGGGCATGCGGGTGGTTCTGGGTGGAAGC
CACAGTAGACAGGACCACAGGACAGCAAATATCTAGTGATGAGGAGGACGAGGACGACGA
TACAGGACAGGATATGGTCGATTTTATTGATGATAGGTATGCTGGGGATGGACAGGAAGG
TGCGCCTGAACTGTACAGACAACAGGAGGTACAAGATGACGAGGCAATTGTGCAGGCCCT
AAAACGAAAGTACATAGCTAGCCCGTTGTCCGCCGGGCTATGTATAGACAAAGAACTGAG
TCCACGGCTGGATGCCATAACCATAGGTAGACGCTCACAAAGGGCAAAACGTAGACTGTT
TGAGCTGCAGGACAGTGGTTATGGCAATACGCAAGTGGATACTGACGCGACAGAGGACCA
GGTAGTAGGGGAACGCAAAGGGGACCCAGGCACGGGGGGGGTACGGGAAGCTGAGGAGGA
GGAGGAGCGTCAGGGGGGGGATGGTGCACCAGGTGCAGAGACAGCTTCAACACAGGCGGG
TACAGAGGCTGTGTTAGCGCTGCTAAAGACAAGTAACCTAAGGGCGACGCTGCTAGGTAA
GTTTAAAGACTTATTTGGGCTGTCATATATGGAGCTGGTACGGCAGTTTAAAAGTAATAA
GACAACATGTATAGACTGGGTAATTTGTGCATTTGGCGTGTATTGTACAGTTGCAGAGGG
CGTAAAGACCCTTATACAGCAACACTGCCAATATGCACATATACAGGTGCAAACCTGCAG
ATGGGGAATGGTGGTCCTAATGCTAGTACGATATAACTGTGCCAAAAACAGAGATACAGT
TGCAAAATGTATGGGCGGGCTGCTAAATATACCAGAGCACCATATGCTTATTGAACCGCC
CAAAATTAGATATCCACCAGCAGCACTATATTGGTACAAAGCAGGCATGGGCAATGCCAG
TGAAATAATTGGAGAGACGCCAGAATGGATAGTAAGACAAACAGTAGTAGGCCACAGTTT
AGAGGAATGTCAATTTCAACTATCAGTAATGGTGCAATGGGCCTATGACCATGACATAAC
AGATGAAAGCATATTGGCATATGAATATGCACGATTGGCAGATGTAGATGGAAATGCAGC
GGCATTTTTAGCAAGCAATTGCCAGGCAAAATATGTAAAGGACGCATGCACTATGTGCAG
GCACTACAAACGGGCAGAGCAGGCCCAAATGACAATGTCACAGTGGATAACCTTTAGAAG
TGCAAAAATATCAGAGGAGGGGGACTGGAGGCCAATAGTGCGGTACCTAAGACATCAGGA
CATAGAGTTTATTTCATTTGTAATCGCACTAAAAAACCTACTAAAGGGAATACCAAAAAA
ATGTTGTATAGTTATTTATGGGCCTGCAGACACAGGCAAATCATACTTTTGTATGAGCTT
GCTACGCTTCCTGGGAGGGGTAGTTATTTCATATGCCAATTCAAGCAGCCATTTTTGGCT
GCAGCCATTATGTGATGCAAAAATAGGATTAATAGACGATGTGACACCCCAGTGCTGGAG

TTACATAGATACATACCTACGAAACGCGCTGGACGGAAACCAGGTATGCATAGACAGAAA
ACACAGGCCATTGCTGCAACTAAAATGTCCACCACTATTAATGACAACAAATACCAATCC
TTTAGAGGAAGACAGGTGGAAATTTTTACGTAGTAGACTGCAAATGTTTACCTTTAAAAA
CGCATTTCCCGTGAATCAGAAAGGGGACCCATTGTATATACTAAATGATGCAAATTGGAA
ATGCTTCTTCCAAAGGTTGTGGGCTCGGTTAGACTTACACGAGCAGGACGACCAGGAGGA
CAATGGAGACATTGGCCAGCCGTTTAGATGTGTGCCAGGAGAAGTTGCTAGAACTGTATG
AAAAGGATAGTGACCAGCTGCAGGACCAAATATTGCACTGGCACCTTGTGCGATGGGAAA
ATGTTATTTTATATAAGGCACGTGAAGCGGGACATTTGCACCTAAACCACCAGGTGGTGC
CAGTGCAATTGATTGCAAAAGCAAAAGCATGTAAAGCTATTGAAGTGCAGCTTGCATTAA
AGACATTGCTAAAAAGCCCCTATGGCACGGAACCGTGGACATTGCGTGACACAAGCCAGG
AAATGTGGGACACAGAGCCTAAGCAATGCTGGAAAAAAAGGGGCTACACCGTAGACGTTA
AGTTTGATGGCGAGGAATCCAAAACTATGTGCTACACATGCTGGAGGGATATATATGTGC
AAAGCATTGCTGATGATACATGGGACAAAGTAACGGGCCATGTGGACCATGCAGGACTAT
ATTATGTGCACGAAGGGACACGTGTAACCTATGTTAAGTTTTCACAGGAAGCACAGACAT
ATGGGAGCACTGGCACCTGGGAGGTGCATGTGGGAGGACACATTATTTATGATGCATTCG
ACCCCTCTGTGTCTAGCACCCAGGACACCGAGCAAGAACCCCTATCCACTACTAGACCTA
CTGACCCATCGCACACCGACCAACCCGACGCCACCACCCCCACCCCCGTGCTGGGCCCCG
CGCCGACCTTCGTGCCGCCGCCGCCTAAGCGTCAGCGACTCGGATACGGACCTCAGCACA
CAGAGCAACCGGACTCAACAGGGGACAGGGGTCAGCGGCTGGCACGTGACAACGCAAGGG
ACCAGTGTAACCCTGACGGCGCGAACCAGCACAGGAACATCAGTGTCGGTCACGGTGCAC
CTGTAATACACCTCCGAGGTGATCCAAATAAGTTAAAGTGTTTAAGATATAGACTAAAAA
AGCATACACCTGTATTATTTACCAAAGCATCATCCACCTGGCGATGGTCAACGTGCAGTA
GCCCCACCGATGCAAATTCTGCCTTTGTAACATTGTGGCATGATAGTGTAGAGCAAAGAG
CAAAGTTCTTAGCTACGGTGACTCTGCCTAAGGACATTGTGGCTGTACCTGGGTACATGA
CTATGTTTGCATAATAGTGGCCTGTGTATATTGTATACTTGTATATTTGTATACCCATTG
TACAGTGCGGAACATTAAGGGCACGTAGGCACGTGTGTATACTACACCCTGGAGATTTTT
ATTTGCTGGCTATCTGTTTGGACACACCTGCTGCTATTGCTTATTTGTTTCTGGCTTTCT
ATGCTGTCCTGCTTTACTGCATTTCTTGTTTTTTTTTTGCTGTGTATATAGCCCTTCTAA
TAGTATACACACAGGCAGTGCGGTCCCTTGACCAACTGTGACCTTACCACACAACCAGCC
AAATCTGCTGCTCAATTGTATATATTCCCTGTGTGTCATAGTGTGCAACTATGTATCCTG
TAGTGGTGCGTGATAAGGGGGGTGATACAGAATATCCTGCTGTACTGTGGACACCCCCAAG
ATGACAAACTGTTGTTATTGCTGCTGGTGTTAGTTTGTCTTTTGGTGTTTTTTAGTTACC
TTCGTTTGTTGCAGTAGTTTTTACCTTTTGTATGTACATGTACTAGGGTGGGTTTTATAT
TATTGGGTGTTGTGGGTTATATTTACATATTTTGTGGTTTTTACATTTCATTTACATATT
TTGCGATAAATACTGTTAATATGCATCGATCCAAGCGACGCAAGCGTGCCTCCACCACAC
AACTATATCAAACCTGCAAAGTGGCAGGTACCTGCCCCCCTGATGTTATTAACAAGGTTG
AACACACCACCATTGCAGATAAAATATTACAATGGGGCAGCTTAGGTGTGTTTTTTGGGG
GGTTGGGAATTGGTACTGGGTCGGGCACTGGTGGGCGCACTGGGTATATACCCATTGGAA
CCCGGCCCCCTACTGTTGTGGATGTAGGCCCTCCAGCCCGCCCCCCTGTTGTTATTGAGC
CAATTGGGGCGTCTGAACCGTCCATTGTCACCTTAGTGGAGGATTCCAGTATTGTTGCCT
CCGGTGCCCCGCATCCCACCTTTACAGGAACAGATGGGTTTGAGGTCACCACTGCCTCTA
CCACAACCCCTGCTGTGTTGGATATAACCCCTTCAGGTGGCACTGTGCAGGTTAGTAGCA
GTAGCTTCACCAATCCATTATTTACTGAGCCAGCATTAGTAGAGCCTCCACAAACAGGTG
AGGTCTCAGGACATATATTAATTACTACACCTACAGCTGGATCTCATGGCTATGAGGAAA
TACCTATGCAAACCCTTGCTGTGCAGGGCACTGGGGAGGAGCCTATAAGCAGTACCCCAC
TTCCGGGGGTACGACGTGTGGCCGGGCCACGGTTGTATAGTAGGGCCTATCAACAAATTA
ATGTACGGGACCCCACCTTTATGTCTGAACCTGCAACATTTGTTACCTATGACAATCCTG
TATATGATCCAGAGGAAACAATTATTTTTGAGCGTCCCAGCATTCAGCAGGCCCCTGATC
CTGACTTTTTAGATATTGTGGCACTTCACAGGCCTGCCTTGGCAGCACGTAAGGGCACTG
TACGGTACAGTCGTCTGGGTCAGCGGGCTACATTAAAAACCCGAAGTGGCAAACGTATAG
GTGCACGGGTTCATTTTTACCATGATATAAGTCCAATTACTCCTGCTGAAGAGTTAGAAA
TGCAACCTCTGGTGTCTGACACCCCTACTTATCTTAATTCCTTATATGATATTTATGCTG
ACACCGAGACGCCTGGGTCGCGTCAGGGCACCTTAGCTTCTATACACACATCCACACCTT
TGCAGGCGCCTTCATTGGCTGCATCATCCACTGTCTCCACAGTATCGCACACTACTGTTC
CCCTGTCTACAAGCTTTGATGTCCCTGTTTTTAGTGGCCCTGACAGTGCTCTTCCCGATT

CCCACACTGCTTGGCCCGTGCCCCCTACCCCTCCTGGTCACCCCACAGGCGATGTGCTTG
TGCACGGGTCTACATATTATTTATTGCCCTCATATGTGTTGCTTCCTAGGAAACGTAAAC
GTTTTCCATCTTTTTTTGCAGATGGCATTGTGGAAGCCTAGTGACAACAAAGTATACCTG
CCTCCCGCCCCTGTGCCAAAGGTTCTGAGCACCGACGACTATGTATCAAGAACAAACATT
TATTATTATGCAGGCAGTTCCCGATTGCTGACCGTAGGTCATCCATATTATCCTATCAAA
AAGCAAAGTGGACGGAACCCTATAGTGGTGCCCAAAGTGTCTGGATACCAATATAGGGTA
TTTAGGGTTCGTTTGCCTGATCCCAACAAATTTGGCCTTCCTGATGCATCGTTATATAAT
CCAGACTCCCAGCGTCTTGTCTGGGCCTGTACGGGTGTTGAGGTTGGTAGGGGGCAGCCA
TTGGGGGTAGGGGTTAGTGGCCACCCCTTATATAATAGGTTGTATGACACTGAAAACACT
AATTTATTTGATGTTGTTCCTGGGGACGACAACCGGGACAATATGTCCATGGATTATAAA
CAAACTCAGTTATGTATTATTGGCTGTAAACCGCCATTGGGCGAACACTGGGCAAAAGGC
ACTCCATGCAATACCTCCAATGTCACTGCAGGGGATTGTCCTCCTTTGGAACTCACATCT
TCCACAATACAGGATGGGGACATGATTGATACAGGGTTTGGTGCTCTTGACTTTGCATCA
CTGCAAACAAATAAATCGGATGTGCCTTTAGATATAGTTACCACCACATGCAAGTACCCA
GATTTTTTGCAGATGGCAGCTGAGCCTTATGGTGATTTAATGTTTTTTTCATTACGCAGG
GAACAAATGTTTGTTAGGCATTTTTACAACAGGGCCGGTACCATGGGGGAGGATGTTCCG
GAAGGGTTTTTACTAAAGGGGCCTACTACTACATCCCGCGCCTCTGCTACAAGCTCACTA
TATGGGTCCACGCCTAGCGGGTCAATGGTTACTTCAGAGTCTCAGTTGTTTAACAAGCCC
TACTGGCTACAACGTGCACAGGGCCGAAACAATGGTATCTGCTGGGGCAATCAGGTATTT
GTCACTGTGGTAGATACCACACGCAGCACCAATATGACTATTTGTGCCACGCAAACAGAA
AATGCCACATACAAAGCTTCCAATTTTAAGGAATACCTGCGCCACGGAGAGGAATTTGAT
TTGCAATTTATTTTTCAGCTATGTGTTATTAATTTAACTGCCGAGGTTATGGCCTACCTA
CATAGTATGAATGCTGCTTTACTAGAGGACTGGAATTTTGGGTCGTTGCCCCCCCCTACC
GCCAGTCTTGGCGATACCTATAGGTTTTTACAGTCCCAGGCCATAACATGTCAAAAGCCC
TCCGCGGCGCCTGCAAAGAAAAAGGACCCTTACGATGGGCTTACGTTTTGGGATGTGGAT
TTATCAGAGCGCTTTTCCCTTGAGCTTGACCAGTTTCCCTTGGGTAGGAAGTTTTTGCTA
CAACATCGTACACGTGTGCGGTCCTCCCCTGTGTCCCGTAAGCGTCCTGCACCATCGTCC
ACCCCACCAGCCACAAAGCGTAAACGGTCCAAGCGTTCCTAACTGTGTTGCTGCTGTGGG
TTGGGTGTACCTGTTGTTGTGTATATAATGTGTGTTGTACTGCATGTATGTACGTTGGTT
GTATGTGCTGTGACTGTGTGTGTATGGGGCGGGACCCCCCCCTGGTATGTATGTTTCCTG
ATGCATGTTTGTGTGCATGTCTTATATATTAATAAATGTGTGTCATGTGTGGTTTGTGTC
CGGTTGCGCCTTGTGAGTAACTATACATTTATGTTGTGTAGTCCAGTCCTTGTGGCCCGG
TCATGCATACCCCCGCCCTTATAACACAGCGACCGATTTCGGTCGCTGCTGGTTTCGGTC
GTCCACAACATGGCGCCTTGTTGGCACTCTTTGCATAACAGTTAATCCTTTCCGCGCCAC
TTTTTTGTTTAAATGCAGGTGTGCATGCCTTTCTAAGGTGTGCCTGGCAGTTATTTGGCA
GGATATAAATGCATTTCCAAATGCATTTCTTAATCTATATTGTCTATGTTACTCACACTT
CAATAAACTATACTTTTAGGCACATAGTTTTATGCTGACTACTTTCACTTAAGTACCTAT
TTGGCAGGCAAACCTGCTTTGCTATTCAACAATGTGTCTTATATAATTGTTCAACAAATT
CAAACATGTGTTTACCACACCTATTCCGGTCGCTCCCACTACACATCTTACTCATTATTA
TAAATTATAATGGTTTTATTACAAAATAGGAGGGACCGAAAACGGTCCAACCGAA
>gi|71726685|gb|DQ080079.1|_Human_papillomavirus_type_68a,_complete_genome
ATGGCGCTATTTCACAACCCTGAGGAACGGCCATACAAATTGCCAGACCTGTGCAGGACA
TTGGACACTACATTGCATGACGTTACAATAGACTGTGTCTATTGCAGAAGGCAACTACAA
CGGACAGAGGTATATGAATTTGCCTTTAGTGACCTATGTGTAGTGTATAGAGACGGGGTA
CCATTTGCTGCATGCCAATCATGTATTAAATTTTATGCTAAAATACGGGAACTACGATAT
TACTCGGAATCGGTGTATGCAACTACATTAGAAACCATAACTAATACAAAGTTATATAAT
TTATTGATAAGGTGCATGAGTTGCCTGAAACCATTGTGTCCAGCAGAAAAACTAAGGCAC
CTAACAACAAAACGAAGATTACATAAAATAGCAGGAAACTTTACAGGACAGTGTCGGCAC
TGCTGGACCAGTAAGCGAGAGGACCGCAGACGCATACGTCAAGAAACACAAGTTTAAGTA
ACTATGCATGGACCAAAGCCCACCGTGCAGGAAATTGTGTTAGAGCTATGTCCATACAAT
GAAATACAGCCGGTTGACCTTGTATGTCACGAGCAATTAGGAGATTCAGACGATGAAATA
GATGAACCCGACCATGCAGTTAATCACCACCAACATCTACTACTAGCCAGACGGGACGAA
CAACAGCGTCACAGAATTCAGTGTCTGTGTTGTAAGTGTAACAAGGCACTGCAACTAGTA
GTAGAAGCGTCGCGGGACAACCTGCGGACACTACAACAGCTGTTTATGGACTCACTAAAT
TTTGTGTGTCCGTGGTGTGCAACTGAAACCCAGTAATCTGCAATGGCCAATTGTGAAGGT

ACCGATGGGGACGGGACGGGGTGTAACGGATGGTTTTTTGTAGAAGCAATAGTAGATAAA
CAAACAGGTGACACAGTCTCAGAGGATGAGGACGAAAACGCAACAGATACAGGTTCAGAC
ATGGTAGATTTTATTGATGATTCTACACATATTTGTATACAGGCAGAGCGTGAGACAGCA
CAGGTACTTTTGAATATGCAAGAGGCCCAAAGGGATGCACAAAGGGTGCGTGCCCTAAAA
CGAAAGTATACAGACAGTATAGAAAGCAGTCCTTTAGCAAAGTCGCCATTACAGGAATTA
TCACTAAATGTAAGCAGTACACAGGCAAGACAACCGGCGTATACAGTGCCGGACAGCGGC
TATGGCAATATGGAAGTGGAAACTAACTCGGAGGTAACTGTAGCACCTAATATAAATGGG
GAGGATGGGGAAAATGAAGGGGAAAATGGCGACAGTATACGGGAGGACTGTAGTAGTGTG
GACAGTGCTATAGATAGTGAAAACCAGGATCCTAAATCACCTACTACCCAACTTAAAGTA
TTATTACAATGTAACAATAAAAAGGCTGCAATGTTAACAGAATTTAAAAAAGTATATGGA
TTGTCATTTAATGACCTGGTACGTACATTTAAAAGTGATAAAACCACATGTACAGACTGG
GTAGCAGCAATATTTGGAGTAAATCCAACCATTGCGGAAGGGTTTAAAACACTAATTAAA
CAATATGCATTGTATACCCATATACAATGTTTAGATACAAAAACTGGAATATTAATACTA
ATGCTAATAAGATACAAATGTGGAAAAAATAGAATAACAGTAGGAAAAGGATTGAGTACA
TTGTTGCATGTTCCAGACAGCTGTATGCTTTTGCAGCCACCAAAATTACGTAGCCCTGTT
GCAGCATTATATTGGTATAGAACAGGAATATCTAATATTAGTGAGGTGTGTGGCGACACG
CCGGAATGGATAAAAAGATTAACCATAATACAACATGGAATAGATGATAGTGTATTTGAT
TTATCAGACATGGTACAATGGGCATTTGATAATGAGTTAACAGATGACAGTGATATAGCA
TTTCAATATGCTATGTTAGCAGATTGTAATAGTAATGCTGCAGCGTTTTTAAAAAGCAAC
TGTCAAGCAAAATATGTAAAAGATTGTGCAACAATGTGTAGACATTACAAACGGGCGCAA
AAACGACAAATGACAATGCCACAATGGATTAAATTTAGATGCAGTAAATGTGACGAAGGC
GGTGATTGGCGCCCAATTGTACAATTTTTAAGATATCAAGGACTGGAATTTATAACATTT
TTATGTGCATTAAAAGATTTTTTAAAAGGCACGCCAAAACGAAATTGTATAGTTATACAT
GGGCCGCCAAATACAGGCAAGTCATATTTTTGCATGAGCCTTATACATTTTTTACAAGGC
ACAATAATTTCATATGTAAATTCAGCAAGTCACTTTTGGTTAGAGCCACTTGCAGATGCA
AAAATAGCCATGCTAGATGACGCAACAGGTACATGCTGGTCATATTTTGATAATTACATG
AGAAATGCATTAGATGGTAACCCAATAAGTTTAGATAGAAAACACAGACACCTAATACAA
ATAAAGTGTCCACCAATGCTAATAACATCCAATACTAACCCTGTAGAAGACAATAGGTGG
CCGTATTTACATAGTAGACTAACCGTGTTTAAATTTCCTAATGCATTTCCATTTGACCAA
AACAGGAACCCAGTGTATACAATCAATGATAAAAACTGGAAATGTTTTTTTGAAAAGACT
TGGTGCAGATTAGACTTGCAGCAGGACGAGGATGAAGGAGACAATGATGGAAACACTTTC
CCAACGTTTAAATGTGTTACAGGAGAAAATATTAGAACATTATGAACAGGACAGTAAATG
TATACAGGACCATATTAACTATTGGAATTGTGTGCGACTGGAAAATGCAATATATTATGC
AGCACGAGAACGTGGTATGCATAATATTGACCACCAGGTGGTGCCGCCTGTAAACATTTC
AAAAACTAAAGCATATCAAGCTATTGAACTGCAGATGGCACTAGAGAGCCTTGCTAAAAC
TGCATATAGTGCAGAGGAGTGGACATTAAGGGACACAAGTAATGAACTATGGCATACAAA
GCCAAAGCAATGTTTTAAAAAACATGGTGTTACAGTGGAAGTATGGTATGATGGGGACAA
GAGTAACTCAATGCATTATGTAGTGTGGGGTACAATTTACTTTAAAAACAGTACAGACAC
ATGGTGTAAAACCCAAGGGCGTGTGGATTACTGGGGTGTATATTATATGTATGAACAACA
AAAAACCTATTACGAAAGGTTTATGCAGGATGCACAACTATATGGGACTAGTGGAAAATG
GGACGTGCATTATAATGGCAACATAATCCATTGTCCTGACTCTATGTGCAGTACCACTGA
CGGAAAAGTATCCACTACTGAATCTGTTGCCGACCTACAGAACACCACCGCGACCCATAC
CACCGCAGGTGCCCCATGCACCAAAAAAACAAACTCGACGTCGTCTCGGAAGTATCCCAG
ACAGTGCGGAATCACTGAGCCCTCTGAGCCCGACAACGTGTCCGTGGACGGTGTCGACCT
CCCACTCCTCAGTAGAAGTGCAGGCCAAAACAAAAGACGGAGCCTTTGTTGTGGTGACAC
TACACCTATAGTGCATTTAAAAGGTGACAAAAATGGATTAAAATGTCTTAGGTATAGGTT
GCAAAAACATAACGCTTTGTATGACAATATATCATGTACATGGCATTGGATAAGGGGTAG
GGGATCAACCAATACAGGTATATTGACTGTAACATATGTTTCAGAAGCACAACGTGACAA
GTTTTTGGAAACTGTTAAACTACCATCTAGTGTAACTGTGTCATTGGGGTATATGACATT
ATAAGTGTGTATTGACCCACACTGTACACTATATGTATATTGTATACATATGCTTGTATT
GGTATTTTTGGTGTGGTTTTGTGTATGCATGTATATATGTTGCACTGTCCCGCTTTTGCA
GTCTGTGCATGTGTGTGTGTATGTGTGGATATTTGTGTTTGTGTTTATATTAGTTAGAAC
TACACCATTTGAGGTGTTTGCTGTATACCTACTTTTTTTTATACTGCCTATGTGGTTATT
ACACAGTCTTACTCTTAATACTATACAGTAACTGTTATAGTGTGCATTTGTATTGTTGTT
TATTTGTATAAATAAATATGGTTTCACACCGTGCTGCCAGGCGCAAGCGTGCATCTGCAA

CTGACTTATATAAAACATGTAAACAATCAGGGACATGTCCTTCTGATGTTATAAATAAGG
TTGAAGGTACTACACTTGCAGACAAAATATTACAATGGACAAGTTTAGGTATATTTTTGG
GTGGCCTGGGTATAGGTACTGGCTCTGGCACCGGAGGTCGTGCTGGGTACATTCCTTTAG
GTGGAAAACCTAATACTGTTGTGGATGTGTCGCCTGCACGTCCACCTGTGGTTATTGAAC
CTGTGGGTCCTACGGAACCCTCCATTGTGCAATTGGTGGAGGATTCCAGTGTTATTACAT
CTGGGACACCAGTACCAACATTTACAGGCACCTCTGGGTTTGAAATTACATCTTCGTCTA
CCACTACACCGGCAGTTTTAGACATTACCCCTTCGTCCGGGTCTGTGCAGGTAAGCAGTA
CTAGTTTTACTAACCCTGCATTTACAGACCCGACTATAATAGAAGTGCCACAAACAGGTG
AGGTGTCTGGTAATGTGTTTGTAAGTACCCCTACATCAGGAACACATGGATATGAGGAAA
TACCTATGCAGGTATTTGCAACACATGGCACTGGTACAGAACCTATTAGTAGTACTCCTA
TACCTGGGGTTAGTCGTGTGGCAGGGCCACGTTTATATAGTAGGGCACATCAACAGGTTC
GTGTTAGTAATTTTGATTTTGTAACGCACCCTTCATCATTTGTAACATTTGATAATCCTG
CTTTTGAGCCTGTTGATACTACTCTTACATATGAACCTGCTGACATAGCTCCTGATCCGG
ATTTTCTGGACATTGTTCGTTTACATAGGCCTGCCTTAACATCCCGAAGAGGAACAGTAC
GTTTTAGCAGAGTAGGCAAAAAGGCGACCATGTTTACACGCCGAGGTACACAAATTGGGG
CACAGGTGCACTATTATCATGATATTAGTAACATTACCCCTGCTGACAGCATTGAACTAC
AACCATTGGTTGCCCCTGAGCAGGCGGACCCTATGGATAACTTATATGATATATATGCAC
CTGATACTGACAATACTACAGTATTAGATACTGCATTTCATAATGCTACATTTACTACTC
GTTCCCACATATCAGTTCCTTCATTGGCTTCTGCTGCATCCACTACATATACTAATACTA
CCATTCCTCTTGGTACTGCTTGGAACACGCCTGTAAATACTGGTCCTGATGTTGTATTAC
CATCTACAACCCCACAGTTGCCTTTAACACCCTCTACTCCAATTGATACAACCTTTGCCA
TAACTATATATGGTTCCAATTATTATTTATTACCATTGTTATTCTTTTTATTAAAAAAAC
GTAAACACCTTCCTTATTTTTTTACAGATGGCATTGTGGCGAGCTAGCGACAACATGGTG
TATTTGCCTCCCCCCTCAGTGGCGAAGGTTGTCAATACAGATGATTATGTGACACGCACT
GGCATGTATTACTATGCTGGTACATCTAGGTTATTAACTGTAGGCCATCCATATTTTAAG
GTTCCTATGTCTGGGGGCCGCAAGCAGGGCATTCCTAAGGTGTCTGCATATCAATACAGA
GTGTTTAGGGTTACCTTACCTGATCCTAATAAATTTAGTGTTCCTGAGTCTACATTATAT
AATCCAGATACACAGCGCATGGTATGGGCCTGTGTTGGTGTTGAAATAGGTAGGGGGCAG
CCATTGGGCGTTGGCCTTAGTGGGCATCCACTATATAATAGGCTGGATGATACTGAAAAT
TCCCCGTTTTCCTCTAATAAAAATCCTAAAGACAGTAGGGACAATGTTGCAGTGGACTGT
AAACAAACACAGCTGTGTATTATAGGCTGTGTTCCTGCTATTGGCGAGCACTGGGCCAAA
GGTAAATCTTGTAAGCCTACCAATGTACAACAAGGGGACTGTCCCCCATTGGAATTGGTA
AATACTCCTATTGAGGATGGCGATATGATTGATACAGGATATGGTGCTATGGACTTTGGT
ACATTACAAGAAACGAAAAGCGAGGTACCTTTGGATATATGTCAATCTGTTTGCAAATAT
CCTGACTATTTGCAAATGTCTGCAGATGTGTATGGAGACAGTATGTTTTTTTGTTTACGT
AGGGAACAGTTATTTGCCAGGCATTTTTGGAATAGGGGAGGCATGGTAGGGGACACTATT
CCCACTGACATGTATATTAAGGGCACTGACATTCGTGAAACTCCTAGTAGTTATGTGTAT
GCCCCCTCGCCTAGCGGGTCTATGGTGTCCTCTGACTCCCAGTTATTTAACAAGCCCTAT
TGGCTGCACAAGGCACAGGGACACAACAATGGTATTTGTTGGCATAATCAATTATTTCTT
ACCGTTGTGGATACAACGCGCAGTACTAATTTTACATTGTCCACTACTACAGACTCTACT
GTACCAGCTGTGTATGATTCTAATAAATTTAAGGAATATGTTAGGCATGTTGAGGAATAT
GATTTGCAGTTTATATTTCAGTTGTGTACTATAACATTATCCACTGATGTAATGTCATAT
ATACATACTATGAATCCTGCTATTTTGGATGATTGGAATTTTGGTGTTGCCCCTCCACCA
TCTGCTAGTCTTGTAGATACATACCGCTACCTACAATCAGCAGCAATTACATGTCAAAAG
GACGCCCCTGCACCTGTTAAAAAAGATCCCTATGATGGTCTTAACTTTTGGAATGTGGAT
TTAAAGGAAAAGTTTAGTTCTGAACTGGACCAATTCCCATTAGGACGCAAATTTCTGTTA
CAGGCAGGTGTTCGCAGACGGCCCACCATAGGCCCTCGTAAACGCACTGCCACTGCAGCT
ACCACATCTACCTCTAAACACAAACGTAAACGTGTGTCAAAATAATTGTTATATGTCTTG
TTTTGTATGTTGTTTGTCTGTGTGGTTGTATATGTGTCATGTTGGTGTTGGTATGTTGTG
TATGTGCATGTATGTGTATGTGTATATGTGTATGTTTGCAAGTATGTGTGTATAATCTGT
TTTTGTTAATAAAGTATGTATGTCCGTGTCCTTTGTGGTTGCACCCTGTGACTAACATAT
GTCCTTGTTTTACATATAATAGGACTGCAACATTTCATACATAATTTGTAGCCCTACCCT
AAGGTGTGTTACATTATATGCAATATATTTATGGTTGCAAACTATACCGTGTGGCCATTT
TGTATGGCCGGGTTGTGTGCGACCGCTTTCGGTCGTTGGTACTATTTGCTTTTAGATAGT
ATTAAAACCTATGTGTTTCAGCAAAAACATGTTTTACCTTGAGTTACCCACATAGTTGGC

ACCAGTAACAGTCGTACTGGCGCACCTTAGTTAGTCATCATCCTGTCCAGGTGCACTGCA
ACAATAGTTTGGCAACCAACGTATCTCCTCCCTTGTAATAAAACTGCTTTTAGGCATAAA
GTTTTAACTGTTTTTGCTTGCCTAATAGCATAGTTGGCCAGTATAACTACTTTTGCATTC
AAGAATCTGTCTGGTAGTGTAAGTTATACAGTGACTAATACTACATCCATAAATTTGTGC
AACCGAAAAAGGTTGGGCACACATACCAATACTTTTACTTATAACATTTTACAATTATTC
TATATAAAAAAAGGGTGGGACCGAAAACGGTCACGACCGAAAACGGTGTATATAAAGCTG
AACACAGCAGTTCTCTATACTA
>gi|2911565|emb|Y15176.1|_Human_papillomavirus_type_80_E6,_E7,_E1,_E2,_E4,_L2,_a
nd_L1_genes
CAAGACACTTGGCAGTACATTTTCTTGGAACACAGCACCGATAACGGTAAGATTATATCT
GTGAACCGTAGGCGGTACTTTCTGATTGGTTTGGATGATAGTAGTTAACAACAATCACTC
TTTATAAATAAATGTAACCGCCTGCGTTACCTTACTTGATCTAAATACAATATGCTGACT
AAACTTTTTAGAGAGCTATATGGATAGGCCAAAGCCTTCTTCTGTGCAGGAGCTTGCAGA
CACTCTTTGTATACCTTTAGTAGATATTTTATTGCCGTGTAGGTTTTGTCAAAGATTTTT
AACTTATATAGAGTTAGTAAGTTTTGATCGCAAATGTTTGCAGTTGATTTGGACTGAAGA
AGACTTTGTCTTTGCTTGTTGTAGCAGTTGTGCTTTTGCTACTGCACAATTTGAATTTTC
TACATTTTATGAACAGTCAGTGTGTGGTTGGGAGATTGAGGTAGTTGAGCGAAAAGCTGT
TGGAGACATTACTATTCGTTGCAAATTTTGTTTAAAGAAATTAGATTTAATAGAAAAGTT
AAATATTTGTTACAACCAGCAGCAATTCCATAAGGTAAGGCGCAATTGGAAAGGATTGTG
TAGACATTGTGGGTCGATAGAATGATTGGGAAAGAAGCTACTATACCAGATATAGAGCTT
GAGCTGCAAGAGCTTGTCCAGCCCACTGACCTGCATTGCTACGAAGAGTTAAGTGAAGAA
GAGACAGAGGAGGAGCCACAACATATTCCATACAAGATTGTAGCTCCGTGTTGCTTCTGT
GAATCCAAGCTTCGACTAATAGTGGTTGCAACTTCTTTTGGAATTCGCGCACAAGAAGAG
TTACTATTTGGAGAAGTTAAGTTGGTGTGTCCAGGGTGTCGAGAGAAACTTCGCCATGTC
TGACGAAAAAGGTACTAAATATGATCCTAAGGAAGGATGTAGTGAATGGTTTGATCTAGA
AGCAGAATGCTCTGATGCTAGTTTAGATGGTGATTTGGAAAAGTTATTTGAAGAAAATAC
AGATACTGACATTTCTGACTTAATAGATAATGAGGACACTATACAGGGGAACTCCCGCGA
ATTATTATGCCAGCAAGAAAGTGAGGAAAGCGAGCAACAGATACACTGGCTAAAACGAAA
GTATATCAGTTCACAAGAGGTTTTGCAGCTTAGTCCTCGTCTGCAGTCTATTTCCATTTC
GCCACAGCATAAATCTAAAAGGAGATTATTTGAACAAGACAGCGGACTAGAACTGTCATT
TAATGAAGCTCAAGATTTTACTCAGCAGACTGTGGAGGTACCGGCGACCGATTCTGTGCC
GCCAGAACAGGGTGCCAAGGGACTGGGAATTGTTAAAGATCTTCTAAAATGTAGCAATGT
AAAAGCAATGTTATTAGCTAAGTTTAAAGAAGCTTTTGGAGTGGGATATATGGAATTAAC
TAGACAATATAGAAGCAGCAAGACATGCTGCAGAGACTGGGTATTAACTGTGTATGCTGT
ACAAGACGAACTATTAGAAAGTTCCAAACAACTTTTAATTCAGCATTGTGCTTATATTTG
GTTGCATCAAATACCTCCTATGTGTTTATATTTATTGTGTTTTAATGTTGGAAAAAGTAG
AGAAACAGTATTAAGATTGCTAACTAATCTTTTGCAAGTATCAGAAGTACAAATAATAGC
TGAACCGCCAAAACTTAGAAGTACATTGTCAGCATTATTTTGGTATAAAGGTAGTATGAA
TCCTAATGTTTATGCTCATGGAGAGTATCCTGAATGGATAATGACGCAGACAATGATAAG
CCATCATACAGCAGAAGCTACACAGTTTGATTTATCTACTATGGTACAGTATGCATATGA
TAATGAGTTAACAGATGAAGCAGAAATTGCCTATCATTATGCCAAGCTTGCAGATACAGA
TGCTAATGCTAGAGCATTTTTACAACATAATAGTCAGGCAAGATTGGTAAAGGACTGTGC
AATTATGGTTAGACATTATAGACGAGGTGAAATGAAAGAAATGTCAATGTCTGCATGGAT
ACATAAAAAATTAGCGGTTGTGGAAGGGGAAGGACATTGGTCAGATATTGTAAAGTTTAT
TAGATATCAAGAGATTAATTTTATTCAGTTTTTAAATGCGTTTAAATTGTTTTTACACAA
TACTCCAAAGAAAAGCTGCTTGCTATTTTATGGGCCTCCTGACACAGGGAAATCTATGTT
TACTATGTCTTTAATAAAGCTTTTAAAGGGAAAGGTTTTGTCATTTGCGAATTATAAAAG
TACCTTTTGGCTTCAGCCTGTTGCAGACACAAAAGTGGCATTGATAGATGATGTAACATA
TGTATGTTGGGATTATATAGAACAGTATTTAAGAAATGCATTAGATGGTAATACTGTTTG
TTTAGATATGAAACATAGGGCGCCATGTCAAATTAGGTTTCCACCATTAATGTTAACGTC
CAACATTGATATTATGAAAGAAGAAAAATATAGATATTTATACAGCAGGATTCAAGCGTT
TGCATTTCCACATAAGTTTCCTTTTGATAGTGATAATAAGCCTCAATTTAAACTTACTGA
CCAAAGCTGGAAATCTTTTTTTGAAAGGCTTTGGAGACAGTTAGATCTCAGTGATCACGA
AGACGAGGGAGACGATGGATACTCTCAGCGAACGTTTCAATGCACTACAAGAGAATCTCA
TGGACATTTATGAGTCAGGTCGAGACGACATAGAAACTCAAATAATGCATTGGCAATATT

TGAGGCAGGAACAAGTCTTATTTTATTTTGCCAGACAGCATGGAGTTATGCGTTTAGGAT
ATCAACCTGTACCCCCTTTAGCCACCAGTGAGGCTAAAGCTAAAGACGCTATTGGCATGG
TGATCTTATTGCAAAGTTTACAAAAATCACAGTATGGCAAGGAGCCATGGACATTAACAC
AAACTAGTTTGGAGACTGTAAGAAGCGCACCTGCAAACTGCTTTAAAAAAGGTCCACAAA
ATATTGAGGTCATGTTTGACAATGATCCTGAAAACCTTATGGTTTATACTGTCTGGTCCC
ACATTTATTATCAGACTTTAGATGATACATGGAAGAAAGTTGAGGGGAAAGTTGACTATC
ATGGTGCATATTACTTGGAAGGAACTTATAAAAACTATTACATTCAGTTTGAAATTGATG
CTGCCAGATTTGGCAAAACTGGAATCTGGGAAGTGCATGTTAATGAGGACACTATCTTTG
CTCCTGTTACTAGCTCTTCGCCGGCAGCTGGAGAGGGGACAGCAACCTCCATCAACCCCG
CATCCCAATCGCCGGCCAACGGACAGCCCACTGCCACTGCCACCTCCGTGTCCACCAGAA
AACGGACACCACCACGAACCGAAAGCAGACGGTACAAGCGAAAAGCATCTAGCCCTACAA
CCACCACCACCCGGAGGCAAAAGAGACAAGGACAAAGACAAGAAGACTCAACAAGGCGAT
CAAGGTCCACCTCAAGGGGGAGACAACAAATCCCCAGGGGAGGGGACCGGCGCAGACGGC
GACGATCCCGAGAAACCTCCACCTCCTCCGAGCGGGGAAGGGGAGGGAGAAGTAGAAGGG
GGCCCACAACACGATCCCAATCAAGGTCCCTCTCAAGATCCAGATCCCGATCCAAATCGA
GATCAGGAGGGTCTTCTTCAGGGGGTGGCATTGCGCCTGCAGACGTGGGAAGTTCAGTTC
AATCACTTGGTAGACAACATAATGGGCGACTTGAAAGATTACTGGAAGACGCTAGGGACC
CCCCAGTAATTGTGCTACGTGGAGATGCTAACAAATTGAAATGCTTTCGCTTCAGAGCTA
AGAAAAAGTATAGGGACTTAGTTAAATATTATAGTACCACATGGTCATGGGTAGGGGGTA
CAACTAATGATAGAATTGGACGTTCACGGATGTTACTTGCATTTACTTCAAACACTGAGA
GACAGCTTTTTATTAACATAATGAAATTACCACCAGGTGTTGATTGGTCATTAGGATCTT
TAGATGATTTGTAGCCCATGTGCCTGTAATTAACTAACAAGCGCTATTTTTTGCTTTTGC
TACTAACATTTACTAACATTCCTCATGGCCCGTGCACGTAGAGTAAAACGTGCATCTGTA
ACTGACATCTACAGGGGTTGCAAGCAAGCAGGTACTTGCCCCCCTGATGTTATTAATAAA
GTGGAACAAACAACCATTGCAGATCAAATTTTAAAGTATGGAAGTGGTGCTGTTTTTTTT
GGTGGGCTGGGAATCAGTACAGGCCGTGGTTCAGGTGGTGCAACAGGATATATTCCTTTG
GGAGAAGGCCCTGGGGTGCGCGTAGGAGGCGCCCCCACCATAATTCGCCCTGGGGTCACA
CCTGAACTTATTGGTCCAGCGGATGTAATACCAATTGACACAGTCACACCAATTGACCCT
ACAGCACCTAGTATTGTCACAATAACTGACAGCAGTGCTGTTGACCTTTTACCTGAATTA
GAAACAATTGCAGAAATACACCCTGTGCCAACAGATACTGTGGATATTGATACTCCTGTT
GTTACAGGAGGTCGGGATTCCAGCGCTATTTTGGAGGTTGCTGATCCCAGTCCTCCTGCA
CGAACAAGAGTGTCTCGCACACAATACCATAACCCTGCATTTCAGGTTCTAACTGAGTCT
ACACCTTTATCTGGTGAATCTGCTTTATCAGACCATGTTATTGTTTTTGAAGGCACTGGA
GGCCAAAATATAGGTGGTTCTCGCAGCACAGCTATAGAACCAGCACAAGAAAGTTTTGAA
ATGCAAACATGGCCTAGTAGATATAGTTTTGAAATAGAGGAAGGTACACCACCTAGGTCT
AGCACTCCTGTTCAAAGAGCAGTTCAGTCTCTTTCAAGTCTTCGAAGAGCTTTATATAAC
CGAAGATTAACTGAACAAGTAGCTGTTACAGATCCACTGTTTTTAGGAAGGCCATCACGC
TTGGTACGATTTCAGTTTGACAACCCTGCCTTTGAGGAGGAGGTTACACAAACATTTGAA
AGAGATTTAGAGTCATTTGAGGAGCCACCTGATAGACAGTTTCTAGATGTAGTTCGTTTG
GGTAGACCTACATATTCTGAAACTCCTGAAGGCTATGTCCGTGTCAGTAGACTTGGTAGA
AGAGCCACTATAAGGACCAGAAGCGGTGCACAAGTTGGAGCTCAGGTTCATTTTTATAGA
GATCTTAGCACTATAGACTCTGAAGCATTGGAAATGCAATTGTTAGGAGAACACTCAGGG
GATAGCACCATTGTACAGGCTCCTATGGAGAGTTCATTTATTGATATAAATATTGATGAG
CCTGAGCCATTAAATGTAGGCCTGCAGGAAACAACTGAAGCAGATGATATAGATTATAAT
TCTGCTGATTTATTACTAGAAGATCATATAGAAGATTTTAGCGGGTCTCAATTAGTATTT
GGTAATGCAAGGCGTAGTACTACTACATACACAGTTCCTCGATTTGAGTCTCCTAGAAAT
ACTGGATTTTATATTCAGGATGTTCAGGGCTATAATGTAGCTTATCCAGAGTCCCGTGAC
ACCACAGAAATAATTTTACCACAATCAGACACACCAACTGTAGTGATTAATTTTGAAGAT
GCTGGTGGAGATTATTATTTACACCCAAGTTTAAAAAGACGTAAACGAAAACGCAAATAT
TTGTAATTGTTTTACAGATGACCTTGTGGCTTCCGGCGACGGGTAAAGTGTATTTACCAC
CAACACCACCTGTAGCACGTGTACAAAGCACCGATGAATATGTGGAAAGAACTAATGTAT
TTTACCATGCCATGAGTGATCGCCTTTTAACAGTTGGGCATCCCTATTTTGATGTTAGAT
CTTCTGATGGAGCTAAAATTGATGTTCCCAAAGTGTCTGGAAATCAATACAGGGCATTTA
GGGTTACATTTCCTGATCCAAATAGATTTGCTTTAGCAGATATGTCTGTTTATAATCCAG
AAAAAGAAAGGTTGGTTTGGGCCTGTGCAGGCCTAGAAATAGGCAGAGGACAACCATTAG

GTGTAGGCACATCAGGCCACCCTTTATTTAATAAAGTTAAGGACACAGAAAATAATAGTG
GCTACCAGGGAGGTTCTACTGATGACAGACAGAATACTTCCTTTGATCCAAAACAGGTTC
AAATGTTTATAGTAGGATGTGTTCCCTGTTTAGGGGAACATTGGGATAGAGCTCCTGTTT
GTGCATCAGAAGAAAATAACCAAGCTGGAAAATGTCCTCCATTAGAACTTAAAAATACTG
TGATTGAAGATGGAGACATGTTTGATATAGGCTTTGGTAATATAAATAACAAAGTGTTAT
CGGTTACTAAGTCAGATGTCAGTTTAGACATAGTGAATGAAACCTGCAAATATCCAGATT
TTTTAACTATGGCCAATGATGTATATGGCGATGCATGTTTCTTTTTTGCAAGACGAGAGC
AATGTTATGCTAGACATTATTTTGTTAGAGGAGGTGCAGTAGGCGATGCTATTCCTGACG
AAGCTGTCAATCAGGATCATAACTTTTATTTACCAGCACAAACAGGCCAGCAACAACGTG
TTTTAGCAAATTCCACATATTTTCCTACTGTTAGTGGCTCTTTAGTAACTTCAGATGCTC
AATTATTTAACAGACCGTTTTGGTTACGAAGGGCACAAGGCCACAACAATGGAATATTGT
GGGGGAATCAAATGTTTATTACTGTTGCTGATAATACAAGAAACACTAATTTCACCATAA
GTGTTACTTCTGATGGTAGTACTATAACTGAATATAATACACAAAATATTAGAGAATTTT
TAAGGCATGTAGAAGAATATCAATTAGCTATTATATTACAACTATGTAAAGTGCCTTTAA
AGGCTGAGGTATTAACACAGATAAATGCAATGAATGCAGGTATTTTAGAAGAGTGGCAAC
TAGGGTTTGTTCCCACACCAGACAATGCTGTGCATGATATTTACAGATATATTGACTCTA
AAGCTACTAAATGTCCTGATGCAGTTGCTCCAAAGGACAAAGAAGACCCATTTGGAAAAT
ATACATTTTGGAACGTAGATTTAACAGAAAAATTGTCTTTAGATTTAGATCAGTATCCTT
TAGGAAGGAAATTTATATTTCAGGCAGGTTTACAAAGACGTCCCAAAACCATCAGATCTT
CTGTAAAAGTTTCTAAAGGTACCAAACGCAAACGCACATGACCGATTTCGGTTTCTAATA
AACAAATAAACTAATAAGGTATGTGAAGTATTTTTTACCATGTTCGTGACTAAACCGTAT
AGGTCATTGCCAACAACCGCACCCGGTTAATCAGATATAAAGTGCACCTGGTGCGATTTT
ATCACCGCTTTTGTGGAAGCACCCGAGGCGCCCGCCAGAACTGCTGC
>gi|2911558|emb|Y15175.1|_Human_papillomavirus_type_77_E6,_E7,_E1,_E2,_E4,_L2,_a
nd_L1_genes
TATAAACTATAACCTTTATATTAAAAAGTAGGGAGGGACCGAAAACGGTACGACCGAATG
AGAGACATATAAAAAGGCGTCAGTACAGCCTGGCAGTAGCTATGTCTACAAGTGATGGAC
GTCCAAAAAACATATTCCTGTTGTGCAGAGACAGTGGAATACCGTTCGACGACCTTCGCC
TACTGTGTGTGTTCTGCACAAAAGAATTAACTACTGCTGAACTGGCAGCATTTACAATTA
GAGAATTAAATGTGGTGTGGAGAGGTGGGGCGCCGTACGGTGCATGTGCACGGTGTTTAC
TGCTATATGGCATTGAGCGGCGCCTGAAACACTGGGAGTATTCATACTACGTGGACGGCG
TGGAGGCAGAAACAAAAGAATCCATAGATACCCAGCAAATCAGATGCTACATGTGTCACA
AACCACTCACCAAAGAGGAAAAGGACAAACATCGAAACGAAAAGCGAAGACTACATAGAA
TCTCCGGACACTGGAGAGGGAGTTGTCTGCATTGTTGGTCACGATGCATGGGCCAAAGCC
GACAGTAAAAGATATTGAATTGGATCTTGCACCAGAGGCCGTCCCTTTACTATGCAATGA
GCAATTAGACAGCTCAGATGAAGAAGATTGTATAGATGTTGTGGAACCAGCACAACAGGC
GTACAGGGTGGTAACTTTGTGTACAAAGTGTAGTTCAACACTGCGACTGGTGGTAGAGAG
CAGCGAAGCAGATATAAGGGCATTCCAGGAGCTCCTACTACGCACATTGAAGATCGTGTG
TCCTCGCTGTGCGTAACGGGTCATGGCTGATAACTCAGGTACAGAGGGGGAGGAGGAGGA
CTGTTCAGAGGCGGAACGGGCTGGGGGATGGTTTATAGTTGAGGCCATTGTAGATAAACG
TACGGGGGACACAATATCCAGTGATGAGGATGAGGAGGGTGAGGGTGAAGACATGGTAGA
CTTTATAGACGATAGACCTATAGGGGACGGACAGGAAGTAGCACAGGAACTGTTGCTGCA
GCAAGCAGCTGCGGATGACGATGTAGCAGTGCAGGCTGTAAAACGAAAGTTTACTCACAG
TCCCTATTTCAGCCCTTTGTGTGTGGATCCCAGCATAGAACATGAGTTAAGTCCCAGGCT
AGATGCCATAAAACTGGGCCGAGAGTCCGCAAAGGCCAAGCGGAGGCTATTCCAACTACC
GGACAGTGGGTATGGCCAAACACAGGTGGATACGGACTCGGGACCAAGCCAGGTACAAGA
TATTTGTGAGGCGGGAGGACAAGATGGCCGCCAAACCAATACGGAGGGGAGTGGTACAGA
GCAAGGGGAAAATGTCAGCCGAGAGGAGGAGCGTGCAGGAGGGGATGGGGAGGAATCGCA
ACCCCTAGGTGCAGAAACAGAGAGAGGAGCATGTGGTGTGTTGTCTATACTGAAAGCTAG
TAATCAGAGAGCAACTCTACTAGGTAAGTTTAAAGAGCAATTTGGACTGGGATATAATGA
ACTGGTAAGGCATTTTAAAAGTAATAGGACAGCATGTGCAGATTGGGTAGTGTGTGTATT
TGGGGTATACTGCACAGTGGCTGAGGGTATAAAACAGTTAATACAGCCACTATGCGATTA
TGCACACATTCAAGTGCTACCCTGTCAGTGGGGGATGACAGTGTTGATGCTACTGCGGTA
CAAACGTGCCAAAAACAGAGAGACAGTTGCAAAGGGCTTAAGCACGTTATTAAATGTGCC
AGAAAGCCATATGTTGATTGAACCACCCAAGTTAAGGAGTGGCCCAGCAGCATTGTATTG

GTATAAGACTGGTATGTCTAATATTAGTGAAGTGTATGGAGACACTCCGGATTGGATAGT
AAGACAAACAATTGTGGGACATGCTTTAGAAGAGACACAGTTTCGGTTATCAGACATGGT
ACAGTGGGCATACGATCATGACATAACAGACGAAGGAACGTTGGCATATGAGTATGCATT
AATAGCTGAGTTCGACGCTAATGCAGCAGCATTTCTTGCCAGCAATTGTCAGGCCAAATA
TGTGAGGGACGCCTGCACAATGTGTAGACATTACAAACGGGGAGAGCAGGCACGTATGAC
TATGTCCGAGTGGATAAAATTCAGAAGTGATAAAATACAGGGCGACGGGAACTGGAAACC
CATAGTACAGTACTTAAGATATCAGGATGTAGAATTTGTACCATTTTTATGTGCCTTAAA
GTCTTTTCTACAAGGCATACCCAAAAAAAGCTGTTTAGTCTTTTATGGGCCTGCGGACAC
AGGAAAGTCATATTTTTGCATGAGTTTGCTAAGATTTATGGGCGGTGCTGTTATTTCATA
TGCTAATTCAACCAGCCATTTTTGGCTGCAGCCATTGTCTGAAGCCAAAATGGGTTTACT
GGACGATGCAACCAGTCAATGTTGGAACTATATAGATACATATTTGAGGAATGCATTAGA
TGGGAACGTAATATGTATTGATAGAAAACATAGGTCCCTGTTACAACTAAAATGCCCGCC
ACTTTTAATAACCACCAATGTAAATCCATTGGAGGATGAGAGATGGAAGTATTTGCGCAG
CAGACTGCAGGTGTTTACATTTAAAAACAAATTTCCAGTAACAAGTAGTGGAGACCCACT
TTATACACTAAATGATCAAAATTGGAAATCGTTTTTTCAAAGGTTATGGGCACGTTTAAG
ACTTACCGATCCTGACGACGAGGAGGAAAATGGAGAACCTAGCGAGCCGTTTAGATGCGT
GCCAGGACAAAATGCTAGAACTCTATGAAAGAGATAGCGACAAACTTGAGGACCAGATAA
TGCACTGGTGTCTTATGCGCCTAGAGAGTGTGTTGTTGTATAAAGCAAGAGAATGTGGAA
TGACACATATAGGCCACCAGGTGGTGCCAACACTTAGTGTAGCCAAAGCAAAGGCATGCA
GTGCCATTGAAATGCATGTAGCCTTACAACAATTGCAACGAAGTGCATATGCACATGAAC
CATGGACACTACGGGACACCTCACGAGAAATGTGGGATGCAGCCCCCAAGAGGTGCTGGA
AAAAAAGAGGAGTGACTGTGGAAGTTAGATATGATGGAGACGAGACCAAAGCAATGTGCT
ATGTACTATGGAAGGAACTAATAGTACAAAATGTTAGTGATGACCAGTGGGTAAAAGTAG
CAGGTCACGTCTCATACGAGGGTCTATACTATGTGCATGAAGAGGAAAAGGTGTTTTACG
TGAAATTCAAAACGGACGCTCGTGTGTATGGGGAAACAGGCAGATGGGAGGTGCATGTGG
GAGGCAAAGTCATTCACCACAATGCATTTGACCCTGTATCTAGCACACGAGAAGTACCCG
CTACTGGACCTTTGTACCCAGAGTACAACACCACCCACGCGCCCACCCAAGCCCCGTTGG
GGCTTGAGGAGGGACCGGAACGGAAACGACGCAGGCCTGAAGCAGTCGGGCCGGGGCAGC
AGCAGCAGCAGCAGCAGCAGCAGCAGCAGCAACAGTGGCCCCCGACCCAGACCCCTGCCG
CGAAAGCCAGTGACCGAACGGGTGGACCATTGGACAGTAACAGGACCCGGGACTGTGACT
CTACAAGTCAAAACTCCATCAGGACACCAAGTGACCTTGACTGTGCACCTGTAATACACC
TACGAGGTGACCCAAACAGTTTAAAATGTTTTCGATATAGATTGAAAAATGGTAAAAAAG
ATCTGTATTGTAGGGCCTCGTCCACGTGGCGGTGGTCCTGCGAACCAGAAAACCAAGCAG
CGTTTGTAACAATATGGTACACCAGTTATACACAGCGCGATGAATTTTTGGCCACTGTCA
AAATACCACCAAATATGCAGGCCATTTTAGGCCACATGTCTGTATTTACATAGAACTGTG
CTACACTGCTATTTAGCCCTGGATTTCTATTTGTGCAGGCTGTATTTTTGGGTGTATTTT
GTGTTGCTGCTGTGTCTGTGCTGGCTGTGTGTGCTTTCTGCACTAACCTGCTATCTGGCT
TTTGTGTTGTGTTTGTATTTAGTATTGGTGGCTTTATATTTACAGGTTGTTTCTCGCATT
GCACGCAATAATCAGTAACACTACCATGTATCCTATCATAATTACCGATGGGGATGGGGA
CCGTCCTGTGTTGTTGTTTGAGCCGGGGGATGTATATGTGTTGGGTTTGTTAATACTGTT
TGTATGCTTATTATTGTTTATTGTATATAGACACTTGGGATTATTGTAATGTTGTATGCT
GCACTTATAGATGTGGTGTACTTGTATTTCATACGTGGCCATTGGATTGTGTATGTGTAC
TTGCTTTTGTACCACTTTTTTTGTTTTGTGTTTTTGTTTTTTTCTTTGCAGTTGAATAAA
GCAACCATGGTGGCACATCGTGCAAGGCGTCGCAAGCGTGCATCCGCCACAGAGCTTTAT
AAAACCTGCAAAGCAGCAGGCACATGCCCCCCTGATGTTATTCCCAAGGTTGAGGGCACT
ACGCTGGCTGACAGGATATTACAATGGGGCAGTCTTGGTGTTTATTTAGGCGGGTTGGGT
ATTGGCACTGGGACAGGCACTGGGGGTCGCACTGGCTATGTTCCTATCGGCACCCGGCCT
GGAACTGTAGTTGATGTGTCTGTTCCTACCAGGCCTCCTGTGGTTGTGGAGCCTGTGGGC
CCTACGGATCCCTCCATTGTTACTCTGTTGGAGGAATCTAGTGTAATTGATTCGGGTGCT
TCTATACCCACCTTTACTGGTACTTCTGGATTTGAGATAACCTCCTCTGCCACAACCACT
CCTGCTGTATTAGACATTACCCCTGCAGGTGACACTGTAGTAGTTACTAGCACAAACTTC
ACTAATCCTTTGTATACTGAGCCGTCACTGGTAGAAGTGCCACAAACTGGAGAAATTTCT
GGACATTTGTTGGTTAGCACAGCCACTTCGGGTACCCACGGGTATGAGGAAATTCCCATG
GATACGTTTGCCACCTCTGGAACGGGGTCTGAGCCCATCAGTAGTACTCCTGTACCTGGT
GTTAGTAGGGTTGCAGGTCCACGCCTATATGGCAAGGCCATGACACAGGTTCGGGTGCCT

GATCCTGCCTTTTTGTCACGTCCCTCTTCTTTTGTGACATTTGACAATCCTGTGTATGAT
CCCGGGGATGAAACCATTATTTTTGAGCGTCCCTCGCCTGGTACTCGTGTGCCCGACCCC
GATTTTCTTGATATTGTCAGGCTGCATAGGCCTGCCTTAACATCTCGCAGGGGCACTGTG
CGCTTCAGTAGAGTAGGTCAAAAGTTTAGCATGCGTACTCGCAGCGGCACACAAATTGGT
GCCAGGGTCCACTATTATCATGACCTTAGTCCCATTACTCACACCGAGGACATAGAGCTA
GAACCACTACTCCCTCCTGCAGATTCTGCTGCTGAGGATTCATTATATGATGTGTATGCT
GATGTAGACGATGCTGACGTGGCCTTTACGAACAGTGGTCGCAATCTTACCTCTTTTGGG
GGTCGCGGTGCATCGTCCTCCCTCCCTTCTGCACTGTCCACTAAGATTGGCAATGTAACA
ATTCCATTTATATCTCCGGTGGATGTGCATTTACACACTGGCCCCGACATTGTTTTGCCT
TCTTCTGCACAATGGCCTTTTGTTCCTGTATTACCTGCTGACACAACACATTATGTGTAC
ATTGATGGAGGCAATTTTTATTTGTGGCCTGTTACCTTTTCTGTGTCTCGGAAACGTCGC
CGTAAACGTCTTTCATATTTTTTTGCAGATGGCACTGTGGCGCTCTAGTGACAACCTGGT
GTACCTGCCTCCCACCCCAGTATCCAAAGTTATCAGCACGGACGACTATGTGACACGCAC
AAATGTGTATTATTATGCAGGCAGTTCTCGCCTGTTAACTGTAGGTCATCCCTATTTTGC
TATTCCCAAAACTTCTGGTACTAAGGTGGATGTGCCTAAGGTATCTGCATTTCAGTATAG
AGTATTTCGTGTCCGTCTGCCAGACCCTAATAAGTTTGGTTTGCCTGATGCTCGTATATA
TAACCCTGAGGCCGAACGTTTGGTTTGGGCCTGCACTGGTGTTGAGGTGGGCCGCGGACA
ACCTCTAGGCGTGGGGTTAAGTGGACACCCTCTGTATAACAAACTGAATGACACTGAAAA
CTCCAACATTGCACATGCTGACAATAGTCCTGACTCCCGGGACAACATTTCTGTTGACTG
TAAGCAAACACAACTGTGCATACTGGGCTGTACGCCCCCCATGGGGGAATACTGGGGTAA
GGGTACCCCTTGTGCACGTACTAATACTACCCCAGGAGACTGTCCTCCCTTGGAGTTAAT
GACATCTTATATTCAGGATGGCGACATGGTGGATACCGGGTATGGTGCCATGGACTTTAC
TGCCCTGCAATTTAATAAGTCTGACGTGCCCCTTGATATTTGCCAGTCTATTTGCAAATA
TCCCGATTATTTGGGCATGGCTGCCGACCCGTATGGCGATAGCATGTTCTTTTTCCTCCG
TCGGGAACAACTGTTTGCCAGACACTTTTTCAATCGTGCGGGTGATGTTGGAGACAAAAT
TCCAGAATCTTTGTACCTCAAAGGGAGTAGCGGGCGTGAGACTCCCGGCAGTGCTATATA
CAGCCCCACACCTAGTGGGTCTATGGTGACCTCTGAGGCACAAATATTCAATAAGCCTTA
CTGGCTACAGCAAGCTCAGGGACACAACAATGGCATATGCTGGGGAAATCAAGTGTTTTT
AACTGTGGTAGACACCACACGCAGCACAAATATGTCTCTCAGTGCCTCCACTGAGTCTCA
AACCCCATCTACCTATGATGCCACTAAAATAAAGGAATATTTAAGGCATGGGGAGGAATA
TGATTTGCAGTTTATCTTTCAGTTGTGTAAAGTAACATTAACACCTGAAATTATGGCCTA
TATTCACACTATGAACACTGCTTTATTGGAGGATTGGAATTTTGGACTGACATTGCCACC
TTCTACTAGTCTGGAAGACACATATAGGTTTGTAACGTCCTCTGCCATTACATGTCAAAA
GGATGTGGCCCCCACTGAAAAGCAGGACCCTTACGCAAAGCTTAATTTTTGGGATGTGGA
TTTAAAGGATAGGTTTACTTTGGATTTGTCACAGTTTCCTCTGGGACGCAAGTTTTTATT
ACAGATTGGTGCGCGACGTCGTTCGGTGGTTCCCTCTCGCAAACGCCGGGCGCCCACCCC
CTCCCCAGCTTCGACCAAACGGAAGCGCTCCAAAAAGTGACCCCATTGTTGTTTGTATGT
GTATGTATGTTTACGACAACTATGTATGTGTACTGTGTATTGCATGTGTTTTGTATGGTA
TGTGTGTGTATACAACAACTATGTATGTATGTGTATATGTGTTAATAAACTGTGTCACAT
AGTTATATATTTTATACATATAATTGCTTGCTCTGTGAGTAAGAATAAAACTTGTAGGTC
AGAAGACCCAATTCGGTCTAAGATGGCCGCCTGTGCAGGTGTGCAAACCACTAATTAGTC
ATATTATTCAATTTCCTGCAACATGCCGTCTCACGCACCTTTTTGGCAGCACTTTTCTGC
TTTCCACTGTTTATTTTATTGCTGTATCATTCATCTTGGCTTGCCACCACATACACATTG
CAATTTTGCTGCTTCTAGGCACCAAGTTATTATGACTACTTTCACATAATTACTGTCTTG
GCTGCCTTTTCTAGGTTATCTTTCCAATAAAACATGTTTGCAAATCCCCACCTTTACATT
GTGTTTCCATGACACATCTAATCCGGTCGCTGCTTGCTTTCCAACCCTAATTAATGCGGT
TGCCTCACCTGTCTTTCTAACCATAAT
>gi|2911551|emb|Y15174.1|_Human_papillomavirus_type_76_E6,_E7,_E1,_E2,_E4,_L2,_a
nd_L1_genes
CAGATTTGTTCCAACAACGATTTTGGCGCGAACATTTTGGCAGCAGAAGAGCGACGGTAA
CGGTAAGTTTCAACCGGGTGCGGTCAAAACAACTTACTCATCATAATGGTTGCCAACAAC
AATCATCGAAACAGATCTTTATGTAACCACATAAGTTGTTTTCACTCTTTTATTCTTGGA
AAGAATAGATACAGGACACATGGCTAGACCTGCTAAGGTATGTGAGCTTAGCCAACTCTT
AAATATACCCATTTCTTCTATGCTGATACCCTGTAACTTTTGCACAGGGTTTCTGACCTA
TTGGGAACTCTTAGAGTTTGATTATAAGGACTTCCATTTAGTGTGGAAGGACGGATTTTG

CTTTGGTTGCTGTGCTAAGTGTGCTTTTGCATCTGCCTACCACGAGTTTACACAGTACCA
TCAGGAAACTGTGGTAGGCATAGAAATAGAAGGCAGAGCTGCAGAAAATATAGCGAACAT
TATAATAAGGTGTCAATTTTGTTTGAAACGCCTGGATTTATTTGAAAAACTTGATCATTG
TGCACAGCAAAGAGAGTTTCACAAAGTTAGAAATAGGTGGAAAGGGGTGTGTAGACATTG
CAGAGTAATAGAATGATTGGGAAAGAGGCTACATTACCTGACATTGTGCTACAAGAAGAG
TTTGGCCAGCCCATTGACCTGCAATGCTACGAGAACTTACCAGCTGAAGTGCCAGCTGAA
CAAGACACAGAGGAGGAGCTTATCCAAGGCATCCCTTACAAAGTGATTGCTTGCTGTGGC
GGCGGATGCGGTTCTAGACTTCGTGTGTTTGTGCTGGCTACTGATCGGGCCATTCGAGAA
TTTCAATCTTTACTGCTAGAGGAGTTGCAATTCCTGTGTCCGGAGTGTCGTGAAGAGATT
CGCAATGGCGGACGATAAAGGTACTGACCCTAAAGAAGGGTGTAGTGAATGGTTCTTAGA
TAATGAAGCAGACTGTAGTGATTTAGAAAATGACCTTGAGCAGTTATTTGATGAAAGTCC
ACAGTCCAATATATCAGAATTAATAGATGATGATGCGGACGTGGAGCAGGGAAATTCGCG
AGATCTGCTTCGCCAGCAGGAATTTGAGGAAAGCGCGGAGCAAGTGCAAAAGTTAAAACG
AAAGTATTTCAGTCCTAAAGCAGTGCAACAACTTAGTCCACGGTTGCAGTCTTTGTCTAT
ATCTCCGCAACAAAAGTCTAAACGGAGGCTGTTTGAGCAGGACAGCGGGCTGGAACTGTC
GGGTTGTGAACAAACTTTGTCTAATGAAGTTGAAGATATTACTACGGAGCTGGAGGTACC
AGCGGTGCCGCCGGCAGAGCAGGGTGGCCAGGGATCGGGAAATTTGCATTATAAAGAATT
AATGCGATGCAGCAATAGTCGTGCAAGATTATTAAGTAAAGTCAAAGAATATTTTGGTGT
AGGATTTTATGAGTTAGCTAGGCAATATAAAAGTGATAAAACATGTTGCAGAGACTGGGT
AGTTGGAGCCTATGGAGTGCGGGAGGAATTGCTAGAAGGTGCAAAACAATTACTTATTAA
TCATTGTTCTTATGTGTGGATTAACATAAATGGGATTATGTCATTATTCTTGCTGTGTTT
TAACAATGCTAAAAGCAGAGAAACTGTGGGAAGATTGCTGATGTCAATGCTTGATGTACA
ATTATTGCAACTAATATGTGAACCTCCCAAACTTAGAAGTGTGGTGTCAGCATTATATTG
GTATAAAGGTAGTATGGACTCATCTGTGTATGCTCACGGAACCTATCCTGATTGGATAGT
AAATCAGACCATGTTAACACATCAGGCTGCAGCAGAAGCTGTGCAATTTGACTTATCTGA
AATGATACAATGGGCCTATGATACAGATCTCACAGATGAAGCTGACATTGCATATGGATA
TGCTAAGATGGCTGAGTCTGACTCAAATGCCAGAGCTTGGCTAGCACATAATAGTCAAGC
AAAATTTGTAAGAGAATGTGCACAAATGGTCAGACATTATAGACGGGGAGAGATGAGAGA
TATGAGTATATCAGAGTGGATTCATTATAGGATAGAACAAGTAGAGGGTGAAGGTCATTG
GTCTGAAATTGTAAAGTTTATTAGATATCAAGGAATAAATTTTATATTATTCCTTGATGC
ATTTAAGCAATTTTTGCATGGGAAACCCAAAAAAAGTTGTTTATTAATTTATGGACCGCC
CGATTGTGGTAAATCTATGTTTGCAATGTCATTAATAAGAGTGTTGAGAGGGAGGGTAAT
ATCATTTGTAAATGCAAAAAGTCAATTTTGGTTATCACCTCTTGCAGAATGTAAAATAGG
TTTATTAGATGATGCCACTGATCCATGTTGGCAATATATAGATACATACTTAAGAAATGG
CATTGATGGTAATATTGTAAGTGTGGACTGTAAACATAAAACTCCATTACAAATTAGATT
TCCTCCTCTTTTAATTACATCAAATTATAACATCAAGGACAATGATAAATACAGATACCT
GTATAGCAGAATTGTGATATTTGAGTTTAAACATAAGTTTCCATTTAAAGAGGATGGTTC
ACCTGAATTTCTCCTTACTGACCAAAGCTGGAAATCTTTTTTTAAAAGGCTTTGGAATCA
ATTAGATCTCAGTGACCCTGAAGACGAGGCAGAGAATGGAGGCACTCAACGATCGTTTCA
ATGCACTACAAGACAAATTGATGGACATTTATGAATCAGGGAAAGATGATCTTGAAACAC
AAATAGAACATTGGAAATTGTTAAGACAAGAACAAGTGTTGTATTATTATGCACGCAAAA
AAGGCGTGTTAAGACTTGGGTATCAACCTATTCCCACACTTGCAACCTCCGAAACTAAGG
CTAAAGAAGCAATTGGAATGATGCTTACACTGGAGAGTTTGCAAAAATCTCCATTTGGAA
AGGAACCATGGACTTTAGTAAATACCAGTCTTGAAACATATCGCACCCCACCTATAAACT
GTTTTAAAAAGGGGCCTGTAAATATAGAAGTAAGATTTGATGAAGATCCTGAAAATATAA
TGTTGTATACATCTTGGACATATATTTACTTTCTAGATGCAGAAGATCAATGGCAAAAAG
TAAAAGGGGAAGTGGACTATTTAGGTGCTTTTTATAAGGATGGGCCACATAAACATTATT
ACATAAACTTCAATGATGATGCCCTTAGATATGGGACATCAGGCCAGTGGGAAGTAAGAA
TTAACAACGAAACTGTGTTTGCTCCTGTCACTAGCTCCACACCACCGGCCGCGGAGCTAC
GGGGATCCTCCAACGCCAGCCCCGTTCCCGCCCCCGACCACACCAGGTCCCAGACGCCCA
CCAGCACCACAGGAACCACCACAACCTACAGCACCACCAGCGTTAGCACCAAGGGAGCAC
CAGAACTCGCATCCAAAGCCAACACCAGGAAAAGAAGGTACGGAAGAAAAGACTCTAGCC
CTACAGCCACCTCCAGGAAAGAGGTCTCGCGGAGACGCTCCCGGTCCAGCACCAGGACCC
GGGCAACCTCCAGAGCAAGGGCCATCAGAGTCAGATCCCAAAGACCCAGCCGATCCAGAT
CGCGATCCAGATCCCGAGAATCCAGAGGATCCAGAGGATCCAGAGGGTCCAGCAGCTCCA

GAGGATCCTCCAGTCGCAGGGGCCAGCGGGGGCGGGGAGGGGGAGGTCGAAGGGCATCCA
CACACACACCCACCCCCAGTCCCCACAAACGGTCACAACGGAGAAGTAGGTCCCGAGAAC
GGGGAACCGGAGCCATTTCTAACAGGTCTGGCATCTCGCCTGACAAGGTGGGATCTGCAG
TACAAACAGTTAGTGGACGACATCTTGGAAGACTTGGAAGGTTACTGGCAGAAGCTAGCG
ATCCTCCAGTAATACTTTTACGAGGTGATCCAAATGTGTTGAAATGCTTTAGATACAGAG
ATAAAAAAAATAAGAAGGGATTAGTAAAATGTTATAGTACCACATGGTCCTGGGTGCATG
CAGACGGTAATGAGAGAATAGGTAGAGCTAGAATGCTTTTAGGTTTTACTTCTCAGCAGC
ATAGAGCACAGTATATTAGTTCCATGAAATTGCCTAAAGGTGTAGAGTGGTCCTATGGTA
ACTTTGATAGGTTATAACATTAATTAACCTACTAACCTTGCTTGCACTACTAATATATTA
ACATTTTATATTTTTTTACTATGGTCCGTGCTCGCAGAACAAAAAGAGATTCTGTAACAC
ACATTTATCAAAGCTGCAAAGCAGCTGGAACCTGTCCTCCGGATGTTTTAAATAAAGTGG
AACAAACAACAATTGCTGACCAGATTTTAAAATATGGCAGCAGTGGTGTTTTCTTTGGTG
GTTTGGGTATAGGCACAGGTCGTGGAACCGGAGGCAGTACTGGCTATGTGCCATTAGGTG
AGGGCCCCGCCATTCGTGTTGGGGGCACTCCAAATGTCATTCGGCCTAGTGTGATACCCG
AGGCTATTGGGCCTGTTGATATCATACCTATTGACACTGTCAATCCAATTGACCCAACTG
CATCATCTGTGGTCCCACTCACTGAAACAGGACCTGATTTGCTGCCTGGGGAAATTGAGA
CAATTGCAGAAGTGAACCCTGCCCCAGATATTCCTAGAGTTGACACATCTGTTGTGACAA
CAAGTCGTGGCACTAGTGCAGTGCTTGAAGTTGCTCCTGAACCAGTACCTCCTACACGGA
CACGTATATCACGAACACAATATCACAATCCTTCTTTTCAAGTACTTACTGAATCCACAC
CAACACTAGGGGAATCCGCATTGTCAGACCAAATTGTAGTTACCAGTGGCTCAGGTGGCC
AGGCCATTGGTGGTTCCAGATATGTGGAAATAGAGCTGGAGGAAATACCAAGTAGATATA
CATTTGAAATAGAAGAACCCACCCCTCCAAGACGCACAAGCACCCCTGTTCAAAGAATTA
CACAAGGTATTAGTAATATTAGAAGGTCTCTATATAATAGAAGACTGACACAGCAGGTTA
ATGTTCAGGATCCTTTATTTTTACAACAACCTTCACGTTTGGTACGTTTTACGTTTGATA
ATCCCGCTTTTGAAGATGAGGTTACACAAATATTTGAAAGAGATGTAGCTACTGTTGAGG
AGCCACCTAATAGAGACTTTTTGGATATAGCTAAACTAAGTCGTCCACTATATTCAGACA
CTCCACAAGGTTATGTACGTGTTAGTCGTCTTGGTAACAGAGCCTCTATAAGGACTAGAA
GTGGTGCTACTGTAGGGGCTCAGGTACATTTCTATAAAGACCTTAGTACTATAGATGCAG
AAGAATCAATAGAATTGTCACTGCTTGGTGAGCATTCAGGTGATGCCTCTATAGTCCAAG
GCCCTGTAGAAAGTACATTTGTTACATCAGATCTACAAGAATTGCCTGTTAATATTGTAG
AGGAAACAGAACCCGAGTTTCATTCTGATGATTTATTGCTTGATGAACAAAATGAGGATT
TTAGTGGATCTCAGTTAGTGTATGGTGCAGGCAGACGGTCCATTACATACACAGTGCCTA
GATTTACAACACCCCGAGCAGATACATTTTATGTGCAGGACTTAGAGGGTTATACAGTGT
CATATCCAGAAAGAAGGGATTATCCAGAAATAATTTATCCTCAACCTGACCTTCCTGCTG
TTGTTATACATACATCTGATACATCTGGGGATTTCTATTTACATCCTAGCCTACGTAGGC
GTAAACGTAAACGAGCTTATTTATAATATTTTTTTCAGATGACTTCGCTTTGGTTACCAG
CAACTGGTAAGGTTTATCTTCCACCATCAACACCTGTGGCCAGGGTTCAAAGCACGGATG
AATACATTGAAAGAACAGACATCTACTATCATGCAAATAGCGATCGCCTCCTTACTGTAG
GCCACCCATATTTTGATGTTAGAGACACTGGGGATTCAACAAAAATACAGGTTCCTAAAG
TATCAGGGAATCAGTTTAGAGCATTTAGATTACTACTACCAGATCCCAATAGATTCGCCT
TGGTAGACATGAATGTCTATAATCCTGAGAAGGAAAGACTGGTTTGGGCCTGTAGAGGTT
TGGAAATAGGTCGCGGACAACCCCTAGGAGTAGGGTCTACAGGTCATCCCCTATTTAATA
AAGTGAAGGATACGGAAAATGCTAATAATTATATAGTAACATCTAAGGATGATAGGCAGG
ACACCTCATTTGATCCTAAACAGGTTCAAATGTTTATTATTGGCTGCGCACCGTGCATAG
GTGAGCACTGGGATGCAGCCAAGCCCTGTGATGCTGACAGAGGGGTAGGCAAATGTCCAC
CTTTGGAACTGGTAAATACTGTAATAGAAGATGGAGATATGGTGGATATAGGTTTTGGAA
ATATAAATAATAAAACCCTGTCAGCAAATAAGTCAGATGTCAGTTTAGATATAGTTAATA
ATATTTGTAAGTATCCAGACTTTTTAAAAATGGCCAATGACATATATGGAGACTCCTGTT
TTTTTTATGCTAGACGGGAGCAATGTTATGCTAGACATTTTTTTGTTAGAGGAGGTAATG
TAGGAGATGCTATTCCTGATGCTGCAGTGGGTCAGGACAATAACTTTGTGTTGCCTGCAG
CTGTTGGACAGGCCCAAAACACTTTGGGTAGCTCTATTTACGTGCCTACCGTTAGTGGTT
CTTTGGTATCCACAGATGCACAATTATTTAATAGGCCCTTTTGGCTACAACGAGCACAGG
GTCATAACAATGGCATTTGTTGGGAAAATCAATTATTTGTTACTGTTGTTGATAATACTA
GGAATACTAATTTTACCATAAGTGTTACCACAGATGGTCAAACACCAACTGATTATGATA
GTACTAAAATCAGAGAGTTTTTAAGACATGTGGAGGAATATGAACTATCAGTTATTTTAC

AATTGTGTAAAGTACCTTTAGTGCCAGAAGTACTTGCACAAATCAATGCAATGAATTCCA
ATATATTAGAAAATTGGCAATTAGGTTTTGTGCCTACACCTGACAATCCCATTCATGACA
CCTATAGATATTTAACATCCCAGGCCACTCGATGTCCAGATAAACAGCCCGCCCCTGAAA
AAAAGGATCCATATGAGCAATACAATTTTTGGCAAGTGGATCTAACTGAGAAATTGTCAT
TAGATTTGGATCAATATTCCTTAGGACGCAAGTTTCTGTTTCAAGCAGGCCTGCGACGGG
CTTCCAGAGTATCCAGGACAGCCACTGTCCGCACAGCATCCAGGGGAACCAAACGCAAAC
GTTCATGACCGTTTTCGGTCACTGGGTCTCATAATAAAATATTTTATAAACTCTTTTGGT
ATGTCAAGCATGTTTTAACCGAGTTCGTGACTAAGATTGATTAAGGCCATCTGCAACCGC
ACCCGGTTAATCAGATTATAAAGGAGCGCCAGGGTTTACCTCAAGCAACTTGGCAGTTAC
AACTTGTCTTCTGCCAGAAGGTCTGTCTGCCAACTTCCTTTCCAATAAC
>gi|2911544|emb|Y15173.1|_Human_papillomavirus_type_75_E6,_E7,_E1,_E2,_E4,_L2,_a
nd_L1_genes
AACCATTTTGGCGCGAACATTTTGGCAGCAGAAGAGCAACGATAACGGTAAGTTTCAACC
GGGCGCGGTCAAAACTACTTACTCATCATTATTGTTGCCAACAACAATCACCGAAACAGA
TATTTATGTAACCACTTGTGTTGTTACCACTTGTGTTGTTTTCACTCTTTTATTCTTGGA
AAGAATACAGACAGAACACATGGCTAGACCTGCTAAGGTATGTGAGCTTAGCCAACTCTT
AAATATACCTATTTCACAAATTTTGTTACCCTGTAACTTTTGCACAGGGTTTCTGACCAC
TTGGGAACTCCTAGAGTTTGATTATAAGGACTTCCACTTGGTGTGGAAGGACGGATTTTG
CTTTGGTTGCTGCTCAAAGTGTGCCTTTGCATCGGCCTATCACGAGTTTACACAGTATCA
TCAGGAGACCGTGGTAGGCATAGAAATAGAAGGCAGAGCAGCAGAAAGTATACATAATCT
TATTGTAAGGTGCCAGTTTTGTCTGAAACGTTTGGATATATTTGAAAAACTTGATAATTG
TGCACAGCACAGAGAATTCCACAAAGTTAGAAATAGGTGGAAAGGGGTGTGTAGACATTG
CAGAGTGATAGAATGATTGGGAAACAGGCTACATTACCTGACATTGTGCTACAAGAAGAG
TTTGGCCAGCCCATTGACCTGCAATGCTACGAGGATTTAGCAGCTGAAGTGCCAGCTGAA
CAACACTCAGAGGAGGAGCTTATTCAAGGCATCCCTTACAAAGTGATTGCTTGCTGTGGC
GGCGGATGCGGTTCTCGACTTCGTGTGTTTGTGCTTGCTACTGATCGGGCTATTCGAGAA
TTTCAAACTTTGCTGTTGGAGGAGCTGCAATTCCTGTGTCCTGAGTGTCGTGAAGAAATT
CGACATGGCGGACGATAAAGGTACTGACCCTAAAGAAGGGTGTAGTGAATGGTTCTTAGA
TAATGAAGCAGACTGTAGTGATTTAGAAAATGACCTTGAGCAGTTATTTGATGAAAATCC
ACAGTCCAATATATCAGAATTAATAGATGATGATGAGGACGTGGAGCAGGGAAATTCGCG
AGATCTGTTTCGCCTGCAGGAATTTGAGGAAAGCGCGGAGCAAGTGCAAATGTTAAAACG
AAAGTATTTCAGTCCTAAAGCTGTTCAACAACTTAGTCCACGGTTGCAGTCTTTGTCCAT
ATCTCCGCAACAAAAGTCTAAACGGAGGCTGTTTGAGCAGGACAGCGGGCTGGAACTGTC
GGGTTGTGAACAAACGTTGTCTAATGAAGTTGAAGATATTACTACGGAGCTGGAGGTACC
GGCGGTGCAGCCGGCAGAGCAGGGTGGCCAGGGACTGGGAAATTTGCACTATAAAGAGTT
AATGCGATGCAGTAATAGTCGTGCCAGATTATTAAGTAAAGTTAAAGAATATTTTGGTGT
AGGGTTTTATGAGTTAGCTAGGCAATATAAAAGTAATAAAACGTGCTGTAGAGACTGGGT
TATTGGAGCGTATGGTGTGCGGGAGGAATTGCTGGAAGGTGCAAAACAATTACTTCTAAA
TCATTGTTCTTATGTGTGGATTAATATGAATGGGATTATGTCATTATTCTTGTTGTGTTT
TAACAATGCAAAAAGCAGAGAAACTGTGGGAAGATTACTAATGTCTATGCTTGATGTACA
ATTATTGCAATTAATATGTGAACCTCCCAAACTTAGAAGTGTGGTGTCAGCATTATATTG
GTATAAAGGTAGTATGGACTCATCTGTGTATGCTCACGGAACGTATCCTGATTGGATTGT
GAATCAGACCATGTTAACACATCAGGCTGCAGCAGAAGCTGTGCAATTTGATTTGTCTCA
AATGATACAATGGGCTTATGATACTGATCTCACAGATGAAGCTGACATTGCATATGGATA
TGCAAAGATGGCTGAGTCTGATTCAAATGCCAGAGCTTGGCTAGCACATAATAGTCAAGC
AAAATTTGTAAGAGAATGTGCACAAATGGTCAGACATTATCGAAGAGGGGAAATGAGAGA
TATGAGTATCTCAGAATGGATTCATTACAGAATAGAACAGGTGGAAGGCGAAGGTCATTG
GTCTGAAATAGTTAAGTTCATTAGATTCCAAGAAATAAATTTTATATTATTTCTTGATGC
ATTCAAACAATTCTTGCATGGTAAACCAAAAAAAAGTTGTTTATTAATATATGGACCGCC
TGACTGTGGAAAATCTATGTTTGCAATGTCATTAATAAGAGTGCTAAAAGGGAGAGTAAT
ATCATTTGTTAATGCAAAAAGTCAATTTTGGTTATCGCCTCTTGCAGAATGTAAAATAGG
ATTATTAGATGATGCCACAGATCCATGTTGGCAATATATAGATGCATATTTAAGAAATGG
AATTGATGGTAATATTGTAAGTGTGGACTGTAAACATAAAACACCATTACAAATTAGATT
TCCTCCTCTTTTAATTACATCAAATTATAATATTAAGGACAATGATAAATATAGATACTT
GTATAGCAGGATTGTGATATTTGAGTTTAAGCATAAGTTTCCATTTAAAGAGGATGGTTC

ACCAGAATTTCTTCTTACTGACCAAAGCTGGAAATCTTTTTTTAAAAGGCTTTGGAGTCA
ATTAGAACTGAGTGATCCTGAAGACGAGGCAGACAATGGAGGCACTCAACGATCGTTTCA
ATGCACTACAAGACAAATTGATGGACATTTATGAATCAGGGAAAGATGATCTTGAAACTC
AAATAGAGCACTGGAAATGTTTAAGACAAGAACAAGTGTTGTATTTTTATGCACGTAAAA
AAGGCATACTAAGAATTGGGTATCAACCTGTACCCACACTTGCAGTATCTGAAACTAAGG
CAAAAGAAGCAATTGGAATGATGCTTACACTGGAGAGTTTGCAAAAATCTCCATTTGGAA
AAGAATCATGGACCTTAGTAAATACGAGTCTTGAAACATTTCGTACCCCACCTGCAAACT
GTTTCAAAAAGGGGCCTATTAATATAGAAGTAAGATTTGATGGAGATCCTGAAAATATAA
TGTTGTATACAGCATGGACACATATTTACTTTCTAGATGCAGAAGAACATTGGCAAAAAG
TAAAAGGGGAAGTGGACTATCTAGGTGCTTTTTATAAGGATGGGCCACATAAACATTACT
ATATACACTTCAATGATGATGCCCTTAGATATGGGACTTCTGGACAATGGGAAGTAAGAA
TTAACAACGAAACTGTGTTTACTCCTGTTACTAGCTCCACACCACCGTCCGCGGAGCTAC
GGGGATCCGCCAACACCAGCCCCGTTCCCGCCGCCGACCACACCAGGTCCCAGACGCCCA
CCAGCACCACAGGAACCACCACCACCTACAGCACCTCCACCGTTAGCACCAAGAGACCAC
CAGAACTCACATCCAAAGCCAACACCAGGAAAGGAAGGTACGGAAGAAAAGACTCTAGCC
CTACAGCCACCTCCAGGAAAGAGGTCTCGCGGAGACGGTCTCGGTCCAGAACCAGGACCC
GGGGACGGGACGCGAGCACAAGGGCCATCAGAGTCAGATCCCAAAAACCCAGCCGATCCA
GATCGCGATCCAGATCCAGAGAATCCCGAGGATCCAGAGGATCCAGCAGCTCCAGAGGAT
CCTCCCGCAGGGGCGAGCGGGGGCGGGGAGGGCGAGGCGGAGGGGCATCCACCCCCCACA
AACGGGCACAAAGGAGAAGTAGGTCCCGAGGACGAGGATCAGGAGCCATTTCTAACGGGT
CTGGCATCGCGCCTGACAAAGTGGGATCAACAGTACAAACAGTTAGTGGAAAACATCTTG
GACGACTTGGAAGGTTACTGGCAGAAGCTAGCGATCCTCCAGTAATACTTTTAAGAGGCG
ATCCAAACATACTAAAATGCTTTAGGTACAGAGACAAGAAAAACAGAAAGGGATTGGTTA
AATGTTATAGTACTACATGGTCTTGGGTGCATGCAGATGGCAATGAAAGAATAGGTAGAG
CTAGGATGCTTTTAGGGTTTACTTCTGAGCAGAATAGAGCAGAATATATTAATTCTATGA
AATTGCCTAAAGGTGTGGAATGGTCCTACGGTAACTTTGATAGGCTCTAACATGTTTTAA
CATACTAACTTGCTTGCACTACTAACATATTAATATTTTATACTTTTTTACTATGGTTCG
TGCCCGCAGAACAAAAAGAGATTCTGTAACCCACATTTACCAAAGCTGCAAAGCAGCTGG
AACCTGTCCTCCAGATGTAGTTAATAAAGTGGAGCAAACAACAATTGCTGACCAGATTCT
GAAATATGGCAGCAGTGGTGTTTTCTTTGGTGGTTTGGGTATAAGCACAGGTCGTGGAAC
TGGAGGCAGTACTGGCTATGTGCCATTGGGTGAGGGCCCCGGCATTCGTGTTGGGGGCAC
TCCAAATGTTATTCGGCCTAGTGTAATACCCGAGGCTATTGGGCCTGTCGATATCATTCC
TATTGACACTGTCAATCCAATTGACCCAACTGCATCATCTGTGGTCCCACTCACTGAAAC
AGGACCTGATTTGTTGCCTGGGGAAATTGAGACAATTGCAGAAGTGAACCCTGCCCCAGA
TATTCCTAGAGTTGACACATCTGTTGTTACATCTAGCAGTGGCACTAGTGCAGTGCTTGA
AGTTGCTCCTGAACCAGTACCTCCTACACGAACACGTATTTCAAGAACACAATATCACAA
TCCTTCCTTTCAGGTGCTCACTGAATCCACACCAACATTGGGGGAGTCTGCATTATCAGA
CCAAATCGTGGTCACCAATGGCTCTGGTGGGCAAGCCATTGGTGGTTCCCGCACTGTGGA
AATAGAATTAGAGGAAATACCAAGCAGATACACATTTGAAATAGAAGAACCCACCCCTCC
AAGACGCACAAGCACTCCTGTTCAAAGAATTACACAGGGAATTAGTAACCTTAGAAGGTC
TTTATATAATAGGAGACTCACACAGCAGATTAATGTGCAAGATCCTTTATTTTTACAACA
ACCATCACGTTTAGTACGTTTCACATTTGATAATCCTGCCTTTGAGGATGAAGTTACACA
AATATTTGAAAGAGATGTGGCTACTATTGAGGAACCCCCAAATAGGGATTTTTTAGATAT
AGCAAAGCTCAGTCGTCCAGTCTATTCAGATACTCCACAGGGTTATGTACGTGTTAGTCG
ACTAGGTAACAGAGCATCTATTAGAACCAGGAGTGGGGCTACTGTAGGAGCTCAGGTTCA
TTTTTATACAGATTTAAGTACTATAGATACAGAGGAATCAATAGAATTATCACTACTTGG
AGAACATTCTGGTGATGCTTCTATAGTACAAGGCCCTGTAGAAAGTACATTTGTAACATC
AGATTTACAAGAGTTACCTGTCAATATTGTAGAGGAAACTGAACCTGAATTTCATTCTGA
TGATTTATTGCTTGATGAACAAAATGAGGATTTTAGTGGATCGCAATTGGTATATGGATC
TGGCAGACGGTCCATTACATATACAGTGCCGAGATTCAGTACACCACGTGCAGATACATT
TTATGTGCAGGATTTGGAGGGCTACACTGTTTCATATCCAGAAAGAAGGGATTATCCAGA
AATAATTTATCCTCAACCAGACCTACCTGCTGTTGTTATACACACATCTGATACTTCTGG
GGATTTCTATTTACATCCTAGTTTACGAAGGCGTAAACGTAAACGAGCTTATTTATAATA
TTTTTTTCAGATGACTTCGCTTTGGTTACCTACAACTGGTAAGGTTTATCTTCCACCATC
AACACCTGTGGCCAGGGTGCAAAGCACGGATGAATACATTCAAAGAACAGACATCTACTA

TCATGCCAATAGTGATCGCCTTCTCACTGTAGGTCATCCATATTTTGATGTTAGAGACAC
TGGAGATTCAACAAAAATATTAGTGCCTAAAGTTTCAGGAAATCAGTTTAGAGCATTTAG
ACTGCTATTACCAGATCCCAATAGATTTGCATTAGTAGATATGAATGTCTACAATCCAGA
AAAAGAAAGATTAGTATGGGCTTGCAGAGGCTTGGAAATAGGTCGAGGACAGCCTTTAGG
GGTAGGTTCTACAGGCCATCCTTTGTTCAATAAAGTAAAGGATACTGAAAATTCAAATAA
TTATATAACAATGTCTAAAGATGATAGGCAGGACACCTCGTTTGACCCTAAGCAGGTTCA
AATGTTTATTATTGGCTGTGCACCTTGTATAGGGGAGCACTGGGATGCTGCCAAACCCTG
TGACGCTGACAAAGGAGACGGTAAATGTCCACCTTTAGAATTAGTAAATACAGTTATTGA
GGATGGGGATATGGTGGATATAGGTTTTGGTAACATAAATAATAAAACCTTGTCAGCAAA
TAAATCAGATGTCAGTTTGGATATAGTTAATAACATTTGTAAGTATCCAGACTTCCTTAA
AATGGCCAATGACATATATGGGGACTCCTGTTTTTTTTATGCCAGACGGGAACAATGTTA
TGCTAGACACTTTTTTGTTAGGGGAGGCAATGTAGGCGATGCAATTCCTAATGCTGCAGT
GGGTCAGGACAATAATTTTATGTTACCTGCAGCCGCTGGGCAGGCTCAAAACACTTTGGG
CAACTCTATTTATGTTCCCACGGTCAGTGGTTCTTTGGTGTCCACAGATGCTCAATTATT
TAACAGGCCATTTTGGCTGCAACGAGCACAGGGTCATAACAATGGTATATGTTGGGAAAA
TCAATTATTTGTTACTGTTGTTGACAATACTAGAAATACCAATTTTACCATAAGTGTTAC
CACAGATGGACAAACACCTACTGATTATGACAGTGGTAAAATACGAGAGTTTTTAAGACA
TGTTGAGGAATATGAATTATCAGTTATTTTACAATTATGTAGAGTGCCTTTAGAGCCTGA
GGTACTTGCACAAATTAATGCAATGAATCCAAATATATTGGAAAATTGGCAATTAGGCTT
TGTACCTTCGCCTGACAATCCCATCCATGACACTTATAGATATTTAACATCGCAGGCTAC
TCGATGTCCAGATAAACAGCCTGCCCCTGAAAAGAAAGATCCTTATGAGCAATATACTTT
CTGGCCTGTGGATCTAACTGAAAAATTATCGTTGGATTTGGATCAATACTCGTTAGGACG
CAAATTCCTTTTTCAGGCAGGCTTACGAAGGGCTTCCAAGGTAACCAGAACAGCCAGTGT
CCGTTCATCGTCCAGGGGAACCAAACGAAAACGCACATGACCGTTTTCGGTCACTGGGTC
TTATAATAAAATATTTTATAAACTCTTGTGGTATGTCAAGCATGTTTTAACCGAGTTCGT
GACTAAGATTGATTAAAGCCACCTGCAACCGCACCCGGTTAATCAGATTATAAAGGAGCG
CCAAGGTTTACCTCAAGCAGCTTGGCAGTTACAACGTGTTTCCTGCCAGAAGTGTGTGTC
TGCCAACTTCCCTGCCAATAACCGAATTTGTTCCAAC
>gi|1491692|emb|X94165.1|_Human_papillomavirus_type_73_E6,_E7,_E1,_E2,_E4,_L2,_a
nd_L1_genes
ACTATAATGTACTATTAAAAAAAAGGGTGTAACCGAAAACGGTTTCAACCGAAATCGGTG
CATATAAAAGTAGGAAAGCAAAAAACGCTACAGATTGGGAAATGCTGTTTCCCAATTCAG
AAGAACGACCATACAAGCTACAAGCGTTATGTGACGAAGTGAATATTTCTATACATGATA
TAAACCTGGACTGTGTGTTTTGCCAACGTGGACTGTACAGATCTGAGGTATATGATTTTG
CATTTAGTGATTTGTGTATTGTATATAGAAAGGATAAACCATATGGTGTATGTCAACCGT
GTTTAAAATTTTATTCTAAAATTAGAGAGTATAGGCGATATAGACAATCAGTATATGGCA
CTACGTTAGAAAATTTAACTAACAAACAGTTATGTAATATTTTAATAAGGTGCGGAAAAT
GCCAAAAACCATTATGTCCACTGGAAAAGCAAAAGCATGTAGATGAAAAAAAACGGTTTC
ATCAAATAGCAGAACAGTGGACCGGACGCTGTACACGGTGCTGGAGACCATCTGCAACTG
TGGTGTAAGATGCATGGAAAAAAAACAACCTTGCAGGACATTACTTTAGACCTGAAACCA
ACAACCGAAATTGACCTTACATGTTACGAGTCATTGGACAACTCAGAGGATGAGGATGAA
ACAGACAGCCATCTAGACAGACAAGCTGAACGAGAGTGTTACAGAATAGTTACTGACTGC
ACGAAGTGTCAGTGCACAGTATGCCTTGCCATTGAAAGCAACAAAGCTGATTTAAGAGTG
ATAGAAGAGTTGCTTATGGGTACACTAGGTATTGTGTGCCCCAACTGTTCCAGAAACCTA
TAAAAGAAGATGGCTGATTCAGGTAATTGGGAAGGGAGGTGTACGGGATGGTTTAATGTA
GAAGCCATTGTAGAAAGAAAAACAGGGGATCCAATTCCAGAGGATGAAAATTATGATGGA
GGGGATACAGATGAGTCGGAAATGGGGGATTTTATTGATAATGCACATATACCAAATATA
TATGCACAACAGGAAATTGCACAGGCATTGTATCAGTCACAGCAAGCAAATGCAGACAAT
GAGGCTATACGTGTTCTAAAACGAAAGTTTACAGGTAGTCCTGGCGGTAGCCCAGATATG
AAAAGAGATGAATTCATAGACAAACAGCTTAGTCCACAAATAAATGTATTGTCAATAAGT
AGCGGTAGAAGTACATCTAAACGAAGACTGTTTGAGGAGCAGGACAGTGGATATGGCAAT
ACTGAAGTGGAAACTTACGAGACAGAGGTACCGGGACTTGGGGCAGGGGTAGGGTGTTTA
CAAAATGTTAATGAAGAAGGCAACCAAATTGTGTCGCCACGTGAAAGCAGTAGTGGGTCC
AGTAGCATTTCAAATATGGATATAGAAACAGAGAGCACACCTATAACAGATATTACAAAT
TTATTACAAAGGAATAATGCAAAAGCAGCATTGCTAGCAAAATTTAAAGAAGTATATGGG

TTAAGTTATATGGAATTAGTTAGACCATATAAAAGTGATAAAACACATTGCCAAGATTGG
GTGTGTGCTGTGTTTGGTGTAATACCCTCACTTGCAGAAAGTTTAAAATCCTTACTAACA
CAGTATTGTATGTATATACATTTGCAGTGTTTAACATGTACATGGGGCATAATAGTGTTA
GTATTAGTAAGATTTAAGTGCAATAAAAATAGACTAACAGTGCAAAAATTATTAAGTAGT
TTATTAAATGTAACACAAGAACGCATGTTAATTGAACCTCCAAGACTACGAAGTACACCA
TGTGCATTATATTGGTATAGAACTAGTTTATCAAATATTAGTGAAATAGTAGGAGACACA
CCTGAGTGGATTAAAAGACAAACGTTAGTGCAGCATAGTTTAGATGATAGTCAATTTGAC
CTATCTCAAATGATACAGTGGGCATTTGATAATGATATAACAGACGACTGTGAAATAGCA
TATAAATATGCATTATTAGGCAATGTAGACAGTAATGCAGCTGCATTTTTAAAAAGTAAT
GCACAAGCAAAATATGTAAAAGACTGTGGTACAATGTGCAGACATTATAAAGCAGCAGAA
CGTAAACAAATGTCAATGGCACAATGGATACAACATAGATGTGATTTAACTAATGATGGT
GGTAATTGGAAAGATATTGTGCTATTCCTAAGATATCAAAATGTAGAATTTATGCCTTTT
TTAATTACATTAAAACAATTTTTAAAAGGTATTCCCAAACAAAACTGTATAGTATTATAT
GGACCGCCAGATACAGGAAAATCACATTTTGGAATGAGTTTAATTAAATTTATACAAGGT
GTAGTTATTTCGTATGTAAATTCAACTAGTCATTTTTGGTTATCACCCTTAGCTGATGCA
AAAATGGCATTATTAGATGATGCAACACCTGGATGCTGGACGTACATAGACAAATATTTA
AGAAATGCATTAGATGGTAATCCTATATGTTTAGATAGAAAACATAAAAAATTTATTACAA
GTTAAATGCCCTCCATTACTGATAACATCAAATACAAATCCTAAAGCAGATGATACTTGG
AAATATTTACATAGTAGAATTAAGGTGTTTACTTTTTTAAATCCATTTCCATTTGACAGT
AATGGGAACCCACTATACCAACTTACTAATGAAAACTGGAAAGCATTTTTTACAAAAACG
TGGTCAAAACTAGATTTAACAGAGGACGACGACAAGGAAAATGATGGAGACACTGTGCAA
ACGTTTAAGTGCGTGTCAGGACGCAATCCTAGAACTGTATGAACGTGACAGTGTACACCT
AAGTGATCATATTGATCATTGGAAACACGTGCGACATGAAAATGTATTATTACATAAAGC
ACGTGAAATGGGACTGCAAACTGTTAACAATCAAGCGGTGCCAAGCCTTGCAGTATCACG
ATCCAAAGGGTATAATGCAATTGAAATGCAAATAGCACTAGAAAGTTTAAATGAATCTTT
GTATAACACAGAGGAATGGACATTGCAACATACAAGTTGGGAACTGTGGGTTACAGAACC
TAAACAATGTTTTAAAAAGGATGGAAAAACAGTAGAGGTTAGATATGACTGTGAAAAGGA
CAATAGCATGCAATATGTATTTTGGACACATATATATTGTTGGTATGAAGGGGGGTGGGC
AAAGGTAGGTAGCAAAATAGATTATAATGGTATATATTATGAAACAGATGATGAGGAAAA
GGTATACTATACAAGATTTGATACAGATGCAAAACGGTACGGGGTAAAAGGCATATGGGA
AGTACATATGGGTGGTCAGGTAATATGTTGTGCTCCTGTATCTAGCGCCTGTGAAGTATC
CATTCCTGAAATTGTTAACCCACTGCACACCACAACCACCAACACCACCACCACCTGCAC
CAACGTTGACACCGGTGTGCCATCACGGAAACGGCAAAGACAGTGTGACTCGGACCAGAG
GCCCCTGGATTGTTTGCATAACCTACATCCCACCACAGAGTCCTGTACCCAGTGTACTAC
ACATAATGTTGCGCCAATAGTGCATTTAAAAGGTGACAAAAACAGCTTAAAATGTTTTAG
ATATAGATTGCATAAAGGCTATTCACATTTATTTAAAAATGTAACAACAACATGGCATTG
GACCAATACTACAAATAGTAAATGTGGTGTAATAACATTAATGTTTACAACTGTATTGCA
ACAACAACATTTTTTACAACATGTAAAAATACCACAAACTATTGTAGTTACATCAGGATA
CATGTCTTTGTAACATTGGTTACACAGTATATATGATTCTTTGTATATTTGTATTTTTGT
TTTGTGTTGGCTTTTGTTTGTGCTTGTGTGTGTCGCTTGCAGTGTCTGTGTATATTTACC
CATGGTTATTGGTATTGATTATAATAACCTTTATACATGTATCACAATCATTGTTAAAAG
TATTTTTTTTATATGTTTTGGTATTTTATATTCCTATGGCACTTGTACATTACCATGCTA
CATTACAAATAACATAAACAATTTTACATATATAATAAACTGCCTAATATTTTTAGTGTA
CCATGCGTCGCAAGCGTGACACACACATACGAAAAAAACGTGCATCTGCAACACAATTAT
ATAAAACATGTAAACAAGCAGGTACGTGCCCTCCTGATGTAATTCCCAAGGTTGAAGGTA
GTACTATAGCTGATAATATATTAAAATATGGTAGTATTGGAGTTTTTTTTGGGGGATTGG
GAATAGGTAGTGGGTCTGGATCAGGGGGGCGTACTGGATACGTTCCATTATCTACAGGCA
CACCATCTAAACCAGTTGAAATTCCATTACAACCTATACGACCATCAGTTGTTACGTCTG
TTGGGCCTTCAGATTCTTCTATTGTTTCATTAGTGGAAGAATCAAGTTTTATAGAGTCAG
GTATACCTGGTCCTACATCTATAGTGCCTTCTACTTCAGGGTTTGATATTACAACTTCTG
TAAACAGTACACCTGCTATTATAGATGTATCTGCTATTAGTGATACTACACAAATATCTG
TTACAACATTTAAAAATCCAACCTTTACTGACCCATCTGTGTTGCAACCTCCTCCACCCT
TAGAAGCCTCTGGCAGACTTTTATTTTCAAATGACACTGTAACTACCCATTCATATGAAA
ATATACCTCTTGACACATTTGTAGTTACAACAGACCACAATAGTATTGTTAGTAGTACGC
CCATCCCAGGGAGGCAACCTGCTGCACGCTTAGGATTATATGGACGTGCAATACAACAGG

TTAAGGTTGTAGACCCTGCGTTTTTAACTACGCCTACACGTTTAGTAACATATGACAACC
CTGCCTTTGAAGGCCTGCAGGATACAACATTAGAGTTTCAGCACAGTGACTTGCATAATG
CTCCTGATTCTGATTTTTAGATATTGTAAAATTACATAGGCCTGCTTTAACCTCTAGAA
AAACAGGCATACGTGTTAGTAGATTGGGACAACGTGCAACACTTTCTACTAGAAGTGGCA
AACGTATAGGTGCTAAAGTACATTTTTATCATGATATAAGTCCTATACCTACTAATGATA
TTGAAATGCAACCTTTAGTTACACCACAAACACCTAGTATAGTAACTGGTAGTAGTATTA
ATGATGGGTTATATGATGTGTTTTTAGACAATGATGTAGAAGAGACTGTACTACAACAAA
CATATACACCTACAAGTATACATAGTAATAGTTTAGTTAGTAGTGATATTTCTACTGCAA
CTGCAAATACAACTATTCCTTTTAGTACTGGGTTAGACACACATCCTGGTCCAGATATTG
CTTTACCACTACCTTCTACAGAAACTATTTTTACACCAATAGTGCCATTACAGCCTGCTG
GTCCTATATATATATTTATGGGTCAGGTTTTATATTACACCCTAGTTATTATTTGTTAAAGC
GCAAACGTAAACGTCTGTCATATTCTTTTACAGATGTGGCGACCTACTGATGCAAAGGTA
TACCTGCCCCCTGTGTCTGTGTCTAAGGTTGTAAGCACAGATGAATATGTAACAAGAACA
AATATATATTATTATGCAGGTAGCACACGTTTGTTGGCTGTGGGACACCCATATTTTCCT
ATCAAGGATTCTCAAAAACGTAAAACCATAGTTCCTAAAGTTTCAGGTTTGCAATACAGG
GTGTTTAGGCTTCGTTTACCAGATCCTAATAAATTTGGATTTCCAGATGCATCCTTTTAT
AATCCTGATAAGGAGCGCCTAGTATGGGCCTGTTCTGGTGTGGAGGTTGGACGTGGACAA
CCCTTAGGTATAGGTACTAGTGGCAATCCATTTATGAATAAATTAGATGATACTGAAAAT
GCTCCTAAATACATTGCTGGACAAAATACAGATGGTAGAGAATGTATGTCAGTGGATTAT
AAACAAACACAGTTGTGTATTTTAGGTTGTAGGCCTCCCTTAGGGGAACATTGGGGTCCA
GGCACGCCATGTACTTCACAAACTGTTAATACTGGTGATTGTCCCCCACTGGAATTAAAG
AACACCCCTATACAGGATGGTGATATGATAGATGTTGGCTTTGGAGCCATGGATTTTAAA
GCTTTACAAGCAAATAAAAGTGATGTACCTATTGATATTTCTAACACTACCTGTAAATAC
CCAGATTATTTAGGCATGGCTGCTGATCCCTATGGTGATTCCATGTGGTTTTATCTTCGT
AGGGAACAAATGTTTGTTCGACACTTATTTAACAGGGCTGGTGATACCGGTGATAAAATC
CCAGATGACCTAATGATTAAAGGCACAGGCAATACTGCAACACCATCCAGTTGTGTTTTT
TATCCTACACCTAGTGGTTCCATGGTTTCTTCAGATGCACAGTTGTTTAATAAACCTTAT
TGGTTGCAAAAGGCACAGGGACAAAATAATGGTATTTGTTGGCATAATCAATTATTTTTA
ACTGTTGTAGATACTACTAGAAGCACTAATTTTTCTGTATGTGTAGGTACACAGGCTAGT
AGCTCTACTACAACGTATGCCAACTCTAATTTTAAGGAATATTTAAGACATGCAGAAGAG
TTTGATTTACAGTTTGTTTTTCAGTTATGTAAAATTAGTTTAACTACTGAGGTAATGACA
TATATACATTCTATGAATTCTACTATATTGGAAGAGTGGAATTTTGGTCTTACCCCACCA
CCGTCAGGTACTTTAGAGGAAACATATAGATATGTAACATCACAGGCTATTAGTTGCCAA
CGTCCTCAACCTCCTAAAGAAACAGAGGACCCATATGCCAAGCTATCCTTTTGGGATGTA
GATCTTAAGGAAAAGTTTTCTGCAGAATTAGACCAGTTTCCTTTGGGAAGAAAATTTTTA
TTACAACTTGGTATGCGTGCACGTCCTAAGTTACAAGCTTCTAAACGTTCTGCATCTGCT
ACCACAAGTGCCACACCTAAGAAAAAACGTGCTAAACGTATTTAATAAGTGTAATGTGTA
TGTGTTGTTTGTTGTATGTTACATGTGTTTTGTATGTTTGTTTGTTGTATGTTAACTGTT
TACTAATACTGTGTGTATGTTTATGTACATGTGTATAACTGTTTGTTTATATATATGTAT
GTATTTGTGTGTATGTGTATGTGTATGTGTATGTGTAGTAATGTTTGTATGTATGTTTAA
TAAAGTTTATATGTGTGTTGTGTGGGTGGTTTACTTGACTACTGTGCTTCCATTTTGTAT
AGTCGCCATTTTACATGCATTAAGGTAAAAAGGGCAACCGATTTCGGTTGCACAGTAAAA
CATGTTTTAATGTGTTTTGCTGTTGTAGCAAAATAGTTGTACTGTTTTTGGCTTCCTGCA
GGCAACTTGGCAGGGTTTGTTTCCTTAACATGTTCATCCCACGCAAGGTTATAAAGGTAA
AAGGCGCCACCTGGCAGTTACTCATTTGTCTGCAATTATTTAAACAATGTCTTGCACACA
CATTTTTTACCCACCCTATCATAAAATTGCTTTTAAGCACATACCTATACTATGTACACA
GTGTACTCTTGGCAGAACATTGTTTTTTAAATGCCAAGTAATTGTTTTATAAATGAGTAA
TAACGTGTTACTCATACTGCACCTAAAAAGTTAAACCTATTTGGATCACACAAATGCCAA
TTTATTTCTTATTACAAATA
>gi|557236|emb|X77858.1|_Human_papilloma_virus_type_59,_complete_viral_genome
GTTAAGACCGAAAACGGTGCATATAAAGGTAGTTGAAAAGAAAAGGGCAACGGCATGGCA
CGCTTTGAGGATCCTACACAACGACCATACAAACTGCCTGATTTGAGCACAACATTGAAT
ATTCCTCTGCATGATATTCGCATCAATTGTGTGTTTTGCAAAGGGGAACTGCAAGAAAGA
GAGGTATTTGAATTTGCTTTTAATGACTTATTTATAGTGTATAGAGACTGTACACCGTAT
GCAGCGTGTCTGAAATGCATTTCATTTTATGCAAGAGTAAGAGAATTAAGATATTATAGA

GATTCCGTGTATGGAGAAACATTAGAGGCTGAAACCAAGACACCGTTACATGAGCTGCTG
ATACGCTGTTATAGATGCCTAAAACCTCTATGTCCAACAGATAAATTAAAGCATATAACT
GAAAAAAGAAGATTCCATAATATAGCTGGAATATATACAGGACAGTGTCGTGGGTGTCGG
ACCCGAGCAAGACACCTAAGACAGCAACGACAAGCGCGTAGTGAAACACTGGTGTAAAAC
AATGCATGGACCAAAAGCAACACTTTGTGACATTGTTTTAGATTTGGAACCACAAAATTA
TGAGGAAGTTGACCTTGTGTGCTACGAGCAATTACCTGACTCCGACTCCGAGAATGAAAA
AGATGAACCAGATGGAGTTAATCATCCTTTGCTACTAGCTAGACGAGCTGAACCACAGCG
TCACAACATTGTGTGTGTGTGTTGTAAGTGTAATAATCAACTTCAGCTAGTAGTAGAAAC
CTCGCAAGACGGATTGCGAGCCTTACAGCAGCTGTTTATGGACACACTATCCTTTGTGTG
TCCTTTGTGTGCAGCAAACCAGTAACCTGCAATGGCCGATTCGGAAGGTACAGATGGGGA
AGGGACGGGGTGCAATGGATGGTTTTTTGTGCAGGCAATAGTAGATAAAAAAACAGGTGA
CAAAATTTCAGATGACGAGGATGAAAATGCAACAGATACAGGTTCAGACTTGGTAGATTT
TATTGATGATACCACAACAATTTGTGTACAGGCAGAGCGCGAGACAGCACAGGCCTTGTT
TAATGTGCAGGAAGCCCAAAGGGATGCACGGGAAATGCATGTTTTAAAACGAAAGTTTGG
GTGCAGTATAGAAAACAGTAGTGAGAAAGCGGCGGCAGGAAAAAAAGCTAAGTCACCATT
ACAAGAAATATCAGTAAATGTTAACCACCCAAAAGTAAAAAGAAGGTTAATAACAGTGCC
AGACAGCGGCTATGGCTATTCTGAAGTGGAAATGCTCGAGACTCAGGTAACCGTGGAGAA
TACTGGAAATGGGGATAGCAATGGCAGTGTTTGTAGCGACAGTCAAATAGACTGTAGCGA
CAGCAGTAACATGGATGTTGAAAACATAGTTCCAACATCCCCCACTAATCAATTGTTACA
GTTATTACATAGCAAAAATAAGAAAGCAGCTATGTATGCAAAATTTAAAGAATTGTATGG
GTTATCATTTCAAGATTTGGTTAGGACATTTAAAAGTGACAGAACTACCTGTAGCGATTG
GGTAACCGCCATTTTTGGTGTTAATCCAACTGTAGCAGAAGGATTTAAAACATTAATACA
ACCCTATGTGCTATATGCACATATACAATGCTTAGATTGTGCATGGGGAGTAGTAATATT
AGCATTATTAAGATATAAATGTGGAAAAAATAGAATAACAGTTGCAAAAGGACTTAGCAC
ATTACTACATGTACCAGATACGTGCATGTTAATTGAACCACCCAAATTGCGTAGTGGTGT
TGCAGCACTATATTGGTACAGAACAGGAATGTCCAATATTAGTGAAGTTATAGGGGAAAC
GCCCGAATGGATACAAAGACTAACAATTATACAACATGGAGTTGATGATAGCGTGTTTGA
CCTGTCAGAAATGATACAATGGGCGTTTGATAATGACCTAACAGATGAAAGTGATATTGC
ATATGAATATGCATTAATAGCAGATAGTAATAGTAACGCCGCTGCATTTTTAAAAAGCAA
CTGCCAGGCAAAATACCTAAAAGATTGTGCAGTTATGTGTAGGCATTATAAAAGAGCACA
AAAAAGACAAATGAGTATGTCACAGTGGATAAAATGGAGATGTGATAAAATAGAAGAGGG
GGGAGATTGGAAACCCATAGTACAATTTTTAAGATATCAAGGAGTAGAATTTATAACGTT
TTTATGTGCATTAAAAGATTTTTTAAAAGGTACCCCAAAAAGAAATTGCATTGTGCTGTG
TGGGCCAGCAAATACAGGCAAGTCATACTTTGGAATGAGCCTGCTACATTTTTTACAAGG
AACTGTAATTTCACATGTAAATTCAAATAGTCACTTTTGGCTAGAACCTTTAACAGATCG
TAAATTAGCTATGCTAGACGATGCAACAGATAGTTGTTGGACATATTTTGATACATATAT
GCGAAATGCTTTGGATGGCAATCCTATAAGTGTAGATAGAAAGCATAGGCACCTAGTACA
AATTAAATGTCCACCAATGCTTATTACATCAAATACAAATCCAGTTACAGATAACAGGTG
GCCATATTTAAATAGCAGATTAATGGTATTTAAATTTCCAAACAAATTGCCATTTGACAA
AAATAGAAATCCAGTATATACAATTAATGACAGAAACTGGAAATGTTTTTTTGAAAGGAC
GTGGTGCAGATTAGATTTGAACGAGGAAGAGGAAGATGCAGACAGTGATGGACACCCTTT
CGCAGCGTTTAAGTGTGTTACAGGATCAAATATTAGAACATTATGAAAACGATAGTAAAG
ACATTAATGAACACATAAACTATTGGAAACTGGTGCGTATGGAAAATGTAATTTTATTTG
CAGCAAGAGAGAACAATATACATACATTAAACCACCAGGTGGTGCCAACGTTTTTGGTGT
CTAAAAACAAGGCATGTGAAGCTATTGAACTGCAGTCAAACCGTACTTCCACTGTAATGC
CCTGTTTTTTAAAACATTTTTTAGGTGCTGTTTGCCATAGTTCTTGGCATGTTTCTTGCA
TTGTCCATTGCTCATTTTTAAACTCAGTTTGTGCCAAACTCTCTAACGCCATCTGCAGCA
AGGAAAACACAATGCATTACACAAGCTGGACATTTATATATTATGTAAATGATGTAGGAC
AGTGGTGTAAAACCACAGGAAATGTGGACTTTTGGGGACTATATTATAAAGTGGAAGAGG
AACAGGTGTACTATGTAAAATTTATACATGATGCCAAAAAATATGGGACTACAGACAAGT
GGGAAGTGCATTATAATGGCAAGGTTATTGATTGTTATGACTCTATGTGCAGTACCAGTG
ACGAGCAAGTATCCACTGCTGGATCTTCTGAGCAACTATCATACCCCTCCGCAACGCCCC
CCGAAGCCACGTACTTGGGCCCCCAAACGTGGAACCGTCAGACGAAGACTGGAAAGCGAC
CAAGACAGTGTGGATACACACAGCACCCTCAGTCTACCAGCGTGTCAGTGGACTACTGTG
ACAACCCAGTCGTCCGTTTGCATCCAGGCAACAACCCGCGACGGCACATCCCTTGCAGTA

ACACTACGCCTATAATACACTTAAAAGGTGACAAAAATGGCCTTAAGTGTTTAAGGTATA
GATTAAGAAAAGTACACTGGTTATTTGAAAATATTTCCTCTACCTGGCATTGGACAGGAA
ACAGAGGATCAGCCAAAACAGGCATTTTAACATTAACATATACAAGCGAAACACAACGCA
ATGAATTTTTAGATACTGTAAAAATTCCTAATAGTGTACAAATACATGTTGGGTATATGA
GTGTGTAATGGTTGTTATGCAAATGTAACACAAGCCAATACTGCTGCTATATTGTATAGC
TGAGGAAATGATAACCCTTGTATTTGTGTGTTGTGTTTGTGTTTGCTTGTGTGTGTGTTG
CAATGTCCCGCTTCTGCAATCTGTCTATATGTGTGCATATACATGGTTACTAGTATTTGT
GTATATTGTGGTTATCACCTCCTCATATGAGTGTTTTTTACTATATATATTGTTTTTTAT
AATTCCACTGTTACTACTATATGCCCATGCAATACTGTCCATACAATAATTGCTGTATAT
TGTAAATTACATTGCACTGTATTGTACAGTATATTTTAAACACATTATTATTTTTGTTAG
GTGTTGGTTTTGTTACATTTATAATAAAACATGGTTTCCCATCGTGCTGCTCGTCGTAAA
CGTGCCTCAGCAACAGACTTATATAAAACTTGCAAGCAGGCAGGTACATGCCCTTCTGAT
GTTATTAATAAAGTTGAAGGTACAACTTTAGCTGATAAAATATTGCAGTGGACCAGCCTA
GGAATATTTTTAGGTGGACTAGGTATTGGTACTGGATCTGGTACCGGTGGCAGAACAGGG
TACATACCTTTAGGGGGGCGTACAAACACTATAGTAGATGTATCGCCTGCTAAACCACCA
GTAGTTATTGAACCTGTTGGACCTACAGATCCATCTATAGTTACATTAGTTGAGGATTCT
AGTGTTATAACATCTGGAGCCCCTGCCCCAACATTTACAGGTACTTCAGGATTTGAAATA
TCTACCTCTAGTACAACAACACCAGCTGTTTTGGATATAACCCCAACCTCTTCTGTTCAA
ATTAGTAGCTCTAGTTTTATAAATCCTGCATTTACAGACCCTTCTGTCATTGAGGTTCCC
CAAACAGGTGAAATTTCTGGTAATATATTAATTAGTACCCCTACCTCTGGTGCACATGGC
TATGAAGAAATTCCAATGCAAACGTTTGCTACGGAAGGTACTGGTTTGGAACCCATTAGC
AGTACCCCCAATCCAACAGTACGTCGTGTGGCTGGACCTAGATTGTACAGTAGGGCTAAT
CAACAAGTTCGGGTGTCTAACGCTGACTTTTTAACACGTCCATCCACATTTGTTACATAT
GATAACCCTGCTTATGATCCAATTGATACTACATTAACTTTTGACCCCTCATCAGAGGTT
CCAGACCCGGACTTTATGGATATAGTTCGTTTGCATAGGCCTGCATTAACATCCAGACGC
AGCACTGTAAGGTTTAGTAGGCTAGGACAACGGGCAACCATGTTTACCCGTAGTGGTAAA
CAAATTGGGGCCCGTGTACATTTTTATCATGATATAAGCCCTATACCACATGCTGAAGAT
ATTGAATTGCAACCTCTTGTTTCTTCCCAGGCTGCTACTGATGATATATATGATATATAT
GCAGATATTACAGATGAAGCACCTACTAGTACTGCCAACACTGCATTTACAATTCCTAAA
TCTTCTTTTCAAAGTTTGTCATTAACACGGTCGGCATCTAGCACCTTTTCAAATGTAACT
GTTCCTTTGGCTACTGCCTGGGATGTTCCTGTAAATACAGGACCCGATATAGTTTTACCT
AATACTAATATTGTTGAACCCACTTATTCTACTACACCCTTTACCACCATACAGTCTATT
AATATAGAAGGCACAAATTATTTTTTATGGCCTATATATTATTTTTTACCTCGTAAACGT
AAACGTGTTCCCTATTTTTTTACAGATGGCTCTATGGCGTTCTAGTGACAACAAGGTGTA
TCTACCTCCACCTTCGGTAGCTAAGGTTGTCAGCACTGATGAGTATGTCACCCGTACCAG
TATTTTCTACCACGCAGGCAGTTCCAGACTTCTTACAGTTGGACATCCATATTTTAAAGT
ACCTAAAGGTGGTAATGGTAGACAGGATGTTCCTAAGGTGTCTGCATATCAATACAGAGT
ATTTAGGGTTAAGTTACCTGATCCCAATAAATTTGGCCTTCCAGATAACACAGTATATGA
TCCTAACTCTCAACGCTTGGTCTGGGCCTGTGTAGGTGTTGAAATCGGTCGGGGCCAACC
TTTAGGGGTAGGACTCAGTGGTCATCCATTATATAATAAATTGGATGACACTGAAAACTC
TCATGTAGCATCTGCTGTTGATACCAAAGATACACGTGATAATGTATCTGTGGATTATAA
ACAAACTCAGCTGTGTATTATTGGCTGTGTACCTGCCATTGGAGAACACTGGACAAAGGG
CACTGCTTGTAAGCCTACTACTGTGGTTCAGGGCGATTGTCCTCCACTAGAATTAATAAA
TACACCAATTGAAGATGGTGATATGGTAGACACAGGATATGGGGCTATGGACTTTAAATT
GTTGCAGGATAACAAAAGTGAAGTACCATTGGATATTTGTCAGTCTATTTGTAAATATCC
TGATTATTTACAAATGTCAGCAGATGCTTATGGAGACAGTATGTTTTTTTGTTTAAGGCG
AGAACAGGTTTTTGCCAGACATTTTTGGAATAGATCTGGTACTATGGGTGATCAACTTCC
TGAATCACTATATATTAAAGGTACTGACATACGTGCCAACCCAGGCAGTTATTTATATTC
CCCTTCCCCAAGTGGGTCTGTGGTTACTTCTGATTCACAATTATTTAATAAACCATATTG
GCTGCACAAGGCTCAGGGTTTAAACAATGGTATATGTTGGCACAATCAATTGTTTTTAAC
AGTTGTAGATACTACTCGCAGCACCAATCTTTCTGTGTGTGCTTCTACTACTTCTTCTAT
TCCTAATGTATACACACCTACCAGTTTTAAAGAATATGCCAGACATGTGGAGGAATTTGA
TTTGCAGTTTATATTTCAACTGTGTAAAATAACATTAACTACAGAGGTAATGTCATACAT
TCATAATATGAATACCACTATTTTGGAGGATTGGAATTTTGGTGTTACACCACCTCCTAC
TGCTAGTTTAGTTGACACATACCGTTTTGTTCAATCTGCTGCTGTAACTTGTCAAAAGGA

CACCGCACCGCCAGTTAAACAGGACCCTTATGACAAACTAAAGTTTTGGCCTGTAGATCT
TAAGGAAAGGTTTTCTGCAGATCTTGATCAGTTTCCTTTGGGACGTAAATTTTTATTGCA
ATTAGGAGCTAGACCTAAGCCCACTATAGGCCCACGCAAACGTGCAGCGCCTGCCCCTAC
CTCTACCCCATCACCAAAACGTGTTAAGCGTCGCAAGTCTTCCAGAAAATAGTGTTGTTT
GTTATGTGTTTGTATGTGTGCATGTTGTATGTTTTGTATTGTTTGCCTGTTTGTATGTTG
TGTATATGTACATGTTTGTTTGTCTGCTGTATGTGTGTATTTGTTTTTGTACATAATAAA
GTATGCATGACAGTTTCATGTGTGGTTGCACCCAATGAGTAAGGTACTGTCCCTTTATTG
TTTCTTTGTCCTTATTACACATTATTACACATTGCCCTACTTACATAGGTGTGTTTGTTC
CTTCATTTTGTCCTGAATGTCCAGTTTTGCATTTGCACATTATATGGCGTCCATTTTATC
CTTTAAATCCTCCATTTTGCTGTGCAACCGTTTTCGGTTACCTTGGTTTAACCTTACCTT
TTTGAACAATTAATCTGTTTAAACATCAGCAAAACAGTTAATCCCCATCTTGTTTCCTCC
TACACGCCTAGACTACTAACACAACTTACAAACGCCAAATAGTTAGTCATCATCCTGTCC
AGGTGCACTCTAACAATACTTGCATAACTTTGGTGGCGCCCTTGTTAATAAAACAGCTTT
TAGGCACATATTTTCACTGTTTTTACTACTTTAATTGCATAATTGGCTTGCAAAACTACT
GTGCAATCCAAGAATGTGTCTATAATTTATTGTAAAAAACATGACTAAGGTTTTTGTCAT
TGTTAAGCAACCGAAAAAGGTCGGGCAAGTACATGCACACTTTCTACTTATTACTTTTTA
CAATCATAGTAATAAAAAAGGGTGTAACCGAAAACG
>gi|397053|emb|X74483.1|_Human_papillomavirus_type_56_genomic_DNA
GAAAGTTTCAATCATACTTTTATATATTGGGAGTGACCGAAAAGGGTTTAAGACCGAAAA
CGGTACATATAAAAGGCAGCTTATTCTGTGTGGACATATCCATGGAGCCACAATTCAACA
ATCCACAGGAACGTCCACGAAGCCTGCACCACTTGAGTGAGGTATTAGAAATACCTTTAA
TTGATCTTAGATTATCATGTGTATATTGCAAAAAAGAACTAACACGTGCTGAGGTATATA
ATTTTGCATGCACTGAATTAAAATTAGTGTATAGGGATGATTTTCCTTATGCAGTGTGCA
GAGTATGTTTATTGTTTTATAGTAAAGTTAGAAAATATAGGTATTATGACTATTCAGTGT
ATGGAGCTACACTAGAAAGTATAACTAAAAAACAGTTATGTGATTTATTAATAAGGTGCT
ACAGATGTCAAAGTCCGTTAACTCCGGAGGAAAAGCAATTGCATTGTGACAGAAAAAGAC
GATTTCATCTAATAGCACATGGTTGGACCGGGTCATGTTTGGGGTGCTGGAGACAAACAT
CTAGAGAACCTAGAGAATCTACAGTATAATCATGCATGGTAAAGTACCAACGCTGCAAGA
CGTTGTATTAGAACTAACACCTCAAACAGAAATTGACCTACAGTGCAATGAGCAATTGGA
CAGCTCAGAGGATGAGGATGAGGATGAAGTAGACCATTTGCAGGAGCGGCCACAGCAAGC
TAGACAAGCTAAACAACATACGTGTTACCTAATACACGTACCTTGTTGTGAGTGTAAGTT
TGTGGTGCAGTTGGACATTCAGAGTACCAAAGAGGACCTGCGTGTTGTACAACAGCTGCT
TATGGGTGCGTTAACAGTAACGTGCCCACTCTGCGCATCAAGTAACTAACTGCAATGGCG
TCACCTGAAGGTACAGATGGGGAGGGGAAGGGATGTTGTGGATGGTTTGAAGTAGAGGCA
ATTGTAGAAAAAAAAACAGGAGATAAAATATCAGATGATGAAAGTGACGAGGAGGATGAA
ATAGATACAGATTTAGATGGATTTATAGACGATTCATATATACAAAATATACAGGCAGAC
GCAGAAACAGTCAACAATTGTTGCAAGTACAAACAGCACATGCAGATAAACAGACGTTGC
AAAAACTAAAACGAAAGTATATAGCTAGTCCATTAAGGGATATTAGTAATCAGCAAACTG
TGTGCCGGGAAGGAGTAAAACGGAGGCTTATTTTATCAGACCTACAAGACAGCGGGTATG
GCAATACATTGGAAACTCTGGAAACACCAGAACAGGTAGATGAAGAGGTACAGGGACGTG
GGTGCGGGAATACACAAAATGGAGGCTCACAAAACAGTACCTATAGTAACAATAGTGAGG
ACTCTGTAATACATATGGATATTGATAGAAACAATGAAACGCCAACACAACAATTGCAGG
ACTTGTTTAAAAGTAGCAATTTACAAGGTAAATTATATTATAAATTTAAAGAAGTGTATG
GTATTCCATTTTCAGAATTGGTGCGTACGTTTAAAAGTGATAGTACATGTTGCAATGATT
GGATATGTGCTATATTTGGTGTTAATGAAACATTAGCCGAGGCACTAAAAACTATAATAA
AACCACACTGTATGTATTATCATATGCAATGTTTAACATGTACATGGGGGGTTATAGTAA
TGATGCTAATTAGATATACATGTGGCAAAAACAGAAAAACAATTGCAAAAGCATTAAGCT
CAATATTAAATGTACCACAGGAGCAAATGTTAATTCAACCACCAAAAATACGAAGTCCTG
CTGTAGCTTTATATTTTTATAAAACAGCAATGTCAAATATTAGTGATGTGTATGGAGACA
CACCAGAATGGATACAAAGACAAACACAATTGCAACACAGTTTACAGGATAGTCAATTTG
AATTATCTAAAATGGTGCAGTGGGCATTTGATAATGAAGTAACAGATGATAGCCAAATTG
CGTTTCAATATGCACAATTAGCAGATGTAGACAGCAATGCACAAGCCTTTTTAAAAAGCA
ATATGCAGGCAAAATATGTAAAGGATTGTGGAATAATGTGTAGACATTATAAAAGGGCAC
AACAGCAACAAATGAATATGTGCCAGTGGATAAAGCACATATGTAGTAAAACAGATGAAG
GGGGTGATTGGAAACCCATTGTACAATTTTTAAGATATCAAGGGGTCGATTTCATTTCAT

TTCTAAGTTACTTTAAATTATTTCTACAAGGAACACCTAAACATAACTGTTTGGTACTTT
GTGGACCGCCAAATACAGGTAAATCATGCTTTGCTATGAGTCTTATAAAGTTTTTTCAAG
GGTCTGTCATTTCATTTGTGAATTCACAAAGCCACTTTTGGTTGCAGCCATTAGACAATG
CTAAACTTGGGTTGTTGGATGATGCAACAGAAATATGTTGGAAATATATAGACGATTATT
TAAGGAATTTGGTAGATGGAAATCCTATAAGTTTAGATAGAAAACATAAACAATTAGTAC
AAATAAAATGTCCACCATTACTAATTACAACCAATATAAATCCTATGCTAGATGCTAAAT
TACGATATTTACACAGTAGAATGTTAGTGTTTCAGTTTCAAAATCCATTTCCATTAGATA
ATAATGGTAATCCTGTATATGAATTAAGTAATGTAAACTGGAAATGTTTCTTTACAAGGA
CGTGGTCCAGATTAAATTTGGATAACGACGAGGACAAAGAAAACAATGGAGACGCTTTCC
CAACGTTTAAATGCGTGCCAGAACAAAATACTAGACTGTTTTGAAAAAAGATAGTAGATG
TATTGCAGATCATATAGAATATTGGAAAGCTGTGCGACATGAAAATGTGCTATACTATAA
AGCAAGAGAAAATGACATTACTGTACTAAACCACCAGATGGTGCCTTGTTTACAAGTATG
TAAAGCAAAAGCATGTAGTGCAATAGAAGTGCAAATAGCACTGGAATCATTAAGTACAAC
AATATATAACAATGAAGAGTGGACATTAAGAGACACATGCGAGGAACTATGGCTTACTGA
ACCTAAAAAATGCTTTAAAAAAGAAGGACAACATATAGAAGTATGGTTTGATGGTAGTAA
AAACAATTGTATGCAATATGTAGCCTGGAAATATATATATTACAATGGAGATTGTGGGTG
GCAAAAAGTGTGTTCTGGGGTAGACTATAGAGGTATATATTATGTACATGATGGCCACAA
AACATACTACACAGACTTTGAACAAGAGGCCAAAAAATTTGGGTGTAAAAACATATGGGA
AGTACATATGGAAAATGAGAGTATTTATTGTCCTGACTCTGTGTCTAGTACCTGTAGATA
CAACGTATCCCCTGTTGAAACTGTTAACGAATACAACACCCACAAGACCACCACCACCAC
CTCCACGTCCGTGGGCAACCAAGACGCCGCAGTATCCCACAGACCAGGAAAACGACCCAG
ACTACGGGAATCAGAATTTGACTCCTCCAGAGAGTCCCACGCAAAGTGTGTCACAACACA
CACACACATCAGCGACACAGACAATACCGACAGTAGAAGTAGAAGTATCAACAACAACAA
CCACCCTGGTGATAAGACTACGCCTGTAGTACATTTAAAAGGTGAACCTAACAGATTAAA
ATGTTGTAGATATCGATTTCAAAAATATAAAACATTGTTTGTGGATGTAACATCAACATA
TCATTGGACAAGTACAGACAATAAAAATTATAGCATAATTACAATTATATATAAGGATGA
AACACAACGAAACAGCTTTTTAAGTCATGTAAAAATTCCAGTAGTGTACAGGTTAGTTTG
GGACAAATGAGTTTTCCATAAAGTGCTGTATATATTGTATATACATTTGTGTTATTGTAA
CACACAAATACGTGAAGTGTACCTGCCATACATTGCTGCTACGCATATATATTGCAACCA
TTGATTTTTGTGTTATTGGTGTGTTTGCGCTTTGCTTTTGTGTTTGTTTGCTTGTGTGTC
ATGTTGTCCCGCTTTTGCTATCTGCCTCTGTGTTTTCCAGTTGTATATTATTAATAATAT
TGTTTTGGTTTGTTATAGCCACATCCTTTTTTAATACATTTATAATATTTTTGATATTTT
TTTACTGTCCTGTGCTGTGTATATATTTACATGCTTTGTGGATAATAAATAATATGTAAA
TGTAGTAGTACTGTTACTACTATGGTTGCCCACCGTGCCACACGACGCAAACGCGCATCT
GCAACACAACTATATAAAACATGTAAGTTGTCTGGTACATGTCCAGAGGATGTTGTTAAT
AAAATAGAGCAAAAAACATGGGCTGATAAAATATTGCAATGGGGAAGTTTATTTACATAT
TTTGGAGGCCTTGGCATTGGTACAGGAACTGGGTCTGGGGGTCGTGCAGGCTATGTTCCA
TTGGGGTCTAGGCCTTCCACAATAGTTGATGTAACTCCGGCGCGACCACCTATTGTTGTG
GAATCCGTAGGGCCTACAGACCCTTCCATTGTTACATTAGTTGAGGAGTCCAGTGTTATA
GAATCTGGTGCAGGGATTCCTAATTTTACTGGGTCTGGGGGATTTGAAATTACATCCTCA
TCAACAACTACACCTGCCGTGTTGGATATTACACCAACCTCTAGTACTGTACATGTCAGT
AGTACCCATATAACCAATCCGTTATTTATTGATCCCCCTGTTATTGAGGCCCCACAAACA
GGCGAGGTGTCTGGCAATATTTTAATTAGCACACCCACATCTGGTATACATAGCTATGAA
GAAATACCTATGCAAACATTTGCTGTTCACGGTTCTGGTACAGAACCTATTAGTAGTACT
CCTATTCCAGGCTTTAGGCGTATTGCAGCTCCTAGATTATATAGAAAAGCATTTCAGCAG
GTTAAGGTAACTGACCCTGCATTTCTTGATAGACCTGCAACATTAGTATCTGCTGATAAT
CCACTTTTTGAAGGTACTGACACATCTTTAGCTTTTTCTCCGTCGGGTGTGGCTCCTGAC
CCTGATTTTATGAATATAGTAGCATTACATAGGCCTGCATTTACTACACGTAGGGGTGGT
GTACGTTTTAGTAGGCTTGGCAGAAAGGCTACTATACAAACACGTAGAGGCACACAAATA
GGTGCCCGTGTGCATTATTATTATGATATAAGTCCTATTGCACAGGCTGAGGAAATTGAA
ATGCAGCCATTATTGTCTGCAAATAATTCATTTGATGGCCTATATGATATTTATGCAAAT
ATAGATGATGAAGCACCTGGTTTGTCTAGCCAGTCAGTTGCTACACCTTCTGCACACTTA
CCTATAAAGCCTTCCACATTGTCTTTTGCTAGTAACACCACTAATGTAACTGCCCCTTTA
GGTAATGTGTGGGAAACACCATTTTATTCAGGTCCTGACATAGTGTTGCCTACAGGCCCC
AGTACGTGGCCCTTTGTTCCTCAGTCTCCTTATGATGTTACCCATGATGTATATATACAG

GGATCCTCCTTTGCATTATGGCCTGTGTATTTTTTTAGACGTAGGCGCCGTAAACGTATT
CCCTATTTTTTTGCAGATGGCGACGTGGCGGCCTAGTGAAAATAAGGTGTATCTACCTCC
AACACCTGTTTCAAAGGTTGTGGCAACGGATTCCTATGTAAAACGCACTAGTATATTTTA
TCATGCAGGCAGTTCACGATTGCTTGCCGTAGGACATCCCTATTACTCTGTGACTAAGGA
CAATACCAAAACAAACATTCCCAAAGTTAGTGCATATCAATATAGGGTATTTAGGGTACG
GTTGCCCGACCCTAATAAGTTTGGGCTTCCAGATACTAATATTTATAATCCGGACCAGGA
ACGGTTAGTGTGGGCATGTGTAGGTTTGGAGGTAGGCCGCGGACAGCCTTTAGGTGCTGG
GCTAAGTGGCCATCCATTGTTTAATAGGCTGGATGATACTGAAAGTTCCAATTTAGCAAA
TAATAATGTTATAGAAGATAGTAGGGACAATATATCAGTTGATGGCAAGCAAACACAGTT
GTGTATTGTTGGATGTACTCCCGCTATGGGTGAACATTGGACTAAAGGTGCTGTGTGTAA
GTCCACACAAGTTACCACAGGGGACTGCCCGCCTCTTGCATTAATTAATACACCTATAGA
GGATGGGGACATGATAGACACAGGATTTGGCGCTATGGACTTTAAGGTGTTGCAGGAATC
TAAGGCTGAGGTACCTTTAGACATTGTACAATCCACCTGTAAATATCCTGACTATTTAAA
AATGTCTGCAGATGCCTATGGTGATTCTATGTGGTTTTACTTACGCAGGGAACAATTATT
TGCCAGACATTATTTTAATAGGGCTGGTAAAGTTGGGGAAACAATACCTGCAGAGTTATA
TTTAAAGGGTAGCAATGGTAGAGAACCCCCTCCGAGTTCTGTATATGTTGCTACGCCTAG
TGGGTCTATGATTACGTCTGAGGCACAGTTATTTAATAAACCTTATTGGTTGCAACGTGC
CCAAGGCCATAATAATGGCATTTGCTGGGGTAATCAATTATTTGTTACTGTAGTAGATAC
TACTAGAAGTACTAACATGACTATTAGTACTGCTACAGAACAGTTAAGTAAATATGATGC
ACGAAAAATTAATCAGTACCTTAGACATGTGGAGGAATATGAATTACAATTTGTTTTTCA
ATTATGCAAAATTACTTTGTCTGCAGAGGTTATGGCATATTTACATAATATGAATGCTAA
CCTACTGGAGGACTGGAATATTGGGTTATCCCCGCCAGTGGCCACCAGCCTAGAAGATAA
ATATAGATATGTTAGAAGCACAGCTATAACATGTCAACGGGAACAGCCACCAACAGAAAA
ACAGGACCCATTAGCTAAATATAAATTTTGGGATGTTAACTTACAGGACAGTTTTTCTAC
AGACCTGGATCAATTTCCACTGGGTAGAAAATTTTTAATGCAACTGGGCACTAGGTCAAA
GCCTGCTGTAGCTACCTCTAAAAAGCGATCTGCTCCTACCTCCACCTCTACACCAGCAAA
ACGTAAAAGGCGGTAGTGTGTTGTTGTGTGTTTGTGTAACTGTGTTTGTGTGTTGTATAT
ATGGTATGTTTGTGTATGTGCTTTATTTTATACTTTGTATGTGTATGTTGTGTTTGTGTA
AATGTTTGTGTGAAATGTTTGTGTGTGTATTCATTGTATGTATGACTGTATATATGTGTA
ATGTTTGTGTGTCTGTAATAAACATGAATGAGTGCTTTTACGCGTGGTTGCATAAACTAA
GGTGTGTCATTATTGTGGCTTTTGTTTTGTAAGTTATTGTGTACAGTGTACTATGTGTAT
TGTGCATACATATATATACCATAACATACTCCATTTTGTTGTTTTTCCGCCATTTTGTAC
ATGCAACCGAATTCGGTTGCATGGCCTAGTGCCATTATTTAAACTAAAAGGAATTCGGTT
GCATGGCCTAGTGCCATTATTTAAACCAAAAGGCCCTTTTCAGCAGAACAGTTAATCCTT
TGGCATATTGCCGTTTCCTGTGTTTTATACTTGAATTATGTACAGTACCGCACCCTGTAT
TACTCACAGGTACTATGACTGCCAACTATGCTTTTATCTGCATACTTTAGTGCTGTTGGG
CACACATTTTTATACATGTGTCTGCAACTTTGGTGTTTTGGCTTGCAGAATACACTATGT
AGGCCAAGTATCTGTCAGTATCTGTTTTGCAAACATGTAACATACAATTACTCATTTTTT
AAAACCGTTTACGGTCGTGCAAAAACAGGTTTCTTTTAATTGTT
>gi|397038|emb|X74481.1|_Human_papillomavirus_type_52_genomic_DNA
TAAATTATAATCTTATACTAGTAAAAAATAGGGTGTAACCGAAAACGGTCAGACCGAAAC
CGGTGTATATATATAGAACACAGTGTAGCTAACGCACGGCCATGTTTGAGGATCCAGCAA
CACGACCCCGGACCCTGCACGAATTGTGTGAGGTGCTGGAAGAATCGGTGCATGAAATAA
GGCTGCAGTGTGTGCAGTGCAAAAAAGAGCTACAACGAAGAGAGGTATACAAGTTTCTAT
TTACAGATTTACGAATAGTATATAGAGACAATAATCCATATGGCGTGTGTATTATGTGCC
TACGCTTTTTATCTAAGATAAGTGAATATAGGCATTATCAATATTCACTGTATGGGAAAA
CATTAGAAGAGAGGGTAAAAAAACCATTAAGTGAAATAACTATTAGATGTATAATTTGTC
AAACGCCATTATGTCCTGAAGAAAAAGAAAGACATGTTAATGCAAACAAGCGATTTCATA
ATATTATGGGTCGTTGGACAGGGCGCTGTTCAGAGTGTTGGAGACCCCGACCTGTGACCC
AAGTGTAACGTCATGCGTGGAGACAAAGCAACTATAAAAGATTATATATTAGATCTGCAA
CCTGAAACAACTGACCTACACTGCTATGAGCAATTAGGTGACAGCTCAGATGAGGAGGAT
ACAGATGGTGTGGACCGGCCAGATGGACAAGCAGAACAAGCCACAAGCAATTACTACATT
GTGACATATTGTCACAGTTGTGATAGCACACTACGGCTATGCATTCATAGCACTGCGACG
GACCTTCGTACTCTACAGCAAATGCTGTTGGGCACATTACAAGTTGTGTGCCCCGGCTGT
GCACGGCTATAAACAACCCTGCAATGGAGGACCCTGAAGGTACAGAGGGCGAAAGGGAGG

GATGTACAGGCTGGTTTGAAGTAGAGGCAATAATAGAAAAACAAACAGGAGATAACATTT
CAGAGGACGAGGATGAAAATGCATATGATAGTGGAACAGATCTAATAGATTTTATAGATG
ATTCAAATATAAATAATGAACAGGCAGAACATGAGGCAGCCCGGGCATTGTTTAATGCAC
AGGAAGGGGAGGATGATTTACATGCTGTGTCTGCAGTAAAACGAAAGTTTACAAGCAGTC
CGGAAAGTGCTGGGCAAGATGGTGTAGAAAAACATGGTAGTCCGCGTGCAAAACACATTT
GTGTAAATACAGAGTGTGTTTTACCAAAACGCAAACCATGTCACGTAGAAGACAGCGGCT
ATGGCAATAGTGAAGTGGAAGCGCAGCAGATGGCAGACCAGGTAGACGGGCAAAATGGCG
ACTGGCAAAGTAACAGTAGTCAATCAAGTGGGGTGGGGGCTAGTAATTCAGATGTAAGTT
GTACTAGTATAGAGGACAATGAGGAAAATAGTAATAGAACGCTAAAAAGCATACAAAATA
TTATGTGCGAAAATAGCATAAAAACAACTGTATTATTTAAATTTAAAGAAACATATGGTG
TTAGCTTTATGGAATTAGTAAGACCATTTAAAAGTAATAGAAGTAGTTGTACAGATTGGT
GTATTATAGGAATGGGAGTAACACCATCAGTTGCAGAAGGATTAAAAGTATTAATACAGC
CCTATAGCATATATGCCCATTTGCAATGTTTAACATGTGACAGAGGCGTGCTTATACTGC
TGCTAATTAGGTTTAAATGTGGAAAAAACAGATTAACAGTGTCCAAACTAATGTCACAGC
TGTTAAATATACCAGAAACACATATGGTAATAGAACCACCAAAATTACGAAGTGCTACCT
GTGCATTATATTGGTATAGAACAGGTTTGTCTAATATTAGTGAGGTATATGGTACCACCC
CAGAATGGATAGAACAACAAACAGTATTACAGCATAGCTTTGACAATAGCATATTCGATT
TTGGAGAAATGGTGCAATGGGCATATGATCATGATATAACAGATGATAGTGACATAGCAT
ATAAATATGCACAGTTAGCAGATGTAAATAGCAATGCTGCAGCATTCCTAAAAAGCAATT
CGCAAGCAAAAATAGTAAAGGACTGTGCAACCATGTGTAGACATTATAAACGGGCAGAAA
GAAAACATATGAATATTGGACAATGGATACAGTATAGATGTGATAGAATAGATGATGGTG
GAGATTGGAGGCCTATAGTAAGATTTTTAAGATATCAAGACATAGAATTTACAGCCTTTT
TAGACGCATTTAAAAAATTTTTAAAAGGTATACCTAAAAAAAATTGTTTAGTATTATATG
GACCTGCAAACACAGGAAAATCATATTTTGGAATGAGTTTAATTAGGTTCTTAAGTGGAT
GTGTAATATCCTATGTAAACTCAAAAAGCCATTTTTGGCTACAACCATTAACAGATGCAA
AAGTGGGTATGATAGATGATGTAACACCTATATGTTGGACATATATAGATGATTATATGA
GAAATGCACTGGATGGAAATGATATATCAGTAGATGTAAAGCATAGAGCCTTAGTACAAA
TAAAATGCCCACCATTAATTTTAACAACAAATACAAATGCAGGAACAGATCCTAGGTGGC
CATATTTACATAGTAGATTGGTTGTGTTTCATTTCAAAAACCCATTTCCATTTGATGAAA
ATGGCAATCCTATATATGAAATTAACAACGAAAATTGGAAATCCTTTTTCTCAAGGACGT
GGTGCAAATTAGATTTAATACAGGAAGAGGACAAGGAAAACGATGGAGTCGATACCGGCA
CGTTTAAATGCAGTGCAGGAAAAAATACTAGATCTATACGAAGCTGATAGTAATGACCTA
AACGCACAAATTGAACATTGGAAATTGACTCGAATGGAATGTGTTTTGTTTTACAAAGCA
AAGGAACTGGGAATAACTCATATAGGCCACCAGGTGGTGCCACCAATGGCAGTGTCTAAG
GCAAAGGCCTGCCAAGCTATTGAACTACAATTGGCATTGGAGGCATTAAACAAAACACAA
TATAGCACAGATGGATGGACATTACAACAAACAAGTCTAGAAATGTGGCGTGCAGAACCA
CAAAAATACTTTAAAAAACATGGGTATACAATAACAGTGCAATACGATAATGATAAAAAC
AATACTATGGATTATACAAACTGGAAGGAAATTTATTTACTTGGTGAGTGTGAATGTACA
ATTGTAGAAGGACAAGTAGATTACTATGGGTTATATTATTGGTGTGATGGAGAAAAAATA
TATTTTGTAAAATTTAGTAACGATGCAAAGCAATATTGTGTAACAGGAGTATGGGAAGTA
CATGTGGGTGGTCAGGTAATTGTTTGTCCTGCATCTGTATCTAGTAACGAAGTATCCACT
ACTGAAACTGCTGTCCACCTATGCACCGAAACCTCCAAGACCTCCGCAGTGTCCGTGGGT
GCCAAAGACACACACCTACAACCACCACAGAAACGACGACGACCAGACGTCACAGACTCC
AGAAACACCAAGTACCCCAACAACCTTTTGCGGGGACAACAATCCGTGGACAGTACTACA
CGGGGACTCGTCACTGCAACTGAGTGCACAAACAAAGGACGGGTTGCACATACAACTTGT
ACTGCACCTATAATACACCTAAAAGGTGATCCTAATAGTTTAAAATGTTTAAGATATAGG
GTAAAAACACATAAAAGTTTGTATGTTCAAATTTCATCTACCTGGCATTGGACCAGTAAT
GAATGTACAAATAATAAACTAGGTATTGTAACAATAACGTACAGTGATGAAACACAACGT
CAACAATTTTTAAAAACTGTTAAAATACCAAATACTGTGCAAGTTATACAAGGTGTCATG
TCATTGTGATATTTGTACATATGTATATATGTATATGTGTATGGTAAACACCCAACACAA
GCCAATATTGCTGCTATTGTGTATATATAACAATGTTAGGATTATTTGTATTTTGTTTTA
TTTTGCTTATGGTGTTTTGTGCAGTGCTTAGGCCGCTCTTGCTATCTATATCGGTGTATG
CGCAGGTGTTGGTGCTGGTGCTTTTGCTATGGGTATCTATTGGGTCACCATTTAAAGTGT
TTTTTTTGTACCTACTGTTTTTATATTTTCCAATGTTTTGTATTCACTGTCATGCACAGT
ATTTGGCACAACTGCAATAACTGTACATGTAGATTGGCTACATGCATATATGCAAAATAT

ACTTTTTCACTTTTGTAGTTTGTCTAATAAATACTTTTATATTTTTTAATAGCTTGTCGC
AATGAGATACAGACGGTCTACACGGCACAAACGTGCTTCTGCAACACAGCTATATCAAAC
ATGCAAAGCCTCTGGCACCTGCCCCCCCGATGTTATTCCTAAAGTGGAAGGCACAACTAT
TGCAGATCAACTTTTAAAATATGGCAGCCTAGGGGTGTTTTTTGGAGGTTTGGGTATAGG
TACAGGTGCAGGCTCTGGTGGTAGGGCAGGCTATGTGCCATTGTCCACTCGTCCTCCCAC
TAGTAGTATTACCACGTCCACCATTCGTCCCCCTGTAACTGTAGAACCCATTGGTCCCTT
AGAACCATCTATAGTTTCTATGATAGAAGAAACAACATTTATTGAGTCTGGCGCACCTGC
TCCATCTATTCCATCAGCAACAGGGTTTGATGTTACAACATCTGCAAATAATACTCCTGC
AATAATTAATGTAACATCTATAGGTGAATCATCTGTACAATCAGTTTCTACACATTTAAA
TCCTACATTCACTGAACCATCTATAATACAGCCCCCGGCACCTGCAGAAGCATCTGGTCA
TGTATTGTTTTCTAGTCCAACTATTAGTACACACACCTATGAAGAAATCCCTATGGATAC
ATTTGTTACCTCTACTGACAGCAGCAGTGTAACAAGTAGTACACCTATTCCAGGGTCTCG
CCCTACGACACGCCTTGGTTTATATAGCCGTGCCACACAACAGGTTAAGGTAGTCGACCC
TGCTTTTATGTCATCACCACAGAAATTAGTAACATATAACAATCCTGTTTTTGAGGGCGT
TGATACAGATGAAACTATAATTTTTGATCGTTCACAACTTTTACCTGCACCGGATCCTGA
TTTTTTAGACATTATAGCTTTGCATAGGCCTGCATTAACCTCTCGAAGAGGTACTGTTAG
GTTTAGCAGGCTTGGTAATAAGGCCACCCTACGTACACGTAGTGGAAAACAAATTGGGGC
ACGGGTACATTATTATCATGATATTAGTCCTATCCAGCCTGCTGAAGTTCAGGAAGACAT
AGAATTGCAACCTTTATTACCACAGTCTGTGTCCCCTTACACTATTAATGATGGTTTGTA
TGATGTGTATGCAGATTCTTTGCAGCAACCCACGTTTCACTTACCTTCCACACTTTCTAC
CCATAATAATACTTTCACTGTACCTATTAATAGTGGTATTGACTTTGTATATCAACCCAC
TATGTCCATTGAGTCAGGTCCTGACATTCCATTACCTTCGTTACCCACACATACTCCTTT
TGTTCCTATAGCCCCTACAGCTCCATCTACATCTATTATTGTTGATGGTACAGATTTTAT
TTTACATCCTAGTTATTTTTTACTACGTCGCAGGCGTAAACGTTTTCCATATTTTTTTAC
AGATGTCCGTGTGGCGGCCTAGTGAGGCCACTGTGTACCTGCCTCCTGTACCTGTCTCTA
AGGTTGTAAGCACTGATGAGTATGTGTCTCGCACAAGCATCTATTATTATGCAGGCAGTT
CTCGATTACTAACAGTAGGACATCCCTATTTTTCTATTAAAAACACCAGTAGTGGTAATG
GTAAAAAAGTTTTAGTTCCCAAGGTGTCTGGCCTGCAATACAGGGTATTTAGAATTAAAT
TGCCGGACCCTAATAAATTTGGTTTTCCAGATACATCTTTTTATAACCCAGAAACCCAAA
GGTTGGTGTGGGCCTGTACAGGCTTGGAAATTGGTAGGGGACAGCCTTTAGGTGTGGGTA
TTAGTGGGCATCCTTTATTAAACAAGTTTGATGATACTGAAACCAGTAACAAATATGCTG
GTAAACCTGGTATAGATAATAGGGAATGTTTATCTATGGATTATAAGCAGACTCAGTTAT
GCATTTTAGGATGCAAACCTCCTATAGGTGAACATTGGGGTAAGGGAACCCCTTGTAATA
ATAATTCAGGAAATCCTGGGGATTGTCCTCCCCTACAGCTCATTAACAGTGTAATACAGG
ATGGGGACATGGTAGATACAGGATTTGGTTGCATGGATTTTAATACCTTGCAAGCTAGTA
AAAGTGATGTGCCCATTGATATATGTAGCAGTGTATGTAAGTATCCAGATTATTTGCAAA
TGGCTAGCGAGCCATATGGTGACAGTTTGTTCTTTTTTCTTAGACGTGAGCAAATGTTTG
TTAGACACTTTTTTAATAGGGCCGGTACCTTAGGTGACCCTGTGCCAGGTGATTTATATA
TACAAGGGTCTAACTCTGGCAATACTGCCACTGTACAAAGCAGTGCTTTTTTTCCTACTC
CTAGTGGTTCTATGGTAACCTCAGAATCCCAATTATTTAATAAACCGTACTGGTTACAAC
GTGCGCAGGGCCACAATAATGGCATATGTTGGGGCAATCAGTTGTTTGTCACAGTTGTGG
ATACCACTCGTAGCACTAACATGACTTTATGTGCTGAGGTTAAAAAGGAAAGCACATATA
AAAATGAAAATTTTAAGGAATACCTTCGTCATGGCGAGGAATTTGATTTACAATTTATTT
TTCAATTGTGCAAAATTACATTAACAGCTGATGTTATGACATACATTCATAAGATGGATG
CCACTATTTTAGAGGACTGGCAATTTGGCCTTACCCCACCACCGTCTGCATCTTTGGAGG
ACACATACAGATTTGTCACTTCTACTGCTATAACTTGTCAAAAAAACACACCACCTAAAG
GAAAGGAAGATCCTTTAAAGGACTATATGTTTTGGGAGGTGGATTTAAAAGAAAAGTTTT
CTGCAGATTTAGATCAGTTTCCTTTAGGTAGGAAGTTTTTGTTACAGGCAGGGCTACAGG
CTAGGCCCAAACTAAAACGCCCTGCATCATCGGCCCCACGTACCTCCACAAAGAAGAAAA
AGGTTAAAAGGTAACCATTGTCTGTTGGGTAATTGTCTGTGTCATGTATGTGTTGTGTAT
GTCAAACACAGGTTAAAAGGTAACCATTGTTTGTTATGTAATTGTTTTGTGTGTGTACTG
TGTTGTTTGCATGTTATGTATGTGTGTGCATGTTTGTTGTATTTGTCAGTTCCTGTATGT
ATGTTTTGTGTATGTATTAATAAAGTACTGTATTTACTAAACTATTTATAGTAGTCTTAT
GTTATGTTATGGTTGCACCCACATGAGTAACAATACAGTTGCTCCTAATCTATTGCATCT
CCTGCCCTACCCTGTGTCCCCTGCCCTACCCTGTGTCCTACTTTGTTACACTACTAATTA

```
GCCTTATACTCTCCATTTTGTACCATTTTGTACTATCCACCATTTTAAATCCTAACCGAA
TTCGGTTGGTCTTGGCACAACTTTGGTTGTCCTTGGCACAGTAACAACTATTTTTATATA
AGTTTCAGCAAACTGCTTAATCCTTTGGTTTCCTGCAGTCCACTGGTCTACACTTGTTGT
CCCGCCTAAACTGACTTCTTGCTGACTCACAGGTCCTGCAGTGCAGCTAAACAATACATT
GCCTAACATTGCATGTTTTAAACTGCTTTTAGGCACATATTTTATTTAAACTTTCAATGC
ACTAATTACAGTGTTGGCTTACACAAGTACATCCTACGCCAAATATGTCTTGTAAAACAT
GATTAAATACTGTTACTCACCAGGTGTGCACTACACGACCGGTTACGGTTACCGTACCCA
CAACCACTTTTTTTTATAATTA
>gi|397022|emb|X74479.1|_Human_papillomavirus_type_45_genomic_DNA
AATACTTTTAACAATTATACTACATAAAAAAGGGTGTAACCGAAAACGGTTGCAACCAAA
AACGGTGCATATAAAAGCTTTGTGGAAAAGTGCATTACAGGATGGCGCGCTTTGACGATC
CAAAGCAACGACCCTACAAGCTACCAGATTTGTGCACAGAATTGAATACATCACTACAAG
ACGTATCTATTGCCTGTGTATATTGCAAAGCAACATTGGAACGCACAGAGGTATATCAAT
TTGCTTTTAAAGATTTATGTATAGTGTATAGAGACTGTATAGCATATGCTGCATGCCATA
AATGTATAGACTTTTATTCCAGAATTAGAGAATTAAGATATTATTCAAACTCTGTATATG
GAGAGACACTGGAAAAAATAACTAATACAGAGTTGTATAATTTGTTAATAAGGTGCCTGC
GGTGCCAGAAACCATTGAACCCAGCAGAAAAACGTAGACACCTTAAGGACAAACGAAGAT
TTCACAGCATAGCTGGACAGTACCGAGGGCAGTGTAATACATGTTGTGACCAGGCACGGC
AAGAAAGACTTCGCAGACGTAGGGAAACACAAGTATAGCAATAAGTATGCATGGACCCCG
GGAAACACTGCAAGAAATTGTATTGCATTTGGAACCTCAGAATGAATTAGATCCTGTTGA
CCTGTTGTGTTACGAGCAATTAAGCGAGTCAGAGGAGGAAAACGATGAAGCAGATGGAGT
TAGTCATGCACAACTACCAGCCCGACGAGCCGAACCACAGCGTCACAAAATTTTGTGTGT
ATGTTGTAAGTGTGACGGCAGAATTGAGCTTACAGTAGAGAGCTCGGCAGAGGACCTTAG
AACACTACAGCAGCTGTTTTTGAGCACCTTGTCCTTTGTGTGTCCGTGGTGTGCAACTAA
CCAATAATCTACAATGGCGGATCCAGAAGGTACCGACGGGGAGGGAACGGGGTGTAATGG
CTGGTTCTTTGTAGAAACAATTGTAGAGAAAAAAACAGGGGATGTAATATCAGATGATGA
GGATGAAACTGCAACAGATACAGGGTCGGATATGGTAGATTTTATTGACACACAATTATC
CATTTGTGAACAGGCAGAGCAAGAGACAGCACAGGCATTGTTCCATGCGCAGGAAGTTCA
GAATGATGCACAGGTGTTGCATCTTTTAAAACGAAAGTTTGCAGGAGGCAGCAAGGAAAA
CAGTCCATTAGGGGAGCAGCTAAGTGTGGATACGGATCTAAGTCCACGGTTACAAGAAAT
TTCATTAAATAGTGGGCACAAAAAAGCAAAACGACGGTTGTTTACAATATCAGATAGTGG
CTATGGCTGTTCTGAAGTGGAAGCTGCAGAGACTCAGGTAACTGTAAACACTAATGCGGA
AAATGGCGGCAGTGTACATAGTACACAAAGTAGTGGTGGGGATAGTAGTGACAATGCAGA
AAATGTAGATCCGCATTGCAGTATTACAGAACTAAAGGAGCTATTACAAGCAAGTAACAA
AAAGGCTGCAATGCTGGCAGTATTTAAAGACATATATGGGCTGTCATTTACGGATTTGGT
TAGAAATTTTAAAAGTGATAAAACAACATGTACAGATTGGGTAATGGCTATATTTGGAGT
TAATCCAACGGTAGCAGAAGGCTTTAAAACATTAATTAAACCAGCAACGTTATACGCCCA
TATCCAATGTTTAGATTGTAAATGGGGAGTATTAATATTAGCTTTATTAAGATATAAATG
TGGCAAAAATAGACTAACTGTTGCAAAAGGCTTAAGCACATTGTTGCACGTACCTGAAAC
ATGTATGTTAATTGAACCACCAAAATTGCGAAGTAGTGTTGCAGCATTATACTGGTATAG
AACAGGTATATCCAATATTAGTGAAGTAAGTGGAGACACACCTGAGTGGATACAAAGACT
GACAATTATTCAACATGGTATTGACGATAGTAATTTTGATTTGTCAGACATGGTGCAATG
GGCATTTGATAATGACCTTACAGATGAAAGTGATATGGCATTTCAATATGCCCAATTAGC
AGACTGCAACAGTAATGCAGCTGCATTTTTAAAAAGTAACTGCCAAGCCAAATATTTAAA
AGATTGTGCTGTAATGTGTAGACATTATAAAAGAGCACAAAAACGCCAAATGAATATGTC
TCAATGGATTAAATATAGATGTTCCAAAATAGATGAAGGTGGGGATTGGAGACCCATAGT
ACAATTCCTAAGATATCAGGGAGTAGAATTTATTAGCTTTTTAAGGGCACTAAAGGAATT
TCTTAAAGGAACACCAAAAAAAAATTGTATACTGTTATATGGACCTGCAAATACAGGAAA
ATCGTATTTTGGAATGAGTTTTATACATTTCCTACAAGGTGCAATAATATCATTTGTAAA
TTCAAACAGCCATTTTTGGTTAGAACCGTTAGCAGATACTAAGGTAGCCATGTTGGATGA
TGCCACACACACGTGTTGGACATATTTTGATAATTATATGAGAAATGCATTAGATGGTAA
TCCTATAAGTATAGACAGAAAGCATAAACCATTATTACAGCTAAAATGTCCTCCAATCCT
ATTAACATCCAATATTGATCCAGCAAAAGATAATAAATGGCCATATTTAGAAAGTAGGGT
GACGGTATTTACATTTCCACATGCATTTCCATTTGATAAAAATGGTAATCCAGTATATGA
AATAAATGATAAAAAATTGGAAATGTTTTTTTGAAAGGACATGGTCCAGATTAGATTTGCA
```

CGAGGACGATGAAGATGCAGACACCGAAGGAATCCCTTTCGGAACGTTTAAGTGCGTTAC
AGGACAAAATACTAGACCACTATGAAAATGACAGTAAAGACATAAACAGCCAAATAAGTT
ATTGGCAACTTATACGTTTGGAAAATGCAATACTATTTACAGCAAGGGAACATGGTATTA
CCAAACTAAACCACCAGGTGGTGCCTCCTATTAACATTTCAAAAAGCAAAGCACATAAAG
CTATTGAACTGCAAATGGCCTTAAAGGGCCTTGCACAAAGCAAGTATAACAATGAGGAAT
GGACACTGCAAGATACATGCGAGGAACTATGGAATACAGAACCGTCGCAGTGTTTTAAAA
AAGGCGGTAAAACCGTGCACGTATACTTTGATGGCAACAAGGACAACTGTATGAACTATG
TAGTATGGGACAGTATATATTATATAACTGAGACAGGGATATGGGACAAAACAGCAGCAT
GTGTTAGCTATTGGGGTGTATATTATATAAAAGATGGAGATACCACATATTATGTACAAT
TTAAAAGCGAATGTGAGAAATATGGAAATAGTAATACGTGGGAAGTACAATATGGGGGCA
ATGTAATTGATTGTAATGACTCTATGTGCAGTACCAGTGACGACACGGTATCCGCTACTC
AGATTGTTAGACAGCTACAACACGCCTCCACGTCGACCCCCAAAACCGCATCCGTGGGCA
CCCCAAAACCCCACATCCAGACGCCGGCTACTAAGCGACCTAGACAGTGTGGACTCACAG
AGCAGCACCACGGACGTGTCAACACCCACGTGCACAACCCGCTCCTGTGTTCAAGTACAA
GTAACAACAAAAGAAGGAAAGTGTGTAGTGGTAACACTACGCCTATAATACACTTAAAAG
GTGACAAAAACAGTTTGAAATGTTTAAGATATAGGCTACGCAAATATGCAGACCATTACT
CAGAAATATCCTCCACCTGGCATTGGACAGGTTGTAATAAAAACACTGGTATATTAACTG
TAACATATAATAGTGAGGTACAAAGAAATACCTTTTTGGATGTAGTTACTATTCCTAACA
GTGTACAAATCTCGGTGGGATACATGACTATATGAATCTGTATATTGTATACAGTATGTA
ACATTACTATGCTATCTTTAGTGTTTTTATTGTGCTTTTCTGTGTGCCTTTATGTGTGCT
GCAATGTCCCGCTTGTGCAGTCTGTCTATGTGTGTGCTTTTGCTTGGTTGTTGGTGTTTC
TTTTTATAGTTGTTATTACATCCCCATTAACAGCATTTGCTGTATACATTTGTTGCTATT
TACTACCTATGTTTGTATTACATATGCATGCTTTACACACCATACAATAATTACTATAAT
GTACAGTACAGTGTAACATACCTGTGATGTGCATGTTGTTGTATTTTTGTATTTTTGTAT
TTTTGTATTTTTGTATTTTATATGTTTAATAAACCATGGTATCCCACCGTGCAGCACGTC
GCAAGCGGGCCTCTGCAACTGACTTATATAGAACATGTAAGCAATCCGGTACGTGCCCCC
CTGATGTTATTAACAAAGTGGAAGGCACAACCTTAGCTGATAAAATTTTACAGTGGTCTA
GCCTTGGGATATTTTTGGGTGGCCTTGGCATTGGTACCGGCAGTGGTTCTGGAGGCCGTA
CGGGCTATGTACCCTTAGGGGGCAGGTCTAATACTGTTGTGGATGTTGGCCCCACTAGGC
CACCTGTGGTTATTGAACCTGTAGGGCCTACTGATCCATCTATTGTTACGTTGGTAGAGG
ATTCCAGTGTTGTTGCCTCTGGTGCTCCGGTTCCCACATTTACCGGAACCTCTGGGTTTG
AAATTACGTCTTCTGGTACTACCACACCAGCTGTGTTGGACATCACACCTACCGTGGACT
CTGTTTCTATTTCGTCAACTAGTTTTACAAATCCTGCATTTTCTGATCCCTCTATTATTG
AGGTGCCCCAAACAGGGGAGGTATCAGGTAATATATTTGTTGGTACACCAACATCGGGCA
GCCATGGATATGAGGAAATACCTTTACAAACATTTGCATCTTCTGGGTCAGGTACGGAAC
CCATTAGTAGTACCCCCCTCCCTACTGTGCGGCGGGTACGGGGTCCCCGCCTGTATAGTA
GGGCTAATCAACAGGTCCGTGTGTCCACCTCACAGTTTTTAACACATCCCTCATCGTTGG
TTACATTTGATAATCCAGCTTATGAGCCCCTGGACACCACACTATCCTTTGAGCCTACCA
GTAATGTTCCTGATTCCGATTTTATGGATATTATTCGTTTGCATAGGCCAGCATTATCCT
CTAGACGTGGCACTGTTAGATTTAGTAGATTGGGTCAAAGGGCAACCATGTTTACACGTA
GTGGTAAACAAATAGGGGGTAGGGTACATTTTTACCATGATATAAGCCCCATTGCTGCTA
CAGAGGAAATTGAATTGCAGCCTTTAATTAGTGCTACAAATGATAGTGACCTGTTTGATG
TATATGCAGACTTCCCACCTCCTGCGTCCACTACACCTAGCACTATACACAAATCATTTA
CATATCCAAAGTATTCCTTGACCATGCCTTCTACTGCTGCATCCTCTTACAGTAATGTTA
CAGTACCATTAACATCTGCATGGGATGTACCTATATATACTGGCCCGGACATTATATTGC
CATCCCATACTCCTATGTGGCCTAGTACATCTCCTACCAATGCTTCCACCACCACCTATA
TAGGTATTCATGGCACACAATATTATTTATGGCCATGGTATTATTATTTTCCTAAAAAAC
GTAAACGTATTCCCTATTTTTTTGCAGATGGCTTTGTGGCGGCCTAGTGACAGTACGGTA
TATCTTCCACCACCTTCTGTGGCCAGAGTTGTCAGCACTGATGATTATGTGTCTCGCACA
AGCATATTTTATCATGCAGGCAGTTCCCGATTATTAACTGTAGGCAATCCATATTTTAGG
GTTGTACCTAATGGTGCAGGTAATAAACAGGCTGTTCCTAAGGTATCCGCATATCAGTAT
AGGGTGTTTAGAGTAGCTTTACCCGATCCTAATAAATTTGGATTACCTGATTCTACTATA
TATAATCCTGAAACACAACGTTTGGTTTGGGCATGTGTAGGTATGGAAATTGGTCGTGGG
CAGCCTTTAGGTATTGGCCTAAGTGGCCATCCATTTTATAATAAATTGGATGATACAGAA
AGTGCTCATGCAGCTACAGCTGTTATTACGCAGGATGTTAGGGATAATGTGTCAGTTGAT

TATAAGCAAACACAGCTGTGTATTTTAGGTTGTGTACCTGCTATTGGTGAGCACTGGGCC
AAGGGCACACTTTGTAAACCTGCACAATTGCAACCTGGTGACTGTCCTCCTTTGGAACTT
AAAAACACCATTATTGAGGATGGTGATATGGTGGATACAGGTTATGGGGCAATGGATTTT
AGTACATTGCAGGATACAAAGTGCGAGGTTCCATTAGACATTTGTCAATCCATCTGTAAA
TATCCAGATTATTTGCAAATGTCTGCTGATCCCTATGGGGATTCTATGTTTTTTTGCCTA
CGCCGTGAACAACTGTTTGCAAGACATTTTTGGAATAGGGCAGGTGTTATGGGTGACACA
GTACCTACGGACCTATATATTAAAGGCACTAGCGCTAATATGCGTGAAACCCCTGGCAGT
TGTGTGTATTCCCCTTCTCCCAGTGGCTCTATTATTACTTCTGATTCTCAATTATTTAAT
AAGCCATATTGGTTACATAAGGCCCAGGGCCATAACAATGGTATTTGTTGGCATAATCAG
TTGTTTGTTACTGTAGTGGACACTACCCGCAGTACTAATTTAACATTATGTGCCTCTACA
CAAAATCCTGTGCCAAGTACATATGACCCTACTAAGTTTAAGCAGTATAGTAGACATGTG
GAGGAATATGATTTACAGTTTATTTTTCAGTTGTGCACTATTACTTTAACTGCAGAGGTT
ATGTCATATATCCATAGTATGAATAGTAGTATATTAGAAAATTGGAATTTTGGTGTCCCT
CCACCACCTACTACAAGTTTGGTGGATACATATCGTTTTGTGCAATCAGTTGCTGTTACC
TGTCAAAAGGATACTACACCTCCAGAAAAGCAGGATCCATATGATAAATTAAAGTTTTGG
ACTGTTGACCTAAAGGAAAAATTTTCCTCCGATTTGGATCAATATCCCCTTGGTCGAAAG
TTTTTAGTTCAGGCTGGGTTACGTCGTAGGCCTACCATAGGACCTCGTAAGCGTCCTGCT
GCTTCCACGTCTACTGCATCTACTGCATCTAGGCCTGCCAAACGTGTACGTATACGTAGT
AAGAAATAATATGTTAGCACATATATGTATGTTTGTATGTATGGTTTTGTATGTTGTATG
TATGTATGTATTTGTGTGATATATTACTGTATTTTGTTTGTTTGCGTGCGTGTATGTATG
AATGTGCCTTGTGGCATGTATGGTGTTACTGTACATAATTGTGGTATTAAATAAAGTATG
CTAATAGTGTTGTGTAGGGTTGCACCCTTGTGAGTAACAATACTATTTGTGTGTATGTGT
ATTGCTTTGTACCCTATATTCTTTCCTGTATTTCAAGTTATAAACTTGCATACTACACAG
CATCCATTTTACTTATAATCCTCCATTTTGCTGTGCAACCGATTTCGGTTGCCTGTGGCT
TATATGTGACCTTTTAAACATAATACCTAAACTGGCACATTTACAACCCCTACATAGTTT
AACCTACTGGCGCGCCTTCTTGGCGTACATGTGGCACACCTGGTATTAGTCATTTTCCTG
TCCAGGTGTACTAAAACAATGGCTTGCACAACTGTATCCACACCCTATGTAATAAAACTG
CTTTTAGGCACATATTTTAGTCTGTTTTTACCTGTGCTAATTGTATAATTGGCGTGTAGA
ACCACTTTCTTATCCAACAATCTGTCTACTTGTTACATAAACTATAAACTGACTCACTTA
TACATACATAGTTTATGCAACCGAAAAAGGTTGGGCCCTATAACACATACCTTTTCTT
>gi|397014|emb|X74478.1|_Human_papillomavirus_type_40_genomic_DNA
TTAATAACAATTGTACTGTTAGTAAAGGGTGTAACCGAAAACGGTCCGACCGAAAGCGGT
ACATATAAATTCCAACCCAAAAAACCTGCTTTGGGGCCAGTATGTCTGCACGGTGCGGCT
CCCAGGCCAGGACCCTGTATGAACTGTGTGACCAGTGCAATATTACATTGCCTACGTTGC
AAATTGATTGTGTGTTTTGCAAGACGGTCCTAAAAACAGCTGAGGTACTGGCCTTTGCCT
TTAGAGAGTTATATGTTGTGTGGCGCGACGACTTTCCACACGCCGCATGTCCACGGTGCC
TGGACCTGCACGGAAAAGTAAACCAATACAGAAACTTTAGATACGCAGCCTATGCACCAA
CCGTGGAAGAAGAGACAGGATTAACCATTTTACAAGTAAGGATTAGATGCTGCAAGTGCC
ACAAGCCTTTGTCTCCCGTGGAAAAAACCAACCATATTGTAAAGAAGACGCAATTCTTTA
AATTAAAAGATTCGTGGACAGGGTACTGTCTACATTGCTGGAAGAAATGCATGGAGAAAG
GCCAACGCTCGGAGACATTGTGTTAAACCTGCACCCTGAACCTGTATGTCTAAACTGCAA
CGAGCAATTAGACAGCTCAGACTCAGAAGATGACCATGAACAGGACCAACTAGACAGCTT
ACACAGTAGAGAGCGTGAGCAACCCACGCAACAGGACCTGCAAGTAAATTTGCAATCATT
TAAAGTAGTAACTCGGTGTGTATTTTGTCAGTGTTTGGTGCGCTTAGCAGTGCATTGTTC
CATCACTGATATAACACAGTTCCAGCAGTTGCTGATGGGCACATTACATATAGTGTGCCC
CAACTGTGCAGCTACAGAGTGACAACAATGGCAGACTCTCCAGGTACAGAGGACGGGGGG
GCTGGGTGCTCAGGATGGTTTGTAGTAGAAGCTGTAGTGGATAAACAAACGGGGGATGCT
GTATCGGAAGATGAGGATGAGGAGGACATAGAGGATAGTGGATTTGATATGATAGATTTT
ATTGATAATAGTGTTGTGGCAGAGGAACATGTAGAACTAAGTAATGCACAGGCACTTTTA
CATGTACAGCAGACATGTGCAGATGCTGCTGACCTGTGCGAGTTAAAACGAAAGTACATT
AGCCCATATGTAAGTCCTATACAATACTCAGAACCGTCTATAGACGGGGACCTAAGTCCA
AGGCTGCATGCAATACGGCTTGGCGGCGGCCAAAAGGCTAAACGGCGGTTGTTCCAGCGT
GTGGAGCAAAGGGACAGTGGCTATGGCTATTCTGAAGTGGAAACAACAGAGAGACAGGTA
GAGACAGAACATGGCGGACCGGAAGATACCGTGGGGGGTAGTGGGAGGGTGACCACAGAT
GAGGCGGAAGCAGTAGAGGTTGTGGAAGACGGCAGTCATGTTATAGACCACTGTAGTCCG

CGCACACAACTAATAGAGCTGTTTAAATGCAAGGACCTAAATGCTAAGCTGTATGGTAAG
TTTAAAGAGCTTTATGGAGTGGGGTTTGGAGACCTGGTAAGACAGTTTAAAAGTGATAAA
TCCACGTGTACCGATTGGGTGTATGCTGTGTTCGGGGTTAATCCCACCATAGCCGAGGGC
TTTCATACACTGCTGAAAAGGCAGGCATTATATTTACATACCCAATGGACGTCATGCAAA
TGGGGTATGGTGTTGCTTGCATTGTGTAGATATAAGGTGGGTAAAAATAGGGAAACAGTT
GTTAGACAGCTATCCAAAATGTTAAATGTACCTGACAACCAGATACTGGTACAACCGCCT
AAATTACAAAGTCCGCCTGCAGCATTATTTTGGTTTAGAGCAGGAATGGGTAATGGGAGC
GAGGTGTCCGGCACAACACCGGAATGGATAGCTAAACAAACTATGTTAGAACACAGCTTT
GCTGACACACAGTTTAGCCTAACAGACATGGTGCAGTGGGCATATGATAACGGGCATACA
GATGAGTGTGAAATAGCATACTATTATGCACAAAGAGCAGACGTGGATGCAAATGCAGCA
GCATTCCTAAAAAGTAACAATCAGGCCAAATACGTTAGGGATTGTGCATCCATGTGCAAA
CACTATAGGTTAGCAGAAATGAGGCGCATGTCAATGGCTGAGTGGATAAAGCATAGAGGG
GAGAAGTGTGATGAAGGCGACTGGAAACCTATAGTTAAATTATTACGCTATCAACATATA
GATATAATAGTTTTTTTAGCTGCATTAAAAAAATGGCTACAGGGTATACCTAAAAAAAAT
TGCATTTGTATTGTGGGTCCTCCAGACACAGGAAAGTCATGTTTTGGAATGAGCCTGATG
CACTTTATGCAAGGTACAATAATATCATATGTTAATTCCTGTAGCCATTTTTGGTTGCAA
TCATTGGCAGATGCAAAGGTAGCTATGCTAGATGATGTTACAGCGGCATGCTGGGGGTAT
ATGGATACACACATGAGGAACTTATTAGATGGTAACCCAACCAGCATAGATAGAAAACAT
AAACCGTTAGCAGTAATTAAGTGCCCTCCGTTACTGTTAACGTCCAATATAAATATAACA
CAGGACAGTAAGTACCAATATTTACAAAGTAGGGTTCAAGTGTTTGAATTTCCAAATCCA
TTTCCATTTGACAGCAACGGCAATGCTGTGTATGAATTAAATGATGCAAATTGGAACTCC
TTTTTTAAAAGGTTGGCATCCAGTTTAGAGCTGCAAACACCTGGGGACGAGGATGGAGAA
TCTAGCCAGGCGCCTAGATTTGTGCCAGGAACAGTTGTTAGAACTGTATGAACAAAACAG
CAAAGAGCTACAGCAACATATATTGCACTGGAAATATATACGTTATGAAAGTGCAATATA
TTATACAGCAAGACAAATGGGCATTAAACATTTAGGCCACCAGGTGGTGCCAAGTTTAGA
TGTTTCAAAGGCTAAAGCCCATGCAGCAATTGAAATGCAAATGTGTTTAGAATCTTTGCA
AAACACTGAATATAATGTAGAGCCATGGACGCTGCAGGACACAAGTCAAGAATTATGGCT
TGCAGAACCTAAGAAATGTTTTAAAAAAGGTGGCAAAACAGTGGAAGTTAGATTTGACTG
CAATGAAACAAATGCAATGCATTATACACTGTGGACCACAGTATATGTACAGGTGGATGA
TGCATGGACAAAGGTAAAAGGCCAGGTGGACTACAAAGGCCTCTCATATACAGTGCACGG
GTGCACAACATATTATGTAGACTTTGCAAAGGAAGCACAAACGTATGGGAAAACAAATAG
GTGGACTGTTATTGTGGGTTCACACGTTATATGTTCTCCTAGTACTATCGAGGAACACGG
ACTACCCATTGTTGAGACTGCTGACGCCAGACCCCCGACCACTGCCACCGACACCCCCGA
CGCCCCCGCCACAGCGACCACCGAAACGGTCGGCCCCGCCCAGGCACCGCCCAGAAAGCG
ACGAAGAAACGGACACCTGCCCATCACCACTACTGTGGGCAAATCACTCGGAGGAGAGTA
CGTGGACACTGCAGACAGAACACGCACGCCTGACCCTGAAAGCAACAACGGGCACAGGAA
CTGTGGTGGAGGTTCTTCTACACCTATAATACAATTAGAAGGTGAAGCCAATTGCTTAAA
GTGTTTTAGATATAGACTAGGAAAAGTGTCACATTTATTTGTAATTCCTCAACTACATG
GAGGTGGACCACTGAATCCAGAACCGAGAAAAATGCTATAATAACGTTAACATATAGTAG
TGTACAGCAACGGTCTGACTTCTTAGCTATTGTAAAAATACCAAAAACAATAAAACATAG
CTTGGGTATGTTAACACTGATGTAATATATGTATATATGTATATATATGGAACACCATCT
GTGAAGGGTATTGTCTAGCACGTACCACACAACCAGCCAATCTGCTGCTATTATTGTTAA
TAGAGTCTACGCTTGGCATACTAGTAGTCTATATTGTTTTTTTACTTATTGCACTGTTGA
TCTGTGTGTGTGTCCCTAATCTGTATGTGTGGAGCTCTATACTGTCTGCTATCTCCATTT
TGTGCTTTGTAACATGGGGTGCACTAACATCTATTACTAACCTGTTTATCCTAATACTCT
TAGTGTGGTATTTGCCTGCGGTGGTCCTTCACACATTTATTATATATCACATACAGAACC
AATTGTAACATGTTGACCTGTCAATTGGATGATGGTGATACCTGGATGGCATTATGGATG
TTACTTGCGTTTATAACTGTGTTGTTACTGTTATTGGTGTTTCATTGTAGAACCTTATAT
TTATATAGGTATACCAAGTAATACTTTGTGTAGTAAATAAACATTTTTTTATATTACAGC
GTCTTGTAATGGTGTCCAGCAGGCCCCGTAGGCGCAAGCGGGCGTCAGCTACTCAGTTAT
ATCAAACATGCAAGGCGGCGGGCACCTGTCCACCTGATGTTGTTCATAAGGTGGAGCAAA
CAACCGTTGCAGATCAAATTTTAAAATGGGGGAGCATGGGAGTATTTTTTGGCGGGCTTG
GCATAGGTTCCGGCTCTGGCACAGGGGGCAGGGCTGGCTATGTGCCTTTGTCTACAGGTT
CCCGGGCTGTCCCTCCTAAATCTTTGGTACCTGACGTTGTTGCTAGGCCACCTGTGGTTG
TGGATACTGTTGCACCCTCCGATCCGTCCATTGTATCTTTAATTGAGGAAAGTAGTATTA

TTCAGTCAGGTGCCCCGTCCCTTACTATTCCAACAGAGGGTGGGTTTTCAGTAACGTCCT
CCGGTACAGATGTGCCTGCCATTTTAGATGTGTCGTCCACAAATACCGTGCATGTGACAG
CCACCACACATCACAATCCTGTCTTTACTGATCCCTCGGTTGTGCAGCCCATCCCACCTG
TGGAGGCCGGTGGTCGCCTCATAGTGTCGCATTCCACTATTACTACTAGTGCTGCTGAGG
AAATACCTTTGGACACGTTTGTTGTCCATAGTGATCCATTGTCCAGTACACCCGTTCCTG
GTACGTCTGGACGGCCCAGGTTGGGACTGTACAGTAAGGCCTTGCAGCAGGTGGAAATTG
TGGACCCTGCCTTTCTGTCTACCCCTCAGCGTTTAATTACATATGACAATCCAGTGTTTG
AGAACGTGGATGATACATTGCAGTTTGAGCAGCCATCCATACATGACGCTCCGGACCCTG
CGTTTATGGACATCATTACCTTGCACAGGCCTGCATTAACGTCTCGGCGGGGTGTCATAC
GCTTTAGCAGGGTGGGTCAACGGGGCACCATGTACACACGGCGAGGGACCCGCATTGGGG
GTCGTGTACACTTTTTTAGGGACATTAGTCCTATTGGTGCAGCTGATGATATTGAATTGC
ACCCTCTAGTGGCCTCGGCACCACATACACTGGAGACACCACATACACTAGAGACACCAC
TGGACACTACTGATGCCCTGTTTGATGTGTATGCAGACATGGATACTATAGATGATGATG
CAGCATATGCTACATTCTCATTACATCCTGCCGATTCTACTCGTATATCTAACACATCCA
TACCTCTTGCCACGGTTTCTGACACATTATTAACATCTGGTCCTGACATAGTGTTTCCTT
CTATTCCTGCAGGTACACCATATTTGCCTGTGTCACCTTCTATACCTGCCATATCTGTAC
TAATTCACGGTACAGATTATTATTTGCATCCTGCATACTATTTAAGAAAACGCCGAAAGC
GCATATTAGCACATCAGTATGTGGCAACTTAATGAAAATCAAGTATATTTACCACCGCCA
ACGCCTGTTGCTACCATTGTTAGCACAGATGAGTATGTGCAACGCACCAGTTTATATTAT
CATGCTGGTAGTGCCAGGTTACTGACTATAGGACATCCATACTTTGAGTTAAAAAAACCC
AATGGTGACATTTCAGTGCCTAAGGTTTCTGGACATCAATACAGGGTATTTAGGGTACGT
TTGCCTGACCCGAATAAGTTTGGTTTATCTGACACCTCCTTGTTTAATTCTGAAACGCAG
CGCCTTGTGTGGGCATGTGTGGGTGTGGAGGTCGGCCGTGGCCAGCCCCTAGGGGTTGGT
GTTAGTGGCCATCCATACTTTAATAAGGATGAGGATGTGGAAAACTCATCTGCCTATGGC
ACAGGTCCGGGGCAGGATAGTAGGGAAAATGTAGCTATGGATTATAAACAGACACAGTTA
TGTATGTTGGGCTGCACACCCCCAATTGGGGAATATTGGGGTAAGGGCACTCCGTGCAAT
GCTTCTCGGGTAACCCTTGGGGACTGTCCTGTATTAGAATTAAAAACTGAGGTTATTCAG
GATGGCGACATGGTGGATACTGGCTTTGGTGCTATGGATTTTGCTTCCTTGCAGGCCAAT
AAAAGTGATGTGCCATTGGATTTATGCACATCTATTAGTAAATATCCAGATTATTTGGGA
ATGGCTGCAGAACCGTATGGAAATAGTTTATTTTTTTTTCTACGCAGGGAACAAATGTTT
GTTAGGCACTTTTTTAATAGGGCAGGTACTACTGGTGATAGTGTCCCAACTGACTTATAT
ATAACAGGTACATCTGGTCGGACTCCTATTGCAGGCAGTATTTATTACTCCACACCAAGT
GGATCCTTGGTTACCTCTGATTCTCAGATATTTAACAAGCCATTGTGGATACAAAAGGCC
CAGGGCCATAACAATGGCATATGTTTTGGCAATCAGTTATTTGTTACAGTTGTAGACACC
ACTCGTAGCACTAATTTAACCTTATGTGCTGCCACACAGTCCCCCACACCAACCCCATAT
AATAACAGTAATTTCAAGGAATATTTGCGTCATGGGGAGGAGTTTGATTTGCAGTTTATT
TTTCAGTTATGTGTAATTACCTTAAATGCAGAGGTTATGACATATATTCATGCAATGGAT
CCTACGTTGTTGGAGGATTGGAACTTTAAAATTGCTCCTCCAGCCTCTGCATCCTTAGAG
GATACATATAGGTTCCTTACCAACAAGGCTATTGCCTGTCAGCGCGATGCGCCCCCCAAG
GTACGGGAGGATCCATATAAAAAATATAAATTTTGGGATGTCAATTTAACAGAAAGATTT
TCTTCCCAATTAGATCAATTTCCATTAGGACGTAAGTTCCTTATGCAGGCTGGTGTACGT
GCAGGGCCTAGGTTTAAATCCAGGAAGCGCCCTGCCCCTTCCTCGTCTTCCTCTTCTAAG
CCAGTCACCCCCAAACGTAAAAAAACAAAGCGATGAGTATGGCTGCATATGTGTATTCTG
GGGAATACACCCTTGCACTCCCGTATGTGTTGGTACTTTGGAATGTATGTATGTATTGTT
TGTGTATGTTTTGTGTTTGTTGTGTATGTGTCCGTATGGTACTGTGTATGTATTGTTTGT
ATGCTGTATACCTACGTTGTGGTTGTGTGTTTAATAAAGCTATATGTGTGGTGTGGTGTG
GTATGGCAGTGTCATGGCGTCTTGTTCAGTGTTGACCATGTACTTTTTGTGTCTAAATCC
TCCATTTTGTACTGCGCGACCGCTTTCGGTCGCGGTTGGCACACACATACATTTGTCAGC
AAATTTTTATTGCATGTTACACATTGTTAGGTCAACATGCCCTGCCAAAAATGTAGTATT
AGGGTGAGGTTTGGCACACCTTTGATTGCACTTTTCCTGTACTGTTACTCATCTTATTGT
ATGCACTTCCAAACATGCTTTTAGGCACATAGTTTTGCCTAATAAAAAGTTAGCTAATAG
CAGTTTTGGCAAAACATAACACTTGTGGTTAACATAGTACATATTACTCATGGTGCACAC
CTGCGGACCGCTTTCGGTTGCTACATGTTTTATTACTTTCTACTTATTA
>gi|397005|emb|X74462.1|_Human_papillomavirus_type_3_genomic_DNA
TCTAACTATAATTATAAATAACAATGCACATAATAAAAAGTAGGGAGTAACCGAAAACGG

TACGACCGAATGGGGTACATATAAAAGGAGGCACATAATGCATGGCAGTAGCCATGTCTA
TGGATGCAAACTGCCCAAAAAACATATTTCTACTGTGCAGAAACACCGGAATAGGATTTG
ACGACCTTCGCCTGCACTGCATATTCTGTACGAAACAGCTGACTACAACTGAACTACAAG
CATTTGCATTACGGGAACTGAATGTGGTGTGGAGAAGGGGAGCGCCCTACGGTGCTTGTG
CACGGTGTTTACTTGTAGAGGGCATTGCACGACGCCTAAAATATTGGGAATATTCATATT
ATGTATCTGGCGTGGAAGAAGAGACAAAACAATCAATAGATACACAGCAAATTAGATGCT
ACATGTGTCACAAACCACTGGTAAAGGAAGAGAAGGACAGACACCGCAACGAAAAGCGAA
GACTGCACAAAATATCTGGTCATTGGAGGGGGAGCTGTCAGTACTGCTGGTCACGATGCA
CGGTCCGCATCCCACGATAAAAGATATAGAATTGAGTCTTGCACCAGAGGACGTCCCTGC
ACTATGCAATGTGCAATTAGATGAAGATGAGTATATAAATGCTGTGGAACCAGCGCAACA
AGCGTATTGTGTAGTCACAGTGTGTCCGAAGTGTAGTTCACAACTTCGACTGGTGGTAGA
GTGCAGCCACGCAGATATAAGGGCCTTCGAGCAGCTTCTGCTGGGCACACTGACGGTTGT
GTGTCCCCGCTGCGTGTAACAGGACATGGATGATACTTCAGGTACAGAGGGGGAATGTTC
CGAGTTGGAACGGGCTGGAGGATGGTTTATGGTAGAGGCAATAGTAGACAGGCGGACGGG
CGATACAGTGTCAAGCGATGAGGATGAGGAGGAGGACGGAGGGGAAGATTTAGTGGATTT
CATAGATGATAGGCCTGTAGGGGACGGACAGGAAGTGGCACAGGAACTGTTGCTGCAGCA
AGCAGCTGCGGATGACGATGTAGAAGTGCAGACAGTAAAACGAAAGTTTGCTCCCAGTCC
GTATTTTAGCCCTGTGTGTGTACATCCCAGCATAGAAAATGAGCTAAGTCCGAGGCTAGA
TGCAATAAAGCTGGGGAGACAAACATCAAAAGCCAAACGCCGGCTATTTGAGCTACCGGA
CAGTGGGTATGGCCAAACACAGGTGGATACGGAATCGGGACCAAAACAGGTACAGGACAT
TTGTAAGACAAGCCAACAAGATGGCTGCCAGGGTGCGGATGAGGGGAGAGGTAGGAATGT
GGGGGGAAATGGCAGCCAGGAGGAGGAGCGTGCAGGAGGGGATGGGGAGGAATCGCAGAC
TGAGAGTGTACAGACAGATACGACAGCCTGTGGAGTGTTGGCAATATTAAAAGCTAGCAA
TCACAAAGCAACGCTACTGGGTAAGTTTAAAGAACAATTTGGGTTAGGATTTAATGAACT
GATTAGACACTTTAAAAGTAACAAAACAGTATGTAGCGATTGGGTGGTATGTGTGTTTGG
TGTATACTGTACATTGGCAGAAAGCTTTAAGACGCTAATACAACCACAGTGCGAATATGC
ACATATACAGGTACTATCCTGTCAATGGGGCATGACAGTGTTAACGTTGGTACGGTTCAA
ACGGGCCAAAAACAGAGAGACGGTGGCTAAAGGTTTCAGCACTTTGCTAAATGTGCCAGA
AAACCACATGTTAATAGAGCCACCAAAATTAAGAAGCGCTCCAGCAGCGCTGTACTGGTT
CAAAACAAGCCTATCAAATTGTAGCGAGGTGTTTGGGGAAACACCAGAGTGGATAGTTAG
GCAGACAGTGGTGGGACATGCATTAGAGGAAGCGCAGTTCAGTCTGTCAGAAATGGTGCA
GTACGCATATGACCACGACATAACAGATGAAAGCACGTTGGCATATGAATATGCACTACA
AGCAGATACAGATGCAAATGCAGCAGCGTTCCTAGCTAGCAATTGTCAGGCAAAATATGT
AAAGGACGCATGCACAATGTGCAGACATTACAAAAGAGGTGAACAGGCCCGAATGAACAT
GTCAGAATGGATAAAGTTTAGAGGAGATAAAATACAGGGGGATGGCGATTGGAAACCAAT
AGTACAGTATTTAAGGTACCAGGACGTAGAATTTATACCATTTCTATGCGCTCTGAAATC
ATTCCTACAAGGAATACCAAAAAAAAGTTGTATAGTGTTTTATGGACCAGCAGATACTGG
GAAGTCATACTTTTGCATGAGCCTGTTGAAATTTCTGGGCGGGGTAGTTATATCTTATGC
CAATTCCAGCAGCCATTTTTGGTTGCAACCATTAGCAGAAGCCAAGATAGGTTTGCTGGA
CGATGCAACTAGTCAGTGTTGGTGTTATATAGACACGTATTTAAGAAATGCTTTAGATGG
AAACCAGGTGTGCATAGATAGAAAGCATAGGGCCTTGCTACAACTGAAATGTCCTCCGTT
ATTGATAACAACTAATATAAATCCTTTGGGGGATGAAAGATGGAAGTATCTGCGCAGCAG
ACTGCAGGTGTTTACATTTAACAACAAATTTCCATTAACTACACAAGGAGAGCCACTGTA
TACATTAAATGATCAAAACTGGAAATCCTTTTTTCAAAGGTTATGGGCACGTTTAAACCT
TACCGATCCTGAAGACGAGGAGGACAATGGAAACACTAGCGAACCGTTTAGATGTGTGCC
AGGACAAAATACTAGAACTGTATGAAAAGGATAGCGACAAACTTGAGGACCAAATAATGC
ATTGGCAATTGATGCGGTTAGAGCAAGCTTTGTTGTACAAAGCAAGGGAATGTGGATTAA
CACACATTGGCCACCAGGTGGTGCCACCTCTTAGTGTAACCAAAGCAAAGGCACGCAGTG
CCATTGAAGTGCATGTATCTTTGCAACAATTACAGCACAGTGCACATGCACAAGACCCCT
GGACACTGCGAGACACGTCACGGGAAATGTGGGACACAGTTCCCAAGAAGTGCTGGAAAA
AAAGAGGTTTAACTGTGGAAGTCAGATATGATGGAGACGAAAACAAAGCAATGTGTTATG
TACAATGGAGGGAAATAATTGTGCAGAACTATACAGATGATAACTGGGTGAAGGTGGCAG
GACTGGTGTCTCATGAGGGTCTATATTACATGCACGAAGGACAGAAAACTTTTTATGTAA
AATTTAAAGATGATGCGCGCGTGTATGGGGACACAGGAACATGGGACGTACATGTGGGAG
GCAAAGTAATTCACCACGATTCATTTGACCCTGTATCTAGCACACGAGAGATACCCGCTC

CTGGACCTCTGTACGCCTGTACCACCCAAGCGCCCACCCAAGCCCAGGTGGGCGCGTCCG
AAGGACCGGAGCAAAAGCGACAGCGACTCGAGACGGTCTACGGGGAGCAGCAGCAGCAAC
AGCAGCAGCAACAGCAACAGCAACAACATACCCAAACCCCCGCCCCGCAAACCACTGAAC
GAGCACGTCAACCATTGGACACTGACAGGACCCGGGACCGTGACACTACGTGTCCACACC
CCATCGGGCATCGAAGTGACCCTGACTGTGTGCCTGTAATACACCTAAGAGGTGATCCTA
ACTGTTTAAAATGTTTTAGATATAGGTTAAACAAAGGTAAAAATAAGTTATATTCAAGGA
CCTCTTCCACATGGAGGTGGTCCTGTGAATCAGAAAATCAGTGTGCGTACGTAACCATTT
GGTATACAAGTTATGGTCAGCGGGAAGCATTTTTGTCCACCGTAAAAGTGCCACCAGGTA
TTCAAGTGATACTGGGACACATGTCAATGTTCACATAATTGTGTCCCCGCATTGTACAGT
CTGGATTACTATTTGTGCAGGCTTTCTCTGTGGGTGTATTTTGTGCTGCTGCTGTGTTTG
TTTTGGCTGTGTGTGCTTCCTGCGCTAACGTGCTATCTGGCAATTGTGCTTTGTGTGTAC
TTGGTCCTGATAGCATTGTATTTACAAATTGTATCACGCATTGTACAGAATAACACATAG
GTTTTACTATGTATCCTCTGGTACTCACAGACAACAATGGCGACCATCTTGTCTTGTTTG
TTGAGCCTGGAGACGTGTACATATTATTGCTGTTTATGTTAGCTGTCATACTTACATTGT
TTATTATGTATAGACATCTGGGACTCCTGTAAGGTTGTAGTTGCAGGTCACCTGTATGTA
TTCTTCCTTGATGTATATGCCCTAGTGTGGTATTGTACCACCGTCTTTTATACTGCTATG
TTTTTTTTTACAGTTCAATAAAGCAACCATGGTGGCACATCGTGCAAGGCGTCGCAAGCG
TGCATCTGCCACACAGCTTTATAGAACCTGCAAGGCCGCAGGCACATGTCCCCCTGATGT
TATTCCCAAAGTTGAGGGCACCACTTTGGCCGATCGTATTTTGCAATGGGGTAGCTTGGG
TGTTTATTTGGGGGGTCTGGGCATTGGTACTGGATCCGGAACTGGGGGGCGCACAGGGTA
TGCGCCAATTAGTACACGGCCTGGTACTGTTGTTGATGTTAGTGTTCCTGCAAAACCTCC
TGTGGTAATTGAGCCTGTGGGGCCATCGGACCCCTCCATTGTTAACCTATTGGAAGACTC
CAGTATTATTAATTCCGGGTCCACCATACCGACCTTTACTGGTACTGATGGATTCGAAGT
TATTTCTTCAGCCACAACTACCCCTGCTGTATTAGATATTACACCTGCCAGTGACAATGT
GGTGGTTAGTAGTACCAATTTTAGCAATCCAGCTTTTACAGAACCTTCCCTGTTGGAGGT
TCCTCAGAATGGTGAGGTTTCAGGGCACATACTTATTAGCACCCCCACATCTGGTACACA
TGGTTATGAAGAAATTCCTATGGAAACCTTTGCTTCGCCAGGTACGGGAACTGAACCTAT
TAGTAGCACCCCTGTACCTGGTGTAAGTAGAATTGCAGGTCCCCGCCTATATAGCAAAGC
TGTCACACAGGTTAAGGTAACAGATCCTGCTTTCTTGACCCGTCCTCGCTCGTTAATGAC
ATTTGACAATCCTGTGTTTGAGCCAGAAGATGAGACTATAATATTTGAACGTCCGTACTC
TCCCTCACAGGTGCCTGACTCTGACTTCCTTGACATTTTACGTTTGCACAGGCCTGCTTT
AACTTCTCGTAGGGGTACTGTGCGTTACAGTAGGGTAGGCCAAAAATTAAGCATGCGCAC
TCGCAGTGGCAAGGGTCTTGGTGCTCGAGTGCATTATTATCAAGATTTAAGCCCCATAGG
TCCTACGGAGGACATTGAAATGGAACCCTTGATTGCTCCTGCATCTGCCTCAGCCTATGA
CTCTCTGTATGATGTGTATGCAGATGTGGACGATGCTGACATAGGTTTTACATCTGGAGG
TCGTAGTGACACTCTGTCTAGAGGCCGTGCTACAGTGTCCCCCCTGTCCTCCACTCTGTC
CACAAAGTATGGCAATGTCACCATTCCCTTTGTGTCTCCTGTGGATGTGCCTTTACAACC
TGGGCCTGATATTTTACTGCCTGCATCAGCTCAGTGGCCGTTTGTTCCCTTGTCTCCTGT
TGACACAACTCATTATGTCTACATAGATGGCGGGGATTTTTATCTATGGCCTGTCACCTT
CTTTTTGCCCCGACGTCGTCGCCGTAAACGTGTCTCATATTTTCTTGCAGATGGCACTGT
GGCGCTCTAGTGACAACCTGGTGTACCTGCCTCCTACCCCTGTTTCCAAGGTTCTCAGCA
CGGACGACTATGTGACACGCACCAACATTTATTATTATGCAGGCAGTTCTCGCTTGCTGA
CCGTGGGTCATCCTTATTTTGCTATCCCCAAATCTTCTAATTCCAAGATGGATATTCCTA
AGGTGTCCGCCTTTCAATATAGAGTGTTTAGGGTGCGGTTGCCCGACCCAAATAAGTTTG
GCCTACCAGATGCACGCATATATAACCCAGACGCCGAAAGGCTGGTCTGGGCTTGCACTG
GGGTTGAGGTAGGCCGCGGGCTGCCTTTGGGTGTAGGCCTCAGTGGACATCCTCTTTATA
ACAAGCTAGATGACACTGAAAACTCTAACATAGCACATGGGGACATAGGTAAAGATTCCC
GGGACAACATATCTGTTGACAATAAGCAAACGCAGCTATGTATTGTGGGTTGTACCCCAC
CTATGGGGGAGCATTGGGGCAAAGGAACACCATGTAAGCAGAATGCGTCACCGGGTGATT
GTCCTCCTCTAGAGCTTATTACTGCACCTATACAAGATGGCGATATGGTGGACACAGGTT
ATGGTGCCATGGACTTTGGTAACTTGCAGTCCAATAAGTCAGACGTGCCATTAGATATTT
GCCAGACCACCTGCAAATATCCTGATTATTTGGGTATGGCCGCTGAGCCCTATGGCGACA
GCATGTTTTTTTATTTGCGAAAGGAGCAGTTGTTTGCAAGACATTTTCTTAACAGAGCTG
GTATGGCTGGAGACACCGTGCCTGACGCGTTGTACATTAAAGGTGACAGTCAGAGCGGCG
GTCGGGATAAAATTGGTAGTGCTGTGTACTGTCCTACCCCTAGTGGGTCCATGGTAACAT

CTGAAACGCAGCTATTCAATAAGCCATATTGGCTGCGGCGTGCTCAGGGACACAATAATG
GTATATGTTGGGCCAACCAATTGTTTGTGACTGTGGTGGATACCACACGTAGTACTAATA
TGACATTGTGTGTTTCTACTGAAACCTCGGCTACATATGATGCTACTAAATTTAAAGAGT
ATTTAAGACACGGGGAGGAATATGATTTACAGTTTATATTCCAGTTGTGCAAAGTTACAT
TAACTCCTGAAATTATGGCCTATTTACACACAATGAACAGTACTTTGTTGGAGGATTGGA
ACTTTGGGTTAACCTTGCCACCGTCCACTAGCTTGGAGGACACCTATAGATTTTTAACTT
CCTCTGCCATTACCTGCCAGAAAGATGCACCTCCCACTGAGAAGCAAGACCCCTACGCCA
AACTAAACTTTTGGGATGTAGATCTTAAGGATCGTTTTTCCCTGGATCTTTCGCAGTTCC
CCCTTGGCAGGAAATTTCTCATGCAGCTCGGTGTAGGTACCCGCTCTAGTATATCTGTTC
GTAAACGCTCGGCGACAACCACATCTAGAACAGCTGCTGCAAAAAGGAAGCGCACCAAAA
AATAGCCACATTTGTGTTTTGTATGTGTAACCTGTGTGTATGTTTTTTATGTATGTACTG
TGTGTGTAATGTGTACTGTCTGTGCTATGTGTTTGTACGTTATTATGTTGTGTGTATGTG
TCAATAAACTGTGTCACATAGTTTTATATTTTTTAATTTTTGTAATTACTGTTCCTGTGA
GTAAGAAAGGTAATTCTGGGTCATGCGACCGATTTCGGTTCTCAAAATGGCCGCCTTTGC
AGGTGTGCACACAAACAATTAGTCATACTGATCTATATCCTGCGACCTGCCTTGTCACGC
ATAGTTTTGGCTGTGATATTATCTTTTCTATAGTTTATTTTATTGCTGCATCATTCTCCC
TGGCACGTCTATCTGTCTCCATTGCAAATTAACAGCTTCTGGGCACTAACTTATTATGAC
TACTTTCACATAATTACTGTCTTGGCTGCGTTTTCTAGTCTGCCTTGCCAATATGTGCTT
CCAAATCTCCACCAAGACACACCTAATCCGGTCGCTGCTTGCTTTCTAGCCATAATTTAT
GCAGTTGCTACACGTTCCTT
>gi|396973|emb|X74474.1|_Human_papillomavirus_type_30_genomic_DNA
TGAAAGTTACAAGCATAGTTTATAGAAAGGGAGTGACCGAAATAGGTTTTCAACCGAAAA
CGGTACATATAAAAGCACTGTACCAAACGGACAGTGTACCCATGGCTTTCAAATTTGAAA
ATACAGGCGAGCGCCCACGTACTGTGCACCATCTTTGTGAGGTACAAGAAACATCGTTGC
TGGAGCTACAGCTACAGTGTGTATATTGCAAGAAGGAATTATCCAGCTCAGAGGTATATA
ATTTTGCATGTAAAGATTTAAGACTGGTATATAGGGAGGACAGCCCATATGCAGTGTGCA
ATTTCTGTTTATTATTTTATAGTAAAGTAAGAAAGATTAGACATTACAACTATTCATTGT
ATGGGGCAAGCCTAGTGGCATTAACTAAAAAAGAGTTATTTGATTTATTAATAAGGTGCT
ACAGATGTCAACAGCCGTTGACACCAGAGGAAAAACAGTTACACTGTGAATATAAGAAAC
GGTTTCACAGAATATCACGTACGTGGACCGGGTTATGTCTGCAATGCTGGAGACACACAA
CGTCCACTGAGACAGCAGTATAATCATGCACGGTAAAGTAACAACTATTCCAGAATATAT
TTTGGACCTTGTACCGCAAACTGAAATTGACCTGCATTGCTATGAGCAATTGAACAGCTC
AGAGGAAGAGGATGAGGATGAAGTAGACAATTTACAGAAGCAGCCACAGCAAGCTAGACA
AGAAGAACAACATCCTTGTTACCTAATTAACACACAGTGTTGTAGGTGTGCGTCTGCGGT
GCAGTTGGCTGTTCAGAGTCCCACAAAGGAGCTGCGTGCCCTACAACAGATGCTTATGGG
CGCACTGGAGCTAGTGTGTCCCCTGTGTGCAACAAGGCGGTAAACGGCAATGGCGTCACC
TGAAGGTACAGATGATGAGGGAGGGGGGTGCACGGGATGGTTTCATGTTGAGGCTGTAGT
CAAGAAACGTACGGGAGATATAATATCAGAGGACGAAACAGAAGAAGATGAGGGCACAGC
ATCAGATTTAGATGGGTTTCTAGACAATAGTAATGTAATAACAACACAGGCAGACAGGGA
GACAGCTCAGCAGTTATTGCATGCCCAAAACACATATGCAGATACACAGACGCTGCACAA
TTTAAAACGAAAGTATTTAGGCAGTCCGTTGGGAGACATTAGTAATCAGCAATTTGTGTG
CCGGGAAGGAGTAAAACGAAGGATAATTGATACAGACGTGGCCGACAGCGGGTATGGCAA
TACTTTGGAAGTGGAAGCAACGCAACAGGTACAGGATAATACGTATGGTAGTGGTAAACA
GCAAGATGGAGGCTCACAGACTAGTGTGTGTAGCAGGGAAAACAGCATAGAAGCAGACAG
TGATATGGATATAGGCGCCACGCCACCACAGCAAATACAAGAATTATTAAAATCTAGTAA
TGTACAGGCAAAGCTGTGTTATAAATTTAAAGAGTTATTTGGAATTCCATTTTCAGAATT
GGTACGTACATTTAAAAGTGACAGCACATGTTGTCATGACTGGATATGTGCCATGTTTGG
TGTTAATGAAACCTTAGCAGAGGCATTAAAAACAATTATTAAATCACAGTGCATGTATTA
CCATATACAGTGTTTAACGTGTACATGGGGCGTTGTAATATTAATGTTAATTAGATATAC
ATGTGGAAAAAATAGAAAAACAATTATAAAATCACTAAGTTCAATTGTAAATGTGCCCAG
CGAACAAATGCTTGTGCAGCCACCTAAAATACGTAGCCCTGCCGTTGCATTATACTTTTA
CAAAACTGCAATGTCTAATATTAGTGACATATATGGTGAGACACCAGAATGGATACAGCG
ACAAACACAAATACAGCACAGTTTTCAGGACTGCCAATTTGAACTGTCGAAAATGGTGCA
ATGGGCATTTGATAATGACGTAACAGATGACAGTGACATTGCATTTTACTATGCACAGTT
AGCAGATGTAGATAGTAATGCACAGGCTTTTTTAAAAAGCAATATGCAGGCCAAATATGT

AAAGGATTGTGGAATAATGTGCAGACACTATAAAAGAGCACAACAACAACAAATGAACAT
GAAACAATGGATTACACACATATGTAGTAAAGTAGATGAGGGCGGGGACTGGAGGCCAAT
TGTACAGTTTTTAAGATATCAAGGGGTCGACTTCATTTCATTTTTAAGTTATTTTAAATT
ATTTTTACGAGGCACACCAAAACACAACTGCCTAGTATTATATGGACCACCAAACACAGG
AAAGTCATGTTTTGCTATGAGCCTTATACAATTTTTTCAGGGGTCTGTTATTTCATATGT
AAATTCACACAGTCACTTTTGGTTACAACCGTTAGACAATGCTAAATTGGGAATGCTAGA
TGATGCCACAGATGCATGCTGGAGATATATAGATGAATATATGCGAAATTTATTAGATGG
GAATCCTGTAAGTTTAGATAGAAAACATAAACAACTAGTACAAATAAAATGTCCACCAGT
AATAATAACAACAAATATTAATCCACTGCACGATGCAAAATTGCAGTACTTGCACAGTAG
AATACATGTGGTGCCATTTCTAAATCCATTTCCAATTGATACAAATGGTAATCCTGTATA
TCAATTAAATAATGTAAACTGGAAATGTTTTTTTGAAAGGACATGGTCCAGATTAGATTT
GAACAACGACGAAGACAAGGAAAACCATGGAGACTCTATGCCAACGTTTAGATGCGTGCC
AGGAGAAAATTCTAGACTGTTTTGAAAACGATAGTAAAAAAATTGAAGACCATATAGTGT
ACTGGAAAGCTGTACGACATGAAAATGTTGTATTATATAAAGCAAGACAAAATAATATTA
CTAAACTACGCCACCAGGTGGTGCCATGTTTACAAGTGTGTAAAGCAAAGGCATGTGTTG
CTATAGAAATACAAATGGCATTGGAATCATTATATAAAACAGAGTATAAAGTGGAGGAGT
GGACATTAAAAGATGTATGTGAAAATATGTGGCATACAGCACCAAAACAGTGTTTTAAAA
AAAGTGGAAAACGTATAGAAGTGTGGTTTGATGGGAAAAAAGACAATCGAACTGAATATG
TTGTGTGGCAATGGGTGTATTACTGTGGGGACAATGGGTGGACTAAAGTGCCTTCTGTAG
TAGATTACAAAGGTATATATTATGTACATGACGGTAACAAAGTATATTATACAGACTTTA
ATGACGAGGCAGTAAAGTATGGGTATAAAGGCACATGGGAAGTGCATATGGGAAATGAAA
GTATTTATTGTCCCGACTCTGTGTCTAGTACCCTCAGATCCAACGTATCCCCTGTTGAAA
CTGTTGTCGAATACAACACCTACAACACCTATCAAACCCCCACCACCTCCACGCCCGTGG
GCGCCAACGAAGCCGCGTCCTCCGCACGGCCGGGAAAACGTCCTAGAACCACAGAGCCCG
ACAGTACAGACACCACCAGACAGTCCGCTGCCAGAGAGTCCCACGCAAACCGTGTCAACA
CAAACAACACAAACAACAGACAGTGCCTTGGTGGAGCTACATGTTACAACACAGAAGTCG
ACGGTGGTTATAAAACTACACCTGTAGTGCATTTAAAAGGTGAACCAAACAGATTAAAGT
GTTTAAGATATAGGTGTCAAAAACATAAGCACCTATTTGTAAATATATCGTCTACATATC
ATTGGACCAATACACATACAGAGTACAGCTACATTACTGTTGTATATAAAGATGAGACCC
AGCGTGCCAACTTTTTAAATGTTGTAAAAATACCACCCAGTATAAAAATTGTAATGGGAC
ATATGACAGGTGTTGATATGTAACAACACATATGTTGTAATTGGTACAACAAACATGTAA
TATTGTATTCCACATGTAAATATTATTGTGTACTATAGTTATACAACAACAAACGCCATA
TTTTGCTGCTACGCCTGTTATATATTGCAACCATTGTTTTATTTGTTAGTTTTGTGTTTT
TGCTTTGCTGTTTTGTGTGCCTGTGTGTCTGCCTATATGTGCCGCTTTTGCTGTCTGCCT
CGTTGTTTTCTACCTGTTTATTACTTATAATATTGTTTTGGTTTGTTGTTGCATCGTCCT
ACATTACTGCATTTACTATATTTTTGCTGTTTTTTTATATACCTTTATTACTTGTATATG
CCCATGCTGTGTGGTTAATAAACACACAATAAATTGTTTTGTAACATATTACACTTTTGC
TTTGTGTAGGGCGTGTACTATGGTCGCCCACCGGGCACGCAGACGAAAACGGGCCTCTGC
TACTCAGCTGTATCAAACGTGCAAGCAGGCTGGCACATGTCCATCTGATGTTATTAACAA
AATTGAACACACAACTTTGGCAGATAAGATATTACAATGGGGTAGTTTGTTTACATTTTT
TGGTAATTTAGGTATAGGCACTGGTGCAGGCTCTGGGGGGCGTGCTGGTTATGTTCCGTT
AGGTACACGGCCTACAACAGTTGTGGACGCATCCCCTGCTAGGCCACCTATTGTTGTTGA
ATCTGTGGGTCCTACGGACCCTTCCATTGTTACACTGGTTGAGGAGTCTAGTGTGGTTAA
TGCTGGAGCATCATTCCCTAATTTTACAGGCACTGCAGGCTTTGAGGTTACGTCTTCCTC
TACCACAACACCTGCTGTATTAGACATTACACCTACCACTGGCTCTGTCCATGTTAGTAG
TACCCATTTTACCAATCCCTCCTTTGTTGAACCCCCAGTTATTGAGGTTCCACAAACTGG
GGAGGTATCCGGCCATATATTGGTTAGCACTCCCACATCTGGTGTACATAGTTATGAAGA
AATACCTATGCAAACATTTGCTGTGCATGGTACAGGCACTGAACCTATTAGTAGTACCCC
TATTCCTGGGTTACGACGTATAGCTGCCCCTAGATTGTATCAAAGGGCCTTTCAGCAGGT
AAAGGTCACTGACCCCACATTCCTTACCAAACCTGAAACATTAATTACTGTGGATAATCC
AGTCTTTGAGGATGCTGACACAACTTTAACCTTTTCACCATCGGGTGTGGCACCTGATCC
TGACTTTTTAGATATTGTGGCTTTACACAGACCTGCCTTTACAACACGTAGGGGTGGTGT
GCGTTTTAGTAGGCTTGGTACAAAGGCCACAATGCGCACAAGAAGTGGCAAACAAATAGG
TGCTCGTGTACATTATTATTATGATGTGAGTCCTATTGCACACACTGAGGAAATTGAAAT
GCAGCCATTATTGTCTGCAAATAATTCATTTGATGGCCTATATGATATTTATGCAAACCT

TGATGATGAGGCGCCAGTGTCATCTCACCTATCCATTGCTACACCGTCCCGGTTGCCTAC
CAACACTGTTCCTTTGTCCTTTAGTAGTCAAACTACCAATGTTACTATACCTTTGGGTAA
ATATTGGGATGTTCCTATTTATTCGGGCCCCGATATAGTATTGCCTACTGGTCCTACCAC
ATGGCCCTATGCCCCTCAGGCCCCATTTGACACTACACATGATGTGGTTATACATGGATC
TACATTTGCTTTATGGCCTGTATACTTTTTAAGGCGTAGGCGTCGTAAACATGTTCCCTA
TTTTCTTGCAGATGGCGGTGTGGCGGCCTAGTGAAACCAAGGTTTACCTGCCTCCTACAC
CTGTATCAAAGGTGGTACCAACAGATGCATATGTAAAGCGCACCAATATATTTTATCATG
CAGGCAGCTCACGTTTGCTTGCTGTTGGACATCCATATTATTCTATTTCTAAGGCTGGTA
ATTCCAAAACAGATGTTCCCAAGGTGTCTGCATTTCAGTATAGGGTCTTTAGGGTCCGCT
TGCCCGATCCCAATAAGTTTGGGTTACCTGATACCAATGTATTTAATCCTGAGCAGGAGC
GGCTGGTATGGGCCTGTGTGGGCCTGGAAATAGGCCGTGGCCAACCTTTAGGTGTTGGTG
TTAGTGGCAATCCTTTATTTAATAAATTGGATGACACTGAAAGTTCCACTATAGCTAATC
AGGATACAGCAGAGGATAGTAGGGACAACATTTCTGTTGATCCAAAGCAAACCCAATTGT
GTATTATTGGGTGCACTCCTGCTATAGGAGAACATTGGGCTAAAGGCACTGCCTGTCGTT
CTGCGCCCCCTGCACAAGGGGATTGTCCTCCCTTGGAACTTGTTAATTCTCCTATACAGG
ATGGCGACATGGTTGATATTGGGTTTGGTGCCATGGATTTTAAAACATTACAGGAATCTA
AATCAGATGTGCCTTTAGATATTTCACAATCCACCTGTAAATATCCTGATTATTTAAAAA
TGAGTGCAGACGCCTATGGCGATTCTATGTGGTTCTATTTGCGTAGGGAACAGTTATTTG
CTAGGCACTACTTTAATAGGGCAGGTGCTATTGGTGAACAATTACCTAGCACATTATATA
TAAAAGGTACAAATAACAGGGATCCCCCGCCAAGCTCAGTATATGTTGCTACTCCTAGTG
GGTCTATGGTAACCTCTGAGGCTCAGTTGTTTAATAAACCTTACTGGTTGCAACGCGCAC
AGGGACACAATAATGGCATTTGTTGGGGCAACCAGGTATTTGTTACTGTTGTGGACACCA
CTAGGAACACAAACATGACTATATCTGCAACCACACAAACGTTATCCACATATAATTCAA
GCCAAATTAAACAGTATGTAAGACATGTAGAGGAATATGAATTACAGTTTGTGTTTCAAC
TGTGTAAAATTTCACTGTCTGCAGAAACTATGGCCTATTTACATACTATGAACTCCACTT
TACTTGAGGGCTGGAATATTGGATTGTCACCCCCAGCTGCCACAAGCTTAGAGGACAAAT
ACAGATATGTTAAAAGCCTTGCTATAACCTGTCAAAAGGATCAGCCTCCTGCTGAAAAAG
AGGACCCACTAGCTAAATATAAGTTTTGGGATGTTAACTTACAGGACAGTTTTTCTGCTG
ACCTTGACCAATTCCCACTGGGCAGAAAGTTTTTAATGCAACTTGGGGTTCGTACTAAAC
CTTCTACTACTACTAAAAAACGCTCGGCCCCCAGTTCCTCTACCTCTACACCATCAGCCA
AACGCAAGCGGCGGTGATTGTTTGTGTCTGTGTATGTTTCCTTACTATTTATTGTGCATG
AATGTATGTTTTTGTATGGATGTATGTTTTGTTTATATGTTTCTGTATGACTGTATGTAT
GTGTAATGACTGTATGTATGTAATAAATATGAATGAGTCTTACTTTTACGTGTGGTTACA
TAAACTAAGGTGCGGTTGTGTCCCTAGGCATTTAGGTAGCAATTTAGGTGGCGTCCCTAT
GTCCTCCACCCTTTTTGGTTGTTGCACACCACTGTGCAGTTACTTTTTATATTTATATTA
TACCACCACAGTAGTGTCCATTTTATGCATTTGTGCCCTCCATTTTACCTTTCAACCGAT
TTCGGTTTCCTGGCATGTATGAGTGTTTTTTATATGTACATGCCAAAAGTACATTCAGCA
AAACACTTAATCCACTGGCATGGTGCCGTTTCCTGCACTTTATGTTTTTTTTTGCAACGT
AAGACGCCTCGCCTTATTAGTCATATATGTATGCTGCCAACTATGCTTTTATCAGCATAC
TTTACTGCTGTTGGGCATAAGTTTTTATTGCAAACATCTGCAACACAATGATTTGGCTTG
CAGCATATATTTTGGTAGCCAACTATGTGTCTTGTAAAGCAGGTGTGTAAAACCTTACTC
ATATCCCACAACCGGTTACGGTTTTGCAGCAACAAGTTTATTTTATAATTAT
>gi|396964|emb|X74473.1|_Human_papillomavirus_type_27_genomic_DNA
TATGTGGTTTATAATATATAACTATAATCCTTTATTTAAAAATAGGGTGTAACCGAAAAC
GGTCCGACCGAAATCGGTCGTATAAAAACAGGAGCAGGATGCGCACAAGGGCAGGGATGT
CAGAAGAGAATCCATGCCCTAGGAACATCTTTTTGCTTTGCAAACAGTATGGTCTGGAGC
TAGAGGATTTGAGATTGCTCTGCGTGTATTGCAGACGAGCGCTTTCAGACGCTGATGTAT
TGGCATTTGCAATAAAAGAACTGTCTGTAGTGTGGAGAAAGGGCTTCCCTTTTGGAGCAT
GTGGAAAATGCCTGATTGCAGCAGGAAAACTTAGACAATACAGACATTGGCATTACTCAT
GCTACGGAGACACAGTGGAGACCGAGACAGGAATACCCATACCTCAGCTGTTTATGAGAT
GCTATATCTGCCATAAGCCCCTGAGCTGGGAGGAGAAGGAGGCATTACTGGTTGGAAACA
AGCGATTCCACAACATATCCGGCCGGTGGACGGGACACTGCATGCAGTGCGGGTCAACAT
GCACGGCACCCGACCCAGCCTCGCGGACATTACATTAATATTGGAAGAAATACCCGAAAT
TATTGACCTACATTGCGACGAGCAATTTGACAGCTCAGAAGAAGAGAATAACCATCAACT
GACAGAACCAGCTGTGCAGGCCTACGGGGTGGTAACAACCTGCTGCAAGTGCGGCAGAGC

CGTCCGGCTGGTGGTTGAGTGCGGACCAGAAGACATAAGAGATCTGGAACAGCTGTTTCT
GAAGACGCTGAATCTAGTGTGCCCCCACTGCGCGTAGCGTTATGGAGGATTCCGAAGGTA
CCGACGGGACAGAGGAGGACGGGTGCCGGGCAGGAGGGTGGTTCCATGTGGAGGCCATTA
TAACACACGGCCAGAGGCAGGTATCCAGTGACGAGGATGAGGACTGCACAGAAACAGGGG
AGGATGTAGACTTCATAGACAATAGGGTTCCCGGAGATGGGCAGGAAATTCCCTTGCAGC
TATATACACAGCAAATCGCACAGGATGACGAAGCAACAGTGCAGGCCCTAAAACGAAAGT
TCGTGGCTAGTCCTTTGTCTGCATGCTCATGCATAGAGAATGATTTAAGTCCCAGATTAG
ATGCAATCTCCCTAAACAGAAAGTCAGAAAAGGCAAAGCGGCGCCTATTCGAGACAGAAC
CACCAGACAGTGGGTATGGCAATACGCAGATGGTTGTTGGAACACCAGAGGAGGTAACGG
GGGATGAGAACAGCGAAGGGGGGCGGCCGGTGGAGGATAAGGAGGAGGAGCGTCAAGGGG
GAGACGGAGAGGCAGACCTAACTGTACAAACTCCACAGTCAGGAACAGATGCGGCGGGTA
GCGTGCTGACCTTACTGAGAAGTAGCAATCTGAAGGCGACGTTACTGAGTAAGTTTAAGG
AACTGTTCGGGGTGGGATATTATGAACTGGTAAGACAGTTCAAGAGCAGCAAGACAGCAT
GTGCAGACTGGGTTGTCTGTGCCTTTGGCGTGTACTATGCTGTAGCAGAGGGTATCAAAC
AATTAATACAGCCACATACGCAATATGCACACATACAGGTACTGACCTGCTCGTGGGGCA
TGGTGGTCTTTATGCTGCTACGATACAACTGTGCAAAGAACAGGGACACGGTGTCCAAGA
ACATGAGCATGCTGTTAAACATCCCTGAAAAGCATATGCTCATAGAACCACCAAAACTAA
GAAGTACCCCTGCTGCCTTGTATTGGTACAAGACGGCTATGGGTAACGGAAGTGAGGTAT
ATGGGGAAACACCAGAATGGATTGTAAGACAGACGTTGGTAGGACATAGTATGGAAGATG
AGCAGTTTAGACTATCTGTCATGGTACAATTTGCATACGACCATGACATTGTAGAGGAAA
GTGTGCTGGCATTCGAGTATGCACAGCTCGCAGATGTAGATGCCAATGCAGCAGCATTTC
TAAACAGTAACTGCCAGGCCAAGTACGTGAAGGACGCAGTGACAATGTGCAGACACTACA
AACGTGCAGAGAGGGCACAGATGAGTATGTCCCAGTGGATAACATTCAGGGGAAATAAAG
TATTAGAGGAAGGCGACTGGAAGCCCATAGTAAAGTTTTAAGGCACCAAGGGGTAGAGT
TTGTGTCGTTCCTTGCCGCCTTTAAATTATTTTTAAAAGGCGTGCCAAAGAAAAATTGTA
TAGTGTTTTATGGACCTGCAGACACAGGCAAGTCATATTTCTGCATGAGCTTGTTGCAGT
TCCTAGGTGGCGCTGTTATCTCATATGCTAATTCCAGCAGCCATTTTTGGCTTCAGCCTT
TATCTGATAGTAAGATAGGGTTACTGGACGATGCAACACCCCAGTGTTGGAGCTATATAG
ACACATATTTAAGGAATTTGCTGGATGGAAACCCAGTAAGCATAGACAGAAAACATAAAA
CCCTGCTGCAGCTTAAATGTCCACCCTTGATGATTACAACCAACATTAATCCCCTTGAGG
AGGACAGATGGAAATATTTGCGCAGCAGGCTAACACTGTTTACATTTAACAATCCATTTC
CTTTTGCAAGCCCGGGGGAACCCCTGTATCCAATAAATAATGCAAACTGGAAATGCTTTT
TCCAAAGGTCGTGGTCCCGCTTAGACCTAAACAGTCCAGAGGAGCAGGACGACAATGGAA
ACACTAGCGAACCGTTTAGATGCGTGCCAGGAGACGTTGCTAGAACTTTATGAAAAAGAT
AGCAACAAACTTGAGGATCAGATTAAGCATTGGGCGCAGGTTCGGCTAGAAAATGTCATG
CTGTTTAAGGCCCGGGAATGTGGAATGACACGAGTCGGCTGTACAACAGTGCCCGCCCTC
ACCGTGTCAAAGGCTAAGGCATGTCAGGCCATAGAGGTACAGCTGGCATTACAGACATTG
ATGCAGAGTGCCTATAGCACGGAGGCATGGACCTTACGAGACACGTGTCTGGAGATGTGG
GATGCACCTCCAAAGAAATGCTGGAAAAAGAAAGGACTGTCAGTATTAGTGAAATTTGAT
GGCAGCAGTGACAGAGACATGATTTATACAGGCTGGCACCACATATATGTGCAGGATGCT
AACGATGACACCTGGCACAAGGTTCCAGGGAAGGTGGACGAACTGGGATTATATTATGAG
CACGATGGTGTGCGTGTTAATTATGTGGACTTTGGAACTGAGTCCTTGACCTATGGGGTC
ACTGGGACGTGGGAGGTGCACGTGGCTGGACGTGTTATTCACCATACATCTGCATCTGTG
TCTAGCACCCAGGCCACCGCCTCGGACGACGAACCACTATCCCCTATTAGACTTGCTGTA
TCCCCAGTCCCAGCCCCAGCATCAGCAGCATCAGCAAGAACAGGAACAGCTCCGCCCACA
AACTTGCTGTGCACCGCCCCGGCGCCACCGAGTCCGCCGGCCAAGCGCCAGCGGGTCATC
GTCGGACAGCAGCATCTCCGGCCCGACTCTACGCGAACGGTCGGAGAGGGGCAAGTGGAG
TGTTACGACAAAAGGAGCATCAGTAACACTAACAGCACAGCTCCCCGGTGGGACCACGGT
GACACTGACACTGTGCCTGTAATCCATTTGCGAGGTGATGCAAATTGTTTAAAGTGCTTC
CGATACAGGGTACAAAAACATAAAGACAAGCTGTATGACAGGGTGTCCTCCACGTGGCAC
TGGGCCGGTGGAAAGTGTGATAAGACAGCCTTTGTGACAGTGTGGTACACCAGTGTGGAG
CAGCGTAAAGAGTTCCTGACAAGGGTCAACATACCTAAGGGGGTGATAGCATTGCCAGGG
TATATGTCTGCCTTTGTATAGACCTACATGCCTGTATAGAACGTATGGTCCAGAACATTT
CAAGGCCTGCTTAGTCAACACAGCCCTGGACTACTTCCTTTGCGTGGTGGCAGGGTGGAC
ACATCTGCTTGTGCTACTGCTATTCCTTTGGCTCTCTCAACTAACTGCTCTTGTGGCCTT

TTTGGTGTTCTTTTTCTGTGTCTATCTAGGGTTGTGGTTGATATACGTGCAGGCCTTTTG
GTTTTTACAATAATTGTGACATATCGCCACCATTTGCTGCTAACCTGTATATATAGTTTT
ACCACTTGTGTTATTTGCAATGTACCCTGTTGTGTATAAAGGGTCGGAGGGGACATATCC
TGTGATATTGTGGGGTCGTGATGATATTGACTGTCTGTTGGTGATTCTCACCCTCATTGG
CTTATTATTGTTGTTGTTTTATGTCCGTTTGATCCAACACACCTAACACCCCTCACCCCT
TTTGATACATTATCTCTTTTCATACATTGTTTTTTTTGTACTTGCTGCGTTGTAATAAAC
TTGCACCCATGCCTCGTGCCAAGCGTCGGAAACGCGCCTCCCCCACCGACCTCTATCGTA
CATGCAAGCAGGCAGGTACCTGCCCCCCAGACATTATTCCAAGGCTAGAACAAAACACCT
TGGCAGATAAAATATTAAAGTGGGGCAGCCTAGGGGTCTTCTTTGGCGGTCTTGGTATAG
GCACTGGCAGTGGCACGGGGGGACGTACCGGGTATATTCCTGTAGGTACCAGGCCGACCA
CTGTGGTTGATATTGGTGTGGCACCCAAGCCACCTGTTGTTATTGAACCTGTGGGGGCCT
CAGAGCCCTCTATAGTCACTTTGGTAGAGGACTCCAGCATCATAAATGCAGGCGCTTCCC
ATCCCACCTTCACTGGTACAGGTGGGTTCGAGGTTACAACTTCCACCGTTACAGACCCCG
CTGTCTTGGATATCACCCCCTCAGGTACCAGTGTGCAGGTCAGCAGCAGTAGCTTTTTGA
ACCCACTATACACTGAACCCGCAATTGTGGAGGCTCCTCAAACAGGGGAGGTATCTGGCC
ATGTACTTGTTAGTACAGCCACTTCAGGGTCTCACGGCTATGAGGAGATACCAATGCAGA
CCTTTGCTACGTCAGGTGGAAGTGGTCAAGAGCCCATAAGTAGCACACCCCTTCCTGGCG
TGCGTAGGGTTGCAGGGCCCCGCTTGTACAGTAGGGCAAATCAGCAGGTGCAAGTCAGGG
ATCCTGCGTTTCTGGAAAGGCCTGCTGATTTGGTAACATTTGACAACCCTGTGTATGACC
CAGAGGAGACCATAATATTTCAGCATCCAGACTTTCATGAGCCACCGGATCCTGATTTCT
TGGACATTGTGGCTTTGCATCGTCCTGCCCTTACGTCCAGACAAGGCACAGTCCGTTTCA
GTAGATTGGGACGCAGGGCCACACTTCGCACCCGTAGTGGTAAACAGATTGGGGCTCGGG
TGCACTTCTATCATGATATCAGCCCTGTCGTCCCTGATGAATTGGAGATGGAGCCATTGT
TACCCCCAGCTTCTACTGTAGGATCAGATGTTTTATATGATGTTTATGCTGATCCTGATG
TCCTGCAGCCATTGGACGATTACTACCCAGCCCCTCGCGGCTCCCTGGCTAATACCACGG
TATCTGCCTCCTCTGCATCTACACTGCGAGGGTCCACTACAGCCCCTCTCTCGGGTGGTG
TTGATGTGCCTGTGTATACTGGGCCTGATATTGAACCACCCGTTGTTCCTGGTTTGGGGC
CCCTCATTCCTGTGGCTCCATCGTTGCCATCCTCTGTTTACATATTTGGGGGGGATTATT
ATTTACTGCCAAGTTATATCCTGTGGCCTAAACGACGTAAACGTGTCAACTATTTCTTTG
CAGATGGCTTTGTGGCGGCCTAATGAAAGCAAGGTATACCTACCTCCAACACCTGTTTCA
AAGGTGATCAGTACGGATGTCTATGTCACGCGGACGAATGTCTATTACCATGGTGGCAGT
TCTAGGCTCCTCACTGTCGGCCACCCATATTATTCTATAAAGAAGGGTAGCAATAATAGG
TTGGCAGTGCCTAAGGTGTCCGGCTACCAATACCGTGTATTTCACGTTAAGCTGCCAGAT
CCCAATAAATTTGGCCTGCCTGATGCTGACCTATATGATCCAGACACTCAAAGACTACTG
TGGGCGTGCGTGGGAGTAGAGGTGGGCCGAGGGCAGCCTTTAGGTGTGGGTGTGTCTGGA
CACCCATATTATAATAGGCAGGATGACACTGAAAATGCACACACACTTGATTCAGCTGAG
GATGGAAGGGAAAATATTTCCATGGATTATAAACAGACCCAGCTGTTTATTCTTGGCTGT
AAACCCTCTATTGGTGAGCACTGGTCCAAGGGAACCACCTGTAATGGGTCCTCTGCTGCT
GGTGACTGCCCGCCCCTCCAGTTTACTAATTCAACTATTGAGGATGGGGATATGGTTGAA
ACAGGGTTTGGTGCATTGGATTTCGCCACTCTGCAGTCCAATAGGTCTGATGTTCCTTTG
GATATTTGTACAAACGTCTGTAAATATCCAGATTACCTGAAAATGGCTGCAGAGCCTTAT
GGTGATTCCATGTTCTTTTCGCTGCGTAGAGAACAGATGTTCACCCGTCATTTTTTCAAT
AGGGCTGGTAAGATGGGTGACACAATCCCAGATGAGTTGTACATTAAAAGTACCACTATC
TCGGACCCCGGCAGTCATGTGTATACCTCCACTCCTAGTGGCTCTATGGTGTCCTCTGAA
CAGCAATTGTTTAATAAGCCCTACTGGCTACGGAGGGCCCAGGGACATAATAATGGTATG
TGCTGGGGCAATCGGATCTTTCTGACTGTGGTGGACACCACACGGAGTACCAATGTCTCT
CTGTGTGCAGCTGAGGTGTCTGATAATACTAATTATAAAGCTACGAATTTTAAGGAATAC
CTCAGGCATATGGAGGAGTATGATTTGCAGTTCATTTTCCAACTGTGCAAAATAACCCTC
ACTCCTGAGATAATGGCCTACATACATAATATGGATCCCCAGTTGTTGGAGGACTGGAAC
TTCGGTGTACCCCCCCCGCCGTCTGCCAGTTTGCAGGACACTTATAGATATTTGCAGTCC
CAGGCTATTACGTGTCAGAAACCTACGCCCCCTAAGACCCCTACAGATCCCTATGCCAAC
ATGACCTTCTGGGATGTGGACCTACGGAAAGTTTTTCTATGGATCTGGACCAATTTCCT
TTGGGTCGCAAGTTCTTATTGCAGCGGGGGACGACGCCCACCGTGTCTCGAAAACGCACC
GCTGTTGGGCGCGGCCACTAGTAAACGCAAACGGGTGAGGCGTTACGTGTGAGTGTCTCG
ATAATTTCCTCTGTCTACCTTTTACATAATATTTGGTGTTGTTTGTGCTTATGTTTGTGT

TGTTGTTTGTACGTATGTTACATGTATACATGTATGGTATGTATCCCCTCCCGTATGAAT
AAACGTGTGTCATGTGTTGTGTGTTCTGTAACTGTTCTTTGTGGTGCACGGGTTTCTGCA
CCCTATTGTCCTTTGTGTAGCCCCCAGTTTCATGCGACCGTTTTCGGTTGCGTGCAGTTT
CGGTCGGCGCCGTTGCCAGCACAGCTTAATCCTTTAATTGCTCACATCCTAAAGTGTTAG
CTGTGCCAGCAACAATGAGTTTGGATTTTTGGTTGTTTAATGCTTTTTCTTTTTAGTTTT
TCCTTTCTCTGTGCCAGGCGCGAGAGGGTGTGTGCATTCCTAGGCTGATTATGGTTCTGT
GTTGGCACAGATTTCTACTGCGTCTGCAGGAAAACCTGCAGCAACAGCACTTTGGGCGCG
TCGTTTCTGCAGCCAACTTTCACTTGCCAACTTGTCTTGCCGCGCATTCCAAGAAACACA
CCTATTCCGGTCGCAATGTCTAC
>gi|396948|emb|X74471.1|_Human_papillomavirus_type_25_genomic_DNA
TAACGGTAAGTCTATTGATACGGGCGCGGTTAAATTATTACTCATTCGTACTTGTTGCTG
CCAACAATCAGCATTAGTAACTTGTTTCTGCCTGTATCGTTATCGACACAGGTGTGTTAT
ACATATATATATATATATATATATATATATATATATATATATATTATATATATACACGTAGA
CACTGCAGCATTAGGACTTATGGCAACTGCAAATGCTGAACAGAGCATAGGACCACCAGA
GCAAGCGCAGGTTATACAGCCACCATTGCCAGCAACAATTACTGATCTAGCAGCTTTATT
GGAAATTCCATTAGATGATTGCTTAGTACCTTGCAACTTCTGTGGCAACTTTCTAACATA
TTTAGAGATCTGTGAGTTTGATGAGAAAAGACTTAGTTTGATTTGGAAAGAATATCTTGT
GTATGCCTGCTGTCGCTGTTGTTGCACAGCAACTGCCACATTTGAATTTAATGAATTTTA
CGAAAGCACTGTAACAGGTAGGGAAATTGAAGACGTTACAGGTAAATCAATTTTTGACAT
AGATGTTAGATGTCAAACGTGCATGAAATATCTTGATGCAATTGAAAAGCTTGATATTTG
TGGCAGAAGACGTCCTTTCCATCTAGTAAGAGGCTCTTGGAAAGGAATCTGTAGGCTGTG
TAAGCATTTCTATAATGATTGGTAAGGAGGTCACATTGCAAGATTTTACATTAGAGTTAA
GTGAATTGCAGCCTGAGGTACAACCAGTTGACCTGTTTTGTGAAGAGGAGTTGCCGGCTG
AGCATCAGGAAACAGAGGAGGAGCCTGCTATTGACAGAACTCCATACAAAGTTGTTGCGC
CTTGTGGTTGCTGCGAGGTCAAGCTTCGCATCTTTGTGAAAGCCACTGATTTTGGTATTA
GAACACTACAAAACCTTCTAATTGAAGAACTGCAGCTGTTGTGTCCGGAGTGTCGCGGGA
ACTGCAAACATGGCGGATCCTAAAGGTAGTACATCTAAAGAAGGGTTTAATGATTGGTGT
ATTTTGGAAGCTGAATGTAGTGATATAGACAATGATTTGGAACAATTATTTGATCAAGAT
ACAGACTCAGATATTTCAGACTTATTAGATGAGAATGACGTGGAACAGGGCAATTCTCGG
GAACTATTTCATCTACAAGAGTGTCAGGAAAGCGAGGAGCAATTGCAAAAACTAAAACGA
AAGTACTTAAGTCCAAAAGCTGTCGCACAGCTTAGTCCGCGATTCGAAAGTATTTCTTTG
TCACCTCAGCAGAAGTCTAAACGAAGGCTCTTTGCAGAGCAGGACAGCGGGCTCGAATTG
ACTTTAACAAATGAAGCTGAAGATGTTTCTCCTGAGGTGGAGGTACCGGCTTTAAACTCT
CAGCCGGTAGCTGAGGGACAATCAGGGGACATAGACATAAGTTATACAGCATTATTGCGT
GCCAGCAATAATAAAGCAATATTAATGGCAAAATTTAAAGAGGCTTTTGGGGTAGGGTTT
AATGATCTGACACGTCAATTTAAGAGTTACAAAACCTGTTGTAATGCTTGGGTTATTTCT
GTATATGCAGTGCATGATGACCTTATAGAAAGTTCAAAGCAGCTTTTGCAACAGCATTGT
GACTATGTGTGGATCCGTGGGATAGGAGCCATGTCATTGTTTTTAGTTTGTTTTAAGGCG
GGAAAAAATCGTGGTACTGTTCATAAATTGATGACAACTATGTTAAATGTGCATGAAAAG
CAAATATTATCGGAACCACCAAAATTAAGAAATGTTGCTGCAGCACTGTTTTGGTATAAA
GGATCAATGGGTTCCGGAGTATTTACATATGGCTCATATCCAGATTGGATAGCCCACCAA
ACAATATTGGGCCATCAAAGCGCTGAAGCTAGTACATTTGATCTATCGGACATGGTTCAA
TGGGCATTTGATAACAATTATTTAGACGAAGCAGATATTGCATATCAATATGCTAAATTG
GCGCCAGACAATAGCAATGCTGTTGCATGGCTCGCACATAATAATCAAGCCAAATTTGTG
CGAGAATGTGCATCCATGGTGAGATTTTATAAAAAGGGTCAGATGAAAGAAATGAGTATG
TCTGAATGGATTTATACTAAAATTCATGAAGTGGAAGGAGAGGGTCAATGGTCCACCATT
GTACAATTTTTAAGGTATCAGCAAGTCAACTTTATAATGTTTTTAGCTGCCTTAAAAGAT
TTACTGCACTCTGTTCCGAAACGAAATTGTATACTTTTTTATGGACCCCCCAATACGGGG
AAATCAGCTTTTACTATGTCATTGATAAAAGTGTTAAAGGGTAGGGTATTATCATTCTGT
AATTCCAAAAGTCAATTTTGGTTACAACCGTTGTCAGAATGTAAAATAGCTTTGCTAGAT
GATGTCACAGACCCCTGTTGGGTGTACATGGACACATATCTGAGAAATGGCTTAGATGGA
CATTATGTGTCATTAGATTGTAAACACAAGGCACCAATGCAGACAAAATTTCCTGCACTA
TTGCTTACATCCAATATAAATGTGCACAATGAAGTGAATTATAGATATCTGCACAGTAGA
ATAAAAGGATTTGAGTTTCCAAATCCTTTTCCAATGAAAGCAGACAATACTCCCCAGTTT
GATTTAACTGACCAAAGCTGGAAATCTTTTTTTACAAGGCTTTGGCATCAATTAGACCTG

AGTGACCAAGAAGACGAGGGCGAAAATGGAGAATCTCAGCGAGCGTTTCAATGTTCTACA
AGATCAGCTAATGAACATTTATGAAACTGCAGCACAAACCCTTGAGGCACAAATTGAGCA
TTGGCAGATTTTGCGAAGAGAAGCTGTGCTACTATATTTTGCTAGGCAAAAGGGTGTTAC
ACGGCTTGGATATCAACCTGTACCTGCCTTAATGGTGTCTGAAGCAAAAGCTAAGGAAGC
TATAGGGATGGTGCTGCAACTGCAGTCATTACAAAAGTCTGAATTTGGAAAAGAGCCATG
GTCATTGGTTGACACCAGTACAGAGACTTATAAAAGTCCACCAGAAAACCATTTCAAAAA
AGGCCCAATGCCTATAGAGGTCATTTATGACAAAGATGCAGATAATGCCAATGCTTATAC
CATGTGGAGATATATTTATTATGTGGATGATGATGACAAATGGCATAAAAGTGCAAGCGG
GGTGAACCACACAGGCATATATTTTATGCACGGAAGCTTTAGACACTATTATGTGTTATT
TGCTGATGATGCTCGTAGATATAGCAATACTGGACATTGGGAAGTAAAAGTTAATAAGGA
CACTGTGTTTACTCCTGTCACCAGCTCCACCCCCCCCGAGTCACCAGGAGGACAAGCAGA
CTCAAACACCTCCTCCAAGACCCCCACCACTGACACCGCGTCCAGACTCTCGCCCACAGG
CTCCGGAGAACGGTCACAACAAACCAGCACCAAGGGACGGAGGTACGAACGGAGGCCCTC
CAGCAGGACACGACGACAGCAAGCCCAAGCGCGCCAGAGGCGATCAAGGTCCAAGTCCCG
GTCCCGGTCCCGGTCCCAGTCCCGCTCCCGTATCCGATCGAGGTCGCGGTCGCGGTCGCG
GTCTGAATCTCAGTCGTCTAAGCGGCGATCAAGATCCAGATCCAGAAGAAAAACCTCAGC
CACCAGAGGGAGAGGTCCAGGGTCACCCACAACCACCACCAGTGACCGAGCCGCAAGGTC
ACCTTCCACCACCTCCTCTGCCACCTCCCAACGGTCACAACGATCGCGATCCAGGGCAGG
GAGCAGCAGGGGGGGCCGCGGCAGGGGCGGGAGACGACGACACAGATTGTCTGAATCCCC
CACCTCCAAGCGATCACGAAGAGAGTCAGGGTCTGTTAGGCTCCATGGCGTCTCTGCTGA
CGCAGTGGGAACATCAGTTCACACAGTTAGTTCAAGACATACAGGAAGACTTGGAAGATT
ATTGGAAGAGGCTCTTGATCCCCCAGTAATTTTAGTTAGAGGGGATGCTAACACGCTTCG
AAGCTTTCGCAACAGGGCAAAGCATATGTATACTGGGCTATTTAGCTCATTTAGTACGGC
CTGGTCGTGGGTGGCTGGAGATGGCATTGAGCGTCTAGGCAGGTCCAGAATGCTCATTAG
CTTTATTTCCAACAGTCAGAGAAAACATTTTGATGATGCTGTGAGATATCCGAAGGGAGT
TGACCGGTCATTTGGATCATTTGATAGCCTTTAACCTACTAACATTAGGCTTTGCTACTA
ACACACTAACATATCTTAACCATTTATTTTGTTTTATATTTTTATGCTATGGCGCGCG
CCAGGCGGGTTAAGCGAGACTCTGCCACCAATATATACAGAACCTGCAAACAAGCAGGCA
CTTGTCCTCCTGATGTACTAAATAAAGTGGAAAATACTACAATAGCTGACAAAATTTTAC
AATATGGCAGTGCTGGTGTGTTTTTCGGGGGTTTGGGCATAAGCACTGGAAAAGGCACAG
GAGGAACAACAGGGTATGTACCTTTGGGAGAAGGTCCGATACGTGTTGGTGGAACCCCCA
CGGTTATTAGACCTTCTTTAGTCCCAGACACTATTGGACCATCTGACATAATACCTGTTG
ACACTCTTAATCCAGTGGAACCCACATCCTCGTCTATTGTCCCACTCACAGAGTCTTCAG
GTCCTGACCTTTTGCCTGGTGAGGTGGAGACAATCGCAGAAATACATCCTGGGCCTGTTG
TACCCTCCACTGACACCCCGGTGACAACAACTTCTAGAGGTGCCAGCGCAGTTTTAGAGG
TTGCACCAGAGCCCACCCCGCCATCGCGCGTCAGAGTTAGTGGCACACAATATCATAATC
CATCATTTCAGGTTATAACTGAATCCACACCTGCTCAAGGTGAAAGTTCACTGGCTGATC
ACATTTTGGTTACTTCTGGTTCTGGAGGGCAAACAATTGGTGGCACTGCCAGTGACCTAA
TTGAACTACAAGAGTTTCCCACTCGCTATTCATTTGAAATAGATGAACCCACACCTCCAA
GACAAAGTAGTACACCTCTTCAAAGGATTAGGACTGCATTAAGACGTAGAGGAGGATTAA
CAAATAGACGATTGGTGCAACAGGTACCTGTAGAAGACCCTTTATTTTTGTCTCAGCCAT
CACGGCTAGTACGGTTTCAGTTTGACAATCCAGTATTTGAGGACGAAGTTACACAAATTT
TTGAGCAGGATTTAAATGATTTTCAGGAGCCTCCTGACAGGGATTTTTTAGATATTCGCT
CATTAGGAAGGCCACAATATTCTGAAACTCCGGCTGGCTATGTACGGGTCAGTCGCCTTG
GTCAAAGACGTACCATTCGCACTCGATCTGGGGCTCAAATAGGCTCACAAGTTCATTTTT
ATAGAGACTTAAGCAGTATAAATACTGAAGATCCTATTGAGCTACAGTTGTTAGGTCAGC
ATTCGGGTGATGCTACAATAGTTCAAGGCCTCACAGAAAGTACCTTTGTAGATGTTAATG
TAGATGAAAATCCATTAGCTGAAGATTTAGTATTTCTGCACACTCTGATGATTTACTTT
TGGATGAAGCTAATGAAGATTTTAGTGGTTCCCAGTTGGTTGTGGGTGGTCGGCGCTCCA
CTTCTACTTACACTGTGCCTCGTGTTGAAACTACACGCTCTGCATCATATTATACCCAAG
ATATTCAGGGATACTATGTGTCCTATCCTGAGGATAGAGATACTAGTAAGGACATTATTT
ACCCTATGCCTGACCTCCCGGTGGTCATTATACACACATATGACACCAGTGGTGATTTTT
ATTTGCATCCCAGTCTGACTACAAGGCGCAGACGCAAAAGAAAATATTTATAATTTTTTC
TTTTACAGATGGCAGTTTGGCAAGCAGCTAGTGGTAAAGTGTACCTTCCACCATCTACAC
CTGTTGCCAGGGTACAAAGCACGGATGAATATGTGCAAAGAACTAACATCTATTATCATG

CCTATAGTGACCGCCTATTAACTGTTGGTCACCCATATTTTAATGTGTACAACGTCCAAG
GCTCTAAATTGCAAATTCCAAAAGTGTCAGGAAATCAACACAGAGTTTTTAGGTTAAAAT
TACCAGATCCCAATCGTTTTGCTCTTGCAGACATGTCAGTTTACAATCCTGACAAAGAAA
GGCTGGTTTGGGCCTGCAGAGGTATTGAAATAGGACGTGGGCAACCTTTAGGTGTGGGAA
GTGTGGGTCACCCATTGTTTAACAAGGTTGGCGACACAGAAAATCCAAATTCTTATAAAG
CTAGTTCTACAGATGACAGGCAAAATGTATCATTTGACCCCAAACAACTACAGATGTTTA
TTATAGGCTGTGCTCCATGTATAGGGGAACATTGGGATAAAGCATTACCTTGTGATGATG
GCAATATTCAACAAGGGTCATGCCCTCCAATAGAATTAATTAATTCTGTCATTGAAGATG
GGGATATGGCAGACATTGGCTATGGTAATTTAAATTTTAAAGCATTACAGCAAAACAGAG
CTGACGTAAGTCTGGATATAGTTAACGAAACCTGTAAATATCCAGACTTTCTTAAAATGC
AAAATGATGTGTATGGGGATTCCTGCTTTTTTTATGCACGGCGGGAACAATGTTATGCCA
GACATTTTTTTGTTAGAGGGGGCAAAACGGGTGACGATATTCCAGCTGGACAAATTGATG
AAGGAAGCATGAAAAATGCATTTTACATACCACCTAACAGTAGTCAGGCTCAATATAATA
ATCTAGGTAACTCAATGTATTTCCCAACAGTCAGTGGCTCATTGGTATCCAGTGATGCTC
AATTATTCAATAGGCCATTTTGGTTACAGCGAGCACAAGGTCATAACAATGGTATATGCT
GGTTTAATCAGCTATTTGTCACTGTGGTAGACAACACACGCAACACTAATTTCAGCATAT
CAATCAATTCAGATGGAACAGATGTTTCCAAAATCACTGATTATAATTCTCAAAAATTTA
CAGAATATTTGAGACATGTAGAAGAATATGAGTTATCATTAATATTACAACTTTGCAAAG
TACCGTTGAAGGCAGAAATATTGGCTCAGATTAATGCAATGAATTCCAACATTTTAGAAG
AATGGCAATTAGGATTCGTTCCTGCACCGGACAATTCTATTCAGGATACTTATCGCTACA
TTGATTCTTTAGCCACACGTTGTCCAGATAAAAATCCTCCTAAGGAAAAAGTAGATCCTT
ATAAAAATTTACACTTTTGGGATGTAGATTTAACAGAACGCCTTTCTTTAGATTTAGATC
AATATTCACTGGGACGTAAGTTTTTATTTCAGGCTGGTTTGCAGCAAACAACCGTAAACG
GTACAAAAACAGTTTCCTCCCGAATATCTACTAGGGGAATAAAAAGAAAACGTAAAAATT
AGAAATTACCGCTTTCGATACAATAAAGTCAACTTTTGCACAGTATTCAAGGAATGTTTA
TTTGTGCTGACTAACTAGGATACCAACCGCACCCGATACATAAAAGGTGAGTTATGTGCC
AAATCAGATACAGTCTGTGCCAAACTCAGGCAGCTGCTCGCCAGATGCGTATCGTCTTTA
GTGTTTTGACACGCTCGGATTAGGACGTTCGCCAATGGAATACTAATATTGCCAATCGCT
TTCGGCTCTTTGTTTGGCAGAGCTCAAGACCGT
>gi|396940|emb|X74470.1|_Human_papillomavirus_type_19_genomic_DNA
GGTAAGTTTATTGATACGGGCGCGGTTAGAAGTTACTCATTCGGTGCTTGTTGTTGCCAA
CAATCAGCGTTATGAACTTGTTTCTGCCTGTATCGGTATCGACACAGGTATTATATATAT
ATATATATATATATATATATATATATATATATATATATATATATATACACACACAGATAC
ATTTTGCAGCTGCAAACTTATGGCTAACGCACAGGCTACAGAAGAAGAGATAGAAATTGT
AGAAGAGGGAACTACTGCACCACAGGTCACAGAGCCACCATTACCAGCAACAATTGCTGG
ATTAGCAGCATTGCTAGAAATACCGTTGGATGACTGTTTAGTGCCTTGTAATTTCTGTGG
CAAGTTTTTATCACATTTAGAAGCGTGCGAATTTGATGATAAAAGACTTAGTTTGATTTG
GAAAGGTCATCTTGTGTATGCTTGCTGTCGCTGGTGTTGCACAGCAACTGCAACATTTGA
ATTTAATGAGTTCTATGAGCATACTGTAACAGGTAGAGAAATTGAGTTTGTAACAGGTAA
ATCTGTCTTTGACATTGATGTTAGATGTCAAAATTGCATGAGATATCTTGATTCAATTGA
AAAGCTTGATATCTGTGGAAGAAGACTTCCTTTTCATAAAGTAAGAGACTCTTGGAAAGG
GATCTGTAGGCTGTGTAAGCATTTCTATAATGATTGGTAAAGAGGTGATATTGCAAGACA
TTGTATTAGAATTAAGTGAGTTGCAGCCTGAGGTACAACCAGTTGACCTGTTTTGTGAAG
AGGAGTTACCGACCGAACAGCAGGAAACAGAGGAGGAGCCTGCTATTGAAAGATCTGCGT
ACAAAGTTGTTGTACTTTGTGGCTGCTGCAAGGTGAAGCTTCGCATCTTTGTGAAAGCCA
CGCAATTTGGTATTAGAACCCTACAGGACATCCTGATTGAAGAATTGCAACTGTTGTGCC
CGGAGTGCCGTGGGAACTGCAATCATGGCGGAGTCTAAAGGTAGTACATCTAAAGAAGGG
TTTGGTGATTGGTGTATTTTGGAAGCTGAATGTAGTGATGTAGAAAATGATTTGGAAAAA
TTATTTGATGAAGATACAGACTCAGATATTTCAGACTTATTAGATGATAATGACTTGGAG
CAGGGTAACTCTCGGGAACTATTTCATCAACAAGAGTGTCAGGAAAGCGAAGAGCATTTG
CAAAAACTAAAACGAAAGTACTTAAGTCCCAAAGCTGTCGCACAGCTTAGTCCGCGATTT
GAAAGTATTTCTTTATCACCTCAGCAGAAGTCTAAACGAAGACTTTTTGCAGAGCAGGAC
AGCGGACTCGAGTTGACTTTAACAAATGAAGCTGAAGATGTTTCTTCTGAGGTGGAGGTA
CCGGCTTTAGATTCTCAGCCGGTAGCTGGGGAACAATCAGGGGACATAGACATACATTTT
ACAGCATTATTGCGTGCAAACAATAACAGAGCAATTTTAATGGCAAAATTCAAGGAAGCG

TTTGGGGTAGGGTTTTATGACTTAACACGCCAATTTAAAAGTTATAAAACATGCTGTAAT
GCTTGGGTTATATCTGTGTATGCAGTTCATGATGATCTGCTTGAAAGCTCAAAACAGCTG
TTGCAACAGCATTGTGACTATGTGTGGATTAGACAGACAGCAGCAATGTCATTGTTTTTA
TTGTGCTTTAAAGTGGGAAAAAAACCGTGGCACGGTGCATAAGTTAATGATGTCTATGTTA
AATGTACATGAAAAACAAATATTATCTGAGCCACCTAAACTGAGAAATACTGCTGCTGCG
TTATTTTGGTATAAGGGCTGTATGGGATCTGGAGGGTTTACTTATGGTCCATACCCAGAT
TGGATAGCACAACAAACAATATTAGGTCATCAAAATGCTGAAGCAAGTAGTTTTGATTTG
TCTGAAATGATTCAATGGGCATTTGATAACAACCACATGGACGAATCAGATATTGCGTAT
CAATATGCAAAATTAGCACCAGAAAACAGCAATGCTGTGGCATGGCTTGCACATAATAAT
CAAGCTAGATTTGTTAGAGAATGTGCGGCAATGGTGCGTTTTTATAAAAAAGGTCAAATG
AAAGAAATGAGCATGTCTGAGTGGATTTATGCTAGAATCCATGAGGTGGACGGAGAAGGA
CACTGGTCCACTATTGCTAAATTTTTAAGATATCAACAAGTAAATTTTATAATGTTTTTA
GCAGCCCTAAAAGATTTATTGCATGCAGTGCCAAAACGAAATTGTATACTAATTTATGGT
CCTCCTAATACTGGCAAATCAGCCTTTACTATGTCACTAATAAGAGTGTTAAAAGGCAGA
GTAATATCGTTTGTAAACTCCAAAAGTCAGTTTTGGTTACAACCTTTGTCAGAGTGTAAA
ATAGCACTCTTAGATGACGTCACTGATCCGTGTTGGATTTACATGGATACATATTTGCGA
AATGGTTTAGATGGGCATGTTGTGTCTTTGGATTGCAAACATAAAGCCCCCATTCAAACT
AAATTTCCTGCGCTTTTACTTACATCTAATATAAATGTACATAATGAGGTTAATTATAGA
TACTTACACAGCAGAATACAAGGATTTGAGTTTCCAAACCCATTTCCTATGAAAGCAGAC
AATACACCTCAATTTGAACTTACTGACCAAAGCTGGAAATCTTTTTTTACAAGGCTTTGG
CAACAATTAGAACTGAGTGACCACGAAGAGGAGGGCGAAAATGGAGAATCTCAGCGAACG
TTTCAATGTTCTACAAGATCAGCTAATGAACATTTATGAATCTGCAGCAGAAACCCTTGA
GTCACAAATTGAACACTGGCAAATTTTGCGAAAAGAAGCTGTACTACTATATTTTGCTAG
GCGAAAGGGTGTTACGCGAATTGGATATCAACCTGTTCCCACGTTAGCAGTGTCTGAAGC
AAAAGCTAAGGAAGCGATAGGGATGGTACTGCAGCTGCAGTCATTACAAAAATCTGAATA
TGGAACTGAGCCTTGGTCCTTGGTTGACACCAGTGCAGAGACGTATAGAAGTGCTCCAGA
AAATTATTTTAAAAAGGGTCCAATGCCTATAGAGGTTATATATGACAAAGATGCAGATAA
TGCCAATTTGTATACCATGTGGAAATTTGTGTATTACGTGGATGAGGATGACAATTGGCA
TAAAAGTGAAAGTGGGGTAAATCATACTGGTTTATATTTTATGCAAGGAAACTTTAGACA
CTATTATGTGTTATTTGCTGATGATGCACGTAAATATAGTGCAACTGGACATTGGGAAGT
AAAAGTTAATAAGGAAACTGTGTTTACTCCTGTCACCAGCTCAACACCCCCCGACTCACC
AGGAGGACAAAGAGACCCAAACACCTCCTCCAAGACCCCCACCACCACCACTGACTCCGC
GTCCAGACTCTCGCCCACAGCCTCCAGAGAACAGTCACAACAAACCAACACCAAAGGACG
GAGGTACGAGAGACGACCTTCCAGCAGGACCCGACGACAAACCCAAACGCGCCAGAAACG
ATCAAGGTCCAAATCCAAGTCCCGGTCGCGGTCGCGGTCGCGGTCTCTTTCGTCTAACCG
GCGATCACGATCCAAATCCAGAAGAAAGGCCTCCACCACTAGAGGGAGAGGTCGAGGGTC
ACCCACCGCCACCAGTGACCAATCCTCCAGGTCACCCTCAGCCACTTCCTCCACAACCTC
CTTGCGATCAAGAGGGAGCAGCAGGGTCGGGCGCAGCAGGGGGGGGCGCAGCAGGGTCGG
GCGCAGTAGGGGGAGAGGGAAACGATCCAGAGAGTCACCATCCCCCACCAACACCAAACG
GTCACGAAGACAGTCAGGGTCTTCTAGGCTCCATGGCGTCTCTGCTGACGCAGTGGGAAC
ATCAGTTCACACGGTTAGTGGAAGAAATACAGGAAGACTTGGAAGATTACTGGAAGAGGC
TCTTGATCCCCCAGTAATTTTAGTTAGGGGGGAACCTAACACGCTACGTAGCTTTCGCAA
CAGAGCTAAGCACATGTATCGAGGGCTTTTTAGCTCATTTAGCACTGCATGGTCATGGGT
GGCTGGAGATGGAATTGAGCGTCTAGGCAGGACCAGAATGCTCATTAGTTTTGTCTCCTT
TAATCAACGAAAGCACTTTGATGATACAGTAAGGTATCCTAAAGGTGTGGACCGATCGTT
TGGCTCATTTGATAGCCTTTAACATACTAACTGCTTTTTTTGCTACTAACACAATAACTT
ACCTATATATATTTTTTTACTGCAATGGCGCGCGCCAGGCGTACTAAGCGAGATTCTGCT
ACCAATATATACAGAACCTGCAAACAAGCAGGTACCTGTCCTCCTGATGTAATCAATAAA
GTAGAACAAACTACAATAGCTGACAAAATCTTGCAATATGGCAGTGCTGGCGTCTTTTTT
GGGGGTTTGGGCATAAGTACTGGCAAGGGCACAGGAGGAGCAACTGGTTACGTGCCTTTG
GGGGAGGGTCCAGTACGTGTTGGTGGTACTGCAACGGTGATCAGGCCTTCGTTGGTTCCA
GACACCATTGGACCATCAGACATAATACCTGTGGACACCCTGAATCCAGTAGAGCCCACT
ACCTCCTCTATTGTTCCACTAACAGAGGCTTCAGGTTCTGACCTGTTACCTGGAGAAGTG
GAAACCATTGCAGAGGTGCACCCTACACCTAGTATACCCTCAACAGATACCCCAGTGACC
ACAACTTCAAGTGGTGCTAGTGCTGTTTTAGAAGTTGCCCCAGAACCTGTTCCTCCATCA

CGTGTAAGAGTTACTCGCACACAATATCATAATCCTTCCTTTCAAATATTAACTGAATCT
ACTCCAACACAGGGTGAAAGCTCCTTGGCTGATCATATTCTGGTCACTTCAGGTTCTGGA
GGACAAACAATTGGTAGTTCTGGCAGTGATTTAATAGAACTTCAAGAATTTCCTACTCGT
TATTCTTTTGAGATAGAAGAACCTACACCTCCACGCCAAAGTAGTACTCCAATTCAAAGA
CTTAGAACTGCATTTAGGCGAAGGGGAGGATTAACAAATAGGCGTTTAGTACAACAAGTA
GCTGTAGATGATCCCATATTCTTAACTCAGCCTTCAAGGTTAGTTTCTTTTCAGTTTGAT
AATCCTGCATTTGAAGAAGAAGTAACACAAATATTTGAACAAGATTTAGATAATTTTCGG
GAGCCACCTAATAGGGATTTTTTGGATGTGCAAACTTTAGGTAGGCCACAATATTCAGAA
ACACCATCTGGTTACATCAGAGTTAGTCGCCTAGGCCAAAGGCGAACCATTCGCACTCGC
TCTGGAGCACAGATAGGTTCACAAGTTCATTTCTATAGAGACTTAAGTACTATAGACTCG
GAAGATCCTATAGAGCTACAATTGTTAGGTCAGCATTCTGGTGACGCTTCAATAGTTCAA
GGTAATACAGAAAGCACATTTATAAATATTAATATTGATGAAAATCCATTAGCTGAAGAT
TATAGTATTACTGCTAACTCAGAAGATTTGCTTTTAGATGAAGCACAGGAAGACTTTAGT
GGGTCACAGTTAGTAGTTGGTGGGCGCCGTTCTACTTCCACATATACAGTTCCCCAATTT
GAAACTACAAGGTCTGGATCATATTATACACAAGACACTAAAGGCTATTATGTTGCATAT
CCTGAAGATAGAAGTACTAGTAAGGATATAATTTATCCCATGCCTGACTTGCCTGTGGTT
ATTATACATACATATGACACCAGTGGTGATTTTTACTTACATCCCAGCCTTCGCAAGCGA
TTCAAACGAAAACGTAAATATTTATAATTTTCTTTTGCAGATGGCAGTATGGCAAGCAGC
TAGTGGTAAGGTATACCTTCCACCATCTACACCAGTTGCCAGGGTACAAAGCACGGATGA
ATATGTTCAAAGGACAAATATCTACTATCATGCTTATAGTGACCGCCTACTCACTGTTGG
TCACCCATATTTTAATGTTTATAATGTTGCAGGATCAAAATTAGAAATTCCAAAAGTTTC
AGGAAATCAACACAGGGTTTTTAGATTAAAACTACCAGACCCTAATCGCTTTGCACTTGC
TGATATGTCAGTGTATAATCCTGATAAAGAAAGGTTAGTGTGGGGCTGCAGAGGAATTGA
AATAGGTAGAGGGCAACCACTAGGTGTAGGTAGTGTAGGACATCCATTGTTTAACAAAGT
AGGGGATACAGAGAACCCTAACTCATATAAGGGCACTTCTACTGATGATAGGCAAAATGT
ATCATTTGACCCTAAACAGCTACAAATGTTTATTATAGGCTGTGCTCCCTGTATAGGGGA
ACACTGGGATAAAGCATTACCATGTGCTGAGCAAGATATTCCTCAGGGATCCTGTCCTCC
TATAGAGTTAATTAACTCAGTTATTGAAGATGGAGACATGGCAGATATTGGCTATGGCAA
TTTAAATTTTAAGGCCTTACAACAAAACAGATCTGATGTCAGTTTAGATATAGTAAATGA
AACTTGTAAATATCCAGATTTCTTAAAGATGCAAAATGATGTGTATGGTGATTCCTGCTT
TTTTTATGCAAGGCGAGAGCAATGTTATGCTAGACATTTTTTTGTTCGTGGAGGCAAGAC
AGGTGATGACATTCCAGCAGGACAAATCGATGAGGGAAGCATGAAAAATACATACTACAT
ACCTCCTAACAATAGTCAGCAACAATATACTAATTTAGGAAATGCCATGTATTTCCCAAC
TGTTAGTGGCTCATTAGTATCCAGTGATGCTCAATTGTTTAACAGGCCATTTTGGTTGCA
GCGCGCACAAGGTCATAACAATGGCATATGCTGGTTTAATCAGCTATTTGTCACAGTAGT
AGACAACACGCGTAACACTAATTTTAGTATATCAGTTAATTCAGATGGAACAGATGTTGC
TAAAATTGCAGATTATAATTCTGCAAACTTTAAAGAATACTTAAGACATGTAGAAGAATA
TGAAATATCTTTAATTTTACAATTATGTAAAATACCTTTGAAAGCAGAAGTTCTGGCACA
AATCAATGCAATGAATTCTAACATATTGGAAGAATGGCAATTAGGTTTCGTGCCTGCACC
CGATAATCCTATTCAGGACACTTATAGATATATAGATTCTTTAGCTACTAGATGCCCTGA
CAAAAATCCTCCTAAGGAAAAAGTAGATCCTTATAAAAACTTACACTTTTGGAATGTAGA
TTTATCAGAACGCCTCTCTTTAGATTTAGATCAATATGCTCTTGGCCGCAAGTTTTTATT
TCAGGCTGGTTTGCAACAGGCAACCGTAAACGGTACAAAAACTATATCTTCACGGGTCTC
CAGCAGAGGAACTAAAAGAAAGCGTAAAAATTAAATTTGTTCGTTTTCGTTACAATAAAG
TCAACTTTTGCACAGTATTCAAGGAATGTTTATTTACTATGACTAACTAAGAAACGAACC
GCACCCGATACATAAAGGTGAGTTATGTGCCAAATCAGATACAGTCTGAGCCGCATCAGG
CACAGCAGCTGGCCAGATCTGATCTTCGTTGTTTTAACACGCTCGGATTAGGACTCTCGC
CAATGGAATCATAATCTTGCCAATCTCTTTTGGCACTGCACTTGGCAAAGGTAAGGACCG
TTAAC
>gi|396932|emb|X74469.1|_Human_papillomavirus_type_17_genomic_DNA
CTGGAGCCAAGGTTTTGGCAGAACACCATCTTGGAACAGCACCGATAACGGTAAGATTAT
ATCTTGGAACCGTAGGCGGTACTTTCTGATTGGTTTGGCTGATAGTAGTTAACAACAATC
TTCTCTCATAAATATATGTGACCGCCTTCGTTACCTTAAATGATCTACATACAATATGAG
AGCTCTTACTTAAGAGCATATGGATAGGCCAAAACCTCAAACAGTGAGGGAGCTTGCTGA
TACCTTGTGTATTCCATTAGTGGATATTTTATTACCTTGCAGATTTTGTAATAGGTTTTT

AGCTTACATAGAATTGGTGGCGTTTGATTTAAAAGGTTTGCAGTTAATTTGGACTGAAGA
AGATTTTGTGTTTGCCTGTTGCAGTAGTTGTGCGTATGCTACAGCACAGTATGAATTTTC
TAAGTTTTATGAACAATCAGTGAGTGGAAGGGAGTTAGAGGAAATAGAGCACAAGCCAAT
AGGGGAAATACCTATTCGCTGCAAGTTTTGTTTAAAGAAATTGGATTTACTAGAGAAGTT
AGACACTTGTTATAGACATCAGCAGTTTCATAAGGTTAGACGCAATTGGAAAGGCTTGTG
CAGACATTGTGGGTCGATAGGATGATTGGGAAAGAAGCTACAATACCAGATATAGTGCTT
GAGCTGCAACAGCTTGTCCAGCCCACTGACCTGCATTGCTACGAAGAGTTAAGTGAAGAA
GAGACAGAGACAGAGGAGGAGCCTCGTCGTATACCATACAAGATTGTAGCTCCGTGCTGC
TTTTGTGGTTCTAAGCTACGGCTTATTGTTCTTGCAACGCACGCTGGAATTCGTTCACAA
GAGGAGCTTTTATTAGGTGAAGTACAGTTGGTGTGTCCTAACTGCAGAGAGAAGCTTCGC
CATGACTGACGACAACAAAGGTACCAAATTTGATCCTAAAGAAGGATGTAGTCAGTGGTG
TATACTAGAAGCAGAATGTTCTGACAATAGTTTAGATGGTGATTTGGAAAAGTTATTTGA
AGAAGGTACAGATACTGAAATTTCTGACTTAATAGATGATGAGGACATTATACAGGGAAA
CTCCCGCGAATTGTTATGCCAGCAAGAAAGTGAGGAAAGCGAGCAACAGATACAATTGCT
AAAACGAAAGTATTTGAGTTCACAAGAGGTTTTGCAGCTCAGTCCGCGCCTGCAGTCTAT
CACTATATCGCCACAGCATAAGTCTAAAAGGAGATTATTTGAACGAGACAGCGGACTAGA
ACTGTCATTTAATGAAGCTGAAGATCTTACTCAGCAGACTTTGGAGGTGCAGGAGGTATC
GGCGACCGGTTCTGTACCGGCAGAACAGGGTGTCAAGGGATTGGGAATTGTTAAAGACCT
TTTAAAATGTAGTAATGTGAAAGCTATGTTATTGGCCAAATTCAAAGAAGCATTTGGAGT
GGGATATATGGATTTAACCAGACAGTATAAAAGTAGTAAGACATGCTGTAGAGATTGGGT
AGTTACATTGTATGCAGTACAGGATGAGTTGATAGAAAGCTCCAAACAATTGCTGCTGCA
ACATTGTGCTTATATATGGCTACAACATATGTCACCTATGTGTTTATATTTATTATGTTT
TAATGTCGGAAAAAGTAGGGAAACTGTATCACGATTGCTTATGAATATTCTGCAAGTAGC
AGAGGTACAAATGTTAGCAGAACCTCCAAAATTGAGAAGCATGTTGTCAGCATTATTTTG
GTATAAAGGAAGTATGAATCCAAATGTGTATGCCCACGGTGAATATCCTGAGTGGATTTT
AACACAAACTATGATTAATCATCAAACAGCACAGGCAACACAATTCGATCTATCTACCAT
GATACAATTTGCTTATGATAACGAATACCTTCAAGAAGATGAAATAGCTTATCATTATGC
TAAATTAGCAGATACAGATGCTAATGCACGAGCATTTTTACAGCATAATAGTCAAGCACG
GTTTGTAAAAGAATGTGCAATAATGGTGAGACATTATAAGCGTGGAGAAATGAAGGAAAT
GAGCATTTCTACGTGGGTACATAGAAAATTATTAGTTGTTGAAGGAGATGGACATTGGTC
TGATATAGTAAAATTTATTAGATATCAGGACATTAATTTTATTAGGTTTTTAGATATATT
TAAATCATTTCTGCACAATAAACCAAAAAAAAACTGTATATTAATTCATGGCCCACCAGA
TACAGGTAAATCTATGTTTACAATGTCTCTTATAAAAGTGTTGAAAGGCAAAGTGTTATC
ATTTGCAAATTGTAGAAGCAATTTCTGGCTTCAGCCATTAGCAGACACTAAGCTTGCATT
AATTGATGATGTGACATTTGTATGTTGGGACTATATAGATCAATATTTAAGAAATGGATT
GGATGGTAATGTTGTGTGTTTAGATTTGAAACATAGAGCGCCATGTCAAATTAAATTTCC
ACCATTACTATTAACATCTAATATTGATGTAATGAAAGAAGACAAATATAGATATTTACA
CAGTAGAATTCAAAGCTTTGCTTTTCCAAATAAGTTTCCGTTTGATAATAACAATATGCC
ACAATTTCGACTTACTGACCAAAGCTGGAAATCTTTTTTTGAAAGGCTTTGGCATCAGTT
AGATCTGAGTGATCAAGAAGAAGAGGGAGACGATGGACAATCTCAGCGAACGTTTCAATG
TACTGCAAGAGAACCTAATGGACATTTATGAGTCAGGTCAAGAAGATATAGAGACTCAAA
TAAAACACTGGCAATTATTAAGACAGGAACAAGTACTGTTTTACTATGCCAGAAAAAATG
GAGTGATGCGTGTAGGTTATCAACCTGTGCCTCCTTTAGCCACCAGTGAGGCTAAAGCAA
AGGATGCTATTGGCATGGTTTTGTTATTGCAGAGCTTGCAAAAATCACCGTATGGCAAAG
AGCCATGGACGCTAACACAAACTAGTTTGGAGACTGTGCGAAGTCCACCCGCAAACTGTT
TTAAAAAGGGTCCTCAAAACATTGAAGTTATGTTTGACAATGACCCTGAAAATCTTATGT
CATATACAGTGTGGTCATTTATTTATTACCAAAATTTAGATGACACCTGGAATAAAGTTG
AGGGCCGTGTTGACTATCATGGTGCATATTATATGGAAGGCTCTCTAAAAGTGTATTATA
TTCAATTTGAAGTGGATGCTGCCAGGTTTGGCAAAACTGGACGTTGGGAAGTGCATGTTA
ATGAGGACACTATCTTTGCTCCTGTTACTAGCTCTTCGCCGGCAGCTGGAGAAGGGACCG
ACGCGTCCCCCATCAACGCCGCATCCCGGTCGTCACCAGCAAGGGGACTCTCTGCCACCT
CCGTGTCCACCCGAACCACACAACGGACATCACCACGGCGATACAGGCGGAAAGCGTCTA
GCCCTACAGCCACCACCACCCGGCACAAAAGACAAGACATCAGACGATCAAGGTCCACCT
CACGGGGGAGACAAGCAATCTCCAGGGGAGGGGAGCGACGCCAGCGGAGACGAGAACGCT
CCTACTCCCGAGACTCCTCAAGATCCCCCAACAGGGGAAGGGGAGGGAGCAGTGGGGGGC

CCACAACACGGTCCCAGTCGCGGTCCCTCTCCCGATCCCGATCGCGGTCACGATCGCGAT
CCAGAGGGTCTTCTGCCGGGGGTGGCGTTGCGCCTGAGCAAGTGGGAAAATCAGTTCGAT
CAGTTGGTAGAAACCCTGGTGGGCGACTTACAAGATTATTGGAAGAAGCTAGGGATCCCC
CAGTAATTTTGCTGCGCGGCGAAGCTAACAAACTGAAATGCTTTCGATATAGAGCAAAAA
AGCGATATGGCAGTTTAGTTAAATATTACAGCACTACATGGTCATGGGTGGGTGCAAATA
CTAATGACAGAATAGGTAGATCAAGAATGTTACTAGCATTTAACACATATGATGAAAGAG
AATTGTTTATCCAAAAAATGAAGCTACCACCAGGTGTTGATTGGTCACTAGGACATCTAG
ATGATTTATAGGCATTACTTTTTAACATTACTAACCTTGCTAAAGCTTGCTTTTGCTAC
TAACACTTTTAACGTTCCCATGGCTCGCTCAAGACGCATAAAGCGTGCCTCTGTAACTG
ACATCTACAGGGGTTGCAAGCAGGCTGGTACTTGCCCCCCTGATGTTATTAATAAAGTGG
AACAGACTACAATAGCAGATAAAATTTTAAAATATGGTAGTTCTGGTGTTTTTTTTGGTG
GGCTGGGCATTAGCACAGGTCGTGGCACGGGTGGGGCAACAGGTTATTTCCCTTTGGGTG
AAGGGCCTGGGGTACGCGTAGGTGGCGCCCCCACTATAGTTCGCCCTGGGGTCATACCTG
AACTCATTGGCCCAGCGGATGTAATACCTATTGATACAGTCACTCCAATTGACCCCGCAG
CACCTAGTATTGTTACAATCACAGACAGCAGTGCTGTTGACCTATTACCCACTGAATTGG
AAACCATTGCAGAAATACATCCTGTGCCTACAGATAATTTAGATATTGACACTCCTGTTG
TTTCAGGAGGCAGGGATTCCAGTGCTGTTTTAGAGGTTGCTGATCCTAGTCCCCCTGTAA
GAACAAGGGTGTCTCGAACACAGTATCACAACCCATCATTTCAAGTAATTACTGAATCTA
CACCTTTATCTGGAGAATCAGCTATGGCGGATCATGTTTTAGTGTTCGAAGGTTTTGGTG
GACAAAACATAGGAGGTTCCAGGAATGCAGCCATTGATACAGCACAGGAGAGCTTTGAAA
TGCAATCCTGGCCTAGCAGATATAGCTTTGAATTAGAAGAAGGCACACCTCCTAGAACAA
GTACTCCAGTTCAACGTGCAGTAGAATCACTATCAAGCTTAAGAAGAGCTTTATACAATA
GACGATTGACTGAACAAGTTGCAGTGACAGATCCACTTTTTTTAAGTAGGCCTTCACGCT
TGGTCCAATTTCAGTTCGATAATCCTGCCTTTGAGGAAGAAGTTACACAGCTGTTTGAAA
GAGATATTGAAGCAGTGGAGGAACCTCCTGATAGACAGTTTTTAGATGTTGTGCGCCTAG
GAAGGCCTACATATTCTGAAACACCTCAGGGTTATTTACGAGTCAGTAGACTAGGTAGAA
GAGCCAGCATTCGTACTCGCAGTGGAGCACAAGTAGGAGCTCAGGTACATTTTTATAGAG
ATGTTAGCACCATCGATTCAGATGCCTTAGAAATGCAGTTATTGGGGGAACATTCTGGTG
ACACTACCATAGTTCAGGGTCCTGTAGAAAGTTCCTTTGTAGACATTAATATTGATGAAC
CAGGGCCTTTGAATGTAGGCATCCAAGAATCACCACTGGCTGACACTATAGAAGAAGATT
TCAATTCTGCAGATTTGTTACTGGAAGATGCTGTAGATGACTTTAGTGGGTCTCAGCTGG
TATTTGGCAATCCTCGCCGCAGCACAACATCTGTAACTGTCCCCCGGTTTGAAACACCTA
GGGACACTGGCTTTTACATACATGACACTCAGGGATACACAGTAGCATATCCAGAGTCAC
GTGACACCACTGAAATAATTCTTCCACATCCTGATACACCAACTGTAGTAATTAAATTTG
CAGAAGCAGGAGGCAGATTTTTATTTACACCCTAGTTTAAAGAAACGAAAAAGAAAACGA
AAATATTTGTAATTGTTTTGCAGATGACATTGTGGCTGCCAACGACCGGTAAAGTATACT
TGCCTCCAACACCACCAGTAGCCCGAGTACAAAGCACGGATGAGTATGTGGAAAGAACAA
ATATTTTTTACCATGCTATGAGTGATCGTCTCCTAACTGTGGGACACCCATTTTATGATG
TAAGATCTACTGATGGATTAAGAATAGAAGTCCCTAAAGTTTCTGGAAATCAGTATAGAG
CCTTCAGAGTCACATTACCAGATCCTAATAAGTTTGCCTTGGCAGATATGTCAGTTTACA
ATCCTGAAAAGGAAAGATTAGTTTGGGCATGTGCAGGCCTTGAGATAGGACGAGGACAGC
CATTAGGTGTAGGCACTACAGGACATCCCTTGTTTAATAAGTTAAGAGACACTGAAAATA
ACAGTAGCTATCAAGGTGGATCTACTGATGATAGACAAAACACGTCATTTGACCCTAAAC
AAGTGCAGATGTTTGTTGTAGGCTGTGTACCTTGTATTGGAGAACATTGGGACAGGGCTC
CTGTATGTGAAAATGAACAAAACAATCAAACAGGCCTGTGTCCACCATTGGAATTAAAAA
ACACTGTTATCGAAGATGGTGACATGGTTGACATAGGCTTTGGAAACATTAATAACAAAG
TGCTTTCATTTAATAAATCAGATGTAAGTTTAGATATAGTTAATGAAACATGCAAATATC
CTGATTTTTTAAGCATGGCAAATGATGTTTATGGTGATGCATGTTTCTTTTTCGCCAGAC
GAGAGCAGTGCTATGCTAGGCATTATTTTGTGAGGGGAGGTAATGTAGGTGATGCTGTTC
CAGATGGTTCTGTAAATCAGGATCACAAATTTTATTTACCAGCTCAAACTGGCCAACAAC
AGCGCACTTTGGGTAATTCCACTTATTTTCCAACTGTAAGTGGTTCTTTAGTAACATCTG
ATGCCCAGCTTTTTAATAGACCATTCTGGTTACGTAGAGCACAAGGACACAATAATGGTA
TTTTATGGGGGAATCAGATATTTGTGACTGTAGCTGACAACACTAGGAACACAAATTTTT
CTATTAGCGTGTCTACAGAAGCTGGGGCTGTTACAGAATATAATTCTCAAAATATCAGAG
AGTATTTAAGACATGTAGAAGAGTATCAGCTATCTTTTATTTTACAATTATGTAAAATAC

CTTTAAAAGCTGAAGTTTTAACTCAAATTAATGCAATGAACTCAGGAATTCTGGAAGACT
GGCAGTTAGGATTTGTGCCTACACCAGATAACCCAGTGCATGATATATATAGGTACATTA
ATTCTAAAGCCACAAAATGTCCTGATGCAGTTGTAGAGAAAGAAAAAGAAGACCCTTTTG
CAAAGTATACATTTTGGAATGTAAATCTTACTGAGAAATTATCATTAGATTTAGATCAGT
ATCCCCTAGGACGAAAATTTATTTTTCAGTCAGGTTTGCAGGCAAGGCCCAGAACTATTC
GGACCTCTGTAAAAGTGCCCAAGGGTATTAAACGCAAACGTTCATGACCGCTTTCGGTCT
CTCAATAAACAAATAAACCAAAATGGTATGTGAAGTATTTTTTACCATGTTCGTGACTAA
ACCGTCTTCGTCAACGCCAGAAACCGCACCCGGTTAATCAGATTATAAATGCACCTGGTG
CGATTTTATCACTGCTTTTGTGGAAGCACCGCAGGCGCCCGCCGAAA
>gi|396924|emb|X74468.1|_Human_papillomavirus_type_15_genomic_DNA
CAAGTAACTTGGCAGAACATTTTCTTGGAAGACAGCACCGATAACGGTAAGATTATATCT
TTGAACCGTAGGCGGTTCTTTCTGATTGGTTTGGCTGATAGTAGTTAACAACAATCACTC
TTTATAAATATATGTAACCGCCTGCGTTACTTTATTTAATCTACATACAATATGCTGAGT
AAACTATTTAGAGAGCTATATGGATAGGCCAAAGCCTTTTTCTGTGCAGCAGCTTGCAGA
CACTCTGTGTATACCTTTAGTAGATATATTATTGCCTTGTAGATTTTGTCAGAGATTTTT
AACATATATAGAATTAGTAAGTTTGAATCGTAAAGGTCTGCAGTTAATTTGGACTGAGGA
AGATTTTGTTTTTGCCTGTTGTTCTAGTTGTGCATTTGCTACAGCGCAGTTTGAATTTTC
TAACTTTTATGAACAGTCGGTGTGTAGTTGGGAAATAGAGATAGTAGAACAGAAGCCTGT
TGGAGATATTATTATTCGCTGCAAATTTTGTCTGAAGAAATTAGATTTAATTGAAAAGTT
AGATATTTGTTACAAAGAGGAGCAATTCCACAAGGTTAGACGCAATTGGAAAGGATTGTG
TAGACATTGTAGGGCGATAGAATGATTGGGAAAGAAGCTACTATACCAGATATAGTGCTT
GAGCTGCAAGAGCTTGTCCAGCCCACTGACCTGCATTGCTACGAAGAGTTAAGTGAAGAA
GAGACAGAGGAGGAGCCACGATTTATTCCTTACAAGATTGTAGTTCCGTGTTGCTTTTGT
GATTCCAAGCTTCGACTTATAGTGGTTGCAACTCCATTTGGAATTCGCTCACAACAAGAC
TTATTATTGGAAGAAGTTAAGTTGGTGTGTCCAGGGTGTCGAGAGAAGCTTCGCCATGTC
TGATGACAAAGGTACATATGATCCTAAAGAAGGCTGTAGTGATTGGTTTGTTCTAGAAGC
AGAATGCTCTGATGCTAGTTTAGATGGTGATTTGGAAAAGTTATTTGAAGAAGGTACAGA
TACTGACATTTCTGACTTAATAGATAATGAGGACACTGTACAGGGGAACTCCCGCGAATT
ATTATGCCAGCAAGAAAGTGAGGAAAGCGAGCAACAAATACACTGGCTAAAACGAAAGTA
TATCAGTTCACAAGAGGTTTTGCAGCTTAGCCCTCGCCTGCAGTGTATATCTATTTCGCC
ACAGCATAAGTCTAAAAGGAGATTATTTGAACAAGACAGCGGACTAGAACTATCATTTAA
TGAAGCTCAAGATTTTACTCAGCAGACTTTGGAGGTACCGGCGACCGATGTTGTGCCGCA
GGGTGCCAAGGGACTGGGCATTGTTAAAGATCTTCTTAAATGTAACAATGTGAAAGCAAT
GTTATTAGCTAAATTTAAAGAAGCGTTTGGAGTGGGGTTTATGGAATTAACTAGACAATA
TAAAAGCAGCAAAACATGCTGTAGAGACTGGGTACTGACTGTTTATGCTGTACAAGATGA
GCTGTTAGAAAGTTCTAAACAATTGTTGATTCAACACTGTGCATATATTTGGTTACATCA
AATACCCCCTATGTGCTTATATTTATTGTGCTTTAATGTTGGTAAAAGTAGAGAAACAGT
ATTAAGATTACTTACGAATTTGTTACAAGTATCTGAAATACAAATAATAGCAGAACCACC
AAAGCTTCGAAGTACACTGTCAGCATTATTTTGGTATAAAGGAAGTATGAATCCAAATGT
TTATGCTCATGGAGAATATCCTGAGTGGATAATGACACAAACAATGATAAATCACCAAAC
AGCAGAAGCTACACAGTTTGATTTATCTACTATGGTACAATATGCATATGATAATGAATT
GTCAGAAGAAGCAGAAATTGCTTGGCATTATGCAAAATTAGCAGATACAGATGCAAACGC
GAGAGCATTTTTACAGCATAACAGTCAGGCAAGACTTGTCAAAGACTGTGCAATAATGGT
TAGACACTATAGACGGGGAGAAATGAAAGAAATGTCTATGTCATCATGGATACATAAAAA
GTTATTGGTTGTTGAAGGAGAAGGACATTGGTCAGATATTGTAAAGTTTGTCAGATACCA
AGATATAAATTTTATACAATTTTTAGATTCATTTAAAAGCTTTTTACATAATACTCCTAA
AAAAAGTTGTATGTTAATATATGGTCCACCTGACACAGGAAAATCCATGTTTACTATGTC
ATTAATAAAAGTCTTAAAGGGTAAAGTTTTGTCATTTGCAAATTATAAAAGTACATTTTG
GCTTCAACCTGTGGCAGATACAAAAATAGCTTTAATAGATGATGTAACTTATGTTTGTTG
GGATTATATAGATCAATATTTAAGAAATGCATTGGACGGTGGTGTTGTTTGTTTAGATAT
GAAACACAGGGCGCCATGTCAAATAAGGTTTCCACCATTAATGCTAACTTCTAACATTGA
TATCATGAAAGAAGAAAGGTATAAATATTTACGCAGTAGAGTGCAAGCTTTTGCATTTCC
ACATAAGTTTCCTTTTGATAGTGATAATAATCCACAATTTAAACTTACTGACCAAAGCTG
GAAATCTTTTTTTGAAAGGCTTTGGAGACAGTTAGAGCTCAGTGATCAAGAAGACGAGGG
AGACGATGGATACTCTCAGCGAACGTTTCAATGCACTACAAGAGAATCTAATGGACATTT

ATGAGTCAGGTCGAGACGACATAGAAACTCAAATATTGCACTGGCAATATTTGAGGCAAG
AACAAGTATTATTCTATTTTGCCAGGAAACATGGAGTTATGCGTTTAGATATCAGCCTGT
ACCCCCTTTAGCCACCAGTGAGACCAAAGCGAAAGATGCTATTGGTATGGTGATTCTGTT
ACAAAGTTTGCAGAAGTCAGCATATGGCAAGGAGCCATGGACACTAACACAGACTAGTTT
GGAGACTGTGAGAAGTGCACCTGCAAACTGCTTTAAAAAAGGGCCACAGAATATTGAAGT
TATGTTTGATAAGGATCCTGAAAACATTATGGTATACACTGTTTGGACATACATTTATTA
CCAGACTTTAGATGACACATGGAACAAGGTGGAAGGAAAAATTGACTATCATGGCGCATA
TTATTTGGAAGGAACTCTTAAAGTTTATTACATACAGTTTGAAGTTGATGCAGCCAGGTT
TGGCAAAACTGGAATCTGGGAGGTGCATGTTAATGAGGACACTATCTTTGCTCCTGTTAC
TAGCTCTTCGCCGGCAGCTGGAGAAGGGGCAACCTCCATCGACTCCGCACCCGAATCGCC
GGCCAACAGACAGCTTTCTTCCACCTCCGTGTCCTCCAGAAAACGGACACCACCACGAAC
CGAAGCCAGACGCTACAACCGAAAAGAATCTAGCCCTACAACCACCACCACCCGGAGGCA
GAAAAGACAAGGACAAAGACAAGAAGACACAGCAAGGCGATCAAGGTCCACCTCAAGGGG
GAGACAAGAAATCTCCAGGGGAGGGAACCAGCGCCGACGGCGACGATCCCGAGAAACCTC
CATCTCCCCCGCCTGGGGAAGGGGAGGGAGAAGTAGAAGGGGGCCCACAACAAGGTCCCA
ATCGAAGTCCCTCTCACGATCCCGATCCCGATCCAAGTCGAGATCACGAGGGTCTTCTCC
ACGGGGTGGCATCTCGCCTGCAGACGTGGGAAGCTCAGTTCGATCACTTGGTAGAAAACA
TACTGGGCGACTTGAAAGATTACTGGAAGAGGCTAGGGACCCCCCAGTAATCTTGCTGCG
CGGTGATGCTAACAAATTAAAATGCTTTCGCTTTAGGGCAAAGAAAAAGTATCAGGATTT
AGTAAAATACTATAGCACCACGTGGTCCTGGGTAGGGGGTACAAGTAATGATAGAATTGG
ACGCTCACGATTGTTACTGGCATTCTCTTCCAACACCGAAAGAGAGCTCTTTATCAAAAT
AATGAAACTGCCACCAGGCGTTGATTGGTCGCTAGGATATTTAGATGATTTATGATTTTT
GTGCTTTTTAATCAACTAACAGTAGTGTTTTTTTATTGCTTTTGCTACTAACACTATACT
AACATTCCCATGGCCCGTGCACGCAGAGTAAAACGTGCATCTGTAACTGACATTTACAGG
GGGTGCAAACAAGCAGGTACCTGCCCCCCTGATGTTCTTAATAAAGTGGAACAAACAACT
ATTGCAGATAAAATTTTGAAATATGGAAGTGCTGCTGTATTTTTTGGTGGGCTGGGAATT
GGTACAGGCCGTGGTTCAGGTGGTGCTACAGGTTATGTTCCTTTGGGAGAAGGCCCTGGT
GTGCGCGTAGGAGGCACCCCTACCATTGTTCGCCCTGGGGTCACACCTGAACTTATTGGT
CCAGCGGATGTAATACCAATTGATACAGTCACACCAATTGACCCCGCAGCACCTAGTATT
GTCACAATAACTGACAGCAGCGCTGTTGACCTTTTACCTGAATTGGAGACAATTGCAGAA
ATACATCCTGTACCAACAGATAATGTAGATATTGATACTCCTGTTGTTACAGGAGGTCGG
GATTCCAGCGCTATTTTAGAAGTTGCTGATCCCAGTCCTCCTGTACGAACAAGAGTGTCT
CGCACACAATATCATAACCCATCATTTCAAATTATTACTGAGTCTACACCTTTGTCTGGC
GAATCTGCACTAGCAGATCACGTTATTGTTTTTGAAGGTAGTGGAGGTCAAAATATAGGC
GGTTCTCGCAGTGCTGCTTTGGATGCAGCACAGGAAAGTTTTGAAATGCAAACATGGCCT
AGTAGATACAGTTTTGAAATACAAGAAGGTACACCACCAAGATCTAGCACTCCTGTTCAA
AGAGCAGTACAATCCCTTTCTAGTCTTAGAAGGGCTTTATATAACAGACGGCTGACTGAA
CAAGTAGCTGTAACAGACCCTTTATTCTTGGGACGACCCTCACGCTTGGTGCAATTTCAA
TTTGACAATCCAACATTTGAAGAAGAAGTAACACAAACTTTTGAAAGAGATGTTGAAGCA
TTTGAAGAGCCTCCAGATAGGCAATTTCTAGATGTAGTTCGCCTAGGAAGGCCTACTTAT
TCTGAAACACCACAAGGTTATGTGCGTGTAAGTAGACTTGGTAGACGGGCCACAATTAGA
ACCAGAAGTGGTGCACAAGTTGGTGCTCAAGTACATTTCTATAGGGATTTAAGTACAATT
GATTCTGAAGCATTAGAAATGCAATTACTAGGAGAACATTCAGGTGATAGTACCATTGTT
CAGGCTCCTATGGAAAGTTCATTTATAGATATAAATATTGATGAGCCTGATTCATTACAT
GTGGGCCTACAGGACAGTACTGAAGCAGATGACATTGATTACAATTCTGCTGATCTTCTT
TTAGAAGATAATATAGAAGATTTTAGTGGTTCTCATTTGGTGTTTGGCAATACACGGCGC
AGCACTACAACATATACAGTACCTAGATTTGAATCACCTAGAAATACTGGGTTTTACATA
CAAGATGTGCATGGGTATAATGTAGCCTATCCGGAGTCCCGTGATACTACCGAAATAATA
TTACCACAATCTGACACGCCAACTGTAGTTATAAATTTTGAAGAGGCAGGTGGAGACTAT
TATTTACATCCAAGCTTAAAAACACGTAAACGAAAACGCAAATATTTGTAATTGTTTTAC
AGATGACATTGTGGCTACCGACGACGGGTAAAGTATATTTGCCACCAACACCACCTGTAG
CACGTGTACAAAGCACCGATGAATATGTGGAGAGAACTAATGTATTTTACCATGCAATGA
GTGACCGTCTGTTAACAGTAGGGCATCCCTACTTTGATGTTAGATCTGTTAATGGAGGTA
GCATAGAAGTTCCTAAAGTGTCTGGAAATCAATATAGAGCATTTAGGGTTACTTTTCCAG
ATCCTAATAGATTTGCATTAGCAGACATGTCTGTCTATAATCCAGAAAAAGAAAGGTTGG

TTTGGGCCTGTGTAGGCCTTGAAATAGGTAGAGGACAACCATTAGGAGTTGGTACTTCAG
GCCATCCTTTATTCAACAAAGTAAAAGATACAGAGAATAACAGTAATTATCAAGGCAACT
CTACTGATGACAGGCAAAATACATCTTTTGACCCAAAGCAGGTTCAAATGTTTGTAGTAG
GCTGTGTTCCATGTTTAGGCGAACACTGGGATAGAGCTCTTGTTTGTGAATCAGAGAGAA
ATAATCAGGCGGGAAAATGTCCTCCTTTGGAACTTAAAAATACAGTTATTGAAGATGGCG
ACATGTTTGATATAGGTTTTGGTAACATTAATAACAAGGCCTTATCAGTTACTAAGTCAG
ATGTGAGTCTGGATATAGTGAATGAAACTTGCAAATATCCAGATTTTTTAACTATGGCAA
ATGATGTGTATGGAGACGCTTGTTTTTTTTTTGCAAGACGAGAACAGTGCTATGCTAGAC
ATTACTTTGTCAGAGGAGGTGCAGTAGGTGATGCTCTTCCTGATGCAGCTGTCAATCAAG
ATCATAATTTTTATTTACCAGCACAATCAACCCAACAACAAAATAACTTAGCAAATTCTA
CTTACTTTCCCACAGTTAGTGGTTCTTTAGTGACATCTGATGCTCAGCTGTTTAATAGAC
CGTTTTGGTTAAGAAGAGCTCAAGGGCATAACAATGGCATACTTTGGGGTAATCAGATGT
TTATTACTGTTGCAGATAACACAAGGAATACAAATTTTACTATTAGTGTTACCTCTGATG
GTAATGCCATAAATGAATATAATTCACAAAATATCAGAGAATTTTTAAGACATGTGGAAG
AATATCAGTTATCTATTATTTTGCAATTGTGTAAAATACCTTTAAAAGCTGAGGTATTAA
CACAAATTAATGCTATGAATTCAGGTATTTTAGAAGACTGGCAACTAGGGTTTGTTCCTA
CACCAGACAACGCTGTACAAGATATTTATAGATATATTGACTCTAAGGCAACTAAATGTC
CTGATGCTGTACAACCAAAGGACAAAGAAGACCCATTTGGAAAGTATACATTTTGGAATG
TAGATTTAACAGAAAAGTTATCTTTAGATTTGGATCAATATCCTTTGGGAAGAAAATTTA
TATTTCAAGCAGGTTTGCAACGTCGCCCCAGAACTATTAAATCCTCTGTAAAAGTTTCTA
AAGGTACTAAACGCAAACGTACATGACCGATTTCGGTCGCTAATAAACAAGTAAACCAAT
AAGGTATGTGAAGTATTTTTTACCATGTTCGTGACTAAACCGCATAGGTCATTGCCAACA
ACCGCACCCGGTTAATCAGATATAAAATGCACCTGGTGCGATTTTATCACCGCTTTTGTG
GAAGCACCCGAGGCGCCCGCCAGAACTGCTGC
>gi|396918|emb|X74467.1|_Human_papillomavirus_type_14D_genomic_DNA
AACGGTAAGTTATTCTGCACCGGGTGCGGTCACTGTATTACTCACTATGTGGTTGTTGTT
GCCAACTACCATTGCTGATAGCATGTTTTTGCCTGTAACGTTATCGACACATACATATCT
ATGTATATATATATATATATATATATATATATATATATATATATATATACTACAGAAAAAAC
AGAGAATGCAGACTCATGGCGACAACTGACTCTTCAACAGACAGTGCAGATGAAGGTCCT
TCTCCTAAGAGTAACTATTGTGATAGCACAGAAACCAAATCTTCTTTTATAGAGCCACCA
TTACCTGCAACTATATTTGGCTTAGCAAACCTATTGGAAATACCACTAGATGATTGTTTA
GTACCTTGTAACTTTTGTGGTAATTTTTTGACTCATTTAGAAGTCTGTGAATTTGATGAG
AAAAAACTAAGTCTAATTTGGAAAGGTCATTGTGTATTTGCTTGTTGCCGTGTATGCTGC
ACAGCAACAGCAACGTATGAGTTTAATGAATTTTATGAGAGTACTGTTGAAGGCAGAGAA
ATAGAGAGTGTAACAGGCAAATCTATTTTTGATGTTGATGTCAGGTGCTATACCTGCATG
AAATTTTTAGATTCAATTGAAAAGCTTCGCATCTTTATAACTGCTACAGAATTTGCTCTT
AGAACCTTCCAGAACCTGTTATTTGAACAACTGCAGCTGTTGTGTCCTGAGTGCCGTGGG
AACTGCAAACATGGCGGATCCTAAAGGTAGTACATCTAAAGACGGGTTGGATGATTGGTG
TATTGTGGAAGCTGAATGTAGCGATATAGAAAATGATTTGGAAGAATTATTTGACAGAGA
TACAGACTCAGATATTTCAGAATTATTAGATGATAATGATGACTTGGACCAGGGAAATTC
TCGGGAACTATTTCATCAACAAGAGAGTAAGGAAAGCGAGGAGCACTTGCAAAAACTAAA
ACGAAAGTACTTGAGTCCTCAAGCTATCGCACAGCTTAGTCCGCGACTTGAAAGTATAAC
ATTGTCACCTCAGCAGAAGTCTAAACGAAGGCTCTTTGCAGAGCAGGACAGCGGGTTGGA
GTTAACTCTTACAAATGAAGCTGAAGATGTTTCTTCTGAGGTGGAGGTACCGGCTCTAGA
CTCTCAGCCGGTTGCTGAGGCACAAATAGGAACAGTAGACATTCATTATACAGAATTATT
ACGTGCCAGCAACAATAAGGCAATTCTTATGGCAAAATTTAAGGAGGCTTTTGGGGTAGG
CTTTAATGATTTGACACGTCAGTTTAAAAGTTACAAAACCTGCTGTAATCATTGGGTTCT
GTCTGTATATGCAGTGCATGATGATCTTCTTGAAAGCTCAAAGAAGTTATTGCAACAGCA
TTGTGATTATGTATGGATACGTGGGATAGCTGCTATGTCATTATTTTTATTGTGTTTCAA
AGTGGGAAAAAAATCGTGGGACAGTACATAAATTAATGACCTCAATGTTAAATGTGCATGA
AAAGCAAATATTGTCTGAGCCTCCAAAGCTACGAAATGTTGCTGCTGCATTGTTCTGGTA
TAAAGGTGCAATGGGGTCAGGGACATTTACTTATGGTCCCTACCCTGATTGGATGGCACA
TCAAACTATTGTTGGCCATCAAAGTACAGAAGCAAATGCATTTGATATGTCTGTTATGGT
GCAGTGGGCATTTGATAACAATTATTTAGATGAAGCTGATATAGCCTATCAATATGCTAA
GTTAGCACCAGAAGATAGTAATGCTGTGGCCTGGCTTGCCCATAATAATCAGGCCAGGTT

TGTTAGAGAATGTGCATCTATGGTTAGATTTTATAAAAAAGGTCAAATGAAAGAAATGTC
TATGTCAGAATGGATACATACTAGAATAACTGAAGTAGAAGGAGAAGGTCATTGGTCAAC
AATAGCAAAATTTCTTAGATATCAACAAGTAAACTTTATAATGTTTTTAGCTGCTTTGAA
AGATATGCTACATTCAGTTCCCAAACGTAATTGTATATTAATATATGGTCCTCCAAATAC
TGGGAAGTCAGCATTTACCATGTCTTTAATTCGTGTGTTAAGAGGAAGGGTGCTTTCATT
TGTTAATTCTAAAAGCCAATTTTGGCTGCAACCAATGTCAGAGTGTAAAATAGCTTTAAT
TGATGATGTCACAGATCCATGTTGGTTGTATATGGACACTTATTTGAGGAATGGCCTTGA
TGGTCATTATGTTTCTTTAGATTGCAAACATAAAGCACCGATACAAACTAAATTTCCTGC
ACTATTACTTACATCTAATATTAATGTACACAATGAAATAACGTATAGATATTTGCATAG
TAGAATTAAGGGATTTGAATTTCCAAATCCATTTCCAATGAAAGCAGACAATACACCTGA
ATTTGAACTCACTGACCAAAGCTGGAAATCTTTCTTTACAAGGCTTTGGAATCAATTAGA
GCTGAGTGACCAAGAAGACGAGGGAGACAATGGAGAATCTCAGCGACCGTTTCAATGCTC
TGCAAGATCAGCTAATGAACATTTATGAGACTGCAGCAAACACACTTGAGTCGCAAATTG
AGCATTGGCAAACTCTTCGAAAAGAAGCTGTGCTACTATATTTTGCTAGGCAAAATGGTG
TGACACGACTTGGATACCAAGTTGTGCCTACATTAGCCATTTCAGAAGCAAAAGCCAAGC
AGGCCATAGGGATGGTGCTGCAGTTGCAATCACTGCAAAAGTCTCAGTTTGGCAGTGAAC
CATGGTCACTGGTTGATACCAGTGGAGAAACATTTAGAAGTGCTCCAGAAAATCATTTCA
AAAAGGGTCCAGTATCAGTAGAGGTGATTTATGATAACGATAAAGACAATGCAAATGCTT
ATACTATGTGGAAGCACATATATTACCAGGATGATGACGAACAGTGGCATAAAAGTGCAA
GCGGGGTCAACCACACAGGCATATATTATATGCAAGGAACCTTTAGAAACTACTATGTTT
TGTTTGCTGATGATGCAACTAGATATAGTAAAACTGGACATTGGGAAGTTAAAGTTAATA
AGGAAACTGTGTTTACTCCTGTCACCAGCTCCACCCCTCCCGAGTCACCAGGAGGACAAG
CAGACTCAAACACCTCCTCCAAGACCCCCACCACCGCCACTGACTCCACGTCCAGACTCT
CGCCCGCAGATTCCAGAAAACAGTCACAACAAGCCAACACCAAAGGAAGAAGGTACGGAC
GCAGACCGTCCAGTAGGACCCGGCGAACGACCGAAACGCGGCAGAGGCGGAGATCGAGGT
CCAAGTCCAGGTCGCGGTCGAGGTCGCGGTCTCGGCTCCGATCTAGATCCCGGTCGCAAT
CGTCTGAGCGGCGGTCTCGGTACCGATCAAGATCCAGATCCAGACAAAAAGAAGTGTCCA
GAATCACAACCACCACCAGAGGGAGAGGTCGAGGGTCATCCTCCACCTCCTCCAAACGGT
CACAACGGGCACGAGGAAGGGGCCGTGGGGGGAGCAGGGGGAGACGGTCATCCTCCACCT
CCCCCACCTCCTCCAAACGGTCACGACGAGAGTCAGAGTCTTCTAGGCAGCGTGGCATCT
CTCCTAGTGACGTGGGAAAGTCACTTCAATCAGTTAGTTCAAGAAATACAGGAAGACTTG
GAAGGTTACTGGACGAAGCTCTCGATCCCCCAGTAATCTTAGTCAGGGGGGACCCTAACA
CGCTACGATGCTTTCGCAATAGAGCTAAGCAAAAGTTTACAGGGCTTTACAGGGCCTTTA
GCACGGCTTGGTCGTGGGTGGCTGGAGATGGCACTGAGCGTCTAGGCAGGTCCAGAATGC
TCATTAGCTTTTTCTCCTTTAACCAGAGAAGAGATTTTGATCAGACTGTTAAGTACCCGA
AAGGAGTGGACCGGTCGTTTGGCTCATTTGATAGCCTATAACACCCCTAACATACTAACA
TAATAGCTTGCTACTAACATCTAACATTTATTGCATTTTTGCTTTTTGTTTGCATTATTT
TAATGCTATGGCGCGTGCTAGGCGAGTCAAGCGTGACTCTGCTACTAACATTTACAGAAC
CTGCAAGCAAGCAGGCACGTGTCCTCCTGATGTCATTAATAAAGTTGAAAGCACAACTAT
TGCTGATAAAATTTTGCAGTATGGTAGTGCTGGTGTTTTTTTTGGGGGTTTGGGCATAAG
CACTGGAAAAGGTACAGGAGGTACCACAGGCTATGTGCCTTTGGGAGAGGGCCCAGCAGT
ACGTGTTGGTGGTGCGCCAACAATTATCAGACCTGCTCTGGTCCCAGACACCATTGGTCC
ATCAGATATTATACCTGTGGACACCTTAGATCCAGTGGAGCCTACGACCTCTTCTATTGT
TCCACTCACGGATTCCACAGGACCAGACCTTTTGCCTGGCGAGGTGGAAACTATTGCAGA
GGTGCATCCTGGCCCGTCTAGGCCTCCTACTGACACTCCTGTCACAACTAGTACAGGAGG
CTCCAGTGCTATATTAGAAGTAGCACCGGAACCTACTCCGCCCTCACGTGTTAGGGTGAC
CCGGACACAATATCATAATCCCTCCTTTCAAGTAATTACCGAATCCACCCCTACCACAGG
TGAAAGTTCATTAGCAGACAATATATTGGTTACCTCTGGTTCTGGGGGACAAACTATTGG
AGGCGCTACACCTGAACTTATAGAACTTCAAGAGTTACCATCTAGATATTCATTTGAAAT
CGAAGAACCAACACCCCCTAGAAGAACTAGTACCCCATTACAAAGGATACAGACAGCTAT
AAGAAGGAGGGGTGGGCTTACAAATAGGCGCTTAGTCCAACAAGTTTCTGTAGAAAACCC
CTTATTTTTAACAAGACCATCTAGACTAGTGCAATTTCAGTTTGATAATCCAGCATTTGA
GGAGGAAGTAACACAAATATTTGAACAAGATATTGAAGATTTTAATGAGCCTCCAGACAG
AGATTTTCTAGATGTTCAAAGGCTGGGTAGGCCCCAATATTCAGAAACTCCAGCAGGGTA
TCTCCGAGTTAGTCGTCTTGGGCAAAGGCGGACTATACGCACTCGTTCTGGAGCACAAAT

TGGGTCTCAAGTTCATTTTTATAGAGATCTAAGTAGTATAAACACAGAAGATCCTATTGA
GCTTCAATTATTAGGTCAGCATTCTGGGGATGCTACTATTGTCCAAGGTCCAGTTGAAAG
CACATTTGTAGACATAAATGTAGATGAAAATCCACTTTCTGAGGATTTTAGTGCACATTC
TGATGACTTGCTTTTAGATGAAGCTAATGAAGACTTTAGTGGCTCTCAATTAGTTGTGGG
TAATCGACGCTCAACATCTTCATATACCGTCCCTCGTTTTGAAACAACCAGATCTGGGTC
ATATTATGCACAGGATACAAAAGGTTATTATGTAGCTTATCCTGAGGATAGGGACATTAG
CATGGATATTATTTATCCTACCCCAGAGTTGCCTGTTGTCATTATTCACACATATGATAC
AAGTGGTGATTTTTACCTGCATCCTAGTCTTCACAAAAGACTCAAAAGAAAACGAAAATA
TTTGTAACTTTTTCTTTTACAGATGGCAGTTTGGCAAGCAGCTAGTGGTAAGGTTTACCT
TCCACCATCTACACCAGTTGCCAGGGTCCAAAGTACGGACGAATATGTGCAAAGGACTAA
CATCTATTATCATGCATACAGTGACAGATTATTAACTGTTGGTCATCCATATTTCAATAT
ATATGACGTGCAAAGTGCTAAGATAAAAGTACCAAAAGTATCTGGAAATCAACATAGGGT
TTTCAGACTAAAGTTGCCAGACCCTAATCGATTTGCATTAGCTGACATGTCTGTTTATAA
TCCAGATAAAGAAAGACTGGTTTGGGCATGCAGAGGTATAGAAATAGGCAGAGGACAACC
TTTAGGTGTAGGTAGTGTAGGACATCCATTATTTAATAAGGTTGGTGATACAGAAAATCC
CAACTCATACAGGCAACAAGCTAACTCCACTGATGACAGACAAAATGTGTCATTTGATCC
TAAGCAACTGCAAATGTTTATAATAGGCTGTGCACCTTGCATGGGGGAACATTGGGATAG
GGCCTTGCCATGTGTAGAAGATAAACCACCCCCTGGTTCTTGCCCTCCAATTGAATTAAA
AAATACAGTGATTGAAGATGGTGACATGGCAGATATAGGCTATGGAAATTTAAATTTTAA
GGCATTACAAGAAAATAGATCTGATGTAAGTTTGGATATAGTTAATGAAATTTGCAAATA
TCCAGACTTTCTGAAAATGCAAAATGATGTATATGGAGATTCCTGCTTTTTTATGCACG
CAGGGAACAATGTTATGCCAGACACTTTTTTGTTAGAGGGGGTAAGACAGGAGATGACAT
ACCAGCAGCACAAGTTGATGAGGGTAGCCTAAAGAATGTTTATTACATTCCACCAATGAC
AAATCAACCACAAAACAATATTGGCAATGCCATGTATTTCCCAACTGTCAGTGGCTCATT
GGTATCCAGTGATGCTCAACTGTTCAATAGACCATTTTGGTTACAGCGCGCACAAGGCCA
CAATAATGGTATTTGTTGGTTTAATCAGTTATTTGTTACTGTTGTGGACAACACACGTAA
CACAAATTTTAGTATATCAGTTAGTTCAGAAAACACTGAGGTATCCAAAATTGACAATTA
TACCTCTCAGAAATTTCAAGAATATTTAAGACATGTAGAAGAATATGAAATGTCTCTAAT
TTTACAACTATGTAAAATACCTTTAACAGCTGAAGTCTTAGCTCAAATTAATGCAATGAA
TTCTAATATTTTAGAGGAGTGGCAATTAGGATTTGTACCTGCACCAGACAATCCTATTCA
TGATACATACAGATATATTGAGTCTGCAGCGACTAGGTGTCCTGATAAAAATCCTCCTAA
AGAAAGAGAAGATCCTTATAAAAACTTTAACTTTTGGAATGTAGATTTAACAGAGAGACT
ATCTTTAGACCTAGATCAATATTCTCTTGGGAGAAAATTTTTATTTCAGGCAGGTTTGCA
GCAATCGACCGTTAACGGTACAAAAACAGTTTCGACTAGGGGATCCATCAAGGGTATTAA
ACGAAAACGCAAGAATTAGACATTATCGATTTCGGTGCAATAAAGTCAACTTTTACACAG
TATTCAAGGAATGTTTATTCACTCTGACTAAGCAAATATGAGCCGCGCCCGATACATAAA
GGTGCCAAATGAGGTGAGTTGTTTGCCAGAAGAGGTCAGAGCCAACTCAGGTTTGCGCCA
GATCAGATACAGCGCGAGCCGCGTTGGATCAAGCTACATCGTCTGAACACGCAAAAGACT
CAAGGAAATGTAAGTGTGCCAGTCTATTGTGTTCGAATTTGGCAAAGTTGAAGACCGTT
>gi|396910|emb|X74466.1|_Human_papillomavirus_type_12_genomic_DNA
TTGTACCAGGTGCGGTACGATTTCCCAATAGCACATTATACTAGATTGTTGTTGCCAACT
ACCATCATCAGTTTCAAGTTTTTGCCTGTATCGTTTTCGTATCATACTAATTCTGTATAT
AATTAAATAAATAAATAAATATATATATATATATATATATAATGTATAAGGCTTGGTTCTTT
TGCAATGTGATTGGGACAAATGGCACAGCAGGCCGATCAGCAGACAGTGACAGACAGTAC
GCCTGAGCTGCCCACAACTATTAAAGAGTTAGCTGACCTTTTAGATATACCTTTAGTTGA
CTGTTTGGTACCTTGCAATTTTTGCGGAAAGTTCTTAGATTTTCTGGAAGTGTGTGATTT
TGACAAAAAGCAGCTAACACTAATTTGGAAAGGTCATTTTGTTACTGCTTGCTGTCGAAG
TTGTTGCGCAGCTACTGCAATATATGAATTTAATGAATTTTATCAACAAACAGTGCTAGG
TAGAGATATAGAGCTTGCTACTGGAAAATCTATATTTGACTTAAAGATAAGGTGTCAGAC
GTGCTTGTCATTTTTAGATACAATTGAAAAGTTAGACAGCTGTGGTCGGGGCCTTCCGTT
CCACAAGGTTAGAGACAGGTGGAAGGGAATTTGCAGACAGTGCAAGCATTTGTATCTGAA
TAATGATCGGTAAAGAGGTCACCGTGCAAGATTTTACCTTGGAGCTTAGTGAGCTGCAGC
CTGAAGTGTTACCAGTTGACCTGCTTTGTGAAGAGGAATTACCAAACGAGCAGGAAACGG
AGGAGGAGTCAGATATCGACAGGACTGTATTCAAAATCATTGCACCGTGTGGCTGCAGCT
CCTGTGAGGTCAACCTTCGTATTTTTGTCAACGCAACTGATACTGGCATTAGGACCCTAC

AGGACCTGCTGATCAGTGACCTGCAGCTGCTGTGCCCAGAGTGCCGTGGTAACTGCAAAC
ATGGCGGATTCTAAAGGTAGTACCTCTAAAGAAGGGTTAAGTGATTGGTGTATTTTGGAA
GCAGAATGTAGTGATTTAGAGAATGATTTTGAACAGTTATTTGAGCAAGATACAGACTCC
GATGTATCGGACTTGCTAGATAATGGTGAACTTGAACAGGGGAATTCTCTGGAACTATTT
CATCAACAAGAGTGTGAGCAAAGCGAGGAGCAATTACAAATTCTAAAACGAAAGTATCTT
AGTCCAAAAGCTGTCGCGCAGCTTAGTCCGCGTCTCGAGTCGATATCGTTGTCACCTCAG
CAGAAATCGAAACGAAGGCTCTTTGCAGAGCAGGACAGCGGACTCGAGCTATCTCTAAAC
AATGAAGCTGAAGATGTTTCTCCTGAGGTGGAGGTACCGGCTATAGACTCTCGGCCGGTA
GATGAGGGAGGATCAGGGGCCATAGATATTGATTATCTGTCATTGCTGCGTAGTAGCAAT
ATTAAAGCCACGTTAATGGCAAAATTCAAAGAGTCATTTGGGGTAGGCTTTAATGAATTG
ACTCGCCAGTTTAAAAGTTACAAAACCTGTTGTAACGATTGGGTTTTAGCTGTGTATGCA
GTTCATGATGATCTATTTGAAAGCTCAAAGCAGCTGTTGCAACAGCATTGTGACTATATC
TGGGTCCGTGGGATAGGAGCTATGACACTTTACCTATTGTGTTTCAAAGCGGGAAAAAAT
CGCGGTACTGTGCATAAGTTAATGACATCAATGCTAAATGTGCAAGAACAGCAGATTTTG
TCTGAGCCTCCTAAGTTAAGAAATACAGCTGCTGCATTGTTCTGGTACAAAGGTGGTATG
GGGTCAGGCGCATTTACCCATGGCACATATCCTGATTGGATTGCACATCAAACAATTTTG
GGCCATCAAAATGCTGAAGCAAGCACATTTGATTTTTCAGCCATGGTCCAATGGGCCTTC
GATAATAATTACTTAGAAGAACCAGATATAGCTTATCAATATGCCAAGCTTGCACCAGAA
GATAGCAATGCAGTAGCATGGCTAGCACATAATCAACAAGCTAAATTTGTAAGAGAGTGT
GCAGCAATGGTACGGTTTTATAAAAAAGGACAAATGAAAGAAATGAGTATGTCAGAGTGG
ATACACACAAAAATTAATGAAGTAGAAGGTGAAGGGCATTGGTCAGATATAGTAAAATTT
TTAAGATATCAAGATGTAAACTTTATTACCTTTTTGGCAGCATTTAAAAACTTTTTGCAT
GCTGTACCAAAACACAATTGTATTCTTATATATGGGCCCCCTAACTCTGGAAAGTCATCA
TTTGCAATGTCACTGATAAAAGTTTTAAAAGGTAGAGTGTTGTCATTTGTGAATTCCAAA
AGTCAATTTTGGTTACAACCTCTTGGAGAAAGTAAAATTGCATTGTTGGATGATGTTACA
GACCCTTGCTGGGTATATATAGACACATATTTAAGAAATGGCTTAGACGGCCATTTTGTT
TCTTTAGATTGTAAATATAAAGCGCCCGTACAAATTAAGTTTCCTCCATTGTTACTCACA
TCTAATATTAATGTTCATGGAGAAACAAATTATAGATATTTACATAGTAGGATAAAGGGA
TTTGAATTTCCACATCCCTTTCCTATGAAACCAGACAATACACCTCAGTTTCAGTTAACT
GACCAAAGCTGGAAATCTTTTTTTGAAAGGCTTTGGACACAATTAGACCTGAGTGACCAA
GAAGAGGAGGGCCAACATGGAGAATCTCAGCGAGCGTTTCAATGTTCTGCAAGATCAGCT
AATGAACATATATGAAGCTGCAGAACATACACTTGAGACACAGATTGCCCATTGGACACT
TTTGCGAAGAGAAGCTGTTTTGCTTTATTATGCTAGGCAAAAGGGTATTACAAGGCTTGG
ATACCAACCGGTGCCTACATTGGCAGTTTCTGAAGCAAAAGCAAAAGAGGCTATAGGGAT
AATGCTGCAATTGCAATCTCTACAGAAGTCTGAGTATGCTTCGGAGAACTGGACATTAGT
GGACACAAGTGCAGAAACGTATAACAATGTTCCAGAACATCACTTTAAAAAGGGTCCAGT
GCCTGTGGAGGTCATTTATGATAAGGAGCCAGAAAATGCAAATGTGTATACAATGTGGAA
GTATGTGTATTATATGGACCCAGAGGATGTATGGCATAAAACCACAAGTGGTGTAAATCA
GACGGGCATTTACTATTTACATGGAGACTTTAAACACTATTATGTACTTTTTGCTGATGG
TGCACGAATGTATAGTAAAACTGGACAATGGGAAGTTAAGGTTAATAAGGAAACTGTGTT
TGCTCCTGTCACCAGCTCCACACCACCCGGGTCACCAGGACAAAGAGACCCAGACGCCAC
CAGCAAGACCCCCGCCACCTCCTCCGACTCCACGACCAGATCCAGTGACAAACAGTCACA
ACAAGCCGACCCCAGGAGGAAAGGATACGGACGACGACCATCTAGCAGAACAAGGCGACA
GGAAACGCAGCAAAGGCGATCAAGGTCGCGATACCGCTCCCAGTCTAACTCCCGGTCGCG
CTCCCAGTCCCAAACCAGGGCCCTTGGCGCCACCTCCGTATCCAGGTCCTCCAGGTCCCC
GTCGGTCACACAGATTAGGAACCGGAGGTCGCGATCGCAATCCAGAGGAAGGGGGGGTCG
AGGGTCATCCACCGACACCACCACTAAGCGGCGGAGATCCAGATCATCCTCCTCCAACAC
CAGAAAACGGTCACAACGGGGAGAAAGAGGACGGGGAGAAAGGGGCGGTAGGGGGAAGCG
ACGAGACCGATCATCCTCCACCTCCCCCACCCCAAAACGATCCCGAGCAGGGTCTAGGTC
TAGCAGGCAACGTGGCGTGTCTCCTGAGCAAGTGGGAAGATCTCTTCAATCTGTTAGTTC
AAAACATAGAGGACGACTTGGGAGATTATTGGAAGAAGCTCTCGACCCCCCAGTGATCAT
TTGCAAAGGGGGGGCAAACACGCTAAAATGCTTTCGCAACAGAGCTAGACACAAATATAC
AGGGCTGTTTAAGGCTTTTAGCACAACATGGTCATGGGTGGCTGGAGATAGCACTGAGCG
TCTAGGCAGGCCCAGAATGCTCATTAGCTTTACTTCAACTAATCAGAGAAAGGATTTTGA
TGAGACTGTGAAATATCCGAAGGGAGTTGAAACTGCATATGGCAACTTAGACAGCCTATA

ACATAACTAACTTTGCTTTGCTACTAACCACACTAACAATTTTTTACATTTATTTTTAC
GTTTTTATTATTGCTATGGCGCGTGCTAAGCGGGTCAAGCGAGACTCTGTTACTCATATA
TATCAAACCTGCAAACAAGCAGGCACTTGCCCCCCTGATGTTCTCAATAAAGTGGAGCAA
ACAACAGTTGCTGACAATATTTTGAAATATGGTAGTGGTGGTGTCTTTTTTGGAGGCCTC
GGTATTGGTACCGGTCGCGGTACTGGAGGTGTCACTGGATACAGACCTCTACCTGAAGGG
CCCGGTATCCGTGTTGGAGGGACTCCCACGGTTGTAAGGCCTTCACTTGTTCCTGAATCT
GTGGGTCCAGCAGATATATTACCAATAGACACTATCGATCCTGTGGAGCCCACTGCTTCC
TCGGTAGTTCCATTAACTGAATCCTCAGCAACTGATCTACTTCCAGGAGAAGTTGAGACA
ATAGCTGAGATTAATCCTGTTTCAGAGGGCCCTACGATTGATTCACCTGTTGTGACAACA
AGCAGAGGCTCCAGTGCAATATTGGAAGTTGCACCAGACCCTATACCTCCAACACGTGTT
AGAGTGGCACGCACACAGTACCATAATCCTGCTTTTCAAATTATAACAGAGTCCACACCT
GCTCAAGGTGAAACATCTTTGGCAGACCACATCCTGGTCACATCTGGCTCAGGAGGTCAA
ACTATAGGTAGTGACATAACAGACATTATTGAATTACAGGAAATACCCAGTAGATACTCC
TTTGAAATAGAAGAGCCAACCCCCCCCCGGCAAAGCAGCACTCCACTTCAGAGGACACAA
ACCACTGGCCGACGTAGAGGAGTGTCCCTAACAAATAGAAGGCTAGTACAACAAGTGCAA
GTTGATAATCCTTTATTTATAGATAAACCTTCTAAGTTAGTACGCTTTTCATTTGATAAC
CCTGTATTTGAGGAGGATATAACAAATATTTTTGAACAGGACTTAGAAACATTTGAGGAG
CCACCTGATAGGGATTTCCTTGATATTAAAAAGCTAAGTCGACCTCAATACTCTACAACA
CCTGCTGGATATGTTAGGGTCAGTAGGCTAGGCACGCGGGGTACCATTCGCACACGTTCC
GGAGCTCAAATAGGATCACAGGTTCATTTTTATAGAGACTTAAGTTCTATTGATTCAGAA
GACCCTATTGAGCTACAATTATTAGGCCAACATTCAGGTGATGCAACCATAGTGCAAGGC
ACTGTTGAAAGTACATTTGTAGATATGGACATAGCGGAAGATCCTTTGTCTGAAAGTATT
GAGGCTCATTCTGATGACCTATTACTTGATGAAGCTGTGGAGGATTTTAGCGGGTCACAA
TTAGTTATTGGAAACAGACGCAGCACAACATCATACACTGTGCCCCGTTTTGAAACTACA
AGGAGCAGTTCTTATTATGTGCAAGATACACAAGGTTATTATGTGGCTTATCCTGAACAT
AGAAATACTGCAGAAATCATTTATCCAACACCAGATATACCTGTGGTAGTTATACATACA
CATGATAATAGTGGTGACTTTTATTTACATCCTAGCCTGCGGAGGCGCAAGCGTAAAAGA
AAATATTTGTGATTTACTTGCAGATGGCTGTGTGGCAAGCGGCCCATGGTAAGGTCTATC
TACCACCATCAACACCAGTGGCCAGGGTGCAAAGCACGGATGAATACATTCAAAGGACTA
ACATCTACTATCACGCCAATACTGACAGACTACTCACTGTAGGACATCCATATTTCAATG
TTTATGATAACACTGGTAAAAAATTGGAGGTTCCTAAAGTGTCAGGAAATCAACACAGAG
TATTTCGCCTCAAATTGCCAGATCCAAACAGATTTGCTTTAGCTGACATGTCTGTATATA
ATCCTGATAGGGAAAGGTTGGTCTGGGCCTGCAGAGGATTGGAAATAAGTAGGGGTCAGC
CTTTAGGCGTTGGAAGTACTGGACATCCCTATTTTAACAAAATTAAAGACACGGAAAACT
CAAATAACTATGCCACAGGCAGTAAGGATGATAGACAGAACACATCATTTGATCCTAAAC
AAATCCAAATGTTTATAGTGGGCTGTACACCTTGTGTGGGAGAACACTGGGAGAAAGCCT
TACCCTGCGGGGATGCACCAGCTGAAAATGGTGTTTGCCCTCCCATAGAGTTAAAGAACA
CTTTCATTGAAGATGGTGACATGGCAGATATTGGTTTTGGCAACATGAATTTTAAAACAT
TGCAACAAAACAGATCTGATGTCAGCCTTGACATAGTGAATGAAACTTGCAAATATCCAG
ATTTTTTGAAAATGCAAAACGATGTATATGGAGATGCTTGCTTTTTTTATGCTCGTAGAG
AGCAGTGTTATGCCAGACATTTCTTTGTGAGAGGAGGTAAAACAGGTGATGACATTCCTG
ACGCACAAATTGATGATGGCAATATGAAAAATCAGTATTACATTCCTGGAGCTCAGGATC
AATCTCAAAAGGATATAGGTAATGCGATGTATTTCCCAACTGTAAGTGGCTCATTAGTTT
CTAGTGATGCTCAGTTGTTTAATAGGCCCTTCTGGCTTCAAAGAGCACAGGGTCATAATA
ATGGCATCCTGTGGGCTAATCAGATGTTTGTCACAGTTGTAGACAATACACGAAACACCA
ATTTCAGTATATCTATTTACAGTGATAATCAAAATGTACACGACATTCCAAATTATGATT
CTCAAAAATTTAGAGAATATTTAAGACATGTAGAGGAATATGAAATTTCTCTTATTTTAC
AATTATGTAAAGTTCCTTTAAAGGCAGAAGTGTTAGCACAGATAAATGCAATGAACTCTT
CTTTGCTGGAGGACTGGCAATTAGGCTTCGTGCCAACTCCTGACAATCCTATTCATGACA
CATACAGATATATCGAATCTCTGGCTACTAGGTGCCCTGATAAAAATCCTCCAAAAGAAA
AGCCGGACCCTTATGATGGCTTAAGTTTTTGGACTGTAGATATGACTGAGAGACTTTCTT
TAGACCTGGATCAGTATTCCTTAGGGCGCAAGTTCTTATTCCAGGCTGGCCTCCAACAAA
CGACCGTTAACGGTACAACAAAATCATCAAGCTATAGAAGTTCCATAAGGGGGACCAAAA
GAAAACGCAAAAACTAAATGTACCGAATTTGGTACAATTACCTCAACTTTTGCACAGTAT
TCAAGGAATGTTTGTTTACTCTGACTAAGTCTAACTCTACCAAGGAAACAGACCGCGCCC

GGTACATAAAGGTGAGTTGGTGCCAAATTAGTCTCACTTTGTTGCCAGAACATACCGTGT
TCGTCCTAACATGCTCGGATTAGGTCGACCGCCAAAGGACCTTTGGTTTGCCAAATAGCT
TACAGCAGCTCAGTTCTGGCACATTTGTGGACCGATAACGGTAAGTCTCATTC
>gi|312100|emb|X70829.1|_Human_papillomavirus_type_65_complete_genome
ATTATTAATGATAGTTGGCAACAACCATCATCTAAAGTAATATATAACCGGAAGAGATAC
ATATAAAAAGACACATTGTATTCCTGATAAATCCCATCCAGATGGCAGATGGCAGACCTG
CAGCTTTGGACGACTTCTGCAGACGATTTGATATTTCTTTTTTTGATTTGCATCTTACTT
GTATTTTTTGTTCTCATACTGTTGATCTGCAAGATCTTGCTTCGTTTTATCTTAAGAAGC
TTAGTTTAGTTTTCAGGGGGGGTTGCTACTATGCATGCTGTTCTGAATGCTTACGTTTAA
GTGCAAGATTTGAGCAAGAGAATTATTTTCAGTGTTCTATAAAAGCTGTTAATTTGGAAG
AAGTAGCTCAAAGGAAAATTAAGGAGATTTGCATCAGATGTATATGTTGTTTGAGACTAC
TAGACATTGTTGAGAAATTAGACTTGCTGTACTCTGACCAGGCCTGCTACTTAATAAGGG
GTTTGTGGAGGGGCTATTGCAGAAATTGCATTAGGAAACAATGAGAGGAGCAGCACCCAG
GGTTGCAGATCTTAATTTAGAATTAAATGACTTGGTGTTGCCTATAAACCTGCTGAGTGA
GGAGGTCTTGCAACCTTCAGATGATGAGTCTGAGGCTCCAGAGGAGGAGCTTTTTCCTTT
TAGAATAGACACCTGTTGCTATAGATGTGAAGTTAATGTAAGGATTACTCTGTTTGCTGT
GGAATTTGGACTTCGAGCGTTGGAACAACTCATAGTGGACGGAAAGCTGACGTTTTGCTG
TACTACTTGTGCAAGAACCTTAAGAAATGGCAGATAAAGGTACAGAAAATTTTGACTTAG
AAGGGAGTAGTTGGTATATTGTGCATGAAGCAGAATGCACAGACAGTATAGACACTTTGG
AGGATTTGTGCGACGAAAGCGATTCAAATGTTTCCAATTTAATTGATGACGATGTGGTTG
ATCAGGGGAATTCCCTGGCACTGTACAATGCAAAAATAACTGATGATTGTGATAATGCAA
TAGCACACCTAAAACGAAAGTATAACAAAAGTCCAGAGCAGGCAGTTGCTGAATTGAGCC
CTCAGTTGCAGGCTGTGAAAATTACTCCTGAAAGAAACAGCAAAAGGAGATTATTTCAGG
AGGACAGTGGGATATTTGAAGATGAAGCTGAAAATTCTCTTACACAGGTAGAATCTAACA
GCCAGACTGGTGGCAACAGCCAAGATGGCGGAGGAGATATTAACTTACTGTTGTTACAAA
CTAGTAATAGGCGTGCTACAATGCTTGCAAAATTTAAAGATTGGTATGGGGTTTCATATA
ATGAAATAACTAGAGTTTATAAAAGTGATAAGTCTTGTAGTGATAATTGGGTTATAGTTA
TCTTTCGAGCTGCTGTTGAAGTTTTGGAAAGCTCTAAAATTGTTTTGCAACAGCACTGTA
CATATATTCAAGTTAAAATATTTGGATTTTCAGCTTTATATTTATTACAATTCAAAAGTG
CGAAAAGCAGAGAAACGGTACAAAAGTTAATGTGTTCTATGTTGAATATTCAAGAGTTTC
AAATATTGACTGACCCTCCTAAGTTACGAAGTGTGCCTACAGCTTTATACTTCTATAAGC
AAGCCATGTTAACAGAGAGCTTTGTTTTCGGACAGACACCAGATTGGATTGCAAAGCAAA
CCTTAGTAAGCCATCAAGCAGCAACTACTGCAGAAACATTCGAATTATCAAAAATGGTTC
AGTGGGCTTATGATAATAATCTGCTGGAAGAATGTGATATTGCGTATCATTATGCTATGT
ATGCTGATGAAGATGCTAATGCTGCAGCCTATTTAAAGAGTAATAATCAAGTAAAACATG
TTAGAGATTGCAGTACAATGGTTAGAATGTATAAAAGATATGAAATGAGGGATATGTCAA
TGTCAGAATGGATTTATAAATGTTGTGATGAATGCACCGAAGAAGGTGATTGGAAACCTA
TTTCTCAATTTTTAAAATATCAAGGTGTCAATATTCTTTCTTTTCTTATAGTTCTAAAGT
CTTTTTTAAAAGGTATTCCTAAGAAAAATTGCATAGTCATTCATGGCCCACCAGATACTG
GAAAATCTCTGTTTTGTTATTCTCTAGTGAAATTTCTTAAAGGTAAAGTAGTATCCTATG
TGAATCGAAGTAGCCACTTCTGGTTGCAGCCTTTAATGGATTGTAAGGTAGGATTTATGG
ATGATGCAACCTATGTATGCTGGACATACATAGATCAGAATTTGAGGAATGCATTAGATG
GAAATCCAATGTGTATAGATGCAAAACATAGAGCTCCACAGCAATTAAAATTACCTCCTA
TGTTAATAACATCAAATATTGATGTGAAACAGGAACAATCATTAATGTATTTGCATAGTA
GGGTCCAGTGTTTTAGTTTTCCTAATAAAATGCCTTTTTTAGATGATGGTTCTCCCATGT
ATACATTTACTGACGCAACGTGGAAATCTTTTTTCCAAAAGCTTGGCAGACAATTAGAGC
TAACAGATCCTGAAGAGGAAAGCAATGGAGTCCCTAGTCGCGCGTTTCGATGCACTTCAA
GAAGCAATTCTGACTCATATTGAGTCTCAGGACGATACTTTGGAATCCCAAATTCGATAT
TGGGAAAATATCAGAAAAGAAAATGCAATAATGCATTTTGCACGAAAACAAGGCCTAACA
AAATTAGGTCTGCAACCACTTCCCACATTAGCAGTAACTGAATATAATGCAAAACAAGCA
ATACAAATACATTTGACTTTGCAGTCATTATTAAAGTCTCCTTATGGATCAGAGCGTTGG
ACTCTGCCAGAAGTTAGTGCAGAACTGATTAATACTGCTCCACAGAACTGTCTAAAGAAA
GGAGGTTATGATGTGTCTGTATGGTTTGATAATGATAGATATAATGCTATGGTGTATACA
AATTGGGATTATCTATATTATCAAGATGTCAATGAAATATGGCATAAAGTTAAAGGTGAA
GTGGATTATGATGGCTTATACTTTACAGACCATACAGGAGAACGTGCATACTTTACTCTG

TTTAGCACAGATGCTCACAGATTTAGCAGAACTGGACTATGGACTGTGCATTTTAAAACA
CAAGTTATTTCCTCCTCTGTTGTCAGCTCAACAAACACCCCCTCCTTCGACTTTGAGGAA
CAACAACTACCCGGCCCTCAACACCCACCTACACCGAGCTTACCCAGGCGAGCCCTTGT
GGTAGGGGGAAATCGAGGGAATCTCAACCGACCTCCACAACGTCCCCCGAAACCTCGGGG
CTACGAGTACGACGAGGACGACGACAAAGAAAATCAGGGCCCGGGCCAGGAGAGACCCCC
AGCAAAAGAAGAAGAGGAGGAGGGAGAGGAGGAGGAGAGACCAGATTGGAGTCTGCGCCA
TCTCCTGGGGAAGTGGGAATCAGACATAGAACAGTTGAAAGACAAGGTCTGTCGCGACTT
GGACAACTACAAGCTGAAGCTAGGGATCCACCCATGATATTGTTAAAGGGCACAGCAAAT
TCGTTGAAATGTTGGAGATATAGGAAACAAAATTCCAGTAACTGTGGGTTTCTCTTTATG
AGCACTGTTTGGAACTGGGTTGGTGATGTGTCAGAAAATCACAGTCGCATGTTAATTGCT
TTTAAAAGTCCTGGTCAGAGAGACTCATTTGTTAAACACAATCTATTCCCAAAACTCTGT
ACATATACCTATGGTTCTTTAAATAGCTTATAAAATGCAAGCCTCACGCAGAACAAAAAG
AGACTCAATACCAAACCTCTATGCAAAATGTCAATTATCTGGCAACTGTCTTCCCGATGT
AAAAAATAAAGTAGAAGCTGATACCCTTGCTGATCGTTTGCTCAGATGGTTGGGAAGTGT
CATATACTTAGGAGGTTTGGGAATTGGGACGGGAAGAGGCAGTGGAGGGTCTTCTGGTTA
TAATCCACTGGGAGCCCCTAGCAGAGTAACACCTAGTGGAACTGTTATAAGACCAACAGT
CCCTGTAGAAGGTCTGGGGCCTAGTGAAATCATCCCTGTAGATGTTGTAAACCCTGGCAG
TTCTTCTGTGGTTCCTTTAGAGGATTTAACAGTTCCAGAAGTCACCATAGATAGCGGGGA
GGTGGGAGGAGGTGGTCTTCATCCTTCTGAGATAGATGTTGTCACTTCCTCAGACCCTAT
TTCAGATGTCACTGGTACAAGTAGCCACCCTACAATAATATCTGGCGAAGATAACGCCAT
TGCAGTCCTAGATGTTTCTCCAACAGAGCCCCCTACCAAACGCATAGCTTTAGGAACTAG
GGGCGCAACATCAACACCACATATAAGTGTAATATCAGGCACGACCGAATTCGGTCAGTC
TTCAGACCTAAATGTGTTTGTTAATGCCACATTTTCTGGAGATTCTATTGGATATACAGA
AGAAATTCCTTTAGAAGAGTTGAATACTATCCAGCAATTTGAAATAGAAACCCCTCCAAA
AACTAGCACACCACGTGAGACTATTGGACGTGCTTTGGAAAGAGCGCGAGATCTTTATAA
CAGAAGAGTGCAGCAGATAGCCACTAGAAATCCAGCAATGCTAGGACAGCCTTCCCGCGC
AATAGTTTTTGGATTTGAAAATCCCGCCTTTGACGCTGACATCACTCAAGTATTTGAGCG
GGACTTGGAACAGGTTGCAGCAGCTCCAGATGCTGATTTTGCTGATATAGTCAGAATAGG
GCGTCCAAGATTTTCTCAGACAGACACTGGGCAAATTAGAATTAGCAGACTTGGGCGCCG
AGGAACTATTAAAACTAGAAGTGGTTTGCAAATTGGTCAGGCTGTGCATTTTTATTATGA
CTTGAGTACAATAGACACTGCTGATGCAATTGAGCTATCTACACTTGGTCAGCATTCAGG
AGAACAAAGCATTGTAGATGCAATGATAGAGAGCAGTTTCGTAGATCCCTTTGAAACTCC
TGATCCTACATACACAGAAGAGCAACAGCTTCTAGATCCCCTTACAGAGGATTTTAGCAA
CTCTCACTTGGTTTTAACTAGCAGTAGACGTGGATCTTCATTCAGTATCCCCACTATTCC
TCCTGGTTTAGGCTTGAGAATTTATGTTGATGATGTAGGCTCCGATTTATTTGTATCTTA
CCCAGAAACAAGAGTTATACCTGCGGGAGGCTTACCTACTGAGCCTTTTACCCCGTTAGA
GCCTCCATTTTTTTCAGAGTTTTACAGTTCTGACTTTGTATATCGTCCTAGTTTGTATCG
CAAGAAACGAAAACGATCAGATATATTTTAATTTTTTGCAGGAACATGGCGAGTTGGTTA
TCTGCAAAGGGTAAAGTGTACCTACCTCCCGCTCAACCTGTGGCAAGAGTTTTGGAAACT
GACGAATATATCACTGGAACATCTCTTTATTTCCACGCTGGAACAGAAAGGCTTTTAACT
GTAGGCCATCCTTATTTTCCAGTGAAAGATGTGCAGGATCAACACAAGGTTTTAGTTCCT
AAAGTTTCTGGAAGTCAGTATAGAGTTTTCAGATTCTATTTACCAGATCCTAATAGGTTT
GCATTGATTGATAATGGTTTCTATGATTCTGACCATGAACGTCTTGTTTGGAGACTCAGA
GGAATAGAAATAGGTAGGGGTGGTCCCCTTGGGATTGGAACAACAGGCCATCCTTTATAT
AATAAGTTTGGAGATTCAGAAAATCCTAATGGATACAGGAAACAATCAGATGACAACAGA
CAGGATGTTTCTTTAGATCCAAAACAGACACAAATGTTTATCATAGGTTGCACTCCTGCT
ATTGGGGAACATTGGGATAAGGCTGAGCCTTGTGCAAGTCCTGTTCCTCAACCAGGAGAT
TGTCCTCCTATAGAGCTTGTGAACACATATATTGAGGATGGGGATATGTGTGATATTGGT
TTTGGAGCTTTTAATTTTAAAGCTTTGCAGCAGGATAAATCTAGCGCTCCATTGGATGTA
GTTGCTACAATGTGCAAATGGCCAGATTTTCTAAAAATGAGTAAAGATGTCTATGGAGAT
AGCTTATTCTTTTATGGTAGGCGAGAACAGTTATATGCCAGGCACTTTTTTGTTAGAGCA
GGAGCAATGGGAGATGCACTACCAGAACCTTTCGAGGTTAAAACTGATTACTGGATTCCT
GCTCAAGAAGGTCAGGATCAGAATACTTTAGGTCCACATATATATATATAGGCACTCCTAGT
GGATCTTTAGTTTCAAGTGAGTCCCAGTTATTTAATCGACCGTATTGGTTAAACAGAGCT
CAGGGTACAAATAATGGAATATGCTGGGATAATCAATTATTTGTGACACTTGTTGACAAT

ACTCATAATACTAATTTTACTATTTCTGTAAAAACAGAAGCAGCTGATGAATCCTATAAA
TATAAAGCAGGTGATTTTAAACAGTATTTAAGACACATTGAGGAGTTTGAAATGGAATTT
ATTTTTCAGCTTTGCACAGTTCCTCTTACAGCCGATGTCATGGCTCACTTAAATGTAATG
AATCCTAATATCTTAGACAATTGGCAATTAAATTTTGTGCCCCCTCCTCCTTCAGGAATT
GAGGATCAATATAGATTTATACAATCCAGAGCTACGAGATGTCCTACTCAGTCTCCCAGT
ACTGAAAAGGAAGACCCTTATAAAGATTTATCATTTTGGGGTGTAGATTTAACAGAAAGA
TTTTCCAGTGAACTTAGCCAATTTTCCTTAGGGAGACGCTTTTTATATCAAAGTGGTTTA
ATTAATGGAACACTTAAAAGAAAACGTACTATTAATTCTCAAGCTCCAACTAGTATTAAA
CGTTCTGCCAAACGAAAACGGTCTATTAAACAGTAGATTAATCCTTATAAGAATGCATTT
GTTCTGCCAATAGAAACTTAAACTGAGCCAATTATTATATAATCTTTGGAAACGTGTATG
TATTTGCTTTTTCTGTGAGAAACTTGAGTAAAATAAACTGCCACATTCTTGCCACTGTCC
ATATTGTCTGACTCATTGGTCAACATGTGTGTCCGCACCCCAGTAATTACCTGGATCGCC
TCCACATCCGTCGGATGCGCGCCAAAAGAGGAACGTCCCCTTCTATTTTTCTAAAAAATT
ACCGTTTCTGCAGCTGCAAAATTGTGTAAGACCGTTATCGTTCCCTGTAACCTTGGCACA
AAAGGTGAGTGAAAGTTTTATAGTAACGTTTATGAGTCAATTTGTCTGGCGGCGCTGAAC
GAATTTGGCTGTCAGCCTTTGCACCGGGAGTGGTAGAAAATAGTTTCT
>gi|60882|emb|X55965.1|_Human_papillomavirus_type_57_complete_DNA
TAATATATAACTATAATCCTTCATTCAAAAAATAGGGCGTAACCGAAAACGGTCAGACCG
AAAACGGTCGTATAAAAACAGGAGCGCCATGTACAAGGGCAGGGATGTCTGAAGAAAATC
CATGCCCTAGGAACATCTTTCTGCTGTGCAGAGAGTATGGTTTGGAGCTAGAGGATTTGA
GAATACTGTGCGTGTATTGCAAGCGGCCGTTATCAGACGCTGATGTGCTGGCATTTGCAG
TAAAGGAACTGTTTGTAGTGTGGAGAAAGGGATTCCCTTATGGAGCATGTGAAAAATGCT
TAATTGCAGCAGCAAAACTTAGACAATACAGGCACTGGCATTACTCATGCTACGGAGACA
CAGTGGAGACCGAGACAGGAATACCCATACCTCAGCTGTTTATGAGATGCTATATCTGCC
ATAAGCCCCTGTGTTGGGAGGAGAAGGAGGCATTACTGGTCGGCAACAAGCGGTTCCACA
AGATATCAGGCCAGTGGACCGGACATTGCATGAACTGTGCGCCAAGATGCATGGAGAACG
CCCCAGCCTTGAGGACATCACACTAATATTGGAAGAAATACCCGAAATTGTTGACCTACA
TTGCGACGAGCAATTTGACAACTCAGAAGAAGATACTAACTATCAACTGACAGAACCAGC
TGTGCAGGCCTACGGGGTGGTAACGACGTGCTGTAAGTGCCACAGTACAGTCCGGCTGGT
GGTTGAGTGCGGAGCGGCGGACATAAGGCATCTGGAGCAGCTGTTCCTGAATACGTTGAC
CATAGTGTGCCCCCGCTGCGTATAGCGTCATGGAGGATTCGGAAGGTACCGACGGGACCG
ATGAGGACGGGTGCCGGGCAGGGGGGTGGTTCCATGTGGAAGCCATAATAACTCATGGCC
AGAGTCAGGTATCCAGTGACGAGGATGAGGATGAAACAGAAACAAGGGAGGATTTAGACT
TCATAGACAATAGGGTTCCCGGAGATGGGCAGGAAGTTCCCTTGCAGCTATATGCACAAC
AAATCGCCCAGGATGACGAAGCAACAGTGCAGGCCCTAAAACGAAAGTTTGTGGCGAGTC
CTTTGTCTGCATGCTCATGCATAGAGAATGATTTGAGTCCCAGATTAGATGCAATCTCGC
TAAACAGAAAGTCAGAAAAAGCGAAGAGACGCTTATTCGAGACAGAGCCACCAGACAGTG
GGTATGGCAATACGCAGATGGTTGTTGGAACGCCAGAAGAGGTAACAGGGGAAGACAACA
GTCAGGGGGGGCGGCCGGTGGACGTTAGGGAGGAGGAGCGTCAAGGGGGGGACGGAGAGG
CAGATCTAACTGTACACACTCCACAGTCAGGAACAGACGCGGCGGGTAGCGTGCTGACGT
TACTGAAAAGTAGCAATCTGAAGGCGACGTTACTGAGTAAGTTCAAGGAGCTATACGGGG
TGGGATATTACGAACTGGTCAGACAGTTCAAGAGCAGCAGGACAGCATGCGCAGACTGGG
TAGTATGTGCCTTTCGTGTGTATTATGCAGTGGCAGAGGGTATAAAACAGCTGATACAGC
CACACACGCAATATGCACACATACAGATACAAACCAGTTCCTGGGGAATGGTGGTTTTCA
TGTTGTTGCGCTACAACTGTGCAAAGAACAGGGACACCGTCTCCAAGAACATGAGCATGC
TGTTAAACATTCCCGAGAAGCATATGCTCATAGAACCACCAAAACTAAGAAGTACACCTG
CTGCCTTGTACTGGTACAAGACATCCATGGGTAATGGGAGTGAGGTCTATGGAGAGACAC
CAGAATGGATTGTGAGACAGACACTGATAGGACACAGTATGGAGGATGAGCAGTTCAAAT
TATCTGTTATGGTGCAGTACGCATATGACCATGACATAACGGACGAGAGCGCACTGGCAT
TTGAGTACGCACAGCTGGCGGACGTGGACGCCAATGCGGCAGCATTTCTAAACAGCAATT
GCCAGGCCAAATACCTAAAAGACGCGGTGACAATGTGCAGACACTACAAGCGTGCAGAGA
GGGAACAAATGAGTATGAGCCAGTGGATCACATTCAGAGGGAGTAAGATATCAGAGGAAG
GAGATTGGAAGCCTATAGTGAAGTTTTTAAGGCATCAGGGGGTAGAGTTCGTGTCATTCC
TTGCCGCCTTTAAGTCATTTCTAAAAGGCGTGCCCAAGAAGAATTGCATAGTGTTTTATG
GACCTGCAGATACAGGCAAATCATATTTTTGCATGAGTCTTTTGCAGTTCCTAGGTGGCG

CTGTAATATCATATGCCAATTCCAGTAGCCACTTTTGGCTGCAGCCCTTAGCTGACAGTA
AGATAGGGTTACTGGACGATGCGACGGCCCAGTGCTGGACGTATATTGATACATATCTTA
GGAACCTACTAGATGGCAATCCCTTCAGCATAGACAGGAAACATAAGACCCTGCTGCAGA
TAAAATGTCCTCCACTGATGATAACAACCAACATAAATCCTTTAGAGGAGGACAGGTGGA
AGTATTTGCGCAGCAGAGTAACACTGTTTAAGTTTACCAACCCATTTCCCTTCGCAAGTC
CCGGGGAGCCCTTATACCCTATAAATAATGCAAACTGGAAATGCTTTTTCCAAAGGTCGT
GGTCCCGCCTAGACCTAAACAGTCCAGAGGATCAGGAAGACAATGGAAACACTGGCGAGC
CGTTTAGATGCGTGCCAGGAGACGTTGCTAGAACTGTATGAAAAAGATAGCAACAAACTT
GAGGACCAAATTAAACATTGGGCGCAGGTCCGGCTAGAAAATGTAATGTTGTTTAAGGCT
CGGGAATGTGGAATGACACGAGTCGGCTGTACAACAGTGCCCGCCCTCACCGTGTCGAAA
GCTAAGGCTTGTCAGGCCATAGAGGTTCAGCTGGCATTACAGACATTGATGCAGAGTGCA
TATAGCACGGAGGCATGGACCCTGCGAGACACGTGCCTGGAGATGTGGGAAGCACCTCCA
AAGAGATGCTGGAAAAAGAAAGGACAATCAGTATTAGTAAAGTTTGATGGCAGCTGTGAC
AGAGACATGATATACACGGGCTGGGGCCATATATATGTGCAGGACATTAACGATGATACC
TGGCATAAGGTGCCCGGGCAGGTGGACGAACTGGGACTATTTTATGTGCACGACGGCGTA
CGTGTAAATTATGTGGACTTTGGAATAGAGGCCCTGACCTATGGGGTTACTGGGACGTGG
GAGGTGCAGGTCGGGGGGCGTGTTATTTATCATACATCCGCATCTGTGTCTAGTACCCAG
GCCGCCACCTCGGACGACGACACACTATCCCCTCTTAGATCTGCTGCGGCCGCAGTCACA
GCCACAGCCACAGCCACAACAGCAGTCCCGCCCACACTCCAGGACTCCGCCCAGGCGCCA
TCGAGTCCGCCACCCAAGCGCCAGCGGGTCATCGTCGGACAGCAGTGGCAACAGCCCGAC
TCTACGCGGAAGGTCAGAGAAGGGCAGGTGGAGTGTCAAAACGACAGGAGCATCCGTAAC
CCTGACAGCACAGACCCCCGGCGGGGCCACAGTGACCTTGACGCTGTGCCTGTGATCCAC
CTGCAAGGTGAAGCAAACTGTTTAAAGTGCTTCAGATACAGGGTGCAAAAACATAAAGAC
GTACTGTTTGTGAAGGCATCCTCCACCTGGCATTGGGCGTGTGGGAATGGTGACAAGACT
GCCTTTGTAACATTGTGGTACAAAAGTCAGGAACAGCGTGCAGAATTCCTTACAAGGGTC
CATCTACCCAAAGGGGTGAAGGCACTGCCAGGGTATATGTCTGCATTTGTATAAATGTAT
AGTCTGTAACATAATACACAGGTCCGCAACATTTCACAGCCTGTATCATCAACACAGCCC
TGGACTACTTTCTCTGCGTGGTGTCAGGGTGGTCCCATCTGCTGGTACTGTTGCTCTTCC
TTTGGCTCTCTCAACTAACCCCTCTGGTAGCCTATCTGGTGTTCTTCTTCTGTGTCTATA
TAGGGCTGTGGTTGATATTTTTGCAGGCCTTGTGGTTTCTACAATAGTTGATATTACATT
GTGACCAACTGCTGCTACCCTGTATATACCTCCCCCCTGTATACTGCAATGTATCCTGCT
GTGTACAAGGGCACGGGCGGGTCGTATCCCATTGTCCTGTGGGGCCCTGATGATGTGGAT
TGCTTGTTGATTATTCTTTTGTTGATTGCCATTTTGTTGTTGATGCTGTACGTCCGTCTG
CTGCAGTAGTGTACATACTCATTGTGTTTTTGCTACATACTCATTTTGATACATTTTTAT
CGGTGTGCACCTGATTGCCTTTGTATTGCTACGTGTCAATAAACTTATTGCCATGTCACC
ACGTGCAAAGCGTCGGAAGCGCGCCTCCCCCACTGACTTGTATCGGACATGCAAGCAGGC
TGGAACGTGCCCCCCTGATATCATACCTAGGGTGGAACAGGACACATTAGCTGATAGGAT
ACTCAAATGGGGCAGCCTGGGGGTCTTTTTCGGTGGCCTCGGTATAGGTACTGGAAGCGG
CACTGGGGGACGCACAGGCTACATACCAGTGGGCACCAGACCAACAACTGTCGTTGATGT
AGGACTGGCGCCAAGGCCACCTGTAGTAATAGAACCTGTGGGGGCGTCTGAACCATCTAT
TGTTAATTTGGTGGAGGATTCTAGCATCATTAATGCTGGGTCCTCTCATCCAACCTTTAC
CGGTACTGGTGGGTTTGAGGTTACCACCTCAACGGTGACTGACCCTGCAGTCCTGGACAT
CACTCCTTCGGGTAATGGGGTGCAGGTTAGCAGCAGTAGCTTTGTGAATCCTCTCTTCAC
TGACCCTGCTATTATTGAGGCTCCCCAGGCTGGGGAGGTTACAGGGCATGTGCTTGTTAG
CACTGCCACATCAGGGTCCCACGGCTTCGAGGAAATACCAATGCAGACCTTTGCGACCTC
TGGTGGGGATGGAGGAGAGCCCATAAGTAGCACACCTGTCCCAGGCGTGCGCAGGGTTGC
TGGGCCCCGCCTTTATAGTAGGGCTAATCAGCAGGTGCGGGTCCGGGACCCTGCCTTTAT
TGACCGTCCTGCGGATTTGGTGACATTTGACAACCCTGTCTATGACCCCGAGGAAACTAT
AATATTTCAGCATCCAGGCTTGCATGAGCCACCGGACCCAGACTTCCTGGACATAGTGTC
ACTGCACCGCCCTGCCCTCACATCCACCCGGCAGGGTACTGTCCGCTTCAGTCGGTTGGG
ACGCCGGGCCACGCTTCGCACGCGTAGTGGTAAACAAATTGGGGCTAGGGTACACTTCTA
TCATGATATCAGCCCTGTCGCTCCCGAGGAATTGGAGATGGAGCCGCTGTTACCCCCCAC
GTCGGAGCCCCTATATGACATATATGCCGAGTCGGATTTCTTGCAACCCTTAGATTCGGA
TGTCCCCGCGGCCCCTCGAGGTACCCTTTCCCTGGCAGACACTGCAGTGTCTGCATCCAC
CGCTTCTACGTTGCGGGGGGCCACCACTGTTCCCCTGTCAGGTGGTGTGGATGTGCCTGT

GTATACCGGTCCTGATATTGACCCGTCTGTAGGCCCTGGTATGGGACCGCTGGTGCCTGT
GATACCAGCCATACCATCCTCTGTGTACATAGTTGGGGGTGATTACTACTTGCTGCCAAG
TTATGTTCTGTGGCCTAAACGACGTAAACGTGTGCACTATTTCTTTGCAGATGGCTATGT
GGCGGCCTAATGAAAGCAAGGTATACCTGCCTCCAACACCTGTCTCAAAGGTGCTCAGTA
CGGATGTCTATGTCACGCGGACGAATGTTTATTATCATGGTGGGAGCTCTCGGCTCCTCA
CAGTAGGCCATCCATATTATTCTATAAAAAAAAGTGGCAATAATAAGGTGTCTGTGCCCA
AGGTATCGGGCTACCAGTACCGTGTGTTCCATGTGAAGCTGCCGGACCCTAATAAGTTTG
GTCTGCCTGATGCCAACCTCTATGATCCCGACACCCAGCGTCTGCTGTGGGCCTGTGTCG
GCGTTGAGGTGGGTCGTGGCCAGCCTCTGGGTGTAGGGATATCCGGCCACCCTTATTATA
ACAAACAGGATGATACTGAAAATTCACACAATCCCGACGCAGCTGATGATGGGAGGGAGT
ATATATCCATGGATTATAAACAGACACAGCTGTTTATTTTGGGTTGCAAGCCCCCTATAG
GTGAGCATTGGTCCAAGGGCACTACCTGCAGCGGGTCTTCTGCTGTTGGTGACTGTCCCC
CCCTGCAGTTTACAAATACCACTATTGAAGATGGGGATATGGTTGAAACCGGGTTCGGGG
CGCTGGATTTTGCCGCTCTACAGTCCAACAAATCAGATGTCCCCTTGGATATCTGTACTA
ACATATGTAAATATCCAGACTATCTGAAGATGGCTGCAGACCCTTATGGCGATTCTATGT
TCTTTTCCCTGCGCAGGGAGCAAATGTTCACTCGGCATTTTTTCAATCGGGGTGGGTCGA
TGGGTGACGCCCTCCCGGATGAGCTATATGTCAAGAGTTCTACCGTCCAGACCCCCGGTA
GTTATGTTTATACCTCCACTCCCAGTGGCTCTATGGTATCCTCTGAACAGCAGTTATTTA
ACAAGCCTTACTGGCTGCGGAGGGCCCAGGGACATAACAATGGCATGTGCTGGGGCAATC
GGATCTTCCTAACAGTGGTGGACACCACGCGCAGCACAAATGTCTCTTTGTGTGCCACTG
TAACCACAGAAACTAATTATAAAGCCTCCAATTATAAGGAATACCTTAGGCATATGGAGG
AATATGATTTGCAGTTCATTTTTCAACTGTGCAAAATAACACTCACCCCCGAGATAATGG
CATACATACATAACATGGATGCGCGGTTGCTAGAGGACTGGAACTTTGGTGTCCCCCCAC
CCCCGTCCGCCAGCCTGCAGGACACCTACAGGTATTTGCAATCCCAAGCGATAACATGTC
AGAAGCCCACACCCCCTAAGACCCCTACTGATCCCTATGCAACCATGACATTCTGGGATG
TGGATCTCAGTGAAAGTTTTTCCATGGATCTGGACCAATTCCCCCTGGGACGCAAGTTTT
TATTGCAGCGGGGGGCCACCCCCACTGTGTCTCGAAAACGCGCCGCTGCAACTGCAGCGG
CGCCCACTGCTAAACGCAAAAAGGTCAGGCGATAGTGATTCTGTGTCTGCCTCATTTCCT
TTGCTCTACTTTTGTATATGTACATATGTTTCAGTGTTGTCTGTGTGTTTGTGTTTGTGT
GTGTTGTCTGTTATTATGTTTGCATGTACACATGTCGGTACACATGTCTGGTATGTATTC
CTCCCATATGAATAAACGTGTGTCATGTGTTGTGTGTCCTGTTGCACTCTGTAATTGTCC
CCGCTGCATGGTTTGCACACTGTGGCCTGTATGTAGCCCCCTGGTAGATACGACCGTTTT
CGGTTGCGTGCAGTTTCGGTCGGCGCTGCTGCCAGCACACTCATATCCTTTAATCCTTTA
ATTGCTTTAATCCTTTCACTTTTTTACTGTGCCAACTAAAATGATTTTGCTTTTTGATTG
TTTTGTGTCTGCATTAATGCAGTTTTTCTTTTTCCAGTGCCAGACCGCGTGTGGGCGTGC
ACATTCCTACATAGATTATCTTCCTGTGTTGGCGGGGATTTTCCCTGCGTCTGCAGAAAA
ACCTGCCACCACAGCACCTTGGGCGCGTCGTTTTTGCAGCCAACTTTCCCTTGCCAAGTT
GTCTTGCCGCGCATTCCAAGAAACACACCTATTCCGGTCGCAATCTCTACTATGTGGTTT
A
>gi|40804528|emb|AJ620211.1|_Human_papillomavirus_type_94_complete_genome
TAATGTAGAGAATAAGAAATAGGGAGGGACCGAATACGGTGCGACCGAAAGGGGTACATA
TAAAACAAGGCACTTACAGACTGGCAGACGCCATGTCTATGGGTGCACAAGAACCCAGAA
ACATATTCCTATTGTGTAGGAACTGTGGAATATCATTTGAGGACCTTCGCCTGTGCTGCG
TGTTCTGCACCAAACAGCTGACCGTAGCCGAATTGACTGCATTTGCATTAAGAGAATTGA
ATTTGGTGTGGAAAGCGGGAGTGCCATACGGTGCCTGTGCCCGGTGTTTACTTCTGCAGG
GCATTGCACGGCGCCTAAAATATTGGCAATATTCATATTATGTAGAAGGCGTGGAAGAGG
AGACCAAGGAATCTATAAATACACAGCAGATCAGATGCTACACGTGCCACAAACCGCTTG
TAAAGGAAGAAAAGGACAGACATCGTAACGAACGGCGACGACTGCACAAAATATCAGGGT
ACTGGAGAGGGTGTTGTGCTTATTGCTGGACACGATGCACGGTCCGCATCCCACAGTAAA
AGATATAGAATTGAGTCTTGCACCAGAGGACATCCCTGTAGTATGCAATGTGCAATTAGA
TGAAGAAGATTATACAGATGTGGTGGAACCAGCACAACAAGCGTATAGGGTGGTAACAGT
ATGTCCAAAGTGTAGTTTACCACTGCGACTGGTGGTAGAGTGCAGCCACGCAGATATAAG
GGCACTGGAGCGTCTGCTGCTAGGCACATTGAAGCTCGTGTGTCCTCGCTGCGTGTAACA
GGACATGGACGATAATACAGGTACAGAGGGGGGCGAATGTTCCGAGGCGGAACGGGCGGG
AGGATGGTTTATAGTGGAAGCCATTGTAGACAGGCGGACAGGCGATCCCGTATCCAGTGA

TGATGATGAGGATGAGGATGAAGAAGGGGGAGATTGTGTAGACTTCATAGATGATACCAG
GTCTGTAGGGGATGGACAGGAAGTGGCACGGGAACTGTTCCTGCAGCAAGCAGCTGCGGA
TGACGATATAGCTGTGCAGACTGTAAAACGAAAGTTTGCACCTAGTCCTTATTTCAGCCC
TGTGTGTGAACAAGCCAGCATAGAACATGAACTAAGTCCAAGGCTAGACGCCATAAAGCT
GGGGAGACAATCAGCAAAGGCTAAACGTCGTCTGTTTGAGCTACCGGACAGTGGGTATGG
CCAAACACAGGTGGATACGGAATCGGGACCAAAACAGGTACAGGGCAGCAATGAGACGCA
AGATGGCCGCCAGGATGATGAGGAGGGGAGTGTGGTACAGAGCAGATGTGAGATGGGCAA
TCAAAATGGCCGCCAGAACAGTACAGAGGGGAGCGGGAGGAATGTGGGGGACCATGGCAG
CCAAGAGGAGGAGCGTGCAGGAGGGGATGGGGAGGAATCGGAGTCCCACAGCATAGACAC
TGGCAGGGGAGCAGGCGGCGTGTTAGCAATATTAAAAGCCAGCAATCATAAAGCAACTTT
ACTGGGTAAGTTCAAAGAACAATTTGGATTGGGATATAATGAACTTATTAGACACTTTAA
AAGTGATAGAACAGCATGTGTAGATTGGGTGGTGTGTGTATTTGGGGTGTATTGCACGGT
GGCAGAGGGCATAAAGACCCTTATACAGCCATTGTGTGATTATGCACACATACAAGTGCT
ACCATGCCAGTGGGGAATGACAGTGTTTATGCTGGTACGGTACAAACGTGCCAAGAACAG
AGAAACAGTAGCAAAAGGCTTAAGCACATTATTGAATGTACCAGAAAGCCAGATGTTAAT
TGAACCACCAAAATTAAGGAGTGGTCCAGCTGCGCTGTATTGGTACAAGACCAGCATGTC
CAGCTGTAGTGATGTGTATGGAGAGACACCAGAGTGGATAGTTAGGCAAACAATGGTGGG
ACATGCAATGGAGGATGCGCAGTTTACCCTTTCAGAGATGATACAGTGGGCATATGATCA
CGATATTACAGATGAGAGCACTCTGGCATACGAGTATGCACTGATTGCTGACACAGATGC
CAATGCTGCTGCCTTTCTTGCTAGCAACTGTCAAGCAAAGTATCTAAAGGATGCATGCAC
AATGTGCAGACATTATAAAAGAGGAGAACAGGCGCGTATGAGTATGTCAGAATGGATACG
GTTCAGAGGCGACAAAGTACAGGGAGATGGAGATTGGAAGCCCATAGTCCAATTTTTAAG
ATATCAGGACGTTGAATTTATACCATTCTTATGTGCCTTTAAAACATTCCTTCAAGGAAT
TCGAATTCCAAAGAAAAGTTGTATAGTGTTTTATGGTCCAGCAGACACCGGAAAATCATA
CTTTTGCATGAGCTTACTTAGATTCCTGGGGGGTGCTGTTATATCATATGCAAATTCAAG
CAGCCATTTTTGGTTACAACCATTATCGGAAGCCAAAATAGGGCTGTTAGACGATGCAAC
CACTCAGTGTTGGAACTATGTAGATACTTATTTAAGAAATGCCTTAGATGGTAACCAAGT
GTGTATAGACAGGAAGCATAGGGCCTTGCTACAGCTAAAATGCCCTCCGCTATTAATAAC
AACAAATGTGAATCCACTGGCGGATGAAAAATGGAAGTATTTGCGCAGCAGGTTGCAGCT
CTTCACATTTAAAAATCCTTTTCCAGTGACAGCACAAGGAGAACCACTGTATACATTAAA
TGATCAAAATTGGAAATGCTTTTTTCGAAGGTTATGGGCACGTTTAAGCCTTACCGATCC
TGAAGACGAGGATGAAAATGGAAACCCTAGCGAACCGTTTAGATGCGTGCCAGGACAAAA
TGCTAGAACTTTATGAAAAGGATAGCAACAAACTTGAGGACCAGATCACGCATTGGCACC
TATTGCGTGTAGAAAATGCTTTGCTGTACAAAGCAAGAGAATGTGGACTGACACATATTG
GCCATCAGGTGGTGCCACCTCTTAGTGTAACTAAAGCCAAGGCACGCAATGCCATTGAAG
TGCATGTAGCTTTACAGCAATTACAAGACAGTGCCTATGCACATGAATCCTGGACATTGA
GAGACACATCACGTGAAATGTGGGACGCGGCCCCTAAAGGGTGCTGGAAAAAAAGAGGGA
TAACTGTGGAAGTAAGATATGATGGAGACGAATCTAAAGCCATGTGCTATGTACAATGGA
GGGATATTTTTGTGCAGAATTATAGTGACGATAGATGGGTAAAGGTGTCAGGCCACGTCT
CACACGAGGGACTATATTACACACATGAAAATGAAAACATATTTTATGCGAATTTCAAGG
ATGACGCGTTTGTATACGGGGAAACAGGTAAATGGGAGGTGCACGTGGGAGGCAAAGTAA
TTCACCACCATGCATTTGACCCTGTATCTAGCACACGAGAAATATCCGCTCCTGGACCTG
TGTGCACCAGCAACTCCACCCCAGCGCCCACCAAAGCCCAGGTGGGCGCGACCGAGGAAC
CGGAACAAAAGCGACAGCGATTCGAGGCGGTCGACGGACAGCAGCAGCGGCAGCGACAAG
GGTCCCAAGATCCCACCCAGAAAGCCTGTGAACGAGCGGGTGGACCATTGGACAGTGACA
GCACCCGGGTGTGTGACACTCGACGTGCATTCCCCGTCTGGCCTCCAAGTGACCCTGACT
GTGCACCTGTAATACACCTAAGAGGTGATCCTAACTGTTTAAAATGTTTTAGATATAGAT
TACATCACGGGAAAAGGAAACTATACTCACGGTCCACCTCCACATGGAGGTGGTCCTGTG
AATCAGAAAATCAAGCAGCTTTTGTAACAATCTGGTACACAAGCGAAGCACAGCGTGCAG
AATTTCTCAATGTTGTAAAAGTACCCCCTGGCATACAAGTAATTTTGGGTTATATGTCAA
TCTTCTAATTGTCTGTATTGTGACAGTGTATTATCCTGGATTTTTATTTGTGTAGACTAC
ATTGCTGGGCTTTTTTTGTGCTGCTGCTGTGTCTGTTTTGGCTGAGTGTGCTTCCTGCGC
TGACGTGCTATTTGGCAATTGTGCTCTGTGTGTACCTGGGCCTGGTAGCATTGTATTTTC
AAATTGTGTCACGTATACTTCAAAACACATAGGTCTCACAATGTATCCTCTTGTATTAAG
AGATAACACTGGTGACCATACTGTATTGTTGTTTGAGCCCGGAGATGTGTATTTACTGCT

GTGTATAATATTGTTTGTACTTATGGCATTGTTTGTTTTATATAGACACCTGGGTGTATT
GTGATATGGATGTAGACGCCTGTGCATAATCTAATGTTTTGCTGTGTGTACCCTTATGTG
CTATTGTAGCACCCTTGTTTTTTTTATTCTTTTGTTTTTTACAGTTAAATAAAGCAACCA
TGGTGGCACATCGTGCAAGGCGTCGCAAGCGTGCATCCGCCACACAGCTTTATAGGACCT
GCAAAGCCTCAGGCACATGCCCCCCAGATGTTATTCCCAAAGTGGAAGGCACCACTTTGG
CAGATAAATTACTGCAGTGGGGTAGTCTTGGTATATTTCTGGGTGGTTTAGGCATTGGCA
CCGGGTCTGGTACTGGGGGACGCACAGGGTATGTTCCTGTCAGTACTCGGCCTGGTACTG
TTGTAGATGTTAGTGTTCCTGTCAAGCCACCTGTGGTGATTGACCCTGTGGGTCCGTCTG
ATCCTTCCATAGTAAATTTGTTAGAGGACTCTAGTATTGTAAATGCTGGGTCCACCATTC
CTACATTTTCTGGTACTGGTGGCTTTGAAGTGACCTCTTCTGCCACCACTACCCCTGCTG
TGTTGGATATTACACCTGCTGGTGATAATGTAGTGATTAGTAGTACAAGCTTTACCAATC
CTGCATATACAGAGCCATCTCTTGTGGAAGTTCCACAGAGTGGTGAAGTGTCGGGACACA
TTCTCATAAGCACACCTACAGCTGGTACCCATGGTTACGAGGAAATCCCAATGGACACTT
TTGCTTCGTCGGGCACTGGCACGGAACCCATCAGTAGTACTCCTGTACCTGGTGTTAGTA
GGATTGCAGGCCCACGCTTATATGGCAAGGCTACCACGCAGGTCAAAGTGTCAGATCCTG
CATTTCTGTCACGTCCGTCCACTTTTTTAACATTTGATAATCCTGCATTTGAGCCAGAGG
ATGAAACCATTATATTTGAACGTCCGTACTCTCCTTCGCGAGTTCCGGACCCTGATTTCC
TTGACATTGTGCGTTTGCATCGCCCTGCATTGACATCTCGCAGGGGTGCTGTGCGTTTCA
GCCGTCTGGGTCAAAAGTTTAGCATGCGTACGCGCAGTGGCAAAAATATCGGGGCCCGGG
TGCATTACTACCATGACCTCAGTCCTATTGCCGCCACCGAGGAAATTGAAATGGAACCTT
TACTTGCCCCAGCAGATCCTGACACTCTATATGACATCTTTGCTGATGTGGATGATGGTG
ATGTTGCCTTTACAAATGGCACTCGAAGTGTCACACCGGCACGGAGCTATAGTACTGCTT
CCCCTTTGTCCTCTACACTTTCAACTAAGTTTGGCAATGTCACCATCCCATTTGTGTCTC
CTGTTGATGTAACTTTACATACCGGGCCTGACATTATATTACCCACCTCGGCGCAGTGGC
CTTATGTGCCTTTAACACCTGCTGACACAACACATTATGTTTACATAGATGGAGGGGATT
TTTATCTCTGGCCTGTTACCTTTTACTTGTCCCGACGTCGTCGTCGTAAACGTGTTTAAT
ATTGTCTTGCAGATGGCACTCTGGCGCCCTAGTGACAACTTGGTGTACCTGCCTCCTACT
CCCGTGTCCAAAGTTCTCAGCACGGACGACTATGTGACACGCACCAACATTTATTATTAT
GCAGGCACTTCTCGGTTGCTTACTGTAGGTCATCCTTATTTTCCTATACCTAAATCATCT
AACAATAAGGTAGATGTTCCCAAAGTATCTGCATATCAGTATAGGGTGTTTCGGGTGCGG
TTGCCTGACCCCAATAAGTTTGGCCTGCCTGACGCCCGCATATATAATCCTGACGCCGAG
CGCTTAGTCTGGGCTTGCACTGGGGTTGAGATAGGTCGCGGGCTGCCCTTGGGTGTGGGC
CTCAGTGGACACCCTTTATACAATAAGCTGGATGACACAGAAAACTCCAACATAGCACAT
GGGAATGTTGGTCAGGATTCACGGGACAATATTTCTGTGGATAATAAGCAAACTCAGTTA
TGTATTCTTGGTTGTACACCTCCCATGGGGGAGCATTGGGGCAAGGGGACCCCGTGCAAT
AGGTCGGCGCCTGCACCGGGCGACTGCCCTCCTCTGGAGCTTGTGACTTCAGTTATTCAA
GATGGCGATATGGTGGACACTGGCTATGGTGCTATGGACTTCACTGCATTACAGTTAAAT
AAGTCTGACGTGCCTATAGATATTTGCCAGTCCACTTGTAAATACCCTGATTATTTGGGC
ATGGCAGCAGAGCCTTATGGCGACAGCATGTTTTTTTATTTGCGCAGAGAGCAACTGTTT
GCAAGACATTTTTTCAATAGAGCCAGTGCAGTGGGAGACACCATTCCTGACACTCTAATA
TTGAAGTCGGCCGGTGGTGACCAAAACGTTGGTAGTGCTGTGTATAGCCCCACTCCCAGT
GGGTCCATGGTAACATCAGAGGCTCAATTATTTAATAAGCCATATTGGCTGAAGCGGGCT
CAGGGGCACAATAATGGTATTTGCTGGGGCAACCAAATGTTTGTGACTGTGGTGGACACC
ACACGCAGCACAAATTTGTCCCTGTGCGTCCCTTCTGATGCCTCCACCGCCACTACGTAT
GATGCTACAAAATTTAAAGAATTTTTGAGGCACGGAGAAGAGTATGACTTGCAGTTTATT
TTTCAGCTGTGTAAAGTGACTTTAACCCCCGAAATTATGGCCTATTTACACACCATGAAC
AGTAGTTTACTAGAAGATTGGAACTTTGGGTTGACTCTGCCGCCGTCCACTAGCTTGGAG
GACACTTATAGGTTCTTGTCGTCCTCCGCAATAACTTGTCAGAAGGAGGCACCCCCCACT
GAAAAGCAAGATCCCTATGCAAAACTAAACTTTTGGGACGTGGATCTGAAGGATAGGTTT
ACCCTAGACCTGTCTCAATTTCCCCTGGGCAGAAAGTTTTTGCTGCAGCTAGGTGTACGT
TCTCGCTCCGCCATCTCTGTCCGCAAACGCTCGGCGACCGCCGCGTCAGGATCCACCGCT
GCAAAAAGAAAAAGAACCAAGAAATGACCCCATGTTTGTGTTTTTATGTATGTGCATATT
TGTGTATGTTGTTTGTCTGGTAATTGGTGTATGTTATGTGTACGCTTGTATGTATTTACT
GTGTATGTATTGTACTGGAATGCATGTGTGGATGTGTAAATAAAGTTTGTCACATAGTTT
TATATTTTTAATTTGTGTAATTGCTGTTCCTGTGAGTAAGTAAGGTAGTTCTAGGTCAG

AGGACCGATTTCGGTTCAAAATGGCCGCCTTTGCAGGTGTGCAAACCACCAATTAGTCAT
ACTGATCCATATCCTGCGACCTGCCCTGTCACGCATGTGTTTTTGGCTGGCATTGTATGG
TTTCTATTATTTATTTTACTGCTGCATCATGCTTTCTGGCACGGCAAAATGTCTCCATTG
CAATTTTAACTGCTTCTGGGCACCAACTTATTATGACTACTTTCACATAATTGCTGTCTT
GGCTGCGTTTTCTAGGTTGCCCTGCCAATAAAATGTGCTTCCAAATCTCCACCAAGACAC
ACCTAATCCGGTCGCTGCTTGCTTTCTAGCTTTAATTAATGCAGTTGCTACACGTCTCTT
TCCAACTATAATTATAAACTA
>gi|40804520|emb|AJ620210.1|_Human_papillomavirus_type_95_E6_gene,_E7_gene,_E1_gene,_E2_gene,_E4_gene,_L2_gene_and_L1_gene
AACATTGTTTTCCTTAATAATAGTTGGCAACAATCATCTCTTATAATAAAATATCACCGG
AAGAGATACATATAAAAAGAGAGCGGTTCTTGCAGAAATTCTCTTTCCAGATGGCAAATG
GCAGACCTACAACCTTGGACGAGTACTGCAGGAGATTTGACATTTCTTTTTTTGATTTGC
GTCTTCCTTGTATTTTTTGCTTTCATCCGGTTGATCTTGCAGAGCTTGCTTCGTTTTATA
TTAAGAAATTAAGTTTGGTTTTTAGGGGTAGCTGTTATTATGCATGCTGTTCTCAATGTT
TAAGATTAAGTGCAAAGTTTGAGCAAGAGAATTATTTTCAGTGTTCTGTATATGCTACTG
GACTGGAAGATTTAGTAGGACAAAAAATCAGGGATATATGTGTAAGATGTTTATGCTGTC
TTAAACTATTAGACATAGTAGAAAAACTTGACCTGTTATACAGTGGTGAGGTGTGCTATT
TAATAAGGGGTTTGTGGAGGGGCTATTGCAGAAACTGTATTAAGAAACAATGAGAGGAGC
AGCACCATCTATTGCAGATGTTGATTTAAACCTCCATGAGTTAGTTGTTCCTGCAAATTT
GCTGAGCGATGAGGTCTTGCAACTCTCTGAGGAGGAGGATGAAGAAAGAGAGGAGGAGCT
TTTACCATTTAGAATAGATACCTGTTGTTATAATTGTGAAGCAAATGTAAGAATCACTTT
GTATGCTGTTGCATTTGGACTACGAGTCGTGGAACAACTTCTTCTGGAAGGGAAGGTGAT
ATTTTGCTGTGTTGGGTGTGCAAGAAATCACAGCAGAAATGGCAGATAAAGGTACTGAAA
ATTTTGACGCTTTGGAAGGGAGTAGTTGGTATTTTGTTGAGGAAGCAGAATGCATAGACA
GCATAGATACTGTGGATGAGTTATTCGACGAAAGTACTGACGGTTCAAATATATCAAATT
TGATTGATGACGATGAAGTTGATCAGGGAAATTCCCTGGCACTGTACAATACACAAATAA
CTGATGCTTGCGAAAATGCTATAGCTGCCCTAAAACGAAAGTATACCAAAAGTCCACAGC
AGGCAGTTGCCGAATTGAGTCCGCAGTTGCAGGCTGTGAGAATTACTCCTGACAGAAAGA
GCAAAAGGAGATTATTTGAGGACAGTGGGATTTGTGAAGATGAAGCTGAAAATTCTGTTG
CACAGGTAGTCTCTGATAAAGAGGAAGCTGGCACAGCCAGTCAAGATGGCGCAGGGGATA
TTAACCTTTTGTTATTGCAGGACAATAACCGAAGGGCAACTATGCTTGCCAAATTTAAAG
ATTGGTATGGAGTTTCATATACTGAAATAACAAGATTATATAAAAGTGATAAATCTTGTA
GTGATAATTGGGTTGTTGTTATATTTAAAGCTCCTGTAGAGGTATTGGAAAGTTCTAAGA
TCGTCCTTCAACAGCATTGCCAATACATTCAAGTAAAGATATTTGGATTTTCTGCTCTTT
ATTTATTACAATTTAAGAGTTCCAAAAGTAGAGAGACTGTTTATAAGTTATTATGTTCGT
TGTTAAATATACAAGAGTTTCAAATTCTAGCTGATCCTCCAAAATTACGAAGCGTTCCTG
CAGCTTTATATTTTTATAAACATGCTTTGTTAACTGAATGCTTTGTATATGGACAAACAC
CGGATTGGATCGCTAAGCAGACAATAGTGAGCCATCAGTCTGCTACAACTGCAGAAACAT
TTGAGTTGTCAAGAATGGTTCAGTGGGCATATGATAATAACCATTTAGAAGAATGTGATA
TAGCTTATCATTATGCTTTATATGCAGATGAGGATGCTAATGCTGCAGCATATTTGAAAA
GCAATAATCAAGTAAAACATGTAAAAGATTGTAGTACAATGGTAAGGATGTATAAAAGAT
ATGAAATGAGAGAAATGTCCATGTCAGAATGGATTCATAAATGCTGTAATGAATGTCCTG
AAGAGGGAGATTGGAAACCAATCTCTCACTTTTTAAAATATCAAGGTGTCAATATTCTTT
CATTTCTTATAGTATTAAAATCATTTTTAAAAGGTATTCCAAAGAAAAATTGTATTTTAA
TTCATGGACCACCAGACACAGGAAAATCATTGTTTTGTTACTCATTGATAAAGTTTTTAA
GGGGAAAAGTAGTGTCATATGTGAATAGAACTAGTCAATTCTGGTTGCAACCACTTATGG
ATGGTAAAATAGGCTTTCTGGATGATGCAACATATGTGTGTTGGACATATATAGATCAAA
ATCTTAGAAATGCTTTAGATGGTAATCCTATGAGTATAGATGCTAAACATAGAGCACCTC
AACAATTAAAATTACCTCCAATGTTAATAACTTCTAATATTAATGTTAAACAAGAGCAAA
CATTAATGTATTTGCATAGTAGGGTACAATCTTTTGAGTTCCCTAACAAAATGCCTTTTT
TAGATGATGGTTCCCCTTTATATACTTTTACTGATGCTACTTGGAAATCTTTTTTCGAAA
AGCTTGGCAGACAATTAGATCTATCAGAGCCTGAAGAGGAAAACAATGGAGTCCTTAGTC
GCACGTTTCGATGCACTTCAAGAAGAAATTCTGACTCATATTGAGTCAGGAAATACAACT
TTGGAATCCCAAATCAAATATTGGGAGAATGTAAGAAAAGAAAATGCTATCATGCATTAT
GCACGAAAACAAGGGCTGACTAAATTGGGTCTTCAACCCCTTCCTTCACTTTTAGCAAGT

GAATACAATGCAAAACAAGCAATACAGATACAGCTAACATTACTATCATTATTAAAGTCG
CCCTATGCTTCTGAACCTTGGACATTACCAGAAGTCAGTGCAGAATTGATTAACACGCCC
CCTCAGAACGTTTTAAAAAAGGGGGGGTATGATGTAACTGTGTGGTTTGATGACGATAGA
AACAATACAATGGTATATACAAACTGGACTGCTTTGTATTATCAAGATGCAAATGAAATA
TGGCATAAAGTAAAGGGTGAAGTGGATTATAATGGTCTGTTCTTTACAGACCATACAGGA
GAGCGTGCATATTTTACATTGTTTAGCACAGATGCTGAAAGATATAGCCAGACTGGACTA
TGGACTGTGCATTTTAAAACCCAGGTTATTTCCTCCTCTGTTGTTAGCTCTACAAACCCC
TCCTCCTTCGACTTCGAGGAACAACTACCCGGGCCCTCCACCAGCAACACCAAAACTACC
AAGCAGACGTCCCCTCGGGGAAGGGGATCGCAGTCGAGGGAACTACAACCATCCTCCACA
ACGTCCCCTGAAGGGAAGGGGCTACGAGTACGACGACGACGAGGACAAGGAGAATCAGGG
TCCGGGACAAGAGAAACCCCCAGCAAAAGAAGAAGAGGAGGAGGAGGAAGAGGAGAAAAG
GAATTTGGATCTGCACCAACTCCTTCAGAAGTGGGATCAAGACATCGACAAGTTGAAACG
AAAGGTCTGTCACGACTTGGACAATTACAAGCAGACGCTAGGGATCCGCCAATGATTATG
TTAAAAGGCCATGCAAATTCATTGAAATGCTGGAGATATAGAAAACTAACTTCAAATTCT
TGTGGCTTCCTTTATATGAGTACTGTTTGGAACTGGGTTGGGGATTCCTCAGAAAATCAT
AGTCGAATGTTAATAGCTTTCACCAGTACAGACCAGAGAGATTACTTTGTAAAGCATCAC
TTCTTCCCCAGACAGTGCACATATACATTTGGTTCTCTGAACAGCCTGTAAAATGAATTC
TGCACGGAGAGTAAAGCGAGACTCAGTCCCTAATCTTTATGCAAAATGTCAACTTTCAGG
CGATTGCCTTCCTGATGTAAAAAATAAAGTAGAAGCTAACACTATTGCTGACCGTTTACT
GAGATGGTTGGGAAGTATCATATACTTGGGTGGGCTGGGCATAGGTACTGGCAGAGGGAG
TGGGGGGTCTTCTGGGTATAATCCACTAGCGACCCCAAGTAGAGTTACCCCTAGTGGAAC
TCTAGTAAGACCCACAGTTCCAGTAGAAGGTCTGGGCCCGGTTGAAATAGTTCCTGTTGA
TGCAGTAGATCCTGCAGGCTCCTCCATTGTCCCGCTCACTGATGTGTCTGTTCCCGAGGT
GGTTGTGGATGGCGAAGGTGGGGCAATAGATTTAGGACCACCCGAAGATACAGGAGTCAC
ATCTCGCCCTCCTGAAATATTAACAACTCCAGACCCTGTTTCAGATGTTTCAGGTACCAA
CAGCCACCCAACAATAATTTCAGGAGAAGATAACACAATAGCTGTGTTAGACATATCTCC
AATAGAGCCCCCCACAAAACGTATAGCCTTGGGCACACGTGGCCAGTCATCAACGCCACA
CATAAGTGTCATATCAGGCTCGACCGATATCGGTCAGTCTTCAGATATCAATGTATTTGT
TGATGCACAGTTTTCAGGAGATTCTATTGGTTATACAGAGGAAATCCCCTTGCAGGATCT
GAACACTATCCAGGAATTTGAAATAGAAACCCCTCCAAAAACCAGTACTCCACGTGACAC
AATAGCCCGCGCAATAGGGAGGGCGCGAGAATTATATAATAGAAGAGTGCAACAAATTCA
AACTAGGAATCCCGCTCTACTAGCTCAACCTTCCCGCGCGATAGTGTTTGGTTTTGAAAA
TCCCGCCTTTGATGCTGACATCACTCAGGTATTTCAGCAAGATCTGGCACAAGTTGCAGC
AGCTCCTGATCCAGATTTTGCTGACATTGTCACAATAGGTCGCCCACAATTTTCAGAAAC
AGAGAGTGGACAAATTCGTGTTAGTAGATTAGGACGCAGGGGCACCATACAGACTAGAAG
TGGTATACAAATTGGTCAAGCAGTACATTTCTATTATGATTTAAGCACCATTGATACAGC
AGATGCAATAGAATTGTCTACTTTAGGACAACATTCAGGAGACCAGAGTATCGTAGATGC
AATGGCAGAAAGTAGCTTGATAGATCCTTTCGCTACACCTGATACCACTGTTGCAGAGGA
ACAGCAATTATTGGATCCTCAAACTGAGGATTTTAGTAATTCTCATTTAGTACTAACTAC
TAGTAACAGAGGTTCAACTTTGACTATTCCTACTATTCCTCCAGGAATTGGTCTACGAAT
TTATGTAGATGATATTGGTTCTGACTTATTTGTTTCTTATCCAGAATCAAGCTTAATTCC
TCCTGGAGGATTACCAACTGAACCATTTTTCCCTCTGAAGCCAGCATTATTAACAGATTT
TTACAGTGATTTCACTTATTATCCTAGCTTATATCGCAAAAAACGAAAACGATCAGATCT
GTTCTGACTTTGTTTTTTTACAGAAAAAATGACGAGTTGGTTGTCTACTACTGGTAAGGTG
TACCTGCCTCCTGCACAACCTGTGGCAAGAGTTCTGGAAACAGATGAATATATTACTGGA
ACATCACTATACTTCCACGCTGGAACAGAAAGACTTTTAACTGTAGGCCATCCTTATTTC
CCAGTGAAAGATGTAGGTGAACCTAAGAAAGTTTTAGTTCCAAAGGTATCTGGAAGTCAA
TATAGAGTCTTTAGGTTTCAATTGCCAGATCCTAACAGATTTGCTTTGATAGATAATGGC
TTTTATGACTCTGACCATGAAAGGTTAGTATGGCGTCTAAGAGGAATTGAAATAGGCCGA
GGTGGACCTTTAGGAATAGGAACAACTGGTCATCCTTTATTTAATAAATATGGAGACACT
GAAAATCCAAATGCATATAAGACACCAACAGATGATAATAGACAGGATGTCTCATTAGAC
CCTAAACAGACCCAAATGTTCATAATTGGTTGTATTCCAGCTATTGGGGAGCACTGGGAT
AAAGCTGAGGCATGCAGAGAACCAGCACCTCAGAAGGGTGATTGCCCACCTATAGAACTT
GTAAACACATACATTGAGGATGGGGATATGTGTGATATAGGCTTTGGAGCTTTTAACTTT
AAAGCTTTGCAGGATGATAAATCCAGTGCACCATTAGATGTAGTTGGTACTTTGTGTAAA

TGGCCTGACTTCTTAAAGATGAGTAAGGACATTTATGGTGACAGTTTATTCTTCTTTGGC
CGAAGGGAACAGCTTTATGCAAGACACTTTTTTGTTAGAGCTGGGACAATGGGTGATGCG
TTACCAGAGCCTTTTGAAGTGAAGTCTGATTACATAATTGCTGCTCAGAGTAACCAAGAA
CAAAATAATCTTGGCCCTCACATTTATTTTGGAACTCCTAGCGGTTCTCTTGTATCAAGT
GAATCTCAGCTTTTTAACCGACCGTATTGGTTAAACAGAGCTCAGGGAACCAATAATGGT
ATTTGTTGGGATAATCAGCTTTTTGTAACTCTGGTTGATAATACCCATAACACTAACTTT
ACTATTTCTGTAAAGCATGAAGGAGAGGATAACAATTATACATATAAAGCAAATGATTTT
AAACAGTATTTAAGACATATTGAGGAATTTGAAATGGAGTTTATCTTTCAGCTTTGCAGA
GTGCCTCTTACAGCAGATGTAATGGCTCATTTGAATGTCATGAACCCAAATATATTAGAT
AATTGGCAATTGAATTTTGTTCCCCCACCACCTTCTGGTATTGAGGATCAATACAGATTC
ATTACATCCAGAGCTACTCGTTGTCCAACTCAAAATCCACCAGCAGAAAGAGAAGACCCT
TTTAAAGATTTGTCCTTTTGGACAGTTGATCTTTCTGAGCGTTTTTCTTCAGAATTAAGC
CAATTTTCATTAGGAAGGCGGTTTCTCTATCAGAGTGGATTAATAAATGGATCTCGTAAA
AGACAAAGAGCTATTACTTCTCAAACTGCAACAGGCACAAAACGTTCTGCCAAACGAAAA
CGTTTATCTAAGTAACCATGTAAATACATAATGGAATGTATTATTTCTGCCAGGAAAAAG
TGCATATTGAGCCAATTATTATATAACCTATTGAAGGATAAAAATTATAAATCTTTTAAA
TGTGAATGCAATAAACTATACCTCCAATTACTATTGTGTCCATGCTTTGTGACTCATGTG
GTCAATATTGTGTCCGCACCCTGATTACTTGGACTGCCTCCACATTAAGTAAGACGCTGG
CGAAGAAAAGAGACGCTCCTTATTTTTTTTGGAAAAAATATTGAATTTGGCATTATTAGG
CGGGAAACAGGACCGGATTCGTTCGCTATTTCCAGGAGTTAAAGGTAGGTGAAAGTTTTA
CTACACCGATTATGAGTCAATTTGTCTGGCGGCGAAGAACGAATCTGGCTGCCTGCCTTT
GCACCGGGAGTGGTTGT
>gi|40804509|emb|AJ620209.1|_Human_papillomavirus_type_81_complete_genome
CTTCCTTTCTTTTATAATATTTTATAACAATCATACATATAATAAAGTAGGGAGGTACCG
AAAACGGTCGACCGGGAAGGATACATATAAAAGCAAACTGTATGGTCAGCACAGCAGTGA
TGTCGCTGGGGCCAGCAAATCCTACCAATTTGTTTTTGCTGTGTAAGGAGTGTGAAGTGG
ACCTAGACGACCTGCAACTGACCTGCATATTTTGCAAAAAGGAATTAACAGTAGGTGAAT
TGCTGTCCTTTGCAATAAGAGAGCTAAACTTGGTGTGGCGAAAGAACTGGCCATTTGGAG
TATGCGCAGCGTGCTTGTGCAGAGAAGCTAAAGTACGGGAGCTGCGGCGGTGGCAATATT
CGTGCTTCGGACCAACAGTAGAAGAGGAGACAGGGCAACCGCTTGCACAAATATATATAC
GATGCCATGCATGCTATAAACCGTTAACATACCAAGAAAAGGAGTATCTTGTGACAGGCC
TAATCCACTTTCATAAAATAGCTGGAGAGTGGATGGGAAAGTGCTGCCATTGTAGAGGAG
CATGCATGGCCAGGCAGCAACGATAAAGGACATTGTCCTAGATGAGTATCCTGATGTGAT
TGACCTACATTGCCACGAGCAATTGCTAGACAGCTCAGAGGAAGAGGTGGATGTGGATGA
TAGGGACAGTGGTGAGCAACTAGTAGAACAAGCACAGCAGGCCTATAGGGTGGTTACTAC
GTGTGGTATTTGTAGGTGTTTAGTTAGGCTGGTGGTGTTAAGTGGAGACGCAGACCTGAG
GCTGCTGCAACAACTACTGACGGATACACTGTCCATAGTGTGTCCTGGCTGCGCATAAGT
GACATGGCTGATGTGGAAGGTACAGAGGATGGGGACGGGGCGGAGGCCGCGGACAGGGCG
GGAGGGTGGTTTATGGTGGAGGCTGTTGTAGAAAAAACCACAGGGTATCAGGAATCAAGT
GATGAAGAGGAAGATAGTGCAGATACAGGAGAGGATCTAGTAGATTTCATAGATACACGA
TATCCAGGGGATGGGCAGGAAGTGCCGTTAGAACTGTTTGTTCAACAAAATGCACAGGAT
GACGCTGCAGCGGTGCATGCACTAAAACGAAAGTATATACATAGTCCCGCTGCAAGCTCG
TGTGTGTCTTGGGTGGACCATGAACTAAGTCCTCGGTTGGACGCCATAAGCCTAGACCGG
GGGCACGAAAGGGCCCGAAGGCGGTTGTTTGACCAGGACAGTGGGTATGGCCATACGCAG
GTGGATATTGGAACATCCGAAGGACAGGTACCGGGGGACGTGCAACAAACAGTAGAAAAT
GTAGAGCAGGAAGTGCCGGAGGAAGTGCGGGGGGGGGGGGATGGGGAGCCTGCACCTACT
GTGCATTTACAACAAACCCAGGAAGGTACAGCAAGTATAGTAGAGCTGTTGAAGGCCAGA
AACTTGCGAGCCACGTTGCTAAGTAAGTTTAAGGAATTGTATGGGCTGGCATTTGGGGAG
CTGGTAAGACAATTTAAAAGTGATAAATCAACATGTACAGATTGGGTGGTATGTGCATTT
GGTGTGTATTATGCAGTGGCGGAGGCCCTAAAAACATTAATACAACCACTGTGCCATTAT
GCGCATATACAAACGCAAACAAGCCAATGGGGCATGGTGCAGTTAATGCTAATTAGATTT
AAGTGTGGCAAAAGTAGGGACACAGTGGCGCATTGTATTGGAACATTACTAAATGTATCA
GAAAAACAAATGTTAATAGAGCCACCAAAAATTAAAAGCACGCCATGTGCATTATACTGG
TATAAAACAGCAATGGGAAATGCAAGTGAAACAGTAGGGGAAACACCAGAATGGATAGTA
AGGCAAACAGTTGTGGGACATGCAATGCAGGAAACACAGTTTAGCCTGTCTGTAATGGTG

CAGTGGGCATACGACCATGATATAACAGATGAAAGCCAGCTGGCATATGAGTATGCATTA
CTAGGCCATGAGGACCCTAATGCAGCTGCCTTTTTAGCAAGCAACTGCCAAGCCAGGTAT
ATAAAAGATGCCATTACCATGTGCAGACACTACAAAAGAGCCGAACAGGCACGCATGTCT
ATGGCACAGTGGATAGCACATAGAAGCCTAAAGGTACCTGATACAGGGGATTGGAAACCA
ATTGTAAGGTTCCTAAGGTTTCAAAAAATAGAATTTATGACATTTATGGGTGCATTTAAA
ATGTGGCTAAAGGGAATACCAAAAAGAAGTTGCATAGTTATACATGGGCCAAGTGATACA
GGCAAATCATTGTTTTGCATGAGTTTAGTGCAATTTCTAGGGGGTGCAGTTATTTCCTAC
GTAAATGCATCAAGTCATTTTTGGCTATCACCATTAGCAGATGCAAAGGTGGGACTGTTA
GATGATGCCACACACCCATGTTGGGTGTACATAGATACACATTTACGTAGTGTGGTAGAT
GGTAACTTAATAAGTATGGACAGAAAACATAGGAATTTAGCACAACTAAAATGCCCCCCA
TTACTGATTACAACTAACATAAATCCATTAGAAGACGTTACCCTAAAATATTTACATAGT
AGAATGGCAGTGTTTAGCTTTATGTACAAGTGTCCGTTAGATGACAATGGGGACCCAGTG
TATAAATTTAATAATGAAAACTGGAAATCATTCTTCCAAAGGTCCTGGGCACGTTTAGAG
GTTAATCCAGAGGAGGAGGAGGAGGAGGAGGAGGAGAATGGCAACACTAGCCGACCGTTT
AGATGCATGCCAGGAGACGCTAATAGACCTTTATGAAAAAGATAGCAACAAGCTTGAGGA
TCAAATAAAGCACTGGCATTGCATACGTCTGGAAAATGCTATATTGTTTAAGGCAAGACA
AGCAGGACTTACACACTTGGGCCACCAGGTGGTGCCAACACTTAATGTTACAAAAGGAAA
AGCCCACCAGGCTATAGCAGTGCATTTATCGTTACAAAGTTTATATGCAAGCGAGTATAA
AGATGAACCATGGACACTACAAAACACGTCACTGGACATGTGGAATACACAGCCAAAGGG
ATGCTGGAAGAAAAAAGGACGGACAGTAACAGTAAAATATGATTGTGAGGACTCCAAAAA
AATGGAGTATGTAAACTGGGGGCATATATATGTGCAAAGCCCACATACAGAGCAATGGCA
CAGGGTGCATGGACAGGTCAGCTACGACGGGCTGTACTACGAACTGGAGGGCCACAAACA
GTACTATGTGCAATTTGCACAGGAGGCCAGAAAATATGGGGAGCACAACAAATGGGAGGT
ACATTGGGGTAACACAGTAATTTATGAACCATGCAACTCTGTATCTAGCACCCAGGACAC
CCTGCGAGAAGTATCCCCTGCTGAAGCTGCTGACCGACTGCTGCACGCCCCCGAACCCGC
CACCAGTACCCAGCACGTGGGCCCCGCCAAGACCACCAAGGCCCCAGTGCAGGCGCCGCC
TTGCAAGCGACAGCGAGTCAACCGGGACGGAGGGCGGCAGCCCGACACTGCACCACCGAA
CATCTGGGTGGACAATAACAAATCAGGGGCCGACGGTTACCCTCACAGCACAGAAACACG
GAACTTCAGTGACGGTGACACTGCACCTGTAATACATTTGCGAGGTGATGCAAACAAATT
AAAGTGTCTTAGATATAGACTACGAAGCTCTGTACCACATTTATTTGTAAACGCGTCCTC
CACATGGCATTGGACCTGTGGAGAGGCAACGCAAAAGTGTGCATTTGTAACAATATGGTA
TGCTAATGAAGACCAACGTGCTAAATTTTTACAACGTGTGACAATACCAAAGGGCATACA
AGCCATGCAGGGCTTTATGTCTATGTGTTTGTAAAAACATATTTGTGTATAAATGTGTAC
TGTAACATACATATGTATATGGTGAAATGGTATAGTGAAACTTATTGTTCTTGCTGTATT
TGAGCCAGGTTTATACATTGTGGTACCACACCTGCATTGTGCAACCGTTATATAATAATC
CTGGATGACCTGTTGTGTTTAATATCTGGGTGGTGTTATGTGCTTTTGCTGCTTATTCTA
TTCTGGCTTTCCTATCTGTCTGCCTTAACTGCATTTGCTGTGTTTTTTTGTATTATATAT
ATTGGCCTGTTGTGTGTGTATTTGCAGGTTTTGTGGTACATAGGCAGTATATAAACCCAC
CAACCAACCAGCCAATCACTGCTGCTACAGTGTATATAGGTAGTTAGTATGTATCCTGTT
ATGGTCCGCACCCATACTGGGGGCACAGATATAGTGGTGTTTGACATGGATAATAGTGGG
TTATGGTTTTTGTTGTGTATTATACTTGCCTTAATACTTTTTGTAGTGTATAAACTGTTA
CAACTGTAACTGCACATTGTTTTGTACTATAATTTTGCTACATATTTTTTAATAAATACA
TTGTATACTCTATTGTATACCATGCCAAAGGTATTGCACCGTCGCAAGCGTGCATCTGTC
ACAGACCTGTACCGCTCCTGTAAGGTATCTGGGACATGTCCCTCCGATGTAGTACCAAAG
GTTGAGGGTAATACATTGGCAGACAAAATATTAAAATGGGCCAGTTTAGGTGTTTTTTTT
GGGGGTCTTGGAATTGGTACGGCCTCTGGCTCCGGTGGACGCACGGGCTACATCCCTATA
GGTGGCCGTCCCCCTTCTGTAGTTGATATAGGTCCTGTGTCCCGTCCTCCTGTAGTTATT
GACCCGGTGGGGGCGGCGGATCCTTCTATTGTTACTCTTGTAGAGGAGTCCAGTGTTATT
GAGGCTGGGGCCACACATCCCAACTTTACAGGGACTTCTGGGTTTGAAATTACCACTTCC
TCTACTACCACCCCTGCTGTCCTAGATATTACTCCCACAGGTACTACAGTGCAGGTTAGT
AGCACTAACTTTTTAAATCCCTTATATACTGAGCCTTCCATAATTGACCCTCCTCAAACT
GGGGAAATCACAGGCCATGTTTTTACAAGTACACCCACTGCTGGGTCCCATAGTTACGAG
GAAATACCTATGGTAACCTTTGCATCTAATACTGGTACTGGTGGTGAACCCATAAGTAGT
ACACCTATGCCTGGTGTACGCCGTCTTGCAGGGCCCCGCCTTAGATTATATACCAAGGCG
ACCCAACAGGTTCCTGTACGTGACCCTGCCTTTGTATCCCATCCTGCATCTTTTGCAACA

TTTGACAATCCTGTGTATGATCCAGAGGAAACTATTATTTTTGAACATCCTAGTATATAC
CAACCACCTGATCCTGACTTTTTGGACATTGTTCAATTGCACAGGCCTGCTTTAACTGCC
CGACAGGGTACTGTTCGGGTTAGTCGTATTGGTCAGCGGGCCTCCTTACGTACCCGCAGT
GGTAAACATATTGGTGCTCGGGTACACTTTTATCATGATATTAGTCCTATACCCACGGAG
GCTGATATTGAGCTACAGCCCTTGCTGCCACCTTCCTCTGTGTCTTCTGCAGATGCCCTT
TATGATGTATATGCAGATGACCAACATTTAGATGCTGTTTTGCAGTCTGTGCCCTCTTTG
TCTTCCAGGTCCTCTGTTTCAGGCTCTGCCACTTTATCTGCTACCTCTGTGGCCTCCTCA
TATAATACCACCGTTCCTTTGTCTTCTGGGTTTACCATCCCTGCCTCTACTGGGCCTGAT
GTAGAATTGCCTTATGCTCCTTTAGCCCCTTCTATAGTCCCTTCGTTTCCTGCAACTACC
CCGTACGCCATATATGTTGTTGGCACTGATTTTTACTTGTTTCCTTCTTATATATTCTTC
CCTAAAAAACATAAACGTATTCACTATTCTTTTACAGATGGCTATGTGGCGGCCTGGTGA
CGGCAAGGTATACCTGCCTCCCACACCTGTGTCTAAAGTTATCAGTACGGACGCCTATGT
AACCCGCACCAACCTTTTTTATTATGGGGGCAGTTCCCGCCTTCTTACTGTAGGGCATCC
ATATTGTACATTAACTATTGGTACCCAAGGAAAGCGTTCCACTATTCCCAAGGTGTCTGG
GTATCAGTACCGGGTGTTTCGTGTGAAGCTCCCTGACCCTAATAAGTTTGCTTTACCTGA
TGGTACCTTATATAACCCAGACACCGAACGCATGGTATGGGCTTGCAGGGGCATTGAGGT
AGGCCGCGGACAGCCTTTGGGCGTAGGTACTAGTGGTCACCCATTATATAATCGCTTGGA
TGATACAGAAAACACTGCTTATTTGGCTGCTGCCAGTGAGGACAGTCGTGACAATATTTC
AGTTGATTATAAGCAAACACAATTATTAATTATTGGTTGCAAGCCCCCCATTGGGGAGCA
CTGGGCCCGGGGTACCTTTTGTGCTAATGTTGCTCCAGCACAAAACGAATGCCCCCCCTT
GGAGTTTAAAAATACAACCATTCAGGATGGGGACATGGTGGAAACTGGCTATGGGGCCAT
TGATTTTAAGGCCCTGCAGGAAAATAAATCGGAGGTGCCCTTGGATATTTGCAATACCAC
CTGTAAGTATCCAGATTATTTACAAATGGCTGCTGAGCCATATGGGGATTGCATGTTTTT
TTGTCTACGTCGGGAGCAAATGTTTGCAAGGCACTTTTTTAATAGACATGGCACTATGGG
CGAGGCCTTACCTGCAGACTTATATATGAAGGGTGCTGCAGGCAGTGACAGAATAACCCC
CGGGAGTTATATTTATGCCCCTACACCTAGTGGGTCTATGGTATCCTCTGATTCCCAGCT
GTTTAATAAGCCTTATTGGCTACAACGGGCACAGGGCCATAATAATGGCATTTGTTGGTT
TAATGAAATGTTTGTTACAGTGGTGGATACTACCAGAAGCACCAATTTTACTATTTGCAC
AGCTACATCTGCTGCTGCAGAATACAAGGCCTCTAACTTTAAGGAATTTCTGCGCCATAC
AGAGGAATATGATTTGCAGTTTATTTTCCAATTATGTAAAATACAGTTAACACCAGAAAT
TATGGCCTACTTACATAATATGAACAAGGCACTGTTGGATGATTGGAATTTTGGTGTGTT
GCCACCTCCTTCCACCAGTTTAGATGACACATATCGCTTTTTACAGTCTCGGGCCATTAC
CTGTCAAAAGGGTGCTGCTGCCCCTGCGCCCAAAGAGGACCCTTATGCCGACATGTCATT
TTGGACAGTTGACCTTAAGGACAAGTTGTCTACTGATTTAGATCAGTTTCCTTTGGGTCG
CAAGTTTTTGTTGCAGTCTGGGTCCCGCACCCGGTCTTTTGCTGTGTCCCGTAAGCGTCC
TGCGTCTGCCAGGACCCCCACTACCTCTGCTAAGCGAAAACGTAAAAAATAGTAGTATGC
TTGTTTGTGCTGTGTGCTTGTGTGTATGTATTTGCATTGTTTTGTGTTGTTTGTATTATA
TGTGCTTTGTATGTTGTGTTGTATTTATATGTGTATTGTATGCGTGCTGCCCTTTTGCCC
CTACACTGTTGTTTGTATATTTGTGGAATGTGTGTGTTGTGTATACATTATTAAATTGTG
TGTCACACTATGTAGCATGTCCGGTGCACCCTGTGAGTAAGTGTGTGTGTGTTTGTTTAT
GTGTTTGTACGCCTTGCACACTTTGTATTTCCTTACCACTCCTTGCAGTAGTGCCTCCAT
TTTACATACCTCCATTTTAAACCGAAACGTTGCAGGACCGTTTTCGGTCGCCCGCCTTTT
CGGTTTCAATAGTGGCACATTGCCACACTGTACACAAAGCGTTTTTAATCCTTAGGCAAC
TCATTTCCTGCATTGGCAGCATGCTCTGTTCCAAACCTGGGTGTGCCTGACACATTGTTG
GCTTTCTAAACTGTTTTGCACACAATTTATACATTTCACTTACTCATTTTGCAATAAAAC
TGCTTTTAGGCACATTCTTTGTGCTGACTACTATGTGGTAATTGCTGGTTTGGCTTTTTT
ACATGTTTTACACGCCAACAATATGTCATGTAAACTTAGGCAAGCTTTGTGCAAACCTGT
TAAACATACCAGGTGCACTGATGTGTAAGC
>gi|40804474|emb|AJ620205.1|_Human_papillomavirus_type_43_complete_genome
CTAACAATTATTATACTTGTAGTTTAAGGTGGGACCGAAAACGGTCCGACCGAAAGCGGT
ACATATATAAACCACCCAAAAACCATAGCTTGTGGGGCATAATGACTGCACGTAGCTGCT
CCCAAAACGCACGGACTATATTTGAGTTGTGTGATGAGTGTAACATAACTTTGCCTACTC
TGCAAATTGGGTGCATATTTTGCAAGAAGTGGTTACTTACCACGGAAGTATTATCGTTTG
CATTTAGAGATTTAAAGGTTGTGTGGCGCGACGGATATCCGTTTGCTGCATGCTTGGCCT
GTCTACAGTTTCATGGAAAAATAAGTCAATATAGGCACTTTGACTACGCAGCATATGCAG

ATACTGTAGAAGAAGAAACTAAGCAAACAGTGTTTGATTTGTGCATTAGATGCTGTAAGT
GCCACAAGCCATTATCACCAGTGGAAAAAGTACAGCATATTGTGCAAAAGGCACAATTCT
TTAAAATACATAGCGTGTGGAAAGGATACTGCCTACATTGCTGGAAATCATGCATGGAAA
AAAGCCGACGATCAGAGACTATGTGCTAACTATGCAACCAGAACCTAGAAGCTTAACCTG
TAACGAGCAATTAGACAGCTCAGACTCAGAGGATGAGCGTGAGCAACCCACGCAACAGGA
CCAGCAAGTGAATCTACAAGTTTACAGAGTTGTAACAGAGTGCACATCCTGTCTGTGTGT
AATTCGACTTGTTGTGCAGTGCTCTGACAGTGACATCAAGAAGCTAGAAGACCTGCTGTT
GGGCACATTAAAAATTGTGTGCCCACTGTGTACCACCACAGCGGTGTGACTACCATGGCT
GATAAACAAGGTACAGAGGATGAGGGGTTGGGATGCTCAGGCTGGTTTATAGTAGAGGCA
ACAGTGGACAAAACAACAGAAAACAATATATCGGAAGATGAGATTGAGGACGAGGAAGAG
GAAGATAGTGGGTTTGATATGGTAGATTTTATTAATAATACATTAGAAGACAGTTGTACA
GACCACAGCAGTGCGCAGGCTCTGTTAAATGCACAACAAGCGGATGCTGATGCTGCTATA
GTGCAGGAGTTAAAACGAAAGTACATGAGTCCCTATGTAAGTCCTATACACTGTTTACAG
GAATCTGTAGACAGAGATCTAAGTCCCAGGTTGCATGCAATAAAGATTGGAGGCGGACAA
AAAGCAAAAAGGAGACTGTTTCACGTAACAGAGCAGCGGGATAGTACCAATGGCAATACT
GAAGTGGAAGCAAGAGAGACACAGGTAGAGGTGGAATATGGCGGACCGGAACGCCATATG
GGGGCCAATAAAGGGTGTGGGAGGGGGAGTAGTAGTGTGGCGGAAGCTGTAGAGGTTGTA
GAGGAAGCGGGTGCAACAAACAGTAGCCAAGACATAGGAGAGCCAAGCCCACGCACGCGT
ATAATAGAATTGTTTAAAGACAAGGATGTTACTGTAAAACTGTTAGGTAAGTTTAAAGAA
TTATTTGGTGTGGGGTTTAATGATTTAGTAAGACAATTTAGGAGTGACAAATCAACATGT
ACCGACTGGGTATATAGTGTATTTGGTGTTAATCCATCCATATCAGAAGGATTTCACATG
TTATTAAAAGAGCATACCCTATACCTACATACCCAGTGGGTGACCTGTAGATGGGGAATG
GTATTGCTTGCATTATGTAGATATAAGGTTGCAAAAAATAGAAGTACAATAGTAAGACAG
CTAGCACAAATGTTAAATGTGCCTGTACAACAAATTTTAATTCAACCACCAAAATTACAA
AGTGCACCTGCAGCACTATTTTGGTTTAGGTCTAGTATGGGAAATGGTAGTGAGGTAACA
GGCACAACACCAGAATGGATAAGCAGACAAACCATGCTGGAACATAGTTTTGCAGATACA
CAGTTTAGTTTAACTGATATGGTGCAATGGGCATATGATAACGGATATACAGAGGAATAT
GATATAGCATATTATTATGCACAAAGAGGTGATATAGATGCAAATGCAGCTGCATTTTTA
AAAAGTAACATGCAAGCTAGATATGTTAGGGATTGTGCATGCATGTGCAAACATTACAAG
TTAGCAGAAATGAAAAAAATGTCAATGGCGGAGTGGATTAAACATAGAGGGGGGAAGTGT
AATGATGGAGACTGGAAACCTATAGTAAAATTTTTAAAATATCAACATATAGACATTATA
GCATTTCTAGGTGCTTTAAAAAAATGGTTACATGGTATACCAAAAAAGAATTGCATTTGC
ATAATAGGTCCGCCGGACACAGGCAAATCATGCTTTGGAATGAGTTTAATGAAATTTTTA
GGCGGTACCATATTATCATATGTAAATGCAAGCAGCCATTTTTGGTTACAGCCATTAGTG
GATGCAAAAGTGGCCATGTTAGATGATGTTACAGCAGGCTGTTGGACATACATGGATATG
CATATGAGAAACCTATTAGATGGAAATCCTACAAGTATTGATAGAAAGCATAGAGCACTC
ACAGTAATAAAGTGTCCTCCATTATTGCTAACATCCAATTTGGATATTAGTACAGAAGAC
AAGTATAAATATTTAAGAAGTAGAATTACAACATTTACATTTCCTAATACATTTCCATTT
GATACTAATGGCAATGCCATTTATGAGTTAAATGATGAAAATTGGAACTCCTTTTTTAAA
AGGTTGGCATCAAGCTTAGAACTGGACACTGCAGAGGACGAAAATGGAGACACTAGCCAG
GCGACTAGATATGTGCCAGGAACAGTTGTTAGAACTGTATGAAAAAGACAGCAAAACTCT
GGAGGACCATGTGTTGCATTGGAAATATATACGTTATGAGTGTGCATTATATTATAAAGC
AAGAGAGGCGGGCATTAAACATCTAGGCCACCAGGTGGTGCCACAGCTAGAAGTATCAAG
ACAAAAAGCATATTTGGCTATTGAATTACAAATGTCATTAGAAGCATTATTACAAACTGA
ATATAGCCTAGAACCGTGGACACTACAGGACACCAGTCAAGAAGTGTGGCTTACAGAACC
ACAAAAATGCTTTAAAAAAAGAGGACAGACAGTAGAAGTCAGATATGACTGCAATCCTGC
AAATGCAATGCATTACACCCTATGGTCAGACATATATGTACCTTTGAATAGCACATGGCT
AAAAGTTTCTGGCCATGTGGACTATGAAGGCCTATCATACACAGTATGTGGGCAAAAGCA
CTATTATGTAGAATTTCATAAAGAGGCACGAACGTATGGGGAAACTGGACAATGGAACGT
TGTTATGGGCTCAAACGTCATATATTCTCCTGCATCTGTATCTAGTACCGTCAGTGAAGT
ATCCTCTGTTGCGTCTACTGAATCTGACACCGGACCAGCGACCACCGTCCCAGATTCCAC
GTGCACCCAAAAAGCAGACTGCCAGGAGCAAGCGCCGCCTAGAAAGCGAGTGCGATTCGA
CCCCCACACTACCCCGATCGCCGACTTGGCCAGAACTGTTGGAAGAGGGTCAGTGGACAG
TTCAGACTCGCGACTCGTGCCTAAGCATACGGACCACCACCCTAGAGGGCACAACCGTGG
AAGTCACACTACGCCTATAATACAGTTACAAGGTGAAGCAAATGCACTGAAATGTTTTAG

ATACAGATTGAATAAACATAAACATTTGTTTGCTGATGTGTCATCCACATGGCGATGGAC
AACCGAGTGTAATAATAAAACTAATACTGCATTAATTACATTAACATATATTAGTGAACA
ACAGAGGGCTGACTTTTTGTCTCGTGTTAAAATACCTATAACTATTAAACAGTGCCTGGG
AGCATTAACAATGATGTAAATCATGTATGTATGTATGTATATTTGTATAGAGGAACTGTA
ACTGTGATAAAAGGGGGGGTATTGTACCCCAACCACACAACCAGCCAACCTGCTGCTATT
ATTGTTTATAGATGACAAGCTTGGCATATTAACACTTAACATTTTTATTTGCATTCTTGC
TGTATTGGTGTGCGCATGGGTAACGGACAACGTGGTGTGGGCTTCTGTGCTTGCATGTGC
TTCTATCTTTTATTTTTTGTCTTGGGGCGTCCTTACATCACCTATTAACGTTTTTATATT
GGCGTTGTTAGTGTGGTATTTGCCAGCCCTTTTATTGCATTGGTATATTATATCTGCTAT
TGCATAACAGTAAATATGTTAACCTGTACATTTGAAGATGGTGATACATGGATGTTTTTA
TGGTTACTTGTAACAGTTATAACTGTATTAATATTGGTGTTAGCGTTTCATTACAGAACA
CTTAAGTTGCATCAGTCCTCCAGCAAATAAACAATCCTTTATATAATCACTTGTAAACAT
GGTGTCTCATACACATAAAAGGCGCAAACGGGCATCAGCTACACAATTATATCAAACATG
CAAGGCTGCTGGCACATGTCCCTCGGATGTAATTAATAAGGTTGAGCATACTACAATAGC
AGATCAGATATTAAAATGGGCGAGCATGGGAGTGTATTTTGGAGGGTTGGGTATTGGAAC
AGGCTCAGGAACTGGAGGCAGAACAGGCTATGTCCCTCTAACAACAGGTCGTACGGGTAT
TGTCCCTAAGGTGACTGCAGAGCCTGGAGTAGTGTCACGTCCTCCTATTGTTGTAGAATC
TGTTGCTCCAACTGATCCTTCTATTGTGTCCTTAATTGAGGAATCAAGCATAATTCAGTC
CGGGGCTCCTATTACCAATATTCCATCACATGGTGGCTTTGAGGTAACCTCCTCTGGATC
AGAGGTTCCTGCAATTTTAGATGTTTCCCCATCTACTTCAGTGCATATTACTACATCTAC
ACATTTAAATCCTGCATTTACTGATCCTACTATTGTACAGCCAACCCCCCCAGTTGAGGC
TGGGGGACGTATTATAATATCTCACTCCACTGTTACTGCTGATAGTGCTGAACAAATTCC
TATGGATACGTTTGTTATACACAGCGATCCTACCACTAGCACACCTATTCCAGGCACTGC
CCCACGACCTCGTTTGGGCCTGTACAGTAAGGCATTGCAGCAGGTGGAAATTGTTGACCC
TACATTTTTGTCCTCGCCACAACGTTTAATTACATATGACAATCCTGTATTTGAGGATCC
TAATGCTACATTAACATTTGAACAGCCTACAGTACATGAAGCTCCTGATTCTAGGTTTAT
GGATATAGTTACTTTACATAGACCTGCATTAACATCCCGACGAGGTATAGTTAGATTTAG
TAGGGTGGGTGCGCGCGGTACTATGTATACTCGCAGTGGTATACGTATTGGGGGTCGTGT
ACACTTTTTTACAGATATTAGTTCCATACCCACAGAGGAATCAATAGAATTGCAGCCCCT
AGGACGTTCCCAGTCCTTTCCTACTGTTTCTGATACTAGTGATTTATATGATATATATGC
AGATGAGAATCTGTTAAATAATGATATTAGTTTTACTGACACACACGTGTCCCTACAGAA
TTCTACTAAGGTTGTTAATACAGCTGTGCCACTTGCAACTGTACCTGATATTTATGCACA
AACGGGGCCTGACATAAGCTTTCCTACTATTCCTATTCACATTCCATATATTCCTGTGTC
CCCATCTATTTCCCCTCAGTCTGTTTCCATACATGGCACTGATTTTTATTTGCATCCTTC
ATTGTGGCATTTGGGCAAACGCCGTAAACGCTTTTCATATTTTTTTACAGATAACTATGT
GGCGGCTTAATGACAACAAGGTTTACCTGCCTCCTCCAGGGCCTATAGCATCTATTGTGA
GCACAGATGAATATGTGCAACGCACCAACTTATTTTATTATGCTGGCAGTTCACGTTTGC
TTGCAGTGGGTCACCCATATTTCCCCCTTAAAAATTCCTCTGGTAAAATAACTGTACCTA
AGGTTTCTGGTTATCAATACAGAGTATTTAGAGTTAAATTGCCTGACCCTAATAAATTTG
GCTTTTCAGAAACAACACTGGTTACATCAGACACTCAGCGTTTAGTCTGGGGATGCGTAG
GAGTTGAAATTGGTAGAGGACAACCTTTAGGTGTTGGAATAAGTGGCCATCCGTATTTAA
ATAAGTATGATGACACTGAAAACCCGTCTGGGTATGGCACATCGCCGGGACAAGATAACA
GAGAAAATGTAGCAATGGATTATAAACAAACACAGCTGTGTATTGTTGGCTGTACACCTC
CTATGGGTGAATATTGGGGTCAGGGTGTGCCTTGCAACGCATCAGGTGTTACCCAAGGTG
ATTGTCCTGTAATAGAATTAAAAAGTGAAGTTATACAGGATGGTGACATGGTAGATACAG
GATTTGGTGCAATGGATTTTGCTTCCCTACAGGCCAGTAAAAGTGATGTACCCTTAGACC
TGGTTAATACTAAAAGTAAATATCCTGATTATTTGGGAATGGCAGCAGAGCCTTATGGGA
ATAGTTTGTTTTTTTTTCTACGCCGGGAACAAATGTTCCTTAGACATTTTTTTAATAAAG
CTGGTAAAACTGGCGACGTTGTGCCTTCCGATATGTATATTGCTGGCTCTAATACCAGGT
CCAAAATTGCAGATAGTATATATTTTTCTACACCCAGTGGGTCTTTGGTTACTTCTGATT
CTCAATTGTTTAACAAACCCTTATGGATACAAAAGGCCCAGGGACATAATAATGGCATTT
GTTTTGGGAATCAGTTGTTTGTTACAGTGGTAGATACCACTCGTAGTACAAACTTAACGT
TATGTGCCTCTACTGACCCTACTGTGCCCAGTACATATGACAATGCAAAGTTTAAGGAAT
ACCTGCGGCATGTGGAAGAATATGATCTGCAGTTTATATTTCAATTATGCATAATAACGC
TAAACCCAGAGGTTATGACATATATTCATACTATGGATCCCACATTATTAGAGGACTGGA

ATTTTGGTGTGTCCCCACCTGCCTCTGCTTCTTTGGAAGATACTTATCGCTTTTTGTCTA
ACAAGGCCATTGCATGTCAAAAAAATGCTCCCCCAAAAGAACGGGAGGATCCCTATAAAA
AGTATACATTTTGGGATATAAATCTTACAGAAAAGTTTTCTGCACAACTTACCCAGTTTC
CCTTAGGGCGCAAATTTGTTATGCAGGCGGGTTTGCGTCCCAAACCTAAATTAAAAACTG
TAAAGCGTTCTGCACCATCCTCCTCTACGTCTGCCCCTGCCTCTAAACGCAAAAAAACTA
AGCGATAACATATGTGTGTGTGTTTTGTACCTGTATGGGTTGTTTTTGCTTGTTGTGTAT
GTATGGTGTGTTTGTGTTACGTACTTGTTTGTGTATGTGTGTATATGTGTTGTATGTATT
GTTGCAATGACAAATAGAAATGTTTGTGTGTATATGTGTATGTTGTATACATTAATAAAG
ATGTTTGTGTGTGGTGTGGTTTGTGGTTGCACCCTATGAGTAACTATGTGTGGTGTTATG
TGTTTATTACCTTTTCCTTTTCCTGTGTTTTTGAGTTTACTATTTGTAACTGGCTTTGGT
TTTAGTAATGGTTACTGTTGTGGGTATGTAAAATGGCGTCTGAACGTCTTCCATTTTGTT
TTTGCAACCGATTTCGGTCGCGTTTGGCACCCTTATTCATGGTGTCAGCATAATTTGCAA
AGGTTACTGTGCCCCTGCCAAAATATGTTGGCACACTTTTTATCCCTTTGGCGTCAAGGT
GTGGCTTCCTACGCCCACATTCCACTTTTACTATACAAATGTACCTTTTCACCTCTATGT
TTTATTACCTTTTTATTCTTTTTGACATATATATTTGTTTTTCATAGTTACTAATAGCTT
TTTTGGCTCACATAAATGAACTGCAGTCATAAAATATGTATGAGTAAGTGTTGTGGTAAG
CACACCTGCAGACCGAAACCGGTCCTTACACACCTTACATTCTTTGTGGTTATTA
>gi|14475578|emb|AJ400628.2|_Human_papillomavirus_type_87_(candidate)_complete_g
enome
CAACAATCATCTATTTTAAAAATAAGGAGGACCGAAAACGGTACGACCGAAAAGGATACA
TATATAAGGGGCCCACAGGGCGGTGTATGTGCAACGGGACAGTCATGCCCAACGGACGCT
ACACACCTACCAATATCTTTTTGCTATGTCAGGAATATGAGGTGGACTTTGACAACTTAA
GGCTGCTTTGCATTTTTTGCAAGGAAGAATTAACAGAAAGCGAATTGCTGGCGTTTGCAT
TAAGGGAATTATTGCTGGTGTGGAGATTTGGATTTCCTTATGGAGTGTGCCTACAATGCC
TATTCAGAGAAGCCAAGAAACGTGAGCTTCGTCACTGGGAGTACTCCAGCTACGGACCCA
CAGTAGAAGAAGAAACAGGTGTAGCATTGGCACTTCAAAATATAAGGTGCCATGCATGCT
GCAAACCATTGTGCTGGCCCGAGAAGGAGCATATGATACACGCGAATCTGCACTTCCACA
AAATAGCTGGGAAGTGGACAGGAAAGTGCAGCAACTGTAGGGCAATATGCATGGCCAGAC
GCCGACTATAAAAGATATTATTATATCTGAAATTCCAGATGTAGTTAACCTACATTGTGA
CGAGCAATTGTTAGACAGCTCAGAGGAGGAGGACAATAGGGATTGTGTGCGTGACCAACC
TGCAGAACCAGCACAGCTGGCCCATAGGGTGTTAACAGAGTGTGGGTTGTGCAGACGTCC
AGTTAGGCTGGTGGTGCTTTGTGGAGCTGGAGACCTAAGGCAGCTACAGCAGCTTATGGT
GGACGCGGTGGCCATTGTGTGTCCTGGTTGTGCATAACATGGCAGAGTCACCTGAAGGTA
CAGATGAGGGACCGGGGGGGGGGCAGGGAGGTGGTTTGTGGTGGAAGCCATGGTACATCA
CACAGCTAATACAGATGTGTCCAGTGATGAGGACGAATCAGTAAGTGATAGGGGAGAGGA
TATGGTAGACTTTATAGATAATACAAGACATCCAGGGGACGGACAGGAAGTGCCGTTAGA
CCTTTATGTTCAACAAACAATGCAGGATGACGCTGCAACGGTGCATGCCCTAAAACGAAA
GTATATTGGAAGCCCGGCAGCTAGATCCTGTGCATCCTGCGTGGATAATGAACTAAGCCC
ACGTTTGGATGCAATATGTTTACAGAAAAGGCACGACAAAGCCAGGAGAAGGTTGTTTGA
ACAAGACAGTGGGTATGGCAATACGCAGGTGGATATTGGAACATCCGAAAGCCAGGTACA
AGGGGAACAAAACAACAGTGGGGGGGGCGACGTAACACAAGACGTGGTAGAGGAGGAACG
AGGGGGGGGGGATGGGGAACCGCAACCACATGTAAATACAGAGCACATACAGCAGACAGA
GGGCACACCAGACGCATTAGACCTGCTACAGGTTAGTAACCTACGGGTTAAGCTACTAGC
AAAGTTTAAGGAACTATTTGGGTTATCATTTATGGATTTAGTAAGACAATTTAAAAGTAA
TAAGTCAACGTGTGGCGACTGGGTAGTAGGGGCGTTTGGGGTGTATTATGCAGTTGCAGA
AGCAGCGAAAACATTGCTGCAGCCTGTGTGTGATTATGCACACATACAAACATTAACAAG
TGAGTGGGGAATGGTAATGCTGCTACTATTACGGTTTAAGTGCAACAAAAGTAGGGAAAC
AGTGGCACACTGTATAGGCACTATATTAAATATTCCAGAGAAACGTATGCTAATAGAACC
GCCAAAGCAACGTAGTGGGCCATGTGCACTATATTGGTACAAAACAGCCATGAGCAATGC
AAGTGAGGTGTTTGGGGAAACGCCGGACTGGATAGTTAGACAAACAGTAATAGGACATGC
AATGGGAGAAACACAATTTAGCTTATCAGTACTGGTGCAGTGGGCGTATGACAATGAAAT
AACTGAGGAAAGTGAGCTAGCCTATGAATATGCACAACTGGGAAATGAGGATGCAAATGC
TGCTGCATTTTTAGCAAGTAATTGTCAGGCAAGATATATTAAAGATGCAATTACAATGGT
AAGACATTACAGACGTGCAGAACAGGCACGTATGACCATGTCACAATGGATTGCATACAG
GGGGCGCAAGGTGGCTGACACAGGCGACTGGAGACATATAGTAAAACTGCTAAGATATCA

AGGAATAGAATTTATTAGTTTTATGGAAGCGTTAAAGCACTTCCTAAAGGGAACCCCCAA
AAAAAGCTGCCTAGTGTTTTATGGACCTAGTGACACAGGGAAGTCAATGTTTTGCATGAG
CCTATTAAAGTATCTAGGTGGAGCAGTAATATCCTTTGTAAATTCTACTAGCCATTTTTG
GTTGTCGCCATTGGTAGACACCAAAATAGGGTTGCTGGACGATGCTACACAGCAGTGTTG
GGTATATATGGATACCTATTTACGAACAGTGCTGGATGGCAATACCATGAGCATAGACAG
AAAACATAAAACCCTACAACAGCTAAAATGCCCACCATTAATGATAACCACAAATGTAAA
TATAGAGGCAGATGACAGTTTAAAGTATTTACGGAGCAGAATGGTGGTGTTTCCGTTTCT
ACACAAGTGCCCACTAGACAGCAATGGAGACCCAGTGTATAAATTAAATAATGAAAATTG
GAAATCCTTTTTTCAAAGGTCGTGGGCACGATTAGACTTAACCCAGGAGGAGGAGGAGGA
GGAGGCGGACAATGGATGCACTAGCCGCCCGTTTAGATGCGTGCCAGGACAAGTTAATAG
ACCTCTATGAAAAAGATAGCAACAAGCTTGAGGATCAATTATTGCATTGGCAGTACACGC
GTCTGGAAAAAGCTATGTTATTTAAGGCAAGAGAAACAGGACTAACACATATAGGCCACC
AGGTGGTGCCACCACTTAGTGTAACAAAAGAAAAGGCCCGGCAAGCTATAACAGTGCATT
TATCTCTACAAAGCCTAAATAACAGTGAATTTAGGCACGAGCCATGGACATTGCAGGACA
CATCATTGGACATGTGGAATGTGTCCCCAAAGGGGTGCTGGAAAAAACAAGGACGTCCTA
TCAGAGTAAAATATGATGGTGAAGATGACAAAGAAATGGAATATGTCAGCTGGGGGTATA
TATATATACAGTGTGCTAGCACTGATACATGGCTTAAGGTGCCTGGACAGATTAGCCACC
AAGGGTTATATTATGAACTGGAAGGCTGTAAACGGTACTATGTTGACTTTGCAAAGGAGG
CAAAACACTATGGGGCAAAAAACATGTGGGAGGTGCATGTGGGAAGCACAATTATTTACC
ATACATGCGAATCTGTATCCAGTACTCAGGGAGGCGTGTCAGAAGTATCCACTGCTGCAA
CTGCTGCAAAGTTATACCACACCACCACCGCGCCCACCCCTACCGCCACCAAGAAAGCCT
CCCAGGTGCAGGCGCCGCCTGTTAAGCGACAGCGACTTGGAGGAGACTGCACCCAGCAGC
CCGATTCTACACAGGACAAGCGACCATCCGTGGACAGTTGCTACACACGGACAAACAATA
ACCCTAACAACACACAGTGGACACGGGACAACTGTATCAGTAACGGTGCACCTGTCCTGC
ACCTAAAAGGTGACCCAAACCGTCTTAAGTGCTTTAGATATAGGTTACAGCAATCAGTGC
CCCAATTGTTTGATAAAGCATCGTCCACGTGGCGGTGGACCTGTGGGGGGACGGACGAAA
AATACTCCTTTGTAACATTGTGGTACAAGGACACTGAACAACGCAAACAGTTTTTGGCAC
GTGTAAATATACCTAAAGGGATTGTGGCTACACAGGGACATATGTCTATGTGTGTATAAA
GCATTGTGCATAATACTGTATACATTGGTGTACCTGTATGTAACCTAGCTGTATAATACA
TTTTGCATACCTGACATATACAGCACAACCCTGGACCTTATTGTATGGGCCGTGCATGCA
TGGTGTAATGTGTTGCTGCTGCTAATTCTGTTTTGGCTTTCCCAACTATCTGCCTTTGTT
GCTTTTCTTGTGTTTGTGTGTTTTATCTATTTAGGGTTGTGGTTTTTGTATATGCAGGTA
CTGTGGTACATTGAAGTGTTATAAGTAAATCACACAACCAGCCAACCTACTGCTGCTACA
TATCCTTGTACAGCCCCTACCCTATACAGTATGTATCCATTACAGGTGCGGGGACCCCAA
GGTGGATATGATATAGTATTGTTTGAGCGTGGAGATGTTGGTATATTGGCATTAATTTTG
TTACTGATTGCTATTATCTTAATTTTGTTATACATACGCATTTTGCATTTATAATATTCT
GTATTGTAATTTTGTATTGTTTGTAATAAACTGGTACCATGCCAAAATCCCGTCGCAAAC
GTGCCTCTGCAACAGACCTGTACCGCACCTGCAAGGTTACAGGCACATGTCCTGCAGATG
TGGTTCCTAAGGTGGAGGGGGATACATTGGCTGATCGTATTCTTAAGTGGGCCAGCCTGG
GTGTCTTCTTCGGTGGCCTTGGCATTGGCACCTCATCGGGCACTGGTGGCCGCACTGGCT
ATATACCCTTGGGAACACGGCCCCCAACCGTGGTTGATGTAGGGCCTACCGCCCGCCCAC
CTGTGGTTATTGAACCTGTGGGGGCCGCGGATCCATCCATTGTTACATTGGTGGAGGATT
CTAGTGTTATTAATTCTGGGGCCCCGTTTCCTAATTTTACTGGAACAGGTGGGTTTGAGG
TTACCACCTCTTCTACCACCACCCCTGCAGTATTGGATATTACTCCCTCCGGGTCGTCTG
TGCAAGTTAGTAGTACAAGTTATATTAATCCCTTATTTACTGAACCTTCTATTATAGAGG
TTCCCCAGGCTGGTGACATTGCTGGCCATGTATTGTCTGGTACAGCTACCTCGGGTACCC
ATACGTTTGAGGAAATTCCTATGCAAACCTTTGCTGTACAAGGGGGGACAGGTCAGGAAC
CCATAAGCAGTACACCTATTCCTGGGGTGCGGCGCCTTGCAGGGCCTCGCTTAAATTTGT
ACAGCCGTGTTACCCAGCAGGTTCCTGTTACTGACTCTGCATTTTTATCACGTCCTGAAT
CATTTGTTACCTTTCATAACCCTATGTATGACCCAGAAGAAACTATTATATTTGAGCATC
CTAGTTTGCATGCCCCTCCTGATCCAGACTTTCTGGATATTGTTACCTTGCATAGGCCTG
CCTTAACAGCACGTAGGTCAGGTGTTAGATATAGTCGTATAGGTCAAAGGGCATCTATGC
GCACTCGCAGTGGCAAACATATTGGGGCCCGTGTACATTTTTATCATGACATAAGTCCTA
TTGCACCGGCGGACAGTATTGAACTGCAGCCCTTGGTGTCCTCCACTGCCCCCAGTGATG
ATACTTTATATGATATATATGCTGATGATACCCATTTGTCCTCTGTGTTACGGGGGCCCT

CTCTTCCATCATTGCACAGCTCTACCCCTACAGCATCTACAAAGGTGTCGGCCACGTCCA
TACTATCCTCTTCCCATTATGACAATGTAACAGTCCCCTTGTCTGCAACAGATGTGCCCC
TATATACAGGTCCCGATATTGATCATTCCATGGCCCCGTCCCCCACGCCCTTTGTTCCTG
TGGTTCCTAGCACTGCCCCATATTCTGTATATATACAGGGGTCTGATTATTATTTACTAC
CTAATTATATATTTTTTCCTAAGCGACGTAAACGTGTGCCCTATTCTTTTTCAGATGGCT
TTGTGGCGGCCTGGTGATGGCAAGGTTTACCTGCCCCCAACTCCCGTGTCTAAGGTTATC
AGCACGGATCGTTATGTATCCCGCACCAACTTATTTTATTATGGTGGCAGTTCTCGCCTG
CTTACTGTGGGTCACCCTTACTATCCAGTTACTGTTACCACCCCTGGTCAGAACAAGAAA
TCCAATATTCCAAAGGTGTCTGGCTATCAGTACAGGGTGTTTCGGGTGCGTTTGCCTGAC
CCTAATAAATTTGGCCTTCCTGACGCCCAGTTATATAATCCTGACACCGAGCGCCTGGTC
TGGGCCTGTAGGGGTCTAGAGGTGGGCCGTGGCCAGCCGCTCGGTGTTGGTACAAGTGGC
CATCCGTTATACAATAGACTGGATGACACAGAAAACACCCCCTTTCTTACTACTGAAAAC
ACTGATAGTAGGGACAATGTGTCTGTGGACTACAAGCAAACCCAGTTGTTAATTGTAGGC
TGTAAGCCCTCTATAGGGGAGCATTGGAGTAAGGGTACTGTTTGTGCCAATGTACAGCCC
AAGACAGGGGATTGTCCACCATTGCAATTTACTAATTCTACTATTCAGGATGGCGACATG
GTTGAGGCAGGTTATGGTGCTATTGATTTCGAAAAGTTACAGGAAAACAAGTCCGAGGTG
CCTTTAGACATTTGTACTACTGTTTGCAAGTATCCTGACTATTTGCAAATGGCTGCTGAG
CCCTATGGCGATTGCATGTTCTTTTGTCTTAGGAGGGAGCAAATGTTTGCCCGCCACTAT
TTTAACAGGCAGGGTACTATGGGCGAGGAGGTCCCTGACACCTATTTTTTGAAGGGGGCG
TCGTCTGGCCCTAGGGCCTCTTTAAGCAGTTATGTGTATGCCCCTACCCCTAGTGGCTCT
ATGGTATCGTCTGATTCCCAGTTGTTTAACAAACCCTATTGGCTGCAGCGGGCCCAGGGC
CACAATAATGGTATTTGCTGGTTTAATCAGTTGTTTGTAACGGTTGTTGATACTACTCGC
AGTACCAATTTTACTATTAGTGCTGCCACTCAAACAACCACTGAATATGACCCCACAAAG
TTTAAGGAATATTTAAGGCATGTGGAGGAATATGATTTACAGTTTATTTTTCAATTGTGT
AAGGTCCGTCTGACCCCTGAAGTAATGGCCTATTTACATACTATGAATGAATCCCTTTTG
GATGAGTGGAATTTTGGGGTTGTGCCCCCTCCTTCCACTAGTTTAGATGATACATACCGA
TACCTTCAGTCCCGTGCCATTACATGTCAAAAGGGGTCTGCAGCCACAAAGCCTAAGGAG
GATCCATATACTGGCATGCTGTTTTGGGAGGTAGATTTAAAAGACAAGTTGTCCACTGAT
TTAGACCAATTTCCTTTGGGCCGCAAGTTTTTGTTGCAGTCCGCCCCTAGGGTGTCCCGT
GTGTCCCGCAAGCGTCCCGCCTCTACCTCTACTGCCTCTACCTCTAAACGTCGCAAAGCC
AAAAAGTAGTGTGGTGTGTGTGCTATATTGTATGTCTGTTTGGTATGTACTTTTTATGTT
TTGTATGTATTGTGTTTGTTTGTATATGCTTTGTATGTATTGTGCAGTGACCTGTATGTG
CCCCTGTCTTGTTGTCCTTGTACTGTTGTGTATGTTTGTGGTGTGTATGTATTTATGGAA
TGTGTGTTATGTTATGGATCAATAAATTGTGTGTCATTGTGGGTTACATGTCCGGCTGCA
CCCTGTGAGTAAGTATGCACCTTGCACACACCTTGTACACCACAACATATCCTCCATTTC
CTGCAAATAGTTTGTATCTCTACCTCCATTTTGTGAGCGACCGAATTCGGTCTCCCGCCT
TTTCGGTCTGTGTTATGGCGCTATTCCAGTACAACTAATCCTTTGGCAACCCTACATCCT
GCCTAGGCAGCTGCAAACGTTCCACACCTAGGTGTGTCTGACATCTCTTTCACTATATAA
TAAAATATTATGTTGTAGTTTATAGGTTTTACTTACTCATCTTCAATAAATATGCTTTTA
GGCAATACTTTGGCTTACAACTTTCCCTTAAGTGCATAGTTGGCTGCCTTAGTACATTGT
ACTGCCAGGTTTCACTTCCATAAACTGCATACTTGTAACACTATCCTGCCAAGTTTCTGT
CTTATAAACCTTAAACAATACTTATGTAACACACACAAATCGGTTGCTGTATTACTCATA
CCTTGTCATTATACTTAA
>gi|39932599|gb|AY395706.1|_Human_papillomavirus_type_62_isolate_Qv18091,_comple
te_genome
ATGACTGCAGGGCCAGCAAGGCCCACGAACCTGTTTTTGCTGTGTAAGGAGTATGATGTG
GACCTGGACGACCTGCACCTAACCTGCATATTTTGCAAAACAGACCTGTCCGCCGGTGAA
CTGCTGTCCTTTGCAATACGGGAGCTACACGTGGTGCGGCGAAAGGACTGGCCATTTGGA
GTATGCGCAGCATGCCTATTGAGAGAGATTAAAGTAAGAGAGCTACGGCGGTGGCAGCAC
TCATGCTTTGGAATAACAGTAGAAGAGGAAACAGGCAGTCCGCTTGCACAAATATATATT
AGATGCCATGCATGCTGTATGCCATTAACCTGTCAGGAAAAGGAGTATCAGGTAGAGAGG
GGAGTGCACTTCCACAAAATAGCAGGAGTGTGGACAGGACGGTACCGCAATTGTAGAGGA
GTATGCACGGCCAGGCAGCAACTATAAAGGACATTGTCCTACAAGAGCGTCCCGATGTGG
TTGACCTACATTGCCACGAGCAATTGTTAGACAGCTCAGAGGAAGAGGTGGATGATAGGG
ACAGTGCTGAGCAACCTGTAGAACGGGCACAGCAGGCCCATAGGGTGGTTACTACGTGTG

GTATTTGCAACTGTTTAGTTAGGTTGGTGGTGTTAAGTGGAGACGCAGAACTCAGACGGC
TGCAACAACTACTGACGGATGCACTGGAGATAGTGTGTCCCGGCTGCGCATAAGTGACAT
GGCCGATGCGGAAGGTACCAATGGCGGGGATGGGATAGATGGTACAAACGGGGCCGGAGG
GTGGTTTATGGTGGAAGTCATAGTAGAACAAACAACAGGGTACCAGGTGTCCAGTGATGA
AGATGAAAACAGTATAGACACAGGGGAAGATCTGGTAGACTTTATAGACAATACCAGACA
TCCCGGGGACGGGCAGGAAGTGCCGTTAGAATTGTTTGTTCAACAAAATGCACGGGATGA
CGCTGCAGCTGTGCAGGCACTAAAACGAAAGTATACATATAGTCCTGCAACAAGCTCATG
TGTATCCTGGGTGGACTGTGAATTGAGCCCGCGGTTGAACGCAATAAGCCTAGACCGGGG
ACACGAAAGGGCCAGAAGGAGGTTGTTTGACCAAGACAGTGGGTATGGCCATACGCAGGT
GGATACGGGACCATCCGAAGGACAGGTACCGGGGGATACACAACAAACAGATGAAAACGT
AGAGCAGGAAGTGCAAGAGGATGTAAGGGGGGGGGGGGATGGGGAACCGGAACGCAGTGT
AGATGTGCAACAATCGCAGGAACGCACAGCAAGTATTGTAGAGCTGTTAAAAACCAGTAA
CCTGCGAGCAACACTGCTAAGTAAGTTTAAGGAGCTGTATGGGCTGGCATTTGGAGAGCT
GGTAAGACAATTTAAAAGTGATAAATCAGTGTGTGGGGACTGGGTTGTATGTGCCTTTGG
TGTATACCATGCAGTGGCAGAGGCTATTAAAACATTAATACAGCCTGTGTGCCTATATGC
CCATATACAAAGCCAAACCTGTTCGTGGGGTATGGTAATATTAATGCTTATTAGATTTAA
ATGTAGTAAAAGCAGGGAAACAGTGGCCAAATGCATTGGCACATTGCTAAATGTGCCTGA
CAAGCAAATGTTAATTGAACCACCAAAAATTAGAAGCGCGCCCTGCGCACTATATTGGTT
TAGGACGGCCATGGGGAATGCAAGTGAAATATTAGGGGAAACACCTGAGTGGATAACAAG
ACAAACAGTTGTGGGACATGCAATGCAAGAAACACAATTTAGCCTATCAGTGCTTGTACA
GTGGGCATATGATAATGATATAGTGGACGAAAGTGACCTAGCATATCAATACGCATTGCT
GGGCAATGACGACCCCAATGCAGCAGCCTTTTTAGCAAGCAACTGCCAAGCAAAGTATAT
AAAGGATGCAATTACAATGTGTAAATATTATAAACGGGCAGAACAAAAACGTATGTCTAT
GGCACAGTGGATAGCACATAGAAGCCAAAAGGTAGCTGATACAGGGGACTGGAGACCTAT
AGTACGGTTTTTAAGGTATCAAAAAATAGAATTTGTGACATTTATGAGTGCACTAAAAAT
GTTTTTAAGAAATATACCCAAAAAAAGCTGTATAGTAATATATGGGCCAAGTGATACAGG
GAAATCATTGTTTTGCATGAGCCTATTAAAGTTTTTAGGAGGGGCAGTAATATCCTATGT
GAATTCCACCAGCCACTTTTGGTTATCCCCCCTAACAGATGCTAAAGTGGGGCTACTGGA
CGATGCCACGTACCCATGCTGGGTATATATAGATACACATTTACGGAGCGTGCTGGATGG
AAACCAAATAAGCATAGATAGAAAACATAAAAACCTGACACAGATACAATGTCCTCCCTT
ATTTATTACCACCAATATAAATCCTTTGGAAGACGCAAATCTAAAATACCTGCATAGTAG
AATTGCAGTATTCCACTTTATGTACAAGTGTCCATTGGATGACAAGGGGGACCCAGTGTA
TCAATTTAATAATGAAAACTGGAAATCCTTTTTTCAAAGGTCCTGGGCACAAATAGAGGG
TACTCCTGAGGAGGAGGAGGAGGAGGAGGAGGAGGACAATGGCAACACTGGCCGACCGTT
TAGATGCGTGCCAGGAGAAACTAATAGACTTTTATGAAAAGGATAGCGACAAGCTTGATG
ACCAAGTAAAACACTGGCAGTGCATACGCCTGGAACATGCCATTTTATTTAAGGCAAGAC
AAGCAGGACTAACACAGTTGGGCCACCAGGTGGTGCCAGCACTTAGTGTTACAAAAGGAA
AAGCCCACCTGGCAATAGAAGTGCATTTAGCATTGAAAAGTTTATATGCAAGTGTGTATA
AAGACGAACCATGGACATTGCAAAGCACGTCTTTGGACATGTGGAACGCAAAGCCACAAA
AATGCTGGAAGAAAAAAGGACGAACAGTTACAGTTAAATTTGATTGTGAGGACTCTAAAA
TAATGGAGTATGTAAGCTGGGGGTACATATATGTGCAAACAGATACCGAGCAATGGTGTA
AAGTACAAGGAAAGGTAAGCTATGAAGGGCTATACTACGAACTGGAGGGCCACAGACACT
ACTATGTAGTATTTGCAGACGAGGCTAAAAAATATGGGGACCTTGACAAATGGGAGGTCC
ATTGGGGGAACACAGTGATTTATGAACCATGCAACTCTGTATCTAGCACCCAGGACATTG
TGCGAGAGATACCCCCTGCTGAAACTACTAGACTGCTACACACCCCCGAACCCACCACCA
GTGCCCAGCACGTGGGCACCACCCAGACCACCAAAGCCACCCCGGTGCCGACGCCGCCTT
GTAAGCGACAGCGAGTCCACGGACACGGAGCGGAGCGGCAGCCCACCGTTACAGAAGCGG
ACAGCAGGGTCGTGGACAATAACGAGTACGGGACCAACAGTTACCCTGACAGCACAAAAG
GACGACACGTGTGTGACGGTGGCGCTGCACCTGTAATACACCTGCGAGGTGAACCAAACA
AATTAAAGTGTCTTAGATATAGATTACGCGGGTCTGTACCACATTTATTTGTAAAGGCGT
CCTCCACGTGGCATTGGACCTGTGGGGACAGCACAGAAAAGGATGCATTTGTAACATTTT
GGTATGCCAATATAGAACAACGCACAGAATTCCTGCAACGTGTAACAATACCAAAAGGCA
TACAAGCAATGCAAGGGTATATGACCATGTGTATGTAACCATATATATATGTACATATAT
ATTACTGTAATCACATATAGCTACGTGTGTAAACAATATTGTTTGTGCTGTATCTGAGCC
AGGTTTATACAGTAAGGTACCACACCTGCATTGTGCAAACGTTACCAATCCTGGATAACT

TGCTATGTTGTATATGTGGGTGGTGTGATGTGCTTTTGCTGCTTATACTTTTCTGGCTTT
CCCAGCTGTCTGCCCTAACTGCCTTTGCTGTGTTTTTTGTATTATATATGTGGGATTTG
GGCTTTTGTATTTGCAGGTTATGTGGTACATTGGTATGCTGTAAACCACAACCAGCCAAC
CACTGCTGCTATTGTGTATATACATAGTAGGTGTATTTGCAGGTGTTTAGTAGGTATGCA
TCCTGTTATGGTACGCTCCTATGACGGGGGCACCGATATTGTGGTGTTGGGGTCTGATGA
CAGTGGTCTACTGTTTTTATTGTGCCTCATAGTGGCATTAATACTGTTTGCTATATACAA
GTTGTTGCAATTGTAACTACCAGCGTTGTACATACCATATTTCTGTTATTGTTGCTTTAT
TTTTTATATTTTGTATGTTGCAATAAATACATTTATACATTGGCTTTCAATATGCCCAAG
GTACTGCATCGTCGCAAGCGTGCATCTGCCACTGACCTTTATCGTACCTGCAAAGTATCA
GGCACGTGTCCTTCTGATGTCATTCCCAAGGTGGAAGGCAATACATTGGCAGATAAAATA
TTAAAGTGGGCCAGCCTTGGGGTATTTTTTGGGGGTCTAGGTATTGGTACGGCCTCTGGC
ACGGGTGGGCGCACCGGCTACATACCTATAGGGGGCCGGCCCCCGTCTGTGGTGGACATT
GGCCCCGTGTCCCGCCCCCCTGTGGTTATTGAACCTGTGGGCGCTACGGATCCATCTATT
GTTACCCTTGTTGAGGATTCCAGTATTATTGAGGCCGGGGCTGTGCATCCCAATTTTACA
GGGTCCTCAGGGTTTGAGGTCACCACATCCTCTACCGCCACCCCTGCTGTTTTGGACATT
TCCCCTACCGGTACCACTGTACAGGTTAGCAGTACCAACTTTTTAAATCCTGCATATACT
GAGCCATCCATTATAGACCCTCCCCAAACTGGTGAGCTTTCGGGTCATGTGCTTACCAGT
ACACCCACAGCTGGGTCACACAGTTATGAGGAAATCCCTATGGTTACCTTTGCATCAAAT
GCTGGTACAGGCTCTGAACCTATTAGTAGCACCCCATTGCCTGGTGTGCGGCGCGTTGCA
GGGCCCCGCCTTGGTTTATACACAAAGGCCACTCAGCAGGTGCCTGTGGCTGACCCTGCC
TTTGTGTCTCGTCCTGCATCCTTTGCAACATTCGACAATCCTATATATGACCCCGAGGAA
ACTATTATTTTTGAGCACCCCAGTATTTACACACCGCCTGATCCTGACTTTTTAGATATT
GTGACTTTACACAGGCCTGCTTTAACCTCTCGCCAGGGCACTGTACGCCTTAGTCGTGTT
GGCCAGCGTGCGTCATTACGCACCCGTAGTGGTAAGCGTATTGGGGCCAGGGTACACTTT
TACCATGACATCAGCCCTATACCCAGTACCACCACTGGTGACATTGAGTTGCAGCCCTTG
CTGCCTTCTGGCTCCTCCTCTGCTGATACATTGTATGATGTATATGCAGATGACCAGCAC
CTTGATGCTGTATTACAGTCTGTGCCTTCTATGTCTTCCAGGCCCTTGGTTCCCAGCAAT
GCCACCATATCTGCCACGTCTGTGGCATCCTCTCATACTAATGTCACTGTACCATTATCC
ACTGGGTTGTCCGTTCCAGCGTCCACTGGCCCTGATGTGGAATTGCCTCAGTTTTCTGTA
CCTGTGTCTGTGCTAACGCCTTCCTTCCCTGCAACTACCCCATATTCTATATATATTGTA
GGTTCTGATTATTATATTGTTCCCTTCCTATATTTTTTTCCCAAAAAAACATAAACGTCTT
CACTATTTTTTTACAGATGGCTATGTGGCGGCCTGGTGACGGCAAGGTATACCTGCCTCC
CACTCCTGTTTCTAAGGTTCTCAGTACGGACACCTATGTTACACGCACCAACCTTTTTTA
TTATGGGGGCAGCTCCCGCCTTCTTACTGTGGGACATCCATATTGTACTTTACAGGTTGG
CCAGGGTAAACGGGCCACCATTCCTAAGGTGTCTGGGTATCAGTACAGGGTGTTTCGTGT
GAAATTACCAGACCCTAATAAATTTACTTTACCTGATGCAACCTTATATAATCCAGACAC
GGAACGCATGGTATGGGCCTGCAGGGGCATTGAGGTCGGCCGTGGGCAGCCACTGGGTGT
TGGCACCAGTGGCCACCCGTTATATAACAGGTTGGATGATACAGAAAATACCTCTTTGTT
GGCTGCTGCTAATGATGACAGTCGGGACAATATCTCTGTGGATTATAAGCAGACCCAGTT
GTTAATTGTGGGGTGTAAGCCCCCTATAGGAGAGCACTGGACCAAAGGTACCTTATGCCC
CAATGCTGCCCCGGCCCCCACGGAATGCCCTCCGTTGGAATTTAAAAATACAACTATTCA
GGATGGTGACATGGTAGAAACGGGCTATGGGGCTATTGATTTCAAAAGCTTGCAGGAAAG
TAAATCTGAGGTGCCCTTGGATATATGCACCTCCACATGTAAATATCCTGACTATTTGCA
AATGGCTGCAGAACCTTATGGCGATTGTATGTTTTTTTGTTTGCGCCGTGAGCAAATGTT
TGCCAGACATTTCTTTAACAGACATGGCACTATGGGCGAGGCCCTGCCCACTGACCTGTA
TATGAAAGGTACCCCAGGCAGTGACAGACAAATGCCGGGCAGTTACATTTATGCCCCCAC
TCCTAGTGGGTCTATGGTGTCGTCTGATTCTCAGTTGTTTAATAAGCCATATTGGCTGCA
GCGGGCGCAGGGCCATAATAATGGTATTTGTTGGTTTAATGAACTGTTTGTTACTGTGGT
GGATACTACTAGGAGTACTAATTTTACTATTTGTACCGCCTCCACTGCTGCAGCAGAATA
CAAGGCTACCAACTTTAGGGAATTTTTGCGACACACGGAGGAATTTGATTTGCAATTTAT
ATTTCAATTGTGCAAAATACAGTTAACCCCCGAAATCATGGCCTACCTGCATAATATGAA
CAAGGACCTTTTGGATGACTGGAACTTTGGGGTTTTACCTCCCCCTTCCACTAGTTTAGA
TGAGACATATCACTATTTGCAGTCTCGGGCTATTACATGTCAAAAGGGGGCTGCTTCCCC
GTCCCCCAAGGTGGACCCGTATGCGCAAATGACATTTTGGACTGTGGATCTTAAGGACAA
GTTGTCTACTGATTTGGACCAGTTTCCTTTGGGGCGTAAGTTCTTGCTACAAGCAGGGTC

TCGCCCTCGGTCTGTTGCTGTGTCCCGTAAGCGTTCTGCACCTACTAAGCAGTCCCCTAC
CTCTGCTAAACGAAAGCGCAGGAAGTAACTGTATGTATGGTATGTGTGTGTGTGCTCTGT
ACTGTGTTGTGCGCGTTTGTCAGTGTGTTTTGTTATATGTATGTATGGCCTGTTTGTATA
TGTTGTGTATGTACCCCCCTCCTCCCGCTGTTGTATATGTATTTATGGAATGTATGTGTT
GTTTGTGCATTATTAAACTGTGTGTCACTGTGCCTAATGTCCGGTGCGCCCTGTGAGTAA
GTGTGTGTGTGTGTATTTGTTTGTACGCCTTGCACACTATGTTATTTCCTTACCACTCCT
TACATTAGTGCCTCCATTTTGTGTGCTACATTTTGTATGACCCCATTGTGTTACCCCGAC
CGTTTTCGGTTGCGCGCCTTTTTGGTTTCTATAGTGGCACACTGCCACACTGTACACAAA
AGGTTTCTAATCCTTAGGCAACTCATTTCCTGCGTTGGCAGCATACTGTGTTTTACACCT
GGGCGTGCCTGACATATTCTTGGCGGCCTACAACTGTTTTGCACACAAATTATACAGTGC
ACTTACTCATTTTGCAATAAAACTGCTTTTAGGCACATTCTTTGTGCTGACTACCATGTC
CTAATTGCTGGTTTGGCTTTTATAAACTTTCTACAAGCCAACTATGTGTCCTATAAACTA
AGCATGCTTTGTGCAAACCTGTTTTAACATACAAGGTGGGGCAATGTGTAAGTCTACTTT
TCCCTTATAATAATTTACAACAATCATCTGTATAATAAAGTAGGGAGGTACCGAAAACGG
TCGACCGGGAAGGGTACATATAAAAGCAAACCGTGTGGTCAGCACAGTAGCA
>gi|4574720|gb|AF131950.1|_Human_papillomavirus_candHPV85,_complete_genome
CTTATACTTTTTTATAATTTTATTACTGAAAAGGGTGTAACCGAAAACGGTCATGACCGA
AATCGGTGCATATATATAACCCAGCTTACAGTACATGTAATAGGATGGCTGAATTTGGCA
ATCCAGCTACCCGACCCTACAAACTACCAGACCTATGCAACACACTGGACACATCACTGC
AAGATATAGAAATAAGCTGTGTATATTGTAAAAGCGTCCTGCAGCGAACAGAGGTATATG
AATTTGCCTTTGCTGATTTATTTGTAGTATATAGAGACGGTATACCATATGCAGCATGTC
AGAACTGTTTAATGTTTTATTCTAAAATAAGAGAACTAAGATATTATAGCGACTCTGTGT
ATGGGGAAACGTTAGAAAAACTAACAAATAGCAATATATATGATATATTAATAAGGTGTT
TACGGTGTCAAAAACCTTTATGTCCGGCAGAAAAACTAAAACACCTTAATGAAAAACGAA
GATTCCATAAAATAGCAGGGAAATATAGAGGACAGTGTCGCCGGTGCATGACGCGTGCAC
AAGAACAGCAAGGCAGCCGCAGAGAAACACAAGTATAAGGGTCGCTATGCATGGACCAAA
ACCTACTGTACATGACATTGTGTTAGATTTGGAACCATATAATGAAGTGCAAGAGGTTGA
CCTGTATTGCTATGAGGAATTAAACAACTCAGAGGAAGAAATAGATGAACCAGATAATGC
AATTAATCACCGACAACCACTACTAGCCAGACGAGAAGAACTACAGCGTCACACTATTTG
CTGCGTGTGTTGTAAATGTGAGGCCAGTCTGCAGTTAGTGGTGGAAAGTTCAGCAGCCGA
TCTACGAGATCTCCAGCAGCTGTTTTTGGGCACGCTATCATTTTTGTGTCCACTGTGTGC
AGTACTGCGGTAAGCTGCAATGGCCGACCCTGAAGGTACAGATGGGGATGGGACGGGATG
TAATGGTTGGTTTTTTGTGCAGGCAATAGTAGACAAAAAAACAGGTGACACCATATCAGA
GGATGAGGACGAGGACGGGACAGATACAGGTTCAGACTTGGTAGATTTTATTGACACTAG
TAATACAAATTATATGCAGGCAGATAGAGAGGCAGCACAAGCGCTATTACATGCACAGGA
AGTAGAAACTGATACAAAGCTATTACATGCCTTAAAACGAAAGTATGGAGCACACAGCAC
AGAAAACAGTCCATGTCGTGATACAGCAAGTATACATAGCAATTTAAGCTCACCATTACA
AGAAATATCGTTAAATAGCTATAATAATACAGCTAAACGAAGGCTGTGTTCAGTGCCAGA
CAGCGGCTATGGCAATACTCAAGTGGAAACTCTGCAGACTCAGGTAACCCTAGATACAAA
TGTGTTTGGGGATGGGAAAAATGGCGACGGTTTAAATAGTGAGGCATGTAGTACAGATAA
TGAAATGGATATAGAAAATCAAAATCCAAACTCACCAATGACACAAATTGTGTCCTTATT
AAAAGTAAATAATAAAAAAGCAGCAATATTGGCTAAATTTAAAGAAACATATGGATTGTC
ATTTACAGATTTGGTACGAACATTTAAAAGTGATAAAACAACATGTACAGATTGGGTAGC
CGCTATTTGTGGAGTAAATCCAAATATTGCAGAAGGATTTAAAACGTTAATACAACCATA
TGTGTTATATGCACATATACAATGCATGGATTGTTCATGGGGAGTATTTATATTAGCTTT
ATTAAGATATAAATGTGGCAAAAATAGACTAACTGTAGCTAAAGGACTAAGTACATTACT
ACATGTACCAGATACCCATATGTTAATTGAGCCACCAAAACTGCGTAGCAGTTGTGCAGC
ACTATATTGGTATAGGACAGGAATATCAAATATTAGTGAAGTAACAGGAGACACACCTGA
GTGGATACAAAGACAAACTATTATACAACATGGTATAGATGACAGTGTATTTGACCTGTC
AGAAATGATACAGTGGGCGTTTGATAATGACTATATAGATGAAAGTGACATAGCATATGA
ATATGCACAGTTAGCTGATTGTAATAGTAATGCAGCAGCATTTTTAAAAAGTAATTGTCA
AGCTAAATACTTACGAGATTGTGCAGTTATGTGTAGACATTATAAAAGGGCACAACGAAA
GCAAATGAATATGTCACAATGGATTAGTTATAGATGTGACAAAATTGATGATGGAGGTGA
TTGGAAACCTATTGTACAATTTTTAAGATTTCAAGGAATAGAGTTTATTACATTTTTAAG
AGCATTTAAAGACTTTTTAAAAGGAACACCAAAAAAAAATTGTATTGTTATATATGGACC

AGCAAATACAGGCAAATCATATTTTTGTATGAGTCTTATACAATTTTTACATGGTACAGT
GTTGTCATTTGTAAATTCAAACAGCCATTTTTGGCTGGAACCGTTAACAGACACTAAGAT
AGCTATGGTAGATGATGCAACACCAACATGCTGGTCCTATTTTGATAATTATATGAGAAA
TGCATTAGATGGCAATCCAATAAGTATAGATAGAAAACATAAACATTTAATACAAATGAA
GTGTCCACCAATGCTAATTACATCTAATACAAATCCTGCAACAGATGATAGATGGCCTTA
TTTACGAAGTAGGGTAACAGTATTTACATTTCCACATACATTTCCATTTGATAGCAATGG
CAATCCAGTGTATGACATAAATGATAAAAATTGGAAATGTTTTTTTAAAGGACGTGGTC
CAGATTAGATTTGCACCAGGAAGAGGAGGATACAGAAAATGATGGATGCCCTATCCCAAC
GTTTAAATGTGTTACAGGAAAAGATTCTAGAACATTACGAGACTGATAGTACAGACATAT
GTGATCAAATAGACTATTGGAAGTGTGTACGTCTGGAAAATGCAATATATTATGCAGCAA
GGGAGCATGGTCTTAAAACAATAAACCACCACGTGGTACCAACATTTCAAATTTCAAAAA
GCAAAGCACATGAAGCAATTGAACTGCAGATGGCACTAGAGAGTCTTGCTAAATCAGAGT
TTAAAAATGAACTGTGGACATTGCAAGATACGTGCCAAGAACTATATCAAACACCTCCAC
AACAGTGTTTTAAAAAACAGGGACAAACCGTCGAAGTACGGTATGATGGAGACAAAGACA
ATACTATGCATTATACATCTTGGGACTATATATATTATGTAACAGAAGGTGATAAGTGGT
GTAAAACCAAAGGTTATGTGAATTATTGTGGATTGTATTATATAAAGGAGGGACAACAAA
CATATTATGTACAGTTTAAATGTGATGCACAACAATATGGACAGAGCGGTAAATGGGAAG
TGTGGTATAATGGTAAAAAAATTGAATGTGTAAATACCTGTGACAAAATAATACAATGTT
CTGAATCTATGTACAGTACCTGTGACGAGACAGTATCCGCTACTGCAATTGCTAGAGAAT
TACAACACCCCACCACACCGTATACCGAAGCCACCACCGTGTGCACCCAAAAGAGCGGGG
GTTCGGCGCCGACTAGAAACCCATTCAGACATTGTGGATTCACAGAAACAAGTGAAGTCG
ACGGACTGTCCGTGGACCACCTCAACAACCCCCTGCTTAGTGCACCTGCAGGCAACAACG
GGTTCAGGAAGCACAATAGTGGTGACACTACGCCTATAGTGCACCTAAAAGGTGATAAAA
ATAGATTAAAGTGTTTGCGATACAGGCTACAAAAGTTCCATAATTTATATACACAAATAT
CATGTACGTGGCATTGGATAGGTGGTAAAGAAACAACAAGGACTGGTATATTAACATTGG
CATATGCAAATGAAACCCAAAGACAAAAATTTTTGGATGTTGTAAAAATACCTAATACTG
TTAATGTATCCCTTGGTTATATGACATTGTAATAGGGGTATGTTAATTGCTGTATTTTTG
TTGTGCTTTTGTGTGTGCCTATATGTGTGTTGCACTATTCCGCTTTTTCCTGCTACTAAT
GTGTGTGCGCATGCTTGGCTACTTGTGTTTTTGTTAATAGTTTTTATTACTACCCCTTTT
ACTGCATTTATTGTATATATATATTTTTTTTATATTGCCAATGTTTTTATTGCATTTGCAC
AGTGTACATACATTTGACTAAGTACTATATAACCTGTATTGTACAGGACATTACATTGGT
AGTGTAATACCTTTTTGTAATAAACATGGTATCCCATCGTGCTGCCCGTCGAAAGCGTGC
ATCTGCAACGGATTTGTATAAAACATGTAAACAATCGGGTACATGCCCTCCAGATGTTAT
TAATAAGGTTGAAGGTACTACTCTTGCAGATAAACTATTACAATGGACTAGTTTGGGAAT
ATTTTTAGGAGGCCTTGGAATAGGTACTGGTAGTGGCACAGGGGGGCGTACCGGCTATAT
TCCATTGGGTGGCAGACCTAATACTGTTGTTGATGTGTCTCCTGCAAGGCCACCTGTTGT
AATTGAATCTGTGGGGCCTTCAGATCCATCTATTGTTACATTAGTTGAAGAGTCCAGTAT
TGTTACATCTGGAGCTCCTGTTCCTACATTTACAGGCACATCTGGATTTGAAATTACATC
CTCTGCAACAACCACCCCTGCAGTATTAGACATTACACCTGCTTCTGGGTCTGTGCAACT
AAGTAGCACTAGTTTTACCAATCCTGCATTTACCGACCCTTCGGTTATTGAGGTTCCTCA
AACAGGGGAGGTGTCTGGAGATATTTTTATTACTACCCCTACATCTGGGACACATGGATA
CGAGGAAATTCCAATGCACACATTTGCAACACAGGGCAGAGGCACCGAACCTATTAGTAG
TACCCCTATTCCTGGTGTTAGGCGTGTGGCAGGACCTAGATTATATAGTCAAGCGTATCA
ACAAGTTAAAATAACTAATTCAGACTTTATATCCCGTCCATCTACGTTGGTTACATTTAC
CAATCCTGCATATGAGCCTATAGACACTACATTAACCTTTTCACCACAGGATGTTGTGCC
TGATCCTGATTTTATGGATATTGTTCGTTTACATAGGCCTGCCTTAACATCCAGACGTGG
TACAGTTAGATTTAGTAGATTGGGTAAAAAACTAACTATGTCTACTCGCAGTGGTAAACA
AATAGGTGCCCAGGTGCATTATTATCATGATATTAGTCCTATTTCACATATAGGTGAAAG
TATTGAAATGCAGCCTTTACTGCCTGATGCAGCAGTTACTGCTGACACTAATGGCCTCTT
TGACATTTATGCTGACACTGATATTGATAATAATGCCATGCTATATGATAGAAATATTTC
TGATGTTACACAACCTACCACTTCAACTATATCTAGTGTATCCTCTCGTTATAGTAATAC
TACTATTCCTTTAGCAACATCTTGGGATGTTCCTGTTCATACAGGGCCTGACATGACATT
ACCTACTACTATACCCCAGTGGCCTAATATAGTACCTTTACTGCCTAATAATACACATTC
AGTTGTACTTCAGGGAACAAACTATTATTTATGGCCTAATTATTATTTTATTTTCAAAAA
ACGTAAACGTGTTCCCTATTTTCTTACAGATGGCTTTGTGGCGTTCTAGTGACAGCAAGG

TATACCTTCCACCACCTTCAGTGGCTAAAGTTGTCAACACAGACGATTATGTAACACGTA
CCAGTACATTTTATCATGCTGGCAGCTCTAGGCTTCTAACCGTTGGACATCCATACTATA
AAGTTACCTCAAATGGAGGCCGCAAGCAAGACATTCCTAAAGTGTCTGCCTATCAGTATC
GAGTGTTTCGGGTTACATTACCTGACCCTAATAAATTTGGTCTTCCAGACACTAATATAT
ATAATCCTGAAACACAACGTTTAGTATGGGCCTGTGTTGGCATGGAGGTAGGTCGTGGGC
AGCCTTTAGGTGTTAGCCTTAGTGGTCATCCCTTTTACAACAAATTGGATGACACAGAAA
ATTCCCATGTTGCTACTTCTGTAGTTACACACGACACTAGAGATAATGTGTCAGTGGATT
ATAAACAAACCCAGTTATGTATTATTGGTTGTGTTCCTGCTATTGGGGAACATTGGGCTA
AGGGTACTGCCTGTAAGCCCGGTGCTGTGCAAACAGGTGACTGTCCTCCATTAGAACTAG
TAAATACACCTATTGAGGATGGTGATATGATTGATACTGGCTATGGTGCTATGGACTTTA
GTACTTTGCAGGATAATAAAAGTGAGGTTCCCTTAGACATTTGTCAATCTATTTGTAAAT
ATCCAGACTATTTGCAAATGTCTGCAGATGCATATGGGGACAGTATGTTTTTTTGTTTAC
GACGCGAACAGTTATTTGCACGCCATTTTTGGAACAGGGGAGGCACTATAGGAGATGCAG
TACCGGAAACATTATATATAAAAGGTACTAATGATAGGGCAACACCTGGAAGCTGTATTT
ATTCTCCATCACCTAGTGGGTCTATGGTATCTTCAGATGCACAAATGTTTAATAAGCCTT
ATTGGCTGCACAAAGCCCAGGGACAAAATAATGGCATTTGCTGGCACAACCAATTATTTA
TAACTGTGGTAGACACAACACGTAGTACCAATCTTACCTTATCTACTGCAACTACTAATC
CAGTTCCATCTATATATGAACCTTCTAAATTTAAGGAATACACACGCCATGTAGAGGAAT
ATGATTTACAATTTATATTTCAATTGTGTAAAATTACACTTACTACTGATGTTATGTCTT
ATATACATAACATGGATCCTACTATTTTAGATAGTTGGAATTTTGGTGTTAGTCCTCCCC
CATCTGCTAGCTTAGTAGATACATATAGGTTTTTACAGTCATCTGCCATTACATGTCAGA
AGGATGTGGTTGTTCCACAAAAAAAGGATCCATATGAAAAATTAAAGTTTTGGAATGTGG
ATCTTAAAGAACATTTTTCATCTGATTTAGATCAGTTTCCTTTAGGACGTAAGTTTTTAT
TACAGGCTGGGTTACGACCTAAACCCACCATAGGCCCTAGGAAACGTGTTGCTTCTACTT
CTACTGCTACTAGGCCCTCCAAACGGAAACGTACTGCTAAATAATGTGTTGTGTATAATT
GTGTTGTTTGTTTTGTTATATGTGTGTTCCTTATATGTTGTGTGCATGTTTGTATGGTTA
TGTGTATGTTTGTATCATGTGTAAGTAATGTGTGTATTGTATGTGTTTAATAAAGTATGT
GTGTTAGTTTCGTGTGTGGTTGCACCCAAATGAGTAAGAGACTGTCCCTTTATTATCCCT
TGTATCCTGTGTATCCTGTGTGCCCTTTGTTCCTACTTTATATAGGGTGTGTTATATGTT
ATACATTATTTATAATACACTTTGTAGCGTCCATTTTATCCATTTGTATCCTACAAGCCT
CCATTTTACTATGCAACCGATATCGGTTGCCTTTGACACACCTAACATTATACATTCTTA
TTGTTACATTATTTAAAGTTAAATCCTTTTTGGCTCTGTTTAAGGCGCCAGTGAGCCAGT
GGCGCACCTTATATTACTCATCATCCTGTCCAGGTGCACTGCAACAATGGTTTGCAAAAC
CTTGTTTTACCCTTACATAATAAAACTGCTTTTAGGCACATATTTTCACTGTTTTTACTT
GCTTTAATTACACTATTGGCCTGTACAACTACTTTTAGATTCAAGAATGTGTCTTGTAGG
TTATATACCTGTCACTGATTCATACTTTTATTGCAACCGATTTCGGTTGGCCAAATCCTT
ACACATTATATA
>gi|27462483|gb|AF436130.1|_Human_papillomavirus_type_74_subtype_AE10,_complete_
genome
ATGGAAAGTGCAAATGCCTCCACGTCTGCAAAAAGTATAGACCAGTTGTGCAAGGACTGC
AACATTCCTATGCACAATTTGCAAATTTCATGCGTGTTTTGCAGGAAAACCTTGTCTTGT
CCAGAGGTATATTCCTTTGCCTATAAAAATTTGTATATAGTATACCGAGGAAACTTTCCA
TTTGCAGCGTGCGCCATTTGCTTAGAACTACAAGGTAAAGTAAATCAGTTTAGACACTTC
AACTACGCGGGATATGCGTTAACAGTGGAAGAAGAAACAAAGCAGTCAATTTTGGATGTG
CTAATACGCTGCTACCTGTGCCATAAGCCGCTGTGCCACGTGGAAAAAGTACGGCACATT
TTGGAAAAGGCAAGATTTATTAAACTAGGCGACACCTGGAAAGGGCGCTGCTTACATTGC
TGGACAACATGCATGGAAAATATCCTACCTTAAAGGAAATTGTGTTACAGCTACAACCTG
ACCCTGTAGGCCTACTTTGCAATGAGCAATTAGACAGCTCAGAAGAGGAGGTGGATGAAC
TAGCCACGCAAGCCACGCAACATACACAGCCTTACCAAATAGTAACCTGCTGTTGTGTGT
GTAATCGCAGCCTTCGGCTTGTTGTGCAGTGTACAGGACCAGACATCAACAATCTACATA
CGCTTCTACTGGGTACACTGAGCCTAGTGTGTCCTCTGTGCGCGCCCAAAACGTAACGAC
ATGGCGGAAAATACAGGTACAGAGGGGACGGGGTGCTCAGGGTGGTTTTTAGTAGAAGCA
ATAGTAGAGCATACAACAGGACAGCAGATTTCAGAGGATGAGGATGAAACAGTAGAGGAT
AATGGGTCTGATATGGTAGACTTTATAGATGACAGGCCTATTACACACAATTCCTTGGAA
GCACAGGCATTGTTAAACGAGCAGGAGGCGGATGCTCATTATGCAGCTGTGCAGGACCTA

AAACGAAAGTATTTAGGTAGTCCATATGTTAGTCCTTTGGGTAATATTGAGCAGTCAGTA
GACTGCGACATAAGTCCCCGGCTGGACGCTATAAAATTAAGTAAAAACCCTAAAAAGGTA
AAGCGACGGCTGTTTGAAACAAAGGAAATAACGGACAGTGGATATGGCAATTCTGAAGTG
GAAACTCAAACGCAGGTAGAGAGCAATGGCGAACCGGAAGGTTGCGGGGGAGGTGGACAG
GGAAGGGACACAGAGGGGGAGGAAACGGAAGTACAAACAGATAGCGACACACAACAGCAG
ACAGCAACCACGCGGATACTAGAACTATTAAAATGTAAGGATGTAAGGGCTACACTTCTT
GGTAAGTTTAAAGACTGTTATGGGTTATCCTATACTGATTTAATTAGACAGTTTAAAAGC
AACAAATCAACATGTGGGCATTGGGTGGTAGCAGCATTCGGGGTACATCATAGTGTAGCA
GACGCTTTTCAAAACCTAATACAGCCTGTAACAACATATAGCCATATACAATGGCTTACA
AATGCATGGGGAATGGTGCTGCTAGCATTATTAACATTTAAAGTAAATAAAAACAGATGT
ACCGTGGCACGAATGCTGGCAACATTGCTTAACATTCCTGAGGACCACATGCTAATAGAG
CCACCAAAAATACAAAGCACTGTGGCGGCACTATATTGGTTTAGGAGTAGTTTATCGAAT
GCCAGTATAGTACAGGGAGACACACCAGACTGGATAACAAGGCAAACCATTGTGGAACAT
GGTATTGCAGACAATCAATTTAAATTAGCAGACATGGTACAATGGGCATATGATAATGAC
CTGTGTGATGAAAGTGAGATTGCCTTTGACTATGCACAACGAGCAGACATAGATGCAAAT
GCAAGGGCATTTTTAAATAGTAATTGTCAGGCAAAATATGTAAAAGACTGTGCCACTATG
TGTAAGCATTATAAAAATGCAGAAATGAAAAAAATGAATATGAAACAATGGATACATTAC
AGGAGTAGTAAAGTTGAAGAGGCAGGAAATTGGAAACCAATAGTACAATTTTTAAAACAT
CAAAATATAGAATTTATTCCATTTCTAAGTAAATTAAAGCTGTGGCTGCATGGTACACCA
AAGAAAAACTGTATTGCAATAGTGGGACCACCAGACACAGGCAAATCATGTTTTTGCATG
AGTTTAATTAAATTTCTAGGAGGTACTGTAATAAGTTATGTAAATTCAAGTAGTCACTTT
TGGCTTCAACCGTTATGCAATGCAAAGGTAGCGTTATTAGATGATGCAACACAATCCTGC
TGGGTATATATAGATACATATATGAGAAACCTATTAGATGGGAATCCAATGAGTATTGAT
AGAAAGCATAAATCATTGGCATTAATAAAATGTCCGCCATTATTAATAACATCTAACATA
GACATTACTAAAGACGAGAAATATAAATACCTATTTAGCAGGGTGTCAGTATTTACATTT
CCAAATCCATTCCCTTTTGACAGAAATGGGAATGCAGTATATGACCTTTGTGAATCAAAC
TGGAAATGTTTCTTTACAAGATTATCAGCAAGCCTAGATATACAAAACTCAGAGGACGAG
GACGATGGAGACAATAGCCAAGCATTTAGATGTGTGCCAGGAACAGTTGTTAGAACTGTA
TGAAGAAAATAGTAATAACCTTGAAAAGCATATACAACATTGGAAATGTGTACGGCACGA
AAGTGTACTGCTACACAAAGCAAGGCAAATGGGCCTACACCATATTGGAATGCAAGTGGT
GCCCCCACTAGCAGTGTCACAAACCAAGGGACATGAGGCAATTGAAATGCAAATGACATT
AGAGACATTACTAAAGTCCGCCTATGGTATGGAGCCATGGACATTGCAAGACACAAGTCG
GGAAATGTGGTTAGCCCCTCCAAAATACTGTTTTAAAAAACAGGGACAAACTGTGGAAGT
AAAATATGACTGCAAGGCAGACAATATTATGGAATATGTTATGTGGAAATTTATATATAT
ACTGGACGGCGACGAATGGGTAAAGGTGGAAGGACAAGTTGACTATAGGGGGCTGTATTA
TGTACAGGGAGGGTATAAAACATACTACACAAATTTTGAAACTGAGGCTAAAAAATATGG
GAATACGTTGCAGTGGGAAGTGTGTGTTGGCAGCACAGTTATATGTTCTCCTGCATCTGT
ATCTAGTACTGTGCAAGACGTATCCATTGCTGGGCCTGCTTCATACCCCACCACCACCAC
CTTTGCACAGGCCGCCCCAACAGTGTCCACTACCGCCGCCGAGGATCACGTGGACGCGCC
GCCCAGTAAAAGACCCCGAGGACGTTCCACCAACACCAACACCACCAACAACACCAACAA
CACCAGAAGCACCGTGTGTATCACAAACGGAGACTCCGTGGACAGTGCAAACAACAACAT
CATCGCTAACCATTACAACAATAACAAAGGACGGGACAACTGTTACTGTCGAGCTACGCC
TATAGTTCAATTACAAGGTGACCCAAATTGTTTAAAGTGTTTTAGATACAGATTACATGA
CAAACATAAACACCTGTTTTTGTCAGCATCCTCCACATGGCATTGGACCAGTCCCACTAT
ACCCACAAAACATGCACTAGTAACACTAACATATGCTAATGAACAACAGCGACAACAGTT
TTTAGATACTGTAAGACTACCTACAACAATTAGTTATAAGTTGGGATATATGTCATTACA
ATTGTTGTAATAGTGCATTGTAAATATTGTATGTATTGTACATGGACCTAATACCTATAG
ATGGTACTATAGGAACAACCAGCACCTCCATACTGCCAGTTGTAATAGCTGTTTTTGTAT
GTATTGTTAGCATTGTATTAATTATAGCAATAACTGATTTTTTTGTATATACATCCATAC
TGGTACTAACACTCATTCTATATTTATTGCTTTGGCTTTTACTAACATCTGCTTTGCAGT
TTTATACACTAACCCTGTGTGTGTGCTATCTACCTGCGTTTTCTTTGCATTTATATATTT
TACATAATTTAGAGTAACTATTACAATGCTAACATGTACATTGGATGATGGTGATACATG
GTTGTTATTATGGTTGTTAACTGCACTTATCGTAGCTATTCTTGCATTATTGTTAATGCA
TTTCAAGGGTGTAACGCCTTCTGTATGCAGTAAATAAGTGTTTTTATACATGCTGTGTAT
GGTGTAATTATGGCACATAGTAGGGCTCGTCGGCGTAAACGTGCATCGGCCACCCAATTA

TATCAAACCTGTAAGGTGTCAGGTACATGTCCCTCTGATATTATACCTAAAATTGAGCAT
AATACTATTGCAGATCAAATATTAAAGTGGGGAAGTTTAGGGGTGTTTTTTGGGGGGCTT
GGCATTGGTACAGGCTCTGGTACCGGTGGCAGGACCGGTTACATTCCTTTACAGTCTGCC
CCACGCCCTGACATTCCTTCTGGGCCTGCTGCACGTCCGCCCATTATTGTTGATACAGTT
GGCCCTGGTGACCCCTCTATAGTGTCCTTAGTTGAAGAATCTGCTATTATTAATTCTGGG
GCTCCTGAGGTGGTGCCCCCTTCACATGGGGGGTTTGAAATTACTACATCTGAATCAACC
ACACCAGCTATATTAGATGTCTCTGTTACAACACACAACACTACCTCTACAAGTGTATTT
AGAAATCCCTCGTTTACTGACCCTTCTGTTATACAGTCTCAACCCCCCGTAGAGGCTGGT
GGTCATGTGCTGATTTCCTCCTCTACTGTTTCTTCCCATCCAGTAGAGGAAATACCTTTA
GATACATTTATTGTCTCCTCCTCTGATAGTAATCCTGCATCCAGTACTCCTATTCCTACA
GCTGCTACACGTCCTCGTATTGGTTTATATAGTAGGGCCCTGCATCAAGTACAGGTCACG
GATCCTGCTTTTTTGTCCAGTCCTCAACGCCTTATAACCTTTGATAATCCAGTATATGAA
GGTGAGGATGTTAGTTTGCAATTTCAACACAATACAATACATAATCCTCCTGATGATGCC
TTTATGGACATTATTCGCCTACACAGACCTGCTATAACGTCTCGACGTGGTCTTGTACGG
TTTAGTAGAATTGGGCAACGGGGGTCTATGCATACGCGCAGTGGTAAGCATATAGGTGGA
CGGGTGCATTTTTTTCAAGACATTTCTCCTATATCTGCTGCAGAATCTGCTGCGGAAGAA
ATAGAGCTGCACCCTCTTGTGGCTCATGCACAGGATAGCAGTGGCTTATTTGATATTTAT
GCAGAACCTGACCTTGAGGTTATGGAGGAACCAGTTCCATTGTCGTTTTCAACATCCACA
CCCTTTCAGCGGTCGGCTGTGTCAGCCACATGGGGCAATACTACTGTCCCCCTTTCATTG
CCAGGTGACATGTTTATACAGCCTGGTCCTGACATAATGTTTCCAACTGCATCTTCTACA
ACGCCCTATAGTCCTGTTACTCCTGCTTTACCTACAGGTCCTGTTTTTATAAATGGTTCT
ACATTTTATCTATATCCATCATGGTATTTTGCACGCAAACGCCGTAAACATGTTCCCTTG
TTTTTTACAGATGTGGCGGCCTAGTGACAACCAGGTTTATGTGCCTCCTCCCGCCCCTGT
ATCCAAGGTCATTACCACGGATGCTTATGTTACACGCACCAACATCTTTTATCATGCTAG
CAGTTCTAGACTACTTGCTGTAGGAAATCCCTATTTCCCTATAAAACAGGTTAACAAAAC
AGTTGTTCCTAAAGTGTCTGGATATCAATTTAGGGTGTTTAAGGTGCTTTTGCCAGATCC
CAATAAATTTGCTTTACCTGACACTTCGTTATTTGACCCCTCCAGTCAACGTTTGGTATG
GGCCTGTACGGGTTTGGAGGTAGGTAGGGGACAGCCTTTAGGTATTGGCATTAGTGGTCA
CCCCTTATTAAATAAATTAGATGATGTGGAAAACTCCGCTAGTTATGCAGCCAATCCTGG
TCAGGATAACAGGGTTAATGTTGCTATGGATTATAAGCAAACACAGTTATGCATGGTAGG
CTGTGCTCCCCCGTTGGGTGAACATTGGGGAAAAGGCAAGCAATGTTCTAATGTTACTGT
AAAGCCAGGCGATTGCCCTGCATTAGAATTAATTACTAGTGTAATTCAGGATGGTGATAT
GGTAGATACAGGCTTTGGTGCCATGAATTTTGCTGAATTGCAGCCTCTTAAATCTGATGT
TCCTTTGGACATATGTAACACTACATGCAAATATCCTGATTATTTACAAATGGCTGCAGA
TCCATATGGGGATAGATTGTTTTTTTACTTACGCAAGGAACAAATGTTTGCAAGGCACTT
TTTTAATAGGGCTGGAACAGTGGGTGAGGACATTCCCCAGGACCTATATATTAAGGGTAC
TACACGTGGTACACTTGCCAATGCTATTTACTTTAATACCCCCAGTGGTTCTCTTGTGTC
TTCTGAAACACAGTTGTTTAATAAGCCCTTTTGGCTACAAAAGGCGCAAGGCCACAATAA
TGGCATTTGTTGGGGTAATCAATTGTTTGTTACAGTTGTGGATACCACACGTAGTACTAA
CATGACAGTGTGTGCGCCTACTACACAATCCCCTCCTGCTGCTACATATACTAGTACAGA
CTACAAACAATACATGAGACATGTGGAGGAATTTGATTTGCAATTTATTTTTCAGTTATG
TAGTATTAAATTAACTGCTGAGGTTATGGCCTATATTCATACTATGAATCCTACAGTTTT
AGAAGACTGGAACTTTGGCCTAACACCTCCCCCCAATGGTACTTTAGAAGACACCTACAG
ATATGTGCAGTCTCAGGCTATTGCATGTCAAAAGCCTACTCCTGATAAAGCAAAGCCTAA
TCCCTATGCCAATTTAAGTTTTTGGGAAGTAAATCTTAAGGAAAAGTTTTCTAGTGAGTT
GGATCAATACCCCCTTGGTAGAAAGTTTTTATTGCAAACAGGCGTGCAGGCGCGCTCTTC
GGTTCGTGTAGGTAAAAAGCGTTCGGCCCCTACAGCCCCCTCCTCAACCACTAAACAGAA
ACGCTCTAGGAAACGATAGCATGCTTTTGTTTTGTGTGTTATATGTTGTTGCATGGTATA
TGTGTGTTGTATTATATTGTGTGTCATATGTTGTTGTATGGTTTATGTTGTATGTACTTG
TGTGTCATATTGTATGTGTCATGTTGTGTAATGTTGTATGTACTGCCTGTATGTGTTTAC
TGTTGTATGTACTGCATGTATGTGTTTGTTGTGTACAATAAACTTTATCTATCCCTGTGT
CCTGTTTTACCCTGTACGTGTGTGTCTTGTCTTATAATTCTATATACCTAATTTACCTTT
TTATAACCCTTTCCCTCCATTTTTAGTGCAACCGTTTTCGGTTATTACTCATCTCTCCAC
ATCCTGTAACCAATTTGTTGCAGCAAGCATTTTATGTAATCCTATATACTGTTTGCCAAG
GTCATACTGTGTTGCCAAGTACACACCTGGTGCACAATTAGGCGCGGTTTTGGCATCCAC

ATAATACATTCCTAATCTATATAACACTCCTGTCTGCCTGTTTAACAATTGGGTACTTTG
TTAATACATTTTTGTAGTTGGTAGCCAACTTTTAAAAGCATTTTTGGCTGCCAGTACCAC
ATTTTTCTAAAGTTACTGTTAGGTTCATAAAATGAGTAACCTAAGGTTACACACCTGCAA
CCGGTATCGGTTAACACACGCCCTCTGCACTTCCTTATTATACATAATAACAATTTTACC
TTTTTGAAAAATAGGAGGAACCGAAATCGGTTCCAACCGAAAACGGTTATATATAAACCA
GCCCAAAAATTAAGCAAGCGGGGCATA
>gi|6970418|dbj|AB027020.1|_Human_papillomavirus_type_69_DNA,_complete_genome
CTTTTAACAATCATAGTTTTATAAAAGGGTGTAACCGAAGCGGTTTTAACCGAAAACGGT
GCATATAAAAGTAAAAGACACAGCCATACACAAAACCAGCTATGTTTCAAGATCCCAGAG
AAAGACCACGAACGATACATGAACTATGTGAAGCTTTGAATACACCTTTGCAATCTTTGC
AGGTACAGTGTGTATATTGCAAGAAAACATTAGAATGGGCAGATGTATATAACTTTGCAA
TATGTGATTTAAGAATAGTGTATAGAAATGATAGTGCATATGGTGCATGTAAAAAATGTA
TAATATTCTATTCAAAAATAATAGAATATAGACGCTACACATCGTCTGTGTATGGTGCAA
CACTGGAAGCGCGTCCTAAACGAAGTTTGTGTAATTTGTTAATAAGGTGTCATAGATGCC
AAATACCATTGGGACCGGAAGAAAAACAGAGAATTGTGGATGAAAAGCGACGGTTCCATG
AAATAGCAGGGTACTGGAAAGGGTTGTGCACAAACTGCTGGAGACCAAGGCGCGAAGCAA
CAGAAACACAAGTATAAATAACAATGCATGGAGACACAATTAATATACAGGATGTTATAT
TAGATTTGGTGCCGCAACCCGAAATTGACCTACAGTGTTACGAACAATTGGACTATGAAC
AATTTGACAGCTCAGAGGAGGATGAAACAGATAATGTGCGTAACCAGCAAGCCAGACAAG
CTGAACAAGAAGCGTGTTATAGAATAGAAGCGGAATGTTGTGTATGTAATAGTATAGTGC
AGCTAGCTGTACTAAGCAGTCGACAGAACGTCCGAGCGGTGGAGCAGCTGCTGATGGGCG
ACGTGAGTTTGGTGTGCCACCAGTGTGCTACATACTAAACCTGCAATGGACTGCGAAGGT
ACAGATGGGGAGGGGTTGGGGTGTACAGGGTGGTTTTCAGTAGAAGCAATAGTAGAAAAA
CATACAGGAGAAACAATATCAGAAGATGAAATAGAGTATAGTAGCGATACAGGATCAGAC
CTAATTGGATTTATAGATGATAGTAATATTAGTGATGGGGCAGAGCAACAGGTAGCGCAG
GCATTGTTTCAGGCACAAGAAACACAAGCAAATAAGAAGGCAGTGCGTGCATTAAAACGA
AAGTTACTAGGTAGTCAGAACAGCCCGTTGCAAGACATAACAAATCAAAGCAACAGTCAG
CAGAGCACAGACGAAGTAAACAATTTACAGGCTAAAAGGAGAGCAGTGGACAGTGTTCCG
GACAGCGGGTATGGCTATACTGAAGTGGAAACTCTTACGCCAGTACAGGTAGATAAACAC
AATGAACAAAATGGCGATAGTGTGTGTAGTCAGGGGGGGTCAAGTGGATCTGTATCAGAC
ATGGAGGTGGACATAGGAGCACAGGCAAGTAGTGTAACCAAAATATGTGAATTGTTAAAA
TGTAGTAATGTAAAAGCAGCATTATTAAGCAAATTTAAAACAGTATATGGTGTAAGTTAT
ACAGAATTAGTACGAGTGTTTAAAAGTGATAAAACATGCTGTTCAGATTGGGTATGTGCA
GCCTTTGGTGTGGCAGGCTCTGTAGCAGAAAGTTTAAAAACACTAATACAACCATATTGT
TTATACTATCATATTCAATGCTTAACATGTAATTGGGGAGTACTACCACTAATGCTAATA
CGATTTACATGTGCCAAAAATAGAGCAACAATTAAAAAATGCCTGTGTACGTTACTGAAT
GTGCCAGACACACAGTTGTTAATAGAACCACCAAAATTACGTAGTACAGCAGTTGCATTA
TATTTTTATAAAACAGGATTATCAAACATAAGTGAAACACATGGGGACACACCAGAATGG
ATAGTAAGACAAACACAATTAGAACATAGTTTTGAAGATACAATATTTGATTTATCAAAA
ATGGTGCAATGGGCATTTGATCATGACATAACAGATGACAGTGAAATTGCATTTAAATAT
GCACAATTGGCAGACATAGAAAGTAACGCTGCAGCATTTTTAAAAAGTAACTGTCAGGCA
AAATATGTAAAAGACTGTGCAACTATGACAAGACATTATAAAAGAGCACAAAAACGATCC
ATGGGCATGTCACAATGGTTACAACATAGGTGTTCAAAAATAGAAGATGGAGGAACGTGG
AAGGACATAGCAAGATTTCTAAGATATCAAAATGTTAATTTTATTTATTTTTTGCAAGTA
TTAAAACAGTTTTTAAAAGGAACACCTAAACACAACTGTATAGTAATATATGGGCCACCA
AATACTGGTAAGTCACAGTTCGCAATGAGTTTTATAAAATTTGTGCAAGGGTCTGTCATT
TCATATGTAAACTCTAACAGTCACTTTTGGCTGCAGCCATTAGAAGATGCAAAGGTGGCA
TTATTAGATGATGCTACATATGGCTGCTGGCTATACATAGATAAATACCTACGTAATTTT
TTAGATGGCAATCCCTGTTGTATAGATAGAAAACATAGAAGTTTAATACAAGTAAGATGT
CCACCATTAATAATTACCTCAAACATTAATCCACAAGACGACAATTCATTAATGTATTTA
CATAGTAGAGTAACAGTTATACCATTTCCAAATACATTTCCATTTGACAGCAATGGGAAT
CCTGTATATGAATTGACTGATGTAAATTGGAAAAGTTTTTTTTCCACCACTTGGTCCAGA
TTAGATTTGGAGGAGGACGCGGACAAAGAAAATGGAGAACCTTTGCCAGCGTTTAAATGC
GTGCCAGGAGAAAATACTAGACTATTATGAACTGGACAGTGACAAATTAAGTGATCAAAT
TGACTATTGGAAACTGGTGCGATATGAATGTGCAATATTTTATAAAGCTCGTGAAGGAA

TATGCAATGTATTAACCACCAGGTGGTGCCATCGACAACAGTGTGTAAAGAAAAAGCTTG
GCAGGCAATTGAATTACATATAGCATTACAGTCTTTAATGAATTCAGAATATGGAAAAGA
GAAATGGACTATGAGAGACACGTGTTATGAATTGTATGTGACAGAGCCAAAACACTGTTT
TAAAAAAGAAGGGACAACAGTAACAGTTGTGTTTGATTGTAATAAGGAAAATACAATGGA
CTATATTAGGTGGACATCTGTGTATTATAAAACAGATATGGGATGGTGTAAAACAAATGG
AGAAGTGGATTATAAGGGGATATACTATATGCATGGGTGTTGTAAACAGTACTATGTGGA
CTTTAAACAAGAGGCGTTAAAATATGGGACTGGTTTGCAATGGGATGTACATGTGTGTGG
TCAGGTAATATGTTGTCCTGAATTTGTATCTAGTACCTCCGGGGACCAAATATCCACTGC
TCAAACTGCTGAACCACTATCAAACAACACCACCCAGACCCCCGAAACCACCTGTGTGCC
CCGGTGCACCGAAGAAACCCCGGCGCCAGAGACAGCAAAGCGACGACGACTTAGTGGACC
CAACACCCCTGTCACCACCACCAGTGTTACAAGGCCCAGAAGTTCCTTGGAGCATACAGA
CAACGTGTTACACAGTAACAGTGGAAGCAACAACCCGGGAAGGGGCACGTGTAGTGACCA
GACTGTGTTTATAGTACATTTAAAAGGTGATACAAATTGTTTAAAATGTTTAAGGTATAG
ATTTAAAAAACATAAGGGATTGTATTGTAAAGTGTCATCTACCTGGCATTGGACCAGTAA
TACTAACCAAGGCATTGTAACAATTACATTTGATAGTGAAACACAACGTGCTAGTTTTCT
TACAACTGTTAAGATACCACAGAGTATAACATCAACATTGGGTATAATGTCATTGTAATC
TATAGTGTAACATATATACATCCCACAAGCCAATATGTGCTGCTAACTGTATATACATAA
CCATTTTACTACTTGTAATTGTGTTTGTGCTTTGCGTGTGTGTGTGTTTGTTGCTGTGTC
GTTTGTTGCCGCTTTTGCTATCTATACACGTGTTTGCAGCCCATTTATTAATAATTATAT
GTTTTTGGTTTGTGGTTTCAACATCATTTACAGCTACGTTTTTGTATATATTTGTTTGT
TTTATATACCTGCATTTTTGTTACATTTTTATGCTGTAATTTTACTACCTAATGGTTTAT
AAATAAACACTTCATAATGGTGGCTGTTCGTGCATCACGACGCAAACGAGCATCAGCAAC
AGACCTTTACAAAACCTGTAAAGCAGCTGGCACATGCCCTCCTGATGTTATACCTAAAAT
TGAAGGCTCTACTCTTGCTGATAAAATTTTACAATGGAGTGGGTTGGGAATATTTCTTGG
TGGACTTGGTATTGGCACAGGAACAGGTACTGGTGGGCGTACTGGGTATATTCCTCTGGG
TGGTGGTGGTAGGCCTTCTGTGGTTGATATTGGCCCTACTAGGCCTCCTATAATAATTGA
ACCTGTGGGCCCAACTGAACCTTCCATAGTTACTTTAGTAGAAGAATCAAGTATTATACA
ATCTGGATCTCCATTTCCTAATTTTAGTGGTGGGGATGGGTTTGAGGTCACTACCTCCTC
TACTACAACCCCTGCTGTGTTAGATATTACCCCCTCCCCTGGAACTGTACACGTTACCAG
CACAAATATACAAAACCCATTATATATTGAACCACCTGTAGATATTCCTCAGTCTGGAGA
GGCTTTAGGACACATTTTTACTAGCACATCTACTGCCGGTACACACAGTTATGAAGAAAT
ACCTATGGAGGTGTTTGCATCTAATACTAGTTCCGGGTCAAAACCTATTAGTAGTACCCC
TATACCTGGCATACGTAGGGTTGCTGCTCCTAGATTATATAGCAAGGCCTATCAACAGGT
TAAGGTCACTGACCCCAATTTTATTTCTAAGCCTTCCACATTTATTACCTTTGATAATCC
TGCATATGAACCTATGGACACTACCCTCACATTTTCTGCAGATAGCCACGTGGCCCCTGA
TCCTGATTTTTTGGATATTATTGCATTGCATCGCCCTGCTCTTACTTCTCGCAGAGGCAC
TGTAAGGTTTAGCAGGTTAGGTCAAAAGGCCACACTTAAAACACGTAGTGGTAAACAAAT
TGGAGCTAAGGTACATTATTATCATGACATAAGTCCTATACATGCTACAGAGGAAGCTAT
TGAATTACAACCTTTAATTACTTCAGAACAGCACTCTACACCATTGTTTGATGTTTATGC
TGATGCTGACCCTGCCCCTACATTTACATTTCCATCTACTACACCTACTACAATCCCTAG
ATTTTCTTCTACAATATTTTCAACCACTAGTAGTGCACCCTTAAATGTTACCATACCTCT
ATCTACATCATTTGATATACCTATATATAACGGCCCTGACATTTATGCTCCTGTACCTTC
TTCCACATGGCCTTATATTCCACCTCCACCCACCACCATGTCACATTCCGTAGTTGCACA
AGGGGGTAATTATTATTTATGGCCTTATATTTATTTAATTCATAAACGCCGCCGTAAACG
TGTGCCTTGTTTTTTTTCAGATGGCCTTGCGGCGTACTAGTGACAGCAAGGTATATCTGC
CTCCTACCCCTGTGTCCCGGGTTGTCAGACGGATGAATATGTACACGCACCGGATATATT
ACTATGCAGGCAGCTCTCGATTATTAACTTTGGGTCATCCCTATTTTCCAATTCCTAAAT
CTGGTTCAACAGCAGAAATTCCTAAAGTGTCTGCTTACCAATATAGGGTTTTTCGTGTTC
ACCTTCCAGACCCTAACAAATTTGGGTTGCCTGACCCCCAGTTATATAATCCCGAAACAG
AACGGTTGGTATGGGCCTGTGTTGGTGTAGAGGTTGGCAGGGGACAACCGTTGGGTGTTG
GGCTAAGTGGGCATCCTTTATTTAATAAATTGGATGACACTGAAAATTCACATCTTGCCA
CTGCTAATGCAGACACTGATAATAGGGACAACGTGTGTGTAGATAATAAACAAACTCAGT
TATGTATTATTGGATGTACCCCTCCTTTAGGTGAACACTGGGGCGTTGGCACTGTATGTA
AAAATGCACAGTCTCAGGTACAGCGTGGCGATTGTCCACCTTTGGAACTTATTTCCAGTG
TTATTGAGGATGGTGATATGATTGATACTGGCTTTGGTGCCATGGATTTTACAGCTTTGC

AAGCTACAAAATGTGATGTGCCACTTGATATTAATCAATCTATTTGCAAATATCCAGATT
ACCTTAAAATGTCTGCAGATACGTATGGAAACAGCATGTTTTTTTTTCTTCGTCGGGAAC
AATTGTTTGCCAGACATTTTTTTAATAAAGCTGGCACTATAGGTGATTCTGTACCAGTAT
CTATGTATATTAAAGGCGCTGGTCAAGGTCGTGAACCTCCAACTACTTCTATATACTCAC
GTACGCCTAGTGGCTCCATGGTGACCTCTGATGCTCAATTGTTTAATAAACCTTACTGGT
TACAGCGTGCCCAGGGTCATAATAATGGCATTTGTTGGGGCAACCAATTGTTTGTTACTT
GTGTAGATACTACCCGCAGTACCAACCTCACTATTAGTACTGTATCTGCACAATCTGCAT
CTGCCACTTTTAAACCATCAGATTATAAGCAGTTTATAAGGCATGGTGAGGAATATGAAT
TACAGTTTATATTTCAATTGTGTAAAATTACTCTTACCACTGATGTAATGGCCTATATCC
ATACAATGAATTCTACTATTTTGGAAAATTGGAATTTTGGCCTTACCTTGCCTCCTACTG
CTAGTTTGGAAGATGCATATAGGTTTATTAAAAATTCAGCTACTACATGTCAACGCGATG
CCCCTGCACAGCCCAAGGAGGATCCATTTAGTAAATTAAAATTTTGGGACGTTGATCTTA
AAGAAAAGTTTTCTATTGATTTAGATCAATTTCCACTGGGCAGAAAATTTATGCTGCAGG
CTGGTATACAGCGACGTCCTAAGTTAGGCACCAAACGTCCAGCTTCTTCCTTATCTGCAT
CTTCCTCCTCTACCACACGTAAAAAACGCAAACTTACTAAGTAATTCCACATGGTTGTGT
CAGTGTATAGTTTTGTATGTGTGTAATGTAAGGTGTGTTGTGTTTGTATGTATAATTGTA
TGTTTCCTGTGTGTGTTTGTATGTATGTATGTATGATTATGTGTTTTCCTGTTTGTGTGG
TTTACTGCATGCTTGTTATTTAATAAACAATGAATGTGTTGTATTTGTTATTTCTTCCTC
CGTAGGGTATAGGGAAGTAACTTAGGTAGTGTTTTAGGTGGTGTTCCTTTTTGCGAACCG
AATTCGGTTGCATTCAACTTTATATTATATTTGCAGGAACGGTTAATCCTTTGGCACAC
AACACATCCTGCCTCCTACGCTTGGCTTGCCTAGACGTGGCTCACCTACATTACTCACAT
ATCTTTTAACAATAGTTGCATACGCACTTTTTTTTGATTATAATAAAACTGCTGTTAGGC
ACATATTTTTATTGTATACATGGGTTTTACTAAGTGCATAGTTGGCTAGCAGTTTTACTG
TTTTATGCCAACTATATGTCTGACACATTCCTTTTAACAATAAAACTGCTGTTAGGCACA
TATTTTATAAGCTACTTAGACACAGGTGTGGTAACAACCGAAATGTGTTTTGCCTTTACA
TGCAACTTTCTTTCTAATAA
>gi|22023568|gb|AF419318.1|_Human_papillomavirus_type_91,_complete_genome
ATGAGTAAGTACTGTGTTAGGCACACCTGCAGACCGAAACCGGTCGCTGCACACCTTACA
TTCTTTGTGGTTATTAGTAACAATTATTATGCTTGTACTAAAGGGAGTAACCGAAAACGG
TCCGACCGAAAGCGGTACATATATAAACCACCCAAAAAACATAGCTTGTGGGGCATAATG
TCTGCACGTAGCTGCTCCCAAAACGCTAAGACCATATTTGAGTTGTGTGACCAGTGTAAC
ATAACTCTGCCTACTCTGCAAATTAGTTGTATATTTTGCAAGAAGTGGTTGACAACCACG
GAAGTGTTGTCGTTTGCATTTAGGGATTTAAAGGTTGTGTGGCGCGACAGTTATCCGTTT
GCAGCGTGTTTGGCCTGCTTAAAATTTTATGGTAAAATAAGCCAATATAGGCATTTTAAG
TACGCGGCATTAGCAGTAACAGTAGAGGAAGAAACACAACAAACTGTATTCGAGTTGTGC
ATTCGCTGTTGTAAGTGCCACAAGCCATTGTCGCCTATAGAAAAGGTGCAGCACATTGTG
CAAAAAGCCCACTTTTTTTACATAAGTAACGCGTGGAAGGGATATTGCCTACACTGCTGG
AAGTCATGTATGGAAAAGAGCCGACGCTCAGAGACTATGTGCTAACCTTGCAACCAGAAC
CTAGAAGTTTAACCTGTGACGAGCAATTAGACAGCTCAGACTCAGAGGATGAGCGTGAGC
AATCCACGCAACAGGACCAGCAAGTGGACCTACAAGTTTACAAAGTTGTAACCGAGTGCA
CCTCTTGTTATTGTTCAATTCGTCTTGTTGTGAAGTGCTCAAGCAGTGACATCAAGACGC
TGGAGGAGCTGCTGTTGGGGACATTACAAATTGTGTGTCCCTTGTGCACTACAGCTGTGT
GACTGCAATGGCTGATAAACAAGGTACAGAGGATGAGGGGTTGGGATGTTCAGGCTGGTT
TATAGTAGAGGCCACAGTAGACAAAACCACGGAAAATGATATATCAGAAGATGAAATTGA
GGAGGAAGAGGATAGTGGGTTTGACATGGTAGATTTTATTAATAATACATTAGAGGACAG
CTGTGCAGAACAAAGCTGTGCGCAGGCCCTGTTGAATAAACAGCAAGCGGATGCTGATGC
TGCTATAGTACAGGAGCTAAAACGAAAGTATATTAGTCCATATGTAAGTCCTATACAGTG
TTTACAGGAGTCTGTGGACAGGGACCTAAGTCCTAGGTTGCATGCCATAAAGATAGGAGG
CGGACAAAAGGCAAAAAGGAGACTGTTTGAACCGACAGAGCAGCGGCATAGTAACTATGG
CAATTGTGAAGTGGAAGCAACTGAGACACAGGTAGATGTGGAAAATGGCGGACCGGAACG
CCATATGGGGGCCATACAACATTGTGGGAGGGGGGGGAGTAGTGTGGCGGAAGCAGTGCA
AGTAGTAGAGGAAGAGGGTGTAACAAACAATAGCCAGACTACAGGAGAGCAAAGCCCACG
CACGCGTATTGTAGAATTATTTAAAGACAAAGATGTTACTGTGAAACTCTTAGGTAAGTT
TAAAGACTTATTTGGCGTGGGATTTAATGATTTGGTAAGACAGTTTAAAAGTGATAAATC
AACATGTACTGATTGGGTATATAGTGTATTTGGTGTTAATCCATCAATAGCAGAAGGATT

CCATATGTTATTAAAAGAACAAACATTATACCTACATACCCAATGGGTTACATGCCGATG
GGGGATGGTGTTGCTTGCATTGTGTAGATATAAGGTTGCCAAAAATCGCAGTACAGTCGG
TAGACAGCTTGCACAAATGTTAAATGTACCTGTGCAACAAATATTGATACAGCCTCCAAA
ATTACAAAGCGCTCCAGCAGCACTGTTTTGGTTTAGAGCCGGTATGGGAAATGGTAGTGA
AGTATCGGGCACAACACCGGAATGGATAAGTAGACAGACAGTGTTAGAACACAGTTTTGC
AGACACACAGTTTAGTTTAACAAATATGGTGCAATGGGCATATGATAATGGATATACAGA
AGAATGTGACATAGCTTATTATTATGCACAACTAGGAGACACGGATGCAAATGCCGCTGC
ATTTTTAAAAAGTAATATGCAAGCTAGGTATGTTAGAGATTGTGCATGTATGTGCAAACA
TTATAAGCTAGCAGAAATGAAAAAAAATGTCTATGGCAGAGTGGATTAAGCATAGAGGAGA
GAAGTGTAATGATGGGGACTGGAAACCTATAATACGATTTTTAAGATATCAGCATATAGA
TATTATTACATTTTTAGCAGCCTTAAAAAAATGGCTGCATGGCATACCAAAAAAAAATTG
TATTTGCATAATAGGGCCGCCCGACACAGGTAAATCAAGTTTTGGAATGAGTTTAATGAA
GTTCCTAGGCGGCACTATGTTATCATATGTAAATTCAAGTAGCCATTTTTGGTTACAGTC
CTTAGTAGATGCAAAGGCAGCCATGTTAGATGATGTTACAGCAGCATGTTGGAATTACAT
GGATATGCATATGCGAAATTTATTAGACGGCAATCTTACAAGTATTGATAGAAAGCATAA
AGCATTAGCAGTTATAAAGTGTCCTCCGTTATTACTAACATCCAACATGGATATTAATAC
AGATGATAAGTATAAGTATTTAAAAAGTAGAATTACAACATTTACATTTCCTAATGCATT
TCCATTTGATACTAATGGCAATGCAATTTATGAGTTCAATGATGAAAATTGGAACCCCTT
TTTTAAAAGGTTGGCATCAAGTCTAGAACTGGAAACTGCAGAGGACGAAAATGGAGACAC
TAGCCAGGCGTCTAGATATGTGCCAGGAACAGTTGTTAGAACTGTATGAAAAAGACAGCA
GAGATCTGCAGGACCATGTGTTGCATTGGAAATGTATACGTTATGAGTGTGCATTATATT
ATAAAGCAAGAGAAATGGGCATCAAACATCTAGGCCACCAGGTGGTGCCAAAGCTAGAAG
TATCACGAGCAAAAGCACATGTGGCAATTGAAATGCAATTATCATTAGAATCATTATTAC
AAACTGAATATAGCATAGAGCCGTGGACACTACAGGACACATGTCAAGAACTATGGCACA
CGGAACCAAAAAAAATGTTTTAAAAAACGGGGACAGACAGTAGAAGTGAGATTTGACTGCA
ACCCTGAAAATACAATGCAATACACCTTATGGTCAGAAATATATGTACCCGTGAATGACA
CGTGGGTAAAAGTACATGGCCATGTGGACTATAAAGGCCTTTCATATACTGTGTGTGGGC
AAAAAACATATTATGTAGATTTTATTAAGGAGGCACAAACGTATGGGGATACTGGACAAT
GGAACGTTGTTGTGGGCTCAAACGTCATATCTTCTCCTGCATCTGTATCTAGTACTGTCA
CCGAAGTATCCTCTGTTGCGTCTACTGAACCCGACTCCAGAACAGCGACCACCGTCCCAG
ATTCCACGTGCACCCAAAAAGACTACGAGGAGCAAGCGCCGCCTAGAAAGCGAGTGCGAT
TCGAGCCCCACACAACCCAGATCCCCTGTACCTTGGCCAGAACTGTTGGAGGAGAGCCAT
TGGACAGTAGAGACCCACGAATCATCCCTAAGCATTCGAACCACAACCAAGGAGGGCACA
GTGGTGGAAGTAACGCTACGCCTATAGTTCAATTACAAGGTGATGCTAACTCACTAAAAT
GTTTTAGATACAGATTGGGCAAATATAAATATTTGTATGTTGATGTGTCATCTACATGGC
GATGGACAACAGAATCTAATAAACAAAACAGTGCATTAATTACATTAACATATGCTAGTG
TACAACAAAGGGAGGCGTTTTTAGCTAATGTAAAAATTCCCACAACAATTAAACATTGTT
TAGGATTTTTAACAATTATGTAAATAGTACCCTATGAATTTGTAAAACTACCTTGTATAT
TGCTTGTATAGTGGAACTGTTACTGTGACGTTAAGGGTGTTGTAACACTCTAACCACACA
ACCAGCCAACCTGCTGCTATTTTTGTTAATAGATGTAAAGCTTGGCATTTTATTAGTTAA
TGTGCTTGTTTGCATAATTGCTGTATTGGTGTGCGCATGGTTTGCGGACAACCTGGTGTG
GGCTTCTGTGCTTGCTTCAGCTTCTATACTTTACTTTTTATCTTGGTCTGCACTTATATC
ACTTACTAACGTGTTTATATTAATACTTGTAGTGTGGTTTGTACCGGCCCTTTTATTACA
TTTACATATTACATATTACATTGCAAATTGAAACACAGCAATTGTAAATACATTGGTGTG
CATATATTTGTACCTAGCATTATGTTAACTTGTCAATTTGATGATGGTGATTCATGGATG
TTGTTATGGCTACTTGTAACATTTATAACTGTATTGTTGATGTTATTAGTGTTTCATTAT
AGGACAGTTAAGCTGCTGCCCTCCAGCAAATAAACAAATTAATTACTGTGTTTATATAAA
CATGGTTTCTTATACACTTAAAAGGCGCAAGCGCGCGTCTGCTACACAATTATATCAAAC
ATGCAAGGCTGCAGGCACTTGTCCCTCTGATGTAATTAACAAGGTTGAGCATACTACAAT
AGCAGATCAAATATTAAAATGGGCTAGCATGGGTGTGTATTTTGGAGGGTTAGGCATTGG
AACTGGCTCTGGAAGTGGAGGAAGAACAGGCTATGTACCTATTCCAACAGGTCGCACAGG
TACTGTTCCTAAAGTTAGTGCAGAGCCTGGGGTAGTGTCACGTCCTCCAGTTGTAATAGA
ACCTGTGGCTCCAACCGATCCTTCCATTGTTTCATTAATTGAAGAATCAAGTATAATTGA
GTCCGGGGCTCCAATCACAACTATACCATCCCATGGTGGTTTTGAGGTAACATCATCTGG
GTCAGATGTCCCAGCCATTTTAGATGTGTCACCAACAACATCTGTGCATGTTACCACCAC

TACACATTTAAATCCTGCATTTACTGATCCTGCAATTGTACAACCCACCCCACCACTTGA
GGCTGGGGGACGTATTATAATTTCCCAATCTACTATTACAGCCCATAGTGCAGAAGACAT
TCCTATGGATACATTTGTTGTGCACAGTGATCCACTAACTAGTACGCCTATACCTGGTAA
TGCCTCACGACCTCGTTTAGGCCTTTACAGTAAGGCTTTACAGCAAATAGAAATTGTAGA
CCCTGCCTTTTTGTCCTCCCCCCAACGTTTAATTACATATGACAATCCTGTGTTTGAAGA
TCCAGATGCTACATTAGCATTTGAGCAGCCAACAGTACATGAGGCCCCTGACTCTAACTT
TATGGATATAGTAACGTTACATAGACCTGCATTAACATCTAGACGTGGTCTTGTTAGATT
TAGTAGGGTGGGCGCACGTGGTACCATGTATACACGTAGTGGTATTCGTATTGGGGGTCG
TGTACACTTTTTTAAAGATATTAGTCCAATTGCAACACAGGAATCTATTGAATTACAGCC
CCTAGGACATTCTACTGTTAATGTTCCTGATGCCACTGACTTGTATGATATTTATGCAGA
TGATAGCATTTTAGATGTACCTCATACCAACACCAATGTGTCTACACTGCCTAACACAAC
AAAAATTGTTAACACTACAGTCCCACTTGCAACAGTACCTGATATATATACACAAGCAGG
ACCTGATATTACATTTCCTACAGTGCCACTTAGTGTCCCCTATGTGCCATTGTCACCGTC
TGTTACACCTTCTGCAGTATCAATATATGGCACAGATTTTTATTTACATCCTTCATTGTT
GCATTTTGGCAAACGCCGTAAACGTATTTCATATTTTTTTGCAGATAACTATGTGGCGGC
TTAATGACAACAAAGTATACCTGCCTCCTCCAGGCCCTATAGCATCTATTGTAAGCACAG
ATGAATATGTGCAGCGCACCAACTTATTTTACCATGCTGGCAGTTCCCGTTTACTGGCTG
TGGGCCACCCTTTTTTTCCTATAAAAAATAATTCTGGTAAAGTAATTGTTCCTAAAGTTT
CAGGTCACCAATATAGGGTGTTTAGAGTTAAATTACCAGATCCTAATAAATTTGGTTTTT
CAGAAACAACATTAGTTACCTCAGACACCCAGCGTTTAGTATGGGGTTGTGTGGGAGTTG
AAATTGGTAGAGGACAACCCTTAGGTGTTGGAATAAGTGGCCATCCCTATTTAAATAAGT
ATGATGACATTGAAAACCCGTCTGGGTATGGCACATCACCAGGACAAGATAACAGAGAAA
ACGTAGCAATGGATTATAAACAAACACAGCTATGTATTGTTGGCTGTACACCTCCCATGG
GCGAATATTGGGGCAAGGGAGTTCCTTGTAGCACCTCAGGTATTACACAAGGTGATTGTC
CTGTAATAGAATTAAAAAGTGAAGTTATAGAGGATGGTGATATGGTAGATACAGGGTTTG
GTGCACTTGATTTTGCATCCTTACAAGCCAGTAAAAGTGACGTGCCCTTAGATTTATGTA
ATACTAAAAGCAAATATCCAGATTATTTAGGAATGGCAGCCGAGCCATACGGAAATAGTT
TATTTTTCTTTTTACGTAGGGAACAAATGTTTGTTAGACATTTCTTTAATAGGGCTGGTA
CTACAGGCGATGCTGTCCCTAAGGATTTGTATATTGCAGGAACAGGCAACAGGGCGAACA
TAGCAGGCAGTATATATTATTCTACTCCTAGTGGGTCTTTAGTAACTTCAGATTCTCAAT
TGTTTAACAAACCGCTTTGGATGCAAAAAGCACAGGGGCATAATAACGGCATCTGTTTTG
GTAACCAGGTGTTTGTAACTGTTGTGGATACAACTCGCAGCACTAACTTAACCTTGTGTG
CATCCACTGAGTCTGTGCTACCTACTACATATGACAACACAAAGTTCAAAGAATATTTAA
GGCATGCAGAAGAATTTGATTTACAGTTTATATTTCAATTATGCATTATAACACTTAATC
CAGAGGTAATGACATACATCCACACTATGGATGCATCATTATTAGAAGACTGGAACTTTG
GGGTATCCCCTCCAGCCACGGCATCACTAGAGGATACTTATCGCTTTTTGGCTAATAAGG
CAATTGCCTGTCAAAAAAATGTCCCACCAAAAGCCAAAGAGGACCCATACAAAAACTATA
CTTTTTGGGATGTGGATCTTACCGAAAGGTTTTCTGCACAACTTACTCAATTTCCATTAG
GACGCAAATTTGTTATGCAGGCTGGATTACGTCCTAGGCCTAAATTAAAATCTGGGAAGC
GTGCAGCACCATCCTCCAGTTCAGCGCCTGCCTCCAAACGAAAGAAAACTAAGCGATAAC
TATTATATTACTGTATGTTGTGTATGTGCATTTGTGTATTGTATGTGGTTTGTATACATG
TGTGTGTGTATTTATGTATTTATACATGTGTGTTATGTGTGCTGTATGGAATGAGGAATA
TGTGTATGTGTGTATGTGTTGCAGCTTAATAAACATTTTTGAGTGTGGTGTGGTCCATGG
TTGCACCCAGTGAGTAACTATGTGTTTCTTTCCTGTTTTGTGTTATGAATGTCCAGCGTT
GGGGCAGCGTTGGGGTTTAGGTCTGGTGGTAGTCATGGCGTCTGTTTGGTTACAAAAAGT
CATTTTTATTACTGTGCAACCGATTTCGGTCGTGTTTGGCAACGTAGGTATTGTGTCAGC
ATAATTGCAAGGTTACATTGACCTGCCAAAATATGTTGGCACAGTATAATTTCCCTTTGG
CGCCAAGGTGTGGCTTCCTACGCCTACATTCCACTTTTACTATAAATTTTACCTTTTTAC
CTTTGTGTTGTCTAAGGTTTTATTACTTTTAACCACATACATGTTTTATGGCTTTGCTTA
AAAGCTTTTTTGGCTCACATAACAATAAAGCACCCATAAAACATGT
>gi|333050|gb|M74117.1|PPH35CG_Human_papillomavirus_type_35_complete_genome
CCCTATAAAAAAAACAGGGAGTGACCGAAAACGGTCGTACCGAAAACGGTTGCCATAAAA
GCAGAAGTGCACAAAAAAGCAGAAGTGGACAGACATTGTAAGGTGCGGTATGTTTCAGGA
CCCAGCTGAACGACCTTACAAACTGCATGATTTGTGCAACGAGGTAGAAGAAAGCATCCA
TGAAATTTGTTTGAATTGTGTATACTGCAAACAAGAATTACAGCGGAGTGAGGTATATGA

CTTTGCATGCTATGATTTGTGTATAGTATATAGAGAAGGCCAGCCATATGGAGTATGCAT
GAAATGTTTAAAATTTTATTCAAAAATAAGTGAATATAGATGGTATAGATATAGTGTGTA
TGGAGAAACGTTAGAAAAACAATGCAACAAACAGTTATGTCATTTATTAATTAGGTGTAT
TACATGTCAAAAACCGCTGTGTCCAGTTGAAAAGCAAAGACATTTAGAAGAAAAAAAACG
ATTCCATAACATCGGTGGACGGTGGACAGGTCGGTGTATGTCCTGTTGGAAACCAACACG
TAGAGAAACCGAGGTGTAATCATGCATGGAGAAATAACTACATTGCAAGACTATGTTTTA
GATTTGGAACCCGAGGCAACTGACCTATACTGTTATGAGCAATTGTGTGACAGCTCAGAG
GAGGAGGAAGATACTATTGACGGTCCAGCTGGACAAGCAAAACCAGACACCTCCAATTAT
AATATTGTAACGTCCTGTTGTAAATGTGAGGCGACACTACGTCTGTGTGTACAGAGCACA
CACATTGACATACGTAAATTGGAAGATTTATTAATGGGCACATTTGGAATAGTGTGCCCC
GGCTGTTCACAGAGAGCATAATCTACAATGGCTGATCCTGCAGGTACAGATGAAGGGGAG
GGGACGGGATGTAATGGATGGTTTTTTGTAGAAGCAGTAGTTAGTAGACGTACGGGATCC
AGTGTAGAGGACGAAAATGAAGATGACTGTGACAGGGGGGAGGATATGGTGGACTTTATA
AATGATACAGATATATTAAACATACAGGCAGAAACAGAGACAGCACAAGCATTATTTCAT
GCACAGGAGGAGCAAACACACAAAGAGGCTGTACAGGTCCTAAAACGAAAGTATGCTAGT
AGTCCACTTAGCAGCGTGAGCTTATGTGTTAATAATAACATAAGTCCACGTTTAAAAGCT
ATTTGCATTGAAAATAAAAATACAGCAGCAAAGCGACGATTATTTGAACTACCAGACAGC
GGTTATGGCAATTCTGAAGTGGAAATACACGAGATACAACAGGTAGAGGGGCATGATACA
GTTGAACAATGTAGTATGGGCAGTGGGGATAGTATAACCTCTAGTAGCGATGAAAGACAT
GATGAGACTCCAACGCGAGACATAATACAAATACTAAAATGTAGTAATGCAAACGCAGCT
ATGTTGGCTAAATTTAAAGAACTATTTGGTATTAGTTTTACAGAACTTATTAGACCATTT
AAGAGTGATAAATCCACATGTACAGATTGGTGTGTGGCCGCATTTGGAATAGCCCCAAGT
GTGGCGAACTTTAAACATATAACATATGTATACATATACAATGTTTATCGTGTTCATGGG
GCTATGGTAATTCTAGCATTATTACGATTTAAAGTCGAAAAACGAGAACAACAATTGAAA
ACTATTGATGCTAAATTGCTATGTATTTCAGCTGCAAGTATGCTAATACAACCACCAAAA
TTACGTAGTACCCCAGCTGCGTTATATTGGTTTAAAACAGCAATGTCAAATATTAGTGAG
GTTGATGGAGAAACACCAGAATGGATTCAAAGACAAACAGTATTACAGCATAGTTTTAAT
GATGCAATATTTGACCTATCTGAAATGGTACAATGGGCATATGACAATGATTTTATAGAT
GATAGTGATATAGCATATAAATATGCACAATTGGCAGAAACTAATAGTAATGCATGTGCT
TTTTTAAAAAGTAATTCGCAAGCTAAAATTGTAAAAGATTGTGCAACAATGTGTAGACAT
TATAAACGAGCTGAAAAAAGAGAAATGACAATGTCACAGTGGATTAAAAGGCGATGTGCA
CAGGTGGACGATGACGGTGACTGGAGGGACATAGTACGATTTTTAAGATATCAACAAGTA
GATTTTGTGGCATTTTTATCTGCACTAAAAAATTTTTTACATGGTGTGCCTAAAAAAAAT
TGCATACTAATATATGGAGCACCAAACACAGGTAAATCATTATTTGGAATGAGTCTAATG
CATTTCTTACAAGGAGCTATTATATCCTATGTAAATTCTAAAAGCCATTTTTGGTTGCAG
CCATTATATGATGCCAAAATAGCTATGTTAGATGATGCTACATCGCCATGTGGCATATAT
AGACCAATATTTAAGAAATGCACTAGATGGAAATCCTATATTTCATTTAGATGTAAAGCA
TTAAGCATAGTGCATATAATGCCCACCTTTACTTATTACATCAATATAAATGCAGGCAAA
GATGACAGGTGGCCATACTTACATAGCAGGGTAGTGGTCTTTACATTTCACAATGAATTC
CCATTTGATAAAAATGGAAACCCAGAGTATGGGCTTAATGATAAAAACTGGAAATCCTTT
TTCTCAAGGACGTGGTGCAGATTAAATTTGCACGAGGAAGAGGTCAAAGAAAATGATGGA
GACGCTTTCCCAGCGTTTAAGTGTGTGTCAGGACAAAATACTAGAACATTACGAGACTGA
TAGCACATGTTTGTCTGATCACATACAGTATTGGAAACTGATTCGTCTTGAATGTGCAGT
ATTTTATAAAGCAAGAGAAATGGGAATTAAAACTCTTAACCACCAAGTGGTTCCAACGCA
GGCCATTTCAAAAGCCAAAGCAATGCAAGCAATTGAACTGCAATTAATGTTAGAGACATT
AAATACAACTGAGTATAGCACAGAGGACTGGACACTGCAAGAAACAAGTATTGAACTATA
TACAACAGTTCCTACAAGATGTTTAAAAAAAGATGTTTATACTGTGGAAGCACAATTTGA
TGGTGATAAACAAAATACTATGCATTATACTAATTGGACACATATATATATATTAGAGGA
CAGTATATGTACTGTTGTAAAGGGACTGGTAAATTATAAAGGTATTTATTATGTGCATCA
GGGTGTAGAAACATATTATGTTACTTTTAGGGAAGAGGCTAAAAAGTATGGAAAAAAAAA
TATATGGGAAGTGCATGTGGGTGGTCAGGTAATTGTTTGTCCTGAATCTGTATTTAGCAG
CACAGAACTATCCACTGCTGAAATTGCTACACAGCTACACGCCTACAACACCACCGAGAC
CCATACCAAAGCCTGCTCCGTGGGCACCACAGAAACCCAGAAGACAAATCACAAACGACT
TCGAGGGGGTACCGAGCTCCCCTACAACCCCACCAAGCGAGTGCGACTCAGTGCCGTGGA
CAGTGTTGACAGAGGGGTCTACTCTACATCTGACTGCACAAACAAAGACCGGTGTGGTAG

TTGTAGTACAACTACACCTATAGTACATTTAAAAGGTGATGCAAATACATTAAAGTGTTC
AAGATATAGATTGGGTAAATATAAAGCATTGTATCAAGATGCTTCATCTACATGGAGATG
GACATGTACAAACGATAAAAAACAAATAGCAATTGTAACATTAACTTACACAACAGAATA
TCAAAGGGATAAATTTTTAACTACAGTAAAAATACCTAACACAGTTACAGTGTCTAAAGG
ATATATGTCTATATGATAGACCTTACAGCTTCCAGTACTGTGTTGCTGTGCTTTTTGTTG
TGCTTTTGTGTGCTTTTGTGCTTGTGTCTGCTTGTACGTTCGCTATTGCTATCTGTGTCA
TTATACTCAGCATTAATATTACTGGTTTTAATACTGTGGGTTACTGTAGCAACACCACTA
CTTGCTTTTGTTGTTTCTTGCTTTTGTATATACCTATGGATGATTAACGCTCATGCACAA
TATTTGGCAGTACAGTAATTGTATACAAACATTGTGTTTGGTACTGTGTAACATGTGTGT
ATGGTGGTTTTATTTTTTGTTGTTCATTGTATATTTTGTTTTTTTACTGTTTTTAAACAT
TTTTATTTCTGTGTTTTTAATAAATTGATCACATGGTATAACCATGCGACACAAAAGGTC
TACAAAACGTGTTAAACGTGCATCTGCAACACAACTATATCGTACTTGCAAAGCTGCAGG
AACTTGTCCACCAGATGTTATACCTAAGGTTGAGGGTAATACTGTTGCTGATCAAATTTT
AAAATATGGCAGCATGGCTGTGTTTTTTGGGGGGTTAGGAATTGGTTCTGGATCTGGCAC
AGGTGGAAGATCTGGATATGTTCCACTGGGTACAACACCTCCAACGGCTGCCACAAACAT
TCCTATACGACCCCCTGTAACTGTGGAAAGTATACCATTAGACACAATTGGCCCTTTAGA
TTCTTCTATAGTGTCATTAGTAGAGGAAACTAGTTTTATTGAGTCTGGTGCCCCTGTTGT
TACACCAAGGGTCCCACCTACAACAGGTTTTACAATAACCACATCTACAGATACCACACC
TGCTATTTTAGATGTGACATCCATAAGTACACATGATAATCCTACTTTCACTGATCCTTC
TGTTTTACACCCACCCACGCCTGCAGAAACTTCAGGTCATTTTGTACTTTCATCATCTTC
TATTAGTACACATAATTATGAAGAAATCCCTATGGATACTTTTATTGTTTCCACAGACAG
CAATAATATAACTAATAGCACGCCTATTCCAGGGTCTCGCCCTACGACACGCCTAGGATT
ATATAGTAAAGGTACCCAGCAGGTTAAGGTTGTTGACCCTGCCTTTATGACTTCTCCTGC
AAAACTTATTACATATGATAATCCTGCATATGAAGGCCTTAACCCTGATACAACCTTACA
ATTTGAGCATGAGGATATTAGCTTAGCTCCGGATCCTGACTTTATGGACATTATAGCTTT
ACATAGGCCTGCACTAACATCTAGGAAAGGCACTATTAGATATAGTAGAGTAGGTAATAA
ACGTACTATGCATACACGAAGTGGAAAAGCTATAGGGGCACGGGTACATTATTATCAGGA
TTTAAGTAGTATTACTGAAGATATAGAATTACAACCCTTACAACATGTACCATCCTCTTT
ACCACATACCACTGTTTCAACATCATTAAATGATGGTATGTTTGATATTTATGCTCCTAT
AGATACTGAGGAAGATATTATATTTTCAGCATCTTCTAACAATACTTTATATACTACATC
TAACACTGCATATGTTCCTAGCAATACTACTATACCATTAAGTAGTGGCTATGATATTCC
TATAACAGCAGGGCCAGACATTGTATTTAACTCTAATACTATTACTAACTCTGTACTACC
GGTACCCACAGGTCCTATATATTCTATTATTGCAGATGGGGGTGACTTTTATTTACACCC
TAGTTATTATTTATTAAAACGACGTCGTAAAGCTATCCCATATTTTTTTGCAGATGTCTC
TGTGGCGGTCTAACGAAGCCACTGTCTACCTGCCTCCAGTGTCAGTGTCTAAGGTTGTTA
GCACTGATGAATATGTAACACGCACAAACATCTACTATCATGCAGGCAGTTCTAGGCTAT
TAGCTGTGGGTCACCCATACTATGCTATTAAAAAACAAGATTCTAATAAAATAGCAGTAC
CCAAGGTATCTGGTTTGCAATACAGAGTATTTAGAGTAAAATTACCAGATCCTAATAAGT
TTGGATTTCCAGACACATCATTTTATGATCCCTGCCTCCAGCGTTTGGTTTGGGCCTGTA
CAGGAGTTGAAGTAGGTCGTGGTCAGCCATTAGGAGTAGGTATTAGTGGTCATCCTTTAT
TAAATAAATTGGATGATACTGAAAATCTTAATAAATATGTTGGTAACTCTGGTAACTCTG
GTACAGATAACAGGGAATGCATTTCTATGGATTATAAACAAACACAATTGTGTTTAATAG
GTTGTAGGCCTCCTATAGGTGAACATTGGGGAAAAGGCACACCTTGTAATGCTAACCAGG
TAAAAGCAGGAGAATGTCCTCCTTTGGAGTTACTAAACACTGTACTACAAGACGGGGACA
TGGTAGACACAGGATTTGGTGCAATGGATTTTACTACATTACAAGCTAATAAAAGTGATG
TTCCCCTAGATATATGCAGTTCCATTTGCAAATATCCTGATTATCTAAAAATGGTTTCTG
AGCCATATGGAGATATGTTATTTTTTTATTTACGTAGGGAGCAAATGTTTGTTAGACATT
TATTTAATAGGGCTGGAACTGTAGGTGAAACAGTACCTGCAGACCTATATATTAAGGGTA
CCACTGGCACATTGCCTAGTACTAGTTATTTTCCTACTCCTAGTGGCTCTATGGTAACCT
CCGATGCACAAATATTTAATAAACCATATTGGTTGCAACGTGCACAAGGCCATAATAATG
GTATTTGTTGGAGTAACCAATTGTTTGTTACTGTAGTTGATACAACCCGTAGTACAAATA
TGTCTGTGTGTTCTGCTGTGTCTTCTAGTGACAGTACATATAAAAATGACAATTTTAAGG
AATATTTAAGGCATGGTGAAGAATATGATTTACAGTTTATTTTTCAGTTATGTAAAATAA
CACTAACAGCAGATGTTATGACATATATTCATAGTATGAACCCGTCCATTTTAGAGGATT
GGAATTTTGGCCTTACACCACCGCCTTCTGGTACCTTAGAGGACACATATCGCTATGTAA

CATCACAGGCTGTAACTTGTCAAAAACCCAGTGCACCAAAACCTAAAGATGATCCATTAA
AAAATTATACTTTTTGGGAGGTTGATTTAAAGGAAAAGTTTTCTGCAGACTTAGATCAGT
TTCCGTTGGGCCGTAAATTTTTGTTACAAGCAGGACTAAAGGCCAGGCCTAATTTTAGAT
TAGGCAGGCGTGCAGCTCCAGCATCTACATCTAAAAAATCTTCTACTAAACGTAGAAAAG
TAAAAAGTTAATGTGTAAATGTGTATGCATGTATACTGTGTGTTATGTGTTGTAGTGCTT
GTATATATATTATGTGTTGTGGTGCCTGTTTGTGTTGTACATGGCGTGTAAATGTGTGTA
TAATATTGTGCAATGTGTTGTACGTGGGTGTTTTTGTACTTAGTGTGTAGTAGTTCAGTA
GCCATAAAGTGATGTGTGTGTTTATAATTAACACTGTATTGTTGTATGACTATGGTGCAC
CGATATGAGCTTACATAATTACATGACAGCTATATTGTGTATATAAATAATCTACCTCCA
TTTTGTGTGTTAGTGTCCTTTACATTACCTTTCAACCGATTTCGGTTGCTGTTGGTAAGC
TTTATATGTTTTTTACAAAAACATTCCTACCTCAGCAGAACACTTAATCCTTGTGTTCCT
GATATATATTGTTTGCCAACTTTATATTGGCTTTTGCCAATCTTTAAACTTGATTCATCT
TGCAGTATTAGTCATTTTTCATACTTGTGGTCCACCCACACTTGTAACACTTGTAACAGT
GCTTTTAGGCACATATTTTTTGCATTTCTAAAGGGCTTTAATTGCACACCTTGGCTTTAC
ATATTATGTGTGTTTGCCAACACCACCCTACACATCCTGCCAACTTTAAGTTAAAACATG
CATGTAAAACATTACTCACTGTATTACACATTGTTATATGCACACAGGTGTGTCCAACCG
ATTTGGATTACAGTTTTATAAGCATTTCTTTTTATTATAGTTAGTAACAAT
>gi|15741127|gb|AF349909.1|AF349909_Human_papillomavirus_type_86_candidate,_comp
lete_genome
ATGCCCAGAGGATGCCACACTCCTACAAATATTTTTGTGCTATGTCAGGAATATGAGGTG
GATTTTGACAGCCTAAGACTGCTTTGCATCTTTTGCAAGAATGAATTAACAGAAGGCGAA
TTGCTGTCGTTTGCACTAAAGGAATTATTACTGGTCTGGAAACACGGATTTCCTCATGGC
GTGTGCCTAAAATGCATATGCAGAGAAGCCAAGGTACGTGAGCTACGACACTGGGAGCAC
TCCAGCTACGGATCCACAGTGGAACAAGACACAGGCGTATCATTGGCACACTTAAATATA
AGGTGCCATGCGTGCTTTAAACCATTGTGCTGGCCAGAGAAGCTGCATATGGTGGAATTA
AATCTGCATTTTCATAAAATAGCAGGCCAGTGGACGGGAAAGTGCAGCAACTGTAGAGCA
ACATGCATGGCCAGACGCCGACCATAAAAGATATAGTGTTATGTGAAATACCAGATGTAG
TTAACCTACATTGTGACGAGCAATTGTTAGACAGCTCAGAGGAGGAGGATAATAGGGATT
GTGACCAACCTGCAGAACCAGCACAGCTGGCCTATAGGGTGTTAACAGAATGTGGGTTGT
GTAGACGTCCACTTAAGCTGGTGGTGCGTTGTGGAGCTGAAGACCTAAGGCAGCTACAGC
AGCTTATGGTGGACGCAGTGGCCCTTGTGTGTCCAGGCTGTGCATAACATGGCAGAGTCT
CCTGAAGGTACAGACGGCGGGACGGGGGGGGGGGCGGGAGGGTGGTTTGTGGTAGAAGCC
ATGGTACACCATACAGCAAATACAGATGTGTCTAGTGATGAGGACGAATCTGCACCTGAT
ACAGGAGAGGATATGGTAGATTTTGTAGATAACACTAGATATCCAGGGGACGGACAGGAA
GTGCCGTTAGGTCTATATGTTCAACAAACAATGCAGGATGACGCTGCAACGGTGCAAGCC
CTAAAACGAAAGTTCATGGGAAGCCCGGCAGCTAGTTCCTGTGCATCCTGGGTGAATAAC
GAATTAAGCCCACGTTTGGATGCAATATGTTTACAGCAAAGGCACGACAAGGCCAGGAGA
CGGTTGTTTGAACAAGACAGTGGGTATGGCAATACGCAGGTGGATATTGGAACGTCCGAA
AGCCAGGTACCAGGGGAACATAACAACAGCGGGGGGGGCGACGTAACACAGGACGTTATA
GAGGAGGAACGGGGGGGGGGGGACGGGGAACCTGGGCCACATGTAGATACAGAACACACA
CAGCAGAAGGAGGGCACGCCCGATGCACTAGACCTGCTACAGGTTAGTAACCTACGGGTA
CAGCTATTTACTAAGTTTAAGGAGCTATTTGGACTATCATTTAAGGACCTAGTAAGACAG
TTCAAAAGTGATAGGTCCACATGTGCAGAGTGGGTAGTAGGGGCATTTGGGGTGTATTAT
GCAGTGGCAGAAGCAGCAAAAACATTATTGCAGCCAGTATGTGAGTATGCACACATACAA
ACATTAACAAGTGAATGGGGAATGGTAATGCTGCTACTGCTACGGTTTAAGTGTAACAAA
AGCAGAGAAACCGTAGCAAAGTGCCTAGGAACCATACTAAATATTCCAGAAAAGCGTATG
CTAATAGAACCCCCAAAACAACGCAGTGCGCCATGTGCACTGTATTGGTACAAGACAGCT
ATGGGCAATGCCAGTGAGGTGTTTGGGGACACGCCGGACTGGATAGTAAGACAAACAGTT
ATTGGACATGCAATGGGAGAAACACAATTTAGCCTATCTGTGTTAGTGCAGTGGGCATAT
GACAATGAAATAACAGATGACAGCGAGCTAGCCTATGAATATGCAAAATTGGGAAATGAG
GACCCAAATGCAGCCGCCTTCTTAGCAAGCAATTGTCAGGCAAGATATATTAAGGATGCA
ATAACAATGGTTAGACATTATAGACGTGCAGAACAGGCACGTATGAGTATGTCACAATGG
ATTGCACATAGGGGGCGCAAGGTGGCTGACACAGGCGACTGGAGACATATAGTAAAACTA
CTAAGGTTTCAGGGCATAGAATTTATTAGCTTTATGGAAGCATTAAAGCAATTCTTAAAG
GGAACACCAAAGAAAAGCTGCCTAGTATTTTATGGACCCAGCGACACAGGGAAATCATTG

TTTTGCATGAGCCTATTAAGGTATTTAGGTGGAGCAGTAATATCCTTTGTAAATTCCACC
AGCCATTTTTGGTTGTCGCCATTGGTAGATGCTAAAATAGGACTGTTGGACGATGCAACA
CAGCAGTGCTGGGTATACATAGATACCTATCTAAGAACTGTACTAGATGGCAATACAATG
AGCATAGACAGAAAACATAAAAACCTACAACAGCTGAAATGTCCACCATTAATGATAACA
ACAAATGTAAATATTGCAGCAGATGACACCTTTAAATATCTTAGAAGTCGAATGGTCGTC
TTTCCGTTTCTACAAAAGTGCCCACTAGATAGCAATGGAGAACCAGTGTATAAACTAAAT
AATGAAAATTGGAAATCCTTTTTTCAAAGGTCGTGGGCACGATTAGACTTAACCCAGGAG
GAGGAGGAGGAGGAGGAGGAGGCTGACAATGGATACACTAGCCGATCGTTTAGATGCGTG
CCAGGACAAGTTACTAGACCTTTATGAAAAGGATAGCAACACTCTTGAGGATCAATTATT
GCATTGGCAATACACACGCCTGGAAAAAGCTATATTATTTAAGGCAAGAGAAGCAGGACT
AACACATATAGGCCACCAGGTGGTGCCTGCAAGTAGTGTAACAAAGGAAAAGGCCCGGCA
AGCTATAACAGTGCATTTGTCTTTGCAAAGCTTAAATAACAGTGAGTTTAAACACGAGCC
ATGGACATTGCAGGACACGTCATTGGACATGTGGAATGTCCCCCCGAAAGGGTGTTGGAA
AAAAGGCGGACGCCCCATCAGAGTAAAATACGATGGAGAAGATGACAAAGAAATGGAATA
TGTCAGCTGGGGGTTTATATATATATATTGTGGTAGTGATGAAACATGGCATAAGGTGCC
TGGTAAGATTAGCCATGCAGGGTGTTACTATGAACTGGAAGGCCGCAAACACTACTATGT
TGACTTTGGAAAGGAGGCAAAACAATATGGGGTAAAAAACATGTGGGAGGTGCATGTGGG
AGGCACAATTATTCACCATGCATGCGACTCTGTATCCAGTACTCAGGGAGGAGTGCCGAT
ATCCACTGCTGAAACTGCTGCACAGTCGCTGTACACCACCACCGCGCCCACCCCCGCCGC
CACCAAGAAAACCCCCCAGGTGCAGGCGCCGCCTGCTAAGCGACAGCGGCTCGGAAGCGA
CGGCATACAGCAGCCCGACTCTACAGGAGACAAGCGAGCATTCGTGGACAGTTGCCTCCC
AAGGACACAAAATAACCCTGACATCCCACAGTGGTCGCGGGACAACAGTAACAGTCACGG
TGCACCTGTCATTCACTTAAAAGGTGACCCCAACCGTCTTAAATGTTTGAGATACCGATT
GCAGCAATCTGTTCCCCAATTGTTTGAAAGAGCATCGTCCACGTGGCGGTGGACCTGTGG
GGGGTCAGAGGACAAACAATCATTTGTAACATTGTGGTATAGGGATACGGAACAACGCCA
ACAGTTTCTAGCACGTGTAAATATACCTAAAGGAATTGTGGTAACACAAGGCATTATGTC
AATGTGCATATAATAGTAGTTGTTTGTAAACATTATATCCTGTATGTGAGCCAATTGTAG
CAATTGTGTGTATTACATACCTGGTATATACTGCATCCTGGAACCTATTGTATGGGCCAT
CCATGTATGGTGTACTGTGCTGCTGCTGCTAATTCTCTTTTGGCTTTCACACCTATCTGC
TCTGGTTGCTTTTGTTGTGTTTGTGTGTTTTATATATTTTGGGTTCTGGCTACTGTATAT
ACAGGTACTGTGGTACATTGGATTGTTATAATCACACAACCAGCCATCTACTGCTGCTAC
ACTACCTTGTACAGCCCCTTCCCCATACAGTATGTATCCCCTACAGGTACGGGGTCCCCA
GGGTGGATATGATATTGTGTTGTTTGACCGTGGGGATATGAGCATATTGGTTTTGTTATT
ACTATTGATTGCTATTATTTTGCTGTTGTTATTCATACGCATGTTACATGTGTAGGTGTG
TATAGTTTTGTATTTTGGTTTGTAATAAACTGTTGGTACCATGCCTAAGGTCAAATCCCG
TCGCAAGCGTGCCTCTGCCACGGACCTTTATCGCACCTGCAAGGCCTCTGGCACATGCCC
TGCTGATGTGGTTCCTAAAGTGGAGGGCGACACATTGGCGGATCGCATATTAAAATGGGC
CAGTTTGGGTGTCTTTTTTGGGGGTCTTGGTATTGGTACCTCCTCGGGGTCCGGCGGCCG
TACTGGCTATGTTCCATTGGGTACCCGGCCCCCTACAGTAGTGGATGTGGGGCCCACTGC
CCGTCCTCCTGTGGTCATTGAACCTGTGGGTGCTACTGACCCGTCCATTGTTACATTGGT
GGAGGATTCTAGTGTTATTAATGCAGGGGCCCCGTTACCCAACTTTACTGGAACGGGCGG
GTTTGAAATTACCACATCGTCCACCACTACCCCTGCAGTATTAGACATTACCCCGTCAGG
GTCCTCTGTGCAAGTTAGTAGCACTAGTTATATTAATCCATTGTTTACTGAGCCATCTAT
TATAGAGGTTCCACAGGCGGGTGACATTACTGGACATGTATTGTCTAGTACTGCCACCTC
AGGGACCCACACATATGAAGAAATACCTATGCAAACATTTGCTGTACAGGGGGGAACAGG
GCATGAACCCATAAGTAGTACACCTATACCTGGGGTGCGGCGCCTTGCAGGGCCTCGCTT
AAATTTGTACAGCCGTGCTACCCAGCAGGTTCCTGTAGCTGACTCTGCATTTTTATCGCG
TCCTGACTCCTTTGTTACCTTTGATAACCCTGTGTATGACCCAGATGAAACCATTATATT
TGAGCATCCTAGTTTACATGCTCCGCCCGATCCTGACTTTCTAGATATTGTTACCTTGCA
TAGGCCTGCCTTAACTGCACGTAGGTCAGGTGTTAGATATAGTCGCATAGGTCAAAGGGC
CTCCATGCGCACACGCAGTGGTAAACATATTGGGGCTCGTGTGCATTTTTATCATGACTT
AAGTCCTATTGCACAGGCTGAGGATATTGAACTGCAGCCGTTGGGTGCTACCACATCCTC
TGGCCAGGCCTCTGGCCAGGATACCTTATATGACATTTATGCAGATGATACACACCTATC
CTCTATATTACAGGAGCCTTCCGTTCCCTCATTGCATTCCACCACGCCTGTATCCTCTGC
TACAGTGTCCGCCACATCTATGGTATCTTCCACATATGATAATGTTACTGTTCCCTTATC

```
ATCTGCAACCGAGGTTCCCTTATATACTGGTCCTGATATTGATCATGCCATAGCCCCATC
GCCCACACCTTTACCTCCTGTGGTTCCCAGCACTACCCCATATGCCATTTATATACAGGG
ATCTGATTACTATTTACTGCCTAATTATATATTTTTTCCACGAAAGCGTAAACGTGTGCA
CTATTCTTTTTCAGATGGCTTTGTGGCGGCCTGGTGACGGCAAGGTTTACCTGCCCCCCA
CTCCCGTGTCTAAGGTTCTCAGCACGGATCGTTATGTATCTCGTACCAACCTATTTTATT
ATGGTGGTAGTTCCCGCTTGCTTACTGTGGCCATCCATATTATCCTGTTACTGTTTCCT
CCAGCCCTGGACAAAACAACAAAAAGGCCAATATTCCCAAGGTTTCGGGGTATCAATACA
GGGTTTTTAGGGTGCGACTGCCTGATCCTAATAAGTTTGGCCTTCCTGACGCCCAATTAT
ATAATCCAGACACTGAGCGCCTGGTATGGGCCTGTAGGGGCCTTGAGGTAGGACGTGGCC
AGCCCTTGGGTGTAGGTGCCAGTGGCCATCCGTTATATAATAGGCTTGATGACACTGAAA
ACACCTCCTTTCTTACTACTGAAAACGTAGACAGTAGGGACAATGTGTCTGTGGACTACA
AGCAAACCCAGGTATTAATTGTAGGCTGTAAACCATCCATTGGTGAGCACTGGGGTAAAG
GCACAGTGTGTTCTAATGTGCAGCCTAAACCAGGGGACTGCCCCCCATTACAGTTTACCA
ATTCCACAATTCAGGATGGTGACATGGTGGAGGCAGGCTATGGTGCTATGGATTTTGACA
AGCTGCAGGAAAACAAGTCTGAGGTGCCTTTAGACATTTGTGCCACTGTCTGTAAATATC
CTGATTATTTACAAATGGCTGCTGAGCCCTATGGCGATTGCATGTTTTTTTGTTTAAGGC
GAGAACAAATGTTTGCACGCCATTTTTTTAATAGGCAGGGTGTTATGGGTGAACCAGTTC
CTGAAACTTATTATTTAAAGGGCTCCTCAGGCCCCCGAGGATCCCTTAGCAGTTATGTAT
ATGCACCCACCCCTAGTGGCTCTATGGTGTCCTCGGATTCCCAATTGTTCAATAAGCCAT
ATTGGTTACAGAAGGCGCAGGGACACAATAATAGTATTTGCTGGTTTAACCAGCTGTTTG
TTACTGTGGTCGACACCACCCGCAGTACTAATTTTACTATTAGTGCCGCTACCCAGAAGG
CCTCTGAATATGACCCCTCTAAGTTTAATGAATATCTAAGGCATGCAGAGGAATATGATT
TGCAATTTATTTTTCAATTGTGTAAGGTCCGTCTTACCCCAGAGATAATGTCCTATTTAC
ATTCTATGAATGAGTCCCTTTTGGATGAGTGGAATTTTGGGGTTGTGCCCCCTCCCTCCA
CAAGTTTGGAGGATACCTACCGTTTCCTGCAGTCCCGTGCCATTACGTGCCAAAAAGGGG
CTACTGCACCCACAGCCTAAAGAGGATCCCTATGCTGGCATGGTGTTTTGGGAGGTTGACC
TTAAGGACAAGTTGTCCACTGATTTGGACCAATTTCCACTGGGCCGCAAGTTTCTATTGC
AGTCGGCCCCACGGGTCTCGCATGTGTCCCGCAAGCGTCCCGCATCTACCTCTACTGCCT
CCTCCTCCAAACGTCGCAAAACTAAAAAATAGTGTGTGTGTGCTGTACTGTCTTGTTTGT
ATGTGTGTCTGTATTGTGTACTGTTGTTGGTTGCACTGTGTTGTGTATATGTATGTGTAT
ATGTATGTGTTTTTTGTATGTGTTGTGTAATGTCCTTTATGTGCCCCTGTATTGTTGTGC
TTTTGTCTTGTTGTGTATTTTGGTGTGTATGTATTTATGGAATGTGTGTTATGTTGTGGA
TCAGTAAAGTATGTGTGTCATTGTGGGGTTCATGTCCGGCAGCACCCTGTGAGTAAGTGT
GCATTTGTACACACCTTGCACTCGGCTACATATCCACCATTTCCTGCAGTTATTTTGTAC
CCGTACTCCATTTTGTATGCGACCGAAATCGGTCTCCCGCCTTTTCGGTCTGTGTTATGG
CACCTTCCCAGGTACACCTAATCCTTTGGCAGCCCTACATCCTGCGTAGGCAGCTGCAAA
CCTTCCACACCTAGGTGTGTCTGACAGCTCTATACATAAAGTAATAAAATATGGTTTGTA
GTTTATAGGTTTTACTTACTCATCTGCAAGAAATATGCTTTTAGGCAATGCTTTGGCTAC
CAACTTTCCCTTAAGTGCATAGTTGGCAGTCTGTACTACATTGTACTGCCAAGTTTCTGT
CCTATAAACCTTTAAACAACTTATGTAACCAACACAACTCGGTCGCTGTATTATTCATAG
TTCCTTCTTTATACTTAACAACAATCATGTAGTTAAAAAATAAGGAGGGACCGAAAACGG
TGCGACCGAAAGGGATACATATATAAGGGCAGCTAAGGGCAGTGGAAAAGCAACGGACA
GCC
>gi|6002612|gb|AF092932.1|AF092932_Human_papillomavirus_type_6,_complete_genome
GTTAATAACAATCTTGGTTTTAAAAAATAGGAGGGACCGAAAACGGTTCAACCGAAAACG
GTTGTATATAAACCAGCCCTAAAATTTAGCAAACGAGGCATTATGGAAAGTGCAAATGCC
TCCACGTCTGCAACGACCATAGACCAGTTGTGCAAGACGTTTAATCTATCTATGCATACG
TTGCAAATTAATTGTGTGTTTTGCAAGAATGCACTGACCACTGCAGAGATTTATTCATAT
GCATATAAACAGCTAAAGGTCCTGTTTCGAGGCGGCTATCCATATGCAGCCTGCGCGTGC
TGCCTAGAATTTCATGGAAAAATTAACCAATATAGACACTTTGATTATGCTGGATATGCA
ACAACTGTTGAAGAAGAAACTAAACAAGACATTTTAGACGTGCTAATTCGGTGCTACCTG
TGTCACAAACCGCTGTGTGAAGTAGAAAAGGTAAAACATATACTAACCAAGGCACGGTTC
ATAAAGCTAAATTGTACGTGGAAGGGTCGCTGCCTACACTGCTGGACAACATGCATGGAA
GACATGTTACCCTAAAGGATATTGTATTAGACCTGCAACCTCCAGACCCTGTAGGGTTAC
ATTGCTATGAGCAATTAGTAGACAGCTCAGAAGATGAGGTGGACGAAGTGGACGGACAAG
```

ATTCACAACCTTTAAAACAACATTACCAAATAGTGACCTGTTGCTGTGGATGTGACAGCA
ACGTTCGACTGGTTGTGCAGTGTACAGAAACAGACATCAGAGAAGTGCAACAGCTTCTGT
TGGGAACACTAAACATAGTGTGTCCCATCTGCGCACCGAAGACATAACAACGATGGCGGA
CGATTCAGGTACAGAAAATGAGGGGTCTGGGTGTACAGGATGGTTTATGGTAGAAGCTAT
AGTGCAACACCCAACAGGTACACAAATATCAGACGATGAGGATGAGGAGGTGGAGGACAG
TGGGTATGACATGGTGGACTTTATTGATGACAGCAATATTACACACAATTCCTTGGAAGC
ACAGGCATTGTTTAACAGGCAGGAGGCGGACACCCATTATGCGACTGTGCAGGACCTAAA
ACGAAAGTATTTAGGTAGTCCATATGTTAGTCCTATAAACACTATAGCCGAGGCAGTGGA
AAGTGAAATAAGTCCACGATTGGACGCCATTAAACTTACAAGACAGCCAAAAAAGGTAAA
GCGACGGCTGTTTCAAACCAGGGAACTAACGGACAGTGGATATGGCTATTCTGAAGTGGA
AGCTGGAACGGGAACGCAGGTAGAGAAACATGGCGTACCGGAAAATGGGGGAGATGGTCA
GGAAAAGGACACAGGAAGGGACATAGAGGGGGAGGAACATACAGAGGCGGAAGCGCCCAC
AAACAGTGTACGGGAGCATGCAGGCACAGCAGGAATATTGGAATTGCTAAAATGTAAAGA
TTTACGGGCAGCATTACTTGGTAAGTTTAAAGAATGCTTTGGGCTGTCTTTTATAGATTT
AATTAGGCCATTTAAAAGTGATAAAACAACATGTGCAGATTGGGTGGTAGCAGGATTTGG
TATACATCATAGCATATCAGAGGCATTTCAAAAATTAATTGAGCCATTAAGTTTATATGC
ACATATACAATGGCTAACAAATGCATGGGGAATGGTATTGTTAGTATTATTAAGATTTAA
AGTAAATAAAAGTAGAAGTACCGTTGCACGTACACTTGCAACGCTATTAAATATACCTGA
AAATCAAATGTTAATAGAGCCACCAAAAATACAAAGTGGTGTTGCAGCCCTGTATTGGTT
TCGTACAGGTATATCAAATGCCAGTACAGTTATAGGGGAAGCACCAGAATGGATAACACG
CCAAACTGTTATTGAACATGGGTTGGCAGACAGTCAGTTTAAATTAACAGAAATGGTGCA
GTGGGCATATGATAATGACATATGCGAGGAGAGTGAAATTGCATTTGAATATGCACAAAG
GGGAGATTTTGATTCTAATGCACGAGCATTTTTAAATAGCAATATGCAGGCAAAATATGT
GAAAGATTGTGCAACTATGTGTAGACATTATAAACATGCAGAAATGAGGAAGATGTCTAT
AAAACAATGGATAAAACATAGGGGTTCTAAAATAGAAGGCACAGGAAATTGGAAACCAAT
TGTACAATTTCTACGACATCAAAATATAGAATTTATTCCATTTTTAAGTAAATTTAAATT
ATGGCTGCACGGTACGCCAAAAAAAAACTGCATAGCCATAGTAGGCCCTCCAGATACTGG
GAAATCGTACTTTTGTATGAGTTTAATAAGCTTTTTAGGAGGTACAGTTATTAGTCATGT
AAATTCCAGCAGCCATTTTTGGCTGCAACCGTTAGTAGATGCTAAGGTAGCATTGCTAGA
TGATGCAACACAGCCATGTTGGATATATATGGATACATATATGAGAAATTTGTTAGATGG
TAATCCTATGAGTATTGACAGAAAGCATAAAGCATTGACATTAATTAAATGTCCACCTCT
GCTAGTAACATCCAACATAGATATTACCAAAGAAGAGAAATATAAGTATTTACATACTAG
AGTAACAACATTTACATTTCCAAATCCATTCCCTTTTGACAGAAATGGGAATGCAGTGTA
TGAACTGTCAAATGCAAACTGGAAATGTTTTTTTGAAAGACTGTCGTCAAGCCTAGACAT
TCAGGATTCAGAGGACGAGGAAGATGGAAGCAATAGCCAAGCGTTTAGATGCGTGCCAGG
AACAGTTGTTAGAACTTTATGAAGAAAACAGTACTGACCTAAACAAACATGTACTGCATT
GGAAATGCATAAGACATGAAAGTGTATTATTATATAAAGCAAAACAAATGGGCCTAAGCC
ACATAGGAATGCAAGTAGTGCCACCATTAAAGGTGTCCGAAGCAAAAGGACATAATGCCA
TTGAAATGCAAATGCATTTAGAATCATTATTAAAGACTGAGTATAGTATGGAACCGTGGA
CATTACAAGAAACAAGTTATGAAATGTGGCAAACACCACCTAAACGCTGTTTTAAAAAAC
GGGGCAAAACTGTAGAAGTTAAATTTGATGGCTGTGCAAACAATACAATGGATTATGTGG
TATGGACAGATGTGTATGTGCAGGACACTGACTCCTGGGTAAAGGTGCATAGTATGGTAG
ATGCTAAGGGTATATATTACACATGTGGACAATTTAAAACATATTATGTAAACTTTGTAA
AAGAGGCAGAAAAGTATGGGAGCACCAAACAATGGGAAGTATGTTATGGCAGCACAGTTA
TATGTTCTCCTGCATCTGTATCTAGCACTACACAAGAAGTATCCATTCCTGAATCTACTA
CATACACCCCCGCACAGACCTCCACCCCTGTGTCCTCAAGCACCCAGGAAGACGCAGTGC
AAACGCCGCCTAGAAAACGAGCACGAGGAGTCCAACAGTCACCTTGCAACGCCTTGTGTG
TGGCCCACATTGGACCCGTGGACAGTGGAAACCACAACGTCATCACTAACAATCACGACC
AGCACCAAAGAAGGAACAACAGTAACAGTTCAGCTACGCCTATAGTGCAATTTCAAGGTG
AATCTAATTGTTTAAAGTGTTTTAGATATAGGCTAAATGACAAACACAGACATTTATTTG
ATTTAATATCATCAACGTGGCACTGGGCCTCCTCAAAGGCACCACATAAACATGCCATTG
TAACTGTAACATATCATAGTGAGGAACAAAGGCAACAGTTTTTAAATGTTGTAAAAATAC
CCCCTACTATTAGACACAAACTGGGGTTTATGTCAATGCACCTATTGTAATTTGTATATA
TGTAAATGTGTAAATATATGGTATTGGTGTAATACAACTGTACATGTATGGAAGTGGTAC
CTGTACAAATAGCTGCAGGAACAACCAGCACATTAATACTGCCTGTTATAATTGCATTTG

TTGTATGTTTTGTTAGCATCATACTTATTGTATGGATATCTGACTTTATTGTGTACACAT
CTGTGCTAGTACTAACACTGCTTTTATACTTACTATTGTGGCTGCTATTAACAACCCCCT
TGCAATTTTTCCTACTAACTTTACTTGTGTGTTACTGTCCCGCTTGTTATATACACCACT
ACATTGTGAACACACAGCAATGATGCTAACATGTCAATTTAATGATGGAGATACCTGGCT
GGGTTTGTGGTTGTTATGTGCCTTTATTGTAGGGGTGTTGGGGTTATTATTAATGCACTA
TAGAGCTGTACAAGGCGATAAACACACCAAATGTAACAAGTGTAACAAACACAACTGTAA
TGATGATTATGTAACTATGCATTATGATACTGCTGGTGATTATATATATATGAATTAGAG
TAAAACTTTTTTTATATTTGTAACAGTGTATGCTTTGTATACCATGGCACATAGTAGGGC
CCGACGACGCAAGCGTGCGTCAGCTACACAGCTATATCAAACATGTAAACTCACTGGAAC
ATGCCCCCCAGATGTAATTCCTAAGGTGGAACACAACACCATTGCAGATCAAATATTAAA
ATGGGGGAGTTTGGGGGTGTTTTTTGGAGGGTTGGGTATAGGCACCGGTTCCGGCACTGG
GGGTCGTACTGGCTATGTTCCCTTAGGAACTTCTGCAAAACCTTCTATTACTAGTGGGCC
TATGGCTCGTCCTCCTGTGGTGGTGGAGCCTGTGGCCCCTTCGGATCCATCCATTGTGTC
TTTAATTGAAGAATCGGCAATCATTAACGCAGGGGCGCCTGAAATTGTGCCCCCTGCACA
CGGTGGGTTTACAATTACATCCTCTGAAACAACTACCCCTGCAATATTGGATGTATCAGT
TACTAGTCATACTACTACTAGTATATTTAGAAATCCTGTCTTTACAGAACCTTCTGTAAC
ACAACCCCAACCACCCGTGGAGGCTAATGGACATATATTAATTTCTGCACCCACTATAAC
GTCACACCCTATAGAGGAAATTCCTTTAGATACTTTTGTGATATCCTCTAGTGATAGCGG
TCCTACATCCAGTACCCCTGTTCCTGGTACTGCACCTCGGCCTCGTGTGGGCCTATATAG
TCGTGCATTGCACCAGGTGCAGGTTACAGACCCTGCATTTCTTTCCACTCCTCAACGCTT
AATTACATATGATAACCCTGTATATGAAGGGGAGGATGTTAGTGTACAATTTAGTCATGA
TTCTATACACAATGCACCTGATGAGGCTTTTATGGACATAATTCGTTTGCACAGACCTGC
TATTGCGTCCCGACGTGGCCTTGTGCGGTACAGTCGCATTGGACAACGGGGGTCTATGCA
CACTCGCAGCGGAAAGCACATAGGGGCCCGCATTCATTATTTTTATGATATTTCACCTAT
TGCACAAGCTGCAGAAGAAATAGAAATGCACCCTCTTGTGGCTGCACAGGAAGATACATT
TGATATTTATGCTGAATCTTTTGAACCTGACATTAACCCTACCCAACACCCTGTTACAAA
TATATCAGATACATATTTAACTTCCACACCTAATACAGTTACACAACCGTGGGGTAACAC
CACAGTTCCATTGTCAATTCCTAATGACCTGTTTTTACAGTCTGGCCCTGATATAACTTT
TCCTACTGCACCTATGGGAACACCCTTTAGTCCTGTAACTCCTGCTTTACCTACAGGCCC
TGTTTTCATTACAGGTTCTGGATTTTATTTGCATCCTGCATGGTATTTTGCACGTAAACG
CCGTAAACGTATTCCCTTATTTTTTTCAGATGTGGCGGCCTAGCGACAGCACAGTATATG
TGCCTCCTCCTAACCCTGTATCCAAAGTTGTTGCCACGGATGCTTATGTTACTCGCACCA
ACATATTTTATCATGCCAGCAGTTCTAGACTTCTTGCAGTGGGTCATCCTTATTTTTCCA
TAAAACGGGCTAACAAAACTGTTGTGCCAAAGGTGTCAGGATATCAATACAGGGTATTTA
AGGTGGTGTTACCAGATCCTAACAAATTTGCATTGCCTGACTCGTCTCTTTTTGATCCCA
CAACACAACGTTTGGTATGGGCATGCACAGGCCTAGAGGTGGGCAGGGGACAGCCATTAG
GTGTGGGTGTAAGTGGACATCCTTTCCTAAATAAATATGATGATGTTGAAAATTCAGGGA
GTGGTGGTAACCCTGGACAGGATAACAGGGTTAATGTTGGTATGGATTATAAACAAACAC
AATTATGCATGGTTGGATGTGCCCCCCCCTTTGGGCGAGCATTGGGGTAAAGGTAAACAGT
GTACTAATACACCTGTACAGGCTGGTGACTGCCCGCCCTTAGAACTTATTACCAGTGTTA
TACAGGATGGCGATATGGTTGACACAGGCTTTGGTGCTATGAATTTTGCTGATTTGCAGA
CCAATAAATCAGATGTTCCTATTTACATATGTGGCACTACATGTAAATATCCAGATTATT
TACAAATGGCTGCAGACCCATATGGTGATAGATTATTTTTTTTTCTACGGAAGGAACAAA
TGTTTGCCAGACATTTTTTTAACAGGGCTGGCGAGGTGGGGGAACCTGTGCCTGATACTC
TTATAATTAAGGGTAGTGGAAATCGAACGTCTGTAGGGAGTAGTATATATGTTAACACCC
CAAGCGGCTCTTTGGTGTCCTCTGAGGCACAATTGTTTAATAAGCCATATTGGCTACAAA
AAGCCCAGGGACATAACAATGGTATTTGTTGGGGTAATCAACTGTTTGTTACTGTGGTAG
ATACCACACGCAGTACCAACATGACATTATGTGCATCCGTAACTACATCTTCCACATACA
CCAATTCTGATTATAAAGAGTACATGCGTCATGTGGAAGAGTATGATTTACAATTTATTT
TTCAATTATGTAGCATTACATTGTCTGCTGAAGTAATGGCCTATATTCACACAATGAATC
CCTCTGTTTTGGAAGACTGGAACTTTGGGTTATCGCCTCCCCCAAATGGTACATTAGAAG
ATACCTATAGGTATGTGCAGTCACAGGCCATTACCTGTCAAAAGCCCACTCCTGAAAAGG
AAAAGCCAGATCCCTATAAGAACCTTAGTTTTTGGGAGGTTAATTTAAAAGAAAAGTTTT
CTAGTGAATTGGATCAGTATCCTTTGGGACGCAAGTTTTTGTTACAAAGTGGATATAGGG
GACGGTCCTCTATTCGTACCGGTGTTAAGCGCCCTGCTGTTTCCAAAGCCTCTGCTGCCC

CTAAACGTAAGCGCGCCAAAACCAAAAGGTAATATATGTGTATATGTACTGTTATATATA
TGTGTGTATGTACTGTTATGTATATGTGTTTATGTACTGTTATATGTGTGTATATATGTG
TATGTGTGTATATGTATATGTATGTGTTGTGTATATATATGTGTGTGTGTGTTATGTGTG
TAATGTAATTTATTTGTGTAATGTGTATGTGTGTTTATGTGCAATAAACAATTAACTACA
TTATTGTATATCTTGTTACACCCTGTGACTCAGTGGCTGTTGCACGCGTTTTGGTTTGCA
CGCGCCTTACACACATAAGTAATATACATGCACAATATATATATTTTTGTTTCCAATAAT
ATATTTTTATATTTGCAACCGTTTTCGGTTGCCCTTGGCATACACTTTCCACCAATTTGT
TACAACGTGTTGCCTGTTAATCCTATATATTTTGTGCCAGGTACACATTGCCCTGCCAAG
TTGATTGCCAAGTGCATCATATCCTGCCAACCACACACCTGGCGCCAGGGTGCGGTATTG
CCTTACTCATATGTTTATTGCCACTGCAATAAACCTGTCTTTGTGTTATACTTTTATGCA
CTGTAGCCAACTCTTAAAAGCATTTTTGGCTTGTAGCAGCACATTTTTTTGCTCTTACTG
TTTGGTATACAATAACATAAAAATGAGTAACCTAAGGTCACACACCTGCAACCGGTTTCG
GTTATCCACACCCTACATATTTCCTTCTTATA
>gi|5059324|gb|AF151983.1|AF151983_Human_papillomavirus_type_83,_complete_genome
ATGTCAGGAGTGCGGTACCCCACCAACATATTTCTGCTGTGCAAAGACTGCGAGGTGGAT
TTGGAAGACTTACGACTGATTTGCATTTATTGCACAAATGAATTAACAACAGCAGAAGTG
CTGTCCTTTGCATGGAAGGAGCTGTGTATTAAGTGGGACCACGAACTGCCCTACGGAGCG
TGTGCACAGTGTCTACGTAAAGCAGCAAAGGTGCGAGAGCTACGGCACTGGAGCCACTCA
AGCTACGGGGCAACAGTAGAAGAAGAAACAAAAACACCACTGGCACAGCTGTATATACGA
TGCCATATGTGCCTGAAGCCGTTGAGTAGTCAAGAAAAAGAATATTTGGTGCAAACAGGT
GATAGACTTCATAATATAGCAGGACAGTGGACAGGACGGTGCTGCCATTGTAGGGCACCA
TGCACGGCCATACAGCAACAATAGCAGAGATTGTACTAGAAGAAATACCAGATATAGTTG
ACCTATATTGTAATGAACAAGGTATAGACAGCTCAGAGGAGGAGGATGATAGGGACTGTG
GCGTGCGTGACCAACTGGCAGAACAAGCAAAGCAGGCCTATAGGGTGTTAACAGTGTGTG
GTATGTGTGGCCAGGCACTTAGACTAGCAGTGCTGTGTGAAGACGCAGACCTAAAACGTC
TACAGGACCTTTTGGTGAATGCAGTGGATATAGTGTGTCCCGGCTGTGCATAAGGACATG
GCGGATACGGAAGGTACAGATACTGGGACTGGGGAATTTAATACAGTAACCGGGGCAGGA
GGGTGGTTTATGGTGGAAGCAATAGTGGACAAAACAACAGGAGATACAGTGTCAAGTGAT
GAGGACGAGGAGCTAACAGATAATGGAGAAGACCTTGTAGACTTTATAGACAGACACCCG
GGGGATGGGCAGGAAGTTCCGTTAGAGCTCTTTGTTCAACAAACTGCAGAGGATGACGCT
GCAATAGTGCAGGCAGTAAAACGAAAGTTTGTATGTAGTCCTGCATCAAGTTCCTGTGCA
ACATGGGTGGACAGTGAACTAAGTCCCCGTTTGGACGCTATAAACTTAAATAGAAGACAG
GAAAAGGCTAGACGGAGGTTGTTTGAGCAGGACAGTGGGTATGGCAATACGCAGGTGGAA
ACTGGAACATCAGAAAGTCAGGTACCCGGGGAAAATGAAGGCGTGGGGGGGGCTGTGGCG
GCACAGGAAACGAGGGGGGAGGGGCGGGAGGGGGGCGACGGCGAATCACAACCGCCTGTG
CAGGTACAGCAAACACAGGACAGAGCGCCAGAACTACTGGAGCTGTTTAAATGCAGTAAT
GTAAGAGCCGCCATCTTAAGTAAGTTTAAAGATTTATTTGGTCTATCATTTTATGATCTA
GTTAGACAGTTTAAAAGTGATAAATCCATATGTGGAGACTGGGTGGTGTGTGCATTTGGG
GTGTACTATGCCGTAGCAGAAGCTGTGAAAACGTTGCTGCAGCCACAATGTTTATATGCA
CATATACAGGTGCAAGCCAGCCAATGGGGAATGGTAGTACTAATGCTACTAAGATTTAAA
TGTGGCAAAAGCCGGGAAACAGTGGCTAAATACATGGGTACCGTACTAAATGTACCCGAA
AAGCATATGCTAATAGAACCACCAAAAATACGTAGCGGGCCATGTGCACTATACTGGTAT
AGAACAGCTATGGGGAATGCCTCAGAAGTACTTGGGGAGACCCCAGAGTGGATTGTGCGA
CAAACAGTAGTAGGACATGCAATGGGAGAAGCCCAATTTAGTTTATCAATGCTTGTGCAG
TGGGCATATGATAACGATATCCAGGAGGAAAGCGACCTAGCATACGGGTATGCACAGTTG
GGAAATACAGATCCCAATGCAGCTGCATTTCTAGCAAGTAATTGTCAGGCAAAATATATT
AAGGATGCAATGACAATGTGTAGGTTGTATCGGCGAGCGGAACAATCCAGAATGAGTATG
GCACAGTGGATAGCGCATAGGGGGCGTAAGGTAGCAGACACAGGAGATTGGAAACATATT
GTGAAATTTTTAAAATATCAAAATGTGGAATTTATTTCATTTATTAGTGCATTTAAATTG
TTTTTAAAGGGAGTGCCTAAAAAAAGCTGTTTAGTATTCTATGGACCTAGTGACACAGGG
AAATCATTGTTTTGTATGAGCCTTTTGCAATACCTAGGGGGCGCAGTTATATCTTATGTA
AATTCAAGCAGCCATTTTTGGTTGTCCCCATTAGCAGATGCTAAAATAGGATTGCTAGAT
GATGCAACAGCGCAATGCTGGACCTACATAGATGTATATTTGCGCAGCATATTAGATGGG
AATCCAACAAGCATTGATAGAAAACATAGAACACTGACACAGCTAAAGTGCCCACCACTA
ATGATAACTACTAATGTAGATCCACTGGCAGATGAGTCCCTAAAGTATCAACGCAGTAGG

ATTACAGTGTTTAAATTTTTAAACAAATGTCCGGTAACCAACAGCGGGGAACTAGTGTAC
ACACTAAATAACGAAACCTGGAAATCATTTTTCCAAAGGTCCTGGGCACGTTTAGAATTG
CATCAAGAGGAGGAGGAGGAGGAGGAGGAGGAGGAGGATGGCATCGCTAGCCGACCGTTTAGA
TGCGTGCCAGGAGACGCTAATAGACCTTTATGAAAAAGATAGCAACAAGTTGGAGGACCA
GTTGTTGCATTGGCAATGTGTGCGTTTGGAACATGCAATATTATTTAGGGCAAAGGAAGC
AGGACATGCAAAAGTAGGCCACCAGGTGGTGCCACCACTTCATGTAACCAAATCAAAGGC
CTGTCAGGCCATTGCAGTCCATTTATCGTTGCAAAGCCTAAATGACAGTGACTTTAAGCA
CGAACCATGGACACTGCAGGACACATCTATGGAAATGTGGAACACACCCCCGCAGGGGTG
CTGGAAGAAAAAGGGACGCACAGTAACAGTGAGATTTGATAAAAGTGACTGTAATGAAAT
GGACTATGTAAGCTGGGGGCACATATATGTATATACGGAGGAAACAAGGACATGGCACAA
AGTGTCGGGGGGTGTGGATTACGCGGGACTGTATTATGAGGTGCAGGGACAGAAACAGTA
CTATGTGGAGTTTGTAAAGGAGGCCCAAAAATATGGGACGCAAAACATGTGGGAGGTGCA
TGTGGGCAACAATGTTATTCACCAACCATGCGACTCTGTATCTAGCACCCAGGACACCGT
GTGGGAAGTACCCCTTGCTGCAACTAGTACCAGGCTACCACACACCGCAGACCCCACCGC
CAGCACCCAGGTGCTGGGCGCCCCCCAGAGCACCACCCAAGTGCAGACGCCGCCTGTCAA
GCGACAGCGACTCCTCTGGGACGGACAGCAGCAGCCCGACTCAACCCAGACAGAGCACCA
AAAGCACGTGGACAGTGGTGCCTACGGACACAGCAGTGACAGTGTCTGCACAAACGCCAC
AAGGGACACAGTGAACCGTTACCATACACCTGTAATCCACCTACAAGGTGAACCAAATAA
ACTAAAGTGCTTTAGGTACAGATTACATAAATCAGTACCAACATTGTTTGTAAGGGCCAC
CTCCACATGGCATTGGACAAGTGGGGATGCCGCCACTAAAGCAGCATTTGTAACCTTATG
CTATGCAAGTATAGAGCAACGGGAGCTATTTCTAACCAGGGTTACAATTCCAAAGGGTAT
AAAGGCAATGCAAGGATATATGTCTATGTGCTTTTAACTGTACATATGTATATATATATA
TAGTGTGTAATACGGGTATGTAACCAATAGTGTATAAGTGTGTTTGCAAATACCTGAGCC
ACTATACACCACACCATATACCAGTACAACCCTGGATTACATATTGTGCTTGATTTGTTT
GTGGTGTCATATTCTTGTGCTTCTAATTTGCTTCTGGCTTTCCTACCTTTCTGCATTTGT
GGCATTTGTTGTGTTTATGTGTATATTGTATTTGGGGTTGTTATTGCTATATGTACAAGT
ATTGTGGTGTATAGGGTTTATATAACCACACAACCAGCCATTCACTGCTGCTACCTGTAT
ATACGTATTCCCTGTGTATTGTACAATCACTATGTATCCATTGGAGGCTAGAGGTGGGGG
GCAGGCATATGATTTAATTGTTATTGGCCGGGACGAAGTGGGATTACTGCTTATTATTTT
AATTCTCATCGTAATTATTATTATTTTGCTGTACATGCGGCTACTACATATGTAACCACC
TCCCCTTCCTGTATACCACATGTAGGGTATTGTTTTGTACTGTTTGTTTTTTACTTTCTT
ATTGGCAATAAACATAGTTATATATACAATGTCCCATGTGCATAGGCGGCGCAAAAGGGC
GTCTGCAACTGATTTGTACCGTACCTGTAAGGTTGCAGGTACATGCCCCCCTGATATAGT
CCCTAAAATTGAAGGTGACACATGGGCCGATAGGTTTTTAAAGTGGGCAAGCCTGGGGCT
GTTCTTTGGACGGCTTGGCATTGGTACGTCCTCTGGCACCGGTGGTCGCACAGGCTATGT
GCCCCTAGGTACCCGTCCTCCCACGGTGGTTGACGTAGGGCCCACAGCGCGCCCCCCTGT
GGTCATTGAACCTGTGGGTGCCACTGAACCATCCATTGTTAACCTTGTGGAGGATTCTAG
TGTTATTGAGTCTGGGGCTACTATACCTACCTTTACGGGCACACATGGCTTTGAGGTTAC
TAGTTCCTCCACTACAACCCCCGCAGTATTGGATATAACGCCGTCGTCCGCCACGGTACA
GGTTAGCAGCTCTAGTTTTACCAATCCTTTATTTACTGAGCCGTCCATAATTGAGCCTCC
ACAACATGGTGACATTACAGGACATGTGTTTACTAGTACCGCTACATCTGGTACTCACGG
GTTTGAGGAAATTCCAATGCAAACATTTGCAACCTCTGGGGGTACAGGCCAGGAACCTAT
TAGTAGTACTCCTATTCCTGGGGTACGCCGTGTTGCGGGTCCTCGCCTAGGCCTTTACAG
CCGTGCCACATCTCAGGTTAAGGTGCCTGATTCTGCCTTTATGTCCCGCCCTGCATCTTT
TGTAACATATGACAATCCTGTATTTGATCCTGATGAAACTATTATATTTGAACACCCTAG
TTTACATAATCCACCCGATCCTGATTTTCTGGATATTGTTTCATTACATAGGCCTGCCTT
AACAGCCAGACAAGGTACTGTGCGGGTGAGTCGTGTTGGCCAAAGGGCTACCTTGCGTAC
CCGTAGTGGCAAACAAATCGGGGCCCGTGTACATTTCTATCATGATATTAGCCCCATTCC
GCCCACGGAGTCCATTGAGCTACAGCCCTTGGAACCCTCCGTGGCAAACACCTCCTTATA
TGACATATATGCAGATGATGTTTTTTTGGAGTCCTCGGTGCCCCTGCGGCCATCCCCCTC
ATCCACCTCTGTGTCCTTGCAGTCCACTAATGTTTCTGCTACCTCTGCTGTGACTGCACA
GTATGACAATGTTACAGTGCCACTGTCCCCTGGATTGGATGTACCTGCCCTTTCAGGCCC
TGACATACACGGTGCTGCTCATATGCCTGCAGACCCTTCTGTTCCTGTGCCATCTAATAT
TCCTATTCAGTCTGTGTTTATAGACGGTACTGACTATTATTTATTGCCTAACTACATTTT
TTTCCCTAAAAAACGTAAACGTGTGCACTATTCTTTTGCAGATGGCTATGTGGCGGCCTG

GTGACGGCAAGACGTACTTGCCTCCCACTCCTGTGTCTAAGGTTATCAGTACGGACCGTT
ATGTTACACGCACCAACCTCTTTTATTACGGTGGCAGCTCCAGACTTCTTACCGTAGGAC
ATCCATATTATCCTGTACAGGTTAATGGTCAAGGAAAAAAAGCCACTATCCCCAAGGTTT
CTGGCTACCAATATAGGGTGTTTCGCATTAGATTACCTGACCCTAATAAGTTTAGCCTTC
CTGATACGTCCCTATATAATCCTGACACGGAACGCATGGTATGGGCATGCCGCGGTATAG
AGGTTGGTCGTGGTCAGCCTCTTGGCGTGGGCACTAGTGGCCATCCCCTATATAATCGGC
TGGATGACACTGAAAACACACCATTGCTTGCTGCTGCAGACACAGATAGGCGTGACAATG
TCTCAGTGGATTACAAACAAACGCAGCTTATTATCATTGGGTGTAAGCCCCCAATAGGGG
AACACTGGGCAAAGGGTACTGTGTGTTCCGGTGTGTCCCCGCAGCGTGGCGATTGTCCCC
CCCTACAGTTTGTAAATTCTACTATACAGGATGGCGACATGGTGGAAACAGGCTATGGTG
CCATGGATTTTGCCACCTTACAGGAATCTAAGTCTGAGGTTCCTATTGATATCTGCACTG
CCACCTGTAAATATCCTGATTATTTACAAATGGCTGCGGAACCATATGGTGATTGTATGT
TCTTTTGCCTCCGTAGGGAACAAATGTTTGCACGACATTTTTTAATAGGCATGGCACTA
TGGGCGAGGTGCTGCCTACCTCTTATTATATTCCAGGCACATCTGCTAATAGTAGGAACA
CTCTTACCAGCTACATCTATGCTCCTACTCCTAGTGGCTCCCTGGTATCGTCCGATTCCC
AACTATTTAATAAGCCCTACTGGCTGCATCGTGCCCAGGGACATAATAATGGCATTTGTT
GGTTTAATGAGTTATTTGTTACAGTTGTAGATACTACCCGCAGTACCAATATTACTATTT
CAGCTGCTGCTACACAGGCTAATGAATACACAGCCTCTAACTTTAAGGAATACCTCCGCC
ACACAGAGGAATATGACTTACAGGTTATATTGCAACTTTGCAAAATACATCTGACCCCTG
AAATTATGGCATACCTACATAGTATGAATGAACATTTATTAGATGAGTGGAATTTTGGCG
TGTTACCGCCTCCCTCCACCAGCCTTGATGATACCTATCGCTATCTGCAGTCCCGTGCTA
TTACCTGCCAAAAGGGTCCTTCCGCCCCTGCCCCTAAAAAGGATCCTTATGATGGCCTTG
TATTTTGGGAGGTTGATTTAAAGGACAAACTATCCACAGATTTAGATCAGTTTCCTTTGG
GCCGTAAGTTTCTTTTGCAACTGGGTCCCCGTTCTGTGTCTGTGTCTCGTAAGCGCCCTG
CCTCTACCGCCCCGTCTGCCCCTTCTAAGAAAAAGGTTAAAAGGCGCAAGTAACATATGT
GTGTGTGTGTTTGTGTGTATATCTGTGTTTGTGCAGTGTTATGCCTATATGCGGTGGTAT
GCCTTTGTGTTATGTATGTGGCAGTACCGTTGTATGTGTTTGTGTATTATATGTTTGTGC
ATGTATCTGTGTTTTGTCTGTGTAGTATGTGTATGTATCTGTGTGTTTTGTATGTGTATA
CATTGTGGAATGTATGTGTTGTGTGTGCTTTAATAAACTGTGTGTCATTGTGTTGCCGTG
GACTAGTGTCCGGCTGCGCCCCGTGCAGTGTCCGGCTGCACCCCATGAGTAACCCCTCCA
TGGCACCTATGTCCTGCACCACCCTATAGTTCCTGACCCCGTAGGTTACTAGTTCCTTTT
GTGTTGGCGCGACCGGTTTCGGTCTCCCGCCTTTTCGGTCTTTGCAGTGGCACGTTGCCA
TTACACACAATGGTAAGTAATCCCTTATTAACTCATATCCTGCATACTTTCTGTGCCAAC
TATTTGCACCTGGGAGTGCCTGACAGTTTGTTGGCTGCACAATATGATTTTTTGACAGGC
TTATATTGTACTTGTACTTCTTTTGCAATAAAACTGCTTTTAGGCACATACTTTTCATGA
CTACTTTCCCCTAAGTACTGTTTTGGACATGTAGACTATTGCAGTAGCCAACTATGTGTC
TATCCAGTAATCCTGCAGTTCTAAACTTGTTGCACACACCCAGGTCGGTTACTACCTACA
CAGCACTTTCCTTTGTAATAATTTATAACAATCACATAGTAAAAAAGTAGGGTGTAACCG
AAATCGGTCGGACCGAGGGGTATACATATAAAAGGGAAGTGTAGGCTCAGCACAGCCAGG
AGCT
>gi|12958167|gb|AF293960.1|AF293960_Human_papillomavirus_type_84,_complete_genom
e
ATGCCCAACGGACGCTACCACCCCACCAATATTTTTGTGCTGTGCCAGGAATACGAGGTG
GAGTTCGACGACCTACGATTAATTTGCATCTTTTGCAAGGAAGAATTAACGGAAGGCGAA
GTGCTGGCCTTTGCAGTAAAGGAATTACTAATTGTTTGGAGGTATAATTTCCCTCATGGG
GTGTGCATGAAATGCTTATGCAGGGAAGCCAAAGTACGTGAGCTACGCCACTGGGATTAC
TCCAGCTTTGGACCAACAGTGGAAGAAGAAACAGGATTACCACTTGCACAAATAAATATA
AGGTGCCACGCGTGCTGCAAGCCATTGTGCTATCAGGAAAAGGAGTATATGGTGGAATTG
CAGTTGCTATTCCACAAAATAGCTGGACAGTGGACAGGGAAGTGCTGCAACTGTAGGGTA
ACATGCGCGGCCAGACGCCAACGTTAAAGGATATTATTTTAACAGATATACCAGATGTAG
TTAGTTTATACTGTGACGAGCAATTGTTAGACAGCTCAGAGGAGGAGGATAATGGGGATT
GCTTGCGTGACCAACCTGCAAAACCAGCACAGCTGGCCTATAGGGTGGTAACAGAGTGTG
GGTTGTGTAGCCGTCCAGTTAGGCTGGCGGTGCTTTGTGGAGTGGAAGACCTCAGAAAGC
TGCAGCAGCTTATAGTGGAAGCAGTGGCCATAGTGTGTCCCGGCTGTGCATAATATGGCA
GAGTCACCTGAAGGTACCGATGGGGGGACTGGCGGGGGGGCTGGGGGATGGTTTGTGGTG

GAAGCCATGGTACACCAGGCAAACACCGATGTGTCTAGCGATGAGGACGAAACACGTATA
GATACAGGAGAAGATTTGGTAGACTTTATAGATGATACCAGACATCCAGGGGATGGACAG
GAAGTGCCGTTAGATTTGTATGTTCAACAAACAATACAGGATGACGCTGCAACGGTGCAA
GCCCTAAAACGAAAGTTTATGGGAAGCCCAGCAACCAGTTCCTGCGCATCCTGGGTGGAT
AATGAACTAAGTCCACGGTTGAATGCAATATGTTTGCAGAAAAGGCACGACAAGGCCAGG
AGAAGGTTGTTTGACCAGGACAGTGGGTATGGCAATACGCAGGTGGATATCGGAACATCC
GAGAGTCAGGTACCAGGGGGAACCACTAACAGCGGGGGGGGAGACGTAACACAGGACGTT
GTAGAGGAGGAACGCAGGGGGGGGGATGGGGAAACACAGCCACATGCAAACACGCAGCAC
ACACAGCAGACGGAAGGCACACCAGACGCATTAGACCTGCTGCAGGTTAGTAACCTAAGG
GTAAAACTATTAGGAAAATTTAAGGAACTATTTGGGCTGTCCTTTATGGATCTGGTAAGA
CAATTTAAAAGTAATAAGTCAACATGTGGAGACTGGGTGGTGGGGGCATTCGGGGTGTAC
CATGCAGTGGCAGAAGCAGCAAAAACACTGCTACAGCCTGTGTGTGAGTATGCACATATC
CAAACATTAACTAGTGAATGGGGAATGGTAATGCTGCTGCTACTGCGCTTTAAGTGTAAC
AAAAGCAGGGAAACAGTAGCGCACTGTATAGGTGGCATTTTAAATGTACCCGAAAAACGT
ATGCTAATAGAGCCCCCAAAGCAACGCAGCGGGCCATGTGCACTATATTGGTATAGAACA
GCAATGGGCAATGCATGTGAAGTGTTAGGGGAAACACCAGACTGGATAGTACGACAAACT
GTAATTGGGCATGCAATGGGGGAAACGCAGTTTAGTTTATCAAAACTAGTGCAGTGGGCA
TATGACAATGACATTACAGATGAAAGTGAACTGGCCTATGAATATGCACAATTGGGAACA
GAGGAGCCAAATGCAGCCGCCTTCTTAGCAAGCAATTGTCAGGCACGATATATAAAGGAT
GCAATGATAATGTGCAGACATTATAGACGTGCAGAACAGACACGTATGAGTATGTCACAA
TGGATAACATATAGGGGGCGCAAGGTAGCTGATACAGGCGATTGGAGGCATATAGTAAAA
CTATTAAGATATCAAGGAATAGAATTTATTAGCTTTATGACAGCATTAAAGCAATTCCTA
AAGGGCACACCAAAAAAAGGCTGTTTGGTATTTTATGGACCCAGCGATACAGGCAAATCG
CTGTTTTGCATGAGTTTAATAAACTATTTAGGTGGAACAGTTATATCCTTTGTAAATTCT
ACTAGTCATTTTTGGCTGTCGCCACTGGCAGATGCTAAAATAGGATTGCTAGACGACGCC
ACATATCAATGCTGGATATATATGGATACATATTTAAGAAGTGTGCTAGATGGCAATGTA
ATAAGCGTAGACAGAAAACATAAAAACCTAGTACAGTTAAAATGTCCTCCGTTACTAATA
ACAACAAACATAAATCCAGAAACTGATGACACATTTAAATACTTACGCAGCCGAATGGTT
ATTTTTCCCTTTTTAAACAAGTGCCCACTGGATGCCAATGGAGACCCAGTGTATCAATTA
AATAATGAAAATTGGAAATCCTTTTTTCGAAGGTCGTGGGCACGCTTAGACTTAACCCAG
GAGGAGGAGGAGGAGGAGGAGTCCCAAAATGGAAACCCTAGCCGACCGTTTAGATGCGTG
CCAGGAGAAGCTAATAGACCTTTATGAAAAAGATAGCAACAAGCTTGAGGACCAGTTAAT
GCATTGGTACTACACGCGCCTGGAACAAGCTATGCTGTTTAAGGCAAGAGAAGCAGGACT
AAGACACATAGGCCACCAGGTGGTGCCCACACTTAGTGTGACAAAAGAAAAAGCTCGGCA
GGCCATAATGGTGCATTTATCTTTGCAAAGCCTAAATAATAGTGCATTTAAGCACGAGCC
ATGGACATTGCAGGACACATCATTGAACATGTGGACAGTAGCCCCAAAAGGGTGTTGGAA
GAAACATGGACAGCCCATCAGAGTAAAATATGATGGAGAAGATGATAAAGAAATGGAATA
TGTAAACTGGGGGTTTATATATGTACATTGTGCCAGTGAGGACACCTGGTATAAGGTACC
TGGACAGATTAGCAACAGAGGGCTGTATTATGAACTGCAAGGCTGCAAACATTACTATGT
GGAATTTGCAAAGGAGGCAAAACAGTATGGGGTAAAAAACATATGGGAGGTGTATATGGG
AGGCAAAATAATTTACCATGCATGCGACTCTGTATCCAGCACCCAGGACGCCGTGCCAGA
AGTACCCACTGCTGAAACTGCTGCACAGCTGCACCACACCACCACCGCGCCCACCCCCAC
CACCCAGCGCTGCACCAAGACTGCCCCCCAGGTGCAGGCGCCGCCTGCTAAGCGACAGCG
ACTCGGAGGGGACACAGTTCAGCAGCCCGACTCTACACAAGGACACCGGCCGGTTGACAG
TTGCAGCACAAGGACAAACAATAACTGTGACAGCCCAAAACGGCCACGGGAACACAGTAA
CTGTGACAGTGCACCTGTCCTACACCTAAAAGGTCAATCCAATAGCCTTAAGTGCTTTCG
ATATAGGTTACACCAGTCGGTGCCTGACCTGTTTGAAAGGGCATCGTCCACGTGGAAGTG
GACCTGCGGGGGGGAGGGCGACAAAACATCATATGTAACACTATGGTATAAAAGTACGGA
CCAACGCAAACAGCTCTTGGCACGTGTACATATACCAAAAGGCATTGTAGCCACACTTGG
TAGTATGTCTATGGTTATGTAAACAGTACCCATTGTATATGTATAGTGTAAATGCTGTAT
TACTGAGCCATTTGTAACAATTGTGTATGTCCTACATACCTGGCAAATAGGGCACAACCC
TGGAACCTATTGTGTGGGCAGTGCATGTATGGTGTAATGTGTTGCTGCTGCTAGTTCTTT
TCTGGCTTTCCCACCTATCTGCTTTTGTTGCATTTGTTGTGTTTGTGTGTGTTTTATATT
TTGGGTTGTTGCTGTTATATTTGCAGGTGCTGTGGTACATTGAATTGTTATAACCACACA
ACCAGCCAACGACTGCTGCTACATATCCCTGTACAGTCCCGGTCCTACCTATTATGTATC

CCATGCAATTACGGGGTCCCCAGGGTGGGTATGACATAGTGGTGTTTGAGCGTGGTGATG
TAGGTCTTTTTACTATTATTTTGCTTTTAATTGTTATTATTTTACTTTGGCTGTGCCACC
GTGTTATGCAGTTTTAAAGTGCTTTTGTATTTTTGTACATACTATAATAAACACTTGGTA
CCATGCCCAAGGTTCTTAAACGTCGCAAGCGTGCTTCTGCCACGGACCTTTATCGCACCT
GCAAGGCCACTGGAACACGTCCTGCTGATGTTATTCCTAAGGTGGAAGGGGATACATGGG
CGGATCGCTTTTTAAAATGGGCCAGCCTGGGTGTGTTTTTTGGTGGTCTGGGTATTGGCA
CATCCTCTGGCACGGGTGGCCGTACGGGCTATATACCCCTTGGTACCCGGCCTCCTACTG
TGGTTGATGTAGGCCCTACGGCCCGTCCGCCCGTTGTCATTGAACCCGTTGGCGCTGCAG
ACCCTTCAATTGTTACCTTGGTGGAGGATTCCAGTGTTATTAATGCCGGGGCCCCTTTTC
CTAACTTTACGGGTACTGGTGGGTTTGAGGTCACCACATCCTCTATTACCACACCTGCGG
TTTTAGACATCACCCCCTCGGGGTCGTCTGTGCAGGTCAGTAGTACCAGTTATGTTAACC
CGCTGTTTACTGAGCCTTCTATTATTGAGCCTCCCCAGGCAGGGGACATTACTGGTCATG
TGTTGTCTAGTACAGCCACGTCCGGGTCACATACCTACGAAGAAATCCCCATGCAAACCT
TTGCCGTGCAGGGGGGTACGGGCCTAGAACCCATAAGTAGTACACCCACACCTGGGGTGC
GGCGCCTTGCAGGGCCTCGCTTAAATTTATACAGTCGTGCCACTCAACAGGTTCCTGTGG
CTGACACTGCATTCTTATCACGTCCTGAATCATTTGTTACCTTTGACAACCCTGTGTTTG
ACCCCGAGGAAACAATTATATTTGAACATCCTAGTTTACATGCGCCACCCGATCCTGACT
TTCTGGATATTGTCACTTTACATAGGCCGGCGTTAACTGCACGTAGGTCAGGTGTACGGT
TCAGTCGCATAGGCCAAAGGGCGTCTATGCGCACACGCAGTGGTAAACATATTGGGGCCC
GTGTTCATTTTTATCATGACCTTAGCCCTATACCACATCTTGAGGATATTGAGCTGCAGC
CCCTGGTATCATCCTCTGCTGTGCCTACGGATTCATTATATGATATATATGCAGATGATG
CCCATTTGTCCTCTGTATTGCGTCCCCCTTCCGTTTCTGCCCTACGTCCAGCTTCCCCCT
TTGCCTCTGCAGACCTTTCTGCCACCTCCATAACCGCCTCCACATATGATAATGTCACTG
TCCCGTTGTTTTCTGGCACCGATGTGCCCGTCTATACAGGCCCTGATATTGACCATTCTG
CTGCTCCCTCCGCACCTCCCTTTGTTCCTGTTATTCCTAGCACAACCCCCATATGCTATTT
ATATCCTGGGGTCAGATTATTATTTGCTTCCTAATTATATATTTTTTCCTAAAAAGCGTA
AACGTGTGCCCTATTCTTTTTCAGATGGCTTTGTGGCGGCCTGGTGACGGCAAGGTATAT
CTGCCCCCCACTCCCGTGTCTAAGGTTATCAGCACGGATCGTTATGTCTCTCGCACCAAC
TTATTTTATTATGGTGGTAGTTCTCGCCTGCTTACTGTGGGACATCCATATTATTCTGTT
CCTGTGTCTACCCCTGGGCAAAACAACAAAAAGGCCACTATCCCCAAGGTTTCTGGGTAT
CAATACAGGGTGTTTAGGGTCCATTTACCTGACCCCAATAAGTTTGGTCTTCCGGATGCA
CAATTATATAATCCTGACACCGAGCGCCTTGTGTGGGCTTGTAGGGGTGTTGAGGTAGGC
CGCGGGCAGCCTTTAGGCGTTGGCACTAGTGGCCACCCTTTATACAATAGGCTTGATGAC
ACTGAAAACACCCCTTTTCTTGCTGCTGGGGACACTGACAGTAGGGATAATGTTTCTGTG
GATTATAAACAAACACAGCTGTTAATTATAGGCTGTAAGCCGTCTATTGGGGAACATTGG
GGTAAGGGTACTGTATGTACTAATGTGCAATATCGTGCGGGTGATTGCCCCCCCTTTGCAG
TTCACTAATTCTACCATTGAGGATGGTGATATGGTTGAGGCTGGCTATGGTGCTATTGAT
TTTGCCACACTGCAGGAAAGTAAGTCAGAGGTGCCTTTGGATCTCTGCACCACTACCTGT
AAATATCCTGATTATTTACAAATGGCTGCAGAGCCGTATGGCGATTGTATGTTTTTTTGT
CTTCGGCGGGAGCAAATGTTTGCTAGGCATTTTTTTAACAGGCAGGGTACTATGGGTGAG
GAGGTGCCTCAGTCTTTTTACCTTAAGGGGACCTCCTCCCGGGCAACCCTTAGCAGTTCT
GTGTATGCCCCTACACCCAGTGGCTCTATGGTGTCCTCGGATTCCCAATTGTTTAATAAG
CCATACTGGTTGCAAAAGGCCCAGGGTCATAACAATGGTATATGCTGGTTTAATCAATTG
TTTGTCACGGTGGTAGATACCACCCGCAGCACCAATTTTACTATTAGTGCTGCTACCAAC
ACCGAATCAGAATATAAACCTACCAATTTTAAGGAATACCTAAGACATGTGGAGGAATAT
GATTTGCAGTTTATATTCCAGTTGTGTAAGGTCCGTCTGACTCCAGAGGTCATGTCCTAT
TTACATACTATGAATGACTCCTTATTAGATGAGTGGAATTTTGGTGTTGTGCCCCCCTCCC
TCCACAAGTTTAGATGATACCTATAGGTACTTGCAGTCTCGCGCCATTACTTGCCAAAAG
GGGGCCGCCGCCGCCAAGCCTAAGGACGATCCTTATGCTGGCATGTCCTTTTGGGATGTA
GATTTAAAGGACAAGTTTTCTACTGATTTGGATCAGTATCCCTTGGGTCGCAAGTTTTTA
TTGCAGTCTGCCCCACGTTCCACCCTGGTGTCCCGTAAACGTACAGCGTCTGCCTCTACC
CCCCCTGCCTCCAAACGGCGTAAGGCCAAAAAGTAATATGTGTCTGTTTGTTTGGTGTCT
GTGTCTGTTTGTTTGGTGTCTGTACAATCTCTGTGTGTGCCATTTTGTATGTGTTTTGTA
CTGTTCTGTATGTGCCCTGTTATGTATAATGTGTATGTATTTATGGAATGCGTGCCCCTG
TATGTTGTGTATGTTGTGTACATGTATTTGTGGAATGTGTGTCATGTTATGGATCAATAA

ATTGTGTGTCATTGTGGGTTTCATGTCCGGCTGCACCCTGTGAGTAAGTGTGCACCATGT
ACACGCCTGGGTAGGTCTACTGTTTCCTTTAAGGGTATAAAGCCTCCATTTTGTATGCAA
CCGTTTTCGGTCTCCCGCCTTTTCGGTCTGTGGTATGGCACTGTCCCAGGTACAGCTAAT
CCTTTGGCAGCCCAACATCCTGCGTAGGCAGCTGCAGACGTCCGCACCCAGGTGTGTCTG
TCAGCTATTTCAATATGCTAATAAATGTATTGTCTACAGTTTATAGGTTTACTTACTCAT
CTGCAAAAAATATGCTTTTAGGCAGTTTTTTGGCCTACAACTTTCCCTTAAGTGCATAGT
TGGCAGGCCGTGCACACTGTGCTGCCAGGTTTCTGTCTTGTAAAATACCACAATACTTAT
GTAACACACCCGTTTCGGTTGCTATGTTATTCATACTATTTTCTTATACTTTACAACAAT
GATACAGTAAAAAAATAGGGAGGGACCGAATTCGGTCCCACCGAAAGGGATACATATATA
AAGGGCAGCAAACGGTACCCGGACAGCC
>gi|333245|gb|M62849.1|PPHT39_Human_papillomavirus_ORFs
CTTATAACATTTTATAAGTATCTTGTTTAAAAAAAGGGAGTAACCGAAAACGGTCAGGAC
CGAAATCGGTGGATATAAAACGCAGTCACAGTTTCTGTCCATACCGATGGCGCGATTTCA
CAATCCTGCAGAACGGCCATACAAATTGCCAGACCTGTGCACAACGCTGGACACCACCTT
GCAGGACATTACAATAGCCTGTGTCTATTGCAGACGACCACTACAGCAAACCGAGGTATA
TGAATTTGCATTTAGTGATTTATATGTAGTATATAGGGACGGGGAACCACTAGCTGCATG
CCAATCATGTATAAAATTTTATGCTAAAATACGGGAGCTACGATATTACTCGGACTCGGT
GTATGCAACTACATTAGAAAATATAACTAATACAAAGTTATATAATTTATTAATAAGGTG
CATGTGTTGTCTGAAACCGCTGTGTCCAGCAGAAAAATTAAGACACCTAAATAGCAAACG
AAGATTTCATAAAATAGCAGGAAGCTATACAGGACAGTGTCGACGGTGCTGGACCACAAA
ACGGGAGGACCGCAGACTAACACGAAGAGAAACCCAAGTATAACATCAGATATGCGTGGA
CCAAAGCCCACCTTGCAGGAAATTGTATTAGATTTATGTCCTTACAATGAAATACAGCCG
GTTGACCTTGTATGTCACGAGCAATTAGGAGAGTCAGAGGATGAAATAGATGAACCCGAC
CATGCAGTTAATCACCAACATCAACTACTAGCCAGACGGGATGAACCACAGCGTCACACA
ATACAGTGTTCGTGTTGTAAGTGTAACAACACACTGCAGCTGGTAGTAGAAGCCTCACGG
GATACTCTGCGACAACTACAGCAGCTGTTTATGGACTCACTAGGATTTGTGTGTCCGTGG
TGTGCAACTGCAAACCAGTAACCTGCTATGGCCAATCGTGAAGGTACAGACGGGGATGGG
TCGGGATGTAACGGATGGTTTCTAGTACAGGCAATAGTAGATAAACAAACAGGCGACACA
GTGTCGGAGGATGAGGATGAAAATGCAACAGATACAGGTTCAGACCTGGCAGACTTTATT
GATGATTCCACAGATATTTGTGTACAGGCAGAGCGTGAGACAGCACAGGTACTTTTACAT
ATGCAAGAGGCCCAAAGGGATGCACAAGCAGTGCGTGCCTTAAAACGAAAGTATACAGAC
AGCAGTGGCGACACTAGACCGTATGGAAAAAAAGTAGGCAGGAATACCAGGGGAACACTA
CAGGAAATTTCATTAAATGTAAGCAGTACGCAGGCAACACAAACGGTGTATTCCGTGCCA
GACAGCGGATATGGCAATATGGAAGTGGAAACAGCTGAAGTGGAGGAGGTAACTGTAGCA
ACTAATACAAATGGGGATGCTGAAGGGGAACATGGCGGCAGTGTACGGGAGGAGTGCAGT
AGTGTGGATAGTGCTATAGATAGTGAAAACCAGGATCCCAAATCTCCAACTGCACAAATT
AAATTATTGTTACAATCCAATAACAAAAAGGCTGCAATGCTAACACAATTTAAAGAAACA
TATGGACTATCCTTTACTGACCTGGTACGTACGTTTAAAAGTGATAAAACAACATGTACA
GACTGGGTGGCAGCCATATTTGGAGTACATCCAACTATTGCAGAAGGATTTAAAACATTA
ATCAACAAATATGCCTTATATACACATATACAAAGCTTAGACACAAAACAAGGAGTACTA
ATTTTAATGCTAATAAGATATACATGTGGAAAAAATAGGGTTACTGTAGGAAAGGGATTA
AGTACATTGTTACATGTTCCAGAAAGTTGTATGCTTCTGGAGCCTCCTAAACTGCGCAGC
CCTGTAGCAGCACTATATTGGTATCGCACAGGTATATCCAATATTAGTGTGGTAACAGGG
GATACGCCAGAATGGATACAACGATTAACTGTTATACAACATGGAATAGATGATAGTGTA
TTTGACCTATCGGACATGGTACAATGGGCATTTGACAATGAATATACTGATGAAAGTGAC
ATAGCATTTAATTATGCAATGTTAGCAGATTGTAACAGTAATGCTGCAGCCTTTTTAAAA
AGTAACTGCCAGGCAAAATATGTAAAAGATTGTGCAACAATGTGTAAACATTACAAGCGA
GCACAAAAAAGGCAAATGTCCATGTCTCAATGGATAAAATTTAGGTGTAGTAAATGTGAT
GAAGGCGGGGACTGGAGACCCATAGTACAATTCTTAAGATATCAAGGAATAGAATTTATA
TCCTTTTTATGTGCATTAAAGGAATTTTTAAAGGGTACTCCCAAAAAAAACTGTATAGTT
ATATATGGACCTGCGAATACAGGAAAGTCACATTTTTGTATGAGCCTTATGCATTTTTTA
CAGGGCACAGTTATTTCATATGTAAACTCCACCAGCCACTTTTGGCTAGAACCACTTGCA
GATGCAAAACTAGCAATGTTAGATGATGCAACCGGTACCTGCTGGTCATATTTCGATAAT
TATATGAGAAATGCATTAGATGGGTATGCAATAAGTTTAGATAGGAAATATAAAAGTTTA
CTACAAATGAAATGTCCACCATTATTAATAACCTCCAATACCAATCCTGTGGAAGACGAT

AGGTGGCCATATTTACGTAGTAGGCTAACAGTGTTTAAATTTCCTAATGCATTTCCATTT
GACCAAAACAGGAATCCAGTGTACACAATCAATGATAAAAACTGGAAATGTTTTTTTGAA
AAGACTTGGTGCAGATTAGACTTGCAGCAGGACGAGGATGAAGGAGACAATGATGAAAAC
ACTTTCACAACGTTTAAATGTGTTACAGGACAAAATACTAGAATACTATGAACAAGACAG
TAAATCAATATATGATCAAATTAATTATTGGAAATGTGTGCGAATGGAAAATGCAATATT
TTATGCAGCACGAGAACGTGGCATGCATACTATTGACCACCAGGTGGTGCCAACCATAAA
CATTTCAAAATGTAAAGCATATCAAGCTATTGAACTGCAGATGGCACTAGAAAGTGTTGC
ACAAACTGAATACAATACAGAGGAGTGGACATTAAAAGACACTAGTAATGAACTGTGGCA
TACACAGCCAAAACAATGTTTTAAAAAACAAGGAACTACAGTGGAGGTGTGGTATGATGG
GGACAAATGTAATGCTATGAACTATGTATTATGGGGTGCTATATATTATAAAAATAATAT
AGACATATGGTGTAAAACAGAAGGGTGTGTGGACTATTGGGGTATATATTATATGAACGA
GCACCTAAAAGTATACTATGAAGTGTTTATTCAAGATGCGGAAAGGTATGGGACTAGTGG
CAAATGGGAAGTGCATTATAATGGCAACATAATTCATTGTCCTGACTCTATGTGCAGTAC
CAGTGACGGATCGGTACCCACTACTGAACTTACTACCGAATTATCAAACACCACCGCGAC
CCATTCCACCGCAACAACCCCATGCACCCAAAAAACAATCCCGCCGCCGTCTCGAAAGCG
ACCTCGACAGTGTGCAGTCACAGAGCCCACTGAGCCCGACGGAGTGTCCCTGGACCATCT
TAACAACCCACTCCACAGTAACAGTACAGGCCACAACACAAGACGGTACCTCAGTTGTGG
TAACACTACGCCTATAATACATTTAAAAGGTGACAAAAATGGTTTAAAATGTTTAAGATA
TAGACTACAAAAATATGACACATTGTTTGAAAATATTTCATGTACCTGGCATTGGATACG
GGGTAAGGGAACCAAAAACGCTGGCATATTAACTGTTACATATGCCACAGAGTCACAACG
CCAAAAATTTTTGGACACTGTTAAAATACCTTCTAGTGTACATGTTTCATTGGGTTACAT
GACATTGTAAAGTATACTATGGATATTGTGTATGTATATTGTATACATACTACATAGATG
ATATTATTGGTATTTTTGGTGTGGTTTGGTGTGTGTATATATATATGTTGCAATGTCCCG
CTTTTGCCGTCTGTGCATGTGTGTGCGTATGTGTGGATAATTGTGTTTGTGTTTATTCTT
ATACGTACCACACCATTGGAGGTGTTTTTTGTATATTTACTATTTTTTGTATTGCCCATG
TGGTTGTTGCATAGACTGGCAATGGATATGATATAGTACTGTATATGTATGTGCATTGTG
CATAACTACTGTACATAGCTTTTTATATTTTTTTTTGTTACTAATAAACATGGTTTCCCA
CCGTGCTGCCAGGCGTAAGCGTGCATCTGCAACTGACCTATATAGAACCTGTAAACAATC
GGGTACCTGTCCACCAGACGTTGTTGATAAAGTTGAGGGTACTACACTTGCTGACAAAAT
TTTACAGTGGACTAGTTTAGGTATATTTTTGGGTGGGTTAGGCATAGGCACAGGTACTGG
TACTGGGGGACGCACAGGATATATACCCCTGGGGGGTAGGCCTAATACTGTTGTAGATGT
GTCTCCTGCACGTCCACCTGTAGTTATTGAACCTGTTGGTCCTTCTGAGCCATCTATTGT
GCAATTGGTGGAGGACTCAAGTGTTATAACCTCTGGAACACCAGTACCAACATTTACAGG
CACCTCTGGATTTGAAATTACTTCTTCTTCTACTACTACGCCTGCGGTATTGGATATTAC
ACCCTCCTCTGGGTCTGTACAAATAACCTCTACTAGTTATACTAACCCTGCCTTTACGGA
TCCTTCCTTAATTGAGGTTCCCCAAACAGGTGAAACCTCGGGTAATATATTTGTCAGTAC
CCCTACATCAGGTACACATGGCTATGAGGAAATACCTATGGAAGTGTTTGCCACACATGG
CACAGGTACCGAACCTATTAGCAGCACACCTACACCTGGAATCAGTCGTGTGGCAGGACC
ACGTTTATATAGTAGAGCACATCAGCAGGTTCGTGTTAGTAATTTTGATTTTGTAACTCA
CCCTTCATCATTTGTAACATTTGATAATCCTGCTTTTGAGCCTGTTGATACTACATTAAC
ATATGAAGCTGCTGACATAGCTCCAGATCCGGATTTTCTGGACATTGTTCGTTTACATAG
GCCTGCCTTAACCTCGCGTAAAGGAACAGTAAGGTTTAGTAGGCTTGGCAAAAAGGCTAC
CATGGTTACCCGGCGTGGCACACAAATTGGAGCGCAAGTACATTATTACCATGACATTAG
TAGTATTGCTCCTGCTGAAAGCATTGAATTACAGCCCCTAGTTCACGCTGAGCCCTCTGA
TGCTTCAGATGCATTATTTGATATATATGCTGATGTGGACAATAACACATATTTAGATAC
TGCATTTAATAATACAAGGGATTCGGGCACTACATATAACACAGGCTCACTACCTTCTGT
GGCTTCTTCAGCATCTACTAAATATGCCAATACAACTATTCCTTTTAGTACCTCATGGAA
TATGCCTGTAAATACTGGTCCTGATATTGCTTTACCAAGTACTACTCCACAGTTGCCATT
GGTGCCTTCTGGACCAATAGACACAACATATGCAATAACCATTCAGGGTTCCAATTATTA
TTTGTTGCCATTATTGTATTTTTTCCTAAAAAAACGTAAACGTATTCCCTATTTTTTTTC
AGATGGCTATGTGGCGGTCTAGTGACAGCATGGTGTATTTGCCTCCACCTTCTGTGGCGA
AGGTTGTCAATACTGATGATTATGTTACACGCACAGGCATATATTATTATGCTGGCAGCT
CTAGATTATTAACAGTAGGACATCCATATTTTAAAGTGGGTATGAATGGTGGTCGCAAGC
AGGACATTCCAAAGGTGTCTGCATATCAATATAGGGTATTTCGCGTGACATTGCCCGATC
CTAATAAATTCAGTATTCCAGATGCATCCTTATATAATCCAGAAACACAACGTTTAGTAT

GGGCTTGTGTAGGGGTGGAGGTGGGCAGGGGCCAGCCATTGGGTGTTGGTATTAGTGGAC
ACCCATTATATAATAGACAGGATGATACTGAAAACTCACCATTTTCATCAACCACCAATA
AGGACAGTAGGGATAATGTGTCTGTGGATTATAAACAGACACAGTTGTGCATTATAGGCT
GTGTTCCCGCCATTGGGGAGCACTGGGGTAAGGGAAAGGCATGCAAGCCCAATAATGTAT
CTACGGGGGACTGTCCTCCTTTGGAACTAGTAAACACCCCTATTGAGGATGGTGATATGA
TTGATACTGGCTATGGAGCTATGGACTTTGGTGCATTGCAGGAAACCAAAAGTGAGGTGC
CTTTAGATATTTGTCAATCCATTTGTAAATATCCTGATTATTTGCAAATGTCTGCAGATG
TGTATGGGGACAGTATGTTCTTCTGTTTACGTAGGGAACAACTGTTTGCAAGACATTTTT
GGAATCGTGGTGGTATGGTGGGTGACGCCATTCCTGCCCAATTGTATATTAAGGGCACAG
ATATACGTGCAAACCCCGGTAGTTCTGTATACTGCCCCTCTCCCAGCGGTTCCATGGTAA
CCTCTGATTCCCAGTTATTTAATAAGCCTTATTGGCTACATAAGGCCCAGGGCCACAACA
ATGGTATATGTTGGCATAATCAATTATTTCTTACTGTTGTGGACACTACCCGTAGTACCA
ACTTTACATTATCTACCTCTATAGAGTCTTCCATACCTTCTACATATGATCCTTCTAAGT
TTAAGGAATATACCAGGCACGTGGAGGAGTATGATTTACAATTTATATTTCAACTGTGTA
CTGTCACATTAACAACTGATGTTATGTCTTATATTCACACTATGAATTCCTCTATATTGG
ACAATTGGAATTTTGCTGTAGCTCCTCCACCATCTGCCAGTTTGGTAGACACTTACAGAT
ACCTACAGTCTGCAGCCATTACATGTCAAAAGGATGCTCCAGCACCTGAAAAGAAAGATC
CATATGACGGTCTAAAGTTTTGGAATGTTGACTTAAGGGAAAAGTTTAGTTTGGAACTTG
ATCAATTCCCTTTGGGACGTAAATTTTTGTTGCAGGCCAGGGTCCGCAGGCGCCCTACTA
TAGGTCCCCGAAAGCGGCCTGCTGCATCCACTTCCTCGTCCTCAGCTACTAAACACAAAC
GTAAACGTGTGTCTAAATAATGCATGTGTATGCCTTGTTATGTGTGTGTATGTTGTTTGT
TTCCTTATGTGTTGAGTGTATATGTGTATGTTTGTAGGTATGTGTGTATATGTTTTTGTT
AATAAAGTATGTATGACAGTTTCATGTGTGATTGCACACCCTGTGACTAACAGTGTATTT
GTTTTACATATAATAGGTCTGCAACATTTCATACATAATCTATATGCCCTACCCTAAGGT
GTGTTTACTACCTAATATGTAATTTTTACATTGTTGTATGCGTTTCTACATTTTATACTT
CGCCATTTTGTGGCGACCGAAGTCGGTCGTGGGTTGAGCATTTTTTTAAACTAGTGGAA
ACCACCTTTCTCAGCAAAAACATGTCTTTACCTTAGGTTCACCCTGCATAGTTGGCACTG
GTAACAGTTTTACTGGCGCGCCTTATTACTCATCATCCTGTCCAGGTGCACTGCAACAAT
ACTTTGGCAACATCCATATCTCCACCCTATGTAATAAAACTGCTTTTAGGCATATATTTT
AGCTGTTTTTACTTGCTTAATTAAATAGTTGGCCTGTATAACTACTTTTTGATTCAGGAA
TGTGTCTTACAGTATAAGTTATACAAGTGACTAATGTAGCACACAATAGTTTATGCAACC
GAAATAGGTTGGGCATACATACCTATACTTTTA
>gi|6970427|dbj|AB027021.1|_Human_papillomavirus_type_82_DNA,_complete_genome
ATACTTTACAATTATCTTGTAAAAAGTAGGGTGTAACCGAAAAGGGTTATGACCGAAAAC
GGTGCATATAAAAGCAAGCACGAAAAATACATAAGAACACCATGTTTGAAGACATAAGAG
AAAGACCACGAACGCTGCACGAATTATGTGAAGCCTGCAATACGTCTATGCACAATATTC
AGGTATTGTGTGTATATTGTAAAAAGGAGTTGTGTAGAGCAGATGTGTATAATGTAGCAT
TTACAGAACTTAGGATTGTATATAGGGACAATACGCCATATGCAGCATGCAAAAAAATGCC
TAATGTTTTATTCTAGAATTAGAGAATACAGAAGGTATAGTAGGTCTGTGTATGGTGCTA
CATTAGAGGCCATTACTAACAAAAGTTTATATGAATTATTAATAAGGTGTCATAGATGTC
AGAGACCACTTGGGCCTGAAGAAAAGCAAAAGGTGGTGGACGACAAAAAAAGGTTTCATG
AAATAGCGGGACGCTGGACGGGACAGTGTGCAAATTGCAGAAAACCACCAAGACAACGTA
GTGAAACCCAGGTGTAATAACGCCATGCGTGGTAATGTACCACAATTAAAGGACATAGTG
TTGGAGTTAACACCACAACCTGAAATTGACTTGCAATGCTACGAGCAATTTGACAGCTCA
GACGAGGAGGATGAAGTAGATAATATGCGTGACCAGCCAGCCAGACAAGCTGGACAGGAT
ACGTGTTACAGAATTAAAGTGCACTGTTGCAGGTGTTCGAGTGTTGTACAGCTCGCAGTG
GAAAGCAGTGGAGACAGCCTTCGCATATTTCAGCAAATGTTACTGGGCGACCTAAGCCTG
GTGTGCCCGTGGTGTGCGACCAACTAACATCGGCAATGGACAGTGAAGGTACAGAGGATG
AGGGGGCGGGGTGTACCGGGTGGTTTTATGTGGAGGCAGTAGTTGACAAAAAAACGGGAG
ATAATATATCAGACGATGAGGAGGAAGATACAAATGATACAGGGTCTGATATAATAGATT
TTATAGATACAAGTAATAGTATTTGTAGTCAGGCGGAACGGGAGACAGCACAGGCGTTGT
TGCAGGTCCAAGAAACACAGGCACACAAAGAGGCTGTGCAGCATCTAAAACGAAAGTTTC
TAGGCAGTCCGCGGAGCAGCCCATTAAAAGACATTACAAATCAAAACACACAAAGCAATA
GTCAGCAACAACCAAAACAGGCAAACCTTCAACACGGTAAAAGGAGATTACTGGACAGTT
ATCCGGACAGTGGATATGGCAATACACAAGTGGAGACTGTGGAAGGACCCTTACAGGTAG

ATGGGCAAAATGACGGGTCACAACATAGTATGTGTAGTGGCGGGGGGAGCAGCGACAGAA
GTACAGAGATAGACCTGGAAACAAACGAAAATGCTACCAATGTAGGACTAAACAGTATAT
GTGCAGTGTTAAAATGTAGCAATGCAAAAGCAATGTTTATGGCAAAATTTAAAGAACTGT
ATGGTGTTAGTTATAATGAGTTGGTAAGGGTATTTAAAAGTGATAAAACATGCTGCACGG
ACTGGGTATGTGCCTTATTTGGGGTATCGCCAATGGTGGCAGAAAATTTAAAAACCCTAA
TTCAGCCATTTTGTATGTACTACCATATACAATGCCTATCATGTGATTGGGGTACTATTG
TGCTACTGTTAGCTAGATTTACATGTGCCAAAAACAGACTTACCATTGCCAAATGTTTAG
GTACATTAGTAAATATTCCACAATCACAAATGTTTATAGAACCACCTAAGCTACGTAGTA
CAGCCGTTGCATTGTACTTTTATAGAACAGGAATATCAAACATTAGTAGCACATATGGCG
AAACACCAGAATGGATTACAAGACAAACACAACTACAGCACAGTTTTGATGATAGCACGT
TTGAACTATCGCAAATGGTACAATGGGCATTTGACCATGATGTAGTAGACGATAGTGAAA
TAGCATTTTACTATGCACAATTGGCTGATACAGATAGCAATGCAGCAGCGTTTCTAAAAA
GTAATTGCCAAGCAAAGTATGTAAAAGACTGTGGGACTATGACACGGCACTACAAACGAG
CACAAAGAAAATCACTAACAATGTCAGCATGGATTAGGTATAGATGTGACAAAGTGCAAG
ACGGGGGTAACTGGAGGGAAATAGCTAAATTTTTGCGATACCAGGGTATTAACTTTATGT
ATTTTATACAAACATTTAAATTGTTTTTAAAAGGAACACCAAAACACAACTGCATAGTAA
TACAGGGCCCACCAAACACAGGGAAATCACAATTTGCAATGAGTTTAATTAGATTTTTGC
AAGGGTGCGTTATTTCATATGTAAATTCAGGTAGTCATTTTTGGTTACAGCCACTAGAGG
ATGCCAAGGTAGCATTATTAGATGATGCCACATATGGCTGTTGGACATATATAGACCAGT
ACCTAAGAAATTTCCTAAATGGTAATCCATGTAGTATTGATAGGAAACACAGAAGCCTGC
TGCAAATTGTATGCCCACCATTGCTTATTACCTCAAATATCAATCCAAAAGAAGATCCAA
ATTTAATGTATTTACATAGTAGAGTGACAGTATTTCAATTTTTAAATGCATTTCCATTTG
ACCCCCATGGGAATCCTGTATATGCACTAAATGATGTAAACTGGAAAAAATTTTTTTCCA
CCACCTGGTCCAGATTAGATTTGGAGGAGGAAGAGGACAAAGAAAATGGAGACCCTATGT
CATCGTTTAAATGTGTGCCAGGAGAAAATACTAGACTGTTATGAACTGGACAGTGATAAA
TTAGTAGATCAAATTAATTATTGGACGTTGGTACGATATGAATGTGCTATGTTTTATACT
GCACGAGAAAGAAACATGCAAACCCTTAACCATCAAGTAGTACCAGCATCGGCAGTATCA
AAACAAAAGGCCTGCCAAGCCATTGAAATGCATATGGCATTAGAATCGCTAAACAAATCT
GAATATAATATGGAACCATGGACAATGCGGGACACATGCTATGAACTATGGGGCGAGGCC
CCTAAACATTGTTTCAAAAAAGGGGGAAAAACTGTAACAGTGATGTTTGATGGGAATAAG
GACAATACAATGGACTATACATGTTGGACATATGTGTATATATATAAAGAGGATAGATGG
GTAAAAACACAGGGAAACGTGGACTATACAGGTATTTATTACAAACCAGATGTAAACAAA
GAATATTATGTAACATTTAAAGATGAAGCAAAAAAATATGGGGCACAACAATGGGAGGTA
TATATGTGTGGCAATGTAATAACATGTCCTGAATATGTATCTAGTACCTACAGCACCCCG
TCACCCTCTACTACAACTGTTGAACAGCTATCGAACACCCCCACGACCAATACCTATTCC
GCCTCCGCGGGCACCACAGAAGCCCAGACACCACAACAACGAAAACGACAGCGACTTGCT
GAGCCCGACTCCTCCACAGTCACCCCGCTGTCCGTGGACACCTGCGACCACCAAATACAC
TGTGGAACTGCAGGCCCAAACACCGGAGGGCACCTCAGTGCAACTAAAACTGCGTTTATA
GTTCATTTAAAAGGTGCAACAAATTGTTTAAAATGTTTAAGATACAGATTTGCAAAACAT
AGAAATTTGTTTAAAGAAGTGTCATCTACCTGGCATTGGACCAGTAATACAAAAGCAGGC
ATTGTTACAATTACATTTGACAGTGCACACCAACGTCAAAAGTTTATTGATACTGTTAAA
GTACCATCAAGTGTGACAGTGTCATTGGGTATTATGACAGTGTAAATATATGTATTGTAC
ATATATAGGTTTACAAGCCAATATGTGCTGCTAAGTGTATATAGTTACTCGCAACCATTG
CGGTGTTTTTGGTGTGTTTATTTGTGCTTTGCGTGTGTGTGTGTCTTGTGTTGTGTTGTT
TGTTGCCGCTATTGCAATCCCAATATGTGTTTGCAGCAGCCTTATTACTAATTATATGTT
TTTGGTTTGTGGTTGCCACATCACCTTTAACTACATTTACTGTATACTTGATTTGTTTTT
ATATTCCATGCTTTTTATTACATTTATATACCTTTTCTATTTTGCAACTTACACAATAAA
ACTTTGTATGTTTTTGTATTATATACAATGGTGGCTGCACGTGCACGGCGGCGTAAGCGA
GCATCTGTAACACAATTATATTCCACATGCAAAGCTGCTGGTACATGTCCTCCTGATGTT
ATTCCTAAGGTAAAGGGCACTACATTAGCTGATAAAATATTACAATGGAGTGGTTTGGGT
ATATTTTTGGGGGGTTTGGGTATTGGCACCGGGTCTGGCACTGGGGGCAGAACTGGGTAC
ATTCCTTTAGGTGGTGGTGGACGGCCTGGTGTTGTAGATATTGCTCCGGCCAGGCCTCCA
ATTATTATTGAACCTGTAGCCCCTACTGAACCTTCTATTGTTAACCTTGTTGAGGATTCT
AGTATTATTAATTCAGGCTCTACTATACCTACCTTTACTGGTACTGATGGCTTTGAAATT
ACTTCCTCTTCTACAACAACTCCTGCGGTTTTAGACATTACACCTTCCGCTGGTACAGTA

CATGTTACAAGCACTAACATTGAAAATCCCTTATATATTGAACCTCCATTTATTGAGGCA
CCACAATCAGGTGAGGTATCTGGACATATATTTACCAGTACCCCTACGTCTGGCACCCAT
GGATATGAGGAAATACCTATGGAGGTATTTGCCTCCAATGTTACTACTGGTAAGGAACCC
ATTAGCAGTACACCCACCCCTGGGGTGCGCCGCATTGCTGCACCTCGTTTATATAGCAGG
GCATTTTCACAGGTTAAGGTTACTAATCCAGACTTTATTAGTAAGCCCTCTACATTTGTT
ACTTTTGATAATCCTGCTTTTGAACCTGCTGATACATCATTGTCCTTTGAGGAACCTACT
GATGTTGCACCAGATCCTGATTTTTTAGATATTATTAAACTGCACCGCCCTGCGCTGACA
TCTCGCAGAGGTACAGTGCGGTTTAGTAGATTGGGTCAAAAGGCCACCATACGCACACGT
AGTGGCAAACAAATAGGTGCTCGTGGACATTATTATCATGATATAAGTAGTATTGCACCT
GCTGAGGAACTTGAAATGCAGCCTTTACTTTCCCCTTCTACTAATAATTATAGTTATGAC
ATTTATGCTGATTTAAATGAAGCTGAAACAGGTTTTATGCAGCCTACACAAACCACACCT
ATGCTTCGCTCTCCATTTTCTCCTTTGTCTACACAACTACCTTCATTGTCTTCCTCTGTT
TCTTCATCTTATGCTAATGTTACTATCCCTTTTTCTACCACATATAATGTTCCTATTCAT
ACGGGTCCTGATGTTGTGTTACCTACTTCACCCACTGTGTGGCCCTTTATTCCACACACA
TCTATTGACACACAACATGCTATTGTTATACAAGGTGGGGATTACTACTTTGTGGCCGTA
TACATATTTGTTACGCAAACGCCGTAAACGTATACCCTATTTTTTTGCAGATGGCTTTGT
GGCGTACTAATGACAGCAAAGTGTATTTACCACCTGCACCAGTGTCACGCATTGTCAACA
CAGAAGAATATATAACCCGCACCGGCATATATTATTATGCAGGCAGTTCCAGACTTATTA
CCTTAGGACATCCATATTTTTCAATACCCAAAACCAATACACGTGCTGAAATACCTAAGG
TATCTGCCTTTCAGTATAGGGTGTTTAGGGTACAGTTACCTGACCCCAACAAATTTGGTC
TTCCTGATCCTAATTTGTTTAATCCAGACACAGATCGTTTGGTGTGGGGATGTGTTGGTG
TTGAAGTAGGTAGGGGTCAGCCGTTAGGTGTTGGCCTTAGTGGTCATCCTTTATTTAATA
AGTATGATGATACTGAAAACTCTAGGTTTGCCAATGGCAACGACCAACAGGATGTTAGGG
ACAACATATCTGTGGACAACAAACAAACTCAGTTATGCATTATAGGCTGCGCTCCTCCTA
TTGGGGAACACTGGGCCACAGGTACCACATGTAAAAACGTACCTGTACCTCAGGGTGACT
GTCCACCTTTGGAACTTGTGTCTACTGTCATTGAGGATGGCGATATGGTGGACACTGGTT
TTGGGGCCATGGATTTTGCTAATTTACAAGCTACTAAATCAGATGTTCCATTGGATATTG
CTCAGTCTGTGTGTAAATACCCTGATTACTTAAAAATGTCAGCAGATACATATGGCAATT
CTATGTTTTTTCATTTACGCAGGGAGCAAATATTTGCTAGGCATTACTATAATAAGGCTG
GTGTGGTTGGTGATGCCATTCCAGACAAGGCTTATATTAAGGGTACTGGTGCTGGCCGCG
ACCCTATTAGTAGTTATATTTATTCAGCTACTCCCAGTGGTTCTATGATAACCTCTGATT
CTCAGATTTTTAATAAGCCTTATTGGTTGCATCGCGCCCAGGGCCACAATAATGGCATTT
GCTGGAATAATCAGCTTTTTATTACTTGTGTTGACACTACTAAAAGTACCAATTTAACCA
TTAGCACTGCTGTTACTCCATCTGTTGCACAAACATTTACTCCAGCAAACTTTAAGCAGT
ACATTAGGCATGGGGAAGAATATGAATTGCAATTTATATTTCAATTGTGTAAAATCACTT
TAACTACTGAAATTATGGCTTACCTGCACACCATGGATTCTACAATTTTAGAACAGTGGA
ATTTTGGATTAACATTGCCCCCCTCCGCTAGTTTGGAGGATGCCTATCGATTTGTAAAAA
ATGCAGCAACATCCTGTCACAAGGACAGTCCTCCACAGGCTAAAGAAGACCCTTTGGCAA
AATATAAATTTTGGAATGTAGACCTTAAGGAACGCTTTTCTTTGGATTTGGATCAGTTTG
CATTGGGTCGCAAGTTTTTATTACAAATCGGTGCCCAACGCAAACCCAGACCAGGCCTTA
AAAGGCCTGCCCCATCCTCTTCCGCTTCCTCGTCTGCCAAACGTAAAAGGGTTAAAAAT
AGGTTCGCCCTGCCTATGTATGTGTTTGTGTGTGTGTGTGTGTGTGTGTGTGCGTGTT
GTGTGTATTTGTGTGTGTATGTTTATGTATATTTTTCTGTATGTTTTGCTGTATGGTTTC
TGTGTGGTTTACTAATAAAGTATGTATGTGTGTGTGTTTTTGCAATGTGTTGTGTTTTTG
CATGACTGCATATGTATGACATAAAAAAGTGTTTGTAATAATTACATTATCCCTGTAGGT
TAAGGGTGGTGTTTAGGTGGCGTCCCTATTGCCCTACCCATATTTGTGGCTTGCAGCACA
CTTGTATATATATGTTCTTGCTGTATTGCATGTACCACAGGATTCCATTTTGTTTTTTCC
TGCAGCCATTTTGTGGCGACCGATTTCGGTTGCTTAAAAATGACTACATTTGCCAACTTT
TGAACCACACTACCTATGTTTTTTGGCATAACCCTTAATTCTTTTGGCATGGTATGTACA
TCCCGCCCGCCCACGCCTTGTTCCTGGCGCGCCTTCCTGGCGCATGTCATACACACCACA
TTACTCATTTGTACTTAAACAATTGTTGGCACAATATTTTTCCGCCCTGTAATAATTTAT
ATGCTTATAGGCATGTATTTTTTGGCTTATTTGCCTTTATTAATTGCATACTTGGCAGG
TCATGAACTAAATGTCTCTGCCAAGTTTCTATCCTACCTATAAAATGTATTACTCATCTG
CAGGTGTGCTACACAACTGTGTTACCCAACCGATTTGTGTTTTGCCTATGCTTGCAACAT
TTTTTCTTATA

>gi|333087|gb|M62877.1|PPHDNA_Human_papilloma_virus_type_51_genomic_DNA,_partial_sequence
AACAATTATCTTGTAAAAACTAGGGTGTAACCGAAAAGGGTTATGACCGAAAACGGTGCA
TATAAAAGTGCAGTGGTAAAAGTATAGAAGAACACCATGTTCGAAGACAAGAGGGAAAGA
CCACGAACGCTGCATGAATTATGTGAAGCTTTGAACGTTTCTATGCACAATATACAGGTA
GTGTGTGTGTATTGTAAAAAGGAATTATGTAGAGCAGATGTATATAATGTAGCATTTACT
GAAATTAAGATTGTATATAGGGATAATAATCCATATGCAGTATGCAAACAATGTTTACTG
TTTTATTCAAAAATTAGAGAGTATAGACGTTATAGCAGGTCTGTGTATGGTACTACATTA
GAGGCAATTACTAAAAAAAGCTTATATGATTTATCGATAAGGTGTCATAGATGTCAAAGA
CCACTTGGGCCTGAAGAAAAGCAAAAATTGGTGGACGAAAAAAAAAGGTTCCATGAAATA
GCGGGACGTTGGACGGGGCAATGCGCTAATTGCTGGCAACGTACACGACAACGTAACGAA
ACCCAAGTGTAATAAAGCCATGCGTGGTAATGTACCACAATTAAAAGATGTAGTATTGCA
TTTAACACCACAGACTGAAATTGACTTGCAATGCTACGAGCAATTTGACAGCTCAGAGGA
GGAGGATGAAGTAGATAATATGCGTGACCAGCTACCAGAAAGACGGGCTGGACAGGCTAC
GTGTTACAGAATTGAAGCTCCGTGTTGCAGGTGTTCAAGTGTAGTACAACTGGCAGTGGA
AAGCAGTGGAGACACCCTTCGCGTTGTACAGCAGATGTTAATGGGCGAACTAAGCCTGGT
TTGCCCGTGTTGTGCGAACAACTAGCAACGGCGATGGACTGTGAAGGTACAGAGGATGAG
GGGGCGGGGTGTAATGGGTGGTTTTTTGTTGAAGCAATAGTAGAAAAAAAAAACAGGAGAT
AATGTTTCGGATGATGAGGATGAAAATGCAGATGATACAGGATCTGATTTAATAAACTTT
ATAGATAGTGAAACTAGTATTTGCAGTCAGGCGGAACAGGAGACAGCACGGGCGTTGTTT
CAGGCCCAAGAATTACAGGCAAACAAAGAGGCTGTGCATCAGTTAAAACGAAAGTTTCTA
GTCAGCCCGCGAAGCAGCCCATTAGGAGACATTACAAATCAAAACAACACACACAGCCAT
AGTCAGGCAAACGAGTCACAAGTTAAAAGGAGATTACTGGACAGTTATCCGGACAGCGGA
TATGGCAATACACAAGTGGAAACTGTGGAAGCAACGTTGCAGGTAGATGGGCAACATGGC
GGTTCACAGAACAGTGTGTGTAGTAGCGGGGGGGGCAGTGTTATGGATGTGGAAACAACA
GAAAGCTGTGCAAATGTAGAACTAAACAGTATATGTGAAGTATTAAAAAGCAGTAATGCA
AAAGCAACGTTAATGGCAAAATTTAAAGAGTTGTATGGTATTAGTTATAATGAGTTGGTA
CGGGTGTTTAAAAGTGATAAAACATGTTGTATAGATTGGGTTTGTGCATTGTTTGGCGTT
TCCCCAATGGTAGCAGAAAATTTAAAAACACTAATTAAGCCATTTTGCATGTACTACCAT
ATACAATGTTTATCATGTGATTGGGGCACCATTGTATTAATGCTAATTAGGTTTTCATGT
GCAAAAAACAGAACAACAATTGCTAAGTGTTTAAGTACATTAGTAAATATCCCACAATCA
CAAATGTTTATAGAACCACCAAAATTACGTAGTACACCTGTGGCATTATATTTTTATAGA
ACAGGCATATCAAACATTAGCAATACATATGGAGAGACACCTGAATGGATTACACGACAA
ACGCAACTACAACATAGTTTTGAGGATAGTACCTTTGAATTATCACAAATGGTGCAATGG
GCATTTGACCATGAAGTATTAGATGATAGTGAAATAGCATTTCATTATGCACAATTAGCA
GATATAGATAGTAATGCTGCAGCGTTTTTAAAGAGTAATTGCCAAGCAAAATATGTAAAA
GATTGTGGGACCATGGCACGGCATTACAAACGAGCACAAAGAAAATCATTATCTATGTCA
GCCTGGATAAGGTATAGATGTGATAGAGCAAAGGATGGAGGCAACTGGAGAGAAATTGCT
AAATTTTTAAGATATCAAGGTGTAAACTTTATGTCCTTTATTCAAATGTTTAAACAGTTT
TTAAAAGGAACACCAAAACACAATTGCATAGTCATATATGGCCCACCAAACACAGGCAAG
TCATTATTTGCAATGAGCCTAATGAAGTTTATGCAAGGGTCCATTATTTCATATGTAAAC
TCTGGTAGTCATTTTTGGTTACAGCCACTAGAGGATGCTAAAATAGCATTGTTAGATGAT
GCTACGTATGGGTGTTGGACATATATTGATCAGTATTTAAGAAACTTTTTAGATGGTAAT
CCATGTAGTATAGATAGAAAACATAGGAGTTTAATACAATTAGTATGTCCACCATTACTA
ATAACGTCAAACATAAATCCACAAGAGGATGCAAACCTAATGTATTTACATACAAGGGTA
ACAGTATTAAAGTTTTTAAATACATTTCCATTTGATAACAATGGGAATGCTGTGTATACA
TTGAATGATGAAAATTGGAAAAATTTTTTTTCCACCACATGGTCCAGATTAGATTTGGAG
GAGGAAGAGGACAAAGAAAATGGAGACCCTATGCCACCGTTTAAATGTGTGCCAGGAGAA
AATACTAGACTGTTATGAACTGGACAGTGATAAATTAGTAGATCAAATTAACTATTGGAC
ATTGTTACGATATGAAGCTGCTATGTTTTATGCAGCACGGGAAAGAAACTTACGAACAAT
CAATCACCAGGTAGTACCAGCAACAACAGTATCAAAACAAAAGGCCTGTCAAGCAATTGA
AATGCACATGGCCTTACAATCGCTTAACAAATCAGACTATAACATGGAACCATGGACAAT
GCGGGAGACATGTTATGAACTATGGTGTGTGGCTCCCAAGCAATGTTTCAAAAAGGGGGG
CATAACTGTAACAGTTATATTTGATGGAAATAAGGACAATGCAATGGACTATACAAGCTG
GAAATTTATATATATATATGATAATGATAAGTGGGTAAAGACAAATGGAAATGTGGACTA

TACGGGTATATATTACACTGTAAATTCAAAAAAAGAATATTATGTACAGTTTAAAGATGA
AGCCAAAATATATGGGGCACAACAGTGGGAGGTCTATATGTATGGTACTGTAATAACATG
TCCTGAATATGTATCTAGTACCTGCAGCGACGCGTTATCCACTACTACAACTGTTGAACA
ACTATCAAACACCCCAACGACCAATCCCCTTACCACCTGCGTGGGCGCCAAAGAAGCCCA
GACACAACAGCGAAAACGACAGCGACTTACTGAGCCCGACTCCTCCACAATCTCCCCACT
GTCCGTGGACAATACAAACAACCAAATACACTGTGGAAGTGGAAGCACTAACACTGGAGG
GCACCAAAGTGCAACTCAGACTGCGTTTATAGTGCATTTAAAAGGTGATACAAATTGTTT
AAAATGTTTTAGATACAGATTTACAAAACACAAAGGGTTATATAAAAACGTATCCTCAAC
CTGGCATTGGACCAGTAATACTAAAACAGGCATTGTTACCATTGTGTTTGACAGTGCACA
TCAACGGGAAACATTTATAAAAACCATTAAAGTACCCCCAAGTGTAACACTGTCATTGGG
AATTATGACACTGTAACTAGTGTAATATATGTATTGTACATATATACTGTCACAAGCCAA
TATGTGCTGCTAATTGTATAGACATATTGTAACCATTGCAGTGTTTATTATTTTGCTATT
TGTGCTTTGCTTGTGTGTGTGTCTTGTGTTGTGTTGTTTGTTGCCGCTACTGCTGTCCCA
ATACGTGTTTGCAGCTGCCTTATTATTAATTTTATGTTTTTGGTTTGTTGTTGCAACATC
CCAATTAACTACATTTTTTGTATATTTGATTTTTTTTTACTTACCTTGTTTACTTTTACA
TCTATATACATTTTTACTTTTGCAATAAACTTGTTATATTTTTGTGATTAAATATGGTGG
CTACACGTGCACGGCGTCGGAAGCGAGCATCTGTAACACAATTATATTCTACATGCAAAG
CTGCTGGTACATGTCCTCCTGATGTTGTGAATAAGGTTGAAGGTACTACATTGGCCGATA
AAATATTACAGTGGAGTGGGTTGGGTATATTTTTGGGTGGCCTAGGTATTGGTACTGGGT
CTGGATCTGGGGGGCGTACTGGATATATCCCTTTAGGTGGTGGGGGTCGCCCAGGCGTGG
TGGATATTGCTCCTGCAAGGCCACCTATTATAATTGACCTATGGCACCATACTGAACCTT
CTATAGTAAATTTGGTTGAGGACTCTAGTATTATTCAGTCTGGGTCTCCTATACCTACCT
TTACTGGTACCGATGGCTTTGAAATTACTTCATCTTCCACAACAACCCCTGCTGTGTTGG
ACATCACCCCATCTGCTGGTACTGTACATGTTTCTAGTACTAACATTGAAAATCCTTTAT
ATATTGAACCTCCATCCATTGAGGCTCCACAATCTGGAGAAGTGTCAGATATATATTTAC
TAGTACACTACTCTGGTACTCATGGGTATGAAGAAATACCTATGGAAGTGTTTGCATCCA
ATGTCAGTACTGGTACTGAACCTATTAGCAGCACACCTACTCCAGGGGTTAGTCGCATAG
CTGCTCCCCGCTTGTATAGTAAGTCCTACACACAGGTTAAAGTTACAAATCCTGATTTTA
TTAGTAAGCCATCCACATTTGTTACATTTAATAATCCTGCTTTTGAGCCTATTGACACAT
CCATAACTTTTGAGGAACCTGATGCTGTTGCACCTGATCCTGATTTTCTGGATATTATTA
CACTGCACCGCCCTGCCCTTACATCTCGTAGAGGCACAGTACGCTTTAGTAGGTTAGGTC
AAAAGGCCACCATGCGCACTCGTAGTGGCAAACAAATTGGTGCTCGTGTACATTATTATC
ATGATATTAGTAGAATTGCACCAGCTGATGAACTTGAAATGCAGCCTTTACTTTCACCTT
CTAATAATTATAGTTATGACATTTATGCTGATTTAGATGAAGCTGAAACAGGTTTTATAC
AGCCCACACACACCACACCTATGTCACACTCCTCTTTGTCTAGGCAGTTGCCCTCCTTAT
CTTCATCTATGTCTTCATCTTATGCAAATGTTACTATTCCATTTTCAACTACATATTCTG
TTCCTATTCATACAGGGCCTGATGTGGTATTGCCCACATCTCCTACAGTATGGCCTTATG
TTCCCCACACTTCCATTGACACCAAGCATTCTATTGTTATACTAGGTGGGGATTACTATT
TGTGGCCCTATACACATTTACTACGCAAACGCCGTAAACGTATACCCTATTTTTTTACAG
ATGGCATTGTGGCGCACTAATGACAGCAAGGTGTATTTGCCACCTGCACCTGTGTCTCGA
ATTGTGAATACAGAAGAATATATCACACGCACCGGCATATATTACTATGCAGGCAGTTCC
AGACTAATAACATTAGGACATCCCTATTTTCCAATACCTAAAACCTCAACGCGTGCTGCT
ATTCCTAAAGTATCTGCATTTCAATACAGGGTATTTAGGGTACAGTTACCAGATCCTAAC
AAGTTTGGACTCCCGGATCCAAATTTATATAATCCAGACACAGATAGGTTGGTGTGGGGT
TGTGTGGGCGTTGAGGTGGGCAGAGGACAGCCCCTTGGTGTTGGCCTTAGTGGTCATCCC
TTATTTAATAAATATGATGACACAGAAAATTCACGCATAGCAAATGGCAATGCACAACAA
GATGTTAGAGATAACACATCTGTTGACAACAAACAGACTCAGTTATGTATAATAGGCTGT
GCTCCACCTATTGGGGAACACTGGGGTATTGGCACTACATGCAAAAACACACCTGTACCT
CCAGGAGACTGCCCCCCCCTGGAACTTGTATCCTCTGTCATTCAGGATGGCGATATGATT
GATACAGGGTTTGGAGCTATGGATTTCGCTGCCCTACAGGCCACCAAATCAGACGTCCCT
TTGGATATTTCACAGTCTGTTTGTAAATATCCTGATTATTTAAAAATGTCTGCAGACACA
TATGGTAATTCCATGTTTTTTCATTTACGCAGGGAGCAAATCTTTGCTAGGCACTATTAT
AATAAACTTGTAGGTGTTGGGGAAGACATTCCTAACGATTATTATATTAAGGGTAGTGGT
AATGGCCGTGACCCTATAGAAAGTTATATATACTCTGCTACTCCCAGTGGGTCTATGATA
ACATCTGATTCTCAAATTTTTAATAAGCCTTATTGGCTCCACCGTGCGCAGGGTCACAAT

AATGGCATTTGCTGGAACAATCAGCTTTTTATTACCTGTGTTGATACTACCAGAAGTACA
AATTTAACTATTAGCACTGCCACTGCTGCGGTTTCCCCAACATTTACTCCAAGTAACTTT
AAGCAATATATTAGGCATGGGGAAGAGTATGAATTGCAATTTATTTTTCAATTATGTAAA
ATTACTTTAACTACAGAGGTAATGGCTTATTTACACACAATGGATCCTACCATTCTTGAA
CAGTGGAATTTTGGATTAACATTACCTCCGTCTGCTAGTTTGGAGGATGCATATAGGTTT
GTTAGAAATGCAGCTACTAGCTGTCAAAAGGACACCCCTCCACAGGCTAAGCCAGATCCT
TTGGCCAAATATAAATTTTGGGATGTTGATTTAAAGGAACGATTTTCTTTAGATTTAGAC
CAATTTGCATTGGGTCGCAAGTTTTTGTTGCAGGTTGGCGTACAACGCAAGCCCAGACCA
GGCCTTAAACGCCCGGCCTCATCGGCATCCTCTTCCTCTTCCTCTTCAGCCAAACGTAAA
CGTGTTAAAAAGTAATGTATGTTAGTTTTTGTATGCTTGTGCACACTGTTGTATGCCTGT
ATGTATATGTTTGTGTATGTACTGTATGTGTTTTTGTGTGTGTGTGTTGTTGTTCCTG
TATGTATGAGTTATGTATGTTTATTATTAATAAACTATGTGGTGTGTGTGTGTGTGTTTT
TGCATGACTGCATTTGTATGACATGTACGGGTGTATGTGGGTATTACATTATCCCCGTAG
GTCAAGGGTGGTGTTTCGGTGGCGTCCCTATTGCCCTACCCATTTTTTGCAGCACAACAG
TTTATATTTGTGCTATTTAGTTATACTTTGTAGCTTCCATTTTGTTACAGCTGCAGCCAT
TTTGAGTGCAACCGATTTCGGTTCGTGTACTTTTAGTATATTTGCCAAGTTTTAAACCAC
AACTGCCAGTTGTTTTTGGCATAAACCATCATTTTTTTATGACATAGTGCATACATCCGC
CCGCCCACGCCTTGTACTTGGCGCGCCTTACCGGCGCTAGTCATACAACCTATTAGTCAT
TTGTACTTTAACAATTGTTGGCACACTGTTTTCCGCCCTATAATAATTTAACTGCTTATA
GGCATGTATTTTTTGGCATATTTTATCTTACTAATTGCATAGTTGGCAGGTCAAATACTA
TGTTTTTAGTGCCAAGTTTCTATCCTACTTATAAACCATCTTACTCATATGCAGGTGTGC
TACACAAATGTGTTACCTAACCGATTTGTGTTCTGCCTATGCTTGCAACATTTTTTCTTA
TAACATTT
>gi|333062|gb|M32305.1|PPH47CG_Human_papillomavirus_type_47_(HPV-47)_+-
sense_strand
AACGGTAAGTTTGCATTAATGTACCAGGTGCGGTACAGATCATTTCACAATGGATATTAT
TGTTGCCAACTACCATAGTCATAATCAAGTTCTTGCCTGTATCGTTTTCGTACCTTACCT
ACAGTATTTTATATTAATATATAAATAAATAAATATATAAATGTGTATTTATTTCTCAGG
CTCAGTTCTTTGCAATTATTAAGACAAATGGCTCAGAAGGCTTTGGAACAGACTACAGTT
AAAGAGGAAAAGCTAGAACTACCTACTACTATTAGAGGCTTAGCTCAATTGTTAGACATA
CCTTTAGTAGATTGTTTGCTACCTTGCAACTTTTGTGGCAGATTTCTTGACTATTTAGAA
GTTTGTGAATTTGATTATAAAAAGCTTACTTTAATTTGGAAAGACTACAGTGTTTATGCC
TGCTGCCGTTTGTGCTGCTCAGCAACTGCCACATATGAATTTAATGTTTTTTATCAACAA
ACAGTGTTAGGTAGAGATATTGAGCTAGCTACAGGCCTTTCCATTTTTGAGATTGACATA
AGGTGTCATACCTGCCTGTCATTTCTTGACATTATTGAAAAGTTAGATAGCTGTGGAAGA
GGACTTCCCTTTCACAAAGTAAGAAACGCCTGGAAGGGTGTTTGTAGGCAGTGTAAGCAT
TTTTACAATGATTGGTAAAGAGGTCACCGTGCGAGATATTGTTCTGGAGTTAAGTGAGGT
TCAACCTGAAGTATTACCAGTTGACCTGTTTTGCGACGAGGAATTACCAAATGAACAACA
GGCGGAGGAGGAGCTAGACATCGACAGAGTCGTTTTCAAAGTGATTGCACCGTGCGGTTG
CAGCTGCTGCGAGGTCAAGCTTCGCATTTTTGTGAACGCAACAAACCGTGGCATCAGGAC
ATTTCAGGAACTTTTGACTGGTGATCTGCAGCTCCTCTGCCCAGAGTGCCGTGGGAACTG
CAAACATGGCGGATTCTAAAGGTAGTACATCTAAAGAAGGGTTTGGTGATTGGTGTATTT
TGGAAGCTGACTGTAGTGATGTTGAGGATGATTTGGGACAATTATTTGAGAGAGATACAG
ACTCAGATATCTCGGACCTGTTAGACAATTGTGACCTGGATCAGGGCAATTCACGGGAAC
TATTTCATCAACAGGAGTGTAAGCAAAGCGAGGAGCAATTACAAAAACTAAAACGAAAGT
ATCTTAGTCCAAAAGCTGTCGCGCAGCTTAGTCCGCGTCTTGAGTCAATTTCATTGTCAC
CTCAGCAGAAATCCAAGAGAAGGCTCTTTGCAGAGCAAGACAGCGGACTCGAGTTAACCT
TTAACAATGAAGCTGAAGATGTTACTCCTGAGGTGGAGGTACCGGCTATAGACTCTCGGC
CGGATGATGATGAGGGAGGATCAGGGGATGTAGATATTCATTATACAGCATTGTTGCGTT
CCAGCAACCAAAAGGCCACATTACTGGCAAAATTCAAACAAGCGTTTGGGGTAGGCTTTA
ATGAATTGACAAGACAATTCAAAAGCTACAAAACCTGCTGTAATCATTGGGTTGTATCCG
TATATGCAGTCCATGATGATCTATTTGAAAGCTCAAAGCAGCTGTTGCAACAGCATTGTG
ACTATATATGGGTCCGTGGGATAGATGCAATGTCATTATATCTATTGTGTTTTAAGGCGG
GAAAAAATCGTGGGACAGTTCATAAGCTAATTACCACAATGTTAAATGTGCATGAGCAAC
AGATATTGTCTGAGCCTCCAAAGTTAAGAAATACAGCTGCTGCATTATTTTGGTACAAAG

GATGTATGGGACCTGGAGTGTTCACCCACGGTCCTTACCCTGAATGGATTGCACAATTAA
CCATTTTGGGCCATAAGAGTGCTGAGGCAAGTGCGTTTGATCTGTCAGTCATGGTTCAAT
GGGCATTTGATAACAATCTGTTTGAGGAGGCAGACATTGCATACGGATATGCAAGACTGG
CACCAGAGGATAGCAATGCAGTTGCATGGCTTGCACATAATAACCAAGCTAAATATGTTA
GAGAATGTGCTATGATGGTTCGATACTACAAAAAGGGGCAAATGAGAGATATGAGCATGT
CTGAGTGGATATATACAAGGATACATGAAGTAGAGGGAGAAGGACAGTGGTCTAGCATTG
TTAAATTTTTAAGATATCAAGAAATAAATTTTATTTCATTTTTGGCTGCTTTAAAAGATT
TATTACATTCAGTACCTAAACGCAATTGTATTTTATTCCATGGCCCTCCAAATACAGGAA
AGTCATCGTTTGGAATGTCCTTAATAAAAGTTCTAAGGGGGAGAGTATTATCATTTGTAA
ACTCCAAAAGTCAGTTTTGGTTGCAGCCTCTTGGAGAATGTAAAATAGCATTATTAGATG
ATGTTACAGATCCATGTTGGGTGTATATGGATCAATATTTAAGAAATGGGTTAGATGGGC
ATTTTGTGTCTTTGGATTGTAAATATAGAGCACCCATGCAAACAAAGTTTCCACCTTTAA
TACTTACATCTAATATTAATGTACATGCAGAGACCAATTATAGATACCTACATAGTAGAA
TTAAGGGTTTTGAATTTAAAAATCCATTTCCTATGAAAGCAGATAATACACCTCAATTTG
AGTTAACTGACCAAAGCTGGAAATCTTTTTTTACAAGGCTTTGGACACACTTAGACCTGA
GTGACCAAGAAGACGAGGGCGAACATGGAGAATCTCAGCGAGCGTTTCAATGCTCTGCAA
GAACAGCTAATGAACATTTATGAAGCTGCAGAACAGACATTAAAGGCACAAATTTTACAT
TGGCAGACATTGCGAAAAGAAGCTGTGACACTCTACTTTGCTAGGCAGAAAGGCATAAAT
AGGTTGGGATACCAACCAGTGCCTGCATTAGCAATATCTGAGGCAAGGGCCAAAGAGGCT
ATATATATGGTGTTGCAGTTAGAGTCGCTACAAAAATCAGCGTTTGCTTTGGAGCCTTGG
ACCTTAGTGGACACTAGTACAGAGACTTTTAAGAGTGCTCCAGAAAATCATTTTAAAAAG
GGGCCTGTACCTGTGGAGGTGATATATGACAAAGATGAAGCAAATGCTAATTTGTATACT
ATGTGGACATTTGTGTATTACATGGATTCAGATGATGTGTGGCATAAGACAACAAGTGGG
GTCAATCAAACTGGCATTTACTACCTATATGGAACATTTAAACACTATTATGTGTTATTT
GCTGATGATGCAAAGAGATATAGTGCTACTGGAGAATGGGAAGTTAAAGTTAATAAGGAA
ACTGTGTTTACTCCTGTCACTAGCTCCACACCACCAGGGTCACCAGGAGGACAAACAGAC
CCAGACACCTCCTCCAAGACCCCCACCACCACCACAGCCGCCACTGACACCTCGCCCAGA
CGCCAATCCATCAATAAACAGTCACAACAAACCGAAACCAAACGAAGAGGGTACGGACGG
AGACCATCAAGCAGAACAAGGCGACCGCAAACGCACCAAAGGCGATCCAGATCCAGATCC
CGGTCGCGGTCCAGTTCTCAAACCCACTCTTCCACCACCACCACCACCACCACCTACAGG
TCCAGGTCTACGTCGCTCAACAAGACTCGTGCTCGTTCCAGGTCAAGGTCCACCTCCAGA
TCTACCAGCACCACCAGTAGAAGGGGAGGTAGAGGGTCATCCACAAGGCAAAGATCGCGA
TCACCCTCCACCTACACCTCAAAACGGTCACGGGAAGGAAACACAAGGGGCAGAGGGAGG
GGGAGACAAGGGAGAGCAGGGAGCAGTGGGGGGAGAGAGCAGCGACGGAGAAGGAGATCA
TTCTCAACCTCCCCTGACTCCTCCAAACGAGTCAGACGGGAGTCTCCTAAATACCGTGGC
GTGTCTCCTAGCGAGGTGGGAAAGCAACTTCGATCAGTTGGTGCAAAACATTCAGGGCGA
CTTGGAAGGTTATTGGAGGAAGCTAGGGACCCCCCAGTAATTCTTGTGCGAGGGGACGCA
AACACATTAAAATGCTTTCGCAACAGAGCAAGGAACAAATATAGAGGGCTTTTTAGATCA
TTCAGCACTACATTTTCCTGGGTAGCTGGAGATAGCATTGAGCGTCTAGGCAGGTCCAGA
ATGCTCATTAGCTTTTCCTGCCTCACTCAGAGAAGGGATTTTGATGATGCTGTCAAATAT
CCAAAAGGAGTCGAGTGGTCATATGGTAGTCTTGATAGCCTTTAACAAGCATTAACGCTG
CTTTGCTACTAACTGCTATTAACAACCACAGCTTTTTTTTTACGTTTTTTTATTTTACTG
ATTTTGTACTGCAATGGCGCGTGCTAGAAGGGTCAAACGTGACTCTGTAACACATATATA
TCAGACCTGCAAACAGGCAGGCACTTGCCCCTCGGACGTTGTTAATAAAGTTGAGCAAAC
AACAGTTGCTGACAATATTTTGAAATATGGCAGTGCTGGTGTCTTTTTTGGAGGCCTTGG
CATAGGAACAGGCCGAGGGACTGGGGGTGCTACTGGGTACGTGCCACTTGGGGAAGGTCC
TGGTGTCCGTGTGGGAGGAACCCCAACGGTTGTAAGGCCTTCTCTTGTTCCTGAAGCAAT
TGGACCAGTTGATATTTTACCCATTGACACAATCGCACCTGTCGAGCCTACTGCTTCATC
TTTAGTCCCATTAACAGAGTCGTCTGGTGCTGATTTACTTCCCGGTGAAGTTGAAACTAT
AGCCGAAATACATCCTATTCCTGAAGGTCCGACAATCGACTCCCCTGTAGTCACCACAAC
GACAGGTTCCAGTGCTGTTCTGGAAGTGGCTCCAGAACCTGTACCCCCTACACGTGTTAG
AATTGCTAGAACACAATATCATAATCCCTCTTTTCAGATACTCACTGAATCAACACCTGC
GCAGGGCGAGAGTTCTCTTGCTGACCATATTTTGGTCACCTCAGGGTCTGGTGGACAAAG
GATAGGCGGTGATATAACAGACGAAATTGAACTTACTGAGTTTCCAAGCAGATATACATT
TGAAATAGAAGAACCCACCCCTCCACGAAAAAGTAGCACACCATTACAAACTGTAGCCTC

TGCAGTAAGGCGACGGGGCTTCTCATTAACAAATAGAAGATTGGTACAACAAGTAGCTGT
AGACAATCCTTTATTTTTAAGTCAACCTTCTAAGATGGTAAGATTCTCATTTGACAATCC
AGCTTTTGAAGAAGAGGTTACCAATATTTTTGAACAGGATGTTAACAGCTTTGAAGAACC
TCCAGACAGGGATTTTCTTGATATTAAACAATTGGGCCGTCCTCAATATTCTACAACACC
AGCAGGTTATATTAGGGTAAGCAGACTAGGAACTCGAGGCACCATTCGCACTCGTTCTGG
TGCACAAATAGGTTCTCAGGTACACTTTTATAGAGATTTAAGTTCTATAAATACTGAGGA
TCCAATAGAACTACAGCTTTTAGGGCAGCATTCTGGAGATGCTACTATTGTTCAAGGTCC
TGTAGAAAGCACATTTATAGATATGGACATTGCTGAAAACCCTTTATCTGAAACAATAGA
TGCTTCATCTAATGATTTACTTTTGGATGAGACTGTGGAGGATTTTAGTGGGTCCCAATT
AGTAATTGGAAATCGAAGGAGTACAACATCATATACTGTTCCCAGATTTGAGACTACTAG
AAGTAGTTCCTATTATGTTCAAGACACAGATGGTTATTATGTTGCTTACCCAGAGTCACG
GGACACTATTGATATTATTTACCCTACACCTGAATTACCTGTAGTTGTCATTCACACCCA
TGACAATTCTGGAGACTTTTACTTACATCCTAGTCTTAGAAGGCGTAAGCGTAAAAGAAA
ATATTTGTGATTTGCATTGCAGATGGCAGTGTGGCACTCGGCTAACGGTAAAGTATACCT
TCCTCCATCAACACCAGTGGCCAGGGTTCAAAGCACGGATGAATACATACAAAGGACTAA
TATCTATTATCATGCAAATACTGACCGCCTTTTAACAGTAGGACATCCATATTTCAATGT
ATACAATAATAATGGAACTACATTAGAGGTTCCAAAAGTATCAGGTAATCAGCATAGGGT
GTTTCGCTTAAAATTGCCAGATCCTAATAGATTTGCTCTAGCGGACATGTCTGTATACAA
CCCTGACAAAGAACGCTTGGTGTGGGCCTGCAGGGGTCTAGAAATTGGAAGGGGTCAACC
TTTAGGTGTTGGCAGTACTGGTCACCCATATTTTAATAAGGTAAAAGATACAGAAAACAG
TAATTCCTATATCACAAACTCAAAAGATGACAGACAAGACACCTCTTTTGATCCTAAACA
AATACAGATGTTTATTGTGGGCTGCACTCCATGTATTGGCGAACACTGGGATAAGGCAGA
GCCTTGTGGGGAACAGCAAACTGGTCTTTGTCCTCCTATTGAATTAAAAAACACATACAT
TCAGGATGGCGACATGGCAGACATTGGTTTTGGCAACATTAATTTCAAGGCCTTACAACA
CAGTAGGTCTGATGTTAGTCTTGACATTGTAAATGAAACTTGCAAGTACCCGGATTTTCT
CAAAATGCAAAATGATGTTTATGGGGATGCTTGCTTTTTTTATGCTCGTAGAGAGCAATG
TTATGCCAGACATTTTTTTGTTAGAGGGGGAAAAACAGGTGATGACATACCAGGAGCACA
GGTTGGCAATGGTAATATGAAAAATCAATTTTACATTCCTGGTGCTACGGGTCAGGCTCA
GAGCACTATAGGTAATGCCATGTATTTCCCAACTGTCAGTGGCTCACTAGTCTCTAGTGA
TGCTCAACTGTTTAACAGGCCATTCTGGCTCCAAAGGGCTCAGGGTCATAATAATGGCAT
TCTGTGGGCTAATCAAATGTTTGTCACAGTTGTAGACAACACAAGAAATACAAATTTCAG
CATCTCTGTTTACTCTCAGGCAGGGGACATAAAGGATATACAGGATTATAATGCAGACAA
TTTTAGAGAGTATCAAAGACATGTGGAGGAATATGAAATTTCTGTAATATTACAATTGTG
CAAAGTTCCTTTAAAAGCAGAAGTTTTAGCACAAATTAATGCCATGAATTCGTCTCTTTT
AGAGGAATGGCAGTTAGGATTTGTGCCTACTCCAGACAACCCTATTCAGGATACATATAG
ATATCTAGAATCTTTGGCCACTAGGTGTCCTGAAAAGTCTCCTCCAAAAGAGAAGGTTGA
CCCCTACAAAGGTTTAAACTTTTGGGATGTCGATATGACAGAGCGCCTTTCCCTGGATTT
AGATCAATATTCATTAGGTAGAAAGTTCTTATTCCAGGCTGGATTACAGCAGACGACCGT
AAACGGTACAAAAACAACTCCTTACAGGGGGTCCATCAGAGGAACAAAGCGCAAACGAAA
AAATTGAAGATGACCGTTTTCGGTACAGATTGTTTAACTTTTACACAGTATTCAAGGAAT
GTCTGTTTACTGTGACTAAGTGTAACTCTGCCAAAGAAACAACCGCACCCGGTACACGTA
TTCAGCTTGTTGCCAAAACAGATAAGCTTGGCAGTCAGAACACACCGTGTTCGTCGCAAC
ACGCTCGGATTAGGTCTTCTGCCAAAAGAAATTTAATCTTGTTATCGTTTTTGGCGATCA
CATTTGGCACCGCGGGCAGCTGTTTTGGCACTACAAGACAACCGTT
>gi|3228267|dbj|D21208.1|_Human_papillomavirus_type_67_complete_genome
TTATAATCCTGTGTTTAAAAAAGTAGGTGTAACCGAAAACGGTTTGACCGAAAACGGTAA
ATATATAAAGCACACCAGTGTCCACCTACAGTGCAAGAAATATGTTTCAGGACACAGACG
AAAAACCACGCAACCTGCACGAATTGTGTGAAGCTTTGGAAACCACGGTGCATGAAATAA
GTTTGCCCTGCGTGCAGTGCAAAAAAACTTTGGACAGAAACGAGGTATATGACTTTTTAT
TTACTGATTTGAAAATAGTATACAGATGTGGGAATCCGTATGGGGTATGTAAGCAATGCC
TAAGACTGTTATCAAAAGTTAGTGAATATAGATATTTTAACTATTCAGTATATGGAAATA
CTTTAGAAGAAATTGTGCATAAACCATTAAATGAAATTACAATACGATGTATAACATGTC
AAAGACCATTGTGTCCACAAGAAAAGCAAAGGCATGTTGATCGCAAAAAGCGATTTCATA
ATATTTCCAATCGTTGGACAGGACGGTGTTCAGTGTGTTGGAGACCTCAACGAACGCAGA
CCCAGGTATAATATTTGAAGCCATGCGTGGAGACAAAGCTACATTGCAAGATTATATACT

AGATTTAAAACCCGAAACAACTGACCTATACTGCTATGAGCAATTGCATGACAGCTCAGA
TGAGGAGGATGAAGTAGGCATGGACCGGCCAGATGGACAAGCAAAACCAGATACAACCAA
TTACCATATTGTTACTGTGTGTAACATCTGTGAGTGCACTTTGCGTTTGTGCATTCACAG
TACAGCAGCAGACGTCCGAACAATCCAGCAGATGCTTATGAACACACTAGGAATAGTGTG
TCCCACCTGTGCACAACAATAAACATCATCTGAAATGGAGGACCCGGAAGGTACAGACGG
GGAACAGGGGGGGTGTACAGGATGGTTTAGTGTAGAAGCAATAATTGAAAAAAGAACAGG
AGATAAGGTATCAGAGGATGAGGATGAGGATGCATCTGACACTGGTAGTGATTTAATTGG
CTTTATAGATGATACACATATAGAAAACATACAGGCAGATACACAGGCAGCCCGCGCATT
ATTTAATTTACAGGAAGAGGAGGATGACCTAAACGCTGTGAGTGCACTAAAACGAAAGTT
TACAGGCATACAGGCATGTGGTGGTAATAGTAATGGTATAGAAAACCAAGCATGTACAGC
CGCAAAACGCAGAGCATACGACATAGAAGACAGCGGATATGGCAATACTGAAGTGGAAAC
TCAAGAGACAATGGTGCAGGTAGAGGGGCAAAATGGCGATATGCAGTGCAGTAGTCAGTG
TAGTACGGGGGCAAGTAGTACGGGAAACAGTGTAGATATGCAATCGGAAAGTAATAGTAG
TCAGGAACAAAGCATGCCATTGCAAACTGTGGAAAATATTATGCATGTAAATAATATAAA
GGCAACGTTAATGCATAAATTTAAGGAAGCATATGGGGTAACGTTTACACAGCTAATAAG
ACCATTTAAAAGTGACAGGACAAGCTGTACAGATTGGTGTGTTACAGCGTTTGGAATAAC
ACCATCAGTAGCAGAAAGTTTAAAGGTGTTAATCAAGCCACAAACGTTGTACACACATTT
ACAATGTTTAACCTGTGACAGAGGTATTATTATATTATTATTAGTAAGGTTTAAATGTGC
AAAAAACAGGTTAACAGTGTCAAAATTAATGTCTAATTTATTATCAATTCCTGAAACGCA
TATGATAATTGAACCACCAAAAATACGGAGTACAACGTGTGCACTATATTGGTTTAGAAC
AGGAATGTCAAATATAAGTGAAGTAAGTGGCAAACACCAGAATGGATAGAAAGACTAAC
GGTACTGCAACATAGTTTTGATGATACTATATTTGATTTAGGAGAAATGGTGCAATGGGC
ATATGACAATGACATAACAGATGATAGTGAAATAGCATATCAATATGCAATGCTGGCAGA
TGTAAACAGTAATGCCGCAGCATTTTTAAAAAGTAATTCGCAAGCCAAAATAGTAAAGGA
TTGTGGTACCATGTGCAGACACTATAAAAGAGCAGAAAAACGAAAAATGACAATAGGACA
ATGGATACAAGCTAGATGTGAAAAAGTAAATGATGGAGGAGACTGGAGAACAATAGTAAA
GCTATTAAGATACCAAAATGTAGAATTTACACAGTTTTTAGCAACATTTAAAAAGTTTTT
AAAAGGTATACCTAAAAAAAGTTGTATGGTAATATGCGGACCACCAAACACAGGGAAAAC
ATATTTTGCAATGAGTTTAATCCACTTTTTACAAGGATGTGTAATATCATATGTAAACGC
CAAAAGTCATTTTTGGCTACAACCATTGTCAGATGCAAAAATAGGAATGATAGATGATGT
TACAGCAATATGCTGGACATACATAGATGATTATTTAAGAAATGCACTTGATGGAAATGA
TATTTCAATAGATGTAAAGCATAAAGCGTTAGTACAGCTAAAATGTCCACCATTACTATT
AACTTCCAACATAGATGTAGCAACAGATTCAAGATGGCCGTTTTTACATAGTAGAGTAGT
AGTGTTTAGATTTAACAATCCATTTCCGTTTGATGAAAATGGCAATCCAGTATATAACTT
AAATGACGAAAATTGGAAATCCTTTTTCTCAAGGACGTGGTGCCAATTAGATTTAATTGA
GGAAGAGGACAAGGAAAACCATGGAGGAAATTGCAACACGTTTAAATGCAGTGCAGGAGA
AAATTCTAGATGTATACGAAGCTAATAAAAAAGACTTATGTACACAAATTGAACATTGGA
GACTGAGACGCATTGAATGTGCTTTGTATTATAAAGCTAAAGAACTGGGACTGGCACGGA
TAGGCCACCAGGTGGTGCCACCTATGTCAGTTACAAAGGCCAAAGCCTGTCAAATAATTG
AACTGCAAATGGCATTACAAACATTAAATGAAACACAATATAGTAATGATGAATGGACAT
TGCAAAGTACCAGCCTAGAACTATGGTTATGTGAACCAAAAAAATGTTTAAAAAAAAAAG
GAGAGACTGTAACAGTGCAATACGATAATGATAAAGAAAATACAATGGAATATACAAGTT
GGAATGAAATATATATAGTAAAGGACTCTGTGTGGACAGTGGTAGTGGGAAAGGTGGACT
ATATGGGACTATATTATATATATGAAACAGAAAAAACGTATTATGTAAAGTTTGAAACCG
ATGCTAAAAAGTATTCAAAACAAAACATTTGGGAAGTGCATGTGGGTGCTCAGGTAATTG
TTTGTCCTACATCTATGTTTAGTAACGAAATATCCACTACTGAGGTTGCTGCCCGGATAC
CACACGCCACAGAAACGTATACCCCTGCCACCACCGCGTGCACCAAAGAAAAACAGGCGC
CTGCCAAACGACGACGACCTGACGTCCCAGACTTCTGCAACAACTCCCAGCACCCCACAA
AGCTATTGTGCAGACAACGGTCCGTGGACTGTACATCGGTGGGGCTCGTCCCTGGACCTC
AGTGCACAAACAAAGGACGGAGTGTGTGTGCACCTAACACTGCACCTATAGTGCATTTAA
AAGGTGACCCTAATAGTTTAAAGTGTTTAAGATATAGATTAAAATGTAATACAGATTTAT
ATTGTCATATGTCATCCACATGGCATTGGACCAGTGGAAAACATACAAAAACTAAAGAAG
CTATTGTTACTGTTACATATGCCAGTGAAACACAACGACAACAGTTTTTAAACTCTGTAC
GCATACCCCCCACTGTGCAAATTGTTTGTGGTATAATGTCATTGTAATATATTGTATATT
ACTACATTACACACAAGCCAATATGTGCTGCTAAGTTGTATATATAGGTATACTATACAA

TGCTGGCCATTTTTGTATTTGCATTTGTGTTGCTGTTGTGCTTTTGTATTGTGCTACGTC
CGCTTTTGCTTTCCATATATGTGTATGCATTATTGCTGGTGCTGGTGCTGGTGCTATGGG
GTTTTATAGGTTCCCCCTTACGGGTATTTTTGGCATATCTTATATTTTTGTACCTACCTA
TGATGTGTATTCATTTACATGCACAGTATATTGTTTCCTAATACTTTGTATACTGTATGT
ATTTGTACATATTTATTGTTTTGTGCACAGTGTGGTACAATGGCATACTTTTTATATTTA
CTAATAAACCATTTTTTTATTTTTACTAGTGTGTACAATGAGACACAAACGATCTACAAG
GCGCAAACGTGCCTCTGCAACACAACTATATCAAACATGCAAGGCGGCAGGCACCTGTCC
CCCGGATGTTATTCCTAAAGTGGAAAGAACCACCATTGCAGATCAAATTCTTAAATTTGG
CAGTTTAGGTGTTTTTTTTGGAGGCCTTGGTATTGGTACTGGGTCTGGTACGGGTGGTAG
AACAGGTTATGTCCCCTTATCTACACGGCCTCCCACTGCATCTGCGCCTACATCTACAAT
ACGACCCCCTGTATCTGTTGATACTGTAGGGCCCCTAGACTCTTCTATTGTGTCTATGAT
AGAAGAAACAAGTTTTATTGAGTCAGGTGCACCTGCACCATCTATTCCAACAGCATCTGG
ATTTGATGTTGCAACTTCTGCAGACAATACTCCTGCAATTATTAATGTTTCATCTATTGG
TGAATCTTCTGTACAATCTGTTACCACCCATTTAAATCCCACTTTTACAGAACCTTCAGT
ACTCCGCCCATTTTCATCTTCTGAAGCCTCTGGTCATTTAATATTTTCTACACCCACTAT
TAGTACACATTCATATGAGGACATTCCTATGGATACCTTTATTGTTTCTACTACTAGTGA
CAATGTAACTAGTAGCACCCCTATTCCTAGGCCTCGCCCCACGGCACGCCTTGGTTTATA
TAGTAAAGGCACACAACAAGTTAAGGTTGTTGACCCTGCTTTTTTGACATCGCCTCGTAG
ATTAATTACATTTGATAATCCTGCATTTCAACCTACAGAACCTGATGAAACACTTTACTT
TCAACACCAAGACATTTCACCTGCACCTGACCCTGACTTTTTAGACATAGTTGCATTACA
TAGGCCTGCTTTAACATCTCGCAAAGGCACTATTAGATTTAGTAGGTTAGGTAGTAAGGC
TACAATGAAAACACGTAGTGGCAAACAAATTGGTGCTCGGGTGCATTATTATCAGGATTT
AAGTCCTATAGTACCTGCTGACTCCATAGAACTACAGCCATTATCTCGCCCTGTGTCTTC
TGCTTCCCATTCTATTAATGATGGTTTATATGATGTATATATGGATCCTGACACACCCTT
TCCACAGCCATCAATATCCTATTCATTACATAGTCCTCAGACCACCAATGTTACTGTCCC
CTTAAGTAGTGGTTTTGATTTTCCATTTTCCTCTACTGTACCATTACAGCCAGGTCCCGA
TATTGTGTCCCCTGTAGCCCCTACATACACTCCTTTTGTACCTGTTATACCTACATCTCC
CTTTAACAATGTACTTGTTTATGGTAGTGATTTTATTTTACACCCTAGTTATTTTTTACG
TCGCAGGCGTAAACGGTTTCCATATTTTTTTGCAGATGTCCGTGTGGCGGCCTAGTGAGG
CCACTGTGTACCTGCCTCCTGTGCCTGTATCTAAGGTTGTAAGCACTGATGAATATGTGT
CACGCACAAGCATTTACTATTACGCTGGTAGCTCCAGACTTTTAGCTGTAGGCCATCCTT
ACTTTTCCATTCCTAATCCCTCCAACACTAAAAAGGTGTTAGTGCCCAAGGTGTCAGGTT
TGCAGTATAGGGTATTTAGGGTTCGTTTACCAGATCCCAACAAGTTTGGATTTCCAGACA
CATCTTTTTATAACCCTGACACACAGCGCTTAGTATGGGCTTGTGTTGGTATTGAAATAG
GGCGAGGACAGCCTTTGGGTGTTGGTATTAGTGGACATCCATTACTTAATAAGTTTGATG
ATACTGAAACAAATAATAAATACCCTAGCCAGCCTGGTACTGATAATAGGGAATGCTTGT
CTATGGATGCTAAGCAAACCCAGCTATGTATTATTGGTTGTAAACCTCCTACAGGTGAAC
ACTGGGGTAAAGGTACCCCGTGTAGTGGTAACAGTAATGGGCCAGGTGCCTGTCCCCCTT
TGGAACTTATGAATACTGTTATTGAGGATGGGGACATGATTGATACTGGTTTTGGCTGCA
TGGATTTTAAATCTTTGCAGGCCAATAAAAGTGATGTTCCGTTAGATATATGTACCAGTA
TTTGTAAATATCCTGATTATCTTGGTATGGCTAGCGAGGCTTATGGGGACAGTTTATTTT
TCTTCCTGCGACGTGAGCAAATGTTTGTTAGGCATTTGTTTAACAGAGCAGGTAAATTAG
GGGAGGATGTACCTACAGATTTGTATTTTAAGGGATCTGCAAACACTTCTGCACTGCAAA
CCTCAGCTTTTTTTCCTACTCCCAGTGGCTCTATGGTTTCCTCAGAATCTCAATTATTTA
ATAAACCATATTGGTTACAACGCGCACAAGGTCATAACAATGGTATATGCTGGGGTAATC
AAATATTTGTTACTGTTGTAGACACTACACGTAGTACCAACATGACTTTATGTTCTGAGG
AAAAATCAGAGGCTACATACAAAAATGAAAACTTTAAGGAATACCTTAGACATGTGGAAG
AATATGATTTGCAGTTTATATTTCAGCTGTGCAAAATATCCCTTACTGCAAATGTTATGC
AATACATACACACCATGAATCCAGATATATTAGAGGACTGGCAATTTGGCCTTACACCAC
CTCCTTCAGGTAATTTACAGGACACATATAGATTTGTTACCTCGCAGGCTATTACCTGTC
AAAAAACATCCCCTCCAACAGCAAAGGAAGATCCTCTTAAAAAGTACAGTTTTTGGGAAA
TCAATTTAAAGGAAAAATTTTCTGCAGATTTAGATCAATTTCCCTTAGGTAGAAAATTTT
TATTGCAGGCAGGTTTTACAGCTAAACCTAAACTAAAACGTTCTTCACCTTCTTCTTCCT
CTTCCTCCTCTGCAAAACGTAAAAAGGTTAAAAGGTAATTGTATGACTGTTGTGTGTTTG
TATTGTTTGTATGTATGATTTGTATGTTTGTGTAATGTGTATGTTATGTGTATGCATGTT

CCCTGTGTCTGTCAATTTCCTGTTTTTGTTAATAAACATTACATACAATGTTAACTATAT
TGTTGTGTCGTGTGGTTGCACCCAATGAGTAAGGTACTGTCCCTATATCCCTGCCCATGC
CATGCCCTTGCCTATCCCTGTATAATTATATGTAGCATACTGTGTTTGGTATATTTATTA
CTTTTACTTATAACATTACAGTCTCCATTTTGGGTTACTACTCCATTTTATATCTGTAAC
CGGTTTCGGTTGCTGTTGGCATAGGGTTTTGGTATTCTAGGTTTCTTGCCAACATTGTTA
ATCTTTGTCTTCCTGCAGTGCAATGCCTTTACTTGCTGTTGCTCTCCCGCCTAAATTGAC
TCATGGTATTGCAGTGCAACACGCCCTTTTACATGTTTTAAACTGCTTTTAGGCACATAT
TTTTACCATGTTTTTATTGCTTAATTGCACTTTTGGCTTGCACAGCACTATTTTATTGGG
TTTATGTTACTATTGTAAGCCAACATACACCTTGTAAAATGATTCAAACCCTTACTCACA
CAGGTGTGTATGCACCGGTTTGGGTTAACATGTTCCAACCATATTGTTACATTATTATAA
A
>gi|1173493|gb|U21941.1|HPU21941_Human_papillomavirus_type_70,_complete_genome
CTTATAACATTTTACAATCATAATTTAAAAAAAGGGAGGCACCGAAAACGGTCACGACCG
AAAACGGTGTATATAAAACCATGCAAAAGTTGCTTGCCCATACGGAATGGCGCGATTTCC
CAATCCTGCAGAACGGCCATACAAATTGCCTGACCTGTGCACGGCGCTGGACACTACATT
GCACGACATTACAATAGACTGTGTCTATTGTAAAACACAGCTACAGCAAACAGAGGTATA
TGAATTTGCATTTAGTGATTTATTTATAGTATATAGAAACGGGGAGCCATATGCTGCATG
CCAAAAATGTATTAAATTTCATGCTAAAGTAAGGGAACTACGGCATTATTCGAACTCGGT
GTATGCAACAACTTTGGAAAGCATAACTAATACCAAGTTATATAATTTATCAATAAGGTG
CATGAGTTGCCTGAAACCATTGTGTCCAGCAGAAAAATTAAGGCATGTTAATACCAAAAG
AAGATTTCACCAAATAGCAGGAAGCTATACAGGACAGTGCCGACACTGCTGGACCAGCAA
CCGGGAGGACCGCAGACGTATACGAAGAGAAACACAAGTATAAATATAAATATGCATGGA
CCACGGCCGACATTGCAAGAGATTGTTTTAGATTTATATCCATACAATGAAATACAGCCG
GTCGACCTTGTATGTCACGAGCAATTAGAAGATTCAGACAATGAAACAGATGAACCCGAC
CATGTAGTTAATCACCAACAACAACTACTAGCCAGACGGGAAGAACCACAGCGTCACAAA
ATACAGTGTATGTGTTGTAAGTGTAATACTACACTGCACTTAGTAGTAGAAGCCTCACAA
GAGAACCTGCGATCTCTACTGCAGCTGTTTATGGAGACACTGTCATTTGTGTGTCCCTGG
TGTGCATCGGGAACCCAGTAACCTGCAATGGCCAATTGTGAAGGTACAGATGGGGATGGG
TCGGGATGTAACGGATGGTTCCTAGTACAGGCAATAGTAGATAAACAAACGGGCGACACT
GTGTCAGAGGACGAGGACGAAAATGCAACAGATACAGGTTCAGACTTGGCAGACTTTATT
GATGATACTACAGATATTTGTGTACAGGCAGAGCGCGAGACAGCACAGGTACTGTATAAT
ATGCAAGAGGCCCAAAGGGATGCACAATCAGTGCGTGCCTTAAAACGAAAGTATGGAGGG
AGCAATCTAAATAAAAGTCCTTGTGCAAAACCGCCAGGCGTACATAGGGAACAAAGGGTA
ACACTACAAGAGCTCCCGGTAAACATATGCAATAAACAGGCAAGAACAAACGTGTATTCA
GTACCAGACAGCGGCTATGGCAATATGGAAGTGGAAACAGCTGAAGTGGAGGTAACTGTA
GTAAATAATACAAATGGGGAAGAGGAAGGGGAAAATGGCGGGGAAAATGGCGGCAGCATA
CGGGAGGAGTGCAGTAGTGTAGACAGTGCTATTGATAGTGAGAATCAAGATCCACAGTCA
CCTACTGCACAGCTAAAAACAGTATTACAGGCTAATAACCAAAAAGCCATACTACTATCA
CAATTTAAACACACACATATGGATTAGCATTTAACGACCTGGTACGTACATTTAAAAGTGAT
AAAACCATATGTACTGACTGGGTAGCAGCAATATGTGGAGTAAATCCCACCATAGCAGAA
GGCTTTAAAACACTAATTCAGCCATATGCGTTATATACACATATACAGTGTTTGGATACC
AAATATGGAGTGTATATACTACTATTAATTAGATATAAATGTGGAAAAAACAGGATAACA
GTAGGCAAAGGATTAAGTAAATTATTACATGTGCCAGAAAGTTGTATGCTAATTGAACCA
CCTAAATTGCGTAGCCCTGTTGCAGCACTGTATTGGTATAGAACTGGAATGTCTAATATA
AGTGAAGTGTCAGGTACTACGCCAGAATGGATACAGCGATTAACAGTAATACAGCATGGA
ATAGATGACAGTGTATTTGACCTGTCTGATATGGTACAATGGGCATTTGATAATGATGTA
ACAGAAGACAGTGACATAGCATATGGATATGCATTATTAGCAGATAGTAATAGTAATGCT
GCAGCATTTTTAAAAAGTAACTGCCAGGCAAAATATGTACGCGACTGTGCTACAATGTGC
AGACATTATAAAAGGGCACAAAAAAAACAAATGACTATGGCGCAATGGATTAGGTTTAGA
TGTGATAAATGTGACGATGGGGGCGACTGGCGACCAATAGTGCAATTTCTAAGGTATCAA
GGGGTAGAATTTATAACCTTTTTGTGTGCATTTAAGGAGTTTTTAAAGGGCACCCCAAAG
AAAAATTGCATAGTAATACAGGGACCACCAAACACAGGCAAGTCATACTTTTGTATGAGT
TTAATGCACTTTTTACAAGGTACAGTAATTTCATATGTAAATTCCACTAGTCATTTTTGG
TTAGAGCCACTTGCAGATGCAAAGGTAGCAATGTTGGATGATGCCACAGGCACATGCTGG
TCATATTTCGATACGTATATGAGAAATGCATTAGATGGAAATCCTATAAGCCTTGACAGA

AAACATAGACATTTAATACAAATTAAGTGTCCACCCATATTAATAACATCCAATACCAAT
CCTGTAGAGGAAAATAGGTGGCCATACCTAACTAGCAGACTAACAGTGTTTACATTTCCT
AATGCATTCCCATTTGACCAAAACAGGAATCCAGTGTACACAATCAATAATAAAAACTGG
AAAAGTTTTTTCCAAAAGACTTGGTGCAAATTAGACTTGCAGCAGGACGAGGATGAAGGA
GACAATGATGGAAACACTATCCCAACGTTTAAATGCGTTACAGGAGAAAATACTAGAACA
TTATGAACAGGACAGTAAACTAATATATGATCAAATCAATTATTGGAAATATGTGCGACT
GGAAAATGCAATATTTTATGCAGCACGGGAACGTGGCATGCATACTATAGACCACCAGGT
GGTGCCACCAGGCACTACTTCAAAAGCAAAAGCATATCAAGCTATTGAACTGCAGATGGC
CCTAGAGAGCCTTGCACAAACTGACTTTAATAAAGAGGAGTGGACATTAAAGGACACAAG
TAATGAAATGTGGCAGACAAAGCCAAAACAATGTTTTAAAAAAAAAGGTGTTACAGTGGA
GGTGTGGTACGATGGAAACAAGGACAATTCTATGCATTATGTAGTGTGGGGAGCAATATA
TTATAAAACACATACAGACACGTGGTGTAAAACAGAAGGGTATGTGGATTACTGGGGTAT
ATATTATGTGCACGAGCAGCATAAGACATATTATGAAGTGTTTAAGCAGGATGCACAAAT
GTATGGGACTAGCGGAAAATGGGAAGTGCATTGTAATGGCAACATAATTCATTGTCCTGA
CTCTATGTACAGTACCAGTGACGACACAGTACCCACTACTGAGCTTACTGCAGAACTACA
ACACACCACCCCGGCCCATACCGCCGCAACAACCCCATGCACCAAAAAAACTAAGTCGGC
GCCGTCTTGCAAGTGTGGAGTCTCCAGACCCTCAGAAACAGACGGAGTGTTCGTGGACCT
TGTTACAAGTAAAGGCTGCAACAAACGACGGCACCAGTGTTGTGGTGACACTACACCTAT
AGTGCATTTAAAAGGTGACAAAAATGGTTTAAAGTGTCTTAGGTATCGATTGCGAAAATT
TAATTCATTGTATGAAAATATTTCATGTACTTGGCATTGGATAGGGGGCAAGGGAAGTAA
ACATACAGGTATACTAACTGTAACATATACTACTGAAGCACAACGCCAAAAATTTTTGGA
AACTGTTAGAATTCCACCTAGTGTACATGTATCTGTGGGATATATGACATTGTAACAGCA
CATGCTGTATGTATATTGTATACATATCAATGATTGCATTGGTGTTTTTGGTGTGGTTTG
CTGTATGCTTATATATATGTTGCAGTGTCCCGCTTTTGCCGTCTGTGCATTTGTGTGCGT
ATATGTGGCTACTTTTATTTGTGTTTATTGTTGTACATACCACACCATTGCAAATGTTTT
GTATATATTTACTATTTTTTATATTGCCTATGTGGTTTTTACACATCCTTTCAGTATATG
CTTAAGTTGTGTTGCTGCATAGTGTATTGTACATTACTTGTTTTTACATTTATATTGTAC
CAATAAACATGGTTTCTAGCCGTGCGTCCAGGCGTAAGCGTGCATCTGCAACAGACATAT
ATAAAACCTGCAAGCAATCAGGCACATGTCCGCCTGATGTTGTTAATAAGGTGGAGGGTA
CCACACTGGCTGATAGGTTTTTACAATGGGCTAGTTTAGGTATTTTTTTGGGTGGTTTGG
GAATCGGTACGGGTACTGGTACTGGGGGCCGCACAGGGTACATTCCTTTGGGGGGTAGGC
CTAGTACAGTTGTAGATGTTACCCCTGCACGTCCTCCTGTGGTTATAGAACCTGTAGGAC
CTACAGAACCTTCTATTGTTCAGTTGGTAGAGGAATCTAGTGTTGTTTCCTCTGGTACAC
CCATCCCTACTTTTACAGGCACATCTGGGTTTGAAATTACATCTTCTGCAACCACAACAC
CTGCTGTATTAGATATTACCCCTGCTTCTGGGTCTGTTCAAATTAGTACCACTAGTTATA
CCAATCCTGCATTTGCTGATCCATCGTTAATTGAGGTTCCACAAACAGGTGAGGTGTCAG
GCAATATATTTGTTACTACTCCAACATCTGGAACACATGGATATGAAGAAATTCCTATGC
AGGTTTTTGCCTCACATGGAACAGGCACAGAACCTATTAGTAGTACTCCTGTTCCTGGTG
TTAGTCGTGTGGCAGGCCCACGTTTATATAGTAGGGCCTATCATCAGGTTCGTGTTAATA
ATTTTGATTTTGTAACCCGCCCTTCATCTTTTGTAACATTTGACAATCCAGCTTTTGAGC
CTGGTGATACATCCTTAACATTTGAACCTGCTGACACAGCTCCTGATCCAGATTTTCTGG
ACATTGTTCGTTTACATCGGCCTGCTTTAACCTCACGACGCGGAACAGTACGCTTTAGTA
GGCTTGGTAAAAAGGCCACAATGTTTACCCGGCGGGGTACACAAATTGGGGCACAGGTTC
ATTATTATCATGATATTAGTAACATTACTGCAACAGAAGACATTGAGATGCAACCTTTAC
TTACCTCTGAATCTACAGATGGTTTATATGATATATATGCAGATGCAGATATAGATAATG
CAATGTTACATACTACTTCTCATACAGGTTCTACAGGACCTAGGTCCCATCTTTCATTTC
CTTCTATACCTTCTACAGTGTCTACAAAATATAGTAATACAACCATTCCATTTACTACTT
CTTGGGACATACCTGTAACCACTGGCCCTGACATAGTTTTACCTACTGCATCCCCCAATT
TGCCCTTTGTCCCTCCTACATCTATAGATACCACAGTTGCAATAGCCATTCAGGGCTCCA
ATTATTATTTATTGCCTTTATTATATTATTTTCTAAAGAAACGTAAACGTATTCCCTATT
TTTTTACAGATGGCTTTGTGGCGGTCTAGTGACAACACGGTGTATTTGCCACCCCCTTCT
GTGGCGAAGGTTGTCAATACAGATGATTATGTAACACGTACAGGCATATATTATTATGCT
GGAAGCTCTCGCTTATTAACAGTAGGGCATCCTTATTTTAAGGTACCTGTAAATGGTGGC
CGCAAGCAGGAAATACCTAAGGTGTCTGCATATCAGTATAGGGTATTTAGGGTATCCCTA
CCTGATCCTAATAAGTTTGGCCTTCCGGATCCTTCCCTTTATAATCCTGACACACAACGC

CTGGTATGGGCCTGTATAGGTGTGGAAATTGGTAGAGGCCAGCCATTGGGCGTTGGCGTT
AGTGGACATCCTTTATATAATAGATTGGATGATACTGAAAATTCTCATTTTTCCTCTGCT
GTTAGTACACAGGACAGTAGGGACAATGTGTCTGTGGACTATAAGCAAACACAGTTATGT
ATTATAGGCTGTGTTCCTGCTATGGGAGAGCACTGGGCTAAGGGCAAGGCCTGTAAGTCC
ACTCAACAGGGCGATTGTCCACCATTAGAATTAGTTAATACTGCAATTGAGGATGGCGAT
ATGATAGATACAGGCTATGGTGCCATGGACTTTCGTACATTGCAGGAAACCAAAAGTGAG
GTACCACTAGATATTTGCCAATCCGTGTGTAAATATCCTGATTATTTGCAGATGTCTGCT
GATGTATATGGGGACAGTATGTTTTTTTGTTTGCGCAAGGAACAGTTGTTTGCCAGGCAC
TTTTGGAATAGAGGTGGCATGGTGGGCGACACAATACCTTCAGAGTTATATATTAAAGGC
ACGGATATACGTGAGCGTCCTGGTACTCATGTATATTCCCCTTCCCCAAGTGGCTCTATG
GTCTCTTCTGATTCCCAGTTGTTTAATAAGCCCTATTGGTTGCATAAGGCCCAGGGACAC
AATAATGGCATTTGTTGGCATAACCAGTTGTTTATTACTGTGGTGGACACTACACGTAGT
ACTAATTTTACATTGTCTGCCTGCACCGAAACGGCCATACCTGCTGTATATAGCCCTACA
AAGTTTAAGGAATATACTAGGCATGTGGAGGAATATGATTTACAATTTATATTTCAATTG
TGTACTATCACATTAACTGCTGACGTTATGGCCTACATCCATACTATGAATCCTGCAATT
TTGGACAATTGGAATATAGGAGTTACCCCTCCACCATCTGCAAGCTTGGTGGACACGTAT
AGGTATTTACAATCAGCAGCTATAGCATGTCAAAAGGATGCTCCTACACCTGAAAAAAAG
GATCCCTATGACGATTTAAAATTTTGGAATGTTGATTTAAAGGAAAAGTTTAGTACAGAA
CTAGATCAGTTTCCTTTGGGGCGCAAATTTTTACTACAGGTAGGGGCTCGCAGACGTCCT
ACTATAGGCCCTCGCAAACGCCCTGCGTCAGCTAAATCGTCTTCCTCAGCCTCTAAACAC
AAACGGAAACGTGTGTCCAAGTAATGTATGTATGTTGTATGCTGTGTATTATTGTACTAT
TACATATTTGTGTTTTTATGTTGTATGCTTGCACACTGTTTACATATTTGTGTTTGTATG
TTGTATGCTTGCACACTGTACTGTATATGTTTGTCCTGGTACATATTTGTGGTTGTATGT
GTATATGTTGCGTGCTATGTGTATGTTTTAGAAGTATGTGTGTATGTATGTTTTTGTTAA
TAAAGTATGTATGGAGGTTTCATTTGTGGTTGCACCCTGTGACTAAGGTGTTGTCCCTGT
TTTACATATAATAGGAGTGTGATTACCAACATTTCCTACATAATTTTATGCCCTACCCTA
AGGTGTGTGTATACCATTTGTAGTTTATACATTTATATTTTATAGTGGGTTACCTGTATA
CAGCAACGGCCATTTTGTGTGAAACCGTTTTCGGTTGCATTTGGCTTTGTACCATCAGTT
ACCCTTATAAACCTTTTGTATCAGCAAAAACATGTCCTGTAACCTAAGTTCACCTACATA
CTTGGCACTACTAACAGTTTTAGTGGCGCACCTACACTTAGTCATCATCCTGTCCAGGTG
CACTACAACAATGCTTTGGCAACCTTATGCACCTCCACCCTGTCTAATAAAGTGCTTTTA
GGCATGTATTTTACCTGTTTTTACTTACCTAAGAGCATAGTTGGCCTGTATAACAGCTTT
TACATCCAAGAATGTGTCGTTTGGTGCAAGTTATATTTTGTGACTAATATTTTTACAGAC
CTGTGTGCAACCGAAATAGGTTGGCAGACATTCCTATACTTTTA
>gi|333074|gb|M12737.1|PPH8CG_Human_papillomavirus_type_8,_complete_genome
AACGGTAAGTTTCATCAGTGTACCAGGTGCGGTATGAAAATTTCTTAATCATAAGTTGTT
ATTGCCAACAACCATCGTCTATAGCATGTTTTTGCCTGTATCGTTTTCGATCACACCATA
TTGTATATTAAATAAATAAATAAATATATATATATATATTGTTACAATGCTGTGACTTGTGC
AATTTTCCTAAGCAAATGGACGGGCAGGACAAGGCTTCATATTTAGACACTAATAAGGAC
GAGCTACCCTCTACTATTAAAGAGTTAGCTGCGGCTTTAGGTATTCCATTGCAGGACTGT
TCAGTACCGTGCAACTTTTGTGGTAACTTTTTGGATTTCTTAGAACTGTGTGAGTTTGAC
AAAAAGAGACTGTGCCTAATTTGGAAAAATTACGTTGTTACTGCGTGTTGTCGTTGTTGT
TGTGTAGCAACCGCAACGTTTGAATTTAATGAATATTATGAGCAAACTGTGCTAGGCAGA
GATATTGAATTAGCTACAGGACGTTCAATTTTTGAGATAGACGTTAGGTGTCAAAACTGC
TTGTCATTTTTGGATATCATAGAGAAATTAGATTGCTGTGGGAGAGGCCGTCCCTTTCAT
AAAGTTAGAGGAGGCTGGAAAGGAGTTTGCAGGCTTTGTAAGCATTTGTATCATGATTGG
TAAAGAGGTCACTGTGCAAGATTTTGTGTTGAAGTTAAGTGAGATACAACCTGAAGTGTT
ACCAGTTGACCTGCTTTGTGAAGAGGAATTACCAAACGAACAGGAAACGGAGGAGGAGCT
AGACATCGAAAGAACTGTATTCAAAATTGTTGCACCGTGTGGCTGCAGCTGCTGTCAGGT
CAAGCTACGTCTTTTTGTCAACGCAACTGATTCGGGTATCAGGACCTTTCAAGAATTGCT
GTTCAGAGACCTACAGCTTCTGTGTCCTGAGTGCCGCGGTAACTGCAAACATGGCGGATC
ATAAAGGTAGTACATCTAAAGAAGGGTTAAGTGAGTGGTGTATTTTGGAAGCTGAATGTA
GTGATGTAGACAATGATTTTGAACAATTATTTGAGCGAGATACAGACTCAGATATTTCGG
ACTTATTAGATAATTGTGACCTGGATCAGGGAAATTCTCTGGAACTATTTCATCAACAGG
AGTGTGAGCAGAGCGAGGAGCAATTACAAAAACTAAAACGAAAGTATCTTAGTCCTAAAG

CTGTCGCGCAGCTCAGTCCGCGGCTCCAGTCAATATCACTGTCACCTCAGCAGAAATCCA
AGCGAAGGCTCTTTGCAGAGCAGGACAGCGGAGTCGAGCTAACTCTTAACAATGAAGCTG
AAGATGTTTCTCATGAGGTGGAGGTACCGGCTATAGACTCTCGGCCGGAAGATGAGGGAG
GATCAGGGGCTTTAGATATTGACTATACAGCATTGTTGCGGTCCAGCAACACAAAGGCCA
CATTAATGGCAAAATTTAAAGAGGCATTTGGGGATGGCTTTAATGAACTAACACGCCAAT
TTAAAAGTTACAAAACTTGCTGTAACTATTGGGTTGTAGCTGCTTATGCAGTGCATGATG
TATATGAAAGCTCAAAGCAGTTATTGCAACAGCATTGTGATTATATTTGGGTTAGAAGTA
TAGCTGCAATTACATTATATCTATTGTCTTTTAAGGCGGGAAAAAAATCGCGGTACTGTGC
ATAAACTAATGACCTCAATGCTAAATGTGCAAGAGCAGCAGATATTGTCTGAGCCTCCTA
AATTGAGACACACTGCTGCTGCTTTGTTTTGGTATAAAGGAGGAATGGGGACAGGAACAT
TCACGTATGGTTCATACCCTGATTGGATTGCACATCAAACAATTCTTGGCCATCAAAGTG
CTGAAGCAAGCACCTTTGATTTTTCTGTAATGGTACAATGGGCATTTGATAATAATCATT
TTGAGGAGGCCGACATTGCTTATGGATATGCAAAACTAGCCCCAGAAGATGCTAATGCTG
TAGCCTGGCTTGCTCATAATAGCCAAGCTAAATTTGTAAGAGAGTGTGCAGCAATGGTTC
GATTTTACAAAAGGGGGCAAATGAGAGAAATGACCATGTCTGAGTGGATATATACAAGGA
TCAATGAGGTTGAAGGAGAGGGGCATTGGTCTTCCATAGTAAAATTTGTAAGGTACCAGG
GTATAAATTTTATTACATTTTTAGCTGCCTTAAAAGATTTTTTGCATTCCGTACCTAAGC
GAAACTGTTTGTTAATCTATGGTCCACCTAATACTGGGAAATCTACATTTGCTATGTCAT
TAATACAGGTGCTGAAGGGTAGAGTATTATCATATGTAAATTCCAAAAGCCAATTTTGGT
TACAACCTCTTGGTGACTGTAAAATAGCACTGTTAGATGATGTGACAGACCCCTGCTGGC
TGTACATGGATACATTTCTGCGAAATGGATTAGATGGGCATGTTGTGTCATTAGATTGTA
AATATAAAGCACCCATGCAAATTAAATTTCCTCCCCTTTTGTTAACTTCCAATATTAACC
TGCATGAGGAGGCTAACTATAGATATTTACATAGCAGAGTCAGAGGATTTGAATTTCCAA
ATCCTTTTCCAATGAAACCAGACAATACACCTGAATTTGAATTAACTGACCAAAGCTGGA
AATCTTTTTTTGCAAGGCTTTGGACACAATTAGAGCTGAGTGATCAAGAAGACGAGGGCG
AACATGGAGAATCTCAGCGAGCGTTTCAATGTTCTGCAAGATCAGCTAATGAACATTTAT
GAAGCTGCAGAACAAACACTTGAGGCACAGATTGCGCATTGGCTGCTTTTGCGAAAAGAA
GCTGTGTTGCTATATTTCGCTAGGCAAAAAGGCATCACCAGGATTGGATATCAACCTGTG
CCGCCACTAGCAGTGTCTGAAGCAAAAGCCAAGCAGGCTATAGGAATAATGTTGCAGCTG
CAGTCACTACAGAAATCTGAGTTTGCAGATGAGCCTTGGACATTAGTGGACACAAGTATA
GAGACTTATAAGAATGCTCCAGAAAATCATTTTAAAAAAGGCGCCACACCTGTGGAGGTG
ATATATGATAAACAGCCTGACAATGCCAATGTATACACTATGTGGAAGCACATTTACTAC
ACTGATGCAGACGATAAGTGGCACAAAACCACCAGTGGGGTTAACCAGACTGGCATATAC
TATATGCAAGGCAGCTTCAGACATTATTATGTTGTGTTTGCTGATGATGCACGTAGATAT
AGTGCTACTGGAGAATGGGAAGTAAAAATTAATAAGGACACTGTGTTTGCTCCTGTTACC
AGCTCCACCCCCCCCGGATCACCACCAGGACAAGCAGACACAGACACCGCCGCCAAGACC
CCCACCACCTCCGCTGACTCCACGTCCAGACAGCAGCGGTCCCCTGCAAAACAGCCACAA
CAAACCGAAACCAAAGGACGAAGGTACGGGAGACGGCCGTCCAGCAGAACAAGACCGCAA
AAAGAGCAGAGGCGATCAAGGTCGCGACACCGCACCAGGTCTCGCTCCCGGTCGCTCTCC
CGGGTTAGGGCCGTTGGCTCCACCACCGTATCCAGGTCCAGGTCCTCGTCGCTCACCAAG
GCAGTTCGGCCCCGGTCCAGATCGCGATCCAGAGGACGGGCTACAGCCACCTCTAGGCGA
AGGGCAGGTCGAGGGTCACCCAGGCGACGGCGATCAACCTCAAGGTCACCCTCCACCAAC
ACCTTCAAACGGTCACAAAGGGGAGGAGGGAGACGGGGAAGAGGAAGGGGCAGTAGGGGG
AGACGGGAACGATCATCCTCCACCTCCCCCACCCCCACCAAACGTTCAAGAGGGGAGTCT
TCTAGGTTGCGTGGGGTCTCTCCTTCTGAAGTGGGAAGATCAGTTCAATCTGTTAGTGCA
AAACATACAGGGCGACTTGGAAGACTACTGGACGAAGCTATCGACCCCCCAGTAATATTG
GTTCGAGGGGAGGCAAACACATTAAAATGCTTTCGCAACAGAGCTAGAGTTAGATATAGA
GGACTGTTCAAATACTTTAGCACCACGTGGTCATGGGTGGCCGGCGATAGCACTGAGCGT
CTAGGCAGGTCCAGAATGCTCATTCTGTTTACTTCAGCTGGCCAAAGAAAGGACTTTGAT
GAGACTGTGAAATACCCGAAGGGAGTTGATACATCTTATGGTAACCTGGACAGTCTATAA
CATTACTAACGCTGCCTTGCTACTAACACACTAACATATTCCCATTTGCTTTTTACTATA
TTTTTTAATTGTATACTGCTATGGCGCGTGCTAGACGGGTCAAGAGAGACTCTGTCACAC
ACATTTATCAAACCTGCAAACAGGCAGGTACATGCCCCCCTGATGTTATTAATAAAGTTG
AGCAAACAACAGTTGCTGACAATATTTTAAAATATGGCAGTGCTGGTGTATTTTTTGGAG
GCCTTGGTATAGGTACCGGCCGTGGTACAGGGGGTGTTACTGGGTACACGCCATTAAGTG

AGGGGCCTGGTATCCGTGTTGGAAATACTCCCACAGTGGTACGGCCCTCACTTGTTCCAG
AAGCGGTAGGTCCTATGGACATACTGCCAATCGACACTATTGACCCTGTAGAGCCTTCAG
TCTCCTCTGTGGTGCCACTCACTGAATCTTCAGGAGCCGACCTACTTCCAGGGGAAGTTG
AGACAATTGCTGAAATTCACCCTGTACCTGAAGGCCCCACCATTGATTCACCTGTGGTGA
CGACAAGCAAAGGGTCAAGTGCCATTTTAGAGGTGGCTCCTGAGCCAACCCCTCCTACCC
GTGTCAGAGTTTCACGTACACAATATCACAACCCTTCTTTTCAAATCATAACTGATTCCA
CACCTACACAAGGAGAAAGTTCTCTAGCAGACCACATTTTAGTGACTTCTGGCTCTGGAG
GCCAGACTATAGGTAGTGACATAACAGATGTCATAGAACTGCAAGAATTTCCTAGTAGGT
ATTCATTTGAGATTGATGAACCTACACCACCACGGCAAAGTAGCACACCTATTGAGAGAC
CTCAAGTAGTTGGTAGACGTAGAGGTATTTCTTTAACAAATAGACGACTGATACAGCAGG
TGGCTGTTGAGGATCCTTTGTTTTTATCAAAACCTTCCAAATTAGTAAGGTTCAGTTTTG
ATAATCCTGTATTTGAAGAGGAGGTCACTAATATATTTGAGCAAGATGTGGACATGGTCG
AGGAGCCTCCAGATAGAGATTTTCTTGATGTTCGGCAGCTAGGGCGCCCACAATACTCTA
CTACACCAGCTGGCTATGTCAGAGTTAGCAGGTTAGGCACACGTGGCACCATTCGCACAC
GTTCTGGTGCACAAATTGGGTCGCAGGTACATTTTTACAGGGATTTAAGTTCAATCAATA
CAGAAGATCCTATAGAACTCCAACTATTAGGTCAACATTCTGGGGATTCTACAATAGTAC
AGGGTCCTGTGGAAAGCACTTTTGTAAATGTTGATATTTCTGAAAATCCTTTATCTGAAA
GTATTCAGGCATTTTCAGATGATTTACTACTGGATGAAACTGTAGAGGATTTTAGTGGGT
CCCAACTGGTAATAGGTAATAGAAGAAGTACAACTTCTTATACTGTTCCTAGATTTGAAA
CAACAAGAAGTGGCTCTTATTATGTGCAAGACACTAAAGGTTATTATGTGGCTTATCCTG
AAAGTCGCAATAATGAGGAAATAATTTACCCTACACCTGATCTTCCGGTGGTAATAATCC
ACACACATGATAACAGTGGTGACTTCTTCTTACATCCTAGCCTTCGCAGACGCAAACGTA
AAAGAAAATATTTGTGATTTTGCATTACAGATGGCAGTGTGGCAATCGGCTACCGGTAAG
GTTTATCTGCCTCCTTCAACACCAGTGGCCAGGGTGCAAAGCACGGATGAATACATTCAA
AGAACTAACATCTACTATCACGCCAATACTGACAGACTGCTCACTGTAGGACATCCATAT
TTCAATGTTTACAACAATAATGGTGACACATTACAGGTTCCCAAAGTATCGGGAAATCAA
CACAGGGTCTTTCGCTTAAAGTTACCAGATCCAAATAGGTTTGCACTGGCAGATATGTCT
GTATACAATCCAGACAAAGAAAGGTTGGTATGGGCTTGCAGAGGCTTAGAAATCAGTAGG
GGACAACCATTAGGTGTTGGGAGCACCGGCCATCCCTATTTTAATAAAGTGAAAGACACT
GAAAACAGCAATTCATACACCACAACATCTACAGATGACAGACAAAATACTTCCTTTGAT
CCTAAGCAAATACAAATGTTCATTGTGGGTTGCACACCCTGCATTGGTGAGCATTGGGAA
AAAGCCATTCCATGTGCAGAGGACCAACAGCAAGGTCTGTGCCCACCCATTGAACTAAAA
AATACAGTTATTGAAGATGGGGACATGGCAGATATTGGTTTTGGTAATATGAACTTTAAG
ACTTTACAACAGAACAGATCTGATGTAAGTCTCGACATAGTAAATGAGATATGCAAATAT
CCTGATTTTTTGAAAATGCAGAATGATGTGTATGGTGATGCCTGTTTCTTTTATGCACGA
AGGGAACAGTGCTATGCTAGACATTTTTTTGTGAGAGGGGGTAAGACAGGTGATGACATA
CCTGCTGCTCAAATTGATGATGGCATGATGAAAAATCAATATTACATTCCTGGTGGACAA
GATCAATCACAAAAGGATATAGGTAATGCTATGTATTTCCCAACCGTCAGTGGCTCACTT
GTTTCAAGTGATGCTCAATTGTTTAACAGGCCTTTCTGGCTGCAGCGTGCCCAGGGTCAT
AATAATGGCATTCTCTGGGCTAATCAAATGTTTGTCACTGTGGTAGACAACACGCGAAAC
ACCAATTTTAGTATTTCAGTTTACACTGAAAATGGGGAACTTAAGAACATCACAGACTAT
AAATCAACCCAGTTCAGAGAATATCTGAGACATGTAGAAGAATATGAAATTTCCCTCATA
TTACAATTGTGTAAGATACCACTAAAGGCTGATGTTTTAGCACAAATCAATGCCATGAAT
TCATCACTACTTGAGGAATGGCAACTGGGATTTGTACCTACCCCTGATACTCCAATTCAT
GACACCTACAGATATATTGATTCTCTTGCCACACGTTGTCCTGATAAAAGTCCCCCTAAG
GAAAAGCCTGATCCATATGCAAAGTTTAACTTCTGGAATGTGGACCTTACAGAACGACTT
TCCCTGGATTTGGATCAATATTCATTAGGCAGGAAGTTCTTGTTTCAAGCAGGTTTGCAA
CAGACGACCGTAAATGGTACAAAATCTATATCTAGGGGCTCCGTCAGGGGCACAAAACGA
AAACGGAAAAATTAGATTGTACCGTTTTCGGTACAAAACCATAAACTTTTACACAGTATT
CAAGGAATGTTTGTTTATTCTGACTCAGCATCACTCTACCTAAGAAACCGACCGCACCCG
GTACATAAAGGTGAGTAGTTGCCAAAACAGACTCAGTTTAGTGCCAGAATAGACCATGTT
CGTTCAAACATGCTCGGATTAGGTCGCCTGCCAAGGAAGTATTGATCTTGCCAATCTATT
TTGGCAGCGCTTTTGGCATCTCCAACGGACCGTT
>gi|1020290|gb|U31794.1|HPU31794_Human_papillomavirus_type_66,_complete_genome
GAAAGTTTCAATCATACTTTATTATATTGGGAGTAACCGAAATGGGTTTAGGACCGAAAA

CGGTACATATAAAAGGCAGCCTGTTGTGCCTGTAGATATCCATGGATTCCATATTCAGCA
ATACACAGGAACGTCCACGAAGCCTGCACCATCTGAGCGAGGTATTACAAATACCTTTAC
TTGATCTTAGATTATCATGTGTATACTGCAAAAAGGAACTTACAAGTTTAGAGCTATATA
GGTTTGCATGTATTGAGTTAAAACTAGTATATAGAAACAATTGGCCATATGCAGTATGTA
GGGTATGTTTATTGTTTTATAGTAAGGTTAGAAAATATAGGTACTATAAATATTCAGTGT
ATGGGGCAACATTAGAAAGTATAACTAAAAAACAGTTATCTGATTTATCAATAAGGTGCT
ACCGATGTCAATGTCCGTTAACACCGGAGGAAAAACAATTGCACTGTGAACATAAAAGAC
GATTTCATTATATAGCATATGCATGGACCGGGTCATGTTTGCAGTGTTGGAGACATACGA
GTAGACAAGCTACAGAATCTACAGTATAACCATGCATGGTAAAGTACCAACGTTGCAAGA
GGTTATATTAGAACTTGCACCGCAAACGGAAATTGACCTACAATGCAATGAGCAATTGGA
CAGCTCAGAGGATGAGGATGAGGATGAAATAGACCATTTGCTGGAGCGGCCACAGCAAGC
TAGACAAGCTGAACAACATAAGTGTTACCTAATTCACGTACCTTGTTGTAAGTGTGAGTT
GGTGGTGCAGTTGGACATTCAGAGTACCAAAGAGGAGCTACGTGTGGTACAACAGCTGCT
TATGGGTGCGTTAACAGTAACGTGCCCACTCTGCGCATCATCTAAATAACTGCAATGGCA
TCACCTGAAGGTACAGATGGGGAGGGGATGGGATGTTGTGGATGGTTTCAGGTAGAAGCA
ATTGTAGAAAGAAAAACGGGGGATACAATATCAGATGATGAAAGCGAGGAGGAGAATGAA
ACAGATACAGATGTAGATGGATTTATAGACAATACACTTATAAACAATACACAGGAAGAC
AGGGAGACAGCTCAACAATTATTGCAAGTACAAACAGCACATGCAGATGCACAGACGTTG
CAAAAACTAAAACGAAAGTATATAGGTAGTCCCTTAAGTGATATTAGTAATCAGCAAACT
GTGTACCGAGAGGAAGTAAAACGAAGGCTAATATTATCAGAAGACAGCGGGTATGGCAAT
ACATTGGAAACATTGGAAACATCACAACAGGTAGAATACGAAAAGGGAAATGGGTGCGGG
AGCTCACAAAATGGAGGCTCGCAAAACAGTAATTGTAGTGAGCACTCGGTATCAAATATG
GATATAGATACAAATATGGAAACACCAACACACCAATTGCAGGAACTATTTAAAAGTAGT
AACGTACAAGGAAGATTACATTTTAAATTTAAAGAAGTGTATGGAGTGCCATATACAGAG
TTGGTGCGAACATTTAAAAGCGATAGTACATGTTGTAACGATTGGATATGTGCAATATTT
GGCGTTAATGAAACATTAGCAGAGGCGTTAAAAACTATACTAAAACCACAATGTGTGTAC
TATCATATGCAATGCTTAACATGTTCATGGGGAGTAATTGTAATGATGCTAATTAGATAT
ATATGTGGAAAAAATAGAAAAACAATTACAAAATCGCTAAGCTCAATTTTAAATGTACCA
CAAGAGCAAATGTTAATTCAACCACCAAAACTACGAAGTCCTGCTGTAGCATTATATTTT
TATAAAACAGCAATGTCAAATATTAGTGAGGTGTATGGGGAAACACCAGAATGGATACAA
AGACAGACACAATTGCAACACAGTTTACAAGACAATCAATTTGAATTGTCTAAAATGGTA
CAGTGGGCATTTGATAATGAAGTAACAGATGATAGCCAAATTGCCTTTTTATATGCACAA
CTAGCAGACATAGATAGCAATGCACAAGCATTTTTAAAAAGTAATATGCAAGCAAAATAT
GTAAAGGATTGTGGAATAATGTGTAGACATTACAAAAGGGCACAGCAACAGCAAATGAAT
ATGTGCCAGTGGATAAAGCATATATGTAGTAAAGTAGATGAAGGGGGTGATTGGAAACCC
ATTGTGCAATTTTTACGATATCAAGGGGTCGACTTCATTTCATTTTTAAGTTATTTTAAA
TTATTTTTACAAGGAACGCCTAAACATAATTGTTTGGTACTGTGTGGACCACCAAATACA
GGTAAATCATGTTTTGCTATGAGCCTTATAAATTTTTTCCAAGGGTCAGTCATTTCATTT
GTTAATTCACAAAGCCACTTTTGGTTACAGCCACTAGACAATGCCAAATTAGGTTTGCTG
GATGATGCAACAGATACGTGTTGGAGATACATAGATGATTATCTAAGAAATTTATTAGAT
GGGAATCCCATAAGTTTAGATAGGAAACATAAACAATTAGTACAAATAAAATGTCCTCCA
GTTATTATTACAACTAATGTAAATCCTATGCAAGATGCAAAATTAAGATATTTACACAGT
AGAATTTCAGTGTTTAAGTTTGAAAATCCATTTCCATTAGATAACAATGGTAATCCTGTG
TATGAATTAAGTAATGTAAATTGGAAATGTTTTTTTGAAAGGACATGGTCCAGATTAAAT
TTGGATAACGACGAGGACAAAGAAAACAATGGAGACTCTATCCCAACGTTTAGATGCGTG
CCAGAACAAAATACTAGACTGTTATGAAAAAGATAGTAAATGCATTATAGATCACATAGA
CTATTGGAAAGCTGTACGACATGAATATGTATTATATTATAAAGCAAGAGAAAATGACAT
TAATGTACTAAACCACCAGATGGTGCCCTCTTTACAAGTGTGTAAAGCAAAAGCATGTAG
TGCAATAGAATTACAAATAGCACTGGAAGCAATAAGTAACACAATATATAAAAATGAAGA
GTGGACATTACGTGATACATGTGATGAACTGTGGCGCACGGAGCCTAAAAACTGTTTTAA
AAAAGAAGGACAACACATAGAAGTGTGGTTTGATGGTAACAAAAATAATTGTATGGAATA
TGTGGTGTGGAAATTTATATATTATAATGGAGAGTGTGGGTGGTGTAAAGTGTCATCAGG
GGTGGATTACAGAGGCATATATTATATGCATGATGGCCACAAAACATATTACACAGACTT
TGAACAGGAGGCCAAAAAATATGGGTGTACAAACATATGGGAAGTACATATGGAAACCGA
GAGTATTTACTGTCCTGACTCTGTGTCTAGTACCTGTAGATACAACGTACCCCCTGTTGA

GACTGTTAACGAATACAACAACCACAGGACCACCACCACCGCCTCCACCTTTGTGGGCGC
CCAAGACGCCGCGGTATCCCACAGACCAGGAAAACGACCCAGAGCAAGTGAATCAGAACC
TGACTCCTCCAGAGAGTCCTACGCACACTGTGTCACAACAGACACAGACATCAGTAACAA
CGCCAACAGTAGAAGTCCACGTATCAACACACAAAGCCACTGTGGTGATAAAACTACGCC
TGTAATCCATTTAAAAGGTGAAGCTAATAGATTAAAGTGTTGTAGATACAGATTTCAAAA
ATATAAAACATTATTTACAGATGTAACAACAACATATCATTGGACAAGTACAGATAATAA
AGACAGTAGTATTATTACAATATTATATAAAGATGAAACACAACGGGACACCTTTTTAAA
TGTTGTAAAAATACCACCTAGTGTACAGGTTATTTGGGACAAATGAGTTGTCCATAAAG
TGTTGTATATATTGTATATACATATGTGTTATTGTAACACTGGTACAGGTGAAGTGTAAT
TGCCATACATTGCTGCTAAGCATATATATTGCACCCATTAATTGTATTTGGTATATTATG
TGTTATTGTAACACTGGGAAAGGTAACGTGTAATCGCCATATATTGCAACCATTGATTTT
TGTGTAATTTGTGTGTTTGCGCTTTGCTTTTGTGTTTGTCTGTGTGTGTGCCATTTTGTC
CCGCTTTTGCTATCTGCATCTTTATTTACAAGTTGTCTTATACTAATTATTTTATTTTGG
TTTGTTGTGGCTACATCATTTTTTGATACTTTTATACTGTTTTTACTATTTTTTTATATA
CCTACACTGTGTATATATTGCCATGCTTTGTGGTTAATAAACCATTTGTAACAGTAGTAA
TTTTTGCTACTATGGTTGCCCACCGTGCCACACGACGCAAACGCGCATCTGCCACACAAT
TATATAAAACATGCAAATTATCTGGTACATGTCCTGAGGATGTTATTAATAAGGTGGAGC
AAAAAACATGGGCTGATAGGATTTTACAATGGGGAAGTTTATTTACATATTTTGGGGGGC
TTGGCATTGGTACTGGGTCTGGGTCGGGTGGTCGGGCGGGCTATGTTCCCTTAGGCTCTA
GGCCTTCTACTATAGTTGATGTCACTCCTGCACGACCACCTATTGTGGTGGAGTCAGTTG
GGCCTACAGATCCTTCTATTGTTACACTGGTAGAAGAATCTAGTGTTATTAACTCAGGGG
CTGGTGTTCCCAATTTTACTGGGTCAGGGGGATTTGAAGTTACATCCTCTTCCACAACCA
CACCTGCTGTGTTGGATATTACACCCACATCTAGTACTGTACATGTAAGTAGTACTACTA
TAACAAACCCACTATATATTGATCCTCCAGTAATTGAGGCTCCACAAACTGGAGAGGTAT
CTGGTAATATTTTGATTAGCACTCCTACATCTGGAATACATAGCTATGAGGAAATACCTA
TGCAAACATTTGCTATACACGGTACTGGCAACGAACCTATTAGTAGTACCCCTATTCCAG
GTTTTAGACGCCTTGCTGCTCCCAGGTTATATAGTAGGGCTTTTCAGCAGGTTAGGGTCA
CTGACCCAGCATTTTTGGACAACCCCACAACATTAATATCTGCTGATAATCCTGTTTTTG
AAGGTGCTGACACAACGTTGACCTTTTCTCCCTCGGGTGTGGCTCCTGATCCTGATTTTA
TGGATATAGTTGCATTACATAGGCCTGCATTTACTACACGTAGAACAGGTGTGCGTTTTA
GTAGGCTAGGCAAAAAGGCTACCATGCAAACACGTAGGGGTACGCAAATAGGTGCTCGTG
TGCATTATTATTATGATATAAGTCCTATTGCACAGGCTGATGAAATTGAAATGCAGCCAT
TATTGTCTACAGACAATTCATTTGATGGCCTATATGATATTTATGCAAATATTGATGATG
AGGCACCCATTTCATTTCGTCAGTCTGGTGCTACACCTTCTGCACAATTACCTATTAAAC
CTTCTACATTATCCTTTGCTAGTAACACAGCTAATGTTACTGCCCCTTTGGGAAATGTTT
GGGAAACACCATTTTATTCAGGTCCTGATATAGTTTTACCTACAGGCCCCAGTACTTGGC
CCTTCGTACCTCAGTCTCCTTCTGATGTTACACATGATGTATATATACAGGGAGCTACAT
TTGCACTATGGCCTGTATATTTTTTTAAACGTAGGCGCCGTAAACGTATTCCCTATTTTT
TTGCAGATGGCGATGTGGCGGCCTAGTGACAATAAGGTGTACCTACCTCCAACACCTGTT
TCAAAGGTTGTGGCAACGGATACATATGTAAAACGTACCAGTATATTTTATCATGCAGGT
AGCTCTAGGTTGCTTGCTGTTGGCCATCCTTATTACTCTGTTTCCAAATCTGGTACCAAA
ACAAACATCCCTAAAGTTAGTGCATATCAGTATAGAGTGTTTAGGGTACGGTTGCCTGAT
CCTAATAAGTTTGGCCTTCCTGATCCATCTTTCTATAATCCTGACCAGGAACGTTTGGTA
TGGGCCTGTGTAGGTTTGGAGGTAGGCCGAGGTCAACCTTTAGGTGCTGGGTTAAGTGGT
CATCCATTATTTAATAGGCTGGATGACACTGAGGTCTCTAATTTAGCAGGTAATAATGTT
ATAGAAGATAGCCGGGACAATATATCTGTTGATTGTAAACAAACCCAGTTATGTATTGTG
GGATGTGCACCAGCATTAGGGGAACATTGGACTAAGGGCGCGGTGTGTAAGTCTACACCA
GGTAATACAGGGGATTGTCCACCTCTTGCATTAGTTAATACCCCGATAGAGGACGGTGAC
ATGGTGGACACCGGGTTTGGTGCAATGGACTTTAAGCTATTACAGGAATCAAAGGCTGAG
GTGCCATTGGACATTGTACAATCTACATGTAAATATCCTGATTATTTAAAAATGTCTGCA
GATGCCTATGGGGATTCTATGTGGTTTTACTTACGCAGGGAACAATTGTTTGCCAGACAT
TACTTTAATAGGGCAGGTAATGTTGGGGAAGCCATTCCTACAGATTTGTATTGGAAGGGT
GGCAATGGCAGGGACCCTCCTCCCAGTTCTGTATATGTTGCTACTCCTAGTGGGTCCATG
ATTACCTCTGAGGCCCAATTATTTAATAAACCTTATTGGTTGCAACGTGCACAGGGCCAT
AATAATGGCATATGCTGGGGTAATCAGGTATTTGTTACTGTTGTGGATACTACCAGAAGC

ACCAACATGACTATTAATGCAGCTAAAAGCACATTAACTAAATATGATGCCCGTGAAATC
AATCAATACCTTCGCCATGTGGAGGAATATGAACTACAGTTTGTGTTTCAACTTTGTAAA
ATAACCTTAACTGCAGAAGTTATGGCATATTTGCATAATATGAATAATACTTTATTAGAC
GATTGGAATATTGGCTTATCCCCACCAGTTGCAACTAGCTTAGAGGATAAATATAGGTAT
ATTAAAAGCACAGCTATTACATGTCAGAGGGAACAGCCCCCTGCAGAAAAGCAGGATCCC
CTGGCTAAATATAAGTTTTGGGAAGTTAATTTACAGGACAGCTTTTCTGCAGACCTGGAT
CAGTTTCCTTTGGGTAGAAAATTTTTAATGCAACTAGGCCCTAGACCCCCTAGACCCAAG
GCTAGTGTATCTGCCTCTAAAAGGCGGGCGGCTCCTACCTCTTCCTCTTCTTCACCAGCT
AAACGTAAAAAACGATAGTTGTGTGTTGTGTGTTGTATGTATTGTATGGTTGTGCTTGTA
CTGTATGTTTTTGTGTATGTTTATGTATTTTATAATTGTGTATGTGCTATGTGTATGTAT
GACTGTATGTATGTGTAATGTTTTGTGTGTATGTAATAAACATGCATGGTTACTTTTACG
CGTGGTTGCATAAACTAAGGTGCGGTAGTATCCTTGGGCAGTGTGTGTCAGGTTAGGTGG
TGTTCCTTACTGTTTAATGTTATATTAAATAGGTTGTTTGTATGCACTATAGTAACACAC
CAAACTCCATTTTAGTGCTGTACGCCATTTTATGCATGCAACCGAATTCGGTTGCCTAGC
CTTTTGTCCTTATTTAAACCCAAAACGACTTTTCAGCAAAACAGTTAATCCTTTGGCATA
TTGCCGTTTCCTGTTGTATGATTCAGGTATGTACACTGCCTTACCCTGTATTACTCACCT
GTATTTCTGTGCCAACTATGCTTTTATCTGCATACTTTGGCGCTGTTGGGCATATGTTTT
TATGCAGGTGTTTGCAATATATTTTGTTGGCGTGTAGCCCTTATTGTATAAGCCAAGTAT
CTGTCTTGCAAATATGTAACCATATACTTACTCATTTTACAAAACCGTTTACGGTCGTGC
TAAAACAGGTTTCTTTTAATTGTT
>gi|1020266|gb|U31791.1|HPU31791_Human_papillomavirus_type_55,_complete_genome
TAATAATAATGTAAGCTTTAGAAAAGAGGAGGGACCGAATTCGGTTCCAACCGAAAACGG
TTATATAAAAACCAGCCCAAAAATTAAGCAAGCGGGGCATAATGGAAAGTGCAAATGCCT
CCACGGCTGCACAAAGTATAGACCAGTTGTGCAAGGAGTGCAACATTCCTATGCACCATT
TGCAAATTTTATGCGTGTTTTGCAGAAAAACCTTGACCACTGCAGAGGTTTATTCATTCG
CATATAAACAGTTATATGTAGTGTTTCGAGGTAACTTTCCATTTGCAGCCTGTGCCATTT
GTTTAGAACTACAAGGTAAGGTCAATCAATTTAGGCATTTTGACTTCGCGGGATATGCAT
TAACAGTGGAAGAAGAGACAAAGCAGTCAATTTTGGACGTGTTCATACGCTGTTATTTGT
GCCACAAACCATTGTGCCACGTGGAAAAGGTGCGCCACATATTGGAGAAGGCGCGATTTA
TTAAATTACAGAATACCTGGAAGGGTCGCTGCTTCCATTGCTGGACATCATGCATGGAAA
CTATCCTACCTTGAAGGAAATTGTTTTAGAGCTGGATCCTCCTGACCCTGTAGGCCTACA
TTGCAATGAGCAATTAGACAGCTCAGAGGATGAGGTGGATGAACTAGCCACGCAGGCCAC
GCAAGACGTTACACAGCCTTACCAAATAGTAACCACCTGTGGTACATGTAACCGGAACGT
TCGGCTGGTTGTGCAGTGCACAGGAACAGACATCTGTCAACTACATACGCTTCTGCTGGG
TTCACTGGAAATACTGTGTCCTGTGTGTGCGCCCAAAAACTAACAACGATGGCAGACAAT
ACAGGTACAGAGGGGACGGGGTGCTCAGGATGGTTCTTAGTAGAGGCTATAGTGGAGAAA
ACAACTGGGCAACAAATATCTGAGGATGAGGATGAGGCAGTGGACGATAGTGGGTTGGAT
ATGGTGGACTTTATAGATGACAGGCCTATTACACACAATTCCATGGAAGCACAGGCATTG
TTAAACGAGCAGGAGGCGGATGCTCATTATGCGGCTGTGCAGGACCTAAAACGAAAGTAT
TTAGGTAGTCCATATGTGAGTCCTTTAAGCAATATTAAGGAGGCAGTGGAGTGTGACATT
AGCCCACGGTTGGATGCCATAAAATTAAGTAGACAACCCAAAAAGGTAAAGCGGCGGCTG
TTTGAAAGACCAGAATTAACGGACAGTGGATATGGCAATATTGATGTGGAAGCTGAAACG
CAGGTAGAGAGAAATGGCGAACCGGAAGATTGTGGGGGAGGTGGACAAGGAAGGGACACA
GAGGAGGGGGTGGAACAGGTGGAAACGGAAGTGCAGACACATAGCGACACACAGCTGCAC
ACCGAGACCACGCGGGTAGTAGAACTGTTAAAATGTAAGAATATACGGGCTACACTGCTT
GGTAAGTTTAAGGACTGTTATGGGTTATCATATACAGATTTAATTAGACAATTTAAAAGT
GACAAGACAACATGTGGGGACTGGGTGATTGCAGCATTTGGGGTGCACCATAGTGTATCG
GAGGCGTTTCAAAATTTAATACAACCAGTAACAACATATAGCCACATACAATGGCTTACA
AATGCATGGGGAATGGTATTACTGGCATTATTACGGTTTAAGGTAAATAAAAACAGATGT
ACAGTGGCACGCATGATGGCAACACGTTTAAATATACCTGAGGATCATATGTTAATTGAA
CCTCCTAAAATACAAAGCGGTGTTGCAGCATTATATTGGTTTAGAAGTAGTATATCCAAT
GCCAGTATAGTTACTGGAGAAACGCCTGAATGGATAACAAGGCAAACCATTGTAGAACAT
GGGCTTGGTGACAACCAATTCAAATTAACAGAAATGGTGCAATGGGCATATGATAATGAC
TTTTGTGAGGAAAGTGAAATTGCATTTGAATATGCACAACGTGCAGATATAGATGCAAAT
GCAAGAGCATTTTTAAATAGTAATTGTCAGGCAAAATATGTAAAAGATTGTGCTACAATG

TGCAAGCACTATAAAACTGCAGAAATGAAAAAAATGAGTATGAAACAGTGGATAAAATTT
AGGAGCAGTAAATATGAAGAAACAGGCAATTGGAAACCAATAGTTCAATTTTTAAGATAT
CAAAATATTGAATTCATTCCATTTTTAACTAAATTGAAAATGTGGCTGCATGGGACACCT
AAAAAAAACTGTATTGCAATTGTAGGACCACCAGACACAGGGAAATCGTGTTTCTGTATG
AGTTTAATTAAATTTTTAGGAGGCACTGTAATTAGTTATGTAAACTCTAGTAGTCACTTT
TGGCTACAGCCCCTATGCAATGCAAAAGTAGCATTGTTAGACGATGTAACACAATCGTGC
TGGGTATATATGGATACATACATGAGAAACCTATTAGATGGAAACCCTATGACTATTGAC
AGAAAACACAAATCATTAGCATTAATAAAATGTCCGCCATTAATAGTAACATCTAACATA
GACATTACTAAAGAAGATAAATATAAATATTTATGTAGCAGGGTAACATTATTTACATTT
CCAAATCCATTCCCCTTTGACAGAAATGGGAATGCACTATATGACCTGTGTGAATCAAAC
TGGAAATGTTTCTTTGCAAGATTATCAACAAGTCTAGATATACAAACATCTGAGGACGAG
GACGATGGAGACAATAGCCAAGCATTTAGATGTGTGCCAGGAACAGTTGTTAGAACTGTA
TGAAGAAAATAGTAATAACCTTACAAAACATATACAACATTGGAAATGCATAAGATATGA
ATGTGTGTTATTACACAAAGCAAAGCAAATGGGCCTAAACCACATTGGAATGCAAGTGGT
GCCAGCATTAACAGTGTCACAGACAAAGGGACACCAGGCCATTGAAATGCAAATGACATT
AGAAACACTATTAAACTCTGACTATGGTATGGAACCATGGACATTGCAAGACACAAGTCG
TGAAATGTGGTTAACAGCACCCAAATACTGTTTTAAAAAACAGGGACAAACTGTGGAAGT
AAAATATGACTGCAATGCAGACAACATTATGGAGTATGTATCGTGGAAATATATTTATGT
GCATGACACAGACAAATGGGTAAAAGTGACAGGACACATAGACTATAAAGGGTTGTATTA
TGTACATGGCGGGCATAAAACCTATTATACCAATTTTGAAAAAGAGGCTAAAAAATATGG
GAACTCATTACAATGGGAGGTCTGTATTGGTAGCAGTGTCATATGTTCTCCTGCATCTAT
ATCTAGTACTGTGCAAGACGTATCCATTGCTGGGCCTGCTTCACACACCTCCTCCTCCAC
CACCACCACCCTTGCACAGGCCTCACCTGCACTGCCCACCTGCACCTCCGAGGAACGCGT
GGACCCGCCGCCATGTAAACGACCCCGAGGACCCACCACAAACACCAACAACGCCAGGGA
CACCGTCAGTGTCCGACACAGCGACTCCGTGGACAGTACAAACAACAACATCTACCCTAA
CAGTTACAACAGTAACAAAGGACGGGACAACAATTTTTGTACAGCTACGCCTGTAGTTCA
ATTACAAGGTGATCCTAATTGTTTAAAGTGTTTAAGATATAGATTACATGCAAAGCATAA
AACATTGTTTGTAGCAGCATCGTCCACATGGCGCTGGACATGTTCAGATACTTCCAGTAA
ACATGCACTGGTAACATTAACATATGTTAATGAAGAGCAACGTGAGCAGTTTTTAAACAC
TGTACGGTTACCTCCAACAGTTACATATAAAGTTGGATATATGTCTTTACAATTGTTATA
ATGCTTATTGTATATATTTAATTGTATAAATTGTACATGGAACATATACCTATAGATGGT
ACTATAGGGGCAACCAGCACATCATTACTGCCAGTTGTACTTGCCCTGTTTGTATGTTTT
GTTAGCATTGTATTAATTATTTACATTTCTGATTTTATAGTGTACACATCTATATTGGTA
CTAACCTTACTGCTATATATGTTACTTTGGCTTTTACTAACCACTGCCCTGCAATTTTAT
GTACTAACACTGTGTGTCTGCTTTTTTCCTGCGTGGTATCTACATTTCTATATTGTACAT
ACACAAGAATAACTATTACAATGCTAACATGTACCTTTGATGATGGTGATACATGGTTGT
TATTATGGTTTATATTAACCTTAATTGTTACCATTGTGGCATTGTTATTAATGCACTTTA
AATCTGTACCATGTGTTACATGCAGTAAATAAGTGTTTGTAAACGTGGTGTGGTTGGTAT
AATTATGGCACACAGCAGGGCACGTAGACGTAAACGTGCCTCAGCTACCCAATTATATCA
AACCTGTAAGGCTGCAGGCACCTGTCCATCTGATATTATTCCTAAAGTTGAACATAACAC
TATTGCTGATCAAATATTAAAGTGGGGCAGTTTGGGTGTTTTTTTTGGAGGACTGGGAAT
TGGTACAGGCTCTGGCACAGGTGGTCGAACAGGATATATTCCTTTGCAATCTACCCCACG
TCCTGAGATTCCCTCTGGCCCTACCACAAGGCCACCTATACTTGTTGATACAGTTGCACC
TGGTGACCCCTCTATTGTTTCTTTGGTTGAAGAATCTGCTATTATAAATTCAGGAGCTCC
GGAATTGGTCCCTCCGTCCCATGGTGGCTTTGAGATTACTACATCAGAATCCACCACACC
AGCCATATTAGATGTGTCTGTTACAACACATACTACCTCTACAAGTGTATTTAGAAACCC
TAGCTTTGCAGACCCATCTGTTGTACAGTCCCAGCCAGCCGTTGAAGCTGGTGGACACAT
ACTTATCTCTACCTCAACCATATCGTCCCACCCTGTAGAGGAAATACCATTAGATACATT
TATAGTATCTTCTTCAGATAGTAATCCTGCATCTAGCACTCCCATTCCAGCATCTGGTGC
ACGACCACGTATTGGCCTATACAGTAAGGCTTTACACCAGGTACAGGTAACGGATCCCGC
CTTTTTGTCCTCTCCCCAACGCCTTATAACATTTGATAATCCTGCATATGAAGGGGAAGA
TGTTAGTTTGGAGTTTGCACACAATACTATACATCAGCCTCCAGATGATGCGTTTATGGA
TATTATACGATTGCACAGACCAGCTATACAATCTCGGCGTGGTCGTGTGCGGTTTAGTAG
AATAGGACAACGGGGGTCTATGTACACACGTAGTGGTAAACATATTGGTGGCAGGATACA
TTTCTATCAAGACATATCTCCTATATCTGCTGCTGCAGAAGAAATAGAACTGCACCCTCT

TGTGGCCACTGCACACGATACTAGCCTGTTTGATATTTATGCAGAACCTGACCCTGACTT
TACAGAAGAACCTGTTCCATTGTCTTTTTCCACCTCCACGCCCTTTCAGCGGTCTTCTGT
GTCAGCCACCCCATGGGGCAATACTACTGTCCCTCTTTCATTACCTGGTGACATGTTTGT
ACAGCCTGGTCCTGACATAATATTTCCTACTGCATCCACTACAACTCCCTATAGTCCTGT
CACTCCTGCTTTACCTACAGGTCCTGTTTTTATAAGTGGTGCTACATTTTATTTATATCC
TGCATGGTATTTTGCACGCAAACGCCGTAAACGTGTTTCCTTGTTTTTTGCAGATGTGGC
GGCCTAGTGAAAACCAGGTATATGTGCCTCCTCCCGCCCCAGTATCCAAAGTAATAACTA
CGGATGCCTATGTCAAACGCACCAACATAGTTTACCATGCTAGCAGTTCTAGACTTCTTG
CTGTAGGCAACCCTTATTTTGCCATACGACCAGCAAACAAGACACTTGTGCCTAAAGTTT
CAGGATTTCAATATAGGGTTTTTAAGGTGGTATTGCCAGACCCTAATAAATTTGCATTAC
CTGACACATCTATATATGACCCTACTACGCAACGCCTGGTATGGGCCTGCATTGGGTTGG
AAGTGGGTAGAGGCCAGCCCCTGGGTGTTGGTATTAGTGGGCATCCATTATTAAACAAAT
TGGATGATGTGGAAAATTCTGCTAGTTATGCAGCCAGTCCAGGTCAGGACAACAGGGTAA
ATGTGGCCATGGACTATAAACAAACACAATTATGTTTAGTTGGCTGTGCACCCCCATTGG
GGGAGCATTGGGGTAAGGGCAAGCCATGTAATAATGGTAGTGTAAATAGCGGAGACTGCC
CTCCCCTGGAATTAATTACTAGTGTTATCGAAGATGGTGATATGGTGGACACTGGTTTTG
GAGCCATGAATTTCGCTGAATTGCAACCAAATAAATCTGATGTTCCCTTAGATATATGCA
CTGCTACATGCAAATATCCTGACTATTTACAAATGGCTGCTGATCCATATGGGGACAGAT
TGTTTTTTTACTTACGAAAGGAACAAATGTTTGCAAGACATTTTTTTAATAGGGCGGGAA
CGGTTGGAGAGGACATTCCACAGGATTTGGTTTTTAAAGGTGCTACTAAAAGTACAGTTC
CTAATGCCATATACTTTAATACACCCAGTGGGTCTCTTGTATCTTCTGAAACCCAATTAT
TTAATAAGCCTTTTTGGTTGCAAAGGGCGCAGGGCCACAATAATGGTATTTGTTGGGGGA
ATCAGTTATTTGTTACTGTTGTAGATACTACACGTAGTACAAACATGACAATATGTGCTG
CTACAACTCAGTCTCCATCTACAACATATAATAGTACAGAATATAAACAATACATGCGAC
ATGTTGAGGAGTTTGACTTACAGTTTATGTTTCAATTATGTAGTATTACCTTAACTGCTG
AGGTAATGGCCTATTTACATACCATGAATCCTGGTATTTTGGAACAGTGGAACTTTGGGT
TGTCGCCACCCCCAAATGGTACCTTAGAAGACAAATACAGATATGTGCAGTCACAGGCCA
TTACATGTCAAAAGCCTCCCCCTGAAAAGGCAAAGCAGGACCCCTATGCAAAATTAAGTT
TTTGGGAGGTAGATCTCAGAGAAAAGTTTTCTAGTGAGTTAGATCAATATCCCCTTGGTA
GGAAATTTTTATTGCAAACGGGTGTGCAGGCCCGGTCCTCTGTTCGTGTGGGTAGAAAAC
GTCCTGCATCTGCAGCCACTTCATCCTCCAGTAAACCCAAACGGTCTAGGAAGAAGTAGT
ATGTGTTATTGTTTTGTTTGTGTATGTGTGTCATATGTTATTGTTTGTTATATGTGTTGT
ATTGTATATATGTTGTATGTCTATGTTGTGTAATGTTGTATGTATGCATGTGTGTGTTGT
ATTTAATAAACTTAATCTGTGTGTCCTGTTCCACCCCATGAGTAAGTGTTGTAGTATTGT
GTGTTATGTCTGGTGTATACAATATACAACATATGTACAGCCATGTTGTTTTATAAAACA
CATTTTTAAACATATTCCTCCATTTTGGGTGCAACCGTTTTCGGTTGTTACTCATCACCC
ACATCCTGTAACCAATTTGTTGTAGCAAGCAAAATATTTAATCTTATCTATAGTTGCCAA
AACTTTACTATGTTACTAAGTACACACCTGGCGCACAGCTAGGCGCGGTTTGGCAACTAC
ACAATACATTCCTAATCTATATAACACTGCTGTCTACTTGTTTAACAATCGTGTGCTGGT
AGCCAACTTTTAAAAGCATTTTTGGCTACTAACACTACATTTTTGTACAGTTACTGTTAG
TTTTATAAAATGAGTAACCTAAGGTCACACACCTGCAACCGGTATCGGTTGAAACACACC
CTGTACATTTCCTTATTATAGT
>gi|1020242|gb|U31788.1|HPU31788_Human_papillomavirus_type_44,_complete_genome
TTAATAATAATCTAACCTTTACAAAAAAGAGGAGGAACCGAATTCGGTTCCAACCGAAAA
CGGTTATATAAAAACCAGCCCAAAAATTAAGCAAGCGGGGCATAATGGAAAGTGCAAATG
CCTCCACGTCTGCACAAAGTATAGACCAGTTGTGCAAGGAGTGCAACATTCCTATGCACA
ATCTGCAAATTTTATGCGTGTTTTGCAGAAAAACGTTAAGTACTGCAGAGGTTTATTCAT
TCGCATATAAACAGTTATATGTAGTGTACCGAGGAAACTTTCCATTTGCAGCCTGTGCCA
TTTGTTTAGAACTACAAGGTAAGGTCAATCAATTTAGGCATTTTAACTACGCGGGATATG
CAGTAACAGTGGAAGAAGAAACAAATAAGTCAATTCTGGACGTGCTGATACGCTGCTATT
TGTGCCACAAACCATTGTGCCACGTGGAAAAGGTGCGCCACATATTGGACAAGGCGCGAT
TCATTAAATTACAAGATACCTGGAAGGGTCGCTGCTTCCATTGTTGGACATCATGCATGG
AAACTATACTACCTTAAAGGAAATTGTTTTACAGCTGGAACCTCCTGACCCTGTAGGCCT
ACATTGCAATGAGCAATTAGACAGCTCAGAAGATGAGGTGGATGAACTAGCCACGCAAGC
CACGCAAGACGTTACACAGCCTTACCAAATAGTAACCACCTGTGGTACATGTAGTCGGAA

GGTTCGGCTGGTTGTGCAGTGCACAGGAACAGACATCCATCACCTACATACGCTTCTGCT
GGGTTCACTGGATATATTGTGTCCTGTGTGTGCGCCCAAAACCTAACAACGATGGCTGAC
AATACAGGTACAGAGGGAACGGGATGCTCAGGATGGTTTCTAGTAGAGGCTATAGTGGAG
AACACAACCGGGCAACAAATATCAGAGGATGAGGATGAGGCAGTGGAGGATAGTGGGTTG
GATATGGTGGACTTTATAGATGACAGGCCTATTACACACAATTCCATGGAAGCACAGGCA
TTGTTAAACGAGCAGGAGGCGGATGCTCATTATGCGGCTGTGCAGGACCTAAAACGAAAG
TATTTAGGTAGTCCATATGTTAGTCCTTTAAGTAATATTGAGCAGGCAGTGGAGTGTGAC
ATTAGCCCACGGCTGGACGCTATAACATTAAGTAGACAACCAAAAAAAGTAAAGCGACGG
CTGTTTGACAGACCAGAATTAACGGACAGTGGATATGGCAATACTGAAGTGGAAGCTGAA
ACGCAGGTAGAGAGAAATGGCGAACCGGAAGATTGTGGGGGAGGTGGACAAGGAAGGGAC
ACAGAGGGGGTGGAACAGGTGGAAACGGAAGTGCAGACACATAGCAACACACAACAGCAC
ACCGGGACCACGCGGGTACTAGAACTATTGAAATGTAAGAATATAAGGGCTACACTGCTT
GGTAAGTTTAAGGATTGCTATGGGTTATCATATACAGATTTAATTAGACAATTTAAAAGT
GACAAGACAACATGTGGGGACTGGGTAATTGCAGCCTTTGGGGTGCACCATAGTGTGTCA
GAGGCGTTTCAAAATTTAATACAGCCAGTAACAACATATAGCCACATACAATGGCTTACA
AATGCATGGGGAATGGTCCTACTGGCATTAGTAAGGTTTAAGGTAAATAAAAACAGATGT
ACAGTGGCACGTATGATGGCAACCCGTTTAAATATACCTGAGGACCACATGTTAATTGAA
CCTCCTAAAATACAAAGCGGTGTTGCAGCGTTATATTGGTTTAGAAGTGGTATATCCAAT
GCCAGTATAGTAACTGGAGAAACACCGGAATGGATAACAAGGCAAACCATTGTAGAACAT
GGGCTTGCAGACAACCAATTTAAATTAGCAGACATGGTTCAATGGGCATATGATAATGAC
TTTTGTGAGGAAAGTGAAATTGCATTTGAATATGCACAACGTGCAGATATAGATGCCAAT
GCCAGAGCATTCCTAAATAGTAATTGTCAGGCAAAATATGTAAAAGACTGTGCCACAATG
TGCAAGCACTATAAAACTGCAGAAATGAAAAAAATGAATATGAAACAGTGGATAAAATTT
AGGAGCAGTAAATTTGAAGACACAGGAAATTGGAAACCAATAGTGCAATTTTTAAGACAC
CAAAACATAGAATTTATTCCGTTTTTAACTAAATTAAAGATGTGGCTGCATGGTACACCA
AAAAAAAACTGTATTGCAATAGTGGGCCCACCAGACACAGGTAAATCGTGTTTTTGTATG
AGTTTAATTAAATTCTTAGGAGGCACTGTAATTAGTTATGTAAACTCCAGCAGTCACTTT
TGGCTACAGCCCTTATGCAATGCAAAAGTAGCATTATTAGATGATGTAACCCAATCCTGC
TGGGTATATATGGATACATATATGAGAAACCTATTAGATGGAAACCCTATGACCATTGAC
AGAAAACACAAATCATTAGCATTAATAAAATGTCCGCCTTTAATAGTAACATCAAACATA
GACATTACTAAAGAAGAGAAATACAAATATTTATGTAGCAGGGTAACATTATTTACATTT
CCAAATCCATTCCCCTTTGACAGAAATGGGAATGCACTATATGACCTGTGTGAAACAAAC
TGGAAATGTTTCTTTGCAAGATTATCATCAAGTCTAGATATACAAACATCAGAGGACGAG
GACGATGGAGACAATAGCCAAGCATTTAGATGTGTGCCAGGAACAGTTGTTAGAACTGTA
TGAAGAAAATAGTAATAAACTTACAAAACATATACAACATTGGAAATGTATACGATATGA
ATGTGTGTTACTACACAAAGCTAAGCAAATGGGCCTGAACCACATTGGAATGCAAGTGGT
GCCAGCATTAGCAGTGTCACAGACAAAGGGACACCAGGCAATTGAAATGCAAATGACATT
AGAAACATTACTAAACTCTGACTATGGTACGGAACCATGGACATTGCAAGAGACAAGTCG
GGAAATGTGGTTAACACCACCCAAATATTGCTTTAAAAAGCAGGGACAAACTGTGGAAGT
AAAATTTGACTGCAATGCAGACAATGCAATGGAGTATGTATGGTGGAAAGTCATTTATGT
ATTTGACACAGACAAATGGGTAAAAGTGACAGGACACATAGACTATAAAGGGTTGTATTA
TGTACATGGTGGGCATAAAACCTATTATACAAATTTTGAAAAGGAGGCCGAAAAATATGG
GAACTCTTTACAATGGGAGGTATGTATTGGCAGCAGTATCATATGTTCTCCTGCATCTAT
ATCTAGTACTGTGCAAGACGTATCCATTGCTGGGCCTGCTTCACACTCCTCCTCCTCCAC
CACCACCACCCTTGCACAGGCCTCATCCACACTGCCCATTGGCACCGCCGAGGACTGCGT
GGACGCGCCGCCATGTAAACGACCCCGAGGACCCCCCACAAACACCAACAACGCCAGAAA
CACCGTCTGTGTCAGAAACAGCGACTCCGTGGACAGTACAAACAACAACATCCTCCCTAA
CAGTTACAACAGTAACAAAGGACGGGACAACAATTATTGTACAGCTACGCCTGTAGTTCA
ATTACAAGGTGATGCTAATTGTTTAAAGTGTTTAAGATATAGATTACATGCAAAGTATAA
AACATTGTTTGTAGCAGCATCGTCCACATGGCGCTGGACATGTTCAGATACATCCAGTAA
TGCACTGGTAACATTAACATATGTTGATGAACAGCAACGCCAGCAGTTTTTAAACACTGT
AAAGTTACCACCAAAAGTTACATATAAAGTTGGATATATGTCTTTACAATTGTTATAATG
TGTGTTGTATATATCTAATTGTATATATTGTACATGGAACACATACCTATAGATGCTACT
ATAGGGGCAACCAGCACATCATTACTGCCAGTTGTAATTGCCCTGTTTGTATGCTTTGTT
AGCATTGTATTAATTATTTGTATTTCTGATTTTATAGTGTACACATCTATATTGGTACTA

ACCTTACTGCTATATCTGTTACTTTGGCTTTTACTAACCTCTGCCCTGCAATTTTATTTA
CTAACACTGTGTGTCTGCTTTTTTCCTGCGTGGTATATACATTTCCATATTGTACATACA
CAACAAGAATAACTATTACAATGCTAACATGTACGTTTGATGATGGTGATACATGGCTGT
TATTGTGGTTGTTATTAACATTAATTGTTACCATTATAGCATTGTTATTAATGCATTTAA
AAACTGTACAATGCGTTACATGCAGTAAATAAGTATTTGTATATTTGGTGTGTATTGTAT
AAATATGGCACACAGTAGGGCACGTAGACGTAAACGTGCATCTGCTACCCAATTATATCA
AACATGTAAGGCTGCAGGCACCTGTCCCTCTGATATTATTCCTAAGGTGGAACATAACAC
TATTGCAGATCAGATATTAAAGTGGGGCAGTTTGGGGGTTTTTTTGGGGGACTGGGGAT
TGGTACAGGCTCTGGCACAGGCGGTAGAACAGGGTATATACCTTTACAATCCACCCCGCG
TCCTGACATTCCCTCTGTACCTACCGCAAGGCCACCTATACTTGTTGATACTGTTGCACC
TGGGGACCCGTCCATTGTATCCTTGGTTGAAGAATCTGCTATTATAAATTCGGGGGCCCC
GGAATTGGTCCCTCCTTCCCATGCAGGATTTGAAATCACTACATCTGAATCTACCACACC
AGCTATATTAGATGTGTCTGTCACCACACATACTACCTCTACAAGTGTATTTAAAAACCC
TAGCTTTGCTGACCCATCTGTTGTACAGTCGCAGCCTGCTGTTGAAGCTGGTGGCCACAT
ACTTATCTCTACCTCATCTATATCGTCCCACCCTGTAGAAGAAATACCTTTGGATACATT
TATAGTATCTTCCTCTGATAGTAATCCTGCATCTAGCACTCCCATTCCAGCATCTGGTGC
ACGGCCGCGTATTGGCCTATACAGTAAGGCTTTGCACCAGGTACAGGTAACGGATCCTGC
CTTTTTGTCCTCTCCCCAGCGCCTAATAACATTTGATAATCCTGCATATGAAGGGGAGGA
TGTTACTTTACACTTTGCACACAATACTATACATGAACCTCCAGATGATGCGTTTATGGA
TATTATACGATTGCACAGACCGGCTATACAGTCCAGGCGTGGTCGTGTGCGGTTTAGTAG
AATTGGACAACGAGGGTCTATGTACACACGTAGTGGCAAACATATTGGTGGCAGGATACA
TTTCTATCAAGACATTTCTCCTATATCTGCTGCTGCAGAAGAAATAGAACTGCACCCCCT
TGTGGCCACTGCACAGGATAGTGGCCTGTTTGATATTTATGCAGAACCTGACCCTGATGT
TACAGAAGAACCTGTTTCATTGTCTTTTTCTACCTCCACACCCTTTCAGCGGTCTTCTGT
GTCAGCCACCCCCATGGGGCAATACTACTGTCCCTCTTTCATTACCTGCTGACATGTTTGT
ACAGCCTGGTCCTGACATAATCTTTCCTACTGCATCCACTACAACTCCCTATAGTCCTGT
CACTCCTGCTTTACCTACAGGTCCTGTTTTTATAAGTGGTGCTGCATTTTATTTATATCC
TACATGGTATTTTGCACGCAAACGCCGTAAACGTGTTTCCTTGTTTTTTGCAGATGTGGC
GGCCTAGTGAAAACCAGGTATATGTGCCTCCTCCCGCCCCAGTATCCAAAGTAATACCTA
CGGATGCCTATGTCAAACGCACCAACATATATTACCATGCTAGCAGTTCTAGACTTCTTG
CTGTGGGCAACCCTTATTTTGCCATACGACCAGCAAACAAGACACTTGTGCCTAAGGTTT
CGGGATTTCAATATAGGGTTTTTAAGATGGTATTGCCAGACCCTAATAAATTTGCCTTAC
CTGACACATCTATATATGACCCCACTACGCAACGCCTGGTATGGGCCTGCATCGGGCTGG
AGGTAGGTAGAGGACAGCCCTTAGGTGTTGGTATTAGTGGGCATCCATTATTAAATAAAT
TGGATGATGTAGAAAATTCAGCTAGTTATGCAGCCGGTCCGGGTCAGGATAACAGGGTAA
ATGTGGCCATGGACTATAAACAAACACAATTATGTTTGGTTGGCTGTGCACCCCCGTTAG
GTGAGCATTGGGGTAAAGGCAAGCAGTGTAATAATGTTAGTGTTAAGGATGGGGACTGCC
CTCCCTTGGAATTAATTACTAGTGTAATTGAGGATGGTGATATGGTGGACACTGGTTTTG
GAGCCATGAATTTTGCTGAATTGCAGCCAAATAAATCTGATGTTCCATTAGATATATGCA
CTGCTACATGTAAATATCCTGACTATTTACAAATGGCTGCAGATCCATATGGGGACAGAT
TGTTTTTTTACTTACGAAAGGAACAGATGTTTGCCAGACATTTTTTTAATAGGGCTGGAA
CAGTTGGTGAGGACGTTTCCCAGGATCTGGTTATTAAAAGTGCTAGTAAAAATACTGTTC
CTAATGCTATATACTTTAATACACCCAGTGGTTCTCTTGTATCTTCTGAAACCCAATTAT
TTAATAAGCCTTTTTGGTTGCAAAAGGCGCAGGGCCACAATAATGGTATTTGTTGGGGAA
ATCAGTTATTTGTTACTGTTGTAGATACTACCCGTAGTACAAACATGACAATATGTGCTG
CCACTACACAGTCCCCTCCGTCTACATATACTAGTGAACAATATAAGCAATACATGCGAC
ATGTTGAGGAGTTTGACTTACAATTTATGTTTCAATTATGTAGTATTACCTTAACGGCGG
AGGTAATGGCCTATCTTCATACTATGAATGCTGGTATTTTAGAACAGTGGAACTTTGGGT
TGTCGCCGCCCCCAAATGGTACCTTAGAGGACAAATACAGATATGTGCAGTCCCAGGCCA
TTACATGTCAAAAGCCACCCCCTGAAAAGGCAAAGCAGGACCCCTATGCAAAATTAAGTT
TTTGGGAGGTGGATCTTAGAGAAAAGTTTTCTAGTGAGTTGGATCAATATCCCCTTGGTA
GAAAATTTTTATTACAAACGGGTGTGCAGGCCCGTTCCTCTGTTCGTGTGGGTAGGAAAC
GTCCTGCGTCTGCAGCCACTTCCTCCAGTAAACAAAAACGGTCTAGGAAGAAGTAGTATG
TGTTATTGTTTTGTTTGTATGTGTGTCATATGTTATTGTGTTATATATGTGTTGTGTTGT
ATATATGTTGTATGTGTATGTTGTGTAATGTTGTCTGTAATGGAATGCATGTGTGTGTTG

TACATAATAAACTTAATCTGTGTGTCCTGTTCCACCCCATGAGTAAGTGTTGTAGTGTTG
TGTTCTATGTTTGGTATATATAATATATAACATATGTACAGCCATGTTAGTTTTTAAACA
TATTCCTCCATTTTGGGTGCAACCGTTTTCGGTTGTTCATTTTGGGTGCAACCGTTTTCG
GTTGTTACTCATTACCCACATCCTGTACCCAATTTGTTATAGCAAGCAAAATATTTAATC
ATCTCTGCCAGAACTTTATTATGTTACTAAGTACACACCTGGCGCACAGCTAGGCGCGGT
TTGGCAACTACACAATACATTCCTAATCTCTATACTACTGCTGTCTCGTTTGTGAACAAT
AGTGCGCTGGTAGCCAACTTTTTAAAAGCATTTTTGGCTACTAGCACTGCATTTTTGTAC
AGTTACTGTTGGTTTTATAAAATGAGTAACCTAAGGTCACACACCTGCGACCGGTATCGG
TTGACACACACCCTGTACACTTCCTTATCATAG
>gi|1020234|gb|U31787.1|HPU31787_Human_papillomavirus_type_38,_complete_genome
CATCTTTGGCAGACGAAGTGCACCGATAACGGTAAGACTTTTCTCTTTTAACCGTAGGCG
TTGGTTTATTATTCCTGGCAACAATGGTGGTTAACAACCATCACACGTAATCGGTACAAG
CAACCGCTTGTGGTAGTAAAATGAATTAAAAAAAAAAACAAGGATATATTTAAGGGGCCT
GTAAGCTTGGGATGTATTCATGGAACTACCAAAACCTCAAACTGTGCAGCAGCTCAGTGA
TAAGTTAACAGTTCCTGTAGAGGATCTGTTATTACCCTGTAGATTCTGCAACAGTTTCCT
CACGTACATTGAATTACGTGAGTTTGATTACAAGAACTTACAGTTAATCTGGACTCAAGA
GGATTTTGTTTTTGCATGTTGTAGCAGTTGTGCTTATGCTTCTGCTCAATATGAATGTCA
GCAGTTTTATGAATTAACTGTCTTTGGCCGTGAAATTGAACAGGTGGAGCAACAGACAAT
AGGCCTTATTGTTATAAGGTGTCAGTATTGTTTAAAGTGTCTTGATTTGATAGAAAAATT
AGATATCTGTTGCTCTCATCAAGCATTTCACAAGGTTAGAGGCAATTGGAAAGGAAGGTG
CAGGCATTGCAAAGCAATAGAATGATTGGGAAACAAGCTACTCTTCGTGATATAGTTCTT
GAAGAGCTTGTCCAGCCCATTGACCTGCATTGCCACGAGGAGTTGCCTGATCTTCCAGAG
GATATTGAAGCATCAGTGGTAGAGGAGGAGCCAGCATACACCCCATACAAAATCATAGTT
CTTTGTGGGGGTTGTGAAGTAAGGCTAAAACTATACGTGTGGGCCACCGACGCTGGGATT
CGGAATCTGCAAGATTGTTTGCTGGGCGACGTAAGGCTTCTGTGTCCCACCTGTCGAGAA
GACATTCGCAATGGCGGACGATAAAGGTACTGATCCTAAAGAAGGCTGTAGTGATTTTAT
ATATTTAGAAGCTGAATGCTCTGACATTAGTGACTTAGATAATGATTTGGAAACATTATT
GGAAGAAGGTGCGGGATCCGATATTTCTGACTTAATAAATGATGAGGTTGTTGAGCAGGG
AAATTCCCGCGAATTATTATGTCAACAAGAGAGAGAGGAGAGCGAACTGCAGGTTCAATA
TCTAAAACGAAAGTGTTTCAGTCCGAAAGCTGTTCAGGAGCTTAGTCCTCGTCTGCAGTC
TATGAATATATCTTCAGAGCATAAATCTAAAAGGAGATTATTTGTGGAGCAAGACAGTGG
ACTGGAGCTATCTCTAAATGAAGCTGAAGATTCTACTCAAGAGTTGGAGGTACCGGCGAG
CGCTCCAGCGCCGGCAGCAGAGGGTGATATAGGGCTGGGTACTGTAAGAGATCTTTTAAG
GAGCAGTAACAGCAGAGCAACACTGTTAAGCAAATTTAAAGACTCGTTTGGGGTCAGCTT
TACAGAACTGACAAGACAATATAAAAGCAATAAAACGTGTTGCCACCATTGGGTCTTGGC
AGTGTATGCTGCTAAGGATGACTTGATAGATGCGTCCAAACAATTGTTACAGCAGCATTG
TTTTTATATATGGCTTCAATCATTTTGTCCCATGTCACTTTATTTATGTTGCTTTAATGT
TGGTAAAAGTAGAGACACTGTTGTAAGACTAATAGCTACATTATTACAGGTGCATGAAAA
TCATATATTGTCAGAGCCACCAAAAAATAGAAGTATTCCAGCAGCGTTATTTTGGTATAA
AGGAAGTTTGAATAGTAATGTGTTTTGTTTTGGTGAAGCTCCTGATTGGATTCTATCACA
AACAATGATACAGCATCAAACTGCTGACACTTTGCAGTTTGACTTGTCTCGAATGATTCA
ATGGGCCTATGATAATGATCATATAGACGAAAGCATTATAGCTTATCAATATGCTAAATT
AGCAGATATTGATAGTAATGCTAAAGCTTTTTTAGCTCATAACAGCCAAGTTAAATATGT
TAAAGAGTGTGCTTTAATGGTAAGATATTATAAAAGAGGAGAGATGAAAGAAATGTCTAT
TTCTGCTTGGATTCATCACTGCATATCTAAAGTTGAAGGAGAAGGCAATTGGCAGCATAT
TGTTAGGTTTATTAGATACCAAAATTTGAATTTTATTATGTTTCTAGATAAGTTTCGGAC
CTTTTTAAAAAATCTGCCAAAAAAAAATTGTTTATTAATATATGGTCCTCCTGACACAGG
AAAGTCTATGTTTGCAATGTCACTTATTAAACTATTGAAAGGTAGTGTAGTATCTTTTGC
TAATTCGAAAAGTCAATTTTGGTTACAGCCACTAGCTGATGGGAAAATTGGTTTATTGGA
TGATGCAACTGATGTGTGTTGGCAGTATATAGATTCTTTTCTTAGAAATGGTTTAGATGG
TAATTTAGTGTCGTTAGATATAAAACATAAAGCACCTTGTCAAATGAAATTTCCTCCATT
AATTATTACTTCCAATATTAATTTATTAAAAGAGGAACGATACAGATTTTTACACAGTAG
AGTAACACAAATTGATTTTCCAAATAAGTTTCCCTTTGACTCAGATAATAAGCCTTTGTT
TGAACTTACTGATCAAAGCTGGGCATCTTTCTTTAAAAGGCTGTGGACACAATTAGAGCT
CAGTGATCAAGAAGACGAGGGAGACAATGGAAACTCTCAGCGCACGTTTCACTGTACTGC

AAGAGAAGTTAATGGACATATATGAATCAGGTGTAGAGGACCTGGATACACAAATTCAGC
ATTGGCAGCTTTTAAGACAAGAGCAAATTATTTATCACTATGCAAGGAGACATGGTGTTA
CTCGATTGGGCTATCAACCTGTACCTTCTTTGGCAAGTTCAGAAGCCAAAGCAAAAGATG
CCATTTCTATGGTCCTTTTACTTGAAAGCCTGAAAAAATCCAAATATGCAGATGAACAAT
GGACATTAGCTCAAACTAGCCTGGAGGCTGTTCGCAGCCCTCCTGCAGACTGTTTTAAAA
AAGGACCTAAAAATATTGAAGTTGTATTTGATGGTGACCCTGAAAATCTTATGTCATATA
CTGTGTGGACATATATATATTACCTGACAGATGAGGACATATGGGAAAAAGTGGAAGGCC
ATGTGGATTATACAGGAGCCTATTATTATGAGGGCAAATTAAAGGTGTATTATTTAAAAT
TTGAAAATGATGCTAAACGATATGGTGTCACAGGATTATGGGAAGTACATGTTAATAAAG
ACACTGTGTTTACCCCCGTTACCAGTTCTACGCCGCCAGTTGGAGACTCCACCGACTCCG
CATCCAGGGCGGCACTCCCCGAGCCTTCCACCTCCGTGTCCCCCGAACGGCCACCATCCC
AAACAGCACGGCGATACGGGAGAAAAGCATCTAGCCCTTCAACCACCTCCCGCAGGCAAA
GGAAAGGACAAAGAGAAACCACAGGCACCCAAAGGAGAAGAAAAAGCAGATCAAGGTCCC
GAAGCACCAACAGGGGAGGGAGGGACACCCGGCGATCCTCCTCCAGAGGATCCTCAGTCT
CCCCCACCAGGGGAAGGAGAAGGGGAGGAGGGGACAGCAGAAGGCGGGGGCCGGTCACCC
GCTCGAGATCAAGATCCCTCTCACGAGCCTCTTCTGCAGGGGGTGGCATATCGCCTGACA
AAGTGGGAACGGCAGTTCGATCAGTTGGTAGACAAAGTGGTGGAAGACTTACGCGGCTAC
TGGCAGACGCTGCAGACCCCCCAGTAATATTGTTACGTGGAGATGCCAATACCTTAAAAT
GCTATCGCTATCGATTTAGAAAAAAACATGCTGGTGGCTTTCGCTTTGTTAGCACAACAT
GGTCATGGATAGGAGATGCATCAAATGATCGCATAGGGCGCTCACGAATGCTTCTAGCTT
TTTATTCAGAATCACAAAGAGAAAAGTTTATACAGACTATGAAATTACCTACAGGTGTAG
AGTGGTCATTAGGACAATTTGATGATTTATAGAATAATCTATAAGAGATATTTTTTATAT
ATTATGTAACCTTTTTTACTAACAATACTGCTTTGCTACTAACACTATGGTTCGAGCACG
TAGAACCAAACGTGCATCTGTTACTGATATATACAGGGGCTGCAAGGCTTCTAATACTTG
CCCTCCTGATGTAATCAATAAAGTGGAACAATCAACAATAGCAGATAAGATTTTAAAATA
TGGTAGTGCTGCTGTCTTTTTTGGTGGGCTGGGTATTAGCACTGGTCGTGGTACAGGCGG
TGCTACAGGCTATGTGCCTTTGGGGCAAGGACCTGGAGTGCGAGTGGGTGGCGCCCCCAC
AGTGGTCCGCCCCGGGGTGATACCTGAAGTAATTGGACCAACCGAACTGATACCTATTGA
CTCAGTCACACCAATTGACCCTACAGCACCTTCAATTGTGTCATTAACTGACAGTAGTGC
TGTTGACCTTTTACCTGGAGAGGTTGAAACTATTGCAGAAGTTCATCCTGGCCCTATAGA
CCCTATAGAAATTGATACCCCTGTTGTGAGTGGAGGCCGCAATACCAATGCTATATTGGA
AGTGGCTGACCCTCATCCACCCACTAGAGCTACTGTTAGCAGAACTCAATATAATAATCC
TGCTTTCCAAATAATTTCTGAAGTAATCCCTACCTCTGGAGAGTCTTCTCTTGCAGATCA
CGTGTTAGTGTCTGAAGGGTCTGGTGGCCAGCAGATAGGAGGTACCAGAACAGCAGAAGA
AATTGAGTTGCAGCCTTTGTTATCTAGATATAGTTTTGAAATTGAGGAGCCAACACCACC
GCGAAGAACTAGCACCCCCTTACAAAGAGCAAGACAACAGTTTTCATCATTACGCAGAGC
ATTATATAATAGAAGGCTAACTGAGCAAGTGGGTGTCACTGACCCTTTATTTTTCACATC
ACCTTCCAAATTGGTGCGTTTCCAATTTGACAATCCTGTATTTGATGAACAAGTAACACA
GATATTTGAGCAGGACATAGCAGACTTTGAGGAACCACCCGATAGACAGTTTTTGGATGT
GGTTAAATTAGGTAGGCCAACATTAACTGAGTCTGCAGAGGGATATGTTAGAGTGAGTCG
TTTGGGAAGACGGGGAACGATCCGAACACGCAGTGGTACACAAATAGGATCACAAGTACA
TTTCTATAGGGATTTAAGTACAATTAATACAGAAGAACCCTTAGAAATGCAGTTATTGGG
TGAGCATTCAGGTGATGCTTCAATTGTACAAGGTCCTGTAGAAAGCACTTTAGTGGATGT
AAATGTGACTGAGGTTCCTGAAGGTGTTCTTACAGAAACTTCTATGGATCCAGATACTTT
TAATTCAGAGGATTTATTACTGGATGATGCTATAGAAGACTTCAGCGGATCTCAGTTAGT
TGTAGGAACTCCACGCAGATCCACTACGTCAATCACTGTACCTAGATTTCAGACTCCTCA
AAATCCTACCATATATTATCAGGATATACAGGGGTATCATGTTTCATATCCCGAAAGCAG
AGAAAGACCCGCCATTATTTATCCTACACCCGATATTCCTACAGTAGTTATACATGTTGC
TGATTCCTCTGGAGATTTTTATTTACATCCCAGTTTACGATGGCGACGGCGCAAACGCAA
ATATTTATAATGTTTTTCAGATGACACTTTGGCTTCCTGCATCTGGTAAAATATACTTGC
CACCAACACCTCCAGTTGCGCGCGTTCAAAGCACGGATGAATATGTGGAACGAACAGACA
TCTATTACCATGCAACTAGCGATCGCCTATTAACAGTAGGCCATCCATATTTTGATGTCA
GATCACAGGATGGTCAAAAAATTGAAGTTCCTAAGGTGTCAGGAAATCAATATAGGTCAT
TTCGGGTAACCTTTCCAGATCCCAATAAGTTTGCTTTGGCAGACATGTCTGTTTATGATC
CAGATAAATATAGGCTGGTGTGGGCCTGCAAAGGCCTTGAAATAGGCCGAGGACAGCCAT

TAGGAGTTGGAACTACAGGACATCCTCTATTTAATAAAGTAAGAGATACTGAAAACTCCA
GTAATTATCAAAACACATCTACTGATGACAGACAAAATACCTCTTTTGATCCTAAACAGG
TGCAAATGTTTATAATAGGCTGCACTCCTTGTCTAGGAGAATACTGGGATAAAGCACCTG
TATGTGATAATGCAGGGGACCAGACAGGCCTATGTCCTCCACTAGAATTGAAAAATAGTG
TAATTGAAGACGGAGACATGTTTGATATAGGATTTGGCAATATAAACAACAAAACTCTAT
CCTTTAATAGATCTGATGTAAGTTTGGATATTGTTAATGAAACCTGCAAATACCCAGATT
TTCTTACCATGTCTAATGATGTTTATGGTGATTCCTGCTTCTTTTTTGTGCGACGGGAGC
AATGCTATGCCAGACATTATTTTGTTCGAGGTGGTGCAGTGGGTGACGCTATTCCAGATG
GTACTGTCAACCAAAATCATAATTATTATTTACCTGCAAAAAATGGACAGGGTCAACGCA
CTTTAGGGAACTCTACGTATTTTCCAACAGTTAGTGGATCCTTGGTGACGTCTGATGCTC
AGTTATTTAATAGACCATTTTGGTTACAAAGAGCACAAGGCCACAATAATGGTATTTTAT
GGGGCAATCAAATGTTTGTTACAGTCGCTGATAATACCCGAAATACAAACTTTACAATCA
GTGTATCCACTGAAAACGGGGGTGCTCAAGAATATGATTCTGCAAATATTAGAGAATATT
TAAGACATGTTGAGGAATACCAATTGTCATTTATATTGCAATTGTGTAAGGTTCCTTTAA
ATGCTGAAGTGCTGACACAGATTAATGCTATGAATTCTGGAATATTAGAAAATTGGCAAT
TAGGCTTTGTACCCACCCCAGACAATTCTGTACACGATACATATCGTTACATAACATCTA
AAGCAACTAAATGTCCAGATGCAGTGCCTGAAACAGAAAAAGAAGATCCCTTTGGTCAAT
ATACATTTTGGAATGTGGACATGACTGAAAAATTGTCTCTAGATTTGGATCAATATCCTT
TGGGGCGCAAATTTTTATTCCAAGCAGGTTTACAAACAGCACGAACACGTGCTGTCAAAC
GGCCGTTAGTAAGAAAATCTTCCAAATCTGTAAAACGCAAAAGGACCCAGTAACCGTTTT
CGGTCGCCCAATAAAATTTATTAACTAATGTGGTATGTGAAGCATTTTTTGACCTTCTTT
GTGACTAAACCGAACAAGTCAACACCAGTAACCGCGCCCGGTTAATCAGATTATAAATTC
CTGAAGGGCAGATTTCAATCAGTGCAGATATCATCTAGCACCTGCAGCAACCGCCAAGAC
TTTGCCAGGACTTGGCAGAA
>gi|1020226|gb|U31786.1|HPU31786_Human_papillomavirus_type_37,_complete_genome
AGCCAAGAATATTTGGCAGAACATTTTCTTGGAAGACAACCGATAACGGTAAGATTGTAA
TCTTTCAACCGTAGGCGGTACTTTCTGATTGGTTTGGCCGATTGTAGCTAACAACAATCT
TTCTTCATAAATACATGTAACCGCCTGCGTTAACTTACATGATCTAAATAAATATGATGA
GCAATACTTAAGAGAATATATGGCTAGGCCTAAGCCTCAATCTGTTCAACAGCTTGCAGA
TACTTTATGTATACCTTTAGTAGATGTTTTACTGCCTTGCAGATTTTGTTATAGATTCTT
AGCATATATAGAATTGATCGCATTTGATCGAAAAGGTCTTCAACTAATTTGGACCGAAGA
AGATTTAGTGTATGCGTGCTGTACTAGCTGTGCCTATGCTACAGCACAGTTTGAATTTAC
CAGTTTCTATGAGCACTCAGTTAGTGGGAGGGAGATAGAAGAGATAGAGCAAAAGCCAAT
AGGAGAAATAGCCATACGCTGCAAATTTTGCTTAAAGTTATTGGATTTGTTAGAGAAGTT
GGAGACTTGCTATACTCAGCAACAATTTCACAAGGTTAGGCGCAATTGGAAAGGCTTGTG
TAGACATTGTGGGTCGATAGGATGATTGGGAAAGAAGCTACAATACCAGAAATAGTGCTT
GAGCTGCAAGAGCTTGTCCAGCCCACTGCTGACCTGCATTGTTACGAAGAGTTGAGTGAA
GAAGAGACAGAGGAGGAGCGTCCTCACATCCCTTACAAGATTGTAGCTCCGTGCTGCTTT
TGTGGTTCTAAACTACGACTGATAGTTGTTGCAACGCCTATTGGAATTAGATCACAAGAA
GAGCTATTACTTGGTGAAGTGCAGCTGGTTTGTCCAAACTGTCGGGGGAAGCTTCGCCAT
GACTGACGACACGAAAGGTACAAAATTTGATCCTAAAGAAGGATGTAGTGATTGGTTTGT
GCTAGAAGCAGAATGCTCTGACAATAGTTTAGATGGTGATTTGGAAAAGTTATTTGAAGA
AGGGAATGATACTGACATTTCTGATTTAATAGATGATGAGGACACTGTTCAGGGAAATTC
CCGCGAATTGTTATGCCAGCAACAAAGTGAGGAAAGCGAGCAACAAATACATTTGCTAAA
ACGAAAGTATTTCAGTTCACAAGAGATTCTGCAGTTAAGTCCTCGTCTGCAGTCTATTAC
TATTTCGCCACAGCATAAGTCTAAAAGGAGATTATTTGAAGGAGACAGCGGACTAGAACT
GTCATTTAATGAAGCTGAAGATTTTACTCAGCAGACTTTGGAGGTGCAGGAGGTATCGGC
ATCCGGCTCTGAGCCGGCAGACCAGGGTGCCAAGGGACTGGGCATTGTTAAAGACCTTCT
TAAATGTAGTAATGTTAAAGCTATGTTGTTAGCAAAATTTAAAGAAGCATTTGGAGTTGG
CTTTATGGAACTTACTAGGCAATATAAAAGTTGTAAAACATGTTGCAGAGATTGGGTTGT
AACGTTGTATGCAGTTCAAGATGAACTGATAGAAAGCTCCAAACAGCTGTTGCTTCAACA
CTGTGCTTATATATGGTTGCAGCATATGCCTCCAATGTGTTTATATTTATTGTGTTTTAA
TGTGGGTAAAAGTAGAGAAACTGTTTTTAGACTGCTAATGAATTTATTGCAAGTAGCAGA
AGTACAAATATTGGCTGAACCTCCAAAGCTTCGGAGCACATTATCTGCACTGTTTTGGTA
TAAAGGTAGCATGAATCCAAATGTCTATGCACATGGTGAATATCCTGAGTGGATTATGAC

ACAAACCATGATCAATCACCAATCAGCAGAAGCTACACAATTTGATTTATCCACTATGAT
ACAATATGCATATGACAATGATTTAATAAATGAAGATGAAATTGCTTATAATTATGCCAA
ATTAGCAGATACAGACGCTAATGCCAGAGCTTTTTACAGCACAATAGTCAAGCCAGATT
CGTTAGAGAATGTGCACTAATGGTTAGATATTACAAACGAGGTGAAATGAAAGATATGAG
CATATCTGCCTGGATACATAATAAAATGTTAGTTGTGGAAGGCGAAGGACATTGGTCTGA
TATTGTAAAGTTTGTAAGATTCCAAGATATCAATTTTATAAGGTTTCTAGATGTCTTTAA
ATCATTTTTGCATAACACTCCTAAAAAGAATTGTCTTTTATTTTATGGTCCACCTGATAC
AGGCAAATCAATGTTTACTATGTCTTTAATTAAAGTGTTAAAAGGAAAAGTTTTATCCTT
TGCAAATTATAAAAGTAATTTTTGGTTGCAGCCGTTGGCAGATACTAAAATTGCTTTAAT
AGATGACGTCACGCATGTGTGTTGGGATTACATAGATCAATATTTAAGAAATGGATTGGA
TGGTAATTTTGTTTGTTTAGACCTAAAACATAGAGCGCCATGTCAAATTAAGTTTCCACC
ATTATTACTGACTTCCAATATGGATATTATGAAGGAAGAAAGGTATAGATATTTACATAG
CAGGGTGCATGCTTTTGCATTTCCAAATAAGTTTCCTTTTGATAGTAACAATAAGCCACA
ATTTCGACTTACTGACCAAAGCTGGAAATCTTTTTTTGAAAGGCTTTGGAAACAGTTAGA
TCTCAGTGACCAAGAAGACGAGGGAGACGATGGACACACTCAGCGATCGTTTCAATGCAC
TGCAAGAGAACCTAATGGACATTTATGAGTCAGGTCGAGATGACCTAGAGACCCAAATTA
TGCATTGGCAACTTCTAAGGCAGGAGCAGATCCTGTTTCATTATGCCAGAAAAAAATGGAG
TCATGCGTTTAGGATATCAACCTGTACCTCCTTTAGCCACCAGTGAAGCTAAAGCAAAAG
ATGCAATTGGCATGGTTATATTATTAGAAAGTTTACAACAGTCTGCTTATGGTAAAGAGT
CCTGGACACTTACACAAACTAGTTTGGAGACCGTGAGGAGTCCACCTGCAAATTGTTTTA
AAAAGGGCCCTCAGAACATTGAAGTGATGTTTGACAATGACCCTGAAAATCTAATGGTGT
ATACTGCCTGGTCATTTATTTATTATCAGACTGTAGATGACACGTGGAACAAGGTTGAGG
GACATGTTGACTACTATGGTGCATATTATTTTGAAGGAGATTTAAAAGTCTATTATATAC
AATTTGAAGGTGATGCTGCCAGGTTTAGCAAAACTGGACGCTGGGAAGTACATGTTAACA
AGGACACTATCTTTGCTCCTGTTACTAGCTCTTCGCCGGCAGCTGGAGAAGGGACAGACG
GGGCAGCCTCCGTCCACACCGTATCCGGGTCGCCGCTCGCACGGGGATTCTCTACCACCT
CCGTGTCCACCAGAAAACGGACACCACCACGGCGATACAGAAGAAAAGCATCTAGCCCTA
CAACCACCGCCGCCCGGCAAAAAAGACAAGGAGAAGACACCGCAACAAGGCGATCAAGGT
CCACCTCCCGGGGGAAACAAGCAACCTCCAGGGGAGGGGACCGACGCAGACGGAGACGAG
AACGCTCCTACTCCCGAGACACCTCCAGTTCCCCCGACAGGGGAAGGGGAGGGAGAAGTA
GAGGGGGGCCCGAGACACGATCCCAATCAAGGTCCCTCTCACGATCCCGGTCGCGGTCGC
GATCCAGAGGGTCTTCTTCCAGGGGTGGCGTTGCGCCTGACGCAGTGGGAAAGTCAGTTC
GAACAGTTGGTAGAGACCATAGTGGACGACTTAAAAGATTACTGGACGAAGCTAGGGATC
CCCCAGTAATTGTGCTGCGTGGTGATGCTAACAAATTAAAATGCTATCGCTATAGAGCTA
AGAAAAAGCATGGAAACCTAGTTAAGTACTACAGTACCACGTGGTCATGGGTTGGGGGCA
GCACCAATGATAGAATTGGAAGGTCACGCATGTTACTTGCATTTCAATCCAATACAGAAA
GAGAGTTGTTTTTAAAAACTATGAAATTACCACCAGGAGTTGATTGGTCACTGGGTCATT
TAGATGAATTGTGAAAACAGCTTTTTTATAACAAACTAACATTGCTTTTGCTTTTGCTAC
TAACCTACTAACGTTCCAATGGCTCGCGCACGTCGTACCAAACGTGCGTCTGTAACTGAC
ATTTACAGGGGTTGCAAGCAGGCCGGCACTTGCCCCCCCGATGTAATTAATAAAGTGGAA
CAAACAACAATTGCAGACAAAATTTTGAAGTATGGTGGTGCTGGTGTTTTTTTGGTGGG
CTTGGGATTAGCACCGGCCGAGGAACAGGTGGTGCTACAGGATATGTCCCTTTGGGGGAA
GGCCCTGGAGTGCGCGTAGGAGGCGCACCCACCATTGTTCGCCCTGGGGTCATACCTGAA
TTGATTGGGCCAGCAGATGTAATACCTATTGACACAGTCACTCCAATTGACCCCGCAGCA
CCCAGTATTGTCACAATTACAGACAGTAGTGCTGTTGACCTTTTACCTAATGAAATAGAA
ACAATTGCAGAAGTGCATCCTGTGCCAACAGACAATTTGGATATTGATACTCCTGTAGTT
ACAGGAGGCCGGGATTCCAGCGCTGTTTTGGAAGTTGCTGATCCTAGTCCCCCTGTGCGA
ACAAGAGTTTCCAGAACACAATATCATAATCCTTCTTTTCAAATAATAACTGAATCTACA
CCTTTAGCAGGAGAATCTGCTTTAGCTGACCATGTTATTGTTTTTGAAGGCACTGGAGGA
CAAAATATAGGTGGTTCTCGAAATGCAACTATAGAAACAGCTCAAGAAAGTTTTGAAATG
CAAAGTTGGCCGAGTAGGTATAGTTTTGAAATAGAAGAAGGAACACCTCCTAGATCTAGC
ACACCAGTACAAAGAGCAGTACAATCACTCTCTAGTTTAAGACGGGCATTGTATAATAGG
AGATTAACAGAACAGGTAGCAGTCACGGATCCTTTATTCTTGAGTAGACCCTCACAATTA
GTACAGTTTCAGTTTGACAATCCTGCATTTGAAGAAGAAGTAACTCAAATATTTGAGAGG
GATTTAGAGGCTGTAGAAGAACCTCCAGATAGACAGTTTTTGGATGTTATTCGCTTAGGT

AGACCTACTGTTGCTGAAACACCACAAGCGTATTTAAGAGTAAGCAGATTAGGACGTCGT
GCTACCATCCGTACTCGTAGTGGAGCACAGGTGGGGGCTCAGGTACATTTTTATAGAGAT
TTAAGTACTATAGATTCTGATGCCCTAGAAATGCAATTATTAGGAGAACATTCAGGTGAT
ACTACTATAGTACAAGGACCTGTAGAAAGTTCATTTGTTGATATAAATATTGATGAACCA
GGTCCCTTAAATATAGGGCAACAAGAGTCTACTATGGCAGATGACACAGATTTTAATTCT
GCAGATTTATTGTTAGAGGATGCTGTAGAAGACTTCTCAGGATCTCAGTTGGTTTTTGGA
ACCTCACGCCGCAGTACAAATTCTATCACAATACCTAGATTTGAAACTCCAAGAGATACT
GGATTTTATATACAAGATATTCAAGGTTACAATGTAGCCTATCCTGAGTCACGTGACACA
ACACAAGTTATCTTGCCACAACCTGAAACACCAACTGTAGTTATTAGATTTGGAGAGGCA
GGTACAGACTATTATTTACATCCTAGCTTAAAAAAGAAAAAGAGAAAACGCAAATATTTA
TAATTGTTTTTACAGATGACTTTGTGGCTGCCAGCGACGGGTAAAGTATACTTGCCTCCA
ACACCACCAGTAGCCCGGGTGCAAAGCACGGATGATTATGTGGAAAGAACAAATGTATTC
TATCATGCCATGAGCGATCGTCTCCTAACTGTAGGACACCCATATTATGATGTAAGATCT
AGTGATGGCTTAAAAATCGAGGTTCCTAAAGTATCTGGAAATCAATACAGAGCTTTTAGG
GTTAGGTTGCCAGATCCAAATAAATTTGCTTTAGCAGATATGTCAGTATATAATCCAGAA
AAGGAAAGGTTGGTGTGGGCCTGTGCGGGCTTGGAGATAGGCCGAGGGCAACCACTTGGA
GTAGGAACGACAGGTCACCCTTTATTTAATAAATTAAGGGACACTGAGAATAATAGTAAT
TACCAAGGGGGGTCACGGGATGATAGACAAAACACATCATTTGATCCAAAACAAGTACAG
ATGTTTGTGGTTGGATGTGTGCCATGCATGGGTGAACATTGGGATAAAGCACCAGTTTGT
GCATCAGAGGAAAATAATCAGACAGGACAGTGTCCACCACTTGAATTAAAAAACACAGTG
ATTGAAGATGGGGACATGTTTGATATAGGGTTCGGAAATATTAACAATAAGGTTCTCTCT
ACTAATAAATCAGATGTTAGTTTAGATATAGTAAATGAAATATGCAAATACCCTGATTTT
TTAACAATGGCTAATGATGTTTATGGGGATGCATGTTTCTTTTTTGCTAGGAGAGAACAA
TGTTATGCCAGACATTATTTTGTAAGAGGGGGAAATGTAGGTGATGCTATTCCCGATGGC
ACTGTTAATCAGGACCACAAATATTACTTACCTGCCAAATCAGACCAGCAGCAGTATCTG
TTAGGCAATTCTACCTATTTTCCCACTGTTAGTGGATCTTTAGTAACATCTGATGCTCAG
CTCTTTAACAGGCCTTTTTGGTTACGCAGAGCTCAAGGTCACAACAATGGCATTTTATGG
GGTAATCAAATGTTTATCACAGTTGCTGATAATACACGGAACACAAACTTTTCTATTAGT
GTGTCTACTGACAATGGCGAAGTTACAGAATATAATTCTCAAACACTCAGAGAATACCTA
AGACATGTTGAAGAATACCAGCTTTCAATTATTTTACAACTTTGTAAAGTTCCTTTAAAG
GCTGAGGTTTTAACTCAGATAAATGCAATGAATTCTGGTATATTGGAAGAGTGGCAATTA
GGATTTGTACCTACTCCAGATAATTCAGTACATGACCTTTATAGGTACATTAATTCAAAG
GCTACCAAGTGTCCTGATGCAGTTGTTGAAAAAGAAAAGGAAGATCCCTTTGCAAAATAT
ACATTTTGGAATGTAGATTTAACTGAAAAATTATCATTGGATTTAGATCAATATCCTTTA
GGGAGGAAATTCATCTTTCAGTCGGGATTGCAAAGTAGACCTAGAATTGTTCGATCGTCT
GTAAAAGTGTCTAAAGGTACAAAGCGTAAACGGTCGTGACCGTTTTCGGTTTCCAATAAA
CAAATAAACCAATAAGGTATGTGAAGCATTTTTTACCATGTTCGTGACTAAACCATATAA
GTCAACGCCAACAACCGCACCCGGTTTAATCAGATATAAAACACCTGGTGCGATTTTATC
AGAGCTTTTGTGGAAGCACCTGAGGCGACCGCCAGAACTGC
>gi|1020218|gb|U31785.1|HPU31785_Human_papillomavirus_type_36,_complete_genome
GTAAGTTATTTAATTTATGTACCAGGTGCGGTACTGGAATTTCACAATAATAATTGTTGT
TGCCAACTACCATTGCTATATTCAAGTTTTTGCCTGTATCGTTTTCGTATCATGTGAATA
ATATACTGTATAGTATAAATAAATAAATAAATATATATATATATGCTTCAAAGGTTGGGT
TTTTTAATAATTAAGGCAAATGGCAGAGCAAGCCTCCGAACAGCAGAATATTACAGAAAA
AGAAAAAGAACAGCTGCCTTTAACTATTAAGGGCCTGTCAGAATCATTAGGCATTCCGTT
TGTAGACTGTCTAATACCTTGTAACTTTTGTGGCAAATTTTTAGATTATTTAGAAGCTTG
TGAGTTTGAGGTTAAAAAGCTTAGTTTAATTTGGAAGGATTACTGTGTATTTGCCTGCTG
TCGTGTTTGTTGTGGTGCAACAGCCACATATGAGTTTAATCAGTTTTATCAGCAGACAGT
ATTAGGAAGAGATATTGAATTGGCTGCAGGTCGCTCCATATTTGAAATTGACATTAGGTG
TCAGACGTGCTTAGCTTTTCTTGACATAATTGAGAAGTTAGATTGTTGTGGAAGAGGACT
TCCCTTTCATAGAGTCAGAAACGCCTGGAAGGGAATCTGTAGGCAGTGTAAGCATTTTTA
TAATGATTGGTAAAGAGGTCACCGTGCAAGATATTGTTCTGGAGCTCAGTGAGGTGCAGC
CTGAAGTACTACCAGTTGACCTGTTTTGTGAAGAGGAATTACCAAACGAGGATACGGAGG
AGGAGCTTGACACCGAAAGAATCGTTTTCAAAGTCATTGCACCGTGTGGTTGCAGCCACT
GTGAGGTCAAGCTCCGCGTTTTTGTCCAAGCTACAGAATTTGGCATCAGAGCATTTCAAC

AGTTGCTGACCGGTGACCTGCAGCTTCTGTGTCCCGAGTGTCGTGGGAACTGCGAACATG
GCGGATCCTAAAGGTAGTACATCTAAAGAAGGGTTTGGTGATTGGTGTATTTTGGAAGCT
GACTGTAGTGATATAGAAAATGATATGGAACAATTATTTGAAAGAGATACAGATTCTGAT
ATTTCGGACTTAATAGATGATTGTGACCTGGAACAGGGAAATTCTTTGGAACTATTTCAC
CAACAGGAGTGTAAGCAGAGCGAGGAGCAATTACAAAAACTAAAACGAAAGTGTCTTAGT
CCAAAAGCTGTCGCACAGCTTAGTCCGCGACTTCAGTCAATTTCATTGTCACCTCAGCAG
AAGTCTAAGCGAAGGCTCTTTGCAGAGCAGGACAGCGGAGTCGAGCTGACCTTAAACAAT
GAAGCTGAAGATGTTACTACTGAGGTGGAGGTACCGGCTATAGACTCTCGGCCGGATGAC
GAGGGAGGATCAGGGGATGTAGATATACATTATCTTTCACTGTTGCGTTCCAGCAACAAA
AAAGCCACATTAATGGCAAAATTTAAAGCAGCGTTTGGGGTAGGCTTTAATGAATTGACA
CGTCAATTCAAAAGTCACAAAACCTGCTGTAATCATTGGGTTGTCTCTGTTTATGCAGTC
CATGATGATCTATTTGAAAGTTCAAAGCAGTTGTTGCAACAGCATTGTGACTATTTATGG
GTTCGTGGGATAGATGCAATGTCATTATATCTATTGTGTTTTAAGGCGGGAAAAAAATCGC
GGGACAGTGCATAAGTTGATGACTTCAATGTTAAATGTGCATGAACAGCAGATTTTGTCT
GAGCCTCCAAAGTTAAGAAATACTGCTGCTGCATTGTTTTGGTACAAAGGCTGTATGGGA
TCGGGGGTGTTCAGTTATGGGCCATATCCTGATTGGATTGCACAACAGACTATATTAGGT
CACAATAATGCTGAAGCAAGCACCTTTGATTTTTCACAGATGGTACAATGGGCCTTTGAT
AATCAGTTAGTTGATGAAGGAGATATTGCATATCGATATGCAAGGCTTGCACCAGAAGAT
GCCAATGCTGTTGCATGGCTTGCACATAATAGTCAAGCTAAATTTGTAAGAGAGTGTGCA
GCAATGGTGCGCTTTTATAAAAAAGGTCAAATGAGGGATATGTCCATGTCGGAATGGATC
TATACTAAAATACATGAGGTAGAAGGTGAAGGTCACTGGTCAGATATAGTAAAATTTTTA
AGATATCAGGAGGTAAACTTTATAATGTTTTTGGCTGCATTCAAAGATTTTTTGCACTCA
AAGCCAAAAAAAAATTGTATTTTAATTCATGGCCCTCCTAATTCAGGAAAGTCATCATTT
GCAATGTCATTAATAAGGGTATTAAAAGGCAGAGTATTATCTTTTGTAAATTCAAAAAGC
CAATTTTGGTTGCAACCCCTTTCTGAATGTAAAATAGCATTGATTGATGATGTAACTGAC
CCCTGTTGGTTATATATGGATAATTATCTAAGAAACGGTTTAGATGGGCATTATGTCTCA
TTGGATTGTAAATACAAAGCTCCAATGCAAACAAAGTTTCCTCCATTATTACTAACATCT
AATATAAATGTGCATGAGGAAGCTAATTACAGATACTTACACAGTAGAATTAAAGGATTT
GCATTTCCAAATCCATTCCCAATGAAATCAGACGATACACCTCAGTTTGAGTTAACTGAC
CAAAGCTGGAAATCTTTTTTTGAAAGGCTTTGGACACAATTAGAACTCAGTGACCAAGAA
GACGAGGGCGAAAATGGAGAATCTCAGCGAGCGTTTCAATGCTCTGCAGGATCTGCTAAT
GAACATTTATGAAGCTGCAGAACAGACACTTGAGGCACAGATAAAACACTGGCAAACCTT
GCGACAAGAAGCTGTTTTGCTCTACTTTGCTAGGCAGAGAGGTGTGACAAGGCTTGGATA
TCAACCTGTGCCTGTAAAAGCTGTATCTGAAGCAAAAGCTAAAGAGGCTATAGCAATGGT
GCTGCAGCTTCAGTCTCTACAAACGTCTGAATATGCATCTGAAACTTGGACATTAGTTGA
CACCAGTATAGAAACTTTTAGAAGCGCTCCAGACGGTCACTTTAAAAAGGGTCCAGTGCC
TGTAGAAGTGATTTATGACAATGATGCAGATAATGCCAATTTGTACACTATGTGGACATA
CGTGTATTATATGGAAGACGATGTGTGGCATAAAGCCAGAAGTGGGGTCAATGAGACTGG
CATATATTATTTACAAGGAACATTTAAATATTACTATGTACTATTTGCTGACGATGCACG
TAAATATAGTCAAACTGGACAATGGGAAGTGAAAGTTAATAAGGAAACTGTCTTTGCCCC
TGTCACCAGCTCCACCCCTCCAGGGTCGCCAGGAGGACAAGCAGACACAAACGCCTCCTC
CAAGACCTCCACCACCACCACAGCCACCGTTGACTCCACGACCAAGCAGCTCACCACATC
AGAACAGCCACAACAAACCGAAACCAAAGGAAGAAAGTACGGACGGAGGCCCTCCAGCAG
GACAAGGAGACCGCAAGCGAAGCAAAGGCGATCAAGGTCCAGACACCGATCCTCTAGGTC
CCGATCGCGGTCCCAGTCCCGGTCCCACACCCCAACCACTCGGTCTGCCACCACCCGGTC
TAGGTCCCCGTCGCTCGCCAAGACTGGGGTCCAGCGGGTATCAACCAGATCACGATCCAG
AAGCACCTCTAGAAGGGGAGGTAGAAGGCGGAGGTCACGGTCACCATCCACCTCCTCCTC
CACCACCACCACCAACAAACGGTCACGAGTGCGGGCCGAAACCACAGGGTCCAGAGGGGC
GCGAGGGGGTAGAGGGGCCAGGGGTGGAGCGGTGGGGGGCGGCGACGAGGACGATCATC
CTCCTCCACCTCCCCCGCCCACAAACGGTCAAGAGAGCACTCTGTTAGGAGCCGTGGCGT
GTCTCCTGACCAAGTGGGAAAGTCACTTCGATCTGTTAGTTCAAAACATACAGGACGACT
TGGAAGATTACTGGAAGAAGCTCTCGATCCCCCAGTGATCCTTGTTAGAGGGGAGGCAAA
TACACTAAAATGCTTTCGCAACAGAGCTAAGATAAAATACATGGGACTGTATAGGTCATT
TAGTACAACTTGGTCATGGGTGGCAGGAGATGGCACTGAGCGTCTAGGCAGGCCCAGAAT
GCTCATTAGCTTTTCGTCCTACAATCAGAGAAGGGATTTTGATGACGTGGTGAGATACCC

GAAAGGAGTTGAAAAATCATATGGCAACCTTGACAGTCTCTAACCACTAATGCTATGCTG
CTTTGCTACTAACAACACTAACAAATTAGCTTTTTATACTTTTTTACTTTTGTACCTGCA
ATGGCGCGTGCTAAAAGGGTCAAGCGAGACTCTGTAACACATATATACCAGACCTGCAAA
CAAGCAGGCACATGCCCCCCTGATGTTGTGAATAAAGTGGAACAAACAACAGTTGCTGAC
AATATTTTGAAATATGGCAGTGCTGGTGTCTTTTTTGGTGGCCTTGGCATTGGTTCGGGC
CGAGGTACTGGGGGTGCTACCGGGTACGTGCCACTTAGTGAAGGTCCTGGTATCCGTGTC
GGAGGTACCCCCACGGTTGTAAGGCCTTCATTAGTTCCTGAAGCAATTGGGCCAGTCGAT
ATTTTGCCCATTGATACAATTGATCCTGTGGAGCCTACAGCATCGTCCGTGGTTCCTCTT
ACTGAATCCACTGGACCTGATTTACTTCCAGGTGAGGTGGAAACAATAGCTGAAATTCAT
CCTGTTGCTGAAGGCCCATCAGTTGATACCCCTGTGGTTACCACAAGCACGGGTTCCAGT
GCTGTTTTGGAAGTTGCTCCAGAGCCTATACCTCCAACACGGGTTAGAATCTCACGTACA
CAATATCACAATCCTTCCTTTCAAATTATTACAGAATCAACACCTGCACAGGGTGAAAGC
TCTCTTGCAGATCACATTTTAGTGACATCAGGGTCCGGGGGACAAAGAATAGGGGCTGAT
ATTACTGATGAAATTGAACTTCAAGAACTTCCTAGTAGATATACTTTTGAAAATGAAGAA
CCAACTCCACCTAGACGTAGCAGCACACCTTTACAGGCCACACGAGCTGCAGGTAGACGG
AGAGGTGTTTCTTTAACTAATAGACGTCTAGTACAGCAGGTACCTGTAGAAAATCCTTTG
TTTTTAACTCAACCCTCTCGATTGGTGCGTTTTGCTTTTGAAAATCCTGCATTTGAAGAA
GAAGTTACAAATATATTTGAACATGATGTAGATGCTTTTGAAGAACCACCAGATAGGGAT
TTTCTTGATGTCCAACGGTTGGGTCGTCCCCAATATTCTACAACTCCTGCAGGGTATGTG
AGGGTTAGTAGATTAGGTACTCGTGCAACCATTCGCACGCGGTCTGGTGCACAAATAGGG
TCACAGGTGCACTTTTATAGGGATCTCAGTTCCATTAATACTGAAGATCCTATAGAATTG
CAGTTGTTGGGGCAACATTCGGGGGATGCTAGTATAGTTCAAGGCCCTGTGGAAAGCACA
TTTATAGACGTAAATGTGTCTGAAAATCCTTTGTCTGAAAGTGTGGAAGCCTTTTCTGAT
GATTTATTGCTGGATGAAGCTGTGGAGGATTTTAGTGGGTCACAATTAGTTATTGGTAAT
AGAAGAAGTACCACTTCTTACACTGTTCCCAGATTTGAAACTACTAGGAGTGGTTCTTAT
TATGTCCAGGACAGTAAAGGATATTATGTTGCATACCCAGAATCTCGCAATAATGCAGAA
ATAATTTATCCTACACCTGATATACCTGTAGTGGTAATACACACTCATGACAATACAGGT
GACTTTTATTTACATCCTAGTCTTCGATGGCGCAAACGTAAAAGAAAATATTTGTGATTT
TGCATTGCAGATGGCAGTGTGGCATTCGGCTAATGGTAAAGTATACCTTCCTCCATCGAC
ACCGGTTGCCAGGGTGCAAAGCACGGATGAATATATACAAAGGACAAATATTTATTATCA
TGCATTCAGCGACAGACTACTGACTGTAGGCCATCCATACTTCAATGTTTACGATATTAC
TGGTAACAAATTAGAAGTCCCTAAAGTGTCTGGTAACCAACACAGGGTTTTTCGCTTGAA
ATTACCTGATCCTAATAGATTTGCATTAGCTGACATGTCAGTTTATAACCCAGATAAAGA
ACGGTTGGTGTGGAGCTGTAGGGGCCTTGAAATAGGTAGAGGACAACCGTTAGGTGTTGG
CAGTACGGGACACCCACTATTTAATAAGTTGAAAGACACTGAAAACAGTAATTCATATAT
AAAATCTTCTAAAGATGATAGACAGGATACATCATTTGATCCCAAACAAATTCAAATGTT
TATCGTGGGATGCACACCTTGTATAGGTGAACATTGGGACAAGGCTATACCTTGTGAAAA
GGAGCGCCAGGACAACAGACTATGCCCACCAATTGAATTGAAAACTACTTATATAGAAGA
TGGCGACATGGCAGATATAGGTTTTGGAAATTTAAACTTCAAAAATCTGCAAGAGAGTAG
GTCAGATGTAAGTTTGGATATAGTTAATGAAACCTGCAAATATCCTGACTTCTTAAAAAT
GCAGAATGATGTTTATGGAGATGCCTGTTTTTTTTATGCTCGCAGAGAGCAATGTTATGC
CAGACATTTTTTTGTCCGTGGAGGTAAAACGGGTGACGACATCCCTGACGCCAGAATTGA
TAATGGGACTTTTAAGAATCAGTTTTTCATTCCTGGGGCTGACGGCCAAGATCAAAAGAC
CATAGGAAATGCCATGTATTACCCAACTGTTAGTGGCTCATTGGTGTCTAGTGATGCTCA
ATTGTTTAACAGGCCCTTCTGGCTCCAGCGAGCACAAGGTCATAATAATGGCATTCTGTG
GGCTAATCAGATGTTTATTACAGTGGTAGACAACACACGAAATACTAACTTTAGTATTTC
AATATATAACAATAATGGGGCACTAAAGGACATCAATGATTACACTGCAGAGCAATTTAG
AGAATATCAAAGGCACGTGGAGGAATATGAAATTTCATTAATATTACAGCTATGTAAGGT
TCCTCTGAAGGCAGAAGTATTGGCTCAGATAAATGCTATGAATTCTTCTTTATTGGAAGA
TTGGCAGTTAGGTTTTGTACCTACTCCAGATAACCCTATTCAAGACACCTATCGATATAT
TGATTCATTAGCCACTCGCTGTCCTGATAAAACCCCCCCTAAGGAAAAAGAGGATCCCTA
CAAGGGGTTAAAGTTCTGGGATGTGGATCTTACTGAACGGTTGTCACTAGATTTGGATCA
ATACTCTTTAGGTAGAAAGTTTCTATTTCAAGCCGGCTTACAGCAGACGACCGTAAGCGG
TACAAAATCAGTGTCTTATCGAGGGTTCACCAGAGGAACCAAGCGCAAGCGAAAACAGTA
ATATGACCGTTTTTGGTACAGATTTATAAACTTTTACACAGTATTCAAGGAATGTTTGTT

TACTCTGACTAAGTATAACTCTACCAAGGGAACCGACCGCACCCGGTACAGTCAACGATA
CTGCTGCCAATATAGACTCTGTTTAGTGCCAGAACATATCATCTTGGAAGCAGATCGACC
GTGTTCGTTGTAACACGCTCGGATTAGAGACATTGCCAAGGAAGATTTAATCTACAATCG
CTGTTGGCAATCGCTTTTGGCTGAGATAGCTGACCGGTAACG
>gi|1020210|gb|U31784.1|HPU31784_Human_papillomavirus_type_29,_complete_genome
TATAAACTATCATCTTCATAATAAAAAGTAGGGAGGGACCGAAAACGGTACGACCGAATG
GGGTACATATAAAAAGACATCACTGCAGCGTGGCAGAAGCCATGTCCAGAGGTGATGGCT
ATCCAAAAAATATATTCCTGTTGTGCAGAGACAGTGGAGTACCATTTGAGGACCTTCGCC
TACAGTGTGTTTTCTGCACGAAAGAGCTAACCAGCCCAGAACTGGCAGCATTTTGCATTC
GGGAATTAAATGTGGTGTGGAAAAGTGGAGCTCCGTACGGTGCATGTGCACGCTGCTTAT
TGTTTGAAGGCATAAAGCGGCGCCTAAAATACTGGCAGTATTCTTGTTTTGTGGAAGGCG
TGGAAGCGGAGACAAACGAGTCCATATATACACAGCTAATTCGCTGCTACATGTGCCACA
AGCCACTTGTCAGAGAGGAAAAAGACAAACACCGAAACGAAAAGCGAAGACTACACAAAA
TTTCTGGATACTGGAGAGGGAGTTGCCTGTATTGTTGGTCACGATGCATGGGCCAAAGCC
CACGGTAAAAGATATTGAATTGGATCTTGCACCAGAGGCCGTACCTTTAGTATGCAATGA
GCAATTAGACAGCTCAGATGAAGAAGATTGTATAGATGTTGTGGAACCAGCACAACAGGC
GTATAGGGTGGTAACTTTGTGTACAAAGTGTAGTACAACACTGCGACTGGTGGTAGAGAG
CAGCGAAGCAGATATAAGGGCATTCCAGGAGCTCCTACTACGCACATTGAAGATCGTGTG
TCCTCGCTGTGCGTAACTGGACATGGCCGATAACTCAGGTACAGAGGGGGAGGAGGAGGA
CTGTTCTGAGGCGGAACGGGCTGGAGGATGGTTCATGGTAGAGGCTATAGTAGACAGACG
GACAGGGGACACAATATCCAGTGACGAGGATGAGGAGGATGAGGGTGAAGACATGGTAGA
CTTTATAGATGATAGACCTATAGGGGACGGACAGGAAGTAGCACAGGAACTGTTGCTGCA
GCAAGCAGCTGCGGATGACGATGAAGCAGTGCACACTGTAAAACGAAAGTTTGCTCCCAG
TCCCTATTTCAGCCCTGTGTGTGTGCCCAGCATAGAACATGAGCTAAGTCCCAGGCTAGA
CGCCATAAAGCTGGGACGGCAGTCCTCTAAAGCCAAACGGAGGCTATTCCAACTACCGGA
CAGTGGGTATGGCCAAACACAGGTGGATACGGACACGGGACCAAGCCAGGTACAAGATGG
TTGCGAGACGGGTGATCAAAATGGCCGACAGCAGTATAAGGAGGGGAGTGGTACAAAGGA
TGGGGAAAATGGCAGCCAAGAGGAGGAGCGTGCAGGAGGGGATGGGGAGGAATCGCAACC
TCTGAGTACAGAAACAGAGAAAGGAGCATGTGGTGTGTTGTCTATACTGAAAGCTAGTAA
TCAGAAAGCAACCCTACTAGGTAAGTTTAAAGAACAATTTGGACTTGGATATAATGAATT
GGTTAGGCATTTTAAAAGTAGTAGGACAGCATGTGTGGATTGGGTAGTGTGTGTGTTTGG
GGTGTACTGCACTGTGGCCGAGGGCATAAAACAGTTGATACAGCCACTATGTGAGTATGC
ACATATACAAGTGCTACCCTGTCAATGGGGAATGACAGTGTTAATGCTGGTGCGGTACAA
ACGTGCCAAGAATAGGGAGACAGTAGCAAAAGGTCTTAGTACTTTATTAAATGTACCAGA
AAGCCATATGTTAATTGAGCCACCTAAACTAAGAAGTAGTCCAGCAGCATTGTATTGGTA
CAAAACTAGTATGTCCAATATTAGTGATGTGTATGGCGAGACACCTGAATGGATAGTAAG
ACAGACAATGGTAGGTCACGCATTACAAGAAGTACAGTTCAGTTTATCTGAAATGGTACA
ATGGGCATATGATCATGATATAACAGATGAAGGTACCTTGGCATACGAGTATGCATTGAT
AGCAGATGTAGACTCTAATGCTGCAGCTTTTCTTGCCAGCAATTGTCAAGCTAAATATGT
AAAGGATGCTTGCACAATGTGCAGACATTACAAACGGGGTGAGCAGGCACGAATGTCCAT
GTCTGAGTGGATACGGTTTAGAAGCAACAAAGTACAGGGAGAGGGGGACTGGAAACCAAT
AGTACACTTTTTAAGATACCAAAATGTAGAATTTATACCATTTCTGTGTGCCTTTAAGTT
ATTCCTACAAGGCATACCCAAGAAAAGCTGTTTAGTGTTTTATGGACCTGCAGACACAGG
GAAGTCATATTTTTGCATGAGTCTGCTAAAATTTATGGGCGGTGTTGTAATTTCATATGC
GAATTCACACAGCCATTTTTGGCTGCAGCCATTGTCTGAAGCTAAAATGGGTCTGCTAGA
CGATGCAACAAGCCAATGTTGGAGTTATGTAGACACATATTTAAGAAATGCATTGGATGG
GAACGTAATGTGCATAGATAGAAAACACAGGTCCCTACTACAACTCAAATGCCCTCCACT
ACTAATAACTACCAATGTGAATCCGTTGGAGGATGACAGATGGAAGTATTTGCGCAGCAG
ACTGCAGGTATTCACATTCAGCAATCCATGTCCATTAACAAGTAAAGGAGAGCCAGTTTA
TACACTAAATGATCAAAATTGGAAATCATTTTTTCAAAGGTTATGGGCACGTTTAAGCCT
TACCGACCCTGACGACGAGGAGGAAAATGGAGAACCTAGCGAACCGTTTAGATGCGTGCC
AGGACAAAATACTAGAACTGTATGAAAGAGATAGCGACAAACTTGAGGACCAGATCACGC
ATTGGTATCTTATGCGTGTAGAGAGTGCGTTGTATTATAAAGCAAGAGAATGTGGAATGA
CACGTATAGGCCACCAGGTGGTGCCAACACTTAGTGTAGCTAAAGCTAAAGCATGCAGTG
CTATTGAAATGCATGTAGCTTTACAACAATTGCAACAAAGTGCATATGGAAAGGAACCAT

GGACACTTCGGGACACTTCACGAGAAATGTGGGACGCAGTACCAAAGAGGTGCTGGAAAA
AAAGAGGAGTGACTGTGGAAGTTAGATATGATGGAGACGAGACTAAAGCAATGTGCCATG
TACTGTGGAAGGACATAATTGTACAAAACCTTAGTGATGACCAGTGGGTTAAAGTTAAAG
GTCAAGTCTCATATGAGGGGCTATATTATGTGCACGAAGACGTAAAAGTGTTTTACGTGA
AATTCCATAAAGACGCACGTGTGTATGGGGAAACAGGCATATGGGAGGTGCATGTGGGAG
GCAAAGTAATTCATCACAATGCATTTGACCCTGTATCTAGCACACAAGAAGTACCCGCTA
CTGGACCTCTATACGCCTCCCACAACACCACCCGCTCGCCCACCCAAGCCCCGTTGGGGC
CTGAGGAGGGACAGGAACGGAAACGACGCAGGCTTGAAGCAGTCGGGCCTGGGCCACAGC
AGCAGCAGCAGCAGCAGCACCAGCAGCAGCAGCAGCAACAGACCCCGACCCACACCCCCT
CCACGCAAGCCTGTGCACGAACGGGTGGACCAGTGGACAGTAACAGGACCCGGGACTGTG
ACTCTACAAGTCAAAACCCCTACCGGCACCCAAGTGATCCTGACTGTGCACCTGTAATAC
ACTTACGAGGTGACCCAAACAGTTTAAAATGTTTTAGATATAGGTTACAAAACGGAAAAA
AAGGGTTGTACTGTAAAGCATCGTCCACGTGGCGGTGGTCCTGTGAACCAGAAAATCAAT
CAGCATTTGTAACAATATGGTACACAAGTGTTACACAGCGAGCCGAATTTTTGGCTAATG
TTAAAATACCACCAGGTATGCAGGCCATTTTAGGCCATATGTCTGTGTTTTGACTACTGT
GCCACAACGTGTAGCAGCCTGGATTTTTATCTGTGTCGACTGTCTCTGTGGGTGTATTTT
GTGTTGCTTCTGTGTCTTTTCTGGCTATCTGTGCTTCCTGCGCTTACTTGCTACTTGGCC
ATTGTGTTGTGTTTATACCTAGGATTGGTGGCACTATATTTACAAGTTGTGCAGCACATT
GCACGAAACACTTAGGCTATCATGTATCCTATAATAATTATAGATGGGTATGGGGATCGT
ACTGTATTGCTGTTTGAGCCAAGGGACGTGTATGTGTTGGGATTGTTAATACTAATGGTA
TGCCTGTTGTTATTTATAGTTTATAGACATTTGGGATTATTATAACCTGTATATAACCTG
TATTTGTACATATACATGTATTTTATATGTGGCTGTGGTAATACGTGTATTGTATACATG
GCCATACAATTGTGCATGTGTTTTTAAAGTTCTACCACTTTTTTTGTTTTTTTTGTTGTT
CCTTTGTTTTTACAGTTCAATAAAGCAACCATGGTGGCACATCGTGCAAGGCGTCGCAAG
CGTGCATCCGCCACAGAGCTTTATAAAACCTGCAAAGTTGCAGGCACATGCCCCCCTGAT
GTTATTCCAAAAGTTGAGGGCACCACACTGGCCGACAGGATATTGCAATGGGGCAGTCTA
GGTGTCTATTTGGGTGGGTTAGGTATCGGTACTGGGTCTGGCACTGGAGGTCGCACAGGT
TATGTCCCTGTCGGCACTCGGCCAGGCACTGTTGTGGATGTTAGTATTCCTACGCGGCCT
CCTGTGGTTATTGAGCCTGTGGGCCCTTCTGATCCTTCTATTGTTACCCTGTTAGAAGAA
TCCAGTGTAATTAATTCGGGTGCTACCATACCCACCTTTACTGGTACATCCGGGTTTGAG
ATAACATCATCTGCCACAACTACCCCGGCTGTGTTAGATATAACCCCTGCTGGTGACAAT
GTAGTCATTACTAGCACAAACTTTAATAATCCTTTATTCACCGAGCCTTCACTCTTGGAA
ATTCCACAAACTGGAGAAACTTCTGGACGTGTCCTGGTGGGCACACCCACCTCGGGTGTC
CACGGGTATGAAGAAATACCCATGGACACGTTTGCCACCTCTGGAACTGGGTTAGAGCCT
ATTAGCAGCACTCCCGTCCCTGGTGTCAGCAGGGTTGCAGGTCCCCGCCTCTATGGCAAG
GCCCTAACACAGGTTAGGGTGTCTGATCCTGCGTTTTTGACTCAGCCTTCTTCGTTTGTA
ACCTTTGATAATCCTGTGTATGATCCTGAGGATGAAACTATTATTTTTGAGCGTCCTTCT
CCCGGCACTCGTGTGCCTGATCCCGATTTTATGGATATTGTTAAGCTGCATAGGCCCGCA
TTAACATCTCGCAGGGGCACGGTGCGCTTCAGTCGCGTTGGTCAGAAGTTTAGCATGCGC
ACTCGCAGTGGCACAAACATAGGTGCCAGGGTTCACTATTATCATGACCTGAGTCCCATA
CTTCCCACCGAGGACATAGAGTTGGAACCACTGCTCCCCCCTGCAGATCCCACTGCTGAG
GAGTCTCTGTATGATATATATGCTGATGTGGACGAGGCTGACATGGCTTTTACAGGCGGT
GGTCGCGGCGCCACCACTTACGGGGGTCGCATTACTCCATCTGTATTTTCCTCCACACTG
TCTACGAGGTATGGCAATGTCACTATTCCATTCGTGTCGCCAGTTGATGTGCCTTTACAC
ACGGGCCCTGATATTATTCTGCCCTCCTCTGCACAATGGCCTTTTGTTCCTGTAGCACCC
GCAGACACGACACATTATGTGTACATTGATGGAGGGGATTATTTTTGTGGCCTGTTACC
TTTCCTGTGTCCCGAAAACGTCGCCGTAAACGTCTTTCATATTTCTTGCAGATGGCTTT
GTGGCGCTCTAGTGACAACCTGGTGTACCTGCCTCCCACCCCAGTCTCAAAAGTTATCAG
CACGGACGACTATGTGACACGCACAAATATTTATTATTATGCAGGCAGTTCTCGCCTGCT
CACTGTGGGTCATCCACATTATTCAATTCCCAAATCCTCTGGTAATAAGGTAGATGTGCC
TAAGGTGTCTGCATTTCAGTACAGGGTTTTCCGTGTGCGTTTGCCTGACCCTAATAAGTT
TGGTTTGCCCGATGCCCGCATATATAACCCTGAGGCAGAACGTTTGGTGTGGGCCTGCAC
TGGTGTGGAGGTAGGTCGAGGGCAACCTCTCGGTGTCGGGTTGAGTGGACACCCCTCTGTA
TAACAAACTGAATGACACAGAAAACTCTAATATTGCACATGCTGAAAATGGTCAGGATTC
CAGGGACAACATTGCTGTTGACTATAAGCAAACACAACTGTGCATTCTGGGCTGTACGCC

TCCCATGGGCGAACACTGGGGTAAGGGCACTGTGTGTGCACGCACTAGTTCCGCTGCTGG
TGATTGCCCCCCCCCTGGAGTTAATGACCACACATATTGAGGATGGCGATATGGTGGATAC
CGGGTACGGTGCCATGGACTTTGCTGCTCTGCAAGTTAATAAGTCTGATGTGCCCCTTGA
TATTTGCCAGTCTACGTGTAAATATCCTGACTACTTAGGCATGGCTGCTGACCCCTATGG
CGACAGCATGTTTTTTTTTCTGCGTAGGGAACAACTGTTTGCCAGGCACTTCTTTAATCG
TGCTGGTGTAGTAGGGGACAAAATCCCAGATTCCTTGTACTTAAAGGGTAACAACGGGCG
AGAAACTCCTGGCAGTGCCATATACAGTCCCACACCTAGTGGGTCCATGGTAACGTCTGA
GGCTCAAATATTTAATAAGCCTTACTGGCTACAGCAGGCCCAGGGACACAACAATGGTAT
ATGCTGGGCCAATCAGGTATTTTTAACTGTGGTGGACACCACACGCAGCACCAATATGTC
GTTGTGTGCTACCACAGAGTCTCAACCGTTGACCACTTATGATGCTACCAAGATTAAAGA
ATATTTGAGACATGGGGAGGAATATGATTTGCAGTTTATTTTCCAGTTGTGTAAAGTTAC
ATTGACACCTGAAATTATGGCTTACCTTCATACTATGAACAGTGCCTTACTTGAAGACTG
GAATTTTGGATTGACATTGCCACCTTCCACTAGCTTGGAAGACACGTATAGGTTTGTAAC
ATCCTCTGCCATAACTTGTCAAAAAGATTTGGCCCCTACAGAAAAGCAGGATCCGTATGC
AAAGCTAAATTTCTGGGATGTAGATTTAAAGGATAGATTTACCCTGGATTTGTCACAGTT
TCCCCTGGGACGTAAATTTTTATTACAGATCGGTGCGCGCCGGCGTTCAGTAGTCCCCTC
CAGAAAGCGCCGAACGACCACCACGGCCCCCACCCCTGCAAAGCGAAAACGCTCGAAAAA
GTAACCCCAGTGTTGTTGTGTGCTGTATGTTGTGTAATGTAATGTGTGTATGTATTTATT
ACCATATGTGTTTGTATGTCTGTATGTCTGTACAATGTATGTATGTATACTTCAACTATG
TATGTGTGGATGTATAAATAAAGTATGTCACATAGTTTTATATTTTATACATATAATTGT
TTGCTGAGTAAGAAGTTAAGGTATAGGTCAGGGGACCGATTTCGGTCTAAAATGGCCGCC
GGTGCAGGTGTGCACACCACTAATTACTCATATTATTCAATTTCCTGCGACATGCCGTCT
CACGCACAGTTTTGGCAGCAATTTTTTGCTTTCCACTGTTTATTTTACTGCTGTATCATT
CTTCTTGGCAAGTTTGCACATATACATTGCAAATTCGCTGCTTCTGGGCACCAACTTATT
ATGACTACTTTCACATAATTACTGTCTTGGCCCAGTTTTCTAAGTTATCTTGCCAATAAA
ACGTGTTTGCAAATCTCCACCTTAACAATGTGTTTCCATGACACACCTAATCCGGTCGCT
GCTTGCTTTCTAACCTTAATTAATGCAGCTGCCACACCTGTCTTTCTAACTATAAT
>gi|1020202|gb|U31783.1|HPU31783_Human_papillomavirus_type_28,_complete_genome
TAAATAATAATGAACAGAAGCAAAAGTAGGGAGGGACCGAAAACGGTACGACCGAATGGG
GTACATATAAAAAGAGGCTTACATTGAATGGCAGAAGCTCAATGGATGACCAACGGCCTA
AAAACATATTCCTATTGTGCAGAGACAGTGGAATATCATTTGATGACCTCCGTTTGCACT
GCATATTCTGCGCCAAAGTGCTGACCACAGCGGAGCTATCAGCATTTGCATTAAGAGAAC
TAAATGTGGTGTGGAGAACGGGAGCGCCCTATGGTGCCTGTGCCCGGTGTTTACTACTGC
AGGGCATTGTGCGACGCCTGAAGCACTGGGACTATTCATTGTATGTGGAAGGCGTGGAAG
AGGAGACAAAACAATCTATAGATACACAGCAAGTGAGATGCTACATGTGTCACAAACCAC
TGGTTAAAGAAGAAAAGGACAGACACAGGAACGAACGGCGACGACTGCACTGCATAGCTG
GCTACTGGAGAGGGAGCTGTCAATACTGCTGGTTAAGATGCACGGTCCGCATCCCACAGT
AAAAGATATAGAATTGAGTCTTGCACCAGAGGACGTCCCAGTGCAATGCAATGTGCAATT
AGATGAAGAAGATTATACAAATGTGGAGGAACCAGCACAACAAGCGTATAGGGTGGTAAC
ACTGTGTCCAAAGTGTAGTTCACCACTGCGACTGGTGGTAGAGTGCAGCCACGCAGATAT
AAGGGCATTGGAGCAGCTCCTGTTGGGCACACTGACGGTCGTGTGCCCTCGCTGCGTGTA
ACTGGACATGGATGATACTTCAGGTACAGAGGGGGACGAATGTTCAGAGTTGGAACGGGC
TGGGGGATGGTTTATGGTAGAGGCCATAGTAGACAGGCGGACGGGCGATAAGCCATCTAG
TGATGAGGATGAGGACGAGGATGCAGACGAAGGGGAAGATTTTGTAGATTTTATAGATGA
CAGGCCTGTAGGGGATGGACAGGAAGTGGCACAGGAACTGTTGCTGCAGCAAGCAGCTGC
AGATGATGATGTAGCAGTTCAGGCTGTAAAACGAAAGTTTGCTCCCAGTCCGTATTTCAG
TCCTGTGTGTATGCAACCCAGCATAGAAAATGAACTCAGTCCGAGGCTAGATGCCATAAA
GCTGGGGAGACAGTCGGGGACAGCCAAACGCCGGCTATTCCAGCTACCGGACAGTGGGTA
TGGCCAAACACAGGTGGATACGGAATCGGGACCACTGCAGGTACAGGACATTTGTGAGAC
GGGGACACAAGATGGCCGCCAGGACGCGGATGAGGGGAGCGGTAGGAATGTGGGGGGAAA
TGGCGGCCAGGAGGAGGAGCGTGCAGGAGGGGATGGGGAGGAATCGCAGACTCAGGGTGT
ACAAACAGACAAGGCAGCCTGTGGAGTGTTGGCAATATTAAGAGCTAGCAATCAAAAAGC
TACGCTACTCGGTAAGTTCAAAGAACAATTTGGGTTGGGATTTAATGAACTTATCCGACA
CTTCAAAAGTAGCAAAACCGTTTGCCTAGATTGGGTGGTATGCGTGTTTGGGGTATACTG
TACATTGGCAGAGGGCATTAAAACATTGATACAGCCTCAGTGTGACTATGCACACATACA

GGTGCTGTCCTGCCAATGGGGGATGACAGTGCTGATGCTGGTACGGTACAAACGTGCCAA
AAACAGAGAAACTGTGGCTAAAGGATTAAGCACATTACTAAATGTGCCAGAGAGCCACAT
GTTGATAGAACCACCTAAACTAAGAAGCGGCCCAGCAGCGCTGTATTGGTATAAGACAGC
CATGTCAAACTGTAGTGATGTGTATGGGGAAACACCAGAGTGGATAGTGAGACAAACGAT
GGTTGGACATGCACTAGAGGAAGCACAGTTTAGTCTGTCGGAGATGGTACAGTATGCATA
CGACCATGACATTACAGATGAGAGCATGCTGGCATTTGAATATGCACTACTAGCAGATAC
AGATGCCAATGCAGCCGCGTTCCTAAGTAGCAATTGTCAGGCAAAATATGTAAAGGACGC
TTGCACAATGTGCAGACACTACAAAAGAGGTGAACAGGCGCGCATGAACATGTCAGAATG
GATATGGTTCAGAGGTGATAAAGTACAGGGCGATGGGGACTGGAAACCCATAGTACAATT
TTTAAGATATCACGATGTGGAATTTATACCATTCCTGTGTGCCTTTAAAACCTTCCTGCA
AGGAATTCCAAAAAAAAGTTGTTTAGTGTTCTATGGTCCAGCAGATACAGGAAAGTCATA
TTTCTGCATGAGTCTATTAAGATTTCTAGGTGGGGTGGTAATATCTTATGCCAATTCAAA
CAGCCATTTTTGGTTGCAACCGTTAGCTGATGCCAAAATAGGGTTGCTAGACGATGCCAC
CAGCCAGTGCTGGTGCTACATAGATACGTATTTAAGAAATGCATTAGATGGAAACCAGGT
TTGCATAGACAGGAAGCATAGAGCACTGCTACAGCTGAAATGTCCTCCTCTATTAATAAC
AACAAATATAAATCCACTAGAGGATGACAGATGGAAGTATCTGCGCAGCAGAGTACAACT
GTTTACATTCAAAAACAAATTTCCATTAACAACACAAGGAGAACCACTGTATACACTTAA
TGATCAAAATTGGAAATGCTTTTTTCGAAGGTTGTGGGCACGTTTAAGCCTTACCGATCC
TGACGACGAGGAGGAAAATGGAAACCCTAGCGAACCGTTTAGATGTGTGCCAGGACAAAA
TGCTAGAACTTTATGAAAAGGATAGCAACAAACTTGAGGACCAGATAATGCATTGGCAAT
TGATGCGTGTAGAAAATGCTTTGCTGTACAAAGCAAGGGAATGTGGATTGACACATATTG
GCCATCAGGTGGTGCCACCTCTTAGTGTAACTAAAGCTAAGGCACGCAGTGCCATTGAAG
TGCATGTAGCTTTGCTACAATTACAAGAAAGTGCATATGCACAAGACTCATGGACACTGC
GAGACACATCACGAGAAATGTGGGACACAGTACCCAAGAAGTGCTGGAAAAAAAGAGGTG
TAACCGTGGAAGTTAGATATGATGGAGACGAAACCAAATCCATGTGTTATGTACACTGGA
GAGACATATTTACTCAGAACTATAGTGATGATAAATGGGTAAAGGTGGCAGGACACGTGT
CATATGAGGGACTGTATTACATACACGAAGGAGAACAAACATTTTATGTCAAATTCAAAG
ATGACGCGTACGTGTATGGGGAAACAGGAAAATGGGAGGTACATGTGGGAGGCAAAGTAA
TTCACCACCATGCATTTGACCCCGTATCTAGCACACGAGAAATACCCGCTGCTGGACCTT
TGTGCACCGGTGACACCACCAAGGCGTCGACCGAAACCTCGGTGGGCGCGACCGAAGGAC
CGCAGCAAAAACGACAGCGACTCGAGACACTCAACTGGGAGCAGCAGCAGCGACAGTACC
CCCAAACCCCCTCCACGCAAACCACTGAACGAGCAAGTCAACCACTGGACGTTACAAGGA
CCAGCGACTGTGACACTACATGTCCATACACCGTCGGGCACCCAAGTGACCCTGACTGTG
CACCTGTAGTACACCTAAAAGGTGATCCTAATTGCTTGAAATGTTTTAGATATAGGTTAC
ACAAAGGGAAAAGAAAACTGTATTGTAAAACCTCTTCCACATGGCGGTGGAGCTGTGAAT
CAGAAAATCAAGCAGCTTTTGTAACTATTTGGTATACAAGTTATTCACAGCGTAATGAAT
TCTTGTCCACTGTAAAGGTACCACCTGGTATACAGGTAATATTGGGACATATGTCAATGT
TTGTATAATTATCGAACCGTGTGGTGATACAGTGTGCAGACTGGATACTTATTTGTGCAG
GCTACATTTTTGGGCTTATTTTGTGCTGCTGCTGTGTTCGTTTTGGCTGTGTGTGCTTCC
TGCGCTACCTTGCTATCTGGCAATTGTGCTGTGTTTGTACCTGGGCCTGGTAGCATTATA
TTTGCGAATTGTATCACATATTGTACAAATCAATACATAAGCCTTGCTATGTATCCTTTG
GTGCTAAGGGACAACAATGGCGATCATACTGTCCTGTTTCTGGAGCCAGATGACGTGTAT
CTATTGTTGTTGTTTATATTTTTTGTTATACTTGCATTGTTTATCTTGTATAGACATTTG
GGTTTTTTGTAACTGTGTAGTTGTGTGTTAGTTGGTCACCACAATGTATTTTTAAGTACT
GTGTATATCCCAGTGTGCTATTGAGCCACTGCCTTTTCTACACTTGTTTTTTTTTTTTAC
AGTTCAATAAAGCAACCATGGTGGCACATCGTGCAAGGCGTCGCAAGCGTGCATCCGCCA
CACAGCTTTATAGGACCTGCAAGGCCGCAGGCACATGCCCCCCTGATGTTATTCCAAAAG
TGGAGGGCACTACTCTGGCAGATCGTATTTTGCAGTGGGGCGGGCTGGGTATTTATTTGG
GGGGTCTGGGGATTGGTACCGGATCTGGGACAGGGGGTCGCACGGGATATGTTCCTCTTA
GCACACGGCCTGGGACTGTTGTTGATGTTAGTGTCCCTGCTAGGCCTCCTGTGGTAATTG
AACCTGTAGGACCATCTGACCCCTCCATTGTAAACTTATTGGAGGACTCCAGTATTATCA
ATTCGGGTTCCACCGTTCCAACTTTTTCTGGTACTGGTGGCTTTGAGGTGACTTCTTCAG
CCACAACTACCCCTGCTGTGTTGGACATCACCCCTGCCACTGACAATGTGGTCATTAGTA
GTTCCAACTTTACCAACCCTGCCTTTACTGAACCCTCGTTGTTAGAGGTTCCCCAAAATG
GTGAAGTGTCGGGACACATTCTTGTTAGTACCCCCACGGCTGGTACACATAGTTATGAAG

AAATACCTATGGAGACATTTGCTTCGCCAGGTACTGGCAATGAACCTATTAGTAGCACCC
CTGTACCTGGTGTAAGTAGGATCGCTGGACCCCGTCTATATGCCAAAGCTGTCACACAGG
TTAAAGTAACAGATCCTGCTTTCTTGTCTCGTCCCACCTCATTGGTTACTTTTGACAATC
CTGCATTTGAGCCGGGGGATGAAACTATTATATTTGAACGCCCATACCCTCCCTCACAAG
TGCCTGACCCAGATTTTATGGACATCATACGTTTGCATAGGCCTGCCTTAACATCTCGCA
GGGGTACTGTGCGTTTCAGTCGCCTCGGTACAAAACTTAGCATGCACACTCGCAGTGGCA
AGGGTATAGGAGCTAGGGTACATTATTATCAGGACCTAAGCCCTATAGGCCCCACGGAAG
ACATTGAAATGGAGCCTTTGCTTGCTCCTGCTGAAAACGCTGCTGGGGATTCTATTTATG
ATGTGTTTGCGGACGTGGAGGACGCTGACATAGCTTTTACTGGTAGAAGTCGCAGCGCCA
CGTCATCTCGGGGATACACTACGGTGTCCCCACTGTCTTCCACACTAACTACCAAGTATG
GCAATGTCACCATTCCCTTTGTGTCTCCTGTGGATGTGCACTTACACCCTGGGCCTGATA
TTATAACGCCTGCGTCTACGCAGTGGCCGTTTGTGCCCTTGGTTCCTGCTGACACAACCC
ATTATGTGTACATAGATGGCGGGGATTTTTATTTATGGCCTGTTACCCTGTTTGTACCCC
GACGTCGTCGCCGTAAACGTCTCTCATATTTTCTTGCAGATGGCACTGTGGCGCTCTAGT
GACAACCTGGTGTACCTGCCTCCCACCCCTGTTTCCAAAGTTCTCAGCACGGACGACTAT
GTGACACGCACCAATATTTATTATTATGCAGGCACTTCTCGGTTGCTGACCGTGGGTCAT
CCTTATTTTCCCATTCCTAAATCATCCACTAACAAAGCAGATGTGCCCAAAGTGTCCGCC
TTTCAGTATAGGGTATTCCGGGTGCGGTTGCCTGACCCAAATAAGTTTGGCCTGCCAGAT
GCACGCATATATAACCCTGATGCAGAAAGACTGGTCTGGGCTTGCACTGGGGTTGAGGTA
GGCCGCGGGCAGCCTTTGGGTGTCGGCCTCAGTGGACATCCTCTCTATAACAAGCTAGAT
GACACTGAAAACTCTAACATAGCACATGGGGACATTGGACAGGATTCCCGAGACAACATA
TCTCTTGATAATAAGCAGACGCAGCTATGTATTGTTGGTTGCACCCCTCCAATGGGGGAG
CATTGGGGCAAGGGGACCCCATGTCGTCAAAATACACAACAGGGAGATTGCCCTCCGTTG
GAGCTTATAACTGCCCCTATTCAGGATGGTGATATGGTGGACACTGGCTTTGGTGCAATG
GACTTTGCTGTATTACAGGCCAATAAATCGGACGTGCCCTTAGATATTTGCCAGTCCACT
TGCAAATATCCTGATTATTTAGGCATGGCCGCGGAGCCGTATGGCGACAGCATGTTTTTT
TACTTGCGAAAGGAACAACTTTTCGCTCGGCATTTTTTTAATAGAGCTGGTGTTGCTGGG
GACACCATTCCTGAAACATTGTATATTAAAGGTCAGGGCAACGGGAGGGATGTGATTGGT
AGTGCTGTGTATAGTCCTACACCTAGTGGATCTATGATCTCCTCTGAGGCCCAGCTTTTC
AATAAGCCATATTGGCTGCGGCGTGCTCAGGGACACAATAATGGTATCTGTTGGGCCAAC
CAATTGTTTGTAACTGTAGTGGATACTACACGCAGTACAAACATGACGTTGTGTGTTTCT
ACTGACTCTTCAGCTACGTACGATGCTAGTAAATTTAAGGAATACTTAAGGCACGGGGAG
GAGTACGATTTGCAGTTTATATTCCAGTTGTGTAAAGTAACCTTGACCCCTGATATTATG
GCATATTTACATACCATGAACAATAGTTTATTGGAGGACTGGAACTTTGGGTTGACTTTA
CCACCATCCACTAGCTTGGAGGACACGTATAGGTTCATATCTTCCTCTGCCATTACCTGT
CAAAAGGATGCTTCCCCCACTACCAAGGAAGACCCTTACGCTAAACTAAACTTTTGGGAA
GTGGATCTTAAGGATCGCTTTTCTCTTGATCTATCGCAATTCCCTCTGGGAAGGAAATTT
TTAATGCAGTTGGGTGTAGGTGCCCGCTCCAGTGTCTCCGTTCGCAAACGTCCAGCGTCC
ACCACGAGAGGATCGTCTGCTGCAAAACGAAAACGCGCCAAAAAGTAGCACTACTGTGTT
TTGTATGTTTGCCTGTTGTTGTCCTGTGTGTCAGTGTATGGGCGTCCCTGATATGTGTTT
GTACAAAGTATGGTGTATGTATTTGTGTATGCATGTAATGTGGTTATAACTGTGTTTTGT
ATGTTTGCCTGTTGTTGTCCTGTGTGTCAGTGTGTGGGCGTCCTGCTATGTGCTTGTACA
AAGTATGGTGTATGTATTTGTGTATGCATGTAATGTGGTTATAAGTTATCATAATAAACT
GTGTCACATAGTTTTATATTTTTTAATTTCTGTAATTGCTGATCCTGTGAGTAAGAAAGG
TGGTTCCAGGTCAAGCGACCGATATCGGTATCCAAAATGGCCGCCTATGCAGGTGTGTTC
CAGGTGTGCTTAATACCAATTAGTCATACAGATCCATATCCTGCAAACCTGCCCTGTCTC
GCAAGTTTTTGGCTGTGATACTATCTTTCTATAGTTTATTTCATTTGCTGTATCATTCTT
TCTGGCACGTCAAAATGTCTCCATTGCAAATTAACTGCTCCTGGGCACCAACTTATTATG
ACTACTTTCACATAATTACTATCTTGGCTGGGTTTTATAGGTTGCCTTGCCAATAGAATG
TGCTTCCAAATCTCCACCAAGACACACCTAATCCGGTCGCTGCTTGCTTTCTAGCCATAA
ATTAAGCAGTTGCTACACGTTTCTTTCCAACTATAATTA
>gi|1020186|gb|U31781.1|HPU31781_Human_papillomavirus_type_23,_complete_genome
AGCAGATACCATCAGCACCTGGAGCGACCGCCAAGACTTCGCCAACTTGGCAGAACATTT
GTTGGCAAGAAAAGAGCACCGATAACGGTAAGAACTTTTATTTTTTGACCGTAGGCGTTC
ATTTACTAACCTTGGCAACAATTGTGGTTAACAACAATCATAAGCCAATAATACATGCAA

CCGCTTGTGGTAATTTATTATGCAGACTGTGCATTATTTAAGTAGGATGTGCTACACCAA
ATTATTGATGGACTCGACGCGACCACTGACGGTACAGCAACTTAGTGATAAGTTGACAGT
ACCAGTGGTAGATCTCTTGCTACCTTGCAGATTTTGTTCTAGGTTTCTTACCTATTTAGA
GTTGCGAGAATTTGATTATAAACATTTGCAGTTAATCTGGACAGAAGAAGATTTTGTATT
TGCATGCTGCAGTGGCTGTGCTTATGCTTCTGCTCAATTTGAAATTCAACAATTTTATCA
GCTAACTGTGTATGGTCGTGAAATTGAGCAGGAGGAGCAACGACCTATAGGCCAAATTTG
TATTAGGTGTCAGTATTGTTTGAAGTCTCTCGATTTGATAGAAAAGCTAGATATCTGTAG
TTTTAATCAACCATTTCACAAGGTTAGAAATCATTGGAAGGGAAGGTGCAGGCATTGTAA
GGAAATAGAATGATTGGGAAACAAGCTACTCTTCGTGATATAGTTCTTGAAGAGCTTGTC
CAGCCCATTGACCTGCATTGCCACGAGGAGCTCACTGAAGAGGTAGAAGAAGCAGTCGTA
GAGGAGGAGCCTGAATACACTCCTTACAAGATCATCGTAGTTTGTGGAGGCTGTGAGACA
CAGTTAAAGCTTTACGTGCTAGCCACAGATTTTGGAATTCGCTCGTTCCAAGCATCTTTG
CTAGAAAACGTGAAGCTGGTGTGTCCTGCCTGTCGAGAAGACATTCGCAATGGACGACGA
TAAAGGTACTGATACTGCTAAAGAAGGCTGTAGTACTTGGTGCTTATTAGAGGCTGCTTG
TTCTGATGATAGTGACCTAGATGATAGTTTGGAGAAATTATTTGAAGAGAATGCAGAGTC
AGATGTGTCTGATTTAATAAATGATGATGATAATGCTGCTCAGGGAAATTCCCGCGAATT
GCTATGTCAACAGGAGAGTGAGGAATGCGAGCAGCAAATACAATACCTAAAACGAAAGTA
TAATATCAGTCCAGAGGCTGTTCAGCAGCTTAGTCCACGTCTACAGTCTTTGAATTTGTC
GCCTGGGCATAAATCTAAAAGGAGATTGTTTGTGGAGCAAGACAGCGGACTGGAGTTATC
TCTAAATGAAGTTGAAGATTTTACTCAAGAGTTGGAGGTACCGGCGAGCGCTCCAGGGCC
GGCAGCCCAGGGTGGAGTAGGGCTGGGACATATTGAAAGTTTGTTAAGATGTAAAAATGC
TAAAGCAGTGTTGCTACATAAATTTAAGGAAGGTTTTGGAATTAGTTATAATGAGCTTAC
CAGACAGTTTAAAAGCAATAAGACCTGCTGTAAACATTGGGTATTGGCCATATATGGTGC
AAAAGAAGAGCTCATAGATGCGTCTAAGCAATTGTTACAACAGCACTGTTCTTATATTTG
GTTGCAGACATACACACCTATGTCACTTTATTTATGTTGCTTTAATGTTGCAAAAAGTAG
AGAAACAGTTGTAAAATTATTGATTTCTATGCTGCAAATACATGAAAATCATATATTATC
AGAACCTCCGAAAAACAGAAGTGTACCTGTAGCTTTATTTTGGTATAAAGGCAGTATGAA
CCCTAATGTATATGCATTTGGTGAGTATCCTGAGTGGATTGTGACACAAACCATGATACA
ACATCAAACTGCTGACAGTATACAATTTGATTTGTCTCGTATGATTCAATGGGCCTACGA
TAATGATCATCTTGACGAATGTAGTATTGCTTATAACTATGCAAAATTGGCTGACACAGA
CAGCAATGCAAGAGCTTTTTTAGCTCAAAATAGCCAAGCAAAACATGTAAGAGATTGTGC
ACAGATGGTTAAGCATTATAAAAGAGGTGAAATGCGAGAAATGACTATTTCTGCATGGGT
ACATCATTGCATATCTAGAATTGAAGGTGATGGACAATGGCAAGATATTGTTAAATTTTT
GCGCTATCAGGGATTAAACTTCATTGTATTTTTAGATAAATTTAGAACGTTTTTACAGAA
TTTTCCAAAAAAAAATTGTTTGTTAATATATGGGCCTCCAGACACAGGCAAATCAATGTT
TACTATGTCTTTAATGAAAGCACTAAGAGGTCAAGTAATATCGTTTGCAAATTCTAAAAG
CCAATTTTGGCTGCAACCATTAGCTGATGCAAAGATCGCCTTATTAGATGATGCAACAGA
AGTTTGTTGGCAATATATTGATATGTTTCTTCGAAATGGATTGGATGGTAATGTAGTGTC
GTTGGATATGAAACATAGAGCACCATGTCAAATGAAATTTCCACCATTAATTATTACATC
TAATATTAGCCTTAAGAAAGAAAAGAAGTTTCCTTACTTGCATAGTAGAATATATGAATT
TGAATTTCCAAACAGATTTCCATTTGATTCAGATGATAAACCTTTGTTTAAACTTACTGA
CCAAAGCTGGGCGTCTTTTTTTAAAAGGCTTTGGATACAATTAGGACTCAGTGACCAAGA
GGACGAGGGAGAGGATGGAAGCACTCAGCGAACGTTTCAGTGCACTACAAGACAAGTTAA
TGGACCTGTATGAATCAGGTTTAGAGGATCTTGAAACTCAAATACAGCATTGGAAACTCT
TAAGACAAGAACAAATTTTATTGTATTATGCTCGAAAACGTGGAATTATGCGTTTGGGGT
ACCAGCCGGTACCTCCTCTGGCAACATCAGAAATTAAAGCAAAAGATGCTATAGCAATTG
GAATTTTGCTGGAAAGTTTACAAAAATCCAAATATGCAGATGAGCCATGGACATTAGTTG
AGACTAGCTTGGAGACAATTAGAAGTCCACCAGTAGATTGCTTTAAAAAGGGACCTAAAA
CAGTGGAGGTGTATTTTGATGGAGATCCTGAAAATGTTATGCCATATACAGTATGGTCTT
ATATTTACTATCAAACTGATGAGGACACTTGGGAAAAGGTTGAAGGACATGTGGATTATA
CAGGAGCTTATTTTTATGAGGGCCAACTTAAAAACTATTACATTAAATTTGAAGCAGATG
CAAAGCGCTTTGGTACTACAGGAATGTGGGAAGTACATGTTAATAAAGATACTGTCTTTA
CCCCTGTTACTAGTTCTACGCCGCCAGTTGGAGACGCCTCCAACAACGCCGTTCCCGAAG
CATCTACCACCTCCTTGTCCTCCCCACAACGGTCACCATCCACCAACCGCCGATACGGCC
GAAAAGCATCTAGCCCTACAGCCACCACCAGGAGGCAAAAAAGACAAGGAAAAGAAACCC

TCACCAGGCGAAGAAAAACCAGATCAAGGTCCCGGAGCAGAGAGCAACGGGGGGGGAGGG
AAACCCAAAGATCCTCCTCCAGAGGAGCCTCAAAATCCCCCCGGCGGGGAGGGAGAAGTG
GAGGGGGGCCCCTCACCCGCTCCAGATCAAGATCCAGATCACCAGAGTCTGTTACAGGGG
GTGGCGTTGCACCTAGTGAAGTGGGAGCGTCACTTCGATCAGTTAGTAGACACAGTAGTG
GAAGACTTGCGCAACTATTGGATGCAGCTAAAGACCCCCCAGTAATATTGCTGCGCGGCG
GTGCAAATACATTAAAATGCTATCGCTATAGGTTTAGAAAAAAGCATGCTGGTAAATTTT
ATTATGTTAGCACAACGTGGTCATGGATTGGGGGTCATTCTACTGATAGAGTAGGGCGTG
CAAGGATGTTAATAGCATTTCATTCTAATCATGAAAGGGAAAAATGTATTCAAGAAATGA
AGTTACCTTTAGGAGTAGATTGGTCCTATGGACAATTTGATGATTTATAACCTGCTTTTT
ATTTAACACACTAACATTGCCTATTGCTATTTTTTTACTAACTTATATTGCTATATTGCT
ACTAACATTATGGTACGGGCGCAAAGAACTAAGCGAGCGTCTGTTACTGATATATACAAA
GGCTGTAAAGCCTCTGGGACTTGTCCCCCTGATGTACTAAATAAAGTGGAACAAAATACA
CTTGCTGATAAAATACTTAAATATGGCAGTGTTGGTGTGTTTTTTGGTGGACTTGGAATT
GGTACAGGTAAGGGTACCGGTGGTGCCACGGGGTACGTCCCATTGCGACCTGGAGTACGA
GTGGGCGGTACTCCTACAGTGGTCCGCCCTGCAGTCATACCTGAAATAATTGGACCAACT
GAATTAATACCAGTTGACTCAATAGCACCAATTGACCCCGAAGCACCATCAATAGTCTCA
TTAACAGACAGTGGCGCAGCTGCTGACCTTTTCCCCAGTGAAGCAGAAACTATTGCAGAG
GTACATCCTACACCTGTAGACATAGGAATTGATACACCTATTGTAGCTGGAGGCCGTGAC
GCCATTTTAGAGGTGGTAGATACTAATCCTCCAACAAGGTTCAGTGTAACAAGAACACAA
TATGATAATCCATCTTTTCAAATAATTTCAGAATCCACACCTATCACAGGTGAGGCATCC
CTTGCTGATCATGTATTTGTGTTTGAAGGTTCTGGAGGTCAGCACGTAGGAGCGGTAACT
GAAGAGATTGAATTAGATACATATCCTTCCAGATATTCCTTTGAAATTGAGGAAGCTACA
CCACCACGCAGAACTAGTACTCCCATTGAAAGAATAAGTCAGGAATTCAGGAACCTACGT
AGAGCACTGTATAACAGGCGCTTAACAGAACAGGTTCAAGTAAAAAACCCTTTATTTTTA
ACTACTCCATCTAAACTTGTAAGATTTCAATTTGATAATCCTGTGTTTGATGAAGAGGTC
ACACAAATATTTGAAAGAGATGTTGCTGAAGTGGAGGAACCTCCAGATAGGGACTTTTTA
GATATAGACAGATTAGGAAGACCATTATTAACAGAATCCACTGAAGGCCGTATTAGATTA
AGTAGGTTAGGTCAAAGGGCTTCCATTCAAACACGCAGTGGAACACGTGTTGGTTCACGT
GTACACTTCTATACAGATTTAAGCACTATTAATACAGAAGAACCTATAGAATTAGAATTA
TTAGGCGAGCATTCTGGAGATGCATCAGTTATTGAGGAACCTCTGCAAAGCACTGTAATA
GATATGAACTTAGATGATGTTGAGGCTATTCAGGATACTATAGATACTGCAGATGATTAT
AACTCTGCAGATCTTTTATTGGACAATGCAATTGAAGAATTTAATAATTCTCAATTAGTG
TTTGGCACTTCTGATAGATCTTCGTCTGCATATTCTATACCACGGTTTGAATCCCCTAGA
GAAACAATTGTATATGTTCAAGATATAGAAGGTAATCAGGTAATTTATCCTGGGCCCACA
GAAAGGCCAACTATAATATTTCCCTTACCTAGTGCCCCTGCTGTAGTCATACACACATTG
GACAAGTCTTTTGATTATTACTTACATCCCAGCTTAAGAAAGAAAAGGCGCAAACGCAAA
TATTTATAATGTTTTTCAGATGACCCTCTGGCTTCCAGCTTCTGGTAAGATATATTTACC
TCCTACGCCACCTGTAGCCCGAGTGCAGAGTACGGATGAATATGTGGAAAGAACTGACAT
CTATTACCATGCAACTAGTGATCGATTACTAACTGTAGGCCACCCATATTTTGATGTTAG
ATCACCGGATGGTAGTAAAATAGATGTACCAAAGGTTTCAGGGAATCAATTCAGGGCCTT
TAGAGTTACATTTCCAGACCCTAATAAGTTTGCATTAGCAGACATGACTATCTATGATCC
TGATAAATACAGGTTGGTGTGGGCCTGCGCAGGACTTGAAATCGGCCGCGGCCAACCTTT
AGGGGTCGGCAGTACAGGACACCCGCTATTTAATAAGCTCCGTGATGCAGAAAATTCTAG
TGAACGTCAGGAAGGTACTGTAGATGACAGAAGAAATATCTCATTTGATCCTAAGCAAGT
ACAGATGTTTATAATTGGTTGCACACCGTGCTTAGGTGAATATTGGGATACAGCTCCTGT
CTGTAAAGATGCAGGTAGCCAACTAGGGTTGTGTCCTCCTTTAGAATTAAAAAACAGTGT
TATAGAAGATGGGGACATGTTCGACATTGGCTTTGGTAATATCAATAATAAAACATTATC
CTTTAATAGATCAGATGTTAGTTTAGATCTTGTAAATGAGGTTTGCAAATATCCAGACTT
TTTGACTATGTCAAATGATGTATATGGAGATGCCTGTTTTTTTTGTGCCCGAAGAGAGCA
ATGCTATGCCAGGCACTATTTTGTTCGAGGCGGTGTAGTAGGAGATGCAATACCTGATGG
TGCAGTTCAACAGGATCACAAATATTATTTACCTGCAGACCAACAAAACACTTTAGAAAA
CTCACTTTATTTTCCTACTGTCAGTGGATCTTTGGTAACTTCTGATTCTCAACTTTTTAA
TAGACCATTTTGGTTAAAACGTGCTCAAGGCCATAACAATGGTATTTTATGGAACAACCA
GATGTTTGTGACTGTAGCAGATAATACACGTAATACAAACTTTAGTATCAGTGTTACCAA
TGACAGCAGTTTAGAAAAGTATGATGCCACTAAAATTAGAGAGTTTACAAGACATGTTGA

AGAATACCAACTTTCTTTTATACTACAGTTGTGCAGGATACCTTTAAAGGCCGAGGTCTT
AACACAAATTAATGCCATGAATTCAGATATTTTAGAGAATTGGCAGTTAGGGTTTGTTCC
TACACCAGATAATGCAGTTCATGACACATACAGATATTTGGCTTCAAAGGCCACAAAATG
TCCAGATGCAGTACCTGACACGCAAAAAGAGGATCCTTTTGGAAAGTATTCATTTTGGAA
TGTTGATATGACAGAAAAATTGTCTCTAGACCTAGATCAATATCCCTTAGGCCGTAAGTT
TCTGTTTCAAATTGGAGTGCAGCGTGTACGGTCCGGTACCAAACGGCCTGCAACTCGAAA
AGTGACCAAAACTGTCAAAAGGAAAAAAGTGCAATTGTAACCGATATCGGTCGCCAATAA
AATATGTTAACTAATCTGGTATGTGAAGTATTTTTTAACCGTCTTTGTGACTAAACCGAA
CAAGTCAACACCAGCAACCGCACCCGTTTCCACATTATAAATTCCTCGAGGTAAGATTAT
GATC
>gi|1020178|gb|U31780.1|HPU31780_Human_papillomavirus_type_22,_complete_genome
CCGCCAAAGCTTTGCCAGGTCTTGGCAGAACATTTGCTGGCAAAGACTGCACCGATAACG
GTAAGAACTTTTAATTTTTAACCGTAGGCGGTTATTTGTTATTCGTAGCAACAATTGTGG
TTAACAACAATCTCCTGCCAGAATATACATGCAACCGCTTGTGGTAATTTATGCACTGCT
TGCATATTATTTAAGTAGGATGGGCTGCTATTCTGTATTCATGGCTTTGCAAAGACCACT
GACAGTACAGCAACTTAGTGATAAGTTGACTGTACCTGTAGTAGATCTTTTGCTACCTTG
TAGATTCTGCAGTAGGTTTTTAACCTATTTGGAATTGCGGCAATTTGATTATAAGAATTT
GCAATTAATTTGGACAGACGAGGACTTTGTGTTTGCATGTTGCAGCGGCTGTGCCTACGC
TTCAGCCCAATTTGAATTTCAGCAGTATTATCAAGTTACTTTGTATGGTCGTGAAATTGA
GCAAGAAGAACAACGACCTGTAGGCCAAATTTATATGAGATGTCAATATTGCTTGAAGTC
TCTTGATTTGCTAGAAAAGTTAGATATCTGCTGTTCCAATCAACCATTTCACAAGGTTAG
AGATCATTGGAAGGGAAGGTGCAGGCACTGTAAAGCAATAGAATGATTGGGAAACAAGCT
ACTCTGTGTGATATAGTTCTTGAAGAGCTTGTCCTGCCCATTGACCTGCATTGCCACGAG
GAGCTGCCTGAACTTCCAGAAGAGTTAGAAGAATCAGTGGTAGAGGAGGAGCCTGAGTAC
ACTCCTTACAAGATTGTAGTATATTGTGGGGGTTGTGATACAAAGCTGAAGCTGTATATA
CTAGCAACTCTCTCTGGAATTCGCGACTTTCAAACATCTCTACTTGGACCTGTAAAACTT
TTGTGTCCCACCTGTCGAGAAGAGATTCGCAATGGACGACGATAAAGGTACTGACACAAC
TGATGCTAAAGAAGGATGTAGTGGTTGGTTTATGTTAGAAGCTGCGTGCTCAGATGATAG
TGACTTAGATAATAGTTTGGAAAAGTTATTTGAAGATGGTACAGAGTCAGATGTATCTGA
TTTAATAAATGATGATGATACTGCTGCTCAGGGAAATTCCCGCGAATTGCTATGTCAACA
GCAAAGTGAGGAATGTGAGCAGCAGATTCAATATCTAAAACGAAAGTATTTCAGTCCAAA
GGCTGTTCAGCAGCTAAGTCCACGTCTGCAGTCTATGAATATTTCGCCTGGGCATAAATC
TAAAAGGAGATTATTTGTGGAGCACGACAGCGGACTGGAGTGTTCCCTAAATGAAGCTGA
AGATCTTACTGAAGAGGTGGAGGTACCGGCGAGCGCTCCAGCGCCGGCAGCACAGGGTGG
TGTAGGGTCGGGACATTACACCAGTTTGTTAAGATGCAACAATGTAAAGGCAGTATTGCT
GGGAAAATTTAAAGACGCATTTGGAGTGAGCTATAATGAGCTGACTAGACAATTTAGAAG
TAATAAGACTTGCTGTAAGCATTGGGTATTGGCCATATATGCTGCTAAAGATGAATTAAT
AGATGCGTCCAAACAATTGTTACAACAGCATTGTACCTATTTGTGGTTGCAAACATTCTC
ACCCATGTCATTATATTTATGTTGTTTTAATGTTGGGAAAAGTAGAGAAACTGTGATGCG
ATTGTTATCTTCCATGTTACAAGTTAATGAGAATCATATTTTATCAGAACCTCCAAAAAT
CAGAAGTATGATAGCTGCTTTATTTTGGTATAAAGGTAGTATGAATCCAAATGTCTATGC
ATTTGGAGAGTATCCTGAGTGGATTATGACACAGACTATGATACATCACCAAACTGCTGA
CAGTGTACAATTTGACCTGTCTGAAATGATACAATGGGCTTATGATCAAGATTATGTTGA
TGAATGTACTATTGCATACCAGTATGCTAGATTGGCTGATAGTAATAGTAATGCCAGAGC
ATTTTTAGCTCATAATAGTCAAGCCAAATATGTTAGAGAATGTGCTCAAATGGTTAGATA
TTATAAACGTGGAGAAATGCGAGATATGTCAATTTCTGCATGGATACATCATTGTATATC
AAAGATAGAAGGCGATGGTCACTGGCAAGATATTGTTAAATTTTTGCGATACCAAGGGTT
AAATTTTATAGTGTTTTTAGATAAATTTAGAACATTTCTAAAAAATTTTCCAAAGAAAAA
TTGTTTGTTAATATGTGGTCCTCCGGATACAGGAAAATCTATGTTTAGCATGTCATTAAT
GAAAGCATTAAGAGGACAGGTAGTTTCATTTGCAAATTCTAAAAGTCATTTTTGGCTACA
GCCTTTAGCAGATGCAAAACTGGCTTTATTAGATGATGCTACAGAAGTTTGCTGGCAATA
TATTGATGCTTTCTTAAGAAATGGATTAGATGGTAACATGGTATCTTTAGATATGAAACA
TAGAGCTCCATGTCAAATGAAATTTCCACCTCTTATTATAACATCTAACATTAGTTTAAA
AAAAGAAAAAAAAATTTCCCTATTTACATAGTAGAATATATGAATTTGAGTTTCCTAACAA
ATTTCCCTTTGACGCAAATGATACACCTCTGTTTAAACTTACTGACCAAAGCTGGGCGTC

TTTTTTTAAAAGGCTTTGGACACAATTAGAACTGAGTGATCAAGAAGAAGAGGGAGAAAA
TGGAGAAACTCAGCGAACGTTTCAGTGCACTACAAGAGAAGTTAATGGACTTATATGAAT
CAGGTGTAGAGGATCTTGAAACCCAAATTCAACATTGGAAATTATTAAGACAAGAACAAG
TGTTATTTTATTATGCAAGGAGACATGGGATATTGCGTTTGGGGTACCAACCAGTACCCA
CTCTGGCAACTTCAGAGAGTAAAGCAAAAGATGCTATAGCCATGGGACTATTGCTGGAAA
GCTTACAAAAATCACAATATGCAGAGGAACCGTGGACCTTAGTAGAAACTAGTTTGGAGA
CAGTTAAAAGCCCTCCAGCAGACTGTTTTAAAAAAGGACCTAAATCTGTGGAAGTGTACT
TTGATGGAGATCCTGAAAATGTAATGTCTTATACAGTGTGGTCATACATTTATTATCAGA
CTGATGATGAGTCATGGGAAAAGGTGGAAGGTCATGTGGACTATACAGGAGCTTACTATA
TAGAAGGGACCTTTAAAACCTATTATATTAAATTTGAAACAGATGCTAAACGATATGGTA
CAACAGGACATTGGGAGGTGCATGTTAATAAAGATACTGTGTTTACCCCTGTTACCAGTT
CTACGCCGCCAGTTGGAGTCGCCTCCCAGAACTCCGCACCCGAACCGGCATCCACCTCCG
ACTCCCCACAACGGTCATCACAAGTCACCCACCGATACGGCCGAAAAGCATCTAGTCCTA
CAATCACCACCATCAGGAGGCAAAAAAGGCGAGAGAGACAAAGACAAGAAACCCCAACAA
GGCGAAGAAAAACCAGATCAAGGTCCCGAAGCACCGAGCAGCGGGGAGGGAGGGCCACCA
GACGATCCCTCTCCAGAGAATCCGCAGAATCCCCCAGGCGGGGAGGGAGAGGTGGAGGGG
GCCCCCTCACCAGGTCCCGCTCAAGGTCGCGATCCCGTACACGAGAGTCTGTTGACGGGG
GTGGCGTCGCGCCTGACGAAGTGGGAGCAACACTTCGATCAATTGGTAGACAGCATAGTG
GGCGACTTGCGCAATTACTGGACGCAGCTAAAGACCCCCCAGTAATTCTGCTACGCGGTG
CAGCAAATACATTAAAATGCTATCGCTATAGATTTAGAAAGAAACATGCTGGAAGCTTCC
AATTTATTAGTACAACGTGGTCCTGGGTAGGGGGGCATACAACCGATAGAATCGGGCGCT
CTAGGATACTAATATCATTTCATACAGATAGGGAAAGAGAGAAGTGCTTGCAACAAATGA
AACTTCCTTTAGGTGTAGAATGGTCATATGGCCAGTTTGATGATTTATAAACTGCTTTTT
TACTAACACACTAACATTGCCTATTTATACTAACCTATTTGCTTGCTACTAACAAAATGG
CGCGAGCGCGAAGAACAAAGCGAGCGTCAGTAACTGACATTTATAAAGGCTGTAAGGCCT
CTGGGACTTGTCCCCCTGATGTTATTAATAAAGTGGAACAAAATACACTTGCTGATAAAA
TTTTAAAGTATGGCAGTGTTGGTGTGTTTTTTGGTGGTCTTGGTATAAGTACAGGTAAGG
GTACCGGTGGTCCTACAGGCTATATTCCTTTAGGTCAAGGTCCTGGAGTGCGTGTGGGCG
CCACTCCCACAGTGGTCCGCCCCGGGGTCATACCTGAAATAATTGGACCAACTGAATTAA
TACCAGTTGACTCAGTAACACCAATTGACCCTGCAGCACCATCCATAGTGACATTAACAG
ACAGTAGTGCAGGTGCTGACCTTTTACCTGGTGAAGTTGAAACTATTGCAGAAGTACATC
CGGTCCCAATAGACAATGTGGAACTTGACACACCTTTAGTTTCTGGGGACCGTCACGCCA
TTTTGGAGGTGACTGATGCTAATCCCCCTTTTAGGCGCACGGTTACCCGGACACAATATC
ATAATCCTGCTTTTGAAATTATTTCAGAGTCTACACCATTAATAGGTGAATCTACACCCT
CTGACCATGTTTTTGTTTTTGAAGGCTCGGGAGGTGTACAGGTAGGGGATGCTAATGAAA
GCATTGAATTGGATACTTTTCCTTCTAGATATAGTTTTGACATTGAGGAGCCAACCCCTC
CTCGTAGAGTTAGTACACCAATTGAAAGAATCAGTCAGGAATTTAGAACTTTAAGAAGAG
CCTTATACAACAGAAGATTAACAGAACAGGTCCAAGTAAGAGACCCCTTGTTTATTCGAT
CCCCGTCCAGGCTTGTGAGATTTCAATTTGATAATCCAGTATTCGATGAGGAAGTTACAC
AAATATTTGAAAGAGATGTAGCTGCAGTAGAAGAACCACCAGACAGGGATTTTTTAGATA
TTGAAAGACTTGGAAGGCCTATACTAACAGAAACTGCAGAAGGCCGTGTTCGTGTCAGCA
GGTTAGGGCAACGTGCATCGCTGAGCACACGCAGCGGCGCACGTGTAGGTGCTAGAGTGC
ATTTCTTTACAGATATTAGCACTATTAATGCAGAAGAGCCCATTGAATTAGAATTATTAG
GTGAGCATTCTGGCGACAGCTCTGTAGTACAAGAACCATTTGAAAGCACAATATTGGATG
TCAATATTGACAACATACCTGAAAGTTTGGATACAAACATAGCAGAAACATCTGTAGACT
ATGATTCTGCTGATTTGTTATTAGACAACGGTGTGGAGGACTTTAGTAGGTCACAATTGG
TAATAGGTCCTTCAGATAGATCACTTCCATCTATTACTGTTCCACAATTTGAATCCCCTA
GAGAAACCATTGTGTACATACAAGACATAGAGGGTAATACAGTTGTATATCCTAAATATG
AAGAAAGGCCAACTATTATATTACCTACACCCTCGGGGCCTGCTATAATTCAATCACCTA
CACATTCCTCCTTTGACTATTATTTACATCCTAGCTTGCGAAGGAAAAAACGCAAACGCA
AATATTTATAATGTTTTTCAGATGACCCTCTGGCTTCCAACTTCGGGTAAGATATATTTG
CCTCCTACGCCACCGGTAGCCCGAGTACAAAACACGGACGAGTATGTGGAGAGGACTGAC
ATCTATTACCATGCTATAAGTGACCGTTTATTAACTGTAGGACATCCTTACTTTGATGTT
AGATCATCAGATGGAGCAAAAATAGAGGTCCCTAAAGTGTCTGGAAATCAGTTTAGGGCT
TTTAGAGTAACATTTCCAGATCCTAACAAATTTGCTTTGGGAGATATGACAATCCATGAT

CCCGAAAGGTATAGATTAGTATGGGCTTGTAAAGGGTTAGAAATAGGAAGAGGACAGCCC
TTAGGTGTAGGTACCACAGGTCATCCATTATTTAATAAATTACATGATACTGAAAACCCT
ACTGAACGCCAGGAAGGAACATCAGATGATAGAAGAAATGTTTCTTTTGATCCTAAACAG
GTTCAAATGTTTATCATTGGATGTATACCGTGTTTAGGTGAATATTGGGATAAAGCTCCT
GTTTGTGAAGATGCAGGCAGTCAGGTAGGATTATGTCCTCCACTAGAATTAAAAAATGGT
GTTATAGAGGATGGAGATATGTTTGATATAGGATTTGGAAATATAAATAATAAAACACTA
TCATTTAATAGATCTGATGTAAGCTTAGACATTGTAAATGAAATCTGTAAATATCCTGAT
TTTCTTACAATGTCAAATGATGTCTATGGCGACTCATGCTTTTTTTGTGCACGTAGGGAG
CAATGTTATGCACGACACAATTTTGTACGTGGTGGTCTTGTTGGTGATGCTATACCAGAT
GATGCAGTTCAACAAGATCATAAATATTACTTGCCTGCAGCTTCACAGACTGCTTTAGAA
AACTCCACTTACTTTCCAACCGTTAGTGGTTCTTTAGTAACCTCTGATGCCCAACTATTC
AACAGGCCTTTTTGGTTGAAGCGCGCGCAGGGCCATAATAATGGTATTTTGTGGAACAAC
CAAATGTTTGTAACAGTAGCTGATAATACCCGTAACACTAATTTTTCTATTAGTGTGGCA
AGTGACGGCACCACAGTTAATTATGATGCTAAAAAAATCAGAGAATTTATGCGCCATGTG
GAAGAATACCAATTATCCTTTATTTTGCAGCTATGTAGAATACCATTAGAAGCAGAGGTA
TTAACTCAAATTAATGCCATGAATCATGGCATTTTAGAAAATTGGCAACTAGGCTTTGTA
CCTACACCAGACAATTCTGTCCATGATACTTATAGGTATTTACAATCTAAAGCTACAAAA
TGTCCTGATGCTGTACCTGACACACAAAAGGAAGATCCCTTTGGTCAATATACTTTTTGG
AATGTAGACATGTCTGAAAAGTTATCATTGGATTTAGATCAGTATCCACTGGGTCGTAAA
TTTTTATTTCAATCTGGGTTACAACGTGCAAGGGCCAGTGCCAGGGTCAGTGTGAAACGT
TCTGCTACGCGGAAAACGTCTAAAACTGTAAAACGAAGGAAACTTACCTCTTAACCGTTT
TCGGTTGCTTTAATAAAATCTATTAACTAATCTGGTATGTGAAGCATTTTTTGACCACCT
TTGTGACTAAACCGAACAAGTCAACACCAGCAACCGCACCCGGTTTTTACATTATAAATT
CCTCGAGGTAAGATAACCATCAGTAGATACCATCGGCACCTGGAGCAA
>gi|1020170|gb|U31779.1|HPU31779_Human_papillomavirus_type_21,_complete_genome
ACGGTAAGTTATGCACCGGGTGCGGTCGAATTATTACTCATTCGATAGTTGTTGTTGCCA
GCTACCATTTAGGACAGCATGTTTTTGCCTGTAACGTTATCGACACATACTCACACCATA
TATATATATATATATATATATATATATATATATATATATATATTCATATATACATACTAG
GGAAGATGCCCTAGTACTCATGGCTGACTCTTCAACAGACAGTGCTGACGAAGGTCCTTC
TCCTAAGCGTAGACATTTAGAAGAAGAAAATACATCTAGCTTTTTAGAGCCACCATTACC
AGCTACAATTCGTGACCTAGCCAATCTGTTAGAGATACCATTGGATGATTGTTTAGTACC
TTGTAACTTTTGCGGTAATTTTCTTACTCATTTAGAAGTTTGTGAGTTTGATGAGAAAAA
GCTTAGTTTACTTTGGAAAGATCATTGTGTGTTTGCCTGTTGTCGTGTTTGTTGCGCAGC
AACAGCGACATATGAATATAATGAATTTTATGAATCTACTGTTGTAGGTAGAGATATAGA
AGAAATAACAGGCAAATCTATTTTTGATATTGATGTCAGGTGCTACAATTGCATGAAATT
TTTAGACTCAATAGAAAAGCTAGACATTTGTGGTAGGAAGTTTTTTTTTCATAAAGTGAG
AGGCTCTTGGAAAGGAATCTGTAGGCTGTGTAAGCATTTTCAATAATGATTGGTAAAGAG
GTCACATTGCAAGATATTGTTCTGGAGTTAAATGAATTGCAGCCTGAGGTACAACCAGTT
GACCTGTTTTGTGAAGAGGAGTTACCGAGCGAGCAGCAGGAAACAGAGGAGGAGCTACCA
GAAAGGACCGCGTACAAAGTTGTTACACCTTGCGGCTGCTGCAAGGTCAAGCTTCGCATC
TTTGTAAACGCTACACAATTTGCTATTAGAACATTTCAGAATCTGCTGTTTGAAGAATTG
CAGCTGTTGTGTCCTGAGTGCCGCGGAAACTGCAAACATGGCGGATCCTAAAGGTAGTAC
ATCTAAAGAAGGGTTGGAGGATTGGTGTATTGTGGAAGCTGAATGTAGCGATGTAGAAAA
TGATTTGGAAGAATTATTTGACAGAGATACAGACTCAGATATTTCAGAATTGTTAGATGA
TAATGACCTGGAGCAGGGAAATTCGCGGGAACTATTTCACCAGCAAGAGAGTAAGGAAAG
CGAGGAGCAATTACAAAAACTAAAACGAAAGTACTTAAGTCCTAAAGCTGTCGCACAGCT
CAGTCCGCGACTCGAAAGTATAACGCTGTCACCTCAGCAGAAGTCTAAACGAAGGCTCTT
TGCAGAGCAGGACAGCGGGCTCGAGTGTACTCTTACAAATGAAGAAGATGTTTCTTCTGA
GGTGGAGGTACCGGCTCTAGACTCTCAGCCGGTTGCTGAGGCACAATTAGGAACAGTAGA
CATTCATTATAAAGAGTTATTACGTGCCAGCAACAATAAGGCGATTCTTATGGCAAAATT
TAAAGAGTTTTTTGGGGTAGGATTTAATGATCTGACACGCCAATTTAAAAGTTACAAAAC
CTGTTGTAATGCTTGGGTTCTGTCTGTATATGCAGTTCATGATGATCTTCTTGAAAGCTC
AAAGCAGTTATTGCAACAGCATTGTGATTATATATGGATACGTGGGATAGGAGCAATGTC
ATTGTTTTTGTTATGTTTTAAAGTTGGAAAAAATCGTGGGACTGTGCATAAGTTGATGAC
TGCAATGTTAAATGTGCATGAAAAGCAGATCATATCTGAGCCACCAAAATTAAGAAATGT

TGCTGCTGCATTGTTTTGGTATAAGGGTGCGATGGGGTCTGGAGCATTTACTTATGGACC
TTATCCTGATTGGATTGCCCAGCAAACAATTGTTGGTCATCAAAGTACAGAAGCCAGTGC
ATTTGATATGTCTGCAATGGTTCAATGGGCGTTTGATAATAACTATTTAGATGAAGCTGA
TATAGCCTATCAATATGCTAAGCTAGCACCAGAAGATAGTAATGCTGTAGCATGGCTTGC
ACATAATAATCAGGCCAGATATGTTAGAGAAGTTGCATCTATGGTAAGATTTTATAAAAA
AGGACAAATGAAAGAAATGTCTATGTCAGAGTGGATACATACTAGAATTAATGAAGTAGA
AGGAGAAGGACATTGGTCAACTATAGCAAAGTTCCTTAGATATCAGCAAGTAAATTTTAT
AATGTTTCTAGCAGCATTAAAAGACATGCTACATTCAGTTCCTAAACGTAATTGTATATT
GATTTATGGTCCCCCTAACACTGGAAAGTCAGCATTTACTATGTCTTTAATTCATGTACT
AAGAGGGAGGGTGCTATCATTTGTGAATTCCAAAAGCCAGTTTTGGCTGCAGCCAATGTC
AGAATGTAAAATAGCATTAATTGATGATGTGACAGATCCATGCTGGATATATATGGATAC
TTATTTAAGAAATGGCCTAGATGGTCATGTTGTATCATTAGACTGCAAACATAAAGCACC
GATGCAAACCAAATTTCCTGCATTACTACTTACATCTAATATCAATGTGCATAATGAAGT
TAATTATAGATATTTGCATAGCAGGATTAAAGGCTTTGAATTCCCAAATCCATTTCCCAT
GAAAGCAGACAATACCCCTGAATTTGAGCTTACTGACCAAAGCTGGAAATCTTTTTTTAC
AAGGCTTTGGAATCAATTAGAGCTGAGTGACCAAGAAGACGAGGGAGAAAATGGAGAATC
TCAGCGATCGTTTCAATGTTCTGCAAGATCAGCTAATGAACATTTATGAGTCTGCAGCAA
ACACTATTGAGTCGCAAATTGAGCATTGGCAAACACTGCGAAAAGAAGCTGTGCTGCTTT
ATTTTGCTAGGCAAAAGGGTGTGACACGGCTTGGATATCAATATGTACCTCCATTAGCAG
TTTCAGAATCAAGAGCTAAACAGGCTATAGGGATGATGCTGCAGTTGCAATCATTGCAAA
AATCTGAATATGCAAAGGAACCATGGTCACTGGTAGATACCAGTGCAGAGACATTTAGAA
GCCCTCCTGAAAATCATTTCAAAAAAGGGCCAGTGTCAGTTGAGGTTATTTATGATAACG
ATAAAGACAATGCTAATGCTTACACCATGTGGAGATATGTTTATTACGTGGATGATGACG
ACCAATGGCATAAAAGTCCAAGCGGTGTCAACCACACAGGCATATATTTTATGCAAGGAA
CTTTTAGACACTACTATGTTTTATTTGCTGATGATGCAAGTAGATATAGCAGAACTGGAC
ATTGGGAAGTTAACGTTAATAAGGAAACTGTGTTTGCTCCTGTCACCAGCTCCACCCCAC
CCGACTCACCAGGAGGACAAGCAGACTCAAACACCTCCTCCACGACCCCCGCCACCACCA
CTGACTCCACGTCCAGACTCTCGTCCACCAGAAAACAGTCACAACAAACCAACACCAAAG
GAAGAAGGTACGGACGGAGACCGTCCAGTAGGACCCGGCGAACGACCCAAACGCATCAAA
GGCGGCGATCGAGGTCCAAGTCCAGGTCGCGGTCGCGGTCGCGGTCGCGGCTCCGATCCC
GATCCCGGTCCCGATCCCGGTCCTATTCCCGGTCCCGGTCTCAATCGTCTGACCAGCCGC
AATACCGATTCAGATCCGGAGGGCAAGTGTCCCTCATCACTACCGCCACCACCACCACCA
CCACCGCAACCAACTACTCCACCAGAGGGTCAGGGCGAGGGTCATCCTCCACCTCCTCCT
CCACCTCCAAACGGCCACGACGGCCACGAGGAGGGGCCATTGGAGGGAGCAGTGGGAGGG
GGAGACGGTCATCCTCCACCTCCCCCAGCCCCTCCAAACGGTCACGAGGAAAGTCAGAGT
CTGTTAGGCAACGTGGCATCTCTCCTGACGACGTGGGAAAGTCTCTTCAATCAGTTAGTA
CAAGAAATACAGGTCGACTTGGAAGATTACTGGACGAAGCTCTCGATCCCCCAGTAATCT
TAGTCAGGGGGGAACCCAATACGCTAAAATGCTTTCGCAATAGAGCCAAGCTTAAATACG
CAGGGTTGTATAAGGCTTTCAGTACGGCCTGGTCGTGGGTGGCTGGAGATGGTACTGAGC
GTCTAGGCAGGTCCAGAATGCTCATTAGCTTCTTCTCCTTTGAGCAAAGAAAAGATTTTG
ATAAGACTGTTAAATATCCGAAAGGTGTTGACCGGTCGTATGGTTCCTTTGATAGCCTAT
AGCAGCCTTTAACATACTAACTATAGCTCTGCTACTAACATATTAACACTTTTTGATTAT
ATATTTTTTTTTATTTTTATTTTTATGCTATGGCGCGTGCTAAGCGAGTCAAGCGAGAC
TCTGCTACTAATATTTACAGAACCTGCAAACAAGCAGGCACATGTCCCCCTGATGTTATT
AATAAAGTTGAAAGCACAACTATTGCTGATAAAATATTGCAGTATGGTAGTGCTGGTGTT
TTTTTCGGGGGGCTGGGCATAAGCACTGGAAAAGGTACAGGCGGTACCACAGGTTATGTG
CCTTTGGGAGAAGGTCCTGCAGTCCGTGTTGGCAATGCTCCTACGGTCATTAGACCTGCA
TTGGTCCCTGACACCATTGGCCCGTCTGATATTATTCCTGTGGACACCTTAAATCCAGTG
GAGCCCACAACTTCCTCTATTGTTCCACTCACAGACTCTACAGGCCCAGATCTGTTACCT
GGAGAAGTGGAAACTATTGCAGAAATACATCCTGGTCCGACCAGGCCTCCACCTGACACT
GCAGTCACTACTAGTACAAATGGTTCTAGTGCTGTTTTAGAAGTAGCACCAGAGCCTACC
CCTCCTTCTCGTGTTAGAGTAACCAGAACACAATATCATAATCCATCTTTTCAAGTAATA
ACTGAATCAACTCCTACTACAGGCGAAAGTTCTTTAGCAGATCATATATTAGTAACATCA
GGGACTGGGGGACAAACTATAGGGGGCAGTACACCTGAACTCATAGAACTCCAGGACTTT
CCTTCTAGATATTCATTTGAAATTGAGGAGCCAACACCTCCTAGAAGAACTAGTACACCC

ATTCAAAGAATTCAAAATATTATAAGGAGAAGGGGTGGCGGGCTCACAAATAGGCGTTTG
GTTCAACAGGTTAATGTAGAGAATCCTTTGTTTGTATCCAGGCCTTCTAGATTAGTGCAG
TTTCAATTTGATAACCCTGCATTTGAAGAAGAAGTGACACAAATATTTGAGCAAGATATT
GATACTTTCAATGAACCACCAGATAGAGACTTTTTAGATATTAAAACACTTGGTAGGCCT
CAATACTCAGAAACCCCTGCAGGTTACGTGAGAGTTAGTCGTCTTGGTAAACGAGGAACT
ATTCGTACTCGTTCAGGAACACAAATTGGTTCTCAGGTCCATTTTTACAGGGACCTTAGC
ACCATTAACACAGAGGACCCTATTGAACTTCAATTATTGGGTGAGCATTCTGGCGATGCT
ACAATTGTCCAGGGTCCAGTTGAAAGCACATTTATTGATATTAACGTTGATGAAAACCCT
CTTTCTGAAGATTTTAGTGCACATTCAGATGATTTACTTCTAGATGAGGCAAATGAAGAT
TTTAGTGGTTCCCAATTAGTGGTTGGAGGCCGCCGCTCCACTTCTTCTTATACTGTTCCA
CGTTTTGAAACTACTAGATCTGGTTCTTATTACGTGCAGGACACCAAGGGCTATTATGTA
GCCTATCCTGAAGATCGAGACACTAGTACAGATATAATCTATCCAACACCAGATTTGCCA
GTTGTAATCATACACACATTTGATACAAGCGGTGATTTTTACTTACATCCGAGTCTTAGC
AGAAAATTTAAGAGAAGAAGGAAATATTTGTAACCTTTTCTTTTGCAGATGGCAGTTTGG
CAAGCAGCTAGTGGTAAGGTTTACCTTCCACCGTCTACACCAGTTGCCAGGGTCCAAAGC
ACGGATGAATATGTACAAAGAACAAACATCTACTATCATGCATATAGTGATCGCTTATTA
ACTGTTGGTCATCCATATTTTAATGTCTATGACGTCAATAGTGCTAAGATAAAAGTACCT
AAAGTATCTGGGAATCAACACAGGGTATTCAGACTCAAATTGCCAGATCCTAATAGATTT
GCACTTGCAGATATGTCTGTATACAATCCAGACAAGGAAAGATTAGTTTGGGCCTGCAGA
GGTATAGAAATAGGAAGAGGGCAACCCTTGGGGGTGGGAAGTGTAGGTCACCCTTTATTT
AATAAAGTTGGGGACACAGAAAATCCTAGTTCATACAAAACTCAACCAAATTCTACTGAT
GATAGACAAAATGTATCATTTGATCCCAAACAACTACAAATGTTTATAATAGGCTGTGCA
CCTTGCTTAGGAGAACATTGGGATAAAGCTATCCCATGTGCAACTGACAATCCACCTCCA
GGATCGTGCCCTCCGATTGAATTAATTAATTCAGCAATACAAGATGGCGATATGGCAGAT
ATAGGATATGGCAATCTAAATTTCAAAGCCTTACAACAAAATAGGTCTGATGTTAGTTTA
GACATAGTTAATGAAACGTGTAAGTATCCAGACTTCTTAAAAATGCAAAATGATGTGTAT
GGAGATTCATGTTTCTTTTATGCACGCAGAGAGCAATGTTATGCCAGACACTTCTTTGTT
AGAGGGGGCAAAACAGGAGATGACATACCCGCAGGACAAATTGATGAGGGTAGTATGAAG
AATGCATATTACATTCCACCAATGAATGATCAAGCACAGTACAAGATTGGTAACTCCATG
TATTTCCCAACTGTCAGTGGCTCATTGGTGTCTAGTGACGCTCAATTGTTTAACAGGCCA
TTTTGGCTACAGCGTGCACAAGGCCATAATAATGGCATATGTTGGTTTAATCAATTATTT
GTTACAGTAGTAGACAACACTCGTAACACAAACTTTAGTATTTCAGTAAATCCTGAGAAT
GCAGACGTGTCTAAAATTGAAAATTATAAAGCCGAGAGCTTTCAAGAATATTTAAGACAC
GTTGAAGAATATGAACTTTCTTTAATTTTACAATTATGTAAAGTTCCTTTAACAGCAGAA
GTCTTAGCTCAAATTAATGCAATGAATGCAAATATTTTAGAAGAATGGCAGTTAGGATTT
GTTCCTGCCCCAGACAATCCTATTCATGATACATATAGATACATTGACTCTGCAGCTACT
AGATGTCCTGATAAAAACCCTCCAAAAGAACGAGAAGATCCTTATAAAAATATGAAATTT
TGGGATGTAGATTTAACAGAACGGTTGTCTCTAGACTTAGATCAATATTCTCTTGGAAGA
AAATTTTTATTTCAAGCAGGTTTGCAGCAGACGACCGTTAACGGTACAAAGACACTTTCT
TCAAGGGTATCTACCAGAGGAATTAAACGAAAACGCAAAAAATTAGACATGACCGTTTTCG
GTACAATAAAGTCAACTTTTACACAGTATTCAAGGAATGTTTATTTACTCTGACTAAGCA
AAATACCAACCGCGCCCGACACATAAAGGTGAGTTGTGAGCCAAATGAGGTGAGTTGTAA
GCCAAAAGAGGTCAGAGCCAAGTCTGTTCTGAGCCAGATCAGATACTACGCGCGCCAGAG
TTGGATCACATCTCGTTGTTCTAACACGCTAAGGACTCAAGGAAATGTAAGTCTGCCAAT
CGATTTTGGCTCGTGTTTTGGCAGAAGTTAGGACCGTTA
>gi|1020162|gb|U31778.1|HPU31778_Human_papillomavirus_type_20,_complete_genome
TCGGGCGCGGTCATACATTACTCATTTGGTAGTTGTTGTTGCCAGCTACCATCAAGCATA
GCATGTTTTTGCCTGTAACGTTATCGGCACAGTGATTAATATATATATATATATATATAT
ATATATATATATATATATATATATATATATATAGATACATATAGACAGATATCATAGAGCTA
ATGCAGAGAGTGCAGGCACATGGCTACACCTCCTTCTTCAGAAGACAGCGCTGATGAAGG
ACCATCTAATATTGGAGAGGCAAAACCTCCAATCTTAGAGCCACCATTGCCTGCAACAAT
CTGTGGCCTAGCAAAACTTTTAGAAATACCGCTAGATGATTGTTTGATACCTTGTAACTT
CTGCGGTAATTTCCTTACACATTTAGAAGTTTGTGAGTTTGATGAGAAGAAGCTTACTTT
AATTTGGAAAGATCATTTGGTTTTTGCATGCTGTCGTGTTTGCTGCTCGGCAACAGCGAC
ATATGAGTTTAATCAATTTTATGAGAGTACTGTTTTAGGCAGAGACATAGAGCAAGTAAC

AGGCAAATCTGTTTTTGATATAGATGTCAGGTGCTACACCTGTATGAAATTTTTAGACTC
AATTGAAAAGCTAGACATCTGTGGCAGAAAGCGTCCATTTTATTTAGTGAGAGGCTCTTG
GAAAGGAATCTGTAGGCTGTGTAAGCATTTTCAATAATGATTGGTAAAGAGGTCACATTG
CAAGATATTGTGCTGGAGTTAAATGAATTGCAGCCTGAGGTTCAACCAGTTGACCTGTTT
TGTGAAGAGGAGTTACCGAACGAGCAGCAGGAGAGAGAGGAGGAGCCTCAGATTGAAAGA
GCCTCATACAAAGTTGTTGCACCTTGCGGCTGCTGCAAGGTGAAACTTCGCATCTTTATA
AGCGCTACAGAATTTGCTATTAGAAGCTTTCAACAATTGCTGATTGACGAGCTGCAGCTG
TTGTGTCCTGACTGTCGCGGGAACTGCAAACATGGCGGATCCTAAAGGTAGTACATCTAA
AGACGGGTTGGATGATTGGTGTATTGTTGAAGCTGAATGTAGCGATGTAGACAATGATTT
GGAAGAATTATTTGACAGAGATACAGACTCAGATATTTCAGAATTATTAGATGATAATGA
CCTCGAGCAGGGCAATTCTCGGGAACTATTTCATCAACAAGAGTGTAAGGACAGCGAGGA
GCAATTACAAAAACTAAAACGAAAGTACATAAGTCCAAAAGCTATTGCACAGCTTAGTCC
GCGACTTGAAAGTATTTCACTGTCACCACAGCAGAAGTCAAAACGAAGGCTTTTTGCAGA
GCAGGACAGCGGGCTCGAGTTAACTCTTACAAATGAAGCTGAAGATGTTTCTTCTGAGGT
GGAGGAGGTACCGGCCCTAGACTCTCAGCCGGTTGCTGAGGGACACTTAGGAACAGTAGA
CATTCATTATACAGAATTATTGCGTGCCAGTAACCATAAGGCAATTTTGTTGGCAAAATT
TAAGGAGGCTTTTGGGATAGGGTTTAATGATTTGACACGTCAATTTAAAAGTTACAAAAC
CTGCTGTAATGATTGGGTTCTATCTGTGTATGCAGTTCATGAGGATCTTCTTGAAAGCTC
AAAGCAGTTATTGCAACAGCATTGTGATTATATATGGATCCGTGGGATAGCAGCAATGTC
ATTGTTTCTATTGTGTTTTAAAGCAGGAAAAAAATCGTGGGACTGTGCATAAATTAATGAC
ATCAATGTTGAATGTGCATGAAAAGCAAATATTGTCTGAGCCTCCAAAATTAAGAAATGT
TGCTGCTGCTTTATTTTGGTATAAAGGTGCAATGGGGTCCGGAGCATTTTCTCATGGTCC
ATATCCTAACTGGATGGCACAGCAAACTATTGTTGGTCATCAGAGCACAGAAGCCAGTGC
TTTTGACTTGTCTGAAATGATTCAGTGGGCATTTGACCATAATTATCTAGATGAGGCTGA
TATAGCCTTTCAGTATGCTAAGCTAGCACCAGAAAATAGTAATGCTGTAGCATGGCTTGC
ACATAATAACCAAGCAAGGTTTGTTAGAGAATGTGCATCAATGGTCAGGTTTTATAAAAA
AGGTCAAATGAAAGAAATGAGCATGTCAGAATGGATTTATGCCAGAATTAATGAAGTAGA
AGGCGAAGGACATTGGTCATCTATTGCTAAATTTCTTAGATATCAGCAAGTAAATGTTAT
AATGTTTTTAGCTGCTTTGAAAGATATGCTGCATTCTGTACCTAAACATAACTGTATATT
AATACATGGCCCACCTAATACTGGAAAATCTGCATTCACTATGTCATTGATACATGTGTT
AAAGGGAAGGGTATTGTCCTTTGTAAATTCTAAAAGCCAATTCTGGTTACAACCAATGTC
AGAAACTAAAATAGCATTAATTGATGACGTAACTGATCCTTGCTGGGTTTATATGGATAC
ATATTTAAGAAATGGCTTAGATGGACATTATGTCTCACTAGATTGCAAGCATAAAGCACC
AATTCAAACAAAATTTCCTGCATTACTGCTTACCTCTAATATTAATGTTCATAATGAAGT
TAACTATAGATATTTACATAGTAGAATTAAAGGATTTGAATTTCCAAATCCATTTCCAAT
GAAACCAGACAATACCCCTGAGTTTGAGCTTACTGACCAAAGCTGGAAATCTTTTTTTAC
AAGGCTTTGGAAGCAATTAGAGCTGAGTGACCAAGAAGACGAGGGAGAAAATGGAGAATC
TCAGCAAGCGTTTCAATGCTCTGCAAGATCAGCTAATGAACATTTATGAGTCTGCACCAG
ACACTCTTGAGTCGCAAATTGAGCACTGGCAAACCCTGCGAAAAGAAGCTGTGCTACTAT
ATTTTGCTAGGCAACATGGTATCAGCAGGGTTGGATATCAACCTGTGCCTGTATTAGCTG
TGTCAGAAGCCAAAGCTAAACAGGCTATAGGAATGGTATTAAGGTTACAATCATTGCAAA
AATCTGAATATGGAAGTGAACCATGGTCTTTGGTAGATGCAAGTGCAGAGACATTTAGAA
GCCCGCCAGAAAATCACTTTAAAAAAGGTCCGATTTCAGTAGAGGTCATATATGACAAAG
ATAAAGACAATGCCAATGCTTATACCATGTGGAGATTTGTTTATTACCAAGATGATGACG
ACAAGTGGCACAAAAGTGCTAGTGGTGTTAACCAAACAGGCATATATTTTATGCAAGGAA
CATTTAGACACTACTATGTTTTGTTTGCTGATGATGCGAGTAGATATAGTACAACTGGAC
AATGGGAAGTGAAAGTTAATAAGGAAACTGTGTTTGCTCCTGTCACCAGCTCCACCCCCC
CCGACTCACCAGGAGGACAAGCAGACTCAAACGCCTCCTCCCAGACCCCCGCCACCACCA
CTGACTCCACGACCAGACAGTCGCCCAGAAAACAGTCACAACAAACCAACACCAAAGGGA
GAAGGTACGGACGGAGACCTTCCAGTAGGACAAGGCGAACAACCCAAACGCGCCAGAGGC
GACGGTCCAGGTCAAAGTCCAAGTCCAAGTCCAGGTCGCGGTCGAGGTCGCGGCACCGGT
CTCGGTCTCGGTCTCGGTCTGAATCGCCGCGCCGGCGGTCTCGGTACCGATCACGATCCG
GATCCAGAGGGAGAGTCGCCCTCCGCGCCATTACCACCACCACCACAACCACCACCAGAC
GGGCAGGTGGAGGGTCACCCACCTCCACCTCCTCCACCACCTCACAACGGTCGCGACAGC
TGCGGGGAGGGGGCCGTGGGGGGAGCAGACAAAGAGCAAGGGGAAGGCGATCATCATCCA

CCTCCCCCACCCCCTCAAAACGGTCACGAGGGGAGTCAGAGTCTGTTAGGCAACATGGCA
TCTCTCCTTCTGACGTGGGAACAGCAGTTTACACAGTTAGTTCAAGACATACAGGAAGAC
TTGGAAGATTACTGGATGAAGCTCTCGATCCCCCAGTGATTTAGTTAGGGGAGAGCCTA
ATACGCTTAAGTGCTTTCGCAATAGGGCCAAACAAAGATATACAGGGCTGTATAAGTCTT
TTAGCACGGCCTGGTCGTGGGTGGCTGGAGATGGCACGGAGCGTCTAGGCAGGTCCAGAA
TGCTCATTAGCTTTATATCCTTCAGTCAAAGAAAAGATTTTGATGAGACTGTGAAATATC
CGAAGGGGGTTGACCGGTCGTTTGGTTCATTTGACAGCTTATAGCAACCTAACCTTCTAA
CCACTGCATGCTACTAACACACTAACATTTTTTAATTTTTATTAATATTTTTTATTTGCT
ATGGCGCGCGCTAAGCGAGTCAAGCGGGACTCTGCTACTAACATATACAGAACCTGCAAA
CAAGCAGGTACTTGTCCTCCTGATGTTATAAATAAAGTGGAAAGCACAACTATTGCTGAT
AAAATTTTGCAGTATGGTAGTGCTGGTGTTTTTTTTGGGGGATTAGGCATAAGCACTGGA
AAAGGTACAGGAGGAACCACAGGTTATGTGCCTTTGGGAGAAGGCCCATCGGTGCGTGTT
GGTGGTACACCTACAGTCATACGACCTGCTTTGGTCCCAGACACCATCGGCCCCTCCGAT
ATTATACCTGTGGACACCTTAAATCCGGTGGAGCCTTCTACCTCTTCTATTGTTCCACTT
ACAGAATCCACAGGACCAGATCTTTTACCTGGTGAAGTGGAAACTATTGCAGAAATACAT
CCAGGCCCCTCAAGGCCACCAACTGATACACCAGTTACATCTACTACCAGTGGTTCTAGT
GCAGTTCTAGAGGTAGCACCAGAACCAACACCTCCAGCTCGTGTCAGAGTCAGCCGCACC
CAGTATCATAACCCATCATTTCAAATAATAACTGAATCAACACCAACATTGGGGGAAAGC
TCATTAGCGGATCATATAGTAGTGACATCTGGTTCTGGGGGCCAAGCAATTGGGGGGATG
ACACCTGAACTTATAGAGCTTCAGGATTTCCCATCAAGGTATTCATTTGAAATAGAAGAG
CCAACCCCTCCTAGAAGAACTAGCACACCTATGCAAAGACTTCAAAATGTGTTCAGGCGT
AGAGGAGGCCTTACTAACAGAAGATTAGTTCAACAAGTGCCTGTAGACAATCCATTATTT
TTGACACAACCTTCTAGATTGGTCCGGTTTCAGTTTGATAACCCGGTTTTTGAGGAAGAA
GTTACTCAAATATTTGAACAAGATTTAGACACTTTTAATGAGCCCCCAGACAGAGACTTT
TTGGATGTTCAGAGTTTAGGCAGGCCTCAATACTCAGAAACTCCTGCAGGTTATGTGCGG
GTCAGCCGTGCAGGTCAACGAAGGACTATCAGAACTCGTTCTGGAGCACAAATAGGGTCT
CAAGTGCACTTTTATAGAGATCTCAGTAGTATTGATACAGAAGATCCTATTGAACTGCAG
TTGTTGGGTCAGCATTCTGGCGATGCAACTATTGTCCAAGGTCCAGTAGAAAGCACTTTT
GTTGATATCAATGTAGATGAAAACCCACTTTCAGAAATCAGTGCATATTCTGATGATTTA
CTTTTAGATGAAGCTAATGAAGACTTTAGTGGCTCTCAGTTAGTTGTAGGGGGAAGGCGT
TCTACATCTACATACACTGTTCCTCACTTTGAAACTACTAGATCTAGCTCTTACTATGTA
CAAGATACAAAGGGGTATTATGTAGCATATCCTGAAGATAGAGATGTTAGTAAGGACATT
ATTTATCCTAATCCAGATTTACCAGTGGTCATTATTCACACATATGACACAAGTGGAGAT
TTTTATTTACATCCAAGTCTTACTAAAAGATTAAAAAGAAAAAGGAAATATTTGTAACTT
TTTCTTTTGCAGATGGCAGTTTGGCAAGCAGCTAGTGGTAAGGTGTACCTTCCACCATCT
ACACCAGTTGCCAGGGTCCAAAGTACGGATGAATATGTGCAAAGGACTAACATATACTAT
CATGCATACAGTGATCGCCTACTAACTGTTGGTCATCCATATTTTAATATATATGACATC
CAAGGCACTAAGATAAAAGTCCCTAAGGTTTCTGGAAATCAGCACAGAGTGTTTAGGTTA
AAACTACCAGATCCCAACAGATTTGCATTAGCAGATATGTCTGTGTATAACCCAGATAAA
GAAAGATTGGTCTGGGGCTGTAGAGGTATAGAAATAGGTAGAGGACAGCCATTAGGCGTT
GGAAGTGTAGGTCATCCATTATTTAATAAACTTGGTGACACAGAAAACCCTAATTCATAT
AAAGGGAATTCAACTGATGATAGACAAAATGTATCTTTTGACCCTAAACAACTACAAATG
TTTATAATAGGCTGTGCCCCATGTTTAGGAGAACATTGGGACAGGGCTTTACCATGTGCA
GACGACGTTCCAAACCCAGGTTCATGCCCTCCAATAGAATTAAAAAATACAGCAATACAA
GATGGCGATATGGCAGATATAGGATATGGCAACCTAAATTTTAAAGCATTACAAGAAAAC
AGAGCAGATGTAAGTTTGGATATTGTTAATGAGACCTGTAAATATCCAGACTTTTTAAAA
ATGCAGAATGATGTTTATGGAGATTCCTGCTTTTTTTATGCTCGGCGGGAACAATGTTAT
GCTAGACACTTTTTTGTACGTGGGGGCAAAACAGGAGATGATATACCTGCAGGACAAATT
GATGAAGGTAGCATGAAGAATGCATTCTACATTCCACCTGTGAATAATCAGGCACAGAAC
AACCTAGGTAATTCAATGTATTTCCCAACTGTCAGTGGCTCATTGGTGTCTAGTGATGCT
CAATTGTTTAATAGGCCATTTTGGCTGCAGCGCGCACAGGGCCACAACAATGGCATCTGC
TGGTTCAATCAACTATTTGTTACTGTAGTAGATAATACTCGAAATACAAATTTTAGCATA
TCAGTTCATTCAGAAAACACTGATGTTTCTAAAATTCAAAATTATGATTCTCAGAAATTT
CAAGAATATTTAAGACACGTAGAAGAATATGAAATTTCATTAATTTTACAGCTCTGTAAA
GTTCCTTTAACAGCTGAAGTTTTAGCTCAAATTAATGCTATGAATTCAAATATATTAGAG

GAGTGGCAGTTAGGATTCGTTCCTGCACCGGATAATCCTATCCACGATACATACAGATAT
ATTAATTCTGCAGCTACTAGATGTCCTGATAAAAATCCTCCAAAAGAAAGAGAAGATCCT
TACAAGGATCTAAACTTTTGGAATGTTGACCTATCAGAAAGATTATCCTTAGAATTGGAT
CAATATTCTTTAGGACGCAAATTCTTATTTCAAGCAGGTTTACAACAAGCGACCGTAAAC
GGTACAAAAACTGTATCTTCAAAGTTATCTACTAGGGGCGTCAAACGAAAACGCAAACAA
TAAACCCGACCGTTTTCGGTACAATAAAGTCAACTTTTACACGGTATTCAAGGAATGTTT
ATTTACTCTGACTAACTAAGATACCAACCGCACCCGACACATAAAGGTGAGTTGTGTGCC
AAATGAGGTGAGTTGTGAGCCAGAAGAGATCACAGCCAAGTCAGGCTTGAGCCAGATCAG
ATACACTGCGTGCCAGAGTTGGCTCAAACTTCATCGTCCCAACACGTTCGGAACAGGAGG
AAATGTAAGGCTGCCAACGCTTTTGGCTCTTCTTTTTGGCACAGCAGAAGACCGTTAACG
GTAAGTTTTTATTTGTA
>gi|333211|gb|M73236.1|PPHPAPV42A_Human_papillomavirus_ORF_E6,_ORF_E7,_ORF_E1,_O
RF_E2,_ORF_E4,_ORF_E5,_ORF_L2,_and_ORF_L1_genes,_complete_cds
CTTATTATAAACTACAATCCTGGCTTTGAAAAATAAGGGAGTAACCGAATTCGGTTCAAC
CGAAACCGGTACATATATAAACCACCCAAAGTAGTGGTCCCAGTTAAGGCAGAATGTCAG
GTACATCTGCCTCATCACAGCCACGCACATTATACCAATTGTGTAAGGAATTTGGGCTGA
CATTGCGGAATTTACAGATTTCCTGCATTTGGTGCAAAAAGCACTTAACAGGCGCAGAGG
TGCTCGCGTACCATTTTAAAGATTTGGTAGTGGTGTGGAGGAAGGACTTTCCATATGCTG
CATGTGCATTTTGTTTAGAATTTAATTCTAAAATTTGTGCACTGCGACACTACGAAAGAT
CAGCATTTTGGTATACAGTGGAGAAAGAAACTGGACTACTTTTAGAAGAACAACAAATTA
GATGTGCCTTGTGTCAAAAGCCGTTATCACAGAGCGAAAAAAACCATCATATTGATACAG
GTACAAGATTTCAATTTATATTGTGTCAGTGGACGGGTCGGTGTACGCATTGCAGAGGAC
AATGCGTGGAGAGACGCCTACCCTAAAGGACATTGTTTTGTTTGACATACCAACGTGTGA
GACACCCATTGACCTGTATTGCTATGAACAATTGGACAGCTCAGATGAAGATGACCAAGC
CAAACAGGACATACAGCGTTACAGAATACTGTGTGTGTGTACACAGTGTTACAAGTCTGT
TAAACTCGTTGTGCAGTGTACAGAGGCGGACATAAGAAACCTGCAACAGATGCTTTTGGG
CACACTGGATATTGTGTGTCCTTTGTGTGCCCGCGTGGAGTAACTGCAATGGCGGATGAT
ACAGGTACAGAGGAGGGGCTAGGGTGTTCTGGATGGTTTTGTGTAGAAGCTATAGTAGAC
AAAACAACAGAAAATGCTATTTCAGATGACGAGGACGAAAATGTAGACGATAGTGGGTTA
GATCTTGTGGATTTTGTAGATAATAGTACAGTAATACATACAAAGCAGGTACATGCACAA
GCCTTATTAAATAAACAACAAGCACATGCAGATCAGGAGGCAGTACAGGCACTAAAACGA
AAGCTATTAGGCAGTCCATATGAAAGCCCTGTCAGTGATTCACAGCACAGCATAGACAAC
GAACTAAGTCCTAGGCTTGGCGGTTTAACGCTATGTCGGGGGTCCCAAGGGGCCAAACGA
CGATTATTCCAGTCACTGGAAAATCGAGACAGTGGATATGGCTATTCTGAAGTGGAAGTA
CAGCAGACACAGGTAGAACACGGACATGGCGCCGTACATGGGACTATGGGTAACGGGGGG
GCAGTGGGTAGTGAACTTGGGGTGCAGGAAAATGAAGAAGGTAGTACTACAAGTACGCCT
ACAACAAGGGTGGTAGAATTACTTAAGTGTAAGAACCTGCATGCAACATTGTTAGGTAAG
TTTAAAGAATTGTTTGGAGTGTCATTTGGCGATTTAGTAAGACAGTTTAAAAGTGACAAA
AGCAGTTGTACAGACTGGGTTATTGCAGCATTTGGGGTTAATCATAGTATTGCAGAAGGG
TTTAATACATTAATTAAAGCAGATTCACTATATACACATATACAATGGCTAACCTGTACG
TGGGGCATGGTGTTATTAATGCTAATTAGATTTAAATGTGGAAAAAATCGTACTACAGTG
TCCAAAGGCCTTAGTAAATTATTAAACATACCTACAAATCAATTATTAATAGAGCCACCT
CGGTTACAAAGTGTGGCTGCCGCCATATACTGGTTTAGATCAGGAATATCTAATGCTAGC
ATTGTAACCGGAGACACACCAGAGTGGATTCAAAGACAAACAATTTTAGAACATTGTTTT
GCAGATGCCCAATTTAATTTAACAGAAATGGTGCAATGGGCATATGATAATGATATTACT
GAAGACAGTGACATTGCATATGAATATGCACAACGGGCAGACAGGGATAGCAATGCTGCT
GCATTTTTAAAAAGTAACTGCCAGGCAAAATATGTAAAAGATTGTGGCGTCATGTGCAGA
CATTATAAAAAAGCACAAATGAGACGTATGTCTATGGGTGCATGGATAAAACATAGAAGT
GCCAAGATAGGGGATAGTGGAGATTGGAAACCTATAGTAAAATTTATTAGATATCAACAA
ATTGATTTTTTAGCATTTATGTCTGCATTTAAAAAGTTTTTACATAATATACCTAAAAAA
AGTTGTTTAGTGTTAATTGGTCCTCCAAATACAGGAAAATCACAGTTTGGAATGAGTTTA
ATAAACTTCTTAGCAGGAACTGTAATATCATTTGTAAATTCACATAGCCATTTTTGGCTG
CAGCCATTGGACAGTGCAAAAATAGCTATGCTGGATGATGCAACTCCACCATGTTGGACA
TATTTAGATATATATTTAAGAAATTTATTAGATGGCAATCCATGCAGTATAGATAGAAAA
CATAAAGCATTAACAGTTGTTAAGTGCCCACCATTACTTATAACATCAAATACAGATATT

AGAACAAATGACAAATGGAAATACCTATACAGCAGAGTTAGTTTATTTGAATTTCCAAAT
CCATTTCCATTAGATACAAATGGAAATCCTGTATATGAATTAAATGACAAAAATTGGAAA
TCATTTTTTCAAAGGTTGTGGTCCAGCTTAGAATTTCAAGAATCAGAGGACGAGGAAGAC
TATGGAGAGACTGGCCAAACGTTTAGATGCGTGCCAGGAACAGTTGTTAGAACTGTATGA
GGAAAATAGTAGGGATTTACAAAAACATATTGAACATTGGAAATGTTTACGTATGGAGGC
AGTGGTATTGTATAAGGCCCGTGAAATGGGCTTTGCAAATATAGGACATCAAATAGTACC
AACATTGGAAACATGTAGAGCCAAGGCCCACATGGCAATTGAAATACACTTGGCATTAGA
GACATTATTGCAGTCCTCGTATGGTAAAGAACCATGGACATTGCAAGAAACAAGTAATGA
ACTGTGGCTTACGAATCCTAAAAAATGTTTTAAAAAACAAGGACGTACCGTGGAGGTTAT
ATTTGATGGAAAACAGGACAATGCAATGCATTATACAGCATGGACATATATATATATACA
AACTGTGCAAGGTACATGGTGTAAAGTACAAGGACACGTTTGCCATGCAGGACTATATTA
TATTGTGGAAAATATGAAACAGTTTTATTGTAATTTTAAAGAGGAGGCAAAAAAATATGG
GGTAACAGACCAATGGGAGGTACATGATGGCAATCAGGTGATTGTTTCTCCTGCACCCAT
ATCTAGCACCACATCCACCGACGCAGAGATACCCTCTACTGGATCTACTAAGTTGGTACA
ACAAGTGTGCACCACAAACCCATTGCACACCACAACGTCCATTGACAACCACCACGCAGA
CTGTACAGACGGAACAGCATACAACGTGCCCATCCAAACCTCACCGCCACGAAAACGATA
CAGACAGTGTGGACAGTCGCCATCACAGCACCTGCAGCACTCAAACCCCAGCATCCCCAG
CATCCCCAGCGCATCCGTGGACCCTGGATTGTGTGGGGTCAGAACTAACAGTGAAAACTG
TAACAAGCGACGGAACCACTGTGGAAGTCAGGCTACGCCTGTAATTCATTTACAAGGTGA
CCCTAATTGCCTAAAATGCCTACGATTTAGGCTAAAAAGAAATTGTTCACATTTATTTAC
ACAGGTGTCATCTACATGGCATTTAACAGAAAATGATTGTACACGTGACACTAAAACTGG
TATAATAACAATACATTATTATGATGAAGCACAAAGAAATTTATTTTTAAATACTGTAAA
AATACCTTCTGGGATAAAATCCTGTATTGGATATATGTCTATGTTACAGTTTATATGATT
AGTTGTATATGTGTATAAACAGTTATAGGACTTCAATACTGTGACTCCACAACGTGTGGG
ACAACCGGCCAGAAACTGCTGCTTTTATTGTTTATAGTTGTTGGTGCGTGTGTTGTGTGT
GTGTGGATTAGTTTACAAAATTATCCATATCCTGTATGGGCCTCTTGCCTTGCTAGCTAC
CTAACATTGGTGCTATTATCATGGTTGCAGGTACTAACATACTTTGACTATTTTTTTCTA
TGTTTAATCATTCTTGGTATTCCTTCTGTCTTACTAACATTACTAATACATTTAGCAATA
CAATAACACATATTAGTTTAGGTGTGTGTGTGTGGTGTGCATGTGATTTGTACATGGTTG
TACATATATAATACCAATTATTGTTTGGCTACTATTTTCATTTATAGCCACACTGCTGTT
TTGCATATTGGTATTACAAACATATAAACTGTTACCATACGTATATACAGTGCTGTAAAT
AAACTTTTGTTATATTGTGTGTACTTCTTTTGTGCTATTACAATGCCACCACAACGGTCC
CGCAGACGAAAGCGGGCCTCTGCCACACAATTATATCAAACGTGTAAGGCCTCAGGGACA
TGTCCTCCAGATGTTATTCCCAAAGTTGAAGGAACCACATTGGCAGATAAAATTTTACAA
TGGGGTAGTTTAGGCGTGTTTTTTGGGGGGTTGGGAATTGGCACTGGTGCAGGTACGGGT
GGGCGCACGGGCTATGTGCCTCTGGGAACAAGGCCTCCTGTAATTGCTGAACCAGGACCT
GCAGTACGCCCACCAATAGCTGTTGACACCGTGGGGCCATCTGATCCTTCTATTGTTTCC
TTATTAGAAGAGTCATCAGTTATTGATGCAGGAATAACAGTACCTGATATTACTTCTCAT
GGAGGTTTTAATATTACTACATCTACTGGTGGGCCTGCCTCAACGCCTGCTATATTAGAT
ATCTCCCCTCCCACTAATACTATACGTGTCACAACAACTACATCTACCAATCCTTTATAT
ATTGATCCTTTTACATTGCAGCCGCCATTGCCAGCAGAGGTTAATGGGCGCCTATTAATA
TCTACTCCTACCATCACACCCCACTCATATGAAGAAATACCAATGGACACGTTTGTTGTA
TCTACAGATACAACTAACACATTTACTAGTACTCCCATTCCTGGCCCTCGGTCGTCTGCA
CGCCTGGGGTTATATTCTAGAGCAACGCAACAACGTCCAGTTACTACCAGTGCATTTTTA
ACATCTCCTGCACGGTTGGTTACTTATGACAATCCAGCCTATGAAGGACTTACGGAGGAT
ACATTAGTATTTGAACATCCATCCATTCATACTGCACCTGACCCTGATTTCATGGATATA
GTTGCATTGCATCGTCCTATGTTATCATCCAAACAGGGTAGTGTACGTGTTAGTAGAATT
GGACAAAGGCTGTCTATGCAGACACGTCGCGGACCCGTTTTGGGTCACGTGTACACTTT
TTTCATGACCTTAGCCCTATTACACACTCTTCAGAAACTATTGAATTACAGCCTTTATCT
GCTTCTTCAGTATCTGCAGCCTCCAATATTAATGATGGGTTATTTGATATTTATGTTGAT
ACTAGTGATGTAAATGTTACAAATACCACTTCCTCTATACCTATGCATGGTTTTGCTACC
CCCCGTTTGTCCACTACATCTTTCCCTACATTACCTAGCATGTCTACACATTCTGCCAAT
ACCACCATACCTTTTTCGTTTCCTGCCACTGTGCATGTGGGCCCTGATTTATCTGTTGTG
GACCACCCATGGGACAGTACCCCAACGTCTGTAATGCCTCAGGGTAACTTTGTAATGGTA
TCAGGATGGGATTTTATATTGCATCCTAGTTATTTTTGGCGTAGGCGCCGTAAACCTGTA

CCATATTTTTTTGCAGATGTCCGTGTGGCGGCCTAGTGACAACAAGGTTTATCTACCTCC
TCCTCCTGTTTCCAAGGTGGTCAGCACTGATGAATATGTGCAACGCACCAACTACTTTTA
CCATGCCAGCAGTTCTAGGCTATTGGTTGTTGGTCACCCTTATTACTCTATTACAAAAAG
GCCAAATAAGACATCTATCCCCAAAGTGTCTGGTTTACAGTACAGAGTATTTAGAGTTAG
GCTCCCTGATCCTAATAAGTTTACATTGCCTGAAACTAATTTATATAACCCAGAGACACA
GCGCATGGTGTGGGCCTGTGTGGGGCTAGAAGTAGGTCGTGGACAGCCTTTGGGCGTTGG
TATTAGTGGCCATCCATTATTGAATAAGTTGGATGATACTGAAAATGCGCCTACATATGG
TGGAGGCCCTGGTACAGACAATAGGGAAAATGTTTCTATGGATTATAAACAAACACAGTT
GTGTTTAGTTGGCTGTAAACCTGCCATAGGGGAGCACTGGGGTAAAGGTACTGCCTGTAC
ACCACAGTCCAATGGTGACTGCCCACCATTAGAATTAAAAAATAGTTTTATTCAGGATGG
GGATATGGTGGATGTAGGGTTTGGGGCACTAGATTTTGGTGCTTTACAATCCTCCAAAGC
TGAGGTACCTTTGGATATTGTAAATTCAATTACTAAATATCCTGATTACTTAAAAATGTC
TGCTGAGGCCTATGGTGACAGTATGTTTTTCTTTTTAAGGCGAGAACAAATGTTTGTTCG
TCATTTGTTTAATAGGGCTGGCGCAATTGGTGAACCTGTACCTGATGAACTGTATACCAA
GGCTGCTAATAATGCATCTGGCAGACATAATTTAGGTAGTAGTATTTATTATCCTACCCC
TAGTGGTTCTATGGTAACATCTGATGCACAACTATTTAATAAACCATATTGGTTACAACA
AGCACAAGGACACAATAATGGTATATGTTGGGGAAATCAGCTATTTTTAACTGTGGTTGA
TACTACCCGTAGTACTAACATGACTTTGTGTGCCACTGCAACATCTGGTGATACATATAC
AGCTGCTAATTTTAAGGAATATTTAAGACATGCTGAAGAATATGATGTGCAATTTATATT
TCAATTGTGTAAAATAACATTAACTGTTGAAGTTATGTCATATATACACAATATGAATCC
TAACATATTAGAGGAGTGGAATGTTGGTGTTGCACCACCACCTTCAGGAACTTTAGAAGA
TAGTTATAGGTATGTACAATCAGAAGCTATTCGCTGTCAGGCTAAGGTAACAACGCCAGA
AAAAAAGGATCCTTATTCAGACTTTTGGTTTTGGGAGGTAAATTTATCTGAAAAGTTTTC
TACTGATTTAGATCAATTTCCTTTAGGTAGAAAGTTTTTACTGCAGGCCGGGTTGCGTGC
AAGGCCTAAACTGTCTGTAGGTAAACGAAAGGCGTCTACAGCTAAATCTGTTTCTTCAGC
TAAACGTAAGAAAACACACAAATAGATGTATGTAGTAATGTTATGATACATATTTATGTT
ATTTATTTGTGTACTGTGTTAATAAACTACTTTTTATATGTTGTGTGTTCTCCATTTTGT
TTTTTGTACTCCATTTTGTTTCTAGACCGATTTCGGTTGTATCTGGCCTGTTACCAGGTG
CATTGGCCATGTTTCCTAACATTTTGCAAACCTATTCACTTTTTAAATTTATAAATGCAA
TATGTGCTGCCAACTGTTTTATGGCACGTATGTTCTGCCAACGTACACTCCCTAATTCCT
TTACATAACACACACGCCTTTGCACAGGCATGTGCACAAAGGTTGGCAAAGGTTAGCATA
TCTCTGCAGTTACCCATTTCCTTTTTCCTTTTTTTTATGTATGAGTAACTTAATTGTTAT
ATGTAATAAAAAAGCTTTTAGGCACATATTTTCAGTGTTGGCATACACATTTACAAGTTA
CCTTGGCTTAAACAAGTAAAGTTATTTGTCACTGTTGACACATTACTCATATATATAATT
TGTTTTTAACATGCAGGTGGCAACCGAAACCGGTACATAAATCCTTCTTATTCTTTT
>gi|333049|gb|M12732.1|PPH33CG_Human_papillomavirus_type_33,_complete_genome
GTAAACTATAATGCCAAGTTTTAAAAAAGTAGGGTGTAACCGAAAGCGGTTCAACCGAAA
ACGGTGCATATATAAAGCAAACATTTTGCAGTAAGGTACTGCACGACTATGTTTCAAGAC
ACTGAGGAAAAACCACGAACATTGCATGATTTGTGCCAAGCATTGGAGACAACTATACAC
AACATTGAACTACAGTGCGTGGAATGCAAAAAACCTTTGCAACGATCTGAGGTATATGAT
TTTGCATTTGCAGATTTAACAGTTGTATATAGAGAGGGAAATCCATTTGGAATATGTAAA
CTGTGTTTGCGGTTCTTATCTAAAATTAGTGAATATAGACATTATAATTATTCTGTATAT
GGAAATACATTAGAACAAACAGTTAAAAAACCTTTAAATGAAATATTAATTAGGTGTATT
ATATGTCAAAGACCTTTGTGTCCTCAAGAAAAAAAACGACATGTGGATTTAAACAAACGA
TTTCATAATATTTCGGGTCGTTGGGCAGGGCGCTGTGCGGCGTGTTGGAGGTCCCGACGT
AGAGAAACTGCACTGTGACGTGTAAAAACGCCATGAGAGGACACAAGCCAACGTTAAAGG
AATATGTTTTAGATTTATATCCTGAACCAACTGACCTATACTGCTATGAGCAATTAAGTG
ACAGCTCAGATGAGGATGAAGGCTTGGACCGGCCAGATGGACAAGCACAACCAGCCACAG
CTGATTACTACATTGTAACCTGTTGTCACACTTGTAACACCACAGTTCGTTTATGTGTCA
ACAGTACAGCAAGTGACCTACGAACCATACAGCAACTACTTATGGGCACAGTGAATATTG
TGTGCCCTACCTGTGCACAACAATAAACATCATCTACAATGGCCGATCCTGAAGGTACAA
ATGGGGCTGGGATGGGGTGTACTGGTTGGTTTGAGGTAGAAGCAGTCATAGAGAGAAGAA
CAGGAGATAATATTTCAGAAGATGAGGATGAAACAGCAGATGACAGTGGCACGGATTTAC
TAGAGTTTATAGATGATTCTATGGAAAATAGTATACAGGCAGACACAGAGGCAGCCCGGG
CATTGTTTAATATACAGGAAGGGGAGGATGATTTAAATGCTGTGTGTGCACTAAAACGAA

AGTTTGCCGCATGTTCACAAAGTGCTGCGGAGGACGTTGTTGATCGTGCTGCAAACCCGT
GTAGAACGTCTATTAATAAAAATAAAGAATGCACATACAGAAAACGAAAAATAGATGAGC
TAGAAGACAGCGGATATGGCAATACTGAAGTGGAAACTCAGCAGATGGTACAACAGGTAG
AAAGTCAAAATGGCGACACAAACTTAAATGACTTAGAATCTAGTGGGGTGGGGGATGATT
CAGAAGTAAGCTGTGAGACAAATGTAGATAGCTGTGAAAATGTTACGTTGCAGGAAATTA
GTAATGTTCTACATAGTAGTAATACAAAAGCAAATATATTATATAAATTTAAAGAGGCCT
ATGGAATAAGTTTTATGGAATTAGTAAGACCATTTAAAAGTGATAAAACAAGCTGTACAG
ATTGGTGTATAACAGGATATGGAATTAGTCCATCAGTAGCAGAAAGTTTAAAAGTATTAA
TTAAACAGCATAGTTTGTATACTCATTTACAATGTTTAACTTGCGATAGAGGAATAATAA
TATTATTGTTAATTAGATTTAGGTGTAGCAAAAACAGGTTAACAGTAGCAAAACTAATGA
GTAATTTATTATCAATACCTGAAACATGTATGGTTATAGAGCCACCAAAATTACGGAGCC
AAACATGTGCATTGTATTGGTTTAGAACAGCAATGTCAAACATTAGTGATGTACAAGGTA
CAACACCTGAATGGATAGATAGACTAACTGTTTTACAACATAGCTTTAATGATAATATAT
TTGATTTAAGTGAAATGGTACAGTGGGCATATGATAACGAGTTAACGGACGATAGTGACA
TTGCATATTATTATGCACAACTTGCAGATTCAAATAGTAATGCTGCTGCATTTTTAAAAA
GTAACTCACAAGCAAAAATAGTAAAGGACTGTGGAATAATGTGTAGACATTATAAAAAAG
CAGAAAAACGTAAAATGTCAATAGGACAATGGATACAAAGTAGATGTGAAAAAACAAATG
ATGGAGGAAATTGGAGACCAATAGTACAGTTGTTAAGATATCAAAACATTGAATTTACAG
CATTTTTAGGTGCATTTAAAAAGTTTTTAAAAGGTATACCAAAAAAAAGCTGTATGCTAA
TTTGTGGACCAGCAAATACAGGAAAGTCATATTTTGGAATGAGTTTAATACAGTTTTTAA
AAGGGTGTGTTATATCATGTGTAAATTCTAAAAGTCACTTTTGGTTGCAGCCATTATCAG
ATGCAAAAATAGGAATGATAGATGATGTAACGCCAATAAGTTGGACATATATAGATGATT
ACATGAGAAATGCGTTAGATGGAAATGAAATTTCAATAGATGTGAAACATAGGGCATTAG
TGCAATTAAAATGTCCACCACTGCTTCTTACCTCAAATACAAATGCAGGCACAGACTCTA
GATGGCCATATTTACATAGTAGATTAACAGTATTTGAATTTAAAAATCCATTCCCATTTG
ATGAAAATGGTAACCCAGTGTATGCAATAAATGATGAAAATTGGAAATCCTTTTTCTCAA
GGACGTGGTGCAAATTAGATTTAATAGAGGAAGAGGACAAGGAAAACCATGGAGGAAATA
TCAGCACGTTTAAATGCAGTGCAGGAGAAAATACTAGATCTTTACGAAGCTGATAAAACT
GATTTACCATCACAAATTGAACATTGGAAACTGATACGCATGGAGTGTGCTTTATTGTAT
ACAGCCAAACAAATGGGATTTTCACATTTATGCCACCAGGTGGTGCCTTCTTTGTTAGCA
TCAAAGACCAAAGCATTTCAAGTAATTGAACTACAAATGGCATTAGAGACATTAAGTAAA
TCACAGTATAGTACAAGCCAATGGACATTGCAACAAACAAGCTTAGAGGTGTGGCTTTGT
GAACCACCAAAATGTTTTAAAAAACAAGGAGAAACAGTAACTGTGCAATATGACAATGAC
AAAAAAAATACAATGGATTATACAAACTGGGGTGAAATATATATTATAGAGGAAGATACA
TGTACTATGGTTACAGGGAAAGTAGATTATATAGGTATGTATTATATACATAACTGTGAA
AAGGTATATTTTAAATATTTTAAAGAGGATGCTGCAAAGTATTCTAAAACACAAATGTGG
GAAGTACATGTGGGTGGTCAGGTAATTGTTTGTCCTACGTCTATATCTAGCAACCAAATA
TCCACTACTGAAACTGCTGACATACAGACAGACAACGATAACCGACCACCACAAGCAGCG
GCCAAACGACGACGACCTGCAGACACCACAGACACCGCCCAGCCCCTTACAAAGCTGTTC
TGTGCAGACCCCGCCTTGGACAATAGAACAGCACGTACTGCAACTAACTGCACAAACAAG
CAGCGGACTGTGTGTAGTTCTAACGTTGCACCTATAGTGCATTTAAAAGGTGAATCAAAT
AGTTTAAAATGTTTAAGATACAGATTAAAACCTTATAAAGAGTTGTATAGTTCTATGTCA
TCCACCTGGCATTGGACCAGTGACAACAAAAATAGTAAAAATGGAATTGTAACTGTAACA
TTTGTAACTGAACAGCAACAACAAATGTTTTTAGGTACCGTAAAAATACCACCTACTGTG
CAAATAAGTACTGGATTTATGACATTATAAGTGTACATCACAAGCCAATATGTGCTGCTA
ATTGTATATAACCATGATATTTGTTTTTGTATTATGTTTTATATTGTTTTTATGCTTATC
CTTATTATTACGTCCTTTAATACTTTCCATTTCTACCTATGCTTGGTTGCTGGTGTTGGT
ATTGCTGCTTTGGGTGTTTGTGGGATCTCCTTTAAAAATTTTTTTTTGCTATTTGTTGTT
TTTATATTTACCAATGATGTGTATTAATTTTCATGCACAGCATATGACACAACAAGAGTA
ATGTATATACATGTATATATTGTTTGTATATATGTGCACATGGTGGTGTTTTAACATTGT
TGTTGTTATTTTAGTTTTTTTTTTTTTGTATTACTAATAAATACCTTTATATTTTAGCAG
TGTATTATTATGAGACACAAACGATCTACAAGGCGCAAGCGTGCATCTGCAACACAACTA
TACCAAACATGCAAGGCCACAGGCACCTGCCCACCCGATGTTATTCCTAAAGTGGAAGGA
AGTACCATAGCAGATCAAATTCTTAAATATGGCAGTTTAGGGGTTTTTTTTGGTGGTTTA
GGTATTGGCACAGGCTCTGGTTCAGGTGGAAGGACTGGCTATGTACCTATTGGTACTGAC

CCACCTACAGCTGCAATCCCCTTGCAGCCTATACGTCCTCCGGTTACTGTAGACACTGTT
GGACCTTTAGACTCGTCTATAGTGTCATTAATAGAAGAAACAAGTTTTATAGAGGCAGGT
GCACCAGCCCCATCTATTCCTACACCATCAGGTTTTGATGTTACTACATCTGCAGATACT
ACACCTGCAATTATTAATGTTTCATCTGTTGGGGAGTCATCTATTCAAACTATTTCTACA
CATTTAAATCCCACATTTACTGAACCATCTGTACTACACCCTCCAGCGCCTGCAGAAGCC
TCTGGACATTTTATATTTCTTCCCCTACTGTTAGCACACAAAGTTATGAAAACATACCA
ATGGATACCTTTGTTGTTTCCACAGACAGTAGTAATGTAACATCAAGCACGCCCATTCCA
GGGTCTCGCCCTGTGGCACGCCTTGGTTTATATAGTCGCAATACCCAACAGGTTAAGGTT
GTTGACCCTGCTTTTTTAACATCGCCTCATAAACTTATAACATATGATAATCCTGCATTT
GAAAGCTTTGACCCTGAAGACACATTACAATTTCAACATAGTGATATATCACCTGCTCCT
GATCCTGACTTTCTAGATATTATTGCATTACATAGGCCTGCTATTACATCTCGTAGACAT
ACTGTGCGTTTTAGTAGAGTAGGTCAAAAAGCCACACTTAAAACTCGCAGTGGTAAACAA
ATTGGAGCTAGAATACATTATTATCAGGATTTAAGTCCTATTGTGCCTTTAGACCACACC
GTGCCAAATGAACAATATGAATTACAGCCTTTACATGATACTTCTACATCGTCTTATAGT
ATTAATGATGGTTTGTATGATGTTTATGCTGACGATGTGGATAATGTACACACCCCAATG
CAACACTCATACAGTACGTTTGCAACAACACGTACCAGCAATGTGTCTATACCTTTAAAT
ACAGGATTTGATACTCCTGTTATGTCTGGCCCTGATATACCTTCCCCTTTATTTCCCACA
TCTAGCCCATTTGTTCCTATTTCGCCTTTTTTTCCTTTTGACACCATTGTTGTAGACGGT
GCTGACTTTGTTTTACATCCTAGTTATTTTATTTTACGTCGCAGGCGTAAACGTTTTCCA
TATTTTTTTACAGATGTCCGTGTGGCGGCCTAGTGAGGCCACAGTGTACCTGCCTCCTGT
ACCTGTATCTAAAGTTGTCAGCACTGATGAATATGTGTCTCGCACAAGCATTTATTATTA
TGCTGGTAGTTCCAGACTTCTTGCTGTTGGCCATCCATATTTTTCTATTAAAAATCCTAC
TAACGCTAAAAAATTATTGGTACCCAAAGTATCAGGCTTGCAATATAGGGTTTTTAGGGT
CCGTTTACCAGATCCTAATAAATTTGGATTTCCTGACACCTCCTTTTATAACCCTGATAC
ACAACGATTAGTATGGGCATGTGTAGGCCTTGAAATAGGTAGAGGGCAGCCATTAGGCGT
TGGCATAAGTGGTCATCCTTTATTAAACAAATTTGATGACACTGAAACCGGTAACAAGTA
TCCTGGACAACCGGGTGCTGATAATAGGGAATGTTTATCCATGGATTATAAACAAACACA
GTTATGTTTACTTGGATGTAAGCCTCCAACAGGGGAACATTGGGGTAAAGGTGTTGCTTG
TACTAATGCAGCACCTGCCAATGATTGTCCACCTTTAGAACTTATAAATACTATTATTGA
GGATGGTGATATGGTGGACACAGGATTTGGTTGCATGGATTTTAAAACATTGCAGGCTAA
TAAAAGTGATGTTCCTATTGATATTTGTGGCAGTACATGCAAATATCCAGATTATTTAAA
AATGACTAGTGAGCCTTATGGTGATAGTTTATTTTTCTTTCTTCGACGTGAACAAATGTT
TGTAAGACACTTTTTTAATAGGGCTGGTACATTAGGAGAGGCTGTTCCCGATGACCTGTA
CATTAAAGGTTCAGGAACTACTGCCTCTATTCAAAGCAGTGCTTTTTTTCCCACTCCTAG
TGGATCAATGGTTACTTCCGAATCTCAGTTATTTAATAAGCCATATTGGCTACAACGTGC
ACAAGGTCATAATAATGGTATTTGTTGGGGCAATCAGGTATTTGTTACTGTGGTAGATAC
CACTCGCAGTACTAATATGACTTTATGCACACAAGTAACTAGTGACAGTACATATAAAAA
TGAAAATTTTAAAGAATATATAAGACATGTTGAAGAATATGATCTACAGTTTGTTTTTCA
ACTATGCAAAGTTACCTTAACTGCAGAAGTTATGACATATATTCATGCTATGAATCCAGA
TATTTTAGAAGATTGGCAATTTGGTTTAACACCTCCTCCATCTGCTAGTTTACAGGATAC
CTATAGGTTTGTTACCTCTCAGGCTATTACGTGTCAAAAAACAGTACCTCCAAAGGAAAA
GGAAGACCCCTTAGGTAAATATACATTTTGGGAAGTGGATTTAAAGGAAAAATTTTCAGC
AGATTTAGATCAGTTTCCTTTGGGACGCAAGTTTTTATTACAGGCAGGTCTTAAAGCAAA
ACCTAAACTTAAACGTGCAGCCCCCACATCCACCCGCACATCGTCTGCAAAACGCAAAAA
GGTTAAAAAATAACACTTTGTGTAATTGTGTTATGTTGTTGTTTTGTTCTGTCTATGTAC
TTTGTGTTGTTGTGTTGTGTTGTTGTTTGTTTTTTGTGTATGTGTTACAATGTATGTTAT
GTTGTATGTTACTGTGTTTGTTTTATGTGTACTTGTTTGTGTGCATGTTCTATGTACTTG
TCAGTTTCCTGTTTGTGTATATGTTAATAAAACATTGTGTGTATTTGTTAAACTATTTGT
ATGTATGTTATGTATATGGGTGTACCTATATGAGTAAGGAGTTGTATTGCTTGCCCTACC
CTGCATTGCAATGTACCTACCTTTATTTCCCTATATTTGTAGTACCTACATGTTTAGTAT
TGCTTTACCTTTTGACATACTAGTGTCCATATTGTACAATTTCCTCCATTTTGTATGCCT
AACCGTTTTCGGTTACTTGGCATACATACCCTATGACATTGGCAGAACAGTTAATCCTTT
TCTTTCCTGCACTGTGTTTGTCTGTACTTGCTGCATTGGCATACATACCCTATGACATTG
GCAGAACAGTTAATCCTTTTCTTTCCTGCACTGTGTTTGTCTGTACTTGCTGCATTGACT
CATATATACATGCAGTGCAATTGCAAAATACTTAATTGTACTAATAGTTTACACATGCTT

TTAGGCACATATTTTTACTTTACTTTCAAACCTTAAGTGCAGTTTTGGCTTACACAATTG
CTTTGTATGCCAAACTATGCCTTGTAAAAGTGAGTCACTACCTGTTTATTACCAGGTGTG
GACTAACCGTTTTAGGTCATATTGGTCATTTATAATCTTTTATATAATA
>gi|333048|gb|J04353.1|PPH31A_Human_papillomavirus_type_31_(HPV-
31)_complete_genome
TAATAATAATAATCTTAGTATAAAAAAGTAGGGAGTGACCGAAAGTGGTGAACCGAAAAC
GGTTGGTATATAAAGCACATAGTATTTTGTGCAAACCTACAGACGCCATGTTCAAAAATC
CTGCAGAAAGACCTCGGAAATTGCATGAACTAAGCTCGGCATTGGAAATACCCTACGATG
AACTAAGATTGAATTGTGTCTACTGCAAAGGTCAGTTAACAGAAACAGAGGTATTAGATT
TTGCATTTACAGATTTAACAATAGTATATAGGGACGACACACCACACGGAGTGTGTACAA
AATGTTTAAGATTTTATTCAAAAGTAAGTGAATTTAGATGGTATAGATATAGTGTGTATG
GAACAACATTAGAAAAATTGACAAACAAAGGTATATGTGATTTGTTAATTAGGTGTATAA
CGTGTCAAAGACCGTTGTGTCCAGAAGAAAAACAAAGACATTTGGATAAAAAGAAACGAT
TCCACAACATAGGAGGAAGGTGGACAGGACGTTGCATAGCATGTTGGAGAAGACCTCGTA
CTGAAACCCAAGTGTAAACATGCGTGGAGAAACACCTACGTTGCAAGACTATGTGTTAGA
TTTGCAACCTGAGGCAACTGACCTCCACTGTTATGAGCAATTACCCGACAGCTCAGATGA
GGAGGATGTCATAGACAGTCCAGCTGGACAAGCAGAACCGGACACATCCAATTACAATAT
CGTTACCTTTTGTTGTCAGTGTAAGTCTACACTTCGTTTGTGTGTACAGAGCACACAAGT
AGATATTCGCATATTGCAAGAGCTGTTAATGGGCTCATTTGGAATCGTGTGCCCCAACTG
TTCTACTAGACTGTAACTACAATGGCTGATCCAGCAGGTACAGATGGGGAGGGGACGGGA
TGCAATGGTTGGTTTTATGTAGAAGCAGTAATTGACAGACAGACAGGGGACAACATTTCA
GAGGACGAAAATGAAGACAGTAGTGATACTGGGGAGGATATGGTTGACTTTATTGACAAT
TGTAATGTATACAACAATCAGGCAGAAGCAGAGACAGCACAGGCATTGTTTCATGCACAG
GAAGCGGAGGAACATGCAGAGGCTGTGCAGGTTCTAAAACGAAAGTATGTAGGTAGTCCT
TTAAGTGATATTAGTAGTTGTGTGGATTATAATATTAGTCCACGGTTAAAAGCTATATGC
ATAGAAAATAACAGTAAAACAGCAAAACGAAGACTCTTTGAACTTCCAGACAGCGGGTAT
GGCAATACTGAAGTGGAAACGCAGCAGATGGTACAGGTAGAGGAGCAACAAACAACATTA
AGTTGTAATGGTAGTGACGGGACACATAGTGAACGAGAGAATGAAACTCCAACACGTAAT
ATATTGCAAGTGTTAAAAACTAGCAATGGTAAAGCTGCTATGTTAGGTAAATTTAAAGAA
TTATATGGTGTAAGTTTTATGGAACTAATTAGGCCATTTCAAAGCAATAAAAGCACATGT
ACTGATTGGTGTGTAGCTGCGTTTGGAGTTACAGGTACAGTTGCAGAAGGATTTAAAACC
CTATTGCAACCATATTGTTTGTATTGCCATTTACAAAGTTTAGCATGTTCCTGGGGCATG
GTTATGTTAATGCTTGTGAGATTTAAATGTGCAAAAAATAGAATAACAATTGAAAAATTA
TTAGAAAAATTATTGTGTATATCTACAAATTGTATGTTAATTCAGCCACCCAAATTACGT
AGCACAGCTGCAGCATTATATTGGTACAGAACAGGAATGTCAAACATTAGCGATGTATAT
GGTGAAACACCAGAATGGATAGAAAGACAAACAGTATTACAGCATAGTTTTAATGACACA
ACATTTGATTTGTCCCAAATGGTACAATGGGCATATGACAATGATGTTATGGATGATAGT
GAAATTGCCTATAAATATGCACAATTAGCTGACAGTGATAGTAATGCATGTGCATTTTTA
AAAAGTAATTCGCAGGCAAAAATAGTTAAAGATTGTGGAACAATGTGTAGACATTATAAA
CGAGCAGAAAAACGACAAATGTCCATGGGACAGTGGATTAAAAGTAGATGTGACAAAGTT
AGTGACGAAGGTGACTGGAGGGACATAGTAAAGTTTTTAAGATATCAACAAATAGAATTT
GTGTCATTTTTATCTGCATTAAAGCTGTTTTTAAAAGGAGTGCCAAAGAAAAACTGTATT
TTAATACATGGTGCACCTAATACAGGTAAATCATATTTTGGAATGAGCCTTATTAGCTTT
TTACAAGGATGTATAATATCATATGCAAATTCAAAAAGTCATTTTTGGTTACAACCACTG
GCTGATGCTAAAATAGGCATGTTAGATGATGCTACAACGCCATGTTGGCATTATATAGAC
AATTACCTACGAAATGCACTAGATGGCAACCCTGTATCTATAGATGTAAAGCATAAAGCT
TTAATGCAGTTAAAATGTCCTCCTTTATTGATTACATCTAATATAAATGCAGGTAAGGAT
GACAGATGGCCATACCTACATAGCAGACTGGTGGTTTTTACATTTCCAAATCCATTTCCA
TTTGACAAAAACGGAAATCCAGTATATGAATTAAGTGATAAAAACTGGAAATCCTTTTTC
TCAAGGACGTGGTGCAGATTAAATTTGCACGAGGAAGAGGACAAAGAAAACGATGGAGAC
TCTTTCTCAACGTTTAAATGTGTGTCAGGACAAAATATTAGAACATTATGAAAATGATAG
TAAACGACTTTGTGATCATATAGACTATTGGAAACATATTCGACTTGAATGTGTATTAAT
GTATAAAGCAAGAGAAATGGGAATACACAGTATTAACCACCAGGTGGTGCCAGCGTTGTC
AGTATCAAAGGCCAAAGCCTTACAAGCTATTGAACTACAAATGATGTTGGAAACATTAAA
TAACACTGAATACAAAAATGAGGACTGGACAATGCAGCAAACAAGTCTTGAACTGTATTT

AACTGCACCTACAGGGTGTTTAAAAAAACATGGATATACTGTAGAGGTGCAATTTGATGG
TGATGTACACAACACCATGCATTATACTAACTGGAAATTTATATACCTATGTATAGATGG
CCAATGTACTGTTGTGGAAGGGCAAGTTAATTGTAAGGGCATTTATTATGTACATGAAGG
ACATATAACATATTTTGTAAATTTTACAGAAGAGGCAAAAAAATATGGGACTGGTAAAAA
ATGGGAAGTGCATGCGGGTGGTCAGGTAATTGTTTTTCCTGAATCTGTATTTAGCAGTGA
CGAAATATCCTTTGCTGGGATTGTTACAAAGCTACCAACAGCCAACAACACCACCACATC
GAATTCCAAAACCTGCGCCTTGGGCACCAGTGAAGGTGTGCGGCGGGCGACGACGTCTAC
TAAGCGACCAAGAACAGAGCCAGAGCACAGAAACACCCACCACCCCAACAAGTTGTTGCG
AGGCGACTCCGTGGACAGTGTCAACTGTGGGGTTATCAGTGCAGCTGCATGCACAAACCA
AACAAGGGCTGTCAGTTGTCCTGCAACTACACCTATAATACACTTAAAAGGTGATGCAAA
TATATTAAAATGTTTAAGATATAGGCTGTCAAAATATAAACAATTGTATGAACAAGTGTC
ATCTACATGGCATTGGACATGTACAGATGGAAAACATAAAAATGCTATTGTAACCTTAAC
ATATATAAGTACATCACAAAGAGACGATTTTTTAAATACTGTAAAAATACCTAACACAGT
ATCAGTGTCAACAGGATATATGACTATTTAGCCTAATGATTGAACTAAATATTTCTACAG
TAAGCATTGTGCTATGCTTTTTGCTTTGCTTTTGTGTGCTACTATTTGTGTGTCTTGTCA
TACGTCCACTTGTGCTGTCTGTGTCGGTATATGCAACACTACTATTATTAATTGTGATTT
TATGGGTTATTGCAACCTCTCCATTACGTTGTTTTTGTATATATGTTGTGTTTATATATA
TTCCATTATTTGTAATTCATACACATGCATCTTTTTTAAGTCAACAGTAACTTTTTTACT
TGTGTATACTGTTGTTTGTATTGGTATTGGTATTGGTATTGGTATTGGTATTGGTATAAT
AAACTTTTTTACTTTTTTTTTATTATTACCATGCGGTCCAAACGCTCTACAAAACGCACT
AAACGTGCGTCTGCTACACAATTATATCAAACATGTAAAGCAGCAGGTACTTGTCCATCA
GACGTTATACCTAAAATAGAACATACTACCATTGCAGACCAAATATTAAGGTATGGTAGT
ATGGGTGTTTTTTTTGGTGGGTTGGGTATTGGGTCCGGCTCTGGTACTGGGGGTCGCACT
GGATATGTCCCTCTTAGTACACGTCCTTCTACAGTATCTGAGGCAAGTATACCTATTAGA
CCACCAGTTAGCATTGACCCTGTAGGTCCCTTGGACCCCTCTATAGTAAGTCTTGTTGAA
GAATCTGGAATTGTTGATGTTGGTGCCCCTGCTCCTATACCACACCCTCCTACAACATCT
GGGTTTGACATTGCTACAACTGCAGACACAACACCTGCAATTTTAGATGTAACAAGTGTT
AGCACACATGAAAATCCTACTTTTACTGATCCATCTGTATTGCAGCCTCCTACACCTGCA
GAAACATCAGGTCATTTACTACTTTCATCATCATCTATTAGCACACATAATTATGAGGAA
ATACCTATGGATACATTTATTGTTTCTACTAATAATGAAAACATAACAAGTAGCACACCC
ATTCCAGGGGTGCGCCGTCCTGCACGTTTAGGGTTATATAGTAAGGCTACACAACAAGTA
AAAGTTATTGATCCAACGTTTCTTAGTGCTCCAAAACAGCTAATTACATATGAAAACCCT
GCCTATGAAACTGTAAATGCTGAAGAATCTTTATACTTTTCCAATACATCGCATAATATA
GCCCCTGATCCCGACTTTCTAGATATTATAGCATTACATAGGCCTGCCCTTACCTCACGT
AGGAACACTGTTAGATATAGTAGACTAGGTAATAAACAAACTTTGCGCACTCGTAGTGGT
GCTACTATTGGTGCAAGGGTGCATTATTATTATGATATTAGTAGTATTAATCCTGCAGGT
GAAAGTATTGAAATGCAACCTTTAGGGGCGTCTGCAACTACTACTTCTACTTTAAATGAT
GGCTTATATGACATTTATGCAGACACTGATTTTACTGTGGATACACCTGCCACACATAAT
GTTTCCCCTTCTACTGCTGTACAGTCCACATCTGCTGTGTCTGCCTATGTACCTACAAAT
ACCACTGTGCCACTAAGTACAGGTTTTGACATTCCCATATTTTCTGGGCCTGATGTACCT
ATAGAGCATGCACCTACACAGGTTTTCCCATTTCCTTTGGCCCCTACAACGCCACAAGTG
TCTATTTTTGTTGATGGGGGTGATTTTTATTTGCACCCTAGTTATTATATGTTAAAACGT
CGACGTAAACGTGTATCATATTTTTTTACAGATGTCTCTGTGGCGGCCTAGCGAGGCTAC
TGTCTACTTACCACCTGTCCCAGTGTCTAAAGTTGTAAGCACGGATGAATATGTAACACG
AACCAACATATATTATCACGCAGGCAGTGCTAGGCTGCTTACAGTAGGCCATCCATATTA
TTCCATACCTAAATCTGACAATCCTAAAAAAATAGTTGTACCAAAGGTGTCAGGATTACA
ATATAGGGTATTTAGGGTTCGTTTACCAGATCCAAACAAATTTGGATTTCCTGATACATC
TTTTTATAATCCTGAAACTCAACGCTTAGTTTGGGCCTGTGTTGGTTTAGAGGTAGGTCG
CGGGCAGCCATTAGGTGTAGGTATTAGTGGTCATCCATTATTAAATAAATTTGATGACAC
TGAAAACTCTAATAGATATGCCGGTGGTCCTGGCACTGATAATAGGGAATGTATATCAAT
GGATTATAAACAAACACAACTGTGTTTACTTGGTTGCAAACCACCTATTGGAGAGCATTG
GGGTAAAGGTAGTCCTTGTAGTAACAATGCTATTACCCCTGGTGATTGTCCTCCATTAGA
ATTAAAAAATTCAGTTATACAAGATGGGGATATGGTTGATACAGGCTTTGGAGCTATGGA
TTTTACTGCTTTACAAGACACTAAAAGTAATGTTCCTTTGGACATTTGTAATTCTATTTG
TAAATATCCAGATTATCTTAAAATGGTTGCTGAGCCATATGGCGATACATTATTTTTTTA

TTTACGTAGGGAACAAATGTTTGTAAGGCATTTTTTAATAGATCAGGCACGGTTGGTGA
ATCGGTCCCTACTGACTTATATATTAAAGGCTCCGGTTCAACAGCTACTTTAGCTAACAG
TACATACTTTCCTACACCTAGCGGCTCCATGGTTACTTCAGATGCACAAATTTTTAATAA
ACCATATTGGATGCAACGTGCTCAGGGACACAATAATGGTATTTGTTGGGGCAATCAGTT
ATTTGTTACTGTGGTAGATACCACACGTAGTACCAATATGTCTGTTTGTGCTGCAATTGC
AAACAGTGATACTACATTTAAAAGTAGTAATTTTAAAGAGTATTTAAGACATGGTGAGGA
ATTTGATTTACAATTTATATTTCAGTTATGCAAAATAACATTATCTGCAGACATAATGAC
ATATATTCACAGTATGAATCCTGCTATTTTGGAAGATTGGAATTTTGGATTGACCACACC
TCCCTCAGGTTCTTTGGAGGATACCTATAGGTTTGTCACCTCACAGGCCATTACATGTCA
AAAAACTGCCCCCCAAAAGCCCAAGGAAGATCCATTTAAAGATTATGTATTTTGGGAGGT
TAATTTAAAAGAAAAGTTTTCTGCAGATTTAGATCAGTTTCCACTGGGTCGCAAATTTTT
ATTACAGGCAGGATATAGGGCACGTCCTAAATTTAAAGCAGGTAAACGTAGTGCACCCTC
AGCATCTACCACTACACCAGCAAAACGTAAAAAAACTAAAAAGTAATGGATGTGTATGTA
ATACATGTGTCTGTATGTGTATGTGCTTGTGCTGTATTGTATATGTGTGTGTTTGTGTGT
TATATATGGTATATGTATGTTTATGTATGCGTGTGTACTTGTATATATGTATAGTATGTT
ATGTGTGTATGTATGCTATGTATGTTAATAAATATGTGTATACCTGTGTGTGTTGTGTAT
GTTGTCCTTATATACACCCTATTAGTAACATACTATTACTATTTTATAAACTATTGTTCC
TACTTGTTCCTACTTGTTCCTGCTCCTCCCAATAGTCATGTACTTATTTCTGCCTATAAT
TTAGGTGTCACGCCATAGTAAAAGTTGTACACCCGGTCCGTTTTTTGCAACTAAAGCTAC
TCCATTTTGATTTTATGCAGCCATTTTAAATCCCTAACCGTTTTCGGTTGCATTGTTTAA
ACATGCTAGTACAACTATGCTGATGCAGTAGTTCTGCGGTTTTTGGTTTCCTGAATACTA
GTTTTTGCCAACATTCTGGCTTGTAGTTTCCTGCCTAACACACCTTGCCAACATATAATC
CAGTCCAACTTTGCAATTATACTATGAATCATGTTTGTTTAAATACAACTGTAGTTCAAC
TATGTGTCATGCACATATATTATATTATCCTACACACCTTAAACTGCTTTTAGGCACATA
TTTTGTAGATTATCTATATCCTTGATTGCAGTGCTGGCTTTTGCACATGTTTAAACTGCC
AAGGTTGTGTCATGCATTATAAATAAGTTGTATGTTACTCATATAATTAATTGCATATAG
GTATTACACCGTTTTCGGTTACAGTTTTACAAGCAATTGTTCTTTTTATACT
>gi|333026|gb|M14119.1|PPH11_Human_papillomavirus_type_11_(HPV-
11)_complete_genome
CTTAATAACAATCTTAGTTTAAAAAAGAGGAGGGACCGAAAACGGTTCAACCGAAAACGGTTATATATAA
ACCAGCCCAAAAAATTAGCAGACGAGGCATTATGGAAAGTAAAGATGCCTCCACGTCTGCAACATCTATA
GACCAGTTGTGCAAGACGTTTAATCTTTCTTTGCACACTCTGCAAATTCAGTGCGTGTTTTGCAGGAATG
CACTGACCACCGCAGAGATATATGCATATGCCTATAAGAACCTAAAGGTTGTGTGGCGAGACAACTTTCC
CTTTGCAGCGTGTGCCTGTTGCTTAGAACTGCAAGGGAAAATTAACCAATATAGACACTTTAATTATGCT
GCATATGCACCTACAGTAGAAGAAGAAACCAATGAAGATATTTTAAAAGTGTTAATTCGTTGTTACCTGT
GTCACAAGCCGTTGTGTGAAATAGAAAAACTAAAGCACATATTGGGAAAGGCACGCTTCATAAAACTAAA
TAACCAGTGGAAGGGTCGTTGCTTACACTGCTGGACAACATGCATGGAAGACTTGTTACCCTAAAGGATA
TAGTACTAGACCTGCAGCCTCCTGACCCTGTAGGGTTACATTGCTATGAGCAATTAGAAGACAGCTCAGA
AGATGAGGTGGACAAGGTGGACAAACAAGACGCACAACCTTTAACACAACATTACCAAATACTGACCTGT
TGCTGTGGATGTGACAGCAACGTCCGACTGGTTGTGGAGTGCACAGACGGAGACATCAGACAACTACAAG
ACCTTTTGCTGGGCACACTAAATATTGTGTGTCCCATCTGCGCACCAAAACCATAACAAGGATGGCGGAC
GATTCAGGTACAGAAAATGAGGGGTCGGGGTGTACAGGATGGTTTATGGTAGAAGCCATAGTAGAGCACA
CTACAGGTACACAAATATCAGAAGATGAGGAAGAGGAGGTGGAGGACAGTGGGTATGACATGGTGGACTT
TATTGATGACAGGCATATTACACAAAATTCTGTGGAAGCACAGGCATTGTTTAATAGGCAGGAGGCGGAT
GCTCATTATGCGACTGTGCAGGACCTAAAACGAAAGTATTTAGGCAGTCCATATGTAAGTCCTATAAGCA
ATGTAGCTAATGCAGTAGAAAGTGAGATAAGTCCACGGTTAGACGCCATTAAACTTACAACACAGCCAAA
AAAGGTAAAGCGACGGCTGTTTGAAACACGGGAATTAACGGACAGTGGATATGGCTATTCTGAAGTGGAA
GCTGCAACGCAGGTAGAGAAACATGGCGACCCGGAAAATGGGGGAGATGGTCAGGAAAGGGACACAGGGA
GGGACATAGAGGGTGAGGGGGTGGAACATAGAGAGGCGGAAGCAGTAGACGACAGCACCCGAGAGCATGC
AGACACATCAGGAATATTAGAATTACTAAAATGTAAGGATATACGATCTACATTACATGGTAAGTTTAAA
GACTGCTTTGGGCTGTCATTTGTTGATTTAATTAGGCCATTTAAAAGTGATAGAACCACATGTGCCGATT
GGGTGGTTGCAGGATTTGGTATACATCATAGCATAGCAGATGCATTTCAAAAGTTAATTGAGCCATTAAG
TTTATATGCACATATACAATGGCTTACAAATGCATGGGGAATGGTACTATTAGTATTAATAAGGTTTAAA
GTAAATAAGAGCAGATGTACCGTGGCACGTACATTAGGTACGTTATTAAATATACCTGAAAATCACATGT
TAATTGAGCCTCCTAAAATACAAAGTGGCGTACGAGCCCTGTATTGGTTTAGGACAGGCATTTCAAATGC

AAGTACAGTTATAGGGGAGGCGCCGGAATGGATAACGCGCCAGACCGTTATTGAACATAGTTTGGCTGAC
AGTCAATTTAAATTAACTGAAATGGTGCAGTGGGCATATGATAATGATATTTGTGAAGAAAGTGAGATAG
CATTTGAATATGCACAGCGTGGAGACTTTGACTCCAATGCAAGGGCCTTTTTAAATAGTAATATGCAGGC
TAAATATGTAAAAGATTGTGCAATTATGTGCAGACATTATAAACATGCAGAAATGAAAAAGATGTCTATT
AAACAATGGATTAAGTATAGGGGTACTAAAGTTGACAGTGTAGGTAACTGGAAGCCAATTGTGCAGTTTC
TAAGACATCAAAACATAGAATTTATTCCATTTTTAAGCAAACTAAAATTATGGCTGCACGGAACGCCCAA
AAAAAATTGTATAGCCATTGTAGGGCCACCTGACACTGGGAAGTCGTGCTTTTGCATGAGTTTAATTAAG
TTTTTGGGGGGAACAGTTATTAGTTATGTTAATTCCTGCAGCCATTTCTGGCTACAGCCACTAACGGATG
CAAAAGTGGCATTATTGGATGATGCCACACAACCATGTTGGACATATATGGATACATATATGAGAAACCT
ATTAGATGGTAATCCTATGAGCATAGATAGAAAACATAGAGCATTAACATTAATTAAGTGTCCACCGCTA
CTGGTTACATCAAATATAGACATTAGCAAAGAGGAGAAATACAAATATTTACATAGTAGAGTTACCACAT
TTACATTTCCAAATCCATTCCCCTTTGACAGAAATGGGAATGCAGTATATGAACTATCAGATGCAAACTG
GAAATGTTTCTTTGAAAGACTGTCGTCCAGCCTAGACATTGAGGATTCAGAGGACGAGGAAGATGGAAGC
AATAGCCAAGCGTTTAGATGCGTGCCAGGATCAGTTGTTAGAACTTTATGAAGAAAACAGTATTGATATA
CACAAACACATTATGCATTGGAAATGCATACGATTGGAAAGTGTATTACTACACAAAGCAAAACAAATGG
GCCTGAGCCACATCGGGTTACAAGTAGTACCACCATTAACTGTGTCAGAGACTAAAGGACATAATGCTAT
TGAAATGCAAATGCATTTAGAATCCTTAGCAAAAACTCAGTATGGTGTGGAACCTTGGACATTACAGGAC
ACCAGTTATGAAATGTGGCTAACACCACCCAAACGGTGCTTTAAAAAACAGGGAAATACTGTGGAGGTAA
AATTTGATGGCTGTGAAGACAATGTAATGGAGTATGTGGTATGGACACATATATACCTGCAGGACAACGA
CTCATGGGTAAAAGTAACTAGTTCCGTAGATGCCAAGGGCATATATTATACATGTGGACAATTTAAAACA
TATTATGTAAATTTTAATAAAGAGGCACAAAAGTATGGTAGTACCAATCATTGGGAAGTATGTTATGGCA
GCACAGTTATATGTTCTCCTGCATCTGTATCTAGCACTGTACGAGAAGTATCCATTGCTGAACCTACTAC
ATACACCCCCGCACAGACCACCGCCCCTACAGTGTCCGCCTGCACCACGGAAGACGGCGTGTCGGCGCCG
CCTAGGAAGCGAGCACGTGGACCGTCCACTAACAACACCCTGTGTGTGGCCAACATCAGATCCGTGGACA
GTACAATCAACAACATCGTCACTGACAATTACAACAAGCACCAAAGAAGGAACAACTGTCACAGTGCAGC
TACGCCTATAGTGCAACTGCAAGGTGATTCCAATTGTTTAAAATGTTTTAGATATAGACTGAATGACAAA
TATAAACATTTGTTTGAATTAGCATCTTCAACGTGGCATTGGGCCTCACCTGAGGCACCACATAAAAATG
CAATTGTAACATTAACATATAGCAGTGAGGAACAACGTCAGCAATTTTTAAACAGTGTAAAAATACCACC
CACCATTAGGCATAAGGTGGGGTTTATGTCATTACATTTATTGTAACCATTACACCTGTATATATGTATA
TGTGTACATAACATACGTGTATGGAGGTAGTGCCTGTACAAATTGCTGCAGCAACAACTACAACATTGAT
ATTGCCTGTTGTTATTGCATTTGCAGTATGTATTCTTAGTATTGTACTTATAATATTAATATCTGATTTT
GTAGTATATACATCTGTGCTGGTACTAACACTTCTTTTATATTTGCTTTTGTGGCTTTTATTAACAACCC
CTTTGCAATTCTTTTTACTAACACTGTGTGTGTGCTATTTTCCTGCCTTTTATATACACATATACATTGT
GCAAACGCAACAATAATGGTGATGTTAACCTGTCACTTAAATGATGGTGATACATGGTTGTTTCTGTGGT
TGTTTACTGCATTTGTTGTAGCTGTACTTGGATTGTTGTTACTACATTACAGGGCTGTACATGGTACTGA
AAAAACTAAATGTGCTAAGTGTAAATCAAACCGCAATACTACTGTGGATTATGTGTATATGTCACATGGT
GATAATGGAGATTATGTGTACATGAACTAGAGTAAACCTTTTTTATACAGTGTGTGGTGTACGTTAGTTA
TATATAATGAAACCTAGGGCACGCAGACGTAAACGTGCGTCAGCCACACAACTATATCAAACATGCAAGG
CCACTGGTACATGTCCCCCAGATGTAATTCCTAAAGTTGAACATACTACTATTGCAGATCAAATATTAAA
ATGGGGAAGCTTAGGGGTTTTTTTTGGTGGGTTAGGTATTGGTACAGGGGCTGGTAGTGGCGGTCGTGCA
GGGTATATACCCTTGGGAAGCTCTCCCAAGCCTGCTATTACTGGGGGGCCAGCAGCACGTCCGCCAGTGC
TTGTGGAGCCTGTTGCCCCTTCCGATCCCTCCATTGTGTCCTTAATTGAGGAGTCTGCTATTATTAATGC
TGGTGCACCTGAGGTGGTACCCCCTACACAGGGTGGCTTTACTATAACATCATCTGAATCGACTACACCT
GCTATTTTAGATGTGTCTGTTACCAATCACACTACCACTAGTGTGTTTCAAAATCCCCTGTTTACAGAAC
CGTCTGTAATACAGCCCCAACCACCTGTGGAGGCCAGTGGTCACATACTTATATCTGCCCCAACAATAAC
ATCCCAACATGTAGAAGACATTCCACTAGACACTTTTGTTGTATCCTCTAGTGATAGTGGACCTACATCC
AGTACTCCTCTTCCTCGTGCTTTTCCTCGGCCTCGGGTGGGTTTGTATAGTCGTGCCTTACAGCAGGTAC
AGGTTACGGACCCCGCGTTTTTGTCCACGCCACAGCGATTGGTAACTTATGACAACCCTGTCTATGAAGG
AGAAGATGTAAGTTTACAATTTACCCATGAGTCTATCCACAATGCACCTGATGAAGCATTTATGGATATT
ATTAGACTACATAGACCAGCTATAACGTCCAGACGGGGTCTTGTGCGTTTTAGTCGCATTGGGCAACGGG
GGTCCATGTACACACGCAGTGGACAACATATAGGTGCCCGCATACATTATTTTCAGGACATTTCACCAGT
TACACAAGCTGCAGAGGAAATAGAACTGCACCCTCTAGTGGCTGCAGAAAATGACACGTTTGATATTTAT
GCTGAACCATTTGACCCTATCCCTGACCCTGTCCAACATTCTGTTACACAGTCTTATCTTACCTCCACAC
CTAATACCCTTTCACAATCGTGGGGTAATACCACAGTCCCATTGTCAATCCCTAGTGACTGGTTTGTGCA
GTCTGGGCCTGACATAACTTTTCCTACTGCATCTATGGGAACACCCTTTAGTCCTGTAACTCCTGCTTTA

CCTACAGGCCCTGTTTTTATTACAGGTTCTGACTTCTATTTGCATCCTACATGGTACTTTGCACGCAGAC
GCCGTAAACGTATTCCCTTATTTTTTACAGATGTGGCGGCCTAGCGACAGCACAGTATATGTGCCTCCTC
CCAACCCTGTATCCAAGGTTGTTGCCACGGATGCGTATGTTAAACGCACCAACATATTTTATCATGCCAG
CAGTTCTAGACTCCTTGCTGTGGGACATCCATATTACTCTATCAAAAAAGTTAACAAAACAGTTGTACCA
AAGGTGTCTGGATATCAATATAGAGTGTTTAAGGTAGTGTTGCCAGATCCTAACAAGTTTGCATTACCTG
ATTCATCCCTGTTTGACCCCACTACACAGCGTTTAGTATGGGCGTGCACAGGGTTGGAGGTAGGCAGGGG
TCAACCTTTAGGCGTTGGTGTTAGTGGGCATCCATTGCTAAACAAATATGATGATGTAGAAAATAGTGGT
GGGTATGGTGGTAATCCTGGTCAGGATAATAGGGTTAATGTAGGTATGGATTATAAACAAACCCAGCTAT
GTATGGTGGGCTGTGCTCCACCGTTAGGTGAACATTGGGGTAAGGGTACACAATGTTCAAATACCTCTGT
ACAAAATGGTGACTGCCCCCCGTTGGAACTTATTACCAGTGTTATACAGGATGGGGACATGGTTGATACA
GGCTTTGGTGCTATGAATTTTGCAGACTTACAAACCAATAAATCGGATGTTCCCCTTGATATTTGTGGAA
CTGTCTGCAAATATCCTGATTATTTGCAAATGGCTGCAGACCCTTATGGTGATAGGTTGTTTTTTTATTT
GCGAAAGGAACAAATGTTTGCTAGACACTTTTTTAATAGGGCCGGTACTGTGGGGGAACCTGTGCCTGAT
GACCTGTTGGTAAAAGGGGGTAATAACAGATCATCTGTAGCTAGTAGTATTTATGTACATACACCTAGTG
GCTCATTGGTGTCTTCAGAGGCTCAATTATTTAATAAACCATATTGGCTTCAAAAGGCTCAGGGACATAA
CAATGGTATTTGCTGGGGAAACCACTTGTTTGTTACTGTGGTAGATACCACACGCAGTACAAATATGACA
CTATGTGCATCTGTGTCTAAATCTGCTACATACACTAATTCAGATTATAAGGAATACATGCGCCATGTGG
AGGAGTTTGATTTACAGTTTATTTTTCAATTGTGTAGCATTACATTATCTGCAGAAGTCATGGCCTATAT
ACACACAATGAATCCTTCTGTTTTGGAGGACTGGAACTTTGGTTTATCGCCTCCACCAAATGGTACACTG
GAGGATACTTATAGATATGTACAGTCACAGGCCATTACCTGTCAGAAACCCACACCTGAAAAAGAAAAAC
AGGATCCCTATAAGGATATGAGTTTTTGGGAGGTTAACTTAAAAGAAAAGTTTTCAAGTGAATTAGATCA
GTTTCCCCTTGGACGTAAGTTTTTATTGCAAAGTGGATATCGAGGACGGACGTCTGCTCGTACAGGTATA
AAGCGCCCAGCTGTGTCTAAGCCCTCTACAGCCCCCAAACGAAAACGTACCAAAACCAAAAAGTAATATA
TGTGTGTCAGTGTGTTGTGTTATTTATATGTTGTTGTAGTGTGTATATGTTTCTTGTATTGTGTATATGT
GTATATGTTTGTGTATATGTGTATGTTATGTATGTTATGTTGTTATGTATGTTTGTGTGTTTAGTGTGTG
TATATATTTGTGGAATGTGTATGTATGTTTTTGTGCAATAAACAATTATTATGTGTGTCCTGTTACACCC
AGTGACTAAGTTGTGTTTTGCACGCGCCGTTTGTGTTGCCTTCATATTATATTATATATATTTGTAATAT
ACCTATACTATGTTACCCCCCCCCACTTGCAACCGTTTTCGGTTGCCCTTACATACACTTACCTCAAATT
TGTTATAACGTGTTTTGTACTAATCCCATATGTTGTGTGCCAAGGTACATATTGCCCTGCCAAGTATCTT
GCCAACAACACACCTGGCCAGGGCGCGGTATTGCATGACTAATGTACAATAAACCTGTCGGTTTGTACAA
TGTTGTGGATTGCAGCCAAAGGTTAAAAGCATTTTTGGCTTCTAGCTGAACATTTTTGTACCCTTAGTAT
ATTATGCACAATACCCACAAAATGAGTAACCTAAGGTCACACACCTGCAACCGGTTTCGGTTACCCACAC
CCTACATATTTCCTTCTTATA

>HPV8_Beta_333074_nt557_E6_AOS|1
TCATAGAGAAATTAGATTGCTGTGGGAGAGGCCGTCCCTTTCATAAAGTTAGAGGAGGCT
>HPV8_Beta_333074_nt549_E6_AOS|1
TTTGGATATCATAGAGAAATTAGATTGCTGTGGGAGAGGCCGTCCCTTTCATAAAGTTAG
>HPV8_Beta_333074_nt536_E6_AOS|1
ACTGCTTGTCATTTTTGGATATCATAGAGAAATTAGATTGCTGTGGGAGAGGCCGTCCCT
>HPV8_Beta_333074_nt529_E6_AOS|1
TGTCAAAACTGCTTGTCATTTTTGGATATCATAGAGAAATTAGATTGCTGTGGGAGAGGC
>HPV8_Beta_333074_nt355_E6_AOS|1
TTTGACAAAAAGAGACTGTGCCTAATTTGGAAAAATTACGTTGTTACTGCGTGTTGTCGT
>HPV8_Beta_333074_nt348_E6_AOS|1
GTGTGAGTTTGACAAAAAGAGACTGTGCCTAATTTGGAAAAATTACGTTGTTACTGCGTG
>HPV51_Alpha_333087_nt490_E6_AOS|1
TGGACGGGGCAATGCGCTAATTGCTGGCAACGTACACGACAACGTAACGAAACCCAAGTG
>HPV51_Alpha_333087_nt149_E6_AOS|1
CTTTGAACGTTTCTATGCACAATATACAGGTAGTGTGTGTGTATTGTAAAAAGGAATTAT
>HPV20_Beta_1020162_nt210_E6_AOS|1
CTCCTTCTTCAGAAGACAGCGCTGATGAAGGACCATCTAATATTGGAGAGGCAAAACCTC
>HPV20_Beta_1020162_nt296_E6_AOS|1
ACAATCTGTGGCCTAGCAAAACTTTTAGAAATACCGCTAGATGATTGTTTGATACCTTGT
>HPV20_Beta_1020162_nt289_E6_AOS|1
GCCTGCAACAATCTGTGGCCTAGCAAAACTTTTAGAAATACCGCTAGATGATTGTTTGAT
>HPV20_Beta_1020162_nt203_E6_AOS|1
GCTACACCTCCTTCTTCAGAAGACAGCGCTGATGAAGGACCATCTAATATTGGAGAGGCA
>HPV21_Beta_1020170_nt284_E6_AOS|1
TTAGAGCCACCATTACCAGCTACAATTCGTGACCTAGCCAATCTGTTAGAGATACCATTG
>HPV21_Beta_1020170_nt277_E6_AOS|1
TAGCTTTTTAGAGCCACCATTACCAGCTACAATTCGTGACCTAGCCAATCTGTTAGAGAT
>HPV22_Beta_1020178_nt480_E6_AOS|1
AGCAAGAAGAACAACGACCTGTAGGCCAAATTTATATGAGATGTCAATATTGCTTGAAGT
>HPV22_Beta_1020178_nt302_E6_AOS|1
AGATTCTGCAGTAGGTTTTTAACCTATTTGGAATTGCGGCAATTTGATTATAAGAATTTG
>HPV22_Beta_1020178_nt286_E6_AOS|1
TCTTTTGCTACCTTGTAGATTCTGCAGTAGGTTTTTAACCTATTTGGAATTGCGGCAATT
>HPV22_Beta_1020178_nt277_E6_AOS|1
TGTAGTAGATCTTTTGCTACCTTGTAGATTCTGCAGTAGGTTTTTAACCTATTTGGAATT
>HPV23_Beta_1020186_nt463_E6_AOS|1
AATTCAACAATTTTATCAGCTAACTGTGTATGGTCGTGAAATTGAGCAGGAGGAGCAACG
>HPV23_Beta_1020186_nt444_E6_AOS|1
ATGCTTCTGCTCAATTTGAAATTCAACAATTTTATCAGCTAACTGTGTATGGTCGTGAAA
>HPV23_Beta_1020186_nt346_E6_AOS|1
TCTTACCTATTTAGAGTTGCGAGAATTTGATTATAAACATTTGCAGTTAATCTGGACAGA
>HPV23_Beta_1020186_nt339_E6_AOS|1
CTAGGTTTCTTACCTATTTAGAGTTGCGAGAATTTGATTATAAACATTTGCAGTTAATCT
>HPV23_Beta_1020186_nt332_E6_AOS|1
TTTTGTTCTAGGTTTCTTACCTATTTAGAGTTGCGAGAATTTGATTATAAACATTTGCAG
>HPV28_Alpha_1020202_nt153_E6_AOS|1
ATATCATTTGATGACCTCCGTTTGCACTGCATATTCTGCGCCAAAGTGCTGACCACAGCG
>HPV28_Alpha_1020202_nt190_E6_AOS|1
GCGCCAAAGTGCTGACCACAGCGGAGCTATCAGCATTTGCATTAAGAGAACTAAATGTGG
>HPV28_Alpha_1020202_nt172_E6_AOS|1
GTTTGCACTGCATATTCTGCGCCAAAGTGCTGACCACAGCGGAGCTATCAGCATTTGCAT
>HPV28_Alpha_1020202_nt165_E6_AOS|1
GACCTCCGTTTGCACTGCATATTCTGCGCCAAAGTGCTGACCACAGCGGAGCTATCAGCA
>HPV28_Alpha_1020202_nt144_E6_AOS|1

GACAGTGGAATATCATTTGATGACCTCCGTTTGCACTGCATATTCTGCGCCAAAGTGCTG
>HPV29_Alpha_1020210_nt158_E6_AOS|1
AGTACCATTTGAGGACCTTCGCCTACAGTGTGTTTTCTGCACGAAAGAGCTAACCAGCCC
>HPV29_Alpha_1020210_nt280_E6_AOS|1
GTGCATGTGCACGCTGCTTATTGTTTGAAGGCATAAAGCGGCGCCTAAAATACTGGCAGT
>HPV29_Alpha_1020210_nt261_E6_AOS|1
AAAAGTGGAGCTCCGTACGGTGCATGTGCACGCTGCTTATTGTTTGAAGGCATAAAGCGG
>HPV29_Alpha_1020210_nt191_E6_AOS|1
TTTCTGCACGAAAGAGCTAACCAGCCCAGAACTGGCAGCATTTTGCATTCGGGAATTAAA
>HPV29_Alpha_1020210_nt184_E6_AOS|1
AGTGTGTTTTCTGCACGAAAGAGCTAACCAGCCCAGAACTGGCAGCATTTTGCATTCGGG
>HPV36_Beta_1020218_nt480_E6_AOS|1
TATTAGGAAGAGATATTGAATTGGCTGCAGGTCGCTCCATATTTGAAATTGACATTAGGT
>HPV36_Beta_1020218_nt207_E6_AOS|1
AGCAAGCCTCCGAACAGCAGAATATTACAGAAAAAGAAAAAGAACAGCTGCCTTTAACTA
>HPV36_Beta_1020218_nt200_E6_AOS|1
ATGGCAGAGCAAGCCTCCGAACAGCAGAATATTACAGAAAAAGAAAAAGAACAGCTGCCT
>HPV37_Beta_1020226_nt308_E6_AOS|1
ATAGAATTGATCGCATTTGATCGAAAAGGTCTTCAACTAATTTGGACCGAAGAAGATTTA
>HPV37_Beta_1020226_nt301_E6_AOS|2
AGCATATATAGAATTGATCGCATTTGATCGAAAAGGTCTTCAACTAATTTGGACCGAAGA
>HPV38_Beta_1020234_nt441_E6_AOS|1
TCTTTGGCCGTGAAATTGAACAGGTGGAGCAACAGACAATAGGCCTTATTGTTATAAGGT
>HPV38_Beta_1020234_nt434_E6_AOS|1
TTAACTGTCTTTGGCCGTGAAATTGAACAGGTGGAGCAACAGACAATAGGCCTTATTGTT
>HPV38_Beta_1020234_nt423_E6_AOS|1
AGTTTTATGAATTAACTGTCTTTGGCCGTGAAATTGAACAGGTGGAGCAACAGACAATAG
>HPV38_Beta_1020234_nt228_E6_AOS|1
AGCAGCTCAGTGATAAGTTAACAGTTCCTGTAGAGGATCTGTTATTACCCTGTAGATTCT
>HPV44_Alpha_1020242_nt363_E6_AOS|1
GTAACAGTGGAAGAAGAAACAAATAAGTCAATTCTGGACGTGCTGATACGCTGCTATTTG
>HPV44_Alpha_1020242_nt378_E6_AOS|1
GAAACAAATAAGTCAATTCTGGACGTGCTGATACGCTGCTATTTGTGCCACAAACCATTG
>HPV44_Alpha_1020242_nt371_E6_AOS|1
GGAAGAAGAAACAAATAAGTCAATTCTGGACGTGCTGATACGCTGCTATTTGTGCCACAA
>HPV44_Alpha_1020242_nt356_E6_AOS|1
ATATGCAGTAACAGTGGAAGAAGAAACAAATAAGTCAATTCTGGACGTGCTGATACGCTG
>HPV44_Alpha_1020242_nt338_E6_AOS|1
GCATTTTAACTACGCGGGATATGCAGTAACAGTGGAAGAAGAAACAAATAAGTCAATTCT
>HPV55_Alpha_1020266_nt376_E6_AOS|1
AGACAAAGCAGTCAATTTTGGACGTGTTCATACGCTGTTATTTGTGCCACAAACCATTGT
>HPV66_Alpha_1020290_nt229_E6_AOS|1
TAGAGCTATATAGGTTTGCATGTATTGAGTTAAAACTAGTATATAGAAACAATTGGCCAT
>HPV66_Alpha_1020290_nt222_E6_AOS|1
ACAAGTTTAGAGCTATATAGGTTTGCATGTATTGAGTTAAAACTAGTATATAGAAACAAT
>HPV66_Alpha_1020290_nt215_E6_AOS|1
GGAACTTACAAGTTTAGAGCTATATAGGTTTGCATGTATTGAGTTAAAACTAGTATATAG
>HPV66_Alpha_1020290_nt208_E6_AOS|1
GCAAAAAGGAACTTACAAGTTTAGAGCTATATAGGTTTGCATGTATTGAGTTAAAACTAG
>HPV70_Alpha_1173493_nt330_E6_AOS|1
TAAGGGAACTACGGCATTATTCGAACTCGGTGTATGCAACAACTTTGGAAAGCATAACTA
>HPV70_Alpha_1173493_nt342_E6_AOS|1
GGCATTATTCGAACTCGGTGTATGCAACAACTTTGGAAAGCATAACTAATACCAAGTTAT
>HPV70_Alpha_1173493_nt263_E6_AOS|1
TTTATAGTATATAGAAACGGGGAGCCATATGCTGCATGCCAAAAATGTATTAAATTTCAT

>HPV70_Alpha_1173493_nt174_E6_AOS|1
CTACATTGCACGACATTACAATAGACTGTGTCTATTGTAAAACACAGCTACAGCAAACAG
>HPV71_Alpha_12084981_nt93_E6_AOS|1
TATAAAGCAATGCTTGGTTCAGCAGATTTAGCTATGTCCAGCGGGGACGCATACCCCACC
>HPV71_Alpha_12084981_nt86_E6_AOS|1
GTGCATATATAAAGCAATGCTTGGTTCAGCAGATTTAGCTATGTCCAGCGGGGACGCATA
>HPV84_Alpha_12958167_nt141_E6_AOS|2
GGAATTACTAATTGTTTGGAGGTATAATTTCCCTCATGGGGTGTGCATGAAATGCTTATG
>HPV84_Alpha_12958167_nt151_E6_AOS|1
ATTGTTTGGAGGTATAATTTCCCTCATGGGGTGTGCATGAAATGCTTATGCAGGGAAGCC
>HPV84_Alpha_12958167_nt134_E6_AOS|1
CAGTAAAGGAATTACTAATTGTTTGGAGGTATAATTTCCCTCATGGGGTGTGCATGAAAT
>HPV107_Beta_126131393_nt531_E6_AOS|1
AGGTAAGCAGATTGAGGAGATCACTCAGCAAAGTATTGGATTTATTAATATTAGATGTGT
>HPV107_Beta_126131393_nt421_E6_AOS|2
AAAAATTTACAATTAATTTGGCATGAAAATTTAGTATATGGTTGTTGTTGCAGCTGTGCT
>HPVcand87_Alpha_14475578_nt253_E6_AOS|1
TGCTGGTGTGGAGATTTGGATTTCCTTATGGAGTGTGCCTACAATGCCTATTCAGAGAAG
>HPVcand87_Alpha_14475578_nt420_E6_AOS|1
TGCAAACCATTGTGCTGGCCCGAGAAGGAGCATATGATACACGCGAATCTGCACTTCCAC
>HPVcand87_Alpha_14475578_nt246_E6_AOS|1
GAATTATTGCTGGTGTGGAGATTTGGATTTCCTTATGGAGTGTGCCTACAATGCCTATTC
>HPVcand87_Alpha_14475578_nt239_E6_AOS|1
ATTAAGGGAATTATTGCTGGTGTGGAGATTTGGATTTCCTTATGGAGTGTGCCTACAATG
>HPVcand87_Alpha_14475578_nt124_E6_AOS|1
CACCTACCAATATCTTTTTGCTATGTCAGGAATATGAGGTGGACTTTGACAACTTAAGGC
>HPVcand87_Alpha_14475578_nt92_E6_AOS|1
CAACGGGACAGTCATGCCCAACGGACGCTACACACCTACCAATATCTTTTTGCTATGTCA
>HPV72_Alpha_1491683_nt438_E6_AOS|1
GAAAAGGAATATCAGGTGCAGACAGGAATCCACTTCCACAAGATAAGCGGACTGTGGACG
>HPV72_Alpha_1491683_nt431_E6_AOS|1
CTGTCAGGAAAAGGAATATCAGGTGCAGACAGGAATCCACTTCCACAAGATAAGCGGACT
>HPV72_Alpha_1491683_nt424_E6_AOS|1
CCCTAAGCTGTCAGGAAAAGGAATATCAGGTGCAGACAGGAATCCACTTCCACAAGATAA
>HPV72_Alpha_1491683_nt416_E6_AOS|1
CTGCAAACCCCTAAGCTGTCAGGAAAAGGAATATCAGGTGCAGACAGGAATCCACTTCCA
>HPV72_Alpha_1491683_nt324_E6_AOS|1
CTACGATACTGGACGTATTCGGGCTACGGACCCACTGTGGAACAGGAAACAGGCAAATCA
>HPV72_Alpha_1491683_nt137_E6_AOS|1
GTTGCTGTGCAAGGAAATTGAGGTGGACCTAGAAGATTTACGGATTACCTGCATATTTTG
>HPV73_Alpha_1491692_nt143_E6_AOS|1
AGCGTTATGTGACGAAGTGAATATTTCTATACATGATATAAACCTGGACTGTGTGTTTTG
>HPV73_Alpha_1491692_nt325_E6_AOS|1
GAGAGTATAGGCGATATAGACAATCAGTATATGGCACTACGTTAGAAAATTTAACTAACA
>HPV73_Alpha_1491692_nt316_E6_AOS|1
CTAAAATTAGAGAGTATAGGCGATATAGACAATCAGTATATGGCACTACGTTAGAAAATT
>HPV73_Alpha_1491692_nt136_E6_AOS|1
AGCTACAAGCGTTATGTGACGAAGTGAATATTTCTATACATGATATAAACCTGGACTGTG
>HPV73_Alpha_1491692_nt129_E6_AOS|1
CCATACAAGCTACAAGCGTTATGTGACGAAGTGAATATTTCTATACATGATATAAACCTG
>HPVcand86_Alpha_15741127_nt149_E6_AOS|1
TACTGGTCTGGAAACACGGATTTCCTCATGGCGTGTGCCTAAAATGCATATGCAGAGAAG
>HPVcand86_Alpha_15741127_nt142_E6_AOS|1
GAATTATTACTGGTCTGGAAACACGGATTTCCTCATGGCGTGTGCCTAAAATGCATATGC
>HPVcand86_Alpha_15741127_nt135_E6_AOS|1

ACTAAAGGAATTATTACTGGTCTGGAAACACGGATTTCCTCATGGCGTGTGCCTAAAATG
>HPV88_Gamma_167600365_nt342_E6_AOS|1
AGGATTTCCTAAGAAAGAATCTAGCTGATGTGGTATGCCGATGCTATGAATGTATGTCAT
>HPV88_Gamma_167600365_nt353_E6_AOS|1
AGAAAGAATCTAGCTGATGTGGTATGCCGATGCTATGAATGTATGTCATTGCTTGATTTA
>HPV88_Gamma_167600365_nt287_E6_AOS|1
GCTAGGTATGAGGCTGAACGCTTTTACCGCTGCTCTGTAAAGGGAATTCATTTTGAGGAT
>HPV88_Gamma_167600365_nt280_E6_AOS|1
GCTTTCTGCTAGGTATGAGGCTGAACGCTTTTACCGCTGCTCTGTAAAGGGAATTCATTT
>HPV88_Gamma_167600365_nt273_E6_AOS|1
GTTCTAGGCTTTCTGCTAGGTATGAGGCTGAACGCTTTTACCGCTGCTCTGTAAAGGGAA
>HPV88_Gamma_167600365_nt266_E6_AOS|1
GTCCCTTGTTCTAGGCTTTCTGCTAGGTATGAGGCTGAACGCTTTTACCGCTGCTCTGTA
>HPV88_Gamma_167600365_nt259_E6_AOS|1
GTGTTGTGTCCCTTGTTCTAGGCTTTCTGCTAGGTATGAGGCTGAACGCTTTTACCGCTG
>HPV88_Gamma_167600365_nt252_E6_AOS|1
GTTTTGCGTGTTGTGTCCCTTGTTCTAGGCTTTCTGCTAGGTATGAGGCTGAACGCTTTT
>HPV88_Gamma_167600365_nt106_E6_AOS|1
ACGGCTAGATGACTTCTGTTCGATTTTTAATATTTCTCTTTTTGATGTGCATCTTCCTTG
>HPV88_Gamma_167600365_nt93_E6_AOS|1
CTTATCTACCAAAACGGCTAGATGACTTCTGTTCGATTTTTAATATTTCTCTTTTTGATG
>HPV110_Beta_186461194_nt239_E6_AOS|1
TATTAATACCGTGTAGATTTTGTACTAGGTTTTTATCATATATAGAATTATTAGGTTTTG
>HPV110_Beta_186461194_nt225_E6_AOS|1
TCCTGTTTTAGACATATTAATACCGTGTAGATTTTGTACTAGGTTTTTATCATATATAGA
>HPV110_Beta_186461194_nt218_E6_AOS|1
TATGCATTCCTGTTTTAGACATATTAATACCGTGTAGATTTTGTACTAGGTTTTTATCAT
>HPV110_Beta_186461194_nt208_E6_AOS|1
GCAGATACATTATGCATTCCTGTTTTAGACATATTAATACCGTGTAGATTTTGTACTAGG
>HPV111_Beta_186461202_nt463_E6_AOS|1
ATTGTTTGAAAAAATTGGATCTGGTAGAGAAGCTAGGAGTGTGCTACAAACAACAATATT
>HPV111_Beta_186461202_nt470_E6_AOS|1
GAAAAAATTGGATCTGGTAGAGAAGCTAGGAGTGTGCTACAAACAACAATATTTTCATAG
>HPV111_Beta_186461202_nt379_E6_AOS|1
AATATTTTGAATACTCTGTTGTAGGAAAACAGATAGAAACTGTAGCAAACCAGCCTATTG
>HPVFA75/KI88-03_Unclassified_206902429_nt411_E6_AOS|1
GAGCAGGTGACTCAACAGTCTGTAGGCTTTATTACTATCAGATGCATTTATTGTTTAAAG
>HPVFA75/KI88-03_Unclassified_206902429_nt426_E6_AOS|1
CAGTCTGTAGGCTTTATTACTATCAGATGCATTTATTGTTTAAAGCATCTAGACCTTTTA
>HPVFA75/KI88-03_Unclassified_206902429_nt419_E6_AOS|1
GACTCAACAGTCTGTAGGCTTTATTACTATCAGATGCATTTATTGTTTAAAGCATCTAGA
>HPV91_Alpha_22023568_nt81_E6_AOS|1
CAATTATTATGCTTGTACTAAAGGGAGTAACCGAAAACGGTCCGACCGAAAGCGGTACAT
>HPV91_Alpha_22023568_nt94_E6_AOS|1
TGTACTAAAGGGAGTAACCGAAAACGGTCCGACCGAAAGCGGTACATATATAAACCACCC
>HPV91_Alpha_22023568_nt74_E6_AOS|1
TTAGTAACAATTATTATGCTTGTACTAAAGGGAGTAACCGAAAACGGTCCGACCGAAAGC
>HPV91_Alpha_22023568_nt52_E6_AOS|1
CACCTTACATTCTTTGTGGTTATTAGTAACAATTATTATGCTTGTACTAAAGGGAGTAAC
>HPV91_Alpha_22023568_nt45_E6_AOS|1
CGCTGCACACCTTACATTCTTTGTGGTTATTAGTAACAATTATTATGCTTGTACTAAAGG
>HPV91_Alpha_22023568_nt38_E6_AOS|1
AACCGGTCGCTGCACACCTTACATTCTTTGTGGTTATTAGTAACAATTATTATGCTTGTA
>HPV91_Alpha_22023568_nt31_E6_AOS|1
AGACCGAAACCGGTCGCTGCACACCTTACATTCTTTGTGGTTATTAGTAACAATTATTAT

>HPV91_Alpha_22023568_nt24_E6_AOS|1
CACCTGCAGACCGAAACCGGTCGCTGCACACCTTACATTCTTTGTGGTTATTAGTAACAA
>HPV91_Alpha_22023568_nt17_E6_AOS|1
TTAGGCACACCTGCAGACCGAAACCGGTCGCTGCACACCTTACATTCTTTGTGGTTATTA
>HPV91_Alpha_22023568_nt10_E6_AOS|1
TACTGTGTTAGGCACACCTGCAGACCGAAACCGGTCGCTGCACACCTTACATTCTTTGTG
>HPVcand89_Alpha_22095322_nt66_E6_AOS|1
GGATCTACGGCTTACATGCATATATTGCACCAAGGAGCTTACAACATCAGAAGTGCTGTC
>HPVcand89_Alpha_22095322_nt59_E6_AOS|1
ATTTAGAGGATCTACGGCTTACATGCATATATTGCACCAAGGAGCTTACAACATCAGAAG
>HPVcand89_Alpha_22095322_nt52_E6_AOS|1
GACGTGGATTTAGAGGATCTACGGCTTACATGCATATATTGCACCAAGGAGCTTACAACA
>HPV90_Alpha_22138122_nt242_E6_AOS|1
CTAGCTATGCACCCACAGTGGAATATGAAACAGGAGTGCCTATTTTAGAATTGCTTATTA
>HPV90_Alpha_22138122_nt267_E6_AOS|1
TGAAACAGGAGTGCCTATTTTAGAATTGCTTATTAGATGCTACCTGTGCCATAAACCACT
>HPV90_Alpha_22138122_nt235_E6_AOS|1
CAGTATTCTAGCTATGCACCCACAGTGGAATATGAAACAGGAGTGCCTATTTTAGAATTG
>HPV90_Alpha_22138122_nt183_E6_AOS|1
ATGTCCTGCGTGTCTGGAAGCAGCCGGGAAACTAAGGCAATTTAGATACTGGCAGTATTC
>HPV90_Alpha_22138122_nt167_E6_AOS|1
ATTTTCCATTTGCAGCATGTCCTGCGTGTCTGGAAGCAGCCGGGAAACTAAGGCAATTTA
>HPV90_Alpha_22138122_nt160_E6_AOS|1
AGAAAGCATTTTCCATTTGCAGCATGTCCTGCGTGTCTGGAAGCAGCCGGGAAACTAAGG
>HPV58_Alpha_222386_nt279_E6_AOS|1
GAAATCCATTTGCAGTATGTAAAGTGTGCTTACGATTGCTATCTAAAATAAGTGAGTATA
>HPV58_Alpha_222386_nt272_E6_AOS|1
AGAGATGGAAATCCATTTGCAGTATGTAAAGTGTGCTTACGATTGCTATCTAAAATAAGT
>HPV58_Alpha_222386_nt265_E6_AOS|1
AGTGTATAGAGATGGAAATCCATTTGCAGTATGTAAAGTGTGCTTACGATTGCTATCTAA
>HPV58_Alpha_222386_nt175_E6_AOS|1
TGTGCATGAAATCGAATTGAAATGCGTTGAATGCAAAAAGACTTTGCAGCGATCTGAGGT
>HPV58_Alpha_222386_nt168_E6_AOS|1
AGACATCTGTGCATGAAATCGAATTGAAATGCGTTGAATGCAAAAAGACTTTGCAGCGAT
>HPV58_Alpha_222386_nt161_E6_AOS|1
GCGTTGGAGACATCTGTGCATGAAATCGAATTGAAATGCGTTGAATGCAAAAAGACTTTG
>HPV58_Alpha_222386_nt154_E6_AOS|1
GTGTCAGGCGTTGGAGACATCTGTGCATGAAATCGAATTGAAATGCGTTGAATGCAAAAA
>HPV58_Alpha_222386_nt147_E6_AOS|1
ATGATTTGTGTCAGGCGTTGGAGACATCTGTGCATGAAATCGAATTGAAATGCGTTGAAT
>HPV74_Alpha_27462483_nt350_E6_AOS|2
TACGGCACATTTTGGAAAAGGCAAGATTTATTAAACTAGGCGACACCTGGAAAGGGCGCT
>HPV92_Beta_27531786_nt349_E6_AOS|1
GATGCTATAGAAAGGGACTCAGGTAGTCTTTTGTGTTGTTTAACTGTTAGATGTAAGTTT
>HPV92_Beta_27531786_nt342_E6_AOS|1
AGGAAAGGATGCTATAGAAAGGGACTCAGGTAGTCTTTTGTGTTGTTTAACTGTTAGATG
>HPV92_Beta_27531786_nt112_E6_AOS|1
AAACCTCCTTCGGTGCAGGAACTTAGAAGACAGTTAGATATTCCATTGGAGGACATTTTA
>HPV75_Beta_2911544_nt470_E6_AOS|1
ATACATAATCTTATTGTAAGGTGCCAGTTTTGTCTGAAACGTTTGGATATATTTGAAAAA
>HPV75_Beta_2911544_nt463_E6_AOS|1
AGAAAGTATACATAATCTTATTGTAAGGTGCCAGTTTTGTCTGAAACGTTTGGATATATT
>HPV75_Beta_2911544_nt456_E6_AOS|1
GAGCAGCAGAAAGTATACATAATCTTATTGTAAGGTGCCAGTTTTGTCTGAAACGTTTGG
>HPV75_Beta_2911544_nt449_E6_AOS|1

GAAGGCAGAGCAGCAGAAAGTATACATAATCTTATTGTAAGGTGCCAGTTTTGTCTGAAA
>HPV75_Beta_2911544_nt441_E6_AOS|1
TAGAAATAGAAGGCAGAGCAGCAGAAAGTATACATAATCTTATTGTAAGGTGCCAGTTTT
>HPV76_Beta_2911551_nt474_E6_AOS|1
CGAACATTATAATAAGGTGTCAATTTTGTTTGAAACGCCTGGATTTATTTGAAAAACTTG
>HPV76_Beta_2911551_nt467_E6_AOS|1
AATATAGCGAACATTATAATAAGGTGTCAATTTTGTTTGAAACGCCTGGATTTATTTGAA
>HPV76_Beta_2911551_nt460_E6_AOS|1
TGCAGAAAATATAGCGAACATTATAATAAGGTGTCAATTTTGTTTGAAACGCCTGGATTT
>HPV76_Beta_2911551_nt453_E6_AOS|1
GCAGAGCTGCAGAAAATATAGCGAACATTATAATAAGGTGTCAATTTTGTTTGAAACGCC
>HPV76_Beta_2911551_nt446_E6_AOS|1
ATAGAAGGCAGAGCTGCAGAAAATATAGCGAACATTATAATAAGGTGTCAATTTTGTTTG
>HPV77_Alpha_2911558_nt161_E6_AOS|1
ACCGTTCGACGACCTTCGCCTACTGTGTGTGTTCTGCACAAAAGAATTAACTACTGCTGA
>HPV77_Alpha_2911558_nt300_E6_AOS|1
CTGCTATATGGCATTGAGCGGCGCCTGAAACACTGGGAGTATTCATACTACGTGGACGGC
>HPV77_Alpha_2911558_nt293_E6_AOS|1
GTGTTTACTGCTATATGGCATTGAGCGGCGCCTGAAACACTGGGAGTATTCATACTACGT
>HPV77_Alpha_2911558_nt198_E6_AOS|1
ACAAAAGAATTAACTACTGCTGAACTGGCAGCATTTACAATTAGAGAATTAAATGTGGTG
>HPV77_Alpha_2911558_nt191_E6_AOS|1
GTTCTGCACAAAAGAATTAACTACTGCTGAACTGGCAGCATTTACAATTAGAGAATTAAA
>HPV77_Alpha_2911558_nt184_E6_AOS|1
TGTGTGTGTTCTGCACAAAAGAATTAACTACTGCTGAACTGGCAGCATTTACAATTAGAG
>HPV77_Alpha_2911558_nt154_E6_AOS|1
GTGGAATACCGTTCGACGACCTTCGCCTACTGTGTGTGTTCTGCACAAAAGAATTAACTA
>HPV80_Beta_2911565_nt422_E6_AOS|1
ACATTTTATGAACAGTCAGTGTGTGGTTGGGAGATTGAGGTAGTTGAGCGAAAAGCTGTT
>HPV80_Beta_2911565_nt318_E6_AOS|1
TAAGTTTTGATCGCAAATGTTTGCAGTTGATTTGGACTGAAGAAGACTTTGTCTTTGCTT
>HPV80_Beta_2911565_nt311_E6_AOS|1
GAGTTAGTAAGTTTTGATCGCAAATGTTTGCAGTTGATTTGGACTGAAGAAGACTTTGTC
>HPV80_Beta_2911565_nt303_E6_AOS|1
CTTATATAGAGTTAGTAAGTTTTGATCGCAAATGTTTGCAGTTGATTTGGACTGAAGAAG
>HPV80_Beta_2911565_nt291_E6_AOS|1
AAAGATTTTTAACTTATATAGAGTTAGTAAGTTTTGATCGCAAATGTTTGCAGTTGATTT
>HPV80_Beta_2911565_nt282_E6_AOS|1
GGTTTTGTCAAAGATTTTTAACTTATATAGAGTTAGTAAGTTTTGATCGCAAATGTTTGC
>HPVRTRX7_Unclassified_30315616_nt530_E6_AOS|1
ATTGAAAATCAGGTGTCAGACTTGCTTATCATTTTTGAGCACCATTGAAAAATTAGATAG
>HPVRTRX7_Unclassified_30315616_nt435_E6_AOS|1
TGTGTAGCTACTGCAACTTTCGAATTCAATGAATATTACCAGCAAACAGTTTTAGGTAGA
>HPVRTRX7_Unclassified_30315616_nt428_E6_AOS|1
AAGTTGTTGTGTAGCTACTGCAACTTTCGAATTCAATGAATATTACCAGCAAACAGTTTT
>HPV65_Gamma_312100_nt417_E6_AOS|1
CTACTAGACATTGTTGAGAAATTAGACTTGCTGTACTCTGACCAGGCCTGCTACTTAATA
>HPV65_Gamma_312100_nt410_E6_AOS|1
TTTGAGACTACTAGACATTGTTGAGAAATTAGACTTGCTGTACTCTGACCAGGCCTGCTA
>HPV65_Gamma_312100_nt403_E6_AOS|1
TATGTTGTTTGAGACTACTAGACATTGTTGAGAAATTAGACTTGCTGTACTCTGACCAGG
>HPV65_Gamma_312100_nt396_E6_AOS|1
AGATGTATATGTTGTTTGAGACTACTAGACATTGTTGAGAAATTAGACTTGCTGTACTCT
>HPV65_Gamma_312100_nt389_E6_AOS|1
TTGCATCAGATGTATATGTTGTTTGAGACTACTAGACATTGTTGAGAAATTAGACTTGCT

>HPV65_Gamma_312100_nt382_E6_AOS|1
AGGAGATTTGCATCAGATGTATATGTTGTTTGAGACTACTAGACATTGTTGAGAAATTAG
>HPV65_Gamma_312100_nt375_E6_AOS|1
AAAATTAAGGAGATTTGCATCAGATGTATATGTTGTTTGAGACTACTAGACATTGTTGAG
>HPV65_Gamma_312100_nt368_E6_AOS|1
TCAAAGGAAAATTAAGGAGATTTGCATCAGATGTATATGTTGTTTGAGACTACTAGACAT
>HPV11_Alpha_333026_nt452_E6_AOS|1
AAAGCACATATTGGGAAAGGCACGCTTCATAAAACTAAATAACCAGTGGAAGGGTCGTTG
>HPV11_Alpha_333026_nt445_E6_AOS|1
AAAAACTAAAGCACATATTGGGAAAGGCACGCTTCATAAAACTAAATAACCAGTGGAAGG
>HPV11_Alpha_333026_nt438_E6_AOS|1
GAAATAGAAAAACTAAAGCACATATTGGGAAAGGCACGCTTCATAAAACTAAATAACCAG
>HPV11_Alpha_333026_nt431_E6_AOS|1
GTTGTGTGAAATAGAAAAACTAAAGCACATATTGGGAAAGGCACGCTTCATAAAACTAAA
>HPV11_Alpha_333026_nt373_E6_AOS|1
AAGAAACCAATGAAGATATTTTAAAAGTGTTAATTCGTTGTTACCTGTGTCACAAGCCGT
>HPV11_Alpha_333026_nt295_E6_AOS|1
CCTGTTGCTTAGAACTGCAAGGGAAAATTAACCAATATAGACACTTTAATTATGCTGCAT
>HPV31_Alpha_333048_nt146_E6_AOS|1
TGAACTAAGCTCGGCATTGGAAATACCCTACGATGAACTAAGATTGAATTGTGTCTACTG
>HPV31_Alpha_333048_nt267_E6_AOS|1
TATAGGGACGACACACCACACGGAGTGTGTACAAAATGTTTAAGATTTTATTCAAAAGTA
>HPV31_Alpha_333048_nt139_E6_AOS|1
AATTGCATGAACTAAGCTCGGCATTGGAAATACCCTACGATGAACTAAGATTGAATTGTG
>HPV31_Alpha_333048_nt132_E6_AOS|1
CCTCGGAAATTGCATGAACTAAGCTCGGCATTGGAAATACCCTACGATGAACTAAGATTG
>HPV31_Alpha_333048_nt125_E6_AOS|1
AGAAAGACCTCGGAAATTGCATGAACTAAGCTCGGCATTGGAAATACCCTACGATGAACT
>HPV31_Alpha_333048_nt118_E6_AOS|1
ATCCTGCAGAAAGACCTCGGAAATTGCATGAACTAAGCTCGGCATTGGAAATACCCTACG
>HPV31_Alpha_333048_nt111_E6_AOS|1
TTCAAAAATCCTGCAGAAAGACCTCGGAAATTGCATGAACTAAGCTCGGCATTGGAAATA
>HPV33_Alpha_333049_nt300_E6_AOS|1
ACTGTGTTTGCGGTTCTTATCTAAAATTAGTGAATATAGACATTATAATTATTCTGTATA
>HPV33_Alpha_333049_nt279_E6_AOS|1
AAATCCATTTGGAATATGTAAACTGTGTTTGCGGTTCTTATCTAAAATTAGTGAATATAG
>HPV33_Alpha_333049_nt272_E6_AOS|1
GAGAGGGAAATCCATTTGGAATATGTAAACTGTGTTTGCGGTTCTTATCTAAAATTAGTG
>HPV33_Alpha_333049_nt265_E6_AOS|1
GTATATAGAGAGGGAAATCCATTTGGAATATGTAAACTGTGTTTGCGGTTCTTATCTAAA
>HPV33_Alpha_333049_nt258_E6_AOS|1
AACAGTTGTATATAGAGAGGGAAATCCATTTGGAATATGTAAACTGTGTTTGCGGTTCTT
>HPV33_Alpha_333049_nt251_E6_AOS|1
CAGATTTAACAGTTGTATATAGAGAGGGAAATCCATTTGGAATATGTAAACTGTGTTTGC
>HPV33_Alpha_333049_nt174_E6_AOS|1
TATACACAACATTGAACTACAGTGCGTGGAATGCAAAAAACCTTTGCAACGATCTGAGGT
>HPV33_Alpha_333049_nt167_E6_AOS|1
AGACAACTATACACAACATTGAACTACAGTGCGTGGAATGCAAAAAACCTTTGCAACGAT
>HPV33_Alpha_333049_nt160_E6_AOS|1
GCATTGGAGACAACTATACACAACATTGAACTACAGTGCGTGGAATGCAAAAAACCTTTG
>HPV35_Alpha_333050_nt466_E6_AOS|1
AGAAGAAAAAAAACGATTCCATAACATCGGTGGACGGTGGACAGGTCGGTGTATGTCCTG
>HPV35_Alpha_333050_nt445_E6_AOS|1
AGTTGAAAAGCAAAGACATTTAGAAGAAAAAAAACGATTCCATAACATCGGTGGACGGTG
>HPV35_Alpha_333050_nt259_E6_AOS|1

GTGTATAGTATATAGAGAAGGCCAGCCATATGGAGTATGCATGAAATGTTTAAAATTTTA
>HPV35_Alpha_333050_nt248_E6_AOS|1
TGCTATGATTTGTGTATAGTATATAGAGAAGGCCAGCCATATGGAGTATGCATGAAATGT
>HPV35_Alpha_333050_nt240_E6_AOS|1
ACTTTGCATGCTATGATTTGTGTATAGTATATAGAGAAGGCCAGCCATATGGAGTATGCA
>HPV47_Beta_333062_nt243_E6_AOS|1
AGAGGAAAAGCTAGAACTACCTACTACTATTAGAGGCTTAGCTCAATTGTTAGACATACC
>HPV47_Beta_333062_nt251_E6_AOS|1
AGCTAGAACTACCTACTACTATTAGAGGCTTAGCTCAATTGTTAGACATACCTTTAGTAG
>HPV47_Beta_333062_nt236_E6_AOS|1
CAGTTAAAGAGGAAAAGCTAGAACTACCTACTACTATTAGAGGCTTAGCTCAATTGTTAG
>HPV47_Beta_333062_nt229_E6_AOS|1
CAGACTACAGTTAAAGAGGAAAAGCTAGAACTACCTACTACTATTAGAGGCTTAGCTCAA
>HPV47_Beta_333062_nt222_E6_AOS|1
TTTGGAACAGACTACAGTTAAAGAGGAAAAGCTAGAACTACCTACTACTATTAGAGGCTT
>HPV42_Alpha_333211_nt456_E6_AOS|1
GAAAAAAACCATCATATTGATACAGGTACAAGATTTCAATTTATATTGTGTCAGTGGACG
>HPV42_Alpha_333211_nt449_E6_AOS|1
ACAGAGCGAAAAAAACCATCATATTGATACAGGTACAAGATTTCAATTTATATTGTGTCA
>HPV42_Alpha_333211_nt442_E6_AOS|1
CGTTATCACAGAGCGAAAAAAACCATCATATTGATACAGGTACAAGATTTCAATTTATAT
>HPV39_Alpha_333245_nt174_E6_AOS|1
CCACCTTGCAGGACATTACAATAGCCTGTGTCTATTGCAGACGACCACTACAGCAAACCG
>HPV39_Alpha_333245_nt162_E6_AOS|1
CAACGCTGGACACCACCTTGCAGGACATTACAATAGCCTGTGTCTATTGCAGACGACCAC
>HPV93_Beta_37089393_nt376_E6_AOS|1
GCTGGGCAATAGAAGTTAAGGAAAAAAAGCCACTCTCTGAAATTATAGTGAGGTGTCGCC
>HPV93_Beta_37089393_nt369_E6_AOS|1
GTTGTTGGCTGGGCAATAGAAGTTAAGGAAAAAAAGCCACTCTCTGAAATTATAGTGAGG
>HPV93_Beta_37089393_nt362_E6_AOS|1
TGATAGTGTTGTTGGCTGGGCAATAGAAGTTAAGGAAAAAAAGCCACTCTCTGAAATTAT
>HPV93_Beta_37089393_nt355_E6_AOS|1
ACTTGAATGATAGTGTTGTTGGCTGGGCAATAGAAGTTAAGGAAAAAAAGCCACTCTCTG
>HPV93_Beta_37089393_nt348_E6_AOS|1
GATAATTACTTGAATGATAGTGTTGTTGGCTGGGCAATAGAAGTTAAGGAAAAAAAGCCA
>HPV12_Beta_396910_nt614_E6_AOS|1
GACAGGTGGAAGGGAATTTGCAGACAGTGCAAGCATTTGTATCTGAATAATGATCGGTAA
>HPV12_Beta_396910_nt607_E6_AOS|1
GGTTAGAGACAGGTGGAAGGGAATTTGCAGACAGTGCAAGCATTTGTATCTGAATAATGA
>HPV12_Beta_396910_nt474_E6_AOS|1
TGCTAGGTAGAGATATAGAGCTTGCTACTGGAAAATCTATATTTGACTTAAAGATAAGGT
>HPV14D_Beta_396918_nt260_E6_AOS|1
GTGATAGCACAGAAACCAAATCTTCTTTTATAGAGCCACCATTACCTGCAACTATATTTG
>HPV14D_Beta_396918_nt380_E6_AOS|1
GTAATTTTTTGACTCATTTAGAAGTCTGTGAATTTGATGAGAAAAAACTAAGTCTAATTT
>HPV14D_Beta_396918_nt302_E6_AOS|1
TACCTGCAACTATATTTGGCTTAGCAAACCTATTGGAAATACCACTAGATGATTGTTTAG
>HPV14D_Beta_396918_nt287_E6_AOS|1
TTATAGAGCCACCATTACCTGCAACTATATTTGGCTTAGCAAACCTATTGGAAATACCAC
>HPV14D_Beta_396918_nt280_E6_AOS|1
TCTTCTTTTATAGAGCCACCATTACCTGCAACTATATTTGGCTTAGCAAACCTATTGGAA
>HPV14D_Beta_396918_nt246_E6_AOS|1
TAAGAGTAACTATTGTGATAGCACAGAAACCAAATCTTCTTTTATAGAGCCACCATTACC
>HPV14D_Beta_396918_nt239_E6_AOS|1
CTTCTCCTAAGAGTAACTATTGTGATAGCACAGAAACCAAATCTTCTTTTATAGAGCCAC

>HPV15_Beta_396924_nt436_E6_AOS|1
GTCGGTGTGTAGTTGGGAAATAGAGATAGTAGAACAGAAGCCTGTTGGAGATATTATTAT
>HPV15_Beta_396924_nt447_E6_AOS|1
GTTGGGAAATAGAGATAGTAGAACAGAAGCCTGTTGGAGATATTATTATTCGCTGCAAAT
>HPV15_Beta_396924_nt429_E6_AOS|1
ATGAACAGTCGGTGTGTAGTTGGGAAATAGAGATAGTAGAACAGAAGCCTGTTGGAGATA
>HPV15_Beta_396924_nt422_E6_AOS|1
AACTTTTATGAACAGTCGGTGTGTAGTTGGGAAATAGAGATAGTAGAACAGAAGCCTGTT
>HPV15_Beta_396924_nt415_E6_AOS|1
ATTTTCTAACTTTTATGAACAGTCGGTGTGTAGTTGGGAAATAGAGATAGTAGAACAGAA
>HPV17_Beta_396932_nt447_E6_AOS|1
GAAGGGAGTTAGAGGAAATAGAGCACAAGCCAATAGGGGAAATACCTATTCGCTGCAAGT
>HPV17_Beta_396932_nt527_E6_AOS|1
TTACTAGAGAAGTTAGACACTTGTTATAGACATCAGCAGTTTCATAAGGTTAGACGCAAT
>HPV17_Beta_396932_nt519_E6_AOS|1
AATTGGATTTACTAGAGAAGTTAGACACTTGTTATAGACATCAGCAGTTTCATAAGGTTA
>HPV17_Beta_396932_nt440_E6_AOS|2
GTGAGTGGAAGGGAGTTAGAGGAAATAGAGCACAAGCCAATAGGGGAAATACCTATTCGC
>HPV17_Beta_396932_nt432_E6_AOS|1
AACAATCAGTGAGTGGAAGGGAGTTAGAGGAAATAGAGCACAAGCCAATAGGGGAAATAC
>HPV17_Beta_396932_nt422_E6_AOS|1
AAGTTTTATGAACAATCAGTGAGTGGAAGGGAGTTAGAGGAAATAGAGCACAAGCCAATA
>HPV17_Beta_396932_nt288_E6_AOS|1
GTAATAGGTTTTTAGCTTACATAGAATTGGTGGCGTTTGATTTAAAAGGTTTGCAGTTAA
>HPV17_Beta_396932_nt281_E6_AOS|2
AGATTTTGTAATAGGTTTTTAGCTTACATAGAATTGGTGGCGTTTGATTTAAAAGGTTTG
>HPV19_Beta_396940_nt491_E6_AOS|1
TTCTATGAGCATACTGTAACAGGTAGAGAAATTGAGTTTGTAACAGGTAAATCTGTCTTT
>HPV19_Beta_396940_nt484_E6_AOS|1
TAATGAGTTCTATGAGCATACTGTAACAGGTAGAGAAATTGAGTTTGTAACAGGTAAATC
>HPV19_Beta_396940_nt474_E6_AOS|1
CATTTGAATTTAATGAGTTCTATGAGCATACTGTAACAGGTAGAGAAATTGAGTTTGTAA
>HPV19_Beta_396940_nt361_E6_AOS|2
CAAGTTTTTATCACATTTAGAAGCGTGCGAATTTGATGATAAAAGACTTAGTTTGATTTG
>HPV19_Beta_396940_nt354_E6_AOS|1
TCTGTGGCAAGTTTTTATCACATTTAGAAGCGTGCGAATTTGATGATAAAAGACTTAGTT
>HPV19_Beta_396940_nt288_E6_AOS|1
CAACAATTGCTGGATTAGCAGCATTGCTAGAAATACCGTTGGATGACTGTTTAGTGCCTT
>HPV25_Beta_396948_nt456_E6_AOS|1
CCACATTTGAATTTAATGAATTTTACGAAAGCACTGTAACAGGTAGGGAAATTGAAGACG
>HPV25_Beta_396948_nt469_E6_AOS|1
TAATGAATTTTACGAAAGCACTGTAACAGGTAGGGAAATTGAAGACGTTACAGGTAAATC
>HPV25_Beta_396948_nt345_E6_AOS|1
GCAACTTTCTAACATATTTAGAGATCTGTGAGTTTGATGAGAAAAGACTTAGTTTGATTT
>HPV25_Beta_396948_nt338_E6_AOS|1
TTCTGTGGCAACTTTCTAACATATTTAGAGATCTGTGAGTTTGATGAGAAAAGACTTAGT
>HPV25_Beta_396948_nt266_E6_AOS|1
TTGCCAGCAACAATTACTGATCTAGCAGCTTTATTGGAAATTCCATTAGATGATTGCTTA
>HPV25_Beta_396948_nt259_E6_AOS|1
GCCACCATTGCCAGCAACAATTACTGATCTAGCAGCTTTATTGGAAATTCCATTAGATGA
>HPV25_Beta_396948_nt252_E6_AOS|1
TTATACAGCCACCATTGCCAGCAACAATTACTGATCTAGCAGCTTTATTGGAAATTCCAT
>HPV25_Beta_396948_nt212_E6_AOS|1
AATGCTGAACAGAGCATAGGACCACCAGAGCAAGCGCAGGTTATACAGCCACCATTGCCA
>HPV27_Alpha_396964_nt213_E6_AOS|1

AGACGAGCGCTTTCAGACGCTGATGTATTGGCATTTGCAATAAAAGAACTGTCTGTAGTG
>HPV27_Alpha_396964_nt199_E6_AOS|1
TCTGCGTGTATTGCAGACGAGCGCTTTCAGACGCTGATGTATTGGCATTTGCAATAAAAG
>HPV30_Alpha_396973_nt133_E6_AOS|1
GCCCACGTACTGTGCACCATCTTTGTGAGGTACAAGAAACATCGTTGCTGGAGCTACAGC
>HPV30_Alpha_396973_nt140_E6_AOS|1
TACTGTGCACCATCTTTGTGAGGTACAAGAAACATCGTTGCTGGAGCTACAGCTACAGTG
>HPV3_Alpha_397005_nt164_E6_AOS|1
CACCGGAATAGGATTTGACGACCTTCGCCTGCACTGCATATTCTGTACGAAACAGCTGAC
>HPV3_Alpha_397005_nt205_E6_AOS|1
TCTGTACGAAACAGCTGACTACAACTGAACTACAAGCATTTGCATTACGGGAACTGAATG
>HPV3_Alpha_397005_nt196_E6_AOS|1
ACTGCATATTCTGTACGAAACAGCTGACTACAACTGAACTACAAGCATTTGCATTACGGG
>HPV3_Alpha_397005_nt189_E6_AOS|1
CGCCTGCACTGCATATTCTGTACGAAACAGCTGACTACAACTGAACTACAAGCATTTGCA
>HPV3_Alpha_397005_nt173_E6_AOS|1
AGGATTTGACGACCTTCGCCTGCACTGCATATTCTGTACGAAACAGCTGACTACAACTGA
>HPV3_Alpha_397005_nt148_E6_AOS|1
TTCTACTGTGCAGAAACACCGGAATAGGATTTGACGACCTTCGCCTGCACTGCATATTCT
>HPV40_Alpha_397014_nt310_E6_AOS|1
ACGGAAAAGTAAACCAATACAGAAACTTTAGATACGCAGCCTATGCACCAACCGTGGAAG
>HPV40_Alpha_397014_nt303_E6_AOS|1
GACCTGCACGGAAAAGTAAACCAATACAGAAACTTTAGATACGCAGCCTATGCACCAACC
>HPV40_Alpha_397014_nt296_E6_AOS|2
GTGCCTGGACCTGCACGGAAAAGTAAACCAATACAGAAACTTTAGATACGCAGCCTATGC
>HPV40_Alpha_397014_nt289_E6_AOS|1
GTCCACGGTGCCTGGACCTGCACGGAAAAGTAAACCAATACAGAAACTTTAGATACGCAG
>HPV40_Alpha_397014_nt282_E6_AOS|1
GCCGCATGTCCACGGTGCCTGGACCTGCACGGAAAAGTAAACCAATACAGAAACTTTAGA
>HPV40_Alpha_397014_nt275_E6_AOS|1
TCCACACGCCGCATGTCCACGGTGCCTGGACCTGCACGGAAAAGTAAACCAATACAGAAA
>HPV45_Alpha_397022_nt498_E6_AOS|1
CAGTACCGAGGGCAGTGTAATACATGTTGTGACCAGGCACGGCAAGAAAGACTTCGCAGA
>HPV52_Alpha_397038_nt368_E6_AOS|1
AGAGAGGGTAAAAAAACCATTAAGTGAAATAACTATTAGATGTATAATTTGTCAAACGCC
>HPV52_Alpha_397038_nt361_E6_AOS|1
CATTAGAAGAGAGGGTAAAAAAACCATTAAGTGAAATAACTATTAGATGTATAATTTGTC
>HPV52_Alpha_397038_nt354_E6_AOS|1
GGGAAAACATTAGAAGAGAGGGTAAAAAAACCATTAAGTGAAATAACTATTAGATGTATA
>HPV52_Alpha_397038_nt347_E6_AOS|1
ACTGTATGGGAAAACATTAGAAGAGAGGGTAAAAAAACCATTAAGTGAAATAACTATTAG
>HPV52_Alpha_397038_nt340_E6_AOS|1
AATATTCACTGTATGGGAAAACATTAGAAGAGAGGGTAAAAAAACCATTAAGTGAAATAA
>HPV52_Alpha_397038_nt333_E6_AOS|1
CATTATCAATATTCACTGTATGGGAAAACATTAGAAGAGAGGGTAAAAAAACCATTAAGT
>HPV52_Alpha_397038_nt326_E6_AOS|1
ATATAGGCATTATCAATATTCACTGTATGGGAAAACATTAGAAGAGAGGGTAAAAAAACC
>HPV52_Alpha_397038_nt319_E6_AOS|1
TAAGTGAATATAGGCATTATCAATATTCACTGTATGGGAAAACATTAGAAGAGAGGGTAA
>HPV52_Alpha_397038_nt305_E6_AOS|1
CTTTTTATCTAAGATAAGTGAATATAGGCATTATCAATATTCACTGTATGGGAAAACATT
>HPV52_Alpha_397038_nt298_E6_AOS|1
GCCTACGCTTTTTATCTAAGATAAGTGAATATAGGCATTATCAATATTCACTGTATGGGA
>HPV56_Alpha_397053_nt268_E6_AOS|2
TGTATAGGGATGATTTTCCTTATGCAGTGTGCAGAGTATGTTTATTGTTTTATAGTAAAG

>HPV56_Alpha_397053_nt261_E6_AOS|2
AAATTAGTGTATAGGGATGATTTTCCTTATGCAGTGTGCAGAGTATGTTTATTGTTTTAT
>HPVcand62_Alpha_39932599_nt188_E6_AOS|1
CAGCATGCCTATTGAGAGAGATTAAAGTAAGAGAGCTACGGCGGTGGCAGCACTCATGCT
>HPVcand62_Alpha_39932599_nt205_E6_AOS|1
GAGATTAAAGTAAGAGAGCTACGGCGGTGGCAGCACTCATGCTTTGGAATAACAGTAGAA
>HPVcand62_Alpha_39932599_nt198_E6_AOS|1
ATTGAGAGAGATTAAAGTAAGAGAGCTACGGCGGTGGCAGCACTCATGCTTTGGAATAAC
>HPV43_Alpha_40804474_nt304_E6_AOS|1
TACAGTTTCATGGAAAAATAAGTCAATATAGGCACTTTGACTACGCAGCATATGCAGATA
>HPV43_Alpha_40804474_nt316_E6_AOS|1
GAAAAATAAGTCAATATAGGCACTTTGACTACGCAGCATATGCAGATACTGTAGAAGAAG
>HPV43_Alpha_40804474_nt297_E6_AOS|1
GCCTGTCTACAGTTTCATGGAAAAATAAGTCAATATAGGCACTTTGACTACGCAGCATAT
>HPV43_Alpha_40804474_nt290_E6_AOS|1
ATGCTTGGCCTGTCTACAGTTTCATGGAAAAATAAGTCAATATAGGCACTTTGACTACGC
>HPV81_Alpha_40804509_nt434_E6_AOS|1
CTATAAACCGTTAACATACCAAGAAAAGGAGTATCTTGTGACAGGCCTAATCCACTTTCA
>HPV81_Alpha_40804509_nt427_E6_AOS|1
ATGCATGCTATAAACCGTTAACATACCAAGAAAAGGAGTATCTTGTGACAGGCCTAATCC
>HPV81_Alpha_40804509_nt319_E6_AOS|1
GCAGAGAAGCTAAAGTACGGGAGCTGCGGCGGTGGCAATATTCGTGCTTCGGACCAACAG
>HPV81_Alpha_40804509_nt102_E6_AOS|1
ATGGTCAGCACAGCAGTGATGTCGCTGGGGCCAGCAAATCCTACCAATTTGTTTTTGCTG
>HPV95_Gamma_40804520_nt360_E6_AOS|2
GGACTGGAAGATTTAGTAGGACAAAAAATCAGGGATATATGTGTAAGATGTTTATGCTGT
>HPV95_Gamma_40804520_nt390_E6_AOS|1
AGGGATATATGTGTAAGATGTTTATGCTGTCTTAAACTATTAGACATAGTAGAAAAACTT
>HPV95_Gamma_40804520_nt383_E6_AOS|1
AAAAATCAGGGATATATGTGTAAGATGTTTATGCTGTCTTAAACTATTAGACATAGTAGA
>HPV95_Gamma_40804520_nt376_E6_AOS|1
TAGGACAAAAAATCAGGGATATATGTGTAAGATGTTTATGCTGTCTTAAACTATTAGACA
>HPV95_Gamma_40804520_nt369_E6_AOS|1
GATTTAGTAGGACAAAAAATCAGGGATATATGTGTAAGATGTTTATGCTGTCTTAAACTA
>HPV95_Gamma_40804520_nt353_E6_AOS|1
TGCTACTGGACTGGAAGATTTAGTAGGACAAAAAATCAGGGATATATGTGTAAGATGTTT
>HPV95_Gamma_40804520_nt346_E6_AOS|1
CTGTATATGCTACTGGACTGGAAGATTTAGTAGGACAAAAAATCAGGGATATATGTGTAA
>HPV94_Alpha_40804528_nt175_E6_AOS|1
GCTGCGTGTTCTGCACCAAACAGCTGACCGTAGCCGAATTGACTGCATTTGCATTAAGAG
>HPV94_Alpha_40804528_nt183_E6_AOS|1
TTCTGCACCAAACAGCTGACCGTAGCCGAATTGACTGCATTTGCATTAAGAGAATTGAAT
>HPV94_Alpha_40804528_nt138_E6_AOS|1
AGGAACTGTGGAATATCATTTGAGGACCTTCGCCTGTGCTGCGTGTTCTGCACCAAACAG
>HPV94_Alpha_40804528_nt130_E6_AOS|1
TATTGTGTAGGAACTGTGGAATATCATTTGAGGACCTTCGCCTGTGCTGCGTGTTCTGCA
>HPVcand85_Alpha_4574720_nt141_E6_AOS|2
AAACTACCAGACCTATGCAACACACTGGACACATCACTGCAAGATATAGAAATAAGCTGT
>HPVcand85_Alpha_4574720_nt404_E6_AOS|1
TATATTAATAAGGTGTTTACGGTGTCAAAAACCTTTATGTCCGGCAGAAAAACTAAAACA
>HPVcand85_Alpha_4574720_nt397_E6_AOS|1
TATATGATATATTAATAAGGTGTTTACGGTGTCAAAAACCTTTATGTCCGGCAGAAAAAC
>HPVcand85_Alpha_4574720_nt312_E6_AOS|1
ATGTTTTATTCTAAAATAAGAGAACTAAGATATTATAGCGACTCTGTGTATGGGGAAACG
>HPVcand85_Alpha_4574720_nt151_E6_AOS|1

ACCTATGCAACACACTGGACACATCACTGCAAGATATAGAAATAAGCTGTGTATATTGTA
>HPV96_Beta_50253426_nt260_E6_AOS|1
TGTTGGCAATTACAACCTGCCACTCATAAACAAGTAACCGCCTGCGTTTCTAGGACATTT
>HPV96_Beta_50253426_nt253_E6_AOS|1
GAGAGGTTGTTGGCAATTACAACCTGCCACTCATAAACAAGTAACCGCCTGCGTTTCTAG
>HPV96_Beta_50253426_nt225_E6_AOS|1
CTCTCTCGGTTGCCAAAAGTTTTATCTTGAGAGGTTGTTGGCAATTACAACCTGCCACTC
>HPV96_Beta_50253426_nt218_E6_AOS|1
TTCGCAACTCTCTCGGTTGCCAAAAGTTTTATCTTGAGAGGTTGTTGGCAATTACAACCT
>HPV96_Beta_50253426_nt210_E6_AOS|1
CGGTAAGTTTCGCAACTCTCTCGGTTGCCAAAAGTTTTATCTTGAGAGGTTGTTGGCAAT
>HPV96_Beta_50253426_nt141_E6_AOS|2
TTTGCCAAAAGCAGTTTAAACCTGCCAAGAAAAGCCCGCCAAAACGTCTGCCAACTTTGG
>HPV96_Beta_50253426_nt116_E6_AOS|1
CCTGGAACAACACCGATCCAGTGGCTTTGCCAAAAGCAGTTTAAACCTGCCAAGAAAAGC
>HPV96_Beta_50253426_nt109_E6_AOS|1
AGTCACTCCTGGAACAACACCGATCCAGTGGCTTTGCCAAAAGCAGTTTAAACCTGCCAA
>HPV96_Beta_50253426_nt102_E6_AOS|1
TCCTGGCAGTCACTCCTGGAACAACACCGATCCAGTGGCTTTGCCAAAAGCAGTTTAAAC
>HPV96_Beta_50253426_nt94_E6_AOS|1
GTATCTGATCCTGGCAGTCACTCCTGGAACAACACCGATCCAGTGGCTTTGCCAAAAGCA
>HPV83_Alpha_5059324_nt192_E6_AOS|1
TCTACGTAAAGCAGCAAAGGTGCGAGAGCTACGGCACTGGAGCCACTCAAGCTACGGGGC
>HPV83_Alpha_5059324_nt257_E6_AOS|1
TAGAAGAAGAAACAAAAACACCACTGGCACAGCTGTATATACGATGCCATATGTGCCTGA
>HPV83_Alpha_5059324_nt250_E6_AOS|1
GCAACAGTAGAAGAAGAAACAAAAACACCACTGGCACAGCTGTATATACGATGCCATATG
>HPV83_Alpha_5059324_nt185_E6_AOS|1
CACAGTGTCTACGTAAAGCAGCAAAGGTGCGAGAGCTACGGCACTGGAGCCACTCAAGCT
>HPV83_Alpha_5059324_nt178_E6_AOS|1
GCGTGTGCACAGTGTCTACGTAAAGCAGCAAAGGTGCGAGAGCTACGGCACTGGAGCCAC
>HPV59_Alpha_557236_nt372_E6_AOS|1
TAGATGCCTAAAACCTCTATGTCCAACAGATAAATTAAAGCATATAACTGAAAAAAGAAG
>HPV59_Alpha_557236_nt365_E6_AOS|1
GCTGTTATAGATGCCTAAAACCTCTATGTCCAACAGATAAATTAAAGCATATAACTGAAA
>HPV6_Alpha_6002612_nt440_E6_AOS|1
AAGTAGAAAAGGTAAAACATATACTAACCAAGGCACGGTTCATAAAGCTAAATTGTACGT
>HPV57_Alpha_60882_nt499_E6_AOS|1
CCGGACATTGCATGAACTGTGCGCCAAGATGCATGGAGAACGCCCCAGCCTTGAGGACAT
>HPV57_Alpha_60882_nt506_E6_AOS|1
TTGCATGAACTGTGCGCCAAGATGCATGGAGAACGCCCCAGCCTTGAGGACATCACACTA
>HPV57_Alpha_60882_nt492_E6_AOS|1
CAGTGGACCGGACATTGCATGAACTGTGCGCCAAGATGCATGGAGAACGCCCCAGCCTTG
>HPV69_Alpha_6970418_nt275_E6_AOS|1
TGCATATGGTGCATGTAAAAAATGTATAATATTCTATTCAAAAATAATAGAATATAGACG
>HPV69_Alpha_6970418_nt268_E6_AOS|1
ATGATAGTGCATATGGTGCATGTAAAAAATGTATAATATTCTATTCAAAAATAATAGAAT
>HPV69_Alpha_6970418_nt261_E6_AOS|2
TATAGAAATGATAGTGCATATGGTGCATGTAAAAAATGTATAATATTCTATTCAAAAATA
>HPV69_Alpha_6970418_nt254_E6_AOS|1
AATAGTGTATAGAAATGATAGTGCATATGGTGCATGTAAAAAATGTATAATATTCTATTC
>HPV82_Alpha_6970427_nt498_E6_AOS|1
ACGGGACAGTGTGCAAATTGCAGAAAACCACCAAGACAACGTAGTGAAACCCAGGTGTAA
>HPV82_Alpha_6970427_nt491_E6_AOS|1
ACGCTGGACGGGACAGTGTGCAAATTGCAGAAAACCACCAAGACAACGTAGTGAAACCCA

>HPV82_Alpha_6970427_nt263_E6_AOS|1
TAGGGACAATACGCCATATGCAGCATGCAAAAAATGCCTAATGTTTTATTCTAGAATTAG
>HPV82_Alpha_6970427_nt249_E6_AOS|1
CTTAGGATTGTATATAGGGACAATACGCCATATGCAGCATGCAAAAAATGCCTAATGTTT
>HPV82_Alpha_6970427_nt242_E6_AOS|1
TACAGAACTTAGGATTGTATATAGGGACAATACGCCATATGCAGCATGCAAAAAATGCCT
>HPV82_Alpha_6970427_nt154_E6_AOS|1
CCTGCAATACGTCTATGCACAATATTCAGGTATTGTGTGTATATTGTAAAAAGGAGTTGT
>HPV82_Alpha_6970427_nt139_E6_AOS|1
ACGAATTATGTGAAGCCTGCAATACGTCTATGCACAATATTCAGGTATTGTGTGTATATT
>HPV68a_Alpha_71726685_nt103_E6_AOS|1
TGCAGAAGGCAACTACAACGGACAGAGGTATATGAATTTGCCTTTAGTGACCTATGTGTA
>HPV97_Alpha_71726694_nt342_E6_AOS|1
AGCAGACAAATATAAACATCTTAAAGACAAACGACGATTCCACCACATATCTGGTTATTA
>HPV97_Alpha_71726694_nt352_E6_AOS|1
TATAAACATCTTAAAGACAAACGACGATTCCACCACATATCTGGTTATTATAGAGGCCAG
>HPV97_Alpha_71726694_nt335_E6_AOS|1
TGAATCCAGCAGACAAATATAAACATCTTAAAGACAAACGACGATTCCACCACATATCTG
>HPV106_Alpha_71726710_nt16_E6_AOS|1
AGAGATATGTTGCTTCAGCACAAAATAGCCATGACCAACGGAGACTGCTGTCCAAAGAAT
>HPV106_Alpha_71726710_nt286_E6_AOS|1
TCCAGCTATGCACCCACAGTGGAGTATGAAACAGGGATACCAATACTAGAGCTGTTTATT
>HPV106_Alpha_71726710_nt279_E6_AOS|1
GGACTACTCCAGCTATGCACCCACAGTGGAGTATGAAACAGGGATACCAATACTAGAGCT
>HPV106_Alpha_71726710_nt243_E6_AOS|1
GAAAGCAGCTGGAAAACTTAGACAATTTAGATACTGGGACTACTCCAGCTATGCACCCAC
>HPV106_Alpha_71726710_nt9_E6_AOS|1
ATATAAAAGAGATATGTTGCTTCAGCACAAAATAGCCATGACCAACGGAGACTGCTGTCC
>HPV102_Alpha_71726718_nt240_E6_AOS|1
CAGCTACGGCGCAACGGTTGAAGAAGAAACCCGCACAGCATTACCACAGCTGTATATGAG
>HPV102_Alpha_71726718_nt233_E6_AOS|1
ACTACTCCAGCTACGGCGCAACGGTTGAAGAAGAAACCCGCACAGCATTACCACAGCTGT
>HPV102_Alpha_71726718_nt226_E6_AOS|1
CACTGGAACTACTCCAGCTACGGCGCAACGGTTGAAGAAGAAACCCGCACAGCATTACCA
>HPV102_Alpha_71726718_nt215_E6_AOS|1
GGGAACTACGGCACTGGAACTACTCCAGCTACGGCGCAACGGTTGAAGAAGAAACCCGCA
>HPV13_Alpha_85827580_nt291_E6_AOS|1
CTTGTGCATGCTGCTTAGAAATACAAGGAAAGATTAACCAGTTTAGGCATTTTGACTTCG
>HPV13_Alpha_85827580_nt284_E6_AOS|1
TTTGCGGCTTGTGCATGCTGCTTAGAAATACAAGGAAAGATTAACCAGTTTAGGCATTTT
>HPV13_Alpha_85827580_nt277_E6_AOS|1
GTTTCCATTTGCGGCTTGTGCATGCTGCTTAGAAATACAAGGAAAGATTAACCAGTTTAG
>HPV13_Alpha_85827580_nt270_E6_AOS|1
GAGGATCGTTTCCATTTGCGGCTTGTGCATGCTGCTTAGAAATACAAGGAAAGATTAACC
>HPV13_Alpha_85827580_nt255_E6_AOS|1
TATATATAGTGTGGCGAGGATCGTTTCCATTTGCGGCTTGTGCATGCTGCTTAGAAATAC
>HPV13_Alpha_85827580_nt248_E6_AOS|1
AAGAGTTTATATATAGTGTGGCGAGGATCGTTTCCATTTGCGGCTTGTGCATGCTGCTTA
>HPV13_Alpha_85827580_nt240_E6_AOS|1
TTCAGTATAAGAGTTTATATATAGTGTGGCGAGGATCGTTTCCATTTGCGGCTTGTGCAT
>HPV13_Alpha_85827580_nt220_E6_AOS|1
CACGGCAGAGGTTTATTCATTTCAGTATAAGAGTTTATATATAGTGTGGCGAGGATCGTT
>HPV2_Alpha_9626032_nt177_E6_AOS|1
ATTTGCGATTGCTCTGTGTATGGTGCAAACGGCCGTTATCAGAGGCTGACATATGGGCAT
>HPV2_Alpha_9626032_nt191_E6_AOS|1

TGTGTATGGTGCAAACGGCCGTTATCAGAGGCTGACATATGGGCATTTGCAATAAAAGAA
>HPV2_Alpha_9626032_nt184_E6_AOS|1
ATTGCTCTGTGTATGGTGCAAACGGCCGTTATCAGAGGCTGACATATGGGCATTTGCAAT
>HPV2_Alpha_9626032_nt170_E6_AOS|1
CTAGAGGATTTGCGATTGCTCTGTGTATGGTGCAAACGGCCGTTATCAGAGGCTGACATA
>HPV41_Nu_9626041_nt304_E6_AOS|1
AAGGGGGGCCAACGGGTATTTGTTCTCAGTGTACTAGAGTGCTTGCAAGGCTGGAGTTCA
>HPV41_Nu_9626041_nt293_E6_AOS|1
ATCCTGGGGAGAAGGGGGGCCAACGGGTATTTGTTCTCAGTGTACTAGAGTGCTTGCAAG
>HPV41_Nu_9626041_nt277_E6_AOS|1
AGAGCTGTCTGTACTTATCCTGGGGAGAAGGGGGGCCAACGGGTATTTGTTCTCAGTGTA
>HPV41_Nu_9626041_nt270_E6_AOS|1
TTCGACCAGAGCTGTCTGTACTTATCCTGGGGAGAAGGGGGGCCAACGGGTATTTGTTCT
>HPV41_Nu_9626041_nt259_E6_AOS|1
ATATTTACGCTTTCGACCAGAGCTGTCTGTACTTATCCTGGGGAGAAGGGGGGCCAACGG
>HPV41_Nu_9626041_nt242_E6_AOS|1
GATCCTTAGCGTATTGGATATTTACGCTTTCGACCAGAGCTGTCTGTACTTATCCTGGGG
>HPV41_Nu_9626041_nt233_E6_AOS|1
TTGCTATAAGATCCTTAGCGTATTGGATATTTACGCTTTCGACCAGAGCTGTCTGTACTT
>HPV41_Nu_9626041_nt224_E6_AOS|1
CTGCCATTATTGCTATAAGATCCTTAGCGTATTGGATATTTACGCTTTCGACCAGAGCTG
>HPV41_Nu_9626041_nt157_E6_AOS|1
AGAAGCCACATACCATACGGGAGTTGTGTTTGGCGCAGCAGATAACTTATCCATGCATAC
>HPV41_Nu_9626041_nt150_E6_AOS|1
GAGACGCAGAAGCCACATACCATACGGGAGTTGTGTTTGGCGCAGCAGATAACTTATCCA
>HPV1_Mu_9626063_nt416_E6_AOS|1
AAACTGAGTGTTGCTGAAAAATTGGAGGTTGTGTCAAACGGAGAAAGAGTGCATAGAGTT
>HPV1_Mu_9626063_nt409_E6_AOS|1
CATAAAAAAACTGAGTGTTGCTGAAAAATTGGAGGTTGTGTCAAACGGAGAAAGAGTGCA
>HPV1_Mu_9626063_nt117_E6_AOS|1
TCCGGACCGTCAGACAGCTTTCCGAAAGCCTCTGTATCCCATATATTGATGTTTTATTGC
>HPV1_Mu_9626063_nt110_E6_AOS|1
ACACCAATCCGGACCGTCAGACAGCTTTCCGAAAGCCTCTGTATCCCATATATTGATGTT
>HPV18_Alpha_9626069_nt142_E6_AOS|1
AGCTACCTGATCTGTGCACGGAACTGAACACTTCACTGCAAGACATAGAAATAACCTGTG
>HPV4_Gamma_9626597_nt202_E6_AOS|1
TCGATCTTGCGGATCTTGCTTTATTCTATCTTAAGAAACTTAGTTTAGTATTTAGAGGAA
>HPV63_Mu_9626605_nt124_E6_AOS|1
CGGTTCGGGATCTGAGTTCTGCTCTCCGTATCCCATTTATTGATTTGGTTGTTCCTTGCA
>HPV63_Mu_9626605_nt214_E6_AOS|1
AAAAATTGCTGTTTGATTATTTTGACTTGCATCTTATCTGGCGAGATAATTTCGTGTTTG
>HPV63_Mu_9626605_nt117_E6_AOS|1
GTACATTCGGTTCGGGATCTGAGTTCTGCTCTCCGTATCCCATTTATTGATTTGGTTGTT
>HPV16_Alpha_9627100_nt484_E6_AOS|1
TATAAGGGGTCGGTGGACCGGTCGATGTATGTCTTGTTGCAGATCATCAAGAACACGTAG
>HPV5_Beta_9627145_nt429_E6_AOS|2
GCTGTGGCGCCACTGCAACTTATGAATTTAACCAATTTTATGAGCAGACAGTGTTAGGAA
>HPV5_Beta_9627145_nt419_E6_AOS|1
TGTCGCGTATGCTGTGGCGCCACTGCAACTTATGAATTTAACCAATTTTATGAGCAGACA
>HPV5_Beta_9627145_nt251_E6_AOS|1
GAATTACCTTTAAGTATTAGAGACTTAGCTGAAGCCTTAGGCATCCCTGTGATTGATTGT
>HPV5_Beta_9627145_nt244_E6_AOS|1
TAAGGCAGAATTACCTTTAAGTATTAGAGACTTAGCTGAAGCCTTAGGCATCCCTGTGAT
>HPV5_Beta_9627145_nt237_E6_AOS|1
AAAAAGATAAGGCAGAATTACCTTTAAGTATTAGAGACTTAGCTGAAGCCTTAGGCATCC

>HPV10_Alpha_9627257_nt146_E6_AOS|1
TAGAAATTGTGGAATACCTTTGGAGGACCTTCGCCTGTGCTGTATATTTTGCACAAAACA
>HPV10_Alpha_9627257_nt192_E6_AOS|1
TTTTGCACAAAACAGCTGACCGCAGCGGAATTGGCAGCATTTGCACTTAGAGAATTATAT
>HPV10_Alpha_9627257_nt185_E6_AOS|1
CTGTATATTTTGCACAAAACAGCTGACCGCAGCGGAATTGGCAGCATTTGCACTTAGAGA
>HPV10_Alpha_9627257_nt139_E6_AOS|1
TTTTGTGTAGAAATTGTGGAATACCTTTGGAGGACCTTCGCCTGTGCTGTATATTTTGCA
>HPV26_Alpha_9627305_nt240_E6_AOS|1
TGACCTAAGAGTAGTATATAGAGATAGGAGTCCGTATGCTGCATGCAAAAGATGTGTAAT
>HPV26_Alpha_9627305_nt255_E6_AOS|1
ATATAGAGATAGGAGTCCGTATGCTGCATGCAAAAGATGTGTAATATTTTATTCAAAAAT
>HPV26_Alpha_9627305_nt248_E6_AOS|1
GAGTAGTATATAGAGATAGGAGTCCGTATGCTGCATGCAAAAGATGTGTAATATTTTATT
>HPV26_Alpha_9627305_nt233_E6_AOS|1
CAATTTGTGACCTAAGAGTAGTATATAGAGATAGGAGTCCGTATGCTGCATGCAAAAGAT
>HPV26_Alpha_9627305_nt226_E6_AOS|1
AATTTTGCAATTTGTGACCTAAGAGTAGTATATAGAGATAGGAGTCCGTATGCTGCATGC
>HPV26_Alpha_9627305_nt103_E6_AOS|1
GAGGATCCTAGAGAACGACCCAGAACGCTACATGAGCTATGTGAAAGCTTGAATACTACT
>HPV32_Alpha_9627327_nt439_E6_AOS|1
CAAGTGAGAAAGATCATCATATTTATAACGGACGGCATTTCAGATTCATTTTAAATAGGT
>HPV32_Alpha_9627327_nt432_E6_AOS|1
TTATCGCCAAGTGAGAAAGATCATCATATTTATAACGGACGGCATTTCAGATTCATTTTA
>HPV32_Alpha_9627327_nt425_E6_AOS|1
AAAGCCTTTATCGCCAAGTGAGAAAGATCATCATATTTATAACGGACGGCATTTCAGATT
>HPV32_Alpha_9627327_nt169_E6_AOS|1
CCCTGCGGAATTTACAAATCTGCTGTATTTGGTGTAAAAACCACTTAACCAGTGCTGAAG
>HPV32_Alpha_9627327_nt151_E6_AOS|1
GCAAAGATTTTGGGCTGACCCTGCGGAATTTACAAATCTGCTGTATTTGGTGTAAAAACC
>HPV34_Alpha_9627334_nt141_E6_AOS|1
CCAGCCTTATGTGAAGAGGTCAACATTTCAATACATGAAATAGAATTGGACTGTGTGTAT
>HPV49_Beta_9627363_nt411_E6_AOS|1
CTAATTATCACCAAGAAATTGTCGTAGGCATCGAAATAGAAGGACGAGCAGCGGCTAATA
>HPV49_Beta_9627363_nt461_E6_AOS|2
GCGGCTAATATTGCTGAGATAGTAGTCAGATGTCTCATTTGCCTTAAGAGGCTAGATTTG
>HPV49_Beta_9627363_nt454_E6_AOS|1
ACGAGCAGCGGCTAATATTGCTGAGATAGTAGTCAGATGTCTCATTTGCCTTAAGAGGCT
>HPV49_Beta_9627363_nt447_E6_AOS|1
TAGAAGGACGAGCAGCGGCTAATATTGCTGAGATAGTAGTCAGATGTCTCATTTGCCTTA
>HPV49_Beta_9627363_nt393_E6_AOS|2
CAGCATATCACGAGTTTACTAATTATCACCAAGAAATTGTCGTAGGCATCGAAATAGAAG
>HPV49_Beta_9627363_nt386_E6_AOS|1
TATAGATCAGCATATCACGAGTTTACTAATTATCACCAAGAAATTGTCGTAGGCATCGAA
>HPV49_Beta_9627363_nt379_E6_AOS|1
TTGTGCCTATAGATCAGCATATCACGAGTTTACTAATTATCACCAAGAAATTGTCGTAGG
>HPV49_Beta_9627363_nt372_E6_AOS|1
GTGCAGCTTGTGCCTATAGATCAGCATATCACGAGTTTACTAATTATCACCAAGAAATTG
>HPV49_Beta_9627363_nt365_E6_AOS|1
GGTTGTTGTGCAGCTTGTGCCTATAGATCAGCATATCACGAGTTTACTAATTATCACCAA
>HPV49_Beta_9627363_nt358_E6_AOS|1
TGTCTTTGGTTGTTGTGCAGCTTGTGCCTATAGATCAGCATATCACGAGTTTACTAATTA
>HPV53_Alpha_9627377_nt311_E6_AOS|1
TTTGCTATTTTATAGTAAGGTCCGAAAATTAAGATATTACAATTGTTCAGTGTACGGGGC
>HPV53_Alpha_9627377_nt342_E6_AOS|1

AGATATTACAATTGTTCAGTGTACGGGGCTAGCCTGGAAGCACTAACTAAAAAAAAGTTA
>HPV53_Alpha_9627377_nt335_E6_AOS|1
AAAATTAAGATATTACAATTGTTCAGTGTACGGGGCTAGCCTGGAAGCACTAACTAAAAA
>HPV53_Alpha_9627377_nt328_E6_AOS|1
AGGTCCGAAAATTAAGATATTACAATTGTTCAGTGTACGGGGCTAGCCTGGAAGCACTAA
>HPV53_Alpha_9627377_nt321_E6_AOS|2
TATAGTAAGGTCCGAAAATTAAGATATTACAATTGTTCAGTGTACGGGGCTAGCCTGGAA
>HPV7_Alpha_9627389_nt296_E6_AOS|1
GTGTTTAGAATTTTATGGAAAAGTGAATCAGTATAGGAACTTTAGATACGCTGCATATGC
>HPV7_Alpha_9627389_nt289_E6_AOS|1
GTGTAAAGTGTTTAGAATTTTATGGAAAAGTGAATCAGTATAGGAACTTTAGATACGCTG
>HPV7_Alpha_9627389_nt282_E6_AOS|1
GCAGCGTGTGTAAAGTGTTTAGAATTTTATGGAAAAGTGAATCAGTATAGGAACTTTAGA
>HPV7_Alpha_9627389_nt106_E6_AOS|1
CTGCACGTTGCGGCTCCACAGCTAGAACTTTATTTGAATTATGTGACCAGTGCAATATAA
>HPV9_Beta_9627396_nt420_E6_AOS|1
CGTCTGCACAGTTAGAATTTACACATTTTTTTCAATTTGCTGTAGTTGGAAAAGATATAG
>HPV9_Beta_9627396_nt460_E6_AOS|1
TGTAGTTGGAAAAGATATAGAAACTGTAGAAGGAACAGCTATTGGAAATATTTGTATTAG
>HPV9_Beta_9627396_nt453_E6_AOS|1
AATTTGCTGTAGTTGGAAAAGATATAGAAACTGTAGAAGGAACAGCTATTGGAAATATTT
>HPV9_Beta_9627396_nt446_E6_AOS|1
TTTTTTCAATTTGCTGTAGTTGGAAAAGATATAGAAACTGTAGAAGGAACAGCTATTGGA
>HPV9_Beta_9627396_nt439_E6_AOS|1
TACACATTTTTTTCAATTTGCTGTAGTTGGAAAAGATATAGAAACTGTAGAAGGAACAGC
>HPV9_Beta_9627396_nt432_E6_AOS|1
TAGAATTTACACATTTTTTTCAATTTGCTGTAGTTGGAAAAGATATAGAAACTGTAGAAG
>HPV54_Alpha_9628437_nt314_E6_AOS|1
GCACGGGCAAATAAATTATAGAAGGCATCGCGACCGTGCGTGCCTGTGGGAAACAGTGGA
>HPV54_Alpha_9628437_nt307_E6_AOS|1
TAGAACTGCACGGGCAAATAAATTATAGAAGGCATCGCGACCGTGCGTGCCTGTGGGAAA
>HPV54_Alpha_9628437_nt300_E6_AOS|1
CTGTGCCTAGAACTGCACGGGCAAATAAATTATAGAAGGCATCGCGACCGTGCGTGCCTG
>HPV24_Unclassified_9628486_nt231_E6_AOS|1
TAGAACTTAGTAGATTATTAAATATACCATTAGACGATTGTGTTGTACCATGTAACTTTT
>HPV24_Unclassified_9628486_nt395_E6_AOS|1
GTTGCTACAGCAGCATTTGAATTTGAAAATTATTTTGTAGAGAGTGTGATAGGTTGGGAA
>HPV24_Unclassified_9628486_nt385_E6_AOS|1
ATATTGTTGTGTTGCTACAGCAGCATTTGAATTTGAAAATTATTTTGTAGAGAGTGTGAT
>HPV24_Unclassified_9628486_nt378_E6_AOS|1
GTTGTAGATATTGTTGTGTTGCTACAGCAGCATTTGAATTTGAAAATTATTTTGTAGAGA
>HPV24_Unclassified_9628486_nt224_E6_AOS|1
TCAGTGTTAGAACTTAGTAGATTATTAAATATACCATTAGACGATTGTGTTGTACCATGT
>HPV24_Unclassified_9628486_nt217_E6_AOS|1
ACCTCAGTCAGTGTTAGAACTTAGTAGATTATTAAATATACCATTAGACGATTGTGTTGT
>HPV48_Gamma_9628542_nt214_E6_AOS|1
ATCTTGCTTCTTTTCATAACAAACGACTGTCTGTAATTTGGAGAGATAATACACCATTTG
>HPV50_Gamma_9628550_nt402_E6_AOS|1
GAAAATTGTATGTGTTTGTTGGATTATATTGAAAAGTACGATTGTATTTGTCACGGTGGT
>HPV50_Gamma_9628550_nt426_E6_AOS|1
TATATTGAAAAGTACGATTGTATTTGTCACGGTGGTTATTTTCATTTAGTTAGAGGTAAT
>HPV50_Gamma_9628550_nt419_E6_AOS|1
GTTGGATTATATTGAAAAGTACGATTGTATTTGTCACGGTGGTTATTTTCATTTAGTTAG
>HPV50_Gamma_9628550_nt412_E6_AOS|1
TGTGTTTGTTGGATTATATTGAAAAGTACGATTGTATTTGTCACGGTGGTTATTTTCATT

>HPV50_Gamma_9628550_nt395_E6_AOS|1
TCGCTGTGAAAATTGTATGTGTTTGTTGGATTATATTGAAAAGTACGATTGTATTTGTCA
>HPV60_Gamma_9628566_nt383_E6_AOS|1
TCCTTTGAGTTCTCTTTCTGTACGCTGTTTTGATTGCCTTACATTGTTATCTTTTGCAGA
>HPV60_Gamma_9628566_nt282_E6_AOS|1
GCTTGTTGTACTCCGTGTCTTAGACTTACTGCTAAATTTGAAGCTGAAAACTATTTTCAG
>HPV61_Alpha_9628574_nt292_E6_AOS|1
TGGCACGTGAAGTAAAAGTGAGGGAGCTGCGACATTGGGACCATTCCTGTTACGGACCCA
>HPV61_Alpha_9628574_nt318_E6_AOS|1
CTGCGACATTGGGACCATTCCTGTTACGGACCCACTGTGGAACAGACAACAGGACGGTCA
>HPV61_Alpha_9628574_nt285_E6_AOS|1
CCATGCTTGGCACGTGAAGTAAAAGTGAGGGAGCTGCGACATTGGGACCATTCCTGTTAC
>HPV61_Alpha_9628574_nt277_E6_AOS|1
TATGCGCACCATGCTTGGCACGTGAAGTAAAAGTGAGGGAGCTGCGACATTGGGACCATT
>HPV61_Alpha_9628574_nt106_E6_AOS|1
GACCGTGCAATCCAACTAATATTTTCCTGCTCTGCAAGGACTACGAGGTGGACTTTGAGG
>HPV8_Beta_333074_nt557_E6_AOS_rc|1
AGCCTCCTCTAACTTTATGAAAGGGACGGCCTCTCCCACAGCAATCTAATTTCTCTATGA
>HPV8_Beta_333074_nt549_E6_AOS_rc|1
CTAACTTTATGAAAGGGACGGCCTCTCCCACAGCAATCTAATTTCTCTATGATATCCAAA
>HPV8_Beta_333074_nt536_E6_AOS_rc|1
AGGGACGGCCTCTCCCACAGCAATCTAATTTCTCTATGATATCCAAAAATGACAAGCAGT
>HPV8_Beta_333074_nt529_E6_AOS_rc|1
GCCTCTCCCACAGCAATCTAATTTCTCTATGATATCCAAAAATGACAAGCAGTTTTGACA
>HPV8_Beta_333074_nt355_E6_AOS_rc|1
ACGACAACACGCAGTAACAACGTAATTTTTCCAAATTAGGCACAGTCTCTTTTTGTCAAA
>HPV8_Beta_333074_nt348_E6_AOS_rc|1
CACGCAGTAACAACGTAATTTTTCCAAATTAGGCACAGTCTCTTTTTGTCAAACTCACAC
>HPV51_Alpha_333087_nt490_E6_AOS_rc|1
CACTTGGGTTTCGTTACGTTGTCGTGTACGTTGCCAGCAATTAGCGCATTGCCCCGTCCA
>HPV51_Alpha_333087_nt149_E6_AOS_rc|1
ATAATTCCTTTTTACAATACACACACACTACCTGTATATTGTGCATAGAAACGTTCAAAG
>HPV20_Beta_1020162_nt210_E6_AOS_rc|1
GAGGTTTTGCCTCTCCAATATTAGATGGTCCTTCATCAGCGCTGTCTTCTGAAGAAGGAG
>HPV20_Beta_1020162_nt296_E6_AOS_rc|1
ACAAGGTATCAAACAATCATCTAGCGGTATTTCTAAAAGTTTTGCTAGGCCACAGATTGT
>HPV20_Beta_1020162_nt289_E6_AOS_rc|1
ATCAAACAATCATCTAGCGGTATTTCTAAAAGTTTTGCTAGGCCACAGATTGTTGCAGGC
>HPV20_Beta_1020162_nt203_E6_AOS_rc|1
TGCCTCTCCAATATTAGATGGTCCTTCATCAGCGCTGTCTTCTGAAGAAGGAGGTGTAGC
>HPV21_Beta_1020170_nt284_E6_AOS_rc|1
CAATGGTATCTCTAACAGATTGGCTAGGTCACGAATTGTAGCTGGTAATGGTGGCTCTAA
>HPV21_Beta_1020170_nt277_E6_AOS_rc|1
ATCTCTAACAGATTGGCTAGGTCACGAATTGTAGCTGGTAATGGTGGCTCTAAAAAGCTA
>HPV22_Beta_1020178_nt480_E6_AOS_rc|1
ACTTCAAGCAATATTGACATCTCATATAAATTTGGCCTACAGGTCGTTGTTCTTCTTGCT
>HPV22_Beta_1020178_nt302_E6_AOS_rc|1
CAAATTCTTATAATCAAATTGCCGCAATTCCAAATAGGTTAAAAACCTACTGCAGAATCT
>HPV22_Beta_1020178_nt286_E6_AOS_rc|1
AATTGCCGCAATTCCAAATAGGTTAAAAACCTACTGCAGAATCTACAAGGTAGCAAAAGA
>HPV22_Beta_1020178_nt277_E6_AOS_rc|1
AATTCCAAATAGGTTAAAAACCTACTGCAGAATCTACAAGGTAGCAAAAGATCTACTACA
>HPV23_Beta_1020186_nt463_E6_AOS_rc|1
CGTTGCTCCTCCTGCTCAATTTCACGACCATACACAGTTAGCTGATAAAATTGTTGAATT
>HPV23_Beta_1020186_nt444_E6_AOS_rc|1

TTTCACGACCATACACAGTTAGCTGATAAAATTGTTGAATTTCAAATTGAGCAGAAGCAT
>HPV23_Beta_1020186_nt346_E6_AOS_rc|1
TCTGTCCAGATTAACTGCAAATGTTTATAATCAAATTCTCGCAACTCTAAATAGGTAAGA
>HPV23_Beta_1020186_nt339_E6_AOS_rc|1
AGATTAACTGCAAATGTTTATAATCAAATTCTCGCAACTCTAAATAGGTAAGAAACCTAG
>HPV23_Beta_1020186_nt332_E6_AOS_rc|1
CTGCAAATGTTTATAATCAAATTCTCGCAACTCTAAATAGGTAAGAAACCTAGAACAAAA
>HPV28_Alpha_1020202_nt153_E6_AOS_rc|1
CGCTGTGGTCAGCACTTTGGCGCAGAATATGCAGTGCAAACGGAGGTCATCAAATGATAT
>HPV28_Alpha_1020202_nt190_E6_AOS_rc|1
CCACATTTAGTTCTCTTAATGCAAATGCTGATAGCTCCGCTGTGGTCAGCACTTTGGCGC
>HPV28_Alpha_1020202_nt172_E6_AOS_rc|1
ATGCAAATGCTGATAGCTCCGCTGTGGTCAGCACTTTGGCGCAGAATATGCAGTGCAAAC
>HPV28_Alpha_1020202_nt165_E6_AOS_rc|1
TGCTGATAGCTCCGCTGTGGTCAGCACTTTGGCGCAGAATATGCAGTGCAAACGGAGGTC
>HPV28_Alpha_1020202_nt144_E6_AOS_rc|1
CAGCACTTTGGCGCAGAATATGCAGTGCAAACGGAGGTCATCAAATGATATTCCACTGTC
>HPV29_Alpha_1020210_nt158_E6_AOS_rc|1
GGGCTGGTTAGCTCTTTCGTGCAGAAAACACACTGTAGGCGAAGGTCCTCAAATGGTACT
>HPV29_Alpha_1020210_nt280_E6_AOS_rc|1
ACTGCCAGTATTTTAGGCGCCGCTTTATGCCTTCAAACAATAAGCAGCGTGCACATGCAC
>HPV29_Alpha_1020210_nt261_E6_AOS_rc|1
CCGCTTTATGCCTTCAAACAATAAGCAGCGTGCACATGCACCGTACGGAGCTCCACTTTT
>HPV29_Alpha_1020210_nt191_E6_AOS_rc|1
TTTAATTCCCGAATGCAAAATGCTGCCAGTTCTGGGCTGGTTAGCTCTTTCGTGCAGAAA
>HPV29_Alpha_1020210_nt184_E6_AOS_rc|1
CCCGAATGCAAAATGCTGCCAGTTCTGGGCTGGTTAGCTCTTTCGTGCAGAAAACACACT
>HPV36_Beta_1020218_nt480_E6_AOS_rc|1
ACCTAATGTCAATTTCAAATATGGAGCGACCTGCAGCCAATTCAATATCTCTTCCTAATA
>HPV36_Beta_1020218_nt207_E6_AOS_rc|1
TAGTTAAAGGCAGCTGTTCTTTTTCTTTTTCTGTAATATTCTGCTGTTCGGAGGCTTGCT
>HPV36_Beta_1020218_nt200_E6_AOS_rc|1
AGGCAGCTGTTCTTTTTCTTTTTCTGTAATATTCTGCTGTTCGGAGGCTTGCTCTGCCAT
>HPV37_Beta_1020226_nt308_E6_AOS_rc|1
TAAATCTTCTTCGGTCCAAATTAGTTGAAGACCTTTTCGATCAAATGCGATCAATTCTAT
>HPV37_Beta_1020226_nt301_E6_AOS_rc|2
TCTTCGGTCCAAATTAGTTGAAGACCTTTTCGATCAAATGCGATCAATTCTATATATGCT
>HPV38_Beta_1020234_nt441_E6_AOS_rc|1
ACCTTATAACAATAAGGCCTATTGTCTGTTGCTCCACCTGTTCAATTTCACGGCCAAAGA
>HPV38_Beta_1020234_nt434_E6_AOS_rc|1
AACAATAAGGCCTATTGTCTGTTGCTCCACCTGTTCAATTTCACGGCCAAAGACAGTTAA
>HPV38_Beta_1020234_nt423_E6_AOS_rc|1
CTATTGTCTGTTGCTCCACCTGTTCAATTTCACGGCCAAAGACAGTTAATTCATAAAACT
>HPV38_Beta_1020234_nt228_E6_AOS_rc|1
AGAATCTACAGGGTAATAACAGATCCTCTACAGGAACTGTTAACTTATCACTGAGCTGCT
>HPV44_Alpha_1020242_nt363_E6_AOS_rc|1
CAAATAGCAGCGTATCAGCACGTCCAGAATTGACTTATTTGTTTCTTCTTCCACTGTTAC
>HPV44_Alpha_1020242_nt378_E6_AOS_rc|1
CAATGGTTTGTGGCACAAATAGCAGCGTATCAGCACGTCCAGAATTGACTTATTTGTTTC
>HPV44_Alpha_1020242_nt371_E6_AOS_rc|1
TTGTGGCACAAATAGCAGCGTATCAGCACGTCCAGAATTGACTTATTTGTTTCTTCTTCC
>HPV44_Alpha_1020242_nt356_E6_AOS_rc|1
CAGCGTATCAGCACGTCCAGAATTGACTTATTTGTTTCTTCTTCCACTGTTACTGCATAT
>HPV44_Alpha_1020242_nt338_E6_AOS_rc|1
AGAATTGACTTATTTGTTTCTTCTTCCACTGTTACTGCATATCCCGCGTAGTTAAAATGC

>HPV55_Alpha_1020266_nt376_E6_AOS_rc|1
ACAATGGTTTGTGGCACAAATAACAGCGTATGAACACGTCCAAAATTGACTGCTTTGTCT
>HPV66_Alpha_1020290_nt229_E6_AOS_rc|1
ATGGCCAATTGTTTCTATATACTAGTTTTAACTCAATACATGCAAACCTATATAGCTCTA
>HPV66_Alpha_1020290_nt222_E6_AOS_rc|1
ATTGTTTCTATATACTAGTTTTAACTCAATACATGCAAACCTATATAGCTCTAAACTTGT
>HPV66_Alpha_1020290_nt215_E6_AOS_rc|1
CTATATACTAGTTTTAACTCAATACATGCAAACCTATATAGCTCTAAACTTGTAAGTTCC
>HPV66_Alpha_1020290_nt208_E6_AOS_rc|1
CTAGTTTTAACTCAATACATGCAAACCTATATAGCTCTAAACTTGTAAGTTCCTTTTTGC
>HPV70_Alpha_1173493_nt330_E6_AOS_rc|1
TAGTTATGCTTTCCAAAGTTGTTGCATACACCGAGTTCGAATAATGCCGTAGTTCCCTTA
>HPV70_Alpha_1173493_nt342_E6_AOS_rc|1
ATAACTTGGTATTAGTTATGCTTTCCAAAGTTGTTGCATACACCGAGTTCGAATAATGCC
>HPV70_Alpha_1173493_nt263_E6_AOS_rc|1
ATGAAATTTAATACATTTTTGGCATGCAGCATATGGCTCCCCGTTTCTATATACTATAAA
>HPV70_Alpha_1173493_nt174_E6_AOS_rc|1
CTGTTTGCTGTAGCTGTGTTTTACAATAGACACAGTCTATTGTAATGTCGTGCAATGTAG
>HPV71_Alpha_12084981_nt93_E6_AOS_rc|1
GGTGGGGTATGCGTCCCCGCTGGACATAGCTAAATCTGCTGAACCAAGCATTGCTTTATA
>HPV71_Alpha_12084981_nt86_E6_AOS_rc|1
TATGCGTCCCCGCTGGACATAGCTAAATCTGCTGAACCAAGCATTGCTTTATATATGCAC
>HPV84_Alpha_12958167_nt141_E6_AOS_rc|2
CATAAGCATTTCATGCACACCCCATGAGGGAAATTATACCTCCAAACAATTAGTAATTCC
>HPV84_Alpha_12958167_nt151_E6_AOS_rc|1
GGCTTCCCTGCATAAGCATTTCATGCACACCCCATGAGGGAAATTATACCTCCAAACAAT
>HPV84_Alpha_12958167_nt134_E6_AOS_rc|1
ATTTCATGCACACCCCATGAGGGAAATTATACCTCCAAACAATTAGTAATTCCTTTACTG
>HPV107_Beta_126131393_nt531_E6_AOS_rc|1
ACACATCTAATATTAATAAATCCAATACTTTGCTGAGTGATCTCCTCAATCTGCTTACCT
>HPV107_Beta_126131393_nt421_E6_AOS_rc|2
AGCACAGCTGCAACAACAACCATATACTAAATTTTCATGCCAAATTAATTGTAAATTTTT
>HPVcand87_Alpha_14475578_nt253_E6_AOS_rc|1
CTTCTCTGAATAGGCATTGTAGGCACACTCCATAAGGAAATCCAAATCTCCACACCAGCA
>HPVcand87_Alpha_14475578_nt420_E6_AOS_rc|1
GTGGAAGTGCAGATTCGCGTGTATCATATGCTCCTTCTCGGGCCAGCACAATGGTTTGCA
>HPVcand87_Alpha_14475578_nt246_E6_AOS_rc|1
GAATAGGCATTGTAGGCACACTCCATAAGGAAATCCAAATCTCCACACCAGCAATAATTC
>HPVcand87_Alpha_14475578_nt239_E6_AOS_rc|1
CATTGTAGGCACACTCCATAAGGAAATCCAAATCTCCACACCAGCAATAATTCCCTTAAT
>HPVcand87_Alpha_14475578_nt124_E6_AOS_rc|1
GCCTTAAGTTGTCAAAGTCCACCTCATATTCCTGACATAGCAAAAAGATATTGGTAGGTG
>HPVcand87_Alpha_14475578_nt92_E6_AOS_rc|1
TGACATAGCAAAAAGATATTGGTAGGTGTGTAGCGTCCGTTGGGCATGACTGTCCCGTTG
>HPV72_Alpha_1491683_nt438_E6_AOS_rc|1
CGTCCACAGTCCGCTTATCTTGTGGAAGTGGATTCCTGTCTGCACCTGATATTCCTTTTC
>HPV72_Alpha_1491683_nt431_E6_AOS_rc|1
AGTCCGCTTATCTTGTGGAAGTGGATTCCTGTCTGCACCTGATATTCCTTTTCCTGACAG
>HPV72_Alpha_1491683_nt424_E6_AOS_rc|1
TTATCTTGTGGAAGTGGATTCCTGTCTGCACCTGATATTCCTTTTCCTGACAGCTTAGGG
>HPV72_Alpha_1491683_nt416_E6_AOS_rc|1
TGGAAGTGGATTCCTGTCTGCACCTGATATTCCTTTTCCTGACAGCTTAGGGGTTTGCAG
>HPV72_Alpha_1491683_nt324_E6_AOS_rc|1
TGATTTGCCTGTTTCCTGTTCCACAGTGGGTCCGTAGCCCGAATACGTCCAGTATCGTAG
>HPV72_Alpha_1491683_nt137_E6_AOS_rc|1

CAAAATATGCAGGTAATCCGTAAATCTTCTAGGTCCACCTCAATTTCCTTGCACAGCAAC
>HPV73_Alpha_1491692_nt143_E6_AOS_rc|1
CAAAACACACAGTCCAGGTTTATATCATGTATAGAAATATTCACTTCGTCACATAACGCT
>HPV73_Alpha_1491692_nt325_E6_AOS_rc|1
TGTTAGTTAAATTTTCTAACGTAGTGCCATATACTGATTGTCTATATCGCCTATACTCTC
>HPV73_Alpha_1491692_nt316_E6_AOS_rc|1
AATTTTCTAACGTAGTGCCATATACTGATTGTCTATATCGCCTATACTCTCTAATTTTAG
>HPV73_Alpha_1491692_nt136_E6_AOS_rc|1
CACAGTCCAGGTTTATATCATGTATAGAAATATTCACTTCGTCACATAACGCTTGTAGCT
>HPV73_Alpha_1491692_nt129_E6_AOS_rc|1
CAGGTTTATATCATGTATAGAAATATTCACTTCGTCACATAACGCTTGTAGCTTGTATGG
>HPVcand86_Alpha_15741127_nt149_E6_AOS_rc|1
CTTCTCTGCATATGCATTTTAGGCACACGCCATGAGGAAATCCGTGTTTCCAGACCAGTA
>HPVcand86_Alpha_15741127_nt142_E6_AOS_rc|1
GCATATGCATTTTAGGCACACGCCATGAGGAAATCCGTGTTTCCAGACCAGTAATAATTC
>HPVcand86_Alpha_15741127_nt135_E6_AOS_rc|1
CATTTTAGGCACACGCCATGAGGAAATCCGTGTTTCCAGACCAGTAATAATTCCTTTAGT
>HPV88_Gamma_167600365_nt342_E6_AOS_rc|1
ATGACATACATTCATAGCATCGGCATACCACATCAGCTAGATTCTTTCTTAGGAAATCCT
>HPV88_Gamma_167600365_nt353_E6_AOS_rc|1
TAAATCAAGCAATGACATACATTCATAGCATCGGCATACCACATCAGCTAGATTCTTTCT
>HPV88_Gamma_167600365_nt287_E6_AOS_rc|1
ATCCTCAAAATGAATTCCCTTTACAGAGCAGCGGTAAAAGCGTTCAGCCTCATACCTAGC
>HPV88_Gamma_167600365_nt280_E6_AOS_rc|1
AAATGAATTCCCTTTACAGAGCAGCGGTAAAAGCGTTCAGCCTCATACCTAGCAGAAAGC
>HPV88_Gamma_167600365_nt273_E6_AOS_rc|1
TTCCCTTTACAGAGCAGCGGTAAAAGCGTTCAGCCTCATACCTAGCAGAAAGCCTAGAAC
>HPV88_Gamma_167600365_nt266_E6_AOS_rc|1
TACAGAGCAGCGGTAAAAGCGTTCAGCCTCATACCTAGCAGAAAGCCTAGAACAAGGGAC
>HPV88_Gamma_167600365_nt259_E6_AOS_rc|1
CAGCGGTAAAAGCGTTCAGCCTCATACCTAGCAGAAAGCCTAGAACAAGGGACACAACAC
>HPV88_Gamma_167600365_nt252_E6_AOS_rc|1
AAAAGCGTTCAGCCTCATACCTAGCAGAAAGCCTAGAACAAGGGACACAACACGCAAAAC
>HPV88_Gamma_167600365_nt106_E6_AOS_rc|1
CAAGGAAGATGCACATCAAAAAGAGAAATATTAAAAATCGAACAGAAGTCATCTAGCCGT
>HPV88_Gamma_167600365_nt93_E6_AOS_rc|1
CATCAAAAAGAGAAATATTAAAAATCGAACAGAAGTCATCTAGCCGTTTTGGTAGATAAG
>HPV110_Beta_186461194_nt239_E6_AOS_rc|1
CAAAACCTAATAATTCTATATATGATAAAAACCTAGTACAAAATCTACACGGTATTAATA
>HPV110_Beta_186461194_nt225_E6_AOS_rc|1
TCTATATATGATAAAAACCTAGTACAAAATCTACACGGTATTAATATGTCTAAAACAGGA
>HPV110_Beta_186461194_nt218_E6_AOS_rc|1
ATGATAAAAACCTAGTACAAAATCTACACGGTATTAATATGTCTAAAACAGGAATGCATA
>HPV110_Beta_186461194_nt208_E6_AOS_rc|1
CCTAGTACAAAATCTACACGGTATTAATATGTCTAAAACAGGAATGCATAATGTATCTGC
>HPV111_Beta_186461202_nt463_E6_AOS_rc|1
AATATTGTTGTTTGTAGCACACTCCTAGCTTCTCTACCAGATCCAATTTTTTCAAACAAT
>HPV111_Beta_186461202_nt470_E6_AOS_rc|1
CTATGAAAATATTGTTGTTTGTAGCACACTCCTAGCTTCTCTACCAGATCCAATTTTTTC
>HPV111_Beta_186461202_nt379_E6_AOS_rc|1
CAATAGGCTGGTTTGCTACAGTTTCTATCTGTTTTCCTACAACAGAGTATTCAAAATATT
>HPVFA75/KI88-03_Unclassified_206902429_nt411_E6_AOS_rc|1
CTTTAAACAATAAATGCATCTGATAGTAATAAAGCCTACAGACTGTTGAGTCACCTGCTC
>HPVFA75/KI88-03_Unclassified_206902429_nt426_E6_AOS_rc|1
TAAAAGGTCTAGATGCTTTAAACAATAAATGCATCTGATAGTAATAAAGCCTACAGACTG

>HPVFA75/KI88-03_Unclassified_206902429_nt419_E6_AOS_rc|1
TCTAGATGCTTTAAACAATAAATGCATCTGATAGTAATAAAGCCTACAGACTGTTGAGTC
>HPV91_Alpha_22023568_nt81_E6_AOS_rc|1
ATGTACCGCTTTCGGTCGGACCGTTTTCGGTTACTCCCTTTAGTACAAGCATAATAATTG
>HPV91_Alpha_22023568_nt94_E6_AOS_rc|1
GGGTGGTTTATATATGTACCGCTTTCGGTCGGACCGTTTTCGGTTACTCCCTTTAGTACA
>HPV91_Alpha_22023568_nt74_E6_AOS_rc|1
GCTTTCGGTCGGACCGTTTTCGGTTACTCCCTTTAGTACAAGCATAATAATTGTTACTAA
>HPV91_Alpha_22023568_nt52_E6_AOS_rc|1
GTTACTCCCTTTAGTACAAGCATAATAATTGTTACTAATAACCACAAAGAATGTAAGGTG
>HPV91_Alpha_22023568_nt45_E6_AOS_rc|1
CCTTTAGTACAAGCATAATAATTGTTACTAATAACCACAAAGAATGTAAGGTGTGCAGCG
>HPV91_Alpha_22023568_nt38_E6_AOS_rc|1
TACAAGCATAATAATTGTTACTAATAACCACAAAGAATGTAAGGTGTGCAGCGACCGGTT
>HPV91_Alpha_22023568_nt31_E6_AOS_rc|1
ATAATAATTGTTACTAATAACCACAAAGAATGTAAGGTGTGCAGCGACCGGTTTCGGTCT
>HPV91_Alpha_22023568_nt24_E6_AOS_rc|1
TTGTTACTAATAACCACAAAGAATGTAAGGTGTGCAGCGACCGGTTTCGGTCTGCAGGTG
>HPV91_Alpha_22023568_nt17_E6_AOS_rc|1
TAATAACCACAAAGAATGTAAGGTGTGCAGCGACCGGTTTCGGTCTGCAGGTGTGCCTAA
>HPV91_Alpha_22023568_nt10_E6_AOS_rc|1
CACAAAGAATGTAAGGTGTGCAGCGACCGGTTTCGGTCTGCAGGTGTGCCTAACACAGTA
>HPVcand89_Alpha_22095322_nt66_E6_AOS_rc|1
GACAGCACTTCTGATGTTGTAAGCTCCTTGGTGCAATATATGCATGTAAGCCGTAGATCC
>HPVcand89_Alpha_22095322_nt59_E6_AOS_rc|1
CTTCTGATGTTGTAAGCTCCTTGGTGCAATATATGCATGTAAGCCGTAGATCCTCTAAAT
>HPVcand89_Alpha_22095322_nt52_E6_AOS_rc|1
TGTTGTAAGCTCCTTGGTGCAATATATGCATGTAAGCCGTAGATCCTCTAAATCCACGTC
>HPV90_Alpha_22138122_nt242_E6_AOS_rc|1
TAATAAGCAATTCTAAAATAGGCACTCCTGTTTCATATTCCACTGTGGGTGCATAGCTAG
>HPV90_Alpha_22138122_nt267_E6_AOS_rc|1
AGTGGTTTATGGCACAGGTAGCATCTAATAAGCAATTCTAAAATAGGCACTCCTGTTTCA
>HPV90_Alpha_22138122_nt235_E6_AOS_rc|1
CAATTCTAAAATAGGCACTCCTGTTTCATATTCCACTGTGGGTGCATAGCTAGAATACTG
>HPV90_Alpha_22138122_nt183_E6_AOS_rc|1
GAATACTGCCAGTATCTAAATTGCCTTAGTTTCCCGGCTGCTTCCAGACACGCAGGACAT
>HPV90_Alpha_22138122_nt167_E6_AOS_rc|1
TAAATTGCCTTAGTTTCCCGGCTGCTTCCAGACACGCAGGACATGCTGCAAATGGAAAAT
>HPV90_Alpha_22138122_nt160_E6_AOS_rc|1
CCTTAGTTTCCCGGCTGCTTCCAGACACGCAGGACATGCTGCAAATGGAAAATGCTTTCT
>HPV58_Alpha_222386_nt279_E6_AOS_rc|1
TATACTCACTTATTTTAGATAGCAATCGTAAGCACACTTTACATACTGCAAATGGATTTC
>HPV58_Alpha_222386_nt272_E6_AOS_rc|1
ACTTATTTTAGATAGCAATCGTAAGCACACTTTACATACTGCAAATGGATTTCCATCTCT
>HPV58_Alpha_222386_nt265_E6_AOS_rc|1
TTAGATAGCAATCGTAAGCACACTTTACATACTGCAAATGGATTTCCATCTCTATACACT
>HPV58_Alpha_222386_nt175_E6_AOS_rc|1
ACCTCAGATCGCTGCAAAGTCTTTTTGCATTCAACGCATTTCAATTCGATTTCATGCACA
>HPV58_Alpha_222386_nt168_E6_AOS_rc|1
ATCGCTGCAAAGTCTTTTTGCATTCAACGCATTTCAATTCGATTTCATGCACAGATGTCT
>HPV58_Alpha_222386_nt161_E6_AOS_rc|1
CAAAGTCTTTTTGCATTCAACGCATTTCAATTCGATTTCATGCACAGATGTCTCCAACGC
>HPV58_Alpha_222386_nt154_E6_AOS_rc|1
TTTTTGCATTCAACGCATTTCAATTCGATTTCATGCACAGATGTCTCCAACGCCTGACAC
>HPV58_Alpha_222386_nt147_E6_AOS_rc|1

ATTCAACGCATTTCAATTCGATTTCATGCACAGATGTCTCCAACGCCTGACACAAATCAT
>HPV74_Alpha_27462483_nt350_E6_AOS_rc|2
AGCGCCCTTTCCAGGTGTCGCCTAGTTTAATAAATCTTGCCTTTTCCAAAATGTGCCGTA
>HPV92_Beta_27531786_nt349_E6_AOS_rc|1
AAACTTACATCTAACAGTTAAACAACACAAAAGACTACCTGAGTCCCTTTCTATAGCATC
>HPV92_Beta_27531786_nt342_E6_AOS_rc|1
CATCTAACAGTTAAACAACACAAAAGACTACCTGAGTCCCTTTCTATAGCATCCTTTCCT
>HPV92_Beta_27531786_nt112_E6_AOS_rc|1
TAAAATGTCCTCCAATGGAATATCTAACTGTCTTCTAAGTTCCTGCACCGAAGGAGGTTT
>HPV75_Beta_2911544_nt470_E6_AOS_rc|1
TTTTTCAAATATATCCAAACGTTTCAGACAAAACTGGCACCTTACAATAAGATTATGTAT
>HPV75_Beta_2911544_nt463_E6_AOS_rc|1
AATATATCCAAACGTTTCAGACAAAACTGGCACCTTACAATAAGATTATGTATACTTTCT
>HPV75_Beta_2911544_nt456_E6_AOS_rc|1
CCAAACGTTTCAGACAAAACTGGCACCTTACAATAAGATTATGTATACTTTCTGCTGCTC
>HPV75_Beta_2911544_nt449_E6_AOS_rc|1
TTTCAGACAAAACTGGCACCTTACAATAAGATTATGTATACTTTCTGCTGCTCTGCCTTC
>HPV75_Beta_2911544_nt441_E6_AOS_rc|1
AAAACTGGCACCTTACAATAAGATTATGTATACTTTCTGCTGCTCTGCCTTCTATTTCTA
>HPV76_Beta_2911551_nt474_E6_AOS_rc|1
CAAGTTTTTCAAATAAATCCAGGCGTTTCAAACAAAATTGACACCTTATTATAATGTTCG
>HPV76_Beta_2911551_nt467_E6_AOS_rc|1
TTCAAATAAATCCAGGCGTTTCAAACAAAATTGACACCTTATTATAATGTTCGCTATATT
>HPV76_Beta_2911551_nt460_E6_AOS_rc|1
AAATCCAGGCGTTTCAAACAAAATTGACACCTTATTATAATGTTCGCTATATTTTCTGCA
>HPV76_Beta_2911551_nt453_E6_AOS_rc|1
GGCGTTTCAAACAAAATTGACACCTTATTATAATGTTCGCTATATTTTCTGCAGCTCTGC
>HPV76_Beta_2911551_nt446_E6_AOS_rc|1
CAAACAAAATTGACACCTTATTATAATGTTCGCTATATTTTCTGCAGCTCTGCCTTCTAT
>HPV77_Alpha_2911558_nt161_E6_AOS_rc|1
TCAGCAGTAGTTAATTCTTTTGTGCAGAACACACACAGTAGGCGAAGGTCGTCGAACGGT
>HPV77_Alpha_2911558_nt300_E6_AOS_rc|1
GCCGTCCACGTAGTATGAATACTCCCAGTGTTTCAGGCGCCGCTCAATGCCATATAGCAG
>HPV77_Alpha_2911558_nt293_E6_AOS_rc|1
ACGTAGTATGAATACTCCCAGTGTTTCAGGCGCCGCTCAATGCCATATAGCAGTAAACAC
>HPV77_Alpha_2911558_nt198_E6_AOS_rc|1
CACCACATTTAATTCTCTAATTGTAAATGCTGCCAGTTCAGCAGTAGTTAATTCTTTTGT
>HPV77_Alpha_2911558_nt191_E6_AOS_rc|1
TTTAATTCTCTAATTGTAAATGCTGCCAGTTCAGCAGTAGTTAATTCTTTTGTGCAGAAC
>HPV77_Alpha_2911558_nt184_E6_AOS_rc|1
CTCTAATTGTAAATGCTGCCAGTTCAGCAGTAGTTAATTCTTTTGTGCAGAACACACACA
>HPV77_Alpha_2911558_nt154_E6_AOS_rc|1
TAGTTAATTCTTTTGTGCAGAACACACACAGTAGGCGAAGGTCGTCGAACGGTATTCCAC
>HPV80_Beta_2911565_nt422_E6_AOS_rc|1
AACAGCTTTTCGCTCAACTACCTCAATCTCCCAACCACACACTGACTGTTCATAAAATGT
>HPV80_Beta_2911565_nt318_E6_AOS_rc|1
AAGCAAAGACAAAGTCTTCTTCAGTCCAAATCAACTGCAAACATTTGCGATCAAAACTTA
>HPV80_Beta_2911565_nt311_E6_AOS_rc|1
GACAAAGTCTTCTTCAGTCCAAATCAACTGCAAACATTTGCGATCAAAACTTACTAACTC
>HPV80_Beta_2911565_nt303_E6_AOS_rc|1
CTTCTTCAGTCCAAATCAACTGCAAACATTTGCGATCAAAACTTACTAACTCTATATAAG
>HPV80_Beta_2911565_nt291_E6_AOS_rc|1
AAATCAACTGCAAACATTTGCGATCAAAACTTACTAACTCTATATAAGTTAAAAATCTTT
>HPV80_Beta_2911565_nt282_E6_AOS_rc|1
GCAAACATTTGCGATCAAAACTTACTAACTCTATATAAGTTAAAAATCTTTGACAAAACC

>HPVRTRX7_Unclassified_30315616_nt530_E6_AOS_rc|1
CTATCTAATTTTTCAATGGTGCTCAAAAATGATAAGCAAGTCTGACACCTGATTTTCAAT
>HPVRTRX7_Unclassified_30315616_nt435_E6_AOS_rc|1
TCTACCTAAAACTGTTTGCTGGTAATATTCATTGAATTCGAAAGTTGCAGTAGCTACACA
>HPVRTRX7_Unclassified_30315616_nt428_E6_AOS_rc|1
AAAACTGTTTGCTGGTAATATTCATTGAATTCGAAAGTTGCAGTAGCTACACAACAACTT
>HPV65_Gamma_312100_nt417_E6_AOS_rc|1
TATTAAGTAGCAGGCCTGGTCAGAGTACAGCAAGTCTAATTTCTCAACAATGTCTAGTAG
>HPV65_Gamma_312100_nt410_E6_AOS_rc|1
TAGCAGGCCTGGTCAGAGTACAGCAAGTCTAATTTCTCAACAATGTCTAGTAGTCTCAAA
>HPV65_Gamma_312100_nt403_E6_AOS_rc|1
CCTGGTCAGAGTACAGCAAGTCTAATTTCTCAACAATGTCTAGTAGTCTCAAACAACATA
>HPV65_Gamma_312100_nt396_E6_AOS_rc|1
AGAGTACAGCAAGTCTAATTTCTCAACAATGTCTAGTAGTCTCAAACAACATATACATCT
>HPV65_Gamma_312100_nt389_E6_AOS_rc|1
AGCAAGTCTAATTTCTCAACAATGTCTAGTAGTCTCAAACAACATATACATCTGATGCAA
>HPV65_Gamma_312100_nt382_E6_AOS_rc|1
CTAATTTCTCAACAATGTCTAGTAGTCTCAAACAACATATACATCTGATGCAAATCTCCT
>HPV65_Gamma_312100_nt375_E6_AOS_rc|1
CTCAACAATGTCTAGTAGTCTCAAACAACATATACATCTGATGCAAATCTCCTTAATTTT
>HPV65_Gamma_312100_nt368_E6_AOS_rc|1
ATGTCTAGTAGTCTCAAACAACATATACATCTGATGCAAATCTCCTTAATTTTCCTTTGA
>HPV11_Alpha_333026_nt452_E6_AOS_rc|1
CAACGACCCTTCCACTGGTTATTTAGTTTTATGAAGCGTGCCTTTCCCAATATGTGCTTT
>HPV11_Alpha_333026_nt445_E6_AOS_rc|1
CCTTCCACTGGTTATTTAGTTTTATGAAGCGTGCCTTTCCCAATATGTGCTTTAGTTTTT
>HPV11_Alpha_333026_nt438_E6_AOS_rc|1
CTGGTTATTTAGTTTTATGAAGCGTGCCTTTCCCAATATGTGCTTTAGTTTTTCTATTTC
>HPV11_Alpha_333026_nt431_E6_AOS_rc|1
TTTAGTTTTATGAAGCGTGCCTTTCCCAATATGTGCTTTAGTTTTTCTATTTCACACAAC
>HPV11_Alpha_333026_nt373_E6_AOS_rc|1
ACGGCTTGTGACACAGGTAACAACGAATTAACACTTTTAAAATATCTTCATTGGTTTCTT
>HPV11_Alpha_333026_nt295_E6_AOS_rc|1
ATGCAGCATAATTAAAGTGTCTATATTGGTTAATTTTCCCTTGCAGTTCTAAGCAACAGG
>HPV31_Alpha_333048_nt146_E6_AOS_rc|1
CAGTAGACACAATTCAATCTTAGTTCATCGTAGGGTATTTCCAATGCCGAGCTTAGTTCA
>HPV31_Alpha_333048_nt267_E6_AOS_rc|1
TACTTTTGAATAAAATCTTAAACATTTTGTACACACTCCGTGTGGTGTGTCGTCCCTATA
>HPV31_Alpha_333048_nt139_E6_AOS_rc|1
CACAATTCAATCTTAGTTCATCGTAGGGTATTTCCAATGCCGAGCTTAGTTCATGCAATT
>HPV31_Alpha_333048_nt132_E6_AOS_rc|1
CAATCTTAGTTCATCGTAGGGTATTTCCAATGCCGAGCTTAGTTCATGCAATTTCCGAGG
>HPV31_Alpha_333048_nt125_E6_AOS_rc|1
AGTTCATCGTAGGGTATTTCCAATGCCGAGCTTAGTTCATGCAATTTCCGAGGTCTTTCT
>HPV31_Alpha_333048_nt118_E6_AOS_rc|1
CGTAGGGTATTTCCAATGCCGAGCTTAGTTCATGCAATTTCCGAGGTCTTTCTGCAGGAT
>HPV31_Alpha_333048_nt111_E6_AOS_rc|1
TATTTCCAATGCCGAGCTTAGTTCATGCAATTTCCGAGGTCTTTCTGCAGGATTTTTGAA
>HPV33_Alpha_333049_nt300_E6_AOS_rc|1
TATACAGAATAATTATAATGTCTATATTCACTAATTTTAGATAAGAACCGCAAACACAGT
>HPV33_Alpha_333049_nt279_E6_AOS_rc|1
CTATATTCACTAATTTTAGATAAGAACCGCAAACACAGTTTACATATTCCAAATGGATTT
>HPV33_Alpha_333049_nt272_E6_AOS_rc|1
CACTAATTTTAGATAAGAACCGCAAACACAGTTTACATATTCCAAATGGATTTCCCTCTC
>HPV33_Alpha_333049_nt265_E6_AOS_rc|1

TTTAGATAAGAACCGCAAACACAGTTTACATATTCCAAATGGATTTCCCTCTCTATATAC
>HPV33_Alpha_333049_nt258_E6_AOS_rc|1
AAGAACCGCAAACACAGTTTACATATTCCAAATGGATTTCCCTCTCTATATACAACTGTT
>HPV33_Alpha_333049_nt251_E6_AOS_rc|1
GCAAACACAGTTTACATATTCCAAATGGATTTCCCTCTCTATATACAACTGTTAAATCTG
>HPV33_Alpha_333049_nt174_E6_AOS_rc|1
ACCTCAGATCGTTGCAAAGGTTTTTTGCATTCCACGCACTGTAGTTCAATGTTGTGTATA
>HPV33_Alpha_333049_nt167_E6_AOS_rc|1
ATCGTTGCAAAGGTTTTTTGCATTCCACGCACTGTAGTTCAATGTTGTGTATAGTTGTCT
>HPV33_Alpha_333049_nt160_E6_AOS_rc|1
CAAAGGTTTTTTGCATTCCACGCACTGTAGTTCAATGTTGTGTATAGTTGTCTCCAATGC
>HPV35_Alpha_333050_nt466_E6_AOS_rc|1
CAGGACATACACCGACCTGTCCACCGTCCACCGATGTTATGGAATCGTTTTTTTTCTTCT
>HPV35_Alpha_333050_nt445_E6_AOS_rc|1
CACCGTCCACCGATGTTATGGAATCGTTTTTTTTCTTCTAAATGTCTTTGCTTTTCAACT
>HPV35_Alpha_333050_nt259_E6_AOS_rc|1
TAAAATTTTAAACATTTCATGCATACTCCATATGGCTGGCCTTCTCTATATACTATACAC
>HPV35_Alpha_333050_nt248_E6_AOS_rc|1
ACATTTCATGCATACTCCATATGGCTGGCCTTCTCTATATACTATACACAAATCATAGCA
>HPV35_Alpha_333050_nt240_E6_AOS_rc|1
TGCATACTCCATATGGCTGGCCTTCTCTATATACTATACACAAATCATAGCATGCAAAGT
>HPV47_Beta_333062_nt243_E6_AOS_rc|1
GGTATGTCTAACAATTGAGCTAAGCCTCTAATAGTAGTAGGTAGTTCTAGCTTTTCCTCT
>HPV47_Beta_333062_nt251_E6_AOS_rc|1
CTACTAAAGGTATGTCTAACAATTGAGCTAAGCCTCTAATAGTAGTAGGTAGTTCTAGCT
>HPV47_Beta_333062_nt236_E6_AOS_rc|1
CTAACAATTGAGCTAAGCCTCTAATAGTAGTAGGTAGTTCTAGCTTTTCCTCTTTAACTG
>HPV47_Beta_333062_nt229_E6_AOS_rc|1
TTGAGCTAAGCCTCTAATAGTAGTAGGTAGTTCTAGCTTTTCCTCTTTAACTGTAGTCTG
>HPV47_Beta_333062_nt222_E6_AOS_rc|1
AAGCCTCTAATAGTAGTAGGTAGTTCTAGCTTTTCCTCTTTAACTGTAGTCTGTTCCAAA
>HPV42_Alpha_333211_nt456_E6_AOS_rc|1
CGTCCACTGACACAATATAAATTGAAATCTTGTACCTGTATCAATATGATGGTTTTTTTC
>HPV42_Alpha_333211_nt449_E6_AOS_rc|1
TGACACAATATAAATTGAAATCTTGTACCTGTATCAATATGATGGTTTTTTTCGCTCTGT
>HPV42_Alpha_333211_nt442_E6_AOS_rc|1
ATATAAATTGAAATCTTGTACCTGTATCAATATGATGGTTTTTTTCGCTCTGTGATAACG
>HPV39_Alpha_333245_nt174_E6_AOS_rc|1
CGGTTTGCTGTAGTGGTCGTCTGCAATAGACACAGGCTATTGTAATGTCCTGCAAGGTGG
>HPV39_Alpha_333245_nt162_E6_AOS_rc|1
GTGGTCGTCTGCAATAGACACAGGCTATTGTAATGTCCTGCAAGGTGGTGTCCAGCGTTG
>HPV93_Beta_37089393_nt376_E6_AOS_rc|1
GGCGACACCTCACTATAATTTCAGAGAGTGGCTTTTTTTCCTTAACTTCTATTGCCCAGC
>HPV93_Beta_37089393_nt369_E6_AOS_rc|1
CCTCACTATAATTTCAGAGAGTGGCTTTTTTTCCTTAACTTCTATTGCCCAGCCAACAAC
>HPV93_Beta_37089393_nt362_E6_AOS_rc|1
ATAATTTCAGAGAGTGGCTTTTTTTCCTTAACTTCTATTGCCCAGCCAACAACACTATCA
>HPV93_Beta_37089393_nt355_E6_AOS_rc|1
CAGAGAGTGGCTTTTTTTCCTTAACTTCTATTGCCCAGCCAACAACACTATCATTCAAGT
>HPV93_Beta_37089393_nt348_E6_AOS_rc|1
TGGCTTTTTTTCCTTAACTTCTATTGCCCAGCCAACAACACTATCATTCAAGTAATTATC
>HPV12_Beta_396910_nt614_E6_AOS_rc|1
TTACCGATCATTATTCAGATACAAATGCTTGCACTGTCTGCAAATTCCCTTCCACCTGTC
>HPV12_Beta_396910_nt607_E6_AOS_rc|1
TCATTATTCAGATACAAATGCTTGCACTGTCTGCAAATTCCCTTCCACCTGTCTCTAACC

>HPV12_Beta_396910_nt474_E6_AOS_rc|1
ACCTTATCTTTAAGTCAAATATAGATTTTCCAGTAGCAAGCTCTATATCTCTACCTAGCA
>HPV14D_Beta_396918_nt260_E6_AOS_rc|1
CAAATATAGTTGCAGGTAATGGTGGCTCTATAAAAGAAGATTTGGTTTCTGTGCTATCAC
>HPV14D_Beta_396918_nt380_E6_AOS_rc|1
AAATTAGACTTAGTTTTTCTCATCAAATTCACAGACTTCTAAATGAGTCAAAAAATTAC
>HPV14D_Beta_396918_nt302_E6_AOS_rc|1
CTAAACAATCATCTAGTGGTATTTCCAATAGGTTTGCTAAGCCAAATATAGTTGCAGGTA
>HPV14D_Beta_396918_nt287_E6_AOS_rc|1
GTGGTATTTCCAATAGGTTTGCTAAGCCAAATATAGTTGCAGGTAATGGTGGCTCTATAA
>HPV14D_Beta_396918_nt280_E6_AOS_rc|1
TTCCAATAGGTTTGCTAAGCCAAATATAGTTGCAGGTAATGGTGGCTCTATAAAAGAAGA
>HPV14D_Beta_396918_nt246_E6_AOS_rc|1
GGTAATGGTGGCTCTATAAAAGAAGATTTGGTTTCTGTGCTATCACAATAGTTACTCTTA
>HPV14D_Beta_396918_nt239_E6_AOS_rc|1
GTGGCTCTATAAAAGAAGATTTGGTTTCTGTGCTATCACAATAGTTACTCTTAGGAGAAG
>HPV15_Beta_396924_nt436_E6_AOS_rc|1
ATAATAATATCTCCAACAGGCTTCTGTTCTACTATCTCTATTTCCCAACTACACACCGAC
>HPV15_Beta_396924_nt447_E6_AOS_rc|1
ATTTGCAGCGAATAATAATATCTCCAACAGGCTTCTGTTCTACTATCTCTATTTCCCAAC
>HPV15_Beta_396924_nt429_E6_AOS_rc|1
TATCTCCAACAGGCTTCTGTTCTACTATCTCTATTTCCCAACTACACACCGACTGTTCAT
>HPV15_Beta_396924_nt422_E6_AOS_rc|1
AACAGGCTTCTGTTCTACTATCTCTATTTCCCAACTACACACCGACTGTTCATAAAAGTT
>HPV15_Beta_396924_nt415_E6_AOS_rc|1
TTCTGTTCTACTATCTCTATTTCCCAACTACACACCGACTGTTCATAAAAGTTAGAAAAT
>HPV17_Beta_396932_nt447_E6_AOS_rc|1
ACTTGCAGCGAATAGGTATTTCCCCTATTGGCTTGTGCTCTATTTCCTCTAACTCCCTTC
>HPV17_Beta_396932_nt527_E6_AOS_rc|1
ATTGCGTCTAACCTTATGAAACTGCTGATGTCTATAACAAGTGTCTAACTTCTCTAGTAA
>HPV17_Beta_396932_nt519_E6_AOS_rc|1
TAACCTTATGAAACTGCTGATGTCTATAACAAGTGTCTAACTTCTCTAGTAAATCCAATT
>HPV17_Beta_396932_nt440_E6_AOS_rc|2
GCGAATAGGTATTTCCCCTATTGGCTTGTGCTCTATTTCCTCTAACTCCCTTCCACTCAC
>HPV17_Beta_396932_nt432_E6_AOS_rc|1
GTATTTCCCCTATTGGCTTGTGCTCTATTTCCTCTAACTCCCTTCCACTCACTGATTGTT
>HPV17_Beta_396932_nt422_E6_AOS_rc|1
TATTGGCTTGTGCTCTATTTCCTCTAACTCCCTTCCACTCACTGATTGTTCATAAAACTT
>HPV17_Beta_396932_nt288_E6_AOS_rc|1
TTAACTGCAAACCTTTTAAATCAAACGCCACCAATTCTATGTAAGCTAAAAACCTATTAC
>HPV17_Beta_396932_nt281_E6_AOS_rc|2
CAAACCTTTTAAATCAAACGCCACCAATTCTATGTAAGCTAAAAACCTATTACAAAATCT
>HPV19_Beta_396940_nt491_E6_AOS_rc|1
AAAGACAGATTTACCTGTTACAAACTCAATTTCTCTACCTGTTACAGTATGCTCATAGAA
>HPV19_Beta_396940_nt484_E6_AOS_rc|1
GATTTACCTGTTACAAACTCAATTTCTCTACCTGTTACAGTATGCTCATAGAACTCATTA
>HPV19_Beta_396940_nt474_E6_AOS_rc|1
TTACAAACTCAATTTCTCTACCTGTTACAGTATGCTCATAGAACTCATTAAATTCAAATG
>HPV19_Beta_396940_nt361_E6_AOS_rc|2
CAAATCAAACTAAGTCTTTTATCATCAAATTCGCACGCTTCTAAATGTGATAAAAACTTG
>HPV19_Beta_396940_nt354_E6_AOS_rc|1
AACTAAGTCTTTTATCATCAAATTCGCACGCTTCTAAATGTGATAAAAACTTGCCACAGA
>HPV19_Beta_396940_nt288_E6_AOS_rc|1
AAGGCACTAAACAGTCATCCAACGGTATTTCTAGCAATGCTGCTAATCCAGCAATTGTTG
>HPV25_Beta_396948_nt456_E6_AOS_rc|1

CGTCTTCAATTTCCCTACCTGTTACAGTGCTTTCGTAAAATTCATTAAATTCAAATGTGG
>HPV25_Beta_396948_nt469_E6_AOS_rc|1
GATTTACCTGTAACGTCTTCAATTTCCCTACCTGTTACAGTGCTTTCGTAAAATTCATTA
>HPV25_Beta_396948_nt345_E6_AOS_rc|1
AAATCAAACTAAGTCTTTTCTCATCAAACTCACAGATCTCTAAATATGTTAGAAAGTTGC
>HPV25_Beta_396948_nt338_E6_AOS_rc|1
ACTAAGTCTTTTCTCATCAAACTCACAGATCTCTAAATATGTTAGAAAGTTGCCACAGAA
>HPV25_Beta_396948_nt266_E6_AOS_rc|1
TAAGCAATCATCTAATGGAATTTCCAATAAAGCTGCTAGATCAGTAATTGTTGCTGGCAA
>HPV25_Beta_396948_nt259_E6_AOS_rc|1
TCATCTAATGGAATTTCCAATAAAGCTGCTAGATCAGTAATTGTTGCTGGCAATGGTGGC
>HPV25_Beta_396948_nt252_E6_AOS_rc|1
ATGGAATTTCCAATAAAGCTGCTAGATCAGTAATTGTTGCTGGCAATGGTGGCTGTATAA
>HPV25_Beta_396948_nt212_E6_AOS_rc|1
TGGCAATGGTGGCTGTATAACCTGCGCTTGCTCTGGTGGTCCTATGCTCTGTTCAGCATT
>HPV27_Alpha_396964_nt213_E6_AOS_rc|1
CACTACAGACAGTTCTTTTATTGCAAATGCCAATACATCAGCGTCTGAAAGCGCTCGTCT
>HPV27_Alpha_396964_nt199_E6_AOS_rc|1
CTTTTATTGCAAATGCCAATACATCAGCGTCTGAAAGCGCTCGTCTGCAATACACGCAGA
>HPV30_Alpha_396973_nt133_E6_AOS_rc|1
GCTGTAGCTCCAGCAACGATGTTTCTTGTACCTCACAAAGATGGTGCACAGTACGTGGGC
>HPV30_Alpha_396973_nt140_E6_AOS_rc|1
CACTGTAGCTGTAGCTCCAGCAACGATGTTTCTTGTACCTCACAAAGATGGTGCACAGTA
>HPV3_Alpha_397005_nt164_E6_AOS_rc|1
GTCAGCTGTTTCGTACAGAATATGCAGTGCAGGCGAAGGTCGTCAAATCCTATTCCGGTG
>HPV3_Alpha_397005_nt205_E6_AOS_rc|1
CATTCAGTTCCCGTAATGCAAATGCTTGTAGTTCAGTTGTAGTCAGCTGTTTCGTACAGA
>HPV3_Alpha_397005_nt196_E6_AOS_rc|1
CCCGTAATGCAAATGCTTGTAGTTCAGTTGTAGTCAGCTGTTTCGTACAGAATATGCAGT
>HPV3_Alpha_397005_nt189_E6_AOS_rc|1
TGCAAATGCTTGTAGTTCAGTTGTAGTCAGCTGTTTCGTACAGAATATGCAGTGCAGGCG
>HPV3_Alpha_397005_nt173_E6_AOS_rc|1
TCAGTTGTAGTCAGCTGTTTCGTACAGAATATGCAGTGCAGGCGAAGGTCGTCAAATCCT
>HPV3_Alpha_397005_nt148_E6_AOS_rc|1
AGAATATGCAGTGCAGGCGAAGGTCGTCAAATCCTATTCCGGTGTTTCTGCACAGTAGAA
>HPV40_Alpha_397014_nt310_E6_AOS_rc|1
CTTCCACGGTTGGTGCATAGGCTGCGTATCTAAAGTTTCTGTATTGGTTTACTTTTCCGT
>HPV40_Alpha_397014_nt303_E6_AOS_rc|1
GGTTGGTGCATAGGCTGCGTATCTAAAGTTTCTGTATTGGTTTACTTTTCCGTGCAGGTC
>HPV40_Alpha_397014_nt296_E6_AOS_rc|2
GCATAGGCTGCGTATCTAAAGTTTCTGTATTGGTTTACTTTTCCGTGCAGGTCCAGGCAC
>HPV40_Alpha_397014_nt289_E6_AOS_rc|1
CTGCGTATCTAAAGTTTCTGTATTGGTTTACTTTTCCGTGCAGGTCCAGGCACCGTGGAC
>HPV40_Alpha_397014_nt282_E6_AOS_rc|1
TCTAAAGTTTCTGTATTGGTTTACTTTTCCGTGCAGGTCCAGGCACCGTGGACATGCGGC
>HPV40_Alpha_397014_nt275_E6_AOS_rc|1
TTTCTGTATTGGTTTACTTTTCCGTGCAGGTCCAGGCACCGTGGACATGCGGCGTGTGGA
>HPV45_Alpha_397022_nt498_E6_AOS_rc|1
TCTGCGAAGTCTTTCTTGCCGTGCCTGGTCACAACATGTATTACACTGCCCTCGGTACTG
>HPV52_Alpha_397038_nt368_E6_AOS_rc|1
GGCGTTTGACAAATTATACATCTAATAGTTATTTCACTTAATGGTTTTTTTACCCTCTCT
>HPV52_Alpha_397038_nt361_E6_AOS_rc|1
GACAAATTATACATCTAATAGTTATTTCACTTAATGGTTTTTTTACCCTCTCTTCTAATG
>HPV52_Alpha_397038_nt354_E6_AOS_rc|1
TATACATCTAATAGTTATTTCACTTAATGGTTTTTTTACCCTCTCTTCTAATGTTTTCCC

>HPV52_Alpha_397038_nt347_E6_AOS_rc|1
CTAATAGTTATTTCACTTAATGGTTTTTTTACCCTCTCTTCTAATGTTTTCCCATACAGT
>HPV52_Alpha_397038_nt340_E6_AOS_rc|1
TTATTTCACTTAATGGTTTTTTTACCCTCTCTTCTAATGTTTTCCCATACAGTGAATATT
>HPV52_Alpha_397038_nt333_E6_AOS_rc|1
ACTTAATGGTTTTTTTACCCTCTCTTCTAATGTTTTCCCATACAGTGAATATTGATAATG
>HPV52_Alpha_397038_nt326_E6_AOS_rc|1
GGTTTTTTTACCCTCTCTTCTAATGTTTTCCCATACAGTGAATATTGATAATGCCTATAT
>HPV52_Alpha_397038_nt319_E6_AOS_rc|1
TTACCCTCTCTTCTAATGTTTTCCCATACAGTGAATATTGATAATGCCTATATTCACTTA
>HPV52_Alpha_397038_nt305_E6_AOS_rc|1
AATGTTTTCCCATACAGTGAATATTGATAATGCCTATATTCACTTATCTTAGATAAAAAG
>HPV52_Alpha_397038_nt298_E6_AOS_rc|1
TCCCATACAGTGAATATTGATAATGCCTATATTCACTTATCTTAGATAAAAAGCGTAGGC
>HPV56_Alpha_397053_nt268_E6_AOS_rc|2
CTTTACTATAAAACAATAAACATACTCTGCACACTGCATAAGGAAAATCATCCCTATACA
>HPV56_Alpha_397053_nt261_E6_AOS_rc|2
ATAAAACAATAAACATACTCTGCACACTGCATAAGGAAAATCATCCCTATACACTAATTT
>HPVcand62_Alpha_39932599_nt188_E6_AOS_rc|1
AGCATGAGTGCTGCCACCGCCGTAGCTCTCTTACTTTAATCTCTCTCAATAGGCATGCTG
>HPVcand62_Alpha_39932599_nt205_E6_AOS_rc|1
TTCTACTGTTATTCCAAAGCATGAGTGCTGCCACCGCCGTAGCTCTCTTACTTTAATCTC
>HPVcand62_Alpha_39932599_nt198_E6_AOS_rc|1
GTTATTCCAAAGCATGAGTGCTGCCACCGCCGTAGCTCTCTTACTTTAATCTCTCTCAAT
>HPV43_Alpha_40804474_nt304_E6_AOS_rc|1
TATCTGCATATGCTGCGTAGTCAAAGTGCCTATATTGACTTATTTTTCCATGAAACTGTA
>HPV43_Alpha_40804474_nt316_E6_AOS_rc|1
CTTCTTCTACAGTATCTGCATATGCTGCGTAGTCAAAGTGCCTATATTGACTTATTTTTC
>HPV43_Alpha_40804474_nt297_E6_AOS_rc|1
ATATGCTGCGTAGTCAAAGTGCCTATATTGACTTATTTTTCCATGAAACTGTAGACAGGC
>HPV43_Alpha_40804474_nt290_E6_AOS_rc|1
GCGTAGTCAAAGTGCCTATATTGACTTATTTTTCCATGAAACTGTAGACAGGCCAAGCAT
>HPV81_Alpha_40804509_nt434_E6_AOS_rc|1
TGAAAGTGGATTAGGCCTGTCACAAGATACTCCTTTTCTTGGTATGTTAACGGTTTATAG
>HPV81_Alpha_40804509_nt427_E6_AOS_rc|1
GGATTAGGCCTGTCACAAGATACTCCTTTTCTTGGTATGTTAACGGTTTATAGCATGCAT
>HPV81_Alpha_40804509_nt319_E6_AOS_rc|1
CTGTTGGTCCGAAGCACGAATATTGCCACCGCCGCAGCTCCCGTACTTTAGCTTCTCTGC
>HPV81_Alpha_40804509_nt102_E6_AOS_rc|1
CAGCAAAAACAAATTGGTAGGATTTGCTGGCCCCAGCGACATCACTGCTGTGCTGACCAT
>HPV95_Gamma_40804520_nt360_E6_AOS_rc|2
ACAGCATAAACATCTTACACATATATCCCTGATTTTTTGTCCTACTAAATCTTCCAGTCC
>HPV95_Gamma_40804520_nt390_E6_AOS_rc|1
AAGTTTTTCTACTATGTCTAATAGTTTAAGACAGCATAAACATCTTACACATATATCCCT
>HPV95_Gamma_40804520_nt383_E6_AOS_rc|1
TCTACTATGTCTAATAGTTTAAGACAGCATAAACATCTTACACATATATCCCTGATTTTT
>HPV95_Gamma_40804520_nt376_E6_AOS_rc|1
TGTCTAATAGTTTAAGACAGCATAAACATCTTACACATATATCCCTGATTTTTTGTCCTA
>HPV95_Gamma_40804520_nt369_E6_AOS_rc|1
TAGTTTAAGACAGCATAAACATCTTACACATATATCCCTGATTTTTTGTCCTACTAAATC
>HPV95_Gamma_40804520_nt353_E6_AOS_rc|1
AAACATCTTACACATATATCCCTGATTTTTTGTCCTACTAAATCTTCCAGTCCAGTAGCA
>HPV95_Gamma_40804520_nt346_E6_AOS_rc|1
TTACACATATATCCCTGATTTTTTGTCCTACTAAATCTTCCAGTCCAGTAGCATATACAG
>HPV94_Alpha_40804528_nt175_E6_AOS_rc|1

CTCTTAATGCAAATGCAGTCAATTCGGCTACGGTCAGCTGTTTGGTGCAGAACACGCAGC
>HPV94_Alpha_40804528_nt183_E6_AOS_rc|1
ATTCAATTCTCTTAATGCAAATGCAGTCAATTCGGCTACGGTCAGCTGTTTGGTGCAGAA
>HPV94_Alpha_40804528_nt138_E6_AOS_rc|1
CTGTTTGGTGCAGAACACGCAGCACAGGCGAAGGTCCTCAAATGATATTCCACAGTTCCT
>HPV94_Alpha_40804528_nt130_E6_AOS_rc|1
TGCAGAACACGCAGCACAGGCGAAGGTCCTCAAATGATATTCCACAGTTCCTACACAATA
>HPVcand85_Alpha_4574720_nt141_E6_AOS_rc|2
ACAGCTTATTTCTATATCTTGCAGTGATGTGTCCAGTGTGTTGCATAGGTCTGGTAGTTT
>HPVcand85_Alpha_4574720_nt404_E6_AOS_rc|1
TGTTTTAGTTTTTCTGCCGGACATAAAGGTTTTTGACACCGTAAACACCTTATTAATATA
>HPVcand85_Alpha_4574720_nt397_E6_AOS_rc|1
GTTTTTCTGCCGGACATAAAGGTTTTTGACACCGTAAACACCTTATTAATATATCATATA
>HPVcand85_Alpha_4574720_nt312_E6_AOS_rc|1
CGTTTCCCCATACACAGAGTCGCTATAATATCTTAGTTCTCTTATTTTAGAATAAAACAT
>HPVcand85_Alpha_4574720_nt151_E6_AOS_rc|1
TACAATATACACAGCTTATTTCTATATCTTGCAGTGATGTGTCCAGTGTGTTGCATAGGT
>HPV96_Beta_50253426_nt260_E6_AOS_rc|1
AAATGTCCTAGAAACGCAGGCGGTTACTTGTTTATGAGTGGCAGGTTGTAATTGCCAACA
>HPV96_Beta_50253426_nt253_E6_AOS_rc|1
CTAGAAACGCAGGCGGTTACTTGTTTATGAGTGGCAGGTTGTAATTGCCAACAACCTCTC
>HPV96_Beta_50253426_nt225_E6_AOS_rc|1
GAGTGGCAGGTTGTAATTGCCAACAACCTCTCAAGATAAAACTTTTGGCAACCGAGAGAG
>HPV96_Beta_50253426_nt218_E6_AOS_rc|1
AGGTTGTAATTGCCAACAACCTCTCAAGATAAAACTTTTGGCAACCGAGAGAGTTGCGAA
>HPV96_Beta_50253426_nt210_E6_AOS_rc|1
ATTGCCAACAACCTCTCAAGATAAAACTTTTGGCAACCGAGAGAGTTGCGAAACTTACCG
>HPV96_Beta_50253426_nt141_E6_AOS_rc|2
CCAAAGTTGGCAGACGTTTTGGCGGGCTTTTCTTGGCAGGTTTAAACTGCTTTTGGCAAA
>HPV96_Beta_50253426_nt116_E6_AOS_rc|1
GCTTTTCTTGGCAGGTTTAAACTGCTTTTGGCAAAGCCACTGGATCGGTGTTGTTCCAGG
>HPV96_Beta_50253426_nt109_E6_AOS_rc|1
TTGGCAGGTTTAAACTGCTTTTGGCAAAGCCACTGGATCGGTGTTGTTCCAGGAGTGACT
>HPV96_Beta_50253426_nt102_E6_AOS_rc|1
GTTTAAACTGCTTTTGGCAAAGCCACTGGATCGGTGTTGTTCCAGGAGTGACTGCCAGGA
>HPV96_Beta_50253426_nt94_E6_AOS_rc|1
TGCTTTTGGCAAAGCCACTGGATCGGTGTTGTTCCAGGAGTGACTGCCAGGATCAGATAC
>HPV83_Alpha_5059324_nt192_E6_AOS_rc|1
GCCCCGTAGCTTGAGTGGCTCCAGTGCCGTAGCTCTCGCACCTTTGCTGCTTTACGTAGA
>HPV83_Alpha_5059324_nt257_E6_AOS_rc|1
TCAGGCACATATGGCATCGTATATACAGCTGTGCCAGTGGTGTTTTTGTTTCTTCTTCTA
>HPV83_Alpha_5059324_nt250_E6_AOS_rc|1
CATATGGCATCGTATATACAGCTGTGCCAGTGGTGTTTTTGTTTCTTCTTCTACTGTTGC
>HPV83_Alpha_5059324_nt185_E6_AOS_rc|1
AGCTTGAGTGGCTCCAGTGCCGTAGCTCTCGCACCTTTGCTGCTTTACGTAGACACTGTG
>HPV83_Alpha_5059324_nt178_E6_AOS_rc|1
GTGGCTCCAGTGCCGTAGCTCTCGCACCTTTGCTGCTTTACGTAGACACTGTGCACACGC
>HPV59_Alpha_557236_nt372_E6_AOS_rc|1
CTTCTTTTTTCAGTTATATGCTTTAATTTATCTGTTGGACATAGAGGTTTTAGGCATCTA
>HPV59_Alpha_557236_nt365_E6_AOS_rc|1
TTTCAGTTATATGCTTTAATTTATCTGTTGGACATAGAGGTTTTAGGCATCTATAACAGC
>HPV6_Alpha_6002612_nt440_E6_AOS_rc|1
ACGTACAATTTAGCTTTATGAACCGTGCCTTGGTTAGTATATGTTTTACCTTTTCTACTT
>HPV57_Alpha_60882_nt499_E6_AOS_rc|1
ATGTCCTCAAGGCTGGGGCGTTCTCCATGCATCTTGGCGCACAGTTCATGCAATGTCCGG

>HPV57_Alpha_60882_nt506_E6_AOS_rc|1
TAGTGTGATGTCCTCAAGGCTGGGGCGTTCTCCATGCATCTTGGCGCACAGTTCATGCAA
>HPV57_Alpha_60882_nt492_E6_AOS_rc|1
CAAGGCTGGGGCGTTCTCCATGCATCTTGGCGCACAGTTCATGCAATGTCCGGTCCACTG
>HPV69_Alpha_6970418_nt275_E6_AOS_rc|1
CGTCTATATTCTATTATTTTGAATAGAATATTATACATTTTTTACATGCACCATATGCA
>HPV69_Alpha_6970418_nt268_E6_AOS_rc|1
ATTCTATTATTTTGAATAGAATATTATACATTTTTTACATGCACCATATGCACTATCAT
>HPV69_Alpha_6970418_nt261_E6_AOS_rc|2
TATTTTTGAATAGAATATTATACATTTTTTACATGCACCATATGCACTATCATTTCTATA
>HPV69_Alpha_6970418_nt254_E6_AOS_rc|1
GAATAGAATATTATACATTTTTTACATGCACCATATGCACTATCATTTCTATACACTATT
>HPV82_Alpha_6970427_nt498_E6_AOS_rc|1
TTACACCTGGGTTTCACTACGTTGTCTTGGTGGTTTTCTGCAATTTGCACACTGTCCCGT
>HPV82_Alpha_6970427_nt491_E6_AOS_rc|1
TGGGTTTCACTACGTTGTCTTGGTGGTTTTCTGCAATTTGCACACTGTCCCGTCCAGCGT
>HPV82_Alpha_6970427_nt263_E6_AOS_rc|1
CTAATTCTAGAATAAAACATTAGGCATTTTTTGCATGCTGCATATGGCGTATTGTCCCTA
>HPV82_Alpha_6970427_nt249_E6_AOS_rc|1
AAACATTAGGCATTTTTTGCATGCTGCATATGGCGTATTGTCCCTATATACAATCCTAAG
>HPV82_Alpha_6970427_nt242_E6_AOS_rc|1
AGGCATTTTTTGCATGCTGCATATGGCGTATTGTCCCTATATACAATCCTAAGTTCTGTA
>HPV82_Alpha_6970427_nt154_E6_AOS_rc|1
ACAACTCCTTTTTACAATATACACACAATACCTGAATATTGTGCATAGACGTATTGCAGG
>HPV82_Alpha_6970427_nt139_E6_AOS_rc|1
AATATACACACAATACCTGAATATTGTGCATAGACGTATTGCAGGCTTCACATAATTCGT
>HPV68a_Alpha_71726685_nt103_E6_AOS_rc|1
TACACATAGGTCACTAAAGGCAAATTCATATACCTCTGTCCGTTGTAGTTGCCTTCTGCA
>HPV97_Alpha_71726694_nt342_E6_AOS_rc|1
TAATAACCAGATATGTGGTGGAATCGTCGTTTGTCTTTAAGATGTTTATATTTGTCTGCT
>HPV97_Alpha_71726694_nt352_E6_AOS_rc|1
CTGGCCTCTATAATAACCAGATATGTGGTGGAATCGTCGTTTGTCTTTAAGATGTTTATA
>HPV97_Alpha_71726694_nt335_E6_AOS_rc|1
CAGATATGTGGTGGAATCGTCGTTTGTCTTTAAGATGTTTATATTTGTCTGCTGGATTCA
>HPV106_Alpha_71726710_nt16_E6_AOS_rc|1
ATTCTTTGGACAGCAGTCTCCGTTGGTCATGGCTATTTTGTGCTGAAGCAACATATCTCT
>HPV106_Alpha_71726710_nt286_E6_AOS_rc|1
AATAAACAGCTCTAGTATTGGTATCCCTGTTTCATACTCCACTGTGGGTGCATAGCTGGA
>HPV106_Alpha_71726710_nt279_E6_AOS_rc|1
AGCTCTAGTATTGGTATCCCTGTTTCATACTCCACTGTGGGTGCATAGCTGGAGTAGTCC
>HPV106_Alpha_71726710_nt243_E6_AOS_rc|1
GTGGGTGCATAGCTGGAGTAGTCCCAGTATCTAAATTGTCTAAGTTTTCCAGCTGCTTTC
>HPV106_Alpha_71726710_nt9_E6_AOS_rc|1
GGACAGCAGTCTCCGTTGGTCATGGCTATTTTGTGCTGAAGCAACATATCTCTTTTATAT
>HPV102_Alpha_71726718_nt240_E6_AOS_rc|1
CTCATATACAGCTGTGGTAATGCTGTGCGGGTTTCTTCTTCAACCGTTGCGCCGTAGCTG
>HPV102_Alpha_71726718_nt233_E6_AOS_rc|1
ACAGCTGTGGTAATGCTGTGCGGGTTTCTTCTTCAACCGTTGCGCCGTAGCTGGAGTAGT
>HPV102_Alpha_71726718_nt226_E6_AOS_rc|1
TGGTAATGCTGTGCGGGTTTCTTCTTCAACCGTTGCGCCGTAGCTGGAGTAGTTCCAGTG
>HPV102_Alpha_71726718_nt215_E6_AOS_rc|1
TGCGGGTTTCTTCTTCAACCGTTGCGCCGTAGCTGGAGTAGTTCCAGTGCCGTAGTTCCC
>HPV13_Alpha_85827580_nt291_E6_AOS_rc|1
CGAAGTCAAAATGCCTAAACTGGTTAATCTTTCCTTGTATTTCTAAGCAGCATGCACAAG
>HPV13_Alpha_85827580_nt284_E6_AOS_rc|1

AAAATGCCTAAACTGGTTAATCTTTCCTTGTATTTCTAAGCAGCATGCACAAGCCGCAAA
>HPV13_Alpha_85827580_nt277_E6_AOS_rc|1
CTAAACTGGTTAATCTTTCCTTGTATTTCTAAGCAGCATGCACAAGCCGCAAATGGAAAC
>HPV13_Alpha_85827580_nt270_E6_AOS_rc|1
GGTTAATCTTTCCTTGTATTTCTAAGCAGCATGCACAAGCCGCAAATGGAAACGATCCTC
>HPV13_Alpha_85827580_nt255_E6_AOS_rc|1
GTATTTCTAAGCAGCATGCACAAGCCGCAAATGGAAACGATCCTCGCCACACTATATATA
>HPV13_Alpha_85827580_nt248_E6_AOS_rc|1
TAAGCAGCATGCACAAGCCGCAAATGGAAACGATCCTCGCCACACTATATATAAACTCTT
>HPV13_Alpha_85827580_nt240_E6_AOS_rc|1
ATGCACAAGCCGCAAATGGAAACGATCCTCGCCACACTATATATAAACTCTTATACTGAA
>HPV13_Alpha_85827580_nt220_E6_AOS_rc|1
AACGATCCTCGCCACACTATATATAAACTCTTATACTGAAATGAATAAACCTCTGCCGTG
>HPV2_Alpha_9626032_nt177_E6_AOS_rc|1
ATGCCCATATGTCAGCCTCTGATAACGGCCGTTTGCACCATACACAGAGCAATCGCAAAT
>HPV2_Alpha_9626032_nt191_E6_AOS_rc|1
TTCTTTTATTGCAAATGCCCATATGTCAGCCTCTGATAACGGCCGTTTGCACCATACACA
>HPV2_Alpha_9626032_nt184_E6_AOS_rc|1
ATTGCAAATGCCCATATGTCAGCCTCTGATAACGGCCGTTTGCACCATACACAGAGCAAT
>HPV2_Alpha_9626032_nt170_E6_AOS_rc|1
TATGTCAGCCTCTGATAACGGCCGTTTGCACCATACACAGAGCAATCGCAAATCCTCTAG
>HPV41_Nu_9626041_nt304_E6_AOS_rc|1
TGAACTCCAGCCTTGCAAGCACTCTAGTACACTGAGAACAAATACCCGTTGGCCCCCCTT
>HPV41_Nu_9626041_nt293_E6_AOS_rc|1
CTTGCAAGCACTCTAGTACACTGAGAACAAATACCCGTTGGCCCCCCTTCTCCCCAGGAT
>HPV41_Nu_9626041_nt277_E6_AOS_rc|1
TACACTGAGAACAAATACCCGTTGGCCCCCCTTCTCCCCAGGATAAGTACAGACAGCTCT
>HPV41_Nu_9626041_nt270_E6_AOS_rc|1
AGAACAAATACCCGTTGGCCCCCCTTCTCCCCAGGATAAGTACAGACAGCTCTGGTCGAA
>HPV41_Nu_9626041_nt259_E6_AOS_rc|1
CCGTTGGCCCCCCTTCTCCCCAGGATAAGTACAGACAGCTCTGGTCGAAAGCGTAAATAT
>HPV41_Nu_9626041_nt242_E6_AOS_rc|1
CCCCAGGATAAGTACAGACAGCTCTGGTCGAAAGCGTAAATATCCAATACGCTAAGGATC
>HPV41_Nu_9626041_nt233_E6_AOS_rc|1
AAGTACAGACAGCTCTGGTCGAAAGCGTAAATATCCAATACGCTAAGGATCTTATAGCAA
>HPV41_Nu_9626041_nt224_E6_AOS_rc|1
CAGCTCTGGTCGAAAGCGTAAATATCCAATACGCTAAGGATCTTATAGCAATAATGGCAG
>HPV41_Nu_9626041_nt157_E6_AOS_rc|1
GTATGCATGGATAAGTTATCTGCTGCGCCAAACACAACTCCCGTATGGTATGTGGCTTCT
>HPV41_Nu_9626041_nt150_E6_AOS_rc|1
TGGATAAGTTATCTGCTGCGCCAAACACAACTCCCGTATGGTATGTGGCTTCTGCGTCTC
>HPV1_Mu_9626063_nt416_E6_AOS_rc|1
AACTCTATGCACTCTTTCTCCGTTTGACACAACCTCCAATTTTTCAGCAACACTCAGTTT
>HPV1_Mu_9626063_nt409_E6_AOS_rc|1
TGCACTCTTTCTCCGTTTGACACAACCTCCAATTTTTCAGCAACACTCAGTTTTTTTATG
>HPV1_Mu_9626063_nt117_E6_AOS_rc|1
GCAATAAAACATCAATATATGGGATACAGAGGCTTTCGGAAAGCTGTCTGACGGTCCGGA
>HPV1_Mu_9626063_nt110_E6_AOS_rc|1
AACATCAATATATGGGATACAGAGGCTTTCGGAAAGCTGTCTGACGGTCCGGATTGGTGT
>HPV18_Alpha_9626069_nt142_E6_AOS_rc|1
CACAGGTTATTTCTATGTCTTGCAGTGAAGTGTTCAGTTCCGTGCACAGATCAGGTAGCT
>HPV4_Gamma_9626597_nt202_E6_AOS_rc|1
TTCCTCTAAATACTAAACTAAGTTTCTTAAGATAGAATAAAGCAAGATCCGCAAGATCGA
>HPV63_Mu_9626605_nt124_E6_AOS_rc|1
TGCAAGGAACAACCAAATCAATAAATGGGATACGGAGAGCAGAACTCAGATCCCGAACCG

>HPV63_Mu_9626605_nt214_E6_AOS_rc|1
CAAACACGAAATTATCTCGCCAGATAAGATGCAAGTCAAAATAATCAAACAGCAATTTTT
>HPV63_Mu_9626605_nt117_E6_AOS_rc|1
AACAACCAAATCAATAAATGGGATACGGAGAGCAGAACTCAGATCCCGAACCGAATGTAC
>HPV16_Alpha_9627100_nt484_E6_AOS_rc|1
CTACGTGTTCTTGATGATCTGCAACAAGACATACATCGACCGGTCCACCGACCCCTTATA
>HPV5_Beta_9627145_nt429_E6_AOS_rc|2
TTCCTAACACTGTCTGCTCATAAAATTGGTTAAATTCATAAGTTGCAGTGGCGCCACAGC
>HPV5_Beta_9627145_nt419_E6_AOS_rc|1
TGTCTGCTCATAAAATTGGTTAAATTCATAAGTTGCAGTGGCGCCACAGCATACGCGACA
>HPV5_Beta_9627145_nt251_E6_AOS_rc|1
ACAATCAATCACAGGGATGCCTAAGGCTTCAGCTAAGTCTCTAATACTTAAAGGTAATTC
>HPV5_Beta_9627145_nt244_E6_AOS_rc|1
ATCACAGGGATGCCTAAGGCTTCAGCTAAGTCTCTAATACTTAAAGGTAATTCTGCCTTA
>HPV5_Beta_9627145_nt237_E6_AOS_rc|1
GGATGCCTAAGGCTTCAGCTAAGTCTCTAATACTTAAAGGTAATTCTGCCTTATCTTTTT
>HPV10_Alpha_9627257_nt146_E6_AOS_rc|1
TGTTTTGTGCAAAATATACAGCACAGGCGAAGGTCCTCCAAAGGTATTCCACAATTTCTA
>HPV10_Alpha_9627257_nt192_E6_AOS_rc|1
ATATAATTCTCTAAGTGCAAATGCTGCCAATTCCGCTGCGGTCAGCTGTTTTGTGCAAAA
>HPV10_Alpha_9627257_nt185_E6_AOS_rc|1
TCTCTAAGTGCAAATGCTGCCAATTCCGCTGCGGTCAGCTGTTTTGTGCAAAATATACAG
>HPV10_Alpha_9627257_nt139_E6_AOS_rc|1
TGCAAAATATACAGCACAGGCGAAGGTCCTCCAAAGGTATTCCACAATTTCTACACAAAA
>HPV26_Alpha_9627305_nt240_E6_AOS_rc|1
ATTACACATCTTTTGCATGCAGCATACGGACTCCTATCTCTATATACTACTCTTAGGTCA
>HPV26_Alpha_9627305_nt255_E6_AOS_rc|1
ATTTTTGAATAAAATATTACACATCTTTTGCATGCAGCATACGGACTCCTATCTCTATAT
>HPV26_Alpha_9627305_nt248_E6_AOS_rc|1
AATAAAATATTACACATCTTTTGCATGCAGCATACGGACTCCTATCTCTATATACTACTC
>HPV26_Alpha_9627305_nt233_E6_AOS_rc|1
ATCTTTTGCATGCAGCATACGGACTCCTATCTCTATATACTACTCTTAGGTCACAAATTG
>HPV26_Alpha_9627305_nt226_E6_AOS_rc|1
GCATGCAGCATACGGACTCCTATCTCTATATACTACTCTTAGGTCACAAATTGCAAAATT
>HPV26_Alpha_9627305_nt103_E6_AOS_rc|1
AGTAGTATTCAAGCTTTCACATAGCTCATGTAGCGTTCTGGGTCGTTCTCTAGGATCCTC
>HPV32_Alpha_9627327_nt439_E6_AOS_rc|1
ACCTATTTAAAATGAATCTGAAATGCCGTCCGTTATAAATATGATGATCTTTCTCACTTG
>HPV32_Alpha_9627327_nt432_E6_AOS_rc|1
TAAAATGAATCTGAAATGCCGTCCGTTATAAATATGATGATCTTTCTCACTTGGCGATAA
>HPV32_Alpha_9627327_nt425_E6_AOS_rc|1
AATCTGAAATGCCGTCCGTTATAAATATGATGATCTTTCTCACTTGGCGATAAAGGCTTT
>HPV32_Alpha_9627327_nt169_E6_AOS_rc|1
CTTCAGCACTGGTTAAGTGGTTTTTACACCAAATACAGCAGATTTGTAAATTCCGCAGGG
>HPV32_Alpha_9627327_nt151_E6_AOS_rc|1
GGTTTTTACACCAAATACAGCAGATTTGTAAATTCCGCAGGGTCAGCCCAAAATCTTTGC
>HPV34_Alpha_9627334_nt141_E6_AOS_rc|1
ATACACACAGTCCAATTCTATTTCATGTATTGAAATGTTGACCTCTTCACATAAGGCTGG
>HPV49_Beta_9627363_nt411_E6_AOS_rc|1
TATTAGCCGCTGCTCGTCCTTCTATTTCGATGCCTACGACAATTTCTTGGTGATAATTAG
>HPV49_Beta_9627363_nt461_E6_AOS_rc|2
CAAATCTAGCCTCTTAAGGCAAATGAGACATCTGACTACTATCTCAGCAATATTAGCCGC
>HPV49_Beta_9627363_nt454_E6_AOS_rc|1
AGCCTCTTAAGGCAAATGAGACATCTGACTACTATCTCAGCAATATTAGCCGCTGCTCGT
>HPV49_Beta_9627363_nt447_E6_AOS_rc|1

TAAGGCAAATGAGACATCTGACTACTATCTCAGCAATATTAGCCGCTGCTCGTCCTTCTA
>HPV49_Beta_9627363_nt393_E6_AOS_rc|2
CTTCTATTTCGATGCCTACGACAATTTCTTGGTGATAATTAGTAAACTCGTGATATGCTG
>HPV49_Beta_9627363_nt386_E6_AOS_rc|1
TTCGATGCCTACGACAATTTCTTGGTGATAATTAGTAAACTCGTGATATGCTGATCTATA
>HPV49_Beta_9627363_nt379_E6_AOS_rc|1
CCTACGACAATTTCTTGGTGATAATTAGTAAACTCGTGATATGCTGATCTATAGGCACAA
>HPV49_Beta_9627363_nt372_E6_AOS_rc|1
CAATTTCTTGGTGATAATTAGTAAACTCGTGATATGCTGATCTATAGGCACAAGCTGCAC
>HPV49_Beta_9627363_nt365_E6_AOS_rc|1
TTGGTGATAATTAGTAAACTCGTGATATGCTGATCTATAGGCACAAGCTGCACAACAACC
>HPV49_Beta_9627363_nt358_E6_AOS_rc|1
TAATTAGTAAACTCGTGATATGCTGATCTATAGGCACAAGCTGCACAACAACCAAAGACA
>HPV53_Alpha_9627377_nt311_E6_AOS_rc|1
GCCCCGTACACTGAACAATTGTAATATCTTAATTTTCGGACCTTACTATAAAATAGCAAA
>HPV53_Alpha_9627377_nt342_E6_AOS_rc|1
TAACTTTTTTTAGTTAGTGCTTCCAGGCTAGCCCCGTACACTGAACAATTGTAATATCT
>HPV53_Alpha_9627377_nt335_E6_AOS_rc|1
TTTTTAGTTAGTGCTTCCAGGCTAGCCCCGTACACTGAACAATTGTAATATCTTAATTTT
>HPV53_Alpha_9627377_nt328_E6_AOS_rc|1
TTAGTGCTTCCAGGCTAGCCCCGTACACTGAACAATTGTAATATCTTAATTTTCGGACCT
>HPV53_Alpha_9627377_nt321_E6_AOS_rc|2
TTCCAGGCTAGCCCCGTACACTGAACAATTGTAATATCTTAATTTTCGGACCTTACTATA
>HPV7_Alpha_9627389_nt296_E6_AOS_rc|1
GCATATGCAGCGTATCTAAAGTTCCTATACTGATTCACTTTTCCATAAAATTCTAAACAC
>HPV7_Alpha_9627389_nt289_E6_AOS_rc|1
CAGCGTATCTAAAGTTCCTATACTGATTCACTTTTCCATAAAATTCTAAACACTTTACAC
>HPV7_Alpha_9627389_nt282_E6_AOS_rc|1
TCTAAAGTTCCTATACTGATTCACTTTTCCATAAAATTCTAAACACTTTACACACGCTGC
>HPV7_Alpha_9627389_nt106_E6_AOS_rc|1
TTATATTGCACTGGTCACATAATTCAAATAAAGTTCTAGCTGTGGAGCCGCAACGTGCAG
>HPV9_Beta_9627396_nt420_E6_AOS_rc|1
CTATATCTTTTCCAACTACAGCAAATTGAAAAAAATGTGTAAATTCTAACTGTGCAGACG
>HPV9_Beta_9627396_nt460_E6_AOS_rc|1
CTAATACAAATATTTCCAATAGCTGTTCCTTCTACAGTTTCTATATCTTTTCCAACTACA
>HPV9_Beta_9627396_nt453_E6_AOS_rc|1
AAATATTTCCAATAGCTGTTCCTTCTACAGTTTCTATATCTTTTCCAACTACAGCAAATT
>HPV9_Beta_9627396_nt446_E6_AOS_rc|1
TCCAATAGCTGTTCCTTCTACAGTTTCTATATCTTTTCCAACTACAGCAAATTGAAAAAA
>HPV9_Beta_9627396_nt439_E6_AOS_rc|1
GCTGTTCCTTCTACAGTTTCTATATCTTTTCCAACTACAGCAAATTGAAAAAAATGTGTA
>HPV9_Beta_9627396_nt432_E6_AOS_rc|1
CTTCTACAGTTTCTATATCTTTTCCAACTACAGCAAATTGAAAAAAATGTGTAAATTCTA
>HPV54_Alpha_9628437_nt314_E6_AOS_rc|1
TCCACTGTTTCCCACAGGCACGCACGGTCGCGATGCCTTCTATAATTTATTTGCCCGTGC
>HPV54_Alpha_9628437_nt307_E6_AOS_rc|1
TTTCCCACAGGCACGCACGGTCGCGATGCCTTCTATAATTTATTTGCCCGTGCAGTTCTA
>HPV54_Alpha_9628437_nt300_E6_AOS_rc|1
CAGGCACGCACGGTCGCGATGCCTTCTATAATTTATTTGCCCGTGCAGTTCTAGGCACAG
>HPV24_Unclassified_9628486_nt231_E6_AOS_rc|1
AAAAGTTACATGGTACAACACAATCGTCTAATGGTATATTTAATAATCTACTAAGTTCTA
>HPV24_Unclassified_9628486_nt395_E6_AOS_rc|1
TTCCCAACCTATCACACTCTCTACAAAATAATTTTCAAATTCAAATGCTGCTGTAGCAAC
>HPV24_Unclassified_9628486_nt385_E6_AOS_rc|1
ATCACACTCTCTACAAAATAATTTTCAAATTCAAATGCTGCTGTAGCAACACAACAATAT

>HPV24_Unclassified_9628486_nt378_E6_AOS_rc|1
TCTCTACAAAATAATTTTCAAATTCAAATGCTGCTGTAGCAACACAACAATATCTACAAC
>HPV24_Unclassified_9628486_nt224_E6_AOS_rc|1
ACATGGTACAACACAATCGTCTAATGGTATATTTAATAATCTACTAAGTTCTAACACTGA
>HPV24_Unclassified_9628486_nt217_E6_AOS_rc|1
ACAACACAATCGTCTAATGGTATATTTAATAATCTACTAAGTTCTAACACTGACTGAGGT
>HPV48_Gamma_9628542_nt214_E6_AOS_rc|1
CAAATGGTGTATTATCTCTCCAAATTACAGACAGTCGTTTGTTATGAAAAGAAGCAAGAT
>HPV50_Gamma_9628550_nt402_E6_AOS_rc|1
ACCACCGTGACAAATACAATCGTACTTTTCAATATAATCCAACAAACACATACAATTTTC
>HPV50_Gamma_9628550_nt426_E6_AOS_rc|1
ATTACCTCTAACTAAATGAAAATAACCACCGTGACAAATACAATCGTACTTTTCAATATA
>HPV50_Gamma_9628550_nt419_E6_AOS_rc|1
CTAACTAAATGAAAATAACCACCGTGACAAATACAATCGTACTTTTCAATATAATCCAAC
>HPV50_Gamma_9628550_nt412_E6_AOS_rc|1
AATGAAAATAACCACCGTGACAAATACAATCGTACTTTTCAATATAATCCAACAAACACA
>HPV50_Gamma_9628550_nt395_E6_AOS_rc|1
TGACAAATACAATCGTACTTTTCAATATAATCCAACAAACACATACAATTTTCACAGCGA
>HPV60_Gamma_9628566_nt383_E6_AOS_rc|1
TCTGCAAAAGATAACAATGTAAGGCAATCAAAACAGCGTACAGAAAGAGAACTCAAAGGA
>HPV60_Gamma_9628566_nt282_E6_AOS_rc|1
CTGAAAATAGTTTTCAGCTTCAAATTTAGCAGTAAGTCTAAGACACGGAGTACAACAAGC
>HPV61_Alpha_9628574_nt292_E6_AOS_rc|1
TGGGTCCGTAACAGGAATGGTCCCAATGTCGCAGCTCCCTCACTTTTACTTCACGTGCCA
>HPV61_Alpha_9628574_nt318_E6_AOS_rc|1
TGACCGTCCTGTTGTCTGTTCCACAGTGGGTCCGTAACAGGAATGGTCCCAATGTCGCAG
>HPV61_Alpha_9628574_nt285_E6_AOS_rc|1
GTAACAGGAATGGTCCCAATGTCGCAGCTCCCTCACTTTTACTTCACGTGCCAAGCATGG
>HPV61_Alpha_9628574_nt277_E6_AOS_rc|1
AATGGTCCCAATGTCGCAGCTCCCTCACTTTTACTTCACGTGCCAAGCATGGTGCGCATA
>HPV61_Alpha_9628574_nt106_E6_AOS_rc|1
CCTCAAAGTCCACCTCGTAGTCCTTGCAGAGCAGGAAAATATTAGTTGGATTGCACGGTC
>HPV82_Alpha_6970427_nt133_E6_VE|1
ACGCTGCACGAATTATGTGAAGCCTGCAATACGTCTATGCACAATATTCAGGTATTGTGT
>HPV106_Alpha_71726710_nt242_E6_VE|1
CTGAAAGCAGCTGGAAAACTTAGACAATTTAGATACTGGGACTACTCCAGCTATGCACCC
>HPV35_Alpha_333050_nt111_E6_VE|1
ATGTTTCAGGACCCAGCTGAACGACCTTACAAACTGCATGATTTGTGCAACGAGGTAGAA
>HPV92_Beta_27531786_nt107_E6_VE|1
ATGGCAAAACCTCCTTCGGTGCAGGAACTTAGAAGACAGTTAGATATTCCATTGGAGGAC
>HPV43_Alpha_40804474_nt223_E6_VE|1
ACGGAAGTATTATCGTTTGCATTTAGAGATTTAAAGGTTGTGTGGCGCGACGGATATCCG
>HPV40_Alpha_397014_nt343_E6_VE|1
TACGCAGCCTATGCACCAACCGTGGAAGAAGAGACAGGATTAACCATTTTACAAGTAAGG
>HPVcand87_Alpha_14475578_nt448_E6_VE|1
GAGCATATGATACACGCGAATCTGCACTTCCACAAAATAGCTGGGAAGTGGACAGGAAAG
>HPV70_Alpha_1173493_nt108_E6_VE|1
ATGGCGCGATTTCCCAATCCTGCAGAACGGCCATACAAATTGCCTGACCTGTGCACGGCG
>HPV90_Alpha_22138122_nt32_E6_VE|1
ATCTTCCTATTGTGCAAAGTCTATGAACTGGACCTGCAGGACCTGAACATAACCTGCGTG
>HPV38_Beta_1020234_nt561_E6_VE|1
CAAGCATTTCACAAGGTTAGAGGCAATTGGAAAGGAAGGTGCAGGCATTGCAAAGCAATA
>HPV91_Alpha_22023568_nt332_E6_VE|1
AAGGTTGTGTGGCGCGACAGTTATCCGTTTGCAGCGTGTTTGGCCTGCTTAAAATTTTAT
>HPV51_Alpha_333087_nt398_E6_VE|1

ATAAGGTGTCATAGATGTCAAAGACCACTTGGGCCTGAAGAAAAGCAAAAATTGGTGGAC
>HPV45_Alpha_397022_nt103_E6_VE|1
ATGGCGCGCTTTGACGATCCAAAGCAACGACCCTACAAGCTACCAGATTTGTGCACAGAA
>HPV40_Alpha_397014_nt403_E6_VE|1
ATTAGATGCTGCAAGTGCCACAAGCCTTTGTCTCCCGTGGAAAAAACCAACCATATTGTA
>HPV20_Beta_1020162_nt441_E6_VE|1
GTTTTTGCATGCTGTCGTGTTTGCTGCTCGGCAACAGCGACATATGAGTTTAATCAATTT
>HPV8_Beta_333074_nt602_E6_VE|1
AAAGTTAGAGGAGGCTGGAAAGGAGTTTGCAGGCTTTGTAAGCATTTGTATCATGATTGG
>HPV32_Alpha_9627327_nt163_E6_VE|1
GGGCTGACCCTGCGGAATTTACAAATCTGCTGTATTTGGTGTAAAAACCACTTAACCAGT
>HPV33_Alpha_333049_nt110_E6_VE|1
ATGTTTCAAGACACTGAGGAAAAACCACGAACATTGCATGATTTGTGCCAAGCATTGGAG
>HPV34_Alpha_9627334_nt433_E6_VE|1
CTGTGTCCATTGGAAAAGCAAAGGCATGTAGATGAAAACAAACGGTTTCACCAAATAGCG
>HPV82_Alpha_6970427_nt433_E6_VE|1
GGGCCTGAAGAAAAGCAAAAGGTGGTGGACGACAAAAAAAGGTTTCATGAAATAGCGGGA
>HPV47_Beta_333062_nt479_E6_VE|1
CAAACAGTGTTAGGTAGAGATATTGAGCTAGCTACAGGCCTTTCCATTTTTGAGATTGAC
>HPV94_Alpha_40804528_nt244_E6_VE|1
TTGGTGTGGAAAGCGGGAGTGCCATACGGTGCCTGTGCCCGGTGTTTACTTCTGCAGGGC
>HPV59_Alpha_557236_nt446_E6_VE|1
GCTGGAATATATACAGGACAGTGTCGTGGGTGTCGGACCCGAGCAAGACACCTAAGACAG
>HPV44_Alpha_1020242_nt106_E6_VE|1
ATGGAAAGTGCAAATGCCTCCACGTCTGCACAAAGTATAGACCAGTTGTGCAAGGAGTGC
>HPV93_Beta_37089393_nt466_E6_VE|1
AAGCTTGATATCTGTGGTAGATCTCTATTGTTTTACAAAGTGAGGAGGGGCTGGAAAGGA
>HPV20_Beta_1020162_nt201_E6_VE|1
ATGGCTACACCTCCTTCTTCAGAAGACAGCGCTGATGAAGGACCATCTAATATTGGAGAG
>HPV38_Beta_1020234_nt261_E6_VE|1
GAGGATCTGTTATTACCCTGTAGATTCTGCAACAGTTTCCTCACGTACATTGAATTACGT
>HPV27_Alpha_396964_nt400_E6_VE|1
ATACCTCAGCTGTTTATGAGATGCTATATCTGCCATAAGCCCCTGAGCTGGGAGGAGAAG
>HPV41_Nu_9626041_nt343_E6_VE|1
GTGCTTGCAAGGCTGGAGTTCACTGCACGGCACGAAGTGTCTTGTGCAGCCAGCCGTCTG
>HPV54_Alpha_9628437_nt433_E6_VE|1
CCTTTGTGCAACGTGGAAAAGCAACGGCACGTAGATTACAACAGGCGATTCCACTGTGTT
>HPV15_Beta_396924_nt201_E6_VE|1
ATGGATAGGCCAAAGCCTTTTTCTGTGCAGCAGCTTGCAGACACTCTGTGTATACCTTTA
>HPV96_Beta_50253426_nt210_E6_VE|1
ACGGTAAGTTTCGCAACTCTCTCGGTTGCCAAAAGTTTTATCTTGAGAGGTTGTTGGCAA
>HPV10_Alpha_9627257_nt403_E6_VE|1
AGATGCTACATGTGTCACAAACCGCTGGTAAGGGAAGAAAAAGACAGACATCGTAACGAA
>HPV5_Beta_9627145_nt381_E6_VE|1
CTTAGTCTAATTTGGAAAGATTATTGTGTGTTTGCGTGCTGTCGCGTATGCTGTGGCGCC
>HPV41_Nu_9626041_nt373_E6_VE|1
CACGAAGTGTCTTGTGCAGCCAGCCGTCTGCCGCACTTTATAGGACAGAGCCTCAGCGAC
>HPV7_Alpha_9627389_nt223_E6_VE|1
GAGGTGCTGGCCTTTGCATTTAGAGAGTTATATGTAGTGTGGCGCAACGACTTTCCTTTT
>HPV95_Gamma_40804520_nt112_E6_VE|1
ATGGCAAATGGCAGACCTACAACCTTGGACGAGTACTGCAGGAGATTTGACATTTCTTTT
>HPV72_Alpha_1491683_nt403_E6_VE|1
AGGTGCCATGCATGCTGCAAACCCCTAAGCTGTCAGGAAAAGGAATATCAGGTGCAGACA
>HPV67_Alpha_3228267_nt163_E6_VE|1
ACCACGGTGCATGAAATAAGTTTGCCCTGCGTGCAGTGCAAAAAAACTTTGGACAGAAAC

```
>HPV90_Alpha_22138122_nt302_E6_VE|1
AGATGCTACCTGTGCCATAAACCACTGTGCAGTGAGGAAAAGGAACGGCTTATCAGTGAA
>HPV28_Alpha_1020202_nt343_E6_VE|1
TATGTGGAAGGCGTGGAAGAGGAGACAAAACAATCTATAGATACACAGCAAGTGAGATGC
>HPV11_Alpha_333026_nt493_E6_VE|1
AACCAGTGGAAGGGTCGTTGCTTACACTGCTGGACAACATGCATGGAAGACTTGTTACCC
>HPV80_Beta_2911565_nt351_E6_VE|1
TGGACTGAAGAAGACTTTGTCTTTGCTTGTTGTAGCAGTTGTGCTTTTGCTACTGCACAA
>HPV68a_Alpha_71726685_nt32_E6_VE|1
CCATACAAATTGCCAGACCTGTGCAGGACATTGGACACTACATTGCATGACGTTACAATA
>HPV6_Alpha_6002612_nt494_E6_VE|1
TGTACGTGGAAGGGTCGCTGCCTACACTGCTGGACAACATGCATGGAAGACATGTTACCC
>HPV63_Mu_9626605_nt253_E6_VE|1
TGGCGAGATAATTTCGTGTTTGCTTGTTGTCAGTGCTGTGCTAGGCATGTTAGTCTGCTT
>HPV43_Alpha_40804474_nt133_E6_VE|1
CGGACTATATTTGAGTTGTGTGATGAGTGTAACATAACTTTGCCTACTCTGCAAATTGGG
>HPV34_Alpha_9627334_nt103_E6_VE|1
ATGTTTTTTCCCAATCCTGAGGAACGGCCATACAAGCTACCAGCCTTATGTGAAGAGGTC
>HPV69_Alpha_6970418_nt313_E6_VE|1
TCAAAAATAATAGAATATAGACGCTACACATCGTCTGTGTATGGTGCAACACTGGAAGCG
>HPV51_Alpha_333087_nt98_E6_VE|1
ATGTTCGAAGACAAGAGGGAAAGACCACGAACGCTGCATGAATTATGTGAAGCTTTGAAC
>HPV29_Alpha_1020210_nt373_E6_VE|1
ACAAACGAGTCCATATATACACAGCTAATTCGCTGCTACATGTGCCACAAGCCACTTGTC
>HPV102_Alpha_71726718_nt32_E6_VE|1
TTTGTGCTGTGCAAAGAATGTGAAGTGGATTTGGAGGACTTACGACTGCTTTGCATTTAT
>HPV23_Beta_1020186_nt261_E6_VE|1
CGACCACTGACGGTACAGCAACTTAGTGATAAGTTGACAGTACCAGTGGTAGATCTCTTG
>HPV84_Alpha_12958167_nt272_E6_VE|1
ACAGGATTACCACTTGCACAAATAAATATAAGGTGCCACGCGTGCTGCAAGCCATTGTGC
>HPV71_Alpha_12084981_nt263_E6_VE|1
AGGAGCTGAAAGTTGTGTGGAGAAGTGGTTTTCCGTTTGCTGCATGTGCCTGCTGTTTGG
>HPV91_Alpha_22023568_nt302_E6_VE|1
GAAGTGTTGTCGTTTGCATTTAGGGATTTAAAGGTTGTGTGGCGCGACAGTTATCCGTTT
>HPV37_Beta_1020226_nt561_E6_VE|1
CAACAATTTCACAAGGTTAGGCGCAATTGGAAAGGCTTGTGTAGACATTGTGGGTCGATA
>HPV84_Alpha_12958167_nt212_E6_VE|1
AAAGTACGTGAGCTACGCCACTGGGATTACTCCAGCTTTGGACCAACAGTGGAAGAAGAA
>HPV95_Gamma_40804520_nt172_E6_VE|1
TTTGATTTGCGTCTTCCTTGTATTTTTTGCTTTCATCCGGTTGATCTTGCAGAGCTTGCT
>HPV21_Beta_1020170_nt201_E6_VE|1
ATGGCTGACTCTTCAACAGACAGTGCTGACGAAGGTCCTTCTCCTAAGCGTAGACATTTA
>HPV4_Gamma_9626597_nt103_E6_VE|1
ATGGCAGATGGCAGACCTGCAACCTTGGACGACTTCTGCAGACGATTCGACATTTCCTTT
>HPV12_Beta_396910_nt381_E6_VE|1
CTAATTTGGAAAGGTCATTTTGTTACTGCTTGCTGTCGAAGTTGTTGCGCAGCTACTGCA
>HPV32_Alpha_9627327_nt313_E6_VE|1
TATTCTAAAGTGTGTGCACTGCGACACTACGACAGATCAGCATTTTGGCATACAGTAGAA
>HPV22_Beta_1020178_nt240_E6_VE|1
CTGACAGTACAGCAACTTAGTGATAAGTTGACTGTACCTGTAGTAGATCTTTTGCTACCT
>HPV20_Beta_1020162_nt231_E6_VE|1
GCTGATGAAGGACCATCTAATATTGGAGAGGCAAAACCTCCAATCTTAGAGCCACCATTG
>HPV63_Mu_9626605_nt283_E6_VE|1
CAGTGCTGTGCTAGGCATGTTAGTCTGCTTGAGTTTATGCTTTATTATCAGGAGTCTTTC
>HPVFA75/KI88-03_Unclassified_206902429_nt493_E6_VE|1
```

TTAGACAATTGTGCTAGGCATCAGCAGTTTTATAAGGTGAGAGGTAATTGGAAGGGATTG
>HPV81_Alpha_40804509_nt253_E6_VE|1
GCAATAAGAGAGCTAAACTTGGTGTGGCGAAAGAACTGGCCATTTGGAGTATGCGCAGCG
>HPV1_Mu_9626063_nt105_E6_VE|1
ATGGCGACACCAATCCGGACCGTCAGACAGCTTTCCGAAAGCCTCTGTATCCCATATATT
>HPV107_Beta_126131393_nt620_E6_VE|1
GAGAAGTTAGACAATTGTGCTAGGCATCAGCAGTTTTTTAAGGTGAGAGGTAATTGGAAG
>HPV22_Beta_1020178_nt390_E6_VE|1
GTGTTTGCATGTTGCAGCGGCTGTGCCTACGCTTCAGCCCAATTTGAATTTCAGCAGTAT
>HPV33_Alpha_333049_nt470_E6_VE|1
TTAAACAAACGATTTCATAATATTTCGGGTCGTTGGGCAGGGCGCTGTGCGGCGTGTTGG
>HPVFA75/KI88-03_Unclassified_206902429_nt163_E6_VE|1
ATGGAGAGGCCTAGAACAGTACAAGTTCTGAGTCGGCATTTAGAAATACCAATAGAAGAT
>HPV96_Beta_50253426_nt270_E6_VE|1
TTACAACCTGCCACTCATAAACAAGTAACCGCCTGCGTTTCTAGGACATTTTATATACAG
>HPV20_Beta_1020162_nt621_E6_VE|1
TGTGGCAGAAAGCGTCCATTTTATTTAGTGAGAGGCTCTTGGAAAGGAATCTGTAGGCTG
>HPV41_Nu_9626041_nt403_E6_VE|1
CCGCACTTTATAGGACAGAGCCTCAGCGACCTTGAGGTGAGGTGTGTGAGGTGCCTAGCT
>HPV18_Alpha_9626069_nt406_E6_VE|1
TTATTAATAAGGTGCCTGCGGTGCCAGAAACCGTTGAATCCAGCAGAAAAACTTAGACAC
>HPV93_Beta_37089393_nt136_E6_VE|1
GCAGTAAGTAAACCTCAGACCATTACAGAGCTTGCTAGGTGTCTTGGTATACCGTTAGAA
>HPV51_Alpha_333087_nt458_E6_VE|1
GAAAAAAAAAGGTTCCATGAAATAGCGGGACGTTGGACGGGGCAATGCGCTAATTGCTGG
>HPV65_Gamma_312100_nt103_E6_VE|1
ATGGCAGATGGCAGACCTGCAGCTTTGGACGACTTCTGCAGACGATTTGATATTTCTTTT
>HPV90_Alpha_22138122_nt362_E6_VE|1
GGCAAACGGTTCCATAAAATAGCTGGGCTTTGGCGAGGCGCTTGCCTGCAGTGTTGGAAA
>HPV53_Alpha_9627377_nt403_E6_VE|1
TCTGATTTATCAATAAGGTGCTACAGATGTCAACATCCGTTGACACCAGAGGAAAAACAG
>HPV67_Alpha_3228267_nt103_E6_VE|1
ATGTTTCAGGACACAGACGAAAAACCACGCAACCTGCACGAATTGTGTGAAGCTTTGGAA
>HPV54_Alpha_9628437_nt463_E6_VE|1
GTAGATTACAACAGGCGATTCCACTGTGTTAGAGGCTATTGGAAGGGTAGGTGTCTACAT
>HPV8_Beta_333074_nt392_E6_VE|1
TACGTTGTTACTGCGTGTTGTCGTTGTTGTTGTGTAGCAACCGCAACGTTTGAATTTAAT
>HPVcand89_Alpha_22095322_nt392_E6_VE|1
ACAGGAAAGTGTTGCAACTGCAGGGTAATATGCACGGCCGCACGCCGACCATCCAGGATA
>HPV80_Beta_2911565_nt201_E6_VE|1
ATGGATAGGCCAAAGCCTTCTTCTGTGCAGGAGCTTGCAGACACTCTTTGTATACCTTTA
>HPV5_Beta_9627145_nt411_E6_VE|1
TTTGCGTGCTGTCGCGTATGCTGTGGCGCCACTGCAACTTATGAATTTAACCAATTTTAT
>HPV93_Beta_37089393_nt376_E6_VE|1
GGCTGGGCAATAGAAGTTAAGGAAAAAAAGCCACTCTCTGAAATTATAGTGAGGTGTCGC
>HPV49_Beta_9627363_nt501_E6_VE|1
TGCCTTAAGAGGCTAGATTTGTTGGAAAAGCTTGATATTTGTGCACAGCACAGAGAGTTT
>HPV40_Alpha_397014_nt223_E6_VE|1
GAGGTACTGGCCTTTGCCTTTAGAGAGTTATATGTTGTGTGGCGCGACGACTTTCCACAC
>HPV21_Beta_1020170_nt441_E6_VE|1
GATCATTGTGTGTTTGCCTGTTGTCGTGTTTGTTGCGCAGCAACAGCGACATATGAATAT
>HPV30_Alpha_396973_nt163_E6_VE|1
GTACAAGAAACATCGTTGCTGGAGCTACAGCTACAGTGTGTATATTGCAAGAAGGAATTA
>HPV17_Beta_396932_nt351_E6_VE|1
TGGACTGAAGAAGATTTTGTGTTTGCCTGTTGCAGTAGTTGTGCGTATGCTACAGCACAG

>HPV43_Alpha_40804474_nt403_E6_VE|1
TGCATTAGATGCTGTAAGTGCCACAAGCCATTATCACCAGTGGAAAAAGTACAGCATATT
>HPV41_Nu_9626041_nt433_E6_VE|1
CTTGAGGTGAGGTGTGTGAGGTGCCTAGCTCTTCTACAATCTGTGGAAAAGGATTACATA
>HPV82_Alpha_6970427_nt313_E6_VE|1
TCTAGAATTAGAGAATACAGAAGGTATAGTAGGTCTGTGTATGGTGCTACATTAGAGGCC
>HPVRTRX7_Unclassified_30315616_nt325_E6_VE|1
TGCAACTTCTGTGGAAGGTTCTTAGATTTTTTGGAGGTTTGCGAGTTTGACAAAAAGCAG
>HPV111_Beta_186461202_nt157_E6_VE|1
ATGGATAGGCCAAAGCCTAGGACAGTAAAGGAACTAGCAGACACACTAGCAATTCCTGTA
>HPV14D_Beta_396918_nt447_E6_VE|1
GTCATTGTGTATTTGCTTGTTGCCGTGTATGCTGCACAGCAACAGCAACGTATGAGTTTA
>HPV91_Alpha_22023568_nt182_E6_VE|1
TCTGCACGTAGCTGCTCCCAAAACGCTAAGACCATATTTGAGTTGTGTGACCAGTGTAAC
>HPV52_Alpha_397038_nt103_E6_VE|1
ATGTTTGAGGATCCAGCAACACGACCCCGGACCCTGCACGAATTGTGTGAGGTGCTGGAA
>HPV56_Alpha_397053_nt103_E6_VE|1
ATGGAGCCACAATTCAACAATCCACAGGAACGTCCACGAAGCCTGCACCACTTGAGTGAG
>HPV23_Beta_1020186_nt501_E6_VE|1
GAAATTGAGCAGGAGGAGCAACGACCTATAGGCCAAATTTGTATTAGGTGTCAGTATTGT
>HPV110_Beta_186461194_nt173_E6_VE|1
ATGGCCAGGCCAAAGCCTTTGACAGTACAACAGCTTGCAGATACATTATGCATTCCTGTT
>HPV31_Alpha_333048_nt109_E6_VE|1
ATGTTCAAAAATCCTGCAGAAAGACCTCGGAAATTGCATGAACTAAGCTCGGCATTGGAA
>HPV25_Beta_396948_nt591_E6_VE|1
CTTGATATTTGTGGCAGAAGACGTCCTTTCCATCTAGTAAGAGGCTCTTGGAAAGGAATC
>HPV22_Beta_1020178_nt360_E6_VE|1
TTGCAATTAATTTGGACAGACGAGGACTTTGTGTTTGCATGTTGCAGCGGCTGTGCCTAC
>HPV66_Alpha_1020290_nt493_E6_VE|1
ATAGCATATGCATGGACCGGGTCATGTTTGCAGTGTTGGAGACATACGAGTAGACAAGCT
>HPV55_Alpha_1020266_nt103_E6_VE|1
ATGGAAAGTGCAAATGCCTCCACGGCTGCACAAAGTATAGACCAGTTGTGCAAGGAGTGC
>HPV42_Alpha_333211_nt259_E6_VE|1
AAAGATTTGGTAGTGGTGTGGAGGAAGGACTTTCCATATGCTGCATGTGCATTTTGTTTA
>HPV66_Alpha_1020290_nt403_E6_VE|1
GATTTATCAATAAGGTGCTACCGATGTCAATGTCCGTTAACACCGGAGGAAAAACAATTG
>HPV18_Alpha_9626069_nt466_E6_VE|1
CTTAATGAAAAACGACGATTTCACAACATAGCTGGGCACTATAGAGGCCAGTGCCATTCG
>HPV22_Beta_1020178_nt570_E6_VE|1
TGCTGTTCCAATCAACCATTTCACAAGGTTAGAGATCATTGGAAGGGAAGGTGCAGGCAC
>HPV102_Alpha_71726718_nt362_E6_VE|1
GATAGGTTTCATAAAATAGCAGGGGAGTGGACAGGAAAGTGCTGCAACTGTAGGGTATCA
>HPV82_Alpha_6970427_nt103_E6_VE|1
ATGTTTGAAGACATAAGAGAAAGACCACGAACGCTGCACGAATTATGTGAAGCCTGCAAT
>HPV47_Beta_333062_nt419_E6_VE|1
GCCTGCTGCCGTTTGTGCTGCTCAGCAACTGCCACATATGAATTTAATGTTTTTTATCAA
>HPV23_Beta_1020186_nt291_E6_VE|1
AAGTTGACAGTACCAGTGGTAGATCTCTTGCTACCTTGCAGATTTTGTTCTAGGTTTCTT
>HPV88_Gamma_167600365_nt198_E6_VE|1
CAGTTGGGTTCGTTCTATCAGAAACAGCTTAGTTTGGTCTGGAGGAGCGGTGCTTGTTTT
>HPVcand85_Alpha_4574720_nt496_E6_VE|1
GCAGGGAAATATAGAGGACAGTGTCGCCGGTGCATGACGCGTGCACAAGAACAGCAAGGC
>HPV19_Beta_396940_nt441_E6_VE|1
GCTTGCTGTCGCTGGTGTTGCACAGCAACTGCAACATTTGAATTTAATGAGTTCTATGAG
>HPV54_Alpha_9628437_nt403_E6_VE|1

ATATTTATAAGGTGCTGGCTGTGCCATAAACCTTTGTGCAACGTGGAAAAGCAACGGCAC
>HPV30_Alpha_396973_nt493_E6_VE|1
ATATCACGTACGTGGACCGGGTTATGTCTGCAATGCTGGAGACACACAACGTCCACTGAG
>HPV31_Alpha_333048_nt469_E6_VE|1
AAAAAGAAACGATTCCACAACATAGGAGGAAGGTGGACAGGACGTTGCATAGCATGTTGG
>HPVcand86_Alpha_15741127_nt182_E6_VE|1
GTGTGCCTAAAATGCATATGCAGAGAAGCCAAGGTACGTGAGCTACGACACTGGGAGCAC
>HPV35_Alpha_333050_nt471_E6_VE|1
GAAAAAAAACGATTCCATAACATCGGTGGACGGTGGACAGGTCGGTGTATGTCCTGTTGG
>HPV38_Beta_1020234_nt441_E6_VE|1
GTCTTTGGCCGTGAAATTGAACAGGTGGAGCAACAGACAATAGGCCTTATTGTTATAAGG
>HPV77_Alpha_2911558_nt253_E6_VE|1
GTGGTGTGGAGAGGTGGGGCGCCGTACGGTGCATGTGCACGGTGTTTACTGCTATATGGC
>HPV25_Beta_396948_nt231_E6_VE|1
GGACCACCAGAGCAAGCGCAGGTTATACAGCCACCATTGCCAGCAACAATTACTGATCTA
>HPV14D_Beta_396918_nt477_E6_VE|1
GCTGCACAGCAACAGCAACGTATGAGTTTAATGAATTTTATGAGAGTACTGTTGAAGGCA
>HPV26_Alpha_9627305_nt428_E6_VE|1
GGGCCAGAAGAAAAACAAAGAATTGTGGATGAAAAGCGACGATTTCACGAAATAGCAGGG
>HPV28_Alpha_1020202_nt253_E6_VE|1
TGGAGAACGGGAGCGCCCTATGGTGCCTGTGCCCGGTGTTTACTACTGCAGGGCATTGTG
>HPV33_Alpha_333049_nt140_E6_VE|1
ACATTGCATGATTTGTGCCAAGCATTGGAGACAACTATACACAACATTGAACTACAGTGC
>HPV42_Alpha_333211_nt139_E6_VE|1
CAGCCACGCACATTATACCAATTGTGTAAGGAATTTGGGCTGACATTGCGGAATTTACAG
>HPV96_Beta_50253426_nt360_E6_VE|1
GATTTAAAGACAGTGCAGCAACTTAGAAGTCATTTGGGCATTCCTACTGAAGACCTGTTG
>HPV96_Beta_50253426_nt150_E6_VE|1
AAGCAGTTTAAACCTGCCAAGAAAAGCCCGCCAAAACGTCTGCCAACTTTGGAACCGTTA
>HPV54_Alpha_9628437_nt133_E6_VE|1
CCGCGTACTCTAGCTGATTTGTGCAAGGTATGCAATATTCCTATGCATAGTTTGCAACTT
>HPV83_Alpha_5059324_nt32_E6_VE|1
TTTCTGCTGTGCAAAGACTGCGAGGTGGATTTGGAAGACTTACGACTGATTTGCATTTAT
>HPV58_Alpha_222386_nt141_E6_VE|1
ACATTGCATGATTTGTGTCAGGCGTTGGAGACATCTGTGCATGAAATCGAATTGAAATGC
>HPVcand89_Alpha_22095322_nt362_E6_VE|1
GAGCGATTTCATAAAATATCTGGAGAGTGGACAGGAAAGTGTTGCAACTGCAGGGTAATA
>HPV52_Alpha_397038_nt163_E6_VE|1
GAATCGGTGCATGAAATAAGGCTGCAGTGTGTGCAGTGCAAAAAAGAGCTACAACGAAGA
>HPV69_Alpha_6970418_nt403_E6_VE|1
ATAAGGTGTCATAGATGCCAAATACCATTGGGACCGGAAGAAAAACAGAGAATTGTGGAT
>HPV94_Alpha_40804528_nt304_E6_VE|1
ATTGCACGGCGCCTAAAATATTGGCAATATTCATATTATGTAGAAGGCGTGGAAGAGGAG
>HPV3_Alpha_397005_nt433_E6_VE|1
AAACCACTGGTAAAGGAAGAGAAGGACAGACACCGCAACGAAAAGCGAAGACTGCACAAA
>HPV61_Alpha_9628574_nt373_E6_VE|1
CGGTCATTAGCTGAACTATATATACGGTGCCATGCATGCAGCAAACCGTTAAGTATACAG
>HPV54_Alpha_9628437_nt253_E6_VE|1
GACCTATTTGTGGTGTGGAGACACGGCTTTCCACATGCTGCATGTGCACTGTGCCTAGAA
>HPV19_Beta_396940_nt291_E6_VE|1
ACAATTGCTGGATTAGCAGCATTGCTAGAAATACCGTTGGATGACTGTTTAGTGCCTTGT
>HPV77_Alpha_2911558_nt223_E6_VE|1
CTGGCAGCATTTACAATTAGAGAATTAAATGTGGTGTGGAGAGGTGGGGCGCCGTACGGT
>HPVcand62_Alpha_39932599_nt152_E6_VE|1
GTGGTGCGGCGAAAGGACTGGCCATTTGGAGTATGCGCAGCATGCCTATTGAGAGAGATT

>HPV95_Gamma_40804520_nt442_E6_VE|1
GAAAAACTTGACCTGTTATACAGTGGTGAGGTGTGCTATTTAATAAGGGGTTTGTGGAGG
>HPVcand87_Alpha_14475578_nt328_E6_VE|1
CTTCGTCACTGGGAGTACTCCAGCTACGGACCCACAGTAGAAGAAGAAACAGGTGTAGCA
>HPV81_Alpha_40804509_nt493_E6_VE|1
CATAAAATAGCTGGAGAGTGGATGGGAAAGTGCTGCCATTGTAGAGGAGCATGCATGGCC
>HPV88_Gamma_167600365_nt228_E6_VE|1
AGTTTGGTCTGGAGGAGCGGTGCTTGTTTTGCGTGTTGTGTCCCTTGTTCTAGGCTTTCT
>HPV3_Alpha_397005_nt283_E6_VE|1
GCGCCCTACGGTGCTTGTGCACGGTGTTTACTTGTAGAGGGCATTGCACGACGCCTAAAA
>HPV60_Gamma_9628566_nt103_E6_VE|1
ATGCAGATGGAAGAAGACAGGTTTCCAACAACAGTGGCTGATTATTGCTCTGAATTTGAT
>HPV42_Alpha_333211_nt499_E6_VE|1
ATATTGTGTCAGTGGACGGGTCGGTGTACGCATTGCAGAGGACAATGCGTGGAGAGACGC
>HPV16_Alpha_9627100_nt84_E6_VE|1
ATGCACCAAAAGAGAACTGCAATGTTTCAGGACCCACAGGAGCGACCCAGAAAGTTACCA
>HPV43_Alpha_40804474_nt493_E6_VE|1
AGCGTGTGGAAAGGATACTGCCTACATTGCTGGAAATCATGCATGGAAAAAAGCCGACGA
>HPV16_Alpha_9627100_nt474_E6_VE|1
AGATTCCATAATATAAGGGGTCGGTGGACCGGTCGATGTATGTCTTGTTGCAGATCATCA
>HPV16_Alpha_9627100_nt444_E6_VE|1
GAAAAGCAAAGACATCTGGACAAAAAGCAAAGATTCCATAATATAAGGGGTCGGTGGACC
>HPVcand86_Alpha_15741127_nt212_E6_VE|1
AAGGTACGTGAGCTACGACACTGGGAGCACTCCAGCTACGGATCCACAGTGGAACAAGAC
>HPV61_Alpha_9628574_nt253_E6_VE|1
TGGAGACATAACTGGCCATTTGGAGTATGCGCACCATGCTTGGCACGTGAAGTAAAAGTG
>HPV3_Alpha_397005_nt403_E6_VE|1
ACACAGCAAATTAGATGCTACATGTGTCACAAACCACTGGTAAAGGAAGAGAAGGACAGA
>HPV7_Alpha_9627389_nt403_E6_VE|1
ATAAGATGCTGCAAATGCCACAAACCATTGTCTCCTGTGGAAAAAAACCAACCACATTGTT
>HPV11_Alpha_333026_nt103_E6_VE|1
ATGGAAAGTAAAGATGCCTCCACGTCTGCAACATCTATAGACCAGTTGTGCAAGACGTTT
>HPVRTRX7_Unclassified_30315616_nt595_E6_VE|1
GGTCGAGGCCTTCCGTTCCACAAAGTGAGAGACGGCTGGAAAGGAGTGTGCAGGCACTGT
>HPV106_Alpha_71726710_nt392_E6_VE|1
CGGCTAATAACAGAAGGCAAACGGTTCCATAAAATAGCCGGGCACTGGCGAGGCGCTTGC
>HPV9_Beta_9627396_nt351_E6_VE|1
CACAAGTGTTTACAGCTTATTTGGACAGAGGAGGATTTGGTGTATGGACTCTGTAGTAGC
>HPV40_Alpha_397014_nt493_E6_VE|1
TCGTGGACAGGGTACTGTCTACATTGCTGGAAGAAATGCATGGAGAAAGGCCAACGCTCG
>HPV3_Alpha_397005_nt253_E6_VE|1
CGGGAACTGAATGTGGTGTGGAGAAGGGGAGCGCCCTACGGTGCTTGTGCACGGTGTTTA
>HPV12_Beta_396910_nt231_E6_VE|1
ACAGACAGTACGCCTGAGCTGCCCACAACTATTAAAGAGTTAGCTGACCTTTTAGATATA
>HPV90_Alpha_22138122_nt272_E6_VE|1
ACAGGAGTGCCTATTTTAGAATTGCTTATTAGATGCTACCTGTGCCATAAACCACTGTGC
>HPV63_Mu_9626605_nt133_E6_VE|1
GATCTGAGTTCTGCTCTCCGTATCCCATTTATTGATTTGGTTGTTCCTTGCAATTTTTGC
>HPV45_Alpha_397022_nt403_E6_VE|1
TTGTTAATAAGGTGCCTGCGGTGCCAGAAACCATTGAACCCAGCAGAAAAACGTAGACAC
>HPV61_Alpha_9628574_nt223_E6_VE|1
GCGTTTGCACTAAAGGAGCTAAGCATTGTGTGGAGACATAACTGGCCATTTGGAGTATGC
>HPV67_Alpha_3228267_nt133_E6_VE|1
AACCTGCACGAATTGTGTGAAGCTTTGGAAACCACGGTGCATGAAATAAGTTTGCCCTGC
>HPV24_Unclassified_9628486_nt561_E6_VE|1

GAATTATTTCATAAAGTGAGGAGGGGCTGGAAAGGACTCTGTAGGCAGTGTAAGCAGATA
>HPV36_Beta_1020218_nt411_E6_VE|1
TTTGCCTGCTGTCGTGTTTGTTGTGGTGCAACAGCCACATATGAGTTTAATCAGTTTTAT
>HPVcand85_Alpha_4574720_nt136_E6_VE|1
CCCTACAAACTACCAGACCTATGCAACACACTGGACACATCACTGCAAGATATAGAAATA
>HPV58_Alpha_222386_nt111_E6_VE|1
ATGTTCCAGGACGCAGAGGAGAAACCACGGACATTGCATGATTTGTGTCAGGCGTTGGAG
>HPV16_Alpha_9627100_nt114_E6_VE|1
GACCCACAGGAGCGACCCAGAAAGTTACCACAGTTATGCACAGAGCTGCAAACAACTATA
>HPVcand85_Alpha_4574720_nt196_E6_VE|1
AGCTGTGTATATTGTAAAAGCGTCCTGCAGCGAACAGAGGTATATGAATTTGCCTTTGCT
>HPV69_Alpha_6970418_nt433_E6_VE|1
GGACCGGAAGAAAAACAGAGAATTGTGGATGAAAAGCGACGGTTCCATGAAATAGCAGGG
>HPV42_Alpha_333211_nt169_E6_VE|1
GAATTTGGGCTGACATTGCGGAATTTACAGATTTCCTGCATTTGGTGCAAAAAGCACTTA
>HPV69_Alpha_6970418_nt493_E6_VE|1
TACTGGAAAGGGTTGTGCACAAACTGCTGGAGACCAAGGCGCGAAGCAACAGAAACACAA
>HPV71_Alpha_12084981_nt173_E6_VE|1
ACCAGTACGACGTGGACCTGCAGGACCTGAACCTAACCTGCATATTCTGCAGAACAATTA
>HPV41_Nu_9626041_nt493_E6_VE|1
TTGCGGGAAGACTTGTCTGTGCATAGAATTGGCGGGATCTGGAGGGGAACTTGTGTTCGA
>HPV72_Alpha_1491683_nt343_E6_VE|1
TCGGGCTACGGACCCACTGTGGAACAGGAAACAGGCAAATCATTAGCAGAACTATATATA
>HPV56_Alpha_397053_nt133_E6_VE|1
CGTCCACGAAGCCTGCACCACTTGAGTGAGGTATTAGAAATACCTTTAATTGATCTTAGA
>HPV18_Alpha_9626069_nt136_E6_VE|1
CCCTACAAGCTACCTGATCTGTGCACGGAACTGAACACTTCACTGCAAGACATAGAAATA
>HPV48_Gamma_9628542_nt463_E6_VE|1
GATTTTCTGTTAATTCGCGGGACTTGGAGAGGTGTTTGTAGAAATTGTATCAGCCATGAG
>HPV39_Alpha_333245_nt198_E6_VE|1
GCCTGTGTCTATTGCAGACGACCACTACAGCAAACCGAGGTATATGAATTTGCATTTAGT
>HPV68a_Alpha_71726685_nt122_E6_VE|1
CGGACAGAGGTATATGAATTTGCCTTTAGTGACCTATGTGTAGTGTATAGAGACGGGGTA
>HPV65_Gamma_312100_nt463_E6_VE|1
GCCTGCTACTTAATAAGGGGTTTGTGGAGGGGCTATTGCAGAAATTGCATTAGGAAACAA
>HPVRTRX7_Unclassified_30315616_nt565_E6_VE|1
TTGAGCACCATTGAAAAATTAGATAGCTGTGGTCGAGGCCTTCCGTTCCACAAAGTGAGA
>HPV36_Beta_1020218_nt591_E6_VE|1
GGAAGAGGACTTCCCTTTCATAGAGTCAGAAACGCCTGGAAGGGAATCTGTAGGCAGTGT
>HPV19_Beta_396940_nt261_E6_VE|1
CCACAGGTCACAGAGCCACCATTACCAGCAACAATTGCTGGATTAGCAGCATTGCTAGAA
>HPV96_Beta_50253426_nt180_E6_VE|1
CCAAAACGTCTGCCAACTTTGGAACCGTTAACGGTAAGTTTCGCAACTCTCTCGGTTGCC
>HPV52_Alpha_397038_nt133_E6_VE|1
ACCCTGCACGAATTGTGTGAGGTGCTGGAAGAATCGGTGCATGAAATAAGGCTGCAGTGT
>HPV59_Alpha_557236_nt56_E6_VE|1
ATGGCACGCTTTGAGGATCCTACACAACGACCATACAAACTGCCTGATTTGAGCACAACA
>HPV53_Alpha_9627377_nt433_E6_VE|1
CAACATCCGTTGACACCAGAGGAAAAACAGTTACACTGTGACTATAAGAAACGGTTTCAC
>HPV83_Alpha_5059324_nt92_E6_VE|1
TGCACAAATGAATTAACAACAGCAGAAGTGCTGTCCTTTGCATGGAAGGAGCTGTGTATT
>HPV77_Alpha_2911558_nt373_E6_VE|1
ACAAAAGAATCCATAGATACCCAGCAAATCAGATGCTACATGTGTCACAAACCACTCACC
>HPV15_Beta_396924_nt561_E6_VE|1
GAGCAATTCCACAAGGTTAGACGCAATTGGAAAGGATTGTGTAGACATTGTAGGGCGATA

>HPV43_Alpha_40804474_nt373_E6_VE|1
GAAGAAACTAAGCAAACAGTGTTTGATTTGTGCATTAGATGCTGTAAGTGCCACAAGCCA
>HPV61_Alpha_9628574_nt343_E6_VE|1
TACGGACCCACTGTGGAACAGACAACAGGACGGTCATTAGCTGAACTATATATACGGTGC
>HPVcand86_Alpha_15741127_nt272_E6_VE|1
ACAGGCGTATCATTGGCACACTTAAATATAAGGTGCCATGCGTGCTTTAAACCATTGTGC
>HPV6_Alpha_6002612_nt104_E6_VE|1
ATGGAAAGTGCAAATGCCTCCACGTCTGCAACGACCATAGACCAGTTGTGCAAGACGTTT
>HPV47_Beta_333062_nt509_E6_VE|1
GCTACAGGCCTTTCCATTTTTGAGATTGACATAAGGTGTCATACCTGCCTGTCATTTCTT
>HPV34_Alpha_9627334_nt463_E6_VE|1
GATGAAAACAAACGGTTTCACCAAATAGCGGATCAGTGGACCGGACGCTGTACACAGTGC
>HPV76_Beta_2911551_nt201_E6_VE|1
ATGGCTAGACCTGCTAAGGTATGTGAGCTTAGCCAACTCTTAAATATACCCATTTCTTCT
>HPV1_Mu_9626063_nt255_E6_VE|1
AGAGACAATTTGGTGTTTGGATGCTGTCAAGGGTGTGCTAGAACTGTTAGCCTATTGGAG
>HPV106_Alpha_71726710_nt272_E6_VE|1
AGATACTGGGACTACTCCAGCTATGCACCCACAGTGGAGTATGAAACAGGGATACCAATA
>HPV5_Beta_9627145_nt591_E6_VE|1
GGCAGAGGCCTTCCCTTTCATAAGGTGAGGAACGCCTGGAAGGGAATCTGTAGGCAGTGT
>HPV16_Alpha_9627100_nt204_E6_VE|1
AAGCAACAGTTACTGCGACGTGAGGTATATGACTTTGCTTTTCGGGATTTATGCATAGTA
>HPV18_Alpha_9626069_nt106_E6_VE|1
ATGGCGCGCTTTGAGGATCCAACACGGCGACCCTACAAGCTACCTGATCTGTGCACGGAA
>HPV93_Beta_37089393_nt106_E6_VE|1
ATGGTATATAAGGCAGACATCTTAATGGCGGCAGTAAGTAAACCTCAGACCATTACAGAG
>HPV88_Gamma_167600365_nt438_E6_VE|1
AGGGGTGAGTGTTTCCATCTTGTGAGAAACACTTGGAGGGGAACGTGCAGAAATTGTTGT
>HPV26_Alpha_9627305_nt458_E6_VE|1
GAAAAGCGACGATTTCACGAAATAGCAGGGCAGTGGAAAGGGTTGTGTACAAATTGTTGG
>HPV38_Beta_1020234_nt231_E6_VE|1
CAGCTCAGTGATAAGTTAACAGTTCCTGTAGAGGATCTGTTATTACCCTGTAGATTCTGC
>HPV21_Beta_1020170_nt291_E6_VE|1
CCACCATTACCAGCTACAATTCGTGACCTAGCCAATCTGTTAGAGATACCATTGGATGAT
>HPV65_Gamma_312100_nt433_E6_VE|1
GAGAAATTAGACTTGCTGTACTCTGACCAGGCCTGCTACTTAATAAGGGGTTTGTGGAGG
>HPV10_Alpha_9627257_nt253_E6_VE|1
TTGGTGTGGAGAGCGGGAGTGCCATACGGTGCCTGTGCACGGTGTTTACTCCTACAGGGC
>HPV72_Alpha_1491683_nt373_E6_VE|1
ACAGGCAAATCATTAGCAGAACTATATATAAGGTGCCATGCATGCTGCAAACCCCTAAGC
>HPV52_Alpha_397038_nt463_E6_VE|1
GCAAACAAGCGATTTCATAATATTATGGGTCGTTGGACAGGGCGCTGTTCAGAGTGTTGG
>HPV73_Alpha_1491692_nt103_E6_VE|1
ATGCTGTTTCCCAATTCAGAAGAACGACCATACAAGCTACAAGCGTTATGTGACGAAGTG
>HPV39_Alpha_333245_nt468_E6_VE|1
CTAAATAGCAAACGAAGATTTCATAAAATAGCAGGAAGCTATACAGGACAGTGTCGACGG
>HPV25_Beta_396948_nt411_E6_VE|1
GAATATCTTGTGTATGCCTGCTGTCGCTGTTGTTGCACAGCAACTGCCACATTTGAATTT
>HPV68a_Alpha_71726685_nt92_E6_VE|1
GACTGTGTCTATTGCAGAAGGCAACTACAACGGACAGAGGTATATGAATTTGCCTTTAGT
>HPV32_Alpha_9627327_nt343_E6_VE|1
GACAGATCAGCATTTTGGCATACAGTAGAACAAGAAACAGGACTACTGTTGGAAGAACAA
>HPV12_Beta_396910_nt531_E6_VE|1
AGGTGTCAGACGTGCTTGTCATTTTTAGATACAATTGAAAAGTTAGACAGCTGTGGTCGG
>HPV14D_Beta_396918_nt207_E6_VE|1

CTGACTCTTCAACAGACAGTGCAGATGAAGGTCCTTCTCCTAAGAGTAACTATTGTGATA
>HPVcand87_Alpha_14475578_nt208_E6_VE|1
GAATTAACAGAAAGCGAATTGCTGGCGTTTGCATTAAGGGAATTATTGCTGGTGTGGAGA
>HPV88_Gamma_167600365_nt258_E6_VE|1
GCGTGTTGTGTCCCTTGTTCTAGGCTTTCTGCTAGGTATGAGGCTGAACGCTTTTACCGC
>HPV68a_Alpha_71726685_nt302_E6_VE|1
TTATTGATAAGGTGCATGAGTTGCCTGAAACCATTGTGTCCAGCAGAAAAACTAAGGCAC
>HPV110_Beta_186461194_nt323_E6_VE|1
TGGACTGAGGAAGATTTTGTTTTTGCATGCTGTAGTAGTTGTGCTCATGCTACAGCGCAA
>HPV102_Alpha_71726718_nt212_E6_VE|1
GTGCGGGAACTACGGCACTGGAACTACTCCAGCTACGGCGCAACGGTTGAAGAAGAAACC
>HPV12_Beta_396910_nt591_E6_VE|1
GGCCTTCCGTTCCACAAGGTTAGAGACAGGTGGAAGGGAATTTGCAGACAGTGCAAGCAT
>HPV82_Alpha_6970427_nt403_E6_VE|1
ATAAGGTGTCATAGATGTCAGAGACCACTTGGGCCTGAAGAAAAGCAAAAGGTGGTGGAC
>HPV29_Alpha_1020210_nt463_E6_VE|1
AAGCGAAGACTACACAAAATTTCTGGATACTGGAGAGGGAGTTGCCTGTATTGTTGGTCA
>HPV8_Beta_333074_nt272_E6_VE|1
GCGGCTTTAGGTATTCCATTGCAGGACTGTTCAGTACCGTGCAACTTTTGTGGTAACTTT
>HPV7_Alpha_9627389_nt493_E6_VE|1
TCGTGGACAGGGTACTGTCTGCACTGTTGGAAGAAATGCATGGAGAAAGGCCAACGCTCG
>HPV37_Beta_1020226_nt531_E6_VE|1
TTAGAGAAGTTGGAGACTTGCTATACTCAGCAACAATTTCACAAGGTTAGGCGCAATTGG
>HPV74_Alpha_27462483_nt2_E6_VE|1
ATGGAAAGTGCAAATGCCTCCACGTCTGCAAAAAGTATAGACCAGTTGTGCAAGGACTGC
>HPV92_Beta_27531786_nt437_E6_VE|1
AAATTGGCAGTTTGTGCATCAGGCATTCCTTTTGATAGAGTTAGAGGAGCTTGGAAGGCA
>HPV34_Alpha_9627334_nt403_E6_VE|1
TTAATAAGGTGCGGAAAATGCCAAAAACCACTGTGTCCATTGGAAAAGCAAAGGCATGTA
>HPV56_Alpha_397053_nt433_E6_VE|1
AGTCCGTTAACTCCGGAGGAAAAGCAATTGCATTGTGACAGAAAAAGACGATTTCATCTA
>HPV39_Alpha_333245_nt138_E6_VE|1
CCATACAAATTGCCAGACCTGTGCACAACGCTGGACACCACCTTGCAGGACATTACAATA
>HPV49_Beta_9627363_nt201_E6_VE|1
ATGGCTAGACCTGTTAAGGTATGTGAGCTAGCCCACCACTTAAATATACCTATTTGGGAA
>HPV25_Beta_396948_nt291_E6_VE|1
GCAGCTTTATTGGAAATTCCATTAGATGATTGCTTAGTACCTTGCAACTTCTGTGGCAAC
>HPVcand85_Alpha_4574720_nt166_E6_VE|1
CTGGACACATCACTGCAAGATATAGAAATAAGCTGTGTATATTGTAAAAGCGTCCTGCAG
>HPV22_Beta_1020178_nt450_E6_VE|1
TATCAAGTTACTTTGTATGGTCGTGAAATTGAGCAAGAAGAACAACGACCTGTAGGCCAA
>HPVcand87_Alpha_14475578_nt478_E6_VE|1
CACAAAATAGCTGGGAAGTGGACAGGAAAGTGCAGCAACTGTAGGGCAATATGCATGGCC
>HPV58_Alpha_222386_nt471_E6_VE|1
TTAAACAAAAGGTTTCATAATATTTCGGGTCGTTGGACAGGGCGCTGTGCAGTGTGTTGG
>HPV65_Gamma_312100_nt253_E6_VE|1
TTCAGGGGGGGTTGCTACTATGCATGCTGTTCTGAATGCTTACGTTTAAGTGCAAGATTT
>HPV42_Alpha_333211_nt109_E6_VE|1
GGCAGAATGTCAGGTACATCTGCCTCATCACAGCCACGCACATTATACCAATTGTGTAAG
>HPV63_Mu_9626605_nt103_E6_VE|1
ATGGACCTGACATCTGTACATTCGGTTCGGGATCTGAGTTCTGCTCTCCGTATCCCATTT
>HPV56_Alpha_397053_nt463_E6_VE|1
CATTGTGACAGAAAAAGACGATTTCATCTAATAGCACATGGTTGGACCGGGTCATGTTTG
>HPV36_Beta_1020218_nt381_E6_VE|1
CTTAGTTTAATTTGGAAGGATTACTGTGTATTTGCCTGCTGTCGTGTTTGTTGTGGTGCA

>HPV71_Alpha_12084981_nt503_E6_VE|1
TAGCAGGCCATTGGCGCGGTGCTTGCCTACAGTGTTGGAAACCATGCGAGGCCAACAATG
>HPV80_Beta_2911565_nt561_E6_VE|1
CAGCAATTCCATAAGGTAAGGCGCAATTGGAAAGGATTGTGTAGACATTGTGGGTCGATA
>HPV9_Beta_9627396_nt381_E6_VE|1
GAGGATTTGGTGTATGGACTCTGTAGTAGCTGTGCTTATGCGTCTGCACAGTTAGAATTT
>HPV81_Alpha_40804509_nt283_E6_VE|1
AAGAACTGGCCATTTGGAGTATGCGCAGCGTGCTTGTGCAGAGAAGCTAAAGTACGGGAG
>HPV81_Alpha_40804509_nt163_E6_VE|1
TGTAAGGAGTGTGAAGTGGACCTAGACGACCTGCAACTGACCTGCATATTTTGCAAAAAG
>HPV8_Beta_333074_nt542_E6_VE|1
TTGTCATTTTTGGATATCATAGAGAAATTAGATTGCTGTGGGAGAGGCCGTCCCTTTCAT
>HPV76_Beta_2911551_nt531_E6_VE|1
CTTGATCATTGTGCACAGCAAAGAGAGTTTCACAAAGTTAGAAATAGGTGGAAAGGGGTG
>HPV59_Alpha_557236_nt326_E6_VE|1
GAGGCTGAAACCAAGACACCGTTACATGAGCTGCTGATACGCTGTTATAGATGCCTAAAA
>HPV39_Alpha_333245_nt108_E6_VE|1
ATGGCGCGATTTCACAATCCTGCAGAACGGCCATACAAATTGCCAGACCTGTGCACAACG
>HPV53_Alpha_9627377_nt193_E6_VE|1
CAACTTGGCTGTGTGTTCTGCAAGAAGGCATTGACAGCGTCAGAGGTATATAATTTTGCA
>HPV59_Alpha_557236_nt296_E6_VE|1
TATAGAGATTCCGTGTATGGAGAAACATTAGAGGCTGAAACCAAGACACCGTTACATGAG
>HPV84_Alpha_12958167_nt92_E6_VE|1
TTTTGCAAGGAAGAATTAACGGAAGGCGAAGTGCTGGCCTTTGCAGTAAAGGAATTACTA
>HPV97_Alpha_71726694_nt362_E6_VE|1
CTTAAAGACAAACGACGATTCCACCACATATCTGGTTATTATAGAGGCCAGTGCAATTCG
>HPV102_Alpha_71726718_nt272_E6_VE|1
CGCACAGCATTACCACAGCTGTATATGAGATGCTATATGTGCCTGAAGCCGTTGTGTAGT
>HPV10_Alpha_9627257_nt373_E6_VE|1
ACCAAACAATCTATATATACACAGCTGATCAGATGCTACATGTGTCACAAACCGCTGGTA
>HPV26_Alpha_9627305_nt488_E6_VE|1
CAGTGGAAAGGGTTGTGTACAAATTGTTGGAGACCAAGGCGCCAAACAGAAACACAAGTG
>HPV97_Alpha_71726694_nt32_E6_VE|1
CCCTACAAGTTGCCAGACCTGTGCACAGAATTGAATACTTCATTACCTGAAATAGAGATA
>HPV107_Beta_126131393_nt320_E6_VE|1
CAGCTTACACAGCATCTTAATATACCAGTGGAAGACTTACTGGTACCTTGCAAATTTTGC
>HPV26_Alpha_9627305_nt98_E6_VE|1
ATGTTCGAGGATCCTAGAGAACGACCCAGAACGCTACATGAGCTATGTGAAAGCTTGAAT
>HPV97_Alpha_71726694_nt392_E6_VE|1
TCTGGTTATTATAGAGGCCAGTGCAATTCGTGTTACAACCAATCACGGCAAGAAAGGCTT
>HPV67_Alpha_3228267_nt463_E6_VE|1
CGCAAAAAGCGATTTCATAATATTTCCAATCGTTGGACAGGACGGTGTTCAGTGTGTTGG
>HPV23_Beta_1020186_nt471_E6_VE|1
CAATTTTATCAGCTAACTGTGTATGGTCGTGAAATTGAGCAGGAGGAGCAACGACCTATA
>HPV5_Beta_9627145_nt561_E6_VE|1
CTTGACATTATAGAAAAGTTAGATTGCTGTGGCAGAGGCCTTCCCTTTCATAAGGTGAGG
>HPV92_Beta_27531786_nt257_E6_VE|1
GAGAATTATGCTTATGCTTGCTGTGGTGCTTGTGCTAAACAAGTAGCAGCAATAGAAACA
>HPV83_Alpha_5059324_nt212_E6_VE|1
GTGCGAGAGCTACGGCACTGGAGCCACTCAAGCTACGGGGCAACAGTAGAAGAAGAAACA
>HPV12_Beta_396910_nt561_E6_VE|1
ACAATTGAAAAGTTAGACAGCTGTGGTCGGGGCCTTCCGTTCCACAAGGTTAGAGACAGG
>HPVcand86_Alpha_15741127_nt362_E6_VE|1
AATCTGCATTTTCATAAAATAGCAGGCCAGTGGACGGGAAAGTGCAGCAACTGTAGAGCA
>HPV15_Beta_396924_nt351_E6_VE|1

TGGACTGAGGAAGATTTTGTTTTTGCCTGTTGTTCTAGTTGTGCATTTGCTACAGCGCAG
>HPV32_Alpha_9627327_nt133_E6_VE|1
AGTACATTATACCAATTGTGCAAAGATTTTGGGCTGACCCTGCGGAATTTACAAATCTGC
>HPV30_Alpha_396973_nt433_E6_VE|1
CAGCCGTTGACACCAGAGGAAAAACAGTTACACTGTGAATATAAGAAACGGTTTCACAGA
>HPV77_Alpha_2911558_nt133_E6_VE|1
ATATTCCTGTTGTGCAGAGACAGTGGAATACCGTTCGACGACCTTCGCCTACTGTGTGTG
>HPV73_Alpha_1491692_nt463_E6_VE|1
GATGAAAAAAAACGGTTTCATCAAATAGCAGAACAGTGGACCGGACGCTGTACACGGTGC
>HPV94_Alpha_40804528_nt424_E6_VE|1
AAGGAAGAAAAGGACAGACATCGTAACGAACGGCGACGACTGCACAAAATATCAGGGTAC
>HPV45_Alpha_397022_nt433_E6_VE|1
CCATTGAACCCAGCAGAAAAACGTAGACACCTTAAGGACAAACGAAGATTTCACAGCATA
>HPV97_Alpha_71726694_nt2_E6_VE|1
ATGGCGCGATTTGAAGATCCAAGCAAACGGCCCTACAAGTTGCCAGACCTGTGCACAGAA
>HPVcand89_Alpha_22095322_nt272_E6_VE|1
AACACACCGTTGGCCCAGCTGTATATGAGATGCTATATATGCTGTAAGCCGTTAGGCAGC
>HPV8_Beta_333074_nt572_E6_VE|1
GATTGCTGTGGGAGAGGCCGTCCCTTTCATAAAGTTAGAGGAGGCTGGAAAGGAGTTTGC
>HPV61_Alpha_9628574_nt463_E6_VE|1
CACTTCCACTATATAGCTGGACAGTGGACGGGAAGGTGTTGCCAGTGTAGAGGGCCATGC
>HPV71_Alpha_12084981_nt473_E6_VE|1
TAATTACAGAAGGCAGGCGATTTCATAAAATAGCAGGCCATTGGCGCGGTGCTTGCCTAC
>HPV2_Alpha_9626032_nt390_E6_VE|1
ATACCTCAGCTGTTTATGAGATGCTATATTTGCCATAAGCCCCTGAGCTGGGAGGAGAAG
>HPV107_Beta_126131393_nt650_E6_VE|1
CAGTTTTTTAAGGTGAGAGGTAATTGGAAGGGATTGTGTAGACATTGTGAGGCGATAGAA
>HPV72_Alpha_1491683_nt463_E6_VE|1
GGAATCCACTTCCACAAGATAAGCGGACTGTGGACGGGAAGGTGCTGCCAGTGTAGAGGG
>HPV69_Alpha_6970418_nt463_E6_VE|1
GAAAAGCGACGGTTCCATGAAATAGCAGGGTACTGGAAAGGGTTGTGCACAAACTGCTGG
>HPV17_Beta_396932_nt561_E6_VE|1
CAGCAGTTTCATAAGGTTAGACGCAATTGGAAAGGCTTGTGCAGACATTGTGGGTCGATA
>HPV38_Beta_1020234_nt351_E6_VE|1
TGGACTCAAGAGGATTTTGTTTTTGCATGTTGTAGCAGTTGTGCTTATGCTTCTGCTCAA
>HPV59_Alpha_557236_nt476_E6_VE|1
TGTCGGACCCGAGCAAGACACCTAAGACAGCAACGACAAGCGCGTAGTGAAACACTGGTG
>HPV68a_Alpha_71726685_nt2_E6_VE|1
ATGGCGCTATTTCACAACCCTGAGGAACGGCCATACAAATTGCCAGACCTGTGCAGGACA
>HPVcand87_Alpha_14475578_nt388_E6_VE|1
TTGGCACTTCAAAATATAAGGTGCCATGCATGCTGCAAACCATTGTGCTGGCCCGAGAAG
>HPV72_Alpha_1491683_nt253_E6_VE|1
ATTGTGTGGCGGGACAACTGGCCATTTGGAGTCTGCGCACCATGCCTTGCAAGAGCAACT
>HPV84_Alpha_12958167_nt2_E6_VE|1
ATGCCCAACGGACGCTACCACCCCACCAATATTTTTGTGCTGTGCCAGGAATACGAGGTG
>HPVcand62_Alpha_39932599_nt362_E6_VE|1
GGAGTGCACTTCCACAAAATAGCAGGAGTGTGGACAGGACGGTACCGCAATTGTAGAGGA
>HPV82_Alpha_6970427_nt133_E6_VE_rc|1
ACACAATACCTGAATATTGTGCATAGACGTATTGCAGGCTTCACATAATTCGTGCAGCGT
>HPV106_Alpha_71726710_nt242_E6_VE_rc|1
GGGTGCATAGCTGGAGTAGTCCCAGTATCTAAATTGTCTAAGTTTTCCAGCTGCTTTCAG
>HPV35_Alpha_333050_nt111_E6_VE_rc|1
TTCTACCTCGTTGCACAAATCATGCAGTTTGTAAGGTCGTTCAGCTGGGTCCTGAAACAT
>HPV92_Beta_27531786_nt107_E6_VE_rc|1
GTCCTCCAATGGAATATCTAACTGTCTTCTAAGTTCCTGCACCGAAGGAGGTTTTGCCAT

>HPV43_Alpha_40804474_nt223_E6_VE_rc|1
CGGATATCCGTCGCGCCACACAACCTTTAAATCTCTAAATGCAAACGATAATACTTCCGT
>HPV40_Alpha_397014_nt343_E6_VE_rc|1
CCTTACTTGTAAAATGGTTAATCCTGTCTCTTCTTCCACGGTTGGTGCATAGGCTGCGTA
>HPVcand87_Alpha_14475578_nt448_E6_VE_rc|1
CTTTCCTGTCCACTTCCCAGCTATTTGTGGAAGTGCAGATTCGCGTGTATCATATGCTC
>HPV70_Alpha_1173493_nt108_E6_VE_rc|1
CGCCGTGCACAGGTCAGGCAATTTGTATGGCCGTTCTGCAGGATTGGGAAATCGCGCCAT
>HPV90_Alpha_22138122_nt32_E6_VE_rc|1
CACGCAGGTTATGTTCAGGTCCTGCAGGTCCAGTTCATAGACTTTGCACAATAGGAAGAT
>HPV38_Beta_1020234_nt561_E6_VE_rc|1
TATTGCTTTGCAATGCCTGCACCTTCCTTTCCAATTGCCTCTAACCTTGTGAAATGCTTG
>HPV91_Alpha_22023568_nt332_E6_VE_rc|1
ATAAAATTTTAAGCAGGCCAAACACGCTGCAAACGGATAACTGTCGCGCCACACAACCTT
>HPV51_Alpha_333087_nt398_E6_VE_rc|1
GTCCACCAATTTTTGCTTTTCTTCAGGCCCAAGTGGTCTTTGACATCTATGACACCTTAT
>HPV45_Alpha_397022_nt103_E6_VE_rc|1
TTCTGTGCACAAATCTGGTAGCTTGTAGGGTCGTTGCTTTGGATCGTCAAAGCGCGCCAT
>HPV40_Alpha_397014_nt403_E6_VE_rc|1
TACAATATGGTTGGTTTTTCCACGGGAGACAAAGGCTTGTGGCACTTGCAGCATCTAAT
>HPV20_Beta_1020162_nt441_E6_VE_rc|1
AAATTGATTAAACTCATATGTCGCTGTTGCCGAGCAGCAAACACGACAGCATGCAAAAAC
>HPV8_Beta_333074_nt602_E6_VE_rc|1
CCAATCATGATACAAATGCTTACAAAGCCTGCAAACTCCTTTCCAGCCTCCTCTAACTTT
>HPV32_Alpha_9627327_nt163_E6_VE_rc|1
ACTGGTTAAGTGGTTTTTACACCAAATACAGCAGATTTGTAAATTCCGCAGGGTCAGCCC
>HPV33_Alpha_333049_nt110_E6_VE_rc|1
CTCCAATGCTTGGCACAAATCATGCAATGTTCGTGGTTTTTCCTCAGTGTCTTGAAACAT
>HPV34_Alpha_9627334_nt433_E6_VE_rc|1
CGCTATTTGGTGAAACCGTTTGTTTTCATCTACATGCCTTTGCTTTTCCAATGGACACAG
>HPV82_Alpha_6970427_nt433_E6_VE_rc|1
TCCCGCTATTTCATGAAACCTTTTTTTGTCGTCCACCACCTTTTGCTTTTCTTCAGGCCC
>HPV47_Beta_333062_nt479_E6_VE_rc|1
GTCAATCTCAAAAATGGAAAGGCCTGTAGCTAGCTCAATATCTCTACCTAACACTGTTTG
>HPV94_Alpha_40804528_nt244_E6_VE_rc|1
GCCCTGCAGAAGTAAACACCGGGCACAGGCACCGTATGGCACTCCCGCTTTCCACACCAA
>HPV59_Alpha_557236_nt446_E6_VE_rc|1
CTGTCTTAGGTGTCTTGCTCGGGTCCGACACCCACGACACTGTCCTGTATATATTCCAGC
>HPV44_Alpha_1020242_nt106_E6_VE_rc|1
GCACTCCTTGCACAACTGGTCTATACTTTGTGCAGACGTGGAGGCATTTGCACTTTCCAT
>HPV93_Beta_37089393_nt466_E6_VE_rc|1
TCCTTTCCAGCCCCTCCTCACTTTGTAAAACAATAGAGATCTACCACAGATATCAAGCTT
>HPV20_Beta_1020162_nt201_E6_VE_rc|1
CTCTCCAATATTAGATGGTCCTTCATCAGCGCTGTCTTCTGAAGAAGGAGGTGTAGCCAT
>HPV38_Beta_1020234_nt261_E6_VE_rc|1
ACGTAATTCAATGTACGTGAGGAAACTGTTGCAGAATCTACAGGGTAATAACAGATCCTC
>HPV27_Alpha_396964_nt400_E6_VE_rc|1
CTTCTCCTCCCAGCTCAGGGGCTTATGGCAGATATAGCATCTCATAAACAGCTGAGGTAT
>HPV41_Nu_9626041_nt343_E6_VE_rc|1
CAGACGGCTGGCTGCACAAGACACTTCGTGCCGTGCAGTGAACTCCAGCCTTGCAAGCAC
>HPV54_Alpha_9628437_nt433_E6_VE_rc|1
AACACAGTGGAATCGCCTGTTGTAATCTACGTGCCGTTGCTTTTCCACGTTGCACAAAGG
>HPV15_Beta_396924_nt201_E6_VE_rc|1
TAAAGGTATACACAGAGTGTCTGCAAGCTGCTGCACAGAAAAAGGCTTTGGCCTATCCAT
>HPV96_Beta_50253426_nt210_E6_VE_rc|1

TTGCCAACAACCTCTCAAGATAAAACTTTTGGCAACCGAGAGAGTTGCGAAACTTACCGT
>HPV10_Alpha_9627257_nt403_E6_VE_rc|1
TTCGTTACGATGTCTGTCTTTTTCTTCCCTTACCAGCGGTTTGTGACACATGTAGCATCT
>HPV5_Beta_9627145_nt381_E6_VE_rc|1
GGCGCCACAGCATACGCGACAGCACGCAAACACACAATAATCTTTCCAAATTAGACTAAG
>HPV41_Nu_9626041_nt373_E6_VE_rc|1
GTCGCTGAGGCTCTGTCCTATAAAGTGCGGCAGACGGCTGGCTGCACAAGACACTTCGTG
>HPV7_Alpha_9627389_nt223_E6_VE_rc|1
AAAAGGAAAGTCGTTGCGCCACACTACATATAACTCTCTAAATGCAAAGGCCAGCACCTC
>HPV95_Gamma_40804520_nt112_E6_VE_rc|1
AAAAGAAATGTCAAATCTCCTGCAGTACTCGTCCAAGGTTGTAGGTCTGCCATTTGCCAT
>HPV72_Alpha_1491683_nt403_E6_VE_rc|1
TGTCTGCACCTGATATTCCTTTTCCTGACAGCTTAGGGGTTTGCAGCATGCATGGCACCT
>HPV67_Alpha_3228267_nt163_E6_VE_rc|1
GTTTCTGTCCAAAGTTTTTTTGCACTGCACGCAGGGCAAACTTATTTCATGCACCGTGGT
>HPV90_Alpha_22138122_nt302_E6_VE_rc|1
TTCACTGATAAGCCGTTCCTTTTCCTCACTGCACAGTGGTTTATGGCACAGGTAGCATCT
>HPV28_Alpha_1020202_nt343_E6_VE_rc|1
GCATCTCACTTGCTGTGTATCTATAGATTGTTTTGTCTCCTCTTCCACGCCTTCCACATA
>HPV11_Alpha_333026_nt493_E6_VE_rc|1
GGGTAACAAGTCTTCCATGCATGTTGTCCAGCAGTGTAAGCAACGACCCTTCCACTGGTT
>HPV80_Beta_2911565_nt351_E6_VE_rc|1
TTGTGCAGTAGCAAAAGCACAACTGCTACAACAAGCAAAGACAAAGTCTTCTTCAGTCCA
>HPV68a_Alpha_71726685_nt32_E6_VE_rc|1
TATTGTAACGTCATGCAATGTAGTGTCCAATGTCCTGCACAGGTCTGGCAATTTGTATGG
>HPV6_Alpha_6002612_nt494_E6_VE_rc|1
GGGTAACATGTCTTCCATGCATGTTGTCCAGCAGTGTAGGCAGCGACCCTTCCACGTACA
>HPV63_Mu_9626605_nt253_E6_VE_rc|1
AAGCAGACTAACATGCCTAGCACAGCACTGACAACAAGCAAACACGAAATTATCTCGCCA
>HPV43_Alpha_40804474_nt133_E6_VE_rc|1
CCCAATTTGCAGAGTAGGCAAAGTTATGTTACACTCATCACACAACTCAAATATAGTCCG
>HPV34_Alpha_9627334_nt103_E6_VE_rc|1
GACCTCTTCACATAAGGCTGGTAGCTTGTATGGCCGTTCCTCAGGATTGGGAAAAAACAT
>HPV69_Alpha_6970418_nt313_E6_VE_rc|1
CGCTTCCAGTGTTGCACCATACACAGACGATGTGTAGCGTCTATATTCTATTATTTTTGA
>HPV51_Alpha_333087_nt98_E6_VE_rc|1
GTTCAAAGCTTCACATAATTCATGCAGCGTTCGTGGTCTTTCCCTCTTGTCTTCGAACAT
>HPV29_Alpha_1020210_nt373_E6_VE_rc|1
GACAAGTGGCTTGTGGCACATGTAGCAGCGAATTAGCTGTGTATATATGGACTCGTTTGT
>HPV102_Alpha_71726718_nt32_E6_VE_rc|1
ATAAATGCAAAGCAGTCGTAAGTCCTCCAAATCCACTTCACATTCTTTGCACAGCACAAA
>HPV23_Beta_1020186_nt261_E6_VE_rc|1
CAAGAGATCTACCACTGGTACTGTCAACTTATCACTAAGTTGCTGTACCGTCAGTGGTCG
>HPV84_Alpha_12958167_nt272_E6_VE_rc|1
GCACAATGGCTTGCAGCACGCGTGGCACCTTATATTTATTTGTGCAAGTGGTAATCCTGT
>HPV71_Alpha_12084981_nt263_E6_VE_rc|1
CCAAACAGCAGGCACATGCAGCAAACGGAAAACCACTTCTCCACACAACTTTCAGCTCCT
>HPV91_Alpha_22023568_nt302_E6_VE_rc|1
AAACGGATAACTGTCGCGCCACACAACCTTTAAATCCCTAAATGCAAACGACAACACTTC
>HPV37_Beta_1020226_nt561_E6_VE_rc|1
TATCGACCCACAATGTCTACACAAGCCTTTCCAATTGCGCCTAACCTTGTGAAATTGTTG
>HPV84_Alpha_12958167_nt212_E6_VE_rc|1
TTCTTCTTCCACTGTTGGTCCAAAGCTGGAGTAATCCCAGTGGCGTAGCTCACGTACTTT
>HPV95_Gamma_40804520_nt172_E6_VE_rc|1
AGCAAGCTCTGCAAGATCAACCGGATGAAAGCAAAAAATACAAGGAAGACGCAAATCAAA

>HPV21_Beta_1020170_nt201_E6_VE_rc|1
TAAATGTCTACGCTTAGGAGAAGGACCTTCGTCAGCACTGTCTGTTGAAGAGTCAGCCAT
>HPV4_Gamma_9626597_nt103_E6_VE_rc|1
AAAGGAAATGTCGAATCGTCTGCAGAAGTCGTCCAAGGTTGCAGGTCTGCCATCTGCCAT
>HPV12_Beta_396910_nt381_E6_VE_rc|1
TGCAGTAGCTGCGCAACAACTTCGACAGCAAGCAGTAACAAAATGACCTTTCCAAATTAG
>HPV32_Alpha_9627327_nt313_E6_VE_rc|1
TTCTACTGTATGCCAAAATGCTGATCTGTCGTAGTGTCGCAGTGCACACACTTTAGAATA
>HPV22_Beta_1020178_nt240_E6_VE_rc|1
AGGTAGCAAAAGATCTACTACAGGTACAGTCAACTTATCACTAAGTTGCTGTACTGTCAG
>HPV20_Beta_1020162_nt231_E6_VE_rc|1
CAATGGTGGCTCTAAGATTGGAGGTTTTGCCTCTCCAATATTAGATGGTCCTTCATCAGC
>HPV63_Mu_9626605_nt283_E6_VE_rc|1
GAAAGACTCCTGATAATAAAGCATAAACTCAAGCAGACTAACATGCCTAGCACAGCACTG
>HPVFA75/KI88-03_Unclassified_206902429_nt493_E6_VE_rc|1
CAATCCCTTCCAATTACCTCTCACCTTATAAAACTGCTGATGCCTAGCACAATTGTCTAA
>HPV81_Alpha_40804509_nt253_E6_VE_rc|1
CGCTGCGCATACTCCAAATGGCCAGTTCTTTCGCCACACCAAGTTTAGCTCTCTTATTGC
>HPV1_Mu_9626063_nt105_E6_VE_rc|1
AATATATGGGATACAGAGGCTTTCGGAAAGCTGTCTGACGGTCCGGATTGGTGTCGCCAT
>HPV107_Beta_126131393_nt620_E6_VE_rc|1
CTTCCAATTACCTCTCACCTTAAAAAACTGCTGATGCCTAGCACAATTGTCTAACTTCTC
>HPV22_Beta_1020178_nt390_E6_VE_rc|1
ATACTGCTGAAATTCAAATTGGGCTGAAGCGTAGGCACAGCCGCTGCAACATGCAAACAC
>HPV33_Alpha_333049_nt470_E6_VE_rc|1
CCAACACGCCGCACAGCGCCCTGCCCAACGACCCGAAATATTATGAAATCGTTTGTTTAA
>HPVFA75/KI88-03_Unclassified_206902429_nt163_E6_VE_rc|1
ATCTTCTATTGGTATTTCTAAATGCCGACTCAGAACTTGTACTGTTCTAGGCCTCTCCAT
>HPV96_Beta_50253426_nt270_E6_VE_rc|1
CTGTATATAAAATGTCCTAGAAACGCAGGCGGTTACTTGTTTATGAGTGGCAGGTTGTAA
>HPV20_Beta_1020162_nt621_E6_VE_rc|1
CAGCCTACAGATTCCTTTCCAAGAGCCTCTCACTAAATAAAATGGACGCTTTCTGCCACA
>HPV41_Nu_9626041_nt403_E6_VE_rc|1
AGCTAGGCACCTCACACACCTCACCTCAAGGTCGCTGAGGCTCTGTCCTATAAAGTGCGG
>HPV18_Alpha_9626069_nt406_E6_VE_rc|1
GTGTCTAAGTTTTTCTGCTGGATTCAACGGTTTCTGGCACCGCAGGCACCTTATTAATAA
>HPV93_Beta_37089393_nt136_E6_VE_rc|1
TTCTAACGGTATACCAAGACACCTAGCAAGCTCTGTAATGGTCTGAGGTTTACTTACTGC
>HPV51_Alpha_333087_nt458_E6_VE_rc|1
CCAGCAATTAGCGCATTGCCCCGTCCAACGTCCCGCTATTTCATGGAACCTTTTTTTTTC
>HPV65_Gamma_312100_nt103_E6_VE_rc|1
AAAAGAAATATCAAATCGTCTGCAGAAGTCGTCCAAAGCTGCAGGTCTGCCATCTGCCAT
>HPV90_Alpha_22138122_nt362_E6_VE_rc|1
TTTCCAACACTGCAGGCAAGCGCCTCGCCAAAGCCCAGCTATTTTATGGAACCGTTTGCC
>HPV53_Alpha_9627377_nt403_E6_VE_rc|1
CTGTTTTTCCTCTGGTGTCAACGGATGTTGACATCTGTAGCACCTTATTGATAAATCAGA
>HPV67_Alpha_3228267_nt103_E6_VE_rc|1
TTCCAAAGCTTCACACAATTCGTGCAGGTTGCGTGGTTTTTCGTCTGTGTCCTGAAACAT
>HPV54_Alpha_9628437_nt463_E6_VE_rc|1
ATGTAGACACCTACCCTTCCAATAGCCTCTAACACAGTGGAATCGCCTGTTGTAATCTAC
>HPV8_Beta_333074_nt392_E6_VE_rc|1
ATTAAATTCAAACGTTGCGGTTGCTACACAACAACAACGACAACACGCAGTAACAACGTA
>HPVcand89_Alpha_22095322_nt392_E6_VE_rc|1
TATCCTGGATGGTCGGCGTGCGGCCGTGCATATTACCCTGCAGTTGCAACACTTTCCTGT
>HPV80_Beta_2911565_nt201_E6_VE_rc|1

TAAAGGTATACAAAGAGTGTCTGCAAGCTCCTGCACAGAAGAAGGCTTTGGCCTATCCAT
>HPV5_Beta_9627145_nt411_E6_VE_rc|1
ATAAAATTGGTTAAATTCATAAGTTGCAGTGGCGCCACAGCATACGCGACAGCACGCAAA
>HPV93_Beta_37089393_nt376_E6_VE_rc|1
GCGACACCTCACTATAATTTCAGAGAGTGGCTTTTTTTCCTTAACTTCTATTGCCCAGCC
>HPV49_Beta_9627363_nt501_E6_VE_rc|1
AAACTCTCTGTGCTGTGCACAAATATCAAGCTTTTCCAACAAATCTAGCCTCTTAAGGCA
>HPV40_Alpha_397014_nt223_E6_VE_rc|1
GTGTGGAAAGTCGTCGCGCCACACAACATATAACTCTCTAAAGGCAAAGGCCAGTACCTC
>HPV21_Beta_1020170_nt441_E6_VE_rc|1
ATATTCATATGTCGCTGTTGCTGCGCAACAAACACGACAACAGGCAAACACACAATGATC
>HPV30_Alpha_396973_nt163_E6_VE_rc|1
TAATTCCTTCTTGCAATATACACACTGTAGCTGTAGCTCCAGCAACGATGTTTCTTGTAC
>HPV17_Beta_396932_nt351_E6_VE_rc|1
CTGTGCTGTAGCATACGCACAACTACTGCAACAGGCAAACACAAAATCTTCTTCAGTCCA
>HPV43_Alpha_40804474_nt403_E6_VE_rc|1
AATATGCTGTACTTTTTCCACTGGTGATAATGGCTTGTGGCACTTACAGCATCTAATGCA
>HPV41_Nu_9626041_nt433_E6_VE_rc|1
TATGTAATCCTTTTCCACAGATTGTAGAAGAGCTAGGCACCTCACACACCTCACCTCAAG
>HPV82_Alpha_6970427_nt313_E6_VE_rc|1
GGCCTCTAATGTAGCACCATACACAGACCTACTATACCTTCTGTATTCTCTAATTCTAGA
>HPVRTRX7_Unclassified_30315616_nt325_E6_VE_rc|1
CTGCTTTTTGTCAAACTCGCAAACCTCCAAAAAATCTAAGAACCTTCCACAGAAGTTGCA
>HPV111_Beta_186461202_nt157_E6_VE_rc|1
TACAGGAATTGCTAGTGTGTCTGCTAGTTCCTTTACTGTCCTAGGCTTTGGCCTATCCAT
>HPV14D_Beta_396918_nt447_E6_VE_rc|1
TAAACTCATACGTTGCTGTTGCTGTGCAGCATACACGGCAACAAGCAAATACACAATGAC
>HPV91_Alpha_22023568_nt182_E6_VE_rc|1
GTTACACTGGTCACACAACTCAAATATGGTCTTAGCGTTTTGGGAGCAGCTACGTGCAGA
>HPV52_Alpha_397038_nt103_E6_VE_rc|1
TTCCAGCACCTCACACAATTCGTGCAGGGTCCGGGGTCGTGTTGCTGGATCCTCAAACAT
>HPV56_Alpha_397053_nt103_E6_VE_rc|1
CTCACTCAAGTGGTGCAGGCTTCGTGGACGTTCCTGTGGATTGTTGAATTGTGGCTCCAT
>HPV23_Beta_1020186_nt501_E6_VE_rc|1
ACAATACTGACACCTAATACAAATTTGGCCTATAGGTCGTTGCTCCTCCTGCTCAATTTC
>HPV110_Beta_186461194_nt173_E6_VE_rc|1
AACAGGAATGCATAATGTATCTGCAAGCTGTTGTACTGTCAAAGGCTTTGGCCTGGCCAT
>HPV31_Alpha_333048_nt109_E6_VE_rc|1
TTCCAATGCCGAGCTTAGTTCATGCAATTTCCGAGGTCTTTCTGCAGGATTTTTGAACAT
>HPV25_Beta_396948_nt591_E6_VE_rc|1
GATTCCTTTCCAAGAGCCTCTTACTAGATGGAAAGGACGTCTTCTGCCACAAATATCAAG
>HPV22_Beta_1020178_nt360_E6_VE_rc|1
GTAGGCACAGCCGCTGCAACATGCAAACACAAAGTCCTCGTCTGTCCAAATTAATTGCAA
>HPV66_Alpha_1020290_nt493_E6_VE_rc|1
AGCTTGTCTACTCGTATGTCTCCAACACTGCAAACATGACCCGGTCCATGCATATGCTAT
>HPV55_Alpha_1020266_nt103_E6_VE_rc|1
GCACTCCTTGCACAACTGGTCTATACTTTGTGCAGCCGTGGAGGCATTTGCACTTTCCAT
>HPV42_Alpha_333211_nt259_E6_VE_rc|1
TAAACAAAATGCACATGCAGCATATGGAAAGTCCTTCCTCCACACCACTACCAAATCTTT
>HPV66_Alpha_1020290_nt403_E6_VE_rc|1
CAATTGTTTTTCCTCCGGTGTTAACGGACATTGACATCGGTAGCACCTTATTGATAAATC
>HPV18_Alpha_9626069_nt466_E6_VE_rc|1
CGAATGGCACTGGCCTCTATAGTGCCCAGCTATGTTGTGAAATCGTCGTTTTTCATTAAG
>HPV22_Beta_1020178_nt570_E6_VE_rc|1
GTGCCTGCACCTTCCCTTCCAATGATCTCTAACCTTGTGAAATGGTTGATTGGAACAGCA

>HPV102_Alpha_71726718_nt362_E6_VE_rc|1
TGATACCCTACAGTTGCAGCACTTTCCTGTCCACTCCCCTGCTATTTTATGAAACCTATC
>HPV82_Alpha_6970427_nt103_E6_VE_rc|1
ATTGCAGGCTTCACATAATTCGTGCAGCGTTCGTGGTCTTTCTCTTATGTCTTCAAACAT
>HPV47_Beta_333062_nt419_E6_VE_rc|1
TTGATAAAAAACATTAAATTCATATGTGGCAGTTGCTGAGCAGCACAAACGGCAGCAGGC
>HPV23_Beta_1020186_nt291_E6_VE_rc|1
AAGAAACCTAGAACAAAATCTGCAAGGTAGCAAGAGATCTACCACTGGTACTGTCAACTT
>HPV88_Gamma_167600365_nt198_E6_VE_rc|1
AAAACAAGCACCGCTCCTCCAGACCAAACTAAGCTGTTTCTGATAGAACGAACCCAACTG
>HPVcand85_Alpha_4574720_nt496_E6_VE_rc|1
GCCTTGCTGTTCTTGTGCACGCGTCATGCACCGGCGACACTGTCCTCTATATTTCCCTGC
>HPV19_Beta_396940_nt441_E6_VE_rc|1
CTCATAGAACTCATTAAATTCAAATGTTGCAGTTGCTGTGCAACACCAGCGACAGCAAGC
>HPV54_Alpha_9628437_nt403_E6_VE_rc|1
GTGCCGTTGCTTTTCCACGTTGCACAAAGGTTTATGGCACAGCCAGCACCTTATAAATAT
>HPV30_Alpha_396973_nt493_E6_VE_rc|1
CTCAGTGGACGTTGTGTGTCTCCAGCATTGCAGACATAACCCGGTCCACGTACGTGATAT
>HPV31_Alpha_333048_nt469_E6_VE_rc|1
CCAACATGCTATGCAACGTCCTGTCCACCTTCCTCCTATGTTGTGGAATCGTTTCTTTTT
>HPVcand86_Alpha_15741127_nt182_E6_VE_rc|1
GTGCTCCCAGTGTCGTAGCTCACGTACCTTGGCTTCTCTGCATATGCATTTTAGGCACAC
>HPV35_Alpha_333050_nt471_E6_VE_rc|1
CCAACAGGACATACACCGACCTGTCCACCGTCCACCGATGTTATGGAATCGTTTTTTTTC
>HPV38_Beta_1020234_nt441_E6_VE_rc|1
CCTTATAACAATAAGGCCTATTGTCTGTTGCTCCACCTGTTCAATTTCACGGCCAAAGAC
>HPV77_Alpha_2911558_nt253_E6_VE_rc|1
GCCATATAGCAGTAAACACCGTGCACATGCACCGTACGGCGCCCCACCTCTCCACACCAC
>HPV25_Beta_396948_nt231_E6_VE_rc|1
TAGATCAGTAATTGTTGCTGGCAATGGTGGCTGTATAACCTGCGCTTGCTCTGGTGGTCC
>HPV14D_Beta_396918_nt477_E6_VE_rc|1
TGCCTTCAACAGTACTCTCATAAAATTCATTAAACTCATACGTTGCTGTTGCTGTGCAGC
>HPV26_Alpha_9627305_nt428_E6_VE_rc|1
CCCTGCTATTTCGTGAAATCGTCGCTTTTCATCCACAATTCTTTGTTTTTCTTCTGGCCC
>HPV28_Alpha_1020202_nt253_E6_VE_rc|1
CACAATGCCCTGCAGTAGTAAACACCGGGCACAGGCACCATAGGGCGCTCCCGTTCTCCA
>HPV33_Alpha_333049_nt140_E6_VE_rc|1
GCACTGTAGTTCAATGTTGTGTATAGTTGTCTCCAATGCTTGGCACAAATCATGCAATGT
>HPV42_Alpha_333211_nt139_E6_VE_rc|1
CTGTAAATTCCGCAATGTCAGCCCAAATTCCTTACACAATTGGTATAATGTGCGTGGCTG
>HPV96_Beta_50253426_nt360_E6_VE_rc|1
CAACAGGTCTTCAGTAGGAATGCCCAAATGACTTCTAAGTTGCTGCACTGTCTTTAAATC
>HPV96_Beta_50253426_nt150_E6_VE_rc|1
TAACGGTTCCAAAGTTGGCAGACGTTTTGGCGGGCTTTTCTTGGCAGGTTTAAACTGCTT
>HPV54_Alpha_9628437_nt133_E6_VE_rc|1
AAGTTGCAAACTATGCATAGGAATATTGCATACCTTGCACAAATCAGCTAGAGTACGCGG
>HPV83_Alpha_5059324_nt32_E6_VE_rc|1
ATAAATGCAAATCAGTCGTAAGTCTTCCAAATCCACCTCGCAGTCTTTGCACAGCAGAAA
>HPV58_Alpha_222386_nt141_E6_VE_rc|1
GCATTTCAATTCGATTTCATGCACAGATGTCTCCAACGCCTGACACAAATCATGCAATGT
>HPVcand89_Alpha_22095322_nt362_E6_VE_rc|1
TATTACCCTGCAGTTGCAACACTTTCCTGTCCACTCTCCAGATATTTTATGAAATCGCTC
>HPV52_Alpha_397038_nt163_E6_VE_rc|1
TCTTCGTTGTAGCTCTTTTTTGCACTGCACACACTGCAGCCTTATTTCATGCACCGATTC
>HPV69_Alpha_6970418_nt403_E6_VE_rc|1

ATCCACAATTCTCTGTTTTTCTTCCGGTCCCAATGGTATTTGGCATCTATGACACCTTAT
>HPV94_Alpha_40804528_nt304_E6_VE_rc|1
CTCCTCTTCCACGCCTTCTACATAATATGAATATTGCCAATATTTTAGGCGCCGTGCAAT
>HPV3_Alpha_397005_nt433_E6_VE_rc|1
TTTGTGCAGTCTTCGCTTTTCGTTGCGGTGTCTGTCCTTCTCTTCCTTTACCAGTGGTTT
>HPV61_Alpha_9628574_nt373_E6_VE_rc|1
CTGTATACTTAACGGTTTGCTGCATGCATGGCACCGTATATATAGTTCAGCTAATGACCG
>HPV54_Alpha_9628437_nt253_E6_VE_rc|1
TTCTAGGCACAGTGCACATGCAGCATGTGGAAAGCCGTGTCTCCACACCACAAATAGGTC
>HPV19_Beta_396940_nt291_E6_VE_rc|1
ACAAGGCACTAAACAGTCATCCAACGGTATTTCTAGCAATGCTGCTAATCCAGCAATTGT
>HPV77_Alpha_2911558_nt223_E6_VE_rc|1
ACCGTACGGCGCCCCACCTCTCCACACCACATTTAATTCTCTAATTGTAAATGCTGCCAG
>HPVcand62_Alpha_39932599_nt152_E6_VE_rc|1
AATCTCTCTCAATAGGCATGCTGCGCATACTCCAAATGGCCAGTCCTTTCGCCGCACCAC
>HPV95_Gamma_40804520_nt442_E6_VE_rc|1
CCTCCACAAACCCCTTATTAAATAGCACACCTCACCACTGTATAACAGGTCAAGTTTTTC
>HPVcand87_Alpha_14475578_nt328_E6_VE_rc|1
TGCTACACCTGTTTCTTCTTCTACTGTGGGTCCGTAGCTGGAGTACTCCCAGTGACGAAG
>HPV81_Alpha_40804509_nt493_E6_VE_rc|1
GGCCATGCATGCTCCTCTACAATGGCAGCACTTTCCCATCCACTCTCCAGCTATTTTATG
>HPV88_Gamma_167600365_nt228_E6_VE_rc|1
AGAAAGCCTAGAACAAGGGACACAACACGCAAAACAAGCACCGCTCCTCCAGACCAAACT
>HPV3_Alpha_397005_nt283_E6_VE_rc|1
TTTTAGGCGTCGTGCAATGCCCTCTACAAGTAAACACCGTGCACAAGCACCGTAGGGCGC
>HPV60_Gamma_9628566_nt103_E6_VE_rc|1
ATCAAATTCAGAGCAATAATCAGCCACTGTTGTTGGAAACCTGTCTTCTTCCATCTGCAT
>HPV42_Alpha_333211_nt499_E6_VE_rc|1
GCGTCTCTCCACGCATTGTCCTCTGCAATGCGTACACCGACCCGTCCACTGACACAATAT
>HPV16_Alpha_9627100_nt84_E6_VE_rc|1
TGGTAACTTTCTGGGTCGCTCCTGTGGGTCCTGAAACATTGCAGTTCTCTTTTGGTGCAT
>HPV43_Alpha_40804474_nt493_E6_VE_rc|1
TCGTCGGCTTTTTTCCATGCATGATTTCCAGCAATGTAGGCAGTATCCTTTCCACACGCT
>HPV16_Alpha_9627100_nt474_E6_VE_rc|1
TGATGATCTGCAACAAGACATACATCGACCGGTCCACCGACCCCTTATATTATGGAATCT
>HPV16_Alpha_9627100_nt444_E6_VE_rc|1
GGTCCACCGACCCCTTATATTATGGAATCTTTGCTTTTTGTCCAGATGTCTTTGCTTTTC
>HPVcand86_Alpha_15741127_nt212_E6_VE_rc|1
GTCTTGTTCCACTGTGGATCCGTAGCTGGAGTGCTCCCAGTGTCGTAGCTCACGTACCTT
>HPV61_Alpha_9628574_nt253_E6_VE_rc|1
CACTTTTACTTCACGTGCCAAGCATGGTGCGCATACTCCAAATGGCCAGTTATGTCTCCA
>HPV3_Alpha_397005_nt403_E6_VE_rc|1
TCTGTCCTTCTCTTCCTTTACCAGTGGTTTGTGACACATGTAGCATCTAATTTGCTGTGT
>HPV7_Alpha_9627389_nt403_E6_VE_rc|1
AACAATGTGGTTGGTTTTTTCCACAGGAGACAATGGTTTGTGGCATTTGCAGCATCTTAT
>HPV11_Alpha_333026_nt103_E6_VE_rc|1
AAACGTCTTGCACAACTGGTCTATAGATGTTGCAGACGTGGAGGCATCTTTACTTTCCAT
>HPVRTRX7_Unclassified_30315616_nt595_E6_VE_rc|1
ACAGTGCCTGCACACTCCTTTCCAGCCGTCTCTCACTTTGTGGAACGGAAGGCCTCGACC
>HPV106_Alpha_71726710_nt392_E6_VE_rc|1
GCAAGCGCCTCGCCAGTGCCCGGCTATTTTATGGAACCGTTTGCCTTCTGTTATTAGCCG
>HPV9_Beta_9627396_nt351_E6_VE_rc|1
GCTACTACAGAGTCCATACACCAAATCCTCCTCTGTCCAAATAAGCTGTAAACACTTGTG
>HPV40_Alpha_397014_nt493_E6_VE_rc|1
CGAGCGTTGGCCTTTCTCCATGCATTTCTTCCAGCAATGTAGACAGTACCCTGTCCACGA

>HPV3_Alpha_397005_nt253_E6_VE_rc|1
TAAACACCGTGCACAAGCACCGTAGGGCGCTCCCCTTCTCCACACCACATTCAGTTCCCG
>HPV12_Beta_396910_nt231_E6_VE_rc|1
TATATCTAAAAGGTCAGCTAACTCTTTAATAGTTGTGGGCAGCTCAGGCGTACTGTCTGT
>HPV90_Alpha_22138122_nt272_E6_VE_rc|1
GCACAGTGGTTTATGGCACAGGTAGCATCTAATAAGCAATTCTAAAATAGGCACTCCTGT
>HPV63_Mu_9626605_nt133_E6_VE_rc|1
GCAAAAATTGCAAGGAACAACCAAATCAATAAATGGGATACGGAGAGCAGAACTCAGATC
>HPV45_Alpha_397022_nt403_E6_VE_rc|1
GTGTCTACGTTTTTCTGCTGGGTTCAATGGTTTCTGGCACCGCAGGCACCTTATTAACAA
>HPV61_Alpha_9628574_nt223_E6_VE_rc|1
GCATACTCCAAATGGCCAGTTATGTCTCCACACAATGCTTAGCTCCTTTAGTGCAAACGC
>HPV67_Alpha_3228267_nt133_E6_VE_rc|1
GCAGGGCAAACTTATTTCATGCACCGTGGTTTCCAAAGCTTCACACAATTCGTGCAGGTT
>HPV24_Unclassified_9628486_nt561_E6_VE_rc|1
TATCTGCTTACACTGCCTACAGAGTCCTTTCCAGCCCCTCCTCACTTTATGAAATAATTC
>HPV36_Beta_1020218_nt411_E6_VE_rc|1
ATAAAACTGATTAAACTCATATGTGGCTGTTGCACCACAACAAACACGACAGCAGGCAAA
>HPVcand85_Alpha_4574720_nt136_E6_VE_rc|1
TATTTCTATATCTTGCAGTGATGTGTCCAGTGTGTTGCATAGGTCTGGTAGTTTGTAGGG
>HPV58_Alpha_222386_nt111_E6_VE_rc|1
CTCCAACGCCTGACACAAATCATGCAATGTCCGTGGTTTCTCCTCTGCGTCCTGGAACAT
>HPV16_Alpha_9627100_nt114_E6_VE_rc|1
TATAGTTGTTTGCAGCTCTGTGCATAACTGTGGTAACTTTCTGGGTCGCTCCTGTGGGTC
>HPVcand85_Alpha_4574720_nt196_E6_VE_rc|1
AGCAAAGGCAAATTCATATACCTCTGTTCGCTGCAGGACGCTTTTACAATATACACAGCT
>HPV69_Alpha_6970418_nt433_E6_VE_rc|1
CCCTGCTATTTCATGGAACCGTCGCTTTTCATCCACAATTCTCTGTTTTTCTTCCGGTCC
>HPV42_Alpha_333211_nt169_E6_VE_rc|1
TAAGTGCTTTTTGCACCAAATGCAGGAAATCTGTAAATTCCGCAATGTCAGCCCAAATTC
>HPV69_Alpha_6970418_nt493_E6_VE_rc|1
TTGTGTTTCTGTTGCTTCGCGCCTTGGTCTCCAGCAGTTTGTGCACAACCCTTTCCAGTA
>HPV71_Alpha_12084981_nt173_E6_VE_rc|1
TAATTGTTCTGCAGAATATGCAGGTTAGGTTCAGGTCCTGCAGGTCCACGTCGTACTGGT
>HPV41_Nu_9626041_nt493_E6_VE_rc|1
TCGAACACAAGTTCCCCTCCAGATCCCGCCAATTCTATGCACAGACAAGTCTTCCCGCAA
>HPV72_Alpha_1491683_nt343_E6_VE_rc|1
TATATATAGTTCTGCTAATGATTTGCCTGTTTCCTGTTCCACAGTGGGTCCGTAGCCCGA
>HPV56_Alpha_397053_nt133_E6_VE_rc|1
TCTAAGATCAATTAAAGGTATTTCTAATACCTCACTCAAGTGGTGCAGGCTTCGTGGACG
>HPV18_Alpha_9626069_nt136_E6_VE_rc|1
TATTTCTATGTCTTGCAGTGAAGTGTTCAGTTCCGTGCACAGATCAGGTAGCTTGTAGGG
>HPV48_Gamma_9628542_nt463_E6_VE_rc|1
CTCATGGCTGATACAATTTCTACAAACACCTCTCCAAGTCCCGCGAATTAACAGAAAATC
>HPV39_Alpha_333245_nt198_E6_VE_rc|1
ACTAAATGCAAATTCATATACCTCGGTTTGCTGTAGTGGTCGTCTGCAATAGACACAGGC
>HPV68a_Alpha_71726685_nt122_E6_VE_rc|1
TACCCCGTCTCTATACACTACACATAGGTCACTAAAGGCAAATTCATATACCTCTGTCCG
>HPV65_Gamma_312100_nt463_E6_VE_rc|1
TTGTTTCCTAATGCAATTTCTGCAATAGCCCCTCCACAAACCCCTTATTAAGTAGCAGGC
>HPVRTRX7_Unclassified_30315616_nt565_E6_VE_rc|1
TCTCACTTTGTGGAACGGAAGGCCTCGACCACAGCTATCTAATTTTTCAATGGTGCTCAA
>HPV36_Beta_1020218_nt591_E6_VE_rc|1
ACACTGCCTACAGATTCCCTTCCAGGCGTTTCTGACTCTATGAAAGGGAAGTCCTCTTCC
>HPV19_Beta_396940_nt261_E6_VE_rc|1

TTCTAGCAATGCTGCTAATCCAGCAATTGTTGCTGGTAATGGTGGCTCTGTGACCTGTGG
>HPV96_Beta_50253426_nt180_E6_VE_rc|1
GGCAACCGAGAGAGTTGCGAAACTTACCGTTAACGGTTCCAAAGTTGGCAGACGTTTTGG
>HPV52_Alpha_397038_nt133_E6_VE_rc|1
ACACTGCAGCCTTATTTCATGCACCGATTCTTCCAGCACCTCACACAATTCGTGCAGGGT
>HPV59_Alpha_557236_nt56_E6_VE_rc|1
TGTTGTGCTCAAATCAGGCAGTTTGTATGGTCGTTGTGTAGGATCCTCAAAGCGTGCCAT
>HPV53_Alpha_9627377_nt433_E6_VE_rc|1
GTGAAACCGTTTCTTATAGTCACAGTGTAACTGTTTTTCCTCTGGTGTCAACGGATGTTG
>HPV83_Alpha_5059324_nt92_E6_VE_rc|1
AATACACAGCTCCTTCCATGCAAAGGACAGCACTTCTGCTGTTGTTAATTCATTTGTGCA
>HPV77_Alpha_2911558_nt373_E6_VE_rc|1
GGTGAGTGGTTTGTGACACATGTAGCATCTGATTTGCTGGGTATCTATGGATTCTTTTGT
>HPV15_Beta_396924_nt561_E6_VE_rc|1
TATCGCCCTACAATGTCTACACAATCCTTTCCAATTGCGTCTAACCTTGTGGAATTGCTC
>HPV43_Alpha_40804474_nt373_E6_VE_rc|1
TGGCTTGTGGCACTTACAGCATCTAATGCACAAATCAAACACTGTTTGCTTAGTTTCTTC
>HPV61_Alpha_9628574_nt343_E6_VE_rc|1
GCACCGTATATATAGTTCAGCTAATGACCGTCCTGTTGTCTGTTCCACAGTGGGTCCGTA
>HPVcand86_Alpha_15741127_nt272_E6_VE_rc|1
GCACAATGGTTTAAAGCACGCATGGCACCTTATATTTAAGTGTGCCAATGATACGCCTGT
>HPV6_Alpha_6002612_nt104_E6_VE_rc|1
AAACGTCTTGCACAACTGGTCTATGGTCGTTGCAGACGTGGAGGCATTTGCACTTTCCAT
>HPV47_Beta_333062_nt509_E6_VE_rc|1
AAGAAATGACAGGCAGGTATGACACCTTATGTCAATCTCAAAAATGGAAAGGCCTGTAGC
>HPV34_Alpha_9627334_nt463_E6_VE_rc|1
GCACTGTGTACAGCGTCCGGTCCACTGATCCGCTATTTGGTGAAACCGTTTGTTTTCATC
>HPV76_Beta_2911551_nt201_E6_VE_rc|1
AGAAGAAATGGGTATATTTAAGAGTTGGCTAAGCTCACATACCTTAGCAGGTCTAGCCAT
>HPV1_Mu_9626063_nt255_E6_VE_rc|1
CTCCAATAGGCTAACAGTTCTAGCACACCCTTGACAGCATCCAAACACCAAATTGTCTCT
>HPV106_Alpha_71726710_nt272_E6_VE_rc|1
TATTGGTATCCCTGTTTCATACTCCACTGTGGGTGCATAGCTGGAGTAGTCCCAGTATCT
>HPV5_Beta_9627145_nt591_E6_VE_rc|1
ACACTGCCTACAGATTCCCTTCCAGGCGTTCCTCACCTTATGAAAGGGAAGGCCTCTGCC
>HPV16_Alpha_9627100_nt204_E6_VE_rc|1
TACTATGCATAAATCCCGAAAAGCAAAGTCATATACCTCACGTCGCAGTAACTGTTGCTT
>HPV18_Alpha_9626069_nt106_E6_VE_rc|1
TTCCGTGCACAGATCAGGTAGCTTGTAGGGTCGCCGTGTTGGATCCTCAAAGCGCGCCAT
>HPV93_Beta_37089393_nt106_E6_VE_rc|1
CTCTGTAATGGTCTGAGGTTTACTTACTGCCGCCATTAAGATGTCTGCCTTATATACCAT
>HPV88_Gamma_167600365_nt438_E6_VE_rc|1
ACAACAATTTCTGCACGTTCCCCTCCAAGTGTTTCTCACAAGATGGAAACACTCACCCCT
>HPV26_Alpha_9627305_nt458_E6_VE_rc|1
CCAACAATTTGTACACAACCCTTTCCACTGCCCTGCTATTTCGTGAAATCGTCGCTTTTC
>HPV38_Beta_1020234_nt231_E6_VE_rc|1
GCAGAATCTACAGGGTAATAACAGATCCTCTACAGGAACTGTTAACTTATCACTGAGCTG
>HPV21_Beta_1020170_nt291_E6_VE_rc|1
ATCATCCAATGGTATCTCTAACAGATTGGCTAGGTCACGAATTGTAGCTGGTAATGGTGG
>HPV65_Gamma_312100_nt433_E6_VE_rc|1
CCTCCACAAACCCCTTATTAAGTAGCAGGCCTGGTCAGAGTACAGCAAGTCTAATTTCTC
>HPV10_Alpha_9627257_nt253_E6_VE_rc|1
GCCCTGTAGGAGTAAACACCGTGCACAGGCACCGTATGGCACTCCCGCTCTCCACACCAA
>HPV72_Alpha_1491683_nt373_E6_VE_rc|1
GCTTAGGGGTTTGCAGCATGCATGGCACCTTATATATAGTTCTGCTAATGATTTGCCTGT

>HPV52_Alpha_397038_nt463_E6_VE_rc|1
CCAACACTCTGAACAGCGCCCTGTCCAACGACCCATAATATTATGAAATCGCTTGTTTGC
>HPV73_Alpha_1491692_nt103_E6_VE_rc|1
CACTTCGTCACATAACGCTTGTAGCTTGTATGGTCGTTCTTCTGAATTGGGAAACAGCAT
>HPV39_Alpha_333245_nt468_E6_VE_rc|1
CCGTCGACACTGTCCTGTATAGCTTCCTGCTATTTTATGAAATCTTCGTTTGCTATTTAG
>HPV25_Beta_396948_nt411_E6_VE_rc|1
AAATTCAAATGTGGCAGTTGCTGTGCAACAACAGCGACAGCAGGCATACACAAGATATTC
>HPV68a_Alpha_71726685_nt92_E6_VE_rc|1
ACTAAAGGCAAATTCATATACCTCTGTCCGTTGTAGTTGCCTTCTGCAATAGACACAGTC
>HPV32_Alpha_9627327_nt343_E6_VE_rc|1
TTGTTCTTCCAACAGTAGTCCTGTTTCTTGTTCTACTGTATGCCAAAATGCTGATCTGTC
>HPV12_Beta_396910_nt531_E6_VE_rc|1
CCGACCACAGCTGTCTAACTTTTCAATTGTATCTAAAAATGACAAGCACGTCTGACACCT
>HPV14D_Beta_396918_nt207_E6_VE_rc|1
TATCACAATAGTTACTCTTAGGAGAAGGACCTTCATCTGCACTGTCTGTTGAAGAGTCAG
>HPVcand87_Alpha_14475578_nt208_E6_VE_rc|1
TCTCCACACCAGCAATAATTCCCTTAATGCAAACGCCAGCAATTCGCTTTCTGTTAATTC
>HPV88_Gamma_167600365_nt258_E6_VE_rc|1
GCGGTAAAAGCGTTCAGCCTCATACCTAGCAGAAAGCCTAGAACAAGGGACACAACACGC
>HPV68a_Alpha_71726685_nt302_E6_VE_rc|1
GTGCCTTAGTTTTTCTGCTGGACACAATGGTTTCAGGCAACTCATGCACCTTATCAATAA
>HPV110_Beta_186461194_nt323_E6_VE_rc|1
TTGCGCTGTAGCATGAGCACAACTACTACAGCATGCAAAAACAAAATCTTCCTCAGTCCA
>HPV102_Alpha_71726718_nt212_E6_VE_rc|1
GGTTTCTTCTTCAACCGTTGCGCCGTAGCTGGAGTAGTTCCAGTGCCGTAGTTCCCGCAC
>HPV12_Beta_396910_nt591_E6_VE_rc|1
ATGCTTGCACTGTCTGCAAATTCCCTTCCACCTGTCTCTAACCTTGTGGAACGGAAGGCC
>HPV82_Alpha_6970427_nt403_E6_VE_rc|1
GTCCACCACCTTTTGCTTTTCTTCAGGCCCAAGTGGTCTCTGACATCTATGACACCTTAT
>HPV29_Alpha_1020210_nt463_E6_VE_rc|1
TGACCAACAATACAGGCAACTCCCTCTCCAGTATCCAGAAATTTTGTGTAGTCTTCGCTT
>HPV8_Beta_333074_nt272_E6_VE_rc|1
AAAGTTACCACAAAAGTTGCACGGTACTGAACAGTCCTGCAATGGAATACCTAAAGCCGC
>HPV7_Alpha_9627389_nt493_E6_VE_rc|1
CGAGCGTTGGCCTTTCTCCATGCATTTCTTCCAACAGTGCAGACAGTACCCTGTCCACGA
>HPV37_Beta_1020226_nt531_E6_VE_rc|1
CCAATTGCGCCTAACCTTGTGAAATTGTTGCTGAGTATAGCAAGTCTCCAACTTCTCTAA
>HPV74_Alpha_27462483_nt2_E6_VE_rc|1
GCAGTCCTTGCACAACTGGTCTATACTTTTTGCAGACGTGGAGGCATTTGCACTTTCCAT
>HPV92_Beta_27531786_nt437_E6_VE_rc|1
TGCCTTCCAAGCTCCTCTAACTCTATCAAAAGGAATGCCTGATGCACAAACTGCCAATTT
>HPV34_Alpha_9627334_nt403_E6_VE_rc|1
TACATGCCTTTGCTTTTCCAATGGACACAGTGGTTTTTGGCATTTTCCGCACCTTATTAA
>HPV56_Alpha_397053_nt433_E6_VE_rc|1
TAGATGAAATCGTCTTTTTCTGTCACAATGCAATTGCTTTTCCTCCGGAGTTAACGGACT
>HPV39_Alpha_333245_nt138_E6_VE_rc|1
TATTGTAATGTCCTGCAAGGTGGTGTCCAGCGTTGTGCACAGGTCTGGCAATTTGTATGG
>HPV49_Beta_9627363_nt201_E6_VE_rc|1
TTCCCAAATAGGTATATTTAAGTGGTGGGCTAGCTCACATACCTTAACAGGTCTAGCCAT
>HPV25_Beta_396948_nt291_E6_VE_rc|1
GTTGCCACAGAAGTTGCAAGGTACTAAGCAATCATCTAATGGAATTTCCAATAAAGCTGC
>HPVcand85_Alpha_4574720_nt166_E6_VE_rc|1
CTGCAGGACGCTTTTACAATATACACAGCTTATTTCTATATCTTGCAGTGATGTGTCCAG
>HPV22_Beta_1020178_nt450_E6_VE_rc|1

TTGGCCTACAGGTCGTTGTTCTTCTTGCTCAATTTCACGACCATACAAAGTAACTTGATA
>HPVcand87_Alpha_14475578_nt478_E6_VE_rc|1
GGCCATGCATATTGCCCTACAGTTGCTGCACTTTCCTGTCCACTTCCCAGCTATTTTGTG
>HPV58_Alpha_222386_nt471_E6_VE_rc|1
CCAACACACTGCACAGCGCCCTGTCCAACGACCCGAAATATTATGAAACCTTTTGTTTAA
>HPV65_Gamma_312100_nt253_E6_VE_rc|1
AAATCTTGCACTTAAACGTAAGCATTCAGAACAGCATGCATAGTAGCAACCCCCCCTGAA
>HPV42_Alpha_333211_nt109_E6_VE_rc|1
CTTACACAATTGGTATAATGTGCGTGGCTGTGATGAGGCAGATGTACCTGACATTCTGCC
>HPV63_Mu_9626605_nt103_E6_VE_rc|1
AAATGGGATACGGAGAGCAGAACTCAGATCCCGAACCGAATGTACAGATGTCAGGTCCAT
>HPV56_Alpha_397053_nt463_E6_VE_rc|1
CAAACATGACCCGGTCCAACCATGTGCTATTAGATGAAATCGTCTTTTTCTGTCACAATG
>HPV36_Beta_1020218_nt381_E6_VE_rc|1
TGCACCACAACAAACACGACAGCAGGCAAATACACAGTAATCCTTCCAAATTAAACTAAG
>HPV71_Alpha_12084981_nt503_E6_VE_rc|1
CATTGTTGGCCTCGCATGGTTTCCAACACTGTAGGCAAGCACCGCGCCAATGGCCTGCTA
>HPV80_Beta_2911565_nt561_E6_VE_rc|1
TATCGACCCACAATGTCTACACAATCCTTTCCAATTGCGCCTTACCTTATGGAATTGCTG
>HPV9_Beta_9627396_nt381_E6_VE_rc|1
AAATTCTAACTGTGCAGACGCATAAGCACAGCTACTACAGAGTCCATACACCAAATCCTC
>HPV81_Alpha_40804509_nt283_E6_VE_rc|1
CTCCCGTACTTTAGCTTCTCTGCACAAGCACGCTGCGCATACTCCAAATGGCCAGTTCTT
>HPV81_Alpha_40804509_nt163_E6_VE_rc|1
CTTTTTGCAAAATATGCAGGTCAGTTGCAGGTCGTCTAGGTCCACTTCACACTCCTTACA
>HPV8_Beta_333074_nt542_E6_VE_rc|1
ATGAAAGGGACGGCCTCTCCCACAGCAATCTAATTTCTCTATGATATCCAAAAATGACAA
>HPV76_Beta_2911551_nt531_E6_VE_rc|1
CACCCCTTTCCACCTATTTCTAACTTTGTGAAACTCTCTTTGCTGTGCACAATGATCAAG
>HPV59_Alpha_557236_nt326_E6_VE_rc|1
TTTTAGGCATCTATAACAGCGTATCAGCAGCTCATGTAACGGTGTCTTGGTTTCAGCCTC
>HPV39_Alpha_333245_nt108_E6_VE_rc|1
CGTTGTGCACAGGTCTGGCAATTTGTATGGCCGTTCTGCAGGATTGTGAAATCGCGCCAT
>HPV53_Alpha_9627377_nt193_E6_VE_rc|1
TGCAAAATTATATACCTCTGACGCTGTCAATGCCTTCTTGCAGAACACACAGCCAAGTTG
>HPV59_Alpha_557236_nt296_E6_VE_rc|1
CTCATGTAACGGTGTCTTGGTTTCAGCCTCTAATGTTTCTCCATACACGGAATCTCTATA
>HPV84_Alpha_12958167_nt92_E6_VE_rc|1
TAGTAATTCCTTTACTGCAAAGGCCAGCACTTCGCCTTCCGTTAATTCTTCCTTGCAAAA
>HPV97_Alpha_71726694_nt362_E6_VE_rc|1
CGAATTGCACTGGCCTCTATAATAACCAGATATGTGGTGGAATCGTCGTTTGTCTTTAAG
>HPV102_Alpha_71726718_nt272_E6_VE_rc|1
ACTACACAACGGCTTCAGGCACATATAGCATCTCATATACAGCTGTGGTAATGCTGTGCG
>HPV10_Alpha_9627257_nt373_E6_VE_rc|1
TACCAGCGGTTTGTGACACATGTAGCATCTGATCAGCTGTGTATATATAGATTGTTTGGT
>HPV26_Alpha_9627305_nt488_E6_VE_rc|1
CACTTGTGTTTCTGTTTGGCGCCTTGGTCTCCAACAATTTGTACACAACCCTTTCCACTG
>HPV97_Alpha_71726694_nt32_E6_VE_rc|1
TATCTCTATTTCAGGTAATGAAGTATTCAATTCTGTGCACAGGTCTGGCAACTTGTAGGG
>HPV107_Beta_126131393_nt320_E6_VE_rc|1
GCAAAATTTGCAAGGTACCAGTAAGTCTTCCACTGGTATATTAAGATGCTGTGTAAGCTG
>HPV26_Alpha_9627305_nt98_E6_VE_rc|1
ATTCAAGCTTTCACATAGCTCATGTAGCGTTCTGGGTCGTTCTCTAGGATCCTCGAACAT
>HPV97_Alpha_71726694_nt392_E6_VE_rc|1
AAGCCTTTCTTGCCGTGATTGGTTGTAACACGAATTGCACTGGCCTCTATAATAACCAGA

>HPV67_Alpha_3228267_nt463_E6_VE_rc|1
CCAACACACTGAACACCGTCCTGTCCAACGATTGGAAATATTATGAAATCGCTTTTTGCG
>HPV23_Beta_1020186_nt471_E6_VE_rc|1
TATAGGTCGTTGCTCCTCCTGCTCAATTTCACGACCATACACAGTTAGCTGATAAAATTG
>HPV5_Beta_9627145_nt561_E6_VE_rc|1
CCTCACCTTATGAAAGGGAAGGCCTCTGCCACAGCAATCTAACTTTTCTATAATGTCAAG
>HPV92_Beta_27531786_nt257_E6_VE_rc|1
TGTTTCTATTGCTGCTACTTGTTTAGCACAAGCACCACAGCAAGCATAAGCATAATTCTC
>HPV83_Alpha_5059324_nt212_E6_VE_rc|1
TGTTTCTTCTTCTACTGTTGCCCCGTAGCTTGAGTGGCTCCAGTGCCGTAGCTCTCGCAC
>HPV12_Beta_396910_nt561_E6_VE_rc|1
CCTGTCTCTAACCTTGTGGAACGGAAGGCCCCGACCACAGCTGTCTAACTTTTCAATTGT
>HPVcand86_Alpha_15741127_nt362_E6_VE_rc|1
TGCTCTACAGTTGCTGCACTTTCCCGTCCACTGGCCTGCTATTTTATGAAAATGCAGATT
>HPV15_Beta_396924_nt351_E6_VE_rc|1
CTGCGCTGTAGCAAATGCACAACTAGAACAACAGGCAAAAACAAAATCTTCCTCAGTCCA
>HPV32_Alpha_9627327_nt133_E6_VE_rc|1
GCAGATTTGTAAATTCCGCAGGGTCAGCCCAAAATCTTTGCACAATTGGTATAATGTACT
>HPV30_Alpha_396973_nt433_E6_VE_rc|1
TCTGTGAAACCGTTTCTTATATTCACAGTGTAACTGTTTTTCCTCTGGTGTCAACGGCTG
>HPV77_Alpha_2911558_nt133_E6_VE_rc|1
CACACACAGTAGGCGAAGGTCGTCGAACGGTATTCCACTGTCTCTGCACAACAGGAATAT
>HPV73_Alpha_1491692_nt463_E6_VE_rc|1
GCACCGTGTACAGCGTCCGGTCCACTGTTCTGCTATTTGATGAAACCGTTTTTTTTCATC
>HPV94_Alpha_40804528_nt424_E6_VE_rc|1
GTACCCTGATATTTTGTGCAGTCGTCGCCGTTCGTTACGATGTCTGTCCTTTTCTTCCTT
>HPV45_Alpha_397022_nt433_E6_VE_rc|1
TATGCTGTGAAATCTTCGTTTGTCCTTAAGGTGTCTACGTTTTTCTGCTGGGTTCAATGG
>HPV97_Alpha_71726694_nt2_E6_VE_rc|1
TTCTGTGCACAGGTCTGGCAACTTGTAGGGCCGTTTGCTTGGATCTTCAAATCGCGCCAT
>HPVcand89_Alpha_22095322_nt272_E6_VE_rc|1
GCTGCCTAACGGCTTACAGCATATATAGCATCTCATATACAGCTGGGCCAACGGTGTGTT
>HPV8_Beta_333074_nt572_E6_VE_rc|1
GCAAACTCCTTTCCAGCCTCCTCTAACTTTATGAAAGGGACGGCCTCTCCCACAGCAATC
>HPV61_Alpha_9628574_nt463_E6_VE_rc|1
GCATGGCCCTCTACACTGGCAACACCTTCCCGTCCACTGTCCAGCTATATAGTGGAAGTG
>HPV71_Alpha_12084981_nt473_E6_VE_rc|1
GTAGGCAAGCACCGCGCCAATGGCCTGCTATTTTATGAAATCGCCTGCCTTCTGTAATTA
>HPV2_Alpha_9626032_nt390_E6_VE_rc|1
CTTCTCCTCCCAGCTCAGGGGCTTATGGCAAATATAGCATCTCATAAACAGCTGAGGTAT
>HPV107_Beta_126131393_nt650_E6_VE_rc|1
TTCTATCGCCTCACAATGTCTACACAATCCCTTCCAATTACCTCTCACCTTAAAAAACTG
>HPV72_Alpha_1491683_nt463_E6_VE_rc|1
CCCTCTACACTGGCAGCACCTTCCCGTCCACAGTCCGCTTATCTTGTGGAAGTGGATTCC
>HPV69_Alpha_6970418_nt463_E6_VE_rc|1
CCAGCAGTTTGTGCACAACCCTTTCCAGTACCCTGCTATTTCATGGAACCGTCGCTTTTC
>HPV17_Beta_396932_nt561_E6_VE_rc|1
TATCGACCCACAATGTCTGCACAAGCCTTTCCAATTGCGTCTAACCTTATGAAACTGCTG
>HPV38_Beta_1020234_nt351_E6_VE_rc|1
TTGAGCAGAAGCATAAGCACAACTGCTACAACATGCAAAAACAAAATCCTCTTGAGTCCA
>HPV59_Alpha_557236_nt476_E6_VE_rc|1
CACCAGTGTTTCACTACGCGCTTGTCGTTGCTGTCTTAGGTGTCTTGCTCGGGTCCGACA
>HPV68a_Alpha_71726685_nt2_E6_VE_rc|1
TGTCCTGCACAGGTCTGGCAATTTGTATGGCCGTTCCTCAGGGTTGTGAAATAGCGCCAT
>HPVcand87_Alpha_14475578_nt388_E6_VE_rc|1

CTTCTCGGGCCAGCACAATGGTTTGCAGCATGCATGGCACCTTATATTTTGAAGTGCCAA
>HPV72_Alpha_1491683_nt253_E6_VE_rc|1
AGTTGCTCTTGCAAGGCATGGTGCGCAGACTCCAAATGGCCAGTTGTCCCGCCACACAAT
>HPV84_Alpha_12958167_nt2_E6_VE_rc|1
CACCTCGTATTCCTGGCACAGCACAAAAATATTGGTGGGGTGGTAGCGTCCGTTGGGCAT
>HPVcand62_Alpha_39932599_nt362_E6_VE_rc|1
TCCTCTACAATTGCGGTACCGTCCTGTCCACACTCCTGCTATTTTGTGGAAGTGCACTCC
>HPV8_Beta_333074_nt846_E7_AOS|1
TACGTCTTTTTGTCAACGCAACTGATTCGGGTATCAGGACCTTTCAAGAATTGCTGTTCA
>HPV51_Alpha_333087_nt756_E7_AOS|1
CAAGTGTAGTACAACTGGCAGTGGAAAGCAGTGGAGACACCCTTCGCGTTGTACAGCAGA
>HPV51_Alpha_333087_nt716_E7_AOS|1
GCTACGTGTTACAGAATTGAAGCTCCGTGTTGCAGGTGTTCAAGTGTAGTACAACTGGCA
>HPV20_Beta_1020162_nt893_E7_AOS|1
TCTTTATAAGCGCTACAGAATTTGCTATTAGAAGCTTTCAACAATTGCTGATTGACGAGC
>HPV20_Beta_1020162_nt881_E7_AOS|1
TGAAACTTCGCATCTTTATAAGCGCTACAGAATTTGCTATTAGAAGCTTTCAACAATTGC
>HPV22_Beta_1020178_nt847_E7_AOS|1
ACTCTCTCTGGAATTCGCGACTTTCAAACATCTCTACTTGGACCTGTAAAACTTTTGTGT
>HPV22_Beta_1020178_nt841_E7_AOS|2
CTAGCAACTCTCTCTGGAATTCGCGACTTTCAAACATCTCTACTTGGACCTGTAAAACTT
>HPV22_Beta_1020178_nt835_E7_AOS|1
TATATACTAGCAACTCTCTCTGGAATTCGCGACTTTCAAACATCTCTACTTGGACCTGTA
>HPV22_Beta_1020178_nt829_E7_AOS|2
AAGCTGTATATACTAGCAACTCTCTCTGGAATTCGCGACTTTCAAACATCTCTACTTGGA
>HPV22_Beta_1020178_nt823_E7_AOS|1
AAGCTGAAGCTGTATATACTAGCAACTCTCTCTGGAATTCGCGACTTTCAAACATCTCTA
>HPV22_Beta_1020178_nt817_E7_AOS|1
GATACAAAGCTGAAGCTGTATATACTAGCAACTCTCTCTGGAATTCGCGACTTTCAAACA
>HPV22_Beta_1020178_nt811_E7_AOS|1
GGTTGTGATACAAAGCTGAAGCTGTATATACTAGCAACTCTCTCTGGAATTCGCGACTTT
>HPV22_Beta_1020178_nt800_E7_AOS|1
TATATTGTGGGGGTTGTGATACAAAGCTGAAGCTGTATATACTAGCAACTCTCTCTGGAA
>HPV22_Beta_1020178_nt794_E7_AOS|1
TTGTAGTATATTGTGGGGGTTGTGATACAAAGCTGAAGCTGTATATACTAGCAACTCTCT
>HPV23_Beta_1020186_nt877_E7_AOS|1
ATTCGCTCGTTCCAAGCATCTTTGCTAGAAAACGTGAAGCTGGTGTGTCCTGCCTGTCGA
>HPV23_Beta_1020186_nt867_E7_AOS|1
AGATTTTGGAATTCGCTCGTTCCAAGCATCTTTGCTAGAAAACGTGAAGCTGGTGTGTCC
>HPV23_Beta_1020186_nt857_E7_AOS|1
TGCTAGCCACAGATTTTGGAATTCGCTCGTTCCAAGCATCTTTGCTAGAAAACGTGAAGC
>HPV23_Beta_1020186_nt847_E7_AOS|1
AAGCTTTACGTGCTAGCCACAGATTTTGGAATTCGCTCGTTCCAAGCATCTTTGCTAGAA
>HPV23_Beta_1020186_nt837_E7_AOS|1
GACACAGTTAAAGCTTTACGTGCTAGCCACAGATTTTGGAATTCGCTCGTTCCAAGCATC
>HPV23_Beta_1020186_nt827_E7_AOS|1
GAGGCTGTGAGACACAGTTAAAGCTTTACGTGCTAGCCACAGATTTTGGAATTCGCTCGT
>HPV28_Alpha_1020202_nt573_E7_AOS|1
ACGTCCCAGTGCAATGCAATGTGCAATTAGATGAAGAAGATTATACAAATGTGGAGGAAC
>HPV28_Alpha_1020202_nt715_E7_AOS|1
AGATATAAGGGCATTGGAGCAGCTCCTGTTGGGCACACTGACGGTCGTGTGCCCTCGCTG
>HPV36_Beta_1020218_nt861_E7_AOS|1
TTTGTCCAAGCTACAGAATTTGGCATCAGAGCATTTCAACAGTTGCTGACCGGTGACCTG
>HPV36_Beta_1020218_nt853_E7_AOS|1
TCCGCGTTTTTGTCCAAGCTACAGAATTTGGCATCAGAGCATTTCAACAGTTGCTGACCG

>HPV36_Beta_1020218_nt847_E7_AOS|1
TCAAGCTCCGCGTTTTTGTCCAAGCTACAGAATTTGGCATCAGAGCATTTCAACAGTTGC
>HPV37_Beta_1020226_nt803_E7_AOS|1
TAGTTGTTGCAACGCCTATTGGAATTAGATCACAAGAAGAGCTATTACTTGGTGAAGTGC
>HPV37_Beta_1020226_nt812_E7_AOS|1
CAACGCCTATTGGAATTAGATCACAAGAAGAGCTATTACTTGGTGAAGTGCAGCTGGTTT
>HPV37_Beta_1020226_nt794_E7_AOS|1
TACGACTGATAGTTGTTGCAACGCCTATTGGAATTAGATCACAAGAAGAGCTATTACTTG
>HPV37_Beta_1020226_nt785_E7_AOS|1
GTTCTAAACTACGACTGATAGTTGTTGCAACGCCTATTGGAATTAGATCACAAGAAGAGC
>HPV38_Beta_1020234_nt752_E7_AOS|1
CAGCATACACCCCATACAAAATCATAGTTCTTTGTGGGGGTTGTGAAGTAAGGCTAAAAC
>HPV66_Alpha_1020290_nt592_E7_AOS|1
GTTGCAAGAGGTTATATTAGAACTTGCACCGCAAACGGAAATTGACCTACAATGCAATGA
>HPV66_Alpha_1020290_nt585_E7_AOS|1
TACCAACGTTGCAAGAGGTTATATTAGAACTTGCACCGCAAACGGAAATTGACCTACAAT
>HPV103_Unclassified_109390382_nt171_E7_AOS|1
GTCATTGTGTATGTATGTGGTAGCCACGGAGGAGGGCATTAGAAACTTTGAACAACAGCT
>HPV103_Unclassified_109390382_nt165_E7_AOS|1
TGAGAAGTCATTGTGTATGTATGTGGTAGCCACGGAGGAGGGCATTAGAAACTTTGAACA
>HPV103_Unclassified_109390382_nt159_E7_AOS|1
GTATTGTGAGAAGTCATTGTGTATGTATGTGGTAGCCACGGAGGAGGGCATTAGAAACTT
>HPV103_Unclassified_109390382_nt153_E7_AOS|1
TTGCAAGTATTGTGAGAAGTCATTGTGTATGTATGTGGTAGCCACGGAGGAGGGCATTAG
>HPV103_Unclassified_109390382_nt147_E7_AOS|1
GACAGATTGCAAGTATTGTGAGAAGTCATTGTGTATGTATGTGGTAGCCACGGAGGAGGG
>HPV101_Unclassified_109390389_nt151_E7_AOS|2
TGTGGGCAGTGTCACAAAACCTTAGCATTACATGTACTAGCTACGGACGGGGCAATAAGG
>HPV101_Unclassified_109390389_nt162_E7_AOS|1
TCACAAAACCTTAGCATTACATGTACTAGCTACGGACGGGGCAATAAGGTCTGTGGAACA
>HPV70_Alpha_1173493_nt804_E7_AOS|1
TAATACTACACTGCACTTAGTAGTAGAAGCCTCACAAGAGAACCTGCGATCTCTACTGCA
>HPV71_Alpha_12084981_nt739_E7_AOS|1
CTTGTGGTGGAAAGCGACGAAGCAGACATAAGAGCGCTTCAACAGCTGTTACTGGGCACA
>HPV71_Alpha_12084981_nt729_E7_AOS|1
TGCAGTTAGGCTTGTGGTGGAAAGCGACGAAGCAGACATAAGAGCGCTTCAACAGCTGTT
>HPV84_Alpha_12958167_nt437_E7_AOS|1
GCCAACGTTAAAGGATATTATTTTAACAGATATACCAGATGTAGTTAGTTTATACTGTGA
>HPV84_Alpha_12958167_nt427_E7_AOS|1
GCGGCCAGACGCCAACGTTAAAGGATATTATTTTAACAGATATACCAGATGTAGTTAGTT
>HPV107_Beta_126131393_nt885_E7_AOS|1
TGTCCAGTGAAACTTCGTCTCTACGTTTTTGCAACCCATTTTGGCATTCGGGCATTGCAG
>HPV107_Beta_126131393_nt879_E7_AOS|1
TCTGGGTGTCCAGTGAAACTTCGTCTCTACGTTTTTGCAACCCATTTTGGCATTCGGGCA
>HPV107_Beta_126131393_nt873_E7_AOS|1
CCTTGTTCTGGGTGTCCAGTGAAACTTCGTCTCTACGTTTTTGCAACCCATTTTGGCATT
>HPV107_Beta_126131393_nt867_E7_AOS|1
GTAGTACCTTGTTCTGGGTGTCCAGTGAAACTTCGTCTCTACGTTTTTGCAACCCATTTT
>HPV107_Beta_126131393_nt861_E7_AOS|2
AAAATTGTAGTACCTTGTTCTGGGTGTCCAGTGAAACTTCGTCTCTACGTTTTTGCAACC
>HPV72_Alpha_1491683_nt623_E7_AOS|1
TCAGAGTCAGAGGATGAGAGGGACGGTGTTGGTGTGCAGGAGCAACTTGTAGAACAAGCA
>HPV72_Alpha_1491683_nt617_E7_AOS|1
TCAGAGTCAGAGTCAGAGGATGAGAGGGACGGTGTTGGTGTGCAGGAGCAACTTGTAGAA
>HPV72_Alpha_1491683_nt607_E7_AOS|1

ACTAGACAGCTCAGAGTCAGAGTCAGAGGATGAGAGGGACGGTGTTGGTGTGCAGGAGCA
>HPV73_Alpha_1491692_nt719_E7_AOS|1
GCACGAAGTGTCAGTGCACAGTATGCCTTGCCATTGAAAGCAACAAAGCTGATTTAAGAG
>HPV73_Alpha_1491692_nt736_E7_AOS|1
ACAGTATGCCTTGCCATTGAAAGCAACAAAGCTGATTTAAGAGTGATAGAAGAGTTGCTT
>HPVcand86_Alpha_15741127_nt431_E7_AOS|1
CCAGACGCCGACCATAAAAGATATAGTGTTATGTGAAATACCAGATGTAGTTAACCTACA
>HPVcand86_Alpha_15741127_nt441_E7_AOS|1
ACCATAAAAGATATAGTGTTATGTGAAATACCAGATGTAGTTAACCTACATTGTGACGAG
>HPV88_Gamma_167600365_nt644_E7_AOS|1
ATACCAGTTGCGCCTTCTGTGAAGCAGGTGTAAGGGTATTTGTTCTAGCTTCACCAGCCG
>HPV110_Beta_186461194_nt742_E7_AOS|1
CTTGTGGCCTTTGTGGTGCGCGACTTCGGCTTTTTGTGCTTGCAACAGTTTTTGGAATTC
>HPV110_Beta_186461194_nt733_E7_AOS|1
TTGTTGCTGCTTGTGGCCTTTGTGGTGCGCGACTTCGGCTTTTTGTGCTTGCAACAGTTT
>HPV110_Beta_186461194_nt724_E7_AOS|1
CTTACAAGATTGTTGCTGCTTGTGGCCTTTGTGGTGCGCGACTTCGGCTTTTTGTGCTTG
>HPV111_Beta_186461202_nt760_E7_AOS|1
TGCTACGGAACTTGGAATAAGAGCCCAACAAGAACTATTACTGGGTGAAGTACAGCTTGT
>HPV111_Beta_186461202_nt752_E7_AOS|1
TACGTCTTTGCTACGGAACTTGGAATAAGAGCCCAACAAGAACTATTACTGGGTGAAGTA
>HPV111_Beta_186461202_nt744_E7_AOS|1
TTCGCTTATACGTCTTTGCTACGGAACTTGGAATAAGAGCCCAACAAGAACTATTACTGG
>HPV111_Beta_186461202_nt736_E7_AOS|1
TACAAGACTTCGCTTATACGTCTTTGCTACGGAACTTGGAATAAGAGCCCAACAAGAACT
>HPVFA75/KI88-03_Unclassified_206902429_nt765_E7_AOS|2
TTCGACTATTTGTGATTGCTACCTTCTTTGGAATCCGCACACAGCAAGAACTTTTAATTC
>HPVFA75/KI88-03_Unclassified_206902429_nt758_E7_AOS|1
GCTCGCCTTCGACTATTTGTGATTGCTACCTTCTTTGGAATCCGCACACAGCAAGAACTT
>HPVFA75/KI88-03_Unclassified_206902429_nt751_E7_AOS|1
TTGTGGCGCTCGCCTTCGACTATTTGTGATTGCTACCTTCTTTGGAATCCGCACACAGCA
>HPVFA75/KI88-03_Unclassified_206902429_nt744_E7_AOS|1
GTGGTGGTTGTGGCGCTCGCCTTCGACTATTTGTGATTGCTACCTTCTTTGGAATCCGCA
>HPVFA75/KI88-03_Unclassified_206902429_nt737_E7_AOS|1
GCTCCTTGTGGTGGTTGTGGCGCTCGCCTTCGACTATTTGTGATTGCTACCTTCTTTGGA
>HPVFA75/KI88-03_Unclassified_206902429_nt730_E7_AOS|1
GATAGTTGCTCCTTGTGGTGGTTGTGGCGCTCGCCTTCGACTATTTGTGATTGCTACCTT
>HPV91_Alpha_22023568_nt746_E7_AOS|1
GGACCTACAAGTTTACAAAGTTGTAACCGAGTGCACCTCTTGTTATTGTTCAATTCGTCT
>HPVcand89_Alpha_22095322_nt604_E7_AOS|1
GTGTGTGCTATCGTGCAGTTAGGTTAGTAGTATTGTCTGACGACGCACATCTAAGACATC
>HPV90_Alpha_22138122_nt439_E7_AOS|1
CAACCTTAAAGGACATATTTCTAGAGCTGGATCAGCCTGATGCGGTTGACCTACATTGTA
>HPV58_Alpha_222386_nt783_E7_AOS|1
CAACAGTACAACAACCGACGTACGAACCCTACAGCAGCTGCTTATGGGCACATGTACCAT
>HPV58_Alpha_222386_nt768_E7_AOS|1
GGTTCGTTTGTGTATCAACAGTACAACAACCGACGTACGAACCCTACAGCAGCTGCTTAT
>HPV58_Alpha_222386_nt744_E7_AOS|1
TTGTTGTTACACTTGTGGCACCACGGTTCGTTTGTGTATCAACAGTACAACAACCGACGT
>HPV74_Alpha_27462483_nt631_E7_AOS|1
GTACAGGACCAGACATCAACAATCTACATACGCTTCTACTGGGTACACTGAGCCTAGTGT
>HPV74_Alpha_27462483_nt646_E7_AOS|1
TCAACAATCTACATACGCTTCTACTGGGTACACTGAGCCTAGTGTGTCCTCTGTGCGCGC
>HPV74_Alpha_27462483_nt559_E7_AOS|1
AACATACACAGCCTTACCAAATAGTAACCTGCTGTTGTGTGTGTAATCGCAGCCTTCGGC

>HPV92_Beta_27531786_nt662_E7_AOS|1
TTCCTCGTGTGGTGGCTGCGGAATTAAGCTTCGAATTTTTGCATCGTGTACCCAATTTGG
>HPV92_Beta_27531786_nt654_E7_AOS|1
AAGATAGTTTCCTCGTGTGGTGGCTGCGGAATTAAGCTTCGAATTTTTGCATCGTGTACC
>HPV75_Beta_2911544_nt847_E7_AOS|1
ACTTTGCTGTTGGAGGAGCTGCAATTCCTGTGTCCTGAGTGTCGTGAAGAAATTCGACAT
>HPV75_Beta_2911544_nt839_E7_AOS|1
AATTTCAAACTTTGCTGTTGGAGGAGCTGCAATTCCTGTGTCCTGAGTGTCGTGAAGAAA
>HPV75_Beta_2911544_nt830_E7_AOS|1
CTATTCGAGAATTTCAAACTTTGCTGTTGGAGGAGCTGCAATTCCTGTGTCCTGAGTGTC
>HPV75_Beta_2911544_nt815_E7_AOS|1
TTGCTACTGATCGGGCTATTCGAGAATTTCAAACTTTGCTGTTGGAGGAGCTGCAATTCC
>HPV75_Beta_2911544_nt808_E7_AOS|1
TTTGTGCTTGCTACTGATCGGGCTATTCGAGAATTTCAAACTTTGCTGTTGGAGGAGCTG
>HPV76_Beta_2911551_nt846_E7_AOS|1
ATCTTTACTGCTAGAGGAGTTGCAATTCCTGTGTCCGGAGTGTCGTGAAGAGATTCGCAA
>HPV80_Beta_2911565_nt797_E7_AOS|1
TAATAGTGGTTGCAACTTCTTTTGGAATTCGCGCACAAGAAGAGTTACTATTTGGAGAAG
>HPVRTRX7_Unclassified_30315616_nt862_E7_AOS|1
CGTGTTTTTGTCAACGCAACTGAACTTGGTATTAGAACCCTGCAGGAACTACTGACCAGC
>HPVRTRX7_Unclassified_30315616_nt882_E7_AOS|1
TGAACTTGGTATTAGAACCCTGCAGGAACTACTGACCAGCGATCTGCAGCTGCTGTGTCC
>HPVRTRX7_Unclassified_30315616_nt875_E7_AOS|1
ACGCAACTGAACTTGGTATTAGAACCCTGCAGGAACTACTGACCAGCGATCTGCAGCTGC
>HPV65_Gamma_312100_nt702_E7_AOS|1
GGATTACTCTGTTTGCTGTGGAATTTGGACTTCGAGCGTTGGAACAACTCATAGTGGACG
>HPV65_Gamma_312100_nt684_E7_AOS|1
GATGTGAAGTTAATGTAAGGATTACTCTGTTTGCTGTGGAATTTGGACTTCGAGCGTTGG
>HPV67_Alpha_3228267_nt720_E7_AOS|1
ATTACCATATTGTTACTGTGTGTAACATCTGTGAGTGCACTTTGCGTTTGTGCATTCACA
>HPV67_Alpha_3228267_nt725_E7_AOS|1
CATATTGTTACTGTGTGTAACATCTGTGAGTGCACTTTGCGTTTGTGCATTCACAGTACA
>HPV67_Alpha_3228267_nt715_E7_AOS|1
AACCAATTACCATATTGTTACTGTGTGTAACATCTGTGAGTGCACTTTGCGTTTGTGCAT
>HPV67_Alpha_3228267_nt710_E7_AOS|1
GATACAACCAATTACCATATTGTTACTGTGTGTAACATCTGTGAGTGCACTTTGCGTTTG
>HPV67_Alpha_3228267_nt705_E7_AOS|1
AACCAGATACAACCAATTACCATATTGTTACTGTGTGTAACATCTGTGAGTGCACTTTGC
>HPV11_Alpha_333026_nt739_E7_AOS|1
GTGCACAGACGGAGACATCAGACAACTACAAGACCTTTTGCTGGGCACACTAAATATTGT
>HPV11_Alpha_333026_nt728_E7_AOS|1
CTGGTTGTGGAGTGCACAGACGGAGACATCAGACAACTACAAGACCTTTTGCTGGGCACA
>HPV31_Alpha_333048_nt696_E7_AOS|1
AACCGGACACATCCAATTACAATATCGTTACCTTTTGTTGTCAGTGTAAGTCTACACTTC
>HPV31_Alpha_333048_nt743_E7_AOS|1
AAGTCTACACTTCGTTTGTGTGTACAGAGCACACAAGTAGATATTCGCATATTGCAAGAG
>HPV31_Alpha_333048_nt733_E7_AOS|1
TTGTCAGTGTAAGTCTACACTTCGTTTGTGTGTACAGAGCACACAAGTAGATATTCGCAT
>HPV31_Alpha_333048_nt720_E7_AOS|1
TCGTTACCTTTTGTTGTCAGTGTAAGTCTACACTTCGTTTGTGTGTACAGAGCACACAAG
>HPV31_Alpha_333048_nt709_E7_AOS|1
CAATTACAATATCGTTACCTTTTGTTGTCAGTGTAAGTCTACACTTCGTTTGTGTGTACA
>HPV33_Alpha_333049_nt763_E7_AOS|1
CAGTTCGTTTATGTGTCAACAGTACAGCAAGTGACCTACGAACCATACAGCAACTACTTA
>HPV33_Alpha_333049_nt746_E7_AOS|1

TCACACTTGTAACACCACAGTTCGTTTATGTGTCAACAGTACAGCAAGTGACCTACGAAC
>HPV33_Alpha_333049_nt736_E7_AOS|1
TAACCTGTTGTCACACTTGTAACACCACAGTTCGTTTATGTGTCAACAGTACAGCAAGTG
>HPV35_Alpha_333050_nt757_E7_AOS|1
CTACGTCTGTGTGTACAGAGCACACACATTGACATACGTAAATTGGAAGATTTATTAATG
>HPV35_Alpha_333050_nt752_E7_AOS|1
CGACACTACGTCTGTGTGTACAGAGCACACACATTGACATACGTAAATTGGAAGATTTAT
>HPV47_Beta_333062_nt874_E7_AOS|1
GAACGCAACAAACCGTGGCATCAGGACATTTCAGGAACTTTTGACTGGTGATCTGCAGCT
>HPV47_Beta_333062_nt867_E7_AOS|1
TTTTTGTGAACGCAACAAACCGTGGCATCAGGACATTTCAGGAACTTTTGACTGGTGATC
>HPV42_Alpha_333211_nt491_E7_AOS|1
TCAATTTATATTGTGTCAGTGGACGGGTCGGTGTACGCATTGCAGAGGACAATGCGTGGA
>HPV42_Alpha_333211_nt481_E7_AOS|1
GTACAAGATTTCAATTTATATTGTGTCAGTGGACGGGTCGGTGTACGCATTGCAGAGGAC
>HPV39_Alpha_333245_nt804_E7_AOS|1
TAACAACACACTGCAGCTGGTAGTAGAAGCCTCACGGGATACTCTGCGACAACTACAGCA
>HPV93_Beta_37089393_nt684_E7_AOS|1
AGATTATTGTGTTCTGCGGCGGAGGCTGTGGATCCCGACTTCGAATATTTGTTGCTGCCA
>HPV93_Beta_37089393_nt674_E7_AOS|1
ACAGCTTTCAAGATTATTGTGTTCTGCGGCGGAGGCTGTGGATCCCGACTTCGAATATTT
>HPV12_Beta_396910_nt860_E7_AOS|1
TATTTTTGTCAACGCAACTGATACTGGCATTAGGACCCTACAGGACCTGCTGATCAGTGA
>HPV12_Beta_396910_nt880_E7_AOS|1
ATACTGGCATTAGGACCCTACAGGACCTGCTGATCAGTGACCTGCAGCTGCTGTGCCCAG
>HPV12_Beta_396910_nt872_E7_AOS|1
CGCAACTGATACTGGCATTAGGACCCTACAGGACCTGCTGATCAGTGACCTGCAGCTGCT
>HPV15_Beta_396924_nt798_E7_AOS|1
TATAGTGGTTGCAACTCCATTTGGAATTCGCTCACAACAAGACTTATTATTGGAAGAAGT
>HPV15_Beta_396924_nt811_E7_AOS|1
ACTCCATTTGGAATTCGCTCACAACAAGACTTATTATTGGAAGAAGTTAAGTTGGTGTGT
>HPV17_Beta_396932_nt810_E7_AOS|1
TCTTGCAACGCACGCTGGAATTCGTTCACAAGAGGAGCTTTTATTAGGTGAAGTACAGTT
>HPV17_Beta_396932_nt801_E7_AOS|2
GCTTATTGTTCTTGCAACGCACGCTGGAATTCGTTCACAAGAGGAGCTTTTATTAGGTGA
>HPV17_Beta_396932_nt792_E7_AOS|1
TAAGCTACGGCTTATTGTTCTTGCAACGCACGCTGGAATTCGTTCACAAGAGGAGCTTTT
>HPV19_Beta_396940_nt904_E7_AOS|1
AATTTGGTATTAGAACCCTACAGGACATCCTGATTGAAGAATTGCAACTGTTGTGCCCGG
>HPV19_Beta_396940_nt898_E7_AOS|1
CCACGCAATTTGGTATTAGAACCCTACAGGACATCCTGATTGAAGAATTGCAACTGTTGT
>HPV19_Beta_396940_nt892_E7_AOS|1
TGAAAGCCACGCAATTTGGTATTAGAACCCTACAGGACATCCTGATTGAAGAATTGCAAC
>HPV25_Beta_396948_nt888_E7_AOS|1
GATTTTGGTATTAGAACACTACAAAACCTTCTAATTGAAGAACTGCAGCTGTTGTGTCCG
>HPV25_Beta_396948_nt882_E7_AOS|1
GCCACTGATTTTGGTATTAGAACACTACAAAACCTTCTAATTGAAGAACTGCAGCTGTTG
>HPV25_Beta_396948_nt872_E7_AOS|1
CTTTGTGAAAGCCACTGATTTTGGTATTAGAACACTACAAAACCTTCTAATTGAAGAACT
>HPV30_Alpha_396973_nt579_E7_AOS|1
TAACAACTATTCCAGAATATATTTTGGACCTTGTACCGCAAACTGAAATTGACCTGCATT
>HPV30_Alpha_396973_nt572_E7_AOS|1
GGTAAAGTAACAACTATTCCAGAATATATTTTGGACCTTGTACCGCAAACTGAAATTGAC
>HPV3_Alpha_397005_nt624_E7_AOS|1
AAGATGAGTATATAAATGCTGTGGAACCAGCGCAACAAGCGTATTGTGTAGTCACAGTGT

>HPV40_Alpha_397014_nt752_E7_AOS|1
TGTTTGGTGCGCTTAGCAGTGCATTGTTCCATCACTGATATAACACAGTTCCAGCAGTTG
>HPV40_Alpha_397014_nt743_E7_AOS|1
TTTTGTCAGTGTTTGGTGCGCTTAGCAGTGCATTGTTCCATCACTGATATAACACAGTTC
>HPV45_Alpha_397022_nt615_E7_AOS|1
AAATTGTATTGCATTTGGAACCTCAGAATGAATTAGATCCTGTTGACCTGTTGTGTTACG
>HPV45_Alpha_397022_nt793_E7_AOS|1
TGACGGCAGAATTGAGCTTACAGTAGAGAGCTCGGCAGAGGACCTTAGAACACTACAGCA
>HPV52_Alpha_397038_nt752_E7_AOS|1
TACGGCTATGCATTCATAGCACTGCGACGGACCTTCGTACTCTACAGCAAATGCTGTTGG
>HPV52_Alpha_397038_nt747_E7_AOS|1
CACACTACGGCTATGCATTCATAGCACTGCGACGGACCTTCGTACTCTACAGCAAATGCT
>HPV56_Alpha_397053_nt592_E7_AOS|1
GCTGCAAGACGTTGTATTAGAACTAACACCTCAAACAGAAATTGACCTACAGTGCAATGA
>HPV56_Alpha_397053_nt585_E7_AOS|1
TACCAACGCTGCAAGACGTTGTATTAGAACTAACACCTCAAACAGAAATTGACCTACAGT
>HPVcand62_Alpha_39932599_nt645_E7_AOS|1
GCAGAACTCAGACGGCTGCAACAACTACTGACGGATGCACTGGAGATAGTGTGTCCCGGC
>HPVcand62_Alpha_39932599_nt602_E7_AOS|1
TATTTGCAACTGTTTAGTTAGGTTGGTGGTGTTAAGTGGAGACGCAGAACTCAGACGGCT
>HPV43_Alpha_40804474_nt736_E7_AOS|1
GCAGTGCTCTGACAGTGACATCAAGAAGCTAGAAGACCTGCTGTTGGGCACATTAAAAAT
>HPV43_Alpha_40804474_nt680_E7_AOS|1
GTTTACAGAGTTGTAACAGAGTGCACATCCTGTCTGTGTGTAATTCGACTTGTTGTGCAG
>HPV43_Alpha_40804474_nt671_E7_AOS|1
AATCTACAAGTTTACAGAGTTGTAACAGAGTGCACATCCTGTCTGTGTGTAATTCGACTT
>HPV43_Alpha_40804474_nt666_E7_AOS|1
AAGTGAATCTACAAGTTTACAGAGTTGTAACAGAGTGCACATCCTGTCTGTGTGTAATTC
>HPV81_Alpha_40804509_nt726_E7_AOS|1
GTATTTGTAGGTGTTTAGTTAGGCTGGTGGTGTTAAGTGGAGACGCAGACCTGAGGCTGC
>HPV81_Alpha_40804509_nt773_E7_AOS|1
GACCTGAGGCTGCTGCAACAACTACTGACGGATACACTGTCCATAGTGTGTCCTGGCTGC
>HPV81_Alpha_40804509_nt545_E7_AOS|1
CATGGCCAGGCAGCAACGATAAAGGACATTGTCCTAGATGAGTATCCTGATGTGATTGAC
>HPV95_Gamma_40804520_nt756_E7_AOS|1
AACTTCTTCTGGAAGGGAAGGTGATATTTTGCTGTGTTGGGTGTGCAAGAAATCACAGCA
>HPV95_Gamma_40804520_nt762_E7_AOS|1
TTCTGGAAGGGAAGGTGATATTTTGCTGTGTTGGGTGTGCAAGAAATCACAGCAGAAATG
>HPV95_Gamma_40804520_nt679_E7_AOS|1
TACCTGTTGTTATAATTGTGAAGCAAATGTAAGAATCACTTTGTATGCTGTTGCATTTGG
>HPVcand85_Alpha_4574720_nt793_E7_AOS|1
TAAATGTGAGGCCAGTCTGCAGTTAGTGGTGGAAAGTTCAGCAGCCGATCTACGAGATCT
>HPVcand85_Alpha_4574720_nt804_E7_AOS|1
CCAGTCTGCAGTTAGTGGTGGAAAGTTCAGCAGCCGATCTACGAGATCTCCAGCAGCTGT
>HPV96_Beta_50253426_nt954_E7_AOS|1
CCGGCTGAAAATCTACGTTTCTGCTACACAGTTTGGAATTCGTTCTTTAGAAGACTTGCT
>HPV96_Beta_50253426_nt961_E7_AOS|1
AAAATCTACGTTTCTGCTACACAGTTTGGAATTCGTTCTTTAGAAGACTTGCTGCTAGAA
>HPV96_Beta_50253426_nt949_E7_AOS|1
GATTCCCGGCTGAAAATCTACGTTTCTGCTACACAGTTTGGAATTCGTTCTTTAGAAGAC
>HPV96_Beta_50253426_nt944_E7_AOS|1
GCTGTGATTCCCGGCTGAAAATCTACGTTTCTGCTACACAGTTTGGAATTCGTTCTTTAG
>HPV96_Beta_50253426_nt939_E7_AOS|1
TGGAGGCTGTGATTCCCGGCTGAAAATCTACGTTTCTGCTACACAGTTTGGAATTCGTTC
>HPV96_Beta_50253426_nt846_E7_AOS|1

```
AGAGTTGACAGAAGAGCAGTCAGAAAACCTGTCAGAATCTACAGTAGCAGAGGTGGAGCC
>HPV96_Beta_50253426_nt839_E7_AOS|1
GTGACGAAGAGTTGACAGAAGAGCAGTCAGAAAACCTGTCAGAATCTACAGTAGCAGAGG
>HPV83_Alpha_5059324_nt440_E7_AOS|1
AATAGCAGAGATTGTACTAGAAGAAATACCAGATATAGTTGACCTATATTGTAATGAACA
>HPV83_Alpha_5059324_nt450_E7_AOS|1
ATTGTACTAGAAGAAATACCAGATATAGTTGACCTATATTGTAATGAACAAGGTATAGAC
>HPV83_Alpha_5059324_nt430_E7_AOS|1
ATACAGCAACAATAGCAGAGATTGTACTAGAAGAAATACCAGATATAGTTGACCTATATT
>HPV59_Alpha_557236_nt746_E7_AOS|1
AAGTGTAATAATCAACTTCAGCTAGTAGTAGAAACCTCGCAAGACGGATTGCGAGCCTTA
>HPV6_Alpha_6002612_nt742_E7_AOS|1
GTACAGAAACAGACATCAGAGAAGTGCAACAGCTTCTGTTGGGAACACTAAACATAGTGT
>HPV57_Alpha_60882_nt728_E7_AOS|1
TGCGGAGCGGCGGACATAAGGCATCTGGAGCAGCTGTTCCTGAATACGTTGACCATAGTG
>HPV69_Alpha_6970418_nt816_E7_AOS|1
GCGGTGGAGCAGCTGCTGATGGGCGACGTGAGTTTGGTGTGCCACCAGTGTGCTACATAC
>HPV69_Alpha_6970418_nt808_E7_AOS|1
ACGTCCGAGCGGTGGAGCAGCTGCTGATGGGCGACGTGAGTTTGGTGTGCCACCAGTGTG
>HPV69_Alpha_6970418_nt790_E7_AOS|1
TACTAAGCAGTCGACAGAACGTCCGAGCGGTGGAGCAGCTGCTGATGGGCGACGTGAGTT
>HPV82_Alpha_6970427_nt804_E7_AOS|1
CATATTTCAGCAAATGTTACTGGGCGACCTAAGCCTGGTGTGCCCGTGGTGTGCGACCAA
>HPV82_Alpha_6970427_nt758_E7_AOS|1
CGAGTGTTGTACAGCTCGCAGTGGAAAGCAGTGGAGACAGCCTTCGCATATTTCAGCAAA
>HPV82_Alpha_6970427_nt718_E7_AOS|1
GATACGTGTTACAGAATTAAAGTGCACTGTTGCAGGTGTTCGAGTGTTGTACAGCTCGCA
>HPV82_Alpha_6970427_nt697_E7_AOS|1
CCAGCCAGACAAGCTGGACAGGATACGTGTTACAGAATTAAAGTGCACTGTTGCAGGTGT
>HPV68a_Alpha_71726685_nt702_E7_AOS|1
CAAGGCACTGCAACTAGTAGTAGAAGCGTCGCGGGACAACCTGCGGACACTACAACAGCT
>HPV97_Alpha_71726694_nt514_E7_AOS|1
AAATTATATTGCATTTGCAACCCCAAAATGAAATAAATTCGGCTGACCTTCAATGTTATG
>HPV97_Alpha_71726694_nt499_E7_AOS|1
AACCAACATTACAAGAAATTATATTGCATTTGCAACCCCAAAATGAAATAAATTCGGCTG
>HPV106_Alpha_71726710_nt480_E7_AOS|1
CAACCAACCTTAAAGGACATCCTGTTAGAATTGGATGAGCCAGATGTTGTTGACCTGCAT
>HPV102_Alpha_71726718_nt428_E7_AOS|1
CCACGCAGCAACGATAGCAGATATAGTTTTAGAAGAATTACCAGATGTAATTGACCTGTA
>HPV102_Alpha_71726718_nt440_E7_AOS|1
GATAGCAGATATAGTTTTAGAAGAATTACCAGATGTAATTGACCTGTATTGTAATGAGCA
>HPV13_Alpha_85827580_nt656_E7_AOS|1
CCACGCAACATAGCACACTATTACAATGCTACCAAATACTAACGTCCTGTAGTAAATGTT
>HPV13_Alpha_85827580_nt732_E7_AOS|1
GGTGGTGGAGTGTACAGGACCTGACATTCACGACCTACACGACCTACTGCTGGGCACGCT
>HPV13_Alpha_85827580_nt673_E7_AOS|1
CTATTACAATGCTACCAAATACTAACGTCCTGTAGTAAATGTTGTAGCAACGTCCGGCTG
>HPV2_Alpha_9626032_nt724_E7_AOS|1
GTTGAGTGCGGACAAGCAGACCTAAGAGAGCTGGAACAGCTGTTCTTGAAGACGCTGACT
>HPV2_Alpha_9626032_nt736_E7_AOS|1
CAAGCAGACCTAAGAGAGCTGGAACAGCTGTTCTTGAAGACGCTGACTCTAGTGTGCCCT
>HPV41_Nu_9626041_nt865_E7_AOS|1
AGCAAGCAACACTGGGATACGGAATCTACAGGCACTCCTGGTCAACAGTCACCTTGACCT
>HPV41_Nu_9626041_nt855_E7_AOS|1
TTGTATGCGTAGCAAGCAACACTGGGATACGGAATCTACAGGCACTCCTGGTCAACAGTC
```

>HPV41_Nu_9626041_nt845_E7_AOS|1
ATCATACGATTTGTATGCGTAGCAAGCAACACTGGGATACGGAATCTACAGGCACTCCTG
>HPV41_Nu_9626041_nt835_E7_AOS|1
TTGCCAGGCTATCATACGATTTGTATGCGTAGCAAGCAACACTGGGATACGGAATCTACA
>HPV41_Nu_9626041_nt816_E7_AOS|1
TAGAGAGTCAGTGTCCATTTTGCCAGGCTATCATACGATTTGTATGCGTAGCAAGCAACA
>HPV41_Nu_9626041_nt806_E7_AOS|1
TTATACCTGGTAGAGAGTCAGTGTCCATTTTGCCAGGCTATCATACGATTTGTATGCGTA
>HPV41_Nu_9626041_nt796_E7_AOS|1
CAGGAGAACATTATACCTGGTAGAGAGTCAGTGTCCATTTTGCCAGGCTATCATACGATT
>HPV41_Nu_9626041_nt786_E7_AOS|1
TCCCCACACCCAGGAGAACATTATACCTGGTAGAGAGTCAGTGTCCATTTTGCCAGGCTA
>HPV41_Nu_9626041_nt776_E7_AOS|1
GTGCAGCAAGTCCCCACACCCAGGAGAACATTATACCTGGTAGAGAGTCAGTGTCCATTT
>HPV41_Nu_9626041_nt766_E7_AOS|1
GCGGGAACAGGTGCAGCAAGTCCCCACACCCAGGAGAACATTATACCTGGTAGAGAGTCA
>HPV1_Mu_9626063_nt657_E7_AOS|1
GCAACCTTATGCTGTCGTTGCTTCCTGTGCCTATTGCGAGAAACTGGTTCGATTGACCGT
>HPV1_Mu_9626063_nt684_E7_AOS|1
TGCCTATTGCGAGAAACTGGTTCGATTGACCGTCCTCGCGGATCACAGCGCCATTAGACA
>HPV1_Mu_9626063_nt675_E7_AOS|1
TGCTTCCTGTGCCTATTGCGAGAAACTGGTTCGATTGACCGTCCTCGCGGATCACAGCGC
>HPV1_Mu_9626063_nt666_E7_AOS|1
TGCTGTCGTTGCTTCCTGTGCCTATTGCGAGAAACTGGTTCGATTGACCGTCCTCGCGGA
>HPV1_Mu_9626063_nt648_E7_AOS|1
GTCGCCTCAGCAACCTTATGCTGTCGTTGCTTCCTGTGCCTATTGCGAGAAACTGGTTCG
>HPV18_Alpha_9626069_nt810_E7_AOS|1
TAGTAGTAGAAAGCTCAGCAGACGACCTTCGAGCATTCCAGCAGCTGTTTCTGAACACCC
>HPV18_Alpha_9626069_nt802_E7_AOS|1
AATTGAGCTAGTAGTAGAAAGCTCAGCAGACGACCTTCGAGCATTCCAGCAGCTGTTTCT
>HPV18_Alpha_9626069_nt795_E7_AOS|1
AAGCCAGAATTGAGCTAGTAGTAGAAAGCTCAGCAGACGACCTTCGAGCATTCCAGCAGC
>HPV18_Alpha_9626069_nt788_E7_AOS|1
AAGTGTGAAGCCAGAATTGAGCTAGTAGTAGAAAGCTCAGCAGACGACCTTCGAGCATTC
>HPV4_Gamma_9626597_nt720_E7_AOS|1
CTGCTGAGCTCGGACTACGGACCTTGGAACAACTTCTTGTAGAAGGAAAGCTGACGTTTT
>HPV4_Gamma_9626597_nt708_E7_AOS|1
TTACATTGTATGCTGCTGAGCTCGGACTACGGACCTTGGAACAACTTCTTGTAGAAGGAA
>HPV4_Gamma_9626597_nt686_E7_AOS|1
AGATGTGAAGTTGCTGTAAGAATTACATTGTATGCTGCTGAGCTCGGACTACGGACCTTG
>HPV63_Mu_9626605_nt697_E7_AOS|1
CGATCAGCTGTTAAGGCTGACCTGCGTTTCTGACCTGTCTACTCTTACGCGTCTGGAGGA
>HPV63_Mu_9626605_nt690_E7_AOS|1
GTTTGTGCGATCAGCTGTTAAGGCTGACCTGCGTTTCTGACCTGTCTACTCTTACGCGTC
>HPV63_Mu_9626605_nt683_E7_AOS|1
CCTTGTGGTTTGTGCGATCAGCTGTTAAGGCTGACCTGCGTTTCTGACCTGTCTACTCTT
>HPV63_Mu_9626605_nt676_E7_AOS|1
TGTGCTTCCTTGTGGTTTGTGCGATCAGCTGTTAAGGCTGACCTGCGTTTCTGACCTGTC
>HPV63_Mu_9626605_nt669_E7_AOS|1
ATGCAATTGTGCTTCCTTGTGGTTTGTGCGATCAGCTGTTAAGGCTGACCTGCGTTTCTG
>HPV63_Mu_9626605_nt662_E7_AOS|1
TATCCATATGCAATTGTGCTTCCTTGTGGTTTGTGCGATCAGCTGTTAAGGCTGACCTGC
>HPV63_Mu_9626605_nt655_E7_AOS|1
GTCTGAATATCCATATGCAATTGTGCTTCCTTGTGGTTTGTGCGATCAGCTGTTAAGGCT
>HPV63_Mu_9626605_nt648_E7_AOS|1

AGGAGGAGTCTGAATATCCATATGCAATTGTGCTTCCTTGTGGTTTGTGCGATCAGCTGT
>HPV16_Alpha_9627100_nt744_E7_AOS|1
TGACTCTACGCTTCGGTTGTGCGTACAAAGCACACACGTAGACATTCGTACTTTGGAAGA
>HPV5_Beta_9627145_nt795_E7_AOS|1
AACGAAAGGATCTCTTACAAAGTTATAGCTCCGTGCGGTTGCAGGAACTGTGAGGTCAAG
>HPV5_Beta_9627145_nt788_E7_AOS|1
GCCTGACAACGAAAGGATCTCTTACAAAGTTATAGCTCCGTGCGGTTGCAGGAACTGTGA
>HPV10_Alpha_9627257_nt551_E7_AOS|1
GATATCGAATTGAGTCTTGCACCAGAGGATATCCCTGTATGCAATGTGCAATTAGATGAA
>HPV26_Alpha_9627305_nt803_E7_AOS|1
TTCGAGTGCTGGAGCAGATGTTAATGGAAGACGTGTCCTTGGTGTGCCATCAGTGTGCTG
>HPV26_Alpha_9627305_nt782_E7_AOS|1
TGCAGAGCAGTCGACAGAACGTTCGAGTGCTGGAGCAGATGTTAATGGAAGACGTGTCCT
>HPV32_Alpha_9627327_nt560_E7_AOS|1
ATTATTTTGTATGACCTGCCAACGTGTGACCCGACAACGTGCGACACACCGCCGGTTGAC
>HPV32_Alpha_9627327_nt641_E7_AOS|2
GACACCTCAGATGAAGATGATGAAGACGATGACCAACCTATAAAACAGGACATACAACGT
>HPV34_Alpha_9627334_nt741_E7_AOS|2
TGTGTCTTACCATTGAGAGCACACACGCTGACCTATTAGTGTTAGAAGACCTGCTTATGG
>HPV34_Alpha_9627334_nt731_E7_AOS|1
CAGTCCACAGTGTGTCTTACCATTGAGAGCACACACGCTGACCTATTAGTGTTAGAAGAC
>HPV34_Alpha_9627334_nt721_E7_AOS|2
CAGCAGATGTCAGTCCACAGTGTGTCTTACCATTGAGAGCACACACGCTGACCTATTAGT
>HPV34_Alpha_9627334_nt711_E7_AOS|1
TTACTGATTGCAGCAGATGTCAGTCCACAGTGTGTCTTACCATTGAGAGCACACACGCTG
>HPV34_Alpha_9627334_nt695_E7_AOS|1
GCCTGGTACAGAATAGTTACTGATTGCAGCAGATGTCAGTCCACAGTGTGTCTTACCATT
>HPV49_Beta_9627363_nt805_E7_AOS|1
CTGCGAGTCTTCGTGTTAGCCACTGACGCTGCTATTAGAAGTTTCCAAGAACTGCTTCTG
>HPV49_Beta_9627363_nt798_E7_AOS|1
TGCCAGACTGCGAGTCTTCGTGTTAGCCACTGACGCTGCTATTAGAAGTTTCCAAGAACT
>HPV49_Beta_9627363_nt791_E7_AOS|1
GATGCGGTGCCAGACTGCGAGTCTTCGTGTTAGCCACTGACGCTGCTATTAGAAGTTTCC
>HPV53_Alpha_9627377_nt581_E7_AOS|2
GTACCAACACTTCCACAATATATTATAGAACTTATACCACAAACTGAGATTGACCTGCAA
>HPV53_Alpha_9627377_nt574_E7_AOS|1
CGGTAACGTACCAACACTTCCACAATATATTATAGAACTTATACCACAAACTGAGATTGA
>HPV7_Alpha_9627389_nt758_E7_AOS|1
GTTCGCCTAGTAGTCCATTGTACTGCTACTGATATAAGGCAGGTTCATCAGTTGCTGATG
>HPV9_Beta_9627396_nt833_E7_AOS|2
CAAATTTAGGAATTCGAGCGCAACAGGAACTTTTGCTGGGTGATATACAACTGGTGTGTC
>HPV9_Beta_9627396_nt824_E7_AOS|1
TGCTTGCTACAAATTTAGGAATTCGAGCGCAACAGGAACTTTTGCTGGGTGATATACAAC
>HPV9_Beta_9627396_nt815_E7_AOS|1
GTTTATACGTGCTTGCTACAAATTTAGGAATTCGAGCGCAACAGGAACTTTTGCTGGGTG
>HPV9_Beta_9627396_nt800_E7_AOS|1
GCGGTGCAAGACTTCGTTTATACGTGCTTGCTACAAATTTAGGAATTCGAGCGCAACAGG
>HPV54_Alpha_9628437_nt634_E7_AOS|1
AGAAGATGAGACAGCAGTAACACAACCTGACAAACAAGCATTTAAGGTGTTAAGCCAGTG
>HPV54_Alpha_9628437_nt706_E7_AOS|1
TTGTAAGACAGTACGGCTATGTGTGTATAGCACGCACACAGGCATAAGGGTACTGCAGGA
>HPV54_Alpha_9628437_nt648_E7_AOS|1
CAGTAACACAACCTGACAAACAAGCATTTAAGGTGTTAAGCCAGTGTGGAGGTGTGTGTT
>HPV24_Unclassified_9628486_nt726_E7_AOS|1
GGTGGAGCCTGAAAGAAGAGCTTACAAAATAATACTTTGTTGCGGCGGCGGTTGTGGAAC

>HPV24_Unclassified_9628486_nt645_E7_AOS|1
ACAAGACATTGTCTTAGAGTTGACTGAGCCACAGACTGTTGATTTGCACTGTGAAGAGGA
>HPV24_Unclassified_9628486_nt635_E7_AOS|1
AGGTCACTCTACAAGACATTGTCTTAGAGTTGACTGAGCCACAGACTGTTGATTTGCACT
>HPV48_Gamma_9628542_nt734_E7_AOS|1
TGAAAGAGCTTTGTTTATTTTGTCCTGCGTGCTCCAGACAACTTCCCCGCAATGGCAGAT
>HPV48_Gamma_9628542_nt725_E7_AOS|1
AACTGTTACTGAAAGAGCTTTGTTTATTTTGTCCTGCGTGCTCCAGACAACTTCCCCGCA
>HPV48_Gamma_9628542_nt716_E7_AOS|1
GTTTTGAGCAACTGTTACTGAAAGAGCTTTGTTTATTTTGTCCTGCGTGCTCCAGACAAC
>HPV48_Gamma_9628542_nt707_E7_AOS|1
GAATACGTTGTTTTGAGCAACTGTTACTGAAAGAGCTTTGTTTATTTTGTCCTGCGTGCT
>HPV48_Gamma_9628542_nt652_E7_AOS|1
AAGTGTCACAATTGTGGCTGTCGCATAAGGGTGACAGTCGCTGCAACTGAATTTGGAATA
>HPV48_Gamma_9628542_nt643_E7_AOS|1
ATAGACTCCAAGTGTCACAATTGTGGCTGTCGCATAAGGGTGACAGTCGCTGCAACTGAA
>HPV48_Gamma_9628542_nt634_E7_AOS|1
CCGTACCGGATAGACTCCAAGTGTCACAATTGTGGCTGTCGCATAAGGGTGACAGTCGCT
>HPV48_Gamma_9628542_nt625_E7_AOS|1
GAGTTTTGTCCGTACCGGATAGACTCCAAGTGTCACAATTGTGGCTGTCGCATAAGGGTG
>HPV50_Gamma_9628550_nt643_E7_AOS|1
ATTGATTCTACGTGTAAGCATTGTCATTGTCGCGTTAGACTGTGTTGTGTCGCCACCGAC
>HPV50_Gamma_9628550_nt634_E7_AOS|1
CCATTCAAGATTGATTCTACGTGTAAGCATTGTCATTGTCGCGTTAGACTGTGTTGTGTC
>HPV50_Gamma_9628550_nt625_E7_AOS|1
GAAAGGTCACCATTCAAGATTGATTCTACGTGTAAGCATTGTCATTGTCGCGTTAGACTG
>HPV50_Gamma_9628550_nt616_E7_AOS|1
GCAGAATCTGAAAGGTCACCATTCAAGATTGATTCTACGTGTAAGCATTGTCATTGTCGC
>HPV60_Gamma_9628566_nt552_E7_AOS|1
ATAACCTTGATGTAAATTTGGAGGAGTTGGTTTTACCTGTCAGTCTTTTAGCTGATGAGG
>HPV60_Gamma_9628566_nt542_E7_AOS|1
CCTAATGTTAATAACCTTGATGTAAATTTGGAGGAGTTGGTTTTACCTGTCAGTCTTTTA
>HPV60_Gamma_9628566_nt532_E7_AOS|1
TGGTAATCAGCCTAATGTTAATAACCTTGATGTAAATTTGGAGGAGTTGGTTTTACCTGT
>HPV61_Alpha_9628574_nt547_E7_AOS|1
CATAGTCCTTGAAGAGCGTCCTGAGGTGGTTGACCTACATTGCAATGAGCAGTTATTAGA
>HPV61_Alpha_9628574_nt601_E7_AOS|1
ATTAGACAGCTCAGAGTCAGAGGAGGAGGATAGTGTGCGTGAGCAACTTGTTGAACAAGC
>HPV8_Beta_333074_nt846_E7_AOS_rc|1
TGAACAGCAATTCTTGAAAGGTCCTGATACCCGAATCAGTTGCGTTGACAAAAAGACGTA
>HPV51_Alpha_333087_nt756_E7_AOS_rc|1
TCTGCTGTACAACGCGAAGGGTGTCTCCACTGCTTTCCACTGCCAGTTGTACTACACTTG
>HPV51_Alpha_333087_nt716_E7_AOS_rc|1
TGCCAGTTGTACTACACTTGAACACCTGCAACACGGAGCTTCAATTCTGTAACACGTAGC
>HPV20_Beta_1020162_nt893_E7_AOS_rc|1
GCTCGTCAATCAGCAATTGTTGAAAGCTTCTAATAGCAAATTCTGTAGCGCTTATAAAGA
>HPV20_Beta_1020162_nt881_E7_AOS_rc|1
GCAATTGTTGAAAGCTTCTAATAGCAAATTCTGTAGCGCTTATAAAGATGCGAAGTTTCA
>HPV22_Beta_1020178_nt847_E7_AOS_rc|1
ACACAAAAGTTTTACAGGTCCAAGTAGAGATGTTTGAAAGTCGCGAATTCCAGAGAGAGT
>HPV22_Beta_1020178_nt841_E7_AOS_rc|2
AAGTTTTACAGGTCCAAGTAGAGATGTTTGAAAGTCGCGAATTCCAGAGAGAGTTGCTAG
>HPV22_Beta_1020178_nt835_E7_AOS_rc|1
TACAGGTCCAAGTAGAGATGTTTGAAAGTCGCGAATTCCAGAGAGAGTTGCTAGTATATA
>HPV22_Beta_1020178_nt829_E7_AOS_rc|2

TCCAAGTAGAGATGTTTGAAAGTCGCGAATTCCAGAGAGAGTTGCTAGTATATACAGCTT
>HPV22_Beta_1020178_nt823_E7_AOS_rc|1
TAGAGATGTTTGAAAGTCGCGAATTCCAGAGAGAGTTGCTAGTATATACAGCTTCAGCTT
>HPV22_Beta_1020178_nt817_E7_AOS_rc|1
TGTTTGAAAGTCGCGAATTCCAGAGAGAGTTGCTAGTATATACAGCTTCAGCTTTGTATC
>HPV22_Beta_1020178_nt811_E7_AOS_rc|1
AAAGTCGCGAATTCCAGAGAGAGTTGCTAGTATATACAGCTTCAGCTTTGTATCACAACC
>HPV22_Beta_1020178_nt800_E7_AOS_rc|1
TTCCAGAGAGAGTTGCTAGTATATACAGCTTCAGCTTTGTATCACAACCCCCACAATATA
>HPV22_Beta_1020178_nt794_E7_AOS_rc|1
AGAGAGTTGCTAGTATATACAGCTTCAGCTTTGTATCACAACCCCCACAATATACTACAA
>HPV23_Beta_1020186_nt877_E7_AOS_rc|1
TCGACAGGCAGGACACACCAGCTTCACGTTTTCTAGCAAAGATGCTTGGAACGAGCGAAT
>HPV23_Beta_1020186_nt867_E7_AOS_rc|1
GGACACACCAGCTTCACGTTTTCTAGCAAAGATGCTTGGAACGAGCGAATTCCAAAATCT
>HPV23_Beta_1020186_nt857_E7_AOS_rc|1
GCTTCACGTTTTCTAGCAAAGATGCTTGGAACGAGCGAATTCCAAAATCTGTGGCTAGCA
>HPV23_Beta_1020186_nt847_E7_AOS_rc|1
TTCTAGCAAAGATGCTTGGAACGAGCGAATTCCAAAATCTGTGGCTAGCACGTAAAGCTT
>HPV23_Beta_1020186_nt837_E7_AOS_rc|1
GATGCTTGGAACGAGCGAATTCCAAAATCTGTGGCTAGCACGTAAAGCTTTAACTGTGTC
>HPV23_Beta_1020186_nt827_E7_AOS_rc|1
ACGAGCGAATTCCAAAATCTGTGGCTAGCACGTAAAGCTTTAACTGTGTCTCACAGCCTC
>HPV28_Alpha_1020202_nt573_E7_AOS_rc|1
GTTCCTCCACATTTGTATAATCTTCTTCATCTAATTGCACATTGCATTGCACTGGGACGT
>HPV28_Alpha_1020202_nt715_E7_AOS_rc|1
CAGCGAGGGCACACGACCGTCAGTGTGCCCAACAGGAGCTGCTCCAATGCCCTTATATCT
>HPV36_Beta_1020218_nt861_E7_AOS_rc|1
CAGGTCACCGGTCAGCAACTGTTGAAATGCTCTGATGCCAAATTCTGTAGCTTGGACAAA
>HPV36_Beta_1020218_nt853_E7_AOS_rc|1
CGGTCAGCAACTGTTGAAATGCTCTGATGCCAAATTCTGTAGCTTGGACAAAAACGCGGA
>HPV36_Beta_1020218_nt847_E7_AOS_rc|1
GCAACTGTTGAAATGCTCTGATGCCAAATTCTGTAGCTTGGACAAAAACGCGGAGCTTGA
>HPV37_Beta_1020226_nt803_E7_AOS_rc|1
GCACTTCACCAAGTAATAGCTCTTCTTGTGATCTAATTCCAATAGGCGTTGCAACAACTA
>HPV37_Beta_1020226_nt812_E7_AOS_rc|1
AAACCAGCTGCACTTCACCAAGTAATAGCTCTTCTTGTGATCTAATTCCAATAGGCGTTG
>HPV37_Beta_1020226_nt794_E7_AOS_rc|1
CAAGTAATAGCTCTTCTTGTGATCTAATTCCAATAGGCGTTGCAACAACTATCAGTCGTA
>HPV37_Beta_1020226_nt785_E7_AOS_rc|1
GCTCTTCTTGTGATCTAATTCCAATAGGCGTTGCAACAACTATCAGTCGTAGTTTAGAAC
>HPV38_Beta_1020234_nt752_E7_AOS_rc|1
GTTTTAGCCTTACTTCACAACCCCCACAAAGAACTATGATTTTGTATGGGGTGTATGCTG
>HPV66_Alpha_1020290_nt592_E7_AOS_rc|1
TCATTGCATTGTAGGTCAATTTCCGTTTGCGGTGCAAGTTCTAATATAACCTCTTGCAAC
>HPV66_Alpha_1020290_nt585_E7_AOS_rc|1
ATTGTAGGTCAATTTCCGTTTGCGGTGCAAGTTCTAATATAACCTCTTGCAACGTTGGTA
>HPV103_Unclassified_109390382_nt171_E7_AOS_rc|1
AGCTGTTGTTCAAAGTTTCTAATGCCCTCCTCCGTGGCTACCACATACATACACAATGAC
>HPV103_Unclassified_109390382_nt165_E7_AOS_rc|1
TGTTCAAAGTTTCTAATGCCCTCCTCCGTGGCTACCACATACATACACAATGACTTCTCA
>HPV103_Unclassified_109390382_nt159_E7_AOS_rc|1
AAGTTTCTAATGCCCTCCTCCGTGGCTACCACATACATACACAATGACTTCTCACAATAC
>HPV103_Unclassified_109390382_nt153_E7_AOS_rc|1
CTAATGCCCTCCTCCGTGGCTACCACATACATACACAATGACTTCTCACAATACTTGCAA

>HPV103_Unclassified_109390382_nt147_E7_AOS_rc|1
CCCTCCTCCGTGGCTACCACATACATACACAATGACTTCTCACAATACTTGCAATCTGTC
>HPV101_Unclassified_109390389_nt151_E7_AOS_rc|2
CCTTATTGCCCCGTCCGTAGCTAGTACATGTAATGCTAAGGTTTTGTGACACTGCCCACA
>HPV101_Unclassified_109390389_nt162_E7_AOS_rc|1
TGTTCCACAGACCTTATTGCCCCGTCCGTAGCTAGTACATGTAATGCTAAGGTTTTGTGA
>HPV70_Alpha_1173493_nt804_E7_AOS_rc|1
TGCAGTAGAGATCGCAGGTTCTCTTGTGAGGCTTCTACTACTAAGTGCAGTGTAGTATTA
>HPV71_Alpha_12084981_nt739_E7_AOS_rc|1
TGTGCCCAGTAACAGCTGTTGAAGCGCTCTTATGTCTGCTTCGTCGCTTTCCACCACAAG
>HPV71_Alpha_12084981_nt729_E7_AOS_rc|1
AACAGCTGTTGAAGCGCTCTTATGTCTGCTTCGTCGCTTTCCACCACAAGCCTAACTGCA
>HPV84_Alpha_12958167_nt437_E7_AOS_rc|1
TCACAGTATAAACTAACTACATCTGGTATATCTGTTAAAATAATATCCTTTAACGTTGGC
>HPV84_Alpha_12958167_nt427_E7_AOS_rc|1
AACTAACTACATCTGGTATATCTGTTAAAATAATATCCTTTAACGTTGGCGTCTGGCCGC
>HPV107_Beta_126131393_nt885_E7_AOS_rc|1
CTGCAATGCCCGAATGCCAAAATGGGTTGCAAAAACGTAGAGACGAAGTTTCACTGGACA
>HPV107_Beta_126131393_nt879_E7_AOS_rc|1
TGCCCGAATGCCAAAATGGGTTGCAAAAACGTAGAGACGAAGTTTCACTGGACACCCAGA
>HPV107_Beta_126131393_nt873_E7_AOS_rc|1
AATGCCAAAATGGGTTGCAAAAACGTAGAGACGAAGTTTCACTGGACACCCAGAACAAGG
>HPV107_Beta_126131393_nt867_E7_AOS_rc|1
AAAATGGGTTGCAAAAACGTAGAGACGAAGTTTCACTGGACACCCAGAACAAGGTACTAC
>HPV107_Beta_126131393_nt861_E7_AOS_rc|2
GGTTGCAAAAACGTAGAGACGAAGTTTCACTGGACACCCAGAACAAGGTACTACAATTTT
>HPV72_Alpha_1491683_nt623_E7_AOS_rc|1
TGCTTGTTCTACAAGTTGCTCCTGCACACCAACACCGTCCCTCTCATCCTCTGACTCTGA
>HPV72_Alpha_1491683_nt617_E7_AOS_rc|1
TTCTACAAGTTGCTCCTGCACACCAACACCGTCCCTCTCATCCTCTGACTCTGACTCTGA
>HPV72_Alpha_1491683_nt607_E7_AOS_rc|1
TGCTCCTGCACACCAACACCGTCCCTCTCATCCTCTGACTCTGACTCTGAGCTGTCTAGT
>HPV73_Alpha_1491692_nt719_E7_AOS_rc|1
CTCTTAAATCAGCTTTGTTGCTTTCAATGGCAAGGCATACTGTGCACTGACACTTCGTGC
>HPV73_Alpha_1491692_nt736_E7_AOS_rc|1
AAGCAACTCTTCTATCACTCTTAAATCAGCTTTGTTGCTTTCAATGGCAAGGCATACTGT
>HPVcand86_Alpha_15741127_nt431_E7_AOS_rc|1
TGTAGGTTAACTACATCTGGTATTTCACATAACACTATATCTTTTATGGTCGGCGTCTGG
>HPVcand86_Alpha_15741127_nt441_E7_AOS_rc|1
CTCGTCACAATGTAGGTTAACTACATCTGGTATTTCACATAACACTATATCTTTTATGGT
>HPV88_Gamma_167600365_nt644_E7_AOS_rc|1
CGGCTGGTGAAGCTAGAACAAATACCCTTACACCTGCTTCACAGAAGGCGCAACTGGTAT
>HPV110_Beta_186461194_nt742_E7_AOS_rc|1
GAATTCCAAAAACTGTTGCAAGCACAAAAAGCCGAAGTCGCGCACCACAAAGGCCACAAG
>HPV110_Beta_186461194_nt733_E7_AOS_rc|1
AAACTGTTGCAAGCACAAAAAGCCGAAGTCGCGCACCACAAAGGCCACAAGCAGCAACAA
>HPV110_Beta_186461194_nt724_E7_AOS_rc|1
CAAGCACAAAAAGCCGAAGTCGCGCACCACAAAGGCCACAAGCAGCAACAATCTTGTAAG
>HPV111_Beta_186461202_nt760_E7_AOS_rc|1
ACAAGCTGTACTTCACCCAGTAATAGTTCTTGTTGGGCTCTTATTCCAAGTTCCGTAGCA
>HPV111_Beta_186461202_nt752_E7_AOS_rc|1
TACTTCACCCAGTAATAGTTCTTGTTGGGCTCTTATTCCAAGTTCCGTAGCAAAGACGTA
>HPV111_Beta_186461202_nt744_E7_AOS_rc|1
CCAGTAATAGTTCTTGTTGGGCTCTTATTCCAAGTTCCGTAGCAAAGACGTATAAGCGAA
>HPV111_Beta_186461202_nt736_E7_AOS_rc|1

AGTTCTTGTTGGGCTCTTATTCCAAGTTCCGTAGCAAAGACGTATAAGCGAAGTCTTGTA
>HPVFA75/KI88-03_Unclassified_206902429_nt765_E7_AOS_rc|2
GAATTAAAAGTTCTTGCTGTGTGCGGATTCCAAAGAAGGTAGCAATCACAAATAGTCGAA
>HPVFA75/KI88-03_Unclassified_206902429_nt758_E7_AOS_rc|1
AAGTTCTTGCTGTGTGCGGATTCCAAAGAAGGTAGCAATCACAAATAGTCGAAGGCGAGC
>HPVFA75/KI88-03_Unclassified_206902429_nt751_E7_AOS_rc|1
TGCTGTGTGCGGATTCCAAAGAAGGTAGCAATCACAAATAGTCGAAGGCGAGCGCCACAA
>HPVFA75/KI88-03_Unclassified_206902429_nt744_E7_AOS_rc|1
TGCGGATTCCAAAGAAGGTAGCAATCACAAATAGTCGAAGGCGAGCGCCACAACCACCAC
>HPVFA75/KI88-03_Unclassified_206902429_nt737_E7_AOS_rc|1
TCCAAAGAAGGTAGCAATCACAAATAGTCGAAGGCGAGCGCCACAACCACCACAAGGAGC
>HPVFA75/KI88-03_Unclassified_206902429_nt730_E7_AOS_rc|1
AAGGTAGCAATCACAAATAGTCGAAGGCGAGCGCCACAACCACCACAAGGAGCAACTATC
>HPV91_Alpha_22023568_nt746_E7_AOS_rc|1
AGACGAATTGAACAATAACAAGAGGTGCACTCGGTTACAACTTTGTAAACTTGTAGGTCC
>HPVcand89_Alpha_22095322_nt604_E7_AOS_rc|1
GATGTCTTAGATGTGCGTCGTCAGACAATACTACTAACCTAACTGCACGATAGCACACAC
>HPV90_Alpha_22138122_nt439_E7_AOS_rc|1
TACAATGTAGGTCAACCGCATCAGGCTGATCCAGCTCTAGAAATATGTCCTTTAAGGTTG
>HPV58_Alpha_222386_nt783_E7_AOS_rc|1
ATGGTACATGTGCCCATAAGCAGCTGCTGTAGGGTTCGTACGTCGGTTGTTGTACTGTTG
>HPV58_Alpha_222386_nt768_E7_AOS_rc|1
ATAAGCAGCTGCTGTAGGGTTCGTACGTCGGTTGTTGTACTGTTGATACACAAACGAACC
>HPV58_Alpha_222386_nt744_E7_AOS_rc|1
ACGTCGGTTGTTGTACTGTTGATACACAAACGAACCGTGGTGCCACAAGTGTAACAACAA
>HPV74_Alpha_27462483_nt631_E7_AOS_rc|1
ACACTAGGCTCAGTGTACCCAGTAGAAGCGTATGTAGATTGTTGATGTCTGGTCCTGTAC
>HPV74_Alpha_27462483_nt646_E7_AOS_rc|1
GCGCGCACAGAGGACACACTAGGCTCAGTGTACCCAGTAGAAGCGTATGTAGATTGTTGA
>HPV74_Alpha_27462483_nt559_E7_AOS_rc|1
GCCGAAGGCTGCGATTACACACACAACAGCAGGTTACTATTTGGTAAGGCTGTGTATGTT
>HPV92_Beta_27531786_nt662_E7_AOS_rc|1
CCAAATTGGGTACACGATGCAAAAATTCGAAGCTTAATTCCGCAGCCACCACACGAGGAA
>HPV92_Beta_27531786_nt654_E7_AOS_rc|1
GGTACACGATGCAAAAATTCGAAGCTTAATTCCGCAGCCACCACACGAGGAAACTATCTT
>HPV75_Beta_2911544_nt847_E7_AOS_rc|1
ATGTCGAATTTCTTCACGACACTCAGGACACAGGAATTGCAGCTCCTCCAACAGCAAAGT
>HPV75_Beta_2911544_nt839_E7_AOS_rc|1
TTTCTTCACGACACTCAGGACACAGGAATTGCAGCTCCTCCAACAGCAAAGTTTGAAATT
>HPV75_Beta_2911544_nt830_E7_AOS_rc|1
GACACTCAGGACACAGGAATTGCAGCTCCTCCAACAGCAAAGTTTGAAATTCTCGAATAG
>HPV75_Beta_2911544_nt815_E7_AOS_rc|1
GGAATTGCAGCTCCTCCAACAGCAAAGTTTGAAATTCTCGAATAGCCCGATCAGTAGCAA
>HPV75_Beta_2911544_nt808_E7_AOS_rc|1
CAGCTCCTCCAACAGCAAAGTTTGAAATTCTCGAATAGCCCGATCAGTAGCAAGCACAAA
>HPV76_Beta_2911551_nt846_E7_AOS_rc|1
TTGCGAATCTCTTCACGACACTCCGGACACAGGAATTGCAACTCCTCTAGCAGTAAAGAT
>HPV80_Beta_2911565_nt797_E7_AOS_rc|1
CTTCTCCAAATAGTAACTCTTCTTGTGCGCGAATTCCAAAAGAAGTTGCAACCACTATTA
>HPVRTRX7_Unclassified_30315616_nt862_E7_AOS_rc|1
GCTGGTCAGTAGTTCCTGCAGGGTTCTAATACCAAGTTCAGTTGCGTTGACAAAAACACG
>HPVRTRX7_Unclassified_30315616_nt882_E7_AOS_rc|1
GGACACAGCAGCTGCAGATCGCTGGTCAGTAGTTCCTGCAGGGTTCTAATACCAAGTTCA
>HPVRTRX7_Unclassified_30315616_nt875_E7_AOS_rc|1
GCAGCTGCAGATCGCTGGTCAGTAGTTCCTGCAGGGTTCTAATACCAAGTTCAGTTGCGT

>HPV65_Gamma_312100_nt702_E7_AOS_rc|1
CGTCCACTATGAGTTGTTCCAACGCTCGAAGTCCAAATTCCACAGCAAACAGAGTAATCC
>HPV65_Gamma_312100_nt684_E7_AOS_rc|1
CCAACGCTCGAAGTCCAAATTCCACAGCAAACAGAGTAATCCTTACATTAACTTCACATC
>HPV67_Alpha_3228267_nt720_E7_AOS_rc|1
TGTGAATGCACAAACGCAAAGTGCACTCACAGATGTTACACACAGTAACAATATGGTAAT
>HPV67_Alpha_3228267_nt725_E7_AOS_rc|1
TGTACTGTGAATGCACAAACGCAAAGTGCACTCACAGATGTTACACACAGTAACAATATG
>HPV67_Alpha_3228267_nt715_E7_AOS_rc|1
ATGCACAAACGCAAAGTGCACTCACAGATGTTACACACAGTAACAATATGGTAATTGGTT
>HPV67_Alpha_3228267_nt710_E7_AOS_rc|1
CAAACGCAAAGTGCACTCACAGATGTTACACACAGTAACAATATGGTAATTGGTTGTATC
>HPV67_Alpha_3228267_nt705_E7_AOS_rc|1
GCAAAGTGCACTCACAGATGTTACACACAGTAACAATATGGTAATTGGTTGTATCTGGTT
>HPV11_Alpha_333026_nt739_E7_AOS_rc|1
ACAATATTTAGTGTGCCCAGCAAAAGGTCTTGTAGTTGTCTGATGTCTCCGTCTGTGCAC
>HPV11_Alpha_333026_nt728_E7_AOS_rc|1
TGTGCCCAGCAAAAGGTCTTGTAGTTGTCTGATGTCTCCGTCTGTGCACTCCACAACCAG
>HPV31_Alpha_333048_nt696_E7_AOS_rc|1
GAAGTGTAGACTTACACTGACAACAAAAGGTAACGATATTGTAATTGGATGTGTCCGGTT
>HPV31_Alpha_333048_nt743_E7_AOS_rc|1
CTCTTGCAATATGCGAATATCTACTTGTGTGCTCTGTACACACAAACGAAGTGTAGACTT
>HPV31_Alpha_333048_nt733_E7_AOS_rc|1
ATGCGAATATCTACTTGTGTGCTCTGTACACACAAACGAAGTGTAGACTTACACTGACAA
>HPV31_Alpha_333048_nt720_E7_AOS_rc|1
CTTGTGTGCTCTGTACACACAAACGAAGTGTAGACTTACACTGACAACAAAAGGTAACGA
>HPV31_Alpha_333048_nt709_E7_AOS_rc|1
TGTACACACAAACGAAGTGTAGACTTACACTGACAACAAAAGGTAACGATATTGTAATTG
>HPV33_Alpha_333049_nt763_E7_AOS_rc|1
TAAGTAGTTGCTGTATGGTTCGTAGGTCACTTGCTGTACTGTTGACACATAAACGAACTG
>HPV33_Alpha_333049_nt746_E7_AOS_rc|1
GTTCGTAGGTCACTTGCTGTACTGTTGACACATAAACGAACTGTGGTGTTACAAGTGTGA
>HPV33_Alpha_333049_nt736_E7_AOS_rc|1
CACTTGCTGTACTGTTGACACATAAACGAACTGTGGTGTTACAAGTGTGACAACAGGTTA
>HPV35_Alpha_333050_nt757_E7_AOS_rc|1
CATTAATAAATCTTCCAATTTACGTATGTCAATGTGTGTGCTCTGTACACACAGACGTAG
>HPV35_Alpha_333050_nt752_E7_AOS_rc|1
ATAAATCTTCCAATTTACGTATGTCAATGTGTGTGCTCTGTACACACAGACGTAGTGTCG
>HPV47_Beta_333062_nt874_E7_AOS_rc|1
AGCTGCAGATCACCAGTCAAAAGTTCCTGAAATGTCCTGATGCCACGGTTTGTTGCGTTC
>HPV47_Beta_333062_nt867_E7_AOS_rc|1
GATCACCAGTCAAAAGTTCCTGAAATGTCCTGATGCCACGGTTTGTTGCGTTCACAAAAA
>HPV42_Alpha_333211_nt491_E7_AOS_rc|1
TCCACGCATTGTCCTCTGCAATGCGTACACCGACCCGTCCACTGACACAATATAAATTGA
>HPV42_Alpha_333211_nt481_E7_AOS_rc|1
GTCCTCTGCAATGCGTACACCGACCCGTCCACTGACACAATATAAATTGAAATCTTGTAC
>HPV39_Alpha_333245_nt804_E7_AOS_rc|1
TGCTGTAGTTGTCGCAGAGTATCCCGTGAGGCTTCTACTACCAGCTGCAGTGTGTTGTTA
>HPV93_Beta_37089393_nt684_E7_AOS_rc|1
TGGCAGCAACAAATATTCGAAGTCGGGATCCACAGCCTCCGCCGCAGAACACAATAATCT
>HPV93_Beta_37089393_nt674_E7_AOS_rc|1
AAATATTCGAAGTCGGGATCCACAGCCTCCGCCGCAGAACACAATAATCTTGAAAGCTGT
>HPV12_Beta_396910_nt860_E7_AOS_rc|1
TCACTGATCAGCAGGTCCTGTAGGGTCCTAATGCCAGTATCAGTTGCGTTGACAAAAATA
>HPV12_Beta_396910_nt880_E7_AOS_rc|1

CTGGGCACAGCAGCTGCAGGTCACTGATCAGCAGGTCCTGTAGGGTCCTAATGCCAGTAT
>HPV12_Beta_396910_nt872_E7_AOS_rc|1
AGCAGCTGCAGGTCACTGATCAGCAGGTCCTGTAGGGTCCTAATGCCAGTATCAGTTGCG
>HPV15_Beta_396924_nt798_E7_AOS_rc|1
ACTTCTTCCAATAATAAGTCTTGTTGTGAGCGAATTCCAAATGGAGTTGCAACCACTATA
>HPV15_Beta_396924_nt811_E7_AOS_rc|1
ACACACCAACTTAACTTCTTCCAATAATAAGTCTTGTTGTGAGCGAATTCCAAATGGAGT
>HPV17_Beta_396932_nt810_E7_AOS_rc|1
AACTGTACTTCACCTAATAAAAGCTCCTCTTGTGAACGAATTCCAGCGTGCGTTGCAAGA
>HPV17_Beta_396932_nt801_E7_AOS_rc|2
TCACCTAATAAAAGCTCCTCTTGTGAACGAATTCCAGCGTGCGTTGCAAGAACAATAAGC
>HPV17_Beta_396932_nt792_E7_AOS_rc|1
AAAAGCTCCTCTTGTGAACGAATTCCAGCGTGCGTTGCAAGAACAATAAGCCGTAGCTTA
>HPV19_Beta_396940_nt904_E7_AOS_rc|1
CCGGGCACAACAGTTGCAATTCTTCAATCAGGATGTCCTGTAGGGTTCTAATACCAAATT
>HPV19_Beta_396940_nt898_E7_AOS_rc|1
ACAACAGTTGCAATTCTTCAATCAGGATGTCCTGTAGGGTTCTAATACCAAATTGCGTGG
>HPV19_Beta_396940_nt892_E7_AOS_rc|1
GTTGCAATTCTTCAATCAGGATGTCCTGTAGGGTTCTAATACCAAATTGCGTGGCTTTCA
>HPV25_Beta_396948_nt888_E7_AOS_rc|1
CGGACACAACAGCTGCAGTTCTTCAATTAGAAGGTTTTGTAGTGTTCTAATACCAAAATC
>HPV25_Beta_396948_nt882_E7_AOS_rc|1
CAACAGCTGCAGTTCTTCAATTAGAAGGTTTTGTAGTGTTCTAATACCAAAATCAGTGGC
>HPV25_Beta_396948_nt872_E7_AOS_rc|1
AGTTCTTCAATTAGAAGGTTTTGTAGTGTTCTAATACCAAAATCAGTGGCTTTCACAAAG
>HPV30_Alpha_396973_nt579_E7_AOS_rc|1
AATGCAGGTCAATTTCAGTTTGCGGTACAAGGTCCAAAATATATTCTGGAATAGTTGTTA
>HPV30_Alpha_396973_nt572_E7_AOS_rc|1
GTCAATTTCAGTTTGCGGTACAAGGTCCAAAATATATTCTGGAATAGTTGTTACTTTACC
>HPV3_Alpha_397005_nt624_E7_AOS_rc|1
ACACTGTGACTACACAATACGCTTGTTGCGCTGGTTCCACAGCATTTATATACTCATCTT
>HPV40_Alpha_397014_nt752_E7_AOS_rc|1
CAACTGCTGGAACTGTGTTATATCAGTGATGGAACAATGCACTGCTAAGCGCACCAAACA
>HPV40_Alpha_397014_nt743_E7_AOS_rc|1
GAACTGTGTTATATCAGTGATGGAACAATGCACTGCTAAGCGCACCAAACACTGACAAAA
>HPV45_Alpha_397022_nt615_E7_AOS_rc|1
CGTAACACAACAGGTCAACAGGATCTAATTCATTCTGAGGTTCCAAATGCAATACAATTT
>HPV45_Alpha_397022_nt793_E7_AOS_rc|1
TGCTGTAGTGTTCTAAGGTCCTCTGCCGAGCTCTCTACTGTAAGCTCAATTCTGCCGTCA
>HPV52_Alpha_397038_nt752_E7_AOS_rc|1
CCAACAGCATTTGCTGTAGAGTACGAAGGTCCGTCGCAGTGCTATGAATGCATAGCCGTA
>HPV52_Alpha_397038_nt747_E7_AOS_rc|1
AGCATTTGCTGTAGAGTACGAAGGTCCGTCGCAGTGCTATGAATGCATAGCCGTAGTGTG
>HPV56_Alpha_397053_nt592_E7_AOS_rc|1
TCATTGCACTGTAGGTCAATTTCTGTTTGAGGTGTTAGTTCTAATACAACGTCTTGCAGC
>HPV56_Alpha_397053_nt585_E7_AOS_rc|1
ACTGTAGGTCAATTTCTGTTTGAGGTGTTAGTTCTAATACAACGTCTTGCAGCGTTGGTA
>HPVcand62_Alpha_39932599_nt645_E7_AOS_rc|1
GCCGGGACACACTATCTCCAGTGCATCCGTCAGTAGTTGTTGCAGCCGTCTGAGTTCTGC
>HPVcand62_Alpha_39932599_nt602_E7_AOS_rc|1
AGCCGTCTGAGTTCTGCGTCTCCACTTAACACCACCAACCTAACTAAACAGTTGCAAATA
>HPV43_Alpha_40804474_nt736_E7_AOS_rc|1
ATTTTTAATGTGCCCAACAGCAGGTCTTCTAGCTTCTTGATGTCACTGTCAGAGCACTGC
>HPV43_Alpha_40804474_nt680_E7_AOS_rc|1
CTGCACAACAAGTCGAATTACACACAGACAGGATGTGCACTCTGTTACAACTCTGTAAAC

>HPV43_Alpha_40804474_nt671_E7_AOS_rc|1
AAGTCGAATTACACACAGACAGGATGTGCACTCTGTTACAACTCTGTAAACTTGTAGATT
>HPV43_Alpha_40804474_nt666_E7_AOS_rc|1
GAATTACACACAGACAGGATGTGCACTCTGTTACAACTCTGTAAACTTGTAGATTCACTT
>HPV81_Alpha_40804509_nt726_E7_AOS_rc|1
GCAGCCTCAGGTCTGCGTCTCCACTTAACACCACCAGCCTAACTAAACACCTACAAATAC
>HPV81_Alpha_40804509_nt773_E7_AOS_rc|1
GCAGCCAGGACACACTATGGACAGTGTATCCGTCAGTAGTTGTTGCAGCAGCCTCAGGTC
>HPV81_Alpha_40804509_nt545_E7_AOS_rc|1
GTCAATCACATCAGGATACTCATCTAGGACAATGTCCTTTATCGTTGCTGCCTGGCCATG
>HPV95_Gamma_40804520_nt756_E7_AOS_rc|1
TGCTGTGATTTCTTGCACACCCAACACAGCAAAATATCACCTTCCCTTCCAGAAGAAGTT
>HPV95_Gamma_40804520_nt762_E7_AOS_rc|1
CATTTCTGCTGTGATTTCTTGCACACCCAACACAGCAAAATATCACCTTCCCTTCCAGAA
>HPV95_Gamma_40804520_nt679_E7_AOS_rc|1
CCAAATGCAACAGCATACAAAGTGATTCTTACATTTGCTTCACAATTATAACAACAGGTA
>HPVcand85_Alpha_4574720_nt793_E7_AOS_rc|1
AGATCTCGTAGATCGGCTGCTGAACTTTCCACCACTAACTGCAGACTGGCCTCACATTTA
>HPVcand85_Alpha_4574720_nt804_E7_AOS_rc|1
ACAGCTGCTGGAGATCTCGTAGATCGGCTGCTGAACTTTCCACCACTAACTGCAGACTGG
>HPV96_Beta_50253426_nt954_E7_AOS_rc|1
AGCAAGTCTTCTAAAGAACGAATTCCAAACTGTGTAGCAGAAACGTAGATTTTCAGCCGG
>HPV96_Beta_50253426_nt961_E7_AOS_rc|1
TTCTAGCAGCAAGTCTTCTAAAGAACGAATTCCAAACTGTGTAGCAGAAACGTAGATTTT
>HPV96_Beta_50253426_nt949_E7_AOS_rc|1
GTCTTCTAAAGAACGAATTCCAAACTGTGTAGCAGAAACGTAGATTTTCAGCCGGGAATC
>HPV96_Beta_50253426_nt944_E7_AOS_rc|1
CTAAAGAACGAATTCCAAACTGTGTAGCAGAAACGTAGATTTTCAGCCGGGAATCACAGC
>HPV96_Beta_50253426_nt939_E7_AOS_rc|1
GAACGAATTCCAAACTGTGTAGCAGAAACGTAGATTTTCAGCCGGGAATCACAGCCTCCA
>HPV96_Beta_50253426_nt846_E7_AOS_rc|1
GGCTCCACCTCTGCTACTGTAGATTCTGACAGGTTTTCTGACTGCTCTTCTGTCAACTCT
>HPV96_Beta_50253426_nt839_E7_AOS_rc|1
CCTCTGCTACTGTAGATTCTGACAGGTTTTCTGACTGCTCTTCTGTCAACTCTTCGTCAC
>HPV83_Alpha_5059324_nt440_E7_AOS_rc|1
TGTTCATTACAATATAGGTCAACTATATCTGGTATTTCTTCTAGTACAATCTCTGCTATT
>HPV83_Alpha_5059324_nt450_E7_AOS_rc|1
GTCTATACCTTGTTCATTACAATATAGGTCAACTATATCTGGTATTTCTTCTAGTACAAT
>HPV83_Alpha_5059324_nt430_E7_AOS_rc|1
AATATAGGTCAACTATATCTGGTATTTCTTCTAGTACAATCTCTGCTATTGTTGCTGTAT
>HPV59_Alpha_557236_nt746_E7_AOS_rc|1
TAAGGCTCGCAATCCGTCTTGCGAGGTTTCTACTACTAGCTGAAGTTGATTATTACACTT
>HPV6_Alpha_6002612_nt742_E7_AOS_rc|1
ACACTATGTTTAGTGTTCCCAACAGAAGCTGTTGCACTTCTCTGATGTCTGTTTCTGTAC
>HPV57_Alpha_60882_nt728_E7_AOS_rc|1
CACTATGGTCAACGTATTCAGGAACAGCTGCTCCAGATGCCTTATGTCCGCCGCTCCGCA
>HPV69_Alpha_6970418_nt816_E7_AOS_rc|1
GTATGTAGCACACTGGTGGCACACCAAACTCACGTCGCCCATCAGCAGCTGCTCCACCGC
>HPV69_Alpha_6970418_nt808_E7_AOS_rc|1
CACACTGGTGGCACACCAAACTCACGTCGCCCATCAGCAGCTGCTCCACCGCTCGGACGT
>HPV69_Alpha_6970418_nt790_E7_AOS_rc|1
AACTCACGTCGCCCATCAGCAGCTGCTCCACCGCTCGGACGTTCTGTCGACTGCTTAGTA
>HPV82_Alpha_6970427_nt804_E7_AOS_rc|1
TTGGTCGCACACCACGGGCACACCAGGCTTAGGTCGCCCAGTAACATTTGCTGAAATATG
>HPV82_Alpha_6970427_nt758_E7_AOS_rc|1

TTTGCTGAAATATGCGAAGGCTGTCTCCACTGCTTTCCACTGCGAGCTGTACAACACTCG
>HPV82_Alpha_6970427_nt718_E7_AOS_rc|1
TGCGAGCTGTACAACACTCGAACACCTGCAACAGTGCACTTTAATTCTGTAACACGTATC
>HPV82_Alpha_6970427_nt697_E7_AOS_rc|1
ACACCTGCAACAGTGCACTTTAATTCTGTAACACGTATCCTGTCCAGCTTGTCTGGCTGG
>HPV68a_Alpha_71726685_nt702_E7_AOS_rc|1
AGCTGTTGTAGTGTCCGCAGGTTGTCCCGCGACGCTTCTACTACTAGTTGCAGTGCCTTG
>HPV97_Alpha_71726694_nt514_E7_AOS_rc|1
CATAACATTGAAGGTCAGCCGAATTTATTTCATTTTGGGGTTGCAAATGCAATATAATTT
>HPV97_Alpha_71726694_nt499_E7_AOS_rc|1
CAGCCGAATTTATTTCATTTTGGGGTTGCAAATGCAATATAATTTCTTGTAATGTTGGTT
>HPV106_Alpha_71726710_nt480_E7_AOS_rc|1
ATGCAGGTCAACAACATCTGGCTCATCCAATTCTAACAGGATGTCCTTTAAGGTTGGTTG
>HPV102_Alpha_71726718_nt428_E7_AOS_rc|1
TACAGGTCAATTACATCTGGTAATTCTTCTAAAACTATATCTGCTATCGTTGCTGCGTGG
>HPV102_Alpha_71726718_nt440_E7_AOS_rc|1
TGCTCATTACAATACAGGTCAATTACATCTGGTAATTCTTCTAAAACTATATCTGCTATC
>HPV13_Alpha_85827580_nt656_E7_AOS_rc|1
AACATTTACTACAGGACGTTAGTATTTGGTAGCATTGTAATAGTGTGCTATGTTGCGTGG
>HPV13_Alpha_85827580_nt732_E7_AOS_rc|1
AGCGTGCCCAGCAGTAGGTCGTGTAGGTCGTGAATGTCAGGTCCTGTACACTCCACCACC
>HPV13_Alpha_85827580_nt673_E7_AOS_rc|1
CAGCCGGACGTTGCTACAACATTTACTACAGGACGTTAGTATTTGGTAGCATTGTAATAG
>HPV2_Alpha_9626032_nt724_E7_AOS_rc|1
AGTCAGCGTCTTCAAGAACAGCTGTTCCAGCTCTCTTAGGTCTGCTTGTCCGCACTCAAC
>HPV2_Alpha_9626032_nt736_E7_AOS_rc|1
AGGGCACACTAGAGTCAGCGTCTTCAAGAACAGCTGTTCCAGCTCTCTTAGGTCTGCTTG
>HPV41_Nu_9626041_nt865_E7_AOS_rc|1
AGGTCAAGGTGACTGTTGACCAGGAGTGCCTGTAGATTCCGTATCCCAGTGTTGCTTGCT
>HPV41_Nu_9626041_nt855_E7_AOS_rc|1
GACTGTTGACCAGGAGTGCCTGTAGATTCCGTATCCCAGTGTTGCTTGCTACGCATACAA
>HPV41_Nu_9626041_nt845_E7_AOS_rc|1
CAGGAGTGCCTGTAGATTCCGTATCCCAGTGTTGCTTGCTACGCATACAAATCGTATGAT
>HPV41_Nu_9626041_nt835_E7_AOS_rc|1
TGTAGATTCCGTATCCCAGTGTTGCTTGCTACGCATACAAATCGTATGATAGCCTGGCAA
>HPV41_Nu_9626041_nt816_E7_AOS_rc|1
TGTTGCTTGCTACGCATACAAATCGTATGATAGCCTGGCAAAATGGACACTGACTCTCTA
>HPV41_Nu_9626041_nt806_E7_AOS_rc|1
TACGCATACAAATCGTATGATAGCCTGGCAAAATGGACACTGACTCTCTACCAGGTATAA
>HPV41_Nu_9626041_nt796_E7_AOS_rc|1
AATCGTATGATAGCCTGGCAAAATGGACACTGACTCTCTACCAGGTATAATGTTCTCCTG
>HPV41_Nu_9626041_nt786_E7_AOS_rc|1
TAGCCTGGCAAAATGGACACTGACTCTCTACCAGGTATAATGTTCTCCTGGGTGTGGGGA
>HPV41_Nu_9626041_nt776_E7_AOS_rc|1
AAATGGACACTGACTCTCTACCAGGTATAATGTTCTCCTGGGTGTGGGGACTTGCTGCAC
>HPV41_Nu_9626041_nt766_E7_AOS_rc|1
TGACTCTCTACCAGGTATAATGTTCTCCTGGGTGTGGGGACTTGCTGCACCTGTTCCCGC
>HPV1_Mu_9626063_nt657_E7_AOS_rc|1
ACGGTCAATCGAACCAGTTTCTCGCAATAGGCACAGGAAGCAACGACAGCATAAGGTTGC
>HPV1_Mu_9626063_nt684_E7_AOS_rc|1
TGTCTAATGGCGCTGTGATCCGCGAGGACGGTCAATCGAACCAGTTTCTCGCAATAGGCA
>HPV1_Mu_9626063_nt675_E7_AOS_rc|1
GCGCTGTGATCCGCGAGGACGGTCAATCGAACCAGTTTCTCGCAATAGGCACAGGAAGCA
>HPV1_Mu_9626063_nt666_E7_AOS_rc|1
TCCGCGAGGACGGTCAATCGAACCAGTTTCTCGCAATAGGCACAGGAAGCAACGACAGCA

>HPV1_Mu_9626063_nt648_E7_AOS_rc|1
CGAACCAGTTTCTCGCAATAGGCACAGGAAGCAACGACAGCATAAGGTTGCTGAGGCGAC
>HPV18_Alpha_9626069_nt810_E7_AOS_rc|1
GGGTGTTCAGAAACAGCTGCTGGAATGCTCGAAGGTCGTCTGCTGAGCTTTCTACTACTA
>HPV18_Alpha_9626069_nt802_E7_AOS_rc|1
AGAAACAGCTGCTGGAATGCTCGAAGGTCGTCTGCTGAGCTTTCTACTACTAGCTCAATT
>HPV18_Alpha_9626069_nt795_E7_AOS_rc|1
GCTGCTGGAATGCTCGAAGGTCGTCTGCTGAGCTTTCTACTACTAGCTCAATTCTGGCTT
>HPV18_Alpha_9626069_nt788_E7_AOS_rc|1
GAATGCTCGAAGGTCGTCTGCTGAGCTTTCTACTACTAGCTCAATTCTGGCTTCACACTT
>HPV4_Gamma_9626597_nt720_E7_AOS_rc|1
AAAACGTCAGCTTTCCTTCTACAAGAAGTTGTTCCAAGGTCCGTAGTCCGAGCTCAGCAG
>HPV4_Gamma_9626597_nt708_E7_AOS_rc|1
TTCCTTCTACAAGAAGTTGTTCCAAGGTCCGTAGTCCGAGCTCAGCAGCATACAATGTAA
>HPV4_Gamma_9626597_nt686_E7_AOS_rc|1
CAAGGTCCGTAGTCCGAGCTCAGCAGCATACAATGTAATTCTTACAGCAACTTCACATCT
>HPV63_Mu_9626605_nt697_E7_AOS_rc|1
TCCTCCAGACGCGTAAGAGTAGACAGGTCAGAAACGCAGGTCAGCCTTAACAGCTGATCG
>HPV63_Mu_9626605_nt690_E7_AOS_rc|1
GACGCGTAAGAGTAGACAGGTCAGAAACGCAGGTCAGCCTTAACAGCTGATCGCACAAAC
>HPV63_Mu_9626605_nt683_E7_AOS_rc|1
AAGAGTAGACAGGTCAGAAACGCAGGTCAGCCTTAACAGCTGATCGCACAAACCACAAGG
>HPV63_Mu_9626605_nt676_E7_AOS_rc|1
GACAGGTCAGAAACGCAGGTCAGCCTTAACAGCTGATCGCACAAACCACAAGGAAGCACA
>HPV63_Mu_9626605_nt669_E7_AOS_rc|1
CAGAAACGCAGGTCAGCCTTAACAGCTGATCGCACAAACCACAAGGAAGCACAATTGCAT
>HPV63_Mu_9626605_nt662_E7_AOS_rc|1
GCAGGTCAGCCTTAACAGCTGATCGCACAAACCACAAGGAAGCACAATTGCATATGGATA
>HPV63_Mu_9626605_nt655_E7_AOS_rc|1
AGCCTTAACAGCTGATCGCACAAACCACAAGGAAGCACAATTGCATATGGATATTCAGAC
>HPV63_Mu_9626605_nt648_E7_AOS_rc|1
ACAGCTGATCGCACAAACCACAAGGAAGCACAATTGCATATGGATATTCAGACTCCTCCT
>HPV16_Alpha_9627100_nt744_E7_AOS_rc|1
TCTTCCAAAGTACGAATGTCTACGTGTGTGCTTTGTACGCACAACCGAAGCGTAGAGTCA
>HPV5_Beta_9627145_nt795_E7_AOS_rc|1
CTTGACCTCACAGTTCCTGCAACCGCACGGAGCTATAACTTTGTAAGAGATCCTTTCGTT
>HPV5_Beta_9627145_nt788_E7_AOS_rc|1
TCACAGTTCCTGCAACCGCACGGAGCTATAACTTTGTAAGAGATCCTTTCGTTGTCAGGC
>HPV10_Alpha_9627257_nt551_E7_AOS_rc|1
TTCATCTAATTGCACATTGCATACAGGGATATCCTCTGGTGCAAGACTCAATTCGATATC
>HPV26_Alpha_9627305_nt803_E7_AOS_rc|1
CAGCACACTGATGGCACACCAAGGACACGTCTTCCATTAACATCTGCTCCAGCACTCGAA
>HPV26_Alpha_9627305_nt782_E7_AOS_rc|1
AGGACACGTCTTCCATTAACATCTGCTCCAGCACTCGAACGTTCTGTCGACTGCTCTGCA
>HPV32_Alpha_9627327_nt560_E7_AOS_rc|1
GTCAACCGGCGGTGTGTCGCACGTTGTCGGGTCACACGTTGGCAGGTCATACAAAATAAT
>HPV32_Alpha_9627327_nt641_E7_AOS_rc|2
ACGTTGTATGTCCTGTTTTATAGGTTGGTCATCGTCTTCATCATCTTCATCTGAGGTGTC
>HPV34_Alpha_9627334_nt741_E7_AOS_rc|2
CCATAAGCAGGTCTTCTAACACTAATAGGTCAGCGTGTGTGCTCTCAATGGTAAGACACA
>HPV34_Alpha_9627334_nt731_E7_AOS_rc|1
GTCTTCTAACACTAATAGGTCAGCGTGTGTGCTCTCAATGGTAAGACACACTGTGGACTG
>HPV34_Alpha_9627334_nt721_E7_AOS_rc|2
ACTAATAGGTCAGCGTGTGTGCTCTCAATGGTAAGACACACTGTGGACTGACATCTGCTG
>HPV34_Alpha_9627334_nt711_E7_AOS_rc|1

CAGCGTGTGTGCTCTCAATGGTAAGACACACTGTGGACTGACATCTGCTGCAATCAGTAA
>HPV34_Alpha_9627334_nt695_E7_AOS_rc|1
AATGGTAAGACACACTGTGGACTGACATCTGCTGCAATCAGTAACTATTCTGTACCAGGC
>HPV49_Beta_9627363_nt805_E7_AOS_rc|1
CAGAAGCAGTTCTTGGAAACTTCTAATAGCAGCGTCAGTGGCTAACACGAAGACTCGCAG
>HPV49_Beta_9627363_nt798_E7_AOS_rc|1
AGTTCTTGGAAACTTCTAATAGCAGCGTCAGTGGCTAACACGAAGACTCGCAGTCTGGCA
>HPV49_Beta_9627363_nt791_E7_AOS_rc|1
GGAAACTTCTAATAGCAGCGTCAGTGGCTAACACGAAGACTCGCAGTCTGGCACCGCATC
>HPV53_Alpha_9627377_nt581_E7_AOS_rc|2
TTGCAGGTCAATCTCAGTTTGTGGTATAAGTTCTATAATATATTGTGGAAGTGTTGGTAC
>HPV53_Alpha_9627377_nt574_E7_AOS_rc|1
TCAATCTCAGTTTGTGGTATAAGTTCTATAATATATTGTGGAAGTGTTGGTACGTTACCG
>HPV7_Alpha_9627389_nt758_E7_AOS_rc|1
CATCAGCAACTGATGAACCTGCCTTATATCAGTAGCAGTACAATGGACTACTAGGCGAAC
>HPV9_Beta_9627396_nt833_E7_AOS_rc|2
GACACACCAGTTGTATATCACCCAGCAAAAGTTCCTGTTGCGCTCGAATTCCTAAATTTG
>HPV9_Beta_9627396_nt824_E7_AOS_rc|1
GTTGTATATCACCCAGCAAAAGTTCCTGTTGCGCTCGAATTCCTAAATTTGTAGCAAGCA
>HPV9_Beta_9627396_nt815_E7_AOS_rc|1
CACCCAGCAAAAGTTCCTGTTGCGCTCGAATTCCTAAATTTGTAGCAAGCACGTATAAAC
>HPV9_Beta_9627396_nt800_E7_AOS_rc|1
CCTGTTGCGCTCGAATTCCTAAATTTGTAGCAAGCACGTATAAACGAAGTCTTGCACCGC
>HPV54_Alpha_9628437_nt634_E7_AOS_rc|1
CACTGGCTTAACACCTTAAATGCTTGTTTGTCAGGTTGTGTTACTGCTGTCTCATCTTCT
>HPV54_Alpha_9628437_nt706_E7_AOS_rc|1
TCCTGCAGTACCCTTATGCCTGTGTGCGTGCTATACACACATAGCCGTACTGTCTTACAA
>HPV54_Alpha_9628437_nt648_E7_AOS_rc|1
AACACACACCTCCACACTGGCTTAACACCTTAAATGCTTGTTTGTCAGGTTGTGTTACTG
>HPV24_Unclassified_9628486_nt726_E7_AOS_rc|1
GTTCCACAACCGCCGCCGCAACAAAGTATTATTTGTAAGCTCTTCTTTCAGGCTCCACC
>HPV24_Unclassified_9628486_nt645_E7_AOS_rc|1
TCCTCTTCACAGTGCAAATCAACAGTCTGTGGCTCAGTCAACTCTAAGACAATGTCTTGT
>HPV24_Unclassified_9628486_nt635_E7_AOS_rc|1
AGTGCAAATCAACAGTCTGTGGCTCAGTCAACTCTAAGACAATGTCTTGTAGAGTGACCT
>HPV48_Gamma_9628542_nt734_E7_AOS_rc|1
ATCTGCCATTGCGGGGAAGTTGTCTGGAGCACGCAGGACAAAATAAACAAAGCTCTTTCA
>HPV48_Gamma_9628542_nt725_E7_AOS_rc|1
TGCGGGGAAGTTGTCTGGAGCACGCAGGACAAAATAAACAAAGCTCTTTCAGTAACAGTT
>HPV48_Gamma_9628542_nt716_E7_AOS_rc|1
GTTGTCTGGAGCACGCAGGACAAAATAAACAAAGCTCTTTCAGTAACAGTTGCTCAAAAC
>HPV48_Gamma_9628542_nt707_E7_AOS_rc|1
AGCACGCAGGACAAAATAAACAAAGCTCTTTCAGTAACAGTTGCTCAAAACAACGTATTC
>HPV48_Gamma_9628542_nt652_E7_AOS_rc|1
TATTCCAAATTCAGTTGCAGCGACTGTCACCCTTATGCGACAGCCACAATTGTGACACTT
>HPV48_Gamma_9628542_nt643_E7_AOS_rc|1
TTCAGTTGCAGCGACTGTCACCCTTATGCGACAGCCACAATTGTGACACTTGGAGTCTAT
>HPV48_Gamma_9628542_nt634_E7_AOS_rc|1
AGCGACTGTCACCCTTATGCGACAGCCACAATTGTGACACTTGGAGTCTATCCGGTACGG
>HPV48_Gamma_9628542_nt625_E7_AOS_rc|1
CACCCTTATGCGACAGCCACAATTGTGACACTTGGAGTCTATCCGGTACGGACAAAACTC
>HPV50_Gamma_9628550_nt643_E7_AOS_rc|1
GTCGGTGGCGACACAACACAGTCTAACGCGACAATGACAATGCTTACACGTAGAATCAAT
>HPV50_Gamma_9628550_nt634_E7_AOS_rc|1
GACACAACACAGTCTAACGCGACAATGACAATGCTTACACGTAGAATCAATCTTGAATGG

>HPV50_Gamma_9628550_nt625_E7_AOS_rc|1
CAGTCTAACGCGACAATGACAATGCTTACACGTAGAATCAATCTTGAATGGTGACCTTTC
>HPV50_Gamma_9628550_nt616_E7_AOS_rc|1
GCGACAATGACAATGCTTACACGTAGAATCAATCTTGAATGGTGACCTTTCAGATTCTGC
>HPV60_Gamma_9628566_nt552_E7_AOS_rc|1
CCTCATCAGCTAAAAGACTGACAGGTAAAACCAACTCCTCCAAATTTACATCAAGGTTAT
>HPV60_Gamma_9628566_nt542_E7_AOS_rc|1
TAAAAGACTGACAGGTAAAACCAACTCCTCCAAATTTACATCAAGGTTATTAACATTAGG
>HPV60_Gamma_9628566_nt532_E7_AOS_rc|1
ACAGGTAAAACCAACTCCTCCAAATTTACATCAAGGTTATTAACATTAGGCTGATTACCA
>HPV61_Alpha_9628574_nt547_E7_AOS_rc|1
TCTAATAACTGCTCATTGCAATGTAGGTCAACCACCTCAGGACGCTCTTCAAGGACTATG
>HPV61_Alpha_9628574_nt601_E7_AOS_rc|1
GCTTGTTCAACAAGTTGCTCACGCACACTATCCTCCTCCTCTGACTCTGAGCTGTCTAAT
>HPVcand87_Alpha_14475578_nt588_E7_VE|1
AACCTACATTGTGACGAGCAATTGTTAGACAGCTCAGAGGAGGAGGACAATAGGGATTGT
>HPV88_Gamma_167600365_nt683_E7_VE|1
TTTGTTCTAGCTTCACCAGCCGGAATTCGCACGCTTCAGCAGCTTTTACTTGCAGAGATT
>HPV36_Beta_1020218_nt664_E7_VE|1
ATGATTGGTAAAGAGGTCACCGTGCAAGATATTGTTCTGGAGCTCAGTGAGGTGCAGCCT
>HPV92_Beta_27531786_nt550_E7_VE|1
ATTGTGCTGGATCTGCAAGACCTTGTCCAGCCCATTGACCTGCATTGTGACGAAGACTTG
>HPV59_Alpha_557236_nt663_E7_VE|1
GATGAACCAGATGGAGTTAATCATCCTTTGCTACTAGCTAGACGAGCTGAACCACAGCGT
>HPV82_Alpha_6970427_nt596_E7_VE|1
ATAGTGTTGGAGTTAACACCACAACCTGAAATTGACTTGCAATGCTACGAGCAATTTGAC
>HPV52_Alpha_397038_nt704_E7_VE|1
ACAAGCAATTACTACATTGTGACATATTGTCACAGTTGTGATAGCACACTACGGCTATGC
>HPV17_Beta_396932_nt653_E7_VE|1
ATAGTGCTTGAGCTGCAACAGCTTGTCCAGCCCACTGACCTGCATTGCTACGAAGAGTTA
>HPV88_Gamma_167600365_nt653_E7_VE|1
TGCGCCTTCTGTGAAGCAGGTGTAAGGGTATTTGTTCTAGCTTCACCAGCCGGAATTCGC
>HPV4_Gamma_9626597_nt732_E7_VE|1
GGACTACGGACCTTGGAACAACTTCTTGTAGAAGGAAAGCTGACGTTTTGCTGCACCGCT
>HPV93_Beta_37089393_nt732_E7_VE|1
TTTGTTGCTGCCACAGAATTTGGAGTTCGTTGCTTTCAGCAATTGCTAGTTAAGGAGTTG
>HPV41_Nu_9626041_nt825_E7_VE|1
CAGTGTCCATTTTGCCAGGCTATCATACGATTTGTATGCGTAGCAAGCAACACTGGGATA
>HPV34_Alpha_9627334_nt582_E7_VE|1
ATTGTGTTAGATCTGAAACCAACGACCGAGACTGACCTTACATGTTACGAGTCATTAGAC
>HPV58_Alpha_222386_nt725_E7_VE|1
GCTAATTACTACATTGTAACTTGTTGTTACACTTGTGGCACCACGGTTCGTTTGTGTATC
>HPV16_Alpha_9627100_nt623_E7_VE|1
GATCTCTACTGTTATGAGCAATTAAATGACAGCTCAGAGGAGGAGGATGAAATAGATGGT
>HPV47_Beta_333062_nt819_E7_VE|1
AAAGTGATTGCACCGTGCGGTTGCAGCTGCTGCGAGGTCAAGCTTCGCATTTTTGTGAAC
>HPV95_Gamma_40804520_nt591_E7_VE|1
CCTGCAAATTTGCTGAGCGATGAGGTCTTGCAACTCTCTGAGGAGGAGGATGAAGAAAGA
>HPV83_Alpha_5059324_nt541_E7_VE|1
GGCGTGCGTGACCAACTGGCAGAACAAGCAAAGCAGGCCTATAGGGTGTTAACAGTGTGT
>HPV7_Alpha_9627389_nt588_E7_VE|1
AGTTTAAGTTGCAACGAGCAATTAGACAGCTCAGACTCAGAAGATGACCATGAACAAGAC
>HPV59_Alpha_557236_nt753_E7_VE|1
AATAATCAACTTCAGCTAGTAGTAGAAACCTCGCAAGACGGATTGCGAGCCTTACAGCAG
>HPV82_Alpha_6970427_nt656_E7_VE|1

AGCTCAGACGAGGAGGATGAAGTAGATAATATGCGTGACCAGCCAGCCAGACAAGCTGGA
>HPV103_Unclassified_109390382_nt182_E7_VE|1
ATGTATGTGGTAGCCACGGAGGAGGGCATTAGAAACTTTGAACAACAGCTGTGTGGAAAC
>HPV63_Mu_9626605_nt675_E7_VE|1
ATTGTGCTTCCTTGTGGTTTGTGCGATCAGCTGTTAAGGCTGACCTGCGTTTCTGACCTG
>HPV102_Alpha_71726718_nt481_E7_VE|1
GACCTGTATTGTAATGAGCAAGCAATAGACAGCTCAGAGGAGGAGGACGAGAGGGATTGT
>HPV41_Nu_9626041_nt915_E7_VE|1
CACCTTGACCTCGCTTGTCACGCCTGTGTCGAGCAGAATGGCGTCCAGGGTCTCAGACAC
>HPV2_Alpha_9626032_nt590_E7_VE|1
ATTGTTGACCTACATTGCGACGAGCAATTTGACAGCTCAGAAGAAGAGAATAACCATCAA
>HPV70_Alpha_1173493_nt743_E7_VE|1
CAACTACTAGCCAGACGGGAAGAACCACAGCGTCACAAAATACAGTGTATGTGTTGTAAG
>HPV48_Gamma_9628542_nt728_E7_VE|1
CTGTTACTGAAAGAGCTTTGTTTATTTTGTCCTGCGTGCTCCAGACAACTTCCCCGCAAT
>HPV42_Alpha_333211_nt567_E7_VE|1
AAGGACATTGTTTTGTTTGACATACCAACGTGTGAGACACCCATTGACCTGTATTGCTAT
>HPV106_Alpha_71726710_nt559_E7_VE|1
AGCTCAGAAGAGGAGGATAATAGGGAGGACGGTGAGCAACCTACAGAACCAGCACAGCAG
>HPV42_Alpha_333211_nt657_E7_VE|1
CAAGCCAAACAGGACATACAGCGTTACAGAATACTGTGTGTGTGTACACAGTGTTACAAG
>HPV23_Beta_1020186_nt701_E7_VE|1
ATAGTTCTTGAAGAGCTTGTCCAGCCCATTGACCTGCATTGCCACGAGGAGCTCACTGAA
>HPV13_Alpha_85827580_nt593_E7_VE|1
GGTCTACATTGCAATGAGCAATTAGACAGCTCAGAAGACGAGGTGGACGAACAAGCCACG
>HPV32_Alpha_9627327_nt681_E7_VE|1
ATAAAACAGGACATACAACGTTACAGAATAGTGTGTGGTTGTACACAGTGTGGACGGTCA
>HPV71_Alpha_12084981_nt590_E7_VE|1
CAGCCAGAGGTTGTTGACCTGTATTGTCACGAGCAATTTGCCAGCTCAGACGAGGAAGAT
>HPV106_Alpha_71726710_nt529_E7_VE|1
GTTGACCTGCATTGTAACGAGCAATTTGCCAGCTCAGAAGAGGAGGATAATAGGGAGGAC
>HPVcand89_Alpha_22095322_nt541_E7_VE|1
ATGCATGAGCAACAGCAACTGGCAGAACGAGCACAGCAGGCCTTTAGGGTGGTAACTACG
>HPV74_Alpha_27462483_nt460_E7_VE|1
ATTGTGTTACAGCTACAACCTGACCCTGTAGGCCTACTTTGCAATGAGCAATTAGACAGC
>HPV80_Beta_2911565_nt623_E7_VE|1
ATGATTGGGAAAGAAGCTACTATACCAGATATAGAGCTTGAGCTGCAAGAGCTTGTCCAG
>HPV84_Alpha_12958167_nt484_E7_VE|1
AGTTTATACTGTGACGAGCAATTGTTAGACAGCTCAGAGGAGGAGGATAATGGGGATTGC
>HPV23_Beta_1020186_nt671_E7_VE|1
ATGATTGGGAAACAAGCTACTCTTCGTGATATAGTTCTTGAAGAGCTTGTCCAGCCCATT
>HPV70_Alpha_1173493_nt713_E7_VE|1
GAACCCGACCATGTAGTTAATCACCAACAACAACTACTAGCCAGACGGGAAGAACCACAG
>HPV60_Gamma_9628566_nt738_E7_VE|1
CTTCGTCAACTATTGCTTCAAGAAGTGTTCCTGACCTGTTTGAGGTGCTCCAGATCCCTT
>HPV73_Alpha_1491692_nt611_E7_VE|1
ATTGACCTTACATGTTACGAGTCATTGGACAACTCAGAGGATGAGGATGAAACAGACAGC
>HPV69_Alpha_6970418_nt685_E7_VE|1
GAAACAGATAATGTGCGTAACCAGCAAGCCAGACAAGCTGAACAAGAAGCGTGTTATAGA
>HPV68a_Alpha_71726685_nt635_E7_VE|1
CATCTACTACTAGCCAGACGGGACGAACAACAGCGTCACAGAATTCAGTGTCTGTGTTGT
>HPV34_Alpha_9627334_nt612_E7_VE|1
ACTGACCTTACATGTTACGAGTCATTAGACAACTCAGAGGATGAGGATGAAACAGACAGC
>HPV96_Beta_50253426_nt764_E7_VE|1
ATGATTGGGAAAGAAGCAACTATACCAGACATAAAGCTGGAGCTGCAAGACCTTGTCCTG

>HPV50_Gamma_9628550_nt638_E7_VE|1
TTCAAGATTGATTCTACGTGTAAGCATTGTCATTGTCGCGTTAGACTGTGTTGTGTCGCC
>HPV65_Gamma_312100_nt582_E7_VE|1
CCTATAAACCTGCTGAGTGAGGAGGTCTTGCAACCTTCAGATGATGAGTCTGAGGCTCCA
>HPV21_Beta_1020170_nt947_E7_VE|1
CTGTTTGAAGAATTGCAGCTGTTGTGTCCTGAGTGCCGCGGAAACTGCAAACATGGCGGA
>HPV31_Alpha_333048_nt651_E7_VE|1
AGCTCAGATGAGGAGGATGTCATAGACAGTCCAGCTGGACAAGCAGAACCGGACACATCC
>HPV103_Unclassified_109390382_nt212_E7_VE|1
AGAAACTTTGAACAACAGCTGTGTGGAAACCTGGATATTGTGTGTCCAGCCTGTGCAAAG
>HPV31_Alpha_333048_nt591_E7_VE|1
TATGTGTTAGATTTGCAACCTGAGGCAACTGACCTCCACTGTTATGAGCAATTACCCGAC
>HPV65_Gamma_312100_nt612_E7_VE|1
CAACCTTCAGATGATGAGTCTGAGGCTCCAGAGGAGGAGCTTTTTCCTTTTAGAATAGAC
>HPV107_Beta_126131393_nt799_E7_VE|1
TTACCAGAGCTTCCAGATGCTACTAGTGAAGAATCAGAGGAGGAGCCTGAACGCATCCCT
>HPV88_Gamma_167600365_nt623_E7_VE|1
GAGCGAGAACCTTTCCAAATAGATACCAGTTGCGCCTTCTGTGAAGCAGGTGTAAGGGTA
>HPV107_Beta_126131393_nt709_E7_VE|1
ATGATTGGGAAAGAAGTAACTATTCCAGATATAGTTCTGGAAGAGCTGCAAGAGCTTGCT
>HPV50_Gamma_9628550_nt668_E7_VE|1
CATTGTCGCGTTAGACTGTGTTGTGTCGCCACCGACGCAGCTATCCGTGTGTTTGAGTGC
>HPV36_Beta_1020218_nt694_E7_VE|1
ATTGTTCTGGAGCTCAGTGAGGTGCAGCCTGAAGTACTACCAGTTGACCTGTTTTGTGAA
>HPV32_Alpha_9627327_nt591_E7_VE|1
CCGACAACGTGCGACACACCGCCGGTTGACCTGTATTGTTATGAACAATTTGACACCTCA
>HPV6_Alpha_6002612_nt562_E7_VE|1
ATTGTATTAGACCTGCAACCTCCAGACCCTGTAGGGTTACATTGCTATGAGCAATTAGTA
>HPV18_Alpha_9626069_nt651_E7_VE|1
ATTCCGGTTGACCTTCTATGTCACGAGCAATTAAGCGACTCAGAGGAAGAAAACGATGAA
>HPV49_Beta_9627363_nt644_E7_VE|1
ATAATACTACAAGAAGAGTTTGGCCAGCCCATTGACCTGCAATGCTACGAGAATCTAACA
>HPV60_Gamma_9628566_nt618_E7_VE|1
TCACCCGATGGTGATCCTGAGGAGGAGGAGCACTATCCCTATACAATAGACACCTGTTGC
>HPV60_Gamma_9628566_nt588_E7_VE|1
CCTGTCAGTCTTTTAGCTGATGAGGAGTTGTCACCCGATGGTGATCCTGAGGAGGAGGAG
>HPV61_Alpha_9628574_nt639_E7_VE|1
CGTGAGCAACTTGTTGAACAAGCACAGCAGGCCTACAGGGTGGTTACTACCTGTGGCATT
>HPV93_Beta_37089393_nt762_E7_VE|1
TGCTTTCAGCAATTGCTAGTTAAGGAGTTGGATCTTCTGTGTCCTAACTGCCGAAACAGC
>HPV58_Alpha_222386_nt635_E7_VE|1
GACCTATTCTGCTATGAGCAATTATGTGACAGCTCAGACGAGGATGAAATAGGCTTGGAC
>HPV101_Unclassified_109390389_nt212_E7_VE|1
TCTGTGGAACAACTGCTGTGTGGAGACCTGGAATTGGTGTGTGCAAGCTGCATAAGAGCC
>HPV29_Alpha_1020210_nt645_E7_VE|1
GAACCAGCACAACAGGCGTATAGGGTGGTAACTTTGTGTACAAAGTGTAGTACAACACTG
>HPV9_Beta_9627396_nt644_E7_VE|1
ATGATTGGGAAAGAAGCTACTATACCAGAGGTGGTTCTAGAACTGCAAGAGCTTGTCCAA
>HPV6_Alpha_6002612_nt592_E7_VE|1
GTAGGGTTACATTGCTATGAGCAATTAGTAGACAGCTCAGAAGATGAGGTGGACGAAGTG
>HPV17_Beta_396932_nt623_E7_VE|1
ATGATTGGGAAAGAAGCTACAATACCAGATATAGTGCTTGAGCTGCAACAGCTTGTCCAG
>HPV94_Alpha_40804528_nt666_E7_VE|1
CCAAAGTGTAGTTTACCACTGCGACTGGTGGTAGAGTGCAGCCACGCAGATATAAGGGCA
>HPV88_Gamma_167600365_nt713_E7_VE|1

```
ACGCTTCAGCAGCTTTTACTTGCAGAGATTTCCATAAGTTGCCCTGGGTGTTCCAGGAAC
>HPV21_Beta_1020170_nt767_E7_VE|1
GAGGTACAACCAGTTGACCTGTTTTGTGAAGAGGAGTTACCGAGCGAGCAGCAGGAAACA
>HPV21_Beta_1020170_nt737_E7_VE|1
ATTGTTCTGGAGTTAAATGAATTGCAGCCTGAGGTACAACCAGTTGACCTGTTTTGTGAA
>HPV58_Alpha_222386_nt695_E7_VE|1
GGGCCAGATGGACAAGCACAACCGGCCACAGCTAATTACTACATTGTAACTTGTTGTTAC
>HPV95_Gamma_40804520_nt741_E7_VE|1
CTACGAGTCGTGGAACAACTTCTTCTGGAAGGGAAGGTGATATTTTGCTGTGTTGGGTGT
>HPV43_Alpha_40804474_nt591_E7_VE|1
AGCTTAACCTGTAACGAGCAATTAGACAGCTCAGACTCAGAGGATGAGCGTGAGCAACCC
>HPV92_Beta_27531786_nt520_E7_VE|1
ATGATTGGGAAACAGGCTACTATACCAGATATTGTGCTGGATCTGCAAGACCTTGTCCAG
>HPV71_Alpha_12084981_nt650_E7_VE|1
AATAGGGTGGACGGTGAGCAACCCACAGAACCAGCACAGCAGGCATATAGGGTGGTTTCA
>HPV1_Mu_9626063_nt620_E7_VE|1
CCTCCTGATGACATAGAGGAGGAGTTAGTGTCGCCTCAGCAACCTTATGCTGTCGTTGCT
>HPV73_Alpha_1491692_nt641_E7_VE|1
AACTCAGAGGATGAGGATGAAACAGACAGCCATCTAGACAGACAAGCTGAACGAGAGTGT
>HPV67_Alpha_3228267_nt654_E7_VE|1
AGCTCAGATGAGGAGGATGAAGTAGGCATGGACCGGCCAGATGGACAAGCAAAACCAGAT
>HPV28_Alpha_1020202_nt519_E7_VE|1
ATGCACGGTCCGCATCCCACAGTAAAAGATATAGAATTGAGTCTTGCACCAGAGGACGTC
>HPV2_Alpha_9626032_nt620_E7_VE|1
GACAGCTCAGAAGAAGAGAATAACCATCAACTGACAGAACCAGATGTGCAGGCCTACGGG
>HPV110_Beta_186461194_nt595_E7_VE|1
ATGATTGGGAAACAAGCTACTATACCAGACATTGTTCTGGAATTGCAAGAGCTTGTTCAG
>HPV15_Beta_396924_nt623_E7_VE|1
ATGATTGGGAAAGAAGCTACTATACCAGATATAGTGCTTGAGCTGCAAGAGCTTGTCCAG
>HPV81_Alpha_40804509_nt603_E7_VE|1
GACCTACATTGCCACGAGCAATTGCTAGACAGCTCAGAGGAAGAGGTGGATGTGGATGAT
>HPV11_Alpha_333026_nt561_E7_VE|1
ATAGTACTAGACCTGCAGCCTCCTGACCCTGTAGGGTTACATTGCTATGAGCAATTAGAA
>HPV67_Alpha_3228267_nt744_E7_VE|1
AACATCTGTGAGTGCACTTTGCGTTTGTGCATTCACAGTACAGCAGCAGACGTCCGAACA
>HPV103_Unclassified_109390382_nt122_E7_VE|2
CAGGATAAGTGTCCTTACCGGGTGGTGACAGATTGCAAGTATTGTGAGAAGTCATTGTGT
>HPV48_Gamma_9628542_nt608_E7_VE|1
GATGCTACTGCAGAGGAGGAGTTTTGTCCGTACCGGATAGACTCCAAGTGTCACAATTGT
>HPV20_Beta_1020162_nt758_E7_VE|1
GAGGTTCAACCAGTTGACCTGTTTTGTGAAGAGGAGTTACCGAACGAGCAGCAGGAGAGA
>HPV54_Alpha_9628437_nt627_E7_VE|1
TCTGATGCAGAAGATGAGACAGCAGTAACACAACCTGACAAACAAGCATTTAAGGTGTTA
>HPV71_Alpha_12084981_nt680_E7_VE|1
CCAGCACAGCAGGCATATAGGGTGGTTTCATACTGTGGTAGGTGCTGTCGTGCAGTTAGG
>HPV51_Alpha_333087_nt621_E7_VE|1
ATTGACTTGCAATGCTACGAGCAATTTGACAGCTCAGAGGAGGAGGATGAAGTAGATAAT
>HPV67_Alpha_3228267_nt774_E7_VE|1
ATTCACAGTACAGCAGCAGACGTCCGAACAATCCAGCAGATGCTTATGAACACACTAGGA
>HPV88_Gamma_167600365_nt593_E7_VE|1
TCATCTGATGAGGAGACCCAGGAGGAGGAGGAGCGAGAACCTTTCCAAATAGATACCAGT
>HPV1_Mu_9626063_nt740_E7_VE|1
AGACAGCTGGAGGAACTCCTTCTGCGATCTTTGAACATCGTGTGCCCACTGTGCACCCTA
>HPV48_Gamma_9628542_nt668_E7_VE|1
GGCTGTCGCATAAGGGTGACAGTCGCTGCAACTGAATTTGGAATACGTTGTTTTGAGCAA
```

>HPV83_Alpha_5059324_nt481_E7_VE|1
GACCTATATTGTAATGAACAAGGTATAGACAGCTCAGAGGAGGAGGATGATAGGGACTGT
>HPVFA75/KI88-03_Unclassified_206902429_nt606_E7_VE|1
ATTGTTCTGGAACTGCAAGAGCTTGTCCAGCCCATTGACCTGCATTGCTACGAAGAATTA
>HPV73_Alpha_1491692_nt671_E7_VE|1
CATCTAGACAGACAAGCTGAACGAGAGTGTTACAGAATAGTTACTGACTGCACGAAGTGT
>HPV69_Alpha_6970418_nt625_E7_VE|1
ATTGACCTACAGTGTTACGAACAATTGGACTATGAACAATTTGACAGCTCAGAGGAGGAT
>HPV24_Unclassified_9628486_nt683_E7_VE|1
GTTGATTTGCACTGTGAAGAGGAGTTGCCAGAGCAGGATACGGAGGTGGAGCCTGAAAGA
>HPV35_Alpha_333050_nt653_E7_VE|1
AGCTCAGAGGAGGAGGAAGATACTATTGACGGTCCAGCTGGACAAGCAAAACCAGACACC
>HPV27_Alpha_396964_nt660_E7_VE|1
CTGACAGAACCAGCTGTGCAGGCCTACGGGGTGGTAACAACCTGCTGCAAGTGCGGCAGA
>HPV42_Alpha_333211_nt627_E7_VE|1
GAACAATTGGACAGCTCAGATGAAGATGACCAAGCCAAACAGGACATACAGCGTTACAGA
>HPV93_Beta_37089393_nt552_E7_VE|1
ATGATTGGAAAAGAGGTCACTCTGCAAGACATTGTGTTAGGGTTGACACCACCAGAGCCT
>HPV67_Alpha_3228267_nt624_E7_VE|1
GACCTATACTGCTATGAGCAATTGCATGACAGCTCAGATGAGGAGGATGAAGTAGGCATG
>HPV80_Beta_2911565_nt653_E7_VE|1
ATAGAGCTTGAGCTGCAAGAGCTTGTCCAGCCCACTGACCTGCATTGCTACGAAGAGTTA
>HPVcand89_Alpha_22095322_nt631_E7_VE|1
GTAGTATTGTCTGACGACGCACATCTAAGACATCTACAACAGCTGCTGCTGAATGACCTG
>HPV93_Beta_37089393_nt642_E7_VE|1
ACCGAGCAGGATACGGAGGAGGAGCCTGCAAGAACAGCTTTCAAGATTATTGTGTTCTGC
>HPVRTRX7_Unclassified_30315616_nt728_E7_VE|1
GAAGTGATACCAGTTGACCTGCTTTGTGAAGAGGAATTACCAAACGAACAGGAAACGGAG
>HPV49_Beta_9627363_nt674_E7_VE|1
ATTGACCTGCAATGCTACGAGAATCTAACAGCTGAAGCGCCAGCTGAACAAGAGTTGGAG
>HPVcand85_Alpha_4574720_nt828_E7_VE|1
AGTTCAGCAGCCGATCTACGAGATCTCCAGCAGCTGTTTTTGGGCACGCTATCATTTTTG
>HPV35_Alpha_333050_nt623_E7_VE|1
GACCTATACTGTTATGAGCAATTGTGTGACAGCTCAGAGGAGGAGGAAGATACTATTGAC
>HPV69_Alpha_6970418_nt655_E7_VE|1
TATGAACAATTTGACAGCTCAGAGGAGGATGAAACAGATAATGTGCGTAACCAGCAAGCC
>HPV93_Beta_37089393_nt612_E7_VE|1
GTTGCATTGAATTGTGAAGAGGAGTTACCAACCGAGCAGGATACGGAGGAGGAGCCTGCA
>HPV4_Gamma_9626597_nt582_E7_VE|1
CCAGCAAACCTGCTGAGTGAGGAGGTCTTGCAATCTTCAGATGATGAGTATGAGATTACA
>HPV22_Beta_1020178_nt644_E7_VE|1
ATGATTGGGAAACAAGCTACTCTGTGTGATATAGTTCTTGAAGAGCTTGTCCTGCCCATT
>HPVcand86_Alpha_15741127_nt484_E7_VE|1
AACCTACATTGTGACGAGCAATTGTTAGACAGCTCAGAGGAGGAGGATAATAGGGATTGT
>HPV77_Alpha_2911558_nt555_E7_VE|1
ATTGAATTGGATCTTGCACCAGAGGCCGTCCCTTTACTATGCAATGAGCAATTAGACAGC
>HPV32_Alpha_9627327_nt531_E7_VE|1
ATGCGTGGAAACGCACCAACGCTAAAGGACATTATTTTGTATGACCTGCCAACGTGTGAC
>HPVcand89_Alpha_22095322_nt601_E7_VE|1
TGTGGTGTGTGCTATCGTGCAGTTAGGTTAGTAGTATTGTCTGACGACGCACATCTAAGA
>HPV81_Alpha_40804509_nt663_E7_VE|1
AGGGACAGTGGTGAGCAACTAGTAGAACAAGCACAGCAGGCCTATAGGGTGGTTACTACG
>HPV52_Alpha_397038_nt644_E7_VE|1
AGCTCAGATGAGGAGGATACAGATGGTGTGGACCGGCCAGATGGACAAGCAGAACAAGCC
>HPV110_Beta_186461194_nt625_E7_VE|1

ATTGTTCTGGAATTGCAAGAGCTTGTTCAGCCCACTGCTGACCTGCATTGCTACGAGGAG
>HPV19_Beta_396940_nt751_E7_VE|1
GAGGTACAACCAGTTGACCTGTTTTGTGAAGAGGAGTTACCGACCGAACAGCAGGAAACA
>HPV55_Alpha_1020266_nt591_E7_VE|1
GTAGGCCTACATTGCAATGAGCAATTAGACAGCTCAGAGGATGAGGTGGATGAACTAGCC
>HPV59_Alpha_557236_nt633_E7_VE|1
TTACCTGACTCCGACTCCGAGAATGAAAAAGATGAACCAGATGGAGTTAATCATCCTTTG
>HPV26_Alpha_9627305_nt677_E7_VE|1
GAAACAGATAATATGCGTGACCAGCAGGCCAGACAAGCTGGACAAGAAGTGTGTTACAGA
>HPV43_Alpha_40804474_nt561_E7_VE|1
TATGTGCTAACTATGCAACCAGAACCTAGAAGCTTAACCTGTAACGAGCAATTAGACAGC
>HPV13_Alpha_85827580_nt563_E7_VE|1
ATTGTTTTAGACCTGACTCCTGACCCTGTAGGTCTACATTGCAATGAGCAATTAGACAGC
>HPV54_Alpha_9628437_nt717_E7_VE|1
GTACGGCTATGTGTGTATAGCACGCACACAGGCATAAGGGTACTGCAGGAACTGCTTCAT
>HPV1_Mu_9626063_nt710_E7_VE|1
TTGACCGTCCTCGCGGATCACAGCGCCATTAGACAGCTGGAGGAACTCCTTCTGCGATCT
>HPV37_Beta_1020226_nt623_E7_VE|1
ATGATTGGGAAAGAAGCTACAATACCAGAAATAGTGCTTGAGCTGCAAGAGCTTGTCCAG
>HPV92_Beta_27531786_nt580_E7_VE|1
CCCATTGACCTGCATTGTGACGAAGACTTGTCAGAAAATCAGGAGGAGGAGCCTGCACCT
>HPV107_Beta_126131393_nt739_E7_VE|1
ATAGTTCTGGAAGAGCTGCAAGAGCTTGCTCAGCCCATTGACCTGCATTGTTACGAAGAG
>HPV83_Alpha_5059324_nt511_E7_VE|1
AGCTCAGAGGAGGAGGATGATAGGGACTGTGGCGTGCGTGACCAACTGGCAGAACAAGCA
>HPV107_Beta_126131393_nt769_E7_VE|1
CAGCCCATTGACCTGCATTGTTACGAAGAGTTACCAGAGCTTCCAGATGCTACTAGTGAA
>HPV6_Alpha_6002612_nt622_E7_VE|1
GACAGCTCAGAAGATGAGGTGGACGAAGTGGACGGACAAGATTCACAACCTTTAAAACAA
>HPV27_Alpha_396964_nt630_E7_VE|1
GACAGCTCAGAAGAAGAGAATAACCATCAACTGACAGAACCAGCTGTGCAGGCCTACGGG
>HPV82_Alpha_6970427_nt626_E7_VE|1
ATTGACTTGCAATGCTACGAGCAATTTGACAGCTCAGACGAGGAGGATGAAGTAGATAAT
>HPV90_Alpha_22138122_nt454_E7_VE|1
ATATTTCTAGAGCTGGATCAGCCTGATGCGGTTGACCTACATTGTAACGAGCAATTAGCC
>HPV60_Gamma_9628566_nt648_E7_VE|1
CACTATCCCTATACAATAGACACCTGTTGCAAACCTTGTGGAGCAGGTGTTAGATTTACT
>HPV76_Beta_2911551_nt644_E7_VE|1
ATTGTGCTACAAGAAGAGTTTGGCCAGCCCATTGACCTGCAATGCTACGAGAACTTACCA
>HPV76_Beta_2911551_nt674_E7_VE|1
ATTGACCTGCAATGCTACGAGAACTTACCAGCTGAAGTGCCAGCTGAACAAGACACAGAG
>HPV8_Beta_333074_nt714_E7_VE|1
GAAGTGTTACCAGTTGACCTGCTTTGTGAAGAGGAATTACCAAACGAACAGGAAACGGAG
>HPV28_Alpha_1020202_nt669_E7_VE|1
CCAAAGTGTAGTTCACCACTGCGACTGGTGGTAGAGTGCAGCCACGCAGATATAAGGGCA
>HPV63_Mu_9626605_nt735_E7_VE|1
TCTACTCTTACGCGTCTGGAGGAGCTGCTGTTAGGCTCACTGAGGATCGTGTGTCCCCTG
>HPV35_Alpha_333050_nt683_E7_VE|1
GGTCCAGCTGGACAAGCAAAACCAGACACCTCCAATTATAATATTGTAACGTCCTGTTGT
>HPV47_Beta_333062_nt699_E7_VE|1
ATTGTTCTGGAGTTAAGTGAGGTTCAACCTGAAGTATTACCAGTTGACCTGTTTTGCGAC
>HPV90_Alpha_22138122_nt484_E7_VE|1
GTTGACCTACATTGTAACGAGCAATTAGCCAGCTCAGAAGAGGAGGATAATAGGGAGGAC
>HPV2_Alpha_9626032_nt650_E7_VE|1
CTGACAGAACCAGATGTGCAGGCCTACGGGGTGGTAACTACCTGCTGTAAGTGTGGCAGA

>HPV102_Alpha_71726718_nt571_E7_VE|1
CAGGCCTATAGGGTGGTAACAAAGTGTGGTATGTGTGGCCAGTCACTGAGACTGGCAGTG
>HPV51_Alpha_333087_nt651_E7_VE|1
AGCTCAGAGGAGGAGGATGAAGTAGATAATATGCGTGACCAGCTACCAGAAAGACGGGCT
>HPVcand62_Alpha_39932599_nt454_E7_VE|1
ATTGTCCTACAAGAGCGTCCCGATGTGGTTGACCTACATTGCCACGAGCAATTGTTAGAC
>HPV97_Alpha_71726694_nt607_E7_VE|1
GAAACAGATGATGTTAATCATCGACAACTACCAGCCCGACGAGCTGAACCACAGCGTCAC
>HPV47_Beta_333062_nt669_E7_VE|1
ATGATTGGTAAAGAGGTCACCGTGCGAGATATTGTTCTGGAGTTAAGTGAGGTTCAACCT
>HPV94_Alpha_40804528_nt606_E7_VE|1
GAAGATTATACAGATGTGGTGGAACCAGCACAACAAGCGTATAGGGTGGTAACAGTATGT
>HPV16_Alpha_9627100_nt773_E7_VE|1
AGCACACACGTAGACATTCGTACTTTGGAAGACCTGTTAATGGGCACACTAGGAATTGTG
>HPV58_Alpha_222386_nt665_E7_VE|1
AGCTCAGACGAGGATGAAATAGGCTTGGACGGGCCAGATGGACAAGCACAACCGGCCACA
>HPV50_Gamma_9628550_nt698_E7_VE|1
ACCGACGCAGCTATCCGTGTGTTTGAGTGCCTTCTTCAATCAGAATTTTCTTTTCTGTGT
>HPVcand85_Alpha_4574720_nt768_E7_VE|1
CGTCACACTATTTGCTGCGTGTGTTGTAAATGTGAGGCCAGTCTGCAGTTAGTGGTGGAA
>HPV32_Alpha_9627327_nt771_E7_VE|1
GACATACAACAGCTGCATCAGATGCTTCTGGACACACTGGGCATTGTGTGTCCATTGTGT
>HPV31_Alpha_333048_nt621_E7_VE|1
GACCTCCACTGTTATGAGCAATTACCCGACAGCTCAGATGAGGAGGATGTCATAGACAGT
>HPV18_Alpha_9626069_nt741_E7_VE|1
GCCCGACGAGCCGAACCACAACGTCACACAATGTTGTGTATGTGTTGTAAGTGTGAAGCC
>HPV76_Beta_2911551_nt614_E7_VE|1
ATGATTGGGAAAGAGGCTACATTACCTGACATTGTGCTACAAGAAGAGTTTGGCCAGCCC
>HPV74_Alpha_27462483_nt490_E7_VE|1
GGCCTACTTTGCAATGAGCAATTAGACAGCTCAGAAGAGGAGGTGGATGAACTAGCCACG
>HPV26_Alpha_9627305_nt647_E7_VE|1
TATGAACAATTTGACAGCTCAGATGAGGATGAAACAGATAATATGCGTGACCAGCAGGCC
>HPV59_Alpha_557236_nt693_E7_VE|1
CTACTAGCTAGACGAGCTGAACCACAGCGTCACAACATTGTGTGTGTGTGTTGTAAGTGT
>HPVFA75/KI88-03_Unclassified_206902429_nt636_E7_VE|1
CCCATTGACCTGCATTGCTACGAAGAATTACCAGAGCTTCCAGATGTTCCTAGTGAAGAT
>HPV12_Beta_396910_nt724_E7_VE|1
GAAGTGTTACCAGTTGACCTGCTTTGTGAAGAGGAATTACCAAACGAGCAGGAAACGGAG
>HPV110_Beta_186461194_nt685_E7_VE|1
TTAACTGAAGAAGAAACAGAGGAGGAGTCACGTCATACACCTTACAAGATTGTTGCTGCT
>HPV35_Alpha_333050_nt743_E7_VE|1
AAATGTGAGGCGACACTACGTCTGTGTGTACAGAGCACACACATTGACATACGTAAATTG
>HPV16_Alpha_9627100_nt683_E7_VE|1
CCAGCTGGACAAGCAGAACCGGACAGAGCCCATTACAATATTGTAACCTTTTGTTGCAAG
>HPV63_Mu_9626605_nt645_E7_VE|1
GAGGAGGAGGAGTCTGAATATCCATATGCAATTGTGCTTCCTTGTGGTTTGTGCGATCAG
>HPV48_Gamma_9628542_nt638_E7_VE|1
TACCGGATAGACTCCAAGTGTCACAATTGTGGCTGTCGCATAAGGGTGACAGTCGCTGCA
>HPV54_Alpha_9628437_nt657_E7_VE|1
CAACCTGACAAACAAGCATTTAAGGTGTTAAGCCAGTGTGGAGGTGTGTGTTGTAAGACA
>HPV41_Nu_9626041_nt885_E7_VE|1
CGGAATCTACAGGCACTCCTGGTCAACAGTCACCTTGACCTCGCTTGTCACGCCTGTGTC
>HPV15_Beta_396924_nt653_E7_VE|1
ATAGTGCTTGAGCTGCAAGAGCTTGTCCAGCCCACTGACCTGCATTGCTACGAAGAGTTA
>HPV45_Alpha_397022_nt738_E7_VE|1

CCAGCCCGACGAGCCGAACCACAGCGTCACAAAATTTTGTGTGTATGTTGTAAGTGTGAC
>HPV63_Mu_9626605_nt615_E7_VE|1
GATCTAAATTGTTATGAGGATATACCTGCTGAGGAGGAGGAGTCTGAATATCCATATGCA
>HPV45_Alpha_397022_nt708_E7_VE|1
GAAGCAGATGGAGTTAGTCATGCACAACTACCAGCCCGACGAGCCGAACCACAGCGTCAC
>HPV24_Unclassified_9628486_nt713_E7_VE|1
GAGCAGGATACGGAGGTGGAGCCTGAAAGAAGAGCTTACAAAATAATACTTTGTTGCGGC
>HPV44_Alpha_1020242_nt594_E7_VE|1
GTAGGCCTACATTGCAATGAGCAATTAGACAGCTCAGAAGATGAGGTGGATGAACTAGCC
>HPV36_Beta_1020218_nt724_E7_VE|1
GAAGTACTACCAGTTGACCTGTTTTGTGAAGAGGAATTACCAAACGAGGATACGGAGGAG
>HPV43_Alpha_40804474_nt621_E7_VE|1
TCAGACTCAGAGGATGAGCGTGAGCAACCCACGCAACAGGACCAGCAAGTGAATCTACAA
>HPV75_Beta_2911544_nt644_E7_VE|1
ATTGTGCTACAAGAAGAGTTTGGCCAGCCCATTGACCTGCAATGCTACGAGGATTTAGCA
>HPV54_Alpha_9628437_nt747_E7_VE|1
GGCATAAGGGTACTGCAGGAACTGCTTCATCAGGACGCACTGCAAATAGTGTGTCCCACC
>HPVcand86_Alpha_15741127_nt544_E7_VE|1
GACCAACCTGCAGAACCAGCACAGCTGGCCTATAGGGTGTTAACAGAATGTGGGTTGTGT
>HPV103_Unclassified_109390382_nt152_E7_VE|1
GATTGCAAGTATTGTGAGAAGTCATTGTGTATGTATGTGGTAGCCACGGAGGAGGGCATT
>HPV4_Gamma_9626597_nt522_E7_VE|1
ATGAGAGGAGCAGCGCCCACGGTTGCAGATCTTAATTTAGAACTAAATGACTTAGTGTTA
>HPV39_Alpha_333245_nt653_E7_VE|1
ATACAGCCGGTTGACCTTGTATGTCACGAGCAATTAGGAGAGTCAGAGGATGAAATAGAT
>HPV95_Gamma_40804520_nt561_E7_VE|1
GTTGATTTAAACCTCCATGAGTTAGTTGTTCCTGCAAATTTGCTGAGCGATGAGGTCTTG
>HPV91_Alpha_22023568_nt667_E7_VE|1
AGTTTAACCTGTGACGAGCAATTAGACAGCTCAGACTCAGAGGATGAGCGTGAGCAATCC
>HPV101_Unclassified_109390389_nt62_E7_VE|1
CCTGCAAACTTATTATGTGATGAATTAGTAACCGAGGAGGAGGAGCAGCAGGACGAGCAA
>HPV37_Beta_1020226_nt683_E7_VE|1
CCCACTGCTGACCTGCATTGTTACGAAGAGTTGAGTGAAGAAGAGACAGAGGAGGAGCGT
>HPV33_Alpha_333049_nt694_E7_VE|1
CCAGATGGACAAGCACAACCAGCCACAGCTGATTACTACATTGTAACCTGTTGTCACACT
>HPV38_Beta_1020234_nt653_E7_VE|1
ATAGTTCTTGAAGAGCTTGTCCAGCCCATTGACCTGCATTGCCACGAGGAGTTGCCTGAT
>HPV32_Alpha_9627327_nt741_E7_VE|1
GTTAAACTTGTTGTCAGTAGTACAGGCGCGGACATACAACAGCTGCATCAGATGCTTCTG
>HPV26_Alpha_9627305_nt617_E7_VE|1
ATTGACCTACGCTGCTACGAACAATTGGACTATGAACAATTTGACAGCTCAGATGAGGAT
>HPV95_Gamma_40804520_nt711_E7_VE|1
AGAATCACTTTGTATGCTGTTGCATTTGGACTACGAGTCGTGGAACAACTTCTTCTGGAA
>HPV39_Alpha_333245_nt743_E7_VE|1
CAACTACTAGCCAGACGGGATGAACCACAGCGTCACACAATACAGTGTTCGTGTTGTAAG
>HPV33_Alpha_333049_nt664_E7_VE|1
AGCTCAGATGAGGATGAAGGCTTGGACCGGCCAGATGGACAAGCACAACCAGCCACAGCT
>HPV96_Beta_50253426_nt794_E7_VE|1
ATAAAGCTGGAGCTGCAAGACCTTGTCCTGCCCATTGACCTGCATTGTGACGAAGAGTTG
>HPV96_Beta_50253426_nt824_E7_VE|1
CCCATTGACCTGCATTGTGACGAAGAGTTGACAGAAGAGCAGTCAGAAAACCTGTCAGAA
>HPVcand89_Alpha_22095322_nt481_E7_VE|1
AGCCTACAATGTGAGGAAGAAATGCTAGACAGCTCAGATGAGGAAGATGCTAGGGATTGT
>HPV101_Unclassified_109390389_nt92_E7_VE|1
ACCGAGGAGGAGGAGCAGCAGGACGAGCAAGCTTTAAGCCCTTATAGAATTGTGACAGAT

>HPV102_Alpha_71726718_nt541_E7_VE|1
GTGCGTGACCAACTGGCAGAACAAGCAAAGCAGGCCTATAGGGTGGTAACAAAGTGTGGT
>HPV52_Alpha_397038_nt764_E7_VE|1
ATTCATAGCACTGCGACGGACCTTCGTACTCTACAGCAAATGCTGTTGGGCACATTACAA
>HPVcand85_Alpha_4574720_nt738_E7_VE|1
CCACTACTAGCCAGACGAGAAGAACTACAGCGTCACACTATTTGCTGCGTGTGTTGTAAA
>HPV10_Alpha_9627257_nt675_E7_VE|1
AAGTGTAGTTTACCACTGCGACTGGTGGTAGAGTGCAGCCACGCAGATATAAGGGCACTG
>HPV94_Alpha_40804528_nt516_E7_VE|1
ATGCACGGTCCGCATCCCACAGTAAAAGATATAGAATTGAGTCTTGCACCAGAGGACATC
>HPV37_Beta_1020226_nt653_E7_VE|1
ATAGTGCTTGAGCTGCAAGAGCTTGTCCAGCCCACTGCTGACCTGCATTGTTACGAAGAG
>HPV33_Alpha_333049_nt634_E7_VE|1
GACCTATACTGCTATGAGCAATTAAGTGACAGCTCAGATGAGGATGAAGGCTTGGACCGG
>HPV48_Gamma_9628542_nt518_E7_VE|1
ATGAGGGGAGATAAGGCTACTATTCCTGACATAGAGTTAGAAGAGCTTGTTTTGCCTGCT
>HPV1_Mu_9626063_nt680_E7_VE|1
TCCTGTGCCTATTGCGAGAAACTGGTTCGATTGACCGTCCTCGCGGATCACAGCGCCATT
>HPV42_Alpha_333211_nt597_E7_VE|1
TGTGAGACACCCATTGACCTGTATTGCTATGAACAATTGGACAGCTCAGATGAAGATGAC
>HPVcand87_Alpha_14475578_nt648_E7_VE|1
GTGCGTGACCAACCTGCAGAACCAGCACAGCTGGCCCATAGGGTGTTAACAGAGTGTGGG
>HPV24_Unclassified_9628486_nt743_E7_VE|1
AGAGCTTACAAAATAATACTTTGTTGCGGCGGCGGTTGTGGAACCCGGCTTCGATTATTT
>HPV52_Alpha_397038_nt614_E7_VE|1
GACCTACACTGCTATGAGCAATTAGGTGACAGCTCAGATGAGGAGGATACAGATGGTGTG
>HPV110_Beta_186461194_nt655_E7_VE|1
CCCACTGCTGACCTGCATTGCTACGAGGAGTTAACTGAAGAAGAAACAGAGGAGGAGTCA
>HPVcand85_Alpha_4574720_nt798_E7_VE|1
TGTGAGGCCAGTCTGCAGTTAGTGGTGGAAAGTTCAGCAGCCGATCTACGAGATCTCCAG
>HPV101_Unclassified_109390389_nt182_E7_VE|1
CATGTACTAGCTACGGACGGGGCAATAAGGTCTGTGGAACAACTGCTGTGTGGAGACCTG
>HPV111_Beta_186461202_nt609_E7_VE|1
GTAGTTCTTGAGCTGCAAGAGCTTGTCCAACCCACTGACCTGCATTGCTACGAAGAATTG
>HPV40_Alpha_397014_nt558_E7_VE|1
ATTGTGTTAAACCTGCACCCTGAACCTGTATGTCTAAACTGCAACGAGCAATTAGACAGC
>HPV61_Alpha_9628574_nt579_E7_VE|1
GACCTACATTGCAATGAGCAGTTATTAGACAGCTCAGAGTCAGAGGAGGAGGATAGTGTG
>HPV40_Alpha_397014_nt648_E7_VE|1
CAACTAGACAGCTTACACAGTAGAGAGCGTGAGCAACCCACGCAACAGGACCTGCAAGTA
>HPV40_Alpha_397014_nt588_E7_VE|1
TGTCTAAACTGCAACGAGCAATTAGACAGCTCAGACTCAGAAGATGACCATGAACAGGAC
>HPV67_Alpha_3228267_nt684_E7_VE|1
GACCGGCCAGATGGACAAGCAAAACCAGATACAACCAATTACCATATTGTTACTGTGTGT
>HPV68a_Alpha_71726685_nt755_E7_VE|1
CAACAGCTGTTTATGGACTCACTAAATTTTGTGTGTCCGTGGTGTGCAACTGAAACCCAG
>HPV11_Alpha_333026_nt591_E7_VE|1
GTAGGGTTACATTGCTATGAGCAATTAGAAGACAGCTCAGAAGATGAGGTGGACAAGGTG
>HPVcand85_Alpha_4574720_nt708_E7_VE|1
GAACCAGATAATGCAATTAATCACCGACAACCACTACTAGCCAGACGAGAAGAACTACAG
>HPVcand87_Alpha_14475578_nt618_E7_VE|1
AGCTCAGAGGAGGAGGACAATAGGGATTGTGTGCGTGACCAACCTGCAGAACCAGCACAG
>HPV54_Alpha_9628437_nt687_E7_VE|1
AGCCAGTGTGGAGGTGTGTGTTGTAAGACAGTACGGCTATGTGTGTATAGCACGCACACA
>HPV1_Mu_9626063_nt650_E7_VE|1

TCGCCTCAGCAACCTTATGCTGTCGTTGCTTCCTGTGCCTATTGCGAGAAACTGGTTCGA
>HPVcand62_Alpha_39932599_nt484_E7_VE|1
GACCTACATTGCCACGAGCAATTGTTAGACAGCTCAGAGGAAGAGGTGGATGATAGGGAC
>HPV68a_Alpha_71726685_nt545_E7_VE|1
ATACAGCCGGTTGACCTTGTATGTCACGAGCAATTAGGAGATTCAGACGATGAAATAGAT
>HPVFA75/KI88-03_Unclassified_206902429_nt576_E7_VE|1
ATGATTGGGAAAGAAGTTACTATTCCAGATATTGTTCTGGAACTGCAAGAGCTTGTCCAG
>HPV16_Alpha_9627100_nt653_E7_VE|1
AGCTCAGAGGAGGAGGATGAAATAGATGGTCCAGCTGGACAAGCAGAACCGGACAGAGCC
>HPVRTRX7_Unclassified_30315616_nt698_E7_VE|1
TTTATTTTGGAGCTTAGTGAGGTGCAGCCTGAAGTGATACCAGTTGACCTGCTTTGTGAA
>HPV24_Unclassified_9628486_nt623_E7_VE|1
ATGATTGGAAAAGAGGTCACTCTACAAGACATTGTCTTAGAGTTGACTGAGCCACAGACT
>HPV41_Nu_9626041_nt795_E7_VE|1
CCCAGGAGAACATTATACCTGGTAGAGAGTCAGTGTCCATTTTGCCAGGCTATCATACGA
>HPV47_Beta_333062_nt729_E7_VE|1
GAAGTATTACCAGTTGACCTGTTTTGCGACGAGGAATTACCAAATGAACAACAGGCGGAG
>HPV31_Alpha_333048_nt681_E7_VE|1
CCAGCTGGACAAGCAGAACCGGACACATCCAATTACAATATCGTTACCTTTTGTTGTCAG
>HPV41_Nu_9626041_nt855_E7_VE|1
TTTGTATGCGTAGCAAGCAACACTGGGATACGGAATCTACAGGCACTCCTGGTCAACAGT
>HPV59_Alpha_557236_nt783_E7_VE|1
TCGCAAGACGGATTGCGAGCCTTACAGCAGCTGTTTATGGACACACTATCCTTTGTGTGT
>HPV103_Unclassified_109390382_nt242_E7_VE|1
CTGGATATTGTGTGTCCAGCCTGTGCAAAGCAACGCCAGCGAAATGGAGGACAAAGACAC
>HPV81_Alpha_40804509_nt693_E7_VE|1
GCACAGCAGGCCTATAGGGTGGTTACTACGTGTGGTATTTGTAGGTGTTTAGTTAGGCTG
>HPVcand89_Alpha_22095322_nt571_E7_VE|1
GCACAGCAGGCCTTTAGGGTGGTAACTACGTGTGGTGTGTGCTATCGTGCAGTTAGGTTA
>HPV7_Alpha_9627389_nt558_E7_VE|1
ATCGTGTTAGACTTGCAACCCGAACCAGTAAGTTTAAGTTGCAACGAGCAATTAGACAGC
>HPV25_Beta_396948_nt736_E7_VE|1
GAGGTACAACCAGTTGACCTGTTTTGTGAAGAGGAGTTGCCGGCTGAGCATCAGGAAACA
>HPV97_Alpha_71726694_nt637_E7_VE|1
CCAGCCCGACGAGCTGAACCACAGCGTCACACAATTGTGTGTATGTGTTGTAAATGTGAG
>HPV52_Alpha_397038_nt674_E7_VE|1
GACCGGCCAGATGGACAAGCAGAACAAGCCACAAGCAATTACTACATTGTGACATATTGT
>HPV42_Alpha_333211_nt747_E7_VE|1
GCGGACATAAGAAACCTGCAACAGATGCTTTTGGGCACACTGGATATTGTGTGTCCTTTG
>HPV22_Beta_1020178_nt674_E7_VE|1
ATAGTTCTTGAAGAGCTTGTCCTGCCCATTGACCTGCATTGCCACGAGGAGCTGCCTGAA
>HPV84_Alpha_12958167_nt544_E7_VE|1
TTGCGTGACCAACCTGCAAAACCAGCACAGCTGGCCTATAGGGTGGTAACAGAGTGTGGG
>HPV68a_Alpha_71726685_nt575_E7_VE|1
CAATTAGGAGATTCAGACGATGAAATAGATGAACCCGACCATGCAGTTAATCACCACCAA
>HPV35_Alpha_333050_nt593_E7_VE|1
TATGTTTTAGATTTGGAACCCGAGGCAACTGACCTATACTGTTATGAGCAATTGTGTGAC
>HPV72_Alpha_1491683_nt675_E7_VE|1
GAACAAGCACAGCAGGCCTACGGGGTGGTTACTACCTGTGGCAGGTGCTACCGTCCAGTT
>HPV63_Mu_9626605_nt705_E7_VE|1
CTGTTAAGGCTGACCTGCGTTTCTGACCTGTCTACTCTTACGCGTCTGGAGGAGCTGCTG
>HPV7_Alpha_9627389_nt798_E7_VE|1
CAGGTTCATCAGTTGCTGATGGGCACACTAAATATAGTGTGCCCCAACTGTGCAGCTACA
>HPV4_Gamma_9626597_nt642_E7_VE|1
GAGGAGGAGTCGGTGGTTCCATTTAGAATAGACACCTGTTGCTATAGATGTGAAGTTGCT

>HPV5_Beta_9627145_nt724_E7_VE|1
GAAGTGCTACCAGTTGACCTGTTTTGTGAAGAGGAATTACCAAACGAGCAGGAAACGGAG
>HPV24_Unclassified_9628486_nt833_E7_VE|1
CTGCAAGACTTACTGCTAGAAGAGGTGGTCATTTTGTGTCCCGACTGCCGTAACAGCGAT
>HPV60_Gamma_9628566_nt558_E7_VE|1
CTTGATGTAAATTTGGAGGAGTTGGTTTTACCTGTCAGTCTTTTAGCTGATGAGGAGTTG
>HPV65_Gamma_312100_nt522_E7_VE|1
ATGAGAGGAGCAGCACCCAGGGTTGCAGATCTTAATTTAGAATTAAATGACTTGGTGTTG
>HPVcand87_Alpha_14475578_nt588_E7_VE_rc|1
ACAATCCCTATTGTCCTCCTCCTCTGAGCTGTCTAACAATTGCTCGTCACAATGTAGGTT
>HPV88_Gamma_167600365_nt683_E7_VE_rc|1
AATCTCTGCAAGTAAAAGCTGCTGAAGCGTGCGAATTCCGGCTGGTGAAGCTAGAACAAA
>HPV36_Beta_1020218_nt664_E7_VE_rc|1
AGGCTGCACCTCACTGAGCTCCAGAACAATATCTTGCACGGTGACCTCTTTACCAATCAT
>HPV92_Beta_27531786_nt550_E7_VE_rc|1
CAAGTCTTCGTCACAATGCAGGTCAATGGGCTGGACAAGGTCTTGCAGATCCAGCACAAT
>HPV59_Alpha_557236_nt663_E7_VE_rc|1
ACGCTGTGGTTCAGCTCGTCTAGCTAGTAGCAAAGGATGATTAACTCCATCTGGTTCATC
>HPV82_Alpha_6970427_nt596_E7_VE_rc|1
GTCAAATTGCTCGTAGCATTGCAAGTCAATTTCAGGTTGTGGTGTTAACTCCAACACTAT
>HPV52_Alpha_397038_nt704_E7_VE_rc|1
GCATAGCCGTAGTGTGCTATCACAACTGTGACAATATGTCACAATGTAGTAATTGCTTGT
>HPV17_Beta_396932_nt653_E7_VE_rc|1
TAACTCTTCGTAGCAATGCAGGTCAGTGGGCTGGACAAGCTGTTGCAGCTCAAGCACTAT
>HPV88_Gamma_167600365_nt653_E7_VE_rc|1
GCGAATTCCGGCTGGTGAAGCTAGAACAAATACCCTTACACCTGCTTCACAGAAGGCGCA
>HPV4_Gamma_9626597_nt732_E7_VE_rc|1
AGCGGTGCAGCAAAACGTCAGCTTTCCTTCTACAAGAAGTTGTTCCAAGGTCCGTAGTCC
>HPV93_Beta_37089393_nt732_E7_VE_rc|1
CAACTCCTTAACTAGCAATTGCTGAAAGCAACGAACTCCAAATTCTGTGGCAGCAACAAA
>HPV41_Nu_9626041_nt825_E7_VE_rc|1
TATCCCAGTGTTGCTTGCTACGCATACAAATCGTATGATAGCCTGGCAAAATGGACACTG
>HPV34_Alpha_9627334_nt582_E7_VE_rc|1
GTCTAATGACTCGTAACATGTAAGGTCAGTCTCGGTCGTTGGTTTCAGATCTAACACAAT
>HPV58_Alpha_222386_nt725_E7_VE_rc|1
GATACACAAACGAACCGTGGTGCCACAAGTGTAACAACAAGTTACAATGTAGTAATTAGC
>HPV16_Alpha_9627100_nt623_E7_VE_rc|1
ACCATCTATTTCATCCTCCTCCTCTGAGCTGTCATTTAATTGCTCATAACAGTAGAGATC
>HPV47_Beta_333062_nt819_E7_VE_rc|1
GTTCACAAAAATGCGAAGCTTGACCTCGCAGCAGCTGCAACCGCACGGTGCAATCACTTT
>HPV95_Gamma_40804520_nt591_E7_VE_rc|1
TCTTTCTTCATCCTCCTCCTCAGAGAGTTGCAAGACCTCATCGCTCAGCAAATTTGCAGG
>HPV83_Alpha_5059324_nt541_E7_VE_rc|1
ACACACTGTTAACACCCTATAGGCCTGCTTTGCTTGTTCTGCCAGTTGGTCACGCACGCC
>HPV7_Alpha_9627389_nt588_E7_VE_rc|1
GTCTTGTTCATGGTCATCTTCTGAGTCTGAGCTGTCTAATTGCTCGTTGCAACTTAAACT
>HPV59_Alpha_557236_nt753_E7_VE_rc|1
CTGCTGTAAGGCTCGCAATCCGTCTTGCGAGGTTTCTACTACTAGCTGAAGTTGATTATT
>HPV82_Alpha_6970427_nt656_E7_VE_rc|1
TCCAGCTTGTCTGGCTGGCTGGTCACGCATATTATCTACTTCATCCTCCTCGTCTGAGCT
>HPV103_Unclassified_109390382_nt182_E7_VE_rc|1
GTTTCCACACAGCTGTTGTTCAAAGTTTCTAATGCCCTCCTCCGTGGCTACCACATACAT
>HPV63_Mu_9626605_nt675_E7_VE_rc|1
CAGGTCAGAAACGCAGGTCAGCCTTAACAGCTGATCGCACAAACCACAAGGAAGCACAAT
>HPV102_Alpha_71726718_nt481_E7_VE_rc|1

ACAATCCCTCTCGTCCTCCTCCTCTGAGCTGTCTATTGCTTGCTCATTACAATACAGGTC
>HPV41_Nu_9626041_nt915_E7_VE_rc|1
GTGTCTGAGACCCTGGACGCCATTCTGCTCGACACAGGCGTGACAAGCGAGGTCAAGGTG
>HPV2_Alpha_9626032_nt590_E7_VE_rc|1
TTGATGGTTATTCTCTTCTTCTGAGCTGTCAAATTGCTCGTCGCAATGTAGGTCAACAAT
>HPV70_Alpha_1173493_nt743_E7_VE_rc|1
CTTACAACACATACACTGTATTTTGTGACGCTGTGGTTCTTCCCGTCTGGCTAGTAGTTG
>HPV48_Gamma_9628542_nt728_E7_VE_rc|1
ATTGCGGGGAAGTTGTCTGGAGCACGCAGGACAAAATAAACAAAGCTCTTTCAGTAACAG
>HPV42_Alpha_333211_nt567_E7_VE_rc|1
ATAGCAATACAGGTCAATGGGTGTCTCACACGTTGGTATGTCAAACAAAACAATGTCCTT
>HPV106_Alpha_71726710_nt559_E7_VE_rc|1
CTGCTGTGCTGGTTCTGTAGGTTGCTCACCGTCCTCCCTATTATCCTCCTCTTCTGAGCT
>HPV42_Alpha_333211_nt657_E7_VE_rc|1
CTTGTAACACTGTGTACACACACACAGTATTCTGTAACGCTGTATGTCCTGTTTGGCTTG
>HPV23_Beta_1020186_nt701_E7_VE_rc|1
TTCAGTGAGCTCCTCGTGGCAATGCAGGTCAATGGGCTGGACAAGCTCTTCAAGAACTAT
>HPV13_Alpha_85827580_nt593_E7_VE_rc|1
CGTGGCTTGTTCGTCCACCTCGTCTTCTGAGCTGTCTAATTGCTCATTGCAATGTAGACC
>HPV32_Alpha_9627327_nt681_E7_VE_rc|1
TGACCGTCCACACTGTGTACAACCACACACTATTCTGTAACGTTGTATGTCCTGTTTTAT
>HPV71_Alpha_12084981_nt590_E7_VE_rc|1
ATCTTCCTCGTCTGAGCTGGCAAATTGCTCGTGACAATACAGGTCAACAACCTCTGGCTG
>HPV106_Alpha_71726710_nt529_E7_VE_rc|1
GTCCTCCCTATTATCCTCCTCTTCTGAGCTGGCAAATTGCTCGTTACAATGCAGGTCAAC
>HPVcand89_Alpha_22095322_nt541_E7_VE_rc|1
CGTAGTTACCACCCTAAAGGCCTGCTGTGCTCGTTCTGCCAGTTGCTGTTGCTCATGCAT
>HPV74_Alpha_27462483_nt460_E7_VE_rc|1
GCTGTCTAATTGCTCATTGCAAAGTAGGCCTACAGGGTCAGGTTGTAGCTGTAACACAAT
>HPV80_Beta_2911565_nt623_E7_VE_rc|1
CTGGACAAGCTCTTGCAGCTCAAGCTCTATATCTGGTATAGTAGCTTCTTTCCCAATCAT
>HPV84_Alpha_12958167_nt484_E7_VE_rc|1
GCAATCCCCATTATCCTCCTCCTCTGAGCTGTCTAACAATTGCTCGTCACAGTATAAACT
>HPV23_Beta_1020186_nt671_E7_VE_rc|1
AATGGGCTGGACAAGCTCTTCAAGAACTATATCACGAAGAGTAGCTTGTTTCCCAATCAT
>HPV70_Alpha_1173493_nt713_E7_VE_rc|1
CTGTGGTTCTTCCCGTCTGGCTAGTAGTTGTTGTTGGTGATTAACTACATGGTCGGGTTC
>HPV60_Gamma_9628566_nt738_E7_VE_rc|1
AAGGGATCTGGAGCACCTCAAACAGGTCAGGAACACTTCTTGAAGCAATAGTTGACGAAG
>HPV73_Alpha_1491692_nt611_E7_VE_rc|1
GCTGTCTGTTTCATCCTCATCCTCTGAGTTGTCCAATGACTCGTAACATGTAAGGTCAAT
>HPV69_Alpha_6970418_nt685_E7_VE_rc|1
TCTATAACACGCTTCTTGTTCAGCTTGTCTGGCTTGCTGGTTACGCACATTATCTGTTTC
>HPV68a_Alpha_71726685_nt635_E7_VE_rc|1
ACAACACAGACACTGAATTCTGTGACGCTGTTGTTCGTCCCGTCTGGCTAGTAGTAGATG
>HPV34_Alpha_9627334_nt612_E7_VE_rc|1
GCTGTCTGTTTCATCCTCATCCTCTGAGTTGTCTAATGACTCGTAACATGTAAGGTCAGT
>HPV96_Beta_50253426_nt764_E7_VE_rc|1
CAGGACAAGGTCTTGCAGCTCCAGCTTTATGTCTGGTATAGTTGCTTCTTTCCCAATCAT
>HPV50_Gamma_9628550_nt638_E7_VE_rc|1
GGCGACACAACACAGTCTAACGCGACAATGACAATGCTTACACGTAGAATCAATCTTGAA
>HPV65_Gamma_312100_nt582_E7_VE_rc|1
TGGAGCCTCAGACTCATCATCTGAAGGTTGCAAGACCTCCTCACTCAGCAGGTTTATAGG
>HPV21_Beta_1020170_nt947_E7_VE_rc|1
TCCGCCATGTTTGCAGTTTCCGCGGCACTCAGGACACAACAGCTGCAATTCTTCAAACAG

```
>HPV31_Alpha_333048_nt651_E7_VE_rc|1
GGATGTGTCCGGTTCTGCTTGTCCAGCTGGACTGTCTATGACATCCTCCTCATCTGAGCT
>HPV103_Unclassified_109390382_nt212_E7_VE_rc|1
CTTTGCACAGGCTGGACACACAATATCCAGGTTTCCACACAGCTGTTGTTCAAAGTTTCT
>HPV31_Alpha_333048_nt591_E7_VE_rc|1
GTCGGGTAATTGCTCATAACAGTGGAGGTCAGTTGCCTCAGGTTGCAAATCTAACACATA
>HPV65_Gamma_312100_nt612_E7_VE_rc|1
GTCTATTCTAAAGGAAAAAGCTCCTCCTCTGGAGCCTCAGACTCATCATCTGAAGGTTG
>HPV107_Beta_126131393_nt799_E7_VE_rc|1
AGGGATGCGTTCAGGCTCCTCCTCTGATTCTTCACTAGTAGCATCTGGAAGCTCTGGTAA
>HPV88_Gamma_167600365_nt623_E7_VE_rc|1
TACCCTTACACCTGCTTCACAGAAGGCGCAACTGGTATCTATTTGGAAAGGTTCTCGCTC
>HPV107_Beta_126131393_nt709_E7_VE_rc|1
AGCAAGCTCTTGCAGCTCTTCCAGAACTATATCTGGAATAGTTACTTCTTTCCCAATCAT
>HPV50_Gamma_9628550_nt668_E7_VE_rc|1
GCACTCAAACACACGGATAGCTGCGTCGGTGGCGACACAACACAGTCTAACGCGACAATG
>HPV36_Beta_1020218_nt694_E7_VE_rc|1
TTCACAAAACAGGTCAACTGGTAGTACTTCAGGCTGCACCTCACTGAGCTCCAGAACAAT
>HPV32_Alpha_9627327_nt591_E7_VE_rc|1
TGAGGTGTCAAATTGTTCATAACAATACAGGTCAACCGGCGGTGTGTCGCACGTTGTCGG
>HPV6_Alpha_6002612_nt562_E7_VE_rc|1
TACTAATTGCTCATAGCAATGTAACCCTACAGGGTCTGGAGGTTGCAGGTCTAATACAAT
>HPV18_Alpha_9626069_nt651_E7_VE_rc|1
TTCATCGTTTTCTTCCTCTGAGTCGCTTAATTGCTCGTGACATAGAAGGTCAACCGGAAT
>HPV49_Beta_9627363_nt644_E7_VE_rc|1
TGTTAGATTCTCGTAGCATTGCAGGTCAATGGGCTGGCCAAACTCTTCTTGTAGTATTAT
>HPV60_Gamma_9628566_nt618_E7_VE_rc|1
GCAACAGGTGTCTATTGTATAGGGATAGTGCTCCTCCTCCTCAGGATCACCATCGGGTGA
>HPV60_Gamma_9628566_nt588_E7_VE_rc|1
CTCCTCCTCCTCAGGATCACCATCGGGTGACAACTCCTCATCAGCTAAAAGACTGACAGG
>HPV61_Alpha_9628574_nt639_E7_VE_rc|1
AATGCCACAGGTAGTAACCACCCTGTAGGCCTGCTGTGCTTGTTCAACAAGTTGCTCACG
>HPV93_Beta_37089393_nt762_E7_VE_rc|1
GCTGTTTCGGCAGTTAGGACACAGAAGATCCAACTCCTTAACTAGCAATTGCTGAAAGCA
>HPV58_Alpha_222386_nt635_E7_VE_rc|1
GTCCAAGCCTATTTCATCCTCGTCTGAGCTGTCACATAATTGCTCATAGCAGAATAGGTC
>HPV101_Unclassified_109390389_nt212_E7_VE_rc|1
GGCTCTTATGCAGCTTGCACACACCAATTCCAGGTCTCCACACAGCAGTTGTTCCACAGA
>HPV29_Alpha_1020210_nt645_E7_VE_rc|1
CAGTGTTGTACTACACTTTGTACACAAAGTTACCACCCTATACGCCTGTTGTGCTGGTTC
>HPV9_Beta_9627396_nt644_E7_VE_rc|1
TTGGACAAGCTCTTGCAGTTCTAGAACCACCTCTGGTATAGTAGCTTCTTTCCCAATCAT
>HPV6_Alpha_6002612_nt592_E7_VE_rc|1
CACTTCGTCCACCTCATCTTCTGAGCTGTCTACTAATTGCTCATAGCAATGTAACCCTAC
>HPV17_Beta_396932_nt623_E7_VE_rc|1
CTGGACAAGCTGTTGCAGCTCAAGCACTATATCTGGTATTGTAGCTTCTTTCCCAATCAT
>HPV94_Alpha_40804528_nt666_E7_VE_rc|1
TGCCCTTATATCTGCGTGGCTGCACTCTACCACCAGTCGCAGTGGTAAACTACACTTTGG
>HPV88_Gamma_167600365_nt713_E7_VE_rc|1
GTTCCTGGAACACCCAGGGCAACTTATGGAAATCTCTGCAAGTAAAAGCTGCTGAAGCGT
>HPV21_Beta_1020170_nt767_E7_VE_rc|1
TGTTTCCTGCTGCTCGCTCGGTAACTCCTCTTCACAAAACAGGTCAACTGGTTGTACCTC
>HPV21_Beta_1020170_nt737_E7_VE_rc|1
TTCACAAAACAGGTCAACTGGTTGTACCTCAGGCTGCAATTCATTTAACTCCAGAACAAT
>HPV58_Alpha_222386_nt695_E7_VE_rc|1
```

GTAACAACAAGTTACAATGTAGTAATTAGCTGTGGCCGGTTGTGCTTGTCCATCTGGCCC
>HPV95_Gamma_40804520_nt741_E7_VE_rc|1
ACACCCAACACAGCAAAATATCACCTTCCCTTCCAGAAGAAGTTGTTCCACGACTCGTAG
>HPV43_Alpha_40804474_nt591_E7_VE_rc|1
GGGTTGCTCACGCTCATCCTCTGAGTCTGAGCTGTCTAATTGCTCGTTACAGGTTAAGCT
>HPV92_Beta_27531786_nt520_E7_VE_rc|1
CTGGACAAGGTCTTGCAGATCCAGCACAATATCTGGTATAGTAGCCTGTTTCCCAATCAT
>HPV71_Alpha_12084981_nt650_E7_VE_rc|1
TGAAACCACCCTATATGCCTGCTGTGCTGGTTCTGTGGGTTGCTCACCGTCCACCCTATT
>HPV1_Mu_9626063_nt620_E7_VE_rc|1
AGCAACGACAGCATAAGGTTGCTGAGGCGACACTAACTCCTCCTCTATGTCATCAGGAGG
>HPV73_Alpha_1491692_nt641_E7_VE_rc|1
ACACTCTCGTTCAGCTTGTCTGTCTAGATGGCTGTCTGTTTCATCCTCATCCTCTGAGTT
>HPV67_Alpha_3228267_nt654_E7_VE_rc|1
ATCTGGTTTTGCTTGTCCATCTGGCCGGTCCATGCCTACTTCATCCTCCTCATCTGAGCT
>HPV28_Alpha_1020202_nt519_E7_VE_rc|1
GACGTCCTCTGGTGCAAGACTCAATTCTATATCTTTTACTGTGGGATGCGGACCGTGCAT
>HPV2_Alpha_9626032_nt620_E7_VE_rc|1
CCCGTAGGCCTGCACATCTGGTTCTGTCAGTTGATGGTTATTCTCTTCTTCTGAGCTGTC
>HPV110_Beta_186461194_nt595_E7_VE_rc|1
CTGAACAAGCTCTTGCAATTCCAGAACAATGTCTGGTATAGTAGCTTGTTTCCCAATCAT
>HPV15_Beta_396924_nt623_E7_VE_rc|1
CTGGACAAGCTCTTGCAGCTCAAGCACTATATCTGGTATAGTAGCTTCTTTCCCAATCAT
>HPV81_Alpha_40804509_nt603_E7_VE_rc|1
ATCATCCACATCCACCTCTTCCTCTGAGCTGTCTAGCAATTGCTCGTGGCAATGTAGGTC
>HPV11_Alpha_333026_nt561_E7_VE_rc|1
TTCTAATTGCTCATAGCAATGTAACCCTACAGGGTCAGGAGGCTGCAGGTCTAGTACTAT
>HPV67_Alpha_3228267_nt744_E7_VE_rc|1
TGTTCGGACGTCTGCTGCTGTACTGTGAATGCACAAACGCAAAGTGCACTCACAGATGTT
>HPV103_Unclassified_109390382_nt122_E7_VE_rc|2
ACACAATGACTTCTCACAATACTTGCAATCTGTCACCACCCGGTAAGGACACTTATCCTG
>HPV48_Gamma_9628542_nt608_E7_VE_rc|1
ACAATTGTGACACTTGGAGTCTATCCGGTACGGACAAAACTCCTCCTCTGCAGTAGCATC
>HPV20_Beta_1020162_nt758_E7_VE_rc|1
TCTCTCCTGCTGCTCGTTCGGTAACTCCTCTTCACAAAACAGGTCAACTGGTTGAACCTC
>HPV54_Alpha_9628437_nt627_E7_VE_rc|1
TAACACCTTAAATGCTTGTTTGTCAGGTTGTGTTACTGCTGTCTCATCTTCTGCATCAGA
>HPV71_Alpha_12084981_nt680_E7_VE_rc|1
CCTAACTGCACGACAGCACCTACCACAGTATGAAACCACCCTATATGCCTGCTGTGCTGG
>HPV51_Alpha_333087_nt621_E7_VE_rc|1
ATTATCTACTTCATCCTCCTCCTCTGAGCTGTCAAATTGCTCGTAGCATTGCAAGTCAAT
>HPV67_Alpha_3228267_nt774_E7_VE_rc|1
TCCTAGTGTGTTCATAAGCATCTGCTGGATTGTTCGGACGTCTGCTGCTGTACTGTGAAT
>HPV88_Gamma_167600365_nt593_E7_VE_rc|1
ACTGGTATCTATTTGGAAAGGTTCTCGCTCCTCCTCCTCCTGGGTCTCCTCATCAGATGA
>HPV1_Mu_9626063_nt740_E7_VE_rc|1
TAGGGTGCACAGTGGGCACACGATGTTCAAAGATCGCAGAAGGAGTTCCTCCAGCTGTCT
>HPV48_Gamma_9628542_nt668_E7_VE_rc|1
TTGCTCAAAACAACGTATTCCAAATTCAGTTGCAGCGACTGTCACCCTTATGCGACAGCC
>HPV83_Alpha_5059324_nt481_E7_VE_rc|1
ACAGTCCCTATCATCCTCCTCCTCTGAGCTGTCTATACCTTGTTCATTACAATATAGGTC
>HPVFA75/KI88-03_Unclassified_206902429_nt606_E7_VE_rc|1
TAATTCTTCGTAGCAATGCAGGTCAATGGGCTGGACAAGCTCTTGCAGTTCCAGAACAAT
>HPV73_Alpha_1491692_nt671_E7_VE_rc|1
ACACTTCGTGCAGTCAGTAACTATTCTGTAACACTCTCGTTCAGCTTGTCTGTCTAGATG

>HPV69_Alpha_6970418_nt625_E7_VE_rc|1
ATCCTCCTCTGAGCTGTCAAATTGTTCATAGTCCAATTGTTCGTAACACTGTAGGTCAAT
>HPV24_Unclassified_9628486_nt683_E7_VE_rc|1
TCTTTCAGGCTCCACCTCCGTATCCTGCTCTGGCAACTCCTCTTCACAGTGCAAATCAAC
>HPV35_Alpha_333050_nt653_E7_VE_rc|1
GGTGTCTGGTTTTGCTTGTCCAGCTGGACCGTCAATAGTATCTTCCTCCTCCTCTGAGCT
>HPV27_Alpha_396964_nt660_E7_VE_rc|1
TCTGCCGCACTTGCAGCAGGTTGTTACCACCCCGTAGGCCTGCACAGCTGGTTCTGTCAG
>HPV42_Alpha_333211_nt627_E7_VE_rc|1
TCTGTAACGCTGTATGTCCTGTTTGGCTTGGTCATCTTCATCTGAGCTGTCCAATTGTTC
>HPV93_Beta_37089393_nt552_E7_VE_rc|1
AGGCTCTGGTGGTGTCAACCCTAACACAATGTCTTGCAGAGTGACCTCTTTTCCAATCAT
>HPV67_Alpha_3228267_nt624_E7_VE_rc|1
CATGCCTACTTCATCCTCCTCATCTGAGCTGTCATGCAATTGCTCATAGCAGTATAGGTC
>HPV80_Beta_2911565_nt653_E7_VE_rc|1
TAACTCTTCGTAGCAATGCAGGTCAGTGGGCTGGACAAGCTCTTGCAGCTCAAGCTCTAT
>HPVcand89_Alpha_22095322_nt631_E7_VE_rc|1
CAGGTCATTCAGCAGCAGCTGTTGTAGATGTCTTAGATGTGCGTCGTCAGACAATACTAC
>HPV93_Beta_37089393_nt642_E7_VE_rc|1
GCAGAACACAATAATCTTGAAAGCTGTTCTTGCAGGCTCCTCCTCCGTATCCTGCTCGGT
>HPVRTRX7_Unclassified_30315616_nt728_E7_VE_rc|1
CTCCGTTTCCTGTTCGTTTGGTAATTCCTCTTCACAAAGCAGGTCAACTGGTATCACTTC
>HPV49_Beta_9627363_nt674_E7_VE_rc|1
CTCCAACTCTTGTTCAGCTGGCGCTTCAGCTGTTAGATTCTCGTAGCATTGCAGGTCAAT
>HPVcand85_Alpha_4574720_nt828_E7_VE_rc|1
CAAAAATGATAGCGTGCCCAAAAACAGCTGCTGGAGATCTCGTAGATCGGCTGCTGAACT
>HPV35_Alpha_333050_nt623_E7_VE_rc|1
GTCAATAGTATCTTCCTCCTCCTCTGAGCTGTCACACAATTGCTCATAACAGTATAGGTC
>HPV69_Alpha_6970418_nt655_E7_VE_rc|1
GGCTTGCTGGTTACGCACATTATCTGTTTCATCCTCCTCTGAGCTGTCAAATTGTTCATA
>HPV93_Beta_37089393_nt612_E7_VE_rc|1
TGCAGGCTCCTCCTCCGTATCCTGCTCGGTTGGTAACTCCTCTTCACAATTCAATGCAAC
>HPV4_Gamma_9626597_nt582_E7_VE_rc|1
TGTAATCTCATACTCATCATCTGAAGATTGCAAGACCTCCTCACTCAGCAGGTTTGCTGG
>HPV22_Beta_1020178_nt644_E7_VE_rc|1
AATGGGCAGGACAAGCTCTTCAAGAACTATATCACACAGAGTAGCTTGTTTCCCAATCAT
>HPVcand86_Alpha_15741127_nt484_E7_VE_rc|1
ACAATCCCTATTATCCTCCTCCTCTGAGCTGTCTAACAATTGCTCGTCACAATGTAGGTT
>HPV77_Alpha_2911558_nt555_E7_VE_rc|1
GCTGTCTAATTGCTCATTGCATAGTAAAGGGACGGCCTCTGGTGCAAGATCCAATTCAAT
>HPV32_Alpha_9627327_nt531_E7_VE_rc|1
GTCACACGTTGGCAGGTCATACAAAATAATGTCCTTTAGCGTTGGTGCGTTTCCACGCAT
>HPVcand89_Alpha_22095322_nt601_E7_VE_rc|1
TCTTAGATGTGCGTCGTCAGACAATACTACTAACCTAACTGCACGATAGCACACACCACA
>HPV81_Alpha_40804509_nt663_E7_VE_rc|1
CGTAGTAACCACCCTATAGGCCTGCTGTGCTTGTTCTACTAGTTGCTCACCACTGTCCCT
>HPV52_Alpha_397038_nt644_E7_VE_rc|1
GGCTTGTTCTGCTTGTCCATCTGGCCGGTCCACACCATCTGTATCCTCCTCATCTGAGCT
>HPV110_Beta_186461194_nt625_E7_VE_rc|1
CTCCTCGTAGCAATGCAGGTCAGCAGTGGGCTGAACAAGCTCTTGCAATTCCAGAACAAT
>HPV19_Beta_396940_nt751_E7_VE_rc|1
TGTTTCCTGCTGTTCGGTCGGTAACTCCTCTTCACAAAACAGGTCAACTGGTTGTACCTC
>HPV55_Alpha_1020266_nt591_E7_VE_rc|1
GGCTAGTTCATCCACCTCATCCTCTGAGCTGTCTAATTGCTCATTGCAATGTAGGCCTAC
>HPV59_Alpha_557236_nt633_E7_VE_rc|1

CAAAGGATGATTAACTCCATCTGGTTCATCTTTTTCATTCTCGGAGTCGGAGTCAGGTAA
>HPV26_Alpha_9627305_nt677_E7_VE_rc|1
TCTGTAACACACTTCTTGTCCAGCTTGTCTGGCCTGCTGGTCACGCATATTATCTGTTTC
>HPV43_Alpha_40804474_nt561_E7_VE_rc|1
GCTGTCTAATTGCTCGTTACAGGTTAAGCTTCTAGGTTCTGGTTGCATAGTTAGCACATA
>HPV13_Alpha_85827580_nt563_E7_VE_rc|1
GCTGTCTAATTGCTCATTGCAATGTAGACCTACAGGGTCAGGAGTCAGGTCTAAAACAAT
>HPV54_Alpha_9628437_nt717_E7_VE_rc|1
ATGAAGCAGTTCCTGCAGTACCCTTATGCCTGTGTGCGTGCTATACACACATAGCCGTAC
>HPV1_Mu_9626063_nt710_E7_VE_rc|1
AGATCGCAGAAGGAGTTCCTCCAGCTGTCTAATGGCGCTGTGATCCGCGAGGACGGTCAA
>HPV37_Beta_1020226_nt623_E7_VE_rc|1
CTGGACAAGCTCTTGCAGCTCAAGCACTATTTCTGGTATTGTAGCTTCTTTCCCAATCAT
>HPV92_Beta_27531786_nt580_E7_VE_rc|1
AGGTGCAGGCTCCTCCTCCTGATTTTCTGACAAGTCTTCGTCACAATGCAGGTCAATGGG
>HPV107_Beta_126131393_nt739_E7_VE_rc|1
CTCTTCGTAACAATGCAGGTCAATGGGCTGAGCAAGCTCTTGCAGCTCTTCCAGAACTAT
>HPV83_Alpha_5059324_nt511_E7_VE_rc|1
TGCTTGTTCTGCCAGTTGGTCACGCACGCCACAGTCCCTATCATCCTCCTCCTCTGAGCT
>HPV107_Beta_126131393_nt769_E7_VE_rc|1
TTCACTAGTAGCATCTGGAAGCTCTGGTAACTCTTCGTAACAATGCAGGTCAATGGGCTG
>HPV6_Alpha_6002612_nt622_E7_VE_rc|1
TTGTTTTAAAGGTTGTGAATCTTGTCCGTCCACTTCGTCCACCTCATCTTCTGAGCTGTC
>HPV27_Alpha_396964_nt630_E7_VE_rc|1
CCCGTAGGCCTGCACAGCTGGTTCTGTCAGTTGATGGTTATTCTCTTCTTCTGAGCTGTC
>HPV82_Alpha_6970427_nt626_E7_VE_rc|1
ATTATCTACTTCATCCTCCTCGTCTGAGCTGTCAAATTGCTCGTAGCATTGCAAGTCAAT
>HPV90_Alpha_22138122_nt454_E7_VE_rc|1
GGCTAATTGCTCGTTACAATGTAGGTCAACCGCATCAGGCTGATCCAGCTCTAGAAATAT
>HPV60_Gamma_9628566_nt648_E7_VE_rc|1
AGTAAATCTAACACCTGCTCCACAAGGTTTGCAACAGGTGTCTATTGTATAGGGATAGTG
>HPV76_Beta_2911551_nt644_E7_VE_rc|1
TGGTAAGTTCTCGTAGCATTGCAGGTCAATGGGCTGGCCAAACTCTTCTTGTAGCACAAT
>HPV76_Beta_2911551_nt674_E7_VE_rc|1
CTCTGTGTCTTGTTCAGCTGGCACTTCAGCTGGTAAGTTCTCGTAGCATTGCAGGTCAAT
>HPV8_Beta_333074_nt714_E7_VE_rc|1
CTCCGTTTCCTGTTCGTTTGGTAATTCCTCTTCACAAAGCAGGTCAACTGGTAACACTTC
>HPV28_Alpha_1020202_nt669_E7_VE_rc|1
TGCCCTTATATCTGCGTGGCTGCACTCTACCACCAGTCGCAGTGGTGAACTACACTTTGG
>HPV63_Mu_9626605_nt735_E7_VE_rc|1
CAGGGGACACACGATCCTCAGTGAGCCTAACAGCAGCTCCTCCAGACGCGTAAGAGTAGA
>HPV35_Alpha_333050_nt683_E7_VE_rc|1
ACAACAGGACGTTACAATATTATAATTGGAGGTGTCTGGTTTTGCTTGTCCAGCTGGACC
>HPV47_Beta_333062_nt699_E7_VE_rc|1
GTCGCAAAACAGGTCAACTGGTAATACTTCAGGTTGAACCTCACTTAACTCCAGAACAAT
>HPV90_Alpha_22138122_nt484_E7_VE_rc|1
GTCCTCCCTATTATCCTCCTCTTCTGAGCTGGCTAATTGCTCGTTACAATGTAGGTCAAC
>HPV2_Alpha_9626032_nt650_E7_VE_rc|1
TCTGCCACACTTACAGCAGGTAGTTACCACCCCGTAGGCCTGCACATCTGGTTCTGTCAG
>HPV102_Alpha_71726718_nt571_E7_VE_rc|1
CACTGCCAGTCTCAGTGACTGGCCACACATACCACACTTTGTTACCACCCTATAGGCCTG
>HPV51_Alpha_333087_nt651_E7_VE_rc|1
AGCCCGTCTTTCTGGTAGCTGGTCACGCATATTATCTACTTCATCCTCCTCCTCTGAGCT
>HPVcand62_Alpha_39932599_nt454_E7_VE_rc|1
GTCTAACAATTGCTCGTGGCAATGTAGGTCAACCACATCGGGACGCTCTTGTAGGACAAT

>HPV97_Alpha_71726694_nt607_E7_VE_rc|1
GTGACGCTGTGGTTCAGCTCGTCGGGCTGGTAGTTGTCGATGATTAACATCATCTGTTTC
>HPV47_Beta_333062_nt669_E7_VE_rc|1
AGGTTGAACCTCACTTAACTCCAGAACAATATCTCGCACGGTGACCTCTTTACCAATCAT
>HPV94_Alpha_40804528_nt606_E7_VE_rc|1
ACATACTGTTACCACCCTATACGCTTGTTGTGCTGGTTCCACCACATCTGTATAATCTTC
>HPV16_Alpha_9627100_nt773_E7_VE_rc|1
CACAATTCCTAGTGTGCCCATTAACAGGTCTTCCAAAGTACGAATGTCTACGTGTGTGCT
>HPV58_Alpha_222386_nt665_E7_VE_rc|1
TGTGGCCGGTTGTGCTTGTCCATCTGGCCCGTCCAAGCCTATTTCATCCTCGTCTGAGCT
>HPV50_Gamma_9628550_nt698_E7_VE_rc|1
ACACAGAAAAGAAAATTCTGATTGAAGAAGGCACTCAAACACACGGATAGCTGCGTCGGT
>HPVcand85_Alpha_4574720_nt768_E7_VE_rc|1
TTCCACCACTAACTGCAGACTGGCCTCACATTTACAACACACGCAGCAAATAGTGTGACG
>HPV32_Alpha_9627327_nt771_E7_VE_rc|1
ACACAATGGACACACAATGCCCAGTGTGTCCAGAAGCATCTGATGCAGCTGTTGTATGTC
>HPV31_Alpha_333048_nt621_E7_VE_rc|1
ACTGTCTATGACATCCTCCTCATCTGAGCTGTCGGGTAATTGCTCATAACAGTGGAGGTC
>HPV18_Alpha_9626069_nt741_E7_VE_rc|1
GGCTTCACACTTACAACACATACACAACATTGTGTGACGTTGTGGTTCGGCTCGTCGGGC
>HPV76_Beta_2911551_nt614_E7_VE_rc|1
GGGCTGGCCAAACTCTTCTTGTAGCACAATGTCAGGTAATGTAGCCTCTTTCCCAATCAT
>HPV74_Alpha_27462483_nt490_E7_VE_rc|1
CGTGGCTAGTTCATCCACCTCCTCTTCTGAGCTGTCTAATTGCTCATTGCAAAGTAGGCC
>HPV26_Alpha_9627305_nt647_E7_VE_rc|1
GGCCTGCTGGTCACGCATATTATCTGTTTCATCCTCATCTGAGCTGTCAAATTGTTCATA
>HPV59_Alpha_557236_nt693_E7_VE_rc|1
ACACTTACAACACACACACACAATGTTGTGACGCTGTGGTTCAGCTCGTCTAGCTAGTAG
>HPVFA75/KI88-03_Unclassified_206902429_nt636_E7_VE_rc|1
ATCTTCACTAGGAACATCTGGAAGCTCTGGTAATTCTTCGTAGCAATGCAGGTCAATGGG
>HPV12_Beta_396910_nt724_E7_VE_rc|1
CTCCGTTTCCTGCTCGTTTGGTAATTCCTCTTCACAAAGCAGGTCAACTGGTAACACTTC
>HPV110_Beta_186461194_nt685_E7_VE_rc|1
AGCAGCAACAATCTTGTAAGGTGTATGACGTGACTCCTCCTCTGTTTCTTCTTCAGTTAA
>HPV35_Alpha_333050_nt743_E7_VE_rc|1
CAATTTACGTATGTCAATGTGTGTGCTCTGTACACACAGACGTAGTGTCGCCTCACATTT
>HPV16_Alpha_9627100_nt683_E7_VE_rc|1
CTTGCAACAAAAGGTTACAATATTGTAATGGGCTCTGTCCGGTTCTGCTTGTCCAGCTGG
>HPV63_Mu_9626605_nt645_E7_VE_rc|1
CTGATCGCACAAACCACAAGGAAGCACAATTGCATATGGATATTCAGACTCCTCCTCCTC
>HPV48_Gamma_9628542_nt638_E7_VE_rc|1
TGCAGCGACTGTCACCCTTATGCGACAGCCACAATTGTGACACTTGGAGTCTATCCGGTA
>HPV54_Alpha_9628437_nt657_E7_VE_rc|1
TGTCTTACAACACACACCTCCACACTGGCTTAACACCTTAAATGCTTGTTTGTCAGGTTG
>HPV41_Nu_9626041_nt885_E7_VE_rc|1
GACACAGGCGTGACAAGCGAGGTCAAGGTGACTGTTGACCAGGAGTGCCTGTAGATTCCG
>HPV15_Beta_396924_nt653_E7_VE_rc|1
TAACTCTTCGTAGCAATGCAGGTCAGTGGGCTGGACAAGCTCTTGCAGCTCAAGCACTAT
>HPV45_Alpha_397022_nt738_E7_VE_rc|1
GTCACACTTACAACATACACACAAAATTTTGTGACGCTGTGGTTCGGCTCGTCGGGCTGG
>HPV63_Mu_9626605_nt615_E7_VE_rc|1
TGCATATGGATATTCAGACTCCTCCTCCTCAGCAGGTATATCCTCATAACAATTTAGATC
>HPV45_Alpha_397022_nt708_E7_VE_rc|1
GTGACGCTGTGGTTCGGCTCGTCGGGCTGGTAGTTGTGCATGACTAACTCCATCTGCTTC
>HPV24_Unclassified_9628486_nt713_E7_VE_rc|1

GCCGCAACAAAGTATTATTTTGTAAGCTCTTCTTTCAGGCTCCACCTCCGTATCCTGCTC
>HPV44_Alpha_1020242_nt594_E7_VE_rc|1
GGCTAGTTCATCCACCTCATCTTCTGAGCTGTCTAATTGCTCATTGCAATGTAGGCCTAC
>HPV36_Beta_1020218_nt724_E7_VE_rc|1
CTCCTCCGTATCCTCGTTTGGTAATTCCTCTTCACAAAACAGGTCAACTGGTAGTACTTC
>HPV43_Alpha_40804474_nt621_E7_VE_rc|1
TTGTAGATTCACTTGCTGGTCCTGTTGCGTGGGTTGCTCACGCTCATCCTCTGAGTCTGA
>HPV75_Beta_2911544_nt644_E7_VE_rc|1
TGCTAAATCCTCGTAGCATTGCAGGTCAATGGGCTGGCCAAACTCTTCTTGTAGCACAAT
>HPV54_Alpha_9628437_nt747_E7_VE_rc|1
GGTGGGACACACTATTTGCAGTGCGTCCTGATGAAGCAGTTCCTGCAGTACCCTTATGCC
>HPVcand86_Alpha_15741127_nt544_E7_VE_rc|1
ACACAACCCACATTCTGTTAACACCCTATAGGCCAGCTGTGCTGGTTCTGCAGGTTGGTC
>HPV103_Unclassified_109390382_nt152_E7_VE_rc|1
AATGCCCTCCTCCGTGGCTACCACATACATACACAATGACTTCTCACAATACTTGCAATC
>HPV4_Gamma_9626597_nt522_E7_VE_rc|1
TAACACTAAGTCATTTAGTTCTAAATTAAGATCTGCAACCGTGGGCGCTGCTCCTCTCAT
>HPV39_Alpha_333245_nt653_E7_VE_rc|1
ATCTATTTCATCCTCTGACTCTCCTAATTGCTCGTGACATACAAGGTCAACCGGCTGTAT
>HPV95_Gamma_40804520_nt561_E7_VE_rc|1
CAAGACCTCATCGCTCAGCAAATTTGCAGGAACAACTAACTCATGGAGGTTTAAATCAAC
>HPV91_Alpha_22023568_nt667_E7_VE_rc|1
GGATTGCTCACGCTCATCCTCTGAGTCTGAGCTGTCTAATTGCTCGTCACAGGTTAAACT
>HPV101_Unclassified_109390389_nt62_E7_VE_rc|1
TTGCTCGTCCTGCTGCTCCTCCTCCTCGGTTACTAATTCATCACATAATAAGTTTGCAGG
>HPV37_Beta_1020226_nt683_E7_VE_rc|1
ACGCTCCTCCTCTGTCTCTTCTTCACTCAACTCTTCGTAACAATGCAGGTCAGCAGTGGG
>HPV33_Alpha_333049_nt694_E7_VE_rc|1
AGTGTGACAACAGGTTACAATGTAGTAATCAGCTGTGGCTGGTTGTGCTTGTCCATCTGG
>HPV38_Beta_1020234_nt653_E7_VE_rc|1
ATCAGGCAACTCCTCGTGGCAATGCAGGTCAATGGGCTGGACAAGCTCTTCAAGAACTAT
>HPV32_Alpha_9627327_nt741_E7_VE_rc|1
CAGAAGCATCTGATGCAGCTGTTGTATGTCCGCGCCTGTACTACTGACAACAAGTTTAAC
>HPV26_Alpha_9627305_nt617_E7_VE_rc|1
ATCCTCATCTGAGCTGTCAAATTGTTCATAGTCCAATTGTTCGTAGCAGCGTAGGTCAAT
>HPV95_Gamma_40804520_nt711_E7_VE_rc|1
TTCCAGAAGAAGTTGTTCCACGACTCGTAGTCCAAATGCAACAGCATACAAAGTGATTCT
>HPV39_Alpha_333245_nt743_E7_VE_rc|1
CTTACAACACGAACACTGTATTGTGTGACGCTGTGGTTCATCCCGTCTGGCTAGTAGTTG
>HPV33_Alpha_333049_nt664_E7_VE_rc|1
AGCTGTGGCTGGTTGTGCTTGTCCATCTGGCCGGTCCAAGCCTTCATCCTCATCTGAGCT
>HPV96_Beta_50253426_nt794_E7_VE_rc|1
CAACTCTTCGTCACAATGCAGGTCAATGGGCAGGACAAGGTCTTGCAGCTCCAGCTTTAT
>HPV96_Beta_50253426_nt824_E7_VE_rc|1
TTCTGACAGGTTTTCTGACTGCTCTTCTGTCAACTCTTCGTCACAATGCAGGTCAATGGG
>HPVcand89_Alpha_22095322_nt481_E7_VE_rc|1
ACAATCCCTAGCATCTTCCTCATCTGAGCTGTCTAGCATTTCTTCCTCACATTGTAGGCT
>HPV101_Unclassified_109390389_nt92_E7_VE_rc|1
ATCTGTCACAATTCTATAAGGGCTTAAAGCTTGCTCGTCCTGCTGCTCCTCCTCCTCGGT
>HPV102_Alpha_71726718_nt541_E7_VE_rc|1
ACCACACTTTGTTACCACCCTATAGGCCTGCTTTGCTTGTTCTGCCAGTTGGTCACGCAC
>HPV52_Alpha_397038_nt764_E7_VE_rc|1
TTGTAATGTGCCCAACAGCATTTGCTGTAGAGTACGAAGGTCCGTCGCAGTGCTATGAAT
>HPVcand85_Alpha_4574720_nt738_E7_VE_rc|1
TTTACAACACACGCAGCAAATAGTGTGACGCTGTAGTTCTTCTCGTCTGGCTAGTAGTGG

>HPV10_Alpha_9627257_nt675_E7_VE_rc|1
CAGTGCCCTTATATCTGCGTGGCTGCACTCTACCACCAGTCGCAGTGGTAAACTACACTT
>HPV94_Alpha_40804528_nt516_E7_VE_rc|1
GATGTCCTCTGGTGCAAGACTCAATTCTATATCTTTTACTGTGGGATGCGGACCGTGCAT
>HPV37_Beta_1020226_nt653_E7_VE_rc|1
CTCTTCGTAACAATGCAGGTCAGCAGTGGGCTGGACAAGCTCTTGCAGCTCAAGCACTAT
>HPV33_Alpha_333049_nt634_E7_VE_rc|1
CCGGTCCAAGCCTTCATCCTCATCTGAGCTGTCACTTAATTGCTCATAGCAGTATAGGTC
>HPV48_Gamma_9628542_nt518_E7_VE_rc|1
AGCAGGCAAAACAAGCTCTTCTAACTCTATGTCAGGAATAGTAGCCTTATCTCCCCTCAT
>HPV1_Mu_9626063_nt680_E7_VE_rc|1
AATGGCGCTGTGATCCGCGAGGACGGTCAATCGAACCAGTTTCTCGCAATAGGCACAGGA
>HPV42_Alpha_333211_nt597_E7_VE_rc|1
GTCATCTTCATCTGAGCTGTCCAATTGTTCATAGCAATACAGGTCAATGGGTGTCTCACA
>HPVcand87_Alpha_14475578_nt648_E7_VE_rc|1
CCCACACTCTGTTAACACCCTATGGGCCAGCTGTGCTGGTTCTGCAGGTTGGTCACGCAC
>HPV24_Unclassified_9628486_nt743_E7_VE_rc|1
AAATAATCGAAGCCGGGTTCCACAACCGCCGCCGCAACAAAGTATTATTTTGTAAGCTCT
>HPV52_Alpha_397038_nt614_E7_VE_rc|1
CACACCATCTGTATCCTCCTCATCTGAGCTGTCACCTAATTGCTCATAGCAGTGTAGGTC
>HPV110_Beta_186461194_nt655_E7_VE_rc|1
TGACTCCTCCTCTGTTTCTTCTTCAGTTAACTCCTCGTAGCAATGCAGGTCAGCAGTGGG
>HPVcand85_Alpha_4574720_nt798_E7_VE_rc|1
CTGGAGATCTCGTAGATCGGCTGCTGAACTTTCCACCACTAACTGCAGACTGGCCTCACA
>HPV101_Unclassified_109390389_nt182_E7_VE_rc|1
CAGGTCTCCACACAGCAGTTGTTCCACAGACCTTATTGCCCCGTCCGTAGCTAGTACATG
>HPV111_Beta_186461202_nt609_E7_VE_rc|1
CAATTCTTCGTAGCAATGCAGGTCAGTGGGTTGGACAAGCTCTTGCAGCTCAAGAACTAC
>HPV40_Alpha_397014_nt558_E7_VE_rc|1
GCTGTCTAATTGCTCGTTGCAGTTTAGACATACAGGTTCAGGGTGCAGGTTTAACACAAT
>HPV61_Alpha_9628574_nt579_E7_VE_rc|1
CACACTATCCTCCTCCTCTGACTCTGAGCTGTCTAATAACTGCTCATTGCAATGTAGGTC
>HPV40_Alpha_397014_nt648_E7_VE_rc|1
TACTTGCAGGTCCTGTTGCGTGGGTTGCTCACGCTCTCTACTGTGTAAGCTGTCTAGTTG
>HPV40_Alpha_397014_nt588_E7_VE_rc|1
GTCCTGTTCATGGTCATCTTCTGAGTCTGAGCTGTCTAATTGCTCGTTGCAGTTTAGACA
>HPV67_Alpha_3228267_nt684_E7_VE_rc|1
ACACACAGTAACAATATGGTAATTGGTTGTATCTGGTTTTGCTTGTCCATCTGGCCGGTC
>HPV68a_Alpha_71726685_nt755_E7_VE_rc|1
CTGGGTTTCAGTTGCACACCACGGACACACAAAATTTAGTGAGTCCATAAACAGCTGTTG
>HPV11_Alpha_333026_nt591_E7_VE_rc|1
CACCTTGTCCACCTCATCTTCTGAGCTGTCTTCTAATTGCTCATAGCAATGTAACCCTAC
>HPVcand85_Alpha_4574720_nt708_E7_VE_rc|1
CTGTAGTTCTTCTCGTCTGGCTAGTAGTGGTTGTCGGTGATTAATTGCATTATCTGGTTC
>HPVcand87_Alpha_14475578_nt618_E7_VE_rc|1
CTGTGCTGGTTCTGCAGGTTGGTCACGCACACAATCCCTATTGTCCTCCTCCTCTGAGCT
>HPV54_Alpha_9628437_nt687_E7_VE_rc|1
TGTGTGCGTGCTATACACACATAGCCGTACTGTCTTACAACACACACCTCCACACTGGCT
>HPV1_Mu_9626063_nt650_E7_VE_rc|1
TCGAACCAGTTTCTCGCAATAGGCACAGGAAGCAACGACAGCATAAGGTTGCTGAGGCGA
>HPVcand62_Alpha_39932599_nt484_E7_VE_rc|1
GTCCCTATCATCCACCTCTTCCTCTGAGCTGTCTAACAATTGCTCGTGGCAATGTAGGTC
>HPV68a_Alpha_71726685_nt545_E7_VE_rc|1
ATCTATTTCATCGTCTGAATCTCCTAATTGCTCGTGACATACAAGGTCAACCGGCTGTAT
>HPVFA75/KI88-03_Unclassified_206902429_nt576_E7_VE_rc|1

CTGGACAAGCTCTTGCAGTTCCAGAACAATATCTGGAATAGTAACTTCTTTCCCAATCAT
>HPV16_Alpha_9627100_nt653_E7_VE_rc|1
GGCTCTGTCCGGTTCTGCTTGTCCAGCTGGACCATCTATTTCATCCTCCTCCTCTGAGCT
>HPVRTRX7_Unclassified_30315616_nt698_E7_VE_rc|1
TTCACAAAGCAGGTCAACTGGTATCACTTCAGGCTGCACCTCACTAAGCTCCAAAATAAA
>HPV24_Unclassified_9628486_nt623_E7_VE_rc|1
AGTCTGTGGCTCAGTCAACTCTAAGACAATGTCTTGTAGAGTGACCTCTTTTCCAATCAT
>HPV41_Nu_9626041_nt795_E7_VE_rc|1
TCGTATGATAGCCTGGCAAAATGGACACTGACTCTCTACCAGGTATAATGTTCTCCTGGG
>HPV47_Beta_333062_nt729_E7_VE_rc|1
CTCCGCCTGTTGTTCATTTGGTAATTCCTCGTCGCAAAACAGGTCAACTGGTAATACTTC
>HPV31_Alpha_333048_nt681_E7_VE_rc|1
CTGACAACAAAAGGTAACGATATTGTAATTGGATGTGTCCGGTTCTGCTTGTCCAGCTGG
>HPV41_Nu_9626041_nt855_E7_VE_rc|1
ACTGTTGACCAGGAGTGCCTGTAGATTCCGTATCCCAGTGTTGCTTGCTACGCATACAAA
>HPV59_Alpha_557236_nt783_E7_VE_rc|1
ACACACAAAGGATAGTGTGTCCATAAACAGCTGCTGTAAGGCTCGCAATCCGTCTTGCGA
>HPV103_Unclassified_109390382_nt242_E7_VE_rc|1
GTGTCTTTGTCCTCCATTTCGCTGGCGTTGCTTTGCACAGGCTGGACACACAATATCCAG
>HPV81_Alpha_40804509_nt693_E7_VE_rc|1
CAGCCTAACTAAACACCTACAAATACCACACGTAGTAACCACCCTATAGGCCTGCTGTGC
>HPVcand89_Alpha_22095322_nt571_E7_VE_rc|1
TAACCTAACTGCACGATAGCACACACCACACGTAGTTACCACCCTAAAGGCCTGCTGTGC
>HPV7_Alpha_9627389_nt558_E7_VE_rc|1
GCTGTCTAATTGCTCGTTGCAACTTAAACTTACTGGTTCGGGTTGCAAGTCTAACACGAT
>HPV25_Beta_396948_nt736_E7_VE_rc|1
TGTTTCCTGATGCTCAGCCGGCAACTCCTCTTCACAAAACAGGTCAACTGGTTGTACCTC
>HPV97_Alpha_71726694_nt637_E7_VE_rc|1
CTCACATTTACAACACATACACACAATTGTGTGACGCTGTGGTTCAGCTCGTCGGGCTGG
>HPV52_Alpha_397038_nt674_E7_VE_rc|1
ACAATATGTCACAATGTAGTAATTGCTTGTGGCTTGTTCTGCTTGTCCATCTGGCCGGTC
>HPV42_Alpha_333211_nt747_E7_VE_rc|1
CAAAGGACACACAATATCCAGTGTGCCCAAAAGCATCTGTTGCAGGTTTCTTATGTCCGC
>HPV22_Beta_1020178_nt674_E7_VE_rc|1
TTCAGGCAGCTCCTCGTGGCAATGCAGGTCAATGGGCAGGACAAGCTCTTCAAGAACTAT
>HPV84_Alpha_12958167_nt544_E7_VE_rc|1
CCCACACTCTGTTACCACCCTATAGGCCAGCTGTGCTGGTTTTGCAGGTTGGTCACGCAA
>HPV68a_Alpha_71726685_nt575_E7_VE_rc|1
TTGGTGGTGATTAACTGCATGGTCGGGTTCATCTATTTCATCGTCTGAATCTCCTAATTG
>HPV35_Alpha_333050_nt593_E7_VE_rc|1
GTCACACAATTGCTCATAACAGTATAGGTCAGTTGCCTCGGGTTCCAAATCTAAAACATA
>HPV72_Alpha_1491683_nt675_E7_VE_rc|1
AACTGGACGGTAGCACCTGCCACAGGTAGTAACCACCCCGTAGGCCTGCTGTGCTTGTTC
>HPV63_Mu_9626605_nt705_E7_VE_rc|1
CAGCAGCTCCTCCAGACGCGTAAGAGTAGACAGGTCAGAAACGCAGGTCAGCCTTAACAG
>HPV7_Alpha_9627389_nt798_E7_VE_rc|1
TGTAGCTGCACAGTTGGGGCACACTATATTTAGTGTGCCCATCAGCAACTGATGAACCTG
>HPV4_Gamma_9626597_nt642_E7_VE_rc|1
AGCAACTTCACATCTATAGCAACAGGTGTCTATTCTAAATGGAACCACCGACTCCTCCTC
>HPV5_Beta_9627145_nt724_E7_VE_rc|1
CTCCGTTTCCTGCTCGTTTGGTAATTCCTCTTCACAAAACAGGTCAACTGGTAGCACTTC
>HPV24_Unclassified_9628486_nt833_E7_VE_rc|1
ATCGCTGTTACGGCAGTCGGGACACAAAATGACCACCTCTTCTAGCAGTAAGTCTTGCAG
>HPV60_Gamma_9628566_nt558_E7_VE_rc|1
CAACTCCTCATCAGCTAAAAGACTGACAGGTAAAACCAACTCCTCCAAATTTACATCAAG

>HPV65_Gamma_312100_nt522_E7_VE_rc|1
CAACACCAAGTCATTTAATTCTAAATTAAGATCTGCAACCCTGGGTGCTGCTCCTCTCAT
>HPV20_Beta_1020162_nt7671_AOS|1
AACAGGAGGAAATGTAAGGCTGCCAACGCTTTTGGCTCTTCTTTTTGGCACAGCAGAAGA
>HPV20_Beta_1020162_nt7644_AOS|1
CAAACTTCATCGTCCCAACACGTTCGGAACAGGAGGAAATGTAAGGCTGCCAACGCTTTT
>HPV20_Beta_1020162_nt5062_AOS|1
GCACACCTATGCAAAGACTTCAAAATGTGTTCAGGCGTAGAGGAGGCCTTACTAACAGAA
>HPV20_Beta_1020162_nt4799_AOS|1
TGCAGTTCTAGAGGTAGCACCAGAACCAACACCTCCAGCTCGTGTCAGAGTCAGCCGCAC
>HPV20_Beta_1020162_nt4740_AOS|1
CCAGGCCCCTCAAGGCCACCAACTGATACACCAGTTACATCTACTACCAGTGGTTCTAGT
>HPV20_Beta_1020162_nt4733_AOS|1
AATACATCCAGGCCCCTCAAGGCCACCAACTGATACACCAGTTACATCTACTACCAGTGG
>HPV20_Beta_1020162_nt217_AOS|1
TCAGAAGACAGCGCTGATGAAGGACCATCTAATATTGGAGAGGCAAAACCTCCAATCTTA
>HPV20_Beta_1020162_nt179_AOS|1
AATGCAGAGAGTGCAGGCACATGGCTACACCTCCTTCTTCAGAAGACAGCGCTGATGAAG
>HPV20_Beta_1020162_nt170_AOS|1
CATAGAGCTAATGCAGAGAGTGCAGGCACATGGCTACACCTCCTTCTTCAGAAGACAGCG
>HPV20_Beta_1020162_nt155_AOS|1
CATATAGACAGATATCATAGAGCTAATGCAGAGAGTGCAGGCACATGGCTACACCTCCTT
>HPV21_Beta_1020170_nt7013_AOS|3
TGAGAATGCAGACGTGTCTAAAATTGAAAATTATAAAGCCGAGAGCTTTCAAGAATATTT
>HPV21_Beta_1020170_nt7006_AOS|2
TAAATCCTGAGAATGCAGACGTGTCTAAAATTGAAAATTATAAAGCCGAGAGCTTTCAAG
>HPV21_Beta_1020170_nt6999_AOS|2
ATTTCAGTAAATCCTGAGAATGCAGACGTGTCTAAAATTGAAAATTATAAAGCCGAGAGC
>HPV21_Beta_1020170_nt5549_AOS|1
ATTTATTGATATTAACGTTGATGAAAACCCTCTTTCTGAAGATTTTAGTGCACATTCAGA
>HPV21_Beta_1020170_nt5162_AOS|1
TCAACAGGTTAATGTAGAGAATCCTTTGTTTGTATCCAGGCCTTCTAGATTAGTGCAGTT
>HPV21_Beta_1020170_nt282_AOS|1
TTTAGAGCCACCATTACCAGCTACAATTCGTGACCTAGCCAATCTGTTAGAGATACCATT
>HPV21_Beta_1020170_nt273_AOS|1
ATCTAGCTTTTTAGAGCCACCATTACCAGCTACAATTCGTGACCTAGCCAATCTGTTAGA
>HPV21_Beta_1020170_nt266_AOS|1
AAAATACATCTAGCTTTTTAGAGCCACCATTACCAGCTACAATTCGTGACCTAGCCAATC
>HPV21_Beta_1020170_nt257_AOS|1
TAGAAGAAGAAAATACATCTAGCTTTTTAGAGCCACCATTACCAGCTACAATTCGTGACC
>HPV22_Beta_1020178_nt6715_AOS|1
TGGCAAGTGACGGCACCACAGTTAATTATGATGCTAAAAAAATCAGAGAATTTATGCGCC
>HPV22_Beta_1020178_nt3899_AOS|1
TCTAGGATACTAATATCATTTCATACAGATAGGGAAAGAGAGAAGTGCTTGCAACAAATG
>HPV22_Beta_1020178_nt3889_AOS|1
AATCGGGCGCTCTAGGATACTAATATCATTTCATACAGATAGGGAAAGAGAGAAGTGCTT
>HPV22_Beta_1020178_nt3877_AOS|1
TACAACCGATAGAATCGGGCGCTCTAGGATACTAATATCATTTCATACAGATAGGGAAAG
>HPV22_Beta_1020178_nt845_AOS|1
AACTCTCTCTGGAATTCGCGACTTTCAAACATCTCTACTTGGACCTGTAAAACTTTTGTG
>HPV22_Beta_1020178_nt837_AOS|1
ATACTAGCAACTCTCTCTGGAATTCGCGACTTTCAAACATCTCTACTTGGACCTGTAAAA
>HPV22_Beta_1020178_nt413_AOS|1
CCTACGCTTCAGCCCAATTTGAATTTCAGCAGTATTATCAAGTTACTTTGTATGGTCGTG
>HPV22_Beta_1020178_nt406_AOS|1

GGCTGTGCCTACGCTTCAGCCCAATTTGAATTTCAGCAGTATTATCAAGTTACTTTGTAT
>HPV22_Beta_1020178_nt181_AOS|1
GCATATTATTTAAGTAGGATGGGCTGCTATTCTGTATTCATGGCTTTGCAAAGACCACTG
>HPV23_Beta_1020186_nt5544_AOS|1
TTACCTAGTGCCCCTGCTGTAGTCATACACACATTGGACAAGTCTTTTGATTATTACTTA
>HPV23_Beta_1020186_nt5536_AOS|1
TATTTCCCTTACCTAGTGCCCCTGCTGTAGTCATACACACATTGGACAAGTCTTTTGATT
>HPV23_Beta_1020186_nt5529_AOS|1
ACTATAATATTTCCCTTACCTAGTGCCCCTGCTGTAGTCATACACACATTGGACAAGTCT
>HPV23_Beta_1020186_nt5258_AOS|1
ACCTCTGCAAAGCACTGTAATAGATATGAACTTAGATGATGTTGAGGCTATTCAGGATAC
>HPV23_Beta_1020186_nt5246_AOS|1
AGTTATTGAGGAACCTCTGCAAAGCACTGTAATAGATATGAACTTAGATGATGTTGAGGC
>HPV23_Beta_1020186_nt3888_AOS|1
GAGTAGGGCGTGCAAGGATGTTAATAGCATTTCATTCTAATCATGAAAGGGAAAAATGTA
>HPV23_Beta_1020186_nt3870_AOS|1
GGGGTCATTCTACTGATAGAGTAGGGCGTGCAAGGATGTTAATAGCATTTCATTCTAATC
>HPV23_Beta_1020186_nt3108_AOS|1
CAGTATGGTCTTATATTTACTATCAAACTGATGAGGACACTTGGGAAAAGGTTGAAGGAC
>HPV23_Beta_1020186_nt1400_AOS|2
GGCTGGGACATATTGAAAGTTTGTTAAGATGTAAAAATGCTAAAGCAGTGTTGCTACATA
>HPV23_Beta_1020186_nt209_AOS|1
TGCATTATTTAAGTAGGATGTGCTACACCAAATTATTGATGGACTCGACGCGACCACTGA
>HPV28_Alpha_1020202_nt7201_AOS|1
CCACGAGAGGATCGTCTGCTGCAAAACGAAAACGCGCCAAAAAGTAGCACTACTGTGTTT
>HPV28_Alpha_1020202_nt4240_AOS|2
ACAATGTATTTTTAAGTACTGTGTATATCCCAGTGTGCTATTGAGCCACTGCCTTTTCTA
>HPV28_Alpha_1020202_nt4222_AOS|1
GTGTGTTAGTTGGTCACCACAATGTATTTTTAAGTACTGTGTATATCCCAGTGTGCTATT
>HPV28_Alpha_1020202_nt4101_AOS|1
GATCATACTGTCCTGTTTCTGGAGCCAGATGACGTGTATCTATTGTTGTTGTTTATATTT
>HPV28_Alpha_1020202_nt4050_AOS|1
CAATACATAAGCCTTGCTATGTATCCTTTGGTGCTAAGGGACAACAATGGCGATCATACT
>HPV28_Alpha_1020202_nt3090_AOS|1
AAACCAAATCCATGTGTTATGTACACTGGAGAGACATATTTACTCAGAACTATAGTGATG
>HPV28_Alpha_1020202_nt1472_AOS|1
GGTTGGGATTTAATGAACTTATCCGACACTTCAAAAGTAGCAAAACCGTTTGCCTAGATT
>HPV28_Alpha_1020202_nt68_AOS|1
AAAAAGAGGCTTACATTGAATGGCAGAAGCTCAATGGATGACCAACGGCCTAAAAACATA
>HPV29_Alpha_1020210_nt5616_AOS|1
TCGCCAGTTGATGTGCCTTTACACACGGGCCCTGATATTATTCTGCCCTCCTCTGCACAA
>HPV29_Alpha_1020210_nt6608_AOS|1
TAGTAGGGGACAAAATCCCAGATTCCTTGTACTTAAAGGGTAACAACGGGCGAGAAACTC
>HPV29_Alpha_1020210_nt6601_AOS|1
GCTGGTGTAGTAGGGGACAAAATCCCAGATTCCTTGTACTTAAAGGGTAACAACGGGCGA
>HPV29_Alpha_1020210_nt6594_AOS|1
TAATCGTGCTGGTGTAGTAGGGGACAAAATCCCAGATTCCTTGTACTTAAAGGGTAACAA
>HPV29_Alpha_1020210_nt5609_AOS|1
ATTCGTGTCGCCAGTTGATGTGCCTTTACACACGGGCCCTGATATTATTCTGCCCTCCTC
>HPV29_Alpha_1020210_nt5602_AOS|1
CTATTCCATTCGTGTCGCCAGTTGATGTGCCTTTACACACGGGCCCTGATATTATTCTGC
>HPV29_Alpha_1020210_nt4180_AOS|2
GATTGTTAATACTAATGGTATGCCTGTTGTTATTTATAGTTTATAGACATTTGGGATTAT
>HPV29_Alpha_1020210_nt4031_AOS|1
TTTATACCTAGGATTGGTGGCACTATATTTACAAGTTGTGCAGCACATTGCACGAAACAC

>HPV29_Alpha_1020210_nt2580_AOS|1
ACTGCAGGTATTCACATTCAGCAATCCATGTCCATTAACAAGTAAAGGAGAGCCAGTTTA
>HPV29_Alpha_1020210_nt2090_AOS|1
GAATGTCCATGTCTGAGTGGATACGGTTTAGAAGCAACAAAGTACAGGGAGAGGGGGACT
>HPV36_Beta_1020218_nt7389_AOS|1
AGTGTCTTATCGAGGGTTCACCAGAGGAACCAAGCGCAAGCGAAAACAGTAATATGACCG
>HPV36_Beta_1020218_nt6951_AOS|1
TAGTATTTCAATATATAACAATAATGGGGCACTAAAGGACATCAATGATTACACTGCAGA
>HPV36_Beta_1020218_nt6695_AOS|2
ACGACATCCCTGACGCCAGAATTGATAATGGGACTTTTAAGAATCAGTTTTTCATTCCTG
>HPV36_Beta_1020218_nt5518_AOS|1
CATTTATAGACGTAAATGTGTCTGAAAATCCTTTGTCTGAAAGTGTGGAAGCCTTTTCTG
>HPV36_Beta_1020218_nt3618_AOS|1
CAAGACTGGGGTCCAGCGGGTATCAACCAGATCACGATCCAGAAGCACCTCTAGAAGGGG
>HPV36_Beta_1020218_nt3611_AOS|1
CGCTCGCCAAGACTGGGGTCCAGCGGGTATCAACCAGATCACGATCCAGAAGCACCTCTA
>HPV36_Beta_1020218_nt260_AOS|2
TAACTATTAAGGGCCTGTCAGAATCATTAGGCATTCCGTTTGTAGACTGTCTAATACCTT
>HPV36_Beta_1020218_nt253_AOS|1
CTGCCTTTAACTATTAAGGGCCTGTCAGAATCATTAGGCATTCCGTTTGTAGACTGTCTA
>HPV36_Beta_1020218_nt183_AOS|1
TTAATAATTAAGGCAAATGGCAGAGCAAGCCTCCGAACAGCAGAATATTACAGAAAAAGA
>HPV37_Beta_1020226_nt6568_AOS|1
TACCTGCCAAATCAGACCAGCAGCAGTATCTGTTAGGCAATTCTACCTATTTTCCCACTG
>HPV37_Beta_1020226_nt6538_AOS|2
GCACTGTTAATCAGGACCACAAATATTACTTACCTGCCAAATCAGACCAGCAGCAGTATC
>HPV37_Beta_1020226_nt6312_AOS|2
GACATGTTTGATATAGGGTTCGGAAATATTAACAATAAGGTTCTCTCTACTAATAAATCA
>HPV37_Beta_1020226_nt6084_AOS|2
TTTAATAAATTAAGGGACACTGAGAATAATAGTAATTACCAAGGGGGGTCACGGGATGAT
>HPV37_Beta_1020226_nt5579_AOS|1
AACACAAGTTATCTTGCCACAACCTGAAACACCAACTGTAGTTATTAGATTTGGAGAGGC
>HPV37_Beta_1020226_nt5337_AOS|1
CCAGGTCCCTTAAATATAGGGCAACAAGAGTCTACTATGGCAGATGACACAGATTTTAAT
>HPV37_Beta_1020226_nt4929_AOS|1
GAACAGGTAGCAGTCACGGATCCTTTATTCTTGAGTAGACCCTCACAATTAGTACAGTTT
>HPV37_Beta_1020226_nt2743_AOS|1
CCTAGAGACCCAAATTATGCATTGGCAACTTCTAAGGCAGGAGCAGATCCTGTTTCATTA
>HPV37_Beta_1020226_nt2736_AOS|1
GAGATGACCTAGAGACCCAAATTATGCATTGGCAACTTCTAAGGCAGGAGCAGATCCTGT
>HPV37_Beta_1020226_nt415_AOS|1
TTTACCAGTTTCTATGAGCACTCAGTTAGTGGGAGGGAGATAGAAGAGATAGAGCAAAAG
>HPV38_Beta_1020234_nt7129_AOS|2
TGCTGTCAAACGGCCGTTAGTAAGAAAATCTTCCAAATCTGTAAAACGCAAAAGGACCCA
>HPV38_Beta_1020234_nt7118_AOS|1
GCACGAACACGTGCTGTCAAACGGCCGTTAGTAAGAAAATCTTCCAAATCTGTAAAACGC
>HPV38_Beta_1020234_nt5426_AOS|1
CGTCAATCACTGTACCTAGATTTCAGACTCCTCAAAATCCTACCATATATTATCAGGATA
>HPV38_Beta_1020234_nt5413_AOS|1
CGCAGATCCACTACGTCAATCACTGTACCTAGATTTCAGACTCCTCAAAATCCTACCATA
>HPV38_Beta_1020234_nt5269_AOS|1
TTAGTGGATGTAAATGTGACTGAGGTTCCTGAAGGTGTTCTTACAGAAACTTCTATGGAT
>HPV38_Beta_1020234_nt5262_AOS|1
AAGCACTTTAGTGGATGTAAATGTGACTGAGGTTCCTGAAGGTGTTCTTACAGAAACTTC
>HPV38_Beta_1020234_nt4509_AOS|1

AATTGATACCCCTGTTGTGAGTGGAGGCCGCAATACCAATGCTATATTGGAAGTGGCTGA
>HPV38_Beta_1020234_nt4492_AOS|1
CCTATAGACCCTATAGAAATTGATACCCCTGTTGTGAGTGGAGGCCGCAATACCAATGCT
>HPV38_Beta_1020234_nt3858_AOS|1
CATCAAATGATCGCATAGGGCGCTCACGAATGCTTCTAGCTTTTTATTCAGAATCACAAA
>HPV38_Beta_1020234_nt185_AOS|1
CTTGGGATGTATTCATGGAACTACCAAAACCTCAAACTGTGCAGCAGCTCAGTGATAAGT
>HPV44_Alpha_1020242_nt6716_AOS|2
GCTGCCACTACACAGTCCCCTCCGTCTACATATACTAGTGAACAATATAAGCAATACATG
>HPV55_Alpha_1020266_nt6494_AOS|1
ATTCCACAGGATTTGGTTTTTAAAGGTGCTACTAAAAGTACAGTTCCTAATGCCATATAC
>HPV55_Alpha_1020266_nt6483_AOS|3
TTGGAGAGGACATTCCACAGGATTTGGTTTTTAAAGGTGCTACTAAAAGTACAGTTCCTA
>HPV55_Alpha_1020266_nt6474_AOS|1
CGGGAACGGTTGGAGAGGACATTCCACAGGATTTGGTTTTTAAAGGTGCTACTAAAAGTA
>HPV66_Alpha_1020290_nt7066_AOS|2
CCCCTAGACCCAAGGCTAGTGTATCTGCCTCTAAAAGGCGGGCGGCTCCTACCTCTTCCT
>HPV66_Alpha_1020290_nt7410_AOS|1
AGGTTGTTTGTATGCACTATAGTAACACACCAAACTCCATTTTAGTGCTGTACGCCATTT
>HPV66_Alpha_1020290_nt7059_AOS|1
CCTAGACCCCCTAGACCCAAGGCTAGTGTATCTGCCTCTAAAAGGCGGGCGGCTCCTACC
>HPV66_Alpha_1020290_nt7052_AOS|1
ACTAGGCCCTAGACCCCCTAGACCCAAGGCTAGTGTATCTGCCTCTAAAAGGCGGGCGGC
>HPV66_Alpha_1020290_nt6681_AOS|2
GCTAAAAGCACATTAACTAAATATGATGCCCGTGAAATCAATCAATACCTTCGCCATGTG
>HPV66_Alpha_1020290_nt6179_AOS|1
AGGTAATACAGGGGATTGTCCACCTCTTGCATTAGTTAATACCCCGATAGAGGACGGTGA
>HPV66_Alpha_1020290_nt6171_AOS|2
TCTACACCAGGTAATACAGGGGATTGTCCACCTCTTGCATTAGTTAATACCCCGATAGAG
>HPV66_Alpha_1020290_nt249_AOS|1
GTATTGAGTTAAAACTAGTATATAGAAACAATTGGCCATATGCAGTATGTAGGGTATGTT
>HPV103_Unclassified_109390382_nt6816_AOS|1
TACTTTGCAAGTTTCAACAACAAGATTACTCAGCCTTCCAGCAACCGAATTAGTTATCTG
>HPV103_Unclassified_109390382_nt6476_AOS|1
TAGACGGGGGGTTTAATCTATGGCATTACCTGTGTTGCACATGCACTTTATATTTTTGTT
>HPV103_Unclassified_109390382_nt6190_AOS|1
GGTTTGTACCACCGCCTCCTTCTTCTATTGGAGATACATACAGATTTTTAACATCTTTAG
>HPV103_Unclassified_109390382_nt5349_AOS|3
TATGTTAAAACAGGAAAGGAAAACAGACAAAGTGTCTCTATGGACCCAAAGCAAACTCAG
>HPV103_Unclassified_109390382_nt5338_AOS|1
ATCCTAATAGTTATGTTAAAACAGGAAAGGAAAACAGACAAAGTGTCTCTATGGACCCAA
>HPV103_Unclassified_109390382_nt4028_AOS|1
AATATTGGGGAATCTATACCATTAGAAACATTTGTGGAAACACAGGGGTCTGCCACATTT
>HPV103_Unclassified_109390382_nt4002_AOS|1
GTGCTGTAACAGAAGGATCTGTTGTCAATATTGGGGAATCTATACCATTAGAAACATTTG
>HPV103_Unclassified_109390382_nt3992_AOS|1
TTTATAGGGGGTGCTGTAACAGAAGGATCTGTTGTCAATATTGGGGAATCTATACCATTA
>HPV103_Unclassified_109390382_nt3346_AOS|1
AATAGCTTACATGGATGTACAACCGAAAAAGCGACAAAAACGGGATACTGTGGAAAATAT
>HPV103_Unclassified_109390382_nt3254_AOS|1
GATTCATTTTAAGCCAATAATGCTGCCAATGGTCCCCTGTATATTATCCTAACCCTTACA
>HPV101_Unclassified_109390389_nt6831_AOS|1
CTGCCAATTTTGCCAGCAGGTATGTTCATGTTTTAACAAATGTATTACTCATGTGTTTTG
>HPV101_Unclassified_109390389_nt6652_AOS|1
TATGTAGCATGTCAACATTGTCGTTGTATGAGTTATTTCTTCTGACTCAGGGCAGTATTT

>HPV101_Unclassified_109390389_nt4092_AOS|1
GGTTATTAGTATAGGTGAATCTATACCTTTAGACACATTTGTTGAAACAGGAGGGTCTTC
>HPV101_Unclassified_109390389_nt3337_AOS|1
TTGTAAATAGCATCACATCTGCCTCTTGTGATTTTGTGTACCTTGTATATTATACTTCTT
>HPV101_Unclassified_109390389_nt3330_AOS|1
TGCCTATTTGTAAATAGCATCACATCTGCCTCTTGTGATTTTGTGTACCTTGTATATTAT
>HPV101_Unclassified_109390389_nt3225_AOS|1
ATAATAGCCAACGGGAAAGGTTTTTACATACTGTGAAGTTACCAAAGGGTACACAGTTTT
>HPV101_Unclassified_109390389_nt3218_AOS|1
TTTAGAAATAATAGCCAACGGGAAAGGTTTTTACATACTGTGAAGTTACCAAAGGGTACA
>HPV101_Unclassified_109390389_nt2669_AOS|1
GATGCAGCAAATGAATGGCAAGTACGAACTGAAAGTGAAATAATATCTAATTCTGTTACC
>HPV101_Unclassified_109390389_nt980_AOS|2
TACTGGAGGCAGCAAAAACATTACTGCAACCACATTGTGAATACCTGCAAATTATAAATC
>HPV101_Unclassified_109390389_nt903_AOS|1
ATTAACAAGACCATTCATAAGTAATAAAAGCTGTTGCTGCAGTTGGATAGCGGGGATATT
>HPV70_Alpha_1173493_nt7811_AOS|1
ATGTGTCGTTTGGTGCAAGTTATATTTTGTGACTAATATTTTTACAGACCTGTGTGCAAC
>HPV70_Alpha_1173493_nt5310_AOS|1
CTACAGGACCTAGGTCCCATCTTTCATTTCCTTCTATACCTTCTACAGTGTCTACAAAAT
>HPV70_Alpha_1173493_nt5272_AOS|1
AGATAATGCAATGTTACATACTACTTCTCATACAGGTTCTACAGGACCTAGGTCCCATCT
>HPV70_Alpha_1173493_nt1486_AOS|1
AAGATCCACAGTCACCTACTGCACAGCTAAAAACAGTATTACAGGCTAATAACCAAAAAG
>HPV70_Alpha_1173493_nt1479_AOS|1
GAGAATCAAGATCCACAGTCACCTACTGCACAGCTAAAAACAGTATTACAGGCTAATAAC
>HPV70_Alpha_1173493_nt1471_AOS|1
TTGATAGTGAGAATCAAGATCCACAGTCACCTACTGCACAGCTAAAAACAGTATTACAGG
>HPV70_Alpha_1173493_nt1267_AOS|1
AAGAGCTCCCGGTAAACATATGCAATAAACAGGCAAGAACAAACGTGTATTCAGTACCAG
>HPV70_Alpha_1173493_nt1260_AOS|2
ACACTACAAGAGCTCCCGGTAAACATATGCAATAAACAGGCAAGAACAAACGTGTATTCA
>HPV70_Alpha_1173493_nt1247_AOS|1
GGAACAAAGGGTAACACTACAAGAGCTCCCGGTAAACATATGCAATAAACAGGCAAGAAC
>HPV70_Alpha_1173493_nt1237_AOS|1
GCGTACATAGGGAACAAAGGGTAACACTACAAGAGCTCCCGGTAAACATATGCAATAAAC
>HPV71_Alpha_12084981_nt7864_AOS|1
AATACCCATTGTTTGGGGCATATAGTTGTGCTGACTATGTTCGCCTAAGTACGTTTTTGG
>HPV71_Alpha_12084981_nt7842_AOS|1
ATACATGTGTTACTCACCGTGCAATACCCATTGTTTGGGGCATATAGTTGTGCTGACTAT
>HPV71_Alpha_12084981_nt6548_AOS|1
CAGAGCCATTGGTGATCGCATGTTTTTCTTGCGCCGAGAGCAATGTTTTAAGACATTTTA
>HPV71_Alpha_12084981_nt5507_AOS|1
ATTATTCCAATGATACTGTCATTCAACCTACTTCTGTGTCCAGCAGGCCTACACCTACTA
>HPV71_Alpha_12084981_nt4272_AOS|1
TTTAGTGTTGGTGTGTCTTATGTTGCTTCTGTTTTTGTTACGGTTGATGCAGTAGGTACC
>HPV71_Alpha_12084981_nt4035_AOS|1
TTTTTGTTACTGTGTTTCTTGGGTTTCTAGCACTATATATACAGGCAGCAGCGTCCCTTA
>HPV71_Alpha_12084981_nt4028_AOS|1
TTAATTTTTTTGTTACTGTGTTTCTTGGGTTTCTAGCACTATATATACAGGCAGCAGCG
>HPV71_Alpha_12084981_nt3186_AOS|1
TTACACGATGGGATACGTGTAGACTGTGTATTATTCTCCAAGGAAGCAGTAATATATGGG
>HPV107_Beta_126131393_nt7432_AOS|1
ATTATATATCGCTGTCGGTGCCAAACAAGACAAAGACTTGGCTGGATCTCGAGGAAACAC
>HPV107_Beta_126131393_nt6610_AOS|1

CAACTAATCAAGATCACAAATACTATATAGCACCTAAGAGTGGACAATCCCAAAGTCCTT
>HPV107_Beta_126131393_nt5546_AOS|1
CTACCACTTCTGTCACTGTACCCAGATTTAGTTCTCCAAGGGAAACTACTTTATATGTGC
>HPV107_Beta_126131393_nt5407_AOS|1
AGTACACTGGTGGATATTAATGTAAGGGATACTCCAGAATTGCTTGAAAGTTCTGAATTT
>HPV107_Beta_126131393_nt3976_AOS|1
AAGCACAAAGGAAACTATAAGTATTTTAGTAGTACCTGGTCCTGGATAGGGGAACATAGT
>HPV107_Beta_126131393_nt3969_AOS|1
ACGCAAAAAGCACAAAGGAAACTATAAGTATTTTAGTAGTACCTGGTCCTGGATAGGGGA
>HPV107_Beta_126131393_nt901_AOS|1
TCTCTACGTTTTTGCAACCCATTTTGGCATTCGGGCATTGCAGGATCTTTTGCTTCAAGA
>HPV107_Beta_126131393_nt506_AOS|1
AAATCATTTTGAATTTCAGGTTGTAGGTAAGCAGATTGAGGAGATCACTCAGCAAAGTAT
>HPV107_Beta_126131393_nt295_AOS|1
TGGACAGGCCAAGGACAATCGTACAGCTTACACAGCATCTTAATATACCAGTGGAAGACT
>HPV107_Beta_126131393_nt288_AOS|1
ATGCTCATGGACAGGCCAAGGACAATCGTACAGCTTACACAGCATCTTAATATACCAGTG
>HPV84_Alpha_12958167_nt7808_AOS|1
CCCGTTTCGGTTGCTATGTTATTCATACTATTTTCTTATACTTTACAACAATGATACAGT
>HPV84_Alpha_12958167_nt4166_AOS|1
TTAATTGTTATTATTTTACTTTGGCTGTGCCACCGTGTTATGCAGTTTTAAAGTGCTTTT
>HPV84_Alpha_12958167_nt4151_AOS|1
ACTATTATTTTGCTTTTAATTGTTATTATTTTACTTTGGCTGTGCCACCGTGTTATGCAG
>HPV84_Alpha_12958167_nt3767_AOS|1
GTAAATGCTGTATTACTGAGCCATTTGTAACAATTGTGTATGTCCTACATACCTGGCAAA
>HPV84_Alpha_12958167_nt3715_AOS|1
CTTGGTAGTATGTCTATGGTTATGTAAACAGTACCCATTGTATATGTATAGTGTAAATGC
>HPV84_Alpha_12958167_nt3707_AOS|1
TAGCCACACTTGGTAGTATGTCTATGGTTATGTAAACAGTACCCATTGTATATGTATAGT
>HPV84_Alpha_12958167_nt3700_AOS|2
GGCATTGTAGCCACACTTGGTAGTATGTCTATGGTTATGTAAACAGTACCCATTGTATAT
>HPV84_Alpha_12958167_nt3622_AOS|1
AAAACATCATATGTAACACTATGGTATAAAAGTACGGACCAACGCAAACAGCTCTTGGCA
>HPV84_Alpha_12958167_nt2433_AOS|1
TTTAAATACTTACGCAGCCGAATGGTTATTTTTCCCTTTTTAAACAAGTGCCCACTGGAT
>HPV84_Alpha_12958167_nt2426_AOS|1
TGACACATTTAAATACTTACGCAGCCGAATGGTTATTTTTCCCTTTTTAAACAAGTGCCC
>HPVcand87_Alpha_14475578_nt5486_AOS|1
CCTACAGCATCTACAAAGGTGTCGGCCACGTCCATACTATCCTCTTCCCATTATGACAAT
>HPVcand87_Alpha_14475578_nt6572_AOS|2
CCCTGACACCTATTTTTTGAAGGGGGCGTCGTCTGGCCCTAGGGCCTCTTTAAGCAGTTA
>HPVcand87_Alpha_14475578_nt3861_AOS|1
ACATTGGTGTACCTGTATGTAACCTAGCTGTATAATACATTTTGCATACCTGACATATAC
>HPVcand87_Alpha_14475578_nt3854_AOS|1
ACTGTATACATTGGTGTACCTGTATGTAACCTAGCTGTATAATACATTTTGCATACCTGA
>HPVcand87_Alpha_14475578_nt3845_AOS|1
GTGCATAATACTGTATACATTGGTGTACCTGTATGTAACCTAGCTGTATAATACATTTTG
>HPVcand87_Alpha_14475578_nt2525_AOS|1
AGGCAGATGACAGTTTAAAGTATTTACGGAGCAGAATGGTGGTGTTTCCGTTTCTACACA
>HPV72_Alpha_1491683_nt7927_AOS|1
TTACAAACGTGTTAAAACATTCTACTCCGGTCGCTCCCCTATGTCTCATGGTTTTATAGT
>HPV72_Alpha_1491683_nt7920_AOS|2
AAGCCCTTTACAAACGTGTTAAAACATTCTACTCCGGTCGCTCCCCTATGTCTCATGGTT
>HPV72_Alpha_1491683_nt7261_AOS|1
TACCTCGACCCCCGCCCCTACTAAACGTAAAAAGCGCAAAAAGTAACATGTCATACTGTT

>HPV72_Alpha_1491683_nt7254_AOS|1
CAAGCTCTACCTCGACCCCCGCCCCTACTAAACGTAAAAAGCGCAAAAAGTAACATGTCA
>HPV72_Alpha_1491683_nt6830_AOS|1
GCCACAGCGTCCTCTGTATCAGAATATACAGCTTCTAATTTTCGTGAGTATCTTCGCCAC
>HPV72_Alpha_1491683_nt5628_AOS|1
CATTGATTTGTCCTTTGCTCCTGCCCCTGTACCTACAATGCCTCTTGTACCCTCTACGCA
>HPV72_Alpha_1491683_nt5497_AOS|1
CGTGCTTCTGTGTCTTCTACATCATTGCACAGCCCGTCCCTGTCTGCAGCGTCTGCTGTT
>HPV72_Alpha_1491683_nt5398_AOS|1
TCATCTGATACTATTGAAATGCAATCCTTGGCCTCCTCTACGCAGCCAGACATAACATAT
>HPV72_Alpha_1491683_nt4200_AOS|2
ACGGATGGGGGTGCATTAATTGTACTACGAGACGATAATTGTGGATTGTGGTTCTTCTTG
>HPV72_Alpha_1491683_nt128_AOS|1
AATATTTGGTTGCTGTGCAAGGAAATTGAGGTGGACCTAGAAGATTTACGGATTACCTGC
>HPV73_Alpha_1491692_nt7206_AOS|1
TTATATGTGTGTTGTGTGGGTGGTTTACTTGACTACTGTGCTTCCATTTTGTATAGTCGC
>HPV73_Alpha_1491692_nt6277_AOS|1
CTGGTGATACCGGTGATAAAATCCCAGATGACCTAATGATTAAAGGCACAGGCAATACTG
>HPV73_Alpha_1491692_nt4315_AOS|1
AGGCACACCATCTAAACCAGTTGAAATTCCATTACAACCTATACGACCATCAGTTGTTAC
>HPV73_Alpha_1491692_nt4308_AOS|1
TATCTACAGGCACACCATCTAAACCAGTTGAAATTCCATTACAACCTATACGACCATCAG
>HPV73_Alpha_1491692_nt4300_AOS|2
CGTTCCATTATCTACAGGCACACCATCTAAACCAGTTGAAATTCCATTACAACCTATACG
>HPV73_Alpha_1491692_nt1319_AOS|1
ACAAAATGTTAATGAAGAAGGCAACCAAATTGTGTCGCCACGTGAAAGCAGTAGTGGGTC
>HPV73_Alpha_1491692_nt1311_AOS|1
GGGTGTTTACAAAATGTTAATGAAGAAGGCAACCAAATTGTGTCGCCACGTGAAAGCAGT
>HPV73_Alpha_1491692_nt1304_AOS|1
AGGGGTAGGGTGTTTACAAAATGTTAATGAAGAAGGCAACCAAATTGTGTCGCCACGTGA
>HPV73_Alpha_1491692_nt1295_AOS|1
ACTTGGGGCAGGGGTAGGGTGTTTACAAAATGTTAATGAAGAAGGCAACCAAATTGTGTC
>HPV73_Alpha_1491692_nt734_AOS|1
CACAGTATGCCTTGCCATTGAAAGCAACAAAGCTGATTTAAGAGTGATAGAAGAGTTGCT
>HPVcand86_Alpha_15741127_nt7836_AOS|1
ACTCGGTCGCTGTATTATTCATAGTTCCTTCTTTATACTTAACAACAATCATGTAGTTAA
>HPVcand86_Alpha_15741127_nt6700_AOS|2
TAGTGCCGCTACCCAGAAGGCCTCTGAATATGACCCCTCTAAGTTTAATGAATATCTAAG
>HPVcand86_Alpha_15741127_nt5377_AOS|1
TCCACCACGCCTGTATCCTCTGCTACAGTGTCCGCCACATCTATGGTATCTTCCACATAT
>HPVcand86_Alpha_15741127_nt5368_AOS|1
TCATTGCATTCCACCACGCCTGTATCCTCTGCTACAGTGTCCGCCACATCTATGGTATCT
>HPVcand86_Alpha_15741127_nt5344_AOS|1
ATATTACAGGAGCCTTCCGTTCCCTCATTGCATTCCACCACGCCTGTATCCTCTGCTACA
>HPVcand86_Alpha_15741127_nt5337_AOS|1
ATCCTCTATATTACAGGAGCCTTCCGTTCCCTCATTGCATTCCACCACGCCTGTATCCTC
>HPVcand86_Alpha_15741127_nt5329_AOS|1
ACACACCTATCCTCTATATTACAGGAGCCTTCCGTTCCCTCATTGCATTCCACCACGCCT
>HPVcand86_Alpha_15741127_nt5322_AOS|1
AGATGATACACACCTATCCTCTATATTACAGGAGCCTTCCGTTCCCTCATTGCATTCCAC
>HPVcand86_Alpha_15741127_nt1560_AOS|2
AGCAGAGAAACCGTAGCAAAGTGCCTAGGAACCATACTAAATATTCCAGAAAAGCGTATG
>HPV88_Gamma_167600365_nt6130_AOS|1
AGAGGGAAATAAATACTTATTGCCACCTACAAATGCTGTGAATTTTAAAATGGGATCTCA
>HPV88_Gamma_167600365_nt5089_AOS|1

CCTGGCGCAGCAATAAAAGCTTTTGTAGATGATTATGCAGATATTATAGTTTCATATCCT
>HPV88_Gamma_167600365_nt5024_AOS|1
GTAATAGTCACTTATTTGTGCATTTTGAGGAAGAGGATGAACTTATATCTGTTCCTACTC
>HPV88_Gamma_167600365_nt4847_AOS|1
TTAGTGCAATTGAACCTATAGAGCCTGATGTTATTGAGCTTTCTAATTTAGGGGAGTTTA
>HPV88_Gamma_167600365_nt4540_AOS|2
TATAATCGTTACATTCAACAAATCAGAACAGACAATGTTGACTTTTTAACCCGGCCTTCA
>HPV88_Gamma_167600365_nt4533_AOS|1
AAGCTTATATAATCGTTACATTCAACAAATCAGAACAGACAATGTTGACTTTTTAACCCG
>HPV88_Gamma_167600365_nt4526_AOS|1
GGGCCAGAAGCTTATATAATCGTTACATTCAACAAATCAGAACAGACAATGTTGACTTTT
>HPV88_Gamma_167600365_nt3715_AOS|1
CCTTGATTCATTGTAACTATGTATAAAACCAGAAGTAAACGTGACACTGCTGAGAATTTG
>HPV88_Gamma_167600365_nt3683_AOS|1
TTGCCAAAGGGTACAACCTACGCATTTGGTTACCTTGATTCATTGTAACTATGTATAAAA
>HPV88_Gamma_167600365_nt2902_AOS|1
GTACTTTGATAATGATCGAGAAAACAGCTTTCCCTACACACAATGGGAACATATTTATTA
>HPV110_Beta_186461194_nt5522_AOS|1
ATCTAGAGACAACACTGAAATTATACTCCCACTACCTGATACTCCAACTGTGGTCATACA
>HPV110_Beta_186461194_nt5286_AOS|1
GACGAGCCAGATCCTTTAAATGTTGGGCAGTTTGAATCAATGGAAATAGAAGAACCAGAT
>HPV110_Beta_186461194_nt5279_AOS|1
TAATATAGACGAGCCAGATCCTTTAAATGTTGGGCAGTTTGAATCAATGGAAATAGAAGA
>HPV110_Beta_186461194_nt760_AOS|2
GCGACTTCGGCTTTTTGTGCTTGCAACAGTTTTTGGAATTCGTACTCAGCAAGAAATTTT
>HPV110_Beta_186461194_nt495_AOS|1
GATCTAATAGAGAAGCTAGAAGTTTGTTGTAAGCATCAGCAATTTCATAGGGTAAGAGGC
>HPV110_Beta_186461194_nt413_AOS|1
AAATGGACGTGAAATAGAAATAGTAGAGCAAAAAGGAATTGGGGAAGTGGTTGTGCGTTG
>HPV110_Beta_186461194_nt406_AOS|1
ATTCTGTAAATGGACGTGAAATAGAAATAGTAGAGCAAAAAGGAATTGGGGAAGTGGTTG
>HPV110_Beta_186461194_nt399_AOS|1
TATGAACATTCTGTAAATGGACGTGAAATAGAAATAGTAGAGCAAAAAGGAATTGGGGAA
>HPV110_Beta_186461194_nt389_AOS|1
TACAAGGTTTTATGAACATTCTGTAAATGGACGTGAAATAGAAATAGTAGAGCAAAAAGG
>HPV110_Beta_186461194_nt198_AOS|1
CAACAGCTTGCAGATACATTATGCATTCCTGTTTTAGACATATTAATACCGTGTAGATTT
>HPV111_Beta_186461202_nt5484_AOS|1
AATATTATTTTTCCTCATCCAGAAGCTCCCACAGTTGTGATTCACATATCAGATACTTCT
>HPV111_Beta_186461202_nt5258_AOS|1
CAGACCGGAAACCCCTCCATTAGAAGATGAAATAGATTTAATTCAGAAGATTTGTTATT
>HPV111_Beta_186461202_nt2691_AOS|1
TCAGATTGAACATTGGCAAGCATTGAGACAAGAGCAAGTTTTATTGTACTTTGCTCGAAA
>HPV111_Beta_186461202_nt1951_AOS|1
TTATATTCGAGGGGAGATGCGAGAGATGAGTATGTCAACTTGGATTCACAAACAATTATT
>HPV111_Beta_186461202_nt1589_AOS|1
CGATTATTAAGCACAATATTACAAGTATCAGAAGTGCAGGTATTGGCAGATCCTCCTAAA
>HPV111_Beta_186461202_nt487_AOS|1
AGAGAAGCTAGGAGTGTGCTACAAACAACAATATTTTCATAGAGTTAGGGATAATTGGAA
>HPV111_Beta_186461202_nt480_AOS|2
ATCTGGTAGAGAAGCTAGGAGTGTGCTACAAACAACAATATTTTCATAGAGTTAGGGATA
>HPV111_Beta_186461202_nt399_AOS|1
TAGGAAAACAGATAGAAACTGTAGCAAACCAGCCTATTGGAAATATTACTGTTAGGTGTC
>HPVFA75/KI88-03_Unclassified_206902429_nt7261_AOS|1
TATAATCTGCTTATAAATCGCAGCCGGTACTTCACCTTACAAAGACTTGGCAGTCTGTCT

>HPVFA75/KI88-03_Unclassified_206902429_nt5141_AOS|1
TACAGGGACCTTAGTACAATTAACACTGAGGAGCCCATAGAATTACAACTTTTAGGAGAA
>HPVFA75/KI88-03_Unclassified_206902429_nt3791_AOS|1
TAAAAGGCATAAGGGTGGCTACAAATATGTCAGTAGTACTTGGTCATGGATCACAGAACA
>HPVFA75/KI88-03_Unclassified_206902429_nt3784_AOS|1
GGGTGCATAAAAGGCATAAGGGTGGCTACAAATATGTCAGTAGTACTTGGTCATGGATCA
>HPVFA75/KI88-03_Unclassified_206902429_nt1776_AOS|1
AAACTATGATTAGCCATCAATCTTTGGAGGCCACGCAATTTTCTTTATCTGACATGATAC
>HPVFA75/KI88-03_Unclassified_206902429_nt1644_AOS|1
AAGTAAATGAAATTCAAACATTGTCTGAACCACCTAAGTGGCGTAGTGTAATAGCGGCAT
>HPVFA75/KI88-03_Unclassified_206902429_nt1626_AOS|1
TACTATCTTCTATTTTGCAAGTAAATGAAATTCAAACATTGTCTGAACCACCTAAGTGGC
>HPVFA75/KI88-03_Unclassified_206902429_nt129_AOS|1
TTACTTGAAGAATATTAGAGACACATATACTGATGGAGAGGCCTAGAACAGTACAAGTTC
>HPVFA75/KI88-03_Unclassified_206902429_nt122_AOS|1
TATAAAATTACTTGAAGAATATTAGAGACACATATACTGATGGAGAGGCCTAGAACAGTA
>HPV91_Alpha_22023568_nt7701_AOS|1
GATTTCGGTCGTGTTTGGCAACGTAGGTATTGTGTCAGCATAATTGCAAGGTTACATTGA
>HPV91_Alpha_22023568_nt7596_AOS|1
TTTGTGTTATGAATGTCCAGCGTTGGGGCAGCGTTGGGGTTTAGGTCTGGTGGTAGTCAT
>HPV91_Alpha_22023568_nt7586_AOS|1
TCTTTCCTGTTTTGTGTTATGAATGTCCAGCGTTGGGGCAGCGTTGGGGTTTAGGTCTGG
>HPV91_Alpha_22023568_nt7579_AOS|1
ATGTGTTTCTTTCCTGTTTTGTGTTATGAATGTCCAGCGTTGGGGCAGCGTTGGGGTTTA
>HPV91_Alpha_22023568_nt5992_AOS|2
ACTGGCTGTGGGCCACCCTTTTTTTCCTATAAAAAATAATTCTGGTAAAGTAATTGTTCC
>HPV91_Alpha_22023568_nt3950_AOS|1
CCTTGTATATTGCTTGTATAGTGGAACTGTTACTGTGACGTTAAGGGTGTTGTAACACTC
>HPV91_Alpha_22023568_nt3605_AOS|1
AGTAGAGACCCACGAATCATCCCTAAGCATTCGAACCACAACCAAGGAGGGCACAGTGGT
>HPV91_Alpha_22023568_nt3595_AOS|1
GCCATTGGACAGTAGAGACCCACGAATCATCCCTAAGCATTCGAACCACAACCAAGGAGG
>HPV91_Alpha_22023568_nt3588_AOS|1
GAGGAGAGCCATTGGACAGTAGAGACCCACGAATCATCCCTAAGCATTCGAACCACAACC
>HPV91_Alpha_22023568_nt3581_AOS|1
ACTGTTGGAGGAGAGCCATTGGACAGTAGAGACCCACGAATCATCCCTAAGCATTCGAAC
>HPVcand89_Alpha_22095322_nt7658_AOS|1
CACTTTACACAAAGACTTGTAATCTTTTATACTTCCTGCGTAGGCGGCTACATGTTCCAC
>HPVcand89_Alpha_22095322_nt7642_AOS|1
TGTTATGGCGCCATGCCACTTTACACAAAGACTTGTAATCTTTTATACTTCCTGCGTAGG
>HPVcand89_Alpha_22095322_nt7555_AOS|1
ATGAGATATGTATTCGGCCATTTCCTTTCCCACCCAGCGTCCATTTTGAGTTCAGCGACC
>HPVcand89_Alpha_22095322_nt7547_AOS|1
TGCGCCCTATGAGATATGTATTCGGCCATTTCCTTTCCCACCCAGCGTCCATTTTGAGTT
>HPVcand89_Alpha_22095322_nt6560_AOS|3
GGACCTGCCAAAAACCTTGTATATGCCTGGGTCTGCTGCTAATAACAGAAATACACTTAC
>HPVcand89_Alpha_22095322_nt5480_AOS|2
ATACCACCTTTTCTGGCCCGACCGTTACTGCTACATCTGCTGTATCGTCTCAATACACAA
>HPVcand89_Alpha_22095322_nt4329_AOS|1
ATACAATAAATACATTGTTGCAATGACCCACCCTGTGCGGCGTCGTAAACGGGCTTCGGT
>HPVcand89_Alpha_22095322_nt307_AOS|1
TATGCTGTAAGCCGTTAGGCAGCCAGGAAAAGGACTATATGGTGTATTTTAACGAGCGAT
>HPVcand89_Alpha_22095322_nt62_AOS|1
AGAGGATCTACGGCTTACATGCATATATTGCACCAAGGAGCTTACAACATCAGAAGTGCT
>HPVcand89_Alpha_22095322_nt55_AOS|1

TGGATTTAGAGGATCTACGGCTTACATGCATATATTGCACCAAGGAGCTTACAACATCAG
>HPV90_Alpha_22138122_nt7261_AOS|1
CCCTCTACCGCCCCCTCTACAAAACGTAAACGGTCCAAACGTTCCTAAGCATGTTATGTG
>HPV90_Alpha_22138122_nt7254_AOS|1
TCCCGCACCCTCTACCGCCCCCTCTACAAAACGTAAACGGTCCAAACGTTCCTAAGCATG
>HPV90_Alpha_22138122_nt6603_AOS|1
AATTCCCGATGGCTTTGTGCTAAAAGGAACATCTTCTACCTCTCGTGGCACTCCTACAAG
>HPV90_Alpha_22138122_nt6596_AOS|1
GCGACGCAATTCCCGATGGCTTTGTGCTAAAAGGAACATCTTCTACCTCTCGTGGCACTC
>HPV90_Alpha_22138122_nt5543_AOS|1
GACTGCATCGTCTGCCCTATCATCTGCAGCATCCAATACCACTGTGCCTCTATCTACAGG
>HPV90_Alpha_22138122_nt3920_AOS|1
TATTGTATTCCTTACTGTGTTTCTAGGCCTCCTGATCATATACATACAGGCGTTGCAGTC
>HPV90_Alpha_22138122_nt3679_AOS|1
CTTCCTATCAACCATAACCTTACCTAAAGACATTGAAGCAGTACTGGGGTACATGACTAT
>HPV90_Alpha_22138122_nt3671_AOS|1
AGGGCAACCTTCCTATCAACCATAACCTTACCTAAAGACATTGAAGCAGTACTGGGGTAC
>HPV90_Alpha_22138122_nt3664_AOS|1
GGAACAAAGGGCAACCTTCCTATCAACCATAACCTTACCTAAAGACATTGAAGCAGTACT
>HPV90_Alpha_22138122_nt1277_AOS|1
AATCAGGTACAGATGCTATCCTGTCTCTGCTTAAAACAAGTAACCTAAGGGCCACATTAC
>HPV58_Alpha_222386_nt7059_AOS|2
TTAAAGCAAAGCCCAGACTAAAACGTTCGGCCCCTACTACCCGTGCACCATCCACCAAAC
>HPV58_Alpha_222386_nt5506_AOS|1
ATGTCTAGTCCATTTATTCCTATATCTCCACTAACTCCTTTTAATACCATAATTGTGGAT
>HPV58_Alpha_222386_nt5486_AOS|1
TTGCATCTTCTGTAACATCTATGTCTAGTCCATTTATTCCTATATCTCCACTAACTCCTT
>HPV58_Alpha_222386_nt5449_AOS|1
GACACTCCTCTTGTGTCATTGGAACCTGGTCCAGACATTGCATCTTCTGTAACATCTATG
>HPV58_Alpha_222386_nt5351_AOS|1
CTGATACTATACATGATTTTCAGAGTCCTCTGCACTCACATACGTCCTTTGCCACCACAC
>HPV58_Alpha_222386_nt5219_AOS|1
AAGACTTAAGTCCCATACAGCCTGTCCAGGAACAGGTACAACAGCAGCAACAATTTGAAT
>HPV58_Alpha_222386_nt5210_AOS|1
ATTACTACCAAGACTTAAGTCCCATACAGCCTGTCCAGGAACAGGTACAACAGCAGCAAC
>HPV58_Alpha_222386_nt5203_AOS|1
AAAGTACATTACTACCAAGACTTAAGTCCCATACAGCCTGTCCAGGAACAGGTACAACAG
>HPV58_Alpha_222386_nt3460_AOS|2
AGAGACAACACCCAGTACTCCACAAAGTATACAGACTGCGCCGTGGACAGTAGACCACGA
>HPV58_Alpha_222386_nt3444_AOS|1
CGATTTACCAGACTCCAGAGACAACACCCAGTACTCCACAAAGTATACAGACTGCGCCGT
>HPV74_Alpha_27462483_nt7051_AOS|1
ACAGCCCCCTCCTCAACCACTAAACAGAAACGCTCTAGGAAACGATAGCATGCTTTTGTT
>HPV74_Alpha_27462483_nt7044_AOS|1
GGCCCCTACAGCCCCCTCCTCAACCACTAAACAGAAACGCTCTAGGAAACGATAGCATGC
>HPV74_Alpha_27462483_nt7037_AOS|2
AGCGTTCGGCCCCTACAGCCCCCTCCTCAACCACTAAACAGAAACGCTCTAGGAAACGAT
>HPV74_Alpha_27462483_nt7030_AOS|1
GGTAAAAAGCGTTCGGCCCCTACAGCCCCCTCCTCAACCACTAAACAGAAACGCTCTAGG
>HPV74_Alpha_27462483_nt4705_AOS|1
CTGTTATACAGTCTCAACCCCCCGTAGAGGCTGGTGGTCATGTGCTGATTTCCTCCTCTA
>HPV74_Alpha_27462483_nt4698_AOS|1
GACCCTTCTGTTATACAGTCTCAACCCCCCGTAGAGGCTGGTGGTCATGTGCTGATTTCC
>HPV74_Alpha_27462483_nt4683_AOS|1
AATCCCTCGTTTACTGACCCTTCTGTTATACAGTCTCAACCCCCCGTAGAGGCTGGTGGT

>HPV74_Alpha_27462483_nt4142_AOS|1
TCAAGGGTGTAACGCCTTCTGTATGCAGTAAATAAGTGTTTTTATACATGCTGTGTATGG
>HPV74_Alpha_27462483_nt2989_AOS|1
TTTATATATATACTGGACGGCGACGAATGGGTAAAGGTGGAAGGACAAGTTGACTATAGG
>HPV92_Beta_27531786_nt5260_AOS|2
TACGTTCGTAAATATTGATTTGGAAGAGTTACCTAATTTAGAGGAAAATGTACACCTAGA
>HPV92_Beta_27531786_nt5154_AOS|1
ATTTTTTTAGGGATATTAGCAGTATTGATACTCAACCTTCTATTGAACTGCAAGTTCTTG
>HPV92_Beta_27531786_nt3875_AOS|1
GAATAGGACGCTCTAGAATGATTATTAGCTTTACCAGTAAATCTCAAAGACAAATGTTTT
>HPV92_Beta_27531786_nt3868_AOS|1
AACGATAGAATAGGACGCTCTAGAATGATTATTAGCTTTACCAGTAAATCTCAAAGACAA
>HPV92_Beta_27531786_nt3041_AOS|1
TTATGGATGGATTAAATAAACAATACTATATCAGATTTGCTCAAGACGCACGCAGATTTA
>HPV92_Beta_27531786_nt3033_AOS|1
TGCCTATTTTATGGATGGATTAAATAAACAATACTATATCAGATTTGCTCAAGACGCACG
>HPV92_Beta_27531786_nt354_AOS|1
ATAGAAAGGGACTCAGGTAGTCTTTTGTGTTGTTTAACTGTTAGATGTAAGTTTTGTTTA
>HPV92_Beta_27531786_nt308_AOS|1
AGAAACAAAACATTTTTATGAGTATAGTGTACAAGGAAAGGATGCTATAGAAAGGGACTC
>HPV75_Beta_2911544_nt3900_AOS|2
ATCCAAACATACTAAAATGCTTTAGGTACAGAGACAAGAAAAACAGAAAGGGATTGGTTA
>HPV75_Beta_2911544_nt7200_AOS|3
CAAATTCCTTTTTCAGGCAGGCTTACGAAGGGCTTCCAAGGTAACCAGAACAGCCAGTGT
>HPV75_Beta_2911544_nt4776_AOS|1
ATTGGGGGAGTCTGCATTATCAGACCAAATCGTGGTCACCAATGGCTCTGGTGGGCAAGC
>HPV75_Beta_2911544_nt3889_AOS|1
TTTAAGAGGCGATCCAAACATACTAAAATGCTTTAGGTACAGAGACAAGAAAAACAGAAA
>HPV75_Beta_2911544_nt471_AOS|1
ACATAATCTTATTGTAAGGTGCCAGTTTTGTCTGAAACGTTTGGATATATTTGAAAAACT
>HPV75_Beta_2911544_nt464_AOS|1
AAAGTATACATAATCTTATTGTAAGGTGCCAGTTTTGTCTGAAACGTTTGGATATATTTG
>HPV75_Beta_2911544_nt457_AOS|1
GCAGCAGAAAGTATACATAATCTTATTGTAAGGTGCCAGTTTTGTCTGAAACGTTTGGAT
>HPV76_Beta_2911551_nt4801_AOS|1
AACACTAGGGGAATCCGCATTGTCAGACCAAATTGTAGTTACCAGTGGCTCAGGTGGCCA
>HPV76_Beta_2911551_nt3933_AOS|1
ATGTGTTGAAATGCTTTAGATACAGAGATAAAAAAAATAAGAAGGGATTAGTAAAATGTT
>HPV76_Beta_2911551_nt471_AOS|1
AGCGAACATTATAATAAGGTGTCAATTTTGTTTGAAACGCCTGGATTTATTTGAAAAACT
>HPV76_Beta_2911551_nt464_AOS|1
AAAATATAGCGAACATTATAATAAGGTGTCAATTTTGTTTGAAACGCCTGGATTTATTTG
>HPV76_Beta_2911551_nt457_AOS|1
GCTGCAGAAAATATAGCGAACATTATAATAAGGTGTCAATTTTGTTTGAAACGCCTGGAT
>HPV77_Alpha_2911558_nt6600_AOS|2
TCCAGAATCTTTGTACCTCAAAGGGAGTAGCGGGCGTGAGACTCCCGGCAGTGCTATATA
>HPV77_Alpha_2911558_nt6593_AOS|2
ACAAAATTCCAGAATCTTTGTACCTCAAAGGGAGTAGCGGGCGTGAGACTCCCGGCAGTG
>HPV77_Alpha_2911558_nt5590_AOS|1
TATCTCCGGTGGATGTGCATTTACACACTGGCCCCGACATTGTTTTGCCTTCTTCTGCAC
>HPV77_Alpha_2911558_nt5583_AOS|1
CCATTTATATCTCCGGTGGATGTGCATTTACACACTGGCCCCGACATTGTTTTGCCTTCT
>HPV77_Alpha_2911558_nt5489_AOS|1
GAACAGTGGTCGCAATCTTACCTCTTTTGGGGGTCGCGGTGCATCGTCCTCCCTCCCTTC
>HPV77_Alpha_2911558_nt5482_AOS|1

CCTTTACGAACAGTGGTCGCAATCTTACCTCTTTTGGGGGTCGCGGTGCATCGTCCTCCC
>HPV77_Alpha_2911558_nt5474_AOS|1
TGACGTGGCCTTTACGAACAGTGGTCGCAATCTTACCTCTTTTGGGGGTCGCGGTGCATC
>HPV77_Alpha_2911558_nt346_AOS|1
CTACGTGGACGGCGTGGAGGCAGAAACAAAAGAATCCATAGATACCCAGCAAATCAGATG
>HPV77_Alpha_2911558_nt327_AOS|1
AACACTGGGAGTATTCATACTACGTGGACGGCGTGGAGGCAGAAACAAAAGAATCCATAG
>HPV80_Beta_2911565_nt3917_AOS|1
GGACGTTCACGGATGTTACTTGCATTTACTTCAAACACTGAGAGACAGCTTTTTATTAAC
>HPV80_Beta_2911565_nt5460_AOS|2
GGTAATGCAAGGCGTAGTACTACTACATACACAGTTCCTCGATTTGAGTCTCCTAGAAAT
>HPV80_Beta_2911565_nt5453_AOS|1
AGTATTTGGTAATGCAAGGCGTAGTACTACTACATACACAGTTCCTCGATTTGAGTCTCC
>HPV80_Beta_2911565_nt3927_AOS|1
GGATGTTACTTGCATTTACTTCAAACACTGAGAGACAGCTTTTTATTAACATAATGAAAT
>HPV80_Beta_2911565_nt1628_AOS|1
TATTAAGATTGCTAACTAATCTTTTGCAAGTATCAGAAGTACAAATAATAGCTGAACCGC
>HPV80_Beta_2911565_nt1489_AOS|1
GTGTATGCTGTACAAGACGAACTATTAGAAAGTTCCAAACAACTTTTAATTCAGCATTGT
>HPV80_Beta_2911565_nt1482_AOS|1
ATTAACTGTGTATGCTGTACAAGACGAACTATTAGAAAGTTCCAAACAACTTTTAATTCA
>HPV80_Beta_2911565_nt448_AOS|1
TGGGAGATTGAGGTAGTTGAGCGAAAAGCTGTTGGAGACATTACTATTCGTTGCAAATTT
>HPV80_Beta_2911565_nt440_AOS|2
TGTGTGGTTGGGAGATTGAGGTAGTTGAGCGAAAAGCTGTTGGAGACATTACTATTCGTT
>HPV80_Beta_2911565_nt311_AOS|1
AGTTAGTAAGTTTTGATCGCAAATGTTTGCAGTTGATTTGGACTGAAGAAGACTTTGTCT
>HPVRTRX7_Unclassified_30315616_nt6990_AOS|3
TATATCTAGTGAAAATCAAGATATACAGCAAATACAATCATATGACTCACAAAAGTTTAG
>HPVRTRX7_Unclassified_30315616_nt5521_AOS|1
CGACCATCATACAAGGTACTGTGGAAAGTACGTTCATAGACATGGATACAGCAGAAAATC
>HPVRTRX7_Unclassified_30315616_nt5122_AOS|1
TTGGCAGGCGTAGGGGGCCTTCTCTAACAAATAGAAGGTTAGTTCAGCAAGTACAAGTAG
>HPVRTRX7_Unclassified_30315616_nt5110_AOS|1
GAGCACTATCCATTGGCAGGCGTAGGGGGCCTTCTCTAACAAATAGAAGGTTAGTTCAGC
>HPVRTRX7_Unclassified_30315616_nt5095_AOS|1
GCACACCACTTCAAAGAGCACTATCCATTGGCAGGCGTAGGGGGCCTTCTCTAACAAATA
>HPVRTRX7_Unclassified_30315616_nt5087_AOS|1
GCGCAGTAGCACACCACTTCAAAGAGCACTATCCATTGGCAGGCGTAGGGGGCCTTCTCT
>HPVRTRX7_Unclassified_30315616_nt3798_AOS|1
AAAGAGGAAGTAGGAGCTGTAGGGGGTGCGGTAGGGGAACAGGAAAGCGACGGGGTAGAT
>HPVRTRX7_Unclassified_30315616_nt3625_AOS|1
GAGTCGGAGGTCAGAATCCAGATCACAATCCCAGAAGTACCTCCAGCCGAAGGCAGGTCG
>HPVRTRX7_Unclassified_30315616_nt3618_AOS|1
AGACTAGGAGTCGGAGGTCAGAATCCAGATCACAATCCCAGAAGTACCTCCAGCCGAAGG
>HPVRTRX7_Unclassified_30315616_nt3610_AOS|1
GGTCTCCAAGACTAGGAGTCGGAGGTCAGAATCCAGATCACAATCCCAGAAGTACCTCCA
>HPV65_Gamma_312100_nt6175_AOS|2
TTCCTGCTCAAGAAGGTCAGGATCAGAATACTTTAGGTCCACATATATATATAGGCACTC
>HPV65_Gamma_312100_nt6168_AOS|1
TACTGGATTCCTGCTCAAGAAGGTCAGGATCAGAATACTTTAGGTCCACATATATATATA
>HPV65_Gamma_312100_nt5090_AOS|1
CCACTATTCCTCCTGGTTTAGGCTTGAGAATTTATGTTGATGATGTAGGCTCCGATTTAT
>HPV65_Gamma_312100_nt5079_AOS|1
ATTCAGTATCCCCACTATTCCTCCTGGTTTAGGCTTGAGAATTTATGTTGATGATGTAGG

>HPV65_Gamma_312100_nt5072_AOS|1
GATCTTCATTCAGTATCCCCACTATTCCTCCTGGTTTAGGCTTGAGAATTTATGTTGATG
>HPV65_Gamma_312100_nt5038_AOS|1
AACTCTCACTTGGTTTTAACTAGCAGTAGACGTGGATCTTCATTCAGTATCCCCACTATT
>HPV65_Gamma_312100_nt5031_AOS|1
TTTTAGCAACTCTCACTTGGTTTTAACTAGCAGTAGACGTGGATCTTCATTCAGTATCCC
>HPV65_Gamma_312100_nt3680_AOS|2
AGACTCATTTGTTAAACACAATCTATTCCCAAAACTCTGTACATATACCTATGGTTCTTT
>HPV65_Gamma_312100_nt350_AOS|1
AATTTGGAAGAAGTAGCTCAAAGGAAAATTAAGGAGATTTGCATCAGATGTATATGTTGT
>HPV67_Alpha_3228267_nt7475_AOS|1
TCTAGGTTTCTTGCCAACATTGTTAATCTTTGTCTTCCTGCAGTGCAATGCCTTTACTTG
>HPV67_Alpha_3228267_nt7466_AOS|1
TTTTGGTATTCTAGGTTTCTTGCCAACATTGTTAATCTTTGTCTTCCTGCAGTGCAATGC
>HPV67_Alpha_3228267_nt7459_AOS|1
CATAGGGTTTTGGTATTCTAGGTTTCTTGCCAACATTGTTAATCTTTGTCTTCCTGCAGT
>HPV67_Alpha_3228267_nt5406_AOS|1
TAGTGGTTTTGATTTTCCATTTTCCTCTACTGTACCATTACAGCCAGGTCCCGATATTGT
>HPV67_Alpha_3228267_nt5381_AOS|1
CCACCAATGTTACTGTCCCCTTAAGTAGTGGTTTTGATTTTCCATTTTCCTCTACTGTAC
>HPV67_Alpha_3228267_nt5370_AOS|1
TAGTCCTCAGACCACCAATGTTACTGTCCCCTTAAGTAGTGGTTTTGATTTTCCATTTTC
>HPV67_Alpha_3228267_nt5363_AOS|1
CATTACATAGTCCTCAGACCACCAATGTTACTGTCCCCTTAAGTAGTGGTTTTGATTTTC
>HPV67_Alpha_3228267_nt5356_AOS|1
TCCTATTCATTACATAGTCCTCAGACCACCAATGTTACTGTCCCCTTAAGTAGTGGTTTT
>HPV67_Alpha_3228267_nt5349_AOS|1
ATCAATATCCTATTCATTACATAGTCCTCAGACCACCAATGTTACTGTCCCCTTAAGTAG
>HPV67_Alpha_3228267_nt5316_AOS|1
ATATATGGATCCTGACACACCCTTTCCACAGCCATCAATATCCTATTCATTACATAGTCC
>HPV11_Alpha_333026_nt4950_AOS|1
ATATCTGCCCCAACAATAACATCCCAACATGTAGAAGACATTCCACTAGACACTTTTGTT
>HPV11_Alpha_333026_nt4942_AOS|1
ACATACTTATATCTGCCCCAACAATAACATCCCAACATGTAGAAGACATTCCACTAGACA
>HPV11_Alpha_333026_nt4934_AOS|1
CAGTGGTCACATACTTATATCTGCCCCAACAATAACATCCCAACATGTAGAAGACATTCC
>HPV11_Alpha_333026_nt4852_AOS|1
CCAATCACACTACCACTAGTGTGTTTCAAAATCCCCTGTTTACAGAACCGTCTGTAATAC
>HPV11_Alpha_333026_nt4343_AOS|1
AATGGAGATTATGTGTACATGAACTAGAGTAAACCTTTTTTATACAGTGTGTGGTGTACG
>HPV11_Alpha_333026_nt4258_AOS|1
ACATGGTACTGAAAAAACTAAATGTGCTAAGTGTAAATCAAACCGCAATACTACTGTGGA
>HPV11_Alpha_333026_nt3126_AOS|1
ACATATATACCTGCAGGACAACGACTCATGGGTAAAAGTAACTAGTTCCGTAGATGCCAA
>HPV11_Alpha_333026_nt446_AOS|1
AAACTAAAGCACATATTGGGAAAGGCACGCTTCATAAAACTAAATAACCAGTGGAAGGGT
>HPV11_Alpha_333026_nt439_AOS|1
AATAGAAAAACTAAAGCACATATTGGGAAAGGCACGCTTCATAAAACTAAATAACCAGTG
>HPV11_Alpha_333026_nt425_AOS|1
AAGCCGTTGTGTGAAATAGAAAAACTAAAGCACATATTGGGAAAGGCACGCTTCATAAAA
>HPV31_Alpha_333048_nt7740_AOS|2
TTTTGTAGATTATCTATATCCTTGATTGCAGTGCTGGCTTTTGCACATGTTTAAACTGCC
>HPV31_Alpha_333048_nt7529_AOS|1
AGTTCTGCGGTTTTTGGTTTCCTGAATACTAGTTTTTGCCAACATTCTGGCTTGTAGTTT
>HPV31_Alpha_333048_nt7421_AOS|1

TTTTTGCAACTAAAGCTACTCCATTTTGATTTTATGCAGCCATTTTAAATCCCTAACCGT
>HPV31_Alpha_333048_nt7414_AOS|1
GGTCCGTTTTTTGCAACTAAAGCTACTCCATTTTGATTTTATGCAGCCATTTTAAATCCC
>HPV31_Alpha_333048_nt7407_AOS|1
TACACCCGGTCCGTTTTTTGCAACTAAAGCTACTCCATTTTGATTTTATGCAGCCATTTT
>HPV31_Alpha_333048_nt7400_AOS|1
AAAGTTGTACACCCGGTCCGTTTTTTGCAACTAAAGCTACTCCATTTTGATTTTATGCAG
>HPV31_Alpha_333048_nt7330_AOS|1
TACTTGTTCCTGCTCCTCCCAATAGTCATGTACTTATTTCTGCCTATAATTTAGGTGTCA
>HPV31_Alpha_333048_nt7322_AOS|1
CTTGTTCCTACTTGTTCCTGCTCCTCCCAATAGTCATGTACTTATTTCTGCCTATAATTT
>HPV31_Alpha_333048_nt7309_AOS|1
ACTATTGTTCCTACTTGTTCCTACTTGTTCCTGCTCCTCCCAATAGTCATGTACTTATTT
>HPV31_Alpha_333048_nt7299_AOS|1
TATTTTATAAACTATTGTTCCTACTTGTTCCTACTTGTTCCTGCTCCTCCCAATAGTCAT
>HPV33_Alpha_333049_nt5467_AOS|1
CATTTGTTCCTATTTCGCCTTTTTTTCCTTTTGACACCATTGTTGTAGACGGTGCTGACT
>HPV33_Alpha_333049_nt5459_AOS|1
ATCTAGCCCATTTGTTCCTATTTCGCCTTTTTTTCCTTTTGACACCATTGTTGTAGACGG
>HPV33_Alpha_333049_nt3904_AOS|1
TTATTACGTCCTTTAATACTTTCCATTTCTACCTATGCTTGGTTGCTGGTGTTGGTATTG
>HPV33_Alpha_333049_nt3891_AOS|1
ATGCTTATCCTTATTATTACGTCCTTTAATACTTTCCATTTCTACCTATGCTTGGTTGCT
>HPV33_Alpha_333049_nt1391_AOS|1
TGTGAGACAAATGTAGATAGCTGTGAAAATGTTACGTTGCAGGAAATTAGTAATGTTCTA
>HPV33_Alpha_333049_nt1384_AOS|1
AGTAAGCTGTGAGACAAATGTAGATAGCTGTGAAAATGTTACGTTGCAGGAAATTAGTAA
>HPV33_Alpha_333049_nt1377_AOS|1
ATTCAGAAGTAAGCTGTGAGACAAATGTAGATAGCTGTGAAAATGTTACGTTGCAGGAAA
>HPV33_Alpha_333049_nt1370_AOS|1
GGGGATGATTCAGAAGTAAGCTGTGAGACAAATGTAGATAGCTGTGAAAATGTTACGTTG
>HPV33_Alpha_333049_nt170_AOS|1
AACTATACACAACATTGAACTACAGTGCGTGGAATGCAAAAAACCTTTGCAACGATCTGA
>HPV33_Alpha_333049_nt160_AOS|2
CATTGGAGACAACTATACACAACATTGAACTACAGTGCGTGGAATGCAAAAAACCTTTGC
>HPV35_Alpha_333050_nt7790_AOS|1
TGTCCAACCGATTTGGATTACAGTTTTATAAGCATTTCTTTTTATTATAGTTAGTAACAA
>HPV35_Alpha_333050_nt7782_AOS|1
CACAGGTGTGTCCAACCGATTTGGATTACAGTTTTATAAGCATTTCTTTTTATTATAGTT
>HPV35_Alpha_333050_nt7544_AOS|1
TAAACTTGATTCATCTTGCAGTATTAGTCATTTTTCATACTTGTGGTCCACCCACACTTG
>HPV35_Alpha_333050_nt5390_AOS|1
ATGATATTCCTATAACAGCAGGGCCAGACATTGTATTTAACTCTAATACTATTACTAACT
>HPV35_Alpha_333050_nt5379_AOS|1
AAGTAGTGGCTATGATATTCCTATAACAGCAGGGCCAGACATTGTATTTAACTCTAATAC
>HPV35_Alpha_333050_nt5362_AOS|1
AATACTACTATACCATTAAGTAGTGGCTATGATATTCCTATAACAGCAGGGCCAGACATT
>HPV35_Alpha_333050_nt5168_AOS|1
GTATTACTGAAGATATAGAATTACAACCCTTACAACATGTACCATCCTCTTTACCACATA
>HPV35_Alpha_333050_nt3942_AOS|1
ACTGTAGCAACACCACTACTTGCTTTTGTTGTTTCTTGCTTTTGTATATACCTATGGATG
>HPV47_Beta_333062_nt7658_AOS|1
TTGTTATCGTTTTTGGCGATCACATTTGGCACCGCGGGCAGCTGTTTTGGCACTACAAGA
>HPV47_Beta_333062_nt7644_AOS|1
AAAGAAATTTAATCTTGTTATCGTTTTTGGCGATCACATTTGGCACCGCGGGCAGCTGTT

>HPV47_Beta_333062_nt6964_AOS|1
TCTGTTTACTCTCAGGCAGGGGACATAAAGGATATACAGGATTATAATGCAGACAATTTT
>HPV47_Beta_333062_nt6954_AOS|1
TTTCAGCATCTCTGTTTACTCTCAGGCAGGGGACATAAAGGATATACAGGATTATAATGC
>HPV47_Beta_333062_nt6944_AOS|1
GAAATACAAATTTCAGCATCTCTGTTTACTCTCAGGCAGGGGACATAAAGGATATACAGG
>HPV47_Beta_333062_nt5758_AOS|1
CGGGACACTATTGATATTATTTACCCTACACCTGAATTACCTGTAGTTGTCATTCACACC
>HPV47_Beta_333062_nt5540_AOS|2
ATATGGACATTGCTGAAAACCCTTTATCTGAAACAATAGATGCTTCATCTAATGATTTAC
>HPV47_Beta_333062_nt4972_AOS|1
GGACAAAGGATAGGCGGTGATATAACAGACGAAATTGAACTTACTGAGTTTCCAAGCAGA
>HPV47_Beta_333062_nt2934_AOS|1
GAGGCTATATATATGGTGTTGCAGTTAGAGTCGCTACAAAAATCAGCGTTTGCTTTGGAG
>HPV47_Beta_333062_nt2925_AOS|1
AGGGCCAAAGAGGCTATATATATGGTGTTGCAGTTAGAGTCGCTACAAAAATCAGCGTTT
>HPV8_Beta_333074_nt6929_AOS|1
AAATGGGGAACTTAAGAACATCACAGACTATAAATCAACCCAGTTCAGAGAATATCTGAG
>HPV8_Beta_333074_nt6913_AOS|2
TTTCAGTTTACACTGAAAATGGGGAACTTAAGAACATCACAGACTATAAATCAACCCAGT
>HPV8_Beta_333074_nt5718_AOS|1
AAATAATTTACCCTACACCTGATCTTCCGGTGGTAATAATCCACACACATGATAACAGTG
>HPV8_Beta_333074_nt5711_AOS|1
AATGAGGAAATAATTTACCCTACACCTGATCTTCCGGTGGTAATAATCCACACACATGAT
>HPV8_Beta_333074_nt5701_AOS|1
AAGTCGCAATAATGAGGAAATAATTTACCCTACACCTGATCTTCCGGTGGTAATAATCCA
>HPV8_Beta_333074_nt4842_AOS|1
AAATCATAACTGATTCCACACCTACACAAGGAGAAAGTTCTCTAGCAGACCACATTTTAG
>HPV8_Beta_333074_nt514_AOS|1
AGATAGACGTTAGGTGTCAAAACTGCTTGTCATTTTTGGATATCATAGAGAAATTAGATT
>HPV8_Beta_333074_nt350_AOS|1
TGAGTTTGACAAAAAGAGACTGTGCCTAATTTGGAAAAATTACGTTGTTACTGCGTGTTG
>HPV8_Beta_333074_nt336_AOS|1
TTCTTAGAACTGTGTGAGTTTGACAAAAAGAGACTGTGCCTAATTTGGAAAAATTACGTT
>HPV8_Beta_333074_nt149_AOS|1
ATATATATTGTTACAATGCTGTGACTTGTGCAATTTTCCTAAGCAAATGGACGGGCAGGA
>HPV51_Alpha_333087_nt6553_AOS|1
GCACTGCCACTGCTGCGGTTTCCCCAACATTTACTCCAAGTAACTTTAAGCAATATATTA
>HPV51_Alpha_333087_nt7439_AOS|1
CAACTGCCAGTTGTTTTTGGCATAAACCATCATTTTTTTATGACATAGTGCATACATCCG
>HPV51_Alpha_333087_nt7397_AOS|1
TCGGTTCGTGTACTTTTAGTATATTTGCCAAGTTTTAAACCACAACTGCCAGTTGTTTTT
>HPV51_Alpha_333087_nt7333_AOS|1
TATTTAGTTATACTTTGTAGCTTCCATTTTGTTACAGCTGCAGCCATTTTGAGTGCAACC
>HPV51_Alpha_333087_nt7316_AOS|1
ACAGTTTATATTTGTGCTATTTAGTTATACTTTGTAGCTTCCATTTTGTTACAGCTGCAG
>HPV51_Alpha_333087_nt5906_AOS|1
AAATTCACGCATAGCAAATGGCAATGCACAACAAGATGTTAGAGATAACACATCTGTTGA
>HPV51_Alpha_333087_nt3729_AOS|1
AACATTTATAAAAACCATTAAAGTACCCCCAAGTGTAACACTGTCATTGGGAATTATGAC
>HPV51_Alpha_333087_nt3722_AOS|1
AACGGGAAACATTTATAAAAACCATTAAAGTACCCCCAAGTGTAACACTGTCATTGGGAA
>HPV51_Alpha_333087_nt3701_AOS|1
TTGTGTTTGACAGTGCACATCAACGGGAAACATTTATAAAAACCATTAAAGTACCCCCAA
>HPV51_Alpha_333087_nt2540_AOS|2

ACAAGAGGATGCAAACCTAATGTATTTACATACAAGGGTAACAGTATTAAAGTTTTTAAA
>HPV42_Alpha_333211_nt7478_AOS|1
GTATCTGGCCTGTTACCAGGTGCATTGGCCATGTTTCCTAACATTTTGCAAACCTATTCA
>HPV42_Alpha_333211_nt7090_AOS|1
TATGTACAATCAGAAGCTATTCGCTGTCAGGCTAAGGTAACAACGCCAGAAAAAAAGGAT
>HPV42_Alpha_333211_nt5963_AOS|1
TGGTTGTTGGTCACCCTTATTACTCTATTACAAAAAGGCCAAATAAGACATCTATCCCCA
>HPV42_Alpha_333211_nt5603_AOS|1
CCCTACATTACCTAGCATGTCTACACATTCTGCCAATACCACCATACCTTTTTCGTTTCC
>HPV42_Alpha_333211_nt5035_AOS|1
TTGTATCTACAGATACAACTAACACATTTACTAGTACTCCCATTCCTGGCCCTCGGTCGT
>HPV42_Alpha_333211_nt4856_AOS|1
AGATATCTCCCCTCCCACTAATACTATACGTGTCACAACAACTACATCTACCAATCCTTT
>HPV42_Alpha_333211_nt4389_AOS|1
TTATATTGTGTGTACTTCTTTTGTGCTATTACAATGCCACCACAACGGTCCCGCAGACGA
>HPV42_Alpha_333211_nt4382_AOS|1
ACTTTTGTTATATTGTGTGTACTTCTTTTGTGCTATTACAATGCCACCACAACGGTCCCG
>HPV42_Alpha_333211_nt4110_AOS|1
GTACTAACATACTTTGACTATTTTTTTCTATGTTTAATCATTCTTGGTATTCCTTCTGTC
>HPV39_Alpha_333245_nt7375_AOS|1
AAGGTGTGTTTACTACCTAATATGTAATTTTTACATTGTTGTATGCGTTTCTACATTTTA
>HPV39_Alpha_333245_nt6450_AOS|1
TTCCTGCCCAATTGTATATTAAGGGCACAGATATACGTGCAAACCCCGGTAGTTCTGTAT
>HPV39_Alpha_333245_nt5491_AOS|1
TTACCAAGTACTACTCCACAGTTGCCATTGGTGCCTTCTGGACCAATAGACACAACATAT
>HPV39_Alpha_333245_nt5377_AOS|1
AACACAGGCTCACTACCTTCTGTGGCTTCTTCAGCATCTACTAAATATGCCAATACAACT
>HPV39_Alpha_333245_nt5370_AOS|1
TACATATAACACAGGCTCACTACCTTCTGTGGCTTCTTCAGCATCTACTAAATATGCCAA
>HPV39_Alpha_333245_nt5348_AOS|1
ATAATACAAGGGATTCGGGCACTACATATAACACAGGCTCACTACCTTCTGTGGCTTCTT
>HPV39_Alpha_333245_nt5341_AOS|1
GCATTTAATAATACAAGGGATTCGGGCACTACATATAACACAGGCTCACTACCTTCTGTG
>HPV39_Alpha_333245_nt5245_AOS|1
GAATTACAGCCCCTAGTTCACGCTGAGCCCTCTGATGCTTCAGATGCATTATTTGATATA
>HPV39_Alpha_333245_nt1211_AOS|1
CACTAGACCGTATGGAAAAAAAGTAGGCAGGAATACCAGGGGAACACTACAGGAAATTTC
>HPV93_Beta_37089393_nt4383_AOS|1
TAGATACCATAGCTCCTGTAGAACCATCCTCATCTTCAATTGTTCCATTAACAGAAACTT
>HPV93_Beta_37089393_nt402_AOS|1
AGCCACTCTCTGAAATTATAGTGAGGTGTCGCCATTGTATGAAAAAATTAGATCAAATTG
>HPV93_Beta_37089393_nt395_AOS|1
GAAAAAAAGCCACTCTCTGAAATTATAGTGAGGTGTCGCCATTGTATGAAAAAATTAGAT
>HPV93_Beta_37089393_nt388_AOS|1
AGTTAAGGAAAAAAAGCCACTCTCTGAAATTATAGTGAGGTGTCGCCATTGTATGAAAAA
>HPV93_Beta_37089393_nt350_AOS|1
AATTACTTGAATGATAGTGTTGTTGGCTGGGCAATAGAAGTTAAGGAAAAAAAGCCACTC
>HPV93_Beta_37089393_nt90_AOS|1
ATAAGTATTGATATATGGTATATAAGGCAGACATCTTAATGGCGGCAGTAAGTAAACCTC
>HPV93_Beta_37089393_nt83_AOS|1
CCTGTTTATAAGTATTGATATATGGTATATAAGGCAGACATCTTAATGGCGGCAGTAAGT
>HPV93_Beta_37089393_nt75_AOS|1
TCGTAGCACCTGTTTATAAGTATTGATATATGGTATATAAGGCAGACATCTTAATGGCGG
>HPV12_Beta_396910_nt6365_AOS|2
TGCGGGGATGCACCAGCTGAAAATGGTGTTTGCCCTCCCATAGAGTTAAAGAACACTTTC

>HPV12_Beta_396910_nt6358_AOS|3
CTTACCCTGCGGGGATGCACCAGCTGAAAATGGTGTTTGCCCTCCCATAGAGTTAAAGAA
>HPV12_Beta_396910_nt6351_AOS|2
AGAAAGCCTTACCCTGCGGGGATGCACCAGCTGAAAATGGTGTTTGCCCTCCCATAGAGT
>HPV12_Beta_396910_nt5094_AOS|1
GTGCAAGTTGATAATCCTTTATTTATAGATAAACCTTCTAAGTTAGTACGCTTTTCATTT
>HPV12_Beta_396910_nt5087_AOS|1
ACAACAAGTGCAAGTTGATAATCCTTTATTTATAGATAAACCTTCTAAGTTAGTACGCTT
>HPV12_Beta_396910_nt5032_AOS|1
GGACACAAACCACTGGCCGACGTAGAGGAGTGTCCCTAACAAATAGAAGGCTAGTACAAC
>HPV12_Beta_396910_nt5021_AOS|1
TCCACTTCAGAGGACACAAACCACTGGCCGACGTAGAGGAGTGTCCCTAACAAATAGAAG
>HPV12_Beta_396910_nt5014_AOS|1
GCAGCACTCCACTTCAGAGGACACAAACCACTGGCCGACGTAGAGGAGTGTCCCTAACAA
>HPV12_Beta_396910_nt5007_AOS|1
CGGCAAAGCAGCACTCCACTTCAGAGGACACAAACCACTGGCCGACGTAGAGGAGTGTCC
>HPV12_Beta_396910_nt4868_AOS|1
TGAAACATCTTTGGCAGACCACATCCTGGTCACATCTGGCTCAGGAGGTCAAACTATAGG
>HPV14D_Beta_396918_nt5444_AOS|1
AGGATAGGGACATTAGCATGGATATTATTTATCCTACCCCAGAGTTGCCTGTTGTCATTA
>HPV14D_Beta_396918_nt5362_AOS|1
TATACCGTCCCTCGTTTTGAAACAACCAGATCTGGGTCATATTATGCACAGGATACAAAA
>HPV14D_Beta_396918_nt5347_AOS|1
CGCTCAACATCTTCATATACCGTCCCTCGTTTTGAAACAACCAGATCTGGGTCATATTAT
>HPV14D_Beta_396918_nt5340_AOS|2
TAATCGACGCTCAACATCTTCATATACCGTCCCTCGTTTTGAAACAACCAGATCTGGGTC
>HPV14D_Beta_396918_nt5326_AOS|1
CAATTAGTTGTGGGTAATCGACGCTCAACATCTTCATATACCGTCCCTCGTTTTGAAACA
>HPV14D_Beta_396918_nt4834_AOS|1
GTCCAACAAGTTTCTGTAGAAAACCCCTTATTTTAACAAGACCATCTAGACTAGTGCAA
>HPV14D_Beta_396918_nt4598_AOS|1
CCGAATCCACCCCTACCACAGGTGAAAGTTCATTAGCAGACAATATATTGGTTACCTCTG
>HPV14D_Beta_396918_nt4591_AOS|1
GTAATTACCGAATCCACCCCTACCACAGGTGAAAGTTCATTAGCAGACAATATATTGGTT
>HPV14D_Beta_396918_nt4580_AOS|2
CCTCCTTTCAAGTAATTACCGAATCCACCCCTACCACAGGTGAAAGTTCATTAGCAGACA
>HPV14D_Beta_396918_nt2184_AOS|1
GTTGTATATGGACACTTATTTGAGGAATGGCCTTGATGGTCATTATGTTTCTTTAGATTG
>HPV15_Beta_396924_nt5489_AOS|1
TGAATCACCTAGAAATACTGGGTTTTACATACAAGATGTGCATGGGTATAATGTAGCCTA
>HPV15_Beta_396924_nt6555_AOS|1
TACCAGCACAATCAACCCAACAACAAAATAACTTAGCAAATTCTACTTACTTTCCCACAG
>HPV15_Beta_396924_nt4900_AOS|2
ATAACAGACGGCTGACTGAACAAGTAGCTGTAACAGACCCTTTATTCTTGGGACGACCCT
>HPV15_Beta_396924_nt3923_AOS|1
TCTCTTCCAACACCGAAAGAGAGCTCTTTATCAAAATAATGAAACTGCCACCAGGCGTTG
>HPV15_Beta_396924_nt3911_AOS|1
TGTTACTGGCATTCTCTTCCAACACCGAAAGAGAGCTCTTTATCAAAATAATGAAACTGC
>HPV15_Beta_396924_nt3904_AOS|1
TCACGATTGTTACTGGCATTCTCTTCCAACACCGAAAGAGAGCTCTTTATCAAAATAATG
>HPV15_Beta_396924_nt3897_AOS|1
TGGACGCTCACGATTGTTACTGGCATTCTCTTCCAACACCGAAAGAGAGCTCTTTATCAA
>HPV15_Beta_396924_nt3890_AOS|1
ATAGAATTGGACGCTCACGATTGTTACTGGCATTCTCTTCCAACACCGAAAGAGAGCTCT
>HPV15_Beta_396924_nt441_AOS|1

GTGTAGTTGGGAAATAGAGATAGTAGAACAGAAGCCTGTTGGAGATATTATTATTCGCTG
>HPV15_Beta_396924_nt434_AOS|1
AGTCGGTGTGTAGTTGGGAAATAGAGATAGTAGAACAGAAGCCTGTTGGAGATATTATTA
>HPV17_Beta_396932_nt5118_AOS|1
AAACACCTCAGGGTTATTTACGAGTCAGTAGACTAGGTAGAAGAGCCAGCATTCGTACTC
>HPV17_Beta_396932_nt5510_AOS|1
GAAACACCTAGGGACACTGGCTTTTACATACATGACACTCAGGGATACACAGTAGCATAT
>HPV17_Beta_396932_nt4842_AOS|1
GCACACCTCCTAGAACAAGTACTCCAGTTCAACGTGCAGTAGAATCACTATCAAGCTTAA
>HPV17_Beta_396932_nt4719_AOS|1
TAGTGTTCGAAGGTTTGGTGGACAAAACATAGGAGGTTCCAGGAATGCAGCCATTGATA
>HPV17_Beta_396932_nt3933_AOS|1
TAGCATTTAACACATATGATGAAAGAGAATTGTTTATCCAAAAAATGAAGCTACCACCAG
>HPV17_Beta_396932_nt3926_AOS|1
ATGTTACTAGCATTTAACACATATGATGAAAGAGAATTGTTTATCCAAAAAATGAAGCTA
>HPV17_Beta_396932_nt1825_AOS|1
ACAGCACAGGCAACACAATTCGATCTATCTACCATGATACAATTTGCTTATGATAACGAA
>HPV17_Beta_396932_nt1809_AOS|1
TATGATTAATCATCAAACAGCACAGGCAACACAATTCGATCTATCTACCATGATACAATT
>HPV17_Beta_396932_nt830_AOS|1
TCGTTCACAAGAGGAGCTTTTATTAGGTGAAGTACAGTTGGTGTGTCCTAACTGCAGAGA
>HPV17_Beta_396932_nt805_AOS|1
TTGTTCTTGCAACGCACGCTGGAATTCGTTCACAAGAGGAGCTTTTATTAGGTGAAGTAC
>HPV19_Beta_396940_nt5585_AOS|1
ACAGTTAGTAGTTGGTGGGCGCCGTTCTACTTCCACATATACAGTTCCCCAATTTGAAAC
>HPV19_Beta_396940_nt5272_AOS|1
ATTCAGAAACACCATCTGGTTACATCAGAGTTAGTCGCCTAGGCCAAAGGCGAACCATTC
>HPV19_Beta_396940_nt4150_AOS|1
AAGCACTTTGATGATACAGTAAGGTATCCTAAAGGTGTGGACCGATCGTTTGGCTCATTT
>HPV19_Beta_396940_nt488_AOS|1
AGTTCTATGAGCATACTGTAACAGGTAGAGAAATTGAGTTTGTAACAGGTAAATCTGTCT
>HPV19_Beta_396940_nt219_AOS|1
AGAAGAAGAGATAGAAATTGTAGAAGAGGGAACTACTGCACCACAGGTCACAGAGCCACC
>HPV19_Beta_396940_nt212_AOS|1
AGGCTACAGAAGAAGAGATAGAAATTGTAGAAGAGGGAACTACTGCACCACAGGTCACAG
>HPV19_Beta_396940_nt205_AOS|1
AACGCACAGGCTACAGAAGAAGAGATAGAAATTGTAGAAGAGGGAACTACTGCACCACAG
>HPV19_Beta_396940_nt198_AOS|1
TATGGCTAACGCACAGGCTACAGAAGAAGAGATAGAAATTGTAGAAGAGGGAACTACTGC
>HPV19_Beta_396940_nt191_AOS|1
GCAAACTTATGGCTAACGCACAGGCTACAGAAGAAGAGATAGAAATTGTAGAAGAGGGAA
>HPV19_Beta_396940_nt183_AOS|1
TTGCAGCTGCAAACTTATGGCTAACGCACAGGCTACAGAAGAAGAGATAGAAATTGTAGA
>HPV25_Beta_396948_nt3784_AOS|1
AGCAGGGGGGGCCGCGGCAGGGGCGGGAGACGACGACACAGATTGTCTGAATCCCCCACC
>HPV25_Beta_396948_nt5666_AOS|1
GAAACTACACGCTCTGCATCATATTATACCCAAGATATTCAGGGATACTATGTGTCCTAT
>HPV25_Beta_396948_nt5037_AOS|1
CAAGACAAAGTAGTACACCTCTTCAAAGGATTAGGACTGCATTAAGACGTAGAGGAGGAT
>HPV25_Beta_396948_nt4737_AOS|1
TTGTACCCTCCACTGACACCCCGGTGACAACAACTTCTAGAGGTGCCAGCGCAGTTTTAG
>HPV25_Beta_396948_nt3802_AOS|1
AGGGGCGGGAGACGACGACACAGATTGTCTGAATCCCCCACCTCCAAGCGATCACGAAGA
>HPV25_Beta_396948_nt3777_AOS|1
AGGGAGCAGCAGGGGGGGCCGCGGCAGGGGCGGGAGACGACGACACAGATTGTCTGAATC

>HPV25_Beta_396948_nt475_AOS|1
TTTTACGAAAGCACTGTAACAGGTAGGGAAATTGAAGACGTTACAGGTAAATCAATTTTT
>HPV25_Beta_396948_nt203_AOS|1
CAACTGCAAATGCTGAACAGAGCATAGGACCACCAGAGCAAGCGCAGGTTATACAGCCAC
>HPV25_Beta_396948_nt196_AOS|1
CTTATGGCAACTGCAAATGCTGAACAGAGCATAGGACCACCAGAGCAAGCGCAGGTTATA
>HPV25_Beta_396948_nt189_AOS|1
ATTAGGACTTATGGCAACTGCAAATGCTGAACAGAGCATAGGACCACCAGAGCAAGCGCA
>HPV27_Alpha_396964_nt5475_AOS|1
ACGATTACTACCCAGCCCCTCGCGGCTCCCTGGCTAATACCACGGTATCTGCCTCCTCTG
>HPV27_Alpha_396964_nt5893_AOS|1
CTGTCGGCCACCCATATTATTCTATAAAGAAGGGTAGCAATAATAGGTTGGCAGTGCCTA
>HPV27_Alpha_396964_nt5468_AOS|1
CCATTGGACGATTACTACCCAGCCCCTCGCGGCTCCCTGGCTAATACCACGGTATCTGCC
>HPV27_Alpha_396964_nt4216_AOS|1
CGTGATGATATTGACTGTCTGTTGGTGATTCTCACCCTCATTGGCTTATTATTGTTGTTG
>HPV30_Alpha_396973_nt7587_AOS|1
GTTTCCTGCACTTTATGTTTTTTTTTGCAACGTAAGACGCCTCGCCTTATTAGTCATATA
>HPV30_Alpha_396973_nt7376_AOS|1
CTATGTCCTCCACCCTTTTTGGTTGTTGCACACCACTGTGCAGTTACTTTTTATATTTAT
>HPV30_Alpha_396973_nt7144_AOS|1
CAAGCGGCGGTGATTGTTTGTGTCTGTGTATGTTTCCTTACTATTTATTGTGCATGAATG
>HPV30_Alpha_396973_nt7137_AOS|1
CCAAACGCAAGCGGCGGTGATTGTTTGTGTCTGTGTATGTTTCCTTACTATTTATTGTGC
>HPV30_Alpha_396973_nt6823_AOS|1
TACTATGAACTCCACTTTACTTGAGGGCTGGAATATTGGATTGTCACCCCCAGCTGCCAC
>HPV30_Alpha_396973_nt4719_AOS|1
TGGCTCTGTCCATGTTAGTAGTACCCATTTTACCAATCCCTCCTTTGTTGAACCCCCAGT
>HPV30_Alpha_396973_nt4712_AOS|1
CTACCACTGGCTCTGTCCATGTTAGTAGTACCCATTTTACCAATCCCTCCTTTGTTGAAC
>HPV30_Alpha_396973_nt3139_AOS|1
TTACTGTGGGGACAATGGGTGGACTAAAGTGCCTTCTGTAGTAGATTACAAAGGTATATA
>HPV30_Alpha_396973_nt1356_AOS|1
GGAAAACAGCATAGAAGCAGACAGTGATATGGATATAGGCGCCACGCCACCACAGCAAAT
>HPV30_Alpha_396973_nt260_AOS|1
AGACTGGTATATAGGGAGGACAGCCCATATGCAGTGTGCAATTTCTGTTTATTATTTTAT
>HPV3_Alpha_397005_nt5431_AOS|1
GATGCTGACATAGGTTTTACATCTGGAGGTCGTAGTGACACTCTGTCTAGAGGCCGTGCT
>HPV3_Alpha_397005_nt4264_AOS|1
TCCTTGATGTATATGCCCTAGTGTGGTATTGTACCACCGTCTTTTATACTGCTATGTTTT
>HPV3_Alpha_397005_nt4247_AOS|1
TCACCTGTATGTATTCTTCCTTGATGTATATGCCCTAGTGTGGTATTGTACCACCGTCTT
>HPV3_Alpha_397005_nt4240_AOS|2
TTGCAGGTCACCTGTATGTATTCTTCCTTGATGTATATGCCCTAGTGTGGTATTGTACCA
>HPV3_Alpha_397005_nt4228_AOS|1
GTAAGGTTGTAGTTGCAGGTCACCTGTATGTATTCTTCCTTGATGTATATGCCCTAGTGT
>HPV3_Alpha_397005_nt4218_AOS|1
TGGGACTCCTGTAAGGTTGTAGTTGCAGGTCACCTGTATGTATTCTTCCTTGATGTATAT
>HPV3_Alpha_397005_nt4134_AOS|1
TGTTTGTTGAGCCTGGAGACGTGTACATATTATTGCTGTTTATGTTAGCTGTCATACTTA
>HPV3_Alpha_397005_nt4127_AOS|1
CTTGTCTTGTTTGTTGAGCCTGGAGACGTGTACATATTATTGCTGTTTATGTTAGCTGTC
>HPV3_Alpha_397005_nt4119_AOS|1
GCGACCATCTTGTCTTGTTTGTTGAGCCTGGAGACGTGTACATATTATTGCTGTTTATGT
>HPV3_Alpha_397005_nt2174_AOS|1

GGTACCAGGACGTAGAATTTATACCATTTCTATGCGCTCTGAAATCATTCCTACAAGGAA
>HPV40_Alpha_397014_nt7524_AOS|1
TTCAGTGTTGACCATGTACTTTTTGTGTCTAAATCCTCCATTTTGTACTGCGCGACCGCT
>HPV40_Alpha_397014_nt7517_AOS|1
CGTCTTGTTCAGTGTTGACCATGTACTTTTTGTGTCTAAATCCTCCATTTTGTACTGCGC
>HPV40_Alpha_397014_nt7510_AOS|1
GTCATGGCGTCTTGTTCAGTGTTGACCATGTACTTTTTGTGTCTAAATCCTCCATTTTGT
>HPV40_Alpha_397014_nt7502_AOS|1
ATGGCAGTGTCATGGCGTCTTGTTCAGTGTTGACCATGTACTTTTTGTGTCTAAATCCTC
>HPV40_Alpha_397014_nt5895_AOS|1
AGGTTACTGACTATAGGACATCCATACTTTGAGTTAAAAAAACCCAATGGTGACATTTCA
>HPV40_Alpha_397014_nt4030_AOS|1
TGTCCCTAATCTGTATGTGTGGAGCTCTATACTGTCTGCTATCTCCATTTTGTGCTTTGT
>HPV40_Alpha_397014_nt4022_AOS|1
TGTGTGTGTGTCCCTAATCTGTATGTGTGGAGCTCTATACTGTCTGCTATCTCCATTTTG
>HPV40_Alpha_397014_nt3791_AOS|1
GGTCTGACTTCTTAGCTATTGTAAAAATACCAAAAACAATAAAACATAGCTTGGGTATGT
>HPV40_Alpha_397014_nt285_AOS|1
CATGTCCACGGTGCCTGGACCTGCACGGAAAAGTAAACCAATACAGAAACTTTAGATACG
>HPV45_Alpha_397022_nt7514_AOS|1
TAAACATAATACCTAAACTGGCACATTTACAACCCCTACATAGTTTAACCTACTGGCGCG
>HPV45_Alpha_397022_nt7098_AOS|1
TCTACTGCATCTAGGCCTGCCAAACGTGTACGTATACGTAGTAAGAAATAATATGTTAGC
>HPV45_Alpha_397022_nt5352_AOS|1
ATTCCTTGACCATGCCTTCTACTGCTGCATCCTCTTACAGTAATGTTACAGTACCATTAA
>HPV45_Alpha_397022_nt5342_AOS|1
TATCCAAAGTATTCCTTGACCATGCCTTCTACTGCTGCATCCTCTTACAGTAATGTTACA
>HPV45_Alpha_397022_nt5335_AOS|1
ATTTACATATCCAAAGTATTCCTTGACCATGCCTTCTACTGCTGCATCCTCTTACAGTAA
>HPV45_Alpha_397022_nt5328_AOS|1
ACAAATCATTTACATATCCAAAGTATTCCTTGACCATGCCTTCTACTGCTGCATCCTCTT
>HPV45_Alpha_397022_nt1443_AOS|1
TGTAGATCCGCATTGCAGTATTACAGAACTAAAGGAGCTATTACAAGCAAGTAACAAAAA
>HPV45_Alpha_397022_nt1436_AOS|1
CAGAAAATGTAGATCCGCATTGCAGTATTACAGAACTAAAGGAGCTATTACAAGCAAGTA
>HPV45_Alpha_397022_nt1429_AOS|1
GACAATGCAGAAAATGTAGATCCGCATTGCAGTATTACAGAACTAAAGGAGCTATTACAA
>HPV52_Alpha_397038_nt7837_AOS|1
GCCAAATATGTCTTGTAAAACATGATTAAATACTGTTACTCACCAGGTGTGCACTACACG
>HPV52_Alpha_397038_nt7597_AOS|1
ACAGTAACAACTATTTTTATATAAGTTTCAGCAAACTGCTTAATCCTTTGGTTTCCTGCA
>HPV52_Alpha_397038_nt7582_AOS|1
TTGGTTGTCCTTGGCACAGTAACAACTATTTTTATATAAGTTTCAGCAAACTGCTTAATC
>HPV52_Alpha_397038_nt5495_AOS|1
CTTCGTTACCCACACATACTCCTTTTGTTCCTATAGCCCCTACAGCTCCATCTACATCTA
>HPV52_Alpha_397038_nt5482_AOS|1
GACATTCCATTACCTTCGTTACCCACACATACTCCTTTTGTTCCTATAGCCCCTACAGCT
>HPV52_Alpha_397038_nt5472_AOS|1
GTCAGGTCCTGACATTCCATTACCTTCGTTACCCACACATACTCCTTTTGTTCCTATAGC
>HPV52_Alpha_397038_nt5465_AOS|1
CCATTGAGTCAGGTCCTGACATTCCATTACCTTCGTTACCCACACATACTCCTTTTGTTC
>HPV52_Alpha_397038_nt5348_AOS|1
ATGCAGATTCTTTGCAGCAACCCACGTTTCACTTACCTTCCACACTTTCTACCCATAATA
>HPV52_Alpha_397038_nt4982_AOS|1
CTTTTATGTCATCACCACAGAAATTAGTAACATATAACAATCCTGTTTTTGAGGGCGTTG

>HPV52_Alpha_397038_nt329_AOS|1
AGGCATTATCAATATTCACTGTATGGGAAAACATTAGAAGAGAGGGTAAAAAAACCATTA
>HPV56_Alpha_397053_nt7708_AOS|1
TTTGGTGTTTTGGCTTGCAGAATACACTATGTAGGCCAAGTATCTGTCAGTATCTGTTTT
>HPV56_Alpha_397053_nt7585_AOS|1
TATACTTGAATTATGTACAGTACCGCACCCTGTATTACTCACAGGTACTATGACTGCCAA
>HPV56_Alpha_397053_nt7578_AOS|1
TGTGTTTTATACTTGAATTATGTACAGTACCGCACCCTGTATTACTCACAGGTACTATGA
>HPV56_Alpha_397053_nt1052_AOS|1
TTCATATATACAAAATATACAGGCAGACGCAGAAACAGTCAACAATTGTTGCAAGTACAA
>HPV56_Alpha_397053_nt1045_AOS|1
TAGACGATTCATATATACAAAATATACAGGCAGACGCAGAAACAGTCAACAATTGTTGCA
>HPV56_Alpha_397053_nt253_AOS|1
TGAATTAAAATTAGTGTATAGGGATGATTTTCCTTATGCAGTGTGCAGAGTATGTTTATT
>HPV56_Alpha_397053_nt66_AOS|1
ATATAAAAGGCAGCTTATTCTGTGTGGACATATCCATGGAGCCACAATTCAACAATCCAC
>HPVcand62_Alpha_39932599_nt7255_AOS|1
CCTACCTCTGCTAAACGAAAGCGCAGGAAGTAACTGTATGTATGGTATGTGTGTGTGTGC
>HPVcand62_Alpha_39932599_nt5645_AOS|1
GTCTGTGCTAACGCCTTCCTTCCCTGCAACTACCCCATATTCTATATATATTGTAGGTTC
>HPVcand62_Alpha_39932599_nt5558_AOS|1
TAATGTCACTGTACCATTATCCACTGGGTTGTCCGTTCCAGCGTCCACTGGCCCTGATGT
>HPVcand62_Alpha_39932599_nt5551_AOS|1
CTCATACTAATGTCACTGTACCATTATCCACTGGGTTGTCCGTTCCAGCGTCCACTGGCC
>HPVcand62_Alpha_39932599_nt5544_AOS|1
GCATCCTCTCATACTAATGTCACTGTACCATTATCCACTGGGTTGTCCGTTCCAGCGTCC
>HPVcand62_Alpha_39932599_nt5520_AOS|2
GCCACCATATCTGCCACGTCTGTGGCATCCTCTCATACTAATGTCACTGTACCATTATCC
>HPVcand62_Alpha_39932599_nt4186_AOS|1
TTGGGGTCTGATGACAGTGGTCTACTGTTTTTATTGTGCCTCATAGTGGCATTAATACTG
>HPVcand62_Alpha_39932599_nt3433_AOS|1
GGACAATAACGAGTACGGGACCAACAGTTACCCTGACAGCACAAAAGGACGACACGTGTG
>HPVcand62_Alpha_39932599_nt1259_AOS|1
TAGATGTGCAACAATCGCAGGAACGCACAGCAAGTATTGTAGAGCTGTTAAAAACCAGTA
>HPVcand62_Alpha_39932599_nt1246_AOS|1
CCGGAACGCAGTGTAGATGTGCAACAATCGCAGGAACGCACAGCAAGTATTGTAGAGCTG
>HPV43_Alpha_40804474_nt7556_AOS|1
TGGTTTTAGTAATGGTTACTGTTGTGGGTATGTAAAATGGCGTCTGAACGTCTTCCATTT
>HPV43_Alpha_40804474_nt6554_AOS|1
GACGTTGTGCCTTCCGATATGTATATTGCTGGCTCTAATACCAGGTCCAAAATTGCAGAT
>HPV43_Alpha_40804474_nt6547_AOS|2
AACTGGCGACGTTGTGCCTTCCGATATGTATATTGCTGGCTCTAATACCAGGTCCAAAAT
>HPV43_Alpha_40804474_nt6537_AOS|1
AAGCTGGTAAAACTGGCGACGTTGTGCCTTCCGATATGTATATTGCTGGCTCTAATACCA
>HPV43_Alpha_40804474_nt4313_AOS|1
CAGAACACTTAAGTTGCATCAGTCCTCCAGCAAATAAACAATCCTTTATATAATCACTTG
>HPV43_Alpha_40804474_nt4287_AOS|1
TTAATATTGGTGTTAGCGTTTCATTACAGAACACTTAAGTTGCATCAGTCCTCCAGCAAA
>HPV43_Alpha_40804474_nt4089_AOS|1
TTATTTTTTGTCTTGGGGCGTCCTTACATCACCTATTAACGTTTTTATATTGGCGTTGTT
>HPV43_Alpha_40804474_nt4071_AOS|1
TGCATGTGCTTCTATCTTTTATTTTTTGTCTTGGGGCGTCCTTACATCACCTATTAACGT
>HPV43_Alpha_40804474_nt3877_AOS|1
TATATTTGTATAGAGGAACTGTAACTGTGATAAAAGGGGGGGTATTGTACCCCAACCACA
>HPV81_Alpha_40804509_nt7474_AOS|1

ATTGTATGCGTGCTGCCCTTTTGCCCCTACACTGTTGTTTGTATATTTGTGGAATGTGTG
>HPV81_Alpha_40804509_nt7328_AOS|1
CCAGGACCCCCACTACCTCTGCTAAGCGAAAACGTAAAAAATAGTAGTATGCTTGTTTGT
>HPV81_Alpha_40804509_nt6381_AOS|1
TGCTAATGTTGCTCCAGCACAAAACGAATGCCCCCCCTTGGAGTTTAAAAATACAACCAT
>HPV81_Alpha_40804509_nt5765_AOS|1
CGCCATATATGTTGTTGGCACTGATTTTTACTTGTTTCCTTCTTATATATTCTTCCCTAA
>HPV81_Alpha_40804509_nt5752_AOS|1
CAACTACCCCGTACGCCATATATGTTGTTGGCACTGATTTTTACTTGTTTCCTTCTTATA
>HPV81_Alpha_40804509_nt5744_AOS|1
GTTTCCTGCAACTACCCCGTACGCCATATATGTTGTTGGCACTGATTTTTACTTGTTTCC
>HPV81_Alpha_40804509_nt5737_AOS|1
TCCCTTCGTTTCCTGCAACTACCCCGTACGCCATATATGTTGTTGGCACTGATTTTTACT
>HPV81_Alpha_40804509_nt924_AOS|1
GTTGTAGAAAAAACCACAGGGTATCAGGAATCAAGTGATGAAGAGGAAGATAGTGCAGAT
>HPV81_Alpha_40804509_nt917_AOS|1
GGAGGCTGTTGTAGAAAAAACCACAGGGTATCAGGAATCAAGTGATGAAGAGGAAGATAG
>HPV95_Gamma_40804520_nt7122_AOS|1
CATTAAGTAAGACGCTGGCGAAGAAAAGAGACGCTCCTTATTTTTTTTGGAAAAAATATT
>HPV95_Gamma_40804520_nt7027_AOS|1
GCAATAAACTATACCTCCAATTACTATTGTGTCCATGCTTTGTGACTCATGTGGTCAATA
>HPV95_Gamma_40804520_nt6822_AOS|2
ATAAATGGATCTCGTAAAAGACAAAGAGCTATTACTTCTCAAACTGCAACAGGCACAAAA
>HPV95_Gamma_40804520_nt6815_AOS|1
TGGATTAATAAATGGATCTCGTAAAAGACAAAGAGCTATTACTTCTCAAACTGCAACAGG
>HPV95_Gamma_40804520_nt6803_AOS|2
TCTCTATCAGAGTGGATTAATAAATGGATCTCGTAAAAGACAAAGAGCTATTACTTCTCA
>HPV95_Gamma_40804520_nt6205_AOS|1
CTGATTACATAATTGCTGCTCAGAGTAACCAAGAACAAAATAATCTTGGCCCTCACATTT
>HPV95_Gamma_40804520_nt422_AOS|1
AAACTATTAGACATAGTAGAAAAACTTGACCTGTTATACAGTGGTGAGGTGTGCTATTTA
>HPV95_Gamma_40804520_nt415_AOS|1
CTGTCTTAAACTATTAGACATAGTAGAAAAACTTGACCTGTTATACAGTGGTGAGGTGTG
>HPV95_Gamma_40804520_nt408_AOS|1
GTTTATGCTGTCTTAAACTATTAGACATAGTAGAAAAACTTGACCTGTTATACAGTGGTG
>HPV94_Alpha_40804528_nt5600_AOS|2
ACCGGGCCTGACATTATATTACCCACCTCGGCGCAGTGGCCTTATGTGCCTTTAACACCT
>HPV94_Alpha_40804528_nt7013_AOS|1
CTTGGAGGACACTTATAGGTTCTTGTCGTCCTCCGCAATAACTTGTCAGAAGGAGGCACC
>HPV94_Alpha_40804528_nt6577_AOS|2
ACACCATTCCTGACACTCTAATATTGAAGTCGGCCGGTGGTGACCAAAACGTTGGTAGTG
>HPV94_Alpha_40804528_nt6570_AOS|2
GTGGGAGACACCATTCCTGACACTCTAATATTGAAGTCGGCCGGTGGTGACCAAAACGTT
>HPV94_Alpha_40804528_nt6555_AOS|1
AATAGAGCCAGTGCAGTGGGAGACACCATTCCTGACACTCTAATATTGAAGTCGGCCGGT
>HPV94_Alpha_40804528_nt5484_AOS|1
GAAGTGTCACACCGGCACGGAGCTATAGTACTGCTTCCCCTTTGTCCTCTACACTTTCAA
>HPV94_Alpha_40804528_nt5469_AOS|1
TTACAAATGGCACTCGAAGTGTCACACCGGCACGGAGCTATAGTACTGCTTCCCCTTTGT
>HPV94_Alpha_40804528_nt5461_AOS|1
TGTTGCCTTTACAAATGGCACTCGAAGTGTCACACCGGCACGGAGCTATAGTACTGCTTC
>HPV94_Alpha_40804528_nt5454_AOS|1
ATGGTGATGTTGCCTTTACAAATGGCACTCGAAGTGTCACACCGGCACGGAGCTATAGTA
>HPV94_Alpha_40804528_nt4247_AOS|1
ACCTGGGTGTATTGTGATATGGATGTAGACGCCTGTGCATAATCTAATGTTTTGCTGTGT

>HPVcand85_Alpha_4574720_nt7507_AOS|1
ATTATTTAAAGTTAAATCCTTTTTGGCTCTGTTTAAGGCGCCAGTGAGCCAGTGGCGCAC
>HPVcand85_Alpha_4574720_nt7500_AOS|1
TTGTTACATTATTTAAAGTTAAATCCTTTTTGGCTCTGTTTAAGGCGCCAGTGAGCCAGT
>HPVcand85_Alpha_4574720_nt3992_AOS|1
CACTATTCCGCTTTTTCCTGCTACTAATGTGTGTGCGCATGCTTGGCTACTTGTGTTTTT
>HPVcand85_Alpha_4574720_nt3551_AOS|1
ACTAGAAACCCATTCAGACATTGTGGATTCACAGAAACAAGTGAAGTCGACGGACTGTCC
>HPVcand85_Alpha_4574720_nt3538_AOS|1
GGGTTCGGCGCCGACTAGAAACCCATTCAGACATTGTGGATTCACAGAAACAAGTGAAGT
>HPVcand85_Alpha_4574720_nt3312_AOS|1
AGTTTAAATGTGATGCACAACAATATGGACAGAGCGGTAAATGGGAAGTGTGGTATAATG
>HPVcand85_Alpha_4574720_nt1395_AOS|1
GAAAAATGGCGACGGTTTAAATAGTGAGGCATGTAGTACAGATAATGAAATGGATATAGA
>HPVcand85_Alpha_4574720_nt1383_AOS|1
GTTTGGGGATGGGAAAAATGGCGACGGTTTAAATAGTGAGGCATGTAGTACAGATAATGA
>HPVcand85_Alpha_4574720_nt1368_AOS|1
CCTAGATACAAATGTGTTTGGGGATGGGAAAAATGGCGACGGTTTAAATAGTGAGGCATG
>HPVcand85_Alpha_4574720_nt1360_AOS|2
CAGGTAACCCTAGATACAAATGTGTTTGGGGATGGGAAAAATGGCGACGGTTTAAATAGT
>HPV96_Beta_50253426_nt4204_AOS|1
TGGTAGCTTTGATAGTCTTTAACTACTAACATTTCATTTTTTTATACTAATAACTAACCC
>HPV96_Beta_50253426_nt4195_AOS|1
TTGGTCCTTTGGTAGCTTTGATAGTCTTTAACTACTAACATTTCATTTTTTTATACTAAT
>HPV96_Beta_50253426_nt3060_AOS|2
AATTAAGAGACTCACCTTACAAAGATGAACCTTGGACATTGATTAACACCAGTTTAGAAA
>HPV96_Beta_50253426_nt3048_AOS|1
TTCAACTAGAGAAATTAAGAGACTCACCTTACAAAGATGAACCTTGGACATTGATTAACA
>HPV96_Beta_50253426_nt1650_AOS|1
AACTGCATATGGAGTTAAAGAGGAATTATTAGAAGGATGTAAGCAACTGTTACAAGAACA
>HPV96_Beta_50253426_nt696_AOS|1
GTATCAAGAAAACTTTTATTTAGTAAGAGGAGGTTGGAAAGGCATTTGCAGATATTGTAG
>HPV96_Beta_50253426_nt484_AOS|1
TTAAATCTTATTTGGAAGGAAGGTTATGCATATGCGTGTTGTTTCAATTGTGCTAGAACA
>HPV83_Alpha_5059324_nt6565_AOS|1
TTATATTCCAGGCACATCTGCTAATAGTAGGAACACTCTTACCAGCTACATCTATGCTCC
>HPV83_Alpha_5059324_nt6558_AOS|1
CCTCTTATTATATTCCAGGCACATCTGCTAATAGTAGGAACACTCTTACCAGCTACATCT
>HPV83_Alpha_5059324_nt5608_AOS|1
GCAGACCCTTCTGTTCCTGTGCCATCTAATATTCCTATTCAGTCTGTGTTTATAGACGGT
>HPV83_Alpha_5059324_nt5599_AOS|1
CATATGCCTGCAGACCCTTCTGTTCCTGTGCCATCTAATATTCCTATTCAGTCTGTGTTT
>HPV83_Alpha_5059324_nt4164_AOS|1
TGGCCGGGACGAAGTGGGATTACTGCTTATTATTTTAATTCTCATCGTAATTATTATTAT
>HPV83_Alpha_5059324_nt4152_AOS|1
TTTAATTGTTATTGGCCGGGACGAAGTGGGATTACTGCTTATTATTTTAATTCTCATCGT
>HPV83_Alpha_5059324_nt4139_AOS|1
GGCAGGCATATGATTTAATTGTTATTGGCCGGGACGAAGTGGGATTACTGCTTATTATTT
>HPV83_Alpha_5059324_nt4106_AOS|1
TCACTATGTATCCATTGGAGGCTAGAGGTGGGGGGCAGGCATATGATTTAATTGTTATTG
>HPV83_Alpha_5059324_nt3859_AOS|1
ACCAGTACAACCCTGGATTACATATTGTGCTTGATTTGTTTGTGGTGTCATATTCTTGTG
>HPV83_Alpha_5059324_nt3822_AOS|1
TTTGCAAATACCTGAGCCACTATACACCACACCATATACCAGTACAACCCTGGATTACAT
>HPV59_Alpha_557236_nt6416_AOS|1

TTCCTGAATCACTATATATTAAAGGTACTGACATACGTGCCAACCCAGGCAGTTATTTAT
>HPV59_Alpha_557236_nt5491_AOS|1
CTACACCCTTTACCACCATACAGTCTATTAATATAGAAGGCACAAATTATTTTTATGGC
>HPV59_Alpha_557236_nt5476_AOS|1
AACCCACTTATTCTACTACACCCTTTACCACCATACAGTCTATTAATATAGAAGGCACAA
>HPV59_Alpha_557236_nt5456_AOS|1
ACCTAATACTAATATTGTTGAACCCACTTATTCTACTACACCCTTTACCACCATACAGTC
>HPV59_Alpha_557236_nt5328_AOS|1
ACAATTCCTAAATCTTCTTTTCAAAGTTTGTCATTAACACGGTCGGCATCTAGCACCTTT
>HPV59_Alpha_557236_nt5321_AOS|1
TGCATTTACAATTCCTAAATCTTCTTTTCAAAGTTTGTCATTAACACGGTCGGCATCTAG
>HPV59_Alpha_557236_nt5313_AOS|1
GCCAACACTGCATTTACAATTCCTAAATCTTCTTTTCAAAGTTTGTCATTAACACGGTCG
>HPV59_Alpha_557236_nt5300_AOS|2
ACCTACTAGTACTGCCAACACTGCATTTACAATTCCTAAATCTTCTTTTCAAAGTTTGTC
>HPV59_Alpha_557236_nt5293_AOS|1
ATGAAGCACCTACTAGTACTGCCAACACTGCATTTACAATTCCTAAATCTTCTTTTCAAA
>HPV59_Alpha_557236_nt1414_AOS|1
ACATCCCCCACTAATCAATTGTTACAGTTATTACATAGCAAAAATAAGAAAGCAGCTATG
>HPV6_Alpha_6002612_nt7533_AOS|1
TGGCTGTTGCACGCGTTTTGGTTTGCACGCGCCTTACACACATAAGTAATATACATGCAC
>HPV6_Alpha_6002612_nt7504_AOS|1
TGTATATCTTGTTACACCCTGTGACTCAGTGGCTGTTGCACGCGTTTTGGTTTGCACGCG
>HPV6_Alpha_6002612_nt7491_AOS|1
TTAACTACATTATTGTATATCTTGTTACACCCTGTGACTCAGTGGCTGTTGCACGCGTTT
>HPV6_Alpha_6002612_nt6604_AOS|2
AATTAAGGGTAGTGGAAATCGAACGTCTGTAGGGAGTAGTATATATGTTAACACCCCAAG
>HPV6_Alpha_6002612_nt5334_AOS|1
TATGCACACTCGCAGCGGAAAGCACATAGGGGCCCGCATTCATTATTTTTATGATATTTC
>HPV6_Alpha_6002612_nt4639_AOS|1
CCCTTAGGAACTTCTGCAAAACCTTCTATTACTAGTGGGCCTATGGCTCGTCCTCCTGTG
>HPV6_Alpha_6002612_nt4211_AOS|1
TGTTATGTGCCTTTATTGTAGGGGTGTTGGGGTTATTATTAATGCACTATAGAGCTGTAC
>HPV6_Alpha_6002612_nt3489_AOS|1
TTGGACCCGTGGACAGTGGAAACCACAACGTCATCACTAACAATCACGACCAGCACCAAA
>HPV6_Alpha_6002612_nt3128_AOS|1
GATGTGTATGTGCAGGACACTGACTCCTGGGTAAAGGTGCATAGTATGGTAGATGCTAAG
>HPV57_Alpha_60882_nt6960_AOS|2
CCCCGTCCGCCAGCCTGCAGGACACCTACAGGTATTTGCAATCCCAAGCGATAACATGTC
>HPV57_Alpha_60882_nt7430_AOS|1
GTAATTGTCCCCGCTGCATGGTTTGCACACTGTGGCCTGTATGTAGCCCCCTGGTAGATA
>HPV57_Alpha_60882_nt7220_AOS|2
AAGGTCAGGCGATAGTGATTCTGTGTCTGCCTCATTTCCTTTGCTCTACTTTTGTATATG
>HPV57_Alpha_60882_nt4317_AOS|1
TATCGGTGTGCACCTGATTGCCTTTGTATTGCTACGTGTCAATAAACTTATTGCCATGTC
>HPV57_Alpha_60882_nt4310_AOS|1
ACATTTTTATCGGTGTGCACCTGATTGCCTTTGTATTGCTACGTGTCAATAAACTTATTG
>HPV57_Alpha_60882_nt4230_AOS|1
ATTTTGTTGTTGATGCTGTACGTCCGTCTGCTGCAGTAGTGTACATACTCATTGTGTTTT
>HPV57_Alpha_60882_nt4120_AOS|2
ATACTGCAATGTATCCTGCTGTGTACAAGGGCACGGGCGGGTCGTATCCCATTGTCCTGT
>HPV57_Alpha_60882_nt4061_AOS|1
CAATAGTTGATATTACATTGTGACCAACTGCTGCTACCCTGTATATACCTCCCCCCTGTA
>HPV57_Alpha_60882_nt4048_AOS|1
CTTGTGGTTTCTACAATAGTTGATATTACATTGTGACCAACTGCTGCTACCCTGTATATA

>HPV69_Alpha_6970418_nt7417_AOS|1
TGGCTCACCTACATTACTCACATATCTTTTAACAATAGTTGCATACGCACTTTTTTTTGA
>HPV69_Alpha_6970418_nt7409_AOS|1
CCTAGACGTGGCTCACCTACATTACTCACATATCTTTTAACAATAGTTGCATACGCACTT
>HPV69_Alpha_6970418_nt7402_AOS|1
TGGCTTGCCTAGACGTGGCTCACCTACATTACTCACATATCTTTTAACAATAGTTGCATA
>HPV69_Alpha_6970418_nt6352_AOS|1
ACCAGTATCTATGTATATTAAAGGCGCTGGTCAAGGTCGTGAACCTCCAACTACTTCTAT
>HPV69_Alpha_6970418_nt6345_AOS|2
ATTCTGTACCAGTATCTATGTATATTAAAGGCGCTGGTCAAGGTCGTGAACCTCCAACTA
>HPV69_Alpha_6970418_nt6337_AOS|1
TATAGGTGATTCTGTACCAGTATCTATGTATATTAAAGGCGCTGGTCAAGGTCGTGAACC
>HPV69_Alpha_6970418_nt5350_AOS|1
TTTGATATACCTATATATAACGGCCCTGACATTTATGCTCCTGTACCTTCTTCCACATGG
>HPV69_Alpha_6970418_nt5309_AOS|1
GTAGTGCACCCTTAAATGTTACCATACCTCTATCTACATCATTTGATATACCTATATATA
>HPV69_Alpha_6970418_nt5302_AOS|1
ACCACTAGTAGTGCACCCTTAAATGTTACCATACCTCTATCTACATCATTTGATATACCT
>HPV69_Alpha_6970418_nt5295_AOS|1
ATTTTCAACCACTAGTAGTGCACCCTTAAATGTTACCATACCTCTATCTACATCATTTGA
>HPV82_Alpha_6970427_nt7511_AOS|1
ACCTATGTTTTTTGGCATAACCCTTAATTCTTTTGGCATGGTATGTACATCCCGCCCGCC
>HPV82_Alpha_6970427_nt4842_AOS|1
CCTCGTTTATATAGCAGGGCATTTTCACAGGTTAAGGTTACTAATCCAGACTTTATTAGT
>HPV82_Alpha_6970427_nt4835_AOS|1
TGCTGCACCTCGTTTATATAGCAGGGCATTTTCACAGGTTAAGGTTACTAATCCAGACTT
>HPV82_Alpha_6970427_nt2884_AOS|1
GAGAAAGAAACATGCAAACCCTTAACCATCAAGTAGTACCAGCATCGGCAGTATCAAAAC
>HPV82_Alpha_6970427_nt2877_AOS|1
ACTGCACGAGAAAGAAACATGCAAACCCTTAACCATCAAGTAGTACCAGCATCGGCAGTA
>HPV82_Alpha_6970427_nt2869_AOS|1
TGTTTTATACTGCACGAGAAAGAAACATGCAAACCCTTAACCATCAAGTAGTACCAGCAT
>HPV82_Alpha_6970427_nt1366_AOS|1
GAGCAGCGACAGAAGTACAGAGATAGACCTGGAAACAAACGAAAATGCTACCAATGTAGG
>HPV82_Alpha_6970427_nt503_AOS|1
CAGTGTGCAAATTGCAGAAAACCACCAAGACAACGTAGTGAAACCCAGGTGTAATAACGC
>HPV82_Alpha_6970427_nt493_AOS|1
CTGGACGGGACAGTGTGCAAATTGCAGAAAACCACCAAGACAACGTAGTGAAACCCAGGT
>HPV68a_Alpha_71726685_nt6285_AOS|1
GTAGGGGACACTATTCCCACTGACATGTATATTAAGGGCACTGACATTCGTGAAACTCCT
>HPV68a_Alpha_71726685_nt6278_AOS|1
AGGCATGGTAGGGGACACTATTCCCACTGACATGTATATTAAGGGCACTGACATTCGTGA
>HPV68a_Alpha_71726685_nt5355_AOS|1
AGTTGCCTTTAACACCCTCTACTCCAATTGATACAACCTTTGCCATAACTATATATGGTT
>HPV68a_Alpha_71726685_nt5347_AOS|1
AACCCCACAGTTGCCTTTAACACCCTCTACTCCAATTGATACAACCTTTGCCATAACTAT
>HPV68a_Alpha_71726685_nt5329_AOS|1
TGTTGTATTACCATCTACAACCCCACAGTTGCCTTTAACACCCTCTACTCCAATTGATAC
>HPV68a_Alpha_71726685_nt5209_AOS|1
ATTTACTACTCGTTCCCACATATCAGTTCCTTCATTGGCTTCTGCTGCATCCACTACATA
>HPV68a_Alpha_71726685_nt5202_AOS|1
ATGCTACATTTACTACTCGTTCCCACATATCAGTTCCTTCATTGGCTTCTGCTGCATCCA
>HPV68a_Alpha_71726685_nt5193_AOS|1
CATTTCATAATGCTACATTTACTACTCGTTCCCACATATCAGTTCCTTCATTGGCTTCTG
>HPV68a_Alpha_71726685_nt3453_AOS|1

CAACGTGTCCGTGGACGGTGTCGACCTCCCACTCCTCAGTAGAAGTGCAGGCCAAAACAA
>HPV68a_Alpha_71726685_nt1336_AOS|1
GTGAAAACCAGGATCCTAAATCACCTACTACCCAACTTAAAGTATTATTACAATGTAACA
>HPV97_Alpha_71726694_nt7616_AOS|1
CCTGTGCAACTACTTTTACATCCAACAATCTGTCTACTCTTCTTATAAAGTATAAAATGA
>HPV97_Alpha_71726694_nt7417_AOS|1
TCTCACATACAATAGTCATACATATTTTCACCACTGGCACGTATTTTGGCGCACACTTTG
>HPV97_Alpha_71726694_nt7410_AOS|1
ATGTTAATCTCACATACAATAGTCATACATATTTTCACCACTGGCACGTATTTTGGCGCA
>HPV97_Alpha_71726694_nt6277_AOS|1
ACAGAGCTGGAACAATGGGTGACACTATTCCACAGGAGTTATATATTAAAGGAACTGGTA
>HPV97_Alpha_71726694_nt5350_AOS|1
TAGTACTTCTGTATGGCCTTTTATAACACCTACATCTACGTCCACGACACACTATATAGG
>HPV97_Alpha_71726694_nt5341_AOS|1
ATTACCTTCTAGTACTTCTGTATGGCCTTTTATAACACCTACATCTACGTCCACGACACA
>HPV97_Alpha_71726694_nt5334_AOS|1
ATATTGTATTACCTTCTAGTACTTCTGTATGGCCTTTTATAACACCTACATCTACGTCCA
>HPV97_Alpha_71726694_nt4890_AOS|1
CTGCCTTTAATCCTATTGACTCTACATTAACATTTGAACCTACTAGTAATGTTCCCGATT
>HPV97_Alpha_71726694_nt3688_AOS|1
TATTAACTGTAATGTATGATAGTGAGCATCAACGAACCAAATTTTTGGATAGTGTTCCTA
>HPV97_Alpha_71726694_nt3224_AOS|1
ACATGAAAGTAAAAAATATGGTAACAGTAATAAGTGGGAAGTGCATTTTGGGGGCGTTGT
>HPV106_Alpha_71726710_nt7526_AOS|1
ATTTATGTTGTGTAGTCCAGTCCTTGTGGCCCGGTCATGCATACCCCCGCCCTTATAACA
>HPV106_Alpha_71726710_nt7516_AOS|1
GTAACTATACATTTATGTTGTGTAGTCCAGTCCTTGTGGCCCGGTCATGCATACCCCCGC
>HPV106_Alpha_71726710_nt7509_AOS|1
CTTGTGAGTAACTATACATTTATGTTGTGTAGTCCAGTCCTTGTGGCCCGGTCATGCATA
>HPV106_Alpha_71726710_nt6592_AOS|2
ATGTTCCGGAAGGGTTTTTACTAAAGGGGCCTACTACTACATCCCGCGCCTCTGCTACAA
>HPV106_Alpha_71726710_nt5560_AOS|2
AGTATCGCACACTACTGTTCCCCTGTCTACAAGCTTTGATGTCCCTGTTTTTAGTGGCCC
>HPV106_Alpha_71726710_nt5552_AOS|1
GTCTCCACAGTATCGCACACTACTGTTCCCCTGTCTACAAGCTTTGATGTCCCTGTTTTT
>HPV106_Alpha_71726710_nt5545_AOS|1
ATCCACTGTCTCCACAGTATCGCACACTACTGTTCCCCTGTCTACAAGCTTTGATGTCCC
>HPV106_Alpha_71726710_nt5533_AOS|1
ATTGGCTGCATCATCCACTGTCTCCACAGTATCGCACACTACTGTTCCCCTGTCTACAAG
>HPV106_Alpha_71726710_nt5367_AOS|1
TAAGTCCAATTACTCCTGCTGAAGAGTTAGAAATGCAACCTCTGGTGTCTGACACCCCTA
>HPV106_Alpha_71726710_nt3874_AOS|1
GTGTATACTACACCCTGGAGATTTTTATTTGCTGGCTATCTGTTTGGACACACCTGCTGC
>HPV102_Alpha_71726718_nt5688_AOS|2
CCCAATCTGTGTTTGTGGATGGTACAGACTATTTTTTATTGCCTAATTACTTATTCTTTC
>HPV102_Alpha_71726718_nt5655_AOS|1
TGCTGCATGTTTCCCAGTCCTCTAATGTTCCTTCCCAATCTGTGTTTGTGGATGGTACAG
>HPV102_Alpha_71726718_nt5456_AOS|1
AATGGCCACTTGGACTCCTTGGCGTCTCCACATGTTTCATATACTTCTTCCACGTCTGTT
>HPV102_Alpha_71726718_nt4263_AOS|1
GAGGCTTATGCACATGTAGACTCCTTACTGTGTTCCTTTTGTATTCCCATACATTTTTGA
>HPV102_Alpha_71726718_nt4168_AOS|1
CCGTCCTATGATGTGATTGTTTTTGGGCAAGCTGATACTGGATTACTGTTAATAATTTTA
>HPV102_Alpha_71726718_nt4161_AOS|1
GGGGGGGCCGTCCTATGATGTGATTGTTTTTGGGCAAGCTGATACTGGATTACTGTTAAT

>HPV102_Alpha_71726718_nt4146_AOS|1
ATTGGAAACACGACGGGGGGGGCCGTCCTATGATGTGATTGTTTTTGGGCAAGCTGATAC
>HPV102_Alpha_71726718_nt4139_AOS|1
TGTATCCATTGGAAACACGACGGGGGGGGCCGTCCTATGATGTGATTGTTTTTGGGCAAG
>HPV102_Alpha_71726718_nt3615_AOS|1
GTGCGGGGAGACAGCCACTAAAGCAACCTTTGTTACCTTGCGGTATAGCAGTGTAGAACA
>HPV102_Alpha_71726718_nt3406_AOS|1
AAACAACATCAAAAGCGGCTGGACACTAGTACCCACGGATTCTACGGTGACACTGAGTGC
>HPV13_Alpha_85827580_nt7631_AOS|1
TTGCTAGGGCGCGGTTATATATGTTTACTAAATCTTACTAATCTTTCTATCACTCATTTT
>HPV13_Alpha_85827580_nt7556_AOS|1
ACGCTGTTTATATAATCTTACCTACCGCCTGCCAAAATTATCCACCGCTTGCCAAAATCA
>HPV13_Alpha_85827580_nt4886_AOS|1
AAAGTGGTGCACACGTGTTTATATCGCCATCTACTATTTCCCCTCATTCTACAGAAGACA
>HPV13_Alpha_85827580_nt4879_AOS|1
TCTATTGAAAGTGGTGCACACGTGTTTATATCGCCATCTACTATTTCCCCTCATTCTACA
>HPV13_Alpha_85827580_nt4872_AOS|1
TCAACCTTCTATTGAAAGTGGTGCACACGTGTTTATATCGCCATCTACTATTTCCCCTCA
>HPV13_Alpha_85827580_nt4865_AOS|1
CACAATCTCAACCTTCTATTGAAAGTGGTGCACACGTGTTTATATCGCCATCTACTATTT
>HPV13_Alpha_85827580_nt3968_AOS|1
TAATTGCACTTACAGTGTGTGTAGTTAGCATTATAACAATATTGTGGATATCAGAGTTTT
>HPV2_Alpha_9626032_nt4623_AOS|1
TTCGCGACCCACCACTGTAGTTGACATTGGTCCAACGCCCAGGCCGCCTGTTATCATTGA
>HPV2_Alpha_9626032_nt4220_AOS|2
CATGATGATGTTCAATGTCTGTTGGTGATTCTTATCCTAATCGCCTTTTTATTGTTGATG
>HPV2_Alpha_9626032_nt4210_AOS|1
ACTGTGGGGTCATGATGATGTTCAATGTCTGTTGGTGATTCTTATCCTAATCGCCTTTTT
>HPV2_Alpha_9626032_nt4178_AOS|1
TATAAGGGATCTGAGGGAACATATCCTGTGGTACTGTGGGGTCATGATGATGTTCAATGT
>HPV2_Alpha_9626032_nt4105_AOS|1
CCATACGTTGCTGCTAGCTTGTATACATAGTCTATATACCCATTGTGTGAGATTTGCAAT
>HPV2_Alpha_9626032_nt4098_AOS|1
TATTTCGCCATACGTTGCTGCTAGCTTGTATACATAGTCTATATACCCATTGTGTGAGAT
>HPV2_Alpha_9626032_nt4091_AOS|1
TCGTTATTATTTCGCCATACGTTGCTGCTAGCTTGTATACATAGTCTATATACCCATTGT
>HPV2_Alpha_9626032_nt4084_AOS|1
ACCATAGTCGTTATTATTTCGCCATACGTTGCTGCTAGCTTGTATACATAGTCTATATAC
>HPV2_Alpha_9626032_nt4077_AOS|1
GGTTTTTACCATAGTCGTTATTATTTCGCCATACGTTGCTGCTAGCTTGTATACATAGTC
>HPV41_Nu_9626041_nt6645_AOS|3
TTCTAAGTTTTTTGAGTTTTTAAGGCACACCGAGGAGTTTCAGCTTGCCTTTATTCTACA
>HPV41_Nu_9626041_nt6603_AOS|2
TACCATCAGTGTTCCTGAGGGGGATGCTTCTTCATATAACAATTCTAAGTTTTTTGAGTT
>HPV41_Nu_9626041_nt3682_AOS|1
TAAGGTACAAATGGAAAAACAAGTATAGCGGTGACATAATGTATCTGGGGACTACTTTCA
>HPV41_Nu_9626041_nt3148_AOS|1
ATTATATTACACCAACAGATGAATGGTATAAAGCTAGAGGTGGCATTGATGACACTGGTA
>HPV41_Nu_9626041_nt2640_AOS|1
GGCAACCGTTATATTACATTGATGGTTATACATGGAACTCTTTTTTTAGGAAATTTTGGC
>HPV41_Nu_9626041_nt2632_AOS|1
AGGAAATGGGCAACCGTTATATTACATTGATGGTTATACATGGAACTCTTTTTTTAGGAA
>HPV41_Nu_9626041_nt2578_AOS|1
ATATCTGCAAACCAGAACAATGTATGTGTACTTTAACAAGCCATTTCCTCTTAAAGGAAA
>HPV41_Nu_9626041_nt2569_AOS|1

TGAATTCAAATATCTGCAAACCAGAACAATGTATGTGTACTTTAACAAGCCATTTCCTCT
>HPV41_Nu_9626041_nt2561_AOS|1
CGATTAGATGAATTCAAATATCTGCAAACCAGAACAATGTATGTGTACTTTAACAAGCCA
>HPV41_Nu_9626041_nt1591_AOS|1
TAGTGAGATAAGTGCTATGGAGGTTTTGCTGCGACAACAATGCCAATTTTTATACATTGA
>HPV1_Mu_9626063_nt7292_AOS|1
TTATTTGTTTAGACTCCGGTGCATTATACACAAGTGTGCATTTTTTGTGTTCTCTGATTG
>HPV1_Mu_9626063_nt7272_AOS|1
TATTCCAGATTGTTGAGGATTTATTTGTTTAGACTCCGGTGCATTATACACAAGTGTGCA
>HPV1_Mu_9626063_nt7265_AOS|1
CTGCATATATTCCAGATTGTTGAGGATTTATTTGTTTAGACTCCGGTGCATTATACACAA
>HPV1_Mu_9626063_nt6565_AOS|2
GTAAAGTAAAGTTAACTCCCGAAAATCTAGCCTACATTCATACAATGGACCCTAATATTT
>HPV1_Mu_9626063_nt5769_AOS|2
GGAATAACGGGCCACCCTCTTTTAAATAAGTTAGATGATGCAGAAAATCCAACAAATTAT
>HPV1_Mu_9626063_nt5578_AOS|2
TTGAGATCTCCAGTAATCAAACTGTAACTATACCAAAAGTGTCACCAAATGCATTTAGAG
>HPV1_Mu_9626063_nt5255_AOS|1
GATACTAGCTTGCATGTATATTACCCAAATTCTTCTAAAGGGACTCCAATAATTAATCCT
>HPV1_Mu_9626063_nt5157_AOS|1
TGGGCGAAAATTTGCAACTTACTATTACTAACTCAGAGGGTGAGGTTTCTATACTAGATT
>HPV1_Mu_9626063_nt5119_AOS|1
ATATTCAGAAGAAGAGCTTTTAGACACAAACGAAAGTGTGGGCGAAAATTTGCAACTTAC
>HPV1_Mu_9626063_nt3748_AOS|1
TTTGCCCAGATCAGTGTCTGTGTTTTTGGGACAGTTTAATGGGTCTTAAAATTAATGGAA
>HPV18_Alpha_9626069_nt5352_AOS|1
ATTTTTTAAATATTCGCCCACTATATCTTCTGCCTCTTCCTATAGTAATGTAACGGTCCC
>HPV18_Alpha_9626069_nt5359_AOS|1
AAATATTCGCCCACTATATCTTCTGCCTCTTCCTATAGTAATGTAACGGTCCCTTTAACC
>HPV18_Alpha_9626069_nt5345_AOS|1
CCTTTGCATTTTTTAAATATTCGCCCACTATATCTTCTGCCTCTTCCTATAGTAATGTAA
>HPV18_Alpha_9626069_nt5338_AOS|1
ACTACCTCCTTTGCATTTTTTAAATATTCGCCCACTATATCTTCTGCCTCTTCCTATAGT
>HPV18_Alpha_9626069_nt5331_AOS|1
GCGTTCTACTACCTCCTTTGCATTTTTTAAATATTCGCCCACTATATCTTCTGCCTCTTC
>HPV18_Alpha_9626069_nt5324_AOS|1
TACCATCGCGTTCTACTACCTCCTTTGCATTTTTTAAATATTCGCCCACTATATCTTCTG
>HPV18_Alpha_9626069_nt1507_AOS|1
GCACAATTAAAAGACTTGTTAAAAGTAAACAATAAACAAGGAGCTATGTTAGCAGTATTT
>HPV18_Alpha_9626069_nt1479_AOS|1
AGAAAATGTAAATCCACAATGTACCATAGCACAATTAAAAGACTTGTTAAAAGTAAACAA
>HPV4_Gamma_9626597_nt6989_AOS|1
AATTATATAAGATTTGCAAACGTGTATGTATCTGTTTTTGTGAACTATAGTGAAATAAAC
>HPV4_Gamma_9626597_nt6804_AOS|2
TGGTTCTCTAAAACGTAAAAGAATAATAAGTTCTTCTCATGCACAAACTAATACCAAACG
>HPV4_Gamma_9626597_nt6192_AOS|2
TATTGGTGCTCAAAACCAACAAGATCAGTACACTTTAGGACCTCATATTTATGTAGGGAC
>HPV4_Gamma_9626597_nt5220_AOS|2
GCCATTTGTTCCTCTAGAACCAGCTTTGTTATCTGATATATTTAGTACGGATTTTGTATA
>HPV4_Gamma_9626597_nt4544_AOS|1
ATCGTGCAATAGGAAGAGCACGGGATTTATATAATAGAAGGGTTCAGCAAATACCTACTA
>HPV4_Gamma_9626597_nt3693_AOS|1
GCTTTTGTAAAACACAACCTTTTTCCTAAACTGTGTACATATACCTACGGCTCATTGAAT
>HPV4_Gamma_9626597_nt3685_AOS|1
AAAGAGACGCTTTTGTAAAACACAACCTTTTTCCTAAACTGTGTACATATACCTACGGCT

>HPV4_Gamma_9626597_nt3678_AOS|1
ACTGACCAAAGAGACGCTTTTGTAAAACACAACCTTTTTCCTAAACTGTGTACATATACC
>HPV4_Gamma_9626597_nt3670_AOS|1
TTGATAGCACTGACCAAAGAGACGCTTTTGTAAAACACAACCTTTTTCCTAAACTGTGTA
>HPV4_Gamma_9626597_nt3649_AOS|1
ATAGTCGCATGCTTATTGCATTTGATAGCACTGACCAAAGAGACGCTTTTGTAAAACACA
>HPV63_Mu_9626605_nt6415_AOS|2
TAAATGTTCTTAACAAAGCAACCCCTGAGACTTATGATAGCGCAGATTATAATGAGTATA
>HPV63_Mu_9626605_nt6407_AOS|1
GATGAATATAAATGTTCTTAACAAAGCAACCCCTGAGACTTATGATAGCGCAGATTATAA
>HPV63_Mu_9626605_nt5232_AOS|1
TTGGATCACCTACTATAAATCCTACTGATCTTGACATTCCATTAATTATTATACATTTAG
>HPV63_Mu_9626605_nt5225_AOS|1
GCTAGTGTTGGATCACCTACTATAAATCCTACTGATCTTGACATTCCATTAATTATTATA
>HPV63_Mu_9626605_nt4424_AOS|1
TTTATAGATGTAGATACGCCGGGTCAAATAGTAGGACAAGAAATACCACTAGTTAATTTT
>HPV63_Mu_9626605_nt4417_AOS|1
TAATATTTTTATAGATGTAGATACGCCGGGTCAAATAGTAGGACAAGAAATACCACTAGT
>HPV63_Mu_9626605_nt3091_AOS|1
TGTTTTATTTAGAATATGATGGTGTCAAAAACTACTATGTTGACTTTCAAGAAGAGGCCA
>HPV63_Mu_9626605_nt3064_AOS|1
CAGCTAGTGATGTAGATGTTCATGGTGTGTTTTATTTAGAATATGATGGTGTCAAAAACT
>HPV63_Mu_9626605_nt3051_AOS|1
AGATGGAGAAAAGCAGCTAGTGATGTAGATGTTCATGGTGTGTTTTATTTAGAATATGAT
>HPV16_Alpha_9627100_nt7744_AOS|1
CATAAGGTTTAAACTTCTAAGGCCAACTAAATGTCACCCTAGTTCATACATGAACTGTGT
>HPV16_Alpha_9627100_nt7072_AOS|2
ATTTACATTAGGAAAACGAAAAGCTACACCCACCACCTCATCTACCTCTACAACTGCTAA
>HPV16_Alpha_9627100_nt6912_AOS|1
CTTGTCAAAAACATACACCTCCAGCACCTAAAGAAGATGATCCCCTTAAAAAATACACTT
>HPV16_Alpha_9627100_nt6893_AOS|1
TTTGTAACCCAGGCAATTGCTTGTCAAAAACATACACCTCCAGCACCTAAAGAAGATGAT
>HPV16_Alpha_9627100_nt6445_AOS|1
TGTACCAGACGATTTATACATTAAAGGCTCTGGGTCTACTGCAAATTTAGCCAGTTCAAA
>HPV16_Alpha_9627100_nt6437_AOS|1
GGTGAAAATGTACCAGACGATTTATACATTAAAGGCTCTGGGTCTACTGCAAATTTAGCC
>HPV16_Alpha_9627100_nt5494_AOS|1
ACTGACCAAGCTCCTTCATTAATTCCTATAGTTCCAGGGTCTCCACAATATACAATTATT
>HPV16_Alpha_9627100_nt5487_AOS|1
TAATATAACTGACCAAGCTCCTTCATTAATTCCTATAGTTCCAGGGTCTCCACAATATAC
>HPV16_Alpha_9627100_nt3973_AOS|1
ATAATATTGGTATTACTATTGTGGATAACAGCAGCCTCTGCGTTTAGGTGTTTTATTGTA
>HPV5_Beta_9627145_nt5089_AOS|1
GTACTCCATTGCCACGCAATCAATCTGTAGGCCGTAGGAGGGGTTTCTCTTTGACTAATA
>HPV5_Beta_9627145_nt6974_AOS|1
TTTCAGTATTTCTGTATATAATCAGGCTGGAGCACTAAAAGATGTTGCAGACTATAATGC
>HPV5_Beta_9627145_nt5159_AOS|1
ACAGCAGGTACAAGTGGACAATCCATTGTTTCTAACTCAACCATCTAAGTTAGTTCGTTT
>HPV5_Beta_9627145_nt4026_AOS|1
TGTCAAAGGGGCGGCTAACACACTGAAAAATGTCCGCAACAGAGCTAAAATTAAATACAT
>HPV5_Beta_9627145_nt4019_AOS|1
TAATCATTGTCAAAGGGGCGGCTAACACACTGAAAAATGTCCGCAACAGAGCTAAAATTA
>HPV5_Beta_9627145_nt2365_AOS|1
CCCTTTCAGAGTGCAAGATAGCTCTATTGGATGATGTAACAGACCCTTGTTGGATATACA
>HPV5_Beta_9627145_nt2174_AOS|1

CCAAAATATAAACTTTATTGTATTCCTAACTGCATTAAAAGAATTCCTACACTCAGTGCC
>HPV5_Beta_9627145_nt215_AOS|1
AACACCAACAGAAACTGACAGAAAAAGATAAGGCAGAATTACCTTTAAGTATTAGAGACT
>HPV5_Beta_9627145_nt208_AOS|1
GGAGCCGAACACCAACAGAAACTGACAGAAAAAGATAAGGCAGAATTACCTTTAAGTATT
>HPV10_Alpha_9627257_nt7080_AOS|3
TTTTGTCCTCTTCAGCCATTACTTGTCAGAAAGATACACCCCCCACCGAGAAGCAGGATC
>HPV10_Alpha_9627257_nt6618_AOS|3
TTGGAGACGCCATCCCAGACACTTTCATATTAAAGAGCAACGGTGGGGGGCGAGACGTTG
>HPV10_Alpha_9627257_nt6609_AOS|2
CTAGTGCTGTTGGAGACGCCATCCCAGACACTTTCATATTAAAGAGCAACGGTGGGGGGC
>HPV10_Alpha_9627257_nt6600_AOS|3
TTAATCGGGCTAGTGCTGTTGGAGACGCCATCCCAGACACTTTCATATTAAAGAGCAACG
>HPV10_Alpha_9627257_nt5534_AOS|1
GTAGTACCACACAGTCCAGGGGATATAATACCACTTCCCCTTTGTCTTCTACACTTTCCA
>HPV10_Alpha_9627257_nt5527_AOS|1
GGATATCGTAGTACCACACAGTCCAGGGGATATAATACCACTTCCCCTTTGTCTTCTACA
>HPV10_Alpha_9627257_nt5520_AOS|2
TACAGAGGGATATCGTAGTACCACACAGTCCAGGGGATATAATACCACTTCCCCTTTGTC
>HPV10_Alpha_9627257_nt5513_AOS|1
TTGCTTTTACAGAGGGATATCGTAGTACCACACAGTCCAGGGGATATAATACCACTTCCC
>HPV10_Alpha_9627257_nt5506_AOS|1
GGTGATGTTGCTTTTACAGAGGGATATCGTAGTACCACACAGTCCAGGGGATATAATACC
>HPV10_Alpha_9627257_nt5499_AOS|1
TGATGATGGTGATGTTGCTTTTACAGAGGGATATCGTAGTACCACACAGTCCAGGGGATA
>HPV26_Alpha_9627305_nt7307_AOS|1
TATTTGTCATTTGTTTTACCCCTGAGGTAATGGGAGGAACCTTAGGTGGTGTCCCTTATA
>HPV26_Alpha_9627305_nt7291_AOS|1
CATGTCTTTACTACACTATTTGTCATTTGTTTTACCCCTGAGGTAATGGGAGGAACCTTA
>HPV26_Alpha_9627305_nt7283_AOS|1
CATGGTTACATGTCTTTACTACACTATTTGTCATTTGTTTTACCCCTGAGGTAATGGGAG
>HPV26_Alpha_9627305_nt7275_AOS|1
TCTTCATGCATGGTTACATGTCTTTACTACACTATTTGTCATTTGTTTTACCCCTGAGGT
>HPV26_Alpha_9627305_nt5390_AOS|1
ATCCACCTCATTTGAACTACCTGTATATAGTGGGTCAGACATTTACACGCCCACATCTTC
>HPV26_Alpha_9627305_nt5377_AOS|1
TTACTGTGCCTTTATCCACCTCATTTGAACTACCTGTATATAGTGGGTCAGACATTTACA
>HPV26_Alpha_9627305_nt5316_AOS|1
TTACCAGTTCCAAGATATGCCTCCAATGTGTTTTCCTCTATTAATACTTCCACTACCAAT
>HPV26_Alpha_9627305_nt5309_AOS|1
TACAACATTACCAGTTCCAAGATATGCCTCCAATGTGTTTTCCTCTATTAATACTTCCAC
>HPV26_Alpha_9627305_nt5302_AOS|1
ATTCCCCTACAACATTACCAGTTCCAAGATATGCCTCCAATGTGTTTTCCTCTATTAATA
>HPV26_Alpha_9627305_nt5291_AOS|1
GCGCATGTCATATTCCCCTACAACATTACCAGTTCCAAGATATGCCTCCAATGTGTTTTC
>HPV32_Alpha_9627327_nt7703_AOS|1
GCATAGCTTTGCATTTACTTATTTCCTTCTTCCTTTGTTCATGTTATGACTACTGTATTT
>HPV32_Alpha_9627327_nt7687_AOS|1
TGTTTTAGAAAGGTTGGCATAGCTTTGCATTTACTTATTTCCTTCTTCCTTTGTTCATGT
>HPV32_Alpha_9627327_nt4954_AOS|1
ATACAGTGATCCTTTTACTTTACGGCCTTCTTTGCCAGTAGAGGGTAATGGTAGACTCTT
>HPV32_Alpha_9627327_nt4946_AOS|1
AATCCAGTATACAGTGATCCTTTTACTTTACGGCCTTCTTTGCCAGTAGAGGGTAATGGT
>HPV32_Alpha_9627327_nt4927_AOS|1
TGCATCTACTACGTCCCACAATCCAGTATACAGTGATCCTTTTACTTTACGGCCTTCTTT

>HPV32_Alpha_9627327_nt4909_AOS|1
CACCAATACTATAAGGGTTGCATCTACTACGTCCCACAATCCAGTATACAGTGATCCTTT
>HPV32_Alpha_9627327_nt4313_AOS|1
ATGGCGATGTGTGGCTGCTGCTGTGGTTTTTAGCCACAGTGTTTATTTGTATATTAGTAC
>HPV32_Alpha_9627327_nt4298_AOS|1
ATTGTACTTTAGAAAATGGCGATGTGTGGCTGCTGCTGTGGTTTTTAGCCACAGTGTTTA
>HPV32_Alpha_9627327_nt4289_AOS|1
ATATGGTACATTGTACTTTAGAAAATGGCGATGTGTGGCTGCTGCTGTGGTTTTTAGCCA
>HPV32_Alpha_9627327_nt4282_AOS|1
CGTGATAATATGGTACATTGTACTTTAGAAAATGGCGATGTGTGGCTGCTGCTGTGGTTT
>HPV34_Alpha_9627334_nt7302_AOS|1
TTCGGTTGCCTATACTATAACATGTTTTGGTCTGTTTGTGCTGTTTTAGCACAAAGGTTT
>HPV34_Alpha_9627334_nt4466_AOS|1
TGCCAGGTCCTACCTCCATAGTGCCTTCTAGTTCTGGATTTAATGTAACAACATCTGTGG
>HPV34_Alpha_9627334_nt4457_AOS|1
AAGCTGGTGTGCCAGGTCCTACCTCCATAGTGCCTTCTAGTTCTGGATTTAATGTAACAA
>HPV34_Alpha_9627334_nt4338_AOS|1
AACTACTCCATCTAGACCAGTTGAAATTCCTTTGCAACCTACCAGACCTCCTGTTATTAC
>HPV34_Alpha_9627334_nt4325_AOS|1
TGCCATTACCCACAACTACTCCATCTAGACCAGTTGAAATTCCTTTGCAACCTACCAGAC
>HPV34_Alpha_9627334_nt4316_AOS|1
CAGGATATGTGCCATTACCCACAACTACTCCATCTAGACCAGTTGAAATTCCTTTGCAAC
>HPV34_Alpha_9627334_nt3909_AOS|1
ATGCATGGTTATTGCTTTTGATTATAATTACTTTTTTACATGTGTCCCAATCCCTGTTAA
>HPV34_Alpha_9627334_nt3138_AOS|1
CATTTGTGTATTATTGGTTGGAAGGCAAGTGGTATAAAGTGAGTAGCCATGTAGATTATA
>HPV34_Alpha_9627334_nt739_AOS|1
GTGTGTCTTACCATTGAGAGCACACACGCTGACCTATTAGTGTTAGAAGACCTGCTTATG
>HPV34_Alpha_9627334_nt317_AOS|1
AAGGTTAGACAATATAGAAGATATAACCAATCAGTGTATGGACGGACGTTAGAGAATTTA
>HPV49_Beta_9627363_nt7123_AOS|1
ATGCCCTGACAAACAACCTGCTCCAGAAAGGAAAGATCCATATGAGCAGTATAACTTTTG
>HPV49_Beta_9627363_nt6649_AOS|2
TAACAATTACATATTACCTGCAGCAAGTCAACAGGCCCAAAATACTCTTGGCAGCTCCAT
>HPV49_Beta_9627363_nt6639_AOS|1
TAGGTCAGGATAACAATTACATATTACCTGCAGCAAGTCAACAGGCCCAAAATACTCTTG
>HPV49_Beta_9627363_nt5574_AOS|1
CCACATTTACTGTACCCCGCTTCTCTACTCCCAGATCTGATACCTTTTATGTACAAGATT
>HPV49_Beta_9627363_nt470_AOS|1
TTGCTGAGATAGTAGTCAGATGTCTCATTTGCCTTAAGAGGCTAGATTTGTTGGAAAAGC
>HPV49_Beta_9627363_nt413_AOS|1
ATTATCACCAAGAAATTGTCGTAGGCATCGAAATAGAAGGACGAGCAGCGGCTAATATTG
>HPV49_Beta_9627363_nt406_AOS|1
TTTACTAATTATCACCAAGAAATTGTCGTAGGCATCGAAATAGAAGGACGAGCAGCGGCT
>HPV53_Alpha_9627377_nt7601_AOS|1
TTTAATGTTTTTTTGTTTTATACACTATGCCTTACCTATTAGTCACTTACTGTAGGCTGC
>HPV53_Alpha_9627377_nt7445_AOS|1
ACATATAGTATAGCTTATAGGTATCCATTTTAGTTTGGCGCCTTTTTGGTACCCTCCATT
>HPV53_Alpha_9627377_nt7121_AOS|1
TCAGCCCCCTCCTCCAAGCGCAAACGCAAATAGTATGTTGTGTGTCTGTATTGTGTATGT
>HPV53_Alpha_9627377_nt7114_AOS|1
ATCTACCTCAGCCCCCTCCTCCAAGCGCAAACGCAAATAGTATGTTGTGTGTCTGTATTG
>HPV53_Alpha_9627377_nt7106_AOS|1
TCTACTACATCTACCTCAGCCCCCTCCTCCAAGCGCAAACGCAAATAGTATGTTGTGTGT
>HPV53_Alpha_9627377_nt5495_AOS|1

ATGTAGTGTTGCCCACAGGACCCCCTACATGGCCTTATGCACCTCAATCTCCTTTTGATA
>HPV53_Alpha_9627377_nt1374_AOS|1
AGGGTCAGGAAGTGATATGGATGTGGATAGACAGGATATAATGCCACTGCAACAAATACA
>HPV53_Alpha_9627377_nt1003_AOS|1
AGGAAAGCACCGAATCTGATTTGGATGGGTTTATAGACAATAGTAATATAATATCTACAC
>HPV53_Alpha_9627377_nt996_AOS|1
ACAGACGAGGAAAGCACCGAATCTGATTTGGATGGGTTTATAGACAATAGTAATATAATA
>HPV53_Alpha_9627377_nt304_AOS|1
ATTCTGTTTGCTATTTTATAGTAAGGTCCGAAAATTAAGATATTACAATTGTTCAGTGTA
>HPV7_Alpha_9627389_nt7661_AOS|1
TATAAATCCTTCAATTTCCACCCATAACCGTTTCCAGTCTCGGTTGGCAAGTCACCATGT
>HPV7_Alpha_9627389_nt7654_AOS|1
ATAACTATATAAATCCTTCAATTTCCACCCATAACCGTTTCCAGTCTCGGTTGGCAAGTC
>HPV7_Alpha_9627389_nt5532_AOS|1
AATAATACACCAACTTCTACCTATTCCTTGTATCCAGGTAATTCTACACGCATAGCAAAT
>HPV7_Alpha_9627389_nt4044_AOS|1
TGCATGCATATACCTGATTTCTGTGTCTGGAGCTCTTTGATGGCCACTATTTCTATTCTT
>HPV7_Alpha_9627389_nt3461_AOS|1
TATACAACGACAGCCACGCACCGCCCCGAAAGCGAAGGAGAGACGGAGACTTGTCCATCA
>HPV7_Alpha_9627389_nt1393_AOS|1
AAGTGCTAGAAGAAAGCAGTGATGTTATACAGCAACTTAGTCCGCGTACACAGGTGGTAG
>HPV7_Alpha_9627389_nt1375_AOS|1
TTGAAACGGAAGCGGTTGAAGTGCTAGAAGAAAGCAGTGATGTTATACAGCAACTTAGTC
>HPV7_Alpha_9627389_nt1368_AOS|1
GCGACAGTTGAAACGGAAGCGGTTGAAGTGCTAGAAGAAAGCAGTGATGTTATACAGCAA
>HPV7_Alpha_9627389_nt1360_AOS|2
GGAGGGCTGCGACAGTTGAAACGGAAGCGGTTGAAGTGCTAGAAGAAAGCAGTGATGTTA
>HPV7_Alpha_9627389_nt1343_AOS|1
TATAGAGGGGGGCAGTGGGAGGGCTGCGACAGTTGAAACGGAAGCGGTTGAAGTGCTAGA
>HPV9_Beta_9627396_nt7206_AOS|1
GAAAACGTCCTATTAAAACATCTGTTAAAACATCTAAAAATGCTAAGAGAAGGCGAACCT
>HPV9_Beta_9627396_nt6606_AOS|1
AAAGTGATCAACAACAACGAACAATAGCTAATTCCACCTACTATCCTACAGTAAGTGGGT
>HPV9_Beta_9627396_nt6591_AOS|1
TCTTTTTGCCAGCAAAAAGTGATCAACAACAACGAACAATAGCTAATTCCACCTACTATC
>HPV9_Beta_9627396_nt4763_AOS|1
TGGGGGCCAGCTAGTAGGTGGTCCTAGGGAATCATACACAGCATCTTCTGAAAACATAGA
>HPV9_Beta_9627396_nt3918_AOS|1
TAGGGGAAGACAGTTGTGATAGAGTTGGAAGAGCGCGAATGATTTTAGCCTTTGACACAT
>HPV9_Beta_9627396_nt3831_AOS|1
ACGCCAATGTGCTTAAGTGCTATCGCTTTCGGGAACGCAAAAAAAAAAGAGGCTTAGTAA
>HPV9_Beta_9627396_nt825_AOS|1
CTTGCTACAAATTTAGGAATTCGAGCGCAACAGGAACTTTTGCTGGGTGATATACAACTG
>HPV9_Beta_9627396_nt789_AOS|1
GGCTGTGGTTGCGGTGCAAGACTTCGTTTATACGTGCTTGCTACAAATTTAGGAATTCGA
>HPV9_Beta_9627396_nt768_AOS|1
ACTCCCTACAAGATCGTAGCTGGCTGTGGTTGCGGTGCAAGACTTCGTTTATACGTGCTT
>HPV54_Alpha_9628437_nt7521_AOS|1
CAGAACAACTTCACTTTCCATTGTGTAAAAACACGCCCTTTGCATTCCAACATTGATTTA
>HPV54_Alpha_9628437_nt7511_AOS|1
TTATAACTGTCAGAACAACTTCACTTTCCATTGTGTAAAAACACGCCCTTTGCATTCCAA
>HPV54_Alpha_9628437_nt4184_AOS|1
ATAAATATTGTGTAGATATATTTGTGTATTATGGCTAAAGCAAGAGCCCCACGCCGGAAA
>HPV54_Alpha_9628437_nt3162_AOS|1
GAACACAAAGTGTATTATGTGGATTTTAAAAAGGAAGCATCTAAGTATGGGGAATATGGA

>HPV54_Alpha_9628437_nt3153_AOS|1
ATGGATGCAGAACACAAAGTGTATTATGTGGATTTTAAAAAGGAAGCATCTAAGTATGGG
>HPV54_Alpha_9628437_nt2990_AOS|1
TGGTTGCTTAAAAAGACGTGGACAAACAGTAGATGTTATATTTGATGGGCATCAGGATAA
>HPV54_Alpha_9628437_nt1334_AOS|1
GATAGCCTAGGAAGGCAGCAAATTACAGAATTAATACATAACACAAATATTCGTGTAGCA
>HPV54_Alpha_9628437_nt1315_AOS|1
ATCTGGGGAAACAACTACAGATAGCCTAGGAAGGCAGCAAATTACAGAATTAATACATAA
>HPV54_Alpha_9628437_nt201_AOS|1
TTTGCAAGAAGACGGTGTGTACAGCAGAGATTTATGCATTTCAATATAAGGACCTATTTG
>HPV24_Unclassified_9628486_nt6245_AOS|1
TGGTGATAATAATGATGCTGGTAGATGTCCACCTATTAAGTTAGTAAATTCAGTAATTCA
>HPV24_Unclassified_9628486_nt6237_AOS|2
AGGTGTGCTGGTGATAATAATGATGCTGGTAGATGTCCACCTATTAAGTTAGTAAATTCA
>HPV24_Unclassified_9628486_nt1745_AOS|1
CAAATGCTGCTACCTTTACACATGGGTCTTATCCTAAATGGATTATAGAACAAACACTGA
>HPV24_Unclassified_9628486_nt1629_AOS|1
ACAAACTGTTATAAGGTTATTAGTGTCTATGTTATATGTGGCAGAGGAGCAAATTTTATC
>HPV24_Unclassified_9628486_nt1571_AOS|1
TTACAGAAATGTCTGCAATGTTTTTATATCTGTTGTGTTTCAAGGCTGGCAAAAATAGAC
>HPV24_Unclassified_9628486_nt441_AOS|1
GATAGGTTGGGAAATAGAACAAAAAGAAAATACACCTCTTTCGGACATTATTGTAAGGTG
>HPV24_Unclassified_9628486_nt434_AOS|1
AGAGTGTGATAGGTTGGGAAATAGAACAAAAAGAAAATACACCTCTTTCGGACATTATTG
>HPV24_Unclassified_9628486_nt427_AOS|1
TTTGTAGAGAGTGTGATAGGTTGGGAAATAGAACAAAAAGAAAATACACCTCTTTCGGAC
>HPV24_Unclassified_9628486_nt419_AOS|1
AAAATTATTTGTAGAGAGTGTGATAGGTTGGGAAATAGAACAAAAAGAAAATACACCTC
>HPV24_Unclassified_9628486_nt193_AOS|1
ATCTTGATGGCTCAACCAGGTAAACCTCAGTCAGTGTTAGAACTTAGTAGATTATTAAAT
>HPV48_Gamma_9628542_nt6713_AOS|1
AGATCTACAAAGCGTAGGAGAGTAAGATAGTAAAGATGTAGAATGTTTACATTATTAATA
>HPV48_Gamma_9628542_nt5284_AOS|1
CGTGCAAGAAACGGATGTATATTTTTATACAAGCACTGAAAGACTTTTAATAGTTGGTAA
>HPV48_Gamma_9628542_nt5113_AOS|1
TTATTTGATAGTTACTCAGACTTTGCTTTAGATCCCTTTTTTATTCCACGTAAGAAACGA
>HPV48_Gamma_9628542_nt5101_AOS|1
AATTGGTTCCCTTTATTTGATAGTTACTCAGACTTTGCTTTAGATCCCTTTTTTATTCCA
>HPV48_Gamma_9628542_nt4718_AOS|1
TAACTATAGGCCCTAAAGTACATTATTATATGGATTTGTCTGCAATATCAACAGAAGCTA
>HPV48_Gamma_9628542_nt4228_AOS|1
GATTCTTATACACAAACTATCTTGCAAACACAGGTAAACCCATTTTTAAATAATGCTATT
>HPV48_Gamma_9628542_nt4217_AOS|1
AGTTGTTTTATGATTCTTATACACAAACTATCTTGCAAACACAGGTAAACCCATTTTTAA
>HPV48_Gamma_9628542_nt2990_AOS|1
GATCTAAATGGTGATTTTGTATACTTTACTTTATTTCAACCTGATGCTGTAAAATATGGA
>HPV48_Gamma_9628542_nt2327_AOS|1
TAACAACTGATGAATCTCTATTTTATTTGAGAAGTAGACTTACGTGTTTCAATTTTCCGA
>HPV48_Gamma_9628542_nt2319_AOS|1
TGTTAATGTAACAACTGATGAATCTCTATTTTATTTGAGAAGTAGACTTACGTGTTTCAA
>HPV50_Gamma_9628550_nt6292_AOS|1
AAGAAAGATGTCAATCCTTTGGATCCTTTAAATGTAGCAAGTTCTTATATGTACTCAAAG
>HPV50_Gamma_9628550_nt5145_AOS|1
TGTTTTAGATTATTCAGATTATGATTTACATCCAGGTCTTCTTCCAAAAAGAAGACGCAT
>HPV50_Gamma_9628550_nt5096_AOS|1

TAGATGCATCCTTGATAGTAAAATCAAATGTTTCTGAACAGCCCCTTTTTGTTTTAGATT
>HPV50_Gamma_9628550_nt4938_AOS|1
CGATGTTGAACATAATTTTACAGAGTCTCATTTAGTTATACCAGCTACTGATGAAGAAAA
>HPV50_Gamma_9628550_nt4929_AOS|1
ATTAACAGACGATGTTGAACATAATTTTACAGAGTCTCATTTAGTTATACCAGCTACTGA
>HPV50_Gamma_9628550_nt4274_AOS|1
TTAATCCCTTCATAAATCCAGACATAAATAATGTTAATGTATTAGTTGACCCCAGCTTTG
>HPV50_Gamma_9628550_nt4251_AOS|1
AGCAGCAACTTTTGAAACACAGATTAATCCCTTCATAAATCCAGACATAAATAATGTTAA
>HPV50_Gamma_9628550_nt4244_AOS|1
CTACTATAGCAGCAACTTTTGAAACACAGATTAATCCCTTCATAAATCCAGACATAAATA
>HPV50_Gamma_9628550_nt1337_AOS|1
GTACACAAAATTGGATTATTGTAGTTTTTGCCTGTGCAGAAGATTTAATTGAAGCTTCTA
>HPV60_Gamma_9628566_nt5555_AOS|1
AGACAGTGGTTCAAATAATATTACTGTACCTAAATGTAGTGGAAATCAGTTTAGAGTTAT
>HPV60_Gamma_9628566_nt5049_AOS|1
AAACTACAAATGAAGGCCTAATACCTGACAATATACTAGAGGATGAATTTACAGAAAACT
>HPV60_Gamma_9628566_nt5042_AOS|1
TTAACTGAAACTACAAATGAAGGCCTAATACCTGACAATATACTAGAGGATGAATTTACA
>HPV60_Gamma_9628566_nt4903_AOS|1
AACTATTGGTCAGAAAGTTCACTTTTATTTTGATATTAGTGATATTCCAGCTGCAGAAAC
>HPV60_Gamma_9628566_nt3700_AOS|2
ATACCACACAACGAGATGTTTTTATGAAATTAGTAACTTTGCCAAGAGGTTGTACATATA
>HPV60_Gamma_9628566_nt3684_AOS|1
TTAGTTGTTTTTAATGATACCACACAACGAGATGTTTTTATGAAATTAGTAACTTTGCCA
>HPV60_Gamma_9628566_nt3659_AOS|1
ACCAGAATCTTCTAGACATAAATTGTTAGTTGTTTTTAATGATACCACACAACGAGATGT
>HPV60_Gamma_9628566_nt3650_AOS|1
CATAGACGTACCAGAATCTTCTAGACATAAATTGTTAGTTGTTTTTAATGATACCACACA
>HPV61_Alpha_9628574_nt7738_AOS|1
GTGTTTGACAGGTTGCTAACACACACAGTTTGGAACAATGCTCCATCTATCTTTTTTGAC
>HPV61_Alpha_9628574_nt7533_AOS|1
TATTGTTGCACGCCTTATTTTGTGCAGCTTCCCCATTTCTGTGTTCCTTGTGTTCGGGTA
>HPV61_Alpha_9628574_nt4298_AOS|1
AATACTGTTGTACAGGCTTTTACATTAGGTGGTGTACCTTGTGTGTTTTCCCTCCCCACA
>HPV61_Alpha_9628574_nt4291_AOS|1
TTATTGCAATACTGTTGTACAGGCTTTTACATTAGGTGGTGTACCTTGTGTGTTTTCCCT
>HPV61_Alpha_9628574_nt4243_AOS|1
TGGATCAGCAAAACTGCGGATTGTTATTTTTGTTTTTGCTATTACTGCTTATTGCAATAC
>HPV61_Alpha_9628574_nt4234_AOS|1
TACTGGTTGTGGATCAGCAAAACTGCGGATTGTTATTTTTGTTTTTGCTATTACTGCTTA
>HPV61_Alpha_9628574_nt4221_AOS|1
CAGGGGTTTGCATTACTGGTTGTGGATCAGCAAAACTGCGGATTGTTATTTTTGTTTTTG
>HPV61_Alpha_9628574_nt4187_AOS|1
TTGCAATATGTATCCCCTTGTAGTTAGTTCCCCTCAGGGGTTTGCATTACTGGTTGTGGA
>HPV61_Alpha_9628574_nt3134_AOS|1
CAGAAACTGATCTGTGGTATAAGGTTCCTGGAAAAGTGTCATATAAGGGCCTATATTATG
>HPV61_Alpha_9628574_nt3127_AOS|1
CAAAGCACAGAAACTGATCTGTGGTATAAGGTTCCTGGAAAAGTGTCATATAAGGGCCTA
>HPV20_Beta_1020162_nt7671_AOS_rc|1
TCTTCTGCTGTGCCAAAAAGAAGAGCCAAAAGCGTTGGCAGCCTTACATTTCCTCCTGTT
>HPV20_Beta_1020162_nt7644_AOS_rc|1
AAAAGCGTTGGCAGCCTTACATTTCCTCCTGTTCCGAACGTGTTGGGACGATGAAGTTTG
>HPV20_Beta_1020162_nt5062_AOS_rc|1
TTCTGTTAGTAAGGCCTCCTCTACGCCTGAACACATTTTGAAGTCTTTGCATAGGTGTGC

>HPV20_Beta_1020162_nt4799_AOS_rc|1
GTGCGGCTGACTCTGACACGAGCTGGAGGTGTTGGTTCTGGTGCTACCTCTAGAACTGCA
>HPV20_Beta_1020162_nt4740_AOS_rc|1
ACTAGAACCACTGGTAGTAGATGTAACTGGTGTATCAGTTGGTGGCCTTGAGGGGCCTGG
>HPV20_Beta_1020162_nt4733_AOS_rc|1
CCACTGGTAGTAGATGTAACTGGTGTATCAGTTGGTGGCCTTGAGGGGCCTGGATGTATT
>HPV20_Beta_1020162_nt217_AOS_rc|1
TAAGATTGGAGGTTTTGCCTCTCCAATATTAGATGGTCCTTCATCAGCGCTGTCTTCTGA
>HPV20_Beta_1020162_nt179_AOS_rc|1
CTTCATCAGCGCTGTCTTCTGAAGAAGGAGGTGTAGCCATGTGCCTGCACTCTCTGCATT
>HPV20_Beta_1020162_nt170_AOS_rc|1
CGCTGTCTTCTGAAGAAGGAGGTGTAGCCATGTGCCTGCACTCTCTGCATTAGCTCTATG
>HPV20_Beta_1020162_nt155_AOS_rc|1
AAGGAGGTGTAGCCATGTGCCTGCACTCTCTGCATTAGCTCTATGATATCTGTCTATATG
>HPV21_Beta_1020170_nt7013_AOS_rc|3
AAATATTCTTGAAAGCTCTCGGCTTTATAATTTTCAATTTTAGACACGTCTGCATTCTCA
>HPV21_Beta_1020170_nt7006_AOS_rc|2
CTTGAAAGCTCTCGGCTTTATAATTTTCAATTTTAGACACGTCTGCATTCTCAGGATTTA
>HPV21_Beta_1020170_nt6999_AOS_rc|2
GCTCTCGGCTTTATAATTTTCAATTTTAGACACGTCTGCATTCTCAGGATTTACTGAAAT
>HPV21_Beta_1020170_nt5549_AOS_rc|1
TCTGAATGTGCACTAAAATCTTCAGAAAGAGGGTTTTCATCAACGTTAATATCAATAAAT
>HPV21_Beta_1020170_nt5162_AOS_rc|1
AACTGCACTAATCTAGAAGGCCTGGATACAAACAAAGGATTCTCTACATTAACCTGTTGA
>HPV21_Beta_1020170_nt282_AOS_rc|1
AATGGTATCTCTAACAGATTGGCTAGGTCACGAATTGTAGCTGGTAATGGTGGCTCTAAA
>HPV21_Beta_1020170_nt273_AOS_rc|1
TCTAACAGATTGGCTAGGTCACGAATTGTAGCTGGTAATGGTGGCTCTAAAAAGCTAGAT
>HPV21_Beta_1020170_nt266_AOS_rc|1
GATTGGCTAGGTCACGAATTGTAGCTGGTAATGGTGGCTCTAAAAAGCTAGATGTATTTT
>HPV21_Beta_1020170_nt257_AOS_rc|1
GGTCACGAATTGTAGCTGGTAATGGTGGCTCTAAAAAGCTAGATGTATTTTCTTCTTCTA
>HPV22_Beta_1020178_nt6715_AOS_rc|1
GGCGCATAAATTCTCTGATTTTTTAGCATCATAATTAACTGTGGTGCCGTCACTTGCCA
>HPV22_Beta_1020178_nt3899_AOS_rc|1
CATTTGTTGCAAGCACTTCTCTCTTTCCCTATCTGTATGAAATGATATTAGTATCCTAGA
>HPV22_Beta_1020178_nt3889_AOS_rc|1
AAGCACTTCTCTCTTTCCCTATCTGTATGAAATGATATTAGTATCCTAGAGCGCCCGATT
>HPV22_Beta_1020178_nt3877_AOS_rc|1
CTTTCCCTATCTGTATGAAATGATATTAGTATCCTAGAGCGCCCGATTCTATCGGTTGTA
>HPV22_Beta_1020178_nt845_AOS_rc|1
CACAAAAGTTTTACAGGTCCAAGTAGAGATGTTTGAAAGTCGCGAATTCCAGAGAGAGTT
>HPV22_Beta_1020178_nt837_AOS_rc|1
TTTTACAGGTCCAAGTAGAGATGTTTGAAAGTCGCGAATTCCAGAGAGAGTTGCTAGTAT
>HPV22_Beta_1020178_nt413_AOS_rc|1
CACGACCATACAAAGTAACTTGATAATACTGCTGAAATTCAAATTGGGCTGAAGCGTAGG
>HPV22_Beta_1020178_nt406_AOS_rc|1
ATACAAAGTAACTTGATAATACTGCTGAAATTCAAATTGGGCTGAAGCGTAGGCACAGCC
>HPV22_Beta_1020178_nt181_AOS_rc|1
CAGTGGTCTTTGCAAAGCCATGAATACAGAATAGCAGCCCATCCTACTTAAATAATATGC
>HPV23_Beta_1020186_nt5544_AOS_rc|1
TAAGTAATAATCAAAAGACTTGTCCAATGTGTGTATGACTACAGCAGGGGCACTAGGTAA
>HPV23_Beta_1020186_nt5536_AOS_rc|1
AATCAAAAGACTTGTCCAATGTGTGTATGACTACAGCAGGGGCACTAGGTAAGGGAAATA
>HPV23_Beta_1020186_nt5529_AOS_rc|1

AGACTTGTCCAATGTGTGTATGACTACAGCAGGGGCACTAGGTAAGGGAAATATTATAGT
>HPV23_Beta_1020186_nt5258_AOS_rc|1
GTATCCTGAATAGCCTCAACATCATCTAAGTTCATATCTATTACAGTGCTTTGCAGAGGT
>HPV23_Beta_1020186_nt5246_AOS_rc|1
GCCTCAACATCATCTAAGTTCATATCTATTACAGTGCTTTGCAGAGGTTCCTCAATAACT
>HPV23_Beta_1020186_nt3888_AOS_rc|1
TACATTTTTCCCTTTCATGATTAGAATGAAATGCTATTAACATCCTTGCACGCCCTACTC
>HPV23_Beta_1020186_nt3870_AOS_rc|1
GATTAGAATGAAATGCTATTAACATCCTTGCACGCCCTACTCTATCAGTAGAATGACCCC
>HPV23_Beta_1020186_nt3108_AOS_rc|1
GTCCTTCAACCTTTTCCCAAGTGTCCTCATCAGTTTGATAGTAAATATAAGACCATACTG
>HPV23_Beta_1020186_nt1400_AOS_rc|2
TATGTAGCAACACTGCTTTAGCATTTTTACATCTTAACAAACTTTCAATATGTCCCAGCC
>HPV23_Beta_1020186_nt209_AOS_rc|1
TCAGTGGTCGCGTCGAGTCCATCAATAATTTGGTGTAGCACATCCTACTTAAATAATGCA
>HPV28_Alpha_1020202_nt7201_AOS_rc|1
AAACACAGTAGTGCTACTTTTTGGCGCGTTTTCGTTTTGCAGCAGACGATCCTCTCGTGG
>HPV28_Alpha_1020202_nt4240_AOS_rc|2
TAGAAAAGGCAGTGGCTCAATAGCACACTGGGATATACACAGTACTTAAAAATACATTGT
>HPV28_Alpha_1020202_nt4222_AOS_rc|1
AATAGCACACTGGGATATACACAGTACTTAAAAATACATTGTGGTGACCAACTAACACAC
>HPV28_Alpha_1020202_nt4101_AOS_rc|1
AAATATAAACAACAACAATAGATACACGTCATCTGGCTCCAGAAACAGGACAGTATGATC
>HPV28_Alpha_1020202_nt4050_AOS_rc|1
AGTATGATCGCCATTGTTGTCCCTTAGCACCAAAGGATACATAGCAAGGCTTATGTATTG
>HPV28_Alpha_1020202_nt3090_AOS_rc|1
CATCACTATAGTTCTGAGTAAATATGTCTCTCCAGTGTACATAACACATGGATTTGGTTT
>HPV28_Alpha_1020202_nt1472_AOS_rc|1
AATCTAGGCAAACGGTTTTGCTACTTTTGAAGTGTCGGATAAGTTCATTAAATCCCAACC
>HPV28_Alpha_1020202_nt68_AOS_rc|1
TATGTTTTTAGGCCGTTGGTCATCCATTGAGCTTCTGCCATTCAATGTAAGCCTCTTTTT
>HPV29_Alpha_1020210_nt5616_AOS_rc|1
TTGTGCAGAGGAGGGCAGAATAATATCAGGGCCCGTGTGTAAAGGCACATCAACTGGCGA
>HPV29_Alpha_1020210_nt6608_AOS_rc|1
GAGTTTCTCGCCCGTTGTTACCCTTTAAGTACAAGGAATCTGGGATTTTGTCCCCTACTA
>HPV29_Alpha_1020210_nt6601_AOS_rc|1
TCGCCCGTTGTTACCCTTTAAGTACAAGGAATCTGGGATTTTGTCCCCTACTACACCAGC
>HPV29_Alpha_1020210_nt6594_AOS_rc|1
TTGTTACCCTTTAAGTACAAGGAATCTGGGATTTTGTCCCCTACTACACCAGCACGATTA
>HPV29_Alpha_1020210_nt5609_AOS_rc|1
GAGGAGGGCAGAATAATATCAGGGCCCGTGTGTAAAGGCACATCAACTGGCGACACGAAT
>HPV29_Alpha_1020210_nt5602_AOS_rc|1
GCAGAATAATATCAGGGCCCGTGTGTAAAGGCACATCAACTGGCGACACGAATGGAATAG
>HPV29_Alpha_1020210_nt4180_AOS_rc|2
ATAATCCCAAATGTCTATAAACTATAAATAACAACAGGCATACCATTAGTATTAACAATC
>HPV29_Alpha_1020210_nt4031_AOS_rc|1
GTGTTTCGTGCAATGTGCTGCACAACTTGTAAATATAGTGCCACCAATCCTAGGTATAAA
>HPV29_Alpha_1020210_nt2580_AOS_rc|1
TAAACTGGCTCTCCTTTACTTGTTAATGGACATGGATTGCTGAATGTGAATACCTGCAGT
>HPV29_Alpha_1020210_nt2090_AOS_rc|1
AGTCCCCCTCTCCCTGTACTTTGTTGCTTCTAAACCGTATCCACTCAGACATGGACATTC
>HPV36_Beta_1020218_nt7389_AOS_rc|1
CGGTCATATTACTGTTTTCGCTTGCGCTTGGTTCCTCTGGTGAACCCTCGATAAGACACT
>HPV36_Beta_1020218_nt6951_AOS_rc|1
TCTGCAGTGTAATCATTGATGTCCTTTAGTGCCCCATTATTGTTATATATTGAAATACTA

>HPV36_Beta_1020218_nt6695_AOS_rc|2
CAGGAATGAAAAACTGATTCTTAAAAGTCCCATTATCAATTCTGGCGTCAGGGATGTCGT
>HPV36_Beta_1020218_nt5518_AOS_rc|1
CAGAAAAGGCTTCCACACTTTCAGACAAAGGATTTTCAGACACATTTACGTCTATAAATG
>HPV36_Beta_1020218_nt3618_AOS_rc|1
CCCCTTCTAGAGGTGCTTCTGGATCGTGATCTGGTTGATACCCGCTGGACCCCAGTCTTG
>HPV36_Beta_1020218_nt3611_AOS_rc|1
TAGAGGTGCTTCTGGATCGTGATCTGGTTGATACCCGCTGGACCCCAGTCTTGGCGAGCG
>HPV36_Beta_1020218_nt260_AOS_rc|2
AAGGTATTAGACAGTCTACAAACGGAATGCCTAATGATTCTGACAGGCCCTTAATAGTTA
>HPV36_Beta_1020218_nt253_AOS_rc|1
TAGACAGTCTACAAACGGAATGCCTAATGATTCTGACAGGCCCTTAATAGTTAAAGGCAG
>HPV36_Beta_1020218_nt183_AOS_rc|1
TCTTTTTCTGTAATATTCTGCTGTTCGGAGGCTTGCTCTGCCATTTGCCTTAATTATTAA
>HPV37_Beta_1020226_nt6568_AOS_rc|1
CAGTGGGAAAATAGGTAGAATTGCCTAACAGATACTGCTGCTGGTCTGATTTGGCAGGTA
>HPV37_Beta_1020226_nt6538_AOS_rc|2
GATACTGCTGCTGGTCTGATTTGGCAGGTAAGTAATATTTGTGGTCCTGATTAACAGTGC
>HPV37_Beta_1020226_nt6312_AOS_rc|2
TGATTTATTAGTAGAGAGAACCTTATTGTTAATATTTCCGAACCCTATATCAAACATGTC
>HPV37_Beta_1020226_nt6084_AOS_rc|2
ATCATCCCGTGACCCCCCTTGGTAATTACTATTATTCTCAGTGTCCCTTAATTTATTAAA
>HPV37_Beta_1020226_nt5579_AOS_rc|1
GCCTCTCCAAATCTAATAACTACAGTTGGTGTTTCAGGTTGTGGCAAGATAACTTGTGTT
>HPV37_Beta_1020226_nt5337_AOS_rc|1
ATTAAAATCTGTGTCATCTGCCATAGTAGACTCTTGTTGCCCTATATTTAAGGGACCTGG
>HPV37_Beta_1020226_nt4929_AOS_rc|1
AAACTGTACTAATTGTGAGGGTCTACTCAAGAATAAAGGATCCGTGACTGCTACCTGTTC
>HPV37_Beta_1020226_nt2743_AOS_rc|1
TAATGAAACAGGATCTGCTCCTGCCTTAGAAGTTGCCAATGCATAATTTGGGTCTCTAGG
>HPV37_Beta_1020226_nt2736_AOS_rc|1
ACAGGATCTGCTCCTGCCTTAGAAGTTGCCAATGCATAATTTGGGTCTCTAGGTCATCTC
>HPV37_Beta_1020226_nt415_AOS_rc|1
CTTTTGCTCTATCTCTTCTATCTCCCTCCCACTAACTGAGTGCTCATAGAAACTGGTAAA
>HPV38_Beta_1020234_nt7129_AOS_rc|2
TGGGTCCTTTTGCGTTTTACAGATTTGGAAGATTTTCTTACTAACGGCCGTTTGACAGCA
>HPV38_Beta_1020234_nt7118_AOS_rc|1
GCGTTTTACAGATTTGGAAGATTTTCTTACTAACGGCCGTTTGACAGCACGTGTTCGTGC
>HPV38_Beta_1020234_nt5426_AOS_rc|1
TATCCTGATAATATATGGTAGGATTTTGAGGAGTCTGAAATCTAGGTACAGTGATTGACG
>HPV38_Beta_1020234_nt5413_AOS_rc|1
TATGGTAGGATTTTGAGGAGTCTGAAATCTAGGTACAGTGATTGACGTAGTGGATCTGCG
>HPV38_Beta_1020234_nt5269_AOS_rc|1
ATCCATAGAAGTTTCTGTAAGAACACCTTCAGGAACCTCAGTCACATTTACATCCACTAA
>HPV38_Beta_1020234_nt5262_AOS_rc|1
GAAGTTTCTGTAAGAACACCTTCAGGAACCTCAGTCACATTTACATCCACTAAAGTGCTT
>HPV38_Beta_1020234_nt4509_AOS_rc|1
TCAGCCACTTCCAATATAGCATTGGTATTGCGGCCTCCACTCACAACAGGGGTATCAATT
>HPV38_Beta_1020234_nt4492_AOS_rc|1
AGCATTGGTATTGCGGCCTCCACTCACAACAGGGGTATCAATTTCTATAGGGTCTATAGG
>HPV38_Beta_1020234_nt3858_AOS_rc|1
TTTGTGATTCTGAATAAAAAGCTAGAAGCATTCGTGAGCGCCCTATGCGATCATTTGATG
>HPV38_Beta_1020234_nt185_AOS_rc|1
ACTTATCACTGAGCTGCTGCACAGTTTGAGGTTTTGGTAGTTCCATGAATACATCCCAAG
>HPV44_Alpha_1020242_nt6716_AOS_rc|2

CATGTATTGCTTATATTGTTCACTAGTATATGTAGACGGAGGGGACTGTGTAGTGGCAGC
>HPV55_Alpha_1020266_nt6494_AOS_rc|1
GTATATGGCATTAGGAACTGTACTTTTAGTAGCACCTTTAAAAACCAAATCCTGTGGAAT
>HPV55_Alpha_1020266_nt6483_AOS_rc|3
TAGGAACTGTACTTTTAGTAGCACCTTTAAAAACCAAATCCTGTGGAATGTCCTCTCCAA
>HPV55_Alpha_1020266_nt6474_AOS_rc|1
TACTTTTAGTAGCACCTTTAAAAACCAAATCCTGTGGAATGTCCTCTCCAACCGTTCCCG
>HPV66_Alpha_1020290_nt7066_AOS_rc|2
AGGAAGAGGTAGGAGCCGCCCGCCTTTTAGAGGCAGATACACTAGCCTTGGGTCTAGGGG
>HPV66_Alpha_1020290_nt7410_AOS_rc|1
AAATGGCGTACAGCACTAAAATGGAGTTTGGTGTGTTACTATAGTGCATACAAACAACCT
>HPV66_Alpha_1020290_nt7059_AOS_rc|1
GGTAGGAGCCGCCCGCCTTTTAGAGGCAGATACACTAGCCTTGGGTCTAGGGGGTCTAGG
>HPV66_Alpha_1020290_nt7052_AOS_rc|1
GCCGCCCGCCTTTTAGAGGCAGATACACTAGCCTTGGGTCTAGGGGGTCTAGGGCCTAGT
>HPV66_Alpha_1020290_nt6681_AOS_rc|2
CACATGGCGAAGGTATTGATTGATTTCACGGGCATCATATTTAGTTAATGTGCTTTTAGC
>HPV66_Alpha_1020290_nt6179_AOS_rc|1
TCACCGTCCTCTATCGGGGTATTAACTAATGCAAGAGGTGGACAATCCCCTGTATTACCT
>HPV66_Alpha_1020290_nt6171_AOS_rc|2
CTCTATCGGGGTATTAACTAATGCAAGAGGTGGACAATCCCCTGTATTACCTGGTGTAGA
>HPV66_Alpha_1020290_nt249_AOS_rc|1
AACATACCCTACATACTGCATATGGCCAATTGTTTCTATATACTAGTTTTAACTCAATAC
>HPV103_Unclassified_109390382_nt6816_AOS_rc|1
CAGATAACTAATTCGGTTGCTGGAAGGCTGAGTAATCTTGTTGTTGAAACTTGCAAAGTA
>HPV103_Unclassified_109390382_nt6476_AOS_rc|1
AACAAAAATATAAAGTGCATGTGCAACACAGGTAATGCCATAGATTAAACCCCCCGTCTA
>HPV103_Unclassified_109390382_nt6190_AOS_rc|1
CTAAAGATGTTAAAAATCTGTATGTATCTCCAATAGAAGAAGGAGGCGGTGGTACAAACC
>HPV103_Unclassified_109390382_nt5349_AOS_rc|3
CTGAGTTTGCTTTGGGTCCATAGAGACACTTTGTCTGTTTTCCTTTCCTGTTTTAACATA
>HPV103_Unclassified_109390382_nt5338_AOS_rc|1
TTGGGTCCATAGAGACACTTTGTCTGTTTTCCTTTCCTGTTTTAACATAACTATTAGGAT
>HPV103_Unclassified_109390382_nt4028_AOS_rc|1
AAATGTGGCAGACCCCTGTGTTTCCACAAATGTTTCTAATGGTATAGATTCCCCAATATT
>HPV103_Unclassified_109390382_nt4002_AOS_rc|1
CAAATGTTTCTAATGGTATAGATTCCCCAATATTGACAACAGATCCTTCTGTTACAGCAC
>HPV103_Unclassified_109390382_nt3992_AOS_rc|1
TAATGGTATAGATTCCCCAATATTGACAACAGATCCTTCTGTTACAGCACCCCCTATAAA
>HPV103_Unclassified_109390382_nt3346_AOS_rc|1
ATATTTTCCACAGTATCCCGTTTTTGTCGCTTTTTCGGTTGTACATCCATGTAAGCTATT
>HPV103_Unclassified_109390382_nt3254_AOS_rc|1
TGTAAGGGTTAGGATAATATACAGGGGACCATTGGCAGCATTATTGGCTTAAAATGAATC
>HPV101_Unclassified_109390389_nt6831_AOS_rc|1
CAAAACACATGAGTAATACATTTGTTAAAACATGAACATACCTGCTGGCAAAATTGGCAG
>HPV101_Unclassified_109390389_nt6652_AOS_rc|1
AAATACTGCCCTGAGTCAGAAGAAATAACTCATACAACGACAATGTTGACATGCTACATA
>HPV101_Unclassified_109390389_nt4092_AOS_rc|1
GAAGACCCTCCTGTTTCAACAAATGTGTCTAAAGGTATAGATTCACCTATACTAATAACC
>HPV101_Unclassified_109390389_nt3337_AOS_rc|1
AAGAAGTATAATATACAAGGTACACAAAATCACAAGAGGCAGATGTGATGCTATTTACAA
>HPV101_Unclassified_109390389_nt3330_AOS_rc|1
ATAATATACAAGGTACACAAAATCACAAGAGGCAGATGTGATGCTATTTACAAATAGGCA
>HPV101_Unclassified_109390389_nt3225_AOS_rc|1
AAAACTGTGTACCCTTTGGTAACTTCACAGTATGTAAAAACCTTTCCCGTTGGCTATTAT

>HPV101_Unclassified_109390389_nt3218_AOS_rc|1
TGTACCCTTTGGTAACTTCACAGTATGTAAAAACCTTTCCCGTTGGCTATTATTTCTAAA
>HPV101_Unclassified_109390389_nt2669_AOS_rc|1
GGTAACAGAATTAGATATTATTTCACTTTCAGTTCGTACTTGCCATTCATTTGCTGCATC
>HPV101_Unclassified_109390389_nt980_AOS_rc|2
GATTTATAATTTGCAGGTATTCACAATGTGGTTGCAGTAATGTTTTTGCTGCCTCCAGTA
>HPV101_Unclassified_109390389_nt903_AOS_rc|1
AATATCCCCGCTATCCAACTGCAGCAACAGCTTTTATTACTTATGAATGGTCTTGTTAAT
>HPV70_Alpha_1173493_nt7811_AOS_rc|1
GTTGCACACAGGTCTGTAAAAATATTAGTCACAAAATATAACTTGCACCAAACGACACAT
>HPV70_Alpha_1173493_nt5310_AOS_rc|1
ATTTTGTAGACACTGTAGAAGGTATAGAAGGAAATGAAAGATGGGACCTAGGTCCTGTAG
>HPV70_Alpha_1173493_nt5272_AOS_rc|1
AGATGGGACCTAGGTCCTGTAGAACCTGTATGAGAAGTAGTATGTAACATTGCATTATCT
>HPV70_Alpha_1173493_nt1486_AOS_rc|1
CTTTTTGGTTATTAGCCTGTAATACTGTTTTTAGCTGTGCAGTAGGTGACTGTGGATCTT
>HPV70_Alpha_1173493_nt1479_AOS_rc|1
GTTATTAGCCTGTAATACTGTTTTTAGCTGTGCAGTAGGTGACTGTGGATCTTGATTCTC
>HPV70_Alpha_1173493_nt1471_AOS_rc|1
CCTGTAATACTGTTTTTAGCTGTGCAGTAGGTGACTGTGGATCTTGATTCTCACTATCAA
>HPV70_Alpha_1173493_nt1267_AOS_rc|1
CTGGTACTGAATACACGTTTGTTCTTGCCTGTTTATTGCATATGTTTACCGGGAGCTCTT
>HPV70_Alpha_1173493_nt1260_AOS_rc|2
TGAATACACGTTTGTTCTTGCCTGTTTATTGCATATGTTTACCGGGAGCTCTTGTAGTGT
>HPV70_Alpha_1173493_nt1247_AOS_rc|1
GTTCTTGCCTGTTTATTGCATATGTTTACCGGGAGCTCTTGTAGTGTTACCCTTTGTTCC
>HPV70_Alpha_1173493_nt1237_AOS_rc|1
GTTTATTGCATATGTTTACCGGGAGCTCTTGTAGTGTTACCCTTTGTTCCCTATGTACGC
>HPV71_Alpha_12084981_nt7864_AOS_rc|1
CCAAAAACGTACTTAGGCGAACATAGTCAGCACAACTATATGCCCCAAACAATGGGTATT
>HPV71_Alpha_12084981_nt7842_AOS_rc|1
ATAGTCAGCACAACTATATGCCCCAAACAATGGGTATTGCACGGTGAGTAACACATGTAT
>HPV71_Alpha_12084981_nt6548_AOS_rc|1
TAAAATGTCTTAAAACATTGCTCTCGGCGCAAGAAAAACATGCGATCACCAATGGCTCTG
>HPV71_Alpha_12084981_nt5507_AOS_rc|1
TAGTAGGTGTAGGCCTGCTGGACACAGAAGTAGGTTGAATGACAGTATCATTGGAATAAT
>HPV71_Alpha_12084981_nt4272_AOS_rc|1
GGTACCTACTGCATCAACCGTAACAAAAACAGAAGCAACATAAGACACACCAACACTAAA
>HPV71_Alpha_12084981_nt4035_AOS_rc|1
TAAGGGACGCTGCTGCCTGTATATATAGTGCTAGAAACCCAAGAAACACAGTAACAAAAA
>HPV71_Alpha_12084981_nt4028_AOS_rc|1
CGCTGCTGCCTGTATATATAGTGCTAGAAACCCAAGAAACACAGTAACAAAAAAAATTAA
>HPV71_Alpha_12084981_nt3186_AOS_rc|1
CCCATATATTACTGCTTCCTTGGAGAATAATACACAGTCTACACGTATCCCATCGTGTAA
>HPV107_Beta_126131393_nt7432_AOS_rc|1
GTGTTTCCTCGAGATCCAGCCAAGTCTTTGTCTTGTTTGGCACCGACAGCGATATATAAT
>HPV107_Beta_126131393_nt6610_AOS_rc|1
AAGGACTTTGGGATTGTCCACTCTTAGGTGCTATATAGTATTTGTGATCTTGATTAGTTG
>HPV107_Beta_126131393_nt5546_AOS_rc|1
GCACATATAAAGTAGTTTCCCTTGGAGAACTAAATCTGGGTACAGTGACAGAAGTGGTAG
>HPV107_Beta_126131393_nt5407_AOS_rc|1
AAATTCAGAACTTTCAAGCAATTCTGGAGTATCCCTTACATTAATATCCACCAGTGTACT
>HPV107_Beta_126131393_nt3976_AOS_rc|1
ACTATGTTCCCCTATCCAGGACCAGGTACTACTAAAATACTTATAGTTTCCTTTGTGCTT
>HPV107_Beta_126131393_nt3969_AOS_rc|1

TCCCCTATCCAGGACCAGGTACTACTAAAATACTTATAGTTTCCTTTGTGCTTTTTGCGT
>HPV107_Beta_126131393_nt901_AOS_rc|1
TCTTGAAGCAAAAGATCCTGCAATGCCCGAATGCCAAAATGGGTTGCAAAAACGTAGAGA
>HPV107_Beta_126131393_nt506_AOS_rc|1
ATACTTTGCTGAGTGATCTCCTCAATCTGCTTACCTACAACCTGAAATTCAAAATGATTT
>HPV107_Beta_126131393_nt295_AOS_rc|1
AGTCTTCCACTGGTATATTAAGATGCTGTGTAAGCTGTACGATTGTCCTTGGCCTGTCCA
>HPV107_Beta_126131393_nt288_AOS_rc|1
CACTGGTATATTAAGATGCTGTGTAAGCTGTACGATTGTCCTTGGCCTGTCCATGAGCAT
>HPV84_Alpha_12958167_nt7808_AOS_rc|1
ACTGTATCATTGTTGTAAAGTATAAGAAAATAGTATGAATAACATAGCAACCGAAACGGG
>HPV84_Alpha_12958167_nt4166_AOS_rc|1
AAAAGCACTTTAAAACTGCATAACACGGTGGCACAGCCAAAGTAAAATAATAACAATTAA
>HPV84_Alpha_12958167_nt4151_AOS_rc|1
CTGCATAACACGGTGGCACAGCCAAAGTAAAATAATAACAATTAAAAGCAAAATAATAGT
>HPV84_Alpha_12958167_nt3767_AOS_rc|1
TTTGCCAGGTATGTAGGACATACACAATTGTTACAAATGGCTCAGTAATACAGCATTTAC
>HPV84_Alpha_12958167_nt3715_AOS_rc|1
GCATTTACACTATACATATACAATGGGTACTGTTTACATAACCATAGACATACTACCAAG
>HPV84_Alpha_12958167_nt3707_AOS_rc|1
ACTATACATATACAATGGGTACTGTTTACATAACCATAGACATACTACCAAGTGTGGCTA
>HPV84_Alpha_12958167_nt3700_AOS_rc|2
ATATACAATGGGTACTGTTTACATAACCATAGACATACTACCAAGTGTGGCTACAATGCC
>HPV84_Alpha_12958167_nt3622_AOS_rc|1
TGCCAAGAGCTGTTTGCGTTGGTCCGTACTTTTATACCATAGTGTTACATATGATGTTTT
>HPV84_Alpha_12958167_nt2433_AOS_rc|1
ATCCAGTGGGCACTTGTTTAAAAAGGGAAAAATAACCATTCGGCTGCGTAAGTATTTAAA
>HPV84_Alpha_12958167_nt2426_AOS_rc|1
GGGCACTTGTTTAAAAAGGGAAAAATAACCATTCGGCTGCGTAAGTATTTAAATGTGTCA
>HPVcand87_Alpha_14475578_nt5486_AOS_rc|1
ATTGTCATAATGGGAAGAGGATAGTATGGACGTGGCCGACACCTTTGTAGATGCTGTAGG
>HPVcand87_Alpha_14475578_nt6572_AOS_rc|2
TAACTGCTTAAAGAGGCCCTAGGGCCAGACGACGCCCCCTTCAAAAAATAGGTGTCAGGG
>HPVcand87_Alpha_14475578_nt3861_AOS_rc|1
GTATATGTCAGGTATGCAAAATGTATTATACAGCTAGGTTACATACAGGTACACCAATGT
>HPVcand87_Alpha_14475578_nt3854_AOS_rc|1
TCAGGTATGCAAAATGTATTATACAGCTAGGTTACATACAGGTACACCAATGTATACAGT
>HPVcand87_Alpha_14475578_nt3845_AOS_rc|1
CAAAATGTATTATACAGCTAGGTTACATACAGGTACACCAATGTATACAGTATTATGCAC
>HPVcand87_Alpha_14475578_nt2525_AOS_rc|1
TGTGTAGAAACGGAAACACCACCATTCTGCTCCGTAAATACTTTAAACTGTCATCTGCCT
>HPV72_Alpha_1491683_nt7927_AOS_rc|1
ACTATAAAACCATGAGACATAGGGGAGCGACCGGAGTAGAATGTTTTAACACGTTTGTAA
>HPV72_Alpha_1491683_nt7920_AOS_rc|2
AACCATGAGACATAGGGGAGCGACCGGAGTAGAATGTTTTAACACGTTTGTAAAGGGCTT
>HPV72_Alpha_1491683_nt7261_AOS_rc|1
AACAGTATGACATGTTACTTTTTGCGCTTTTTACGTTTAGTAGGGGCGGGGGTCGAGGTA
>HPV72_Alpha_1491683_nt7254_AOS_rc|1
TGACATGTTACTTTTTGCGCTTTTTACGTTTAGTAGGGGCGGGGGTCGAGGTAGAGCTTG
>HPV72_Alpha_1491683_nt6830_AOS_rc|1
GTGGCGAAGATACTCACGAAAATTAGAAGCTGTATATTCTGATACAGAGGACGCTGTGGC
>HPV72_Alpha_1491683_nt5628_AOS_rc|1
TGCGTAGAGGGTACAAGAGGCATTGTAGGTACAGGGGCAGGAGCAAAGGACAAATCAATG
>HPV72_Alpha_1491683_nt5497_AOS_rc|1
AACAGCAGACGCTGCAGACAGGGACGGGCTGTGCAATGATGTAGAAGACACAGAAGCACG

>HPV72_Alpha_1491683_nt5398_AOS_rc|1
ATATGTTATGTCTGGCTGCGTAGAGGAGGCCAAGGATTGCATTTCAATAGTATCAGATGA
>HPV72_Alpha_1491683_nt4200_AOS_rc|2
CAAGAAGAACCACAATCCACAATTATCGTCTCGTAGTACAATTAATGCACCCCCATCCGT
>HPV72_Alpha_1491683_nt128_AOS_rc|1
GCAGGTAATCCGTAAATCTTCTAGGTCCACCTCAATTTCCTTGCACAGCAACCAAATATT
>HPV73_Alpha_1491692_nt7206_AOS_rc|1
GCGACTATACAAAATGGAAGCACAGTAGTCAAGTAAACCACCCACACAACACACATATAA
>HPV73_Alpha_1491692_nt6277_AOS_rc|1
CAGTATTGCCTGTGCCTTTAATCATTAGGTCATCTGGGATTTTATCACCGGTATCACCAG
>HPV73_Alpha_1491692_nt4315_AOS_rc|1
GTAACAACTGATGGTCGTATAGGTTGTAATGGAATTTCAACTGGTTTAGATGGTGTGCCT
>HPV73_Alpha_1491692_nt4308_AOS_rc|1
CTGATGGTCGTATAGGTTGTAATGGAATTTCAACTGGTTTAGATGGTGTGCCTGTAGATA
>HPV73_Alpha_1491692_nt4300_AOS_rc|2
CGTATAGGTTGTAATGGAATTTCAACTGGTTTAGATGGTGTGCCTGTAGATAATGGAACG
>HPV73_Alpha_1491692_nt1319_AOS_rc|1
GACCCACTACTGCTTTCACGTGGCGACACAATTTGGTTGCCTTCTTCATTAACATTTTGT
>HPV73_Alpha_1491692_nt1311_AOS_rc|1
ACTGCTTTCACGTGGCGACACAATTTGGTTGCCTTCTTCATTAACATTTTGTAAACACCC
>HPV73_Alpha_1491692_nt1304_AOS_rc|1
TCACGTGGCGACACAATTTGGTTGCCTTCTTCATTAACATTTTGTAAACACCCTACCCCT
>HPV73_Alpha_1491692_nt1295_AOS_rc|1
GACACAATTTGGTTGCCTTCTTCATTAACATTTTGTAAACACCCTACCCCTGCCCCAAGT
>HPV73_Alpha_1491692_nt734_AOS_rc|1
AGCAACTCTTCTATCACTCTTAAATCAGCTTTGTTGCTTTCAATGGCAAGGCATACTGTG
>HPVcand86_Alpha_15741127_nt7836_AOS_rc|1
TTAACTACATGATTGTTGTTAAGTATAAAGAAGGAACTATGAATAATACAGCGACCGAGT
>HPVcand86_Alpha_15741127_nt6700_AOS_rc|2
CTTAGATATTCATTAAACTTAGAGGGGTCATATTCAGAGGCCTTCTGGGTAGCGGCACTA
>HPVcand86_Alpha_15741127_nt5377_AOS_rc|1
ATATGTGGAAGATACCATAGATGTGGCGGACACTGTAGCAGAGGATACAGGCGTGGTGGA
>HPVcand86_Alpha_15741127_nt5368_AOS_rc|1
AGATACCATAGATGTGGCGGACACTGTAGCAGAGGATACAGGCGTGGTGGAATGCAATGA
>HPVcand86_Alpha_15741127_nt5344_AOS_rc|1
TGTAGCAGAGGATACAGGCGTGGTGGAATGCAATGAGGGAACGGAAGGCTCCTGTAATAT
>HPVcand86_Alpha_15741127_nt5337_AOS_rc|1
GAGGATACAGGCGTGGTGGAATGCAATGAGGGAACGGAAGGCTCCTGTAATATAGAGGAT
>HPVcand86_Alpha_15741127_nt5329_AOS_rc|1
AGGCGTGGTGGAATGCAATGAGGGAACGGAAGGCTCCTGTAATATAGAGGATAGGTGTGT
>HPVcand86_Alpha_15741127_nt5322_AOS_rc|1
GTGGAATGCAATGAGGGAACGGAAGGCTCCTGTAATATAGAGGATAGGTGTGTATCATCT
>HPVcand86_Alpha_15741127_nt1560_AOS_rc|2
CATACGCTTTTCTGGAATATTTAGTATGGTTCCTAGGCACTTTGCTACGGTTTCTCTGCT
>HPV88_Gamma_167600365_nt6130_AOS_rc|1
TGAGATCCCATTTTAAAATTCACAGCATTTGTAGGTGGCAATAAGTATTTATTTCCCTCT
>HPV88_Gamma_167600365_nt5089_AOS_rc|1
AGGATATGAAACTATAATATCTGCATAATCATCTACAAAAGCTTTTATTGCTGCGCCAGG
>HPV88_Gamma_167600365_nt5024_AOS_rc|1
GAGTAGGAACAGATATAAGTTCATCCTCTTCCTCAAAATGCACAAATAAGTGACTATTAC
>HPV88_Gamma_167600365_nt4847_AOS_rc|1
TAAACTCCCCTAAATTAGAAAGCTCAATAACATCAGGCTCTATAGGTTCAATTGCACTAA
>HPV88_Gamma_167600365_nt4540_AOS_rc|2
TGAAGGCCGGGTTAAAAAGTCAACATTGTCTGTTCTGATTTGTTGAATGTAACGATTATA
>HPV88_Gamma_167600365_nt4533_AOS_rc|1

CGGGTTAAAAAGTCAACATTGTCTGTTCTGATTTGTTGAATGTAACGATTATATAAGCTT
>HPV88_Gamma_167600365_nt4526_AOS_rc|1
AAAAGTCAACATTGTCTGTTCTGATTTGTTGAATGTAACGATTATATAAGCTTCTGGCCC
>HPV88_Gamma_167600365_nt3715_AOS_rc|1
CAAATTCTCAGCAGTGTCACGTTTACTTCTGGTTTTATACATAGTTACAATGAATCAAGG
>HPV88_Gamma_167600365_nt3683_AOS_rc|1
TTTTATACATAGTTACAATGAATCAAGGTAACCAAATGCGTAGGTTGTACCCTTTGGCAA
>HPV88_Gamma_167600365_nt2902_AOS_rc|1
TAATAAATATGTTCCCATTGTGTGTAGGGAAAGCTGTTTTCTCGATCATTATCAAAGTAC
>HPV110_Beta_186461194_nt5522_AOS_rc|1
TGTATGACCACAGTTGGAGTATCAGGTAGTGGGAGTATAATTTCAGTGTTGTCTCTAGAT
>HPV110_Beta_186461194_nt5286_AOS_rc|1
ATCTGGTTCTTCTATTTCCATTGATTCAAACTGCCCAACATTTAAAGGATCTGGCTCGTC
>HPV110_Beta_186461194_nt5279_AOS_rc|1
TCTTCTATTTCCATTGATTCAAACTGCCCAACATTTAAAGGATCTGGCTCGTCTATATTA
>HPV110_Beta_186461194_nt760_AOS_rc|2
AAAATTTCTTGCTGAGTACGAATTCCAAAAACTGTTGCAAGCACAAAAAGCCGAAGTCGC
>HPV110_Beta_186461194_nt495_AOS_rc|1
GCCTCTTACCCTATGAAATTGCTGATGCTTACAACAAACTTCTAGCTTCTCTATTAGATC
>HPV110_Beta_186461194_nt413_AOS_rc|1
CAACGCACAACCACTTCCCCAATTCCTTTTTGCTCTACTATTTCTATTTCACGTCCATTT
>HPV110_Beta_186461194_nt406_AOS_rc|1
CAACCACTTCCCCAATTCCTTTTTGCTCTACTATTTCTATTTCACGTCCATTTACAGAAT
>HPV110_Beta_186461194_nt399_AOS_rc|1
TTCCCCAATTCCTTTTTGCTCTACTATTTCTATTTCACGTCCATTTACAGAATGTTCATA
>HPV110_Beta_186461194_nt389_AOS_rc|1
CCTTTTTGCTCTACTATTTCTATTTCACGTCCATTTACAGAATGTTCATAAAACCTTGTA
>HPV110_Beta_186461194_nt198_AOS_rc|1
AAATCTACACGGTATTAATATGTCTAAAACAGGAATGCATAATGTATCTGCAAGCTGTTG
>HPV111_Beta_186461202_nt5484_AOS_rc|1
AGAAGTATCTGATATGTGAATCACAACTGTGGGAGCTTCTGGATGAGGAAAAATAATATT
>HPV111_Beta_186461202_nt5258_AOS_rc|1
AATAACAAATCTTCTGAATTAAAATCTATTTCATCTTCTAATGGAGGGGTTTCCGGTCTG
>HPV111_Beta_186461202_nt2691_AOS_rc|1
TTTCGAGCAAAGTACAATAAAACTTGCTCTTGTCTCAATGCTTGCCAATGTTCAATCTGA
>HPV111_Beta_186461202_nt1951_AOS_rc|1
AATAATTGTTTGTGAATCCAAGTTGACATACTCATCTCTCGCATCTCCCCTCGAATATAA
>HPV111_Beta_186461202_nt1589_AOS_rc|1
TTTAGGAGGATCTGCCAATACCTGCACTTCTGATACTTGTAATATTGTGCTTAATAATCG
>HPV111_Beta_186461202_nt487_AOS_rc|1
TTCCAATTATCCCTAACTCTATGAAAATATTGTTGTTTGTAGCACACTCCTAGCTTCTCT
>HPV111_Beta_186461202_nt480_AOS_rc|2
TATCCCTAACTCTATGAAAATATTGTTGTTTGTAGCACACTCCTAGCTTCTCTACCAGAT
>HPV111_Beta_186461202_nt399_AOS_rc|1
GACACCTAACAGTAATATTTCCAATAGGCTGGTTTGCTACAGTTTCTATCTGTTTTCCTA
>HPVFA75/KI88-03_Unclassified_206902429_nt7261_AOS_rc|1
AGACAGACTGCCAAGTCTTTGTAAGGTGAAGTACCGGCTGCGATTTATAAGCAGATTATA
>HPVFA75/KI88-03_Unclassified_206902429_nt5141_AOS_rc|1
TTCTCCTAAAAGTTGTAATTCTATGGGCTCCTCAGTGTTAATTGTACTAAGGTCCCTGTA
>HPVFA75/KI88-03_Unclassified_206902429_nt3791_AOS_rc|1
TGTTCTGTGATCCATGACCAAGTACTACTGACATATTTGTAGCCACCCTTATGCCTTTTA
>HPVFA75/KI88-03_Unclassified_206902429_nt3784_AOS_rc|1
TGATCCATGACCAAGTACTACTGACATATTTGTAGCCACCCTTATGCCTTTTATGCACCC
>HPVFA75/KI88-03_Unclassified_206902429_nt1776_AOS_rc|1
GTATCATGTCAGATAAAGAAAATTGCGTGGCCTCCAAAGATTGATGGCTAATCATAGTTT

>HPVFA75/KI88-03_Unclassified_206902429_nt1644_AOS_rc|1
ATGCCGCTATTACACTACGCCACTTAGGTGGTTCAGACAATGTTTGAATTTCATTTACTT
>HPVFA75/KI88-03_Unclassified_206902429_nt1626_AOS_rc|1
GCCACTTAGGTGGTTCAGACAATGTTTGAATTTCATTTACTTGCAAAATAGAAGATAGTA
>HPVFA75/KI88-03_Unclassified_206902429_nt129_AOS_rc|1
GAACTTGTACTGTTCTAGGCCTCTCCATCAGTATATGTGTCTCTAATATTCTTCAAGTAA
>HPVFA75/KI88-03_Unclassified_206902429_nt122_AOS_rc|1
TACTGTTCTAGGCCTCTCCATCAGTATATGTGTCTCTAATATTCTTCAAGTAATTTTATA
>HPV91_Alpha_22023568_nt7701_AOS_rc|1
TCAATGTAACCTTGCAATTATGCTGACACAATACCTACGTTGCCAAACACGACCGAAATC
>HPV91_Alpha_22023568_nt7596_AOS_rc|1
ATGACTACCACCAGACCTAAACCCCAACGCTGCCCCAACGCTGGACATTCATAACACAAA
>HPV91_Alpha_22023568_nt7586_AOS_rc|1
CCAGACCTAAACCCCAACGCTGCCCCAACGCTGGACATTCATAACACAAAACAGGAAAGA
>HPV91_Alpha_22023568_nt7579_AOS_rc|1
TAAACCCCAACGCTGCCCCAACGCTGGACATTCATAACACAAAACAGGAAAGAAACACAT
>HPV91_Alpha_22023568_nt5992_AOS_rc|2
GGAACAATTACTTTACCAGAATTATTTTTATAGGAAAAAAAGGGTGGCCCACAGCCAGT
>HPV91_Alpha_22023568_nt3950_AOS_rc|1
GAGTGTTACAACACCCTTAACGTCACAGTAACAGTTCCACTATACAAGCAATATACAAGG
>HPV91_Alpha_22023568_nt3605_AOS_rc|1
ACCACTGTGCCCTCCTTGGTTGTGGTTCGAATGCTTAGGGATGATTCGTGGGTCTCTACT
>HPV91_Alpha_22023568_nt3595_AOS_rc|1
CCTCCTTGGTTGTGGTTCGAATGCTTAGGGATGATTCGTGGGTCTCTACTGTCCAATGGC
>HPV91_Alpha_22023568_nt3588_AOS_rc|1
GGTTGTGGTTCGAATGCTTAGGGATGATTCGTGGGTCTCTACTGTCCAATGGCTCTCCTC
>HPV91_Alpha_22023568_nt3581_AOS_rc|1
GTTCGAATGCTTAGGGATGATTCGTGGGTCTCTACTGTCCAATGGCTCTCCTCCAACAGT
>HPVcand89_Alpha_22095322_nt7658_AOS_rc|1
GTGGAACATGTAGCCGCCTACGCAGGAAGTATAAAAGATTACAAGTCTTTGTGTAAAGTG
>HPVcand89_Alpha_22095322_nt7642_AOS_rc|1
CCTACGCAGGAAGTATAAAAGATTACAAGTCTTTGTGTAAAGTGGCATGGCGCCATAACA
>HPVcand89_Alpha_22095322_nt7555_AOS_rc|1
GGTCGCTGAACTCAAAATGGACGCTGGGTGGGAAAGGAAATGGCCGAATACATATCTCAT
>HPVcand89_Alpha_22095322_nt7547_AOS_rc|1
AACTCAAAATGGACGCTGGGTGGGAAAGGAAATGGCCGAATACATATCTCATAGGGCGCA
>HPVcand89_Alpha_22095322_nt6560_AOS_rc|3
GTAAGTGTATTTCTGTTATTAGCAGCAGACCCAGGCATATACAAGGTTTTTGGCAGGTCC
>HPVcand89_Alpha_22095322_nt5480_AOS_rc|2
TTGTGTATTGAGACGATACAGCAGATGTAGCAGTAACGGTCGGGCCAGAAAAGGTGGTAT
>HPVcand89_Alpha_22095322_nt4329_AOS_rc|1
ACCGAAGCCCGTTTACGACGCCGCACAGGGTGGGTCATTGCAACAATGTATTTATTGTAT
>HPVcand89_Alpha_22095322_nt307_AOS_rc|1
ATCGCTCGTTAAAATACACCATATAGTCCTTTTCCTGGCTGCCTAACGGCTTACAGCATA
>HPVcand89_Alpha_22095322_nt62_AOS_rc|1
AGCACTTCTGATGTTGTAAGCTCCTTGGTGCAATATATGCATGTAAGCCGTAGATCCTCT
>HPVcand89_Alpha_22095322_nt55_AOS_rc|1
CTGATGTTGTAAGCTCCTTGGTGCAATATATGCATGTAAGCCGTAGATCCTCTAAATCCA
>HPV90_Alpha_22138122_nt7261_AOS_rc|1
CACATAACATGCTTAGGAACGTTTGGACCGTTTACGTTTTGTAGAGGGGGCGGTAGAGGG
>HPV90_Alpha_22138122_nt7254_AOS_rc|1
CATGCTTAGGAACGTTTGGACCGTTTACGTTTTGTAGAGGGGGCGGTAGAGGGTGCGGGA
>HPV90_Alpha_22138122_nt6603_AOS_rc|1
CTTGTAGGAGTGCCACGAGAGGTAGAAGATGTTCCTTTTAGCACAAAGCCATCGGGAATT
>HPV90_Alpha_22138122_nt6596_AOS_rc|1

GAGTGCCACGAGAGGTAGAAGATGTTCCTTTTAGCACAAAGCCATCGGGAATTGCGTCGC
>HPV90_Alpha_22138122_nt5543_AOS_rc|1
CCTGTAGATAGAGGCACAGTGGTATTGGATGCTGCAGATGATAGGGCAGACGATGCAGTC
>HPV90_Alpha_22138122_nt3920_AOS_rc|1
GACTGCAACGCCTGTATGTATATGATCAGGAGGCCTAGAAACACAGTAAGGAATACAATA
>HPV90_Alpha_22138122_nt3679_AOS_rc|1
ATAGTCATGTACCCCAGTACTGCTTCAATGTCTTTAGGTAAGGTTATGGTTGATAGGAAG
>HPV90_Alpha_22138122_nt3671_AOS_rc|1
GTACCCCAGTACTGCTTCAATGTCTTTAGGTAAGGTTATGGTTGATAGGAAGGTTGCCCT
>HPV90_Alpha_22138122_nt3664_AOS_rc|1
AGTACTGCTTCAATGTCTTTAGGTAAGGTTATGGTTGATAGGAAGGTTGCCCTTTGTTCC
>HPV90_Alpha_22138122_nt1277_AOS_rc|1
GTAATGTGGCCCTTAGGTTACTTGTTTTAAGCAGAGACAGGATAGCATCTGTACCTGATT
>HPV58_Alpha_222386_nt7059_AOS_rc|2
GTTTGGTGGATGGTGCACGGGTAGTAGGGGCCGAACGTTTTAGTCTGGGCTTTGCTTTAA
>HPV58_Alpha_222386_nt5506_AOS_rc|1
ATCCACAATTATGGTATTAAAAGGAGTTAGTGGAGATATAGGAATAAATGGACTAGACAT
>HPV58_Alpha_222386_nt5486_AOS_rc|1
AAGGAGTTAGTGGAGATATAGGAATAAATGGACTAGACATAGATGTTACAGAAGATGCAA
>HPV58_Alpha_222386_nt5449_AOS_rc|1
CATAGATGTTACAGAAGATGCAATGTCTGGACCAGGTTCCAATGACACAAGAGGAGTGTC
>HPV58_Alpha_222386_nt5351_AOS_rc|1
GTGTGGTGGCAAAGGACGTATGTGAGTGCAGAGGACTCTGAAAATCATGTATAGTATCAG
>HPV58_Alpha_222386_nt5219_AOS_rc|1
ATTCAAATTGTTGCTGCTGTTGTACCTGTTCCTGGACAGGCTGTATGGGACTTAAGTCTT
>HPV58_Alpha_222386_nt5210_AOS_rc|1
GTTGCTGCTGTTGTACCTGTTCCTGGACAGGCTGTATGGGACTTAAGTCTTGGTAGTAAT
>HPV58_Alpha_222386_nt5203_AOS_rc|1
CTGTTGTACCTGTTCCTGGACAGGCTGTATGGGACTTAAGTCTTGGTAGTAATGTACTTT
>HPV58_Alpha_222386_nt3460_AOS_rc|2
TCGTGGTCTACTGTCCACGGCGCAGTCTGTATACTTTGTGGAGTACTGGGTGTTGTCTCT
>HPV58_Alpha_222386_nt3444_AOS_rc|1
ACGGCGCAGTCTGTATACTTTGTGGAGTACTGGGTGTTGTCTCTGGAGTCTGGTAAATCG
>HPV74_Alpha_27462483_nt7051_AOS_rc|1
AACAAAAGCATGCTATCGTTTCCTAGAGCGTTTCTGTTTAGTGGTTGAGGAGGGGGCTGT
>HPV74_Alpha_27462483_nt7044_AOS_rc|1
GCATGCTATCGTTTCCTAGAGCGTTTCTGTTTAGTGGTTGAGGAGGGGGCTGTAGGGGCC
>HPV74_Alpha_27462483_nt7037_AOS_rc|2
ATCGTTTCCTAGAGCGTTTCTGTTTAGTGGTTGAGGAGGGGGCTGTAGGGGCCGAACGCT
>HPV74_Alpha_27462483_nt7030_AOS_rc|1
CCTAGAGCGTTTCTGTTTAGTGGTTGAGGAGGGGGCTGTAGGGGCCGAACGCTTTTTACC
>HPV74_Alpha_27462483_nt4705_AOS_rc|1
TAGAGGAGGAAATCAGCACATGACCACCAGCCTCTACGGGGGGTTGAGACTGTATAACAG
>HPV74_Alpha_27462483_nt4698_AOS_rc|1
GGAAATCAGCACATGACCACCAGCCTCTACGGGGGGTTGAGACTGTATAACAGAAGGGTC
>HPV74_Alpha_27462483_nt4683_AOS_rc|1
ACCACCAGCCTCTACGGGGGGTTGAGACTGTATAACAGAAGGGTCAGTAAACGAGGGATT
>HPV74_Alpha_27462483_nt4142_AOS_rc|1
CCATACACAGCATGTATAAAAACACTTATTTACTGCATACAGAAGGCGTTACACCCTTGA
>HPV74_Alpha_27462483_nt2989_AOS_rc|1
CCTATAGTCAACTTGTCCTTCCACCTTTACCCATTCGTCGCCGTCCAGTATATATATAAA
>HPV92_Beta_27531786_nt5260_AOS_rc|2
TCTAGGTGTACATTTTCCTCTAAATTAGGTAACTCTTCCAAATCAATATTTACGAACGTA
>HPV92_Beta_27531786_nt5154_AOS_rc|1
CAAGAACTTGCAGTTCAATAGAAGGTTGAGTATCAATACTGCTAATATCCCTAAAAAAAT

>HPV92_Beta_27531786_nt3875_AOS_rc|1
AAAACATTTGTCTTTGAGATTTACTGGTAAAGCTAATAATCATTCTAGAGCGTCCTATTC
>HPV92_Beta_27531786_nt3868_AOS_rc|1
TTGTCTTTGAGATTTACTGGTAAAGCTAATAATCATTCTAGAGCGTCCTATTCTATCGTT
>HPV92_Beta_27531786_nt3041_AOS_rc|1
TAAATCTGCGTGCGTCTTGAGCAAATCTGATATAGTATTGTTTATTTAATCCATCCATAA
>HPV92_Beta_27531786_nt3033_AOS_rc|1
CGTGCGTCTTGAGCAAATCTGATATAGTATTGTTTATTTAATCCATCCATAAAATAGGCA
>HPV92_Beta_27531786_nt354_AOS_rc|1
TAAACAAAACTTACATCTAACAGTTAAACAACACAAAAGACTACCTGAGTCCCTTTCTAT
>HPV92_Beta_27531786_nt308_AOS_rc|1
GAGTCCCTTTCTATAGCATCCTTTCCTTGTACACTATACTCATAAAAATGTTTTGTTTCT
>HPV75_Beta_2911544_nt3900_AOS_rc|2
TAACCAATCCCTTTCTGTTTTTCTTGTCTCTGTACCTAAAGCATTTTAGTATGTTTGGAT
>HPV75_Beta_2911544_nt7200_AOS_rc|3
ACACTGGCTGTTCTGGTTACCTTGGAAGCCCTTCGTAAGCCTGCCTGAAAAAGGAATTTG
>HPV75_Beta_2911544_nt4776_AOS_rc|1
GCTTGCCCACCAGAGCCATTGGTGACCACGATTTGGTCTGATAATGCAGACTCCCCCAAT
>HPV75_Beta_2911544_nt3889_AOS_rc|1
TTTCTGTTTTTCTTGTCTCTGTACCTAAAGCATTTTAGTATGTTTGGATCGCCTCTTAAA
>HPV75_Beta_2911544_nt471_AOS_rc|1
AGTTTTTCAAATATATCCAAACGTTTCAGACAAAACTGGCACCTTACAATAAGATTATGT
>HPV75_Beta_2911544_nt464_AOS_rc|1
CAAATATATCCAAACGTTTCAGACAAAACTGGCACCTTACAATAAGATTATGTATACTTT
>HPV75_Beta_2911544_nt457_AOS_rc|1
ATCCAAACGTTTCAGACAAAACTGGCACCTTACAATAAGATTATGTATACTTTCTGCTGC
>HPV76_Beta_2911551_nt4801_AOS_rc|1
TGGCCACCTGAGCCACTGGTAACTACAATTTGGTCTGACAATGCGGATTCCCCTAGTGTT
>HPV76_Beta_2911551_nt3933_AOS_rc|1
AACATTTTACTAATCCCTTCTTATTTTTTTATCTCTGTATCTAAAGCATTTCAACACAT
>HPV76_Beta_2911551_nt471_AOS_rc|1
AGTTTTTCAAATAAATCCAGGCGTTTCAAACAAAATTGACACCTTATTATAATGTTCGCT
>HPV76_Beta_2911551_nt464_AOS_rc|1
CAAATAAATCCAGGCGTTTCAAACAAAATTGACACCTTATTATAATGTTCGCTATATTTT
>HPV76_Beta_2911551_nt457_AOS_rc|1
ATCCAGGCGTTTCAAACAAAATTGACACCTTATTATAATGTTCGCTATATTTTCTGCAGC
>HPV77_Alpha_2911558_nt6600_AOS_rc|2
TATATAGCACTGCCGGGAGTCTCACGCCCGCTACTCCCTTTGAGGTACAAAGATTCTGGA
>HPV77_Alpha_2911558_nt6593_AOS_rc|2
CACTGCCGGGAGTCTCACGCCCGCTACTCCCTTTGAGGTACAAAGATTCTGGAATTTTGT
>HPV77_Alpha_2911558_nt5590_AOS_rc|1
GTGCAGAAGAAGGCAAAACAATGTCGGGGCCAGTGTGTAAATGCACATCCACCGGAGATA
>HPV77_Alpha_2911558_nt5583_AOS_rc|1
AGAAGGCAAAACAATGTCGGGGCCAGTGTGTAAATGCACATCCACCGGAGATATAAATGG
>HPV77_Alpha_2911558_nt5489_AOS_rc|1
GAAGGGAGGGAGGACGATGCACCGCGACCCCCAAAAGAGGTAAGATTGCGACCACTGTTC
>HPV77_Alpha_2911558_nt5482_AOS_rc|1
GGGAGGACGATGCACCGCGACCCCCAAAAGAGGTAAGATTGCGACCACTGTTCGTAAAGG
>HPV77_Alpha_2911558_nt5474_AOS_rc|1
GATGCACCGCGACCCCCAAAAGAGGTAAGATTGCGACCACTGTTCGTAAAGGCCACGTCA
>HPV77_Alpha_2911558_nt346_AOS_rc|1
CATCTGATTTGCTGGGTATCTATGGATTCTTTTGTTTCTGCCTCCACGCCGTCCACGTAG
>HPV77_Alpha_2911558_nt327_AOS_rc|1
CTATGGATTCTTTTGTTTCTGCCTCCACGCCGTCCACGTAGTATGAATACTCCCAGTGTT
>HPV80_Beta_2911565_nt3917_AOS_rc|1

GTTAATAAAAAGCTGTCTCTCAGTGTTTGAAGTAAATGCAAGTAACATCCGTGAACGTCC
>HPV80_Beta_2911565_nt5460_AOS_rc|2
ATTTCTAGGAGACTCAAATCGAGGAACTGTGTATGTAGTAGTACTACGCCTTGCATTACC
>HPV80_Beta_2911565_nt5453_AOS_rc|1
GGAGACTCAAATCGAGGAACTGTGTATGTAGTAGTACTACGCCTTGCATTACCAAATACT
>HPV80_Beta_2911565_nt3927_AOS_rc|1
ATTTCATTATGTTAATAAAAAGCTGTCTCTCAGTGTTTGAAGTAAATGCAAGTAACATCC
>HPV80_Beta_2911565_nt1628_AOS_rc|1
GCGGTTCAGCTATTATTTGTACTTCTGATACTTGCAAAAGATTAGTTAGCAATCTTAATA
>HPV80_Beta_2911565_nt1489_AOS_rc|1
ACAATGCTGAATTAAAAGTTGTTTGGAACTTTCTAATAGTTCGTCTTGTACAGCATACAC
>HPV80_Beta_2911565_nt1482_AOS_rc|1
TGAATTAAAAGTTGTTTGGAACTTTCTAATAGTTCGTCTTGTACAGCATACACAGTTAAT
>HPV80_Beta_2911565_nt448_AOS_rc|1
AAATTTGCAACGAATAGTAATGTCTCCAACAGCTTTTCGCTCAACTACCTCAATCTCCCA
>HPV80_Beta_2911565_nt440_AOS_rc|2
AACGAATAGTAATGTCTCCAACAGCTTTTCGCTCAACTACCTCAATCTCCCAACCACACA
>HPV80_Beta_2911565_nt311_AOS_rc|1
AGACAAAGTCTTCTTCAGTCCAAATCAACTGCAAACATTTGCGATCAAAACTTACTAACT
>HPVRTRX7_Unclassified_30315616_nt6990_AOS_rc|3
CTAAACTTTTGTGAGTCATATGATTGTATTTGCTGTATATCTTGATTTTCACTAGATATA
>HPVRTRX7_Unclassified_30315616_nt5521_AOS_rc|1
GATTTTCTGCTGTATCCATGTCTATGAACGTACTTTCCACAGTACCTTGTATGATGGTCG
>HPVRTRX7_Unclassified_30315616_nt5122_AOS_rc|1
CTACTTGTACTTGCTGAACTAACCTTCTATTTGTTAGAGAAGGCCCCCTACGCCTGCCAA
>HPVRTRX7_Unclassified_30315616_nt5110_AOS_rc|1
GCTGAACTAACCTTCTATTTGTTAGAGAAGGCCCCCTACGCCTGCCAATGGATAGTGCTC
>HPVRTRX7_Unclassified_30315616_nt5095_AOS_rc|1
TATTTGTTAGAGAAGGCCCCCTACGCCTGCCAATGGATAGTGCTCTTTGAAGTGGTGTGC
>HPVRTRX7_Unclassified_30315616_nt5087_AOS_rc|1
AGAGAAGGCCCCCTACGCCTGCCAATGGATAGTGCTCTTTGAAGTGGTGTGCTACTGCGC
>HPVRTRX7_Unclassified_30315616_nt3798_AOS_rc|1
ATCTACCCCGTCGCTTTCCTGTTCCCCTACCGCACCCCCTACAGCTCCTACTTCCTCTTT
>HPVRTRX7_Unclassified_30315616_nt3625_AOS_rc|1
CGACCTGCCTTCGGCTGGAGGTACTTCTGGGATTGTGATCTGGATTCTGACCTCCGACTC
>HPVRTRX7_Unclassified_30315616_nt3618_AOS_rc|1
CCTTCGGCTGGAGGTACTTCTGGGATTGTGATCTGGATTCTGACCTCCGACTCCTAGTCT
>HPVRTRX7_Unclassified_30315616_nt3610_AOS_rc|1
TGGAGGTACTTCTGGGATTGTGATCTGGATTCTGACCTCCGACTCCTAGTCTTGGAGACC
>HPV65_Gamma_312100_nt6175_AOS_rc|2
GAGTGCCTATATATATATGTGGACCTAAAGTATTCTGATCCTGACCTTCTTGAGCAGGAA
>HPV65_Gamma_312100_nt6168_AOS_rc|1
TATATATATATGTGGACCTAAAGTATTCTGATCCTGACCTTCTTGAGCAGGAATCCAGTA
>HPV65_Gamma_312100_nt5090_AOS_rc|1
ATAAATCGGAGCCTACATCATCAACATAAATTCTCAAGCCTAAACCAGGAGGAATAGTGG
>HPV65_Gamma_312100_nt5079_AOS_rc|1
CCTACATCATCAACATAAATTCTCAAGCCTAAACCAGGAGGAATAGTGGGGATACTGAAT
>HPV65_Gamma_312100_nt5072_AOS_rc|1
CATCAACATAAATTCTCAAGCCTAAACCAGGAGGAATAGTGGGGATACTGAATGAAGATC
>HPV65_Gamma_312100_nt5038_AOS_rc|1
AATAGTGGGGATACTGAATGAAGATCCACGTCTACTGCTAGTTAAAACCAAGTGAGAGTT
>HPV65_Gamma_312100_nt5031_AOS_rc|1
GGGATACTGAATGAAGATCCACGTCTACTGCTAGTTAAAACCAAGTGAGAGTTGCTAAAA
>HPV65_Gamma_312100_nt3680_AOS_rc|2
AAAGAACCATAGGTATATGTACAGAGTTTTGGGAATAGATTGTGTTTAACAAATGAGTCT

>HPV65_Gamma_312100_nt350_AOS_rc|1
ACAACATATACATCTGATGCAAATCTCCTTAATTTTCCTTTGAGCTACTTCTTCCAAATT
>HPV67_Alpha_3228267_nt7475_AOS_rc|1
CAAGTAAAGGCATTGCACTGCAGGAAGACAAAGATTAACAATGTTGGCAAGAAACCTAGA
>HPV67_Alpha_3228267_nt7466_AOS_rc|1
GCATTGCACTGCAGGAAGACAAAGATTAACAATGTTGGCAAGAAACCTAGAATACCAAAA
>HPV67_Alpha_3228267_nt7459_AOS_rc|1
ACTGCAGGAAGACAAAGATTAACAATGTTGGCAAGAAACCTAGAATACCAAAACCCTATG
>HPV67_Alpha_3228267_nt5406_AOS_rc|1
ACAATATCGGGACCTGGCTGTAATGGTACAGTAGAGGAAAATGGAAAATCAAAACCACTA
>HPV67_Alpha_3228267_nt5381_AOS_rc|1
GTACAGTAGAGGAAAATGGAAAATCAAAACCACTACTTAAGGGGACAGTAACATTGGTGG
>HPV67_Alpha_3228267_nt5370_AOS_rc|1
GAAAATGGAAAATCAAAACCACTACTTAAGGGGACAGTAACATTGGTGGTCTGAGGACTA
>HPV67_Alpha_3228267_nt5363_AOS_rc|1
GAAAATCAAAACCACTACTTAAGGGGACAGTAACATTGGTGGTCTGAGGACTATGTAATG
>HPV67_Alpha_3228267_nt5356_AOS_rc|1
AAAACCACTACTTAAGGGGACAGTAACATTGGTGGTCTGAGGACTATGTAATGAATAGGA
>HPV67_Alpha_3228267_nt5349_AOS_rc|1
CTACTTAAGGGGACAGTAACATTGGTGGTCTGAGGACTATGTAATGAATAGGATATTGAT
>HPV67_Alpha_3228267_nt5316_AOS_rc|1
GGACTATGTAATGAATAGGATATTGATGGCTGTGGAAAGGGTGTGTCAGGATCCATATAT
>HPV11_Alpha_333026_nt4950_AOS_rc|1
AACAAAAGTGTCTAGTGGAATGTCTTCTACATGTTGGGATGTTATTGTTGGGGCAGATAT
>HPV11_Alpha_333026_nt4942_AOS_rc|1
TGTCTAGTGGAATGTCTTCTACATGTTGGGATGTTATTGTTGGGGCAGATATAAGTATGT
>HPV11_Alpha_333026_nt4934_AOS_rc|1
GGAATGTCTTCTACATGTTGGGATGTTATTGTTGGGGCAGATATAAGTATGTGACCACTG
>HPV11_Alpha_333026_nt4852_AOS_rc|1
GTATTACAGACGGTTCTGTAAACAGGGGATTTTGAAACACACTAGTGGTAGTGTGATTGG
>HPV11_Alpha_333026_nt4343_AOS_rc|1
CGTACACCACACACTGTATAAAAAAGGTTTACTCTAGTTCATGTACACATAATCTCCATT
>HPV11_Alpha_333026_nt4258_AOS_rc|1
TCCACAGTAGTATTGCGGTTTGATTTACACTTAGCACATTTAGTTTTTCAGTACCATGT
>HPV11_Alpha_333026_nt3126_AOS_rc|1
TTGGCATCTACGGAACTAGTTACTTTTACCCATGAGTCGTTGTCCTGCAGGTATATATGT
>HPV11_Alpha_333026_nt446_AOS_rc|1
ACCCTTCCACTGGTTATTTAGTTTTATGAAGCGTGCCTTTCCCAATATGTGCTTTAGTTT
>HPV11_Alpha_333026_nt439_AOS_rc|1
CACTGGTTATTTAGTTTTATGAAGCGTGCCTTTCCCAATATGTGCTTTAGTTTTTCTATT
>HPV11_Alpha_333026_nt425_AOS_rc|1
TTTTATGAAGCGTGCCTTTCCCAATATGTGCTTTAGTTTTTCTATTTCACACAACGGCTT
>HPV31_Alpha_333048_nt7740_AOS_rc|2
GGCAGTTTAAACATGTGCAAAAGCCAGCACTGCAATCAAGGATATAGATAATCTACAAAA
>HPV31_Alpha_333048_nt7529_AOS_rc|1
AAACTACAAGCCAGAATGTTGGCAAAAACTAGTATTCAGGAAACCAAAAACCGCAGAACT
>HPV31_Alpha_333048_nt7421_AOS_rc|1
ACGGTTAGGGATTTAAAATGGCTGCATAAAATCAAAATGGAGTAGCTTTAGTTGCAAAAA
>HPV31_Alpha_333048_nt7414_AOS_rc|1
GGGATTTAAAATGGCTGCATAAAATCAAAATGGAGTAGCTTTAGTTGCAAAAAACGGACC
>HPV31_Alpha_333048_nt7407_AOS_rc|1
AAAATGGCTGCATAAAATCAAAATGGAGTAGCTTTAGTTGCAAAAAACGGACCGGGTGTA
>HPV31_Alpha_333048_nt7400_AOS_rc|1
CTGCATAAAATCAAAATGGAGTAGCTTTAGTTGCAAAAAACGGACCGGGTGTACAACTTT
>HPV31_Alpha_333048_nt7330_AOS_rc|1

TGACACCTAAATTATAGGCAGAAATAAGTACATGACTATTGGGAGGAGCAGGAACAAGTA
>HPV31_Alpha_333048_nt7322_AOS_rc|1
AAATTATAGGCAGAAATAAGTACATGACTATTGGGAGGAGCAGGAACAAGTAGGAACAAG
>HPV31_Alpha_333048_nt7309_AOS_rc|1
AAATAAGTACATGACTATTGGGAGGAGCAGGAACAAGTAGGAACAAGTAGGAACAATAGT
>HPV31_Alpha_333048_nt7299_AOS_rc|1
ATGACTATTGGGAGGAGCAGGAACAAGTAGGAACAAGTAGGAACAATAGTTTATAAAATA
>HPV33_Alpha_333049_nt5467_AOS_rc|1
AGTCAGCACCGTCTACAACAATGGTGTCAAAAGGAAAAAAAGGCGAAATAGGAACAAATG
>HPV33_Alpha_333049_nt5459_AOS_rc|1
CCGTCTACAACAATGGTGTCAAAAGGAAAAAAAGGCGAAATAGGAACAAATGGGCTAGAT
>HPV33_Alpha_333049_nt3904_AOS_rc|1
CAATACCAACACCAGCAACCAAGCATAGGTAGAAATGGAAAGTATTAAAGGACGTAATAA
>HPV33_Alpha_333049_nt3891_AOS_rc|1
AGCAACCAAGCATAGGTAGAAATGGAAAGTATTAAAGGACGTAATAATAAGGATAAGCAT
>HPV33_Alpha_333049_nt1391_AOS_rc|1
TAGAACATTACTAATTTCCTGCAACGTAACATTTTCACAGCTATCTACATTTGTCTCACA
>HPV33_Alpha_333049_nt1384_AOS_rc|1
TTACTAATTTCCTGCAACGTAACATTTTCACAGCTATCTACATTTGTCTCACAGCTTACT
>HPV33_Alpha_333049_nt1377_AOS_rc|1
TTTCCTGCAACGTAACATTTTCACAGCTATCTACATTTGTCTCACAGCTTACTTCTGAAT
>HPV33_Alpha_333049_nt1370_AOS_rc|1
CAACGTAACATTTTCACAGCTATCTACATTTGTCTCACAGCTTACTTCTGAATCATCCCC
>HPV33_Alpha_333049_nt170_AOS_rc|1
TCAGATCGTTGCAAAGGTTTTTTGCATTCCACGCACTGTAGTTCAATGTTGTGTATAGTT
>HPV33_Alpha_333049_nt160_AOS_rc|2
GCAAAGGTTTTTTGCATTCCACGCACTGTAGTTCAATGTTGTGTATAGTTGTCTCCAATG
>HPV35_Alpha_333050_nt7790_AOS_rc|1
TTGTTACTAACTATAATAAAAAGAAATGCTTATAAAACTGTAATCCAAATCGGTTGGACA
>HPV35_Alpha_333050_nt7782_AOS_rc|1
AACTATAATAAAAAGAAATGCTTATAAAACTGTAATCCAAATCGGTTGGACACACCTGTG
>HPV35_Alpha_333050_nt7544_AOS_rc|1
CAAGTGTGGGTGGACCACAAGTATGAAAAATGACTAATACTGCAAGATGAATCAAGTTTA
>HPV35_Alpha_333050_nt5390_AOS_rc|1
AGTTAGTAATAGTATTAGAGTTAAATACAATGTCTGGCCCTGCTGTTATAGGAATATCAT
>HPV35_Alpha_333050_nt5379_AOS_rc|1
GTATTAGAGTTAAATACAATGTCTGGCCCTGCTGTTATAGGAATATCATAGCCACTACTT
>HPV35_Alpha_333050_nt5362_AOS_rc|1
AATGTCTGGCCCTGCTGTTATAGGAATATCATAGCCACTACTTAATGGTATAGTAGTATT
>HPV35_Alpha_333050_nt5168_AOS_rc|1
TATGTGGTAAAGAGGATGGTACATGTTGTAAGGGTTGTAATTCTATATCTTCAGTAATAC
>HPV35_Alpha_333050_nt3942_AOS_rc|1
CATCCATAGGTATATACAAAAGCAAGAAACAACAAAAGCAAGTAGTGGTGTTGCTACAGT
>HPV47_Beta_333062_nt7658_AOS_rc|1
TCTTGTAGTGCCAAAACAGCTGCCCGCGGTGCCAAATGTGATCGCCAAAAACGATAACAA
>HPV47_Beta_333062_nt7644_AOS_rc|1
AACAGCTGCCCGCGGTGCCAAATGTGATCGCCAAAAACGATAACAAGATTAAATTTCTTT
>HPV47_Beta_333062_nt6964_AOS_rc|1
AAAATTGTCTGCATTATAATCCTGTATATCCTTTATGTCCCCTGCCTGAGAGTAAACAGA
>HPV47_Beta_333062_nt6954_AOS_rc|1
GCATTATAATCCTGTATATCCTTTATGTCCCCTGCCTGAGAGTAAACAGAGATGCTGAAA
>HPV47_Beta_333062_nt6944_AOS_rc|1
CCTGTATATCCTTTATGTCCCCTGCCTGAGAGTAAACAGAGATGCTGAAATTTGTATTTC
>HPV47_Beta_333062_nt5758_AOS_rc|1
GGTGTGAATGACAACTACAGGTAATTCAGGTGTAGGGTAAATAATATCAATAGTGTCCCG

>HPV47_Beta_333062_nt5540_AOS_rc|2
GTAAATCATTAGATGAAGCATCTATTGTTTCAGATAAAGGGTTTTCAGCAATGTCCATAT
>HPV47_Beta_333062_nt4972_AOS_rc|1
TCTGCTTGGAAACTCAGTAAGTTCAATTTCGTCTGTTATATCACCGCCTATCCTTTGTCC
>HPV47_Beta_333062_nt2934_AOS_rc|1
CTCCAAAGCAAACGCTGATTTTTGTAGCGACTCTAACTGCAACACCATATATATAGCCTC
>HPV47_Beta_333062_nt2925_AOS_rc|1
AAACGCTGATTTTTGTAGCGACTCTAACTGCAACACCATATATATAGCCTCTTTGGCCCT
>HPV8_Beta_333074_nt6929_AOS_rc|1
CTCAGATATTCTCTGAACTGGGTTGATTTATAGTCTGTGATGTTCTTAAGTTCCCCATTT
>HPV8_Beta_333074_nt6913_AOS_rc|2
ACTGGGTTGATTTATAGTCTGTGATGTTCTTAAGTTCCCCATTTTCAGTGTAAACTGAAA
>HPV8_Beta_333074_nt5718_AOS_rc|1
CACTGTTATCATGTGTGTGGATTATTACCACCGGAAGATCAGGTGTAGGGTAAATTATTT
>HPV8_Beta_333074_nt5711_AOS_rc|1
ATCATGTGTGTGGATTATTACCACCGGAAGATCAGGTGTAGGGTAAATTATTTCCTCATT
>HPV8_Beta_333074_nt5701_AOS_rc|1
TGGATTATTACCACCGGAAGATCAGGTGTAGGGTAAATTATTTCCTCATTATTGCGACTT
>HPV8_Beta_333074_nt4842_AOS_rc|1
CTAAAATGTGGTCTGCTAGAGAACTTTCTCCTTGTGTAGGTGTGGAATCAGTTATGATTT
>HPV8_Beta_333074_nt514_AOS_rc|1
AATCTAATTTCTCTATGATATCCAAAAATGACAAGCAGTTTTGACACCTAACGTCTATCT
>HPV8_Beta_333074_nt350_AOS_rc|1
CAACACGCAGTAACAACGTAATTTTTCCAAATTAGGCACAGTCTCTTTTTGTCAAACTCA
>HPV8_Beta_333074_nt336_AOS_rc|1
AACGTAATTTTTCCAAATTAGGCACAGTCTCTTTTTGTCAAACTCACACAGTTCTAAGAA
>HPV8_Beta_333074_nt149_AOS_rc|1
TCCTGCCCGTCCATTTGCTTAGGAAAATTGCACAAGTCACAGCATTGTAACAATATATAT
>HPV51_Alpha_333087_nt6553_AOS_rc|1
TAATATATTGCTTAAAGTTACTTGGAGTAAATGTTGGGGAAACCGCAGCAGTGGCAGTGC
>HPV51_Alpha_333087_nt7439_AOS_rc|1
CGGATGTATGCACTATGTCATAAAAAAATGATGGTTTATGCCAAAAACAACTGGCAGTTG
>HPV51_Alpha_333087_nt7397_AOS_rc|1
AAAAACAACTGGCAGTTGTGGTTTAAAACTTGGCAAATATACTAAAAGTACACGAACCGA
>HPV51_Alpha_333087_nt7333_AOS_rc|1
GGTTGCACTCAAAATGGCTGCAGCTGTAACAAAATGGAAGCTACAAAGTATAACTAAATA
>HPV51_Alpha_333087_nt7316_AOS_rc|1
CTGCAGCTGTAACAAAATGGAAGCTACAAAGTATAACTAAATAGCACAAATATAAACTGT
>HPV51_Alpha_333087_nt5906_AOS_rc|1
TCAACAGATGTGTTATCTCTAACATCTTGTTGTGCATTGCCATTTGCTATGCGTGAATTT
>HPV51_Alpha_333087_nt3729_AOS_rc|1
GTCATAATTCCCAATGACAGTGTTACACTTGGGGGTACTTTAATGGTTTTTATAAATGTT
>HPV51_Alpha_333087_nt3722_AOS_rc|1
TTCCCAATGACAGTGTTACACTTGGGGGTACTTTAATGGTTTTTATAAATGTTTCCCGTT
>HPV51_Alpha_333087_nt3701_AOS_rc|1
TTGGGGGTACTTTAATGGTTTTTATAAATGTTTCCCGTTGATGTGCACTGTCAAACACAA
>HPV51_Alpha_333087_nt2540_AOS_rc|2
TTTAAAAACTTTAATACTGTTACCCTTGTATGTAAATACATTAGGTTTGCATCCTCTTGT
>HPV42_Alpha_333211_nt7478_AOS_rc|1
TGAATAGGTTTGCAAAATGTTAGGAAACATGGCCAATGCACCTGGTAACAGGCCAGATAC
>HPV42_Alpha_333211_nt7090_AOS_rc|1
ATCCTTTTTTTCTGGCGTTGTTACCTTAGCCTGACAGCGAATAGCTTCTGATTGTACATA
>HPV42_Alpha_333211_nt5963_AOS_rc|1
TGGGGATAGATGTCTTATTTGGCCTTTTTGTAATAGAGTAATAAGGGTGACCAACAACCA
>HPV42_Alpha_333211_nt5603_AOS_rc|1

GGAAACGAAAAAGGTATGGTGGTATTGGCAGAATGTGTAGACATGCTAGGTAATGTAGGG
>HPV42_Alpha_333211_nt5035_AOS_rc|1
ACGACCGAGGGCCAGGAATGGGAGTACTAGTAAATGTGTTAGTTGTATCTGTAGATACAA
>HPV42_Alpha_333211_nt4856_AOS_rc|1
AAAGGATTGGTAGATGTAGTTGTTGTGACACGTATAGTATTAGTGGGAGGGGAGATATCT
>HPV42_Alpha_333211_nt4389_AOS_rc|1
TCGTCTGCGGGACCGTTGTGGTGGCATTGTAATAGCACAAAAGAAGTACACACAATATAA
>HPV42_Alpha_333211_nt4382_AOS_rc|1
CGGGACCGTTGTGGTGGCATTGTAATAGCACAAAAGAAGTACACACAATATAACAAAAGT
>HPV42_Alpha_333211_nt4110_AOS_rc|1
GACAGAAGGAATACCAAGAATGATTAAACATAGAAAAAAATAGTCAAAGTATGTTAGTAC
>HPV39_Alpha_333245_nt7375_AOS_rc|1
TAAAATGTAGAAACGCATACAACAATGTAAAAATTACATATTAGGTAGTAAACACACCTT
>HPV39_Alpha_333245_nt6450_AOS_rc|1
ATACAGAACTACCGGGGTTTGCACGTATATCTGTGCCCTTAATATACAATTGGGCAGGAA
>HPV39_Alpha_333245_nt5491_AOS_rc|1
ATATGTTGTGTCTATTGGTCCAGAAGGCACCAATGGCAACTGTGGAGTAGTACTTGGTAA
>HPV39_Alpha_333245_nt5377_AOS_rc|1
AGTTGTATTGGCATATTTAGTAGATGCTGAAGAAGCCACAGAAGGTAGTGAGCCTGTGTT
>HPV39_Alpha_333245_nt5370_AOS_rc|1
TTGGCATATTTAGTAGATGCTGAAGAAGCCACAGAAGGTAGTGAGCCTGTGTTATATGTA
>HPV39_Alpha_333245_nt5348_AOS_rc|1
AAGAAGCCACAGAAGGTAGTGAGCCTGTGTTATATGTAGTGCCCGAATCCCTTGTATTAT
>HPV39_Alpha_333245_nt5341_AOS_rc|1
CACAGAAGGTAGTGAGCCTGTGTTATATGTAGTGCCCGAATCCCTTGTATTATTAAATGC
>HPV39_Alpha_333245_nt5245_AOS_rc|1
TATATCAAATAATGCATCTGAAGCATCAGAGGGCTCAGCGTGAACTAGGGGCTGTAATTC
>HPV39_Alpha_333245_nt1211_AOS_rc|1
GAAATTTCCTGTAGTGTTCCCCTGGTATTCCTGCCTACTTTTTTTCCATACGGTCTAGTG
>HPV93_Beta_37089393_nt4383_AOS_rc|1
AAGTTTCTGTTAATGGAACAATTGAAGATGAGGATGGTTCTACAGGAGCTATGGTATCTA
>HPV93_Beta_37089393_nt402_AOS_rc|1
CAATTTGATCTAATTTTTTCATACAATGGCGACACCTCACTATAATTTCAGAGAGTGGCT
>HPV93_Beta_37089393_nt395_AOS_rc|1
ATCTAATTTTTTCATACAATGGCGACACCTCACTATAATTTCAGAGAGTGGCTTTTTTTC
>HPV93_Beta_37089393_nt388_AOS_rc|1
TTTTTCATACAATGGCGACACCTCACTATAATTTCAGAGAGTGGCTTTTTTTCCTTAACT
>HPV93_Beta_37089393_nt350_AOS_rc|1
GAGTGGCTTTTTTTCCTTAACTTCTATTGCCCAGCCAACAACACTATCATTCAAGTAATT
>HPV93_Beta_37089393_nt90_AOS_rc|1
GAGGTTTACTTACTGCCGCCATTAAGATGTCTGCCTTATATACCATATATCAATACTTAT
>HPV93_Beta_37089393_nt83_AOS_rc|1
ACTTACTGCCGCCATTAAGATGTCTGCCTTATATACCATATATCAATACTTATAAACAGG
>HPV93_Beta_37089393_nt75_AOS_rc|1
CCGCCATTAAGATGTCTGCCTTATATACCATATATCAATACTTATAAACAGGTGCTACGA
>HPV12_Beta_396910_nt6365_AOS_rc|2
GAAAGTGTTCTTTAACTCTATGGGAGGGCAAACACCATTTTCAGCTGGTGCATCCCCGCA
>HPV12_Beta_396910_nt6358_AOS_rc|3
TTCTTTAACTCTATGGGAGGGCAAACACCATTTTCAGCTGGTGCATCCCCGCAGGGTAAG
>HPV12_Beta_396910_nt6351_AOS_rc|2
ACTCTATGGGAGGGCAAACACCATTTTCAGCTGGTGCATCCCCGCAGGGTAAGGCTTTCT
>HPV12_Beta_396910_nt5094_AOS_rc|1
AAATGAAAAGCGTACTAACTTAGAAGGTTTATCTATAAATAAAGGATTATCAACTTGCAC
>HPV12_Beta_396910_nt5087_AOS_rc|1
AAGCGTACTAACTTAGAAGGTTTATCTATAAATAAAGGATTATCAACTTGCACTTGTTGT

>HPV12_Beta_396910_nt5032_AOS_rc|1
GTTGTACTAGCCTTCTATTTGTTAGGGACACTCCTCTACGTCGGCCAGTGGTTTGTGTCC
>HPV12_Beta_396910_nt5021_AOS_rc|1
CTTCTATTTGTTAGGGACACTCCTCTACGTCGGCCAGTGGTTTGTGTCCTCTGAAGTGGA
>HPV12_Beta_396910_nt5014_AOS_rc|1
TTGTTAGGGACACTCCTCTACGTCGGCCAGTGGTTTGTGTCCTCTGAAGTGGAGTGCTGC
>HPV12_Beta_396910_nt5007_AOS_rc|1
GGACACTCCTCTACGTCGGCCAGTGGTTTGTGTCCTCTGAAGTGGAGTGCTGCTTTGCCG
>HPV12_Beta_396910_nt4868_AOS_rc|1
CCTATAGTTTGACCTCCTGAGCCAGATGTGACCAGGATGTGGTCTGCCAAAGATGTTTCA
>HPV14D_Beta_396918_nt5444_AOS_rc|1
TAATGACAACAGGCAACTCTGGGGTAGGATAAATAATATCCATGCTAATGTCCCTATCCT
>HPV14D_Beta_396918_nt5362_AOS_rc|1
TTTTGTATCCTGTGCATAATATGACCCAGATCTGGTTGTTTCAAAACGAGGGACGGTATA
>HPV14D_Beta_396918_nt5347_AOS_rc|1
ATAATATGACCCAGATCTGGTTGTTTCAAAACGAGGGACGGTATATGAAGATGTTGAGCG
>HPV14D_Beta_396918_nt5340_AOS_rc|2
GACCCAGATCTGGTTGTTTCAAAACGAGGGACGGTATATGAAGATGTTGAGCGTCGATTA
>HPV14D_Beta_396918_nt5326_AOS_rc|1
TGTTTCAAAACGAGGGACGGTATATGAAGATGTTGAGCGTCGATTACCCACAACTAATTG
>HPV14D_Beta_396918_nt4834_AOS_rc|1
TTGCACTAGTCTAGATGGTCTTGTTAAAAATAAGGGGTTTTCTACAGAAACTTGTTGGAC
>HPV14D_Beta_396918_nt4598_AOS_rc|1
CAGAGGTAACCAATATATTGTCTGCTAATGAACTTTCACCTGTGGTAGGGGTGGATTCGG
>HPV14D_Beta_396918_nt4591_AOS_rc|1
AACCAATATATTGTCTGCTAATGAACTTTCACCTGTGGTAGGGGTGGATTCGGTAATTAC
>HPV14D_Beta_396918_nt4580_AOS_rc|2
TGTCTGCTAATGAACTTTCACCTGTGGTAGGGGTGGATTCGGTAATTACTTGAAAGGAGG
>HPV14D_Beta_396918_nt2184_AOS_rc|1
CAATCTAAAGAAACATAATGACCATCAAGGCCATTCCTCAAATAAGTGTCCATATACAAC
>HPV15_Beta_396924_nt5489_AOS_rc|1
TAGGCTACATTATACCCATGCACATCTTGTATGTAAAACCCAGTATTTCTAGGTGATTCA
>HPV15_Beta_396924_nt6555_AOS_rc|1
CTGTGGGAAAGTAAGTAGAATTTGCTAAGTTATTTTGTTGTTGGGTTGATTGTGCTGGTA
>HPV15_Beta_396924_nt4900_AOS_rc|2
AGGGTCGTCCCAAGAATAAAGGGTCTGTTACAGCTACTTGTTCAGTCAGCCGTCTGTTAT
>HPV15_Beta_396924_nt3923_AOS_rc|1
CAACGCCTGGTGGCAGTTTCATTATTTTGATAAAGAGCTCTCTTTCGGTGTTGGAAGAGA
>HPV15_Beta_396924_nt3911_AOS_rc|1
GCAGTTTCATTATTTTGATAAAGAGCTCTCTTTCGGTGTTGGAAGAGAATGCCAGTAACA
>HPV15_Beta_396924_nt3904_AOS_rc|1
CATTATTTTGATAAAGAGCTCTCTTTCGGTGTTGGAAGAGAATGCCAGTAACAATCGTGA
>HPV15_Beta_396924_nt3897_AOS_rc|1
TTGATAAAGAGCTCTCTTTCGGTGTTGGAAGAGAATGCCAGTAACAATCGTGAGCGTCCA
>HPV15_Beta_396924_nt3890_AOS_rc|1
AGAGCTCTCTTTCGGTGTTGGAAGAGAATGCCAGTAACAATCGTGAGCGTCCAATTCTAT
>HPV15_Beta_396924_nt441_AOS_rc|1
CAGCGAATAATAATATCTCCAACAGGCTTCTGTTCTACTATCTCTATTTCCCAACTACAC
>HPV15_Beta_396924_nt434_AOS_rc|1
TAATAATATCTCCAACAGGCTTCTGTTCTACTATCTCTATTTCCCAACTACACACCGACT
>HPV17_Beta_396932_nt5118_AOS_rc|1
GAGTACGAATGCTGGCTCTTCTACCTAGTCTACTGACTCGTAAATAACCCTGAGGTGTTT
>HPV17_Beta_396932_nt5510_AOS_rc|1
ATATGCTACTGTGTATCCCTGAGTGTCATGTATGTAAAAGCCAGTGTCCCTAGGTGTTTC
>HPV17_Beta_396932_nt4842_AOS_rc|1

TTAAGCTTGATAGTGATTCTACTGCACGTTGAACTGGAGTACTTGTTCTAGGAGGTGTGC
>HPV17_Beta_396932_nt4719_AOS_rc|1
TATCAATGGCTGCATTCCTGGAACCTCCTATGTTTTGTCCACCAAAACCTTCGAACACTA
>HPV17_Beta_396932_nt3933_AOS_rc|1
CTGGTGGTAGCTTCATTTTTTGGATAAACAATTCTCTTTCATCATATGTGTTAAATGCTA
>HPV17_Beta_396932_nt3926_AOS_rc|1
TAGCTTCATTTTTTGGATAAACAATTCTCTTTCATCATATGTGTTAAATGCTAGTAACAT
>HPV17_Beta_396932_nt1825_AOS_rc|1
TTCGTTATCATAAGCAAATTGTATCATGGTAGATAGATCGAATTGTGTTGCCTGTGCTGT
>HPV17_Beta_396932_nt1809_AOS_rc|1
AATTGTATCATGGTAGATAGATCGAATTGTGTTGCCTGTGCTGTTTGATGATTAATCATA
>HPV17_Beta_396932_nt830_AOS_rc|1
TCTCTGCAGTTAGGACACACCAACTGTACTTCACCTAATAAAAGCTCCTCTTGTGAACGA
>HPV17_Beta_396932_nt805_AOS_rc|1
GTACTTCACCTAATAAAAGCTCCTCTTGTGAACGAATTCCAGCGTGCGTTGCAAGAACAA
>HPV19_Beta_396940_nt5585_AOS_rc|1
GTTTCAAATTGGGGAACTGTATATGTGGAAGTAGAACGGCGCCCACCAACTACTAACTGT
>HPV19_Beta_396940_nt5272_AOS_rc|1
GAATGGTTCGCCTTTGGCCTAGGCGACTAACTCTGATGTAACCAGATGGTGTTTCTGAAT
>HPV19_Beta_396940_nt4150_AOS_rc|1
AAATGAGCCAAACGATCGGTCCACACCTTTAGGATACCTTACTGTATCATCAAAGTGCTT
>HPV19_Beta_396940_nt488_AOS_rc|1
AGACAGATTTACCTGTTACAAACTCAATTTCTCTACCTGTTACAGTATGCTCATAGAACT
>HPV19_Beta_396940_nt219_AOS_rc|1
GGTGGCTCTGTGACCTGTGGTGCAGTAGTTCCCTCTTCTACAATTTCTATCTCTTCTTCT
>HPV19_Beta_396940_nt212_AOS_rc|1
CTGTGACCTGTGGTGCAGTAGTTCCCTCTTCTACAATTTCTATCTCTTCTTCTGTAGCCT
>HPV19_Beta_396940_nt205_AOS_rc|1
CTGTGGTGCAGTAGTTCCCTCTTCTACAATTTCTATCTCTTCTTCTGTAGCCTGTGCGTT
>HPV19_Beta_396940_nt198_AOS_rc|1
GCAGTAGTTCCCTCTTCTACAATTTCTATCTCTTCTTCTGTAGCCTGTGCGTTAGCCATA
>HPV19_Beta_396940_nt191_AOS_rc|1
TTCCCTCTTCTACAATTTCTATCTCTTCTTCTGTAGCCTGTGCGTTAGCCATAAGTTTGC
>HPV19_Beta_396940_nt183_AOS_rc|1
TCTACAATTTCTATCTCTTCTTCTGTAGCCTGTGCGTTAGCCATAAGTTTGCAGCTGCAA
>HPV25_Beta_396948_nt3784_AOS_rc|1
GGTGGGGGATTCAGACAATCTGTGTCGTCGTCTCCCGCCCCTGCCGCGGCCCCCCCTGCT
>HPV25_Beta_396948_nt5666_AOS_rc|1
ATAGGACACATAGTATCCCTGAATATCTTGGGTATAATATGATGCAGAGCGTGTAGTTTC
>HPV25_Beta_396948_nt5037_AOS_rc|1
ATCCTCCTCTACGTCTTAATGCAGTCCTAATCCTTTGAAGAGGTGTACTACTTTGTCTTG
>HPV25_Beta_396948_nt4737_AOS_rc|1
CTAAAACTGCGCTGGCACCTCTAGAAGTTGTTGTCACCGGGGTGTCAGTGGAGGGTACAA
>HPV25_Beta_396948_nt3802_AOS_rc|1
TCTTCGTGATCGCTTGGAGGTGGGGGATTCAGACAATCTGTGTCGTCGTCTCCCGCCCCT
>HPV25_Beta_396948_nt3777_AOS_rc|1
GATTCAGACAATCTGTGTCGTCGTCTCCCGCCCCTGCCGCGGCCCCCCCTGCTGCTCCCT
>HPV25_Beta_396948_nt475_AOS_rc|1
AAAAATTGATTTACCTGTAACGTCTTCAATTTCCCTACCTGTTACAGTGCTTTCGTAAAA
>HPV25_Beta_396948_nt203_AOS_rc|1
GTGGCTGTATAACCTGCGCTTGCTCTGGTGGTCCTATGCTCTGTTCAGCATTTGCAGTTG
>HPV25_Beta_396948_nt196_AOS_rc|1
TATAACCTGCGCTTGCTCTGGTGGTCCTATGCTCTGTTCAGCATTTGCAGTTGCCATAAG
>HPV25_Beta_396948_nt189_AOS_rc|1
TGCGCTTGCTCTGGTGGTCCTATGCTCTGTTCAGCATTTGCAGTTGCCATAAGTCCTAAT

>HPV27_Alpha_396964_nt5475_AOS_rc|1
CAGAGGAGGCAGATACCGTGGTATTAGCCAGGGAGCCGCGAGGGGCTGGGTAGTAATCGT
>HPV27_Alpha_396964_nt5893_AOS_rc|1
TAGGCACTGCCAACCTATTATTGCTACCCTTCTTTATAGAATAATATGGGTGGCCGACAG
>HPV27_Alpha_396964_nt5468_AOS_rc|1
GGCAGATACCGTGGTATTAGCCAGGGAGCCGCGAGGGGCTGGGTAGTAATCGTCCAATGG
>HPV27_Alpha_396964_nt4216_AOS_rc|1
CAACAACAATAATAAGCCAATGAGGGTGAGAATCACCAACAGACAGTCAATATCATCACG
>HPV30_Alpha_396973_nt7587_AOS_rc|1
TATATGACTAATAAGGCGAGGCGTCTTACGTTGCAAAAAAAAACATAAAGTGCAGGAAAC
>HPV30_Alpha_396973_nt7376_AOS_rc|1
ATAAATATAAAAAGTAACTGCACAGTGGTGTGCAACAACCAAAAAGGGTGGAGGACATAG
>HPV30_Alpha_396973_nt7144_AOS_rc|1
CATTCATGCACAATAAATAGTAAGGAAACATACACAGACACAAACAATCACCGCCGCTTG
>HPV30_Alpha_396973_nt7137_AOS_rc|1
GCACAATAAATAGTAAGGAAACATACACAGACACAAACAATCACCGCCGCTTGCGTTTGG
>HPV30_Alpha_396973_nt6823_AOS_rc|1
GTGGCAGCTGGGGGTGACAATCCAATATTCCAGCCCTCAAGTAAAGTGGAGTTCATAGTA
>HPV30_Alpha_396973_nt4719_AOS_rc|1
ACTGGGGGTTCAACAAAGGAGGGATTGGTAAAATGGGTACTACTAACATGGACAGAGCCA
>HPV30_Alpha_396973_nt4712_AOS_rc|1
GTTCAACAAAGGAGGGATTGGTAAAATGGGTACTACTAACATGGACAGAGCCAGTGGTAG
>HPV30_Alpha_396973_nt3139_AOS_rc|1
TATATACCTTTGTAATCTACTACAGAAGGCACTTTAGTCCACCCATTGTCCCCACAGTAA
>HPV30_Alpha_396973_nt1356_AOS_rc|1
ATTTGCTGTGGTGGCGTGGCGCCTATATCCATATCACTGTCTGCTTCTATGCTGTTTTCC
>HPV30_Alpha_396973_nt260_AOS_rc|1
ATAAAATAATAAACAGAAATTGCACACTGCATATGGGCTGTCCTCCCTATATACCAGTCT
>HPV3_Alpha_397005_nt5431_AOS_rc|1
AGCACGGCCTCTAGACAGAGTGTCACTACGACCTCCAGATGTAAAACCTATGTCAGCATC
>HPV3_Alpha_397005_nt4264_AOS_rc|1
AAAACATAGCAGTATAAAAGACGGTGGTACAATACCACACTAGGGCATATACATCAAGGA
>HPV3_Alpha_397005_nt4247_AOS_rc|1
AAGACGGTGGTACAATACCACACTAGGGCATATACATCAAGGAAGAATACATACAGGTGA
>HPV3_Alpha_397005_nt4240_AOS_rc|2
TGGTACAATACCACACTAGGGCATATACATCAAGGAAGAATACATACAGGTGACCTGCAA
>HPV3_Alpha_397005_nt4228_AOS_rc|1
ACACTAGGGCATATACATCAAGGAAGAATACATACAGGTGACCTGCAACTACAACCTTAC
>HPV3_Alpha_397005_nt4218_AOS_rc|1
ATATACATCAAGGAAGAATACATACAGGTGACCTGCAACTACAACCTTACAGGAGTCCCA
>HPV3_Alpha_397005_nt4134_AOS_rc|1
TAAGTATGACAGCTAACATAAACAGCAATAATATGTACACGTCTCCAGGCTCAACAAACA
>HPV3_Alpha_397005_nt4127_AOS_rc|1
GACAGCTAACATAAACAGCAATAATATGTACACGTCTCCAGGCTCAACAAACAAGACAAG
>HPV3_Alpha_397005_nt4119_AOS_rc|1
ACATAAACAGCAATAATATGTACACGTCTCCAGGCTCAACAAACAAGACAAGATGGTCGC
>HPV3_Alpha_397005_nt2174_AOS_rc|1
TTCCTTGTAGGAATGATTTCAGAGCGCATAGAAATGGTATAAATTCTACGTCCTGGTACC
>HPV40_Alpha_397014_nt7524_AOS_rc|1
AGCGGTCGCGCAGTACAAAATGGAGGATTTAGACACAAAAAGTACATGGTCAACACTGAA
>HPV40_Alpha_397014_nt7517_AOS_rc|1
GCGCAGTACAAAATGGAGGATTTAGACACAAAAAGTACATGGTCAACACTGAACAAGACG
>HPV40_Alpha_397014_nt7510_AOS_rc|1
ACAAAATGGAGGATTTAGACACAAAAAGTACATGGTCAACACTGAACAAGACGCCATGAC
>HPV40_Alpha_397014_nt7502_AOS_rc|1

GAGGATTTAGACACAAAAAGTACATGGTCAACACTGAACAAGACGCCATGACACTGCCAT
>HPV40_Alpha_397014_nt5895_AOS_rc|1
TGAAATGTCACCATTGGGTTTTTTAACTCAAAGTATGGATGTCCTATAGTCAGTAACCT
>HPV40_Alpha_397014_nt4030_AOS_rc|1
ACAAAGCACAAAATGGAGATAGCAGACAGTATAGAGCTCCACACATACAGATTAGGGACA
>HPV40_Alpha_397014_nt4022_AOS_rc|1
CAAAATGGAGATAGCAGACAGTATAGAGCTCCACACATACAGATTAGGGACACACACACA
>HPV40_Alpha_397014_nt3791_AOS_rc|1
ACATACCCAAGCTATGTTTTATTGTTTTTGGTATTTTTACAATAGCTAAGAAGTCAGACC
>HPV40_Alpha_397014_nt285_AOS_rc|1
CGTATCTAAAGTTTCTGTATTGGTTTACTTTTCCGTGCAGGTCCAGGCACCGTGGACATG
>HPV45_Alpha_397022_nt7514_AOS_rc|1
CGCGCCAGTAGGTTAAACTATGTAGGGGTTGTAAATGTGCCAGTTTAGGTATTATGTTTA
>HPV45_Alpha_397022_nt7098_AOS_rc|1
GCTAACATATTATTTCTTACTACGTATACGTACACGTTTGGCAGGCCTAGATGCAGTAGA
>HPV45_Alpha_397022_nt5352_AOS_rc|1
TTAATGGTACTGTAACATTACTGTAAGAGGATGCAGCAGTAGAAGGCATGGTCAAGGAAT
>HPV45_Alpha_397022_nt5342_AOS_rc|1
TGTAACATTACTGTAAGAGGATGCAGCAGTAGAAGGCATGGTCAAGGAATACTTTGGATA
>HPV45_Alpha_397022_nt5335_AOS_rc|1
TTACTGTAAGAGGATGCAGCAGTAGAAGGCATGGTCAAGGAATACTTTGGATATGTAAAT
>HPV45_Alpha_397022_nt5328_AOS_rc|1
AAGAGGATGCAGCAGTAGAAGGCATGGTCAAGGAATACTTTGGATATGTAAATGATTTGT
>HPV45_Alpha_397022_nt1443_AOS_rc|1
TTTTTGTTACTTGCTTGTAATAGCTCCTTTAGTTCTGTAATACTGCAATGCGGATCTACA
>HPV45_Alpha_397022_nt1436_AOS_rc|1
TACTTGCTTGTAATAGCTCCTTTAGTTCTGTAATACTGCAATGCGGATCTACATTTTCTG
>HPV45_Alpha_397022_nt1429_AOS_rc|1
TTGTAATAGCTCCTTTAGTTCTGTAATACTGCAATGCGGATCTACATTTTCTGCATTGTC
>HPV52_Alpha_397038_nt7837_AOS_rc|1
CGTGTAGTGCACACCTGGTGAGTAACAGTATTTAATCATGTTTTACAAGACATATTTGGC
>HPV52_Alpha_397038_nt7597_AOS_rc|1
TGCAGGAAACCAAAGGATTAAGCAGTTTGCTGAAACTTATATAAAAATAGTTGTTACTGT
>HPV52_Alpha_397038_nt7582_AOS_rc|1
GATTAAGCAGTTTGCTGAAACTTATATAAAAATAGTTGTTACTGTGCCAAGGACAACCAA
>HPV52_Alpha_397038_nt5495_AOS_rc|1
TAGATGTAGATGGAGCTGTAGGGGCTATAGGAACAAAAGGAGTATGTGTGGGTAACGAAG
>HPV52_Alpha_397038_nt5482_AOS_rc|1
AGCTGTAGGGGCTATAGGAACAAAAGGAGTATGTGTGGGTAACGAAGGTAATGGAATGTC
>HPV52_Alpha_397038_nt5472_AOS_rc|1
GCTATAGGAACAAAAGGAGTATGTGTGGGTAACGAAGGTAATGGAATGTCAGGACCTGAC
>HPV52_Alpha_397038_nt5465_AOS_rc|1
GAACAAAAGGAGTATGTGTGGGTAACGAAGGTAATGGAATGTCAGGACCTGACTCAATGG
>HPV52_Alpha_397038_nt5348_AOS_rc|1
TATTATGGGTAGAAAGTGTGGAAGGTAAGTGAAACGTGGGTTGCTGCAAAGAATCTGCAT
>HPV52_Alpha_397038_nt4982_AOS_rc|1
CAACGCCCTCAAAAACAGGATTGTTATATGTTACTAATTTCTGTGGTGATGACATAAAAG
>HPV52_Alpha_397038_nt329_AOS_rc|1
TAATGGTTTTTTTACCCTCTCTTCTAATGTTTTCCCATACAGTGAATATTGATAATGCCT
>HPV56_Alpha_397053_nt7708_AOS_rc|1
AAAACAGATACTGACAGATACTTGGCCTACATAGTGTATTCTGCAAGCCAAAACACCAAA
>HPV56_Alpha_397053_nt7585_AOS_rc|1
TTGGCAGTCATAGTACCTGTGAGTAATACAGGGTGCGGTACTGTACATAATTCAAGTATA
>HPV56_Alpha_397053_nt7578_AOS_rc|1
TCATAGTACCTGTGAGTAATACAGGGTGCGGTACTGTACATAATTCAAGTATAAAACACA

>HPV56_Alpha_397053_nt1052_AOS_rc|1
TTGTACTTGCAACAATTGTTGACTGTTTCTGCGTCTGCCTGTATATTTTGTATATATGAA
>HPV56_Alpha_397053_nt1045_AOS_rc|1
TGCAACAATTGTTGACTGTTTCTGCGTCTGCCTGTATATTTTGTATATATGAATCGTCTA
>HPV56_Alpha_397053_nt253_AOS_rc|1
AATAAACATACTCTGCACACTGCATAAGGAAAATCATCCCTATACACTAATTTTAATTCA
>HPV56_Alpha_397053_nt66_AOS_rc|1
GTGGATTGTTGAATTGTGGCTCCATGGATATGTCCACACAGAATAAGCTGCCTTTTATAT
>HPVcand62_Alpha_39932599_nt7255_AOS_rc|1
GCACACACACACATACCATACATACAGTTACTTCCTGCGCTTTCGTTTAGCAGAGGTAGG
>HPVcand62_Alpha_39932599_nt5645_AOS_rc|1
GAACCTACAATATATATAGAATATGGGGTAGTTGCAGGGAAGGAAGGCGTTAGCACAGAC
>HPVcand62_Alpha_39932599_nt5558_AOS_rc|1
ACATCAGGGCCAGTGGACGCTGGAACGGACAACCCAGTGGATAATGGTACAGTGACATTA
>HPVcand62_Alpha_39932599_nt5551_AOS_rc|1
GGCCAGTGGACGCTGGAACGGACAACCCAGTGGATAATGGTACAGTGACATTAGTATGAG
>HPVcand62_Alpha_39932599_nt5544_AOS_rc|1
GGACGCTGGAACGGACAACCCAGTGGATAATGGTACAGTGACATTAGTATGAGAGGATGC
>HPVcand62_Alpha_39932599_nt5520_AOS_rc|2
GGATAATGGTACAGTGACATTAGTATGAGAGGATGCCACAGACGTGGCAGATATGGTGGC
>HPVcand62_Alpha_39932599_nt4186_AOS_rc|1
CAGTATTAATGCCACTATGAGGCACAATAAAAACAGTAGACCACTGTCATCAGACCCCAA
>HPVcand62_Alpha_39932599_nt3433_AOS_rc|1
CACACGTGTCGTCCTTTTGTGCTGTCAGGGTAACTGTTGGTCCCGTACTCGTTATTGTCC
>HPVcand62_Alpha_39932599_nt1259_AOS_rc|1
TACTGGTTTTTAACAGCTCTACAATACTTGCTGTGCGTTCCTGCGATTGTTGCACATCTA
>HPVcand62_Alpha_39932599_nt1246_AOS_rc|1
CAGCTCTACAATACTTGCTGTGCGTTCCTGCGATTGTTGCACATCTACACTGCGTTCCGG
>HPV43_Alpha_40804474_nt7556_AOS_rc|1
AAATGGAAGACGTTCAGACGCCATTTTACATACCCACAACAGTAACCATTACTAAAACCA
>HPV43_Alpha_40804474_nt6554_AOS_rc|1
ATCTGCAATTTTGGACCTGGTATTAGAGCCAGCAATATACATATCGGAAGGCACAACGTC
>HPV43_Alpha_40804474_nt6547_AOS_rc|2
ATTTTGGACCTGGTATTAGAGCCAGCAATATACATATCGGAAGGCACAACGTCGCCAGTT
>HPV43_Alpha_40804474_nt6537_AOS_rc|1
TGGTATTAGAGCCAGCAATATACATATCGGAAGGCACAACGTCGCCAGTTTTACCAGCTT
>HPV43_Alpha_40804474_nt4313_AOS_rc|1
CAAGTGATTATATAAAGGATTGTTTATTTGCTGGAGGACTGATGCAACTTAAGTGTTCTG
>HPV43_Alpha_40804474_nt4287_AOS_rc|1
TTTGCTGGAGGACTGATGCAACTTAAGTGTTCTGTAATGAAACGCTAACACCAATATTAA
>HPV43_Alpha_40804474_nt4089_AOS_rc|1
AACAACGCCAATATAAAAACGTTAATAGGTGATGTAAGGACGCCCCAAGACAAAAAATAA
>HPV43_Alpha_40804474_nt4071_AOS_rc|1
ACGTTAATAGGTGATGTAAGGACGCCCCAAGACAAAAAATAAAAGATAGAAGCACATGCA
>HPV43_Alpha_40804474_nt3877_AOS_rc|1
TGTGGTTGGGGTACAATACCCCCCCTTTTATCACAGTTACAGTTCCTCTATACAAATATA
>HPV81_Alpha_40804509_nt7474_AOS_rc|1
CACACATTCCACAAATATACAAACAACAGTGTAGGGGCAAAAGGGCAGCACGCATACAAT
>HPV81_Alpha_40804509_nt7328_AOS_rc|1
ACAAACAAGCATACTACTATTTTTTACGTTTTCGCTTAGCAGAGGTAGTGGGGGTCCTGG
>HPV81_Alpha_40804509_nt6381_AOS_rc|1
ATGGTTGTATTTTTAAACTCCAAGGGGGGGCATTCGTTTTGTGCTGGAGCAACATTAGCA
>HPV81_Alpha_40804509_nt5765_AOS_rc|1
TTAGGGAAGAATATATAAGAAGGAAACAAGTAAAAATCAGTGCCAACAACATATATGGCG
>HPV81_Alpha_40804509_nt5752_AOS_rc|1

TATAAGAAGGAAACAAGTAAAAATCAGTGCCAACAACATATATGGCGTACGGGGTAGTTG
>HPV81_Alpha_40804509_nt5744_AOS_rc|1
GGAAACAAGTAAAAATCAGTGCCAACAACATATATGGCGTACGGGGTAGTTGCAGGAAAC
>HPV81_Alpha_40804509_nt5737_AOS_rc|1
AGTAAAAATCAGTGCCAACAACATATATGGCGTACGGGGTAGTTGCAGGAAACGAAGGGA
>HPV81_Alpha_40804509_nt924_AOS_rc|1
ATCTGCACTATCTTCCTCTTCATCACTTGATTCCTGATACCCTGTGGTTTTTCTACAAC
>HPV81_Alpha_40804509_nt917_AOS_rc|1
CTATCTTCCTCTTCATCACTTGATTCCTGATACCCTGTGGTTTTTCTACAACAGCCTCC
>HPV95_Gamma_40804520_nt7122_AOS_rc|1
AATATTTTTTCCAAAAAAAATAAGGAGCGTCTCTTTTCTTCGCCAGCGTCTTACTTAATG
>HPV95_Gamma_40804520_nt7027_AOS_rc|1
TATTGACCACATGAGTCACAAAGCATGGACACAATAGTAATTGGAGGTATAGTTTATTGC
>HPV95_Gamma_40804520_nt6822_AOS_rc|2
TTTTGTGCCTGTTGCAGTTTGAGAAGTAATAGCTCTTTGTCTTTTACGAGATCCATTTAT
>HPV95_Gamma_40804520_nt6815_AOS_rc|1
CCTGTTGCAGTTTGAGAAGTAATAGCTCTTTGTCTTTTACGAGATCCATTTATTAATCCA
>HPV95_Gamma_40804520_nt6803_AOS_rc|2
TGAGAAGTAATAGCTCTTTGTCTTTTACGAGATCCATTTATTAATCCACTCTGATAGAGA
>HPV95_Gamma_40804520_nt6205_AOS_rc|1
AAATGTGAGGGCCAAGATTATTTTGTTCTTGGTTACTCTGAGCAGCAATTATGTAATCAG
>HPV95_Gamma_40804520_nt422_AOS_rc|1
TAAATAGCACACCTCACCACTGTATAACAGGTCAAGTTTTTCTACTATGTCTAATAGTTT
>HPV95_Gamma_40804520_nt415_AOS_rc|1
CACACCTCACCACTGTATAACAGGTCAAGTTTTTCTACTATGTCTAATAGTTTAAGACAG
>HPV95_Gamma_40804520_nt408_AOS_rc|1
CACCACTGTATAACAGGTCAAGTTTTTCTACTATGTCTAATAGTTTAAGACAGCATAAAC
>HPV94_Alpha_40804528_nt5600_AOS_rc|2
AGGTGTTAAAGGCACATAAGGCCACTGCGCCGAGGTGGGTAATATAATGTCAGGCCCGGT
>HPV94_Alpha_40804528_nt7013_AOS_rc|1
GGTGCCTCCTTCTGACAAGTTATTGCGGAGGACGACAAGAACCTATAAGTGTCCTCCAAG
>HPV94_Alpha_40804528_nt6577_AOS_rc|2
CACTACCAACGTTTTGGTCACCACCGGCCGACTTCAATATTAGAGTGTCAGGAATGGTGT
>HPV94_Alpha_40804528_nt6570_AOS_rc|2
AACGTTTTGGTCACCACCGGCCGACTTCAATATTAGAGTGTCAGGAATGGTGTCTCCCAC
>HPV94_Alpha_40804528_nt6555_AOS_rc|1
ACCGGCCGACTTCAATATTAGAGTGTCAGGAATGGTGTCTCCCACTGCACTGGCTCTATT
>HPV94_Alpha_40804528_nt5484_AOS_rc|1
TTGAAAGTGTAGAGGACAAAGGGGAAGCAGTACTATAGCTCCGTGCCGGTGTGACACTTC
>HPV94_Alpha_40804528_nt5469_AOS_rc|1
ACAAAGGGGAAGCAGTACTATAGCTCCGTGCCGGTGTGACACTTCGAGTGCCATTTGTAA
>HPV94_Alpha_40804528_nt5461_AOS_rc|1
GAAGCAGTACTATAGCTCCGTGCCGGTGTGACACTTCGAGTGCCATTTGTAAAGGCAACA
>HPV94_Alpha_40804528_nt5454_AOS_rc|1
TACTATAGCTCCGTGCCGGTGTGACACTTCGAGTGCCATTTGTAAAGGCAACATCACCAT
>HPV94_Alpha_40804528_nt4247_AOS_rc|1
ACACAGCAAAACATTAGATTATGCACAGGCGTCTACATCCATATCACAATACACCCAGGT
>HPVcand85_Alpha_4574720_nt7507_AOS_rc|1
GTGCGCCACTGGCTCACTGGCGCCTTAAACAGAGCCAAAAAGGATTTAACTTTAAATAAT
>HPVcand85_Alpha_4574720_nt7500_AOS_rc|1
ACTGGCTCACTGGCGCCTTAAACAGAGCCAAAAAGGATTTAACTTTAAATAATGTAACAA
>HPVcand85_Alpha_4574720_nt3992_AOS_rc|1
AAAAACACAAGTAGCCAAGCATGCGCACACACATTAGTAGCAGGAAAAAGCGGAATAGTG
>HPVcand85_Alpha_4574720_nt3551_AOS_rc|1
GGACAGTCCGTCGACTTCACTTGTTTCTGTGAATCCACAATGTCTGAATGGGTTTCTAGT

>HPVcand85_Alpha_4574720_nt3538_AOS_rc|1
ACTTCACTTGTTTCTGTGAATCCACAATGTCTGAATGGGTTTCTAGTCGGCGCCGAACCC
>HPVcand85_Alpha_4574720_nt3312_AOS_rc|1
CATTATACCACACTTCCCATTTACCGCTCTGTCCATATTGTTGTGCATCACATTTAAACT
>HPVcand85_Alpha_4574720_nt1395_AOS_rc|1
TCTATATCCATTTCATTATCTGTACTACATGCCTCACTATTTAAACCGTCGCCATTTTTC
>HPVcand85_Alpha_4574720_nt1383_AOS_rc|1
TCATTATCTGTACTACATGCCTCACTATTTAAACCGTCGCCATTTTTCCCATCCCCAAAC
>HPVcand85_Alpha_4574720_nt1368_AOS_rc|1
CATGCCTCACTATTTAAACCGTCGCCATTTTTCCCATCCCCAAACACATTTGTATCTAGG
>HPVcand85_Alpha_4574720_nt1360_AOS_rc|2
ACTATTTAAACCGTCGCCATTTTTCCCATCCCCAAACACATTTGTATCTAGGGTTACCTG
>HPV96_Beta_50253426_nt4204_AOS_rc|1
GGGTTAGTTATTAGTATAAAAAAATGAAATGTTAGTAGTTAAAGACTATCAAAGCTACCA
>HPV96_Beta_50253426_nt4195_AOS_rc|1
ATTAGTATAAAAAAATGAAATGTTAGTAGTTAAAGACTATCAAAGCTACCAAAGGACCAA
>HPV96_Beta_50253426_nt3060_AOS_rc|2
TTTCTAAACTGGTGTTAATCAATGTCCAAGGTTCATCTTTGTAAGGTGAGTCTCTTAATT
>HPV96_Beta_50253426_nt3048_AOS_rc|1
TGTTAATCAATGTCCAAGGTTCATCTTTGTAAGGTGAGTCTCTTAATTTCTCTAGTTGAA
>HPV96_Beta_50253426_nt1650_AOS_rc|1
TGTTCTTGTAACAGTTGCTTACATCCTTCTAATAATTCCTCTTTAACTCCATATGCAGTT
>HPV96_Beta_50253426_nt696_AOS_rc|1
CTACAATATCTGCAAATGCCTTTCCAACCTCCTCTTACTAAATAAAAGTTTTCTTGATAC
>HPV96_Beta_50253426_nt484_AOS_rc|1
TGTTCTAGCACAATTGAAACAACACGCATATGCATAACCTTCCTTCCAAATAAGATTTAA
>HPV83_Alpha_5059324_nt6565_AOS_rc|1
GGAGCATAGATGTAGCTGGTAAGAGTGTTCCTACTATTAGCAGATGTGCCTGGAATATAA
>HPV83_Alpha_5059324_nt6558_AOS_rc|1
AGATGTAGCTGGTAAGAGTGTTCCTACTATTAGCAGATGTGCCTGGAATATAATAAGAGG
>HPV83_Alpha_5059324_nt5608_AOS_rc|1
ACCGTCTATAAACACAGACTGAATAGGAATATTAGATGGCACAGGAACAGAAGGGTCTGC
>HPV83_Alpha_5059324_nt5599_AOS_rc|1
AAACACAGACTGAATAGGAATATTAGATGGCACAGGAACAGAAGGGTCTGCAGGCATATG
>HPV83_Alpha_5059324_nt4164_AOS_rc|1
ATAATAATAATTACGATGAGAATTAAAATAATAAGCAGTAATCCCACTTCGTCCCGGCCA
>HPV83_Alpha_5059324_nt4152_AOS_rc|1
ACGATGAGAATTAAAATAATAAGCAGTAATCCCACTTCGTCCCGGCCAATAACAATTAAA
>HPV83_Alpha_5059324_nt4139_AOS_rc|1
AAATAATAAGCAGTAATCCCACTTCGTCCCGGCCAATAACAATTAAATCATATGCCTGCC
>HPV83_Alpha_5059324_nt4106_AOS_rc|1
CAATAACAATTAAATCATATGCCTGCCCCCCACCTCTAGCCTCCAATGGATACATAGTGA
>HPV83_Alpha_5059324_nt3859_AOS_rc|1
CACAAGAATATGACACCACAAACAAATCAAGCACAATATGTAATCCAGGGTTGTACTGGT
>HPV83_Alpha_5059324_nt3822_AOS_rc|1
ATGTAATCCAGGGTTGTACTGGTATATGGTGTGGTGTATAGTGGCTCAGGTATTTGCAAA
>HPV59_Alpha_557236_nt6416_AOS_rc|1
ATAAATAACTGCCTGGGTTGGCACGTATGTCAGTACCTTTAATATATAGTGATTCAGGAA
>HPV59_Alpha_557236_nt5491_AOS_rc|1
GCCATAAAAAATAATTTGTGCCTTCTATATTAATAGACTGTATGGTGGTAAAGGGTGTAG
>HPV59_Alpha_557236_nt5476_AOS_rc|1
TTGTGCCTTCTATATTAATAGACTGTATGGTGGTAAAGGGTGTAGTAGAATAAGTGGGTT
>HPV59_Alpha_557236_nt5456_AOS_rc|1
GACTGTATGGTGGTAAAGGGTGTAGTAGAATAAGTGGGTTCAACAATATTAGTATTAGGT
>HPV59_Alpha_557236_nt5328_AOS_rc|1

AAAGGTGCTAGATGCCGACCGTGTTAATGACAAACTTTGAAAAGAAGATTTAGGAATTGT
>HPV59_Alpha_557236_nt5321_AOS_rc|1
CTAGATGCCGACCGTGTTAATGACAAACTTTGAAAAGAAGATTTAGGAATTGTAAATGCA
>HPV59_Alpha_557236_nt5313_AOS_rc|1
CGACCGTGTTAATGACAAACTTTGAAAAGAAGATTTAGGAATTGTAAATGCAGTGTTGGC
>HPV59_Alpha_557236_nt5300_AOS_rc|2
GACAAACTTTGAAAAGAAGATTTAGGAATTGTAAATGCAGTGTTGGCAGTACTAGTAGGT
>HPV59_Alpha_557236_nt5293_AOS_rc|1
TTTGAAAAGAAGATTTAGGAATTGTAAATGCAGTGTTGGCAGTACTAGTAGGTGCTTCAT
>HPV59_Alpha_557236_nt1414_AOS_rc|1
CATAGCTGCTTTCTTATTTTTGCTATGTAATAACTGTAACAATTGATTAGTGGGGGATGT
>HPV6_Alpha_6002612_nt7533_AOS_rc|1
GTGCATGTATATTACTTATGTGTGTAAGGCGCGTGCAAACCAAAACGCGTGCAACAGCCA
>HPV6_Alpha_6002612_nt7504_AOS_rc|1
CGCGTGCAAACCAAAACGCGTGCAACAGCCACTGAGTCACAGGGTGTAACAAGATATACA
>HPV6_Alpha_6002612_nt7491_AOS_rc|1
AAACGCGTGCAACAGCCACTGAGTCACAGGGTGTAACAAGATATACAATAATGTAGTTAA
>HPV6_Alpha_6002612_nt6604_AOS_rc|2
CTTGGGGTGTTAACATATATACTACTCCCTACAGACGTTCGATTTCCACTACCCTTAATT
>HPV6_Alpha_6002612_nt5334_AOS_rc|1
GAAATATCATAAAAATAATGAATGCGGGCCCCTATGTGCTTTCCGCTGCGAGTGTGCATA
>HPV6_Alpha_6002612_nt4639_AOS_rc|1
CACAGGAGGACGAGCCATAGGCCCACTAGTAATAGAAGGTTTTGCAGAAGTTCCTAAGGG
>HPV6_Alpha_6002612_nt4211_AOS_rc|1
GTACAGCTCTATAGTGCATTAATAATAACCCCAACACCCCTACAATAAAGGCACATAACA
>HPV6_Alpha_6002612_nt3489_AOS_rc|1
TTTGGTGCTGGTCGTGATTGTTAGTGATGACGTTGTGGTTTCCACTGTCCACGGGTCCAA
>HPV6_Alpha_6002612_nt3128_AOS_rc|1
CTTAGCATCTACCATACTATGCACCTTTACCCAGGAGTCAGTGTCCTGCACATACACATC
>HPV57_Alpha_60882_nt6960_AOS_rc|2
GACATGTTATCGCTTGGGATTGCAAATACCTGTAGGTGTCCTGCAGGCTGGCGGACGGGG
>HPV57_Alpha_60882_nt7430_AOS_rc|1
TATCTACCAGGGGGCTACATACAGGCCACAGTGTGCAAACCATGCAGCGGGGACAATTAC
>HPV57_Alpha_60882_nt7220_AOS_rc|2
CATATACAAAAGTAGAGCAAAGGAAATGAGGCAGACACAGAATCACTATCGCCTGACCTT
>HPV57_Alpha_60882_nt4317_AOS_rc|1
GACATGGCAATAAGTTTATTGACACGTAGCAATACAAAGGCAATCAGGTGCACACCGATA
>HPV57_Alpha_60882_nt4310_AOS_rc|1
CAATAAGTTTATTGACACGTAGCAATACAAAGGCAATCAGGTGCACACCGATAAAAATGT
>HPV57_Alpha_60882_nt4230_AOS_rc|1
AAAACACAATGAGTATGTACACTACTGCAGCAGACGGACGTACAGCATCAACAACAAAAT
>HPV57_Alpha_60882_nt4120_AOS_rc|2
ACAGGACAATGGGATACGACCCGCCCGTGCCCTTGTACACAGCAGGATACATTGCAGTAT
>HPV57_Alpha_60882_nt4061_AOS_rc|1
TACAGGGGGGAGGTATATACAGGGTAGCAGCAGTTGGTCACAATGTAATATCAACTATTG
>HPV57_Alpha_60882_nt4048_AOS_rc|1
TATATACAGGGTAGCAGCAGTTGGTCACAATGTAATATCAACTATTGTAGAAACCACAAG
>HPV69_Alpha_6970418_nt7417_AOS_rc|1
TCAAAAAAAAGTGCGTATGCAACTATTGTTAAAAGATATGTGAGTAATGTAGGTGAGCCA
>HPV69_Alpha_6970418_nt7409_AOS_rc|1
AAGTGCGTATGCAACTATTGTTAAAAGATATGTGAGTAATGTAGGTGAGCCACGTCTAGG
>HPV69_Alpha_6970418_nt7402_AOS_rc|1
TATGCAACTATTGTTAAAAGATATGTGAGTAATGTAGGTGAGCCACGTCTAGGCAAGCCA
>HPV69_Alpha_6970418_nt6352_AOS_rc|1
ATAGAAGTAGTTGGAGGTTCACGACCTTGACCAGCGCCTTTAATATACATAGATACTGGT

>HPV69_Alpha_6970418_nt6345_AOS_rc|2
TAGTTGGAGGTTCACGACCTTGACCAGCGCCTTTAATATACATAGATACTGGTACAGAAT
>HPV69_Alpha_6970418_nt6337_AOS_rc|1
GGTTCACGACCTTGACCAGCGCCTTTAATATACATAGATACTGGTACAGAATCACCTATA
>HPV69_Alpha_6970418_nt5350_AOS_rc|1
CCATGTGGAAGAAGGTACAGGAGCATAAATGTCAGGGCCGTTATATATAGGTATATCAAA
>HPV69_Alpha_6970418_nt5309_AOS_rc|1
TATATATAGGTATATCAAATGATGTAGATAGAGGTATGGTAACATTTAAGGGTGCACTAC
>HPV69_Alpha_6970418_nt5302_AOS_rc|1
AGGTATATCAAATGATGTAGATAGAGGTATGGTAACATTTAAGGGTGCACTACTAGTGGT
>HPV69_Alpha_6970418_nt5295_AOS_rc|1
TCAAATGATGTAGATAGAGGTATGGTAACATTTAAGGGTGCACTACTAGTGGTTGAAAAT
>HPV82_Alpha_6970427_nt7511_AOS_rc|1
GGCGGGCGGGATGTACATACCATGCCAAAAGAATTAAGGGTTATGCCAAAAAACATAGGT
>HPV82_Alpha_6970427_nt4842_AOS_rc|1
ACTAATAAAGTCTGGATTAGTAACCTTAACCTGTGAAAATGCCCTGCTATATAAACGAGG
>HPV82_Alpha_6970427_nt4835_AOS_rc|1
AAGTCTGGATTAGTAACCTTAACCTGTGAAAATGCCCTGCTATATAAACGAGGTGCAGCA
>HPV82_Alpha_6970427_nt2884_AOS_rc|1
GTTTTGATACTGCCGATGCTGGTACTACTTGATGGTTAAGGGTTTGCATGTTTCTTTCTC
>HPV82_Alpha_6970427_nt2877_AOS_rc|1
TACTGCCGATGCTGGTACTACTTGATGGTTAAGGGTTTGCATGTTTCTTTCTCGTGCAGT
>HPV82_Alpha_6970427_nt2869_AOS_rc|1
ATGCTGGTACTACTTGATGGTTAAGGGTTTGCATGTTTCTTTCTCGTGCAGTATAAAACA
>HPV82_Alpha_6970427_nt1366_AOS_rc|1
CCTACATTGGTAGCATTTTCGTTTGTTTCCAGGTCTATCTCTGTACTTCTGTCGCTGCTC
>HPV82_Alpha_6970427_nt503_AOS_rc|1
GCGTTATTACACCTGGGTTTCACTACGTTGTCTTGGTGGTTTTCTGCAATTTGCACACTG
>HPV82_Alpha_6970427_nt493_AOS_rc|1
ACCTGGGTTTCACTACGTTGTCTTGGTGGTTTTCTGCAATTTGCACACTGTCCCGTCCAG
>HPV68a_Alpha_71726685_nt6285_AOS_rc|1
AGGAGTTTCACGAATGTCAGTGCCCTTAATATACATGTCAGTGGGAATAGTGTCCCCTAC
>HPV68a_Alpha_71726685_nt6278_AOS_rc|1
TCACGAATGTCAGTGCCCTTAATATACATGTCAGTGGGAATAGTGTCCCCTACCATGCCT
>HPV68a_Alpha_71726685_nt5355_AOS_rc|1
AACCATATATAGTTATGGCAAAGGTTGTATCAATTGGAGTAGAGGGTGTTAAAGGCAACT
>HPV68a_Alpha_71726685_nt5347_AOS_rc|1
ATAGTTATGGCAAAGGTTGTATCAATTGGAGTAGAGGGTGTTAAAGGCAACTGTGGGGTT
>HPV68a_Alpha_71726685_nt5329_AOS_rc|1
GTATCAATTGGAGTAGAGGGTGTTAAAGGCAACTGTGGGGTTGTAGATGGTAATACAACA
>HPV68a_Alpha_71726685_nt5209_AOS_rc|1
TATGTAGTGGATGCAGCAGAAGCCAATGAAGGAACTGATATGTGGGAACGAGTAGTAAAT
>HPV68a_Alpha_71726685_nt5202_AOS_rc|1
TGGATGCAGCAGAAGCCAATGAAGGAACTGATATGTGGGAACGAGTAGTAAATGTAGCAT
>HPV68a_Alpha_71726685_nt5193_AOS_rc|1
CAGAAGCCAATGAAGGAACTGATATGTGGGAACGAGTAGTAAATGTAGCATTATGAAATG
>HPV68a_Alpha_71726685_nt3453_AOS_rc|1
TTGTTTTGGCCTGCACTTCTACTGAGGAGTGGGAGGTCGACACCGTCCACGGACACGTTG
>HPV68a_Alpha_71726685_nt1336_AOS_rc|1
TGTTACATTGTAATAATACTTTAAGTTGGGTAGTAGGTGATTTAGGATCCTGGTTTTCAC
>HPV97_Alpha_71726694_nt7616_AOS_rc|1
TCATTTTATACTTTATAAGAAGAGTAGACAGATTGTTGGATGTAAAAGTAGTTGCACAGG
>HPV97_Alpha_71726694_nt7417_AOS_rc|1
CAAAGTGTGCGCCAAAATACGTGCCAGTGGTGAAAATATGTATGACTATTGTATGTGAGA
>HPV97_Alpha_71726694_nt7410_AOS_rc|1

TGCGCCAAAATACGTGCCAGTGGTGAAAATATGTATGACTATTGTATGTGAGATTAACAT
>HPV97_Alpha_71726694_nt6277_AOS_rc|1
TACCAGTTCCTTTAATATATAACTCCTGTGGAATAGTGTCACCCATTGTTCCAGCTCTGT
>HPV97_Alpha_71726694_nt5350_AOS_rc|1
CCTATATAGTGTGTCGTGGACGTAGATGTAGGTGTTATAAAAGGCCATACAGAAGTACTA
>HPV97_Alpha_71726694_nt5341_AOS_rc|1
TGTGTCGTGGACGTAGATGTAGGTGTTATAAAAGGCCATACAGAAGTACTAGAAGGTAAT
>HPV97_Alpha_71726694_nt5334_AOS_rc|1
TGGACGTAGATGTAGGTGTTATAAAAGGCCATACAGAAGTACTAGAAGGTAATACAATAT
>HPV97_Alpha_71726694_nt4890_AOS_rc|1
AATCGGGAACATTACTAGTAGGTTCAAATGTTAATGTAGAGTCAATAGGATTAAAGGCAG
>HPV97_Alpha_71726694_nt3688_AOS_rc|1
TAGGAACACTATCCAAAAATTTGGTTCGTTGATGCTCACTATCATACATTACAGTTAATA
>HPV97_Alpha_71726694_nt3224_AOS_rc|1
ACAACGCCCCCAAAATGCACTTCCCACTTATTACTGTTACCATATTTTTTACTTTCATGT
>HPV106_Alpha_71726710_nt7526_AOS_rc|1
TGTTATAAGGGCGGGGGTATGCATGACCGGGCCACAAGGACTGGACTACACAACATAAAT
>HPV106_Alpha_71726710_nt7516_AOS_rc|1
GCGGGGGTATGCATGACCGGGCCACAAGGACTGGACTACACAACATAAATGTATAGTTAC
>HPV106_Alpha_71726710_nt7509_AOS_rc|1
TATGCATGACCGGGCCACAAGGACTGGACTACACAACATAAATGTATAGTTACTCACAAG
>HPV106_Alpha_71726710_nt6592_AOS_rc|2
TTGTAGCAGAGGCGCGGGATGTAGTAGTAGGCCCCTTTAGTAAAAACCCTTCCGGAACAT
>HPV106_Alpha_71726710_nt5560_AOS_rc|2
GGGCCACTAAAAACAGGGACATCAAAGCTTGTAGACAGGGGAACAGTAGTGTGCGATACT
>HPV106_Alpha_71726710_nt5552_AOS_rc|1
AAAAACAGGGACATCAAAGCTTGTAGACAGGGGAACAGTAGTGTGCGATACTGTGGAGAC
>HPV106_Alpha_71726710_nt5545_AOS_rc|1
GGGACATCAAAGCTTGTAGACAGGGGAACAGTAGTGTGCGATACTGTGGAGACAGTGGAT
>HPV106_Alpha_71726710_nt5533_AOS_rc|1
CTTGTAGACAGGGGAACAGTAGTGTGCGATACTGTGGAGACAGTGGATGATGCAGCCAAT
>HPV106_Alpha_71726710_nt5367_AOS_rc|1
TAGGGGTGTCAGACACCAGAGGTTGCATTTCTAACTCTTCAGCAGGAGTAATTGGACTTA
>HPV106_Alpha_71726710_nt3874_AOS_rc|1
GCAGCAGGTGTGTCCAAACAGATAGCCAGCAAATAAAAATCTCCAGGGTGTAGTATACAC
>HPV102_Alpha_71726718_nt5688_AOS_rc|2
GAAAGAATAAGTAATTAGGCAATAAAAAATAGTCTGTACCATCCACAAACACAGATTGGG
>HPV102_Alpha_71726718_nt5655_AOS_rc|1
CTGTACCATCCACAAACACAGATTGGGAAGGAACATTAGAGGACTGGGAAACATGCAGCA
>HPV102_Alpha_71726718_nt5456_AOS_rc|1
AACAGACGTGGAAGAAGTATATGAAACATGTGGAGACGCCAAGGAGTCCAAGTGGCCATT
>HPV102_Alpha_71726718_nt4263_AOS_rc|1
TCAAAAATGTATGGGAATACAAAAGGAACACAGTAAGGAGTCTACATGTGCATAAGCCTC
>HPV102_Alpha_71726718_nt4168_AOS_rc|1
TAAAATTATTAACAGTAATCCAGTATCAGCTTGCCCAAAAACAATCACATCATAGGACGG
>HPV102_Alpha_71726718_nt4161_AOS_rc|1
ATTAACAGTAATCCAGTATCAGCTTGCCCAAAAACAATCACATCATAGGACGGCCCCCCC
>HPV102_Alpha_71726718_nt4146_AOS_rc|1
GTATCAGCTTGCCCAAAAACAATCACATCATAGGACGGCCCCCCCCGTCGTGTTTCCAAT
>HPV102_Alpha_71726718_nt4139_AOS_rc|1
CTTGCCCAAAAACAATCACATCATAGGACGGCCCCCCCCGTCGTGTTTCCAATGGATACA
>HPV102_Alpha_71726718_nt3615_AOS_rc|1
TGTTCTACACTGCTATACCGCAAGGTAACAAAGGTTGCTTTAGTGGCTGTCTCCCCGCAC
>HPV102_Alpha_71726718_nt3406_AOS_rc|1
GCACTCAGTGTCACCGTAGAATCCGTGGGTACTAGTGTCCAGCCGCTTTTGATGTTGTTT

>HPV13_Alpha_85827580_nt7631_AOS_rc|1
AAAATGAGTGATAGAAAGATTAGTAAGATTTAGTAAACATATATAACCGCGCCCTAGCAA
>HPV13_Alpha_85827580_nt7556_AOS_rc|1
TGATTTTGGCAAGCGGTGGATAATTTTGGCAGGCGGTAGGTAAGATTATATAAACAGCGT
>HPV13_Alpha_85827580_nt4886_AOS_rc|1
TGTCTTCTGTAGAATGAGGGGAAATAGTAGATGGCGATATAAACACGTGTGCACCACTTT
>HPV13_Alpha_85827580_nt4879_AOS_rc|1
TGTAGAATGAGGGGAAATAGTAGATGGCGATATAAACACGTGTGCACCACTTTCAATAGA
>HPV13_Alpha_85827580_nt4872_AOS_rc|1
TGAGGGGAAATAGTAGATGGCGATATAAACACGTGTGCACCACTTTCAATAGAAGGTTGA
>HPV13_Alpha_85827580_nt4865_AOS_rc|1
AAATAGTAGATGGCGATATAAACACGTGTGCACCACTTTCAATAGAAGGTTGAGATTGTG
>HPV13_Alpha_85827580_nt3968_AOS_rc|1
AAAACTCTGATATCCACAATATTGTTATAATGCTAACTACACACACTGTAAGTGCAATTA
>HPV2_Alpha_9626032_nt4623_AOS_rc|1
TCAATGATAACAGGCGGCCTGGGCGTTGGACCAATGTCAACTACAGTGGTGGGTCGCGAA
>HPV2_Alpha_9626032_nt4220_AOS_rc|2
CATCAACAATAAAAAGGCGATTAGGATAAGAATCACCAACAGACATTGAACATCATCATG
>HPV2_Alpha_9626032_nt4210_AOS_rc|1
AAAAAGGCGATTAGGATAAGAATCACCAACAGACATTGAACATCATCATGACCCCACAGT
>HPV2_Alpha_9626032_nt4178_AOS_rc|1
ACATTGAACATCATCATGACCCCACAGTACCACAGGATATGTTCCCTCAGATCCCTTATA
>HPV2_Alpha_9626032_nt4105_AOS_rc|1
ATTGCAAATCTCACACAATGGGTATATAGACTATGTATACAAGCTAGCAGCAACGTATGG
>HPV2_Alpha_9626032_nt4098_AOS_rc|1
ATCTCACACAATGGGTATATAGACTATGTATACAAGCTAGCAGCAACGTATGGCGAAATA
>HPV2_Alpha_9626032_nt4091_AOS_rc|1
ACAATGGGTATATAGACTATGTATACAAGCTAGCAGCAACGTATGGCGAAATAATAACGA
>HPV2_Alpha_9626032_nt4084_AOS_rc|1
GTATATAGACTATGTATACAAGCTAGCAGCAACGTATGGCGAAATAATAACGACTATGGT
>HPV2_Alpha_9626032_nt4077_AOS_rc|1
GACTATGTATACAAGCTAGCAGCAACGTATGGCGAAATAATAACGACTATGGTAAAAACC
>HPV41_Nu_9626041_nt6645_AOS_rc|3
TGTAGAATAAAGGCAAGCTGAAACTCCTCGGTGTGCCTTAAAAACTCAAAAAACTTAGAA
>HPV41_Nu_9626041_nt6603_AOS_rc|2
AACTCAAAAAACTTAGAATTGTTATATGAAGAAGCATCCCCCTCAGGAACACTGATGGTA
>HPV41_Nu_9626041_nt3682_AOS_rc|1
TGAAAGTAGTCCCCAGATACATTATGTCACCGCTATACTTGTTTTTCCATTTGTACCTTA
>HPV41_Nu_9626041_nt3148_AOS_rc|1
TACCAGTGTCATCAATGCCACCTCTAGCTTTATACCATTCATCTGTTGGTGTAATATAAT
>HPV41_Nu_9626041_nt2640_AOS_rc|1
GCCAAAATTTCCTAAAAAAAGAGTTCCATGTATAACCATCAATGTAATATAACGGTTGCC
>HPV41_Nu_9626041_nt2632_AOS_rc|1
TTCCTAAAAAAAGAGTTCCATGTATAACCATCAATGTAATATAACGGTTGCCCATTTCCT
>HPV41_Nu_9626041_nt2578_AOS_rc|1
TTTCCTTTAAGAGGAAATGGCTTGTTAAAGTACACATACATTGTTCTGGTTTGCAGATAT
>HPV41_Nu_9626041_nt2569_AOS_rc|1
AGAGGAAATGGCTTGTTAAAGTACACATACATTGTTCTGGTTTGCAGATATTTGAATTCA
>HPV41_Nu_9626041_nt2561_AOS_rc|1
TGGCTTGTTAAAGTACACATACATTGTTCTGGTTTGCAGATATTTGAATTCATCTAATCG
>HPV41_Nu_9626041_nt1591_AOS_rc|1
TCAATGTATAAAAATTGGCATTGTTGTCGCAGCAAAACCTCCATAGCACTTATCTCACTA
>HPV1_Mu_9626063_nt7292_AOS_rc|1
CAATCAGAGAACACAAAAAATGCACACTTGTGTATAATGCACCGGAGTCTAAACAAATAA
>HPV1_Mu_9626063_nt7272_AOS_rc|1

TGCACACTTGTGTATAATGCACCGGAGTCTAAACAAATAAATCCTCAACAATCTGGAATA
>HPV1_Mu_9626063_nt7265_AOS_rc|1
TTGTGTATAATGCACCGGAGTCTAAACAAATAAATCCTCAACAATCTGGAATATATGCAG
>HPV1_Mu_9626063_nt6565_AOS_rc|2
AAATATTAGGGTCCATTGTATGAATGTAGGCTAGATTTTCGGGAGTTAACTTTACTTTAC
>HPV1_Mu_9626063_nt5769_AOS_rc|2
ATAATTTGTTGGATTTTCTGCATCATCTAACTTATTTAAAAGAGGGTGGCCCGTTATTCC
>HPV1_Mu_9626063_nt5578_AOS_rc|2
CTCTAAATGCATTTGGTGACACTTTTGGTATAGTTACAGTTTGATTACTGGAGATCTCAA
>HPV1_Mu_9626063_nt5255_AOS_rc|1
AGGATTAATTATTGGAGTCCCTTTAGAAGAATTTGGGTAATATACATGCAAGCTAGTATC
>HPV1_Mu_9626063_nt5157_AOS_rc|1
AATCTAGTATAGAAACCTCACCCTCTGAGTTAGTAATAGTAAGTTGCAAATTTTCGCCCA
>HPV1_Mu_9626063_nt5119_AOS_rc|1
GTAAGTTGCAAATTTTCGCCCACACTTTCGTTTGTGTCTAAAAGCTCTTCTTCTGAATAT
>HPV1_Mu_9626063_nt3748_AOS_rc|1
TTCCATTAATTTTAAGACCCATTAAACTGTCCCAAAAACACAGACACTGATCTGGGCAAA
>HPV18_Alpha_9626069_nt5352_AOS_rc|1
GGGACCGTTACATTACTATAGGAAGAGGCAGAAGATATAGTGGGCGAATATTTAAAAAAT
>HPV18_Alpha_9626069_nt5359_AOS_rc|1
GGTTAAAGGGACCGTTACATTACTATAGGAAGAGGCAGAAGATATAGTGGGCGAATATTT
>HPV18_Alpha_9626069_nt5345_AOS_rc|1
TTACATTACTATAGGAAGAGGCAGAAGATATAGTGGGCGAATATTTAAAAAATGCAAAGG
>HPV18_Alpha_9626069_nt5338_AOS_rc|1
ACTATAGGAAGAGGCAGAAGATATAGTGGGCGAATATTTAAAAAATGCAAAGGAGGTAGT
>HPV18_Alpha_9626069_nt5331_AOS_rc|1
GAAGAGGCAGAAGATATAGTGGGCGAATATTTAAAAAATGCAAAGGAGGTAGTAGAACGC
>HPV18_Alpha_9626069_nt5324_AOS_rc|1
CAGAAGATATAGTGGGCGAATATTTAAAAAATGCAAAGGAGGTAGTAGAACGCGATGGTA
>HPV18_Alpha_9626069_nt1507_AOS_rc|1
AAATACTGCTAACATAGCTCCTTGTTTATTGTTTACTTTTAACAAGTCTTTTAATTGTGC
>HPV18_Alpha_9626069_nt1479_AOS_rc|1
TTGTTTACTTTTAACAAGTCTTTTAATTGTGCTATGGTACATTGTGGATTTACATTTTCT
>HPV4_Gamma_9626597_nt6989_AOS_rc|1
GTTTATTTCACTATAGTTCACAAAAACAGATACATACACGTTTGCAAATCTTATATAATT
>HPV4_Gamma_9626597_nt6804_AOS_rc|2
CGTTTGGTATTAGTTTGTGCATGAGAAGAACTTATTATTCTTTTACGTTTTAGAGAACCA
>HPV4_Gamma_9626597_nt6192_AOS_rc|2
GTCCCTACATAAATATGAGGTCCTAAAGTGTACTGATCTTGTTGGTTTTGAGCACCAATA
>HPV4_Gamma_9626597_nt5220_AOS_rc|2
TATACAAAATCCGTACTAAATATATCAGATAACAAAGCTGGTTCTAGAGGAACAAATGGC
>HPV4_Gamma_9626597_nt4544_AOS_rc|1
TAGTAGGTATTTGCTGAACCCTTCTATTATATAAATCCCGTGCTCTTCCTATTGCACGAT
>HPV4_Gamma_9626597_nt3693_AOS_rc|1
ATTCAATGAGCCGTAGGTATATGTACACAGTTTAGGAAAAAGGTTGTGTTTTACAAAAGC
>HPV4_Gamma_9626597_nt3685_AOS_rc|1
AGCCGTAGGTATATGTACACAGTTTAGGAAAAAGGTTGTGTTTTACAAAAGCGTCTCTTT
>HPV4_Gamma_9626597_nt3678_AOS_rc|1
GGTATATGTACACAGTTTAGGAAAAAGGTTGTGTTTTACAAAAGCGTCTCTTTGGTCAGT
>HPV4_Gamma_9626597_nt3670_AOS_rc|1
TACACAGTTTAGGAAAAAGGTTGTGTTTTACAAAAGCGTCTCTTTGGTCAGTGCTATCAA
>HPV4_Gamma_9626597_nt3649_AOS_rc|1
TGTGTTTTACAAAAGCGTCTCTTTGGTCAGTGCTATCAAATGCAATAAGCATGCGACTAT
>HPV63_Mu_9626605_nt6415_AOS_rc|2
TATACTCATTATAATCTGCGCTATCATAAGTCTCAGGGGTTGCTTTGTTAAGAACATTTA

>HPV63_Mu_9626605_nt6407_AOS_rc|1
TTATAATCTGCGCTATCATAAGTCTCAGGGGTTGCTTTGTTAAGAACATTTATATTCATC
>HPV63_Mu_9626605_nt5232_AOS_rc|1
CTAAATGTATAATAATTAATGGAATGTCAAGATCAGTAGGATTTATAGTAGGTGATCCAA
>HPV63_Mu_9626605_nt5225_AOS_rc|1
TATAATAATTAATGGAATGTCAAGATCAGTAGGATTTATAGTAGGTGATCCAACACTAGC
>HPV63_Mu_9626605_nt4424_AOS_rc|1
AAAATTAACTAGTGGTATTTCTTGTCCTACTATTTGACCCGGCGTATCTACATCTATAAA
>HPV63_Mu_9626605_nt4417_AOS_rc|1
ACTAGTGGTATTTCTTGTCCTACTATTTGACCCGGCGTATCTACATCTATAAAAATATTA
>HPV63_Mu_9626605_nt3091_AOS_rc|1
TGGCCTCTTCTTGAAAGTCAACATAGTAGTTTTTGACACCATCATATTCTAAATAAAACA
>HPV63_Mu_9626605_nt3064_AOS_rc|1
AGTTTTTGACACCATCATATTCTAAATAAAACACACCATGAACATCTACATCACTAGCTG
>HPV63_Mu_9626605_nt3051_AOS_rc|1
ATCATATTCTAAATAAAACACACCATGAACATCTACATCACTAGCTGCTTTTCTCCATCT
>HPV16_Alpha_9627100_nt7744_AOS_rc|1
ACACAGTTCATGTATGAACTAGGGTGACATTTAGTTGGCCTTAGAAGTTTAAACCTTATG
>HPV16_Alpha_9627100_nt7072_AOS_rc|2
TTAGCAGTTGTAGAGGTAGATGAGGTGGTGGGTGTAGCTTTTCGTTTTCCTAATGTAAAT
>HPV16_Alpha_9627100_nt6912_AOS_rc|1
AAGTGTATTTTTTAAGGGGATCATCTTCTTTAGGTGCTGGAGGTGTATGTTTTTGACAAG
>HPV16_Alpha_9627100_nt6893_AOS_rc|1
ATCATCTTCTTTAGGTGCTGGAGGTGTATGTTTTTGACAAGCAATTGCCTGGGTTACAAA
>HPV16_Alpha_9627100_nt6445_AOS_rc|1
TTTGAACTGGCTAAATTTGCAGTAGACCCAGAGCCTTTAATGTATAAATCGTCTGGTACA
>HPV16_Alpha_9627100_nt6437_AOS_rc|1
GGCTAAATTTGCAGTAGACCCAGAGCCTTTAATGTATAAATCGTCTGGTACATTTTCACC
>HPV16_Alpha_9627100_nt5494_AOS_rc|1
AATAATTGTATATTGTGGAGACCCTGGAACTATAGGAATTAATGAAGGAGCTTGGTCAGT
>HPV16_Alpha_9627100_nt5487_AOS_rc|1
GTATATTGTGGAGACCCTGGAACTATAGGAATTAATGAAGGAGCTTGGTCAGTTATATTA
>HPV16_Alpha_9627100_nt3973_AOS_rc|1
TACAATAAAACACCTAAACGCAGAGGCTGCTGTTATCCACAATAGTAATACCAATATTAT
>HPV5_Beta_9627145_nt5089_AOS_rc|1
TATTAGTCAAAGAGAAACCCCTCCTACGGCCTACAGATTGATTGCGTGGCAATGGAGTAC
>HPV5_Beta_9627145_nt6974_AOS_rc|1
GCATTATAGTCTGCAACATCTTTTAGTGCTCCAGCCTGATTATATACAGAAATACTGAAA
>HPV5_Beta_9627145_nt5159_AOS_rc|1
AAACGAACTAACTTAGATGGTTGAGTTAGAAACAATGGATTGTCCACTTGTACCTGCTGT
>HPV5_Beta_9627145_nt4026_AOS_rc|1
ATGTATTTAATTTTAGCTCTGTTGCGGACATTTTTCAGTGTGTTAGCCGCCCCTTTGACA
>HPV5_Beta_9627145_nt4019_AOS_rc|1
TAATTTTAGCTCTGTTGCGGACATTTTTCAGTGTGTTAGCCGCCCCTTTGACAATGATTA
>HPV5_Beta_9627145_nt2365_AOS_rc|1
TGTATATCCAACAAGGGTCTGTTACATCATCCAATAGAGCTATCTTGCACTCTGAAAGGG
>HPV5_Beta_9627145_nt2174_AOS_rc|1
GGCACTGAGTGTAGGAATTCTTTTAATGCAGTTAGGAATACAATAAAGTTTATATTTTGG
>HPV5_Beta_9627145_nt215_AOS_rc|1
AGTCTCTAATACTTAAAGGTAATTCTGCCTTATCTTTTTCTGTCAGTTTCTGTTGGTGTT
>HPV5_Beta_9627145_nt208_AOS_rc|1
AATACTTAAAGGTAATTCTGCCTTATCTTTTTCTGTCAGTTTCTGTTGGTGTTCGGCTCC
>HPV10_Alpha_9627257_nt7080_AOS_rc|3
GATCCTGCTTCTCGGTGGGGGGTGTATCTTTCTGACAAGTAATGGCTGAAGAGGACAAAA
>HPV10_Alpha_9627257_nt6618_AOS_rc|3

CAACGTCTCGCCCCCCACCGTTGCTCTTTAATATGAAAGTGTCTGGGATGGCGTCTCCAA
>HPV10_Alpha_9627257_nt6609_AOS_rc|2
GCCCCCCACCGTTGCTCTTTAATATGAAAGTGTCTGGGATGGCGTCTCCAACAGCACTAG
>HPV10_Alpha_9627257_nt6600_AOS_rc|3
CGTTGCTCTTTAATATGAAAGTGTCTGGGATGGCGTCTCCAACAGCACTAGCCCGATTAA
>HPV10_Alpha_9627257_nt5534_AOS_rc|1
TGGAAAGTGTAGAAGACAAAGGGGAAGTGGTATTATATCCCCTGGACTGTGTGGTACTAC
>HPV10_Alpha_9627257_nt5527_AOS_rc|1
TGTAGAAGACAAAGGGGAAGTGGTATTATATCCCCTGGACTGTGTGGTACTACGATATCC
>HPV10_Alpha_9627257_nt5520_AOS_rc|2
GACAAAGGGGAAGTGGTATTATATCCCCTGGACTGTGTGGTACTACGATATCCCTCTGTA
>HPV10_Alpha_9627257_nt5513_AOS_rc|1
GGGAAGTGGTATTATATCCCCTGGACTGTGTGGTACTACGATATCCCTCTGTAAAAGCAA
>HPV10_Alpha_9627257_nt5506_AOS_rc|1
GGTATTATATCCCCTGGACTGTGTGGTACTACGATATCCCTCTGTAAAAGCAACATCACC
>HPV10_Alpha_9627257_nt5499_AOS_rc|1
TATCCCCTGGACTGTGTGGTACTACGATATCCCTCTGTAAAAGCAACATCACCATCATCA
>HPV26_Alpha_9627305_nt7307_AOS_rc|1
TATAAGGGACACCACCTAAGGTTCCTCCCATTACCTCAGGGGTAAAACAAATGACAAATA
>HPV26_Alpha_9627305_nt7291_AOS_rc|1
TAAGGTTCCTCCCATTACCTCAGGGGTAAAACAAATGACAAATAGTGTAGTAAAGACATG
>HPV26_Alpha_9627305_nt7283_AOS_rc|1
CTCCCATTACCTCAGGGGTAAAACAAATGACAAATAGTGTAGTAAAGACATGTAACCATG
>HPV26_Alpha_9627305_nt7275_AOS_rc|1
ACCTCAGGGGTAAAACAAATGACAAATAGTGTAGTAAAGACATGTAACCATGCATGAAGA
>HPV26_Alpha_9627305_nt5390_AOS_rc|1
GAAGATGTGGGCGTGTAAATGTCTGACCCACTATATACAGGTAGTTCAAATGAGGTGGAT
>HPV26_Alpha_9627305_nt5377_AOS_rc|1
TGTAAATGTCTGACCCACTATATACAGGTAGTTCAAATGAGGTGGATAAAGGCACAGTAA
>HPV26_Alpha_9627305_nt5316_AOS_rc|1
ATTGGTAGTGGAAGTATTAATAGAGGAAAACACATTGGAGGCATATCTTGGAACTGGTAA
>HPV26_Alpha_9627305_nt5309_AOS_rc|1
GTGGAAGTATTAATAGAGGAAAACACATTGGAGGCATATCTTGGAACTGGTAATGTTGTA
>HPV26_Alpha_9627305_nt5302_AOS_rc|1
TATTAATAGAGGAAAACACATTGGAGGCATATCTTGGAACTGGTAATGTTGTAGGGGAAT
>HPV26_Alpha_9627305_nt5291_AOS_rc|1
GAAAACACATTGGAGGCATATCTTGGAACTGGTAATGTTGTAGGGGAATATGACATGCGC
>HPV32_Alpha_9627327_nt7703_AOS_rc|1
AAATACAGTAGTCATAACATGAACAAAGGAAGAAGGAAATAAGTAAATGCAAAGCTATGC
>HPV32_Alpha_9627327_nt7687_AOS_rc|1
ACATGAACAAAGGAAGAAGGAAATAAGTAAATGCAAAGCTATGCCAACCTTTCTAAAACA
>HPV32_Alpha_9627327_nt4954_AOS_rc|1
AAGAGTCTACCATTACCCTCTACTGGCAAAGAAGGCCGTAAAGTAAAAGGATCACTGTAT
>HPV32_Alpha_9627327_nt4946_AOS_rc|1
ACCATTACCCTCTACTGGCAAAGAAGGCCGTAAAGTAAAAGGATCACTGTATACTGGATT
>HPV32_Alpha_9627327_nt4927_AOS_rc|1
AAAGAAGGCCGTAAAGTAAAAGGATCACTGTATACTGGATTGTGGGACGTAGTAGATGCA
>HPV32_Alpha_9627327_nt4909_AOS_rc|1
AAAGGATCACTGTATACTGGATTGTGGGACGTAGTAGATGCAACCCTTATAGTATTGGTG
>HPV32_Alpha_9627327_nt4313_AOS_rc|1
GTACTAATATACAAATAAACACTGTGGCTAAAAACCACAGCAGCAGCCACACATCGCCAT
>HPV32_Alpha_9627327_nt4298_AOS_rc|1
TAAACACTGTGGCTAAAAACCACAGCAGCAGCCACACATCGCCATTTTCTAAAGTACAAT
>HPV32_Alpha_9627327_nt4289_AOS_rc|1
TGGCTAAAAACCACAGCAGCAGCCACACATCGCCATTTTCTAAAGTACAATGTACCATAT

>HPV32_Alpha_9627327_nt4282_AOS_rc|1
AAACCACAGCAGCAGCCACACATCGCCATTTTCTAAAGTACAATGTACCATATTATCACG
>HPV34_Alpha_9627334_nt7302_AOS_rc|1
AAACCTTTGTGCTAAAACAGCACAAACAGACCAAAACATGTTATAGTATAGGCAACCGAA
>HPV34_Alpha_9627334_nt4466_AOS_rc|1
CCACAGATGTTGTTACATTAAATCCAGAACTAGAAGGCACTATGGAGGTAGGACCTGGCA
>HPV34_Alpha_9627334_nt4457_AOS_rc|1
TTGTTACATTAAATCCAGAACTAGAAGGCACTATGGAGGTAGGACCTGGCACACCAGCTT
>HPV34_Alpha_9627334_nt4338_AOS_rc|1
GTAATAACAGGAGGTCTGGTAGGTTGCAAAGGAATTTCAACTGGTCTAGATGGAGTAGTT
>HPV34_Alpha_9627334_nt4325_AOS_rc|1
GTCTGGTAGGTTGCAAAGGAATTTCAACTGGTCTAGATGGAGTAGTTGTGGGTAATGGCA
>HPV34_Alpha_9627334_nt4316_AOS_rc|1
GTTGCAAAGGAATTTCAACTGGTCTAGATGGAGTAGTTGTGGGTAATGGCACATATCCTG
>HPV34_Alpha_9627334_nt3909_AOS_rc|1
TTAACAGGGATTGGGACACATGTAAAAAAGTAATTATAATCAAAAGCAATAACCATGCAT
>HPV34_Alpha_9627334_nt3138_AOS_rc|1
TATAATCTACATGGCTACTCACTTTATACCACTTGCCTTCCAACCAATAATACACAAATG
>HPV34_Alpha_9627334_nt739_AOS_rc|1
CATAAGCAGGTCTTCTAACACTAATAGGTCAGCGTGTGTGCTCTCAATGGTAAGACACAC
>HPV34_Alpha_9627334_nt317_AOS_rc|1
TAAATTCTCTAACGTCCGTCCATACACTGATTGGTTATATCTTCTATATTGTCTAACCTT
>HPV49_Beta_9627363_nt7123_AOS_rc|1
CAAAAGTTATACTGCTCATATGGATCTTTCCTTTCTGGAGCAGGTTGTTTGTCAGGGCAT
>HPV49_Beta_9627363_nt6649_AOS_rc|2
ATGGAGCTGCCAAGAGTATTTTGGGCCTGTTGACTTGCTGCAGGTAATATGTAATTGTTA
>HPV49_Beta_9627363_nt6639_AOS_rc|1
CAAGAGTATTTTGGGCCTGTTGACTTGCTGCAGGTAATATGTAATTGTTATCCTGACCTA
>HPV49_Beta_9627363_nt5574_AOS_rc|1
AATCTTGTACATAAAAGGTATCAGATCTGGGAGTAGAGAAGCGGGGTACAGTAAATGTGG
>HPV49_Beta_9627363_nt470_AOS_rc|1
GCTTTTCCAACAAATCTAGCCTCTTAAGGCAAATGAGACATCTGACTACTATCTCAGCAA
>HPV49_Beta_9627363_nt413_AOS_rc|1
CAATATTAGCCGCTGCTCGTCCTTCTATTTCGATGCCTACGACAATTTCTTGGTGATAAT
>HPV49_Beta_9627363_nt406_AOS_rc|1
AGCCGCTGCTCGTCCTTCTATTTCGATGCCTACGACAATTTCTTGGTGATAATTAGTAAA
>HPV53_Alpha_9627377_nt7601_AOS_rc|1
GCAGCCTACAGTAAGTGACTAATAGGTAAGGCATAGTGTATAAAACAAAAAAACATTAAA
>HPV53_Alpha_9627377_nt7445_AOS_rc|1
AATGGAGGGTACCAAAAAGGCGCCAAACTAAAATGGATACCTATAAGCTATACTATATGT
>HPV53_Alpha_9627377_nt7121_AOS_rc|1
ACATACACAATACAGACACACAACATACTATTTGCGTTTGCGCTTGGAGGAGGGGGCTGA
>HPV53_Alpha_9627377_nt7114_AOS_rc|1
CAATACAGACACACAACATACTATTTGCGTTTGCGCTTGGAGGAGGGGGCTGAGGTAGAT
>HPV53_Alpha_9627377_nt7106_AOS_rc|1
ACACACAACATACTATTTGCGTTTGCGCTTGGAGGAGGGGGCTGAGGTAGATGTAGTAGA
>HPV53_Alpha_9627377_nt5495_AOS_rc|1
TATCAAAAGGAGATTGAGGTGCATAAGGCCATGTAGGGGGTCCTGTGGGCAACACTACAT
>HPV53_Alpha_9627377_nt1374_AOS_rc|1
TGTATTTGTTGCAGTGGCATTATATCCTGTCTATCCACATCCATATCACTTCCTGACCCT
>HPV53_Alpha_9627377_nt1003_AOS_rc|1
GTGTAGATATTATATTACTATTGTCTATAAACCCATCCAAATCAGATTCGGTGCTTTCCT
>HPV53_Alpha_9627377_nt996_AOS_rc|1
TATTATATTACTATTGTCTATAAACCCATCCAAATCAGATTCGGTGCTTTCCTCGTCTGT
>HPV53_Alpha_9627377_nt304_AOS_rc|1

TACACTGAACAATTGTAATATCTTAATTTTCGGACCTTACTATAAAATAGCAAACAGAAT
>HPV7_Alpha_9627389_nt7661_AOS_rc|1
ACATGGTGACTTGCCAACCGAGACTGGAAACGGTTATGGGTGGAAATTGAAGGATTTATA
>HPV7_Alpha_9627389_nt7654_AOS_rc|1
GACTTGCCAACCGAGACTGGAAACGGTTATGGGTGGAAATTGAAGGATTTATATAGTTAT
>HPV7_Alpha_9627389_nt5532_AOS_rc|1
ATTTGCTATGCGTGTAGAATTACCTGGATACAAGGAATAGGTAGAAGTTGGTGTATTATT
>HPV7_Alpha_9627389_nt4044_AOS_rc|1
AAGAATAGAAATAGTGGCCATCAAAGAGCTCCAGACACAGAAATCAGGTATATGCATGCA
>HPV7_Alpha_9627389_nt3461_AOS_rc|1
TGATGGACAAGTCTCCGTCTCTCCTTCGCTTTCGGGGCGGTGCGTGGCTGTCGTTGTATA
>HPV7_Alpha_9627389_nt1393_AOS_rc|1
CTACCACCTGTGTACGCGGACTAAGTTGCTGTATAACATCACTGCTTTCTTCTAGCACTT
>HPV7_Alpha_9627389_nt1375_AOS_rc|1
GACTAAGTTGCTGTATAACATCACTGCTTTCTTCTAGCACTTCAACCGCTTCCGTTTCAA
>HPV7_Alpha_9627389_nt1368_AOS_rc|1
TTGCTGTATAACATCACTGCTTTCTTCTAGCACTTCAACCGCTTCCGTTTCAACTGTCGC
>HPV7_Alpha_9627389_nt1360_AOS_rc|2
TAACATCACTGCTTTCTTCTAGCACTTCAACCGCTTCCGTTTCAACTGTCGCAGCCCTCC
>HPV7_Alpha_9627389_nt1343_AOS_rc|1
TCTAGCACTTCAACCGCTTCCGTTTCAACTGTCGCAGCCCTCCCACTGCCCCCCTCTATA
>HPV9_Beta_9627396_nt7206_AOS_rc|1
AGGTTCGCCTTCTCTTAGCATTTTTAGATGTTTTAACAGATGTTTTAATAGGACGTTTTC
>HPV9_Beta_9627396_nt6606_AOS_rc|1
ACCCACTTACTGTAGGATAGTAGGTGGAATTAGCTATTGTTCGTTGTTGTTGATCACTTT
>HPV9_Beta_9627396_nt6591_AOS_rc|1
GATAGTAGGTGGAATTAGCTATTGTTCGTTGTTGTTGATCACTTTTTGCTGGCAAAAAGA
>HPV9_Beta_9627396_nt4763_AOS_rc|1
TCTATGTTTTCAGAAGATGCTGTGTATGATTCCCTAGGACCACCTACTAGCTGGCCCCCA
>HPV9_Beta_9627396_nt3918_AOS_rc|1
ATGTGTCAAAGGCTAAAATCATTCGCGCTCTTCCAACTCTATCACAACTGTCTTCCCCTA
>HPV9_Beta_9627396_nt3831_AOS_rc|1
TTACTAAGCCTCTTTTTTTTTTGCGTTCCCGAAAGCGATAGCACTTAAGCACATTGGCGT
>HPV9_Beta_9627396_nt825_AOS_rc|1
CAGTTGTATATCACCCAGCAAAAGTTCCTGTTGCGCTCGAATTCCTAAATTTGTAGCAAG
>HPV9_Beta_9627396_nt789_AOS_rc|1
TCGAATTCCTAAATTTGTAGCAAGCACGTATAAACGAAGTCTTGCACCGCAACCACAGCC
>HPV9_Beta_9627396_nt768_AOS_rc|1
AAGCACGTATAAACGAAGTCTTGCACCGCAACCACAGCCAGCTACGATCTTGTAGGGAGT
>HPV54_Alpha_9628437_nt7521_AOS_rc|1
TAAATCAATGTTGGAATGCAAAGGGCGTGTTTTTACACAATGGAAAGTGAAGTTGTTCTG
>HPV54_Alpha_9628437_nt7511_AOS_rc|1
TTGGAATGCAAAGGGCGTGTTTTTACACAATGGAAAGTGAAGTTGTTCTGACAGTTATAA
>HPV54_Alpha_9628437_nt4184_AOS_rc|1
TTTCCGGCGTGGGGCTCTTGCTTTAGCCATAATACACAAATATATCTACACAATATTTAT
>HPV54_Alpha_9628437_nt3162_AOS_rc|1
TCCATATTCCCCATACTTAGATGCTTCCTTTTTAAAATCCACATAATACACTTTGTGTTC
>HPV54_Alpha_9628437_nt3153_AOS_rc|1
CCCATACTTAGATGCTTCCTTTTTAAAATCCACATAATACACTTTGTGTTCTGCATCCAT
>HPV54_Alpha_9628437_nt2990_AOS_rc|1
TTATCCTGATGCCCATCAAATATAACATCTACTGTTTGTCCACGTCTTTTTAAGCAACCA
>HPV54_Alpha_9628437_nt1334_AOS_rc|1
TGCTACACGAATATTTGTGTTATGTATTAATTCTGTAATTTGCTGCCTTCCTAGGCTATC
>HPV54_Alpha_9628437_nt1315_AOS_rc|1
TTATGTATTAATTCTGTAATTTGCTGCCTTCCTAGGCTATCTGTAGTTGTTTCCCCAGAT

>HPV54_Alpha_9628437_nt201_AOS_rc|1
CAAATAGGTCCTTATATTGAAATGCATAAATCTCTGCTGTACACACCGTCTTCTTGCAAA
>HPV24_Unclassified_9628486_nt6245_AOS_rc|1
TGAATTACTGAATTTACTAACTTAATAGGTGGACATCTACCAGCATCATTATTATCACCA
>HPV24_Unclassified_9628486_nt6237_AOS_rc|2
TGAATTTACTAACTTAATAGGTGGACATCTACCAGCATCATTATTATCACCAGCACACCT
>HPV24_Unclassified_9628486_nt1745_AOS_rc|1
TCAGTGTTTGTTCTATAATCCATTTAGGATAAGACCCATGTGTAAAGGTAGCAGCATTTG
>HPV24_Unclassified_9628486_nt1629_AOS_rc|1
GATAAAATTTGCTCCTCTGCCACATATAACATAGACACTAATAACCTTATAACAGTTTGT
>HPV24_Unclassified_9628486_nt1571_AOS_rc|1
GTCTATTTTTGCCAGCCTTGAAACACAACAGATATAAAAACATTGCAGACATTTCTGTAA
>HPV24_Unclassified_9628486_nt441_AOS_rc|1
CACCTTACAATAATGTCCGAAAGAGGTGTATTTTCTTTTTGTTCTATTTCCCAACCTATC
>HPV24_Unclassified_9628486_nt434_AOS_rc|1
CAATAATGTCCGAAAGAGGTGTATTTTCTTTTTGTTCTATTTCCCAACCTATCACACTCT
>HPV24_Unclassified_9628486_nt427_AOS_rc|1
GTCCGAAAGAGGTGTATTTTCTTTTTGTTCTATTTCCCAACCTATCACACTCTCTACAAA
>HPV24_Unclassified_9628486_nt419_AOS_rc|1
GAGGTGTATTTTCTTTTTGTTCTATTTCCCAACCTATCACACTCTCTACAAAATAATTTT
>HPV24_Unclassified_9628486_nt193_AOS_rc|1
ATTTAATAATCTACTAAGTTCTAACACTGACTGAGGTTTACCTGGTTGAGCCATCAAGAT
>HPV48_Gamma_9628542_nt6713_AOS_rc|1
TATTAATAATGTAAACATTCTACATCTTTACTATCTTACTCTCCTACGCTTTGTAGATCT
>HPV48_Gamma_9628542_nt5284_AOS_rc|1
TTACCAACTATTAAAAGTCTTTCAGTGCTTGTATAAAAATATACATCCGTTTCTTGCACG
>HPV48_Gamma_9628542_nt5113_AOS_rc|1
TCGTTTCTTACGTGGAATAAAAAAGGGATCTAAAGCAAAGTCTGAGTAACTATCAAATAA
>HPV48_Gamma_9628542_nt5101_AOS_rc|1
TGGAATAAAAAAGGGATCTAAAGCAAAGTCTGAGTAACTATCAAATAAAGGGAACCAATT
>HPV48_Gamma_9628542_nt4718_AOS_rc|1
TAGCTTCTGTTGATATTGCAGACAAATCCATATAATAATGTACTTTAGGGCCTATAGTTA
>HPV48_Gamma_9628542_nt4228_AOS_rc|1
AATAGCATTATTTAAAAATGGGTTTACCTGTGTTTGCAAGATAGTTTGTGTATAAGAATC
>HPV48_Gamma_9628542_nt4217_AOS_rc|1
TTAAAAATGGGTTTACCTGTGTTTGCAAGATAGTTTGTGTATAAGAATCATAAAACAACT
>HPV48_Gamma_9628542_nt2990_AOS_rc|1
TCCATATTTTACAGCATCAGGTTGAAATAAAGTAAAGTATACAAAATCACCATTTAGATC
>HPV48_Gamma_9628542_nt2327_AOS_rc|1
TCGGAAAATTGAAACACGTAAGTCTACTTCTCAAATAAAATAGAGATTCATCAGTTGTTA
>HPV48_Gamma_9628542_nt2319_AOS_rc|1
TTGAAACACGTAAGTCTACTTCTCAAATAAAATAGAGATTCATCAGTTGTTACATTAACA
>HPV50_Gamma_9628550_nt6292_AOS_rc|1
CTTTGAGTACATATAAGAACTTGCTACATTTAAAGGATCCAAAGGATTGACATCTTTCTT
>HPV50_Gamma_9628550_nt5145_AOS_rc|1
ATGCGTCTTCTTTTTGGAAGAAGACCTGGATGTAAATCATAATCTGAATAATCTAAAACA
>HPV50_Gamma_9628550_nt5096_AOS_rc|1
AATCTAAAACAAAAAGGGGCTGTTCAGAAACATTTGATTTACTATCAAGGATGCATCTA
>HPV50_Gamma_9628550_nt4938_AOS_rc|1
TTTTCTTCATCAGTAGCTGGTATAACTAAATGAGACTCTGTAAAATTATGTTCAACATCG
>HPV50_Gamma_9628550_nt4929_AOS_rc|1
TCAGTAGCTGGTATAACTAAATGAGACTCTGTAAAATTATGTTCAACATCGTCTGTTAAT
>HPV50_Gamma_9628550_nt4274_AOS_rc|1
CAAAGCTGGGGTCAACTAATACATTAACATTATTTATGTCTGGATTTATGAAGGGATTAA
>HPV50_Gamma_9628550_nt4251_AOS_rc|1

TTAACATTATTTATGTCTGGATTTATGAAGGGATTAATCTGTGTTTCAAAAGTTGCTGCT
>HPV50_Gamma_9628550_nt4244_AOS_rc|1
TATTTATGTCTGGATTTATGAAGGGATTAATCTGTGTTTCAAAAGTTGCTGCTATAGTAG
>HPV50_Gamma_9628550_nt1337_AOS_rc|1
TAGAAGCTTCAATTAAATCTTCTGCACAGGCAAAAACTACAATAATCCAATTTTGTGTAC
>HPV60_Gamma_9628566_nt5555_AOS_rc|1
ATAACTCTAAACTGATTTCCACTACATTTAGGTACAGTAATATTATTTGAACCACTGTCT
>HPV60_Gamma_9628566_nt5049_AOS_rc|1
AGTTTTCTGTAAATTCATCCTCTAGTATATTGTCAGGTATTAGGCCTTCATTTGTAGTTT
>HPV60_Gamma_9628566_nt5042_AOS_rc|1
TGTAAATTCATCCTCTAGTATATTGTCAGGTATTAGGCCTTCATTTGTAGTTTCAGTTAA
>HPV60_Gamma_9628566_nt4903_AOS_rc|1
GTTTCTGCAGCTGGAATATCACTAATATCAAAATAAAAGTGAACTTTCTGACCAATAGTT
>HPV60_Gamma_9628566_nt3700_AOS_rc|2
TATATGTACAACCTCTTGGCAAAGTTACTAATTTCATAAAAACATCTCGTTGTGTGGTAT
>HPV60_Gamma_9628566_nt3684_AOS_rc|1
TGGCAAAGTTACTAATTTCATAAAAACATCTCGTTGTGTGGTATCATTAAAAACAACTAA
>HPV60_Gamma_9628566_nt3659_AOS_rc|1
ACATCTCGTTGTGTGGTATCATTAAAAACAACTAACAATTTATGTCTAGAAGATTCTGGT
>HPV60_Gamma_9628566_nt3650_AOS_rc|1
TGTGTGGTATCATTAAAAACAACTAACAATTTATGTCTAGAAGATTCTGGTACGTCTATG
>HPV61_Alpha_9628574_nt7738_AOS_rc|1
GTCAAAAAAGATAGATGGAGCATTGTTCCAAACTGTGTGTGTTAGCAACCTGTCAAACAC
>HPV61_Alpha_9628574_nt7533_AOS_rc|1
TACCCGAACACAAGGAACACAGAAATGGGGAAGCTGCACAAAATAAGGCGTGCAACAATA
>HPV61_Alpha_9628574_nt4298_AOS_rc|1
TGTGGGGAGGGAAAACACACAAGGTACACCACCTAATGTAAAAGCCTGTACAACAGTATT
>HPV61_Alpha_9628574_nt4291_AOS_rc|1
AGGGAAAACACACAAGGTACACCACCTAATGTAAAAGCCTGTACAACAGTATTGCAATAA
>HPV61_Alpha_9628574_nt4243_AOS_rc|1
GTATTGCAATAAGCAGTAATAGCAAAAACAAAAATAACAATCCGCAGTTTTGCTGATCCA
>HPV61_Alpha_9628574_nt4234_AOS_rc|1
TAAGCAGTAATAGCAAAAACAAAAATAACAATCCGCAGTTTTGCTGATCCACAACCAGTA
>HPV61_Alpha_9628574_nt4221_AOS_rc|1
CAAAAACAAAAATAACAATCCGCAGTTTTGCTGATCCACAACCAGTAATGCAAACCCCTG
>HPV61_Alpha_9628574_nt4187_AOS_rc|1
TCCACAACCAGTAATGCAAACCCCTGAGGGGAACTAACTACAAGGGGATACATATTGCAA
>HPV61_Alpha_9628574_nt3134_AOS_rc|1
CATAATATAGGCCCTTATATGACACTTTTCCAGGAACCTTATACCACAGATCAGTTTCTG
>HPV61_Alpha_9628574_nt3127_AOS_rc|1
TAGGCCCTTATATGACACTTTTCCAGGAACCTTATACCACAGATCAGTTTCTGTGCTTTG
>HPV8_Beta_333074_nt6932_L1_AOS|1
ATGGGGAACTTAAGAACATCACAGACTATAAATCAACCCAGTTCAGAGAATATCTGAGAC
>HPV8_Beta_333074_nt6473_L1_AOS|1
ACTTTAAGACTTTACAACAGAACAGATCTGATGTAAGTCTCGACATAGTAAATGAGATAT
>HPV8_Beta_333074_nt6360_L1_AOS|1
AAAAGCCATTCCATGTGCAGAGGACCAACAGCAAGGTCTGTGCCCACCCATTGAACTAAA
>HPV51_Alpha_333087_nt6559_L1_AOS|1
GCCACTGCTGCGGTTTCCCCAACATTTACTCCAAGTAACTTTAAGCAATATATTAGGCAT
>HPV51_Alpha_333087_nt6048_L1_AOS|1
CACACCTGTACCTCCAGGAGACTGCCCCCCCCTGGAACTTGTATCCTCTGTCATTCAGGA
>HPV51_Alpha_333087_nt6041_L1_AOS|1
GCAAAAACACACCTGTACCTCCAGGAGACTGCCCCCCCCTGGAACTTGTATCCTCTGTCA
>HPV20_Beta_1020162_nt6739_L1_AOS|1
AATGCATTCTACATTCCACCTGTGAATAATCAGGCACAGAACAACCTAGGTAATTCAATG

>HPV20_Beta_1020162_nt6956_L1_AOS|1
GCATATCAGTTCATTCAGAAAACACTGATGTTTCTAAAATTCAAAATTATGATTCTCAGA
>HPV20_Beta_1020162_nt6944_L1_AOS|1
ATACAAATTTTAGCATATCAGTTCATTCAGAAAACACTGATGTTTCTAAAATTCAAAATT
>HPV20_Beta_1020162_nt6936_L1_AOS|1
TACTCGAAATACAAATTTTAGCATATCAGTTCATTCAGAAAACACTGATGTTTCTAAAAT
>HPV22_Beta_1020178_nt7135_L1_AOS|2
AAACGTTCTGCTACGCGGAAAACGTCTAAAACTGTAAAACGAAGGAAACTTACCTCTTAA
>HPV22_Beta_1020178_nt7128_L1_AOS|1
CAGTGTGAAACGTTCTGCTACGCGGAAAACGTCTAAAACTGTAAAACGAAGGAAACTTAC
>HPV22_Beta_1020178_nt7121_L1_AOS|1
CCAGGGTCAGTGTGAAACGTTCTGCTACGCGGAAAACGTCTAAAACTGTAAAACGAAGGA
>HPV22_Beta_1020178_nt7114_L1_AOS|1
GCCAGTGCCAGGGTCAGTGTGAAACGTTCTGCTACGCGGAAAACGTCTAAAACTGTAAAA
>HPV22_Beta_1020178_nt7107_L1_AOS|2
TGCAAGGGCCAGTGCCAGGGTCAGTGTGAAACGTTCTGCTACGCGGAAAACGTCTAAAAC
>HPV23_Beta_1020186_nt7100_L1_AOS|2
CAGCGTGTACGGTCCGGTACCAAACGGCCTGCAACTCGAAAAGTGACCAAAACTGTCAAA
>HPV23_Beta_1020186_nt7120_L1_AOS|2
CAAACGGCCTGCAACTCGAAAAGTGACCAAAACTGTCAAAAGGAAAAAAGTGCAATTGTA
>HPV23_Beta_1020186_nt7113_L1_AOS|1
CCGGTACCAAACGGCCTGCAACTCGAAAAGTGACCAAAACTGTCAAAAGGAAAAAAGTGC
>HPV23_Beta_1020186_nt7093_L1_AOS|1
TGGAGTGCAGCGTGTACGGTCCGGTACCAAACGGCCTGCAACTCGAAAAGTGACCAAAAC
>HPV23_Beta_1020186_nt7086_L1_AOS|1
TTCAAATTGGAGTGCAGCGTGTACGGTCCGGTACCAAACGGCCTGCAACTCGAAAAGTGA
>HPV23_Beta_1020186_nt6046_L1_AOS|1
TGCAGAAAATTCTAGTGAACGTCAGGAAGGTACTGTAGATGACAGAAGAAATATCTCATT
>HPV28_Alpha_1020202_nt6496_L1_AOS|1
CAACTTTTCGCTCGGCATTTTTTTAATAGAGCTGGTGTTGCTGGGGACACCATTCCTGAA
>HPV28_Alpha_1020202_nt7188_L1_AOS|1
ACGTCCAGCGTCCACCACGAGAGGATCGTCTGCTGCAAAACGAAAACGCGCCAAAAAGTA
>HPV28_Alpha_1020202_nt7181_L1_AOS|1
TTCGCAAACGTCCAGCGTCCACCACGAGAGGATCGTCTGCTGCAAAACGAAAACGCGCCA
>HPV28_Alpha_1020202_nt6503_L1_AOS|1
TCGCTCGGCATTTTTTTAATAGAGCTGGTGTTGCTGGGGACACCATTCCTGAAACATTGT
>HPV29_Alpha_1020210_nt7244_L1_AOS|1
CGTTCAGTAGTCCCCTCCAGAAAGCGCCGAACGACCACCACGGCCCCCACCCCTGCAAAG
>HPV29_Alpha_1020210_nt7264_L1_AOS|1
AAAGCGCCGAACGACCACCACGGCCCCCACCCCTGCAAAGCGAAAACGCTCGAAAAAGTA
>HPV29_Alpha_1020210_nt7257_L1_AOS|1
CCTCCAGAAAGCGCCGAACGACCACCACGGCCCCCACCCCTGCAAAGCGAAAACGCTCGA
>HPV29_Alpha_1020210_nt7237_L1_AOS|1
GCGCCGGCGTTCAGTAGTCCCCTCCAGAAAGCGCCGAACGACCACCACGGCCCCCACCCC
>HPV29_Alpha_1020210_nt6611_L1_AOS|1
GTAGGGGACAAAATCCCAGATTCCTTGTACTTAAAGGGTAACAACGGGCGAGAAACTCCT
>HPV29_Alpha_1020210_nt6604_L1_AOS|1
TGGTGTAGTAGGGGACAAAATCCCAGATTCCTTGTACTTAAAGGGTAACAACGGGCGAGA
>HPV29_Alpha_1020210_nt6597_L1_AOS|1
ATCGTGCTGGTGTAGTAGGGGACAAAATCCCAGATTCCTTGTACTTAAAGGGTAACAACG
>HPV29_Alpha_1020210_nt6588_L1_AOS|2
ACTTCTTTAATCGTGCTGGTGTAGTAGGGGACAAAATCCCAGATTCCTTGTACTTAAAGG
>HPV36_Beta_1020218_nt6692_L1_AOS|1
GGTGACGACATCCCTGACGCCAGAATTGATAATGGGACTTTTAAGAATCAGTTTTTCATT
>HPV36_Beta_1020218_nt6701_L1_AOS|1

ATCCCTGACGCCAGAATTGATAATGGGACTTTTAAGAATCAGTTTTTCATTCCTGGGGCT
>HPV37_Beta_1020226_nt7176_L1_AOS|1
TAGACCTAGAATTGTTCGATCGTCTGTAAAAGTGTCTAAAGGTACAAAGCGTAAACGGTC
>HPV37_Beta_1020226_nt7162_L1_AOS|1
TCGGGATTGCAAAGTAGACCTAGAATTGTTCGATCGTCTGTAAAAGTGTCTAAAGGTACA
>HPV37_Beta_1020226_nt7155_L1_AOS|1
CTTTCAGTCGGGATTGCAAAGTAGACCTAGAATTGTTCGATCGTCTGTAAAAGTGTCTAA
>HPV37_Beta_1020226_nt7147_L1_AOS|1
AAATTCATCTTTCAGTCGGGATTGCAAAGTAGACCTAGAATTGTTCGATCGTCTGTAAAA
>HPV37_Beta_1020226_nt7140_L1_AOS|1
AGGGAGGAAATTCATCTTTCAGTCGGGATTGCAAAGTAGACCTAGAATTGTTCGATCGTC
>HPV37_Beta_1020226_nt6528_L1_AOS|1
TATTCCCGATGGCACTGTTAATCAGGACCACAAATATTACTTACCTGCCAAATCAGACCA
>HPV37_Beta_1020226_nt6225_L1_AOS|1
TAAAGCACCAGTTTGTGCATCAGAGGAAAATAATCAGACAGGACAGTGTCCACCACTTGA
>HPV38_Beta_1020234_nt7123_L1_AOS|1
GAACACGTGCTGTCAAACGGCCGTTAGTAAGAAAATCTTCCAAATCTGTAAAACGCAAAA
>HPV38_Beta_1020234_nt7116_L1_AOS|2
ACAGCACGAACACGTGCTGTCAAACGGCCGTTAGTAAGAAAATCTTCCAAATCTGTAAAA
>HPV38_Beta_1020234_nt7109_L1_AOS|1
TTTACAAACAGCACGAACACGTGCTGTCAAACGGCCGTTAGTAAGAAAATCTTCCAAATC
>HPV38_Beta_1020234_nt6918_L1_AOS|1
CCAGACAATTCTGTACACGATACATATCGTTACATAACATCTAAAGCAACTAAATGTCCA
>HPV38_Beta_1020234_nt6911_L1_AOS|2
ACCCACCCCAGACAATTCTGTACACGATACATATCGTTACATAACATCTAAAGCAACTAA
>HPV38_Beta_1020234_nt6904_L1_AOS|1
GCTTTGTACCCACCCCAGACAATTCTGTACACGATACATATCGTTACATAACATCTAAAG
>HPV38_Beta_1020234_nt6480_L1_AOS|1
GGTACTGTCAACCAAAATCATAATTATTATTTACCTGCAAAAAATGGACAGGGTCAACGC
>HPV38_Beta_1020234_nt6470_L1_AOS|1
TATTCCAGATGGTACTGTCAACCAAAATCATAATTATTATTTACCTGCAAAAAATGGACA
>HPV55_Alpha_1020266_nt6709_L1_AOS|2
CAATATGTGCTGCTACAACTCAGTCTCCATCTACAACATATAATAGTACAGAATATAAAC
>HPV55_Alpha_1020266_nt6717_L1_AOS|1
GCTGCTACAACTCAGTCTCCATCTACAACATATAATAGTACAGAATATAAACAATACATG
>HPV55_Alpha_1020266_nt6702_L1_AOS|1
AACATGACAATATGTGCTGCTACAACTCAGTCTCCATCTACAACATATAATAGTACAGAA
>HPV55_Alpha_1020266_nt6498_L1_AOS|1
CCACAGGATTTGGTTTTTAAAGGTGCTACTAAAAGTACAGTTCCTAATGCCATATACTTT
>HPV55_Alpha_1020266_nt6491_L1_AOS|1
GGACATTCCACAGGATTTGGTTTTTAAAGGTGCTACTAAAAGTACAGTTCCTAATGCCAT
>HPV55_Alpha_1020266_nt6477_L1_AOS|1
GGAACGGTTGGAGAGGACATTCCACAGGATTTGGTTTTTAAAGGTGCTACTAAAAGTACA
>HPV55_Alpha_1020266_nt6470_L1_AOS|1
TAGGGCGGGAACGGTTGGAGAGGACATTCCACAGGATTTGGTTTTTAAAGGTGCTACTAA
>HPV66_Alpha_1020290_nt7064_L1_AOS|1
GACCCCCTAGACCCAAGGCTAGTGTATCTGCCTCTAAAAGGCGGGCGGCTCCTACCTCTT
>HPV66_Alpha_1020290_nt7057_L1_AOS|2
GGCCCTAGACCCCCTAGACCCAAGGCTAGTGTATCTGCCTCTAAAAGGCGGGCGGCTCCT
>HPV66_Alpha_1020290_nt7050_L1_AOS|1
GCAACTAGGCCCTAGACCCCCTAGACCCAAGGCTAGTGTATCTGCCTCTAAAAGGCGGGC
>HPV66_Alpha_1020290_nt6861_L1_AOS|2
CCCACCAGTTGCAACTAGCTTAGAGGATAAATATAGGTATATTAAAAGCACAGCTATTAC
>HPV66_Alpha_1020290_nt6854_L1_AOS|1
GCTTATCCCCACCAGTTGCAACTAGCTTAGAGGATAAATATAGGTATATTAAAAGCACAG

>HPV66_Alpha_1020290_nt6847_L1_AOS|2
AATATTGGCTTATCCCCACCAGTTGCAACTAGCTTAGAGGATAAATATAGGTATATTAAA
>HPV103_Unclassified_109390382_nt6381_L1_AOS|2
TTTATCTCAAACTGGGGTTAGCTCGTCTTCTACCTCCTCTAGAAAGCGGTCTCGTGTAGA
>HPV103_Unclassified_109390382_nt6212_L1_AOS|1
CTTCTATTGGAGATACATACAGATTTTTAACATCTTTAGCCACACGCTGCCCTGCTGCAG
>HPV103_Unclassified_109390382_nt6195_L1_AOS|2
TGTACCACCGCCTCCTTCTTCTATTGGAGATACATACAGATTTTTAACATCTTTAGCCAC
>HPV103_Unclassified_109390382_nt5992_L1_AOS|1
GTAAATTTTACAATAAATGTTAAGTCAGACGCTGGCGTTGCACAGCAATATGCTGCCACC
>HPV103_Unclassified_109390382_nt5819_L1_AOS|1
ACCAAAATACAGCCTCCTCGTCTGTTTACTATGCAACCCCCAGTGGATCACTGGTTTCTA
>HPV103_Unclassified_109390382_nt5809_L1_AOS|1
TCTGACAAAGACCAAAATACAGCCTCCTCGTCTGTTTACTATGCAACCCCCAGTGGATCA
>HPV103_Unclassified_109390382_nt5796_L1_AOS|1
TTTACAGGGGGCGTCTGACAAAGACCAAAATACAGCCTCCTCGTCTGTTTACTATGCAAC
>HPV103_Unclassified_109390382_nt5738_L1_AOS|1
GTGTCATGGGCGACAGTATACCTGAGGCTGAATTTGAGGGTGTTAAGCACACCTATATTT
>HPV103_Unclassified_109390382_nt5724_L1_AOS|1
TTTTGCTAGGGATGGTGTCATGGGCGACAGTATACCTGAGGCTGAATTTGAGGGTGTTAA
>HPV101_Unclassified_109390389_nt6520_L1_AOS|1
GTCCTTCTACGCCTACCAGGCCTACCTCCAGGTCTTCCAAACGCCGACGCAGGCAAGTTT
>HPV101_Unclassified_109390389_nt6193_L1_AOS|1
TGCAGCTCTGTAAAGTGTCTCTTACTCCCGATGTATTGGCACACATACACTTAATGAATC
>HPV101_Unclassified_109390389_nt6186_L1_AOS|1
CTCATTGTGCAGCTCTGTAAAGTGTCTCTTACTCCCGATGTATTGGCACACATACACTTA
>HPV101_Unclassified_109390389_nt6179_L1_AOS|1
AATTTCTCTCATTGTGCAGCTCTGTAAAGTGTCTCTTACTCCCGATGTATTGGCACACAT
>HPV101_Unclassified_109390389_nt5813_L1_AOS|1
TTATTTCACCCATAATGGCACTGTTGGTGACAGTGTTCCAGAGGAGGGGTTTGAGGGTAA
>HPV101_Unclassified_109390389_nt4975_L1_AOS|1
GCATCCAAGTCACAGACGCAAACGCAAGCGCAAACGGTCTGGTTTTTAATGTTTTGCAGA
>HPV101_Unclassified_109390389_nt4968_L1_AOS|1
TTTATTTGCATCCAAGTCACAGACGCAAACGCAAGCGCAAACGGTCTGGTTTTTAATGTT
>HPV101_Unclassified_109390389_nt4961_L1_AOS|1
GATACATTTTATTTGCATCCAAGTCACAGACGCAAACGCAAGCGCAAACGGTCTGGTTTT
>HPV101_Unclassified_109390389_nt4954_L1_AOS|1
TGCATTTGATACATTTTATTTGCATCCAAGTCACAGACGCAAACGCAAGCGCAAACGGTC
>HPV101_Unclassified_109390389_nt4925_L1_AOS|1
GAACCTCCTATTGTTGTGGATGGTTTTGATGCATTTGATACATTTTATTTGCATCCAAGT
>HPV70_Alpha_1173493_nt6356_L1_AOS|1
GGCACTTTTGGAATAGAGGTGGCATGGTGGGCGACACAATACCTTCAGAGTTATATATTA
>HPV71_Alpha_12084981_nt6854_L1_AOS|1
CCATCTGTGCTACCAAAACTGTTGAGTCTACATATAAAGCCTCTAGTTTCATGGAATATT
>HPV71_Alpha_12084981_nt6847_L1_AOS|1
AATATGTCCATCTGTGCTACCAAAACTGTTGAGTCTACATATAAAGCCTCTAGTTTCATG
>HPV71_Alpha_12084981_nt6652_L1_AOS|1
TTAAAGGTTCACCTTCTACCTTTCGTGCTTCCCCTACAAGCTCTCTTTATGCATCCACAC
>HPV71_Alpha_12084981_nt6363_L1_AOS|1
TGGCGCTTCAGCTGCCGCTGGTAGTTGCCCCCCACTTGAACTTGCCAGTACTGTTATACA
>HPV71_Alpha_12084981_nt6356_L1_AOS|1
CATGCAGTGGCGCTTCAGCTGCCGCTGGTAGTTGCCCCCCACTTGAACTTGCCAGTACTG
>HPV107_Beta_126131393_nt7242_L1_AOS|1
TGCCTCTCGAAAACGTGCAGCGCCGACCACCAAATCGGTTGCAAAGCATAGCAAACGAAA
>HPV107_Beta_126131393_nt7235_L1_AOS|1

CACTACCTGCCTCTCGAAAACGTGCAGCGCCGACCACCAAATCGGTTGCAAAGCATAGCA
>HPV107_Beta_126131393_nt7228_L1_AOS|1
CAAACAACACTACCTGCCTCTCGAAAACGTGCAGCGCCGACCACCAAATCGGTTGCAAAG
>HPV107_Beta_126131393_nt7221_L1_AOS|2
CCTATTCCAAACAACACTACCTGCCTCTCGAAAACGTGCAGCGCCGACCACCAAATCGGT
>HPV107_Beta_126131393_nt7214_L1_AOS|1
GTAAATTCCTATTCCAAACAACACTACCTGCCTCTCGAAAACGTGCAGCGCCGACCACCA
>HPV107_Beta_126131393_nt6639_L1_AOS|1
AGCACCTAAGAGTGGACAATCCCAAAGTCCTTTGGGTAATTCTATTTACTATCCCACCGT
>HPV107_Beta_126131393_nt6617_L1_AOS|1
ATCAAGATCACAAATACTATATAGCACCTAAGAGTGGACAATCCCAAAGTCCTTTGGGTA
>HPV107_Beta_126131393_nt6610_L1_AOS|1
ACAACTAATCAAGATCACAAATACTATATAGCACCTAAGAGTGGACAATCCCAAAGTCCT
>HPV107_Beta_126131393_nt6603_L1_AOS|2
TGATACAACAACTAATCAAGATCACAAATACTATATAGCACCTAAGAGTGGACAATCCCA
>HPV107_Beta_126131393_nt6596_L1_AOS|1
CTATACCTGATACAACAACTAATCAAGATCACAAATACTATATAGCACCTAAGAGTGGAC
>HPV84_Alpha_12958167_nt6476_L1_AOS|1
GTGAGGAGGTGCCTCAGTCTTTTTACCTTAAGGGGACCTCCTCCCGGGCAACCCTTAGCA
>HPVcand87_Alpha_14475578_nt5598_L1_AOS|1
ATTGATCATTCCATGGCCCCGTCCCCCACGCCCTTTGTTCCTGTGGTTCCTAGCACTGCC
>HPVcand87_Alpha_14475578_nt5614_L1_AOS|1
CCCCGTCCCCCACGCCCTTTGTTCCTGTGGTTCCTAGCACTGCCCCATATTCTGTATATA
>HPVcand87_Alpha_14475578_nt5607_L1_AOS|1
TCCATGGCCCCGTCCCCCACGCCCTTTGTTCCTGTGGTTCCTAGCACTGCCCCATATTCT
>HPVcand87_Alpha_14475578_nt5591_L1_AOS|1
TCCCGATATTGATCATTCCATGGCCCCGTCCCCCACGCCCTTTGTTCCTGTGGTTCCTAG
>HPVcand87_Alpha_14475578_nt5584_L1_AOS|1
ATACAGGTCCCGATATTGATCATTCCATGGCCCCGTCCCCCACGCCCTTTGTTCCTGTGG
>HPVcand87_Alpha_14475578_nt5577_L1_AOS|1
CCCCTATATACAGGTCCCGATATTGATCATTCCATGGCCCCGTCCCCCACGCCCTTTGTT
>HPV72_Alpha_1491683_nt6832_L1_AOS|1
CCACAGCGTCCTCTGTATCAGAATATACAGCTTCTAATTTTCGTGAGTATCTTCGCCACA
>HPV72_Alpha_1491683_nt7247_L1_AOS|1
TGCCCCACCAAGCTCTACCTCGACCCCCGCCCCTACTAAACGTAAAAAGCGCAAAAAGTA
>HPV72_Alpha_1491683_nt6825_L1_AOS|1
TGTACTGCCACAGCGTCCTCTGTATCAGAATATACAGCTTCTAATTTTCGTGAGTATCTT
>HPV72_Alpha_1491683_nt6815_L1_AOS|1
TGTAACTATTTGTACTGCCACAGCGTCCTCTGTATCAGAATATACAGCTTCTAATTTTCG
>HPV72_Alpha_1491683_nt6597_L1_AOS|1
CTACCAGCCAGTTTATATCTTAAAGGTGCCTCGGGTAGCGACAGGGTGACACCTGGTAGT
>HPV72_Alpha_1491683_nt6590_L1_AOS|1
TGAGGCACTACCAGCCAGTTTATATCTTAAAGGTGCCTCGGGTAGCGACAGGGTGACACC
>HPV73_Alpha_1491692_nt6515_L1_AOS|1
CTGTATGTGTAGGTACACAGGCTAGTAGCTCTACTACAACGTATGCCAACTCTAATTTTA
>HPV73_Alpha_1491692_nt6508_L1_AOS|1
AATTTTTCTGTATGTGTAGGTACACAGGCTAGTAGCTCTACTACAACGTATGCCAACTCT
>HPV73_Alpha_1491692_nt6010_L1_AOS|1
TGTACTTCACAAACTGTTAATACTGGTGATTGTCCCCCACTGGAATTAAAGAACACCCCT
>HPVcand86_Alpha_15741127_nt6699_L1_AOS|1
ATTAGTGCCGCTACCCAGAAGGCCTCTGAATATGACCCCTCTAAGTTTAATGAATATCTA
>HPVcand86_Alpha_15741127_nt6493_L1_AOS|1
ATTTAAAGGGCTCCTCAGGCCCCCGAGGATCCCTTAGCAGTTATGTATATGCACCCACCC
>HPV88_Gamma_167600365_nt6759_L1_AOS|1
CTGCCCACAAAACGTGTCAGGACAGTTGCCAATAGTTCTGTTAGAAATACTTCCAGAACA

>HPV88_Gamma_167600365_nt6768_L1_AOS|1
AAACGTGTCAGGACAGTTGCCAATAGTTCTGTTAGAAATACTTCCAGAACAGTAAAAAGA
>HPV88_Gamma_167600365_nt6752_L1_AOS|1
TGGTTTGCTGCCCACAAAACGTGTCAGGACAGTTGCCAATAGTTCTGTTAGAAATACTTC
>HPV88_Gamma_167600365_nt6445_L1_AOS|2
ACGAATTGGAAGCTATATTCCAGCTTTGTAGAGTTCCTTTGGAGCCTGATATTTTAGCTC
>HPV88_Gamma_167600365_nt6173_L1_AOS|1
TTTTAAAATGGGATCTCATGTTTATTTCCCTACTGCTAGTGGATCACTTACTACTAGTGA
>HPV88_Gamma_167600365_nt6166_L1_AOS|1
CTGTGAATTTTAAAATGGGATCTCATGTTTATTTCCCTACTGCTAGTGGATCACTTACTA
>HPV88_Gamma_167600365_nt6158_L1_AOS|1
TACAAATGCTGTGAATTTTAAAATGGGATCTCATGTTTATTTCCCTACTGCTAGTGGATC
>HPV88_Gamma_167600365_nt6150_L1_AOS|1
TTGCCACCTACAAATGCTGTGAATTTTAAAATGGGATCTCATGTTTATTTCCCTACTGCT
>HPV88_Gamma_167600365_nt6143_L1_AOS|1
ATACTTATTGCCACCTACAAATGCTGTGAATTTTAAAATGGGATCTCATGTTTATTTCCC
>HPV88_Gamma_167600365_nt6132_L1_AOS|1
GAGGGAAATAAATACTTATTGCCACCTACAAATGCTGTGAATTTTAAAATGGGATCTCAT
>HPV110_Beta_186461194_nt6515_L1_AOS|1
TCATAACTATTTCTTGCCAGCAAAGCAAGATCAACAACAACGCACACTAGCAAACTCTAC
>HPV110_Beta_186461194_nt6508_L1_AOS|1
ATCAAAATCATAACTATTTCTTGCCAGCAAAGCAAGATCAACAACAACGCACACTAGCAA
>HPV111_Beta_186461202_nt6757_L1_AOS|2
ATCAATTGTCTTTGATTCTTCAACTATGTAAAGTTTCTTTAGTTCCAGAAGTTTTATCTC
>HPV111_Beta_186461202_nt6466_L1_AOS|1
ATTATCTGCCAGCACAGGGTAATCAACAACAAAACATTTTGGGTAACTCCATTTATTATC
>HPV111_Beta_186461202_nt6445_L1_AOS|1
CAGTGCAACAAAATCACAATTATTATCTGCCAGCACAGGGTAATCAACAACAAAACATTT
>HPV111_Beta_186461202_nt6436_L1_AOS|1
CAGATGCAGCAGTGCAACAAAATCACAATTATTATCTGCCAGCACAGGGTAATCAACAAC
>HPVFA75/KI88-03_Unclassified_206902429_nt7084_L1_AOS|2
AAATCATTAAAGCGTAGCAGATCTGTTTCTCGGGGTACTGCAAAACGACGACGGACATAA
>HPVFA75/KI88-03_Unclassified_206902429_nt7075_L1_AOS|1
ACCAGGGTCAAATCATTAAAGCGTAGCAGATCTGTTTCTCGGGGTACTGCAAAACGACGA
>HPVFA75/KI88-03_Unclassified_206902429_nt7068_L1_AOS|1
ACAGAACACCAGGGTCAAATCATTAAAGCGTAGCAGATCTGTTTCTCGGGGTACTGCAAA
>HPVFA75/KI88-03_Unclassified_206902429_nt7061_L1_AOS|1
CAGGATTACAGAACACCAGGGTCAAATCATTAAAGCGTAGCAGATCTGTTTCTCGGGGTA
>HPVFA75/KI88-03_Unclassified_206902429_nt6902_L1_AOS|1
GATTTATAAATTCCTCAGCCACTAAATGTCCAGATAAGGTTCCTGCAAAAGATAGAGAGG
>HPVFA75/KI88-03_Unclassified_206902429_nt6670_L1_AOS|2
ACCATTTGTGTACCTTCAGATAATGGTGCTATAACTGAGTATGATTCTAGCAAATTTAGA
>HPVFA75/KI88-03_Unclassified_206902429_nt6663_L1_AOS|1
TAACTTTACCATTTGTGTACCTTCAGATAATGGTGCTATAACTGAGTATGATTCTAGCAA
>HPVFA75/KI88-03_Unclassified_206902429_nt6650_L1_AOS|2
ACACTAGAAACACTAACTTTACCATTTGTGTACCTTCAGATAATGGTGCTATAACTGAGT
>HPVFA75/KI88-03_Unclassified_206902429_nt6643_L1_AOS|1
GCTGATAACACTAGAAACACTAACTTTACCATTTGTGTACCTTCAGATAATGGTGCTATA
>HPVFA75/KI88-03_Unclassified_206902429_nt6397_L1_AOS|1
TATTTTACCAGAGGAGGTAATGTTGGAGACGCTACTCCAGATGGAGCAAATAATCAAGAT
>HPV91_Alpha_22023568_nt5981_L1_AOS|2
CAGTTCCCGTTTACTGGCTGTGGGCCACCCTTTTTTTCCTATAAAAAATAATTCTGGTAA
>HPV91_Alpha_22023568_nt5744_L1_AOS|1
GTGCCATTGTCACCGTCTGTTACACCTTCTGCAGTATCAATATATGGCACAGATTTTTAT
>HPV91_Alpha_22023568_nt5726_L1_AOS|1

CCACTTAGTGTCCCCTATGTGCCATTGTCACCGTCTGTTACACCTTCTGCAGTATCAATA
>HPV91_Alpha_22023568_nt5700_L1_AOS|2
GACCTGATATTACATTTCCTACAGTGCCACTTAGTGTCCCCTATGTGCCATTGTCACCGT
>HPV91_Alpha_22023568_nt5693_L1_AOS|1
CAAGCAGGACCTGATATTACATTTCCTACAGTGCCACTTAGTGTCCCCTATGTGCCATTG
>HPV91_Alpha_22023568_nt5683_L1_AOS|1
TATATATACACAAGCAGGACCTGATATTACATTTCCTACAGTGCCACTTAGTGTCCCCTA
>HPV91_Alpha_22023568_nt5673_L1_AOS|1
CAGTACCTGATATATATACACAAGCAGGACCTGATATTACATTTCCTACAGTGCCACTTA
>HPV91_Alpha_22023568_nt5642_L1_AOS|1
AAAATTGTTAACACTACAGTCCCACTTGCAACAGTACCTGATATATATACACAAGCAGGA
>HPV91_Alpha_22023568_nt5627_L1_AOS|1
CTGCCTAACACAACAAAAATTGTTAACACTACAGTCCCACTTGCAACAGTACCTGATATA
>HPVcand89_Alpha_22095322_nt6573_L1_AOS|1
AACCTTGTATATGCCTGGGTCTGCTGCTAATAACAGAAATACACTTACTAGCTATGTATA
>HPVcand89_Alpha_22095322_nt6554_L1_AOS|1
TGGGTGAGGACCTGCCAAAAACCTTGTATATGCCTGGGTCTGCTGCTAATAACAGAAATA
>HPV90_Alpha_22138122_nt6189_L1_AOS|1
CCAATTTATATGATGTTGTGCCTGGCGATGACACCCGGGACAATCTTACTATGGACTATA
>HPV58_Alpha_222386_nt5791_L1_AOS|1
TTTCCATCAAAAGTCCCAATAACAATAAAAAAGTATTAGTTCCCAAGGTATCAGGCTTAC
>HPV74_Alpha_27462483_nt6609_L1_AOS|1
TGTGTGCGCCTACTACACAATCCCCTCCTGCTGCTACATATACTAGTACAGACTACAAAC
>HPV74_Alpha_27462483_nt6109_L1_AOS|1
TAATGTTACTGTAAAGCCAGGCGATTGCCCTGCATTAGAATTAATTACTAGTGTAATTCA
>HPV74_Alpha_27462483_nt6102_L1_AOS|1
AATGTTCTAATGTTACTGTAAAGCCAGGCGATTGCCCTGCATTAGAATTAATTACTAGTG
>HPV92_Beta_27531786_nt7104_L1_AOS|1
ATCTGTTAATGGTCTTAAAAGAACTAGAAGCAGTTCTCAAAGAGGTACTAAACGAAAAAG
>HPV92_Beta_27531786_nt7116_L1_AOS|1
TCTTAAAAGAACTAGAAGCAGTTCTCAAAGAGGTACTAAACGAAAAAGAAAAAGTAACTA
>HPV92_Beta_27531786_nt7097_L1_AOS|1
CTAATACATCTGTTAATGGTCTTAAAAGAACTAGAAGCAGTTCTCAAAGAGGTACTAAAC
>HPV92_Beta_27531786_nt7090_L1_AOS|1
GGATTAACTAATACATCTGTTAATGGTCTTAAAAGAACTAGAAGCAGTTCTCAAAGAGGT
>HPV92_Beta_27531786_nt6709_L1_AOS|1
CCTAAGGAAGGTGGTCAAATAACCGACTATGATTCACAAAAGATTAGAGAATACACTAGA
>HPV92_Beta_27531786_nt6434_L1_AOS|1
GTGTTGTGGGTGATACCATCCCAGATGCAGTTGTAAATGAAGACCATAACTTTATGTTAC
>HPV92_Beta_27531786_nt6427_L1_AOS|1
CGCGGTGGTGTTGTGGGTGATACCATCCCAGATGCAGTTGTAAATGAAGACCATAACTTT
>HPV92_Beta_27531786_nt6420_L1_AOS|1
GTTTGTGCGCGGTGGTGTTGTGGGTGATACCATCCCAGATGCAGTTGTAAATGAAGACCA
>HPV75_Beta_2911544_nt7203_L1_AOS|1
AATTCCTTTTTCAGGCAGGCTTACGAAGGGCTTCCAAGGTAACCAGAACAGCCAGTGTCC
>HPV75_Beta_2911544_nt6633_L1_AOS|1
CCGCTGGGCAGGCTCAAAACACTTTGGGCAACTCTATTTATGTTCCCACGGTCAGTGGTT
>HPV75_Beta_2911544_nt6624_L1_AOS|1
TACCTGCAGCCGCTGGGCAGGCTCAAAACACTTTGGGCAACTCTATTTATGTTCCCACGG
>HPV76_Beta_2911551_nt6655_L1_AOS|1
CTGCAGCTGTTGGACAGGCCCAAAACACTTTGGGTAGCTCTATTTACGTGCCTACCGTTA
>HPV76_Beta_2911551_nt7242_L1_AOS|1
CAAGCAGGCCTGCGACGGGCTTCCAGAGTATCCAGGACAGCCACTGTCCGCACAGCATCC
>HPV76_Beta_2911551_nt7234_L1_AOS|1
TTCTGTTTCAAGCAGGCCTGCGACGGGCTTCCAGAGTATCCAGGACAGCCACTGTCCGCA

>HPV76_Beta_2911551_nt6118_L1_AOS|1
GTTTGGAAATAGGTCGCGGACAACCCCTAGGAGTAGGGTCTACAGGTCATCCCCTATTTA
>HPV77_Alpha_2911558_nt6602_L1_AOS|1
CCAGAATCTTTGTACCTCAAAGGGAGTAGCGGGCGTGAGACTCCCGGCAGTGCTATATAC
>HPV77_Alpha_2911558_nt7240_L1_AOS|1
CAAACGCCGGGCGCCCACCCCCTCCCCAGCTTCGACCAAACGGAAGCGCTCCAAAAAGTG
>HPV77_Alpha_2911558_nt7230_L1_AOS|1
TTCCCTCTCGCAAACGCCGGGCGCCCACCCCCTCCCCAGCTTCGACCAAACGGAAGCGCT
>HPV77_Alpha_2911558_nt7223_L1_AOS|1
TCGGTGGTTCCCTCTCGCAAACGCCGGGCGCCCACCCCCTCCCCAGCTTCGACCAAACGG
>HPV77_Alpha_2911558_nt7214_L1_AOS|1
CGACGTCGTTCGGTGGTTCCCTCTCGCAAACGCCGGGCGCCCACCCCCTCCCCAGCTTCG
>HPV77_Alpha_2911558_nt6587_L1_AOS|1
GTTGGAGACAAAATTCCAGAATCTTTGTACCTCAAAGGGAGTAGCGGGCGTGAGACTCCC
>HPV77_Alpha_2911558_nt6579_L1_AOS|1
CGGGTGATGTTGGAGACAAAATTCCAGAATCTTTGTACCTCAAAGGGAGTAGCGGGCGTG
>HPV80_Beta_2911565_nt6571_L1_AOS|1
TACCAGCACAAACAGGCCAGCAACAACGTGTTTTAGCAAATTCCACATATTTTCCTACTG
>HPV80_Beta_2911565_nt6772_L1_AOS|1
TCACCATAAGTGTTACTTCTGATGGTAGTACTATAACTGAATATAATACACAAAATATTA
>HPV80_Beta_2911565_nt6763_L1_AOS|2
ACACTAATTTCACCATAAGTGTTACTTCTGATGGTAGTACTATAACTGAATATAATACAC
>HPV80_Beta_2911565_nt6756_L1_AOS|1
ACAAGAAACACTAATTTCACCATAAGTGTTACTTCTGATGGTAGTACTATAACTGAATAT
>HPV80_Beta_2911565_nt6749_L1_AOS|1
TGATAATACAAGAAACACTAATTTCACCATAAGTGTTACTTCTGATGGTAGTACTATAAC
>HPV80_Beta_2911565_nt6562_L1_AOS|1
ACTTTTATTTACCAGCACAAACAGGCCAGCAACAACGTGTTTTAGCAAATTCCACATATT
>HPVRTRX7_Unclassified_30315616_nt7253_L1_AOS|1
CCCGATAAAAATCCACCAAAGGAAAAACCTGATCCTTATGAAAAATTAACATTCTGGAAT
>HPVRTRX7_Unclassified_30315616_nt6984_L1_AOS|1
GTATATCTATATCTAGTGAAAATCAAGATATACAGCAAATACAATCATATGACTCACAAA
>HPV65_Gamma_312100_nt6179_L1_AOS|1
CTGCTCAAGAAGGTCAGGATCAGAATACTTTAGGTCCACATATATATATATAGGCACTCCTA
>HPV65_Gamma_312100_nt6172_L1_AOS|1
TGGATTCCTGCTCAAGAAGGTCAGGATCAGAATACTTTAGGTCCACATATATATATATAGGC
>HPV65_Gamma_312100_nt6165_L1_AOS|1
TGATTACTGGATTCCTGCTCAAGAAGGTCAGGATCAGAATACTTTAGGTCCACATATATA
>HPV65_Gamma_312100_nt6158_L1_AOS|1
TTAAAACTGATTACTGGATTCCTGCTCAAGAAGGTCAGGATCAGAATACTTTAGGTCCAC
>HPV65_Gamma_312100_nt6151_L1_AOS|2
TTCGAGGTTAAAACTGATTACTGGATTCCTGCTCAAGAAGGTCAGGATCAGAATACTTTA
>HPV65_Gamma_312100_nt6144_L1_AOS|1
AGAACCTTTCGAGGTTAAAACTGATTACTGGATTCCTGCTCAAGAAGGTCAGGATCAGAA
>HPV65_Gamma_312100_nt5585_L1_AOS|1
TGATTGATAATGGTTTCTATGATTCTGACCATGAACGTCTTGTTTGGAGACTCAGAGGAA
>HPV65_Gamma_312100_nt5576_L1_AOS|2
GGTTTGCATTGATTGATAATGGTTTCTATGATTCTGACCATGAACGTCTTGTTTGGAGAC
>HPV67_Alpha_3228267_nt6906_L1_AOS|1
ACATCCCCTCCAACAGCAAAGGAAGATCCTCTTAAAAAGTACAGTTTTTGGGAAATCAAT
>HPV67_Alpha_3228267_nt6895_L1_AOS|1
CCTGTCAAAAAACATCCCCTCCAACAGCAAAGGAAGATCCTCTTAAAAAGTACAGTTTTT
>HPV67_Alpha_3228267_nt6760_L1_AOS|1
CCCTTACTGCAAATGTTATGCAATACATACACACCATGAATCCAGATATATTAGAGGACT
>HPV67_Alpha_3228267_nt6753_L1_AOS|1

AAAATATCCCTTACTGCAAATGTTATGCAATACATACACACCATGAATCCAGATATATTA
>HPV67_Alpha_3228267_nt6745_L1_AOS|1
AGCTGTGCAAAATATCCCTTACTGCAAATGTTATGCAATACATACACACCATGAATCCAG
>HPV67_Alpha_3228267_nt6738_L1_AOS|1
ATATTTCAGCTGTGCAAAATATCCCTTACTGCAAATGTTATGCAATACATACACACCATG
>HPV67_Alpha_3228267_nt6437_L1_AOS|1
AGATTTGTATTTTAAGGGATCTGCAAACACTTCTGCACTGCAAACCTCAGCTTTTTTTCC
>HPV67_Alpha_3228267_nt5754_L1_AOS|1
CATCCTTACTTTTCCATTCCTAATCCCTCCAACACTAAAAAGGTGTTAGTGCCCAAGGTG
>HPV11_Alpha_333026_nt7176_L1_AOS|2
GATATCGAGGACGGACGTCTGCTCGTACAGGTATAAAGCGCCCAGCTGTGTCTAAGCCCT
>HPV11_Alpha_333026_nt7193_L1_AOS|1
TCTGCTCGTACAGGTATAAAGCGCCCAGCTGTGTCTAAGCCCTCTACAGCCCCCAAACGA
>HPV11_Alpha_333026_nt7186_L1_AOS|1
ACGGACGTCTGCTCGTACAGGTATAAAGCGCCCAGCTGTGTCTAAGCCCTCTACAGCCCC
>HPV11_Alpha_333026_nt6787_L1_AOS|1
GACACTATGTGCATCTGTGTCTAAATCTGCTACATACACTAATTCAGATTATAAGGAATA
>HPV31_Alpha_333048_nt6981_L1_AOS|1
CACGTCCTAAATTTAAAGCAGGTAAACGTAGTGCACCCTCAGCATCTACCACTACACCAG
>HPV31_Alpha_333048_nt6974_L1_AOS|1
TATAGGGCACGTCCTAAATTTAAAGCAGGTAAACGTAGTGCACCCTCAGCATCTACCACT
>HPV31_Alpha_333048_nt6967_L1_AOS|2
GGCAGGATATAGGGCACGTCCTAAATTTAAAGCAGGTAAACGTAGTGCACCCTCAGCATC
>HPV33_Alpha_333049_nt7012_L1_AOS|1
TAAAGCAAAACCTAAACTTAAACGTGCAGCCCCCACATCCACCCGCACATCGTCTGCAAA
>HPV35_Alpha_333050_nt6970_L1_AOS|1
GCCGTAAATTTTTGTTACAAGCAGGACTAAAGGCCAGGCCTAATTTTAGATTAGGCAGGC
>HPV35_Alpha_333050_nt6992_L1_AOS|1
AGGACTAAAGGCCAGGCCTAATTTTAGATTAGGCAGGCGTGCAGCTCCAGCATCTACATC
>HPV35_Alpha_333050_nt6982_L1_AOS|1
TGTTACAAGCAGGACTAAAGGCCAGGCCTAATTTTAGATTAGGCAGGCGTGCAGCTCCAG
>HPV35_Alpha_333050_nt6963_L1_AOS|1
CCGTTGGGCCGTAAATTTTTGTTACAAGCAGGACTAAAGGCCAGGCCTAATTTTAGATTA
>HPV35_Alpha_333050_nt5966_L1_AOS|1
TCTTAATAAATATGTTGGTAACTCTGGTAACTCTGGTACAGATAACAGGGAATGCATTTC
>HPV35_Alpha_333050_nt5957_L1_AOS|1
TACTGAAAATCTTAATAAATATGTTGGTAACTCTGGTAACTCTGGTACAGATAACAGGGA
>HPV35_Alpha_333050_nt5714_L1_AOS|1
CCCATACTATGCTATTAAAAAACAAGATTCTAATAAAATAGCAGTACCCAAGGTATCTGG
>HPV47_Beta_333062_nt6949_L1_AOS|1
TACAAATTTCAGCATCTCTGTTTACTCTCAGGCAGGGGACATAAAGGATATACAGGATTA
>HPV47_Beta_333062_nt6964_L1_AOS|1
CTCTGTTTACTCTCAGGCAGGGGACATAAAGGATATACAGGATTATAATGCAGACAATTT
>HPV47_Beta_333062_nt6956_L1_AOS|1
TTCAGCATCTCTGTTTACTCTCAGGCAGGGGACATAAAGGATATACAGGATTATAATGCA
>HPV42_Alpha_333211_nt6618_L1_AOS|1
CTGGCGCAATTGGTGAACCTGTACCTGATGAACTGTATACCAAGGCTGCTAATAATGCAT
>HPV42_Alpha_333211_nt7265_L1_AOS|1
CCTAAACTGTCTGTAGGTAAACGAAAGGCGTCTACAGCTAAATCTGTTTCTTCAGCTAAA
>HPV42_Alpha_333211_nt6645_L1_AOS|1
ATGAACTGTATACCAAGGCTGCTAATAATGCATCTGGCAGACATAATTTAGGTAGTAGTA
>HPV42_Alpha_333211_nt6638_L1_AOS|1
GTACCTGATGAACTGTATACCAAGGCTGCTAATAATGCATCTGGCAGACATAATTTAGGT
>HPV39_Alpha_333245_nt7078_L1_AOS|2
CTATAGGTCCCCGAAAGCGGCCTGCTGCATCCACTTCCTCGTCCTCAGCTACTAAACACA

>HPV39_Alpha_333245_nt7071_L1_AOS|1
CGCCCTACTATAGGTCCCCGAAAGCGGCCTGCTGCATCCACTTCCTCGTCCTCAGCTACT
>HPV39_Alpha_333245_nt7063_L1_AOS|1
TCCGCAGGCGCCCTACTATAGGTCCCCGAAAGCGGCCTGCTGCATCCACTTCCTCGTCCT
>HPV39_Alpha_333245_nt6856_L1_AOS|1
CTGTAGCTCCTCCACCATCTGCCAGTTTGGTAGACACTTACAGATACCTACAGTCTGCAG
>HPV39_Alpha_333245_nt6658_L1_AOS|2
CCAACTTTACATTATCTACCTCTATAGAGTCTTCCATACCTTCTACATATGATCCTTCTA
>HPV39_Alpha_333245_nt6455_L1_AOS|1
TGCCCAATTGTATATTAAGGGCACAGATATACGTGCAAACCCCGGTAGTTCTGTATACTG
>HPV39_Alpha_333245_nt6448_L1_AOS|2
CCATTCCTGCCCAATTGTATATTAAGGGCACAGATATACGTGCAAACCCCGGTAGTTCTG
>HPV39_Alpha_333245_nt6441_L1_AOS|2
GGTGACGCCATTCCTGCCCAATTGTATATTAAGGGCACAGATATACGTGCAAACCCCGGT
>HPV39_Alpha_333245_nt6433_L1_AOS|1
GTATGGTGGGTGACGCCATTCCTGCCCAATTGTATATTAAGGGCACAGATATACGTGCAA
>HPV39_Alpha_333245_nt6426_L1_AOS|1
CGTGGTGGTATGGTGGGTGACGCCATTCCTGCCCAATTGTATATTAAGGGCACAGATATA
>HPV93_Beta_37089393_nt7102_L1_AOS|1
ATCAGGCAAATCTTGTACAATCACCAGCTAAACGGTCATCATCTATTTCTCGAGGAACTA
>HPV93_Beta_37089393_nt6942_L1_AOS|1
ACTTATCGATATATTAATAGTTTAGCTACACCATGTCCCGATAAGGTTCCACCAAAGGAA
>HPV93_Beta_37089393_nt6935_L1_AOS|2
ACAGAGTACTTATCGATATATTAATAGTTTAGCTACACCATGTCCCGATAAGGTTCCACC
>HPV93_Beta_37089393_nt6928_L1_AOS|1
ATCCGTTACAGAGTACTTATCGATATATTAATAGTTTAGCTACACCATGTCCCGATAAGG
>HPV93_Beta_37089393_nt6921_L1_AOS|2
CCAGACAATCCGTTACAGAGTACTTATCGATATATTAATAGTTTAGCTACACCATGTCCC
>HPV93_Beta_37089393_nt6158_L1_AOS|1
TGCACCTAAATGTGATGGAGCCAATGACCAAAATGGGATTTGCCCACCTATACAATTAGT
>HPV93_Beta_37089393_nt6151_L1_AOS|1
GGGATGTTGCACCTAAATGTGATGGAGCCAATGACCAAAATGGGATTTGCCCACCTATAC
>HPV12_Beta_396910_nt6897_L1_AOS|1
ACCAATTTCAGTATATCTATTTACAGTGATAATCAAAATGTACACGACATTCCAAATTAT
>HPV12_Beta_396910_nt6373_L1_AOS|1
ATGCACCAGCTGAAAATGGTGTTTGCCCTCCCATAGAGTTAAAGAACACTTTCATTGAAG
>HPV14D_Beta_396918_nt6445_L1_AOS|1
TAGCCTAAAGAATGTTTATTACATTCCACCAATGACAAATCAACCACAAAACAATATTGG
>HPV14D_Beta_396918_nt6672_L1_AOS|1
GTATATCAGTTAGTTCAGAAAACACTGAGGTATCCAAAATTGACAATTATACCTCTCAGA
>HPV14D_Beta_396918_nt6458_L1_AOS|1
GTTTATTACATTCCACCAATGACAAATCAACCACAAAACAATATTGGCAATGCCATGTAT
>HPV14D_Beta_396918_nt5997_L1_AOS|1
ATCCCAACTCATACAGGCAACAAGCTAACTCCACTGATGACAGACAAAATGTGTCATTTG
>HPV14D_Beta_396918_nt5982_L1_AOS|1
TTGGTGATACAGAAAATCCCAACTCATACAGGCAACAAGCTAACTCCACTGATGACAGAC
>HPV15_Beta_396924_nt6558_L1_AOS|1
CCAGCACAATCAACCCAACAACAAAATAACTTAGCAAATTCTACTTACTTTCCCACAGTT
>HPV15_Beta_396924_nt6547_L1_AOS|1
ATTTTATTTACCAGCACAATCAACCCAACAACAAAATAACTTAGCAAATTCTACTTACT
>HPV17_Beta_396932_nt6751_L1_AOS|1
TAGCTGACAACACTAGGAACACAAATTTTTCTATTAGCGTGTCTACAGAAGCTGGGGCTG
>HPV17_Beta_396932_nt6581_L1_AOS|2
AGCTCAAACTGGCCAACAACAGCGCACTTTGGGTAATTCCACTTATTTTCCAACTGTAAG
>HPV17_Beta_396932_nt6574_L1_AOS|1

ATTTACCAGCTCAAACTGGCCAACAACAGCGCACTTTGGGTAATTCCACTTATTTTCCAA
>HPV17_Beta_396932_nt6565_L1_AOS|1
ACAAATTTTATTTACCAGCTCAAACTGGCCAACAACAGCGCACTTTGGGTAATTCCACTT
>HPV17_Beta_396932_nt6558_L1_AOS|1
CAGGATCACAAATTTTATTTACCAGCTCAAACTGGCCAACAACAGCGCACTTTGGGTAAT
>HPV17_Beta_396932_nt6070_L1_AOS|1
TAGGCACTACAGGACATCCCTTGTTTAATAAGTTAAGAGACACTGAAAATAACAGTAGCT
>HPV19_Beta_396940_nt6704_L1_AOS|2
AAAAATACATACTACATACCTCCTAACAATAGTCAGCAACAATATACTAATTTAGGAAAT
>HPV25_Beta_396948_nt7158_L1_AOS|1
GTTCCTGCACCGGACAATTCTATTCAGGATACTTATCGCTACATTGATTCTTTAGCCACA
>HPV25_Beta_396948_nt7147_L1_AOS|1
AATTAGGATTCGTTCCTGCACCGGACAATTCTATTCAGGATACTTATCGCTACATTGATT
>HPV25_Beta_396948_nt7061_L1_AOS|2
AATATTACAACTTTGCAAAGTACCGTTGAAGGCAGAAATATTGGCTCAGATTAATGCAAT
>HPV25_Beta_396948_nt7054_L1_AOS|2
TATCATTAATATTACAACTTTGCAAAGTACCGTTGAAGGCAGAAATATTGGCTCAGATTA
>HPV25_Beta_396948_nt7047_L1_AOS|1
TATGAGTTATCATTAATATTACAACTTTGCAAAGTACCGTTGAAGGCAGAAATATTGGCT
>HPV25_Beta_396948_nt6737_L1_AOS|1
TGCATTTTACATACCACCTAACAGTAGTCAGGCTCAATATAATAATCTAGGTAACTCAAT
>HPV27_Alpha_396964_nt5524_L1_AOS|1
CTGCCTCCTCTGCATCTACACTGCGAGGGTCCACTACAGCCCCTCTCTCGGGTGGTGTTG
>HPV27_Alpha_396964_nt5669_L1_AOS|1
ATCCTCTGTTTACATATTTGGGGGGGATTATTATTTACTGCCAAGTTATATCCTGTGGCC
>HPV27_Alpha_396964_nt5641_L1_AOS|1
CCCTCATTCCTGTGGCTCCATCGTTGCCATCCTCTGTTTACATATTTGGGGGGGATTATT
>HPV27_Alpha_396964_nt5632_L1_AOS|1
GTTTGGGGCCCCTCATTCCTGTGGCTCCATCGTTGCCATCCTCTGTTTACATATTTGGGG
>HPV27_Alpha_396964_nt5624_L1_AOS|1
TGTTCCTGGTTTGGGGCCCCTCATTCCTGTGGCTCCATCGTTGCCATCCTCTGTTTACAT
>HPV27_Alpha_396964_nt5608_L1_AOS|1
ATATTGAACCACCCGTTGTTCCTGGTTTGGGGCCCCTCATTCCTGTGGCTCCATCGTTGC
>HPV27_Alpha_396964_nt5595_L1_AOS|1
TATACTGGGCCTGATATTGAACCACCCGTTGTTCCTGGTTTGGGGCCCCTCATTCCTGTG
>HPV27_Alpha_396964_nt5581_L1_AOS|1
TTGATGTGCCTGTGTATACTGGGCCTGATATTGAACCACCCGTTGTTCCTGGTTTGGGGC
>HPV27_Alpha_396964_nt5560_L1_AOS|1
CAGCCCCTCTCTCGGGTGGTGTTGATGTGCCTGTGTATACTGGGCCTGATATTGAACCAC
>HPV27_Alpha_396964_nt5510_L1_AOS|1
TAATACCACGGTATCTGCCTCCTCTGCATCTACACTGCGAGGGTCCACTACAGCCCCTCT
>HPV30_Alpha_396973_nt7098_L1_AOS|2
AAACGCTCGGCCCCCAGTTCCTCTACCTCTACACCATCAGCCAAACGCAAGCGGCGGTGA
>HPV30_Alpha_396973_nt7090_L1_AOS|1
CTACTAAAAAACGCTCGGCCCCCAGTTCCTCTACCTCTACACCATCAGCCAAACGCAAGC
>HPV30_Alpha_396973_nt7082_L1_AOS|1
TTCTACTACTACTAAAAAACGCTCGGCCCCCAGTTCCTCTACCTCTACACCATCAGCCAA
>HPV30_Alpha_396973_nt7075_L1_AOS|1
CTAAACCTTCTACTACTACTAAAAAACGCTCGGCCCCCAGTTCCTCTACCTCTACACCAT
>HPV30_Alpha_396973_nt7066_L1_AOS|1
GGGTTCGTACTAAACCTTCTACTACTACTAAAAAACGCTCGGCCCCCAGTTCCTCTACCT
>HPV30_Alpha_396973_nt6841_L1_AOS|1
TACTTGAGGGCTGGAATATTGGATTGTCACCCCCAGCTGCCACAAGCTTAGAGGACAAAT
>HPV30_Alpha_396973_nt6833_L1_AOS|1
CTCCACTTTACTTGAGGGCTGGAATATTGGATTGTCACCCCCAGCTGCCACAAGCTTAGA

>HPV30_Alpha_396973_nt6826_L1_AOS|2
CTATGAACTCCACTTTACTTGAGGGCTGGAATATTGGATTGTCACCCCCAGCTGCCACAA
>HPV30_Alpha_396973_nt6819_L1_AOS|1
TTACATACTATGAACTCCACTTTACTTGAGGGCTGGAATATTGGATTGTCACCCCCAGCT
>HPV30_Alpha_396973_nt6655_L1_AOS|1
ACACCACTAGGAACACAAACATGACTATATCTGCAACCACACAAACGTTATCCACATATA
>HPV3_Alpha_397005_nt7029_L1_AOS|1
ATTACCTGCCAGAAAGATGCACCTCCCACTGAGAAGCAAGACCCCTACGCCAAACTAAAC
>HPV3_Alpha_397005_nt7178_L1_AOS|1
TACCCGCTCTAGTATATCTGTTCGTAAACGCTCGGCGACAACCACATCTAGAACAGCTGC
>HPV3_Alpha_397005_nt7154_L1_AOS|1
ATTTCTCATGCAGCTCGGTGTAGGTACCCGCTCTAGTATATCTGTTCGTAAACGCTCGGC
>HPV3_Alpha_397005_nt7147_L1_AOS|1
GCAGGAAATTTCTCATGCAGCTCGGTGTAGGTACCCGCTCTAGTATATCTGTTCGTAAAC
>HPV3_Alpha_397005_nt6772_L1_AOS|1
GTACTAATATGACATTGTGTGTTTCTACTGAAACCTCGGCTACATATGATGCTACTAAAT
>HPV3_Alpha_397005_nt6523_L1_AOS|1
ATTTTCTTAACAGAGCTGGTATGGCTGGAGACACCGTGCCTGACGCGTTGTACATTAAAG
>HPV40_Alpha_397014_nt6569_L1_AOS|2
CTACTGGTGATAGTGTCCCAACTGACTTATATATAACAGGTACATCTGGTCGGACTCCTA
>HPV45_Alpha_397022_nt7077_L1_AOS|1
TGCTGCTTCCACGTCTACTGCATCTACTGCATCTAGGCCTGCCAAACGTGTACGTATACG
>HPV45_Alpha_397022_nt7089_L1_AOS|1
GTCTACTGCATCTACTGCATCTAGGCCTGCCAAACGTGTACGTATACGTAGTAAGAAATA
>HPV45_Alpha_397022_nt7070_L1_AOS|1
AGCGTCCTGCTGCTTCCACGTCTACTGCATCTACTGCATCTAGGCCTGCCAAACGTGTAC
>HPV52_Alpha_397038_nt6462_L1_AOS|1
GTGCCAGGTGATTTATATATACAAGGGTCTAACTCTGGCAATACTGCCACTGTACAAAGC
>HPV52_Alpha_397038_nt6455_L1_AOS|1
TGACCCTGTGCCAGGTGATTTATATATACAAGGGTCTAACTCTGGCAATACTGCCACTGT
>HPV52_Alpha_397038_nt6448_L1_AOS|1
CCTTAGGTGACCCTGTGCCAGGTGATTTATATATACAAGGGTCTAACTCTGGCAATACTG
>HPV52_Alpha_397038_nt6441_L1_AOS|1
GCCGGTACCTTAGGTGACCCTGTGCCAGGTGATTTATATATACAAGGGTCTAACTCTGGC
>HPV56_Alpha_397053_nt6120_L1_AOS|1
AGTCCACACAAGTTACCACAGGGGACTGCCCGCCTCTTGCATTAATTAATACACCTATAG
>HPV56_Alpha_397053_nt7010_L1_AOS|1
ACTAGGTCAAAGCCTGCTGTAGCTACCTCTAAAAAGCGATCTGCTCCTACCTCCACCTCT
>HPV56_Alpha_397053_nt7002_L1_AOS|2
AACTGGGCACTAGGTCAAAGCCTGCTGTAGCTACCTCTAAAAAGCGATCTGCTCCTACCT
>HPV56_Alpha_397053_nt6626_L1_AOS|1
AGTACTGCTACAGAACAGTTAAGTAAATATGATGCACGAAAAATTAATCAGTACCTTAGA
>HPV56_Alpha_397053_nt6409_L1_AOS|1
TGCAGAGTTATATTTAAAGGGTAGCAATGGTAGAGAACCCCCTCCGAGTTCTGTATATGT
>HPV56_Alpha_397053_nt6387_L1_AOS|2
GTAAAGTTGGGGAAACAATACCTGCAGAGTTATATTTAAAGGGTAGCAATGGTAGAGAAC
>HPV56_Alpha_397053_nt6108_L1_AOS|1
GTGCTGTGTGTAAGTCCACACAAGTTACCACAGGGGACTGCCCGCCTCTTGCATTAATTA
>HPV56_Alpha_397053_nt5509_L1_AOS|1
GTATATATACAGGGATCCTCCTTTGCATTATGGCCTGTGTATTTTTTTAGACGTAGGCGC
>HPV56_Alpha_397053_nt5502_L1_AOS|1
CCATGATGTATATATACAGGGATCCTCCTTTGCATTATGGCCTGTGTATTTTTTTAGACG
>HPV56_Alpha_397053_nt5495_L1_AOS|1
ATGTTACCCATGATGTATATATACAGGGATCCTCCTTTGCATTATGGCCTGTGTATTTTT
>HPVcand62_Alpha_39932599_nt6590_L1_AOS|1

ACTGACCTGTATATGAAAGGTACCCCAGGCAGTGACAGACAAATGCCGGGCAGTTACATT
>HPVcand62_Alpha_39932599_nt6583_L1_AOS|1
CCTGCCCACTGACCTGTATATGAAAGGTACCCCAGGCAGTGACAGACAAATGCCGGGCAG
>HPV43_Alpha_40804474_nt6971_L1_AOS|1
GTCCCCACCTGCCTCTGCTTCTTTGGAAGATACTTATCGCTTTTTGTCTAACAAGGCCAT
>HPV43_Alpha_40804474_nt6541_L1_AOS|2
CTGGTAAAACTGGCGACGTTGTGCCTTCCGATATGTATATTGCTGGCTCTAATACCAGGT
>HPV81_Alpha_40804509_nt6384_L1_AOS|2
CTAATGTTGCTCCAGCACAAAACGAATGCCCCCCCTTGGAGTTTAAAAATACAACCATTC
>HPV81_Alpha_40804509_nt6359_L1_AOS|2
CACTGGGCCCGGGGTACCTTTTGTGCTAATGTTGCTCCAGCACAAAACGAATGCCCCCCC
>HPV95_Gamma_40804520_nt6821_L1_AOS|1
TAATAAATGGATCTCGTAAAAGACAAAGAGCTATTACTTCTCAAACTGCAACAGGCACAA
>HPV95_Gamma_40804520_nt6812_L1_AOS|1
AGAGTGGATTAATAAATGGATCTCGTAAAAGACAAAGAGCTATTACTTCTCAAACTGCAA
>HPV95_Gamma_40804520_nt6650_L1_AOS|1
AATACAGATTCATTACATCCAGAGCTACTCGTTGTCCAACTCAAAATCCACCAGCAGAAA
>HPV95_Gamma_40804520_nt6232_L1_AOS|1
AACCAAGAACAAAATAATCTTGGCCCTCACATTTATTTTGGAACTCCTAGCGGTTCTCTT
>HPV95_Gamma_40804520_nt6224_L1_AOS|1
CTCAGAGTAACCAAGAACAAAATAATCTTGGCCCTCACATTTATTTTGGAACTCCTAGCG
>HPV95_Gamma_40804520_nt6217_L1_AOS|1
ATTGCTGCTCAGAGTAACCAAGAACAAAATAATCTTGGCCCTCACATTTATTTTGGAACT
>HPV95_Gamma_40804520_nt6210_L1_AOS|1
TTACATAATTGCTGCTCAGAGTAACCAAGAACAAAATAATCTTGGCCCTCACATTTATTT
>HPV95_Gamma_40804520_nt6203_L1_AOS|1
AGTCTGATTACATAATTGCTGCTCAGAGTAACCAAGAACAAAATAATCTTGGCCCTCACA
>HPV95_Gamma_40804520_nt6196_L1_AOS|1
GAAGTGAAGTCTGATTACATAATTGCTGCTCAGAGTAACCAAGAACAAAATAATCTTGGC
>HPV94_Alpha_40804528_nt6559_L1_AOS|1
AGAGCCAGTGCAGTGGGAGACACCATTCCTGACACTCTAATATTGAAGTCGGCCGGTGGT
>HPV94_Alpha_40804528_nt6552_L1_AOS|1
TTTCAATAGAGCCAGTGCAGTGGGAGACACCATTCCTGACACTCTAATATTGAAGTCGGC
>HPVcand85_Alpha_4574720_nt5995_L1_AOS|2
CAGAAAATTCCCATGTTGCTACTTCTGTAGTTACACACGACACTAGAGATAATGTGTCAG
>HPV96_Beta_50253426_nt7334_L1_AOS|1
AGGGGTTACTGTAACCGGGAGGGCTACAACCTCAAGAGGTACAAAACGAAAACGACGCTG
>HPV96_Beta_50253426_nt7326_L1_AOS|1
GTTAACAGAGGGGTTACTGTAACCGGGAGGGCTACAACCTCAAGAGGTACAAAACGAAAA
>HPV96_Beta_50253426_nt7319_L1_AOS|1
CAAAAGAGTTAACAGAGGGGTTACTGTAACCGGGAGGGCTACAACCTCAAGAGGTACAAA
>HPV96_Beta_50253426_nt7312_L1_AOS|1
TGCAAAACAAAAGAGTTAACAGAGGGGTTACTGTAACCGGGAGGGCTACAACCTCAAGAG
>HPV96_Beta_50253426_nt7303_L1_AOS|1
AAGCCAACCTGCAAAACAAAAGAGTTAACAGAGGGGTTACTGTAACCGGGAGGGCTACAA
>HPV96_Beta_50253426_nt6751_L1_AOS|2
GTTCCATTTACACTCCCACAGTCAGTGGATCTTTAGTTTCTACAGATGCACAGATTTTCA
>HPV96_Beta_50253426_nt6714_L1_AOS|1
AAGGCTGCCAGTGATCAAAACAGAGATACAATGGCAAGTTCCATTTACACTCCCACAGTC
>HPV96_Beta_50253426_nt6707_L1_AOS|1
TTATTTAAAGGCTGCCAGTGATCAAAACAGAGATACAATGGCAAGTTCCATTTACACTCC
>HPV96_Beta_50253426_nt6700_L1_AOS|1
ACCATTATTATTTAAAGGCTGCCAGTGATCAAAACAGAGATACAATGGCAAGTTCCATTT
>HPV96_Beta_50253426_nt6693_L1_AOS|1
GAAGACAACCATTATTATTTAAAGGCTGCCAGTGATCAAAACAGAGATACAATGGCAAGT

>HPV83_Alpha_5059324_nt6567_L1_AOS|2
TATATTCCAGGCACATCTGCTAATAGTAGGAACACTCTTACCAGCTACATCTATGCTCCT
>HPV83_Alpha_5059324_nt6560_L1_AOS|1
CTCTTATTATATTCCAGGCACATCTGCTAATAGTAGGAACACTCTTACCAGCTACATCTA
>HPV83_Alpha_5059324_nt6553_L1_AOS|1
TGCCTACCTCTTATTATATTCCAGGCACATCTGCTAATAGTAGGAACACTCTTACCAGCT
>HPV83_Alpha_5059324_nt6546_L1_AOS|1
GAGGTGCTGCCTACCTCTTATTATATTCCAGGCACATCTGCTAATAGTAGGAACACTCTT
>HPV59_Alpha_557236_nt7071_L1_AOS|2
CTGCCCCTACCTCTACCCCATCACCAAAACGTGTTAAGCGTCGCAAGTCTTCCAGAAAAT
>HPV59_Alpha_557236_nt7064_L1_AOS|1
GCAGCGCCTGCCCCTACCTCTACCCCATCACCAAAACGTGTTAAGCGTCGCAAGTCTTCC
>HPV59_Alpha_557236_nt7057_L1_AOS|1
CAAACGTGCAGCGCCTGCCCCTACCTCTACCCCATCACCAAAACGTGTTAAGCGTCGCAA
>HPV59_Alpha_557236_nt6901_L1_AOS|3
CACCGCACCGCCAGTTAAACAGGACCCTTATGACAAACTAAAGTTTTGGCCTGTAGATCT
>HPV59_Alpha_557236_nt6643_L1_AOS|1
TTCTACTACTTCTTCTATTCCTAATGTATACACACCTACCAGTTTTAAAGAATATGCCAG
>HPV59_Alpha_557236_nt6634_L1_AOS|1
TGTGTGTGCTTCTACTACTTCTTCTATTCCTAATGTATACACACCTACCAGTTTTAAAGA
>HPV59_Alpha_557236_nt6627_L1_AOS|1
ATCTTTCTGTGTGTGCTTCTACTACTTCTTCTATTCCTAATGTATACACACCTACCAGTT
>HPV59_Alpha_557236_nt6620_L1_AOS|1
AGCACCAATCTTTCTGTGTGTGCTTCTACTACTTCTTCTATTCCTAATGTATACACACCT
>HPV6_Alpha_6002612_nt7207_L1_AOS|1
CCTCTATTCGTACCGGTGTTAAGCGCCCTGCTGTTTCCAAAGCCTCTGCTGCCCCTAAAC
>HPV6_Alpha_6002612_nt7232_L1_AOS|1
CCCTGCTGTTTCCAAAGCCTCTGCTGCCCCTAAACGTAAGCGCGCCAAAACCAAAAGGTA
>HPV6_Alpha_6002612_nt7223_L1_AOS|1
TGTTAAGCGCCCTGCTGTTTCCAAAGCCTCTGCTGCCCCTAAACGTAAGCGCGCCAAAAC
>HPV6_Alpha_6002612_nt7216_L1_AOS|1
GTACCGGTGTTAAGCGCCCTGCTGTTTCCAAAGCCTCTGCTGCCCCTAAACGTAAGCGCG
>HPV6_Alpha_6002612_nt7200_L1_AOS|1
GGACGGTCCTCTATTCGTACCGGTGTTAAGCGCCCTGCTGTTTCCAAAGCCTCTGCTGCC
>HPV6_Alpha_6002612_nt7193_L1_AOS|1
ATATAGGGGACGGTCCTCTATTCGTACCGGTGTTAAGCGCCCTGCTGTTTCCAAAGCCTC
>HPV57_Alpha_60882_nt6984_L1_AOS|1
ACCTACAGGTATTTGCAATCCCAAGCGATAACATGTCAGAAGCCCACACCCCCTAAGACC
>HPV57_Alpha_60882_nt6977_L1_AOS|1
GCAGGACACCTACAGGTATTTGCAATCCCAAGCGATAACATGTCAGAAGCCCACACCCCC
>HPV57_Alpha_60882_nt6970_L1_AOS|1
CCAGCCTGCAGGACACCTACAGGTATTTGCAATCCCAAGCGATAACATGTCAGAAGCCCA
>HPV57_Alpha_60882_nt6952_L1_AOS|1
TCCCCCCACCCCCGTCCGCCAGCCTGCAGGACACCTACAGGTATTTGCAATCCCAAGCGA
>HPV57_Alpha_60882_nt6540_L1_AOS|1
ATGGGTGACGCCCTCCCGGATGAGCTATATGTCAAGAGTTCTACCGTCCAGACCCCCGGT
>HPV57_Alpha_60882_nt6370_L1_AOS|1
TTGCCGCTCTACAGTCCAACAAATCAGATGTCCCCTTGGATATCTGTACTAACATATGTA
>HPV69_Alpha_6970418_nt6371_L1_AOS|2
TAAAGGCGCTGGTCAAGGTCGTGAACCTCCAACTACTTCTATATACTCACGTACGCCTAG
>HPV82_Alpha_6970427_nt6610_L1_AOS|1
CTGTTACTCCATCTGTTGCACAAACATTTACTCCAGCAAACTTTAAGCAGTACATTAGGC
>HPV82_Alpha_6970427_nt6589_L1_AOS|1
CCAATTTAACCATTAGCACTGCTGTTACTCCATCTGTTGCACAAACATTTACTCCAGCAA
>HPV82_Alpha_6970427_nt6341_L1_AOS|3

GCATTACTATAATAAGGCTGGTGTGGTTGGTGATGCCATTCCAGACAAGGCTTATATTAA
>HPV68a_Alpha_71726685_nt6284_L1_AOS|1
TGGTAGGGGACACTATTCCCACTGACATGTATATTAAGGGCACTGACATTCGTGAAACTC
>HPV68a_Alpha_71726685_nt6512_L1_AOS|1
TTACATTGTCCACTACTACAGACTCTACTGTACCAGCTGTGTATGATTCTAATAAATTTA
>HPV68a_Alpha_71726685_nt6277_L1_AOS|1
GGAGGCATGGTAGGGGACACTATTCCCACTGACATGTATATTAAGGGCACTGACATTCGT
>HPV97_Alpha_71726694_nt6956_L1_AOS|1
CCCCGTCTGCCCCTACTACTTCCAAACCTGCTAAGCGTTTACGTGTCCGTCGTAAAAAAT
>HPV97_Alpha_71726694_nt6530_L1_AOS|1
CTTCTACACAAAATGGCGTAGCTACCACATATGACCCTACAAAATATAAACAGTATAGTA
>HPV97_Alpha_71726694_nt6522_L1_AOS|1
ACTGTGTGCTTCTACACAAAATGGCGTAGCTACCACATATGACCCTACAAAATATAAACA
>HPV106_Alpha_71726710_nt6586_L1_AOS|1
GGGGAGGATGTTCCGGAAGGGTTTTTACTAAAGGGGCCTACTACTACATCCCGCGCCTCT
>HPV106_Alpha_71726710_nt5690_L1_AOS|1
CGATGTGCTTGTGCACGGGTCTACATATTATTTATTGCCCTCATATGTGTTGCTTCCTAG
>HPV106_Alpha_71726710_nt5678_L1_AOS|1
TCACCCCACAGGCGATGTGCTTGTGCACGGGTCTACATATTATTTATTGCCCTCATATGT
>HPV106_Alpha_71726710_nt5671_L1_AOS|1
CTCCTGGTCACCCCACAGGCGATGTGCTTGTGCACGGGTCTACATATTATTTATTGCCCT
>HPV106_Alpha_71726710_nt5664_L1_AOS|2
CCTACCCCTCCTGGTCACCCCACAGGCGATGTGCTTGTGCACGGGTCTACATATTATTTA
>HPV106_Alpha_71726710_nt5656_L1_AOS|1
CCGTGCCCCCTACCCCTCCTGGTCACCCCACAGGCGATGTGCTTGTGCACGGGTCTACAT
>HPV106_Alpha_71726710_nt5620_L1_AOS|1
CTGACAGTGCTCTTCCCGATTCCCACACTGCTTGGCCCGTGCCCCCTACCCCTCCTGGTC
>HPV106_Alpha_71726710_nt5613_L1_AOS|1
AGTGGCCCTGACAGTGCTCTTCCCGATTCCCACACTGCTTGGCCCGTGCCCCCTACCCCT
>HPV106_Alpha_71726710_nt5605_L1_AOS|1
CTGTTTTTAGTGGCCCTGACAGTGCTCTTCCCGATTCCCACACTGCTTGGCCCGTGCCCC
>HPV102_Alpha_71726718_nt5682_L1_AOS|1
GTTCCTTCCCAATCTGTGTTTGTGGATGGTACAGACTATTTTTTATTGCCTAATTACTTA
>HPV102_Alpha_71726718_nt5673_L1_AOS|1
TCCTCTAATGTTCCTTCCCAATCTGTGTTTGTGGATGGTACAGACTATTTTTTATTGCCT
>HPV102_Alpha_71726718_nt5525_L1_AOS|1
ATCTACAAATATTTCAGCTACATCTGTGGTCTCTGCGCGGTATGACAATGTCACAGTACC
>HPV102_Alpha_71726718_nt5518_L1_AOS|2
CTTTGCAATCTACAAATATTTCAGCTACATCTGTGGTCTCTGCGCGGTATGACAATGTCA
>HPV102_Alpha_71726718_nt5511_L1_AOS|1
TCTGTTCCTTTGCAATCTACAAATATTTCAGCTACATCTGTGGTCTCTGCGCGGTATGAC
>HPV102_Alpha_71726718_nt5493_L1_AOS|2
TCATATACTTCTTCCACGTCTGTTCCTTTGCAATCTACAAATATTTCAGCTACATCTGTG
>HPV102_Alpha_71726718_nt5482_L1_AOS|1
CTCCACATGTTTCATATACTTCTTCCACGTCTGTTCCTTTGCAATCTACAAATATTTCAG
>HPV102_Alpha_71726718_nt5475_L1_AOS|1
TTGGCGTCTCCACATGTTTCATATACTTCTTCCACGTCTGTTCCTTTGCAATCTACAAAT
>HPV102_Alpha_71726718_nt5447_L1_AOS|1
TTATGCTGATAATGGCCACTTGGACTCCTTGGCGTCTCCACATGTTTCATATACTTCTTC
>HPV13_Alpha_85827580_nt5868_L1_AOS|1
GCAGTGGGAAATCCTTATTTTCCTATTAAGAAACAAAACAAAACTGTTGTCCCTAAGGTA
>HPV13_Alpha_85827580_nt7147_L1_AOS|2
TTCAGTCTAGGTCACCTATTCGTGTAGGTAGAAAACGTGCTGCATCTACATCTACTGCCA
>HPV13_Alpha_85827580_nt7140_L1_AOS|1
ACAGGCGTTCAGTCTAGGTCACCTATTCGTGTAGGTAGAAAACGTGCTGCATCTACATCT

>HPV13_Alpha_85827580_nt7133_L1_AOS|1
ATTACAAACAGGCGTTCAGTCTAGGTCACCTATTCGTGTAGGTAGAAAACGTGCTGCATC
>HPV13_Alpha_85827580_nt6758_L1_AOS|1
TGCAGCCACTACATCATCTCTTTCAGACACATATAAGGCCACAGAATATAAACAGTACAT
>HPV13_Alpha_85827580_nt6751_L1_AOS|1
CTGTGTGTGCAGCCACTACATCATCTCTTTCAGACACATATAAGGCCACAGAATATAAAC
>HPV13_Alpha_85827580_nt6508_L1_AOS|1
ATTTCTTTAACAGGGCAGGCTCTGTTGGTGAACTAATCCCAGCAGAATTATATGTTAAGG
>HPV2_Alpha_9626032_nt7210_L1_AOS|1
GCAAGCGCGCCGCTGTTTCGGGGACCACGCCGCCCACTAGTAAACGAAAACGGGTAAGGC
>HPV2_Alpha_9626032_nt6787_L1_AOS|1
GTAGCACTAATGTATCTCTGTGTGCCACTGAGGCGTCTGATACTAATTATAAGGCTACCA
>HPV41_Nu_9626041_nt6790_L1_AOS|1
TGTCACTTCACCTCCCAATTCTGTACTGGAGGATCATTATAGGTACATACTGTCCATTGC
>HPV41_Nu_9626041_nt6775_L1_AOS|1
AGACTGGCATTTAGCTGTCACTTCACCTCCCAATTCTGTACTGGAGGATCATTATAGGTA
>HPV41_Nu_9626041_nt6768_L1_AOS|1
TTATTGAAGACTGGCATTTAGCTGTCACTTCACCTCCCAATTCTGTACTGGAGGATCATT
>HPV41_Nu_9626041_nt6662_L1_AOS|1
TTTTTAAGGCACACCGAGGAGTTTCAGCTTGCCTTTATTCTACAGCTGTGTAAGGTAGAC
>HPV41_Nu_9626041_nt6287_L1_AOS|1
TATGGAGACCACATGTTTTTCTATGTGCGGCGTGAAGCTCTGTATGCTAGGCATATAATG
>HPV41_Nu_9626041_nt5779_L1_AOS|1
TCCCAATACCTTTGCATTTTGTGATAAGTCCCTTTTTAACCCTGACAAGGAGCGTCTGGT
>HPV41_Nu_9626041_nt5772_L1_AOS|1
TCCCAAATCCCAATACCTTTGCATTTTGTGATAAGTCCCTTTTTAACCCTGACAAGGAGC
>HPV41_Nu_9626041_nt5694_L1_AOS|1
ACAATATTACTAATGCGGATGGCAAAGAGGTGGTCCCTAAAGTTTCCTCTAATCAGTTCA
>HPV1_Mu_9626063_nt6713_L1_AOS|2
AATGTCCAGAACAGGCGCCTCCTGAGCCCCAGACTGATCCTTATAGTCAATATAAATTCT
>HPV1_Mu_9626063_nt6706_L1_AOS|1
GCAGCAAAATGTCCAGAACAGGCGCCTCCTGAGCCCCAGACTGATCCTTATAGTCAATAT
>HPV1_Mu_9626063_nt6233_L1_AOS|1
AGGAGGCAGTCCCACAAAGCCTGTATTTAACAGCAGATGCTGAACCAAGAACAACTTTAG
>HPV1_Mu_9626063_nt6226_L1_AOS|1
GGTGATAAGGAGGCAGTCCCACAAAGCCTGTATTTAACAGCAGATGCTGAACCAAGAACA
>HPV1_Mu_9626063_nt6219_L1_AOS|1
TTCGGTGGGTGATAAGGAGGCAGTCCCACAAAGCCTGTATTTAACAGCAGATGCTGAACC
>HPV1_Mu_9626063_nt6203_L1_AOS|2
TTTTTACTCGCGGGGGTTCGGTGGGTGATAAGGAGGCAGTCCCACAAAGCCTGTATTTAA
>HPV1_Mu_9626063_nt5440_L1_AOS|1
TGGTTACCAGCGCAGAATAAGTTCTATCTTCCTCCCCAGCCCATCACTAGAATCCTGTCC
>HPV18_Alpha_9626069_nt6649_L1_AOS|1
CTTCTACACAGTCTCCTGTACCTGGGCAATATGATGCTACCAAATTTAAGCAGTATAGCA
>HPV18_Alpha_9626069_nt5455_L1_AOS|1
TATTACATTACCATCTACTACCTCTGTATGGCCCATTGTATCACCCACGGCCCCTGCCTC
>HPV18_Alpha_9626069_nt5448_L1_AOS|1
GTCCTGATATTACATTACCATCTACTACCTCTGTATGGCCCATTGTATCACCCACGGCCC
>HPV18_Alpha_9626069_nt5441_L1_AOS|2
TACACGGGTCCTGATATTACATTACCATCTACTACCTCTGTATGGCCCATTGTATCACCC
>HPV18_Alpha_9626069_nt5434_L1_AOS|1
GCCTGTATACACGGGTCCTGATATTACATTACCATCTACTACCTCTGTATGGCCCATTGT
>HPV4_Gamma_9626597_nt6396_L1_AOS|1
TTACAATTTCTGTGAAGTCAGATGGTGCTAATGACAATTATCAGTATAAAGCTAGTGATT
>HPV4_Gamma_9626597_nt6420_L1_AOS|2

GTGCTAATGACAATTATCAGTATAAAGCTAGTGATTTTAAACAGTACCTCAGACATATAG
>HPV4_Gamma_9626597_nt6413_L1_AOS|1
TCAGATGGTGCTAATGACAATTATCAGTATAAAGCTAGTGATTTTAAACAGTACCTCAGA
>HPV4_Gamma_9626597_nt6406_L1_AOS|1
TGTGAAGTCAGATGGTGCTAATGACAATTATCAGTATAAAGCTAGTGATTTTAAACAGTA
>HPV4_Gamma_9626597_nt6200_L1_AOS|2
GCTCAAAACCAACAAGATCAGTACACTTTAGGACCTCATATTTATGTAGGGACCCCTAGT
>HPV63_Mu_9626605_nt6567_L1_AOS|1
GGATCCAACAATTATCGATTCCTGGCAGTTAACAGTTTCTCAACCTCCTGCAAATGCTAT
>HPV63_Mu_9626605_nt6560_L1_AOS|1
ATAATATGGATCCAACAATTATCGATTCCTGGCAGTTAACAGTTTCTCAACCTCCTGCAA
>HPV63_Mu_9626605_nt6425_L1_AOS|1
TTAACAAAGCAACCCCTGAGACTTATGATAGCGCAGATTATAATGAGTATACTCGCCATG
>HPV63_Mu_9626605_nt6417_L1_AOS|1
AAATGTTCTTAACAAAGCAACCCCTGAGACTTATGATAGCGCAGATTATAATGAGTATAC
>HPV63_Mu_9626605_nt6410_L1_AOS|1
TGAATATAAATGTTCTTAACAAAGCAACCCCTGAGACTTATGATAGCGCAGATTATAATG
>HPV63_Mu_9626605_nt6403_L1_AOS|1
ACCACGATGAATATAAATGTTCTTAACAAAGCAACCCCTGAGACTTATGATAGCGCAGAT
>HPV63_Mu_9626605_nt6396_L1_AOS|1
CAGAGGAACCACGATGAATATAAATGTTCTTAACAAAGCAACCCCTGAGACTTATGATAG
>HPV63_Mu_9626605_nt6206_L1_AOS|2
ATATTGCTGCTAAAGCCGAGCAACCCCAAAATACTATTGCTACAGATAATTATTTTGTAG
>HPV63_Mu_9626605_nt6169_L1_AOS|1
ATTGTTAGTGAAAAAGAGAAAGTCCCTACCAGTGCATATATTGCTGCTAAAGCCGAGCAA
>HPV63_Mu_9626605_nt6162_L1_AOS|1
TGCTGGAATTGTTAGTGAAAAAGAGAAAGTCCCTACCAGTGCATATATTGCTGCTAAAGC
>HPV16_Alpha_9627100_nt7066_L1_AOS|1
AACCAAAATTTACATTAGGAAAACGAAAAGCTACACCCACCACCTCATCTACCTCTACAA
>HPV16_Alpha_9627100_nt7059_L1_AOS|1
AAGGCCAAACCAAAATTTACATTAGGAAAACGAAAAGCTACACCCACCACCTCATCTACC
>HPV16_Alpha_9627100_nt6455_L1_AOS|1
CGATTTATACATTAAAGGCTCTGGGTCTACTGCAAATTTAGCCAGTTCAAATTATTTTCC
>HPV16_Alpha_9627100_nt6448_L1_AOS|1
TACCAGACGATTTATACATTAAAGGCTCTGGGTCTACTGCAAATTTAGCCAGTTCAAATT
>HPV16_Alpha_9627100_nt6441_L1_AOS|2
GAAAATGTACCAGACGATTTATACATTAAAGGCTCTGGGTCTACTGCAAATTTAGCCAGT
>HPV16_Alpha_9627100_nt6434_L1_AOS|2
TGTTGGTGAAAATGTACCAGACGATTTATACATTAAAGGCTCTGGGTCTACTGCAAATTT
>HPV5_Beta_9627145_nt6972_L1_AOS|2
TAATTTCAGTATTTCTGTATATAATCAGGCTGGAGCACTAAAAGATGTTGCAGACTATAA
>HPV5_Beta_9627145_nt6989_L1_AOS|1
TATATAATCAGGCTGGAGCACTAAAAGATGTTGCAGACTATAATGCAGATCAATTTAGAG
>HPV5_Beta_9627145_nt6981_L1_AOS|2
TATTTCTGTATATAATCAGGCTGGAGCACTAAAAGATGTTGCAGACTATAATGCAGATCA
>HPV5_Beta_9627145_nt6965_L1_AOS|1
GAAATACTAATTTCAGTATTTCTGTATATAATCAGGCTGGAGCACTAAAAGATGTTGCAG
>HPV5_Beta_9627145_nt6740_L1_AOS|1
AAATTGACAATGGTACATACAAAAATCAGTTTTACATTCCAGGGGCTGATGGCCAAGCTC
>HPV5_Beta_9627145_nt6731_L1_AOS|1
CACGTGCACAAATTGACAATGGTACATACAAAAATCAGTTTTACATTCCAGGGGCTGATG
>HPV5_Beta_9627145_nt6533_L1_AOS|1
GGAACATGAATTTTAAGGCACTTCAAGATAGTAGATCAGATGTCAGTTTAGACATCGTCA
>HPV5_Beta_9627145_nt6520_L1_AOS|1
GACATAGGTTTTGGGAACATGAATTTTAAGGCACTTCAAGATAGTAGATCAGATGTCAGT

>HPV5_Beta_9627145_nt6513_L1_AOS|1
TATGGCAGACATAGGTTTTGGGAACATGAATTTTAAGGCACTTCAAGATAGTAGATCAGA
>HPV5_Beta_9627145_nt6057_L1_AOS|1
TCATCCTTATTTCAATGTATACAATATTAATGGTGATAAGCTTGAGGTTCCTAAGGTTTC
>HPV26_Alpha_9627305_nt6112_L1_AOS|1
AAATACACAGACACAACGTGGGGATTGCCCCCCCTTAGAATTAATTTCCAGCATTATTGA
>HPV32_Alpha_9627327_nt6673_L1_AOS|1
GTTCCTGAGGACATGTATATAAAAGCTTCTAATGGTGCTTCTGGCAGAAATAATTTAGCT
>HPV32_Alpha_9627327_nt7323_L1_AOS|1
AACAGCATCTTCCAGTCAAAAGTCTTCTTCTCCTGCAAAACGCAGAAAAACACGTAAATA
>HPV32_Alpha_9627327_nt7316_L1_AOS|1
TAAAACGAACAGCATCTTCCAGTCAAAAGTCTTCTTCTCCTGCAAAACGCAGAAAAACAC
>HPV32_Alpha_9627327_nt7309_L1_AOS|1
ACAGCAGTAAAACGAACAGCATCTTCCAGTCAAAAGTCTTCTTCTCCTGCAAAACGCAGA
>HPV32_Alpha_9627327_nt7302_L1_AOS|2
TAAACTTACAGCAGTAAAACGAACAGCATCTTCCAGTCAAAAGTCTTCTTCTCCTGCAAA
>HPV32_Alpha_9627327_nt6656_L1_AOS|1
GAACCCTTGGTGAACCTGTTCCTGAGGACATGTATATAAAAGCTTCTAATGGTGCTTCTG
>HPV34_Alpha_9627334_nt6019_L1_AOS|1
GCACACCATGTGGTACCCAAAATGCTGGGGAATGTCCTCCGCTTGAATTAAAGAACACCA
>HPV34_Alpha_9627334_nt6930_L1_AOS|1
GCCCGTCCTAGGTTACAGGCCTCTAAACGGTCTGCACCTTCATCATCTAGTACAGCACCT
>HPV34_Alpha_9627334_nt6526_L1_AOS|1
CAGTTTGTGTAGGTACACAATCCACAAGTACAACTGCACCATATGCAAACAGTAATTTTA
>HPV34_Alpha_9627334_nt6519_L1_AOS|1
AACTTTTCAGTTTGTGTAGGTACACAATCCACAAGTACAACTGCACCATATGCAAACAGT
>HPV34_Alpha_9627334_nt6512_L1_AOS|1
AAGCACAAACTTTTCAGTTTGTGTAGGTACACAATCCACAAGTACAACTGCACCATATGC
>HPV49_Beta_9627363_nt7264_L1_AOS|1
TACAACGGGCTTCTAGAGTGTCTAAATCCTCTGCTGCTAGAGCTTCCACACGGGGTATTA
>HPV49_Beta_9627363_nt6871_L1_AOS|1
TTAGTGTAAGTACGGATGGCCAGACACCTACAGAATATGACAGTACCAAGGTTAGAGAAT
>HPV49_Beta_9627363_nt6657_L1_AOS|1
TACATATTACCTGCAGCAAGTCAACAGGCCCAAAATACTCTTGGCAGCTCCATCTATTTC
>HPV53_Alpha_9627377_nt7072_L1_AOS|1
CTAAACCGCCTGTATCCTCTAAAAAACGCTCTGCTTCTACTACATCTACCTCAGCCCCCT
>HPV7_Alpha_9627389_nt6169_L1_AOS|1
TGAAGATGTGGAAAACTCGTCTGTATATGGAACAGTACCTGGTCAGGACAGCAGAGAAAA
>HPV7_Alpha_9627389_nt6833_L1_AOS|1
ACACAATCGCCCACACCAACACCATATGACAATAGTAAGTTTAAAGAATATTTACGTCAT
>HPV7_Alpha_9627389_nt6594_L1_AOS|1
GAGACAGTGTTCCAAATGATTTATATATAACAGGTTCATCTAATCGCGCTTCTATTGCAG
>HPV7_Alpha_9627389_nt6587_L1_AOS|1
ACTACTGGAGACAGTGTTCCAAATGATTTATATATAACAGGTTCATCTAATCGCGCTTCT
>HPV7_Alpha_9627389_nt6162_L1_AOS|1
ATAAAGATGAAGATGTGGAAAACTCGTCTGTATATGGAACAGTACCTGGTCAGGACAGCA
>HPV9_Beta_9627396_nt6611_L1_AOS|1
TGATCAACAACAACGAACAATAGCTAATTCCACCTACTATCCTACAGTAAGTGGGTCATT
>HPV9_Beta_9627396_nt6886_L1_AOS|1
ATCAGATTTCATTAATCTTACAGTTGTGTAAAGTGCCTTTAGTAGCTGAAGTATTATCCC
>HPV9_Beta_9627396_nt6604_L1_AOS|1
CAAAAAGTGATCAACAACAACGAACAATAGCTAATTCCACCTACTATCCTACAGTAAGTG
>HPV9_Beta_9627396_nt6597_L1_AOS|1
TTGCCAGCAAAAAGTGATCAACAACAACGAACAATAGCTAATTCCACCTACTATCCTACA
>HPV9_Beta_9627396_nt6590_L1_AOS|1

TTTCTTTTTGCCAGCAAAAAGTGATCAACAACAACGAACAATAGCTAATTCCACCTACTA
>HPV9_Beta_9627396_nt6574_L1_AOS|1
TAAACCAGGATCATAATTTCTTTTTGCCAGCAAAAAGTGATCAACAACAACGAACAATAG
>HPV54_Alpha_9628437_nt6631_L1_AOS|1
GCTACAGCATCCACGCAGGATAGCTTTAATAATTCTGACTTTAGGGAGTATATTAGACAT
>HPV54_Alpha_9628437_nt7047_L1_AOS|1
GCGTGCAGCCCCTTCCTCCTCTAAGGGTACAGCGCGCAAGCGTGCTAAAACTAAAAGGTA
>HPV54_Alpha_9628437_nt7004_L1_AOS|1
TACAGGCTGGCCTACGTGCACGTCCGCGCCTTCGGCCTGTAAAGCGTGCAGCCCCTTCCT
>HPV54_Alpha_9628437_nt6623_L1_AOS|1
CATTGTGTGCTACAGCATCCACGCAGGATAGCTTTAATAATTCTGACTTTAGGGAGTATA
>HPV54_Alpha_9628437_nt6616_L1_AOS|1
AACCTAACATTGTGTGCTACAGCATCCACGCAGGATAGCTTTAATAATTCTGACTTTAGG
>HPV54_Alpha_9628437_nt6420_L1_AOS|1
TGACTTATACATTAAGAAATCCTCAGGTAACCTTGACAGTTCTATTTATGCTGCAACTCC
>HPV24_Unclassified_9628486_nt6249_L1_AOS|1
TGATAATAATGATGCTGGTAGATGTCCACCTATTAAGTTAGTAAATTCAGTAATTCAAGA
>HPV24_Unclassified_9628486_nt6800_L1_AOS|1
AAAATGGCAAAGTAACAGATATTAACGAGTATGATGCTAATAAATTTAGGGAATATCAGA
>HPV24_Unclassified_9628486_nt6785_L1_AOS|1
TAAGTGTATACACTGAAAATGGCAAAGTAACAGATATTAACGAGTATGATGCTAATAAAT
>HPV24_Unclassified_9628486_nt6242_L1_AOS|1
GTGCTGGTGATAATAATGATGCTGGTAGATGTCCACCTATTAAGTTAGTAAATTCAGTAA
>HPV24_Unclassified_9628486_nt6235_L1_AOS|1
GAGAGGTGTGCTGGTGATAATAATGATGCTGGTAGATGTCCACCTATTAAGTTAGTAAAT
>HPV24_Unclassified_9628486_nt6228_L1_AOS|1
AGTAGCTGAGAGGTGTGCTGGTGATAATAATGATGCTGGTAGATGTCCACCTATTAAGTT
>HPV24_Unclassified_9628486_nt6221_L1_AOS|1
ATTGGGAAGTAGCTGAGAGGTGTGCTGGTGATAATAATGATGCTGGTAGATGTCCACCTA
>HPV48_Gamma_9628542_nt6558_L1_AOS|1
CCATACAAAGCTTACAGTTTCTGGACTTTAGATATGACAGAGCGCTTCTCGTCTGATTTA
>HPV48_Gamma_9628542_nt6653_L1_AOS|1
AACTGGTTTGTTAAATGGTAAACGAGCTAGAACAGACTATACAGCTGCAGGATCTAGTAC
>HPV48_Gamma_9628542_nt6550_L1_AOS|1
ACACAGATCCATACAAAGCTTACAGTTTCTGGACTTTAGATATGACAGAGCGCTTCTCGT
>HPV48_Gamma_9628542_nt6543_L1_AOS|1
GAAGAAGACACAGATCCATACAAAGCTTACAGTTTCTGGACTTTAGATATGACAGAGCGC
>HPV48_Gamma_9628542_nt6535_L1_AOS|1
CTGAACCAGAAGAAGACACAGATCCATACAAAGCTTACAGTTTCTGGACTTTAGATATGA
>HPV48_Gamma_9628542_nt6507_L1_AOS|2
AAATCCATGGCTACTAAATGCCCTACTGCTGAACCAGAAGAAGACACAGATCCATACAAA
>HPV48_Gamma_9628542_nt6498_L1_AOS|1
AGGTATATCAAATCCATGGCTACTAAATGCCCTACTGCTGAACCAGAAGAAGACACAGAT
>HPV48_Gamma_9628542_nt6026_L1_AOS|1
TTATATAACTCCTGATTCTGCCGATCAAAACAACAGATCTTCTCATTTAGGATCTTCTGT
>HPV48_Gamma_9628542_nt6018_L1_AOS|1
GGAGTATATTATATAACTCCTGATTCTGCCGATCAAAACAACAGATCTTCTCATTTAGGA
>HPV48_Gamma_9628542_nt5981_L1_AOS|2
AGCTGGCCAAATGGGGGAGCCTATACCAACAGAAAATGGAGTATATTATATAACTCCTGA
>HPV50_Gamma_9628550_nt6294_L1_AOS|1
AGAAAGATGTCAATCCTTTGGATCCTTTAAATGTAGCAAGTTCTTATATGTACTCAAAGG
>HPV50_Gamma_9628550_nt6689_L1_AOS|1
TTTCAGACTGGATTATTAAAGAGAAGAGTTAGAACTGATTACACTGTTGCTACAGTTTCC
>HPV50_Gamma_9628550_nt6325_L1_AOS|1
TGTAGCAAGTTCTTATATGTACTCAAAGGATGATTTTAATCAGTACAGCCGTCATACTGA

>HPV50_Gamma_9628550_nt6318_L1_AOS|1
CTTTAAATGTAGCAAGTTCTTATATGTACTCAAAGGATGATTTTAATCAGTACAGCCGTC
>HPV50_Gamma_9628550_nt6311_L1_AOS|2
TTGGATCCTTTAAATGTAGCAAGTTCTTATATGTACTCAAAGGATGATTTTAATCAGTAC
>HPV50_Gamma_9628550_nt6287_L1_AOS|1
AGTGTTAAGAAAGATGTCAATCCTTTGGATCCTTTAAATGTAGCAAGTTCTTATATGTAC
>HPV50_Gamma_9628550_nt6051_L1_AOS|2
CAGGAGTTTATAATTATGCAGTAAACCCTAATCAACCAGAGCAAAATCGTAGAACCAATA
>HPV50_Gamma_9628550_nt5860_L1_AOS|1
TAAACTACAACAGGACAGAGCAGGGGTCCCTTTAGATATAGTGGATTCAATCAGCTTATG
>HPV50_Gamma_9628550_nt5853_L1_AOS|1
ACTTTCCTAAACTACAACAGGACAGAGCAGGGGTCCCTTTAGATATAGTGGATTCAATCA
>HPV50_Gamma_9628550_nt5734_L1_AOS|1
GGATTTAGCAGAACCTTGTGACAAAAACAGCTTAAATAATGGCAAGTGTCCTCCAATACA
>HPV60_Gamma_9628566_nt6762_L1_AOS|1
GACAATAGATTTGCAAGAACGTTTTTCTAACGAATTATCCCAGTTTTCTCTTGGAAAAAG
>HPV60_Gamma_9628566_nt6226_L1_AOS|1
TATTACTTAGCACCACAAACTGATAAACCTCAAAATAACCTTGGTGGTTATAGTTACTTC
>HPV60_Gamma_9628566_nt6218_L1_AOS|1
ATTCTGGATATTACTTAGCACCACAAACTGATAAACCTCAAAATAACCTTGGTGGTTATA
>HPV61_Alpha_9628574_nt6569_L1_AOS|1
GTGAGGCACTGCCTGACTCTTATTATCTTAAGGGTGCGAATGACAAGGCGGCCCCTGGTA
>HPV8_Beta_333074_nt6932_L1_AOS_rc|1
GTCTCAGATATTCTCTGAACTGGGTTGATTTATAGTCTGTGATGTTCTTAAGTTCCCCAT
>HPV8_Beta_333074_nt6473_L1_AOS_rc|1
ATATCTCATTTACTATGTCGAGACTTACATCAGATCTGTTCTGTTGTAAAGTCTTAAAGT
>HPV8_Beta_333074_nt6360_L1_AOS_rc|1
TTTAGTTCAATGGGTGGGCACAGACCTTGCTGTTGGTCCTCTGCACATGGAATGGCTTTT
>HPV51_Alpha_333087_nt6559_L1_AOS_rc|1
ATGCCTAATATATTGCTTAAAGTTACTTGGAGTAAATGTTGGGGAAACCGCAGCAGTGGC
>HPV51_Alpha_333087_nt6048_L1_AOS_rc|1
TCCTGAATGACAGAGGATACAAGTTCCAGGGGGGGGCAGTCTCCTGGAGGTACAGGTGTG
>HPV51_Alpha_333087_nt6041_L1_AOS_rc|1
TGACAGAGGATACAAGTTCCAGGGGGGGGCAGTCTCCTGGAGGTACAGGTGTGTTTTTGC
>HPV20_Beta_1020162_nt6739_L1_AOS_rc|1
CATTGAATTACCTAGGTTGTTCTGTGCCTGATTATTCACAGGTGGAATGTAGAATGCATT
>HPV20_Beta_1020162_nt6956_L1_AOS_rc|1
TCTGAGAATCATAATTTTGAATTTTAGAAACATCAGTGTTTTCTGAATGAACTGATATGC
>HPV20_Beta_1020162_nt6944_L1_AOS_rc|1
AATTTTGAATTTTAGAAACATCAGTGTTTTCTGAATGAACTGATATGCTAAAATTTGTAT
>HPV20_Beta_1020162_nt6936_L1_AOS_rc|1
ATTTTAGAAACATCAGTGTTTTCTGAATGAACTGATATGCTAAAATTTGTATTTCGAGTA
>HPV22_Beta_1020178_nt7135_L1_AOS_rc|2
TTAAGAGGTAAGTTTCCTTCGTTTTACAGTTTTAGACGTTTTCCGCGTAGCAGAACGTTT
>HPV22_Beta_1020178_nt7128_L1_AOS_rc|1
GTAAGTTTCCTTCGTTTTACAGTTTTAGACGTTTTCCGCGTAGCAGAACGTTTCACACTG
>HPV22_Beta_1020178_nt7121_L1_AOS_rc|1
TCCTTCGTTTTACAGTTTTAGACGTTTTCCGCGTAGCAGAACGTTTCACACTGACCCTGG
>HPV22_Beta_1020178_nt7114_L1_AOS_rc|1
TTTTACAGTTTTAGACGTTTTCCGCGTAGCAGAACGTTTCACACTGACCCTGGCACTGGC
>HPV22_Beta_1020178_nt7107_L1_AOS_rc|2
GTTTTAGACGTTTTCCGCGTAGCAGAACGTTTCACACTGACCCTGGCACTGGCCCTTGCA
>HPV23_Beta_1020186_nt7100_L1_AOS_rc|2
TTTGACAGTTTTGGTCACTTTTCGAGTTGCAGGCCGTTTGGTACCGGACCGTACACGCTG
>HPV23_Beta_1020186_nt7120_L1_AOS_rc|2

TACAATTGCACTTTTTTCCTTTTGACAGTTTTGGTCACTTTTCGAGTTGCAGGCCGTTTG
>HPV23_Beta_1020186_nt7113_L1_AOS_rc|1
GCACTTTTTTCCTTTTGACAGTTTTGGTCACTTTTCGAGTTGCAGGCCGTTTGGTACCGG
>HPV23_Beta_1020186_nt7093_L1_AOS_rc|1
GTTTTGGTCACTTTTCGAGTTGCAGGCCGTTTGGTACCGGACCGTACACGCTGCACTCCA
>HPV23_Beta_1020186_nt7086_L1_AOS_rc|1
TCACTTTTCGAGTTGCAGGCCGTTTGGTACCGGACCGTACACGCTGCACTCCAATTTGAA
>HPV23_Beta_1020186_nt6046_L1_AOS_rc|1
AATGAGATATTTCTTCTGTCATCTACAGTACCTTCCTGACGTTCACTAGAATTTTCTGCA
>HPV28_Alpha_1020202_nt6496_L1_AOS_rc|1
TTCAGGAATGGTGTCCCCAGCAACACCAGCTCTATTAAAAAAATGCCGAGCGAAAAGTTG
>HPV28_Alpha_1020202_nt7188_L1_AOS_rc|1
TACTTTTTGGCGCGTTTTCGTTTTGCAGCAGACGATCCTCTCGTGGTGGACGCTGGACGT
>HPV28_Alpha_1020202_nt7181_L1_AOS_rc|1
TGGCGCGTTTTCGTTTTGCAGCAGACGATCCTCTCGTGGTGGACGCTGGACGTTTGCGAA
>HPV28_Alpha_1020202_nt6503_L1_AOS_rc|1
ACAATGTTTCAGGAATGGTGTCCCCAGCAACACCAGCTCTATTAAAAAAATGCCGAGCGA
>HPV29_Alpha_1020210_nt7244_L1_AOS_rc|1
CTTTGCAGGGGTGGGGGCCGTGGTGGTCGTTCGGCGCTTTCTGGAGGGGACTACTGAACG
>HPV29_Alpha_1020210_nt7264_L1_AOS_rc|1
TACTTTTTCGAGCGTTTTCGCTTTGCAGGGGTGGGGGCCGTGGTGGTCGTTCGGCGCTTT
>HPV29_Alpha_1020210_nt7257_L1_AOS_rc|1
TCGAGCGTTTTCGCTTTGCAGGGGTGGGGGCCGTGGTGGTCGTTCGGCGCTTTCTGGAGG
>HPV29_Alpha_1020210_nt7237_L1_AOS_rc|1
GGGGTGGGGGCCGTGGTGGTCGTTCGGCGCTTTCTGGAGGGGACTACTGAACGCCGGCGC
>HPV29_Alpha_1020210_nt6611_L1_AOS_rc|1
AGGAGTTTCTCGCCCGTTGTTACCCTTTAAGTACAAGGAATCTGGGATTTTGTCCCCTAC
>HPV29_Alpha_1020210_nt6604_L1_AOS_rc|1
TCTCGCCCGTTGTTACCCTTTAAGTACAAGGAATCTGGGATTTTGTCCCCTACTACACCA
>HPV29_Alpha_1020210_nt6597_L1_AOS_rc|1
CGTTGTTACCCTTTAAGTACAAGGAATCTGGGATTTTGTCCCCTACTACACCAGCACGAT
>HPV29_Alpha_1020210_nt6588_L1_AOS_rc|2
CCTTTAAGTACAAGGAATCTGGGATTTTGTCCCCTACTACACCAGCACGATTAAAGAAGT
>HPV36_Beta_1020218_nt6692_L1_AOS_rc|1
AATGAAAAACTGATTCTTAAAAGTCCCATTATCAATTCTGGCGTCAGGGATGTCGTCACC
>HPV36_Beta_1020218_nt6701_L1_AOS_rc|1
AGCCCCAGGAATGAAAAACTGATTCTTAAAAGTCCCATTATCAATTCTGGCGTCAGGGAT
>HPV37_Beta_1020226_nt7176_L1_AOS_rc|1
GACCGTTTACGCTTTGTACCTTTAGACACTTTTACAGACGATCGAACAATTCTAGGTCTA
>HPV37_Beta_1020226_nt7162_L1_AOS_rc|1
TGTACCTTTAGACACTTTTACAGACGATCGAACAATTCTAGGTCTACTTTGCAATCCCGA
>HPV37_Beta_1020226_nt7155_L1_AOS_rc|1
TTAGACACTTTTACAGACGATCGAACAATTCTAGGTCTACTTTGCAATCCCGACTGAAAG
>HPV37_Beta_1020226_nt7147_L1_AOS_rc|1
TTTTACAGACGATCGAACAATTCTAGGTCTACTTTGCAATCCCGACTGAAAGATGAATTT
>HPV37_Beta_1020226_nt7140_L1_AOS_rc|1
GACGATCGAACAATTCTAGGTCTACTTTGCAATCCCGACTGAAAGATGAATTTCCTCCCT
>HPV37_Beta_1020226_nt6528_L1_AOS_rc|1
TGGTCTGATTTGGCAGGTAAGTAATATTTGTGGTCCTGATTAACAGTGCCATCGGGAATA
>HPV37_Beta_1020226_nt6225_L1_AOS_rc|1
TCAAGTGGTGGACACTGTCCTGTCTGATTATTTTCCTCTGATGCACAAACTGGTGCTTTA
>HPV38_Beta_1020234_nt7123_L1_AOS_rc|1
TTTTGCGTTTTACAGATTTGGAAGATTTTCTTACTAACGGCCGTTTGACAGCACGTGTTC
>HPV38_Beta_1020234_nt7116_L1_AOS_rc|2
TTTTACAGATTTGGAAGATTTTCTTACTAACGGCCGTTTGACAGCACGTGTTCGTGCTGT

```
>HPV38_Beta_1020234_nt7109_L1_AOS_rc|1
GATTTGGAAGATTTTCTTACTAACGGCCGTTTGACAGCACGTGTTCGTGCTGTTTGTAAA
>HPV38_Beta_1020234_nt6918_L1_AOS_rc|1
TGGACATTTAGTTGCTTTAGATGTTATGTAACGATATGTATCGTGTACAGAATTGTCTGG
>HPV38_Beta_1020234_nt6911_L1_AOS_rc|2
TTAGTTGCTTTAGATGTTATGTAACGATATGTATCGTGTACAGAATTGTCTGGGGTGGGT
>HPV38_Beta_1020234_nt6904_L1_AOS_rc|1
CTTTAGATGTTATGTAACGATATGTATCGTGTACAGAATTGTCTGGGGTGGGTACAAAGC
>HPV38_Beta_1020234_nt6480_L1_AOS_rc|1
GCGTTGACCCTGTCCATTTTTTGCAGGTAAATAATAATTATGATTTTGGTTGACAGTACC
>HPV38_Beta_1020234_nt6470_L1_AOS_rc|1
TGTCCATTTTTTGCAGGTAAATAATAATTATGATTTTGGTTGACAGTACCATCTGGAATA
>HPV55_Alpha_1020266_nt6709_L1_AOS_rc|2
GTTTATATTCTGTACTATTATATGTTGTAGATGGAGACTGAGTTGTAGCAGCACATATTG
>HPV55_Alpha_1020266_nt6717_L1_AOS_rc|1
CATGTATTGTTTATATTCTGTACTATTATATGTTGTAGATGGAGACTGAGTTGTAGCAGC
>HPV55_Alpha_1020266_nt6702_L1_AOS_rc|1
TTCTGTACTATTATATGTTGTAGATGGAGACTGAGTTGTAGCAGCACATATTGTCATGTT
>HPV55_Alpha_1020266_nt6498_L1_AOS_rc|1
AAAGTATATGGCATTAGGAACTGTACTTTTAGTAGCACCTTTAAAAACCAAATCCTGTGG
>HPV55_Alpha_1020266_nt6491_L1_AOS_rc|1
ATGGCATTAGGAACTGTACTTTTAGTAGCACCTTTAAAAACCAAATCCTGTGGAATGTCC
>HPV55_Alpha_1020266_nt6477_L1_AOS_rc|1
TGTACTTTTAGTAGCACCTTTAAAAACCAAATCCTGTGGAATGTCCTCTCCAACCGTTCC
>HPV55_Alpha_1020266_nt6470_L1_AOS_rc|1
TTAGTAGCACCTTTAAAAACCAAATCCTGTGGAATGTCCTCTCCAACCGTTCCCGCCCTA
>HPV66_Alpha_1020290_nt7064_L1_AOS_rc|1
AAGAGGTAGGAGCCGCCCGCCTTTTAGAGGCAGATACACTAGCCTTGGGTCTAGGGGGTC
>HPV66_Alpha_1020290_nt7057_L1_AOS_rc|2
AGGAGCCGCCCGCCTTTTAGAGGCAGATACACTAGCCTTGGGTCTAGGGGGTCTAGGGCC
>HPV66_Alpha_1020290_nt7050_L1_AOS_rc|1
GCCCGCCTTTTAGAGGCAGATACACTAGCCTTGGGTCTAGGGGGTCTAGGGCCTAGTTGC
>HPV66_Alpha_1020290_nt6861_L1_AOS_rc|2
GTAATAGCTGTGCTTTTAATATACCTATATTTATCCTCTAAGCTAGTTGCAACTGGTGGG
>HPV66_Alpha_1020290_nt6854_L1_AOS_rc|1
CTGTGCTTTTAATATACCTATATTTATCCTCTAAGCTAGTTGCAACTGGTGGGGATAAGC
>HPV66_Alpha_1020290_nt6847_L1_AOS_rc|2
TTTAATATACCTATATTTATCCTCTAAGCTAGTTGCAACTGGTGGGGATAAGCCAATATT
>HPV103_Unclassified_109390382_nt6381_L1_AOS_rc|2
TCTACACGAGACCGCTTTCTAGAGGAGGTAGAAGACGAGCTAACCCCAGTTTGAGATAAA
>HPV103_Unclassified_109390382_nt6212_L1_AOS_rc|1
CTGCAGCAGGGCAGCGTGTGGCTAAAGATGTTAAAAATCTGTATGTATCTCCAATAGAAG
>HPV103_Unclassified_109390382_nt6195_L1_AOS_rc|2
GTGGCTAAAGATGTTAAAAATCTGTATGTATCTCCAATAGAAGAAGGAGGCGGTGGTACA
>HPV103_Unclassified_109390382_nt5992_L1_AOS_rc|1
GGTGGCAGCATATTGCTGTGCAACGCCAGCGTCTGACTTAACATTTATTGTAAAATTTAC
>HPV103_Unclassified_109390382_nt5819_L1_AOS_rc|1
TAGAAACCAGTGATCCACTGGGGGTTGCATAGTAAACAGACGAGGAGGCTGTATTTTGGT
>HPV103_Unclassified_109390382_nt5809_L1_AOS_rc|1
TGATCCACTGGGGGTTGCATAGTAAACAGACGAGGAGGCTGTATTTTGGTCTTTGTCAGA
>HPV103_Unclassified_109390382_nt5796_L1_AOS_rc|1
GTTGCATAGTAAACAGACGAGGAGGCTGTATTTTGGTCTTTGTCAGACGCCCCCTGTAAA
>HPV103_Unclassified_109390382_nt5738_L1_AOS_rc|1
AAATATAGGTGTGCTTAACACCCTCAAATTCAGCCTCAGGTATACTGTCGCCCATGACAC
>HPV103_Unclassified_109390382_nt5724_L1_AOS_rc|1
```

TTAACACCCTCAAATTCAGCCTCAGGTATACTGTCGCCCATGACACCATCCCTAGCAAAA
>HPV101_Unclassified_109390389_nt6520_L1_AOS_rc|1
AAACTTGCCTGCGTCGGCGTTTGGAAGACCTGGAGGTAGGCCTGGTAGGCGTAGAAGGAC
>HPV101_Unclassified_109390389_nt6193_L1_AOS_rc|1
GATTCATTAAGTGTATGTGTGCCAATACATCGGGAGTAAGAGACACTTTACAGAGCTGCA
>HPV101_Unclassified_109390389_nt6186_L1_AOS_rc|1
TAAGTGTATGTGTGCCAATACATCGGGAGTAAGAGACACTTTACAGAGCTGCACAATGAG
>HPV101_Unclassified_109390389_nt6179_L1_AOS_rc|1
ATGTGTGCCAATACATCGGGAGTAAGAGACACTTTACAGAGCTGCACAATGAGAGAAATT
>HPV101_Unclassified_109390389_nt5813_L1_AOS_rc|1
TTACCCTCAAACCCCTCCTCTGGAACACTGTCACCAACAGTGCCATTATGGGTGAAATAA
>HPV101_Unclassified_109390389_nt4975_L1_AOS_rc|1
TCTGCAAAACATTAAAAACCAGACCGTTTGCGCTTGCGTTTGCGTCTGTGACTTGGATGC
>HPV101_Unclassified_109390389_nt4968_L1_AOS_rc|1
AACATTAAAAACCAGACCGTTTGCGCTTGCGTTTGCGTCTGTGACTTGGATGCAAATAAA
>HPV101_Unclassified_109390389_nt4961_L1_AOS_rc|1
AAAACCAGACCGTTTGCGCTTGCGTTTGCGTCTGTGACTTGGATGCAAATAAAATGTATC
>HPV101_Unclassified_109390389_nt4954_L1_AOS_rc|1
GACCGTTTGCGCTTGCGTTTGCGTCTGTGACTTGGATGCAAATAAAATGTATCAAATGCA
>HPV101_Unclassified_109390389_nt4925_L1_AOS_rc|1
ACTTGGATGCAAATAAAATGTATCAAATGCATCAAAACCATCCACAACAATAGGAGGTTC
>HPV70_Alpha_1173493_nt6356_L1_AOS_rc|1
TAATATATAACTCTGAAGGTATTGTGTCGCCCACCATGCCACCTCTATTCCAAAAGTGCC
>HPV71_Alpha_12084981_nt6854_L1_AOS_rc|1
AATATTCCATGAAACTAGAGGCTTTATATGTAGACTCAACAGTTTTGGTAGCACAGATGG
>HPV71_Alpha_12084981_nt6847_L1_AOS_rc|1
CATGAAACTAGAGGCTTTATATGTAGACTCAACAGTTTTGGTAGCACAGATGGACATATT
>HPV71_Alpha_12084981_nt6652_L1_AOS_rc|1
GTGTGGATGCATAAAGAGAGCTTGTAGGGGAAGCACGAAAGGTAGAAGGTGAACCTTTAA
>HPV71_Alpha_12084981_nt6363_L1_AOS_rc|1
TGTATAACAGTACTGGCAAGTTCAAGTGGGGGGCAACTACCAGCGGCAGCTGAAGCGCCA
>HPV71_Alpha_12084981_nt6356_L1_AOS_rc|1
CAGTACTGGCAAGTTCAAGTGGGGGGCAACTACCAGCGGCAGCTGAAGCGCCACTGCATG
>HPV107_Beta_126131393_nt7242_L1_AOS_rc|1
TTTCGTTTGCTATGCTTTGCAACCGATTTGGTGGTCGGCGCTGCACGTTTTCGAGAGGCA
>HPV107_Beta_126131393_nt7235_L1_AOS_rc|1
TGCTATGCTTTGCAACCGATTTGGTGGTCGGCGCTGCACGTTTTCGAGAGGCAGGTAGTG
>HPV107_Beta_126131393_nt7228_L1_AOS_rc|1
CTTTGCAACCGATTTGGTGGTCGGCGCTGCACGTTTTCGAGAGGCAGGTAGTGTTGTTTG
>HPV107_Beta_126131393_nt7221_L1_AOS_rc|2
ACCGATTTGGTGGTCGGCGCTGCACGTTTTCGAGAGGCAGGTAGTGTTGTTTGGAATAGG
>HPV107_Beta_126131393_nt7214_L1_AOS_rc|1
TGGTGGTCGGCGCTGCACGTTTTCGAGAGGCAGGTAGTGTTGTTTGGAATAGGAATTTAC
>HPV107_Beta_126131393_nt6639_L1_AOS_rc|1
ACGGTGGGATAGTAAATAGAATTACCCAAAGGACTTTGGGATTGTCCACTCTTAGGTGCT
>HPV107_Beta_126131393_nt6617_L1_AOS_rc|1
TACCCAAAGGACTTTGGGATTGTCCACTCTTAGGTGCTATATAGTATTTGTGATCTTGAT
>HPV107_Beta_126131393_nt6610_L1_AOS_rc|1
AGGACTTTGGGATTGTCCACTCTTAGGTGCTATATAGTATTTGTGATCTTGATTAGTTGT
>HPV107_Beta_126131393_nt6603_L1_AOS_rc|2
TGGGATTGTCCACTCTTAGGTGCTATATAGTATTTGTGATCTTGATTAGTTGTTGTATCA
>HPV107_Beta_126131393_nt6596_L1_AOS_rc|1
GTCCACTCTTAGGTGCTATATAGTATTTGTGATCTTGATTAGTTGTTGTATCAGGTATAG
>HPV84_Alpha_12958167_nt6476_L1_AOS_rc|1
TGCTAAGGGTTGCCCGGGAGGAGGTCCCCTTAAGGTAAAAAGACTGAGGCACCTCCTCAC

>HPVcand87_Alpha_14475578_nt5598_L1_AOS_rc|1
GGCAGTGCTAGGAACCACAGGAACAAAGGGCGTGGGGACGGGGCCATGGAATGATCAAT
>HPVcand87_Alpha_14475578_nt5614_L1_AOS_rc|1
TATATACAGAATATGGGGCAGTGCTAGGAACCACAGGAACAAAGGGCGTGGGGACGGGG
>HPVcand87_Alpha_14475578_nt5607_L1_AOS_rc|1
AGAATATGGGGCAGTGCTAGGAACCACAGGAACAAAGGGCGTGGGGACGGGGCCATGGA
>HPVcand87_Alpha_14475578_nt5591_L1_AOS_rc|1
CTAGGAACCACAGGAACAAAGGGCGTGGGGACGGGGCCATGGAATGATCAATATCGGGA
>HPVcand87_Alpha_14475578_nt5584_L1_AOS_rc|1
CCACAGGAACAAAGGGCGTGGGGACGGGGCCATGGAATGATCAATATCGGGACCTGTAT
>HPVcand87_Alpha_14475578_nt5577_L1_AOS_rc|1
AACAAAGGGCGTGGGGACGGGGCCATGGAATGATCAATATCGGGACCTGTATATAGGGG
>HPV72_Alpha_1491683_nt6832_L1_AOS_rc|1
TGTGGCGAAGATACTCACGAAAATTAGAAGCTGTATATTCTGATACAGAGGACGCTGTGG
>HPV72_Alpha_1491683_nt7247_L1_AOS_rc|1
TACTTTTTGCGCTTTTTACGTTTAGTAGGGGCGGGGGTCGAGGTAGAGCTTGGTGGGGCA
>HPV72_Alpha_1491683_nt6825_L1_AOS_rc|1
AAGATACTCACGAAAATTAGAAGCTGTATATTCTGATACAGAGGACGCTGTGGCAGTACA
>HPV72_Alpha_1491683_nt6815_L1_AOS_rc|1
CGAAAATTAGAAGCTGTATATTCTGATACAGAGGACGCTGTGGCAGTACAAATAGTTACA
>HPV72_Alpha_1491683_nt6597_L1_AOS_rc|1
ACTACCAGGTGTCACCCTGTCGCTACCCGAGGCACCTTTAAGATATAAACTGGCTGGTAG
>HPV72_Alpha_1491683_nt6590_L1_AOS_rc|1
GGTGTCACCCTGTCGCTACCCGAGGCACCTTTAAGATATAAACTGGCTGGTAGTGCCTCA
>HPV73_Alpha_1491692_nt6515_L1_AOS_rc|1
TAAAATTAGAGTTGGCATACGTTGTAGTAGAGCTACTAGCCTGTGTACCTACACATACAG
>HPV73_Alpha_1491692_nt6508_L1_AOS_rc|1
AGAGTTGGCATACGTTGTAGTAGAGCTACTAGCCTGTGTACCTACACATACAGAAAAATT
>HPV73_Alpha_1491692_nt6010_L1_AOS_rc|1
AGGGGTGTTCTTTAATTCCAGTGGGGGACAATCACCAGTATTAACAGTTTGTGAAGTACA
>HPVcand86_Alpha_15741127_nt6699_L1_AOS_rc|1
TAGATATTCATTAAACTTAGAGGGGTCATATTCAGAGGCCTTCTGGGTAGCGGCACTAAT
>HPVcand86_Alpha_15741127_nt6493_L1_AOS_rc|1
GGGTGGGTGCATATACATAACTGCTAAGGGATCCTCGGGGGCCTGAGGAGCCCTTTAAAT
>HPV88_Gamma_167600365_nt6759_L1_AOS_rc|1
TGTTCTGGAAGTATTTCTAACAGAACTATTGGCAACTGTCCTGACACGTTTTGTGGGCAG
>HPV88_Gamma_167600365_nt6768_L1_AOS_rc|1
TCTTTTTACTGTTCTGGAAGTATTTCTAACAGAACTATTGGCAACTGTCCTGACACGTTT
>HPV88_Gamma_167600365_nt6752_L1_AOS_rc|1
GAAGTATTTCTAACAGAACTATTGGCAACTGTCCTGACACGTTTTGTGGGCAGCAAACCA
>HPV88_Gamma_167600365_nt6445_L1_AOS_rc|2
GAGCTAAAATATCAGGCTCCAAAGGAACTCTACAAAGCTGGAATATAGCTTCCAATTCGT
>HPV88_Gamma_167600365_nt6173_L1_AOS_rc|1
TCACTAGTAGTAAGTGATCCACTAGCAGTAGGGAAATAAACATGAGATCCCATTTTAAAA
>HPV88_Gamma_167600365_nt6166_L1_AOS_rc|1
TAGTAAGTGATCCACTAGCAGTAGGGAAATAAACATGAGATCCCATTTTAAAATTCACAG
>HPV88_Gamma_167600365_nt6158_L1_AOS_rc|1
GATCCACTAGCAGTAGGGAAATAAACATGAGATCCCATTTTAAAATTCACAGCATTTGTA
>HPV88_Gamma_167600365_nt6150_L1_AOS_rc|1
AGCAGTAGGGAAATAAACATGAGATCCCATTTTAAAATTCACAGCATTTGTAGGTGGCAA
>HPV88_Gamma_167600365_nt6143_L1_AOS_rc|1
GGGAAATAAACATGAGATCCCATTTTAAAATTCACAGCATTTGTAGGTGGCAATAAGTAT
>HPV88_Gamma_167600365_nt6132_L1_AOS_rc|1
ATGAGATCCCATTTTAAAATTCACAGCATTTGTAGGTGGCAATAAGTATTTATTTCCCTC
>HPV110_Beta_186461194_nt6515_L1_AOS_rc|1

GTAGAGTTTGCTAGTGTGCGTTGTTGTTGATCTTGCTTTGCTGGCAAGAAATAGTTATGA
>HPV110_Beta_186461194_nt6508_L1_AOS_rc|1
TTGCTAGTGTGCGTTGTTGTTGATCTTGCTTTGCTGGCAAGAAATAGTTATGATTTTGAT
>HPV111_Beta_186461202_nt6757_L1_AOS_rc|2
GAGATAAAACTTCTGGAACTAAAGAAACTTTACATAGTTGAAGAATCAAAGACAATTGAT
>HPV111_Beta_186461202_nt6466_L1_AOS_rc|1
GATAATAAATGGAGTTACCCAAAATGTTTTGTTGTTGATTACCCTGTGCTGGCAGATAAT
>HPV111_Beta_186461202_nt6445_L1_AOS_rc|1
AAATGTTTTGTTGTTGATTACCCTGTGCTGGCAGATAATAATTGTGATTTTGTTGCACTG
>HPV111_Beta_186461202_nt6436_L1_AOS_rc|1
GTTGTTGATTACCCTGTGCTGGCAGATAATAATTGTGATTTTGTTGCACTGCTGCATCTG
>HPVFA75/KI88-03_Unclassified_206902429_nt7084_L1_AOS_rc|2
TTATGTCCGTCGTCGTTTTGCAGTACCCCGAGAAACAGATCTGCTACGCTTTAATGATTT
>HPVFA75/KI88-03_Unclassified_206902429_nt7075_L1_AOS_rc|1
TCGTCGTTTTGCAGTACCCCGAGAAACAGATCTGCTACGCTTTAATGATTTGACCCTGGT
>HPVFA75/KI88-03_Unclassified_206902429_nt7068_L1_AOS_rc|1
TTTGCAGTACCCCGAGAAACAGATCTGCTACGCTTTAATGATTTGACCCTGGTGTTCTGT
>HPVFA75/KI88-03_Unclassified_206902429_nt7061_L1_AOS_rc|1
TACCCCGAGAAACAGATCTGCTACGCTTTAATGATTTGACCCTGGTGTTCTGTAATCCTG
>HPVFA75/KI88-03_Unclassified_206902429_nt6902_L1_AOS_rc|1
CCTCTCTATCTTTTGCAGGAACCTTATCTGGACATTTAGTGGCTGAGGAATTTATAAATC
>HPVFA75/KI88-03_Unclassified_206902429_nt6670_L1_AOS_rc|2
TCTAAATTTGCTAGAATCATACTCAGTTATAGCACCATTATCTGAAGGTACACAAATGGT
>HPVFA75/KI88-03_Unclassified_206902429_nt6663_L1_AOS_rc|1
TTGCTAGAATCATACTCAGTTATAGCACCATTATCTGAAGGTACACAAATGGTAAAGTTA
>HPVFA75/KI88-03_Unclassified_206902429_nt6650_L1_AOS_rc|2
ACTCAGTTATAGCACCATTATCTGAAGGTACACAAATGGTAAAGTTAGTGTTTCTAGTGT
>HPVFA75/KI88-03_Unclassified_206902429_nt6643_L1_AOS_rc|1
TATAGCACCATTATCTGAAGGTACACAAATGGTAAAGTTAGTGTTTCTAGTGTTATCAGC
>HPVFA75/KI88-03_Unclassified_206902429_nt6397_L1_AOS_rc|1
ATCTTGATTATTTGCTCCATCTGGAGTAGCGTCTCCAACATTACCTCCTCTGGTAAAATA
>HPV91_Alpha_22023568_nt5981_L1_AOS_rc|2
TTACCAGAATTATTTTTTATAGGAAAAAAAGGGTGGCCCACAGCCAGTAAACGGGAACTG
>HPV91_Alpha_22023568_nt5744_L1_AOS_rc|1
ATAAAAATCTGTGCCATATATTGATACTGCAGAAGGTGTAACAGACGGTGACAATGGCAC
>HPV91_Alpha_22023568_nt5726_L1_AOS_rc|1
TATTGATACTGCAGAAGGTGTAACAGACGGTGACAATGGCACATAGGGGACACTAAGTGG
>HPV91_Alpha_22023568_nt5700_L1_AOS_rc|2
ACGGTGACAATGGCACATAGGGGACACTAAGTGGCACTGTAGGAAATGTAATATCAGGTC
>HPV91_Alpha_22023568_nt5693_L1_AOS_rc|1
CAATGGCACATAGGGGACACTAAGTGGCACTGTAGGAAATGTAATATCAGGTCCTGCTTG
>HPV91_Alpha_22023568_nt5683_L1_AOS_rc|1
TAGGGGACACTAAGTGGCACTGTAGGAAATGTAATATCAGGTCCTGCTTGTGTATATATA
>HPV91_Alpha_22023568_nt5673_L1_AOS_rc|1
TAAGTGGCACTGTAGGAAATGTAATATCAGGTCCTGCTTGTGTATATATATCAGGTACTG
>HPV91_Alpha_22023568_nt5642_L1_AOS_rc|1
TCCTGCTTGTGTATATATATCAGGTACTGTTGCAAGTGGGACTGTAGTGTTAACAATTTT
>HPV91_Alpha_22023568_nt5627_L1_AOS_rc|1
TATATCAGGTACTGTTGCAAGTGGGACTGTAGTGTTAACAATTTTTGTTGTGTTAGGCAG
>HPVcand89_Alpha_22095322_nt6573_L1_AOS_rc|1
TATACATAGCTAGTAAGTGTATTTCTGTTATTAGCAGCAGACCCAGGCATATACAAGGTT
>HPVcand89_Alpha_22095322_nt6554_L1_AOS_rc|1
TATTTCTGTTATTAGCAGCAGACCCAGGCATATACAAGGTTTTTGGCAGGTCCTCACCCA
>HPV90_Alpha_22138122_nt6189_L1_AOS_rc|1
TATAGTCCATAGTAAGATTGTCCCGGGTGTCATCGCCAGGCACAACATCATATAAATTGG

>HPV58_Alpha_222386_nt5791_L1_AOS_rc|1
GTAAGCCTGATACCTTGGGAACTAATACTTTTTTATTGTTATTGGGACTTTTGATGGAAA
>HPV74_Alpha_27462483_nt6609_L1_AOS_rc|1
GTTTGTAGTCTGTACTAGTATATGTAGCAGCAGGAGGGGATTGTGTAGTAGGCGCACACA
>HPV74_Alpha_27462483_nt6109_L1_AOS_rc|1
TGAATTACACTAGTAATTAATTCTAATGCAGGGCAATCGCCTGGCTTTACAGTAACATTA
>HPV74_Alpha_27462483_nt6102_L1_AOS_rc|1
CACTAGTAATTAATTCTAATGCAGGGCAATCGCCTGGCTTTACAGTAACATTAGAACATT
>HPV92_Beta_27531786_nt7104_L1_AOS_rc|1
CTTTTTCGTTTAGTACCTCTTTGAGAACTGCTTCTAGTTCTTTTAAGACCATTAACAGAT
>HPV92_Beta_27531786_nt7116_L1_AOS_rc|1
TAGTTACTTTTTCTTTTTCGTTTAGTACCTCTTTGAGAACTGCTTCTAGTTCTTTTAAGA
>HPV92_Beta_27531786_nt7097_L1_AOS_rc|1
GTTTAGTACCTCTTTGAGAACTGCTTCTAGTTCTTTTAAGACCATTAACAGATGTATTAG
>HPV92_Beta_27531786_nt7090_L1_AOS_rc|1
ACCTCTTTGAGAACTGCTTCTAGTTCTTTTAAGACCATTAACAGATGTATTAGTTAATCC
>HPV92_Beta_27531786_nt6709_L1_AOS_rc|1
TCTAGTGTATTCTCTAATCTTTTGTGAATCATAGTCGGTTATTTGACCACCTTCCTTAGG
>HPV92_Beta_27531786_nt6434_L1_AOS_rc|1
GTAACATAAAGTTATGGTCTTCATTTACAACTGCATCTGGGATGGTATCACCCACAACAC
>HPV92_Beta_27531786_nt6427_L1_AOS_rc|1
AAAGTTATGGTCTTCATTTACAACTGCATCTGGGATGGTATCACCCACAACACCACCGCG
>HPV92_Beta_27531786_nt6420_L1_AOS_rc|1
TGGTCTTCATTTACAACTGCATCTGGGATGGTATCACCCACAACACCACCGCGCACAAAC
>HPV75_Beta_2911544_nt7203_L1_AOS_rc|1
GGACACTGGCTGTTCTGGTTACCTTGGAAGCCCTTCGTAAGCCTGCCTGAAAAAGGAATT
>HPV75_Beta_2911544_nt6633_L1_AOS_rc|1
AACCACTGACCGTGGGAACATAAATAGAGTTGCCCAAAGTGTTTTGAGCCTGCCCAGCGG
>HPV75_Beta_2911544_nt6624_L1_AOS_rc|1
CCGTGGGAACATAAATAGAGTTGCCCAAAGTGTTTTGAGCCTGCCCAGCGGCTGCAGGTA
>HPV76_Beta_2911551_nt6655_L1_AOS_rc|1
TAACGGTAGGCACGTAAATAGAGCTACCCAAAGTGTTTTGGGCCTGTCCAACAGCTGCAG
>HPV76_Beta_2911551_nt7242_L1_AOS_rc|1
GGATGCTGTGCGGACAGTGGCTGTCCTGGATACTCTGGAAGCCCGTCGCAGGCCTGCTTG
>HPV76_Beta_2911551_nt7234_L1_AOS_rc|1
TGCGGACAGTGGCTGTCCTGGATACTCTGGAAGCCCGTCGCAGGCCTGCTTGAAACAGAA
>HPV76_Beta_2911551_nt6118_L1_AOS_rc|1
TAAATAGGGGATGACCTGTAGACCCTACTCCTAGGGGTTGTCCGCGACCTATTTCCAAAC
>HPV77_Alpha_2911558_nt6602_L1_AOS_rc|1
GTATATAGCACTGCCGGGAGTCTCACGCCCGCTACTCCCTTTGAGGTACAAAGATTCTGG
>HPV77_Alpha_2911558_nt7240_L1_AOS_rc|1
CACTTTTTGGAGCGCTTCCGTTTGGTCGAAGCTGGGGAGGGGGTGGGCGCCCGGCGTTTG
>HPV77_Alpha_2911558_nt7230_L1_AOS_rc|1
AGCGCTTCCGTTTGGTCGAAGCTGGGGAGGGGGTGGGCGCCCGGCGTTTGCGAGAGGGAA
>HPV77_Alpha_2911558_nt7223_L1_AOS_rc|1
CCGTTTGGTCGAAGCTGGGGAGGGGGTGGGCGCCCGGCGTTTGCGAGAGGGAACCACCGA
>HPV77_Alpha_2911558_nt7214_L1_AOS_rc|1
CGAAGCTGGGGAGGGGGTGGGCGCCCGGCGTTTGCGAGAGGGAACCACCGAACGACGTCG
>HPV77_Alpha_2911558_nt6587_L1_AOS_rc|1
GGGAGTCTCACGCCCGCTACTCCCTTTGAGGTACAAAGATTCTGGAATTTTGTCTCCAAC
>HPV77_Alpha_2911558_nt6579_L1_AOS_rc|1
CACGCCCGCTACTCCCTTTGAGGTACAAAGATTCTGGAATTTTGTCTCCAACATCACCCG
>HPV80_Beta_2911565_nt6571_L1_AOS_rc|1
CAGTAGGAAAATATGTGGAATTTGCTAAAACACGTTGTTGCTGGCCTGTTTGTGCTGGTA
>HPV80_Beta_2911565_nt6772_L1_AOS_rc|1

TAATATTTTGTGTATTATATTCAGTTATAGTACTACCATCAGAAGTAACACTTATGGTGA
>HPV80_Beta_2911565_nt6763_L1_AOS_rc|2
GTGTATTATATTCAGTTATAGTACTACCATCAGAAGTAACACTTATGGTGAAATTAGTGT
>HPV80_Beta_2911565_nt6756_L1_AOS_rc|1
ATATTCAGTTATAGTACTACCATCAGAAGTAACACTTATGGTGAAATTAGTGTTTCTTGT
>HPV80_Beta_2911565_nt6749_L1_AOS_rc|1
GTTATAGTACTACCATCAGAAGTAACACTTATGGTGAAATTAGTGTTTCTTGTATTATCA
>HPV80_Beta_2911565_nt6562_L1_AOS_rc|1
AATATGTGGAATTTGCTAAAACACGTTGTTGCTGGCCTGTTTGTGCTGGTAAATAAAAGT
>HPVRTRX7_Unclassified_30315616_nt7253_L1_AOS_rc|1
ATTCCAGAATGTTAATTTTTCATAAGGATCAGGTTTTTCCTTTGGTGGATTTTTATCGGG
>HPVRTRX7_Unclassified_30315616_nt6984_L1_AOS_rc|1
TTTGTGAGTCATATGATTGTATTTGCTGTATATCTTGATTTTCACTAGATATAGATATAC
>HPV65_Gamma_312100_nt6179_L1_AOS_rc|1
TAGGAGTGCCTATATATATATATGTGGACCTAAAGTATTCTGATCCTGACCTTCTTGAGCAG
>HPV65_Gamma_312100_nt6172_L1_AOS_rc|1
GCCTATATATATATATGTGGACCTAAAGTATTCTGATCCTGACCTTCTTGAGCAGGAATCCA
>HPV65_Gamma_312100_nt6165_L1_AOS_rc|1
TATATATGTGGACCTAAAGTATTCTGATCCTGACCTTCTTGAGCAGGAATCCAGTAATCA
>HPV65_Gamma_312100_nt6158_L1_AOS_rc|1
GTGGACCTAAAGTATTCTGATCCTGACCTTCTTGAGCAGGAATCCAGTAATCAGTTTTAA
>HPV65_Gamma_312100_nt6151_L1_AOS_rc|2
TAAAGTATTCTGATCCTGACCTTCTTGAGCAGGAATCCAGTAATCAGTTTTAACCTCGAA
>HPV65_Gamma_312100_nt6144_L1_AOS_rc|1
TTCTGATCCTGACCTTCTTGAGCAGGAATCCAGTAATCAGTTTTAACCTCGAAAGGTTCT
>HPV65_Gamma_312100_nt5585_L1_AOS_rc|1
TTCCTCTGAGTCTCCAAACAAGACGTTCATGGTCAGAATCATAGAAACCATTATCAATCA
>HPV65_Gamma_312100_nt5576_L1_AOS_rc|2
GTCTCCAAACAAGACGTTCATGGTCAGAATCATAGAAACCATTATCAATCAATGCAAACC
>HPV67_Alpha_3228267_nt6906_L1_AOS_rc|1
ATTGATTTCCCAAAAACTGTACTTTTTAAGAGGATCTTCCTTTGCTGTTGGAGGGGATGT
>HPV67_Alpha_3228267_nt6895_L1_AOS_rc|1
AAAAACTGTACTTTTTAAGAGGATCTTCCTTTGCTGTTGGAGGGGATGTTTTTTGACAGG
>HPV67_Alpha_3228267_nt6760_L1_AOS_rc|1
AGTCCTCTAATATATCTGGATTCATGGTGTGTATGTATTGCATAACATTTGCAGTAAGGG
>HPV67_Alpha_3228267_nt6753_L1_AOS_rc|1
TAATATATCTGGATTCATGGTGTGTATGTATTGCATAACATTTGCAGTAAGGGATATTTT
>HPV67_Alpha_3228267_nt6745_L1_AOS_rc|1
CTGGATTCATGGTGTGTATGTATTGCATAACATTTGCAGTAAGGGATATTTTGCACAGCT
>HPV67_Alpha_3228267_nt6738_L1_AOS_rc|1
CATGGTGTGTATGTATTGCATAACATTTGCAGTAAGGGATATTTTGCACAGCTGAAATAT
>HPV67_Alpha_3228267_nt6437_L1_AOS_rc|1
GGAAAAAAAGCTGAGGTTTGCAGTGCAGAAGTGTTTGCAGATCCCTTAAAATACAAATCT
>HPV67_Alpha_3228267_nt5754_L1_AOS_rc|1
CACCTTGGGCACTAACACCTTTTTAGTGTTGGAGGGATTAGGAATGGAAAAGTAAGGATG
>HPV11_Alpha_333026_nt7176_L1_AOS_rc|2
AGGGCTTAGACACAGCTGGGCGCTTTATACCTGTACGAGCAGACGTCCGTCCTCGATATC
>HPV11_Alpha_333026_nt7193_L1_AOS_rc|1
TCGTTTGGGGGCTGTAGAGGGCTTAGACACAGCTGGGCGCTTTATACCTGTACGAGCAGA
>HPV11_Alpha_333026_nt7186_L1_AOS_rc|1
GGGGCTGTAGAGGGCTTAGACACAGCTGGGCGCTTTATACCTGTACGAGCAGACGTCCGT
>HPV11_Alpha_333026_nt6787_L1_AOS_rc|1
TATTCCTTATAATCTGAATTAGTGTATGTAGCAGATTTAGACACAGATGCACATAGTGTC
>HPV31_Alpha_333048_nt6981_L1_AOS_rc|1
CTGGTGTAGTGGTAGATGCTGAGGGTGCACTACGTTTACCTGCTTTAAATTTAGGACGTG

>HPV31_Alpha_333048_nt6974_L1_AOS_rc|1
AGTGGTAGATGCTGAGGGTGCACTACGTTTACCTGCTTTAAATTTAGGACGTGCCCTATA
>HPV31_Alpha_333048_nt6967_L1_AOS_rc|2
GATGCTGAGGGTGCACTACGTTTACCTGCTTTAAATTTAGGACGTGCCCTATATCCTGCC
>HPV33_Alpha_333049_nt7012_L1_AOS_rc|1
TTTGCAGACGATGTGCGGGTGGATGTGGGGGCTGCACGTTTAAGTTTAGGTTTTGCTTTA
>HPV35_Alpha_333050_nt6970_L1_AOS_rc|1
GCCTGCCTAATCTAAAATTAGGCCTGGCCTTTAGTCCTGCTTGTAACAAAAATTTACGGC
>HPV35_Alpha_333050_nt6992_L1_AOS_rc|1
GATGTAGATGCTGGAGCTGCACGCCTGCCTAATCTAAAATTAGGCCTGGCCTTTAGTCCT
>HPV35_Alpha_333050_nt6982_L1_AOS_rc|1
CTGGAGCTGCACGCCTGCCTAATCTAAAATTAGGCCTGGCCTTTAGTCCTGCTTGTAACA
>HPV35_Alpha_333050_nt6963_L1_AOS_rc|1
TAATCTAAAATTAGGCCTGGCCTTTAGTCCTGCTTGTAACAAAAATTTACGGCCCAACGG
>HPV35_Alpha_333050_nt5966_L1_AOS_rc|1
GAAATGCATTCCCTGTTATCTGTACCAGAGTTACCAGAGTTACCAACATATTTATTAAGA
>HPV35_Alpha_333050_nt5957_L1_AOS_rc|1
TCCCTGTTATCTGTACCAGAGTTACCAGAGTTACCAACATATTTATTAAGATTTTCAGTA
>HPV35_Alpha_333050_nt5714_L1_AOS_rc|1
CCAGATACCTTGGGTACTGCTATTTTATTAGAATCTTGTTTTTAATAGCATAGTATGGG
>HPV47_Beta_333062_nt6949_L1_AOS_rc|1
TAATCCTGTATATCCTTTATGTCCCCTGCCTGAGAGTAAACAGAGATGCTGAAATTTGTA
>HPV47_Beta_333062_nt6964_L1_AOS_rc|1
AAATTGTCTGCATTATAATCCTGTATATCCTTTATGTCCCCTGCCTGAGAGTAAACAGAG
>HPV47_Beta_333062_nt6956_L1_AOS_rc|1
TGCATTATAATCCTGTATATCCTTTATGTCCCCTGCCTGAGAGTAAACAGAGATGCTGAA
>HPV42_Alpha_333211_nt6618_L1_AOS_rc|1
ATGCATTATTAGCAGCCTTGGTATACAGTTCATCAGGTACAGGTTCACCAATTGCGCCAG
>HPV42_Alpha_333211_nt7265_L1_AOS_rc|1
TTTAGCTGAAGAAACAGATTTAGCTGTAGACGCCTTTCGTTTACCTACAGACAGTTTAGG
>HPV42_Alpha_333211_nt6645_L1_AOS_rc|1
TACTACTACCTAAATTATGTCTGCCAGATGCATTATTAGCAGCCTTGGTATACAGTTCAT
>HPV42_Alpha_333211_nt6638_L1_AOS_rc|1
ACCTAAATTATGTCTGCCAGATGCATTATTAGCAGCCTTGGTATACAGTTCATCAGGTAC
>HPV39_Alpha_333245_nt7078_L1_AOS_rc|2
TGTGTTTAGTAGCTGAGGACGAGGAAGTGGATGCAGCAGGCCGCTTTCGGGGACCTATAG
>HPV39_Alpha_333245_nt7071_L1_AOS_rc|1
AGTAGCTGAGGACGAGGAAGTGGATGCAGCAGGCCGCTTTCGGGGACCTATAGTAGGGCG
>HPV39_Alpha_333245_nt7063_L1_AOS_rc|1
AGGACGAGGAAGTGGATGCAGCAGGCCGCTTTCGGGGACCTATAGTAGGGCGCCTGCGGA
>HPV39_Alpha_333245_nt6856_L1_AOS_rc|1
CTGCAGACTGTAGGTATCTGTAAGTGTCTACCAAACTGGCAGATGGTGGAGGAGCTACAG
>HPV39_Alpha_333245_nt6658_L1_AOS_rc|2
TAGAAGGATCATATGTAGAAGGTATGGAAGACTCTATAGAGGTAGATAATGTAAAGTTGG
>HPV39_Alpha_333245_nt6455_L1_AOS_rc|1
CAGTATACAGAACTACCGGGGTTTGCACGTATATCTGTGCCCTTAATATACAATTGGGCA
>HPV39_Alpha_333245_nt6448_L1_AOS_rc|2
CAGAACTACCGGGGTTTGCACGTATATCTGTGCCCTTAATATACAATTGGGCAGGAATGG
>HPV39_Alpha_333245_nt6441_L1_AOS_rc|2
ACCGGGGTTTGCACGTATATCTGTGCCCTTAATATACAATTGGGCAGGAATGGCGTCACC
>HPV39_Alpha_333245_nt6433_L1_AOS_rc|1
TTGCACGTATATCTGTGCCCTTAATATACAATTGGGCAGGAATGGCGTCACCCACCATAC
>HPV39_Alpha_333245_nt6426_L1_AOS_rc|1
TATATCTGTGCCCTTAATATACAATTGGGCAGGAATGGCGTCACCCACCATACCACCACG
>HPV93_Beta_37089393_nt7102_L1_AOS_rc|1

TAGTTCCTCGAGAAATAGATGATGACCGTTTAGCTGGTGATTGTACAAGATTTGCCTGAT
>HPV93_Beta_37089393_nt6942_L1_AOS_rc|1
TTCCTTTGGTGGAACCTTATCGGGACATGGTGTAGCTAAACTATTAATATATCGATAAGT
>HPV93_Beta_37089393_nt6935_L1_AOS_rc|2
GGTGGAACCTTATCGGGACATGGTGTAGCTAAACTATTAATATATCGATAAGTACTCTGT
>HPV93_Beta_37089393_nt6928_L1_AOS_rc|1
CCTTATCGGGACATGGTGTAGCTAAACTATTAATATATCGATAAGTACTCTGTAACGGAT
>HPV93_Beta_37089393_nt6921_L1_AOS_rc|2
GGGACATGGTGTAGCTAAACTATTAATATATCGATAAGTACTCTGTAACGGATTGTCTGG
>HPV93_Beta_37089393_nt6158_L1_AOS_rc|1
ACTAATTGTATAGGTGGGCAAATCCCATTTTGGTCATTGGCTCCATCACATTTAGGTGCA
>HPV93_Beta_37089393_nt6151_L1_AOS_rc|1
GTATAGGTGGGCAAATCCCATTTTGGTCATTGGCTCCATCACATTTAGGTGCAACATCCC
>HPV12_Beta_396910_nt6897_L1_AOS_rc|1
ATAATTTGGAATGTCGTGTACATTTTGATTATCACTGTAAATAGATATACTGAAATTGGT
>HPV12_Beta_396910_nt6373_L1_AOS_rc|1
CTTCAATGAAAGTGTTCTTTAACTCTATGGGAGGGCAAACACCATTTTCAGCTGGTGCAT
>HPV14D_Beta_396918_nt6445_L1_AOS_rc|1
CCAATATTGTTTTGTGGTTGATTTGTCATTGGTGGAATGTAATAAACATTCTTTAGGCTA
>HPV14D_Beta_396918_nt6672_L1_AOS_rc|1
TCTGAGAGGTATAATTGTCAATTTTGGATACCTCAGTGTTTTCTGAACTAACTGATATAC
>HPV14D_Beta_396918_nt6458_L1_AOS_rc|1
ATACATGGCATTGCCAATATTGTTTTGTGGTTGATTTGTCATTGGTGGAATGTAATAAAC
>HPV14D_Beta_396918_nt5997_L1_AOS_rc|1
CAAATGACACATTTTGTCTGTCATCAGTGGAGTTAGCTTGTTGCCTGTATGAGTTGGGAT
>HPV14D_Beta_396918_nt5982_L1_AOS_rc|1
GTCTGTCATCAGTGGAGTTAGCTTGTTGCCTGTATGAGTTGGGATTTTCTGTATCACCAA
>HPV15_Beta_396924_nt6558_L1_AOS_rc|1
AACTGTGGGAAAGTAAGTAGAATTTGCTAAGTTATTTGTTGTTGGGTTGATTGTGCTGG
>HPV15_Beta_396924_nt6547_L1_AOS_rc|1
AGTAAGTAGAATTTGCTAAGTTATTTGTTGTTGGGTTGATTGTGCTGGTAAATAAAAAT
>HPV17_Beta_396932_nt6751_L1_AOS_rc|1
CAGCCCCAGCTTCTGTAGACACGCTAATAGAAAAATTTGTGTTCCTAGTGTTGTCAGCTA
>HPV17_Beta_396932_nt6581_L1_AOS_rc|2
CTTACAGTTGGAAAATAAGTGGAATTACCCAAAGTGCGCTGTTGTTGGCCAGTTTGAGCT
>HPV17_Beta_396932_nt6574_L1_AOS_rc|1
TTGGAAAATAAGTGGAATTACCCAAAGTGCGCTGTTGTTGGCCAGTTTGAGCTGGTAAAT
>HPV17_Beta_396932_nt6565_L1_AOS_rc|1
AAGTGGAATTACCCAAAGTGCGCTGTTGTTGGCCAGTTTGAGCTGGTAAATAAAATTTGT
>HPV17_Beta_396932_nt6558_L1_AOS_rc|1
ATTACCCAAAGTGCGCTGTTGTTGGCCAGTTTGAGCTGGTAAATAAAATTTGTGATCCTG
>HPV17_Beta_396932_nt6070_L1_AOS_rc|1
AGCTACTGTTATTTTCAGTGTCTCTTAACTTATTAAACAAGGGATGTCCTGTAGTGCCTA
>HPV19_Beta_396940_nt6704_L1_AOS_rc|2
ATTTCCTAAATTAGTATATTGTTGCTGACTATTGTTAGGAGGTATGTAGTATGTATTTTT
>HPV25_Beta_396948_nt7158_L1_AOS_rc|1
TGTGGCTAAAGAATCAATGTAGCGATAAGTATCCTGAATAGAATTGTCCGGTGCAGGAAC
>HPV25_Beta_396948_nt7147_L1_AOS_rc|1
AATCAATGTAGCGATAAGTATCCTGAATAGAATTGTCCGGTGCAGGAACGAATCCTAATT
>HPV25_Beta_396948_nt7061_L1_AOS_rc|2
ATTGCATTAATCTGAGCCAATATTTCTGCCTTCAACGGTACTTTGCAAAGTTGTAATATT
>HPV25_Beta_396948_nt7054_L1_AOS_rc|2
TAATCTGAGCCAATATTTCTGCCTTCAACGGTACTTTGCAAAGTTGTAATATTAATGATA
>HPV25_Beta_396948_nt7047_L1_AOS_rc|1
AGCCAATATTTCTGCCTTCAACGGTACTTTGCAAAGTTGTAATATTAATGATAACTCATA

>HPV25_Beta_396948_nt6737_L1_AOS_rc|1
ATTGAGTTACCTAGATTATTATATTGAGCCTGACTACTGTTAGGTGGTATGTAAAATGCA
>HPV27_Alpha_396964_nt5524_L1_AOS_rc|1
CAACACCACCCGAGAGAGGGGCTGTAGTGGACCCTCGCAGTGTAGATGCAGAGGAGGCAG
>HPV27_Alpha_396964_nt5669_L1_AOS_rc|1
GGCCACAGGATATAACTTGGCAGTAAATAATAATCCCCCCCAAATATGTAAACAGAGGAT
>HPV27_Alpha_396964_nt5641_L1_AOS_rc|1
AATAATCCCCCCCAAATATGTAAACAGAGGATGGCAACGATGGAGCCACAGGAATGAGGG
>HPV27_Alpha_396964_nt5632_L1_AOS_rc|1
CCCCAAATATGTAAACAGAGGATGGCAACGATGGAGCCACAGGAATGAGGGGCCCCAAAC
>HPV27_Alpha_396964_nt5624_L1_AOS_rc|1
ATGTAAACAGAGGATGGCAACGATGGAGCCACAGGAATGAGGGGCCCCAAACCAGGAACA
>HPV27_Alpha_396964_nt5608_L1_AOS_rc|1
GCAACGATGGAGCCACAGGAATGAGGGGCCCCAAACCAGGAACAACGGGTGGTTCAATAT
>HPV27_Alpha_396964_nt5595_L1_AOS_rc|1
CACAGGAATGAGGGGCCCCAAACCAGGAACAACGGGTGGTTCAATATCAGGCCCAGTATA
>HPV27_Alpha_396964_nt5581_L1_AOS_rc|1
GCCCCAAACCAGGAACAACGGGTGGTTCAATATCAGGCCCAGTATACACAGGCACATCAA
>HPV27_Alpha_396964_nt5560_L1_AOS_rc|1
GTGGTTCAATATCAGGCCCAGTATACACAGGCACATCAACACCACCCGAGAGAGGGGCTG
>HPV27_Alpha_396964_nt5510_L1_AOS_rc|1
AGAGGGGCTGTAGTGGACCCTCGCAGTGTAGATGCAGAGGAGGCAGATACCGTGGTATTA
>HPV30_Alpha_396973_nt7098_L1_AOS_rc|2
TCACCGCCGCTTGCGTTTGGCTGATGGTGTAGAGGTAGAGGAACTGGGGGCCGAGCGTTT
>HPV30_Alpha_396973_nt7090_L1_AOS_rc|1
GCTTGCGTTTGGCTGATGGTGTAGAGGTAGAGGAACTGGGGGCCGAGCGTTTTTTAGTAG
>HPV30_Alpha_396973_nt7082_L1_AOS_rc|1
TTGGCTGATGGTGTAGAGGTAGAGGAACTGGGGGCCGAGCGTTTTTTAGTAGTAGTAGAA
>HPV30_Alpha_396973_nt7075_L1_AOS_rc|1
ATGGTGTAGAGGTAGAGGAACTGGGGGCCGAGCGTTTTTTAGTAGTAGTAGAAGGTTTAG
>HPV30_Alpha_396973_nt7066_L1_AOS_rc|1
AGGTAGAGGAACTGGGGGCCGAGCGTTTTTTAGTAGTAGTAGAAGGTTTAGTACGAACCC
>HPV30_Alpha_396973_nt6841_L1_AOS_rc|1
ATTTGTCCTCTAAGCTTGTGGCAGCTGGGGGTGACAATCCAATATTCCAGCCCTCAAGTA
>HPV30_Alpha_396973_nt6833_L1_AOS_rc|1
TCTAAGCTTGTGGCAGCTGGGGGTGACAATCCAATATTCCAGCCCTCAAGTAAAGTGGAG
>HPV30_Alpha_396973_nt6826_L1_AOS_rc|2
TTGTGGCAGCTGGGGGTGACAATCCAATATTCCAGCCCTCAAGTAAAGTGGAGTTCATAG
>HPV30_Alpha_396973_nt6819_L1_AOS_rc|1
AGCTGGGGGTGACAATCCAATATTCCAGCCCTCAAGTAAAGTGGAGTTCATAGTATGTAA
>HPV30_Alpha_396973_nt6655_L1_AOS_rc|1
TATATGTGGATAACGTTTGTGTGGTTGCAGATATAGTCATGTTTGTGTTCCTAGTGGTGT
>HPV3_Alpha_397005_nt7029_L1_AOS_rc|1
GTTTAGTTTGGCGTAGGGGTCTTGCTTCTCAGTGGGAGGTGCATCTTTCTGGCAGGTAAT
>HPV3_Alpha_397005_nt7178_L1_AOS_rc|1
GCAGCTGTTCTAGATGTGGTTGTCGCCGAGCGTTTACGAACAGATATACTAGAGCGGGTA
>HPV3_Alpha_397005_nt7154_L1_AOS_rc|1
GCCGAGCGTTTACGAACAGATATACTAGAGCGGGTACCTACACCGAGCTGCATGAGAAAT
>HPV3_Alpha_397005_nt7147_L1_AOS_rc|1
GTTTACGAACAGATATACTAGAGCGGGTACCTACACCGAGCTGCATGAGAAATTTCCTGC
>HPV3_Alpha_397005_nt6772_L1_AOS_rc|1
ATTTAGTAGCATCATATGTAGCCGAGGTTTCAGTAGAAACACACAATGTCATATTAGTAC
>HPV3_Alpha_397005_nt6523_L1_AOS_rc|1
CTTTAATGTACAACGCGTCAGGCACGGTGTCTCCAGCCATACCAGCTCTGTTAAGAAAAT
>HPV40_Alpha_397014_nt6569_L1_AOS_rc|2

TAGGAGTCCGACCAGATGTACCTGTTATATATAAGTCAGTTGGGACACTATCACCAGTAG
>HPV45_Alpha_397022_nt7077_L1_AOS_rc|1
CGTATACGTACACGTTTGGCAGGCCTAGATGCAGTAGATGCAGTAGACGTGGAAGCAGCA
>HPV45_Alpha_397022_nt7089_L1_AOS_rc|1
TATTTCTTACTACGTATACGTACACGTTTGGCAGGCCTAGATGCAGTAGATGCAGTAGAC
>HPV45_Alpha_397022_nt7070_L1_AOS_rc|1
GTACACGTTTGGCAGGCCTAGATGCAGTAGATGCAGTAGACGTGGAAGCAGCAGGACGCT
>HPV52_Alpha_397038_nt6462_L1_AOS_rc|1
GCTTTGTACAGTGGCAGTATTGCCAGAGTTAGACCCTTGTATATATAAATCACCTGGCAC
>HPV52_Alpha_397038_nt6455_L1_AOS_rc|1
ACAGTGGCAGTATTGCCAGAGTTAGACCCTTGTATATATAAATCACCTGGCACAGGGTCA
>HPV52_Alpha_397038_nt6448_L1_AOS_rc|1
CAGTATTGCCAGAGTTAGACCCTTGTATATATAAATCACCTGGCACAGGGTCACCTAAGG
>HPV52_Alpha_397038_nt6441_L1_AOS_rc|1
GCCAGAGTTAGACCCTTGTATATATAAATCACCTGGCACAGGGTCACCTAAGGTACCGGC
>HPV56_Alpha_397053_nt6120_L1_AOS_rc|1
CTATAGGTGTATTAATTAATGCAAGAGGCGGGCAGTCCCCTGTGGTAACTTGTGTGGACT
>HPV56_Alpha_397053_nt7010_L1_AOS_rc|1
AGAGGTGGAGGTAGGAGCAGATCGCTTTTTAGAGGTAGCTACAGCAGGCTTTGACCTAGT
>HPV56_Alpha_397053_nt7002_L1_AOS_rc|2
AGGTAGGAGCAGATCGCTTTTTAGAGGTAGCTACAGCAGGCTTTGACCTAGTGCCCAGTT
>HPV56_Alpha_397053_nt6626_L1_AOS_rc|1
TCTAAGGTACTGATTAATTTTTCGTGCATCATATTTACTTAACTGTTCTGTAGCAGTACT
>HPV56_Alpha_397053_nt6409_L1_AOS_rc|1
ACATATACAGAACTCGGAGGGGGTTCTCTACCATTGCTACCCTTTAAATATAACTCTGCA
>HPV56_Alpha_397053_nt6387_L1_AOS_rc|2
GTTCTCTACCATTGCTACCCTTTAAATATAACTCTGCAGGTATTGTTTCCCCAACTTTAC
>HPV56_Alpha_397053_nt6108_L1_AOS_rc|1
TAATTAATGCAAGAGGCGGGCAGTCCCCTGTGGTAACTTGTGTGGACTTACACACAGCAC
>HPV56_Alpha_397053_nt5509_L1_AOS_rc|1
GCGCCTACGTCTAAAAAAATACACAGGCCATAATGCAAAGGAGGATCCCTGTATATATAC
>HPV56_Alpha_397053_nt5502_L1_AOS_rc|1
CGTCTAAAAAAATACACAGGCCATAATGCAAAGGAGGATCCCTGTATATATACATCATGG
>HPV56_Alpha_397053_nt5495_L1_AOS_rc|1
AAAAATACACAGGCCATAATGCAAAGGAGGATCCCTGTATATATACATCATGGGTAACAT
>HPVcand62_Alpha_39932599_nt6590_L1_AOS_rc|1
AATGTAACTGCCCGGCATTTGTCTGTCACTGCCTGGGGTACCTTTCATATACAGGTCAGT
>HPVcand62_Alpha_39932599_nt6583_L1_AOS_rc|1
CTGCCCGGCATTTGTCTGTCACTGCCTGGGGTACCTTTCATATACAGGTCAGTGGGCAGG
>HPV43_Alpha_40804474_nt6971_L1_AOS_rc|1
ATGGCCTTGTTAGACAAAAAGCGATAAGTATCTTCCAAAGAAGCAGAGGCAGGTGGGGAC
>HPV43_Alpha_40804474_nt6541_L1_AOS_rc|2
ACCTGGTATTAGAGCCAGCAATATACATATCGGAAGGCACAACGTCGCCAGTTTTACCAG
>HPV81_Alpha_40804509_nt6384_L1_AOS_rc|2
GAATGGTTGTATTTTTAAACTCCAAGGGGGGGCATTCGTTTTGTGCTGGAGCAACATTAG
>HPV81_Alpha_40804509_nt6359_L1_AOS_rc|2
GGGGGGGCATTCGTTTTGTGCTGGAGCAACATTAGCACAAAAGGTACCCCGGGCCCAGTG
>HPV95_Gamma_40804520_nt6821_L1_AOS_rc|1
TTGTGCCTGTTGCAGTTTGAGAAGTAATAGCTCTTTGTCTTTTACGAGATCCATTTATTA
>HPV95_Gamma_40804520_nt6812_L1_AOS_rc|1
TTGCAGTTTGAGAAGTAATAGCTCTTTGTCTTTTACGAGATCCATTTATTAATCCACTCT
>HPV95_Gamma_40804520_nt6650_L1_AOS_rc|1
TTTCTGCTGGTGGATTTTGAGTTGGACAACGAGTAGCTCTGGATGTAATGAATCTGTATT
>HPV95_Gamma_40804520_nt6232_L1_AOS_rc|1
AAGAGAACCGCTAGGAGTTCCAAAATAAATGTGAGGGCCAAGATTATTTTGTTCTTGGTT

>HPV95_Gamma_40804520_nt6224_L1_AOS_rc|1
CGCTAGGAGTTCCAAAATAAATGTGAGGGCCAAGATTATTTTGTTCTTGGTTACTCTGAG
>HPV95_Gamma_40804520_nt6217_L1_AOS_rc|1
AGTTCCAAAATAAATGTGAGGGCCAAGATTATTTTGTTCTTGGTTACTCTGAGCAGCAAT
>HPV95_Gamma_40804520_nt6210_L1_AOS_rc|1
AAATAAATGTGAGGGCCAAGATTATTTTGTTCTTGGTTACTCTGAGCAGCAATTATGTAA
>HPV95_Gamma_40804520_nt6203_L1_AOS_rc|1
TGTGAGGGCCAAGATTATTTTGTTCTTGGTTACTCTGAGCAGCAATTATGTAATCAGACT
>HPV95_Gamma_40804520_nt6196_L1_AOS_rc|1
GCCAAGATTATTTTGTTCTTGGTTACTCTGAGCAGCAATTATGTAATCAGACTTCACTTC
>HPV94_Alpha_40804528_nt6559_L1_AOS_rc|1
ACCACCGGCCGACTTCAATATTAGAGTGTCAGGAATGGTGTCTCCCACTGCACTGGCTCT
>HPV94_Alpha_40804528_nt6552_L1_AOS_rc|1
GCCGACTTCAATATTAGAGTGTCAGGAATGGTGTCTCCCACTGCACTGGCTCTATTGAAA
>HPVcand85_Alpha_4574720_nt5995_L1_AOS_rc|2
CTGACACATTATCTCTAGTGTCGTGTGTAACTACAGAAGTAGCAACATGGGAATTTTCTG
>HPV96_Beta_50253426_nt7334_L1_AOS_rc|1
CAGCGTCGTTTTCGTTTTGTACCTCTTGAGGTTGTAGCCCTCCCGGTTACAGTAACCCCT
>HPV96_Beta_50253426_nt7326_L1_AOS_rc|1
TTTTCGTTTTGTACCTCTTGAGGTTGTAGCCCTCCCGGTTACAGTAACCCCTCTGTTAAC
>HPV96_Beta_50253426_nt7319_L1_AOS_rc|1
TTTGTACCTCTTGAGGTTGTAGCCCTCCCGGTTACAGTAACCCCTCTGTTAACTCTTTTG
>HPV96_Beta_50253426_nt7312_L1_AOS_rc|1
CTCTTGAGGTTGTAGCCCTCCCGGTTACAGTAACCCCTCTGTTAACTCTTTTGTTTTGCA
>HPV96_Beta_50253426_nt7303_L1_AOS_rc|1
TTGTAGCCCTCCCGGTTACAGTAACCCCTCTGTTAACTCTTTTGTTTTGCAGGTTGGCTT
>HPV96_Beta_50253426_nt6751_L1_AOS_rc|2
TGAAAATCTGTGCATCTGTAGAAACTAAAGATCCACTGACTGTGGGAGTGTAAATGGAAC
>HPV96_Beta_50253426_nt6714_L1_AOS_rc|1
GACTGTGGGAGTGTAAATGGAACTTGCCATTGTATCTCTGTTTTGATCACTGGCAGCCTT
>HPV96_Beta_50253426_nt6707_L1_AOS_rc|1
GGAGTGTAAATGGAACTTGCCATTGTATCTCTGTTTTGATCACTGGCAGCCTTTAAATAA
>HPV96_Beta_50253426_nt6700_L1_AOS_rc|1
AAATGGAACTTGCCATTGTATCTCTGTTTTGATCACTGGCAGCCTTTAAATAATAATGGT
>HPV96_Beta_50253426_nt6693_L1_AOS_rc|1
ACTTGCCATTGTATCTCTGTTTTGATCACTGGCAGCCTTTAAATAATAATGGTTGTCTTC
>HPV83_Alpha_5059324_nt6567_L1_AOS_rc|2
AGGAGCATAGATGTAGCTGGTAAGAGTGTTCCTACTATTAGCAGATGTGCCTGGAATATA
>HPV83_Alpha_5059324_nt6560_L1_AOS_rc|1
TAGATGTAGCTGGTAAGAGTGTTCCTACTATTAGCAGATGTGCCTGGAATATAATAAGAG
>HPV83_Alpha_5059324_nt6553_L1_AOS_rc|1
AGCTGGTAAGAGTGTTCCTACTATTAGCAGATGTGCCTGGAATATAATAAGAGGTAGGCA
>HPV83_Alpha_5059324_nt6546_L1_AOS_rc|1
AAGAGTGTTCCTACTATTAGCAGATGTGCCTGGAATATAATAAGAGGTAGGCAGCACCTC
>HPV59_Alpha_557236_nt7071_L1_AOS_rc|2
ATTTTCTGGAAGACTTGCGACGCTTAACACGTTTTGGTGATGGGGTAGAGGTAGGGGCAG
>HPV59_Alpha_557236_nt7064_L1_AOS_rc|1
GGAAGACTTGCGACGCTTAACACGTTTTGGTGATGGGGTAGAGGTAGGGGCAGGCGCTGC
>HPV59_Alpha_557236_nt7057_L1_AOS_rc|1
TTGCGACGCTTAACACGTTTTGGTGATGGGGTAGAGGTAGGGGCAGGCGCTGCACGTTTG
>HPV59_Alpha_557236_nt6901_L1_AOS_rc|3
AGATCTACAGGCCAAAACTTTAGTTTGTCATAAGGGTCCTGTTTAACTGGCGGTGCGGTG
>HPV59_Alpha_557236_nt6643_L1_AOS_rc|1
CTGGCATATTCTTTAAAACTGGTAGGTGTGTATACATTAGGAATAGAAGAAGTAGTAGAA
>HPV59_Alpha_557236_nt6634_L1_AOS_rc|1

TCTTTAAAACTGGTAGGTGTGTATACATTAGGAATAGAAGAAGTAGTAGAAGCACACACA
>HPV59_Alpha_557236_nt6627_L1_AOS_rc|1
AACTGGTAGGTGTGTATACATTAGGAATAGAAGAAGTAGTAGAAGCACACACAGAAAGAT
>HPV59_Alpha_557236_nt6620_L1_AOS_rc|1
AGGTGTGTATACATTAGGAATAGAAGAAGTAGTAGAAGCACACACAGAAAGATTGGTGCT
>HPV6_Alpha_6002612_nt7207_L1_AOS_rc|1
GTTTAGGGGCAGCAGAGGCTTTGGAAACAGCAGGGCGCTTAACACCGGTACGAATAGAGG
>HPV6_Alpha_6002612_nt7232_L1_AOS_rc|1
TACCTTTTGGTTTTGGCGCGCTTACGTTTAGGGGCAGCAGAGGCTTTGGAAACAGCAGGG
>HPV6_Alpha_6002612_nt7223_L1_AOS_rc|1
GTTTTGGCGCGCTTACGTTTAGGGGCAGCAGAGGCTTTGGAAACAGCAGGGCGCTTAACA
>HPV6_Alpha_6002612_nt7216_L1_AOS_rc|1
CGCGCTTACGTTTAGGGGCAGCAGAGGCTTTGGAAACAGCAGGGCGCTTAACACCGGTAC
>HPV6_Alpha_6002612_nt7200_L1_AOS_rc|1
GGCAGCAGAGGCTTTGGAAACAGCAGGGCGCTTAACACCGGTACGAATAGAGGACCGTCC
>HPV6_Alpha_6002612_nt7193_L1_AOS_rc|1
GAGGCTTTGGAAACAGCAGGGCGCTTAACACCGGTACGAATAGAGGACCGTCCCCTATAT
>HPV57_Alpha_60882_nt6984_L1_AOS_rc|1
GGTCTTAGGGGGTGTGGGCTTCTGACATGTTATCGCTTGGGATTGCAAATACCTGTAGGT
>HPV57_Alpha_60882_nt6977_L1_AOS_rc|1
GGGGGTGTGGGCTTCTGACATGTTATCGCTTGGGATTGCAAATACCTGTAGGTGTCCTGC
>HPV57_Alpha_60882_nt6970_L1_AOS_rc|1
TGGGCTTCTGACATGTTATCGCTTGGGATTGCAAATACCTGTAGGTGTCCTGCAGGCTGG
>HPV57_Alpha_60882_nt6952_L1_AOS_rc|1
TCGCTTGGGATTGCAAATACCTGTAGGTGTCCTGCAGGCTGGCGGACGGGGGTGGGGGGA
>HPV57_Alpha_60882_nt6540_L1_AOS_rc|1
ACCGGGGGTCTGGACGGTAGAACTCTTGACATATAGCTCATCCGGGAGGGCGTCACCCAT
>HPV57_Alpha_60882_nt6370_L1_AOS_rc|1
TACATATGTTAGTACAGATATCCAAGGGGACATCTGATTTGTTGGACTGTAGAGCGGCAA
>HPV69_Alpha_6970418_nt6371_L1_AOS_rc|2
CTAGGCGTACGTGAGTATATAGAAGTAGTTGGAGGTTCACGACCTTGACCAGCGCCTTTA
>HPV82_Alpha_6970427_nt6610_L1_AOS_rc|1
GCCTAATGTACTGCTTAAAGTTTGCTGGAGTAAATGTTTGTGCAACAGATGGAGTAACAG
>HPV82_Alpha_6970427_nt6589_L1_AOS_rc|1
TTGCTGGAGTAAATGTTTGTGCAACAGATGGAGTAACAGCAGTGCTAATGGTTAAATTGG
>HPV82_Alpha_6970427_nt6341_L1_AOS_rc|3
TTAATATAAGCCTTGTCTGGAATGGCATCACCAACCACACCAGCCTTATTATAGTAATGC
>HPV68a_Alpha_71726685_nt6284_L1_AOS_rc|1
GAGTTTCACGAATGTCAGTGCCCTTAATATACATGTCAGTGGGAATAGTGTCCCCTACCA
>HPV68a_Alpha_71726685_nt6512_L1_AOS_rc|1
TAAATTTATTAGAATCATACACAGCTGGTACAGTAGAGTCTGTAGTAGTGGACAATGTAA
>HPV68a_Alpha_71726685_nt6277_L1_AOS_rc|1
ACGAATGTCAGTGCCCTTAATATACATGTCAGTGGGAATAGTGTCCCCTACCATGCCTCC
>HPV97_Alpha_71726694_nt6956_L1_AOS_rc|1
ATTTTTTACGACGGACACGTAAACGCTTAGCAGGTTTGGAAGTAGTAGGGGCAGACGGGG
>HPV97_Alpha_71726694_nt6530_L1_AOS_rc|1
TACTATACTGTTTATATTTTGTAGGGTCATATGTGGTAGCTACGCCATTTTGTGTAGAAG
>HPV97_Alpha_71726694_nt6522_L1_AOS_rc|1
TGTTTATATTTTGTAGGGTCATATGTGGTAGCTACGCCATTTTGTGTAGAAGCACACAGT
>HPV106_Alpha_71726710_nt6586_L1_AOS_rc|1
AGAGGCGCGGGATGTAGTAGTAGGCCCCTTTAGTAAAAACCCTTCCGGAACATCCTCCCC
>HPV106_Alpha_71726710_nt5690_L1_AOS_rc|1
CTAGGAAGCAACACATATGAGGGCAATAAATAATATGTAGACCCGTGCACAAGCACATCG
>HPV106_Alpha_71726710_nt5678_L1_AOS_rc|1
ACATATGAGGGCAATAAATAATATGTAGACCCGTGCACAAGCACATCGCCTGTGGGGTGA

>HPV106_Alpha_71726710_nt5671_L1_AOS_rc|1
AGGGCAATAAATAATATGTAGACCCGTGCACAAGCACATCGCCTGTGGGGTGACCAGGAG
>HPV106_Alpha_71726710_nt5664_L1_AOS_rc|2
TAAATAATATGTAGACCCGTGCACAAGCACATCGCCTGTGGGGTGACCAGGAGGGGTAGG
>HPV106_Alpha_71726710_nt5656_L1_AOS_rc|1
ATGTAGACCCGTGCACAAGCACATCGCCTGTGGGGTGACCAGGAGGGGTAGGGGGCACGG
>HPV106_Alpha_71726710_nt5620_L1_AOS_rc|1
GACCAGGAGGGGTAGGGGGCACGGGCCAAGCAGTGTGGGAATCGGGAAGAGCACTGTCAG
>HPV106_Alpha_71726710_nt5613_L1_AOS_rc|1
AGGGGTAGGGGGCACGGGCCAAGCAGTGTGGGAATCGGGAAGAGCACTGTCAGGGCCACT
>HPV106_Alpha_71726710_nt5605_L1_AOS_rc|1
GGGGCACGGGCCAAGCAGTGTGGGAATCGGGAAGAGCACTGTCAGGGCCACTAAAAACAG
>HPV102_Alpha_71726718_nt5682_L1_AOS_rc|1
TAAGTAATTAGGCAATAAAAAATAGTCTGTACCATCCACAAACACAGATTGGGAAGGAAC
>HPV102_Alpha_71726718_nt5673_L1_AOS_rc|1
AGGCAATAAAAAATAGTCTGTACCATCCACAAACACAGATTGGGAAGGAACATTAGAGGA
>HPV102_Alpha_71726718_nt5525_L1_AOS_rc|1
GGTACTGTGACATTGTCATACCGCGCAGAGACCACAGATGTAGCTGAAATATTTGTAGAT
>HPV102_Alpha_71726718_nt5518_L1_AOS_rc|2
TGACATTGTCATACCGCGCAGAGACCACAGATGTAGCTGAAATATTTGTAGATTGCAAAG
>HPV102_Alpha_71726718_nt5511_L1_AOS_rc|1
GTCATACCGCGCAGAGACCACAGATGTAGCTGAAATATTTGTAGATTGCAAAGGAACAGA
>HPV102_Alpha_71726718_nt5493_L1_AOS_rc|2
CACAGATGTAGCTGAAATATTTGTAGATTGCAAAGGAACAGACGTGGAAGAAGTATATGA
>HPV102_Alpha_71726718_nt5482_L1_AOS_rc|1
CTGAAATATTTGTAGATTGCAAAGGAACAGACGTGGAAGAAGTATATGAAACATGTGGAG
>HPV102_Alpha_71726718_nt5475_L1_AOS_rc|1
ATTTGTAGATTGCAAAGGAACAGACGTGGAAGAAGTATATGAAACATGTGGAGACGCCAA
>HPV102_Alpha_71726718_nt5447_L1_AOS_rc|1
GAAGAAGTATATGAAACATGTGGAGACGCCAAGGAGTCCAAGTGGCCATTATCAGCATAA
>HPV13_Alpha_85827580_nt5868_L1_AOS_rc|1
TACCTTAGGGACAACAGTTTTGTTTTGTTTCTTAATAGGAAAATAAGGATTTCCCACTGC
>HPV13_Alpha_85827580_nt7147_L1_AOS_rc|2
TGGCAGTAGATGTAGATGCAGCACGTTTTCTACCTACACGAATAGGTGACCTAGACTGAA
>HPV13_Alpha_85827580_nt7140_L1_AOS_rc|1
AGATGTAGATGCAGCACGTTTTCTACCTACACGAATAGGTGACCTAGACTGAACGCCTGT
>HPV13_Alpha_85827580_nt7133_L1_AOS_rc|1
GATGCAGCACGTTTTCTACCTACACGAATAGGTGACCTAGACTGAACGCCTGTTTGTAAT
>HPV13_Alpha_85827580_nt6758_L1_AOS_rc|1
ATGTACTGTTTATATTCTGTGGCCTTATATGTGTCTGAAAGAGATGATGTAGTGGCTGCA
>HPV13_Alpha_85827580_nt6751_L1_AOS_rc|1
GTTTATATTCTGTGGCCTTATATGTGTCTGAAAGAGATGATGTAGTGGCTGCACACACAG
>HPV13_Alpha_85827580_nt6508_L1_AOS_rc|1
CCTTAACATATAATTCTGCTGGGATTAGTTCACCAACAGAGCCTGCCCTGTTAAAGAAAT
>HPV2_Alpha_9626032_nt7210_L1_AOS_rc|1
GCCTTACCCGTTTTCGTTTACTAGTGGGCGGCGTGGTCCCCGAAACAGCGGCGCGCTTGC
>HPV2_Alpha_9626032_nt6787_L1_AOS_rc|1
TGGTAGCCTTATAATTAGTATCAGACGCCTCAGTGGCACACAGAGATACATTAGTGCTAC
>HPV41_Nu_9626041_nt6790_L1_AOS_rc|1
GCAATGGACAGTATGTACCTATAATGATCCTCCAGTACAGAATTGGGAGGTGAAGTGACA
>HPV41_Nu_9626041_nt6775_L1_AOS_rc|1
TACCTATAATGATCCTCCAGTACAGAATTGGGAGGTGAAGTGACAGCTAAATGCCAGTCT
>HPV41_Nu_9626041_nt6768_L1_AOS_rc|1
AATGATCCTCCAGTACAGAATTGGGAGGTGAAGTGACAGCTAAATGCCAGTCTTCAATAA
>HPV41_Nu_9626041_nt6662_L1_AOS_rc|1

```
GTCTACCTTACACAGCTGTAGAATAAAGGCAAGCTGAAACTCCTCGGTGTGCCTTAAAAA
>HPV41_Nu_9626041_nt6287_L1_AOS_rc|1
CATTATATGCCTAGCATACAGAGCTTCACGCCGCACATAGAAAAACATGTGGTCTCCATA
>HPV41_Nu_9626041_nt5779_L1_AOS_rc|1
ACCAGACGCTCCTTGTCAGGGTTAAAAAGGGACTTATCACAAAATGCAAAGGTATTGGGA
>HPV41_Nu_9626041_nt5772_L1_AOS_rc|1
GCTCCTTGTCAGGGTTAAAAAGGGACTTATCACAAAATGCAAAGGTATTGGGATTTGGGA
>HPV41_Nu_9626041_nt5694_L1_AOS_rc|1
TGAACTGATTAGAGGAAACTTTAGGGACCACCTCTTTGCCATCCGCATTAGTAATATTGT
>HPV1_Mu_9626063_nt6713_L1_AOS_rc|2
AGAATTTATATTGACTATAAGGATCAGTCTGGGGCTCAGGAGGCGCCTGTTCTGGACATT
>HPV1_Mu_9626063_nt6706_L1_AOS_rc|1
ATATTGACTATAAGGATCAGTCTGGGGCTCAGGAGGCGCCTGTTCTGGACATTTTGCTGC
>HPV1_Mu_9626063_nt6233_L1_AOS_rc|1
CTAAAGTTGTTCTTGGTTCAGCATCTGCTGTTAAATACAGGCTTTGTGGGACTGCCTCCT
>HPV1_Mu_9626063_nt6226_L1_AOS_rc|1
TGTTCTTGGTTCAGCATCTGCTGTTAAATACAGGCTTTGTGGGACTGCCTCCTTATCACC
>HPV1_Mu_9626063_nt6219_L1_AOS_rc|1
GGTTCAGCATCTGCTGTTAAATACAGGCTTTGTGGGACTGCCTCCTTATCACCCACCGAA
>HPV1_Mu_9626063_nt6203_L1_AOS_rc|2
TTAAATACAGGCTTTGTGGGACTGCCTCCTTATCACCCACCGAACCCCCGCGAGTAAAAA
>HPV1_Mu_9626063_nt5440_L1_AOS_rc|1
GGACAGGATTCTAGTGATGGGCTGGGGAGGAAGATAGAACTTATTCTGCGCTGGTAACCA
>HPV18_Alpha_9626069_nt6649_L1_AOS_rc|1
TGCTATACTGCTTAAATTTGGTAGCATCATATTGCCCAGGTACAGGAGACTGTGTAGAAG
>HPV18_Alpha_9626069_nt5455_L1_AOS_rc|1
GAGGCAGGGGCCGTGGGTGATACAATGGGCCATACAGAGGTAGTAGATGGTAATGTAATA
>HPV18_Alpha_9626069_nt5448_L1_AOS_rc|1
GGGCCGTGGGTGATACAATGGGCCATACAGAGGTAGTAGATGGTAATGTAATATCAGGAC
>HPV18_Alpha_9626069_nt5441_L1_AOS_rc|2
GGGTGATACAATGGGCCATACAGAGGTAGTAGATGGTAATGTAATATCAGGACCCGTGTA
>HPV18_Alpha_9626069_nt5434_L1_AOS_rc|1
ACAATGGGCCATACAGAGGTAGTAGATGGTAATGTAATATCAGGACCCGTGTATACAGGC
>HPV4_Gamma_9626597_nt6396_L1_AOS_rc|1
AATCACTAGCTTTATACTGATAATTGTCATTAGCACCATCTGACTTCACAGAAATTGTAA
>HPV4_Gamma_9626597_nt6420_L1_AOS_rc|2
CTATATGTCTGAGGTACTGTTTAAAATCACTAGCTTTATACTGATAATTGTCATTAGCAC
>HPV4_Gamma_9626597_nt6413_L1_AOS_rc|1
TCTGAGGTACTGTTTAAAATCACTAGCTTTATACTGATAATTGTCATTAGCACCATCTGA
>HPV4_Gamma_9626597_nt6406_L1_AOS_rc|1
TACTGTTTAAAATCACTAGCTTTATACTGATAATTGTCATTAGCACCATCTGACTTCACA
>HPV4_Gamma_9626597_nt6200_L1_AOS_rc|2
ACTAGGGGTCCCTACATAAATATGAGGTCCTAAAGTGTACTGATCTTGTTGGTTTTGAGC
>HPV63_Mu_9626605_nt6567_L1_AOS_rc|1
ATAGCATTTGCAGGAGGTTGAGAAACTGTTAACTGCCAGGAATCGATAATTGTTGGATCC
>HPV63_Mu_9626605_nt6560_L1_AOS_rc|1
TTGCAGGAGGTTGAGAAACTGTTAACTGCCAGGAATCGATAATTGTTGGATCCATATTAT
>HPV63_Mu_9626605_nt6425_L1_AOS_rc|1
CATGGCGAGTATACTCATTATAATCTGCGCTATCATAAGTCTCAGGGGTTGCTTTGTTAA
>HPV63_Mu_9626605_nt6417_L1_AOS_rc|1
GTATACTCATTATAATCTGCGCTATCATAAGTCTCAGGGGTTGCTTTGTTAAGAACATTT
>HPV63_Mu_9626605_nt6410_L1_AOS_rc|1
CATTATAATCTGCGCTATCATAAGTCTCAGGGGTTGCTTTGTTAAGAACATTTATATTCA
>HPV63_Mu_9626605_nt6403_L1_AOS_rc|1
ATCTGCGCTATCATAAGTCTCAGGGGTTGCTTTGTTAAGAACATTTATATTCATCGTGGT
```

>HPV63_Mu_9626605_nt6396_L1_AOS_rc|1
CTATCATAAGTCTCAGGGGTTGCTTTGTTAAGAACATTTATATTCATCGTGGTTCCTCTG
>HPV63_Mu_9626605_nt6206_L1_AOS_rc|2
CTACAAAATAATTATCTGTAGCAATAGTATTTTGGGGTTGCTCGGCTTTAGCAGCAATAT
>HPV63_Mu_9626605_nt6169_L1_AOS_rc|1
TTGCTCGGCTTTAGCAGCAATATATGCACTGGTAGGGACTTTCTCTTTTTCACTAACAAT
>HPV63_Mu_9626605_nt6162_L1_AOS_rc|1
GCTTTAGCAGCAATATATGCACTGGTAGGGACTTTCTCTTTTTCACTAACAATTCCAGCA
>HPV16_Alpha_9627100_nt7066_L1_AOS_rc|1
TTGTAGAGGTAGATGAGGTGGTGGGTGTAGCTTTTCGTTTTCCTAATGTAAATTTTGGTT
>HPV16_Alpha_9627100_nt7059_L1_AOS_rc|1
GGTAGATGAGGTGGTGGGTGTAGCTTTTCGTTTTCCTAATGTAAATTTTGGTTTGGCCTT
>HPV16_Alpha_9627100_nt6455_L1_AOS_rc|1
GGAAAATAATTTGAACTGGCTAAATTTGCAGTAGACCCAGAGCCTTTAATGTATAAATCG
>HPV16_Alpha_9627100_nt6448_L1_AOS_rc|1
AATTTGAACTGGCTAAATTTGCAGTAGACCCAGAGCCTTTAATGTATAAATCGTCTGGTA
>HPV16_Alpha_9627100_nt6441_L1_AOS_rc|2
ACTGGCTAAATTTGCAGTAGACCCAGAGCCTTTAATGTATAAATCGTCTGGTACATTTTC
>HPV16_Alpha_9627100_nt6434_L1_AOS_rc|2
AAATTTGCAGTAGACCCAGAGCCTTTAATGTATAAATCGTCTGGTACATTTTCACCAACA
>HPV5_Beta_9627145_nt6972_L1_AOS_rc|2
TTATAGTCTGCAACATCTTTTAGTGCTCCAGCCTGATTATATACAGAAATACTGAAATTA
>HPV5_Beta_9627145_nt6989_L1_AOS_rc|1
CTCTAAATTGATCTGCATTATAGTCTGCAACATCTTTTAGTGCTCCAGCCTGATTATATA
>HPV5_Beta_9627145_nt6981_L1_AOS_rc|2
TGATCTGCATTATAGTCTGCAACATCTTTTAGTGCTCCAGCCTGATTATATACAGAAATA
>HPV5_Beta_9627145_nt6965_L1_AOS_rc|1
CTGCAACATCTTTTAGTGCTCCAGCCTGATTATATACAGAAATACTGAAATTAGTATTTC
>HPV5_Beta_9627145_nt6740_L1_AOS_rc|1
GAGCTTGGCCATCAGCCCCTGGAATGTAAAACTGATTTTGTATGTACCATTGTCAATTT
>HPV5_Beta_9627145_nt6731_L1_AOS_rc|1
CATCAGCCCCTGGAATGTAAAACTGATTTTGTATGTACCATTGTCAATTTGTGCACGTG
>HPV5_Beta_9627145_nt6533_L1_AOS_rc|1
TGACGATGTCTAAACTGACATCTGATCTACTATCTTGAAGTGCCTTAAAATTCATGTTCC
>HPV5_Beta_9627145_nt6520_L1_AOS_rc|1
ACTGACATCTGATCTACTATCTTGAAGTGCCTTAAAATTCATGTTCCCAAAACCTATGTC
>HPV5_Beta_9627145_nt6513_L1_AOS_rc|1
TCTGATCTACTATCTTGAAGTGCCTTAAAATTCATGTTCCCAAAACCTATGTCTGCCATA
>HPV5_Beta_9627145_nt6057_L1_AOS_rc|1
GAAACCTTAGGAACCTCAAGCTTATCACCATTAATATTGTATACATTGAAATAAGGATGA
>HPV26_Alpha_9627305_nt6112_L1_AOS_rc|1
TCAATAATGCTGGAAATTAATTCTAAGGGGGGGCAATCCCCACGTTGTGTCTGTGTATTT
>HPV32_Alpha_9627327_nt6673_L1_AOS_rc|1
AGCTAAATTATTTCTGCCAGAAGCACCATTAGAAGCTTTTATATACATGTCCTCAGGAAC
>HPV32_Alpha_9627327_nt7323_L1_AOS_rc|1
TATTTACGTGTTTTTCTGCGTTTTGCAGGAGAAGAAGACTTTTGACTGGAAGATGCTGTT
>HPV32_Alpha_9627327_nt7316_L1_AOS_rc|1
GTGTTTTTCTGCGTTTTGCAGGAGAAGAAGACTTTTGACTGGAAGATGCTGTTCGTTTTA
>HPV32_Alpha_9627327_nt7309_L1_AOS_rc|1
TCTGCGTTTTGCAGGAGAAGAAGACTTTTGACTGGAAGATGCTGTTCGTTTTACTGCTGT
>HPV32_Alpha_9627327_nt7302_L1_AOS_rc|2
TTTGCAGGAGAAGAAGACTTTTGACTGGAAGATGCTGTTCGTTTTACTGCTGTAAGTTTA
>HPV32_Alpha_9627327_nt6656_L1_AOS_rc|1
CAGAAGCACCATTAGAAGCTTTTATATACATGTCCTCAGGAACAGGTTCACCAAGGGTTC
>HPV34_Alpha_9627334_nt6019_L1_AOS_rc|1

TGGTGTTCTTTAATTCAAGCGGAGGACATTCCCCAGCATTTTGGGTACCACATGGTGTGC
>HPV34_Alpha_9627334_nt6930_L1_AOS_rc|1
AGGTGCTGTACTAGATGATGAAGGTGCAGACCGTTTAGAGGCCTGTAACCTAGGACGGGC
>HPV34_Alpha_9627334_nt6526_L1_AOS_rc|1
TAAAATTACTGTTTGCATATGGTGCAGTTGTACTTGTGGATTGTGTACCTACACAAACTG
>HPV34_Alpha_9627334_nt6519_L1_AOS_rc|1
ACTGTTTGCATATGGTGCAGTTGTACTTGTGGATTGTGTACCTACACAAACTGAAAAGTT
>HPV34_Alpha_9627334_nt6512_L1_AOS_rc|1
GCATATGGTGCAGTTGTACTTGTGGATTGTGTACCTACACAAACTGAAAAGTTTGTGCTT
>HPV49_Beta_9627363_nt7264_L1_AOS_rc|1
TAATACCCCGTGTGGAAGCTCTAGCAGCAGAGGATTTAGACACTCTAGAAGCCCGTTGTA
>HPV49_Beta_9627363_nt6871_L1_AOS_rc|1
ATTCTCTAACCTTGGTACTGTCATATTCTGTAGGTGTCTGGCCATCCGTACTTACACTAA
>HPV49_Beta_9627363_nt6657_L1_AOS_rc|1
GAAATAGATGGAGCTGCCAAGAGTATTTTGGGCCTGTTGACTTGCTGCAGGTAATATGTA
>HPV53_Alpha_9627377_nt7072_L1_AOS_rc|1
AGGGGGCTGAGGTAGATGTAGTAGAAGCAGAGCGTTTTTAGAGGATACAGGCGGTTTAG
>HPV7_Alpha_9627389_nt6169_L1_AOS_rc|1
TTTTCTCTGCTGTCCTGACCAGGTACTGTTCCATATACAGACGAGTTTTCCACATCTTCA
>HPV7_Alpha_9627389_nt6833_L1_AOS_rc|1
ATGACGTAAATATTCTTTAAACTTACTATTGTCATATGGTGTTGGTGTGGGCGATTGTGT
>HPV7_Alpha_9627389_nt6594_L1_AOS_rc|1
CTGCAATAGAAGCGCGATTAGATGAACCTGTTATATATAAATCATTTGGAACACTGTCTC
>HPV7_Alpha_9627389_nt6587_L1_AOS_rc|1
AGAAGCGCGATTAGATGAACCTGTTATATATAAATCATTTGGAACACTGTCTCCAGTAGT
>HPV7_Alpha_9627389_nt6162_L1_AOS_rc|1
TGCTGTCCTGACCAGGTACTGTTCCATATACAGACGAGTTTTCCACATCTTCATCTTTAT
>HPV9_Beta_9627396_nt6611_L1_AOS_rc|1
AATGACCCACTTACTGTAGGATAGTAGGTGGAATTAGCTATTGTTCGTTGTTGTTGATCA
>HPV9_Beta_9627396_nt6886_L1_AOS_rc|1
GGGATAATACTTCAGCTACTAAAGGCACTTTACACAACTGTAAGATTAATGAAATCTGAT
>HPV9_Beta_9627396_nt6604_L1_AOS_rc|1
CACTTACTGTAGGATAGTAGGTGGAATTAGCTATTGTTCGTTGTTGTTGATCACTTTTTG
>HPV9_Beta_9627396_nt6597_L1_AOS_rc|1
TGTAGGATAGTAGGTGGAATTAGCTATTGTTCGTTGTTGTTGATCACTTTTTGCTGGCAA
>HPV9_Beta_9627396_nt6590_L1_AOS_rc|1
TAGTAGGTGGAATTAGCTATTGTTCGTTGTTGTTGATCACTTTTTGCTGGCAAAAAGAAA
>HPV9_Beta_9627396_nt6574_L1_AOS_rc|1
CTATTGTTCGTTGTTGTTGATCACTTTTTGCTGGCAAAAAGAAATTATGATCCTGGTTTA
>HPV54_Alpha_9628437_nt6631_L1_AOS_rc|1
ATGTCTAATATACTCCCTAAAGTCAGAATTATTAAAGCTATCCTGCGTGGATGCTGTAGC
>HPV54_Alpha_9628437_nt7047_L1_AOS_rc|1
TACCTTTTAGTTTTAGCACGCTTGCGCGCTGTACCCTTAGAGGAGGAAGGGGCTGCACGC
>HPV54_Alpha_9628437_nt7004_L1_AOS_rc|1
AGGAAGGGGCTGCACGCTTTACAGGCCGAAGGCGCGGACGTGCACGTAGGCCAGCCTGTA
>HPV54_Alpha_9628437_nt6623_L1_AOS_rc|1
TATACTCCCTAAAGTCAGAATTATTAAAGCTATCCTGCGTGGATGCTGTAGCACACAATG
>HPV54_Alpha_9628437_nt6616_L1_AOS_rc|1
CCTAAAGTCAGAATTATTAAAGCTATCCTGCGTGGATGCTGTAGCACACAATGTTAGGTT
>HPV54_Alpha_9628437_nt6420_L1_AOS_rc|1
GGAGTTGCAGCATAAATAGAACTGTCAAGGTTACCTGAGGATTTCTTAATGTATAAGTCA
>HPV24_Unclassified_9628486_nt6249_L1_AOS_rc|1
TCTTGAATTACTGAATTTACTAACTTAATAGGTGGACATCTACCAGCATCATTATTATCA
>HPV24_Unclassified_9628486_nt6800_L1_AOS_rc|1
TCTGATATTCCCTAAATTTATTAGCATCATACTCGTTAATATCTGTTACTTTGCCATTTT

>HPV24_Unclassified_9628486_nt6785_L1_AOS_rc|1
ATTTATTAGCATCATACTCGTTAATATCTGTTACTTTGCCATTTTCAGTGTATACACTTA
>HPV24_Unclassified_9628486_nt6242_L1_AOS_rc|1
TTACTGAATTTACTAACTTAATAGGTGGACATCTACCAGCATCATTATTATCACCAGCAC
>HPV24_Unclassified_9628486_nt6235_L1_AOS_rc|1
ATTTACTAACTTAATAGGTGGACATCTACCAGCATCATTATTATCACCAGCACACCTCTC
>HPV24_Unclassified_9628486_nt6228_L1_AOS_rc|1
AACTTAATAGGTGGACATCTACCAGCATCATTATTATCACCAGCACACCTCTCAGCTACT
>HPV24_Unclassified_9628486_nt6221_L1_AOS_rc|1
TAGGTGGACATCTACCAGCATCATTATTATCACCAGCACACCTCTCAGCTACTTCCCAAT
>HPV48_Gamma_9628542_nt6558_L1_AOS_rc|1
TAAATCAGACGAGAAGCGCTCTGTCATATCTAAAGTCCAGAAACTGTAAGCTTTGTATGG
>HPV48_Gamma_9628542_nt6653_L1_AOS_rc|1
GTACTAGATCCTGCAGCTGTATAGTCTGTTCTAGCTCGTTTACCATTTAACAAACCAGTT
>HPV48_Gamma_9628542_nt6550_L1_AOS_rc|1
ACGAGAAGCGCTCTGTCATATCTAAAGTCCAGAAACTGTAAGCTTTGTATGGATCTGTGT
>HPV48_Gamma_9628542_nt6543_L1_AOS_rc|1
GCGCTCTGTCATATCTAAAGTCCAGAAACTGTAAGCTTTGTATGGATCTGTGTCTTCTTC
>HPV48_Gamma_9628542_nt6535_L1_AOS_rc|1
TCATATCTAAAGTCCAGAAACTGTAAGCTTTGTATGGATCTGTGTCTTCTTCTGGTTCAG
>HPV48_Gamma_9628542_nt6507_L1_AOS_rc|2
TTTGTATGGATCTGTGTCTTCTTCTGGTTCAGCAGTAGGGCATTTAGTAGCCATGGATTT
>HPV48_Gamma_9628542_nt6498_L1_AOS_rc|1
ATCTGTGTCTTCTTCTGGTTCAGCAGTAGGGCATTTAGTAGCCATGGATTTGATATACCT
>HPV48_Gamma_9628542_nt6026_L1_AOS_rc|1
ACAGAAGATCCTAAATGAGAAGATCTGTTGTTTTGATCGGCAGAATCAGGAGTTATATAA
>HPV48_Gamma_9628542_nt6018_L1_AOS_rc|1
TCCTAAATGAGAAGATCTGTTGTTTTGATCGGCAGAATCAGGAGTTATATAATATACTCC
>HPV48_Gamma_9628542_nt5981_L1_AOS_rc|2
TCAGGAGTTATATAATATACTCCATTTTCTGTTGGTATAGGCTCCCCCATTTGGCCAGCT
>HPV50_Gamma_9628550_nt6294_L1_AOS_rc|1
CCTTTGAGTACATATAAGAACTTGCTACATTTAAAGGATCCAAAGGATTGACATCTTTCT
>HPV50_Gamma_9628550_nt6689_L1_AOS_rc|1
GGAAACTGTAGCAACAGTGTAATCAGTTCTAACTCTTCTCTTTAATAATCCAGTCTGAAA
>HPV50_Gamma_9628550_nt6325_L1_AOS_rc|1
TCAGTATGACGGCTGTACTGATTAAAATCATCCTTTGAGTACATATAAGAACTTGCTACA
>HPV50_Gamma_9628550_nt6318_L1_AOS_rc|1
GACGGCTGTACTGATTAAAATCATCCTTTGAGTACATATAAGAACTTGCTACATTTAAAG
>HPV50_Gamma_9628550_nt6311_L1_AOS_rc|2
GTACTGATTAAAATCATCCTTTGAGTACATATAAGAACTTGCTACATTTAAAGGATCCAA
>HPV50_Gamma_9628550_nt6287_L1_AOS_rc|1
GTACATATAAGAACTTGCTACATTTAAAGGATCCAAAGGATTGACATCTTTCTTAACACT
>HPV50_Gamma_9628550_nt6051_L1_AOS_rc|2
TATTGGTTCTACGATTTTGCTCTGGTTGATTAGGGTTTACTGCATAATTATAAACTCCTG
>HPV50_Gamma_9628550_nt5860_L1_AOS_rc|1
CATAAGCTGATTGAATCCACTATATCTAAAGGGACCCCTGCTCTGTCCTGTTGTAGTTTA
>HPV50_Gamma_9628550_nt5853_L1_AOS_rc|1
TGATTGAATCCACTATATCTAAAGGGACCCCTGCTCTGTCCTGTTGTAGTTTAGGAAAGT
>HPV50_Gamma_9628550_nt5734_L1_AOS_rc|1
TGTATTGGAGGACACTTGCCATTATTTAAGCTGTTTTTGTCACAAGGTTCTGCTAAATCC
>HPV60_Gamma_9628566_nt6762_L1_AOS_rc|1
CTTTTTCCAAGAGAAAACTGGGATAATTCGTTAGAAAAACGTTCTTGCAAATCTATTGTC
>HPV60_Gamma_9628566_nt6226_L1_AOS_rc|1
GAAGTAACTATAACCACCAAGGTTATTTTGAGGTTTATCAGTTTGTGGTGCTAAGTAATA
>HPV60_Gamma_9628566_nt6218_L1_AOS_rc|1

TATAACCACCAAGGTTATTTTGAGGTTTATCAGTTTGTGGTGCTAAGTAATATCCAGAAT
>HPV61_Alpha_9628574_nt6569_L1_AOS_rc|1
TACCAGGGGCCGCCTTGTCATTCGCACCCTTAAGATAATAAGAGTCAGGCAGTGCCTCAC
>HPVcand85_Alpha_4574720_nt6511_L1_VE|1
TCTTCAGATGCACAAATGTTTAATAAGCCTTATTGGCTGCACAAAGCCCAGGGACAAAAT
>HPV88_Gamma_167600365_nt5491_L1_VE|1
AAAATTGAGATTCCTAAGGTGTCTGCATGCCAATATCGAGTGTTTAGGCTGAAATTGCCT
>HPV17_Beta_396932_nt6955_L1_VE|1
GAAGACTGGCAGTTAGGATTTGTGCCTACACCAGATAACCCAGTGCATGATATATATAGG
>HPV60_Gamma_9628566_nt5876_L1_VE|1
ATTGTAGGATGTAAACCAGCCACTGGTGAACACTGGGATATTGCTAAACCTTGTGATCCT
>HPV22_Beta_1020178_nt6593_L1_VE|1
CAACTATTCAACAGGCCTTTTTGGTTGAAGCGCGCGCAGGGCCATAATAATGGTATTTTG
>HPV73_Alpha_1491692_nt6245_L1_VE|1
GAACAAATGTTTGTTCGACACTTATTTAACAGGGCTGGTGATACCGGTGATAAAATCCCA
>HPV13_Alpha_85827580_nt6313_L1_VE|1
GTAATTCAGGATGGTGACATGGTAGATACAGGATTTGGAGCCATGAATTTTGAGGAATTG
>HPV42_Alpha_333211_nt5847_L1_VE|1
TGGCGGCCTAGTGACAACAAGGTTTATCTACCTCCTCCTCCTGTTTCCAAGGTGGTCAGC
>HPV95_Gamma_40804520_nt6119_L1_VE|1
GGCCGAAGGGAACAGCTTTATGCAAGACACTTTTTTGTTAGAGCTGGGACAATGGGTGAT
>HPV3_Alpha_397005_nt6328_L1_VE|1
CCTATACAAGATGGCGATATGGTGGACACAGGTTATGGTGCCATGGACTTTGGTAACTTG
>HPVcand62_Alpha_39932599_nt6678_L1_VE|1
GTGTCGTCTGATTCTCAGTTGTTTAATAAGCCATATTGGCTGCAGCGGGCGCAGGGCCAT
>HPV48_Gamma_9628542_nt5473_L1_VE|2
AATTCAGATAAGGAACGTTTAGTTTGGAAACTTGTTGGTTTGGAAGTAGGCAGAGGGGGT
>HPV92_Beta_27531786_nt5669_L1_VE|1
GTATACTTACCCCCTTCAACACCGGTTGCAAGGGTACAAAGTACGGATGAATTTGTTCAA
>HPV81_Alpha_40804509_nt6765_L1_VE|1
TCCTCTGATTCCCAGCTGTTTAATAAGCCTTATTGGCTACAACGGGCACAGGGCCATAAT
>HPV63_Mu_9626605_nt5672_L1_VE|2
GGTCTTAGAGGCATAGAAATCGGTAGGGGTCAACCATTAGGTGTGGGTATTTCAGGCAAT
>HPV20_Beta_1020162_nt6824_L1_VE|1
TTGGTGTCTAGTGATGCTCAATTGTTTAATAGGCCATTTTGGCTGCAGCGCGCACAGGGC
>HPV37_Beta_1020226_nt5897_L1_VE|1
ATCGAGGTTCCTAAAGTATCTGGAAATCAATACAGAGCTTTTAGGGTTAGGTTGCCAGAT
>HPV36_Beta_1020218_nt6792_L1_VE|1
GCCATGTATTACCCAACTGTTAGTGGCTCATTGGTGTCTAGTGATGCTCAATTGTTTAAC
>HPV95_Gamma_40804520_nt5489_L1_VE|1
GAAAGACTTTTAACTGTAGGCCATCCTTATTTCCCAGTGAAAGATGTAGGTGAACCTAAG
>HPV84_Alpha_12958167_nt6086_L1_VE|1
GCTGGGGACACTGACAGTAGGGATAATGTTTCTGTGGATTATAAACAAACACAGCTGTTA
>HPV40_Alpha_397014_nt6530_L1_VE|1
GAACAAATGTTTGTTAGGCACTTTTTTAATAGGGCAGGTACTACTGGTGATAGTGTCCCA
>HPVFA75/KI88-03_Unclassified_206902429_nt5621_L1_VE|1
ATGTCTCTCTGGCTTCCTGCAAGTGGTAAGGTATATTTGCCACCATCAACTCCAGTAGCT
>HPV56_Alpha_397053_nt5793_L1_VE|1
GCATATCAATATAGGGTATTTAGGGTACGGTTGCCCGACCCTAATAAGTTTGGGCTTCCA
>HPVFA75/KI88-03_Unclassified_206902429_nt6371_L1_VE|1
AGAAGGGAACAGTGCTATGCAAGACATTATTTTACCAGAGGAGGTAATGTTGGAGACGCT
>HPV73_Alpha_1491692_nt6065_L1_VE|1
ACCCCTATACAGGATGGTGATATGATAGATGTTGGCTTTGGAGCCATGGATTTTAAAGCT
>HPV59_Alpha_557236_nt5697_L1_VE|1
ACCAGTATTTTCTACCACGCAGGCAGTTCCAGACTTCTTACAGTTGGACATCCATATTTT

>HPV88_Gamma_167600365_nt6061_L1_VE|1
TTTGGAAAACGTGAGCAGTCGTATGCTCGCCATTTTATGGCTCGTGAAGGTAAAATGGGA
>HPV52_Alpha_397038_nt6586_L1_VE|1
CCGTACTGGTTACAACGTGCGCAGGGCCACAATAATGGCATATGTTGGGGCAATCAGTTG
>HPV10_Alpha_9627257_nt5860_L1_VE|1
CTGCCTCCCACTCCCGTGTCTAAAGTTCTCAGCACGGACGACTATGTGACACGCACCAAC
>HPV33_Alpha_333049_nt5805_L1_VE|1
TATAGGGTTTTAGGGTCCGTTTACCAGATCCTAATAAATTTGGATTTCCTGACACCTCC
>HPV48_Gamma_9628542_nt5533_L1_VE|1
CCTCTTGGCGTAGGATCTACAGGTCATCCTTTACTAAATAAGATAGGGGACACAGAAAAT
>HPV22_Beta_1020178_nt6563_L1_VE|1
GTTAGTGGTTCTTTAGTAACCTCTGATGCCCAACTATTCAACAGGCCTTTTTGGTTGAAG
>HPVcand85_Alpha_4574720_nt5611_L1_VE|1
ATGGCTTTGTGGCGTTCTAGTGACAGCAAGGTATACCTTCCACCACCTTCAGTGGCTAAA
>HPV42_Alpha_333211_nt5877_L1_VE|1
CCTCCTCCTCCTGTTTCCAAGGTGGTCAGCACTGATGAATATGTGCAACGCACCAACTAC
>HPV81_Alpha_40804509_nt6735_L1_VE|1
TATGCCCCTACACCTAGTGGGTCTATGGTATCCTCTGATTCCCAGCTGTTTAATAAGCCT
>HPV2_Alpha_9626032_nt5773_L1_VE|1
TCCACTATTTCTTTGCAGATGGCTTTGTGGCGGCCTAATGAAAGCAAGGTATACCTACCT
>HPV90_Alpha_22138122_nt6003_L1_VE|1
TATAGGGTGTTTAGGGTACGTTTGCCTGATCCCAATAAGTTTGGCCTTCCTGATGCATCG
>HPV26_Alpha_9627305_nt6159_L1_VE|1
TCCAGCATTATTGAGGATGGCGATATGATTGATACAGGCTTTGGAGCTATGGATTTTACT
>HPV33_Alpha_333049_nt5655_L1_VE|1
GTTGTCAGCACTGATGAATATGTGTCTCGCACAAGCATTTATTATTATGCTGGTAGTTCC
>HPV106_Alpha_71726710_nt5990_L1_VE|1
CAATATAGGGTATTTAGGGTTCGTTTGCCTGATCCCAACAAATTTGGCCTTCCTGATGCA
>HPV45_Alpha_397022_nt6551_L1_VE|1
TGGTTACATAAGGCCCAGGGCCATAACAATGGTATTTGTTGGCATAATCAGTTGTTTGTT
>HPV41_Nu_9626041_nt5667_L1_VE|2
CGTTTGCTTACTGTTGGACATCCATTTTACAATATTACTAATGCGGATGGCAAAGAGGTG
>HPV33_Alpha_333049_nt6555_L1_VE|1
AATGGTATTTGTTGGGGCAATCAGGTATTTGTTACTGTGGTAGATACCACTCGCAGTACT
>HPVFA75/KI88-03_Unclassified_206902429_nt6791_L1_VE|2
TCACTGCAACCTGATGTGCTAGCCCAGATCAATGCAATGAATTCAGGTATATTAGAAGAT
>HPV7_Alpha_9627389_nt5979_L1_VE|1
CCTAAAGTGTCTGGACATCAATACAGAGTGTTTAGAGTACGCTTGCCCGACCCTAATAAA
>HPV29_Alpha_1020210_nt6744_L1_VE|1
TACTGGCTACAGCAGGCCCAGGGACACAACAATGGTATATGCTGGGCCAATCAGGTATTT
>HPV48_Gamma_9628542_nt6433_L1_VE|2
AGAATATTAGAGGAGTGGCAATTAGCTTTTGTTCCACCAGCTCCAACGGGCATTGAAGAT
>HPVcand89_Alpha_22095322_nt6668_L1_VE|1
GATTCCCAGTTATTTAATAAGCCTTATTGGCTGCACCGTGCACAGGGCCATAATAATGGT
>HPV44_Alpha_1020242_nt6265_L1_VE|1
GTAATTGAGGATGGTGATATGGTGGACACTGGTTTTGGAGCCATGAATTTTGCTGAATTG
>HPV19_Beta_396940_nt6765_L1_VE|1
GCCATGTATTTCCCAACTGTTAGTGGCTCATTAGTATCCAGTGATGCTCAATTGTTTAAC
>HPV103_Unclassified_109390382_nt5036_L1_VE|1
GAAACGTCTATTTTCTTCCATGCCTCCAGCGACCGCCTGCTTACTGTAGGACACCCATAT
>HPV73_Alpha_1491692_nt5525_L1_VE|1
CTGCCCCCTGTGTCTGTGTCTAAGGTTGTAAGCACAGATGAATATGTAACAAGAACAAAT
>HPV59_Alpha_557236_nt5787_L1_VE|1
GATGTTCCTAAGGTGTCTGCATATCAATACAGAGTATTTAGGGTTAAGTTACCTGATCCC
>HPV52_Alpha_397038_nt5956_L1_VE|1

TGTACAGGCTTGGAAATTGGTAGGGGACAGCCTTTAGGTGTGGGTATTAGTGGGCATCCT
>HPV35_Alpha_333050_nt5635_L1_VE|1
GTTGTTAGCACTGATGAATATGTAACACGCACAAACATCTACTATCATGCAGGCAGTTCT
>HPV58_Alpha_222386_nt5956_L1_VE|1
GGCCTTGAAATAGGTAGGGGACAGCCATTGGGTGTTGGCGTAAGTGGTCATCCTTATTTA
>HPV58_Alpha_222386_nt5686_L1_VE|1
CCTGTGCCTGTGTCTAAGGTTGTAAGCACTGATGAATATGTGTCACGCACAAGCATTTAT
>HPV93_Beta_37089393_nt6601_L1_VE|1
TTTAATAGGCCCTTCTGGCTCCAACGCGCACAAGGTCATAATAATGGTATTTGTTGGGCA
>HPV58_Alpha_222386_nt5716_L1_VE|1
GATGAATATGTGTCACGCACAAGCATTTATTATTATGCTGGCAGTTCCAGACTTTTGGCT
>HPV69_Alpha_6970418_nt6472_L1_VE|1
CCTTACTGGTTACAGCGTGCCCAGGGTCATAATAATGGCATTTGTTGGGGCAACCAATTG
>HPV93_Beta_37089393_nt5671_L1_VE|1
GTATATTTGCCACCTTCAACACCAGTTGCCAGAGTACAAAGCACGGATGAATATGTACAA
>HPV41_Nu_9626041_nt5637_L1_VE|1
ACCAGTACTTTCCTCCATGCTGCCACTGACCGTTTGCTTACTGTTGGACATCCATTTTAC
>HPV38_Beta_1020234_nt6412_L1_VE|1
CGACGGGAGCAATGCTATGCCAGACATTATTTTGTTCGAGGTGGTGCAGTGGGTGACGCT
>HPV102_Alpha_71726718_nt6704_L1_VE|1
CAACTGTTTAATAAGCCTTACTGGCTTCAACGGGCCCAGGGACACAATAATGGCATTTGT
>HPV97_Alpha_71726694_nt6407_L1_VE|1
CAGTTGTTTAATAAGCCATATTGGTTGCATAGGGCCCAGGGCCATAACAATGGTATTTGT
>HPV10_Alpha_9627257_nt6400_L1_VE|1
CCTATTCAGGATGGTGATATGGTGGACACTGGCTATGGGGCCATGGACTTTACTGCTTTA
>HPV38_Beta_1020234_nt5722_L1_VE|1
CGCGTTCAAAGCACGGATGAATATGTGGAACGAACAGACATCTATTACCATGCAACTAGC
>HPVFA75/KI88-03_Unclassified_206902429_nt6521_L1_VE|1
CCTACTGTTAGTGGTTCTCTAGTCTCTTCAGATGCTCAGTTATTTAATAGGCCTTTTTGG
>HPV58_Alpha_222386_nt6586_L1_VE|1
CGTGCACAAGGTCATAACAATGGCATTTGCTGGGGCAATCAGTTATTTGTTACCGTGGTT
>HPV42_Alpha_333211_nt6147_L1_VE|1
CTAGAAGTAGGTCGTGGACAGCCTTTGGGCGTTGGTATTAGTGGCCATCCATTATTGAAT
>HPV41_Nu_9626041_nt5847_L1_VE|1
ATTCGTGGGATTGAGGTTTCTAGGGGACAGCCCTTAGGTATTGGTGTAACAGGGAACCCT
>HPV50_Gamma_9628550_nt5562_L1_VE|1
CCTTTAGGTGTTGGATCTACTGGTCATCCCTTGTTTAATAAGGTTGGAGACACAGAAAAT
>HPV103_Unclassified_109390382_nt5066_L1_VE|1
GACCGCCTGCTTACTGTAGGACACCCATATTTTCCAATTACAAAAGTGGGTGAAGGTACA
>HPV52_Alpha_397038_nt5716_L1_VE|1
GATGAGTATGTGTCTCGCACAAGCATCTATTATTATGCAGGCAGTTCTCGATTACTAACA
>HPV92_Beta_27531786_nt6599_L1_VE|1
CCATATTGGTTACAAAGAGCACAAGGCCACAACAATGGTATTTGCTGGAGTAATGAACTG
>HPV92_Beta_27531786_nt6569_L1_VE|1
GTATCCACTGATGCACAATTATTTAATCGGCCATATTGGTTACAAAGAGCACAAGGCCAC
>HPV21_Beta_1020170_nt6830_L1_VE|1
GGTAACTCCATGTATTTCCCAACTGTCAGTGGCTCATTGGTGTCTAGTGACGCTCAATTG
>HPV9_Beta_9627396_nt5776_L1_VE|1
GTATATTTGCCACCAGCAACACCAGTGGCGAGAGTTCAAAGCACCGATGAATATGTGGAA
>HPV32_Alpha_9627327_nt6443_L1_VE|1
GTTATTCAGGATGGTGATATGGCAGATGTAGGGTTTGGAGCAATGGACTTTAGTGCTTTG
>HPV76_Beta_2911551_nt5830_L1_VE|1
AAGGTTTATCTTCCACCATCAACACCTGTGGCCAGGGTTCAAAGCACGGATGAATACATT
>HPV97_Alpha_71726694_nt6077_L1_VE|1
TTGGAGGATGGCGATATGGTAGATACAGGTTATGGTGCCATGGACTTTAGTACATTACAG

```
>HPV1_Mu_9626063_nt6014_L1_VE|1
GATGGTGACATGATGGATATTGGTTTTGGGGCTATGGATTTTGCTGCTTTACAGCAAGAC
>HPV11_Alpha_333026_nt6702_L1_VE|1
CAAAAGGCTCAGGGACATAACAATGGTATTTGCTGGGGAAACCACTTGTTTGTTACTGTG
>HPV66_Alpha_1020290_nt6608_L1_VE|1
GGCATATGCTGGGGTAATCAGGTATTTGTTACTGTTGTGGATACTACCAGAAGCACCAAC
>HPV34_Alpha_9627334_nt6421_L1_VE|1
AATAAGCCTTATTGGTTGCAAAAGGCCCAGGGACAAAACAATGGCATTTGCTGGCATAAT
>HPV107_Beta_126131393_nt6749_L1_VE|1
TGGTTGAAACGTGCACAAGGCCACAATAATGGTATTCTATGGGGAAACCAAATGTTTGTT
>HPV65_Gamma_312100_nt5447_L1_VE|1
GAAAGGCTTTTAACTGTAGGCCATCCTTATTTTCCAGTGAAAGATGTGCAGGATCAACAC
>HPV30_Alpha_396973_nt6592_L1_VE|1
CAACGCGCACAGGGACACAATAATGGCATTTGTTGGGGCAACCAGGTATTTGTTACTGTT
>HPV34_Alpha_9627334_nt6181_L1_VE|1
ATATGTAAATATCCAGATTATCTAGGCATGGCCGCAGATCCCTATGGCGATTCTATGTGG
>HPV70_Alpha_1173493_nt5921_L1_VE|1
CAGCCATTGGGCGTTGGCGTTAGTGGACATCCTTTATATAATAGATTGGATGATACTGAA
>HPV58_Alpha_222386_nt6616_L1_VE|1
TGGGGCAATCAGTTATTTGTTACCGTGGTTGATACCACTCGTAGCACTAATATGACATTA
>HPV96_Beta_50253426_nt5917_L1_VE|1
GCCAGGGTGCAAAGCACGGACTCCTACATACAAAGAACAAACATCTATTATCATGCTAAT
>HPV96_Beta_50253426_nt6187_L1_VE|1
GGTCGTGGACAGCCATTAGGTGTAGGTACATCAGGACATCCATTATTTAACAAGGTGAAA
>HPV49_Beta_9627363_nt5842_L1_VE|1
AAGGTATATCTACCACCTTCAACACCTGTGGCAAGGGTACAAAGCACGGATGAATACATT
>HPV82_Alpha_6970427_nt5872_L1_VE|1
TGTGTTGGTGTTGAAGTAGGTAGGGGTCAGCCGTTAGGTGTTGGCCTTAGTGGTCATCCT
>HPV50_Gamma_9628550_nt6462_L1_VE|1
ATGGACCCCAGAATTTTGGAAAATTGGCAATTAGCTTATGTTCCACCAGCTCCCAGTGGT
>HPV103_Unclassified_109390382_nt5546_L1_VE|1
TGTGATATTGGCTTTGGTGCAATGAATTTTGCTACATTGCAGGAGGACCATTCAGGTGTA
>HPV53_Alpha_9627377_nt6526_L1_VE|1
GCTACACCTAGTGGGTCTATGATAACTTCAGAGGCTCAATTGTTTAATAAGCCATATTGG
>HPV70_Alpha_1173493_nt6521_L1_VE|1
TTGCATAAGGCCCAGGGACACAATAATGGCATTTGTTGGCATAACCAGTTGTTTATTACT
>HPV34_Alpha_9627334_nt5731_L1_VE|1
CGTTTGCCAGATCCCAATAAATTTGGGTTCCCAGATGCATCTTTTTATAATCCTGACAAG
>HPV96_Beta_50253426_nt5887_L1_VE|1
AAGGTCTATTTACCACCATCAACACCAGTTGCCAGGGTGCAAAGCACGGACTCCTACATA
>HPV17_Beta_396932_nt5755_L1_VE|1
GTATACTTGCCTCCAACACCACCAGTAGCCCGAGTACAAAGCACGGATGAGTATGTGGAA
>HPV82_Alpha_6970427_nt6142_L1_VE|1
TCTACTGTCATTGAGGATGGCGATATGGTGGACACTGGTTTTGGGGCCATGGATTTTGCT
>HPV37_Beta_1020226_nt5987_L1_VE|1
GTATATAATCCAGAAAAGGAAAGGTTGGTGTGGGCCTGTGCGGGCTTGGAGATAGGCCGA
>HPV9_Beta_9627396_nt6676_L1_VE|1
ACTTCAGATGCTCAATTGTTTAATAGGCCATTTTGGCTCCAAAGAGCACAAGGTCACAAC
>HPVRTRX7_Unclassified_30315616_nt6882_L1_VE|1
AGGCCCTTCTGGCTGCAAAGAGCACAGGGTCATAATAATGGCATTCTGTGGGCTAATCAG
>HPV71_Alpha_12084981_nt5807_L1_VE|1
CTAAACGTGTCCACTATTTTTTTGCAGATGGCTTTGTGGCGGCCTAGTGACAGCAAGGTA
>HPV84_Alpha_12958167_nt6596_L1_VE|1
AATAAGCCATACTGGTTGCAAAAGGCCCAGGGTCATAACAATGGTATATGCTGGTTTAAT
>HPV80_Beta_2911565_nt5749_L1_VE|1
```

GTGTATTTACCACCAACACCACCTGTAGCACGTGTACAAAGCACCGATGAATATGTGGAA
>HPV65_Gamma_312100_nt5417_L1_VE|1
GGAACATCTCTTTATTTCCACGCTGGAACAGAAAGGCTTTTAACTGTAGGCCATCCTTAT
>HPV11_Alpha_333026_nt6432_L1_VE|1
ATTTGTGGAACTGTCTGCAAATATCCTGATTATTTGCAAATGGCTGCAGACCCTTATGGT
>HPV74_Alpha_27462483_nt6024_L1_VE|1
GCTATGGATTATAAGCAAACACAGTTATGCATGGTAGGCTGTGCTCCCCCGTTGGGTGAA
>HPV31_Alpha_333048_nt6483_L1_VE|1
CCATATTGGATGCAACGTGCTCAGGGACACAATAATGGTATTTGTTGGGGCAATCAGTTA
>HPV101_Unclassified_109390389_nt5608_L1_VE|1
GTGTCCTCTGTTATTCAGGATGGGGATATGTGTGATATTGGCTTTGGTGCCATGAATTTT
>HPV52_Alpha_397038_nt6406_L1_VE|1
CGTGAGCAAATGTTTGTTAGACACTTTTTTAATAGGGCCGGTACCTTAGGTGACCCTGTG
>HPV22_Beta_1020178_nt6143_L1_VE|1
TGTATACCGTGTTTAGGTGAATATTGGGATAAAGCTCCTGTTTGTGAAGATGCAGGCAGT
>HPV83_Alpha_5059324_nt6673_L1_VE|1
AAGCCCTACTGGCTGCATCGTGCCCAGGGACATAATAATGGCATTTGTTGGTTTAATGAG
>HPV28_Alpha_1020202_nt6311_L1_VE|1
ACTGCCCCTATTCAGGATGGTGATATGGTGGACACTGGCTTTGGTGCAATGGACTTTGCT
>HPV88_Gamma_167600365_nt5431_L1_VE|1
GATCGTCTGTTAACAGTAGACCATCCCTACTATGAAATTAGGAATGATGGAGATCCCAAC
>HPV53_Alpha_9627377_nt6586_L1_VE|1
CTGCAACGTGCCCAGGGACATAATAATGGCATCTGTTGGAACAATCAGTTATTTGTAACT
>HPV82_Alpha_6970427_nt6472_L1_VE|1
ACCTCTGATTCTCAGATTTTTAATAAGCCTTATTGGTTGCATCGCGCCCAGGGCCACAAT
>HPV107_Beta_126131393_nt6539_L1_VE|1
AGGAGAGAACAATGTTATGCTAGACATTATTTCACTAGAGGAGGAGCTGTGGGTGATGCT
>HPVcand62_Alpha_39932599_nt5778_L1_VE|1
ATGGCTATGTGGCGGCCTGGTGACGGCAAGGTATACCTGCCTCCCACTCCTGTTTCTAAG
>HPV8_Beta_333074_nt6812_L1_VE|1
CCTTTCTGGCTGCAGCGTGCCCAGGGTCATAATAATGGCATTCTCTGGGCTAATCAAATG
>HPV17_Beta_396932_nt5935_L1_VE|1
TATAGAGCCTTCAGAGTCACATTACCAGATCCTAATAAGTTTGCCTTGGCAGATATGTCA
>HPV70_Alpha_1173493_nt6491_L1_VE|1
GATTCCCAGTTGTTTAATAAGCCCTATTGGTTGCATAAGGCCCAGGGACACAATAATGGC
>HPV91_Alpha_22023568_nt6316_L1_VE|1
TATAAACAAACACAGCTATGTATTGTTGGCTGTACACCTCCCATGGGCGAATATTGGGGC
>HPV43_Alpha_40804474_nt6328_L1_VE|1
GAAGTTATACAGGATGGTGACATGGTAGATACAGGATTTGGTGCAATGGATTTTGCTTCC
>HPVcand89_Alpha_22095322_nt6698_L1_VE|1
CTGCACCGTGCACAGGGCCATAATAATGGTATTTGTTGGTTTAATGAGTTGTTTGTTACT
>HPV17_Beta_396932_nt6145_L1_VE|1
ACTGATGATAGACAAAACACGTCATTTGACCCTAAACAAGTGCAGATGTTTGTTGTAGGC
>HPV63_Mu_9626605_nt6752_L1_VE|2
TCGGAGCAGCTTGATCAATTTCCTTTAGGCCGCAAATTTTTGTATCAAAGTGGTCTTGCA
>HPV3_Alpha_397005_nt5818_L1_VE|1
AGCACGGACGACTATGTGACACGCACCAACATTTATTATTATGCAGGCAGTTCTCGCTTG
>HPV4_Gamma_9626597_nt5466_L1_VE|1
GAAAGGCTTTTAACTGTAGGCCATCCTTATTTCCAGTGAAAGATGTACAGGAACCTCAC
>HPV12_Beta_396910_nt6805_L1_VE|1
AGGCCCTTCTGGCTTCAAAGAGCACAGGGTCATAATAATGGCATCCTGTGGGCTAATCAG
>HPV37_Beta_1020226_nt6467_L1_VE|1
GCTAGGAGAGAACAATGTTATGCCAGACATTATTTTGTAAGAGGGGGAAATGTAGGTGAT
>HPV48_Gamma_9628542_nt5773_L1_VE|1
GTTATTCAGGATGGTGATATGGGAGATATAGGCTTTGGGGCTGCTAATTTTCCTAAGTTG

>HPV28_Alpha_1020202_nt6671_L1_VE|1
TATTGGCTGCGGCGTGCTCAGGGACACAATAATGGTATCTGTTGGGCCAACCAATTGTTT
>HPV66_Alpha_1020290_nt6578_L1_VE|1
TGGTTGCAACGTGCACAGGGCCATAATAATGGCATATGCTGGGGTAATCAGGTATTTGTT
>HPV4_Gamma_9626597_nt5436_L1_VE|1
GGAACATCTCTGTATTTCCACGCTGGTACAGAAAGGCTTTTAACTGTAGGCCATCCTTAT
>HPV54_Alpha_9628437_nt6515_L1_VE|1
TTTAATAAGCCATACTGGTTACAACGGGCCCAGGGTCAAAACAATGGTATTTGTTGGGGC
>HPV39_Alpha_333245_nt6574_L1_VE|1
TGGCTACATAAGGCCCAGGGCCACAACAATGGTATATGTTGGCATAATCAATTATTTCTT
>HPV22_Beta_1020178_nt6623_L1_VE|1
CGCGCGCAGGGCCATAATAATGGTATTTTGTGGAACAACCAAATGTTTGTAACAGTAGCT
>HPV67_Alpha_3228267_nt5650_L1_VE|1
TACCTGCCTCCTGTGCCTGTATCTAAGGTTGTAAGCACTGATGAATATGTGTCACGCACA
>HPV61_Alpha_9628574_nt6665_L1_VE|1
GTGTCCTCTGATTCCCAATTATTTAATAAGCCATATTGGCTGCAACGTGCCCAGGGCCAC
>HPV84_Alpha_12958167_nt6566_L1_VE|1
TCTATGGTGTCCTCGGATTCCCAATTGTTTAATAAGCCATACTGGTTGCAAAAGGCCCAG
>HPVcand62_Alpha_39932599_nt6438_L1_VE|1
TTGGATATATGCACCTCCACATGTAAATATCCTGACTATTTGCAAATGGCTGCAGAACCT
>HPV101_Unclassified_109390389_nt5638_L1_VE|1
TGTGATATTGGCTTTGGTGCCATGAATTTTGCTGCCTTGCAGGCTGATCACTCAGGGGTA
>HPV39_Alpha_333245_nt6544_L1_VE|1
TCTGATTCCCAGTTATTTAATAAGCCTTATTGGCTACATAAGGCCCAGGGCCACAACAAT
>HPV111_Beta_186461202_nt6553_L1_VE|1
TCAGATGCGCAGCTATTTAATAGACCATTTTGGTTGCAACGTGCACAAGGCCACAACAAT
>HPV68a_Alpha_71726685_nt5669_L1_VE|1
GGCATTCCTAAGGTGTCTGCATATCAATACAGAGTGTTTAGGGTTACCTTACCTGATCCT
>HPV90_Alpha_22138122_nt6753_L1_VE|1
GGCCCTAACAATGGTATCTGCTGGGGTAATCAGCTTTTTGTAACTGTGGTTGATACTACA
>HPV61_Alpha_9628574_nt5765_L1_VE|1
ATGGCTTTGTGGCGGCCTGGTGACGGCAAGGTATACCTGCCTCCCACTCCTGTGTCTAAA
>HPV7_Alpha_9627389_nt6549_L1_VE|1
GAACAAATGTTTGTTAGGCACTTTTTTAATAGGGCAGGAACTACTGGAGACAGTGTTCCA
>HPV93_Beta_37089393_nt6391_L1_VE|1
TTTGCTCGCAGAGAGCAATGTTATGCCAGACACTTTTTTGTAAGAGGTGGCAACGTAGGA
>HPV34_Alpha_9627334_nt5761_L1_VE|1
CCAGATGCATCTTTTTATAATCCTGACAAGGAGCGGTTGGTTTGGGCCTGTGCAGGCGTT
>HPV101_Unclassified_109390389_nt6358_L1_VE|1
GCAGAGCCTCCTAAGGAAAAGGAAGATCCATATGACAAAATGTCTTTTTGGGTGGTTGAT
>HPV51_Alpha_333087_nt6092_L1_VE|1
TCCTCTGTCATTCAGGATGGCGATATGATTGATACAGGGTTTGGAGCTATGGATTTCGCT
>HPV72_Alpha_1491683_nt5764_L1_VE|1
AACGTGTGCCCTATTCTTTTGCAGATGGCTTTGTGGCGGCCTGGTGACGGCAAGGTATAC
>HPV41_Nu_9626041_nt6507_L1_VE|1
CCCTTTTGGCTGCAGAGATCCCAGGGCCATAACAATGGCATACTGTGGCACAACGAGGCC
>HPV26_Alpha_9627305_nt5589_L1_VE|1
ATGGCTTTGTGGCGTACTAGTGACAGCAAGGTATATCTTCCTCCCACCCCTGTGTCTCGG
>HPV11_Alpha_333026_nt6342_L1_VE|1
GTTATACAGGATGGGGACATGGTTGATACAGGCTTTGGTGCTATGAATTTTGCAGACTTA
>HPV6_Alpha_6002612_nt6721_L1_VE|1
AAAGCCCAGGGACATAACAATGGTATTTGTTGGGGTAATCAACTGTTTGTTACTGTGGTA
>HPV57_Alpha_60882_nt5734_L1_VE|1
TGCACTATTTCTTTGCAGATGGCTATGTGGCGGCCTAATGAAAGCAAGGTATACCTGCCT
>HPV110_Beta_186461194_nt6007_L1_VE|1

GGGCAACCTTTAGGGGTAGGTACCAGTGGTCATCCCTTATTTAATAAAGTTAGAGATACT
>HPV73_Alpha_1491692_nt6185_L1_VE|1
GATTATTTAGGCATGGCTGCTGATCCCTATGGTGATTCCATGTGGTTTTATCTTCGTAGG
>HPV83_Alpha_5059324_nt6313_L1_VE|1
GTAAATTCTACTATACAGGATGGCGACATGGTGGAAACAGGCTATGGTGCCATGGATTTT
>HPV52_Alpha_397038_nt6616_L1_VE|1
AATAATGGCATATGTTGGGGCAATCAGTTGTTTGTCACAGTTGTGGATACCACTCGTAGC
>HPV110_Beta_186461194_nt5887_L1_VE|1
TACAGAGCATTCCGGGTGCAATTGCCTGATCCTAATAGGTTTGCATTAGCAGACATGTCT
>HPV3_Alpha_397005_nt6688_L1_VE|1
TATTGGCTGCGGCGTGCTCAGGGACACAATAATGGTATATGTTGGGCCAACCAATTGTTT
>HPVFA75/KI88-03_Unclassified_206902429_nt6101_L1_VE|1
TGTACACCATGTTTAGGAGAATACTGGGATAAAGCTGAGCCATGTGAAAATGCAGGAGAT
>HPV43_Alpha_40804474_nt5818_L1_VE|1
GTGAGCACAGATGAATATGTGCAACGCACCAACTTATTTTATTATGCTGGCAGTTCACGT
>HPV9_Beta_9627396_nt6496_L1_VE|1
GCAAGAAGAGAACAATGTTATGCCAGGCATTATTATGTTAGAGGAGGTTCAGTTGGTGAC
>HPV38_Beta_1020234_nt5782_L1_VE|1
GATCGCCTATTAACAGTAGGCCATCCATATTTTGATGTCAGATCACAGGATGGTCAAAAA
>HPV103_Unclassified_109390382_nt5846_L1_VE|2
TACTATGCAACCCCCAGTGGATCACTGGTTTCTAGTGATTCTCAGTTGTTTGGCAGACCA
>HPV44_Alpha_1020242_nt6625_L1_VE|1
AAGGCGCAGGGCCACAATAATGGTATTTGTTGGGGAAATCAGTTATTTGTTACTGTTGTA
>HPV56_Alpha_397053_nt6513_L1_VE|1
TTTAATAAACCTTATTGGTTGCAACGTGCCCAAGGCCATAATAATGGCATTTGCTGGGGT
>HPV53_Alpha_9627377_nt5656_L1_VE|1
ATGGCGGTGTGGCGGCCTAGTGACAGCAAGGTTTATCTGCCTCCTACCCCTGTATCAAAG
>HPV60_Gamma_9628566_nt6686_L1_VE|2
ATGTCTCAGGCAACAATGTGTCCAACAGATGTTCCTAATACTGAAAGGGAAGACCCTTAT
>HPV68a_Alpha_71726685_nt6389_L1_VE|1
TCCTCTGACTCCCAGTTATTTAACAAGCCCTATTGGCTGCACAAGGCACAGGGACACAAC
>HPV14D_Beta_396918_nt6504_L1_VE|1
GGCAATGCCATGTATTTCCCAACTGTCAGTGGCTCATTGGTATCCAGTGATGCTCAACTG
>HPV38_Beta_1020234_nt5692_L1_VE|1
ATATACTTGCCACCAACACCTCCAGTTGCGCGCGTTCAAAGCACGGATGAATATGTGGAA
>HPV23_Beta_1020186_nt5781_L1_VE|1
GATCGATTACTAACTGTAGGCCACCCATATTTTGATGTTAGATCACCGGATGGTAGTAAA
>HPV35_Alpha_333050_nt6475_L1_VE|1
GTAACCTCCGATGCACAAATATTTAATAAACCATATTGGTTGCAACGTGCACAAGGCCAT
>HPV39_Alpha_333245_nt6064_L1_VE|1
GACAGTAGGGATAATGTGTCTGTGGATTATAAACAGACACAGTTGTGCATTATAGGCTGT
>HPV31_Alpha_333048_nt6543_L1_VE|1
TTTGTTACTGTGGTAGATACCACACGTAGTACCAATATGTCTGTTTGTGCTGCAATTGCA
>HPV59_Alpha_557236_nt6057_L1_VE|1
TATAAACAAACTCAGCTGTGTATTATTGGCTGTGTACCTGCCATTGGAGAACACTGGACA
>HPV60_Gamma_9628566_nt6266_L1_VE|2
CTTGGTGGTTATAGTTACTTCCCTACACCAAGTGGTTCTGTAGCTTCTAGTGATAATCAA
>HPV54_Alpha_9628437_nt6545_L1_VE|1
CAGGGTCAAAACAATGGTATTTGTTGGGGCAATCAGGTGTTTTTAACAGTTGTAGATACC
>HPV74_Alpha_27462483_nt6174_L1_VE|1
GGTGATATGGTAGATACAGGCTTTGGTGCCATGAATTTTGCTGAATTGCAGCCTCTTAAA
>HPV65_Gamma_312100_nt5777_L1_VE|1
GATCCAAAACAGACACAAATGTTTATCATAGGTTGCACTCCTGCTATTGGGGAACATTGG
>HPV51_Alpha_333087_nt6452_L1_VE|1
TATTGGCTCCACCGTGCGCAGGGTCACAATAATGGCATTTGCTGGAACAATCAGCTTTTT

```
>HPV40_Alpha_397014_nt5960_L1_VE|1
CCTAAGGTTTCTGGACATCAATACAGGGTATTTAGGGTACGTTTGCCTGACCCGAATAAG
>HPV23_Beta_1020186_nt6621_L1_VE|1
CGTGCTCAAGGCCATAACAATGGTATTTTATGGAACAACCAGATGTTTGTGACTGTAGCA
>HPV3_Alpha_397005_nt6718_L1_VE|1
AATGGTATATGTTGGGCCAACCAATTGTTTGTGACTGTGGTGGATACCACACGTAGTACT
>HPV37_Beta_1020226_nt5747_L1_VE|1
GTATACTTGCCTCCAACACCACCAGTAGCCCGGGTGCAAAGCACGGATGATTATGTGGAA
>HPV111_Beta_186461202_nt6343_L1_VE|1
GTCTATGGGGACGCTTGTTTCTTTTTTGCCAGAAGGGAGCAATGTTATGCCAGACATTAC
>HPV25_Beta_396948_nt6790_L1_VE|1
AACTCAATGTATTTCCCAACAGTCAGTGGCTCATTGGTATCCAGTGATGCTCAATTATTC
>HPV21_Beta_1020170_nt5960_L1_VE|1
GTTTACCTTCCACCGTCTACACCAGTTGCCAGGGTCCAAAGCACGGATGAATATGTACAA
>HPV103_Unclassified_109390382_nt5486_L1_VE|1
GATATGGGTGCATGTCCTCCTTTAGAGCTTGTTAGTTCCATTATTGAGGATGGGGATATG
>HPV60_Gamma_9628566_nt6236_L1_VE|2
GCACCACAAACTGATAAACCTCAAAATAACCTTGGTGGTTATAGTTACTTCCCTACACCA
>HPV26_Alpha_9627305_nt6489_L1_VE|1
ACTTCGGATGCACAACTATTTAATAAGCCATACTGGTTACAACGTGCACAGGGTCATAAT
>HPV53_Alpha_9627377_nt6556_L1_VE|1
GAGGCTCAATTGTTTAATAAGCCATATTGGCTGCAACGTGCCCAGGGACATAATAATGGC
>HPV1_Mu_9626063_nt5714_L1_VE|1
AGATTAGTTTGGGGCCTAAGAGGGATAGAGATAGGTAGAGGCCAGCCTTTAGGTATAGGA
>HPV40_Alpha_397014_nt6350_L1_VE|1
GAGGTTATTCAGGATGGCGACATGGTGGATACTGGCTTTGGTGCTATGGATTTTGCTTCC
>HPV51_Alpha_333087_nt5972_L1_VE|1
AAACAGACTCAGTTATGTATAATAGGCTGTGCTCCACCTATTGGGGAACACTGGGGTATT
>HPV1_Mu_9626063_nt5984_L1_VE|1
AGGGTGCAAATGATAGAGTCTGTCATAGAAGATGGTGACATGATGGATATTGGTTTTGGG
>HPV40_Alpha_397014_nt6380_L1_VE|1
ACTGGCTTTGGTGCTATGGATTTTGCTTCCTTGCAGGCCAATAAAAGTGATGTGCCATTG
>HPV25_Beta_396948_nt6820_L1_VE|1
TCATTGGTATCCAGTGATGCTCAATTATTCAATAGGCCATTTTGGTTACAGCGAGCACAA
>HPV56_Alpha_397053_nt6483_L1_VE|1
GGGTCTATGATTACGTCTGAGGCACAGTTATTTAATAAACCTTATTGGTTGCAACGTGCC
>HPV65_Gamma_312100_nt5807_L1_VE|1
GGTTGCACTCCTGCTATTGGGGAACATTGGGATAAGGCTGAGCCTTGTGCAAGTCCTGTT
>HPVcand87_Alpha_14475578_nt6443_L1_VE|1
GTTTGCAAGTATCCTGACTATTTGCAAATGGCTGCTGAGCCCTATGGCGATTGCATGTTC
>HPV48_Gamma_9628542_nt6133_L1_VE|1
TTATTTAATAGGCCTTATTGGCTTAGACGAGCCCAGGGAACAAATAATGGCATTTGTTGG
>HPV92_Beta_27531786_nt6869_L1_VE|1
TTGGAGGACTGGCAGTTAGGATTTGTTCCTACTCCAGATAACCCTATTCAGGATGCATAC
>HPV58_Alpha_222386_nt6226_L1_VE|1
ATTGAGGATGGTGACATGGTAGATACAGGGTTTGGATGCATGGACTTTGGTACATTGCAG
>HPV54_Alpha_9628437_nt6275_L1_VE|1
GCTACCTCAATTTGTAAATATCCTGATTACCTTAAAATGGCTGCAGAGGCATATGGGGAC
>HPV10_Alpha_9627257_nt6730_L1_VE|1
GCTCAATTGTTTAATAAGCCATATTGGCTGCGGCGGGCCCAGGGGCACAACAATGGTATA
>HPV80_Beta_2911565_nt6469_L1_VE|1
GCAAGACGAGAGCAATGTTATGCTAGACATTATTTTGTTAGAGGAGGTGCAGTAGGCGAT
>HPV92_Beta_27531786_nt5969_L1_VE|1
CGAGGGCAGCCTTTAGGTGTTGGCACCACAGGTCATCCATTATTTAATAAGGTATTGGAC
>HPV33_Alpha_333049_nt6345_L1_VE|1
```

CGACGTGAACAAATGTTTGTAAGACACTTTTTTAATAGGGCTGGTACATTAGGAGAGGCT
>HPV10_Alpha_9627257_nt6700_L1_VE|1
ACACCCAGTGGGTCCATGGTAACGTCTGAGGCTCAATTGTTTAATAAGCCATATTGGCTG
>HPV38_Beta_1020234_nt6112_L1_VE|1
CCTAAACAGGTGCAAATGTTTATAATAGGCTGCACTCCTTGTCTAGGAGAATACTGGGAT
>HPV66_Alpha_1020290_nt5648_L1_VE|1
ATGGCGATGTGGCGGCCTAGTGACAATAAGGTGTACCTACCTCCAACACCTGTTTCAAAG
>HPV30_Alpha_396973_nt6562_L1_VE|1
GCTCAGTTGTTTAATAAACCTTACTGGTTGCAACGCGCACAGGGACACAATAATGGCATT
>HPV32_Alpha_9627327_nt6803_L1_VE|1
TATTGGTTACAGCAGGCACAAGGCCACAATAATGGTATATGTTGGGGTAATCAAGTGTTT
>HPV2_Alpha_9626032_nt6853_L1_VE|1
AAGGAATATCTCAGGCATATGGAGGAATATGATTTGCAGTTCATCTTCCAACTGTGCAAG
>HPV16_Alpha_9627100_nt6580_L1_VE|1
CGAGCACAGGGCCACAATAATGGCATTTGTTGGGGTAACCAACTATTTGTTACTGTTGTT
>HPV5_Beta_9627145_nt6668_L1_VE|1
CGTAGGGAGCAATGTTATGCCAGACACTTTTTTGTTAGAGGGGGAAAAACTGGTGATGAC
>HPV30_Alpha_396973_nt6532_L1_VE|1
ACTCCTAGTGGGTCTATGGTAACCTCTGAGGCTCAGTTGTTTAATAAACCTTACTGGTTG
>HPV80_Beta_2911565_nt6019_L1_VE|1
TGGGCCTGTGCAGGCCTAGAAATAGGCAGAGGACAACCATTAGGTGTAGGCACATCAGGC
>HPV90_Alpha_22138122_nt5973_L1_VE|1
ATAGTGGTTCCCAAGGTGTCTGGATATCAATATAGGGTGTTTAGGGTACGTTTGCCTGAT
>HPV50_Gamma_9628550_nt5532_L1_VE|1
CTAGTAGGTATAGAAGTCGGGAGAGGTGGGCCTTTAGGTGTTGGATCTACTGGTCATCCC
>HPV111_Beta_186461202_nt6373_L1_VE|1
AGAAGGGAGCAATGTTATGCCAGACATTACTTTGTTAGAGGAGGCAATGTAGGAGATGCA
>HPV29_Alpha_1020210_nt5874_L1_VE|1
GTTATCAGCACGGACGACTATGTGACACGCACAAATATTTATTATTATGCAGGCAGTTCT
>HPV23_Beta_1020186_nt5721_L1_VE|1
CGAGTGCAGAGTACGGATGAATATGTGGAAAGAACTGACATCTATTACCATGCAACTAGT
>HPV42_Alpha_333211_nt5817_L1_VE|1
CTGTACCATATTTTTTTGCAGATGTCCGTGTGGCGGCCTAGTGACAACAAGGTTTATCTA
>HPV96_Beta_50253426_nt6817_L1_VE|1
CCTTTTTGGCTGCAAAGGGCTCAAGGCCATAATAATGGTATTTGCTGGGGTAATCAAATC
>HPV33_Alpha_333049_nt5925_L1_VE|1
GGGCAGCCATTAGGCGTTGGCATAAGTGGTCATCCTTTATTAAACAAATTTGATGACACT
>HPV13_Alpha_85827580_nt6043_L1_VE|1
GGTTTAGAGGTTGGTAGGGGTCAACCCTTAGGTGTTGGTATTAGTGGTCATCCATTATTA
>HPV69_Alpha_6970418_nt6502_L1_VE|1
AATAATGGCATTTGTTGGGGCAACCAATTGTTTGTTACTTGTGTAGATACTACCCGCAGT
>HPV15_Beta_396924_nt5734_L1_VE|1
GTATATTTGCCACCAACACCACCTGTAGCACGTGTACAAAGCACCGATGAATATGTGGAG
>HPV4_Gamma_9626597_nt5796_L1_VE|1
GACCCAAAACAAACACAGATGTTTATTATAGGTTGCACTCCTGCAATAGGTGAACATTGG
>HPV69_Alpha_6970418_nt6442_L1_VE|1
GTGACCTCTGATGCTCAATTGTTTAATAAACCTTACTGGTTACAGCGTGCCCAGGGTCAT
>HPV33_Alpha_333049_nt5625_L1_VE|1
GTGTACCTGCCTCCTGTACCTGTATCTAAAGTTGTCAGCACTGATGAATATGTGTCTCGC
>HPV23_Beta_1020186_nt6891_L1_VE|1
GGGTTTGTTCCTACACCAGATAATGCAGTTCATGACACATACAGATATTTGGCTTCAAAG
>HPV27_Alpha_396964_nt6668_L1_VE|1
TTGTTTAATAAGCCCTACTGGCTACGGAGGGCCCAGGGACATAATAATGGTATGTGCTGG
>HPV55_Alpha_1020266_nt6625_L1_VE|1
AGGGCGCAGGGCCACAATAATGGTATTTGTTGGGGGAATCAGTTATTTGTTACTGTTGTA

```
>HPV61_Alpha_9628574_nt6635_L1_VE|1
ATTTATTCCCCTACGCCTAGTGGCTCTATGGTGTCCTCTGATTCCCAATTATTTAATAAG
>HPV88_Gamma_167600365_nt6721_L1_VE|1
TTTAGCTTAGGTCGCAAATTTTTGTTTCAAAGTGGTTTGCTGCCCACAAAACGTGTCAGG
>HPV14D_Beta_396918_nt6564_L1_VE|1
TTCAATAGACCATTTTGGTTACAGCGCGCACAAGGCCACAATAATGGTATTTGTTGGTTT
>HPV102_Alpha_71726718_nt6464_L1_VE|1
GGTATTTGTAAATATCCTGATTATTTACAAATGGCGGCTGAGCCCTACGGAGACTGTATG
>HPVcand62_Alpha_39932599_nt6708_L1_VE|1
CCATATTGGCTGCAGCGGGCGCAGGGCCATAATAATGGTATTTGTTGGTTTAATGAACTG
>HPV32_Alpha_9627327_nt6173_L1_VE|1
TGTGTGGGTTTGGAGGTTGGCCGTGGACAGCCTTTAGGTGTTGGTCTTAGTGGGCATCCT
>HPV21_Beta_1020170_nt6860_L1_VE|1
GGCTCATTGGTGTCTAGTGACGCTCAATTGTTTAACAGGCCATTTTGGCTACAGCGTGCA
>HPV102_Alpha_71726718_nt6734_L1_VE|1
CGGGCCCAGGGACACAATAATGGCATTTGTTGGTTTAATGAGGTATTTGTAACAGTTGTA
>HPV1_Mu_9626063_nt6374_L1_VE|2
CAGCGATGTCAAGGCCAGAATAATGGCATTTGCTGGAGAAACCAGTTATTTATTACAGTT
>HPV16_Alpha_9627100_nt6220_L1_VE|1
ATTCAGGATGGTGATATGGTTCATACTGGCTTTGGTGCTATGGACTTTACTACATTACAG
>HPV18_Alpha_9626069_nt5941_L1_VE|1
GGTCAGCCTTTAGGTGTTGGCCTTAGTGGGCATCCATTTTATAATAAATTAGATGACACT
>HPV107_Beta_126131393_nt5789_L1_VE|1
ATGTCTTTGTGGCTTCCTGCAACTGGTAAAGTATATTTGCCACCATCAACTCCGGTGGCA
>HPV50_Gamma_9628550_nt6192_L1_VE|1
ATTCGCAGAGCTCAGGGTACGAACAATTGTATTTGTTGGGGTAATGAGGTATTTGTTACA
>HPV66_Alpha_1020290_nt5858_L1_VE|1
GTGTTTAGGGTACGGTTGCCTGATCCTAATAAGTTTGGCCTTCCTGATCCATCTTTCTAT
>HPV57_Alpha_60882_nt6664_L1_VE|1
AAGCCTTACTGGCTGCGGAGGGCCCAGGGACATAACAATGGCATGTGCTGGGGCAATCGG
>HPV50_Gamma_9628550_nt5262_L1_VE|1
TTATACCTTCCACCAAGTACTCCTGTTGCCAGAGTTTTAAGCACCGATGAATATGTGAAA
>HPV97_Alpha_71726694_nt6437_L1_VE|1
AGGGCCCAGGGCCATAACAATGGTATTTGTTGGCATAATCAATTATTTGTTACTGTGGTG
>HPV26_Alpha_9627305_nt6519_L1_VE|1
TACTGGTTACAACGTGCACAGGGTCATAATAATGGTATCTGTTGGGGCAATCAATTGTTT
>HPV82_Alpha_6970427_nt6502_L1_VE|1
TATTGGTTGCATCGCGCCCAGGGCCACAATAATGGCATTTGCTGGAATAATCAGCTTTTT
>HPV75_Beta_2911544_nt5802_L1_VE|1
AAGGTTTATCTTCCACCATCAACACCTGTGGCCAGGGTGCAAAGCACGGATGAATACATT
>HPV32_Alpha_9627327_nt5873_L1_VE|1
ATGTCCGTGTGGCGGCCTAGTGACAACAAGGTTTATCTGCCTCCTCCTCCTGTTTCCAAG
>HPV74_Alpha_27462483_nt6504_L1_VE|1
AAGCCCTTTTGGCTACAAAAGGCGCAAGGCCACAATAATGGCATTTGTTGGGGTAATCAA
>HPV106_Alpha_71726710_nt6200_L1_VE|1
CCTGGGGACGACAACCGGGACAATATGTCCATGGATTATAAACAAACTCAGTTATGTATT
>HPV35_Alpha_333050_nt6505_L1_VE|1
CCATATTGGTTGCAACGTGCACAAGGCCATAATAATGGTATTTGTTGGAGTAACCAATTG
>HPV73_Alpha_1491692_nt6365_L1_VE|1
CCTACACCTAGTGGTTCCATGGTTTCTTCAGATGCACAGTTGTTTAATAAACCTTATTGG
>HPV88_Gamma_167600365_nt5641_L1_VE|1
AGAGGTGGACCACTAAATGTAGGTACTGCAGGTCATCCTTTATTTAACAAAGCTCCAGAT
>HPV29_Alpha_1020210_nt6714_L1_VE|1
ACGTCTGAGGCTCAAATATTTAATAAGCCTTACTGGCTACAGCAGGCCCAGGGACACAAC
>HPV63_Mu_9626605_nt5432_L1_VE|3
```

ATTCTAAGCAGCGATGATTATGTGTCTCGCACCAACATCTTCTATCACGCTACCAGTGAT
>HPV63_Mu_9626605_nt6032_L1_VE|2
GCTCCTTTGGATATTGCAGGCACTGTCTGCAAATATCCAGATTATATTAAAATGGGACAG
>HPV111_Beta_186461202_nt5653_L1_VE|1
GTATATTTGCCACCATCTACACCAGTGGCTCGCGTACAAAGCACCGATGAATATGTGGAA
>HPV106_Alpha_71726710_nt6740_L1_VE|1
CAGGGCCGAAACAATGGTATCTGCTGGGGCAATCAGGTATTTGTCACTGTGGTAGATACC
>HPV18_Alpha_9626069_nt6511_L1_VE|1
GTTACCTCTGACTCCCAGTTGTTTAATAAACCATATTGGTTACATAAGGCACAGGGTCAT
>HPV16_Alpha_9627100_nt5920_L1_VE|1
ACACAGCGGCTGGTTTGGGCCTGTGTAGGTGTTGAGGTAGGTCGTGGTCAGCCATTAGGT
>HPV19_Beta_396940_nt5895_L1_VE|1
TACCTTCCACCATCTACACCAGTTGCCAGGGTACAAAGCACGGATGAATATGTTCAAAGG
>HPV9_Beta_9627396_nt6046_L1_VE|1
TGGGCCTGTAGAGGTATTGAAATAGGCAGAGGACAACCTTTAGGGGTTGGAACATCAGGT
>HPV29_Alpha_1020210_nt6384_L1_VE|1
ATGACCACACATATTGAGGATGGCGATATGGTGGATACCGGGTACGGTGCCATGGACTTT
>HPV107_Beta_126131393_nt6029_L1_VE|1
CCCAATCGATTTGCATTAGCAGACATGTCAGTATACAATCCTGACAAGGAAAGGCTGGTA
>HPV7_Alpha_9627389_nt6369_L1_VE|1
GAAGTTATTGAGGATGGCGACATGGTTGATGCAGGCTTTGGTGCCATGGATTTTGCATCA
>HPV11_Alpha_333026_nt6732_L1_VE|1
TGCTGGGGAAACCACTTGTTTGTTACTGTGGTAGATACCACACGCAGTACAAATATGACA
>HPV25_Beta_396948_nt5920_L1_VE|1
GTGTACCTTCCACCATCTACACCTGTTGCCAGGGTACAAAGCACGGATGAATATGTGCAA
>HPV24_Unclassified_9628486_nt6614_L1_VE|1
GGCAACTCAATGTATTTCCCAACAGTCAGTGGCTCATTGGTATCTAGTGATGCTCAATTA
>HPV54_Alpha_9628437_nt5615_L1_VE|1
ATGTGGCGGCCTAGCGAAAACAAAGTATACCTGCCTCCTACCCCAGTTTCTAAGGTTGTC
>HPV11_Alpha_333026_nt6192_L1_VE|1
AGGGTTAATGTAGGTATGGATTATAAACAAACCCAGCTATGTATGGTGGGCTGTGCTCCA
>HPV1_Mu_9626063_nt6614_L1_VE|1
GACCCTAATATTTTAGAGGATTGGCAACTATCTGTATCTCAACCACCTACCAATCCTCTA
>HPV70_Alpha_1173493_nt6011_L1_VE|1
CAGGACAGTAGGGACAATGTGTCTGTGGACTATAAGCAAACACAGTTATGTATTATAGGC
>HPV39_Alpha_333245_nt6214_L1_VE|1
AACACCCCTATTGAGGATGGTGATATGATTGATACTGGCTATGGAGCTATGGACTTTGGT
>HPV101_Unclassified_109390389_nt5578_L1_VE|1
CCTAAAGGCACCTGTCCTCCTTTACAGTTGGTGTCCTCTGTTATTCAGGATGGGGATATG
>HPV31_Alpha_333048_nt5853_L1_VE|1
TGGGCCTGTGTTGGTTTAGAGGTAGGTCGCGGGCAGCCATTAGGTGTAGGTATTAGTGGT
>HPV44_Alpha_1020242_nt5995_L1_VE|1
GGGCTGGAGGTAGGTAGAGGACAGCCCTTAGGTGTTGGTATTAGTGGGCATCCATTATTA
>HPV23_Beta_1020186_nt5871_L1_VE|1
TTCAGGGCCTTTAGAGTTACATTTCCAGACCCTAATAAGTTTGCATTAGCAGACATGACT
>HPV15_Beta_396924_nt6004_L1_VE|1
TGGGCCTGTGTAGGCCTTGAAATAGGTAGAGGACAACCATTAGGAGTTGGTACTTCAGGC
>HPV101_Unclassified_109390389_nt6148_L1_VE|2
AAGCACTACCTCAGACATGTGGAGGAATTTGAAATTTCTCTCATTGTGCAGCTCTGTAAA
>HPV22_Beta_1020178_nt5963_L1_VE|1
TGGGCTTGTAAAGGGTTAGAAATAGGAAGAGGACAGCCCTTAGGTGTAGGTACCACAGGT
>HPV6_Alpha_6002612_nt6361_L1_VE|1
ATACAGGATGGCGATATGGTTGACACAGGCTTTGGTGCTATGAATTTTGCTGATTTGCAG
>HPV16_Alpha_9627100_nt5950_L1_VE|1
GTTGAGGTAGGTCGTGGTCAGCCATTAGGTGTGGGCATTAGTGGCCATCCTTTATTAAAT

>HPV63_Mu_9626605_nt5822_L1_VE|1
GATGCAAAACAAACCCAAATGTTTCTAATTGGCTGTACACCAGCCACTGGGGAACACTGG
>HPV80_Beta_2911565_nt5989_L1_VE|1
GTTTATAATCCAGAAAAAGAAAGGTTGGTTTGGGCCTGTGCAGGCCTAGAAATAGGCAGA
>HPVcand85_Alpha_4574720_nt6511_L1_VE_rc|1
ATTTTGTCCCTGGGCTTTGTGCAGCCAATAAGGCTTATTAAACATTTGTGCATCTGAAGA
>HPV88_Gamma_167600365_nt5491_L1_VE_rc|1
AGGCAATTTCAGCCTAAACACTCGATATTGGCATGCAGACACCTTAGGAATCTCAATTTT
>HPV17_Beta_396932_nt6955_L1_VE_rc|1
CCTATATATATCATGCACTGGGTTATCTGGTGTAGGCACAAATCCTAACTGCCAGTCTTC
>HPV60_Gamma_9628566_nt5876_L1_VE_rc|1
AGGATCACAAGGTTTAGCAATATCCCAGTGTTCACCAGTGGCTGGTTTACATCCTACAAT
>HPV22_Beta_1020178_nt6593_L1_VE_rc|1
CAAAATACCATTATTATGGCCCTGCGCGCGCTTCAACCAAAAAGGCCTGTTGAATAGTTG
>HPV73_Alpha_1491692_nt6245_L1_VE_rc|1
TGGGATTTTATCACCGGTATCACCAGCCCTGTTAAATAAGTGTCGAACAAACATTTGTTC
>HPV13_Alpha_85827580_nt6313_L1_VE_rc|1
CAATTCCTCAAAATTCATGGCTCCAAATCCTGTATCTACCATGTCACCATCCTGAATTAC
>HPV42_Alpha_333211_nt5847_L1_VE_rc|1
GCTGACCACCTTGGAAACAGGAGGAGGAGGTAGATAAACCTTGTTGTCACTAGGCCGCCA
>HPV95_Gamma_40804520_nt6119_L1_VE_rc|1
ATCACCCATTGTCCCAGCTCTAACAAAAAAGTGTCTTGCATAAAGCTGTTCCCTTCGGCC
>HPV3_Alpha_397005_nt6328_L1_VE_rc|1
CAAGTTACCAAAGTCCATGGCACCATAACCTGTGTCCACCATATCGCCATCTTGTATAGG
>HPVcand62_Alpha_39932599_nt6678_L1_VE_rc|1
ATGGCCCTGCGCCCGCTGCAGCCAATATGGCTTATTAAACAACTGAGAATCAGACGACAC
>HPV48_Gamma_9628542_nt5473_L1_VE_rc|2
ACCCCCTCTGCCTACTTCCAAACCAACAAGTTTCCAAACTAAACGTTCCTTATCTGAATT
>HPV92_Beta_27531786_nt5669_L1_VE_rc|1
TTGAACAAATTCATCCGTACTTTGTACCCTTGCAACCGGTGTTGAAGGGGGTAAGTATAC
>HPV81_Alpha_40804509_nt6765_L1_VE_rc|1
ATTATGGCCCTGTGCCCGTTGTAGCCAATAAGGCTTATTAAACAGCTGGGAATCAGAGGA
>HPV63_Mu_9626605_nt5672_L1_VE_rc|2
ATTGCCTGAAATACCCACACCTAATGGTTGACCCCTACCGATTTCTATGCCTCTAAGACC
>HPV20_Beta_1020162_nt6824_L1_VE_rc|1
GCCCTGTGCGCGCTGCAGCCAAAATGGCCTATTAAACAATTGAGCATCACTAGACACCAA
>HPV37_Beta_1020226_nt5897_L1_VE_rc|1
ATCTGGCAACCTAACCCTAAAAGCTCTGTATTGATTTCCAGATACTTTAGGAACCTCGAT
>HPV36_Beta_1020218_nt6792_L1_VE_rc|1
GTTAAACAATTGAGCATCACTAGACACCAATGAGCCACTAACAGTTGGGTAATACATGGC
>HPV95_Gamma_40804520_nt5489_L1_VE_rc|1
CTTAGGTTCACCTACATCTTTCACTGGGAAATAAGGATGGCCTACAGTTAAAAGTCTTTC
>HPV84_Alpha_12958167_nt6086_L1_VE_rc|1
TAACAGCTGTGTTTGTTTATAATCCACAGAAACATTATCCCTACTGTCAGTGTCCCCAGC
>HPV40_Alpha_397014_nt6530_L1_VE_rc|1
TGGGACACTATCACCAGTAGTACCTGCCCTATTAAAAAAGTGCCTAACAAACATTTGTTC
>HPVFA75/KI88-03_Unclassified_206902429_nt5621_L1_VE_rc|1
AGCTACTGGAGTTGATGGTGGCAAATATACCTTACCACTTGCAGGAAGCCAGAGAGACAT
>HPV56_Alpha_397053_nt5793_L1_VE_rc|1
TGGAAGCCCAAACTTATTAGGGTCGGGCAACCGTACCCTAAATACCCTATATTGATATGC
>HPVFA75/KI88-03_Unclassified_206902429_nt6371_L1_VE_rc|1
AGCGTCTCCAACATTACCTCCTCTGGTAAAATAATGTCTTGCATAGCACTGTTCCCTTCT
>HPV73_Alpha_1491692_nt6065_L1_VE_rc|1
AGCTTTAAAATCCATGGCTCCAAAGCCAACATCTATCATATCACCATCCTGTATAGGGGT
>HPV59_Alpha_557236_nt5697_L1_VE_rc|1

AAAATATGGATGTCCAACTGTAAGAAGTCTGGAACTGCCTGCGTGGTAGAAAATACTGGT
>HPV88_Gamma_167600365_nt6061_L1_VE_rc|1
TCCCATTTTACCTTCACGAGCCATAAAATGGCGAGCATACGACTGCTCACGTTTTCCAAA
>HPV52_Alpha_397038_nt6586_L1_VE_rc|1
CAACTGATTGCCCCAACATATGCCATTATTGTGGCCCTGCGCACGTTGTAACCAGTACGG
>HPV10_Alpha_9627257_nt5860_L1_VE_rc|1
GTTGGTGCGTGTCACATAGTCGTCCGTGCTGAGAACTTTAGACACGGGAGTGGGAGGCAG
>HPV33_Alpha_333049_nt5805_L1_VE_rc|1
GGAGGTGTCAGGAAATCCAAATTTATTAGGATCTGGTAAACGGACCCTAAAAACCCTATA
>HPV48_Gamma_9628542_nt5533_L1_VE_rc|1
ATTTTCTGTGTCCCCTATCTTATTTAGTAAAGGATGACCTGTAGATCCTACGCCAAGAGG
>HPV22_Beta_1020178_nt6563_L1_VE_rc|1
CTTCAACCAAAAAGGCCTGTTGAATAGTTGGGCATCAGAGGTTACTAAAGAACCACTAAC
>HPVcand85_Alpha_4574720_nt5611_L1_VE_rc|1
TTTAGCCACTGAAGGTGGTGGAAGGTATACCTTGCTGTCACTAGAACGCCACAAAGCCAT
>HPV42_Alpha_333211_nt5877_L1_VE_rc|1
GTAGTTGGTGCGTTGCACATATTCATCAGTGCTGACCACCTTGGAAACAGGAGGAGGAGG
>HPV81_Alpha_40804509_nt6735_L1_VE_rc|1
AGGCTTATTAAACAGCTGGGAATCAGAGGATACCATAGACCCACTAGGTGTAGGGGCATA
>HPV2_Alpha_9626032_nt5773_L1_VE_rc|1
AGGTAGGTATACCTTGCTTTCATTAGGCCGCCACAAAGCCATCTGCAAAGAAATAGTGGA
>HPV90_Alpha_22138122_nt6003_L1_VE_rc|1
CGATGCATCAGGAAGGCCAAACTTATTGGGATCAGGCAAACGTACCCTAAACACCCTATA
>HPV26_Alpha_9627305_nt6159_L1_VE_rc|1
AGTAAAATCCATAGCTCCAAAGCCTGTATCAATCATATCGCCATCCTCAATAATGCTGGA
>HPV33_Alpha_333049_nt5655_L1_VE_rc|1
GGAACTACCAGCATAATAATAAATGCTTGTGCGAGACACATATTCATCAGTGCTGACAAC
>HPV106_Alpha_71726710_nt5990_L1_VE_rc|1
TGCATCAGGAAGGCCAAATTTGTTGGGATCAGGCAAACGAACCCTAAATACCCTATATTG
>HPV45_Alpha_397022_nt6551_L1_VE_rc|1
AACAAACAACTGATTATGCCAACAAATACCATTGTTATGGCCCTGGGCCTTATGTAACCA
>HPV41_Nu_9626041_nt5667_L1_VE_rc|2
CACCTCTTTGCCATCCGCATTAGTAATATTGTAAAATGGATGTCCAACAGTAAGCAAACG
>HPV33_Alpha_333049_nt6555_L1_VE_rc|1
AGTACTGCGAGTGGTATCTACCACAGTAACAAATACCTGATTGCCCCAACAAATACCATT
>HPVFA75/KI88-03_Unclassified_206902429_nt6791_L1_VE_rc|2
ATCTTCTAATATACCTGAATTCATTGCATTGATCTGGGCTAGCACATCAGGTTGCAGTGA
>HPV7_Alpha_9627389_nt5979_L1_VE_rc|1
TTTATTAGGGTCGGGCAAGCGTACTCTAAACACTCTGTATTGATGTCCAGACACTTTAGG
>HPV29_Alpha_1020210_nt6744_L1_VE_rc|1
AAATACCTGATTGGCCCAGCATATACCATTGTTGTGTCCCTGGGCCTGCTGTAGCCAGTA
>HPV48_Gamma_9628542_nt6433_L1_VE_rc|2
ATCTTCAATGCCCGTTGGAGCTGGTGGAACAAAAGCTAATTGCCACTCCTCTAATATTCT
>HPVcand89_Alpha_22095322_nt6668_L1_VE_rc|1
ACCATTATTATGGCCCTGTGCACGGTGCAGCCAATAAGGCTTATTAAATAACTGGGAATC
>HPV44_Alpha_1020242_nt6265_L1_VE_rc|1
CAATTCAGCAAAATTCATGGCTCCAAAACCAGTGTCCACCATATCACCATCCTCAATTAC
>HPV19_Beta_396940_nt6765_L1_VE_rc|1
GTTAAACAATTGAGCATCACTGGATACTAATGAGCCACTAACAGTTGGGAAATACATGGC
>HPV103_Unclassified_109390382_nt5036_L1_VE_rc|1
ATATGGGTGTCCTACAGTAAGCAGGCGGTCGCTGGAGGCATGGAAGAAAATAGACGTTTC
>HPV73_Alpha_1491692_nt5525_L1_VE_rc|1
ATTTGTTCTTGTTACATATTCATCTGTGCTTACAACCTTAGACACAGACACAGGGGGCAG
>HPV59_Alpha_557236_nt5787_L1_VE_rc|1
GGGATCAGGTAACTTAACCCTAAATACTCTGTATTGATATGCAGACACCTTAGGAACATC

>HPV52_Alpha_397038_nt5956_L1_VE_rc|1
AGGATGCCCACTAATACCCACACCTAAAGGCTGTCCCCTACCAATTTCCAAGCCTGTACA
>HPV35_Alpha_333050_nt5635_L1_VE_rc|1
AGAACTGCCTGCATGATAGTAGATGTTTGTGCGTGTTACATATTCATCAGTGCTAACAAC
>HPV58_Alpha_222386_nt5956_L1_VE_rc|1
TAAATAAGGATGACCACTTACGCCAACACCCAATGGCTGTCCCCTACCTATTTCAAGGCC
>HPV58_Alpha_222386_nt5686_L1_VE_rc|1
ATAAATGCTTGTGCGTGACACATATTCATCAGTGCTTACAACCTTAGACACAGGCACAGG
>HPV93_Beta_37089393_nt6601_L1_VE_rc|1
TGCCCAACAAATACCATTATTATGACCTTGTGCGCGTTGGAGCCAGAAGGGCCTATTAAA
>HPV58_Alpha_222386_nt5716_L1_VE_rc|1
AGCCAAAAGTCTGGAACTGCCAGCATAATAATAAATGCTTGTGCGTGACACATATTCATC
>HPV69_Alpha_6970418_nt6472_L1_VE_rc|1
CAATTGGTTGCCCCAACAAATGCCATTATTATGACCCTGGGCACGCTGTAACCAGTAAGG
>HPV93_Beta_37089393_nt5671_L1_VE_rc|1
TTGTACATATTCATCCGTGCTTTGTACTCTGGCAACTGGTGTTGAAGGTGGCAAATATAC
>HPV41_Nu_9626041_nt5637_L1_VE_rc|1
GTAAAATGGATGTCCAACAGTAAGCAAACGGTCAGTGGCAGCATGGAGGAAAGTACTGGT
>HPV38_Beta_1020234_nt6412_L1_VE_rc|1
AGCGTCACCCACTGCACCACCTCGAACAAAATAATGTCTGGCATAGCATTGCTCCCGTCG
>HPV102_Alpha_71726718_nt6704_L1_VE_rc|1
ACAAATGCCATTATTGTGTCCCTGGGCCCGTTGAAGCCAGTAAGGCTTATTAAACAGTTG
>HPV97_Alpha_71726694_nt6407_L1_VE_rc|1
ACAAATACCATTGTTATGGCCCTGGGCCCTATGCAACCAATATGGCTTATTAAACAACTG
>HPV10_Alpha_9627257_nt6400_L1_VE_rc|1
TAAAGCAGTAAAGTCCATGGCCCCATAGCCAGTGTCCACCATATCACCATCCTGAATAGG
>HPV38_Beta_1020234_nt5722_L1_VE_rc|1
GCTAGTTGCATGGTAATAGATGTCTGTTCGTTCCACATATTCATCCGTGCTTTGAACGCG
>HPVFA75/KI88-03_Unclassified_206902429_nt6521_L1_VE_rc|1
CCAAAAAGGCCTATTAAATAACTGAGCATCTGAAGAGACTAGAGAACCACTAACAGTAGG
>HPV58_Alpha_222386_nt6586_L1_VE_rc|1
AACCACGGTAACAAATAACTGATTGCCCCAGCAAATGCCATTGTTATGACCTTGTGCACG
>HPV42_Alpha_333211_nt6147_L1_VE_rc|1
ATTCAATAATGGATGGCCACTAATACCAACGCCCAAAGGCTGTCCACGACCTACTTCTAG
>HPV41_Nu_9626041_nt5847_L1_VE_rc|1
AGGGTTCCCTGTTACACCAATACCTAAGGGCTGTCCCCTAGAAACCTCAATCCCACGAAT
>HPV50_Gamma_9628550_nt5562_L1_VE_rc|1
ATTTTCTGTGTCTCCAACCTTATTAAACAAGGGATGACCAGTAGATCCAACACCTAAAGG
>HPV103_Unclassified_109390382_nt5066_L1_VE_rc|1
TGTACCTTCACCCACTTTTGTAATTGGAAAATATGGGTGTCCTACAGTAAGCAGGCGGTC
>HPV52_Alpha_397038_nt5716_L1_VE_rc|1
TGTTAGTAATCGAGAACTGCCTGCATAATAATAGATGCTTGTGCGAGACACATACTCATC
>HPV92_Beta_27531786_nt6599_L1_VE_rc|1
CAGTTCATTACTCCAGCAAATACCATTGTTGTGGCCTTGTGCTCTTTGTAACCAATATGG
>HPV92_Beta_27531786_nt6569_L1_VE_rc|1
GTGGCCTTGTGCTCTTTGTAACCAATATGGCCGATTAAATAATTGTGCATCAGTGGATAC
>HPV21_Beta_1020170_nt6830_L1_VE_rc|1
CAATTGAGCGTCACTAGACACCAATGAGCCACTGACAGTTGGGAAATACATGGAGTTACC
>HPV9_Beta_9627396_nt5776_L1_VE_rc|1
TTCCACATATTCATCGGTGCTTTGAACTCTCGCCACTGGTGTTGCTGGTGGCAAATATAC
>HPV32_Alpha_9627327_nt6443_L1_VE_rc|1
CAAAGCACTAAAGTCCATTGCTCCAAACCCTACATCTGCCATATCACCATCCTGAATAAC
>HPV76_Beta_2911551_nt5830_L1_VE_rc|1
AATGTATTCATCCGTGCTTTGAACCCTGGCCACAGGTGTTGATGGTGGAAGATAAACCTT
>HPV97_Alpha_71726694_nt6077_L1_VE_rc|1

CTGTAATGTACTAAAGTCCATGGCACCATAACCTGTATCTACCATATCGCCATCCTCCAA
>HPV1_Mu_9626063_nt6014_L1_VE_rc|1
GTCTTGCTGTAAAGCAGCAAAATCCATAGCCCCAAAACCAATATCCATCATGTCACCATC
>HPV11_Alpha_333026_nt6702_L1_VE_rc|1
CACAGTAACAAACAAGTGGTTTCCCCAGCAAATACCATTGTTATGTCCCTGAGCCTTTTG
>HPV66_Alpha_1020290_nt6608_L1_VE_rc|1
GTTGGTGCTTCTGGTAGTATCCACAACAGTAACAAATACCTGATTACCCCAGCATATGCC
>HPV34_Alpha_9627334_nt6421_L1_VE_rc|1
ATTATGCCAGCAAATGCCATTGTTTTGTCCCTGGCCTTTTGCAACCAATAAGGCTTATT
>HPV107_Beta_126131393_nt6749_L1_VE_rc|1
AACAAACATTTGGTTTCCCCATAGAATACCATTATTGTGGCCTTGTGCACGTTTCAACCA
>HPV65_Gamma_312100_nt5447_L1_VE_rc|1
GTGTTGATCCTGCACATCTTTCACTGGAAAATAAGGATGGCCTACAGTTAAAAGCCTTTC
>HPV30_Alpha_396973_nt6592_L1_VE_rc|1
AACAGTAACAAATACCTGGTTGCCCCAACAAATGCCATTATTGTGTCCCTGTGCGCGTTG
>HPV34_Alpha_9627334_nt6181_L1_VE_rc|1
CCACATAGAATCGCCATAGGGATCTGCGGCCATGCCTAGATAATCTGGATATTTACATAT
>HPV70_Alpha_1173493_nt5921_L1_VE_rc|1
TTCAGTATCATCCAATCTATTATATAAAGGATGTCCACTAACGCCAACGCCCAATGGCTG
>HPV58_Alpha_222386_nt6616_L1_VE_rc|1
TAATGTCATATTAGTGCTACGAGTGGTATCAACCACGGTAACAAATAACTGATTGCCCCA
>HPV96_Beta_50253426_nt5917_L1_VE_rc|1
ATTAGCATGATAATAGATGTTTGTTCTTTGTATGTAGGAGTCCGTGCTTTGCACCCTGGC
>HPV96_Beta_50253426_nt6187_L1_VE_rc|1
TTTCACCTTGTTAAATAATGGATGTCCTGATGTACCTACACCTAATGGCTGTCCACGACC
>HPV49_Beta_9627363_nt5842_L1_VE_rc|1
AATGTATTCATCCGTGCTTTGTACCCTTGCCACAGGTGTTGAAGGTGGTAGATATACCTT
>HPV82_Alpha_6970427_nt5872_L1_VE_rc|1
AGGATGACCACTAAGGCCAACACCTAACGGCTGACCCCTACCTACTTCAACACCAACACA
>HPV50_Gamma_9628550_nt6462_L1_VE_rc|1
ACCACTGGGAGCTGGTGGAACATAAGCTAATTGCCAATTTTCCAAAATTCTGGGGTCCAT
>HPV103_Unclassified_109390382_nt5546_L1_VE_rc|1
TACACCTGAATGGTCCTCCTGCAATGTAGCAAAATTCATTGCACCAAAGCCAATATCACA
>HPV53_Alpha_9627377_nt6526_L1_VE_rc|1
CCAATATGGCTTATTAAACAATTGAGCCTCTGAAGTTATCATAGACCCACTAGGTGTAGC
>HPV70_Alpha_1173493_nt6521_L1_VE_rc|1
AGTAATAAACAACTGGTTATGCCAACAAATGCCATTATTGTGTCCCTGGGCCTTATGCAA
>HPV34_Alpha_9627334_nt5731_L1_VE_rc|1
CTTGTCAGGATTATAAAAAGATGCATCTGGGAACCCAAATTTATTGGGATCTGGCAAACG
>HPV96_Beta_50253426_nt5887_L1_VE_rc|1
TATGTAGGAGTCCGTGCTTTGCACCCTGGCAACTGGTGTTGATGGTGGTAAATAGACCTT
>HPV17_Beta_396932_nt5755_L1_VE_rc|1
TTCCACATACTCATCCGTGCTTTGTACTCGGGCTACTGGTGGTGTTGGAGGCAAGTATAC
>HPV82_Alpha_6970427_nt6142_L1_VE_rc|1
AGCAAAATCCATGGCCCCAAAACCAGTGTCCACCATATCGCCATCCTCAATGACAGTAGA
>HPV37_Beta_1020226_nt5987_L1_VE_rc|1
TCGGCCTATCTCCAAGCCCGCACAGGCCCACACCAACCTTTCCTTTTCTGGATTATATAC
>HPV9_Beta_9627396_nt6676_L1_VE_rc|1
GTTGTGACCTTGTGCTCTTTGGAGCCAAAATGGCCTATTAAACAATTGAGCATCTGAAGT
>HPVRTRX7_Unclassified_30315616_nt6882_L1_VE_rc|1
CTGATTAGCCCACAGAATGCCATTATTATGACCCTGTGCTCTTTGCAGCCAGAAGGGCCT
>HPV71_Alpha_12084981_nt5807_L1_VE_rc|1
TACCTTGCTGTCACTAGGCCGCCACAAAGCCATCTGCAAAAAAATAGTGGACACGTTTAG
>HPV84_Alpha_12958167_nt6596_L1_VE_rc|1
ATTAAACCAGCATATACCATTGTTATGACCCTGGGCCTTTTGCAACCAGTATGGCTTATT

>HPV80_Beta_2911565_nt5749_L1_VE_rc|1
TTCCACATATTCATCGGTGCTTTGTACACGTGCTACAGGTGGTGTTGGTGGTAAATACAC
>HPV65_Gamma_312100_nt5417_L1_VE_rc|1
ATAAGGATGGCCTACAGTTAAAAGCCTTTCTGTTCCAGCGTGGAAATAAAGAGATGTTCC
>HPV11_Alpha_333026_nt6432_L1_VE_rc|1
ACCATAAGGGTCTGCAGCCATTTGCAAATAATCAGGATATTTGCAGACAGTTCCACAAAT
>HPV74_Alpha_27462483_nt6024_L1_VE_rc|1
TTCACCCAACGGGGGAGCACAGCCTACCATGCATAACTGTGTTTGCTTATAATCCATAGC
>HPV31_Alpha_333048_nt6483_L1_VE_rc|1
TAACTGATTGCCCCAACAAATACCATTATTGTGTCCCTGAGCACGTTGCATCCAATATGG
>HPV101_Unclassified_109390389_nt5608_L1_VE_rc|1
AAAATTCATGGCACCAAAGCCAATATCACACATATCCCCATCCTGAATAACAGAGGACAC
>HPV52_Alpha_397038_nt6406_L1_VE_rc|1
CACAGGGTCACCTAAGGTACCGGCCCTATTAAAAAAGTGTCTAACAAACATTTGCTCACG
>HPV22_Beta_1020178_nt6143_L1_VE_rc|1
ACTGCCTGCATCTTCACAAACAGGAGCTTTATCCCAATATTCACCTAAACACGGTATACA
>HPV83_Alpha_5059324_nt6673_L1_VE_rc|1
CTCATTAAACCAACAAATGCCATTATTATGTCCCTGGGCACGATGCAGCCAGTAGGGCTT
>HPV28_Alpha_1020202_nt6311_L1_VE_rc|1
AGCAAAGTCCATTGCACCAAAGCCAGTGTCCACCATATCACCATCCTGAATAGGGGCAGT
>HPV88_Gamma_167600365_nt5431_L1_VE_rc|1
GTTGGGATCTCCATCATTCCTAATTTCATAGTAGGGATGGTCTACTGTTAACAGACGATC
>HPV53_Alpha_9627377_nt6586_L1_VE_rc|1
AGTTACAAATAACTGATTGTTCCAACAGATGCCATTATTATGTCCCTGGGCACGTTGCAG
>HPV82_Alpha_6970427_nt6472_L1_VE_rc|1
ATTGTGGCCCTGGGCGCGATGCAACCAATAAGGCTTATTAAAAATCTGAGAATCAGAGGT
>HPV107_Beta_126131393_nt6539_L1_VE_rc|1
AGCATCACCCACAGCTCCTCCTCTAGTGAAATAATGTCTAGCATAACATTGTTCTCTCCT
>HPVcand62_Alpha_39932599_nt5778_L1_VE_rc|1
CTTAGAAACAGGAGTGGGAGGCAGGTATACCTTGCCGTCACCAGGCCGCCACATAGCCAT
>HPV8_Beta_333074_nt6812_L1_VE_rc|1
CATTTGATTAGCCCAGAGAATGCCATTATTATGACCCTGGGCACGCTGCAGCCAGAAAGG
>HPV17_Beta_396932_nt5935_L1_VE_rc|1
TGACATATCTGCCAAGGCAAACTTATTAGGATCTGGTAATGTGACTCTGAAGGCTCTATA
>HPV70_Alpha_1173493_nt6491_L1_VE_rc|1
GCCATTATTGTGTCCCTGGGCCTTATGCAACCAATAGGGCTTATTAAACAACTGGGAATC
>HPV91_Alpha_22023568_nt6316_L1_VE_rc|1
GCCCCAATATTCGCCCATGGGAGGTGTACAGCCAACAATACATAGCTGTGTTTGTTTATA
>HPV43_Alpha_40804474_nt6328_L1_VE_rc|1
GGAAGCAAAATCCATTGCACCAAATCCTGTATCTACCATGTCACCATCCTGTATAACTTC
>HPVcand89_Alpha_22095322_nt6698_L1_VE_rc|1
AGTAACAAACAACTCATTAAACCAACAAATACCATTATTATGGCCCTGTGCACGGTGCAG
>HPV17_Beta_396932_nt6145_L1_VE_rc|1
GCCTACAACAAACATCTGCACTTGTTTAGGGTCAAATGACGTGTTTTGTCTATCATCAGT
>HPV63_Mu_9626605_nt6752_L1_VE_rc|2
TGCAAGACCACTTTGATACAAAAATTTGCGGCCTAAAGGAAATTGATCAAGCTGCTCCGA
>HPV3_Alpha_397005_nt5818_L1_VE_rc|1
CAAGCGAGAACTGCCTGCATAATAATAAATGTTGGTGCGTGTCACATAGTCGTCCGTGCT
>HPV4_Gamma_9626597_nt5466_L1_VE_rc|1
GTGAGGTTCCTGTACATCTTTCACTGGAAAATAAGGATGGCCTACAGTTAAAAGCCTTTC
>HPV12_Beta_396910_nt6805_L1_VE_rc|1
CTGATTAGCCCACAGGATGCCATTATTATGACCCTGTGCTCTTTGAAGCCAGAAGGGCCT
>HPV37_Beta_1020226_nt6467_L1_VE_rc|1
ATCACCTACATTTCCCCCTCTTACAAAATAATGTCTGGCATAACATTGTTCTCTCCTAGC
>HPV48_Gamma_9628542_nt5773_L1_VE_rc|1

CAACTTAGGAAAATTAGCAGCCCCAAAGCCTATATCTCCCATATCACCATCCTGAATAAC
>HPV28_Alpha_1020202_nt6671_L1_VE_rc|1
AAACAATTGGTTGGCCCAACAGATACCATTATTGTGTCCCTGAGCACGCCGCAGCCAATA
>HPV66_Alpha_1020290_nt6578_L1_VE_rc|1
AACAAATACCTGATTACCCCAGCATATGCCATTATTATGGCCCTGTGCACGTTGCAACCA
>HPV4_Gamma_9626597_nt5436_L1_VE_rc|1
ATAAGGATGGCCTACAGTTAAAAGCCTTTCTGTACCAGCGTGGAAATACAGAGATGTTCC
>HPV54_Alpha_9628437_nt6515_L1_VE_rc|1
GCCCCAACAAATACCATTGTTTTGACCCTGGGCCCGTTGTAACCAGTATGGCTTATTAAA
>HPV39_Alpha_333245_nt6574_L1_VE_rc|1
AAGAAATAATTGATTATGCCAACATATACCATTGTTGTGGCCCTGGGCCTTATGTAGCCA
>HPV22_Beta_1020178_nt6623_L1_VE_rc|1
AGCTACTGTTACAAACATTTGGTTGTTCCACAAAATACCATTATTATGGCCCTGCGCGCG
>HPV67_Alpha_3228267_nt5650_L1_VE_rc|1
TGTGCGTGACACATATTCATCAGTGCTTACAACCTTAGATACAGGCACAGGAGGCAGGTA
>HPV61_Alpha_9628574_nt6665_L1_VE_rc|1
GTGGCCCTGGGCACGTTGCAGCCAATATGGCTTATTAAATAATTGGGAATCAGAGGACAC
>HPV84_Alpha_12958167_nt6566_L1_VE_rc|1
CTGGGCCTTTTGCAACCAGTATGGCTTATTAAACAATTGGGAATCCGAGGACACCATAGA
>HPVcand62_Alpha_39932599_nt6438_L1_VE_rc|1
AGGTTCTGCAGCCATTTGCAAATAGTCAGGATATTTACATGTGGAGGTGCATATATCCAA
>HPV101_Unclassified_109390389_nt5638_L1_VE_rc|1
TACCCCTGAGTGATCAGCCTGCAAGGCAGCAAAATTCATGGCACCAAAGCCAATATCACA
>HPV39_Alpha_333245_nt6544_L1_VE_rc|1
ATTGTTGTGGCCCTGGGCCTTATGTAGCCAATAAGGCTTATTAAATAACTGGGAATCAGA
>HPV111_Beta_186461202_nt6553_L1_VE_rc|1
ATTGTTGTGGCCTTGTGCACGTTGCAACCAAAATGGTCTATTAAATAGCTGCGCATCTGA
>HPV68a_Alpha_71726685_nt5669_L1_VE_rc|1
AGGATCAGGTAAGGTAACCCTAAACACTCTGTATTGATATGCAGACACCTTAGGAATGCC
>HPV90_Alpha_22138122_nt6753_L1_VE_rc|1
TGTAGTATCAACCACAGTTACAAAAAGCTGATTACCCCAGCAGATACCATTGTTAGGGCC
>HPV61_Alpha_9628574_nt5765_L1_VE_rc|1
TTTAGACACAGGAGTGGGAGGCAGGTATACCTTGCCGTCACCAGGCCGCCACAAAGCCAT
>HPV7_Alpha_9627389_nt6549_L1_VE_rc|1
TGGAACACTGTCTCCAGTAGTTCCTGCCCTATTAAAAAAGTGCCTAACAAACATTTGTTC
>HPV93_Beta_37089393_nt6391_L1_VE_rc|1
TCCTACGTTGCCACCTCTTACAAAAAAGTGTCTGGCATAACATTGCTCTCTGCGAGCAAA
>HPV34_Alpha_9627334_nt5761_L1_VE_rc|1
AACGCCTGCACAGGCCCAAACCAACCGCTCCTTGTCAGGATTATAAAAAGATGCATCTGG
>HPV101_Unclassified_109390389_nt6358_L1_VE_rc|1
ATCAACCACCCAAAAAGACATTTTGTCATATGGATCTTCCTTTTCCTTAGGAGGCTCTGC
>HPV51_Alpha_333087_nt6092_L1_VE_rc|1
AGCGAAATCCATAGCTCCAAACCCTGTATCAATCATATCGCCATCCTGAATGACAGAGGA
>HPV72_Alpha_1491683_nt5764_L1_VE_rc|1
GTATACCTTGCCGTCACCAGGCCGCCACAAAGCCATCTGCAAAAGAATAGGGCACACGTT
>HPV41_Nu_9626041_nt6507_L1_VE_rc|1
GGCCTCGTTGTGCCACAGTATGCCATTGTTATGGCCCTGGGATCTCTGCAGCCAAAAGGG
>HPV26_Alpha_9627305_nt5589_L1_VE_rc|1
CCGAGACACAGGGGTGGGAGGAAGATATACCTTGCTGTCACTAGTACGCCACAAAGCCAT
>HPV11_Alpha_333026_nt6342_L1_VE_rc|1
TAAGTCTGCAAAATTCATAGCACCAAAGCCTGTATCAACCATGTCCCCATCCTGTATAAC
>HPV6_Alpha_6002612_nt6721_L1_VE_rc|1
TACCACAGTAACAAACAGTTGATTACCCCAACAAATACCATTGTTATGTCCCTGGGCTTT
>HPV57_Alpha_60882_nt5734_L1_VE_rc|1
AGGCAGGTATACCTTGCTTTCATTAGGCCGCCACATAGCCATCTGCAAAGAATAGTGCA

>HPV110_Beta_186461194_nt6007_L1_VE_rc|1
AGTATCTCTAACTTTATTAAATAAGGGATGACCACTGGTACCTACCCCTAAAGGTTGCCC
>HPV73_Alpha_1491692_nt6185_L1_VE_rc|1
CCTACGAAGATAAAACCACATGGAATCACCATAGGGATCAGCAGCCATGCCTAAATAATC
>HPV83_Alpha_5059324_nt6313_L1_VE_rc|1
AAAATCCATGGCACCATAGCCTGTTTCCACCATGTCGCCATCCTGTATAGTAGAATTTAC
>HPV52_Alpha_397038_nt6616_L1_VE_rc|1
GCTACGAGTGGTATCCACAACTGTGACAAACAACTGATTGCCCCAACATATGCCATTATT
>HPV110_Beta_186461194_nt5887_L1_VE_rc|1
AGACATGTCTGCTAATGCAAACCTATTAGGATCAGGCAATTGCACCCGGAATGCTCTGTA
>HPV3_Alpha_397005_nt6688_L1_VE_rc|1
AAACAATTGGTTGGCCCAACATATACCATTATTGTGTCCCTGAGCACGCCGCAGCCAATA
>HPVFA75/KI88-03_Unclassified_206902429_nt6101_L1_VE_rc|1
ATCTCCTGCATTTTCACATGGCTCAGCTTTATCCCAGTATTCTCCTAAACATGGTGTACA
>HPV43_Alpha_40804474_nt5818_L1_VE_rc|1
ACGTGAACTGCCAGCATAATAAAATAAGTTGGTGCGTTGCACATATTCATCTGTGCTCAC
>HPV9_Beta_9627396_nt6496_L1_VE_rc|1
GTCACCAACTGAACCTCCTCTAACATAATAATGCCTGGCATAACATTGTTCTCTTCTTGC
>HPV38_Beta_1020234_nt5782_L1_VE_rc|1
TTTTTGACCATCCTGTGATCTGACATCAAAATATGGATGGCCTACTGTTAATAGGCGATC
>HPV103_Unclassified_109390382_nt5846_L1_VE_rc|2
TGGTCTGCCAAACAACTGAGAATCACTAGAAACCAGTGATCCACTGGGGGTTGCATAGTA
>HPV44_Alpha_1020242_nt6625_L1_VE_rc|1
TACAACAGTAACAAATAACTGATTTCCCCAACAAATACCATTATTGTGGCCCTGCGCCTT
>HPV56_Alpha_397053_nt6513_L1_VE_rc|1
ACCCCAGCAAATGCCATTATTATGGCCTTGGGCACGTTGCAACCAATAAGGTTTATTAAA
>HPV53_Alpha_9627377_nt5656_L1_VE_rc|1
CTTTGATACAGGGGTAGGAGGCAGATAAACCTTGCTGTCACTAGGCCGCCACACCGCCAT
>HPV60_Gamma_9628566_nt6686_L1_VE_rc|2
ATAAGGGTCTTCCCTTTCAGTATTAGGAACATCTGTTGGACACATTGTTGCCTGAGACAT
>HPV68a_Alpha_71726685_nt6389_L1_VE_rc|1
GTTGTGTCCCTGTGCCTTGTGCAGCCAATAGGGCTTGTTAAATAACTGGGAGTCAGAGGA
>HPV14D_Beta_396918_nt6504_L1_VE_rc|1
CAGTTGAGCATCACTGGATACCAATGAGCCACTGACAGTTGGGAAATACATGGCATTGCC
>HPV38_Beta_1020234_nt5692_L1_VE_rc|1
TTCCACATATTCATCCGTGCTTTGAACGCGCGCAACTGGAGGTGTTGGTGGCAAGTATAT
>HPV23_Beta_1020186_nt5781_L1_VE_rc|1
TTTACTACCATCCGGTGATCTAACATCAAAATATGGGTGGCCTACAGTTAGTAATCGATC
>HPV35_Alpha_333050_nt6475_L1_VE_rc|1
ATGGCCTTGTGCACGTTGCAACCAATATGGTTTATTAAATATTTGTGCATCGGAGGTTAC
>HPV39_Alpha_333245_nt6064_L1_VE_rc|1
ACAGCCTATAATGCACAACTGTGTCTGTTTATAATCCACAGACACATTATCCCTACTGTC
>HPV31_Alpha_333048_nt6543_L1_VE_rc|1
TGCAATTGCAGCACAAACAGACATATTGGTACTACGTGTGGTATCTACCACAGTAACAAA
>HPV59_Alpha_557236_nt6057_L1_VE_rc|1
TGTCCAGTGTTCTCCAATGGCAGGTACACAGCCAATAATACACAGCTGAGTTTGTTTATA
>HPV60_Gamma_9628566_nt6266_L1_VE_rc|2
TTGATTATCACTAGAAGCTACAGAACCACTTGGTGTAGGGAAGTAACTATAACCACCAAG
>HPV54_Alpha_9628437_nt6545_L1_VE_rc|1
GGTATCTACAACTGTTAAAAACACCTGATTGCCCCAACAAATACCATTGTTTTGACCCTG
>HPV74_Alpha_27462483_nt6174_L1_VE_rc|1
TTTAAGAGGCTGCAATTCAGCAAAATTCATGGCACCAAAGCCTGTATCTACCATATCACC
>HPV65_Gamma_312100_nt5777_L1_VE_rc|1
CCAATGTTCCCCAATAGCAGGAGTGCAACCTATGATAAACATTTGTGTCTGTTTTGGATC
>HPV51_Alpha_333087_nt6452_L1_VE_rc|1

AAAAAGCTGATTGTTCCAGCAAATGCCATTATTGTGACCCTGCGCACGGTGGAGCCAATA
>HPV40_Alpha_397014_nt5960_L1_VE_rc|1
CTTATTCGGGTCAGGCAAACGTACCCTAAATACCCTGTATTGATGTCCAGAAACCTTAGG
>HPV23_Beta_1020186_nt6621_L1_VE_rc|1
TGCTACAGTCACAAACATCTGGTTGTTCCATAAAATACCATTGTTATGGCCTTGAGCACG
>HPV3_Alpha_397005_nt6718_L1_VE_rc|1
AGTACTACGTGTGGTATCCACCACAGTCACAAACAATTGGTTGGCCCAACATATACCATT
>HPV37_Beta_1020226_nt5747_L1_VE_rc|1
TTCCACATAATCATCCGTGCTTTGCACCCGGGCTACTGGTGGTGTTGGAGGCAAGTATAC
>HPV111_Beta_186461202_nt6343_L1_VE_rc|1
GTAATGTCTGGCATAACATTGCTCCCTTCTGGCAAAAAAGAAACAAGCGTCCCCATAGAC
>HPV25_Beta_396948_nt6790_L1_VE_rc|1
GAATAATTGAGCATCACTGGATACCAATGAGCCACTGACTGTTGGGAAATACATTGAGTT
>HPV21_Beta_1020170_nt5960_L1_VE_rc|1
TTGTACATATTCATCCGTGCTTTGGACCCTGGCAACTGGTGTAGACGGTGGAAGGTAAAC
>HPV103_Unclassified_109390382_nt5486_L1_VE_rc|1
CATATCCCCATCCTCAATAATGGAACTAACAAGCTCTAAAGGAGGACATGCACCCATATC
>HPV60_Gamma_9628566_nt6236_L1_VE_rc|2
TGGTGTAGGGAAGTAACTATAACCACCAAGGTTATTTTGAGGTTTATCAGTTTGTGGTGC
>HPV26_Alpha_9627305_nt6489_L1_VE_rc|1
ATTATGACCCTGTGCACGTTGTAACCAGTATGGCTTATTAAATAGTTGTGCATCCGAAGT
>HPV53_Alpha_9627377_nt6556_L1_VE_rc|1
GCCATTATTATGTCCCTGGGCACGTTGCAGCCAATATGGCTTATTAAACAATTGAGCCTC
>HPV1_Mu_9626063_nt5714_L1_VE_rc|1
TCCTATACCTAAAGGCTGGCCTCTACCTATCTCTATCCCTCTTAGGCCCCAAACTAATCT
>HPV40_Alpha_397014_nt6350_L1_VE_rc|1
GGAAGCAAAATCCATAGCACCAAAGCCAGTATCCACCATGTCGCCATCCTGAATAACCTC
>HPV51_Alpha_333087_nt5972_L1_VE_rc|1
AATACCCCAGTGTTCCCCAATAGGTGGAGCACAGCCTATTATACATAACTGAGTCTGTTT
>HPV1_Mu_9626063_nt5984_L1_VE_rc|1
CCCAAAACCAATATCCATCATGTCACCATCTTCTATGACAGACTCTATCATTTGCACCCT
>HPV40_Alpha_397014_nt6380_L1_VE_rc|1
CAATGGCACATCACTTTTATTGGCCTGCAAGGAAGCAAAATCCATAGCACCAAAGCCAGT
>HPV25_Beta_396948_nt6820_L1_VE_rc|1
TTGTGCTCGCTGTAACCAAAATGGCCTATTGAATAATTGAGCATCACTGGATACCAATGA
>HPV56_Alpha_397053_nt6483_L1_VE_rc|1
GGCACGTTGCAACCAATAAGGTTTATTAAATAACTGTGCCTCAGACGTAATCATAGACCC
>HPV65_Gamma_312100_nt5807_L1_VE_rc|1
AACAGGACTTGCACAAGGCTCAGCCTTATCCCAATGTTCCCCAATAGCAGGAGTGCAACC
>HPVcand87_Alpha_14475578_nt6443_L1_VE_rc|1
GAACATGCAATCGCCATAGGGCTCAGCAGCCATTTGCAAATAGTCAGGATACTTGCAAAC
>HPV48_Gamma_9628542_nt6133_L1_VE_rc|1
CCAACAAATGCCATTATTTGTTCCCTGGGCTCGTCTAAGCCAATAAGGCCTATTAAATAA
>HPV92_Beta_27531786_nt6869_L1_VE_rc|1
GTATGCATCCTGAATAGGGTTATCTGGAGTAGGAACAAATCCTAACTGCCAGTCCTCCAA
>HPV58_Alpha_222386_nt6226_L1_VE_rc|1
CTGCAATGTACCAAAGTCCATGCATCCAAACCCTGTATCTACCATGTCACCATCCTCAAT
>HPV54_Alpha_9628437_nt6275_L1_VE_rc|1
GTCCCCATATGCCTCTGCAGCCATTTTAAGGTAATCAGGATATTTACAAATTGAGGTAGC
>HPV10_Alpha_9627257_nt6730_L1_VE_rc|1
TATACCATTGTTGTGCCCCTGGGCCCGCCGCAGCCAATATGGCTTATTAAACAATTGAGC
>HPV80_Beta_2911565_nt6469_L1_VE_rc|1
ATCGCCTACTGCACCTCCTCTAACAAAATAATGTCTAGCATAACATTGCTCTCGTCTTGC
>HPV92_Beta_27531786_nt5969_L1_VE_rc|1
GTCCAATACCTTATTAAATAATGGATGACCTGTGGTGCCAACACCTAAAGGCTGCCCTCG

>HPV33_Alpha_333049_nt6345_L1_VE_rc|1
AGCCTCTCCTAATGTACCAGCCCTATTAAAAAGTGTCTTACAAACATTTGTTCACGTCG
>HPV10_Alpha_9627257_nt6700_L1_VE_rc|1
CAGCCAATATGGCTTATTAAACAATTGAGCCTCAGACGTTACCATGGACCCACTGGGTGT
>HPV38_Beta_1020234_nt6112_L1_VE_rc|1
ATCCCAGTATTCTCCTAGACAAGGAGTGCAGCCTATTATAAACATTTGCACCTGTTTAGG
>HPV66_Alpha_1020290_nt5648_L1_VE_rc|1
CTTTGAAACAGGTGTTGGAGGTAGGTACACCTTATTGTCACTAGGCCGCCACATCGCCAT
>HPV30_Alpha_396973_nt6562_L1_VE_rc|1
AATGCCATTATTGTGTCCCTGTGCGCGTTGCAACCAGTAAGGTTTATTAAACAACTGAGC
>HPV32_Alpha_9627327_nt6803_L1_VE_rc|1
AAACACTTGATTACCCCAACATATACCATTATTGTGGCCTTGTGCCTGCTGTAACCAATA
>HPV2_Alpha_9626032_nt6853_L1_VE_rc|1
CTTGCACAGTTGGAAGATGAACTGCAAATCATATTCCTCCATATGCCTGAGATATTCCTT
>HPV16_Alpha_9627100_nt6580_L1_VE_rc|1
AACAACAGTAACAAATAGTTGGTTACCCCAACAAATGCCATTATTGTGGCCCTGTGCTCG
>HPV5_Beta_9627145_nt6668_L1_VE_rc|1
GTCATCACCAGTTTTTCCCCCTCTAACAAAAAAGTGTCTGGCATAACATTGCTCCCTACG
>HPV30_Alpha_396973_nt6532_L1_VE_rc|1
CAACCAGTAAGGTTTATTAAACAACTGAGCCTCAGAGGTTACCATAGACCCACTAGGAGT
>HPV80_Beta_2911565_nt6019_L1_VE_rc|1
GCCTGATGTGCCTACACCTAATGGTTGTCCTCTGCCTATTTCTAGGCCTGCACAGGCCCA
>HPV90_Alpha_22138122_nt5973_L1_VE_rc|1
ATCAGGCAAACGTACCCTAAACACCCTATATTGATATCCAGACACCTTGGGAACCACTAT
>HPV50_Gamma_9628550_nt5532_L1_VE_rc|1
GGGATGACCAGTAGATCCAACACCTAAAGGCCCACCTCTCCCGACTTCTATACCTACTAG
>HPV111_Beta_186461202_nt6373_L1_VE_rc|1
TGCATCTCCTACATTGCCTCCTCTAACAAAGTAATGTCTGGCATAACATTGCTCCCTTCT
>HPV29_Alpha_1020210_nt5874_L1_VE_rc|1
AGAACTGCCTGCATAATAATAAATATTTGTGCGTGTCACATAGTCGTCCGTGCTGATAAC
>HPV23_Beta_1020186_nt5721_L1_VE_rc|1
ACTAGTTGCATGGTAATAGATGTCAGTTCTTTCCACATATTCATCCGTACTCTGCACTCG
>HPV42_Alpha_333211_nt5817_L1_VE_rc|1
TAGATAAACCTTGTTGTCACTAGGCCGCCACACGGACATCTGCAAAAAAATATGGTACAG
>HPV96_Beta_50253426_nt6817_L1_VE_rc|1
GATTTGATTACCCCAGCAAATACCATTATTATGGCCTTGAGCCCTTTGCAGCCAAAAAGG
>HPV33_Alpha_333049_nt5925_L1_VE_rc|1
AGTGTCATCAAATTTGTTTAATAAAGGATGACCACTTATGCCAACGCCTAATGGCTGCCC
>HPV13_Alpha_85827580_nt6043_L1_VE_rc|1
TAATAATGGATGACCACTAATACCAACACCTAAGGGTTGACCCCTACCAACCTCTAAACC
>HPV69_Alpha_6970418_nt6502_L1_VE_rc|1
ACTGCGGGTAGTATCTACACAAGTAACAAACAATTGGTTGCCCCAACAAATGCCATTATT
>HPV15_Beta_396924_nt5734_L1_VE_rc|1
CTCCACATATTCATCGGTGCTTTGTACACGTGCTACAGGTGGTGTTGGTGGCAAATATAC
>HPV4_Gamma_9626597_nt5796_L1_VE_rc|1
CCAATGTTCACCTATTGCAGGAGTGCAACCTATAATAAACATCTGTGTTTGTTTTGGGTC
>HPV69_Alpha_6970418_nt6442_L1_VE_rc|1
ATGACCCTGGGCACGCTGTAACCAGTAAGGTTTATTAAACAATTGAGCATCAGAGGTCAC
>HPV33_Alpha_333049_nt5625_L1_VE_rc|1
GCGAGACACATATTCATCAGTGCTGACAACTTTAGATACAGGTACAGGAGGCAGGTACAC
>HPV23_Beta_1020186_nt6891_L1_VE_rc|1
CTTTGAAGCCAAATATCTGTATGTGTCATGAACTGCATTATCTGGTGTAGGAACAAACCC
>HPV27_Alpha_396964_nt6668_L1_VE_rc|1
CCAGCACATACCATTATTATGTCCCTGGGCCCTCCGTAGCCAGTAGGGCTTATTAAACAA
>HPV55_Alpha_1020266_nt6625_L1_VE_rc|1

```
TACAACAGTAACAAATAACTGATTCCCCCAACAAATACCATTATTGTGGCCCTGCGCCCT
>HPV61_Alpha_9628574_nt6635_L1_VE_rc|1
CTTATTAAATAATTGGGAATCAGAGGACACCATAGAGCCACTAGGCGTAGGGGAATAAAT
>HPV88_Gamma_167600365_nt6721_L1_VE_rc|1
CCTGACACGTTTTGTGGGCAGCAAACCACTTTGAAACAAAAATTTGCGACCTAAGCTAAA
>HPV14D_Beta_396918_nt6564_L1_VE_rc|1
AAACCAACAAATACCATTATTGTGGCCTTGTGCGCGCTGTAACCAAAATGGTCTATTGAA
>HPV102_Alpha_71726718_nt6464_L1_VE_rc|1
CATACAGTCTCCGTAGGGCTCAGCCGCCATTTGTAAATAATCAGGATATTTACAAATACC
>HPVcand62_Alpha_39932599_nt6708_L1_VE_rc|1
CAGTTCATTAAACCAACAAATACCATTATTATGGCCCTGCGCCCGCTGCAGCCAATATGG
>HPV32_Alpha_9627327_nt6173_L1_VE_rc|1
AGGATGCCCACTAAGACCAACACCTAAAGGCTGTCCACGGCCAACCTCCAAACCCACACA
>HPV21_Beta_1020170_nt6860_L1_VE_rc|1
TGCACGCTGTAGCCAAAATGGCCTGTTAAACAATTGAGCGTCACTAGACACCAATGAGCC
>HPV102_Alpha_71726718_nt6734_L1_VE_rc|1
TACAACTGTTACAAATACCTCATTAAACCAACAAATGCCATTATTGTGTCCCTGGGCCCG
>HPV1_Mu_9626063_nt6374_L1_VE_rc|2
AACTGTAATAAATAACTGGTTTCTCCAGCAAATGCCATTATTCTGGCCTTGACATCGCTG
>HPV16_Alpha_9627100_nt6220_L1_VE_rc|1
CTGTAATGTAGTAAAGTCCATAGCACCAAAGCCAGTATGAACCATATCACCATCCTGAAT
>HPV18_Alpha_9626069_nt5941_L1_VE_rc|1
AGTGTCATCTAATTTATTATAAAATGGATGCCCACTAAGGCCAACACCTAAAGGCTGACC
>HPV107_Beta_126131393_nt5789_L1_VE_rc|1
TGCCACCGGAGTTGATGGTGGCAAATATACTTTACCAGTTGCAGGAAGCCACAAAGACAT
>HPV50_Gamma_9628550_nt6192_L1_VE_rc|1
TGTAACAAATACCTCATTACCCCAACAAATACAATTGTTCGTACCCTGAGCTCTGCGAAT
>HPV66_Alpha_1020290_nt5858_L1_VE_rc|1
ATAGAAAGATGGATCAGGAAGGCCAAACTTATTAGGATCAGGCAACCGTACCCTAAACAC
>HPV57_Alpha_60882_nt6664_L1_VE_rc|1
CCGATTGCCCCAGCACATGCCATTGTTATGTCCCTGGGCCCTCCGCAGCCAGTAAGGCTT
>HPV50_Gamma_9628550_nt5262_L1_VE_rc|1
TTTCACATATTCATCGGTGCTTAAAACTCTGGCAACAGGAGTACTTGGTGGAAGGTATAA
>HPV97_Alpha_71726694_nt6437_L1_VE_rc|1
CACCACAGTAACAAATAATTGATTATGCCAACAAATACCATTGTTATGGCCCTGGGCCCT
>HPV26_Alpha_9627305_nt6519_L1_VE_rc|1
AAACAATTGATTGCCCCAACAGATACCATTATTATGACCCTGTGCACGTTGTAACCAGTA
>HPV82_Alpha_6970427_nt6502_L1_VE_rc|1
AAAAAGCTGATTATTCCAGCAAATGCCATTATTGTGGCCCTGGGCGCGATGCAACCAATA
>HPV75_Beta_2911544_nt5802_L1_VE_rc|1
AATGTATTCATCCGTGCTTTGCACCCTGGCCACAGGTGTTGATGGTGGAAGATAAACCTT
>HPV32_Alpha_9627327_nt5873_L1_VE_rc|1
CTTGGAAACAGGAGGAGGAGGCAGATAAACCTTGTTGTCACTAGGCCGCCACACGGACAT
>HPV74_Alpha_27462483_nt6504_L1_VE_rc|1
TTGATTACCCCAACAAATGCCATTATTGTGGCCTTGCGCCTTTTGTAGCCAAAAGGGCTT
>HPV106_Alpha_71726710_nt6200_L1_VE_rc|1
AATACATAACTGAGTTTGTTTATAATCCATGGACATATTGTCCCGGTTGTCGTCCCCAGG
>HPV35_Alpha_333050_nt6505_L1_VE_rc|1
CAATTGGTTACTCCAACAAATACCATTATTATGGCCTTGTGCACGTTGCAACCAATATGG
>HPV73_Alpha_1491692_nt6365_L1_VE_rc|1
CCAATAAGGTTTATTAAACAACTGTGCATCTGAAGAAACCATGGAACCACTAGGTGTAGG
>HPV88_Gamma_167600365_nt5641_L1_VE_rc|1
ATCTGGAGCTTTGTTAAATAAAGGATGACCTGCAGTACCTACATTTAGTGGTCCACCTCT
>HPV29_Alpha_1020210_nt6714_L1_VE_rc|1
GTTGTGTCCCTGGGCCTGCTGTAGCCAGTAAGGCTTATTAAATATTTGAGCCTCAGACGT
```

>HPV63_Mu_9626605_nt5432_L1_VE_rc|3
ATCACTGGTAGCGTGATAGAAGATGTTGGTGCGAGACACATAATCATCGCTGCTTAGAAT
>HPV63_Mu_9626605_nt6032_L1_VE_rc|2
CTGTCCCATTTTAATATAATCTGGATATTTGCAGACAGTGCCTGCAATATCCAAAGGAGC
>HPV111_Beta_186461202_nt5653_L1_VE_rc|1
TTCCACATATTCATCGGTGCTTTGTACGCGAGCCACTGGTGTAGATGGTGGCAAATATAC
>HPV106_Alpha_71726710_nt6740_L1_VE_rc|1
GGTATCTACCACAGTGACAAATACCTGATTGCCCCAGCAGATACCATTGTTTCGGCCCTG
>HPV18_Alpha_9626069_nt6511_L1_VE_rc|1
ATGACCCTGTGCCTTATGTAACCAATATGGTTTATTAAACAACTGGGAGTCAGAGGTAAC
>HPV16_Alpha_9627100_nt5920_L1_VE_rc|1
ACCTAATGGCTGACCACGACCTACCTCAACACCTACACAGGCCCAAACCAGCCGCTGTGT
>HPV19_Beta_396940_nt5895_L1_VE_rc|1
CCTTTGAACATATTCATCCGTGCTTTGTACCCTGGCAACTGGTGTAGATGGTGGAAGGTA
>HPV9_Beta_9627396_nt6046_L1_VE_rc|1
ACCTGATGTTCCAACCCCTAAAGGTTGTCCTCTGCCTATTTCAATACCTCTACAGGCCCA
>HPV29_Alpha_1020210_nt6384_L1_VE_rc|1
AAAGTCCATGGCACCGTACCCGGTATCCACCATATCGCCATCCTCAATATGTGTGGTCAT
>HPV107_Beta_126131393_nt6029_L1_VE_rc|1
TACCAGCCTTTCCTTGTCAGGATTGTATACTGACATGTCTGCTAATGCAAATCGATTGGG
>HPV7_Alpha_9627389_nt6369_L1_VE_rc|1
TGATGCAAAATCCATGGCACCAAAGCCTGCATCAACCATGTCGCCATCCTCAATAACTTC
>HPV11_Alpha_333026_nt6732_L1_VE_rc|1
TGTCATATTTGTACTGCGTGTGGTATCTACCACAGTAACAAACAAGTGGTTTCCCCAGCA
>HPV25_Beta_396948_nt5920_L1_VE_rc|1
TTGCACATATTCATCCGTGCTTTGTACCCTGGCAACAGGTGTAGATGGTGGAAGGTACAC
>HPV24_Unclassified_9628486_nt6614_L1_VE_rc|1
TAATTGAGCATCACTAGATACCAATGAGCCACTGACTGTTGGGAAATACATTGAGTTGCC
>HPV54_Alpha_9628437_nt5615_L1_VE_rc|1
GACAACCTTAGAAACTGGGGTAGGAGGCAGGTATACTTTGTTTTCGCTAGGCCGCCACAT
>HPV11_Alpha_333026_nt6192_L1_VE_rc|1
TGGAGCACAGCCCACCATACATAGCTGGGTTTGTTTATAATCCATACCTACATTAACCCT
>HPV1_Mu_9626063_nt6614_L1_VE_rc|1
TAGAGGATTGGTAGGTGGTTGAGATACAGATAGTTGCCAATCCTCTAAAATATTAGGGTC
>HPV70_Alpha_1173493_nt6011_L1_VE_rc|1
GCCTATAATACATAACTGTGTTTGCTTATAGTCCACAGACACATTGTCCCTACTGTCCTG
>HPV39_Alpha_333245_nt6214_L1_VE_rc|1
ACCAAAGTCCATAGCTCCATAGCCAGTATCAATCATATCACCATCCTCAATAGGGGTGTT
>HPV101_Unclassified_109390389_nt5578_L1_VE_rc|1
CATATCCCCATCCTGAATAACAGAGGACACCAACTGTAAAGGAGGACAGGTGCCTTTAGG
>HPV31_Alpha_333048_nt5853_L1_VE_rc|1
ACCACTAATACCTACACCTAATGGCTGCCCGCGACCTACCTCTAAACCAACACAGGCCCA
>HPV44_Alpha_1020242_nt5995_L1_VE_rc|1
TAATAATGGATGCCCACTAATACCAACACCTAAGGGCTGTCCTCTACCTACCTCCAGCCC
>HPV23_Beta_1020186_nt5871_L1_VE_rc|1
AGTCATGTCTGCTAATGCAAACTTATTAGGGTCTGGAAATGTAACTCTAAAGGCCCTGAA
>HPV15_Beta_396924_nt6004_L1_VE_rc|1
GCCTGAAGTACCAACTCCTAATGGTTGTCCTCTACCTATTTCAAGGCCTACACAGGCCCA
>HPV101_Unclassified_109390389_nt6148_L1_VE_rc|2
TTTACAGAGCTGCACAATGAGAGAAATTTCAAATTCCTCCACATGTCTGAGGTAGTGCTT
>HPV22_Beta_1020178_nt5963_L1_VE_rc|1
ACCTGTGGTACCTACACCTAAGGGCTGTCCTCTTCCTATTTCTAACCCTTTACAAGCCCA
>HPV6_Alpha_6002612_nt6361_L1_VE_rc|1
CTGCAAATCAGCAAAATTCATAGCACCAAAGCCTGTGTCAACCATATCGCCATCCTGTAT
>HPV16_Alpha_9627100_nt5950_L1_VE_rc|1

ATTTAATAAAGGATGGCCACTAATGCCCACACCTAATGGCTGACCACGACCTACCTCAAC
>HPV63_Mu_9626605_nt5822_L1_VE_rc|1
CCAGTGTTCCCCAGTGGCTGGTGTACAGCCAATTAGAAACATTTGGGTTTGTTTTGCATC
>HPV80_Beta_2911565_nt5989_L1_VE_rc|1
TCTGCCTATTTCTAGGCCTGCACAGGCCCAAACCAACCTTTCTTTTTCTGGATTATAAAC
>HPV1_Mu_9626063_nt5414_L1_Tile|1
ATGTATAATGTTTTTCAGATGGCTGTCTGGTTACCAGCGCAGAATAAGTTCTATCTTCCT
>HPV1_Mu_9626063_nt5419_L1_Tile|1
TAATGTTTTTCAGATGGCTGTCTGGTTACCAGCGCAGAATAAGTTCTATCTTCCTCCCCA
>HPV1_Mu_9626063_nt5434_L1_Tile|1
GGCTGTCTGGTTACCAGCGCAGAATAAGTTCTATCTTCCTCCCCAGCCCATCACTAGAAT
>HPV1_Mu_9626063_nt5439_L1_Tile|1
TCTGGTTACCAGCGCAGAATAAGTTCTATCTTCCTCCCCAGCCCATCACTAGAATCCTGT
>HPV1_Mu_9626063_nt5449_L1_Tile|1
AGCGCAGAATAAGTTCTATCTTCCTCCCCAGCCCATCACTAGAATCCTGTCCACTGATGA
>HPV1_Mu_9626063_nt5454_L1_Tile|1
AGAATAAGTTCTATCTTCCTCCCCAGCCCATCACTAGAATCCTGTCCACTGATGAATATG
>HPV1_Mu_9626063_nt5459_L1_Tile|1
AAGTTCTATCTTCCTCCCCAGCCCATCACTAGAATCCTGTCCACTGATGAATATGTAACC
>HPV1_Mu_9626063_nt5474_L1_Tile|1
CCCCAGCCCATCACTAGAATCCTGTCCACTGATGAATATGTAACCAGAACCAATCTCTTC
>HPV1_Mu_9626063_nt5479_L1_Tile|1
GCCCATCACTAGAATCCTGTCCACTGATGAATATGTAACCAGAACCAATCTCTTCTACCA
>HPV1_Mu_9626063_nt5484_L1_Tile|1
TCACTAGAATCCTGTCCACTGATGAATATGTAACCAGAACCAATCTCTTCTACCATGCAA
>HPV1_Mu_9626063_nt5489_L1_Tile|1
AGAATCCTGTCCACTGATGAATATGTAACCAGAACCAATCTCTTCTACCATGCAACATCT
>HPV1_Mu_9626063_nt5499_L1_Tile|1
CCACTGATGAATATGTAACCAGAACCAATCTCTTCTACCATGCAACATCTGAACGTCTAC
>HPV1_Mu_9626063_nt5504_L1_Tile|1
GATGAATATGTAACCAGAACCAATCTCTTCTACCATGCAACATCTGAACGTCTACTGCTG
>HPV1_Mu_9626063_nt5509_L1_Tile|1
ATATGTAACCAGAACCAATCTCTTCTACCATGCAACATCTGAACGTCTACTGCTGGTCGG
>HPV1_Mu_9626063_nt5514_L1_Tile|1
TAACCAGAACCAATCTCTTCTACCATGCAACATCTGAACGTCTACTGCTGGTCGGACATC
>HPV1_Mu_9626063_nt5529_L1_Tile|1
TCTTCTACCATGCAACATCTGAACGTCTACTGCTGGTCGGACATCCTTTGTTTGAGATCT
>HPV1_Mu_9626063_nt5534_L1_Tile|1
TACCATGCAACATCTGAACGTCTACTGCTGGTCGGACATCCTTTGTTTGAGATCTCCAGT
>HPV1_Mu_9626063_nt5539_L1_Tile|1
TGCAACATCTGAACGTCTACTGCTGGTCGGACATCCTTTGTTTGAGATCTCCAGTAATCA
>HPV1_Mu_9626063_nt5544_L1_Tile|1
CATCTGAACGTCTACTGCTGGTCGGACATCCTTTGTTTGAGATCTCCAGTAATCAAACTG
>HPV1_Mu_9626063_nt5549_L1_Tile|1
GAACGTCTACTGCTGGTCGGACATCCTTTGTTTGAGATCTCCAGTAATCAAACTGTAACT
>HPV1_Mu_9626063_nt5554_L1_Tile|1
TCTACTGCTGGTCGGACATCCTTTGTTTGAGATCTCCAGTAATCAAACTGTAACTATACC
>HPV1_Mu_9626063_nt5559_L1_Tile|1
TGCTGGTCGGACATCCTTTGTTTGAGATCTCCAGTAATCAAACTGTAACTATACCAAAAG
>HPV1_Mu_9626063_nt5564_L1_Tile|1
GTCGGACATCCTTTGTTTGAGATCTCCAGTAATCAAACTGTAACTATACCAAAAGTGTCA
>HPV1_Mu_9626063_nt5579_L1_Tile|1
TTTGAGATCTCCAGTAATCAAACTGTAACTATACCAAAAGTGTCACCAAATGCATTTAGA
>HPV1_Mu_9626063_nt5584_L1_Tile|1
GATCTCCAGTAATCAAACTGTAACTATACCAAAAGTGTCACCAAATGCATTTAGAGTTTT

>HPV1_Mu_9626063_nt5594_L1_Tile|1
AATCAAACTGTAACTATACCAAAAGTGTCACCAAATGCATTTAGAGTTTTTAGGGTGCGT
>HPV1_Mu_9626063_nt5599_L1_Tile|1
AACTGTAACTATACCAAAAGTGTCACCAAATGCATTTAGAGTTTTTAGGGTGCGTTTTGC
>HPV1_Mu_9626063_nt5604_L1_Tile|1
TAACTATACCAAAAGTGTCACCAAATGCATTTAGAGTTTTTAGGGTGCGTTTTGCTGATC
>HPV1_Mu_9626063_nt5609_L1_Tile|1
ATACCAAAAGTGTCACCAAATGCATTTAGAGTTTTTAGGGTGCGTTTTGCTGATCCAAAT
>HPV1_Mu_9626063_nt5614_L1_Tile|1
AAAAGTGTCACCAAATGCATTTAGAGTTTTTAGGGTGCGTTTTGCTGATCCAAATAGATT
>HPV1_Mu_9626063_nt5644_L1_Tile|1
TAGGGTGCGTTTTGCTGATCCAAATAGATTTGCATTTGGGGATAAGGCAATTTTTAATCC
>HPV1_Mu_9626063_nt5729_L1_Tile|1
CTAAGAGGGATAGAGATAGGTAGAGGCCAGCCTTTAGGTATAGGAATAACGGGCCACCCT
>HPV1_Mu_9626063_nt5734_L1_Tile|1
AGGGATAGAGATAGGTAGAGGCCAGCCTTTAGGTATAGGAATAACGGGCCACCCTCTTTT
>HPV1_Mu_9626063_nt5739_L1_Tile|1
TAGAGATAGGTAGAGGCCAGCCTTTAGGTATAGGAATAACGGGCCACCCTCTTTTAAATA
>HPV1_Mu_9626063_nt5744_L1_Tile|1
ATAGGTAGAGGCCAGCCTTTAGGTATAGGAATAACGGGCCACCCTCTTTTAAATAAGTTA
>HPV1_Mu_9626063_nt5754_L1_Tile|1
GCCAGCCTTTAGGTATAGGAATAACGGGCCACCCTCTTTTAAATAAGTTAGATGATGCAG
>HPV1_Mu_9626063_nt5759_L1_Tile|1
CCTTTAGGTATAGGAATAACGGGCCACCCTCTTTTAAATAAGTTAGATGATGCAGAAAAT
>HPV1_Mu_9626063_nt5764_L1_Tile|1
AGGTATAGGAATAACGGGCCACCCTCTTTTAAATAAGTTAGATGATGCAGAAAATCCAAC
>HPV1_Mu_9626063_nt5769_L1_Tile|1
TAGGAATAACGGGCCACCCTCTTTTAAATAAGTTAGATGATGCAGAAAATCCAACAAATT
>HPV1_Mu_9626063_nt5774_L1_Tile|1
ATAACGGGCCACCCTCTTTTAAATAAGTTAGATGATGCAGAAAATCCAACAAATTATATT
>HPV1_Mu_9626063_nt5784_L1_Tile|1
ACCCTCTTTTAAATAAGTTAGATGATGCAGAAAATCCAACAAATTATATTAATACTCATG
>HPV1_Mu_9626063_nt5789_L1_Tile|1
CTTTTAAATAAGTTAGATGATGCAGAAAATCCAACAAATTATATTAATACTCATGCAAAT
>HPV1_Mu_9626063_nt5794_L1_Tile|1
AAATAAGTTAGATGATGCAGAAAATCCAACAAATTATATTAATACTCATGCAAATGGAGA
>HPV1_Mu_9626063_nt5799_L1_Tile|1
AGTTAGATGATGCAGAAAATCCAACAAATTATATTAATACTCATGCAAATGGAGATTCTA
>HPV1_Mu_9626063_nt5809_L1_Tile|1
TGCAGAAAATCCAACAAATTATATTAATACTCATGCAAATGGAGATTCTAGACAAAATAC
>HPV1_Mu_9626063_nt5819_L1_Tile|1
CCAACAAATTATATTAATACTCATGCAAATGGAGATTCTAGACAAAATACTGCTTTTGAT
>HPV1_Mu_9626063_nt5844_L1_Tile|1
CAAATGGAGATTCTAGACAAAATACTGCTTTTGATGCAAAACAGACACAAATGTTCCTCG
>HPV1_Mu_9626063_nt5849_L1_Tile|1
GGAGATTCTAGACAAAATACTGCTTTTGATGCAAAACAGACACAAATGTTCCTCGTCGGC
>HPV1_Mu_9626063_nt5864_L1_Tile|1
AATACTGCTTTTGATGCAAAACAGACACAAATGTTCCTCGTCGGCTGTACTCCTGCTTCA
>HPV1_Mu_9626063_nt5874_L1_Tile|1
TTGATGCAAAACAGACACAAATGTTCCTCGTCGGCTGTACTCCTGCTTCAGGTGAACACT
>HPV1_Mu_9626063_nt5884_L1_Tile|1
ACAGACACAAATGTTCCTCGTCGGCTGTACTCCTGCTTCAGGTGAACACTGGACAAGTAG
>HPV1_Mu_9626063_nt5889_L1_Tile|1
CACAAATGTTCCTCGTCGGCTGTACTCCTGCTTCAGGTGAACACTGGACAAGTAGTCGTT
>HPV1_Mu_9626063_nt5894_L1_Tile|1

ATGTTCCTCGTCGGCTGTACTCCTGCTTCAGGTGAACACTGGACAAGTAGTCGTTGCCCA
>HPV1_Mu_9626063_nt5904_L1_Tile|1
TCGGCTGTACTCCTGCTTCAGGTGAACACTGGACAAGTAGTCGTTGCCCAGGGGAACAAG
>HPV1_Mu_9626063_nt5909_L1_Tile|1
TGTACTCCTGCTTCAGGTGAACACTGGACAAGTAGTCGTTGCCCAGGGGAACAAGTGAAA
>HPV1_Mu_9626063_nt5914_L1_Tile|1
TCCTGCTTCAGGTGAACACTGGACAAGTAGTCGTTGCCCAGGGGAACAAGTGAAACTTGG
>HPV1_Mu_9626063_nt5929_L1_Tile|1
ACACTGGACAAGTAGTCGTTGCCCAGGGGAACAAGTGAAACTTGGGGACTGCCCCAGGGT
>HPV1_Mu_9626063_nt5954_L1_Tile|1
GGGGAACAAGTGAAACTTGGGGACTGCCCCAGGGTGCAAATGATAGAGTCTGTCATAGAA
>HPV1_Mu_9626063_nt5964_L1_Tile|1
TGAAACTTGGGGACTGCCCCAGGGTGCAAATGATAGAGTCTGTCATAGAAGATGGTGACA
>HPV1_Mu_9626063_nt5974_L1_Tile|1
GGACTGCCCCAGGGTGCAAATGATAGAGTCTGTCATAGAAGATGGTGACATGATGGATAT
>HPV1_Mu_9626063_nt6044_L1_Tile|1
GCTATGGATTTTGCTGCTTTACAGCAAGACAAGTCTGATGTCCCTTTAGATGTTGTTCAA
>HPV1_Mu_9626063_nt6049_L1_Tile|1
GGATTTTGCTGCTTTACAGCAAGACAAGTCTGATGTCCCTTTAGATGTTGTTCAAGCAAC
>HPV1_Mu_9626063_nt6079_L1_Tile|1
TGATGTCCCTTTAGATGTTGTTCAAGCAACATGCAAATATCCTGATTATATCAGAATGAA
>HPV1_Mu_9626063_nt6084_L1_Tile|1
TCCCTTTAGATGTTGTTCAAGCAACATGCAAATATCCTGATTATATCAGAATGAACCATG
>HPV1_Mu_9626063_nt6089_L1_Tile|1
TTAGATGTTGTTCAAGCAACATGCAAATATCCTGATTATATCAGAATGAACCATGAAGCC
>HPV1_Mu_9626063_nt6099_L1_Tile|1
TTCAAGCAACATGCAAATATCCTGATTATATCAGAATGAACCATGAAGCCTATGGCAACT
>HPV1_Mu_9626063_nt6104_L1_Tile|1
GCAACATGCAAATATCCTGATTATATCAGAATGAACCATGAAGCCTATGGCAACTCTATG
>HPV1_Mu_9626063_nt6114_L1_Tile|1
AATATCCTGATTATATCAGAATGAACCATGAAGCCTATGGCAACTCTATGTTTTTTTTTG
>HPV1_Mu_9626063_nt6119_L1_Tile|1
CCTGATTATATCAGAATGAACCATGAAGCCTATGGCAACTCTATGTTTTTTTTTGCACGT
>HPV1_Mu_9626063_nt6129_L1_Tile|1
TCAGAATGAACCATGAAGCCTATGGCAACTCTATGTTTTTTTTTGCACGTCGCGAGCAAA
>HPV1_Mu_9626063_nt6134_L1_Tile|1
ATGAACCATGAAGCCTATGGCAACTCTATGTTTTTTTTTGCACGTCGCGAGCAAATGTAT
>HPV1_Mu_9626063_nt6139_L1_Tile|1
CCATGAAGCCTATGGCAACTCTATGTTTTTTTTTGCACGTCGCGAGCAAATGTATACCAG
>HPV1_Mu_9626063_nt6184_L1_Tile|1
GCAAATGTATACCAGGCACTTTTTTACTCGCGGGGGTTCGGTGGGTGATAAGGAGGCAGT
>HPV1_Mu_9626063_nt6194_L1_Tile|1
ACCAGGCACTTTTTTACTCGCGGGGGTTCGGTGGGTGATAAGGAGGCAGTCCCACAAAGC
>HPV1_Mu_9626063_nt6199_L1_Tile|1
GCACTTTTTTACTCGCGGGGGTTCGGTGGGTGATAAGGAGGCAGTCCCACAAAGCCTGTA
>HPV1_Mu_9626063_nt6209_L1_Tile|1
ACTCGCGGGGGTTCGGTGGGTGATAAGGAGGCAGTCCCACAAAGCCTGTATTTAACAGCA
>HPV1_Mu_9626063_nt6224_L1_Tile|1
GTGGGTGATAAGGAGGCAGTCCCACAAAGCCTGTATTTAACAGCAGATGCTGAACCAAGA
>HPV1_Mu_9626063_nt6229_L1_Tile|1
TGATAAGGAGGCAGTCCCACAAAGCCTGTATTTAACAGCAGATGCTGAACCAAGAACAAC
>HPV1_Mu_9626063_nt6239_L1_Tile|1
GCAGTCCCACAAAGCCTGTATTTAACAGCAGATGCTGAACCAAGAACAACTTTAGCAACA
>HPV1_Mu_9626063_nt6244_L1_Tile|1
CCCACAAAGCCTGTATTTAACAGCAGATGCTGAACCAAGAACAACTTTAGCAACAACAAA

>HPV1_Mu_9626063_nt6249_L1_Tile|1
AAAGCCTGTATTTAACAGCAGATGCTGAACCAAGAACAACTTTAGCAACAACAAATTATG
>HPV1_Mu_9626063_nt6254_L1_Tile|1
CTGTATTTAACAGCAGATGCTGAACCAAGAACAACTTTAGCAACAACAAATTATGTAGGC
>HPV1_Mu_9626063_nt6264_L1_Tile|1
CAGCAGATGCTGAACCAAGAACAACTTTAGCAACAACAAATTATGTAGGCACACCAAGTG
>HPV1_Mu_9626063_nt6269_L1_Tile|1
GATGCTGAACCAAGAACAACTTTAGCAACAACAAATTATGTAGGCACACCAAGTGGCTCT
>HPV1_Mu_9626063_nt6279_L1_Tile|1
CAAGAACAACTTTAGCAACAACAAATTATGTAGGCACACCAAGTGGCTCTATGGTTTCAT
>HPV1_Mu_9626063_nt6359_L1_Tile|1
AGATCTTACTGGCTTCAGCGATGTCAAGGCCAGAATAATGGCATTTGCTGGAGAAACCAG
>HPV1_Mu_9626063_nt6369_L1_Tile|1
GGCTTCAGCGATGTCAAGGCCAGAATAATGGCATTTGCTGGAGAAACCAGTTATTTATTA
>HPV1_Mu_9626063_nt6379_L1_Tile|1
ATGTCAAGGCCAGAATAATGGCATTTGCTGGAGAAACCAGTTATTTATTACAGTTGGAGA
>HPV1_Mu_9626063_nt6384_L1_Tile|1
AAGGCCAGAATAATGGCATTTGCTGGAGAAACCAGTTATTTATTACAGTTGGAGATAATA
>HPV1_Mu_9626063_nt6394_L1_Tile|1
TAATGGCATTTGCTGGAGAAACCAGTTATTTATTACAGTTGGAGATAATACCAGAGGAAC
>HPV1_Mu_9626063_nt6409_L1_Tile|1
GAGAAACCAGTTATTTATTACAGTTGGAGATAATACCAGAGGAACAAGTTTATCTATCAG
>HPV1_Mu_9626063_nt6414_L1_Tile|1
ACCAGTTATTTATTACAGTTGGAGATAATACCAGAGGAACAAGTTTATCTATCAGTATGA
>HPV1_Mu_9626063_nt6419_L1_Tile|1
TTATTTATTACAGTTGGAGATAATACCAGAGGAACAAGTTTATCTATCAGTATGAAAAAC
>HPV1_Mu_9626063_nt6424_L1_Tile|1
TATTACAGTTGGAGATAATACCAGAGGAACAAGTTTATCTATCAGTATGAAAAACAATGC
>HPV1_Mu_9626063_nt6429_L1_Tile|1
CAGTTGGAGATAATACCAGAGGAACAAGTTTATCTATCAGTATGAAAAACAATGCAAGTA
>HPV1_Mu_9626063_nt6434_L1_Tile|1
GGAGATAATACCAGAGGAACAAGTTTATCTATCAGTATGAAAAACAATGCAAGTACTACA
>HPV1_Mu_9626063_nt6439_L1_Tile|1
TAATACCAGAGGAACAAGTTTATCTATCAGTATGAAAAACAATGCAAGTACTACATATTC
>HPV1_Mu_9626063_nt6444_L1_Tile|1
CCAGAGGAACAAGTTTATCTATCAGTATGAAAAACAATGCAAGTACTACATATTCCAATG
>HPV1_Mu_9626063_nt6449_L1_Tile|1
GGAACAAGTTTATCTATCAGTATGAAAAACAATGCAAGTACTACATATTCCAATGCTAAT
>HPV1_Mu_9626063_nt6454_L1_Tile|1
AAGTTTATCTATCAGTATGAAAAACAATGCAAGTACTACATATTCCAATGCTAATTTTAA
>HPV1_Mu_9626063_nt6459_L1_Tile|1
TATCTATCAGTATGAAAAACAATGCAAGTACTACATATTCCAATGCTAATTTTAATGATT
>HPV1_Mu_9626063_nt6464_L1_Tile|1
ATCAGTATGAAAAACAATGCAAGTACTACATATTCCAATGCTAATTTTAATGATTTTCTA
>HPV1_Mu_9626063_nt6469_L1_Tile|1
TATGAAAAACAATGCAAGTACTACATATTCCAATGCTAATTTTAATGATTTTCTAAGACA
>HPV1_Mu_9626063_nt6474_L1_Tile|1
AAAACAATGCAAGTACTACATATTCCAATGCTAATTTTAATGATTTTCTAAGACATACTG
>HPV1_Mu_9626063_nt6479_L1_Tile|1
AATGCAAGTACTACATATTCCAATGCTAATTTTAATGATTTTCTAAGACATACTGAAGAA
>HPV1_Mu_9626063_nt6484_L1_Tile|1
AAGTACTACATATTCCAATGCTAATTTTAATGATTTTCTAAGACATACTGAAGAATTTGA
>HPV1_Mu_9626063_nt6489_L1_Tile|1
CTACATATTCCAATGCTAATTTTAATGATTTTCTAAGACATACTGAAGAATTTGATCTTT
>HPV1_Mu_9626063_nt6494_L1_Tile|1

TATTCCAATGCTAATTTTAATGATTTTCTAAGACATACTGAAGAATTTGATCTTTCTTTT
>HPV1_Mu_9626063_nt6499_L1_Tile|1
CAATGCTAATTTTAATGATTTTCTAAGACATACTGAAGAATTTGATCTTTCTTTTATAGT
>HPV1_Mu_9626063_nt6504_L1_Tile|1
CTAATTTTAATGATTTTCTAAGACATACTGAAGAATTTGATCTTTCTTTTATAGTTCAGC
>HPV1_Mu_9626063_nt6509_L1_Tile|1
TTTAATGATTTTCTAAGACATACTGAAGAATTTGATCTTTCTTTTATAGTTCAGCTTTGT
>HPV1_Mu_9626063_nt6519_L1_Tile|1
TTCTAAGACATACTGAAGAATTTGATCTTTCTTTTATAGTTCAGCTTTGTAAAGTAAAGT
>HPV1_Mu_9626063_nt6524_L1_Tile|1
AGACATACTGAAGAATTTGATCTTTCTTTTATAGTTCAGCTTTGTAAAGTAAAGTTAACT
>HPV1_Mu_9626063_nt6529_L1_Tile|1
TACTGAAGAATTTGATCTTTCTTTTATAGTTCAGCTTTGTAAAGTAAAGTTAACTCCCGA
>HPV1_Mu_9626063_nt6539_L1_Tile|1
TTTGATCTTTCTTTTATAGTTCAGCTTTGTAAAGTAAAGTTAACTCCCGAAAATCTAGCC
>HPV1_Mu_9626063_nt6544_L1_Tile|1
TCTTTCTTTTATAGTTCAGCTTTGTAAAGTAAAGTTAACTCCCGAAAATCTAGCCTACAT
>HPV1_Mu_9626063_nt6549_L1_Tile|1
CTTTTATAGTTCAGCTTTGTAAAGTAAAGTTAACTCCCGAAAATCTAGCCTACATTCATA
>HPV1_Mu_9626063_nt6554_L1_Tile|1
ATAGTTCAGCTTTGTAAAGTAAAGTTAACTCCCGAAAATCTAGCCTACATTCATACAATG
>HPV1_Mu_9626063_nt6559_L1_Tile|1
TCAGCTTTGTAAAGTAAAGTTAACTCCCGAAAATCTAGCCTACATTCATACAATGGACCC
>HPV1_Mu_9626063_nt6564_L1_Tile|1
TTTGTAAAGTAAAGTTAACTCCCGAAAATCTAGCCTACATTCATACAATGGACCCTAATA
>HPV1_Mu_9626063_nt6569_L1_Tile|1
AAAGTAAAGTTAACTCCCGAAAATCTAGCCTACATTCATACAATGGACCCTAATATTTTA
>HPV1_Mu_9626063_nt6574_L1_Tile|1
AAAGTTAACTCCCGAAAATCTAGCCTACATTCATACAATGGACCCTAATATTTTAGAGGA
>HPV1_Mu_9626063_nt6579_L1_Tile|1
TAACTCCCGAAAATCTAGCCTACATTCATACAATGGACCCTAATATTTTAGAGGATTGGC
>HPV1_Mu_9626063_nt6594_L1_Tile|1
TAGCCTACATTCATACAATGGACCCTAATATTTTAGAGGATTGGCAACTATCTGTATCTC
>HPV1_Mu_9626063_nt6629_L1_Tile|1
GAGGATTGGCAACTATCTGTATCTCAACCACCTACCAATCCTCTAGAAGATCAATATAGG
>HPV1_Mu_9626063_nt6639_L1_Tile|1
AACTATCTGTATCTCAACCACCTACCAATCCTCTAGAAGATCAATATAGGTTTTTAGGGT
>HPV1_Mu_9626063_nt6644_L1_Tile|1
TCTGTATCTCAACCACCTACCAATCCTCTAGAAGATCAATATAGGTTTTTAGGGTCTTCC
>HPV1_Mu_9626063_nt6649_L1_Tile|1
ATCTCAACCACCTACCAATCCTCTAGAAGATCAATATAGGTTTTTAGGGTCTTCCTTGGC
>HPV1_Mu_9626063_nt6654_L1_Tile|1
AACCACCTACCAATCCTCTAGAAGATCAATATAGGTTTTTAGGGTCTTCCTTGGCAGCAA
>HPV1_Mu_9626063_nt6659_L1_Tile|1
CCTACCAATCCTCTAGAAGATCAATATAGGTTTTTAGGGTCTTCCTTGGCAGCAAAATGT
>HPV1_Mu_9626063_nt6664_L1_Tile|1
CAATCCTCTAGAAGATCAATATAGGTTTTTAGGGTCTTCCTTGGCAGCAAAATGTCCAGA
>HPV1_Mu_9626063_nt6669_L1_Tile|1
CTCTAGAAGATCAATATAGGTTTTTAGGGTCTTCCTTGGCAGCAAAATGTCCAGAACAGG
>HPV1_Mu_9626063_nt6694_L1_Tile|1
AGGGTCTTCCTTGGCAGCAAAATGTCCAGAACAGGCGCCTCCTGAGCCCCAGACTGATCC
>HPV1_Mu_9626063_nt6709_L1_Tile|1
AGCAAAATGTCCAGAACAGGCGCCTCCTGAGCCCCAGACTGATCCTTATAGTCAATATAA
>HPV1_Mu_9626063_nt6719_L1_Tile|1
CCAGAACAGGCGCCTCCTGAGCCCCAGACTGATCCTTATAGTCAATATAAATTCTGGGAA

>HPV1_Mu_9626063_nt6729_L1_Tile|1
CGCCTCCTGAGCCCCAGACTGATCCTTATAGTCAATATAAATTCTGGGAAGTCGATCTCA
>HPV1_Mu_9626063_nt6734_L1_Tile|1
CCTGAGCCCCAGACTGATCCTTATAGTCAATATAAATTCTGGGAAGTCGATCTCACAGAA
>HPV1_Mu_9626063_nt6744_L1_Tile|1
AGACTGATCCTTATAGTCAATATAAATTCTGGGAAGTCGATCTCACAGAAAGGATGTCCG
>HPV1_Mu_9626063_nt6749_L1_Tile|1
GATCCTTATAGTCAATATAAATTCTGGGAAGTCGATCTCACAGAAAGGATGTCCGAACAA
>HPV1_Mu_9626063_nt6754_L1_Tile|1
TTATAGTCAATATAAATTCTGGGAAGTCGATCTCACAGAAAGGATGTCCGAACAATTAGA
>HPV1_Mu_9626063_nt6759_L1_Tile|1
GTCAATATAAATTCTGGGAAGTCGATCTCACAGAAAGGATGTCCGAACAATTAGACCAAT
>HPV1_Mu_9626063_nt6764_L1_Tile|1
TATAAATTCTGGGAAGTCGATCTCACAGAAAGGATGTCCGAACAATTAGACCAATTTCCA
>HPV1_Mu_9626063_nt6769_L1_Tile|1
ATTCTGGGAAGTCGATCTCACAGAAAGGATGTCCGAACAATTAGACCAATTTCCACTAGG
>HPV1_Mu_9626063_nt6774_L1_Tile|1
GGGAAGTCGATCTCACAGAAAGGATGTCCGAACAATTAGACCAATTTCCACTAGGAAGGA
>HPV1_Mu_9626063_nt6779_L1_Tile|1
GTCGATCTCACAGAAAGGATGTCCGAACAATTAGACCAATTTCCACTAGGAAGGAAATTT
>HPV1_Mu_9626063_nt6784_L1_Tile|1
TCTCACAGAAAGGATGTCCGAACAATTAGACCAATTTCCACTAGGAAGGAAATTTCTATA
>HPV1_Mu_9626063_nt6789_L1_Tile|1
CAGAAAGGATGTCCGAACAATTAGACCAATTTCCACTAGGAAGGAAATTTCTATATCAAA
>HPV1_Mu_9626063_nt6794_L1_Tile|1
AGGATGTCCGAACAATTAGACCAATTTCCACTAGGAAGGAAATTTCTATATCAAAGTGGC
>HPV1_Mu_9626063_nt6799_L1_Tile|1
GTCCGAACAATTAGACCAATTTCCACTAGGAAGGAAATTTCTATATCAAAGTGGCATGAC
>HPV1_Mu_9626063_nt6804_L1_Tile|1
AACAATTAGACCAATTTCCACTAGGAAGGAAATTTCTATATCAAAGTGGCATGACACAAC
>HPV1_Mu_9626063_nt6809_L1_Tile|1
TTAGACCAATTTCCACTAGGAAGGAAATTTCTATATCAAAGTGGCATGACACAACGTACT
>HPV1_Mu_9626063_nt6814_L1_Tile|1
CCAATTTCCACTAGGAAGGAAATTTCTATATCAAAGTGGCATGACACAACGTACTGCTAC
>HPV1_Mu_9626063_nt6819_L1_Tile|1
TTCCACTAGGAAGGAAATTTCTATATCAAAGTGGCATGACACAACGTACTGCTACTAGTT
>HPV1_Mu_9626063_nt6824_L1_Tile|1
CTAGGAAGGAAATTTCTATATCAAAGTGGCATGACACAACGTACTGCTACTAGTTCCACC
>HPV1_Mu_9626063_nt6834_L1_Tile|1
AATTTCTATATCAAAGTGGCATGACACAACGTACTGCTACTAGTTCCACCACAAAGCGCA
>HPV1_Mu_9626063_nt6839_L1_Tile|1
CTATATCAAAGTGGCATGACACAACGTACTGCTACTAGTTCCACCACAAAGCGCAAAACA
>HPV1_Mu_9626063_nt6844_L1_Tile|1
TCAAAGTGGCATGACACAACGTACTGCTACTAGTTCCACCACAAAGCGCAAAACAGTGCG
>HPV1_Mu_9626063_nt6849_L1_Tile|1
GTGGCATGACACAACGTACTGCTACTAGTTCCACCACAAAGCGCAAAACAGTGCGTGTAT
>HPV1_Mu_9626063_nt6854_L1_Tile|1
ATGACACAACGTACTGCTACTAGTTCCACCACAAAGCGCAAAACAGTGCGTGTATCTACG
>HPV1_Mu_9626063_nt6859_L1_Tile|1
ACAACGTACTGCTACTAGTTCCACCACAAAGCGCAAAACAGTGCGTGTATCTACGTCAGC
>HPV1_Mu_9626063_nt6864_L1_Tile|1
GTACTGCTACTAGTTCCACCACAAAGCGCAAAACAGTGCGTGTATCTACGTCAGCCAAGC
>HPV1_Mu_9626063_nt6879_L1_Tile|1
CCACCACAAAGCGCAAAACAGTGCGTGTATCTACGTCAGCCAAGCGCAGGCGTAAGGCTT
>HPV1_Mu_9626063_nt6881_L1_Tile|1

ACCACAAAGCGCAAAACAGTGCGTGTATCTACGTCAGCCAAGCGCAGGCGTAAGGCTTAG
>HPV2_Alpha_9626032_nt5908_L1_Tile|1
TCTAGGCTTCTCACTGTGGGTCATCCATATTACTCTATAAAGAAGAGTAATAATAAGGTG
>HPV2_Alpha_9626032_nt5913_L1_Tile|1
GCTTCTCACTGTGGGTCATCCATATTACTCTATAAAGAAGAGTAATAATAAGGTGGCTGT
>HPV2_Alpha_9626032_nt6793_L1_Tile|1
ACTAATGTATCTCTGTGTGCCACTGAGGCGTCTGATACTAATTATAAGGCTACCAATTTT
>HPV2_Alpha_9626032_nt7193_L1_Tile|1
CTATGCCTACCGTGTCTCGCAAGCGCGCCGCTGTTTCGGGGACCACGCCGCCCACTAGTA
>HPV2_Alpha_9626032_nt7198_L1_Tile|1
CCTACCGTGTCTCGCAAGCGCGCCGCTGTTTCGGGGACCACGCCGCCCACTAGTAAACGA
>HPV3_Alpha_397005_nt5878_L1_Tile|1
CTGACCGTGGGTCATCCTTATTTTGCTATCCCCAAATCTTCTAATTCCAAGATGGATATT
>HPV3_Alpha_397005_nt5883_L1_Tile|1
CGTGGGTCATCCTTATTTTGCTATCCCCAAATCTTCTAATTCCAAGATGGATATTCCTAA
>HPV3_Alpha_397005_nt5903_L1_Tile|1
CTATCCCCAAATCTTCTAATTCCAAGATGGATATTCCTAAGGTGTCCGCCTTTCAATATA
>HPV3_Alpha_397005_nt5908_L1_Tile|1
CCCAAATCTTCTAATTCCAAGATGGATATTCCTAAGGTGTCCGCCTTTCAATATAGAGTG
>HPV3_Alpha_397005_nt6273_L1_Tile|1
ATGTAAGCAGAATGCGTCACCGGGTGATTGTCCTCCTCTAGAGCTTATTACTGCACCTAT
>HPV3_Alpha_397005_nt6278_L1_Tile|1
AGCAGAATGCGTCACCGGGTGATTGTCCTCCTCTAGAGCTTATTACTGCACCTATACAAG
>HPV3_Alpha_397005_nt6518_L1_Tile|1
CAAGACATTTTCTTAACAGAGCTGGTATGGCTGGAGACACCGTGCCTGACGCGTTGTACA
>HPV3_Alpha_397005_nt6523_L1_Tile|1
CATTTTCTTAACAGAGCTGGTATGGCTGGAGACACCGTGCCTGACGCGTTGTACATTAAA
>HPV3_Alpha_397005_nt6528_L1_Tile|1
TCTTAACAGAGCTGGTATGGCTGGAGACACCGTGCCTGACGCGTTGTACATTAAAGGTGA
>HPV3_Alpha_397005_nt6533_L1_Tile|1
ACAGAGCTGGTATGGCTGGAGACACCGTGCCTGACGCGTTGTACATTAAAGGTGACAGTC
>HPV3_Alpha_397005_nt6538_L1_Tile|1
GCTGGTATGGCTGGAGACACCGTGCCTGACGCGTTGTACATTAAAGGTGACAGTCAGAGC
>HPV3_Alpha_397005_nt6543_L1_Tile|1
TATGGCTGGAGACACCGTGCCTGACGCGTTGTACATTAAAGGTGACAGTCAGAGCGGCGG
>HPV3_Alpha_397005_nt6553_L1_Tile|1
GACACCGTGCCTGACGCGTTGTACATTAAAGGTGACAGTCAGAGCGGCGGTCGGGATAAA
>HPV3_Alpha_397005_nt6563_L1_Tile|1
CTGACGCGTTGTACATTAAAGGTGACAGTCAGAGCGGCGGTCGGGATAAAATTGGTAGTG
>HPV3_Alpha_397005_nt6783_L1_Tile|1
GACATTGTGTGTTTCTACTGAAACCTCGGCTACATATGATGCTACTAAATTTAAAGAGTA
>HPV3_Alpha_397005_nt6788_L1_Tile|1
TGTGTGTTTCTACTGAAACCTCGGCTACATATGATGCTACTAAATTTAAAGAGTATTTAA
>HPV3_Alpha_397005_nt6793_L1_Tile|1
GTTTCTACTGAAACCTCGGCTACATATGATGCTACTAAATTTAAAGAGTATTTAAGACAC
>HPV3_Alpha_397005_nt7003_L1_Tile|1
ACCTATAGATTTTTAACTTCCTCTGCCATTACCTGCCAGAAAGATGCACCTCCCACTGAG
>HPV3_Alpha_397005_nt7028_L1_Tile|1
CCATTACCTGCCAGAAAGATGCACCTCCCACTGAGAAGCAAGACCCCTACGCCAAACTAA
>HPV3_Alpha_397005_nt7103_L1_Tile|1
ATCTTAAGGATCGTTTTTCCCTGGATCTTTCGCAGTTCCCCCTTGGCAGGAAATTTCTCA
>HPV3_Alpha_397005_nt7118_L1_Tile|1
TTTCCCTGGATCTTTCGCAGTTCCCCCTTGGCAGGAAATTTCTCATGCAGCTCGGTGTAG
>HPV3_Alpha_397005_nt7123_L1_Tile|1
CTGGATCTTTCGCAGTTCCCCCTTGGCAGGAAATTTCTCATGCAGCTCGGTGTAGGTACC

>HPV3_Alpha_397005_nt7138_L1_Tile|1
TTCCCCCTTGGCAGGAAATTTCTCATGCAGCTCGGTGTAGGTACCCGCTCTAGTATATCT
>HPV3_Alpha_397005_nt7153_L1_Tile|1
AAATTTCTCATGCAGCTCGGTGTAGGTACCCGCTCTAGTATATCTGTTCGTAAACGCTCG
>HPV3_Alpha_397005_nt7173_L1_Tile|1
TGTAGGTACCCGCTCTAGTATATCTGTTCGTAAACGCTCGGCGACAACCACATCTAGAAC
>HPV3_Alpha_397005_nt7178_L1_Tile|1
GTACCCGCTCTAGTATATCTGTTCGTAAACGCTCGGCGACAACCACATCTAGAACAGCTG
>HPV4_Gamma_9626597_nt5516_L1_Tile|1
GGAACCTCACAAAGTATTAGTTCCTAAGGTTTCAGGAAGTCAATTTAGAGTGTTTAGATT
>HPV4_Gamma_9626597_nt5541_L1_Tile|1
AAGGTTTCAGGAAGTCAATTTAGAGTGTTTAGATTCAATTTGCCAGACCCAAACAGATTT
>HPV4_Gamma_9626597_nt5556_L1_Tile|1
CAATTTAGAGTGTTTAGATTCAATTTGCCAGACCCAAACAGATTTGCTTTAATTGATAAT
>HPV4_Gamma_9626597_nt5621_L1_Tile|1
TTATGATTCTGATCATGAACGCCTAGTATGGAAACTGAGGGGAATAGAAATAGGAAGAGG
>HPV4_Gamma_9626597_nt5631_L1_Tile|1
GATCATGAACGCCTAGTATGGAAACTGAGGGGAATAGAAATAGGAAGAGGAGGACCGCTT
>HPV4_Gamma_9626597_nt5636_L1_Tile|1
TGAACGCCTAGTATGGAAACTGAGGGGAATAGAAATAGGAAGAGGAGGACCGCTTGGTAT
>HPV4_Gamma_9626597_nt5646_L1_Tile|1
GTATGGAAACTGAGGGGAATAGAAATAGGAAGAGGAGGACCGCTTGGTATAGGTACTACA
>HPV4_Gamma_9626597_nt5671_L1_Tile|1
TAGGAAGAGGAGGACCGCTTGGTATAGGTACTACAGGTCATCCTTTATATAATAAGTTTG
>HPV4_Gamma_9626597_nt6071_L1_Tile|1
CTATGGAGATAGCTTGTTTTTCTTTGGAAGAAGAGAACAACTATATGCCAGACATTTCTT
>HPV4_Gamma_9626597_nt6076_L1_Tile|1
GAGATAGCTTGTTTTTCTTTGGAAGAAGAGAACAACTATATGCCAGACATTTCTTTGTCA
>HPV4_Gamma_9626597_nt6086_L1_Tile|1
GTTTTTCTTTGGAAGAAGAGAACAACTATATGCCAGACATTTCTTTGTCAGAGCAGGCAC
>HPV4_Gamma_9626597_nt6151_L1_Tile|1
GAGATGCTCTACCAGAACCTTTTGAAGCTACCTCAGATTATTTTATTGGTGCTCAAAACC
>HPV4_Gamma_9626597_nt6156_L1_Tile|1
GCTCTACCAGAACCTTTTGAAGCTACCTCAGATTATTTTATTGGTGCTCAAAACCAACAA
>HPV4_Gamma_9626597_nt6161_L1_Tile|1
ACCAGAACCTTTTGAAGCTACCTCAGATTATTTTATTGGTGCTCAAAACCAACAAGATCA
>HPV4_Gamma_9626597_nt6166_L1_Tile|1
AACCTTTTGAAGCTACCTCAGATTATTTTATTGGTGCTCAAAACCAACAAGATCAGTACA
>HPV4_Gamma_9626597_nt6171_L1_Tile|1
TTTGAAGCTACCTCAGATTATTTTATTGGTGCTCAAAACCAACAAGATCAGTACACTTTA
>HPV4_Gamma_9626597_nt6176_L1_Tile|1
AGCTACCTCAGATTATTTTATTGGTGCTCAAAACCAACAAGATCAGTACACTTTAGGACC
>HPV4_Gamma_9626597_nt6181_L1_Tile|1
CCTCAGATTATTTTATTGGTGCTCAAAACCAACAAGATCAGTACACTTTAGGACCTCATA
>HPV4_Gamma_9626597_nt6186_L1_Tile|1
GATTATTTTATTGGTGCTCAAAACCAACAAGATCAGTACACTTTAGGACCTCATATTTAT
>HPV4_Gamma_9626597_nt6191_L1_Tile|1
TTTTATTGGTGCTCAAAACCAACAAGATCAGTACACTTTAGGACCTCATATTTATGTAGG
>HPV4_Gamma_9626597_nt6196_L1_Tile|1
TTGGTGCTCAAAACCAACAAGATCAGTACACTTTAGGACCTCATATTTATGTAGGGACCC
>HPV4_Gamma_9626597_nt6206_L1_Tile|1
AAACCAACAAGATCAGTACACTTTAGGACCTCATATTTATGTAGGGACCCCTAGTGGCTC
>HPV4_Gamma_9626597_nt6211_L1_Tile|1
AACAAGATCAGTACACTTTAGGACCTCATATTTATGTAGGGACCCCTAGTGGCTCTTTAG
>HPV4_Gamma_9626597_nt6216_L1_Tile|1

GATCAGTACACTTTAGGACCTCATATTTATGTAGGGACCCCTAGTGGCTCTTTAGTATCC
>HPV4_Gamma_9626597_nt6221_L1_Tile|1
GTACACTTTAGGACCTCATATTTATGTAGGGACCCCTAGTGGCTCTTTAGTATCCAGTGA
>HPV4_Gamma_9626597_nt6226_L1_Tile|1
CTTTAGGACCTCATATTTATGTAGGGACCCCTAGTGGCTCTTTAGTATCCAGTGAATCCC
>HPV4_Gamma_9626597_nt6356_L1_Tile|1
GTTGTTTGTTACTCTTGTAGATAACACTCATAATACAAACTTTACAATTTCTGTGAAGTC
>HPV4_Gamma_9626597_nt6361_L1_Tile|1
TTGTTACTCTTGTAGATAACACTCATAATACAAACTTTACAATTTCTGTGAAGTCAGATG
>HPV4_Gamma_9626597_nt6366_L1_Tile|1
ACTCTTGTAGATAACACTCATAATACAAACTTTACAATTTCTGTGAAGTCAGATGGTGCT
>HPV4_Gamma_9626597_nt6371_L1_Tile|1
TGTAGATAACACTCATAATACAAACTTTACAATTTCTGTGAAGTCAGATGGTGCTAATGA
>HPV4_Gamma_9626597_nt6376_L1_Tile|1
ATAACACTCATAATACAAACTTTACAATTTCTGTGAAGTCAGATGGTGCTAATGACAATT
>HPV4_Gamma_9626597_nt6381_L1_Tile|1
ACTCATAATACAAACTTTACAATTTCTGTGAAGTCAGATGGTGCTAATGACAATTATCAG
>HPV4_Gamma_9626597_nt6386_L1_Tile|1
TAATACAAACTTTACAATTTCTGTGAAGTCAGATGGTGCTAATGACAATTATCAGTATAA
>HPV4_Gamma_9626597_nt6391_L1_Tile|1
CAAACTTTACAATTTCTGTGAAGTCAGATGGTGCTAATGACAATTATCAGTATAAAGCTA
>HPV4_Gamma_9626597_nt6396_L1_Tile|1
TTTACAATTTCTGTGAAGTCAGATGGTGCTAATGACAATTATCAGTATAAAGCTAGTGAT
>HPV4_Gamma_9626597_nt6401_L1_Tile|1
AATTTCTGTGAAGTCAGATGGTGCTAATGACAATTATCAGTATAAAGCTAGTGATTTTAA
>HPV4_Gamma_9626597_nt6406_L1_Tile|1
CTGTGAAGTCAGATGGTGCTAATGACAATTATCAGTATAAAGCTAGTGATTTTAAACAGT
>HPV4_Gamma_9626597_nt6411_L1_Tile|1
AAGTCAGATGGTGCTAATGACAATTATCAGTATAAAGCTAGTGATTTTAAACAGTACCTC
>HPV4_Gamma_9626597_nt6416_L1_Tile|1
AGATGGTGCTAATGACAATTATCAGTATAAAGCTAGTGATTTTAAACAGTACCTCAGACA
>HPV4_Gamma_9626597_nt6626_L1_Tile|1
TCAATATAGATTTTTGCAATCTAGAGCTACAAGATGCCCTACACAGACCCCTGCAACTGA
>HPV4_Gamma_9626597_nt6631_L1_Tile|1
ATAGATTTTTGCAATCTAGAGCTACAAGATGCCCTACACAGACCCCTGCAACTGAAAAAG
>HPV4_Gamma_9626597_nt6636_L1_Tile|1
TTTTTGCAATCTAGAGCTACAAGATGCCCTACACAGACCCCTGCAACTGAAAAAGAAGAT
>HPV4_Gamma_9626597_nt6641_L1_Tile|1
GCAATCTAGAGCTACAAGATGCCCTACACAGACCCCTGCAACTGAAAAAGAAGATCCATA
>HPV4_Gamma_9626597_nt6646_L1_Tile|1
CTAGAGCTACAAGATGCCCTACACAGACCCCTGCAACTGAAAAAGAAGATCCATATAAAG
>HPV4_Gamma_9626597_nt6651_L1_Tile|1
GCTACAAGATGCCCTACACAGACCCCTGCAACTGAAAAAGAAGATCCATATAAAGATTTG
>HPV4_Gamma_9626597_nt6656_L1_Tile|1
AAGATGCCCTACACAGACCCCTGCAACTGAAAAAGAAGATCCATATAAAGATTTGTCTTT
>HPV4_Gamma_9626597_nt6771_L1_Tile|1
GGCAGGCGGTTTTTATATCAAAGTGGTTTAATTAATGGTTCTCTAAAACGTAAAAGAATA
>HPV4_Gamma_9626597_nt6776_L1_Tile|1
GCGGTTTTTATATCAAAGTGGTTTAATTAATGGTTCTCTAAAACGTAAAAGAATAATAAG
>HPV4_Gamma_9626597_nt6781_L1_Tile|1
TTTTATATCAAAGTGGTTTAATTAATGGTTCTCTAAAACGTAAAAGAATAATAAGTTCTT
>HPV4_Gamma_9626597_nt6786_L1_Tile|1
TATCAAAGTGGTTTAATTAATGGTTCTCTAAAACGTAAAAGAATAATAAGTTCTTCTCAT
>HPV4_Gamma_9626597_nt6791_L1_Tile|1
AAGTGGTTTAATTAATGGTTCTCTAAAACGTAAAAGAATAATAAGTTCTTCTCATGCACA

>HPV4_Gamma_9626597_nt6801_L1_Tile|1
ATTAATGGTTCTCTAAAACGTAAAAGAATAATAAGTTCTTCTCATGCACAAACTAATACC
>HPV4_Gamma_9626597_nt6811_L1_Tile|1
CTCTAAAACGTAAAAGAATAATAAGTTCTTCTCATGCACAAACTAATACCAAACGTTCTG
>HPV4_Gamma_9626597_nt6816_L1_Tile|1
AAACGTAAAAGAATAATAAGTTCTTCTCATGCACAAACTAATACCAAACGTTCTGCCAAA
>HPV5_Beta_9627145_nt6278_L1_Tile|1
CACCCTTATTTCAATAAAGTAAAAGATACAGAAAACAGTAATGCATACATAACATTTTCT
>HPV5_Beta_9627145_nt6283_L1_Tile|1
TTATTTCAATAAAGTAAAAGATACAGAAAACAGTAATGCATACATAACATTTTCTAAAGA
>HPV5_Beta_9627145_nt6513_L1_Tile|1
ATATGGCAGACATAGGTTTTGGGAACATGAATTTTAAGGCACTTCAAGATAGTAGATCAG
>HPV5_Beta_9627145_nt6518_L1_Tile|1
GCAGACATAGGTTTTGGGAACATGAATTTTAAGGCACTTCAAGATAGTAGATCAGATGTC
>HPV5_Beta_9627145_nt6523_L1_Tile|1
CATAGGTTTTGGGAACATGAATTTTAAGGCACTTCAAGATAGTAGATCAGATGTCAGTTT
>HPV5_Beta_9627145_nt6533_L1_Tile|1
GGGAACATGAATTTTAAGGCACTTCAAGATAGTAGATCAGATGTCAGTTTAGACATCGTC
>HPV5_Beta_9627145_nt6538_L1_Tile|1
CATGAATTTTAAGGCACTTCAAGATAGTAGATCAGATGTCAGTTTAGACATCGTCAATGA
>HPV5_Beta_9627145_nt6958_L1_Tile|1
CAACACAAGAAATACTAATTTCAGTATTTCTGTATATAATCAGGCTGGAGCACTAAAAGA
>HPV5_Beta_9627145_nt6963_L1_Tile|1
CAAGAAATACTAATTTCAGTATTTCTGTATATAATCAGGCTGGAGCACTAAAAGATGTTG
>HPV5_Beta_9627145_nt6968_L1_Tile|1
AATACTAATTTCAGTATTTCTGTATATAATCAGGCTGGAGCACTAAAAGATGTTGCAGAC
>HPV5_Beta_9627145_nt6988_L1_Tile|1
TGTATATAATCAGGCTGGAGCACTAAAAGATGTTGCAGACTATAATGCAGATCAATTTAG
>HPV5_Beta_9627145_nt6993_L1_Tile|1
ATAATCAGGCTGGAGCACTAAAAGATGTTGCAGACTATAATGCAGATCAATTTAGAGAAT
>HPV5_Beta_9627145_nt6998_L1_Tile|1
CAGGCTGGAGCACTAAAAGATGTTGCAGACTATAATGCAGATCAATTTAGAGAATATCAA
>HPV5_Beta_9627145_nt7003_L1_Tile|1
TGGAGCACTAAAAGATGTTGCAGACTATAATGCAGATCAATTTAGAGAATATCAAAGACA
>HPV5_Beta_9627145_nt7008_L1_Tile|1
CACTAAAAGATGTTGCAGACTATAATGCAGATCAATTTAGAGAATATCAAAGACATGTAG
>HPV5_Beta_9627145_nt7068_L1_Tile|1
AAGAATATGAAATATCTTTAATTCTACAACTCTGTAAGGTTCCTTTAAAGGCACAGGTAT
>HPV5_Beta_9627145_nt7073_L1_Tile|1
TATGAAATATCTTTAATTCTACAACTCTGTAAGGTTCCTTTAAAGGCACAGGTATTGGCA
>HPV5_Beta_9627145_nt7078_L1_Tile|1
AATATCTTTAATTCTACAACTCTGTAAGGTTCCTTTAAAGGCACAGGTATTGGCACAGAT
>HPV5_Beta_9627145_nt7223_L1_Tile|1
TATATTGACTCTTTGGCTACACGGTGTCCAGATAAGAATCCTCCGAAAGAAAAGGAAGAC
>HPV6_Alpha_6002612_nt5926_L1_Tile|1
CATCCTTATTTTTCCATAAAACGGGCTAACAAAACTGTTGTGCCAAAGGTGTCAGGATAT
>HPV6_Alpha_6002612_nt6131_L1_Tile|1
TAAGTGGACATCCTTTCCTAAATAAATATGATGATGTTGAAAATTCAGGGAGTGGTGGTA
>HPV6_Alpha_6002612_nt6141_L1_Tile|1
TCCTTTCCTAAATAAATATGATGATGTTGAAAATTCAGGGAGTGGTGGTAACCCTGGACA
>HPV6_Alpha_6002612_nt6146_L1_Tile|1
TCCTAAATAAATATGATGATGTTGAAAATTCAGGGAGTGGTGGTAACCCTGGACAGGATA
>HPV6_Alpha_6002612_nt6151_L1_Tile|1
AATAAATATGATGATGTTGAAAATTCAGGGAGTGGTGGTAACCCTGGACAGGATAACAGG
>HPV6_Alpha_6002612_nt6266_L1_Tile|1

CCCCTTTGGGCGAGCATTGGGGTAAAGGTAAACAGTGTACTAATACACCTGTACAGGCTG
>HPV6_Alpha_6002612_nt6561_L1_Tile|1
TAACAGGGCTGGCGAGGTGGGGAACCTGTGCCTGATACTCTTATAATTAAGGGTAGTGG
>HPV6_Alpha_6002612_nt6566_L1_Tile|1
GGGCTGGCGAGGTGGGGAACCTGTGCCTGATACTCTTATAATTAAGGGTAGTGGAAATC
>HPV6_Alpha_6002612_nt6571_L1_Tile|1
GGCGAGGTGGGGAACCTGTGCCTGATACTCTTATAATTAAGGGTAGTGGAAATCGAACG
>HPV6_Alpha_6002612_nt6591_L1_Tile|1
GCCTGATACTCTTATAATTAAGGGTAGTGGAAATCGAACGTCTGTAGGGAGTAGTATATA
>HPV6_Alpha_6002612_nt6596_L1_Tile|1
ATACTCTTATAATTAAGGGTAGTGGAAATCGAACGTCTGTAGGGAGTAGTATATATGTTA
>HPV6_Alpha_6002612_nt6601_L1_Tile|1
CTTATAATTAAGGGTAGTGGAAATCGAACGTCTGTAGGGAGTAGTATATATGTTAACACC
>HPV6_Alpha_6002612_nt6611_L1_Tile|1
AGGGTAGTGGAAATCGAACGTCTGTAGGGAGTAGTATATATGTTAACACCCCAAGCGGCT
>HPV6_Alpha_6002612_nt6616_L1_Tile|1
AGTGGAAATCGAACGTCTGTAGGGAGTAGTATATATGTTAACACCCCAAGCGGCTCTTTG
>HPV6_Alpha_6002612_nt6621_L1_Tile|1
AAATCGAACGTCTGTAGGGAGTAGTATATATGTTAACACCCCAAGCGGCTCTTTGGTGTC
>HPV6_Alpha_6002612_nt6791_L1_Tile|1
GCAGTACCAACATGACATTATGTGCATCCGTAACTACATCTTCCACATACACCAATTCTG
>HPV6_Alpha_6002612_nt6796_L1_Tile|1
ACCAACATGACATTATGTGCATCCGTAACTACATCTTCCACATACACCAATTCTGATTAT
>HPV6_Alpha_6002612_nt6801_L1_Tile|1
CATGACATTATGTGCATCCGTAACTACATCTTCCACATACACCAATTCTGATTATAAAGA
>HPV6_Alpha_6002612_nt6806_L1_Tile|1
CATTATGTGCATCCGTAACTACATCTTCCACATACACCAATTCTGATTATAAAGAGTACA
>HPV6_Alpha_6002612_nt6811_L1_Tile|1
TGTGCATCCGTAACTACATCTTCCACATACACCAATTCTGATTATAAAGAGTACATGCGT
>HPV6_Alpha_6002612_nt6816_L1_Tile|1
ATCCGTAACTACATCTTCCACATACACCAATTCTGATTATAAAGAGTACATGCGTCATGT
>HPV6_Alpha_6002612_nt6821_L1_Tile|1
TAACTACATCTTCCACATACACCAATTCTGATTATAAAGAGTACATGCGTCATGTGGAAG
>HPV6_Alpha_6002612_nt6826_L1_Tile|1
ACATCTTCCACATACACCAATTCTGATTATAAAGAGTACATGCGTCATGTGGAAGAGTAT
>HPV6_Alpha_6002612_nt7171_L1_Tile|1
CGCAAGTTTTGTTACAAAGTGGATATAGGGGACGGTCCTCTATTCGTACCGGTGTTAAG
>HPV6_Alpha_6002612_nt7191_L1_Tile|1
TGGATATAGGGGACGGTCCTCTATTCGTACCGGTGTTAAGCGCCCTGCTGTTTCCAAAGC
>HPV7_Alpha_9627389_nt5904_L1_Tile|1
GCAGGTAGTACCAGGTTATTAACCATAGGACATCCATATTTTGAATTGAAAAAGCCTAAT
>HPV7_Alpha_9627389_nt5909_L1_Tile|1
TAGTACCAGGTTATTAACCATAGGACATCCATATTTTGAATTGAAAAAGCCTAATGGCGA
>HPV7_Alpha_9627389_nt5914_L1_Tile|1
CCAGGTTATTAACCATAGGACATCCATATTTTGAATTGAAAAAGCCTAATGGCGATGTAT
>HPV7_Alpha_9627389_nt5919_L1_Tile|1
TTATTAACCATAGGACATCCATATTTTGAATTGAAAAAGCCTAATGGCGATGTATCGGTG
>HPV7_Alpha_9627389_nt5924_L1_Tile|1
AACCATAGGACATCCATATTTTGAATTGAAAAAGCCTAATGGCGATGTATCGGTGCCTAA
>HPV7_Alpha_9627389_nt5929_L1_Tile|1
TAGGACATCCATATTTTGAATTGAAAAAGCCTAATGGCGATGTATCGGTGCCTAAAGTGT
>HPV7_Alpha_9627389_nt5934_L1_Tile|1
CATCCATATTTTGAATTGAAAAAGCCTAATGGCGATGTATCGGTGCCTAAAGTGTCTGGA
>HPV7_Alpha_9627389_nt5939_L1_Tile|1
ATATTTTGAATTGAAAAAGCCTAATGGCGATGTATCGGTGCCTAAAGTGTCTGGACATCA

>HPV7_Alpha_9627389_nt5944_L1_Tile|1
TTGAATTGAAAAAGCCTAATGGCGATGTATCGGTGCCTAAAGTGTCTGGACATCAATACA
>HPV7_Alpha_9627389_nt6004_L1_Tile|1
GAGTGTTTAGAGTACGCTTGCCCGACCCTAATAAATTTGGATTATCAGACACGTCTTTAT
>HPV7_Alpha_9627389_nt6014_L1_Tile|1
AGTACGCTTGCCCGACCCTAATAAATTTGGATTATCAGACACGTCTTTATTTAATTCTGA
>HPV7_Alpha_9627389_nt6029_L1_Tile|1
CCCTAATAAATTTGGATTATCAGACACGTCTTTATTTAATTCTGAAACCCAACGCCTTGT
>HPV7_Alpha_9627389_nt6584_L1_Tile|1
AGGAACTACTGGAGACAGTGTTCCAAATGATTTATATATAACAGGTTCATCTAATCGCGC
>HPV7_Alpha_9627389_nt6589_L1_Tile|1
CTACTGGAGACAGTGTTCCAAATGATTTATATATAACAGGTTCATCTAATCGCGCTTCTA
>HPV7_Alpha_9627389_nt6594_L1_Tile|1
GGAGACAGTGTTCCAAATGATTTATATATAACAGGTTCATCTAATCGCGCTTCTATTGCA
>HPV7_Alpha_9627389_nt6959_L1_Tile|1
GACATATATACATGCTATGGATTCTTCCTTATTAGATGATTGGAATTTTAAAATTGGTCC
>HPV7_Alpha_9627389_nt6979_L1_Tile|1
ATTCTTCCTTATTAGATGATTGGAATTTTAAAATTGGTCCTCCAGCGTCTGCAACCTTGG
>HPV7_Alpha_9627389_nt6984_L1_Tile|1
TCCTTATTAGATGATTGGAATTTTAAAATTGGTCCTCCAGCGTCTGCAACCTTGGAAGAT
>HPV7_Alpha_9627389_nt7034_L1_Tile|1
CTTGGAAGATACTTATAGGTTTCTTACCAATAAAGCCATAGCATGTCAGCGTGATGCACC
>HPV7_Alpha_9627389_nt7044_L1_Tile|1
ACTTATAGGTTTCTTACCAATAAAGCCATAGCATGTCAGCGTGATGCACCCCCAAAAGAA
>HPV8_Beta_333074_nt6007_L1_Tile|1
TGTTTACAACAATAATGGTGACACATTACAGGTTCCCAAAGTATCGGGAAATCAACACAG
>HPV8_Beta_333074_nt6012_L1_Tile|1
ACAACAATAATGGTGACACATTACAGGTTCCCAAAGTATCGGGAAATCAACACAGGGTCT
>HPV8_Beta_333074_nt6027_L1_Tile|1
ACACATTACAGGTTCCCAAAGTATCGGGAAATCAACACAGGGTCTTTCGCTTAAAGTTAC
>HPV8_Beta_333074_nt6042_L1_Tile|2
CCAAAGTATCGGGAAATCAACACAGGGTCTTTCGCTTAAAGTTACCAGATCCAAATAGGT
>HPV8_Beta_333074_nt6197_L1_Tile|1
GTTGGGAGCACCGGCCATCCCTATTTTAATAAAGTGAAAGACACTGAAAACAGCAATTCA
>HPV8_Beta_333074_nt6202_L1_Tile|2
GAGCACCGGCCATCCCTATTTTAATAAAGTGAAAGACACTGAAAACAGCAATTCATACAC
>HPV8_Beta_333074_nt6207_L1_Tile|1
CCGGCCATCCCTATTTTAATAAAGTGAAAGACACTGAAAACAGCAATTCATACACCACAA
>HPV8_Beta_333074_nt6357_L1_Tile|1
GGGAAAAAGCCATTCCATGTGCAGAGGACCAACAGCAAGGTCTGTGCCCACCCATTGAAC
>HPV8_Beta_333074_nt6362_L1_Tile|2
AAAGCCATTCCATGTGCAGAGGACCAACAGCAAGGTCTGTGCCCACCCATTGAACTAAAA
>HPV8_Beta_333074_nt6367_L1_Tile|1
CATTCCATGTGCAGAGGACCAACAGCAAGGTCTGTGCCCACCCATTGAACTAAAAAATAC
>HPV8_Beta_333074_nt6372_L1_Tile|1
CATGTGCAGAGGACCAACAGCAAGGTCTGTGCCCACCCATTGAACTAAAAAATACAGTTA
>HPV8_Beta_333074_nt6377_L1_Tile|1
GCAGAGGACCAACAGCAAGGTCTGTGCCCACCCATTGAACTAAAAAATACAGTTATTGAA
>HPV8_Beta_333074_nt6382_L1_Tile|2
GGACCAACAGCAAGGTCTGTGCCCACCCATTGAACTAAAAAATACAGTTATTGAAGATGG
>HPV8_Beta_333074_nt6457_L1_Tile|1
TGGTTTTGGTAATATGAACTTTAAGACTTTACAACAGAACAGATCTGATGTAAGTCTCGA
>HPV8_Beta_333074_nt6462_L1_Tile|2
TTGGTAATATGAACTTTAAGACTTTACAACAGAACAGATCTGATGTAAGTCTCGACATAG
>HPV8_Beta_333074_nt6467_L1_Tile|1

AATATGAACTTTAAGACTTTACAACAGAACAGATCTGATGTAAGTCTCGACATAGTAAAT
>HPV8_Beta_333074_nt6472_L1_Tile|1
GAACTTTAAGACTTTACAACAGAACAGATCTGATGTAAGTCTCGACATAGTAAATGAGAT
>HPV8_Beta_333074_nt6477_L1_Tile|1
TTAAGACTTTACAACAGAACAGATCTGATGTAAGTCTCGACATAGTAAATGAGATATGCA
>HPV8_Beta_333074_nt6482_L1_Tile|2
ACTTTACAACAGAACAGATCTGATGTAAGTCTCGACATAGTAAATGAGATATGCAAATAT
>HPV8_Beta_333074_nt6892_L1_Tile|1
CACGCGAAACACCAATTTTAGTATTTCAGTTTACACTGAAAATGGGGAACTTAAGAACAT
>HPV8_Beta_333074_nt6897_L1_Tile|1
GAAACACCAATTTTAGTATTTCAGTTTACACTGAAAATGGGGAACTTAAGAACATCACAG
>HPV8_Beta_333074_nt6902_L1_Tile|2
ACCAATTTTAGTATTTCAGTTTACACTGAAAATGGGGAACTTAAGAACATCACAGACTAT
>HPV8_Beta_333074_nt6907_L1_Tile|1
TTTTAGTATTTCAGTTTACACTGAAAATGGGGAACTTAAGAACATCACAGACTATAAATC
>HPV8_Beta_333074_nt6912_L1_Tile|1
GTATTTCAGTTTACACTGAAAATGGGGAACTTAAGAACATCACAGACTATAAATCAACCC
>HPV8_Beta_333074_nt6917_L1_Tile|1
TCAGTTTACACTGAAAATGGGGAACTTAAGAACATCACAGACTATAAATCAACCCAGTTC
>HPV8_Beta_333074_nt6922_L1_Tile|2
TTACACTGAAAATGGGGAACTTAAGAACATCACAGACTATAAATCAACCCAGTTCAGAGA
>HPV8_Beta_333074_nt6927_L1_Tile|1
CTGAAAATGGGGAACTTAAGAACATCACAGACTATAAATCAACCCAGTTCAGAGAATATC
>HPV8_Beta_333074_nt6932_L1_Tile|1
AATGGGGAACTTAAGAACATCACAGACTATAAATCAACCCAGTTCAGAGAATATCTGAGA
>HPV8_Beta_333074_nt6937_L1_Tile|1
GGAACTTAAGAACATCACAGACTATAAATCAACCCAGTTCAGAGAATATCTGAGACATGT
>HPV8_Beta_333074_nt6942_L1_Tile|2
TTAAGAACATCACAGACTATAAATCAACCCAGTTCAGAGAATATCTGAGACATGTAGAAG
>HPV8_Beta_333074_nt7002_L1_Tile|2
AATATGAAATTTCCCTCATATTACAATTGTGTAAGATACCACTAAAGGCTGATGTTTTAG
>HPV8_Beta_333074_nt7012_L1_Tile|1
TTCCCTCATATTACAATTGTGTAAGATACCACTAAAGGCTGATGTTTTAGCACAAATCAA
>HPV8_Beta_333074_nt7077_L1_Tile|1
TGAATTCATCACTACTTGAGGAATGGCAACTGGGATTTGTACCTACCCCTGATACTCCAA
>HPV8_Beta_333074_nt7082_L1_Tile|2
TCATCACTACTTGAGGAATGGCAACTGGGATTTGTACCTACCCCTGATACTCCAATTCAT
>HPV8_Beta_333074_nt7197_L1_Tile|1
CTAAGGAAAAGCCTGATCCATATGCAAAGTTTAACTTCTGGAATGTGGACCTTACAGAAC
>HPV8_Beta_333074_nt7202_L1_Tile|2
GAAAAGCCTGATCCATATGCAAAGTTTAACTTCTGGAATGTGGACCTTACAGAACGACTT
>HPV8_Beta_333074_nt7207_L1_Tile|1
GCCTGATCCATATGCAAAGTTTAACTTCTGGAATGTGGACCTTACAGAACGACTTTCCCT
>HPV8_Beta_333074_nt7217_L1_Tile|1
TATGCAAAGTTTAACTTCTGGAATGTGGACCTTACAGAACGACTTTCCCTGGATTTGGAT
>HPV8_Beta_333074_nt7222_L1_Tile|2
AAAGTTTAACTTCTGGAATGTGGACCTTACAGAACGACTTTCCCTGGATTTGGATCAATA
>HPV9_Beta_9627396_nt5861_L1_Tile|1
TTAGTGACCGTTTGCTAACAGTGGGTCATCCATATTATGATGTCCGCTCAGGCGACGGAC
>HPV9_Beta_9627396_nt5866_L1_Tile|1
GACCGTTTGCTAACAGTGGGTCATCCATATTATGATGTCCGCTCAGGCGACGGACAAAGG
>HPV9_Beta_9627396_nt5871_L1_Tile|1
TTTGCTAACAGTGGGTCATCCATATTATGATGTCCGCTCAGGCGACGGACAAAGGATTGA
>HPV9_Beta_9627396_nt5881_L1_Tile|1
GTGGGTCATCCATATTATGATGTCCGCTCAGGCGACGGACAAAGGATTGAAGTCCCTAAA

>HPV9_Beta_9627396_nt5901_L1_Tile|1
TGTCCGCTCAGGCGACGGACAAAGGATTGAAGTCCCTAAAGTGTCTGGTAATCAGTATCG
>HPV9_Beta_9627396_nt6181_L1_Tile|1
AACACATCTTTTGACCCCAAACAGGTACAAATGTTCATTATAGGATGTATTCCATGCTTA
>HPV9_Beta_9627396_nt6236_L1_Tile|1
GCTTAGGAGAACACTGGGATAAAGCCAAAGTGTGTGAAAAGGATGCTAATAATCAACTAG
>HPV9_Beta_9627396_nt6241_L1_Tile|1
GGAGAACACTGGGATAAAGCCAAAGTGTGTGAAAAGGATGCTAATAATCAACTAGGCTTA
>HPV9_Beta_9627396_nt6246_L1_Tile|1
ACACTGGGATAAAGCCAAAGTGTGTGAAAAGGATGCTAATAATCAACTAGGCTTATGTCC
>HPV9_Beta_9627396_nt6251_L1_Tile|1
GGGATAAAGCCAAAGTGTGTGAAAAGGATGCTAATAATCAACTAGGCTTATGTCCTCCTA
>HPV9_Beta_9627396_nt6256_L1_Tile|1
AAAGCCAAAGTGTGTGAAAAGGATGCTAATAATCAACTAGGCTTATGTCCTCCTATAGAA
>HPV9_Beta_9627396_nt6261_L1_Tile|1
CAAAGTGTGTGAAAAGGATGCTAATAATCAACTAGGCTTATGTCCTCCTATAGAATTAAG
>HPV9_Beta_9627396_nt6266_L1_Tile|1
TGTGTGAAAAGGATGCTAATAATCAACTAGGCTTATGTCCTCCTATAGAATTAAGAAACA
>HPV9_Beta_9627396_nt6366_L1_Tile|1
AAATATCAACAATAAGGAACTGTCCTTTAATAAGTCTGATGTAAGCTTAGATATTGTTGA
>HPV9_Beta_9627396_nt6371_L1_Tile|1
TCAACAATAAGGAACTGTCCTTTAATAAGTCTGATGTAAGCTTAGATATTGTTGATGAAA
>HPV9_Beta_9627396_nt6521_L1_Tile|1
GGCATTATTATGTTAGAGGAGGTTCAGTTGGTGACGCTGTTCCTGATGGTGCAGTAAACC
>HPV9_Beta_9627396_nt6526_L1_Tile|1
TATTATGTTAGAGGAGGTTCAGTTGGTGACGCTGTTCCTGATGGTGCAGTAAACCAGGAT
>HPV9_Beta_9627396_nt6536_L1_Tile|1
GAGGAGGTTCAGTTGGTGACGCTGTTCCTGATGGTGCAGTAAACCAGGATCATAATTTCT
>HPV9_Beta_9627396_nt6551_L1_Tile|1
GTGACGCTGTTCCTGATGGTGCAGTAAACCAGGATCATAATTTCTTTTTGCCAGCAAAAA
>HPV9_Beta_9627396_nt6556_L1_Tile|1
GCTGTTCCTGATGGTGCAGTAAACCAGGATCATAATTTCTTTTTGCCAGCAAAAAGTGAT
>HPV9_Beta_9627396_nt6586_L1_Tile|1
CATAATTTCTTTTTGCCAGCAAAAAGTGATCAACAACAACGAACAATAGCTAATTCCACC
>HPV9_Beta_9627396_nt6596_L1_Tile|1
TTTTGCCAGCAAAAAGTGATCAACAACAACGAACAATAGCTAATTCCACCTACTATCCTA
>HPV9_Beta_9627396_nt6601_L1_Tile|1
CCAGCAAAAAGTGATCAACAACAACGAACAATAGCTAATTCCACCTACTATCCTACAGTA
>HPV9_Beta_9627396_nt6606_L1_Tile|1
AAAAAGTGATCAACAACAACGAACAATAGCTAATTCCACCTACTATCCTACAGTAAGTGG
>HPV9_Beta_9627396_nt6616_L1_Tile|1
CAACAACAACGAACAATAGCTAATTCCACCTACTATCCTACAGTAAGTGGGTCATTAGTA
>HPV9_Beta_9627396_nt6621_L1_Tile|1
ACAACGAACAATAGCTAATTCCACCTACTATCCTACAGTAAGTGGGTCATTAGTAACTTC
>HPV9_Beta_9627396_nt6756_L1_Tile|1
TCAGATATTTGTTACAGTGGCAGACAATACACGTAACACCAATTTTACCATTAGTGTGTC
>HPV9_Beta_9627396_nt6766_L1_Tile|1
GTTACAGTGGCAGACAATACACGTAACACCAATTTTACCATTAGTGTGTCTACAGAGGCA
>HPV9_Beta_9627396_nt6776_L1_Tile|1
CAGACAATACACGTAACACCAATTTTACCATTAGTGTGTCTACAGAGGCAGCTCAAACAG
>HPV9_Beta_9627396_nt6781_L1_Tile|1
AATACACGTAACACCAATTTTACCATTAGTGTGTCTACAGAGGCAGCTCAAACAGAAGAA
>HPV9_Beta_9627396_nt6786_L1_Tile|1
ACGTAACACCAATTTTACCATTAGTGTGTCTACAGAGGCAGCTCAAACAGAAGAATATAA
>HPV9_Beta_9627396_nt6791_L1_Tile|1

ACACCAATTTTACCATTAGTGTGTCTACAGAGGCAGCTCAAACAGAAGAATATAATGCCA
>HPV9_Beta_9627396_nt6796_L1_Tile|1
AATTTTACCATTAGTGTGTCTACAGAGGCAGCTCAAACAGAAGAATATAATGCCAATAAT
>HPV9_Beta_9627396_nt6801_L1_Tile|1
TACCATTAGTGTGTCTACAGAGGCAGCTCAAACAGAAGAATATAATGCCAATAATATTAG
>HPV9_Beta_9627396_nt6806_L1_Tile|1
TTAGTGTGTCTACAGAGGCAGCTCAAACAGAAGAATATAATGCCAATAATATTAGAGAAT
>HPV9_Beta_9627396_nt6886_L1_Tile|1
TATCAGATTTCATTAATCTTACAGTTGTGTAAAGTGCCTTTAGTAGCTGAAGTATTATCC
>HPV9_Beta_9627396_nt6891_L1_Tile|1
GATTTCATTAATCTTACAGTTGTGTAAAGTGCCTTTAGTAGCTGAAGTATTATCCCAGAT
>HPV9_Beta_9627396_nt7026_L1_Tile|1
TATCTACAGATATATTGATTCAAAAGCCACAAAATGCCCAGATGCTGTTGAGCCTACAGA
>HPV9_Beta_9627396_nt7096_L1_Tile|1
CCCTTTGCCAAATACTCATTTTGGAAAGTGGATCTAACTGAAAGATTATCGTTGGATCTT
>HPV9_Beta_9627396_nt7101_L1_Tile|1
TGCCAAATACTCATTTTGGAAAGTGGATCTAACTGAAAGATTATCGTTGGATCTTGATCA
>HPV9_Beta_9627396_nt7136_L1_Tile|1
AAAGATTATCGTTGGATCTTGATCAATATCCTTTAGGTAGAAAATTTCTTTTTCAAGCTG
>HPV9_Beta_9627396_nt7166_L1_Tile|1
CTTTAGGTAGAAAATTTCTTTTTCAAGCTGGTTTGCAAACACGAAAACGTCCTATTAAAA
>HPV9_Beta_9627396_nt7171_L1_Tile|1
GGTAGAAAATTTCTTTTTCAAGCTGGTTTGCAAACACGAAAACGTCCTATTAAAACATCT
>HPV9_Beta_9627396_nt7176_L1_Tile|1
AAAATTTCTTTTTCAAGCTGGTTTGCAAACACGAAAACGTCCTATTAAAACATCTGTTAA
>HPV9_Beta_9627396_nt7181_L1_Tile|1
TTCTTTTTCAAGCTGGTTTGCAAACACGAAAACGTCCTATTAAAACATCTGTTAAAACAT
>HPV9_Beta_9627396_nt7186_L1_Tile|1
TTTCAAGCTGGTTTGCAAACACGAAAACGTCCTATTAAAACATCTGTTAAAACATCTAAA
>HPV9_Beta_9627396_nt7191_L1_Tile|1
AGCTGGTTTGCAAACACGAAAACGTCCTATTAAAACATCTGTTAAAACATCTAAAAATGC
>HPV9_Beta_9627396_nt7196_L1_Tile|1
GTTTGCAAACACGAAAACGTCCTATTAAAACATCTGTTAAAACATCTAAAAATGCTAAGA
>HPV9_Beta_9627396_nt7201_L1_Tile|1
CAAACACGAAAACGTCCTATTAAAACATCTGTTAAAACATCTAAAAATGCTAAGAGAAGG
>HPV9_Beta_9627396_nt7206_L1_Tile|1
ACGAAAACGTCCTATTAAAACATCTGTTAAAACATCTAAAAATGCTAAGAGAAGGCGAAC
>HPV9_Beta_9627396_nt7210_L1_Tile|1
AAACGTCCTATTAAAACATCTGTTAAAACATCTAAAAATGCTAAGAGAAGGCGAACCTAA
>HPV10_Alpha_9627257_nt6590_L1_Tile|1
CAAGACATTTCTTTAATCGGGCTAGTGCTGTTGGAGACGCCATCCCAGACACTTTCATAT
>HPV10_Alpha_9627257_nt6595_L1_Tile|1
CATTTCTTTAATCGGGCTAGTGCTGTTGGAGACGCCATCCCAGACACTTTCATATTAAAG
>HPV10_Alpha_9627257_nt6600_L1_Tile|1
CTTTAATCGGGCTAGTGCTGTTGGAGACGCCATCCCAGACACTTTCATATTAAAGAGCAA
>HPV10_Alpha_9627257_nt6615_L1_Tile|1
TGCTGTTGGAGACGCCATCCCAGACACTTTCATATTAAAGAGCAACGGTGGGGGGCGAGA
>HPV10_Alpha_9627257_nt6625_L1_Tile|1
GACGCCATCCCAGACACTTTCATATTAAAGAGCAACGGTGGGGGGCGAGACGTTGGTAGT
>HPV10_Alpha_9627257_nt6815_L1_Tile|1
CTGTGGTAGACACGACTCGCAGTACCAATATGTGCTTGTGTGTTCCTTCTGAGGCCTCCC
>HPV10_Alpha_9627257_nt6820_L1_Tile|1
GTAGACACGACTCGCAGTACCAATATGTGCTTGTGTGTTCCTTCTGAGGCCTCCCCTGCC
>HPV10_Alpha_9627257_nt6825_L1_Tile|1
CACGACTCGCAGTACCAATATGTGCTTGTGTGTTCCTTCTGAGGCCTCCCCTGCCACTAC

>HPV10_Alpha_9627257_nt6840_L1_Tile|1
CAATATGTGCTTGTGTGTTCCTTCTGAGGCCTCCCCTGCCACTACGTATGACGCCACCAA
>HPV101_Unclassified_109390389_nt4918_L1_Tile|1
ATGTGTCTGAACCTCCTATTGTTGTGGATGGTTTTGATGCATTTGATACATTTTATTTGC
>HPV101_Unclassified_109390389_nt4923_L1_Tile|1
TCTGAACCTCCTATTGTTGTGGATGGTTTTGATGCATTTGATACATTTTATTTGCATCCA
>HPV101_Unclassified_109390389_nt4928_L1_Tile|1
ACCTCCTATTGTTGTGGATGGTTTTGATGCATTTGATACATTTTATTTGCATCCAAGTCA
>HPV101_Unclassified_109390389_nt4933_L1_Tile|1
CTATTGTTGTGGATGGTTTTGATGCATTTGATACATTTTATTTGCATCCAAGTCACAGAC
>HPV101_Unclassified_109390389_nt4938_L1_Tile|1
GTTGTGGATGGTTTTGATGCATTTGATACATTTTATTTGCATCCAAGTCACAGACGCAAA
>HPV101_Unclassified_109390389_nt4963_L1_Tile|1
ATACATTTTATTTGCATCCAAGTCACAGACGCAAACGCAAGCGCAAACGGTCTGGTTTTT
>HPV101_Unclassified_109390389_nt4968_L1_Tile|1
TTTTATTTGCATCCAAGTCACAGACGCAAACGCAAGCGCAAACGGTCTGGTTTTTAATGT
>HPV101_Unclassified_109390389_nt5008_L1_Tile|1
AACGGTCTGGTTTTTAATGTTTTGCAGATGGCTGGTTTGTGGCTACCTCAGACTGGTAAA
>HPV101_Unclassified_109390389_nt5013_L1_Tile|1
TCTGGTTTTTAATGTTTTGCAGATGGCTGGTTTGTGGCTACCTCAGACTGGTAAAATATA
>HPV101_Unclassified_109390389_nt5018_L1_Tile|1
TTTTTAATGTTTTGCAGATGGCTGGTTTGTGGCTACCTCAGACTGGTAAAATATACCTAC
>HPV101_Unclassified_109390389_nt5043_L1_Tile|1
TTTGTGGCTACCTCAGACTGGTAAAATATACCTACCTCCAGCTCCTCCTGTGTCTAGGAG
>HPV101_Unclassified_109390389_nt5048_L1_Tile|1
GGCTACCTCAGACTGGTAAAATATACCTACCTCCAGCTCCTCCTGTGTCTAGGAGTCTTA
>HPV101_Unclassified_109390389_nt5053_L1_Tile|1
CCTCAGACTGGTAAAATATACCTACCTCCAGCTCCTCCTGTGTCTAGGAGTCTTACCACC
>HPV101_Unclassified_109390389_nt5058_L1_Tile|1
GACTGGTAAAATATACCTACCTCCAGCTCCTCCTGTGTCTAGGAGTCTTACCACCGATGA
>HPV101_Unclassified_109390389_nt5063_L1_Tile|1
GTAAAATATACCTACCTCCAGCTCCTCCTGTGTCTAGGAGTCTTACCACCGATGAATATG
>HPV101_Unclassified_109390389_nt5083_L1_Tile|1
GCTCCTCCTGTGTCTAGGAGTCTTACCACCGATGAATATGTTGTGGAAACATCTGTATTC
>HPV101_Unclassified_109390389_nt5088_L1_Tile|1
TCCTGTGTCTAGGAGTCTTACCACCGATGAATATGTTGTGGAAACATCTGTATTCTTCCA
>HPV101_Unclassified_109390389_nt5118_L1_Tile|1
ATATGTTGTGGAAACATCTGTATTCTTCCATGCATCCAGTGATCGTCTAGTAACTGTGGG
>HPV101_Unclassified_109390389_nt5128_L1_Tile|1
GAAACATCTGTATTCTTCCATGCATCCAGTGATCGTCTAGTAACTGTGGGTCACCCCTAT
>HPV101_Unclassified_109390389_nt5133_L1_Tile|1
ATCTGTATTCTTCCATGCATCCAGTGATCGTCTAGTAACTGTGGGTCACCCCTATTTTCC
>HPV101_Unclassified_109390389_nt5138_L1_Tile|1
TATTCTTCCATGCATCCAGTGATCGTCTAGTAACTGTGGGTCACCCCTATTTTCCCGTTA
>HPV101_Unclassified_109390389_nt5143_L1_Tile|1
TTCCATGCATCCAGTGATCGTCTAGTAACTGTGGGTCACCCCTATTTTCCCGTTATAAAA
>HPV101_Unclassified_109390389_nt5148_L1_Tile|1
TGCATCCAGTGATCGTCTAGTAACTGTGGGTCACCCCTATTTTCCCGTTATAAAACCAGG
>HPV101_Unclassified_109390389_nt5153_L1_Tile|1
CCAGTGATCGTCTAGTAACTGTGGGTCACCCCTATTTTCCCGTTATAAAACCAGGCAATC
>HPV101_Unclassified_109390389_nt5158_L1_Tile|1
GATCGTCTAGTAACTGTGGGTCACCCCTATTTTCCCGTTATAAAACCAGGCAATCACACC
>HPV101_Unclassified_109390389_nt5163_L1_Tile|1
TCTAGTAACTGTGGGTCACCCCTATTTTCCCGTTATAAAACCAGGCAATCACACCGTTGA
>HPV101_Unclassified_109390389_nt5168_L1_Tile|1

TAACTGTGGGTCACCCCTATTTTCCCGTTATAAAACCAGGCAATCACACCGTTGAGGTTC
>HPV101_Unclassified_109390389_nt5178_L1_Tile|1
TCACCCCTATTTTCCCGTTATAAAACCAGGCAATCACACCGTTGAGGTTCCTAAGGTTTC
>HPV101_Unclassified_109390389_nt5183_L1_Tile|1
CCTATTTTCCCGTTATAAAACCAGGCAATCACACCGTTGAGGTTCCTAAGGTTTCTGGCA
>HPV101_Unclassified_109390389_nt5188_L1_Tile|1
TTTCCCGTTATAAAACCAGGCAATCACACCGTTGAGGTTCCTAAGGTTTCTGGCAACCAG
>HPV101_Unclassified_109390389_nt5193_L1_Tile|1
CGTTATAAAACCAGGCAATCACACCGTTGAGGTTCCTAAGGTTTCTGGCAACCAGTTTAG
>HPV101_Unclassified_109390389_nt5198_L1_Tile|1
TAAAACCAGGCAATCACACCGTTGAGGTTCCTAAGGTTTCTGGCAACCAGTTTAGGGCTT
>HPV101_Unclassified_109390389_nt5208_L1_Tile|1
CAATCACACCGTTGAGGTTCCTAAGGTTTCTGGCAACCAGTTTAGGGCTTTTCGCCTACG
>HPV101_Unclassified_109390389_nt5223_L1_Tile|1
GGTTCCTAAGGTTTCTGGCAACCAGTTTAGGGCTTTTCGCCTACGTCTCCCAGATCCCAA
>HPV101_Unclassified_109390389_nt5233_L1_Tile|1
GTTTCTGGCAACCAGTTTAGGGCTTTTCGCCTACGTCTCCCAGATCCCAATAAGTTTGCT
>HPV101_Unclassified_109390389_nt5238_L1_Tile|1
TGGCAACCAGTTTAGGGCTTTTCGCCTACGTCTCCCAGATCCCAATAAGTTTGCTTTAAT
>HPV101_Unclassified_109390389_nt5298_L1_Tile|1
TGACAAACAATTGTATAATGTTGAATCGGAGCGCCTCGTGTGGCGTCTTAGAGGAATTGA
>HPV101_Unclassified_109390389_nt5303_L1_Tile|1
AACAATTGTATAATGTTGAATCGGAGCGCCTCGTGTGGCGTCTTAGAGGAATTGATATTG
>HPV101_Unclassified_109390389_nt5308_L1_Tile|1
TTGTATAATGTTGAATCGGAGCGCCTCGTGTGGCGTCTTAGAGGAATTGATATTGGCAGA
>HPV101_Unclassified_109390389_nt5313_L1_Tile|1
TAATGTTGAATCGGAGCGCCTCGTGTGGCGTCTTAGAGGAATTGATATTGGCAGAGGAGG
>HPV101_Unclassified_109390389_nt5403_L1_Tile|1
TTTATTTAATAAGTTAAAGGATACTGAAAATCCTAATGGATATCAAAAGGGTGGTGCTAA
>HPV101_Unclassified_109390389_nt5408_L1_Tile|1
TTAATAAGTTAAAGGATACTGAAAATCCTAATGGATATCAAAAGGGTGGTGCTAAGGACG
>HPV101_Unclassified_109390389_nt5413_L1_Tile|1
AAGTTAAAGGATACTGAAAATCCTAATGGATATCAAAAGGGTGGTGCTAAGGACGCTAGA
>HPV101_Unclassified_109390389_nt5423_L1_Tile|1
ATACTGAAAATCCTAATGGATATCAAAAGGGTGGTGCTAAGGACGCTAGACAAAATGTTT
>HPV101_Unclassified_109390389_nt5428_L1_Tile|1
GAAAATCCTAATGGATATCAAAAGGGTGGTGCTAAGGACGCTAGACAAAATGTTTCTATG
>HPV101_Unclassified_109390389_nt5433_L1_Tile|1
TCCTAATGGATATCAAAAGGGTGGTGCTAAGGACGCTAGACAAAATGTTTCTATGGATCC
>HPV101_Unclassified_109390389_nt5438_L1_Tile|1
ATGGATATCAAAAGGGTGGTGCTAAGGACGCTAGACAAAATGTTTCTATGGATCCTAAAC
>HPV101_Unclassified_109390389_nt5543_L1_Tile|1
ACTGGGATGTTGCTCCTGCATGTGACAAAAATCCTCCTAAAGGCACCTGTCCTCCTTTAC
>HPV101_Unclassified_109390389_nt5548_L1_Tile|1
GATGTTGCTCCTGCATGTGACAAAAATCCTCCTAAAGGCACCTGTCCTCCTTTACAGTTG
>HPV101_Unclassified_109390389_nt5558_L1_Tile|1
CTGCATGTGACAAAAATCCTCCTAAAGGCACCTGTCCTCCTTTACAGTTGGTGTCCTCTG
>HPV101_Unclassified_109390389_nt5563_L1_Tile|1
TGTGACAAAAATCCTCCTAAAGGCACCTGTCCTCCTTTACAGTTGGTGTCCTCTGTTATT
>HPV101_Unclassified_109390389_nt5568_L1_Tile|1
CAAAAATCCTCCTAAAGGCACCTGTCCTCCTTTACAGTTGGTGTCCTCTGTTATTCAGGA
>HPV101_Unclassified_109390389_nt5658_L1_Tile|1
CATGAATTTTGCTGCCTTGCAGGCTGATCACTCAGGGGTACCCCTAGATATTGTTGCAGA
>HPV101_Unclassified_109390389_nt5663_L1_Tile|1
ATTTTGCTGCCTTGCAGGCTGATCACTCAGGGGTACCCCTAGATATTGTTGCAGATACTT

>HPV101_Unclassified_109390389_nt5668_L1_Tile|1
GCTGCCTTGCAGGCTGATCACTCAGGGGTACCCCTAGATATTGTTGCAGATACTTGTAAA
>HPV101_Unclassified_109390389_nt5678_L1_Tile|1
AGGCTGATCACTCAGGGGTACCCCTAGATATTGTTGCAGATACTTGTAAATGGCCAGACT
>HPV101_Unclassified_109390389_nt5683_L1_Tile|1
GATCACTCAGGGGTACCCCTAGATATTGTTGCAGATACTTGTAAATGGCCAGACTTTCTT
>HPV101_Unclassified_109390389_nt5688_L1_Tile|1
CTCAGGGGTACCCCTAGATATTGTTGCAGATACTTGTAAATGGCCAGACTTTCTTGGTAT
>HPV101_Unclassified_109390389_nt5698_L1_Tile|1
CCCCTAGATATTGTTGCAGATACTTGTAAATGGCCAGACTTTCTTGGTATGGCCAATGAT
>HPV101_Unclassified_109390389_nt5723_L1_Tile|1
GTAAATGGCCAGACTTTCTTGGTATGGCCAATGATAGGTTTGGGGATGGTATGTTTTTCT
>HPV101_Unclassified_109390389_nt5743_L1_Tile|1
GGTATGGCCAATGATAGGTTTGGGGATGGTATGTTTTTCTTTGGCCGCCGCGAGCAGGTG
>HPV101_Unclassified_109390389_nt5748_L1_Tile|1
GGCCAATGATAGGTTTGGGGATGGTATGTTTTTCTTTGGCCGCCGCGAGCAGGTGTATGC
>HPV101_Unclassified_109390389_nt5758_L1_Tile|1
AGGTTTGGGGATGGTATGTTTTTCTTTGGCCGCCGCGAGCAGGTGTATGCACGTCATTAT
>HPV101_Unclassified_109390389_nt5763_L1_Tile|1
TGGGGATGGTATGTTTTTCTTTGGCCGCCGCGAGCAGGTGTATGCACGTCATTATTTCAC
>HPV101_Unclassified_109390389_nt5768_L1_Tile|1
ATGGTATGTTTTTCTTTGGCCGCCGCGAGCAGGTGTATGCACGTCATTATTTCACCCATA
>HPV101_Unclassified_109390389_nt5773_L1_Tile|1
ATGTTTTTCTTTGGCCGCCGCGAGCAGGTGTATGCACGTCATTATTTCACCCATAATGGC
>HPV101_Unclassified_109390389_nt5778_L1_Tile|1
TTTCTTTGGCCGCCGCGAGCAGGTGTATGCACGTCATTATTTCACCCATAATGGCACTGT
>HPV101_Unclassified_109390389_nt5793_L1_Tile|1
CGAGCAGGTGTATGCACGTCATTATTTCACCCATAATGGCACTGTTGGTGACAGTGTTCC
>HPV101_Unclassified_109390389_nt5798_L1_Tile|1
AGGTGTATGCACGTCATTATTTCACCCATAATGGCACTGTTGGTGACAGTGTTCCAGAGG
>HPV101_Unclassified_109390389_nt5808_L1_Tile|1
ACGTCATTATTTCACCCATAATGGCACTGTTGGTGACAGTGTTCCAGAGGAGGGGTTTGA
>HPV101_Unclassified_109390389_nt5818_L1_Tile|1
TTCACCCATAATGGCACTGTTGGTGACAGTGTTCCAGAGGAGGGGTTTGAGGGTAATGGT
>HPV101_Unclassified_109390389_nt5828_L1_Tile|1
ATGGCACTGTTGGTGACAGTGTTCCAGAGGAGGGGTTTGAGGGTAATGGTAAAAAGTATT
>HPV101_Unclassified_109390389_nt5833_L1_Tile|1
ACTGTTGGTGACAGTGTTCCAGAGGAGGGGTTTGAGGGTAATGGTAAAAAGTATTTTATT
>HPV101_Unclassified_109390389_nt5848_L1_Tile|1
GTTCCAGAGGAGGGGTTTGAGGGTAATGGTAAAAAGTATTTTATTGGTGCTGAGCAAGGC
>HPV101_Unclassified_109390389_nt5863_L1_Tile|1
TTTGAGGGTAATGGTAAAAAGTATTTTATTGGTGCTGAGCAAGGCCAGGACCAGCATCAG
>HPV101_Unclassified_109390389_nt5868_L1_Tile|1
GGGTAATGGTAAAAAGTATTTTATTGGTGCTGAGCAAGGCCAGGACCAGCATCAGCTGGC
>HPV101_Unclassified_109390389_nt5878_L1_Tile|1
AAAAAGTATTTTATTGGTGCTGAGCAAGGCCAGGACCAGCATCAGCTGGCTCCCTCTTTA
>HPV101_Unclassified_109390389_nt5883_L1_Tile|1
GTATTTTATTGGTGCTGAGCAAGGCCAGGACCAGCATCAGCTGGCTCCCTCTTTATATTT
>HPV101_Unclassified_109390389_nt5888_L1_Tile|1
TTATTGGTGCTGAGCAAGGCCAGGACCAGCATCAGCTGGCTCCCTCTTTATATTTTGCTA
>HPV101_Unclassified_109390389_nt5893_L1_Tile|1
GGTGCTGAGCAAGGCCAGGACCAGCATCAGCTGGCTCCCTCTTTATATTTTGCTACACCT
>HPV101_Unclassified_109390389_nt5913_L1_Tile|1
CCAGCATCAGCTGGCTCCCTCTTTATATTTTGCTACACCTAGTGGGTCCTTAGCATCTAG
>HPV101_Unclassified_109390389_nt5918_L1_Tile|1

ATCAGCTGGCTCCCTCTTTATATTTTGCTACACCTAGTGGGTCCTTAGCATCTAGTGACT
>HPV101_Unclassified_109390389_nt5928_L1_Tile|1
TCCCTCTTTATATTTTGCTACACCTAGTGGGTCCTTAGCATCTAGTGACTCCCAGTTATT
>HPV101_Unclassified_109390389_nt5933_L1_Tile|1
CTTTATATTTTGCTACACCTAGTGGGTCCTTAGCATCTAGTGACTCCCAGTTATTTAGTA
>HPV101_Unclassified_109390389_nt5958_L1_Tile|1
GTCCTTAGCATCTAGTGACTCCCAGTTATTTAGTAGGCCCTACTGGATTGAACGGGCTCA
>HPV101_Unclassified_109390389_nt5978_L1_Tile|1
CCCAGTTATTTAGTAGGCCCTACTGGATTGAACGGGCTCAGGGTGCTAATAATGCTGTAT
>HPV101_Unclassified_109390389_nt5983_L1_Tile|1
TTATTTAGTAGGCCCTACTGGATTGAACGGGCTCAGGGTGCTAATAATGCTGTATTATGG
>HPV101_Unclassified_109390389_nt5988_L1_Tile|1
TAGTAGGCCCTACTGGATTGAACGGGCTCAGGGTGCTAATAATGCTGTATTATGGAATAA
>HPV101_Unclassified_109390389_nt5993_L1_Tile|1
GGCCCTACTGGATTGAACGGGCTCAGGGTGCTAATAATGCTGTATTATGGAATAATAATG
>HPV101_Unclassified_109390389_nt5998_L1_Tile|1
TACTGGATTGAACGGGCTCAGGGTGCTAATAATGCTGTATTATGGAATAATAATGTATTT
>HPV101_Unclassified_109390389_nt6003_L1_Tile|1
GATTGAACGGGCTCAGGGTGCTAATAATGCTGTATTATGGAATAATAATGTATTTGTTAC
>HPV101_Unclassified_109390389_nt6008_L1_Tile|1
AACGGGCTCAGGGTGCTAATAATGCTGTATTATGGAATAATAATGTATTTGTTACTTTGG
>HPV101_Unclassified_109390389_nt6018_L1_Tile|1
GGGTGCTAATAATGCTGTATTATGGAATAATAATGTATTTGTTACTTTGGTTGATAATAC
>HPV101_Unclassified_109390389_nt6023_L1_Tile|1
CTAATAATGCTGTATTATGGAATAATAATGTATTTGTTACTTTGGTTGATAATACCCACA
>HPV101_Unclassified_109390389_nt6033_L1_Tile|1
TGTATTATGGAATAATAATGTATTTGTTACTTTGGTTGATAATACCCACAATGTGAATTT
>HPV101_Unclassified_109390389_nt6038_L1_Tile|1
TATGGAATAATAATGTATTTGTTACTTTGGTTGATAATACCCACAATGTGAATTTTACTA
>HPV101_Unclassified_109390389_nt6043_L1_Tile|1
AATAATAATGTATTTGTTACTTTGGTTGATAATACCCACAATGTGAATTTTACTATTAAT
>HPV101_Unclassified_109390389_nt6048_L1_Tile|1
TAATGTATTTGTTACTTTGGTTGATAATACCCACAATGTGAATTTTACTATTAATGTTGC
>HPV101_Unclassified_109390389_nt6053_L1_Tile|1
TATTTGTTACTTTGGTTGATAATACCCACAATGTGAATTTTACTATTAATGTTGCTACAG
>HPV101_Unclassified_109390389_nt6058_L1_Tile|1
GTTACTTTGGTTGATAATACCCACAATGTGAATTTTACTATTAATGTTGCTACAGAAGAT
>HPV101_Unclassified_109390389_nt6063_L1_Tile|1
TTTGGTTGATAATACCCACAATGTGAATTTTACTATTAATGTTGCTACAGAAGATAATAT
>HPV101_Unclassified_109390389_nt6068_L1_Tile|1
TTGATAATACCCACAATGTGAATTTTACTATTAATGTTGCTACAGAAGATAATATTCAGT
>HPV101_Unclassified_109390389_nt6073_L1_Tile|1
AATACCCACAATGTGAATTTTACTATTAATGTTGCTACAGAAGATAATATTCAGTCCTAT
>HPV101_Unclassified_109390389_nt6078_L1_Tile|1
CCACAATGTGAATTTTACTATTAATGTTGCTACAGAAGATAATATTCAGTCCTATGATTC
>HPV101_Unclassified_109390389_nt6088_L1_Tile|1
AATTTTACTATTAATGTTGCTACAGAAGATAATATTCAGTCCTATGATTCTGCAAAGTAC
>HPV101_Unclassified_109390389_nt6093_L1_Tile|1
TACTATTAATGTTGCTACAGAAGATAATATTCAGTCCTATGATTCTGCAAAGTACAAGCA
>HPV101_Unclassified_109390389_nt6098_L1_Tile|1
TTAATGTTGCTACAGAAGATAATATTCAGTCCTATGATTCTGCAAAGTACAAGCACTACC
>HPV101_Unclassified_109390389_nt6103_L1_Tile|1
GTTGCTACAGAAGATAATATTCAGTCCTATGATTCTGCAAAGTACAAGCACTACCTCAGA
>HPV101_Unclassified_109390389_nt6108_L1_Tile|1
TACAGAAGATAATATTCAGTCCTATGATTCTGCAAAGTACAAGCACTACCTCAGACATGT

>HPV101_Unclassified_109390389_nt6113_L1_Tile|1
AAGATAATATTCAGTCCTATGATTCTGCAAAGTACAAGCACTACCTCAGACATGTGGAGG
>HPV101_Unclassified_109390389_nt6118_L1_Tile|1
AATATTCAGTCCTATGATTCTGCAAAGTACAAGCACTACCTCAGACATGTGGAGGAATTT
>HPV101_Unclassified_109390389_nt6123_L1_Tile|1
TCAGTCCTATGATTCTGCAAAGTACAAGCACTACCTCAGACATGTGGAGGAATTTGAAAT
>HPV101_Unclassified_109390389_nt6128_L1_Tile|1
CCTATGATTCTGCAAAGTACAAGCACTACCTCAGACATGTGGAGGAATTTGAAATTTCTC
>HPV101_Unclassified_109390389_nt6133_L1_Tile|1
GATTCTGCAAAGTACAAGCACTACCTCAGACATGTGGAGGAATTTGAAATTTCTCTCATT
>HPV101_Unclassified_109390389_nt6138_L1_Tile|1
TGCAAAGTACAAGCACTACCTCAGACATGTGGAGGAATTTGAAATTTCTCTCATTGTGCA
>HPV101_Unclassified_109390389_nt6143_L1_Tile|1
AGTACAAGCACTACCTCAGACATGTGGAGGAATTTGAAATTTCTCTCATTGTGCAGCTCT
>HPV101_Unclassified_109390389_nt6158_L1_Tile|1
TCAGACATGTGGAGGAATTTGAAATTTCTCTCATTGTGCAGCTCTGTAAAGTGTCTCTTA
>HPV101_Unclassified_109390389_nt6163_L1_Tile|1
CATGTGGAGGAATTTGAAATTTCTCTCATTGTGCAGCTCTGTAAAGTGTCTCTTACTCCC
>HPV101_Unclassified_109390389_nt6173_L1_Tile|1
AATTTGAAATTTCTCTCATTGTGCAGCTCTGTAAAGTGTCTCTTACTCCCGATGTATTGG
>HPV101_Unclassified_109390389_nt6178_L1_Tile|1
GAAATTTCTCTCATTGTGCAGCTCTGTAAAGTGTCTCTTACTCCCGATGTATTGGCACAC
>HPV101_Unclassified_109390389_nt6183_L1_Tile|1
TTCTCTCATTGTGCAGCTCTGTAAAGTGTCTCTTACTCCCGATGTATTGGCACACATACA
>HPV101_Unclassified_109390389_nt6193_L1_Tile|1
GTGCAGCTCTGTAAAGTGTCTCTTACTCCCGATGTATTGGCACACATACACTTAATGAAT
>HPV101_Unclassified_109390389_nt6198_L1_Tile|1
GCTCTGTAAAGTGTCTCTTACTCCCGATGTATTGGCACACATACACTTAATGAATCCCTC
>HPV101_Unclassified_109390389_nt6203_L1_Tile|1
GTAAAGTGTCTCTTACTCCCGATGTATTGGCACACATACACTTAATGAATCCCTCTATTC
>HPV101_Unclassified_109390389_nt6208_L1_Tile|1
GTGTCTCTTACTCCCGATGTATTGGCACACATACACTTAATGAATCCCTCTATTCTGGAT
>HPV101_Unclassified_109390389_nt6213_L1_Tile|1
TCTTACTCCCGATGTATTGGCACACATACACTTAATGAATCCCTCTATTCTGGATGAATG
>HPV101_Unclassified_109390389_nt6218_L1_Tile|1
CTCCCGATGTATTGGCACACATACACTTAATGAATCCCTCTATTCTGGATGAATGGCAAT
>HPV101_Unclassified_109390389_nt6223_L1_Tile|1
GATGTATTGGCACACATACACTTAATGAATCCCTCTATTCTGGATGAATGGCAATTAGGT
>HPV101_Unclassified_109390389_nt6238_L1_Tile|1
ATACACTTAATGAATCCCTCTATTCTGGATGAATGGCAATTAGGTATTGCGGCTCCACCG
>HPV101_Unclassified_109390389_nt6243_L1_Tile|1
CTTAATGAATCCCTCTATTCTGGATGAATGGCAATTAGGTATTGCGGCTCCACCGTCTAC
>HPV101_Unclassified_109390389_nt6248_L1_Tile|1
TGAATCCCTCTATTCTGGATGAATGGCAATTAGGTATTGCGGCTCCACCGTCTACCTCTA
>HPV101_Unclassified_109390389_nt6253_L1_Tile|1
CCCTCTATTCTGGATGAATGGCAATTAGGTATTGCGGCTCCACCGTCTACCTCTATTGAA
>HPV101_Unclassified_109390389_nt6263_L1_Tile|1
TGGATGAATGGCAATTAGGTATTGCGGCTCCACCGTCTACCTCTATTGAAACCACTTACA
>HPV101_Unclassified_109390389_nt6268_L1_Tile|1
GAATGGCAATTAGGTATTGCGGCTCCACCGTCTACCTCTATTGAAACCACTTACAGATAT
>HPV101_Unclassified_109390389_nt6273_L1_Tile|1
GCAATTAGGTATTGCGGCTCCACCGTCTACCTCTATTGAAACCACTTACAGATATATTGA
>HPV101_Unclassified_109390389_nt6278_L1_Tile|1
TAGGTATTGCGGCTCCACCGTCTACCTCTATTGAAACCACTTACAGATATATTGATTCTC
>HPV101_Unclassified_109390389_nt6283_L1_Tile|1

ATTGCGGCTCCACCGTCTACCTCTATTGAAACCACTTACAGATATATTGATTCTCTTGCT
>HPV101_Unclassified_109390389_nt6303_L1_Tile|1
CTCTATTGAAACCACTTACAGATATATTGATTCTCTTGCTACAATGTGTCCCACAGCAGA
>HPV101_Unclassified_109390389_nt6343_L1_Tile|1
ACAATGTGTCCCACAGCAGAGCCTCCTAAGGAAAAGGAAGATCCATATGACAAAATGTCT
>HPV101_Unclassified_109390389_nt6383_L1_Tile|1
ATCCATATGACAAAATGTCTTTTTGGGTGGTTGATTTATCAGACAGAATGTCCTCTGAAT
>HPV101_Unclassified_109390389_nt6388_L1_Tile|1
TATGACAAAATGTCTTTTTGGGTGGTTGATTTATCAGACAGAATGTCCTCTGAATTAGGG
>HPV101_Unclassified_109390389_nt6393_L1_Tile|1
CAAAATGTCTTTTTGGGTGGTTGATTTATCAGACAGAATGTCCTCTGAATTAGGGGCCTA
>HPV101_Unclassified_109390389_nt6398_L1_Tile|1
TGTCTTTTTGGGTGGTTGATTTATCAGACAGAATGTCCTCTGAATTAGGGGCCTATCCTC
>HPV101_Unclassified_109390389_nt6403_L1_Tile|1
TTTTGGGTGGTTGATTTATCAGACAGAATGTCCTCTGAATTAGGGGCCTATCCTCTTGGT
>HPV101_Unclassified_109390389_nt6413_L1_Tile|1
TTGATTTATCAGACAGAATGTCCTCTGAATTAGGGGCCTATCCTCTTGGTAGGCGCTTTT
>HPV101_Unclassified_109390389_nt6418_L1_Tile|1
TTATCAGACAGAATGTCCTCTGAATTAGGGGCCTATCCTCTTGGTAGGCGCTTTTTACAT
>HPV101_Unclassified_109390389_nt6423_L1_Tile|1
AGACAGAATGTCCTCTGAATTAGGGGCCTATCCTCTTGGTAGGCGCTTTTTACATCAAAC
>HPV101_Unclassified_109390389_nt6433_L1_Tile|1
TCCTCTGAATTAGGGGCCTATCCTCTTGGTAGGCGCTTTTTACATCAAACAGGGTCTGCC
>HPV101_Unclassified_109390389_nt6453_L1_Tile|1
TCCTCTTGGTAGGCGCTTTTTACATCAAACAGGGTCTGCCCGGCCTGCGCGCCCCCTAAA
>HPV101_Unclassified_109390389_nt6458_L1_Tile|1
TTGGTAGGCGCTTTTTACATCAAACAGGGTCTGCCCGGCCTGCGCGCCCCCTAAAGCGAC
>HPV101_Unclassified_109390389_nt6468_L1_Tile|1
CTTTTTACATCAAACAGGGTCTGCCCGGCCTGCGCGCCCCCTAAAGCGACCCCGTCCTTC
>HPV101_Unclassified_109390389_nt6473_L1_Tile|1
TACATCAAACAGGGTCTGCCCGGCCTGCGCGCCCCCTAAAGCGACCCCGTCCTTCTACGC
>HPV101_Unclassified_109390389_nt6518_L1_Tile|1
CCCGTCCTTCTACGCCTACCAGGCCTACCTCCAGGTCTTCCAAACGCCGACGCAGGCAAG
>HPV101_Unclassified_109390389_nt6523_L1_Tile|1
CCTTCTACGCCTACCAGGCCTACCTCCAGGTCTTCCAAACGCCGACGCAGGCAAGTTTAA
>HPV102_Alpha_71726718_nt5359_L1_Tile|1
ATTAGTCCTATTGCAGGTGCAGAGCATATTGAACTTCGCCCCTTGCAGCCTTCCATTGCT
>HPV102_Alpha_71726718_nt5364_L1_Tile|1
TCCTATTGCAGGTGCAGAGCATATTGAACTTCGCCCCTTGCAGCCTTCCATTGCTACTGC
>HPV102_Alpha_71726718_nt5379_L1_Tile|1
AGAGCATATTGAACTTCGCCCCTTGCAGCCTTCCATTGCTACTGCCCCAGGGGACTCCTT
>HPV102_Alpha_71726718_nt5384_L1_Tile|1
ATATTGAACTTCGCCCCTTGCAGCCTTCCATTGCTACTGCCCCAGGGGACTCCTTATATG
>HPV102_Alpha_71726718_nt5389_L1_Tile|1
GAACTTCGCCCCTTGCAGCCTTCCATTGCTACTGCCCCAGGGGACTCCTTATATGATATT
>HPV102_Alpha_71726718_nt5394_L1_Tile|1
TCGCCCCTTGCAGCCTTCCATTGCTACTGCCCCAGGGGACTCCTTATATGATATTTATGC
>HPV102_Alpha_71726718_nt5399_L1_Tile|1
CCTTGCAGCCTTCCATTGCTACTGCCCCAGGGGACTCCTTATATGATATTTATGCTGATA
>HPV102_Alpha_71726718_nt5404_L1_Tile|1
CAGCCTTCCATTGCTACTGCCCCAGGGGACTCCTTATATGATATTTATGCTGATAATGGC
>HPV102_Alpha_71726718_nt5409_L1_Tile|1
TTCCATTGCTACTGCCCCAGGGGACTCCTTATATGATATTTATGCTGATAATGGCCACTT
>HPV102_Alpha_71726718_nt5414_L1_Tile|1
TTGCTACTGCCCCAGGGGACTCCTTATATGATATTTATGCTGATAATGGCCACTTGGACT

>HPV102_Alpha_71726718_nt5419_L1_Tile|1
ACTGCCCCAGGGGACTCCTTATATGATATTTATGCTGATAATGGCCACTTGGACTCCTTG
>HPV102_Alpha_71726718_nt5429_L1_Tile|1
GGGACTCCTTATATGATATTTATGCTGATAATGGCCACTTGGACTCCTTGGCGTCTCCAC
>HPV102_Alpha_71726718_nt5434_L1_Tile|1
TCCTTATATGATATTTATGCTGATAATGGCCACTTGGACTCCTTGGCGTCTCCACATGTT
>HPV102_Alpha_71726718_nt5444_L1_Tile|1
ATATTTATGCTGATAATGGCCACTTGGACTCCTTGGCGTCTCCACATGTTTCATATACTT
>HPV102_Alpha_71726718_nt5449_L1_Tile|1
TATGCTGATAATGGCCACTTGGACTCCTTGGCGTCTCCACATGTTTCATATACTTCTTCC
>HPV102_Alpha_71726718_nt5454_L1_Tile|1
TGATAATGGCCACTTGGACTCCTTGGCGTCTCCACATGTTTCATATACTTCTTCCACGTC
>HPV102_Alpha_71726718_nt5474_L1_Tile|1
CCTTGGCGTCTCCACATGTTTCATATACTTCTTCCACGTCTGTTCCTTTGCAATCTACAA
>HPV102_Alpha_71726718_nt5484_L1_Tile|1
TCCACATGTTTCATATACTTCTTCCACGTCTGTTCCTTTGCAATCTACAAATATTTCAGC
>HPV102_Alpha_71726718_nt5489_L1_Tile|1
ATGTTTCATATACTTCTTCCACGTCTGTTCCTTTGCAATCTACAAATATTTCAGCTACAT
>HPV102_Alpha_71726718_nt5504_L1_Tile|1
CTTCCACGTCTGTTCCTTTGCAATCTACAAATATTTCAGCTACATCTGTGGTCTCTGCGC
>HPV102_Alpha_71726718_nt5509_L1_Tile|1
ACGTCTGTTCCTTTGCAATCTACAAATATTTCAGCTACATCTGTGGTCTCTGCGCGGTAT
>HPV102_Alpha_71726718_nt5514_L1_Tile|1
TGTTCCTTTGCAATCTACAAATATTTCAGCTACATCTGTGGTCTCTGCGCGGTATGACAA
>HPV102_Alpha_71726718_nt5529_L1_Tile|1
TACAAATATTTCAGCTACATCTGTGGTCTCTGCGCGGTATGACAATGTCACAGTACCACT
>HPV102_Alpha_71726718_nt5534_L1_Tile|1
ATATTTCAGCTACATCTGTGGTCTCTGCGCGGTATGACAATGTCACAGTACCACTCTCTT
>HPV102_Alpha_71726718_nt5539_L1_Tile|1
TCAGCTACATCTGTGGTCTCTGCGCGGTATGACAATGTCACAGTACCACTCTCTTCTGGG
>HPV102_Alpha_71726718_nt5544_L1_Tile|1
TACATCTGTGGTCTCTGCGCGGTATGACAATGTCACAGTACCACTCTCTTCTGGGTTTGA
>HPV102_Alpha_71726718_nt5549_L1_Tile|1
CTGTGGTCTCTGCGCGGTATGACAATGTCACAGTACCACTCTCTTCTGGGTTTGATTTGC
>HPV102_Alpha_71726718_nt5554_L1_Tile|1
GTCTCTGCGCGGTATGACAATGTCACAGTACCACTCTCTTCTGGGTTTGATTTGCCTGCA
>HPV102_Alpha_71726718_nt5564_L1_Tile|1
GGTATGACAATGTCACAGTACCACTCTCTTCTGGGTTTGATTTGCCTGCATATACAGGCC
>HPV102_Alpha_71726718_nt5574_L1_Tile|1
TGTCACAGTACCACTCTCTTCTGGGTTTGATTTGCCTGCATATACAGGCCCTGACATTCC
>HPV102_Alpha_71726718_nt5579_L1_Tile|1
CAGTACCACTCTCTTCTGGGTTTGATTTGCCTGCATATACAGGCCCTGACATTCCAATTG
>HPV102_Alpha_71726718_nt5584_L1_Tile|1
CCACTCTCTTCTGGGTTTGATTTGCCTGCATATACAGGCCCTGACATTCCAATTGCCACT
>HPV102_Alpha_71726718_nt5589_L1_Tile|1
CTCTTCTGGGTTTGATTTGCCTGCATATACAGGCCCTGACATTCCAATTGCCACTCCTAC
>HPV102_Alpha_71726718_nt5594_L1_Tile|1
CTGGGTTTGATTTGCCTGCATATACAGGCCCTGACATTCCAATTGCCACTCCTACTACCA
>HPV102_Alpha_71726718_nt5619_L1_Tile|1
AGGCCCTGACATTCCAATTGCCACTCCTACTACCAGTGTGCTGCATGTTTCCCAGTCCTC
>HPV102_Alpha_71726718_nt5624_L1_Tile|1
CTGACATTCCAATTGCCACTCCTACTACCAGTGTGCTGCATGTTTCCCAGTCCTCTAATG
>HPV102_Alpha_71726718_nt5629_L1_Tile|1
ATTCCAATTGCCACTCCTACTACCAGTGTGCTGCATGTTTCCCAGTCCTCTAATGTTCCT
>HPV102_Alpha_71726718_nt5639_L1_Tile|1

CCACTCCTACTACCAGTGTGCTGCATGTTTCCCAGTCCTCTAATGTTCCTTCCCAATCTG
>HPV102_Alpha_71726718_nt5644_L1_Tile|1
CCTACTACCAGTGTGCTGCATGTTTCCCAGTCCTCTAATGTTCCTTCCCAATCTGTGTTT
>HPV102_Alpha_71726718_nt5649_L1_Tile|1
TACCAGTGTGCTGCATGTTTCCCAGTCCTCTAATGTTCCTTCCCAATCTGTGTTTGTGGA
>HPV102_Alpha_71726718_nt5669_L1_Tile|1
CCCAGTCCTCTAATGTTCCTTCCCAATCTGTGTTTGTGGATGGTACAGACTATTTTTTAT
>HPV102_Alpha_71726718_nt5679_L1_Tile|1
TAATGTTCCTTCCCAATCTGTGTTTGTGGATGGTACAGACTATTTTTTATTGCCTAATTA
>HPV102_Alpha_71726718_nt5684_L1_Tile|1
TTCCTTCCCAATCTGTGTTTGTGGATGGTACAGACTATTTTTTATTGCCTAATTACTTAT
>HPV102_Alpha_71726718_nt5694_L1_Tile|1
ATCTGTGTTTGTGGATGGTACAGACTATTTTTTATTGCCTAATTACTTATTCTTTCCTAA
>HPV102_Alpha_71726718_nt6219_L1_Tile|1
ACAATGTTTCTGTAGATTACAAACAAACCCAACTACTTATTATTGGGTGTAAACCCCCTA
>HPV102_Alpha_71726718_nt6224_L1_Tile|1
GTTTCTGTAGATTACAAACAAACCCAACTACTTATTATTGGGTGTAAACCCCCTATTGGG
>HPV102_Alpha_71726718_nt6559_L1_Tile|1
GCATTTCTTTAACAGACATGGAACCATGGGCGAGGAACTCCCTGCCTCCTATTACATACC
>HPV102_Alpha_71726718_nt6579_L1_Tile|1
GAACCATGGGCGAGGAACTCCCTGCCTCCTATTACATACCTGGCACCTCTGGAAATAATA
>HPV102_Alpha_71726718_nt6584_L1_Tile|1
ATGGGCGAGGAACTCCCTGCCTCCTATTACATACCTGGCACCTCTGGAAATAATAGACAA
>HPV102_Alpha_71726718_nt6589_L1_Tile|1
CGAGGAACTCCCTGCCTCCTATTACATACCTGGCACCTCTGGAAATAATAGACAAACTCC
>HPV102_Alpha_71726718_nt6604_L1_Tile|1
CTCCTATTACATACCTGGCACCTCTGGAAATAATAGACAAACTCCTACCAGTTATGTATA
>HPV102_Alpha_71726718_nt6609_L1_Tile|1
ATTACATACCTGGCACCTCTGGAAATAATAGACAAACTCCTACCAGTTATGTATATGCCC
>HPV102_Alpha_71726718_nt7109_L1_Tile|1
CCATATGATGGCCTTGCTTTTTGGGAGGTGGATCTCAAAGACAAACTGTCTATGGATCTG
>HPV102_Alpha_71726718_nt7114_L1_Tile|1
TGATGGCCTTGCTTTTTGGGAGGTGGATCTCAAAGACAAACTGTCTATGGATCTGGATCA
>HPV103_Unclassified_109390382_nt4981_L1_Tile|1
TCTACCACCTTCTCAGCCAGTGGCTAAAATACTGCCTACTGATGAATACGTTGTGGAAAC
>HPV103_Unclassified_109390382_nt4986_L1_Tile|1
CACCTTCTCAGCCAGTGGCTAAAATACTGCCTACTGATGAATACGTTGTGGAAACGTCTA
>HPV103_Unclassified_109390382_nt4991_L1_Tile|1
TCTCAGCCAGTGGCTAAAATACTGCCTACTGATGAATACGTTGTGGAAACGTCTATTTTC
>HPV103_Unclassified_109390382_nt4996_L1_Tile|1
GCCAGTGGCTAAAATACTGCCTACTGATGAATACGTTGTGGAAACGTCTATTTTCTTCCA
>HPV103_Unclassified_109390382_nt5016_L1_Tile|1
CTACTGATGAATACGTTGTGGAAACGTCTATTTTCTTCCATGCCTCCAGCGACCGCCTGC
>HPV103_Unclassified_109390382_nt5021_L1_Tile|1
GATGAATACGTTGTGGAAACGTCTATTTTCTTCCATGCCTCCAGCGACCGCCTGCTTACT
>HPV103_Unclassified_109390382_nt5031_L1_Tile|1
TTGTGGAAACGTCTATTTTCTTCCATGCCTCCAGCGACCGCCTGCTTACTGTAGGACACC
>HPV103_Unclassified_109390382_nt5076_L1_Tile|1
TTACTGTAGGACACCCATATTTTCCAATTACAAAAGTGGGTGAAGGTACAATTGTTGTAC
>HPV103_Unclassified_109390382_nt5081_L1_Tile|1
GTAGGACACCCATATTTTCCAATTACAAAAGTGGGTGAAGGTACAATTGTTGTACCTAAG
>HPV103_Unclassified_109390382_nt5086_L1_Tile|1
ACACCCATATTTTCCAATTACAAAAGTGGGTGAAGGTACAATTGTTGTACCTAAGGTATC
>HPV103_Unclassified_109390382_nt5091_L1_Tile|1
CATATTTTCCAATTACAAAAGTGGGTGAAGGTACAATTGTTGTACCTAAGGTATCTGGTA

>HPV103_Unclassified_109390382_nt5096_L1_Tile|1
TTTCCAATTACAAAAGTGGGTGAAGGTACAATTGTTGTACCTAAGGTATCTGGTAATCAA
>HPV103_Unclassified_109390382_nt5101_L1_Tile|1
AATTACAAAAGTGGGTGAAGGTACAATTGTTGTACCTAAGGTATCTGGTAATCAATTCAG
>HPV103_Unclassified_109390382_nt5106_L1_Tile|1
CAAAAGTGGGTGAAGGTACAATTGTTGTACCTAAGGTATCTGGTAATCAATTCAGGGCCT
>HPV103_Unclassified_109390382_nt5116_L1_Tile|1
TGAAGGTACAATTGTTGTACCTAAGGTATCTGGTAATCAATTCAGGGCCTTTCACATCCT
>HPV103_Unclassified_109390382_nt5121_L1_Tile|1
GTACAATTGTTGTACCTAAGGTATCTGGTAATCAATTCAGGGCCTTTCACATCCTTTTAC
>HPV103_Unclassified_109390382_nt5141_L1_Tile|1
GTATCTGGTAATCAATTCAGGGCCTTTCACATCCTTTTACCTGATCCTAACAAGTTGGCA
>HPV103_Unclassified_109390382_nt5146_L1_Tile|1
TGGTAATCAATTCAGGGCCTTTCACATCCTTTTACCTGATCCTAACAAGTTGGCACTTGT
>HPV103_Unclassified_109390382_nt5151_L1_Tile|1
ATCAATTCAGGGCCTTTCACATCCTTTTACCTGATCCTAACAAGTTGGCACTTGTGGATT
>HPV103_Unclassified_109390382_nt5161_L1_Tile|1
GGCCTTTCACATCCTTTTACCTGATCCTAACAAGTTGGCACTTGTGGATTCCAATATATA
>HPV103_Unclassified_109390382_nt5166_L1_Tile|1
TTCACATCCTTTTACCTGATCCTAACAAGTTGGCACTTGTGGATTCCAATATATATAATG
>HPV103_Unclassified_109390382_nt5171_L1_Tile|1
ATCCTTTTACCTGATCCTAACAAGTTGGCACTTGTGGATTCCAATATATATAATGCCGAG
>HPV103_Unclassified_109390382_nt5176_L1_Tile|1
TTTACCTGATCCTAACAAGTTGGCACTTGTGGATTCCAATATATATAATGCCGAGTCAGA
>HPV103_Unclassified_109390382_nt5181_L1_Tile|1
CTGATCCTAACAAGTTGGCACTTGTGGATTCCAATATATATAATGCCGAGTCAGAACGGC
>HPV103_Unclassified_109390382_nt5186_L1_Tile|1
CCTAACAAGTTGGCACTTGTGGATTCCAATATATATAATGCCGAGTCAGAACGGCTTGTT
>HPV103_Unclassified_109390382_nt5191_L1_Tile|1
CAAGTTGGCACTTGTGGATTCCAATATATATAATGCCGAGTCAGAACGGCTTGTTTGGAA
>HPV103_Unclassified_109390382_nt5201_L1_Tile|1
CTTGTGGATTCCAATATATATAATGCCGAGTCAGAACGGCTTGTTTGGAAGTTACGGGGT
>HPV103_Unclassified_109390382_nt5206_L1_Tile|1
GGATTCCAATATATATAATGCCGAGTCAGAACGGCTTGTTTGGAAGTTACGGGGTCTGGA
>HPV103_Unclassified_109390382_nt5216_L1_Tile|1
ATATATAATGCCGAGTCAGAACGGCTTGTTTGGAAGTTACGGGGTCTGGATATTGGTCGT
>HPV103_Unclassified_109390382_nt5221_L1_Tile|1
TAATGCCGAGTCAGAACGGCTTGTTTGGAAGTTACGGGGTCTGGATATTGGTCGTGGTGG
>HPV103_Unclassified_109390382_nt5226_L1_Tile|1
CCGAGTCAGAACGGCTTGTTTGGAAGTTACGGGGTCTGGATATTGGTCGTGGTGGACCCC
>HPV103_Unclassified_109390382_nt5231_L1_Tile|1
TCAGAACGGCTTGTTTGGAAGTTACGGGGTCTGGATATTGGTCGTGGTGGACCCCTTAGC
>HPV103_Unclassified_109390382_nt5236_L1_Tile|1
ACGGCTTGTTTGGAAGTTACGGGGTCTGGATATTGGTCGTGGTGGACCCCTTAGCGTTGG
>HPV103_Unclassified_109390382_nt5241_L1_Tile|1
TTGTTTGGAAGTTACGGGGTCTGGATATTGGTCGTGGTGGACCCCTTAGCGTTGGCTCTT
>HPV103_Unclassified_109390382_nt5246_L1_Tile|1
TGGAAGTTACGGGGTCTGGATATTGGTCGTGGTGGACCCCTTAGCGTTGGCTCTTCTGGA
>HPV103_Unclassified_109390382_nt5251_L1_Tile|1
GTTACGGGGTCTGGATATTGGTCGTGGTGGACCCCTTAGCGTTGGCTCTTCTGGACACCC
>HPV103_Unclassified_109390382_nt5261_L1_Tile|1
CTGGATATTGGTCGTGGTGGACCCCTTAGCGTTGGCTCTTCTGGACACCCTTTATTTAAT
>HPV103_Unclassified_109390382_nt5271_L1_Tile|1
GTCGTGGTGGACCCCTTAGCGTTGGCTCTTCTGGACACCCTTTATTTAATAAACTTAAGG
>HPV103_Unclassified_109390382_nt5276_L1_Tile|1

GGTGGACCCCTTAGCGTTGGCTCTTCTGGACACCCTTTATTTAATAAACTTAAGGATACT
>HPV103_Unclassified_109390382_nt5281_L1_Tile|1
ACCCCTTAGCGTTGGCTCTTCTGGACACCCTTTATTTAATAAACTTAAGGATACTGAAAA
>HPV103_Unclassified_109390382_nt5301_L1_Tile|1
CTGGACACCCTTTATTTAATAAACTTAAGGATACTGAAAATCCTAATAGTTATGTTAAAA
>HPV103_Unclassified_109390382_nt5306_L1_Tile|1
CACCCTTTATTTAATAAACTTAAGGATACTGAAAATCCTAATAGTTATGTTAAAACAGGA
>HPV103_Unclassified_109390382_nt5311_L1_Tile|1
TTTATTTAATAAACTTAAGGATACTGAAAATCCTAATAGTTATGTTAAAACAGGAAAGGA
>HPV103_Unclassified_109390382_nt5316_L1_Tile|1
TTAATAAACTTAAGGATACTGAAAATCCTAATAGTTATGTTAAAACAGGAAAGGAAAACA
>HPV103_Unclassified_109390382_nt5321_L1_Tile|1
AAACTTAAGGATACTGAAAATCCTAATAGTTATGTTAAAACAGGAAAGGAAAACAGACAA
>HPV103_Unclassified_109390382_nt5326_L1_Tile|1
TAAGGATACTGAAAATCCTAATAGTTATGTTAAAACAGGAAAGGAAAACAGACAAAGTGT
>HPV103_Unclassified_109390382_nt5331_L1_Tile|1
ATACTGAAAATCCTAATAGTTATGTTAAAACAGGAAAGGAAAACAGACAAAGTGTCTCTA
>HPV103_Unclassified_109390382_nt5336_L1_Tile|1
GAAAATCCTAATAGTTATGTTAAAACAGGAAAGGAAAACAGACAAAGTGTCTCTATGGAC
>HPV103_Unclassified_109390382_nt5341_L1_Tile|1
TCCTAATAGTTATGTTAAAACAGGAAAGGAAAACAGACAAAGTGTCTCTATGGACCCAAA
>HPV103_Unclassified_109390382_nt5346_L1_Tile|1
ATAGTTATGTTAAAACAGGAAAGGAAAACAGACAAAGTGTCTCTATGGACCCAAAGCAAA
>HPV103_Unclassified_109390382_nt5356_L1_Tile|1
TAAAACAGGAAAGGAAAACAGACAAAGTGTCTCTATGGACCCAAAGCAAACTCAGCTATT
>HPV103_Unclassified_109390382_nt5361_L1_Tile|1
CAGGAAAGGAAAACAGACAAAGTGTCTCTATGGACCCAAAGCAAACTCAGCTATTTGTAG
>HPV103_Unclassified_109390382_nt5366_L1_Tile|1
AAGGAAAACAGACAAAGTGTCTCTATGGACCCAAAGCAAACTCAGCTATTTGTAGTAGGC
>HPV103_Unclassified_109390382_nt5406_L1_Tile|1
CTCAGCTATTTGTAGTAGGCTGTACTCCTTGTTTGGGGGAGCATTGGGATGCTGCAAAGC
>HPV103_Unclassified_109390382_nt5451_L1_Tile|1
GGGATGCTGCAAAGCCATGTGCAGATGCTAGGCAGGATATGGGTGCATGTCCTCCTTTAG
>HPV103_Unclassified_109390382_nt5466_L1_Tile|1
CATGTGCAGATGCTAGGCAGGATATGGGTGCATGTCCTCCTTTAGAGCTTGTTAGTTCCA
>HPV103_Unclassified_109390382_nt5471_L1_Tile|1
GCAGATGCTAGGCAGGATATGGGTGCATGTCCTCCTTTAGAGCTTGTTAGTTCCATTATT
>HPV103_Unclassified_109390382_nt5566_L1_Tile|1
AATGAATTTTGCTACATTGCAGGAGGACCATTCAGGTGTACCCTTGGACATTGTAGCTTC
>HPV103_Unclassified_109390382_nt5571_L1_Tile|1
ATTTTGCTACATTGCAGGAGGACCATTCAGGTGTACCCTTGGACATTGTAGCTTCTAAAT
>HPV103_Unclassified_109390382_nt5576_L1_Tile|1
GCTACATTGCAGGAGGACCATTCAGGTGTACCCTTGGACATTGTAGCTTCTAAATGTAAA
>HPV103_Unclassified_109390382_nt5581_L1_Tile|1
ATTGCAGGAGGACCATTCAGGTGTACCCTTGGACATTGTAGCTTCTAAATGTAAATGGCC
>HPV103_Unclassified_109390382_nt5586_L1_Tile|1
AGGAGGACCATTCAGGTGTACCCTTGGACATTGTAGCTTCTAAATGTAAATGGCCTGATT
>HPV103_Unclassified_109390382_nt5591_L1_Tile|1
GACCATTCAGGTGTACCCTTGGACATTGTAGCTTCTAAATGTAAATGGCCTGATTTTCTT
>HPV103_Unclassified_109390382_nt5596_L1_Tile|1
TTCAGGTGTACCCTTGGACATTGTAGCTTCTAAATGTAAATGGCCTGATTTTCTTAGAAT
>HPV103_Unclassified_109390382_nt5631_L1_Tile|1
GTAAATGGCCTGATTTTCTTAGAATGGCAAATGACACATATGGCAATCAAATGTTTTTCT
>HPV103_Unclassified_109390382_nt5636_L1_Tile|1
TGGCCTGATTTTCTTAGAATGGCAAATGACACATATGGCAATCAAATGTTTTTCTTTGGA

>HPV103_Unclassified_109390382_nt5646_L1_Tile|1
TTCTTAGAATGGCAAATGACACATATGGCAATCAAATGTTTTTCTTTGGAAGACGTGAGC
>HPV103_Unclassified_109390382_nt5651_L1_Tile|1
AGAATGGCAAATGACACATATGGCAATCAAATGTTTTTCTTTGGAAGACGTGAGCAGTTG
>HPV103_Unclassified_109390382_nt5656_L1_Tile|1
GGCAAATGACACATATGGCAATCAAATGTTTTTCTTTGGAAGACGTGAGCAGTTGTATGC
>HPV103_Unclassified_109390382_nt5661_L1_Tile|1
ATGACACATATGGCAATCAAATGTTTTTCTTTGGAAGACGTGAGCAGTTGTATGCTCGCC
>HPV103_Unclassified_109390382_nt5671_L1_Tile|1
TGGCAATCAAATGTTTTTCTTTGGAAGACGTGAGCAGTTGTATGCTCGCCACTTTTTTGC
>HPV103_Unclassified_109390382_nt5676_L1_Tile|1
ATCAAATGTTTTTCTTTGGAAGACGTGAGCAGTTGTATGCTCGCCACTTTTTTGCTAGGG
>HPV103_Unclassified_109390382_nt5696_L1_Tile|1
AGACGTGAGCAGTTGTATGCTCGCCACTTTTTTGCTAGGGATGGTGTCATGGGCGACAGT
>HPV103_Unclassified_109390382_nt5701_L1_Tile|1
TGAGCAGTTGTATGCTCGCCACTTTTTTGCTAGGGATGGTGTCATGGGCGACAGTATACC
>HPV103_Unclassified_109390382_nt5716_L1_Tile|1
TCGCCACTTTTTTGCTAGGGATGGTGTCATGGGCGACAGTATACCTGAGGCTGAATTTGA
>HPV103_Unclassified_109390382_nt5721_L1_Tile|1
ACTTTTTTGCTAGGGATGGTGTCATGGGCGACAGTATACCTGAGGCTGAATTTGAGGGTG
>HPV103_Unclassified_109390382_nt5726_L1_Tile|1
TTTGCTAGGGATGGTGTCATGGGCGACAGTATACCTGAGGCTGAATTTGAGGGTGTTAAG
>HPV103_Unclassified_109390382_nt5731_L1_Tile|1
TAGGGATGGTGTCATGGGCGACAGTATACCTGAGGCTGAATTTGAGGGTGTTAAGCACAC
>HPV103_Unclassified_109390382_nt5741_L1_Tile|1
GTCATGGGCGACAGTATACCTGAGGCTGAATTTGAGGGTGTTAAGCACACCTATATTTTA
>HPV103_Unclassified_109390382_nt5746_L1_Tile|1
GGGCGACAGTATACCTGAGGCTGAATTTGAGGGTGTTAAGCACACCTATATTTTACAGGG
>HPV103_Unclassified_109390382_nt5766_L1_Tile|1
CTGAATTTGAGGGTGTTAAGCACACCTATATTTTACAGGGGGCGTCTGACAAAGACCAAA
>HPV103_Unclassified_109390382_nt5771_L1_Tile|1
TTTGAGGGTGTTAAGCACACCTATATTTTACAGGGGGCGTCTGACAAAGACCAAAATACA
>HPV103_Unclassified_109390382_nt5781_L1_Tile|1
TTAAGCACACCTATATTTTACAGGGGGCGTCTGACAAAGACCAAAATACAGCCTCCTCGT
>HPV103_Unclassified_109390382_nt5786_L1_Tile|1
CACACCTATATTTTACAGGGGGCGTCTGACAAAGACCAAAATACAGCCTCCTCGTCTGTT
>HPV103_Unclassified_109390382_nt5791_L1_Tile|1
CTATATTTTACAGGGGGCGTCTGACAAAGACCAAAATACAGCCTCCTCGTCTGTTTACTA
>HPV103_Unclassified_109390382_nt5811_L1_Tile|1
CTGACAAAGACCAAAATACAGCCTCCTCGTCTGTTTACTATGCAACCCCCAGTGGATCAC
>HPV103_Unclassified_109390382_nt5816_L1_Tile|1
AAAGACCAAAATACAGCCTCCTCGTCTGTTTACTATGCAACCCCCAGTGGATCACTGGTT
>HPV103_Unclassified_109390382_nt5821_L1_Tile|1
CCAAAATACAGCCTCCTCGTCTGTTTACTATGCAACCCCCAGTGGATCACTGGTTTCTAG
>HPV103_Unclassified_109390382_nt5826_L1_Tile|1
ATACAGCCTCCTCGTCTGTTTACTATGCAACCCCCAGTGGATCACTGGTTTCTAGTGATT
>HPV103_Unclassified_109390382_nt5836_L1_Tile|1
CTCGTCTGTTTACTATGCAACCCCCAGTGGATCACTGGTTTCTAGTGATTCTCAGTTGTT
>HPV103_Unclassified_109390382_nt5841_L1_Tile|1
CTGTTTACTATGCAACCCCCAGTGGATCACTGGTTTCTAGTGATTCTCAGTTGTTTGGCA
>HPV103_Unclassified_109390382_nt5866_L1_Tile|1
ATCACTGGTTTCTAGTGATTCTCAGTTGTTTGGCAGACCATACTGGATACAAAAGGCTCA
>HPV103_Unclassified_109390382_nt5886_L1_Tile|1
CTCAGTTGTTTGGCAGACCATACTGGATACAAAAGGCTCAGGGCCCTAATAATAGTGTGT
>HPV103_Unclassified_109390382_nt5896_L1_Tile|1

TGGCAGACCATACTGGATACAAAAGGCTCAGGGCCCTAATAATAGTGTGTGTTGGAGAAA
>HPV103_Unclassified_109390382_nt5901_L1_Tile|1
GACCATACTGGATACAAAAGGCTCAGGGCCCTAATAATAGTGTGTGTTGGAGAAATAATT
>HPV103_Unclassified_109390382_nt5941_L1_Tile|1
TGTGTGTTGGAGAAATAATTTATTTATTACATTGGTTGATAACACACACAATGTAAATTT
>HPV103_Unclassified_109390382_nt5946_L1_Tile|1
GTTGGAGAAATAATTTATTTATTACATTGGTTGATAACACACACAATGTAAATTTTACAA
>HPV103_Unclassified_109390382_nt5951_L1_Tile|1
AGAAATAATTTATTTATTACATTGGTTGATAACACACACAATGTAAATTTTACAATAAAT
>HPV103_Unclassified_109390382_nt5956_L1_Tile|1
TAATTTATTTATTACATTGGTTGATAACACACACAATGTAAATTTTACAATAAATGTTAA
>HPV103_Unclassified_109390382_nt5961_L1_Tile|1
TATTTATTACATTGGTTGATAACACACACAATGTAAATTTTACAATAAATGTTAAGTCAG
>HPV103_Unclassified_109390382_nt5966_L1_Tile|1
ATTACATTGGTTGATAACACACACAATGTAAATTTTACAATAAATGTTAAGTCAGACGCT
>HPV103_Unclassified_109390382_nt5971_L1_Tile|1
ATTGGTTGATAACACACACAATGTAAATTTTACAATAAATGTTAAGTCAGACGCTGGCGT
>HPV103_Unclassified_109390382_nt5976_L1_Tile|1
TTGATAACACACACAATGTAAATTTTACAATAAATGTTAAGTCAGACGCTGGCGTTGCAC
>HPV103_Unclassified_109390382_nt5986_L1_Tile|1
ACACAATGTAAATTTTACAATAAATGTTAAGTCAGACGCTGGCGTTGCACAGCAATATGC
>HPV103_Unclassified_109390382_nt5991_L1_Tile|1
ATGTAAATTTTACAATAAATGTTAAGTCAGACGCTGGCGTTGCACAGCAATATGCTGCCA
>HPV103_Unclassified_109390382_nt6016_L1_Tile|1
GTCAGACGCTGGCGTTGCACAGCAATATGCTGCCACCAGCTATAAACATTATCTGCGACA
>HPV103_Unclassified_109390382_nt6026_L1_Tile|1
GGCGTTGCACAGCAATATGCTGCCACCAGCTATAAACATTATCTGCGACATGTAGAGGAA
>HPV103_Unclassified_109390382_nt6031_L1_Tile|1
TGCACAGCAATATGCTGCCACCAGCTATAAACATTATCTGCGACATGTAGAGGAATTTGA
>HPV103_Unclassified_109390382_nt6051_L1_Tile|1
CCAGCTATAAACATTATCTGCGACATGTAGAGGAATTTGAGATTGCTCTTATATTGCAAT
>HPV103_Unclassified_109390382_nt6056_L1_Tile|1
TATAAACATTATCTGCGACATGTAGAGGAATTTGAGATTGCTCTTATATTGCAATTATGC
>HPV103_Unclassified_109390382_nt6061_L1_Tile|1
ACATTATCTGCGACATGTAGAGGAATTTGAGATTGCTCTTATATTGCAATTATGCAAGGT
>HPV103_Unclassified_109390382_nt6071_L1_Tile|1
CGACATGTAGAGGAATTTGAGATTGCTCTTATATTGCAATTATGCAAGGTTCCTCTCACA
>HPV103_Unclassified_109390382_nt6081_L1_Tile|1
AGGAATTTGAGATTGCTCTTATATTGCAATTATGCAAGGTTCCTCTCACAGCTGATGTAT
>HPV103_Unclassified_109390382_nt6086_L1_Tile|1
TTTGAGATTGCTCTTATATTGCAATTATGCAAGGTTCCTCTCACAGCTGATGTATTAGCC
>HPV103_Unclassified_109390382_nt6091_L1_Tile|1
GATTGCTCTTATATTGCAATTATGCAAGGTTCCTCTCACAGCTGATGTATTAGCCCATAT
>HPV103_Unclassified_109390382_nt6096_L1_Tile|1
CTCTTATATTGCAATTATGCAAGGTTCCTCTCACAGCTGATGTATTAGCCCATATACATG
>HPV103_Unclassified_109390382_nt6101_L1_Tile|1
ATATTGCAATTATGCAAGGTTCCTCTCACAGCTGATGTATTAGCCCATATACATGTAATG
>HPV103_Unclassified_109390382_nt6106_L1_Tile|1
GCAATTATGCAAGGTTCCTCTCACAGCTGATGTATTAGCCCATATACATGTAATGGATCC
>HPV103_Unclassified_109390382_nt6116_L1_Tile|1
AAGGTTCCTCTCACAGCTGATGTATTAGCCCATATACATGTAATGGATCCAGCTATAATT
>HPV103_Unclassified_109390382_nt6121_L1_Tile|1
TCCTCTCACAGCTGATGTATTAGCCCATATACATGTAATGGATCCAGCTATAATTGAGGA
>HPV103_Unclassified_109390382_nt6131_L1_Tile|1
GCTGATGTATTAGCCCATATACATGTAATGGATCCAGCTATAATTGAGGACTGGCAGCTA

>HPV103_Unclassified_109390382_nt6136_L1_Tile|1
TGTATTAGCCCATATACATGTAATGGATCCAGCTATAATTGAGGACTGGCAGCTAGGGTT
>HPV103_Unclassified_109390382_nt6141_L1_Tile|1
TAGCCCATATACATGTAATGGATCCAGCTATAATTGAGGACTGGCAGCTAGGGTTTGTAC
>HPV103_Unclassified_109390382_nt6146_L1_Tile|1
CATATACATGTAATGGATCCAGCTATAATTGAGGACTGGCAGCTAGGGTTTGTACCACCG
>HPV103_Unclassified_109390382_nt6156_L1_Tile|1
TAATGGATCCAGCTATAATTGAGGACTGGCAGCTAGGGTTTGTACCACCGCCTCCTTCTT
>HPV103_Unclassified_109390382_nt6161_L1_Tile|1
GATCCAGCTATAATTGAGGACTGGCAGCTAGGGTTTGTACCACCGCCTCCTTCTTCTATT
>HPV103_Unclassified_109390382_nt6166_L1_Tile|1
AGCTATAATTGAGGACTGGCAGCTAGGGTTTGTACCACCGCCTCCTTCTTCTATTGGAGA
>HPV103_Unclassified_109390382_nt6171_L1_Tile|1
TAATTGAGGACTGGCAGCTAGGGTTTGTACCACCGCCTCCTTCTTCTATTGGAGATACAT
>HPV103_Unclassified_109390382_nt6176_L1_Tile|1
GAGGACTGGCAGCTAGGGTTTGTACCACCGCCTCCTTCTTCTATTGGAGATACATACAGA
>HPV103_Unclassified_109390382_nt6181_L1_Tile|1
CTGGCAGCTAGGGTTTGTACCACCGCCTCCTTCTTCTATTGGAGATACATACAGATTTTT
>HPV103_Unclassified_109390382_nt6186_L1_Tile|1
AGCTAGGGTTTGTACCACCGCCTCCTTCTTCTATTGGAGATACATACAGATTTTTAACAT
>HPV103_Unclassified_109390382_nt6201_L1_Tile|1
CACCGCCTCCTTCTTCTATTGGAGATACATACAGATTTTTAACATCTTTAGCCACACGCT
>HPV103_Unclassified_109390382_nt6211_L1_Tile|1
TTCTTCTATTGGAGATACATACAGATTTTTAACATCTTTAGCCACACGCTGCCCTGCTGC
>HPV103_Unclassified_109390382_nt6216_L1_Tile|1
CTATTGGAGATACATACAGATTTTTAACATCTTTAGCCACACGCTGCCCTGCTGCAGAAC
>HPV103_Unclassified_109390382_nt6226_L1_Tile|1
TACATACAGATTTTTAACATCTTTAGCCACACGCTGCCCTGCTGCAGAACCTCCCAAGGA
>HPV103_Unclassified_109390382_nt6241_L1_Tile|1
AACATCTTTAGCCACACGCTGCCCTGCTGCAGAACCTCCCAAGGAAAAACAGGACCCATA
>HPV103_Unclassified_109390382_nt6246_L1_Tile|1
CTTTAGCCACACGCTGCCCTGCTGCAGAACCTCCCAAGGAAAAACAGGACCCATATGATA
>HPV103_Unclassified_109390382_nt6346_L1_Tile|1
CTCTGATCTTGACAGATATCCTTTGGGTCGCAGATTTTTATCTCAAACTGGGGTTAGCTC
>HPV103_Unclassified_109390382_nt6351_L1_Tile|1
ATCTTGACAGATATCCTTTGGGTCGCAGATTTTTATCTCAAACTGGGGTTAGCTCGTCTT
>HPV103_Unclassified_109390382_nt6356_L1_Tile|1
GACAGATATCCTTTGGGTCGCAGATTTTTATCTCAAACTGGGGTTAGCTCGTCTTCTACC
>HPV103_Unclassified_109390382_nt6361_L1_Tile|1
ATATCCTTTGGGTCGCAGATTTTTATCTCAAACTGGGGTTAGCTCGTCTTCTACCTCCTC
>HPV103_Unclassified_109390382_nt6366_L1_Tile|1
CTTTGGGTCGCAGATTTTTATCTCAAACTGGGGTTAGCTCGTCTTCTACCTCCTCTAGAA
>HPV103_Unclassified_109390382_nt6376_L1_Tile|1
CAGATTTTTATCTCAAACTGGGGTTAGCTCGTCTTCTACCTCCTCTAGAAAGCGGTCTCG
>HPV103_Unclassified_109390382_nt6381_L1_Tile|1
TTTTATCTCAAACTGGGGTTAGCTCGTCTTCTACCTCCTCTAGAAAGCGGTCTCGTGTAG
>HPV103_Unclassified_109390382_nt6386_L1_Tile|1
TCTCAAACTGGGGTTAGCTCGTCTTCTACCTCCTCTAGAAAGCGGTCTCGTGTAGAGACC
>HPV103_Unclassified_109390382_nt6391_L1_Tile|1
AACTGGGGTTAGCTCGTCTTCTACCTCCTCTAGAAAGCGGTCTCGTGTAGAGACCCCTAC
>HPV103_Unclassified_109390382_nt6396_L1_Tile|1
GGGTTAGCTCGTCTTCTACCTCCTCTAGAAAGCGGTCTCGTGTAGAGACCCCTACCCGCC
>HPV103_Unclassified_109390382_nt6401_L1_Tile|1
AGCTCGTCTTCTACCTCCTCTAGAAAGCGGTCTCGTGTAGAGACCCCTACCCGCCCAGTA
>HPV103_Unclassified_109390382_nt6406_L1_Tile|1

GTCTTCTACCTCCTCTAGAAAGCGGTCTCGTGTAGAGACCCCTACCCGCCCAGTACGGTC
>HPV103_Unclassified_109390382_nt6411_L1_Tile|1
CTACCTCCTCTAGAAAGCGGTCTCGTGTAGAGACCCCTACCCGCCCAGTACGGTCTGCTA
>HPV103_Unclassified_109390382_nt6416_L1_Tile|1
TCCTCTAGAAAGCGGTCTCGTGTAGAGACCCCTACCCGCCCAGTACGGTCTGCTAAAAAA
>HPV103_Unclassified_109390382_nt6421_L1_Tile|1
TAGAAAGCGGTCTCGTGTAGAGACCCCTACCCGCCCAGTACGGTCTGCTAAAAAACGTAG
>HPV103_Unclassified_109390382_nt6426_L1_Tile|1
AGCGGTCTCGTGTAGAGACCCCTACCCGCCCAGTACGGTCTGCTAAAAAACGTAGACGGG
>HPV103_Unclassified_109390382_nt6431_L1_Tile|1
TCTCGTGTAGAGACCCCTACCCGCCCAGTACGGTCTGCTAAAAAACGTAGACGGGGGGTT
>HPV103_Unclassified_109390382_nt6434_L1_Tile|1
CGTGTAGAGACCCCTACCCGCCCAGTACGGTCTGCTAAAAAACGTAGACGGGGGGTTTAA
>HPV106_Alpha_71726710_nt5675_L1_Tile|1
CTGGTCACCCCACAGGCGATGTGCTTGTGCACGGGTCTACATATTATTTATTGCCCTCAT
>HPV106_Alpha_71726710_nt5685_L1_Tile|1
CACAGGCGATGTGCTTGTGCACGGGTCTACATATTATTTATTGCCCTCATATGTGTTGCT
>HPV106_Alpha_71726710_nt5895_L1_Tile|1
GCAGTTCCCGATTGCTGACCGTAGGTCATCCATATTATCCTATCAAAAAGCAAAGTGGAC
>HPV106_Alpha_71726710_nt5900_L1_Tile|1
TCCCGATTGCTGACCGTAGGTCATCCATATTATCCTATCAAAAAGCAAAGTGGACGGAAC
>HPV106_Alpha_71726710_nt5905_L1_Tile|1
ATTGCTGACCGTAGGTCATCCATATTATCCTATCAAAAAGCAAAGTGGACGGAACCCTAT
>HPV106_Alpha_71726710_nt6270_L1_Tile|1
AACCGCCATTGGGCGAACACTGGGCAAAAGGCACTCCATGCAATACCTCCAATGTCACTG
>HPV106_Alpha_71726710_nt6275_L1_Tile|1
CCATTGGGCGAACACTGGGCAAAAGGCACTCCATGCAATACCTCCAATGTCACTGCAGGG
>HPV106_Alpha_71726710_nt6555_L1_Tile|1
TTAGGCATTTTTACAACAGGGCCGGTACCATGGGGGAGGATGTTCCGGAAGGGTTTTTAC
>HPV106_Alpha_71726710_nt6560_L1_Tile|1
CATTTTTACAACAGGGCCGGTACCATGGGGGAGGATGTTCCGGAAGGGTTTTTACTAAAG
>HPV106_Alpha_71726710_nt6565_L1_Tile|1
TTACAACAGGGCCGGTACCATGGGGGAGGATGTTCCGGAAGGGTTTTTACTAAAGGGGCC
>HPV106_Alpha_71726710_nt6570_L1_Tile|1
ACAGGGCCGGTACCATGGGGGAGGATGTTCCGGAAGGGTTTTTACTAAAGGGGCCTACTA
>HPV106_Alpha_71726710_nt6575_L1_Tile|1
GCCGGTACCATGGGGGAGGATGTTCCGGAAGGGTTTTTACTAAAGGGGCCTACTACTACA
>HPV106_Alpha_71726710_nt6580_L1_Tile|1
TACCATGGGGGAGGATGTTCCGGAAGGGTTTTTACTAAAGGGGCCTACTACTACATCCCG
>HPV106_Alpha_71726710_nt6590_L1_Tile|1
GAGGATGTTCCGGAAGGGTTTTTACTAAAGGGGCCTACTACTACATCCCGCGCCTCTGCT
>HPV106_Alpha_71726710_nt6595_L1_Tile|1
TGTTCCGGAAGGGTTTTTACTAAAGGGGCCTACTACTACATCCCGCGCCTCTGCTACAAG
>HPV106_Alpha_71726710_nt6605_L1_Tile|1
GGGTTTTTACTAAAGGGGCCTACTACTACATCCCGCGCCTCTGCTACAAGCTCACTATAT
>HPV106_Alpha_71726710_nt6610_L1_Tile|1
TTTACTAAAGGGGCCTACTACTACATCCCGCGCCTCTGCTACAAGCTCACTATATGGGTC
>HPV106_Alpha_71726710_nt6995_L1_Tile|1
AATTTTGGGTCGTTGCCCCCCCCTACCGCCAGTCTTGGCGATACCTATAGGTTTTTACAG
>HPV106_Alpha_71726710_nt7070_L1_Tile|1
TGTCAAAAGCCCTCCGCGGCGCCTGCAAAGAAAAAGGACCCTTACGATGGGCTTACGTTT
>HPV106_Alpha_71726710_nt7130_L1_Tile|1
TGGGATGTGGATTTATCAGAGCGCTTTTCCCTTGAGCTTGACCAGTTTCCCTTGGGTAGG
>HPV106_Alpha_71726710_nt7240_L1_Tile|1
TAAGCGTCCTGCACCATCGTCCACCCCACCAGCCACAAAGCGTAAACGGTCCAAGCGTTC

>HPV107_Beta_126131393_nt5829_L1_Tile|1
CACCATCAACTCCGGTGGCACGGGTGCTAAGTACCGATGAGTACGTGGAGCGGACCAATA
>HPV107_Beta_126131393_nt5834_L1_Tile|1
TCAACTCCGGTGGCACGGGTGCTAAGTACCGATGAGTACGTGGAGCGGACCAATATATTT
>HPV107_Beta_126131393_nt5839_L1_Tile|1
TCCGGTGGCACGGGTGCTAAGTACCGATGAGTACGTGGAGCGGACCAATATATTTTATCA
>HPV107_Beta_126131393_nt5849_L1_Tile|1
CGGGTGCTAAGTACCGATGAGTACGTGGAGCGGACCAATATATTTTATCACGCCACTAGT
>HPV107_Beta_126131393_nt5854_L1_Tile|1
GCTAAGTACCGATGAGTACGTGGAGCGGACCAATATATTTTATCACGCCACTAGTGACAG
>HPV107_Beta_126131393_nt5859_L1_Tile|1
GTACCGATGAGTACGTGGAGCGGACCAATATATTTTATCACGCCACTAGTGACAGATTAT
>HPV107_Beta_126131393_nt5864_L1_Tile|1
GATGAGTACGTGGAGCGGACCAATATATTTTATCACGCCACTAGTGACAGATTATTAACA
>HPV107_Beta_126131393_nt6154_L1_Tile|1
TTTGTTCAATAAGGTAAAGGACACTGAAAATACAAATAGCTATCAAAACAGTTCAACAGA
>HPV107_Beta_126131393_nt6159_L1_Tile|1
TCAATAAGGTAAAGGACACTGAAAATACAAATAGCTATCAAAACAGTTCAACAGATGATA
>HPV107_Beta_126131393_nt6164_L1_Tile|1
AAGGTAAAGGACACTGAAAATACAAATAGCTATCAAAACAGTTCAACAGATGATAGACAA
>HPV107_Beta_126131393_nt6274_L1_Tile|1
ACCATGTTTAGGGGAATATTGGGATAGGGCACTGCCATGCGATGATGCTGGTGACCAACA
>HPV107_Beta_126131393_nt6284_L1_Tile|1
GGGGAATATTGGGATAGGGCACTGCCATGCGATGATGCTGGTGACCAACAAGGTAAATGT
>HPV107_Beta_126131393_nt6294_L1_Tile|1
GGGATAGGGCACTGCCATGCGATGATGCTGGTGACCAACAAGGTAAATGTCCGCCCATTG
>HPV107_Beta_126131393_nt6299_L1_Tile|1
AGGGCACTGCCATGCGATGATGCTGGTGACCAACAAGGTAAATGTCCGCCCATTGAATTA
>HPV107_Beta_126131393_nt6304_L1_Tile|1
ACTGCCATGCGATGATGCTGGTGACCAACAAGGTAAATGTCCGCCCATTGAATTAAAAAA
>HPV107_Beta_126131393_nt6314_L1_Tile|1
GATGATGCTGGTGACCAACAAGGTAAATGTCCGCCCATTGAATTAAAAAATTCTTTTATA
>HPV107_Beta_126131393_nt6319_L1_Tile|1
TGCTGGTGACCAACAAGGTAAATGTCCGCCCATTGAATTAAAAAATTCTTTTATAGAAGA
>HPV107_Beta_126131393_nt6364_L1_Tile|1
TTCTTTTATAGAAGATGGGGATATGATAGATATTGGGTTTGGCAATATAAATAACAAGAC
>HPV107_Beta_126131393_nt6374_L1_Tile|1
GAAGATGGGGATATGATAGATATTGGGTTTGGCAATATAAATAACAAGACATTATCAGTA
>HPV107_Beta_126131393_nt6379_L1_Tile|1
TGGGGATATGATAGATATTGGGTTTGGCAATATAAATAACAAGACATTATCAGTAAACAA
>HPV107_Beta_126131393_nt6384_L1_Tile|1
ATATGATAGATATTGGGTTTGGCAATATAAATAACAAGACATTATCAGTAAACAAATCAG
>HPV107_Beta_126131393_nt6389_L1_Tile|1
ATAGATATTGGGTTTGGCAATATAAATAACAAGACATTATCAGTAAACAAATCAGATGTT
>HPV107_Beta_126131393_nt6394_L1_Tile|1
TATTGGGTTTGGCAATATAAATAACAAGACATTATCAGTAAACAAATCAGATGTTAGTTT
>HPV107_Beta_126131393_nt6399_L1_Tile|1
GGTTTGGCAATATAAATAACAAGACATTATCAGTAAACAAATCAGATGTTAGTTTAGATT
>HPV107_Beta_126131393_nt6404_L1_Tile|1
GGCAATATAAATAACAAGACATTATCAGTAAACAAATCAGATGTTAGTTTAGATTTAGTA
>HPV107_Beta_126131393_nt6409_L1_Tile|1
TATAAATAACAAGACATTATCAGTAAACAAATCAGATGTTAGTTTAGATTTAGTAAATGA
>HPV107_Beta_126131393_nt6559_L1_Tile|1
TAGACATTATTTCACTAGAGGAGGAGCTGTGGGTGATGCTATACCTGATACAACAACTAA
>HPV107_Beta_126131393_nt6564_L1_Tile|1

ATTATTTCACTAGAGGAGGAGCTGTGGGTGATGCTATACCTGATACAACAACTAATCAAG
>HPV107_Beta_126131393_nt6579_L1_Tile|1
GAGGAGCTGTGGGTGATGCTATACCTGATACAACAACTAATCAAGATCACAAATACTATA
>HPV107_Beta_126131393_nt6584_L1_Tile|1
GCTGTGGGTGATGCTATACCTGATACAACAACTAATCAAGATCACAAATACTATATAGCA
>HPV107_Beta_126131393_nt6589_L1_Tile|1
GGGTGATGCTATACCTGATACAACAACTAATCAAGATCACAAATACTATATAGCACCTAA
>HPV107_Beta_126131393_nt6594_L1_Tile|1
ATGCTATACCTGATACAACAACTAATCAAGATCACAAATACTATATAGCACCTAAGAGTG
>HPV107_Beta_126131393_nt6599_L1_Tile|1
ATACCTGATACAACAACTAATCAAGATCACAAATACTATATAGCACCTAAGAGTGGACAA
>HPV107_Beta_126131393_nt6609_L1_Tile|1
CAACAACTAATCAAGATCACAAATACTATATAGCACCTAAGAGTGGACAATCCCAAAGTC
>HPV107_Beta_126131393_nt6614_L1_Tile|1
ACTAATCAAGATCACAAATACTATATAGCACCTAAGAGTGGACAATCCCAAAGTCCTTTG
>HPV107_Beta_126131393_nt6624_L1_Tile|1
ATCACAAATACTATATAGCACCTAAGAGTGGACAATCCCAAAGTCCTTTGGGTAATTCTA
>HPV107_Beta_126131393_nt6629_L1_Tile|1
AAATACTATATAGCACCTAAGAGTGGACAATCCCAAAGTCCTTTGGGTAATTCTATTTAC
>HPV107_Beta_126131393_nt6634_L1_Tile|1
CTATATAGCACCTAAGAGTGGACAATCCCAAAGTCCTTTGGGTAATTCTATTTACTATCC
>HPV107_Beta_126131393_nt6639_L1_Tile|1
TAGCACCTAAGAGTGGACAATCCCAAAGTCCTTTGGGTAATTCTATTTACTATCCCACCG
>HPV107_Beta_126131393_nt6654_L1_Tile|1
GACAATCCCAAAGTCCTTTGGGTAATTCTATTTACTATCCCACCGTTAGTGGCTCCTTAG
>HPV107_Beta_126131393_nt6659_L1_Tile|1
TCCCAAAGTCCTTTGGGTAATTCTATTTACTATCCCACCGTTAGTGGCTCCTTAGTTTCT
>HPV107_Beta_126131393_nt6664_L1_Tile|1
AAGTCCTTTGGGTAATTCTATTTACTATCCCACCGTTAGTGGCTCCTTAGTTTCTTCTGA
>HPV107_Beta_126131393_nt6669_L1_Tile|1
CTTTGGGTAATTCTATTTACTATCCCACCGTTAGTGGCTCCTTAGTTTCTTCTGATGCAC
>HPV107_Beta_126131393_nt6699_L1_Tile|1
TTAGTGGCTCCTTAGTTTCTTCTGATGCACAGCTCTTTAACAGACCCTTCTGGTTGAAAC
>HPV107_Beta_126131393_nt6809_L1_Tile|1
ACTGTTGCAGACAATACAAGGAACACCAATTTTACTATAAGTGTGCCTAGTCAAAATGGA
>HPV107_Beta_126131393_nt6839_L1_Tile|1
TTTACTATAAGTGTGCCTAGTCAAAATGGACCCCTTACAGAATATGATGCCAATAATATC
>HPV107_Beta_126131393_nt6844_L1_Tile|1
TATAAGTGTGCCTAGTCAAAATGGACCCCTTACAGAATATGATGCCAATAATATCAGGGA
>HPV107_Beta_126131393_nt6849_L1_Tile|1
GTGTGCCTAGTCAAAATGGACCCCTTACAGAATATGATGCCAATAATATCAGGGAATTTT
>HPV107_Beta_126131393_nt6854_L1_Tile|1
CCTAGTCAAAATGGACCCCTTACAGAATATGATGCCAATAATATCAGGGAATTTTTAAGG
>HPV107_Beta_126131393_nt6879_L1_Tile|1
AATATGATGCCAATAATATCAGGGAATTTTTAAGGCATGTTGAGGAATATCAAATATCTG
>HPV107_Beta_126131393_nt6884_L1_Tile|1
GATGCCAATAATATCAGGGAATTTTTAAGGCATGTTGAGGAATATCAAATATCTGTAATA
>HPV107_Beta_126131393_nt6909_L1_Tile|1
TAAGGCATGTTGAGGAATATCAAATATCTGTAATACTGCAACTTTGTAAAGTGTCTTTAC
>HPV107_Beta_126131393_nt6914_L1_Tile|1
CATGTTGAGGAATATCAAATATCTGTAATACTGCAACTTTGTAAAGTGTCTTTACAACCA
>HPV107_Beta_126131393_nt6919_L1_Tile|1
TGAGGAATATCAAATATCTGTAATACTGCAACTTTGTAAAGTGTCTTTACAACCAGATGT
>HPV107_Beta_126131393_nt6934_L1_Tile|1
ATCTGTAATACTGCAACTTTGTAAAGTGTCTTTACAACCAGATGTGTTAGCCCAAATTAA

>HPV107_Beta_126131393_nt6939_L1_Tile|1
TAATACTGCAACTTTGTAAAGTGTCTTTACAACCAGATGTGTTAGCCCAAATTAATGCTA
>HPV107_Beta_126131393_nt6944_L1_Tile|1
CTGCAACTTTGTAAAGTGTCTTTACAACCAGATGTGTTAGCCCAAATTAATGCTATGAAT
>HPV107_Beta_126131393_nt6954_L1_Tile|1
GTAAAGTGTCTTTACAACCAGATGTGTTAGCCCAAATTAATGCTATGAATTCAGGCATTT
>HPV107_Beta_126131393_nt6959_L1_Tile|1
GTGTCTTTACAACCAGATGTGTTAGCCCAAATTAATGCTATGAATTCAGGCATTTTGGAA
>HPV107_Beta_126131393_nt7124_L1_Tile|1
GAAAAGGAAGATCCTTTTGCTCAATACTTTTTCTGGAGAGTTGATATGACAGAAAAATTA
>HPV107_Beta_126131393_nt7184_L1_Tile|1
TCTTTGGATTTAGATCAGTATCCTTTAGGACGTAAATTCCTATTCCAAACAACACTACCT
>HPV107_Beta_126131393_nt7194_L1_Tile|1
TAGATCAGTATCCTTTAGGACGTAAATTCCTATTCCAAACAACACTACCTGCCTCTCGAA
>HPV107_Beta_126131393_nt7199_L1_Tile|1
CAGTATCCTTTAGGACGTAAATTCCTATTCCAAACAACACTACCTGCCTCTCGAAAACGT
>HPV107_Beta_126131393_nt7209_L1_Tile|1
TAGGACGTAAATTCCTATTCCAAACAACACTACCTGCCTCTCGAAAACGTGCAGCGCCGA
>HPV107_Beta_126131393_nt7219_L1_Tile|1
ATTCCTATTCCAAACAACACTACCTGCCTCTCGAAAACGTGCAGCGCCGACCACCAAATC
>HPV107_Beta_126131393_nt7224_L1_Tile|1
TATTCCAAACAACACTACCTGCCTCTCGAAAACGTGCAGCGCCGACCACCAAATCGGTTG
>HPV107_Beta_126131393_nt7253_L1_Tile|1
AAACGTGCAGCGCCGACCACCAAATCGGTTGCAAAGCATAGCAAACGAAAACGTACTTGA
>HPV11_Alpha_333026_nt5892_L1_Tile|1
CTCCTTGCTGTGGGACATCCATATTACTCTATCAAAAAAGTTAACAAAACAGTTGTACCA
>HPV11_Alpha_333026_nt5897_L1_Tile|1
TGCTGTGGGACATCCATATTACTCTATCAAAAAAGTTAACAAAACAGTTGTACCAAAGGT
>HPV11_Alpha_333026_nt5902_L1_Tile|2
TGGGACATCCATATTACTCTATCAAAAAAGTTAACAAAACAGTTGTACCAAAGGTGTCTG
>HPV11_Alpha_333026_nt6002_L1_Tile|2
TAACAAGTTTGCATTACCTGATTCATCCCTGTTTGACCCCACTACACAGCGTTTAGTATG
>HPV11_Alpha_333026_nt6117_L1_Tile|1
GGGCATCCATTGCTAAACAAATATGATGATGTAGAAAATAGTGGTGGGTATGGTGGTAAT
>HPV11_Alpha_333026_nt6272_L1_Tile|1
TAAGGGTACACAATGTTCAAATACCTCTGTACAAAATGGTGACTGCCCCCCGTTGGAACT
>HPV11_Alpha_333026_nt6277_L1_Tile|1
GTACACAATGTTCAAATACCTCTGTACAAAATGGTGACTGCCCCCCGTTGGAACTTATTA
>HPV11_Alpha_333026_nt6392_L1_Tile|1
TGCAGACTTACAAACCAATAAATCGGATGTTCCCCTTGATATTTGTGGAACTGTCTGCAA
>HPV11_Alpha_333026_nt6547_L1_Tile|1
ATAGGGCCGGTACTGTGGGGGAACCTGTGCCTGATGACCTGTTGGTAAAAGGGGGTAATA
>HPV11_Alpha_333026_nt6557_L1_Tile|1
TACTGTGGGGGAACCTGTGCCTGATGACCTGTTGGTAAAAGGGGGTAATAACAGATCATC
>HPV11_Alpha_333026_nt6572_L1_Tile|1
TGTGCCTGATGACCTGTTGGTAAAAGGGGGTAATAACAGATCATCTGTAGCTAGTAGTAT
>HPV11_Alpha_333026_nt6577_L1_Tile|1
CTGATGACCTGTTGGTAAAAGGGGGTAATAACAGATCATCTGTAGCTAGTAGTATTTATG
>HPV11_Alpha_333026_nt6582_L1_Tile|2
GACCTGTTGGTAAAAGGGGGTAATAACAGATCATCTGTAGCTAGTAGTATTTATGTACAT
>HPV11_Alpha_333026_nt6592_L1_Tile|1
TAAAAGGGGGTAATAACAGATCATCTGTAGCTAGTAGTATTTATGTACATACACCTAGTG
>HPV11_Alpha_333026_nt6607_L1_Tile|1
ACAGATCATCTGTAGCTAGTAGTATTTATGTACATACACCTAGTGGCTCATTGGTGTCTT
>HPV11_Alpha_333026_nt6622_L1_Tile|2

CTAGTAGTATTTATGTACATACACCTAGTGGCTCATTGGTGTCTTCAGAGGCTCAATTAT
>HPV11_Alpha_333026_nt6777_L1_Tile|1
AGTACAAATATGACACTATGTGCATCTGTGTCTAAATCTGCTACATACACTAATTCAGAT
>HPV11_Alpha_333026_nt6782_L1_Tile|2
AAATATGACACTATGTGCATCTGTGTCTAAATCTGCTACATACACTAATTCAGATTATAA
>HPV11_Alpha_333026_nt6787_L1_Tile|1
TGACACTATGTGCATCTGTGTCTAAATCTGCTACATACACTAATTCAGATTATAAGGAAT
>HPV11_Alpha_333026_nt6792_L1_Tile|1
CTATGTGCATCTGTGTCTAAATCTGCTACATACACTAATTCAGATTATAAGGAATACATG
>HPV11_Alpha_333026_nt6797_L1_Tile|1
TGCATCTGTGTCTAAATCTGCTACATACACTAATTCAGATTATAAGGAATACATGCGCCA
>HPV11_Alpha_333026_nt7142_L1_Tile|2
GTTTCCCCTTGGACGTAAGTTTTTATTGCAAAGTGGATATCGAGGACGGACGTCTGCTCG
>HPV11_Alpha_333026_nt7182_L1_Tile|2
CGAGGACGGACGTCTGCTCGTACAGGTATAAAGCGCCCAGCTGTGTCTAAGCCCTCTACA
>HPV11_Alpha_333026_nt7197_L1_Tile|1
GCTCGTACAGGTATAAAGCGCCCAGCTGTGTCTAAGCCCTCTACAGCCCCCAAACGAAAA
>HPV11_Alpha_333026_nt7207_L1_Tile|1
GTATAAAGCGCCCAGCTGTGTCTAAGCCCTCTACAGCCCCCAAACGAAAACGTACCAAAA
>HPV110_Beta_186461194_nt5847_L1_Tile|1
TGGCGCAAAAATAGAAGTCCCTAAGGTATCAGGAAATCAATACAGAGCATTCCGGGTGCA
>HPV110_Beta_186461194_nt6062_L1_Tile|2
ATACTGAAAATTCTAGCAATTATCAAGCAGGCTCTACTGATGACAGGCAGAATACTTCAT
>HPV110_Beta_186461194_nt6167_L1_Tile|1
GTATGGGAGAGCATTGGGACAAAGCTCCTGTTTGTGAATCAGAAGCAGGTAATCAGTTAG
>HPV110_Beta_186461194_nt6172_L1_Tile|1
GGAGAGCATTGGGACAAAGCTCCTGTTTGTGAATCAGAAGCAGGTAATCAGTTAGGTTTA
>HPV110_Beta_186461194_nt6482_L1_Tile|2
GGGATGCAATACCTGATGGTGCAGTGAATCAAAATCATAACTATTTCTTGCCAGCAAAGC
>HPV110_Beta_186461194_nt6487_L1_Tile|1
GCAATACCTGATGGTGCAGTGAATCAAAATCATAACTATTTCTTGCCAGCAAAGCAAGAT
>HPV110_Beta_186461194_nt6507_L1_Tile|1
GAATCAAAATCATAACTATTTCTTGCCAGCAAAGCAAGATCAACAACAACGCACACTAGC
>HPV110_Beta_186461194_nt6512_L1_Tile|1
AAAATCATAACTATTTCTTGCCAGCAAAGCAAGATCAACAACAACGCACACTAGCAAACT
>HPV110_Beta_186461194_nt6517_L1_Tile|1
CATAACTATTTCTTGCCAGCAAAGCAAGATCAACAACAACGCACACTAGCAAACTCTACA
>HPV110_Beta_186461194_nt6532_L1_Tile|1
CCAGCAAAGCAAGATCAACAACAACGCACACTAGCAAACTCTACATATTTTCCAACAGTA
>HPV110_Beta_186461194_nt6537_L1_Tile|1
AAAGCAAGATCAACAACAACGCACACTAGCAAACTCTACATATTTTCCAACAGTAAGTGG
>HPV110_Beta_186461194_nt6557_L1_Tile|1
GCACACTAGCAAACTCTACATATTTTCCAACAGTAAGTGGTTCTCTAGTTACTTCAGATG
>HPV110_Beta_186461194_nt6697_L1_Tile|1
ATAACTGTTGCTGATAATACTCGTAATACTAATTTTTCAATTAGTGTTACTACAGAAGCT
>HPV110_Beta_186461194_nt6702_L1_Tile|2
TGTTGCTGATAATACTCGTAATACTAATTTTTCAATTAGTGTTACTACAGAAGCTGGAGC
>HPV110_Beta_186461194_nt6707_L1_Tile|1
CTGATAATACTCGTAATACTAATTTTTCAATTAGTGTTACTACAGAAGCTGGAGCAACTG
>HPV110_Beta_186461194_nt6712_L1_Tile|1
AATACTCGTAATACTAATTTTTCAATTAGTGTTACTACAGAAGCTGGAGCAACTGAATAC
>HPV110_Beta_186461194_nt6717_L1_Tile|1
TCGTAATACTAATTTTTCAATTAGTGTTACTACAGAAGCTGGAGCAACTGAATACAATTC
>HPV110_Beta_186461194_nt6722_L1_Tile|2
ATACTAATTTTTCAATTAGTGTTACTACAGAAGCTGGAGCAACTGAATACAATTCACAAA

>HPV110_Beta_186461194_nt6727_L1_Tile|1
AATTTTTCAATTAGTGTTACTACAGAAGCTGGAGCAACTGAATACAATTCACAAAATATA
>HPV110_Beta_186461194_nt6732_L1_Tile|1
TTCAATTAGTGTTACTACAGAAGCTGGAGCAACTGAATACAATTCACAAAATATAAGAGA
>HPV110_Beta_186461194_nt6742_L1_Tile|2
GTTACTACAGAAGCTGGAGCAACTGAATACAATTCACAAAATATAAGAGAATTTCTTAGA
>HPV110_Beta_186461194_nt6752_L1_Tile|1
AAGCTGGAGCAACTGAATACAATTCACAAAATATAAGAGAATTTCTTAGACATGTAGAGG
>HPV110_Beta_186461194_nt6772_L1_Tile|1
AATTCACAAAATATAAGAGAATTTCTTAGACATGTAGAGGAATATCAACTTTCTTTAATA
>HPV110_Beta_186461194_nt6777_L1_Tile|1
ACAAAATATAAGAGAATTTCTTAGACATGTAGAGGAATATCAACTTTCTTTAATACTGCA
>HPV110_Beta_186461194_nt6782_L1_Tile|2
ATATAAGAGAATTTCTTAGACATGTAGAGGAATATCAACTTTCTTTAATACTGCAATTAT
>HPV110_Beta_186461194_nt6792_L1_Tile|1
ATTTCTTAGACATGTAGAGGAATATCAACTTTCTTTAATACTGCAATTATGTAAGGTACC
>HPV110_Beta_186461194_nt6802_L1_Tile|2
CATGTAGAGGAATATCAACTTTCTTTAATACTGCAATTATGTAAGGTACCCTTAAAAGCA
>HPV110_Beta_186461194_nt6807_L1_Tile|1
AGAGGAATATCAACTTTCTTTAATACTGCAATTATGTAAGGTACCCTTAAAAGCAGAGGT
>HPV110_Beta_186461194_nt6812_L1_Tile|1
AATATCAACTTTCTTTAATACTGCAATTATGTAAGGTACCCTTAAAAGCAGAGGTTTTAA
>HPV110_Beta_186461194_nt6817_L1_Tile|1
CAACTTTCTTTAATACTGCAATTATGTAAGGTACCCTTAAAAGCAGAGGTTTTAACACAA
>HPV110_Beta_186461194_nt6822_L1_Tile|2
TTCTTTAATACTGCAATTATGTAAGGTACCCTTAAAAGCAGAGGTTTTAACACAAATTAA
>HPV110_Beta_186461194_nt7107_L1_Tile|1
ATTTTTATTTCAGGCTGGTTTACAAAGTCGACCTCGCTCTGTAAGGACATCTGTTAAGGT
>HPV110_Beta_186461194_nt7112_L1_Tile|1
TATTTCAGGCTGGTTTACAAAGTCGACCTCGCTCTGTAAGGACATCTGTTAAGGTCTCTA
>HPV110_Beta_186461194_nt7117_L1_Tile|1
CAGGCTGGTTTACAAAGTCGACCTCGCTCTGTAAGGACATCTGTTAAGGTCTCTAAGGGT
>HPV110_Beta_186461194_nt7127_L1_Tile|1
TACAAAGTCGACCTCGCTCTGTAAGGACATCTGTTAAGGTCTCTAAGGGTACCAAACGCA
>HPV110_Beta_186461194_nt7137_L1_Tile|1
ACCTCGCTCTGTAAGGACATCTGTTAAGGTCTCTAAGGGTACCAAACGCAAACGGTCGTG
>HPV110_Beta_186461194_nt7138_L1_Tile|1
CCTCGCTCTGTAAGGACATCTGTTAAGGTCTCTAAGGGTACCAAACGCAAACGGTCGTGA
>HPV111_Beta_186461202_nt5988_L1_Tile|1
ATTATTTAATAAGGTTCGTGACACTGAAAATTCCAGTAATTATCAAGCTGTGACACAAGA
>HPV111_Beta_186461202_nt5993_L1_Tile|1
TTAATAAGGTTCGTGACACTGAAAATTCCAGTAATTATCAAGCTGTGACACAAGATGATA
>HPV111_Beta_186461202_nt6128_L1_Tile|1
GGGACAAAGCAAAACCCTGTGATGATGCAGGAAACCAGCAAGGCTTATGCCCGCCAATTG
>HPV111_Beta_186461202_nt6133_L1_Tile|1
AAAGCAAAACCCTGTGATGATGCAGGAAACCAGCAAGGCTTATGCCCGCCAATTGAATTA
>HPV111_Beta_186461202_nt6138_L1_Tile|1
AAAACCCTGTGATGATGCAGGAAACCAGCAAGGCTTATGCCCGCCAATTGAATTAAAGAA
>HPV111_Beta_186461202_nt6148_L1_Tile|1
GATGATGCAGGAAACCAGCAAGGCTTATGCCCGCCAATTGAATTAAAGAATTCTGTAATA
>HPV111_Beta_186461202_nt6158_L1_Tile|1
GAAACCAGCAAGGCTTATGCCCGCCAATTGAATTAAAGAATTCTGTAATAGAGGATGGAG
>HPV111_Beta_186461202_nt6168_L1_Tile|1
AGGCTTATGCCCGCCAATTGAATTAAAGAATTCTGTAATAGAGGATGGAGACATGTTTGA
>HPV111_Beta_186461202_nt6288_L1_Tile|1

AGATATTGTCAATGAAGTATGTAAATACCCTGACTTCCTAACTATGGCTAATGACGTCTA
>HPV111_Beta_186461202_nt6293_L1_Tile|1
TTGTCAATGAAGTATGTAAATACCCTGACTTCCTAACTATGGCTAATGACGTCTATGGGG
>HPV111_Beta_186461202_nt6418_L1_Tile|1
AATGTAGGAGATGCAATTCCAGATGCAGCAGTGCAACAAAATCACAATTATTATCTGCCA
>HPV111_Beta_186461202_nt6423_L1_Tile|1
AGGAGATGCAATTCCAGATGCAGCAGTGCAACAAAATCACAATTATTATCTGCCAGCACA
>HPV111_Beta_186461202_nt6433_L1_Tile|1
ATTCCAGATGCAGCAGTGCAACAAAATCACAATTATTATCTGCCAGCACAGGGTAATCAA
>HPV111_Beta_186461202_nt6448_L1_Tile|1
GTGCAACAAAATCACAATTATTATCTGCCAGCACAGGGTAATCAACAACAAAACATTTTG
>HPV111_Beta_186461202_nt6453_L1_Tile|1
ACAAAATCACAATTATTATCTGCCAGCACAGGGTAATCAACAACAAAACATTTTGGGTAA
>HPV111_Beta_186461202_nt6458_L1_Tile|1
ATCACAATTATTATCTGCCAGCACAGGGTAATCAACAACAAAACATTTTGGGTAACTCCA
>HPV111_Beta_186461202_nt6463_L1_Tile|1
AATTATTATCTGCCAGCACAGGGTAATCAACAACAAAACATTTTGGGTAACTCCATTTAT
>HPV111_Beta_186461202_nt6468_L1_Tile|1
TTATCTGCCAGCACAGGGTAATCAACAACAAAACATTTTGGGTAACTCCATTTATTATCC
>HPV111_Beta_186461202_nt6478_L1_Tile|1
GCACAGGGTAATCAACAACAAAACATTTTGGGTAACTCCATTTATTATCCTACTGTTAGT
>HPV111_Beta_186461202_nt6493_L1_Tile|1
CAACAAAACATTTTGGGTAACTCCATTTATTATCCTACTGTTAGTGGTTCTCTTGTTACT
>HPV111_Beta_186461202_nt6633_L1_Tile|1
AATGTTTATAACAGTAGCAGATAATACCAGAAACACTAATTTTACTATAAGCGTATCCAC
>HPV111_Beta_186461202_nt6638_L1_Tile|1
TTATAACAGTAGCAGATAATACCAGAAACACTAATTTTACTATAAGCGTATCCACAGAAG
>HPV111_Beta_186461202_nt6643_L1_Tile|1
ACAGTAGCAGATAATACCAGAAACACTAATTTTACTATAAGCGTATCCACAGAAGCTAAT
>HPV111_Beta_186461202_nt6648_L1_Tile|1
AGCAGATAATACCAGAAACACTAATTTTACTATAAGCGTATCCACAGAAGCTAATGCACA
>HPV111_Beta_186461202_nt6658_L1_Tile|1
ACCAGAAACACTAATTTTACTATAAGCGTATCCACAGAAGCTAATGCACAGCAATATAAT
>HPV111_Beta_186461202_nt6663_L1_Tile|1
AAACACTAATTTTACTATAAGCGTATCCACAGAAGCTAATGCACAGCAATATAATGCTAG
>HPV111_Beta_186461202_nt6668_L1_Tile|1
CTAATTTTACTATAAGCGTATCCACAGAAGCTAATGCACAGCAATATAATGCTAGTAATA
>HPV111_Beta_186461202_nt6673_L1_Tile|1
TTTACTATAAGCGTATCCACAGAAGCTAATGCACAGCAATATAATGCTAGTAATATTAGA
>HPV111_Beta_186461202_nt6678_L1_Tile|1
TATAAGCGTATCCACAGAAGCTAATGCACAGCAATATAATGCTAGTAATATTAGAGAATA
>HPV111_Beta_186461202_nt6748_L1_Tile|1
GTAGAAGAATATCAATTGTCTTTGATTCTTCAACTATGTAAAGTTTCTTTAGTTCCAGAA
>HPV111_Beta_186461202_nt6753_L1_Tile|1
AGAATATCAATTGTCTTTGATTCTTCAACTATGTAAAGTTTCTTTAGTTCCAGAAGTTTT
>HPV111_Beta_186461202_nt6763_L1_Tile|1
TTGTCTTTGATTCTTCAACTATGTAAAGTTTCTTTAGTTCCAGAAGTTTTATCTCAAATC
>HPV111_Beta_186461202_nt6768_L1_Tile|1
TTTGATTCTTCAACTATGTAAAGTTTCTTTAGTTCCAGAAGTTTTATCTCAAATCAATGC
>HPV111_Beta_186461202_nt7053_L1_Tile|1
TTTATTCCAGGCTGGTTTACAAACACGAAAACGGACTTTAAAACCTTCGTCTGTGAAATC
>HPV111_Beta_186461202_nt7058_L1_Tile|1
TCCAGGCTGGTTTACAAACACGAAAACGGACTTTAAAACCTTCGTCTGTGAAATCTACTA
>HPV111_Beta_186461202_nt7063_L1_Tile|1
GCTGGTTTACAAACACGAAAACGGACTTTAAAACCTTCGTCTGTGAAATCTACTAAATCT

>HPV111_Beta_186461202_nt7068_L1_Tile|1
TTTACAAACACGAAAACGGACTTTAAAACCTTCGTCTGTGAAATCTACTAAATCTGCAAA
>HPV111_Beta_186461202_nt7083_L1_Tile|1
ACGGACTTTAAAACCTTCGTCTGTGAAATCTACTAAATCTGCAAAACGCAGACGCACCTA
>HPV111_Beta_186461202_nt7084_L1_Tile|1
CGGACTTTAAAACCTTCGTCTGTGAAATCTACTAAATCTGCAAAACGCAGACGCACCTAA
>HPV12_Beta_396910_nt6165_L1_Tile|1
AATAAGTAGGGGTCAGCCTTTAGGCGTTGGAAGTACTGGACATCCCTATTTTAACAAAAT
>HPV12_Beta_396910_nt6205_L1_Tile|1
CATCCCTATTTTAACAAAATTAAAGACACGGAAAACTCAAATAACTATGCCACAGGCAGT
>HPV12_Beta_396910_nt6220_L1_Tile|1
AAAATTAAAGACACGGAAAACTCAAATAACTATGCCACAGGCAGTAAGGATGATAGACAG
>HPV12_Beta_396910_nt6235_L1_Tile|1
GAAAACTCAAATAACTATGCCACAGGCAGTAAGGATGATAGACAGAACACATCATTTGAT
>HPV12_Beta_396910_nt6330_L1_Tile|1
ACCTTGTGTGGGAGAACACTGGGAGAAAGCCTTACCCTGCGGGGATGCACCAGCTGAAAA
>HPV12_Beta_396910_nt6335_L1_Tile|1
GTGTGGGAGAACACTGGGAGAAAGCCTTACCCTGCGGGGATGCACCAGCTGAAAATGGTG
>HPV12_Beta_396910_nt6340_L1_Tile|1
GGAGAACACTGGGAGAAAGCCTTACCCTGCGGGGATGCACCAGCTGAAAATGGTGTTTGC
>HPV12_Beta_396910_nt6350_L1_Tile|1
GGGAGAAAGCCTTACCCTGCGGGGATGCACCAGCTGAAAATGGTGTTTGCCCTCCCATAG
>HPV12_Beta_396910_nt6355_L1_Tile|1
AAAGCCTTACCCTGCGGGGATGCACCAGCTGAAAATGGTGTTTGCCCTCCCATAGAGTTA
>HPV12_Beta_396910_nt6375_L1_Tile|1
TGCACCAGCTGAAAATGGTGTTTGCCCTCCCATAGAGTTAAAGAACACTTTCATTGAAGA
>HPV12_Beta_396910_nt6380_L1_Tile|1
CAGCTGAAAATGGTGTTTGCCCTCCCATAGAGTTAAAGAACACTTTCATTGAAGATGGTG
>HPV12_Beta_396910_nt6895_L1_Tile|1
AACACCAATTTCAGTATATCTATTTACAGTGATAATCAAAATGTACACGACATTCCAAAT
>HPV12_Beta_396910_nt6900_L1_Tile|1
CAATTTCAGTATATCTATTTACAGTGATAATCAAAATGTACACGACATTCCAAATTATGA
>HPV12_Beta_396910_nt6905_L1_Tile|1
TCAGTATATCTATTTACAGTGATAATCAAAATGTACACGACATTCCAAATTATGATTCTC
>HPV12_Beta_396910_nt6910_L1_Tile|1
ATATCTATTTACAGTGATAATCAAAATGTACACGACATTCCAAATTATGATTCTCAAAAA
>HPV12_Beta_396910_nt6915_L1_Tile|1
TATTTACAGTGATAATCAAAATGTACACGACATTCCAAATTATGATTCTCAAAAATTTAG
>HPV12_Beta_396910_nt6920_L1_Tile|1
ACAGTGATAATCAAAATGTACACGACATTCCAAATTATGATTCTCAAAAATTTAGAGAAT
>HPV12_Beta_396910_nt6925_L1_Tile|1
GATAATCAAAATGTACACGACATTCCAAATTATGATTCTCAAAAATTTAGAGAATATTTA
>HPV12_Beta_396910_nt7075_L1_Tile|1
AACTCTTCTTTGCTGGAGGACTGGCAATTAGGCTTCGTGCCAACTCCTGACAATCCTATT
>HPV12_Beta_396910_nt7100_L1_Tile|1
AATTAGGCTTCGTGCCAACTCCTGACAATCCTATTCATGACACATACAGATATATCGAAT
>HPV12_Beta_396910_nt7105_L1_Tile|1
GGCTTCGTGCCAACTCCTGACAATCCTATTCATGACACATACAGATATATCGAATCTCTG
>HPV12_Beta_396910_nt7200_L1_Tile|1
AAAGCCGGACCCTTATGATGGCTTAAGTTTTTGGACTGTAGATATGACTGAGAGACTTTC
>HPV12_Beta_396910_nt7205_L1_Tile|1
CGGACCCTTATGATGGCTTAAGTTTTTGGACTGTAGATATGACTGAGAGACTTTCTTTAG
>HPV12_Beta_396910_nt7230_L1_Tile|1
TTGGACTGTAGATATGACTGAGAGACTTTCTTTAGACCTGGATCAGTATTCCTTAGGGCG
>HPV12_Beta_396910_nt7260_L1_Tile|1

TTTAGACCTGGATCAGTATTCCTTAGGGCGCAAGTTCTTATTCCAGGCTGGCCTCCAACA
>HPV12_Beta_396910_nt7325_L1_Tile|1
CCGTTAACGGTACAACAAAATCATCAAGCTATAGAAGTTCCATAAGGGGGACCAAAAGAA
>HPV13_Alpha_85827580_nt5873_L1_Tile|1
TGGGAAATCCTTATTTTCCTATTAAGAAACAAAACAAAACTGTTGTCCCTAAGGTATCTG
>HPV13_Alpha_85827580_nt5878_L1_Tile|1
AATCCTTATTTTCCTATTAAGAAACAAAACAAAACTGTTGTCCCTAAGGTATCTGGTTAT
>HPV13_Alpha_85827580_nt5883_L1_Tile|1
TTATTTTCCTATTAAGAAACAAAACAAAACTGTTGTCCCTAAGGTATCTGGTTATCAGTT
>HPV13_Alpha_85827580_nt5888_L1_Tile|1
TTCCTATTAAGAAACAAAACAAAACTGTTGTCCCTAAGGTATCTGGTTATCAGTTTAGGG
>HPV13_Alpha_85827580_nt5893_L1_Tile|1
ATTAAGAAACAAAACAAAACTGTTGTCCCTAAGGTATCTGGTTATCAGTTTAGGGTATTT
>HPV13_Alpha_85827580_nt6253_L1_Tile|1
CAATGTACTGGTGTAAATGTACAACCTGGAGATTGCCCTCCTTTAGAATTAATTAGTAGT
>HPV13_Alpha_85827580_nt6503_L1_Tile|1
CAAGGCATTTCTTTAACAGGGCAGGCTCTGTTGGTGAACTAATCCCAGCAGAATTATATG
>HPV13_Alpha_85827580_nt6508_L1_Tile|1
CATTTCTTTAACAGGGCAGGCTCTGTTGGTGAACTAATCCCAGCAGAATTATATGTTAAG
>HPV13_Alpha_85827580_nt6513_L1_Tile|1
CTTTAACAGGGCAGGCTCTGTTGGTGAACTAATCCCAGCAGAATTATATGTTAAGGGTAG
>HPV13_Alpha_85827580_nt6523_L1_Tile|1
GCAGGCTCTGTTGGTGAACTAATCCCAGCAGAATTATATGTTAAGGGTAGTAATACACTT
>HPV13_Alpha_85827580_nt6528_L1_Tile|1
CTCTGTTGGTGAACTAATCCCAGCAGAATTATATGTTAAGGGTAGTAATACACTTTCTAA
>HPV13_Alpha_85827580_nt6533_L1_Tile|1
TTGGTGAACTAATCCCAGCAGAATTATATGTTAAGGGTAGTAATACACTTTCTAATAGTA
>HPV13_Alpha_85827580_nt6538_L1_Tile|1
GAACTAATCCCAGCAGAATTATATGTTAAGGGTAGTAATACACTTTCTAATAGTATTTAC
>HPV13_Alpha_85827580_nt6543_L1_Tile|1
AATCCCAGCAGAATTATATGTTAAGGGTAGTAATACACTTTCTAATAGTATTTACTATAA
>HPV13_Alpha_85827580_nt6548_L1_Tile|1
CAGCAGAATTATATGTTAAGGGTAGTAATACACTTTCTAATAGTATTTACTATAATACTC
>HPV13_Alpha_85827580_nt6553_L1_Tile|1
GAATTATATGTTAAGGGTAGTAATACACTTTCTAATAGTATTTACTATAATACTCCCAGT
>HPV13_Alpha_85827580_nt6558_L1_Tile|1
ATATGTTAAGGGTAGTAATACACTTTCTAATAGTATTTACTATAATACTCCCAGTGGCTC
>HPV13_Alpha_85827580_nt6563_L1_Tile|1
TTAAGGGTAGTAATACACTTTCTAATAGTATTTACTATAATACTCCCAGTGGCTCTCTTG
>HPV13_Alpha_85827580_nt6743_L1_Tile|1
CTAACATGACTGTGTGTGCAGCCACTACATCATCTCTTTCAGACACATATAAGGCCACAG
>HPV13_Alpha_85827580_nt6748_L1_Tile|1
ATGACTGTGTGTGCAGCCACTACATCATCTCTTTCAGACACATATAAGGCCACAGAATAT
>HPV13_Alpha_85827580_nt6753_L1_Tile|1
TGTGTGTGCAGCCACTACATCATCTCTTTCAGACACATATAAGGCCACAGAATATAAACA
>HPV13_Alpha_85827580_nt6763_L1_Tile|1
GCCACTACATCATCTCTTTCAGACACATATAAGGCCACAGAATATAAACAGTACATGCGA
>HPV13_Alpha_85827580_nt6913_L1_Tile|1
CCTACAATTCTAGAAGACTGGAACTTTGGGCTATCTCCCCCTCCTAATGGAACATTGGAA
>HPV13_Alpha_85827580_nt6928_L1_Tile|1
GACTGGAACTTTGGGCTATCTCCCCCTCCTAATGGAACATTGGAAGACACATATAGATAT
>HPV13_Alpha_85827580_nt6933_L1_Tile|1
GAACTTTGGGCTATCTCCCCCTCCTAATGGAACATTGGAAGACACATATAGATATGTACA
>HPV13_Alpha_85827580_nt6938_L1_Tile|1
TTGGGCTATCTCCCCCTCCTAATGGAACATTGGAAGACACATATAGATATGTACAATCTC

>HPV13_Alpha_85827580_nt6943_L1_Tile|1
CTATCTCCCCCTCCTAATGGAACATTGGAAGACACATATAGATATGTACAATCTCAGGCC
>HPV13_Alpha_85827580_nt6953_L1_Tile|1
CTCCTAATGGAACATTGGAAGACACATATAGATATGTACAATCTCAGGCCATAACGTGTC
>HPV13_Alpha_85827580_nt7003_L1_Tile|1
ATAACGTGTCAAAAGCCTACACCTGATAAAGAAAAACAGGATCCGTATGCGGGTCTTAGT
>HPV13_Alpha_85827580_nt7133_L1_Tile|1
TATTACAAACAGGCGTTCAGTCTAGGTCACCTATTCGTGTAGGTAGAAAACGTGCTGCAT
>HPV13_Alpha_85827580_nt7138_L1_Tile|1
CAAACAGGCGTTCAGTCTAGGTCACCTATTCGTGTAGGTAGAAAACGTGCTGCATCTACA
>HPV13_Alpha_85827580_nt7143_L1_Tile|1
AGGCGTTCAGTCTAGGTCACCTATTCGTGTAGGTAGAAAACGTGCTGCATCTACATCTAC
>HPV13_Alpha_85827580_nt7153_L1_Tile|1
TCTAGGTCACCTATTCGTGTAGGTAGAAAACGTGCTGCATCTACATCTACTGCCACACCT
>HPV13_Alpha_85827580_nt7163_L1_Tile|1
CTATTCGTGTAGGTAGAAAACGTGCTGCATCTACATCTACTGCCACACCTACTACACGTA
>HPV13_Alpha_85827580_nt7168_L1_Tile|1
CGTGTAGGTAGAAAACGTGCTGCATCTACATCTACTGCCACACCTACTACACGTAAAAAA
>HPV13_Alpha_85827580_nt7173_L1_Tile|1
AGGTAGAAAACGTGCTGCATCTACATCTACTGCCACACCTACTACACGTAAAAAAGCTAA
>HPV13_Alpha_85827580_nt7178_L1_Tile|1
GAAAACGTGCTGCATCTACATCTACTGCCACACCTACTACACGTAAAAAAGCTAAAAGGA
>HPV13_Alpha_85827580_nt7183_L1_Tile|1
CGTGCTGCATCTACATCTACTGCCACACCTACTACACGTAAAAAAGCTAAAAGGAAATAA
>HPV14D_Beta_396918_nt5969_L1_Tile|1
ATTATTTAATAAGGTTGGTGATACAGAAAATCCCAACTCATACAGGCAACAAGCTAACTC
>HPV14D_Beta_396918_nt5974_L1_Tile|1
TTAATAAGGTTGGTGATACAGAAAATCCCAACTCATACAGGCAACAAGCTAACTCCACTG
>HPV14D_Beta_396918_nt5979_L1_Tile|1
AAGGTTGGTGATACAGAAAATCCCAACTCATACAGGCAACAAGCTAACTCCACTGATGAC
>HPV14D_Beta_396918_nt5984_L1_Tile|1
TGGTGATACAGAAAATCCCAACTCATACAGGCAACAAGCTAACTCCACTGATGACAGACA
>HPV14D_Beta_396918_nt5989_L1_Tile|1
ATACAGAAAATCCCAACTCATACAGGCAACAAGCTAACTCCACTGATGACAGACAAAATG
>HPV14D_Beta_396918_nt5994_L1_Tile|1
GAAAATCCCAACTCATACAGGCAACAAGCTAACTCCACTGATGACAGACAAAATGTGTCA
>HPV14D_Beta_396918_nt5999_L1_Tile|1
TCCCAACTCATACAGGCAACAAGCTAACTCCACTGATGACAGACAAAATGTGTCATTTGA
>HPV14D_Beta_396918_nt6649_L1_Tile|1
ACAACACACGTAACACAAATTTTAGTATATCAGTTAGTTCAGAAAACACTGAGGTATCCA
>HPV14D_Beta_396918_nt6669_L1_Tile|1
TTTAGTATATCAGTTAGTTCAGAAAACACTGAGGTATCCAAAATTGACAATTATACCTCT
>HPV14D_Beta_396918_nt7099_L1_Tile|1
GTACAAAAACAGTTTCGACTAGGGGATCCATCAAGGGTATTAAACGAAAACGCAAGAATT
>HPV14D_Beta_396918_nt7101_L1_Tile|1
ACAAAAACAGTTTCGACTAGGGGATCCATCAAGGGTATTAAACGAAAACGCAAGAATTAG
>HPV15_Beta_396924_nt6039_L1_Tile|1
ACCATTAGGAGTTGGTACTTCAGGCCATCCTTTATTCAACAAAGTAAAAGATACAGAGAA
>HPV15_Beta_396924_nt6044_L1_Tile|1
TAGGAGTTGGTACTTCAGGCCATCCTTTATTCAACAAAGTAAAAGATACAGAGAATAACA
>HPV15_Beta_396924_nt6049_L1_Tile|1
GTTGGTACTTCAGGCCATCCTTTATTCAACAAAGTAAAAGATACAGAGAATAACAGTAAT
>HPV15_Beta_396924_nt6059_L1_Tile|1
CAGGCCATCCTTTATTCAACAAAGTAAAAGATACAGAGAATAACAGTAATTATCAAGGCA
>HPV15_Beta_396924_nt6064_L1_Tile|1

CATCCTTTATTCAACAAAGTAAAAGATACAGAGAATAACAGTAATTATCAAGGCAACTCT
>HPV15_Beta_396924_nt6069_L1_Tile|1
TTTATTCAACAAAGTAAAAGATACAGAGAATAACAGTAATTATCAAGGCAACTCTACTGA
>HPV15_Beta_396924_nt6314_L1_Tile|1
TAGGTTTTGGTAACATTAATAACAAGGCCTTATCAGTTACTAAGTCAGATGTGAGTCTGG
>HPV15_Beta_396924_nt6319_L1_Tile|1
TTTGGTAACATTAATAACAAGGCCTTATCAGTTACTAAGTCAGATGTGAGTCTGGATATA
>HPV15_Beta_396924_nt6324_L1_Tile|1
TAACATTAATAACAAGGCCTTATCAGTTACTAAGTCAGATGTGAGTCTGGATATAGTGAA
>HPV15_Beta_396924_nt6549_L1_Tile|1
TTTTTATTTACCAGCACAATCAACCCAACAACAAAATAACTTAGCAAATTCTACTTACTT
>HPV15_Beta_396924_nt6554_L1_Tile|1
ATTTACCAGCACAATCAACCCAACAACAAAATAACTTAGCAAATTCTACTTACTTTCCCA
>HPV15_Beta_396924_nt6699_L1_Tile|1
CATACTTTGGGGTAATCAGATGTTTATTACTGTTGCAGATAACACAAGGAATACAAATTT
>HPV15_Beta_396924_nt6709_L1_Tile|1
GGTAATCAGATGTTTATTACTGTTGCAGATAACACAAGGAATACAAATTTTACTATTAGT
>HPV15_Beta_396924_nt6734_L1_Tile|1
CAGATAACACAAGGAATACAAATTTTACTATTAGTGTTACCTCTGATGGTAATGCCATAA
>HPV15_Beta_396924_nt6744_L1_Tile|1
AAGGAATACAAATTTTACTATTAGTGTTACCTCTGATGGTAATGCCATAAATGAATATAA
>HPV15_Beta_396924_nt6759_L1_Tile|1
TACTATTAGTGTTACCTCTGATGGTAATGCCATAAATGAATATAATTCACAAAATATCAG
>HPV16_Alpha_9627100_nt6015_L1_Tile|1
GGATGACACAGAAAATGCTAGTGCTTATGCAGCAAATGCAGGTGTGGATAATAGAGAATG
>HPV16_Alpha_9627100_nt6020_L1_Tile|1
ACACAGAAAATGCTAGTGCTTATGCAGCAAATGCAGGTGTGGATAATAGAGAATGTATAT
>HPV16_Alpha_9627100_nt6025_L1_Tile|1
GAAAATGCTAGTGCTTATGCAGCAAATGCAGGTGTGGATAATAGAGAATGTATATCTATG
>HPV16_Alpha_9627100_nt6035_L1_Tile|1
GTGCTTATGCAGCAAATGCAGGTGTGGATAATAGAGAATGTATATCTATGGATTACAAAC
>HPV16_Alpha_9627100_nt6120_L1_Tile|1
CAAACCACCTATAGGGGAACACTGGGGCAAAGGATCCCCATGTACCAATGTTGCAGTAAA
>HPV16_Alpha_9627100_nt6125_L1_Tile|1
CACCTATAGGGGAACACTGGGGCAAAGGATCCCCATGTACCAATGTTGCAGTAAATCCAG
>HPV16_Alpha_9627100_nt6155_L1_Tile|1
CCCCATGTACCAATGTTGCAGTAAATCCAGGTGATTGTCCACCATTAGAGTTAATAAACA
>HPV16_Alpha_9627100_nt6160_L1_Tile|1
TGTACCAATGTTGCAGTAAATCCAGGTGATTGTCCACCATTAGAGTTAATAAACACAGTT
>HPV16_Alpha_9627100_nt6420_L1_Tile|1
TAATAGGGCTGGTACTGTTGGTGAAAATGTACCAGACGATTTATACATTAAAGGCTCTGG
>HPV16_Alpha_9627100_nt6425_L1_Tile|1
GGGCTGGTACTGTTGGTGAAAATGTACCAGACGATTTATACATTAAAGGCTCTGGGTCTA
>HPV16_Alpha_9627100_nt6440_L1_Tile|1
GTGAAAATGTACCAGACGATTTATACATTAAAGGCTCTGGGTCTACTGCAAATTTAGCCA
>HPV16_Alpha_9627100_nt6445_L1_Tile|1
AATGTACCAGACGATTTATACATTAAAGGCTCTGGGTCTACTGCAAATTTAGCCAGTTCA
>HPV16_Alpha_9627100_nt6450_L1_Tile|1
ACCAGACGATTTATACATTAAAGGCTCTGGGTCTACTGCAAATTTAGCCAGTTCAAATTA
>HPV16_Alpha_9627100_nt6455_L1_Tile|1
ACGATTTATACATTAAAGGCTCTGGGTCTACTGCAAATTTAGCCAGTTCAAATTATTTTC
>HPV16_Alpha_9627100_nt6460_L1_Tile|1
TTATACATTAAAGGCTCTGGGTCTACTGCAAATTTAGCCAGTTCAAATTATTTTCCTACA
>HPV16_Alpha_9627100_nt6640_L1_Tile|1
GATACTACACGCAGTACAAATATGTCATTATGTGCTGCCATATCTACTTCAGAAACTACA

>HPV16_Alpha_9627100_nt6650_L1_Tile|1
GCAGTACAAATATGTCATTATGTGCTGCCATATCTACTTCAGAAACTACATATAAAAATA
>HPV16_Alpha_9627100_nt6655_L1_Tile|1
ACAAATATGTCATTATGTGCTGCCATATCTACTTCAGAAACTACATATAAAAATACTAAC
>HPV16_Alpha_9627100_nt6820_L1_Tile|1
AATTCCACTATTTTGGAGGACTGGAATTTTGGTCTACAACCTCCCCCAGGAGGCACACTA
>HPV16_Alpha_9627100_nt6845_L1_Tile|1
ATTTTGGTCTACAACCTCCCCCAGGAGGCACACTAGAAGATACTTATAGGTTTGTAACCC
>HPV16_Alpha_9627100_nt6850_L1_Tile|1
GGTCTACAACCTCCCCCAGGAGGCACACTAGAAGATACTTATAGGTTTGTAACCCAGGCA
>HPV16_Alpha_9627100_nt6860_L1_Tile|1
CTCCCCCAGGAGGCACACTAGAAGATACTTATAGGTTTGTAACCCAGGCAATTGCTTGTC
>HPV16_Alpha_9627100_nt6865_L1_Tile|1
CCAGGAGGCACACTAGAAGATACTTATAGGTTTGTAACCCAGGCAATTGCTTGTCAAAAA
>HPV16_Alpha_9627100_nt6870_L1_Tile|1
AGGCACACTAGAAGATACTTATAGGTTTGTAACCCAGGCAATTGCTTGTCAAAAACATAC
>HPV16_Alpha_9627100_nt6880_L1_Tile|1
GAAGATACTTATAGGTTTGTAACCCAGGCAATTGCTTGTCAAAAACATACACCTCCAGCA
>HPV16_Alpha_9627100_nt6890_L1_Tile|1
ATAGGTTTGTAACCCAGGCAATTGCTTGTCAAAAACATACACCTCCAGCACCTAAAGAAG
>HPV16_Alpha_9627100_nt6900_L1_Tile|1
AACCCAGGCAATTGCTTGTCAAAAACATACACCTCCAGCACCTAAAGAAGATGATCCCCT
>HPV16_Alpha_9627100_nt6910_L1_Tile|1
ATTGCTTGTCAAAAACATACACCTCCAGCACCTAAAGAAGATGATCCCCTTAAAAAATAC
>HPV16_Alpha_9627100_nt6915_L1_Tile|1
TTGTCAAAAACATACACCTCCAGCACCTAAAGAAGATGATCCCCTTAAAAAATACACTTT
>HPV16_Alpha_9627100_nt6920_L1_Tile|1
AAAAACATACACCTCCAGCACCTAAAGAAGATGATCCCCTTAAAAAATACACTTTTTGGG
>HPV16_Alpha_9627100_nt6930_L1_Tile|1
ACCTCCAGCACCTAAAGAAGATGATCCCCTTAAAAAATACACTTTTTGGGAAGTAAATTT
>HPV16_Alpha_9627100_nt6935_L1_Tile|1
CAGCACCTAAAGAAGATGATCCCCTTAAAAAATACACTTTTTGGGAAGTAAATTTAAAGG
>HPV16_Alpha_9627100_nt7020_L1_Tile|1
GTTTCCTTTAGGACGCAAATTTTTACTACAAGCAGGATTGAAGGCCAAACCAAAATTTAC
>HPV16_Alpha_9627100_nt7025_L1_Tile|1
CTTTAGGACGCAAATTTTTACTACAAGCAGGATTGAAGGCCAAACCAAAATTTACATTAG
>HPV16_Alpha_9627100_nt7030_L1_Tile|1
GGACGCAAATTTTTACTACAAGCAGGATTGAAGGCCAAACCAAAATTTACATTAGGAAAA
>HPV16_Alpha_9627100_nt7035_L1_Tile|1
CAAATTTTTACTACAAGCAGGATTGAAGGCCAAACCAAAATTTACATTAGGAAAACGAAA
>HPV16_Alpha_9627100_nt7040_L1_Tile|1
TTTTACTACAAGCAGGATTGAAGGCCAAACCAAAATTTACATTAGGAAAACGAAAAGCTA
>HPV16_Alpha_9627100_nt7050_L1_Tile|1
AGCAGGATTGAAGGCCAAACCAAAATTTACATTAGGAAAACGAAAAGCTACACCCACCAC
>HPV16_Alpha_9627100_nt7055_L1_Tile|1
GATTGAAGGCCAAACCAAAATTTACATTAGGAAAACGAAAAGCTACACCCACCACCTCAT
>HPV16_Alpha_9627100_nt7065_L1_Tile|1
CAAACCAAAATTTACATTAGGAAAACGAAAAGCTACACCCACCACCTCATCTACCTCTAC
>HPV16_Alpha_9627100_nt7070_L1_Tile|1
CAAAATTTACATTAGGAAAACGAAAAGCTACACCCACCACCTCATCTACCTCTACAACTG
>HPV16_Alpha_9627100_nt7075_L1_Tile|1
TTTACATTAGGAAAACGAAAAGCTACACCCACCACCTCATCTACCTCTACAACTGCTAAA
>HPV16_Alpha_9627100_nt7085_L1_Tile|1
GAAAACGAAAAGCTACACCCACCACCTCATCTACCTCTACAACTGCTAAACGCAAAAAAC
>HPV16_Alpha_9627100_nt7095_L1_Tile|1

AGCTACACCCACCACCTCATCTACCTCTACAACTGCTAAACGCAAAAAACGTAAGCTGTA
>HPV16_Alpha_9627100_nt7096_L1_Tile|1
GCTACACCCACCACCTCATCTACCTCTACAACTGCTAAACGCAAAAAACGTAAGCTGTAA
>HPV17_Beta_396932_nt6085_L1_Tile|1
CATCCCTTGTTTAATAAGTTAAGAGACACTGAAAATAACAGTAGCTATCAAGGTGGATCT
>HPV17_Beta_396932_nt6205_L1_Tile|1
TGTGTACCTTGTATTGGAGAACATTGGGACAGGGCTCCTGTATGTGAAAATGAACAAAAC
>HPV17_Beta_396932_nt6210_L1_Tile|1
ACCTTGTATTGGAGAACATTGGGACAGGGCTCCTGTATGTGAAAATGAACAAAACAATCA
>HPV17_Beta_396932_nt6215_L1_Tile|1
GTATTGGAGAACATTGGGACAGGGCTCCTGTATGTGAAAATGAACAAAACAATCAAACAG
>HPV17_Beta_396932_nt6230_L1_Tile|1
GGGACAGGGCTCCTGTATGTGAAAATGAACAAAACAATCAAACAGGCCTGTGTCCACCAT
>HPV17_Beta_396932_nt6240_L1_Tile|1
TCCTGTATGTGAAAATGAACAAAACAATCAAACAGGCCTGTGTCCACCATTGGAATTAAA
>HPV17_Beta_396932_nt6245_L1_Tile|1
TATGTGAAAATGAACAAAACAATCAAACAGGCCTGTGTCCACCATTGGAATTAAAAAACA
>HPV17_Beta_396932_nt6255_L1_Tile|1
TGAACAAAACAATCAAACAGGCCTGTGTCCACCATTGGAATTAAAAAACACTGTTATCGA
>HPV17_Beta_396932_nt6565_L1_Tile|1
CACAAATTTTATTTACCAGCTCAAACTGGCCAACAACAGCGCACTTTGGGTAATTCCACT
>HPV17_Beta_396932_nt6730_L1_Tile|1
GGGAATCAGATATTTGTGACTGTAGCTGACAACACTAGGAACACAAATTTTTCTATTAGC
>HPV17_Beta_396932_nt6745_L1_Tile|1
GTGACTGTAGCTGACAACACTAGGAACACAAATTTTTCTATTAGCGTGTCTACAGAAGCT
>HPV17_Beta_396932_nt6755_L1_Tile|1
CTGACAACACTAGGAACACAAATTTTTCTATTAGCGTGTCTACAGAAGCTGGGGCTGTTA
>HPV17_Beta_396932_nt7100_L1_Tile|1
ATGTAAATCTTACTGAGAAATTATCATTAGATTTAGATCAGTATCCCCTAGGACGAAAAT
>HPV17_Beta_396932_nt7105_L1_Tile|1
AATCTTACTGAGAAATTATCATTAGATTTAGATCAGTATCCCCTAGGACGAAAATTTATT
>HPV18_Alpha_9626069_nt5436_L1_Tile|1
CCTGTATACACGGGTCCTGATATTACATTACCATCTACTACCTCTGTATGGCCCATTGTA
>HPV18_Alpha_9626069_nt5441_L1_Tile|1
ATACACGGGTCCTGATATTACATTACCATCTACTACCTCTGTATGGCCCATTGTATCACC
>HPV18_Alpha_9626069_nt5446_L1_Tile|1
CGGGTCCTGATATTACATTACCATCTACTACCTCTGTATGGCCCATTGTATCACCCACGG
>HPV18_Alpha_9626069_nt5466_L1_Tile|1
CCATCTACTACCTCTGTATGGCCCATTGTATCACCCACGGCCCCTGCCTCTACACAGTAT
>HPV18_Alpha_9626069_nt5476_L1_Tile|1
CCTCTGTATGGCCCATTGTATCACCCACGGCCCCTGCCTCTACACAGTATATTGGTATAC
>HPV18_Alpha_9626069_nt5481_L1_Tile|1
GTATGGCCCATTGTATCACCCACGGCCCCTGCCTCTACACAGTATATTGGTATACATGGT
>HPV18_Alpha_9626069_nt5486_L1_Tile|1
GCCCATTGTATCACCCACGGCCCCTGCCTCTACACAGTATATTGGTATACATGGTACACA
>HPV18_Alpha_9626069_nt5491_L1_Tile|1
TTGTATCACCCACGGCCCCTGCCTCTACACAGTATATTGGTATACATGGTACACATTATT
>HPV18_Alpha_9626069_nt5496_L1_Tile|1
TCACCCACGGCCCCTGCCTCTACACAGTATATTGGTATACATGGTACACATTATTATTTG
>HPV18_Alpha_9626069_nt5506_L1_Tile|1
CCCCTGCCTCTACACAGTATATTGGTATACATGGTACACATTATTATTTGTGGCCATTAT
>HPV18_Alpha_9626069_nt5511_L1_Tile|1
GCCTCTACACAGTATATTGGTATACATGGTACACATTATTATTTGTGGCCATTATATTAT
>HPV18_Alpha_9626069_nt5976_L1_Tile|1
ATTTTATAATAAATTAGATGACACTGAAAGTTCCCATGCCGCCACGTCTAATGTTTCTGA

>HPV18_Alpha_9626069_nt5981_L1_Tile|1
ATAATAAATTAGATGACACTGAAAGTTCCCATGCCGCCACGTCTAATGTTTCTGAGGACG
>HPV18_Alpha_9626069_nt5996_L1_Tile|1
ACACTGAAAGTTCCCATGCCGCCACGTCTAATGTTTCTGAGGACGTTAGGGACAATGTGT
>HPV18_Alpha_9626069_nt6131_L1_Tile|1
CTGCTTGTAAATCGCGTCCTTTATCACAGGGCGATTGCCCCCCTTTAGAACTTAAAAACA
>HPV18_Alpha_9626069_nt6136_L1_Tile|1
TGTAAATCGCGTCCTTTATCACAGGGCGATTGCCCCCCTTTAGAACTTAAAAACACAGTT
>HPV18_Alpha_9626069_nt6146_L1_Tile|1
GTCCTTTATCACAGGGCGATTGCCCCCCTTTAGAACTTAAAAACACAGTTTTGGAAGATG
>HPV18_Alpha_9626069_nt6421_L1_Tile|1
ACTGTGCCTCAATCCTTATATATTAAAGGCACAGGTATGCCTGCTTCACCTGGCAGCTGT
>HPV18_Alpha_9626069_nt6426_L1_Tile|1
GCCTCAATCCTTATATATTAAAGGCACAGGTATGCCTGCTTCACCTGGCAGCTGTGTGTA
>HPV18_Alpha_9626069_nt6441_L1_Tile|1
TATTAAAGGCACAGGTATGCCTGCTTCACCTGGCAGCTGTGTGTATTCTCCCTCTCCAAG
>HPV18_Alpha_9626069_nt6621_L1_Tile|1
CACTCCCAGTACCAATTTAACAATATGTGCTTCTACACAGTCTCCTGTACCTGGGCAATA
>HPV18_Alpha_9626069_nt6626_L1_Tile|1
CCAGTACCAATTTAACAATATGTGCTTCTACACAGTCTCCTGTACCTGGGCAATATGATG
>HPV18_Alpha_9626069_nt6631_L1_Tile|1
ACCAATTTAACAATATGTGCTTCTACACAGTCTCCTGTACCTGGGCAATATGATGCTACC
>HPV18_Alpha_9626069_nt6636_L1_Tile|1
TTTAACAATATGTGCTTCTACACAGTCTCCTGTACCTGGGCAATATGATGCTACCAAATT
>HPV18_Alpha_9626069_nt6641_L1_Tile|1
CAATATGTGCTTCTACACAGTCTCCTGTACCTGGGCAATATGATGCTACCAAATTTAAGC
>HPV18_Alpha_9626069_nt6651_L1_Tile|1
TTCTACACAGTCTCCTGTACCTGGGCAATATGATGCTACCAAATTTAAGCAGTATAGCAG
>HPV18_Alpha_9626069_nt6791_L1_Tile|1
ATAGTATGAATAGCAGTATTTTAGAGGATTGGAACTTTGGTGTTCCCCCCCCCCCAACTA
>HPV18_Alpha_9626069_nt7078_L1_Tile|1
CCATCTGCCACTACGTCTTCTAAACCTGCCAAGCGTGTGCGTGTACGTGCCAGGAAGTAA
>HPV19_Beta_396940_nt5795_L1_Tile|1
TACTTACATCCCAGCCTTCGCAAGCGATTCAAACGAAAACGTAAATATTTATAATTTTCT
>HPV19_Beta_396940_nt5800_L1_Tile|1
ACATCCCAGCCTTCGCAAGCGATTCAAACGAAAACGTAAATATTTATAATTTTCTTTTGC
>HPV19_Beta_396940_nt6005_L1_Tile|1
CCCATATTTTAATGTTTATAATGTTGCAGGATCAAAATTAGAAATTCCAAAAGTTTCAGG
>HPV19_Beta_396940_nt6010_L1_Tile|1
ATTTTAATGTTTATAATGTTGCAGGATCAAAATTAGAAATTCCAAAAGTTTCAGGAAATC
>HPV19_Beta_396940_nt6360_L1_Tile|1
GAACACTGGGATAAAGCATTACCATGTGCTGAGCAAGATATTCCTCAGGGATCCTGTCCT
>HPV19_Beta_396940_nt6370_L1_Tile|1
ATAAAGCATTACCATGTGCTGAGCAAGATATTCCTCAGGGATCCTGTCCTCCTATAGAGT
>HPV19_Beta_396940_nt6375_L1_Tile|1
GCATTACCATGTGCTGAGCAAGATATTCCTCAGGGATCCTGTCCTCCTATAGAGTTAATT
>HPV19_Beta_396940_nt6380_L1_Tile|1
ACCATGTGCTGAGCAAGATATTCCTCAGGGATCCTGTCCTCCTATAGAGTTAATTAACTC
>HPV19_Beta_396940_nt6385_L1_Tile|1
GTGCTGAGCAAGATATTCCTCAGGGATCCTGTCCTCCTATAGAGTTAATTAACTCAGTTA
>HPV19_Beta_396940_nt6400_L1_Tile|1
TTCCTCAGGGATCCTGTCCTCCTATAGAGTTAATTAACTCAGTTATTGAAGATGGAGACA
>HPV19_Beta_396940_nt6710_L1_Tile|1
TACATACTACATACCTCCTAACAATAGTCAGCAACAATATACTAATTTAGGAAATGCCAT
>HPV19_Beta_396940_nt7125_L1_Tile|1

GGTTTCGTGCCTGCACCCGATAATCCTATTCAGGACACTTATAGATATATAGATTCTTTA
>HPV19_Beta_396940_nt7160_L1_Tile|1
CACTTATAGATATATAGATTCTTTAGCTACTAGATGCCCTGACAAAAATCCTCCTAAGGA
>HPV19_Beta_396940_nt7355_L1_Tile|1
TACAAAAACTATATCTTCACGGGTCTCCAGCAGAGGAACTAAAAGAAAGCGTAAAAATTA
>HPV19_Beta_396940_nt7356_L1_Tile|1
ACAAAAACTATATCTTCACGGGTCTCCAGCAGAGGAACTAAAAGAAAGCGTAAAAATTAA
>HPV20_Beta_1020162_nt6039_L1_Tile|1
CATATTTTAATATATATGACATCCAAGGCACTAAGATAAAAGTCCCTAAGGTTTCTGGAA
>HPV20_Beta_1020162_nt6044_L1_Tile|1
TTTAATATATATGACATCCAAGGCACTAAGATAAAAGTCCCTAAGGTTTCTGGAAATCAG
>HPV20_Beta_1020162_nt6049_L1_Tile|1
TATATATGACATCCAAGGCACTAAGATAAAAGTCCCTAAGGTTTCTGGAAATCAGCACAG
>HPV20_Beta_1020162_nt6259_L1_Tile|1
ATTATTTAATAAACTTGGTGACACAGAAAACCCTAATTCATATAAAGGGAATTCAACTGA
>HPV20_Beta_1020162_nt6264_L1_Tile|1
TTAATAAACTTGGTGACACAGAAAACCCTAATTCATATAAAGGGAATTCAACTGATGATA
>HPV20_Beta_1020162_nt6269_L1_Tile|1
AAACTTGGTGACACAGAAAACCCTAATTCATATAAAGGGAATTCAACTGATGATAGACAA
>HPV20_Beta_1020162_nt6274_L1_Tile|1
TGGTGACACAGAAAACCCTAATTCATATAAAGGGAATTCAACTGATGATAGACAAAATGT
>HPV20_Beta_1020162_nt6399_L1_Tile|1
GGGACAGGGCTTTACCATGTGCAGACGACGTTCCAAACCCAGGTTCATGCCCTCCAATAG
>HPV20_Beta_1020162_nt6739_L1_Tile|1
GAATGCATTCTACATTCCACCTGTGAATAATCAGGCACAGAACAACCTAGGTAATTCAAT
>HPV20_Beta_1020162_nt6744_L1_Tile|1
CATTCTACATTCCACCTGTGAATAATCAGGCACAGAACAACCTAGGTAATTCAATGTATT
>HPV20_Beta_1020162_nt6894_L1_Tile|1
GCATCTGCTGGTTCAATCAACTATTTGTTACTGTAGTAGATAATACTCGAAATACAAATT
>HPV20_Beta_1020162_nt6904_L1_Tile|1
GTTCAATCAACTATTTGTTACTGTAGTAGATAATACTCGAAATACAAATTTTAGCATATC
>HPV20_Beta_1020162_nt6914_L1_Tile|1
CTATTTGTTACTGTAGTAGATAATACTCGAAATACAAATTTTAGCATATCAGTTCATTCA
>HPV20_Beta_1020162_nt6919_L1_Tile|1
TGTTACTGTAGTAGATAATACTCGAAATACAAATTTTAGCATATCAGTTCATTCAGAAAA
>HPV20_Beta_1020162_nt6924_L1_Tile|1
CTGTAGTAGATAATACTCGAAATACAAATTTTAGCATATCAGTTCATTCAGAAAACACTG
>HPV20_Beta_1020162_nt6929_L1_Tile|1
GTAGATAATACTCGAAATACAAATTTTAGCATATCAGTTCATTCAGAAAACACTGATGTT
>HPV20_Beta_1020162_nt6934_L1_Tile|1
TAATACTCGAAATACAAATTTTAGCATATCAGTTCATTCAGAAAACACTGATGTTTCTAA
>HPV20_Beta_1020162_nt6949_L1_Tile|1
AAATTTTAGCATATCAGTTCATTCAGAAAACACTGATGTTTCTAAAATTCAAAATTATGA
>HPV20_Beta_1020162_nt6954_L1_Tile|1
TTAGCATATCAGTTCATTCAGAAAACACTGATGTTTCTAAAATTCAAAATTATGATTCTC
>HPV20_Beta_1020162_nt7249_L1_Tile|1
AAGAGAAGATCCTTACAAGGATCTAAACTTTTGGAATGTTGACCTATCAGAAAGATTATC
>HPV20_Beta_1020162_nt7254_L1_Tile|1
AAGATCCTTACAAGGATCTAAACTTTTGGAATGTTGACCTATCAGAAAGATTATCCTTAG
>HPV20_Beta_1020162_nt7259_L1_Tile|1
CCTTACAAGGATCTAAACTTTTGGAATGTTGACCTATCAGAAAGATTATCCTTAGAATTG
>HPV20_Beta_1020162_nt7384_L1_Tile|1
TACAAAAACTGTATCTTCAAAGTTATCTACTAGGGGCGTCAAACGAAAACGCAAACAATA
>HPV20_Beta_1020162_nt7385_L1_Tile|1
ACAAAAACTGTATCTTCAAAGTTATCTACTAGGGGCGTCAAACGAAAACGCAAACAATAA

>HPV21_Beta_1020170_nt6300_L1_Tile|1
TTAATAAAGTTGGGGACACAGAAAATCCTAGTTCATACAAAACTCAACCAAATTCTACTG
>HPV21_Beta_1020170_nt6305_L1_Tile|1
AAAGTTGGGGACACAGAAAATCCTAGTTCATACAAAACTCAACCAAATTCTACTGATGAT
>HPV21_Beta_1020170_nt6430_L1_Tile|1
AGGAGAACATTGGGATAAAGCTATCCCATGTGCAACTGACAATCCACCTCCAGGATCGTG
>HPV21_Beta_1020170_nt6445_L1_Tile|1
TAAAGCTATCCCATGTGCAACTGACAATCCACCTCCAGGATCGTGCCCTCCGATTGAATT
>HPV21_Beta_1020170_nt6450_L1_Tile|1
CTATCCCATGTGCAACTGACAATCCACCTCCAGGATCGTGCCCTCCGATTGAATTAATTA
>HPV21_Beta_1020170_nt6455_L1_Tile|1
CCATGTGCAACTGACAATCCACCTCCAGGATCGTGCCCTCCGATTGAATTAATTAATTCA
>HPV21_Beta_1020170_nt6460_L1_Tile|1
TGCAACTGACAATCCACCTCCAGGATCGTGCCCTCCGATTGAATTAATTAATTCAGCAAT
>HPV21_Beta_1020170_nt6970_L1_Tile|1
AGTAGACAACACTCGTAACACAAACTTTAGTATTTCAGTAAATCCTGAGAATGCAGACGT
>HPV21_Beta_1020170_nt6980_L1_Tile|1
ACTCGTAACACAAACTTTAGTATTTCAGTAAATCCTGAGAATGCAGACGTGTCTAAAATT
>HPV21_Beta_1020170_nt6985_L1_Tile|1
TAACACAAACTTTAGTATTTCAGTAAATCCTGAGAATGCAGACGTGTCTAAAATTGAAAA
>HPV21_Beta_1020170_nt6990_L1_Tile|1
CAAACTTTAGTATTTCAGTAAATCCTGAGAATGCAGACGTGTCTAAAATTGAAAATTATA
>HPV21_Beta_1020170_nt6995_L1_Tile|1
TTTAGTATTTCAGTAAATCCTGAGAATGCAGACGTGTCTAAAATTGAAAATTATAAAGCC
>HPV21_Beta_1020170_nt7000_L1_Tile|1
TATTTCAGTAAATCCTGAGAATGCAGACGTGTCTAAAATTGAAAATTATAAAGCCGAGAG
>HPV21_Beta_1020170_nt7005_L1_Tile|1
CAGTAAATCCTGAGAATGCAGACGTGTCTAAAATTGAAAATTATAAAGCCGAGAGCTTTC
>HPV21_Beta_1020170_nt7425_L1_Tile|1
GTACAAAGACACTTTCTTCAAGGGTATCTACCAGAGGAATTAAACGAAAACGCAAAAATT
>HPV21_Beta_1020170_nt7427_L1_Tile|1
ACAAAGACACTTTCTTCAAGGGTATCTACCAGAGGAATTAAACGAAAACGCAAAAATTAG
>HPV22_Beta_1020178_nt5883_L1_Tile|1
TTAGAGTAACATTTCCAGATCCTAACAAATTTGCTTTGGGAGATATGACAATCCATGATC
>HPV22_Beta_1020178_nt5893_L1_Tile|1
ATTTCCAGATCCTAACAAATTTGCTTTGGGAGATATGACAATCCATGATCCCGAAAGGTA
>HPV22_Beta_1020178_nt5898_L1_Tile|1
CAGATCCTAACAAATTTGCTTTGGGAGATATGACAATCCATGATCCCGAAAGGTATAGAT
>HPV22_Beta_1020178_nt5903_L1_Tile|1
CCTAACAAATTTGCTTTGGGAGATATGACAATCCATGATCCCGAAAGGTATAGATTAGTA
>HPV22_Beta_1020178_nt5908_L1_Tile|1
CAAATTTGCTTTGGGAGATATGACAATCCATGATCCCGAAAGGTATAGATTAGTATGGGC
>HPV22_Beta_1020178_nt5913_L1_Tile|1
TTGCTTTGGGAGATATGACAATCCATGATCCCGAAAGGTATAGATTAGTATGGGCTTGTA
>HPV22_Beta_1020178_nt5918_L1_Tile|1
TTGGGAGATATGACAATCCATGATCCCGAAAGGTATAGATTAGTATGGGCTTGTAAAGGG
>HPV22_Beta_1020178_nt5923_L1_Tile|1
AGATATGACAATCCATGATCCCGAAAGGTATAGATTAGTATGGGCTTGTAAAGGGTTAGA
>HPV22_Beta_1020178_nt5928_L1_Tile|1
TGACAATCCATGATCCCGAAAGGTATAGATTAGTATGGGCTTGTAAAGGGTTAGAAATAG
>HPV22_Beta_1020178_nt5933_L1_Tile|1
ATCCATGATCCCGAAAGGTATAGATTAGTATGGGCTTGTAAAGGGTTAGAAATAGGAAGA
>HPV22_Beta_1020178_nt6028_L1_Tile|1
ATTATTTAATAAATTACATGATACTGAAAACCCTACTGAACGCCAGGAAGGAACATCAGA
>HPV22_Beta_1020178_nt6033_L1_Tile|1

TTAATAAATTACATGATACTGAAAACCCTACTGAACGCCAGGAAGGAACATCAGATGATA
>HPV22_Beta_1020178_nt6038_L1_Tile|1
AAATTACATGATACTGAAAACCCTACTGAACGCCAGGAAGGAACATCAGATGATAGAAGA
>HPV22_Beta_1020178_nt6043_L1_Tile|1
ACATGATACTGAAAACCCTACTGAACGCCAGGAAGGAACATCAGATGATAGAAGAAATGT
>HPV22_Beta_1020178_nt6288_L1_Tile|1
ATAATAAAACACTATCATTTAATAGATCTGATGTAAGCTTAGACATTGTAAATGAAATCT
>HPV22_Beta_1020178_nt6358_L1_Tile|1
TGATTTTCTTACAATGTCAAATGATGTCTATGGCGACTCATGCTTTTTTTGTGCACGTAG
>HPV22_Beta_1020178_nt6363_L1_Tile|1
TTCTTACAATGTCAAATGATGTCTATGGCGACTCATGCTTTTTTTGTGCACGTAGGGAGC
>HPV22_Beta_1020178_nt6428_L1_Tile|1
TATGCACGACACAATTTTGTACGTGGTGGTCTTGTTGGTGATGCTATACCAGATGATGCA
>HPV22_Beta_1020178_nt6433_L1_Tile|1
ACGACACAATTTTGTACGTGGTGGTCTTGTTGGTGATGCTATACCAGATGATGCAGTTCA
>HPV22_Beta_1020178_nt6443_L1_Tile|1
TTTGTACGTGGTGGTCTTGTTGGTGATGCTATACCAGATGATGCAGTTCAACAAGATCAT
>HPV22_Beta_1020178_nt6453_L1_Tile|1
GTGGTCTTGTTGGTGATGCTATACCAGATGATGCAGTTCAACAAGATCATAAATATTACT
>HPV22_Beta_1020178_nt6458_L1_Tile|1
CTTGTTGGTGATGCTATACCAGATGATGCAGTTCAACAAGATCATAAATATTACTTGCCT
>HPV22_Beta_1020178_nt6473_L1_Tile|1
ATACCAGATGATGCAGTTCAACAAGATCATAAATATTACTTGCCTGCAGCTTCACAGACT
>HPV22_Beta_1020178_nt6498_L1_Tile|1
ATCATAAATATTACTTGCCTGCAGCTTCACAGACTGCTTTAGAAAACTCCACTTACTTTC
>HPV22_Beta_1020178_nt6503_L1_Tile|1
AAATATTACTTGCCTGCAGCTTCACAGACTGCTTTAGAAAACTCCACTTACTTTCCAACC
>HPV22_Beta_1020178_nt6508_L1_Tile|1
TTACTTGCCTGCAGCTTCACAGACTGCTTTAGAAAACTCCACTTACTTTCCAACCGTTAG
>HPV22_Beta_1020178_nt6513_L1_Tile|1
TGCCTGCAGCTTCACAGACTGCTTTAGAAAACTCCACTTACTTTCCAACCGTTAGTGGTT
>HPV22_Beta_1020178_nt6518_L1_Tile|1
GCAGCTTCACAGACTGCTTTAGAAAACTCCACTTACTTTCCAACCGTTAGTGGTTCTTTA
>HPV22_Beta_1020178_nt6573_L1_Tile|1
CTTTAGTAACCTCTGATGCCCAACTATTCAACAGGCCTTTTTGGTTGAAGCGCGCGCAGG
>HPV22_Beta_1020178_nt6683_L1_Tile|1
GATAATACCCGTAACACTAATTTTTCTATTAGTGTGGCAAGTGACGGCACCACAGTTAAT
>HPV22_Beta_1020178_nt6688_L1_Tile|1
TACCCGTAACACTAATTTTTCTATTAGTGTGGCAAGTGACGGCACCACAGTTAATTATGA
>HPV22_Beta_1020178_nt6693_L1_Tile|1
GTAACACTAATTTTTCTATTAGTGTGGCAAGTGACGGCACCACAGTTAATTATGATGCTA
>HPV22_Beta_1020178_nt6698_L1_Tile|1
ACTAATTTTTCTATTAGTGTGGCAAGTGACGGCACCACAGTTAATTATGATGCTAAAAAA
>HPV22_Beta_1020178_nt6703_L1_Tile|1
TTTTTCTATTAGTGTGGCAAGTGACGGCACCACAGTTAATTATGATGCTAAAAAAATCAG
>HPV22_Beta_1020178_nt6708_L1_Tile|1
CTATTAGTGTGGCAAGTGACGGCACCACAGTTAATTATGATGCTAAAAAAATCAGAGAAT
>HPV22_Beta_1020178_nt6718_L1_Tile|1
GGCAAGTGACGGCACCACAGTTAATTATGATGCTAAAAAAATCAGAGAATTTATGCGCCA
>HPV22_Beta_1020178_nt6738_L1_Tile|1
TTAATTATGATGCTAAAAAAATCAGAGAATTTATGCGCCATGTGGAAGAATACCAATTAT
>HPV22_Beta_1020178_nt6763_L1_Tile|1
AGAATTTATGCGCCATGTGGAAGAATACCAATTATCCTTTATTTTGCAGCTATGTAGAAT
>HPV22_Beta_1020178_nt6768_L1_Tile|1
TTATGCGCCATGTGGAAGAATACCAATTATCCTTTATTTTGCAGCTATGTAGAATACCAT

>HPV22_Beta_1020178_nt6773_L1_Tile|1
CGCCATGTGGAAGAATACCAATTATCCTTTATTTTGCAGCTATGTAGAATACCATTAGAA
>HPV22_Beta_1020178_nt6783_L1_Tile|1
AAGAATACCAATTATCCTTTATTTTGCAGCTATGTAGAATACCATTAGAAGCAGAGGTAT
>HPV22_Beta_1020178_nt6788_L1_Tile|1
TACCAATTATCCTTTATTTTGCAGCTATGTAGAATACCATTAGAAGCAGAGGTATTAACT
>HPV22_Beta_1020178_nt6793_L1_Tile|1
ATTATCCTTTATTTTGCAGCTATGTAGAATACCATTAGAAGCAGAGGTATTAACTCAAAT
>HPV22_Beta_1020178_nt6808_L1_Tile|1
GCAGCTATGTAGAATACCATTAGAAGCAGAGGTATTAACTCAAATTAATGCCATGAATCA
>HPV22_Beta_1020178_nt6813_L1_Tile|1
TATGTAGAATACCATTAGAAGCAGAGGTATTAACTCAAATTAATGCCATGAATCATGGCA
>HPV22_Beta_1020178_nt7063_L1_Tile|1
GTATCCACTGGGTCGTAAATTTTTATTTCAATCTGGGTTACAACGTGCAAGGGCCAGTGC
>HPV22_Beta_1020178_nt7083_L1_Tile|1
TTTTATTTCAATCTGGGTTACAACGTGCAAGGGCCAGTGCCAGGGTCAGTGTGAAACGTT
>HPV22_Beta_1020178_nt7088_L1_Tile|1
TTTCAATCTGGGTTACAACGTGCAAGGGCCAGTGCCAGGGTCAGTGTGAAACGTTCTGCT
>HPV22_Beta_1020178_nt7098_L1_Tile|1
GGTTACAACGTGCAAGGGCCAGTGCCAGGGTCAGTGTGAAACGTTCTGCTACGCGGAAAA
>HPV22_Beta_1020178_nt7128_L1_Tile|1
TCAGTGTGAAACGTTCTGCTACGCGGAAAACGTCTAAAACTGTAAAACGAAGGAAACTTA
>HPV23_Beta_1020186_nt5816_L1_Tile|1
TGTTAGATCACCGGATGGTAGTAAAATAGATGTACCAAAGGTTTCAGGGAATCAATTCAG
>HPV23_Beta_1020186_nt5896_L1_Tile|1
CAGACCCTAATAAGTTTGCATTAGCAGACATGACTATCTATGATCCTGATAAATACAGGT
>HPV23_Beta_1020186_nt5971_L1_Tile|1
CAGGACTTGAAATCGGCCGCGGCCAACCTTTAGGGGTCGGCAGTACAGGACACCCGCTAT
>HPV23_Beta_1020186_nt6001_L1_Tile|1
TAGGGGTCGGCAGTACAGGACACCCGCTATTTAATAAGCTCCGTGATGCAGAAAATTCTA
>HPV23_Beta_1020186_nt6006_L1_Tile|1
GTCGGCAGTACAGGACACCCGCTATTTAATAAGCTCCGTGATGCAGAAAATTCTAGTGAA
>HPV23_Beta_1020186_nt6026_L1_Tile|1
GCTATTTAATAAGCTCCGTGATGCAGAAAATTCTAGTGAACGTCAGGAAGGTACTGTAGA
>HPV23_Beta_1020186_nt6031_L1_Tile|1
TTAATAAGCTCCGTGATGCAGAAAATTCTAGTGAACGTCAGGAAGGTACTGTAGATGACA
>HPV23_Beta_1020186_nt6041_L1_Tile|1
CCGTGATGCAGAAAATTCTAGTGAACGTCAGGAAGGTACTGTAGATGACAGAAGAAATAT
>HPV23_Beta_1020186_nt6046_L1_Tile|1
ATGCAGAAAATTCTAGTGAACGTCAGGAAGGTACTGTAGATGACAGAAGAAATATCTCAT
>HPV23_Beta_1020186_nt6051_L1_Tile|1
GAAAATTCTAGTGAACGTCAGGAAGGTACTGTAGATGACAGAAGAAATATCTCATTTGAT
>HPV23_Beta_1020186_nt6166_L1_Tile|1
GGGATACAGCTCCTGTCTGTAAAGATGCAGGTAGCCAACTAGGGTTGTGTCCTCCTTTAG
>HPV23_Beta_1020186_nt6181_L1_Tile|1
TCTGTAAAGATGCAGGTAGCCAACTAGGGTTGTGTCCTCCTTTAGAATTAAAAAACAGTG
>HPV23_Beta_1020186_nt6236_L1_Tile|1
CAGTGTTATAGAAGATGGGGACATGTTCGACATTGGCTTTGGTAATATCAATAATAAAAC
>HPV23_Beta_1020186_nt6246_L1_Tile|1
GAAGATGGGGACATGTTCGACATTGGCTTTGGTAATATCAATAATAAAACATTATCCTTT
>HPV23_Beta_1020186_nt6256_L1_Tile|1
ACATGTTCGACATTGGCTTTGGTAATATCAATAATAAAACATTATCCTTTAATAGATCAG
>HPV23_Beta_1020186_nt6286_L1_Tile|1
ATAATAAAACATTATCCTTTAATAGATCAGATGTTAGTTTAGATCTTGTAAATGAGGTTT
>HPV23_Beta_1020186_nt6476_L1_Tile|1

TGATGGTGCAGTTCAACAGGATCACAAATATTATTTACCTGCAGACCAACAAAACACTTT
>HPV23_Beta_1020186_nt6491_L1_Tile|1
ACAGGATCACAAATATTATTTACCTGCAGACCAACAAAACACTTTAGAAAACTCACTTTA
>HPV23_Beta_1020186_nt6501_L1_Tile|1
AAATATTATTTACCTGCAGACCAACAAAACACTTTAGAAAACTCACTTTATTTTCCTACT
>HPV23_Beta_1020186_nt6506_L1_Tile|1
TTATTTACCTGCAGACCAACAAAACACTTTAGAAAACTCACTTTATTTTCCTACTGTCAG
>HPV23_Beta_1020186_nt6516_L1_Tile|1
GCAGACCAACAAAACACTTTAGAAAACTCACTTTATTTTCCTACTGTCAGTGGATCTTTG
>HPV23_Beta_1020186_nt6521_L1_Tile|1
CCAACAAAACACTTTAGAAAACTCACTTTATTTTCCTACTGTCAGTGGATCTTTGGTAAC
>HPV23_Beta_1020186_nt6526_L1_Tile|1
AAAACACTTTAGAAAACTCACTTTATTTTCCTACTGTCAGTGGATCTTTGGTAACTTCTG
>HPV23_Beta_1020186_nt6646_L1_Tile|1
TTTTATGGAACAACCAGATGTTTGTGACTGTAGCAGATAATACACGTAATACAAACTTTA
>HPV23_Beta_1020186_nt6651_L1_Tile|1
TGGAACAACCAGATGTTTGTGACTGTAGCAGATAATACACGTAATACAAACTTTAGTATC
>HPV23_Beta_1020186_nt6666_L1_Tile|1
TTTGTGACTGTAGCAGATAATACACGTAATACAAACTTTAGTATCAGTGTTACCAATGAC
>HPV23_Beta_1020186_nt6671_L1_Tile|1
GACTGTAGCAGATAATACACGTAATACAAACTTTAGTATCAGTGTTACCAATGACAGCAG
>HPV23_Beta_1020186_nt6676_L1_Tile|1
TAGCAGATAATACACGTAATACAAACTTTAGTATCAGTGTTACCAATGACAGCAGTTTAG
>HPV23_Beta_1020186_nt6681_L1_Tile|1
GATAATACACGTAATACAAACTTTAGTATCAGTGTTACCAATGACAGCAGTTTAGAAAAG
>HPV23_Beta_1020186_nt6686_L1_Tile|1
TACACGTAATACAAACTTTAGTATCAGTGTTACCAATGACAGCAGTTTAGAAAAGTATGA
>HPV23_Beta_1020186_nt6691_L1_Tile|1
GTAATACAAACTTTAGTATCAGTGTTACCAATGACAGCAGTTTAGAAAAGTATGATGCCA
>HPV23_Beta_1020186_nt6696_L1_Tile|1
ACAAACTTTAGTATCAGTGTTACCAATGACAGCAGTTTAGAAAAGTATGATGCCACTAAA
>HPV23_Beta_1020186_nt6701_L1_Tile|1
CTTTAGTATCAGTGTTACCAATGACAGCAGTTTAGAAAAGTATGATGCCACTAAAATTAG
>HPV23_Beta_1020186_nt6706_L1_Tile|1
GTATCAGTGTTACCAATGACAGCAGTTTAGAAAAGTATGATGCCACTAAAATTAGAGAGT
>HPV23_Beta_1020186_nt6711_L1_Tile|1
AGTGTTACCAATGACAGCAGTTTAGAAAAGTATGATGCCACTAAAATTAGAGAGTTTACA
>HPV23_Beta_1020186_nt6716_L1_Tile|1
TACCAATGACAGCAGTTTAGAAAAGTATGATGCCACTAAAATTAGAGAGTTTACAAGACA
>HPV23_Beta_1020186_nt6721_L1_Tile|1
ATGACAGCAGTTTAGAAAAGTATGATGCCACTAAAATTAGAGAGTTTACAAGACATGTTG
>HPV23_Beta_1020186_nt6741_L1_Tile|1
TATGATGCCACTAAAATTAGAGAGTTTACAAGACATGTTGAAGAATACCAACTTTCTTTT
>HPV23_Beta_1020186_nt6761_L1_Tile|1
AGAGTTTACAAGACATGTTGAAGAATACCAACTTTCTTTTATACTACAGTTGTGCAGGAT
>HPV23_Beta_1020186_nt6781_L1_Tile|1
AAGAATACCAACTTTCTTTTATACTACAGTTGTGCAGGATACCTTTAAAGGCCGAGGTCT
>HPV23_Beta_1020186_nt6786_L1_Tile|1
TACCAACTTTCTTTTATACTACAGTTGTGCAGGATACCTTTAAAGGCCGAGGTCTTAACA
>HPV23_Beta_1020186_nt6791_L1_Tile|1
ACTTTCTTTTATACTACAGTTGTGCAGGATACCTTTAAAGGCCGAGGTCTTAACACAAAT
>HPV23_Beta_1020186_nt6796_L1_Tile|1
CTTTTATACTACAGTTGTGCAGGATACCTTTAAAGGCCGAGGTCTTAACACAAATTAATG
>HPV23_Beta_1020186_nt6801_L1_Tile|1
ATACTACAGTTGTGCAGGATACCTTTAAAGGCCGAGGTCTTAACACAAATTAATGCCATG

>HPV23_Beta_1020186_nt6896_L1_Tile|1
TGTTCCTACACCAGATAATGCAGTTCATGACACATACAGATATTTGGCTTCAAAGGCCAC
>HPV23_Beta_1020186_nt6926_L1_Tile|1
CACATACAGATATTTGGCTTCAAAGGCCACAAAATGTCCAGATGCAGTACCTGACACGCA
>HPV23_Beta_1020186_nt6936_L1_Tile|1
TATTTGGCTTCAAAGGCCACAAAATGTCCAGATGCAGTACCTGACACGCAAAAAGAGGAT
>HPV23_Beta_1020186_nt7021_L1_Tile|1
ATGTTGATATGACAGAAAAATTGTCTCTAGACCTAGATCAATATCCCTTAGGCCGTAAGT
>HPV23_Beta_1020186_nt7026_L1_Tile|1
GATATGACAGAAAAATTGTCTCTAGACCTAGATCAATATCCCTTAGGCCGTAAGTTTCTG
>HPV23_Beta_1020186_nt7041_L1_Tile|1
TTGTCTCTAGACCTAGATCAATATCCCTTAGGCCGTAAGTTTCTGTTTCAAATTGGAGTG
>HPV23_Beta_1020186_nt7051_L1_Tile|1
ACCTAGATCAATATCCCTTAGGCCGTAAGTTTCTGTTTCAAATTGGAGTGCAGCGTGTAC
>HPV23_Beta_1020186_nt7061_L1_Tile|1
ATATCCCTTAGGCCGTAAGTTTCTGTTTCAAATTGGAGTGCAGCGTGTACGGTCCGGTAC
>HPV23_Beta_1020186_nt7066_L1_Tile|1
CCTTAGGCCGTAAGTTTCTGTTTCAAATTGGAGTGCAGCGTGTACGGTCCGGTACCAAAC
>HPV23_Beta_1020186_nt7086_L1_Tile|1
TTTCAAATTGGAGTGCAGCGTGTACGGTCCGGTACCAAACGGCCTGCAACTCGAAAAGTG
>HPV23_Beta_1020186_nt7096_L1_Tile|1
GAGTGCAGCGTGTACGGTCCGGTACCAAACGGCCTGCAACTCGAAAAGTGACCAAAACTG
>HPV23_Beta_1020186_nt7106_L1_Tile|1
TGTACGGTCCGGTACCAAACGGCCTGCAACTCGAAAAGTGACCAAAACTGTCAAAAGGAA
>HPV23_Beta_1020186_nt7111_L1_Tile|1
GGTCCGGTACCAAACGGCCTGCAACTCGAAAAGTGACCAAAACTGTCAAAAGGAAAAAAG
>HPV23_Beta_1020186_nt7116_L1_Tile|1
GGTACCAAACGGCCTGCAACTCGAAAAGTGACCAAAACTGTCAAAAGGAAAAAAGTGCAA
>HPV23_Beta_1020186_nt7122_L1_Tile|2
AAACGGCCTGCAACTCGAAAAGTGACCAAAACTGTCAAAAGGAAAAAAGTGCAATTGTAA
>HPV24_Unclassified_9628486_nt6079_L1_Tile|1
ATTATTTAACAAAGTGAATGACACAGAAAACCCTGTATCATATAGGACACAAGCATCGTC
>HPV24_Unclassified_9628486_nt6089_L1_Tile|1
AAAGTGAATGACACAGAAAACCCTGTATCATATAGGACACAAGCATCGTCCACAGATGAT
>HPV24_Unclassified_9628486_nt6094_L1_Tile|1
GAATGACACAGAAAACCCTGTATCATATAGGACACAAGCATCGTCCACAGATGATAGACA
>HPV24_Unclassified_9628486_nt6104_L1_Tile|1
GAAAACCCTGTATCATATAGGACACAAGCATCGTCCACAGATGATAGACAAAATACCTCA
>HPV24_Unclassified_9628486_nt6204_L1_Tile|1
CACCCTGCATAGGAGAACATTGGGAAGTAGCTGAGAGGTGTGCTGGTGATAATAATGATG
>HPV24_Unclassified_9628486_nt6209_L1_Tile|1
TGCATAGGAGAACATTGGGAAGTAGCTGAGAGGTGTGCTGGTGATAATAATGATGCTGGT
>HPV24_Unclassified_9628486_nt6214_L1_Tile|1
AGGAGAACATTGGGAAGTAGCTGAGAGGTGTGCTGGTGATAATAATGATGCTGGTAGATG
>HPV24_Unclassified_9628486_nt6234_L1_Tile|1
CTGAGAGGTGTGCTGGTGATAATAATGATGCTGGTAGATGTCCACCTATTAAGTTAGTAA
>HPV24_Unclassified_9628486_nt6244_L1_Tile|1
TGCTGGTGATAATAATGATGCTGGTAGATGTCCACCTATTAAGTTAGTAAATTCAGTAAT
>HPV24_Unclassified_9628486_nt6249_L1_Tile|1
GTGATAATAATGATGCTGGTAGATGTCCACCTATTAAGTTAGTAAATTCAGTAATTCAAG
>HPV24_Unclassified_9628486_nt6254_L1_Tile|1
AATAATGATGCTGGTAGATGTCCACCTATTAAGTTAGTAAATTCAGTAATTCAAGATGGT
>HPV24_Unclassified_9628486_nt6324_L1_Tile|1
ATATTGGTTATGGGAATTTAAATTTTAGAACACTACAACAAAGTAGATCAGACGTAAGTT
>HPV24_Unclassified_9628486_nt6329_L1_Tile|1

GGTTATGGGAATTTAAATTTTAGAACACTACAACAAAGTAGATCAGACGTAAGTTTGGAT
>HPV24_Unclassified_9628486_nt6334_L1_Tile|1
TGGGAATTTAAATTTTAGAACACTACAACAAAGTAGATCAGACGTAAGTTTGGATATTGT
>HPV24_Unclassified_9628486_nt6339_L1_Tile|1
ATTTAAATTTTAGAACACTACAACAAAGTAGATCAGACGTAAGTTTGGATATTGTAAATG
>HPV24_Unclassified_9628486_nt6344_L1_Tile|1
AATTTTAGAACACTACAACAAAGTAGATCAGACGTAAGTTTGGATATTGTAAATGAAACC
>HPV24_Unclassified_9628486_nt6514_L1_Tile|1
TAAACCAGGGGATGACATACCTGGTGAGCAAATTGATGCAGGGACCTACAAAAATGACTT
>HPV24_Unclassified_9628486_nt6739_L1_Tile|1
ATTGTTCATTACGGTAGTAGATAACACCAGGAACACCAATTTTAGTATAAGTGTATACAC
>HPV24_Unclassified_9628486_nt6774_L1_Tile|1
CCAATTTTAGTATAAGTGTATACACTGAAAATGGCAAAGTAACAGATATTAACGAGTATG
>HPV24_Unclassified_9628486_nt6779_L1_Tile|1
TTTAGTATAAGTGTATACACTGAAAATGGCAAAGTAACAGATATTAACGAGTATGATGCT
>HPV24_Unclassified_9628486_nt6784_L1_Tile|1
TATAAGTGTATACACTGAAAATGGCAAAGTAACAGATATTAACGAGTATGATGCTAATAA
>HPV24_Unclassified_9628486_nt6794_L1_Tile|1
TACACTGAAAATGGCAAAGTAACAGATATTAACGAGTATGATGCTAATAAATTTAGGGAA
>HPV24_Unclassified_9628486_nt6799_L1_Tile|1
TGAAAATGGCAAAGTAACAGATATTAACGAGTATGATGCTAATAAATTTAGGGAATATCA
>HPV24_Unclassified_9628486_nt6804_L1_Tile|1
ATGGCAAAGTAACAGATATTAACGAGTATGATGCTAATAAATTTAGGGAATATCAGAGAC
>HPV24_Unclassified_9628486_nt6809_L1_Tile|1
AAAGTAACAGATATTAACGAGTATGATGCTAATAAATTTAGGGAATATCAGAGACATGTA
>HPV24_Unclassified_9628486_nt6839_L1_Tile|1
AATAAATTTAGGGAATATCAGAGACATGTAGAAGAGTATGAAATTTCACTTATACTGCAG
>HPV24_Unclassified_9628486_nt6844_L1_Tile|1
ATTTAGGGAATATCAGAGACATGTAGAAGAGTATGAAATTTCACTTATACTGCAGCTATG
>HPV24_Unclassified_9628486_nt6854_L1_Tile|1
TATCAGAGACATGTAGAAGAGTATGAAATTTCACTTATACTGCAGCTATGTAAAATCCCT
>HPV24_Unclassified_9628486_nt6864_L1_Tile|1
ATGTAGAAGAGTATGAAATTTCACTTATACTGCAGCTATGTAAAATCCCTTTAAAAGCAG
>HPV24_Unclassified_9628486_nt6869_L1_Tile|1
GAAGAGTATGAAATTTCACTTATACTGCAGCTATGTAAAATCCCTTTAAAAGCAGATGTG
>HPV24_Unclassified_9628486_nt6884_L1_Tile|1
TCACTTATACTGCAGCTATGTAAAATCCCTTTAAAAGCAGATGTGTTAGCACAGATCAAT
>HPV24_Unclassified_9628486_nt6904_L1_Tile|1
TAAAATCCCTTTAAAAGCAGATGTGTTAGCACAGATCAATGCTATGAATCCATCTCTATT
>HPV24_Unclassified_9628486_nt6989_L1_Tile|1
CCTGCACCAGACAATCCTCTTCAAAGTACCTACAGATATATAGAAAGTTTGGCAACACCT
>HPV24_Unclassified_9628486_nt6994_L1_Tile|1
ACCAGACAATCCTCTTCAAAGTACCTACAGATATATAGAAAGTTTGGCAACACCTTGTCC
>HPV24_Unclassified_9628486_nt7119_L1_Tile|1
TATCTGAACGCTTATCTTTGGAACTGGATCAATATTCCTTAGGACGAAAGTTCCTGTTTC
>HPV24_Unclassified_9628486_nt7149_L1_Tile|1
AATATTCCTTAGGACGAAAGTTCCTGTTTCAAGCGGGTCTTGTACAAAAAACATCTAAAA
>HPV24_Unclassified_9628486_nt7154_L1_Tile|1
TCCTTAGGACGAAAGTTCCTGTTTCAAGCGGGTCTTGTACAAAAAACATCTAAAAAAACA
>HPV24_Unclassified_9628486_nt7159_L1_Tile|1
AGGACGAAAGTTCCTGTTTCAAGCGGGTCTTGTACAAAAAACATCTAAAAAAACATCTAA
>HPV24_Unclassified_9628486_nt7164_L1_Tile|1
GAAAGTTCCTGTTTCAAGCGGGTCTTGTACAAAAAACATCTAAAAAAACATCTAATGTAT
>HPV24_Unclassified_9628486_nt7169_L1_Tile|1
TTCCTGTTTCAAGCGGGTCTTGTACAAAAAACATCTAAAAAAACATCTAATGTATCCAAG

>HPV24_Unclassified_9628486_nt7174_L1_Tile|1
GTTTCAAGCGGGTCTTGTACAAAAAACATCTAAAAAAACATCTAATGTATCCAAGGGGAC
>HPV24_Unclassified_9628486_nt7179_L1_Tile|1
AAGCGGGTCTTGTACAAAAAACATCTAAAAAAACATCTAATGTATCCAAGGGGACCAAAC
>HPV24_Unclassified_9628486_nt7184_L1_Tile|1
GGTCTTGTACAAAAAACATCTAAAAAAACATCTAATGTATCCAAGGGGACCAAACGAAAA
>HPV24_Unclassified_9628486_nt7193_L1_Tile|1
CAAAAAACATCTAAAAAAACATCTAATGTATCCAAGGGGACCAAACGAAAACGCACGTAA
>HPV25_Beta_396948_nt6030_L1_Tile|1
TCACCCATATTTTAATGTGTACAACGTCCAAGGCTCTAAATTGCAAATTCCAAAAGTGTC
>HPV25_Beta_396948_nt6035_L1_Tile|1
CATATTTTAATGTGTACAACGTCCAAGGCTCTAAATTGCAAATTCCAAAAGTGTCAGGAA
>HPV25_Beta_396948_nt6040_L1_Tile|1
TTTAATGTGTACAACGTCCAAGGCTCTAAATTGCAAATTCCAAAAGTGTCAGGAAATCAA
>HPV25_Beta_396948_nt6245_L1_Tile|1
TGGGTCACCCATTGTTTAACAAGGTTGGCGACACAGAAAATCCAAATTCTTATAAAGCTA
>HPV25_Beta_396948_nt6250_L1_Tile|1
CACCCATTGTTTAACAAGGTTGGCGACACAGAAAATCCAAATTCTTATAAAGCTAGTTCT
>HPV25_Beta_396948_nt6255_L1_Tile|1
ATTGTTTAACAAGGTTGGCGACACAGAAAATCCAAATTCTTATAAAGCTAGTTCTACAGA
>HPV25_Beta_396948_nt6275_L1_Tile|1
ACACAGAAAATCCAAATTCTTATAAAGCTAGTTCTACAGATGACAGGCAAAATGTATCAT
>HPV25_Beta_396948_nt6280_L1_Tile|1
GAAAATCCAAATTCTTATAAAGCTAGTTCTACAGATGACAGGCAAAATGTATCATTTGAC
>HPV25_Beta_396948_nt6415_L1_Tile|1
GATGATGGCAATATTCAACAAGGGTCATGCCCTCCAATAGAATTAATTAATTCTGTCATT
>HPV25_Beta_396948_nt6735_L1_Tile|1
AAATGCATTTTACATACCACCTAACAGTAGTCAGGCTCAATATAATAATCTAGGTAACTC
>HPV25_Beta_396948_nt6740_L1_Tile|1
CATTTTACATACCACCTAACAGTAGTCAGGCTCAATATAATAATCTAGGTAACTCAATGT
>HPV25_Beta_396948_nt6975_L1_Tile|1
TGGAACAGATGTTTCCAAAATCACTGATTATAATTCTCAAAAATTTACAGAATATTTGAG
>HPV25_Beta_396948_nt6980_L1_Tile|1
CAGATGTTTCCAAAATCACTGATTATAATTCTCAAAAATTTACAGAATATTTGAGACATG
>HPV25_Beta_396948_nt7030_L1_Tile|1
TTGAGACATGTAGAAGAATATGAGTTATCATTAATATTACAACTTTGCAAAGTACCGTTG
>HPV25_Beta_396948_nt7035_L1_Tile|1
ACATGTAGAAGAATATGAGTTATCATTAATATTACAACTTTGCAAAGTACCGTTGAAGGC
>HPV25_Beta_396948_nt7040_L1_Tile|1
TAGAAGAATATGAGTTATCATTAATATTACAACTTTGCAAAGTACCGTTGAAGGCAGAAA
>HPV25_Beta_396948_nt7045_L1_Tile|1
GAATATGAGTTATCATTAATATTACAACTTTGCAAAGTACCGTTGAAGGCAGAAATATTG
>HPV25_Beta_396948_nt7050_L1_Tile|1
TGAGTTATCATTAATATTACAACTTTGCAAAGTACCGTTGAAGGCAGAAATATTGGCTCA
>HPV25_Beta_396948_nt7065_L1_Tile|1
ATTACAACTTTGCAAAGTACCGTTGAAGGCAGAAATATTGGCTCAGATTAATGCAATGAA
>HPV25_Beta_396948_nt7070_L1_Tile|1
AACTTTGCAAAGTACCGTTGAAGGCAGAAATATTGGCTCAGATTAATGCAATGAATTCCA
>HPV25_Beta_396948_nt7075_L1_Tile|1
TGCAAAGTACCGTTGAAGGCAGAAATATTGGCTCAGATTAATGCAATGAATTCCAACATT
>HPV25_Beta_396948_nt7080_L1_Tile|1
AGTACCGTTGAAGGCAGAAATATTGGCTCAGATTAATGCAATGAATTCCAACATTTTAGA
>HPV25_Beta_396948_nt7165_L1_Tile|1
GCACCGGACAATTCTATTCAGGATACTTATCGCTACATTGATTCTTTAGCCACACGTTGT
>HPV25_Beta_396948_nt7170_L1_Tile|1

GGACAATTCTATTCAGGATACTTATCGCTACATTGATTCTTTAGCCACACGTTGTCCAGA
>HPV25_Beta_396948_nt7175_L1_Tile|1
ATTCTATTCAGGATACTTATCGCTACATTGATTCTTTAGCCACACGTTGTCCAGATAAAA
>HPV25_Beta_396948_nt7180_L1_Tile|1
ATTCAGGATACTTATCGCTACATTGATTCTTTAGCCACACGTTGTCCAGATAAAAATCCT
>HPV25_Beta_396948_nt7384_L1_Tile|1
ACAAAAACAGTTTCCTCCCGAATATCTACTAGGGGAATAAAAAGAAAACGTAAAAATTAG
>HPV26_Alpha_9627305_nt5944_L1_Tile|1
ATCCTTTGTTTAATAAGTTGGATGATACCGAAAACTCTCATTTGGCTACTGTAAATGCAG
>HPV26_Alpha_9627305_nt5954_L1_Tile|1
TAATAAGTTGGATGATACCGAAAACTCTCATTTGGCTACTGTAAATGCAGACACTGACAA
>HPV26_Alpha_9627305_nt5959_L1_Tile|1
AGTTGGATGATACCGAAAACTCTCATTTGGCTACTGTAAATGCAGACACTGACAACAGGG
>HPV26_Alpha_9627305_nt5964_L1_Tile|1
GATGATACCGAAAACTCTCATTTGGCTACTGTAAATGCAGACACTGACAACAGGGACAAT
>HPV26_Alpha_9627305_nt5969_L1_Tile|1
TACCGAAAACTCTCATTTGGCTACTGTAAATGCAGACACTGACAACAGGGACAATGTTTC
>HPV26_Alpha_9627305_nt5974_L1_Tile|1
AAAACTCTCATTTGGCTACTGTAAATGCAGACACTGACAACAGGGACAATGTTTCAGTTG
>HPV26_Alpha_9627305_nt5979_L1_Tile|1
TCTCATTTGGCTACTGTAAATGCAGACACTGACAACAGGGACAATGTTTCAGTTGATAAT
>HPV26_Alpha_9627305_nt6059_L1_Tile|1
TATAGGTTGTACACCGCCCTTGGGAGAGCACTGGGGTATTGGCACTATATGTAAAAATAC
>HPV26_Alpha_9627305_nt6094_L1_Tile|1
GTATTGGCACTATATGTAAAAATACACAGACACAACGTGGGGATTGCCCCCCCTTAGAAT
>HPV26_Alpha_9627305_nt6099_L1_Tile|1
GGCACTATATGTAAAAATACACAGACACAACGTGGGGATTGCCCCCCCTTAGAATTAATT
>HPV26_Alpha_9627305_nt6104_L1_Tile|1
TATATGTAAAAATACACAGACACAACGTGGGGATTGCCCCCCCTTAGAATTAATTTCCAG
>HPV26_Alpha_9627305_nt6109_L1_Tile|1
GTAAAAATACACAGACACAACGTGGGGATTGCCCCCCCTTAGAATTAATTTCCAGCATTA
>HPV26_Alpha_9627305_nt6114_L1_Tile|1
AATACACAGACACAACGTGGGGATTGCCCCCCCTTAGAATTAATTTCCAGCATTATTGAG
>HPV26_Alpha_9627305_nt6354_L1_Tile|1
GCCAGACATTTTTATAATAAGGCGGGGGCTGTTGGGGATGCTATACCCACCACTTTGTAT
>HPV26_Alpha_9627305_nt6359_L1_Tile|1
ACATTTTTATAATAAGGCGGGGGCTGTTGGGGATGCTATACCCACCACTTTGTATATTAA
>HPV26_Alpha_9627305_nt6364_L1_Tile|1
TTTATAATAAGGCGGGGGCTGTTGGGGATGCTATACCCACCACTTTGTATATTAAAGGTG
>HPV26_Alpha_9627305_nt6369_L1_Tile|1
AATAAGGCGGGGGCTGTTGGGGATGCTATACCCACCACTTTGTATATTAAAGGTGCTGAA
>HPV26_Alpha_9627305_nt6379_L1_Tile|1
GGGCTGTTGGGGATGCTATACCCACCACTTTGTATATTAAAGGTGCTGAATCAGGCAGGG
>HPV26_Alpha_9627305_nt6389_L1_Tile|1
GGATGCTATACCCACCACTTTGTATATTAAAGGTGCTGAATCAGGCAGGGAGCCCCCTAC
>HPV26_Alpha_9627305_nt6399_L1_Tile|1
CCCACCACTTTGTATATTAAAGGTGCTGAATCAGGCAGGGAGCCCCCTACATCTTCTATT
>HPV26_Alpha_9627305_nt6404_L1_Tile|1
CACTTTGTATATTAAAGGTGCTGAATCAGGCAGGGAGCCCCCTACATCTTCTATTTATTC
>HPV26_Alpha_9627305_nt6409_L1_Tile|1
TGTATATTAAAGGTGCTGAATCAGGCAGGGAGCCCCCTACATCTTCTATTTATTCTGCTA
>HPV26_Alpha_9627305_nt6414_L1_Tile|1
ATTAAAGGTGCTGAATCAGGCAGGGAGCCCCCTACATCTTCTATTTATTCTGCTACACCT
>HPV26_Alpha_9627305_nt6719_L1_Tile|1
TCAGTTGTGTAAAATAACACTTACAACAGATGTTATGGCTTACATACATTTAATGAATGC

>HPV26_Alpha_9627305_nt6724_L1_Tile|1
TGTGTAAAATAACACTTACAACAGATGTTATGGCTTACATACATTTAATGAATGCCTCCA
>HPV26_Alpha_9627305_nt6729_L1_Tile|1
AAAATAACACTTACAACAGATGTTATGGCTTACATACATTTAATGAATGCCTCCATATTG
>HPV26_Alpha_9627305_nt6739_L1_Tile|1
TTACAACAGATGTTATGGCTTACATACATTTAATGAATGCCTCCATATTGGAGGATTGGA
>HPV26_Alpha_9627305_nt6759_L1_Tile|1
TACATACATTTAATGAATGCCTCCATATTGGAGGATTGGAATTTTGGACTAACCTTACCT
>HPV26_Alpha_9627305_nt6974_L1_Tile|1
ATTTCCACTAGGGCGTAAGTTTATGTTACAGGCCGGCATACAACGGCGGCCGAAACTAGG
>HPV26_Alpha_9627305_nt6979_L1_Tile|1
CACTAGGGCGTAAGTTTATGTTACAGGCCGGCATACAACGGCGGCCGAAACTAGGCACCA
>HPV26_Alpha_9627305_nt6984_L1_Tile|1
GGGCGTAAGTTTATGTTACAGGCCGGCATACAACGGCGGCCGAAACTAGGCACCAAACGT
>HPV26_Alpha_9627305_nt6989_L1_Tile|1
TAAGTTTATGTTACAGGCCGGCATACAACGGCGGCCGAAACTAGGCACCAAACGTCCCTT
>HPV26_Alpha_9627305_nt6994_L1_Tile|1
TTATGTTACAGGCCGGCATACAACGGCGGCCGAAACTAGGCACCAAACGTCCCTTATCTT
>HPV27_Alpha_396964_nt5458_L1_Tile|1
GATGTCCTGCAGCCATTGGACGATTACTACCCAGCCCCTCGCGGCTCCCTGGCTAATACC
>HPV27_Alpha_396964_nt5468_L1_Tile|1
AGCCATTGGACGATTACTACCCAGCCCCTCGCGGCTCCCTGGCTAATACCACGGTATCTG
>HPV27_Alpha_396964_nt5483_L1_Tile|1
ACTACCCAGCCCCTCGCGGCTCCCTGGCTAATACCACGGTATCTGCCTCCTCTGCATCTA
>HPV27_Alpha_396964_nt5513_L1_Tile|1
ATACCACGGTATCTGCCTCCTCTGCATCTACACTGCGAGGGTCCACTACAGCCCCTCTCT
>HPV27_Alpha_396964_nt5903_L1_Tile|1
CACCCATATTATTCTATAAAGAAGGGTAGCAATAATAGGTTGGCAGTGCCTAAGGTGTCC
>HPV27_Alpha_396964_nt5918_L1_Tile|1
ATAAAGAAGGGTAGCAATAATAGGTTGGCAGTGCCTAAGGTGTCCGGCTACCAATACCGT
>HPV27_Alpha_396964_nt5923_L1_Tile|1
GAAGGGTAGCAATAATAGGTTGGCAGTGCCTAAGGTGTCCGGCTACCAATACCGTGTATT
>HPV27_Alpha_396964_nt6763_L1_Tile|1
ACGGAGTACCAATGTCTCTCTGTGTGCAGCTGAGGTGTCTGATAATACTAATTATAAAGC
>HPV27_Alpha_396964_nt6773_L1_Tile|1
AATGTCTCTCTGTGTGCAGCTGAGGTGTCTGATAATACTAATTATAAAGCTACGAATTTT
>HPV28_Alpha_1020202_nt5881_L1_Tile|1
TCCTTATTTTCCCATTCCTAAATCATCCACTAACAAAGCAGATGTGCCCAAAGTGTCCGC
>HPV28_Alpha_1020202_nt5886_L1_Tile|1
ATTTTCCCATTCCTAAATCATCCACTAACAAAGCAGATGTGCCCAAAGTGTCCGCCTTTC
>HPV28_Alpha_1020202_nt5891_L1_Tile|1
CCCATTCCTAAATCATCCACTAACAAAGCAGATGTGCCCAAAGTGTCCGCCTTTCAGTAT
>HPV28_Alpha_1020202_nt5901_L1_Tile|1
AATCATCCACTAACAAAGCAGATGTGCCCAAAGTGTCCGCCTTTCAGTATAGGGTATTCC
>HPV28_Alpha_1020202_nt6491_L1_Tile|1
AAGGAACAACTTTTCGCTCGGCATTTTTTTAATAGAGCTGGTGTTGCTGGGGACACCATT
>HPV28_Alpha_1020202_nt6521_L1_Tile|1
AATAGAGCTGGTGTTGCTGGGGACACCATTCCTGAAACATTGTATATTAAAGGTCAGGGC
>HPV28_Alpha_1020202_nt6531_L1_Tile|1
GTGTTGCTGGGGACACCATTCCTGAAACATTGTATATTAAAGGTCAGGGCAACGGGAGGG
>HPV28_Alpha_1020202_nt6536_L1_Tile|1
GCTGGGGACACCATTCCTGAAACATTGTATATTAAAGGTCAGGGCAACGGGAGGGATGTG
>HPV28_Alpha_1020202_nt6541_L1_Tile|1
GGACACCATTCCTGAAACATTGTATATTAAAGGTCAGGGCAACGGGAGGGATGTGATTGG
>HPV28_Alpha_1020202_nt6546_L1_Tile|1

CCATTCCTGAAACATTGTATATTAAAGGTCAGGGCAACGGGAGGGATGTGATTGGTAGTG
>HPV28_Alpha_1020202_nt6746_L1_Tile|1
ACTACACGCAGTACAAACATGACGTTGTGTGTTTCTACTGACTCTTCAGCTACGTACGAT
>HPV28_Alpha_1020202_nt6771_L1_Tile|1
TGTGTGTTTCTACTGACTCTTCAGCTACGTACGATGCTAGTAAATTTAAGGAATACTTAA
>HPV28_Alpha_1020202_nt6776_L1_Tile|1
GTTTCTACTGACTCTTCAGCTACGTACGATGCTAGTAAATTTAAGGAATACTTAAGGCAC
>HPV28_Alpha_1020202_nt6791_L1_Tile|1
TCAGCTACGTACGATGCTAGTAAATTTAAGGAATACTTAAGGCACGGGGAGGAGTACGAT
>HPV28_Alpha_1020202_nt6796_L1_Tile|1
TACGTACGATGCTAGTAAATTTAAGGAATACTTAAGGCACGGGGAGGAGTACGATTTGCA
>HPV28_Alpha_1020202_nt7086_L1_Tile|1
ATCTTAAGGATCGCTTTTCTCTTGATCTATCGCAATTCCCTCTGGGAAGGAAATTTTTAA
>HPV28_Alpha_1020202_nt7106_L1_Tile|1
CTTGATCTATCGCAATTCCCTCTGGGAAGGAAATTTTTAATGCAGTTGGGTGTAGGTGCC
>HPV28_Alpha_1020202_nt7156_L1_Tile|1
TGTAGGTGCCCGCTCCAGTGTCTCCGTTCGCAAACGTCCAGCGTCCACCACGAGAGGATC
>HPV28_Alpha_1020202_nt7186_L1_Tile|1
CAAACGTCCAGCGTCCACCACGAGAGGATCGTCTGCTGCAAAACGAAAACGCGCCAAAAA
>HPV29_Alpha_1020210_nt6329_L1_Tile|1
CACTGTGTGTGCACGCACTAGTTCCGCTGCTGGTGATTGCCCCCCCCTGGAGTTAATGAC
>HPV29_Alpha_1020210_nt6334_L1_Tile|1
TGTGTGCACGCACTAGTTCCGCTGCTGGTGATTGCCCCCCCCTGGAGTTAATGACCACAC
>HPV29_Alpha_1020210_nt6339_L1_Tile|1
GCACGCACTAGTTCCGCTGCTGGTGATTGCCCCCCCCTGGAGTTAATGACCACACATATT
>HPV29_Alpha_1020210_nt6579_L1_Tile|1
TTTGCCAGGCACTTCTTTAATCGTGCTGGTGTAGTAGGGGACAAAATCCCAGATTCCTTG
>HPV29_Alpha_1020210_nt6584_L1_Tile|1
CAGGCACTTCTTTAATCGTGCTGGTGTAGTAGGGGACAAAATCCCAGATTCCTTGTACTT
>HPV29_Alpha_1020210_nt6594_L1_Tile|1
TTTAATCGTGCTGGTGTAGTAGGGGACAAAATCCCAGATTCCTTGTACTTAAAGGGTAAC
>HPV29_Alpha_1020210_nt6599_L1_Tile|1
TCGTGCTGGTGTAGTAGGGGACAAAATCCCAGATTCCTTGTACTTAAAGGGTAACAACGG
>HPV29_Alpha_1020210_nt6604_L1_Tile|1
CTGGTGTAGTAGGGGACAAAATCCCAGATTCCTTGTACTTAAAGGGTAACAACGGGCGAG
>HPV29_Alpha_1020210_nt6609_L1_Tile|1
GTAGTAGGGGACAAAATCCCAGATTCCTTGTACTTAAAGGGTAACAACGGGCGAGAAACT
>HPV29_Alpha_1020210_nt6614_L1_Tile|1
AGGGGACAAAATCCCAGATTCCTTGTACTTAAAGGGTAACAACGGGCGAGAAACTCCTGG
>HPV29_Alpha_1020210_nt6624_L1_Tile|1
ATCCCAGATTCCTTGTACTTAAAGGGTAACAACGGGCGAGAAACTCCTGGCAGTGCCATA
>HPV29_Alpha_1020210_nt6629_L1_Tile|1
AGATTCCTTGTACTTAAAGGGTAACAACGGGCGAGAAACTCCTGGCAGTGCCATATACAG
>HPV29_Alpha_1020210_nt6829_L1_Tile|1
GCACCAATATGTCGTTGTGTGCTACCACAGAGTCTCAACCGTTGACCACTTATGATGCTA
>HPV29_Alpha_1020210_nt6834_L1_Tile|1
AATATGTCGTTGTGTGCTACCACAGAGTCTCAACCGTTGACCACTTATGATGCTACCAAG
>HPV29_Alpha_1020210_nt6844_L1_Tile|1
TGTGTGCTACCACAGAGTCTCAACCGTTGACCACTTATGATGCTACCAAGATTAAAGAAT
>HPV29_Alpha_1020210_nt6849_L1_Tile|1
GCTACCACAGAGTCTCAACCGTTGACCACTTATGATGCTACCAAGATTAAAGAATATTTG
>HPV29_Alpha_1020210_nt6854_L1_Tile|1
CACAGAGTCTCAACCGTTGACCACTTATGATGCTACCAAGATTAAAGAATATTTGAGACA
>HPV29_Alpha_1020210_nt6859_L1_Tile|1
AGTCTCAACCGTTGACCACTTATGATGCTACCAAGATTAAAGAATATTTGAGACATGGGG

>HPV29_Alpha_1020210_nt6959_L1_Tile|1
TACATTGACACCTGAAATTATGGCTTACCTTCATACTATGAACAGTGCCTTACTTGAAGA
>HPV29_Alpha_1020210_nt6984_L1_Tile|1
TACCTTCATACTATGAACAGTGCCTTACTTGAAGACTGGAATTTTGGATTGACATTGCCA
>HPV29_Alpha_1020210_nt7224_L1_Tile|1
TTACAGATCGGTGCGCGCCGGCGTTCAGTAGTCCCCTCCAGAAAGCGCCGAACGACCACC
>HPV29_Alpha_1020210_nt7229_L1_Tile|1
GATCGGTGCGCGCCGGCGTTCAGTAGTCCCCTCCAGAAAGCGCCGAACGACCACCACGGC
>HPV29_Alpha_1020210_nt7249_L1_Tile|1
CAGTAGTCCCCTCCAGAAAGCGCCGAACGACCACCACGGCCCCCACCCCTGCAAAGCGAA
>HPV29_Alpha_1020210_nt7254_L1_Tile|1
GTCCCCTCCAGAAAGCGCCGAACGACCACCACGGCCCCCACCCCTGCAAAGCGAAAACGC
>HPV29_Alpha_1020210_nt7259_L1_Tile|1
CTCCAGAAAGCGCCGAACGACCACCACGGCCCCCACCCCTGCAAAGCGAAAACGCTCGAA
>HPV30_Alpha_396973_nt5772_L1_Tile|1
ACGTTTGCTTGCTGTTGGACATCCATATTATTCTATTTCTAAGGCTGGTAATTCCAAAAC
>HPV30_Alpha_396973_nt5777_L1_Tile|1
TGCTTGCTGTTGGACATCCATATTATTCTATTTCTAAGGCTGGTAATTCCAAAACAGATG
>HPV30_Alpha_396973_nt6032_L1_Tile|1
ATGACACTGAAAGTTCCACTATAGCTAATCAGGATACAGCAGAGGATAGTAGGGACAACA
>HPV30_Alpha_396973_nt6037_L1_Tile|1
ACTGAAAGTTCCACTATAGCTAATCAGGATACAGCAGAGGATAGTAGGGACAACATTTCT
>HPV30_Alpha_396973_nt6042_L1_Tile|2
AAGTTCCACTATAGCTAATCAGGATACAGCAGAGGATAGTAGGGACAACATTTCTGTTGA
>HPV30_Alpha_396973_nt6047_L1_Tile|1
CCACTATAGCTAATCAGGATACAGCAGAGGATAGTAGGGACAACATTTCTGTTGATCCAA
>HPV30_Alpha_396973_nt6087_L1_Tile|1
CAACATTTCTGTTGATCCAAAGCAAACCCAATTGTGTATTATTGGGTGCACTCCTGCTAT
>HPV30_Alpha_396973_nt6092_L1_Tile|1
TTTCTGTTGATCCAAAGCAAACCCAATTGTGTATTATTGGGTGCACTCCTGCTATAGGAG
>HPV30_Alpha_396973_nt6097_L1_Tile|1
GTTGATCCAAAGCAAACCCAATTGTGTATTATTGGGTGCACTCCTGCTATAGGAGAACAT
>HPV30_Alpha_396973_nt6412_L1_Tile|1
CAGTTATTTGCTAGGCACTACTTTAATAGGGCAGGTGCTATTGGTGAACAATTACCTAGC
>HPV30_Alpha_396973_nt6447_L1_Tile|1
TGCTATTGGTGAACAATTACCTAGCACATTATATATAAAAGGTACAAATAACAGGGATCC
>HPV30_Alpha_396973_nt6457_L1_Tile|1
GAACAATTACCTAGCACATTATATATAAAAGGTACAAATAACAGGGATCCCCCGCCAAGC
>HPV30_Alpha_396973_nt6462_L1_Tile|2
ATTACCTAGCACATTATATATAAAAGGTACAAATAACAGGGATCCCCCGCCAAGCTCAGT
>HPV30_Alpha_396973_nt6467_L1_Tile|1
CTAGCACATTATATATAAAAGGTACAAATAACAGGGATCCCCCGCCAAGCTCAGTATATG
>HPV30_Alpha_396973_nt6472_L1_Tile|1
ACATTATATATAAAAGGTACAAATAACAGGGATCCCCCGCCAAGCTCAGTATATGTTGCT
>HPV30_Alpha_396973_nt6482_L1_Tile|2
TAAAAGGTACAAATAACAGGGATCCCCCGCCAAGCTCAGTATATGTTGCTACTCCTAGTG
>HPV30_Alpha_396973_nt6822_L1_Tile|2
ACATACTATGAACTCCACTTTACTTGAGGGCTGGAATATTGGATTGTCACCCCCAGCTGC
>HPV30_Alpha_396973_nt6832_L1_Tile|1
AACTCCACTTTACTTGAGGGCTGGAATATTGGATTGTCACCCCCAGCTGCCACAAGCTTA
>HPV30_Alpha_396973_nt7022_L1_Tile|2
ACCTTGACCAATTCCCACTGGGCAGAAAGTTTTTAATGCAACTTGGGGTTCGTACTAAAC
>HPV30_Alpha_396973_nt7032_L1_Tile|1
ATTCCCACTGGGCAGAAAGTTTTTAATGCAACTTGGGGTTCGTACTAAACCTTCTACTAC
>HPV30_Alpha_396973_nt7077_L1_Tile|1

TAAACCTTCTACTACTACTAAAAAACGCTCGGCCCCCAGTTCCTCTACCTCTACACCATC
>HPV30_Alpha_396973_nt7092_L1_Tile|1
TACTAAAAAACGCTCGGCCCCCAGTTCCTCTACCTCTACACCATCAGCCAAACGCAAGCG
>HPV31_Alpha_333048_nt5633_L1_Tile|1
TGTAACACGAACCAACATATATTATCACGCAGGCAGTGCTAGGCTGCTTACAGTAGGCCA
>HPV31_Alpha_333048_nt5668_L1_Tile|1
GTGCTAGGCTGCTTACAGTAGGCCATCCATATTATTCCATACCTAAATCTGACAATCCTA
>HPV31_Alpha_333048_nt5673_L1_Tile|1
AGGCTGCTTACAGTAGGCCATCCATATTATTCCATACCTAAATCTGACAATCCTAAAAAA
>HPV31_Alpha_333048_nt5678_L1_Tile|1
GCTTACAGTAGGCCATCCATATTATTCCATACCTAAATCTGACAATCCTAAAAAAATAGT
>HPV31_Alpha_333048_nt5683_L1_Tile|1
CAGTAGGCCATCCATATTATTCCATACCTAAATCTGACAATCCTAAAAAAATAGTTGTAC
>HPV31_Alpha_333048_nt5688_L1_Tile|1
GGCCATCCATATTATTCCATACCTAAATCTGACAATCCTAAAAAAATAGTTGTACCAAAG
>HPV31_Alpha_333048_nt5693_L1_Tile|1
TCCATATTATTCCATACCTAAATCTGACAATCCTAAAAAAATAGTTGTACCAAAGGTGTC
>HPV31_Alpha_333048_nt5938_L1_Tile|1
ACACTGAAAACTCTAATAGATATGCCGGTGGTCCTGGCACTGATAATAGGGAATGTATAT
>HPV31_Alpha_333048_nt5943_L1_Tile|1
GAAAACTCTAATAGATATGCCGGTGGTCCTGGCACTGATAATAGGGAATGTATATCAATG
>HPV31_Alpha_333048_nt6028_L1_Tile|1
TACTTGGTTGCAAACCACCTATTGGAGAGCATTGGGGTAAAGGTAGTCCTTGTAGTAACA
>HPV31_Alpha_333048_nt6033_L1_Tile|1
GGTTGCAAACCACCTATTGGAGAGCATTGGGGTAAAGGTAGTCCTTGTAGTAACAATGCT
>HPV31_Alpha_333048_nt6038_L1_Tile|1
CAAACCACCTATTGGAGAGCATTGGGGTAAAGGTAGTCCTTGTAGTAACAATGCTATTAC
>HPV31_Alpha_333048_nt6043_L1_Tile|1
CACCTATTGGAGAGCATTGGGGTAAAGGTAGTCCTTGTAGTAACAATGCTATTACCCCTG
>HPV31_Alpha_333048_nt6063_L1_Tile|1
GGTAAAGGTAGTCCTTGTAGTAACAATGCTATTACCCCTGGTGATTGTCCTCCATTAGAA
>HPV31_Alpha_333048_nt6338_L1_Tile|1
TAATAGATCAGGCACGGTTGGTGAATCGGTCCCTACTGACTTATATATTAAAGGCTCCGG
>HPV31_Alpha_333048_nt6353_L1_Tile|1
GGTTGGTGAATCGGTCCCTACTGACTTATATATTAAAGGCTCCGGTTCAACAGCTACTTT
>HPV31_Alpha_333048_nt6358_L1_Tile|1
GTGAATCGGTCCCTACTGACTTATATATTAAAGGCTCCGGTTCAACAGCTACTTTAGCTA
>HPV31_Alpha_333048_nt6368_L1_Tile|1
CCCTACTGACTTATATATTAAAGGCTCCGGTTCAACAGCTACTTTAGCTAACAGTACATA
>HPV31_Alpha_333048_nt6373_L1_Tile|1
CTGACTTATATATTAAAGGCTCCGGTTCAACAGCTACTTTAGCTAACAGTACATACTTTC
>HPV31_Alpha_333048_nt6378_L1_Tile|1
TTATATATTAAAGGCTCCGGTTCAACAGCTACTTTAGCTAACAGTACATACTTTCCTACA
>HPV31_Alpha_333048_nt6383_L1_Tile|1
TATTAAAGGCTCCGGTTCAACAGCTACTTTAGCTAACAGTACATACTTTCCTACACCTAG
>HPV31_Alpha_333048_nt6393_L1_Tile|1
TCCGGTTCAACAGCTACTTTAGCTAACAGTACATACTTTCCTACACCTAGCGGCTCCATG
>HPV31_Alpha_333048_nt6398_L1_Tile|1
TTCAACAGCTACTTTAGCTAACAGTACATACTTTCCTACACCTAGCGGCTCCATGGTTAC
>HPV31_Alpha_333048_nt6403_L1_Tile|1
CAGCTACTTTAGCTAACAGTACATACTTTCCTACACCTAGCGGCTCCATGGTTACTTCAG
>HPV31_Alpha_333048_nt6568_L1_Tile|1
GTAGTACCAATATGTCTGTTTGTGCTGCAATTGCAAACAGTGATACTACATTTAAAAGTA
>HPV31_Alpha_333048_nt6573_L1_Tile|1
ACCAATATGTCTGTTTGTGCTGCAATTGCAAACAGTGATACTACATTTAAAAGTAGTAAT

>HPV31_Alpha_333048_nt6578_L1_Tile|1
TATGTCTGTTTGTGCTGCAATTGCAAACAGTGATACTACATTTAAAAGTAGTAATTTTAA
>HPV31_Alpha_333048_nt6588_L1_Tile|1
TGTGCTGCAATTGCAAACAGTGATACTACATTTAAAAGTAGTAATTTTAAAGAGTATTTA
>HPV31_Alpha_333048_nt6593_L1_Tile|1
TGCAATTGCAAACAGTGATACTACATTTAAAAGTAGTAATTTTAAAGAGTATTTAAGACA
>HPV31_Alpha_333048_nt6768_L1_Tile|1
GGATTGACCACACCTCCCTCAGGTTCTTTGGAGGATACCTATAGGTTTGTCACCTCACAG
>HPV31_Alpha_333048_nt6828_L1_Tile|1
GCCATTACATGTCAAAAAACTGCCCCCCAAAAGCCCAAGGAAGATCCATTTAAAGATTAT
>HPV31_Alpha_333048_nt6948_L1_Tile|1
GGTCGCAAATTTTTATTACAGGCAGGATATAGGGCACGTCCTAAATTTAAAGCAGGTAAA
>HPV31_Alpha_333048_nt6953_L1_Tile|1
CAAATTTTTATTACAGGCAGGATATAGGGCACGTCCTAAATTTAAAGCAGGTAAACGTAG
>HPV31_Alpha_333048_nt6958_L1_Tile|1
TTTTATTACAGGCAGGATATAGGGCACGTCCTAAATTTAAAGCAGGTAAACGTAGTGCAC
>HPV31_Alpha_333048_nt6963_L1_Tile|1
TTACAGGCAGGATATAGGGCACGTCCTAAATTTAAAGCAGGTAAACGTAGTGCACCCTCA
>HPV31_Alpha_333048_nt6973_L1_Tile|1
GATATAGGGCACGTCCTAAATTTAAAGCAGGTAAACGTAGTGCACCCTCAGCATCTACCA
>HPV31_Alpha_333048_nt6983_L1_Tile|1
ACGTCCTAAATTTAAAGCAGGTAAACGTAGTGCACCCTCAGCATCTACCACTACACCAGC
>HPV31_Alpha_333048_nt6988_L1_Tile|1
CTAAATTTAAAGCAGGTAAACGTAGTGCACCCTCAGCATCTACCACTACACCAGCAAAAC
>HPV31_Alpha_333048_nt7003_L1_Tile|1
GTAAACGTAGTGCACCCTCAGCATCTACCACTACACCAGCAAAACGTAAAAAAACTAAAA
>HPV32_Alpha_9627327_nt5988_L1_Tile|1
GTTCTAGGCTTTTGGCTGTTGGGCATCCATATTATACTATTAAGAAGACACCCAATAGAA
>HPV32_Alpha_9627327_nt5993_L1_Tile|1
AGGCTTTTGGCTGTTGGGCATCCATATTATACTATTAAGAAGACACCCAATAGAACATCT
>HPV32_Alpha_9627327_nt5998_L1_Tile|1
TTTGGCTGTTGGGCATCCATATTATACTATTAAGAAGACACCCAATAGAACATCTATTCC
>HPV32_Alpha_9627327_nt6003_L1_Tile|1
CTGTTGGGCATCCATATTATACTATTAAGAAGACACCCAATAGAACATCTATTCCAAAGG
>HPV32_Alpha_9627327_nt6008_L1_Tile|1
GGGCATCCATATTATACTATTAAGAAGACACCCAATAGAACATCTATTCCAAAGGTGTCT
>HPV32_Alpha_9627327_nt6013_L1_Tile|1
TCCATATTATACTATTAAGAAGACACCCAATAGAACATCTATTCCAAAGGTGTCTGGATT
>HPV32_Alpha_9627327_nt6018_L1_Tile|1
ATTATACTATTAAGAAGACACCCAATAGAACATCTATTCCAAAGGTGTCTGGATTGCAGT
>HPV32_Alpha_9627327_nt6023_L1_Tile|1
ACTATTAAGAAGACACCCAATAGAACATCTATTCCAAAGGTGTCTGGATTGCAGTATAGA
>HPV32_Alpha_9627327_nt6038_L1_Tile|1
CCCAATAGAACATCTATTCCAAAGGTGTCTGGATTGCAGTATAGAGTATTTAGGGTTAGG
>HPV32_Alpha_9627327_nt6263_L1_Tile|1
GGGCCTAGATATGCTGCAGGGCCTGGAACTGATAATAGAGAAAATGTATCTATGGATTGT
>HPV32_Alpha_9627327_nt6338_L1_Tile|1
TGTTTGGTGGGTTGTAAACCTGCCATTGGCGAGCATTGGGGTAAGGGTGCTGCTTGCTCT
>HPV32_Alpha_9627327_nt6358_L1_Tile|1
TGCCATTGGCGAGCATTGGGGTAAGGGTGCTGCTTGCTCTGCACAATCAAATGGCGACTG
>HPV32_Alpha_9627327_nt6378_L1_Tile|1
GTAAGGGTGCTGCTTGCTCTGCACAATCAAATGGCGACTGCCCACCTTTGGAATTACAAA
>HPV32_Alpha_9627327_nt6453_L1_Tile|1
ATGGTGATATGGCAGATGTAGGGTTTGGAGCAATGGACTTTAGTGCTTTGCAAACTTCAA
>HPV32_Alpha_9627327_nt6458_L1_Tile|1

GATATGGCAGATGTAGGGTTTGGAGCAATGGACTTTAGTGCTTTGCAAACTTCAAAAGCT
>HPV32_Alpha_9627327_nt6483_L1_Tile|1
CAATGGACTTTAGTGCTTTGCAAACTTCAAAAGCTGAGGTGCCATTAGATATTATGAACT
>HPV32_Alpha_9627327_nt6488_L1_Tile|1
GACTTTAGTGCTTTGCAAACTTCAAAAGCTGAGGTGCCATTAGATATTATGAACTCCATT
>HPV32_Alpha_9627327_nt6648_L1_Tile|1
ATAGGGCAGGAACCCTTGGTGAACCTGTTCCTGAGGACATGTATATAAAAGCTTCTAATG
>HPV32_Alpha_9627327_nt6653_L1_Tile|1
GCAGGAACCCTTGGTGAACCTGTTCCTGAGGACATGTATATAAAAGCTTCTAATGGTGCT
>HPV32_Alpha_9627327_nt6658_L1_Tile|1
AACCCTTGGTGAACCTGTTCCTGAGGACATGTATATAAAAGCTTCTAATGGTGCTTCTGG
>HPV32_Alpha_9627327_nt6668_L1_Tile|1
GAACCTGTTCCTGAGGACATGTATATAAAAGCTTCTAATGGTGCTTCTGGCAGAAATAAT
>HPV32_Alpha_9627327_nt6673_L1_Tile|1
TGTTCCTGAGGACATGTATATAAAAGCTTCTAATGGTGCTTCTGGCAGAAATAATTTAGC
>HPV32_Alpha_9627327_nt6678_L1_Tile|1
CTGAGGACATGTATATAAAAGCTTCTAATGGTGCTTCTGGCAGAAATAATTTAGCTAGTA
>HPV32_Alpha_9627327_nt6683_L1_Tile|1
GACATGTATATAAAAGCTTCTAATGGTGCTTCTGGCAGAAATAATTTAGCTAGTAGTATT
>HPV32_Alpha_9627327_nt6888_L1_Tile|1
GTACTAACATGACTGTGTGTGCTACTGTAACAACTGAAGACACATACAAGTCTACTAACT
>HPV32_Alpha_9627327_nt6893_L1_Tile|1
AACATGACTGTGTGTGCTACTGTAACAACTGAAGACACATACAAGTCTACTAACTTTAAG
>HPV32_Alpha_9627327_nt6898_L1_Tile|1
GACTGTGTGTGCTACTGTAACAACTGAAGACACATACAAGTCTACTAACTTTAAGGAATA
>HPV32_Alpha_9627327_nt6903_L1_Tile|1
TGTGTGCTACTGTAACAACTGAAGACACATACAAGTCTACTAACTTTAAGGAATATCTAC
>HPV32_Alpha_9627327_nt6908_L1_Tile|1
GCTACTGTAACAACTGAAGACACATACAAGTCTACTAACTTTAAGGAATATCTACGCCAT
>HPV32_Alpha_9627327_nt7008_L1_Tile|1
AAATTACATTATCTGTAGAGGTTATGTCATATATCCACACCATGAATCCTGACATACTAG
>HPV32_Alpha_9627327_nt7038_L1_Tile|1
ATATCCACACCATGAATCCTGACATACTAGACGATTGGAATGTTGGTGTAGCTCCACCGC
>HPV32_Alpha_9627327_nt7113_L1_Tile|1
AAGATAGTTATAGATTTGTGCAGTCTCAGGCCATACGATGTCAAGCTAAGGTAACAGCAC
>HPV32_Alpha_9627327_nt7273_L1_Tile|1
TTTACTGCAAGCTGGGTTACGTGCAAGACCTAAACTTACAGCAGTAAAACGAACAGCATC
>HPV32_Alpha_9627327_nt7278_L1_Tile|1
TGCAAGCTGGGTTACGTGCAAGACCTAAACTTACAGCAGTAAAACGAACAGCATCTTCCA
>HPV32_Alpha_9627327_nt7283_L1_Tile|1
GCTGGGTTACGTGCAAGACCTAAACTTACAGCAGTAAAACGAACAGCATCTTCCAGTCAA
>HPV32_Alpha_9627327_nt7288_L1_Tile|1
GTTACGTGCAAGACCTAAACTTACAGCAGTAAAACGAACAGCATCTTCCAGTCAAAAGTC
>HPV32_Alpha_9627327_nt7298_L1_Tile|1
AGACCTAAACTTACAGCAGTAAAACGAACAGCATCTTCCAGTCAAAAGTCTTCTTCTCCT
>HPV32_Alpha_9627327_nt7325_L1_Tile|1
ACAGCATCTTCCAGTCAAAAGTCTTCTTCTCCTGCAAAACGCAGAAAAACACGTAAATAA
>HPV33_Alpha_333049_nt5725_L1_Tile|1
CTGTTGGCCATCCATATTTTTCTATTAAAAATCCTACTAACGCTAAAAAATTATTGGTAC
>HPV33_Alpha_333049_nt5730_L1_Tile|1
GGCCATCCATATTTTTCTATTAAAAATCCTACTAACGCTAAAAAATTATTGGTACCCAAA
>HPV33_Alpha_333049_nt5735_L1_Tile|1
TCCATATTTTTCTATTAAAAATCCTACTAACGCTAAAAAATTATTGGTACCCAAAGTATC
>HPV33_Alpha_333049_nt5740_L1_Tile|1
ATTTTTCTATTAAAAATCCTACTAACGCTAAAAAATTATTGGTACCCAAAGTATCAGGCT

>HPV33_Alpha_333049_nt5745_L1_Tile|1
TCTATTAAAAATCCTACTAACGCTAAAAAATTATTGGTACCCAAAGTATCAGGCTTGCAA
>HPV33_Alpha_333049_nt5750_L1_Tile|1
TAAAAATCCTACTAACGCTAAAAAATTATTGGTACCCAAAGTATCAGGCTTGCAATATAG
>HPV33_Alpha_333049_nt6385_L1_Tile|1
CTGGTACATTAGGAGAGGCTGTTCCCGATGACCTGTACATTAAAGGTTCAGGAACTACTG
>HPV33_Alpha_333049_nt6390_L1_Tile|1
ACATTAGGAGAGGCTGTTCCCGATGACCTGTACATTAAAGGTTCAGGAACTACTGCCTCT
>HPV33_Alpha_333049_nt6395_L1_Tile|1
AGGAGAGGCTGTTCCCGATGACCTGTACATTAAAGGTTCAGGAACTACTGCCTCTATTCA
>HPV33_Alpha_333049_nt6400_L1_Tile|1
AGGCTGTTCCCGATGACCTGTACATTAAAGGTTCAGGAACTACTGCCTCTATTCAAAGCA
>HPV33_Alpha_333049_nt6405_L1_Tile|1
GTTCCCGATGACCTGTACATTAAAGGTTCAGGAACTACTGCCTCTATTCAAAGCAGTGCT
>HPV33_Alpha_333049_nt6865_L1_Tile|1
CTATTACGTGTCAAAAAACAGTACCTCCAAAGGAAAAGGAAGACCCCTTAGGTAAATATA
>HPV33_Alpha_333049_nt6995_L1_Tile|1
TTTATTACAGGCAGGTCTTAAAGCAAAACCTAAACTTAAACGTGCAGCCCCCACATCCAC
>HPV33_Alpha_333049_nt7000_L1_Tile|1
TACAGGCAGGTCTTAAAGCAAAACCTAAACTTAAACGTGCAGCCCCCACATCCACCCGCA
>HPV33_Alpha_333049_nt7010_L1_Tile|1
TCTTAAAGCAAAACCTAAACTTAAACGTGCAGCCCCCACATCCACCCGCACATCGTCTGC
>HPV34_Alpha_9627334_nt5616_L1_Tile|1
TAGTACACGCTTGCTGGCAGTAGGACATCCCTATTATCCTATAAAGGATACTAATGGGAA
>HPV34_Alpha_9627334_nt5626_L1_Tile|1
TTGCTGGCAGTAGGACATCCCTATTATCCTATAAAGGATACTAATGGGAAACGTAAGATT
>HPV34_Alpha_9627334_nt5631_L1_Tile|1
GGCAGTAGGACATCCCTATTATCCTATAAAGGATACTAATGGGAAACGTAAGATTGCTGT
>HPV34_Alpha_9627334_nt5636_L1_Tile|1
TAGGACATCCCTATTATCCTATAAAGGATACTAATGGGAAACGTAAGATTGCTGTACCTA
>HPV34_Alpha_9627334_nt5646_L1_Tile|1
CTATTATCCTATAAAGGATACTAATGGGAAACGTAAGATTGCTGTACCTAAAGTTTCAGG
>HPV34_Alpha_9627334_nt5866_L1_Tile|1
CCTTTTATGAATAAACTTGAGGATACTGAAAATGCTGCAAAATATATTGGTGGAAACATA
>HPV34_Alpha_9627334_nt5876_L1_Tile|1
ATAAACTTGAGGATACTGAAAATGCTGCAAAATATATTGGTGGAAACATAGCAGATAGTA
>HPV34_Alpha_9627334_nt5881_L1_Tile|1
CTTGAGGATACTGAAAATGCTGCAAAATATATTGGTGGAAACATAGCAGATAGTAGGGAG
>HPV34_Alpha_9627334_nt5886_L1_Tile|1
GGATACTGAAAATGCTGCAAAATATATTGGTGGAAACATAGCAGATAGTAGGGAGTGTAT
>HPV34_Alpha_9627334_nt5901_L1_Tile|1
TGCAAAATATATTGGTGGAAACATAGCAGATAGTAGGGAGTGTATGTCAGTAGATTATAA
>HPV34_Alpha_9627334_nt6011_L1_Tile|1
GGGGTACAGGCACACCATGTGGTACCCAAAATGCTGGGGAATGTCCTCCGCTTGAATTAA
>HPV34_Alpha_9627334_nt6016_L1_Tile|1
ACAGGCACACCATGTGGTACCCAAAATGCTGGGGAATGTCCTCCGCTTGAATTAAAGAAC
>HPV34_Alpha_9627334_nt6026_L1_Tile|1
CATGTGGTACCCAAAATGCTGGGGAATGTCCTCCGCTTGAATTAAAGAACACCACTATAC
>HPV34_Alpha_9627334_nt6266_L1_Tile|1
TTGTTAGACACCTATTTAACAGGGCAGGTACTGTAGGCGATGCTATTCCAGATGACTTAA
>HPV34_Alpha_9627334_nt6276_L1_Tile|1
CCTATTTAACAGGGCAGGTACTGTAGGCGATGCTATTCCAGATGACTTAATGATTAAGGG
>HPV34_Alpha_9627334_nt6281_L1_Tile|1
TTAACAGGGCAGGTACTGTAGGCGATGCTATTCCAGATGACTTAATGATTAAGGGTACAG
>HPV34_Alpha_9627334_nt6506_L1_Tile|1

CTACTAGAAGCACAAACTTTTCAGTTTGTGTAGGTACACAATCCACAAGTACAACTGCAC
>HPV34_Alpha_9627334_nt6511_L1_Tile|1
AGAAGCACAAACTTTTCAGTTTGTGTAGGTACACAATCCACAAGTACAACTGCACCATAT
>HPV34_Alpha_9627334_nt6516_L1_Tile|1
CACAAACTTTTCAGTTTGTGTAGGTACACAATCCACAAGTACAACTGCACCATATGCAAA
>HPV34_Alpha_9627334_nt6536_L1_Tile|1
TAGGTACACAATCCACAAGTACAACTGCACCATATGCAAACAGTAATTTTAAGGAATACC
>HPV34_Alpha_9627334_nt6541_L1_Tile|1
ACACAATCCACAAGTACAACTGCACCATATGCAAACAGTAATTTTAAGGAATACCTCAGA
>HPV34_Alpha_9627334_nt6546_L1_Tile|1
ATCCACAAGTACAACTGCACCATATGCAAACAGTAATTTTAAGGAATACCTCAGACATGC
>HPV34_Alpha_9627334_nt6936_L1_Tile|1
TCCTAGGTTACAGGCCTCTAAACGGTCTGCACCTTCATCATCTAGTACAGCACCTAAGAA
>HPV35_Alpha_333050_nt5690_L1_Tile|1
GTTCTAGGCTATTAGCTGTGGGTCACCCATACTATGCTATTAAAAAACAAGATTCTAATA
>HPV35_Alpha_333050_nt5695_L1_Tile|1
AGGCTATTAGCTGTGGGTCACCCATACTATGCTATTAAAAAACAAGATTCTAATAAAATA
>HPV35_Alpha_333050_nt5700_L1_Tile|1
ATTAGCTGTGGGTCACCCATACTATGCTATTAAAAAACAAGATTCTAATAAAATAGCAGT
>HPV35_Alpha_333050_nt5710_L1_Tile|1
GGTCACCCATACTATGCTATTAAAAAACAAGATTCTAATAAAATAGCAGTACCCAAGGTA
>HPV35_Alpha_333050_nt5720_L1_Tile|1
ACTATGCTATTAAAAAACAAGATTCTAATAAAATAGCAGTACCCAAGGTATCTGGTTTGC
>HPV35_Alpha_333050_nt5725_L1_Tile|1
GCTATTAAAAAACAAGATTCTAATAAAATAGCAGTACCCAAGGTATCTGGTTTGCAATAC
>HPV35_Alpha_333050_nt5730_L1_Tile|1
TAAAAAACAAGATTCTAATAAAATAGCAGTACCCAAGGTATCTGGTTTGCAATACAGAGT
>HPV35_Alpha_333050_nt5735_L1_Tile|1
AACAAGATTCTAATAAAATAGCAGTACCCAAGGTATCTGGTTTGCAATACAGAGTATTTA
>HPV35_Alpha_333050_nt5955_L1_Tile|1
TGATACTGAAAATCTTAATAAATATGTTGGTAACTCTGGTAACTCTGGTACAGATAACAG
>HPV35_Alpha_333050_nt5960_L1_Tile|1
CTGAAAATCTTAATAAATATGTTGGTAACTCTGGTAACTCTGGTACAGATAACAGGGAAT
>HPV35_Alpha_333050_nt5970_L1_Tile|1
TAATAAATATGTTGGTAACTCTGGTAACTCTGGTACAGATAACAGGGAATGCATTTCTAT
>HPV35_Alpha_333050_nt6085_L1_Tile|1
CATTGGGGAAAAGGCACACCTTGTAATGCTAACCAGGTAAAAGCAGGAGAATGTCCTCCT
>HPV35_Alpha_333050_nt6090_L1_Tile|1
GGGAAAAGGCACACCTTGTAATGCTAACCAGGTAAAAGCAGGAGAATGTCCTCCTTTGGA
>HPV35_Alpha_333050_nt6095_L1_Tile|1
AAGGCACACCTTGTAATGCTAACCAGGTAAAAGCAGGAGAATGTCCTCCTTTGGAGTTAC
>HPV35_Alpha_333050_nt6100_L1_Tile|1
ACACCTTGTAATGCTAACCAGGTAAAAGCAGGAGAATGTCCTCCTTTGGAGTTACTAAAC
>HPV35_Alpha_333050_nt6105_L1_Tile|1
TTGTAATGCTAACCAGGTAAAAGCAGGAGAATGTCCTCCTTTGGAGTTACTAAACACTGT
>HPV35_Alpha_333050_nt6110_L1_Tile|1
ATGCTAACCAGGTAAAAGCAGGAGAATGTCCTCCTTTGGAGTTACTAAACACTGTACTAC
>HPV35_Alpha_333050_nt6140_L1_Tile|1
CTCCTTTGGAGTTACTAAACACTGTACTACAAGACGGGGACATGGTAGACACAGGATTTG
>HPV35_Alpha_333050_nt6200_L1_Tile|1
GTGCAATGGATTTTACTACATTACAAGCTAATAAAAGTGATGTTCCCCTAGATATATGCA
>HPV35_Alpha_333050_nt6205_L1_Tile|1
ATGGATTTTACTACATTACAAGCTAATAAAAGTGATGTTCCCCTAGATATATGCAGTTCC
>HPV35_Alpha_333050_nt6210_L1_Tile|1
TTTTACTACATTACAAGCTAATAAAAGTGATGTTCCCCTAGATATATGCAGTTCCATTTG

>HPV35_Alpha_333050_nt6375_L1_Tile|1
TGGAACTGTAGGTGAAACAGTACCTGCAGACCTATATATTAAGGGTACCACTGGCACATT
>HPV35_Alpha_333050_nt6380_L1_Tile|1
CTGTAGGTGAAACAGTACCTGCAGACCTATATATTAAGGGTACCACTGGCACATTGCCTA
>HPV35_Alpha_333050_nt6395_L1_Tile|1
TACCTGCAGACCTATATATTAAGGGTACCACTGGCACATTGCCTAGTACTAGTTATTTTC
>HPV35_Alpha_333050_nt6405_L1_Tile|1
CCTATATATTAAGGGTACCACTGGCACATTGCCTAGTACTAGTTATTTTCCTACTCCTAG
>HPV35_Alpha_333050_nt6410_L1_Tile|1
ATATTAAGGGTACCACTGGCACATTGCCTAGTACTAGTTATTTTCCTACTCCTAGTGGCT
>HPV35_Alpha_333050_nt6570_L1_Tile|1
TACTGTAGTTGATACAACCCGTAGTACAAATATGTCTGTGTGTTCTGCTGTGTCTTCTAG
>HPV35_Alpha_333050_nt6575_L1_Tile|1
TAGTTGATACAACCCGTAGTACAAATATGTCTGTGTGTTCTGCTGTGTCTTCTAGTGACA
>HPV35_Alpha_333050_nt6580_L1_Tile|1
GATACAACCCGTAGTACAAATATGTCTGTGTGTTCTGCTGTGTCTTCTAGTGACAGTACA
>HPV35_Alpha_333050_nt6710_L1_Tile|1
TATGTAAAATAACACTAACAGCAGATGTTATGACATATATTCATAGTATGAACCCGTCCA
>HPV35_Alpha_333050_nt6815_L1_Tile|1
CCTTAGAGGACACATATCGCTATGTAACATCACAGGCTGTAACTTGTCAAAAACCCAGTG
>HPV35_Alpha_333050_nt6840_L1_Tile|1
AACATCACAGGCTGTAACTTGTCAAAAACCCAGTGCACCAAAACCTAAAGATGATCCATT
>HPV35_Alpha_333050_nt6970_L1_Tile|1
GGCCGTAAATTTTTGTTACAAGCAGGACTAAAGGCCAGGCCTAATTTTAGATTAGGCAGG
>HPV35_Alpha_333050_nt6980_L1_Tile|1
TTTTGTTACAAGCAGGACTAAAGGCCAGGCCTAATTTTAGATTAGGCAGGCGTGCAGCTC
>HPV35_Alpha_333050_nt6985_L1_Tile|1
TTACAAGCAGGACTAAAGGCCAGGCCTAATTTTAGATTAGGCAGGCGTGCAGCTCCAGCA
>HPV35_Alpha_333050_nt6990_L1_Tile|1
AGCAGGACTAAAGGCCAGGCCTAATTTTAGATTAGGCAGGCGTGCAGCTCCAGCATCTAC
>HPV35_Alpha_333050_nt6995_L1_Tile|1
GACTAAAGGCCAGGCCTAATTTTAGATTAGGCAGGCGTGCAGCTCCAGCATCTACATCTA
>HPV35_Alpha_333050_nt7000_L1_Tile|1
AAGGCCAGGCCTAATTTTAGATTAGGCAGGCGTGCAGCTCCAGCATCTACATCTAAAAAA
>HPV35_Alpha_333050_nt7005_L1_Tile|1
CAGGCCTAATTTTAGATTAGGCAGGCGTGCAGCTCCAGCATCTACATCTAAAAAATCTTC
>HPV35_Alpha_333050_nt7010_L1_Tile|1
CTAATTTTAGATTAGGCAGGCGTGCAGCTCCAGCATCTACATCTAAAAAATCTTCTACTA
>HPV35_Alpha_333050_nt7015_L1_Tile|1
TTTAGATTAGGCAGGCGTGCAGCTCCAGCATCTACATCTAAAAAATCTTCTACTAAACGT
>HPV35_Alpha_333050_nt7025_L1_Tile|1
GCAGGCGTGCAGCTCCAGCATCTACATCTAAAAAATCTTCTACTAAACGTAGAAAAGTAA
>HPV36_Beta_1020218_nt5967_L1_Tile|1
GATGAATATATACAAAGGACAAATATTTATTATCATGCATTCAGCGACAGACTACTGACT
>HPV36_Beta_1020218_nt5972_L1_Tile|1
ATATATACAAAGGACAAATATTTATTATCATGCATTCAGCGACAGACTACTGACTGTAGG
>HPV36_Beta_1020218_nt5987_L1_Tile|1
AAATATTTATTATCATGCATTCAGCGACAGACTACTGACTGTAGGCCATCCATACTTCAA
>HPV36_Beta_1020218_nt6022_L1_Tile|2
TGACTGTAGGCCATCCATACTTCAATGTTTACGATATTACTGGTAACAAATTAGAAGTCC
>HPV36_Beta_1020218_nt6027_L1_Tile|1
GTAGGCCATCCATACTTCAATGTTTACGATATTACTGGTAACAAATTAGAAGTCCCTAAA
>HPV36_Beta_1020218_nt6047_L1_Tile|1
TGTTTACGATATTACTGGTAACAAATTAGAAGTCCCTAAAGTGTCTGGTAACCAACACAG
>HPV36_Beta_1020218_nt6062_L1_Tile|2

TGGTAACAAATTAGAAGTCCCTAAAGTGTCTGGTAACCAACACAGGGTTTTTCGCTTGAA
>HPV36_Beta_1020218_nt6387_L1_Tile|1
GGTGAACATTGGGACAAGGCTATACCTTGTGAAAAGGAGCGCCAGGACAACAGACTATGC
>HPV36_Beta_1020218_nt6397_L1_Tile|1
GGGACAAGGCTATACCTTGTGAAAAGGAGCGCCAGGACAACAGACTATGCCCACCAATTG
>HPV36_Beta_1020218_nt6402_L1_Tile|2
AAGGCTATACCTTGTGAAAAGGAGCGCCAGGACAACAGACTATGCCCACCAATTGAATTG
>HPV36_Beta_1020218_nt6407_L1_Tile|1
TATACCTTGTGAAAAGGAGCGCCAGGACAACAGACTATGCCCACCAATTGAATTGAAAAC
>HPV36_Beta_1020218_nt6427_L1_Tile|1
GCCAGGACAACAGACTATGCCCACCAATTGAATTGAAAACTACTTATATAGAAGATGGCG
>HPV36_Beta_1020218_nt6487_L1_Tile|1
ACATGGCAGATATAGGTTTTGGAAATTTAAACTTCAAAAATCTGCAAGAGAGTAGGTCAG
>HPV36_Beta_1020218_nt6492_L1_Tile|1
GCAGATATAGGTTTTGGAAATTTAAACTTCAAAAATCTGCAAGAGAGTAGGTCAGATGTA
>HPV36_Beta_1020218_nt6497_L1_Tile|1
TATAGGTTTTGGAAATTTAAACTTCAAAAATCTGCAAGAGAGTAGGTCAGATGTAAGTTT
>HPV36_Beta_1020218_nt6502_L1_Tile|2
GTTTTGGAAATTTAAACTTCAAAAATCTGCAAGAGAGTAGGTCAGATGTAAGTTTGGATA
>HPV36_Beta_1020218_nt6687_L1_Tile|1
AAAACGGGTGACGACATCCCTGACGCCAGAATTGATAATGGGACTTTTAAGAATCAGTTT
>HPV36_Beta_1020218_nt6692_L1_Tile|1
GGGTGACGACATCCCTGACGCCAGAATTGATAATGGGACTTTTAAGAATCAGTTTTTCAT
>HPV36_Beta_1020218_nt6707_L1_Tile|1
TGACGCCAGAATTGATAATGGGACTTTTAAGAATCAGTTTTTCATTCCTGGGGCTGACGG
>HPV36_Beta_1020218_nt6927_L1_Tile|1
GTAGACAACACACGAAATACTAACTTTAGTATTTCAATATATAACAATAATGGGGCACTA
>HPV36_Beta_1020218_nt6942_L1_Tile|2
AATACTAACTTTAGTATTTCAATATATAACAATAATGGGGCACTAAAGGACATCAATGAT
>HPV36_Beta_1020218_nt6947_L1_Tile|1
TAACTTTAGTATTTCAATATATAACAATAATGGGGCACTAAAGGACATCAATGATTACAC
>HPV36_Beta_1020218_nt6952_L1_Tile|1
TTAGTATTTCAATATATAACAATAATGGGGCACTAAAGGACATCAATGATTACACTGCAG
>HPV36_Beta_1020218_nt6962_L1_Tile|2
AATATATAACAATAATGGGGCACTAAAGGACATCAATGATTACACTGCAGAGCAATTTAG
>HPV36_Beta_1020218_nt6967_L1_Tile|1
ATAACAATAATGGGGCACTAAAGGACATCAATGATTACACTGCAGAGCAATTTAGAGAAT
>HPV36_Beta_1020218_nt6987_L1_Tile|1
AAGGACATCAATGATTACACTGCAGAGCAATTTAGAGAATATCAAAGGCACGTGGAGGAA
>HPV36_Beta_1020218_nt7152_L1_Tile|1
GGTTTTGTACCTACTCCAGATAACCCTATTCAAGACACCTATCGATATATTGATTCATTA
>HPV36_Beta_1020218_nt7162_L1_Tile|2
CTACTCCAGATAACCCTATTCAAGACACCTATCGATATATTGATTCATTAGCCACTCGCT
>HPV36_Beta_1020218_nt7167_L1_Tile|1
CCAGATAACCCTATTCAAGACACCTATCGATATATTGATTCATTAGCCACTCGCTGTCCT
>HPV36_Beta_1020218_nt7242_L1_Tile|2
AAGGAAAAAGAGGATCCCTACAAGGGGTTAAAGTTCTGGGATGTGGATCTTACTGAACGG
>HPV36_Beta_1020218_nt7252_L1_Tile|1
AGGATCCCTACAAGGGGTTAAAGTTCTGGGATGTGGATCTTACTGAACGGTTGTCACTAG
>HPV36_Beta_1020218_nt7257_L1_Tile|1
CCCTACAAGGGGTTAAAGTTCTGGGATGTGGATCTTACTGAACGGTTGTCACTAGATTTG
>HPV36_Beta_1020218_nt7382_L1_Tile|2
TACAAAATCAGTGTCTTATCGAGGGTTCACCAGAGGAACCAAGCGCAAGCGAAAACAGTA
>HPV36_Beta_1020218_nt7383_L1_Tile|1
ACAAAATCAGTGTCTTATCGAGGGTTCACCAGAGGAACCAAGCGCAAGCGAAAACAGTAA

>HPV37_Beta_1020226_nt6027_L1_Tile|1
CGGGCTTGGAGATAGGCCGAGGGCAACCACTTGGAGTAGGAACGACAGGTCACCCTTTAT
>HPV37_Beta_1020226_nt6082_L1_Tile|2
TTTATTTAATAAATTAAGGGACACTGAGAATAATAGTAATTACCAAGGGGGGTCACGGGA
>HPV37_Beta_1020226_nt6087_L1_Tile|1
TTAATAAATTAAGGGACACTGAGAATAATAGTAATTACCAAGGGGGGTCACGGGATGATA
>HPV37_Beta_1020226_nt6092_L1_Tile|1
AAATTAAGGGACACTGAGAATAATAGTAATTACCAAGGGGGGTCACGGGATGATAGACAA
>HPV37_Beta_1020226_nt6097_L1_Tile|1
AAGGGACACTGAGAATAATAGTAATTACCAAGGGGGGTCACGGGATGATAGACAAAACAC
>HPV37_Beta_1020226_nt6102_L1_Tile|2
ACACTGAGAATAATAGTAATTACCAAGGGGGGTCACGGGATGATAGACAAAACACATCAT
>HPV37_Beta_1020226_nt6107_L1_Tile|1
GAGAATAATAGTAATTACCAAGGGGGGTCACGGGATGATAGACAAAACACATCATTTGAT
>HPV37_Beta_1020226_nt6227_L1_Tile|1
AAAGCACCAGTTTGTGCATCAGAGGAAAATAATCAGACAGGACAGTGTCCACCACTTGAA
>HPV37_Beta_1020226_nt6232_L1_Tile|1
ACCAGTTTGTGCATCAGAGGAAAATAATCAGACAGGACAGTGTCCACCACTTGAATTAAA
>HPV37_Beta_1020226_nt6247_L1_Tile|1
AGAGGAAAATAATCAGACAGGACAGTGTCCACCACTTGAATTAAAAAACACAGTGATTGA
>HPV37_Beta_1020226_nt6312_L1_Tile|1
GGGACATGTTTGATATAGGGTTCGGAAATATTAACAATAAGGTTCTCTCTACTAATAAAT
>HPV37_Beta_1020226_nt6317_L1_Tile|1
ATGTTTGATATAGGGTTCGGAAATATTAACAATAAGGTTCTCTCTACTAATAAATCAGAT
>HPV37_Beta_1020226_nt6322_L1_Tile|2
TGATATAGGGTTCGGAAATATTAACAATAAGGTTCTCTCTACTAATAAATCAGATGTTAG
>HPV37_Beta_1020226_nt6327_L1_Tile|1
TAGGGTTCGGAAATATTAACAATAAGGTTCTCTCTACTAATAAATCAGATGTTAGTTTAG
>HPV37_Beta_1020226_nt6332_L1_Tile|1
TTCGGAAATATTAACAATAAGGTTCTCTCTACTAATAAATCAGATGTTAGTTTAGATATA
>HPV37_Beta_1020226_nt6337_L1_Tile|1
AAATATTAACAATAAGGTTCTCTCTACTAATAAATCAGATGTTAGTTTAGATATAGTAAA
>HPV37_Beta_1020226_nt6342_L1_Tile|2
TTAACAATAAGGTTCTCTCTACTAATAAATCAGATGTTAGTTTAGATATAGTAAATGAAA
>HPV37_Beta_1020226_nt6527_L1_Tile|1
GCTATTCCCGATGGCACTGTTAATCAGGACCACAAATATTACTTACCTGCCAAATCAGAC
>HPV37_Beta_1020226_nt6532_L1_Tile|1
TCCCGATGGCACTGTTAATCAGGACCACAAATATTACTTACCTGCCAAATCAGACCAGCA
>HPV37_Beta_1020226_nt6542_L1_Tile|2
ACTGTTAATCAGGACCACAAATATTACTTACCTGCCAAATCAGACCAGCAGCAGTATCTG
>HPV37_Beta_1020226_nt6547_L1_Tile|1
TAATCAGGACCACAAATATTACTTACCTGCCAAATCAGACCAGCAGCAGTATCTGTTAGG
>HPV37_Beta_1020226_nt6552_L1_Tile|1
AGGACCACAAATATTACTTACCTGCCAAATCAGACCAGCAGCAGTATCTGTTAGGCAATT
>HPV37_Beta_1020226_nt6562_L1_Tile|2
ATATTACTTACCTGCCAAATCAGACCAGCAGCAGTATCTGTTAGGCAATTCTACCTATTT
>HPV37_Beta_1020226_nt6567_L1_Tile|1
ACTTACCTGCCAAATCAGACCAGCAGCAGTATCTGTTAGGCAATTCTACCTATTTTCCCA
>HPV37_Beta_1020226_nt6572_L1_Tile|1
CCTGCCAAATCAGACCAGCAGCAGTATCTGTTAGGCAATTCTACCTATTTTCCCACTGTT
>HPV37_Beta_1020226_nt6577_L1_Tile|1
CAAATCAGACCAGCAGCAGTATCTGTTAGGCAATTCTACCTATTTTCCCACTGTTAGTGG
>HPV37_Beta_1020226_nt6972_L1_Tile|1
CTACTCCAGATAATTCAGTACATGACCTTTATAGGTACATTAATTCAAAGGCTACCAAGT
>HPV37_Beta_1020226_nt7112_L1_Tile|1

TTATCATTGGATTTAGATCAATATCCTTTAGGGAGGAAATTCATCTTTCAGTCGGGATTG
>HPV37_Beta_1020226_nt7122_L1_Tile|2
ATTTAGATCAATATCCTTTAGGGAGGAAATTCATCTTTCAGTCGGGATTGCAAAGTAGAC
>HPV37_Beta_1020226_nt7127_L1_Tile|1
GATCAATATCCTTTAGGGAGGAAATTCATCTTTCAGTCGGGATTGCAAAGTAGACCTAGA
>HPV37_Beta_1020226_nt7132_L1_Tile|1
ATATCCTTTAGGGAGGAAATTCATCTTTCAGTCGGGATTGCAAAGTAGACCTAGAATTGT
>HPV37_Beta_1020226_nt7137_L1_Tile|1
CTTTAGGGAGGAAATTCATCTTTCAGTCGGGATTGCAAAGTAGACCTAGAATTGTTCGAT
>HPV37_Beta_1020226_nt7142_L1_Tile|2
GGGAGGAAATTCATCTTTCAGTCGGGATTGCAAAGTAGACCTAGAATTGTTCGATCGTCT
>HPV37_Beta_1020226_nt7147_L1_Tile|1
GAAATTCATCTTTCAGTCGGGATTGCAAAGTAGACCTAGAATTGTTCGATCGTCTGTAAA
>HPV37_Beta_1020226_nt7152_L1_Tile|1
TCATCTTTCAGTCGGGATTGCAAAGTAGACCTAGAATTGTTCGATCGTCTGTAAAAGTGT
>HPV37_Beta_1020226_nt7157_L1_Tile|1
TTTCAGTCGGGATTGCAAAGTAGACCTAGAATTGTTCGATCGTCTGTAAAAGTGTCTAAA
>HPV37_Beta_1020226_nt7167_L1_Tile|1
GATTGCAAAGTAGACCTAGAATTGTTCGATCGTCTGTAAAAGTGTCTAAAGGTACAAAGC
>HPV37_Beta_1020226_nt7172_L1_Tile|1
CAAAGTAGACCTAGAATTGTTCGATCGTCTGTAAAAGTGTCTAAAGGTACAAAGCGTAAA
>HPV37_Beta_1020226_nt7181_L1_Tile|1
CCTAGAATTGTTCGATCGTCTGTAAAAGTGTCTAAAGGTACAAAGCGTAAACGGTCGTGA
>HPV38_Beta_1020234_nt6352_L1_Tile|1
TACCCAGATTTTCTTACCATGTCTAATGATGTTTATGGTGATTCCTGCTTCTTTTTTGTG
>HPV38_Beta_1020234_nt6437_L1_Tile|1
ATTATTTTGTTCGAGGTGGTGCAGTGGGTGACGCTATTCCAGATGGTACTGTCAACCAAA
>HPV38_Beta_1020234_nt6442_L1_Tile|2
TTTGTTCGAGGTGGTGCAGTGGGTGACGCTATTCCAGATGGTACTGTCAACCAAAATCAT
>HPV38_Beta_1020234_nt6462_L1_Tile|2
GGGTGACGCTATTCCAGATGGTACTGTCAACCAAAATCATAATTATTATTTACCTGCAAA
>HPV38_Beta_1020234_nt6467_L1_Tile|1
ACGCTATTCCAGATGGTACTGTCAACCAAAATCATAATTATTATTTACCTGCAAAAAATG
>HPV38_Beta_1020234_nt6477_L1_Tile|1
AGATGGTACTGTCAACCAAAATCATAATTATTATTTACCTGCAAAAAATGGACAGGGTCA
>HPV38_Beta_1020234_nt6482_L1_Tile|2
GTACTGTCAACCAAAATCATAATTATTATTTACCTGCAAAAAATGGACAGGGTCAACGCA
>HPV38_Beta_1020234_nt6487_L1_Tile|1
GTCAACCAAAATCATAATTATTATTTACCTGCAAAAAATGGACAGGGTCAACGCACTTTA
>HPV38_Beta_1020234_nt6492_L1_Tile|1
CCAAAATCATAATTATTATTTACCTGCAAAAAATGGACAGGGTCAACGCACTTTAGGGAA
>HPV38_Beta_1020234_nt6497_L1_Tile|1
ATCATAATTATTATTTACCTGCAAAAAATGGACAGGGTCAACGCACTTTAGGGAACTCTA
>HPV38_Beta_1020234_nt6502_L1_Tile|2
AATTATTATTTACCTGCAAAAAATGGACAGGGTCAACGCACTTTAGGGAACTCTACGTAT
>HPV38_Beta_1020234_nt6507_L1_Tile|1
TTATTTACCTGCAAAAAATGGACAGGGTCAACGCACTTTAGGGAACTCTACGTATTTTCC
>HPV38_Beta_1020234_nt6537_L1_Tile|1
ACGCACTTTAGGGAACTCTACGTATTTTCCAACAGTTAGTGGATCCTTGGTGACGTCTGA
>HPV38_Beta_1020234_nt6667_L1_Tile|1
AATCAAATGTTTGTTACAGTCGCTGATAATACCCGAAATACAAACTTTACAATCAGTGTA
>HPV38_Beta_1020234_nt6672_L1_Tile|1
AATGTTTGTTACAGTCGCTGATAATACCCGAAATACAAACTTTACAATCAGTGTATCCAC
>HPV38_Beta_1020234_nt6677_L1_Tile|1
TTGTTACAGTCGCTGATAATACCCGAAATACAAACTTTACAATCAGTGTATCCACTGAAA

>HPV38_Beta_1020234_nt6682_L1_Tile|2
ACAGTCGCTGATAATACCCGAAATACAAACTTTACAATCAGTGTATCCACTGAAAACGGG
>HPV38_Beta_1020234_nt6692_L1_Tile|1
ATAATACCCGAAATACAAACTTTACAATCAGTGTATCCACTGAAAACGGGGGTGCTCAAG
>HPV38_Beta_1020234_nt6702_L1_Tile|2
AAATACAAACTTTACAATCAGTGTATCCACTGAAAACGGGGGTGCTCAAGAATATGATTC
>HPV38_Beta_1020234_nt6797_L1_Tile|1
AATACCAATTGTCATTTATATTGCAATTGTGTAAGGTTCCTTTAAATGCTGAAGTGCTGA
>HPV38_Beta_1020234_nt6802_L1_Tile|2
CAATTGTCATTTATATTGCAATTGTGTAAGGTTCCTTTAAATGCTGAAGTGCTGACACAG
>HPV38_Beta_1020234_nt6807_L1_Tile|1
GTCATTTATATTGCAATTGTGTAAGGTTCCTTTAAATGCTGAAGTGCTGACACAGATTAA
>HPV38_Beta_1020234_nt6897_L1_Tile|1
GCAATTAGGCTTTGTACCCACCCCAGACAATTCTGTACACGATACATATCGTTACATAAC
>HPV38_Beta_1020234_nt6907_L1_Tile|1
TTTGTACCCACCCCAGACAATTCTGTACACGATACATATCGTTACATAACATCTAAAGCA
>HPV38_Beta_1020234_nt6922_L1_Tile|2
GACAATTCTGTACACGATACATATCGTTACATAACATCTAAAGCAACTAAATGTCCAGAT
>HPV38_Beta_1020234_nt6927_L1_Tile|1
TTCTGTACACGATACATATCGTTACATAACATCTAAAGCAACTAAATGTCCAGATGCAGT
>HPV38_Beta_1020234_nt6937_L1_Tile|1
GATACATATCGTTACATAACATCTAAAGCAACTAAATGTCCAGATGCAGTGCCTGAAACA
>HPV38_Beta_1020234_nt6942_L1_Tile|2
ATATCGTTACATAACATCTAAAGCAACTAAATGTCCAGATGCAGTGCCTGAAACAGAAAA
>HPV38_Beta_1020234_nt7092_L1_Tile|1
ATTTTTATTCCAAGCAGGTTTACAAACAGCACGAACACGTGCTGTCAAACGGCCGTTAGT
>HPV38_Beta_1020234_nt7097_L1_Tile|1
TATTCCAAGCAGGTTTACAAACAGCACGAACACGTGCTGTCAAACGGCCGTTAGTAAGAA
>HPV38_Beta_1020234_nt7102_L1_Tile|2
CAAGCAGGTTTACAAACAGCACGAACACGTGCTGTCAAACGGCCGTTAGTAAGAAAATCT
>HPV38_Beta_1020234_nt7107_L1_Tile|1
AGGTTTACAAACAGCACGAACACGTGCTGTCAAACGGCCGTTAGTAAGAAAATCTTCCAA
>HPV38_Beta_1020234_nt7112_L1_Tile|1
TACAAACAGCACGAACACGTGCTGTCAAACGGCCGTTAGTAAGAAAATCTTCCAAATCTG
>HPV38_Beta_1020234_nt7127_L1_Tile|1
CACGTGCTGTCAAACGGCCGTTAGTAAGAAAATCTTCCAAATCTGTAAAACGCAAAAGGA
>HPV38_Beta_1020234_nt7135_L1_Tile|1
GTCAAACGGCCGTTAGTAAGAAAATCTTCCAAATCTGTAAAACGCAAAAGGACCCAGTAA
>HPV39_Alpha_333245_nt6004_L1_Tile|1
CCATTATATAATAGACAGGATGATACTGAAAACTCACCATTTTCATCAACCACCAATAAG
>HPV39_Alpha_333245_nt6009_L1_Tile|1
ATATAATAGACAGGATGATACTGAAAACTCACCATTTTCATCAACCACCAATAAGGACAG
>HPV39_Alpha_333245_nt6014_L1_Tile|1
ATAGACAGGATGATACTGAAAACTCACCATTTTCATCAACCACCAATAAGGACAGTAGGG
>HPV39_Alpha_333245_nt6419_L1_Tile|1
TTTGGAATCGTGGTGGTATGGTGGGTGACGCCATTCCTGCCCAATTGTATATTAAGGGCA
>HPV39_Alpha_333245_nt6424_L1_Tile|1
AATCGTGGTGGTATGGTGGGTGACGCCATTCCTGCCCAATTGTATATTAAGGGCACAGAT
>HPV39_Alpha_333245_nt6429_L1_Tile|1
TGGTGGTATGGTGGGTGACGCCATTCCTGCCCAATTGTATATTAAGGGCACAGATATACG
>HPV39_Alpha_333245_nt6439_L1_Tile|1
GTGGGTGACGCCATTCCTGCCCAATTGTATATTAAGGGCACAGATATACGTGCAAACCCC
>HPV39_Alpha_333245_nt6454_L1_Tile|1
CCTGCCCAATTGTATATTAAGGGCACAGATATACGTGCAAACCCCGGTAGTTCTGTATAC
>HPV39_Alpha_333245_nt6469_L1_Tile|1

ATTAAGGGCACAGATATACGTGCAAACCCCGGTAGTTCTGTATACTGCCCCTCTCCCAGC
>HPV39_Alpha_333245_nt6479_L1_Tile|1
CAGATATACGTGCAAACCCCGGTAGTTCTGTATACTGCCCCTCTCCCAGCGGTTCCATGG
>HPV39_Alpha_333245_nt6489_L1_Tile|1
TGCAAACCCCGGTAGTTCTGTATACTGCCCCTCTCCCAGCGGTTCCATGGTAACCTCTGA
>HPV39_Alpha_333245_nt6499_L1_Tile|1
GGTAGTTCTGTATACTGCCCCTCTCCCAGCGGTTCCATGGTAACCTCTGATTCCCAGTTA
>HPV39_Alpha_333245_nt6619_L1_Tile|1
AATCAATTATTTCTTACTGTTGTGGACACTACCCGTAGTACCAACTTTACATTATCTACC
>HPV39_Alpha_333245_nt6624_L1_Tile|1
ATTATTTCTTACTGTTGTGGACACTACCCGTAGTACCAACTTTACATTATCTACCTCTAT
>HPV39_Alpha_333245_nt6629_L1_Tile|1
TTCTTACTGTTGTGGACACTACCCGTAGTACCAACTTTACATTATCTACCTCTATAGAGT
>HPV39_Alpha_333245_nt6634_L1_Tile|1
ACTGTTGTGGACACTACCCGTAGTACCAACTTTACATTATCTACCTCTATAGAGTCTTCC
>HPV39_Alpha_333245_nt6639_L1_Tile|1
TGTGGACACTACCCGTAGTACCAACTTTACATTATCTACCTCTATAGAGTCTTCCATACC
>HPV39_Alpha_333245_nt6644_L1_Tile|1
ACACTACCCGTAGTACCAACTTTACATTATCTACCTCTATAGAGTCTTCCATACCTTCTA
>HPV39_Alpha_333245_nt6649_L1_Tile|1
ACCCGTAGTACCAACTTTACATTATCTACCTCTATAGAGTCTTCCATACCTTCTACATAT
>HPV39_Alpha_333245_nt6654_L1_Tile|1
TAGTACCAACTTTACATTATCTACCTCTATAGAGTCTTCCATACCTTCTACATATGATCC
>HPV39_Alpha_333245_nt6664_L1_Tile|1
TTTACATTATCTACCTCTATAGAGTCTTCCATACCTTCTACATATGATCCTTCTAAGTTT
>HPV39_Alpha_333245_nt6674_L1_Tile|1
CTACCTCTATAGAGTCTTCCATACCTTCTACATATGATCCTTCTAAGTTTAAGGAATATA
>HPV39_Alpha_333245_nt6679_L1_Tile|1
TCTATAGAGTCTTCCATACCTTCTACATATGATCCTTCTAAGTTTAAGGAATATACCAGG
>HPV39_Alpha_333245_nt6764_L1_Tile|1
TTATATTTCAACTGTGTACTGTCACATTAACAACTGATGTTATGTCTTATATTCACACTA
>HPV39_Alpha_333245_nt6779_L1_Tile|1
GTACTGTCACATTAACAACTGATGTTATGTCTTATATTCACACTATGAATTCCTCTATAT
>HPV39_Alpha_333245_nt6789_L1_Tile|1
ATTAACAACTGATGTTATGTCTTATATTCACACTATGAATTCCTCTATATTGGACAATTG
>HPV39_Alpha_333245_nt6844_L1_Tile|1
AATTGGAATTTTGCTGTAGCTCCTCCACCATCTGCCAGTTTGGTAGACACTTACAGATAC
>HPV39_Alpha_333245_nt6849_L1_Tile|1
GAATTTTGCTGTAGCTCCTCCACCATCTGCCAGTTTGGTAGACACTTACAGATACCTACA
>HPV39_Alpha_333245_nt6854_L1_Tile|1
TTGCTGTAGCTCCTCCACCATCTGCCAGTTTGGTAGACACTTACAGATACCTACAGTCTG
>HPV39_Alpha_333245_nt6869_L1_Tile|1
CACCATCTGCCAGTTTGGTAGACACTTACAGATACCTACAGTCTGCAGCCATTACATGTC
>HPV39_Alpha_333245_nt7054_L1_Tile|1
CAGGCCAGGGTCCGCAGGCGCCCTACTATAGGTCCCCGAAAGCGGCCTGCTGCATCCACT
>HPV39_Alpha_333245_nt7059_L1_Tile|1
CAGGGTCCGCAGGCGCCCTACTATAGGTCCCCGAAAGCGGCCTGCTGCATCCACTTCCTC
>HPV40_Alpha_397014_nt5885_L1_Tile|1
GCTGGTAGTGCCAGGTTACTGACTATAGGACATCCATACTTTGAGTTAAAAAAACCCAAT
>HPV40_Alpha_397014_nt5890_L1_Tile|1
TAGTGCCAGGTTACTGACTATAGGACATCCATACTTTGAGTTAAAAAAACCCAATGGTGA
>HPV40_Alpha_397014_nt5900_L1_Tile|1
TTACTGACTATAGGACATCCATACTTTGAGTTAAAAAAACCCAATGGTGACATTTCAGTG
>HPV40_Alpha_397014_nt5905_L1_Tile|1
GACTATAGGACATCCATACTTTGAGTTAAAAAAACCCAATGGTGACATTTCAGTGCCTAA

>HPV40_Alpha_397014_nt6145_L1_Tile|1
TAAGGATGAGGATGTGGAAAACTCATCTGCCTATGGCACAGGTCCGGGGCAGGATAGTAG
>HPV40_Alpha_397014_nt6160_L1_Tile|1
GGAAAACTCATCTGCCTATGGCACAGGTCCGGGGCAGGATAGTAGGGAAAATGTAGCTAT
>HPV40_Alpha_397014_nt6565_L1_Tile|1
AGGTACTACTGGTGATAGTGTCCCAACTGACTTATATATAACAGGTACATCTGGTCGGAC
>HPV40_Alpha_397014_nt6575_L1_Tile|1
GGTGATAGTGTCCCAACTGACTTATATATAACAGGTACATCTGGTCGGACTCCTATTGCA
>HPV40_Alpha_397014_nt6795_L1_Tile|1
ATTTAACCTTATGTGCTGCCACACAGTCCCCCACACCAACCCCATATAATAACAGTAATT
>HPV40_Alpha_397014_nt6800_L1_Tile|1
ACCTTATGTGCTGCCACACAGTCCCCCACACCAACCCCATATAATAACAGTAATTTCAAG
>HPV40_Alpha_397014_nt6810_L1_Tile|1
CTGCCACACAGTCCCCCACACCAACCCCATATAATAACAGTAATTTCAAGGAATATTTGC
>HPV40_Alpha_397014_nt6990_L1_Tile|1
AAATTGCTCCTCCAGCCTCTGCATCCTTAGAGGATACATATAGGTTCCTTACCAACAAGG
>HPV40_Alpha_397014_nt6995_L1_Tile|1
GCTCCTCCAGCCTCTGCATCCTTAGAGGATACATATAGGTTCCTTACCAACAAGGCTATT
>HPV40_Alpha_397014_nt7000_L1_Tile|1
TCCAGCCTCTGCATCCTTAGAGGATACATATAGGTTCCTTACCAACAAGGCTATTGCCTG
>HPV40_Alpha_397014_nt7005_L1_Tile|1
CCTCTGCATCCTTAGAGGATACATATAGGTTCCTTACCAACAAGGCTATTGCCTGTCAGC
>HPV40_Alpha_397014_nt7010_L1_Tile|1
GCATCCTTAGAGGATACATATAGGTTCCTTACCAACAAGGCTATTGCCTGTCAGCGCGAT
>HPV41_Nu_9626041_nt5337_L1_Tile|1
ATGACAGGCCTTCAGTATTTATTTTTAGCGATGATGGCACTCACATTGTCTATCCTACTA
>HPV41_Nu_9626041_nt5342_L1_Tile|2
AGGCCTTCAGTATTTATTTTTAGCGATGATGGCACTCACATTGTCTATCCTACTAGCACA
>HPV41_Nu_9626041_nt5357_L1_Tile|1
ATTTTTAGCGATGATGGCACTCACATTGTCTATCCTACTAGCACAACAGCCACCACCCCA
>HPV41_Nu_9626041_nt5367_L1_Tile|1
ATGATGGCACTCACATTGTCTATCCTACTAGCACAACAGCCACCACCCCACTCGTGCCTG
>HPV41_Nu_9626041_nt5372_L1_Tile|1
GGCACTCACATTGTCTATCCTACTAGCACAACAGCCACCACCCCACTCGTGCCTGCACAG
>HPV41_Nu_9626041_nt5397_L1_Tile|1
GCACAACAGCCACCACCCCACTCGTGCCTGCACAGCCCAGCGATGTGCCCTACATTGTTG
>HPV41_Nu_9626041_nt5407_L1_Tile|1
CACCACCCCACTCGTGCCTGCACAGCCCAGCGATGTGCCCTACATTGTTGTTGACTTGTA
>HPV41_Nu_9626041_nt5427_L1_Tile|1
CACAGCCCAGCGATGTGCCCTACATTGTTGTTGACTTGTATAGTGGAAGTATGGATTATG
>HPV41_Nu_9626041_nt5432_L1_Tile|1
CCCAGCGATGTGCCCTACATTGTTGTTGACTTGTATAGTGGAAGTATGGATTATGATATA
>HPV41_Nu_9626041_nt5442_L1_Tile|2
TGCCCTACATTGTTGTTGACTTGTATAGTGGAAGTATGGATTATGATATACATCCTAGCC
>HPV41_Nu_9626041_nt5447_L1_Tile|1
TACATTGTTGTTGACTTGTATAGTGGAAGTATGGATTATGATATACATCCTAGCCTGTTG
>HPV41_Nu_9626041_nt5452_L1_Tile|1
TGTTGTTGACTTGTATAGTGGAAGTATGGATTATGATATACATCCTAGCCTGTTGCGCAG
>HPV41_Nu_9626041_nt5467_L1_Tile|1
TAGTGGAAGTATGGATTATGATATACATCCTAGCCTGTTGCGCAGGAAACGTAAAAAACG
>HPV41_Nu_9626041_nt5472_L1_Tile|1
GAAGTATGGATTATGATATACATCCTAGCCTGTTGCGCAGGAAACGTAAAAAACGCAAAC
>HPV41_Nu_9626041_nt5522_L1_Tile|2
AAACGCAAACGTGTTTATTTTTCAGATGGCCGTGTGGCTTCCAGGCCCAAATAGATTTTA
>HPV41_Nu_9626041_nt5527_L1_Tile|1

CAAACGTGTTTATTTTTCAGATGGCCGTGTGGCTTCCAGGCCCAAATAGATTTTACTTAC
>HPV41_Nu_9626041_nt5537_L1_Tile|1
TATTTTTCAGATGGCCGTGTGGCTTCCAGGCCCAAATAGATTTTACTTACCCCCTCAACC
>HPV41_Nu_9626041_nt5542_L1_Tile|2
TTCAGATGGCCGTGTGGCTTCCAGGCCCAAATAGATTTTACTTACCCCCTCAACCTATAC
>HPV41_Nu_9626041_nt5552_L1_Tile|1
CGTGTGGCTTCCAGGCCCAAATAGATTTTACTTACCCCCTCAACCTATACAACGGACATT
>HPV41_Nu_9626041_nt5557_L1_Tile|1
GGCTTCCAGGCCCAAATAGATTTTACTTACCCCCTCAACCTATACAACGGACATTGAACA
>HPV41_Nu_9626041_nt5572_L1_Tile|1
ATAGATTTTACTTACCCCCTCAACCTATACAACGGACATTGAACACAGAGGAATACGTGA
>HPV41_Nu_9626041_nt5577_L1_Tile|1
TTTTACTTACCCCCTCAACCTATACAACGGACATTGAACACAGAGGAATACGTGAGACGC
>HPV41_Nu_9626041_nt5587_L1_Tile|1
CCCCTCAACCTATACAACGGACATTGAACACAGAGGAATACGTGAGACGCACCAGTACTT
>HPV41_Nu_9626041_nt5612_L1_Tile|1
GAACACAGAGGAATACGTGAGACGCACCAGTACTTTCCTCCATGCTGCCACTGACCGTTT
>HPV41_Nu_9626041_nt5622_L1_Tile|2
GAATACGTGAGACGCACCAGTACTTTCCTCCATGCTGCCACTGACCGTTTGCTTACTGTT
>HPV41_Nu_9626041_nt5642_L1_Tile|3
TACTTTCCTCCATGCTGCCACTGACCGTTTGCTTACTGTTGGACATCCATTTTACAATAT
>HPV41_Nu_9626041_nt5647_L1_Tile|1
TCCTCCATGCTGCCACTGACCGTTTGCTTACTGTTGGACATCCATTTTACAATATTACTA
>HPV41_Nu_9626041_nt5657_L1_Tile|1
TGCCACTGACCGTTTGCTTACTGTTGGACATCCATTTTACAATATTACTAATGCGGATGG
>HPV41_Nu_9626041_nt5662_L1_Tile|2
CTGACCGTTTGCTTACTGTTGGACATCCATTTTACAATATTACTAATGCGGATGGCAAAG
>HPV41_Nu_9626041_nt5672_L1_Tile|1
GCTTACTGTTGGACATCCATTTTACAATATTACTAATGCGGATGGCAAAGAGGTGGTCCC
>HPV41_Nu_9626041_nt5682_L1_Tile|2
GGACATCCATTTTACAATATTACTAATGCGGATGGCAAAGAGGTGGTCCCTAAAGTTTCC
>HPV41_Nu_9626041_nt5687_L1_Tile|1
TCCATTTTACAATATTACTAATGCGGATGGCAAAGAGGTGGTCCCTAAAGTTTCCTCTAA
>HPV41_Nu_9626041_nt5692_L1_Tile|1
TTTACAATATTACTAATGCGGATGGCAAAGAGGTGGTCCCTAAAGTTTCCTCTAATCAGT
>HPV41_Nu_9626041_nt5697_L1_Tile|1
AATATTACTAATGCGGATGGCAAAGAGGTGGTCCCTAAAGTTTCCTCTAATCAGTTCAGG
>HPV41_Nu_9626041_nt5707_L1_Tile|1
ATGCGGATGGCAAAGAGGTGGTCCCTAAAGTTTCCTCTAATCAGTTCAGGGCCTTCCGTG
>HPV41_Nu_9626041_nt5722_L1_Tile|2
AGGTGGTCCCTAAAGTTTCCTCTAATCAGTTCAGGGCCTTCCGTGTCCGTTTCCCAAATC
>HPV41_Nu_9626041_nt5727_L1_Tile|1
GTCCCTAAAGTTTCCTCTAATCAGTTCAGGGCCTTCCGTGTCCGTTTCCCAAATCCCAAT
>HPV41_Nu_9626041_nt5732_L1_Tile|1
TAAAGTTTCCTCTAATCAGTTCAGGGCCTTCCGTGTCCGTTTCCCAAATCCCAATACCTT
>HPV41_Nu_9626041_nt5737_L1_Tile|1
TTTCCTCTAATCAGTTCAGGGCCTTCCGTGTCCGTTTCCCAAATCCCAATACCTTTGCAT
>HPV41_Nu_9626041_nt5742_L1_Tile|2
TCTAATCAGTTCAGGGCCTTCCGTGTCCGTTTCCCAAATCCCAATACCTTTGCATTTTGT
>HPV41_Nu_9626041_nt5747_L1_Tile|1
TCAGTTCAGGGCCTTCCGTGTCCGTTTCCCAAATCCCAATACCTTTGCATTTTGTGATAA
>HPV41_Nu_9626041_nt5757_L1_Tile|1
GCCTTCCGTGTCCGTTTCCCAAATCCCAATACCTTTGCATTTTGTGATAAGTCCCTTTTT
>HPV41_Nu_9626041_nt5762_L1_Tile|2
CCGTGTCCGTTTCCCAAATCCCAATACCTTTGCATTTTGTGATAAGTCCCTTTTTAACCC

>HPV41_Nu_9626041_nt5772_L1_Tile|1
TTCCCAAATCCCAATACCTTTGCATTTTGTGATAAGTCCCTTTTTAACCCTGACAAGGAG
>HPV41_Nu_9626041_nt5777_L1_Tile|1
AAATCCCAATACCTTTGCATTTTGTGATAAGTCCCTTTTTAACCCTGACAAGGAGCGTCT
>HPV41_Nu_9626041_nt5782_L1_Tile|2
CCAATACCTTTGCATTTTGTGATAAGTCCCTTTTTAACCCTGACAAGGAGCGTCTGGTCT
>HPV41_Nu_9626041_nt5792_L1_Tile|1
TGCATTTTGTGATAAGTCCCTTTTTAACCCTGACAAGGAGCGTCTGGTCTGGGGTATTCG
>HPV41_Nu_9626041_nt5852_L1_Tile|1
TGGGATTGAGGTTTCTAGGGGACAGCCCTTAGGTATTGGTGTAACAGGGAACCCTTTTTT
>HPV41_Nu_9626041_nt5857_L1_Tile|1
TTGAGGTTTCTAGGGGACAGCCCTTAGGTATTGGTGTAACAGGGAACCCTTTTTTTAATA
>HPV41_Nu_9626041_nt5862_L1_Tile|2
GTTTCTAGGGGACAGCCCTTAGGTATTGGTGTAACAGGGAACCCTTTTTTTAATAAGTTT
>HPV41_Nu_9626041_nt5882_L1_Tile|2
AGGTATTGGTGTAACAGGGAACCCTTTTTTTAATAAGTTTGATGATGCTGAAAATCCCTA
>HPV41_Nu_9626041_nt5887_L1_Tile|1
TTGGTGTAACAGGGAACCCTTTTTTTAATAAGTTTGATGATGCTGAAAATCCCTACAATG
>HPV41_Nu_9626041_nt5892_L1_Tile|1
GTAACAGGGAACCCTTTTTTTAATAAGTTTGATGATGCTGAAAATCCCTACAATGGTATA
>HPV41_Nu_9626041_nt5897_L1_Tile|1
AGGGAACCCTTTTTTTAATAAGTTTGATGATGCTGAAAATCCCTACAATGGTATAAACAA
>HPV41_Nu_9626041_nt5902_L1_Tile|2
ACCCTTTTTTTAATAAGTTTGATGATGCTGAAAATCCCTACAATGGTATAAACAAAAATA
>HPV41_Nu_9626041_nt5907_L1_Tile|1
TTTTTTAATAAGTTTGATGATGCTGAAAATCCCTACAATGGTATAAACAAAAATAACATT
>HPV41_Nu_9626041_nt5912_L1_Tile|1
TAATAAGTTTGATGATGCTGAAAATCCCTACAATGGTATAAACAAAAATAACATTACTGA
>HPV41_Nu_9626041_nt5917_L1_Tile|1
AGTTTGATGATGCTGAAAATCCCTACAATGGTATAAACAAAAATAACATTACTGACCAAG
>HPV41_Nu_9626041_nt5922_L1_Tile|2
GATGATGCTGAAAATCCCTACAATGGTATAAACAAAAATAACATTACTGACCAAGGTTCA
>HPV41_Nu_9626041_nt5927_L1_Tile|1
TGCTGAAAATCCCTACAATGGTATAAACAAAAATAACATTACTGACCAAGGTTCAGACTC
>HPV41_Nu_9626041_nt5932_L1_Tile|1
AAAATCCCTACAATGGTATAAACAAAAATAACATTACTGACCAAGGTTCAGACTCAAGGT
>HPV41_Nu_9626041_nt5937_L1_Tile|1
CCCTACAATGGTATAAACAAAAATAACATTACTGACCAAGGTTCAGACTCAAGGTTGAGC
>HPV41_Nu_9626041_nt5942_L1_Tile|2
CAATGGTATAAACAAAAATAACATTACTGACCAAGGTTCAGACTCAAGGTTGAGCATTGC
>HPV41_Nu_9626041_nt5982_L1_Tile|2
GACTCAAGGTTGAGCATTGCATTTGACCCTAAGCAAACACAGCTGCTGATAGTAGGTGCT
>HPV41_Nu_9626041_nt5987_L1_Tile|1
AAGGTTGAGCATTGCATTTGACCCTAAGCAAACACAGCTGCTGATAGTAGGTGCTAAACC
>HPV41_Nu_9626041_nt5997_L1_Tile|1
ATTGCATTTGACCCTAAGCAAACACAGCTGCTGATAGTAGGTGCTAAACCTGCAAAGGGT
>HPV41_Nu_9626041_nt6002_L1_Tile|2
ATTTGACCCTAAGCAAACACAGCTGCTGATAGTAGGTGCTAAACCTGCAAAGGGTGAGTA
>HPV41_Nu_9626041_nt6017_L1_Tile|1
AACACAGCTGCTGATAGTAGGTGCTAAACCTGCAAAGGGTGAGTACTGGGACGTTGCTGC
>HPV41_Nu_9626041_nt6022_L1_Tile|2
AGCTGCTGATAGTAGGTGCTAAACCTGCAAAGGGTGAGTACTGGGACGTTGCTGCAACAT
>HPV41_Nu_9626041_nt6027_L1_Tile|1
CTGATAGTAGGTGCTAAACCTGCAAAGGGTGAGTACTGGGACGTTGCTGCAACATGTGAA
>HPV41_Nu_9626041_nt6032_L1_Tile|1

AGTAGGTGCTAAACCTGCAAAGGGTGAGTACTGGGACGTTGCTGCAACATGTGAAAACCC
>HPV41_Nu_9626041_nt6057_L1_Tile|1
GAGTACTGGGACGTTGCTGCAACATGTGAAAACCCTCCACTGACCAAAGCAGATGACAAA
>HPV41_Nu_9626041_nt6077_L1_Tile|1
AACATGTGAAAACCCTCCACTGACCAAAGCAGATGACAAATGTCCTGCTCTAGAGCTTAA
>HPV41_Nu_9626041_nt6082_L1_Tile|2
GTGAAAACCCTCCACTGACCAAAGCAGATGACAAATGTCCTGCTCTAGAGCTTAAGTCCT
>HPV41_Nu_9626041_nt6087_L1_Tile|1
AACCCTCCACTGACCAAAGCAGATGACAAATGTCCTGCTCTAGAGCTTAAGTCCTCATAC
>HPV41_Nu_9626041_nt6092_L1_Tile|1
TCCACTGACCAAAGCAGATGACAAATGTCCTGCTCTAGAGCTTAAGTCCTCATACATTGA
>HPV41_Nu_9626041_nt6102_L1_Tile|2
AAAGCAGATGACAAATGTCCTGCTCTAGAGCTTAAGTCCTCATACATTGAGGATGCAGAC
>HPV41_Nu_9626041_nt6112_L1_Tile|1
ACAAATGTCCTGCTCTAGAGCTTAAGTCCTCATACATTGAGGATGCAGACATGAGTGACA
>HPV41_Nu_9626041_nt6122_L1_Tile|2
TGCTCTAGAGCTTAAGTCCTCATACATTGAGGATGCAGACATGAGTGACATAGGCCTGGG
>HPV41_Nu_9626041_nt6127_L1_Tile|1
TAGAGCTTAAGTCCTCATACATTGAGGATGCAGACATGAGTGACATAGGCCTGGGAAACT
>HPV41_Nu_9626041_nt6132_L1_Tile|1
CTTAAGTCCTCATACATTGAGGATGCAGACATGAGTGACATAGGCCTGGGAAACTTGAAT
>HPV41_Nu_9626041_nt6137_L1_Tile|1
GTCCTCATACATTGAGGATGCAGACATGAGTGACATAGGCCTGGGAAACTTGAATTTTTC
>HPV41_Nu_9626041_nt6147_L1_Tile|1
ATTGAGGATGCAGACATGAGTGACATAGGCCTGGGAAACTTGAATTTTTCTACACTGCAG
>HPV41_Nu_9626041_nt6152_L1_Tile|1
GGATGCAGACATGAGTGACATAGGCCTGGGAAACTTGAATTTTTCTACACTGCAGAGAAA
>HPV41_Nu_9626041_nt6157_L1_Tile|1
CAGACATGAGTGACATAGGCCTGGGAAACTTGAATTTTTCTACACTGCAGAGAAACAAAT
>HPV41_Nu_9626041_nt6162_L1_Tile|2
ATGAGTGACATAGGCCTGGGAAACTTGAATTTTTCTACACTGCAGAGAAACAAATCCGAT
>HPV41_Nu_9626041_nt6167_L1_Tile|1
TGACATAGGCCTGGGAAACTTGAATTTTTCTACACTGCAGAGAAACAAATCCGATGCCCC
>HPV41_Nu_9626041_nt6172_L1_Tile|1
TAGGCCTGGGAAACTTGAATTTTTCTACACTGCAGAGAAACAAATCCGATGCCCCATTAG
>HPV41_Nu_9626041_nt6177_L1_Tile|1
CTGGGAAACTTGAATTTTTCTACACTGCAGAGAAACAAATCCGATGCCCCATTAGATATT
>HPV41_Nu_9626041_nt6182_L1_Tile|2
AAACTTGAATTTTTCTACACTGCAGAGAAACAAATCCGATGCCCCATTAGATATTGTGGA
>HPV41_Nu_9626041_nt6187_L1_Tile|1
TGAATTTTTCTACACTGCAGAGAAACAAATCCGATGCCCCATTAGATATTGTGGATTCTA
>HPV41_Nu_9626041_nt6192_L1_Tile|1
TTTTCTACACTGCAGAGAAACAAATCCGATGCCCCATTAGATATTGTGGATTCTATCTGC
>HPV41_Nu_9626041_nt6197_L1_Tile|1
TACACTGCAGAGAAACAAATCCGATGCCCCATTAGATATTGTGGATTCTATCTGCAAATA
>HPV41_Nu_9626041_nt6202_L1_Tile|2
TGCAGAGAAACAAATCCGATGCCCCATTAGATATTGTGGATTCTATCTGCAAATATCCTG
>HPV41_Nu_9626041_nt6207_L1_Tile|1
AGAAACAAATCCGATGCCCCATTAGATATTGTGGATTCTATCTGCAAATATCCTGACTAC
>HPV41_Nu_9626041_nt6257_L1_Tile|1
TCCTGACTACCTGCAAATGATAGAAGAACTATATGGAGACCACATGTTTTTCTATGTGCG
>HPV41_Nu_9626041_nt6267_L1_Tile|1
CTGCAAATGATAGAAGAACTATATGGAGACCACATGTTTTTCTATGTGCGGCGTGAAGCT
>HPV41_Nu_9626041_nt6272_L1_Tile|1
AATGATAGAAGAACTATATGGAGACCACATGTTTTTCTATGTGCGGCGTGAAGCTCTGTA

>HPV41_Nu_9626041_nt6277_L1_Tile|1
TAGAAGAACTATATGGAGACCACATGTTTTTCTATGTGCGGCGTGAAGCTCTGTATGCTA
>HPV41_Nu_9626041_nt6282_L1_Tile|2
GAACTATATGGAGACCACATGTTTTTCTATGTGCGGCGTGAAGCTCTGTATGCTAGGCAT
>HPV41_Nu_9626041_nt6287_L1_Tile|1
ATATGGAGACCACATGTTTTTCTATGTGCGGCGTGAAGCTCTGTATGCTAGGCATATAAT
>HPV41_Nu_9626041_nt6302_L1_Tile|2
GTTTTTCTATGTGCGGCGTGAAGCTCTGTATGCTAGGCATATAATGCAACACGCGGGCAA
>HPV41_Nu_9626041_nt6307_L1_Tile|1
TCTATGTGCGGCGTGAAGCTCTGTATGCTAGGCATATAATGCAACACGCGGGCAAGATGG
>HPV41_Nu_9626041_nt6317_L1_Tile|1
GCGTGAAGCTCTGTATGCTAGGCATATAATGCAACACGCGGGCAAGATGGATGCTGAGCA
>HPV41_Nu_9626041_nt6322_L1_Tile|2
AAGCTCTGTATGCTAGGCATATAATGCAACACGCGGGCAAGATGGATGCTGAGCAATTTC
>HPV41_Nu_9626041_nt6327_L1_Tile|1
CTGTATGCTAGGCATATAATGCAACACGCGGGCAAGATGGATGCTGAGCAATTTCCCACT
>HPV41_Nu_9626041_nt6332_L1_Tile|1
TGCTAGGCATATAATGCAACACGCGGGCAAGATGGATGCTGAGCAATTTCCCACTTCTCT
>HPV41_Nu_9626041_nt6337_L1_Tile|1
GGCATATAATGCAACACGCGGGCAAGATGGATGCTGAGCAATTTCCCACTTCTCTGTACA
>HPV41_Nu_9626041_nt6342_L1_Tile|2
ATAATGCAACACGCGGGCAAGATGGATGCTGAGCAATTTCCCACTTCTCTGTACATAGAC
>HPV41_Nu_9626041_nt6352_L1_Tile|1
ACGCGGGCAAGATGGATGCTGAGCAATTTCCCACTTCTCTGTACATAGACTCCTCTGTAG
>HPV41_Nu_9626041_nt6357_L1_Tile|1
GGCAAGATGGATGCTGAGCAATTTCCCACTTCTCTGTACATAGACTCCTCTGTAGAAGGT
>HPV41_Nu_9626041_nt6362_L1_Tile|2
GATGGATGCTGAGCAATTTCCCACTTCTCTGTACATAGACTCCTCTGTAGAAGGTGAGAA
>HPV41_Nu_9626041_nt6367_L1_Tile|1
ATGCTGAGCAATTTCCCACTTCTCTGTACATAGACTCCTCTGTAGAAGGTGAGAAATTAA
>HPV41_Nu_9626041_nt6372_L1_Tile|1
GAGCAATTTCCCACTTCTCTGTACATAGACTCCTCTGTAGAAGGTGAGAAATTAAATTCC
>HPV41_Nu_9626041_nt6377_L1_Tile|1
ATTTCCCACTTCTCTGTACATAGACTCCTCTGTAGAAGGTGAGAAATTAAATTCCTTGCA
>HPV41_Nu_9626041_nt6387_L1_Tile|1
TCTCTGTACATAGACTCCTCTGTAGAAGGTGAGAAATTAAATTCCTTGCAGCGCACTGAT
>HPV41_Nu_9626041_nt6392_L1_Tile|1
GTACATAGACTCCTCTGTAGAAGGTGAGAAATTAAATTCCTTGCAGCGCACTGATAGGTA
>HPV41_Nu_9626041_nt6397_L1_Tile|1
TAGACTCCTCTGTAGAAGGTGAGAAATTAAATTCCTTGCAGCGCACTGATAGGTATTTCA
>HPV41_Nu_9626041_nt6402_L1_Tile|2
TCCTCTGTAGAAGGTGAGAAATTAAATTCCTTGCAGCGCACTGATAGGTATTTCATGACA
>HPV41_Nu_9626041_nt6407_L1_Tile|1
TGTAGAAGGTGAGAAATTAAATTCCTTGCAGCGCACTGATAGGTATTTCATGACACCCAG
>HPV41_Nu_9626041_nt6417_L1_Tile|1
GAGAAATTAAATTCCTTGCAGCGCACTGATAGGTATTTCATGACACCCAGCGGCTCCCTG
>HPV41_Nu_9626041_nt6422_L1_Tile|2
ATTAAATTCCTTGCAGCGCACTGATAGGTATTTCATGACACCCAGCGGCTCCCTGGTAGC
>HPV41_Nu_9626041_nt6427_L1_Tile|1
ATTCCTTGCAGCGCACTGATAGGTATTTCATGACACCCAGCGGCTCCCTGGTAGCTACTG
>HPV41_Nu_9626041_nt6447_L1_Tile|1
AGGTATTTCATGACACCCAGCGGCTCCCTGGTAGCTACTGAGCAGCAGCTGTTTAACAGG
>HPV41_Nu_9626041_nt6457_L1_Tile|1
TGACACCCAGCGGCTCCCTGGTAGCTACTGAGCAGCAGCTGTTTAACAGGCCCTTTTGGC
>HPV41_Nu_9626041_nt6517_L1_Tile|1

TGCAGAGATCCCAGGGCCATAACAATGGCATACTGTGGCACAACGAGGCCTTTGTAACAT
>HPV41_Nu_9626041_nt6522_L1_Tile|2
AGATCCCAGGGCCATAACAATGGCATACTGTGGCACAACGAGGCCTTTGTAACATTGGTT
>HPV41_Nu_9626041_nt6547_L1_Tile|1
TACTGTGGCACAACGAGGCCTTTGTAACATTGGTTGACACTACCAGGGGAACTAACTTTA
>HPV41_Nu_9626041_nt6552_L1_Tile|1
TGGCACAACGAGGCCTTTGTAACATTGGTTGACACTACCAGGGGAACTAACTTTACCATC
>HPV41_Nu_9626041_nt6557_L1_Tile|1
CAACGAGGCCTTTGTAACATTGGTTGACACTACCAGGGGAACTAACTTTACCATCAGTGT
>HPV41_Nu_9626041_nt6567_L1_Tile|1
TTTGTAACATTGGTTGACACTACCAGGGGAACTAACTTTACCATCAGTGTTCCTGAGGGG
>HPV41_Nu_9626041_nt6577_L1_Tile|1
TGGTTGACACTACCAGGGGAACTAACTTTACCATCAGTGTTCCTGAGGGGGATGCTTCTT
>HPV41_Nu_9626041_nt6582_L1_Tile|2
GACACTACCAGGGGAACTAACTTTACCATCAGTGTTCCTGAGGGGGATGCTTCTTCATAT
>HPV41_Nu_9626041_nt6587_L1_Tile|1
TACCAGGGGAACTAACTTTACCATCAGTGTTCCTGAGGGGGATGCTTCTTCATATAACAA
>HPV41_Nu_9626041_nt6592_L1_Tile|1
GGGGAACTAACTTTACCATCAGTGTTCCTGAGGGGGATGCTTCTTCATATAACAATTCTA
>HPV41_Nu_9626041_nt6597_L1_Tile|1
ACTAACTTTACCATCAGTGTTCCTGAGGGGGATGCTTCTTCATATAACAATTCTAAGTTT
>HPV41_Nu_9626041_nt6602_L1_Tile|2
CTTTACCATCAGTGTTCCTGAGGGGGATGCTTCTTCATATAACAATTCTAAGTTTTTTGA
>HPV41_Nu_9626041_nt6607_L1_Tile|1
CCATCAGTGTTCCTGAGGGGGATGCTTCTTCATATAACAATTCTAAGTTTTTTGAGTTTT
>HPV41_Nu_9626041_nt6627_L1_Tile|1
GATGCTTCTTCATATAACAATTCTAAGTTTTTTGAGTTTTTAAGGCACACCGAGGAGTTT
>HPV41_Nu_9626041_nt6632_L1_Tile|1
TTCTTCATATAACAATTCTAAGTTTTTTGAGTTTTTAAGGCACACCGAGGAGTTTCAGCT
>HPV41_Nu_9626041_nt6637_L1_Tile|1
CATATAACAATTCTAAGTTTTTTGAGTTTTTAAGGCACACCGAGGAGTTTCAGCTTGCCT
>HPV41_Nu_9626041_nt6642_L1_Tile|2
AACAATTCTAAGTTTTTTGAGTTTTTAAGGCACACCGAGGAGTTTCAGCTTGCCTTTATT
>HPV41_Nu_9626041_nt6657_L1_Tile|1
TTTGAGTTTTTAAGGCACACCGAGGAGTTTCAGCTTGCCTTTATTCTACAGCTGTGTAAG
>HPV41_Nu_9626041_nt6662_L1_Tile|2
GTTTTTAAGGCACACCGAGGAGTTTCAGCTTGCCTTTATTCTACAGCTGTGTAAGGTAGA
>HPV41_Nu_9626041_nt6667_L1_Tile|1
TAAGGCACACCGAGGAGTTTCAGCTTGCCTTTATTCTACAGCTGTGTAAGGTAGACCTTA
>HPV41_Nu_9626041_nt6672_L1_Tile|1
CACACCGAGGAGTTTCAGCTTGCCTTTATTCTACAGCTGTGTAAGGTAGACCTTACCCCT
>HPV41_Nu_9626041_nt6677_L1_Tile|1
CGAGGAGTTTCAGCTTGCCTTTATTCTACAGCTGTGTAAGGTAGACCTTACCCCTGAGAA
>HPV41_Nu_9626041_nt6687_L1_Tile|1
CAGCTTGCCTTTATTCTACAGCTGTGTAAGGTAGACCTTACCCCTGAGAATTTGGCTTAC
>HPV41_Nu_9626041_nt6692_L1_Tile|1
TGCCTTTATTCTACAGCTGTGTAAGGTAGACCTTACCCCTGAGAATTTGGCTTACATACA
>HPV41_Nu_9626041_nt6697_L1_Tile|1
TTATTCTACAGCTGTGTAAGGTAGACCTTACCCCTGAGAATTTGGCTTACATACACACAA
>HPV41_Nu_9626041_nt6702_L1_Tile|2
CTACAGCTGTGTAAGGTAGACCTTACCCCTGAGAATTTGGCTTACATACACACAATGGAT
>HPV41_Nu_9626041_nt6707_L1_Tile|1
GCTGTGTAAGGTAGACCTTACCCCTGAGAATTTGGCTTACATACACACAATGGATCCATC
>HPV41_Nu_9626041_nt6712_L1_Tile|1
GTAAGGTAGACCTTACCCCTGAGAATTTGGCTTACATACACACAATGGATCCATCCATTA

>HPV41_Nu_9626041_nt6722_L1_Tile|2
CCTTACCCCTGAGAATTTGGCTTACATACACACAATGGATCCATCCATTATTGAAGACTG
>HPV41_Nu_9626041_nt6732_L1_Tile|1
GAGAATTTGGCTTACATACACACAATGGATCCATCCATTATTGAAGACTGGCATTTAGCT
>HPV41_Nu_9626041_nt6742_L1_Tile|2
CTTACATACACACAATGGATCCATCCATTATTGAAGACTGGCATTTAGCTGTCACTTCAC
>HPV41_Nu_9626041_nt6747_L1_Tile|1
ATACACACAATGGATCCATCCATTATTGAAGACTGGCATTTAGCTGTCACTTCACCTCCC
>HPV41_Nu_9626041_nt6752_L1_Tile|1
CACAATGGATCCATCCATTATTGAAGACTGGCATTTAGCTGTCACTTCACCTCCCAATTC
>HPV41_Nu_9626041_nt6757_L1_Tile|1
TGGATCCATCCATTATTGAAGACTGGCATTTAGCTGTCACTTCACCTCCCAATTCTGTAC
>HPV41_Nu_9626041_nt6762_L1_Tile|2
CCATCCATTATTGAAGACTGGCATTTAGCTGTCACTTCACCTCCCAATTCTGTACTGGAG
>HPV41_Nu_9626041_nt6767_L1_Tile|1
CATTATTGAAGACTGGCATTTAGCTGTCACTTCACCTCCCAATTCTGTACTGGAGGATCA
>HPV41_Nu_9626041_nt6772_L1_Tile|1
TTGAAGACTGGCATTTAGCTGTCACTTCACCTCCCAATTCTGTACTGGAGGATCATTATA
>HPV41_Nu_9626041_nt6777_L1_Tile|1
GACTGGCATTTAGCTGTCACTTCACCTCCCAATTCTGTACTGGAGGATCATTATAGGTAC
>HPV41_Nu_9626041_nt6782_L1_Tile|2
GCATTTAGCTGTCACTTCACCTCCCAATTCTGTACTGGAGGATCATTATAGGTACATACT
>HPV41_Nu_9626041_nt6787_L1_Tile|1
TAGCTGTCACTTCACCTCCCAATTCTGTACTGGAGGATCATTATAGGTACATACTGTCCA
>HPV41_Nu_9626041_nt6792_L1_Tile|1
GTCACTTCACCTCCCAATTCTGTACTGGAGGATCATTATAGGTACATACTGTCCATTGCA
>HPV41_Nu_9626041_nt6797_L1_Tile|1
TTCACCTCCCAATTCTGTACTGGAGGATCATTATAGGTACATACTGTCCATTGCAACTAA
>HPV41_Nu_9626041_nt6802_L1_Tile|2
CTCCCAATTCTGTACTGGAGGATCATTATAGGTACATACTGTCCATTGCAACTAAATGTC
>HPV41_Nu_9626041_nt6807_L1_Tile|1
AATTCTGTACTGGAGGATCATTATAGGTACATACTGTCCATTGCAACTAAATGTCCCTCT
>HPV41_Nu_9626041_nt6812_L1_Tile|1
TGTACTGGAGGATCATTATAGGTACATACTGTCCATTGCAACTAAATGTCCCTCTAAGGA
>HPV41_Nu_9626041_nt6822_L1_Tile|2
GATCATTATAGGTACATACTGTCCATTGCAACTAAATGTCCCTCTAAGGATGCAGATGAT
>HPV41_Nu_9626041_nt6832_L1_Tile|1
GGTACATACTGTCCATTGCAACTAAATGTCCCTCTAAGGATGCAGATGATACCTCCACTG
>HPV41_Nu_9626041_nt6837_L1_Tile|1
ATACTGTCCATTGCAACTAAATGTCCCTCTAAGGATGCAGATGATACCTCCACTGACCCA
>HPV41_Nu_9626041_nt6872_L1_Tile|1
TGCAGATGATACCTCCACTGACCCATACAAAGATCTTAAGTTTTGGGAGGTTGATCTACG
>HPV41_Nu_9626041_nt6877_L1_Tile|1
ATGATACCTCCACTGACCCATACAAAGATCTTAAGTTTTGGGAGGTTGATCTACGGGATC
>HPV41_Nu_9626041_nt6887_L1_Tile|1
CACTGACCCATACAAAGATCTTAAGTTTTGGGAGGTTGATCTACGGGATCGTATGACAGA
>HPV41_Nu_9626041_nt6927_L1_Tile|1
CTACGGGATCGTATGACAGAGCAATTGGACCAGACTCCCCTTGGCAGGAAGTTTTTGTTT
>HPV41_Nu_9626041_nt6932_L1_Tile|1
GGATCGTATGACAGAGCAATTGGACCAGACTCCCCTTGGCAGGAAGTTTTTGTTTCAAAC
>HPV41_Nu_9626041_nt6942_L1_Tile|2
ACAGAGCAATTGGACCAGACTCCCCTTGGCAGGAAGTTTTTGTTTCAAACTGGTATCACT
>HPV41_Nu_9626041_nt6947_L1_Tile|1
GCAATTGGACCAGACTCCCCTTGGCAGGAAGTTTTTGTTTCAAACTGGTATCACTCAGTC
>HPV41_Nu_9626041_nt6952_L1_Tile|1

TGGACCAGACTCCCCTTGGCAGGAAGTTTTTGTTTCAAACTGGTATCACTCAGTCATCAT
>HPV41_Nu_9626041_nt6962_L1_Tile|2
TCCCCTTGGCAGGAAGTTTTTGTTTCAAACTGGTATCACTCAGTCATCATCAAATAAGCG
>HPV41_Nu_9626041_nt6967_L1_Tile|1
TTGGCAGGAAGTTTTTGTTTCAAACTGGTATCACTCAGTCATCATCAAATAAGCGGGTGT
>HPV41_Nu_9626041_nt6972_L1_Tile|1
AGGAAGTTTTTGTTTCAAACTGGTATCACTCAGTCATCATCAAATAAGCGGGTGTCCACG
>HPV41_Nu_9626041_nt6977_L1_Tile|1
GTTTTTGTTTCAAACTGGTATCACTCAGTCATCATCAAATAAGCGGGTGTCCACGCAGTC
>HPV41_Nu_9626041_nt6982_L1_Tile|2
TGTTTCAAACTGGTATCACTCAGTCATCATCAAATAAGCGGGTGTCCACGCAGTCTACTG
>HPV41_Nu_9626041_nt6987_L1_Tile|1
CAAACTGGTATCACTCAGTCATCATCAAATAAGCGGGTGTCCACGCAGTCTACTGCCCTT
>HPV41_Nu_9626041_nt6992_L1_Tile|1
TGGTATCACTCAGTCATCATCAAATAAGCGGGTGTCCACGCAGTCTACTGCCCTTACTAC
>HPV41_Nu_9626041_nt6997_L1_Tile|1
TCACTCAGTCATCATCAAATAAGCGGGTGTCCACGCAGTCTACTGCCCTTACTACCTACA
>HPV41_Nu_9626041_nt7012_L1_Tile|1
CAAATAAGCGGGTGTCCACGCAGTCTACTGCCCTTACTACCTACAGGCGGCCTACTAAGC
>HPV41_Nu_9626041_nt7022_L1_Tile|2
GGTGTCCACGCAGTCTACTGCCCTTACTACCTACAGGCGGCCTACTAAGCGCCGCCGGAA
>HPV41_Nu_9626041_nt7029_L1_Tile|1
ACGCAGTCTACTGCCCTTACTACCTACAGGCGGCCTACTAAGCGCCGCCGGAAGGCTTAA
>HPV42_Alpha_333211_nt5942_L1_Tile|2
CCATGCCAGCAGTTCTAGGCTATTGGTTGTTGGTCACCCTTATTACTCTATTACAAAAAG
>HPV42_Alpha_333211_nt5947_L1_Tile|1
CCAGCAGTTCTAGGCTATTGGTTGTTGGTCACCCTTATTACTCTATTACAAAAAGGCCAA
>HPV42_Alpha_333211_nt5952_L1_Tile|1
AGTTCTAGGCTATTGGTTGTTGGTCACCCTTATTACTCTATTACAAAAAGGCCAAATAAG
>HPV42_Alpha_333211_nt5957_L1_Tile|1
TAGGCTATTGGTTGTTGGTCACCCTTATTACTCTATTACAAAAAGGCCAAATAAGACATC
>HPV42_Alpha_333211_nt5962_L1_Tile|2
TATTGGTTGTTGGTCACCCTTATTACTCTATTACAAAAAGGCCAAATAAGACATCTATCC
>HPV42_Alpha_333211_nt5967_L1_Tile|1
GTTGTTGGTCACCCTTATTACTCTATTACAAAAAGGCCAAATAAGACATCTATCCCCAAA
>HPV42_Alpha_333211_nt5972_L1_Tile|1
TGGTCACCCTTATTACTCTATTACAAAAAGGCCAAATAAGACATCTATCCCCAAAGTGTC
>HPV42_Alpha_333211_nt5977_L1_Tile|1
ACCCTTATTACTCTATTACAAAAAGGCCAAATAAGACATCTATCCCCAAAGTGTCTGGTT
>HPV42_Alpha_333211_nt5982_L1_Tile|2
TATTACTCTATTACAAAAAGGCCAAATAAGACATCTATCCCCAAAGTGTCTGGTTTACAG
>HPV42_Alpha_333211_nt5992_L1_Tile|1
TTACAAAAAGGCCAAATAAGACATCTATCCCCAAAGTGTCTGGTTTACAGTACAGAGTAT
>HPV42_Alpha_333211_nt5997_L1_Tile|1
AAAAGGCCAAATAAGACATCTATCCCCAAAGTGTCTGGTTTACAGTACAGAGTATTTAGA
>HPV42_Alpha_333211_nt6002_L1_Tile|2
GCCAAATAAGACATCTATCCCCAAAGTGTCTGGTTTACAGTACAGAGTATTTAGAGTTAG
>HPV42_Alpha_333211_nt6007_L1_Tile|1
ATAAGACATCTATCCCCAAAGTGTCTGGTTTACAGTACAGAGTATTTAGAGTTAGGCTCC
>HPV42_Alpha_333211_nt6052_L1_Tile|1
TTAGAGTTAGGCTCCCTGATCCTAATAAGTTTACATTGCCTGAAACTAATTTATATAACC
>HPV42_Alpha_333211_nt6217_L1_Tile|1
ATACTGAAAATGCGCCTACATATGGTGGAGGCCCTGGTACAGACAATAGGGAAAATGTTT
>HPV42_Alpha_333211_nt6222_L1_Tile|2
GAAAATGCGCCTACATATGGTGGAGGCCCTGGTACAGACAATAGGGAAAATGTTTCTATG

>HPV42_Alpha_333211_nt6347_L1_Tile|1
AGGTACTGCCTGTACACCACAGTCCAATGGTGACTGCCCACCATTAGAATTAAAAAATAG
>HPV42_Alpha_333211_nt6352_L1_Tile|1
CTGCCTGTACACCACAGTCCAATGGTGACTGCCCACCATTAGAATTAAAAAATAGTTTTA
>HPV42_Alpha_333211_nt6357_L1_Tile|1
TGTACACCACAGTCCAATGGTGACTGCCCACCATTAGAATTAAAAAATAGTTTTATTCAG
>HPV42_Alpha_333211_nt6422_L1_Tile|2
GGATATGGTGGATGTAGGGTTTGGGGCACTAGATTTTGGTGCTTTACAATCCTCCAAAGC
>HPV42_Alpha_333211_nt6442_L1_Tile|2
TTGGGGCACTAGATTTTGGTGCTTTACAATCCTCCAAAGCTGAGGTACCTTTGGATATTG
>HPV42_Alpha_333211_nt6447_L1_Tile|1
GCACTAGATTTTGGTGCTTTACAATCCTCCAAAGCTGAGGTACCTTTGGATATTGTAAAT
>HPV42_Alpha_333211_nt6452_L1_Tile|1
AGATTTTGGTGCTTTACAATCCTCCAAAGCTGAGGTACCTTTGGATATTGTAAATTCAAT
>HPV42_Alpha_333211_nt6457_L1_Tile|1
TTGGTGCTTTACAATCCTCCAAAGCTGAGGTACCTTTGGATATTGTAAATTCAATTACTA
>HPV42_Alpha_333211_nt6462_L1_Tile|2
GCTTTACAATCCTCCAAAGCTGAGGTACCTTTGGATATTGTAAATTCAATTACTAAATAT
>HPV42_Alpha_333211_nt6467_L1_Tile|1
ACAATCCTCCAAAGCTGAGGTACCTTTGGATATTGTAAATTCAATTACTAAATATCCTGA
>HPV42_Alpha_333211_nt6472_L1_Tile|1
CCTCCAAAGCTGAGGTACCTTTGGATATTGTAAATTCAATTACTAAATATCCTGATTACT
>HPV42_Alpha_333211_nt6477_L1_Tile|1
AAAGCTGAGGTACCTTTGGATATTGTAAATTCAATTACTAAATATCCTGATTACTTAAAA
>HPV42_Alpha_333211_nt6612_L1_Tile|1
AATAGGGCTGGCGCAATTGGTGAACCTGTACCTGATGAACTGTATACCAAGGCTGCTAAT
>HPV42_Alpha_333211_nt6622_L1_Tile|2
GCGCAATTGGTGAACCTGTACCTGATGAACTGTATACCAAGGCTGCTAATAATGCATCTG
>HPV42_Alpha_333211_nt6632_L1_Tile|1
TGAACCTGTACCTGATGAACTGTATACCAAGGCTGCTAATAATGCATCTGGCAGACATAA
>HPV42_Alpha_333211_nt6642_L1_Tile|2
CCTGATGAACTGTATACCAAGGCTGCTAATAATGCATCTGGCAGACATAATTTAGGTAGT
>HPV42_Alpha_333211_nt6647_L1_Tile|1
TGAACTGTATACCAAGGCTGCTAATAATGCATCTGGCAGACATAATTTAGGTAGTAGTAT
>HPV42_Alpha_333211_nt6852_L1_Tile|1
AGTACTAACATGACTTTGTGTGCCACTGCAACATCTGGTGATACATATACAGCTGCTAAT
>HPV42_Alpha_333211_nt6857_L1_Tile|1
TAACATGACTTTGTGTGCCACTGCAACATCTGGTGATACATATACAGCTGCTAATTTTAA
>HPV42_Alpha_333211_nt6862_L1_Tile|2
TGACTTTGTGTGCCACTGCAACATCTGGTGATACATATACAGCTGCTAATTTTAAGGAAT
>HPV42_Alpha_333211_nt6877_L1_Tile|1
CTGCAACATCTGGTGATACATATACAGCTGCTAATTTTAAGGAATATTTAAGACATGCTG
>HPV42_Alpha_333211_nt6972_L1_Tile|1
AAAATAACATTAACTGTTGAAGTTATGTCATATATACACAATATGAATCCTAACATATTA
>HPV42_Alpha_333211_nt7057_L1_Tile|1
CACCACCTTCAGGAACTTTAGAAGATAGTTATAGGTATGTACAATCAGAAGCTATTCGCT
>HPV42_Alpha_333211_nt7062_L1_Tile|2
CCTTCAGGAACTTTAGAAGATAGTTATAGGTATGTACAATCAGAAGCTATTCGCTGTCAG
>HPV42_Alpha_333211_nt7067_L1_Tile|1
AGGAACTTTAGAAGATAGTTATAGGTATGTACAATCAGAAGCTATTCGCTGTCAGGCTAA
>HPV42_Alpha_333211_nt7072_L1_Tile|1
CTTTAGAAGATAGTTATAGGTATGTACAATCAGAAGCTATTCGCTGTCAGGCTAAGGTAA
>HPV42_Alpha_333211_nt7077_L1_Tile|1
GAAGATAGTTATAGGTATGTACAATCAGAAGCTATTCGCTGTCAGGCTAAGGTAACAACG
>HPV42_Alpha_333211_nt7082_L1_Tile|2

TAGTTATAGGTATGTACAATCAGAAGCTATTCGCTGTCAGGCTAAGGTAACAACGCCAGA
>HPV42_Alpha_333211_nt7097_L1_Tile|1
ACAATCAGAAGCTATTCGCTGTCAGGCTAAGGTAACAACGCCAGAAAAAAAGGATCCTTA
>HPV42_Alpha_333211_nt7102_L1_Tile|2
CAGAAGCTATTCGCTGTCAGGCTAAGGTAACAACGCCAGAAAAAAAGGATCCTTATTCAG
>HPV42_Alpha_333211_nt7107_L1_Tile|1
GCTATTCGCTGTCAGGCTAAGGTAACAACGCCAGAAAAAAAGGATCCTTATTCAGACTTT
>HPV42_Alpha_333211_nt7247_L1_Tile|1
GGCCGGGTTGCGTGCAAGGCCTAAACTGTCTGTAGGTAAACGAAAGGCGTCTACAGCTAA
>HPV42_Alpha_333211_nt7252_L1_Tile|1
GGTTGCGTGCAAGGCCTAAACTGTCTGTAGGTAAACGAAAGGCGTCTACAGCTAAATCTG
>HPV42_Alpha_333211_nt7257_L1_Tile|1
CGTGCAAGGCCTAAACTGTCTGTAGGTAAACGAAAGGCGTCTACAGCTAAATCTGTTTCT
>HPV42_Alpha_333211_nt7262_L1_Tile|2
AAGGCCTAAACTGTCTGTAGGTAAACGAAAGGCGTCTACAGCTAAATCTGTTTCTTCAGC
>HPV42_Alpha_333211_nt7267_L1_Tile|1
CTAAACTGTCTGTAGGTAAACGAAAGGCGTCTACAGCTAAATCTGTTTCTTCAGCTAAAC
>HPV42_Alpha_333211_nt7272_L1_Tile|1
CTGTCTGTAGGTAAACGAAAGGCGTCTACAGCTAAATCTGTTTCTTCAGCTAAACGTAAG
>HPV42_Alpha_333211_nt7277_L1_Tile|1
TGTAGGTAAACGAAAGGCGTCTACAGCTAAATCTGTTTCTTCAGCTAAACGTAAGAAAAC
>HPV42_Alpha_333211_nt7287_L1_Tile|1
CGAAAGGCGTCTACAGCTAAATCTGTTTCTTCAGCTAAACGTAAGAAAACACACAAATAG
>HPV43_Alpha_40804474_nt5863_L1_Tile|1
GCTGGCAGTTCACGTTTGCTTGCAGTGGGTCACCCATATTTCCCCCTTAAAAATTCCTCT
>HPV43_Alpha_40804474_nt5868_L1_Tile|1
CAGTTCACGTTTGCTTGCAGTGGGTCACCCATATTTCCCCCTTAAAAATTCCTCTGGTAA
>HPV43_Alpha_40804474_nt5873_L1_Tile|1
CACGTTTGCTTGCAGTGGGTCACCCATATTTCCCCCTTAAAAATTCCTCTGGTAAAATAA
>HPV43_Alpha_40804474_nt5878_L1_Tile|1
TTGCTTGCAGTGGGTCACCCATATTTCCCCCTTAAAAATTCCTCTGGTAAAATAACTGTA
>HPV43_Alpha_40804474_nt5883_L1_Tile|1
TGCAGTGGGTCACCCATATTTCCCCCTTAAAAATTCCTCTGGTAAAATAACTGTACCTAA
>HPV43_Alpha_40804474_nt5893_L1_Tile|1
CACCCATATTTCCCCCTTAAAAATTCCTCTGGTAAAATAACTGTACCTAAGGTTTCTGGT
>HPV43_Alpha_40804474_nt5898_L1_Tile|1
ATATTTCCCCCTTAAAAATTCCTCTGGTAAAATAACTGTACCTAAGGTTTCTGGTTATCA
>HPV43_Alpha_40804474_nt6518_L1_Tile|1
TCCTTAGACATTTTTTTAATAAAGCTGGTAAAACTGGCGACGTTGTGCCTTCCGATATGT
>HPV43_Alpha_40804474_nt6528_L1_Tile|1
TTTTTTAATAAAGCTGGTAAAACTGGCGACGTTGTGCCTTCCGATATGTATATTGCTGG
>HPV43_Alpha_40804474_nt6533_L1_Tile|1
TTAATAAAGCTGGTAAAACTGGCGACGTTGTGCCTTCCGATATGTATATTGCTGGCTCTA
>HPV43_Alpha_40804474_nt6538_L1_Tile|1
AAAGCTGGTAAAACTGGCGACGTTGTGCCTTCCGATATGTATATTGCTGGCTCTAATACC
>HPV43_Alpha_40804474_nt6543_L1_Tile|1
TGGTAAAACTGGCGACGTTGTGCCTTCCGATATGTATATTGCTGGCTCTAATACCAGGTC
>HPV43_Alpha_40804474_nt6548_L1_Tile|1
AAACTGGCGACGTTGTGCCTTCCGATATGTATATTGCTGGCTCTAATACCAGGTCCAAAA
>HPV43_Alpha_40804474_nt6553_L1_Tile|1
GGCGACGTTGTGCCTTCCGATATGTATATTGCTGGCTCTAATACCAGGTCCAAAATTGCA
>HPV43_Alpha_40804474_nt6558_L1_Tile|1
CGTTGTGCCTTCCGATATGTATATTGCTGGCTCTAATACCAGGTCCAAAATTGCAGATAG
>HPV43_Alpha_40804474_nt6563_L1_Tile|1
TGCCTTCCGATATGTATATTGCTGGCTCTAATACCAGGTCCAAAATTGCAGATAGTATAT

>HPV43_Alpha_40804474_nt6568_L1_Tile|1
TCCGATATGTATATTGCTGGCTCTAATACCAGGTCCAAAATTGCAGATAGTATATATTTT
>HPV43_Alpha_40804474_nt6573_L1_Tile|1
TATGTATATTGCTGGCTCTAATACCAGGTCCAAAATTGCAGATAGTATATATTTTTCTAC
>HPV43_Alpha_40804474_nt6578_L1_Tile|1
ATATTGCTGGCTCTAATACCAGGTCCAAAATTGCAGATAGTATATATTTTTCTACACCCA
>HPV43_Alpha_40804474_nt6583_L1_Tile|1
GCTGGCTCTAATACCAGGTCCAAAATTGCAGATAGTATATATTTTTCTACACCCAGTGGG
>HPV43_Alpha_40804474_nt6588_L1_Tile|1
CTCTAATACCAGGTCCAAAATTGCAGATAGTATATATTTTTCTACACCCAGTGGGTCTTT
>HPV43_Alpha_40804474_nt6593_L1_Tile|1
ATACCAGGTCCAAAATTGCAGATAGTATATATTTTTCTACACCCAGTGGGTCTTTGGTTA
>HPV43_Alpha_40804474_nt6598_L1_Tile|1
AGGTCCAAAATTGCAGATAGTATATATTTTTCTACACCCAGTGGGTCTTTGGTTACTTCT
>HPV43_Alpha_40804474_nt6753_L1_Tile|1
AGATACCACTCGTAGTACAAACTTAACGTTATGTGCCTCTACTGACCCTACTGTGCCCAG
>HPV43_Alpha_40804474_nt6788_L1_Tile|1
CCTCTACTGACCCTACTGTGCCCAGTACATATGACAATGCAAAGTTTAAGGAATACCTGC
>HPV43_Alpha_40804474_nt6888_L1_Tile|1
ATGCATAATAACGCTAAACCCAGAGGTTATGACATATATTCATACTATGGATCCCACATT
>HPV43_Alpha_40804474_nt6893_L1_Tile|1
TAATAACGCTAAACCCAGAGGTTATGACATATATTCATACTATGGATCCCACATTATTAG
>HPV43_Alpha_40804474_nt6973_L1_Tile|1
TCCCCACCTGCCTCTGCTTCTTTGGAAGATACTTATCGCTTTTTGTCTAACAAGGCCATT
>HPV43_Alpha_40804474_nt7068_L1_Tile|1
GGATCCCTATAAAAAGTATACATTTTGGGATATAAATCTTACAGAAAAGTTTTCTGCACA
>HPV43_Alpha_40804474_nt7193_L1_Tile|1
TAAAAACTGTAAAGCGTTCTGCACCATCCTCCTCTACGTCTGCCCCTGCCTCTAAACGCA
>HPV43_Alpha_40804474_nt7203_L1_Tile|1
AAAGCGTTCTGCACCATCCTCCTCTACGTCTGCCCCTGCCTCTAAACGCAAAAAAACTAA
>HPV44_Alpha_1020242_nt6475_L1_Tile|1
GCTGGAACAGTTGGTGAGGACGTTTCCCAGGATCTGGTTATTAAAAGTGCTAGTAAAAAT
>HPV44_Alpha_1020242_nt6480_L1_Tile|1
AACAGTTGGTGAGGACGTTTCCCAGGATCTGGTTATTAAAAGTGCTAGTAAAAATACTGT
>HPV44_Alpha_1020242_nt6720_L1_Tile|1
TGCCACTACACAGTCCCCTCCGTCTACATATACTAGTGAACAATATAAGCAATACATGCG
>HPV45_Alpha_397022_nt5731_L1_Tile|1
ATTATTAACTGTAGGCAATCCATATTTTAGGGTTGTACCTAATGGTGCAGGTAATAAACA
>HPV45_Alpha_397022_nt5736_L1_Tile|1
TAACTGTAGGCAATCCATATTTTAGGGTTGTACCTAATGGTGCAGGTAATAAACAGGCTG
>HPV45_Alpha_397022_nt5751_L1_Tile|1
CATATTTTAGGGTTGTACCTAATGGTGCAGGTAATAAACAGGCTGTTCCTAAGGTATCCG
>HPV45_Alpha_397022_nt5756_L1_Tile|1
TTTAGGGTTGTACCTAATGGTGCAGGTAATAAACAGGCTGTTCCTAAGGTATCCGCATAT
>HPV45_Alpha_397022_nt5761_L1_Tile|1
GGTTGTACCTAATGGTGCAGGTAATAAACAGGCTGTTCCTAAGGTATCCGCATATCAGTA
>HPV45_Alpha_397022_nt5971_L1_Tile|1
TCCATTTTATAATAAATTGGATGATACAGAAAGTGCTCATGCAGCTACAGCTGTTATTAC
>HPV45_Alpha_397022_nt6136_L1_Tile|1
TAAACCTGCACAATTGCAACCTGGTGACTGTCCTCCTTTGGAACTTAAAAACACCATTAT
>HPV45_Alpha_397022_nt6416_L1_Tile|1
GACACAGTACCTACGGACCTATATATTAAAGGCACTAGCGCTAATATGCGTGAAACCCCT
>HPV45_Alpha_397022_nt6426_L1_Tile|1
CTACGGACCTATATATTAAAGGCACTAGCGCTAATATGCGTGAAACCCCTGGCAGTTGTG
>HPV45_Alpha_397022_nt6936_L1_Tile|1

ATCCATATGATAAATTAAAGTTTTGGACTGTTGACCTAAAGGAAAAATTTTCCTCCGATT
>HPV45_Alpha_397022_nt6941_L1_Tile|1
TATGATAAATTAAAGTTTTGGACTGTTGACCTAAAGGAAAAATTTTCCTCCGATTTGGAT
>HPV45_Alpha_397022_nt6946_L1_Tile|1
TAAATTAAAGTTTTGGACTGTTGACCTAAAGGAAAAATTTTCCTCCGATTTGGATCAATA
>HPV45_Alpha_397022_nt7056_L1_Tile|1
CCATAGGACCTCGTAAGCGTCCTGCTGCTTCCACGTCTACTGCATCTACTGCATCTAGGC
>HPV45_Alpha_397022_nt7091_L1_Tile|1
TCTACTGCATCTACTGCATCTAGGCCTGCCAAACGTGTACGTATACGTAGTAAGAAATAA
>HPV47_Beta_333062_nt6269_L1_Tile|1
ATATTTTAATAAGGTAAAAGATACAGAAAACAGTAATTCCTATATCACAAACTCAAAAGA
>HPV47_Beta_333062_nt6289_L1_Tile|1
ATACAGAAAACAGTAATTCCTATATCACAAACTCAAAAGATGACAGACAAGACACCTCTT
>HPV47_Beta_333062_nt6294_L1_Tile|1
GAAAACAGTAATTCCTATATCACAAACTCAAAAGATGACAGACAAGACACCTCTTTTGAT
>HPV47_Beta_333062_nt6729_L1_Tile|1
AATGGTAATATGAAAAATCAATTTTACATTCCTGGTGCTACGGGTCAGGCTCAGAGCACT
>HPV47_Beta_333062_nt6934_L1_Tile|1
TAGACAACACAAGAAATACAAATTTCAGCATCTCTGTTTACTCTCAGGCAGGGGACATAA
>HPV47_Beta_333062_nt6939_L1_Tile|1
AACACAAGAAATACAAATTTCAGCATCTCTGTTTACTCTCAGGCAGGGGACATAAAGGAT
>HPV47_Beta_333062_nt6944_L1_Tile|1
AAGAAATACAAATTTCAGCATCTCTGTTTACTCTCAGGCAGGGGACATAAAGGATATACA
>HPV47_Beta_333062_nt6949_L1_Tile|1
ATACAAATTTCAGCATCTCTGTTTACTCTCAGGCAGGGGACATAAAGGATATACAGGATT
>HPV47_Beta_333062_nt6954_L1_Tile|1
AATTTCAGCATCTCTGTTTACTCTCAGGCAGGGGACATAAAGGATATACAGGATTATAAT
>HPV47_Beta_333062_nt6964_L1_Tile|1
TCTCTGTTTACTCTCAGGCAGGGGACATAAAGGATATACAGGATTATAATGCAGACAATT
>HPV47_Beta_333062_nt6969_L1_Tile|1
GTTTACTCTCAGGCAGGGGACATAAAGGATATACAGGATTATAATGCAGACAATTTTAGA
>HPV47_Beta_333062_nt6974_L1_Tile|1
CTCTCAGGCAGGGGACATAAAGGATATACAGGATTATAATGCAGACAATTTTAGAGAGTA
>HPV47_Beta_333062_nt6984_L1_Tile|1
GGGGACATAAAGGATATACAGGATTATAATGCAGACAATTTTAGAGAGTATCAAAGACAT
>HPV47_Beta_333062_nt7164_L1_Tile|1
GTGCCTACTCCAGACAACCCTATTCAGGATACATATAGATATCTAGAATCTTTGGCCACT
>HPV47_Beta_333062_nt7179_L1_Tile|1
AACCCTATTCAGGATACATATAGATATCTAGAATCTTTGGCCACTAGGTGTCCTGAAAAG
>HPV47_Beta_333062_nt7184_L1_Tile|1
TATTCAGGATACATATAGATATCTAGAATCTTTGGCCACTAGGTGTCCTGAAAAGTCTCC
>HPV47_Beta_333062_nt7189_L1_Tile|1
AGGATACATATAGATATCTAGAATCTTTGGCCACTAGGTGTCCTGAAAAGTCTCCTCCAA
>HPV47_Beta_333062_nt7204_L1_Tile|1
ATCTAGAATCTTTGGCCACTAGGTGTCCTGAAAAGTCTCCTCCAAAAGAGAAGGTTGACC
>HPV47_Beta_333062_nt7214_L1_Tile|1
TTTGGCCACTAGGTGTCCTGAAAAGTCTCCTCCAAAAGAGAAGGTTGACCCCTACAAAGG
>HPV47_Beta_333062_nt7224_L1_Tile|1
AGGTGTCCTGAAAAGTCTCCTCCAAAAGAGAAGGTTGACCCCTACAAAGGTTTAAACTTT
>HPV47_Beta_333062_nt7264_L1_Tile|1
CCTACAAAGGTTTAAACTTTTGGGATGTCGATATGACAGAGCGCCTTTCCCTGGATTTAG
>HPV48_Gamma_9628542_nt5248_L1_Tile|1
AGTGCACCAGTAGCTCGTGTGTTGCGCACTGATGAATACGTGCAAGAAACGGATGTATAT
>HPV48_Gamma_9628542_nt5253_L1_Tile|1
ACCAGTAGCTCGTGTGTTGCGCACTGATGAATACGTGCAAGAAACGGATGTATATTTTTA

>HPV48_Gamma_9628542_nt5258_L1_Tile|1
TAGCTCGTGTGTTGCGCACTGATGAATACGTGCAAGAAACGGATGTATATTTTTATACAA
>HPV48_Gamma_9628542_nt5263_L1_Tile|1
CGTGTGTTGCGCACTGATGAATACGTGCAAGAAACGGATGTATATTTTTATACAAGCACT
>HPV48_Gamma_9628542_nt5273_L1_Tile|1
GCACTGATGAATACGTGCAAGAAACGGATGTATATTTTTATACAAGCACTGAAAGACTTT
>HPV48_Gamma_9628542_nt5278_L1_Tile|1
GATGAATACGTGCAAGAAACGGATGTATATTTTTATACAAGCACTGAAAGACTTTTAATA
>HPV48_Gamma_9628542_nt5283_L1_Tile|1
ATACGTGCAAGAAACGGATGTATATTTTTATACAAGCACTGAAAGACTTTTAATAGTTGG
>HPV48_Gamma_9628542_nt5288_L1_Tile|1
TGCAAGAAACGGATGTATATTTTTATACAAGCACTGAAAGACTTTTAATAGTTGGTAATC
>HPV48_Gamma_9628542_nt5293_L1_Tile|1
GAAACGGATGTATATTTTTATACAAGCACTGAAAGACTTTTAATAGTTGGTAATCCTTAT
>HPV48_Gamma_9628542_nt5298_L1_Tile|1
GGATGTATATTTTTATACAAGCACTGAAAGACTTTTAATAGTTGGTAATCCTTATTTTGA
>HPV48_Gamma_9628542_nt5303_L1_Tile|1
TATATTTTTATACAAGCACTGAAAGACTTTTAATAGTTGGTAATCCTTATTTTGATGTTG
>HPV48_Gamma_9628542_nt5308_L1_Tile|1
TTTTATACAAGCACTGAAAGACTTTTAATAGTTGGTAATCCTTATTTTGATGTTGAAAAC
>HPV48_Gamma_9628542_nt5313_L1_Tile|1
TACAAGCACTGAAAGACTTTTAATAGTTGGTAATCCTTATTTTGATGTTGAAAACAGAGA
>HPV48_Gamma_9628542_nt5318_L1_Tile|1
GCACTGAAAGACTTTTAATAGTTGGTAATCCTTATTTTGATGTTGAAAACAGAGACACTA
>HPV48_Gamma_9628542_nt5323_L1_Tile|1
GAAAGACTTTTAATAGTTGGTAATCCTTATTTTGATGTTGAAAACAGAGACACTATAACA
>HPV48_Gamma_9628542_nt5328_L1_Tile|1
ACTTTTAATAGTTGGTAATCCTTATTTTGATGTTGAAAACAGAGACACTATAACAGTACC
>HPV48_Gamma_9628542_nt5333_L1_Tile|1
TAATAGTTGGTAATCCTTATTTTGATGTTGAAAACAGAGACACTATAACAGTACCTAAAG
>HPV48_Gamma_9628542_nt5338_L1_Tile|1
GTTGGTAATCCTTATTTTGATGTTGAAAACAGAGACACTATAACAGTACCTAAAGTTTCT
>HPV48_Gamma_9628542_nt5343_L1_Tile|1
TAATCCTTATTTTGATGTTGAAAACAGAGACACTATAACAGTACCTAAAGTTTCTGCTAA
>HPV48_Gamma_9628542_nt5348_L1_Tile|1
CTTATTTTGATGTTGAAAACAGAGACACTATAACAGTACCTAAAGTTTCTGCTAATCAAT
>HPV48_Gamma_9628542_nt5353_L1_Tile|1
TTTGATGTTGAAAACAGAGACACTATAACAGTACCTAAAGTTTCTGCTAATCAATACCGA
>HPV48_Gamma_9628542_nt5373_L1_Tile|1
CACTATAACAGTACCTAAAGTTTCTGCTAATCAATACCGAGTATTTAGATGTAAACTTCC
>HPV48_Gamma_9628542_nt5408_L1_Tile|1
ACCGAGTATTTAGATGTAAACTTCCAGATCCTAACAAGTTTGCTCTAGTTGATAAAAATT
>HPV48_Gamma_9628542_nt5418_L1_Tile|1
TAGATGTAAACTTCCAGATCCTAACAAGTTTGCTCTAGTTGATAAAAATTTATATAATTC
>HPV48_Gamma_9628542_nt5453_L1_Tile|1
TAGTTGATAAAAATTTATATAATTCAGATAAGGAACGTTTAGTTTGGAAACTTGTTGGTT
>HPV48_Gamma_9628542_nt5458_L1_Tile|1
GATAAAAATTTATATAATTCAGATAAGGAACGTTTAGTTTGGAAACTTGTTGGTTTGGAA
>HPV48_Gamma_9628542_nt5463_L1_Tile|1
AAATTTATATAATTCAGATAAGGAACGTTTAGTTTGGAAACTTGTTGGTTTGGAAGTAGG
>HPV48_Gamma_9628542_nt5483_L1_Tile|1
AGGAACGTTTAGTTTGGAAACTTGTTGGTTTGGAAGTAGGCAGAGGGGGTCCTCTTGGCG
>HPV48_Gamma_9628542_nt5488_L1_Tile|1
CGTTTAGTTTGGAAACTTGTTGGTTTGGAAGTAGGCAGAGGGGGTCCTCTTGGCGTAGGA
>HPV48_Gamma_9628542_nt5493_L1_Tile|1

AGTTTGGAAACTTGTTGGTTTGGAAGTAGGCAGAGGGGGTCCTCTTGGCGTAGGATCTAC
>HPV48_Gamma_9628542_nt5498_L1_Tile|1
GGAAACTTGTTGGTTTGGAAGTAGGCAGAGGGGGTCCTCTTGGCGTAGGATCTACAGGTC
>HPV48_Gamma_9628542_nt5503_L1_Tile|1
CTTGTTGGTTTGGAAGTAGGCAGAGGGGGTCCTCTTGGCGTAGGATCTACAGGTCATCCT
>HPV48_Gamma_9628542_nt5508_L1_Tile|1
TGGTTTGGAAGTAGGCAGAGGGGGTCCTCTTGGCGTAGGATCTACAGGTCATCCTTTACT
>HPV48_Gamma_9628542_nt5513_L1_Tile|1
TGGAAGTAGGCAGAGGGGGTCCTCTTGGCGTAGGATCTACAGGTCATCCTTTACTAAATA
>HPV48_Gamma_9628542_nt5518_L1_Tile|1
GTAGGCAGAGGGGGTCCTCTTGGCGTAGGATCTACAGGTCATCCTTTACTAAATAAGATA
>HPV48_Gamma_9628542_nt5523_L1_Tile|1
CAGAGGGGGTCCTCTTGGCGTAGGATCTACAGGTCATCCTTTACTAAATAAGATAGGGGA
>HPV48_Gamma_9628542_nt5588_L1_Tile|1
AAAATCCTAGCTTTTATTTAGGAGAACAAACAAAGGATGAGAGACAAAATGTTTCCATGG
>HPV48_Gamma_9628542_nt5603_L1_Tile|1
ATTTAGGAGAACAAACAAAGGATGAGAGACAAAATGTTTCCATGGATCCTAAGCAGAGTC
>HPV48_Gamma_9628542_nt5613_L1_Tile|1
ACAAACAAAGGATGAGAGACAAAATGTTTCCATGGATCCTAAGCAGAGTCAGATACTAAT
>HPV48_Gamma_9628542_nt5638_L1_Tile|1
GTTTCCATGGATCCTAAGCAGAGTCAGATACTAATAGTTGGTTGTGCTCCAGCCACTGGA
>HPV48_Gamma_9628542_nt5643_L1_Tile|1
CATGGATCCTAAGCAGAGTCAGATACTAATAGTTGGTTGTGCTCCAGCCACTGGAGAATA
>HPV48_Gamma_9628542_nt5648_L1_Tile|1
ATCCTAAGCAGAGTCAGATACTAATAGTTGGTTGTGCTCCAGCCACTGGAGAATACTGGG
>HPV48_Gamma_9628542_nt5653_L1_Tile|1
AAGCAGAGTCAGATACTAATAGTTGGTTGTGCTCCAGCCACTGGAGAATACTGGGATTTA
>HPV48_Gamma_9628542_nt5658_L1_Tile|1
GAGTCAGATACTAATAGTTGGTTGTGCTCCAGCCACTGGAGAATACTGGGATTTAGCTAA
>HPV48_Gamma_9628542_nt5663_L1_Tile|1
AGATACTAATAGTTGGTTGTGCTCCAGCCACTGGAGAATACTGGGATTTAGCTAAACCAT
>HPV48_Gamma_9628542_nt5673_L1_Tile|1
AGTTGGTTGTGCTCCAGCCACTGGAGAATACTGGGATTTAGCTAAACCATGCAATGATTT
>HPV48_Gamma_9628542_nt5698_L1_Tile|1
GAATACTGGGATTTAGCTAAACCATGCAATGATTTGGAAAACGGGGCTGCACCACCAATT
>HPV48_Gamma_9628542_nt5703_L1_Tile|1
CTGGGATTTAGCTAAACCATGCAATGATTTGGAAAACGGGGCTGCACCACCAATTCAACT
>HPV48_Gamma_9628542_nt5708_L1_Tile|1
ATTTAGCTAAACCATGCAATGATTTGGAAAACGGGGCTGCACCACCAATTCAACTAGTTA
>HPV48_Gamma_9628542_nt5713_L1_Tile|1
GCTAAACCATGCAATGATTTGGAAAACGGGGCTGCACCACCAATTCAACTAGTTAATACT
>HPV48_Gamma_9628542_nt5723_L1_Tile|1
GCAATGATTTGGAAAACGGGGCTGCACCACCAATTCAACTAGTTAATACTGTTATTCAGG
>HPV48_Gamma_9628542_nt5728_L1_Tile|1
GATTTGGAAAACGGGGCTGCACCACCAATTCAACTAGTTAATACTGTTATTCAGGATGGT
>HPV48_Gamma_9628542_nt5733_L1_Tile|1
GGAAAACGGGGCTGCACCACCAATTCAACTAGTTAATACTGTTATTCAGGATGGTGATAT
>HPV48_Gamma_9628542_nt5798_L1_Tile|1
ATATAGGCTTTGGGGCTGCTAATTTTCCTAAGTTGATGCAGGATCGTGCTGGCGTCCCTC
>HPV48_Gamma_9628542_nt5803_L1_Tile|1
GGCTTTGGGGCTGCTAATTTTCCTAAGTTGATGCAGGATCGTGCTGGCGTCCCTCTAGAG
>HPV48_Gamma_9628542_nt5808_L1_Tile|1
TGGGGCTGCTAATTTTCCTAAGTTGATGCAGGATCGTGCTGGCGTCCCTCTAGAGTTAAT
>HPV48_Gamma_9628542_nt5813_L1_Tile|1
CTGCTAATTTTCCTAAGTTGATGCAGGATCGTGCTGGCGTCCCTCTAGAGTTAATAGATT

>HPV48_Gamma_9628542_nt5818_L1_Tile|1
AATTTTCCTAAGTTGATGCAGGATCGTGCTGGCGTCCCTCTAGAGTTAATAGATTCTATT
>HPV48_Gamma_9628542_nt5823_L1_Tile|1
TCCTAAGTTGATGCAGGATCGTGCTGGCGTCCCTCTAGAGTTAATAGATTCTATTAGTAT
>HPV48_Gamma_9628542_nt5833_L1_Tile|1
ATGCAGGATCGTGCTGGCGTCCCTCTAGAGTTAATAGATTCTATTAGTATATGGCCAGAT
>HPV48_Gamma_9628542_nt5838_L1_Tile|1
GGATCGTGCTGGCGTCCCTCTAGAGTTAATAGATTCTATTAGTATATGGCCAGATTTTTT
>HPV48_Gamma_9628542_nt5843_L1_Tile|1
GTGCTGGCGTCCCTCTAGAGTTAATAGATTCTATTAGTATATGGCCAGATTTTTTAAAGA
>HPV48_Gamma_9628542_nt5853_L1_Tile|1
CCCTCTAGAGTTAATAGATTCTATTAGTATATGGCCAGATTTTTTAAAGATGACCAAAGA
>HPV48_Gamma_9628542_nt5858_L1_Tile|1
TAGAGTTAATAGATTCTATTAGTATATGGCCAGATTTTTTAAAGATGACCAAAGATATTT
>HPV48_Gamma_9628542_nt5868_L1_Tile|1
AGATTCTATTAGTATATGGCCAGATTTTTTAAAGATGACCAAAGATATTTATGGAGACTC
>HPV48_Gamma_9628542_nt5888_L1_Tile|1
CAGATTTTTTAAAGATGACCAAAGATATTTATGGAGACTCTGTCTTCTTTTTTGGTAAAC
>HPV48_Gamma_9628542_nt5893_L1_Tile|1
TTTTTAAAGATGACCAAAGATATTTATGGAGACTCTGTCTTCTTTTTTGGTAAACGGGAA
>HPV48_Gamma_9628542_nt5898_L1_Tile|1
AAAGATGACCAAAGATATTTATGGAGACTCTGTCTTCTTTTTTGGTAAACGGGAACAATG
>HPV48_Gamma_9628542_nt5903_L1_Tile|1
TGACCAAAGATATTTATGGAGACTCTGTCTTCTTTTTTGGTAAACGGGAACAATGTTATG
>HPV48_Gamma_9628542_nt5918_L1_Tile|1
ATGGAGACTCTGTCTTCTTTTTTGGTAAACGGGAACAATGTTATGCTCGTCATCTTTTTG
>HPV48_Gamma_9628542_nt5928_L1_Tile|1
TGTCTTCTTTTTTGGTAAACGGGAACAATGTTATGCTCGTCATCTTTTTGCAAGAGCTGG
>HPV48_Gamma_9628542_nt5933_L1_Tile|1
TCTTTTTTGGTAAACGGGAACAATGTTATGCTCGTCATCTTTTTGCAAGAGCTGGCCAAA
>HPV48_Gamma_9628542_nt5938_L1_Tile|1
TTTGGTAAACGGGAACAATGTTATGCTCGTCATCTTTTTGCAAGAGCTGGCCAAATGGGG
>HPV48_Gamma_9628542_nt5948_L1_Tile|1
GGGAACAATGTTATGCTCGTCATCTTTTTGCAAGAGCTGGCCAAATGGGGGAGCCTATAC
>HPV48_Gamma_9628542_nt5953_L1_Tile|1
CAATGTTATGCTCGTCATCTTTTTGCAAGAGCTGGCCAAATGGGGGAGCCTATACCAACA
>HPV48_Gamma_9628542_nt5958_L1_Tile|1
TTATGCTCGTCATCTTTTTGCAAGAGCTGGCCAAATGGGGGAGCCTATACCAACAGAAAA
>HPV48_Gamma_9628542_nt5963_L1_Tile|1
CTCGTCATCTTTTTGCAAGAGCTGGCCAAATGGGGGAGCCTATACCAACAGAAAATGGAG
>HPV48_Gamma_9628542_nt5968_L1_Tile|1
CATCTTTTTGCAAGAGCTGGCCAAATGGGGGAGCCTATACCAACAGAAAATGGAGTATAT
>HPV48_Gamma_9628542_nt5973_L1_Tile|1
TTTTGCAAGAGCTGGCCAAATGGGGGAGCCTATACCAACAGAAAATGGAGTATATTATAT
>HPV48_Gamma_9628542_nt5988_L1_Tile|1
CCAAATGGGGGAGCCTATACCAACAGAAAATGGAGTATATTATATAACTCCTGATTCTGC
>HPV48_Gamma_9628542_nt5993_L1_Tile|1
TGGGGGAGCCTATACCAACAGAAAATGGAGTATATTATATAACTCCTGATTCTGCCGATC
>HPV48_Gamma_9628542_nt5998_L1_Tile|1
GAGCCTATACCAACAGAAAATGGAGTATATTATATAACTCCTGATTCTGCCGATCAAAAC
>HPV48_Gamma_9628542_nt6008_L1_Tile|1
CAACAGAAAATGGAGTATATTATATAACTCCTGATTCTGCCGATCAAAACAACAGATCTT
>HPV48_Gamma_9628542_nt6013_L1_Tile|1
GAAAATGGAGTATATTATATAACTCCTGATTCTGCCGATCAAAACAACAGATCTTCTCAT
>HPV48_Gamma_9628542_nt6018_L1_Tile|1

TGGAGTATATTATATAACTCCTGATTCTGCCGATCAAAACAACAGATCTTCTCATTTAGG
>HPV48_Gamma_9628542_nt6023_L1_Tile|1
TATATTATATAACTCCTGATTCTGCCGATCAAAACAACAGATCTTCTCATTTAGGATCTT
>HPV48_Gamma_9628542_nt6028_L1_Tile|1
TATATAACTCCTGATTCTGCCGATCAAAACAACAGATCTTCTCATTTAGGATCTTCTGTG
>HPV48_Gamma_9628542_nt6033_L1_Tile|1
AACTCCTGATTCTGCCGATCAAAACAACAGATCTTCTCATTTAGGATCTTCTGTGTATTT
>HPV48_Gamma_9628542_nt6038_L1_Tile|1
CTGATTCTGCCGATCAAAACAACAGATCTTCTCATTTAGGATCTTCTGTGTATTTTACAA
>HPV48_Gamma_9628542_nt6043_L1_Tile|1
TCTGCCGATCAAAACAACAGATCTTCTCATTTAGGATCTTCTGTGTATTTTACAACACCA
>HPV48_Gamma_9628542_nt6058_L1_Tile|1
AACAGATCTTCTCATTTAGGATCTTCTGTGTATTTTACAACACCAAGTGGATCCTTGAAT
>HPV48_Gamma_9628542_nt6063_L1_Tile|1
ATCTTCTCATTTAGGATCTTCTGTGTATTTTACAACACCAAGTGGATCCTTGAATACTAG
>HPV48_Gamma_9628542_nt6068_L1_Tile|1
CTCATTTAGGATCTTCTGTGTATTTTACAACACCAAGTGGATCCTTGAATACTAGCGATT
>HPV48_Gamma_9628542_nt6178_L1_Tile|1
AATGGCATTTGTTGGGGAAATGAGCTGTTTATTACAGTTTTTGATAATACTCATAATGTG
>HPV48_Gamma_9628542_nt6183_L1_Tile|1
CATTTGTTGGGGAAATGAGCTGTTTATTACAGTTTTTGATAATACTCATAATGTGAATTT
>HPV48_Gamma_9628542_nt6188_L1_Tile|1
GTTGGGGAAATGAGCTGTTTATTACAGTTTTTGATAATACTCATAATGTGAATTTTACTA
>HPV48_Gamma_9628542_nt6193_L1_Tile|1
GGAAATGAGCTGTTTATTACAGTTTTTGATAATACTCATAATGTGAATTTTACTATTAGT
>HPV48_Gamma_9628542_nt6198_L1_Tile|1
TGAGCTGTTTATTACAGTTTTTGATAATACTCATAATGTGAATTTTACTATTAGTGTTAA
>HPV48_Gamma_9628542_nt6203_L1_Tile|1
TGTTTATTACAGTTTTTGATAATACTCATAATGTGAATTTTACTATTAGTGTTAAGAATG
>HPV48_Gamma_9628542_nt6208_L1_Tile|1
ATTACAGTTTTTGATAATACTCATAATGTGAATTTTACTATTAGTGTTAAGAATGATAAA
>HPV48_Gamma_9628542_nt6213_L1_Tile|1
AGTTTTTGATAATACTCATAATGTGAATTTTACTATTAGTGTTAAGAATGATAAAACTGC
>HPV48_Gamma_9628542_nt6218_L1_Tile|1
TTGATAATACTCATAATGTGAATTTTACTATTAGTGTTAAGAATGATAAAACTGCATTAA
>HPV48_Gamma_9628542_nt6223_L1_Tile|1
AATACTCATAATGTGAATTTTACTATTAGTGTTAAGAATGATAAAACTGCATTAACTGAA
>HPV48_Gamma_9628542_nt6228_L1_Tile|1
TCATAATGTGAATTTTACTATTAGTGTTAAGAATGATAAAACTGCATTAACTGAAAACTA
>HPV48_Gamma_9628542_nt6233_L1_Tile|1
ATGTGAATTTTACTATTAGTGTTAAGAATGATAAAACTGCATTAACTGAAAACTACATAG
>HPV48_Gamma_9628542_nt6238_L1_Tile|1
AATTTTACTATTAGTGTTAAGAATGATAAAACTGCATTAACTGAAAACTACATAGATAAT
>HPV48_Gamma_9628542_nt6243_L1_Tile|1
TACTATTAGTGTTAAGAATGATAAAACTGCATTAACTGAAAACTACATAGATAATGGTTA
>HPV48_Gamma_9628542_nt6248_L1_Tile|1
TTAGTGTTAAGAATGATAAAACTGCATTAACTGAAAACTACATAGATAATGGTTACAAAT
>HPV48_Gamma_9628542_nt6253_L1_Tile|1
GTTAAGAATGATAAAACTGCATTAACTGAAAACTACATAGATAATGGTTACAAATATAAT
>HPV48_Gamma_9628542_nt6258_L1_Tile|1
GAATGATAAAACTGCATTAACTGAAAACTACATAGATAATGGTTACAAATATAATAATGC
>HPV48_Gamma_9628542_nt6263_L1_Tile|1
ATAAAACTGCATTAACTGAAAACTACATAGATAATGGTTACAAATATAATAATGCAGATT
>HPV48_Gamma_9628542_nt6268_L1_Tile|1
ACTGCATTAACTGAAAACTACATAGATAATGGTTACAAATATAATAATGCAGATTTTAAA

>HPV48_Gamma_9628542_nt6273_L1_Tile|1
ATTAACTGAAAACTACATAGATAATGGTTACAAATATAATAATGCAGATTTTAAACAATA
>HPV48_Gamma_9628542_nt6278_L1_Tile|1
CTGAAAACTACATAGATAATGGTTACAAATATAATAATGCAGATTTTAAACAATATCTTC
>HPV48_Gamma_9628542_nt6283_L1_Tile|1
AACTACATAGATAATGGTTACAAATATAATAATGCAGATTTTAAACAATATCTTCGACAT
>HPV48_Gamma_9628542_nt6288_L1_Tile|1
CATAGATAATGGTTACAAATATAATAATGCAGATTTTAAACAATATCTTCGACATACAGA
>HPV48_Gamma_9628542_nt6293_L1_Tile|1
ATAATGGTTACAAATATAATAATGCAGATTTTAAACAATATCTTCGACATACAGAAGAAT
>HPV48_Gamma_9628542_nt6298_L1_Tile|1
GGTTACAAATATAATAATGCAGATTTTAAACAATATCTTCGACATACAGAAGAATATGAA
>HPV48_Gamma_9628542_nt6303_L1_Tile|1
CAAATATAATAATGCAGATTTTAAACAATATCTTCGACATACAGAAGAATATGAAATAGA
>HPV48_Gamma_9628542_nt6308_L1_Tile|1
ATAATAATGCAGATTTTAAACAATATCTTCGACATACAGAAGAATATGAAATAGAGTTGG
>HPV48_Gamma_9628542_nt6313_L1_Tile|1
AATGCAGATTTTAAACAATATCTTCGACATACAGAAGAATATGAAATAGAGTTGGTTTTT
>HPV48_Gamma_9628542_nt6318_L1_Tile|1
AGATTTTAAACAATATCTTCGACATACAGAAGAATATGAAATAGAGTTGGTTTTTCAGTT
>HPV48_Gamma_9628542_nt6323_L1_Tile|1
TTAAACAATATCTTCGACATACAGAAGAATATGAAATAGAGTTGGTTTTTCAGTTATGCA
>HPV48_Gamma_9628542_nt6348_L1_Tile|1
AGAATATGAAATAGAGTTGGTTTTTCAGTTATGCAAAGTGAACTTGACTGCAGATGTTCT
>HPV48_Gamma_9628542_nt6353_L1_Tile|1
ATGAAATAGAGTTGGTTTTTCAGTTATGCAAAGTGAACTTGACTGCAGATGTTCTGGCAC
>HPV48_Gamma_9628542_nt6363_L1_Tile|1
GTTGGTTTTTCAGTTATGCAAAGTGAACTTGACTGCAGATGTTCTGGCACATTTACACGT
>HPV48_Gamma_9628542_nt6368_L1_Tile|1
TTTTTCAGTTATGCAAAGTGAACTTGACTGCAGATGTTCTGGCACATTTACACGTTATGA
>HPV48_Gamma_9628542_nt6373_L1_Tile|1
CAGTTATGCAAAGTGAACTTGACTGCAGATGTTCTGGCACATTTACACGTTATGAATCCC
>HPV48_Gamma_9628542_nt6378_L1_Tile|1
ATGCAAAGTGAACTTGACTGCAGATGTTCTGGCACATTTACACGTTATGAATCCCAGAAT
>HPV48_Gamma_9628542_nt6383_L1_Tile|1
AAGTGAACTTGACTGCAGATGTTCTGGCACATTTACACGTTATGAATCCCAGAATATTAG
>HPV48_Gamma_9628542_nt6388_L1_Tile|1
AACTTGACTGCAGATGTTCTGGCACATTTACACGTTATGAATCCCAGAATATTAGAGGAG
>HPV48_Gamma_9628542_nt6398_L1_Tile|1
CAGATGTTCTGGCACATTTACACGTTATGAATCCCAGAATATTAGAGGAGTGGCAATTAG
>HPV48_Gamma_9628542_nt6458_L1_Tile|1
CTTTTGTTCCACCAGCTCCAACGGGCATTGAAGATACTTACAGGTATATCAAATCCATGG
>HPV48_Gamma_9628542_nt6463_L1_Tile|1
GTTCCACCAGCTCCAACGGGCATTGAAGATACTTACAGGTATATCAAATCCATGGCTACT
>HPV48_Gamma_9628542_nt6468_L1_Tile|1
ACCAGCTCCAACGGGCATTGAAGATACTTACAGGTATATCAAATCCATGGCTACTAAATG
>HPV48_Gamma_9628542_nt6473_L1_Tile|1
CTCCAACGGGCATTGAAGATACTTACAGGTATATCAAATCCATGGCTACTAAATGCCCTA
>HPV48_Gamma_9628542_nt6478_L1_Tile|1
ACGGGCATTGAAGATACTTACAGGTATATCAAATCCATGGCTACTAAATGCCCTACTGCT
>HPV48_Gamma_9628542_nt6483_L1_Tile|1
CATTGAAGATACTTACAGGTATATCAAATCCATGGCTACTAAATGCCCTACTGCTGAACC
>HPV48_Gamma_9628542_nt6488_L1_Tile|1
AAGATACTTACAGGTATATCAAATCCATGGCTACTAAATGCCCTACTGCTGAACCAGAAG
>HPV48_Gamma_9628542_nt6493_L1_Tile|1

ACTTACAGGTATATCAAATCCATGGCTACTAAATGCCCTACTGCTGAACCAGAAGAAGAC
>HPV48_Gamma_9628542_nt6503_L1_Tile|1
ATATCAAATCCATGGCTACTAAATGCCCTACTGCTGAACCAGAAGAAGACACAGATCCAT
>HPV48_Gamma_9628542_nt6518_L1_Tile|1
CTACTAAATGCCCTACTGCTGAACCAGAAGAAGACACAGATCCATACAAAGCTTACAGTT
>HPV48_Gamma_9628542_nt6528_L1_Tile|1
CCCTACTGCTGAACCAGAAGAAGACACAGATCCATACAAAGCTTACAGTTTCTGGACTTT
>HPV48_Gamma_9628542_nt6533_L1_Tile|1
CTGCTGAACCAGAAGAAGACACAGATCCATACAAAGCTTACAGTTTCTGGACTTTAGATA
>HPV48_Gamma_9628542_nt6538_L1_Tile|1
GAACCAGAAGAAGACACAGATCCATACAAAGCTTACAGTTTCTGGACTTTAGATATGACA
>HPV48_Gamma_9628542_nt6543_L1_Tile|1
AGAAGAAGACACAGATCCATACAAAGCTTACAGTTTCTGGACTTTAGATATGACAGAGCG
>HPV48_Gamma_9628542_nt6548_L1_Tile|1
AAGACACAGATCCATACAAAGCTTACAGTTTCTGGACTTTAGATATGACAGAGCGCTTCT
>HPV48_Gamma_9628542_nt6553_L1_Tile|1
ACAGATCCATACAAAGCTTACAGTTTCTGGACTTTAGATATGACAGAGCGCTTCTCGTCT
>HPV48_Gamma_9628542_nt6558_L1_Tile|1
TCCATACAAAGCTTACAGTTTCTGGACTTTAGATATGACAGAGCGCTTCTCGTCTGATTT
>HPV48_Gamma_9628542_nt6563_L1_Tile|1
ACAAAGCTTACAGTTTCTGGACTTTAGATATGACAGAGCGCTTCTCGTCTGATTTAAGTC
>HPV48_Gamma_9628542_nt6568_L1_Tile|1
GCTTACAGTTTCTGGACTTTAGATATGACAGAGCGCTTCTCGTCTGATTTAAGTCAGTTT
>HPV48_Gamma_9628542_nt6573_L1_Tile|1
CAGTTTCTGGACTTTAGATATGACAGAGCGCTTCTCGTCTGATTTAAGTCAGTTTTCCTT
>HPV48_Gamma_9628542_nt6578_L1_Tile|1
TCTGGACTTTAGATATGACAGAGCGCTTCTCGTCTGATTTAAGTCAGTTTTCCTTGGGTC
>HPV48_Gamma_9628542_nt6583_L1_Tile|1
ACTTTAGATATGACAGAGCGCTTCTCGTCTGATTTAAGTCAGTTTTCCTTGGGTCGAAAA
>HPV48_Gamma_9628542_nt6588_L1_Tile|1
AGATATGACAGAGCGCTTCTCGTCTGATTTAAGTCAGTTTTCCTTGGGTCGAAAATTTTT
>HPV48_Gamma_9628542_nt6593_L1_Tile|1
TGACAGAGCGCTTCTCGTCTGATTTAAGTCAGTTTTCCTTGGGTCGAAAATTTTTATATC
>HPV48_Gamma_9628542_nt6598_L1_Tile|1
GAGCGCTTCTCGTCTGATTTAAGTCAGTTTTCCTTGGGTCGAAAATTTTTATATCAAACT
>HPV48_Gamma_9628542_nt6603_L1_Tile|1
CTTCTCGTCTGATTTAAGTCAGTTTTCCTTGGGTCGAAAATTTTTATATCAAACTGGTTT
>HPV48_Gamma_9628542_nt6613_L1_Tile|1
GATTTAAGTCAGTTTTCCTTGGGTCGAAAATTTTTATATCAAACTGGTTTGTTAAATGGT
>HPV48_Gamma_9628542_nt6623_L1_Tile|1
AGTTTTCCTTGGGTCGAAAATTTTTATATCAAACTGGTTTGTTAAATGGTAAACGAGCTA
>HPV48_Gamma_9628542_nt6628_L1_Tile|1
TCCTTGGGTCGAAAATTTTTATATCAAACTGGTTTGTTAAATGGTAAACGAGCTAGAACA
>HPV48_Gamma_9628542_nt6633_L1_Tile|1
GGGTCGAAAATTTTTATATCAAACTGGTTTGTTAAATGGTAAACGAGCTAGAACAGACTA
>HPV48_Gamma_9628542_nt6638_L1_Tile|1
GAAAATTTTTATATCAAACTGGTTTGTTAAATGGTAAACGAGCTAGAACAGACTATACAG
>HPV48_Gamma_9628542_nt6648_L1_Tile|1
ATATCAAACTGGTTTGTTAAATGGTAAACGAGCTAGAACAGACTATACAGCTGCAGGATC
>HPV48_Gamma_9628542_nt6653_L1_Tile|1
AAACTGGTTTGTTAAATGGTAAACGAGCTAGAACAGACTATACAGCTGCAGGATCTAGTA
>HPV48_Gamma_9628542_nt6658_L1_Tile|1
GGTTTGTTAAATGGTAAACGAGCTAGAACAGACTATACAGCTGCAGGATCTAGTACCAGA
>HPV48_Gamma_9628542_nt6663_L1_Tile|1
GTTAAATGGTAAACGAGCTAGAACAGACTATACAGCTGCAGGATCTAGTACCAGATCTAC

>HPV48_Gamma_9628542_nt6668_L1_Tile|1
ATGGTAAACGAGCTAGAACAGACTATACAGCTGCAGGATCTAGTACCAGATCTACAAAGC
>HPV48_Gamma_9628542_nt6673_L1_Tile|1
AAACGAGCTAGAACAGACTATACAGCTGCAGGATCTAGTACCAGATCTACAAAGCGTAGG
>HPV48_Gamma_9628542_nt6678_L1_Tile|1
AGCTAGAACAGACTATACAGCTGCAGGATCTAGTACCAGATCTACAAAGCGTAGGAGAGT
>HPV48_Gamma_9628542_nt6683_L1_Tile|1
GAACAGACTATACAGCTGCAGGATCTAGTACCAGATCTACAAAGCGTAGGAGAGTAAGAT
>HPV48_Gamma_9628542_nt6685_L1_Tile|1
ACAGACTATACAGCTGCAGGATCTAGTACCAGATCTACAAAGCGTAGGAGAGTAAGATAG
>HPV49_Beta_9627363_nt5952_L1_Tile|1
AGGACATCCATATTTTGATGTGAGAGATACAGCAGACAATTCTAAAATTTTAGTACCAAA
>HPV49_Beta_9627363_nt5957_L1_Tile|1
ATCCATATTTTGATGTGAGAGATACAGCAGACAATTCTAAAATTTTAGTACCAAAGGTTT
>HPV49_Beta_9627363_nt6167_L1_Tile|1
GTACAACAGGACATCCATTGTTTAACAAAGTCAAAGATACTGAAAATGCTAATAACTATA
>HPV49_Beta_9627363_nt6177_L1_Tile|1
ACATCCATTGTTTAACAAAGTCAAAGATACTGAAAATGCTAATAACTATATAGTAACTTC
>HPV49_Beta_9627363_nt6182_L1_Tile|2
CATTGTTTAACAAAGTCAAAGATACTGAAAATGCTAATAACTATATAGTAACTTCTAAAG
>HPV49_Beta_9627363_nt6292_L1_Tile|1
ATAGGTTGTACTCCTTGTATGGGTGAGTACTGGGACGCTGCTAAACCTTGTGATGCAGAT
>HPV49_Beta_9627363_nt6297_L1_Tile|1
TTGTACTCCTTGTATGGGTGAGTACTGGGACGCTGCTAAACCTTGTGATGCAGATGCTGG
>HPV49_Beta_9627363_nt6332_L1_Tile|1
CTAAACCTTGTGATGCAGATGCTGGTCAGGGTAAATGCCCTCCATTAGAATTAATCAATT
>HPV49_Beta_9627363_nt6337_L1_Tile|1
CCTTGTGATGCAGATGCTGGTCAGGGTAAATGCCCTCCATTAGAATTAATCAATTCAGTT
>HPV49_Beta_9627363_nt6392_L1_Tile|1
CAGTTATACAAGATGGTGATATGATTGATATAGGTTTTGGTAATATCAATAATAAGACAT
>HPV49_Beta_9627363_nt6587_L1_Tile|1
GGCACTTCTTTGTTAGAGGTGGTAATGTAGGGGATGCGATACCCAATACTGCTGTAGGTC
>HPV49_Beta_9627363_nt6592_L1_Tile|1
TTCTTTGTTAGAGGTGGTAATGTAGGGGATGCGATACCCAATACTGCTGTAGGTCAGGAT
>HPV49_Beta_9627363_nt6602_L1_Tile|2
GAGGTGGTAATGTAGGGGATGCGATACCCAATACTGCTGTAGGTCAGGATAACAATTACA
>HPV49_Beta_9627363_nt6607_L1_Tile|1
GGTAATGTAGGGGATGCGATACCCAATACTGCTGTAGGTCAGGATAACAATTACATATTA
>HPV49_Beta_9627363_nt6612_L1_Tile|1
TGTAGGGGATGCGATACCCAATACTGCTGTAGGTCAGGATAACAATTACATATTACCTGC
>HPV49_Beta_9627363_nt6617_L1_Tile|1
GGGATGCGATACCCAATACTGCTGTAGGTCAGGATAACAATTACATATTACCTGCAGCAA
>HPV49_Beta_9627363_nt6622_L1_Tile|2
GCGATACCCAATACTGCTGTAGGTCAGGATAACAATTACATATTACCTGCAGCAAGTCAA
>HPV49_Beta_9627363_nt6627_L1_Tile|1
ACCCAATACTGCTGTAGGTCAGGATAACAATTACATATTACCTGCAGCAAGTCAACAGGC
>HPV49_Beta_9627363_nt6632_L1_Tile|1
ATACTGCTGTAGGTCAGGATAACAATTACATATTACCTGCAGCAAGTCAACAGGCCCAAA
>HPV49_Beta_9627363_nt6647_L1_Tile|1
AGGATAACAATTACATATTACCTGCAGCAAGTCAACAGGCCCAAAATACTCTTGGCAGCT
>HPV49_Beta_9627363_nt6652_L1_Tile|1
AACAATTACATATTACCTGCAGCAAGTCAACAGGCCCAAAATACTCTTGGCAGCTCCATC
>HPV49_Beta_9627363_nt6657_L1_Tile|1
TTACATATTACCTGCAGCAAGTCAACAGGCCCAAAATACTCTTGGCAGCTCCATCTATTT
>HPV49_Beta_9627363_nt6677_L1_Tile|1

GTCAACAGGCCCAAAATACTCTTGGCAGCTCCATCTATTTCCCTACCGTCAGTGGCTCTT
>HPV49_Beta_9627363_nt6822_L1_Tile|2
TCAGCTTTTTATAACAGTGGCTGATAATACCAGAAATACCAATTTTACTATTAGTGTAAG
>HPV49_Beta_9627363_nt6827_L1_Tile|1
TTTTTATAACAGTGGCTGATAATACCAGAAATACCAATTTTACTATTAGTGTAAGTACGG
>HPV49_Beta_9627363_nt6832_L1_Tile|1
ATAACAGTGGCTGATAATACCAGAAATACCAATTTTACTATTAGTGTAAGTACGGATGGC
>HPV49_Beta_9627363_nt6847_L1_Tile|1
AATACCAGAAATACCAATTTTACTATTAGTGTAAGTACGGATGGCCAGACACCTACAGAA
>HPV49_Beta_9627363_nt6852_L1_Tile|1
CAGAAATACCAATTTTACTATTAGTGTAAGTACGGATGGCCAGACACCTACAGAATATGA
>HPV49_Beta_9627363_nt6857_L1_Tile|1
ATACCAATTTTACTATTAGTGTAAGTACGGATGGCCAGACACCTACAGAATATGACAGTA
>HPV49_Beta_9627363_nt6862_L1_Tile|2
AATTTTACTATTAGTGTAAGTACGGATGGCCAGACACCTACAGAATATGACAGTACCAAG
>HPV49_Beta_9627363_nt6867_L1_Tile|1
TACTATTAGTGTAAGTACGGATGGCCAGACACCTACAGAATATGACAGTACCAAGGTTAG
>HPV49_Beta_9627363_nt7052_L1_Tile|1
AATTGGGATTTGTTCCTACCCCTGATAATCCTATACATGACACATATAGGTATCTTACAT
>HPV49_Beta_9627363_nt7057_L1_Tile|1
GGATTTGTTCCTACCCCTGATAATCCTATACATGACACATATAGGTATCTTACATCACAG
>HPV49_Beta_9627363_nt7067_L1_Tile|1
CTACCCCTGATAATCCTATACATGACACATATAGGTATCTTACATCACAGGCAACACGAT
>HPV49_Beta_9627363_nt7072_L1_Tile|1
CCTGATAATCCTATACATGACACATATAGGTATCTTACATCACAGGCAACACGATGCCCT
>HPV49_Beta_9627363_nt7077_L1_Tile|1
TAATCCTATACATGACACATATAGGTATCTTACATCACAGGCAACACGATGCCCTGACAA
>HPV49_Beta_9627363_nt7082_L1_Tile|2
CTATACATGACACATATAGGTATCTTACATCACAGGCAACACGATGCCCTGACAAACAAC
>HPV49_Beta_9627363_nt7087_L1_Tile|1
CATGACACATATAGGTATCTTACATCACAGGCAACACGATGCCCTGACAAACAACCTGCT
>HPV49_Beta_9627363_nt7097_L1_Tile|1
ATAGGTATCTTACATCACAGGCAACACGATGCCCTGACAAACAACCTGCTCCAGAAAGGA
>HPV49_Beta_9627363_nt7112_L1_Tile|1
CACAGGCAACACGATGCCCTGACAAACAACCTGCTCCAGAAAGGAAAGATCCATATGAGC
>HPV49_Beta_9627363_nt7117_L1_Tile|1
GCAACACGATGCCCTGACAAACAACCTGCTCCAGAAAGGAAAGATCCATATGAGCAGTAT
>HPV49_Beta_9627363_nt7122_L1_Tile|2
ACGATGCCCTGACAAACAACCTGCTCCAGAAAGGAAAGATCCATATGAGCAGTATAACTT
>HPV49_Beta_9627363_nt7232_L1_Tile|1
CTTTAGGAAGAAAGTTTTTATTTCAAGCTGGGCTACAACGGGCTTCTAGAGTGTCTAAAT
>HPV49_Beta_9627363_nt7242_L1_Tile|2
AAAGTTTTTATTTCAAGCTGGGCTACAACGGGCTTCTAGAGTGTCTAAATCCTCTGCTGC
>HPV49_Beta_9627363_nt7247_L1_Tile|1
TTTTATTTCAAGCTGGGCTACAACGGGCTTCTAGAGTGTCTAAATCCTCTGCTGCTAGAG
>HPV49_Beta_9627363_nt7252_L1_Tile|1
TTTCAAGCTGGGCTACAACGGGCTTCTAGAGTGTCTAAATCCTCTGCTGCTAGAGCTTCC
>HPV49_Beta_9627363_nt7257_L1_Tile|1
AGCTGGGCTACAACGGGCTTCTAGAGTGTCTAAATCCTCTGCTGCTAGAGCTTCCACACG
>HPV49_Beta_9627363_nt7262_L1_Tile|2
GGCTACAACGGGCTTCTAGAGTGTCTAAATCCTCTGCTGCTAGAGCTTCCACACGGGGTA
>HPV49_Beta_9627363_nt7267_L1_Tile|1
CAACGGGCTTCTAGAGTGTCTAAATCCTCTGCTGCTAGAGCTTCCACACGGGGTATTAAA
>HPV49_Beta_9627363_nt7282_L1_Tile|2
GTGTCTAAATCCTCTGCTGCTAGAGCTTCCACACGGGGTATTAAACGAAAACGGAGATGA

```
>HPV50_Gamma_9628550_nt5232_L1_Tile|1
ATGGCTCATTGGTCCTCAACCTCTGGAAAGTTATACCTTCCACCAAGTACTCCTGTTGCC
>HPV50_Gamma_9628550_nt5237_L1_Tile|1
TCATTGGTCCTCAACCTCTGGAAAGTTATACCTTCCACCAAGTACTCCTGTTGCCAGAGT
>HPV50_Gamma_9628550_nt5242_L1_Tile|2
GGTCCTCAACCTCTGGAAAGTTATACCTTCCACCAAGTACTCCTGTTGCCAGAGTTTTAA
>HPV50_Gamma_9628550_nt5247_L1_Tile|1
TCAACCTCTGGAAAGTTATACCTTCCACCAAGTACTCCTGTTGCCAGAGTTTTAAGCACC
>HPV50_Gamma_9628550_nt5272_L1_Tile|1
CACCAAGTACTCCTGTTGCCAGAGTTTTAAGCACCGATGAATATGTGAAAGAAACTGATG
>HPV50_Gamma_9628550_nt5277_L1_Tile|1
AGTACTCCTGTTGCCAGAGTTTTAAGCACCGATGAATATGTGAAAGAAACTGATGTTTAC
>HPV50_Gamma_9628550_nt5322_L1_Tile|2
GAAACTGATGTTTACTTTCATGCACGCAGTGAGCGTTTACTAATTGTGGGACACCCATAT
>HPV50_Gamma_9628550_nt5347_L1_Tile|1
GCAGTGAGCGTTTACTAATTGTGGGACACCCATATTATGATATAGAAGATGGTGGAGATA
>HPV50_Gamma_9628550_nt5352_L1_Tile|1
GAGCGTTTACTAATTGTGGGACACCCATATTATGATATAGAAGATGGTGGAGATATCAAA
>HPV50_Gamma_9628550_nt5357_L1_Tile|1
TTTACTAATTGTGGGACACCCATATTATGATATAGAAGATGGTGGAGATATCAAAGTTCC
>HPV50_Gamma_9628550_nt5362_L1_Tile|2
TAATTGTGGGACACCCATATTATGATATAGAAGATGGTGGAGATATCAAAGTTCCTAAAG
>HPV50_Gamma_9628550_nt5372_L1_Tile|1
ACACCCATATTATGATATAGAAGATGGTGGAGATATCAAAGTTCCTAAAGTATCAGCAAA
>HPV50_Gamma_9628550_nt5377_L1_Tile|1
CATATTATGATATAGAAGATGGTGGAGATATCAAAGTTCCTAAAGTATCAGCAAATCAAT
>HPV50_Gamma_9628550_nt5397_L1_Tile|1
GGTGGAGATATCAAAGTTCCTAAAGTATCAGCAAATCAATACAGAGTTTTTCGTTGTGAA
>HPV50_Gamma_9628550_nt5482_L1_Tile|2
TAATTGATACTACATTGTATAACTCAGATACAGAACGTTTGGTTTGGAAACTAGTAGGTA
>HPV50_Gamma_9628550_nt5487_L1_Tile|1
GATACTACATTGTATAACTCAGATACAGAACGTTTGGTTTGGAAACTAGTAGGTATAGAA
>HPV50_Gamma_9628550_nt5492_L1_Tile|1
TACATTGTATAACTCAGATACAGAACGTTTGGTTTGGAAACTAGTAGGTATAGAAGTCGG
>HPV50_Gamma_9628550_nt5497_L1_Tile|1
TGTATAACTCAGATACAGAACGTTTGGTTTGGAAACTAGTAGGTATAGAAGTCGGGAGAG
>HPV50_Gamma_9628550_nt5507_L1_Tile|1
AGATACAGAACGTTTGGTTTGGAAACTAGTAGGTATAGAAGTCGGGAGAGGTGGGCCTTT
>HPV50_Gamma_9628550_nt5522_L1_Tile|2
GGTTTGGAAACTAGTAGGTATAGAAGTCGGGAGAGGTGGGCCTTTAGGTGTTGGATCTAC
>HPV50_Gamma_9628550_nt5527_L1_Tile|1
GGAAACTAGTAGGTATAGAAGTCGGGAGAGGTGGGCCTTTAGGTGTTGGATCTACTGGTC
>HPV50_Gamma_9628550_nt5617_L1_Tile|1
AAAATCCTAGCTTTTACTTAGGTCCACAAGAAAAAGATGAAAGACAAAACTTGTCTATAG
>HPV50_Gamma_9628550_nt5622_L1_Tile|2
CCTAGCTTTTACTTAGGTCCACAAGAAAAAGATGAAAGACAAAACTTGTCTATAGATCCT
>HPV50_Gamma_9628550_nt5632_L1_Tile|1
ACTTAGGTCCACAAGAAAAAGATGAAAGACAAAACTTGTCTATAGATCCTAAACAAACAC
>HPV50_Gamma_9628550_nt5642_L1_Tile|2
ACAAGAAAAAGATGAAAGACAAAACTTGTCTATAGATCCTAAACAAACACAGTTATTGAT
>HPV50_Gamma_9628550_nt5647_L1_Tile|1
AAAAAGATGAAAGACAAAACTTGTCTATAGATCCTAAACAAACACAGTTATTGATTGTAG
>HPV50_Gamma_9628550_nt5652_L1_Tile|1
GATGAAAGACAAAACTTGTCTATAGATCCTAAACAAACACAGTTATTGATTGTAGGCTGC
>HPV50_Gamma_9628550_nt5667_L1_Tile|1
```

TTGTCTATAGATCCTAAACAAACACAGTTATTGATTGTAGGCTGCAAACCAGCTGTTGGT
>HPV50_Gamma_9628550_nt5687_L1_Tile|1
AACACAGTTATTGATTGTAGGCTGCAAACCAGCTGTTGGTGAATATTGGGATTTAGCAGA
>HPV50_Gamma_9628550_nt5692_L1_Tile|1
AGTTATTGATTGTAGGCTGCAAACCAGCTGTTGGTGAATATTGGGATTTAGCAGAACCTT
>HPV50_Gamma_9628550_nt5702_L1_Tile|2
TGTAGGCTGCAAACCAGCTGTTGGTGAATATTGGGATTTAGCAGAACCTTGTGACAAAAA
>HPV50_Gamma_9628550_nt5717_L1_Tile|1
AGCTGTTGGTGAATATTGGGATTTAGCAGAACCTTGTGACAAAAACAGCTTAAATAATGG
>HPV50_Gamma_9628550_nt5742_L1_Tile|2
GCAGAACCTTGTGACAAAAACAGCTTAAATAATGGCAAGTGTCCTCCAATACAACTAGTA
>HPV50_Gamma_9628550_nt5747_L1_Tile|1
ACCTTGTGACAAAAACAGCTTAAATAATGGCAAGTGTCCTCCAATACAACTAGTAAACAG
>HPV50_Gamma_9628550_nt5752_L1_Tile|1
GTGACAAAAACAGCTTAAATAATGGCAAGTGTCCTCCAATACAACTAGTAAACAGTTATA
>HPV50_Gamma_9628550_nt5757_L1_Tile|1
AAAAACAGCTTAAATAATGGCAAGTGTCCTCCAATACAACTAGTAAACAGTTATATTCAG
>HPV50_Gamma_9628550_nt5827_L1_Tile|1
TGGGAGATATAGGATTTGGTAATGCAAACTTTCCTAAACTACAACAGGACAGAGCAGGGG
>HPV50_Gamma_9628550_nt5832_L1_Tile|1
GATATAGGATTTGGTAATGCAAACTTTCCTAAACTACAACAGGACAGAGCAGGGGTCCCT
>HPV50_Gamma_9628550_nt5837_L1_Tile|1
AGGATTTGGTAATGCAAACTTTCCTAAACTACAACAGGACAGAGCAGGGGTCCCTTTAGA
>HPV50_Gamma_9628550_nt5847_L1_Tile|1
AATGCAAACTTTCCTAAACTACAACAGGACAGAGCAGGGGTCCCTTTAGATATAGTGGAT
>HPV50_Gamma_9628550_nt5852_L1_Tile|1
AAACTTTCCTAAACTACAACAGGACAGAGCAGGGGTCCCTTTAGATATAGTGGATTCAAT
>HPV50_Gamma_9628550_nt5862_L1_Tile|2
AAACTACAACAGGACAGAGCAGGGGTCCCTTTAGATATAGTGGATTCAATCAGCTTATGG
>HPV50_Gamma_9628550_nt5872_L1_Tile|1
AGGACAGAGCAGGGGTCCCTTTAGATATAGTGGATTCAATCAGCTTATGGCCTGATCTAT
>HPV50_Gamma_9628550_nt5882_L1_Tile|2
AGGGGTCCCTTTAGATATAGTGGATTCAATCAGCTTATGGCCTGATCTATTAAAAATGAC
>HPV50_Gamma_9628550_nt5887_L1_Tile|1
TCCCTTTAGATATAGTGGATTCAATCAGCTTATGGCCTGATCTATTAAAAATGACTAAAG
>HPV50_Gamma_9628550_nt5892_L1_Tile|1
TTAGATATAGTGGATTCAATCAGCTTATGGCCTGATCTATTAAAAATGACTAAAGATGTC
>HPV50_Gamma_9628550_nt5897_L1_Tile|1
TATAGTGGATTCAATCAGCTTATGGCCTGATCTATTAAAAATGACTAAAGATGTCTATGG
>HPV50_Gamma_9628550_nt5902_L1_Tile|2
TGGATTCAATCAGCTTATGGCCTGATCTATTAAAAATGACTAAAGATGTCTATGGGGACC
>HPV50_Gamma_9628550_nt5917_L1_Tile|1
TATGGCCTGATCTATTAAAAATGACTAAAGATGTCTATGGGGACCATGTGTTTTTCTATG
>HPV50_Gamma_9628550_nt5927_L1_Tile|1
TCTATTAAAAATGACTAAAGATGTCTATGGGGACCATGTGTTTTTCTATGCAAAGCAAGA
>HPV50_Gamma_9628550_nt5937_L1_Tile|1
ATGACTAAAGATGTCTATGGGGACCATGTGTTTTTCTATGCAAAGCAAGAGCAATTATAT
>HPV50_Gamma_9628550_nt5977_L1_Tile|1
CAAAGCAAGAGCAATTATATGCCAGACATTTGTTTACCCATGCAGGACCAATTGGGGAAC
>HPV50_Gamma_9628550_nt5982_L1_Tile|2
CAAGAGCAATTATATGCCAGACATTTGTTTACCCATGCAGGACCAATTGGGGAACCTATA
>HPV50_Gamma_9628550_nt5987_L1_Tile|1
GCAATTATATGCCAGACATTTGTTTACCCATGCAGGACCAATTGGGGAACCTATACCTAA
>HPV50_Gamma_9628550_nt5992_L1_Tile|1
TATATGCCAGACATTTGTTTACCCATGCAGGACCAATTGGGGAACCTATACCTAATGTCT

>HPV50_Gamma_9628550_nt5997_L1_Tile|1
GCCAGACATTTGTTTACCCATGCAGGACCAATTGGGGAACCTATACCTAATGTCTCAGGA
>HPV50_Gamma_9628550_nt6002_L1_Tile|2
ACATTTGTTTACCCATGCAGGACCAATTGGGGAACCTATACCTAATGTCTCAGGAGTTTA
>HPV50_Gamma_9628550_nt6007_L1_Tile|1
TGTTTACCCATGCAGGACCAATTGGGGAACCTATACCTAATGTCTCAGGAGTTTATAATT
>HPV50_Gamma_9628550_nt6022_L1_Tile|2
GACCAATTGGGGAACCTATACCTAATGTCTCAGGAGTTTATAATTATGCAGTAAACCCTA
>HPV50_Gamma_9628550_nt6027_L1_Tile|1
ATTGGGGAACCTATACCTAATGTCTCAGGAGTTTATAATTATGCAGTAAACCCTAATCAA
>HPV50_Gamma_9628550_nt6032_L1_Tile|1
GGAACCTATACCTAATGTCTCAGGAGTTTATAATTATGCAGTAAACCCTAATCAACCAGA
>HPV50_Gamma_9628550_nt6037_L1_Tile|1
CTATACCTAATGTCTCAGGAGTTTATAATTATGCAGTAAACCCTAATCAACCAGAGCAAA
>HPV50_Gamma_9628550_nt6042_L1_Tile|2
CCTAATGTCTCAGGAGTTTATAATTATGCAGTAAACCCTAATCAACCAGAGCAAAATCGT
>HPV50_Gamma_9628550_nt6047_L1_Tile|1
TGTCTCAGGAGTTTATAATTATGCAGTAAACCCTAATCAACCAGAGCAAAATCGTAGAAC
>HPV50_Gamma_9628550_nt6057_L1_Tile|1
GTTTATAATTATGCAGTAAACCCTAATCAACCAGAGCAAAATCGTAGAACCAATATTGGT
>HPV50_Gamma_9628550_nt6062_L1_Tile|2
TAATTATGCAGTAAACCCTAATCAACCAGAGCAAAATCGTAGAACCAATATTGGTTCCTA
>HPV50_Gamma_9628550_nt6067_L1_Tile|1
ATGCAGTAAACCCTAATCAACCAGAGCAAAATCGTAGAACCAATATTGGTTCCTACTTAT
>HPV50_Gamma_9628550_nt6072_L1_Tile|1
GTAAACCCTAATCAACCAGAGCAAAATCGTAGAACCAATATTGGTTCCTACTTATATTTT
>HPV50_Gamma_9628550_nt6077_L1_Tile|1
CCCTAATCAACCAGAGCAAAATCGTAGAACCAATATTGGTTCCTACTTATATTTTACTAC
>HPV50_Gamma_9628550_nt6082_L1_Tile|2
ATCAACCAGAGCAAAATCGTAGAACCAATATTGGTTCCTACTTATATTTTACTACACCAA
>HPV50_Gamma_9628550_nt6092_L1_Tile|1
GCAAAATCGTAGAACCAATATTGGTTCCTACTTATATTTTACTACACCAAGTGGATCTTT
>HPV50_Gamma_9628550_nt6097_L1_Tile|1
ATCGTAGAACCAATATTGGTTCCTACTTATATTTTACTACACCAAGTGGATCTTTAAATA
>HPV50_Gamma_9628550_nt6102_L1_Tile|2
AGAACCAATATTGGTTCCTACTTATATTTTACTACACCAAGTGGATCTTTAAATACAAGC
>HPV50_Gamma_9628550_nt6107_L1_Tile|1
CAATATTGGTTCCTACTTATATTTTACTACACCAAGTGGATCTTTAAATACAAGCAGTTC
>HPV50_Gamma_9628550_nt6112_L1_Tile|1
TTGGTTCCTACTTATATTTTACTACACCAAGTGGATCTTTAAATACAAGCAGTTCACAGT
>HPV50_Gamma_9628550_nt6117_L1_Tile|1
TCCTACTTATATTTTACTACACCAAGTGGATCTTTAAATACAAGCAGTTCACAGTTATTT
>HPV50_Gamma_9628550_nt6137_L1_Tile|1
ACCAAGTGGATCTTTAAATACAAGCAGTTCACAGTTATTTAATAGACCATATTGGATTCG
>HPV50_Gamma_9628550_nt6207_L1_Tile|1
GGTACGAACAATTGTATTTGTTGGGGTAATGAGGTATTTGTTACAGTATTTGATAACACC
>HPV50_Gamma_9628550_nt6217_L1_Tile|1
ATTGTATTTGTTGGGGTAATGAGGTATTTGTTACAGTATTTGATAACACCAGAAACATTA
>HPV50_Gamma_9628550_nt6222_L1_Tile|2
ATTTGTTGGGGTAATGAGGTATTTGTTACAGTATTTGATAACACCAGAAACATTAACTTT
>HPV50_Gamma_9628550_nt6227_L1_Tile|1
TTGGGGTAATGAGGTATTTGTTACAGTATTTGATAACACCAGAAACATTAACTTTAATAT
>HPV50_Gamma_9628550_nt6232_L1_Tile|1
GTAATGAGGTATTTGTTACAGTATTTGATAACACCAGAAACATTAACTTTAATATTAGTG
>HPV50_Gamma_9628550_nt6237_L1_Tile|1

GAGGTATTTGTTACAGTATTTGATAACACCAGAAACATTAACTTTAATATTAGTGTTAAG
>HPV50_Gamma_9628550_nt6242_L1_Tile|2
ATTTGTTACAGTATTTGATAACACCAGAAACATTAACTTTAATATTAGTGTTAAGAAAGA
>HPV50_Gamma_9628550_nt6247_L1_Tile|1
TTACAGTATTTGATAACACCAGAAACATTAACTTTAATATTAGTGTTAAGAAAGATGTCA
>HPV50_Gamma_9628550_nt6252_L1_Tile|1
GTATTTGATAACACCAGAAACATTAACTTTAATATTAGTGTTAAGAAAGATGTCAATCCT
>HPV50_Gamma_9628550_nt6257_L1_Tile|1
TGATAACACCAGAAACATTAACTTTAATATTAGTGTTAAGAAAGATGTCAATCCTTTGGA
>HPV50_Gamma_9628550_nt6262_L1_Tile|2
ACACCAGAAACATTAACTTTAATATTAGTGTTAAGAAAGATGTCAATCCTTTGGATCCTT
>HPV50_Gamma_9628550_nt6267_L1_Tile|1
AGAAACATTAACTTTAATATTAGTGTTAAGAAAGATGTCAATCCTTTGGATCCTTTAAAT
>HPV50_Gamma_9628550_nt6272_L1_Tile|1
CATTAACTTTAATATTAGTGTTAAGAAAGATGTCAATCCTTTGGATCCTTTAAATGTAGC
>HPV50_Gamma_9628550_nt6277_L1_Tile|1
ACTTTAATATTAGTGTTAAGAAAGATGTCAATCCTTTGGATCCTTTAAATGTAGCAAGTT
>HPV50_Gamma_9628550_nt6287_L1_Tile|1
TAGTGTTAAGAAAGATGTCAATCCTTTGGATCCTTTAAATGTAGCAAGTTCTTATATGTA
>HPV50_Gamma_9628550_nt6292_L1_Tile|1
TTAAGAAAGATGTCAATCCTTTGGATCCTTTAAATGTAGCAAGTTCTTATATGTACTCAA
>HPV50_Gamma_9628550_nt6297_L1_Tile|1
AAAGATGTCAATCCTTTGGATCCTTTAAATGTAGCAAGTTCTTATATGTACTCAAAGGAT
>HPV50_Gamma_9628550_nt6302_L1_Tile|2
TGTCAATCCTTTGGATCCTTTAAATGTAGCAAGTTCTTATATGTACTCAAAGGATGATTT
>HPV50_Gamma_9628550_nt6307_L1_Tile|1
ATCCTTTGGATCCTTTAAATGTAGCAAGTTCTTATATGTACTCAAAGGATGATTTTAATC
>HPV50_Gamma_9628550_nt6322_L1_Tile|2
TAAATGTAGCAAGTTCTTATATGTACTCAAAGGATGATTTTAATCAGTACAGCCGTCATA
>HPV50_Gamma_9628550_nt6327_L1_Tile|1
GTAGCAAGTTCTTATATGTACTCAAAGGATGATTTTAATCAGTACAGCCGTCATACTGAA
>HPV50_Gamma_9628550_nt6332_L1_Tile|1
AAGTTCTTATATGTACTCAAAGGATGATTTTAATCAGTACAGCCGTCATACTGAAGAATA
>HPV50_Gamma_9628550_nt6337_L1_Tile|1
CTTATATGTACTCAAAGGATGATTTTAATCAGTACAGCCGTCATACTGAAGAATATGAAT
>HPV50_Gamma_9628550_nt6342_L1_Tile|2
ATGTACTCAAAGGATGATTTTAATCAGTACAGCCGTCATACTGAAGAATATGAATTAGAA
>HPV50_Gamma_9628550_nt6347_L1_Tile|1
CTCAAAGGATGATTTTAATCAGTACAGCCGTCATACTGAAGAATATGAATTAGAATTTAT
>HPV50_Gamma_9628550_nt6387_L1_Tile|1
GAATATGAATTAGAATTTATATTTCAGTTATGTAAAGTAGGATTGGATGCTGATATATTA
>HPV50_Gamma_9628550_nt6392_L1_Tile|1
TGAATTAGAATTTATATTTCAGTTATGTAAAGTAGGATTGGATGCTGATATATTAGCTCA
>HPV50_Gamma_9628550_nt6397_L1_Tile|1
TAGAATTTATATTTCAGTTATGTAAAGTAGGATTGGATGCTGATATATTAGCTCATCTTA
>HPV50_Gamma_9628550_nt6402_L1_Tile|2
TTTATATTTCAGTTATGTAAAGTAGGATTGGATGCTGATATATTAGCTCATCTTAATGTA
>HPV50_Gamma_9628550_nt6407_L1_Tile|1
ATTTCAGTTATGTAAAGTAGGATTGGATGCTGATATATTAGCTCATCTTAATGTAATGGA
>HPV50_Gamma_9628550_nt6417_L1_Tile|1
TGTAAAGTAGGATTGGATGCTGATATATTAGCTCATCTTAATGTAATGGACCCCAGAATT
>HPV50_Gamma_9628550_nt6422_L1_Tile|2
AGTAGGATTGGATGCTGATATATTAGCTCATCTTAATGTAATGGACCCCAGAATTTTGGA
>HPV50_Gamma_9628550_nt6427_L1_Tile|1
GATTGGATGCTGATATATTAGCTCATCTTAATGTAATGGACCCCAGAATTTTGGAAAATT

>HPV50_Gamma_9628550_nt6432_L1_Tile|1
GATGCTGATATATTAGCTCATCTTAATGTAATGGACCCCAGAATTTTGGAAAATTGGCAA
>HPV50_Gamma_9628550_nt6437_L1_Tile|1
TGATATATTAGCTCATCTTAATGTAATGGACCCCAGAATTTTGGAAAATTGGCAATTAGC
>HPV50_Gamma_9628550_nt6442_L1_Tile|2
TATTAGCTCATCTTAATGTAATGGACCCCAGAATTTTGGAAAATTGGCAATTAGCTTATG
>HPV50_Gamma_9628550_nt6447_L1_Tile|1
GCTCATCTTAATGTAATGGACCCCAGAATTTTGGAAAATTGGCAATTAGCTTATGTTCCA
>HPV50_Gamma_9628550_nt6472_L1_Tile|1
GAATTTTGGAAAATTGGCAATTAGCTTATGTTCCACCAGCTCCCAGTGGTATAGGCGATA
>HPV50_Gamma_9628550_nt6522_L1_Tile|2
ATAGGCGATACTTACAGATACTTAAAATCTGATGCTACAAAATGTCCTGCAAAAGACAGT
>HPV50_Gamma_9628550_nt6527_L1_Tile|1
CGATACTTACAGATACTTAAAATCTGATGCTACAAAATGTCCTGCAAAAGACAGTAGCGC
>HPV50_Gamma_9628550_nt6532_L1_Tile|1
CTTACAGATACTTAAAATCTGATGCTACAAAATGTCCTGCAAAAGACAGTAGCGCCGAAG
>HPV50_Gamma_9628550_nt6537_L1_Tile|1
AGATACTTAAAATCTGATGCTACAAAATGTCCTGCAAAAGACAGTAGCGCCGAAGTAGTA
>HPV50_Gamma_9628550_nt6542_L1_Tile|2
CTTAAAATCTGATGCTACAAAATGTCCTGCAAAAGACAGTAGCGCCGAAGTAGTAGACCC
>HPV50_Gamma_9628550_nt6547_L1_Tile|1
AATCTGATGCTACAAAATGTCCTGCAAAAGACAGTAGCGCCGAAGTAGTAGACCCTTATA
>HPV50_Gamma_9628550_nt6552_L1_Tile|1
GATGCTACAAAATGTCCTGCAAAAGACAGTAGCGCCGAAGTAGTAGACCCTTATAAAGAA
>HPV50_Gamma_9628550_nt6572_L1_Tile|1
AAAAGACAGTAGCGCCGAAGTAGTAGACCCTTATAAAGAATATACTTTTTGGAATGTCAA
>HPV50_Gamma_9628550_nt6587_L1_Tile|1
CGAAGTAGTAGACCCTTATAAAGAATATACTTTTTGGAATGTCAACCTTACAGAAAAGTT
>HPV50_Gamma_9628550_nt6592_L1_Tile|1
TAGTAGACCCTTATAAAGAATATACTTTTTGGAATGTCAACCTTACAGAAAAGTTTTCGT
>HPV50_Gamma_9628550_nt6597_L1_Tile|1
GACCCTTATAAAGAATATACTTTTTGGAATGTCAACCTTACAGAAAAGTTTTCGTCTGAA
>HPV50_Gamma_9628550_nt6602_L1_Tile|2
TTATAAAGAATATACTTTTTGGAATGTCAACCTTACAGAAAAGTTTTCGTCTGAACTTGA
>HPV50_Gamma_9628550_nt6607_L1_Tile|1
AAGAATATACTTTTTGGAATGTCAACCTTACAGAAAAGTTTTCGTCTGAACTTGATCAGT
>HPV50_Gamma_9628550_nt6612_L1_Tile|1
TATACTTTTTGGAATGTCAACCTTACAGAAAAGTTTTCGTCTGAACTTGATCAGTATGCC
>HPV50_Gamma_9628550_nt6617_L1_Tile|1
TTTTTGGAATGTCAACCTTACAGAAAAGTTTTCGTCTGAACTTGATCAGTATGCCCTAGG
>HPV50_Gamma_9628550_nt6627_L1_Tile|1
GTCAACCTTACAGAAAAGTTTTCGTCTGAACTTGATCAGTATGCCCTAGGACGAAAATTT
>HPV50_Gamma_9628550_nt6642_L1_Tile|2
AAGTTTTCGTCTGAACTTGATCAGTATGCCCTAGGACGAAAATTTTTGTTTCAGACTGGA
>HPV50_Gamma_9628550_nt6647_L1_Tile|1
TTCGTCTGAACTTGATCAGTATGCCCTAGGACGAAAATTTTTGTTTCAGACTGGATTATT
>HPV50_Gamma_9628550_nt6652_L1_Tile|1
CTGAACTTGATCAGTATGCCCTAGGACGAAAATTTTTGTTTCAGACTGGATTATTAAAGA
>HPV50_Gamma_9628550_nt6657_L1_Tile|1
CTTGATCAGTATGCCCTAGGACGAAAATTTTTGTTTCAGACTGGATTATTAAAGAGAAGA
>HPV50_Gamma_9628550_nt6662_L1_Tile|2
TCAGTATGCCCTAGGACGAAAATTTTTGTTTCAGACTGGATTATTAAAGAGAAGAGTTAG
>HPV50_Gamma_9628550_nt6667_L1_Tile|1
ATGCCCTAGGACGAAAATTTTTGTTTCAGACTGGATTATTAAAGAGAAGAGTTAGAACTG
>HPV50_Gamma_9628550_nt6672_L1_Tile|1

CTAGGACGAAAATTTTTGTTTCAGACTGGATTATTAAAGAGAAGAGTTAGAACTGATTAC
>HPV50_Gamma_9628550_nt6677_L1_Tile|1
ACGAAAATTTTTGTTTCAGACTGGATTATTAAAGAGAAGAGTTAGAACTGATTACACTGT
>HPV50_Gamma_9628550_nt6682_L1_Tile|2
AATTTTTGTTTCAGACTGGATTATTAAAGAGAAGAGTTAGAACTGATTACACTGTTGCTA
>HPV50_Gamma_9628550_nt6687_L1_Tile|1
TTGTTTCAGACTGGATTATTAAAGAGAAGAGTTAGAACTGATTACACTGTTGCTACAGTT
>HPV50_Gamma_9628550_nt6692_L1_Tile|1
TCAGACTGGATTATTAAAGAGAAGAGTTAGAACTGATTACACTGTTGCTACAGTTTCCAA
>HPV50_Gamma_9628550_nt6697_L1_Tile|1
CTGGATTATTAAAGAGAAGAGTTAGAACTGATTACACTGTTGCTACAGTTTCCAAACCAA
>HPV50_Gamma_9628550_nt6702_L1_Tile|2
TTATTAAAGAGAAGAGTTAGAACTGATTACACTGTTGCTACAGTTTCCAAACCAAACAAG
>HPV50_Gamma_9628550_nt6707_L1_Tile|1
AAAGAGAAGAGTTAGAACTGATTACACTGTTGCTACAGTTTCCAAACCAAACAAGAGAAA
>HPV50_Gamma_9628550_nt6712_L1_Tile|1
GAAGAGTTAGAACTGATTACACTGTTGCTACAGTTTCCAAACCAAACAAGAGAAAACGTA
>HPV50_Gamma_9628550_nt6720_L1_Tile|1
AGAACTGATTACACTGTTGCTACAGTTTCCAAACCAAACAAGAGAAAACGTACCAGATAA
>HPV51_Alpha_333087_nt5667_L1_Tile|1
ATTTTCCAATACCTAAAACCTCAACGCGTGCTGCTATTCCTAAAGTATCTGCATTTCAAT
>HPV51_Alpha_333087_nt5892_L1_Tile|1
AATATGATGACACAGAAAATTCACGCATAGCAAATGGCAATGCACAACAAGATGTTAGAG
>HPV51_Alpha_333087_nt5897_L1_Tile|1
GATGACACAGAAAATTCACGCATAGCAAATGGCAATGCACAACAAGATGTTAGAGATAAC
>HPV51_Alpha_333087_nt5902_L1_Tile|2
CACAGAAAATTCACGCATAGCAAATGGCAATGCACAACAAGATGTTAGAGATAACACATC
>HPV51_Alpha_333087_nt5907_L1_Tile|1
AAAATTCACGCATAGCAAATGGCAATGCACAACAAGATGTTAGAGATAACACATCTGTTG
>HPV51_Alpha_333087_nt5912_L1_Tile|1
TCACGCATAGCAAATGGCAATGCACAACAAGATGTTAGAGATAACACATCTGTTGACAAC
>HPV51_Alpha_333087_nt6027_L1_Tile|1
GTATTGGCACTACATGCAAAAACACACCTGTACCTCCAGGAGACTGCCCCCCCCTGGAAC
>HPV51_Alpha_333087_nt6032_L1_Tile|1
GGCACTACATGCAAAAACACACCTGTACCTCCAGGAGACTGCCCCCCCCTGGAACTTGTA
>HPV51_Alpha_333087_nt6272_L1_Tile|1
AGGGAGCAAATCTTTGCTAGGCACTATTATAATAAACTTGTAGGTGTTGGGGAAGACATT
>HPV51_Alpha_333087_nt6282_L1_Tile|2
TCTTTGCTAGGCACTATTATAATAAACTTGTAGGTGTTGGGGAAGACATTCCTAACGATT
>HPV51_Alpha_333087_nt6287_L1_Tile|1
GCTAGGCACTATTATAATAAACTTGTAGGTGTTGGGGAAGACATTCCTAACGATTATTAT
>HPV51_Alpha_333087_nt6292_L1_Tile|1
GCACTATTATAATAAACTTGTAGGTGTTGGGGAAGACATTCCTAACGATTATTATATTAA
>HPV51_Alpha_333087_nt6297_L1_Tile|1
ATTATAATAAACTTGTAGGTGTTGGGGAAGACATTCCTAACGATTATTATATTAAGGGTA
>HPV51_Alpha_333087_nt6302_L1_Tile|2
AATAAACTTGTAGGTGTTGGGGAAGACATTCCTAACGATTATTATATTAAGGGTAGTGGT
>HPV51_Alpha_333087_nt6307_L1_Tile|1
ACTTGTAGGTGTTGGGGAAGACATTCCTAACGATTATTATATTAAGGGTAGTGGTAATGG
>HPV51_Alpha_333087_nt6312_L1_Tile|1
TAGGTGTTGGGGAAGACATTCCTAACGATTATTATATTAAGGGTAGTGGTAATGGCCGTG
>HPV51_Alpha_333087_nt6517_L1_Tile|1
CTGTGTTGATACTACCAGAAGTACAAATTTAACTATTAGCACTGCCACTGCTGCGGTTTC
>HPV51_Alpha_333087_nt6522_L1_Tile|2
TTGATACTACCAGAAGTACAAATTTAACTATTAGCACTGCCACTGCTGCGGTTTCCCCAA

>HPV51_Alpha_333087_nt6527_L1_Tile|1
ACTACCAGAAGTACAAATTTAACTATTAGCACTGCCACTGCTGCGGTTTCCCCAACATTT
>HPV51_Alpha_333087_nt6532_L1_Tile|1
CAGAAGTACAAATTTAACTATTAGCACTGCCACTGCTGCGGTTTCCCCAACATTTACTCC
>HPV51_Alpha_333087_nt6537_L1_Tile|1
GTACAAATTTAACTATTAGCACTGCCACTGCTGCGGTTTCCCCAACATTTACTCCAAGTA
>HPV51_Alpha_333087_nt6542_L1_Tile|2
AATTTAACTATTAGCACTGCCACTGCTGCGGTTTCCCCAACATTTACTCCAAGTAACTTT
>HPV51_Alpha_333087_nt6547_L1_Tile|1
AACTATTAGCACTGCCACTGCTGCGGTTTCCCCAACATTTACTCCAAGTAACTTTAAGCA
>HPV51_Alpha_333087_nt6552_L1_Tile|1
TTAGCACTGCCACTGCTGCGGTTTCCCCAACATTTACTCCAAGTAACTTTAAGCAATATA
>HPV51_Alpha_333087_nt6557_L1_Tile|1
ACTGCCACTGCTGCGGTTTCCCCAACATTTACTCCAAGTAACTTTAAGCAATATATTAGG
>HPV51_Alpha_333087_nt6652_L1_Tile|1
ATTATGTAAAATTACTTTAACTACAGAGGTAATGGCTTATTTACACACAATGGATCCTAC
>HPV51_Alpha_333087_nt6667_L1_Tile|1
TTTAACTACAGAGGTAATGGCTTATTTACACACAATGGATCCTACCATTCTTGAACAGTG
>HPV52_Alpha_397038_nt5756_L1_Tile|1
GCAGTTCTCGATTACTAACAGTAGGACATCCCTATTTTTCTATTAAAAACACCAGTAGTG
>HPV52_Alpha_397038_nt5761_L1_Tile|1
TCTCGATTACTAACAGTAGGACATCCCTATTTTTCTATTAAAAACACCAGTAGTGGTAAT
>HPV52_Alpha_397038_nt5771_L1_Tile|1
TAACAGTAGGACATCCCTATTTTTCTATTAAAAACACCAGTAGTGGTAATGGTAAAAAAG
>HPV52_Alpha_397038_nt5776_L1_Tile|1
GTAGGACATCCCTATTTTTCTATTAAAAACACCAGTAGTGGTAATGGTAAAAAAGTTTTA
>HPV52_Alpha_397038_nt5781_L1_Tile|1
ACATCCCTATTTTTCTATTAAAAACACCAGTAGTGGTAATGGTAAAAAAGTTTTAGTTCC
>HPV52_Alpha_397038_nt5791_L1_Tile|1
TTTTCTATTAAAAACACCAGTAGTGGTAATGGTAAAAAAGTTTTAGTTCCCAAGGTGTCT
>HPV52_Alpha_397038_nt6021_L1_Tile|1
AAACAAGTTTGATGATACTGAAACCAGTAACAAATATGCTGGTAAACCTGGTATAGATAA
>HPV52_Alpha_397038_nt6131_L1_Tile|1
GATGCAAACCTCCTATAGGTGAACATTGGGGTAAGGGAACCCCTTGTAATAATAATTCAG
>HPV52_Alpha_397038_nt6156_L1_Tile|1
TTGGGGTAAGGGAACCCCTTGTAATAATAATTCAGGAAATCCTGGGGATTGTCCTCCCCT
>HPV52_Alpha_397038_nt6161_L1_Tile|1
GTAAGGGAACCCCTTGTAATAATAATTCAGGAAATCCTGGGGATTGTCCTCCCCTACAGC
>HPV52_Alpha_397038_nt6166_L1_Tile|1
GGAACCCCTTGTAATAATAATTCAGGAAATCCTGGGGATTGTCCTCCCCTACAGCTCATT
>HPV52_Alpha_397038_nt6171_L1_Tile|1
CCCTTGTAATAATAATTCAGGAAATCCTGGGGATTGTCCTCCCCTACAGCTCATTAACAG
>HPV52_Alpha_397038_nt6176_L1_Tile|1
GTAATAATAATTCAGGAAATCCTGGGGATTGTCCTCCCCTACAGCTCATTAACAGTGTAA
>HPV52_Alpha_397038_nt6436_L1_Tile|1
AATAGGGCCGGTACCTTAGGTGACCCTGTGCCAGGTGATTTATATATACAAGGGTCTAAC
>HPV52_Alpha_397038_nt6446_L1_Tile|1
GTACCTTAGGTGACCCTGTGCCAGGTGATTTATATATACAAGGGTCTAACTCTGGCAATA
>HPV52_Alpha_397038_nt6451_L1_Tile|1
TTAGGTGACCCTGTGCCAGGTGATTTATATATACAAGGGTCTAACTCTGGCAATACTGCC
>HPV52_Alpha_397038_nt6461_L1_Tile|1
CTGTGCCAGGTGATTTATATATACAAGGGTCTAACTCTGGCAATACTGCCACTGTACAAA
>HPV52_Alpha_397038_nt6466_L1_Tile|1
CCAGGTGATTTATATATACAAGGGTCTAACTCTGGCAATACTGCCACTGTACAAAGCAGT
>HPV52_Alpha_397038_nt6471_L1_Tile|1

TGATTTATATATACAAGGGTCTAACTCTGGCAATACTGCCACTGTACAAAGCAGTGCTTT
>HPV52_Alpha_397038_nt6671_L1_Tile|1
GTAGCACTAACATGACTTTATGTGCTGAGGTTAAAAAGGAAAGCACATATAAAAATGAAA
>HPV52_Alpha_397038_nt6781_L1_Tile|1
TTTCAATTGTGCAAAATTACATTAACAGCTGATGTTATGACATACATTCATAAGATGGAT
>HPV52_Alpha_397038_nt6786_L1_Tile|1
ATTGTGCAAAATTACATTAACAGCTGATGTTATGACATACATTCATAAGATGGATGCCAC
>HPV52_Alpha_397038_nt6791_L1_Tile|1
GCAAAATTACATTAACAGCTGATGTTATGACATACATTCATAAGATGGATGCCACTATTT
>HPV52_Alpha_397038_nt6891_L1_Tile|1
ATCTTTGGAGGACACATACAGATTTGTCACTTCTACTGCTATAACTTGTCAAAAAAACAC
>HPV52_Alpha_397038_nt6906_L1_Tile|1
ATACAGATTTGTCACTTCTACTGCTATAACTTGTCAAAAAAACACACCACCTAAAGGAAA
>HPV52_Alpha_397038_nt6911_L1_Tile|1
GATTTGTCACTTCTACTGCTATAACTTGTCAAAAAAACACACCACCTAAAGGAAAGGAAG
>HPV52_Alpha_397038_nt6916_L1_Tile|1
GTCACTTCTACTGCTATAACTTGTCAAAAAAACACACCACCTAAAGGAAAGGAAGATCCT
>HPV52_Alpha_397038_nt6931_L1_Tile|1
ATAACTTGTCAAAAAAACACACCACCTAAAGGAAAGGAAGATCCTTTAAAGGACTATATG
>HPV52_Alpha_397038_nt7041_L1_Tile|1
TCCTTTAGGTAGGAAGTTTTTGTTACAGGCAGGGCTACAGGCTAGGCCCAAACTAAAACG
>HPV52_Alpha_397038_nt7046_L1_Tile|1
TAGGTAGGAAGTTTTTGTTACAGGCAGGGCTACAGGCTAGGCCCAAACTAAAACGCCCTG
>HPV52_Alpha_397038_nt7051_L1_Tile|1
AGGAAGTTTTTGTTACAGGCAGGGCTACAGGCTAGGCCCAAACTAAAACGCCCTGCATCA
>HPV52_Alpha_397038_nt7056_L1_Tile|1
GTTTTTGTTACAGGCAGGGCTACAGGCTAGGCCCAAACTAAAACGCCCTGCATCATCGGC
>HPV52_Alpha_397038_nt7071_L1_Tile|1
AGGGCTACAGGCTAGGCCCAAACTAAAACGCCCTGCATCATCGGCCCCACGTACCTCCAC
>HPV52_Alpha_397038_nt7076_L1_Tile|1
TACAGGCTAGGCCCAAACTAAAACGCCCTGCATCATCGGCCCCACGTACCTCCACAAAGA
>HPV52_Alpha_397038_nt7081_L1_Tile|1
GCTAGGCCCAAACTAAAACGCCCTGCATCATCGGCCCCACGTACCTCCACAAAGAAGAAA
>HPV52_Alpha_397038_nt7086_L1_Tile|1
GCCCAAACTAAAACGCCCTGCATCATCGGCCCCACGTACCTCCACAAAGAAGAAAAAGGT
>HPV52_Alpha_397038_nt7091_L1_Tile|1
AACTAAAACGCCCTGCATCATCGGCCCCACGTACCTCCACAAAGAAGAAAAAGGTTAAAA
>HPV52_Alpha_397038_nt7096_L1_Tile|1
AAACGCCCTGCATCATCGGCCCCACGTACCTCCACAAAGAAGAAAAAGGTTAAAAGGTAA
>HPV53_Alpha_9627377_nt5751_L1_Tile|1
TATATTTTATCATGCTGGAAGCTCTCGCTTGCTTACCGTGGGACATCCTTATTACCCCAT
>HPV53_Alpha_9627377_nt5791_L1_Tile|1
GGACATCCTTATTACCCCATTTCTAAATCTGGTAAAGCAGACATCCCTAAGGTGTCTGCA
>HPV53_Alpha_9627377_nt5796_L1_Tile|1
TCCTTATTACCCCATTTCTAAATCTGGTAAAGCAGACATCCCTAAGGTGTCTGCATTTCA
>HPV53_Alpha_9627377_nt6136_L1_Tile|1
CCCGCTATAGGTGAACACTGGACAAAGGGTACGGCCTGTCGTTCCACACCTACTACAGCG
>HPV53_Alpha_9627377_nt6161_L1_Tile|1
AGGGTACGGCCTGTCGTTCCACACCTACTACAGCGGGCGACTGCCCCCCATTGGAACTTA
>HPV53_Alpha_9627377_nt6176_L1_Tile|1
GTTCCACACCTACTACAGCGGGCGACTGCCCCCCATTGGAACTTATCAATTCACCTATTG
>HPV53_Alpha_9627377_nt6251_L1_Tile|1
TGGACACAGGTTTTGGTGCATTAAACTTTAAAGCTTTGCAGGAATCTAAATCTGATGTGC
>HPV53_Alpha_9627377_nt6441_L1_Tile|1
AGGCGTTATTGGTGAGGAAATACCTAATGACTTATATATTAAGGGTAGTAATGGCAGGGA

>HPV53_Alpha_9627377_nt6456_L1_Tile|1
GGAAATACCTAATGACTTATATATTAAGGGTAGTAATGGCAGGGACCCGCCCCCTAGCTC
>HPV53_Alpha_9627377_nt6461_L1_Tile|1
TACCTAATGACTTATATATTAAGGGTAGTAATGGCAGGGACCCGCCCCCTAGCTCTGTAT
>HPV53_Alpha_9627377_nt6466_L1_Tile|1
AATGACTTATATATTAAGGGTAGTAATGGCAGGGACCCGCCCCCTAGCTCTGTATATGTT
>HPV53_Alpha_9627377_nt6816_L1_Tile|1
TTTACATACTATGAATTCTACCTTACTGGAAGACTGGAATATAGGTTTGTCGCCTCCTGT
>HPV53_Alpha_9627377_nt6821_L1_Tile|1
ATACTATGAATTCTACCTTACTGGAAGACTGGAATATAGGTTTGTCGCCTCCTGTTGCCA
>HPV53_Alpha_9627377_nt6831_L1_Tile|1
TTCTACCTTACTGGAAGACTGGAATATAGGTTTGTCGCCTCCTGTTGCCACTAGCTTAGA
>HPV53_Alpha_9627377_nt6946_L1_Tile|1
CCTCCTGAAAAGCAGGACCCACTATCTAAATATAAATTTTGGGAGGTCAATTTGCAAAAC
>HPV53_Alpha_9627377_nt6966_L1_Tile|1
ACTATCTAAATATAAATTTTGGGAGGTCAATTTGCAAAACAGTTTTTCTGCTGATTTGGA
>HPV53_Alpha_9627377_nt6991_L1_Tile|1
GTCAATTTGCAAAACAGTTTTTCTGCTGATTTGGATCAGTTTCCTCTTGGCAGGAAGTTT
>HPV53_Alpha_9627377_nt6996_L1_Tile|1
TTTGCAAAACAGTTTTTCTGCTGATTTGGATCAGTTTCCTCTTGGCAGGAAGTTTTTAAT
>HPV53_Alpha_9627377_nt7081_L1_Tile|1
CCTGTATCCTCTAAAAAACGCTCTGCTTCTACTACATCTACCTCAGCCCCCTCCTCCAAG
>HPV53_Alpha_9627377_nt7086_L1_Tile|1
ATCCTCTAAAAAACGCTCTGCTTCTACTACATCTACCTCAGCCCCCTCCTCCAAGCGCAA
>HPV54_Alpha_9628437_nt5985_L1_Tile|1
ATGATACTGAAAATGCACCTAAATATGTTGGTGCAGGAGCTGACAATAGGGAAAATGTTA
>HPV54_Alpha_9628437_nt5995_L1_Tile|1
AAATGCACCTAAATATGTTGGTGCAGGAGCTGACAATAGGGAAAATGTTAGCATGGACTA
>HPV54_Alpha_9628437_nt6085_L1_Tile|1
CACACCACCTATAGGTGAACACTGGGCTAAAGGCAACCTATGTACACCTAATACATTGGC
>HPV54_Alpha_9628437_nt6095_L1_Tile|1
ATAGGTGAACACTGGGCTAAAGGCAACCTATGTACACCTAATACATTGGCTGCTGGTGAC
>HPV54_Alpha_9628437_nt6215_L1_Tile|1
TTTGGGGCTATGGATTTTAAAACCCTACAAACCTCAAAAAGTGAGGTACCCCTTGATGTA
>HPV54_Alpha_9628437_nt6220_L1_Tile|1
GGCTATGGATTTTAAAACCCTACAAACCTCAAAAAGTGAGGTACCCCTTGATGTAGCTAC
>HPV54_Alpha_9628437_nt6225_L1_Tile|1
TGGATTTTAAAACCCTACAAACCTCAAAAAGTGAGGTACCCCTTGATGTAGCTACCTCAA
>HPV54_Alpha_9628437_nt6235_L1_Tile|1
AACCCTACAAACCTCAAAAAGTGAGGTACCCCTTGATGTAGCTACCTCAATTTGTAAATA
>HPV54_Alpha_9628437_nt6240_L1_Tile|1
TACAAACCTCAAAAAGTGAGGTACCCCTTGATGTAGCTACCTCAATTTGTAAATATCCTG
>HPV54_Alpha_9628437_nt6250_L1_Tile|1
AAAAAGTGAGGTACCCCTTGATGTAGCTACCTCAATTTGTAAATATCCTGATTACCTTAA
>HPV54_Alpha_9628437_nt6415_L1_Tile|1
ACCTAATGACTTATACATTAAGAAATCCTCAGGTAACCTTGACAGTTCTATTTATGCTGC
>HPV54_Alpha_9628437_nt6585_L1_Tile|1
TTTTAACAGTTGTAGATACCACCCGTAGTACTAACCTAACATTGTGTGCTACAGCATCCA
>HPV54_Alpha_9628437_nt6605_L1_Tile|1
ACCCGTAGTACTAACCTAACATTGTGTGCTACAGCATCCACGCAGGATAGCTTTAATAAT
>HPV54_Alpha_9628437_nt6610_L1_Tile|1
TAGTACTAACCTAACATTGTGTGCTACAGCATCCACGCAGGATAGCTTTAATAATTCTGA
>HPV54_Alpha_9628437_nt6615_L1_Tile|1
CTAACCTAACATTGTGTGCTACAGCATCCACGCAGGATAGCTTTAATAATTCTGACTTTA
>HPV54_Alpha_9628437_nt6620_L1_Tile|1

CTAACATTGTGTGCTACAGCATCCACGCAGGATAGCTTTAATAATTCTGACTTTAGGGAG
>HPV54_Alpha_9628437_nt6625_L1_Tile|1
ATTGTGTGCTACAGCATCCACGCAGGATAGCTTTAATAATTCTGACTTTAGGGAGTATAT
>HPV54_Alpha_9628437_nt6635_L1_Tile|1
ACAGCATCCACGCAGGATAGCTTTAATAATTCTGACTTTAGGGAGTATATTAGACATGTG
>HPV54_Alpha_9628437_nt6725_L1_Tile|1
TTATGTACCATAACCCTTACAGCAGATGTTATGGCCTATATTCATGGAATGAATCCCACT
>HPV54_Alpha_9628437_nt6730_L1_Tile|1
TACCATAACCCTTACAGCAGATGTTATGGCCTATATTCATGGAATGAATCCCACTATTCT
>HPV54_Alpha_9628437_nt6735_L1_Tile|1
TAACCCTTACAGCAGATGTTATGGCCTATATTCATGGAATGAATCCCACTATTCTAGAGG
>HPV54_Alpha_9628437_nt6770_L1_Tile|1
GGAATGAATCCCACTATTCTAGAGGACTGGAACTTTGGTATAACCCCCCCAGCTACAAGT
>HPV54_Alpha_9628437_nt6805_L1_Tile|1
TGGTATAACCCCCCCAGCTACAAGTAGTTTGGAGGACACATATAGGTTTGTACAGTCACA
>HPV54_Alpha_9628437_nt6915_L1_Tile|1
CTTACAGTAAATTTAATTTTTGGACTGTTGACCTTAAGGAACGATTTTCATCTGACCTTG
>HPV54_Alpha_9628437_nt6920_L1_Tile|1
AGTAAATTTAATTTTTGGACTGTTGACCTTAAGGAACGATTTTCATCTGACCTTGACCAG
>HPV54_Alpha_9628437_nt6925_L1_Tile|1
ATTTAATTTTTGGACTGTTGACCTTAAGGAACGATTTTCATCTGACCTTGACCAGTTTCC
>HPV54_Alpha_9628437_nt6930_L1_Tile|1
ATTTTTGGACTGTTGACCTTAAGGAACGATTTTCATCTGACCTTGACCAGTTTCCCTTGG
>HPV54_Alpha_9628437_nt6990_L1_Tile|1
GTCGCAAGTTTTTACTACAGGCTGGCCTACGTGCACGTCCGCGCCTTCGGCCTGTAAAGC
>HPV54_Alpha_9628437_nt6995_L1_Tile|1
AAGTTTTTACTACAGGCTGGCCTACGTGCACGTCCGCGCCTTCGGCCTGTAAAGCGTGCA
>HPV55_Alpha_1020266_nt6470_L1_Tile|1
ATAGGGCGGGAACGGTTGGAGAGGACATTCCACAGGATTTGGTTTTTAAAGGTGCTACTA
>HPV55_Alpha_1020266_nt6480_L1_Tile|1
AACGGTTGGAGAGGACATTCCACAGGATTTGGTTTTTAAAGGTGCTACTAAAAGTACAGT
>HPV55_Alpha_1020266_nt6495_L1_Tile|1
CATTCCACAGGATTTGGTTTTTAAAGGTGCTACTAAAAGTACAGTTCCTAATGCCATATA
>HPV55_Alpha_1020266_nt6705_L1_Tile|1
CATGACAATATGTGCTGCTACAACTCAGTCTCCATCTACAACATATAATAGTACAGAATA
>HPV55_Alpha_1020266_nt6715_L1_Tile|1
TGTGCTGCTACAACTCAGTCTCCATCTACAACATATAATAGTACAGAATATAAACAATAC
>HPV55_Alpha_1020266_nt6720_L1_Tile|1
TGCTACAACTCAGTCTCCATCTACAACATATAATAGTACAGAATATAAACAATACATGCG
>HPV56_Alpha_397053_nt5708_L1_Tile|1
AGGCAGTTCACGATTGCTTGCCGTAGGACATCCCTATTACTCTGTGACTAAGGACAATAC
>HPV56_Alpha_397053_nt5713_L1_Tile|1
GTTCACGATTGCTTGCCGTAGGACATCCCTATTACTCTGTGACTAAGGACAATACCAAAA
>HPV56_Alpha_397053_nt5728_L1_Tile|1
CCGTAGGACATCCCTATTACTCTGTGACTAAGGACAATACCAAAACAAACATTCCCAAAG
>HPV56_Alpha_397053_nt6033_L1_Tile|1
ATATCAGTTGATGGCAAGCAAACACAGTTGTGTATTGTTGGATGTACTCCCGCTATGGGT
>HPV56_Alpha_397053_nt6058_L1_Tile|1
AGTTGTGTATTGTTGGATGTACTCCCGCTATGGGTGAACATTGGACTAAAGGTGCTGTGT
>HPV56_Alpha_397053_nt6068_L1_Tile|1
TGTTGGATGTACTCCCGCTATGGGTGAACATTGGACTAAAGGTGCTGTGTGTAAGTCCAC
>HPV56_Alpha_397053_nt6073_L1_Tile|1
GATGTACTCCCGCTATGGGTGAACATTGGACTAAAGGTGCTGTGTGTAAGTCCACACAAG
>HPV56_Alpha_397053_nt6078_L1_Tile|1
ACTCCCGCTATGGGTGAACATTGGACTAAAGGTGCTGTGTGTAAGTCCACACAAGTTACC

>HPV56_Alpha_397053_nt6083_L1_Tile|1
CGCTATGGGTGAACATTGGACTAAAGGTGCTGTGTGTAAGTCCACACAAGTTACCACAGG
>HPV56_Alpha_397053_nt6108_L1_Tile|1
GGTGCTGTGTGTAAGTCCACACAAGTTACCACAGGGGACTGCCCGCCTCTTGCATTAATT
>HPV56_Alpha_397053_nt6118_L1_Tile|1
GTAAGTCCACACAAGTTACCACAGGGGACTGCCCGCCTCTTGCATTAATTAATACACCTA
>HPV56_Alpha_397053_nt6128_L1_Tile|1
ACAAGTTACCACAGGGGACTGCCCGCCTCTTGCATTAATTAATACACCTATAGAGGATGG
>HPV56_Alpha_397053_nt6368_L1_Tile|1
ACATTATTTTAATAGGGCTGGTAAAGTTGGGGAAACAATACCTGCAGAGTTATATTTAAA
>HPV56_Alpha_397053_nt6383_L1_Tile|1
GGCTGGTAAAGTTGGGGAAACAATACCTGCAGAGTTATATTTAAAGGGTAGCAATGGTAG
>HPV56_Alpha_397053_nt6408_L1_Tile|1
CCTGCAGAGTTATATTTAAAGGGTAGCAATGGTAGAGAACCCCCTCCGAGTTCTGTATAT
>HPV56_Alpha_397053_nt6413_L1_Tile|1
AGAGTTATATTTAAAGGGTAGCAATGGTAGAGAACCCCCTCCGAGTTCTGTATATGTTGC
>HPV56_Alpha_397053_nt6588_L1_Tile|1
ACTGTAGTAGATACTACTAGAAGTACTAACATGACTATTAGTACTGCTACAGAACAGTTA
>HPV56_Alpha_397053_nt6593_L1_Tile|1
AGTAGATACTACTAGAAGTACTAACATGACTATTAGTACTGCTACAGAACAGTTAAGTAA
>HPV56_Alpha_397053_nt6608_L1_Tile|1
AAGTACTAACATGACTATTAGTACTGCTACAGAACAGTTAAGTAAATATGATGCACGAAA
>HPV56_Alpha_397053_nt6613_L1_Tile|1
CTAACATGACTATTAGTACTGCTACAGAACAGTTAAGTAAATATGATGCACGAAAAATTA
>HPV56_Alpha_397053_nt6618_L1_Tile|1
ATGACTATTAGTACTGCTACAGAACAGTTAAGTAAATATGATGCACGAAAAATTAATCAG
>HPV56_Alpha_397053_nt6628_L1_Tile|1
GTACTGCTACAGAACAGTTAAGTAAATATGATGCACGAAAAATTAATCAGTACCTTAGAC
>HPV56_Alpha_397053_nt6633_L1_Tile|1
GCTACAGAACAGTTAAGTAAATATGATGCACGAAAAATTAATCAGTACCTTAGACATGTG
>HPV56_Alpha_397053_nt6773_L1_Tile|1
GAATGCTAACCTACTGGAGGACTGGAATATTGGGTTATCCCCGCCAGTGGCCACCAGCCT
>HPV56_Alpha_397053_nt6778_L1_Tile|1
CTAACCTACTGGAGGACTGGAATATTGGGTTATCCCCGCCAGTGGCCACCAGCCTAGAAG
>HPV56_Alpha_397053_nt6803_L1_Tile|1
TGGGTTATCCCCGCCAGTGGCCACCAGCCTAGAAGATAAATATAGATATGTTAGAAGCAC
>HPV56_Alpha_397053_nt6808_L1_Tile|1
TATCCCCGCCAGTGGCCACCAGCCTAGAAGATAAATATAGATATGTTAGAAGCACAGCTA
>HPV56_Alpha_397053_nt6818_L1_Tile|1
AGTGGCCACCAGCCTAGAAGATAAATATAGATATGTTAGAAGCACAGCTATAACATGTCA
>HPV56_Alpha_397053_nt6988_L1_Tile|1
GAAAATTTTTAATGCAACTGGGCACTAGGTCAAAGCCTGCTGTAGCTACCTCTAAAAAGC
>HPV56_Alpha_397053_nt6993_L1_Tile|1
TTTTTAATGCAACTGGGCACTAGGTCAAAGCCTGCTGTAGCTACCTCTAAAAAGCGATCT
>HPV56_Alpha_397053_nt6998_L1_Tile|1
AATGCAACTGGGCACTAGGTCAAAGCCTGCTGTAGCTACCTCTAAAAAGCGATCTGCTCC
>HPV56_Alpha_397053_nt7013_L1_Tile|1
TAGGTCAAAGCCTGCTGTAGCTACCTCTAAAAAGCGATCTGCTCCTACCTCCACCTCTAC
>HPV56_Alpha_397053_nt7023_L1_Tile|1
CCTGCTGTAGCTACCTCTAAAAAGCGATCTGCTCCTACCTCCACCTCTACACCAGCAAAA
>HPV57_Alpha_60882_nt5879_L1_Tile|1
TCACAGTAGGCCATCCATATTATTCTATAAAAAAAAGTGGCAATAATAAGGTGTCTGTGC
>HPV57_Alpha_60882_nt5884_L1_Tile|1
GTAGGCCATCCATATTATTCTATAAAAAAAAGTGGCAATAATAAGGTGTCTGTGCCCAAG
>HPV57_Alpha_60882_nt5894_L1_Tile|1

CATATTATTCTATAAAAAAAAGTGGCAATAATAAGGTGTCTGTGCCCAAGGTATCGGGCT
>HPV57_Alpha_60882_nt5989_L1_Tile|1
CCTAATAAGTTTGGTCTGCCTGATGCCAACCTCTATGATCCCGACACCCAGCGTCTGCTG
>HPV57_Alpha_60882_nt6094_L1_Tile|1
GTAGGGATATCCGGCCACCCTTATTATAACAAACAGGATGATACTGAAAATTCACACAAT
>HPV57_Alpha_60882_nt6099_L1_Tile|1
GATATCCGGCCACCCTTATTATAACAAACAGGATGATACTGAAAATTCACACAATCCCGA
>HPV57_Alpha_60882_nt6349_L1_Tile|1
ACCGGGTTCGGGGCGCTGGATTTTGCCGCTCTACAGTCCAACAAATCAGATGTCCCCTTG
>HPV57_Alpha_60882_nt6364_L1_Tile|1
CTGGATTTTGCCGCTCTACAGTCCAACAAATCAGATGTCCCCTTGGATATCTGTACTAAC
>HPV57_Alpha_60882_nt6369_L1_Tile|1
TTTTGCCGCTCTACAGTCCAACAAATCAGATGTCCCCTTGGATATCTGTACTAACATATG
>HPV57_Alpha_60882_nt6389_L1_Tile|1
ACAAATCAGATGTCCCCTTGGATATCTGTACTAACATATGTAAATATCCAGACTATCTGA
>HPV57_Alpha_60882_nt6534_L1_Tile|1
TGGGTCGATGGGTGACGCCCTCCCGGATGAGCTATATGTCAAGAGTTCTACCGTCCAGAC
>HPV57_Alpha_60882_nt6539_L1_Tile|1
CGATGGGTGACGCCCTCCCGGATGAGCTATATGTCAAGAGTTCTACCGTCCAGACCCCCG
>HPV57_Alpha_60882_nt6544_L1_Tile|1
GGTGACGCCCTCCCGGATGAGCTATATGTCAAGAGTTCTACCGTCCAGACCCCCGGTAGT
>HPV57_Alpha_60882_nt6549_L1_Tile|1
CGCCCTCCCGGATGAGCTATATGTCAAGAGTTCTACCGTCCAGACCCCCGGTAGTTATGT
>HPV57_Alpha_60882_nt6554_L1_Tile|1
TCCCGGATGAGCTATATGTCAAGAGTTCTACCGTCCAGACCCCCGGTAGTTATGTTTATA
>HPV57_Alpha_60882_nt6559_L1_Tile|1
GATGAGCTATATGTCAAGAGTTCTACCGTCCAGACCCCCGGTAGTTATGTTTATACCTCC
>HPV57_Alpha_60882_nt6564_L1_Tile|1
GCTATATGTCAAGAGTTCTACCGTCCAGACCCCCGGTAGTTATGTTTATACCTCCACTCC
>HPV57_Alpha_60882_nt6754_L1_Tile|1
AGCACAAATGTCTCTTTGTGTGCCACTGTAACCACAGAAACTAATTATAAAGCCTCCAAT
>HPV57_Alpha_60882_nt6759_L1_Tile|1
AAATGTCTCTTTGTGTGCCACTGTAACCACAGAAACTAATTATAAAGCCTCCAATTATAA
>HPV57_Alpha_60882_nt6944_L1_Tile|1
ACTTTGGTGTCCCCCCACCCCCGTCCGCCAGCCTGCAGGACACCTACAGGTATTTGCAAT
>HPV57_Alpha_60882_nt6984_L1_Tile|1
CACCTACAGGTATTTGCAATCCCAAGCGATAACATGTCAGAAGCCCACACCCCCTAAGAC
>HPV58_Alpha_222386_nt5766_L1_Tile|1
ACTTTTGGCTGTTGGCAATCCATATTTTTCCATCAAAAGTCCCAATAACAATAAAAAAGT
>HPV58_Alpha_222386_nt5776_L1_Tile|1
GTTGGCAATCCATATTTTTCCATCAAAAGTCCCAATAACAATAAAAAAGTATTAGTTCCC
>HPV58_Alpha_222386_nt5786_L1_Tile|1
CATATTTTTCCATCAAAAGTCCCAATAACAATAAAAAAGTATTAGTTCCCAAGGTATCAG
>HPV58_Alpha_222386_nt5791_L1_Tile|1
TTTTCCATCAAAAGTCCCAATAACAATAAAAAAGTATTAGTTCCCAAGGTATCAGGCTTA
>HPV58_Alpha_222386_nt5796_L1_Tile|1
CATCAAAAGTCCCAATAACAATAAAAAAGTATTAGTTCCCAAGGTATCAGGCTTACAGTA
>HPV58_Alpha_222386_nt5801_L1_Tile|1
AAAGTCCCAATAACAATAAAAAAGTATTAGTTCCCAAGGTATCAGGCTTACAGTATAGGG
>HPV58_Alpha_222386_nt5806_L1_Tile|1
CCCAATAACAATAAAAAAGTATTAGTTCCCAAGGTATCAGGCTTACAGTATAGGGTCTTT
>HPV58_Alpha_222386_nt6166_L1_Tile|1
GCCTGTAACAATAATGCAGCTGCTACTGATTGTCCTCCATTGGAACTTTTTAATTCTATT
>HPV58_Alpha_222386_nt6431_L1_Tile|1
GGGCTGGAAAACTTGGCGAGGCTGTCCCGGATGACCTTTATATTAAAGGGTCCGGTAATA

>HPV58_Alpha_222386_nt6436_L1_Tile|1
GGAAAACTTGGCGAGGCTGTCCCGGATGACCTTTATATTAAAGGGTCCGGTAATACTGCA
>HPV58_Alpha_222386_nt6441_L1_Tile|1
ACTTGGCGAGGCTGTCCCGGATGACCTTTATATTAAAGGGTCCGGTAATACTGCAGTTAT
>HPV58_Alpha_222386_nt6446_L1_Tile|1
GCGAGGCTGTCCCGGATGACCTTTATATTAAAGGGTCCGGTAATACTGCAGTTATCCAAA
>HPV58_Alpha_222386_nt6456_L1_Tile|1
CCCGGATGACCTTTATATTAAAGGGTCCGGTAATACTGCAGTTATCCAAAGTAGTGCATT
>HPV58_Alpha_222386_nt6461_L1_Tile|1
ATGACCTTTATATTAAAGGGTCCGGTAATACTGCAGTTATCCAAAGTAGTGCATTTTTTC
>HPV58_Alpha_222386_nt6466_L1_Tile|1
CTTTATATTAAAGGGTCCGGTAATACTGCAGTTATCCAAAGTAGTGCATTTTTTCCAACT
>HPV58_Alpha_222386_nt7041_L1_Tile|1
GTTTTTATTACAATCAGGCCTTAAAGCAAAGCCCAGACTAAAACGTTCGGCCCCTACTAC
>HPV58_Alpha_222386_nt7046_L1_Tile|1
TATTACAATCAGGCCTTAAAGCAAAGCCCAGACTAAAACGTTCGGCCCCTACTACCCGTG
>HPV58_Alpha_222386_nt7051_L1_Tile|1
CAATCAGGCCTTAAAGCAAAGCCCAGACTAAAACGTTCGGCCCCTACTACCCGTGCACCA
>HPV58_Alpha_222386_nt7056_L1_Tile|1
AGGCCTTAAAGCAAAGCCCAGACTAAAACGTTCGGCCCCTACTACCCGTGCACCATCCAC
>HPV59_Alpha_557236_nt5647_L1_Tile|1
CTCCACCTTCGGTAGCTAAGGTTGTCAGCACTGATGAGTATGTCACCCGTACCAGTATTT
>HPV59_Alpha_557236_nt5652_L1_Tile|1
CCTTCGGTAGCTAAGGTTGTCAGCACTGATGAGTATGTCACCCGTACCAGTATTTTCTAC
>HPV59_Alpha_557236_nt5662_L1_Tile|2
CTAAGGTTGTCAGCACTGATGAGTATGTCACCCGTACCAGTATTTTCTACCACGCAGGCA
>HPV59_Alpha_557236_nt5677_L1_Tile|1
CTGATGAGTATGTCACCCGTACCAGTATTTTCTACCACGCAGGCAGTTCCAGACTTCTTA
>HPV59_Alpha_557236_nt5682_L1_Tile|2
GAGTATGTCACCCGTACCAGTATTTTCTACCACGCAGGCAGTTCCAGACTTCTTACAGTT
>HPV59_Alpha_557236_nt5722_L1_Tile|2
GTTCCAGACTTCTTACAGTTGGACATCCATATTTTAAAGTACCTAAAGGTGGTAATGGTA
>HPV59_Alpha_557236_nt5727_L1_Tile|1
AGACTTCTTACAGTTGGACATCCATATTTTAAAGTACCTAAAGGTGGTAATGGTAGACAG
>HPV59_Alpha_557236_nt5842_L1_Tile|2
ATCCCAATAAATTTGGCCTTCCAGATAACACAGTATATGATCCTAACTCTCAACGCTTGG
>HPV59_Alpha_557236_nt5862_L1_Tile|2
CCAGATAACACAGTATATGATCCTAACTCTCAACGCTTGGTCTGGGCCTGTGTAGGTGTT
>HPV59_Alpha_557236_nt5972_L1_Tile|1
ATATAATAAATTGGATGACACTGAAAACTCTCATGTAGCATCTGCTGTTGATACCAAAGA
>HPV59_Alpha_557236_nt5977_L1_Tile|1
ATAAATTGGATGACACTGAAAACTCTCATGTAGCATCTGCTGTTGATACCAAAGATACAC
>HPV59_Alpha_557236_nt5982_L1_Tile|2
TTGGATGACACTGAAAACTCTCATGTAGCATCTGCTGTTGATACCAAAGATACACGTGAT
>HPV59_Alpha_557236_nt5987_L1_Tile|1
TGACACTGAAAACTCTCATGTAGCATCTGCTGTTGATACCAAAGATACACGTGATAATGT
>HPV59_Alpha_557236_nt6002_L1_Tile|2
TCATGTAGCATCTGCTGTTGATACCAAAGATACACGTGATAATGTATCTGTGGATTATAA
>HPV59_Alpha_557236_nt6117_L1_Tile|1
AAGGGCACTGCTTGTAAGCCTACTACTGTGGTTCAGGGCGATTGTCCTCCACTAGAATTA
>HPV59_Alpha_557236_nt6127_L1_Tile|1
CTTGTAAGCCTACTACTGTGGTTCAGGGCGATTGTCCTCCACTAGAATTAATAAATACAC
>HPV59_Alpha_557236_nt6417_L1_Tile|1
CTTCCTGAATCACTATATATTAAAGGTACTGACATACGTGCCAACCCAGGCAGTTATTTA
>HPV59_Alpha_557236_nt6422_L1_Tile|2

TGAATCACTATATATTAAAGGTACTGACATACGTGCCAACCCAGGCAGTTATTTATATTC
>HPV59_Alpha_557236_nt6427_L1_Tile|1
CACTATATATTAAAGGTACTGACATACGTGCCAACCCAGGCAGTTATTTATATTCCCCTT
>HPV59_Alpha_557236_nt6432_L1_Tile|1
TATATTAAAGGTACTGACATACGTGCCAACCCAGGCAGTTATTTATATTCCCCTTCCCCA
>HPV59_Alpha_557236_nt6442_L1_Tile|2
GTACTGACATACGTGCCAACCCAGGCAGTTATTTATATTCCCCTTCCCCAAGTGGGTCTG
>HPV59_Alpha_557236_nt6602_L1_Tile|2
AGTTGTAGATACTACTCGCAGCACCAATCTTTCTGTGTGTGCTTCTACTACTTCTTCTAT
>HPV59_Alpha_557236_nt6607_L1_Tile|1
TAGATACTACTCGCAGCACCAATCTTTCTGTGTGTGCTTCTACTACTTCTTCTATTCCTA
>HPV59_Alpha_557236_nt6612_L1_Tile|1
ACTACTCGCAGCACCAATCTTTCTGTGTGTGCTTCTACTACTTCTTCTATTCCTAATGTA
>HPV59_Alpha_557236_nt6617_L1_Tile|1
TCGCAGCACCAATCTTTCTGTGTGTGCTTCTACTACTTCTTCTATTCCTAATGTATACAC
>HPV59_Alpha_557236_nt6627_L1_Tile|1
AATCTTTCTGTGTGTGCTTCTACTACTTCTTCTATTCCTAATGTATACACACCTACCAGT
>HPV59_Alpha_557236_nt6632_L1_Tile|1
TTCTGTGTGTGCTTCTACTACTTCTTCTATTCCTAATGTATACACACCTACCAGTTTTAA
>HPV59_Alpha_557236_nt6637_L1_Tile|1
TGTGTGCTTCTACTACTTCTTCTATTCCTAATGTATACACACCTACCAGTTTTAAAGAAT
>HPV59_Alpha_557236_nt6642_L1_Tile|2
GCTTCTACTACTTCTTCTATTCCTAATGTATACACACCTACCAGTTTTAAAGAATATGCC
>HPV59_Alpha_557236_nt6652_L1_Tile|1
CTTCTTCTATTCCTAATGTATACACACCTACCAGTTTTAAAGAATATGCCAGACATGTGG
>HPV59_Alpha_557236_nt6737_L1_Tile|1
TCAACTGTGTAAAATAACATTAACTACAGAGGTAATGTCATACATTCATAATATGAATAC
>HPV59_Alpha_557236_nt6742_L1_Tile|2
TGTGTAAAATAACATTAACTACAGAGGTAATGTCATACATTCATAATATGAATACCACTA
>HPV59_Alpha_557236_nt6817_L1_Tile|1
ATTTTGGTGTTACACCACCTCCTACTGCTAGTTTAGTTGACACATACCGTTTTGTTCAAT
>HPV59_Alpha_557236_nt6822_L1_Tile|2
GGTGTTACACCACCTCCTACTGCTAGTTTAGTTGACACATACCGTTTTGTTCAATCTGCT
>HPV59_Alpha_557236_nt6827_L1_Tile|1
TACACCACCTCCTACTGCTAGTTTAGTTGACACATACCGTTTTGTTCAATCTGCTGCTGT
>HPV59_Alpha_557236_nt6832_L1_Tile|1
CACCTCCTACTGCTAGTTTAGTTGACACATACCGTTTTGTTCAATCTGCTGCTGTAACTT
>HPV59_Alpha_557236_nt6842_L1_Tile|2
TGCTAGTTTAGTTGACACATACCGTTTTGTTCAATCTGCTGCTGTAACTTGTCAAAAGGA
>HPV59_Alpha_557236_nt6862_L1_Tile|2
ACCGTTTTGTTCAATCTGCTGCTGTAACTTGTCAAAAGGACACCGCACCGCCAGTTAAAC
>HPV59_Alpha_557236_nt6867_L1_Tile|1
TTTGTTCAATCTGCTGCTGTAACTTGTCAAAAGGACACCGCACCGCCAGTTAAACAGGAC
>HPV59_Alpha_557236_nt6882_L1_Tile|2
GCTGTAACTTGTCAAAAGGACACCGCACCGCCAGTTAAACAGGACCCTTATGACAAACTA
>HPV59_Alpha_557236_nt6887_L1_Tile|1
AACTTGTCAAAAGGACACCGCACCGCCAGTTAAACAGGACCCTTATGACAAACTAAAGTT
>HPV59_Alpha_557236_nt6892_L1_Tile|1
GTCAAAAGGACACCGCACCGCCAGTTAAACAGGACCCTTATGACAAACTAAAGTTTTGGC
>HPV59_Alpha_557236_nt6907_L1_Tile|1
CACCGCCAGTTAAACAGGACCCTTATGACAAACTAAAGTTTTGGCCTGTAGATCTTAAGG
>HPV59_Alpha_557236_nt6912_L1_Tile|1
CCAGTTAAACAGGACCCTTATGACAAACTAAAGTTTTGGCCTGTAGATCTTAAGGAAAGG
>HPV59_Alpha_557236_nt7042_L1_Tile|2
CCACTATAGGCCCACGCAAACGTGCAGCGCCTGCCCCTACCTCTACCCCATCACCAAAAC

>HPV59_Alpha_557236_nt7057_L1_Tile|1
GCAAACGTGCAGCGCCTGCCCCTACCTCTACCCCATCACCAAAACGTGTTAAGCGTCGCA
>HPV60_Gamma_9628566_nt5401_L1_Tile|1
TCTTTGGTTGCAGACAGCTGGACAATTGTATCTTCCACCAAGCAAGCCTGTTGCTCGTGT
>HPV60_Gamma_9628566_nt5411_L1_Tile|1
CAGACAGCTGGACAATTGTATCTTCCACCAAGCAAGCCTGTTGCTCGTGTACTCAGTACG
>HPV60_Gamma_9628566_nt5436_L1_Tile|1
CACCAAGCAAGCCTGTTGCTCGTGTACTCAGTACGGATGAATATGTACAGCCAACAAATT
>HPV60_Gamma_9628566_nt5441_L1_Tile|1
AGCAAGCCTGTTGCTCGTGTACTCAGTACGGATGAATATGTACAGCCAACAAATTTAGTT
>HPV60_Gamma_9628566_nt5446_L1_Tile|1
GCCTGTTGCTCGTGTACTCAGTACGGATGAATATGTACAGCCAACAAATTTAGTTTTTCA
>HPV60_Gamma_9628566_nt5451_L1_Tile|1
TTGCTCGTGTACTCAGTACGGATGAATATGTACAGCCAACAAATTTAGTTTTTCATACAG
>HPV60_Gamma_9628566_nt5456_L1_Tile|1
CGTGTACTCAGTACGGATGAATATGTACAGCCAACAAATTTAGTTTTTCATACAGGAACT
>HPV60_Gamma_9628566_nt5461_L1_Tile|1
ACTCAGTACGGATGAATATGTACAGCCAACAAATTTAGTTTTTCATACAGGAACTGACAG
>HPV60_Gamma_9628566_nt5466_L1_Tile|1
GTACGGATGAATATGTACAGCCAACAAATTTAGTTTTTCATACAGGAACTGACAGAATGC
>HPV60_Gamma_9628566_nt5471_L1_Tile|1
GATGAATATGTACAGCCAACAAATTTAGTTTTTCATACAGGAACTGACAGAATGCTAATT
>HPV60_Gamma_9628566_nt5476_L1_Tile|1
ATATGTACAGCCAACAAATTTAGTTTTTCATACAGGAACTGACAGAATGCTAATTGTAGG
>HPV60_Gamma_9628566_nt5486_L1_Tile|1
CCAACAAATTTAGTTTTTCATACAGGAACTGACAGAATGCTAATTGTAGGACACCCTTAT
>HPV60_Gamma_9628566_nt5491_L1_Tile|1
AAATTTAGTTTTTCATACAGGAACTGACAGAATGCTAATTGTAGGACACCCTTATTTTGA
>HPV60_Gamma_9628566_nt5496_L1_Tile|1
TAGTTTTTCATACAGGAACTGACAGAATGCTAATTGTAGGACACCCTTATTTTGATATTA
>HPV60_Gamma_9628566_nt5501_L1_Tile|1
TTTCATACAGGAACTGACAGAATGCTAATTGTAGGACACCCTTATTTTGATATTATAGAC
>HPV60_Gamma_9628566_nt5506_L1_Tile|1
TACAGGAACTGACAGAATGCTAATTGTAGGACACCCTTATTTTGATATTATAGACAGTGG
>HPV60_Gamma_9628566_nt5511_L1_Tile|1
GAACTGACAGAATGCTAATTGTAGGACACCCTTATTTTGATATTATAGACAGTGGTTCAA
>HPV60_Gamma_9628566_nt5516_L1_Tile|1
GACAGAATGCTAATTGTAGGACACCCTTATTTTGATATTATAGACAGTGGTTCAAATAAT
>HPV60_Gamma_9628566_nt5521_L1_Tile|1
AATGCTAATTGTAGGACACCCTTATTTTGATATTATAGACAGTGGTTCAAATAATATTAC
>HPV60_Gamma_9628566_nt5526_L1_Tile|1
TAATTGTAGGACACCCTTATTTTGATATTATAGACAGTGGTTCAAATAATATTACTGTAC
>HPV60_Gamma_9628566_nt5531_L1_Tile|1
GTAGGACACCCTTATTTTGATATTATAGACAGTGGTTCAAATAATATTACTGTACCTAAA
>HPV60_Gamma_9628566_nt5536_L1_Tile|1
ACACCCTTATTTTGATATTATAGACAGTGGTTCAAATAATATTACTGTACCTAAATGTAG
>HPV60_Gamma_9628566_nt5541_L1_Tile|1
CTTATTTTGATATTATAGACAGTGGTTCAAATAATATTACTGTACCTAAATGTAGTGGAA
>HPV60_Gamma_9628566_nt5546_L1_Tile|1
TTTGATATTATAGACAGTGGTTCAAATAATATTACTGTACCTAAATGTAGTGGAAATCAG
>HPV60_Gamma_9628566_nt5551_L1_Tile|1
TATTATAGACAGTGGTTCAAATAATATTACTGTACCTAAATGTAGTGGAAATCAGTTTAG
>HPV60_Gamma_9628566_nt5556_L1_Tile|1
TAGACAGTGGTTCAAATAATATTACTGTACCTAAATGTAGTGGAAATCAGTTTAGAGTTA
>HPV60_Gamma_9628566_nt5561_L1_Tile|1

AGTGGTTCAAATAATATTACTGTACCTAAATGTAGTGGAAATCAGTTTAGAGTTATGCGA
>HPV60_Gamma_9628566_nt5566_L1_Tile|1
TTCAAATAATATTACTGTACCTAAATGTAGTGGAAATCAGTTTAGAGTTATGCGACTACT
>HPV60_Gamma_9628566_nt5571_L1_Tile|1
ATAATATTACTGTACCTAAATGTAGTGGAAATCAGTTTAGAGTTATGCGACTACTATTTC
>HPV60_Gamma_9628566_nt5601_L1_Tile|1
ATCAGTTTAGAGTTATGCGACTACTATTTCCAGATCCAAACAAATTTGCAATGATAGACA
>HPV60_Gamma_9628566_nt5606_L1_Tile|1
TTTAGAGTTATGCGACTACTATTTCCAGATCCAAACAAATTTGCAATGATAGACAGAGCT
>HPV60_Gamma_9628566_nt5611_L1_Tile|1
AGTTATGCGACTACTATTTCCAGATCCAAACAAATTTGCAATGATAGACAGAGCTGTCTT
>HPV60_Gamma_9628566_nt5616_L1_Tile|1
TGCGACTACTATTTCCAGATCCAAACAAATTTGCAATGATAGACAGAGCTGTCTTTAACC
>HPV60_Gamma_9628566_nt5626_L1_Tile|1
ATTTCCAGATCCAAACAAATTTGCAATGATAGACAGAGCTGTCTTTAACCCAGAAAGAGA
>HPV60_Gamma_9628566_nt5631_L1_Tile|1
CAGATCCAAACAAATTTGCAATGATAGACAGAGCTGTCTTTAACCCAGAAAGAGAGAGAT
>HPV60_Gamma_9628566_nt5636_L1_Tile|1
CCAAACAAATTTGCAATGATAGACAGAGCTGTCTTTAACCCAGAAAGAGAGAGATTAGTC
>HPV60_Gamma_9628566_nt5641_L1_Tile|1
CAAATTTGCAATGATAGACAGAGCTGTCTTTAACCCAGAAAGAGAGAGATTAGTCTGGAG
>HPV60_Gamma_9628566_nt5646_L1_Tile|1
TTGCAATGATAGACAGAGCTGTCTTTAACCCAGAAAGAGAGAGATTAGTCTGGAGATTGG
>HPV60_Gamma_9628566_nt5651_L1_Tile|1
ATGATAGACAGAGCTGTCTTTAACCCAGAAAGAGAGAGATTAGTCTGGAGATTGGAAGGC
>HPV60_Gamma_9628566_nt5661_L1_Tile|1
GAGCTGTCTTTAACCCAGAAAGAGAGAGATTAGTCTGGAGATTGGAAGGCCTTGAAATTG
>HPV60_Gamma_9628566_nt5666_L1_Tile|1
GTCTTTAACCCAGAAAGAGAGAGATTAGTCTGGAGATTGGAAGGCCTTGAAATTGGTAGA
>HPV60_Gamma_9628566_nt5671_L1_Tile|1
TAACCCAGAAAGAGAGAGATTAGTCTGGAGATTGGAAGGCCTTGAAATTGGTAGAGGTGG
>HPV60_Gamma_9628566_nt5676_L1_Tile|1
CAGAAAGAGAGAGATTAGTCTGGAGATTGGAAGGCCTTGAAATTGGTAGAGGTGGGCCTT
>HPV60_Gamma_9628566_nt5761_L1_Tile|1
ATTATTTAATAAATATGGAGATACAGAAAATCCTGCCGCATATCCTTTAAAGCAGAATAA
>HPV60_Gamma_9628566_nt5766_L1_Tile|1
TTAATAAATATGGAGATACAGAAAATCCTGCCGCATATCCTTTAAAGCAGAATAATGGTG
>HPV60_Gamma_9628566_nt5771_L1_Tile|1
AAATATGGAGATACAGAAAATCCTGCCGCATATCCTTTAAAGCAGAATAATGGTGATGAC
>HPV60_Gamma_9628566_nt5776_L1_Tile|1
TGGAGATACAGAAAATCCTGCCGCATATCCTTTAAAGCAGAATAATGGTGATGACAATAG
>HPV60_Gamma_9628566_nt5781_L1_Tile|1
ATACAGAAAATCCTGCCGCATATCCTTTAAAGCAGAATAATGGTGATGACAATAGAATGG
>HPV60_Gamma_9628566_nt5786_L1_Tile|1
GAAAATCCTGCCGCATATCCTTTAAAGCAGAATAATGGTGATGACAATAGAATGGATGTT
>HPV60_Gamma_9628566_nt5796_L1_Tile|1
CCGCATATCCTTTAAAGCAGAATAATGGTGATGACAATAGAATGGATGTTTCAATGGATC
>HPV60_Gamma_9628566_nt5806_L1_Tile|1
TTTAAAGCAGAATAATGGTGATGACAATAGAATGGATGTTTCAATGGATCCAAAACAAAT
>HPV60_Gamma_9628566_nt5811_L1_Tile|1
AGCAGAATAATGGTGATGACAATAGAATGGATGTTTCAATGGATCCAAAACAAATGCAAT
>HPV60_Gamma_9628566_nt5816_L1_Tile|1
AATAATGGTGATGACAATAGAATGGATGTTTCAATGGATCCAAAACAAATGCAATTGTTT
>HPV60_Gamma_9628566_nt5831_L1_Tile|1
AATAGAATGGATGTTTCAATGGATCCAAAACAAATGCAATTGTTTATTGTAGGATGTAAA

>HPV60_Gamma_9628566_nt5836_L1_Tile|1
AATGGATGTTTCAATGGATCCAAAACAAATGCAATTGTTTATTGTAGGATGTAAACCAGC
>HPV60_Gamma_9628566_nt5841_L1_Tile|1
ATGTTTCAATGGATCCAAAACAAATGCAATTGTTTATTGTAGGATGTAAACCAGCCACTG
>HPV60_Gamma_9628566_nt5866_L1_Tile|1
GCAATTGTTTATTGTAGGATGTAAACCAGCCACTGGTGAACACTGGGATATTGCTAAACC
>HPV60_Gamma_9628566_nt5881_L1_Tile|1
AGGATGTAAACCAGCCACTGGTGAACACTGGGATATTGCTAAACCTTGTGATCCTGCTCC
>HPV60_Gamma_9628566_nt5886_L1_Tile|1
GTAAACCAGCCACTGGTGAACACTGGGATATTGCTAAACCTTGTGATCCTGCTCCAGCAA
>HPV60_Gamma_9628566_nt5906_L1_Tile|1
CACTGGGATATTGCTAAACCTTGTGATCCTGCTCCAGCAAAAGGATCCTGTCCCCCAATT
>HPV60_Gamma_9628566_nt5911_L1_Tile|1
GGATATTGCTAAACCTTGTGATCCTGCTCCAGCAAAAGGATCCTGTCCCCCAATTAAATT
>HPV60_Gamma_9628566_nt5921_L1_Tile|1
AAACCTTGTGATCCTGCTCCAGCAAAAGGATCCTGTCCCCCAATTAAATTAACACAGTCT
>HPV60_Gamma_9628566_nt5926_L1_Tile|1
TTGTGATCCTGCTCCAGCAAAAGGATCCTGTCCCCCAATTAAATTAACACAGTCTATAAT
>HPV60_Gamma_9628566_nt5941_L1_Tile|1
AGCAAAAGGATCCTGTCCCCCAATTAAATTAACACAGTCTATAATTCAGGACGGTGAAAT
>HPV60_Gamma_9628566_nt5946_L1_Tile|1
AAGGATCCTGTCCCCCAATTAAATTAACACAGTCTATAATTCAGGACGGTGAAATGTGCG
>HPV60_Gamma_9628566_nt5961_L1_Tile|1
CAATTAAATTAACACAGTCTATAATTCAGGACGGTGAAATGTGCGATACAGGCTTTGGAA
>HPV60_Gamma_9628566_nt5966_L1_Tile|1
AAATTAACACAGTCTATAATTCAGGACGGTGAAATGTGCGATACAGGCTTTGGAAATGCA
>HPV60_Gamma_9628566_nt5976_L1_Tile|1
AGTCTATAATTCAGGACGGTGAAATGTGCGATACAGGCTTTGGAAATGCAAATTTTATTA
>HPV60_Gamma_9628566_nt5991_L1_Tile|1
ACGGTGAAATGTGCGATACAGGCTTTGGAAATGCAAATTTTATTACCTTGCAAGAAGATA
>HPV60_Gamma_9628566_nt5996_L1_Tile|1
GAAATGTGCGATACAGGCTTTGGAAATGCAAATTTTATTACCTTGCAAGAAGATAAATCA
>HPV60_Gamma_9628566_nt6011_L1_Tile|1
GGCTTTGGAAATGCAAATTTTATTACCTTGCAAGAAGATAAATCAGGTGTTCCTCTAGAT
>HPV60_Gamma_9628566_nt6016_L1_Tile|1
TGGAAATGCAAATTTTATTACCTTGCAAGAAGATAAATCAGGTGTTCCTCTAGATATTAC
>HPV60_Gamma_9628566_nt6021_L1_Tile|1
ATGCAAATTTTATTACCTTGCAAGAAGATAAATCAGGTGTTCCTCTAGATATTACCAATG
>HPV60_Gamma_9628566_nt6026_L1_Tile|1
AATTTTATTACCTTGCAAGAAGATAAATCAGGTGTTCCTCTAGATATTACCAATGAAATT
>HPV60_Gamma_9628566_nt6031_L1_Tile|1
TATTACCTTGCAAGAAGATAAATCAGGTGTTCCTCTAGATATTACCAATGAAATTTGTAA
>HPV60_Gamma_9628566_nt6036_L1_Tile|1
CCTTGCAAGAAGATAAATCAGGTGTTCCTCTAGATATTACCAATGAAATTTGTAAATATC
>HPV60_Gamma_9628566_nt6056_L1_Tile|1
GGTGTTCCTCTAGATATTACCAATGAAATTTGTAAATATCCTGATTTACTAAAAATGACA
>HPV60_Gamma_9628566_nt6061_L1_Tile|1
TCCTCTAGATATTACCAATGAAATTTGTAAATATCCTGATTTACTAAAAATGACAAAAGA
>HPV60_Gamma_9628566_nt6066_L1_Tile|1
TAGATATTACCAATGAAATTTGTAAATATCCTGATTTACTAAAAATGACAAAAGATATCT
>HPV60_Gamma_9628566_nt6081_L1_Tile|1
AAATTTGTAAATATCCTGATTTACTAAAAATGACAAAAGATATCTATGGAGATGCAGTAT
>HPV60_Gamma_9628566_nt6136_L1_Tile|1
AGTATTTTTCTTTGGTAAACGTGAACAAATTTATTCCAGGCATTATTTTGTAAGAGGAGG
>HPV60_Gamma_9628566_nt6146_L1_Tile|1

TTTGGTAAACGTGAACAAATTTATTCCAGGCATTATTTTGTAAGAGGAGGGATAGATGGA
>HPV60_Gamma_9628566_nt6151_L1_Tile|1
TAAACGTGAACAAATTTATTCCAGGCATTATTTTGTAAGAGGAGGGATAGATGGAGATAG
>HPV60_Gamma_9628566_nt6156_L1_Tile|1
GTGAACAAATTTATTCCAGGCATTATTTTGTAAGAGGAGGGATAGATGGAGATAGTTTGC
>HPV60_Gamma_9628566_nt6166_L1_Tile|1
TTATTCCAGGCATTATTTTGTAAGAGGAGGGATAGATGGAGATAGTTTGCCAGATTCTGG
>HPV60_Gamma_9628566_nt6171_L1_Tile|1
CCAGGCATTATTTTGTAAGAGGAGGGATAGATGGAGATAGTTTGCCAGATTCTGGATATT
>HPV60_Gamma_9628566_nt6181_L1_Tile|1
TTTTGTAAGAGGAGGGATAGATGGAGATAGTTTGCCAGATTCTGGATATTACTTAGCACC
>HPV60_Gamma_9628566_nt6186_L1_Tile|1
TAAGAGGAGGGATAGATGGAGATAGTTTGCCAGATTCTGGATATTACTTAGCACCACAAA
>HPV60_Gamma_9628566_nt6191_L1_Tile|1
GGAGGGATAGATGGAGATAGTTTGCCAGATTCTGGATATTACTTAGCACCACAAACTGAT
>HPV60_Gamma_9628566_nt6196_L1_Tile|1
GATAGATGGAGATAGTTTGCCAGATTCTGGATATTACTTAGCACCACAAACTGATAAACC
>HPV60_Gamma_9628566_nt6201_L1_Tile|1
ATGGAGATAGTTTGCCAGATTCTGGATATTACTTAGCACCACAAACTGATAAACCTCAAA
>HPV60_Gamma_9628566_nt6206_L1_Tile|1
GATAGTTTGCCAGATTCTGGATATTACTTAGCACCACAAACTGATAAACCTCAAAATAAC
>HPV60_Gamma_9628566_nt6211_L1_Tile|1
TTTGCCAGATTCTGGATATTACTTAGCACCACAAACTGATAAACCTCAAAATAACCTTGG
>HPV60_Gamma_9628566_nt6216_L1_Tile|1
CAGATTCTGGATATTACTTAGCACCACAAACTGATAAACCTCAAAATAACCTTGGTGGTT
>HPV60_Gamma_9628566_nt6221_L1_Tile|1
TCTGGATATTACTTAGCACCACAAACTGATAAACCTCAAAATAACCTTGGTGGTTATAGT
>HPV60_Gamma_9628566_nt6226_L1_Tile|1
ATATTACTTAGCACCACAAACTGATAAACCTCAAAATAACCTTGGTGGTTATAGTTACTT
>HPV60_Gamma_9628566_nt6231_L1_Tile|1
ACTTAGCACCACAAACTGATAAACCTCAAAATAACCTTGGTGGTTATAGTTACTTCCCTA
>HPV60_Gamma_9628566_nt6241_L1_Tile|1
ACAAACTGATAAACCTCAAAATAACCTTGGTGGTTATAGTTACTTCCCTACACCAAGTGG
>HPV60_Gamma_9628566_nt6246_L1_Tile|1
CTGATAAACCTCAAAATAACCTTGGTGGTTATAGTTACTTCCCTACACCAAGTGGTTCTG
>HPV60_Gamma_9628566_nt6251_L1_Tile|1
AAACCTCAAAATAACCTTGGTGGTTATAGTTACTTCCCTACACCAAGTGGTTCTGTAGCT
>HPV60_Gamma_9628566_nt6256_L1_Tile|1
TCAAAATAACCTTGGTGGTTATAGTTACTTCCCTACACCAAGTGGTTCTGTAGCTTCTAG
>HPV60_Gamma_9628566_nt6261_L1_Tile|1
ATAACCTTGGTGGTTATAGTTACTTCCCTACACCAAGTGGTTCTGTAGCTTCTAGTGATA
>HPV60_Gamma_9628566_nt6271_L1_Tile|1
TGGTTATAGTTACTTCCCTACACCAAGTGGTTCTGTAGCTTCTAGTGATAATCAACTTTT
>HPV60_Gamma_9628566_nt6276_L1_Tile|1
ATAGTTACTTCCCTACACCAAGTGGTTCTGTAGCTTCTAGTGATAATCAACTTTTTAATA
>HPV60_Gamma_9628566_nt6281_L1_Tile|1
TACTTCCCTACACCAAGTGGTTCTGTAGCTTCTAGTGATAATCAACTTTTTAATAGACCT
>HPV60_Gamma_9628566_nt6286_L1_Tile|1
CCCTACACCAAGTGGTTCTGTAGCTTCTAGTGATAATCAACTTTTTAATAGACCTTATTG
>HPV60_Gamma_9628566_nt6306_L1_Tile|1
TAGCTTCTAGTGATAATCAACTTTTTAATAGACCTTATTGGCTTCACAGAGCCCAAGGTG
>HPV60_Gamma_9628566_nt6376_L1_Tile|1
TATTTGTTGGGGTAACCAATTATTCATTACTATAGTAGATAATACTCGAAATACTAATTT
>HPV60_Gamma_9628566_nt6381_L1_Tile|1
GTTGGGGTAACCAATTATTCATTACTATAGTAGATAATACTCGAAATACTAATTTATCTA

>HPV60_Gamma_9628566_nt6386_L1_Tile|1
GGTAACCAATTATTCATTACTATAGTAGATAATACTCGAAATACTAATTTATCTATTTCA
>HPV60_Gamma_9628566_nt6391_L1_Tile|1
CCAATTATTCATTACTATAGTAGATAATACTCGAAATACTAATTTATCTATTTCAGTTTA
>HPV60_Gamma_9628566_nt6401_L1_Tile|1
ATTACTATAGTAGATAATACTCGAAATACTAATTTATCTATTTCAGTTTACAAACAAGAT
>HPV60_Gamma_9628566_nt6406_L1_Tile|1
TATAGTAGATAATACTCGAAATACTAATTTATCTATTTCAGTTTACAAACAAGATGCTGC
>HPV60_Gamma_9628566_nt6411_L1_Tile|1
TAGATAATACTCGAAATACTAATTTATCTATTTCAGTTTACAAACAAGATGCTGCTATTG
>HPV60_Gamma_9628566_nt6416_L1_Tile|1
AATACTCGAAATACTAATTTATCTATTTCAGTTTACAAACAAGATGCTGCTATTGATAAC
>HPV60_Gamma_9628566_nt6421_L1_Tile|1
TCGAAATACTAATTTATCTATTTCAGTTTACAAACAAGATGCTGCTATTGATAACAGATA
>HPV60_Gamma_9628566_nt6426_L1_Tile|1
ATACTAATTTATCTATTTCAGTTTACAAACAAGATGCTGCTATTGATAACAGATATAAAT
>HPV60_Gamma_9628566_nt6431_L1_Tile|1
AATTTATCTATTTCAGTTTACAAACAAGATGCTGCTATTGATAACAGATATAAATATAAA
>HPV60_Gamma_9628566_nt6476_L1_Tile|1
AGATATAAATATAAACAGGAAGATTTTAGACAGTATTTACGCCATACTGAAGAATATGAA
>HPV60_Gamma_9628566_nt6481_L1_Tile|1
TAAATATAAACAGGAAGATTTTAGACAGTATTTACGCCATACTGAAGAATATGAAGTAGA
>HPV60_Gamma_9628566_nt6486_L1_Tile|1
ATAAACAGGAAGATTTTAGACAGTATTTACGCCATACTGAAGAATATGAAGTAGAGTTAA
>HPV60_Gamma_9628566_nt6491_L1_Tile|1
CAGGAAGATTTTAGACAGTATTTACGCCATACTGAAGAATATGAAGTAGAGTTAATTCTG
>HPV60_Gamma_9628566_nt6496_L1_Tile|1
AGATTTTAGACAGTATTTACGCCATACTGAAGAATATGAAGTAGAGTTAATTCTGAGACT
>HPV60_Gamma_9628566_nt6506_L1_Tile|1
CAGTATTTACGCCATACTGAAGAATATGAAGTAGAGTTAATTCTGAGACTCTGTAAAGTG
>HPV60_Gamma_9628566_nt6511_L1_Tile|1
TTTACGCCATACTGAAGAATATGAAGTAGAGTTAATTCTGAGACTCTGTAAAGTGCCTTT
>HPV60_Gamma_9628566_nt6516_L1_Tile|1
GCCATACTGAAGAATATGAAGTAGAGTTAATTCTGAGACTCTGTAAAGTGCCTTTGAATC
>HPV60_Gamma_9628566_nt6521_L1_Tile|1
ACTGAAGAATATGAAGTAGAGTTAATTCTGAGACTCTGTAAAGTGCCTTTGAATCCAGAT
>HPV60_Gamma_9628566_nt6526_L1_Tile|1
AGAATATGAAGTAGAGTTAATTCTGAGACTCTGTAAAGTGCCTTTGAATCCAGATGTTCT
>HPV60_Gamma_9628566_nt6531_L1_Tile|1
ATGAAGTAGAGTTAATTCTGAGACTCTGTAAAGTGCCTTTGAATCCAGATGTTCTAGCTC
>HPV60_Gamma_9628566_nt6536_L1_Tile|1
GTAGAGTTAATTCTGAGACTCTGTAAAGTGCCTTTGAATCCAGATGTTCTAGCTCATTTA
>HPV60_Gamma_9628566_nt6541_L1_Tile|1
GTTAATTCTGAGACTCTGTAAAGTGCCTTTGAATCCAGATGTTCTAGCTCATTTAAATGT
>HPV60_Gamma_9628566_nt6631_L1_Tile|1
ACTTTCATTTGTACCTCCACCACCACAAGGTATTGAAGATGCTTACAGATATATAATGTC
>HPV60_Gamma_9628566_nt6641_L1_Tile|1
GTACCTCCACCACCACAAGGTATTGAAGATGCTTACAGATATATAATGTCTCAGGCAACA
>HPV60_Gamma_9628566_nt6666_L1_Tile|1
AAGATGCTTACAGATATATAATGTCTCAGGCAACAATGTGTCCAACAGATGTTCCTAATA
>HPV60_Gamma_9628566_nt6671_L1_Tile|1
GCTTACAGATATATAATGTCTCAGGCAACAATGTGTCCAACAGATGTTCCTAATACTGAA
>HPV60_Gamma_9628566_nt6676_L1_Tile|1
CAGATATATAATGTCTCAGGCAACAATGTGTCCAACAGATGTTCCTAATACTGAAAGGGA
>HPV60_Gamma_9628566_nt6691_L1_Tile|1

TCAGGCAACAATGTGTCCAACAGATGTTCCTAATACTGAAAGGGAAGACCCTTATAAACA
>HPV60_Gamma_9628566_nt6696_L1_Tile|1
CAACAATGTGTCCAACAGATGTTCCTAATACTGAAAGGGAAGACCCTTATAAACAGTATA
>HPV60_Gamma_9628566_nt6726_L1_Tile|1
CTGAAAGGGAAGACCCTTATAAACAGTATACATTTTGGACAATAGATTTGCAAGAACGTT
>HPV60_Gamma_9628566_nt6741_L1_Tile|1
CTTATAAACAGTATACATTTTGGACAATAGATTTGCAAGAACGTTTTTCTAACGAATTAT
>HPV60_Gamma_9628566_nt6746_L1_Tile|1
AAACAGTATACATTTTGGACAATAGATTTGCAAGAACGTTTTTCTAACGAATTATCCCAG
>HPV60_Gamma_9628566_nt6751_L1_Tile|1
GTATACATTTTGGACAATAGATTTGCAAGAACGTTTTTCTAACGAATTATCCCAGTTTTC
>HPV60_Gamma_9628566_nt6756_L1_Tile|1
CATTTTGGACAATAGATTTGCAAGAACGTTTTTCTAACGAATTATCCCAGTTTTCTCTTG
>HPV60_Gamma_9628566_nt6761_L1_Tile|1
TGGACAATAGATTTGCAAGAACGTTTTTCTAACGAATTATCCCAGTTTTCTCTTGGAAAA
>HPV60_Gamma_9628566_nt6811_L1_Tile|1
TCTTGGAAAAAGATATTTATATCAGTATGGCTTACTTAACGGACGAAAACGGTCTGCTAG
>HPV60_Gamma_9628566_nt6816_L1_Tile|1
GAAAAAGATATTTATATCAGTATGGCTTACTTAACGGACGAAAACGGTCTGCTAGTAGTT
>HPV60_Gamma_9628566_nt6821_L1_Tile|1
AGATATTTATATCAGTATGGCTTACTTAACGGACGAAAACGGTCTGCTAGTAGTTTTGTA
>HPV60_Gamma_9628566_nt6826_L1_Tile|1
TTTATATCAGTATGGCTTACTTAACGGACGAAAACGGTCTGCTAGTAGTTTTGTAACGAA
>HPV60_Gamma_9628566_nt6831_L1_Tile|1
ATCAGTATGGCTTACTTAACGGACGAAAACGGTCTGCTAGTAGTTTTGTAACGAAGAAAT
>HPV60_Gamma_9628566_nt6836_L1_Tile|1
TATGGCTTACTTAACGGACGAAAACGGTCTGCTAGTAGTTTTGTAACGAAGAAATCAAAA
>HPV60_Gamma_9628566_nt6841_L1_Tile|1
CTTACTTAACGGACGAAAACGGTCTGCTAGTAGTTTTGTAACGAAGAAATCAAAAACTGT
>HPV60_Gamma_9628566_nt6846_L1_Tile|1
TTAACGGACGAAAACGGTCTGCTAGTAGTTTTGTAACGAAGAAATCAAAAACTGTGAAAC
>HPV60_Gamma_9628566_nt6851_L1_Tile|1
GGACGAAAACGGTCTGCTAGTAGTTTTGTAACGAAGAAATCAAAAACTGTGAAACGTAAA
>HPV60_Gamma_9628566_nt6861_L1_Tile|1
GGTCTGCTAGTAGTTTTGTAACGAAGAAATCAAAAACTGTGAAACGTAAAAGAACTAAAT
>HPV60_Gamma_9628566_nt6863_L1_Tile|1
TCTGCTAGTAGTTTTGTAACGAAGAAATCAAAAACTGTGAAACGTAAAAGAACTAAATAA
>HPV61_Alpha_9628574_nt5880_L1_Tile|1
GTTCCCGTCTGCTTACTGTAGGACATCCCTATTGTAGTTTGCAGCTTGATGGGCTGCAGG
>HPV61_Alpha_9628574_nt5890_L1_Tile|1
GCTTACTGTAGGACATCCCTATTGTAGTTTGCAGCTTGATGGGCTGCAGGGCAAGAAAAA
>HPV61_Alpha_9628574_nt5895_L1_Tile|1
CTGTAGGACATCCCTATTGTAGTTTGCAGCTTGATGGGCTGCAGGGCAAGAAAAACACTA
>HPV61_Alpha_9628574_nt5900_L1_Tile|1
GGACATCCCTATTGTAGTTTGCAGCTTGATGGGCTGCAGGGCAAGAAAAACACTATCCCC
>HPV61_Alpha_9628574_nt5910_L1_Tile|1
ATTGTAGTTTGCAGCTTGATGGGCTGCAGGGCAAGAAAAACACTATCCCCAAGGTGTCTG
>HPV61_Alpha_9628574_nt6195_L1_Tile|1
GTCGTGACAATGTGTCTGTTGATTATAAGCAGACCCAGCTACTTATTGTAGGTTGCAAAC
>HPV61_Alpha_9628574_nt6285_L1_Tile|1
CTGCTTGTGCTAACCCCGCGCCCCGGCCCACTGACTGTCCTCCATTGGAATTTACAAATA
>HPV61_Alpha_9628574_nt6550_L1_Tile|1
CAATCGCCAGGGTGTCATGGGTGAGGCACTGCCTGACTCTTATTATCTTAAGGGTGCGAA
>HPV61_Alpha_9628574_nt6585_L1_Tile|1
ACTCTTATTATCTTAAGGGTGCGAATGACAAGGCGGCCCCTGGTAGTTATATTTATTCCC

>HPV61_Alpha_9628574_nt6780_L1_Tile|1
GCAGTACTAATTTAACCATTTGTACTGCTACATCCCCCCCTGTATCTGAATATAAAGCCA
>HPV61_Alpha_9628574_nt6785_L1_Tile|1
ACTAATTTAACCATTTGTACTGCTACATCCCCCCCTGTATCTGAATATAAAGCCACAAGC
>HPV61_Alpha_9628574_nt6790_L1_Tile|1
TTTAACCATTTGTACTGCTACATCCCCCCCTGTATCTGAATATAAAGCCACAAGCTTTAG
>HPV61_Alpha_9628574_nt6795_L1_Tile|1
CCATTTGTACTGCTACATCCCCCCCTGTATCTGAATATAAAGCCACAAGCTTTAGGGAAT
>HPV61_Alpha_9628574_nt6800_L1_Tile|1
TGTACTGCTACATCCCCCCCTGTATCTGAATATAAAGCCACAAGCTTTAGGGAATATTTG
>HPV61_Alpha_9628574_nt6805_L1_Tile|1
TGCTACATCCCCCCCTGTATCTGAATATAAAGCCACAAGCTTTAGGGAATATTTGCGCCA
>HPV61_Alpha_9628574_nt6810_L1_Tile|1
CATCCCCCCCTGTATCTGAATATAAAGCCACAAGCTTTAGGGAATATTTGCGCCATACAG
>HPV61_Alpha_9628574_nt6820_L1_Tile|1
TGTATCTGAATATAAAGCCACAAGCTTTAGGGAATATTTGCGCCATACAGAGGAGTTTGA
>HPV61_Alpha_9628574_nt6985_L1_Tile|1
GGTACCACCACCCTCTACCAGTTTAGAAGACACATATAGGTTTTTGCAGTCCAGAGCTAT
>HPVcand62_Alpha_39932599_nt6163_L1_Tile|1
CAGAAAATACCTCTTTGTTGGCTGCTGCTAATGATGACAGTCGGGACAATATCTCTGTGG
>HPVcand62_Alpha_39932599_nt6168_L1_Tile|1
AATACCTCTTTGTTGGCTGCTGCTAATGATGACAGTCGGGACAATATCTCTGTGGATTAT
>HPVcand62_Alpha_39932599_nt6238_L1_Tile|1
AGTTGTTAATTGTGGGGTGTAAGCCCCCTATAGGAGAGCACTGGACCAAAGGTACCTTAT
>HPVcand62_Alpha_39932599_nt6243_L1_Tile|1
TTAATTGTGGGGTGTAAGCCCCCTATAGGAGAGCACTGGACCAAAGGTACCTTATGCCCC
>HPVcand62_Alpha_39932599_nt6248_L1_Tile|1
TGTGGGGTGTAAGCCCCCTATAGGAGAGCACTGGACCAAAGGTACCTTATGCCCCAATGC
>HPVcand62_Alpha_39932599_nt6253_L1_Tile|1
GGTGTAAGCCCCCTATAGGAGAGCACTGGACCAAAGGTACCTTATGCCCCAATGCTGCCC
>HPVcand62_Alpha_39932599_nt6588_L1_Tile|1
CCCACTGACCTGTATATGAAAGGTACCCCAGGCAGTGACAGACAAATGCCGGGCAGTTAC
>HPVcand62_Alpha_39932599_nt6593_L1_Tile|1
TGACCTGTATATGAAAGGTACCCCAGGCAGTGACAGACAAATGCCGGGCAGTTACATTTA
>HPVcand62_Alpha_39932599_nt6603_L1_Tile|1
ATGAAAGGTACCCCAGGCAGTGACAGACAAATGCCGGGCAGTTACATTTATGCCCCCACT
>HPVcand62_Alpha_39932599_nt7073_L1_Tile|1
TGCTTCCCCGTCCCCCAAGGTGGACCCGTATGCGCAAATGACATTTTGGACTGTGGATCT
>HPVcand62_Alpha_39932599_nt7230_L1_Tile|1
AAGCGTTCTGCACCTACTAAGCAGTCCCCTACCTCTGCTAAACGAAAGCGCAGGAAGTAA
>HPV63_Mu_9626605_nt5372_L1_Tile|1
ATGGCTGTTTGGCTTCCTGCCCAGAATAAGTTTTACCTTCCTACCCAACCGATCACCAAG
>HPV63_Mu_9626605_nt5377_L1_Tile|1
TGTTTGGCTTCCTGCCCAGAATAAGTTTTACCTTCCTACCCAACCGATCACCAAGATTCT
>HPV63_Mu_9626605_nt5392_L1_Tile|1
CCAGAATAAGTTTTACCTTCCTACCCAACCGATCACCAAGATTCTAAGCAGCGATGATTA
>HPV63_Mu_9626605_nt5397_L1_Tile|1
ATAAGTTTTACCTTCCTACCCAACCGATCACCAAGATTCTAAGCAGCGATGATTATGTGT
>HPV63_Mu_9626605_nt5407_L1_Tile|1
CCTTCCTACCCAACCGATCACCAAGATTCTAAGCAGCGATGATTATGTGTCTCGCACCAA
>HPV63_Mu_9626605_nt5412_L1_Tile|1
CTACCCAACCGATCACCAAGATTCTAAGCAGCGATGATTATGTGTCTCGCACCAACATCT
>HPV63_Mu_9626605_nt5417_L1_Tile|1
CAACCGATCACCAAGATTCTAAGCAGCGATGATTATGTGTCTCGCACCAACATCTTCTAT
>HPV63_Mu_9626605_nt5422_L1_Tile|2

GATCACCAAGATTCTAAGCAGCGATGATTATGTGTCTCGCACCAACATCTTCTATCACGC
>HPV63_Mu_9626605_nt5427_L1_Tile|1
CCAAGATTCTAAGCAGCGATGATTATGTGTCTCGCACCAACATCTTCTATCACGCTACCA
>HPV63_Mu_9626605_nt5442_L1_Tile|2
GCGATGATTATGTGTCTCGCACCAACATCTTCTATCACGCTACCAGTGATCGACTGCTCA
>HPV63_Mu_9626605_nt5447_L1_Tile|1
GATTATGTGTCTCGCACCAACATCTTCTATCACGCTACCAGTGATCGACTGCTCATTGTG
>HPV63_Mu_9626605_nt5462_L1_Tile|2
ACCAACATCTTCTATCACGCTACCAGTGATCGACTGCTCATTGTGGGACACCCGCTCTAT
>HPV63_Mu_9626605_nt5467_L1_Tile|1
CATCTTCTATCACGCTACCAGTGATCGACTGCTCATTGTGGGACACCCGCTCTATGAGGT
>HPV63_Mu_9626605_nt5472_L1_Tile|1
TCTATCACGCTACCAGTGATCGACTGCTCATTGTGGGACACCCGCTCTATGAGGTTACCC
>HPV63_Mu_9626605_nt5487_L1_Tile|1
GTGATCGACTGCTCATTGTGGGACACCCGCTCTATGAGGTTACCCGTGCAAATGATAACA
>HPV63_Mu_9626605_nt5492_L1_Tile|1
CGACTGCTCATTGTGGGACACCCGCTCTATGAGGTTACCCGTGCAAATGATAACACTATG
>HPV63_Mu_9626605_nt5497_L1_Tile|1
GCTCATTGTGGGACACCCGCTCTATGAGGTTACCCGTGCAAATGATAACACTATGACTGT
>HPV63_Mu_9626605_nt5512_L1_Tile|1
CCCGCTCTATGAGGTTACCCGTGCAAATGATAACACTATGACTGTGCCTAAAGTTTCTCC
>HPV63_Mu_9626605_nt5517_L1_Tile|1
TCTATGAGGTTACCCGTGCAAATGATAACACTATGACTGTGCCTAAAGTTTCTCCAAATC
>HPV63_Mu_9626605_nt5522_L1_Tile|2
GAGGTTACCCGTGCAAATGATAACACTATGACTGTGCCTAAAGTTTCTCCAAATCAGTAT
>HPV63_Mu_9626605_nt5532_L1_Tile|1
GTGCAAATGATAACACTATGACTGTGCCTAAAGTTTCTCCAAATCAGTATAGAGTCTTTC
>HPV63_Mu_9626605_nt5537_L1_Tile|1
AATGATAACACTATGACTGTGCCTAAAGTTTCTCCAAATCAGTATAGAGTCTTTCGTGTT
>HPV63_Mu_9626605_nt5542_L1_Tile|2
TAACACTATGACTGTGCCTAAAGTTTCTCCAAATCAGTATAGAGTCTTTCGTGTTAGATT
>HPV63_Mu_9626605_nt5547_L1_Tile|1
CTATGACTGTGCCTAAAGTTTCTCCAAATCAGTATAGAGTCTTTCGTGTTAGATTTCCAG
>HPV63_Mu_9626605_nt5577_L1_Tile|1
AGTATAGAGTCTTTCGTGTTAGATTTCCAGATCCTAACCGATTTGCCTTTGGAGATAAGG
>HPV63_Mu_9626605_nt5582_L1_Tile|2
AGAGTCTTTCGTGTTAGATTTCCAGATCCTAACCGATTTGCCTTTGGAGATAAGGATATT
>HPV63_Mu_9626605_nt5622_L1_Tile|2
CCTTTGGAGATAAGGATATTTTTGACCCAGAAACTGAGAGACTAGTTTGGGGTCTTAGAG
>HPV63_Mu_9626605_nt5627_L1_Tile|1
GGAGATAAGGATATTTTTGACCCAGAAACTGAGAGACTAGTTTGGGGTCTTAGAGGCATA
>HPV63_Mu_9626605_nt5632_L1_Tile|1
TAAGGATATTTTTGACCCAGAAACTGAGAGACTAGTTTGGGGTCTTAGAGGCATAGAAAT
>HPV63_Mu_9626605_nt5637_L1_Tile|1
ATATTTTTGACCCAGAAACTGAGAGACTAGTTTGGGGTCTTAGAGGCATAGAAATCGGTA
>HPV63_Mu_9626605_nt5712_L1_Tile|1
GTGTGGGTATTTCAGGCAATCCATTATTAAATAGGTTTGATGATGCTGAAAATCCTAGCA
>HPV63_Mu_9626605_nt5722_L1_Tile|2
TTCAGGCAATCCATTATTAAATAGGTTTGATGATGCTGAAAATCCTAGCAGATATAATAA
>HPV63_Mu_9626605_nt5727_L1_Tile|1
GCAATCCATTATTAAATAGGTTTGATGATGCTGAAAATCCTAGCAGATATAATAATACAC
>HPV63_Mu_9626605_nt5732_L1_Tile|1
CCATTATTAAATAGGTTTGATGATGCTGAAAATCCTAGCAGATATAATAATACACATGCA
>HPV63_Mu_9626605_nt5742_L1_Tile|2
ATAGGTTTGATGATGCTGAAAATCCTAGCAGATATAATAATACACATGCAACTGGTGATA

>HPV63_Mu_9626605_nt5747_L1_Tile|1
TTTGATGATGCTGAAAATCCTAGCAGATATAATAATACACATGCAACTGGTGATAATAGG
>HPV63_Mu_9626605_nt5757_L1_Tile|1
CTGAAAATCCTAGCAGATATAATAATACACATGCAACTGGTGATAATAGGCAAAATGTTG
>HPV63_Mu_9626605_nt5762_L1_Tile|2
AATCCTAGCAGATATAATAATACACATGCAACTGGTGATAATAGGCAAAATGTTGCTTTT
>HPV63_Mu_9626605_nt5792_L1_Tile|1
ACTGGTGATAATAGGCAAAATGTTGCTTTTGATGCAAAACAAACCCAAATGTTTCTAATT
>HPV63_Mu_9626605_nt5852_L1_Tile|1
GGCTGTACACCAGCCACTGGGGAACACTGGTCAATAGCTCGACGCTGTGCAGGAACACAG
>HPV63_Mu_9626605_nt5857_L1_Tile|1
TACACCAGCCACTGGGGAACACTGGTCAATAGCTCGACGCTGTGCAGGAACACAGTTTCA
>HPV63_Mu_9626605_nt5872_L1_Tile|1
GGAACACTGGTCAATAGCTCGACGCTGTGCAGGAACACAGTTTCAGCTTGGAGATTGTCC
>HPV63_Mu_9626605_nt5877_L1_Tile|1
ACTGGTCAATAGCTCGACGCTGTGCAGGAACACAGTTTCAGCTTGGAGATTGTCCTCCTA
>HPV63_Mu_9626605_nt5967_L1_Tile|1
GTGATATGTTTGACATAGGTCTAGGTGCTATGGACTTTGGTTCTTTGCAAGCAAACAAAG
>HPV63_Mu_9626605_nt5972_L1_Tile|1
ATGTTTGACATAGGTCTAGGTGCTATGGACTTTGGTTCTTTGCAAGCAAACAAAGCAGAT
>HPV63_Mu_9626605_nt5977_L1_Tile|1
TGACATAGGTCTAGGTGCTATGGACTTTGGTTCTTTGCAAGCAAACAAAGCAGATGCTCC
>HPV63_Mu_9626605_nt5982_L1_Tile|2
TAGGTCTAGGTGCTATGGACTTTGGTTCTTTGCAAGCAAACAAAGCAGATGCTCCTTTGG
>HPV63_Mu_9626605_nt5992_L1_Tile|1
TGCTATGGACTTTGGTTCTTTGCAAGCAAACAAAGCAGATGCTCCTTTGGATATTGCAGG
>HPV63_Mu_9626605_nt5997_L1_Tile|1
TGGACTTTGGTTCTTTGCAAGCAAACAAAGCAGATGCTCCTTTGGATATTGCAGGCACTG
>HPV63_Mu_9626605_nt6002_L1_Tile|2
TTTGGTTCTTTGCAAGCAAACAAAGCAGATGCTCCTTTGGATATTGCAGGCACTGTCTGC
>HPV63_Mu_9626605_nt6072_L1_Tile|1
ATTATATTAAAATGGGACAGGAAGTACATGGTAATTCTCTGTTTTTCTTTGCTCGCAGAG
>HPV63_Mu_9626605_nt6077_L1_Tile|1
ATTAAAATGGGACAGGAAGTACATGGTAATTCTCTGTTTTTCTTTGCTCGCAGAGAACAA
>HPV63_Mu_9626605_nt6082_L1_Tile|2
AATGGGACAGGAAGTACATGGTAATTCTCTGTTTTTCTTTGCTCGCAGAGAACAAATGTA
>HPV63_Mu_9626605_nt6087_L1_Tile|1
GACAGGAAGTACATGGTAATTCTCTGTTTTTCTTTGCTCGCAGAGAACAAATGTATTTAA
>HPV63_Mu_9626605_nt6112_L1_Tile|1
GTTTTTCTTTGCTCGCAGAGAACAAATGTATTTAAGGCATGTATTTACACATGCTGGAAT
>HPV63_Mu_9626605_nt6117_L1_Tile|1
TCTTTGCTCGCAGAGAACAAATGTATTTAAGGCATGTATTTACACATGCTGGAATTGTTA
>HPV63_Mu_9626605_nt6122_L1_Tile|2
GCTCGCAGAGAACAAATGTATTTAAGGCATGTATTTACACATGCTGGAATTGTTAGTGAA
>HPV63_Mu_9626605_nt6127_L1_Tile|1
CAGAGAACAAATGTATTTAAGGCATGTATTTACACATGCTGGAATTGTTAGTGAAAAAGA
>HPV63_Mu_9626605_nt6132_L1_Tile|1
AACAAATGTATTTAAGGCATGTATTTACACATGCTGGAATTGTTAGTGAAAAAGAGAAAG
>HPV63_Mu_9626605_nt6137_L1_Tile|1
ATGTATTTAAGGCATGTATTTACACATGCTGGAATTGTTAGTGAAAAAGAGAAAGTCCCT
>HPV63_Mu_9626605_nt6142_L1_Tile|2
TTTAAGGCATGTATTTACACATGCTGGAATTGTTAGTGAAAAAGAGAAAGTCCCTACCAG
>HPV63_Mu_9626605_nt6147_L1_Tile|1
GGCATGTATTTACACATGCTGGAATTGTTAGTGAAAAAGAGAAAGTCCCTACCAGTGCAT
>HPV63_Mu_9626605_nt6152_L1_Tile|1

GTATTTACACATGCTGGAATTGTTAGTGAAAAAGAGAAAGTCCCTACCAGTGCATATATT
>HPV63_Mu_9626605_nt6157_L1_Tile|1
TACACATGCTGGAATTGTTAGTGAAAAAGAGAAAGTCCCTACCAGTGCATATATTGCTGC
>HPV63_Mu_9626605_nt6167_L1_Tile|1
GGAATTGTTAGTGAAAAAGAGAAAGTCCCTACCAGTGCATATATTGCTGCTAAAGCCGAG
>HPV63_Mu_9626605_nt6172_L1_Tile|1
TGTTAGTGAAAAAGAGAAAGTCCCTACCAGTGCATATATTGCTGCTAAAGCCGAGCAACC
>HPV63_Mu_9626605_nt6182_L1_Tile|2
AAAGAGAAAGTCCCTACCAGTGCATATATTGCTGCTAAAGCCGAGCAACCCCAAAATACT
>HPV63_Mu_9626605_nt6187_L1_Tile|1
GAAAGTCCCTACCAGTGCATATATTGCTGCTAAAGCCGAGCAACCCCAAAATACTATTGC
>HPV63_Mu_9626605_nt6192_L1_Tile|1
TCCCTACCAGTGCATATATTGCTGCTAAAGCCGAGCAACCCCAAAATACTATTGCTACAG
>HPV63_Mu_9626605_nt6202_L1_Tile|2
TGCATATATTGCTGCTAAAGCCGAGCAACCCCAAAATACTATTGCTACAGATAATTATTT
>HPV63_Mu_9626605_nt6212_L1_Tile|1
GCTGCTAAAGCCGAGCAACCCCAAAATACTATTGCTACAGATAATTATTTTGTAGCTCCC
>HPV63_Mu_9626605_nt6217_L1_Tile|1
TAAAGCCGAGCAACCCCAAAATACTATTGCTACAGATAATTATTTTGTAGCTCCCAGTGG
>HPV63_Mu_9626605_nt6222_L1_Tile|2
CCGAGCAACCCCAAAATACTATTGCTACAGATAATTATTTTGTAGCTCCCAGTGGATCTT
>HPV63_Mu_9626605_nt6227_L1_Tile|1
CAACCCCAAAATACTATTGCTACAGATAATTATTTTGTAGCTCCCAGTGGATCTTTAGTG
>HPV63_Mu_9626605_nt6237_L1_Tile|1
ATACTATTGCTACAGATAATTATTTTGTAGCTCCCAGTGGATCTTTAGTGTCCTCTGATG
>HPV63_Mu_9626605_nt6242_L1_Tile|2
ATTGCTACAGATAATTATTTTGTAGCTCCCAGTGGATCTTTAGTGTCCTCTGATGTGCAA
>HPV63_Mu_9626605_nt6247_L1_Tile|1
TACAGATAATTATTTTGTAGCTCCCAGTGGATCTTTAGTGTCCTCTGATGTGCAAATTTT
>HPV63_Mu_9626605_nt6252_L1_Tile|1
ATAATTATTTTGTAGCTCCCAGTGGATCTTTAGTGTCCTCTGATGTGCAAATTTTTAATA
>HPV63_Mu_9626605_nt6257_L1_Tile|1
TATTTTGTAGCTCCCAGTGGATCTTTAGTGTCCTCTGATGTGCAAATTTTTAATAGGCCC
>HPV63_Mu_9626605_nt6282_L1_Tile|2
TAGTGTCCTCTGATGTGCAAATTTTTAATAGGCCCTATTGGTTACAACGTTCTCAAGGAC
>HPV63_Mu_9626605_nt6342_L1_Tile|2
AGAACAATGGTATCTGTTGGAGAAATGAGTTATTTGTAACTGTAGCTGATAATACCAGAG
>HPV63_Mu_9626605_nt6352_L1_Tile|1
TATCTGTTGGAGAAATGAGTTATTTGTAACTGTAGCTGATAATACCAGAGGAACCACGAT
>HPV63_Mu_9626605_nt6362_L1_Tile|2
AGAAATGAGTTATTTGTAACTGTAGCTGATAATACCAGAGGAACCACGATGAATATAAAT
>HPV63_Mu_9626605_nt6367_L1_Tile|1
TGAGTTATTTGTAACTGTAGCTGATAATACCAGAGGAACCACGATGAATATAAATGTTCT
>HPV63_Mu_9626605_nt6372_L1_Tile|1
TATTTGTAACTGTAGCTGATAATACCAGAGGAACCACGATGAATATAAATGTTCTTAACA
>HPV63_Mu_9626605_nt6377_L1_Tile|1
GTAACTGTAGCTGATAATACCAGAGGAACCACGATGAATATAAATGTTCTTAACAAAGCA
>HPV63_Mu_9626605_nt6387_L1_Tile|1
CTGATAATACCAGAGGAACCACGATGAATATAAATGTTCTTAACAAAGCAACCCCTGAGA
>HPV63_Mu_9626605_nt6392_L1_Tile|1
AATACCAGAGGAACCACGATGAATATAAATGTTCTTAACAAAGCAACCCCTGAGACTTAT
>HPV63_Mu_9626605_nt6402_L1_Tile|2
GAACCACGATGAATATAAATGTTCTTAACAAAGCAACCCCTGAGACTTATGATAGCGCAG
>HPV63_Mu_9626605_nt6407_L1_Tile|1
ACGATGAATATAAATGTTCTTAACAAAGCAACCCCTGAGACTTATGATAGCGCAGATTAT

>HPV63_Mu_9626605_nt6412_L1_Tile|1
GAATATAAATGTTCTTAACAAAGCAACCCCTGAGACTTATGATAGCGCAGATTATAATGA
>HPV63_Mu_9626605_nt6422_L1_Tile|2
GTTCTTAACAAAGCAACCCCTGAGACTTATGATAGCGCAGATTATAATGAGTATACTCGC
>HPV63_Mu_9626605_nt6427_L1_Tile|1
TAACAAAGCAACCCCTGAGACTTATGATAGCGCAGATTATAATGAGTATACTCGCCATGT
>HPV63_Mu_9626605_nt6437_L1_Tile|1
ACCCCTGAGACTTATGATAGCGCAGATTATAATGAGTATACTCGCCATGTGGAGGAATAT
>HPV63_Mu_9626605_nt6447_L1_Tile|1
CTTATGATAGCGCAGATTATAATGAGTATACTCGCCATGTGGAGGAATATGAGTTATCCT
>HPV63_Mu_9626605_nt6452_L1_Tile|1
GATAGCGCAGATTATAATGAGTATACTCGCCATGTGGAGGAATATGAGTTATCCTTTATA
>HPV63_Mu_9626605_nt6457_L1_Tile|1
CGCAGATTATAATGAGTATACTCGCCATGTGGAGGAATATGAGTTATCCTTTATAGTTCA
>HPV63_Mu_9626605_nt6462_L1_Tile|2
ATTATAATGAGTATACTCGCCATGTGGAGGAATATGAGTTATCCTTTATAGTTCAGCTTT
>HPV63_Mu_9626605_nt6492_L1_Tile|1
AATATGAGTTATCCTTTATAGTTCAGCTTTGTAAGGTAAAACTAACACCTGAAAATTTAG
>HPV63_Mu_9626605_nt6497_L1_Tile|1
GAGTTATCCTTTATAGTTCAGCTTTGTAAGGTAAAACTAACACCTGAAAATTTAGCATTT
>HPV63_Mu_9626605_nt6502_L1_Tile|2
ATCCTTTATAGTTCAGCTTTGTAAGGTAAAACTAACACCTGAAAATTTAGCATTTTTGCA
>HPV63_Mu_9626605_nt6537_L1_Tile|1
CACCTGAAAATTTAGCATTTTTGCATAATATGGATCCAACAATTATCGATTCCTGGCAGT
>HPV63_Mu_9626605_nt6542_L1_Tile|2
GAAAATTTAGCATTTTTGCATAATATGGATCCAACAATTATCGATTCCTGGCAGTTAACA
>HPV63_Mu_9626605_nt6547_L1_Tile|1
TTTAGCATTTTTGCATAATATGGATCCAACAATTATCGATTCCTGGCAGTTAACAGTTTC
>HPV63_Mu_9626605_nt6552_L1_Tile|1
CATTTTTGCATAATATGGATCCAACAATTATCGATTCCTGGCAGTTAACAGTTTCTCAAC
>HPV63_Mu_9626605_nt6562_L1_Tile|2
TAATATGGATCCAACAATTATCGATTCCTGGCAGTTAACAGTTTCTCAACCTCCTGCAAA
>HPV63_Mu_9626605_nt6572_L1_Tile|1
CCAACAATTATCGATTCCTGGCAGTTAACAGTTTCTCAACCTCCTGCAAATGCTATAGAG
>HPV63_Mu_9626605_nt6582_L1_Tile|2
TCGATTCCTGGCAGTTAACAGTTTCTCAACCTCCTGCAAATGCTATAGAGGACAAGTATA
>HPV63_Mu_9626605_nt6587_L1_Tile|1
TCCTGGCAGTTAACAGTTTCTCAACCTCCTGCAAATGCTATAGAGGACAAGTATAGATTT
>HPV63_Mu_9626605_nt6592_L1_Tile|1
GCAGTTAACAGTTTCTCAACCTCCTGCAAATGCTATAGAGGACAAGTATAGATTTATTGA
>HPV63_Mu_9626605_nt6597_L1_Tile|1
TAACAGTTTCTCAACCTCCTGCAAATGCTATAGAGGACAAGTATAGATTTATTGAATCAT
>HPV63_Mu_9626605_nt6607_L1_Tile|1
TCAACCTCCTGCAAATGCTATAGAGGACAAGTATAGATTTATTGAATCATTAGCAACAAA
>HPV63_Mu_9626605_nt6612_L1_Tile|1
CTCCTGCAAATGCTATAGAGGACAAGTATAGATTTATTGAATCATTAGCAACAAAATGTC
>HPV63_Mu_9626605_nt6617_L1_Tile|1
GCAAATGCTATAGAGGACAAGTATAGATTTATTGAATCATTAGCAACAAAATGTCCTGAT
>HPV63_Mu_9626605_nt6622_L1_Tile|2
TGCTATAGAGGACAAGTATAGATTTATTGAATCATTAGCAACAAAATGTCCTGATAACGT
>HPV63_Mu_9626605_nt6632_L1_Tile|1
GACAAGTATAGATTTATTGAATCATTAGCAACAAAATGTCCTGATAACGTGCCCCCACCC
>HPV63_Mu_9626605_nt6637_L1_Tile|1
GTATAGATTTATTGAATCATTAGCAACAAAATGTCCTGATAACGTGCCCCCACCCACTCC
>HPV63_Mu_9626605_nt6657_L1_Tile|1

TAGCAACAAAATGTCCTGATAACGTGCCCCCACCCACTCCTACTGATCCTTACAAAGATT
>HPV63_Mu_9626605_nt6662_L1_Tile|2
ACAAAATGTCCTGATAACGTGCCCCCACCCACTCCTACTGATCCTTACAAAGATTTACGT
>HPV63_Mu_9626605_nt6672_L1_Tile|1
CTGATAACGTGCCCCCACCCACTCCTACTGATCCTTACAAAGATTTACGTTTTTGGGATG
>HPV63_Mu_9626605_nt6677_L1_Tile|1
AACGTGCCCCCACCCACTCCTACTGATCCTTACAAAGATTTACGTTTTTGGGATGTAGAC
>HPV63_Mu_9626605_nt6682_L1_Tile|2
GCCCCCACCCACTCCTACTGATCCTTACAAAGATTTACGTTTTTGGGATGTAGACCTCAG
>HPV63_Mu_9626605_nt6687_L1_Tile|1
CACCCACTCCTACTGATCCTTACAAAGATTTACGTTTTTGGGATGTAGACCTCAGTGAGC
>HPV63_Mu_9626605_nt6692_L1_Tile|1
ACTCCTACTGATCCTTACAAAGATTTACGTTTTTGGGATGTAGACCTCAGTGAGCGAATG
>HPV63_Mu_9626605_nt6697_L1_Tile|1
TACTGATCCTTACAAAGATTTACGTTTTTGGGATGTAGACCTCAGTGAGCGAATGTCGGA
>HPV63_Mu_9626605_nt6702_L1_Tile|2
ATCCTTACAAAGATTTACGTTTTTGGGATGTAGACCTCAGTGAGCGAATGTCGGAGCAGC
>HPV63_Mu_9626605_nt6707_L1_Tile|1
TACAAAGATTTACGTTTTTGGGATGTAGACCTCAGTGAGCGAATGTCGGAGCAGCTTGAT
>HPV63_Mu_9626605_nt6712_L1_Tile|1
AGATTTACGTTTTTGGGATGTAGACCTCAGTGAGCGAATGTCGGAGCAGCTTGATCAATT
>HPV63_Mu_9626605_nt6717_L1_Tile|1
TACGTTTTTGGGATGTAGACCTCAGTGAGCGAATGTCGGAGCAGCTTGATCAATTTCCTT
>HPV63_Mu_9626605_nt6722_L1_Tile|2
TTTTGGGATGTAGACCTCAGTGAGCGAATGTCGGAGCAGCTTGATCAATTTCCTTTAGGC
>HPV63_Mu_9626605_nt6732_L1_Tile|1
TAGACCTCAGTGAGCGAATGTCGGAGCAGCTTGATCAATTTCCTTTAGGCCGCAAATTTT
>HPV63_Mu_9626605_nt6737_L1_Tile|1
CTCAGTGAGCGAATGTCGGAGCAGCTTGATCAATTTCCTTTAGGCCGCAAATTTTTGTAT
>HPV63_Mu_9626605_nt6742_L1_Tile|2
TGAGCGAATGTCGGAGCAGCTTGATCAATTTCCTTTAGGCCGCAAATTTTTGTATCAAAG
>HPV63_Mu_9626605_nt6782_L1_Tile|2
CGCAAATTTTTGTATCAAAGTGGTCTTGCACAGCGTTCTGTTCCAAAAACTGTGAATTTC
>HPV63_Mu_9626605_nt6787_L1_Tile|1
ATTTTTGTATCAAAGTGGTCTTGCACAGCGTTCTGTTCCAAAAACTGTGAATTTCAGAAA
>HPV63_Mu_9626605_nt6792_L1_Tile|1
TGTATCAAAGTGGTCTTGCACAGCGTTCTGTTCCAAAAACTGTGAATTTCAGAAAACGTA
>HPV63_Mu_9626605_nt6797_L1_Tile|1
CAAAGTGGTCTTGCACAGCGTTCTGTTCCAAAAACTGTGAATTTCAGAAAACGTAGATCC
>HPV63_Mu_9626605_nt6802_L1_Tile|2
TGGTCTTGCACAGCGTTCTGTTCCAAAAACTGTGAATTTCAGAAAACGTAGATCCTCCAA
>HPV63_Mu_9626605_nt6807_L1_Tile|1
TTGCACAGCGTTCTGTTCCAAAAACTGTGAATTTCAGAAAACGTAGATCCTCCAATACTA
>HPV63_Mu_9626605_nt6832_L1_Tile|1
TGTGAATTTCAGAAAACGTAGATCCTCCAATACTACTGTGGCCAAACGGAGGCGACGGGC
>HPV63_Mu_9626605_nt6836_L1_Tile|1
AATTTCAGAAAACGTAGATCCTCCAATACTACTGTGGCCAAACGGAGGCGACGGGCCTGA
>HPV65_Gamma_312100_nt5552_L1_Tile|1
AGATTCTATTTACCAGATCCTAATAGGTTTGCATTGATTGATAATGGTTTCTATGATTCT
>HPV65_Gamma_312100_nt5567_L1_Tile|1
GATCCTAATAGGTTTGCATTGATTGATAATGGTTTCTATGATTCTGACCATGAACGTCTT
>HPV65_Gamma_312100_nt5582_L1_Tile|2
GCATTGATTGATAATGGTTTCTATGATTCTGACCATGAACGTCTTGTTTGGAGACTCAGA
>HPV65_Gamma_312100_nt5587_L1_Tile|1
GATTGATAATGGTTTCTATGATTCTGACCATGAACGTCTTGTTTGGAGACTCAGAGGAAT

>HPV65_Gamma_312100_nt5592_L1_Tile|1
ATAATGGTTTCTATGATTCTGACCATGAACGTCTTGTTTGGAGACTCAGAGGAATAGAAA
>HPV65_Gamma_312100_nt5597_L1_Tile|1
GGTTTCTATGATTCTGACCATGAACGTCTTGTTTGGAGACTCAGAGGAATAGAAATAGGT
>HPV65_Gamma_312100_nt5612_L1_Tile|1
GACCATGAACGTCTTGTTTGGAGACTCAGAGGAATAGAAATAGGTAGGGGTGGTCCCCTT
>HPV65_Gamma_312100_nt5617_L1_Tile|1
TGAACGTCTTGTTTGGAGACTCAGAGGAATAGAAATAGGTAGGGGTGGTCCCCTTGGGAT
>HPV65_Gamma_312100_nt5622_L1_Tile|2
GTCTTGTTTGGAGACTCAGAGGAATAGAAATAGGTAGGGGTGGTCCCCTTGGGATTGGAA
>HPV65_Gamma_312100_nt5637_L1_Tile|1
TCAGAGGAATAGAAATAGGTAGGGGTGGTCCCCTTGGGATTGGAACAACAGGCCATCCTT
>HPV65_Gamma_312100_nt5642_L1_Tile|2
GGAATAGAAATAGGTAGGGGTGGTCCCCTTGGGATTGGAACAACAGGCCATCCTTTATAT
>HPV65_Gamma_312100_nt5652_L1_Tile|1
TAGGTAGGGGTGGTCCCCTTGGGATTGGAACAACAGGCCATCCTTTATATAATAAGTTTG
>HPV65_Gamma_312100_nt5832_L1_Tile|1
ATTGGGATAAGGCTGAGCCTTGTGCAAGTCCTGTTCCTCAACCAGGAGATTGTCCTCCTA
>HPV65_Gamma_312100_nt6037_L1_Tile|1
AATGAGTAAAGATGTCTATGGAGATAGCTTATTCTTTTATGGTAGGCGAGAACAGTTATA
>HPV65_Gamma_312100_nt6052_L1_Tile|1
CTATGGAGATAGCTTATTCTTTTATGGTAGGCGAGAACAGTTATATGCCAGGCACTTTTT
>HPV65_Gamma_312100_nt6117_L1_Tile|1
GAGCAGGAGCAATGGGAGATGCACTACCAGAACCTTTCGAGGTTAAAACTGATTACTGGA
>HPV65_Gamma_312100_nt6122_L1_Tile|2
GGAGCAATGGGAGATGCACTACCAGAACCTTTCGAGGTTAAAACTGATTACTGGATTCCT
>HPV65_Gamma_312100_nt6127_L1_Tile|1
AATGGGAGATGCACTACCAGAACCTTTCGAGGTTAAAACTGATTACTGGATTCCTGCTCA
>HPV65_Gamma_312100_nt6132_L1_Tile|1
GAGATGCACTACCAGAACCTTTCGAGGTTAAAACTGATTACTGGATTCCTGCTCAAGAAG
>HPV65_Gamma_312100_nt6137_L1_Tile|1
GCACTACCAGAACCTTTCGAGGTTAAAACTGATTACTGGATTCCTGCTCAAGAAGGTCAG
>HPV65_Gamma_312100_nt6142_L1_Tile|2
ACCAGAACCTTTCGAGGTTAAAACTGATTACTGGATTCCTGCTCAAGAAGGTCAGGATCA
>HPV65_Gamma_312100_nt6147_L1_Tile|1
AACCTTTCGAGGTTAAAACTGATTACTGGATTCCTGCTCAAGAAGGTCAGGATCAGAATA
>HPV65_Gamma_312100_nt6157_L1_Tile|1
GGTTAAAACTGATTACTGGATTCCTGCTCAAGAAGGTCAGGATCAGAATACTTTAGGTCC
>HPV65_Gamma_312100_nt6162_L1_Tile|2
AAACTGATTACTGGATTCCTGCTCAAGAAGGTCAGGATCAGAATACTTTAGGTCCACATA
>HPV65_Gamma_312100_nt6167_L1_Tile|1
GATTACTGGATTCCTGCTCAAGAAGGTCAGGATCAGAATACTTTAGGTCCACATATATAT
>HPV65_Gamma_312100_nt6182_L1_Tile|2
GCTCAAGAAGGTCAGGATCAGAATACTTTAGGTCCACATATATATATAGGCACTCCTAGT
>HPV65_Gamma_312100_nt6187_L1_Tile|1
AGAAGGTCAGGATCAGAATACTTTAGGTCCACATATATATATAGGCACTCCTAGTGGATC
>HPV65_Gamma_312100_nt6197_L1_Tile|1
GATCAGAATACTTTAGGTCCACATATATATATAGGCACTCCTAGTGGATCTTTAGTTTCA
>HPV65_Gamma_312100_nt6202_L1_Tile|2
GAATACTTTAGGTCCACATATATATATAGGCACTCCTAGTGGATCTTTAGTTTCAAGTGA
>HPV65_Gamma_312100_nt6207_L1_Tile|1
CTTTAGGTCCACATATATATATAGGCACTCCTAGTGGATCTTTAGTTTCAAGTGAGTCCC
>HPV65_Gamma_312100_nt6337_L1_Tile|1
ATTATTTGTGACACTTGTTGACAATACTCATAATACTAATTTTACTATTTCTGTAAAAAC
>HPV65_Gamma_312100_nt6392_L1_Tile|1

AAAACAGAAGCAGCTGATGAATCCTATAAATATAAAGCAGGTGATTTTAAACAGTATTTA
>HPV65_Gamma_312100_nt6612_L1_Tile|1
ATAGATTTATACAATCCAGAGCTACGAGATGTCCTACTCAGTCTCCCAGTACTGAAAAGG
>HPV65_Gamma_312100_nt6617_L1_Tile|1
TTTATACAATCCAGAGCTACGAGATGTCCTACTCAGTCTCCCAGTACTGAAAAGGAAGAC
>HPV65_Gamma_312100_nt6632_L1_Tile|1
GCTACGAGATGTCCTACTCAGTCTCCCAGTACTGAAAAGGAAGACCCTTATAAAGATTTA
>HPV65_Gamma_312100_nt6752_L1_Tile|1
GGGAGACGCTTTTTATATCAAAGTGGTTTAATTAATGGAACACTTAAAAGAAAACGTACT
>HPV65_Gamma_312100_nt6757_L1_Tile|1
ACGCTTTTTATATCAAAGTGGTTTAATTAATGGAACACTTAAAAGAAAACGTACTATTAA
>HPV65_Gamma_312100_nt6762_L1_Tile|2
TTTTATATCAAAGTGGTTTAATTAATGGAACACTTAAAAGAAAACGTACTATTAATTCTC
>HPV65_Gamma_312100_nt6767_L1_Tile|1
TATCAAAGTGGTTTAATTAATGGAACACTTAAAAGAAAACGTACTATTAATTCTCAAGCT
>HPV65_Gamma_312100_nt6772_L1_Tile|1
AAGTGGTTTAATTAATGGAACACTTAAAAGAAAACGTACTATTAATTCTCAAGCTCCAAC
>HPV65_Gamma_312100_nt6777_L1_Tile|1
GTTTAATTAATGGAACACTTAAAAGAAAACGTACTATTAATTCTCAAGCTCCAACTAGTA
>HPV65_Gamma_312100_nt6782_L1_Tile|2
ATTAATGGAACACTTAAAAGAAAACGTACTATTAATTCTCAAGCTCCAACTAGTATTAAA
>HPV65_Gamma_312100_nt6787_L1_Tile|1
TGGAACACTTAAAAGAAAACGTACTATTAATTCTCAAGCTCCAACTAGTATTAAACGTTC
>HPV66_Alpha_1020290_nt5763_L1_Tile|1
GCTCTAGGTTGCTTGCTGTTGGCCATCCTTATTACTCTGTTTCCAAATCTGGTACCAAAA
>HPV66_Alpha_1020290_nt5778_L1_Tile|1
CTGTTGGCCATCCTTATTACTCTGTTTCCAAATCTGGTACCAAAACAAACATCCCTAAAG
>HPV66_Alpha_1020290_nt6148_L1_Tile|1
TTGGACTAAGGGCGCGGTGTGTAAGTCTACACCAGGTAATACAGGGGATTGTCCACCTCT
>HPV66_Alpha_1020290_nt6153_L1_Tile|1
CTAAGGGCGCGGTGTGTAAGTCTACACCAGGTAATACAGGGGATTGTCCACCTCTTGCAT
>HPV66_Alpha_1020290_nt6168_L1_Tile|1
GTAAGTCTACACCAGGTAATACAGGGGATTGTCCACCTCTTGCATTAGTTAATACCCCGA
>HPV66_Alpha_1020290_nt6183_L1_Tile|1
GTAATACAGGGGATTGTCCACCTCTTGCATTAGTTAATACCCCGATAGAGGACGGTGACA
>HPV66_Alpha_1020290_nt6418_L1_Tile|1
ACATTACTTTAATAGGGCAGGTAATGTTGGGGAAGCCATTCCTACAGATTTGTATTGGAA
>HPV66_Alpha_1020290_nt6428_L1_Tile|1
AATAGGGCAGGTAATGTTGGGGAAGCCATTCCTACAGATTTGTATTGGAAGGGTGGCAAT
>HPV66_Alpha_1020290_nt6438_L1_Tile|1
GTAATGTTGGGGAAGCCATTCCTACAGATTTGTATTGGAAGGGTGGCAATGGCAGGGACC
>HPV66_Alpha_1020290_nt6443_L1_Tile|1
GTTGGGGAAGCCATTCCTACAGATTTGTATTGGAAGGGTGGCAATGGCAGGGACCCTCCT
>HPV66_Alpha_1020290_nt6448_L1_Tile|1
GGAAGCCATTCCTACAGATTTGTATTGGAAGGGTGGCAATGGCAGGGACCCTCCTCCCAG
>HPV66_Alpha_1020290_nt6453_L1_Tile|1
CCATTCCTACAGATTTGTATTGGAAGGGTGGCAATGGCAGGGACCCTCCTCCCAGTTCTG
>HPV66_Alpha_1020290_nt6458_L1_Tile|1
CCTACAGATTTGTATTGGAAGGGTGGCAATGGCAGGGACCCTCCTCCCAGTTCTGTATAT
>HPV66_Alpha_1020290_nt6718_L1_Tile|1
AATCAATCAATACCTTCGCCATGTGGAGGAATATGAACTACAGTTTGTGTTTCAACTTTG
>HPV66_Alpha_1020290_nt6793_L1_Tile|1
TGCAGAAGTTATGGCATATTTGCATAATATGAATAATACTTTATTAGACGATTGGAATAT
>HPV66_Alpha_1020290_nt6798_L1_Tile|1
AAGTTATGGCATATTTGCATAATATGAATAATACTTTATTAGACGATTGGAATATTGGCT

>HPV66_Alpha_1020290_nt6808_L1_Tile|1
ATATTTGCATAATATGAATAATACTTTATTAGACGATTGGAATATTGGCTTATCCCCACC
>HPV66_Alpha_1020290_nt6813_L1_Tile|1
TGCATAATATGAATAATACTTTATTAGACGATTGGAATATTGGCTTATCCCCACCAGTTG
>HPV66_Alpha_1020290_nt6823_L1_Tile|1
GAATAATACTTTATTAGACGATTGGAATATTGGCTTATCCCCACCAGTTGCAACTAGCTT
>HPV66_Alpha_1020290_nt6828_L1_Tile|1
ATACTTTATTAGACGATTGGAATATTGGCTTATCCCCACCAGTTGCAACTAGCTTAGAGG
>HPV66_Alpha_1020290_nt6833_L1_Tile|1
TTATTAGACGATTGGAATATTGGCTTATCCCCACCAGTTGCAACTAGCTTAGAGGATAAA
>HPV66_Alpha_1020290_nt6838_L1_Tile|1
AGACGATTGGAATATTGGCTTATCCCCACCAGTTGCAACTAGCTTAGAGGATAAATATAG
>HPV66_Alpha_1020290_nt6853_L1_Tile|1
TGGCTTATCCCCACCAGTTGCAACTAGCTTAGAGGATAAATATAGGTATATTAAAAGCAC
>HPV66_Alpha_1020290_nt6863_L1_Tile|1
CCACCAGTTGCAACTAGCTTAGAGGATAAATATAGGTATATTAAAAGCACAGCTATTACA
>HPV66_Alpha_1020290_nt7038_L1_Tile|1
GAAAATTTTTAATGCAACTAGGCCCTAGACCCCCTAGACCCAAGGCTAGTGTATCTGCCT
>HPV66_Alpha_1020290_nt7043_L1_Tile|1
TTTTTAATGCAACTAGGCCCTAGACCCCCTAGACCCAAGGCTAGTGTATCTGCCTCTAAA
>HPV66_Alpha_1020290_nt7063_L1_Tile|1
TAGACCCCCTAGACCCAAGGCTAGTGTATCTGCCTCTAAAAGGCGGGCGGCTCCTACCTC
>HPV66_Alpha_1020290_nt7078_L1_Tile|1
CAAGGCTAGTGTATCTGCCTCTAAAAGGCGGGCGGCTCCTACCTCTTCCTCTTCTTCACC
>HPV66_Alpha_1020290_nt7083_L1_Tile|1
CTAGTGTATCTGCCTCTAAAAGGCGGGCGGCTCCTACCTCTTCCTCTTCTTCACCAGCTA
>HPV67_Alpha_3228267_nt5725_L1_Tile|1
GCTGGTAGCTCCAGACTTTTAGCTGTAGGCCATCCTTACTTTTCCATTCCTAATCCCTCC
>HPV67_Alpha_3228267_nt5735_L1_Tile|1
CCAGACTTTTAGCTGTAGGCCATCCTTACTTTTCCATTCCTAATCCCTCCAACACTAAAA
>HPV67_Alpha_3228267_nt5740_L1_Tile|1
CTTTTAGCTGTAGGCCATCCTTACTTTTCCATTCCTAATCCCTCCAACACTAAAAAGGTG
>HPV67_Alpha_3228267_nt5750_L1_Tile|1
TAGGCCATCCTTACTTTTCCATTCCTAATCCCTCCAACACTAAAAAGGTGTTAGTGCCCA
>HPV67_Alpha_3228267_nt5760_L1_Tile|1
TTACTTTTCCATTCCTAATCCCTCCAACACTAAAAAGGTGTTAGTGCCCAAGGTGTCAGG
>HPV67_Alpha_3228267_nt5765_L1_Tile|1
TTTCCATTCCTAATCCCTCCAACACTAAAAAGGTGTTAGTGCCCAAGGTGTCAGGTTTGC
>HPV67_Alpha_3228267_nt5970_L1_Tile|1
TAGTGGACATCCATTACTTAATAAGTTTGATGATACTGAAACAAATAATAAATACCCTAG
>HPV67_Alpha_3228267_nt5975_L1_Tile|1
GACATCCATTACTTAATAAGTTTGATGATACTGAAACAAATAATAAATACCCTAGCCAGC
>HPV67_Alpha_3228267_nt5985_L1_Tile|1
ACTTAATAAGTTTGATGATACTGAAACAAATAATAAATACCCTAGCCAGCCTGGTACTGA
>HPV67_Alpha_3228267_nt6005_L1_Tile|1
CTGAAACAAATAATAAATACCCTAGCCAGCCTGGTACTGATAATAGGGAATGCTTGTCTA
>HPV67_Alpha_3228267_nt6020_L1_Tile|1
AATACCCTAGCCAGCCTGGTACTGATAATAGGGAATGCTTGTCTATGGATGCTAAGCAAA
>HPV67_Alpha_3228267_nt6105_L1_Tile|1
ACCTCCTACAGGTGAACACTGGGGTAAAGGTACCCCGTGTAGTGGTAACAGTAATGGGCC
>HPV67_Alpha_3228267_nt6280_L1_Tile|1
CCGTTAGATATATGTACCAGTATTTGTAAATATCCTGATTATCTTGGTATGGCTAGCGAG
>HPV67_Alpha_3228267_nt6320_L1_Tile|1
ATCTTGGTATGGCTAGCGAGGCTTATGGGGACAGTTTATTTTTCTTCCTGCGACGTGAGC
>HPV67_Alpha_3228267_nt6360_L1_Tile|1

TTTCTTCCTGCGACGTGAGCAAATGTTTGTTAGGCATTTGTTTAACAGAGCAGGTAAATT
>HPV67_Alpha_3228267_nt6385_L1_Tile|1
TTTGTTAGGCATTTGTTTAACAGAGCAGGTAAATTAGGGGAGGATGTACCTACAGATTTG
>HPV67_Alpha_3228267_nt6395_L1_Tile|1
ATTTGTTTAACAGAGCAGGTAAATTAGGGGAGGATGTACCTACAGATTTGTATTTTAAGG
>HPV67_Alpha_3228267_nt6400_L1_Tile|1
TTTAACAGAGCAGGTAAATTAGGGGAGGATGTACCTACAGATTTGTATTTTAAGGGATCT
>HPV67_Alpha_3228267_nt6405_L1_Tile|1
CAGAGCAGGTAAATTAGGGGAGGATGTACCTACAGATTTGTATTTTAAGGGATCTGCAAA
>HPV67_Alpha_3228267_nt6410_L1_Tile|1
CAGGTAAATTAGGGGAGGATGTACCTACAGATTTGTATTTTAAGGGATCTGCAAACACTT
>HPV67_Alpha_3228267_nt6415_L1_Tile|1
AAATTAGGGGAGGATGTACCTACAGATTTGTATTTTAAGGGATCTGCAAACACTTCTGCA
>HPV67_Alpha_3228267_nt6430_L1_Tile|1
GTACCTACAGATTTGTATTTTAAGGGATCTGCAAACACTTCTGCACTGCAAACCTCAGCT
>HPV67_Alpha_3228267_nt6435_L1_Tile|1
TACAGATTTGTATTTTAAGGGATCTGCAAACACTTCTGCACTGCAAACCTCAGCTTTTTT
>HPV67_Alpha_3228267_nt6440_L1_Tile|1
ATTTGTATTTTAAGGGATCTGCAAACACTTCTGCACTGCAAACCTCAGCTTTTTTTCCTA
>HPV67_Alpha_3228267_nt6450_L1_Tile|1
TAAGGGATCTGCAAACACTTCTGCACTGCAAACCTCAGCTTTTTTTCCTACTCCCAGTGG
>HPV67_Alpha_3228267_nt6620_L1_Tile|1
TAGACACTACACGTAGTACCAACATGACTTTATGTTCTGAGGAAAAATCAGAGGCTACAT
>HPV67_Alpha_3228267_nt6625_L1_Tile|1
ACTACACGTAGTACCAACATGACTTTATGTTCTGAGGAAAAATCAGAGGCTACATACAAA
>HPV67_Alpha_3228267_nt6630_L1_Tile|1
ACGTAGTACCAACATGACTTTATGTTCTGAGGAAAAATCAGAGGCTACATACAAAAATGA
>HPV67_Alpha_3228267_nt6735_L1_Tile|1
GTTTATATTTCAGCTGTGCAAAATATCCCTTACTGCAAATGTTATGCAATACATACACAC
>HPV67_Alpha_3228267_nt6750_L1_Tile|1
GTGCAAAATATCCCTTACTGCAAATGTTATGCAATACATACACACCATGAATCCAGATAT
>HPV67_Alpha_3228267_nt6755_L1_Tile|1
AAATATCCCTTACTGCAAATGTTATGCAATACATACACACCATGAATCCAGATATATTAG
>HPV67_Alpha_3228267_nt6760_L1_Tile|1
TCCCTTACTGCAAATGTTATGCAATACATACACACCATGAATCCAGATATATTAGAGGAC
>HPV67_Alpha_3228267_nt6765_L1_Tile|1
TACTGCAAATGTTATGCAATACATACACACCATGAATCCAGATATATTAGAGGACTGGCA
>HPV67_Alpha_3228267_nt6770_L1_Tile|1
CAAATGTTATGCAATACATACACACCATGAATCCAGATATATTAGAGGACTGGCAATTTG
>HPV67_Alpha_3228267_nt6890_L1_Tile|1
CTATTACCTGTCAAAAAACATCCCCTCCAACAGCAAAGGAAGATCCTCTTAAAAAGTACA
>HPV67_Alpha_3228267_nt6895_L1_Tile|1
ACCTGTCAAAAAACATCCCCTCCAACAGCAAAGGAAGATCCTCTTAAAAAGTACAGTTTT
>HPV67_Alpha_3228267_nt7015_L1_Tile|1
AAATTTTTATTGCAGGCAGGTTTTACAGCTAAACCTAAACTAAAACGTTCTTCACCTTCT
>HPV67_Alpha_3228267_nt7020_L1_Tile|1
TTTATTGCAGGCAGGTTTTACAGCTAAACCTAAACTAAAACGTTCTTCACCTTCTTCTTC
>HPV68a_Alpha_71726685_nt5599_L1_Tile|1
TGGTACATCTAGGTTATTAACTGTAGGCCATCCATATTTTAAGGTTCCTATGTCTGGGGG
>HPV68a_Alpha_71726685_nt5979_L1_Tile|1
CTATTGGCGAGCACTGGGCCAAAGGTAAATCTTGTAAGCCTACCAATGTACAACAAGGGG
>HPV68a_Alpha_71726685_nt5999_L1_Tile|1
AAAGGTAAATCTTGTAAGCCTACCAATGTACAACAAGGGGACTGTCCCCCATTGGAATTG
>HPV68a_Alpha_71726685_nt6004_L1_Tile|1
TAAATCTTGTAAGCCTACCAATGTACAACAAGGGGACTGTCCCCCATTGGAATTGGTAAA

>HPV68a_Alpha_71726685_nt6009_L1_Tile|1
CTTGTAAGCCTACCAATGTACAACAAGGGGACTGTCCCCCATTGGAATTGGTAAATACTC
>HPV68a_Alpha_71726685_nt6114_L1_Tile|1
ACTTTGGTACATTACAAGAAACGAAAAGCGAGGTACCTTTGGATATATGTCAATCTGTTT
>HPV68a_Alpha_71726685_nt6254_L1_Tile|1
TTTGCCAGGCATTTTTGGAATAGGGGAGGCATGGTAGGGGACACTATTCCCACTGACATG
>HPV68a_Alpha_71726685_nt6259_L1_Tile|1
CAGGCATTTTTGGAATAGGGGAGGCATGGTAGGGGACACTATTCCCACTGACATGTATAT
>HPV68a_Alpha_71726685_nt6264_L1_Tile|1
ATTTTTGGAATAGGGGAGGCATGGTAGGGGACACTATTCCCACTGACATGTATATTAAGG
>HPV68a_Alpha_71726685_nt6279_L1_Tile|1
GAGGCATGGTAGGGGACACTATTCCCACTGACATGTATATTAAGGGCACTGACATTCGTG
>HPV68a_Alpha_71726685_nt6289_L1_Tile|1
AGGGGACACTATTCCCACTGACATGTATATTAAGGGCACTGACATTCGTGAAACTCCTAG
>HPV68a_Alpha_71726685_nt6294_L1_Tile|1
ACACTATTCCCACTGACATGTATATTAAGGGCACTGACATTCGTGAAACTCCTAGTAGTT
>HPV68a_Alpha_71726685_nt6299_L1_Tile|1
ATTCCCACTGACATGTATATTAAGGGCACTGACATTCGTGAAACTCCTAGTAGTTATGTG
>HPV68a_Alpha_71726685_nt6304_L1_Tile|1
CACTGACATGTATATTAAGGGCACTGACATTCGTGAAACTCCTAGTAGTTATGTGTATGC
>HPV68a_Alpha_71726685_nt6469_L1_Tile|1
TCAATTATTTCTTACCGTTGTGGATACAACGCGCAGTACTAATTTTACATTGTCCACTAC
>HPV68a_Alpha_71726685_nt6489_L1_Tile|1
TGGATACAACGCGCAGTACTAATTTTACATTGTCCACTACTACAGACTCTACTGTACCAG
>HPV68a_Alpha_71726685_nt6504_L1_Tile|1
GTACTAATTTTACATTGTCCACTACTACAGACTCTACTGTACCAGCTGTGTATGATTCTA
>HPV68a_Alpha_71726685_nt6509_L1_Tile|1
AATTTTACATTGTCCACTACTACAGACTCTACTGTACCAGCTGTGTATGATTCTAATAAA
>HPV68a_Alpha_71726685_nt6514_L1_Tile|1
TACATTGTCCACTACTACAGACTCTACTGTACCAGCTGTGTATGATTCTAATAAATTTAA
>HPV68a_Alpha_71726685_nt6624_L1_Tile|1
TGTGTACTATAACATTATCCACTGATGTAATGTCATATATACATACTATGAATCCTGCTA
>HPV68a_Alpha_71726685_nt6729_L1_Tile|1
GTCTTGTAGATACATACCGCTACCTACAATCAGCAGCAATTACATGTCAAAAGGACGCCC
>HPV68a_Alpha_71726685_nt6819_L1_Tile|1
GTCTTAACTTTTGGAATGTGGATTTAAAGGAAAAGTTTAGTTCTGAACTGGACCAATTCC
>HPV68a_Alpha_71726685_nt6824_L1_Tile|1
AACTTTTGGAATGTGGATTTAAAGGAAAAGTTTAGTTCTGAACTGGACCAATTCCCATTA
>HPV68a_Alpha_71726685_nt6839_L1_Tile|1
GATTTAAAGGAAAAGTTTAGTTCTGAACTGGACCAATTCCCATTAGGACGCAAATTTCTG
>HPV68a_Alpha_71726685_nt6844_L1_Tile|1
AAAGGAAAAGTTTAGTTCTGAACTGGACCAATTCCCATTAGGACGCAAATTTCTGTTACA
>HPV68a_Alpha_71726685_nt6849_L1_Tile|1
AAAAGTTTAGTTCTGAACTGGACCAATTCCCATTAGGACGCAAATTTCTGTTACAGGCAG
>HPV68a_Alpha_71726685_nt6859_L1_Tile|1
TTCTGAACTGGACCAATTCCCATTAGGACGCAAATTTCTGTTACAGGCAGGTGTTCGCAG
>HPV68a_Alpha_71726685_nt6864_L1_Tile|1
AACTGGACCAATTCCCATTAGGACGCAAATTTCTGTTACAGGCAGGTGTTCGCAGACGGC
>HPV68a_Alpha_71726685_nt6869_L1_Tile|1
GACCAATTCCCATTAGGACGCAAATTTCTGTTACAGGCAGGTGTTCGCAGACGGCCCACC
>HPV68a_Alpha_71726685_nt6874_L1_Tile|1
ATTCCCATTAGGACGCAAATTTCTGTTACAGGCAGGTGTTCGCAGACGGCCCACCATAGG
>HPV68a_Alpha_71726685_nt6924_L1_Tile|1
CCACCATAGGCCCTCGTAAACGCACTGCCACTGCAGCTACCACATCTACCTCTAAACACA
>HPV68a_Alpha_71726685_nt6929_L1_Tile|1

ATAGGCCCTCGTAAACGCACTGCCACTGCAGCTACCACATCTACCTCTAAACACAAACGT
>HPV69_Alpha_6970418_nt5592_L1_Tile|1
TGTGTCCCGGGTTGTCAGACGGATGAATATGTACACGCACCGGATATATTACTATGCAGG
>HPV69_Alpha_6970418_nt5602_L1_Tile|2
GTTGTCAGACGGATGAATATGTACACGCACCGGATATATTACTATGCAGGCAGCTCTCGA
>HPV69_Alpha_6970418_nt5607_L1_Tile|1
CAGACGGATGAATATGTACACGCACCGGATATATTACTATGCAGGCAGCTCTCGATTATT
>HPV69_Alpha_6970418_nt5612_L1_Tile|1
GGATGAATATGTACACGCACCGGATATATTACTATGCAGGCAGCTCTCGATTATTAACTT
>HPV69_Alpha_6970418_nt5617_L1_Tile|1
AATATGTACACGCACCGGATATATTACTATGCAGGCAGCTCTCGATTATTAACTTTGGGT
>HPV69_Alpha_6970418_nt5652_L1_Tile|1
CAGCTCTCGATTATTAACTTTGGGTCATCCCTATTTTCCAATTCCTAAATCTGGTTCAAC
>HPV69_Alpha_6970418_nt5657_L1_Tile|1
CTCGATTATTAACTTTGGGTCATCCCTATTTTCCAATTCCTAAATCTGGTTCAACAGCAG
>HPV69_Alpha_6970418_nt5662_L1_Tile|2
TTATTAACTTTGGGTCATCCCTATTTTCCAATTCCTAAATCTGGTTCAACAGCAGAAATT
>HPV69_Alpha_6970418_nt5682_L1_Tile|2
CTATTTTCCAATTCCTAAATCTGGTTCAACAGCAGAAATTCCTAAAGTGTCTGCTTACCA
>HPV69_Alpha_6970418_nt5687_L1_Tile|1
TTCCAATTCCTAAATCTGGTTCAACAGCAGAAATTCCTAAAGTGTCTGCTTACCAATATA
>HPV69_Alpha_6970418_nt5917_L1_Tile|1
GACACTGAAAATTCACATCTTGCCACTGCTAATGCAGACACTGATAATAGGGACAACGTG
>HPV69_Alpha_6970418_nt5927_L1_Tile|1
ATTCACATCTTGCCACTGCTAATGCAGACACTGATAATAGGGACAACGTGTGTGTAGATA
>HPV69_Alpha_6970418_nt5932_L1_Tile|1
CATCTTGCCACTGCTAATGCAGACACTGATAATAGGGACAACGTGTGTGTAGATAATAAA
>HPV69_Alpha_6970418_nt6007_L1_Tile|1
ATTATTGGATGTACCCCTCCTTTAGGTGAACACTGGGGCGTTGGCACTGTATGTAAAAAT
>HPV69_Alpha_6970418_nt6012_L1_Tile|1
TGGATGTACCCCTCCTTTAGGTGAACACTGGGGCGTTGGCACTGTATGTAAAAATGCACA
>HPV69_Alpha_6970418_nt6017_L1_Tile|1
GTACCCCTCCTTTAGGTGAACACTGGGGCGTTGGCACTGTATGTAAAAATGCACAGTCTC
>HPV69_Alpha_6970418_nt6167_L1_Tile|1
ATTTTACAGCTTTGCAAGCTACAAAATGTGATGTGCCACTTGATATTAATCAATCTATTT
>HPV69_Alpha_6970418_nt6172_L1_Tile|1
ACAGCTTTGCAAGCTACAAAATGTGATGTGCCACTTGATATTAATCAATCTATTTGCAAA
>HPV69_Alpha_6970418_nt6317_L1_Tile|1
ATTTTTTTAATAAAGCTGGCACTATAGGTGATTCTGTACCAGTATCTATGTATATTAAAG
>HPV69_Alpha_6970418_nt6322_L1_Tile|2
TTTAATAAAGCTGGCACTATAGGTGATTCTGTACCAGTATCTATGTATATTAAAGGCGCT
>HPV69_Alpha_6970418_nt6327_L1_Tile|1
TAAAGCTGGCACTATAGGTGATTCTGTACCAGTATCTATGTATATTAAAGGCGCTGGTCA
>HPV69_Alpha_6970418_nt6332_L1_Tile|1
CTGGCACTATAGGTGATTCTGTACCAGTATCTATGTATATTAAAGGCGCTGGTCAAGGTC
>HPV69_Alpha_6970418_nt6337_L1_Tile|1
ACTATAGGTGATTCTGTACCAGTATCTATGTATATTAAAGGCGCTGGTCAAGGTCGTGAA
>HPV69_Alpha_6970418_nt6342_L1_Tile|2
AGGTGATTCTGTACCAGTATCTATGTATATTAAAGGCGCTGGTCAAGGTCGTGAACCTCC
>HPV69_Alpha_6970418_nt6357_L1_Tile|1
AGTATCTATGTATATTAAAGGCGCTGGTCAAGGTCGTGAACCTCCAACTACTTCTATATA
>HPV69_Alpha_6970418_nt6362_L1_Tile|2
CTATGTATATTAAAGGCGCTGGTCAAGGTCGTGAACCTCCAACTACTTCTATATACTCAC
>HPV69_Alpha_6970418_nt6367_L1_Tile|1
TATATTAAAGGCGCTGGTCAAGGTCGTGAACCTCCAACTACTTCTATATACTCACGTACG

>HPV69_Alpha_6970418_nt6377_L1_Tile|1
GCGCTGGTCAAGGTCGTGAACCTCCAACTACTTCTATATACTCACGTACGCCTAGTGGCT
>HPV69_Alpha_6970418_nt6387_L1_Tile|1
AGGTCGTGAACCTCCAACTACTTCTATATACTCACGTACGCCTAGTGGCTCCATGGTGAC
>HPV69_Alpha_6970418_nt6402_L1_Tile|2
AACTACTTCTATATACTCACGTACGCCTAGTGGCTCCATGGTGACCTCTGATGCTCAATT
>HPV69_Alpha_6970418_nt6677_L1_Tile|1
AATTGTGTAAAATTACTCTTACCACTGATGTAATGGCCTATATCCATACAATGAATTCTA
>HPV69_Alpha_6970418_nt6682_L1_Tile|2
TGTAAAATTACTCTTACCACTGATGTAATGGCCTATATCCATACAATGAATTCTACTATT
>HPV69_Alpha_6970418_nt6717_L1_Tile|1
TATCCATACAATGAATTCTACTATTTTGGAAAATTGGAATTTTGGCCTTACCTTGCCTCC
>HPV69_Alpha_6970418_nt6927_L1_Tile|1
TCAATTTCCACTGGGCAGAAAATTTATGCTGCAGGCTGGTATACAGCGACGTCCTAAGTT
>HPV69_Alpha_6970418_nt6932_L1_Tile|1
TTCCACTGGGCAGAAAATTTATGCTGCAGGCTGGTATACAGCGACGTCCTAAGTTAGGCA
>HPV69_Alpha_6970418_nt6937_L1_Tile|1
CTGGGCAGAAAATTTATGCTGCAGGCTGGTATACAGCGACGTCCTAAGTTAGGCACCAAA
>HPV69_Alpha_6970418_nt6942_L1_Tile|2
CAGAAAATTTATGCTGCAGGCTGGTATACAGCGACGTCCTAAGTTAGGCACCAAACGTCC
>HPV69_Alpha_6970418_nt6947_L1_Tile|1
AATTTATGCTGCAGGCTGGTATACAGCGACGTCCTAAGTTAGGCACCAAACGTCCAGCTT
>HPV69_Alpha_6970418_nt6957_L1_Tile|1
GCAGGCTGGTATACAGCGACGTCCTAAGTTAGGCACCAAACGTCCAGCTTCTTCCTTATC
>HPV69_Alpha_6970418_nt6962_L1_Tile|2
CTGGTATACAGCGACGTCCTAAGTTAGGCACCAAACGTCCAGCTTCTTCCTTATCTGCAT
>HPV69_Alpha_6970418_nt6967_L1_Tile|1
ATACAGCGACGTCCTAAGTTAGGCACCAAACGTCCAGCTTCTTCCTTATCTGCATCTTCC
>HPV70_Alpha_1173493_nt6391_L1_Tile|1
CACAATACCTTCAGAGTTATATATTAAAGGCACGGATATACGTGAGCGTCCTGGTACTCA
>HPV70_Alpha_1173493_nt6396_L1_Tile|1
TACCTTCAGAGTTATATATTAAAGGCACGGATATACGTGAGCGTCCTGGTACTCATGTAT
>HPV70_Alpha_1173493_nt6406_L1_Tile|1
GTTATATATTAAAGGCACGGATATACGTGAGCGTCCTGGTACTCATGTATATTCCCCTTC
>HPV70_Alpha_1173493_nt6586_L1_Tile|1
GGACACTACACGTAGTACTAATTTTACATTGTCTGCCTGCACCGAAACGGCCATACCTGC
>HPV70_Alpha_1173493_nt6591_L1_Tile|1
CTACACGTAGTACTAATTTTACATTGTCTGCCTGCACCGAAACGGCCATACCTGCTGTAT
>HPV70_Alpha_1173493_nt6596_L1_Tile|1
CGTAGTACTAATTTTACATTGTCTGCCTGCACCGAAACGGCCATACCTGCTGTATATAGC
>HPV70_Alpha_1173493_nt6601_L1_Tile|1
TACTAATTTTACATTGTCTGCCTGCACCGAAACGGCCATACCTGCTGTATATAGCCCTAC
>HPV70_Alpha_1173493_nt6606_L1_Tile|1
ATTTTACATTGTCTGCCTGCACCGAAACGGCCATACCTGCTGTATATAGCCCTACAAAGT
>HPV70_Alpha_1173493_nt6611_L1_Tile|1
ACATTGTCTGCCTGCACCGAAACGGCCATACCTGCTGTATATAGCCCTACAAAGTTTAAG
>HPV70_Alpha_1173493_nt6626_L1_Tile|1
ACCGAAACGGCCATACCTGCTGTATATAGCCCTACAAAGTTTAAGGAATATACTAGGCAT
>HPV70_Alpha_1173493_nt6711_L1_Tile|1
TATTTCAATTGTGTACTATCACATTAACTGCTGACGTTATGGCCTACATCCATACTATGA
>HPV70_Alpha_1173493_nt6746_L1_Tile|1
GTTATGGCCTACATCCATACTATGAATCCTGCAATTTTGGACAATTGGAATATAGGAGTT
>HPV70_Alpha_1173493_nt6791_L1_Tile|1
TGGAATATAGGAGTTACCCCTCCACCATCTGCAAGCTTGGTGGACACGTATAGGTATTTA
>HPV70_Alpha_1173493_nt6796_L1_Tile|1

TATAGGAGTTACCCCTCCACCATCTGCAAGCTTGGTGGACACGTATAGGTATTTACAATC
>HPV70_Alpha_1173493_nt6801_L1_Tile|1
GAGTTACCCCTCCACCATCTGCAAGCTTGGTGGACACGTATAGGTATTTACAATCAGCAG
>HPV70_Alpha_1173493_nt6806_L1_Tile|1
ACCCCTCCACCATCTGCAAGCTTGGTGGACACGTATAGGTATTTACAATCAGCAGCTATA
>HPV70_Alpha_1173493_nt6811_L1_Tile|1
TCCACCATCTGCAAGCTTGGTGGACACGTATAGGTATTTACAATCAGCAGCTATAGCATG
>HPV70_Alpha_1173493_nt6831_L1_Tile|1
TGGACACGTATAGGTATTTACAATCAGCAGCTATAGCATGTCAAAAGGATGCTCCTACAC
>HPV70_Alpha_1173493_nt6836_L1_Tile|1
ACGTATAGGTATTTACAATCAGCAGCTATAGCATGTCAAAAGGATGCTCCTACACCTGAA
>HPV70_Alpha_1173493_nt7001_L1_Tile|1
GTAGGGGCTCGCAGACGTCCTACTATAGGCCCTCGCAAACGCCCTGCGTCAGCTAAATCG
>HPV70_Alpha_1173493_nt7016_L1_Tile|1
CGTCCTACTATAGGCCCTCGCAAACGCCCTGCGTCAGCTAAATCGTCTTCCTCAGCCTCT
>HPV70_Alpha_1173493_nt7021_L1_Tile|1
TACTATAGGCCCTCGCAAACGCCCTGCGTCAGCTAAATCGTCTTCCTCAGCCTCTAAACA
>HPV70_Alpha_1173493_nt7026_L1_Tile|1
TAGGCCCTCGCAAACGCCCTGCGTCAGCTAAATCGTCTTCCTCAGCCTCTAAACACAAAC
>HPV70_Alpha_1173493_nt7031_L1_Tile|1
CCTCGCAAACGCCCTGCGTCAGCTAAATCGTCTTCCTCAGCCTCTAAACACAAACGGAAA
>HPV71_Alpha_12084981_nt5907_L1_Tile|1
ACGACTATGTTACCAGAACAAAACTATTTTATTATGCTGGTAGTTCTAGATTACTTACTG
>HPV71_Alpha_12084981_nt5952_L1_Tile|1
CTAGATTACTTACTGTTGGCCATCCATATTTTCCTATTCGCCAGGCAAGTGGTAAAAATC
>HPV71_Alpha_12084981_nt5957_L1_Tile|1
TTACTTACTGTTGGCCATCCATATTTTCCTATTCGCCAGGCAAGTGGTAAAAATCGTATA
>HPV71_Alpha_12084981_nt5967_L1_Tile|1
TTGGCCATCCATATTTTCCTATTCGCCAGGCAAGTGGTAAAAATCGTATAGTTGTCCCCA
>HPV71_Alpha_12084981_nt5972_L1_Tile|1
CATCCATATTTTCCTATTCGCCAGGCAAGTGGTAAAAATCGTATAGTTGTCCCCAAAGTG
>HPV71_Alpha_12084981_nt6072_L1_Tile|1
ACCCAAATAAATTTGGACTACCTGATGCTTCATTATACAATCCCGATACCCAGCGCTTAG
>HPV71_Alpha_12084981_nt6087_L1_Tile|1
GACTACCTGATGCTTCATTATACAATCCCGATACCCAGCGCTTAGTGTGGGCTTGTAAGG
>HPV71_Alpha_12084981_nt6192_L1_Tile|1
GCCATCCATTGTTTAACAAGCTTAATGACACTGAAAATGCCACGCTGTTTGATGTTAATC
>HPV71_Alpha_12084981_nt6197_L1_Tile|1
CCATTGTTTAACAAGCTTAATGACACTGAAAATGCCACGCTGTTTGATGTTAATCCTGGT
>HPV71_Alpha_12084981_nt6207_L1_Tile|1
ACAAGCTTAATGACACTGAAAATGCCACGCTGTTTGATGTTAATCCTGGTGAGGATACTC
>HPV71_Alpha_12084981_nt6222_L1_Tile|2
CTGAAAATGCCACGCTGTTTGATGTTAATCCTGGTGAGGATACTCGGGATAATGTTTCTA
>HPV71_Alpha_12084981_nt6322_L1_Tile|2
GCCTCCCTTAGGTGAACATTGGGCAAAAGGTACTCCATGCAGTGGCGCTTCAGCTGCCGC
>HPV71_Alpha_12084981_nt6347_L1_Tile|1
AAAGGTACTCCATGCAGTGGCGCTTCAGCTGCCGCTGGTAGTTGCCCCCCACTTGAACTT
>HPV71_Alpha_12084981_nt6352_L1_Tile|1
TACTCCATGCAGTGGCGCTTCAGCTGCCGCTGGTAGTTGCCCCCCACTTGAACTTGCCAG
>HPV71_Alpha_12084981_nt6442_L1_Tile|2
CACAGGTTTTGGGGCAATGGATTTTGCAGCCCTGCAAACAAATAGGTCTGACGTTCCCTT
>HPV71_Alpha_12084981_nt6452_L1_Tile|1
GGGGCAATGGATTTTGCAGCCCTGCAAACAAATAGGTCTGACGTTCCCTTGGATATTGTT
>HPV71_Alpha_12084981_nt6467_L1_Tile|1
GCAGCCCTGCAAACAAATAGGTCTGACGTTCCCTTGGATATTGTTACTACAACATGTAAA

>HPV71_Alpha_12084981_nt6517_L1_Tile|1
AACATGTAAATATCAGATTATTTCGAATGGCTGCAGAGCCATTGGTGATCGCATGTTTTT
>HPV71_Alpha_12084981_nt6522_L1_Tile|2
GTAAATATCAGATTATTTCGAATGGCTGCAGAGCCATTGGTGATCGCATGTTTTTCTTGC
>HPV71_Alpha_12084981_nt6532_L1_Tile|1
GATTATTTCGAATGGCTGCAGAGCCATTGGTGATCGCATGTTTTTCTTGCGCCGAGAGCA
>HPV71_Alpha_12084981_nt6547_L1_Tile|1
CTGCAGAGCCATTGGTGATCGCATGTTTTTCTTGCGCCGAGAGCAATGTTTTAAGACATT
>HPV71_Alpha_12084981_nt6552_L1_Tile|1
GAGCCATTGGTGATCGCATGTTTTTCTTGCGCCGAGAGCAATGTTTTAAGACATTTTATA
>HPV71_Alpha_12084981_nt6592_L1_Tile|1
ATGTTTTAAGACATTTTATAATAGGCAGGCACACCTAGGCAGGGTTCCTGATGACTATTA
>HPV71_Alpha_12084981_nt6602_L1_Tile|2
ACATTTTATAATAGGCAGGCACACCTAGGCAGGGTTCCTGATGACTATTATTTAAAGGTT
>HPV71_Alpha_12084981_nt6607_L1_Tile|1
TTATAATAGGCAGGCACACCTAGGCAGGGTTCCTGATGACTATTATTTAAAGGTTCACCT
>HPV71_Alpha_12084981_nt6617_L1_Tile|1
CAGGCACACCTAGGCAGGGTTCCTGATGACTATTATTTAAAGGTTCACCTTCTACCTTTC
>HPV71_Alpha_12084981_nt6632_L1_Tile|1
AGGGTTCCTGATGACTATTATTTAAAGGTTCACCTTCTACCTTTCGTGCTTCCCCTACAA
>HPV71_Alpha_12084981_nt6637_L1_Tile|1
TCCTGATGACTATTATTTAAAGGTTCACCTTCTACCTTTCGTGCTTCCCCTACAAGCTCT
>HPV71_Alpha_12084981_nt6642_L1_Tile|2
ATGACTATTATTTAAAGGTTCACCTTCTACCTTTCGTGCTTCCCCTACAAGCTCTCTTTA
>HPV71_Alpha_12084981_nt6647_L1_Tile|1
TATTATTTAAAGGTTCACCTTCTACCTTTCGTGCTTCCCCTACAAGCTCTCTTTATGCAT
>HPV71_Alpha_12084981_nt6657_L1_Tile|1
AGGTTCACCTTCTACCTTTCGTGCTTCCCCTACAAGCTCTCTTTATGCATCCACACCTAG
>HPV71_Alpha_12084981_nt6777_L1_Tile|1
ACAGGGCACAAACAATGGCATTTGTTGGGCAATCTGCTTTTTGTAACAGTTGTGACACAT
>HPV71_Alpha_12084981_nt6782_L1_Tile|2
GCACAAACAATGGCATTTGTTGGGCAATCTGCTTTTTGTAACAGTTGTGACACATCACGT
>HPV71_Alpha_12084981_nt6787_L1_Tile|1
AACAATGGCATTTGTTGGGCAATCTGCTTTTTGTAACAGTTGTGACACATCACGTAGTAC
>HPV71_Alpha_12084981_nt6792_L1_Tile|1
TGGCATTTGTTGGGCAATCTGCTTTTTGTAACAGTTGTGACACATCACGTAGTACAAATA
>HPV71_Alpha_12084981_nt6802_L1_Tile|2
TGGGCAATCTGCTTTTTGTAACAGTTGTGACACATCACGTAGTACAAATATGTCCATCTG
>HPV71_Alpha_12084981_nt6807_L1_Tile|1
AATCTGCTTTTTGTAACAGTTGTGACACATCACGTAGTACAAATATGTCCATCTGTGCTA
>HPV71_Alpha_12084981_nt6812_L1_Tile|1
GCTTTTTGTAACAGTTGTGACACATCACGTAGTACAAATATGTCCATCTGTGCTACCAAA
>HPV71_Alpha_12084981_nt6817_L1_Tile|1
TTGTAACAGTTGTGACACATCACGTAGTACAAATATGTCCATCTGTGCTACCAAAACTGT
>HPV71_Alpha_12084981_nt6822_L1_Tile|2
ACAGTTGTGACACATCACGTAGTACAAATATGTCCATCTGTGCTACCAAAACTGTTGAGT
>HPV71_Alpha_12084981_nt6827_L1_Tile|1
TGTGACACATCACGTAGTACAAATATGTCCATCTGTGCTACCAAAACTGTTGAGTCTACA
>HPV71_Alpha_12084981_nt6832_L1_Tile|1
CACATCACGTAGTACAAATATGTCCATCTGTGCTACCAAAACTGTTGAGTCTACATATAA
>HPV71_Alpha_12084981_nt6837_L1_Tile|1
CACGTAGTACAAATATGTCCATCTGTGCTACCAAAACTGTTGAGTCTACATATAAAGCCT
>HPV71_Alpha_12084981_nt6852_L1_Tile|1
TGTCCATCTGTGCTACCAAAACTGTTGAGTCTACATATAAAGCCTCTAGTTTCATGGAAT
>HPV71_Alpha_12084981_nt6857_L1_Tile|1

ATCTGTGCTACCAAAACTGTTGAGTCTACATATAAAGCCTCTAGTTTCATGGAATATTTG
>HPV71_Alpha_12084981_nt6862_L1_Tile|2
TGCTACCAAAACTGTTGAGTCTACATATAAAGCCTCTAGTTTCATGGAATATTTGAGACA
>HPV71_Alpha_12084981_nt6867_L1_Tile|1
CCAAAACTGTTGAGTCTACATATAAAGCCTCTAGTTTCATGGAATATTTGAGACATGGAG
>HPV71_Alpha_12084981_nt6872_L1_Tile|1
ACTGTTGAGTCTACATATAAAGCCTCTAGTTTCATGGAATATTTGAGACATGGAGAAGAA
>HPV71_Alpha_12084981_nt6882_L1_Tile|2
CTACATATAAAGCCTCTAGTTTCATGGAATATTTGAGACATGGAGAAGAATTTGATTTGC
>HPV71_Alpha_12084981_nt6887_L1_Tile|1
TATAAAGCCTCTAGTTTCATGGAATATTTGAGACATGGAGAAGAATTTGATTTGCAATTT
>HPV71_Alpha_12084981_nt6967_L1_Tile|1
TAATTTAACAGCTGAAATTATGGCCTACTTACATGGCATGGATGCTACATTACTGGAGGA
>HPV71_Alpha_12084981_nt7092_L1_Tile|1
CTCAGGCCATAACCTGTCAGAAAAACAGTCCTCCTCCTGCAGAAAAAAAGGACCCCTATG
>HPV71_Alpha_12084981_nt7102_L1_Tile|2
AACCTGTCAGAAAAACAGTCCTCCTCCTGCAGAAAAAAAGGACCCCTATGCAGATCTTAC
>HPV71_Alpha_12084981_nt7107_L1_Tile|1
GTCAGAAAAACAGTCCTCCTCCTGCAGAAAAAAAGGACCCCTATGCAGATCTTACATTTT
>HPV71_Alpha_12084981_nt7237_L1_Tile|1
GCAAAGTGGCACCCGCTCGCGGCCTACTGCATTGTCCCGTAAAAGGGTTGCAGCATCTAC
>HPV71_Alpha_12084981_nt7257_L1_Tile|1
GGCCTACTGCATTGTCCCGTAAAAGGGTTGCAGCATCTACCACATCCACCGCCCCCAAAC
>HPV72_Alpha_1491683_nt5899_L1_Tile|1
GGCAGTTCTCGTCTACTAACTGTAGGACATCCTTACTGTGCCATACCTCTCAACGGACAG
>HPV72_Alpha_1491683_nt5909_L1_Tile|1
GTCTACTAACTGTAGGACATCCTTACTGTGCCATACCTCTCAACGGACAGGGCAAAAAAA
>HPV72_Alpha_1491683_nt5914_L1_Tile|1
CTAACTGTAGGACATCCTTACTGTGCCATACCTCTCAACGGACAGGGCAAAAAAAACACC
>HPV72_Alpha_1491683_nt5919_L1_Tile|1
TGTAGGACATCCTTACTGTGCCATACCTCTCAACGGACAGGGCAAAAAAAACACCATTCC
>HPV72_Alpha_1491683_nt6184_L1_Tile|1
ACTTCCTTACTTGTGGCTGACAATTCTGACAGTCGGGACAATGTATCTGTTGACTACAAA
>HPV72_Alpha_1491683_nt6219_L1_Tile|1
GGACAATGTATCTGTTGACTACAAACAGACCCAATTGCTTATTATAGGGTGCAAGCCTCC
>HPV72_Alpha_1491683_nt6229_L1_Tile|1
TCTGTTGACTACAAACAGACCCAATTGCTTATTATAGGGTGCAAGCCTCCCATTGGTGAG
>HPV72_Alpha_1491683_nt6234_L1_Tile|1
TGACTACAAACAGACCCAATTGCTTATTATAGGGTGCAAGCCTCCCATTGGTGAGCATTG
>HPV72_Alpha_1491683_nt6269_L1_Tile|1
GCAAGCCTCCCATTGGTGAGCATTGGACCAAGGGCACTCCTTGTGCAGGCTCTAATTCTC
>HPV72_Alpha_1491683_nt6279_L1_Tile|1
CATTGGTGAGCATTGGACCAAGGGCACTCCTTGTGCAGGCTCTAATTCTCAGCCAACTGA
>HPV72_Alpha_1491683_nt6284_L1_Tile|1
GTGAGCATTGGACCAAGGGCACTCCTTGTGCAGGCTCTAATTCTCAGCCAACTGACTGCC
>HPV72_Alpha_1491683_nt6314_L1_Tile|1
CAGGCTCTAATTCTCAGCCAACTGACTGCCCCCCTTTAGAATTTACAAATTCCACTATAC
>HPV72_Alpha_1491683_nt6564_L1_Tile|1
TTTTTTTAATAGGCAGGGCACAATGGGTGAGGCACTACCAGCCAGTTTATATCTTAAAGG
>HPV72_Alpha_1491683_nt6569_L1_Tile|1
TTAATAGGCAGGGCACAATGGGTGAGGCACTACCAGCCAGTTTATATCTTAAAGGTGCCT
>HPV72_Alpha_1491683_nt6574_L1_Tile|1
AGGCAGGGCACAATGGGTGAGGCACTACCAGCCAGTTTATATCTTAAAGGTGCCTCGGGT
>HPV72_Alpha_1491683_nt6579_L1_Tile|1
GGGCACAATGGGTGAGGCACTACCAGCCAGTTTATATCTTAAAGGTGCCTCGGGTAGCGA

>HPV72_Alpha_1491683_nt6584_L1_Tile|1
CAATGGGTGAGGCACTACCAGCCAGTTTATATCTTAAAGGTGCCTCGGGTAGCGACAGGG
>HPV72_Alpha_1491683_nt6589_L1_Tile|1
GGTGAGGCACTACCAGCCAGTTTATATCTTAAAGGTGCCTCGGGTAGCGACAGGGTGACA
>HPV72_Alpha_1491683_nt6614_L1_Tile|1
ATCTTAAAGGTGCCTCGGGTAGCGACAGGGTGACACCTGGTAGTTATATTTATTCTCCCA
>HPV72_Alpha_1491683_nt6624_L1_Tile|1
TGCCTCGGGTAGCGACAGGGTGACACCTGGTAGTTATATTTATTCTCCCACCCCCAGCGG
>HPV72_Alpha_1491683_nt6629_L1_Tile|1
CGGGTAGCGACAGGGTGACACCTGGTAGTTATATTTATTCTCCCACCCCCAGCGGCTCTA
>HPV72_Alpha_1491683_nt6809_L1_Tile|1
GTACTAATGTAACTATTTGTACTGCCACAGCGTCCTCTGTATCAGAATATACAGCTTCTA
>HPV72_Alpha_1491683_nt6814_L1_Tile|1
AATGTAACTATTTGTACTGCCACAGCGTCCTCTGTATCAGAATATACAGCTTCTAATTTT
>HPV72_Alpha_1491683_nt6819_L1_Tile|1
AACTATTTGTACTGCCACAGCGTCCTCTGTATCAGAATATACAGCTTCTAATTTTCGTGA
>HPV72_Alpha_1491683_nt6824_L1_Tile|1
TTTGTACTGCCACAGCGTCCTCTGTATCAGAATATACAGCTTCTAATTTTCGTGAGTATC
>HPV72_Alpha_1491683_nt6844_L1_Tile|1
TCTGTATCAGAATATACAGCTTCTAATTTTCGTGAGTATCTTCGCCACACTGAGGAATTT
>HPV72_Alpha_1491683_nt7069_L1_Tile|1
ATTACCTGTCAAAAGGGGGCTGCCACCCCTCCTCCTAAAGAAGATCCATATGCTAACTTA
>HPV73_Alpha_1491692_nt5595_L1_Tile|1
ATGCAGGTAGCACACGTTTGTTGGCTGTGGGACACCCATATTTTCCTATCAAGGATTCTC
>HPV73_Alpha_1491692_nt5600_L1_Tile|1
GGTAGCACACGTTTGTTGGCTGTGGGACACCCATATTTTCCTATCAAGGATTCTCAAAAA
>HPV73_Alpha_1491692_nt5610_L1_Tile|1
GTTTGTTGGCTGTGGGACACCCATATTTTCCTATCAAGGATTCTCAAAAACGTAAAACCA
>HPV73_Alpha_1491692_nt5620_L1_Tile|1
TGTGGGACACCCATATTTTCCTATCAAGGATTCTCAAAAACGTAAAACCATAGTTCCTAA
>HPV73_Alpha_1491692_nt5625_L1_Tile|1
GACACCCATATTTTCCTATCAAGGATTCTCAAAAACGTAAAACCATAGTTCCTAAAGTTT
>HPV73_Alpha_1491692_nt5630_L1_Tile|1
CCATATTTTCCTATCAAGGATTCTCAAAAACGTAAAACCATAGTTCCTAAAGTTTCAGGT
>HPV73_Alpha_1491692_nt5850_L1_Tile|1
CATTTATGAATAAATTAGATGATACTGAAAATGCTCCTAAATACATTGCTGGACAAAATA
>HPV73_Alpha_1491692_nt5860_L1_Tile|1
TAAATTAGATGATACTGAAAATGCTCCTAAATACATTGCTGGACAAAATACAGATGGTAG
>HPV73_Alpha_1491692_nt5865_L1_Tile|1
TAGATGATACTGAAAATGCTCCTAAATACATTGCTGGACAAAATACAGATGGTAGAGAAT
>HPV73_Alpha_1491692_nt5870_L1_Tile|1
GATACTGAAAATGCTCCTAAATACATTGCTGGACAAAATACAGATGGTAGAGAATGTATG
>HPV73_Alpha_1491692_nt5875_L1_Tile|1
TGAAAATGCTCCTAAATACATTGCTGGACAAAATACAGATGGTAGAGAATGTATGTCAGT
>HPV73_Alpha_1491692_nt5880_L1_Tile|1
ATGCTCCTAAATACATTGCTGGACAAAATACAGATGGTAGAGAATGTATGTCAGTGGATT
>HPV73_Alpha_1491692_nt5885_L1_Tile|1
CCTAAATACATTGCTGGACAAAATACAGATGGTAGAGAATGTATGTCAGTGGATTATAAA
>HPV73_Alpha_1491692_nt6010_L1_Tile|1
ATGTACTTCACAAACTGTTAATACTGGTGATTGTCCCCCACTGGAATTAAAGAACACCCC
>HPV73_Alpha_1491692_nt6015_L1_Tile|1
CTTCACAAACTGTTAATACTGGTGATTGTCCCCCACTGGAATTAAAGAACACCCCTATAC
>HPV73_Alpha_1491692_nt6255_L1_Tile|1
TTGTTCGACACTTATTTAACAGGGCTGGTGATACCGGTGATAAAATCCCAGATGACCTAA
>HPV73_Alpha_1491692_nt6265_L1_Tile|1

CTTATTTAACAGGGCTGGTGATACCGGTGATAAAATCCCAGATGACCTAATGATTAAAGG
>HPV73_Alpha_1491692_nt6285_L1_Tile|1
ATACCGGTGATAAAATCCCAGATGACCTAATGATTAAAGGCACAGGCAATACTGCAACAC
>HPV73_Alpha_1491692_nt6495_L1_Tile|1
CTACTAGAAGCACTAATTTTTCTGTATGTGTAGGTACACAGGCTAGTAGCTCTACTACAA
>HPV73_Alpha_1491692_nt6500_L1_Tile|1
AGAAGCACTAATTTTTCTGTATGTGTAGGTACACAGGCTAGTAGCTCTACTACAACGTAT
>HPV73_Alpha_1491692_nt6505_L1_Tile|1
CACTAATTTTTCTGTATGTGTAGGTACACAGGCTAGTAGCTCTACTACAACGTATGCCAA
>HPV73_Alpha_1491692_nt6510_L1_Tile|1
ATTTTTCTGTATGTGTAGGTACACAGGCTAGTAGCTCTACTACAACGTATGCCAACTCTA
>HPV73_Alpha_1491692_nt6515_L1_Tile|1
TCTGTATGTGTAGGTACACAGGCTAGTAGCTCTACTACAACGTATGCCAACTCTAATTTT
>HPV73_Alpha_1491692_nt6520_L1_Tile|1
ATGTGTAGGTACACAGGCTAGTAGCTCTACTACAACGTATGCCAACTCTAATTTTAAGGA
>HPV73_Alpha_1491692_nt6525_L1_Tile|1
TAGGTACACAGGCTAGTAGCTCTACTACAACGTATGCCAACTCTAATTTTAAGGAATATT
>HPV73_Alpha_1491692_nt6920_L1_Tile|1
GCACGTCCTAAGTTACAAGCTTCTAAACGTTCTGCATCTGCTACCACAAGTGCCACACCT
>HPV73_Alpha_1491692_nt6925_L1_Tile|1
TCCTAAGTTACAAGCTTCTAAACGTTCTGCATCTGCTACCACAAGTGCCACACCTAAGAA
>HPV74_Alpha_27462483_nt5714_L1_Tile|1
ACTTGCTGTAGGAAATCCCTATTTCCCTATAAAACAGGTTAACAAAACAGTTGTTCCTAA
>HPV74_Alpha_27462483_nt5719_L1_Tile|1
CTGTAGGAAATCCCTATTTCCCTATAAAACAGGTTAACAAAACAGTTGTTCCTAAAGTGT
>HPV74_Alpha_27462483_nt5724_L1_Tile|1
GGAAATCCCTATTTCCCTATAAAACAGGTTAACAAAACAGTTGTTCCTAAAGTGTCTGGA
>HPV74_Alpha_27462483_nt6099_L1_Tile|1
AAGCAATGTTCTAATGTTACTGTAAAGCCAGGCGATTGCCCTGCATTAGAATTAATTACT
>HPV74_Alpha_27462483_nt6104_L1_Tile|1
ATGTTCTAATGTTACTGTAAAGCCAGGCGATTGCCCTGCATTAGAATTAATTACTAGTGT
>HPV74_Alpha_27462483_nt6109_L1_Tile|1
CTAATGTTACTGTAAAGCCAGGCGATTGCCCTGCATTAGAATTAATTACTAGTGTAATTC
>HPV74_Alpha_27462483_nt6374_L1_Tile|1
TGGAACAGTGGGTGAGGACATTCCCCAGGACCTATATATTAAGGGTACTACACGTGGTAC
>HPV74_Alpha_27462483_nt6399_L1_Tile|1
CAGGACCTATATATTAAGGGTACTACACGTGGTACACTTGCCAATGCTATTTACTTTAAT
>HPV74_Alpha_27462483_nt6404_L1_Tile|1
CCTATATATTAAGGGTACTACACGTGGTACACTTGCCAATGCTATTTACTTTAATACCCC
>HPV74_Alpha_27462483_nt6594_L1_Tile|1
AGTACTAACATGACAGTGTGTGCGCCTACTACACAATCCCCTCCTGCTGCTACATATACT
>HPV74_Alpha_27462483_nt6599_L1_Tile|1
TAACATGACAGTGTGTGCGCCTACTACACAATCCCCTCCTGCTGCTACATATACTAGTAC
>HPV74_Alpha_27462483_nt6604_L1_Tile|1
TGACAGTGTGTGCGCCTACTACACAATCCCCTCCTGCTGCTACATATACTAGTACAGACT
>HPV74_Alpha_27462483_nt6609_L1_Tile|1
GTGTGTGCGCCTACTACACAATCCCCTCCTGCTGCTACATATACTAGTACAGACTACAAA
>HPV74_Alpha_27462483_nt6619_L1_Tile|1
CTACTACACAATCCCCTCCTGCTGCTACATATACTAGTACAGACTACAAACAATACATGA
>HPV74_Alpha_27462483_nt6624_L1_Tile|1
ACACAATCCCCTCCTGCTGCTACATATACTAGTACAGACTACAAACAATACATGAGACAT
>HPV74_Alpha_27462483_nt7014_L1_Tile|1
CGCTCTTCGGTTCGTGTAGGTAAAAAGCGTTCGGCCCCTACAGCCCCCTCCTCAACCACT
>HPV74_Alpha_27462483_nt7029_L1_Tile|1
GTAGGTAAAAAGCGTTCGGCCCCTACAGCCCCCTCCTCAACCACTAAACAGAAACGCTCT

>HPV74_Alpha_27462483_nt7034_L1_Tile|1
TAAAAAGCGTTCGGCCCCTACAGCCCCCTCCTCAACCACTAAACAGAAACGCTCTAGGAA
>HPV74_Alpha_27462483_nt7041_L1_Tile|1
CGTTCGGCCCCTACAGCCCCCTCCTCAACCACTAAACAGAAACGCTCTAGGAAACGATAG
>HPV75_Beta_2911544_nt6142_L1_Tile|2
CTTTGTTCAATAAAGTAAAGGATACTGAAAATTCAAATAATTATATAACAATGTCTAAAG
>HPV75_Beta_2911544_nt7112_L1_Tile|1
AAAGAAAGATCCTTATGAGCAATATACTTTCTGGCCTGTGGATCTAACTGAAAAATTATC
>HPV75_Beta_2911544_nt7122_L1_Tile|2
CCTTATGAGCAATATACTTTCTGGCCTGTGGATCTAACTGAAAAATTATCGTTGGATTTG
>HPV75_Beta_2911544_nt7187_L1_Tile|1
ATACTCGTTAGGACGCAAATTCCTTTTTCAGGCAGGCTTACGAAGGGCTTCCAAGGTAAC
>HPV75_Beta_2911544_nt7192_L1_Tile|1
CGTTAGGACGCAAATTCCTTTTTCAGGCAGGCTTACGAAGGGCTTCCAAGGTAACCAGAA
>HPV76_Beta_2911551_nt6035_L1_Tile|1
TACTACCAGATCCCAATAGATTCGCCTTGGTAGACATGAATGTCTATAATCCTGAGAAGG
>HPV76_Beta_2911551_nt6045_L1_Tile|1
TCCCAATAGATTCGCCTTGGTAGACATGAATGTCTATAATCCTGAGAAGGAAAGACTGGT
>HPV76_Beta_2911551_nt6050_L1_Tile|1
ATAGATTCGCCTTGGTAGACATGAATGTCTATAATCCTGAGAAGGAAAGACTGGTTTGGG
>HPV76_Beta_2911551_nt6330_L1_Tile|1
TGATGCTGACAGAGGGGTAGGCAAATGTCCACCTTTGGAACTGGTAAATACTGTAATAGA
>HPV76_Beta_2911551_nt6335_L1_Tile|1
CTGACAGAGGGGTAGGCAAATGTCCACCTTTGGAACTGGTAAATACTGTAATAGAAGATG
>HPV76_Beta_2911551_nt6340_L1_Tile|1
AGAGGGGTAGGCAAATGTCCACCTTTGGAACTGGTAAATACTGTAATAGAAGATGGAGAT
>HPV76_Beta_2911551_nt7145_L1_Tile|1
AGGATCCATATGAGCAATACAATTTTTGGCAAGTGGATCTAACTGAGAAATTGTCATTAG
>HPV76_Beta_2911551_nt7150_L1_Tile|1
CCATATGAGCAATACAATTTTTGGCAAGTGGATCTAACTGAGAAATTGTCATTAGATTTG
>HPV76_Beta_2911551_nt7215_L1_Tile|1
ATATTCCTTAGGACGCAAGTTTCTGTTTCAAGCAGGCCTGCGACGGGCTTCCAGAGTATC
>HPV77_Alpha_2911558_nt5914_L1_Tile|1
TGTTAACTGTAGGTCATCCCTATTTTGCTATTCCCAAAACTTCTGGTACTAAGGTGGATG
>HPV77_Alpha_2911558_nt6284_L1_Tile|1
GGGGGAATACTGGGGTAAGGGTACCCCTTGTGCACGTACTAATACTACCCCAGGAGACTG
>HPV77_Alpha_2911558_nt6289_L1_Tile|1
AATACTGGGGTAAGGGTACCCCTTGTGCACGTACTAATACTACCCCAGGAGACTGTCCTC
>HPV77_Alpha_2911558_nt6304_L1_Tile|1
GTACCCCTTGTGCACGTACTAATACTACCCCAGGAGACTGTCCTCCCTTGGAGTTAATGA
>HPV77_Alpha_2911558_nt6309_L1_Tile|1
CCTTGTGCACGTACTAATACTACCCCAGGAGACTGTCCTCCCTTGGAGTTAATGACATCT
>HPV77_Alpha_2911558_nt6319_L1_Tile|1
GTACTAATACTACCCCAGGAGACTGTCCTCCCTTGGAGTTAATGACATCTTATATTCAGG
>HPV77_Alpha_2911558_nt6564_L1_Tile|1
CACTTTTTCAATCGTGCGGGTGATGTTGGAGACAAAATTCCAGAATCTTTGTACCTCAAA
>HPV77_Alpha_2911558_nt6569_L1_Tile|1
TTTCAATCGTGCGGGTGATGTTGGAGACAAAATTCCAGAATCTTTGTACCTCAAAGGGAG
>HPV77_Alpha_2911558_nt6574_L1_Tile|1
ATCGTGCGGGTGATGTTGGAGACAAAATTCCAGAATCTTTGTACCTCAAAGGGAGTAGCG
>HPV77_Alpha_2911558_nt6579_L1_Tile|1
GCGGGTGATGTTGGAGACAAAATTCCAGAATCTTTGTACCTCAAAGGGAGTAGCGGGCGT
>HPV77_Alpha_2911558_nt6584_L1_Tile|1
TGATGTTGGAGACAAAATTCCAGAATCTTTGTACCTCAAAGGGAGTAGCGGGCGTGAGAC
>HPV77_Alpha_2911558_nt6589_L1_Tile|1

TTGGAGACAAAATTCCAGAATCTTTGTACCTCAAAGGGAGTAGCGGGCGTGAGACTCCCG
>HPV77_Alpha_2911558_nt6594_L1_Tile|1
GACAAAATTCCAGAATCTTTGTACCTCAAAGGGAGTAGCGGGCGTGAGACTCCCGGCAGT
>HPV77_Alpha_2911558_nt6599_L1_Tile|1
AATTCCAGAATCTTTGTACCTCAAAGGGAGTAGCGGGCGTGAGACTCCCGGCAGTGCTAT
>HPV77_Alpha_2911558_nt6604_L1_Tile|1
CAGAATCTTTGTACCTCAAAGGGAGTAGCGGGCGTGAGACTCCCGGCAGTGCTATATACA
>HPV77_Alpha_2911558_nt6804_L1_Tile|1
AGCACAAATATGTCTCTCAGTGCCTCCACTGAGTCTCAAACCCCATCTACCTATGATGCC
>HPV77_Alpha_2911558_nt6809_L1_Tile|1
AAATATGTCTCTCAGTGCCTCCACTGAGTCTCAAACCCCATCTACCTATGATGCCACTAA
>HPV77_Alpha_2911558_nt6814_L1_Tile|1
TGTCTCTCAGTGCCTCCACTGAGTCTCAAACCCCATCTACCTATGATGCCACTAAAATAA
>HPV77_Alpha_2911558_nt6819_L1_Tile|1
CTCAGTGCCTCCACTGAGTCTCAAACCCCATCTACCTATGATGCCACTAAAATAAAGGAA
>HPV77_Alpha_2911558_nt7229_L1_Tile|1
GGTTCCCTCTCGCAAACGCCGGGCGCCCACCCCCTCCCCAGCTTCGACCAAACGGAAGCG
>HPV77_Alpha_2911558_nt7242_L1_Tile|2
AAACGCCGGGCGCCCACCCCCTCCCCAGCTTCGACCAAACGGAAGCGCTCCAAAAAGTGA
>HPV80_Beta_2911565_nt6084_L1_Tile|1
TTTATTTAATAAAGTTAAGGACACAGAAAATAATAGTGGCTACCAGGGAGGTTCTACTGA
>HPV80_Beta_2911565_nt6089_L1_Tile|1
TTAATAAAGTTAAGGACACAGAAAATAATAGTGGCTACCAGGGAGGTTCTACTGATGACA
>HPV80_Beta_2911565_nt6094_L1_Tile|1
AAAGTTAAGGACACAGAAAATAATAGTGGCTACCAGGGAGGTTCTACTGATGACAGACAG
>HPV80_Beta_2911565_nt6099_L1_Tile|1
TAAGGACACAGAAAATAATAGTGGCTACCAGGGAGGTTCTACTGATGACAGACAGAATAC
>HPV80_Beta_2911565_nt6249_L1_Tile|1
AGAAGAAAATAACCAAGCTGGAAAATGTCCTCCATTAGAACTTAAAAATACTGTGATTGA
>HPV80_Beta_2911565_nt6579_L1_Tile|1
ACAAACAGGCCAGCAACAACGTGTTTTAGCAAATTCCACATATTTTCCTACTGTTAGTGG
>HPV80_Beta_2911565_nt6589_L1_Tile|1
CAGCAACAACGTGTTTTAGCAAATTCCACATATTTTCCTACTGTTAGTGGCTCTTTAGTA
>HPV80_Beta_2911565_nt6594_L1_Tile|1
ACAACGTGTTTTAGCAAATTCCACATATTTTCCTACTGTTAGTGGCTCTTTAGTAACTTC
>HPV80_Beta_2911565_nt6734_L1_Tile|1
TGTTTATTACTGTTGCTGATAATACAAGAAACACTAATTTCACCATAAGTGTTACTTCTG
>HPV80_Beta_2911565_nt6744_L1_Tile|1
TGTTGCTGATAATACAAGAAACACTAATTTCACCATAAGTGTTACTTCTGATGGTAGTAC
>HPV80_Beta_2911565_nt6749_L1_Tile|1
CTGATAATACAAGAAACACTAATTTCACCATAAGTGTTACTTCTGATGGTAGTACTATAA
>HPV80_Beta_2911565_nt6754_L1_Tile|1
AATACAAGAAACACTAATTTCACCATAAGTGTTACTTCTGATGGTAGTACTATAACTGAA
>HPV80_Beta_2911565_nt6769_L1_Tile|1
AATTTCACCATAAGTGTTACTTCTGATGGTAGTACTATAACTGAATATAATACACAAAAT
>HPV80_Beta_2911565_nt6774_L1_Tile|1
CACCATAAGTGTTACTTCTGATGGTAGTACTATAACTGAATATAATACACAAAATATTAG
>HPV80_Beta_2911565_nt7139_L1_Tile|1
CTTTAGGAAGGAAATTTATATTTCAGGCAGGTTTACAAAGACGTCCCAAAACCATCAGAT
>HPV80_Beta_2911565_nt7144_L1_Tile|1
GGAAGGAAATTTATATTTCAGGCAGGTTTACAAAGACGTCCCAAAACCATCAGATCTTCT
>HPV80_Beta_2911565_nt7149_L1_Tile|1
GAAATTTATATTTCAGGCAGGTTTACAAAGACGTCCCAAAACCATCAGATCTTCTGTAAA
>HPV80_Beta_2911565_nt7154_L1_Tile|1
TTATATTTCAGGCAGGTTTACAAAGACGTCCCAAAACCATCAGATCTTCTGTAAAAGTTT

>HPV81_Alpha_40804509_nt5775_L1_Tile|1
ATGTTGTTGGCACTGATTTTTACTTGTTTCCTTCTTATATATTCTTCCCTAAAAAACATA
>HPV81_Alpha_40804509_nt5780_L1_Tile|1
GTTGGCACTGATTTTTACTTGTTTCCTTCTTATATATTCTTCCCTAAAAAACATAAACGT
>HPV81_Alpha_40804509_nt5985_L1_Tile|1
CTTACTGTAGGGCATCCATATTGTACATTAACTATTGGTACCCAAGGAAAGCGTTCCACT
>HPV81_Alpha_40804509_nt5990_L1_Tile|1
TGTAGGGCATCCATATTGTACATTAACTATTGGTACCCAAGGAAAGCGTTCCACTATTCC
>HPV81_Alpha_40804509_nt5995_L1_Tile|1
GGCATCCATATTGTACATTAACTATTGGTACCCAAGGAAAGCGTTCCACTATTCCCAAGG
>HPV81_Alpha_40804509_nt6275_L1_Tile|1
CAGTGAGGACAGTCGTGACAATATTTCAGTTGATTATAAGCAAACACAATTATTAATTAT
>HPV81_Alpha_40804509_nt6355_L1_Tile|1
GGGAGCACTGGGCCCGGGGTACCTTTTGTGCTAATGTTGCTCCAGCACAAAACGAATGCC
>HPV81_Alpha_40804509_nt6370_L1_Tile|1
GGGGTACCTTTTGTGCTAATGTTGCTCCAGCACAAAACGAATGCCCCCCCTTGGAGTTTA
>HPV81_Alpha_40804509_nt6375_L1_Tile|1
ACCTTTTGTGCTAATGTTGCTCCAGCACAAAACGAATGCCCCCCCTTGGAGTTTAAAAAT
>HPV81_Alpha_40804509_nt6380_L1_Tile|1
TTGTGCTAATGTTGCTCCAGCACAAAACGAATGCCCCCCCTTGGAGTTTAAAAATACAAC
>HPV81_Alpha_40804509_nt6675_L1_Tile|1
GCAGACTTATATATGAAGGGTGCTGCAGGCAGTGACAGAATAACCCCCGGGAGTTATATT
>HPV81_Alpha_40804509_nt6870_L1_Tile|1
ACTACCAGAAGCACCAATTTTACTATTTGCACAGCTACATCTGCTGCTGCAGAATACAAG
>HPV82_Alpha_6970427_nt5927_L1_Tile|1
ATCCTTTATTTAATAAGTATGATGATACTGAAAACTCTAGGTTTGCCAATGGCAACGACC
>HPV82_Alpha_6970427_nt5932_L1_Tile|1
TTATTTAATAAGTATGATGATACTGAAAACTCTAGGTTTGCCAATGGCAACGACCAACAG
>HPV82_Alpha_6970427_nt5937_L1_Tile|1
TAATAAGTATGATGATACTGAAAACTCTAGGTTTGCCAATGGCAACGACCAACAGGATGT
>HPV82_Alpha_6970427_nt5942_L1_Tile|2
AGTATGATGATACTGAAAACTCTAGGTTTGCCAATGGCAACGACCAACAGGATGTTAGGG
>HPV82_Alpha_6970427_nt5947_L1_Tile|1
GATGATACTGAAAACTCTAGGTTTGCCAATGGCAACGACCAACAGGATGTTAGGGACAAC
>HPV82_Alpha_6970427_nt5952_L1_Tile|1
TACTGAAAACTCTAGGTTTGCCAATGGCAACGACCAACAGGATGTTAGGGACAACATATC
>HPV82_Alpha_6970427_nt5957_L1_Tile|1
AAAACTCTAGGTTTGCCAATGGCAACGACCAACAGGATGTTAGGGACAACATATCTGTGG
>HPV82_Alpha_6970427_nt5962_L1_Tile|2
TCTAGGTTTGCCAATGGCAACGACCAACAGGATGTTAGGGACAACATATCTGTGGACAAC
>HPV82_Alpha_6970427_nt6187_L1_Tile|1
GCCATGGATTTTGCTAATTTACAAGCTACTAAATCAGATGTTCCATTGGATATTGCTCAG
>HPV82_Alpha_6970427_nt6207_L1_Tile|1
ACAAGCTACTAAATCAGATGTTCCATTGGATATTGCTCAGTCTGTGTGTAAATACCCTGA
>HPV82_Alpha_6970427_nt6322_L1_Tile|2
AGGGAGCAAATATTTGCTAGGCATTACTATAATAAGGCTGGTGTGGTTGGTGATGCCATT
>HPV82_Alpha_6970427_nt6327_L1_Tile|1
GCAAATATTTGCTAGGCATTACTATAATAAGGCTGGTGTGGTTGGTGATGCCATTCCAGA
>HPV82_Alpha_6970427_nt6337_L1_Tile|1
GCTAGGCATTACTATAATAAGGCTGGTGTGGTTGGTGATGCCATTCCAGACAAGGCTTAT
>HPV82_Alpha_6970427_nt6352_L1_Tile|1
AATAAGGCTGGTGTGGTTGGTGATGCCATTCCAGACAAGGCTTATATTAAGGGTACTGGT
>HPV82_Alpha_6970427_nt6372_L1_Tile|1
TGATGCCATTCCAGACAAGGCTTATATTAAGGGTACTGGTGCTGGCCGCGACCCTATTAG
>HPV82_Alpha_6970427_nt6562_L1_Tile|2

ATTACTTGTGTTGACACTACTAAAAGTACCAATTTAACCATTAGCACTGCTGTTACTCCA
>HPV82_Alpha_6970427_nt6567_L1_Tile|1
TTGTGTTGACACTACTAAAAGTACCAATTTAACCATTAGCACTGCTGTTACTCCATCTGT
>HPV82_Alpha_6970427_nt6572_L1_Tile|1
TTGACACTACTAAAAGTACCAATTTAACCATTAGCACTGCTGTTACTCCATCTGTTGCAC
>HPV82_Alpha_6970427_nt6577_L1_Tile|1
ACTACTAAAAGTACCAATTTAACCATTAGCACTGCTGTTACTCCATCTGTTGCACAAACA
>HPV82_Alpha_6970427_nt6582_L1_Tile|2
TAAAAGTACCAATTTAACCATTAGCACTGCTGTTACTCCATCTGTTGCACAAACATTTAC
>HPV82_Alpha_6970427_nt6587_L1_Tile|1
GTACCAATTTAACCATTAGCACTGCTGTTACTCCATCTGTTGCACAAACATTTACTCCAG
>HPV82_Alpha_6970427_nt6592_L1_Tile|1
AATTTAACCATTAGCACTGCTGTTACTCCATCTGTTGCACAAACATTTACTCCAGCAAAC
>HPV82_Alpha_6970427_nt6597_L1_Tile|1
AACCATTAGCACTGCTGTTACTCCATCTGTTGCACAAACATTTACTCCAGCAAACTTTAA
>HPV82_Alpha_6970427_nt6607_L1_Tile|1
ACTGCTGTTACTCCATCTGTTGCACAAACATTTACTCCAGCAAACTTTAAGCAGTACATT
>HPV82_Alpha_6970427_nt6612_L1_Tile|1
TGTTACTCCATCTGTTGCACAAACATTTACTCCAGCAAACTTTAAGCAGTACATTAGGCA
>HPV82_Alpha_6970427_nt6702_L1_Tile|2
TCAATTGTGTAAAATCACTTTAACTACTGAAATTATGGCTTACCTGCACACCATGGATTC
>HPV82_Alpha_6970427_nt6707_L1_Tile|1
TGTGTAAAATCACTTTAACTACTGAAATTATGGCTTACCTGCACACCATGGATTCTACAA
>HPV82_Alpha_6970427_nt6712_L1_Tile|1
AAAATCACTTTAACTACTGAAATTATGGCTTACCTGCACACCATGGATTCTACAATTTTA
>HPV82_Alpha_6970427_nt6717_L1_Tile|1
CACTTTAACTACTGAAATTATGGCTTACCTGCACACCATGGATTCTACAATTTTAGAACA
>HPV82_Alpha_6970427_nt6722_L1_Tile|2
TAACTACTGAAATTATGGCTTACCTGCACACCATGGATTCTACAATTTTAGAACAGTGGA
>HPV82_Alpha_6970427_nt6792_L1_Tile|1
AACATTGCCCCCCTCCGCTAGTTTGGAGGATGCCTATCGATTTGTAAAAAATGCAGCAAC
>HPV82_Alpha_6970427_nt6882_L1_Tile|2
TAAAGAAGACCCTTTGGCAAAATATAAATTTTGGAATGTAGACCTTAAGGAACGCTTTTC
>HPV83_Alpha_5059324_nt5868_L1_Tile|1
TCTTACCGTAGGACATCCATATTATCCTGTACAGGTTAATGGTCAAGGAAAAAAAGCCAC
>HPV83_Alpha_5059324_nt5873_L1_Tile|1
CCGTAGGACATCCATATTATCCTGTACAGGTTAATGGTCAAGGAAAAAAAGCCACTATCC
>HPV83_Alpha_5059324_nt5878_L1_Tile|1
GGACATCCATATTATCCTGTACAGGTTAATGGTCAAGGAAAAAAAGCCACTATCCCCAAG
>HPV83_Alpha_5059324_nt6158_L1_Tile|1
CAGACACAGATAGGCGTGACAATGTCTCAGTGGATTACAAACAAACGCAGCTTATTATCA
>HPV83_Alpha_5059324_nt6163_L1_Tile|1
ACAGATAGGCGTGACAATGTCTCAGTGGATTACAAACAAACGCAGCTTATTATCATTGGG
>HPV83_Alpha_5059324_nt6168_L1_Tile|1
TAGGCGTGACAATGTCTCAGTGGATTACAAACAAACGCAGCTTATTATCATTGGGTGTAA
>HPV83_Alpha_5059324_nt6178_L1_Tile|1
AATGTCTCAGTGGATTACAAACAAACGCAGCTTATTATCATTGGGTGTAAGCCCCCAATA
>HPV83_Alpha_5059324_nt6528_L1_Tile|1
TAGGCATGGCACTATGGGCGAGGTGCTGCCTACCTCTTATTATATTCCAGGCACATCTGC
>HPV83_Alpha_5059324_nt6533_L1_Tile|1
ATGGCACTATGGGCGAGGTGCTGCCTACCTCTTATTATATTCCAGGCACATCTGCTAATA
>HPV83_Alpha_5059324_nt6538_L1_Tile|1
ACTATGGGCGAGGTGCTGCCTACCTCTTATTATATTCCAGGCACATCTGCTAATAGTAGG
>HPV83_Alpha_5059324_nt6543_L1_Tile|1
GGGCGAGGTGCTGCCTACCTCTTATTATATTCCAGGCACATCTGCTAATAGTAGGAACAC

>HPV83_Alpha_5059324_nt6548_L1_Tile|1
AGGTGCTGCCTACCTCTTATTATATTCCAGGCACATCTGCTAATAGTAGGAACACTCTTA
>HPV83_Alpha_5059324_nt6553_L1_Tile|1
CTGCCTACCTCTTATTATATTCCAGGCACATCTGCTAATAGTAGGAACACTCTTACCAGC
>HPV83_Alpha_5059324_nt6558_L1_Tile|1
TACCTCTTATTATATTCCAGGCACATCTGCTAATAGTAGGAACACTCTTACCAGCTACAT
>HPV83_Alpha_5059324_nt6563_L1_Tile|1
CTTATTATATTCCAGGCACATCTGCTAATAGTAGGAACACTCTTACCAGCTACATCTATG
>HPV83_Alpha_5059324_nt6573_L1_Tile|1
TCCAGGCACATCTGCTAATAGTAGGAACACTCTTACCAGCTACATCTATGCTCCTACTCC
>HPV83_Alpha_5059324_nt6583_L1_Tile|1
TCTGCTAATAGTAGGAACACTCTTACCAGCTACATCTATGCTCCTACTCCTAGTGGCTCC
>HPV83_Alpha_5059324_nt6593_L1_Tile|1
GTAGGAACACTCTTACCAGCTACATCTATGCTCCTACTCCTAGTGGCTCCCTGGTATCGT
>HPV83_Alpha_5059324_nt6598_L1_Tile|1
AACACTCTTACCAGCTACATCTATGCTCCTACTCCTAGTGGCTCCCTGGTATCGTCCGAT
>HPV83_Alpha_5059324_nt6838_L1_Tile|1
CGCCACACAGAGGAATATGACTTACAGGTTATATTGCAACTTTGCAAAATACATCTGACC
>HPV83_Alpha_5059324_nt6843_L1_Tile|1
CACAGAGGAATATGACTTACAGGTTATATTGCAACTTTGCAAAATACATCTGACCCCTGA
>HPV83_Alpha_5059324_nt6858_L1_Tile|1
CTTACAGGTTATATTGCAACTTTGCAAAATACATCTGACCCCTGAAATTATGGCATACCT
>HPV83_Alpha_5059324_nt7108_L1_Tile|1
AAACTATCCACAGATTTAGATCAGTTTCCTTTGGGCCGTAAGTTTCTTTTGCAACTGGGT
>HPV84_Alpha_12958167_nt6196_L1_Tile|1
ATGTACTAATGTGCAATATCGTGCGGGTGATTGCCCCCCTTTGCAGTTCACTAATTCTAC
>HPV84_Alpha_12958167_nt6201_L1_Tile|1
CTAATGTGCAATATCGTGCGGGTGATTGCCCCCCTTTGCAGTTCACTAATTCTACCATTG
>HPV84_Alpha_12958167_nt6466_L1_Tile|1
GGGTACTATGGGTGAGGAGGTGCCTCAGTCTTTTTACCTTAAGGGGACCTCCTCCCGGGC
>HPV84_Alpha_12958167_nt6471_L1_Tile|1
CTATGGGTGAGGAGGTGCCTCAGTCTTTTTACCTTAAGGGGACCTCCTCCCGGGCAACCC
>HPV84_Alpha_12958167_nt6481_L1_Tile|1
GGAGGTGCCTCAGTCTTTTTACCTTAAGGGGACCTCCTCCCGGGCAACCCTTAGCAGTTC
>HPV84_Alpha_12958167_nt6486_L1_Tile|1
TGCCTCAGTCTTTTTACCTTAAGGGGACCTCCTCCCGGGCAACCCTTAGCAGTTCTGTGT
>HPV84_Alpha_12958167_nt6691_L1_Tile|1
CACCAATTTTACTATTAGTGCTGCTACCAACACCGAATCAGAATATAAACCTACCAATTT
>HPV84_Alpha_12958167_nt6696_L1_Tile|1
ATTTTACTATTAGTGCTGCTACCAACACCGAATCAGAATATAAACCTACCAATTTTAAGG
>HPV84_Alpha_12958167_nt6701_L1_Tile|1
ACTATTAGTGCTGCTACCAACACCGAATCAGAATATAAACCTACCAATTTTAAGGAATAC
>HPV84_Alpha_12958167_nt7076_L1_Tile|1
TTTTTATTGCAGTCTGCCCCACGTTCCACCCTGGTGTCCCGTAAACGTACAGCGTCTGCC
>HPV84_Alpha_12958167_nt7111_L1_Tile|1
GTCCCGTAAACGTACAGCGTCTGCCTCTACCCCCCCTGCCTCCAAACGGCGTAAGGCCAA
>HPV84_Alpha_12958167_nt7116_L1_Tile|1
GTAAACGTACAGCGTCTGCCTCTACCCCCCCTGCCTCCAAACGGCGTAAGGCCAAAAAGT
>HPV84_Alpha_12958167_nt7118_L1_Tile|1
AAACGTACAGCGTCTGCCTCTACCCCCCCTGCCTCCAAACGGCGTAAGGCCAAAAAGTAA
>HPVcand85_Alpha_4574720_nt5721_L1_Tile|1
TGGCAGCTCTAGGCTTCTAACCGTTGGACATCCATACTATAAAGTTACCTCAAATGGAGG
>HPVcand85_Alpha_4574720_nt5736_L1_Tile|1
TCTAACCGTTGGACATCCATACTATAAAGTTACCTCAAATGGAGGCCGCAAGCAAGACAT
>HPVcand85_Alpha_4574720_nt5966_L1_Tile|1

GTCATCCCTTTTACAACAAATTGGATGACACAGAAAATTCCCATGTTGCTACTTCTGTAG
>HPVcand85_Alpha_4574720_nt5971_L1_Tile|1
CCCTTTTACAACAAATTGGATGACACAGAAAATTCCCATGTTGCTACTTCTGTAGTTACA
>HPVcand85_Alpha_4574720_nt5976_L1_Tile|1
TTACAACAAATTGGATGACACAGAAAATTCCCATGTTGCTACTTCTGTAGTTACACACGA
>HPVcand85_Alpha_4574720_nt5981_L1_Tile|1
ACAAATTGGATGACACAGAAAATTCCCATGTTGCTACTTCTGTAGTTACACACGACACTA
>HPVcand85_Alpha_4574720_nt5986_L1_Tile|1
TTGGATGACACAGAAAATTCCCATGTTGCTACTTCTGTAGTTACACACGACACTAGAGAT
>HPVcand85_Alpha_4574720_nt6001_L1_Tile|1
AATTCCCATGTTGCTACTTCTGTAGTTACACACGACACTAGAGATAATGTGTCAGTGGAT
>HPVcand85_Alpha_4574720_nt6131_L1_Tile|1
CCTGTAAGCCCGGTGCTGTGCAAACAGGTGACTGTCCTCCATTAGAACTAGTAAATACAC
>HPVcand85_Alpha_4574720_nt6136_L1_Tile|1
AAGCCCGGTGCTGTGCAAACAGGTGACTGTCCTCCATTAGAACTAGTAAATACACCTATT
>HPVcand85_Alpha_4574720_nt6376_L1_Tile|1
TTTGCACGCCATTTTTGGAACAGGGGAGGCACTATAGGAGATGCAGTACCGGAAACATTA
>HPVcand85_Alpha_4574720_nt6381_L1_Tile|1
ACGCCATTTTTGGAACAGGGGAGGCACTATAGGAGATGCAGTACCGGAAACATTATATAT
>HPVcand85_Alpha_4574720_nt6386_L1_Tile|1
ATTTTTGGAACAGGGGAGGCACTATAGGAGATGCAGTACCGGAAACATTATATATAAAAG
>HPVcand85_Alpha_4574720_nt6391_L1_Tile|1
TGGAACAGGGGAGGCACTATAGGAGATGCAGTACCGGAAACATTATATATAAAAGGTACT
>HPVcand85_Alpha_4574720_nt6396_L1_Tile|1
CAGGGGAGGCACTATAGGAGATGCAGTACCGGAAACATTATATATAAAAGGTACTAATGA
>HPVcand85_Alpha_4574720_nt6401_L1_Tile|1
GAGGCACTATAGGAGATGCAGTACCGGAAACATTATATATAAAAGGTACTAATGATAGGG
>HPVcand85_Alpha_4574720_nt6406_L1_Tile|1
ACTATAGGAGATGCAGTACCGGAAACATTATATATAAAAGGTACTAATGATAGGGCAACA
>HPVcand85_Alpha_4574720_nt6411_L1_Tile|1
AGGAGATGCAGTACCGGAAACATTATATATAAAAGGTACTAATGATAGGGCAACACCTGG
>HPVcand85_Alpha_4574720_nt6416_L1_Tile|1
ATGCAGTACCGGAAACATTATATATAAAAGGTACTAATGATAGGGCAACACCTGGAAGCT
>HPVcand85_Alpha_4574720_nt6421_L1_Tile|1
GTACCGGAAACATTATATATAAAAGGTACTAATGATAGGGCAACACCTGGAAGCTGTATT
>HPVcand85_Alpha_4574720_nt6426_L1_Tile|1
GGAAACATTATATATAAAAGGTACTAATGATAGGGCAACACCTGGAAGCTGTATTTATTC
>HPVcand85_Alpha_4574720_nt6431_L1_Tile|1
CATTATATATAAAAGGTACTAATGATAGGGCAACACCTGGAAGCTGTATTTATTCTCCAT
>HPVcand85_Alpha_4574720_nt6436_L1_Tile|1
TATATAAAAGGTACTAATGATAGGGCAACACCTGGAAGCTGTATTTATTCTCCATCACCT
>HPVcand85_Alpha_4574720_nt6441_L1_Tile|1
AAAAGGTACTAATGATAGGGCAACACCTGGAAGCTGTATTTATTCTCCATCACCTAGTGG
>HPVcand85_Alpha_4574720_nt6446_L1_Tile|1
GTACTAATGATAGGGCAACACCTGGAAGCTGTATTTATTCTCCATCACCTAGTGGGTCTA
>HPVcand85_Alpha_4574720_nt6586_L1_Tile|1
CACAACCAATTATTTATAACTGTGGTAGACACAACACGTAGTACCAATCTTACCTTATCT
>HPVcand85_Alpha_4574720_nt6606_L1_Tile|1
TGTGGTAGACACAACACGTAGTACCAATCTTACCTTATCTACTGCAACTACTAATCCAGT
>HPVcand85_Alpha_4574720_nt6611_L1_Tile|1
TAGACACAACACGTAGTACCAATCTTACCTTATCTACTGCAACTACTAATCCAGTTCCAT
>HPVcand85_Alpha_4574720_nt6616_L1_Tile|1
ACAACACGTAGTACCAATCTTACCTTATCTACTGCAACTACTAATCCAGTTCCATCTATA
>HPVcand85_Alpha_4574720_nt6621_L1_Tile|1
ACGTAGTACCAATCTTACCTTATCTACTGCAACTACTAATCCAGTTCCATCTATATATGA

>HPVcand85_Alpha_4574720_nt6661_L1_Tile|1
CCAGTTCCATCTATATATGAACCTTCTAAATTTAAGGAATACACACGCCATGTAGAGGAA
>HPVcand85_Alpha_4574720_nt6741_L1_Tile|1
TCAATTGTGTAAAATTACACTTACTACTGATGTTATGTCTTATATACATAACATGGATCC
>HPVcand85_Alpha_4574720_nt6746_L1_Tile|1
TGTGTAAAATTACACTTACTACTGATGTTATGTCTTATATACATAACATGGATCCTACTA
>HPVcand85_Alpha_4574720_nt6751_L1_Tile|1
AAAATTACACTTACTACTGATGTTATGTCTTATATACATAACATGGATCCTACTATTTTA
>HPVcand85_Alpha_4574720_nt6756_L1_Tile|1
TACACTTACTACTGATGTTATGTCTTATATACATAACATGGATCCTACTATTTTAGATAG
>HPVcand85_Alpha_4574720_nt6761_L1_Tile|1
TTACTACTGATGTTATGTCTTATATACATAACATGGATCCTACTATTTTAGATAGTTGGA
>HPVcand85_Alpha_4574720_nt6826_L1_Tile|1
GGTGTTAGTCCTCCCCCATCTGCTAGCTTAGTAGATACATATAGGTTTTTACAGTCATCT
>HPVcand85_Alpha_4574720_nt6886_L1_Tile|1
GCCATTACATGTCAGAAGGATGTGGTTGTTCCACAAAAAAAGGATCCATATGAAAAATTA
>HPVcand85_Alpha_4574720_nt6931_L1_Tile|1
CCATATGAAAAATTAAAGTTTTGGAATGTGGATCTTAAAGAACATTTTTCATCTGATTTA
>HPVcand85_Alpha_4574720_nt7011_L1_Tile|1
TAAGTTTTTATTACAGGCTGGGTTACGACCTAAACCCACCATAGGCCCTAGGAAACGTGT
>HPVcand85_Alpha_4574720_nt7026_L1_Tile|1
GGCTGGGTTACGACCTAAACCCACCATAGGCCCTAGGAAACGTGTTGCTTCTACTTCTAC
>HPVcand85_Alpha_4574720_nt7031_L1_Tile|1
GGTTACGACCTAAACCCACCATAGGCCCTAGGAAACGTGTTGCTTCTACTTCTACTGCTA
>HPVcand85_Alpha_4574720_nt7036_L1_Tile|1
CGACCTAAACCCACCATAGGCCCTAGGAAACGTGTTGCTTCTACTTCTACTGCTACTAGG
>HPVcand85_Alpha_4574720_nt7041_L1_Tile|1
TAAACCCACCATAGGCCCTAGGAAACGTGTTGCTTCTACTTCTACTGCTACTAGGCCCTC
>HPVcand85_Alpha_4574720_nt7051_L1_Tile|1
ATAGGCCCTAGGAAACGTGTTGCTTCTACTTCTACTGCTACTAGGCCCTCCAAACGGAAA
>HPVcand85_Alpha_4574720_nt7056_L1_Tile|1
CCCTAGGAAACGTGTTGCTTCTACTTCTACTGCTACTAGGCCCTCCAAACGGAAACGTAC
>HPVcand85_Alpha_4574720_nt7061_L1_Tile|1
GGAAACGTGTTGCTTCTACTTCTACTGCTACTAGGCCCTCCAAACGGAAACGTACTGCTA
>HPVcand86_Alpha_15741127_nt6186_L1_Tile|1
AGTGTGTTCTAATGTGCAGCCTAAACCAGGGGACTGCCCCCCATTACAGTTTACCAATTC
>HPVcand86_Alpha_15741127_nt6486_L1_Tile|1
AACTTATTATTTAAAGGGCTCCTCAGGCCCCCGAGGATCCCTTAGCAGTTATGTATATGC
>HPVcand86_Alpha_15741127_nt6491_L1_Tile|1
ATTATTTAAAGGGCTCCTCAGGCCCCCGAGGATCCCTTAGCAGTTATGTATATGCACCCA
>HPVcand86_Alpha_15741127_nt6701_L1_Tile|1
TTAGTGCCGCTACCCAGAAGGCCTCTGAATATGACCCCTCTAAGTTTAATGAATATCTAA
>HPVcand87_Alpha_14475578_nt5878_L1_Tile|1
CCTGCTTACTGTGGGTCACCCTTACTATCCAGTTACTGTTACCACCCCTGGTCAGAACAA
>HPVcand87_Alpha_14475578_nt5883_L1_Tile|1
TTACTGTGGGTCACCCTTACTATCCAGTTACTGTTACCACCCCTGGTCAGAACAAGAAAT
>HPVcand87_Alpha_14475578_nt5888_L1_Tile|1
GTGGGTCACCCTTACTATCCAGTTACTGTTACCACCCCTGGTCAGAACAAGAAATCCAAT
>HPVcand87_Alpha_14475578_nt5893_L1_Tile|1
TCACCCTTACTATCCAGTTACTGTTACCACCCCTGGTCAGAACAAGAAATCCAATATTCC
>HPVcand87_Alpha_14475578_nt5898_L1_Tile|1
CTTACTATCCAGTTACTGTTACCACCCCTGGTCAGAACAAGAAATCCAATATTCCAAAGG
>HPVcand87_Alpha_14475578_nt5903_L1_Tile|1
TATCCAGTTACTGTTACCACCCCTGGTCAGAACAAGAAATCCAATATTCCAAAGGTGTCT
>HPVcand87_Alpha_14475578_nt5908_L1_Tile|1

AGTTACTGTTACCACCCCTGGTCAGAACAAGAAATCCAATATTCCAAAGGTGTCTGGCTA
>HPVcand87_Alpha_14475578_nt6243_L1_Tile|1
GTAAGCCCTCTATAGGGGAGCATTGGAGTAAGGGTACTGTTTGTGCCAATGTACAGCCCA
>HPVcand87_Alpha_14475578_nt6248_L1_Tile|1
CCCTCTATAGGGGAGCATTGGAGTAAGGGTACTGTTTGTGCCAATGTACAGCCCAAGACA
>HPVcand87_Alpha_14475578_nt6253_L1_Tile|1
TATAGGGGAGCATTGGAGTAAGGGTACTGTTTGTGCCAATGTACAGCCCAAGACAGGGGA
>HPVcand87_Alpha_14475578_nt6268_L1_Tile|1
GAGTAAGGGTACTGTTTGTGCCAATGTACAGCCCAAGACAGGGGATTGTCCACCATTGCA
>HPVcand87_Alpha_14475578_nt6283_L1_Tile|1
TTGTGCCAATGTACAGCCCAAGACAGGGGATTGTCCACCATTGCAATTTACTAATTCTAC
>HPVcand87_Alpha_14475578_nt6288_L1_Tile|1
CCAATGTACAGCCCAAGACAGGGGATTGTCCACCATTGCAATTTACTAATTCTACTATTC
>HPVcand87_Alpha_14475578_nt6558_L1_Tile|1
CTATGGGCGAGGAGGTCCCTGACACCTATTTTTTGAAGGGGGCGTCGTCTGGCCCTAGGG
>HPVcand87_Alpha_14475578_nt6568_L1_Tile|1
GGAGGTCCCTGACACCTATTTTTTGAAGGGGGCGTCGTCTGGCCCTAGGGCCTCTTTAAG
>HPVcand87_Alpha_14475578_nt6583_L1_Tile|1
CTATTTTTTGAAGGGGGCGTCGTCTGGCCCTAGGGCCTCTTTAAGCAGTTATGTGTATGC
>HPVcand87_Alpha_14475578_nt6798_L1_Tile|1
TTAGTGCTGCCACTCAAACAACCACTGAATATGACCCCACAAAGTTTAAGGAATATTTAA
>HPV88_Gamma_167600365_nt5321_L1_Tile|1
GGCAACAACAAACTGGGAAGCTGTTCCTTCCGCCGGCTAAACCTGTGGCAAAAATATTAT
>HPV88_Gamma_167600365_nt5326_L1_Tile|1
CAACAAACTGGGAAGCTGTTCCTTCCGCCGGCTAAACCTGTGGCAAAAATATTATCTACA
>HPV88_Gamma_167600365_nt5336_L1_Tile|1
GGAAGCTGTTCCTTCCGCCGGCTAAACCTGTGGCAAAAATATTATCTACAGATGATTATG
>HPV88_Gamma_167600365_nt5341_L1_Tile|1
CTGTTCCTTCCGCCGGCTAAACCTGTGGCAAAAATATTATCTACAGATGATTATGTTATT
>HPV88_Gamma_167600365_nt5356_L1_Tile|1
GCTAAACCTGTGGCAAAAATATTATCTACAGATGATTATGTTATTGGAACTCAGTTACAT
>HPV88_Gamma_167600365_nt5361_L1_Tile|1
ACCTGTGGCAAAAATATTATCTACAGATGATTATGTTATTGGAACTCAGTTACATTTTCA
>HPV88_Gamma_167600365_nt5366_L1_Tile|1
TGGCAAAAATATTATCTACAGATGATTATGTTATTGGAACTCAGTTACATTTTCATGCTG
>HPV88_Gamma_167600365_nt5376_L1_Tile|1
ATTATCTACAGATGATTATGTTATTGGAACTCAGTTACATTTTCATGCTGGCACTGATCG
>HPV88_Gamma_167600365_nt5381_L1_Tile|1
CTACAGATGATTATGTTATTGGAACTCAGTTACATTTTCATGCTGGCACTGATCGTCTGT
>HPV88_Gamma_167600365_nt5386_L1_Tile|1
GATGATTATGTTATTGGAACTCAGTTACATTTTCATGCTGGCACTGATCGTCTGTTAACA
>HPV88_Gamma_167600365_nt5391_L1_Tile|1
TTATGTTATTGGAACTCAGTTACATTTTCATGCTGGCACTGATCGTCTGTTAACAGTAGA
>HPV88_Gamma_167600365_nt5396_L1_Tile|1
TTATTGGAACTCAGTTACATTTTCATGCTGGCACTGATCGTCTGTTAACAGTAGACCATC
>HPV88_Gamma_167600365_nt5411_L1_Tile|1
TACATTTTCATGCTGGCACTGATCGTCTGTTAACAGTAGACCATCCCTACTATGAAATTA
>HPV88_Gamma_167600365_nt5426_L1_Tile|1
GCACTGATCGTCTGTTAACAGTAGACCATCCCTACTATGAAATTAGGAATGATGGAGATC
>HPV88_Gamma_167600365_nt5436_L1_Tile|1
TCTGTTAACAGTAGACCATCCCTACTATGAAATTAGGAATGATGGAGATCCCAACAAAAT
>HPV88_Gamma_167600365_nt5441_L1_Tile|1
TAACAGTAGACCATCCCTACTATGAAATTAGGAATGATGGAGATCCCAACAAAATTGAGA
>HPV88_Gamma_167600365_nt5446_L1_Tile|1
GTAGACCATCCCTACTATGAAATTAGGAATGATGGAGATCCCAACAAAATTGAGATTCCT

>HPV88_Gamma_167600365_nt5451_L1_Tile|1
CCATCCCTACTATGAAATTAGGAATGATGGAGATCCCAACAAAATTGAGATTCCTAAGGT
>HPV88_Gamma_167600365_nt5456_L1_Tile|1
CCTACTATGAAATTAGGAATGATGGAGATCCCAACAAAATTGAGATTCCTAAGGTGTCTG
>HPV88_Gamma_167600365_nt5461_L1_Tile|1
TATGAAATTAGGAATGATGGAGATCCCAACAAAATTGAGATTCCTAAGGTGTCTGCATGC
>HPV88_Gamma_167600365_nt5516_L1_Tile|1
CATGCCAATATCGAGTGTTTAGGCTGAAATTGCCTGATCCAAATAAATTTGCCTTAATAG
>HPV88_Gamma_167600365_nt5521_L1_Tile|1
CAATATCGAGTGTTTAGGCTGAAATTGCCTGATCCAAATAAATTTGCCTTAATAGATCAG
>HPV88_Gamma_167600365_nt5526_L1_Tile|1
TCGAGTGTTTAGGCTGAAATTGCCTGATCCAAATAAATTTGCCTTAATAGATCAGTCAAT
>HPV88_Gamma_167600365_nt5531_L1_Tile|1
TGTTTAGGCTGAAATTGCCTGATCCAAATAAATTTGCCTTAATAGATCAGTCAATTTTCA
>HPV88_Gamma_167600365_nt5536_L1_Tile|1
AGGCTGAAATTGCCTGATCCAAATAAATTTGCCTTAATAGATCAGTCAATTTTCAATCCT
>HPV88_Gamma_167600365_nt5541_L1_Tile|1
GAAATTGCCTGATCCAAATAAATTTGCCTTAATAGATCAGTCAATTTTCAATCCTGAACG
>HPV88_Gamma_167600365_nt5546_L1_Tile|1
TGCCTGATCCAAATAAATTTGCCTTAATAGATCAGTCAATTTTCAATCCTGAACGTGAAC
>HPV88_Gamma_167600365_nt5551_L1_Tile|1
GATCCAAATAAATTTGCCTTAATAGATCAGTCAATTTTCAATCCTGAACGTGAACGTTTA
>HPV88_Gamma_167600365_nt5556_L1_Tile|1
AAATAAATTTGCCTTAATAGATCAGTCAATTTTCAATCCTGAACGTGAACGTTTAGTTTG
>HPV88_Gamma_167600365_nt5566_L1_Tile|1
GCCTTAATAGATCAGTCAATTTTCAATCCTGAACGTGAACGTTTAGTTTGGAAATTAAAA
>HPV88_Gamma_167600365_nt5571_L1_Tile|1
AATAGATCAGTCAATTTTCAATCCTGAACGTGAACGTTTAGTTTGGAAATTAAAAGGTAT
>HPV88_Gamma_167600365_nt5576_L1_Tile|1
ATCAGTCAATTTTCAATCCTGAACGTGAACGTTTAGTTTGGAAATTAAAAGGTATTCAAA
>HPV88_Gamma_167600365_nt5581_L1_Tile|1
TCAATTTTCAATCCTGAACGTGAACGTTTAGTTTGGAAATTAAAAGGTATTCAAATTGGT
>HPV88_Gamma_167600365_nt5586_L1_Tile|1
TTTCAATCCTGAACGTGAACGTTTAGTTTGGAAATTAAAAGGTATTCAAATTGGTAGAGG
>HPV88_Gamma_167600365_nt5591_L1_Tile|1
ATCCTGAACGTGAACGTTTAGTTTGGAAATTAAAAGGTATTCAAATTGGTAGAGGTGGAC
>HPV88_Gamma_167600365_nt5596_L1_Tile|1
GAACGTGAACGTTTAGTTTGGAAATTAAAAGGTATTCAAATTGGTAGAGGTGGACCACTA
>HPV88_Gamma_167600365_nt5601_L1_Tile|1
TGAACGTTTAGTTTGGAAATTAAAAGGTATTCAAATTGGTAGAGGTGGACCACTAAATGT
>HPV88_Gamma_167600365_nt5616_L1_Tile|1
GAAATTAAAAGGTATTCAAATTGGTAGAGGTGGACCACTAAATGTAGGTACTGCAGGTCA
>HPV88_Gamma_167600365_nt5621_L1_Tile|1
TAAAAGGTATTCAAATTGGTAGAGGTGGACCACTAAATGTAGGTACTGCAGGTCATCCTT
>HPV88_Gamma_167600365_nt5626_L1_Tile|1
GGTATTCAAATTGGTAGAGGTGGACCACTAAATGTAGGTACTGCAGGTCATCCTTTATTT
>HPV88_Gamma_167600365_nt5631_L1_Tile|1
TCAAATTGGTAGAGGTGGACCACTAAATGTAGGTACTGCAGGTCATCCTTTATTTAACAA
>HPV88_Gamma_167600365_nt5636_L1_Tile|1
TTGGTAGAGGTGGACCACTAAATGTAGGTACTGCAGGTCATCCTTTATTTAACAAAGCTC
>HPV88_Gamma_167600365_nt5666_L1_Tile|1
CTGCAGGTCATCCTTTATTTAACAAAGCTCCAGATACTGAAAATCCTAATGATTATCCAC
>HPV88_Gamma_167600365_nt5676_L1_Tile|1
TCCTTTATTTAACAAAGCTCCAGATACTGAAAATCCTAATGATTATCCACCAGCTCAAAC
>HPV88_Gamma_167600365_nt5681_L1_Tile|1

TATTTAACAAAGCTCCAGATACTGAAAATCCTAATGATTATCCACCAGCTCAAACAGATG
>HPV88_Gamma_167600365_nt5731_L1_Tile|1
CAAACAGATGAAGACAGACGTAACGTGTCTATGGACCCCAAACATACCCAATTATTTATA
>HPV88_Gamma_167600365_nt5736_L1_Tile|1
AGATGAAGACAGACGTAACGTGTCTATGGACCCCAAACATACCCAATTATTTATAATTGG
>HPV88_Gamma_167600365_nt5741_L1_Tile|1
AAGACAGACGTAACGTGTCTATGGACCCCAAACATACCCAATTATTTATAATTGGTTGCA
>HPV88_Gamma_167600365_nt5746_L1_Tile|1
AGACGTAACGTGTCTATGGACCCCAAACATACCCAATTATTTATAATTGGTTGCATTCCT
>HPV88_Gamma_167600365_nt5751_L1_Tile|1
TAACGTGTCTATGGACCCCAAACATACCCAATTATTTATAATTGGTTGCATTCCTGCAGA
>HPV88_Gamma_167600365_nt5756_L1_Tile|1
TGTCTATGGACCCCAAACATACCCAATTATTTATAATTGGTTGCATTCCTGCAGATGGTG
>HPV88_Gamma_167600365_nt5761_L1_Tile|1
ATGGACCCCAAACATACCCAATTATTTATAATTGGTTGCATTCCTGCAGATGGTGAATAT
>HPV88_Gamma_167600365_nt5806_L1_Tile|1
GCAGATGGTGAATATTGGGATGTTGCTAAACCATGTGTTGGAGATCAGGCGAATGCAGGA
>HPV88_Gamma_167600365_nt5811_L1_Tile|1
TGGTGAATATTGGGATGTTGCTAAACCATGTGTTGGAGATCAGGCGAATGCAGGAGACTG
>HPV88_Gamma_167600365_nt5816_L1_Tile|1
AATATTGGGATGTTGCTAAACCATGTGTTGGAGATCAGGCGAATGCAGGAGACTGTCCAC
>HPV88_Gamma_167600365_nt5826_L1_Tile|1
TGTTGCTAAACCATGTGTTGGAGATCAGGCGAATGCAGGAGACTGTCCACCTATACAGTT
>HPV88_Gamma_167600365_nt5831_L1_Tile|1
CTAAACCATGTGTTGGAGATCAGGCGAATGCAGGAGACTGTCCACCTATACAGTTAGTTA
>HPV88_Gamma_167600365_nt5836_L1_Tile|1
CCATGTGTTGGAGATCAGGCGAATGCAGGAGACTGTCCACCTATACAGTTAGTTAATGCT
>HPV88_Gamma_167600365_nt5841_L1_Tile|1
TGTTGGAGATCAGGCGAATGCAGGAGACTGTCCACCTATACAGTTAGTTAATGCTGTTAT
>HPV88_Gamma_167600365_nt5846_L1_Tile|1
GAGATCAGGCGAATGCAGGAGACTGTCCACCTATACAGTTAGTTAATGCTGTTATTCAGG
>HPV88_Gamma_167600365_nt5851_L1_Tile|1
CAGGCGAATGCAGGAGACTGTCCACCTATACAGTTAGTTAATGCTGTTATTCAGGACGGA
>HPV88_Gamma_167600365_nt5856_L1_Tile|1
GAATGCAGGAGACTGTCCACCTATACAGTTAGTTAATGCTGTTATTCAGGACGGAACAAT
>HPV88_Gamma_167600365_nt5861_L1_Tile|1
CAGGAGACTGTCCACCTATACAGTTAGTTAATGCTGTTATTCAGGACGGAACAATGGGTG
>HPV88_Gamma_167600365_nt5866_L1_Tile|1
GACTGTCCACCTATACAGTTAGTTAATGCTGTTATTCAGGACGGAACAATGGGTGACACA
>HPV88_Gamma_167600365_nt5901_L1_Tile|1
TCAGGACGGAACAATGGGTGACACAGGTTTTGGGGCAGCAAATTTTAAGAACTTTTTGCA
>HPV88_Gamma_167600365_nt5926_L1_Tile|1
GGTTTTGGGGCAGCAAATTTTAAGAACTTTTTGCAAGACAAAGCAGGTGTTCCTCTGGAT
>HPV88_Gamma_167600365_nt5941_L1_Tile|1
AATTTTAAGAACTTTTTGCAAGACAAAGCAGGTGTTCCTCTGGATATTATTAATGATATA
>HPV88_Gamma_167600365_nt5986_L1_Tile|1
ATTATTAATGATATATGTATGTATCCAGACTTCCTTAAAATGGACAAAGATGTTTATGGC
>HPV88_Gamma_167600365_nt5991_L1_Tile|1
TAATGATATATGTATGTATCCAGACTTCCTTAAAATGGACAAAGATGTTTATGGCGATAA
>HPV88_Gamma_167600365_nt6011_L1_Tile|1
CAGACTTCCTTAAAATGGACAAAGATGTTTATGGCGATAAATGTTTCTTTTTTGGAAAAC
>HPV88_Gamma_167600365_nt6016_L1_Tile|1
TTCCTTAAAATGGACAAAGATGTTTATGGCGATAAATGTTTCTTTTTTGGAAAACGTGAG
>HPV88_Gamma_167600365_nt6031_L1_Tile|1
AAAGATGTTTATGGCGATAAATGTTTCTTTTTTGGAAAACGTGAGCAGTCGTATGCTCGC

>HPV88_Gamma_167600365_nt6041_L1_Tile|1
ATGGCGATAAATGTTTCTTTTTTGGAAAACGTGAGCAGTCGTATGCTCGCCATTTTATGG
>HPV88_Gamma_167600365_nt6051_L1_Tile|1
ATGTTTCTTTTTTGGAAAACGTGAGCAGTCGTATGCTCGCCATTTTATGGCTCGTGAAGG
>HPV88_Gamma_167600365_nt6056_L1_Tile|1
TCTTTTTTGGAAAACGTGAGCAGTCGTATGCTCGCCATTTTATGGCTCGTGAAGGTAAAA
>HPV88_Gamma_167600365_nt6066_L1_Tile|1
AAAACGTGAGCAGTCGTATGCTCGCCATTTTATGGCTCGTGAAGGTAAAATGGGAGATGC
>HPV88_Gamma_167600365_nt6071_L1_Tile|1
GTGAGCAGTCGTATGCTCGCCATTTTATGGCTCGTGAAGGTAAAATGGGAGATGCCATAC
>HPV88_Gamma_167600365_nt6076_L1_Tile|1
CAGTCGTATGCTCGCCATTTTATGGCTCGTGAAGGTAAAATGGGAGATGCCATACCAGAG
>HPV88_Gamma_167600365_nt6081_L1_Tile|1
GTATGCTCGCCATTTTATGGCTCGTGAAGGTAAAATGGGAGATGCCATACCAGAGGGAAA
>HPV88_Gamma_167600365_nt6086_L1_Tile|1
CTCGCCATTTTATGGCTCGTGAAGGTAAAATGGGAGATGCCATACCAGAGGGAAATAAAT
>HPV88_Gamma_167600365_nt6091_L1_Tile|1
CATTTTATGGCTCGTGAAGGTAAAATGGGAGATGCCATACCAGAGGGAAATAAATACTTA
>HPV88_Gamma_167600365_nt6096_L1_Tile|1
TATGGCTCGTGAAGGTAAAATGGGAGATGCCATACCAGAGGGAAATAAATACTTATTGCC
>HPV88_Gamma_167600365_nt6106_L1_Tile|1
GAAGGTAAAATGGGAGATGCCATACCAGAGGGAAATAAATACTTATTGCCACCTACAAAT
>HPV88_Gamma_167600365_nt6116_L1_Tile|1
TGGGAGATGCCATACCAGAGGGAAATAAATACTTATTGCCACCTACAAATGCTGTGAATT
>HPV88_Gamma_167600365_nt6121_L1_Tile|1
GATGCCATACCAGAGGGAAATAAATACTTATTGCCACCTACAAATGCTGTGAATTTTAAA
>HPV88_Gamma_167600365_nt6126_L1_Tile|1
CATACCAGAGGGAAATAAATACTTATTGCCACCTACAAATGCTGTGAATTTTAAAATGGG
>HPV88_Gamma_167600365_nt6131_L1_Tile|1
CAGAGGGAAATAAATACTTATTGCCACCTACAAATGCTGTGAATTTTAAAATGGGATCTC
>HPV88_Gamma_167600365_nt6136_L1_Tile|1
GGAAATAAATACTTATTGCCACCTACAAATGCTGTGAATTTTAAAATGGGATCTCATGTT
>HPV88_Gamma_167600365_nt6141_L1_Tile|1
TAAATACTTATTGCCACCTACAAATGCTGTGAATTTTAAAATGGGATCTCATGTTTATTT
>HPV88_Gamma_167600365_nt6161_L1_Tile|1
CAAATGCTGTGAATTTTAAAATGGGATCTCATGTTTATTTCCCTACTGCTAGTGGATCAC
>HPV88_Gamma_167600365_nt6166_L1_Tile|1
GCTGTGAATTTTAAAATGGGATCTCATGTTTATTTCCCTACTGCTAGTGGATCACTTACT
>HPV88_Gamma_167600365_nt6171_L1_Tile|1
GAATTTTAAAATGGGATCTCATGTTTATTTCCCTACTGCTAGTGGATCACTTACTACTAG
>HPV88_Gamma_167600365_nt6176_L1_Tile|1
TTAAAATGGGATCTCATGTTTATTTCCCTACTGCTAGTGGATCACTTACTACTAGTGATT
>HPV88_Gamma_167600365_nt6181_L1_Tile|1
ATGGGATCTCATGTTTATTTCCCTACTGCTAGTGGATCACTTACTACTAGTGATTCTAAC
>HPV88_Gamma_167600365_nt6186_L1_Tile|1
ATCTCATGTTTATTTCCCTACTGCTAGTGGATCACTTACTACTAGTGATTCTAACTTATT
>HPV88_Gamma_167600365_nt6196_L1_Tile|1
TATTTCCCTACTGCTAGTGGATCACTTACTACTAGTGATTCTAACTTATTTAACAGACCT
>HPV88_Gamma_167600365_nt6201_L1_Tile|1
CCCTACTGCTAGTGGATCACTTACTACTAGTGATTCTAACTTATTTAACAGACCTTATTG
>HPV88_Gamma_167600365_nt6211_L1_Tile|1
AGTGGATCACTTACTACTAGTGATTCTAACTTATTTAACAGACCTTATTGGCTACAGAGA
>HPV88_Gamma_167600365_nt6221_L1_Tile|1
TTACTACTAGTGATTCTAACTTATTTAACAGACCTTATTGGCTACAGAGATCTTTAGGTG
>HPV88_Gamma_167600365_nt6316_L1_Tile|1

GTTACTGTTCTTGATAACAGCAGGAATACTAATTTTACTCTTTCCATTTATAATCAGGGA
>HPV88_Gamma_167600365_nt6321_L1_Tile|1
TGTTCTTGATAACAGCAGGAATACTAATTTTACTCTTTCCATTTATAATCAGGGAGGAGC
>HPV88_Gamma_167600365_nt6326_L1_Tile|1
TTGATAACAGCAGGAATACTAATTTTACTCTTTCCATTTATAATCAGGGAGGAGCTATTG
>HPV88_Gamma_167600365_nt6336_L1_Tile|1
CAGGAATACTAATTTTACTCTTTCCATTTATAATCAGGGAGGAGCTATTGGAAATCAGTA
>HPV88_Gamma_167600365_nt6341_L1_Tile|1
ATACTAATTTTACTCTTTCCATTTATAATCAGGGAGGAGCTATTGGAAATCAGTATACTT
>HPV88_Gamma_167600365_nt6346_L1_Tile|1
AATTTTACTCTTTCCATTTATAATCAGGGAGGAGCTATTGGAAATCAGTATACTTATAAA
>HPV88_Gamma_167600365_nt6351_L1_Tile|1
TACTCTTTCCATTTATAATCAGGGAGGAGCTATTGGAAATCAGTATACTTATAAATCTCA
>HPV88_Gamma_167600365_nt6356_L1_Tile|1
TTTCCATTTATAATCAGGGAGGAGCTATTGGAAATCAGTATACTTATAAATCTCAAGATT
>HPV88_Gamma_167600365_nt6361_L1_Tile|1
ATTTATAATCAGGGAGGAGCTATTGGAAATCAGTATACTTATAAATCTCAAGATTTTACT
>HPV88_Gamma_167600365_nt6366_L1_Tile|1
TAATCAGGGAGGAGCTATTGGAAATCAGTATACTTATAAATCTCAAGATTTTACTCAGTA
>HPV88_Gamma_167600365_nt6371_L1_Tile|1
AGGGAGGAGCTATTGGAAATCAGTATACTTATAAATCTCAAGATTTTACTCAGTATACTC
>HPV88_Gamma_167600365_nt6376_L1_Tile|1
GGAGCTATTGGAAATCAGTATACTTATAAATCTCAAGATTTTACTCAGTATACTCGACAT
>HPV88_Gamma_167600365_nt6381_L1_Tile|1
TATTGGAAATCAGTATACTTATAAATCTCAAGATTTTACTCAGTATACTCGACATGCAGA
>HPV88_Gamma_167600365_nt6386_L1_Tile|1
GAAATCAGTATACTTATAAATCTCAAGATTTTACTCAGTATACTCGACATGCAGAGGAAT
>HPV88_Gamma_167600365_nt6391_L1_Tile|1
CAGTATACTTATAAATCTCAAGATTTTACTCAGTATACTCGACATGCAGAGGAATACGAA
>HPV88_Gamma_167600365_nt6396_L1_Tile|1
TACTTATAAATCTCAAGATTTTACTCAGTATACTCGACATGCAGAGGAATACGAATTGGA
>HPV88_Gamma_167600365_nt6401_L1_Tile|1
ATAAATCTCAAGATTTTACTCAGTATACTCGACATGCAGAGGAATACGAATTGGAAGCTA
>HPV88_Gamma_167600365_nt6406_L1_Tile|1
TCTCAAGATTTTACTCAGTATACTCGACATGCAGAGGAATACGAATTGGAAGCTATATTC
>HPV88_Gamma_167600365_nt6411_L1_Tile|1
AGATTTTACTCAGTATACTCGACATGCAGAGGAATACGAATTGGAAGCTATATTCCAGCT
>HPV88_Gamma_167600365_nt6421_L1_Tile|1
CAGTATACTCGACATGCAGAGGAATACGAATTGGAAGCTATATTCCAGCTTTGTAGAGTT
>HPV88_Gamma_167600365_nt6426_L1_Tile|1
TACTCGACATGCAGAGGAATACGAATTGGAAGCTATATTCCAGCTTTGTAGAGTTCCTTT
>HPV88_Gamma_167600365_nt6431_L1_Tile|1
GACATGCAGAGGAATACGAATTGGAAGCTATATTCCAGCTTTGTAGAGTTCCTTTGGAGC
>HPV88_Gamma_167600365_nt6451_L1_Tile|1
TTGGAAGCTATATTCCAGCTTTGTAGAGTTCCTTTGGAGCCTGATATTTTAGCTCACTTA
>HPV88_Gamma_167600365_nt6456_L1_Tile|1
AGCTATATTCCAGCTTTGTAGAGTTCCTTTGGAGCCTGATATTTTAGCTCACTTAAATGT
>HPV88_Gamma_167600365_nt6566_L1_Tile|1
CTCCTCAGAGTATAGAGGACAATTATAGATATATAACATCTTTGGCAACAAGATGTCCTA
>HPV88_Gamma_167600365_nt6571_L1_Tile|1
CAGAGTATAGAGGACAATTATAGATATATAACATCTTTGGCAACAAGATGTCCTACTGAA
>HPV88_Gamma_167600365_nt6576_L1_Tile|1
TATAGAGGACAATTATAGATATATAACATCTTTGGCAACAAGATGTCCTACTGAAAATCC
>HPV88_Gamma_167600365_nt6581_L1_Tile|1
AGGACAATTATAGATATATAACATCTTTGGCAACAAGATGTCCTACTGAAAATCCTGCTC

>HPV88_Gamma_167600365_nt6596_L1_Tile|1
ATATAACATCTTTGGCAACAAGATGTCCTACTGAAAATCCTGCTCCAGAAATACCTGACC
>HPV88_Gamma_167600365_nt6601_L1_Tile|1
ACATCTTTGGCAACAAGATGTCCTACTGAAAATCCTGCTCCAGAAATACCTGACCCTTAT
>HPV88_Gamma_167600365_nt6606_L1_Tile|1
TTTGGCAACAAGATGTCCTACTGAAAATCCTGCTCCAGAAATACCTGACCCTTATAAAGA
>HPV88_Gamma_167600365_nt6611_L1_Tile|1
CAACAAGATGTCCTACTGAAAATCCTGCTCCAGAAATACCTGACCCTTATAAAGATTATA
>HPV88_Gamma_167600365_nt6621_L1_Tile|1
TCCTACTGAAAATCCTGCTCCAGAAATACCTGACCCTTATAAAGATTATAATTTTTGGTT
>HPV88_Gamma_167600365_nt6626_L1_Tile|1
CTGAAAATCCTGCTCCAGAAATACCTGACCCTTATAAAGATTATAATTTTTGGTTAGTTG
>HPV88_Gamma_167600365_nt6631_L1_Tile|1
AATCCTGCTCCAGAAATACCTGACCCTTATAAAGATTATAATTTTTGGTTAGTTGATTTG
>HPV88_Gamma_167600365_nt6641_L1_Tile|1
CAGAAATACCTGACCCTTATAAAGATTATAATTTTTGGTTAGTTGATTTGCAAGAGAAGT
>HPV88_Gamma_167600365_nt6646_L1_Tile|1
ATACCTGACCCTTATAAAGATTATAATTTTTGGTTAGTTGATTTGCAAGAGAAGTTCTCC
>HPV88_Gamma_167600365_nt6651_L1_Tile|1
TGACCCTTATAAAGATTATAATTTTTGGTTAGTTGATTTGCAAGAGAAGTTCTCCAGTGA
>HPV88_Gamma_167600365_nt6656_L1_Tile|1
CTTATAAAGATTATAATTTTTGGTTAGTTGATTTGCAAGAGAAGTTCTCCAGTGAACTTG
>HPV88_Gamma_167600365_nt6661_L1_Tile|1
AAAGATTATAATTTTTGGTTAGTTGATTTGCAAGAGAAGTTCTCCAGTGAACTTGACCAA
>HPV88_Gamma_167600365_nt6666_L1_Tile|1
TTATAATTTTTGGTTAGTTGATTTGCAAGAGAAGTTCTCCAGTGAACTTGACCAATTTAG
>HPV88_Gamma_167600365_nt6676_L1_Tile|1
TGGTTAGTTGATTTGCAAGAGAAGTTCTCCAGTGAACTTGACCAATTTAGCTTAGGTCGC
>HPV88_Gamma_167600365_nt6681_L1_Tile|1
AGTTGATTTGCAAGAGAAGTTCTCCAGTGAACTTGACCAATTTAGCTTAGGTCGCAAATT
>HPV88_Gamma_167600365_nt6686_L1_Tile|1
ATTTGCAAGAGAAGTTCTCCAGTGAACTTGACCAATTTAGCTTAGGTCGCAAATTTTTGT
>HPV88_Gamma_167600365_nt6691_L1_Tile|1
CAAGAGAAGTTCTCCAGTGAACTTGACCAATTTAGCTTAGGTCGCAAATTTTTGTTTCAA
>HPV88_Gamma_167600365_nt6731_L1_Tile|1
GTCGCAAATTTTTGTTTCAAAGTGGTTTGCTGCCCACAAAACGTGTCAGGACAGTTGCCA
>HPV88_Gamma_167600365_nt6736_L1_Tile|1
AAATTTTTGTTTCAAAGTGGTTTGCTGCCCACAAAACGTGTCAGGACAGTTGCCAATAGT
>HPV88_Gamma_167600365_nt6761_L1_Tile|1
TGCCCACAAAACGTGTCAGGACAGTTGCCAATAGTTCTGTTAGAAATACTTCCAGAACAG
>HPV88_Gamma_167600365_nt6766_L1_Tile|1
ACAAAACGTGTCAGGACAGTTGCCAATAGTTCTGTTAGAAATACTTCCAGAACAGTAAAA
>HPV88_Gamma_167600365_nt6771_L1_Tile|1
ACGTGTCAGGACAGTTGCCAATAGTTCTGTTAGAAATACTTCCAGAACAGTAAAAAGAAA
>HPV88_Gamma_167600365_nt6776_L1_Tile|1
TCAGGACAGTTGCCAATAGTTCTGTTAGAAATACTTCCAGAACAGTAAAAAGAAAACGAA
>HPV88_Gamma_167600365_nt6784_L1_Tile|1
GTTGCCAATAGTTCTGTTAGAAATACTTCCAGAACAGTAAAAAGAAAACGAACACGATAA
>HPVcand89_Alpha_22095322_nt5808_L1_Tile|1
TGTCCAAAGTTTTAAGTACGGACCGCTATGTCCAGCGTACCAACCTGTACTATTATGGAG
>HPVcand89_Alpha_22095322_nt5853_L1_Tile|1
TGTACTATTATGGAGGCAGCTCCCGCCTTATTACAGTTGGCCACCCTTATTATACTGTAC
>HPVcand89_Alpha_22095322_nt5868_L1_Tile|1
GCAGCTCCCGCCTTATTACAGTTGGCCACCCTTATTATACTGTACAGGTCAATGGTGCTA
>HPVcand89_Alpha_22095322_nt5873_L1_Tile|1

TCCCGCCTTATTACAGTTGGCCACCCTTATTATACTGTACAGGTCAATGGTGCTAACAAA
>HPVcand89_Alpha_22095322_nt5883_L1_Tile|1
TTACAGTTGGCCACCCTTATTATACTGTACAGGTCAATGGTGCTAACAAAAAGGCCAACA
>HPVcand89_Alpha_22095322_nt5888_L1_Tile|1
GTTGGCCACCCTTATTATACTGTACAGGTCAATGGTGCTAACAAAAAGGCCAACATACCT
>HPVcand89_Alpha_22095322_nt5898_L1_Tile|1
CTTATTATACTGTACAGGTCAATGGTGCTAACAAAAAGGCCAACATACCTAAGGTATCAG
>HPVcand89_Alpha_22095322_nt6123_L1_Tile|1
TATACAACCGTTTGGATGACACTGAAAATGCTTCCTTATTGGTTGCTGCAGATGTTGACA
>HPVcand89_Alpha_22095322_nt6138_L1_Tile|1
ATGACACTGAAAATGCTTCCTTATTGGTTGCTGCAGATGTTGACAGTAGGGATAATATAT
>HPVcand89_Alpha_22095322_nt6143_L1_Tile|1
ACTGAAAATGCTTCCTTATTGGTTGCTGCAGATGTTGACAGTAGGGATAATATATCTGTT
>HPVcand89_Alpha_22095322_nt6153_L1_Tile|1
CTTCCTTATTGGTTGCTGCAGATGTTGACAGTAGGGATAATATATCTGTTGACTATAAAC
>HPVcand89_Alpha_22095322_nt6158_L1_Tile|1
TTATTGGTTGCTGCAGATGTTGACAGTAGGGATAATATATCTGTTGACTATAAACAAACA
>HPVcand89_Alpha_22095322_nt6273_L1_Tile|1
CTGTGTGCTCTAGTGTTGTGCCTCAGCAGGGGGATTGCCCACCTTTGCAGTTGCTAAATA
>HPVcand89_Alpha_22095322_nt6278_L1_Tile|1
TGCTCTAGTGTTGTGCCTCAGCAGGGGGATTGCCCACCTTTGCAGTTGCTAAATACTACT
>HPVcand89_Alpha_22095322_nt6538_L1_Tile|1
TAATAGGCAGGGTGTTATGGGTGAGGACCTGCCAAAAACCTTGTATATGCCTGGGTCTGC
>HPVcand89_Alpha_22095322_nt6548_L1_Tile|1
GGTGTTATGGGTGAGGACCTGCCAAAAACCTTGTATATGCCTGGGTCTGCTGCTAATAAC
>HPVcand89_Alpha_22095322_nt6553_L1_Tile|1
TATGGGTGAGGACCTGCCAAAAACCTTGTATATGCCTGGGTCTGCTGCTAATAACAGAAA
>HPVcand89_Alpha_22095322_nt6558_L1_Tile|1
GTGAGGACCTGCCAAAAACCTTGTATATGCCTGGGTCTGCTGCTAATAACAGAAATACAC
>HPVcand89_Alpha_22095322_nt6563_L1_Tile|1
GACCTGCCAAAAACCTTGTATATGCCTGGGTCTGCTGCTAATAACAGAAATACACTTACT
>HPVcand89_Alpha_22095322_nt6568_L1_Tile|1
GCCAAAAACCTTGTATATGCCTGGGTCTGCTGCTAATAACAGAAATACACTTACTAGCTA
>HPVcand89_Alpha_22095322_nt6573_L1_Tile|1
AAACCTTGTATATGCCTGGGTCTGCTGCTAATAACAGAAATACACTTACTAGCTATGTAT
>HPVcand89_Alpha_22095322_nt6578_L1_Tile|1
TTGTATATGCCTGGGTCTGCTGCTAATAACAGAAATACACTTACTAGCTATGTATATGCC
>HPVcand89_Alpha_22095322_nt6583_L1_Tile|1
TATGCCTGGGTCTGCTGCTAATAACAGAAATACACTTACTAGCTATGTATATGCCCCAAC
>HPVcand89_Alpha_22095322_nt6598_L1_Tile|1
TGCTAATAACAGAAATACACTTACTAGCTATGTATATGCCCCAACGCCCAGTGGGTCTAT
>HPVcand89_Alpha_22095322_nt6603_L1_Tile|1
ATAACAGAAATACACTTACTAGCTATGTATATGCCCCAACGCCCAGTGGGTCTATGGTTA
>HPVcand89_Alpha_22095322_nt6608_L1_Tile|1
AGAAATACACTTACTAGCTATGTATATGCCCCAACGCCCAGTGGGTCTATGGTTACATCT
>HPVcand89_Alpha_22095322_nt6778_L1_Tile|1
TACCAACCTTACCATTTGTGCTGCTTCCCAGTCTGGCACAGAATACAGTTCTACACGCTT
>HPVcand89_Alpha_22095322_nt6783_L1_Tile|1
ACCTTACCATTTGTGCTGCTTCCCAGTCTGGCACAGAATACAGTTCTACACGCTTTAAGG
>HPVcand89_Alpha_22095322_nt6788_L1_Tile|1
ACCATTTGTGCTGCTTCCCAGTCTGGCACAGAATACAGTTCTACACGCTTTAAGGAATAT
>HPVcand89_Alpha_22095322_nt6793_L1_Tile|1
TTGTGCTGCTTCCCAGTCTGGCACAGAATACAGTTCTACACGCTTTAAGGAATATTTAAG
>HPVcand89_Alpha_22095322_nt6798_L1_Tile|1
CTGCTTCCCAGTCTGGCACAGAATACAGTTCTACACGCTTTAAGGAATATTTAAGACACA

>HPVcand89_Alpha_22095322_nt6858_L1_Tile|1
CTGAGGAATATGACCTACAGTTTATATTCCAACTATGTAAGATACACCTAACGCCTGAGA
>HPVcand89_Alpha_22095322_nt6863_L1_Tile|1
GAATATGACCTACAGTTTATATTCCAACTATGTAAGATACACCTAACGCCTGAGATAATG
>HPVcand89_Alpha_22095322_nt6873_L1_Tile|1
TACAGTTTATATTCCAACTATGTAAGATACACCTAACGCCTGAGATAATGTCCTATTTAC
>HPVcand89_Alpha_22095322_nt6878_L1_Tile|1
TTTATATTCCAACTATGTAAGATACACCTAACGCCTGAGATAATGTCCTATTTACACAAT
>HPVcand89_Alpha_22095322_nt6883_L1_Tile|1
ATTCCAACTATGTAAGATACACCTAACGCCTGAGATAATGTCCTATTTACACAATATGAA
>HPVcand89_Alpha_22095322_nt6888_L1_Tile|1
AACTATGTAAGATACACCTAACGCCTGAGATAATGTCCTATTTACACAATATGAATGACA
>HPVcand89_Alpha_22095322_nt6893_L1_Tile|1
TGTAAGATACACCTAACGCCTGAGATAATGTCCTATTTACACAATATGAATGACACATTG
>HPVcand89_Alpha_22095322_nt6898_L1_Tile|1
GATACACCTAACGCCTGAGATAATGTCCTATTTACACAATATGAATGACACATTGTTAGA
>HPVcand89_Alpha_22095322_nt6903_L1_Tile|1
ACCTAACGCCTGAGATAATGTCCTATTTACACAATATGAATGACACATTGTTAGATGAAT
>HPVcand89_Alpha_22095322_nt7198_L1_Tile|1
ACCTCGCAAGCGTGCTGCCCCCGTGTCTACCTCCAAAGCCCCCAAACGCAAAAAGGCCAA
>HPV90_Alpha_22138122_nt5908_L1_Tile|1
GTTCCCGACTGTTAACTGTTGGCCATCCTTATTTTGCTATCAAAAAGCAATCAGGAAAAA
>HPV90_Alpha_22138122_nt6258_L1_Tile|1
CAGCTATTTATTATAGGGTGCAAACCTCCTTTAGGCGAGCACTGGGCAAAGGGTACCCCA
>HPV90_Alpha_22138122_nt6263_L1_Tile|1
ATTTATTATAGGGTGCAAACCTCCTTTAGGCGAGCACTGGGCAAAGGGTACCCCATGCAA
>HPV90_Alpha_22138122_nt6273_L1_Tile|1
GGGTGCAAACCTCCTTTAGGCGAGCACTGGGCAAAGGGTACCCCATGCAATATGTCTAAT
>HPV90_Alpha_22138122_nt6278_L1_Tile|1
CAAACCTCCTTTAGGCGAGCACTGGGCAAAGGGTACCCCATGCAATATGTCTAATGTACA
>HPV90_Alpha_22138122_nt6283_L1_Tile|1
CTCCTTTAGGCGAGCACTGGGCAAAGGGTACCCCATGCAATATGTCTAATGTACAGGCTG
>HPV90_Alpha_22138122_nt6308_L1_Tile|1
GGGTACCCCATGCAATATGTCTAATGTACAGGCTGGGGATTGCCCTCCTATAGAACTTAA
>HPV90_Alpha_22138122_nt6313_L1_Tile|1
CCCCATGCAATATGTCTAATGTACAGGCTGGGGATTGCCCTCCTATAGAACTTAAATCTT
>HPV90_Alpha_22138122_nt6523_L1_Tile|1
GTGACCTTATGTTTTTCTGTTTACGTAGGGAACAGATGTTTATAAGGCACTTTTATAACA
>HPV90_Alpha_22138122_nt6548_L1_Tile|1
TAGGGAACAGATGTTTATAAGGCACTTTTATAACAGAGCAGGTACTATGGGCGACGCAAT
>HPV90_Alpha_22138122_nt6558_L1_Tile|1
ATGTTTATAAGGCACTTTTATAACAGAGCAGGTACTATGGGCGACGCAATTCCCGATGGC
>HPV90_Alpha_22138122_nt6563_L1_Tile|1
TATAAGGCACTTTTATAACAGAGCAGGTACTATGGGCGACGCAATTCCCGATGGCTTTGT
>HPV90_Alpha_22138122_nt6568_L1_Tile|1
GGCACTTTTATAACAGAGCAGGTACTATGGGCGACGCAATTCCCGATGGCTTTGTGCTAA
>HPV90_Alpha_22138122_nt6573_L1_Tile|1
TTTTATAACAGAGCAGGTACTATGGGCGACGCAATTCCCGATGGCTTTGTGCTAAAAGGA
>HPV90_Alpha_22138122_nt6578_L1_Tile|1
TAACAGAGCAGGTACTATGGGCGACGCAATTCCCGATGGCTTTGTGCTAAAAGGAACATC
>HPV90_Alpha_22138122_nt6583_L1_Tile|1
GAGCAGGTACTATGGGCGACGCAATTCCCGATGGCTTTGTGCTAAAAGGAACATCTTCTA
>HPV90_Alpha_22138122_nt6593_L1_Tile|1
TATGGGCGACGCAATTCCCGATGGCTTTGTGCTAAAAGGAACATCTTCTACCTCTCGTGG
>HPV90_Alpha_22138122_nt6603_L1_Tile|1

GCAATTCCCGATGGCTTTGTGCTAAAAGGAACATCTTCTACCTCTCGTGGCACTCCTACA
>HPV90_Alpha_22138122_nt7058_L1_Tile|1
CCTACAGTCCCAGGCTATTTCCTGTCAAAAACCTTCTGCTGTCCCTACAGAGAACAAGGA
>HPV90_Alpha_22138122_nt7148_L1_Tile|1
GGATTTGTCTGAACGTTTTTCCCTTGAGCTAGACCAATTCCCTCTTGGCAGGAAATTTTT
>HPV90_Alpha_22138122_nt7173_L1_Tile|1
GAGCTAGACCAATTCCCTCTTGGCAGGAAATTTTTGCTGCAGCGTGGCACCCGGGTACGG
>HPV90_Alpha_22138122_nt7178_L1_Tile|1
AGACCAATTCCCTCTTGGCAGGAAATTTTTGCTGCAGCGTGGCACCCGGGTACGGTCGTC
>HPV90_Alpha_22138122_nt7251_L1_Tile|1
AAGCGTCCCGCACCCTCTACCGCCCCCTCTACAAAACGTAAACGGTCCAAACGTTCCTAA
>HPV91_Alpha_22023568_nt5596_L1_Tile|1
ATGTACCTCATACCAACACCAATGTGTCTACACTGCCTAACACAACAAAAATTGTTAACA
>HPV91_Alpha_22023568_nt5601_L1_Tile|1
CCTCATACCAACACCAATGTGTCTACACTGCCTAACACAACAAAAATTGTTAACACTACA
>HPV91_Alpha_22023568_nt5606_L1_Tile|1
TACCAACACCAATGTGTCTACACTGCCTAACACAACAAAAATTGTTAACACTACAGTCCC
>HPV91_Alpha_22023568_nt5611_L1_Tile|1
ACACCAATGTGTCTACACTGCCTAACACAACAAAAATTGTTAACACTACAGTCCCACTTG
>HPV91_Alpha_22023568_nt5616_L1_Tile|1
AATGTGTCTACACTGCCTAACACAACAAAAATTGTTAACACTACAGTCCCACTTGCAACA
>HPV91_Alpha_22023568_nt5621_L1_Tile|1
GTCTACACTGCCTAACACAACAAAAATTGTTAACACTACAGTCCCACTTGCAACAGTACC
>HPV91_Alpha_22023568_nt5626_L1_Tile|1
CACTGCCTAACACAACAAAAATTGTTAACACTACAGTCCCACTTGCAACAGTACCTGATA
>HPV91_Alpha_22023568_nt5631_L1_Tile|1
CCTAACACAACAAAAATTGTTAACACTACAGTCCCACTTGCAACAGTACCTGATATATAT
>HPV91_Alpha_22023568_nt5646_L1_Tile|1
ATTGTTAACACTACAGTCCCACTTGCAACAGTACCTGATATATATACACAAGCAGGACCT
>HPV91_Alpha_22023568_nt5651_L1_Tile|1
TAACACTACAGTCCCACTTGCAACAGTACCTGATATATATACACAAGCAGGACCTGATAT
>HPV91_Alpha_22023568_nt5656_L1_Tile|1
CTACAGTCCCACTTGCAACAGTACCTGATATATATACACAAGCAGGACCTGATATTACAT
>HPV91_Alpha_22023568_nt5666_L1_Tile|1
ACTTGCAACAGTACCTGATATATATACACAAGCAGGACCTGATATTACATTTCCTACAGT
>HPV91_Alpha_22023568_nt5676_L1_Tile|1
GTACCTGATATATATACACAAGCAGGACCTGATATTACATTTCCTACAGTGCCACTTAGT
>HPV91_Alpha_22023568_nt5681_L1_Tile|1
TGATATATATACACAAGCAGGACCTGATATTACATTTCCTACAGTGCCACTTAGTGTCCC
>HPV91_Alpha_22023568_nt5691_L1_Tile|1
ACACAAGCAGGACCTGATATTACATTTCCTACAGTGCCACTTAGTGTCCCCTATGTGCCA
>HPV91_Alpha_22023568_nt5696_L1_Tile|1
AGCAGGACCTGATATTACATTTCCTACAGTGCCACTTAGTGTCCCCTATGTGCCATTGTC
>HPV91_Alpha_22023568_nt5706_L1_Tile|1
GATATTACATTTCCTACAGTGCCACTTAGTGTCCCCTATGTGCCATTGTCACCGTCTGTT
>HPV91_Alpha_22023568_nt5711_L1_Tile|1
TACATTTCCTACAGTGCCACTTAGTGTCCCCTATGTGCCATTGTCACCGTCTGTTACACC
>HPV91_Alpha_22023568_nt5716_L1_Tile|1
TTCCTACAGTGCCACTTAGTGTCCCCTATGTGCCATTGTCACCGTCTGTTACACCTTCTG
>HPV91_Alpha_22023568_nt5726_L1_Tile|1
GCCACTTAGTGTCCCCTATGTGCCATTGTCACCGTCTGTTACACCTTCTGCAGTATCAAT
>HPV91_Alpha_22023568_nt5731_L1_Tile|1
TTAGTGTCCCCTATGTGCCATTGTCACCGTCTGTTACACCTTCTGCAGTATCAATATATG
>HPV91_Alpha_22023568_nt5736_L1_Tile|1
GTCCCCTATGTGCCATTGTCACCGTCTGTTACACCTTCTGCAGTATCAATATATGGCACA

>HPV91_Alpha_22023568_nt5751_L1_Tile|1
TTGTCACCGTCTGTTACACCTTCTGCAGTATCAATATATGGCACAGATTTTTATTTACAT
>HPV91_Alpha_22023568_nt5761_L1_Tile|1
CTGTTACACCTTCTGCAGTATCAATATATGGCACAGATTTTTATTTACATCCTTCATTGT
>HPV91_Alpha_22023568_nt5971_L1_Tile|1
TACCATGCTGGCAGTTCCCGTTTACTGGCTGTGGGCCACCCTTTTTTTCCTATAAAAAAT
>HPV91_Alpha_22023568_nt5976_L1_Tile|1
TGCTGGCAGTTCCCGTTTACTGGCTGTGGGCCACCCTTTTTTTCCTATAAAAAATAATTC
>HPV91_Alpha_22023568_nt5981_L1_Tile|1
GCAGTTCCCGTTTACTGGCTGTGGGCCACCCTTTTTTTCCTATAAAAAATAATTCTGGTA
>HPV91_Alpha_22023568_nt5986_L1_Tile|1
TCCCGTTTACTGGCTGTGGGCCACCCTTTTTTTCCTATAAAAAATAATTCTGGTAAAGTA
>HPV91_Alpha_22023568_nt5991_L1_Tile|1
TTTACTGGCTGTGGGCCACCCTTTTTTTCCTATAAAAAATAATTCTGGTAAAGTAATTGT
>HPV91_Alpha_22023568_nt6006_L1_Tile|1
CCACCCTTTTTTTCCTATAAAAAATAATTCTGGTAAAGTAATTGTTCCTAAAGTTTCAGG
>HPV91_Alpha_22023568_nt6011_L1_Tile|1
CTTTTTTTCCTATAAAAAATAATTCTGGTAAAGTAATTGTTCCTAAAGTTTCAGGTCACC
>HPV91_Alpha_22023568_nt6561_L1_Tile|1
AGATTATTTAGGAATGGCAGCCGAGCCATACGGAAATAGTTTATTTTTCTTTTTACGTAG
>HPV91_Alpha_22023568_nt6651_L1_Tile|1
TAGGGCTGGTACTACAGGCGATGCTGTCCCTAAGGATTTGTATATTGCAGGAACAGGCAA
>HPV91_Alpha_22023568_nt6666_L1_Tile|1
AGGCGATGCTGTCCCTAAGGATTTGTATATTGCAGGAACAGGCAACAGGGCGAACATAGC
>HPV91_Alpha_22023568_nt6671_L1_Tile|1
ATGCTGTCCCTAAGGATTTGTATATTGCAGGAACAGGCAACAGGGCGAACATAGCAGGCA
>HPV91_Alpha_22023568_nt6676_L1_Tile|1
GTCCCTAAGGATTTGTATATTGCAGGAACAGGCAACAGGGCGAACATAGCAGGCAGTATA
>HPV91_Alpha_22023568_nt6681_L1_Tile|1
TAAGGATTTGTATATTGCAGGAACAGGCAACAGGGCGAACATAGCAGGCAGTATATATTA
>HPV91_Alpha_22023568_nt6686_L1_Tile|1
ATTTGTATATTGCAGGAACAGGCAACAGGGCGAACATAGCAGGCAGTATATATTATTCTA
>HPV91_Alpha_22023568_nt6691_L1_Tile|1
TATATTGCAGGAACAGGCAACAGGGCGAACATAGCAGGCAGTATATATTATTCTACTCCT
>HPV91_Alpha_22023568_nt6791_L1_Tile|1
AACCGCTTTGGATGCAAAAAGCACAGGGGCATAATAACGGCATCTGTTTTGGTAACCAGG
>HPV91_Alpha_22023568_nt6826_L1_Tile|1
AACGGCATCTGTTTTGGTAACCAGGTGTTTGTAACTGTTGTGGATACAACTCGCAGCACT
>HPV91_Alpha_22023568_nt6851_L1_Tile|1
TGTTTGTAACTGTTGTGGATACAACTCGCAGCACTAACTTAACCTTGTGTGCATCCACTG
>HPV91_Alpha_22023568_nt6861_L1_Tile|1
TGTTGTGGATACAACTCGCAGCACTAACTTAACCTTGTGTGCATCCACTGAGTCTGTGCT
>HPV91_Alpha_22023568_nt6866_L1_Tile|1
TGGATACAACTCGCAGCACTAACTTAACCTTGTGTGCATCCACTGAGTCTGTGCTACCTA
>HPV91_Alpha_22023568_nt6871_L1_Tile|1
ACAACTCGCAGCACTAACTTAACCTTGTGTGCATCCACTGAGTCTGTGCTACCTACTACA
>HPV91_Alpha_22023568_nt6881_L1_Tile|1
GCACTAACTTAACCTTGTGTGCATCCACTGAGTCTGTGCTACCTACTACATATGACAACA
>HPV91_Alpha_22023568_nt6886_L1_Tile|1
AACTTAACCTTGTGTGCATCCACTGAGTCTGTGCTACCTACTACATATGACAACACAAAG
>HPV91_Alpha_22023568_nt6891_L1_Tile|1
AACCTTGTGTGCATCCACTGAGTCTGTGCTACCTACTACATATGACAACACAAAGTTCAA
>HPV91_Alpha_22023568_nt6896_L1_Tile|1
TGTGTGCATCCACTGAGTCTGTGCTACCTACTACATATGACAACACAAAGTTCAAAGAAT
>HPV91_Alpha_22023568_nt6901_L1_Tile|1

GCATCCACTGAGTCTGTGCTACCTACTACATATGACAACACAAAGTTCAAAGAATATTTA
>HPV91_Alpha_22023568_nt6906_L1_Tile|1
CACTGAGTCTGTGCTACCTACTACATATGACAACACAAAGTTCAAAGAATATTTAAGGCA
>HPV91_Alpha_22023568_nt6911_L1_Tile|1
AGTCTGTGCTACCTACTACATATGACAACACAAAGTTCAAAGAATATTTAAGGCATGCAG
>HPV91_Alpha_22023568_nt6986_L1_Tile|1
AGTTTATATTTCAATTATGCATTATAACACTTAATCCAGAGGTAATGACATACATCCACA
>HPV91_Alpha_22023568_nt7001_L1_Tile|1
TATGCATTATAACACTTAATCCAGAGGTAATGACATACATCCACACTATGGATGCATCAT
>HPV91_Alpha_22023568_nt7006_L1_Tile|1
ATTATAACACTTAATCCAGAGGTAATGACATACATCCACACTATGGATGCATCATTATTA
>HPV91_Alpha_22023568_nt7011_L1_Tile|1
AACACTTAATCCAGAGGTAATGACATACATCCACACTATGGATGCATCATTATTAGAAGA
>HPV91_Alpha_22023568_nt7026_L1_Tile|1
GGTAATGACATACATCCACACTATGGATGCATCATTATTAGAAGACTGGAACTTTGGGGT
>HPV91_Alpha_22023568_nt7051_L1_Tile|1
GATGCATCATTATTAGAAGACTGGAACTTTGGGGTATCCCCTCCAGCCACGGCATCACTA
>HPV91_Alpha_22023568_nt7076_L1_Tile|1
ACTTTGGGGTATCCCCTCCAGCCACGGCATCACTAGAGGATACTTATCGCTTTTTGGCTA
>HPV91_Alpha_22023568_nt7081_L1_Tile|1
GGGGTATCCCCTCCAGCCACGGCATCACTAGAGGATACTTATCGCTTTTTGGCTAATAAG
>HPV91_Alpha_22023568_nt7086_L1_Tile|1
ATCCCCTCCAGCCACGGCATCACTAGAGGATACTTATCGCTTTTTGGCTAATAAGGCAAT
>HPV91_Alpha_22023568_nt7131_L1_Tile|1
GGCTAATAAGGCAATTGCCTGTCAAAAAAATGTCCCACCAAAAGCCAAAGAGGACCCATA
>HPV91_Alpha_22023568_nt7136_L1_Tile|1
ATAAGGCAATTGCCTGTCAAAAAAATGTCCCACCAAAAGCCAAAGAGGACCCATACAAAA
>HPV91_Alpha_22023568_nt7141_L1_Tile|1
GCAATTGCCTGTCAAAAAAATGTCCCACCAAAAGCCAAAGAGGACCCATACAAAAACTAT
>HPV91_Alpha_22023568_nt7146_L1_Tile|1
TGCCTGTCAAAAAAATGTCCCACCAAAAGCCAAAGAGGACCCATACAAAAACTATACTTT
>HPV91_Alpha_22023568_nt7166_L1_Tile|1
CACCAAAAGCCAAAGAGGACCCATACAAAAACTATACTTTTTGGGATGTGGATCTTACCG
>HPV91_Alpha_22023568_nt7171_L1_Tile|1
AAAGCCAAAGAGGACCCATACAAAAACTATACTTTTTGGGATGTGGATCTTACCGAAAGG
>HPV91_Alpha_22023568_nt7316_L1_Tile|1
GGAAGCGTGCAGCACCATCCTCCAGTTCAGCGCCTGCCTCCAAACGAAAGAAAACTAAGC
>HPV92_Beta_27531786_nt5719_L1_Tile|1
ATTTGTTCAAAGGACCAACATCTTTTATCATGCAAATAGTGATCGCCTGCTGACAGTTGG
>HPV92_Beta_27531786_nt5729_L1_Tile|1
AGGACCAACATCTTTTATCATGCAAATAGTGATCGCCTGCTGACAGTTGGACACCCTTAT
>HPV92_Beta_27531786_nt5754_L1_Tile|1
ATAGTGATCGCCTGCTGACAGTTGGACACCCTTATTTGAAGTGAGAAGCTCAGTTGATC
>HPV92_Beta_27531786_nt5759_L1_Tile|1
GATCGCCTGCTGACAGTTGGACACCCTTATTTGAAGTGAGAAGCTCAGTTGATCCACAT
>HPV92_Beta_27531786_nt5764_L1_Tile|1
CCTGCTGACAGTTGGACACCCTTATTTGAAGTGAGAAGCTCAGTTGATCCACATGATGT
>HPV92_Beta_27531786_nt5769_L1_Tile|1
TGACAGTTGGACACCCTTATTTGAAGTGAGAAGCTCAGTTGATCCACATGATGTATTAG
>HPV92_Beta_27531786_nt5774_L1_Tile|1
GTTGGACACCCTTATTTGAAGTGAGAAGCTCAGTTGATCCACATGATGTATTAGTGCCT
>HPV92_Beta_27531786_nt5779_L1_Tile|1
ACACCCTTATTTGAAGTGAGAAGCTCAGTTGATCCACATGATGTATTAGTGCCTAAGGT
>HPV92_Beta_27531786_nt5799_L1_Tile|1
GAAGCTCAGTTGATCCACATGATGTATTAGTGCCTAAGGTGTCAGGGAATCAGTTTAGAG

>HPV92_Beta_27531786_nt6034_L1_Tile|1
AAATCCAAATAAGTACAATACTGGAACAAAGGATGACAGAGTAAACACATCTTTTGATCC
>HPV92_Beta_27531786_nt6039_L1_Tile|1
CAAATAAGTACAATACTGGAACAAAGGATGACAGAGTAAACACATCTTTTGATCCAAAGC
>HPV92_Beta_27531786_nt6044_L1_Tile|1
AAGTACAATACTGGAACAAAGGATGACAGAGTAAACACATCTTTTGATCCAAAGCAAATT
>HPV92_Beta_27531786_nt6049_L1_Tile|1
CAATACTGGAACAAAGGATGACAGAGTAAACACATCTTTTGATCCAAAGCAAATTCAGTT
>HPV92_Beta_27531786_nt6059_L1_Tile|1
ACAAAGGATGACAGAGTAAACACATCTTTTGATCCAAAGCAAATTCAGTTATTTGTTTTA
>HPV92_Beta_27531786_nt6064_L1_Tile|1
GGATGACAGAGTAAACACATCTTTTGATCCAAAGCAAATTCAGTTATTTGTTTTAGGATG
>HPV92_Beta_27531786_nt6069_L1_Tile|1
ACAGAGTAAACACATCTTTTGATCCAAAGCAAATTCAGTTATTTGTTTTAGGATGTACAC
>HPV92_Beta_27531786_nt6084_L1_Tile|1
CTTTTGATCCAAAGCAAATTCAGTTATTTGTTTTAGGATGTACACCATGCTTAGGTGAAC
>HPV92_Beta_27531786_nt6119_L1_Tile|1
GGATGTACACCATGCTTAGGTGAACATTGGGACACAGCCTTACCATGTGCTGAAAAGCAA
>HPV92_Beta_27531786_nt6139_L1_Tile|1
TGAACATTGGGACACAGCCTTACCATGTGCTGAAAAGCAACCAGATACTGGGGGATGCCC
>HPV92_Beta_27531786_nt6154_L1_Tile|1
AGCCTTACCATGTGCTGAAAAGCAACCAGATACTGGGGGATGCCCACCATTAGAGTTAAA
>HPV92_Beta_27531786_nt6159_L1_Tile|1
TACCATGTGCTGAAAAGCAACCAGATACTGGGGGATGCCCACCATTAGAGTTAAAAAACA
>HPV92_Beta_27531786_nt6164_L1_Tile|1
TGTGCTGAAAAGCAACCAGATACTGGGGGATGCCCACCATTAGAGTTAAAAAACACTGTT
>HPV92_Beta_27531786_nt6169_L1_Tile|1
TGAAAAGCAACCAGATACTGGGGGATGCCCACCATTAGAGTTAAAAAACACTGTTATCTC
>HPV92_Beta_27531786_nt6174_L1_Tile|1
AGCAACCAGATACTGGGGGATGCCCACCATTAGAGTTAAAAAACACTGTTATCTCTGATG
>HPV92_Beta_27531786_nt6179_L1_Tile|1
CCAGATACTGGGGGATGCCCACCATTAGAGTTAAAAAACACTGTTATCTCTGATGGAGAT
>HPV92_Beta_27531786_nt6224_L1_Tile|1
ATCTCTGATGGAGATATGGTTGACATAGGCTTCGGTAATATGAATTTTAAGGCCTTATCA
>HPV92_Beta_27531786_nt6229_L1_Tile|1
TGATGGAGATATGGTTGACATAGGCTTCGGTAATATGAATTTTAAGGCCTTATCAGTAAC
>HPV92_Beta_27531786_nt6234_L1_Tile|1
GAGATATGGTTGACATAGGCTTCGGTAATATGAATTTTAAGGCCTTATCAGTAACCAAAT
>HPV92_Beta_27531786_nt6239_L1_Tile|1
ATGGTTGACATAGGCTTCGGTAATATGAATTTTAAGGCCTTATCAGTAACCAAATCTGAC
>HPV92_Beta_27531786_nt6244_L1_Tile|1
TGACATAGGCTTCGGTAATATGAATTTTAAGGCCTTATCAGTAACCAAATCTGACGTAAG
>HPV92_Beta_27531786_nt6249_L1_Tile|1
TAGGCTTCGGTAATATGAATTTTAAGGCCTTATCAGTAACCAAATCTGACGTAAGTTTGG
>HPV92_Beta_27531786_nt6254_L1_Tile|1
TTCGGTAATATGAATTTTAAGGCCTTATCAGTAACCAAATCTGACGTAAGTTTGGATATA
>HPV92_Beta_27531786_nt6259_L1_Tile|1
TAATATGAATTTTAAGGCCTTATCAGTAACCAAATCTGACGTAAGTTTGGATATAGTAGA
>HPV92_Beta_27531786_nt6264_L1_Tile|1
TGAATTTTAAGGCCTTATCAGTAACCAAATCTGACGTAAGTTTGGATATAGTAGACTCCA
>HPV92_Beta_27531786_nt6394_L1_Tile|1
ACGAGAACAATGTTATGTTAGGCATATGTTTGTGCGCGGTGGTGTTGTGGGTGATACCAT
>HPV92_Beta_27531786_nt6399_L1_Tile|1
AACAATGTTATGTTAGGCATATGTTTGTGCGCGGTGGTGTTGTGGGTGATACCATCCCAG
>HPV92_Beta_27531786_nt6439_L1_Tile|1

TGTGGGTGATACCATCCCAGATGCAGTTGTAAATGAAGACCATAACTTTATGTTACCTGC
>HPV92_Beta_27531786_nt6454_L1_Tile|1
CCCAGATGCAGTTGTAAATGAAGACCATAACTTTATGTTACCTGCAGCATCCAGTGACCA
>HPV92_Beta_27531786_nt6459_L1_Tile|1
ATGCAGTTGTAAATGAAGACCATAACTTTATGTTACCTGCAGCATCCAGTGACCAGTCTA
>HPV92_Beta_27531786_nt6469_L1_Tile|1
AAATGAAGACCATAACTTTATGTTACCTGCAGCATCCAGTGACCAGTCTAGAAGTCAAAT
>HPV92_Beta_27531786_nt6474_L1_Tile|1
AAGACCATAACTTTATGTTACCTGCAGCATCCAGTGACCAGTCTAGAAGTCAAATTGCCA
>HPV92_Beta_27531786_nt6479_L1_Tile|1
CATAACTTTATGTTACCTGCAGCATCCAGTGACCAGTCTAGAAGTCAAATTGCCAGTTCT
>HPV92_Beta_27531786_nt6489_L1_Tile|1
TGTTACCTGCAGCATCCAGTGACCAGTCTAGAAGTCAAATTGCCAGTTCTATCTATTTCC
>HPV92_Beta_27531786_nt6499_L1_Tile|1
AGCATCCAGTGACCAGTCTAGAAGTCAAATTGCCAGTTCTATCTATTTCCCTACTGTTAG
>HPV92_Beta_27531786_nt6644_L1_Tile|1
TGGAGTAATGAACTGTTTCTGACAGTTTGTGATAATACCAGGAATACTAACTTTAATATT
>HPV92_Beta_27531786_nt6649_L1_Tile|1
TAATGAACTGTTTCTGACAGTTTGTGATAATACCAGGAATACTAACTTTAATATTAGTGT
>HPV92_Beta_27531786_nt6654_L1_Tile|1
AACTGTTTCTGACAGTTTGTGATAATACCAGGAATACTAACTTTAATATTAGTGTCCCTA
>HPV92_Beta_27531786_nt6659_L1_Tile|1
TTTCTGACAGTTTGTGATAATACCAGGAATACTAACTTTAATATTAGTGTCCCTAAGGAA
>HPV92_Beta_27531786_nt6664_L1_Tile|1
GACAGTTTGTGATAATACCAGGAATACTAACTTTAATATTAGTGTCCCTAAGGAAGGTGG
>HPV92_Beta_27531786_nt6669_L1_Tile|1
TTTGTGATAATACCAGGAATACTAACTTTAATATTAGTGTCCCTAAGGAAGGTGGTCAAA
>HPV92_Beta_27531786_nt6674_L1_Tile|1
GATAATACCAGGAATACTAACTTTAATATTAGTGTCCCTAAGGAAGGTGGTCAAATAACC
>HPV92_Beta_27531786_nt6679_L1_Tile|1
TACCAGGAATACTAACTTTAATATTAGTGTCCCTAAGGAAGGTGGTCAAATAACCGACTA
>HPV92_Beta_27531786_nt6684_L1_Tile|1
GGAATACTAACTTTAATATTAGTGTCCCTAAGGAAGGTGGTCAAATAACCGACTATGATT
>HPV92_Beta_27531786_nt6689_L1_Tile|1
ACTAACTTTAATATTAGTGTCCCTAAGGAAGGTGGTCAAATAACCGACTATGATTCACAA
>HPV92_Beta_27531786_nt6694_L1_Tile|1
CTTTAATATTAGTGTCCCTAAGGAAGGTGGTCAAATAACCGACTATGATTCACAAAAGAT
>HPV92_Beta_27531786_nt6699_L1_Tile|1
ATATTAGTGTCCCTAAGGAAGGTGGTCAAATAACCGACTATGATTCACAAAAGATTAGAG
>HPV92_Beta_27531786_nt6704_L1_Tile|1
AGTGTCCCTAAGGAAGGTGGTCAAATAACCGACTATGATTCACAAAAGATTAGAGAATAC
>HPV92_Beta_27531786_nt6709_L1_Tile|1
CCCTAAGGAAGGTGGTCAAATAACCGACTATGATTCACAAAAGATTAGAGAATACACTAG
>HPV92_Beta_27531786_nt6719_L1_Tile|1
GGTGGTCAAATAACCGACTATGATTCACAAAAGATTAGAGAATACACTAGACATGTTGAA
>HPV92_Beta_27531786_nt6724_L1_Tile|1
TCAAATAACCGACTATGATTCACAAAAGATTAGAGAATACACTAGACATGTTGAAGAATA
>HPV92_Beta_27531786_nt6739_L1_Tile|1
TGATTCACAAAAGATTAGAGAATACACTAGACATGTTGAAGAATATGAAATATCACTAAT
>HPV92_Beta_27531786_nt6894_L1_Tile|1
TTCCTACTCCAGATAACCCTATTCAGGATGCATACAGATTTATTCATTCTAAAGCAACAC
>HPV92_Beta_27531786_nt6899_L1_Tile|1
ACTCCAGATAACCCTATTCAGGATGCATACAGATTTATTCATTCTAAAGCAACACCTTGT
>HPV92_Beta_27531786_nt6904_L1_Tile|1
AGATAACCCTATTCAGGATGCATACAGATTTATTCATTCTAAAGCAACACCTTGTCCAGA

>HPV92_Beta_27531786_nt7059_L1_Tile|1
ATTCATTGGGTAGAAAATTTTTATTTCAAGCTGGATTAACTAATACATCTGTTAATGGTC
>HPV92_Beta_27531786_nt7064_L1_Tile|1
TTGGGTAGAAAATTTTTATTTCAAGCTGGATTAACTAATACATCTGTTAATGGTCTTAAA
>HPV92_Beta_27531786_nt7069_L1_Tile|1
TAGAAAATTTTTATTTCAAGCTGGATTAACTAATACATCTGTTAATGGTCTTAAAAGAAC
>HPV92_Beta_27531786_nt7074_L1_Tile|1
AATTTTTATTTCAAGCTGGATTAACTAATACATCTGTTAATGGTCTTAAAAGAACTAGAA
>HPV92_Beta_27531786_nt7084_L1_Tile|1
TCAAGCTGGATTAACTAATACATCTGTTAATGGTCTTAAAAGAACTAGAAGCAGTTCTCA
>HPV92_Beta_27531786_nt7089_L1_Tile|1
CTGGATTAACTAATACATCTGTTAATGGTCTTAAAAGAACTAGAAGCAGTTCTCAAAGAG
>HPV92_Beta_27531786_nt7094_L1_Tile|1
TTAACTAATACATCTGTTAATGGTCTTAAAAGAACTAGAAGCAGTTCTCAAAGAGGTACT
>HPV92_Beta_27531786_nt7099_L1_Tile|1
TAATACATCTGTTAATGGTCTTAAAAGAACTAGAAGCAGTTCTCAAAGAGGTACTAAACG
>HPV92_Beta_27531786_nt7104_L1_Tile|1
CATCTGTTAATGGTCTTAAAAGAACTAGAAGCAGTTCTCAAAGAGGTACTAAACGAAAAA
>HPV92_Beta_27531786_nt7109_L1_Tile|1
GTTAATGGTCTTAAAAGAACTAGAAGCAGTTCTCAAAGAGGTACTAAACGAAAAAGAAAA
>HPV92_Beta_27531786_nt7114_L1_Tile|1
TGGTCTTAAAAGAACTAGAAGCAGTTCTCAAAGAGGTACTAAACGAAAAAGAAAAAGTAA
>HPV92_Beta_27531786_nt7118_L1_Tile|1
CTTAAAAGAACTAGAAGCAGTTCTCAAAGAGGTACTAAACGAAAAAGAAAAAGTAACTAA
>HPV93_Beta_37089393_nt5771_L1_Tile|1
TAACAGTAGGTCACCCATATTACAATGTTTACAATAATAATGGAACAGTATTGGAAGTAC
>HPV93_Beta_37089393_nt5776_L1_Tile|1
GTAGGTCACCCATATTACAATGTTTACAATAATAATGGAACAGTATTGGAAGTACCAAAG
>HPV93_Beta_37089393_nt6016_L1_Tile|1
AAGGTAAATGATACAGAAAATCCTTTAAATTACAGAACTCAGGCATTTTCTACTGATGAT
>HPV93_Beta_37089393_nt6146_L1_Tile|1
AACACTGGGATGTTGCACCTAAATGTGATGGAGCCAATGACCAAAATGGGATTTGCCCAC
>HPV93_Beta_37089393_nt6151_L1_Tile|1
TGGGATGTTGCACCTAAATGTGATGGAGCCAATGACCAAAATGGGATTTGCCCACCTATA
>HPV93_Beta_37089393_nt6161_L1_Tile|1
CACCTAAATGTGATGGAGCCAATGACCAAAATGGGATTTGCCCACCTATACAATTAGTAA
>HPV93_Beta_37089393_nt6166_L1_Tile|1
AAATGTGATGGAGCCAATGACCAAAATGGGATTTGCCCACCTATACAATTAGTAAACACT
>HPV93_Beta_37089393_nt6176_L1_Tile|1
GAGCCAATGACCAAAATGGGATTTGCCCACCTATACAATTAGTAAACACTGTTATTCAAG
>HPV93_Beta_37089393_nt6206_L1_Tile|1
CTATACAATTAGTAAACACTGTTATTCAAGATGGGGATATGGCAGATATCGGGTATGGTA
>HPV93_Beta_37089393_nt6211_L1_Tile|1
CAATTAGTAAACACTGTTATTCAAGATGGGGATATGGCAGATATCGGGTATGGTAATCTT
>HPV93_Beta_37089393_nt6236_L1_Tile|1
ATGGGGATATGGCAGATATCGGGTATGGTAATCTTAATTTTAAGGCTTTGCAGGAAAATA
>HPV93_Beta_37089393_nt6251_L1_Tile|1
ATATCGGGTATGGTAATCTTAATTTTAAGGCTTTGCAGGAAAATAGATCTGATGTTAGTC
>HPV93_Beta_37089393_nt6256_L1_Tile|1
GGGTATGGTAATCTTAATTTTAAGGCTTTGCAGGAAAATAGATCTGATGTTAGTCTTGAC
>HPV93_Beta_37089393_nt6421_L1_Tile|1
CACTTTTTTGTAAGAGGTGGCAACGTAGGAGATGACATTCCTGGTGAACAAATAGACGCA
>HPV93_Beta_37089393_nt6426_L1_Tile|1
TTTTGTAAGAGGTGGCAACGTAGGAGATGACATTCCTGGTGAACAAATAGACGCAGGCAC
>HPV93_Beta_37089393_nt6431_L1_Tile|1

TAAGAGGTGGCAACGTAGGAGATGACATTCCTGGTGAACAAATAGACGCAGGCACATATA
>HPV93_Beta_37089393_nt6436_L1_Tile|1
GGTGGCAACGTAGGAGATGACATTCCTGGTGAACAAATAGACGCAGGCACATATAAAAAT
>HPV93_Beta_37089393_nt6441_L1_Tile|1
CAACGTAGGAGATGACATTCCTGGTGAACAAATAGACGCAGGCACATATAAAAATGATTT
>HPV93_Beta_37089393_nt6446_L1_Tile|1
TAGGAGATGACATTCCTGGTGAACAAATAGACGCAGGCACATATAAAAATGATTTTTACA
>HPV93_Beta_37089393_nt6681_L1_Tile|1
TGTAGACAACACAAGAAACACAAACTTCAGTATTAGTGTTTATACAGACCCTGGGAAAAT
>HPV93_Beta_37089393_nt6686_L1_Tile|1
ACAACACAAGAAACACAAACTTCAGTATTAGTGTTTATACAGACCCTGGGAAAATAAGGG
>HPV93_Beta_37089393_nt6691_L1_Tile|1
ACAAGAAACACAAACTTCAGTATTAGTGTTTATACAGACCCTGGGAAAATAAGGGATATA
>HPV93_Beta_37089393_nt6696_L1_Tile|1
AAACACAAACTTCAGTATTAGTGTTTATACAGACCCTGGGAAAATAAGGGATATAAATGA
>HPV93_Beta_37089393_nt6891_L1_Tile|1
AGAGGAATGGCAATTAGGGTTTGTCCCAACTCCAGACAATCCGTTACAGAGTACTTATCG
>HPV93_Beta_37089393_nt6896_L1_Tile|1
AATGGCAATTAGGGTTTGTCCCAACTCCAGACAATCCGTTACAGAGTACTTATCGATATA
>HPV93_Beta_37089393_nt6901_L1_Tile|1
CAATTAGGGTTTGTCCCAACTCCAGACAATCCGTTACAGAGTACTTATCGATATATTAAT
>HPV93_Beta_37089393_nt6906_L1_Tile|1
AGGGTTTGTCCCAACTCCAGACAATCCGTTACAGAGTACTTATCGATATATTAATAGTTT
>HPV93_Beta_37089393_nt6911_L1_Tile|1
TTGTCCCAACTCCAGACAATCCGTTACAGAGTACTTATCGATATATTAATAGTTTAGCTA
>HPV93_Beta_37089393_nt6916_L1_Tile|1
CCAACTCCAGACAATCCGTTACAGAGTACTTATCGATATATTAATAGTTTAGCTACACCA
>HPV93_Beta_37089393_nt6921_L1_Tile|1
TCCAGACAATCCGTTACAGAGTACTTATCGATATATTAATAGTTTAGCTACACCATGTCC
>HPV93_Beta_37089393_nt6926_L1_Tile|1
ACAATCCGTTACAGAGTACTTATCGATATATTAATAGTTTAGCTACACCATGTCCCGATA
>HPV93_Beta_37089393_nt6931_L1_Tile|1
CCGTTACAGAGTACTTATCGATATATTAATAGTTTAGCTACACCATGTCCCGATAAGGTT
>HPV93_Beta_37089393_nt6941_L1_Tile|1
GTACTTATCGATATATTAATAGTTTAGCTACACCATGTCCCGATAAGGTTCCACCAAAGG
>HPV93_Beta_37089393_nt6951_L1_Tile|1
ATATATTAATAGTTTAGCTACACCATGTCCCGATAAGGTTCCACCAAAGGAAAAGGAAGA
>HPV93_Beta_37089393_nt7066_L1_Tile|1
GATCTAGATCAATACTCCTTAGGGCGCAAATTTCTGTATCAGGCAAATCTTGTACAATCA
>HPV93_Beta_37089393_nt7076_L1_Tile|1
AATACTCCTTAGGGCGCAAATTTCTGTATCAGGCAAATCTTGTACAATCACCAGCTAAAC
>HPV93_Beta_37089393_nt7086_L1_Tile|1
AGGGCGCAAATTTCTGTATCAGGCAAATCTTGTACAATCACCAGCTAAACGGTCATCATC
>HPV93_Beta_37089393_nt7091_L1_Tile|1
GCAAATTTCTGTATCAGGCAAATCTTGTACAATCACCAGCTAAACGGTCATCATCTATTT
>HPV93_Beta_37089393_nt7096_L1_Tile|1
TTTCTGTATCAGGCAAATCTTGTACAATCACCAGCTAAACGGTCATCATCTATTTCTCGA
>HPV93_Beta_37089393_nt7101_L1_Tile|1
GTATCAGGCAAATCTTGTACAATCACCAGCTAAACGGTCATCATCTATTTCTCGAGGAAC
>HPV93_Beta_37089393_nt7111_L1_Tile|1
AATCTTGTACAATCACCAGCTAAACGGTCATCATCTATTTCTCGAGGAACTAAAAGAAAA
>HPV93_Beta_37089393_nt7121_L1_Tile|1
AATCACCAGCTAAACGGTCATCATCTATTTCTCGAGGAACTAAAAGAAAAAGAAGCAAAT
>HPV93_Beta_37089393_nt7123_L1_Tile|1
TCACCAGCTAAACGGTCATCATCTATTTCTCGAGGAACTAAAAGAAAAAGAAGCAAATAA

>HPV94_Alpha_40804528_nt6540_L1_Tile|1
TTGCAAGACATTTTTTCAATAGAGCCAGTGCAGTGGGAGACACCATTCCTGACACTCTAA
>HPV94_Alpha_40804528_nt6545_L1_Tile|1
AGACATTTTTTCAATAGAGCCAGTGCAGTGGGAGACACCATTCCTGACACTCTAATATTG
>HPV94_Alpha_40804528_nt6550_L1_Tile|1
TTTTTTCAATAGAGCCAGTGCAGTGGGAGACACCATTCCTGACACTCTAATATTGAAGTC
>HPV94_Alpha_40804528_nt6555_L1_Tile|1
TCAATAGAGCCAGTGCAGTGGGAGACACCATTCCTGACACTCTAATATTGAAGTCGGCCG
>HPV94_Alpha_40804528_nt6575_L1_Tile|1
GGAGACACCATTCCTGACACTCTAATATTGAAGTCGGCCGGTGGTGACCAAAACGTTGGT
>HPV94_Alpha_40804528_nt7035_L1_Tile|1
TCTTGTCGTCCTCCGCAATAACTTGTCAGAAGGAGGCACCCCCCACTGAAAAGCAAGATC
>HPV95_Gamma_40804520_nt5534_L1_Tile|1
GTAGGTGAACCTAAGAAAGTTTTAGTTCCAAAGGTATCTGGAAGTCAATATAGAGTCTTT
>HPV95_Gamma_40804520_nt5539_L1_Tile|1
TGAACCTAAGAAAGTTTTAGTTCCAAAGGTATCTGGAAGTCAATATAGAGTCTTTAGGTT
>HPV95_Gamma_40804520_nt5544_L1_Tile|1
CTAAGAAAGTTTTAGTTCCAAAGGTATCTGGAAGTCAATATAGAGTCTTTAGGTTTCAAT
>HPV95_Gamma_40804520_nt5579_L1_Tile|1
CAATATAGAGTCTTTAGGTTTCAATTGCCAGATCCTAACAGATTTGCTTTGATAGATAAT
>HPV95_Gamma_40804520_nt5624_L1_Tile|1
GCTTTGATAGATAATGGCTTTTATGACTCTGACCATGAAAGGTTAGTATGGCGTCTAAGA
>HPV95_Gamma_40804520_nt5629_L1_Tile|1
GATAGATAATGGCTTTTATGACTCTGACCATGAAAGGTTAGTATGGCGTCTAAGAGGAAT
>HPV95_Gamma_40804520_nt5634_L1_Tile|1
ATAATGGCTTTTATGACTCTGACCATGAAAGGTTAGTATGGCGTCTAAGAGGAATTGAAA
>HPV95_Gamma_40804520_nt5654_L1_Tile|1
GACCATGAAAGGTTAGTATGGCGTCTAAGAGGAATTGAAATAGGCCGAGGTGGACCTTTA
>HPV95_Gamma_40804520_nt5804_L1_Tile|1
CAGGATGTCTCATTAGACCCTAAACAGACCCAAATGTTCATAATTGGTTGTATTCCAGCT
>HPV95_Gamma_40804520_nt5839_L1_Tile|1
GTTCATAATTGGTTGTATTCCAGCTATTGGGGAGCACTGGGATAAAGCTGAGGCATGCAG
>HPV95_Gamma_40804520_nt5844_L1_Tile|1
TAATTGGTTGTATTCCAGCTATTGGGGAGCACTGGGATAAAGCTGAGGCATGCAGAGAAC
>HPV95_Gamma_40804520_nt5894_L1_Tile|1
TGCAGAGAACCAGCACCTCAGAAGGGTGATTGCCCACCTATAGAACTTGTAAACACATAC
>HPV95_Gamma_40804520_nt6014_L1_Tile|1
GATGATAAATCCAGTGCACCATTAGATGTAGTTGGTACTTTGTGTAAATGGCCTGACTTC
>HPV95_Gamma_40804520_nt6019_L1_Tile|1
TAAATCCAGTGCACCATTAGATGTAGTTGGTACTTTGTGTAAATGGCCTGACTTCTTAAA
>HPV95_Gamma_40804520_nt6034_L1_Tile|1
ATTAGATGTAGTTGGTACTTTGTGTAAATGGCCTGACTTCTTAAAGATGAGTAAGGACAT
>HPV95_Gamma_40804520_nt6039_L1_Tile|1
ATGTAGTTGGTACTTTGTGTAAATGGCCTGACTTCTTAAAGATGAGTAAGGACATTTATG
>HPV95_Gamma_40804520_nt6044_L1_Tile|1
GTTGGTACTTTGTGTAAATGGCCTGACTTCTTAAAGATGAGTAAGGACATTTATGGTGAC
>HPV95_Gamma_40804520_nt6049_L1_Tile|1
TACTTTGTGTAAATGGCCTGACTTCTTAAAGATGAGTAAGGACATTTATGGTGACAGTTT
>HPV95_Gamma_40804520_nt6054_L1_Tile|1
TGTGTAAATGGCCTGACTTCTTAAAGATGAGTAAGGACATTTATGGTGACAGTTTATTCT
>HPV95_Gamma_40804520_nt6064_L1_Tile|1
GCCTGACTTCTTAAAGATGAGTAAGGACATTTATGGTGACAGTTTATTCTTCTTTGGCCG
>HPV95_Gamma_40804520_nt6069_L1_Tile|1
ACTTCTTAAAGATGAGTAAGGACATTTATGGTGACAGTTTATTCTTCTTTGGCCGAAGGG
>HPV95_Gamma_40804520_nt6074_L1_Tile|1

TTAAAGATGAGTAAGGACATTTATGGTGACAGTTTATTCTTCTTTGGCCGAAGGGAACAG
>HPV95_Gamma_40804520_nt6079_L1_Tile|1
GATGAGTAAGGACATTTATGGTGACAGTTTATTCTTCTTTGGCCGAAGGGAACAGCTTTA
>HPV95_Gamma_40804520_nt6084_L1_Tile|1
GTAAGGACATTTATGGTGACAGTTTATTCTTCTTTGGCCGAAGGGAACAGCTTTATGCAA
>HPV95_Gamma_40804520_nt6094_L1_Tile|1
TTATGGTGACAGTTTATTCTTCTTTGGCCGAAGGGAACAGCTTTATGCAAGACACTTTTT
>HPV95_Gamma_40804520_nt6099_L1_Tile|1
GTGACAGTTTATTCTTCTTTGGCCGAAGGGAACAGCTTTATGCAAGACACTTTTTTGTTA
>HPV95_Gamma_40804520_nt6104_L1_Tile|1
AGTTTATTCTTCTTTGGCCGAAGGGAACAGCTTTATGCAAGACACTTTTTTGTTAGAGCT
>HPV95_Gamma_40804520_nt6109_L1_Tile|1
ATTCTTCTTTGGCCGAAGGGAACAGCTTTATGCAAGACACTTTTTTGTTAGAGCTGGGAC
>HPV95_Gamma_40804520_nt6149_L1_Tile|1
TTTTTTGTTAGAGCTGGGACAATGGGTGATGCGTTACCAGAGCCTTTTGAAGTGAAGTCT
>HPV95_Gamma_40804520_nt6154_L1_Tile|1
TGTTAGAGCTGGGACAATGGGTGATGCGTTACCAGAGCCTTTTGAAGTGAAGTCTGATTA
>HPV95_Gamma_40804520_nt6159_L1_Tile|1
GAGCTGGGACAATGGGTGATGCGTTACCAGAGCCTTTTGAAGTGAAGTCTGATTACATAA
>HPV95_Gamma_40804520_nt6164_L1_Tile|1
GGGACAATGGGTGATGCGTTACCAGAGCCTTTTGAAGTGAAGTCTGATTACATAATTGCT
>HPV95_Gamma_40804520_nt6179_L1_Tile|1
GCGTTACCAGAGCCTTTTGAAGTGAAGTCTGATTACATAATTGCTGCTCAGAGTAACCAA
>HPV95_Gamma_40804520_nt6189_L1_Tile|1
AGCCTTTTGAAGTGAAGTCTGATTACATAATTGCTGCTCAGAGTAACCAAGAACAAAATA
>HPV95_Gamma_40804520_nt6194_L1_Tile|1
TTTGAAGTGAAGTCTGATTACATAATTGCTGCTCAGAGTAACCAAGAACAAAATAATCTT
>HPV95_Gamma_40804520_nt6199_L1_Tile|1
AGTGAAGTCTGATTACATAATTGCTGCTCAGAGTAACCAAGAACAAAATAATCTTGGCCC
>HPV95_Gamma_40804520_nt6209_L1_Tile|1
GATTACATAATTGCTGCTCAGAGTAACCAAGAACAAAATAATCTTGGCCCTCACATTTAT
>HPV95_Gamma_40804520_nt6214_L1_Tile|1
CATAATTGCTGCTCAGAGTAACCAAGAACAAAATAATCTTGGCCCTCACATTTATTTTGG
>HPV95_Gamma_40804520_nt6219_L1_Tile|1
TTGCTGCTCAGAGTAACCAAGAACAAAATAATCTTGGCCCTCACATTTATTTTGGAACTC
>HPV95_Gamma_40804520_nt6224_L1_Tile|1
GCTCAGAGTAACCAAGAACAAAATAATCTTGGCCCTCACATTTATTTTGGAACTCCTAGC
>HPV95_Gamma_40804520_nt6229_L1_Tile|1
GAGTAACCAAGAACAAAATAATCTTGGCCCTCACATTTATTTTGGAACTCCTAGCGGTTC
>HPV95_Gamma_40804520_nt6234_L1_Tile|1
ACCAAGAACAAAATAATCTTGGCCCTCACATTTATTTTGGAACTCCTAGCGGTTCTCTTG
>HPV95_Gamma_40804520_nt6239_L1_Tile|1
GAACAAAATAATCTTGGCCCTCACATTTATTTTGGAACTCCTAGCGGTTCTCTTGTATCA
>HPV95_Gamma_40804520_nt6244_L1_Tile|1
AAATAATCTTGGCCCTCACATTTATTTTGGAACTCCTAGCGGTTCTCTTGTATCAAGTGA
>HPV95_Gamma_40804520_nt6249_L1_Tile|1
ATCTTGGCCCTCACATTTATTTTGGAACTCCTAGCGGTTCTCTTGTATCAAGTGAATCTC
>HPV95_Gamma_40804520_nt6259_L1_Tile|1
TCACATTTATTTTGGAACTCCTAGCGGTTCTCTTGTATCAAGTGAATCTCAGCTTTTTAA
>HPV95_Gamma_40804520_nt6264_L1_Tile|1
TTTATTTTGGAACTCCTAGCGGTTCTCTTGTATCAAGTGAATCTCAGCTTTTTAACCGAC
>HPV95_Gamma_40804520_nt6269_L1_Tile|1
TTTGGAACTCCTAGCGGTTCTCTTGTATCAAGTGAATCTCAGCTTTTTAACCGACCGTAT
>HPV95_Gamma_40804520_nt6274_L1_Tile|1
AACTCCTAGCGGTTCTCTTGTATCAAGTGAATCTCAGCTTTTTAACCGACCGTATTGGTT

>HPV95_Gamma_40804520_nt6364_L1_Tile|1
TTGTTGGGATAATCAGCTTTTTGTAACTCTGGTTGATAATACCCATAACACTAACTTTAC
>HPV95_Gamma_40804520_nt6369_L1_Tile|1
GGGATAATCAGCTTTTTGTAACTCTGGTTGATAATACCCATAACACTAACTTTACTATTT
>HPV95_Gamma_40804520_nt6374_L1_Tile|1
AATCAGCTTTTTGTAACTCTGGTTGATAATACCCATAACACTAACTTTACTATTTCTGTA
>HPV95_Gamma_40804520_nt6384_L1_Tile|1
TTGTAACTCTGGTTGATAATACCCATAACACTAACTTTACTATTTCTGTAAAGCATGAAG
>HPV95_Gamma_40804520_nt6389_L1_Tile|1
ACTCTGGTTGATAATACCCATAACACTAACTTTACTATTTCTGTAAAGCATGAAGGAGAG
>HPV95_Gamma_40804520_nt6394_L1_Tile|1
GGTTGATAATACCCATAACACTAACTTTACTATTTCTGTAAAGCATGAAGGAGAGGATAA
>HPV95_Gamma_40804520_nt6399_L1_Tile|1
ATAATACCCATAACACTAACTTTACTATTTCTGTAAAGCATGAAGGAGAGGATAACAATT
>HPV95_Gamma_40804520_nt6404_L1_Tile|1
ACCCATAACACTAACTTTACTATTTCTGTAAAGCATGAAGGAGAGGATAACAATTATACA
>HPV95_Gamma_40804520_nt6409_L1_Tile|1
TAACACTAACTTTACTATTTCTGTAAAGCATGAAGGAGAGGATAACAATTATACATATAA
>HPV95_Gamma_40804520_nt6414_L1_Tile|1
CTAACTTTACTATTTCTGTAAAGCATGAAGGAGAGGATAACAATTATACATATAAAGCAA
>HPV95_Gamma_40804520_nt6419_L1_Tile|1
TTTACTATTTCTGTAAAGCATGAAGGAGAGGATAACAATTATACATATAAAGCAAATGAT
>HPV95_Gamma_40804520_nt6424_L1_Tile|1
TATTTCTGTAAAGCATGAAGGAGAGGATAACAATTATACATATAAAGCAAATGATTTTAA
>HPV95_Gamma_40804520_nt6429_L1_Tile|1
CTGTAAAGCATGAAGGAGAGGATAACAATTATACATATAAAGCAAATGATTTTAAACAGT
>HPV95_Gamma_40804520_nt6434_L1_Tile|1
AAGCATGAAGGAGAGGATAACAATTATACATATAAAGCAAATGATTTTAAACAGTATTTA
>HPV95_Gamma_40804520_nt6539_L1_Tile|1
AGAGTGCCTCTTACAGCAGATGTAATGGCTCATTTGAATGTCATGAACCCAAATATATTA
>HPV95_Gamma_40804520_nt6544_L1_Tile|1
GCCTCTTACAGCAGATGTAATGGCTCATTTGAATGTCATGAACCCAAATATATTAGATAA
>HPV95_Gamma_40804520_nt6624_L1_Tile|1
CCCCACCACCTTCTGGTATTGAGGATCAATACAGATTCATTACATCCAGAGCTACTCGTT
>HPV95_Gamma_40804520_nt6644_L1_Tile|1
GAGGATCAATACAGATTCATTACATCCAGAGCTACTCGTTGTCCAACTCAAAATCCACCA
>HPV95_Gamma_40804520_nt6649_L1_Tile|1
TCAATACAGATTCATTACATCCAGAGCTACTCGTTGTCCAACTCAAAATCCACCAGCAGA
>HPV95_Gamma_40804520_nt6654_L1_Tile|1
ACAGATTCATTACATCCAGAGCTACTCGTTGTCCAACTCAAAATCCACCAGCAGAAAGAG
>HPV95_Gamma_40804520_nt6659_L1_Tile|1
TTCATTACATCCAGAGCTACTCGTTGTCCAACTCAAAATCCACCAGCAGAAAGAGAAGAC
>HPV95_Gamma_40804520_nt6679_L1_Tile|1
TCGTTGTCCAACTCAAAATCCACCAGCAGAAAGAGAAGACCCTTTTAAAGATTTGTCCTT
>HPV95_Gamma_40804520_nt6694_L1_Tile|1
AAATCCACCAGCAGAAAGAGAAGACCCTTTTAAAGATTTGTCCTTTTGGACAGTTGATCT
>HPV95_Gamma_40804520_nt6699_L1_Tile|1
CACCAGCAGAAAGAGAAGACCCTTTTAAAGATTTGTCCTTTTGGACAGTTGATCTTTCTG
>HPV95_Gamma_40804520_nt6704_L1_Tile|1
GCAGAAAGAGAAGACCCTTTTAAAGATTTGTCCTTTTGGACAGTTGATCTTTCTGAGCGT
>HPV95_Gamma_40804520_nt6709_L1_Tile|1
AAGAGAAGACCCTTTTAAAGATTTGTCCTTTTGGACAGTTGATCTTTCTGAGCGTTTTTC
>HPV95_Gamma_40804520_nt6714_L1_Tile|1
AAGACCCTTTTAAAGATTTGTCCTTTTGGACAGTTGATCTTTCTGAGCGTTTTTCTTCAG
>HPV95_Gamma_40804520_nt6724_L1_Tile|1

TAAAGATTTGTCCTTTTGGACAGTTGATCTTTCTGAGCGTTTTTCTTCAGAATTAAGCCA
>HPV95_Gamma_40804520_nt6734_L1_Tile|1
TCCTTTTGGACAGTTGATCTTTCTGAGCGTTTTTCTTCAGAATTAAGCCAATTTTCATTA
>HPV95_Gamma_40804520_nt6739_L1_Tile|1
TTGGACAGTTGATCTTTCTGAGCGTTTTTCTTCAGAATTAAGCCAATTTTCATTAGGAAG
>HPV95_Gamma_40804520_nt6749_L1_Tile|1
GATCTTTCTGAGCGTTTTTCTTCAGAATTAAGCCAATTTTCATTAGGAAGGCGGTTTCTC
>HPV95_Gamma_40804520_nt6784_L1_Tile|1
ATTTTCATTAGGAAGGCGGTTTCTCTATCAGAGTGGATTAATAAATGGATCTCGTAAAAG
>HPV95_Gamma_40804520_nt6789_L1_Tile|1
CATTAGGAAGGCGGTTTCTCTATCAGAGTGGATTAATAAATGGATCTCGTAAAAGACAAA
>HPV95_Gamma_40804520_nt6794_L1_Tile|1
GGAAGGCGGTTTCTCTATCAGAGTGGATTAATAAATGGATCTCGTAAAAGACAAAGAGCT
>HPV95_Gamma_40804520_nt6799_L1_Tile|1
GCGGTTTCTCTATCAGAGTGGATTAATAAATGGATCTCGTAAAAGACAAAGAGCTATTAC
>HPV95_Gamma_40804520_nt6804_L1_Tile|1
TTCTCTATCAGAGTGGATTAATAAATGGATCTCGTAAAAGACAAAGAGCTATTACTTCTC
>HPV95_Gamma_40804520_nt6809_L1_Tile|1
TATCAGAGTGGATTAATAAATGGATCTCGTAAAAGACAAAGAGCTATTACTTCTCAAACT
>HPV95_Gamma_40804520_nt6819_L1_Tile|1
GATTAATAAATGGATCTCGTAAAAGACAAAGAGCTATTACTTCTCAAACTGCAACAGGCA
>HPV95_Gamma_40804520_nt6829_L1_Tile|1
TGGATCTCGTAAAAGACAAAGAGCTATTACTTCTCAAACTGCAACAGGCACAAAACGTTC
>HPV96_Beta_50253426_nt5987_L1_Tile|1
TGTTAACAGTAGGACATCCTTATTTTGATGTGAGGAAAAATAATGGAGATCATGAAGTGT
>HPV96_Beta_50253426_nt5992_L1_Tile|1
ACAGTAGGACATCCTTATTTTGATGTGAGGAAAAATAATGGAGATCATGAAGTGTTAGTT
>HPV96_Beta_50253426_nt5997_L1_Tile|1
AGGACATCCTTATTTTGATGTGAGGAAAAATAATGGAGATCATGAAGTGTTAGTTCCCAA
>HPV96_Beta_50253426_nt6002_L1_Tile|2
ATCCTTATTTTGATGTGAGGAAAAATAATGGAGATCATGAAGTGTTAGTTCCCAAGGTGT
>HPV96_Beta_50253426_nt6222_L1_Tile|2
ACATCCATTATTTAACAAGGTGAAAGACACGGAAAATCCAAATGGCTATAATACAGGTGG
>HPV96_Beta_50253426_nt6252_L1_Tile|1
GGAAAATCCAAATGGCTATAATACAGGTGGAAAGGATGATAGGGTGAATACATCCTTTGA
>HPV96_Beta_50253426_nt6257_L1_Tile|1
ATCCAAATGGCTATAATACAGGTGGAAAGGATGATAGGGTGAATACATCCTTTGATCCCA
>HPV96_Beta_50253426_nt6367_L1_Tile|1
TGGGACAAGGCCTTACCTTGTGTAGAAAATCCTCCTGATCAGGGAGCGTGTCCACCTCTA
>HPV96_Beta_50253426_nt6377_L1_Tile|1
CCTTACCTTGTGTAGAAAATCCTCCTGATCAGGGAGCGTGTCCACCTCTAGAATTAAAAA
>HPV96_Beta_50253426_nt6382_L1_Tile|2
CCTTGTGTAGAAAATCCTCCTGATCAGGGAGCGTGTCCACCTCTAGAATTAAAAAATACT
>HPV96_Beta_50253426_nt6387_L1_Tile|1
TGTAGAAAATCCTCCTGATCAGGGAGCGTGTCCACCTCTAGAATTAAAAAATACTATTAT
>HPV96_Beta_50253426_nt6487_L1_Tile|1
AATTTTAAAACATTATCAGTCACTAAGTCTGATGTTAGTCTGGATATTGTTAATGAAATT
>HPV96_Beta_50253426_nt6632_L1_Tile|1
CCAGACATATGTTTTGTAGAGGTGGGTCAGTAGGAGACAGTATTCCAGATGATGCAGTTG
>HPV96_Beta_50253426_nt6637_L1_Tile|1
CATATGTTTTGTAGAGGTGGGTCAGTAGGAGACAGTATTCCAGATGATGCAGTTGGAGAA
>HPV96_Beta_50253426_nt6647_L1_Tile|1
GTAGAGGTGGGTCAGTAGGAGACAGTATTCCAGATGATGCAGTTGGAGAAGACAACCATT
>HPV96_Beta_50253426_nt6662_L1_Tile|2
TAGGAGACAGTATTCCAGATGATGCAGTTGGAGAAGACAACCATTATTATTTAAAGGCTG

>HPV96_Beta_50253426_nt6667_L1_Tile|1
GACAGTATTCCAGATGATGCAGTTGGAGAAGACAACCATTATTATTTAAAGGCTGCCAGT
>HPV96_Beta_50253426_nt6672_L1_Tile|1
TATTCCAGATGATGCAGTTGGAGAAGACAACCATTATTATTTAAAGGCTGCCAGTGATCA
>HPV96_Beta_50253426_nt6682_L1_Tile|2
GATGCAGTTGGAGAAGACAACCATTATTATTTAAAGGCTGCCAGTGATCAAAACAGAGAT
>HPV96_Beta_50253426_nt6687_L1_Tile|1
AGTTGGAGAAGACAACCATTATTATTTAAAGGCTGCCAGTGATCAAAACAGAGATACAAT
>HPV96_Beta_50253426_nt6692_L1_Tile|1
GAGAAGACAACCATTATTATTTAAAGGCTGCCAGTGATCAAAACAGAGATACAATGGCAA
>HPV96_Beta_50253426_nt6697_L1_Tile|1
GACAACCATTATTATTTAAAGGCTGCCAGTGATCAAAACAGAGATACAATGGCAAGTTCC
>HPV96_Beta_50253426_nt6702_L1_Tile|2
CCATTATTATTTAAAGGCTGCCAGTGATCAAAACAGAGATACAATGGCAAGTTCCATTTA
>HPV96_Beta_50253426_nt6707_L1_Tile|1
ATTATTTAAAGGCTGCCAGTGATCAAAACAGAGATACAATGGCAAGTTCCATTTACACTC
>HPV96_Beta_50253426_nt6727_L1_Tile|1
GATCAAAACAGAGATACAATGGCAAGTTCCATTTACACTCCCACAGTCAGTGGATCTTTA
>HPV96_Beta_50253426_nt6732_L1_Tile|1
AAACAGAGATACAATGGCAAGTTCCATTTACACTCCCACAGTCAGTGGATCTTTAGTTTC
>HPV96_Beta_50253426_nt6737_L1_Tile|1
GAGATACAATGGCAAGTTCCATTTACACTCCCACAGTCAGTGGATCTTTAGTTTCTACAG
>HPV96_Beta_50253426_nt6742_L1_Tile|2
ACAATGGCAAGTTCCATTTACACTCCCACAGTCAGTGGATCTTTAGTTTCTACAGATGCA
>HPV96_Beta_50253426_nt6747_L1_Tile|1
GGCAAGTTCCATTTACACTCCCACAGTCAGTGGATCTTTAGTTTCTACAGATGCACAGAT
>HPV96_Beta_50253426_nt6757_L1_Tile|1
ATTTACACTCCCACAGTCAGTGGATCTTTAGTTTCTACAGATGCACAGATTTTCAATAGG
>HPV96_Beta_50253426_nt6777_L1_Tile|1
TGGATCTTTAGTTTCTACAGATGCACAGATTTTCAATAGGCCTTTTTGGCTGCAAAGGGC
>HPV96_Beta_50253426_nt6782_L1_Tile|2
CTTTAGTTTCTACAGATGCACAGATTTTCAATAGGCCTTTTTGGCTGCAAAGGGCTCAAG
>HPV96_Beta_50253426_nt6862_L1_Tile|2
TGGGGTAATCAAATCTTTCTCACAGTAATAGATAATACCAGGAATACTAATTTCTGTATC
>HPV96_Beta_50253426_nt6867_L1_Tile|1
TAATCAAATCTTTCTCACAGTAATAGATAATACCAGGAATACTAATTTCTGTATCAGTGT
>HPV96_Beta_50253426_nt6877_L1_Tile|1
TTTCTCACAGTAATAGATAATACCAGGAATACTAATTTCTGTATCAGTGTCTCCTCAAAT
>HPV96_Beta_50253426_nt6882_L1_Tile|2
CACAGTAATAGATAATACCAGGAATACTAATTTCTGTATCAGTGTCTCCTCAAATGATCA
>HPV96_Beta_50253426_nt6887_L1_Tile|1
TAATAGATAATACCAGGAATACTAATTTCTGTATCAGTGTCTCCTCAAATGATCAGGCAT
>HPV96_Beta_50253426_nt6892_L1_Tile|1
GATAATACCAGGAATACTAATTTCTGTATCAGTGTCTCCTCAAATGATCAGGCATTACAG
>HPV96_Beta_50253426_nt6897_L1_Tile|1
TACCAGGAATACTAATTTCTGTATCAGTGTCTCCTCAAATGATCAGGCATTACAGGAATA
>HPV96_Beta_50253426_nt6902_L1_Tile|2
GGAATACTAATTTCTGTATCAGTGTCTCCTCAAATGATCAGGCATTACAGGAATACAATA
>HPV96_Beta_50253426_nt6907_L1_Tile|1
ACTAATTTCTGTATCAGTGTCTCCTCAAATGATCAGGCATTACAGGAATACAATACTGCA
>HPV96_Beta_50253426_nt6912_L1_Tile|1
TTTCTGTATCAGTGTCTCCTCAAATGATCAGGCATTACAGGAATACAATACTGCAAACTT
>HPV96_Beta_50253426_nt6917_L1_Tile|1
GTATCAGTGTCTCCTCAAATGATCAGGCATTACAGGAATACAATACTGCAAACTTTAGAG
>HPV96_Beta_50253426_nt6922_L1_Tile|2

AGTGTCTCCTCAAATGATCAGGCATTACAGGAATACAATACTGCAAACTTTAGAGAATAT
>HPV96_Beta_50253426_nt6927_L1_Tile|1
CTCCTCAAATGATCAGGCATTACAGGAATACAATACTGCAAACTTTAGAGAATATTTGAG
>HPV96_Beta_50253426_nt6932_L1_Tile|1
CAAATGATCAGGCATTACAGGAATACAATACTGCAAACTTTAGAGAATATTTGAGACATG
>HPV96_Beta_50253426_nt6987_L1_Tile|1
ACATGTAGAAGAGTATGAATTATCCTTTATATTACAATTATGTAAAGTTCCATTAGAGCC
>HPV96_Beta_50253426_nt6992_L1_Tile|1
TAGAAGAGTATGAATTATCCTTTATATTACAATTATGTAAAGTTCCATTAGAGCCAGAAG
>HPV96_Beta_50253426_nt6997_L1_Tile|1
GAGTATGAATTATCCTTTATATTACAATTATGTAAAGTTCCATTAGAGCCAGAAGTATTA
>HPV96_Beta_50253426_nt7122_L1_Tile|2
TGACAATCCCATCAATGATACATATAGATACATACATTCAGCAGCCACACGGTGTCCAGA
>HPV96_Beta_50253426_nt7127_L1_Tile|1
ATCCCATCAATGATACATATAGATACATACATTCAGCAGCCACACGGTGTCCAGATAAAA
>HPV96_Beta_50253426_nt7132_L1_Tile|1
ATCAATGATACATATAGATACATACATTCAGCAGCCACACGGTGTCCAGATAAAACTACA
>HPV96_Beta_50253426_nt7182_L1_Tile|2
TAAAACTACACCTAAAGAAAAAGCAGATCCCTTTGCAGGTTATCACTTTTGGGATGTTGA
>HPV96_Beta_50253426_nt7187_L1_Tile|1
CTACACCTAAAGAAAAAGCAGATCCCTTTGCAGGTTATCACTTTTGGGATGTTGATTTGT
>HPV96_Beta_50253426_nt7197_L1_Tile|1
AGAAAAAGCAGATCCCTTTGCAGGTTATCACTTTTGGGATGTTGATTTGTCTGAAAAGTT
>HPV96_Beta_50253426_nt7272_L1_Tile|1
TCAGTATTCTCTGGGACGTAAATTCTTATTTCAAGCCAACCTGCAAAACAAAAGAGTTAA
>HPV96_Beta_50253426_nt7292_L1_Tile|1
AATTCTTATTTCAAGCCAACCTGCAAAACAAAAGAGTTAACAGAGGGGTTACTGTAACCG
>HPV96_Beta_50253426_nt7297_L1_Tile|1
TTATTTCAAGCCAACCTGCAAAACAAAAGAGTTAACAGAGGGGTTACTGTAACCGGGAGG
>HPV96_Beta_50253426_nt7302_L1_Tile|2
TCAAGCCAACCTGCAAAACAAAAGAGTTAACAGAGGGGTTACTGTAACCGGGAGGGCTAC
>HPV96_Beta_50253426_nt7317_L1_Tile|1
AAACAAAAGAGTTAACAGAGGGGTTACTGTAACCGGGAGGGCTACAACCTCAAGAGGTAC
>HPV96_Beta_50253426_nt7322_L1_Tile|2
AAAGAGTTAACAGAGGGGTTACTGTAACCGGGAGGGCTACAACCTCAAGAGGTACAAAAC
>HPV96_Beta_50253426_nt7336_L1_Tile|1
GGGGTTACTGTAACCGGGAGGGCTACAACCTCAAGAGGTACAAAACGAAAACGACGCTGA
>HPV97_Alpha_71726694_nt5612_L1_Tile|1
TCTAGATTATTAGCTGTAGGCAATCCTTATTTTAAAGTTTCTAAAGGTGCAGGTAGTAGG
>HPV97_Alpha_71726694_nt5617_L1_Tile|1
ATTATTAGCTGTAGGCAATCCTTATTTTAAAGTTTCTAAAGGTGCAGGTAGTAGGCAAGA
>HPV97_Alpha_71726694_nt5622_L1_Tile|2
TAGCTGTAGGCAATCCTTATTTTAAAGTTTCTAAAGGTGCAGGTAGTAGGCAAGATGTTC
>HPV97_Alpha_71726694_nt5642_L1_Tile|2
TTTAAAGTTTCTAAAGGTGCAGGTAGTAGGCAAGATGTTCCCAAAGTATCTGCCTACCAA
>HPV97_Alpha_71726694_nt5647_L1_Tile|1
AGTTTCTAAAGGTGCAGGTAGTAGGCAAGATGTTCCCAAAGTATCTGCCTACCAATATAG
>HPV97_Alpha_71726694_nt5657_L1_Tile|1
GGTGCAGGTAGTAGGCAAGATGTTCCCAAAGTATCTGCCTACCAATATAGAGTGTTTAGG
>HPV97_Alpha_71726694_nt5662_L1_Tile|2
AGGTAGTAGGCAAGATGTTCCCAAAGTATCTGCCTACCAATATAGAGTGTTTAGGGTAAC
>HPV97_Alpha_71726694_nt5667_L1_Tile|1
GTAGGCAAGATGTTCCCAAAGTATCTGCCTACCAATATAGAGTGTTTAGGGTAACTTTGC
>HPV97_Alpha_71726694_nt5857_L1_Tile|1
TTTATATAACAAATTAGATGACACTGAAAGTTCACATGCTGCTACAGCTGTTGTTACACA

>HPV97_Alpha_71726694_nt5982_L1_Tile|2
CTGCTATTGGTGAACATTGGGCTAAAGGTACACCATGTAAATCTACTCACATACAGCAAG
>HPV97_Alpha_71726694_nt6017_L1_Tile|1
TGTAAATCTACTCACATACAGCAAGGTGACTGTCCTCCTTTAGAGCTTAAAAATACAGTG
>HPV97_Alpha_71726694_nt6287_L1_Tile|1
GGAACAATGGGTGACACTATTCCACAGGAGTTATATATTAAAGGAACTGGTATGCGTGAA
>HPV97_Alpha_71726694_nt6302_L1_Tile|2
ACTATTCCACAGGAGTTATATATTAAAGGAACTGGTATGCGTGAAACTCCTGGCAGTTTT
>HPV97_Alpha_71726694_nt6517_L1_Tile|1
TTTAACACTGTGTGCTTCTACACAAAATGGCGTAGCTACCACATATGACCCTACAAAATA
>HPV97_Alpha_71726694_nt6522_L1_Tile|2
CACTGTGTGCTTCTACACAAAATGGCGTAGCTACCACATATGACCCTACAAAATATAAAC
>HPV97_Alpha_71726694_nt6712_L1_Tile|1
TGTCCCACCGCCACCCACTACCAGTTTAGTAGATACATATCGCTTTGTACAGTCTGTTGC
>HPV97_Alpha_71726694_nt6717_L1_Tile|1
CACCGCCACCCACTACCAGTTTAGTAGATACATATCGCTTTGTACAGTCTGTTGCTATAA
>HPV97_Alpha_71726694_nt6952_L1_Tile|1
TTCTGCCCCGTCTGCCCCTACTACTTCCAAACCTGCTAAGCGTTTACGTGTCCGTCGTAA
>HPVFA75/KI88-03_Unclassified_206902429_nt5666_L1_Tile|1
TCAACTCCAGTAGCTAAGGTGTTAAGCACTGATGAATATGTAGAAAGAACTAACATATTT
>HPVFA75/KI88-03_Unclassified_206902429_nt6136_L1_Tile|1
TGAGCCATGTGAAAATGCAGGAGATCAACAGGGAAAATGTCCACCTATAGAACTGAAAAA
>HPVFA75/KI88-03_Unclassified_206902429_nt6141_L1_Tile|1
CATGTGAAAATGCAGGAGATCAACAGGGAAAATGTCCACCTATAGAACTGAAAAATTCTG
>HPVFA75/KI88-03_Unclassified_206902429_nt6166_L1_Tile|1
GGGAAAATGTCCACCTATAGAACTGAAAAATTCTGTGATTCAAGATGGCGATATGATGGA
>HPVFA75/KI88-03_Unclassified_206902429_nt6186_L1_Tile|1
AACTGAAAAATTCTGTGATTCAAGATGGCGATATGATGGACATTGGATTTGGAAATATAA
>HPVFA75/KI88-03_Unclassified_206902429_nt6236_L1_Tile|1
GGAAATATAAATTTTAAAACTCTATCCCTGAATAGATCAGATGTTAGCTTAGATTTAGTA
>HPVFA75/KI88-03_Unclassified_206902429_nt6241_L1_Tile|1
TATAAATTTTAAAACTCTATCCCTGAATAGATCAGATGTTAGCTTAGATTTAGTAGACGA
>HPVFA75/KI88-03_Unclassified_206902429_nt6246_L1_Tile|1
ATTTTAAAACTCTATCCCTGAATAGATCAGATGTTAGCTTAGATTTAGTAGACGAAATTG
>HPVFA75/KI88-03_Unclassified_206902429_nt6251_L1_Tile|1
AAAACTCTATCCCTGAATAGATCAGATGTTAGCTTAGATTTAGTAGACGAAATTGCTAAA
>HPVFA75/KI88-03_Unclassified_206902429_nt6391_L1_Tile|1
AAGACATTATTTTACCAGAGGAGGTAATGTTGGAGACGCTACTCCAGATGGAGCAAATAA
>HPVFA75/KI88-03_Unclassified_206902429_nt6396_L1_Tile|1
ATTATTTTACCAGAGGAGGTAATGTTGGAGACGCTACTCCAGATGGAGCAAATAATCAAG
>HPVFA75/KI88-03_Unclassified_206902429_nt6401_L1_Tile|1
TTTACCAGAGGAGGTAATGTTGGAGACGCTACTCCAGATGGAGCAAATAATCAAGATCAT
>HPVFA75/KI88-03_Unclassified_206902429_nt6406_L1_Tile|1
CAGAGGAGGTAATGTTGGAGACGCTACTCCAGATGGAGCAAATAATCAAGATCATAAATT
>HPVFA75/KI88-03_Unclassified_206902429_nt6416_L1_Tile|1
AATGTTGGAGACGCTACTCCAGATGGAGCAAATAATCAAGATCATAAATTTTATTTACCT
>HPVFA75/KI88-03_Unclassified_206902429_nt6421_L1_Tile|1
TGGAGACGCTACTCCAGATGGAGCAAATAATCAAGATCATAAATTTTATTTACCTCCTAA
>HPVFA75/KI88-03_Unclassified_206902429_nt6426_L1_Tile|1
ACGCTACTCCAGATGGAGCAAATAATCAAGATCATAAATTTTATTTACCTCCTAAGTCAT
>HPVFA75/KI88-03_Unclassified_206902429_nt6436_L1_Tile|1
AGATGGAGCAAATAATCAAGATCATAAATTTTATTTACCTCCTAAGTCATCACAAAGTCA
>HPVFA75/KI88-03_Unclassified_206902429_nt6441_L1_Tile|1
GAGCAAATAATCAAGATCATAAATTTTATTTACCTCCTAAGTCATCACAAAGTCAAACAC
>HPVFA75/KI88-03_Unclassified_206902429_nt6446_L1_Tile|1

AATAATCAAGATCATAAATTTTATTTACCTCCTAAGTCATCACAAAGTCAAACACTAGGA
>HPVFA75/KI88-03_Unclassified_206902429_nt6451_L1_Tile|1
TCAAGATCATAAATTTTATTTACCTCCTAAGTCATCACAAAGTCAAACACTAGGAAATTC
>HPVFA75/KI88-03_Unclassified_206902429_nt6456_L1_Tile|1
ATCATAAATTTTATTTACCTCCTAAGTCATCACAAAGTCAAACACTAGGAAATTCAATTT
>HPVFA75/KI88-03_Unclassified_206902429_nt6461_L1_Tile|1
AAATTTTATTTACCTCCTAAGTCATCACAAAGTCAAACACTAGGAAATTCAATTTACTTT
>HPVFA75/KI88-03_Unclassified_206902429_nt6466_L1_Tile|1
TTATTTACCTCCTAAGTCATCACAAAGTCAAACACTAGGAAATTCAATTTACTTTCCTAC
>HPVFA75/KI88-03_Unclassified_206902429_nt6471_L1_Tile|1
TACCTCCTAAGTCATCACAAAGTCAAACACTAGGAAATTCAATTTACTTTCCTACTGTTA
>HPVFA75/KI88-03_Unclassified_206902429_nt6476_L1_Tile|1
CCTAAGTCATCACAAAGTCAAACACTAGGAAATTCAATTTACTTTCCTACTGTTAGTGGT
>HPVFA75/KI88-03_Unclassified_206902429_nt6481_L1_Tile|1
GTCATCACAAAGTCAAACACTAGGAAATTCAATTTACTTTCCTACTGTTAGTGGTTCTCT
>HPVFA75/KI88-03_Unclassified_206902429_nt6491_L1_Tile|1
AGTCAAACACTAGGAAATTCAATTTACTTTCCTACTGTTAGTGGTTCTCTAGTCTCTTCA
>HPVFA75/KI88-03_Unclassified_206902429_nt6641_L1_Tile|1
GTTGCTGATAACACTAGAAACACTAACTTTACCATTTGTGTACCTTCAGATAATGGTGCT
>HPVFA75/KI88-03_Unclassified_206902429_nt6646_L1_Tile|1
TGATAACACTAGAAACACTAACTTTACCATTTGTGTACCTTCAGATAATGGTGCTATAAC
>HPVFA75/KI88-03_Unclassified_206902429_nt6656_L1_Tile|1
AGAAACACTAACTTTACCATTTGTGTACCTTCAGATAATGGTGCTATAACTGAGTATGAT
>HPVFA75/KI88-03_Unclassified_206902429_nt6661_L1_Tile|1
CACTAACTTTACCATTTGTGTACCTTCAGATAATGGTGCTATAACTGAGTATGATTCTAG
>HPVFA75/KI88-03_Unclassified_206902429_nt6666_L1_Tile|1
ACTTTACCATTTGTGTACCTTCAGATAATGGTGCTATAACTGAGTATGATTCTAGCAAAT
>HPVFA75/KI88-03_Unclassified_206902429_nt6676_L1_Tile|1
TTGTGTACCTTCAGATAATGGTGCTATAACTGAGTATGATTCTAGCAAATTTAGAGAATT
>HPVFA75/KI88-03_Unclassified_206902429_nt6681_L1_Tile|1
TACCTTCAGATAATGGTGCTATAACTGAGTATGATTCTAGCAAATTTAGAGAATTTTTAA
>HPVFA75/KI88-03_Unclassified_206902429_nt6691_L1_Tile|1
TAATGGTGCTATAACTGAGTATGATTCTAGCAAATTTAGAGAATTTTTAAGGCACGTGGA
>HPVFA75/KI88-03_Unclassified_206902429_nt6701_L1_Tile|1
ATAACTGAGTATGATTCTAGCAAATTTAGAGAATTTTTAAGGCACGTGGAAGAGTATCAA
>HPVFA75/KI88-03_Unclassified_206902429_nt6706_L1_Tile|1
TGAGTATGATTCTAGCAAATTTAGAGAATTTTTAAGGCACGTGGAAGAGTATCAAATATC
>HPVFA75/KI88-03_Unclassified_206902429_nt6711_L1_Tile|1
ATGATTCTAGCAAATTTAGAGAATTTTTAAGGCACGTGGAAGAGTATCAAATATCTGTAA
>HPVFA75/KI88-03_Unclassified_206902429_nt6766_L1_Tile|1
TGTAATATTACAACTGTGTAAAGTATCACTGCAACCTGATGTGCTAGCCCAGATCAATGC
>HPVFA75/KI88-03_Unclassified_206902429_nt6771_L1_Tile|1
TATTACAACTGTGTAAAGTATCACTGCAACCTGATGTGCTAGCCCAGATCAATGCAATGA
>HPVFA75/KI88-03_Unclassified_206902429_nt6786_L1_Tile|1
AAGTATCACTGCAACCTGATGTGCTAGCCCAGATCAATGCAATGAATTCAGGTATATTAG
>HPVFA75/KI88-03_Unclassified_206902429_nt6861_L1_Tile|1
GATTTGTACCAACTCCTGACAATGCAGTACATGACACCTATAGATTTATAAATTCCTCAG
>HPVFA75/KI88-03_Unclassified_206902429_nt6871_L1_Tile|1
AACTCCTGACAATGCAGTACATGACACCTATAGATTTATAAATTCCTCAGCCACTAAATG
>HPVFA75/KI88-03_Unclassified_206902429_nt6891_L1_Tile|1
ATGACACCTATAGATTTATAAATTCCTCAGCCACTAAATGTCCAGATAAGGTTCCTGCAA
>HPVFA75/KI88-03_Unclassified_206902429_nt6896_L1_Tile|1
ACCTATAGATTTATAAATTCCTCAGCCACTAAATGTCCAGATAAGGTTCCTGCAAAAGAT
>HPVFA75/KI88-03_Unclassified_206902429_nt6901_L1_Tile|1
TAGATTTATAAATTCCTCAGCCACTAAATGTCCAGATAAGGTTCCTGCAAAAGATAGAGA

>HPVFA75/KI88-03_Unclassified_206902429_nt6906_L1_Tile|1
TTATAAATTCCTCAGCCACTAAATGTCCAGATAAGGTTCCTGCAAAAGATAGAGAGGATC
>HPVFA75/KI88-03_Unclassified_206902429_nt6911_L1_Tile|1
AATTCCTCAGCCACTAAATGTCCAGATAAGGTTCCTGCAAAAGATAGAGAGGATCCATTT
>HPVFA75/KI88-03_Unclassified_206902429_nt6926_L1_Tile|1
AAATGTCCAGATAAGGTTCCTGCAAAAGATAGAGAGGATCCATTTGCTCAATATTTCTTT
>HPVFA75/KI88-03_Unclassified_206902429_nt7021_L1_Tile|1
TTTAGATCAATATCCTTTAGGAAGAAAGTTTTTGTTCCAAGCAGGATTACAGAACACCAG
>HPVFA75/KI88-03_Unclassified_206902429_nt7026_L1_Tile|1
ATCAATATCCTTTAGGAAGAAAGTTTTTGTTCCAAGCAGGATTACAGAACACCAGGGTCA
>HPVFA75/KI88-03_Unclassified_206902429_nt7031_L1_Tile|1
TATCCTTTAGGAAGAAAGTTTTTGTTCCAAGCAGGATTACAGAACACCAGGGTCAAATCA
>HPVFA75/KI88-03_Unclassified_206902429_nt7036_L1_Tile|1
TTTAGGAAGAAAGTTTTTGTTCCAAGCAGGATTACAGAACACCAGGGTCAAATCATTAAA
>HPVFA75/KI88-03_Unclassified_206902429_nt7041_L1_Tile|1
GAAGAAAGTTTTTGTTCCAAGCAGGATTACAGAACACCAGGGTCAAATCATTAAAGCGTA
>HPVFA75/KI88-03_Unclassified_206902429_nt7046_L1_Tile|1
AAGTTTTTGTTCCAAGCAGGATTACAGAACACCAGGGTCAAATCATTAAAGCGTAGCAGA
>HPVFA75/KI88-03_Unclassified_206902429_nt7051_L1_Tile|1
TTTGTTCCAAGCAGGATTACAGAACACCAGGGTCAAATCATTAAAGCGTAGCAGATCTGT
>HPVFA75/KI88-03_Unclassified_206902429_nt7071_L1_Tile|1
AGAACACCAGGGTCAAATCATTAAAGCGTAGCAGATCTGTTTCTCGGGGTACTGCAAAAC
>HPVFA75/KI88-03_Unclassified_206902429_nt7081_L1_Tile|1
GGTCAAATCATTAAAGCGTAGCAGATCTGTTTCTCGGGGTACTGCAAAACGACGACGGAC
>HPVRTRX7_Unclassified_30315616_nt6412_L1_Tile|1
GTGTGGGAGAGCATTGGGAAAAGGCCTTACCTTGTGGAGATGCGCCTGCAGATAATGGTA
>HPVRTRX7_Unclassified_30315616_nt6427_L1_Tile|1
GGGAAAAGGCCTTACCTTGTGGAGATGCGCCTGCAGATAATGGTATTTGCCCTCCAATTG
>HPVRTRX7_Unclassified_30315616_nt6447_L1_Tile|1
GGAGATGCGCCTGCAGATAATGGTATTTGCCCTCCAATTGAATTAAAAAACACTTTTATT
>HPVRTRX7_Unclassified_30315616_nt6532_L1_Tile|1
GTTTTGGCAATATGAATTTCAAAACTTTACAGCAGAATAGGGCTGATGTCAGTCTGGATA
>HPVRTRX7_Unclassified_30315616_nt6537_L1_Tile|1
GGCAATATGAATTTCAAAACTTTACAGCAGAATAGGGCTGATGTCAGTCTGGATATAGTT
>HPVRTRX7_Unclassified_30315616_nt6542_L1_Tile|2
TATGAATTTCAAAACTTTACAGCAGAATAGGGCTGATGTCAGTCTGGATATAGTTAATGA
>HPVRTRX7_Unclassified_30315616_nt6547_L1_Tile|1
ATTTCAAAACTTTACAGCAGAATAGGGCTGATGTCAGTCTGGATATAGTTAATGAAACTT
>HPVRTRX7_Unclassified_30315616_nt6987_L1_Tile|1
ATATCTATATCTAGTGAAAATCAAGATATACAGCAAATACAATCATATGACTCACAAAAG
>HPVRTRX7_Unclassified_30315616_nt6997_L1_Tile|1
CTAGTGAAAATCAAGATATACAGCAAATACAATCATATGACTCACAAAAGTTTAGGGAAT
>HPVRTRX7_Unclassified_30315616_nt7002_L1_Tile|2
GAAAATCAAGATATACAGCAAATACAATCATATGACTCACAAAAGTTTAGGGAATATTTA
>HPVRTRX7_Unclassified_30315616_nt7007_L1_Tile|1
TCAAGATATACAGCAAATACAATCATATGACTCACAAAAGTTTAGGGAATATTTAAGGCA
>HPVRTRX7_Unclassified_30315616_nt7012_L1_Tile|1
ATATACAGCAAATACAATCATATGACTCACAAAAGTTTAGGGAATATTTAAGGCACGTAG
>HPVRTRX7_Unclassified_30315616_nt7107_L1_Tile|1
AAGATTCCACTACAAGCAGAAGTTTTAGCACAAATAAATGCAATGAACCCCTCCTTACTA
>HPVRTRX7_Unclassified_30315616_nt7182_L1_Tile|2
GGATTTGTGCCAACTCCCGATAATCCTATCCAGGACACATACAGATTTATTGATTCCTTA
>HPVRTRX7_Unclassified_30315616_nt7197_L1_Tile|1
CCCGATAATCCTATCCAGGACACATACAGATTTATTGATTCCTTAGCTACCAGGTGTCCC
>HPVRTRX7_Unclassified_30315616_nt7202_L1_Tile|2

TAATCCTATCCAGGACACATACAGATTTATTGATTCCTTAGCTACCAGGTGTCCCGATAA
>HPVRTRX7_Unclassified_30315616_nt7207_L1_Tile|1
CTATCCAGGACACATACAGATTTATTGATTCCTTAGCTACCAGGTGTCCCGATAAAAATC
>HPVRTRX7_Unclassified_30315616_nt7212_L1_Tile|1
CAGGACACATACAGATTTATTGATTCCTTAGCTACCAGGTGTCCCGATAAAAATCCACCA
>HPVRTRX7_Unclassified_30315616_nt7217_L1_Tile|1
CACATACAGATTTATTGATTCCTTAGCTACCAGGTGTCCCGATAAAAATCCACCAAAGGA
>HPVRTRX7_Unclassified_30315616_nt7397_L1_Tile|1
AACGACCGTTAACGGTACAAAGCCTTCAACGTATAGAAGTTCTGTACGGGGGATCAAAAG
>HPV1_Mu_9626063_nt5414_L1_Tile_rc|1
AGGAAGATAGAACTTATTCTGCGCTGGTAACCAGACAGCCATCTGAAAAACATTATACAT
>HPV1_Mu_9626063_nt5419_L1_Tile_rc|1
TGGGGAGGAAGATAGAACTTATTCTGCGCTGGTAACCAGACAGCCATCTGAAAAACATTA
>HPV1_Mu_9626063_nt5434_L1_Tile_rc|1
ATTCTAGTGATGGGCTGGGGAGGAAGATAGAACTTATTCTGCGCTGGTAACCAGACAGCC
>HPV1_Mu_9626063_nt5439_L1_Tile_rc|1
ACAGGATTCTAGTGATGGGCTGGGGAGGAAGATAGAACTTATTCTGCGCTGGTAACCAGA
>HPV1_Mu_9626063_nt5449_L1_Tile_rc|1
TCATCAGTGGACAGGATTCTAGTGATGGGCTGGGGAGGAAGATAGAACTTATTCTGCGCT
>HPV1_Mu_9626063_nt5454_L1_Tile_rc|1
CATATTCATCAGTGGACAGGATTCTAGTGATGGGCTGGGGAGGAAGATAGAACTTATTCT
>HPV1_Mu_9626063_nt5459_L1_Tile_rc|1
GGTTACATATTCATCAGTGGACAGGATTCTAGTGATGGGCTGGGGAGGAAGATAGAACTT
>HPV1_Mu_9626063_nt5474_L1_Tile_rc|1
GAAGAGATTGGTTCTGGTTACATATTCATCAGTGGACAGGATTCTAGTGATGGGCTGGGG
>HPV1_Mu_9626063_nt5479_L1_Tile_rc|1
TGGTAGAAGAGATTGGTTCTGGTTACATATTCATCAGTGGACAGGATTCTAGTGATGGGC
>HPV1_Mu_9626063_nt5484_L1_Tile_rc|1
TTGCATGGTAGAAGAGATTGGTTCTGGTTACATATTCATCAGTGGACAGGATTCTAGTGA
>HPV1_Mu_9626063_nt5489_L1_Tile_rc|1
AGATGTTGCATGGTAGAAGAGATTGGTTCTGGTTACATATTCATCAGTGGACAGGATTCT
>HPV1_Mu_9626063_nt5499_L1_Tile_rc|1
GTAGACGTTCAGATGTTGCATGGTAGAAGAGATTGGTTCTGGTTACATATTCATCAGTGG
>HPV1_Mu_9626063_nt5504_L1_Tile_rc|1
CAGCAGTAGACGTTCAGATGTTGCATGGTAGAAGAGATTGGTTCTGGTTACATATTCATC
>HPV1_Mu_9626063_nt5509_L1_Tile_rc|1
CCGACCAGCAGTAGACGTTCAGATGTTGCATGGTAGAAGAGATTGGTTCTGGTTACATAT
>HPV1_Mu_9626063_nt5514_L1_Tile_rc|1
GATGTCCGACCAGCAGTAGACGTTCAGATGTTGCATGGTAGAAGAGATTGGTTCTGGTTA
>HPV1_Mu_9626063_nt5529_L1_Tile_rc|1
AGATCTCAAACAAAGGATGTCCGACCAGCAGTAGACGTTCAGATGTTGCATGGTAGAAGA
>HPV1_Mu_9626063_nt5534_L1_Tile_rc|1
ACTGGAGATCTCAAACAAAGGATGTCCGACCAGCAGTAGACGTTCAGATGTTGCATGGTA
>HPV1_Mu_9626063_nt5539_L1_Tile_rc|1
TGATTACTGGAGATCTCAAACAAAGGATGTCCGACCAGCAGTAGACGTTCAGATGTTGCA
>HPV1_Mu_9626063_nt5544_L1_Tile_rc|1
CAGTTTGATTACTGGAGATCTCAAACAAAGGATGTCCGACCAGCAGTAGACGTTCAGATG
>HPV1_Mu_9626063_nt5549_L1_Tile_rc|1
AGTTACAGTTTGATTACTGGAGATCTCAAACAAAGGATGTCCGACCAGCAGTAGACGTTC
>HPV1_Mu_9626063_nt5554_L1_Tile_rc|1
GGTATAGTTACAGTTTGATTACTGGAGATCTCAAACAAAGGATGTCCGACCAGCAGTAGA
>HPV1_Mu_9626063_nt5559_L1_Tile_rc|1
CTTTTGGTATAGTTACAGTTTGATTACTGGAGATCTCAAACAAAGGATGTCCGACCAGCA
>HPV1_Mu_9626063_nt5564_L1_Tile_rc|1
TGACACTTTTGGTATAGTTACAGTTTGATTACTGGAGATCTCAAACAAAGGATGTCCGAC

>HPV1_Mu_9626063_nt5579_L1_Tile_rc|1
TCTAAATGCATTTGGTGACACTTTTGGTATAGTTACAGTTTGATTACTGGAGATCTCAAA
>HPV1_Mu_9626063_nt5584_L1_Tile_rc|1
AAAACTCTAAATGCATTTGGTGACACTTTTGGTATAGTTACAGTTTGATTACTGGAGATC
>HPV1_Mu_9626063_nt5594_L1_Tile_rc|1
ACGCACCCTAAAAACTCTAAATGCATTTGGTGACACTTTTGGTATAGTTACAGTTTGATT
>HPV1_Mu_9626063_nt5599_L1_Tile_rc|1
GCAAAACGCACCCTAAAAACTCTAAATGCATTTGGTGACACTTTTGGTATAGTTACAGTT
>HPV1_Mu_9626063_nt5604_L1_Tile_rc|1
GATCAGCAAAACGCACCCTAAAAACTCTAAATGCATTTGGTGACACTTTTGGTATAGTTA
>HPV1_Mu_9626063_nt5609_L1_Tile_rc|1
ATTTGGATCAGCAAAACGCACCCTAAAAACTCTAAATGCATTTGGTGACACTTTTGGTAT
>HPV1_Mu_9626063_nt5614_L1_Tile_rc|1
AATCTATTTGGATCAGCAAAACGCACCCTAAAAACTCTAAATGCATTTGGTGACACTTTT
>HPV1_Mu_9626063_nt5644_L1_Tile_rc|1
GGATTAAAAATTGCCTTATCCCCAAATGCAAATCTATTTGGATCAGCAAAACGCACCCTA
>HPV1_Mu_9626063_nt5729_L1_Tile_rc|1
AGGGTGGCCCGTTATTCCTATACCTAAAGGCTGGCCTCTACCTATCTCTATCCCTCTTAG
>HPV1_Mu_9626063_nt5734_L1_Tile_rc|1
AAAAGAGGGTGGCCCGTTATTCCTATACCTAAAGGCTGGCCTCTACCTATCTCTATCCCT
>HPV1_Mu_9626063_nt5739_L1_Tile_rc|1
TATTTAAAAGAGGGTGGCCCGTTATTCCTATACCTAAAGGCTGGCCTCTACCTATCTCTA
>HPV1_Mu_9626063_nt5744_L1_Tile_rc|1
TAACTTATTTAAAAGAGGGTGGCCCGTTATTCCTATACCTAAAGGCTGGCCTCTACCTAT
>HPV1_Mu_9626063_nt5754_L1_Tile_rc|1
CTGCATCATCTAACTTATTTAAAAGAGGGTGGCCCGTTATTCCTATACCTAAAGGCTGGC
>HPV1_Mu_9626063_nt5759_L1_Tile_rc|1
ATTTTCTGCATCATCTAACTTATTTAAAAGAGGGTGGCCCGTTATTCCTATACCTAAAGG
>HPV1_Mu_9626063_nt5764_L1_Tile_rc|1
GTTGGATTTTCTGCATCATCTAACTTATTTAAAAGAGGGTGGCCCGTTATTCCTATACCT
>HPV1_Mu_9626063_nt5769_L1_Tile_rc|1
AATTTGTTGGATTTTCTGCATCATCTAACTTATTTAAAAGAGGGTGGCCCGTTATTCCTA
>HPV1_Mu_9626063_nt5774_L1_Tile_rc|1
AATATAATTTGTTGGATTTTCTGCATCATCTAACTTATTTAAAAGAGGGTGGCCCGTTAT
>HPV1_Mu_9626063_nt5784_L1_Tile_rc|1
CATGAGTATTAATATAATTTGTTGGATTTTCTGCATCATCTAACTTATTTAAAAGAGGGT
>HPV1_Mu_9626063_nt5789_L1_Tile_rc|1
ATTTGCATGAGTATTAATATAATTTGTTGGATTTTCTGCATCATCTAACTTATTTAAAAG
>HPV1_Mu_9626063_nt5794_L1_Tile_rc|1
TCTCCATTTGCATGAGTATTAATATAATTTGTTGGATTTTCTGCATCATCTAACTTATTT
>HPV1_Mu_9626063_nt5799_L1_Tile_rc|1
TAGAATCTCCATTTGCATGAGTATTAATATAATTTGTTGGATTTTCTGCATCATCTAACT
>HPV1_Mu_9626063_nt5809_L1_Tile_rc|1
GTATTTTGTCTAGAATCTCCATTTGCATGAGTATTAATATAATTTGTTGGATTTTCTGCA
>HPV1_Mu_9626063_nt5819_L1_Tile_rc|1
ATCAAAAGCAGTATTTTGTCTAGAATCTCCATTTGCATGAGTATTAATATAATTTGTTGG
>HPV1_Mu_9626063_nt5844_L1_Tile_rc|1
CGAGGAACATTTGTGTCTGTTTTGCATCAAAAGCAGTATTTTGTCTAGAATCTCCATTTG
>HPV1_Mu_9626063_nt5849_L1_Tile_rc|1
GCCGACGAGGAACATTTGTGTCTGTTTTGCATCAAAAGCAGTATTTTGTCTAGAATCTCC
>HPV1_Mu_9626063_nt5864_L1_Tile_rc|1
TGAAGCAGGAGTACAGCCGACGAGGAACATTTGTGTCTGTTTTGCATCAAAAGCAGTATT
>HPV1_Mu_9626063_nt5874_L1_Tile_rc|1
AGTGTTCACCTGAAGCAGGAGTACAGCCGACGAGGAACATTTGTGTCTGTTTTGCATCAA
>HPV1_Mu_9626063_nt5884_L1_Tile_rc|1

CTACTTGTCCAGTGTTCACCTGAAGCAGGAGTACAGCCGACGAGGAACATTTGTGTCTGT
>HPV1_Mu_9626063_nt5889_L1_Tile_rc|1
AACGACTACTTGTCCAGTGTTCACCTGAAGCAGGAGTACAGCCGACGAGGAACATTTGTG
>HPV1_Mu_9626063_nt5894_L1_Tile_rc|1
TGGGCAACGACTACTTGTCCAGTGTTCACCTGAAGCAGGAGTACAGCCGACGAGGAACAT
>HPV1_Mu_9626063_nt5904_L1_Tile_rc|1
CTTGTTCCCCTGGGCAACGACTACTTGTCCAGTGTTCACCTGAAGCAGGAGTACAGCCGA
>HPV1_Mu_9626063_nt5909_L1_Tile_rc|1
TTTCACTTGTTCCCCTGGGCAACGACTACTTGTCCAGTGTTCACCTGAAGCAGGAGTACA
>HPV1_Mu_9626063_nt5914_L1_Tile_rc|1
CCAAGTTTCACTTGTTCCCCTGGGCAACGACTACTTGTCCAGTGTTCACCTGAAGCAGGA
>HPV1_Mu_9626063_nt5929_L1_Tile_rc|1
ACCCTGGGGCAGTCCCCAAGTTTCACTTGTTCCCCTGGGCAACGACTACTTGTCCAGTGT
>HPV1_Mu_9626063_nt5954_L1_Tile_rc|1
TTCTATGACAGACTCTATCATTTGCACCCTGGGGCAGTCCCCAAGTTTCACTTGTTCCCC
>HPV1_Mu_9626063_nt5964_L1_Tile_rc|1
TGTCACCATCTTCTATGACAGACTCTATCATTTGCACCCTGGGGCAGTCCCCAAGTTTCA
>HPV1_Mu_9626063_nt5974_L1_Tile_rc|1
ATATCCATCATGTCACCATCTTCTATGACAGACTCTATCATTTGCACCCTGGGGCAGTCC
>HPV1_Mu_9626063_nt6044_L1_Tile_rc|1
TTGAACAACATCTAAAGGGACATCAGACTTGTCTTGCTGTAAAGCAGCAAAATCCATAGC
>HPV1_Mu_9626063_nt6049_L1_Tile_rc|1
GTTGCTTGAACAACATCTAAAGGGACATCAGACTTGTCTTGCTGTAAAGCAGCAAAATCC
>HPV1_Mu_9626063_nt6079_L1_Tile_rc|1
TTCATTCTGATATAATCAGGATATTTGCATGTTGCTTGAACAACATCTAAAGGGACATCA
>HPV1_Mu_9626063_nt6084_L1_Tile_rc|1
CATGGTTCATTCTGATATAATCAGGATATTTGCATGTTGCTTGAACAACATCTAAAGGGA
>HPV1_Mu_9626063_nt6089_L1_Tile_rc|1
GGCTTCATGGTTCATTCTGATATAATCAGGATATTTGCATGTTGCTTGAACAACATCTAA
>HPV1_Mu_9626063_nt6099_L1_Tile_rc|1
AGTTGCCATAGGCTTCATGGTTCATTCTGATATAATCAGGATATTTGCATGTTGCTTGAA
>HPV1_Mu_9626063_nt6104_L1_Tile_rc|1
CATAGAGTTGCCATAGGCTTCATGGTTCATTCTGATATAATCAGGATATTTGCATGTTGC
>HPV1_Mu_9626063_nt6114_L1_Tile_rc|1
CAAAAAAAAACATAGAGTTGCCATAGGCTTCATGGTTCATTCTGATATAATCAGGATATT
>HPV1_Mu_9626063_nt6119_L1_Tile_rc|1
ACGTGCAAAAAAAAACATAGAGTTGCCATAGGCTTCATGGTTCATTCTGATATAATCAGG
>HPV1_Mu_9626063_nt6129_L1_Tile_rc|1
TTTGCTCGCGACGTGCAAAAAAAAACATAGAGTTGCCATAGGCTTCATGGTTCATTCTGA
>HPV1_Mu_9626063_nt6134_L1_Tile_rc|1
ATACATTTGCTCGCGACGTGCAAAAAAAAACATAGAGTTGCCATAGGCTTCATGGTTCAT
>HPV1_Mu_9626063_nt6139_L1_Tile_rc|1
CTGGTATACATTTGCTCGCGACGTGCAAAAAAAAACATAGAGTTGCCATAGGCTTCATGG
>HPV1_Mu_9626063_nt6184_L1_Tile_rc|1
ACTGCCTCCTTATCACCCACCGAACCCCCGCGAGTAAAAAAGTGCCTGGTATACATTTGC
>HPV1_Mu_9626063_nt6194_L1_Tile_rc|1
GCTTTGTGGGACTGCCTCCTTATCACCCACCGAACCCCCGCGAGTAAAAAAGTGCCTGGT
>HPV1_Mu_9626063_nt6199_L1_Tile_rc|1
TACAGGCTTTGTGGGACTGCCTCCTTATCACCCACCGAACCCCCGCGAGTAAAAAAGTGC
>HPV1_Mu_9626063_nt6209_L1_Tile_rc|1
TGCTGTTAAATACAGGCTTTGTGGGACTGCCTCCTTATCACCCACCGAACCCCCGCGAGT
>HPV1_Mu_9626063_nt6224_L1_Tile_rc|1
TCTTGGTTCAGCATCTGCTGTTAAATACAGGCTTTGTGGGACTGCCTCCTTATCACCCAC
>HPV1_Mu_9626063_nt6229_L1_Tile_rc|1
GTTGTTCTTGGTTCAGCATCTGCTGTTAAATACAGGCTTTGTGGGACTGCCTCCTTATCA

>HPV1_Mu_9626063_nt6239_L1_Tile_rc|1
TGTTGCTAAAGTTGTTCTTGGTTCAGCATCTGCTGTTAAATACAGGCTTTGTGGGACTGC
>HPV1_Mu_9626063_nt6244_L1_Tile_rc|1
TTTGTTGTTGCTAAAGTTGTTCTTGGTTCAGCATCTGCTGTTAAATACAGGCTTTGTGGG
>HPV1_Mu_9626063_nt6249_L1_Tile_rc|1
CATAATTTGTTGTTGCTAAAGTTGTTCTTGGTTCAGCATCTGCTGTTAAATACAGGCTTT
>HPV1_Mu_9626063_nt6254_L1_Tile_rc|1
GCCTACATAATTTGTTGTTGCTAAAGTTGTTCTTGGTTCAGCATCTGCTGTTAAATACAG
>HPV1_Mu_9626063_nt6264_L1_Tile_rc|1
CACTTGGTGTGCCTACATAATTTGTTGTTGCTAAAGTTGTTCTTGGTTCAGCATCTGCTG
>HPV1_Mu_9626063_nt6269_L1_Tile_rc|1
AGAGCCACTTGGTGTGCCTACATAATTTGTTGTTGCTAAAGTTGTTCTTGGTTCAGCATC
>HPV1_Mu_9626063_nt6279_L1_Tile_rc|1
ATGAAACCATAGAGCCACTTGGTGTGCCTACATAATTTGTTGTTGCTAAAGTTGTTCTTG
>HPV1_Mu_9626063_nt6359_L1_Tile_rc|1
CTGGTTTCTCCAGCAAATGCCATTATTCTGGCCTTGACATCGCTGAAGCCAGTAAGATCT
>HPV1_Mu_9626063_nt6369_L1_Tile_rc|1
TAATAAATAACTGGTTTCTCCAGCAAATGCCATTATTCTGGCCTTGACATCGCTGAAGCC
>HPV1_Mu_9626063_nt6379_L1_Tile_rc|1
TCTCCAACTGTAATAAATAACTGGTTTCTCCAGCAAATGCCATTATTCTGGCCTTGACAT
>HPV1_Mu_9626063_nt6384_L1_Tile_rc|1
TATTATCTCCAACTGTAATAAATAACTGGTTTCTCCAGCAAATGCCATTATTCTGGCCTT
>HPV1_Mu_9626063_nt6394_L1_Tile_rc|1
GTTCCTCTGGTATTATCTCCAACTGTAATAAATAACTGGTTTCTCCAGCAAATGCCATTA
>HPV1_Mu_9626063_nt6409_L1_Tile_rc|1
CTGATAGATAAACTTGTTCCTCTGGTATTATCTCCAACTGTAATAAATAACTGGTTTCTC
>HPV1_Mu_9626063_nt6414_L1_Tile_rc|1
TCATACTGATAGATAAACTTGTTCCTCTGGTATTATCTCCAACTGTAATAAATAACTGGT
>HPV1_Mu_9626063_nt6419_L1_Tile_rc|1
GTTTTTCATACTGATAGATAAACTTGTTCCTCTGGTATTATCTCCAACTGTAATAAATAA
>HPV1_Mu_9626063_nt6424_L1_Tile_rc|1
GCATTGTTTTTCATACTGATAGATAAACTTGTTCCTCTGGTATTATCTCCAACTGTAATA
>HPV1_Mu_9626063_nt6429_L1_Tile_rc|1
TACTTGCATTGTTTTTCATACTGATAGATAAACTTGTTCCTCTGGTATTATCTCCAACTG
>HPV1_Mu_9626063_nt6434_L1_Tile_rc|1
TGTAGTACTTGCATTGTTTTTCATACTGATAGATAAACTTGTTCCTCTGGTATTATCTCC
>HPV1_Mu_9626063_nt6439_L1_Tile_rc|1
GAATATGTAGTACTTGCATTGTTTTTCATACTGATAGATAAACTTGTTCCTCTGGTATTA
>HPV1_Mu_9626063_nt6444_L1_Tile_rc|1
CATTGGAATATGTAGTACTTGCATTGTTTTTCATACTGATAGATAAACTTGTTCCTCTGG
>HPV1_Mu_9626063_nt6449_L1_Tile_rc|1
ATTAGCATTGGAATATGTAGTACTTGCATTGTTTTTCATACTGATAGATAAACTTGTTCC
>HPV1_Mu_9626063_nt6454_L1_Tile_rc|1
TTAAAATTAGCATTGGAATATGTAGTACTTGCATTGTTTTTCATACTGATAGATAAACTT
>HPV1_Mu_9626063_nt6459_L1_Tile_rc|1
AATCATTAAAATTAGCATTGGAATATGTAGTACTTGCATTGTTTTTCATACTGATAGATA
>HPV1_Mu_9626063_nt6464_L1_Tile_rc|1
TAGAAAATCATTAAAATTAGCATTGGAATATGTAGTACTTGCATTGTTTTTCATACTGAT
>HPV1_Mu_9626063_nt6469_L1_Tile_rc|1
TGTCTTAGAAAATCATTAAAATTAGCATTGGAATATGTAGTACTTGCATTGTTTTTCATA
>HPV1_Mu_9626063_nt6474_L1_Tile_rc|1
CAGTATGTCTTAGAAAATCATTAAAATTAGCATTGGAATATGTAGTACTTGCATTGTTTT
>HPV1_Mu_9626063_nt6479_L1_Tile_rc|1
TTCTTCAGTATGTCTTAGAAAATCATTAAAATTAGCATTGGAATATGTAGTACTTGCATT
>HPV1_Mu_9626063_nt6484_L1_Tile_rc|1

TCAAATTCTTCAGTATGTCTTAGAAAATCATTAAAATTAGCATTGGAATATGTAGTACTT
>HPV1_Mu_9626063_nt6489_L1_Tile_rc|1
AAAGATCAAATTCTTCAGTATGTCTTAGAAAATCATTAAAATTAGCATTGGAATATGTAG
>HPV1_Mu_9626063_nt6494_L1_Tile_rc|1
AAAAGAAAGATCAAATTCTTCAGTATGTCTTAGAAAATCATTAAAATTAGCATTGGAATA
>HPV1_Mu_9626063_nt6499_L1_Tile_rc|1
ACTATAAAAGAAAGATCAAATTCTTCAGTATGTCTTAGAAAATCATTAAAATTAGCATTG
>HPV1_Mu_9626063_nt6504_L1_Tile_rc|1
GCTGAACTATAAAAGAAAGATCAAATTCTTCAGTATGTCTTAGAAAATCATTAAAATTAG
>HPV1_Mu_9626063_nt6509_L1_Tile_rc|1
ACAAAGCTGAACTATAAAAGAAAGATCAAATTCTTCAGTATGTCTTAGAAAATCATTAAA
>HPV1_Mu_9626063_nt6519_L1_Tile_rc|1
ACTTTACTTTACAAAGCTGAACTATAAAAGAAAGATCAAATTCTTCAGTATGTCTTAGAA
>HPV1_Mu_9626063_nt6524_L1_Tile_rc|1
AGTTAACTTTACTTTACAAAGCTGAACTATAAAAGAAAGATCAAATTCTTCAGTATGTCT
>HPV1_Mu_9626063_nt6529_L1_Tile_rc|1
TCGGGAGTTAACTTTACTTTACAAAGCTGAACTATAAAAGAAAGATCAAATTCTTCAGTA
>HPV1_Mu_9626063_nt6539_L1_Tile_rc|1
GGCTAGATTTTCGGGAGTTAACTTTACTTTACAAAGCTGAACTATAAAAGAAAGATCAAA
>HPV1_Mu_9626063_nt6544_L1_Tile_rc|1
ATGTAGGCTAGATTTTCGGGAGTTAACTTTACTTTACAAAGCTGAACTATAAAAGAAAGA
>HPV1_Mu_9626063_nt6549_L1_Tile_rc|1
TATGAATGTAGGCTAGATTTTCGGGAGTTAACTTTACTTTACAAAGCTGAACTATAAAAG
>HPV1_Mu_9626063_nt6554_L1_Tile_rc|1
CATTGTATGAATGTAGGCTAGATTTTCGGGAGTTAACTTTACTTTACAAAGCTGAACTAT
>HPV1_Mu_9626063_nt6559_L1_Tile_rc|1
GGGTCCATTGTATGAATGTAGGCTAGATTTTCGGGAGTTAACTTTACTTTACAAAGCTGA
>HPV1_Mu_9626063_nt6564_L1_Tile_rc|1
TATTAGGGTCCATTGTATGAATGTAGGCTAGATTTTCGGGAGTTAACTTTACTTTACAAA
>HPV1_Mu_9626063_nt6569_L1_Tile_rc|1
TAAAATATTAGGGTCCATTGTATGAATGTAGGCTAGATTTTCGGGAGTTAACTTTACTTT
>HPV1_Mu_9626063_nt6574_L1_Tile_rc|1
TCCTCTAAAATATTAGGGTCCATTGTATGAATGTAGGCTAGATTTTCGGGAGTTAACTTT
>HPV1_Mu_9626063_nt6579_L1_Tile_rc|1
GCCAATCCTCTAAAATATTAGGGTCCATTGTATGAATGTAGGCTAGATTTTCGGGAGTTA
>HPV1_Mu_9626063_nt6594_L1_Tile_rc|1
GAGATACAGATAGTTGCCAATCCTCTAAAATATTAGGGTCCATTGTATGAATGTAGGCTA
>HPV1_Mu_9626063_nt6629_L1_Tile_rc|1
CCTATATTGATCTTCTAGAGGATTGGTAGGTGGTTGAGATACAGATAGTTGCCAATCCTC
>HPV1_Mu_9626063_nt6639_L1_Tile_rc|1
ACCCTAAAAACCTATATTGATCTTCTAGAGGATTGGTAGGTGGTTGAGATACAGATAGTT
>HPV1_Mu_9626063_nt6644_L1_Tile_rc|1
GGAAGACCCTAAAAACCTATATTGATCTTCTAGAGGATTGGTAGGTGGTTGAGATACAGA
>HPV1_Mu_9626063_nt6649_L1_Tile_rc|1
GCCAAGGAAGACCCTAAAAACCTATATTGATCTTCTAGAGGATTGGTAGGTGGTTGAGAT
>HPV1_Mu_9626063_nt6654_L1_Tile_rc|1
TTGCTGCCAAGGAAGACCCTAAAAACCTATATTGATCTTCTAGAGGATTGGTAGGTGGTT
>HPV1_Mu_9626063_nt6659_L1_Tile_rc|1
ACATTTTGCTGCCAAGGAAGACCCTAAAAACCTATATTGATCTTCTAGAGGATTGGTAGG
>HPV1_Mu_9626063_nt6664_L1_Tile_rc|1
TCTGGACATTTTGCTGCCAAGGAAGACCCTAAAAACCTATATTGATCTTCTAGAGGATTG
>HPV1_Mu_9626063_nt6669_L1_Tile_rc|1
CCTGTTCTGGACATTTTGCTGCCAAGGAAGACCCTAAAAACCTATATTGATCTTCTAGAG
>HPV1_Mu_9626063_nt6694_L1_Tile_rc|1
GGATCAGTCTGGGGCTCAGGAGGCGCCTGTTCTGGACATTTTGCTGCCAAGGAAGACCCT

>HPV1_Mu_9626063_nt6709_L1_Tile_rc|1
TTATATTGACTATAAGGATCAGTCTGGGGCTCAGGAGGCGCCTGTTCTGGACATTTTGCT
>HPV1_Mu_9626063_nt6719_L1_Tile_rc|1
TTCCCAGAATTTATATTGACTATAAGGATCAGTCTGGGGCTCAGGAGGCGCCTGTTCTGG
>HPV1_Mu_9626063_nt6729_L1_Tile_rc|1
TGAGATCGACTTCCCAGAATTTATATTGACTATAAGGATCAGTCTGGGGCTCAGGAGGCG
>HPV1_Mu_9626063_nt6734_L1_Tile_rc|1
TTCTGTGAGATCGACTTCCCAGAATTTATATTGACTATAAGGATCAGTCTGGGGCTCAGG
>HPV1_Mu_9626063_nt6744_L1_Tile_rc|1
CGGACATCCTTTCTGTGAGATCGACTTCCCAGAATTTATATTGACTATAAGGATCAGTCT
>HPV1_Mu_9626063_nt6749_L1_Tile_rc|1
TTGTTCGGACATCCTTTCTGTGAGATCGACTTCCCAGAATTTATATTGACTATAAGGATC
>HPV1_Mu_9626063_nt6754_L1_Tile_rc|1
TCTAATTGTTCGGACATCCTTTCTGTGAGATCGACTTCCCAGAATTTATATTGACTATAA
>HPV1_Mu_9626063_nt6759_L1_Tile_rc|1
ATTGGTCTAATTGTTCGGACATCCTTTCTGTGAGATCGACTTCCCAGAATTTATATTGAC
>HPV1_Mu_9626063_nt6764_L1_Tile_rc|1
TGGAAATTGGTCTAATTGTTCGGACATCCTTTCTGTGAGATCGACTTCCCAGAATTTATA
>HPV1_Mu_9626063_nt6769_L1_Tile_rc|1
CCTAGTGGAAATTGGTCTAATTGTTCGGACATCCTTTCTGTGAGATCGACTTCCCAGAAT
>HPV1_Mu_9626063_nt6774_L1_Tile_rc|1
TCCTTCCTAGTGGAAATTGGTCTAATTGTTCGGACATCCTTTCTGTGAGATCGACTTCCC
>HPV1_Mu_9626063_nt6779_L1_Tile_rc|1
AAATTTCCTTCCTAGTGGAAATTGGTCTAATTGTTCGGACATCCTTTCTGTGAGATCGAC
>HPV1_Mu_9626063_nt6784_L1_Tile_rc|1
TATAGAAATTTCCTTCCTAGTGGAAATTGGTCTAATTGTTCGGACATCCTTTCTGTGAGA
>HPV1_Mu_9626063_nt6789_L1_Tile_rc|1
TTTGATATAGAAATTTCCTTCCTAGTGGAAATTGGTCTAATTGTTCGGACATCCTTTCTG
>HPV1_Mu_9626063_nt6794_L1_Tile_rc|1
GCCACTTTGATATAGAAATTTCCTTCCTAGTGGAAATTGGTCTAATTGTTCGGACATCCT
>HPV1_Mu_9626063_nt6799_L1_Tile_rc|1
GTCATGCCACTTTGATATAGAAATTTCCTTCCTAGTGGAAATTGGTCTAATTGTTCGGAC
>HPV1_Mu_9626063_nt6804_L1_Tile_rc|1
GTTGTGTCATGCCACTTTGATATAGAAATTTCCTTCCTAGTGGAAATTGGTCTAATTGTT
>HPV1_Mu_9626063_nt6809_L1_Tile_rc|1
AGTACGTTGTGTCATGCCACTTTGATATAGAAATTTCCTTCCTAGTGGAAATTGGTCTAA
>HPV1_Mu_9626063_nt6814_L1_Tile_rc|1
GTAGCAGTACGTTGTGTCATGCCACTTTGATATAGAAATTTCCTTCCTAGTGGAAATTGG
>HPV1_Mu_9626063_nt6819_L1_Tile_rc|1
AACTAGTAGCAGTACGTTGTGTCATGCCACTTTGATATAGAAATTTCCTTCCTAGTGGAA
>HPV1_Mu_9626063_nt6824_L1_Tile_rc|1
GGTGGAACTAGTAGCAGTACGTTGTGTCATGCCACTTTGATATAGAAATTTCCTTCCTAG
>HPV1_Mu_9626063_nt6834_L1_Tile_rc|1
TGCGCTTTGTGGTGGAACTAGTAGCAGTACGTTGTGTCATGCCACTTTGATATAGAAATT
>HPV1_Mu_9626063_nt6839_L1_Tile_rc|1
TGTTTTGCGCTTTGTGGTGGAACTAGTAGCAGTACGTTGTGTCATGCCACTTTGATATAG
>HPV1_Mu_9626063_nt6844_L1_Tile_rc|1
CGCACTGTTTTGCGCTTTGTGGTGGAACTAGTAGCAGTACGTTGTGTCATGCCACTTTGA
>HPV1_Mu_9626063_nt6849_L1_Tile_rc|1
ATACACGCACTGTTTTGCGCTTTGTGGTGGAACTAGTAGCAGTACGTTGTGTCATGCCAC
>HPV1_Mu_9626063_nt6854_L1_Tile_rc|1
CGTAGATACACGCACTGTTTTGCGCTTTGTGGTGGAACTAGTAGCAGTACGTTGTGTCAT
>HPV1_Mu_9626063_nt6859_L1_Tile_rc|1
GCTGACGTAGATACACGCACTGTTTTGCGCTTTGTGGTGGAACTAGTAGCAGTACGTTGT
>HPV1_Mu_9626063_nt6864_L1_Tile_rc|1

GCTTGGCTGACGTAGATACACGCACTGTTTTGCGCTTTGTGGTGGAACTAGTAGCAGTAC
>HPV1_Mu_9626063_nt6879_L1_Tile_rc|1
AAGCCTTACGCCTGCGCTTGGCTGACGTAGATACACGCACTGTTTTGCGCTTTGTGGTGG
>HPV1_Mu_9626063_nt6881_L1_Tile_rc|1
CTAAGCCTTACGCCTGCGCTTGGCTGACGTAGATACACGCACTGTTTTGCGCTTTGTGGT
>HPV2_Alpha_9626032_nt5908_L1_Tile_rc|1
CACCTTATTATTACTCTTCTTTATAGAGTAATATGGATGACCCACAGTGAGAAGCCTAGA
>HPV2_Alpha_9626032_nt5913_L1_Tile_rc|1
ACAGCCACCTTATTATTACTCTTCTTTATAGAGTAATATGGATGACCCACAGTGAGAAGC
>HPV2_Alpha_9626032_nt6793_L1_Tile_rc|1
AAAATTGGTAGCCTTATAATTAGTATCAGACGCCTCAGTGGCACACAGAGATACATTAGT
>HPV2_Alpha_9626032_nt7193_L1_Tile_rc|1
TACTAGTGGGCGGCGTGGTCCCCGAAACAGCGGCGCGCTTGCGAGACACGGTAGGCATAG
>HPV2_Alpha_9626032_nt7198_L1_Tile_rc|1
TCGTTTACTAGTGGGCGGCGTGGTCCCCGAAACAGCGGCGCGCTTGCGAGACACGGTAGG
>HPV3_Alpha_397005_nt5878_L1_Tile_rc|1
AATATCCATCTTGGAATTAGAAGATTTGGGGATAGCAAAATAAGGATGACCCACGGTCAG
>HPV3_Alpha_397005_nt5883_L1_Tile_rc|1
TTAGGAATATCCATCTTGGAATTAGAAGATTTGGGGATAGCAAAATAAGGATGACCCACG
>HPV3_Alpha_397005_nt5903_L1_Tile_rc|1
TATATTGAAAGGCGGACACCTTAGGAATATCCATCTTGGAATTAGAAGATTTGGGGATAG
>HPV3_Alpha_397005_nt5908_L1_Tile_rc|1
CACTCTATATTGAAAGGCGGACACCTTAGGAATATCCATCTTGGAATTAGAAGATTTGGG
>HPV3_Alpha_397005_nt6273_L1_Tile_rc|1
ATAGGTGCAGTAATAAGCTCTAGAGGAGGACAATCACCCGGTGACGCATTCTGCTTACAT
>HPV3_Alpha_397005_nt6278_L1_Tile_rc|1
CTTGTATAGGTGCAGTAATAAGCTCTAGAGGAGGACAATCACCCGGTGACGCATTCTGCT
>HPV3_Alpha_397005_nt6518_L1_Tile_rc|1
TGTACAACGCGTCAGGCACGGTGTCTCCAGCCATACCAGCTCTGTTAAGAAAATGTCTTG
>HPV3_Alpha_397005_nt6523_L1_Tile_rc|1
TTTAATGTACAACGCGTCAGGCACGGTGTCTCCAGCCATACCAGCTCTGTTAAGAAAATG
>HPV3_Alpha_397005_nt6528_L1_Tile_rc|1
TCACCTTTAATGTACAACGCGTCAGGCACGGTGTCTCCAGCCATACCAGCTCTGTTAAGA
>HPV3_Alpha_397005_nt6533_L1_Tile_rc|1
GACTGTCACCTTTAATGTACAACGCGTCAGGCACGGTGTCTCCAGCCATACCAGCTCTGT
>HPV3_Alpha_397005_nt6538_L1_Tile_rc|1
GCTCTGACTGTCACCTTTAATGTACAACGCGTCAGGCACGGTGTCTCCAGCCATACCAGC
>HPV3_Alpha_397005_nt6543_L1_Tile_rc|1
CCGCCGCTCTGACTGTCACCTTTAATGTACAACGCGTCAGGCACGGTGTCTCCAGCCATA
>HPV3_Alpha_397005_nt6553_L1_Tile_rc|1
TTTATCCCGACCGCCGCTCTGACTGTCACCTTTAATGTACAACGCGTCAGGCACGGTGTC
>HPV3_Alpha_397005_nt6563_L1_Tile_rc|1
CACTACCAATTTTATCCCGACCGCCGCTCTGACTGTCACCTTTAATGTACAACGCGTCAG
>HPV3_Alpha_397005_nt6783_L1_Tile_rc|1
TACTCTTTAAATTTAGTAGCATCATATGTAGCCGAGGTTTCAGTAGAAACACACAATGTC
>HPV3_Alpha_397005_nt6788_L1_Tile_rc|1
TTAAATACTCTTTAAATTTAGTAGCATCATATGTAGCCGAGGTTTCAGTAGAAACACACA
>HPV3_Alpha_397005_nt6793_L1_Tile_rc|1
GTGTCTTAAATACTCTTTAAATTTAGTAGCATCATATGTAGCCGAGGTTTCAGTAGAAAC
>HPV3_Alpha_397005_nt7003_L1_Tile_rc|1
CTCAGTGGGAGGTGCATCTTTCTGGCAGGTAATGGCAGAGGAAGTTAAAAATCTATAGGT
>HPV3_Alpha_397005_nt7028_L1_Tile_rc|1
TTAGTTTGGCGTAGGGGTCTTGCTTCTCAGTGGGAGGTGCATCTTTCTGGCAGGTAATGG
>HPV3_Alpha_397005_nt7103_L1_Tile_rc|1
TGAGAAATTTCCTGCCAAGGGGGAACTGCGAAAGATCCAGGGAAAAACGATCCTTAAGAT

>HPV3_Alpha_397005_nt7118_L1_Tile_rc|1
CTACACCGAGCTGCATGAGAAATTTCCTGCCAAGGGGGAACTGCGAAAGATCCAGGGAAA
>HPV3_Alpha_397005_nt7123_L1_Tile_rc|1
GGTACCTACACCGAGCTGCATGAGAAATTTCCTGCCAAGGGGGAACTGCGAAAGATCCAG
>HPV3_Alpha_397005_nt7138_L1_Tile_rc|1
AGATATACTAGAGCGGGTACCTACACCGAGCTGCATGAGAAATTTCCTGCCAAGGGGGAA
>HPV3_Alpha_397005_nt7153_L1_Tile_rc|1
CGAGCGTTTACGAACAGATATACTAGAGCGGGTACCTACACCGAGCTGCATGAGAAATTT
>HPV3_Alpha_397005_nt7173_L1_Tile_rc|1
GTTCTAGATGTGGTTGTCGCCGAGCGTTTACGAACAGATATACTAGAGCGGGTACCTACA
>HPV3_Alpha_397005_nt7178_L1_Tile_rc|1
CAGCTGTTCTAGATGTGGTTGTCGCCGAGCGTTTACGAACAGATATACTAGAGCGGGTAC
>HPV4_Gamma_9626597_nt5516_L1_Tile_rc|1
AATCTAAACACTCTAAATTGACTTCCTGAAACCTTAGGAACTAATACTTTGTGAGGTTCC
>HPV4_Gamma_9626597_nt5541_L1_Tile_rc|1
AAATCTGTTTGGGTCTGGCAAATTGAATCTAAACACTCTAAATTGACTTCCTGAAACCTT
>HPV4_Gamma_9626597_nt5556_L1_Tile_rc|1
ATTATCAATTAAAGCAAATCTGTTTGGGTCTGGCAAATTGAATCTAAACACTCTAAATTG
>HPV4_Gamma_9626597_nt5621_L1_Tile_rc|1
CCTCTTCCTATTTCTATTCCCCTCAGTTTCCATACTAGGCGTTCATGATCAGAATCATAA
>HPV4_Gamma_9626597_nt5631_L1_Tile_rc|1
AAGCGGTCCTCCTCTTCCTATTTCTATTCCCCTCAGTTTCCATACTAGGCGTTCATGATC
>HPV4_Gamma_9626597_nt5636_L1_Tile_rc|1
ATACCAAGCGGTCCTCCTCTTCCTATTTCTATTCCCCTCAGTTTCCATACTAGGCGTTCA
>HPV4_Gamma_9626597_nt5646_L1_Tile_rc|1
TGTAGTACCTATACCAAGCGGTCCTCCTCTTCCTATTTCTATTCCCCTCAGTTTCCATAC
>HPV4_Gamma_9626597_nt5671_L1_Tile_rc|1
CAAACTTATTATATATAAAGGATGACCTGTAGTACCTATACCAAGCGGTCCTCCTCTTCCTA
>HPV4_Gamma_9626597_nt6071_L1_Tile_rc|1
AAGAAATGTCTGGCATATAGTTGTTCTCTTCTTCCAAAGAAAAACAAGCTATCTCCATAG
>HPV4_Gamma_9626597_nt6076_L1_Tile_rc|1
TGACAAAGAAATGTCTGGCATATAGTTGTTCTCTTCTTCCAAAGAAAAACAAGCTATCTC
>HPV4_Gamma_9626597_nt6086_L1_Tile_rc|1
GTGCCTGCTCTGACAAAGAAATGTCTGGCATATAGTTGTTCTCTTCTTCCAAAGAAAAAC
>HPV4_Gamma_9626597_nt6151_L1_Tile_rc|1
GGTTTTGAGCACCAATAAAATAATCTGAGGTAGCTTCAAAAGGTTCTGGTAGAGCATCTC
>HPV4_Gamma_9626597_nt6156_L1_Tile_rc|1
TTGTTGGTTTTGAGCACCAATAAAATAATCTGAGGTAGCTTCAAAAGGTTCTGGTAGAGC
>HPV4_Gamma_9626597_nt6161_L1_Tile_rc|1
TGATCTTGTTGGTTTTGAGCACCAATAAAATAATCTGAGGTAGCTTCAAAAGGTTCTGGT
>HPV4_Gamma_9626597_nt6166_L1_Tile_rc|1
TGTACTGATCTTGTTGGTTTTGAGCACCAATAAAATAATCTGAGGTAGCTTCAAAAGGTT
>HPV4_Gamma_9626597_nt6171_L1_Tile_rc|1
TAAAGTGTACTGATCTTGTTGGTTTTGAGCACCAATAAAATAATCTGAGGTAGCTTCAAA
>HPV4_Gamma_9626597_nt6176_L1_Tile_rc|1
GGTCCTAAAGTGTACTGATCTTGTTGGTTTTGAGCACCAATAAAATAATCTGAGGTAGCT
>HPV4_Gamma_9626597_nt6181_L1_Tile_rc|1
TATGAGGTCCTAAAGTGTACTGATCTTGTTGGTTTTGAGCACCAATAAAATAATCTGAGG
>HPV4_Gamma_9626597_nt6186_L1_Tile_rc|1
ATAAATATGAGGTCCTAAAGTGTACTGATCTTGTTGGTTTTGAGCACCAATAAAATAATC
>HPV4_Gamma_9626597_nt6191_L1_Tile_rc|1
CCTACATAAATATGAGGTCCTAAAGTGTACTGATCTTGTTGGTTTTGAGCACCAATAAAA
>HPV4_Gamma_9626597_nt6196_L1_Tile_rc|1
GGGTCCCTACATAAATATGAGGTCCTAAAGTGTACTGATCTTGTTGGTTTTGAGCACCAA
>HPV4_Gamma_9626597_nt6206_L1_Tile_rc|1

GAGCCACTAGGGGTCCCTACATAAATATGAGGTCCTAAAGTGTACTGATCTTGTTGGTTT
>HPV4_Gamma_9626597_nt6211_L1_Tile_rc|1
CTAAAGAGCCACTAGGGGTCCCTACATAAATATGAGGTCCTAAAGTGTACTGATCTTGTT
>HPV4_Gamma_9626597_nt6216_L1_Tile_rc|1
GGATACTAAAGAGCCACTAGGGGTCCCTACATAAATATGAGGTCCTAAAGTGTACTGATC
>HPV4_Gamma_9626597_nt6221_L1_Tile_rc|1
TCACTGGATACTAAAGAGCCACTAGGGGTCCCTACATAAATATGAGGTCCTAAAGTGTAC
>HPV4_Gamma_9626597_nt6226_L1_Tile_rc|1
GGGATTCACTGGATACTAAAGAGCCACTAGGGGTCCCTACATAAATATGAGGTCCTAAAG
>HPV4_Gamma_9626597_nt6356_L1_Tile_rc|1
GACTTCACAGAAATTGTAAAGTTTGTATTATGAGTGTTATCTACAAGAGTAACAAACAAC
>HPV4_Gamma_9626597_nt6361_L1_Tile_rc|1
CATCTGACTTCACAGAAATTGTAAAGTTTGTATTATGAGTGTTATCTACAAGAGTAACAA
>HPV4_Gamma_9626597_nt6366_L1_Tile_rc|1
AGCACCATCTGACTTCACAGAAATTGTAAAGTTTGTATTATGAGTGTTATCTACAAGAGT
>HPV4_Gamma_9626597_nt6371_L1_Tile_rc|1
TCATTAGCACCATCTGACTTCACAGAAATTGTAAAGTTTGTATTATGAGTGTTATCTACA
>HPV4_Gamma_9626597_nt6376_L1_Tile_rc|1
AATTGTCATTAGCACCATCTGACTTCACAGAAATTGTAAAGTTTGTATTATGAGTGTTAT
>HPV4_Gamma_9626597_nt6381_L1_Tile_rc|1
CTGATAATTGTCATTAGCACCATCTGACTTCACAGAAATTGTAAAGTTTGTATTATGAGT
>HPV4_Gamma_9626597_nt6386_L1_Tile_rc|1
TTATACTGATAATTGTCATTAGCACCATCTGACTTCACAGAAATTGTAAAGTTTGTATTA
>HPV4_Gamma_9626597_nt6391_L1_Tile_rc|1
TAGCTTTATACTGATAATTGTCATTAGCACCATCTGACTTCACAGAAATTGTAAAGTTTG
>HPV4_Gamma_9626597_nt6396_L1_Tile_rc|1
ATCACTAGCTTTATACTGATAATTGTCATTAGCACCATCTGACTTCACAGAAATTGTAAA
>HPV4_Gamma_9626597_nt6401_L1_Tile_rc|1
TTAAAATCACTAGCTTTATACTGATAATTGTCATTAGCACCATCTGACTTCACAGAAATT
>HPV4_Gamma_9626597_nt6406_L1_Tile_rc|1
ACTGTTTAAAATCACTAGCTTTATACTGATAATTGTCATTAGCACCATCTGACTTCACAG
>HPV4_Gamma_9626597_nt6411_L1_Tile_rc|1
GAGGTACTGTTTAAAATCACTAGCTTTATACTGATAATTGTCATTAGCACCATCTGACTT
>HPV4_Gamma_9626597_nt6416_L1_Tile_rc|1
TGTCTGAGGTACTGTTTAAAATCACTAGCTTTATACTGATAATTGTCATTAGCACCATCT
>HPV4_Gamma_9626597_nt6626_L1_Tile_rc|1
TCAGTTGCAGGGGTCTGTGTAGGGCATCTTGTAGCTCTAGATTGCAAAAATCTATATTGA
>HPV4_Gamma_9626597_nt6631_L1_Tile_rc|1
CTTTTTCAGTTGCAGGGGTCTGTGTAGGGCATCTTGTAGCTCTAGATTGCAAAAATCTAT
>HPV4_Gamma_9626597_nt6636_L1_Tile_rc|1
ATCTTCTTTTTCAGTTGCAGGGGTCTGTGTAGGGCATCTTGTAGCTCTAGATTGCAAAAA
>HPV4_Gamma_9626597_nt6641_L1_Tile_rc|1
TATGGATCTTCTTTTTCAGTTGCAGGGGTCTGTGTAGGGCATCTTGTAGCTCTAGATTGC
>HPV4_Gamma_9626597_nt6646_L1_Tile_rc|1
CTTTATATGGATCTTCTTTTTCAGTTGCAGGGGTCTGTGTAGGGCATCTTGTAGCTCTAG
>HPV4_Gamma_9626597_nt6651_L1_Tile_rc|1
CAAATCTTTATATGGATCTTCTTTTTCAGTTGCAGGGGTCTGTGTAGGGCATCTTGTAGC
>HPV4_Gamma_9626597_nt6656_L1_Tile_rc|1
AAAGACAAATCTTTATATGGATCTTCTTTTTCAGTTGCAGGGGTCTGTGTAGGGCATCTT
>HPV4_Gamma_9626597_nt6771_L1_Tile_rc|1
TATTCTTTTACGTTTTAGAGAACCATTAATTAAACCACTTTGATATAAAAACCGCCTGCC
>HPV4_Gamma_9626597_nt6776_L1_Tile_rc|1
CTTATTATTCTTTTACGTTTTAGAGAACCATTAATTAAACCACTTTGATATAAAAACCGC
>HPV4_Gamma_9626597_nt6781_L1_Tile_rc|1
AAGAACTTATTATTCTTTTACGTTTTAGAGAACCATTAATTAAACCACTTTGATATAAAA

>HPV4_Gamma_9626597_nt6786_L1_Tile_rc|1
ATGAGAAGAACTTATTATTCTTTTACGTTTTAGAGAACCATTAATTAAACCACTTTGATA
>HPV4_Gamma_9626597_nt6791_L1_Tile_rc|1
TGTGCATGAGAAGAACTTATTATTCTTTTACGTTTTAGAGAACCATTAATTAAACCACTT
>HPV4_Gamma_9626597_nt6801_L1_Tile_rc|1
GGTATTAGTTTGTGCATGAGAAGAACTTATTATTCTTTTACGTTTTAGAGAACCATTAAT
>HPV4_Gamma_9626597_nt6811_L1_Tile_rc|1
CAGAACGTTTGGTATTAGTTTGTGCATGAGAAGAACTTATTATTCTTTTACGTTTTAGAG
>HPV4_Gamma_9626597_nt6816_L1_Tile_rc|1
TTTGGCAGAACGTTTGGTATTAGTTTGTGCATGAGAAGAACTTATTATTCTTTTACGTTT
>HPV5_Beta_9627145_nt6278_L1_Tile_rc|1
AGAAAATGTTATGTATGCATTACTGTTTTCTGTATCTTTTACTTTATTGAAATAAGGGTG
>HPV5_Beta_9627145_nt6283_L1_Tile_rc|1
TCTTTAGAAAATGTTATGTATGCATTACTGTTTTCTGTATCTTTTACTTTATTGAAATAA
>HPV5_Beta_9627145_nt6513_L1_Tile_rc|1
CTGATCTACTATCTTGAAGTGCCTTAAAATTCATGTTCCCAAAACCTATGTCTGCCATAT
>HPV5_Beta_9627145_nt6518_L1_Tile_rc|1
GACATCTGATCTACTATCTTGAAGTGCCTTAAAATTCATGTTCCCAAAACCTATGTCTGC
>HPV5_Beta_9627145_nt6523_L1_Tile_rc|1
AAACTGACATCTGATCTACTATCTTGAAGTGCCTTAAAATTCATGTTCCCAAAACCTATG
>HPV5_Beta_9627145_nt6533_L1_Tile_rc|1
GACGATGTCTAAACTGACATCTGATCTACTATCTTGAAGTGCCTTAAAATTCATGTTCCC
>HPV5_Beta_9627145_nt6538_L1_Tile_rc|1
TCATTGACGATGTCTAAACTGACATCTGATCTACTATCTTGAAGTGCCTTAAAATTCATG
>HPV5_Beta_9627145_nt6958_L1_Tile_rc|1
TCTTTTAGTGCTCCAGCCTGATTATATACAGAAATACTGAAATTAGTATTTCTTGTGTTG
>HPV5_Beta_9627145_nt6963_L1_Tile_rc|1
CAACATCTTTTAGTGCTCCAGCCTGATTATATACAGAAATACTGAAATTAGTATTTCTTG
>HPV5_Beta_9627145_nt6968_L1_Tile_rc|1
GTCTGCAACATCTTTTAGTGCTCCAGCCTGATTATATACAGAAATACTGAAATTAGTATT
>HPV5_Beta_9627145_nt6988_L1_Tile_rc|1
CTAAATTGATCTGCATTATAGTCTGCAACATCTTTTAGTGCTCCAGCCTGATTATATACA
>HPV5_Beta_9627145_nt6993_L1_Tile_rc|1
ATTCTCTAAATTGATCTGCATTATAGTCTGCAACATCTTTTAGTGCTCCAGCCTGATTAT
>HPV5_Beta_9627145_nt6998_L1_Tile_rc|1
TTGATATTCTCTAAATTGATCTGCATTATAGTCTGCAACATCTTTTAGTGCTCCAGCCTG
>HPV5_Beta_9627145_nt7003_L1_Tile_rc|1
TGTCTTTGATATTCTCTAAATTGATCTGCATTATAGTCTGCAACATCTTTTAGTGCTCCA
>HPV5_Beta_9627145_nt7008_L1_Tile_rc|1
CTACATGTCTTTGATATTCTCTAAATTGATCTGCATTATAGTCTGCAACATCTTTTAGTG
>HPV5_Beta_9627145_nt7068_L1_Tile_rc|1
ATACCTGTGCCTTTAAAGGAACCTTACAGAGTTGTAGAATTAAAGATATTTCATATTCTT
>HPV5_Beta_9627145_nt7073_L1_Tile_rc|1
TGCCAATACCTGTGCCTTTAAAGGAACCTTACAGAGTTGTAGAATTAAAGATATTTCATA
>HPV5_Beta_9627145_nt7078_L1_Tile_rc|1
ATCTGTGCCAATACCTGTGCCTTTAAAGGAACCTTACAGAGTTGTAGAATTAAAGATATT
>HPV5_Beta_9627145_nt7223_L1_Tile_rc|1
GTCTTCCTTTTCTTTCGGAGGATTCTTATCTGGACACCGTGTAGCCAAAGAGTCAATATA
>HPV6_Alpha_6002612_nt5926_L1_Tile_rc|1
ATATCCTGACACCTTTGGCACAACAGTTTTGTTAGCCCGTTTTATGGAAAAATAAGGATG
>HPV6_Alpha_6002612_nt6131_L1_Tile_rc|1
TACCACCACTCCCTGAATTTTCAACATCATCATATTTATTTAGGAAAGGATGTCCACTTA
>HPV6_Alpha_6002612_nt6141_L1_Tile_rc|1
TGTCCAGGGTTACCACCACTCCCTGAATTTTCAACATCATCATATTTATTTAGGAAAGGA
>HPV6_Alpha_6002612_nt6146_L1_Tile_rc|1

TATCCTGTCCAGGGTTACCACCACTCCCTGAATTTTCAACATCATCATATTTATTTAGGA
>HPV6_Alpha_6002612_nt6151_L1_Tile_rc|1
CCTGTTATCCTGTCCAGGGTTACCACCACTCCCTGAATTTTCAACATCATCATATTTATT
>HPV6_Alpha_6002612_nt6266_L1_Tile_rc|1
CAGCCTGTACAGGTGTATTAGTACACTGTTTACCTTTACCCCAATGCTCGCCCAAAGGGG
>HPV6_Alpha_6002612_nt6561_L1_Tile_rc|1
CCACTACCCTTAATTATAAGAGTATCAGGCACAGGTTCCCCCACCTCGCCAGCCCTGTTA
>HPV6_Alpha_6002612_nt6566_L1_Tile_rc|1
GATTTCCACTACCCTTAATTATAAGAGTATCAGGCACAGGTTCCCCCACCTCGCCAGCCC
>HPV6_Alpha_6002612_nt6571_L1_Tile_rc|1
CGTTCGATTTCCACTACCCTTAATTATAAGAGTATCAGGCACAGGTTCCCCCACCTCGCC
>HPV6_Alpha_6002612_nt6591_L1_Tile_rc|1
TATATACTACTCCCTACAGACGTTCGATTTCCACTACCCTTAATTATAAGAGTATCAGGC
>HPV6_Alpha_6002612_nt6596_L1_Tile_rc|1
TAACATATATACTACTCCCTACAGACGTTCGATTTCCACTACCCTTAATTATAAGAGTAT
>HPV6_Alpha_6002612_nt6601_L1_Tile_rc|1
GGTGTTAACATATATACTACTCCCTACAGACGTTCGATTTCCACTACCCTTAATTATAAG
>HPV6_Alpha_6002612_nt6611_L1_Tile_rc|1
AGCCGCTTGGGGTGTTAACATATATACTACTCCCTACAGACGTTCGATTTCCACTACCCT
>HPV6_Alpha_6002612_nt6616_L1_Tile_rc|1
CAAAGAGCCGCTTGGGGTGTTAACATATATACTACTCCCTACAGACGTTCGATTTCCACT
>HPV6_Alpha_6002612_nt6621_L1_Tile_rc|1
GACACCAAAGAGCCGCTTGGGGTGTTAACATATATACTACTCCCTACAGACGTTCGATTT
>HPV6_Alpha_6002612_nt6791_L1_Tile_rc|1
CAGAATTGGTGTATGTGGAAGATGTAGTTACGGATGCACATAATGTCATGTTGGTACTGC
>HPV6_Alpha_6002612_nt6796_L1_Tile_rc|1
ATAATCAGAATTGGTGTATGTGGAAGATGTAGTTACGGATGCACATAATGTCATGTTGGT
>HPV6_Alpha_6002612_nt6801_L1_Tile_rc|1
TCTTTATAATCAGAATTGGTGTATGTGGAAGATGTAGTTACGGATGCACATAATGTCATG
>HPV6_Alpha_6002612_nt6806_L1_Tile_rc|1
TGTACTCTTTATAATCAGAATTGGTGTATGTGGAAGATGTAGTTACGGATGCACATAATG
>HPV6_Alpha_6002612_nt6811_L1_Tile_rc|1
ACGCATGTACTCTTTATAATCAGAATTGGTGTATGTGGAAGATGTAGTTACGGATGCACA
>HPV6_Alpha_6002612_nt6816_L1_Tile_rc|1
ACATGACGCATGTACTCTTTATAATCAGAATTGGTGTATGTGGAAGATGTAGTTACGGAT
>HPV6_Alpha_6002612_nt6821_L1_Tile_rc|1
CTTCCACATGACGCATGTACTCTTTATAATCAGAATTGGTGTATGTGGAAGATGTAGTTA
>HPV6_Alpha_6002612_nt6826_L1_Tile_rc|1
ATACTCTTCCACATGACGCATGTACTCTTTATAATCAGAATTGGTGTATGTGGAAGATGT
>HPV6_Alpha_6002612_nt7171_L1_Tile_rc|1
CTTAACACCGGTACGAATAGAGGACCGTCCCCTATATCCACTTTGTAACAAAAACTTGCG
>HPV6_Alpha_6002612_nt7191_L1_Tile_rc|1
GCTTTGGAAACAGCAGGGCGCTTAACACCGGTACGAATAGAGGACCGTCCCCTATATCCA
>HPV7_Alpha_9627389_nt5904_L1_Tile_rc|1
ATTAGGCTTTTTCAATTCAAAATATGGATGTCCTATGGTTAATAACCTGGTACTACCTGC
>HPV7_Alpha_9627389_nt5909_L1_Tile_rc|1
TCGCCATTAGGCTTTTTCAATTCAAAATATGGATGTCCTATGGTTAATAACCTGGTACTA
>HPV7_Alpha_9627389_nt5914_L1_Tile_rc|1
ATACATCGCCATTAGGCTTTTTCAATTCAAAATATGGATGTCCTATGGTTAATAACCTGG
>HPV7_Alpha_9627389_nt5919_L1_Tile_rc|1
CACCGATACATCGCCATTAGGCTTTTTCAATTCAAAATATGGATGTCCTATGGTTAATAA
>HPV7_Alpha_9627389_nt5924_L1_Tile_rc|1
TTAGGCACCGATACATCGCCATTAGGCTTTTTCAATTCAAAATATGGATGTCCTATGGTT
>HPV7_Alpha_9627389_nt5929_L1_Tile_rc|1
ACACTTTAGGCACCGATACATCGCCATTAGGCTTTTTCAATTCAAAATATGGATGTCCTA

>HPV7_Alpha_9627389_nt5934_L1_Tile_rc|1
TCCAGACACTTTAGGCACCGATACATCGCCATTAGGCTTTTTCAATTCAAAATATGGATG
>HPV7_Alpha_9627389_nt5939_L1_Tile_rc|1
TGATGTCCAGACACTTTAGGCACCGATACATCGCCATTAGGCTTTTTCAATTCAAAATAT
>HPV7_Alpha_9627389_nt5944_L1_Tile_rc|1
TGTATTGATGTCCAGACACTTTAGGCACCGATACATCGCCATTAGGCTTTTTCAATTCAA
>HPV7_Alpha_9627389_nt6004_L1_Tile_rc|1
ATAAAGACGTGTCTGATAATCCAAATTTATTAGGGTCGGGCAAGCGTACTCTAAACACTC
>HPV7_Alpha_9627389_nt6014_L1_Tile_rc|1
TCAGAATTAAATAAAGACGTGTCTGATAATCCAAATTTATTAGGGTCGGGCAAGCGTACT
>HPV7_Alpha_9627389_nt6029_L1_Tile_rc|1
ACAAGGCGTTGGGTTTCAGAATTAAATAAAGACGTGTCTGATAATCCAAATTTATTAGGG
>HPV7_Alpha_9627389_nt6584_L1_Tile_rc|1
GCGCGATTAGATGAACCTGTTATATATAAATCATTTGGAACACTGTCTCCAGTAGTTCCT
>HPV7_Alpha_9627389_nt6589_L1_Tile_rc|1
TAGAAGCGCGATTAGATGAACCTGTTATATATAAATCATTTGGAACACTGTCTCCAGTAG
>HPV7_Alpha_9627389_nt6594_L1_Tile_rc|1
TGCAATAGAAGCGCGATTAGATGAACCTGTTATATATAAATCATTTGGAACACTGTCTCC
>HPV7_Alpha_9627389_nt6959_L1_Tile_rc|1
GGACCAATTTTAAAATTCCAATCATCTAATAAGGAAGAATCCATAGCATGTATATATGTC
>HPV7_Alpha_9627389_nt6979_L1_Tile_rc|1
CCAAGGTTGCAGACGCTGGAGGACCAATTTTAAAATTCCAATCATCTAATAAGGAAGAAT
>HPV7_Alpha_9627389_nt6984_L1_Tile_rc|1
ATCTTCCAAGGTTGCAGACGCTGGAGGACCAATTTTAAAATTCCAATCATCTAATAAGGA
>HPV7_Alpha_9627389_nt7034_L1_Tile_rc|1
GGTGCATCACGCTGACATGCTATGGCTTTATTGGTAAGAAACCTATAAGTATCTTCCAAG
>HPV7_Alpha_9627389_nt7044_L1_Tile_rc|1
TTCTTTTGGGGGTGCATCACGCTGACATGCTATGGCTTTATTGGTAAGAAACCTATAAGT
>HPV8_Beta_333074_nt6007_L1_Tile_rc|1
CTGTGTTGATTTCCCGATACTTTGGGAACCTGTAATGTGTCACCATTATTGTTGTAAACA
>HPV8_Beta_333074_nt6012_L1_Tile_rc|1
AGACCCTGTGTTGATTTCCCGATACTTTGGGAACCTGTAATGTGTCACCATTATTGTTGT
>HPV8_Beta_333074_nt6027_L1_Tile_rc|1
GTAACTTTAAGCGAAAGACCCTGTGTTGATTTCCCGATACTTTGGGAACCTGTAATGTGT
>HPV8_Beta_333074_nt6042_L1_Tile_rc|2
ACCTATTTGGATCTGGTAACTTTAAGCGAAAGACCCTGTGTTGATTTCCCGATACTTTGG
>HPV8_Beta_333074_nt6197_L1_Tile_rc|1
TGAATTGCTGTTTTCAGTGTCTTTCACTTTATTAAAATAGGGATGGCCGGTGCTCCCAAC
>HPV8_Beta_333074_nt6202_L1_Tile_rc|2
GTGTATGAATTGCTGTTTTCAGTGTCTTTCACTTTATTAAAATAGGGATGGCCGGTGCTC
>HPV8_Beta_333074_nt6207_L1_Tile_rc|1
TTGTGGTGTATGAATTGCTGTTTTCAGTGTCTTTCACTTTATTAAAATAGGGATGGCCGG
>HPV8_Beta_333074_nt6357_L1_Tile_rc|1
GTTCAATGGGTGGGCACAGACCTTGCTGTTGGTCCTCTGCACATGGAATGGCTTTTTCCC
>HPV8_Beta_333074_nt6362_L1_Tile_rc|2
TTTTAGTTCAATGGGTGGGCACAGACCTTGCTGTTGGTCCTCTGCACATGGAATGGCTTT
>HPV8_Beta_333074_nt6367_L1_Tile_rc|1
GTATTTTTTAGTTCAATGGGTGGGCACAGACCTTGCTGTTGGTCCTCTGCACATGGAATG
>HPV8_Beta_333074_nt6372_L1_Tile_rc|1
TAACTGTATTTTTTAGTTCAATGGGTGGGCACAGACCTTGCTGTTGGTCCTCTGCACATG
>HPV8_Beta_333074_nt6377_L1_Tile_rc|1
TTCAATAACTGTATTTTTTAGTTCAATGGGTGGGCACAGACCTTGCTGTTGGTCCTCTGC
>HPV8_Beta_333074_nt6382_L1_Tile_rc|2
CCATCTTCAATAACTGTATTTTTTAGTTCAATGGGTGGGCACAGACCTTGCTGTTGGTCC
>HPV8_Beta_333074_nt6457_L1_Tile_rc|1

TCGAGACTTACATCAGATCTGTTCTGTTGTAAAGTCTTAAAGTTCATATTACCAAAACCA
>HPV8_Beta_333074_nt6462_L1_Tile_rc|2
CTATGTCGAGACTTACATCAGATCTGTTCTGTTGTAAAGTCTTAAAGTTCATATTACCAA
>HPV8_Beta_333074_nt6467_L1_Tile_rc|1
ATTTACTATGTCGAGACTTACATCAGATCTGTTCTGTTGTAAAGTCTTAAAGTTCATATT
>HPV8_Beta_333074_nt6472_L1_Tile_rc|1
ATCTCATTTACTATGTCGAGACTTACATCAGATCTGTTCTGTTGTAAAGTCTTAAAGTTC
>HPV8_Beta_333074_nt6477_L1_Tile_rc|1
TGCATATCTCATTTACTATGTCGAGACTTACATCAGATCTGTTCTGTTGTAAAGTCTTAA
>HPV8_Beta_333074_nt6482_L1_Tile_rc|2
ATATTTGCATATCTCATTTACTATGTCGAGACTTACATCAGATCTGTTCTGTTGTAAAGT
>HPV8_Beta_333074_nt6892_L1_Tile_rc|1
ATGTTCTTAAGTTCCCCATTTTCAGTGTAAACTGAAATACTAAAATTGGTGTTTCGCGTG
>HPV8_Beta_333074_nt6897_L1_Tile_rc|1
CTGTGATGTTCTTAAGTTCCCCATTTTCAGTGTAAACTGAAATACTAAAATTGGTGTTTC
>HPV8_Beta_333074_nt6902_L1_Tile_rc|2
ATAGTCTGTGATGTTCTTAAGTTCCCCATTTTCAGTGTAAACTGAAATACTAAAATTGGT
>HPV8_Beta_333074_nt6907_L1_Tile_rc|1
GATTTATAGTCTGTGATGTTCTTAAGTTCCCCATTTTCAGTGTAAACTGAAATACTAAAA
>HPV8_Beta_333074_nt6912_L1_Tile_rc|1
GGGTTGATTTATAGTCTGTGATGTTCTTAAGTTCCCCATTTTCAGTGTAAACTGAAATAC
>HPV8_Beta_333074_nt6917_L1_Tile_rc|1
GAACTGGGTTGATTTATAGTCTGTGATGTTCTTAAGTTCCCCATTTTCAGTGTAAACTGA
>HPV8_Beta_333074_nt6922_L1_Tile_rc|2
TCTCTGAACTGGGTTGATTTATAGTCTGTGATGTTCTTAAGTTCCCCATTTTCAGTGTAA
>HPV8_Beta_333074_nt6927_L1_Tile_rc|1
GATATTCTCTGAACTGGGTTGATTTATAGTCTGTGATGTTCTTAAGTTCCCCATTTTCAG
>HPV8_Beta_333074_nt6932_L1_Tile_rc|1
TCTCAGATATTCTCTGAACTGGGTTGATTTATAGTCTGTGATGTTCTTAAGTTCCCCATT
>HPV8_Beta_333074_nt6937_L1_Tile_rc|1
ACATGTCTCAGATATTCTCTGAACTGGGTTGATTTATAGTCTGTGATGTTCTTAAGTTCC
>HPV8_Beta_333074_nt6942_L1_Tile_rc|2
CTTCTACATGTCTCAGATATTCTCTGAACTGGGTTGATTTATAGTCTGTGATGTTCTTAA
>HPV8_Beta_333074_nt7002_L1_Tile_rc|2
CTAAAACATCAGCCTTTAGTGGTATCTTACACAATTGTAATATGAGGGAAATTTCATATT
>HPV8_Beta_333074_nt7012_L1_Tile_rc|1
TTGATTTGTGCTAAAACATCAGCCTTTAGTGGTATCTTACACAATTGTAATATGAGGGAA
>HPV8_Beta_333074_nt7077_L1_Tile_rc|1
TTGGAGTATCAGGGGTAGGTACAAATCCCAGTTGCCATTCCTCAAGTAGTGATGAATTCA
>HPV8_Beta_333074_nt7082_L1_Tile_rc|2
ATGAATTGGAGTATCAGGGGTAGGTACAAATCCCAGTTGCCATTCCTCAAGTAGTGATGA
>HPV8_Beta_333074_nt7197_L1_Tile_rc|1
GTTCTGTAAGGTCCACATTCCAGAAGTTAAACTTTGCATATGGATCAGGCTTTTCCTTAG
>HPV8_Beta_333074_nt7202_L1_Tile_rc|2
AAGTCGTTCTGTAAGGTCCACATTCCAGAAGTTAAACTTTGCATATGGATCAGGCTTTTC
>HPV8_Beta_333074_nt7207_L1_Tile_rc|1
AGGGAAAGTCGTTCTGTAAGGTCCACATTCCAGAAGTTAAACTTTGCATATGGATCAGGC
>HPV8_Beta_333074_nt7217_L1_Tile_rc|1
ATCCAAATCCAGGGAAAGTCGTTCTGTAAGGTCCACATTCCAGAAGTTAAACTTTGCATA
>HPV8_Beta_333074_nt7222_L1_Tile_rc|2
TATTGATCCAAATCCAGGGAAAGTCGTTCTGTAAGGTCCACATTCCAGAAGTTAAACTTT
>HPV9_Beta_9627396_nt5861_L1_Tile_rc|1
GTCCGTCGCCTGAGCGGACATCATAATATGGATGACCCACTGTTAGCAAACGGTCACTAA
>HPV9_Beta_9627396_nt5866_L1_Tile_rc|1
CCTTTGTCCGTCGCCTGAGCGGACATCATAATATGGATGACCCACTGTTAGCAAACGGTC

>HPV9_Beta_9627396_nt5871_L1_Tile_rc|1
TCAATCCTTTGTCCGTCGCCTGAGCGGACATCATAATATGGATGACCCACTGTTAGCAAA
>HPV9_Beta_9627396_nt5881_L1_Tile_rc|1
TTTAGGGACTTCAATCCTTTGTCCGTCGCCTGAGCGGACATCATAATATGGATGACCCAC
>HPV9_Beta_9627396_nt5901_L1_Tile_rc|1
CGATACTGATTACCAGACACTTTAGGGACTTCAATCCTTTGTCCGTCGCCTGAGCGGACA
>HPV9_Beta_9627396_nt6181_L1_Tile_rc|1
TAAGCATGGAATACATCCTATAATGAACATTTGTACCTGTTTGGGGTCAAAAGATGTGTT
>HPV9_Beta_9627396_nt6236_L1_Tile_rc|1
CTAGTTGATTATTAGCATCCTTTTCACACACTTTGGCTTTATCCCAGTGTTCTCCTAAGC
>HPV9_Beta_9627396_nt6241_L1_Tile_rc|1
TAAGCCTAGTTGATTATTAGCATCCTTTTCACACACTTTGGCTTTATCCCAGTGTTCTCC
>HPV9_Beta_9627396_nt6246_L1_Tile_rc|1
GGACATAAGCCTAGTTGATTATTAGCATCCTTTTCACACACTTTGGCTTTATCCCAGTGT
>HPV9_Beta_9627396_nt6251_L1_Tile_rc|1
TAGGAGGACATAAGCCTAGTTGATTATTAGCATCCTTTTCACACACTTTGGCTTTATCCC
>HPV9_Beta_9627396_nt6256_L1_Tile_rc|1
TTCTATAGGAGGACATAAGCCTAGTTGATTATTAGCATCCTTTTCACACACTTTGGCTTT
>HPV9_Beta_9627396_nt6261_L1_Tile_rc|1
CTTAATTCTATAGGAGGACATAAGCCTAGTTGATTATTAGCATCCTTTTCACACACTTTG
>HPV9_Beta_9627396_nt6266_L1_Tile_rc|1
TGTTTCTTAATTCTATAGGAGGACATAAGCCTAGTTGATTATTAGCATCCTTTTCACACA
>HPV9_Beta_9627396_nt6366_L1_Tile_rc|1
TCAACAATATCTAAGCTTACATCAGACTTATTAAAGGACAGTTCCTTATTGTTGATATTT
>HPV9_Beta_9627396_nt6371_L1_Tile_rc|1
TTTCATCAACAATATCTAAGCTTACATCAGACTTATTAAAGGACAGTTCCTTATTGTTGA
>HPV9_Beta_9627396_nt6521_L1_Tile_rc|1
GGTTTACTGCACCATCAGGAACAGCGTCACCAACTGAACCTCCTCTAACATAATAATGCC
>HPV9_Beta_9627396_nt6526_L1_Tile_rc|1
ATCCTGGTTTACTGCACCATCAGGAACAGCGTCACCAACTGAACCTCCTCTAACATAATA
>HPV9_Beta_9627396_nt6536_L1_Tile_rc|1
AGAAATTATGATCCTGGTTTACTGCACCATCAGGAACAGCGTCACCAACTGAACCTCCTC
>HPV9_Beta_9627396_nt6551_L1_Tile_rc|1
TTTTTGCTGGCAAAAAGAAATTATGATCCTGGTTTACTGCACCATCAGGAACAGCGTCAC
>HPV9_Beta_9627396_nt6556_L1_Tile_rc|1
ATCACTTTTTGCTGGCAAAAAGAAATTATGATCCTGGTTTACTGCACCATCAGGAACAGC
>HPV9_Beta_9627396_nt6586_L1_Tile_rc|1
GGTGGAATTAGCTATTGTTCGTTGTTGTTGATCACTTTTTGCTGGCAAAAAGAAATTATG
>HPV9_Beta_9627396_nt6596_L1_Tile_rc|1
TAGGATAGTAGGTGGAATTAGCTATTGTTCGTTGTTGTTGATCACTTTTTGCTGGCAAAA
>HPV9_Beta_9627396_nt6601_L1_Tile_rc|1
TACTGTAGGATAGTAGGTGGAATTAGCTATTGTTCGTTGTTGTTGATCACTTTTTGCTGG
>HPV9_Beta_9627396_nt6606_L1_Tile_rc|1
CCACTTACTGTAGGATAGTAGGTGGAATTAGCTATTGTTCGTTGTTGTTGATCACTTTTT
>HPV9_Beta_9627396_nt6616_L1_Tile_rc|1
TACTAATGACCCACTTACTGTAGGATAGTAGGTGGAATTAGCTATTGTTCGTTGTTGTTG
>HPV9_Beta_9627396_nt6621_L1_Tile_rc|1
GAAGTTACTAATGACCCACTTACTGTAGGATAGTAGGTGGAATTAGCTATTGTTCGTTGT
>HPV9_Beta_9627396_nt6756_L1_Tile_rc|1
GACACACTAATGGTAAAATTGGTGTTACGTGTATTGTCTGCCACTGTAACAAATATCTGA
>HPV9_Beta_9627396_nt6766_L1_Tile_rc|1
TGCCTCTGTAGACACACTAATGGTAAAATTGGTGTTACGTGTATTGTCTGCCACTGTAAC
>HPV9_Beta_9627396_nt6776_L1_Tile_rc|1
CTGTTTGAGCTGCCTCTGTAGACACACTAATGGTAAAATTGGTGTTACGTGTATTGTCTG
>HPV9_Beta_9627396_nt6781_L1_Tile_rc|1

TTCTTCTGTTTGAGCTGCCTCTGTAGACACACTAATGGTAAAATTGGTGTTACGTGTATT
>HPV9_Beta_9627396_nt6786_L1_Tile_rc|1
TTATATTCTTCTGTTTGAGCTGCCTCTGTAGACACACTAATGGTAAAATTGGTGTTACGT
>HPV9_Beta_9627396_nt6791_L1_Tile_rc|1
TGGCATTATATTCTTCTGTTTGAGCTGCCTCTGTAGACACACTAATGGTAAAATTGGTGT
>HPV9_Beta_9627396_nt6796_L1_Tile_rc|1
ATTATTGGCATTATATTCTTCTGTTTGAGCTGCCTCTGTAGACACACTAATGGTAAAATT
>HPV9_Beta_9627396_nt6801_L1_Tile_rc|1
CTAATATTATTGGCATTATATTCTTCTGTTTGAGCTGCCTCTGTAGACACACTAATGGTA
>HPV9_Beta_9627396_nt6806_L1_Tile_rc|1
ATTCTCTAATATTATTGGCATTATATTCTTCTGTTTGAGCTGCCTCTGTAGACACACTAA
>HPV9_Beta_9627396_nt6886_L1_Tile_rc|1
GGATAATACTTCAGCTACTAAAGGCACTTTACACAACTGTAAGATTAATGAAATCTGATA
>HPV9_Beta_9627396_nt6891_L1_Tile_rc|1
ATCTGGGATAATACTTCAGCTACTAAAGGCACTTTACACAACTGTAAGATTAATGAAATC
>HPV9_Beta_9627396_nt7026_L1_Tile_rc|1
TCTGTAGGCTCAACAGCATCTGGGCATTTTGTGGCTTTTGAATCAATATATCTGTAGATA
>HPV9_Beta_9627396_nt7096_L1_Tile_rc|1
AAGATCCAACGATAATCTTTCAGTTAGATCCACTTTCCAAAATGAGTATTTGGCAAAGGG
>HPV9_Beta_9627396_nt7101_L1_Tile_rc|1
TGATCAAGATCCAACGATAATCTTTCAGTTAGATCCACTTTCCAAAATGAGTATTTGGCA
>HPV9_Beta_9627396_nt7136_L1_Tile_rc|1
CAGCTTGAAAAAGAAATTTTCTACCTAAAGGATATTGATCAAGATCCAACGATAATCTTT
>HPV9_Beta_9627396_nt7166_L1_Tile_rc|1
TTTTAATAGGACGTTTTCGTGTTTGCAAACCAGCTTGAAAAAGAAATTTTCTACCTAAAG
>HPV9_Beta_9627396_nt7171_L1_Tile_rc|1
AGATGTTTTAATAGGACGTTTTCGTGTTTGCAAACCAGCTTGAAAAAGAAATTTTCTACC
>HPV9_Beta_9627396_nt7176_L1_Tile_rc|1
TTAACAGATGTTTTAATAGGACGTTTTCGTGTTTGCAAACCAGCTTGAAAAAGAAATTTT
>HPV9_Beta_9627396_nt7181_L1_Tile_rc|1
ATGTTTTAACAGATGTTTTAATAGGACGTTTTCGTGTTTGCAAACCAGCTTGAAAAAGAA
>HPV9_Beta_9627396_nt7186_L1_Tile_rc|1
TTTAGATGTTTTAACAGATGTTTTAATAGGACGTTTTCGTGTTTGCAAACCAGCTTGAAA
>HPV9_Beta_9627396_nt7191_L1_Tile_rc|1
GCATTTTTAGATGTTTTAACAGATGTTTTAATAGGACGTTTTCGTGTTTGCAAACCAGCT
>HPV9_Beta_9627396_nt7196_L1_Tile_rc|1
TCTTAGCATTTTTAGATGTTTTAACAGATGTTTTAATAGGACGTTTTCGTGTTTGCAAAC
>HPV9_Beta_9627396_nt7201_L1_Tile_rc|1
CCTTCTCTTAGCATTTTTAGATGTTTTAACAGATGTTTTAATAGGACGTTTTCGTGTTTG
>HPV9_Beta_9627396_nt7206_L1_Tile_rc|1
GTTCGCCTTCTCTTAGCATTTTTAGATGTTTTAACAGATGTTTTAATAGGACGTTTTCGT
>HPV9_Beta_9627396_nt7210_L1_Tile_rc|1
TTAGGTTCGCCTTCTCTTAGCATTTTTAGATGTTTTAACAGATGTTTTAATAGGACGTTT
>HPV10_Alpha_9627257_nt6590_L1_Tile_rc|1
ATATGAAAGTGTCTGGGATGGCGTCTCCAACAGCACTAGCCCGATTAAAGAAATGTCTTG
>HPV10_Alpha_9627257_nt6595_L1_Tile_rc|1
CTTTAATATGAAAGTGTCTGGGATGGCGTCTCCAACAGCACTAGCCCGATTAAAGAAATG
>HPV10_Alpha_9627257_nt6600_L1_Tile_rc|1
TTGCTCTTTAATATGAAAGTGTCTGGGATGGCGTCTCCAACAGCACTAGCCCGATTAAAG
>HPV10_Alpha_9627257_nt6615_L1_Tile_rc|1
TCTCGCCCCCCACCGTTGCTCTTTAATATGAAAGTGTCTGGGATGGCGTCTCCAACAGCA
>HPV10_Alpha_9627257_nt6625_L1_Tile_rc|1
ACTACCAACGTCTCGCCCCCCACCGTTGCTCTTTAATATGAAAGTGTCTGGGATGGCGTC
>HPV10_Alpha_9627257_nt6815_L1_Tile_rc|1
GGGAGGCCTCAGAAGGAACACACAAGCACATATTGGTACTGCGAGTCGTGTCTACCACAG

>HPV10_Alpha_9627257_nt6820_L1_Tile_rc|1
GGCAGGGGAGGCCTCAGAAGGAACACACAAGCACATATTGGTACTGCGAGTCGTGTCTAC
>HPV10_Alpha_9627257_nt6825_L1_Tile_rc|1
GTAGTGGCAGGGGAGGCCTCAGAAGGAACACACAAGCACATATTGGTACTGCGAGTCGTG
>HPV10_Alpha_9627257_nt6840_L1_Tile_rc|1
TTGGTGGCGTCATACGTAGTGGCAGGGGAGGCCTCAGAAGGAACACACAAGCACATATTG
>HPV101_Unclassified_109390389_nt4918_L1_Tile_rc|1
GCAAATAAAATGTATCAAATGCATCAAAACCATCCACAACAATAGGAGGTTCAGACACAT
>HPV101_Unclassified_109390389_nt4923_L1_Tile_rc|1
TGGATGCAAATAAAATGTATCAAATGCATCAAAACCATCCACAACAATAGGAGGTTCAGA
>HPV101_Unclassified_109390389_nt4928_L1_Tile_rc|1
TGACTTGGATGCAAATAAAATGTATCAAATGCATCAAAACCATCCACAACAATAGGAGGT
>HPV101_Unclassified_109390389_nt4933_L1_Tile_rc|1
GTCTGTGACTTGGATGCAAATAAAATGTATCAAATGCATCAAAACCATCCACAACAATAG
>HPV101_Unclassified_109390389_nt4938_L1_Tile_rc|1
TTTGCGTCTGTGACTTGGATGCAAATAAAATGTATCAAATGCATCAAAACCATCCACAAC
>HPV101_Unclassified_109390389_nt4963_L1_Tile_rc|1
AAAAACCAGACCGTTTGCGCTTGCGTTTGCGTCTGTGACTTGGATGCAAATAAAATGTAT
>HPV101_Unclassified_109390389_nt4968_L1_Tile_rc|1
ACATTAAAAACCAGACCGTTTGCGCTTGCGTTTGCGTCTGTGACTTGGATGCAAATAAAA
>HPV101_Unclassified_109390389_nt5008_L1_Tile_rc|1
TTTACCAGTCTGAGGTAGCCACAAACCAGCCATCTGCAAAACATTAAAAACCAGACCGTT
>HPV101_Unclassified_109390389_nt5013_L1_Tile_rc|1
TATATTTTACCAGTCTGAGGTAGCCACAAACCAGCCATCTGCAAAACATTAAAAACCAGA
>HPV101_Unclassified_109390389_nt5018_L1_Tile_rc|1
GTAGGTATATTTTACCAGTCTGAGGTAGCCACAAACCAGCCATCTGCAAAACATTAAAAA
>HPV101_Unclassified_109390389_nt5043_L1_Tile_rc|1
CTCCTAGACACAGGAGGAGCTGGAGGTAGGTATATTTTACCAGTCTGAGGTAGCCACAAA
>HPV101_Unclassified_109390389_nt5048_L1_Tile_rc|1
TAAGACTCCTAGACACAGGAGGAGCTGGAGGTAGGTATATTTTACCAGTCTGAGGTAGCC
>HPV101_Unclassified_109390389_nt5053_L1_Tile_rc|1
GGTGGTAAGACTCCTAGACACAGGAGGAGCTGGAGGTAGGTATATTTTACCAGTCTGAGG
>HPV101_Unclassified_109390389_nt5058_L1_Tile_rc|1
TCATCGGTGGTAAGACTCCTAGACACAGGAGGAGCTGGAGGTAGGTATATTTTACCAGTC
>HPV101_Unclassified_109390389_nt5063_L1_Tile_rc|1
CATATTCATCGGTGGTAAGACTCCTAGACACAGGAGGAGCTGGAGGTAGGTATATTTTAC
>HPV101_Unclassified_109390389_nt5083_L1_Tile_rc|1
GAATACAGATGTTTCCACAACATATTCATCGGTGGTAAGACTCCTAGACACAGGAGGAGC
>HPV101_Unclassified_109390389_nt5088_L1_Tile_rc|1
TGGAAGAATACAGATGTTTCCACAACATATTCATCGGTGGTAAGACTCCTAGACACAGGA
>HPV101_Unclassified_109390389_nt5118_L1_Tile_rc|1
CCCACAGTTACTAGACGATCACTGGATGCATGGAAGAATACAGATGTTTCCACAACATAT
>HPV101_Unclassified_109390389_nt5128_L1_Tile_rc|1
ATAGGGGTGACCCACAGTTACTAGACGATCACTGGATGCATGGAAGAATACAGATGTTTC
>HPV101_Unclassified_109390389_nt5133_L1_Tile_rc|1
GGAAAATAGGGGTGACCCACAGTTACTAGACGATCACTGGATGCATGGAAGAATACAGAT
>HPV101_Unclassified_109390389_nt5138_L1_Tile_rc|1
TAACGGGAAAATAGGGGTGACCCACAGTTACTAGACGATCACTGGATGCATGGAAGAATA
>HPV101_Unclassified_109390389_nt5143_L1_Tile_rc|1
TTTTATAACGGGAAAATAGGGGTGACCCACAGTTACTAGACGATCACTGGATGCATGGAA
>HPV101_Unclassified_109390389_nt5148_L1_Tile_rc|1
CCTGGTTTTATAACGGGAAAATAGGGGTGACCCACAGTTACTAGACGATCACTGGATGCA
>HPV101_Unclassified_109390389_nt5153_L1_Tile_rc|1
GATTGCCTGGTTTTATAACGGGAAAATAGGGGTGACCCACAGTTACTAGACGATCACTGG
>HPV101_Unclassified_109390389_nt5158_L1_Tile_rc|1

GGTGTGATTGCCTGGTTTTATAACGGGAAAATAGGGGTGACCCACAGTTACTAGACGATC
>HPV101_Unclassified_109390389_nt5163_L1_Tile_rc|1
TCAACGGTGTGATTGCCTGGTTTTATAACGGGAAAATAGGGGTGACCCACAGTTACTAGA
>HPV101_Unclassified_109390389_nt5168_L1_Tile_rc|1
GAACCTCAACGGTGTGATTGCCTGGTTTTATAACGGGAAAATAGGGGTGACCCACAGTTA
>HPV101_Unclassified_109390389_nt5178_L1_Tile_rc|1
GAAACCTTAGGAACCTCAACGGTGTGATTGCCTGGTTTTATAACGGGAAAATAGGGGTGA
>HPV101_Unclassified_109390389_nt5183_L1_Tile_rc|1
TGCCAGAAACCTTAGGAACCTCAACGGTGTGATTGCCTGGTTTTATAACGGGAAAATAGG
>HPV101_Unclassified_109390389_nt5188_L1_Tile_rc|1
CTGGTTGCCAGAAACCTTAGGAACCTCAACGGTGTGATTGCCTGGTTTTATAACGGGAAA
>HPV101_Unclassified_109390389_nt5193_L1_Tile_rc|1
CTAAACTGGTTGCCAGAAACCTTAGGAACCTCAACGGTGTGATTGCCTGGTTTTATAACG
>HPV101_Unclassified_109390389_nt5198_L1_Tile_rc|1
AAGCCCTAAACTGGTTGCCAGAAACCTTAGGAACCTCAACGGTGTGATTGCCTGGTTTTA
>HPV101_Unclassified_109390389_nt5208_L1_Tile_rc|1
CGTAGGCGAAAAGCCCTAAACTGGTTGCCAGAAACCTTAGGAACCTCAACGGTGTGATTG
>HPV101_Unclassified_109390389_nt5223_L1_Tile_rc|1
TTGGGATCTGGGAGACGTAGGCGAAAAGCCCTAAACTGGTTGCCAGAAACCTTAGGAACC
>HPV101_Unclassified_109390389_nt5233_L1_Tile_rc|1
AGCAAACTTATTGGGATCTGGGAGACGTAGGCGAAAAGCCCTAAACTGGTTGCCAGAAAC
>HPV101_Unclassified_109390389_nt5238_L1_Tile_rc|1
ATTAAAGCAAACTTATTGGGATCTGGGAGACGTAGGCGAAAAGCCCTAAACTGGTTGCCA
>HPV101_Unclassified_109390389_nt5298_L1_Tile_rc|1
TCAATTCCTCTAAGACGCCACACGAGGCGCTCCGATTCAACATTATACAATTGTTTGTCA
>HPV101_Unclassified_109390389_nt5303_L1_Tile_rc|1
CAATATCAATTCCTCTAAGACGCCACACGAGGCGCTCCGATTCAACATTATACAATTGTT
>HPV101_Unclassified_109390389_nt5308_L1_Tile_rc|1
TCTGCCAATATCAATTCCTCTAAGACGCCACACGAGGCGCTCCGATTCAACATTATACAA
>HPV101_Unclassified_109390389_nt5313_L1_Tile_rc|1
CCTCCTCTGCCAATATCAATTCCTCTAAGACGCCACACGAGGCGCTCCGATTCAACATTA
>HPV101_Unclassified_109390389_nt5403_L1_Tile_rc|1
TTAGCACCACCCTTTTGATATCCATTAGGATTTTCAGTATCCTTTAACTTATTAAATAAA
>HPV101_Unclassified_109390389_nt5408_L1_Tile_rc|1
CGTCCTTAGCACCACCCTTTTGATATCCATTAGGATTTTCAGTATCCTTTAACTTATTAA
>HPV101_Unclassified_109390389_nt5413_L1_Tile_rc|1
TCTAGCGTCCTTAGCACCACCCTTTTGATATCCATTAGGATTTTCAGTATCCTTTAACTT
>HPV101_Unclassified_109390389_nt5423_L1_Tile_rc|1
AAACATTTTGTCTAGCGTCCTTAGCACCACCCTTTTGATATCCATTAGGATTTTCAGTAT
>HPV101_Unclassified_109390389_nt5428_L1_Tile_rc|1
CATAGAAACATTTTGTCTAGCGTCCTTAGCACCACCCTTTTGATATCCATTAGGATTTTC
>HPV101_Unclassified_109390389_nt5433_L1_Tile_rc|1
GGATCCATAGAAACATTTTGTCTAGCGTCCTTAGCACCACCCTTTTGATATCCATTAGGA
>HPV101_Unclassified_109390389_nt5438_L1_Tile_rc|1
GTTTAGGATCCATAGAAACATTTTGTCTAGCGTCCTTAGCACCACCCTTTTGATATCCAT
>HPV101_Unclassified_109390389_nt5543_L1_Tile_rc|1
GTAAAGGAGGACAGGTGCCTTTAGGAGGATTTTTGTCACATGCAGGAGCAACATCCCAGT
>HPV101_Unclassified_109390389_nt5548_L1_Tile_rc|1
CAACTGTAAAGGAGGACAGGTGCCTTTAGGAGGATTTTTGTCACATGCAGGAGCAACATC
>HPV101_Unclassified_109390389_nt5558_L1_Tile_rc|1
CAGAGGACACCAACTGTAAAGGAGGACAGGTGCCTTTAGGAGGATTTTTGTCACATGCAG
>HPV101_Unclassified_109390389_nt5563_L1_Tile_rc|1
AATAACAGAGGACACCAACTGTAAAGGAGGACAGGTGCCTTTAGGAGGATTTTTGTCACA
>HPV101_Unclassified_109390389_nt5568_L1_Tile_rc|1
TCCTGAATAACAGAGGACACCAACTGTAAAGGAGGACAGGTGCCTTTAGGAGGATTTTTG

>HPV101_Unclassified_109390389_nt5658_L1_Tile_rc|1
TCTGCAACAATATCTAGGGGTACCCCTGAGTGATCAGCCTGCAAGGCAGCAAAATTCATG
>HPV101_Unclassified_109390389_nt5663_L1_Tile_rc|1
AAGTATCTGCAACAATATCTAGGGGTACCCCTGAGTGATCAGCCTGCAAGGCAGCAAAAT
>HPV101_Unclassified_109390389_nt5668_L1_Tile_rc|1
TTTACAAGTATCTGCAACAATATCTAGGGGTACCCCTGAGTGATCAGCCTGCAAGGCAGC
>HPV101_Unclassified_109390389_nt5678_L1_Tile_rc|1
AGTCTGGCCATTTACAAGTATCTGCAACAATATCTAGGGGTACCCCTGAGTGATCAGCCT
>HPV101_Unclassified_109390389_nt5683_L1_Tile_rc|1
AAGAAAGTCTGGCCATTTACAAGTATCTGCAACAATATCTAGGGGTACCCCTGAGTGATC
>HPV101_Unclassified_109390389_nt5688_L1_Tile_rc|1
ATACCAAGAAAGTCTGGCCATTTACAAGTATCTGCAACAATATCTAGGGGTACCCCTGAG
>HPV101_Unclassified_109390389_nt5698_L1_Tile_rc|1
ATCATTGGCCATACCAAGAAAGTCTGGCCATTTACAAGTATCTGCAACAATATCTAGGGG
>HPV101_Unclassified_109390389_nt5723_L1_Tile_rc|1
AGAAAAACATACCATCCCCAAACCTATCATTGGCCATACCAAGAAAGTCTGGCCATTTAC
>HPV101_Unclassified_109390389_nt5743_L1_Tile_rc|1
CACCTGCTCGCGGCGGCCAAAGAAAAACATACCATCCCCAAACCTATCATTGGCCATACC
>HPV101_Unclassified_109390389_nt5748_L1_Tile_rc|1
GCATACACCTGCTCGCGGCGGCCAAAGAAAAACATACCATCCCCAAACCTATCATTGGCC
>HPV101_Unclassified_109390389_nt5758_L1_Tile_rc|1
ATAATGACGTGCATACACCTGCTCGCGGCGGCCAAAGAAAAACATACCATCCCCAAACCT
>HPV101_Unclassified_109390389_nt5763_L1_Tile_rc|1
GTGAAATAATGACGTGCATACACCTGCTCGCGGCGGCCAAAGAAAAACATACCATCCCCA
>HPV101_Unclassified_109390389_nt5768_L1_Tile_rc|1
TATGGGTGAAATAATGACGTGCATACACCTGCTCGCGGCGGCCAAAGAAAAACATACCAT
>HPV101_Unclassified_109390389_nt5773_L1_Tile_rc|1
GCCATTATGGGTGAAATAATGACGTGCATACACCTGCTCGCGGCGGCCAAAGAAAAACAT
>HPV101_Unclassified_109390389_nt5778_L1_Tile_rc|1
ACAGTGCCATTATGGGTGAAATAATGACGTGCATACACCTGCTCGCGGCGGCCAAAGAAA
>HPV101_Unclassified_109390389_nt5793_L1_Tile_rc|1
GGAACACTGTCACCAACAGTGCCATTATGGGTGAAATAATGACGTGCATACACCTGCTCG
>HPV101_Unclassified_109390389_nt5798_L1_Tile_rc|1
CCTCTGGAACACTGTCACCAACAGTGCCATTATGGGTGAAATAATGACGTGCATACACCT
>HPV101_Unclassified_109390389_nt5808_L1_Tile_rc|1
TCAAACCCCTCCTCTGGAACACTGTCACCAACAGTGCCATTATGGGTGAAATAATGACGT
>HPV101_Unclassified_109390389_nt5818_L1_Tile_rc|1
ACCATTACCCTCAAACCCCTCCTCTGGAACACTGTCACCAACAGTGCCATTATGGGTGAA
>HPV101_Unclassified_109390389_nt5828_L1_Tile_rc|1
AATACTTTTTACCATTACCCTCAAACCCCTCCTCTGGAACACTGTCACCAACAGTGCCAT
>HPV101_Unclassified_109390389_nt5833_L1_Tile_rc|1
AATAAAATACTTTTTACCATTACCCTCAAACCCCTCCTCTGGAACACTGTCACCAACAGT
>HPV101_Unclassified_109390389_nt5848_L1_Tile_rc|1
GCCTTGCTCAGCACCAATAAAATACTTTTTACCATTACCCTCAAACCCCTCCTCTGGAAC
>HPV101_Unclassified_109390389_nt5863_L1_Tile_rc|1
CTGATGCTGGTCCTGGCCTTGCTCAGCACCAATAAAATACTTTTTACCATTACCCTCAAA
>HPV101_Unclassified_109390389_nt5868_L1_Tile_rc|1
GCCAGCTGATGCTGGTCCTGGCCTTGCTCAGCACCAATAAAATACTTTTTACCATTACCC
>HPV101_Unclassified_109390389_nt5878_L1_Tile_rc|1
TAAAGAGGGAGCCAGCTGATGCTGGTCCTGGCCTTGCTCAGCACCAATAAAATACTTTTT
>HPV101_Unclassified_109390389_nt5883_L1_Tile_rc|1
AAATATAAAGAGGGAGCCAGCTGATGCTGGTCCTGGCCTTGCTCAGCACCAATAAAATAC
>HPV101_Unclassified_109390389_nt5888_L1_Tile_rc|1
TAGCAAAATATAAAGAGGGAGCCAGCTGATGCTGGTCCTGGCCTTGCTCAGCACCAATAA
>HPV101_Unclassified_109390389_nt5893_L1_Tile_rc|1

AGGTGTAGCAAAATATAAAGAGGGAGCCAGCTGATGCTGGTCCTGGCCTTGCTCAGCACC
>HPV101_Unclassified_109390389_nt5913_L1_Tile_rc|1
CTAGATGCTAAGGACCCACTAGGTGTAGCAAAATATAAAGAGGGAGCCAGCTGATGCTGG
>HPV101_Unclassified_109390389_nt5918_L1_Tile_rc|1
AGTCACTAGATGCTAAGGACCCACTAGGTGTAGCAAAATATAAAGAGGGAGCCAGCTGAT
>HPV101_Unclassified_109390389_nt5928_L1_Tile_rc|1
AATAACTGGGAGTCACTAGATGCTAAGGACCCACTAGGTGTAGCAAAATATAAAGAGGGA
>HPV101_Unclassified_109390389_nt5933_L1_Tile_rc|1
TACTAAATAACTGGGAGTCACTAGATGCTAAGGACCCACTAGGTGTAGCAAAATATAAAG
>HPV101_Unclassified_109390389_nt5958_L1_Tile_rc|1
TGAGCCCGTTCAATCCAGTAGGGCCTACTAAATAACTGGGAGTCACTAGATGCTAAGGAC
>HPV101_Unclassified_109390389_nt5978_L1_Tile_rc|1
ATACAGCATTATTAGCACCCTGAGCCCGTTCAATCCAGTAGGGCCTACTAAATAACTGGG
>HPV101_Unclassified_109390389_nt5983_L1_Tile_rc|1
CCATAATACAGCATTATTAGCACCCTGAGCCCGTTCAATCCAGTAGGGCCTACTAAATAA
>HPV101_Unclassified_109390389_nt5988_L1_Tile_rc|1
TTATTCCATAATACAGCATTATTAGCACCCTGAGCCCGTTCAATCCAGTAGGGCCTACTA
>HPV101_Unclassified_109390389_nt5993_L1_Tile_rc|1
CATTATTATTCCATAATACAGCATTATTAGCACCCTGAGCCCGTTCAATCCAGTAGGGCC
>HPV101_Unclassified_109390389_nt5998_L1_Tile_rc|1
AAATACATTATTATTCCATAATACAGCATTATTAGCACCCTGAGCCCGTTCAATCCAGTA
>HPV101_Unclassified_109390389_nt6003_L1_Tile_rc|1
GTAACAAATACATTATTATTCCATAATACAGCATTATTAGCACCCTGAGCCCGTTCAATC
>HPV101_Unclassified_109390389_nt6008_L1_Tile_rc|1
CCAAAGTAACAAATACATTATTATTCCATAATACAGCATTATTAGCACCCTGAGCCCGTT
>HPV101_Unclassified_109390389_nt6018_L1_Tile_rc|1
GTATTATCAACCAAAGTAACAAATACATTATTATTCCATAATACAGCATTATTAGCACCC
>HPV101_Unclassified_109390389_nt6023_L1_Tile_rc|1
TGTGGGTATTATCAACCAAAGTAACAAATACATTATTATTCCATAATACAGCATTATTAG
>HPV101_Unclassified_109390389_nt6033_L1_Tile_rc|1
AAATTCACATTGTGGGTATTATCAACCAAAGTAACAAATACATTATTATTCCATAATACA
>HPV101_Unclassified_109390389_nt6038_L1_Tile_rc|1
TAGTAAAATTCACATTGTGGGTATTATCAACCAAAGTAACAAATACATTATTATTCCATA
>HPV101_Unclassified_109390389_nt6043_L1_Tile_rc|1
ATTAATAGTAAAATTCACATTGTGGGTATTATCAACCAAAGTAACAAATACATTATTATT
>HPV101_Unclassified_109390389_nt6048_L1_Tile_rc|1
GCAACATTAATAGTAAAATTCACATTGTGGGTATTATCAACCAAAGTAACAAATACATTA
>HPV101_Unclassified_109390389_nt6053_L1_Tile_rc|1
CTGTAGCAACATTAATAGTAAAATTCACATTGTGGGTATTATCAACCAAAGTAACAAATA
>HPV101_Unclassified_109390389_nt6058_L1_Tile_rc|1
ATCTTCTGTAGCAACATTAATAGTAAAATTCACATTGTGGGTATTATCAACCAAAGTAAC
>HPV101_Unclassified_109390389_nt6063_L1_Tile_rc|1
ATATTATCTTCTGTAGCAACATTAATAGTAAAATTCACATTGTGGGTATTATCAACCAAA
>HPV101_Unclassified_109390389_nt6068_L1_Tile_rc|1
ACTGAATATTATCTTCTGTAGCAACATTAATAGTAAAATTCACATTGTGGGTATTATCAA
>HPV101_Unclassified_109390389_nt6073_L1_Tile_rc|1
ATAGGACTGAATATTATCTTCTGTAGCAACATTAATAGTAAAATTCACATTGTGGGTATT
>HPV101_Unclassified_109390389_nt6078_L1_Tile_rc|1
GAATCATAGGACTGAATATTATCTTCTGTAGCAACATTAATAGTAAAATTCACATTGTGG
>HPV101_Unclassified_109390389_nt6088_L1_Tile_rc|1
GTACTTTGCAGAATCATAGGACTGAATATTATCTTCTGTAGCAACATTAATAGTAAAATT
>HPV101_Unclassified_109390389_nt6093_L1_Tile_rc|1
TGCTTGTACTTTGCAGAATCATAGGACTGAATATTATCTTCTGTAGCAACATTAATAGTA
>HPV101_Unclassified_109390389_nt6098_L1_Tile_rc|1
GGTAGTGCTTGTACTTTGCAGAATCATAGGACTGAATATTATCTTCTGTAGCAACATTAA

>HPV101_Unclassified_109390389_nt6103_L1_Tile_rc|1
TCTGAGGTAGTGCTTGTACTTTGCAGAATCATAGGACTGAATATTATCTTCTGTAGCAAC
>HPV101_Unclassified_109390389_nt6108_L1_Tile_rc|1
ACATGTCTGAGGTAGTGCTTGTACTTTGCAGAATCATAGGACTGAATATTATCTTCTGTA
>HPV101_Unclassified_109390389_nt6113_L1_Tile_rc|1
CCTCCACATGTCTGAGGTAGTGCTTGTACTTTGCAGAATCATAGGACTGAATATTATCTT
>HPV101_Unclassified_109390389_nt6118_L1_Tile_rc|1
AAATTCCTCCACATGTCTGAGGTAGTGCTTGTACTTTGCAGAATCATAGGACTGAATATT
>HPV101_Unclassified_109390389_nt6123_L1_Tile_rc|1
ATTTCAAATTCCTCCACATGTCTGAGGTAGTGCTTGTACTTTGCAGAATCATAGGACTGA
>HPV101_Unclassified_109390389_nt6128_L1_Tile_rc|1
GAGAAATTTCAAATTCCTCCACATGTCTGAGGTAGTGCTTGTACTTTGCAGAATCATAGG
>HPV101_Unclassified_109390389_nt6133_L1_Tile_rc|1
AATGAGAGAAATTTCAAATTCCTCCACATGTCTGAGGTAGTGCTTGTACTTTGCAGAATC
>HPV101_Unclassified_109390389_nt6138_L1_Tile_rc|1
TGCACAATGAGAGAAATTTCAAATTCCTCCACATGTCTGAGGTAGTGCTTGTACTTTGCA
>HPV101_Unclassified_109390389_nt6143_L1_Tile_rc|1
AGAGCTGCACAATGAGAGAAATTTCAAATTCCTCCACATGTCTGAGGTAGTGCTTGTACT
>HPV101_Unclassified_109390389_nt6158_L1_Tile_rc|1
TAAGAGACACTTTACAGAGCTGCACAATGAGAGAAATTTCAAATTCCTCCACATGTCTGA
>HPV101_Unclassified_109390389_nt6163_L1_Tile_rc|1
GGGAGTAAGAGACACTTTACAGAGCTGCACAATGAGAGAAATTTCAAATTCCTCCACATG
>HPV101_Unclassified_109390389_nt6173_L1_Tile_rc|1
CCAATACATCGGGAGTAAGAGACACTTTACAGAGCTGCACAATGAGAGAAATTTCAAATT
>HPV101_Unclassified_109390389_nt6178_L1_Tile_rc|1
GTGTGCCAATACATCGGGAGTAAGAGACACTTTACAGAGCTGCACAATGAGAGAAATTTC
>HPV101_Unclassified_109390389_nt6183_L1_Tile_rc|1
TGTATGTGTGCCAATACATCGGGAGTAAGAGACACTTTACAGAGCTGCACAATGAGAGAA
>HPV101_Unclassified_109390389_nt6193_L1_Tile_rc|1
ATTCATTAAGTGTATGTGTGCCAATACATCGGGAGTAAGAGACACTTTACAGAGCTGCAC
>HPV101_Unclassified_109390389_nt6198_L1_Tile_rc|1
GAGGGATTCATTAAGTGTATGTGTGCCAATACATCGGGAGTAAGAGACACTTTACAGAGC
>HPV101_Unclassified_109390389_nt6203_L1_Tile_rc|1
GAATAGAGGGATTCATTAAGTGTATGTGTGCCAATACATCGGGAGTAAGAGACACTTTAC
>HPV101_Unclassified_109390389_nt6208_L1_Tile_rc|1
ATCCAGAATAGAGGGATTCATTAAGTGTATGTGTGCCAATACATCGGGAGTAAGAGACAC
>HPV101_Unclassified_109390389_nt6213_L1_Tile_rc|1
CATTCATCCAGAATAGAGGGATTCATTAAGTGTATGTGTGCCAATACATCGGGAGTAAGA
>HPV101_Unclassified_109390389_nt6218_L1_Tile_rc|1
ATTGCCATTCATCCAGAATAGAGGGATTCATTAAGTGTATGTGTGCCAATACATCGGGAG
>HPV101_Unclassified_109390389_nt6223_L1_Tile_rc|1
ACCTAATTGCCATTCATCCAGAATAGAGGGATTCATTAAGTGTATGTGTGCCAATACATC
>HPV101_Unclassified_109390389_nt6238_L1_Tile_rc|1
CGGTGGAGCCGCAATACCTAATTGCCATTCATCCAGAATAGAGGGATTCATTAAGTGTAT
>HPV101_Unclassified_109390389_nt6243_L1_Tile_rc|1
GTAGACGGTGGAGCCGCAATACCTAATTGCCATTCATCCAGAATAGAGGGATTCATTAAG
>HPV101_Unclassified_109390389_nt6248_L1_Tile_rc|1
TAGAGGTAGACGGTGGAGCCGCAATACCTAATTGCCATTCATCCAGAATAGAGGGATTCA
>HPV101_Unclassified_109390389_nt6253_L1_Tile_rc|1
TTCAATAGAGGTAGACGGTGGAGCCGCAATACCTAATTGCCATTCATCCAGAATAGAGGG
>HPV101_Unclassified_109390389_nt6263_L1_Tile_rc|1
TGTAAGTGGTTTCAATAGAGGTAGACGGTGGAGCCGCAATACCTAATTGCCATTCATCCA
>HPV101_Unclassified_109390389_nt6268_L1_Tile_rc|1
ATATCTGTAAGTGGTTTCAATAGAGGTAGACGGTGGAGCCGCAATACCTAATTGCCATTC
>HPV101_Unclassified_109390389_nt6273_L1_Tile_rc|1

TCAATATATCTGTAAGTGGTTTCAATAGAGGTAGACGGTGGAGCCGCAATACCTAATTGC
>HPV101_Unclassified_109390389_nt6278_L1_Tile_rc|1
GAGAATCAATATATCTGTAAGTGGTTTCAATAGAGGTAGACGGTGGAGCCGCAATACCTA
>HPV101_Unclassified_109390389_nt6283_L1_Tile_rc|1
AGCAAGAGAATCAATATATCTGTAAGTGGTTTCAATAGAGGTAGACGGTGGAGCCGCAAT
>HPV101_Unclassified_109390389_nt6303_L1_Tile_rc|1
TCTGCTGTGGGACACATTGTAGCAAGAGAATCAATATATCTGTAAGTGGTTTCAATAGAG
>HPV101_Unclassified_109390389_nt6343_L1_Tile_rc|1
AGACATTTTGTCATATGGATCTTCCTTTTCCTTAGGAGGCTCTGCTGTGGGACACATTGT
>HPV101_Unclassified_109390389_nt6383_L1_Tile_rc|1
ATTCAGAGGACATTCTGTCTGATAAATCAACCACCCAAAAAGACATTTTGTCATATGGAT
>HPV101_Unclassified_109390389_nt6388_L1_Tile_rc|1
CCCTAATTCAGAGGACATTCTGTCTGATAAATCAACCACCCAAAAAGACATTTTGTCATA
>HPV101_Unclassified_109390389_nt6393_L1_Tile_rc|1
TAGGCCCCTAATTCAGAGGACATTCTGTCTGATAAATCAACCACCCAAAAAGACATTTTG
>HPV101_Unclassified_109390389_nt6398_L1_Tile_rc|1
GAGGATAGGCCCCTAATTCAGAGGACATTCTGTCTGATAAATCAACCACCCAAAAAGACA
>HPV101_Unclassified_109390389_nt6403_L1_Tile_rc|1
ACCAAGAGGATAGGCCCCTAATTCAGAGGACATTCTGTCTGATAAATCAACCACCCAAAA
>HPV101_Unclassified_109390389_nt6413_L1_Tile_rc|1
AAAAGCGCCTACCAAGAGGATAGGCCCCTAATTCAGAGGACATTCTGTCTGATAAATCAA
>HPV101_Unclassified_109390389_nt6418_L1_Tile_rc|1
ATGTAAAAAGCGCCTACCAAGAGGATAGGCCCCTAATTCAGAGGACATTCTGTCTGATAA
>HPV101_Unclassified_109390389_nt6423_L1_Tile_rc|1
GTTTGATGTAAAAAGCGCCTACCAAGAGGATAGGCCCCTAATTCAGAGGACATTCTGTCT
>HPV101_Unclassified_109390389_nt6433_L1_Tile_rc|1
GGCAGACCCTGTTTGATGTAAAAAGCGCCTACCAAGAGGATAGGCCCCTAATTCAGAGGA
>HPV101_Unclassified_109390389_nt6453_L1_Tile_rc|1
TTTAGGGGGCGCGCAGGCCGGGCAGACCCTGTTTGATGTAAAAAGCGCCTACCAAGAGGA
>HPV101_Unclassified_109390389_nt6458_L1_Tile_rc|1
GTCGCTTTAGGGGGCGCGCAGGCCGGGCAGACCCTGTTTGATGTAAAAAGCGCCTACCAA
>HPV101_Unclassified_109390389_nt6468_L1_Tile_rc|1
GAAGGACGGGGTCGCTTTAGGGGGCGCGCAGGCCGGGCAGACCCTGTTTGATGTAAAAAG
>HPV101_Unclassified_109390389_nt6473_L1_Tile_rc|1
GCGTAGAAGGACGGGGTCGCTTTAGGGGGCGCGCAGGCCGGGCAGACCCTGTTTGATGTA
>HPV101_Unclassified_109390389_nt6518_L1_Tile_rc|1
CTTGCCTGCGTCGGCGTTTGGAAGACCTGGAGGTAGGCCTGGTAGGCGTAGAAGGACGGG
>HPV101_Unclassified_109390389_nt6523_L1_Tile_rc|1
TTAAACTTGCCTGCGTCGGCGTTTGGAAGACCTGGAGGTAGGCCTGGTAGGCGTAGAAGG
>HPV102_Alpha_71726718_nt5359_L1_Tile_rc|1
AGCAATGGAAGGCTGCAAGGGGCGAAGTTCAATATGCTCTGCACCTGCAATAGGACTAAT
>HPV102_Alpha_71726718_nt5364_L1_Tile_rc|1
GCAGTAGCAATGGAAGGCTGCAAGGGGCGAAGTTCAATATGCTCTGCACCTGCAATAGGA
>HPV102_Alpha_71726718_nt5379_L1_Tile_rc|1
AAGGAGTCCCCTGGGGCAGTAGCAATGGAAGGCTGCAAGGGGCGAAGTTCAATATGCTCT
>HPV102_Alpha_71726718_nt5384_L1_Tile_rc|1
CATATAAGGAGTCCCCTGGGGCAGTAGCAATGGAAGGCTGCAAGGGGCGAAGTTCAATAT
>HPV102_Alpha_71726718_nt5389_L1_Tile_rc|1
AATATCATATAAGGAGTCCCCTGGGGCAGTAGCAATGGAAGGCTGCAAGGGGCGAAGTTC
>HPV102_Alpha_71726718_nt5394_L1_Tile_rc|1
GCATAAATATCATATAAGGAGTCCCCTGGGGCAGTAGCAATGGAAGGCTGCAAGGGGCGA
>HPV102_Alpha_71726718_nt5399_L1_Tile_rc|1
TATCAGCATAAATATCATATAAGGAGTCCCCTGGGGCAGTAGCAATGGAAGGCTGCAAGG
>HPV102_Alpha_71726718_nt5404_L1_Tile_rc|1
GCCATTATCAGCATAAATATCATATAAGGAGTCCCCTGGGGCAGTAGCAATGGAAGGCTG

>HPV102_Alpha_71726718_nt5409_L1_Tile_rc|1
AAGTGGCCATTATCAGCATAAATATCATATAAGGAGTCCCCTGGGGCAGTAGCAATGGAA
>HPV102_Alpha_71726718_nt5414_L1_Tile_rc|1
AGTCCAAGTGGCCATTATCAGCATAAATATCATATAAGGAGTCCCCTGGGGCAGTAGCAA
>HPV102_Alpha_71726718_nt5419_L1_Tile_rc|1
CAAGGAGTCCAAGTGGCCATTATCAGCATAAATATCATATAAGGAGTCCCCTGGGGCAGT
>HPV102_Alpha_71726718_nt5429_L1_Tile_rc|1
GTGGAGACGCCAAGGAGTCCAAGTGGCCATTATCAGCATAAATATCATATAAGGAGTCCC
>HPV102_Alpha_71726718_nt5434_L1_Tile_rc|1
AACATGTGGAGACGCCAAGGAGTCCAAGTGGCCATTATCAGCATAAATATCATATAAGGA
>HPV102_Alpha_71726718_nt5444_L1_Tile_rc|1
AAGTATATGAAACATGTGGAGACGCCAAGGAGTCCAAGTGGCCATTATCAGCATAAATAT
>HPV102_Alpha_71726718_nt5449_L1_Tile_rc|1
GGAAGAAGTATATGAAACATGTGGAGACGCCAAGGAGTCCAAGTGGCCATTATCAGCATA
>HPV102_Alpha_71726718_nt5454_L1_Tile_rc|1
GACGTGGAAGAAGTATATGAAACATGTGGAGACGCCAAGGAGTCCAAGTGGCCATTATCA
>HPV102_Alpha_71726718_nt5474_L1_Tile_rc|1
TTGTAGATTGCAAAGGAACAGACGTGGAAGAAGTATATGAAACATGTGGAGACGCCAAGG
>HPV102_Alpha_71726718_nt5484_L1_Tile_rc|1
GCTGAAATATTTGTAGATTGCAAAGGAACAGACGTGGAAGAAGTATATGAAACATGTGGA
>HPV102_Alpha_71726718_nt5489_L1_Tile_rc|1
ATGTAGCTGAAATATTTGTAGATTGCAAAGGAACAGACGTGGAAGAAGTATATGAAACAT
>HPV102_Alpha_71726718_nt5504_L1_Tile_rc|1
GCGCAGAGACCACAGATGTAGCTGAAATATTTGTAGATTGCAAAGGAACAGACGTGGAAG
>HPV102_Alpha_71726718_nt5509_L1_Tile_rc|1
ATACCGCGCAGAGACCACAGATGTAGCTGAAATATTTGTAGATTGCAAAGGAACAGACGT
>HPV102_Alpha_71726718_nt5514_L1_Tile_rc|1
TTGTCATACCGCGCAGAGACCACAGATGTAGCTGAAATATTTGTAGATTGCAAAGGAACA
>HPV102_Alpha_71726718_nt5529_L1_Tile_rc|1
AGTGGTACTGTGACATTGTCATACCGCGCAGAGACCACAGATGTAGCTGAAATATTTGTA
>HPV102_Alpha_71726718_nt5534_L1_Tile_rc|1
AAGAGAGTGGTACTGTGACATTGTCATACCGCGCAGAGACCACAGATGTAGCTGAAATAT
>HPV102_Alpha_71726718_nt5539_L1_Tile_rc|1
CCCAGAAGAGAGTGGTACTGTGACATTGTCATACCGCGCAGAGACCACAGATGTAGCTGA
>HPV102_Alpha_71726718_nt5544_L1_Tile_rc|1
TCAAACCCAGAAGAGAGTGGTACTGTGACATTGTCATACCGCGCAGAGACCACAGATGTA
>HPV102_Alpha_71726718_nt5549_L1_Tile_rc|1
GCAAATCAAACCCAGAAGAGAGTGGTACTGTGACATTGTCATACCGCGCAGAGACCACAG
>HPV102_Alpha_71726718_nt5554_L1_Tile_rc|1
TGCAGGCAAATCAAACCCAGAAGAGAGTGGTACTGTGACATTGTCATACCGCGCAGAGAC
>HPV102_Alpha_71726718_nt5564_L1_Tile_rc|1
GGCCTGTATATGCAGGCAAATCAAACCCAGAAGAGAGTGGTACTGTGACATTGTCATACC
>HPV102_Alpha_71726718_nt5574_L1_Tile_rc|1
GGAATGTCAGGGCCTGTATATGCAGGCAAATCAAACCCAGAAGAGAGTGGTACTGTGACA
>HPV102_Alpha_71726718_nt5579_L1_Tile_rc|1
CAATTGGAATGTCAGGGCCTGTATATGCAGGCAAATCAAACCCAGAAGAGAGTGGTACTG
>HPV102_Alpha_71726718_nt5584_L1_Tile_rc|1
AGTGGCAATTGGAATGTCAGGGCCTGTATATGCAGGCAAATCAAACCCAGAAGAGAGTGG
>HPV102_Alpha_71726718_nt5589_L1_Tile_rc|1
GTAGGAGTGGCAATTGGAATGTCAGGGCCTGTATATGCAGGCAAATCAAACCCAGAAGAG
>HPV102_Alpha_71726718_nt5594_L1_Tile_rc|1
TGGTAGTAGGAGTGGCAATTGGAATGTCAGGGCCTGTATATGCAGGCAAATCAAACCCAG
>HPV102_Alpha_71726718_nt5619_L1_Tile_rc|1
GAGGACTGGGAAACATGCAGCACACTGGTAGTAGGAGTGGCAATTGGAATGTCAGGGCCT
>HPV102_Alpha_71726718_nt5624_L1_Tile_rc|1

CATTAGAGGACTGGGAAACATGCAGCACACTGGTAGTAGGAGTGGCAATTGGAATGTCAG
>HPV102_Alpha_71726718_nt5629_L1_Tile_rc|1
AGGAACATTAGAGGACTGGGAAACATGCAGCACACTGGTAGTAGGAGTGGCAATTGGAAT
>HPV102_Alpha_71726718_nt5639_L1_Tile_rc|1
CAGATTGGGAAGGAACATTAGAGGACTGGGAAACATGCAGCACACTGGTAGTAGGAGTGG
>HPV102_Alpha_71726718_nt5644_L1_Tile_rc|1
AAACACAGATTGGGAAGGAACATTAGAGGACTGGGAAACATGCAGCACACTGGTAGTAGG
>HPV102_Alpha_71726718_nt5649_L1_Tile_rc|1
TCCACAAACACAGATTGGGAAGGAACATTAGAGGACTGGGAAACATGCAGCACACTGGTA
>HPV102_Alpha_71726718_nt5669_L1_Tile_rc|1
ATAAAAAATAGTCTGTACCATCCACAAACACAGATTGGGAAGGAACATTAGAGGACTGGG
>HPV102_Alpha_71726718_nt5679_L1_Tile_rc|1
TAATTAGGCAATAAAAAATAGTCTGTACCATCCACAAACACAGATTGGGAAGGAACATTA
>HPV102_Alpha_71726718_nt5684_L1_Tile_rc|1
ATAAGTAATTAGGCAATAAAAAATAGTCTGTACCATCCACAAACACAGATTGGGAAGGAA
>HPV102_Alpha_71726718_nt5694_L1_Tile_rc|1
TTAGGAAAGAATAAGTAATTAGGCAATAAAAAATAGTCTGTACCATCCACAAACACAGAT
>HPV102_Alpha_71726718_nt6219_L1_Tile_rc|1
TAGGGGGTTTACACCCAATAATAAGTAGTTGGGTTTGTTTGTAATCTACAGAAACATTGT
>HPV102_Alpha_71726718_nt6224_L1_Tile_rc|1
CCCAATAGGGGGTTTACACCCAATAATAAGTAGTTGGGTTTGTTTGTAATCTACAGAAAC
>HPV102_Alpha_71726718_nt6559_L1_Tile_rc|1
GGTATGTAATAGGAGGCAGGGAGTTCCTCGCCCATGGTTCCATGTCTGTTAAAGAAATGC
>HPV102_Alpha_71726718_nt6579_L1_Tile_rc|1
TATTATTTCCAGAGGTGCCAGGTATGTAATAGGAGGCAGGGAGTTCCTCGCCCATGGTTC
>HPV102_Alpha_71726718_nt6584_L1_Tile_rc|1
TTGTCTATTATTTCCAGAGGTGCCAGGTATGTAATAGGAGGCAGGGAGTTCCTCGCCCAT
>HPV102_Alpha_71726718_nt6589_L1_Tile_rc|1
GGAGTTTGTCTATTATTTCCAGAGGTGCCAGGTATGTAATAGGAGGCAGGGAGTTCCTCG
>HPV102_Alpha_71726718_nt6604_L1_Tile_rc|1
TATACATAACTGGTAGGAGTTTGTCTATTATTTCCAGAGGTGCCAGGTATGTAATAGGAG
>HPV102_Alpha_71726718_nt6609_L1_Tile_rc|1
GGGCATATACATAACTGGTAGGAGTTTGTCTATTATTTCCAGAGGTGCCAGGTATGTAAT
>HPV102_Alpha_71726718_nt7109_L1_Tile_rc|1
CAGATCCATAGACAGTTTGTCTTTGAGATCCACCTCCCAAAAAGCAAGGCCATCATATGG
>HPV102_Alpha_71726718_nt7114_L1_Tile_rc|1
TGATCCAGATCCATAGACAGTTTGTCTTTGAGATCCACCTCCCAAAAAGCAAGGCCATCA
>HPV103_Unclassified_109390382_nt4981_L1_Tile_rc|1
GTTTCCACAACGTATTCATCAGTAGGCAGTATTTTAGCCACTGGCTGAGAAGGTGGTAGA
>HPV103_Unclassified_109390382_nt4986_L1_Tile_rc|1
TAGACGTTTCCACAACGTATTCATCAGTAGGCAGTATTTTAGCCACTGGCTGAGAAGGTG
>HPV103_Unclassified_109390382_nt4991_L1_Tile_rc|1
GAAAATAGACGTTTCCACAACGTATTCATCAGTAGGCAGTATTTTAGCCACTGGCTGAGA
>HPV103_Unclassified_109390382_nt4996_L1_Tile_rc|1
TGGAAGAAAATAGACGTTTCCACAACGTATTCATCAGTAGGCAGTATTTTAGCCACTGGC
>HPV103_Unclassified_109390382_nt5016_L1_Tile_rc|1
GCAGGCGGTCGCTGGAGGCATGGAAGAAAATAGACGTTTCCACAACGTATTCATCAGTAG
>HPV103_Unclassified_109390382_nt5021_L1_Tile_rc|1
AGTAAGCAGGCGGTCGCTGGAGGCATGGAAGAAAATAGACGTTTCCACAACGTATTCATC
>HPV103_Unclassified_109390382_nt5031_L1_Tile_rc|1
GGTGTCCTACAGTAAGCAGGCGGTCGCTGGAGGCATGGAAGAAAATAGACGTTTCCACAA
>HPV103_Unclassified_109390382_nt5076_L1_Tile_rc|1
GTACAACAATTGTACCTTCACCCACTTTTGTAATTGGAAAATATGGGTGTCCTACAGTAA
>HPV103_Unclassified_109390382_nt5081_L1_Tile_rc|1
CTTAGGTACAACAATTGTACCTTCACCCACTTTTGTAATTGGAAAATATGGGTGTCCTAC

>HPV103_Unclassified_109390382_nt5086_L1_Tile_rc|1
GATACCTTAGGTACAACAATTGTACCTTCACCCACTTTTGTAATTGGAAAATATGGGTGT
>HPV103_Unclassified_109390382_nt5091_L1_Tile_rc|1
TACCAGATACCTTAGGTACAACAATTGTACCTTCACCCACTTTTGTAATTGGAAAATATG
>HPV103_Unclassified_109390382_nt5096_L1_Tile_rc|1
TTGATTACCAGATACCTTAGGTACAACAATTGTACCTTCACCCACTTTTGTAATTGGAAA
>HPV103_Unclassified_109390382_nt5101_L1_Tile_rc|1
CTGAATTGATTACCAGATACCTTAGGTACAACAATTGTACCTTCACCCACTTTTGTAATT
>HPV103_Unclassified_109390382_nt5106_L1_Tile_rc|1
AGGCCCTGAATTGATTACCAGATACCTTAGGTACAACAATTGTACCTTCACCCACTTTTG
>HPV103_Unclassified_109390382_nt5116_L1_Tile_rc|1
AGGATGTGAAAGGCCCTGAATTGATTACCAGATACCTTAGGTACAACAATTGTACCTTCA
>HPV103_Unclassified_109390382_nt5121_L1_Tile_rc|1
GTAAAAGGATGTGAAAGGCCCTGAATTGATTACCAGATACCTTAGGTACAACAATTGTAC
>HPV103_Unclassified_109390382_nt5141_L1_Tile_rc|1
TGCCAACTTGTTAGGATCAGGTAAAAGGATGTGAAAGGCCCTGAATTGATTACCAGATAC
>HPV103_Unclassified_109390382_nt5146_L1_Tile_rc|1
ACAAGTGCCAACTTGTTAGGATCAGGTAAAAGGATGTGAAAGGCCCTGAATTGATTACCA
>HPV103_Unclassified_109390382_nt5151_L1_Tile_rc|1
AATCCACAAGTGCCAACTTGTTAGGATCAGGTAAAAGGATGTGAAAGGCCCTGAATTGAT
>HPV103_Unclassified_109390382_nt5161_L1_Tile_rc|1
TATATATTGGAATCCACAAGTGCCAACTTGTTAGGATCAGGTAAAAGGATGTGAAAGGCC
>HPV103_Unclassified_109390382_nt5166_L1_Tile_rc|1
CATTATATATATTGGAATCCACAAGTGCCAACTTGTTAGGATCAGGTAAAAGGATGTGAA
>HPV103_Unclassified_109390382_nt5171_L1_Tile_rc|1
CTCGGCATTATATATATTGGAATCCACAAGTGCCAACTTGTTAGGATCAGGTAAAAGGAT
>HPV103_Unclassified_109390382_nt5176_L1_Tile_rc|1
TCTGACTCGGCATTATATATATTGGAATCCACAAGTGCCAACTTGTTAGGATCAGGTAAA
>HPV103_Unclassified_109390382_nt5181_L1_Tile_rc|1
GCCGTTCTGACTCGGCATTATATATATTGGAATCCACAAGTGCCAACTTGTTAGGATCAG
>HPV103_Unclassified_109390382_nt5186_L1_Tile_rc|1
AACAAGCCGTTCTGACTCGGCATTATATATATTGGAATCCACAAGTGCCAACTTGTTAGG
>HPV103_Unclassified_109390382_nt5191_L1_Tile_rc|1
TTCCAAACAAGCCGTTCTGACTCGGCATTATATATATTGGAATCCACAAGTGCCAACTTG
>HPV103_Unclassified_109390382_nt5201_L1_Tile_rc|1
ACCCCGTAACTTCCAAACAAGCCGTTCTGACTCGGCATTATATATATTGGAATCCACAAG
>HPV103_Unclassified_109390382_nt5206_L1_Tile_rc|1
TCCAGACCCCGTAACTTCCAAACAAGCCGTTCTGACTCGGCATTATATATATTGGAATCC
>HPV103_Unclassified_109390382_nt5216_L1_Tile_rc|1
ACGACCAATATCCAGACCCCGTAACTTCCAAACAAGCCGTTCTGACTCGGCATTATATAT
>HPV103_Unclassified_109390382_nt5221_L1_Tile_rc|1
CCACCACGACCAATATCCAGACCCCGTAACTTCCAAACAAGCCGTTCTGACTCGGCATTA
>HPV103_Unclassified_109390382_nt5226_L1_Tile_rc|1
GGGGTCCACCACGACCAATATCCAGACCCCGTAACTTCCAAACAAGCCGTTCTGACTCGG
>HPV103_Unclassified_109390382_nt5231_L1_Tile_rc|1
GCTAAGGGGTCCACCACGACCAATATCCAGACCCCGTAACTTCCAAACAAGCCGTTCTGA
>HPV103_Unclassified_109390382_nt5236_L1_Tile_rc|1
CCAACGCTAAGGGGTCCACCACGACCAATATCCAGACCCCGTAACTTCCAAACAAGCCGT
>HPV103_Unclassified_109390382_nt5241_L1_Tile_rc|1
AAGAGCCAACGCTAAGGGGTCCACCACGACCAATATCCAGACCCCGTAACTTCCAAACAA
>HPV103_Unclassified_109390382_nt5246_L1_Tile_rc|1
TCCAGAAGAGCCAACGCTAAGGGGTCCACCACGACCAATATCCAGACCCCGTAACTTCCA
>HPV103_Unclassified_109390382_nt5251_L1_Tile_rc|1
GGGTGTCCAGAAGAGCCAACGCTAAGGGGTCCACCACGACCAATATCCAGACCCCGTAAC
>HPV103_Unclassified_109390382_nt5261_L1_Tile_rc|1

```
ATTAAATAAAGGGTGTCCAGAAGAGCCAACGCTAAGGGGTCCACCACGACCAATATCCAG
>HPV103_Unclassified_109390382_nt5271_L1_Tile_rc|1
CCTTAAGTTTATTAAATAAAGGGTGTCCAGAAGAGCCAACGCTAAGGGGTCCACCACGAC
>HPV103_Unclassified_109390382_nt5276_L1_Tile_rc|1
AGTATCCTTAAGTTTATTAAATAAAGGGTGTCCAGAAGAGCCAACGCTAAGGGGTCCACC
>HPV103_Unclassified_109390382_nt5281_L1_Tile_rc|1
TTTTCAGTATCCTTAAGTTTATTAAATAAAGGGTGTCCAGAAGAGCCAACGCTAAGGGGT
>HPV103_Unclassified_109390382_nt5301_L1_Tile_rc|1
TTTTAACATAACTATTAGGATTTTCAGTATCCTTAAGTTTATTAAATAAAGGGTGTCCAG
>HPV103_Unclassified_109390382_nt5306_L1_Tile_rc|1
TCCTGTTTTAACATAACTATTAGGATTTTCAGTATCCTTAAGTTTATTAAATAAAGGGTG
>HPV103_Unclassified_109390382_nt5311_L1_Tile_rc|1
TCCTTTCCTGTTTTAACATAACTATTAGGATTTTCAGTATCCTTAAGTTTATTAAATAAA
>HPV103_Unclassified_109390382_nt5316_L1_Tile_rc|1
TGTTTTCCTTTCCTGTTTTAACATAACTATTAGGATTTTCAGTATCCTTAAGTTTATTAA
>HPV103_Unclassified_109390382_nt5321_L1_Tile_rc|1
TTGTCTGTTTTCCTTTCCTGTTTTAACATAACTATTAGGATTTTCAGTATCCTTAAGTTT
>HPV103_Unclassified_109390382_nt5326_L1_Tile_rc|1
ACACTTTGTCTGTTTTCCTTTCCTGTTTTAACATAACTATTAGGATTTTCAGTATCCTTA
>HPV103_Unclassified_109390382_nt5331_L1_Tile_rc|1
TAGAGACACTTTGTCTGTTTTCCTTTCCTGTTTTAACATAACTATTAGGATTTTCAGTAT
>HPV103_Unclassified_109390382_nt5336_L1_Tile_rc|1
GTCCATAGAGACACTTTGTCTGTTTTCCTTTCCTGTTTTAACATAACTATTAGGATTTTC
>HPV103_Unclassified_109390382_nt5341_L1_Tile_rc|1
TTTGGGTCCATAGAGACACTTTGTCTGTTTTCCTTTCCTGTTTTAACATAACTATTAGGA
>HPV103_Unclassified_109390382_nt5346_L1_Tile_rc|1
TTTGCTTTGGGTCCATAGAGACACTTTGTCTGTTTTCCTTTCCTGTTTTAACATAACTAT
>HPV103_Unclassified_109390382_nt5356_L1_Tile_rc|1
AATAGCTGAGTTTGCTTTGGGTCCATAGAGACACTTTGTCTGTTTTCCTTTCCTGTTTTA
>HPV103_Unclassified_109390382_nt5361_L1_Tile_rc|1
CTACAAATAGCTGAGTTTGCTTTGGGTCCATAGAGACACTTTGTCTGTTTTCCTTTCCTG
>HPV103_Unclassified_109390382_nt5366_L1_Tile_rc|1
GCCTACTACAAATAGCTGAGTTTGCTTTGGGTCCATAGAGACACTTTGTCTGTTTTCCTT
>HPV103_Unclassified_109390382_nt5406_L1_Tile_rc|1
GCTTTGCAGCATCCCAATGCTCCCCCAAACAAGGAGTACAGCCTACTACAAATAGCTGAG
>HPV103_Unclassified_109390382_nt5451_L1_Tile_rc|1
CTAAAGGAGGACATGCACCCATATCCTGCCTAGCATCTGCACATGGCTTTGCAGCATCCC
>HPV103_Unclassified_109390382_nt5466_L1_Tile_rc|1
TGGAACTAACAAGCTCTAAAGGAGGACATGCACCCATATCCTGCCTAGCATCTGCACATG
>HPV103_Unclassified_109390382_nt5471_L1_Tile_rc|1
AATAATGGAACTAACAAGCTCTAAAGGAGGACATGCACCCATATCCTGCCTAGCATCTGC
>HPV103_Unclassified_109390382_nt5566_L1_Tile_rc|1
GAAGCTACAATGTCCAAGGGTACACCTGAATGGTCCTCCTGCAATGTAGCAAAATTCATT
>HPV103_Unclassified_109390382_nt5571_L1_Tile_rc|1
ATTTAGAAGCTACAATGTCCAAGGGTACACCTGAATGGTCCTCCTGCAATGTAGCAAAAT
>HPV103_Unclassified_109390382_nt5576_L1_Tile_rc|1
TTTACATTTAGAAGCTACAATGTCCAAGGGTACACCTGAATGGTCCTCCTGCAATGTAGC
>HPV103_Unclassified_109390382_nt5581_L1_Tile_rc|1
GGCCATTTACATTTAGAAGCTACAATGTCCAAGGGTACACCTGAATGGTCCTCCTGCAAT
>HPV103_Unclassified_109390382_nt5586_L1_Tile_rc|1
AATCAGGCCATTTACATTTAGAAGCTACAATGTCCAAGGGTACACCTGAATGGTCCTCCT
>HPV103_Unclassified_109390382_nt5591_L1_Tile_rc|1
AAGAAAATCAGGCCATTTACATTTAGAAGCTACAATGTCCAAGGGTACACCTGAATGGTC
>HPV103_Unclassified_109390382_nt5596_L1_Tile_rc|1
ATTCTAAGAAAATCAGGCCATTTACATTTAGAAGCTACAATGTCCAAGGGTACACCTGAA
```

>HPV103_Unclassified_109390382_nt5631_L1_Tile_rc|1
AGAAAAACATTTGATTGCCATATGTGTCATTTGCCATTCTAAGAAAATCAGGCCATTTAC
>HPV103_Unclassified_109390382_nt5636_L1_Tile_rc|1
TCCAAAGAAAAACATTTGATTGCCATATGTGTCATTTGCCATTCTAAGAAAATCAGGCCA
>HPV103_Unclassified_109390382_nt5646_L1_Tile_rc|1
GCTCACGTCTTCCAAAGAAAAACATTTGATTGCCATATGTGTCATTTGCCATTCTAAGAA
>HPV103_Unclassified_109390382_nt5651_L1_Tile_rc|1
CAACTGCTCACGTCTTCCAAAGAAAAACATTTGATTGCCATATGTGTCATTTGCCATTCT
>HPV103_Unclassified_109390382_nt5656_L1_Tile_rc|1
GCATACAACTGCTCACGTCTTCCAAAGAAAAACATTTGATTGCCATATGTGTCATTTGCC
>HPV103_Unclassified_109390382_nt5661_L1_Tile_rc|1
GGCGAGCATACAACTGCTCACGTCTTCCAAAGAAAAACATTTGATTGCCATATGTGTCAT
>HPV103_Unclassified_109390382_nt5671_L1_Tile_rc|1
GCAAAAAAGTGGCGAGCATACAACTGCTCACGTCTTCCAAAGAAAAACATTTGATTGCCA
>HPV103_Unclassified_109390382_nt5676_L1_Tile_rc|1
CCCTAGCAAAAAAGTGGCGAGCATACAACTGCTCACGTCTTCCAAAGAAAAACATTTGAT
>HPV103_Unclassified_109390382_nt5696_L1_Tile_rc|1
ACTGTCGCCCATGACACCATCCCTAGCAAAAAAGTGGCGAGCATACAACTGCTCACGTCT
>HPV103_Unclassified_109390382_nt5701_L1_Tile_rc|1
GGTATACTGTCGCCCATGACACCATCCCTAGCAAAAAAGTGGCGAGCATACAACTGCTCA
>HPV103_Unclassified_109390382_nt5716_L1_Tile_rc|1
TCAAATTCAGCCTCAGGTATACTGTCGCCCATGACACCATCCCTAGCAAAAAAGTGGCGA
>HPV103_Unclassified_109390382_nt5721_L1_Tile_rc|1
CACCCTCAAATTCAGCCTCAGGTATACTGTCGCCCATGACACCATCCCTAGCAAAAAAGT
>HPV103_Unclassified_109390382_nt5726_L1_Tile_rc|1
CTTAACACCCTCAAATTCAGCCTCAGGTATACTGTCGCCCATGACACCATCCCTAGCAAA
>HPV103_Unclassified_109390382_nt5731_L1_Tile_rc|1
GTGTGCTTAACACCCTCAAATTCAGCCTCAGGTATACTGTCGCCCATGACACCATCCCTA
>HPV103_Unclassified_109390382_nt5741_L1_Tile_rc|1
TAAAATATAGGTGTGCTTAACACCCTCAAATTCAGCCTCAGGTATACTGTCGCCCATGAC
>HPV103_Unclassified_109390382_nt5746_L1_Tile_rc|1
CCCTGTAAAATATAGGTGTGCTTAACACCCTCAAATTCAGCCTCAGGTATACTGTCGCCC
>HPV103_Unclassified_109390382_nt5766_L1_Tile_rc|1
TTTGGTCTTTGTCAGACGCCCCCTGTAAAATATAGGTGTGCTTAACACCCTCAAATTCAG
>HPV103_Unclassified_109390382_nt5771_L1_Tile_rc|1
TGTATTTTGGTCTTTGTCAGACGCCCCCTGTAAAATATAGGTGTGCTTAACACCCTCAAA
>HPV103_Unclassified_109390382_nt5781_L1_Tile_rc|1
ACGAGGAGGCTGTATTTTGGTCTTTGTCAGACGCCCCCTGTAAAATATAGGTGTGCTTAA
>HPV103_Unclassified_109390382_nt5786_L1_Tile_rc|1
AACAGACGAGGAGGCTGTATTTTGGTCTTTGTCAGACGCCCCCTGTAAAATATAGGTGTG
>HPV103_Unclassified_109390382_nt5791_L1_Tile_rc|1
TAGTAAACAGACGAGGAGGCTGTATTTTGGTCTTTGTCAGACGCCCCCTGTAAAATATAG
>HPV103_Unclassified_109390382_nt5811_L1_Tile_rc|1
GTGATCCACTGGGGGTTGCATAGTAAACAGACGAGGAGGCTGTATTTTGGTCTTTGTCAG
>HPV103_Unclassified_109390382_nt5816_L1_Tile_rc|1
AACCAGTGATCCACTGGGGGTTGCATAGTAAACAGACGAGGAGGCTGTATTTTGGTCTTT
>HPV103_Unclassified_109390382_nt5821_L1_Tile_rc|1
CTAGAAACCAGTGATCCACTGGGGGTTGCATAGTAAACAGACGAGGAGGCTGTATTTTGG
>HPV103_Unclassified_109390382_nt5826_L1_Tile_rc|1
AATCACTAGAAACCAGTGATCCACTGGGGGTTGCATAGTAAACAGACGAGGAGGCTGTAT
>HPV103_Unclassified_109390382_nt5836_L1_Tile_rc|1
AACAACTGAGAATCACTAGAAACCAGTGATCCACTGGGGGTTGCATAGTAAACAGACGAG
>HPV103_Unclassified_109390382_nt5841_L1_Tile_rc|1
TGCCAAACAACTGAGAATCACTAGAAACCAGTGATCCACTGGGGGTTGCATAGTAAACAG
>HPV103_Unclassified_109390382_nt5866_L1_Tile_rc|1

```
TGAGCCTTTTGTATCCAGTATGGTCTGCCAAACAACTGAGAATCACTAGAAACCAGTGAT
>HPV103_Unclassified_109390382_nt5886_L1_Tile_rc|1
ACACACTATTATTAGGGCCCTGAGCCTTTTGTATCCAGTATGGTCTGCCAAACAACTGAG
>HPV103_Unclassified_109390382_nt5896_L1_Tile_rc|1
TTTCTCCAACACACACTATTATTAGGGCCCTGAGCCTTTTGTATCCAGTATGGTCTGCCA
>HPV103_Unclassified_109390382_nt5901_L1_Tile_rc|1
AATTATTTCTCCAACACACACTATTATTAGGGCCCTGAGCCTTTTGTATCCAGTATGGTC
>HPV103_Unclassified_109390382_nt5941_L1_Tile_rc|1
AAATTTACATTGTGTGTGTTATCAACCAATGTAATAAATAAATTATTTCTCCAACACACA
>HPV103_Unclassified_109390382_nt5946_L1_Tile_rc|1
TTGTAAAATTTACATTGTGTGTGTTATCAACCAATGTAATAAATAAATTATTTCTCCAAC
>HPV103_Unclassified_109390382_nt5951_L1_Tile_rc|1
ATTTATTGTAAAATTTACATTGTGTGTGTTATCAACCAATGTAATAAATAAATTATTTCT
>HPV103_Unclassified_109390382_nt5956_L1_Tile_rc|1
TTAACATTTATTGTAAAATTTACATTGTGTGTGTTATCAACCAATGTAATAAATAAATTA
>HPV103_Unclassified_109390382_nt5961_L1_Tile_rc|1
CTGACTTAACATTTATTGTAAAATTTACATTGTGTGTGTTATCAACCAATGTAATAAATA
>HPV103_Unclassified_109390382_nt5966_L1_Tile_rc|1
AGCGTCTGACTTAACATTTATTGTAAAATTTACATTGTGTGTGTTATCAACCAATGTAAT
>HPV103_Unclassified_109390382_nt5971_L1_Tile_rc|1
ACGCCAGCGTCTGACTTAACATTTATTGTAAAATTTACATTGTGTGTGTTATCAACCAAT
>HPV103_Unclassified_109390382_nt5976_L1_Tile_rc|1
GTGCAACGCCAGCGTCTGACTTAACATTTATTGTAAAATTTACATTGTGTGTGTTATCAA
>HPV103_Unclassified_109390382_nt5986_L1_Tile_rc|1
GCATATTGCTGTGCAACGCCAGCGTCTGACTTAACATTTATTGTAAAATTTACATTGTGT
>HPV103_Unclassified_109390382_nt5991_L1_Tile_rc|1
TGGCAGCATATTGCTGTGCAACGCCAGCGTCTGACTTAACATTTATTGTAAAATTTACAT
>HPV103_Unclassified_109390382_nt6016_L1_Tile_rc|1
TGTCGCAGATAATGTTTATAGCTGGTGGCAGCATATTGCTGTGCAACGCCAGCGTCTGAC
>HPV103_Unclassified_109390382_nt6026_L1_Tile_rc|1
TTCCTCTACATGTCGCAGATAATGTTTATAGCTGGTGGCAGCATATTGCTGTGCAACGCC
>HPV103_Unclassified_109390382_nt6031_L1_Tile_rc|1
TCAAATTCCTCTACATGTCGCAGATAATGTTTATAGCTGGTGGCAGCATATTGCTGTGCA
>HPV103_Unclassified_109390382_nt6051_L1_Tile_rc|1
ATTGCAATATAAGAGCAATCTCAAATTCCTCTACATGTCGCAGATAATGTTTATAGCTGG
>HPV103_Unclassified_109390382_nt6056_L1_Tile_rc|1
GCATAATTGCAATATAAGAGCAATCTCAAATTCCTCTACATGTCGCAGATAATGTTTATA
>HPV103_Unclassified_109390382_nt6061_L1_Tile_rc|1
ACCTTGCATAATTGCAATATAAGAGCAATCTCAAATTCCTCTACATGTCGCAGATAATGT
>HPV103_Unclassified_109390382_nt6071_L1_Tile_rc|1
TGTGAGAGGAACCTTGCATAATTGCAATATAAGAGCAATCTCAAATTCCTCTACATGTCG
>HPV103_Unclassified_109390382_nt6081_L1_Tile_rc|1
ATACATCAGCTGTGAGAGGAACCTTGCATAATTGCAATATAAGAGCAATCTCAAATTCCT
>HPV103_Unclassified_109390382_nt6086_L1_Tile_rc|1
GGCTAATACATCAGCTGTGAGAGGAACCTTGCATAATTGCAATATAAGAGCAATCTCAAA
>HPV103_Unclassified_109390382_nt6091_L1_Tile_rc|1
ATATGGGCTAATACATCAGCTGTGAGAGGAACCTTGCATAATTGCAATATAAGAGCAATC
>HPV103_Unclassified_109390382_nt6096_L1_Tile_rc|1
CATGTATATGGGCTAATACATCAGCTGTGAGAGGAACCTTGCATAATTGCAATATAAGAG
>HPV103_Unclassified_109390382_nt6101_L1_Tile_rc|1
CATTACATGTATATGGGCTAATACATCAGCTGTGAGAGGAACCTTGCATAATTGCAATAT
>HPV103_Unclassified_109390382_nt6106_L1_Tile_rc|1
GGATCCATTACATGTATATGGGCTAATACATCAGCTGTGAGAGGAACCTTGCATAATTGC
>HPV103_Unclassified_109390382_nt6116_L1_Tile_rc|1
AATTATAGCTGGATCCATTACATGTATATGGGCTAATACATCAGCTGTGAGAGGAACCTT
```

>HPV103_Unclassified_109390382_nt6121_L1_Tile_rc|1
TCCTCAATTATAGCTGGATCCATTACATGTATATGGGCTAATACATCAGCTGTGAGAGGA
>HPV103_Unclassified_109390382_nt6131_L1_Tile_rc|1
TAGCTGCCAGTCCTCAATTATAGCTGGATCCATTACATGTATATGGGCTAATACATCAGC
>HPV103_Unclassified_109390382_nt6136_L1_Tile_rc|1
AACCCTAGCTGCCAGTCCTCAATTATAGCTGGATCCATTACATGTATATGGGCTAATACA
>HPV103_Unclassified_109390382_nt6141_L1_Tile_rc|1
GTACAAACCCTAGCTGCCAGTCCTCAATTATAGCTGGATCCATTACATGTATATGGGCTA
>HPV103_Unclassified_109390382_nt6146_L1_Tile_rc|1
CGGTGGTACAAACCCTAGCTGCCAGTCCTCAATTATAGCTGGATCCATTACATGTATATG
>HPV103_Unclassified_109390382_nt6156_L1_Tile_rc|1
AAGAAGGAGGCGGTGGTACAAACCCTAGCTGCCAGTCCTCAATTATAGCTGGATCCATTA
>HPV103_Unclassified_109390382_nt6161_L1_Tile_rc|1
AATAGAAGAAGGAGGCGGTGGTACAAACCCTAGCTGCCAGTCCTCAATTATAGCTGGATC
>HPV103_Unclassified_109390382_nt6166_L1_Tile_rc|1
TCTCCAATAGAAGAAGGAGGCGGTGGTACAAACCCTAGCTGCCAGTCCTCAATTATAGCT
>HPV103_Unclassified_109390382_nt6171_L1_Tile_rc|1
ATGTATCTCCAATAGAAGAAGGAGGCGGTGGTACAAACCCTAGCTGCCAGTCCTCAATTA
>HPV103_Unclassified_109390382_nt6176_L1_Tile_rc|1
TCTGTATGTATCTCCAATAGAAGAAGGAGGCGGTGGTACAAACCCTAGCTGCCAGTCCTC
>HPV103_Unclassified_109390382_nt6181_L1_Tile_rc|1
AAAAATCTGTATGTATCTCCAATAGAAGAAGGAGGCGGTGGTACAAACCCTAGCTGCCAG
>HPV103_Unclassified_109390382_nt6186_L1_Tile_rc|1
ATGTTAAAAATCTGTATGTATCTCCAATAGAAGAAGGAGGCGGTGGTACAAACCCTAGCT
>HPV103_Unclassified_109390382_nt6201_L1_Tile_rc|1
AGCGTGTGGCTAAAGATGTTAAAAATCTGTATGTATCTCCAATAGAAGAAGGAGGCGGTG
>HPV103_Unclassified_109390382_nt6211_L1_Tile_rc|1
GCAGCAGGGCAGCGTGTGGCTAAAGATGTTAAAAATCTGTATGTATCTCCAATAGAAGAA
>HPV103_Unclassified_109390382_nt6216_L1_Tile_rc|1
GTTCTGCAGCAGGGCAGCGTGTGGCTAAAGATGTTAAAAATCTGTATGTATCTCCAATAG
>HPV103_Unclassified_109390382_nt6226_L1_Tile_rc|1
TCCTTGGGAGGTTCTGCAGCAGGGCAGCGTGTGGCTAAAGATGTTAAAAATCTGTATGTA
>HPV103_Unclassified_109390382_nt6241_L1_Tile_rc|1
TATGGGTCCTGTTTTTCCTTGGGAGGTTCTGCAGCAGGGCAGCGTGTGGCTAAAGATGTT
>HPV103_Unclassified_109390382_nt6246_L1_Tile_rc|1
TATCATATGGGTCCTGTTTTTCCTTGGGAGGTTCTGCAGCAGGGCAGCGTGTGGCTAAAG
>HPV103_Unclassified_109390382_nt6346_L1_Tile_rc|1
GAGCTAACCCCAGTTTGAGATAAAAATCTGCGACCCAAAGGATATCTGTCAAGATCAGAG
>HPV103_Unclassified_109390382_nt6351_L1_Tile_rc|1
AAGACGAGCTAACCCCAGTTTGAGATAAAAATCTGCGACCCAAAGGATATCTGTCAAGAT
>HPV103_Unclassified_109390382_nt6356_L1_Tile_rc|1
GGTAGAAGACGAGCTAACCCCAGTTTGAGATAAAAATCTGCGACCCAAAGGATATCTGTC
>HPV103_Unclassified_109390382_nt6361_L1_Tile_rc|1
GAGGAGGTAGAAGACGAGCTAACCCCAGTTTGAGATAAAAATCTGCGACCCAAAGGATAT
>HPV103_Unclassified_109390382_nt6366_L1_Tile_rc|1
TTCTAGAGGAGGTAGAAGACGAGCTAACCCCAGTTTGAGATAAAAATCTGCGACCCAAAG
>HPV103_Unclassified_109390382_nt6376_L1_Tile_rc|1
CGAGACCGCTTTCTAGAGGAGGTAGAAGACGAGCTAACCCCAGTTTGAGATAAAAATCTG
>HPV103_Unclassified_109390382_nt6381_L1_Tile_rc|1
CTACACGAGACCGCTTTCTAGAGGAGGTAGAAGACGAGCTAACCCCAGTTTGAGATAAAA
>HPV103_Unclassified_109390382_nt6386_L1_Tile_rc|1
GGTCTCTACACGAGACCGCTTTCTAGAGGAGGTAGAAGACGAGCTAACCCCAGTTTGAGA
>HPV103_Unclassified_109390382_nt6391_L1_Tile_rc|1
GTAGGGGTCTCTACACGAGACCGCTTTCTAGAGGAGGTAGAAGACGAGCTAACCCCAGTT
>HPV103_Unclassified_109390382_nt6396_L1_Tile_rc|1

GGCGGGTAGGGGTCTCTACACGAGACCGCTTTCTAGAGGAGGTAGAAGACGAGCTAACCC
>HPV103_Unclassified_109390382_nt6401_L1_Tile_rc|1
TACTGGGCGGGTAGGGGTCTCTACACGAGACCGCTTTCTAGAGGAGGTAGAAGACGAGCT
>HPV103_Unclassified_109390382_nt6406_L1_Tile_rc|1
GACCGTACTGGGCGGGTAGGGGTCTCTACACGAGACCGCTTTCTAGAGGAGGTAGAAGAC
>HPV103_Unclassified_109390382_nt6411_L1_Tile_rc|1
TAGCAGACCGTACTGGGCGGGTAGGGGTCTCTACACGAGACCGCTTTCTAGAGGAGGTAG
>HPV103_Unclassified_109390382_nt6416_L1_Tile_rc|1
TTTTTAGCAGACCGTACTGGGCGGGTAGGGGTCTCTACACGAGACCGCTTTCTAGAGGA
>HPV103_Unclassified_109390382_nt6421_L1_Tile_rc|1
CTACGTTTTTTAGCAGACCGTACTGGGCGGGTAGGGGTCTCTACACGAGACCGCTTTCTA
>HPV103_Unclassified_109390382_nt6426_L1_Tile_rc|1
CCCGTCTACGTTTTTTAGCAGACCGTACTGGGCGGGTAGGGGTCTCTACACGAGACCGCT
>HPV103_Unclassified_109390382_nt6431_L1_Tile_rc|1
AACCCCCCGTCTACGTTTTTTAGCAGACCGTACTGGGCGGGTAGGGGTCTCTACACGAGA
>HPV103_Unclassified_109390382_nt6434_L1_Tile_rc|1
TTAAACCCCCCGTCTACGTTTTTTAGCAGACCGTACTGGGCGGGTAGGGGTCTCTACACG
>HPV106_Alpha_71726710_nt5675_L1_Tile_rc|1
ATGAGGGCAATAAATAATATGTAGACCCGTGCACAAGCACATCGCCTGTGGGGTGACCAG
>HPV106_Alpha_71726710_nt5685_L1_Tile_rc|1
AGCAACACATATGAGGGCAATAAATAATATGTAGACCCGTGCACAAGCACATCGCCTGTG
>HPV106_Alpha_71726710_nt5895_L1_Tile_rc|1
GTCCACTTTGCTTTTTGATAGGATAATATGGATGACCTACGGTCAGCAATCGGGAACTGC
>HPV106_Alpha_71726710_nt5900_L1_Tile_rc|1
GTTCCGTCCACTTTGCTTTTTGATAGGATAATATGGATGACCTACGGTCAGCAATCGGGA
>HPV106_Alpha_71726710_nt5905_L1_Tile_rc|1
ATAGGGTTCCGTCCACTTTGCTTTTTGATAGGATAATATGGATGACCTACGGTCAGCAAT
>HPV106_Alpha_71726710_nt6270_L1_Tile_rc|1
CAGTGACATTGGAGGTATTGCATGGAGTGCCTTTTGCCCAGTGTTCGCCCAATGGCGGTT
>HPV106_Alpha_71726710_nt6275_L1_Tile_rc|1
CCCTGCAGTGACATTGGAGGTATTGCATGGAGTGCCTTTTGCCCAGTGTTCGCCCAATGG
>HPV106_Alpha_71726710_nt6555_L1_Tile_rc|1
GTAAAAACCCTTCCGGAACATCCTCCCCCATGGTACCGGCCCTGTTGTAAAAATGCCTAA
>HPV106_Alpha_71726710_nt6560_L1_Tile_rc|1
CTTTAGTAAAAACCCTTCCGGAACATCCTCCCCCATGGTACCGGCCCTGTTGTAAAAATG
>HPV106_Alpha_71726710_nt6565_L1_Tile_rc|1
GGCCCCTTTAGTAAAAACCCTTCCGGAACATCCTCCCCCATGGTACCGGCCCTGTTGTAA
>HPV106_Alpha_71726710_nt6570_L1_Tile_rc|1
TAGTAGGCCCCTTTAGTAAAAACCCTTCCGGAACATCCTCCCCCATGGTACCGGCCCTGT
>HPV106_Alpha_71726710_nt6575_L1_Tile_rc|1
TGTAGTAGTAGGCCCCTTTAGTAAAAACCCTTCCGGAACATCCTCCCCCATGGTACCGGC
>HPV106_Alpha_71726710_nt6580_L1_Tile_rc|1
CGGGATGTAGTAGTAGGCCCCTTTAGTAAAAACCCTTCCGGAACATCCTCCCCCATGGTA
>HPV106_Alpha_71726710_nt6590_L1_Tile_rc|1
AGCAGAGGCGCGGGATGTAGTAGTAGGCCCCTTTAGTAAAAACCCTTCCGGAACATCCTC
>HPV106_Alpha_71726710_nt6595_L1_Tile_rc|1
CTTGTAGCAGAGGCGCGGGATGTAGTAGTAGGCCCCTTTAGTAAAAACCCTTCCGGAACA
>HPV106_Alpha_71726710_nt6605_L1_Tile_rc|1
ATATAGTGAGCTTGTAGCAGAGGCGCGGGATGTAGTAGTAGGCCCCTTTAGTAAAAACCC
>HPV106_Alpha_71726710_nt6610_L1_Tile_rc|1
GACCCATATAGTGAGCTTGTAGCAGAGGCGCGGGATGTAGTAGTAGGCCCCTTTAGTAAA
>HPV106_Alpha_71726710_nt6995_L1_Tile_rc|1
CTGTAAAAACCTATAGGTATCGCCAAGACTGGCGGTAGGGGGGGGCAACGACCCAAAATT
>HPV106_Alpha_71726710_nt7070_L1_Tile_rc|1
AAACGTAAGCCCATCGTAAGGGTCCTTTTTCTTTGCAGGCGCCGCGGAGGGCTTTTGACA

>HPV106_Alpha_71726710_nt7130_L1_Tile_rc|1
CCTACCCAAGGGAAACTGGTCAAGCTCAAGGGAAAAGCGCTCTGATAAATCCACATCCCA
>HPV106_Alpha_71726710_nt7240_L1_Tile_rc|1
GAACGCTTGGACCGTTTACGCTTTGTGGCTGGTGGGGTGGACGATGGTGCAGGACGCTTA
>HPV107_Beta_126131393_nt5829_L1_Tile_rc|1
TATTGGTCCGCTCCACGTACTCATCGGTACTTAGCACCCGTGCCACCGGAGTTGATGGTG
>HPV107_Beta_126131393_nt5834_L1_Tile_rc|1
AAATATATTGGTCCGCTCCACGTACTCATCGGTACTTAGCACCCGTGCCACCGGAGTTGA
>HPV107_Beta_126131393_nt5839_L1_Tile_rc|1
TGATAAAATATATTGGTCCGCTCCACGTACTCATCGGTACTTAGCACCCGTGCCACCGGA
>HPV107_Beta_126131393_nt5849_L1_Tile_rc|1
ACTAGTGGCGTGATAAAATATATTGGTCCGCTCCACGTACTCATCGGTACTTAGCACCCG
>HPV107_Beta_126131393_nt5854_L1_Tile_rc|1
CTGTCACTAGTGGCGTGATAAAATATATTGGTCCGCTCCACGTACTCATCGGTACTTAGC
>HPV107_Beta_126131393_nt5859_L1_Tile_rc|1
ATAATCTGTCACTAGTGGCGTGATAAAATATATTGGTCCGCTCCACGTACTCATCGGTAC
>HPV107_Beta_126131393_nt5864_L1_Tile_rc|1
TGTTAATAATCTGTCACTAGTGGCGTGATAAAATATATTGGTCCGCTCCACGTACTCATC
>HPV107_Beta_126131393_nt6154_L1_Tile_rc|1
TCTGTTGAACTGTTTTGATAGCTATTTGTATTTTCAGTGTCCTTTACCTTATTGAACAAA
>HPV107_Beta_126131393_nt6159_L1_Tile_rc|1
TATCATCTGTTGAACTGTTTTGATAGCTATTTGTATTTTCAGTGTCCTTTACCTTATTGA
>HPV107_Beta_126131393_nt6164_L1_Tile_rc|1
TTGTCTATCATCTGTTGAACTGTTTTGATAGCTATTTGTATTTTCAGTGTCCTTTACCTT
>HPV107_Beta_126131393_nt6274_L1_Tile_rc|1
TGTTGGTCACCAGCATCATCGCATGGCAGTGCCCTATCCCAATATTCCCCTAAACATGGT
>HPV107_Beta_126131393_nt6284_L1_Tile_rc|1
ACATTTACCTTGTTGGTCACCAGCATCATCGCATGGCAGTGCCCTATCCCAATATTCCCC
>HPV107_Beta_126131393_nt6294_L1_Tile_rc|1
CAATGGGCGGACATTTACCTTGTTGGTCACCAGCATCATCGCATGGCAGTGCCCTATCCC
>HPV107_Beta_126131393_nt6299_L1_Tile_rc|1
TAATTCAATGGGCGGACATTTACCTTGTTGGTCACCAGCATCATCGCATGGCAGTGCCCT
>HPV107_Beta_126131393_nt6304_L1_Tile_rc|1
TTTTTAATTCAATGGGCGGACATTTACCTTGTTGGTCACCAGCATCATCGCATGGCAGT
>HPV107_Beta_126131393_nt6314_L1_Tile_rc|1
TATAAAAGAATTTTTTAATTCAATGGGCGGACATTTACCTTGTTGGTCACCAGCATCATC
>HPV107_Beta_126131393_nt6319_L1_Tile_rc|1
TCTTCTATAAAAGAATTTTTTAATTCAATGGGCGGACATTTACCTTGTTGGTCACCAGCA
>HPV107_Beta_126131393_nt6364_L1_Tile_rc|1
GTCTTGTTATTTATATTGCCAAACCCAATATCTATCATATCCCCATCTTCTATAAAAGAA
>HPV107_Beta_126131393_nt6374_L1_Tile_rc|1
TACTGATAATGTCTTGTTATTTATATTGCCAAACCCAATATCTATCATATCCCCATCTTC
>HPV107_Beta_126131393_nt6379_L1_Tile_rc|1
TTGTTTACTGATAATGTCTTGTTATTTATATTGCCAAACCCAATATCTATCATATCCCCA
>HPV107_Beta_126131393_nt6384_L1_Tile_rc|1
CTGATTTGTTTACTGATAATGTCTTGTTATTTATATTGCCAAACCCAATATCTATCATAT
>HPV107_Beta_126131393_nt6389_L1_Tile_rc|1
AACATCTGATTTGTTTACTGATAATGTCTTGTTATTTATATTGCCAAACCCAATATCTAT
>HPV107_Beta_126131393_nt6394_L1_Tile_rc|1
AAACTAACATCTGATTTGTTTACTGATAATGTCTTGTTATTTATATTGCCAAACCCAATA
>HPV107_Beta_126131393_nt6399_L1_Tile_rc|1
AATCTAAACTAACATCTGATTTGTTTACTGATAATGTCTTGTTATTTATATTGCCAAACC
>HPV107_Beta_126131393_nt6404_L1_Tile_rc|1
TACTAAATCTAAACTAACATCTGATTTGTTTACTGATAATGTCTTGTTATTTATATTGCC
>HPV107_Beta_126131393_nt6409_L1_Tile_rc|1

TCATTTACTAAATCTAAACTAACATCTGATTTGTTTACTGATAATGTCTTGTTATTTATA
>HPV107_Beta_126131393_nt6559_L1_Tile_rc|1
TTAGTTGTTGTATCAGGTATAGCATCACCCACAGCTCCTCCTCTAGTGAAATAATGTCTA
>HPV107_Beta_126131393_nt6564_L1_Tile_rc|1
CTTGATTAGTTGTTGTATCAGGTATAGCATCACCCACAGCTCCTCCTCTAGTGAAATAAT
>HPV107_Beta_126131393_nt6579_L1_Tile_rc|1
TATAGTATTTGTGATCTTGATTAGTTGTTGTATCAGGTATAGCATCACCCACAGCTCCTC
>HPV107_Beta_126131393_nt6584_L1_Tile_rc|1
TGCTATATAGTATTTGTGATCTTGATTAGTTGTTGTATCAGGTATAGCATCACCCACAGC
>HPV107_Beta_126131393_nt6589_L1_Tile_rc|1
TTAGGTGCTATATAGTATTTGTGATCTTGATTAGTTGTTGTATCAGGTATAGCATCACCC
>HPV107_Beta_126131393_nt6594_L1_Tile_rc|1
CACTCTTAGGTGCTATATAGTATTTGTGATCTTGATTAGTTGTTGTATCAGGTATAGCAT
>HPV107_Beta_126131393_nt6599_L1_Tile_rc|1
TTGTCCACTCTTAGGTGCTATATAGTATTTGTGATCTTGATTAGTTGTTGTATCAGGTAT
>HPV107_Beta_126131393_nt6609_L1_Tile_rc|1
GACTTTGGGATTGTCCACTCTTAGGTGCTATATAGTATTTGTGATCTTGATTAGTTGTTG
>HPV107_Beta_126131393_nt6614_L1_Tile_rc|1
CAAAGGACTTTGGGATTGTCCACTCTTAGGTGCTATATAGTATTTGTGATCTTGATTAGT
>HPV107_Beta_126131393_nt6624_L1_Tile_rc|1
TAGAATTACCCAAAGGACTTTGGGATTGTCCACTCTTAGGTGCTATATAGTATTTGTGAT
>HPV107_Beta_126131393_nt6629_L1_Tile_rc|1
GTAAATAGAATTACCCAAAGGACTTTGGGATTGTCCACTCTTAGGTGCTATATAGTATTT
>HPV107_Beta_126131393_nt6634_L1_Tile_rc|1
GGATAGTAAATAGAATTACCCAAAGGACTTTGGGATTGTCCACTCTTAGGTGCTATATAG
>HPV107_Beta_126131393_nt6639_L1_Tile_rc|1
CGGTGGGATAGTAAATAGAATTACCCAAAGGACTTTGGGATTGTCCACTCTTAGGTGCTA
>HPV107_Beta_126131393_nt6654_L1_Tile_rc|1
CTAAGGAGCCACTAACGGTGGGATAGTAAATAGAATTACCCAAAGGACTTTGGGATTGTC
>HPV107_Beta_126131393_nt6659_L1_Tile_rc|1
AGAAACTAAGGAGCCACTAACGGTGGGATAGTAAATAGAATTACCCAAAGGACTTTGGGA
>HPV107_Beta_126131393_nt6664_L1_Tile_rc|1
TCAGAAGAAACTAAGGAGCCACTAACGGTGGGATAGTAAATAGAATTACCCAAAGGACTT
>HPV107_Beta_126131393_nt6669_L1_Tile_rc|1
GTGCATCAGAAGAAACTAAGGAGCCACTAACGGTGGGATAGTAAATAGAATTACCCAAAG
>HPV107_Beta_126131393_nt6699_L1_Tile_rc|1
GTTTCAACCAGAAGGGTCTGTTAAAGAGCTGTGCATCAGAAGAAACTAAGGAGCCACTAA
>HPV107_Beta_126131393_nt6809_L1_Tile_rc|1
TCCATTTTGACTAGGCACACTTATAGTAAAATTGGTGTTCCTTGTATTGTCTGCAACAGT
>HPV107_Beta_126131393_nt6839_L1_Tile_rc|1
GATATTATTGGCATCATATTCTGTAAGGGGTCCATTTTGACTAGGCACACTTATAGTAAA
>HPV107_Beta_126131393_nt6844_L1_Tile_rc|1
TCCCTGATATTATTGGCATCATATTCTGTAAGGGGTCCATTTTGACTAGGCACACTTATA
>HPV107_Beta_126131393_nt6849_L1_Tile_rc|1
AAAATTCCCTGATATTATTGGCATCATATTCTGTAAGGGGTCCATTTTGACTAGGCACAC
>HPV107_Beta_126131393_nt6854_L1_Tile_rc|1
CCTTAAAAATTCCCTGATATTATTGGCATCATATTCTGTAAGGGGTCCATTTTGACTAGG
>HPV107_Beta_126131393_nt6879_L1_Tile_rc|1
CAGATATTTGATATTCCTCAACATGCCTTAAAAATTCCCTGATATTATTGGCATCATATT
>HPV107_Beta_126131393_nt6884_L1_Tile_rc|1
TATTACAGATATTTGATATTCCTCAACATGCCTTAAAAATTCCCTGATATTATTGGCATC
>HPV107_Beta_126131393_nt6909_L1_Tile_rc|1
GTAAAGACACTTTACAAAGTTGCAGTATTACAGATATTTGATATTCCTCAACATGCCTTA
>HPV107_Beta_126131393_nt6914_L1_Tile_rc|1
TGGTTGTAAAGACACTTTACAAAGTTGCAGTATTACAGATATTTGATATTCCTCAACATG

>HPV107_Beta_126131393_nt6919_L1_Tile_rc|1
ACATCTGGTTGTAAAGACACTTTACAAAGTTGCAGTATTACAGATATTTGATATTCCTCA
>HPV107_Beta_126131393_nt6934_L1_Tile_rc|1
TTAATTTGGGCTAACACATCTGGTTGTAAAGACACTTTACAAAGTTGCAGTATTACAGAT
>HPV107_Beta_126131393_nt6939_L1_Tile_rc|1
TAGCATTAATTTGGGCTAACACATCTGGTTGTAAAGACACTTTACAAAGTTGCAGTATTA
>HPV107_Beta_126131393_nt6944_L1_Tile_rc|1
ATTCATAGCATTAATTTGGGCTAACACATCTGGTTGTAAAGACACTTTACAAAGTTGCAG
>HPV107_Beta_126131393_nt6954_L1_Tile_rc|1
AAATGCCTGAATTCATAGCATTAATTTGGGCTAACACATCTGGTTGTAAAGACACTTTAC
>HPV107_Beta_126131393_nt6959_L1_Tile_rc|1
TTCCAAAATGCCTGAATTCATAGCATTAATTTGGGCTAACACATCTGGTTGTAAAGACAC
>HPV107_Beta_126131393_nt7124_L1_Tile_rc|1
TAATTTTTCTGTCATATCAACTCTCCAGAAAAAGTATTGAGCAAAAGGATCTTCCTTTTC
>HPV107_Beta_126131393_nt7184_L1_Tile_rc|1
AGGTAGTGTTGTTTGGAATAGGAATTTACGTCCTAAAGGATACTGATCTAAATCCAAAGA
>HPV107_Beta_126131393_nt7194_L1_Tile_rc|1
TTCGAGAGGCAGGTAGTGTTGTTTGGAATAGGAATTTACGTCCTAAAGGATACTGATCTA
>HPV107_Beta_126131393_nt7199_L1_Tile_rc|1
ACGTTTTCGAGAGGCAGGTAGTGTTGTTTGGAATAGGAATTTACGTCCTAAAGGATACTG
>HPV107_Beta_126131393_nt7209_L1_Tile_rc|1
TCGGCGCTGCACGTTTTCGAGAGGCAGGTAGTGTTGTTTGGAATAGGAATTTACGTCCTA
>HPV107_Beta_126131393_nt7219_L1_Tile_rc|1
GATTTGGTGGTCGGCGCTGCACGTTTTCGAGAGGCAGGTAGTGTTGTTTGGAATAGGAAT
>HPV107_Beta_126131393_nt7224_L1_Tile_rc|1
CAACCGATTTGGTGGTCGGCGCTGCACGTTTTCGAGAGGCAGGTAGTGTTGTTTGGAATA
>HPV107_Beta_126131393_nt7253_L1_Tile_rc|1
TCAAGTACGTTTTCGTTTGCTATGCTTTGCAACCGATTTGGTGGTCGGCGCTGCACGTTT
>HPV11_Alpha_333026_nt5892_L1_Tile_rc|1
TGGTACAACTGTTTTGTTAACTTTTTTGATAGAGTAATATGGATGTCCCACAGCAAGGAG
>HPV11_Alpha_333026_nt5897_L1_Tile_rc|1
ACCTTTGGTACAACTGTTTTGTTAACTTTTTTGATAGAGTAATATGGATGTCCCACAGCA
>HPV11_Alpha_333026_nt5902_L1_Tile_rc|2
CAGACACCTTTGGTACAACTGTTTTGTTAACTTTTTTGATAGAGTAATATGGATGTCCCA
>HPV11_Alpha_333026_nt6002_L1_Tile_rc|2
CATACTAAACGCTGTGTAGTGGGGTCAAACAGGGATGAATCAGGTAATGCAAACTTGTTA
>HPV11_Alpha_333026_nt6117_L1_Tile_rc|1
ATTACCACCATACCCACCACTATTTTCTACATCATCATATTTGTTTAGCAATGGATGCCC
>HPV11_Alpha_333026_nt6272_L1_Tile_rc|1
AGTTCCAACGGGGGGCAGTCACCATTTTGTACAGAGGTATTTGAACATTGTGTACCCTTA
>HPV11_Alpha_333026_nt6277_L1_Tile_rc|1
TAATAAGTTCCAACGGGGGGCAGTCACCATTTTGTACAGAGGTATTTGAACATTGTGTAC
>HPV11_Alpha_333026_nt6392_L1_Tile_rc|1
TTGCAGACAGTTCCACAAATATCAAGGGGAACATCCGATTTATTGGTTTGTAAGTCTGCA
>HPV11_Alpha_333026_nt6547_L1_Tile_rc|1
TATTACCCCCTTTTACCAACAGGTCATCAGGCACAGGTTCCCCCACAGTACCGGCCCTAT
>HPV11_Alpha_333026_nt6557_L1_Tile_rc|1
GATGATCTGTTATTACCCCCTTTTACCAACAGGTCATCAGGCACAGGTTCCCCCACAGTA
>HPV11_Alpha_333026_nt6572_L1_Tile_rc|1
ATACTACTAGCTACAGATGATCTGTTATTACCCCCTTTTACCAACAGGTCATCAGGCACA
>HPV11_Alpha_333026_nt6577_L1_Tile_rc|1
CATAAATACTACTAGCTACAGATGATCTGTTATTACCCCCTTTTACCAACAGGTCATCAG
>HPV11_Alpha_333026_nt6582_L1_Tile_rc|2
ATGTACATAAATACTACTAGCTACAGATGATCTGTTATTACCCCCTTTTACCAACAGGTC
>HPV11_Alpha_333026_nt6592_L1_Tile_rc|1

CACTAGGTGTATGTACATAAATACTACTAGCTACAGATGATCTGTTATTACCCCCTTTTA
>HPV11_Alpha_333026_nt6607_L1_Tile_rc|1
AAGACACCAATGAGCCACTAGGTGTATGTACATAAATACTACTAGCTACAGATGATCTGT
>HPV11_Alpha_333026_nt6622_L1_Tile_rc|2
ATAATTGAGCCTCTGAAGACACCAATGAGCCACTAGGTGTATGTACATAAATACTACTAG
>HPV11_Alpha_333026_nt6777_L1_Tile_rc|1
ATCTGAATTAGTGTATGTAGCAGATTTAGACACAGATGCACATAGTGTCATATTTGTACT
>HPV11_Alpha_333026_nt6782_L1_Tile_rc|2
TTATAATCTGAATTAGTGTATGTAGCAGATTTAGACACAGATGCACATAGTGTCATATTT
>HPV11_Alpha_333026_nt6787_L1_Tile_rc|1
ATTCCTTATAATCTGAATTAGTGTATGTAGCAGATTTAGACACAGATGCACATAGTGTCA
>HPV11_Alpha_333026_nt6792_L1_Tile_rc|1
CATGTATTCCTTATAATCTGAATTAGTGTATGTAGCAGATTTAGACACAGATGCACATAG
>HPV11_Alpha_333026_nt6797_L1_Tile_rc|1
TGGCGCATGTATTCCTTATAATCTGAATTAGTGTATGTAGCAGATTTAGACACAGATGCA
>HPV11_Alpha_333026_nt7142_L1_Tile_rc|2
CGAGCAGACGTCCGTCCTCGATATCCACTTTGCAATAAAAACTTACGTCCAAGGGGAAAC
>HPV11_Alpha_333026_nt7182_L1_Tile_rc|2
TGTAGAGGGCTTAGACACAGCTGGGCGCTTTATACCTGTACGAGCAGACGTCCGTCCTCG
>HPV11_Alpha_333026_nt7197_L1_Tile_rc|1
TTTTCGTTTGGGGGCTGTAGAGGGCTTAGACACAGCTGGGCGCTTTATACCTGTACGAGC
>HPV11_Alpha_333026_nt7207_L1_Tile_rc|1
TTTTGGTACGTTTTCGTTTGGGGGCTGTAGAGGGCTTAGACACAGCTGGGCGCTTTATAC
>HPV110_Beta_186461194_nt5847_L1_Tile_rc|1
TGCACCCGGAATGCTCTGTATTGATTTCCTGATACCTTAGGGACTTCTATTTTTGCGCCA
>HPV110_Beta_186461194_nt6062_L1_Tile_rc|2
ATGAAGTATTCTGCCTGTCATCAGTAGAGCCTGCTTGATAATTGCTAGAATTTTCAGTAT
>HPV110_Beta_186461194_nt6167_L1_Tile_rc|1
CTAACTGATTACCTGCTTCTGATTCACAAACAGGAGCTTTGTCCCAATGCTCTCCCATAC
>HPV110_Beta_186461194_nt6172_L1_Tile_rc|1
TAAACCTAACTGATTACCTGCTTCTGATTCACAAACAGGAGCTTTGTCCCAATGCTCTCC
>HPV110_Beta_186461194_nt6482_L1_Tile_rc|2
GCTTTGCTGGCAAGAAATAGTTATGATTTTGATTCACTGCACCATCAGGTATTGCATCCC
>HPV110_Beta_186461194_nt6487_L1_Tile_rc|1
ATCTTGCTTTGCTGGCAAGAAATAGTTATGATTTTGATTCACTGCACCATCAGGTATTGC
>HPV110_Beta_186461194_nt6507_L1_Tile_rc|1
GCTAGTGTGCGTTGTTGTTGATCTTGCTTTGCTGGCAAGAAATAGTTATGATTTTGATTC
>HPV110_Beta_186461194_nt6512_L1_Tile_rc|1
AGTTTGCTAGTGTGCGTTGTTGTTGATCTTGCTTTGCTGGCAAGAAATAGTTATGATTTT
>HPV110_Beta_186461194_nt6517_L1_Tile_rc|1
TGTAGAGTTTGCTAGTGTGCGTTGTTGTTGATCTTGCTTTGCTGGCAAGAAATAGTTATG
>HPV110_Beta_186461194_nt6532_L1_Tile_rc|1
TACTGTTGGAAAATATGTAGAGTTTGCTAGTGTGCGTTGTTGTTGATCTTGCTTTGCTGG
>HPV110_Beta_186461194_nt6537_L1_Tile_rc|1
CCACTTACTGTTGGAAAATATGTAGAGTTTGCTAGTGTGCGTTGTTGTTGATCTTGCTTT
>HPV110_Beta_186461194_nt6557_L1_Tile_rc|1
CATCTGAAGTAACTAGAGAACCACTTACTGTTGGAAAATATGTAGAGTTTGCTAGTGTGC
>HPV110_Beta_186461194_nt6697_L1_Tile_rc|1
AGCTTCTGTAGTAACACTAATTGAAAAATTAGTATTACGAGTATTATCAGCAACAGTTAT
>HPV110_Beta_186461194_nt6702_L1_Tile_rc|2
GCTCCAGCTTCTGTAGTAACACTAATTGAAAAATTAGTATTACGAGTATTATCAGCAACA
>HPV110_Beta_186461194_nt6707_L1_Tile_rc|1
CAGTTGCTCCAGCTTCTGTAGTAACACTAATTGAAAAATTAGTATTACGAGTATTATCAG
>HPV110_Beta_186461194_nt6712_L1_Tile_rc|1
GTATTCAGTTGCTCCAGCTTCTGTAGTAACACTAATTGAAAAATTAGTATTACGAGTATT

>HPV110_Beta_186461194_nt6717_L1_Tile_rc|1
GAATTGTATTCAGTTGCTCCAGCTTCTGTAGTAACACTAATTGAAAAATTAGTATTACGA
>HPV110_Beta_186461194_nt6722_L1_Tile_rc|2
TTTGTGAATTGTATTCAGTTGCTCCAGCTTCTGTAGTAACACTAATTGAAAAATTAGTAT
>HPV110_Beta_186461194_nt6727_L1_Tile_rc|1
TATATTTTGTGAATTGTATTCAGTTGCTCCAGCTTCTGTAGTAACACTAATTGAAAAATT
>HPV110_Beta_186461194_nt6732_L1_Tile_rc|1
TCTCTTATATTTTGTGAATTGTATTCAGTTGCTCCAGCTTCTGTAGTAACACTAATTGAA
>HPV110_Beta_186461194_nt6742_L1_Tile_rc|2
TCTAAGAAATTCTCTTATATTTTGTGAATTGTATTCAGTTGCTCCAGCTTCTGTAGTAAC
>HPV110_Beta_186461194_nt6752_L1_Tile_rc|1
CCTCTACATGTCTAAGAAATTCTCTTATATTTTGTGAATTGTATTCAGTTGCTCCAGCTT
>HPV110_Beta_186461194_nt6772_L1_Tile_rc|1
TATTAAAGAAAGTTGATATTCCTCTACATGTCTAAGAAATTCTCTTATATTTTGTGAATT
>HPV110_Beta_186461194_nt6777_L1_Tile_rc|1
TGCAGTATTAAAGAAAGTTGATATTCCTCTACATGTCTAAGAAATTCTCTTATATTTTGT
>HPV110_Beta_186461194_nt6782_L1_Tile_rc|2
ATAATTGCAGTATTAAAGAAAGTTGATATTCCTCTACATGTCTAAGAAATTCTCTTATAT
>HPV110_Beta_186461194_nt6792_L1_Tile_rc|1
GGTACCTTACATAATTGCAGTATTAAAGAAAGTTGATATTCCTCTACATGTCTAAGAAAT
>HPV110_Beta_186461194_nt6802_L1_Tile_rc|2
TGCTTTTAAGGGTACCTTACATAATTGCAGTATTAAAGAAAGTTGATATTCCTCTACATG
>HPV110_Beta_186461194_nt6807_L1_Tile_rc|1
ACCTCTGCTTTTAAGGGTACCTTACATAATTGCAGTATTAAAGAAAGTTGATATTCCTCT
>HPV110_Beta_186461194_nt6812_L1_Tile_rc|1
TTAAAACCTCTGCTTTTAAGGGTACCTTACATAATTGCAGTATTAAAGAAAGTTGATATT
>HPV110_Beta_186461194_nt6817_L1_Tile_rc|1
TTGTGTTAAAACCTCTGCTTTTAAGGGTACCTTACATAATTGCAGTATTAAAGAAAGTTG
>HPV110_Beta_186461194_nt6822_L1_Tile_rc|2
TTAATTTGTGTTAAAACCTCTGCTTTTAAGGGTACCTTACATAATTGCAGTATTAAAGAA
>HPV110_Beta_186461194_nt7107_L1_Tile_rc|1
ACCTTAACAGATGTCCTTACAGAGCGAGGTCGACTTTGTAAACCAGCCTGAAATAAAAAT
>HPV110_Beta_186461194_nt7112_L1_Tile_rc|1
TAGAGACCTTAACAGATGTCCTTACAGAGCGAGGTCGACTTTGTAAACCAGCCTGAAATA
>HPV110_Beta_186461194_nt7117_L1_Tile_rc|1
ACCCTTAGAGACCTTAACAGATGTCCTTACAGAGCGAGGTCGACTTTGTAAACCAGCCTG
>HPV110_Beta_186461194_nt7127_L1_Tile_rc|1
TGCGTTTGGTACCCTTAGAGACCTTAACAGATGTCCTTACAGAGCGAGGTCGACTTTGTA
>HPV110_Beta_186461194_nt7137_L1_Tile_rc|1
CACGACCGTTTGCGTTTGGTACCCTTAGAGACCTTAACAGATGTCCTTACAGAGCGAGGT
>HPV110_Beta_186461194_nt7138_L1_Tile_rc|1
TCACGACCGTTTGCGTTTGGTACCCTTAGAGACCTTAACAGATGTCCTTACAGAGCGAGG
>HPV111_Beta_186461202_nt5988_L1_Tile_rc|1
TCTTGTGTCACAGCTTGATAATTACTGGAATTTTCAGTGTCACGAACCTTATTAAATAAT
>HPV111_Beta_186461202_nt5993_L1_Tile_rc|1
TATCATCTTGTGTCACAGCTTGATAATTACTGGAATTTTCAGTGTCACGAACCTTATTAA
>HPV111_Beta_186461202_nt6128_L1_Tile_rc|1
CAATTGGCGGGCATAAGCCTTGCTGGTTTCCTGCATCATCACAGGGTTTTGCTTTGTCCC
>HPV111_Beta_186461202_nt6133_L1_Tile_rc|1
TAATTCAATTGGCGGGCATAAGCCTTGCTGGTTTCCTGCATCATCACAGGGTTTTGCTTT
>HPV111_Beta_186461202_nt6138_L1_Tile_rc|1
TTCTTTAATTCAATTGGCGGGCATAAGCCTTGCTGGTTTCCTGCATCATCACAGGGTTTT
>HPV111_Beta_186461202_nt6148_L1_Tile_rc|1
TATTACAGAATTCTTTAATTCAATTGGCGGGCATAAGCCTTGCTGGTTTCCTGCATCATC
>HPV111_Beta_186461202_nt6158_L1_Tile_rc|1

CTCCATCCTCTATTACAGAATTCTTTAATTCAATTGGCGGGCATAAGCCTTGCTGGTTTC
>HPV111_Beta_186461202_nt6168_L1_Tile_rc|1
TCAAACATGTCTCCATCCTCTATTACAGAATTCTTTAATTCAATTGGCGGGCATAAGCCT
>HPV111_Beta_186461202_nt6288_L1_Tile_rc|1
TAGACGTCATTAGCCATAGTTAGGAAGTCAGGGTATTTACATACTTCATTGACAATATCT
>HPV111_Beta_186461202_nt6293_L1_Tile_rc|1
CCCCATAGACGTCATTAGCCATAGTTAGGAAGTCAGGGTATTTACATACTTCATTGACAA
>HPV111_Beta_186461202_nt6418_L1_Tile_rc|1
TGGCAGATAATAATTGTGATTTTGTTGCACTGCTGCATCTGGAATTGCATCTCCTACATT
>HPV111_Beta_186461202_nt6423_L1_Tile_rc|1
TGTGCTGGCAGATAATAATTGTGATTTTGTTGCACTGCTGCATCTGGAATTGCATCTCCT
>HPV111_Beta_186461202_nt6433_L1_Tile_rc|1
TTGATTACCCTGTGCTGGCAGATAATAATTGTGATTTTGTTGCACTGCTGCATCTGGAAT
>HPV111_Beta_186461202_nt6448_L1_Tile_rc|1
CAAAATGTTTTGTTGTTGATTACCCTGTGCTGGCAGATAATAATTGTGATTTTGTTGCAC
>HPV111_Beta_186461202_nt6453_L1_Tile_rc|1
TTACCCAAAATGTTTTGTTGTTGATTACCCTGTGCTGGCAGATAATAATTGTGATTTTGT
>HPV111_Beta_186461202_nt6458_L1_Tile_rc|1
TGGAGTTACCCAAAATGTTTTGTTGTTGATTACCCTGTGCTGGCAGATAATAATTGTGAT
>HPV111_Beta_186461202_nt6463_L1_Tile_rc|1
ATAAATGGAGTTACCCAAAATGTTTTGTTGTTGATTACCCTGTGCTGGCAGATAATAATT
>HPV111_Beta_186461202_nt6468_L1_Tile_rc|1
GGATAATAAATGGAGTTACCCAAAATGTTTTGTTGTTGATTACCCTGTGCTGGCAGATAA
>HPV111_Beta_186461202_nt6478_L1_Tile_rc|1
ACTAACAGTAGGATAATAAATGGAGTTACCCAAAATGTTTTGTTGTTGATTACCCTGTGC
>HPV111_Beta_186461202_nt6493_L1_Tile_rc|1
AGTAACAAGAGAACCACTAACAGTAGGATAATAAATGGAGTTACCCAAAATGTTTTGTTG
>HPV111_Beta_186461202_nt6633_L1_Tile_rc|1
GTGGATACGCTTATAGTAAAATTAGTGTTTCTGGTATTATCTGCTACTGTTATAAACATT
>HPV111_Beta_186461202_nt6638_L1_Tile_rc|1
CTTCTGTGGATACGCTTATAGTAAAATTAGTGTTTCTGGTATTATCTGCTACTGTTATAA
>HPV111_Beta_186461202_nt6643_L1_Tile_rc|1
ATTAGCTTCTGTGGATACGCTTATAGTAAAATTAGTGTTTCTGGTATTATCTGCTACTGT
>HPV111_Beta_186461202_nt6648_L1_Tile_rc|1
TGTGCATTAGCTTCTGTGGATACGCTTATAGTAAAATTAGTGTTTCTGGTATTATCTGCT
>HPV111_Beta_186461202_nt6658_L1_Tile_rc|1
ATTATATTGCTGTGCATTAGCTTCTGTGGATACGCTTATAGTAAAATTAGTGTTTCTGGT
>HPV111_Beta_186461202_nt6663_L1_Tile_rc|1
CTAGCATTATATTGCTGTGCATTAGCTTCTGTGGATACGCTTATAGTAAAATTAGTGTTT
>HPV111_Beta_186461202_nt6668_L1_Tile_rc|1
TATTACTAGCATTATATTGCTGTGCATTAGCTTCTGTGGATACGCTTATAGTAAAATTAG
>HPV111_Beta_186461202_nt6673_L1_Tile_rc|1
TCTAATATTACTAGCATTATATTGCTGTGCATTAGCTTCTGTGGATACGCTTATAGTAAA
>HPV111_Beta_186461202_nt6678_L1_Tile_rc|1
TATTCTCTAATATTACTAGCATTATATTGCTGTGCATTAGCTTCTGTGGATACGCTTATA
>HPV111_Beta_186461202_nt6748_L1_Tile_rc|1
TTCTGGAACTAAAGAAACTTTACATAGTTGAAGAATCAAAGACAATTGATATTCTTCTAC
>HPV111_Beta_186461202_nt6753_L1_Tile_rc|1
AAAACTTCTGGAACTAAAGAAACTTTACATAGTTGAAGAATCAAAGACAATTGATATTCT
>HPV111_Beta_186461202_nt6763_L1_Tile_rc|1
GATTTGAGATAAAACTTCTGGAACTAAAGAAACTTTACATAGTTGAAGAATCAAAGACAA
>HPV111_Beta_186461202_nt6768_L1_Tile_rc|1
GCATTGATTTGAGATAAAACTTCTGGAACTAAAGAAACTTTACATAGTTGAAGAATCAAA
>HPV111_Beta_186461202_nt7053_L1_Tile_rc|1
GATTTCACAGACGAAGGTTTTAAAGTCCGTTTTCGTGTTTGTAAACCAGCCTGGAATAAA

>HPV111_Beta_186461202_nt7058_L1_Tile_rc|1
TAGTAGATTTCACAGACGAAGGTTTTAAAGTCCGTTTTCGTGTTTGTAAACCAGCCTGGA
>HPV111_Beta_186461202_nt7063_L1_Tile_rc|1
AGATTTAGTAGATTTCACAGACGAAGGTTTTAAAGTCCGTTTTCGTGTTTGTAAACCAGC
>HPV111_Beta_186461202_nt7068_L1_Tile_rc|1
TTTGCAGATTTAGTAGATTTCACAGACGAAGGTTTTAAAGTCCGTTTTCGTGTTTGTAAA
>HPV111_Beta_186461202_nt7083_L1_Tile_rc|1
TAGGTGCGTCTGCGTTTTGCAGATTTAGTAGATTTCACAGACGAAGGTTTTAAAGTCCGT
>HPV111_Beta_186461202_nt7084_L1_Tile_rc|1
TTAGGTGCGTCTGCGTTTTGCAGATTTAGTAGATTTCACAGACGAAGGTTTTAAAGTCCG
>HPV12_Beta_396910_nt6165_L1_Tile_rc|1
ATTTTGTTAAAATAGGGATGTCCAGTACTTCCAACGCCTAAAGGCTGACCCCTACTTATT
>HPV12_Beta_396910_nt6205_L1_Tile_rc|1
ACTGCCTGTGGCATAGTTATTTGAGTTTTCCGTGTCTTTAATTTTGTTAAAATAGGGATG
>HPV12_Beta_396910_nt6220_L1_Tile_rc|1
CTGTCTATCATCCTTACTGCCTGTGGCATAGTTATTTGAGTTTTCCGTGTCTTTAATTTT
>HPV12_Beta_396910_nt6235_L1_Tile_rc|1
ATCAAATGATGTGTTCTGTCTATCATCCTTACTGCCTGTGGCATAGTTATTTGAGTTTTC
>HPV12_Beta_396910_nt6330_L1_Tile_rc|1
TTTTCAGCTGGTGCATCCCCGCAGGGTAAGGCTTTCTCCCAGTGTTCTCCCACACAAGGT
>HPV12_Beta_396910_nt6335_L1_Tile_rc|1
CACCATTTTCAGCTGGTGCATCCCCGCAGGGTAAGGCTTTCTCCCAGTGTTCTCCCACAC
>HPV12_Beta_396910_nt6340_L1_Tile_rc|1
GCAAACACCATTTTCAGCTGGTGCATCCCCGCAGGGTAAGGCTTTCTCCCAGTGTTCTCC
>HPV12_Beta_396910_nt6350_L1_Tile_rc|1
CTATGGGAGGGCAAACACCATTTTCAGCTGGTGCATCCCCGCAGGGTAAGGCTTTCTCCC
>HPV12_Beta_396910_nt6355_L1_Tile_rc|1
TAACTCTATGGGAGGGCAAACACCATTTTCAGCTGGTGCATCCCCGCAGGGTAAGGCTTT
>HPV12_Beta_396910_nt6375_L1_Tile_rc|1
TCTTCAATGAAAGTGTTCTTTAACTCTATGGGAGGGCAAACACCATTTTCAGCTGGTGCA
>HPV12_Beta_396910_nt6380_L1_Tile_rc|1
CACCATCTTCAATGAAAGTGTTCTTTAACTCTATGGGAGGGCAAACACCATTTTCAGCTG
>HPV12_Beta_396910_nt6895_L1_Tile_rc|1
ATTTGGAATGTCGTGTACATTTTGATTATCACTGTAAATAGATATACTGAAATTGGTGTT
>HPV12_Beta_396910_nt6900_L1_Tile_rc|1
TCATAATTTGGAATGTCGTGTACATTTTGATTATCACTGTAAATAGATATACTGAAATTG
>HPV12_Beta_396910_nt6905_L1_Tile_rc|1
GAGAATCATAATTTGGAATGTCGTGTACATTTTGATTATCACTGTAAATAGATATACTGA
>HPV12_Beta_396910_nt6910_L1_Tile_rc|1
TTTTTGAGAATCATAATTTGGAATGTCGTGTACATTTTGATTATCACTGTAAATAGATAT
>HPV12_Beta_396910_nt6915_L1_Tile_rc|1
CTAAATTTTTGAGAATCATAATTTGGAATGTCGTGTACATTTTGATTATCACTGTAAATA
>HPV12_Beta_396910_nt6920_L1_Tile_rc|1
ATTCTCTAAATTTTTGAGAATCATAATTTGGAATGTCGTGTACATTTTGATTATCACTGT
>HPV12_Beta_396910_nt6925_L1_Tile_rc|1
TAAATATTCTCTAAATTTTTGAGAATCATAATTTGGAATGTCGTGTACATTTTGATTATC
>HPV12_Beta_396910_nt7075_L1_Tile_rc|1
AATAGGATTGTCAGGAGTTGGCACGAAGCCTAATTGCCAGTCCTCCAGCAAAGAAGAGTT
>HPV12_Beta_396910_nt7100_L1_Tile_rc|1
ATTCGATATATCTGTATGTGTCATGAATAGGATTGTCAGGAGTTGGCACGAAGCCTAATT
>HPV12_Beta_396910_nt7105_L1_Tile_rc|1
CAGAGATTCGATATATCTGTATGTGTCATGAATAGGATTGTCAGGAGTTGGCACGAAGCC
>HPV12_Beta_396910_nt7200_L1_Tile_rc|1
GAAAGTCTCTCAGTCATATCTACAGTCCAAAAACTTAAGCCATCATAAGGGTCCGGCTTT
>HPV12_Beta_396910_nt7205_L1_Tile_rc|1

CTAAAGAAAGTCTCTCAGTCATATCTACAGTCCAAAAACTTAAGCCATCATAAGGGTCCG
>HPV12_Beta_396910_nt7230_L1_Tile_rc|1
CGCCCTAAGGAATACTGATCCAGGTCTAAAGAAAGTCTCTCAGTCATATCTACAGTCCAA
>HPV12_Beta_396910_nt7260_L1_Tile_rc|1
TGTTGGAGGCCAGCCTGGAATAAGAACTTGCGCCCTAAGGAATACTGATCCAGGTCTAAA
>HPV12_Beta_396910_nt7325_L1_Tile_rc|1
TTCTTTTGGTCCCCCTTATGGAACTTCTATAGCTTGATGATTTTGTTGTACCGTTAACGG
>HPV13_Alpha_85827580_nt5873_L1_Tile_rc|1
CAGATACCTTAGGGACAACAGTTTTGTTTTGTTTCTTAATAGGAAAATAAGGATTTCCCA
>HPV13_Alpha_85827580_nt5878_L1_Tile_rc|1
ATAACCAGATACCTTAGGGACAACAGTTTTGTTTTGTTTCTTAATAGGAAAATAAGGATT
>HPV13_Alpha_85827580_nt5883_L1_Tile_rc|1
AACTGATAACCAGATACCTTAGGGACAACAGTTTTGTTTTGTTTCTTAATAGGAAAATAA
>HPV13_Alpha_85827580_nt5888_L1_Tile_rc|1
CCCTAAACTGATAACCAGATACCTTAGGGACAACAGTTTTGTTTTGTTTCTTAATAGGAA
>HPV13_Alpha_85827580_nt5893_L1_Tile_rc|1
AAATACCCTAAACTGATAACCAGATACCTTAGGGACAACAGTTTTGTTTTGTTTCTTAAT
>HPV13_Alpha_85827580_nt6253_L1_Tile_rc|1
ACTACTAATTAATTCTAAAGGAGGGCAATCTCCAGGTTGTACATTTACACCAGTACATTG
>HPV13_Alpha_85827580_nt6503_L1_Tile_rc|1
CATATAATTCTGCTGGGATTAGTTCACCAACAGAGCCTGCCCTGTTAAAGAAATGCCTTG
>HPV13_Alpha_85827580_nt6508_L1_Tile_rc|1
CTTAACATATAATTCTGCTGGGATTAGTTCACCAACAGAGCCTGCCCTGTTAAAGAAATG
>HPV13_Alpha_85827580_nt6513_L1_Tile_rc|1
CTACCCTTAACATATAATTCTGCTGGGATTAGTTCACCAACAGAGCCTGCCCTGTTAAAG
>HPV13_Alpha_85827580_nt6523_L1_Tile_rc|1
AAGTGTATTACTACCCTTAACATATAATTCTGCTGGGATTAGTTCACCAACAGAGCCTGC
>HPV13_Alpha_85827580_nt6528_L1_Tile_rc|1
TTAGAAAGTGTATTACTACCCTTAACATATAATTCTGCTGGGATTAGTTCACCAACAGAG
>HPV13_Alpha_85827580_nt6533_L1_Tile_rc|1
TACTATTAGAAAGTGTATTACTACCCTTAACATATAATTCTGCTGGGATTAGTTCACCAA
>HPV13_Alpha_85827580_nt6538_L1_Tile_rc|1
GTAAATACTATTAGAAAGTGTATTACTACCCTTAACATATAATTCTGCTGGGATTAGTTC
>HPV13_Alpha_85827580_nt6543_L1_Tile_rc|1
TTATAGTAAATACTATTAGAAAGTGTATTACTACCCTTAACATATAATTCTGCTGGGATT
>HPV13_Alpha_85827580_nt6548_L1_Tile_rc|1
GAGTATTATAGTAAATACTATTAGAAAGTGTATTACTACCCTTAACATATAATTCTGCTG
>HPV13_Alpha_85827580_nt6553_L1_Tile_rc|1
ACTGGGAGTATTATAGTAAATACTATTAGAAAGTGTATTACTACCCTTAACATATAATTC
>HPV13_Alpha_85827580_nt6558_L1_Tile_rc|1
GAGCCACTGGGAGTATTATAGTAAATACTATTAGAAAGTGTATTACTACCCTTAACATAT
>HPV13_Alpha_85827580_nt6563_L1_Tile_rc|1
CAAGAGAGCCACTGGGAGTATTATAGTAAATACTATTAGAAAGTGTATTACTACCCTTAA
>HPV13_Alpha_85827580_nt6743_L1_Tile_rc|1
CTGTGGCCTTATATGTGTCTGAAAGAGATGATGTAGTGGCTGCACACACAGTCATGTTAG
>HPV13_Alpha_85827580_nt6748_L1_Tile_rc|1
ATATTCTGTGGCCTTATATGTGTCTGAAAGAGATGATGTAGTGGCTGCACACACAGTCAT
>HPV13_Alpha_85827580_nt6753_L1_Tile_rc|1
TGTTTATATTCTGTGGCCTTATATGTGTCTGAAAGAGATGATGTAGTGGCTGCACACACA
>HPV13_Alpha_85827580_nt6763_L1_Tile_rc|1
TCGCATGTACTGTTTATATTCTGTGGCCTTATATGTGTCTGAAAGAGATGATGTAGTGGC
>HPV13_Alpha_85827580_nt6913_L1_Tile_rc|1
TTCCAATGTTCCATTAGGAGGGGGAGATAGCCCAAAGTTCCAGTCTTCTAGAATTGTAGG
>HPV13_Alpha_85827580_nt6928_L1_Tile_rc|1
ATATCTATATGTGTCTTCCAATGTTCCATTAGGAGGGGGAGATAGCCCAAAGTTCCAGTC

>HPV13_Alpha_85827580_nt6933_L1_Tile_rc|1
TGTACATATCTATATGTGTCTTCCAATGTTCCATTAGGAGGGGGAGATAGCCCAAAGTTC
>HPV13_Alpha_85827580_nt6938_L1_Tile_rc|1
GAGATTGTACATATCTATATGTGTCTTCCAATGTTCCATTAGGAGGGGGAGATAGCCCAA
>HPV13_Alpha_85827580_nt6943_L1_Tile_rc|1
GGCCTGAGATTGTACATATCTATATGTGTCTTCCAATGTTCCATTAGGAGGGGGAGATAG
>HPV13_Alpha_85827580_nt6953_L1_Tile_rc|1
GACACGTTATGGCCTGAGATTGTACATATCTATATGTGTCTTCCAATGTTCCATTAGGAG
>HPV13_Alpha_85827580_nt7003_L1_Tile_rc|1
ACTAAGACCCGCATACGGATCCTGTTTTTCTTTATCAGGTGTAGGCTTTTGACACGTTAT
>HPV13_Alpha_85827580_nt7133_L1_Tile_rc|1
ATGCAGCACGTTTTCTACCTACACGAATAGGTGACCTAGACTGAACGCCTGTTTGTAATA
>HPV13_Alpha_85827580_nt7138_L1_Tile_rc|1
TGTAGATGCAGCACGTTTTCTACCTACACGAATAGGTGACCTAGACTGAACGCCTGTTTG
>HPV13_Alpha_85827580_nt7143_L1_Tile_rc|1
GTAGATGTAGATGCAGCACGTTTTCTACCTACACGAATAGGTGACCTAGACTGAACGCCT
>HPV13_Alpha_85827580_nt7153_L1_Tile_rc|1
AGGTGTGGCAGTAGATGTAGATGCAGCACGTTTTCTACCTACACGAATAGGTGACCTAGA
>HPV13_Alpha_85827580_nt7163_L1_Tile_rc|1
TACGTGTAGTAGGTGTGGCAGTAGATGTAGATGCAGCACGTTTTCTACCTACACGAATAG
>HPV13_Alpha_85827580_nt7168_L1_Tile_rc|1
TTTTTTACGTGTAGTAGGTGTGGCAGTAGATGTAGATGCAGCACGTTTTCTACCTACACG
>HPV13_Alpha_85827580_nt7173_L1_Tile_rc|1
TTAGCTTTTTTACGTGTAGTAGGTGTGGCAGTAGATGTAGATGCAGCACGTTTTCTACCT
>HPV13_Alpha_85827580_nt7178_L1_Tile_rc|1
TCCTTTTAGCTTTTTTACGTGTAGTAGGTGTGGCAGTAGATGTAGATGCAGCACGTTTTC
>HPV13_Alpha_85827580_nt7183_L1_Tile_rc|1
TTATTTCCTTTTAGCTTTTTTACGTGTAGTAGGTGTGGCAGTAGATGTAGATGCAGCACG
>HPV14D_Beta_396918_nt5969_L1_Tile_rc|1
GAGTTAGCTTGTTGCCTGTATGAGTTGGGATTTTCTGTATCACCAACCTTATTAAATAAT
>HPV14D_Beta_396918_nt5974_L1_Tile_rc|1
CAGTGGAGTTAGCTTGTTGCCTGTATGAGTTGGGATTTTCTGTATCACCAACCTTATTAA
>HPV14D_Beta_396918_nt5979_L1_Tile_rc|1
GTCATCAGTGGAGTTAGCTTGTTGCCTGTATGAGTTGGGATTTTCTGTATCACCAACCTT
>HPV14D_Beta_396918_nt5984_L1_Tile_rc|1
TGTCTGTCATCAGTGGAGTTAGCTTGTTGCCTGTATGAGTTGGGATTTTCTGTATCACCA
>HPV14D_Beta_396918_nt5989_L1_Tile_rc|1
CATTTTGTCTGTCATCAGTGGAGTTAGCTTGTTGCCTGTATGAGTTGGGATTTTCTGTAT
>HPV14D_Beta_396918_nt5994_L1_Tile_rc|1
TGACACATTTTGTCTGTCATCAGTGGAGTTAGCTTGTTGCCTGTATGAGTTGGGATTTTC
>HPV14D_Beta_396918_nt5999_L1_Tile_rc|1
TCAAATGACACATTTTGTCTGTCATCAGTGGAGTTAGCTTGTTGCCTGTATGAGTTGGGA
>HPV14D_Beta_396918_nt6649_L1_Tile_rc|1
TGGATACCTCAGTGTTTTCTGAACTAACTGATATACTAAAATTTGTGTTACGTGTGTTGT
>HPV14D_Beta_396918_nt6669_L1_Tile_rc|1
AGAGGTATAATTGTCAATTTTGGATACCTCAGTGTTTTCTGAACTAACTGATATACTAAA
>HPV14D_Beta_396918_nt7099_L1_Tile_rc|1
AATTCTTGCGTTTTCGTTTAATACCCTTGATGGATCCCCTAGTCGAAACTGTTTTTGTAC
>HPV14D_Beta_396918_nt7101_L1_Tile_rc|1
CTAATTCTTGCGTTTTCGTTTAATACCCTTGATGGATCCCCTAGTCGAAACTGTTTTTGT
>HPV15_Beta_396924_nt6039_L1_Tile_rc|1
TTCTCTGTATCTTTTACTTTGTTGAATAAAGGATGGCCTGAAGTACCAACTCCTAATGGT
>HPV15_Beta_396924_nt6044_L1_Tile_rc|1
TGTTATTCTCTGTATCTTTTACTTTGTTGAATAAAGGATGGCCTGAAGTACCAACTCCTA
>HPV15_Beta_396924_nt6049_L1_Tile_rc|1

ATTACTGTTATTCTCTGTATCTTTTACTTTGTTGAATAAAGGATGGCCTGAAGTACCAAC
>HPV15_Beta_396924_nt6059_L1_Tile_rc|1
TGCCTTGATAATTACTGTTATTCTCTGTATCTTTTACTTTGTTGAATAAAGGATGGCCTG
>HPV15_Beta_396924_nt6064_L1_Tile_rc|1
AGAGTTGCCTTGATAATTACTGTTATTCTCTGTATCTTTTACTTTGTTGAATAAAGGATG
>HPV15_Beta_396924_nt6069_L1_Tile_rc|1
TCAGTAGAGTTGCCTTGATAATTACTGTTATTCTCTGTATCTTTTACTTTGTTGAATAAA
>HPV15_Beta_396924_nt6314_L1_Tile_rc|1
CCAGACTCACATCTGACTTAGTAACTGATAAGGCCTTGTTATTAATGTTACCAAAACCTA
>HPV15_Beta_396924_nt6319_L1_Tile_rc|1
TATATCCAGACTCACATCTGACTTAGTAACTGATAAGGCCTTGTTATTAATGTTACCAAA
>HPV15_Beta_396924_nt6324_L1_Tile_rc|1
TTCACTATATCCAGACTCACATCTGACTTAGTAACTGATAAGGCCTTGTTATTAATGTTA
>HPV15_Beta_396924_nt6549_L1_Tile_rc|1
AAGTAAGTAGAATTTGCTAAGTTATTTTGTTGTTGGGTTGATTGTGCTGGTAAATAAAAA
>HPV15_Beta_396924_nt6554_L1_Tile_rc|1
TGGGAAAGTAAGTAGAATTTGCTAAGTTATTTTGTTGTTGGGTTGATTGTGCTGGTAAAT
>HPV15_Beta_396924_nt6699_L1_Tile_rc|1
AAATTTGTATTCCTTGTGTTATCTGCAACAGTAATAAACATCTGATTACCCCAAAGTATG
>HPV15_Beta_396924_nt6709_L1_Tile_rc|1
ACTAATAGTAAAATTTGTATTCCTTGTGTTATCTGCAACAGTAATAAACATCTGATTACC
>HPV15_Beta_396924_nt6734_L1_Tile_rc|1
TTATGGCATTACCATCAGAGGTAACACTAATAGTAAAATTTGTATTCCTTGTGTTATCTG
>HPV15_Beta_396924_nt6744_L1_Tile_rc|1
TTATATTCATTTATGGCATTACCATCAGAGGTAACACTAATAGTAAAATTTGTATTCCTT
>HPV15_Beta_396924_nt6759_L1_Tile_rc|1
CTGATATTTTGTGAATTATATTCATTTATGGCATTACCATCAGAGGTAACACTAATAGTA
>HPV16_Alpha_9627100_nt6015_L1_Tile_rc|1
CATTCTCTATTATCCACACCTGCATTTGCTGCATAAGCACTAGCATTTTCTGTGTCATCC
>HPV16_Alpha_9627100_nt6020_L1_Tile_rc|1
ATATACATTCTCTATTATCCACACCTGCATTTGCTGCATAAGCACTAGCATTTTCTGTGT
>HPV16_Alpha_9627100_nt6025_L1_Tile_rc|1
CATAGATATACATTCTCTATTATCCACACCTGCATTTGCTGCATAAGCACTAGCATTTTC
>HPV16_Alpha_9627100_nt6035_L1_Tile_rc|1
GTTTGTAATCCATAGATATACATTCTCTATTATCCACACCTGCATTTGCTGCATAAGCAC
>HPV16_Alpha_9627100_nt6120_L1_Tile_rc|1
TTTACTGCAACATTGGTACATGGGGATCCTTTGCCCCAGTGTTCCCCTATAGGTGGTTTG
>HPV16_Alpha_9627100_nt6125_L1_Tile_rc|1
CTGGATTTACTGCAACATTGGTACATGGGGATCCTTTGCCCCAGTGTTCCCCTATAGGTG
>HPV16_Alpha_9627100_nt6155_L1_Tile_rc|1
TGTTTATTAACTCTAATGGTGGACAATCACCTGGATTTACTGCAACATTGGTACATGGGG
>HPV16_Alpha_9627100_nt6160_L1_Tile_rc|1
AACTGTGTTTATTAACTCTAATGGTGGACAATCACCTGGATTTACTGCAACATTGGTACA
>HPV16_Alpha_9627100_nt6420_L1_Tile_rc|1
CCAGAGCCTTTAATGTATAAATCGTCTGGTACATTTTCACCAACAGTACCAGCCCTATTA
>HPV16_Alpha_9627100_nt6425_L1_Tile_rc|1
TAGACCCAGAGCCTTTAATGTATAAATCGTCTGGTACATTTTCACCAACAGTACCAGCCC
>HPV16_Alpha_9627100_nt6440_L1_Tile_rc|1
TGGCTAAATTTGCAGTAGACCCAGAGCCTTTAATGTATAAATCGTCTGGTACATTTTCAC
>HPV16_Alpha_9627100_nt6445_L1_Tile_rc|1
TGAACTGGCTAAATTTGCAGTAGACCCAGAGCCTTTAATGTATAAATCGTCTGGTACATT
>HPV16_Alpha_9627100_nt6450_L1_Tile_rc|1
TAATTTGAACTGGCTAAATTTGCAGTAGACCCAGAGCCTTTAATGTATAAATCGTCTGGT
>HPV16_Alpha_9627100_nt6455_L1_Tile_rc|1
GAAAATAATTTGAACTGGCTAAATTTGCAGTAGACCCAGAGCCTTTAATGTATAAATCGT

>HPV16_Alpha_9627100_nt6460_L1_Tile_rc|1
TGTAGGAAAATAATTTGAACTGGCTAAATTTGCAGTAGACCCAGAGCCTTTAATGTATAA
>HPV16_Alpha_9627100_nt6640_L1_Tile_rc|1
TGTAGTTTCTGAAGTAGATATGGCAGCACATAATGACATATTTGTACTGCGTGTAGTATC
>HPV16_Alpha_9627100_nt6650_L1_Tile_rc|1
TATTTTTATATGTAGTTTCTGAAGTAGATATGGCAGCACATAATGACATATTTGTACTGC
>HPV16_Alpha_9627100_nt6655_L1_Tile_rc|1
GTTAGTATTTTTATATGTAGTTTCTGAAGTAGATATGGCAGCACATAATGACATATTTGT
>HPV16_Alpha_9627100_nt6820_L1_Tile_rc|1
TAGTGTGCCTCCTGGGGGAGGTTGTAGACCAAAATTCCAGTCCTCCAAAATAGTGGAATT
>HPV16_Alpha_9627100_nt6845_L1_Tile_rc|1
GGGTTACAAACCTATAAGTATCTTCTAGTGTGCCTCCTGGGGGAGGTTGTAGACCAAAAT
>HPV16_Alpha_9627100_nt6850_L1_Tile_rc|1
TGCCTGGGTTACAAACCTATAAGTATCTTCTAGTGTGCCTCCTGGGGGAGGTTGTAGACC
>HPV16_Alpha_9627100_nt6860_L1_Tile_rc|1
GACAAGCAATTGCCTGGGTTACAAACCTATAAGTATCTTCTAGTGTGCCTCCTGGGGGAG
>HPV16_Alpha_9627100_nt6865_L1_Tile_rc|1
TTTTTGACAAGCAATTGCCTGGGTTACAAACCTATAAGTATCTTCTAGTGTGCCTCCTGG
>HPV16_Alpha_9627100_nt6870_L1_Tile_rc|1
GTATGTTTTTGACAAGCAATTGCCTGGGTTACAAACCTATAAGTATCTTCTAGTGTGCCT
>HPV16_Alpha_9627100_nt6880_L1_Tile_rc|1
TGCTGGAGGTGTATGTTTTTGACAAGCAATTGCCTGGGTTACAAACCTATAAGTATCTTC
>HPV16_Alpha_9627100_nt6890_L1_Tile_rc|1
CTTCTTTAGGTGCTGGAGGTGTATGTTTTTGACAAGCAATTGCCTGGGTTACAAACCTAT
>HPV16_Alpha_9627100_nt6900_L1_Tile_rc|1
AGGGGATCATCTTCTTTAGGTGCTGGAGGTGTATGTTTTTGACAAGCAATTGCCTGGGTT
>HPV16_Alpha_9627100_nt6910_L1_Tile_rc|1
GTATTTTTTAAGGGGATCATCTTCTTTAGGTGCTGGAGGTGTATGTTTTTGACAAGCAAT
>HPV16_Alpha_9627100_nt6915_L1_Tile_rc|1
AAAGTGTATTTTTTAAGGGGATCATCTTCTTTAGGTGCTGGAGGTGTATGTTTTTGACAA
>HPV16_Alpha_9627100_nt6920_L1_Tile_rc|1
CCCAAAAAGTGTATTTTTTAAGGGGATCATCTTCTTTAGGTGCTGGAGGTGTATGTTTTT
>HPV16_Alpha_9627100_nt6930_L1_Tile_rc|1
AAATTTACTTCCCAAAAAGTGTATTTTTTAAGGGGATCATCTTCTTTAGGTGCTGGAGGT
>HPV16_Alpha_9627100_nt6935_L1_Tile_rc|1
CCTTTAAATTTACTTCCCAAAAAGTGTATTTTTTAAGGGGATCATCTTCTTTAGGTGCTG
>HPV16_Alpha_9627100_nt7020_L1_Tile_rc|1
GTAAATTTTGGTTTGGCCTTCAATCCTGCTTGTAGTAAAAATTTGCGTCCTAAAGGAAAC
>HPV16_Alpha_9627100_nt7025_L1_Tile_rc|1
CTAATGTAAATTTTGGTTTGGCCTTCAATCCTGCTTGTAGTAAAAATTTGCGTCCTAAAG
>HPV16_Alpha_9627100_nt7030_L1_Tile_rc|1
TTTTCCTAATGTAAATTTTGGTTTGGCCTTCAATCCTGCTTGTAGTAAAAATTTGCGTCC
>HPV16_Alpha_9627100_nt7035_L1_Tile_rc|1
TTTCGTTTTCCTAATGTAAATTTTGGTTTGGCCTTCAATCCTGCTTGTAGTAAAAATTTG
>HPV16_Alpha_9627100_nt7040_L1_Tile_rc|1
TAGCTTTTCGTTTTCCTAATGTAAATTTTGGTTTGGCCTTCAATCCTGCTTGTAGTAAAA
>HPV16_Alpha_9627100_nt7050_L1_Tile_rc|1
GTGGTGGGTGTAGCTTTTCGTTTTCCTAATGTAAATTTTGGTTTGGCCTTCAATCCTGCT
>HPV16_Alpha_9627100_nt7055_L1_Tile_rc|1
ATGAGGTGGTGGGTGTAGCTTTTCGTTTTCCTAATGTAAATTTTGGTTTGGCCTTCAATC
>HPV16_Alpha_9627100_nt7065_L1_Tile_rc|1
GTAGAGGTAGATGAGGTGGTGGGTGTAGCTTTTCGTTTTCCTAATGTAAATTTTGGTTTG
>HPV16_Alpha_9627100_nt7070_L1_Tile_rc|1
CAGTTGTAGAGGTAGATGAGGTGGTGGGTGTAGCTTTTCGTTTTCCTAATGTAAATTTTG
>HPV16_Alpha_9627100_nt7075_L1_Tile_rc|1

TTTAGCAGTTGTAGAGGTAGATGAGGTGGTGGGTGTAGCTTTTCGTTTTCCTAATGTAAA
>HPV16_Alpha_9627100_nt7085_L1_Tile_rc|1
GTTTTTTGCGTTTAGCAGTTGTAGAGGTAGATGAGGTGGTGGGTGTAGCTTTTCGTTTTC
>HPV16_Alpha_9627100_nt7095_L1_Tile_rc|1
TACAGCTTACGTTTTTTGCGTTTAGCAGTTGTAGAGGTAGATGAGGTGGTGGGTGTAGCT
>HPV16_Alpha_9627100_nt7096_L1_Tile_rc|1
TTACAGCTTACGTTTTTTGCGTTTAGCAGTTGTAGAGGTAGATGAGGTGGTGGGTGTAGC
>HPV17_Beta_396932_nt6085_L1_Tile_rc|1
AGATCCACCTTGATAGCTACTGTTATTTTCAGTGTCTCTTAACTTATTAAACAAGGGATG
>HPV17_Beta_396932_nt6205_L1_Tile_rc|1
GTTTTGTTCATTTTCACATACAGGAGCCCTGTCCCAATGTTCTCCAATACAAGGTACACA
>HPV17_Beta_396932_nt6210_L1_Tile_rc|1
TGATTGTTTTGTTCATTTTCACATACAGGAGCCCTGTCCCAATGTTCTCCAATACAAGGT
>HPV17_Beta_396932_nt6215_L1_Tile_rc|1
CTGTTTGATTGTTTTGTTCATTTTCACATACAGGAGCCCTGTCCCAATGTTCTCCAATAC
>HPV17_Beta_396932_nt6230_L1_Tile_rc|1
ATGGTGGACACAGGCCTGTTTGATTGTTTTGTTCATTTTCACATACAGGAGCCCTGTCCC
>HPV17_Beta_396932_nt6240_L1_Tile_rc|1
TTTAATTCCAATGGTGGACACAGGCCTGTTTGATTGTTTTGTTCATTTTCACATACAGGA
>HPV17_Beta_396932_nt6245_L1_Tile_rc|1
TGTTTTTTAATTCCAATGGTGGACACAGGCCTGTTTGATTGTTTTGTTCATTTTCACATA
>HPV17_Beta_396932_nt6255_L1_Tile_rc|1
TCGATAACAGTGTTTTTTAATTCCAATGGTGGACACAGGCCTGTTTGATTGTTTTGTTCA
>HPV17_Beta_396932_nt6565_L1_Tile_rc|1
AGTGGAATTACCCAAAGTGCGCTGTTGTTGGCCAGTTTGAGCTGGTAAATAAAATTTGTG
>HPV17_Beta_396932_nt6730_L1_Tile_rc|1
GCTAATAGAAAAATTTGTGTTCCTAGTGTTGTCAGCTACAGTCACAAATATCTGATTCCC
>HPV17_Beta_396932_nt6745_L1_Tile_rc|1
AGCTTCTGTAGACACGCTAATAGAAAAATTTGTGTTCCTAGTGTTGTCAGCTACAGTCAC
>HPV17_Beta_396932_nt6755_L1_Tile_rc|1
TAACAGCCCCAGCTTCTGTAGACACGCTAATAGAAAAATTTGTGTTCCTAGTGTTGTCAG
>HPV17_Beta_396932_nt7100_L1_Tile_rc|1
ATTTTCGTCCTAGGGGATACTGATCTAAATCTAATGATAATTTCTCAGTAAGATTTACAT
>HPV17_Beta_396932_nt7105_L1_Tile_rc|1
AATAAATTTTCGTCCTAGGGGATACTGATCTAAATCTAATGATAATTTCTCAGTAAGATT
>HPV18_Alpha_9626069_nt5436_L1_Tile_rc|1
TACAATGGGCCATACAGAGGTAGTAGATGGTAATGTAATATCAGGACCCGTGTATACAGG
>HPV18_Alpha_9626069_nt5441_L1_Tile_rc|1
GGTGATACAATGGGCCATACAGAGGTAGTAGATGGTAATGTAATATCAGGACCCGTGTAT
>HPV18_Alpha_9626069_nt5446_L1_Tile_rc|1
CCGTGGGTGATACAATGGGCCATACAGAGGTAGTAGATGGTAATGTAATATCAGGACCCG
>HPV18_Alpha_9626069_nt5466_L1_Tile_rc|1
ATACTGTGTAGAGGCAGGGGCCGTGGGTGATACAATGGGCCATACAGAGGTAGTAGATGG
>HPV18_Alpha_9626069_nt5476_L1_Tile_rc|1
GTATACCAATATACTGTGTAGAGGCAGGGGCCGTGGGTGATACAATGGGCCATACAGAGG
>HPV18_Alpha_9626069_nt5481_L1_Tile_rc|1
ACCATGTATACCAATATACTGTGTAGAGGCAGGGGCCGTGGGTGATACAATGGGCCATAC
>HPV18_Alpha_9626069_nt5486_L1_Tile_rc|1
TGTGTACCATGTATACCAATATACTGTGTAGAGGCAGGGGCCGTGGGTGATACAATGGGC
>HPV18_Alpha_9626069_nt5491_L1_Tile_rc|1
AATAATGTGTACCATGTATACCAATATACTGTGTAGAGGCAGGGGCCGTGGGTGATACAA
>HPV18_Alpha_9626069_nt5496_L1_Tile_rc|1
CAAATAATAATGTGTACCATGTATACCAATATACTGTGTAGAGGCAGGGGCCGTGGGTGA
>HPV18_Alpha_9626069_nt5506_L1_Tile_rc|1
ATAATGGCCACAAATAATAATGTGTACCATGTATACCAATATACTGTGTAGAGGCAGGGG

>HPV18_Alpha_9626069_nt5511_L1_Tile_rc|1
ATAATATAATGGCCACAAATAATAATGTGTACCATGTATACCAATATACTGTGTAGAGGC
>HPV18_Alpha_9626069_nt5976_L1_Tile_rc|1
TCAGAAACATTAGACGTGGCGGCATGGGAACTTTCAGTGTCATCTAATTTATTATAAAAT
>HPV18_Alpha_9626069_nt5981_L1_Tile_rc|1
CGTCCTCAGAAACATTAGACGTGGCGGCATGGGAACTTTCAGTGTCATCTAATTTATTAT
>HPV18_Alpha_9626069_nt5996_L1_Tile_rc|1
ACACATTGTCCCTAACGTCCTCAGAAACATTAGACGTGGCGGCATGGGAACTTTCAGTGT
>HPV18_Alpha_9626069_nt6131_L1_Tile_rc|1
TGTTTTTAAGTTCTAAAGGGGGGCAATCGCCCTGTGATAAAGGACGCGATTTACAAGCAG
>HPV18_Alpha_9626069_nt6136_L1_Tile_rc|1
AACTGTGTTTTTAAGTTCTAAAGGGGGGCAATCGCCCTGTGATAAAGGACGCGATTTACA
>HPV18_Alpha_9626069_nt6146_L1_Tile_rc|1
CATCTTCCAAAACTGTGTTTTTAAGTTCTAAAGGGGGGCAATCGCCCTGTGATAAAGGAC
>HPV18_Alpha_9626069_nt6421_L1_Tile_rc|1
ACAGCTGCCAGGTGAAGCAGGCATACCTGTGCCTTTAATATATAAGGATTGAGGCACAGT
>HPV18_Alpha_9626069_nt6426_L1_Tile_rc|1
TACACACAGCTGCCAGGTGAAGCAGGCATACCTGTGCCTTTAATATATAAGGATTGAGGC
>HPV18_Alpha_9626069_nt6441_L1_Tile_rc|1
CTTGGAGAGGGAGAATACACACAGCTGCCAGGTGAAGCAGGCATACCTGTGCCTTTAATA
>HPV18_Alpha_9626069_nt6621_L1_Tile_rc|1
TATTGCCCAGGTACAGGAGACTGTGTAGAAGCACATATTGTTAAATTGGTACTGGGAGTG
>HPV18_Alpha_9626069_nt6626_L1_Tile_rc|1
CATCATATTGCCCAGGTACAGGAGACTGTGTAGAAGCACATATTGTTAAATTGGTACTGG
>HPV18_Alpha_9626069_nt6631_L1_Tile_rc|1
GGTAGCATCATATTGCCCAGGTACAGGAGACTGTGTAGAAGCACATATTGTTAAATTGGT
>HPV18_Alpha_9626069_nt6636_L1_Tile_rc|1
AATTTGGTAGCATCATATTGCCCAGGTACAGGAGACTGTGTAGAAGCACATATTGTTAAA
>HPV18_Alpha_9626069_nt6641_L1_Tile_rc|1
GCTTAAATTTGGTAGCATCATATTGCCCAGGTACAGGAGACTGTGTAGAAGCACATATTG
>HPV18_Alpha_9626069_nt6651_L1_Tile_rc|1
CTGCTATACTGCTTAAATTTGGTAGCATCATATTGCCCAGGTACAGGAGACTGTGTAGAA
>HPV18_Alpha_9626069_nt6791_L1_Tile_rc|1
TAGTTGGGGGGGGGGGAACACCAAAGTTCCAATCCTCTAAAATACTGCTATTCATACTAT
>HPV18_Alpha_9626069_nt7078_L1_Tile_rc|1
TTACTTCCTGGCACGTACACGCACACGCTTGGCAGGTTTAGAAGACGTAGTGGCAGATGG
>HPV19_Beta_396940_nt5795_L1_Tile_rc|1
AGAAAATTATAAATATTTACGTTTTCGTTTGAATCGCTTGCGAAGGCTGGGATGTAAGTA
>HPV19_Beta_396940_nt5800_L1_Tile_rc|1
GCAAAAGAAAATTATAAATATTTACGTTTTCGTTTGAATCGCTTGCGAAGGCTGGGATGT
>HPV19_Beta_396940_nt6005_L1_Tile_rc|1
CCTGAAACTTTTGGAATTTCTAATTTTGATCCTGCAACATTATAAACATTAAAATATGGG
>HPV19_Beta_396940_nt6010_L1_Tile_rc|1
GATTTCCTGAAACTTTTGGAATTTCTAATTTTGATCCTGCAACATTATAAACATTAAAAT
>HPV19_Beta_396940_nt6360_L1_Tile_rc|1
AGGACAGGATCCCTGAGGAATATCTTGCTCAGCACATGGTAATGCTTTATCCCAGTGTTC
>HPV19_Beta_396940_nt6370_L1_Tile_rc|1
ACTCTATAGGAGGACAGGATCCCTGAGGAATATCTTGCTCAGCACATGGTAATGCTTTAT
>HPV19_Beta_396940_nt6375_L1_Tile_rc|1
AATTAACTCTATAGGAGGACAGGATCCCTGAGGAATATCTTGCTCAGCACATGGTAATGC
>HPV19_Beta_396940_nt6380_L1_Tile_rc|1
GAGTTAATTAACTCTATAGGAGGACAGGATCCCTGAGGAATATCTTGCTCAGCACATGGT
>HPV19_Beta_396940_nt6385_L1_Tile_rc|1
TAACTGAGTTAATTAACTCTATAGGAGGACAGGATCCCTGAGGAATATCTTGCTCAGCAC
>HPV19_Beta_396940_nt6400_L1_Tile_rc|1

TGTCTCCATCTTCAATAACTGAGTTAATTAACTCTATAGGAGGACAGGATCCCTGAGGAA
>HPV19_Beta_396940_nt6710_L1_Tile_rc|1
ATGGCATTTCCTAAATTAGTATATTGTTGCTGACTATTGTTAGGAGGTATGTAGTATGTA
>HPV19_Beta_396940_nt7125_L1_Tile_rc|1
TAAAGAATCTATATATCTATAAGTGTCCTGAATAGGATTATCGGGTGCAGGCACGAAACC
>HPV19_Beta_396940_nt7160_L1_Tile_rc|1
TCCTTAGGAGGATTTTTGTCAGGGCATCTAGTAGCTAAAGAATCTATATATCTATAAGTG
>HPV19_Beta_396940_nt7355_L1_Tile_rc|1
TAATTTTTACGCTTTCTTTTAGTTCCTCTGCTGGAGACCCGTGAAGATATAGTTTTTGTA
>HPV19_Beta_396940_nt7356_L1_Tile_rc|1
TTAATTTTTACGCTTTCTTTTAGTTCCTCTGCTGGAGACCCGTGAAGATATAGTTTTTGT
>HPV20_Beta_1020162_nt6039_L1_Tile_rc|1
TTCCAGAAACCTTAGGGACTTTTATCTTAGTGCCTTGGATGTCATATATATTAAAATATG
>HPV20_Beta_1020162_nt6044_L1_Tile_rc|1
CTGATTTCCAGAAACCTTAGGGACTTTTATCTTAGTGCCTTGGATGTCATATATATTAAA
>HPV20_Beta_1020162_nt6049_L1_Tile_rc|1
CTGTGCTGATTTCCAGAAACCTTAGGGACTTTTATCTTAGTGCCTTGGATGTCATATATA
>HPV20_Beta_1020162_nt6259_L1_Tile_rc|1
TCAGTTGAATTCCCTTTATATGAATTAGGGTTTTCTGTGTCACCAAGTTTATTAAATAAT
>HPV20_Beta_1020162_nt6264_L1_Tile_rc|1
TATCATCAGTTGAATTCCCTTTATATGAATTAGGGTTTTCTGTGTCACCAAGTTTATTAA
>HPV20_Beta_1020162_nt6269_L1_Tile_rc|1
TTGTCTATCATCAGTTGAATTCCCTTTATATGAATTAGGGTTTTCTGTGTCACCAAGTTT
>HPV20_Beta_1020162_nt6274_L1_Tile_rc|1
ACATTTTGTCTATCATCAGTTGAATTCCCTTTATATGAATTAGGGTTTTCTGTGTCACCA
>HPV20_Beta_1020162_nt6399_L1_Tile_rc|1
CTATTGGAGGGCATGAACCTGGGTTTGGAACGTCGTCTGCACATGGTAAAGCCCTGTCCC
>HPV20_Beta_1020162_nt6739_L1_Tile_rc|1
ATTGAATTACCTAGGTTGTTCTGTGCCTGATTATTCACAGGTGGAATGTAGAATGCATTC
>HPV20_Beta_1020162_nt6744_L1_Tile_rc|1
AATACATTGAATTACCTAGGTTGTTCTGTGCCTGATTATTCACAGGTGGAATGTAGAATG
>HPV20_Beta_1020162_nt6894_L1_Tile_rc|1
AATTTGTATTTCGAGTATTATCTACTACAGTAACAAATAGTTGATTGAACCAGCAGATGC
>HPV20_Beta_1020162_nt6904_L1_Tile_rc|1
GATATGCTAAAATTTGTATTTCGAGTATTATCTACTACAGTAACAAATAGTTGATTGAAC
>HPV20_Beta_1020162_nt6914_L1_Tile_rc|1
TGAATGAACTGATATGCTAAAATTTGTATTTCGAGTATTATCTACTACAGTAACAAATAG
>HPV20_Beta_1020162_nt6919_L1_Tile_rc|1
TTTTCTGAATGAACTGATATGCTAAAATTTGTATTTCGAGTATTATCTACTACAGTAACA
>HPV20_Beta_1020162_nt6924_L1_Tile_rc|1
CAGTGTTTTCTGAATGAACTGATATGCTAAAATTTGTATTTCGAGTATTATCTACTACAG
>HPV20_Beta_1020162_nt6929_L1_Tile_rc|1
AACATCAGTGTTTTCTGAATGAACTGATATGCTAAAATTTGTATTTCGAGTATTATCTAC
>HPV20_Beta_1020162_nt6934_L1_Tile_rc|1
TTAGAAACATCAGTGTTTTCTGAATGAACTGATATGCTAAAATTTGTATTTCGAGTATTA
>HPV20_Beta_1020162_nt6949_L1_Tile_rc|1
TCATAATTTTGAATTTTAGAAACATCAGTGTTTTCTGAATGAACTGATATGCTAAAATTT
>HPV20_Beta_1020162_nt6954_L1_Tile_rc|1
GAGAATCATAATTTTGAATTTTAGAAACATCAGTGTTTTCTGAATGAACTGATATGCTAA
>HPV20_Beta_1020162_nt7249_L1_Tile_rc|1
GATAATCTTTCTGATAGGTCAACATTCCAAAAGTTTAGATCCTTGTAAGGATCTTCTCTT
>HPV20_Beta_1020162_nt7254_L1_Tile_rc|1
CTAAGGATAATCTTTCTGATAGGTCAACATTCCAAAAGTTTAGATCCTTGTAAGGATCTT
>HPV20_Beta_1020162_nt7259_L1_Tile_rc|1
CAATTCTAAGGATAATCTTTCTGATAGGTCAACATTCCAAAAGTTTAGATCCTTGTAAGG

>HPV20_Beta_1020162_nt7384_L1_Tile_rc|1
TATTGTTTGCGTTTTCGTTTGACGCCCCTAGTAGATAACTTTGAAGATACAGTTTTTGTA
>HPV20_Beta_1020162_nt7385_L1_Tile_rc|1
TTATTGTTTGCGTTTTCGTTTGACGCCCCTAGTAGATAACTTTGAAGATACAGTTTTTGT
>HPV21_Beta_1020170_nt6300_L1_Tile_rc|1
CAGTAGAATTTGGTTGAGTTTTGTATGAACTAGGATTTTCTGTGTCCCCAACTTTATTAA
>HPV21_Beta_1020170_nt6305_L1_Tile_rc|1
ATCATCAGTAGAATTTGGTTGAGTTTTGTATGAACTAGGATTTTCTGTGTCCCCAACTTT
>HPV21_Beta_1020170_nt6430_L1_Tile_rc|1
CACGATCCTGGAGGTGGATTGTCAGTTGCACATGGGATAGCTTTATCCCAATGTTCTCCT
>HPV21_Beta_1020170_nt6445_L1_Tile_rc|1
AATTCAATCGGAGGGCACGATCCTGGAGGTGGATTGTCAGTTGCACATGGGATAGCTTTA
>HPV21_Beta_1020170_nt6450_L1_Tile_rc|1
TAATTAATTCAATCGGAGGGCACGATCCTGGAGGTGGATTGTCAGTTGCACATGGGATAG
>HPV21_Beta_1020170_nt6455_L1_Tile_rc|1
TGAATTAATTAATTCAATCGGAGGGCACGATCCTGGAGGTGGATTGTCAGTTGCACATGG
>HPV21_Beta_1020170_nt6460_L1_Tile_rc|1
ATTGCTGAATTAATTAATTCAATCGGAGGGCACGATCCTGGAGGTGGATTGTCAGTTGCA
>HPV21_Beta_1020170_nt6970_L1_Tile_rc|1
ACGTCTGCATTCTCAGGATTTACTGAAATACTAAAGTTTGTGTTACGAGTGTTGTCTACT
>HPV21_Beta_1020170_nt6980_L1_Tile_rc|1
AATTTTAGACACGTCTGCATTCTCAGGATTTACTGAAATACTAAAGTTTGTGTTACGAGT
>HPV21_Beta_1020170_nt6985_L1_Tile_rc|1
TTTTCAATTTTAGACACGTCTGCATTCTCAGGATTTACTGAAATACTAAAGTTTGTGTTA
>HPV21_Beta_1020170_nt6990_L1_Tile_rc|1
TATAATTTTCAATTTTAGACACGTCTGCATTCTCAGGATTTACTGAAATACTAAAGTTTG
>HPV21_Beta_1020170_nt6995_L1_Tile_rc|1
GGCTTTATAATTTTCAATTTTAGACACGTCTGCATTCTCAGGATTTACTGAAATACTAAA
>HPV21_Beta_1020170_nt7000_L1_Tile_rc|1
CTCTCGGCTTTATAATTTTCAATTTTAGACACGTCTGCATTCTCAGGATTTACTGAAATA
>HPV21_Beta_1020170_nt7005_L1_Tile_rc|1
GAAAGCTCTCGGCTTTATAATTTTCAATTTTAGACACGTCTGCATTCTCAGGATTTACTG
>HPV21_Beta_1020170_nt7425_L1_Tile_rc|1
AATTTTTGCGTTTTCGTTTAATTCCTCTGGTAGATACCCTTGAAGAAAGTGTCTTTGTAC
>HPV21_Beta_1020170_nt7427_L1_Tile_rc|1
CTAATTTTTGCGTTTTCGTTTAATTCCTCTGGTAGATACCCTTGAAGAAAGTGTCTTTGT
>HPV22_Beta_1020178_nt5883_L1_Tile_rc|1
GATCATGGATTGTCATATCTCCCAAAGCAAATTTGTTAGGATCTGGAAATGTTACTCTAA
>HPV22_Beta_1020178_nt5893_L1_Tile_rc|1
TACCTTTCGGGATCATGGATTGTCATATCTCCCAAAGCAAATTTGTTAGGATCTGGAAAT
>HPV22_Beta_1020178_nt5898_L1_Tile_rc|1
ATCTATACCTTTCGGGATCATGGATTGTCATATCTCCCAAAGCAAATTTGTTAGGATCTG
>HPV22_Beta_1020178_nt5903_L1_Tile_rc|1
TACTAATCTATACCTTTCGGGATCATGGATTGTCATATCTCCCAAAGCAAATTTGTTAGG
>HPV22_Beta_1020178_nt5908_L1_Tile_rc|1
GCCCATACTAATCTATACCTTTCGGGATCATGGATTGTCATATCTCCCAAAGCAAATTTG
>HPV22_Beta_1020178_nt5913_L1_Tile_rc|1
TACAAGCCCATACTAATCTATACCTTTCGGGATCATGGATTGTCATATCTCCCAAAGCAA
>HPV22_Beta_1020178_nt5918_L1_Tile_rc|1
CCCTTTACAAGCCCATACTAATCTATACCTTTCGGGATCATGGATTGTCATATCTCCCAA
>HPV22_Beta_1020178_nt5923_L1_Tile_rc|1
TCTAACCCTTTACAAGCCCATACTAATCTATACCTTTCGGGATCATGGATTGTCATATCT
>HPV22_Beta_1020178_nt5928_L1_Tile_rc|1
CTATTTCTAACCCTTTACAAGCCCATACTAATCTATACCTTTCGGGATCATGGATTGTCA
>HPV22_Beta_1020178_nt5933_L1_Tile_rc|1

TCTTCCTATTTCTAACCCTTTACAAGCCCATACTAATCTATACCTTTCGGGATCATGGAT
>HPV22_Beta_1020178_nt6028_L1_Tile_rc|1
TCTGATGTTCCTTCCTGGCGTTCAGTAGGGTTTTCAGTATCATGTAATTTATTAAATAAT
>HPV22_Beta_1020178_nt6033_L1_Tile_rc|1
TATCATCTGATGTTCCTTCCTGGCGTTCAGTAGGGTTTTCAGTATCATGTAATTTATTAA
>HPV22_Beta_1020178_nt6038_L1_Tile_rc|1
TCTTCTATCATCTGATGTTCCTTCCTGGCGTTCAGTAGGGTTTTCAGTATCATGTAATTT
>HPV22_Beta_1020178_nt6043_L1_Tile_rc|1
ACATTTCTTCTATCATCTGATGTTCCTTCCTGGCGTTCAGTAGGGTTTTCAGTATCATGT
>HPV22_Beta_1020178_nt6288_L1_Tile_rc|1
AGATTTCATTTACAATGTCTAAGCTTACATCAGATCTATTAAATGATAGTGTTTTATTAT
>HPV22_Beta_1020178_nt6358_L1_Tile_rc|1
CTACGTGCACAAAAAAAGCATGAGTCGCCATAGACATCATTTGACATTGTAAGAAAATCA
>HPV22_Beta_1020178_nt6363_L1_Tile_rc|1
GCTCCCTACGTGCACAAAAAAAGCATGAGTCGCCATAGACATCATTTGACATTGTAAGAA
>HPV22_Beta_1020178_nt6428_L1_Tile_rc|1
TGCATCATCTGGTATAGCATCACCAACAAGACCACCACGTACAAAATTGTGTCGTGCATA
>HPV22_Beta_1020178_nt6433_L1_Tile_rc|1
TGAACTGCATCATCTGGTATAGCATCACCAACAAGACCACCACGTACAAAATTGTGTCGT
>HPV22_Beta_1020178_nt6443_L1_Tile_rc|1
ATGATCTTGTTGAACTGCATCATCTGGTATAGCATCACCAACAAGACCACCACGTACAAA
>HPV22_Beta_1020178_nt6453_L1_Tile_rc|1
AGTAATATTTATGATCTTGTTGAACTGCATCATCTGGTATAGCATCACCAACAAGACCAC
>HPV22_Beta_1020178_nt6458_L1_Tile_rc|1
AGGCAAGTAATATTTATGATCTTGTTGAACTGCATCATCTGGTATAGCATCACCAACAAG
>HPV22_Beta_1020178_nt6473_L1_Tile_rc|1
AGTCTGTGAAGCTGCAGGCAAGTAATATTTATGATCTTGTTGAACTGCATCATCTGGTAT
>HPV22_Beta_1020178_nt6498_L1_Tile_rc|1
GAAAGTAAGTGGAGTTTTCTAAAGCAGTCTGTGAAGCTGCAGGCAAGTAATATTTATGAT
>HPV22_Beta_1020178_nt6503_L1_Tile_rc|1
GGTTGGAAAGTAAGTGGAGTTTTCTAAAGCAGTCTGTGAAGCTGCAGGCAAGTAATATTT
>HPV22_Beta_1020178_nt6508_L1_Tile_rc|1
CTAACGGTTGGAAAGTAAGTGGAGTTTTCTAAAGCAGTCTGTGAAGCTGCAGGCAAGTAA
>HPV22_Beta_1020178_nt6513_L1_Tile_rc|1
AACCACTAACGGTTGGAAAGTAAGTGGAGTTTTCTAAAGCAGTCTGTGAAGCTGCAGGCA
>HPV22_Beta_1020178_nt6518_L1_Tile_rc|1
TAAAGAACCACTAACGGTTGGAAAGTAAGTGGAGTTTTCTAAAGCAGTCTGTGAAGCTGC
>HPV22_Beta_1020178_nt6573_L1_Tile_rc|1
CCTGCGCGCGCTTCAACCAAAAAGGCCTGTTGAATAGTTGGGCATCAGAGGTTACTAAAG
>HPV22_Beta_1020178_nt6683_L1_Tile_rc|1
ATTAACTGTGGTGCCGTCACTTGCCACACTAATAGAAAAATTAGTGTTACGGGTATTATC
>HPV22_Beta_1020178_nt6688_L1_Tile_rc|1
TCATAATTAACTGTGGTGCCGTCACTTGCCACACTAATAGAAAAATTAGTGTTACGGGTA
>HPV22_Beta_1020178_nt6693_L1_Tile_rc|1
TAGCATCATAATTAACTGTGGTGCCGTCACTTGCCACACTAATAGAAAAATTAGTGTTAC
>HPV22_Beta_1020178_nt6698_L1_Tile_rc|1
TTTTTTAGCATCATAATTAACTGTGGTGCCGTCACTTGCCACACTAATAGAAAAATTAGT
>HPV22_Beta_1020178_nt6703_L1_Tile_rc|1
CTGATTTTTTTAGCATCATAATTAACTGTGGTGCCGTCACTTGCCACACTAATAGAAAAA
>HPV22_Beta_1020178_nt6708_L1_Tile_rc|1
ATTCTCTGATTTTTTTAGCATCATAATTAACTGTGGTGCCGTCACTTGCCACACTAATAG
>HPV22_Beta_1020178_nt6718_L1_Tile_rc|1
TGGCGCATAAATTCTCTGATTTTTTTAGCATCATAATTAACTGTGGTGCCGTCACTTGCC
>HPV22_Beta_1020178_nt6738_L1_Tile_rc|1
ATAATTGGTATTCTTCCACATGGCGCATAAATTCTCTGATTTTTTTAGCATCATAATTAA

>HPV22_Beta_1020178_nt6763_L1_Tile_rc|1
ATTCTACATAGCTGCAAAATAAAGGATAATTGGTATTCTTCCACATGGCGCATAAATTCT
>HPV22_Beta_1020178_nt6768_L1_Tile_rc|1
ATGGTATTCTACATAGCTGCAAAATAAAGGATAATTGGTATTCTTCCACATGGCGCATAA
>HPV22_Beta_1020178_nt6773_L1_Tile_rc|1
TTCTAATGGTATTCTACATAGCTGCAAAATAAAGGATAATTGGTATTCTTCCACATGGCG
>HPV22_Beta_1020178_nt6783_L1_Tile_rc|1
ATACCTCTGCTTCTAATGGTATTCTACATAGCTGCAAAATAAAGGATAATTGGTATTCTT
>HPV22_Beta_1020178_nt6788_L1_Tile_rc|1
AGTTAATACCTCTGCTTCTAATGGTATTCTACATAGCTGCAAAATAAAGGATAATTGGTA
>HPV22_Beta_1020178_nt6793_L1_Tile_rc|1
ATTTGAGTTAATACCTCTGCTTCTAATGGTATTCTACATAGCTGCAAAATAAAGGATAAT
>HPV22_Beta_1020178_nt6808_L1_Tile_rc|1
TGATTCATGGCATTAATTTGAGTTAATACCTCTGCTTCTAATGGTATTCTACATAGCTGC
>HPV22_Beta_1020178_nt6813_L1_Tile_rc|1
TGCCATGATTCATGGCATTAATTTGAGTTAATACCTCTGCTTCTAATGGTATTCTACATA
>HPV22_Beta_1020178_nt7063_L1_Tile_rc|1
GCACTGGCCCTTGCACGTTGTAACCCAGATTGAAATAAAAATTTACGACCCAGTGGATAC
>HPV22_Beta_1020178_nt7083_L1_Tile_rc|1
AACGTTTCACACTGACCCTGGCACTGGCCCTTGCACGTTGTAACCCAGATTGAAATAAAA
>HPV22_Beta_1020178_nt7088_L1_Tile_rc|1
AGCAGAACGTTTCACACTGACCCTGGCACTGGCCCTTGCACGTTGTAACCCAGATTGAAA
>HPV22_Beta_1020178_nt7098_L1_Tile_rc|1
TTTTCCGCGTAGCAGAACGTTTCACACTGACCCTGGCACTGGCCCTTGCACGTTGTAACC
>HPV22_Beta_1020178_nt7128_L1_Tile_rc|1
TAAGTTTCCTTCGTTTTACAGTTTTAGACGTTTTCCGCGTAGCAGAACGTTTCACACTGA
>HPV23_Beta_1020186_nt5816_L1_Tile_rc|1
CTGAATTGATTCCCTGAAACCTTTGGTACATCTATTTTACTACCATCCGGTGATCTAACA
>HPV23_Beta_1020186_nt5896_L1_Tile_rc|1
ACCTGTATTTATCAGGATCATAGATAGTCATGTCTGCTAATGCAAACTTATTAGGGTCTG
>HPV23_Beta_1020186_nt5971_L1_Tile_rc|1
ATAGCGGGTGTCCTGTACTGCCGACCCCTAAAGGTTGGCCGCGGCCGATTTCAAGTCCTG
>HPV23_Beta_1020186_nt6001_L1_Tile_rc|1
TAGAATTTTCTGCATCACGGAGCTTATTAAATAGCGGGTGTCCTGTACTGCCGACCCCTA
>HPV23_Beta_1020186_nt6006_L1_Tile_rc|1
TTCACTAGAATTTTCTGCATCACGGAGCTTATTAAATAGCGGGTGTCCTGTACTGCCGAC
>HPV23_Beta_1020186_nt6026_L1_Tile_rc|1
TCTACAGTACCTTCCTGACGTTCACTAGAATTTTCTGCATCACGGAGCTTATTAAATAGC
>HPV23_Beta_1020186_nt6031_L1_Tile_rc|1
TGTCATCTACAGTACCTTCCTGACGTTCACTAGAATTTTCTGCATCACGGAGCTTATTAA
>HPV23_Beta_1020186_nt6041_L1_Tile_rc|1
ATATTTCTTCTGTCATCTACAGTACCTTCCTGACGTTCACTAGAATTTTCTGCATCACGG
>HPV23_Beta_1020186_nt6046_L1_Tile_rc|1
ATGAGATATTTCTTCTGTCATCTACAGTACCTTCCTGACGTTCACTAGAATTTTCTGCAT
>HPV23_Beta_1020186_nt6051_L1_Tile_rc|1
ATCAAATGAGATATTTCTTCTGTCATCTACAGTACCTTCCTGACGTTCACTAGAATTTTC
>HPV23_Beta_1020186_nt6166_L1_Tile_rc|1
CTAAAGGAGGACACAACCCTAGTTGGCTACCTGCATCTTTACAGACAGGAGCTGTATCCC
>HPV23_Beta_1020186_nt6181_L1_Tile_rc|1
CACTGTTTTTTAATTCTAAAGGAGGACACAACCCTAGTTGGCTACCTGCATCTTTACAGA
>HPV23_Beta_1020186_nt6236_L1_Tile_rc|1
GTTTTATTATTGATATTACCAAAGCCAATGTCGAACATGTCCCCATCTTCTATAACACTG
>HPV23_Beta_1020186_nt6246_L1_Tile_rc|1
AAAGGATAATGTTTTATTATTGATATTACCAAAGCCAATGTCGAACATGTCCCCATCTTC
>HPV23_Beta_1020186_nt6256_L1_Tile_rc|1

CTGATCTATTAAAGGATAATGTTTTATTATTGATATTACCAAAGCCAATGTCGAACATGT
>HPV23_Beta_1020186_nt6286_L1_Tile_rc|1
AAACCTCATTTACAAGATCTAAACTAACATCTGATCTATTAAAGGATAATGTTTTATTAT
>HPV23_Beta_1020186_nt6476_L1_Tile_rc|1
AAAGTGTTTTGTTGGTCTGCAGGTAAATAATATTTGTGATCCTGTTGAACTGCACCATCA
>HPV23_Beta_1020186_nt6491_L1_Tile_rc|1
TAAAGTGAGTTTTCTAAAGTGTTTTGTTGGTCTGCAGGTAAATAATATTTGTGATCCTGT
>HPV23_Beta_1020186_nt6501_L1_Tile_rc|1
AGTAGGAAAATAAAGTGAGTTTTCTAAAGTGTTTTGTTGGTCTGCAGGTAAATAATATTT
>HPV23_Beta_1020186_nt6506_L1_Tile_rc|1
CTGACAGTAGGAAAATAAAGTGAGTTTTCTAAAGTGTTTTGTTGGTCTGCAGGTAAATAA
>HPV23_Beta_1020186_nt6516_L1_Tile_rc|1
CAAAGATCCACTGACAGTAGGAAAATAAAGTGAGTTTTCTAAAGTGTTTTGTTGGTCTGC
>HPV23_Beta_1020186_nt6521_L1_Tile_rc|1
GTTACCAAAGATCCACTGACAGTAGGAAAATAAAGTGAGTTTTCTAAAGTGTTTTGTTGG
>HPV23_Beta_1020186_nt6526_L1_Tile_rc|1
CAGAAGTTACCAAAGATCCACTGACAGTAGGAAAATAAAGTGAGTTTTCTAAAGTGTTTT
>HPV23_Beta_1020186_nt6646_L1_Tile_rc|1
TAAAGTTTGTATTACGTGTATTATCTGCTACAGTCACAAACATCTGGTTGTTCCATAAAA
>HPV23_Beta_1020186_nt6651_L1_Tile_rc|1
GATACTAAAGTTTGTATTACGTGTATTATCTGCTACAGTCACAAACATCTGGTTGTTCCA
>HPV23_Beta_1020186_nt6666_L1_Tile_rc|1
GTCATTGGTAACACTGATACTAAAGTTTGTATTACGTGTATTATCTGCTACAGTCACAAA
>HPV23_Beta_1020186_nt6671_L1_Tile_rc|1
CTGCTGTCATTGGTAACACTGATACTAAAGTTTGTATTACGTGTATTATCTGCTACAGTC
>HPV23_Beta_1020186_nt6676_L1_Tile_rc|1
CTAAACTGCTGTCATTGGTAACACTGATACTAAAGTTTGTATTACGTGTATTATCTGCTA
>HPV23_Beta_1020186_nt6681_L1_Tile_rc|1
CTTTTCTAAACTGCTGTCATTGGTAACACTGATACTAAAGTTTGTATTACGTGTATTATC
>HPV23_Beta_1020186_nt6686_L1_Tile_rc|1
TCATACTTTTCTAAACTGCTGTCATTGGTAACACTGATACTAAAGTTTGTATTACGTGTA
>HPV23_Beta_1020186_nt6691_L1_Tile_rc|1
TGGCATCATACTTTTCTAAACTGCTGTCATTGGTAACACTGATACTAAAGTTTGTATTAC
>HPV23_Beta_1020186_nt6696_L1_Tile_rc|1
TTTAGTGGCATCATACTTTTCTAAACTGCTGTCATTGGTAACACTGATACTAAAGTTTGT
>HPV23_Beta_1020186_nt6701_L1_Tile_rc|1
CTAATTTTAGTGGCATCATACTTTTCTAAACTGCTGTCATTGGTAACACTGATACTAAAG
>HPV23_Beta_1020186_nt6706_L1_Tile_rc|1
ACTCTCTAATTTTAGTGGCATCATACTTTTCTAAACTGCTGTCATTGGTAACACTGATAC
>HPV23_Beta_1020186_nt6711_L1_Tile_rc|1
TGTAAACTCTCTAATTTTAGTGGCATCATACTTTTCTAAACTGCTGTCATTGGTAACACT
>HPV23_Beta_1020186_nt6716_L1_Tile_rc|1
TGTCTTGTAAACTCTCTAATTTTAGTGGCATCATACTTTTCTAAACTGCTGTCATTGGTA
>HPV23_Beta_1020186_nt6721_L1_Tile_rc|1
CAACATGTCTTGTAAACTCTCTAATTTTAGTGGCATCATACTTTTCTAAACTGCTGTCAT
>HPV23_Beta_1020186_nt6741_L1_Tile_rc|1
AAAAGAAAGTTGGTATTCTTCAACATGTCTTGTAAACTCTCTAATTTTAGTGGCATCATA
>HPV23_Beta_1020186_nt6761_L1_Tile_rc|1
ATCCTGCACAACTGTAGTATAAAAGAAAGTTGGTATTCTTCAACATGTCTTGTAAACTCT
>HPV23_Beta_1020186_nt6781_L1_Tile_rc|1
AGACCTCGGCCTTTAAAGGTATCCTGCACAACTGTAGTATAAAAGAAAGTTGGTATTCTT
>HPV23_Beta_1020186_nt6786_L1_Tile_rc|1
TGTTAAGACCTCGGCCTTTAAAGGTATCCTGCACAACTGTAGTATAAAAGAAAGTTGGTA
>HPV23_Beta_1020186_nt6791_L1_Tile_rc|1
ATTTGTGTTAAGACCTCGGCCTTTAAAGGTATCCTGCACAACTGTAGTATAAAAGAAAGT

>HPV23_Beta_1020186_nt6796_L1_Tile_rc|1
CATTAATTTGTGTTAAGACCTCGGCCTTTAAAGGTATCCTGCACAACTGTAGTATAAAAG
>HPV23_Beta_1020186_nt6801_L1_Tile_rc|1
CATGGCATTAATTTGTGTTAAGACCTCGGCCTTTAAAGGTATCCTGCACAACTGTAGTAT
>HPV23_Beta_1020186_nt6896_L1_Tile_rc|1
GTGGCCTTTGAAGCCAAATATCTGTATGTGTCATGAACTGCATTATCTGGTGTAGGAACA
>HPV23_Beta_1020186_nt6926_L1_Tile_rc|1
TGCGTGTCAGGTACTGCATCTGGACATTTTGTGGCCTTTGAAGCCAAATATCTGTATGTG
>HPV23_Beta_1020186_nt6936_L1_Tile_rc|1
ATCCTCTTTTTGCGTGTCAGGTACTGCATCTGGACATTTTGTGGCCTTTGAAGCCAAATA
>HPV23_Beta_1020186_nt7021_L1_Tile_rc|1
ACTTACGGCCTAAGGGATATTGATCTAGGTCTAGAGACAATTTTTCTGTCATATCAACAT
>HPV23_Beta_1020186_nt7026_L1_Tile_rc|1
CAGAAACTTACGGCCTAAGGGATATTGATCTAGGTCTAGAGACAATTTTTCTGTCATATC
>HPV23_Beta_1020186_nt7041_L1_Tile_rc|1
CACTCCAATTTGAAACAGAAACTTACGGCCTAAGGGATATTGATCTAGGTCTAGAGACAA
>HPV23_Beta_1020186_nt7051_L1_Tile_rc|1
GTACACGCTGCACTCCAATTTGAAACAGAAACTTACGGCCTAAGGGATATTGATCTAGGT
>HPV23_Beta_1020186_nt7061_L1_Tile_rc|1
GTACCGGACCGTACACGCTGCACTCCAATTTGAAACAGAAACTTACGGCCTAAGGGATAT
>HPV23_Beta_1020186_nt7066_L1_Tile_rc|1
GTTTGGTACCGGACCGTACACGCTGCACTCCAATTTGAAACAGAAACTTACGGCCTAAGG
>HPV23_Beta_1020186_nt7086_L1_Tile_rc|1
CACTTTTCGAGTTGCAGGCCGTTTGGTACCGGACCGTACACGCTGCACTCCAATTTGAAA
>HPV23_Beta_1020186_nt7096_L1_Tile_rc|1
CAGTTTTGGTCACTTTTCGAGTTGCAGGCCGTTTGGTACCGGACCGTACACGCTGCACTC
>HPV23_Beta_1020186_nt7106_L1_Tile_rc|1
TTCCTTTTGACAGTTTTGGTCACTTTTCGAGTTGCAGGCCGTTTGGTACCGGACCGTACA
>HPV23_Beta_1020186_nt7111_L1_Tile_rc|1
CTTTTTCCTTTTGACAGTTTTGGTCACTTTTCGAGTTGCAGGCCGTTTGGTACCGGACC
>HPV23_Beta_1020186_nt7116_L1_Tile_rc|1
TTGCACTTTTTTCCTTTTGACAGTTTTGGTCACTTTTCGAGTTGCAGGCCGTTTGGTACC
>HPV23_Beta_1020186_nt7122_L1_Tile_rc|2
TTACAATTGCACTTTTTTCCTTTTGACAGTTTTGGTCACTTTTCGAGTTGCAGGCCGTTT
>HPV24_Unclassified_9628486_nt6079_L1_Tile_rc|1
GACGATGCTTGTGTCCTATATGATACAGGGTTTTCTGTGTCATTCACTTTGTTAAATAAT
>HPV24_Unclassified_9628486_nt6089_L1_Tile_rc|1
ATCATCTGTGGACGATGCTTGTGTCCTATATGATACAGGGTTTTCTGTGTCATTCACTTT
>HPV24_Unclassified_9628486_nt6094_L1_Tile_rc|1
TGTCTATCATCTGTGGACGATGCTTGTGTCCTATATGATACAGGGTTTTCTGTGTCATTC
>HPV24_Unclassified_9628486_nt6104_L1_Tile_rc|1
TGAGGTATTTTGTCTATCATCTGTGGACGATGCTTGTGTCCTATATGATACAGGGTTTTC
>HPV24_Unclassified_9628486_nt6204_L1_Tile_rc|1
CATCATTATTATCACCAGCACACCTCTCAGCTACTTCCCAATGTTCTCCTATGCAGGGTG
>HPV24_Unclassified_9628486_nt6209_L1_Tile_rc|1
ACCAGCATCATTATTATCACCAGCACACCTCTCAGCTACTTCCCAATGTTCTCCTATGCA
>HPV24_Unclassified_9628486_nt6214_L1_Tile_rc|1
CATCTACCAGCATCATTATTATCACCAGCACACCTCTCAGCTACTTCCCAATGTTCTCCT
>HPV24_Unclassified_9628486_nt6234_L1_Tile_rc|1
TTACTAACTTAATAGGTGGACATCTACCAGCATCATTATTATCACCAGCACACCTCTCAG
>HPV24_Unclassified_9628486_nt6244_L1_Tile_rc|1
ATTACTGAATTTACTAACTTAATAGGTGGACATCTACCAGCATCATTATTATCACCAGCA
>HPV24_Unclassified_9628486_nt6249_L1_Tile_rc|1
CTTGAATTACTGAATTTACTAACTTAATAGGTGGACATCTACCAGCATCATTATTATCAC
>HPV24_Unclassified_9628486_nt6254_L1_Tile_rc|1

ACCATCTTGAATTACTGAATTTACTAACTTAATAGGTGGACATCTACCAGCATCATTATT
>HPV24_Unclassified_9628486_nt6324_L1_Tile_rc|1
AACTTACGTCTGATCTACTTTGTTGTAGTGTTCTAAAATTTAAATTCCCATAACCAATAT
>HPV24_Unclassified_9628486_nt6329_L1_Tile_rc|1
ATCCAAACTTACGTCTGATCTACTTTGTTGTAGTGTTCTAAAATTTAAATTCCCATAACC
>HPV24_Unclassified_9628486_nt6334_L1_Tile_rc|1
ACAATATCCAAACTTACGTCTGATCTACTTTGTTGTAGTGTTCTAAAATTTAAATTCCCA
>HPV24_Unclassified_9628486_nt6339_L1_Tile_rc|1
CATTTACAATATCCAAACTTACGTCTGATCTACTTTGTTGTAGTGTTCTAAAATTTAAAT
>HPV24_Unclassified_9628486_nt6344_L1_Tile_rc|1
GGTTTCATTTACAATATCCAAACTTACGTCTGATCTACTTTGTTGTAGTGTTCTAAAATT
>HPV24_Unclassified_9628486_nt6514_L1_Tile_rc|1
AAGTCATTTTTGTAGGTCCCTGCATCAATTTGCTCACCAGGTATGTCATCCCCTGGTTTA
>HPV24_Unclassified_9628486_nt6739_L1_Tile_rc|1
GTGTATACACTTATACTAAAATTGGTGTTCCTGGTGTTATCTACTACCGTAATGAACAAT
>HPV24_Unclassified_9628486_nt6774_L1_Tile_rc|1
CATACTCGTTAATATCTGTTACTTTGCCATTTTCAGTGTATACACTTATACTAAAATTGG
>HPV24_Unclassified_9628486_nt6779_L1_Tile_rc|1
AGCATCATACTCGTTAATATCTGTTACTTTGCCATTTTCAGTGTATACACTTATACTAAA
>HPV24_Unclassified_9628486_nt6784_L1_Tile_rc|1
TTATTAGCATCATACTCGTTAATATCTGTTACTTTGCCATTTTCAGTGTATACACTTATA
>HPV24_Unclassified_9628486_nt6794_L1_Tile_rc|1
TTCCCTAAATTTATTAGCATCATACTCGTTAATATCTGTTACTTTGCCATTTTCAGTGTA
>HPV24_Unclassified_9628486_nt6799_L1_Tile_rc|1
TGATATTCCCTAAATTTATTAGCATCATACTCGTTAATATCTGTTACTTTGCCATTTTCA
>HPV24_Unclassified_9628486_nt6804_L1_Tile_rc|1
GTCTCTGATATTCCCTAAATTTATTAGCATCATACTCGTTAATATCTGTTACTTTGCCAT
>HPV24_Unclassified_9628486_nt6809_L1_Tile_rc|1
TACATGTCTCTGATATTCCCTAAATTTATTAGCATCATACTCGTTAATATCTGTTACTTT
>HPV24_Unclassified_9628486_nt6839_L1_Tile_rc|1
CTGCAGTATAAGTGAAATTTCATACTCTTCTACATGTCTCTGATATTCCCTAAATTTATT
>HPV24_Unclassified_9628486_nt6844_L1_Tile_rc|1
CATAGCTGCAGTATAAGTGAAATTTCATACTCTTCTACATGTCTCTGATATTCCCTAAAT
>HPV24_Unclassified_9628486_nt6854_L1_Tile_rc|1
AGGGATTTTACATAGCTGCAGTATAAGTGAAATTTCATACTCTTCTACATGTCTCTGATA
>HPV24_Unclassified_9628486_nt6864_L1_Tile_rc|1
CTGCTTTTAAAGGGATTTTACATAGCTGCAGTATAAGTGAAATTTCATACTCTTCTACAT
>HPV24_Unclassified_9628486_nt6869_L1_Tile_rc|1
CACATCTGCTTTTAAAGGGATTTTACATAGCTGCAGTATAAGTGAAATTTCATACTCTTC
>HPV24_Unclassified_9628486_nt6884_L1_Tile_rc|1
ATTGATCTGTGCTAACACATCTGCTTTTAAAGGGATTTTACATAGCTGCAGTATAAGTGA
>HPV24_Unclassified_9628486_nt6904_L1_Tile_rc|1
AATAGAGATGGATTCATAGCATTGATCTGTGCTAACACATCTGCTTTTAAAGGGATTTTA
>HPV24_Unclassified_9628486_nt6989_L1_Tile_rc|1
AGGTGTTGCCAAACTTTCTATATATCTGTAGGTACTTTGAAGAGGATTGTCTGGTGCAGG
>HPV24_Unclassified_9628486_nt6994_L1_Tile_rc|1
GGACAAGGTGTTGCCAAACTTTCTATATATCTGTAGGTACTTTGAAGAGGATTGTCTGGT
>HPV24_Unclassified_9628486_nt7119_L1_Tile_rc|1
GAAACAGGAACTTTCGTCCTAAGGAATATTGATCCAGTTCCAAAGATAAGCGTTCAGATA
>HPV24_Unclassified_9628486_nt7149_L1_Tile_rc|1
TTTTAGATGTTTTTTGTACAAGACCCGCTTGAAACAGGAACTTTCGTCCTAAGGAATATT
>HPV24_Unclassified_9628486_nt7154_L1_Tile_rc|1
TGTTTTTTTAGATGTTTTTTGTACAAGACCCGCTTGAAACAGGAACTTTCGTCCTAAGGA
>HPV24_Unclassified_9628486_nt7159_L1_Tile_rc|1
TTAGATGTTTTTTTAGATGTTTTTTGTACAAGACCCGCTTGAAACAGGAACTTTCGTCCT

>HPV24_Unclassified_9628486_nt7164_L1_Tile_rc|1
ATACATTAGATGTTTTTTAGATGTTTTTTGTACAAGACCCGCTTGAAACAGGAACTTTC
>HPV24_Unclassified_9628486_nt7169_L1_Tile_rc|1
CTTGGATACATTAGATGTTTTTTAGATGTTTTTTGTACAAGACCCGCTTGAAACAGGAA
>HPV24_Unclassified_9628486_nt7174_L1_Tile_rc|1
GTCCCCTTGGATACATTAGATGTTTTTTAGATGTTTTTTGTACAAGACCCGCTTGAAAC
>HPV24_Unclassified_9628486_nt7179_L1_Tile_rc|1
GTTTGGTCCCCTTGGATACATTAGATGTTTTTTAGATGTTTTTTGTACAAGACCCGCTT
>HPV24_Unclassified_9628486_nt7184_L1_Tile_rc|1
TTTTCGTTTGGTCCCCTTGGATACATTAGATGTTTTTTAGATGTTTTTTGTACAAGACC
>HPV24_Unclassified_9628486_nt7193_L1_Tile_rc|1
TTACGTGCGTTTTCGTTTGGTCCCCTTGGATACATTAGATGTTTTTTAGATGTTTTTTG
>HPV25_Beta_396948_nt6030_L1_Tile_rc|1
GACACTTTTGGAATTTGCAATTTAGAGCCTTGGACGTTGTACACATTAAAATATGGGTGA
>HPV25_Beta_396948_nt6035_L1_Tile_rc|1
TTCCTGACACTTTTGGAATTTGCAATTTAGAGCCTTGGACGTTGTACACATTAAAATATG
>HPV25_Beta_396948_nt6040_L1_Tile_rc|1
TTGATTTCCTGACACTTTTGGAATTTGCAATTTAGAGCCTTGGACGTTGTACACATTAAA
>HPV25_Beta_396948_nt6245_L1_Tile_rc|1
TAGCTTTATAAGAATTTGGATTTTCTGTGTCGCCAACCTTGTTAAACAATGGGTGACCCA
>HPV25_Beta_396948_nt6250_L1_Tile_rc|1
AGAACTAGCTTTATAAGAATTTGGATTTTCTGTGTCGCCAACCTTGTTAAACAATGGGTG
>HPV25_Beta_396948_nt6255_L1_Tile_rc|1
TCTGTAGAACTAGCTTTATAAGAATTTGGATTTTCTGTGTCGCCAACCTTGTTAAACAAT
>HPV25_Beta_396948_nt6275_L1_Tile_rc|1
ATGATACATTTTGCCTGTCATCTGTAGAACTAGCTTTATAAGAATTTGGATTTTCTGTGT
>HPV25_Beta_396948_nt6280_L1_Tile_rc|1
GTCAAATGATACATTTTGCCTGTCATCTGTAGAACTAGCTTTATAAGAATTTGGATTTTC
>HPV25_Beta_396948_nt6415_L1_Tile_rc|1
AATGACAGAATTAATTAATTCTATTGGAGGGCATGACCCTTGTTGAATATTGCCATCATC
>HPV25_Beta_396948_nt6735_L1_Tile_rc|1
GAGTTACCTAGATTATTATATTGAGCCTGACTACTGTTAGGTGGTATGTAAAATGCATTT
>HPV25_Beta_396948_nt6740_L1_Tile_rc|1
ACATTGAGTTACCTAGATTATTATATTGAGCCTGACTACTGTTAGGTGGTATGTAAAATG
>HPV25_Beta_396948_nt6975_L1_Tile_rc|1
CTCAAATATTCTGTAAATTTTTGAGAATTATAATCAGTGATTTTGGAAACATCTGTTCCA
>HPV25_Beta_396948_nt6980_L1_Tile_rc|1
CATGTCTCAAATATTCTGTAAATTTTTGAGAATTATAATCAGTGATTTTGGAAACATCTG
>HPV25_Beta_396948_nt7030_L1_Tile_rc|1
CAACGGTACTTTGCAAAGTTGTAATATTAATGATAACTCATATTCTTCTACATGTCTCAA
>HPV25_Beta_396948_nt7035_L1_Tile_rc|1
GCCTTCAACGGTACTTTGCAAAGTTGTAATATTAATGATAACTCATATTCTTCTACATGT
>HPV25_Beta_396948_nt7040_L1_Tile_rc|1
TTTCTGCCTTCAACGGTACTTTGCAAAGTTGTAATATTAATGATAACTCATATTCTTCTA
>HPV25_Beta_396948_nt7045_L1_Tile_rc|1
CAATATTTCTGCCTTCAACGGTACTTTGCAAAGTTGTAATATTAATGATAACTCATATTC
>HPV25_Beta_396948_nt7050_L1_Tile_rc|1
TGAGCCAATATTTCTGCCTTCAACGGTACTTTGCAAAGTTGTAATATTAATGATAACTCA
>HPV25_Beta_396948_nt7065_L1_Tile_rc|1
TTCATTGCATTAATCTGAGCCAATATTTCTGCCTTCAACGGTACTTTGCAAAGTTGTAAT
>HPV25_Beta_396948_nt7070_L1_Tile_rc|1
TGGAATTCATTGCATTAATCTGAGCCAATATTTCTGCCTTCAACGGTACTTTGCAAAGTT
>HPV25_Beta_396948_nt7075_L1_Tile_rc|1
AATGTTGGAATTCATTGCATTAATCTGAGCCAATATTTCTGCCTTCAACGGTACTTTGCA
>HPV25_Beta_396948_nt7080_L1_Tile_rc|1

TCTAAAATGTTGGAATTCATTGCATTAATCTGAGCCAATATTTCTGCCTTCAACGGTACT
>HPV25_Beta_396948_nt7165_L1_Tile_rc|1
ACAACGTGTGGCTAAAGAATCAATGTAGCGATAAGTATCCTGAATAGAATTGTCCGGTGC
>HPV25_Beta_396948_nt7170_L1_Tile_rc|1
TCTGGACAACGTGTGGCTAAAGAATCAATGTAGCGATAAGTATCCTGAATAGAATTGTCC
>HPV25_Beta_396948_nt7175_L1_Tile_rc|1
TTTTATCTGGACAACGTGTGGCTAAAGAATCAATGTAGCGATAAGTATCCTGAATAGAAT
>HPV25_Beta_396948_nt7180_L1_Tile_rc|1
AGGATTTTTATCTGGACAACGTGTGGCTAAAGAATCAATGTAGCGATAAGTATCCTGAAT
>HPV25_Beta_396948_nt7384_L1_Tile_rc|1
CTAATTTTTACGTTTTCTTTTTATTCCCCTAGTAGATATTCGGGAGGAAACTGTTTTTGT
>HPV26_Alpha_9627305_nt5944_L1_Tile_rc|1
CTGCATTTACAGTAGCCAAATGAGAGTTTTCGGTATCATCCAACTTATTAAACAAAGGAT
>HPV26_Alpha_9627305_nt5954_L1_Tile_rc|1
TTGTCAGTGTCTGCATTTACAGTAGCCAAATGAGAGTTTTCGGTATCATCCAACTTATTA
>HPV26_Alpha_9627305_nt5959_L1_Tile_rc|1
CCCTGTTGTCAGTGTCTGCATTTACAGTAGCCAAATGAGAGTTTTCGGTATCATCCAACT
>HPV26_Alpha_9627305_nt5964_L1_Tile_rc|1
ATTGTCCCTGTTGTCAGTGTCTGCATTTACAGTAGCCAAATGAGAGTTTTCGGTATCATC
>HPV26_Alpha_9627305_nt5969_L1_Tile_rc|1
GAAACATTGTCCCTGTTGTCAGTGTCTGCATTTACAGTAGCCAAATGAGAGTTTTCGGTA
>HPV26_Alpha_9627305_nt5974_L1_Tile_rc|1
CAACTGAAACATTGTCCCTGTTGTCAGTGTCTGCATTTACAGTAGCCAAATGAGAGTTTT
>HPV26_Alpha_9627305_nt5979_L1_Tile_rc|1
ATTATCAACTGAAACATTGTCCCTGTTGTCAGTGTCTGCATTTACAGTAGCCAAATGAGA
>HPV26_Alpha_9627305_nt6059_L1_Tile_rc|1
GTATTTTTACATATAGTGCCAATACCCCAGTGCTCTCCCAAGGGCGGTGTACAACCTATA
>HPV26_Alpha_9627305_nt6094_L1_Tile_rc|1
ATTCTAAGGGGGGGCAATCCCCACGTTGTGTCTGTGTATTTTTACATATAGTGCCAATAC
>HPV26_Alpha_9627305_nt6099_L1_Tile_rc|1
AATTAATTCTAAGGGGGGGCAATCCCCACGTTGTGTCTGTGTATTTTTACATATAGTGCC
>HPV26_Alpha_9627305_nt6104_L1_Tile_rc|1
CTGGAAATTAATTCTAAGGGGGGGCAATCCCCACGTTGTGTCTGTGTATTTTTACATATA
>HPV26_Alpha_9627305_nt6109_L1_Tile_rc|1
TAATGCTGGAAATTAATTCTAAGGGGGGGCAATCCCCACGTTGTGTCTGTGTATTTTTAC
>HPV26_Alpha_9627305_nt6114_L1_Tile_rc|1
CTCAATAATGCTGGAAATTAATTCTAAGGGGGGGCAATCCCCACGTTGTGTCTGTGTATT
>HPV26_Alpha_9627305_nt6354_L1_Tile_rc|1
ATACAAAGTGGTGGGTATAGCATCCCCAACAGCCCCCGCCTTATTATAAAAATGTCTGGC
>HPV26_Alpha_9627305_nt6359_L1_Tile_rc|1
TTAATATACAAAGTGGTGGGTATAGCATCCCCAACAGCCCCCGCCTTATTATAAAAATGT
>HPV26_Alpha_9627305_nt6364_L1_Tile_rc|1
CACCTTTAATATACAAAGTGGTGGGTATAGCATCCCCAACAGCCCCCGCCTTATTATAAA
>HPV26_Alpha_9627305_nt6369_L1_Tile_rc|1
TTCAGCACCTTTAATATACAAAGTGGTGGGTATAGCATCCCCAACAGCCCCCGCCTTATT
>HPV26_Alpha_9627305_nt6379_L1_Tile_rc|1
CCCTGCCTGATTCAGCACCTTTAATATACAAAGTGGTGGGTATAGCATCCCCAACAGCCC
>HPV26_Alpha_9627305_nt6389_L1_Tile_rc|1
GTAGGGGGCTCCCTGCCTGATTCAGCACCTTTAATATACAAAGTGGTGGGTATAGCATCC
>HPV26_Alpha_9627305_nt6399_L1_Tile_rc|1
AATAGAAGATGTAGGGGGCTCCCTGCCTGATTCAGCACCTTTAATATACAAAGTGGTGGG
>HPV26_Alpha_9627305_nt6404_L1_Tile_rc|1
GAATAAATAGAAGATGTAGGGGGCTCCCTGCCTGATTCAGCACCTTTAATATACAAAGTG
>HPV26_Alpha_9627305_nt6409_L1_Tile_rc|1
TAGCAGAATAAATAGAAGATGTAGGGGGCTCCCTGCCTGATTCAGCACCTTTAATATACA

>HPV26_Alpha_9627305_nt6414_L1_Tile_rc|1
AGGTGTAGCAGAATAAATAGAAGATGTAGGGGGCTCCCTGCCTGATTCAGCACCTTTAAT
>HPV26_Alpha_9627305_nt6719_L1_Tile_rc|1
GCATTCATTAAATGTATGTAAGCCATAACATCTGTTGTAAGTGTTATTTTACACAACTGA
>HPV26_Alpha_9627305_nt6724_L1_Tile_rc|1
TGGAGGCATTCATTAAATGTATGTAAGCCATAACATCTGTTGTAAGTGTTATTTTACACA
>HPV26_Alpha_9627305_nt6729_L1_Tile_rc|1
CAATATGGAGGCATTCATTAAATGTATGTAAGCCATAACATCTGTTGTAAGTGTTATTTT
>HPV26_Alpha_9627305_nt6739_L1_Tile_rc|1
TCCAATCCTCCAATATGGAGGCATTCATTAAATGTATGTAAGCCATAACATCTGTTGTAA
>HPV26_Alpha_9627305_nt6759_L1_Tile_rc|1
AGGTAAGGTTAGTCCAAAATTCCAATCCTCCAATATGGAGGCATTCATTAAATGTATGTA
>HPV26_Alpha_9627305_nt6974_L1_Tile_rc|1
CCTAGTTTCGGCCGCCGTTGTATGCCGGCCTGTAACATAAACTTACGCCCTAGTGGAAAT
>HPV26_Alpha_9627305_nt6979_L1_Tile_rc|1
TGGTGCCTAGTTTCGGCCGCCGTTGTATGCCGGCCTGTAACATAAACTTACGCCCTAGTG
>HPV26_Alpha_9627305_nt6984_L1_Tile_rc|1
ACGTTTGGTGCCTAGTTTCGGCCGCCGTTGTATGCCGGCCTGTAACATAAACTTACGCCC
>HPV26_Alpha_9627305_nt6989_L1_Tile_rc|1
AAGGGACGTTTGGTGCCTAGTTTCGGCCGCCGTTGTATGCCGGCCTGTAACATAAACTTA
>HPV26_Alpha_9627305_nt6994_L1_Tile_rc|1
AAGATAAGGGACGTTTGGTGCCTAGTTTCGGCCGCCGTTGTATGCCGGCCTGTAACATAA
>HPV27_Alpha_396964_nt5458_L1_Tile_rc|1
GGTATTAGCCAGGGAGCCGCGAGGGGCTGGGTAGTAATCGTCCAATGGCTGCAGGACATC
>HPV27_Alpha_396964_nt5468_L1_Tile_rc|1
CAGATACCGTGGTATTAGCCAGGGAGCCGCGAGGGGCTGGGTAGTAATCGTCCAATGGCT
>HPV27_Alpha_396964_nt5483_L1_Tile_rc|1
TAGATGCAGAGGAGGCAGATACCGTGGTATTAGCCAGGGAGCCGCGAGGGGCTGGGTAGT
>HPV27_Alpha_396964_nt5513_L1_Tile_rc|1
AGAGAGGGGCTGTAGTGGACCCTCGCAGTGTAGATGCAGAGGAGGCAGATACCGTGGTAT
>HPV27_Alpha_396964_nt5903_L1_Tile_rc|1
GGACACCTTAGGCACTGCCAACCTATTATTGCTACCCTTCTTTATAGAATAATATGGGTG
>HPV27_Alpha_396964_nt5918_L1_Tile_rc|1
ACGGTATTGGTAGCCGGACACCTTAGGCACTGCCAACCTATTATTGCTACCCTTCTTTAT
>HPV27_Alpha_396964_nt5923_L1_Tile_rc|1
AATACACGGTATTGGTAGCCGGACACCTTAGGCACTGCCAACCTATTATTGCTACCCTTC
>HPV27_Alpha_396964_nt6763_L1_Tile_rc|1
GCTTTATAATTAGTATTATCAGACACCTCAGCTGCACACAGAGAGACATTGGTACTCCGT
>HPV27_Alpha_396964_nt6773_L1_Tile_rc|1
AAAATTCGTAGCTTTATAATTAGTATTATCAGACACCTCAGCTGCACACAGAGAGACATT
>HPV28_Alpha_1020202_nt5881_L1_Tile_rc|1
GCGGACACTTTGGGCACATCTGCTTTGTTAGTGGATGATTTAGGAATGGGAAAATAAGGA
>HPV28_Alpha_1020202_nt5886_L1_Tile_rc|1
GAAAGGCGGACACTTTGGGCACATCTGCTTTGTTAGTGGATGATTTAGGAATGGGAAAAT
>HPV28_Alpha_1020202_nt5891_L1_Tile_rc|1
ATACTGAAAGGCGGACACTTTGGGCACATCTGCTTTGTTAGTGGATGATTTAGGAATGGG
>HPV28_Alpha_1020202_nt5901_L1_Tile_rc|1
GGAATACCCTATACTGAAAGGCGGACACTTTGGGCACATCTGCTTTGTTAGTGGATGATT
>HPV28_Alpha_1020202_nt6491_L1_Tile_rc|1
AATGGTGTCCCCAGCAACACCAGCTCTATTAAAAAAATGCCGAGCGAAAAGTTGTTCCTT
>HPV28_Alpha_1020202_nt6521_L1_Tile_rc|1
GCCCTGACCTTTAATATACAATGTTTCAGGAATGGTGTCCCCAGCAACACCAGCTCTATT
>HPV28_Alpha_1020202_nt6531_L1_Tile_rc|1
CCCTCCCGTTGCCCTGACCTTTAATATACAATGTTTCAGGAATGGTGTCCCCAGCAACAC
>HPV28_Alpha_1020202_nt6536_L1_Tile_rc|1

CACATCCCTCCCGTTGCCCTGACCTTTAATATACAATGTTTCAGGAATGGTGTCCCCAGC
>HPV28_Alpha_1020202_nt6541_L1_Tile_rc|1
CCAATCACATCCCTCCCGTTGCCCTGACCTTTAATATACAATGTTTCAGGAATGGTGTCC
>HPV28_Alpha_1020202_nt6546_L1_Tile_rc|1
CACTACCAATCACATCCCTCCCGTTGCCCTGACCTTTAATATACAATGTTTCAGGAATGG
>HPV28_Alpha_1020202_nt6746_L1_Tile_rc|1
ATCGTACGTAGCTGAAGAGTCAGTAGAAACACACAACGTCATGTTTGTACTGCGTGTAGT
>HPV28_Alpha_1020202_nt6771_L1_Tile_rc|1
TTAAGTATTCCTTAAATTTACTAGCATCGTACGTAGCTGAAGAGTCAGTAGAAACACACA
>HPV28_Alpha_1020202_nt6776_L1_Tile_rc|1
GTGCCTTAAGTATTCCTTAAATTTACTAGCATCGTACGTAGCTGAAGAGTCAGTAGAAAC
>HPV28_Alpha_1020202_nt6791_L1_Tile_rc|1
ATCGTACTCCTCCCCGTGCCTTAAGTATTCCTTAAATTTACTAGCATCGTACGTAGCTGA
>HPV28_Alpha_1020202_nt6796_L1_Tile_rc|1
TGCAAATCGTACTCCTCCCCGTGCCTTAAGTATTCCTTAAATTTACTAGCATCGTACGTA
>HPV28_Alpha_1020202_nt7086_L1_Tile_rc|1
TTAAAAATTTCCTTCCCAGAGGGAATTGCGATAGATCAAGAGAAAAGCGATCCTTAAGAT
>HPV28_Alpha_1020202_nt7106_L1_Tile_rc|1
GGCACCTACACCCAACTGCATTAAAAATTTCCTTCCCAGAGGGAATTGCGATAGATCAAG
>HPV28_Alpha_1020202_nt7156_L1_Tile_rc|1
GATCCTCTCGTGGTGGACGCTGGACGTTTGCGAACGGAGACACTGGAGCGGGCACCTACA
>HPV28_Alpha_1020202_nt7186_L1_Tile_rc|1
TTTTTGGCGCGTTTTCGTTTTGCAGCAGACGATCCTCTCGTGGTGGACGCTGGACGTTTG
>HPV29_Alpha_1020210_nt6329_L1_Tile_rc|1
GTCATTAACTCCAGGGGGGGGCAATCACCAGCAGCGGAACTAGTGCGTGCACACACAGTG
>HPV29_Alpha_1020210_nt6334_L1_Tile_rc|1
GTGTGGTCATTAACTCCAGGGGGGGGCAATCACCAGCAGCGGAACTAGTGCGTGCACACA
>HPV29_Alpha_1020210_nt6339_L1_Tile_rc|1
AATATGTGTGGTCATTAACTCCAGGGGGGGGCAATCACCAGCAGCGGAACTAGTGCGTGC
>HPV29_Alpha_1020210_nt6579_L1_Tile_rc|1
CAAGGAATCTGGGATTTTGTCCCCTACTACACCAGCACGATTAAAGAAGTGCCTGGCAAA
>HPV29_Alpha_1020210_nt6584_L1_Tile_rc|1
AAGTACAAGGAATCTGGGATTTTGTCCCCTACTACACCAGCACGATTAAAGAAGTGCCTG
>HPV29_Alpha_1020210_nt6594_L1_Tile_rc|1
GTTACCCTTTAAGTACAAGGAATCTGGGATTTTGTCCCCTACTACACCAGCACGATTAAA
>HPV29_Alpha_1020210_nt6599_L1_Tile_rc|1
CCGTTGTTACCCTTTAAGTACAAGGAATCTGGGATTTTGTCCCCTACTACACCAGCACGA
>HPV29_Alpha_1020210_nt6604_L1_Tile_rc|1
CTCGCCCGTTGTTACCCTTTAAGTACAAGGAATCTGGGATTTTGTCCCCTACTACACCAG
>HPV29_Alpha_1020210_nt6609_L1_Tile_rc|1
AGTTTCTCGCCCGTTGTTACCCTTTAAGTACAAGGAATCTGGGATTTTGTCCCCTACTAC
>HPV29_Alpha_1020210_nt6614_L1_Tile_rc|1
CCAGGAGTTTCTCGCCCGTTGTTACCCTTTAAGTACAAGGAATCTGGGATTTTGTCCCCT
>HPV29_Alpha_1020210_nt6624_L1_Tile_rc|1
TATGGCACTGCCAGGAGTTTCTCGCCCGTTGTTACCCTTTAAGTACAAGGAATCTGGGAT
>HPV29_Alpha_1020210_nt6629_L1_Tile_rc|1
CTGTATATGGCACTGCCAGGAGTTTCTCGCCCGTTGTTACCCTTTAAGTACAAGGAATCT
>HPV29_Alpha_1020210_nt6829_L1_Tile_rc|1
TAGCATCATAAGTGGTCAACGGTTGAGACTCTGTGGTAGCACACAACGACATATTGGTGC
>HPV29_Alpha_1020210_nt6834_L1_Tile_rc|1
CTTGGTAGCATCATAAGTGGTCAACGGTTGAGACTCTGTGGTAGCACACAACGACATATT
>HPV29_Alpha_1020210_nt6844_L1_Tile_rc|1
ATTCTTTAATCTTGGTAGCATCATAAGTGGTCAACGGTTGAGACTCTGTGGTAGCACACA
>HPV29_Alpha_1020210_nt6849_L1_Tile_rc|1
CAAATATTCTTTAATCTTGGTAGCATCATAAGTGGTCAACGGTTGAGACTCTGTGGTAGC

>HPV29_Alpha_1020210_nt6854_L1_Tile_rc|1
TGTCTCAAATATTCTTTAATCTTGGTAGCATCATAAGTGGTCAACGGTTGAGACTCTGTG
>HPV29_Alpha_1020210_nt6859_L1_Tile_rc|1
CCCCATGTCTCAAATATTCTTTAATCTTGGTAGCATCATAAGTGGTCAACGGTTGAGACT
>HPV29_Alpha_1020210_nt6959_L1_Tile_rc|1
TCTTCAAGTAAGGCACTGTTCATAGTATGAAGGTAAGCCATAATTTCAGGTGTCAATGTA
>HPV29_Alpha_1020210_nt6984_L1_Tile_rc|1
TGGCAATGTCAATCCAAAATTCCAGTCTTCAAGTAAGGCACTGTTCATAGTATGAAGGTA
>HPV29_Alpha_1020210_nt7224_L1_Tile_rc|1
GGTGGTCGTTCGGCGCTTTCTGGAGGGGACTACTGAACGCCGGCGCGCACCGATCTGTAA
>HPV29_Alpha_1020210_nt7229_L1_Tile_rc|1
GCCGTGGTGGTCGTTCGGCGCTTTCTGGAGGGGACTACTGAACGCCGGCGCGCACCGATC
>HPV29_Alpha_1020210_nt7249_L1_Tile_rc|1
TTCGCTTTGCAGGGGTGGGGGCCGTGGTGGTCGTTCGGCGCTTTCTGGAGGGGACTACTG
>HPV29_Alpha_1020210_nt7254_L1_Tile_rc|1
GCGTTTTCGCTTTGCAGGGGTGGGGGCCGTGGTGGTCGTTCGGCGCTTTCTGGAGGGGAC
>HPV29_Alpha_1020210_nt7259_L1_Tile_rc|1
TTCGAGCGTTTTCGCTTTGCAGGGGTGGGGGCCGTGGTGGTCGTTCGGCGCTTTCTGGAG
>HPV30_Alpha_396973_nt5772_L1_Tile_rc|1
GTTTTGGAATTACCAGCCTTAGAAATAGAATAATATGGATGTCCAACAGCAAGCAAACGT
>HPV30_Alpha_396973_nt5777_L1_Tile_rc|1
CATCTGTTTTGGAATTACCAGCCTTAGAAATAGAATAATATGGATGTCCAACAGCAAGCA
>HPV30_Alpha_396973_nt6032_L1_Tile_rc|1
TGTTGTCCCTACTATCCTCTGCTGTATCCTGATTAGCTATAGTGGAACTTTCAGTGTCAT
>HPV30_Alpha_396973_nt6037_L1_Tile_rc|1
AGAAATGTTGTCCCTACTATCCTCTGCTGTATCCTGATTAGCTATAGTGGAACTTTCAGT
>HPV30_Alpha_396973_nt6042_L1_Tile_rc|2
TCAACAGAAATGTTGTCCCTACTATCCTCTGCTGTATCCTGATTAGCTATAGTGGAACTT
>HPV30_Alpha_396973_nt6047_L1_Tile_rc|1
TTGGATCAACAGAAATGTTGTCCCTACTATCCTCTGCTGTATCCTGATTAGCTATAGTGG
>HPV30_Alpha_396973_nt6087_L1_Tile_rc|1
ATAGCAGGAGTGCACCCAATAATACACAATTGGGTTTGCTTTGGATCAACAGAAATGTTG
>HPV30_Alpha_396973_nt6092_L1_Tile_rc|1
CTCCTATAGCAGGAGTGCACCCAATAATACACAATTGGGTTTGCTTTGGATCAACAGAAA
>HPV30_Alpha_396973_nt6097_L1_Tile_rc|1
ATGTTCTCCTATAGCAGGAGTGCACCCAATAATACACAATTGGGTTTGCTTTGGATCAAC
>HPV30_Alpha_396973_nt6412_L1_Tile_rc|1
GCTAGGTAATTGTTCACCAATAGCACCTGCCCTATTAAAGTAGTGCCTAGCAAATAACTG
>HPV30_Alpha_396973_nt6447_L1_Tile_rc|1
GGATCCCTGTTATTTGTACCTTTTATATATAATGTGCTAGGTAATTGTTCACCAATAGCA
>HPV30_Alpha_396973_nt6457_L1_Tile_rc|1
GCTTGGCGGGGGATCCCTGTTATTTGTACCTTTTATATATAATGTGCTAGGTAATTGTTC
>HPV30_Alpha_396973_nt6462_L1_Tile_rc|2
ACTGAGCTTGGCGGGGGATCCCTGTTATTTGTACCTTTTATATATAATGTGCTAGGTAAT
>HPV30_Alpha_396973_nt6467_L1_Tile_rc|1
CATATACTGAGCTTGGCGGGGGATCCCTGTTATTTGTACCTTTTATATATAATGTGCTAG
>HPV30_Alpha_396973_nt6472_L1_Tile_rc|1
AGCAACATATACTGAGCTTGGCGGGGGATCCCTGTTATTTGTACCTTTTATATATAATGT
>HPV30_Alpha_396973_nt6482_L1_Tile_rc|2
CACTAGGAGTAGCAACATATACTGAGCTTGGCGGGGGATCCCTGTTATTTGTACCTTTTA
>HPV30_Alpha_396973_nt6822_L1_Tile_rc|2
GCAGCTGGGGGTGACAATCCAATATTCCAGCCCTCAAGTAAAGTGGAGTTCATAGTATGT
>HPV30_Alpha_396973_nt6832_L1_Tile_rc|1
TAAGCTTGTGGCAGCTGGGGGTGACAATCCAATATTCCAGCCCTCAAGTAAAGTGGAGTT
>HPV30_Alpha_396973_nt7022_L1_Tile_rc|2

GTTTAGTACGAACCCCAAGTTGCATTAAAAACTTTCTGCCCAGTGGGAATTGGTCAAGGT
>HPV30_Alpha_396973_nt7032_L1_Tile_rc|1
GTAGTAGAAGGTTTAGTACGAACCCCAAGTTGCATTAAAAACTTTCTGCCCAGTGGGAAT
>HPV30_Alpha_396973_nt7077_L1_Tile_rc|1
GATGGTGTAGAGGTAGAGGAACTGGGGGCCGAGCGTTTTTAGTAGTAGTAGAAGGTTTA
>HPV30_Alpha_396973_nt7092_L1_Tile_rc|1
CGCTTGCGTTTGGCTGATGGTGTAGAGGTAGAGGAACTGGGGGCCGAGCGTTTTTAGTA
>HPV31_Alpha_333048_nt5633_L1_Tile_rc|1
TGGCCTACTGTAAGCAGCCTAGCACTGCCTGCGTGATAATATATGTTGGTTCGTGTTACA
>HPV31_Alpha_333048_nt5668_L1_Tile_rc|1
TAGGATTGTCAGATTTAGGTATGGAATAATATGGATGGCCTACTGTAAGCAGCCTAGCAC
>HPV31_Alpha_333048_nt5673_L1_Tile_rc|1
TTTTTTAGGATTGTCAGATTTAGGTATGGAATAATATGGATGGCCTACTGTAAGCAGCCT
>HPV31_Alpha_333048_nt5678_L1_Tile_rc|1
ACTATTTTTTTAGGATTGTCAGATTTAGGTATGGAATAATATGGATGGCCTACTGTAAGC
>HPV31_Alpha_333048_nt5683_L1_Tile_rc|1
GTACAACTATTTTTTTAGGATTGTCAGATTTAGGTATGGAATAATATGGATGGCCTACTG
>HPV31_Alpha_333048_nt5688_L1_Tile_rc|1
CTTTGGTACAACTATTTTTTTAGGATTGTCAGATTTAGGTATGGAATAATATGGATGGCC
>HPV31_Alpha_333048_nt5693_L1_Tile_rc|1
GACACCTTTGGTACAACTATTTTTTTAGGATTGTCAGATTTAGGTATGGAATAATATGGA
>HPV31_Alpha_333048_nt5938_L1_Tile_rc|1
ATATACATTCCCTATTATCAGTGCCAGGACCACCGGCATATCTATTAGAGTTTTCAGTGT
>HPV31_Alpha_333048_nt5943_L1_Tile_rc|1
CATTGATATACATTCCCTATTATCAGTGCCAGGACCACCGGCATATCTATTAGAGTTTTC
>HPV31_Alpha_333048_nt6028_L1_Tile_rc|1
TGTTACTACAAGGACTACCTTTACCCCAATGCTCTCCAATAGGTGGTTTGCAACCAAGTA
>HPV31_Alpha_333048_nt6033_L1_Tile_rc|1
AGCATTGTTACTACAAGGACTACCTTTACCCCAATGCTCTCCAATAGGTGGTTTGCAACC
>HPV31_Alpha_333048_nt6038_L1_Tile_rc|1
GTAATAGCATTGTTACTACAAGGACTACCTTTACCCCAATGCTCTCCAATAGGTGGTTTG
>HPV31_Alpha_333048_nt6043_L1_Tile_rc|1
CAGGGGTAATAGCATTGTTACTACAAGGACTACCTTTACCCCAATGCTCTCCAATAGGTG
>HPV31_Alpha_333048_nt6063_L1_Tile_rc|1
TTCTAATGGAGGACAATCACCAGGGGTAATAGCATTGTTACTACAAGGACTACCTTTACC
>HPV31_Alpha_333048_nt6338_L1_Tile_rc|1
CCGGAGCCTTTAATATATAAGTCAGTAGGGACCGATTCACCAACCGTGCCTGATCTATTA
>HPV31_Alpha_333048_nt6353_L1_Tile_rc|1
AAAGTAGCTGTTGAACCGGAGCCTTTAATATATAAGTCAGTAGGGACCGATTCACCAACC
>HPV31_Alpha_333048_nt6358_L1_Tile_rc|1
TAGCTAAAGTAGCTGTTGAACCGGAGCCTTTAATATATAAGTCAGTAGGGACCGATTCAC
>HPV31_Alpha_333048_nt6368_L1_Tile_rc|1
TATGTACTGTTAGCTAAAGTAGCTGTTGAACCGGAGCCTTTAATATATAAGTCAGTAGGG
>HPV31_Alpha_333048_nt6373_L1_Tile_rc|1
GAAAGTATGTACTGTTAGCTAAAGTAGCTGTTGAACCGGAGCCTTTAATATATAAGTCAG
>HPV31_Alpha_333048_nt6378_L1_Tile_rc|1
TGTAGGAAAGTATGTACTGTTAGCTAAAGTAGCTGTTGAACCGGAGCCTTTAATATATAA
>HPV31_Alpha_333048_nt6383_L1_Tile_rc|1
CTAGGTGTAGGAAAGTATGTACTGTTAGCTAAAGTAGCTGTTGAACCGGAGCCTTTAATA
>HPV31_Alpha_333048_nt6393_L1_Tile_rc|1
CATGGAGCCGCTAGGTGTAGGAAAGTATGTACTGTTAGCTAAAGTAGCTGTTGAACCGGA
>HPV31_Alpha_333048_nt6398_L1_Tile_rc|1
GTAACCATGGAGCCGCTAGGTGTAGGAAAGTATGTACTGTTAGCTAAAGTAGCTGTTGAA
>HPV31_Alpha_333048_nt6403_L1_Tile_rc|1
CTGAAGTAACCATGGAGCCGCTAGGTGTAGGAAAGTATGTACTGTTAGCTAAAGTAGCTG

>HPV31_Alpha_333048_nt6568_L1_Tile_rc|1
TACTTTTAAATGTAGTATCACTGTTTGCAATTGCAGCACAAACAGACATATTGGTACTAC
>HPV31_Alpha_333048_nt6573_L1_Tile_rc|1
ATTACTACTTTTAAATGTAGTATCACTGTTTGCAATTGCAGCACAAACAGACATATTGGT
>HPV31_Alpha_333048_nt6578_L1_Tile_rc|1
TTAAAATTACTACTTTTAAATGTAGTATCACTGTTTGCAATTGCAGCACAAACAGACATA
>HPV31_Alpha_333048_nt6588_L1_Tile_rc|1
TAAATACTCTTTAAAATTACTACTTTTAAATGTAGTATCACTGTTTGCAATTGCAGCACA
>HPV31_Alpha_333048_nt6593_L1_Tile_rc|1
TGTCTTAAATACTCTTTAAAATTACTACTTTTAAATGTAGTATCACTGTTTGCAATTGCA
>HPV31_Alpha_333048_nt6768_L1_Tile_rc|1
CTGTGAGGTGACAAACCTATAGGTATCCTCCAAAGAACCTGAGGGAGGTGTGGTCAATCC
>HPV31_Alpha_333048_nt6828_L1_Tile_rc|1
ATAATCTTTAAATGGATCTTCCTTGGGCTTTTGGGGGGCAGTTTTTTGACATGTAATGGC
>HPV31_Alpha_333048_nt6948_L1_Tile_rc|1
TTTACCTGCTTTAAATTTAGGACGTGCCCTATATCCTGCCTGTAATAAAAATTTGCGACC
>HPV31_Alpha_333048_nt6953_L1_Tile_rc|1
CTACGTTTACCTGCTTTAAATTTAGGACGTGCCCTATATCCTGCCTGTAATAAAAATTTG
>HPV31_Alpha_333048_nt6958_L1_Tile_rc|1
GTGCACTACGTTTACCTGCTTTAAATTTAGGACGTGCCCTATATCCTGCCTGTAATAAAA
>HPV31_Alpha_333048_nt6963_L1_Tile_rc|1
TGAGGGTGCACTACGTTTACCTGCTTTAAATTTAGGACGTGCCCTATATCCTGCCTGTAA
>HPV31_Alpha_333048_nt6973_L1_Tile_rc|1
TGGTAGATGCTGAGGGTGCACTACGTTTACCTGCTTTAAATTTAGGACGTGCCCTATATC
>HPV31_Alpha_333048_nt6983_L1_Tile_rc|1
GCTGGTGTAGTGGTAGATGCTGAGGGTGCACTACGTTTACCTGCTTTAAATTTAGGACGT
>HPV31_Alpha_333048_nt6988_L1_Tile_rc|1
GTTTTGCTGGTGTAGTGGTAGATGCTGAGGGTGCACTACGTTTACCTGCTTTAAATTTAG
>HPV31_Alpha_333048_nt7003_L1_Tile_rc|1
TTTTAGTTTTTTACGTTTTGCTGGTGTAGTGGTAGATGCTGAGGGTGCACTACGTTTAC
>HPV32_Alpha_9627327_nt5988_L1_Tile_rc|1
TTCTATTGGGTGTCTTCTTAATAGTATAATATGGATGCCCAACAGCCAAAAGCCTAGAAC
>HPV32_Alpha_9627327_nt5993_L1_Tile_rc|1
AGATGTTCTATTGGGTGTCTTCTTAATAGTATAATATGGATGCCCAACAGCCAAAAGCCT
>HPV32_Alpha_9627327_nt5998_L1_Tile_rc|1
GGAATAGATGTTCTATTGGGTGTCTTCTTAATAGTATAATATGGATGCCCAACAGCCAAA
>HPV32_Alpha_9627327_nt6003_L1_Tile_rc|1
CCTTTGGAATAGATGTTCTATTGGGTGTCTTCTTAATAGTATAATATGGATGCCCAACAG
>HPV32_Alpha_9627327_nt6008_L1_Tile_rc|1
AGACACCTTTGGAATAGATGTTCTATTGGGTGTCTTCTTAATAGTATAATATGGATGCCC
>HPV32_Alpha_9627327_nt6013_L1_Tile_rc|1
AATCCAGACACCTTTGGAATAGATGTTCTATTGGGTGTCTTCTTAATAGTATAATATGGA
>HPV32_Alpha_9627327_nt6018_L1_Tile_rc|1
ACTGCAATCCAGACACCTTTGGAATAGATGTTCTATTGGGTGTCTTCTTAATAGTATAAT
>HPV32_Alpha_9627327_nt6023_L1_Tile_rc|1
TCTATACTGCAATCCAGACACCTTTGGAATAGATGTTCTATTGGGTGTCTTCTTAATAGT
>HPV32_Alpha_9627327_nt6038_L1_Tile_rc|1
CCTAACCCTAAATACTCTATACTGCAATCCAGACACCTTTGGAATAGATGTTCTATTGGG
>HPV32_Alpha_9627327_nt6263_L1_Tile_rc|1
ACAATCCATAGATACATTTTCTCTATTATCAGTTCCAGGCCCTGCAGCATATCTAGGCCC
>HPV32_Alpha_9627327_nt6338_L1_Tile_rc|1
AGAGCAAGCAGCACCCTTACCCCAATGCTCGCCAATGGCAGGTTTACAACCCACCAAACA
>HPV32_Alpha_9627327_nt6358_L1_Tile_rc|1
CAGTCGCCATTTGATTGTGCAGAGCAAGCAGCACCCTTACCCCAATGCTCGCCAATGGCA
>HPV32_Alpha_9627327_nt6378_L1_Tile_rc|1

TTTGTAATTCCAAAGGTGGGCAGTCGCCATTTGATTGTGCAGAGCAAGCAGCACCCTTAC
>HPV32_Alpha_9627327_nt6453_L1_Tile_rc|1
TTGAAGTTTGCAAAGCACTAAAGTCCATTGCTCCAAACCCTACATCTGCCATATCACCAT
>HPV32_Alpha_9627327_nt6458_L1_Tile_rc|1
AGCTTTTGAAGTTTGCAAAGCACTAAAGTCCATTGCTCCAAACCCTACATCTGCCATATC
>HPV32_Alpha_9627327_nt6483_L1_Tile_rc|1
AGTTCATAATATCTAATGGCACCTCAGCTTTTGAAGTTTGCAAAGCACTAAAGTCCATTG
>HPV32_Alpha_9627327_nt6488_L1_Tile_rc|1
AATGGAGTTCATAATATCTAATGGCACCTCAGCTTTTGAAGTTTGCAAAGCACTAAAGTC
>HPV32_Alpha_9627327_nt6648_L1_Tile_rc|1
CATTAGAAGCTTTTATATACATGTCCTCAGGAACAGGTTCACCAAGGGTTCCTGCCCTAT
>HPV32_Alpha_9627327_nt6653_L1_Tile_rc|1
AGCACCATTAGAAGCTTTTATATACATGTCCTCAGGAACAGGTTCACCAAGGGTTCCTGC
>HPV32_Alpha_9627327_nt6658_L1_Tile_rc|1
CCAGAAGCACCATTAGAAGCTTTTATATACATGTCCTCAGGAACAGGTTCACCAAGGGTT
>HPV32_Alpha_9627327_nt6668_L1_Tile_rc|1
ATTATTTCTGCCAGAAGCACCATTAGAAGCTTTTATATACATGTCCTCAGGAACAGGTTC
>HPV32_Alpha_9627327_nt6673_L1_Tile_rc|1
GCTAAATTATTTCTGCCAGAAGCACCATTAGAAGCTTTTATATACATGTCCTCAGGAACA
>HPV32_Alpha_9627327_nt6678_L1_Tile_rc|1
TACTAGCTAAATTATTTCTGCCAGAAGCACCATTAGAAGCTTTTATATACATGTCCTCAG
>HPV32_Alpha_9627327_nt6683_L1_Tile_rc|1
AATACTACTAGCTAAATTATTTCTGCCAGAAGCACCATTAGAAGCTTTTATATACATGTC
>HPV32_Alpha_9627327_nt6888_L1_Tile_rc|1
AGTTAGTAGACTTGTATGTGTCTTCAGTTGTTACAGTAGCACACACAGTCATGTTAGTAC
>HPV32_Alpha_9627327_nt6893_L1_Tile_rc|1
CTTAAAGTTAGTAGACTTGTATGTGTCTTCAGTTGTTACAGTAGCACACACAGTCATGTT
>HPV32_Alpha_9627327_nt6898_L1_Tile_rc|1
TATTCCTTAAAGTTAGTAGACTTGTATGTGTCTTCAGTTGTTACAGTAGCACACACAGTC
>HPV32_Alpha_9627327_nt6903_L1_Tile_rc|1
GTAGATATTCCTTAAAGTTAGTAGACTTGTATGTGTCTTCAGTTGTTACAGTAGCACACA
>HPV32_Alpha_9627327_nt6908_L1_Tile_rc|1
ATGGCGTAGATATTCCTTAAAGTTAGTAGACTTGTATGTGTCTTCAGTTGTTACAGTAGC
>HPV32_Alpha_9627327_nt7008_L1_Tile_rc|1
CTAGTATGTCAGGATTCATGGTGTGGATATATGACATAACCTCTACAGATAATGTAATTT
>HPV32_Alpha_9627327_nt7038_L1_Tile_rc|1
GCGGTGGAGCTACACCAACATTCCAATCGTCTAGTATGTCAGGATTCATGGTGTGGATAT
>HPV32_Alpha_9627327_nt7113_L1_Tile_rc|1
GTGCTGTTACCTTAGCTTGACATCGTATGGCCTGAGACTGCACAAATCTATAACTATCTT
>HPV32_Alpha_9627327_nt7273_L1_Tile_rc|1
GATGCTGTTCGTTTTACTGCTGTAAGTTTAGGTCTTGCACGTAACCCAGCTTGCAGTAAA
>HPV32_Alpha_9627327_nt7278_L1_Tile_rc|1
TGGAAGATGCTGTTCGTTTTACTGCTGTAAGTTTAGGTCTTGCACGTAACCCAGCTTGCA
>HPV32_Alpha_9627327_nt7283_L1_Tile_rc|1
TTGACTGGAAGATGCTGTTCGTTTTACTGCTGTAAGTTTAGGTCTTGCACGTAACCCAGC
>HPV32_Alpha_9627327_nt7288_L1_Tile_rc|1
GACTTTTGACTGGAAGATGCTGTTCGTTTTACTGCTGTAAGTTTAGGTCTTGCACGTAAC
>HPV32_Alpha_9627327_nt7298_L1_Tile_rc|1
AGGAGAAGAAGACTTTTGACTGGAAGATGCTGTTCGTTTTACTGCTGTAAGTTTAGGTCT
>HPV32_Alpha_9627327_nt7325_L1_Tile_rc|1
TTATTTACGTGTTTTTCTGCGTTTTGCAGGAGAAGAAGACTTTTGACTGGAAGATGCTGT
>HPV33_Alpha_333049_nt5725_L1_Tile_rc|1
GTACCAATAATTTTTTAGCGTTAGTAGGATTTTTAATAGAAAAATATGGATGGCCAACAG
>HPV33_Alpha_333049_nt5730_L1_Tile_rc|1
TTTGGGTACCAATAATTTTTTAGCGTTAGTAGGATTTTTAATAGAAAAATATGGATGGCC

>HPV33_Alpha_333049_nt5735_L1_Tile_rc|1
GATACTTTGGGTACCAATAATTTTTTAGCGTTAGTAGGATTTTTAATAGAAAAATATGGA
>HPV33_Alpha_333049_nt5740_L1_Tile_rc|1
AGCCTGATACTTTGGGTACCAATAATTTTTTAGCGTTAGTAGGATTTTTAATAGAAAAAT
>HPV33_Alpha_333049_nt5745_L1_Tile_rc|1
TTGCAAGCCTGATACTTTGGGTACCAATAATTTTTTAGCGTTAGTAGGATTTTTAATAGA
>HPV33_Alpha_333049_nt5750_L1_Tile_rc|1
CTATATTGCAAGCCTGATACTTTGGGTACCAATAATTTTTTAGCGTTAGTAGGATTTTTA
>HPV33_Alpha_333049_nt6385_L1_Tile_rc|1
CAGTAGTTCCTGAACCTTTAATGTACAGGTCATCGGGAACAGCCTCTCCTAATGTACCAG
>HPV33_Alpha_333049_nt6390_L1_Tile_rc|1
AGAGGCAGTAGTTCCTGAACCTTTAATGTACAGGTCATCGGGAACAGCCTCTCCTAATGT
>HPV33_Alpha_333049_nt6395_L1_Tile_rc|1
TGAATAGAGGCAGTAGTTCCTGAACCTTTAATGTACAGGTCATCGGGAACAGCCTCTCCT
>HPV33_Alpha_333049_nt6400_L1_Tile_rc|1
TGCTTTGAATAGAGGCAGTAGTTCCTGAACCTTTAATGTACAGGTCATCGGGAACAGCCT
>HPV33_Alpha_333049_nt6405_L1_Tile_rc|1
AGCACTGCTTTGAATAGAGGCAGTAGTTCCTGAACCTTTAATGTACAGGTCATCGGGAAC
>HPV33_Alpha_333049_nt6865_L1_Tile_rc|1
TATATTTACCTAAGGGGTCTTCCTTTTCCTTTGGAGGTACTGTTTTTTGACACGTAATAG
>HPV33_Alpha_333049_nt6995_L1_Tile_rc|1
GTGGATGTGGGGGCTGCACGTTTAAGTTTAGGTTTTGCTTTAAGACCTGCCTGTAATAAA
>HPV33_Alpha_333049_nt7000_L1_Tile_rc|1
TGCGGGTGGATGTGGGGGCTGCACGTTTAAGTTTAGGTTTTGCTTTAAGACCTGCCTGTA
>HPV33_Alpha_333049_nt7010_L1_Tile_rc|1
GCAGACGATGTGCGGGTGGATGTGGGGGCTGCACGTTTAAGTTTAGGTTTTGCTTTAAGA
>HPV34_Alpha_9627334_nt5616_L1_Tile_rc|1
TTCCCATTAGTATCCTTTATAGGATAATAGGGATGTCCTACTGCCAGCAAGCGTGTACTA
>HPV34_Alpha_9627334_nt5626_L1_Tile_rc|1
AATCTTACGTTTCCCATTAGTATCCTTTATAGGATAATAGGGATGTCCTACTGCCAGCAA
>HPV34_Alpha_9627334_nt5631_L1_Tile_rc|1
ACAGCAATCTTACGTTTCCCATTAGTATCCTTTATAGGATAATAGGGATGTCCTACTGCC
>HPV34_Alpha_9627334_nt5636_L1_Tile_rc|1
TAGGTACAGCAATCTTACGTTTCCCATTAGTATCCTTTATAGGATAATAGGGATGTCCTA
>HPV34_Alpha_9627334_nt5646_L1_Tile_rc|1
CCTGAAACTTTAGGTACAGCAATCTTACGTTTCCCATTAGTATCCTTTATAGGATAATAG
>HPV34_Alpha_9627334_nt5866_L1_Tile_rc|1
TATGTTTCCACCAATATATTTTGCAGCATTTTCAGTATCCTCAAGTTTATTCATAAAAGG
>HPV34_Alpha_9627334_nt5876_L1_Tile_rc|1
TACTATCTGCTATGTTTCCACCAATATATTTTGCAGCATTTTCAGTATCCTCAAGTTTAT
>HPV34_Alpha_9627334_nt5881_L1_Tile_rc|1
CTCCCTACTATCTGCTATGTTTCCACCAATATATTTTGCAGCATTTTCAGTATCCTCAAG
>HPV34_Alpha_9627334_nt5886_L1_Tile_rc|1
ATACACTCCCTACTATCTGCTATGTTTCCACCAATATATTTTGCAGCATTTTCAGTATCC
>HPV34_Alpha_9627334_nt5901_L1_Tile_rc|1
TTATAATCTACTGACATACACTCCCTACTATCTGCTATGTTTCCACCAATATATTTTGCA
>HPV34_Alpha_9627334_nt6011_L1_Tile_rc|1
TTAATTCAAGCGGAGGACATTCCCCAGCATTTTGGGTACCACATGGTGTGCCTGTACCCC
>HPV34_Alpha_9627334_nt6016_L1_Tile_rc|1
GTTCTTTAATTCAAGCGGAGGACATTCCCCAGCATTTTGGGTACCACATGGTGTGCCTGT
>HPV34_Alpha_9627334_nt6026_L1_Tile_rc|1
GTATAGTGGTGTTCTTTAATTCAAGCGGAGGACATTCCCCAGCATTTTGGGTACCACATG
>HPV34_Alpha_9627334_nt6266_L1_Tile_rc|1
TTAAGTCATCTGGAATAGCATCGCCTACAGTACCTGCCCTGTTAAATAGGTGTCTAACAA
>HPV34_Alpha_9627334_nt6276_L1_Tile_rc|1

CCCTTAATCATTAAGTCATCTGGAATAGCATCGCCTACAGTACCTGCCCTGTTAAATAGG
>HPV34_Alpha_9627334_nt6281_L1_Tile_rc|1
CTGTACCCTTAATCATTAAGTCATCTGGAATAGCATCGCCTACAGTACCTGCCCTGTTAA
>HPV34_Alpha_9627334_nt6506_L1_Tile_rc|1
GTGCAGTTGTACTTGTGGATTGTGTACCTACACAAACTGAAAAGTTTGTGCTTCTAGTAG
>HPV34_Alpha_9627334_nt6511_L1_Tile_rc|1
ATATGGTGCAGTTGTACTTGTGGATTGTGTACCTACACAAACTGAAAAGTTTGTGCTTCT
>HPV34_Alpha_9627334_nt6516_L1_Tile_rc|1
TTTGCATATGGTGCAGTTGTACTTGTGGATTGTGTACCTACACAAACTGAAAAGTTTGTG
>HPV34_Alpha_9627334_nt6536_L1_Tile_rc|1
GGTATTCCTTAAAATTACTGTTTGCATATGGTGCAGTTGTACTTGTGGATTGTGTACCTA
>HPV34_Alpha_9627334_nt6541_L1_Tile_rc|1
TCTGAGGTATTCCTTAAAATTACTGTTTGCATATGGTGCAGTTGTACTTGTGGATTGTGT
>HPV34_Alpha_9627334_nt6546_L1_Tile_rc|1
GCATGTCTGAGGTATTCCTTAAAATTACTGTTTGCATATGGTGCAGTTGTACTTGTGGAT
>HPV34_Alpha_9627334_nt6936_L1_Tile_rc|1
TTCTTAGGTGCTGTACTAGATGATGAAGGTGCAGACCGTTTAGAGGCCTGTAACCTAGGA
>HPV35_Alpha_333050_nt5690_L1_Tile_rc|1
TATTAGAATCTTGTTTTTTAATAGCATAGTATGGGTGACCCACAGCTAATAGCCTAGAAC
>HPV35_Alpha_333050_nt5695_L1_Tile_rc|1
TATTTTATTAGAATCTTGTTTTTTAATAGCATAGTATGGGTGACCCACAGCTAATAGCCT
>HPV35_Alpha_333050_nt5700_L1_Tile_rc|1
ACTGCTATTTTATTAGAATCTTGTTTTTTAATAGCATAGTATGGGTGACCCACAGCTAAT
>HPV35_Alpha_333050_nt5710_L1_Tile_rc|1
TACCTTGGGTACTGCTATTTTATTAGAATCTTGTTTTTTAATAGCATAGTATGGGTGACC
>HPV35_Alpha_333050_nt5720_L1_Tile_rc|1
GCAAACCAGATACCTTGGGTACTGCTATTTTATTAGAATCTTGTTTTTTAATAGCATAGT
>HPV35_Alpha_333050_nt5725_L1_Tile_rc|1
GTATTGCAAACCAGATACCTTGGGTACTGCTATTTTATTAGAATCTTGTTTTTTAATAGC
>HPV35_Alpha_333050_nt5730_L1_Tile_rc|1
ACTCTGTATTGCAAACCAGATACCTTGGGTACTGCTATTTTATTAGAATCTTGTTTTTTA
>HPV35_Alpha_333050_nt5735_L1_Tile_rc|1
TAAATACTCTGTATTGCAAACCAGATACCTTGGGTACTGCTATTTTATTAGAATCTTGTT
>HPV35_Alpha_333050_nt5955_L1_Tile_rc|1
CTGTTATCTGTACCAGAGTTACCAGAGTTACCAACATATTTATTAAGATTTTCAGTATCA
>HPV35_Alpha_333050_nt5960_L1_Tile_rc|1
ATTCCCTGTTATCTGTACCAGAGTTACCAGAGTTACCAACATATTTATTAAGATTTTCAG
>HPV35_Alpha_333050_nt5970_L1_Tile_rc|1
ATAGAAATGCATTCCCTGTTATCTGTACCAGAGTTACCAGAGTTACCAACATATTTATTA
>HPV35_Alpha_333050_nt6085_L1_Tile_rc|1
AGGAGGACATTCTCCTGCTTTTACCTGGTTAGCATTACAAGGTGTGCCTTTTCCCCAATG
>HPV35_Alpha_333050_nt6090_L1_Tile_rc|1
TCCAAAGGAGGACATTCTCCTGCTTTTACCTGGTTAGCATTACAAGGTGTGCCTTTTCCC
>HPV35_Alpha_333050_nt6095_L1_Tile_rc|1
GTAACTCCAAAGGAGGACATTCTCCTGCTTTTACCTGGTTAGCATTACAAGGTGTGCCTT
>HPV35_Alpha_333050_nt6100_L1_Tile_rc|1
GTTTAGTAACTCCAAAGGAGGACATTCTCCTGCTTTTACCTGGTTAGCATTACAAGGTGT
>HPV35_Alpha_333050_nt6105_L1_Tile_rc|1
ACAGTGTTTAGTAACTCCAAAGGAGGACATTCTCCTGCTTTTACCTGGTTAGCATTACAA
>HPV35_Alpha_333050_nt6110_L1_Tile_rc|1
GTAGTACAGTGTTTAGTAACTCCAAAGGAGGACATTCTCCTGCTTTTACCTGGTTAGCAT
>HPV35_Alpha_333050_nt6140_L1_Tile_rc|1
CAAATCCTGTGTCTACCATGTCCCCGTCTTGTAGTACAGTGTTTAGTAACTCCAAAGGAG
>HPV35_Alpha_333050_nt6200_L1_Tile_rc|1
TGCATATATCTAGGGGAACATCACTTTTATTAGCTTGTAATGTAGTAAAATCCATTGCAC

>HPV35_Alpha_333050_nt6205_L1_Tile_rc|1
GGAACTGCATATATCTAGGGGAACATCACTTTTATTAGCTTGTAATGTAGTAAAATCCAT
>HPV35_Alpha_333050_nt6210_L1_Tile_rc|1
CAAATGGAACTGCATATATCTAGGGGAACATCACTTTTATTAGCTTGTAATGTAGTAAAA
>HPV35_Alpha_333050_nt6375_L1_Tile_rc|1
AATGTGCCAGTGGTACCCTTAATATATAGGTCTGCAGGTACTGTTTCACCTACAGTTCCA
>HPV35_Alpha_333050_nt6380_L1_Tile_rc|1
TAGGCAATGTGCCAGTGGTACCCTTAATATATAGGTCTGCAGGTACTGTTTCACCTACAG
>HPV35_Alpha_333050_nt6395_L1_Tile_rc|1
GAAAATAACTAGTACTAGGCAATGTGCCAGTGGTACCCTTAATATATAGGTCTGCAGGTA
>HPV35_Alpha_333050_nt6405_L1_Tile_rc|1
CTAGGAGTAGGAAAATAACTAGTACTAGGCAATGTGCCAGTGGTACCCTTAATATATAGG
>HPV35_Alpha_333050_nt6410_L1_Tile_rc|1
AGCCACTAGGAGTAGGAAAATAACTAGTACTAGGCAATGTGCCAGTGGTACCCTTAATAT
>HPV35_Alpha_333050_nt6570_L1_Tile_rc|1
CTAGAAGACACAGCAGAACACACAGACATATTTGTACTACGGGTTGTATCAACTACAGTA
>HPV35_Alpha_333050_nt6575_L1_Tile_rc|1
TGTCACTAGAAGACACAGCAGAACACACAGACATATTTGTACTACGGGTTGTATCAACTA
>HPV35_Alpha_333050_nt6580_L1_Tile_rc|1
TGTACTGTCACTAGAAGACACAGCAGAACACACAGACATATTTGTACTACGGGTTGTATC
>HPV35_Alpha_333050_nt6710_L1_Tile_rc|1
TGGACGGGTTCATACTATGAATATATGTCATAACATCTGCTGTTAGTGTTATTTTACATA
>HPV35_Alpha_333050_nt6815_L1_Tile_rc|1
CACTGGGTTTTTGACAAGTTACAGCCTGTGATGTTACATAGCGATATGTGTCCTCTAAGG
>HPV35_Alpha_333050_nt6840_L1_Tile_rc|1
AATGGATCATCTTTAGGTTTTGGTGCACTGGGTTTTTGACAAGTTACAGCCTGTGATGTT
>HPV35_Alpha_333050_nt6970_L1_Tile_rc|1
CCTGCCTAATCTAAAATTAGGCCTGGCCTTTAGTCCTGCTTGTAACAAAAATTTACGGCC
>HPV35_Alpha_333050_nt6980_L1_Tile_rc|1
GAGCTGCACGCCTGCCTAATCTAAAATTAGGCCTGGCCTTTAGTCCTGCTTGTAACAAAA
>HPV35_Alpha_333050_nt6985_L1_Tile_rc|1
TGCTGGAGCTGCACGCCTGCCTAATCTAAAATTAGGCCTGGCCTTTAGTCCTGCTTGTAA
>HPV35_Alpha_333050_nt6990_L1_Tile_rc|1
GTAGATGCTGGAGCTGCACGCCTGCCTAATCTAAAATTAGGCCTGGCCTTTAGTCCTGCT
>HPV35_Alpha_333050_nt6995_L1_Tile_rc|1
TAGATGTAGATGCTGGAGCTGCACGCCTGCCTAATCTAAAATTAGGCCTGGCCTTTAGTC
>HPV35_Alpha_333050_nt7000_L1_Tile_rc|1
TTTTTAGATGTAGATGCTGGAGCTGCACGCCTGCCTAATCTAAAATTAGGCCTGGCCTT
>HPV35_Alpha_333050_nt7005_L1_Tile_rc|1
GAAGATTTTTTAGATGTAGATGCTGGAGCTGCACGCCTGCCTAATCTAAAATTAGGCCTG
>HPV35_Alpha_333050_nt7010_L1_Tile_rc|1
TAGTAGAAGATTTTTTAGATGTAGATGCTGGAGCTGCACGCCTGCCTAATCTAAAATTAG
>HPV35_Alpha_333050_nt7015_L1_Tile_rc|1
ACGTTTAGTAGAAGATTTTTTAGATGTAGATGCTGGAGCTGCACGCCTGCCTAATCTAAA
>HPV35_Alpha_333050_nt7025_L1_Tile_rc|1
TTACTTTTCTACGTTTAGTAGAAGATTTTTTAGATGTAGATGCTGGAGCTGCACGCCTGC
>HPV36_Beta_1020218_nt5967_L1_Tile_rc|1
AGTCAGTAGTCTGTCGCTGAATGCATGATAATAAATATTTGTCCTTTGTATATATTCATC
>HPV36_Beta_1020218_nt5972_L1_Tile_rc|1
[truncated: 459,782 more chars]
